# MMpred: functional miRNA – mRNA interaction analyses by miRNA expression prediction

**DOI:** 10.1186/1471-2164-13-620

**Published:** 2012-11-14

**Authors:** Przemyslaw A Stempor, Michael Cauchi, Paul Wilson

**Affiliations:** 1 Cranfield Health, Cranfield University, Vincent Building, Cranfield, UK; 2 Computational Biology, GlaxoSmithKline Medicine Research Centre, Gunnels Wood Road, Stevenage, UK

## Abstract

**Background:**

MicroRNA (miRNA) directed gene repression is an important mechanism of posttranscriptional regulation. Comprehensive analyses of how microRNA influence biological processes requires paired miRNA-mRNA expression datasets. However, a review of both GEO and ArrayExpress repositories revealed few such datasets, which was in stark contrast to the large number of messenger RNA (mRNA) only datasets. It is of interest that numerous primary miRNAs (precursors of microRNA) are known to be co-expressed with coding genes (host genes).

**Results:**

We developed a miRNA-mRNA interaction analyses pipeline. The proposed solution is based on two miRNA expression prediction methods – a scaling function and a linear model. Additionally, miRNA-mRNA anti-correlation analyses are used to determine the most probable miRNA gene targets (*i.e.* the differentially expressed genes under the influence of up- or down-regulated microRNA). Both the consistency and accuracy of the prediction method is ensured by the application of stringent statistical methods. Finally, the predicted targets are subjected to functional enrichment analyses including GO, KEGG and DO, to better understand the predicted interactions.

**Conclusions:**

The MMpred pipeline requires only mRNA expression data as input and is independent of third party miRNA target prediction methods. The method passed extensive numerical validation based on the binding energy between the mature miRNA and 3’ UTR region of the target gene. We report that MMpred is capable of generating results similar to that obtained using paired datasets. For the reported test cases we generated consistent output and predicted biological relationships that will help formulate further testable hypotheses.

## Background

MicroRNAs are short non-coding RNAs that utilise the cellular RNA-induced silencing complex (RISC) to influence gene expression [1]. The biogenesis of those regulatory organic polymers involves nuclear processing of the primary microRNA (pri-miRNA) by Drosha RNase III to precursor sequences (pre-miRNA). Pre-miRNA are in turn processed by Dicer endoribonuclease before being imported into the RISC, or redirected to the nucleus. The primary function of miRNA is believed to be gene repression [2], although gene activation (RNAa) has also been reported [3]. The majority of human coding genes are believed to be regulated by a relatively small set of microRNAs [4,5]. However, for efficient targeting of mRNA transcripts the co-regulation of many miRNAs is required. This many-to-many relationship between microRNA and coding transcripts creates an extensive, robust regulatory network, which is highly influential during cell differentiation and disease processes [6]. This complex regulatory miRNA-mRNA network is further integrated via co-expression of the coding transcripts. That is, the majority of pri-miRNAs are either located within introns or are in close proximity of coding genes, the so called host genes [7,8]. Consequently microRNAs are assumed to share transcription regulatory sites and to be co-expressed with coding mRNA transcripts. Recent surveys indicate that as few as 26% of intergenic mammalian miRNAs are transcribed from their own specific promoters [9]. Lutter *et al.* report that at least 37% of miRNAs are co-located within coding genes [10], while Rodriguez *et al.* state that approximately half of miRNAs are located within introns of coding and non-coding RNA [11]. Furthermore, Kim and Kim report that among microRNAs mapped to ESTs the percentage of intronic and exonic ones are 87% and 13% respectively [12]. The authors also suggest that due to exon pairing/tethering the independent processes of intronic miRNA biogenesis and mRNA splicing may occur in parallel, without affecting each other [12]. Moreover, genomic mapping conducted in support of this report (based on miRBase release 15) indicate that 578 of 940 (61%) human miRNAs share a primary RNA transcript with known coding genes [10]. These findings are further supported by a widely reported coherence of function between miRNA and host genes [8,10,13,14]. It is current opinion that microRNA support host gene function by repressing the expression and increasing decay rate of antagonistically acting genes, or promoting the expression of synergistically acting genes. For example, murine heart-specific gene Myh6 overlaps with miR-208a, which has been reported to negatively regulate the thyroid hormone associated protein and myostatin both of which negatively regulate muscle growth and hypertrophy [15]. Similar antagonistic effects have been shown for miR-346, miR-338 and their corresponding host genes GRID1 and AATK [13,14]. Furthermore, genes that share expression profiles with miRNA have been observed not to encode their respective microRNA seed regions [16], leading to the postulation that host genes have developed evolutionary resistance for miRNA mediated repression and degeneration [10,13]. Moreover, host genes tend to be co-expressed in clusters, which when combined with miRNA expression data create large, significantly correlated expression patterns [8,10,13].

The most significant changes of miRNA repression activity are observed during differentiation process [17,18]. It is believed that functional miRNA-mRNA coherence acts as a stabilizing mechanism that promotes the expression of tissue-specific genes while suppressing the expression of genes specific to stem cells and other tissues. Thus, a miRNA expression profile is tissue specific [10,19].

**Figure 1:**
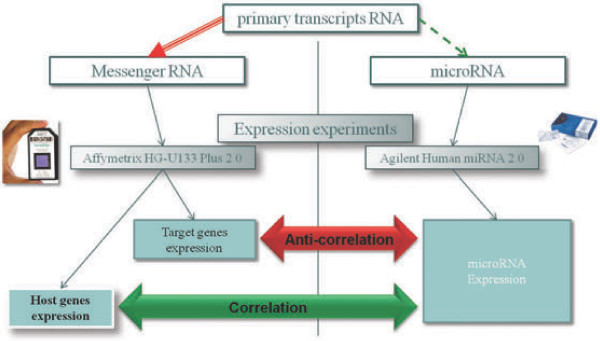
Simple overview of the Predictive Model assumptions.

Combined these observations imply that a miRNA expression profile is positively correlated with it’s host gene mRNA expression profile and anti-correlated with it’s target genes expression profiles. This simple functional model can be further extended to identify functional clusters of miRNA host genes. An intriguing application of this model is that we can use mRNA expression data to predict both miRNA expression and their putative targets (Figure 1).

Performing functional analyses of miRNA-mRNA interactions using standard methodology would require measuring global expression of mRNA and miRNA using two different arrays or RNA-sequencing experiments. Such approach requires a large quantity of purified RNA, increased processing and handling overhead, as well as the additional costs of supporting two different array platforms. Such impediments are reflected in the relatively small number of paired miRNA-mRNA datasets available in public repositories - (*i.e.* there are only nine Agilent Human miRNA Microarray (V2) datasets in GEO [20,21]; see Additional file 1). In contrast, GEO contains an impressive collection of high quality mRNA assays. Currently there are 2,170 datasets (60,334 samples) derived from the Affymetrix Human Genome U133 Plus 2.0 array and 117 datasets (4,642 samples) for Affymetrix Human Exon 1.0 ST, (as of 04/12/2010). Mining these data for host gene – miRNA targets offers a tremendous and immediate source of information regarding both miRNA target identification and regulation networks. In this paper we describe a method that is capable of both identifying putative regulatory clusters and predicting approximate expression levels of miRNAs from mRNA microarray data.

In completing this investigation we have focused on paired Affymetrix Human Exon ST 1.0 – Agilent Human miRNA Microarray 2.0 datasets to build a prediction model, and data derived from the Affymetrix Human Genome U133 Plus 2.0 - Agilent Human miRNA Microarray 2.0 as validation sets.

The initial step of this process involved mapping all of the miRBase human miRNAs to Affymetrix probes. Then, the paired datasets were used to construct two independent, general predictors. A consensus method was then developed to consolidate the predictors’ output and to correlate this with experimental mRNA expression data. This was used to identify putative miRNA interactions with coding genes (targets). Finally overrepresentation of the predicted target genes in different ontologies was estimated using a hypergeometric test to determine functionally annotated clusters of miRNA-genes interactions. The model has been implemented in the R statistical environment and is accessible as a modular, user-friendly analysis pipeline for the prediction of microRNA regulatory mechanisms using HG-U133Plus2 microarray data as input.

## Results

### User input and pre-processing

**Figure 2:**
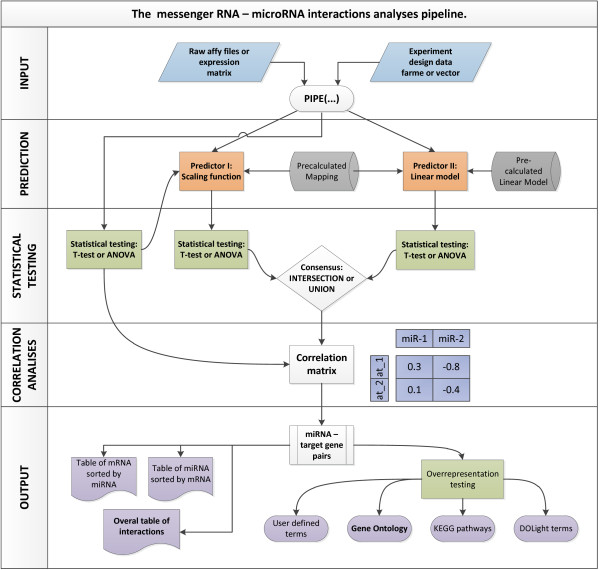
The flowchart presenting the general structure of the pipeline.

Raw microarray intensity values are pre-processed using the Robust Microarray Average (RMA) method [22]. Subsequent t-test or ANOVA statistical testing (this is dependent on the user-defined experimental design table) is performed. To determine a statistical significance (p-value) cut-off an equivalent test is performed on a randomised expression matrix. The randomization is achieved by simple permutation of the experimentally derived data. The lowest p-value statistic observed in the randomly designed experiment is chosen as the p-value cut-off for the experimental data. The pipeline output includes the expression matrix of significantly up-/down-regulated genes along with the estimated p-values and fold change vectors (Figure 2). Furthermore, visualisation of principal components analysis (PCA) scores, hierarchal cluster analysis (HCA) heatmaps and volcano plots are used as quality control assessments of the predicted model.

### The mapping of microRNAs to protein coding “host genes”

The mapping between microRNAs and its host genes was completed using a simple method that utilizes genomic coordinates retrieved from miRBase [23]. Retrieved fields include the Ensembl transcript IDs, and information detailing the nature of overlap (*e.g.* exonic, intronic, 3’UTR, 5’UTR *etc.*), the. This method proved more efficient than other tested approaches – (*e.g.* direct string matching and chromosome location methods; see Methods for further details).

The resulting network comprised 690 mature miRNAs and 544 coding genes connected by 3992 edges. The large number of connections between the nodes supports current opinion of a many-to-many relationship between miRNAs and host genes. 92% of the overlaps (3653) involve intronic sites, while 208 (5%) involve the exons of coding genes. In addition, 97 and 34 (2% and >1%) involve the 5’UTR and 3’UTRs respectively. Sorting the overlaps by DNA strand indicated that 3320 (83%) of the predicted interactions involve the coding strand and 672 (17%) the anti-sense strand.

The microarray platform specific mappings between Affymetrix genes/exons IDs and mature mRNA identifiers represented on the chosen platforms were retrieved and directly incorporated into the pipeline. In the case of Affymetrix Human Genome 133 Plus 2.0 mapping to Agilent Human miRNA Microarray 2.0, 996 probesets corresponding to 483 host genes (1,600 Ensembl transcripts), were identified. A total of 4,857 edges connect the transcripts to 544 pre-microRNAs. This can be further processed to 646 mature miRNAs as represented on the Human miRNA microarray. The second mapping features the same miRNA array platform and Affymetrix Human Exon 1.0 ST array. In this instance 996 probesets representing 14,191 exons (encoding 544 genes), have been identified as in close proximity of pri-miRNA sequences. An estimated 16,851 edges associate these transcripts to 578 pre-microRNAs, (representative of 646 mature miRNAs). Due to the increased genomic coverage and robust expression measurements the HuEx-1.0ST mapping were used to calculate the predictors’ parameters and validate the model. However, because of much larger numbers of HG-U133Plus2 experiments in GEO, this array was selected as the primary input platform for the pipeline.

The mapping is utilised as a binary file when the pipeline is executed. Obviously the mappings can be re-calculated, with new releases of the source databases. A representative section of the mapping table is illustrated in Table 1; the full mapping table is included as Additional file 2.

**Table 1:** The sample of the mapping table containing information from miRBase and Ensembl

**Mirbase_id**	**s.**	**Overlap**	**Evidence**	**Ensembl_gene_id**	**Ensembl_transcript_id**	**Affy_hg_u133_plus_2**	**Affy_hu x _1_0_st_v2**	**Chromosome**	**Start_ position**	**End_ position**	**miR**	miR*
hsa-let-7a-3	+	exon	HGNC_automatic_transcript	ENSG00000197182	ENST00000360737	**232480_at**	3948921	22	46449741	46509808	**hsa-let-7a**	hsa-let-7a*
hsa-let-7a-3	+	exon	Vega_transcript	ENSG00000197182	ENST00000360737	**232480_at**	3948949	22	46449741	46509808	**hsa-let-7a**	hsa-let-7a*
hsa-let-7b	+	exon	HGNC_automatic_transcript	ENSG00000197182	ENST00000360737	**232480_at**	3948921	22	46449741	46509808	**hsa-let-7b**	hsa-let-7b*
hsa-let-7b	+	exon	Vega_transcript	ENSG00000197182	ENST00000360737	**232480_at**	3948949	22	46449741	46509808	**hsa-let-7b**	hsa-let-7b*
hsa-let-7c	+	intron	HGNC_curated_transcript	ENSG00000215386	ENST00000308787	**1559901_s_at**	3915214	21	17442842	17982094	**hsa-let-7c**	hsa-let-7c*
hsa-let-7c	+	intron	HGNC_curated_transcript	ENSG00000215386	ENST00000308787	**1559901_s_at**	3915194	21	17442842	17982094	**hsa-let-7c**	hsa-let-7c*
hsa-let-7c	+	intron	HGNC_curated_transcript	ENSG00000215386	ENST00000308787	**1559901_s_at**	3915317	21	17442842	17982094	**hsa-let-7c**	hsa-let-7c*
hsa-let-7c	+	intron	HGNC_curated_transcript	ENSG00000215386	ENST00000308787	**1559901_s_at**	3915201	21	17442842	17982094	**hsa-let-7c**	hsa-let-7c*
hsa-let-7c	+	intron	HGNC_curated_transcript	ENSG00000215386	ENST00000308787	**1559901_s_at**	3915291	21	17442842	17982094	**hsa-let-7c**	hsa-let-7c*
hsa-let-7c	+	intron	HGNC_curated_transcript	ENSG00000215386	ENST00000308787	**1559901_s_at**	3915202	21	17442842	17982094	**hsa-let-7c**	hsa-let-7c*
hsa-let-7c	+	intron	HGNC_curated_transcript	ENSG00000215386	ENST00000308787	**1559901_s_at**	3915257	21	17442842	17982094	**hsa-let-7c**	hsa-let-7c*
hsa-let-7c	+	intron	HGNC_curated_transcript	ENSG00000215386	ENST00000308787	**1559901_s_at**	3915275	21	17442842	17982094	**hsa-let-7c**	hsa-let-7c*
hsa-let-7c	+	intron	HGNC_automatic_transcript	ENSG00000215386	ENST00000308787	**1559901_s_at**	3915192	21	17442842	17982094	**hsa-let-7c**	hsa-let-7c*
hsa-let-7c	+	intron	Vega_transcript	ENSG00000215386	ENST00000308787	**1559901_s_at**	3915318	21	17442842	17982094	**hsa-let-7c**	hsa-let-7c*
hsa-let-7c	+	intron	Vega_transcript	ENSG00000215386	ENST00000308787	**1559901_s_at**	3915193	21	17442842	17982094	**hsa-let-7c**	hsa-let-7c*
hsa-let-7c	+	intron	Vega_transcript	ENSG00000215386	ENST00000400178	**1559901_s_at**	3915214	21	17442842	17982094	**hsa-let-7c**	hsa-let-7c*

This table is used by the mapping function, essential for both prediction methods.

### Predictor I: Scaling function

Paired microRNA-mRNA dataset “*Array-based bioinformatic analysis on pediatric primary central nervous system germ cell tumors”*[24] has been selected to test linear model assumptions. After obtaining an expression matrix using the RMA method [22], correlation coefficients were calculated for each gene – microRNA interaction (*i.e.* how each row of the miRNA matrix correlates with each row of the mRNA matrix). Correlation values were determined using the Pearson product–moment coefficient, which is generally considered suitable when estimating the linear relationships. Also Spearman’s ρ and Kendall’s τ rank coefficients were used. These methods are sensitive to monotonic association and resistant to outliers. No significant correlation was detected with the unfiltered data using either method. Furthermore, the distribution of correlation coefficients was very close to a standard normal distribution (supported by the Shapiro-Wilk test [25]).

In contrast, when only those miRNAs that had been mapped to the host genes transcripts were used, the correlation coefficient values attained were 0.23 for Pearson’s, 0.22 for Spearman’s and 0.16 for Kendall’s method. This is a significant improvement over non-mapped interactions. The relatively higher value of the Pearson product–moment correlation suggests that the observed correlation in the dataset may be linear in nature. To determine if the mapped genes represent a random sampling of the population of all genes, the Shapiro-Wilk test was performed. The null hypothesis that the sample is derived from a normally distributed population, was rejected with a 99% confidence interval p-value of < 0.0001 (α level 0.05).

**Figure 3:**
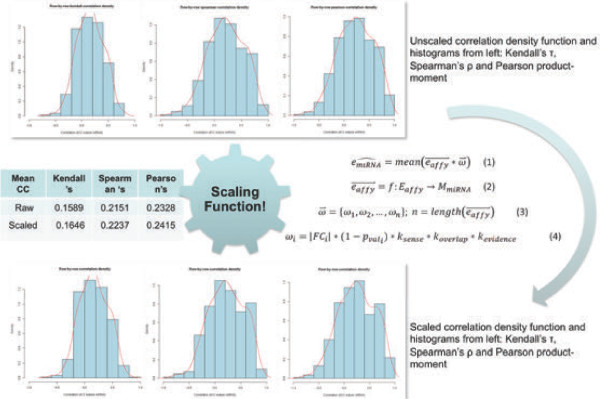
Predictor I – The scaling function.

Consequently a scaling function was introduced to estimate the miRNA expression values from the corresponding host genes’ expression (Figure 3). The main assumption of the model is that the expression of 587 pre-miRNA can be predicted from mRNA expression. However, many microRNA have been mapped to more than one probeset, likewise some Affymetrix probeset IDs correspond to more than one miRNA (*i.e.* a many-to-many relationship). Consequently, miRNA mapping to the sense strand of the intronic regions of coding genes, and those miRNA with experimental evidence are much more relevant to the model. Furthermore, significant differential expression of host gene mRNA transcripts (*i.e.* identified by high absolute fold change in association with a low p-value) indicates a significant change in expression of corresponding microRNA.

Validation of the model indicated that the mean correlation of overlapping miRNA with their host genes is only marginally improved by performing scaling. However, values on the right tail of the probability distribution plot, representing strongly correlated expressions (*i.e.* a Pearson’s correlation coefficient of 0.6 and above), are significantly enriched. This is readily apparent when we compare the respective un-scaled and scaled histograms (Figure 3). Furthermore, the scaling function introduces even stronger deviation from Gaussian distribution. This is reflected the lower p-value obtained from the Shapiro-Wilk normality test. The mean Pearson’s, Spearman and Kendall correlation coefficients achieved after scaling were 0.24, 0.22 and 0.16 respectively.

Finally the predictor uses calculated expression values to build a pseudo-expression matrix. This matrix has exactly the same construction as expression sets obtained from real microarray experiments, but the values are generated in silico, using the linear predictor, rather than experimentally determined expression data.

### Predictor II: Linear model

Despite the satisfactory performance of scaling function predictor several tests indicated that implementing a general linear model might further enhance the predictive power of the model. When applying this approach the coefficients are fitted using least squares method derived from the paired data rather than being arbitrarily chosen. Furthermore, it is also feasible to introduce individual coefficient values for each miRNA to more accurately reflect biological dependencies.

To fit a linear model that correctly optimizes the linear function parameters for each microRNA, an appropriate training dataset was required. The *“Array-based bioinformatic analysis on pediatric primary central nervous system germ cell tumors”* dataset, previously used for validation and evaluation was obviously too small for building a robust model capable of generalization. Consequently it was decided to train the model on a larger dataset and use the smaller dataset for validation. Ideally the training set should comprise >100 paired arrays and provide the best coverage for both coding transcripts and miRNAs. Assessing GEO and ArrayExpress identified only one dataset [26] that met these specifications: *“Integrative genomic profiling of human prostate cancer”* (GSE21032). The raw array data were RMA normalised [22]. Messenger RNA expression indexes were used as independent variable to describe the dependent variable – *i.e.* the miRNA expression. The linear regression coefficients were fitted using the least squares method.

To pair miRBase IDs with their corresponding Affymetrix Human Exon Array host transcripts IDs, the previously used mapping array was extended using HuEx-1.0ST transcript IDs. Since the Human Exon chip is backward compatible with Affymetrix genome chips this operation proved feasible [27-29].

**Figure 4:**
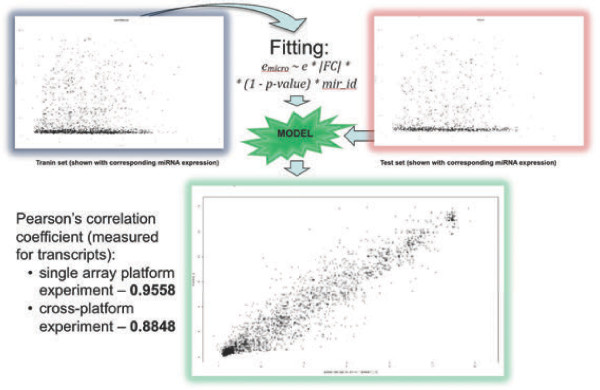
Predictor II – linear model.

In order to optimize the predictor power and avoid over-fitting expression values were split into a training set (2/3 of the data) and a test set (1/3 of data). To minimise any potential bias the composition of both sets was randomized after pairing miRNA expression indexes with their respective mRNA expression values (Figure 4).

After maximizing the prediction power the utility of generalizing predictions on different array experiments and platforms were assessed. On this occasion, the linear models were trained on all available data from the *“Integrative genomic profiling of human prostate cancer”* (GSE21032) dataset (*i.e.* previous training and test set joined together) and validated using the “*Array-based bioinformatic analysis on pediatric primary central nervous system germ cell tumors*” (GSE19350) dataset. The calculated cross-platform correlation was 0.884, which support s the assumption of conservative cross-tissue miRNA-mRNA regulatory mechanisms (*i.e.* the model trained on the prostate cancer dataset was able to precisely predict miRNA expression in brain tissue).

### Correlation analyses

**Figure 5:**
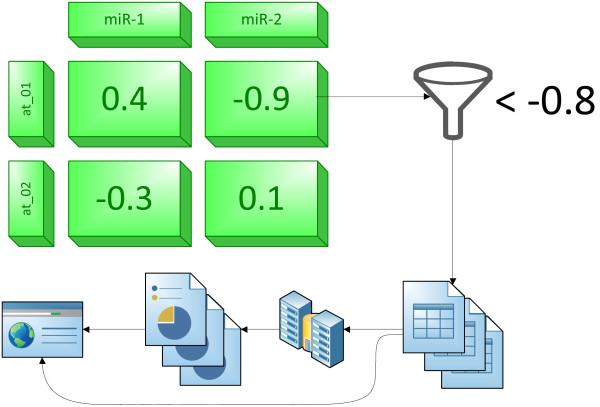
**The flow-chart presenting the idea of filtering putative miRNA target genes.** The predictor output based correlation matrix is filtered by a negative correlation cut-off in order to find putative miRNA-target interactions. These interactions are subsequently used for GO, KEGG, DOlight over-representation testing and creating user-readable HTML output.

Correlation between messenger RNA and microRNA is the corner stone of the pipeline. A positive correlation indicates a host gene relationship while a negative value suggests a target gene relationship. The pipeline utilizes both dependences to extract genes predicted to be influenced by miRNA (*i.e.* in the absence of experimentally estimated miRNA expression data). Each significant predicted miRNA pseudo-expression value is correlated to the significant experimental mRNA expression data creating a correlation matrix (Figure 5). Then a user-determined cut-off filter is applied. The cut-off is a negative number representing strong reversed correlation. The default value of −0.8 was chosen for robust general performance. Typically, if a user is interested in the broad spectrum of processes that may be influenced by miRNA the cut-off should be higher. In contrast a narrow and highly reliable set of predicted interactions is achieved using lower cut-off values.

### Final analyses – GO, KEGG, DOLight and user defined terms overrepresentation testing

Filtering the most anti-correlated expression values generates a list of microRNA – target gene interactions. Depending on the parameters defined by the user and the quality of the input data the length of this list may vary significantly. The pipeline generates three summary lists: (1) influenced genes, sorted by miRNA identified as inducer of coding transcript quantity change, (2) miRNAs sorted by genes they are influencing and (3) all interactions with significance score (*i.e.* the number of anti-correlation values supporting the interaction).

The Affymetrix probe IDs are transformed into user-friendly Entrez IDs, HGNC symbols and gene names, which are also easily integrated into third party tools. Each of the lists is available to the user in either CSV format, or displayed in an HTML report.

The final step of the pipeline performs analyses of gene ontology terms, KEGG pathways, DOLite disease ontology and user defined Entrez terms. In each case a hypergeometric test is applied to those genes predicted to be influenced by miRNA differential expression to evaluate enrichment of each category. Subsequently, the corresponding table of terms with test statistics, pie chart, bar chart, and concept network of interaction and heatmap of most overrepresented genes featured in each of the ontology categories is generated. These tables and plots are incorporated into a final HTML report. The motivation for incorporating such analyses into the pipeline was to facilitate biological interpretation of the output. The lists of miRNAs and differentially repressed mRNAs may by very long; enrichment categories offers the user a consistent, compact output and simplifies assessment of the biological significance of the predicted mRNA – miRNA interactions and direct further validation studies.

Examples of the pipeline results and sample HTML reports (*i.e.* basic output of the pipeline, as well as reports generated by performing case-studies) are provided as supplementary material (Additional files 3 and 4).

### The validation of expression based target prediction and pipeline’s general performance

We experimentally validated the predictive models by correlating the predicted miRNA expressions with the ones obtained from microarrays. To validate if strongly anti-correlated interactions between the predicted miRNA and measured mRNA expressions can identify putative target genes we implemented systematic, numerical method based on the binding energy between the mature miRNA and 3’ UTR region of the gene. The general pipeline performance was assayed by comparing the analyses presented in the GSE19350 validation dataset author’s publication (Wang *et al.*, BMC Genomics. 2010) with the output generated by MMpred. Finally, we applied the analysis pipeline to a number of datasets to further investigate the validity of predicted miRNA-mRNA interaction networks. Two of the completed case studies are provided in Supplementary materials (Additional files 5, 6 and 7).

### The miRNA-target binding energy base validation

**Figure 6:**
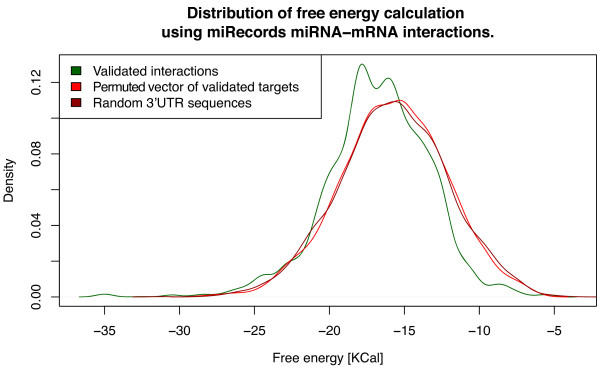
**The distribution of calculated free energies in validated targets set driven from miRecords.** The calculation has been obtained by sampling 3240 sequences of 3’UTR human target genes for optimal miRNA binding free energy. The randomized sampling contains the same number of free energy calculations.

The method we propose is modified “energy walk” procedure described in the paper by Ritchie *et al.*[30], which utilizes the impact of binding energy in proper miRNA-target pairing [31,32]. The experimentally proven miRNA-mRNA interactions from miRecords were sampled in order to calibrate the method. To confirm the significance of results, two random sets of free binding energies were calculated: by permutation of genes name vector (using the same set of 3’UTR sequences) and by substituting the original set of 3’UTR with random gene sequences. The results are shown on Figure 6.

The study of lowest binding energy distributions revealed that using fixed free energy cut-off (−20 Kcal, Ritchie *et al.*) would discard most of validated targets. For this reason we compared the distributions of minimal energy among the miRNA-target pairs rather than the number of high energy binding sites like in original procedure.

To further assay the significance between actual and randomized energy calculation the Welch Two Sample t-test has been performed. The null hypothesis (true difference in means between actual and randomized data is equal to 0) has been rejected with p-value < 2.2e-16 for both randomizations. It should be noted that the randomized samples have the same mean with p-value = 0.9776.

Further, we validated experimentally measured miRNA-mRNA expression anti-correlation as target identification method using the paired microarray dataset “*Comparative genomics matches mutations and cells to generate faithful ependymoma models*” (GSE21687). At first, measured miRNA expression matrix was correlated against mRNA expression matrix. Then the correlations have been filtered using *GetHT* function from MMpred pipeline with correlation cut-off equal −0.6. The predicted interactions were subjected to the same procedure as miRecords interactions. Two randomized energy calculations have been prepared: using permutated vector of predicted targets (Figure 6) and the permutated target sequence (not shown on the figure). The mean of predicted targets is significantly different from the randomized values (p-values of 2.925e-11 and 5.663e-07; Welch Two Sample t-test). Furthermore, the distribution is similar to validated targets (p-value = 0.3748; Welch Two Sample t-test).

**Figure 7:**
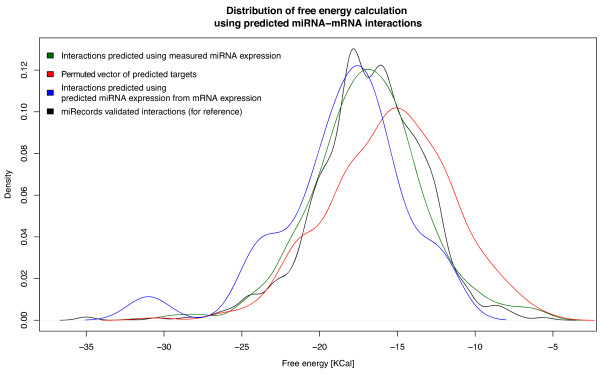
**The distribution of calculated free energies in expression anti-correlation based target predictions.** The calculations have been obtained by sampling sequences of 697 3’UTR human target genes candidates for experimental miRNA expression dataset and 26 for predictor drive miRNA expression (full MMpred pipeline). The randomized sampling contains 697 free energy calculations.

**Figure 8:**
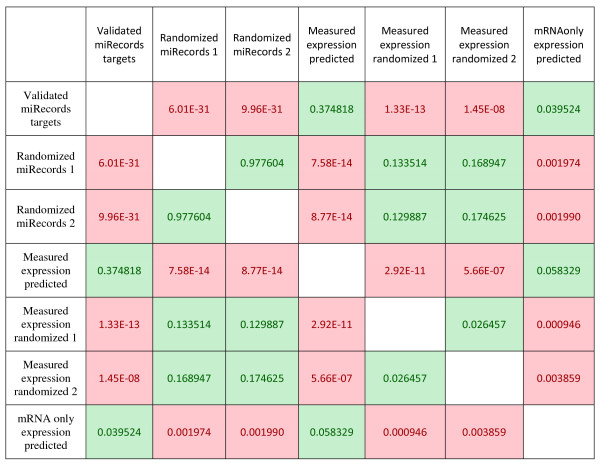
**P-values obtained from Welch Two Sample t-test.** The cases where the null hypothesis has been rejected are marked in red (p-value cut-off equals 0.01), otherwise marked in green.

Finally, to assess both predictive power of miRNA expression predictor and targets predictive capabilities, the full MMpred pipeline has been run on GSE21687 mRNA expression data only, repeating the same free energy calculation procedure (distribution shown on Figure 7 as blue curve). The distribution is similar to both miRecords and experimental data driven distributions (p-values equals 0.03952 and 0.05833) and dissimilar to randomized ones (see Figure 8 for all p-value comparisons). Comprehensive description of the method can be found in Additional file 8.

### General performance and usability

We compared the analyses presented in Wang *et al.* publication (BMC Genomics. 2010), which is citing the GSE19350 dataset, with the output of MMpred. The original analyses have been performed on paired dataset (miRNA expression assayed by microarray), while MMpred used mRNA data only (miRNA expression was predicted). Wang *et al.* picked signature miRNAs and predicted their targets. Of the three intragenic miRNAs listed there MMpred determined hsa-mir-218 to be significantly deregulated. Of the 6 target genes identified for hsa-mir-218 by Wang *et al.* MMpred was able to predict 5. Considering 5 MMpred predictions overlapping and 1 not overlapping with published data, 146 other MMpred predictions and 21835 other possible predictions (based on 21976 protein coding genes represented on HGU-133plus2 microarray; source: Ensembl67) the Fisher's exact test p-value equals 8.52e-11. Moreover, the downstream analyses performed by Wang *et al.* and automated MMpred pipeline output shows significant overlap; *e.g*. MMpred identified 4 out of 5 top KEGG pathways (total of 7 KEGG pathways identified by Wang *et al.*). The presented results were obtained using default MMpred settings and input dataset have never been used for training the predictive model. Re-mapping the miRNAs both to their host genes and to the transcripts represented on mRNA expression arrays using the most recent releases of miRbase and Ensemble databases enhances the prediction power even further. The examples of such analyses are provided in Additional file 8. Presented examples clearly illustrates that MMpred is capable of generating similar results to that obtained using paired datasets (considering the limitation to intragenic miRNA).

## Discussion

The primary objective of the reported model is to facilitate miRNA focussed analyses of the large body of mRNA expression data available in public repositories. Extensive, long term usage of microarray gene expression assays in clinical studies has produced a vast repository of extremely valuable, well-designed datasets. This is in contrast to the very limited miRNA expression datasets available in the public domain. Our model enables inexpensive hypothesis generation regarding miRNA regulatory events, from this vast repository of mRNA expression datasets. The primary assumption implemented in the pipeline is that analyses of correlation between regulatory host genes and miRNAs can be used to predict miRNA regulatory networks. Since the majority of human microRNAs are co-expressed with host genes we propose that expression of these miRNAs is positively correlated to their host transcripts. That is, over-expression of host genes indicates a positive fold change of miRNA copy number and visa-versa. A further assumption is that such microRNAs are expressed in the same quantity and at the same time as their respective host genes (*i.e.* we conveniently neglect the maturation process and post-transcriptional regulation of miRNA, of which little is currently known).

In contrast, miRNAs promote target gene degradation, which is in turn detected as a lower expression signal on mRNA microarrays. These two dependences were used to create a general mathematical model of miRNA expression prediction and to predict regulatory miRNA networks. The model was initially validated using numerical coherence between predicted and experimental data achieving a significant degree of correlation. Subsequent functional hypothesis generation using model predictions was evaluated by completing case studies with three previously reported mRNA expression datasets (GSE11327 [33], GSE11375 [34] and GSE19743 [35]). All illustrated cases indicate that it is feasible to predict what appears to be biologically coherent miRNA-mRNA regulatory networks using only mRNA expression data. Further systematic validation of target prediction was successfully accomplished by analysing the distribution of free binding energy between miRNAs and predicted target’s 3’ UTR region. We showed that the predicted binding energy distribution is similar to energy distribution driven from miRecords [36] validated targets database, and significantly different from randomized one (see Additional file 9 for details).

Possible applications of the pipeline include, miRNA target prediction, constructing putative miRNA regulatory clusters and a cost efficient means of generating a large number of predicted differential miRNA expression profiles from the vast repository of human mRNA data in the public domain.

Methodology similar to MMpred was previously reported. For example, several tools utilises miRNA-targets anti-correlation to rank the computational target predictions (usually sequence matching or homology based) and identify ones, which are most probable to be a true biological hits. The validation is usually performed by experimental assays or measuring the enrichment in overlap between top ranked predictions and validated miRNA targets. A noteworthy example is the HOCTAR method [37,38], which uses large collection of mRNA expression profiles (utilizing both host genes’ correlation and anti-correlation with targets) to score predictions from PicTar, TargetScan, and miRanda. Similar approach is proposed in GenMiR++ method [39], though this method does not utilize host genes interaction and requires paired miRNA-mRNA microarray datasets. Furthermore, a method developed by Ritchie *et al.* uses expression patterns conserved between human and mouse to predict miRNA targets more accurately [30]. Moreover, several assays not focussed on target prediction use similar methodology as a validation technique: *e.g.* the “enrichment score” proposed by Biasiolo *et al.*[40]. Despite several published methods focusing on the correlation of expression patterns we strongly believe that MMpred is a significant improvement and valuable addition to the field. While other methods study large collections of expression experiments and provide general target predictions MMpred focuses on case specific targets, which are under differential control of differentially expressed miRNAs. Furthermore MMpred is independent of both external target predictions and miRNA expression data. The model predicts and functionally annotates dataset specific miRNA regulatory networks using abundant coding gene expression data.

However, before applying the model one must be aware of it’s limitations. In particular, the predictor does not determine if genes connected within the functional category are suppressed by miRNA, or that the suppression normally existing in the control group has been alleviated. The pipeline does identify if the expression of differentially regulated genes is significantly anti-correlated with the expression of one more predicted miRNA. The direction of regulation (*i.e.* up-regulation by lifting miRNA suppression or down-regulation by introducing miRNA suppression and degradation) is determined using fold change calculations.

The functional analyses (*i.e.* GO, KEGG, DO and user determined Entrez terms) are performed using predicted target gene annotation. MicroRNAs are poorly annotated, with no consistent ontology. Many miRNAs are reported to regulate a large numbers of genes so it is very difficult to determine the primary miRNA function. To determine the specific function of miRNA in a given expression set both the combined predictions and overrepresentation testing of significant miRNA targets is required.

Although the gene ID method was chosen as the default pipeline’s mapping generator, other tested methods (*i.e.* direct string matching and genomic location) are also worthy of consideration. Apart from the associated computational complexity a string matching approach would be expected to generate the most accurate results. Moreover, this method generates a number of pre-miRNA sequence overlaps with each gene sequence, which could be used to boost the predictor’s accuracy. However this approach is likely to also produce false negatives, as partial miRNA-mRNA matches may still be co-expressed. Furthermore, the changes that would be incurred with different human genome assembly versions may introduce unwanted variability of mappings.

The validation of predictors indicated that for many intronic miRNAs the linear model predictor performed better, though in a few cases the scaling functions performed best. For that reason we decided to implement both predictors in the pipeline. The number of miRNA predicted to be significantly misregulated after performing auto-generated cut-off may differ considerably for each of the predictors. In certain extreme cases there may be no miRNA found significantly over- or under-expressed by one or both predictors. If only one predictor returns significant miRNAs the pipeline will continue to execute. If both predictors return no significant result further analyses is impossible and the process will terminate. In such scenarios the user would either adjust the cut-off parameter or re-evaluate the experiment design. A union of the predictions is used to report a consensus result. When using the linear model approach fold change values are generally smaller and possibly more likely to reflect experimental fold change. This is due to the specificity of this predictor – that is the linear model uses coefficients fitted using the experimental data, hence making its predictions more accurate. In contrast, the coefficients of the scaling functions are chosen manually and the final coefficient is a product of the multiplication. This approach may overestimate the fold change value of genes/miRNAs with high expression index. Beside linear predictors some higher order predicting methods (*e.g.* Generalized Linear Model (GLM), Neural Networks and Genetic Algorithms) have been tested without any significant improvement to the pipeline’s performance.

**Figure 9:**
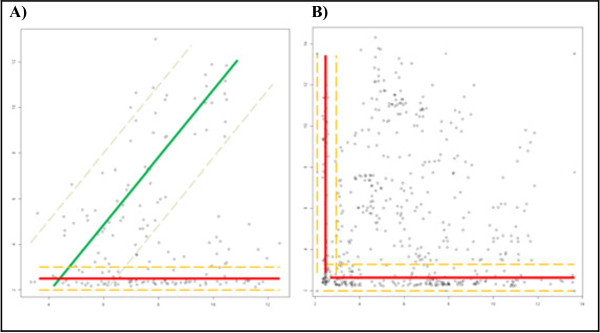
**Correlation box. (A)** Top500 mRNA transcripts ranked by p-value (X-axis) plotted against corresponding miRNA expressions (Y-axis). The group presenting good linear correlation is featured with green regression line, while the group with no expression indexes dependence is featured with red one. **(B)** Messenger RNA transcripts expression index (X-axis) plotted against corresponding exotic miRNA transcripts’ expressions index (Y-axis). No expression indexes dependence is visible on this plot.

The interactions derived from correlation analyses support the biological rational of the predictors. Our first investigation is an assessment of the top 500 mRNA intronic transcripts expression (*“Integrative genomic profiling of human prostate cancer”*, GSE21032 dataset [26]) ranked by the absolute value of fold change plotted against expression indexes of corresponding miRNAs (Figure 9A). The visualized transcripts can be divided into two subsets. The first one presents a strong linear correlation. In the second, increased mRNA expression have not been reflected in a higher miRNA expression index. This perfectly illustrates the biological dependence between microRNA and its host genes (*i.e.* the pri-miRNA transcript must be transcribed along with the mRNA to be processed and then detected on the microarray as mature miRNA). Given this define relationship no miRNA with high expression corresponds to a mRNA with low expression. In contrast there are many observed mRNA with high expression values corresponding to miRNA with very low expression indexes. This likely to occur as posttranscriptional regulation is an important factor both during miRNA transport and maturation, so simply expressing a pre-miRNA transcript does not guarantee it will be processed to the mature form. Such relationships introduce a danger that the model may produce a significant number of false positives, but the risk of false negatives is minimal. Exonic transcripts that overlap with pri-miRNA have been used as negative control. Our rational being that those transcripts are primarily used to generate mRNA, and our model assumes that they are not available for miRNA processing, so the probability of maturating into a functional miRNA is minimal. This is confirmed when observing the scatter plot of expressions (Figure 9B) - where numerous highly expressed miRNAs map to mRNA with very low expression index and also highly expressed mRNA corresponding to miRNA with very low expression indexes. Furthermore, the distribution of intermediate points seems random, as there is no significant linear correlation present in the exonic transcripts.

## Conclusions

We present details of MMpred, a novel and generally applicable mathematical model of miRNA-mRNA interactions predicted from mRNA expression data. The method enables cost and time efficient hypotheses building of both miRNA differential expression and miRNA-mRNA interactions using retrospective analyses of publicly available mRNA microarray datasets. The notable advantage of the model is the creation of case specific predictions of miRNA-mRNA signalling networks from mRNA datasets. Contrary to the approach applied by other miRNA target prediction tools, that aim to find all possible miRNA-target repression interactions, our minimalistic, case specific approach reduces the burden of numerous false discovery rates. Additionally, the fewer number of significant targets returned by the prediction pipeline simplifies associated functional analyses of the predicted networks.

The MMpred pipeline reports the functional enrichment categories of the most likely miRNA-mRNA relationships given the experimentally determined differential gene expression profile. The data are presented in a succinct manner to facilitate testable hypothesis generation of the predicted miRNA-mRNA interaction networks. For example, the comparative burn and blunt injuries case study indicates that miRNAs repressing immune system cells’ metabolic genes are down-regulated in order to relief the metabolic lock of inflammatory response, thus protecting the organism against infections and promoting the regeneration process (see Additional file 5). The predicted signalling mechanism appears biologically meaningful and facilitates the design of further experimental validation studies.

The MMpred model is implemented as an R package and is suitable for further community validation (details in the Supplementary materials). Our validation showed significant prediction power and ability to partially reproduce results obtained by analysing paired expression datasets. The reported case studies indicate that the method predicts biologically coherent miRNA-mRNA networks and that the approach will add value to current miRNA regulatory network analysis efforts. Consequently, we believe MMpred is a useful tool for mining the vast mRNA expression data resources and screening for potential miRNA targets and miRNA-mRNA functional modules.

## Methods

### The mathematical bases of the predictors and correlation analyses

The scaling function predictor can be summarized as set of vector equations and implemented as required in the model:

$e_{m\hat{\text{iR}}\text{NA}}$  Estimated microRNA expression index

$e_{\text{affy}}$   Vector of mRNAs expression indexes

***E***_***affy***_   Expression matrix of mRNA obtained from Affymetrix microarray

***E***_***miRNA***_   Expression matrix of miRNA obtained from Agilent microarray

***FC***_***i***_    Fold change of i-th mRNA present on Affymetrix array

$p_{val_{i}}$   p-value (from Student’s t-test or ANOVA) statistic of i-th mRNA present on Affymetrix array

***k***_***sense***_  Strain dependant coefficient (*1.2* for sense *0.8* for antisense)

***k***_***overlap***_  Overlap dependant coefficient (*2* for intronic *0.8* for exonic, 3’UTR, 5’UTR)

***k***_***evidence***_ Evidence dependant coefficient (*1.2* for experimentally determined *0.8* for predicted)

**Equation 1** - General formula for the scaling function predictor

(1) $$\hat{e_{\text{miRNA}}}=\text{mean}\left({e_{\text{affy}}}^{⇀}*\omega^{⇀}\right)$$

**Equation 2** - Mapping function

(2) $$\vec{e_{\text{affy}}}=f:E_{\text{affy}}\to E_{\text{miRNA}}$$

**Equation 3** - Weight vector

(3) $$\vec{\omega }=\left\{\omega_{1},\omega_{2},\ldots ,\omega_{n}\right\};n=length\left(\vec{e_{\text{affy}}}\right)$$

**Equation 4** - Scaling coefficients determining weight vector elements

(4) $$\omega_{i}=\left|FC_{i}\right|*\left(1-{p_{\text{val}}}_{i}\right)*k_{\text{sense}}*k_{\text{overlap}}*k_{\text{evidence}}$$

**Equation1** represents the general form of the predictor, which calculates the estimated microRNA expression index by averaging elements of the experimentally observed mRNA expression vector multiplied by a weight vector. The expression vector is created by a mapping function, which selects expression values corresponding to host genes from the messenger RNA expression matrix (**Equation2**). Simultaneously a weights vector of the same length is created (**Equation3**). Each value in this vector is calculated by multiplying the absolute fold change (FC) and reverse scaled p-value (1-*Pval*) obtained for each gene during pre-processing in addition to three coefficients (*k*_sense_, *k*_*overlap*_ and *k*_*evidence*_) that combined describe the nature of predicted edge between miRNA and mRNA (**Equation4**). Values for these coefficients have been arbitrarily assigned, using biological knowledge and computational tests performed prior to building the function. For example the intronic regions are extracted from coding sequences during splicing, which theoretically makes them available to the Drosha enzyme. However, both the 3’UTR, and 5’UTR are incorporated into mature mRNA, so they can only be processed to miRNA if the maturation process and transportation of mRNA out of the nucleus is interrupted. These scenarios dictate that the model preferentially promotes intronic sequences.

For linear model predictor the principal mathematical problem encountered while constructing the optimal regression formula was the variable number of the independent values describing each dependent value. The mapping function assigned every miRNA from 1 to 32 mRNA expression indexes. Parameters such as the p-value, fold change and genomic context of transcripts that were used successfully in the previous predictor were again incorporated into the linear model. In addition, the regression model includes additional ordinal (categorical) and continuous descriptive parameters:

***e***_***i***_     Messenger RNA expression values from the microarray experiment

***FC***_***i***_    Fold Change in expression between sample and control

$p_{val_{i}}$   p-value from t-test or ANOVA on mRNA expression data

***overlay***  Categorical parameter of levels: *intron, exon, 3’UTR, 5’UTR*

***strand***   Categorical parameter of levels: *sense* or *antisense*

***evidence*** Categorical parameter e.g. *clone based, curated transcript, automatic transcript*

The following equations describe how starting with the simplest scenario (*i.e.* one microRNA’s expression dependent on only 1 mRNA transcript) we can implement a general regression formula based on these assumptions:

**Equation 5** - The regression formula when predicting the miRNA expression of 1 microRNA when dependent on 1 mRNA transcript

(5) $$\begin{array}{l} E_{\text{micro}}\sim e_{1}*\left|FC_{1}\right|*\left(1-p_{val_{1}}\right)*\text{overla}y_{1}*\text{stran}d_{1}\\ \;*\text{evidence}ve_{1} \end{array}$$

**Equation 6** - The regression formula when predicting the expression of a miRNA when dependent on 2 mRNA transcripts

(6) $$\begin{array}{l} E_{\text{micro}}\sim e_{1}*|FC_{1}|*(1-p_{val_{1}})*\text{overla}y_{1}*\text{stran}d_{1}*\text{evidenc}e_{1}\\ \;+e_{2}*|FC_{2}|*(1-p_{val_{2}})*\text{overla}y_{2}*\text{stran}d_{2}*\text{evidenc}e_{2} \end{array}$$

**Equation 7** - A general regression formula for predicting the expression of a miRNA expression value when dependent on n transcripts

(7) $$\begin{array}{l} E_{\text{micro}}\sim e_{1}*|FC_{1}|*(1-p_{val_{1}})*\text{overla}y_{1}*\text{stran}d_{1}*\text{evidence}e_{1}\\ \;+e_{2}*|FC_{2}|*(1-p_{val_{2}})*\text{overla}y_{2}*\text{stran}d_{2}*\text{evidenc}e_{2}\\ \;+E_{n}*|Fc_{n}|*(1-p_{val_{i}})*\text{overla}y_{i}*\text{stran}d_{i}*\text{evidenc}e_{i} \end{array}$$

Implementing the iterative formula into the linear model is mathematically impossible. Instead the model predicts miRNA expression with each transcript separately and then calculates a median value as the final prediction for each miRNA. However, using the model described by **Equation5** with this method resulted in poor prediction power – the Pearson’s correlation coefficient between the measured values and our predictions was 0.324. As solution the factor containing the names of miRNAs was introduced into the model. This allowed the fitting function to select different linear equation coefficients for unique miRNAs (**Equation 8**).

**Equation 8** - The regression formula for predicting the expression value of a microRNA after introducing miRNAs’ name factor

(8) $$\begin{array}{l} E_{\text{micro}}\sim E*\left|FC\right|*\left(1-p_{val}\right)*\text{overlay}*\text{strand}\\ \;*\text{evidence}*miR_{id} \end{array}$$

This model achieved a high performance, with an estimated correlation value of 0.945 between the experimental values and our predicted values. Additional analyses indicated that miRNAs located on antisense strand, exonic, 3’UTR and 5’UTR are weakly correlated and may introduce noise rather than add to the signal in the model. Pre-filtering these transcripts marginally increased the correlation to 0.949. The ambiguous nature of the evidence (*i.e.* origin of the entry in miRBase) also introduced the noise. Discarding this independent variable (**Equation9**) further increased prediction power to 0.956. This simplification of the model (**Equation10**), based only on mRNA expression values and miRNA ID factor resulted in a correlation coefficient of 0.955. Despite the larger computational complexity the best performing regression formula described by **Equation9** was implemented in the pipeline (Figure 4).

**Equation 9** - Final regression formula characterised by the highest prediction power and moderate resource consumption.

(9) $$E_{\text{micro}}\sim E*\left|FC\right|*\left(1-p_{val}\right)*miR_{id}$$

**Equation 10** - Simplified regression formula for the linear model predictor.

(10) $$E_{\text{micro}}\sim E*miR_{id}$$

Finally the miRNA-mRNA correlation analyses can be simplified to the following formula:

**Equation 11** - The mathematical bases of miRNA-mRNA correlation analyses:

(11) $$\begin{array}{c} e_{\text{hosts}}\overset{\text{correlated}}{↔}e_{\text{miRNA}}\\ e_{\text{targets}}\underset{\text{anti–correlated}}{↔}e_{\text{miRNA}} \end{array}\;\}\Rightarrow e_{\text{hosts}}\overset{\text{anti–correlated}}{↔}e_{\text{targets}}$$

### R/ Bioconductor implementation

Despite the complexity of the model, the R implementation (referred further as the pipeline) has been designed to be simple and user friendly. The pipeline takes as input raw Affimetrix CEL files and experiment design vector (or matrix in case of more complicated ANOVA statistics), which distinguish the biological replicates, time series etc. (e. g. sample versus control in the simplest case). The output is HTML formatted report. This includes output of predictors in tabular form, as well as quality assessment plots on statistical pre-processing and performance of the predictors. Functional analyses presented as hypergeometric test result tables are supported by pie charts, bar plots, interaction concept networks and annotated heatmaps (provided by R/Bioconductor GeneAnswers library). The primary pipeline interface is in the form of a command-line R console, however users with different requirements may use a convenient graphical user interface (GUI) build with GTK+. Most advanced users may benefit on the modular structure of the pipeline, which facilitate applying changes to the components and utilising single modules in third party projects.

An explicit documentation explaining the interfaces, system requirements and implementation structure is available as Additional file 10. The MMpred software implemented as R scripts and distributed under BSD licence is attached as Additional file 11.

### Pre-processing of raw array data in R

The expression matrices for both array types were obtained by performing standard Robust Multi-chip Average procedure [22] – the probes signal was obtained from perfect match (PM) probes; the quantiles method was incorporated for cross-array normalization and MedianPolish for summarization of the results. The BioConductor Affy library was used for processing HG-U133Plus2 chips, and the same functions ported in AgiMicroRna library were incorporated for Agilent miRNA 2.0 arrays.

### Correlation matrix

The idea of creating correlation matrices has been inspired by mathematical procedures present in regression analyses. The independent variables are being correlated against each other to assess their independence. The important differences are that regression analyses method operates on vectors, creates square matrices and aims to minimize the absolute value of correlation: correlation close to 0 indicates that independent variables are not biased to describe each other. The method that we have developed operates on arrays – though can be treated as reducing the dimensionality of the data. The basic assumption is that the expression matrices calculated using every paired dataset have the same number of columns – the same quantity of arrays must be used to assay miRNA and mRNA, and different number of rows – there is much more coding genes than miRNAs. Every row of the miRNA array is correlated against each row of the mRNA array and the correlation coefficient is captured – this way two matrices are collapsed into one, which shares the number of rows with miRNA’s expression matrix. The number of columns is equal to the number of rows present in mRNA array.

The most correlation comprehensive investigation has been made on the *“Integrative genomic profiling of human prostate cancer”* (GSE21032) dataset. 1,411,189 exons are represented on the Affymetrix Human Exon 1.0 ST array. Agilent Human miRNA Microarray 2.0 captures the expression of 821 different miRNAs and control quality sets. In constructing the correlation matrix quality control probesets and viral miRNA have also been correlated to mRNA for negative control. The output matrix, size of 821 × 1,411,189, has captured 1,158,586,169 correlation coefficients.

### The design of microarrays used in our studies

Affymetrix HG-U133 Plus 2.0 and Human Exon 1.0 ST measures messenger RNA expression by in situ oligonucleotide hybridization. The important difference between those platforms is that HuEx-1.0ST measures gene expression at the exon level – each probeset corresponds to a single exon rather than gene. The older platforms, including U133 arrays used probes complementary to the 3’UTR regions only. The new approach requires using the most current, high-density arrays, but should ensure higher precision of expression measurements and allows performing alternative splicing analyses. The manufacturer guarantees that on the genomic level HuEx-1.0ST arrays are fully backward compatible with the U133 family. Since gene mapping between those platforms is possible numerous comparative studies have been performed. The high concordance between HuEx-1.0ST and HG-U133Plus2 platforms is confirmed by many independent research groups [27-29,41]. However, the same groups report no or very low difference in precision of measurement between those platforms [27-29,41], so the only certain advantage of the less cost efficient HuEx-1.0ST arrays for the project is better genomic coverage. The detailed differences in array design are covered in Table 2.

**Table 2:** **The differences between microarray platforms used in the project (*source: Affymetrix and Agilent data sheets*)**

	**Affymetrix human genome U133 Plus 2.0**	**Affymetrix human Exon1.0 ST**	**Agilent human miRNA Microarray 2.0**
**Total features per array**	~ 1 million	> 5.5 million	~ 15,000
**Probe sets**	>54,000	1.4 million	821
**Exon clusters / Transcripts / miRNAs**	~47,400	>1 million	723 human + 76 viral
**Oligonucleotide probe length**	25-mer	25-mer	~ 40–60 nucleotides
**Resolution**	11 pairs/transcript, 16.1 /gene	5.8 /exon, 44.8 /gene	20–40 /sequence
**Feature size**	11 μm	5 μm	65 μm

**Agilent Human miRNA** microarrays utilize similar technology to Affymetrix GeneChips, but measure the abundance of mature microRNA transcripts (both dominant and minor transcripts). This platform contains probes complementary to 723 human microRNAs and 76 human viral microRNAs. The probesets design is based on the miRBase version 10.1. The raw data are extracted as a text (.TXT) file, which can be further processed by Agilent's feature extraction software to a GeneView file or directly analysed by the BioConductor AgiMicroRna library [21]. This platform has been evaluated as one of the most robust and accurate tools for global miRNA expression measurement. It is also characterised by the best human genome coverage [20,42].

### Paired datasets

The paired datasets required for building and testing the model are publically available and have been obtained from Gene Expression Omnibus repository.

*“Array-based bioinformatic analysis on pediatric primary central nervous system germ cell tumors”* (GSE19350) contains 12 Agilent Human miRNA Microarray 2.0 paired with 12 Affymetrix Human Genome U133 Plus 2.0, as well as unused in the model genotyping and analysis of chromosome copy number experiments (Illumina Human 610-Quad v1.0 BeadChip) [20,24,42].

*“Integrative genomic profiling of human prostate cancer”* (GSE21032) dataset includes 743 mRNA (HuEx-1.0ST), miRNA (Agilent Human miRNA Microarray 2.0) and genotyping arrays, of which 280 have been identified as paired miRNA-mRNA (genetic material for both experiments have been isolated from the same sample) [26]. The samples were extracted from both healthy individuals and affected patients from various ethnic origins, as well as from prostate tissue cultures [26].

### The miRNA-target binding energy base validation

The procedure utilizes the Vienna RNA Package version 1.8.5 to calculate minimum free energy of miRNA binding. The 3’UTR sequences are scanned using sliding window of 25bp and 5bp step. Since RNAfold algorithm allows only the calculation of free energy for single stranded RNA molecule, the scanned 25bp fragments of 3’UTR mRNA have been joined with mature miRNA sequence using 8bp artificial inter-linker sequence containing 'X' bases that cannot be paired (as described by Enright *et al.*[43]). The region of lowest free energy is considered to be the optimum binding site.

The validation has been implemented in R language. Mature miRNA sequences have been obtained from miRBase version 17.0 using miRbase.db R library. 3’UTR sequences have been downloaded from Ensembl via biomaRt R interface. For genes with multiple 5’UTR transcripts the longest isoform was selected to ensure the sampling of all possible binding locations. Genes with 3’UTRs shorter that 100bp were discarded from analysis. The free energy calculations have been executed using GeneRfold R interface for Vienna RNA library. The miRecords (version 3, *mirecords.biolead.org/download.php*) have been used as comprehensive collection of validated targets.

## Abbreviations

***pri-miRNA***: **pri**mary **mi**cro **r**ibo**n**ucleic **a**cid; ***pre-miRNA***: **pre**cursor **mi**cro **r**ibo**n**ucleic **a**cid; **RISC**: **R**NA-**i**nduced **s**ilencing **c**omplex; ***Nt***: **n**ucleo**t**ides; **HG-U133Plus2**: Affymetrix GeneChip **H**uman **G**enome **U133 Plus 2**.0 Array; **HuEx-1.0ST**: Affymetrix GeneChip **Hu**man **Ex**on **1.0 ST** Array; ***RMA***: **R**obust **M**ulti-array **A**verage; **GEO**: **G**ene **E**xpression **O**mnibus; ***GO***: **G**ene **O**ntology; ***DO***: **D**isease **O**ntology; ***KEGG***: **K**yoto **E**ncyclopedia of **G**enes and **G**enomes.

## Competing interests

The authors declare that they have no competing interests.

## Authors’ contributions

PS, MC and PW conceived and planned the project. PS developed, implemented and tested the algorithm, plus performed the analyses. PS, MC and PW co-authored the paper. All authors read and approved the final manuscript.

## Supplementary Material

Additional file 1 **Summary of all miRNA datasets performed on popular platforms in GEO (represented by at least 25 arrays, data from July 2011).** Paired datasets are marked in green with mRNA array platform and number of samples stated). Description and remarks: All the data have been derived from NCBI Gene Expression Omnibus (http://www.ncbi.nlm.nih.gov/geo/). The newest and most advanced, 3rd version of Agilent Human miRNA is represented only by 49 samples, gathered in 5 datasets. The older Agilent array (version 2.0, capable of measuring 723 microRNAs), listed currently as most popular global miRNA expression test in GEO is represented only by 17 datasets containing 539 samples. Among 10 major miRNA microarray platforms available in GEO (those platforms are represented by more than 25 arrays) only 23 experiments have been identified as paired miRNA-mRNA. The vast majority of those assays concern large cancer tissue expression studies, so the chance of finding a dataset on different biological subject is relatively low. Click here for file

Additional file 2 **The complete mapping table in CSV format.** Filtered for sense intronic transcripts only. Click here for file

Additional file 3 Sample pipeline outputs in HTML format (compressed file). Click here for file

Additional file 4 Overview of the pipeline outputs (raw MMpred output for both case studies). Click here for file

Additional file 5 The short description of analysed case studies. Click here for file

Additional file 6 Detailed report on case study I: Toll-like 4 receptor activated by Lipopolysaccharide (LPS). Click here for file

Additional file 7 Detailed report on case study II: Comparison of miRNA regulation in human severe blunt trauma and severe burn injury. Click here for file

Additional file 8 Systematic validation of target prediction by the similarity of binding free energy distribution with miRecords. Click here for file

Additional file 9 Examples of MMpred predictions supported by experimental data and mapping against current databases. Click here for file

Additional file 10 The detailed description of software implementation in R language. Click here for file

Additional file 11 The R implementation of the presented method: MMpred. Click here for file
