# Supplementary material for: MMpred: functional miRNA – mRNA interaction analyses by miRNA expression prediction
Source: BMC Genomics. 2012 Nov 14;13:620. doi: 10.1186/1471-2164-13-620 (PMC3562514; doi:10.1186/1471-2164-13-620)
Supplement: Additional file 3 — Sample pipeline outputs in HTML format (compressed file). [file 1471-2164-13-620-S3.ZIP › Bunt182-ttest0.8/REPORT_Fri-23-07-2010_13-26-05.html]

REPORT


## Report of miRNA-mRNA interactions for all arrays. [generated on 2010-07-23 13:26:05]

---

Statistical testing for messanger RNA arrays: 233 genes found significanltly up/downregulated . Details:

| |  | ArrayFile | FunctionalGroup | | --- | --- | --- | | 1 | GSM287288.CEL | 1 | | 2 | GSM287289.CEL | 1 | | 3 | GSM287290.CEL | 1 | | 4 | GSM287291.CEL | 1 | | 5 | GSM287292.CEL | 1 | | 6 | GSM287293.CEL | 1 | | 7 | GSM287294.CEL | 1 | | 8 | GSM287295.CEL | 1 | | 9 | GSM287296.CEL | 2 | | 10 | GSM287297.CEL | 2 | | 11 | GSM287298.CEL | 2 | | 12 | GSM287299.CEL | 2 | | 13 | GSM287300.CEL | 2 | | 14 | GSM287301.CEL | 2 | | 15 | GSM287302.CEL | 2 | | 16 | GSM287304.CEL | 2 | | 17 | GSM287305.CEL | 2 | | 18 | GSM287306.CEL | 2 | | 19 | GSM287307.CEL | 2 | | 20 | GSM287308.CEL | 2 | | 21 | GSM287309.CEL | 2 | | 22 | GSM287310.CEL | 2 | | 23 | GSM287311.CEL | 2 | | 24 | GSM287312.CEL | 2 | | 25 | GSM287313.CEL | 2 | | 26 | GSM287314.CEL | 2 | | 27 | GSM287315.CEL | 2 | | 28 | GSM287316.CEL | 2 | | 29 | GSM287317.CEL | 2 | | 30 | GSM287318.CEL | 2 | | 31 | GSM287319.CEL | 2 | | 32 | GSM287320.CEL | 2 | | 33 | GSM287321.CEL | 2 | | 34 | GSM287322.CEL | 2 | | 35 | GSM287323.CEL | 2 | | 36 | GSM287324.CEL | 2 | | 37 | GSM287325.CEL | 2 | | 38 | GSM287326.CEL | 2 | | 39 | GSM287327.CEL | 2 | | 40 | GSM287328.CEL | 2 | | 41 | GSM287329.CEL | 2 | | 42 | GSM287330.CEL | 2 | | 43 | GSM287331.CEL | 2 | | 44 | GSM287332.CEL | 2 | | 45 | GSM287333.CEL | 2 | | 46 | GSM287334.CEL | 2 | | 47 | GSM287335.CEL | 2 | | 48 | GSM287336.CEL | 2 | | 49 | GSM287337.CEL | 2 | | 50 | GSM287339.CEL | 2 | | 51 | GSM287340.CEL | 2 | | 52 | GSM287341.CEL | 2 | | 53 | GSM287342.CEL | 2 | | 54 | GSM287343.CEL | 2 | | 55 | GSM287344.CEL | 2 | | 56 | GSM287345.CEL | 2 | | 57 | GSM287346.CEL | 2 | | 58 | GSM287347.CEL | 2 | | 59 | GSM287348.CEL | 2 | | 60 | GSM287349.CEL | 2 | | 61 | GSM287350.CEL | 2 | | 62 | GSM287351.CEL | 2 | | 63 | GSM287352.CEL | 2 | | 64 | GSM287353.CEL | 2 | | 65 | GSM287354.CEL | 2 | | 66 | GSM287355.CEL | 2 | | 67 | GSM287356.CEL | 2 | | 68 | GSM287357.CEL | 2 | | 69 | GSM287358.CEL | 2 | | 70 | GSM287359.CEL | 2 | | 71 | GSM287360.CEL | 2 | | 72 | GSM287361.CEL | 2 | | 73 | GSM287362.CEL | 2 | | 74 | GSM287363.CEL | 2 | | 75 | GSM287364.CEL | 2 | | 76 | GSM287365.CEL | 2 | | 77 | GSM287366.CEL | 2 | | 78 | GSM287367.CEL | 2 | | 79 | GSM287368.CEL | 2 | | 80 | GSM287369.CEL | 2 | | 81 | GSM287370.CEL | 2 | | 82 | GSM287371.CEL | 2 | | 83 | GSM287372.CEL | 2 | | 84 | GSM287373.CEL | 2 | | 85 | GSM287374.CEL | 2 | | 86 | GSM287375.CEL | 2 | | 87 | GSM287376.CEL | 2 | | 88 | GSM287377.CEL | 2 | | 89 | GSM287378.CEL | 2 | | 90 | GSM287379.CEL | 2 | | 91 | GSM287380.CEL | 2 | | 92 | GSM287381.CEL | 2 | | 93 | GSM287382.CEL | 2 | | 94 | GSM287383.CEL | 2 | | 95 | GSM287384.CEL | 1 | | 96 | GSM287385.CEL | 2 | | 97 | GSM287386.CEL | 2 | | 98 | GSM287387.CEL | 2 | | 99 | GSM287388.CEL | 2 | | 100 | GSM287389.CEL | 2 | | 101 | GSM287390.CEL | 2 | | 102 | GSM287391.CEL | 2 | | 103 | GSM287392.CEL | 2 | | 104 | GSM287393.CEL | 2 | | 105 | GSM287394.CEL | 2 | | 106 | GSM287395.CEL | 1 | | 107 | GSM287396.CEL | 2 | | 108 | GSM287397.CEL | 2 | | 109 | GSM287398.CEL | 2 | | 110 | GSM287399.CEL | 2 | | 111 | GSM287400.CEL | 2 | | 112 | GSM287401.CEL | 2 | | 113 | GSM287402.CEL | 2 | | 114 | GSM287403.CEL | 2 | | 115 | GSM287404.CEL | 2 | | 116 | GSM287405.CEL | 2 | | 117 | GSM287406.CEL | 1 | | 118 | GSM287407.CEL | 2 | | 119 | GSM287408.CEL | 2 | | 120 | GSM287409.CEL | 2 | | 121 | GSM287410.CEL | 2 | | 122 | GSM287411.CEL | 2 | | 123 | GSM287412.CEL | 2 | | 124 | GSM287413.CEL | 2 | | 125 | GSM287414.CEL | 2 | | 126 | GSM287415.CEL | 2 | | 127 | GSM287416.CEL | 2 | | 128 | GSM287417.CEL | 1 | | 129 | GSM287418.CEL | 2 | | 130 | GSM287419.CEL | 2 | | 131 | GSM287420.CEL | 2 | | 132 | GSM287421.CEL | 2 | | 133 | GSM287422.CEL | 2 | | 134 | GSM287423.CEL | 2 | | 135 | GSM287424.CEL | 2 | | 136 | GSM287425.CEL | 2 | | 137 | GSM287426.CEL | 2 | | 138 | GSM287427.CEL | 2 | | 139 | GSM287428.CEL | 1 | | 140 | GSM287429.CEL | 2 | | 141 | GSM287430.CEL | 2 | | 142 | GSM287431.CEL | 2 | | 143 | GSM287432.CEL | 2 | | 144 | GSM287433.CEL | 2 | | 145 | GSM287434.CEL | 2 | | 146 | GSM287435.CEL | 2 | | 147 | GSM287436.CEL | 2 | | 148 | GSM287437.CEL | 2 | | 149 | GSM287438.CEL | 2 | | 150 | GSM287439.CEL | 1 | | 151 | GSM287440.CEL | 2 | | 152 | GSM287441.CEL | 2 | | 153 | GSM287442.CEL | 2 | | 154 | GSM287443.CEL | 2 | | 155 | GSM287444.CEL | 2 | | 156 | GSM287445.CEL | 2 | | 157 | GSM287446.CEL | 2 | | 158 | GSM287447.CEL | 2 | | 159 | GSM287448.CEL | 2 | | 160 | GSM287449.CEL | 2 | | 161 | GSM287450.CEL | 1 | | 162 | GSM287451.CEL | 2 | | 163 | GSM287452.CEL | 2 | | 164 | GSM287453.CEL | 2 | | 165 | GSM287454.CEL | 2 | | 166 | GSM287455.CEL | 2 | | 167 | GSM287456.CEL | 2 | | 168 | GSM287457.CEL | 2 | | 169 | GSM287458.CEL | 2 | | 170 | GSM287459.CEL | 2 | | 171 | GSM287460.CEL | 2 | | 172 | GSM287461.CEL | 1 | | 173 | GSM287462.CEL | 1 | | 174 | GSM287463.CEL | 1 | | 175 | GSM287464.CEL | 1 | | 176 | GSM287465.CEL | 1 | | 177 | GSM287466.CEL | 1 | | 178 | GSM287467.CEL | 1 | | 179 | GSM287468.CEL | 1 | | 180 | GSM287469.CEL | 1 | | 181 | GSM287470.CEL | 1 | | 182 | GSM287471.CEL | 1 | |

  

Principal component analises:

Heatmap for top 50 geneses from statuistic analises (ordered by p-value):

Voolcano plot with for auto cutoff calculation audit (cutoff shown with red line):

---

Statistical testing for microRNA prediction method I - liniar modeling: 22 genes found significanltly up/downregulated . Details:

Principal component analysis:

Heatmap for top 50 geneses from statuistic analises (ordered by p-value):

Voolcano plot with for auto cutoff calculation audit (cutoff shown with red line):

---

Statistical testing for microRNA prediction method II - scaling function: 22 genes found significanltly up/downregulated . Details:

Principal component analysis:

Heatmap for top 50 geneses from statuistic analises (ordered by p-value):

Voolcano plot with for auto cutoff calculation audit (cutoff shown with red line):

---

Mean anti-correlation detected between mRNA and miRNA = -0.331067 . Details:

Histogram of most anti-correlated miRNA-mRNA pairs - potential mirNA-target intercations:

---
