# Supplementary material for: MMpred: functional miRNA – mRNA interaction analyses by miRNA expression prediction
Source: BMC Genomics. 2012 Nov 14;13:620. doi: 10.1186/1471-2164-13-620 (PMC3562514; doi:10.1186/1471-2164-13-620)
Supplement: Additional file 3 — Sample pipeline outputs in HTML format (compressed file). [file 1471-2164-13-620-S3.ZIP › Burn_dead-alive-control/BuntTtestSvsC_Thu-09-09-2010_02-26-21.html]

REPORT


## Report of miRNA-mRNA interactions for all arrays. [generated on 2010-09-09 02:26:21]

---

Statistical testing for messenger RNA arrays: 27173 genes found significantly up-/down-regulated. Details:

| |  | ArrayFile | FunctionalGroup | | --- | --- | --- | | 1 | GSM493655.CEL | No | | 2 | GSM493656.CEL | No | | 3 | GSM493657.CEL | Yes | | 4 | GSM493658.CEL | Yes | | 5 | GSM493659.CEL | Yes | | 6 | GSM493660.CEL | Yes | | 7 | GSM493661.CEL | Yes | | 8 | GSM493662.CEL | Yes | | 9 | GSM493663.CEL | Yes | | 10 | GSM493664.CEL | Yes | | 11 | GSM493665.CEL | No | | 12 | GSM493666.CEL | No | | 13 | GSM493667.CEL | No | | 14 | GSM493668.CEL | No | | 15 | GSM493669.CEL | Yes | | 16 | GSM493670.CEL | Yes | | 17 | GSM493671.CEL | Yes | | 18 | GSM493672.CEL | Yes | | 19 | GSM493673.CEL | No | | 20 | GSM493674.CEL | No | | 21 | GSM493675.CEL | Yes | | 22 | GSM493676.CEL | Yes | | 23 | GSM493677.CEL | Yes | | 24 | GSM493678.CEL | Yes | | 25 | GSM493679.CEL | Yes | | 26 | GSM493680.CEL | Yes | | 27 | GSM493681.CEL | No | | 28 | GSM493682.CEL | No | | 29 | GSM493683.CEL | Yes | | 30 | GSM493684.CEL | Yes | | 31 | GSM493685.CEL | Yes | | 32 | GSM493686.CEL | Yes | | 33 | GSM493687.CEL | Yes | | 34 | GSM493688.CEL | Yes | | 35 | GSM493689.CEL | Yes | | 36 | GSM493690.CEL | Yes | | 37 | GSM493691.CEL | No | | 38 | GSM493692.CEL | No | | 39 | GSM493693.CEL | Yes | | 40 | GSM493694.CEL | Yes | | 41 | GSM493695.CEL | Yes | | 42 | GSM493696.CEL | Yes | | 43 | GSM493697.CEL | Yes | | 44 | GSM493698.CEL | Yes | | 45 | GSM493699.CEL | Yes | | 46 | GSM493700.CEL | Yes | | 47 | GSM493701.CEL | Yes | | 48 | GSM493702.CEL | Yes | | 49 | GSM493703.CEL | No | | 50 | GSM493704.CEL | No | | 51 | GSM493705.CEL | Yes | | 52 | GSM493706.CEL | Yes | | 53 | GSM493707.CEL | Yes | | 54 | GSM493708.CEL | Yes | | 55 | GSM493709.CEL | Yes | | 56 | GSM493710.CEL | Yes | | 57 | GSM493711.CEL | No | | 58 | GSM493712.CEL | No | | 59 | GSM493713.CEL | Yes | | 60 | GSM493714.CEL | Yes | | 61 | GSM493715.CEL | Yes | | 62 | GSM493716.CEL | Yes | | 63 | GSM493717.CEL | Yes | | 64 | GSM493718.CEL | Yes | | 65 | GSM493719.CEL | Yes | | 66 | GSM493720.CEL | Yes | | 67 | GSM493721.CEL | Yes | | 68 | GSM493722.CEL | Yes | | 69 | GSM493723.CEL | Yes | | 70 | GSM493724.CEL | Yes | | 71 | GSM493725.CEL | Yes | | 72 | GSM493726.CEL | Yes | | 73 | GSM493727.CEL | Yes | | 74 | GSM493728.CEL | Yes | | 75 | GSM493729.CEL | Yes | | 76 | GSM493730.CEL | Yes | | 77 | GSM493731.CEL | Yes | | 78 | GSM493732.CEL | Yes | | 79 | GSM493733.CEL | Yes | | 80 | GSM493734.CEL | Yes | | 81 | GSM493735.CEL | No | | 82 | GSM493736.CEL | No | | 83 | GSM493737.CEL | No | | 84 | GSM493738.CEL | No | | 85 | GSM493739.CEL | Yes | | 86 | GSM493740.CEL | Yes | | 87 | GSM493741.CEL | Yes | | 88 | GSM493742.CEL | Yes | | 89 | GSM493743.CEL | Yes | | 90 | GSM493744.CEL | Yes | | 91 | GSM493745.CEL | Yes | | 92 | GSM493746.CEL | Yes | | 93 | GSM493747.CEL | Yes | | 94 | GSM493748.CEL | Yes | | 95 | GSM493749.CEL | No | | 96 | GSM493750.CEL | No | | 97 | GSM493751.CEL | Yes | | 98 | GSM493752.CEL | Yes | | 99 | GSM493753.CEL | Yes | | 100 | GSM493754.CEL | Yes | | 101 | GSM493755.CEL | No | | 102 | GSM493756.CEL | No | | 103 | GSM493757.CEL | No | | 104 | GSM493758.CEL | No | | 105 | GSM493759.CEL | No | | 106 | GSM493760.CEL | No | | 107 | GSM493761.CEL | Yes | | 108 | GSM493762.CEL | Yes | | 109 | GSM493763.CEL | No | | 110 | GSM493764.CEL | No | | 111 | GSM493765.CEL | Yes | | 112 | GSM493766.CEL | Yes | | 113 | GSM493767.CEL | Yes | | 114 | GSM493768.CEL | Yes | | 115 | GSM493769.CEL | control | | 116 | GSM493770.CEL | control | | 117 | GSM493771.CEL | control | | 118 | GSM493772.CEL | control | | 119 | GSM493773.CEL | control | | 120 | GSM493774.CEL | control | | 121 | GSM493775.CEL | control | | 122 | GSM493776.CEL | control | | 123 | GSM493777.CEL | control | | 124 | GSM493778.CEL | control | | 125 | GSM493779.CEL | control | | 126 | GSM493780.CEL | control | | 127 | GSM493781.CEL | control | | 128 | GSM493782.CEL | control | | 129 | GSM493783.CEL | control | | 130 | GSM493784.CEL | control | | 131 | GSM493785.CEL | control | | 132 | GSM493786.CEL | control | | 133 | GSM493787.CEL | control | | 134 | GSM493788.CEL | control | | 135 | GSM493789.CEL | control | | 136 | GSM493790.CEL | control | | 137 | GSM493791.CEL | control | | 138 | GSM493792.CEL | control | | 139 | GSM493793.CEL | control | | 140 | GSM493794.CEL | control | | 141 | GSM493795.CEL | control | | 142 | GSM493796.CEL | control | | 143 | GSM493797.CEL | control | | 144 | GSM493798.CEL | control | | 145 | GSM493799.CEL | control | | 146 | GSM493800.CEL | control | | 147 | GSM493801.CEL | control | | 148 | GSM493802.CEL | control | | 149 | GSM493803.CEL | control | | 150 | GSM493804.CEL | control | | 151 | GSM493805.CEL | control | | 152 | GSM493806.CEL | control | | 153 | GSM493807.CEL | control | | 154 | GSM493808.CEL | control | | 155 | GSM493809.CEL | control | | 156 | GSM493810.CEL | control | | 157 | GSM493811.CEL | control | | 158 | GSM493812.CEL | control | | 159 | GSM493813.CEL | control | | 160 | GSM493814.CEL | control | | 161 | GSM493815.CEL | control | | 162 | GSM493816.CEL | control | | 163 | GSM493817.CEL | control | | 164 | GSM493818.CEL | control | | 165 | GSM493819.CEL | control | | 166 | GSM493820.CEL | control | | 167 | GSM493821.CEL | control | | 168 | GSM493822.CEL | control | | 169 | GSM493823.CEL | control | | 170 | GSM493824.CEL | control | | 171 | GSM493825.CEL | control | | 172 | GSM493826.CEL | control | | 173 | GSM493827.CEL | control | | 174 | GSM493828.CEL | control | | 175 | GSM493829.CEL | control | | 176 | GSM493830.CEL | control | | 177 | GSM493831.CEL | control | |

  

Principal Component Analyses:

Heatmap for top 50 geneses from statistical analyses (ordered by p-value):

Volcano plot with for auto cut-off calculation audit (cut-off shown with red line):

---

Statistical testing for microRNA prediction method I - scaling function: 125 genes found significantly up-/down-regulated. Details:

Heatmap for top 50 geneses from statistical analyses (ordered by p-value):

Volcano plot with for auto cut-off calculation audit (cut-off shown with red line):

---

Statistical testing for microRNA prediction method II - linear modelling: 160 genes found significantly up-/down-regulated. Details:

Principal Component Analysis:

Heatmap for top 50 geneses from statistical analyses (ordered by p-value):

Volcano plot with for auto cut-off calculation audit (cut-off shown with red line):

---

Mean anti-correlation detected between mRNA and miRNA = -0.312736. Details:

Histogram of most anti-correlated miRNA-mRNA pairs - potential miRNA-target interactions:

---

Total number of 63 miRNAs are predicted to have significantly up-/down-regulated targets. Expend:

| |  | microRNA | NoSuppresedGenes | | --- | --- | --- | | 1 | hsa-miR-1233 | 409 | | 2 | hsa-miR-766 | 323 | | 3 | hsa-miR-571 | 297 | | 4 | hsa-miR-874 | 269 | | 5 | hsa-miR-95 | 251 | | 6 | hsa-miR-591 | 232 | | 7 | hsa-miR-770-5p | 229 | | 8 | hsa-miR-10a | 195 | | 9 | hsa-miR-569 | 143 | | 10 | hsa-mir-10a | 114 | | 11 | hsa-mir-3130-1 | 93 | | 12 | hsa-mir-3130-2 | 93 | | 13 | hsa-mir-3130-3 | 93 | | 14 | hsa-miR-635 | 84 | | 15 | hsa-miR-638 | 83 | | 16 | hsa-miR-623 | 69 | | 17 | hsa-miR-576-5p | 66 | | 18 | hsa-miR-604 | 60 | | 19 | hsa-miR-938 | 60 | | 20 | hsa-miR-765 | 49 | | 21 | hsa-miR-454\* | 48 | | 22 | hsa-miR-627 | 42 | | 23 | hsa-miR-934 | 39 | | 24 | hsa-mir-423 | 38 | | 25 | hsa-miR-593\* | 33 | | 26 | hsa-miR-671-5p | 28 | | 27 | hsa-miR-617 | 25 | | 28 | hsa-miR-190 | 21 | | 29 | hsa-mir-505 | 15 | | 30 | hsa-miR-128 | 14 | | 31 | hsa-miR-636 | 14 | | 32 | hsa-mir-199a-2 | 13 | | 33 | hsa-miR-199a-5p | 13 | | 34 | hsa-mir-214 | 13 | | 35 | hsa-mir-885 | 12 | | 36 | hsa-mir-1224 | 11 | | 37 | hsa-miR-1224-5p | 11 | | 38 | hsa-miR-135a | 10 | | 39 | hsa-mir-628 | 8 | | 40 | hsa-miR-628-5p | 8 | | 41 | hsa-miR-643 | 8 | | 42 | hsa-mir-135b | 7 | | 43 | hsa-mir-149 | 7 | | 44 | hsa-miR-188-5p | 6 | | 45 | hsa-mir-576 | 6 | | 46 | hsa-miR-126\* | 5 | | 47 | hsa-miR-16 | 4 | | 48 | hsa-miR-1236 | 3 | | 49 | hsa-mir-186 | 2 | | 50 | hsa-miR-186 | 2 | | 51 | hsa-mir-28 | 2 | | 52 | hsa-miR-28-5p | 2 | | 53 | hsa-mir-148b | 1 | | 54 | hsa-miR-148b\* | 1 | | 55 | hsa-mir-155 | 1 | | 56 | hsa-miR-15b | 1 | | 57 | hsa-mir-16-2 | 1 | | 58 | hsa-miR-218 | 1 | | 59 | hsa-miR-301a | 1 | | 60 | hsa-mir-454 | 1 | | 61 | hsa-miR-554 | 1 | | 62 | hsa-miR-567 | 1 | | 63 | hsa-miR-619 | 1 | |

---

Total number of 1318 genes are predicted to be under differential miRNA repression. Expend:

| |  | GenSymbols | GeneName | NoTargetingMicroRNA | | --- | --- | --- | --- | | 1 | ATP6V0A2 | ATPase, H+ transporting, lysosomal V0 subunit a2 | 12 | | 2 | SPDEF | SAM pointed domain containing ets transcription factor | 12 | | 3 | CREBZF | CREB/ATF bZIP transcription factor | 11 | | 4 | SMYD2 | SET and MYND domain containing 2 | 11 | | 5 | UPF3A | UPF3 regulator of nonsense transcripts homolog A (yeast) | 11 | | 6 | BRCC3 | BRCA1/BRCA2-containing complex, subunit 3 | 10 | | 7 | HIC2 | hypermethylated in cancer 2 | 10 | | 8 | HLA-DOA | major histocompatibility complex, class II, DO alpha | 10 | | 9 | RNF31 | ring finger protein 31 | 10 | | 10 | C6orf130 | chromosome 6 open reading frame 130 | 9 | | 11 | HFE | hemochromatosis | 9 | | 12 | ACAP1 | ArfGAP with coiled-coil, ankyrin repeat and PH domains 1 | 8 | | 13 | BAT5 | chromosome 5 open reading frame 28 | 8 | | 14 | C5orf28 | HLA-B associated transcript 5 | 8 | | 15 | KREMEN1 | kringle containing transmembrane protein 1 | 8 | | 16 | LRTM2 | leucine-rich repeats and transmembrane domains 2 | 8 | | 17 | PLIN4 | perilipin 4 | 8 | | 18 | PTER | phosphotriesterase related | 8 | | 19 | SGCA | sarcoglycan, alpha (50kDa dystrophin-associated glycoprotein) | 8 | | 20 | SNCG | spermatogenesis associated 21 | 8 | | 21 | SPATA21 | synuclein, gamma (breast cancer-specific protein 1) | 8 | | 22 | ADAMTSL3 | ADAMTS-like 3 | 7 | | 23 | ANAPC4 | anaphase promoting complex subunit 4 | 7 | | 24 | ATR | ataxia telangiectasia and Rad3 related | 7 | | 25 | CASP8AP2 | C-type lectin domain family 14, member A | 7 | | 26 | CLEC14A | caspase 8 associated protein 2 | 7 | | 27 | DNAJC4 | DnaJ (Hsp40) homolog, subfamily C, member 4 | 7 | | 28 | FNBP4 | formiminotransferase cyclodeaminase | 7 | | 29 | FTCD | formin binding protein 4 | 7 | | 30 | GLI4 | GLI family zinc finger 4 | 7 | | 31 | hCG\_2045089 | heterogeneous nuclear ribonucleoprotein A1 | 7 | | 32 | HNRNPA1 | hypothetical LOC286359 | 7 | | 33 | IGF1 | insulin-like growth factor 1 (somatomedin C) | 7 | | 34 | KIF12 | kinesin family member 12 | 7 | | 35 | LANCL1 | LanC lantibiotic synthetase component C-like 1 (bacterial) | 7 | | 36 | MMP11 | matrix metallopeptidase 11 (stromelysin 3) | 7 | | 37 | NFATC4 | nuclear factor of activated T-cells, cytoplasmic, calcineurin-dependent 4 | 7 | | 38 | NR2C1 | nuclear receptor subfamily 2, group C, member 1 | 7 | | 39 | PCYT1B | PC4 and SFRS1 interacting protein 1 | 7 | | 40 | PIK3C2A | Pentatricopeptide repeat domain 3 | 7 | | 41 | PLCB2 | phosphate cytidylyltransferase 1, choline, beta | 7 | | 42 | PRKRA | phosphoinositide-3-kinase, class 2, alpha polypeptide | 7 | | 43 | PSIP1 | phospholipase C, beta 2 | 7 | | 44 | PTCD3 | protein kinase, interferon-inducible double stranded RNA dependent activator | 7 | | 45 | SFRS13A | solute carrier family 4, sodium borate transporter, member 11 | 7 | | 46 | SLC4A11 | sorting nexin 1 | 7 | | 47 | SNX1 | splicing factor, arginine/serine-rich 13A | 7 | | 48 | TRAPPC9 | trafficking protein particle complex 9 | 7 | | 49 | UPRT | uracil phosphoribosyltransferase (FUR1) homolog (S. cerevisiae) | 7 | | 50 | WFDC2 | WAP four-disulfide core domain 2 | 7 | | 51 | A2LD1 | 5-methyltetrahydrofolate-homocysteine methyltransferase | 6 | | 52 | ADSL | acylglycerol kinase | 6 | | 53 | AGK | adenylosuccinate lyase | 6 | | 54 | ANKIB1 | AIG2-like domain 1 | 6 | | 55 | BARX2 | ankyrin repeat and IBR domain containing 1 | 6 | | 56 | BTN3A1 | BARX homeobox 2 | 6 | | 57 | C14orf115 | butyrophilin, subfamily 3, member A1 | 6 | | 58 | C16orf52 | C-type lectin domain family 2, member D | 6 | | 59 | C21orf90 | calcium and integrin binding family member 3 | 6 | | 60 | CALD1 | caldesmon 1 | 6 | | 61 | CCNT2 | carboxypeptidase M | 6 | | 62 | CEMP1 | cementum protein 1 | 6 | | 63 | CHRD | cholinergic receptor, nicotinic, alpha 4 | 6 | | 64 | CHRNA4 | chordin | 6 | | 65 | CIB3 | chromosome 14 open reading frame 115 | 6 | | 66 | CLEC2D | chromosome 16 open reading frame 52 | 6 | | 67 | COG6 | chromosome 21 open reading frame 90 | 6 | | 68 | CPM | component of oligomeric golgi complex 6 | 6 | | 69 | CTU2 | cyclin T2 | 6 | | 70 | DES | cytosolic thiouridylase subunit 2 homolog (S. pombe) | 6 | | 71 | DHRS7C | dehydrogenase/reductase (SDR family) member 7C | 6 | | 72 | DKFZp761P0212 | desmin | 6 | | 73 | DUSP16 | dual-specificity tyrosine-(Y)-phosphorylation regulated kinase 2 | 6 | | 74 | DYRK2 | dual specificity phosphatase 16 | 6 | | 75 | ESR1 | estrogen receptor 1 | 6 | | 76 | FGFR2 | fibroblast growth factor receptor 2 | 6 | | 77 | JMY | HBV preS1-transactivated protein 4 | 6 | | 78 | KANK2 | hypothetical LOC388780 | 6 | | 79 | KCNF1 | hypothetical protein DKFZp761P0212 | 6 | | 80 | LAMA3 | hypothetical protein LOC100288438 | 6 | | 81 | LIAS | hypothetical protein LOC728073 | 6 | | 82 | LOC100288438 | junction mediating and regulatory protein, p53 cofactor | 6 | | 83 | LOC388780 | KN motif and ankyrin repeat domains 2 | 6 | | 84 | LOC728073 | laminin, alpha 3 | 6 | | 85 | LRPPRC | leucine-rich PPR-motif containing | 6 | | 86 | MAP2K3 | lipoic acid synthetase | 6 | | 87 | MDFIC | matrix metallopeptidase 16 (membrane-inserted) | 6 | | 88 | MMP16 | mitogen-activated protein kinase kinase 3 | 6 | | 89 | MTR | MyoD family inhibitor domain containing | 6 | | 90 | NF2 | neurofibromin 2 (merlin) | 6 | | 91 | NYX | nyctalopin | 6 | | 92 | PAQR8 | perilipin 5 | 6 | | 93 | PLIN5 | potassium voltage-gated channel, subfamily F, member 1 | 6 | | 94 | PPP1R16B | progestin and adipoQ receptor family member VIII | 6 | | 95 | PS1TP4 | protein phosphatase 1, regulatory (inhibitor) subunit 16B | 6 | | 96 | PTK2B | PTK2B protein tyrosine kinase 2 beta | 6 | | 97 | RAB40C | RAB40C, member RAS oncogene family | 6 | | 98 | RAPGEF3 | Rap guanine nucleotide exchange factor (GEF) 3 | 6 | | 99 | RBBP4 | retinoblastoma binding protein 4 | 6 | | 100 | RRN3 | RRN3 RNA polymerase I transcription factor homolog (S. cerevisiae) | 6 | | 101 | SEC31A | SEC31 homolog A (S. cerevisiae) | 6 | | 102 | SEMA6B | sema domain, transmembrane domain (TM), and cytoplasmic domain, (semaphorin) 6B | 6 | | 103 | SEPT9 | septin 9 | 6 | | 104 | SLC35A3 | solute carrier family 35 (UDP-N-acetylglucosamine (UDP-GlcNAc) transporter), member A3 | 6 | | 105 | TCERG1 | T cell receptor delta locus | 6 | | 106 | TM6SF2 | tankyrase, TRF1-interacting ankyrin-related ADP-ribose polymerase | 6 | | 107 | TNKS | teashirt zinc finger homeobox 1 | 6 | | 108 | TOMM20 | tetratricopeptide repeat domain 39A | 6 | | 109 | TPPP | transcription elongation regulator 1 | 6 | | 110 | TRD@ | translocase of outer mitochondrial membrane 20 homolog (yeast) | 6 | | 111 | TRIM8 | transmembrane 6 superfamily member 2 | 6 | | 112 | TRMT11 | tripartite motif-containing 8 | 6 | | 113 | TSHZ1 | tRNA methyltransferase 11 homolog (S. cerevisiae) | 6 | | 114 | TTC39A | tubulin polymerization promoting protein | 6 | | 115 | VPS41 | vacuolar protein sorting 41 homolog (S. cerevisiae) | 6 | | 116 | ZAP70 | zeta-chain (TCR) associated protein kinase 70kDa | 6 | | 117 | ZNF226 | zinc finger protein 226 | 6 | | 118 | ABCB1 | A kinase (PRKA) anchor protein 11 | 5 | | 119 | ACADSB | activating transcription factor 7 interacting protein 2 | 5 | | 120 | AKAP11 | acyl-Coenzyme A dehydrogenase, short/branched chain | 5 | | 121 | ALKBH7 | additional sex combs like 1 (Drosophila) | 5 | | 122 | AMIGO2 | adhesion molecule with Ig-like domain 2 | 5 | | 123 | ANKH | alkB, alkylation repair homolog 7 (E. coli) | 5 | | 124 | APLP2 | amyloid beta (A4) precursor-like protein 2 | 5 | | 125 | ARHGAP5 | ankylosis, progressive homolog (mouse) | 5 | | 126 | ARHGEF3 | arrestin, beta 2 | 5 | | 127 | ARRB2 | ATP-binding cassette, sub-family B (MDR/TAP), member 1 | 5 | | 128 | ASXL1 | bromodomain and WD repeat domain containing 1 | 5 | | 129 | ATF7IP2 | BTAF1 RNA polymerase II, B-TFIID transcription factor-associated, 170kDa (Mot1 homolog, S. cerevisiae) | 5 | | 130 | BRWD1 | caprin family member 2 | 5 | | 131 | BTAF1 | CD160 molecule | 5 | | 132 | C10orf18 | CD28 molecule | 5 | | 133 | C19orf12 | CD96 molecule | 5 | | 134 | C1orf174 | cell division cycle 23 homolog (S. cerevisiae) | 5 | | 135 | C1orf9 | chromodomain helicase DNA binding protein 9 | 5 | | 136 | C2orf68 | chromosome 1 open reading frame 174 | 5 | | 137 | C3P1 | chromosome 1 open reading frame 9 | 5 | | 138 | C5orf39 | chromosome 10 open reading frame 18 | 5 | | 139 | C6orf48 | chromosome 19 open reading frame 12 | 5 | | 140 | C9orf5 | chromosome 2 open reading frame 68 | 5 | | 141 | CAPRIN2 | chromosome 5 open reading frame 39 | 5 | | 142 | CD160 | chromosome 6 open reading frame 48 | 5 | | 143 | CD28 | chromosome 9 open reading frame 5 | 5 | | 144 | CD96 | clathrin, light chain (Lca) | 5 | | 145 | CDC23 | claudin 18 | 5 | | 146 | CHD9 | complement component 3 precursor pseudogene | 5 | | 147 | CLDN18 | DEAD (Asp-Glu-Ala-Asp) box polypeptide 24 | 5 | | 148 | CLTA | DiGeorge syndrome critical region gene 5 (non-protein coding) | 5 | | 149 | DCBLD1 | discoidin, CUB and LCCL domain containing 1 | 5 | | 150 | DDX24 | DnaJ (Hsp40) homolog, subfamily A, member 3 | 5 | | 151 | DGCR5 | Enah/Vasp-like | 5 | | 152 | DNAJA3 | engulfment and cell motility 2 | 5 | | 153 | EIF3B | eukaryotic translation initiation factor 3, subunit B | 5 | | 154 | ELMO2 | exportin 4 | 5 | | 155 | EVL | family with sequence similarity 85, member A | 5 | | 156 | FAM85A | far upstream element (FUSE) binding protein 3 | 5 | | 157 | FBRSL1 | fibrosin-like 1 | 5 | | 158 | FUBP3 | FYN oncogene related to SRC, FGR, YES | 5 | | 159 | FYN | glucose-6-phosphate dehydrogenase | 5 | | 160 | G6PD | HAUS augmin-like complex, subunit 3 | 5 | | 161 | HAUS3 | heat shock protein family B (small), member 11 | 5 | | 162 | HEG1 | HEG homolog 1 (zebrafish) | 5 | | 163 | HNRNPR | heterogeneous nuclear ribonucleoprotein D-like | 5 | | 164 | HNRNPU | heterogeneous nuclear ribonucleoprotein R | 5 | | 165 | HNRPDL | heterogeneous nuclear ribonucleoprotein U (scaffold attachment factor A) | 5 | | 166 | HSPB11 | hypothetical LOC221122 | 5 | | 167 | KIAA0748 | KIAA0748 | 5 | | 168 | LOC221122 | lipin 1 | 5 | | 169 | LPIN1 | major facilitator superfamily domain containing 8 | 5 | | 170 | MAN1C1 | mannosidase, alpha, class 1C, member 1 | 5 | | 171 | MDN1 | MDN1, midasin homolog (yeast) | 5 | | 172 | MFSD8 | MKI67 (FHA domain) interacting nucleolar phosphoprotein | 5 | | 173 | MKI67IP | N-acyl phosphatidylethanolamine phospholipase D | 5 | | 174 | NAA35 | N(alpha)-acetyltransferase 35, NatC auxiliary subunit | 5 | | 175 | NAPEPLD | natural cytotoxicity triggering receptor 2 | 5 | | 176 | NCBP2 | nuclear cap binding protein subunit 2, 20kDa | 5 | | 177 | NCR2 | nuclear factor (erythroid-derived 2), 45kDa | 5 | | 178 | NFATC2 | nuclear factor of activated T-cells, cytoplasmic, calcineurin-dependent 2 | 5 | | 179 | NFE2 | nucleolar and coiled-body phosphoprotein 1 | 5 | | 180 | NOLC1 | nucleoporin 35kDa | 5 | | 181 | NUP35 | oxidoreductase NAD-binding domain containing 1 | 5 | | 182 | OSBPL3 | oxysterol binding protein-like 3 | 5 | | 183 | OXNAD1 | PAP associated domain containing 7 | 5 | | 184 | PAPD7 | patched homolog 1 (Drosophila) | 5 | | 185 | PDCD4 | poliovirus receptor | 5 | | 186 | PDCD5 | polymerase (RNA) I polypeptide B, 128kDa | 5 | | 187 | PJA1 | praja ring finger 1 | 5 | | 188 | POLR1B | programmed cell death 4 (neoplastic transformation inhibitor) | 5 | | 189 | PRKAB2 | programmed cell death 5 | 5 | | 190 | PTCH1 | protein kinase, AMP-activated, beta 2 non-catalytic subunit | 5 | | 191 | PVR | pyrin and HIN domain family, member 1 | 5 | | 192 | PYHIN1 | retinitis pigmentosa 1 (autosomal dominant) | 5 | | 193 | RP1 | Rho GTPase activating protein 5 | 5 | | 194 | RPS6KA1 | Rho guanine nucleotide exchange factor (GEF) 3 | 5 | | 195 | SCYL3 | ribosomal protein S6 kinase, 90kDa, polypeptide 1 | 5 | | 196 | SDAD1 | SCY1-like 3 (S. cerevisiae) | 5 | | 197 | SFRS6 | SDA1 domain containing 1 | 5 | | 198 | SH2D1A | SH2 domain containing 1B | 5 | | 199 | SH2D1B | SH2 domain protein 1A | 5 | | 200 | SLC25A45 | signal transducer and activator of transcription 5B | 5 | | 201 | SLC7A6 | solute carrier family 25, member 45 | 5 | | 202 | SMARCA4 | solute carrier family 7 (cationic amino acid transporter, y+ system), member 6 | 5 | | 203 | SMOC1 | sortilin-related VPS10 domain containing receptor 2 | 5 | | 204 | SORCS2 | SPARC related modular calcium binding 1 | 5 | | 205 | STAT5B | splicing factor, arginine/serine-rich 6 | 5 | | 206 | SUSD2 | sushi domain containing 2 | 5 | | 207 | SYN1 | SWI/SNF related, matrix associated, actin dependent regulator of chromatin, subfamily a, member 4 | 5 | | 208 | TMEM218 | synapsin I | 5 | | 209 | TMF1 | TATA element modulatory factor 1 | 5 | | 210 | TNFRSF25 | tetratricopeptide repeat domain 3 | 5 | | 211 | TOX | thymocyte selection-associated high mobility group box | 5 | | 212 | TRAF5 | TNF receptor-associated factor 5 | 5 | | 213 | TRIM33 | transmembrane protein 218 | 5 | | 214 | TRIM68 | tripartite motif-containing 33 | 5 | | 215 | TTC3 | tripartite motif-containing 68 | 5 | | 216 | USP24 | tumor necrosis factor receptor superfamily, member 25 | 5 | | 217 | USP49 | ubiquitin specific peptidase 24 | 5 | | 218 | USPL1 | ubiquitin specific peptidase 49 | 5 | | 219 | XPO4 | ubiquitin specific peptidase like 1 | 5 | | 220 | ZFAND1 | zinc finger protein 146 | 5 | | 221 | ZNF146 | zinc finger protein 567 | 5 | | 222 | ZNF567 | zinc finger protein 831 | 5 | | 223 | ZNF831 | zinc finger, AN1-type domain 1 | 5 | | 224 | ACTL7B | 2-oxoglutarate and iron-dependent oxygenase domain containing 1 | 4 | | 225 | ADA | actin-like 7B | 4 | | 226 | AFG3L1 | adaptor protein, phosphotyrosine interaction, PH domain and leucine zipper containing 1 | 4 | | 227 | AFG3L2 | adenosine deaminase | 4 | | 228 | AGAP4 | ADP-ribosylation factor 3 | 4 | | 229 | ANGEL2 | AFG3 ATPase family gene 3-like 1 (S. cerevisiae) | 4 | | 230 | APBB1IP | AFG3 ATPase family gene 3-like 2 (yeast) | 4 | | 231 | APPL1 | amyloid beta (A4) precursor protein-binding, family B, member 1 interacting protein | 4 | | 232 | ARF3 | angel homolog 2 (Drosophila) | 4 | | 233 | ARTN | ArfGAP with GTPase domain, ankyrin repeat and PH domain 4 | 4 | | 234 | ATP5D | artemin | 4 | | 235 | ATP6V0C | ATP synthase, H+ transporting, mitochondrial F1 complex, delta subunit | 4 | | 236 | BAG5 | ATPase, H+ transporting, lysosomal 16kDa, V0 subunit c | 4 | | 237 | BCL2 | B-cell CLL/lymphoma 2 | 4 | | 238 | BTBD11 | BCL2-associated athanogene 5 | 4 | | 239 | C10orf104 | BTB (POZ) domain containing 11 | 4 | | 240 | C14orf126 | calcium/calmodulin-dependent protein kinase II delta | 4 | | 241 | C16orf80 | cathepsin D | 4 | | 242 | C2orf89 | CD2 molecule | 4 | | 243 | CAMK2D | centrosomal protein 290kDa | 4 | | 244 | CD2 | choroideremia-like (Rab escort protein 2) | 4 | | 245 | CDYL2 | chromodomain protein, Y-like 2 | 4 | | 246 | CEP290 | chromosome 10 open reading frame 104 | 4 | | 247 | CHML | chromosome 14 open reading frame 126 | 4 | | 248 | CHTF8 | chromosome 16 open reading frame 80 | 4 | | 249 | CMTM3 | chromosome 2 open reading frame 89 | 4 | | 250 | COL6A1 | CKLF-like MARVEL transmembrane domain containing 3 | 4 | | 251 | COL7A1 | cleavage and polyadenylation specific factor 6, 68kDa | 4 | | 252 | CPNE6 | collagen, type VI, alpha 1 | 4 | | 253 | CPSF6 | collagen, type VII, alpha 1 | 4 | | 254 | CTSD | copine VI (neuronal) | 4 | | 255 | DAP3 | CTF8, chromosome transmission fidelity factor 8 homolog (S. cerevisiae) | 4 | | 256 | DENND2D | death associated protein 3 | 4 | | 257 | EBAG9 | DENN/MADD domain containing 2D | 4 | | 258 | EHD1 | EH-domain containing 1 | 4 | | 259 | EIF1AX | establishment of cohesion 1 homolog 1 (S. cerevisiae) | 4 | | 260 | EIF3M | estrogen receptor binding site associated, antigen, 9 | 4 | | 261 | ERCC1 | eukaryotic translation initiation factor 1A, X-linked | 4 | | 262 | ESCO1 | eukaryotic translation initiation factor 3, subunit M | 4 | | 263 | FAM179B | excision repair cross-complementing rodent repair deficiency, complementation group 1 (includes overlapping antisense sequence) | 4 | | 264 | FAM35A | family with sequence similarity 179, member B | 4 | | 265 | FLJ35220 | family with sequence similarity 35, member A | 4 | | 266 | FUT7 | fucosyltransferase 7 (alpha (1,3) fucosyltransferase) | 4 | | 267 | GALNTL1 | G-protein signaling modulator 3 (AGS3-like, C. elegans) | 4 | | 268 | GATAD2A | gasdermin B | 4 | | 269 | GCAT | GATA zinc finger domain containing 2A | 4 | | 270 | GEMIN4 | gem (nuclear organelle) associated protein 4 | 4 | | 271 | GIMAP1 | general transcription factor IIIA | 4 | | 272 | GLTSCR2 | glioma tumor suppressor candidate region gene 2 | 4 | | 273 | GOLGA2B | glycine C-acetyltransferase | 4 | | 274 | GOLT1B | golgi transport 1 homolog B (S. cerevisiae) | 4 | | 275 | GPSM3 | golgin A2 family, member B | 4 | | 276 | GRAMD1A | GRAM domain containing 1A | 4 | | 277 | GSDMB | GTPase, IMAP family member 1 | 4 | | 278 | GTF3A | H1 histone family, member O, oocyte-specific | 4 | | 279 | H1FOO | high density lipoprotein binding protein | 4 | | 280 | HDLBP | hypothetical LOC254559 | 4 | | 281 | HLA-DMB | hypothetical protein FLJ35220 | 4 | | 282 | IL11RA | hypothetical protein LOC93444 | 4 | | 283 | IL1RN | interleukin 1 receptor antagonist | 4 | | 284 | KCNQ4 | interleukin 11 receptor, alpha | 4 | | 285 | KIAA1143 | karyopherin alpha 5 (importin alpha 6) | 4 | | 286 | KIAA1539 | KIAA1143 | 4 | | 287 | KIAA1826 | KIAA1539 | 4 | | 288 | KPNA5 | KIAA1826 | 4 | | 289 | LBH | lactate dehydrogenase B | 4 | | 290 | LDHB | large subunit GTPase 1 homolog (S. cerevisiae) | 4 | | 291 | LILRA5 | leukocyte immunoglobulin-like receptor pseudogene 2 | 4 | | 292 | LILRP2 | leukocyte immunoglobulin-like receptor, subfamily A (with TM domain), member 5 | 4 | | 293 | LOC254559 | limb bud and heart development homolog (mouse) | 4 | | 294 | LOC93444 | LUC7-like (S. cerevisiae) | 4 | | 295 | LSG1 | LYR motif containing 4 | 4 | | 296 | LUC7L | MAGI family member, X-linked | 4 | | 297 | LYRM4 | major histocompatibility complex, class II, DM beta | 4 | | 298 | MAGIX | mal, T-cell differentiation protein | 4 | | 299 | MAL | methylcrotonoyl-Coenzyme A carboxylase 2 (beta) | 4 | | 300 | MAPK8IP1 | methylthioribose-1-phosphate isomerase homolog (S. cerevisiae) | 4 | | 301 | MAPKAPK2 | mitochondrial ribosomal protein S9 | 4 | | 302 | MCCC2 | mitogen-activated protein kinase-activated protein kinase 2 | 4 | | 303 | MRI1 | mitogen-activated protein kinase 8 interacting protein 1 | 4 | | 304 | MRPS9 | MTERF domain containing 1 | 4 | | 305 | MTERFD1 | MTERF domain containing 2 | 4 | | 306 | MTERFD2 | MYC binding protein 2 | 4 | | 307 | MYADM | myeloid-associated differentiation marker | 4 | | 308 | MYBL1 | myosin, heavy chain 9, non-muscle | 4 | | 309 | MYCBP2 | necdin-like 2 | 4 | | 310 | MYH9 | nuclear assembly factor 1 homolog (S. cerevisiae) | 4 | | 311 | NAF1 | nuclear VCP-like | 4 | | 312 | NDNL2 | nucleolar complex associated 3 homolog (S. cerevisiae) | 4 | | 313 | NOC3L | nucleolar protein 9 | 4 | | 314 | NOL9 | nucleoporin 133kDa | 4 | | 315 | NUP133 | nucleoporin 54kDa | 4 | | 316 | NUP54 | olfactory receptor, family 10, subfamily D, member 3 pseudogene | 4 | | 317 | NVL | origin recognition complex, subunit 5-like (yeast) | 4 | | 318 | OGFOD1 | paxillin | 4 | | 319 | OR10D3P | phosphatidylglycerophosphate synthase 1 | 4 | | 320 | ORC5L | phosphoribosylformylglycinamidine synthase | 4 | | 321 | PAIP1 | PMS1 postmeiotic segregation increased 1 (S. cerevisiae) | 4 | | 322 | PATZ1 | poly(A) binding protein interacting protein 1 | 4 | | 323 | PFAS | polyhomeotic homolog 3 (Drosophila) | 4 | | 324 | PGS1 | polymerase (RNA) I polypeptide C, 30kDa | 4 | | 325 | PHC3 | polymerase (RNA) I polypeptide E, 53kDa | 4 | | 326 | PMS1 | polymerase (RNA) II (DNA directed) polypeptide B, 140kDa | 4 | | 327 | POLR1C | potassium voltage-gated channel, KQT-like subfamily, member 4 | 4 | | 328 | POLR1E | POZ (BTB) and AT hook containing zinc finger 1 | 4 | | 329 | POLR2B | protease, serine, 23 | 4 | | 330 | PPM1K | protein kinase C, delta | 4 | | 331 | PRKCD | protein phosphatase 1K (PP2C domain containing) | 4 | | 332 | PRSS23 | protein tyrosine phosphatase-like (proline instead of catalytic arginine), member b | 4 | | 333 | PTPLB | protein tyrosine phosphatase, non-receptor type 4 (megakaryocyte) | 4 | | 334 | PTPN4 | RAD17 homolog (S. pombe) | 4 | | 335 | PXN | RAS p21 protein activator 3 | 4 | | 336 | RAD17 | regulatory factor X-associated protein | 4 | | 337 | RARA | retinoblastoma binding protein 9 | 4 | | 338 | RASA3 | retinoic acid receptor, alpha | 4 | | 339 | RBBP9 | rhomboid, veinlet-like 2 (Drosophila) | 4 | | 340 | RBM15 | ribonuclease type III, nuclear | 4 | | 341 | RFXAP | ribosomal protein L12 | 4 | | 342 | RHBDL2 | ribosomal protein L14 | 4 | | 343 | RNASEN | ribosomal protein L22 | 4 | | 344 | RNPC3 | ribosomal protein L3 | 4 | | 345 | RPL12 | ribosomal RNA processing 15 homolog (S. cerevisiae) | 4 | | 346 | RPL14 | RNA-binding region (RNP1, RRM) containing 3 | 4 | | 347 | RPL22 | RNA binding motif protein 15 | 4 | | 348 | RPL3 | SH3-binding domain kinase 1 | 4 | | 349 | RRP15 | solute carrier family 25, member 32 | 4 | | 350 | SAMD3 | solute carrier family 9 (sodium/hydrogen exchanger), member 5 | 4 | | 351 | SBK1 | src kinase associated phosphoprotein 1 | 4 | | 352 | SBNO2 | sterile alpha motif domain containing 3 | 4 | | 353 | SKAP1 | strawberry notch homolog 2 (Drosophila) | 4 | | 354 | SLC25A32 | tafazzin | 4 | | 355 | SLC9A5 | TAR DNA binding protein | 4 | | 356 | TARDBP | torsin family 1, member A (torsin A) | 4 | | 357 | TAZ | transcription factor binding to IGHM enhancer 3 | 4 | | 358 | TFE3 | transmembrane protein 120A | 4 | | 359 | TMEM120A | transmembrane protein 128 | 4 | | 360 | TMEM128 | transmembrane protein 50B | 4 | | 361 | TMEM50B | tripartite motif-containing 25 | 4 | | 362 | TOR1A | tripartite motif-containing 52 | 4 | | 363 | TRIM25 | TruB pseudouridine (psi) synthase homolog 1 (E. coli) | 4 | | 364 | TRIM52 | TWIST neighbor | 4 | | 365 | TRUB1 | ubiquitin-conjugating enzyme E2I (UBC9 homolog, yeast) | 4 | | 366 | TWISTNB | ubiquitin-conjugating enzyme E2Q family member 2 | 4 | | 367 | UBE2I | UDP-N-acetyl-alpha-D-galactosamine:polypeptide N-acetylgalactosaminyltransferase-like 1 | 4 | | 368 | UBE2Q2 | UFM1-specific peptidase 2 | 4 | | 369 | UFSP2 | v-myb myeloblastosis viral oncogene homolog (avian)-like 1 | 4 | | 370 | VEZT | vezatin, adherens junctions transmembrane protein | 4 | | 371 | WNK1 | WNK lysine deficient protein kinase 1 | 4 | | 372 | YLPM1 | YLP motif containing 1 | 4 | | 373 | ZBTB24 | zinc finger and BTB domain containing 24 | 4 | | 374 | ZFP3 | zinc finger protein 140 | 4 | | 375 | ZFP62 | zinc finger protein 211 | 4 | | 376 | ZNF140 | zinc finger protein 22 (KOX 15) | 4 | | 377 | ZNF211 | zinc finger protein 3 homolog (mouse) | 4 | | 378 | ZNF22 | zinc finger protein 302 | 4 | | 379 | ZNF302 | zinc finger protein 383 | 4 | | 380 | ZNF383 | zinc finger protein 529 | 4 | | 381 | ZNF529 | zinc finger protein 566 | 4 | | 382 | ZNF566 | zinc finger protein 62 homolog (mouse) | 4 | | 383 | ZNHIT3 | zinc finger, HIT type 3 | 4 | | 384 | ZYX | zyxin | 4 | | 385 | ABCE1 | activating transcription factor 7 interacting protein | 3 | | 386 | ADRA2C | adaptor-related protein complex 3, mu 2 subunit | 3 | | 387 | AIMP1 | ADP-ribosylation factor-like 5C | 3 | | 388 | ALDH3B1 | adrenergic, alpha-2C-, receptor | 3 | | 389 | ANAPC1 | aldehyde dehydrogenase 3 family, member B1 | 3 | | 390 | ANGPTL4 | aminoacyl tRNA synthetase complex-interacting multifunctional protein 1 | 3 | | 391 | AP3M2 | anaphase promoting complex subunit 1 | 3 | | 392 | APOA5 | angiopoietin-like 4 | 3 | | 393 | APOBEC3F | apolipoprotein A-V | 3 | | 394 | ARHGEF18 | apolipoprotein B mRNA editing enzyme, catalytic polypeptide-like 3F | 3 | | 395 | ARHGEF9 | ATG5 autophagy related 5 homolog (S. cerevisiae) | 3 | | 396 | ARL5C | ATP-binding cassette, sub-family E (OABP), member 1 | 3 | | 397 | ATF7IP | ATPase, Ca++ transporting, plasma membrane 3 | 3 | | 398 | ATG5 | ATPase, H+ transporting, lysosomal 56/58kDa, V1 subunit B1 | 3 | | 399 | ATP2B3 | axin 2 | 3 | | 400 | ATP6V1B1 | baculoviral IAP repeat-containing 7 | 3 | | 401 | AXIN2 | BRCA1 associated protein-1 (ubiquitin carboxy-terminal hydrolase) | 3 | | 402 | BACH2 | BTB and CNC homology 1, basic leucine zipper transcription factor 2 | 3 | | 403 | BAP1 | calcium binding protein 39 | 3 | | 404 | BIRC7 | calcium channel, voltage-dependent, beta 1 subunit | 3 | | 405 | C10orf72 | calcium channel, voltage-dependent, T type, alpha 1I subunit | 3 | | 406 | C11orf46 | carboxylesterase 4, pseudogene | 3 | | 407 | C12orf47 | CD82 molecule | 3 | | 408 | C1orf211 | CD8b molecule | 3 | | 409 | C1orf52 | Cdc42 guanine nucleotide exchange factor (GEF) 9 | 3 | | 410 | C20orf165 | centromere protein C 1 | 3 | | 411 | C2orf3 | centromere protein V | 3 | | 412 | C3orf58 | ceroid-lipofuscinosis, neuronal 6, late infantile, variant | 3 | | 413 | C7orf27 | chemokine (C-C motif) ligand 28 | 3 | | 414 | C9orf86 | chromosome 1 open reading frame 211 | 3 | | 415 | CAB39 | chromosome 1 open reading frame 52 | 3 | | 416 | CACNA1I | chromosome 10 open reading frame 72 | 3 | | 417 | CACNB1 | chromosome 11 open reading frame 46 | 3 | | 418 | CCDC45 | chromosome 12 open reading frame 47 | 3 | | 419 | CCDC85A | chromosome 2 open reading frame 3 | 3 | | 420 | CCL28 | chromosome 20 open reading frame 165 | 3 | | 421 | CCND2 | chromosome 3 open reading frame 58 | 3 | | 422 | CCNDBP1 | chromosome 7 open reading frame 27 | 3 | | 423 | CD82 | chromosome 9 open reading frame 86 | 3 | | 424 | CD8B | citrate lyase beta like | 3 | | 425 | CENPC1 | cleavage stimulation factor, 3' pre-RNA, subunit 1, 50kDa | 3 | | 426 | CENPV | coagulation factor VII (serum prothrombin conversion accelerator) | 3 | | 427 | CES4 | coiled-coil domain containing 45 | 3 | | 428 | CLN6 | coiled-coil domain containing 85A | 3 | | 429 | CLYBL | complement component (3b/4b) receptor 1 (Knops blood group) | 3 | | 430 | CMPK1 | crystallin, zeta (quinone reductase)-like 1 | 3 | | 431 | CR1 | cyclin D-type binding-protein 1 | 3 | | 432 | CRYZL1 | cyclin D2 | 3 | | 433 | CSTF1 | cytidine monophosphate (UMP-CMP) kinase 1, cytosolic | 3 | | 434 | DAB2IP | DAB2 interacting protein | 3 | | 435 | DBNL | dehydrogenase/reductase (SDR family) member 2 | 3 | | 436 | DENND2A | DENN/MADD domain containing 2A | 3 | | 437 | DHRS2 | down-regulator of transcription 1, TBP-binding (negative cofactor 2) | 3 | | 438 | DR1 | drebrin-like | 3 | | 439 | EEF1A1 | E1A binding protein p400 | 3 | | 440 | ENPP3 | ectonucleoside triphosphate diphosphohydrolase 2 | 3 | | 441 | ENTPD2 | ectonucleotide pyrophosphatase/phosphodiesterase 3 | 3 | | 442 | EP400 | erythrocyte membrane protein band 4.1-like 1 | 3 | | 443 | EPB41L1 | eukaryotic translation elongation factor 1 alpha 1 | 3 | | 444 | EXOSC2 | exosome component 2 | 3 | | 445 | F7 | family with sequence similarity 115, member A | 3 | | 446 | FAM115A | family with sequence similarity 117, member B | 3 | | 447 | FAM117B | family with sequence similarity 84, member B | 3 | | 448 | FAM84B | Fc fragment of IgA, receptor for | 3 | | 449 | FCAR | Fc fragment of IgE, high affinity I, receptor for; alpha polypeptide | 3 | | 450 | FCER1A | FK506 binding protein 8, 38kDa | 3 | | 451 | FKBP8 | FLJ41603 protein | 3 | | 452 | FLJ22536 | folate receptor 2 (fetal) | 3 | | 453 | FLJ31356 | forkhead box O1 | 3 | | 454 | FLJ39609 | forkhead box O6 | 3 | | 455 | FLJ41603 | G protein-coupled receptor 172B | 3 | | 456 | FOLR2 | G protein-coupled receptor, family C, group 5, member B | 3 | | 457 | FOXO1 | gap junction protein, beta 1, 32kDa | 3 | | 458 | FOXO6 | gastrulation brain homeobox 2 | 3 | | 459 | GBX2 | gelsolin (amyloidosis, Finnish type) | 3 | | 460 | GDPD1 | general transcription factor IIIC, polypeptide 2, beta 110kDa | 3 | | 461 | GJB1 | glomulin, FKBP associated protein | 3 | | 462 | GLMN | glycerophosphodiester phosphodiesterase domain containing 1 | 3 | | 463 | GNAS | GNAS complex locus | 3 | | 464 | GNB2L1 | golgin A8 family, member B | 3 | | 465 | GOLGA8B | granzyme A (granzyme 1, cytotoxic T-lymphocyte-associated serine esterase 3) | 3 | | 466 | GPR172B | granzyme K (granzyme 3; tryptase II) | 3 | | 467 | GPRC5B | guanine nucleotide binding protein (G protein), beta polypeptide 2-like 1 | 3 | | 468 | GSN | heat shock protein, alpha-crystallin-related, B9 | 3 | | 469 | GTF3C2 | HECT, UBA and WWE domain containing 1 | 3 | | 470 | GZMA | heparan sulfate 6-O-sulfotransferase 1 | 3 | | 471 | GZMK | hepatocellular carcinoma-related HCRP1 | 3 | | 472 | hCG\_2042068 | heterogeneous nuclear ribonucleoprotein A3 | 3 | | 473 | HCRP1 | histamine receptor H2 | 3 | | 474 | HDAC5 | histone deacetylase 5 | 3 | | 475 | HNRNPA3 | HOP homeobox | 3 | | 476 | HOPX | hypothetical LOC100131541 | 3 | | 477 | HRH2 | hypothetical LOC148145 | 3 | | 478 | HS6ST1 | hypothetical LOC283588 | 3 | | 479 | HSPB9 | hypothetical LOC404266 | 3 | | 480 | HUWE1 | hypothetical locus LOC401237 | 3 | | 481 | IFT74 | hypothetical protein FLJ31356 | 3 | | 482 | ITGB4 | hypothetical protein LOC100128198 | 3 | | 483 | ITPR1 | hypothetical protein MGC40069 | 3 | | 484 | JAK1 | inositol 1,4,5-triphosphate receptor, type 1 | 3 | | 485 | KIAA1430 | integrin, beta 4 | 3 | | 486 | KLHL28 | intraflagellar transport 74 homolog (Chlamydomonas) | 3 | | 487 | KRT3 | Janus kinase 1 | 3 | | 488 | KRT31 | kelch-like 28 (Drosophila) | 3 | | 489 | LAX1 | keratin 3 | 3 | | 490 | LDB3 | keratin 31 | 3 | | 491 | LGR4 | KIAA1430 | 3 | | 492 | LINS1 | leucine-rich repeat-containing G protein-coupled receptor 4 | 3 | | 493 | LOC100128198 | leucine rich repeat containing 16B | 3 | | 494 | LOC100293142 | leucine rich repeat neuronal 3 | 3 | | 495 | LOC148145 | leucine zipper transcription factor-like 1 | 3 | | 496 | LOC283588 | LIM domain binding 3 | 3 | | 497 | LOC645744 | lines homolog 1 (Drosophila) | 3 | | 498 | LONP1 | lon peptidase 1, mitochondrial | 3 | | 499 | LRRC16B | lymphocyte antigen 6 complex, locus D | 3 | | 500 | LRRN3 | lymphocyte transmembrane adaptor 1 | 3 | | 501 | LY6D | mastermind-like 2 (Drosophila) | 3 | | 502 | LZTFL1 | MAX binding protein | 3 | | 503 | MAGEE1 | mediator complex subunit 14 | 3 | | 504 | MAML2 | Meis homeobox 3 | 3 | | 505 | MAP4K5 | melanoma antigen family E, 1 | 3 | | 506 | MAPK8 | metallo-beta-lactamase domain containing 2 | 3 | | 507 | MBLAC2 | methylmalonic aciduria (cobalamin deficiency) cblA type | 3 | | 508 | MED14 | mindbomb homolog 2 (Drosophila) | 3 | | 509 | MEIS3 | mitochondrial ribosomal protein S6 | 3 | | 510 | MGC40069 | mitogen-activated protein kinase 8 | 3 | | 511 | MIB2 | mitogen-activated protein kinase kinase kinase kinase 5 | 3 | | 512 | MMAA | MORC family CW-type zinc finger 2 | 3 | | 513 | MNT | musashi homolog 1 (Drosophila) | 3 | | 514 | MORC2 | myelin transcription factor 1 | 3 | | 515 | MRPS6 | neurocalcin delta | 3 | | 516 | MSI1 | neuroguidin, EIF4E binding protein | 3 | | 517 | MYT1 | neutrophil cytosolic factor 4, 40kDa | 3 | | 518 | NCALD | NHS-like 1 | 3 | | 519 | NCF4 | NIN1/RPN12 binding protein 1 homolog (S. cerevisiae) | 3 | | 520 | NCL | NLR family, pyrin domain containing 12 | 3 | | 521 | NCRNA00110 | non-protein coding RNA 110 | 3 | | 522 | NFYC | NSA2 ribosome biogenesis homolog (S. cerevisiae) | 3 | | 523 | NGDN | nuclear casein kinase and cyclin-dependent kinase substrate 1 | 3 | | 524 | NHSL1 | nuclear transcription factor Y, gamma | 3 | | 525 | NLRP12 | nucleolin | 3 | | 526 | NOB1 | nucleophosmin (nucleolar phosphoprotein B23, numatrin) | 3 | | 527 | NPM1 | nucleoporin 214kDa | 3 | | 528 | NSA2 | odz, odd Oz/ten-m homolog 3 (Drosophila) | 3 | | 529 | NUCKS1 | olfactory receptor, family 1, subfamily C, member 1 | 3 | | 530 | NUP214 | olfactory receptor, family 1, subfamily J, member 4 | 3 | | 531 | ODZ3 | oral-facial-digital syndrome 1 | 3 | | 532 | OFD1 | par-6 partitioning defective 6 homolog gamma (C. elegans) | 3 | | 533 | OR1C1 | peptidase (mitochondrial processing) beta | 3 | | 534 | OR1J4 | peptidyl arginine deiminase, type IV | 3 | | 535 | PADI4 | persephin | 3 | | 536 | PARD6G | phosphoinositide-3-kinase adaptor protein 1 | 3 | | 537 | PCDHB18 | phosphoinositide-3-kinase, regulatory subunit 1 (alpha) | 3 | | 538 | PCSK6 | pim-3 oncogene | 3 | | 539 | PHLDA2 | pleckstrin and Sec7 domain containing 4 | 3 | | 540 | PIBF1 | pleckstrin homology-like domain, family A, member 2 | 3 | | 541 | PIK3AP1 | poliovirus receptor related immunoglobulin domain containing | 3 | | 542 | PIK3R1 | polymerase (DNA directed), gamma 2, accessory subunit | 3 | | 543 | PIM3 | PPPDE peptidase domain containing 1 | 3 | | 544 | PMPCB | progesterone immunomodulatory binding factor 1 | 3 | | 545 | POLG2 | proprotein convertase subtilisin/kexin type 6 | 3 | | 546 | PPP1R7 | protein phosphatase 1, regulatory (inhibitor) subunit 7 | 3 | | 547 | PPPDE1 | protein tyrosine phosphatase, non-receptor type 11 | 3 | | 548 | PSD4 | protocadherin beta 18 pseudogene | 3 | | 549 | PSPN | RAB11 family interacting protein 3 (class II) | 3 | | 550 | PTPN11 | RAD50 homolog (S. cerevisiae) | 3 | | 551 | PVRIG | RecQ protein-like 5 | 3 | | 552 | RAB11FIP3 | regulating synaptic membrane exocytosis 3 | 3 | | 553 | RAD50 | reversion-inducing-cysteine-rich protein with kazal motifs | 3 | | 554 | RBM26 | Rho/Rac guanine nucleotide exchange factor (GEF) 18 | 3 | | 555 | RECK | ribosomal protein S4, X-linked | 3 | | 556 | RECQL5 | RNA binding motif protein 26 | 3 | | 557 | RIMS3 | SECIS binding protein 2 | 3 | | 558 | RPS4X | selenium binding protein 1 | 3 | | 559 | SECISBP2 | SET domain containing 4 | 3 | | 560 | SELENBP1 | SET domain containing 5 | 3 | | 561 | SETD4 | sialic acid binding Ig-like lectin 11 | 3 | | 562 | SETD5 | similar to hCG1995469 | 3 | | 563 | SFRS14 | similar to hCG2040019 | 3 | | 564 | SFRS7 | similar to PCAF associated factor 65 beta | 3 | | 565 | SIGLEC11 | single-stranded DNA binding protein 1 | 3 | | 566 | SLC15A4 | slingshot homolog 1 (Drosophila) | 3 | | 567 | SLC16A11 | slit homolog 1 (Drosophila) | 3 | | 568 | SLC22A6 | solute carrier family 15, member 4 | 3 | | 569 | SLC25A38 | solute carrier family 16, member 11 (monocarboxylic acid transporter 11) | 3 | | 570 | SLC4A7 | solute carrier family 22 (organic anion transporter), member 6 | 3 | | 571 | SLC9A3R2 | solute carrier family 25, member 38 | 3 | | 572 | SLIT1 | solute carrier family 4, sodium bicarbonate cotransporter, member 7 | 3 | | 573 | SOCS7 | solute carrier family 9 (sodium/hydrogen exchanger), member 3 regulator 2 | 3 | | 574 | SPAG16 | spectrin, beta, non-erythrocytic 4 | 3 | | 575 | SPSB4 | sperm associated antigen 16 | 3 | | 576 | SPTBN4 | splA/ryanodine receptor domain and SOCS box containing 4 | 3 | | 577 | SSBP1 | splicing factor, arginine/serine-rich 14 | 3 | | 578 | SSH1 | splicing factor, arginine/serine-rich 7, 35kDa | 3 | | 579 | ST3GAL1 | ST3 beta-galactoside alpha-2,3-sialyltransferase 1 | 3 | | 580 | STT3B | STT3, subunit of the oligosaccharyltransferase complex, homolog B (S. cerevisiae) | 3 | | 581 | STX17 | suppressor of cytokine signaling 7 | 3 | | 582 | SYMPK | symplekin | 3 | | 583 | TBX1 | syntaxin 17 | 3 | | 584 | TBX21 | T-box 1 | 3 | | 585 | TDRD3 | T-box 21 | 3 | | 586 | TIGD2 | tigger transposable element derived 2 | 3 | | 587 | TMC5 | TP53 target 5 | 3 | | 588 | TMCO3 | TraB domain containing | 3 | | 589 | TMEM135 | transmembrane and coiled-coil domains 3 | 3 | | 590 | TMEM150C | transmembrane channel-like 5 | 3 | | 591 | TMEM184A | transmembrane protein 135 | 3 | | 592 | TMEM203 | transmembrane protein 150C | 3 | | 593 | TMEM55B | transmembrane protein 184A | 3 | | 594 | TP53TG5 | transmembrane protein 203 | 3 | | 595 | TPPP2 | transmembrane protein 55B | 3 | | 596 | TRABD | tribbles homolog 2 (Drosophila) | 3 | | 597 | TRIB2 | tubulin polymerization-promoting protein family member 2 | 3 | | 598 | TUSC5 | tudor domain containing 3 | 3 | | 599 | UBA7 | tumor suppressor candidate 5 | 3 | | 600 | UFM1 | ubiquitin-fold modifier 1 | 3 | | 601 | UHRF2 | ubiquitin-like modifier activating enzyme 7 | 3 | | 602 | UNC5A | ubiquitin-like with PHD and ring finger domains 2 | 3 | | 603 | UNQ6228 | ubiquitin specific peptidase 28 | 3 | | 604 | UPF1 | unc-5 homolog A (C. elegans) | 3 | | 605 | USP28 | UPF1 regulator of nonsense transcripts homolog (yeast) | 3 | | 606 | UTRN | utrophin | 3 | | 607 | VAMP3 | vesicle-associated membrane protein 3 (cellubrevin) | 3 | | 608 | WIPI2 | WD repeat domain, phosphoinositide interacting 2 | 3 | | 609 | WNK2 | WNK lysine deficient protein kinase 2 | 3 | | 610 | ZCCHC7 | zinc finger protein 14 | 3 | | 611 | ZDHHC1 | zinc finger protein 141 | 3 | | 612 | ZDHHC21 | zinc finger protein 24 | 3 | | 613 | ZNF14 | zinc finger protein 275 | 3 | | 614 | ZNF141 | zinc finger protein 382 | 3 | | 615 | ZNF24 | zinc finger protein 468 | 3 | | 616 | ZNF275 | zinc finger protein 506 | 3 | | 617 | ZNF382 | zinc finger protein 550 | 3 | | 618 | ZNF468 | zinc finger protein 599 | 3 | | 619 | ZNF506 | zinc finger protein 642 | 3 | | 620 | ZNF550 | zinc finger protein 682 | 3 | | 621 | ZNF599 | zinc finger, CCHC domain containing 7 | 3 | | 622 | ZNF642 | zinc finger, DHHC-type containing 1 | 3 | | 623 | ZNF682 | zinc finger, DHHC-type containing 21 | 3 | | 624 | ABHD12 | 1-acylglycerol-3-phosphate O-acyltransferase 2 (lysophosphatidic acid acyltransferase, beta) | 2 | | 625 | ABHD2 | 1-acylglycerol-3-phosphate O-acyltransferase 5 (lysophosphatidic acid acyltransferase, epsilon) | 2 | | 626 | ACACB | 6-phosphofructo-2-kinase/fructose-2,6-biphosphatase 4 | 2 | | 627 | ADCY5 | abhydrolase domain containing 12 | 2 | | 628 | ADIG | abhydrolase domain containing 2 | 2 | | 629 | AGPAT2 | acetyl-Coenzyme A carboxylase beta | 2 | | 630 | AGPAT5 | adenomatosis polyposis coli 2 | 2 | | 631 | AHCYL2 | adenosylhomocysteinase-like 2 | 2 | | 632 | AKR1B1 | adenylate cyclase 5 | 2 | | 633 | AKT1S1 | adipogenin | 2 | | 634 | ALDH6A1 | ADP-ribosylation factor guanine nucleotide-exchange factor 1(brefeldin A-inhibited) | 2 | | 635 | ANKLE2 | AKT1 substrate 1 (proline-rich) | 2 | | 636 | ANKMY2 | aldehyde dehydrogenase 6 family, member A1 | 2 | | 637 | ANKRD60 | aldo-keto reductase family 1, member B1 (aldose reductase) | 2 | | 638 | APC2 | ankyrin repeat and LEM domain containing 2 | 2 | | 639 | AQP2 | ankyrin repeat and MYND domain containing 2 | 2 | | 640 | AQP3 | ankyrin repeat domain 60 | 2 | | 641 | ARFGEF1 | aquaporin 2 (collecting duct) | 2 | | 642 | ARHGAP17 | aquaporin 3 (Gill blood group) | 2 | | 643 | ARHGDIA | asparaginyl-tRNA synthetase | 2 | | 644 | ATP2A3 | ataxin 10 | 2 | | 645 | ATPBD4 | ATP binding domain 4 | 2 | | 646 | ATXN10 | ATPase, Ca++ transporting, ubiquitous | 2 | | 647 | BAIAP2 | B-cell CLL/lymphoma 11A (zinc finger protein) | 2 | | 648 | BBX | baculoviral IAP repeat-containing 3 | 2 | | 649 | BCCIP | BAI1-associated protein 2 | 2 | | 650 | BCL11A | BMS1 homolog, ribosome assembly protein (yeast) | 2 | | 651 | BIRC3 | bobby sox homolog (Drosophila) | 2 | | 652 | BMS1 | brain protein I3 | 2 | | 653 | BRI3 | BRCA2 and CDKN1A interacting protein | 2 | | 654 | C10orf78 | C-type lectin-like 1 | 2 | | 655 | C10orf88 | C-type lectin domain family 16, member A | 2 | | 656 | C16orf63 | cadherin 26 | 2 | | 657 | C17orf57 | calcium binding atopy-related autoantigen 1 | 2 | | 658 | C19orf60 | calmodulin regulated spectrin-associated protein 1 | 2 | | 659 | C19orf70 | calponin 2 | 2 | | 660 | C1orf27 | cAMP responsive element binding protein 1 | 2 | | 661 | C1orf91 | Cas-Br-M (murine) ecotropic retroviral transforming sequence-like 1 | 2 | | 662 | C1orf93 | caspase recruitment domain family, member 10 | 2 | | 663 | C2orf54 | catenin (cadherin-associated protein), alpha 1, 102kDa | 2 | | 664 | C2orf85 | CD177 molecule | 2 | | 665 | C3orf65 | CDK5 regulatory subunit associated protein 2 | 2 | | 666 | C4orf52 | ceroid-lipofuscinosis, neuronal 5 | 2 | | 667 | C5orf24 | chloride channel CLIC-like 1 | 2 | | 668 | C5orf25 | cholecystokinin B receptor | 2 | | 669 | C5orf44 | choline dehydrogenase | 2 | | 670 | CAMSAP1 | chromodomain helicase DNA binding protein 3 | 2 | | 671 | CARD10 | chromosome 1 open reading frame 27 | 2 | | 672 | CARS | chromosome 1 open reading frame 91 | 2 | | 673 | CBARA1 | chromosome 1 open reading frame 93 | 2 | | 674 | CBLL1 | chromosome 10 open reading frame 78 | 2 | | 675 | CCDC111 | chromosome 10 open reading frame 88 | 2 | | 676 | CCKBR | chromosome 16 open reading frame 63 | 2 | | 677 | CD177 | chromosome 17 open reading frame 57 | 2 | | 678 | CDH26 | chromosome 19 open reading frame 60 | 2 | | 679 | CDK5RAP2 | chromosome 19 open reading frame 70 | 2 | | 680 | CHD3 | chromosome 2 open reading frame 54 | 2 | | 681 | CHDH | chromosome 2 open reading frame 85 | 2 | | 682 | CLCC1 | chromosome 3 open reading frame 65 | 2 | | 683 | CLEC16A | chromosome 4 open reading frame 52 | 2 | | 684 | CLECL1 | chromosome 5 open reading frame 24 | 2 | | 685 | CLN5 | chromosome 5 open reading frame 25 | 2 | | 686 | CNDP2 | chromosome 5 open reading frame 44 | 2 | | 687 | CNN2 | ciliary neurotrophic factor receptor | 2 | | 688 | CNTFR | CNDP dipeptidase 2 (metallopeptidase M20 family) | 2 | | 689 | COPS4 | coenzyme Q10 homolog A (S. cerevisiae) | 2 | | 690 | COPS8 | coiled-coil domain containing 111 | 2 | | 691 | COQ10A | COP9 constitutive photomorphogenic homolog subunit 4 (Arabidopsis) | 2 | | 692 | COX4I1 | COP9 constitutive photomorphogenic homolog subunit 8 (Arabidopsis) | 2 | | 693 | CREB1 | CXXC finger 5 | 2 | | 694 | CTNNA1 | cysteinyl-tRNA synthetase | 2 | | 695 | CXXC5 | cytochrome b-561 domain containing 2 | 2 | | 696 | CYB561D2 | cytochrome c oxidase subunit IV isoform 1 | 2 | | 697 | DCAF11 | DDB1 and CUL4 associated factor 11 | 2 | | 698 | DCAF13 | DDB1 and CUL4 associated factor 13 | 2 | | 699 | DCAF8 | DDB1 and CUL4 associated factor 8 | 2 | | 700 | DDX31 | DEAD (Asp-Glu-Ala-Asp) box polypeptide 31 | 2 | | 701 | DERL1 | Der1-like domain family, member 1 | 2 | | 702 | DFFB | dihydrouridine synthase 4-like (S. cerevisiae) | 2 | | 703 | DNM1L | dishevelled, dsh homolog 2 (Drosophila) | 2 | | 704 | DST | DNA fragmentation factor, 40kDa, beta polypeptide (caspase-activated DNase) | 2 | | 705 | DUS4L | dynamin 1-like | 2 | | 706 | DVL2 | dystonin | 2 | | 707 | ECHS1 | EF-hand calcium binding domain 6 | 2 | | 708 | EFCAB6 | enoyl Coenzyme A hydratase, short chain, 1, mitochondrial | 2 | | 709 | EIF2S3 | eukaryotic translation initiation factor 2, subunit 3 gamma, 52kDa | 2 | | 710 | EIF4A2 | eukaryotic translation initiation factor 4A2 | 2 | | 711 | ERBB3 | exosome component 9 | 2 | | 712 | EXOSC9 | exportin 1 (CRM1 homolog, yeast) | 2 | | 713 | FAM126A | FAD1 flavin adenine dinucleotide synthetase homolog (S. cerevisiae) | 2 | | 714 | FAM38A | family with sequence similarity 126, member A | 2 | | 715 | FAM73A | family with sequence similarity 38, member A | 2 | | 716 | FAM83C | family with sequence similarity 73, member A | 2 | | 717 | FARS2 | family with sequence similarity 83, member C | 2 | | 718 | FCER1G | Fc fragment of IgE, high affinity I, receptor for; gamma polypeptide | 2 | | 719 | FIGNL1 | fidgetin-like 1 | 2 | | 720 | FLAD1 | forkhead box P2 | 2 | | 721 | FOXP2 | G protein-coupled receptor 110 | 2 | | 722 | GBAS | G protein-coupled receptor 183 | 2 | | 723 | GLG1 | glioblastoma amplified sequence | 2 | | 724 | GMEB1 | glucocorticoid modulatory element binding protein 1 | 2 | | 725 | GOLGA7 | glucoside xylosyltransferase 1 | 2 | | 726 | GORAB | glutamine and serine rich 1 | 2 | | 727 | GPR110 | glutaminyl-tRNA synthetase | 2 | | 728 | GPR183 | golgi glycoprotein 1 | 2 | | 729 | GRPEL2 | golgin A7 | 2 | | 730 | GRTP1 | golgin, RAB6-interacting | 2 | | 731 | GTPBP4 | granzyme B (granzyme 2, cytotoxic T-lymphocyte-associated serine esterase 1) | 2 | | 732 | GXYLT1 | growth hormone regulated TBC protein 1 | 2 | | 733 | GZMB | GrpE-like 2, mitochondrial (E. coli) | 2 | | 734 | HABP4 | GTP binding protein 4 | 2 | | 735 | HAUS1 | HAUS augmin-like complex, subunit 1 | 2 | | 736 | HCFC2 | heat shock 70kDa protein 14 | 2 | | 737 | HDAC10 | hect domain and RLD 6 | 2 | | 738 | HDAC9 | histone deacetylase 10 | 2 | | 739 | HERC6 | histone deacetylase 9 | 2 | | 740 | HINFP | histone H4 transcription factor | 2 | | 741 | HOXB5 | homeobox B5 | 2 | | 742 | HOXB6 | homeobox B6 | 2 | | 743 | HOXD12 | homeobox D12 | 2 | | 744 | HSPA14 | host cell factor C2 | 2 | | 745 | HVCN1 | hyaluronan binding protein 4 | 2 | | 746 | IMP3 | hydrogen voltage-gated channel 1 | 2 | | 747 | IMPACT | hypothetical LOC100272216 | 2 | | 748 | ING3 | hypothetical LOC399753 | 2 | | 749 | INPP4A | hypothetical LOC731789 | 2 | | 750 | INPP5B | hypothetical protein LOC150759 | 2 | | 751 | IPW | hypothetical protein LOC283875 | 2 | | 752 | ISG20L2 | hypothetical protein LOC286052 | 2 | | 753 | ITGA4 | hypothetical protein LOC286272 | 2 | | 754 | ITPKB | hypothetical protein LOC728449 | 2 | | 755 | KIAA0090 | hypothetical protein, clone pT-Adv JuaX22 | 2 | | 756 | KIAA0226 | IMP3, U3 small nucleolar ribonucleoprotein, homolog (yeast) | 2 | | 757 | KIAA0652 | Impact homolog (mouse) | 2 | | 758 | KIAA1919 | imprinted in Prader-Willi syndrome (non-protein coding) | 2 | | 759 | KLK3 | inhibitor of growth family, member 3 | 2 | | 760 | LEMD1 | inositol 1,4,5-trisphosphate 3-kinase B | 2 | | 761 | LMCD1 | inositol polyphosphate-4-phosphatase, type I, 107kDa | 2 | | 762 | LOC100133790 | inositol polyphosphate-5-phosphatase, 75kDa | 2 | | 763 | LOC100272216 | integrin, alpha 4 (antigen CD49D, alpha 4 subunit of VLA-4 receptor) | 2 | | 764 | LOC100287482 | interferon stimulated exonuclease gene 20kDa-like 2 | 2 | | 765 | LOC150759 | intestinal mucin-like | 2 | | 766 | LOC283875 | kallikrein-related peptidase 3 | 2 | | 767 | LOC286052 | kazrin | 2 | | 768 | LOC286272 | KIAA0090 | 2 | | 769 | LOC728449 | KIAA0226 | 2 | | 770 | LOC731789 | KIAA0652 | 2 | | 771 | LOXHD1 | KIAA1919 | 2 | | 772 | LRP10 | LEM domain containing 1 | 2 | | 773 | LRRC69 | leucine rich repeat containing 69 | 2 | | 774 | LSM14B | LIM and cysteine-rich domains 1 | 2 | | 775 | LSM5 | lipoxygenase homology domains 1 | 2 | | 776 | LTV1 | low density lipoprotein receptor-related protein 10 | 2 | | 777 | MAGEF1 | LSM14B, SCD6 homolog B (S. cerevisiae) | 2 | | 778 | MAN2C1 | LSM5 homolog, U6 small nuclear RNA associated (S. cerevisiae) | 2 | | 779 | MAP3K7 | LTV1 homolog (S. cerevisiae) | 2 | | 780 | MAP4K3 | makorin ring finger protein 2 | 2 | | 781 | MAPK11 | malignant T cell amplified sequence 1 | 2 | | 782 | MBIP | mannosidase, alpha, class 2C, member 1 | 2 | | 783 | MBOAT7 | mannosyl-oligosaccharide glucosidase | 2 | | 784 | MCTS1 | MAP kinase interacting serine/threonine kinase 1 | 2 | | 785 | MED12 | MAP3K12 binding inhibitory protein 1 | 2 | | 786 | METAP1 | matrix metallopeptidase 8 (neutrophil collagenase) | 2 | | 787 | MFNG | MAX gene associated | 2 | | 788 | MGA | mediator complex subunit 12 | 2 | | 789 | MIA3 | melanoma antigen family F, 1 | 2 | | 790 | MID1 | melanoma inhibitory activity family, member 3 | 2 | | 791 | MKNK1 | membrane bound O-acyltransferase domain containing 7 | 2 | | 792 | MKRN2 | methionyl aminopeptidase 1 | 2 | | 793 | MMP8 | MFNG O-fucosylpeptide 3-beta-N-acetylglucosaminyltransferase | 2 | | 794 | MOGS | midline 1 (Opitz/BBB syndrome) | 2 | | 795 | MON2 | mitochondrial ribosomal protein L41 | 2 | | 796 | MRPL41 | mitogen-activated protein kinase 11 | 2 | | 797 | MTMR15 | mitogen-activated protein kinase kinase kinase 7 | 2 | | 798 | MUC13 | mitogen-activated protein kinase kinase kinase kinase 3 | 2 | | 799 | MUDENG | MON2 homolog (S. cerevisiae) | 2 | | 800 | NAA15 | MU-2/AP1M2 domain containing, death-inducing | 2 | | 801 | NARS | mucin 13, cell surface associated | 2 | | 802 | NAT10 | myotubularin related protein 15 | 2 | | 803 | NCRNA00201 | N-acetyltransferase 10 (GCN5-related) | 2 | | 804 | NDUFS7 | N(alpha)-acetyltransferase 15, NatA auxiliary subunit | 2 | | 805 | NFAM1 | NADH dehydrogenase (ubiquinone) Fe-S protein 7, 20kDa (NADH-coenzyme Q reductase) | 2 | | 806 | NFYB | neuro-oncological ventral antigen 2 | 2 | | 807 | NHP2L1 | NFAT activating protein with ITAM motif 1 | 2 | | 808 | NIP7 | NHP2 non-histone chromosome protein 2-like 1 (S. cerevisiae) | 2 | | 809 | NIT1 | nicotinamide N-methyltransferase | 2 | | 810 | NNMT | nitrilase 1 | 2 | | 811 | NOL6 | non-protein coding RNA 201 | 2 | | 812 | NOVA2 | nuclear distribution gene C homolog (A. nidulans) | 2 | | 813 | NUDC | nuclear fragile X mental retardation protein interacting protein 1 | 2 | | 814 | NUFIP1 | nuclear import 7 homolog (S. cerevisiae) | 2 | | 815 | NUP155 | nuclear transcription factor Y, beta | 2 | | 816 | NUP93 | nucleolar protein family 6 (RNA-associated) | 2 | | 817 | ODF2L | nucleoporin 155kDa | 2 | | 818 | OGDHL | nucleoporin 93kDa | 2 | | 819 | OGT | O-linked N-acetylglucosamine (GlcNAc) transferase (UDP-N-acetylglucosamine:polypeptide-N-acetylglucosaminyl transferase) | 2 | | 820 | OR5L2 | olfactory receptor, family 5, subfamily L, member 2 | 2 | | 821 | ORF1 | osteosarcoma amplified 9, endoplasmic reticulum lectin | 2 | | 822 | OS9 | OTU domain containing 6B | 2 | | 823 | OTUD6B | outer dense fiber of sperm tails 2-like | 2 | | 824 | P2RX3 | oxoglutarate dehydrogenase-like | 2 | | 825 | PAN2 | PAN2 poly(A) specific ribonuclease subunit homolog (S. cerevisiae) | 2 | | 826 | PCDHB3 | PDS5, regulator of cohesion maintenance, homolog B (S. cerevisiae) | 2 | | 827 | PCGF3 | peptidylprolyl isomerase domain and WD repeat containing 1 | 2 | | 828 | PDCD6 | peroxisome proliferator-activated receptor gamma, coactivator-related 1 | 2 | | 829 | PDE8A | phenylalanyl-tRNA synthetase 2, mitochondrial | 2 | | 830 | PDS5B | phosphodiesterase 8A | 2 | | 831 | PDSS2 | phospholipid scramblase 3 | 2 | | 832 | PFKFB4 | pleckstrin homology domain containing, family B (evectins) member 1 | 2 | | 833 | PLEKHB1 | pogo transposable element with ZNF domain | 2 | | 834 | PLSCR3 | polycomb group ring finger 3 | 2 | | 835 | POGZ | polymerase (RNA) III (DNA directed) polypeptide B | 2 | | 836 | POLR3B | PR domain containing 9 | 2 | | 837 | PPM1G | prenyl (decaprenyl) diphosphate synthase, subunit 2 | 2 | | 838 | PPP2R5C | programmed cell death 6 | 2 | | 839 | PPRC1 | prostaglandin E receptor 4 (subtype EP4) | 2 | | 840 | PPWD1 | proteasome (prosome, macropain) 26S subunit, non-ATPase, 11 | 2 | | 841 | PRDM9 | protein arginine methyltransferase 1 | 2 | | 842 | PRKACA | protein kinase, cAMP-dependent, catalytic, alpha | 2 | | 843 | PRMT1 | protein phosphatase 1G (formerly 2C), magnesium-dependent, gamma isoform | 2 | | 844 | PRPF19 | protein phosphatase 2, regulatory subunit B', gamma isoform | 2 | | 845 | PSMD11 | protein tyrosine phosphatase, receptor type, J | 2 | | 846 | PTGER4 | protocadherin beta 3 | 2 | | 847 | PTPRJ | PRP19/PSO4 pre-mRNA processing factor 19 homolog (S. cerevisiae) | 2 | | 848 | QARS | purinergic receptor P2X, ligand-gated ion channel, 3 | 2 | | 849 | QDPR | quinoid dihydropteridine reductase | 2 | | 850 | QSER1 | RAB12, member RAS oncogene family | 2 | | 851 | RAB12 | rabaptin, RAB GTPase binding effector protein 2 | 2 | | 852 | RABEP2 | radical S-adenosyl methionine domain containing 1 | 2 | | 853 | RBM10 | RAP1 interacting factor homolog (yeast) | 2 | | 854 | RBM25 | regulator of G-protein signaling 16 | 2 | | 855 | RG9MTD1 | relaxin 2 | 2 | | 856 | RGS16 | retinitis pigmentosa 9 (autosomal dominant) | 2 | | 857 | RIF1 | Rho-associated, coiled-coil containing protein kinase 1 | 2 | | 858 | RLN2 | Rho GDP dissociation inhibitor (GDI) alpha | 2 | | 859 | RNF115 | Rho GTPase activating protein 17 | 2 | | 860 | RNF24 | ribosomal protein L32 pseudogene 3 | 2 | | 861 | ROCK1 | ring finger protein 115 | 2 | | 862 | RP1-21O18.1 | ring finger protein 24 | 2 | | 863 | RP11-144G6.7 | RNA (guanine-9-) methyltransferase domain containing 1 | 2 | | 864 | RP9 | RNA binding motif protein 10 | 2 | | 865 | RPL32P3 | RNA binding motif protein 25 | 2 | | 866 | RPUSD4 | RNA pseudouridylate synthase domain containing 4 | 2 | | 867 | RSAD1 | S100 calcium binding protein A11 | 2 | | 868 | S100A11 | Scm-like with four mbt domains 2 | 2 | | 869 | SAE1 | SEC63 homolog (S. cerevisiae) | 2 | | 870 | SAP130 | septin 1 | 2 | | 871 | SEC63 | SH2B adaptor protein 1 | 2 | | 872 | SEPT1 | signal sequence receptor, delta (translocon-associated protein delta) | 2 | | 873 | SFMBT2 | similar to hCG2038584 | 2 | | 874 | SFRS3 | Sin3A-associated protein, 130kDa | 2 | | 875 | SFRS5 | Sjogren syndrome antigen B (autoantigen La) | 2 | | 876 | SH2B1 | SLAIN motif family, member 1 | 2 | | 877 | SLAIN1 | SMAD family member 5 | 2 | | 878 | SLC25A18 | solute carrier family 25 (mitochondrial carrier), member 18 | 2 | | 879 | SLC26A6 | solute carrier family 26, member 6 | 2 | | 880 | SMAD5 | sorbin and SH3 domain containing 1 | 2 | | 881 | SORBS1 | splicing factor, arginine/serine-rich 3 | 2 | | 882 | SRC | splicing factor, arginine/serine-rich 5 | 2 | | 883 | SSB | stromal antigen 3-like 4 | 2 | | 884 | SSR4 | SUMO1 activating enzyme subunit 1 | 2 | | 885 | STAG3L4 | suppressor of Ty 16 homolog (S. cerevisiae) | 2 | | 886 | SUPT16H | taste receptor, type 2, member 45 | 2 | | 887 | TADA3 | TBC1 domain family, member 10B | 2 | | 888 | TAS2R45 | TBC1 domain family, member 16 | 2 | | 889 | TBC1D10B | testis expressed 10 | 2 | | 890 | TBC1D16 | THAP domain containing 6 | 2 | | 891 | TCEA3 | TIA1 cytotoxic granule-associated RNA binding protein | 2 | | 892 | TCF7 | TNFRSF1A-associated via death domain | 2 | | 893 | TEX10 | transcription elongation factor A (SII), 3 | 2 | | 894 | TFRC | transcription factor 7 (T-cell specific, HMG-box) | 2 | | 895 | TGFB2 | transcriptional adaptor 3 | 2 | | 896 | THAP6 | transferrin receptor (p90, CD71) | 2 | | 897 | TIA1 | transforming growth factor, beta 2 | 2 | | 898 | TIMM23 | translocase of inner mitochondrial membrane 23 homolog (yeast) | 2 | | 899 | TMCC1 | transmembrane and coiled-coil domain family 1 | 2 | | 900 | TMEM126B | transmembrane protein 126B | 2 | | 901 | TRADD | tripartite motif-containing 29 | 2 | | 902 | TRIM29 | TSC22 domain family, member 2 | 2 | | 903 | TSC22D2 | tyrosine 3-monooxygenase/tryptophan 5-monooxygenase activation protein, zeta polypeptide | 2 | | 904 | UBA2 | tyrosyl-tRNA synthetase | 2 | | 905 | UBAP1 | ubiquitin-like modifier activating enzyme 2 | 2 | | 906 | USP14 | ubiquitin associated protein 1 | 2 | | 907 | USP2 | ubiquitin specific peptidase 14 (tRNA-guanine transglycosylase) | 2 | | 908 | USP36 | ubiquitin specific peptidase 2 | 2 | | 909 | USP6 | ubiquitin specific peptidase 36 | 2 | | 910 | VPRBP | ubiquitin specific peptidase 6 (Tre-2 oncogene) | 2 | | 911 | WASF2 | v-erb-b2 erythroblastic leukemia viral oncogene homolog 3 (avian) | 2 | | 912 | WBSCR22 | v-src sarcoma (Schmidt-Ruppin A-2) viral oncogene homolog (avian) | 2 | | 913 | WDR5B | Vpr (HIV-1) binding protein | 2 | | 914 | WDR73 | WAS protein family, member 2 | 2 | | 915 | XPO1 | WD repeat domain 5B | 2 | | 916 | YARS | WD repeat domain 73 | 2 | | 917 | YWHAZ | Williams Beuren syndrome chromosome region 22 | 2 | | 918 | ZAN | zinc finger CCCH-type containing 18 | 2 | | 919 | ZC3H18 | zinc finger protein 148 | 2 | | 920 | ZDHHC5 | zinc finger protein 263 | 2 | | 921 | ZNF148 | zinc finger protein 268 | 2 | | 922 | ZNF263 | zinc finger protein 320 | 2 | | 923 | ZNF268 | zinc finger protein 440 | 2 | | 924 | ZNF320 | zinc finger protein 536 | 2 | | 925 | ZNF440 | zinc finger protein 7 | 2 | | 926 | ZNF536 | zinc finger protein 706 | 2 | | 927 | ZNF7 | zinc finger protein 764 | 2 | | 928 | ZNF706 | zinc finger, DHHC-type containing 5 | 2 | | 929 | ZNF764 | zinc finger, RAN-binding domain containing 2 | 2 | | 930 | ZRANB2 | zonadhesin | 2 | | 931 | ABCD1 | activity-dependent neuroprotector homeobox | 1 | | 932 | ABCD2 | acyl-CoA synthetase short-chain family member 1 | 1 | | 933 | ACOX1 | acyl-Coenzyme A oxidase 1, palmitoyl | 1 | | 934 | ACSS1 | adaptor-related protein complex 3, mu 1 subunit | 1 | | 935 | ADNP | ADP-ribosylation factor 1 | 1 | | 936 | AHCTF1 | Alport syndrome, mental retardation, midface hypoplasia and elliptocytosis chromosomal region gene 1 | 1 | | 937 | ALG8 | anaphase promoting complex subunit 7 | 1 | | 938 | AMMECR1 | anoctamin 10 | 1 | | 939 | ANAPC7 | anoctamin 2 | 1 | | 940 | ANO10 | asparagine-linked glycosylation 8, alpha-1,3-glucosyltransferase homolog (S. cerevisiae) | 1 | | 941 | ANO2 | AT hook containing transcription factor 1 | 1 | | 942 | AP3M1 | ATG2 autophagy related 2 homolog B (S. cerevisiae) | 1 | | 943 | ARF1 | ATP-binding cassette, sub-family D (ALD), member 1 | 1 | | 944 | ATG2B | ATP-binding cassette, sub-family D (ALD), member 2 | 1 | | 945 | ATP10D | ATPase, class V, type 10D | 1 | | 946 | BAT2L2 | bactericidal/permeability-increasing protein | 1 | | 947 | BBS1 | Bardet-Biedl syndrome 1 | 1 | | 948 | BBS9 | Bardet-Biedl syndrome 9 | 1 | | 949 | BICD2 | bicaudal D homolog 2 (Drosophila) | 1 | | 950 | BPI | bromodomain PHD finger transcription factor | 1 | | 951 | BPTF | BTB (POZ) domain containing 18 | 1 | | 952 | BTBD18 | calcineurin binding protein 1 | 1 | | 953 | C10orf4 | calcium binding protein 39-like | 1 | | 954 | C12orf29 | calcium homeostasis endoplasmic reticulum protein | 1 | | 955 | C14orf1 | calcyphosine | 1 | | 956 | C18orf18 | calpain 1, (mu/I) large subunit | 1 | | 957 | C18orf19 | cAMP responsive element binding protein 3-like 2 | 1 | | 958 | C18orf8 | carnitine acetyltransferase | 1 | | 959 | C1orf144 | catechol-O-methyltransferase | 1 | | 960 | C1orf162 | CD27 molecule | 1 | | 961 | C20orf11 | CD58 molecule | 1 | | 962 | C21orf89 | CD74 molecule, major histocompatibility complex, class II invariant chain | 1 | | 963 | C2orf28 | CDC42 small effector 2 | 1 | | 964 | C3orf52 | CDKN2A interacting protein | 1 | | 965 | C5orf33 | chaperonin containing TCP1, subunit 4 (delta) | 1 | | 966 | C7orf46 | chaperonin containing TCP1, subunit 8 (theta)-like 2 | 1 | | 967 | C8orf33 | chemokine (C-C motif) receptor 7 | 1 | | 968 | CAB39L | chemokine (C-X-C motif) receptor 3 | 1 | | 969 | CABIN1 | chemokine (C motif) ligand 1 | 1 | | 970 | CAPN1 | chromosome 1 open reading frame 144 | 1 | | 971 | CAPS | chromosome 1 open reading frame 162 | 1 | | 972 | CCDC115 | chromosome 10 open reading frame 4 | 1 | | 973 | CCDC116 | chromosome 12 open reading frame 29 | 1 | | 974 | CCDC59 | chromosome 14 open reading frame 1 | 1 | | 975 | CCR7 | chromosome 18 open reading frame 18 | 1 | | 976 | CCT4 | chromosome 18 open reading frame 19 | 1 | | 977 | CCT8L2 | chromosome 18 open reading frame 8 | 1 | | 978 | CD27 | chromosome 2 open reading frame 28 | 1 | | 979 | CD58 | chromosome 20 open reading frame 11 | 1 | | 980 | CD74 | chromosome 21 open reading frame 89 | 1 | | 981 | CDA | chromosome 3 open reading frame 52 | 1 | | 982 | CDADC1 | chromosome 5 open reading frame 33 | 1 | | 983 | CDC42SE2 | chromosome 7 open reading frame 46 | 1 | | 984 | CDKN2AIP | chromosome 8 open reading frame 33 | 1 | | 985 | CFB | chromosome X open reading frame 40B | 1 | | 986 | CHCHD3 | clathrin interactor 1 | 1 | | 987 | CHERP | coiled-coil-helix-coiled-coil-helix domain containing 3 | 1 | | 988 | CLINT1 | coiled-coil domain containing 115 | 1 | | 989 | CNIH4 | coiled-coil domain containing 116 | 1 | | 990 | CNTNAP4 | coiled-coil domain containing 59 | 1 | | 991 | COG1 | collagen, type XX, alpha 1 | 1 | | 992 | COG3 | complement factor B | 1 | | 993 | COL20A1 | component of oligomeric golgi complex 1 | 1 | | 994 | COMT | component of oligomeric golgi complex 3 | 1 | | 995 | CORO2A | contactin associated protein-like 4 | 1 | | 996 | CRAMP1L | cornichon homolog 4 (Drosophila) | 1 | | 997 | CRAT | coronin, actin binding protein, 2A | 1 | | 998 | CREB3L2 | Crm, cramped-like (Drosophila) | 1 | | 999 | CST11 | CTD (carboxy-terminal domain, RNA polymerase II, polypeptide A) small phosphatase like 2 | 1 | | 1000 | CTDSPL2 | cullin 2 | 1 | | 1001 | CUL2 | cystatin 11 | 1 | | 1002 | CXCR3 | cytidine and dCMP deaminase domain containing 1 | 1 | | 1003 | CXorf40B | cytidine deaminase | 1 | | 1004 | CYB561D1 | cytochrome b-561 domain containing 1 | 1 | | 1005 | DAG1 | damage-specific DNA binding protein 1, 127kDa | 1 | | 1006 | DCAF7 | DDB1 and CUL4 associated factor 7 | 1 | | 1007 | DDB1 | DEAD (Asp-Glu-Ala-Asp) box polypeptide 1 | 1 | | 1008 | DDX1 | DEAD (Asp-Glu-Ala-Asp) box polypeptide 21 | 1 | | 1009 | DDX21 | DEAD (Asp-Glu-Ala-Asp) box polypeptide 27 | 1 | | 1010 | DDX27 | DEAD (Asp-Glu-Ala-Asp) box polypeptide 46 | 1 | | 1011 | DDX46 | DEAH (Asp-Glu-Ala-His) box polypeptide 15 | 1 | | 1012 | DEPDC7 | DEAH (Asp-Glu-Ala-His) box polypeptide 33 | 1 | | 1013 | DHX15 | DEP domain containing 7 | 1 | | 1014 | DHX33 | dickkopf homolog 2 (Xenopus laevis) | 1 | | 1015 | DKK2 | DNA methyltransferase 1 associated protein 1 | 1 | | 1016 | DMAP1 | DnaJ (Hsp40) homolog, subfamily C, member 24 | 1 | | 1017 | DNAJC24 | dolichyl-phosphate mannosyltransferase polypeptide 1, catalytic subunit | 1 | | 1018 | DPM1 | dolichyl-phosphate mannosyltransferase polypeptide 3 | 1 | | 1019 | DPM3 | dystroglycan 1 (dystrophin-associated glycoprotein 1) | 1 | | 1020 | EDC4 | elastin microfibril interfacer 2 | 1 | | 1021 | EED | embryonic ectoderm development | 1 | | 1022 | EEF2 | endomucin | 1 | | 1023 | EID1 | enhancer of mRNA decapping 4 | 1 | | 1024 | EIF2AK3 | EP300 interacting inhibitor of differentiation 1 | 1 | | 1025 | EIF4G3 | eukaryotic translation elongation factor 2 | 1 | | 1026 | EMCN | eukaryotic translation initiation factor 2-alpha kinase 3 | 1 | | 1027 | EMILIN2 | eukaryotic translation initiation factor 4 gamma, 3 | 1 | | 1028 | EXOSC1 | exosome component 1 | 1 | | 1029 | EXOSC4 | exosome component 4 | 1 | | 1030 | FAM182B | F-box protein 25 | 1 | | 1031 | FAM186B | family with sequence similarity 182, member B | 1 | | 1032 | FBXO25 | family with sequence similarity 186, member B | 1 | | 1033 | FHIT | fibroblast growth factor receptor substrate 2 | 1 | | 1034 | FLJ25006 | fragile histidine triad gene | 1 | | 1035 | FLJ36840 | fragile X mental retardation 1 | 1 | | 1036 | FMR1 | fumarylacetoacetate hydrolase domain containing 2 pseudogene | 1 | | 1037 | FRS2 | G protein-coupled receptor 114 | 1 | | 1038 | GAR1 | G protein-coupled receptor 132 | 1 | | 1039 | GCLC | G protein-coupled receptor 56 | 1 | | 1040 | GFOD2 | GAR1 ribonucleoprotein homolog (yeast) | 1 | | 1041 | GGA2 | glucose-fructose oxidoreductase domain containing 2 | 1 | | 1042 | GLRX | glutamate-cysteine ligase, catalytic subunit | 1 | | 1043 | GPR114 | glutamate receptor, ionotropic, kainate 2 | 1 | | 1044 | GPR132 | glutaminyl-tRNA synthase (glutamine-hydrolyzing)-like 1 | 1 | | 1045 | GPR56 | glutaredoxin (thioltransferase) | 1 | | 1046 | GRIK2 | glutathione S-transferase omega 1 | 1 | | 1047 | GSTO1 | golgi-associated, gamma adaptin ear containing, ARF binding protein 2 | 1 | | 1048 | HAP1 | H6 family homeobox 1 | 1 | | 1049 | HAUS4 | haptoglobin | 1 | | 1050 | hCG\_2008140 | HAUS augmin-like complex, subunit 4 | 1 | | 1051 | HCG18 | HEAT repeat containing 2 | 1 | | 1052 | HEATR2 | helicase with zinc finger | 1 | | 1053 | HELZ | Hermansky-Pudlak syndrome 4 | 1 | | 1054 | HERPUD2 | HERPUD family member 2 | 1 | | 1055 | HMX1 | heterogeneous nuclear ribonucleoprotein D (AU-rich element RNA binding protein 1, 37kDa) | 1 | | 1056 | HNRNPD | heterogeneous nuclear ribonucleoprotein M | 1 | | 1057 | HNRNPM | HLA-B associated transcript 2-like 2 | 1 | | 1058 | HOXD10 | HLA complex group 18 | 1 | | 1059 | HP | homeobox D10 | 1 | | 1060 | HPS4 | huntingtin-associated protein 1 | 1 | | 1061 | IGF2AS | hypothetical LOC100233209 | 1 | | 1062 | IGSF9B | hypothetical LOC100303728 | 1 | | 1063 | IL17RD | hypothetical LOC145837 | 1 | | 1064 | IL18R1 | hypothetical LOC146880 | 1 | | 1065 | IL1R2 | hypothetical LOC150527 | 1 | | 1066 | INPP5A | hypothetical LOC283663 | 1 | | 1067 | INTS10 | hypothetical LOC399904 | 1 | | 1068 | IRAK1BP1 | hypothetical LOC400657 | 1 | | 1069 | KCTD10 | hypothetical LOC440944 | 1 | | 1070 | KHDRBS1 | hypothetical LOC645212 | 1 | | 1071 | KIAA0114 | hypothetical LOC645524 | 1 | | 1072 | KIAA0317 | hypothetical LOC654841 | 1 | | 1073 | KIAA0494 | hypothetical LOC729570 | 1 | | 1074 | KIAA0947 | hypothetical LOC729614 | 1 | | 1075 | KIAA1279 | hypothetical LOC80054 | 1 | | 1076 | KIAA1468 | hypothetical protein LOC149086 | 1 | | 1077 | KIAA1671 | hypothetical protein LOC284669 | 1 | | 1078 | KIF21A | hypothetical protein LOC285463 | 1 | | 1079 | KLF5 | hypothetical protein LOC285857 | 1 | | 1080 | KLHL22 | hypothetical protein LOC728769 | 1 | | 1081 | KLHL35 | immunoglobulin superfamily, member 9B | 1 | | 1082 | KLHL9 | inositol polyphosphate-5-phosphatase, 40kDa | 1 | | 1083 | KRT10 | insulin-like growth factor 2 antisense | 1 | | 1084 | LAPTM5 | integrator complex subunit 10 | 1 | | 1085 | LASS5 | interleukin-1 receptor-associated kinase 1 binding protein 1 | 1 | | 1086 | LCMT1 | interleukin 1 receptor, type II | 1 | | 1087 | LHX4 | interleukin 17 receptor D | 1 | | 1088 | LNPEP | interleukin 18 receptor 1 | 1 | | 1089 | LOC100132352 | kelch-like 22 (Drosophila) | 1 | | 1090 | LOC100233209 | kelch-like 35 (Drosophila) | 1 | | 1091 | LOC100303728 | kelch-like 9 (Drosophila) | 1 | | 1092 | LOC145837 | keratin 10 | 1 | | 1093 | LOC146880 | KH domain containing, RNA binding, signal transduction associated 1 | 1 | | 1094 | LOC149086 | KIAA0114 | 1 | | 1095 | LOC150527 | KIAA0317 | 1 | | 1096 | LOC283663 | KIAA0494 | 1 | | 1097 | LOC284669 | KIAA0947 | 1 | | 1098 | LOC285463 | KIAA1279 | 1 | | 1099 | LOC285857 | KIAA1468 | 1 | | 1100 | LOC399904 | KIAA1671 | 1 | | 1101 | LOC400657 | kinesin family member 21A | 1 | | 1102 | LOC440944 | Kruppel-like factor 5 (intestinal) | 1 | | 1103 | LOC645212 | LAG1 homolog, ceramide synthase 5 | 1 | | 1104 | LOC654841 | leucine carboxyl methyltransferase 1 | 1 | | 1105 | LOC728769 | leucine rich repeat containing 47 | 1 | | 1106 | LOC729234 | leucyl/cystinyl aminopeptidase | 1 | | 1107 | LOC729570 | LIM homeobox 4 | 1 | | 1108 | LOC80054 | Ly1 antibody reactive homolog (mouse) | 1 | | 1109 | LPAR5 | lysophosphatidic acid receptor 5 | 1 | | 1110 | LRRC47 | lysosomal protein transmembrane 5 | 1 | | 1111 | LYAR | major facilitator superfamily domain containing 3 | 1 | | 1112 | MANEAL | malic enzyme 2, NAD(+)-dependent, mitochondrial | 1 | | 1113 | MAPK13 | mannosidase, endo-alpha-like | 1 | | 1114 | MBLAC1 | mediator complex subunit 16 | 1 | | 1115 | ME2 | mediator complex subunit 19 | 1 | | 1116 | MEAF6 | mediator complex subunit 24 | 1 | | 1117 | MED16 | metallo-beta-lactamase domain containing 1 | 1 | | 1118 | MED19 | methylthioadenosine phosphorylase | 1 | | 1119 | MED24 | methyltransferase like 13 | 1 | | 1120 | METTL13 | methyltransferase like 3 | 1 | | 1121 | METTL3 | microsomal glutathione S-transferase 1 | 1 | | 1122 | MFSD3 | mitochondrial ribosomal protein L14 | 1 | | 1123 | MGST1 | mitochondrial ribosomal protein S12 | 1 | | 1124 | MOSC1 | mitochondrial ribosomal protein S2 | 1 | | 1125 | MRPL14 | mitogen-activated protein kinase 13 | 1 | | 1126 | MRPS12 | MOCO sulphurase C-terminal domain containing 1 | 1 | | 1127 | MRPS2 | myomesin family, member 3 | 1 | | 1128 | MTAP | myosin regulatory light chain interacting protein | 1 | | 1129 | MTMR12 | myotubularin related protein 12 | 1 | | 1130 | MYLIP | MYST/Esa1-associated factor 6 | 1 | | 1131 | MYOM3 | N-acetylglucosamine-1-phosphodiester alpha-N-acetylglucosaminidase | 1 | | 1132 | N4BP2 | NCK adaptor protein 1 | 1 | | 1133 | NAGPA | NEDD4 binding protein 2 | 1 | | 1134 | NAP1L4 | nicastrin | 1 | | 1135 | NCK1 | NIMA (never in mitosis gene a)-related kinase 1 | 1 | | 1136 | NCRNA00202 | nitric oxide synthase interacting protein | 1 | | 1137 | NCSTN | nitrilase family, member 2 | 1 | | 1138 | NEK1 | NmrA-like family domain containing 1 | 1 | | 1139 | NFKB2 | non-POU domain containing, octamer-binding | 1 | | 1140 | NFKBIL2 | non-protein coding RNA 202 | 1 | | 1141 | NIT2 | nuclear factor of kappa light polypeptide gene enhancer in B-cells 2 (p49/p100) | 1 | | 1142 | NMRAL1 | nuclear factor of kappa light polypeptide gene enhancer in B-cells inhibitor-like 2 | 1 | | 1143 | NONO | nuclear pore complex interacting protein | 1 | | 1144 | NOSIP | nucleophosmin/nucleoplasmin 2 | 1 | | 1145 | NPIP | nucleosome assembly protein 1-like 4 | 1 | | 1146 | NPM2 | OCIA domain containing 1 | 1 | | 1147 | OCIAD1 | olfactory receptor, family 51, subfamily E, member 2 | 1 | | 1148 | OR51E2 | ORM1-like 2 (S. cerevisiae) | 1 | | 1149 | ORMDL2 | ORM1-like 3 (S. cerevisiae) | 1 | | 1150 | ORMDL3 | partner of NOB1 homolog (S. cerevisiae) | 1 | | 1151 | P2RX1 | patatin-like phospholipase domain containing 7 | 1 | | 1152 | PCGF5 | pentatricopeptide repeat domain 2 | 1 | | 1153 | PCYT1A | peptidylprolyl isomerase (cyclophilin)-like 2 | 1 | | 1154 | PDCD11 | periphilin 1 | 1 | | 1155 | PDCD7 | peroxisomal trans-2-enoyl-CoA reductase | 1 | | 1156 | PDGFC | PHD finger protein 20 | 1 | | 1157 | PECR | PHD finger protein 5A | 1 | | 1158 | PGAP3 | phosphate cytidylyltransferase 1, choline, alpha | 1 | | 1159 | PHF20 | phosphatidic acid phosphatase type 2A | 1 | | 1160 | PHF5A | phosphatidylinositol 4-kinase type 2 beta | 1 | | 1161 | PHTF1 | phosphatidylinositol glycan anchor biosynthesis, class Y | 1 | | 1162 | PI4K2B | phosphoinositide-3-kinase, regulatory subunit 4 | 1 | | 1163 | PIAS2 | phospholipase B domain containing 1 | 1 | | 1164 | PIGY | phosphoribosyl pyrophosphate synthetase 1 | 1 | | 1165 | PIK3R4 | platelet derived growth factor C | 1 | | 1166 | PINK1 | pleckstrin homology domain containing, family A (phosphoinositide binding specific) member 8 | 1 | | 1167 | PKD2 | pleckstrin homology domain containing, family O member 1 | 1 | | 1168 | PLBD1 | PML-RARA regulated adaptor molecule 1 | 1 | | 1169 | PLEKHA8 | polycomb group ring finger 5 | 1 | | 1170 | PLEKHO1 | polycystic kidney disease 2 (autosomal dominant) | 1 | | 1171 | PNO1 | polymerase (DNA directed) iota | 1 | | 1172 | PNPLA7 | polymerase (RNA) II (DNA directed) polypeptide J4, pseudogene | 1 | | 1173 | POLI | post-GPI attachment to proteins 3 | 1 | | 1174 | POLR2J4 | potassium channel tetramerisation domain containing 10 | 1 | | 1175 | PPAP2A | programmed cell death 11 | 1 | | 1176 | PPHLN1 | programmed cell death 7 | 1 | | 1177 | PPIL2 | protease, serine, 2 (trypsin 2) | 1 | | 1178 | PPP1R12A | protein arginine methyltransferase 3 | 1 | | 1179 | PPP1R3D | protein inhibitor of activated STAT, 2 | 1 | | 1180 | PPP2R5B | protein kinase D3 | 1 | | 1181 | PRAM1 | protein phosphatase 1, regulatory (inhibitor) subunit 12A | 1 | | 1182 | PRKD3 | protein phosphatase 1, regulatory (inhibitor) subunit 3D | 1 | | 1183 | PRMT3 | protein phosphatase 2, regulatory subunit B', beta isoform | 1 | | 1184 | PRPF31 | prothymosin, alpha | 1 | | 1185 | PRPF38B | PRP31 pre-mRNA processing factor 31 homolog (S. cerevisiae) | 1 | | 1186 | PRPS1 | PRP38 pre-mRNA processing factor 38 (yeast) domain containing B | 1 | | 1187 | PRSS2 | PTEN induced putative kinase 1 | 1 | | 1188 | PTCD2 | purinergic receptor P2X, ligand-gated ion channel, 1 | 1 | | 1189 | PTMA | putative homeodomain transcription factor 1 | 1 | | 1190 | QRSL1 | queuine tRNA-ribosyltransferase 1 | 1 | | 1191 | QTRT1 | RAB32, member RAS oncogene family | 1 | | 1192 | RAB32 | radial spoke head 6 homolog A (Chlamydomonas) | 1 | | 1193 | RAP2A | ral guanine nucleotide dissociation stimulator-like 4 | 1 | | 1194 | RASSF1 | RAP2A, member of RAS oncogene family | 1 | | 1195 | RBM22 | Ras association (RalGDS/AF-6) domain family member 1 | 1 | | 1196 | RBM33 | ras homolog gene family, member H | 1 | | 1197 | RBMS1 | resistin | 1 | | 1198 | RBMX2 | ribonucleotide reductase M1 | 1 | | 1199 | RETN | ribosomal protein L29 | 1 | | 1200 | RGL4 | ribosomal protein L35 | 1 | | 1201 | RHOH | ribosomal protein L36a | 1 | | 1202 | RNF139 | ribosomal protein L6 | 1 | | 1203 | ROGDI | ribosomal protein S14 | 1 | | 1204 | ROPN1L | ribosomal protein S15 | 1 | | 1205 | RPL29 | ribosomal protein S17 pseudogene 5 | 1 | | 1206 | RPL35 | ribosomal protein S20 | 1 | | 1207 | RPL36A | ribosomal protein S27-like | 1 | | 1208 | RPL6 | ribosomal protein S6 | 1 | | 1209 | RPLP0 | ribosomal protein, large, P0 | 1 | | 1210 | RPS14 | ribosome binding protein 1 homolog 180kDa (dog) | 1 | | 1211 | RPS15 | ring finger protein 139 | 1 | | 1212 | RPS17P5 | RNA binding motif protein 22 | 1 | | 1213 | RPS20 | RNA binding motif protein 33 | 1 | | 1214 | RPS27L | RNA binding motif protein, X-linked 2 | 1 | | 1215 | RPS6 | RNA binding motif, single stranded interacting protein 1 | 1 | | 1216 | RRBP1 | rogdi homolog (Drosophila) | 1 | | 1217 | RRM1 | ropporin 1-like | 1 | | 1218 | RSBN1L | round spermatid basic protein 1-like | 1 | | 1219 | RSPH6A | runt-related transcription factor 1 | 1 | | 1220 | RUNX1 | selenoprotein X, 1 | 1 | | 1221 | SCLT1 | SET and MYND domain containing 1 | 1 | | 1222 | SEPX1 | SET domain containing (lysine methyltransferase) 8 | 1 | | 1223 | SETD8 | SFT2 domain containing 3 | 1 | | 1224 | SFRS16 | SH3-domain GRB2-like endophilin B1 | 1 | | 1225 | SFRS2 | shisa homolog 7 (Xenopus laevis) | 1 | | 1226 | SFT2D3 | sideroflexin 4 | 1 | | 1227 | SFXN4 | signal recognition particle 68kDa | 1 | | 1228 | SH3GLB1 | similar to hCG1989297 | 1 | | 1229 | SHISA7 | single immunoglobulin and toll-interleukin 1 receptor (TIR) domain | 1 | | 1230 | SIGIRR | sirtuin (silent mating type information regulation 2 homolog) 6 (S. cerevisiae) | 1 | | 1231 | SIRT6 | small nuclear RNA activating complex, polypeptide 3, 50kDa | 1 | | 1232 | SLC20A1 | small nuclear RNA activating complex, polypeptide 5, 19kDa | 1 | | 1233 | SLC25A13 | small nucleolar RNA, C/D box 123 | 1 | | 1234 | SLC25A14 | sodium channel and clathrin linker 1 | 1 | | 1235 | SLC25A15 | solute carrier family 20 (phosphate transporter), member 1 | 1 | | 1236 | SLC6A17 | solute carrier family 25 (mitochondrial carrier, brain), member 14 | 1 | | 1237 | SLC7A1 | solute carrier family 25 (mitochondrial carrier; ornithine transporter) member 15 | 1 | | 1238 | SMC3 | solute carrier family 25, member 13 (citrin) | 1 | | 1239 | SMYD1 | solute carrier family 6, member 17 | 1 | | 1240 | SNAPC3 | solute carrier family 7 (cationic amino acid transporter, y+ system), member 1 | 1 | | 1241 | SNAPC5 | sorting nexin 5 | 1 | | 1242 | SNORD123 | speckle-type POZ protein | 1 | | 1243 | SNX5 | spindlin 1 | 1 | | 1244 | SOCS3 | splicing factor, arginine/serine-rich 16 | 1 | | 1245 | SPIN1 | splicing factor, arginine/serine-rich 2 | 1 | | 1246 | SPOP | structural maintenance of chromosomes 3 | 1 | | 1247 | SQRDL | sulfide quinone reductase-like (yeast) | 1 | | 1248 | SRP68 | suppressor of cytokine signaling 3 | 1 | | 1249 | SSX3 | suppressor of defective silencing 3 homolog (S. cerevisiae) | 1 | | 1250 | SUDS3 | synovial sarcoma, X breakpoint 3 | 1 | | 1251 | TCEAL1 | T cell receptor associated transmembrane adaptor 1 | 1 | | 1252 | TEK | TEK tyrosine kinase, endothelial | 1 | | 1253 | TESC | tescalcin | 1 | | 1254 | TGM4 | tetraspanin 14 | 1 | | 1255 | TGM7 | tetraspanin 31 | 1 | | 1256 | TIMM22 | tetratricopeptide repeat domain 30B | 1 | | 1257 | TM4SF5 | tetratricopeptide repeat domain 9 | 1 | | 1258 | TMC8 | topoisomerase (DNA) II beta 180kDa | 1 | | 1259 | TMED10 | transcription elongation factor A (SII)-like 1 | 1 | | 1260 | TMEM108 | transcription termination factor, RNA polymerase I | 1 | | 1261 | TMEM109 | transglutaminase 4 (prostate) | 1 | | 1262 | TMEM69 | transglutaminase 7 | 1 | | 1263 | TMEM85 | translocase of inner mitochondrial membrane 22 homolog (yeast) | 1 | | 1264 | TNRC6A | transmembrane 4 L six family member 5 | 1 | | 1265 | TOP2B | transmembrane channel-like 8 | 1 | | 1266 | TPP1 | transmembrane emp24-like trafficking protein 10 (yeast) | 1 | | 1267 | TRAT1 | transmembrane protein 108 | 1 | | 1268 | TRNT1 | transmembrane protein 109 | 1 | | 1269 | TSPAN14 | transmembrane protein 69 | 1 | | 1270 | TSPAN31 | transmembrane protein 85 | 1 | | 1271 | TTC30B | trinucleotide repeat containing 6A | 1 | | 1272 | TTC9 | tripeptidyl peptidase I | 1 | | 1273 | TTF1 | tRNA nucleotidyl transferase, CCA-adding, 1 | 1 | | 1274 | TUBGCP5 | tubulin, gamma complex associated protein 5 | 1 | | 1275 | TUBGCP6 | tubulin, gamma complex associated protein 6 | 1 | | 1276 | UAP1 | ubinuclein 2 | 1 | | 1277 | UBN2 | ubiquitin specific peptidase 34 | 1 | | 1278 | ULK4 | UDP-N-acteylglucosamine pyrophosphorylase 1 | 1 | | 1279 | UMPS | unc-51-like kinase 4 (C. elegans) | 1 | | 1280 | UPP1 | uncharacterized serine/threonine-protein kinase SgK494 | 1 | | 1281 | USP34 | uridine monophosphate synthetase | 1 | | 1282 | UTP18 | uridine phosphorylase 1 | 1 | | 1283 | UTP23 | UTP18, small subunit (SSU) processome component, homolog (yeast) | 1 | | 1284 | WAC | UTP23, small subunit (SSU) processome component, homolog (yeast) | 1 | | 1285 | WDR18 | WD repeat domain 18 | 1 | | 1286 | WDR38 | WD repeat domain 38 | 1 | | 1287 | WDR4 | WD repeat domain 4 | 1 | | 1288 | WDR81 | WD repeat domain 81 | 1 | | 1289 | WISP2 | WNT1 inducible signaling pathway protein 2 | 1 | | 1290 | XCL1 | WW domain containing adaptor with coiled-coil | 1 | | 1291 | ZACN | zinc activated ligand-gated ion channel | 1 | | 1292 | ZBTB7B | zinc finger and BTB domain containing 7B | 1 | | 1293 | ZC3H12A | zinc finger CCCH-type containing 12A | 1 | | 1294 | ZC3H6 | zinc finger CCCH-type containing 6 | 1 | | 1295 | ZFP106 | zinc finger family member 767 | 1 | | 1296 | ZHX2 | zinc finger protein 106 homolog (mouse) | 1 | | 1297 | ZMAT5 | zinc finger protein 137 | 1 | | 1298 | ZNF137 | zinc finger protein 25 | 1 | | 1299 | ZNF25 | zinc finger protein 33B | 1 | | 1300 | ZNF33B | zinc finger protein 37A | 1 | | 1301 | ZNF37A | zinc finger protein 434 | 1 | | 1302 | ZNF434 | zinc finger protein 438 | 1 | | 1303 | ZNF438 | zinc finger protein 497 | 1 | | 1304 | ZNF497 | zinc finger protein 555 | 1 | | 1305 | ZNF555 | zinc finger protein 564 | 1 | | 1306 | ZNF564 | zinc finger protein 565 | 1 | | 1307 | ZNF565 | zinc finger protein 569 | 1 | | 1308 | ZNF569 | zinc finger protein 652 | 1 | | 1309 | ZNF652 | zinc finger protein 675 | 1 | | 1310 | ZNF675 | zinc finger protein 689 | 1 | | 1311 | ZNF689 | zinc finger protein 70 | 1 | | 1312 | ZNF70 | zinc finger protein 700 | 1 | | 1313 | ZNF700 | zinc finger protein 738 | 1 | | 1314 | ZNF738 | zinc finger protein 80 | 1 | | 1315 | ZNF767 | zinc finger protein 828 | 1 | | 1316 | ZNF80 | zinc finger protein 846 | 1 | | 1317 | ZNF828 | zinc finger, matrin type 5 | 1 | | 1318 | ZNF846 | zinc fingers and homeoboxes 2 | 1 | |

---

Total number of miRNA-mRNA 3693 interactions for given cut-off. Press for ALL:

| |  | miR | EntrezID | Gene | Name | Score | | --- | --- | --- | --- | --- | --- | | 347 | hsa-miR-1233 | 7267 | TTC3 | tetratricopeptide repeat domain 3 | 7 | | 1110 | hsa-miR-569 | 7267 | TTC3 | tetratricopeptide repeat domain 3 | 6 | | 1111 | hsa-miR-766 | 7267 | TTC3 | tetratricopeptide repeat domain 3 | 6 | | 114 | hsa-miR-604 | 6777 | STAT5B | signal transducer and activator of transcription 5B | 5 | | 115 | hsa-miR-938 | 6777 | STAT5B | signal transducer and activator of transcription 5B | 5 | | 348 | hsa-miR-571 | 7267 | TTC3 | tetratricopeptide repeat domain 3 | 5 | | 1112 | hsa-miR-874 | 7267 | TTC3 | tetratricopeptide repeat domain 3 | 5 | | 1153 | hsa-miR-1233 | 6146 | RPL22 | ribosomal protein L22 | 5 | | 1155 | hsa-miR-874 | 6146 | RPL22 | ribosomal protein L22 | 5 | | 2240 | hsa-mir-3130-1 | 10841 | FTCD | formiminotransferase cyclodeaminase | 5 | | 2241 | hsa-mir-3130-2 | 10841 | FTCD | formiminotransferase cyclodeaminase | 5 | | 2242 | hsa-mir-3130-3 | 10841 | FTCD | formiminotransferase cyclodeaminase | 5 | | 2243 | hsa-miR-10a | 10841 | FTCD | formiminotransferase cyclodeaminase | 5 | | 2244 | hsa-miR-770-5p | 10841 | FTCD | formiminotransferase cyclodeaminase | 5 | | 2245 | hsa-miR-95 | 10841 | FTCD | formiminotransferase cyclodeaminase | 5 | | 168 | hsa-miR-874 | 10128 | LRPPRC | leucine-rich PPR-motif containing | 4 | | 229 | hsa-miR-766 | 2534 | FYN | FYN oncogene related to SRC, FGR, YES | 4 | | 367 | hsa-miR-874 | 3178 | HNRNPA1 | heterogeneous nuclear ribonucleoprotein A1 | 4 | | 444 | hsa-miR-593\* | 6175 | RPLP0 | ribosomal protein, large, P0 | 4 | | 626 | hsa-miR-1233 | 8445 | DYRK2 | dual-specificity tyrosine-(Y)-phosphorylation regulated kinase 2 | 4 | | 635 | hsa-miR-1233 | 58487 | CREBZF | CREB/ATF bZIP transcription factor | 4 | | 1176 | hsa-miR-638 | 10938 | EHD1 | EH-domain containing 1 | 4 | | 1515 | hsa-miR-1233 | 171023 | ASXL1 | additional sex combs like 1 (Drosophila) | 4 | | 1948 | hsa-miR-1233 | 55037 | PTCD3 | Pentatricopeptide repeat domain 3 | 4 | | 1952 | hsa-miR-874 | 55037 | PTCD3 | Pentatricopeptide repeat domain 3 | 4 | | 2199 | hsa-miR-1233 | 29121 | CLEC2D | C-type lectin domain family 2, member D | 4 | | 2641 | hsa-miR-1233 | 817 | CAMK2D | calcium/calmodulin-dependent protein kinase II delta | 4 | | 2643 | hsa-miR-874 | 817 | CAMK2D | calcium/calmodulin-dependent protein kinase II delta | 4 | | 66 | hsa-miR-766 | 51275 | C12orf47 | chromosome 12 open reading frame 47 | 3 | | 131 | hsa-miR-635 | 353514 | LILRA5 | leukocyte immunoglobulin-like receptor, subfamily A (with TM domain), member 5 | 3 | | 165 | hsa-miR-1233 | 10128 | LRPPRC | leucine-rich PPR-motif containing | 3 | | 166 | hsa-miR-571 | 10128 | LRPPRC | leucine-rich PPR-motif containing | 3 | | 167 | hsa-miR-766 | 10128 | LRPPRC | leucine-rich PPR-motif containing | 3 | | 179 | hsa-miR-1233 | 64417 | C5orf28 | chromosome 5 open reading frame 28 | 3 | | 183 | hsa-miR-874 | 64417 | C5orf28 | chromosome 5 open reading frame 28 | 3 | | 222 | hsa-miR-766 | 10801 | SEPT9 | septin 9 | 3 | | 228 | hsa-miR-1233 | 2534 | FYN | FYN oncogene related to SRC, FGR, YES | 3 | | 336 | hsa-miR-874 | 81550 | TDRD3 | tudor domain containing 3 | 3 | | 380 | hsa-miR-1233 | 6136 | RPL12 | ribosomal protein L12 | 3 | | 381 | hsa-miR-766 | 6136 | RPL12 | ribosomal protein L12 | 3 | | 421 | hsa-miR-623 | 6159 | RPL29 | ribosomal protein L29 | 3 | | 427 | hsa-miR-766 | 6191 | RPS4X | ribosomal protein S4, X-linked | 3 | | 470 | hsa-miR-1233 | 2971 | GTF3A | general transcription factor IIIA | 3 | | 478 | hsa-miR-638 | 9261 | MAPKAPK2 | mitogen-activated protein kinase-activated protein kinase 2 | 3 | | 521 | hsa-miR-1233 | 9987 | HNRPDL | heterogeneous nuclear ribonucleoprotein D-like | 3 | | 629 | hsa-miR-766 | 8445 | DYRK2 | dual-specificity tyrosine-(Y)-phosphorylation regulated kinase 2 | 3 | | 630 | hsa-miR-874 | 8445 | DYRK2 | dual-specificity tyrosine-(Y)-phosphorylation regulated kinase 2 | 3 | | 698 | hsa-miR-765 | 9968 | MED12 | mediator complex subunit 12 | 3 | | 853 | hsa-miR-635 | 6777 | STAT5B | signal transducer and activator of transcription 5B | 3 | | 1081 | hsa-miR-635 | 2204 | FCAR | Fc fragment of IgA, receptor for | 3 | | 1117 | hsa-miR-765 | 334 | APLP2 | amyloid beta (A4) precursor-like protein 2 | 3 | | 1154 | hsa-miR-617 | 6146 | RPL22 | ribosomal protein L22 | 3 | | 1516 | hsa-miR-569 | 171023 | ASXL1 | additional sex combs like 1 (Drosophila) | 3 | | 1517 | hsa-miR-766 | 171023 | ASXL1 | additional sex combs like 1 (Drosophila) | 3 | | 1588 | hsa-miR-1233 | 26051 | PPP1R16B | protein phosphatase 1, regulatory (inhibitor) subunit 16B | 3 | | 1590 | hsa-miR-766 | 26051 | PPP1R16B | protein phosphatase 1, regulatory (inhibitor) subunit 16B | 3 | | 1591 | hsa-miR-874 | 26051 | PPP1R16B | protein phosphatase 1, regulatory (inhibitor) subunit 16B | 3 | | 1765 | hsa-miR-1233 | 3178 | HNRNPA1 | heterogeneous nuclear ribonucleoprotein A1 | 3 | | 1768 | hsa-miR-766 | 3178 | HNRNPA1 | heterogeneous nuclear ribonucleoprotein A1 | 3 | | 1792 | hsa-miR-571 | 54014 | BRWD1 | bromodomain and WD repeat domain containing 1 | 3 | | 1951 | hsa-miR-766 | 55037 | PTCD3 | Pentatricopeptide repeat domain 3 | 3 | | 1954 | hsa-miR-571 | 394 | ARHGAP5 | Rho GTPase activating protein 5 | 3 | | 1984 | hsa-miR-571 | 4801 | NFYB | nuclear transcription factor Y, beta | 3 | | 2033 | hsa-miR-1233 | 55900 | ZNF302 | zinc finger protein 302 | 3 | | 2034 | hsa-miR-571 | 55900 | ZNF302 | zinc finger protein 302 | 3 | | 2064 | hsa-miR-635 | 54512 | EXOSC4 | exosome component 4 | 3 | | 2196 | hsa-miR-1233 | 56172 | ANKH | ankylosis, progressive homolog (mouse) | 3 | | 2197 | hsa-miR-571 | 56172 | ANKH | ankylosis, progressive homolog (mouse) | 3 | | 2198 | hsa-miR-874 | 56172 | ANKH | ankylosis, progressive homolog (mouse) | 3 | | 2202 | hsa-miR-766 | 29121 | CLEC2D | C-type lectin domain family 2, member D | 3 | | 2321 | hsa-miR-1233 | 60468 | BACH2 | BTB and CNC homology 1, basic leucine zipper transcription factor 2 | 3 | | 2642 | hsa-miR-571 | 817 | CAMK2D | calcium/calmodulin-dependent protein kinase II delta | 3 | | 2681 | hsa-miR-1233 | 5928 | RBBP4 | retinoblastoma binding protein 4 | 3 | | 2682 | hsa-miR-571 | 5928 | RBBP4 | retinoblastoma binding protein 4 | 3 | | 3377 | hsa-miR-1233 | 163081 | ZNF567 | zinc finger protein 567 | 3 | | 1 | hsa-miR-623 | 7318 | UBA7 | ubiquitin-like modifier activating enzyme 7 | 2 | | 3 | hsa-mir-3130-1 | 10406 | WFDC2 | WAP four-disulfide core domain 2 | 2 | | 4 | hsa-mir-3130-2 | 10406 | WFDC2 | WAP four-disulfide core domain 2 | 2 | | 5 | hsa-mir-3130-3 | 10406 | WFDC2 | WAP four-disulfide core domain 2 | 2 | | 9 | hsa-miR-1233 | 172 | AFG3L1 | AFG3 ATPase family gene 3-like 1 (S. cerevisiae) | 2 | | 14 | hsa-miR-1233 | 170575 | GIMAP1 | GTPase, IMAP family member 1 | 2 | | 40 | hsa-miR-1233 | 150864 | FAM117B | family with sequence similarity 117, member B | 2 | | 41 | hsa-miR-571 | 150864 | FAM117B | family with sequence similarity 117, member B | 2 | | 42 | hsa-miR-874 | 150864 | FAM117B | family with sequence similarity 117, member B | 2 | | 57 | hsa-miR-571 | 148266 | ZNF569 | zinc finger protein 569 | 2 | | 64 | hsa-miR-571 | 112487 | C14orf126 | chromosome 14 open reading frame 126 | 2 | | 67 | hsa-miR-1233 | 9093 | DNAJA3 | DnaJ (Hsp40) homolog, subfamily A, member 3 | 2 | | 68 | hsa-miR-569 | 9093 | DNAJA3 | DnaJ (Hsp40) homolog, subfamily A, member 3 | 2 | | 70 | hsa-miR-766 | 9093 | DNAJA3 | DnaJ (Hsp40) homolog, subfamily A, member 3 | 2 | | 71 | hsa-miR-874 | 9093 | DNAJA3 | DnaJ (Hsp40) homolog, subfamily A, member 3 | 2 | | 72 | hsa-miR-1233 | 79707 | NOL9 | nucleolar protein 9 | 2 | | 73 | hsa-miR-569 | 79707 | NOL9 | nucleolar protein 9 | 2 | | 74 | hsa-miR-766 | 79707 | NOL9 | nucleolar protein 9 | 2 | | 84 | hsa-miR-766 | 7705 | ZNF146 | zinc finger protein 146 | 2 | | 85 | hsa-mir-10a | 55180 | LINS1 | lines homolog 1 (Drosophila) | 2 | | 98 | hsa-miR-10a | 54933 | RHBDL2 | rhomboid, veinlet-like 2 (Drosophila) | 2 | | 101 | hsa-miR-635 | 2207 | FCER1G | Fc fragment of IgE, high affinity I, receptor for; gamma polypeptide | 2 | | 102 | hsa-miR-604 | 91662 | NLRP12 | NLR family, pyrin domain containing 12 | 2 | | 103 | hsa-miR-635 | 91662 | NLRP12 | NLR family, pyrin domain containing 12 | 2 | | 104 | hsa-miR-938 | 91662 | NLRP12 | NLR family, pyrin domain containing 12 | 2 | | 116 | hsa-miR-1233 | 10225 | CD96 | CD96 molecule | 2 | | 117 | hsa-miR-569 | 10225 | CD96 | CD96 molecule | 2 | | 118 | hsa-miR-571 | 10225 | CD96 | CD96 molecule | 2 | | 119 | hsa-miR-874 | 10225 | CD96 | CD96 molecule | 2 | | 128 | hsa-miR-766 | 7535 | ZAP70 | zeta-chain (TCR) associated protein kinase 70kDa | 2 | | 130 | hsa-miR-604 | 353514 | LILRA5 | leukocyte immunoglobulin-like receptor, subfamily A (with TM domain), member 5 | 2 | | 132 | hsa-miR-938 | 353514 | LILRA5 | leukocyte immunoglobulin-like receptor, subfamily A (with TM domain), member 5 | 2 | | 142 | hsa-miR-1233 | 5431 | POLR2B | polymerase (RNA) II (DNA directed) polypeptide B, 140kDa | 2 | | 156 | hsa-miR-766 | 58517 | RBM25 | RNA binding motif protein 25 | 2 | | 181 | hsa-miR-571 | 64417 | C5orf28 | chromosome 5 open reading frame 28 | 2 | | 187 | hsa-miR-1233 | 130916 | MTERFD2 | MTERF domain containing 2 | 2 | | 189 | hsa-miR-571 | 130916 | MTERFD2 | MTERF domain containing 2 | 2 | | 191 | hsa-mir-10a | 5565 | PRKAB2 | protein kinase, AMP-activated, beta 2 non-catalytic subunit | 2 | | 221 | hsa-miR-1233 | 10801 | SEPT9 | septin 9 | 2 | | 223 | hsa-miR-1233 | 53335 | BCL11A | B-cell CLL/lymphoma 11A (zinc finger protein) | 2 | | 230 | hsa-miR-874 | 2534 | FYN | FYN oncogene related to SRC, FGR, YES | 2 | | 254 | hsa-miR-623 | 4676 | NAP1L4 | nucleosome assembly protein 1-like 4 | 2 | | 260 | hsa-miR-635 | 8569 | MKNK1 | MAP kinase interacting serine/threonine kinase 1 | 2 | | 270 | hsa-miR-770-5p | 348180 | CTU2 | cytosolic thiouridylase subunit 2 homolog (S. pombe) | 2 | | 271 | hsa-miR-95 | 348180 | CTU2 | cytosolic thiouridylase subunit 2 homolog (S. pombe) | 2 | | 279 | hsa-miR-10a | 79917 | MAGIX | MAGI family member, X-linked | 2 | | 280 | hsa-miR-770-5p | 79917 | MAGIX | MAGI family member, X-linked | 2 | | 281 | hsa-miR-95 | 79917 | MAGIX | MAGI family member, X-linked | 2 | | 316 | hsa-miR-571 | 1122 | CHML | choroideremia-like (Rab escort protein 2) | 2 | | 317 | hsa-miR-591 | 1122 | CHML | choroideremia-like (Rab escort protein 2) | 2 | | 332 | hsa-mir-10a | 84851 | TRIM52 | tripartite motif-containing 52 | 2 | | 335 | hsa-miR-571 | 81550 | TDRD3 | tudor domain containing 3 | 2 | | 349 | hsa-miR-1233 | 23195 | MDN1 | MDN1, midasin homolog (yeast) | 2 | | 350 | hsa-miR-571 | 23195 | MDN1 | MDN1, midasin homolog (yeast) | 2 | | 351 | hsa-miR-874 | 23195 | MDN1 | MDN1, midasin homolog (yeast) | 2 | | 360 | hsa-miR-128 | 374955 | SPATA21 | spermatogenesis associated 21 | 2 | | 361 | hsa-miR-770-5p | 374955 | SPATA21 | spermatogenesis associated 21 | 2 | | 362 | hsa-miR-95 | 374955 | SPATA21 | spermatogenesis associated 21 | 2 | | 382 | hsa-miR-874 | 6136 | RPL12 | ribosomal protein L12 | 2 | | 390 | hsa-miR-635 | 6282 | S100A11 | S100 calcium binding protein A11 | 2 | | 391 | hsa-miR-1233 | 9804 | TOMM20 | translocase of outer mitochondrial membrane 20 homolog (yeast) | 2 | | 393 | hsa-miR-571 | 9804 | TOMM20 | translocase of outer mitochondrial membrane 20 homolog (yeast) | 2 | | 394 | hsa-miR-766 | 9804 | TOMM20 | translocase of outer mitochondrial membrane 20 homolog (yeast) | 2 | | 395 | hsa-miR-874 | 9804 | TOMM20 | translocase of outer mitochondrial membrane 20 homolog (yeast) | 2 | | 399 | hsa-miR-766 | 57062 | DDX24 | DEAD (Asp-Glu-Ala-Asp) box polypeptide 24 | 2 | | 404 | hsa-miR-638 | 10956 | OS9 | osteosarcoma amplified 9, endoplasmic reticulum lectin | 2 | | 405 | hsa-miR-765 | 10956 | OS9 | osteosarcoma amplified 9, endoplasmic reticulum lectin | 2 | | 416 | hsa-miR-604 | 7791 | ZYX | zyxin | 2 | | 417 | hsa-miR-638 | 7791 | ZYX | zyxin | 2 | | 418 | hsa-miR-765 | 7791 | ZYX | zyxin | 2 | | 419 | hsa-miR-938 | 7791 | ZYX | zyxin | 2 | | 432 | hsa-miR-638 | 527 | ATP6V0C | ATPase, H+ transporting, lysosomal 16kDa, V0 subunit c | 2 | | 433 | hsa-miR-765 | 527 | ATP6V0C | ATPase, H+ transporting, lysosomal 16kDa, V0 subunit c | 2 | | 440 | hsa-miR-1233 | 3945 | LDHB | lactate dehydrogenase B | 2 | | 442 | hsa-miR-766 | 3945 | LDHB | lactate dehydrogenase B | 2 | | 443 | hsa-miR-874 | 3945 | LDHB | lactate dehydrogenase B | 2 | | 447 | hsa-miR-604 | 5829 | PXN | paxillin | 2 | | 448 | hsa-miR-638 | 5829 | PXN | paxillin | 2 | | 450 | hsa-miR-938 | 5829 | PXN | paxillin | 2 | | 455 | hsa-miR-135a | 396 | ARHGDIA | Rho GDP dissociation inhibitor (GDI) alpha | 2 | | 456 | hsa-miR-638 | 396 | ARHGDIA | Rho GDP dissociation inhibitor (GDI) alpha | 2 | | 458 | hsa-miR-623 | 10726 | NUDC | nuclear distribution gene C homolog (A. nidulans) | 2 | | 463 | hsa-miR-1236 | 5510 | PPP1R7 | protein phosphatase 1, regulatory (inhibitor) subunit 7 | 2 | | 464 | hsa-miR-593\* | 6122 | RPL3 | ribosomal protein L3 | 2 | | 465 | hsa-miR-766 | 6122 | RPL3 | ribosomal protein L3 | 2 | | 469 | hsa-miR-635 | 9341 | VAMP3 | vesicle-associated membrane protein 3 (cellubrevin) | 2 | | 471 | hsa-miR-874 | 2971 | GTF3A | general transcription factor IIIA | 2 | | 491 | hsa-mir-10a | 9097 | USP14 | ubiquitin specific peptidase 14 (tRNA-guanine transglycosylase) | 2 | | 507 | hsa-miR-1233 | 6059 | ABCE1 | ATP-binding cassette, sub-family E (OABP), member 1 | 2 | | 512 | hsa-miR-766 | 6574 | SLC20A1 | solute carrier family 20 (phosphate transporter), member 1 | 2 | | 517 | hsa-miR-1233 | 23077 | MYCBP2 | MYC binding protein 2 | 2 | | 518 | hsa-miR-766 | 23077 | MYCBP2 | MYC binding protein 2 | 2 | | 522 | hsa-miR-874 | 9987 | HNRPDL | heterogeneous nuclear ribonucleoprotein D-like | 2 | | 532 | hsa-miR-1233 | 158 | ADSL | adenylosuccinate lyase | 2 | | 562 | hsa-miR-1233 | 10915 | TCERG1 | transcription elongation regulator 1 | 2 | | 565 | hsa-miR-766 | 10915 | TCERG1 | transcription elongation regulator 1 | 2 | | 571 | hsa-miR-617 | 11098 | PRSS23 | protease, serine, 23 | 2 | | 578 | hsa-miR-1233 | 28951 | TRIB2 | tribbles homolog 2 (Drosophila) | 2 | | 580 | hsa-miR-766 | 28951 | TRIB2 | tribbles homolog 2 (Drosophila) | 2 | | 601 | hsa-miR-627 | 23523 | CABIN1 | calcineurin binding protein 1 | 2 | | 613 | hsa-miR-638 | 5566 | PRKACA | protein kinase, cAMP-dependent, catalytic, alpha | 2 | | 627 | hsa-miR-569 | 8445 | DYRK2 | dual-specificity tyrosine-(Y)-phosphorylation regulated kinase 2 | 2 | | 628 | hsa-miR-571 | 8445 | DYRK2 | dual-specificity tyrosine-(Y)-phosphorylation regulated kinase 2 | 2 | | 637 | hsa-miR-766 | 58487 | CREBZF | CREB/ATF bZIP transcription factor | 2 | | 639 | hsa-miR-571 | 58487 | CREBZF | CREB/ATF bZIP transcription factor | 2 | | 641 | hsa-mir-10a | 9529 | BAG5 | BCL2-associated athanogene 5 | 2 | | 649 | hsa-miR-604 | 2185 | PTK2B | PTK2B protein tyrosine kinase 2 beta | 2 | | 650 | hsa-miR-627 | 2185 | PTK2B | PTK2B protein tyrosine kinase 2 beta | 2 | | 651 | hsa-miR-638 | 2185 | PTK2B | PTK2B protein tyrosine kinase 2 beta | 2 | | 653 | hsa-miR-938 | 2185 | PTK2B | PTK2B protein tyrosine kinase 2 beta | 2 | | 695 | hsa-miR-593\* | 8662 | EIF3B | eukaryotic translation initiation factor 3, subunit B | 2 | | 696 | hsa-miR-623 | 8662 | EIF3B | eukaryotic translation initiation factor 3, subunit B | 2 | | 703 | hsa-miR-1233 | 8481 | OFD1 | oral-facial-digital syndrome 1 | 2 | | 704 | hsa-miR-874 | 8481 | OFD1 | oral-facial-digital syndrome 1 | 2 | | 719 | hsa-miR-576-5p | 2332 | FMR1 | fragile X mental retardation 1 | 2 | | 726 | hsa-miR-635 | 5937 | RBMS1 | RNA binding motif, single stranded interacting protein 1 | 2 | | 731 | hsa-miR-1233 | 4548 | MTR | 5-methyltetrahydrofolate-homocysteine methyltransferase | 2 | | 734 | hsa-miR-591 | 4548 | MTR | 5-methyltetrahydrofolate-homocysteine methyltransferase | 2 | | 736 | hsa-miR-874 | 4548 | MTR | 5-methyltetrahydrofolate-homocysteine methyltransferase | 2 | | 741 | hsa-miR-593\* | 513 | ATP5D | ATP synthase, H+ transporting, mitochondrial F1 complex, delta subunit | 2 | | 747 | hsa-miR-766 | 22880 | MORC2 | MORC family CW-type zinc finger 2 | 2 | | 749 | hsa-miR-135a | 6901 | TAZ | tafazzin | 2 | | 751 | hsa-miR-638 | 6901 | TAZ | tafazzin | 2 | | 763 | hsa-miR-638 | 22904 | SBNO2 | strawberry notch homolog 2 (Drosophila) | 2 | | 774 | hsa-miR-765 | 63940 | GPSM3 | G-protein signaling modulator 3 (AGS3-like, C. elegans) | 2 | | 785 | hsa-miR-576-5p | 1385 | CREB1 | cAMP responsive element binding protein 1 | 2 | | 793 | hsa-miR-454\* | 2976 | GTF3C2 | general transcription factor IIIC, polypeptide 2, beta 110kDa | 2 | | 794 | hsa-miR-591 | 2976 | GTF3C2 | general transcription factor IIIC, polypeptide 2, beta 110kDa | 2 | | 811 | hsa-miR-766 | 100 | ADA | adenosine deaminase | 2 | | 817 | hsa-miR-576-5p | 905 | CCNT2 | cyclin T2 | 2 | | 836 | hsa-miR-571 | 7181 | NR2C1 | nuclear receptor subfamily 2, group C, member 1 | 2 | | 863 | hsa-miR-604 | 4689 | NCF4 | neutrophil cytosolic factor 4, 40kDa | 2 | | 864 | hsa-miR-635 | 4689 | NCF4 | neutrophil cytosolic factor 4, 40kDa | 2 | | 865 | hsa-miR-938 | 4689 | NCF4 | neutrophil cytosolic factor 4, 40kDa | 2 | | 871 | hsa-miR-604 | 9744 | ACAP1 | ArfGAP with coiled-coil, ankyrin repeat and PH domains 1 | 2 | | 873 | hsa-miR-638 | 9744 | ACAP1 | ArfGAP with coiled-coil, ankyrin repeat and PH domains 1 | 2 | | 874 | hsa-miR-765 | 9744 | ACAP1 | ArfGAP with coiled-coil, ankyrin repeat and PH domains 1 | 2 | | 875 | hsa-miR-938 | 9744 | ACAP1 | ArfGAP with coiled-coil, ankyrin repeat and PH domains 1 | 2 | | 880 | hsa-miR-874 | 80184 | CEP290 | centrosomal protein 290kDa | 2 | | 916 | hsa-mir-199a-2 | 57147 | SCYL3 | SCY1-like 3 (S. cerevisiae) | 2 | | 917 | hsa-mir-214 | 57147 | SCYL3 | SCY1-like 3 (S. cerevisiae) | 2 | | 918 | hsa-miR-199a-5p | 57147 | SCYL3 | SCY1-like 3 (S. cerevisiae) | 2 | | 949 | hsa-miR-1233 | 3676 | ITGA4 | integrin, alpha 4 (antigen CD49D, alpha 4 subunit of VLA-4 receptor) | 2 | | 950 | hsa-miR-766 | 3676 | ITGA4 | integrin, alpha 4 (antigen CD49D, alpha 4 subunit of VLA-4 receptor) | 2 | | 952 | hsa-miR-1233 | 11168 | PSIP1 | PC4 and SFRS1 interacting protein 1 | 2 | | 953 | hsa-miR-569 | 11168 | PSIP1 | PC4 and SFRS1 interacting protein 1 | 2 | | 954 | hsa-miR-766 | 11168 | PSIP1 | PC4 and SFRS1 interacting protein 1 | 2 | | 961 | hsa-miR-604 | 1378 | CR1 | complement component (3b/4b) receptor 1 (Knops blood group) | 2 | | 962 | hsa-miR-635 | 1378 | CR1 | complement component (3b/4b) receptor 1 (Knops blood group) | 2 | | 963 | hsa-miR-938 | 1378 | CR1 | complement component (3b/4b) receptor 1 (Knops blood group) | 2 | | 1038 | hsa-miR-1233 | 65110 | UPF3A | UPF3 regulator of nonsense transcripts homolog A (yeast) | 2 | | 1040 | hsa-miR-571 | 65110 | UPF3A | UPF3 regulator of nonsense transcripts homolog A (yeast) | 2 | | 1067 | hsa-miR-569 | 11119 | BTN3A1 | butyrophilin, subfamily 3, member A1 | 2 | | 1071 | hsa-miR-1233 | 9497 | SLC4A7 | solute carrier family 4, sodium bicarbonate cotransporter, member 7 | 2 | | 1072 | hsa-miR-874 | 9497 | SLC4A7 | solute carrier family 4, sodium bicarbonate cotransporter, member 7 | 2 | | 1077 | hsa-miR-604 | 5606 | MAP2K3 | mitogen-activated protein kinase kinase 3 | 2 | | 1078 | hsa-miR-638 | 5606 | MAP2K3 | mitogen-activated protein kinase kinase 3 | 2 | | 1080 | hsa-miR-938 | 5606 | MAP2K3 | mitogen-activated protein kinase kinase 3 | 2 | | 1083 | hsa-miR-10a | 9048 | ARTN | artemin | 2 | | 1084 | hsa-miR-770-5p | 9048 | ARTN | artemin | 2 | | 1085 | hsa-miR-95 | 9048 | ARTN | artemin | 2 | | 1091 | hsa-miR-638 | 80256 | KIAA1539 | KIAA1539 | 2 | | 1092 | hsa-miR-765 | 80256 | KIAA1539 | KIAA1539 | 2 | | 1107 | hsa-miR-454\* | 10605 | PAIP1 | poly(A) binding protein interacting protein 1 | 2 | | 1109 | hsa-miR-591 | 10605 | PAIP1 | poly(A) binding protein interacting protein 1 | 2 | | 1121 | hsa-miR-623 | 6482 | ST3GAL1 | ST3 beta-galactoside alpha-2,3-sialyltransferase 1 | 2 | | 1142 | hsa-miR-604 | 334 | APLP2 | amyloid beta (A4) precursor-like protein 2 | 2 | | 1144 | hsa-miR-638 | 334 | APLP2 | amyloid beta (A4) precursor-like protein 2 | 2 | | 1145 | hsa-miR-938 | 334 | APLP2 | amyloid beta (A4) precursor-like protein 2 | 2 | | 1152 | hsa-miR-1233 | 10236 | HNRNPR | heterogeneous nuclear ribonucleoprotein R | 2 | | 1163 | hsa-miR-627 | 1107 | CHD3 | chromodomain helicase DNA binding protein 3 | 2 | | 1171 | hsa-miR-766 | 23062 | GGA2 | golgi-associated, gamma adaptin ear containing, ARF binding protein 2 | 2 | | 1177 | hsa-miR-604 | 10938 | EHD1 | EH-domain containing 1 | 2 | | 1178 | hsa-miR-938 | 10938 | EHD1 | EH-domain containing 1 | 2 | | 1181 | hsa-miR-766 | 9987 | HNRPDL | heterogeneous nuclear ribonucleoprotein D-like | 2 | | 1182 | hsa-miR-635 | 51100 | SH3GLB1 | SH3-domain GRB2-like endophilin B1 | 2 | | 1187 | hsa-miR-571 | 8575 | PRKRA | protein kinase, interferon-inducible double stranded RNA dependent activator | 2 | | 1224 | hsa-mir-3130-1 | 3479 | IGF1 | insulin-like growth factor 1 (somatomedin C) | 2 | | 1225 | hsa-mir-3130-2 | 3479 | IGF1 | insulin-like growth factor 1 (somatomedin C) | 2 | | 1226 | hsa-mir-3130-3 | 3479 | IGF1 | insulin-like growth factor 1 (somatomedin C) | 2 | | 1229 | hsa-miR-1233 | 26031 | OSBPL3 | oxysterol binding protein-like 3 | 2 | | 1230 | hsa-miR-569 | 26031 | OSBPL3 | oxysterol binding protein-like 3 | 2 | | 1231 | hsa-miR-571 | 26031 | OSBPL3 | oxysterol binding protein-like 3 | 2 | | 1232 | hsa-miR-766 | 26031 | OSBPL3 | oxysterol binding protein-like 3 | 2 | | 1233 | hsa-miR-874 | 26031 | OSBPL3 | oxysterol binding protein-like 3 | 2 | | 1255 | hsa-miR-1233 | 54674 | LRRN3 | leucine rich repeat neuronal 3 | 2 | | 1257 | hsa-miR-874 | 54674 | LRRN3 | leucine rich repeat neuronal 3 | 2 | | 1263 | hsa-miR-1233 | 545 | ATR | ataxia telangiectasia and Rad3 related | 2 | | 1265 | hsa-miR-569 | 545 | ATR | ataxia telangiectasia and Rad3 related | 2 | | 1266 | hsa-miR-571 | 545 | ATR | ataxia telangiectasia and Rad3 related | 2 | | 1267 | hsa-miR-766 | 545 | ATR | ataxia telangiectasia and Rad3 related | 2 | | 1268 | hsa-miR-874 | 545 | ATR | ataxia telangiectasia and Rad3 related | 2 | | 1281 | hsa-miR-874 | 5243 | ABCB1 | ATP-binding cassette, sub-family B (MDR/TAP), member 1 | 2 | | 1304 | hsa-miR-766 | 10772 | SFRS13A | splicing factor, arginine/serine-rich 13A | 2 | | 1322 | hsa-miR-95 | 887 | CCKBR | cholecystokinin B receptor | 2 | | 1370 | hsa-miR-135a | 10555 | AGPAT2 | 1-acylglycerol-3-phosphate O-acyltransferase 2 (lysophosphatidic acid acyltransferase, beta) | 2 | | 1382 | hsa-miR-1233 | 8718 | TNFRSF25 | tumor necrosis factor receptor superfamily, member 25 | 2 | | 1383 | hsa-miR-766 | 8718 | TNFRSF25 | tumor necrosis factor receptor superfamily, member 25 | 2 | | 1388 | hsa-miR-576-5p | 27072 | VPS41 | vacuolar protein sorting 41 homolog (S. cerevisiae) | 2 | | 1390 | hsa-mir-186 | 3077 | HFE | hemochromatosis | 2 | | 1391 | hsa-mir-423 | 3077 | HFE | hemochromatosis | 2 | | 1392 | hsa-mir-3130-1 | 3077 | HFE | hemochromatosis | 2 | | 1393 | hsa-mir-3130-2 | 3077 | HFE | hemochromatosis | 2 | | 1394 | hsa-mir-3130-3 | 3077 | HFE | hemochromatosis | 2 | | 1395 | hsa-miR-10a | 3077 | HFE | hemochromatosis | 2 | | 1396 | hsa-miR-186 | 3077 | HFE | hemochromatosis | 2 | | 1433 | hsa-miR-604 | 2204 | FCAR | Fc fragment of IgA, receptor for | 2 | | 1434 | hsa-miR-938 | 2204 | FCAR | Fc fragment of IgA, receptor for | 2 | | 1436 | hsa-miR-95 | 3077 | HFE | hemochromatosis | 2 | | 1438 | hsa-mir-3130-1 | 2263 | FGFR2 | fibroblast growth factor receptor 2 | 2 | | 1439 | hsa-mir-3130-2 | 2263 | FGFR2 | fibroblast growth factor receptor 2 | 2 | | 1440 | hsa-mir-3130-3 | 2263 | FGFR2 | fibroblast growth factor receptor 2 | 2 | | 1441 | hsa-miR-10a | 2263 | FGFR2 | fibroblast growth factor receptor 2 | 2 | | 1442 | hsa-miR-770-5p | 2263 | FGFR2 | fibroblast growth factor receptor 2 | 2 | | 1443 | hsa-miR-95 | 2263 | FGFR2 | fibroblast growth factor receptor 2 | 2 | | 1445 | hsa-miR-635 | 23569 | PADI4 | peptidyl arginine deiminase, type IV | 2 | | 1456 | hsa-miR-569 | 10128 | LRPPRC | leucine-rich PPR-motif containing | 2 | | 1464 | hsa-miR-1233 | 29969 | MDFIC | MyoD family inhibitor domain containing | 2 | | 1481 | hsa-miR-770-5p | 3691 | ITGB4 | integrin, beta 4 | 2 | | 1482 | hsa-miR-95 | 3691 | ITGB4 | integrin, beta 4 | 2 | | 1488 | hsa-miR-1233 | 220988 | HNRNPA3 | heterogeneous nuclear ribonucleoprotein A3 | 2 | | 1491 | hsa-miR-623 | 23215 | BAT2L2 | HLA-B associated transcript 2-like 2 | 2 | | 1509 | hsa-miR-1233 | 23360 | FNBP4 | formin binding protein 4 | 2 | | 1513 | hsa-miR-766 | 23360 | FNBP4 | formin binding protein 4 | 2 | | 1521 | hsa-miR-1233 | 23175 | LPIN1 | lipin 1 | 2 | | 1523 | hsa-miR-571 | 23175 | LPIN1 | lipin 1 | 2 | | 1524 | hsa-miR-766 | 23175 | LPIN1 | lipin 1 | 2 | | 1525 | hsa-miR-874 | 23175 | LPIN1 | lipin 1 | 2 | | 1530 | hsa-mir-10a | 51029 | PPPDE1 | PPPDE peptidase domain containing 1 | 2 | | 1544 | hsa-miR-576-5p | 51592 | TRIM33 | tripartite motif-containing 33 | 2 | | 1564 | hsa-miR-1233 | 27250 | PDCD4 | programmed cell death 4 (neoplastic transformation inhibitor) | 2 | | 1566 | hsa-miR-874 | 27250 | PDCD4 | programmed cell death 4 (neoplastic transformation inhibitor) | 2 | | 1576 | hsa-miR-604 | 3557 | IL1RN | interleukin 1 receptor antagonist | 2 | | 1577 | hsa-miR-938 | 3557 | IL1RN | interleukin 1 receptor antagonist | 2 | | 1589 | hsa-miR-569 | 26051 | PPP1R16B | protein phosphatase 1, regulatory (inhibitor) subunit 16B | 2 | | 1598 | hsa-miR-766 | 56252 | YLPM1 | YLP motif containing 1 | 2 | | 1604 | hsa-mir-10a | 8939 | FUBP3 | far upstream element (FUSE) binding protein 3 | 2 | | 1609 | hsa-miR-1233 | 56950 | SMYD2 | SET and MYND domain containing 2 | 2 | | 1611 | hsa-miR-766 | 56950 | SMYD2 | SET and MYND domain containing 2 | 2 | | 1612 | hsa-miR-874 | 56950 | SMYD2 | SET and MYND domain containing 2 | 2 | | 1616 | hsa-miR-770-5p | 1291 | COL6A1 | collagen, type VI, alpha 1 | 2 | | 1617 | hsa-miR-95 | 1291 | COL6A1 | collagen, type VI, alpha 1 | 2 | | 1619 | hsa-miR-766 | 23269 | MGA | MAX gene associated | 2 | | 1626 | hsa-miR-571 | 9747 | FAM115A | family with sequence similarity 115, member A | 2 | | 1627 | hsa-miR-874 | 9747 | FAM115A | family with sequence similarity 115, member A | 2 | | 1634 | hsa-miR-576-5p | 7110 | TMF1 | TATA element modulatory factor 1 | 2 | | 1651 | hsa-mir-10a | 5286 | PIK3C2A | phosphoinositide-3-kinase, class 2, alpha polypeptide | 2 | | 1675 | hsa-miR-1233 | 221443 | C6orf130 | chromosome 6 open reading frame 130 | 2 | | 1677 | hsa-miR-571 | 221443 | C6orf130 | chromosome 6 open reading frame 130 | 2 | | 1688 | hsa-miR-1233 | 6191 | RPS4X | ribosomal protein S4, X-linked | 2 | | 1689 | hsa-miR-874 | 6191 | RPS4X | ribosomal protein S4, X-linked | 2 | | 1692 | hsa-mir-423 | 25803 | SPDEF | SAM pointed domain containing ets transcription factor | 2 | | 1693 | hsa-mir-576 | 25803 | SPDEF | SAM pointed domain containing ets transcription factor | 2 | | 1694 | hsa-miR-16 | 25803 | SPDEF | SAM pointed domain containing ets transcription factor | 2 | | 1699 | hsa-miR-1233 | 10147 | SFRS14 | splicing factor, arginine/serine-rich 14 | 2 | | 1708 | hsa-miR-1233 | 6432 | SFRS7 | splicing factor, arginine/serine-rich 7, 35kDa | 2 | | 1709 | hsa-miR-766 | 6432 | SFRS7 | splicing factor, arginine/serine-rich 7, 35kDa | 2 | | 1727 | hsa-miR-569 | 6964 | TRD@ | T cell receptor delta locus | 2 | | 1728 | hsa-miR-571 | 6964 | TRD@ | T cell receptor delta locus | 2 | | 1731 | hsa-miR-874 | 6964 | TRD@ | T cell receptor delta locus | 2 | | 1751 | hsa-miR-766 | 10147 | SFRS14 | splicing factor, arginine/serine-rich 14 | 2 | | 1758 | hsa-miR-1233 | 57134 | MAN1C1 | mannosidase, alpha, class 1C, member 1 | 2 | | 1760 | hsa-miR-874 | 57134 | MAN1C1 | mannosidase, alpha, class 1C, member 1 | 2 | | 1766 | hsa-miR-569 | 3178 | HNRNPA1 | heterogeneous nuclear ribonucleoprotein A1 | 2 | | 1767 | hsa-miR-571 | 3178 | HNRNPA1 | heterogeneous nuclear ribonucleoprotein A1 | 2 | | 1771 | hsa-mir-3130-1 | 25803 | SPDEF | SAM pointed domain containing ets transcription factor | 2 | | 1772 | hsa-mir-3130-2 | 25803 | SPDEF | SAM pointed domain containing ets transcription factor | 2 | | 1773 | hsa-mir-3130-3 | 25803 | SPDEF | SAM pointed domain containing ets transcription factor | 2 | | 1774 | hsa-miR-10a | 25803 | SPDEF | SAM pointed domain containing ets transcription factor | 2 | | 1789 | hsa-miR-1233 | 51018 | RRP15 | ribosomal RNA processing 15 homolog (S. cerevisiae) | 2 | | 1790 | hsa-miR-571 | 51018 | RRP15 | ribosomal RNA processing 15 homolog (S. cerevisiae) | 2 | | 1791 | hsa-miR-874 | 51018 | RRP15 | ribosomal RNA processing 15 homolog (S. cerevisiae) | 2 | | 1825 | hsa-miR-1233 | 57711 | ZNF529 | zinc finger protein 529 | 2 | | 1826 | hsa-miR-571 | 57711 | ZNF529 | zinc finger protein 529 | 2 | | 1827 | hsa-miR-874 | 57711 | ZNF529 | zinc finger protein 529 | 2 | | 1834 | hsa-miR-1233 | 55876 | GSDMB | gasdermin B | 2 | | 1869 | hsa-miR-1233 | 149628 | PYHIN1 | pyrin and HIN domain family, member 1 | 2 | | 1870 | hsa-miR-569 | 149628 | PYHIN1 | pyrin and HIN domain family, member 1 | 2 | | 1871 | hsa-miR-571 | 149628 | PYHIN1 | pyrin and HIN domain family, member 1 | 2 | | 1872 | hsa-miR-766 | 149628 | PYHIN1 | pyrin and HIN domain family, member 1 | 2 | | 1885 | hsa-mir-3130-1 | 9436 | NCR2 | natural cytotoxicity triggering receptor 2 | 2 | | 1886 | hsa-mir-3130-2 | 9436 | NCR2 | natural cytotoxicity triggering receptor 2 | 2 | | 1887 | hsa-mir-3130-3 | 9436 | NCR2 | natural cytotoxicity triggering receptor 2 | 2 | | 1888 | hsa-miR-10a | 9436 | NCR2 | natural cytotoxicity triggering receptor 2 | 2 | | 1926 | hsa-miR-1233 | 23545 | ATP6V0A2 | ATPase, H+ transporting, lysosomal V0 subunit a2 | 2 | | 1927 | hsa-miR-571 | 23545 | ATP6V0A2 | ATPase, H+ transporting, lysosomal V0 subunit a2 | 2 | | 1928 | hsa-miR-874 | 23545 | ATP6V0A2 | ATPase, H+ transporting, lysosomal V0 subunit a2 | 2 | | 1933 | hsa-miR-1233 | 64710 | NUCKS1 | nuclear casein kinase and cyclin-dependent kinase substrate 1 | 2 | | 1934 | hsa-miR-766 | 64710 | NUCKS1 | nuclear casein kinase and cyclin-dependent kinase substrate 1 | 2 | | 1935 | hsa-miR-874 | 64710 | NUCKS1 | nuclear casein kinase and cyclin-dependent kinase substrate 1 | 2 | | 1941 | hsa-miR-766 | 51466 | EVL | Enah/Vasp-like | 2 | | 1944 | hsa-miR-1233 | 51727 | CMPK1 | cytidine monophosphate (UMP-CMP) kinase 1, cytosolic | 2 | | 1949 | hsa-miR-569 | 55037 | PTCD3 | Pentatricopeptide repeat domain 3 | 2 | | 1950 | hsa-miR-571 | 55037 | PTCD3 | Pentatricopeptide repeat domain 3 | 2 | | 1953 | hsa-miR-1233 | 394 | ARHGAP5 | Rho GTPase activating protein 5 | 2 | | 1955 | hsa-miR-591 | 394 | ARHGAP5 | Rho GTPase activating protein 5 | 2 | | 1976 | hsa-miR-1233 | 51569 | UFM1 | ubiquitin-fold modifier 1 | 2 | | 1999 | hsa-miR-1233 | 79048 | SECISBP2 | SECIS binding protein 2 | 2 | | 2010 | hsa-miR-1233 | 54906 | C10orf18 | chromosome 10 open reading frame 18 | 2 | | 2012 | hsa-miR-576-5p | 54906 | C10orf18 | chromosome 10 open reading frame 18 | 2 | | 2020 | hsa-miR-571 | 54585 | LZTFL1 | leucine zipper transcription factor-like 1 | 2 | | 2035 | hsa-miR-874 | 55900 | ZNF302 | zinc finger protein 302 | 2 | | 2047 | hsa-miR-1233 | 55750 | AGK | acylglycerol kinase | 2 | | 2051 | hsa-miR-1233 | 64682 | ANAPC1 | anaphase promoting complex subunit 1 | 2 | | 2103 | hsa-miR-874 | 9317 | PTER | phosphotriesterase related | 2 | | 2104 | hsa-miR-591 | 64940 | STAG3L4 | stromal antigen 3-like 4 | 2 | | 2130 | hsa-miR-1233 | 64783 | RBM15 | RNA binding motif protein 15 | 2 | | 2131 | hsa-miR-571 | 64783 | RBM15 | RNA binding motif protein 15 | 2 | | 2132 | hsa-miR-591 | 64783 | RBM15 | RNA binding motif protein 15 | 2 | | 2164 | hsa-miR-571 | 7769 | ZNF226 | zinc finger protein 226 | 2 | | 2182 | hsa-miR-1233 | 80063 | ATF7IP2 | activating transcription factor 7 interacting protein 2 | 2 | | 2183 | hsa-miR-569 | 80063 | ATF7IP2 | activating transcription factor 7 interacting protein 2 | 2 | | 2184 | hsa-miR-571 | 80063 | ATF7IP2 | activating transcription factor 7 interacting protein 2 | 2 | | 2185 | hsa-miR-766 | 80063 | ATF7IP2 | activating transcription factor 7 interacting protein 2 | 2 | | 2186 | hsa-miR-874 | 80063 | ATF7IP2 | activating transcription factor 7 interacting protein 2 | 2 | | 2195 | hsa-miR-576-5p | 79872 | CBLL1 | Cas-Br-M (murine) ecotropic retroviral transforming sequence-like 1 | 2 | | 2200 | hsa-miR-569 | 29121 | CLEC2D | C-type lectin domain family 2, member D | 2 | | 2201 | hsa-miR-571 | 29121 | CLEC2D | C-type lectin domain family 2, member D | 2 | | 2209 | hsa-miR-635 | 55002 | TMCO3 | transmembrane and coiled-coil domains 3 | 2 | | 2212 | hsa-miR-638 | 63916 | ELMO2 | engulfment and cell motility 2 | 2 | | 2316 | hsa-mir-10a | 79184 | BRCC3 | BRCA1/BRCA2-containing complex, subunit 3 | 2 | | 2322 | hsa-miR-766 | 60468 | BACH2 | BTB and CNC homology 1, basic leucine zipper transcription factor 2 | 2 | | 2344 | hsa-miR-766 | 55341 | LSG1 | large subunit GTPase 1 homolog (S. cerevisiae) | 2 | | 2372 | hsa-miR-638 | 80305 | TRABD | TraB domain containing | 2 | | 2376 | hsa-miR-591 | 90806 | ANGEL2 | angel homolog 2 (Drosophila) | 2 | | 2379 | hsa-miR-10a | 27147 | DENND2A | DENN/MADD domain containing 2A | 2 | | 2388 | hsa-miR-635 | 84034 | EMILIN2 | elastin microfibril interfacer 2 | 2 | | 2411 | hsa-miR-591 | 92595 | ZNF764 | zinc finger protein 764 | 2 | | 2491 | hsa-miR-1233 | 23731 | C9orf5 | chromosome 9 open reading frame 5 | 2 | | 2493 | hsa-miR-766 | 23731 | C9orf5 | chromosome 9 open reading frame 5 | 2 | | 2505 | hsa-miR-571 | 55591 | VEZT | vezatin, adherens junctions transmembrane protein | 2 | | 2507 | hsa-miR-591 | 55591 | VEZT | vezatin, adherens junctions transmembrane protein | 2 | | 2508 | hsa-miR-569 | 56172 | ANKH | ankylosis, progressive homolog (mouse) | 2 | | 2509 | hsa-miR-766 | 56172 | ANKH | ankylosis, progressive homolog (mouse) | 2 | | 2516 | hsa-miR-1233 | 10194 | TSHZ1 | teashirt zinc finger homeobox 1 | 2 | | 2528 | hsa-miR-623 | 84266 | ALKBH7 | alkB, alkylation repair homolog 7 (E. coli) | 2 | | 2530 | hsa-miR-671-5p | 84266 | ALKBH7 | alkB, alkylation repair homolog 7 (E. coli) | 2 | | 2537 | hsa-miR-1233 | 84172 | POLR1B | polymerase (RNA) I polypeptide B, 128kDa | 2 | | 2538 | hsa-miR-571 | 84172 | POLR1B | polymerase (RNA) I polypeptide B, 128kDa | 2 | | 2540 | hsa-miR-874 | 84172 | POLR1B | polymerase (RNA) I polypeptide B, 128kDa | 2 | | 2552 | hsa-miR-1233 | 83636 | C19orf12 | chromosome 19 open reading frame 12 | 2 | | 2555 | hsa-miR-766 | 83636 | C19orf12 | chromosome 19 open reading frame 12 | 2 | | 2596 | hsa-miR-1233 | 84365 | MKI67IP | MKI67 (FHA domain) interacting nucleolar phosphoprotein | 2 | | 2598 | hsa-miR-766 | 84365 | MKI67IP | MKI67 (FHA domain) interacting nucleolar phosphoprotein | 2 | | 2618 | hsa-miR-604 | 57655 | GRAMD1A | GRAM domain containing 1A | 2 | | 2621 | hsa-miR-938 | 57655 | GRAMD1A | GRAM domain containing 1A | 2 | | 2652 | hsa-miR-591 | 51535 | PPHLN1 | periphilin 1 | 2 | | 2678 | hsa-miR-766 | 10838 | ZNF275 | zinc finger protein 275 | 2 | | 2683 | hsa-miR-766 | 5928 | RBBP4 | retinoblastoma binding protein 4 | 2 | | 2711 | hsa-miR-874 | 58487 | CREBZF | CREB/ATF bZIP transcription factor | 2 | | 2719 | hsa-miR-591 | 25879 | DCAF13 | DDB1 and CUL4 associated factor 13 | 2 | | 2760 | hsa-miR-1233 | 222236 | NAPEPLD | N-acyl phosphatidylethanolamine phospholipase D | 2 | | 2761 | hsa-miR-571 | 222236 | NAPEPLD | N-acyl phosphatidylethanolamine phospholipase D | 2 | | 2764 | hsa-miR-571 | 5599 | MAPK8 | mitogen-activated protein kinase 8 | 2 | | 2817 | hsa-miR-571 | 133746 | JMY | junction mediating and regulatory protein, p53 cofactor | 2 | | 2833 | hsa-miR-874 | 85315 | PAQR8 | progestin and adipoQ receptor family member VIII | 2 | | 2834 | hsa-miR-1233 | 84186 | ZCCHC7 | zinc finger, CCHC domain containing 7 | 2 | | 2844 | hsa-miR-617 | 388228 | SBK1 | SH3-binding domain kinase 1 | 2 | | 2848 | hsa-miR-1233 | 30837 | SOCS7 | suppressor of cytokine signaling 7 | 2 | | 2850 | hsa-miR-766 | 30837 | SOCS7 | suppressor of cytokine signaling 7 | 2 | | 2929 | hsa-miR-1233 | 730094 | C16orf52 | chromosome 16 open reading frame 52 | 2 | | 2930 | hsa-miR-874 | 730094 | C16orf52 | chromosome 16 open reading frame 52 | 2 | | 2931 | hsa-miR-766 | 147138 | TMC8 | transmembrane channel-like 8 | 2 | | 2995 | hsa-miR-1233 | 3841 | KPNA5 | karyopherin alpha 5 (importin alpha 6) | 2 | | 2996 | hsa-miR-571 | 3841 | KPNA5 | karyopherin alpha 5 (importin alpha 6) | 2 | | 2997 | hsa-miR-766 | 3841 | KPNA5 | karyopherin alpha 5 (importin alpha 6) | 2 | | 2998 | hsa-miR-874 | 3841 | KPNA5 | karyopherin alpha 5 (importin alpha 6) | 2 | | 3035 | hsa-miR-1233 | 256471 | MFSD8 | major facilitator superfamily domain containing 8 | 2 | | 3036 | hsa-miR-571 | 256471 | MFSD8 | major facilitator superfamily domain containing 8 | 2 | | 3045 | hsa-miR-591 | 55900 | ZNF302 | zinc finger protein 302 | 2 | | 3061 | hsa-miR-766 | 817 | CAMK2D | calcium/calmodulin-dependent protein kinase II delta | 2 | | 3067 | hsa-miR-571 | 57489 | ODF2L | outer dense fiber of sperm tails 2-like | 2 | | 3092 | hsa-miR-571 | 340481 | ZDHHC21 | zinc finger, DHHC-type containing 21 | 2 | | 3128 | hsa-miR-635 | 220929 | ZNF438 | zinc finger protein 438 | 2 | | 3131 | hsa-miR-591 | 92345 | NAF1 | nuclear assembly factor 1 homolog (S. cerevisiae) | 2 | | 3164 | hsa-miR-571 | 730094 | C16orf52 | chromosome 16 open reading frame 52 | 2 | | 3186 | hsa-mir-149 | 7707 | ZNF148 | zinc finger protein 148 | 2 | | 3187 | hsa-miR-576-5p | 7707 | ZNF148 | zinc finger protein 148 | 2 | | 3333 | hsa-mir-3130-1 | 83999 | KREMEN1 | kringle containing transmembrane protein 1 | 2 | | 3334 | hsa-mir-3130-2 | 83999 | KREMEN1 | kringle containing transmembrane protein 1 | 2 | | 3335 | hsa-mir-3130-3 | 83999 | KREMEN1 | kringle containing transmembrane protein 1 | 2 | | 3355 | hsa-miR-766 | 152926 | PPM1K | protein phosphatase 1K (PP2C domain containing) | 2 | | 3378 | hsa-miR-571 | 163081 | ZNF567 | zinc finger protein 567 | 2 | | 3380 | hsa-miR-874 | 163081 | ZNF567 | zinc finger protein 567 | 2 | | 3426 | hsa-miR-1233 | 11146 | GLMN | glomulin, FKBP associated protein | 2 | | 3427 | hsa-miR-571 | 11146 | GLMN | glomulin, FKBP associated protein | 2 | | 3428 | hsa-miR-874 | 11146 | GLMN | glomulin, FKBP associated protein | 2 | | 3438 | hsa-miR-190 | 5928 | RBBP4 | retinoblastoma binding protein 4 | 2 | | 2 | hsa-mir-423 | 10406 | WFDC2 | WAP four-disulfide core domain 2 | 1 | | 6 | hsa-miR-10a | 10406 | WFDC2 | WAP four-disulfide core domain 2 | 1 | | 7 | hsa-miR-770-5p | 10406 | WFDC2 | WAP four-disulfide core domain 2 | 1 | | 8 | hsa-miR-95 | 10406 | WFDC2 | WAP four-disulfide core domain 2 | 1 | | 10 | hsa-miR-569 | 172 | AFG3L1 | AFG3 ATPase family gene 3-like 1 (S. cerevisiae) | 1 | | 11 | hsa-miR-571 | 172 | AFG3L1 | AFG3 ATPase family gene 3-like 1 (S. cerevisiae) | 1 | | 12 | hsa-miR-766 | 172 | AFG3L1 | AFG3 ATPase family gene 3-like 1 (S. cerevisiae) | 1 | | 13 | hsa-miR-623 | 170575 | GIMAP1 | GTPase, IMAP family member 1 | 1 | | 15 | hsa-miR-190 | 170575 | GIMAP1 | GTPase, IMAP family member 1 | 1 | | 16 | hsa-miR-766 | 170575 | GIMAP1 | GTPase, IMAP family member 1 | 1 | | 17 | hsa-mir-10a | 9946 | CRYZL1 | crystallin, zeta (quinone reductase)-like 1 | 1 | | 18 | hsa-mir-423 | 32 | ACACB | acetyl-Coenzyme A carboxylase beta | 1 | | 19 | hsa-miR-10a | 32 | ACACB | acetyl-Coenzyme A carboxylase beta | 1 | | 20 | hsa-mir-3130-1 | 117286 | CIB3 | calcium and integrin binding family member 3 | 1 | | 21 | hsa-mir-3130-2 | 117286 | CIB3 | calcium and integrin binding family member 3 | 1 | | 22 | hsa-mir-3130-3 | 117286 | CIB3 | calcium and integrin binding family member 3 | 1 | | 23 | hsa-miR-10a | 117286 | CIB3 | calcium and integrin binding family member 3 | 1 | | 24 | hsa-miR-770-5p | 117286 | CIB3 | calcium and integrin binding family member 3 | 1 | | 25 | hsa-miR-95 | 117286 | CIB3 | calcium and integrin binding family member 3 | 1 | | 26 | hsa-miR-10a | 114132 | SIGLEC11 | sialic acid binding Ig-like lectin 11 | 1 | | 27 | hsa-miR-770-5p | 114132 | SIGLEC11 | sialic acid binding Ig-like lectin 11 | 1 | | 28 | hsa-miR-95 | 114132 | SIGLEC11 | sialic acid binding Ig-like lectin 11 | 1 | | 29 | hsa-miR-638 | 23299 | BICD2 | bicaudal D homolog 2 (Drosophila) | 1 | | 30 | hsa-mir-505 | 132243 | H1FOO | H1 histone family, member O, oocyte-specific | 1 | | 31 | hsa-miR-10a | 132243 | H1FOO | H1 histone family, member O, oocyte-specific | 1 | | 32 | hsa-miR-770-5p | 132243 | H1FOO | H1 histone family, member O, oocyte-specific | 1 | | 33 | hsa-miR-95 | 132243 | H1FOO | H1 histone family, member O, oocyte-specific | 1 | | 34 | hsa-miR-95 | 89884 | LHX4 | LIM homeobox 4 | 1 | | 35 | hsa-miR-1233 | 117157 | SH2D1B | SH2 domain containing 1B | 1 | | 36 | hsa-miR-569 | 117157 | SH2D1B | SH2 domain containing 1B | 1 | | 37 | hsa-miR-571 | 117157 | SH2D1B | SH2 domain containing 1B | 1 | | 38 | hsa-miR-766 | 117157 | SH2D1B | SH2 domain containing 1B | 1 | | 39 | hsa-miR-874 | 117157 | SH2D1B | SH2 domain containing 1B | 1 | | 43 | hsa-mir-423 | 6853 | SYN1 | synapsin I | 1 | | 44 | hsa-mir-3130-1 | 6853 | SYN1 | synapsin I | 1 | | 45 | hsa-mir-3130-2 | 6853 | SYN1 | synapsin I | 1 | | 46 | hsa-mir-3130-3 | 6853 | SYN1 | synapsin I | 1 | | 47 | hsa-miR-10a | 6853 | SYN1 | synapsin I | 1 | | 48 | hsa-miR-591 | 124923 | FLJ25006 | uncharacterized serine/threonine-protein kinase SgK494 | 1 | | 49 | hsa-miR-95 | 85445 | CNTNAP4 | contactin associated protein-like 4 | 1 | | 50 | hsa-mir-3130-1 | 100293142 | LOC100293142 | similar to hCG2040019 | 1 | | 51 | hsa-mir-3130-2 | 100293142 | LOC100293142 | similar to hCG2040019 | 1 | | 52 | hsa-mir-3130-3 | 100293142 | LOC100293142 | similar to hCG2040019 | 1 | | 53 | hsa-mir-3130-1 | 124359 | CDYL2 | chromodomain protein, Y-like 2 | 1 | | 54 | hsa-mir-3130-2 | 124359 | CDYL2 | chromodomain protein, Y-like 2 | 1 | | 55 | hsa-mir-3130-3 | 124359 | CDYL2 | chromodomain protein, Y-like 2 | 1 | | 56 | hsa-miR-643 | 124359 | CDYL2 | chromodomain protein, Y-like 2 | 1 | | 58 | hsa-miR-10a | 148645 | C1orf211 | chromosome 1 open reading frame 211 | 1 | | 59 | hsa-miR-770-5p | 148645 | C1orf211 | chromosome 1 open reading frame 211 | 1 | | 60 | hsa-miR-95 | 148645 | C1orf211 | chromosome 1 open reading frame 211 | 1 | | 61 | hsa-miR-10a | 128497 | C20orf165 | chromosome 20 open reading frame 165 | 1 | | 62 | hsa-miR-770-5p | 128497 | C20orf165 | chromosome 20 open reading frame 165 | 1 | | 63 | hsa-miR-95 | 128497 | C20orf165 | chromosome 20 open reading frame 165 | 1 | | 65 | hsa-miR-571 | 163050 | ZNF564 | zinc finger protein 564 | 1 | | 69 | hsa-miR-571 | 9093 | DNAJA3 | DnaJ (Hsp40) homolog, subfamily A, member 3 | 1 | | 75 | hsa-miR-770-5p | 149685 | ADIG | adipogenin | 1 | | 76 | hsa-miR-95 | 149685 | ADIG | adipogenin | 1 | | 77 | hsa-miR-604 | 51125 | GOLGA7 | golgin A7 | 1 | | 78 | hsa-miR-938 | 51125 | GOLGA7 | golgin A7 | 1 | | 79 | hsa-mir-576 | 255374 | MBLAC1 | metallo-beta-lactamase domain containing 1 | 1 | | 80 | hsa-miR-10a | 642976 | NCRNA00110 | non-protein coding RNA 110 | 1 | | 81 | hsa-miR-770-5p | 642976 | NCRNA00110 | non-protein coding RNA 110 | 1 | | 82 | hsa-miR-95 | 642976 | NCRNA00110 | non-protein coding RNA 110 | 1 | | 83 | hsa-miR-569 | 7705 | ZNF146 | zinc finger protein 146 | 1 | | 86 | hsa-miR-1233 | 55180 | LINS1 | lines homolog 1 (Drosophila) | 1 | | 87 | hsa-miR-571 | 55180 | LINS1 | lines homolog 1 (Drosophila) | 1 | | 88 | hsa-miR-604 | 118788 | PIK3AP1 | phosphoinositide-3-kinase adaptor protein 1 | 1 | | 89 | hsa-miR-635 | 118788 | PIK3AP1 | phosphoinositide-3-kinase adaptor protein 1 | 1 | | 90 | hsa-miR-938 | 118788 | PIK3AP1 | phosphoinositide-3-kinase adaptor protein 1 | 1 | | 91 | hsa-miR-571 | 150737 | TTC30B | tetratricopeptide repeat domain 30B | 1 | | 92 | hsa-miR-10a | 9711 | KIAA0226 | KIAA0226 | 1 | | 93 | hsa-miR-638 | 3632 | INPP5A | inositol polyphosphate-5-phosphatase, 40kDa | 1 | | 94 | hsa-miR-10a | 525 | ATP6V1B1 | ATPase, H+ transporting, lysosomal 56/58kDa, V1 subunit B1 | 1 | | 95 | hsa-miR-770-5p | 525 | ATP6V1B1 | ATPase, H+ transporting, lysosomal 56/58kDa, V1 subunit B1 | 1 | | 96 | hsa-miR-95 | 525 | ATP6V1B1 | ATPase, H+ transporting, lysosomal 56/58kDa, V1 subunit B1 | 1 | | 97 | hsa-mir-505 | 54933 | RHBDL2 | rhomboid, veinlet-like 2 (Drosophila) | 1 | | 99 | hsa-miR-770-5p | 54933 | RHBDL2 | rhomboid, veinlet-like 2 (Drosophila) | 1 | | 100 | hsa-miR-95 | 54933 | RHBDL2 | rhomboid, veinlet-like 2 (Drosophila) | 1 | | 105 | hsa-mir-3130-1 | 114043 | C21orf90 | chromosome 21 open reading frame 90 | 1 | | 106 | hsa-mir-3130-2 | 114043 | C21orf90 | chromosome 21 open reading frame 90 | 1 | | 107 | hsa-mir-3130-3 | 114043 | C21orf90 | chromosome 21 open reading frame 90 | 1 | | 108 | hsa-miR-10a | 114043 | C21orf90 | chromosome 21 open reading frame 90 | 1 | | 109 | hsa-miR-770-5p | 114043 | C21orf90 | chromosome 21 open reading frame 90 | 1 | | 110 | hsa-miR-95 | 114043 | C21orf90 | chromosome 21 open reading frame 90 | 1 | | 111 | hsa-miR-95 | 9870 | KIAA0317 | KIAA0317 | 1 | | 112 | hsa-miR-770-5p | 9098 | USP6 | ubiquitin specific peptidase 6 (Tre-2 oncogene) | 1 | | 113 | hsa-miR-95 | 9098 | USP6 | ubiquitin specific peptidase 6 (Tre-2 oncogene) | 1 | | 120 | hsa-mir-3130-1 | 80824 | DUSP16 | dual specificity phosphatase 16 | 1 | | 121 | hsa-mir-3130-2 | 80824 | DUSP16 | dual specificity phosphatase 16 | 1 | | 122 | hsa-mir-3130-3 | 80824 | DUSP16 | dual specificity phosphatase 16 | 1 | | 123 | hsa-miR-10a | 80824 | DUSP16 | dual specificity phosphatase 16 | 1 | | 124 | hsa-miR-770-5p | 80824 | DUSP16 | dual specificity phosphatase 16 | 1 | | 125 | hsa-miR-95 | 80824 | DUSP16 | dual specificity phosphatase 16 | 1 | | 126 | hsa-miR-95 | 353174 | ZACN | zinc activated ligand-gated ion channel | 1 | | 127 | hsa-miR-623 | 7535 | ZAP70 | zeta-chain (TCR) associated protein kinase 70kDa | 1 | | 129 | hsa-miR-619 | 54434 | SSH1 | slingshot homolog 1 (Drosophila) | 1 | | 133 | hsa-miR-638 | 353514 | LILRA5 | leukocyte immunoglobulin-like receptor, subfamily A (with TM domain), member 5 | 1 | | 134 | hsa-mir-423 | 123920 | CMTM3 | CKLF-like MARVEL transmembrane domain containing 3 | 1 | | 135 | hsa-miR-10a | 123920 | CMTM3 | CKLF-like MARVEL transmembrane domain containing 3 | 1 | | 136 | hsa-miR-770-5p | 123920 | CMTM3 | CKLF-like MARVEL transmembrane domain containing 3 | 1 | | 137 | hsa-miR-95 | 123920 | CMTM3 | CKLF-like MARVEL transmembrane domain containing 3 | 1 | | 138 | hsa-miR-627 | 8314 | BAP1 | BRCA1 associated protein-1 (ubiquitin carboxy-terminal hydrolase) | 1 | | 139 | hsa-miR-638 | 8314 | BAP1 | BRCA1 associated protein-1 (ubiquitin carboxy-terminal hydrolase) | 1 | | 140 | hsa-miR-671-5p | 8314 | BAP1 | BRCA1 associated protein-1 (ubiquitin carboxy-terminal hydrolase) | 1 | | 141 | hsa-miR-10a | 164592 | CCDC116 | coiled-coil domain containing 116 | 1 | | 143 | hsa-miR-571 | 5431 | POLR2B | polymerase (RNA) II (DNA directed) polypeptide B, 140kDa | 1 | | 144 | hsa-miR-576-5p | 5431 | POLR2B | polymerase (RNA) II (DNA directed) polypeptide B, 140kDa | 1 | | 145 | hsa-miR-454\* | 57107 | PDSS2 | prenyl (decaprenyl) diphosphate synthase, subunit 2 | 1 | | 146 | hsa-miR-591 | 57107 | PDSS2 | prenyl (decaprenyl) diphosphate synthase, subunit 2 | 1 | | 147 | hsa-miR-10a | 100130417 | FLJ39609 | similar to hCG1995469 | 1 | | 148 | hsa-miR-770-5p | 100130417 | FLJ39609 | similar to hCG1995469 | 1 | | 149 | hsa-miR-95 | 100130417 | FLJ39609 | similar to hCG1995469 | 1 | | 150 | hsa-miR-1233 | 57692 | MAGEE1 | melanoma antigen family E, 1 | 1 | | 151 | hsa-miR-571 | 57692 | MAGEE1 | melanoma antigen family E, 1 | 1 | | 152 | hsa-miR-874 | 57692 | MAGEE1 | melanoma antigen family E, 1 | 1 | | 153 | hsa-miR-766 | 23065 | KIAA0090 | KIAA0090 | 1 | | 154 | hsa-miR-874 | 7634 | ZNF80 | zinc finger protein 80 | 1 | | 155 | hsa-miR-874 | 56252 | YLPM1 | YLP motif containing 1 | 1 | | 157 | hsa-miR-770-5p | 728449 | LOC728449 | hypothetical protein LOC728449 | 1 | | 158 | hsa-miR-95 | 728449 | LOC728449 | hypothetical protein LOC728449 | 1 | | 159 | hsa-miR-1233 | 283588 | LOC283588 | hypothetical LOC283588 | 1 | | 160 | hsa-miR-571 | 283588 | LOC283588 | hypothetical LOC283588 | 1 | | 161 | hsa-miR-874 | 283588 | LOC283588 | hypothetical LOC283588 | 1 | | 162 | hsa-miR-1233 | 84911 | ZNF382 | zinc finger protein 382 | 1 | | 163 | hsa-miR-571 | 84911 | ZNF382 | zinc finger protein 382 | 1 | | 164 | hsa-miR-874 | 84911 | ZNF382 | zinc finger protein 382 | 1 | | 169 | hsa-miR-10a | 148145 | LOC148145 | hypothetical LOC148145 | 1 | | 170 | hsa-miR-770-5p | 148145 | LOC148145 | hypothetical LOC148145 | 1 | | 171 | hsa-miR-95 | 148145 | LOC148145 | hypothetical LOC148145 | 1 | | 172 | hsa-miR-1233 | 22882 | ZHX2 | zinc fingers and homeoboxes 2 | 1 | | 173 | hsa-miR-1233 | 5527 | PPP2R5C | protein phosphatase 2, regulatory subunit B', gamma isoform | 1 | | 174 | hsa-miR-1233 | 10939 | AFG3L2 | AFG3 ATPase family gene 3-like 2 (yeast) | 1 | | 175 | hsa-miR-571 | 10939 | AFG3L2 | AFG3 ATPase family gene 3-like 2 (yeast) | 1 | | 176 | hsa-miR-591 | 10939 | AFG3L2 | AFG3 ATPase family gene 3-like 2 (yeast) | 1 | | 177 | hsa-miR-874 | 10939 | AFG3L2 | AFG3 ATPase family gene 3-like 2 (yeast) | 1 | | 178 | hsa-mir-10a | 64417 | C5orf28 | chromosome 5 open reading frame 28 | 1 | | 180 | hsa-miR-190 | 64417 | C5orf28 | chromosome 5 open reading frame 28 | 1 | | 182 | hsa-miR-766 | 64417 | C5orf28 | chromosome 5 open reading frame 28 | 1 | | 184 | hsa-miR-934 | 64417 | C5orf28 | chromosome 5 open reading frame 28 | 1 | | 185 | hsa-miR-95 | 654841 | LOC654841 | hypothetical LOC654841 | 1 | | 186 | hsa-miR-635 | 9446 | GSTO1 | glutathione S-transferase omega 1 | 1 | | 188 | hsa-miR-569 | 130916 | MTERFD2 | MTERF domain containing 2 | 1 | | 190 | hsa-miR-766 | 130916 | MTERFD2 | MTERF domain containing 2 | 1 | | 192 | hsa-mir-885 | 5565 | PRKAB2 | protein kinase, AMP-activated, beta 2 non-catalytic subunit | 1 | | 193 | hsa-miR-770-5p | 6714 | SRC | v-src sarcoma (Schmidt-Ruppin A-2) viral oncogene homolog (avian) | 1 | | 194 | hsa-miR-95 | 6714 | SRC | v-src sarcoma (Schmidt-Ruppin A-2) viral oncogene homolog (avian) | 1 | | 195 | hsa-miR-10a | 202915 | TMEM184A | transmembrane protein 184A | 1 | | 196 | hsa-miR-770-5p | 202915 | TMEM184A | transmembrane protein 184A | 1 | | 197 | hsa-miR-95 | 202915 | TMEM184A | transmembrane protein 184A | 1 | | 198 | hsa-miR-770-5p | 148103 | ZNF599 | zinc finger protein 599 | 1 | | 199 | hsa-miR-95 | 148103 | ZNF599 | zinc finger protein 599 | 1 | | 200 | hsa-mir-505 | 654429 | LRTM2 | leucine-rich repeats and transmembrane domains 2 | 1 | | 201 | hsa-mir-3130-1 | 654429 | LRTM2 | leucine-rich repeats and transmembrane domains 2 | 1 | | 202 | hsa-mir-3130-2 | 654429 | LRTM2 | leucine-rich repeats and transmembrane domains 2 | 1 | | 203 | hsa-mir-3130-3 | 654429 | LRTM2 | leucine-rich repeats and transmembrane domains 2 | 1 | | 204 | hsa-miR-10a | 654429 | LRTM2 | leucine-rich repeats and transmembrane domains 2 | 1 | | 205 | hsa-miR-128 | 654429 | LRTM2 | leucine-rich repeats and transmembrane domains 2 | 1 | | 206 | hsa-miR-770-5p | 654429 | LRTM2 | leucine-rich repeats and transmembrane domains 2 | 1 | | 207 | hsa-miR-95 | 654429 | LRTM2 | leucine-rich repeats and transmembrane domains 2 | 1 | | 208 | hsa-miR-1233 | 100131541 | UNQ6228 | hypothetical LOC100131541 | 1 | | 209 | hsa-miR-766 | 100131541 | UNQ6228 | hypothetical LOC100131541 | 1 | | 210 | hsa-miR-874 | 100131541 | UNQ6228 | hypothetical LOC100131541 | 1 | | 211 | hsa-miR-571 | 7582 | ZNF33B | zinc finger protein 33B | 1 | | 212 | hsa-miR-623 | 64224 | HERPUD2 | HERPUD family member 2 | 1 | | 213 | hsa-miR-1233 | 128611 | ZNF831 | zinc finger protein 831 | 1 | | 214 | hsa-miR-569 | 128611 | ZNF831 | zinc finger protein 831 | 1 | | 215 | hsa-miR-571 | 128611 | ZNF831 | zinc finger protein 831 | 1 | | 216 | hsa-miR-766 | 128611 | ZNF831 | zinc finger protein 831 | 1 | | 217 | hsa-miR-874 | 128611 | ZNF831 | zinc finger protein 831 | 1 | | 218 | hsa-mir-10a | 132241 | RPL32P3 | ribosomal protein L32 pseudogene 3 | 1 | | 219 | hsa-miR-591 | 132241 | RPL32P3 | ribosomal protein L32 pseudogene 3 | 1 | | 220 | hsa-miR-591 | 9813 | KIAA0494 | KIAA0494 | 1 | | 224 | hsa-mir-505 | 6553 | SLC9A5 | solute carrier family 9 (sodium/hydrogen exchanger), member 5 | 1 | | 225 | hsa-miR-10a | 6553 | SLC9A5 | solute carrier family 9 (sodium/hydrogen exchanger), member 5 | 1 | | 226 | hsa-miR-770-5p | 6553 | SLC9A5 | solute carrier family 9 (sodium/hydrogen exchanger), member 5 | 1 | | 227 | hsa-miR-95 | 6553 | SLC9A5 | solute carrier family 9 (sodium/hydrogen exchanger), member 5 | 1 | | 231 | hsa-mir-423 | 57188 | ADAMTSL3 | ADAMTS-like 3 | 1 | | 232 | hsa-mir-3130-1 | 57188 | ADAMTSL3 | ADAMTS-like 3 | 1 | | 233 | hsa-mir-3130-2 | 57188 | ADAMTSL3 | ADAMTS-like 3 | 1 | | 234 | hsa-mir-3130-3 | 57188 | ADAMTSL3 | ADAMTS-like 3 | 1 | | 235 | hsa-miR-10a | 57188 | ADAMTSL3 | ADAMTS-like 3 | 1 | | 236 | hsa-miR-770-5p | 57188 | ADAMTSL3 | ADAMTS-like 3 | 1 | | 237 | hsa-miR-95 | 57188 | ADAMTSL3 | ADAMTS-like 3 | 1 | | 238 | hsa-mir-505 | 285093 | C2orf85 | chromosome 2 open reading frame 85 | 1 | | 239 | hsa-miR-128 | 285093 | C2orf85 | chromosome 2 open reading frame 85 | 1 | | 240 | hsa-mir-3130-1 | 3909 | LAMA3 | laminin, alpha 3 | 1 | | 241 | hsa-mir-3130-2 | 3909 | LAMA3 | laminin, alpha 3 | 1 | | 242 | hsa-mir-3130-3 | 3909 | LAMA3 | laminin, alpha 3 | 1 | | 243 | hsa-miR-10a | 3909 | LAMA3 | laminin, alpha 3 | 1 | | 244 | hsa-miR-770-5p | 3909 | LAMA3 | laminin, alpha 3 | 1 | | 245 | hsa-miR-95 | 3909 | LAMA3 | laminin, alpha 3 | 1 | | 246 | hsa-mir-3130-1 | 390790 | ARL5C | ADP-ribosylation factor-like 5C | 1 | | 247 | hsa-mir-3130-2 | 390790 | ARL5C | ADP-ribosylation factor-like 5C | 1 | | 248 | hsa-mir-3130-3 | 390790 | ARL5C | ADP-ribosylation factor-like 5C | 1 | | 249 | hsa-mir-148b | 221122 | LOC221122 | hypothetical LOC221122 | 1 | | 250 | hsa-miR-10a | 221122 | LOC221122 | hypothetical LOC221122 | 1 | | 251 | hsa-miR-148b\* | 221122 | LOC221122 | hypothetical LOC221122 | 1 | | 252 | hsa-miR-770-5p | 221122 | LOC221122 | hypothetical LOC221122 | 1 | | 253 | hsa-miR-95 | 221122 | LOC221122 | hypothetical LOC221122 | 1 | | 255 | hsa-miR-571 | 1211 | CLTA | clathrin, light chain (Lca) | 1 | | 256 | hsa-miR-591 | 1211 | CLTA | clathrin, light chain (Lca) | 1 | | 257 | hsa-miR-10a | 100128198 | LOC100128198 | hypothetical protein LOC100128198 | 1 | | 258 | hsa-miR-770-5p | 100128198 | LOC100128198 | hypothetical protein LOC100128198 | 1 | | 259 | hsa-miR-95 | 100128198 | LOC100128198 | hypothetical protein LOC100128198 | 1 | | 261 | hsa-miR-95 | 150527 | LOC150527 | hypothetical LOC150527 | 1 | | 262 | hsa-miR-10a | 403150 | FLJ31356 | hypothetical protein FLJ31356 | 1 | | 263 | hsa-miR-770-5p | 403150 | FLJ31356 | hypothetical protein FLJ31356 | 1 | | 264 | hsa-miR-95 | 403150 | FLJ31356 | hypothetical protein FLJ31356 | 1 | | 265 | hsa-miR-10a | 284669 | LOC284669 | hypothetical protein LOC284669 | 1 | | 266 | hsa-mir-3130-1 | 348180 | CTU2 | cytosolic thiouridylase subunit 2 homolog (S. pombe) | 1 | | 267 | hsa-mir-3130-2 | 348180 | CTU2 | cytosolic thiouridylase subunit 2 homolog (S. pombe) | 1 | | 268 | hsa-mir-3130-3 | 348180 | CTU2 | cytosolic thiouridylase subunit 2 homolog (S. pombe) | 1 | | 269 | hsa-miR-10a | 348180 | CTU2 | cytosolic thiouridylase subunit 2 homolog (S. pombe) | 1 | | 272 | hsa-miR-770-5p | 160365 | CLECL1 | C-type lectin-like 1 | 1 | | 273 | hsa-miR-95 | 160365 | CLECL1 | C-type lectin-like 1 | 1 | | 274 | hsa-miR-10a | 646600 | C3orf65 | chromosome 3 open reading frame 65 | 1 | | 275 | hsa-miR-95 | 646600 | C3orf65 | chromosome 3 open reading frame 65 | 1 | | 276 | hsa-miR-770-5p | 2065 | ERBB3 | v-erb-b2 erythroblastic leukemia viral oncogene homolog 3 (avian) | 1 | | 277 | hsa-miR-95 | 2065 | ERBB3 | v-erb-b2 erythroblastic leukemia viral oncogene homolog 3 (avian) | 1 | | 278 | hsa-mir-423 | 79917 | MAGIX | MAGI family member, X-linked | 1 | | 282 | hsa-miR-1233 | 283130 | SLC25A45 | solute carrier family 25, member 45 | 1 | | 283 | hsa-miR-569 | 283130 | SLC25A45 | solute carrier family 25, member 45 | 1 | | 284 | hsa-miR-571 | 283130 | SLC25A45 | solute carrier family 25, member 45 | 1 | | 285 | hsa-miR-591 | 283130 | SLC25A45 | solute carrier family 25, member 45 | 1 | | 286 | hsa-miR-874 | 283130 | SLC25A45 | solute carrier family 25, member 45 | 1 | | 287 | hsa-miR-95 | 285463 | LOC285463 | hypothetical protein LOC285463 | 1 | | 288 | hsa-mir-3130-1 | 286359 | hCG\_2045089 | hypothetical LOC286359 | 1 | | 289 | hsa-mir-3130-2 | 286359 | hCG\_2045089 | hypothetical LOC286359 | 1 | | 290 | hsa-mir-3130-3 | 286359 | hCG\_2045089 | hypothetical LOC286359 | 1 | | 291 | hsa-miR-10a | 286359 | hCG\_2045089 | hypothetical LOC286359 | 1 | | 292 | hsa-miR-128 | 286359 | hCG\_2045089 | hypothetical LOC286359 | 1 | | 293 | hsa-miR-770-5p | 286359 | hCG\_2045089 | hypothetical LOC286359 | 1 | | 294 | hsa-miR-95 | 286359 | hCG\_2045089 | hypothetical LOC286359 | 1 | | 295 | hsa-miR-770-5p | 128876 | FAM83C | family with sequence similarity 83, member C | 1 | | 296 | hsa-miR-95 | 128876 | FAM83C | family with sequence similarity 83, member C | 1 | | 297 | hsa-miR-770-5p | 283875 | LOC283875 | hypothetical protein LOC283875 | 1 | | 298 | hsa-miR-95 | 283875 | LOC283875 | hypothetical protein LOC283875 | 1 | | 299 | hsa-miR-16 | 285857 | LOC285857 | hypothetical protein LOC285857 | 1 | | 300 | hsa-mir-3130-1 | 728073 | LOC728073 | hypothetical protein LOC728073 | 1 | | 301 | hsa-mir-3130-2 | 728073 | LOC728073 | hypothetical protein LOC728073 | 1 | | 302 | hsa-mir-3130-3 | 728073 | LOC728073 | hypothetical protein LOC728073 | 1 | | 303 | hsa-miR-10a | 728073 | LOC728073 | hypothetical protein LOC728073 | 1 | | 304 | hsa-miR-770-5p | 728073 | LOC728073 | hypothetical protein LOC728073 | 1 | | 305 | hsa-miR-95 | 728073 | LOC728073 | hypothetical protein LOC728073 | 1 | | 306 | hsa-miR-770-5p | 93986 | FOXP2 | forkhead box P2 | 1 | | 307 | hsa-miR-95 | 93986 | FOXP2 | forkhead box P2 | 1 | | 308 | hsa-miR-635 | 4257 | MGST1 | microsomal glutathione S-transferase 1 | 1 | | 309 | hsa-miR-1233 | 119016 | AGAP4 | ArfGAP with GTPase domain, ankyrin repeat and PH domain 4 | 1 | | 310 | hsa-miR-571 | 119016 | AGAP4 | ArfGAP with GTPase domain, ankyrin repeat and PH domain 4 | 1 | | 311 | hsa-miR-766 | 119016 | AGAP4 | ArfGAP with GTPase domain, ankyrin repeat and PH domain 4 | 1 | | 312 | hsa-miR-874 | 119016 | AGAP4 | ArfGAP with GTPase domain, ankyrin repeat and PH domain 4 | 1 | | 313 | hsa-mir-3130-1 | 221 | ALDH3B1 | aldehyde dehydrogenase 3 family, member B1 | 1 | | 314 | hsa-mir-3130-2 | 221 | ALDH3B1 | aldehyde dehydrogenase 3 family, member B1 | 1 | | 315 | hsa-mir-3130-3 | 221 | ALDH3B1 | aldehyde dehydrogenase 3 family, member B1 | 1 | | 318 | hsa-miR-10a | 149086 | LOC149086 | hypothetical protein LOC149086 | 1 | | 319 | hsa-miR-10a | 26188 | OR1C1 | olfactory receptor, family 1, subfamily C, member 1 | 1 | | 320 | hsa-miR-770-5p | 26188 | OR1C1 | olfactory receptor, family 1, subfamily C, member 1 | 1 | | 321 | hsa-miR-95 | 26188 | OR1C1 | olfactory receptor, family 1, subfamily C, member 1 | 1 | | 322 | hsa-mir-3130-1 | 26497 | OR10D3P | olfactory receptor, family 10, subfamily D, member 3 pseudogene | 1 | | 323 | hsa-mir-3130-2 | 26497 | OR10D3P | olfactory receptor, family 10, subfamily D, member 3 pseudogene | 1 | | 324 | hsa-mir-3130-3 | 26497 | OR10D3P | olfactory receptor, family 10, subfamily D, member 3 pseudogene | 1 | | 325 | hsa-miR-95 | 26497 | OR10D3P | olfactory receptor, family 10, subfamily D, member 3 pseudogene | 1 | | 326 | hsa-miR-10a | 26219 | OR1J4 | olfactory receptor, family 1, subfamily J, member 4 | 1 | | 327 | hsa-miR-770-5p | 26219 | OR1J4 | olfactory receptor, family 1, subfamily J, member 4 | 1 | | 328 | hsa-miR-95 | 26219 | OR1J4 | olfactory receptor, family 1, subfamily J, member 4 | 1 | | 329 | hsa-miR-770-5p | 26338 | OR5L2 | olfactory receptor, family 5, subfamily L, member 2 | 1 | | 330 | hsa-miR-95 | 26338 | OR5L2 | olfactory receptor, family 5, subfamily L, member 2 | 1 | | 331 | hsa-miR-10a | 972 | CD74 | CD74 molecule, major histocompatibility complex, class II invariant chain | 1 | | 333 | hsa-mir-885 | 84851 | TRIM52 | tripartite motif-containing 52 | 1 | | 334 | hsa-miR-1233 | 81550 | TDRD3 | tudor domain containing 3 | 1 | | 337 | hsa-miR-604 | 55209 | SETD5 | SET domain containing 5 | 1 | | 338 | hsa-miR-638 | 55209 | SETD5 | SET domain containing 5 | 1 | | 339 | hsa-miR-938 | 55209 | SETD5 | SET domain containing 5 | 1 | | 340 | hsa-miR-571 | 339559 | ZNF642 | zinc finger protein 642 | 1 | | 341 | hsa-miR-591 | 339559 | ZNF642 | zinc finger protein 642 | 1 | | 342 | hsa-miR-874 | 339559 | ZNF642 | zinc finger protein 642 | 1 | | 343 | hsa-miR-571 | 162993 | ZNF846 | zinc finger protein 846 | 1 | | 344 | hsa-miR-10a | 56917 | MEIS3 | Meis homeobox 3 | 1 | | 345 | hsa-miR-770-5p | 56917 | MEIS3 | Meis homeobox 3 | 1 | | 346 | hsa-miR-95 | 56917 | MEIS3 | Meis homeobox 3 | 1 | | 352 | hsa-mir-423 | 83733 | SLC25A18 | solute carrier family 25 (mitochondrial carrier), member 18 | 1 | | 353 | hsa-miR-10a | 83733 | SLC25A18 | solute carrier family 25 (mitochondrial carrier), member 18 | 1 | | 354 | hsa-miR-770-5p | 124989 | C17orf57 | chromosome 17 open reading frame 57 | 1 | | 355 | hsa-miR-95 | 124989 | C17orf57 | chromosome 17 open reading frame 57 | 1 | | 356 | hsa-mir-3130-1 | 374955 | SPATA21 | spermatogenesis associated 21 | 1 | | 357 | hsa-mir-3130-2 | 374955 | SPATA21 | spermatogenesis associated 21 | 1 | | 358 | hsa-mir-3130-3 | 374955 | SPATA21 | spermatogenesis associated 21 | 1 | | 359 | hsa-miR-10a | 374955 | SPATA21 | spermatogenesis associated 21 | 1 | | 363 | hsa-mir-505 | 374955 | SPATA21 | spermatogenesis associated 21 | 1 | | 364 | hsa-miR-623 | 8717 | TRADD | TNFRSF1A-associated via death domain | 1 | | 365 | hsa-miR-671-5p | 8717 | TRADD | TNFRSF1A-associated via death domain | 1 | | 366 | hsa-miR-623 | 11224 | RPL35 | ribosomal protein L35 | 1 | | 368 | hsa-miR-1233 | 4677 | NARS | asparaginyl-tRNA synthetase | 1 | | 369 | hsa-miR-874 | 4677 | NARS | asparaginyl-tRNA synthetase | 1 | | 370 | hsa-miR-766 | 6128 | RPL6 | ribosomal protein L6 | 1 | | 371 | hsa-miR-874 | 10657 | KHDRBS1 | KH domain containing, RNA binding, signal transduction associated 1 | 1 | | 372 | hsa-miR-1233 | 7705 | ZNF146 | zinc finger protein 146 | 1 | | 373 | hsa-miR-571 | 7705 | ZNF146 | zinc finger protein 146 | 1 | | 374 | hsa-miR-874 | 7705 | ZNF146 | zinc finger protein 146 | 1 | | 375 | hsa-miR-593\* | 4841 | NONO | non-POU domain containing, octamer-binding | 1 | | 376 | hsa-miR-1233 | 4869 | NPM1 | nucleophosmin (nucleolar phosphoprotein B23, numatrin) | 1 | | 377 | hsa-miR-874 | 4869 | NPM1 | nucleophosmin (nucleolar phosphoprotein B23, numatrin) | 1 | | 378 | hsa-miR-623 | 4670 | HNRNPM | heterogeneous nuclear ribonucleoprotein M | 1 | | 379 | hsa-miR-623 | 9045 | RPL14 | ribosomal protein L14 | 1 | | 383 | hsa-miR-623 | 3192 | HNRNPU | heterogeneous nuclear ribonucleoprotein U (scaffold attachment factor A) | 1 | | 384 | hsa-miR-1233 | 4691 | NCL | nucleolin | 1 | | 385 | hsa-miR-766 | 4691 | NCL | nucleolin | 1 | | 386 | hsa-miR-874 | 4691 | NCL | nucleolin | 1 | | 387 | hsa-miR-604 | 7534 | YWHAZ | tyrosine 3-monooxygenase/tryptophan 5-monooxygenase activation protein, zeta polypeptide | 1 | | 388 | hsa-miR-938 | 7534 | YWHAZ | tyrosine 3-monooxygenase/tryptophan 5-monooxygenase activation protein, zeta polypeptide | 1 | | 389 | hsa-miR-623 | 10399 | GNB2L1 | guanine nucleotide binding protein (G protein), beta polypeptide 2-like 1 | 1 | | 392 | hsa-miR-190 | 9804 | TOMM20 | translocase of outer mitochondrial membrane 20 homolog (yeast) | 1 | | 396 | hsa-miR-1233 | 57062 | DDX24 | DEAD (Asp-Glu-Ala-Asp) box polypeptide 24 | 1 | | 397 | hsa-miR-569 | 57062 | DDX24 | DEAD (Asp-Glu-Ala-Asp) box polypeptide 24 | 1 | | 398 | hsa-miR-571 | 57062 | DDX24 | DEAD (Asp-Glu-Ala-Asp) box polypeptide 24 | 1 | | 400 | hsa-miR-874 | 57062 | DDX24 | DEAD (Asp-Glu-Ala-Asp) box polypeptide 24 | 1 | | 401 | hsa-miR-604 | 2934 | GSN | gelsolin (amyloidosis, Finnish type) | 1 | | 402 | hsa-miR-635 | 2934 | GSN | gelsolin (amyloidosis, Finnish type) | 1 | | 403 | hsa-miR-938 | 2934 | GSN | gelsolin (amyloidosis, Finnish type) | 1 | | 406 | hsa-miR-765 | 1200 | TPP1 | tripeptidyl peptidase I | 1 | | 407 | hsa-miR-638 | 823 | CAPN1 | calpain 1, (mu/I) large subunit | 1 | | 408 | hsa-miR-636 | 6427 | SFRS2 | splicing factor, arginine/serine-rich 2 | 1 | | 409 | hsa-miR-604 | 1495 | CTNNA1 | catenin (cadherin-associated protein), alpha 1, 102kDa | 1 | | 410 | hsa-miR-938 | 1495 | CTNNA1 | catenin (cadherin-associated protein), alpha 1, 102kDa | 1 | | 411 | hsa-miR-135a | 1509 | CTSD | cathepsin D | 1 | | 412 | hsa-miR-635 | 1509 | CTSD | cathepsin D | 1 | | 413 | hsa-miR-638 | 1509 | CTSD | cathepsin D | 1 | | 414 | hsa-miR-765 | 1509 | CTSD | cathepsin D | 1 | | 415 | hsa-miR-623 | 5757 | PTMA | prothymosin, alpha | 1 | | 420 | hsa-miR-593\* | 6209 | RPS15 | ribosomal protein S15 | 1 | | 422 | hsa-miR-1233 | 10575 | CCT4 | chaperonin containing TCP1, subunit 4 (delta) | 1 | | 423 | hsa-miR-1233 | 1974 | EIF4A2 | eukaryotic translation initiation factor 4A2 | 1 | | 424 | hsa-miR-874 | 1974 | EIF4A2 | eukaryotic translation initiation factor 4A2 | 1 | | 425 | hsa-miR-623 | 5496 | PPM1G | protein phosphatase 1G (formerly 2C), magnesium-dependent, gamma isoform | 1 | | 426 | hsa-miR-627 | 5496 | PPM1G | protein phosphatase 1G (formerly 2C), magnesium-dependent, gamma isoform | 1 | | 428 | hsa-miR-617 | 6224 | RPS20 | ribosomal protein S20 | 1 | | 429 | hsa-miR-1233 | 894 | CCND2 | cyclin D2 | 1 | | 430 | hsa-miR-569 | 894 | CCND2 | cyclin D2 | 1 | | 431 | hsa-miR-766 | 894 | CCND2 | cyclin D2 | 1 | | 434 | hsa-miR-593\* | 6748 | SSR4 | signal sequence receptor, delta (translocon-associated protein delta) | 1 | | 435 | hsa-miR-623 | 6748 | SSR4 | signal sequence receptor, delta (translocon-associated protein delta) | 1 | | 436 | hsa-miR-1233 | 1964 | EIF1AX | eukaryotic translation initiation factor 1A, X-linked | 1 | | 437 | hsa-miR-571 | 1964 | EIF1AX | eukaryotic translation initiation factor 1A, X-linked | 1 | | 438 | hsa-miR-591 | 1964 | EIF1AX | eukaryotic translation initiation factor 1A, X-linked | 1 | | 439 | hsa-miR-766 | 1964 | EIF1AX | eukaryotic translation initiation factor 1A, X-linked | 1 | | 441 | hsa-miR-569 | 3945 | LDHB | lactate dehydrogenase B | 1 | | 445 | hsa-miR-593\* | 4809 | NHP2L1 | NHP2 non-histone chromosome protein 2-like 1 (S. cerevisiae) | 1 | | 446 | hsa-miR-766 | 4809 | NHP2L1 | NHP2 non-histone chromosome protein 2-like 1 (S. cerevisiae) | 1 | | 449 | hsa-miR-765 | 5829 | PXN | paxillin | 1 | | 451 | hsa-miR-1233 | 1892 | ECHS1 | enoyl Coenzyme A hydratase, short chain, 1, mitochondrial | 1 | | 452 | hsa-miR-766 | 1892 | ECHS1 | enoyl Coenzyme A hydratase, short chain, 1, mitochondrial | 1 | | 453 | hsa-miR-1233 | 6741 | SSB | Sjogren syndrome antigen B (autoantigen La) | 1 | | 454 | hsa-miR-766 | 6741 | SSB | Sjogren syndrome antigen B (autoantigen La) | 1 | | 457 | hsa-miR-593\* | 10726 | NUDC | nuclear distribution gene C homolog (A. nidulans) | 1 | | 459 | hsa-miR-1233 | 10054 | UBA2 | ubiquitin-like modifier activating enzyme 2 | 1 | | 460 | hsa-miR-571 | 10054 | UBA2 | ubiquitin-like modifier activating enzyme 2 | 1 | | 461 | hsa-miR-638 | 6238 | RRBP1 | ribosome binding protein 1 homolog 180kDa (dog) | 1 | | 462 | hsa-mir-10a | 5510 | PPP1R7 | protein phosphatase 1, regulatory (inhibitor) subunit 7 | 1 | | 466 | hsa-miR-766 | 1653 | DDX1 | DEAD (Asp-Glu-Ala-Asp) box polypeptide 1 | 1 | | 467 | hsa-miR-1233 | 231 | AKR1B1 | aldo-keto reductase family 1, member B1 (aldose reductase) | 1 | | 468 | hsa-miR-766 | 231 | AKR1B1 | aldo-keto reductase family 1, member B1 (aldose reductase) | 1 | | 472 | hsa-miR-593\* | 79073 | TMEM109 | transmembrane protein 109 | 1 | | 473 | hsa-miR-636 | 1665 | DHX15 | DEAH (Asp-Glu-Ala-His) box polypeptide 15 | 1 | | 474 | hsa-miR-638 | 26020 | LRP10 | low density lipoprotein receptor-related protein 10 | 1 | | 475 | hsa-miR-765 | 26020 | LRP10 | low density lipoprotein receptor-related protein 10 | 1 | | 476 | hsa-miR-576-5p | 7072 | TIA1 | TIA1 cytotoxic granule-associated RNA binding protein | 1 | | 477 | hsa-miR-591 | 7072 | TIA1 | TIA1 cytotoxic granule-associated RNA binding protein | 1 | | 479 | hsa-miR-765 | 9261 | MAPKAPK2 | mitogen-activated protein kinase-activated protein kinase 2 | 1 | | 480 | hsa-miR-604 | 9261 | MAPKAPK2 | mitogen-activated protein kinase-activated protein kinase 2 | 1 | | 481 | hsa-miR-938 | 9261 | MAPKAPK2 | mitogen-activated protein kinase-activated protein kinase 2 | 1 | | 482 | hsa-miR-454\* | 6240 | RRM1 | ribonucleotide reductase M1 | 1 | | 483 | hsa-miR-1233 | 22916 | NCBP2 | nuclear cap binding protein subunit 2, 20kDa | 1 | | 484 | hsa-miR-569 | 22916 | NCBP2 | nuclear cap binding protein subunit 2, 20kDa | 1 | | 485 | hsa-miR-571 | 22916 | NCBP2 | nuclear cap binding protein subunit 2, 20kDa | 1 | | 486 | hsa-miR-766 | 22916 | NCBP2 | nuclear cap binding protein subunit 2, 20kDa | 1 | | 487 | hsa-miR-874 | 22916 | NCBP2 | nuclear cap binding protein subunit 2, 20kDa | 1 | | 488 | hsa-miR-635 | 4659 | PPP1R12A | protein phosphatase 1, regulatory (inhibitor) subunit 12A | 1 | | 489 | hsa-miR-638 | 1265 | CNN2 | calponin 2 | 1 | | 490 | hsa-miR-765 | 1265 | CNN2 | calponin 2 | 1 | | 492 | hsa-miR-454\* | 9097 | USP14 | ubiquitin specific peptidase 14 (tRNA-guanine transglycosylase) | 1 | | 493 | hsa-miR-1233 | 9512 | PMPCB | peptidase (mitochondrial processing) beta | 1 | | 494 | hsa-miR-571 | 9512 | PMPCB | peptidase (mitochondrial processing) beta | 1 | | 495 | hsa-miR-591 | 9512 | PMPCB | peptidase (mitochondrial processing) beta | 1 | | 496 | hsa-miR-604 | 6642 | SNX1 | sorting nexin 1 | 1 | | 497 | hsa-miR-627 | 6642 | SNX1 | sorting nexin 1 | 1 | | 498 | hsa-miR-638 | 6642 | SNX1 | sorting nexin 1 | 1 | | 499 | hsa-miR-765 | 6642 | SNX1 | sorting nexin 1 | 1 | | 500 | hsa-miR-938 | 6642 | SNX1 | sorting nexin 1 | 1 | | 501 | hsa-miR-638 | 7805 | LAPTM5 | lysosomal protein transmembrane 5 | 1 | | 502 | hsa-miR-635 | 9685 | CLINT1 | clathrin interactor 1 | 1 | | 503 | hsa-miR-576-5p | 23394 | ADNP | activity-dependent neuroprotector homeobox | 1 | | 504 | hsa-miR-766 | 5431 | POLR2B | polymerase (RNA) II (DNA directed) polypeptide B, 140kDa | 1 | | 505 | hsa-miR-576-5p | 2631 | GBAS | glioblastoma amplified sequence | 1 | | 506 | hsa-miR-591 | 2631 | GBAS | glioblastoma amplified sequence | 1 | | 508 | hsa-miR-571 | 6059 | ABCE1 | ATP-binding cassette, sub-family E (OABP), member 1 | 1 | | 509 | hsa-miR-766 | 6059 | ABCE1 | ATP-binding cassette, sub-family E (OABP), member 1 | 1 | | 510 | hsa-miR-576-5p | 5527 | PPP2R5C | protein phosphatase 2, regulatory subunit B', gamma isoform | 1 | | 511 | hsa-miR-591 | 11231 | SEC63 | SEC63 homolog (S. cerevisiae) | 1 | | 513 | hsa-miR-1233 | 10412 | NSA2 | NSA2 ribosome biogenesis homolog (S. cerevisiae) | 1 | | 514 | hsa-miR-766 | 10412 | NSA2 | NSA2 ribosome biogenesis homolog (S. cerevisiae) | 1 | | 515 | hsa-miR-874 | 10412 | NSA2 | NSA2 ribosome biogenesis homolog (S. cerevisiae) | 1 | | 516 | hsa-miR-635 | 8672 | EIF4G3 | eukaryotic translation initiation factor 4 gamma, 3 | 1 | | 519 | hsa-miR-571 | 23077 | MYCBP2 | MYC binding protein 2 | 1 | | 520 | hsa-miR-874 | 23077 | MYCBP2 | MYC binding protein 2 | 1 | | 523 | hsa-mir-10a | 10314 | LANCL1 | LanC lantibiotic synthetase component C-like 1 (bacterial) | 1 | | 524 | hsa-miR-1233 | 10314 | LANCL1 | LanC lantibiotic synthetase component C-like 1 (bacterial) | 1 | | 525 | hsa-miR-569 | 10314 | LANCL1 | LanC lantibiotic synthetase component C-like 1 (bacterial) | 1 | | 526 | hsa-miR-571 | 10314 | LANCL1 | LanC lantibiotic synthetase component C-like 1 (bacterial) | 1 | | 527 | hsa-miR-591 | 10314 | LANCL1 | LanC lantibiotic synthetase component C-like 1 (bacterial) | 1 | | 528 | hsa-miR-766 | 10314 | LANCL1 | LanC lantibiotic synthetase component C-like 1 (bacterial) | 1 | | 529 | hsa-miR-874 | 10314 | LANCL1 | LanC lantibiotic synthetase component C-like 1 (bacterial) | 1 | | 530 | hsa-miR-454\* | 10920 | COPS8 | COP9 constitutive photomorphogenic homolog subunit 8 (Arabidopsis) | 1 | | 531 | hsa-miR-591 | 10920 | COPS8 | COP9 constitutive photomorphogenic homolog subunit 8 (Arabidopsis) | 1 | | 533 | hsa-miR-571 | 158 | ADSL | adenylosuccinate lyase | 1 | | 534 | hsa-miR-874 | 158 | ADSL | adenylosuccinate lyase | 1 | | 535 | hsa-mir-10a | 55746 | NUP133 | nucleoporin 133kDa | 1 | | 536 | hsa-miR-1233 | 55746 | NUP133 | nucleoporin 133kDa | 1 | | 537 | hsa-miR-571 | 55746 | NUP133 | nucleoporin 133kDa | 1 | | 538 | hsa-miR-591 | 55746 | NUP133 | nucleoporin 133kDa | 1 | | 539 | hsa-miR-1233 | 9688 | NUP93 | nucleoporin 93kDa | 1 | | 540 | hsa-miR-766 | 9688 | NUP93 | nucleoporin 93kDa | 1 | | 541 | hsa-mir-505 | 1674 | DES | desmin | 1 | | 542 | hsa-mir-454 | 1674 | DES | desmin | 1 | | 543 | hsa-miR-128 | 1674 | DES | desmin | 1 | | 544 | hsa-miR-301a | 1674 | DES | desmin | 1 | | 545 | hsa-miR-770-5p | 1674 | DES | desmin | 1 | | 546 | hsa-miR-95 | 1674 | DES | desmin | 1 | | 547 | hsa-miR-571 | 10523 | CHERP | calcium homeostasis endoplasmic reticulum protein | 1 | | 548 | hsa-miR-770-5p | 4837 | NNMT | nicotinamide N-methyltransferase | 1 | | 549 | hsa-miR-95 | 4837 | NNMT | nicotinamide N-methyltransferase | 1 | | 550 | hsa-miR-766 | 50717 | DCAF8 | DDB1 and CUL4 associated factor 8 | 1 | | 551 | hsa-miR-934 | 50717 | DCAF8 | DDB1 and CUL4 associated factor 8 | 1 | | 552 | hsa-miR-604 | 2539 | G6PD | glucose-6-phosphate dehydrogenase | 1 | | 553 | hsa-miR-635 | 2539 | G6PD | glucose-6-phosphate dehydrogenase | 1 | | 554 | hsa-miR-638 | 2539 | G6PD | glucose-6-phosphate dehydrogenase | 1 | | 555 | hsa-miR-765 | 2539 | G6PD | glucose-6-phosphate dehydrogenase | 1 | | 556 | hsa-miR-938 | 2539 | G6PD | glucose-6-phosphate dehydrogenase | 1 | | 557 | hsa-miR-604 | 1861 | TOR1A | torsin family 1, member A (torsin A) | 1 | | 558 | hsa-miR-635 | 1861 | TOR1A | torsin family 1, member A (torsin A) | 1 | | 559 | hsa-miR-638 | 1861 | TOR1A | torsin family 1, member A (torsin A) | 1 | | 560 | hsa-miR-938 | 1861 | TOR1A | torsin family 1, member A (torsin A) | 1 | | 561 | hsa-mir-10a | 10915 | TCERG1 | transcription elongation regulator 1 | 1 | | 563 | hsa-miR-571 | 10915 | TCERG1 | transcription elongation regulator 1 | 1 | | 564 | hsa-miR-591 | 10915 | TCERG1 | transcription elongation regulator 1 | 1 | | 566 | hsa-miR-874 | 10915 | TCERG1 | transcription elongation regulator 1 | 1 | | 567 | hsa-miR-593\* | 26121 | PRPF31 | PRP31 pre-mRNA processing factor 31 homolog (S. cerevisiae) | 1 | | 568 | hsa-miR-627 | 10014 | HDAC5 | histone deacetylase 5 | 1 | | 569 | hsa-miR-638 | 10014 | HDAC5 | histone deacetylase 5 | 1 | | 570 | hsa-miR-671-5p | 10014 | HDAC5 | histone deacetylase 5 | 1 | | 572 | hsa-miR-1233 | 11044 | PAPD7 | PAP associated domain containing 7 | 1 | | 573 | hsa-miR-569 | 11044 | PAPD7 | PAP associated domain containing 7 | 1 | | 574 | hsa-miR-571 | 11044 | PAPD7 | PAP associated domain containing 7 | 1 | | 575 | hsa-miR-766 | 11044 | PAPD7 | PAP associated domain containing 7 | 1 | | 576 | hsa-miR-874 | 11044 | PAPD7 | PAP associated domain containing 7 | 1 | | 577 | hsa-miR-591 | 11052 | CPSF6 | cleavage and polyadenylation specific factor 6, 68kDa | 1 | | 579 | hsa-miR-569 | 28951 | TRIB2 | tribbles homolog 2 (Drosophila) | 1 | | 581 | hsa-miR-623 | 23644 | EDC4 | enhancer of mRNA decapping 4 | 1 | | 582 | hsa-mir-149 | 9474 | ATG5 | ATG5 autophagy related 5 homolog (S. cerevisiae) | 1 | | 583 | hsa-miR-576-5p | 9474 | ATG5 | ATG5 autophagy related 5 homolog (S. cerevisiae) | 1 | | 584 | hsa-miR-591 | 9474 | ATG5 | ATG5 autophagy related 5 homolog (S. cerevisiae) | 1 | | 585 | hsa-miR-591 | 9255 | AIMP1 | aminoacyl tRNA synthetase complex-interacting multifunctional protein 1 | 1 | | 586 | hsa-miR-604 | 5580 | PRKCD | protein kinase C, delta | 1 | | 587 | hsa-miR-635 | 5580 | PRKCD | protein kinase C, delta | 1 | | 588 | hsa-miR-638 | 5580 | PRKCD | protein kinase C, delta | 1 | | 589 | hsa-miR-938 | 5580 | PRKCD | protein kinase C, delta | 1 | | 590 | hsa-mir-10a | 8658 | TNKS | tankyrase, TRF1-interacting ankyrin-related ADP-ribose polymerase | 1 | | 591 | hsa-miR-1233 | 8658 | TNKS | tankyrase, TRF1-interacting ankyrin-related ADP-ribose polymerase | 1 | | 592 | hsa-miR-190 | 8658 | TNKS | tankyrase, TRF1-interacting ankyrin-related ADP-ribose polymerase | 1 | | 593 | hsa-miR-571 | 8658 | TNKS | tankyrase, TRF1-interacting ankyrin-related ADP-ribose polymerase | 1 | | 594 | hsa-miR-766 | 8658 | TNKS | tankyrase, TRF1-interacting ankyrin-related ADP-ribose polymerase | 1 | | 595 | hsa-miR-874 | 8658 | TNKS | tankyrase, TRF1-interacting ankyrin-related ADP-ribose polymerase | 1 | | 596 | hsa-miR-95 | 11161 | C14orf1 | chromosome 14 open reading frame 1 | 1 | | 597 | hsa-miR-636 | 6742 | SSBP1 | single-stranded DNA binding protein 1 | 1 | | 598 | hsa-mir-10a | 9282 | MED14 | mediator complex subunit 14 | 1 | | 599 | hsa-mir-885 | 9282 | MED14 | mediator complex subunit 14 | 1 | | 600 | hsa-miR-591 | 9282 | MED14 | mediator complex subunit 14 | 1 | | 602 | hsa-miR-576-5p | 8813 | DPM1 | dolichyl-phosphate mannosyltransferase polypeptide 1, catalytic subunit | 1 | | 603 | hsa-miR-593\* | 1327 | COX4I1 | cytochrome c oxidase subunit IV isoform 1 | 1 | | 604 | hsa-miR-623 | 1327 | COX4I1 | cytochrome c oxidase subunit IV isoform 1 | 1 | | 605 | hsa-miR-1233 | 7372 | UMPS | uridine monophosphate synthetase | 1 | | 606 | hsa-miR-1233 | 2308 | FOXO1 | forkhead box O1 | 1 | | 607 | hsa-miR-571 | 2308 | FOXO1 | forkhead box O1 | 1 | | 608 | hsa-miR-874 | 2308 | FOXO1 | forkhead box O1 | 1 | | 609 | hsa-miR-569 | 27250 | PDCD4 | programmed cell death 4 (neoplastic transformation inhibitor) | 1 | | 610 | hsa-miR-766 | 27250 | PDCD4 | programmed cell death 4 (neoplastic transformation inhibitor) | 1 | | 611 | hsa-miR-593\* | 9780 | FAM38A | family with sequence similarity 38, member A | 1 | | 612 | hsa-miR-623 | 9780 | FAM38A | family with sequence similarity 38, member A | 1 | | 614 | hsa-miR-765 | 5566 | PRKACA | protein kinase, cAMP-dependent, catalytic, alpha | 1 | | 615 | hsa-miR-638 | 4817 | NIT1 | nitrilase 1 | 1 | | 616 | hsa-miR-765 | 4817 | NIT1 | nitrilase 1 | 1 | | 617 | hsa-mir-10a | 8697 | CDC23 | cell division cycle 23 homolog (S. cerevisiae) | 1 | | 618 | hsa-miR-1233 | 8697 | CDC23 | cell division cycle 23 homolog (S. cerevisiae) | 1 | | 619 | hsa-miR-454\* | 8697 | CDC23 | cell division cycle 23 homolog (S. cerevisiae) | 1 | | 620 | hsa-miR-571 | 8697 | CDC23 | cell division cycle 23 homolog (S. cerevisiae) | 1 | | 621 | hsa-miR-591 | 8697 | CDC23 | cell division cycle 23 homolog (S. cerevisiae) | 1 | | 622 | hsa-miR-623 | 6428 | SFRS3 | splicing factor, arginine/serine-rich 3 | 1 | | 623 | hsa-miR-571 | 23658 | LSM5 | LSM5 homolog, U6 small nuclear RNA associated (S. cerevisiae) | 1 | | 624 | hsa-miR-591 | 23658 | LSM5 | LSM5 homolog, U6 small nuclear RNA associated (S. cerevisiae) | 1 | | 625 | hsa-mir-10a | 2729 | GCLC | glutamate-cysteine ligase, catalytic subunit | 1 | | 631 | hsa-miR-617 | 8445 | DYRK2 | dual-specificity tyrosine-(Y)-phosphorylation regulated kinase 2 | 1 | | 632 | hsa-miR-10a | 58487 | CREBZF | CREB/ATF bZIP transcription factor | 1 | | 633 | hsa-miR-770-5p | 58487 | CREBZF | CREB/ATF bZIP transcription factor | 1 | | 634 | hsa-miR-95 | 58487 | CREBZF | CREB/ATF bZIP transcription factor | 1 | | 636 | hsa-miR-569 | 58487 | CREBZF | CREB/ATF bZIP transcription factor | 1 | | 638 | hsa-mir-10a | 58487 | CREBZF | CREB/ATF bZIP transcription factor | 1 | | 640 | hsa-miR-934 | 58487 | CREBZF | CREB/ATF bZIP transcription factor | 1 | | 642 | hsa-miR-766 | 9529 | BAG5 | BCL2-associated athanogene 5 | 1 | | 643 | hsa-miR-934 | 9529 | BAG5 | BCL2-associated athanogene 5 | 1 | | 644 | hsa-miR-576-5p | 8453 | CUL2 | cullin 2 | 1 | | 645 | hsa-miR-1233 | 9790 | BMS1 | BMS1 homolog, ribosome assembly protein (yeast) | 1 | | 646 | hsa-miR-766 | 9790 | BMS1 | BMS1 homolog, ribosome assembly protein (yeast) | 1 | | 647 | hsa-miR-593\* | 27339 | PRPF19 | PRP19/PSO4 pre-mRNA processing factor 19 homolog (S. cerevisiae) | 1 | | 648 | hsa-miR-623 | 27339 | PRPF19 | PRP19/PSO4 pre-mRNA processing factor 19 homolog (S. cerevisiae) | 1 | | 652 | hsa-miR-765 | 2185 | PTK2B | PTK2B protein tyrosine kinase 2 beta | 1 | | 654 | hsa-miR-135a | 2185 | PTK2B | PTK2B protein tyrosine kinase 2 beta | 1 | | 655 | hsa-miR-1233 | 9924 | PAN2 | PAN2 poly(A) specific ribonuclease subunit homolog (S. cerevisiae) | 1 | | 656 | hsa-miR-766 | 9924 | PAN2 | PAN2 poly(A) specific ribonuclease subunit homolog (S. cerevisiae) | 1 | | 657 | hsa-mir-10a | 11215 | AKAP11 | A kinase (PRKA) anchor protein 11 | 1 | | 658 | hsa-miR-1233 | 11215 | AKAP11 | A kinase (PRKA) anchor protein 11 | 1 | | 659 | hsa-miR-190 | 11215 | AKAP11 | A kinase (PRKA) anchor protein 11 | 1 | | 660 | hsa-miR-766 | 11215 | AKAP11 | A kinase (PRKA) anchor protein 11 | 1 | | 661 | hsa-miR-934 | 11215 | AKAP11 | A kinase (PRKA) anchor protein 11 | 1 | | 662 | hsa-miR-591 | 6302 | TSPAN31 | tetraspanin 31 | 1 | | 663 | hsa-miR-635 | 7378 | UPP1 | uridine phosphorylase 1 | 1 | | 664 | hsa-miR-1233 | 23229 | ARHGEF9 | Cdc42 guanine nucleotide exchange factor (GEF) 9 | 1 | | 665 | hsa-miR-571 | 23229 | ARHGEF9 | Cdc42 guanine nucleotide exchange factor (GEF) 9 | 1 | | 666 | hsa-miR-766 | 23229 | ARHGEF9 | Cdc42 guanine nucleotide exchange factor (GEF) 9 | 1 | | 667 | hsa-miR-627 | 7318 | UBA7 | ubiquitin-like modifier activating enzyme 7 | 1 | | 668 | hsa-miR-671-5p | 7318 | UBA7 | ubiquitin-like modifier activating enzyme 7 | 1 | | 669 | hsa-miR-627 | 23550 | PSD4 | pleckstrin and Sec7 domain containing 4 | 1 | | 670 | hsa-miR-638 | 23550 | PSD4 | pleckstrin and Sec7 domain containing 4 | 1 | | 671 | hsa-miR-671-5p | 23550 | PSD4 | pleckstrin and Sec7 domain containing 4 | 1 | | 672 | hsa-miR-135a | 9776 | KIAA0652 | KIAA0652 | 1 | | 673 | hsa-miR-638 | 9776 | KIAA0652 | KIAA0652 | 1 | | 674 | hsa-miR-135a | 6195 | RPS6KA1 | ribosomal protein S6 kinase, 90kDa, polypeptide 1 | 1 | | 675 | hsa-miR-604 | 6195 | RPS6KA1 | ribosomal protein S6 kinase, 90kDa, polypeptide 1 | 1 | | 676 | hsa-miR-627 | 6195 | RPS6KA1 | ribosomal protein S6 kinase, 90kDa, polypeptide 1 | 1 | | 677 | hsa-miR-638 | 6195 | RPS6KA1 | ribosomal protein S6 kinase, 90kDa, polypeptide 1 | 1 | | 678 | hsa-miR-938 | 6195 | RPS6KA1 | ribosomal protein S6 kinase, 90kDa, polypeptide 1 | 1 | | 679 | hsa-miR-1233 | 6430 | SFRS5 | splicing factor, arginine/serine-rich 5 | 1 | | 680 | hsa-miR-874 | 6430 | SFRS5 | splicing factor, arginine/serine-rich 5 | 1 | | 681 | hsa-miR-604 | 409 | ARRB2 | arrestin, beta 2 | 1 | | 682 | hsa-miR-627 | 409 | ARRB2 | arrestin, beta 2 | 1 | | 683 | hsa-miR-638 | 409 | ARRB2 | arrestin, beta 2 | 1 | | 684 | hsa-miR-765 | 409 | ARRB2 | arrestin, beta 2 | 1 | | 685 | hsa-miR-938 | 409 | ARRB2 | arrestin, beta 2 | 1 | | 686 | hsa-miR-1233 | 10947 | AP3M2 | adaptor-related protein complex 3, mu 2 subunit | 1 | | 687 | hsa-miR-591 | 10947 | AP3M2 | adaptor-related protein complex 3, mu 2 subunit | 1 | | 688 | hsa-miR-874 | 10947 | AP3M2 | adaptor-related protein complex 3, mu 2 subunit | 1 | | 689 | hsa-miR-623 | 10016 | PDCD6 | programmed cell death 6 | 1 | | 690 | hsa-mir-199a-2 | 51430 | C1orf9 | chromosome 1 open reading frame 9 | 1 | | 691 | hsa-mir-214 | 51430 | C1orf9 | chromosome 1 open reading frame 9 | 1 | | 692 | hsa-miR-199a-5p | 51430 | C1orf9 | chromosome 1 open reading frame 9 | 1 | | 693 | hsa-miR-576-5p | 51430 | C1orf9 | chromosome 1 open reading frame 9 | 1 | | 694 | hsa-miR-591 | 51430 | C1orf9 | chromosome 1 open reading frame 9 | 1 | | 697 | hsa-miR-638 | 9968 | MED12 | mediator complex subunit 12 | 1 | | 699 | hsa-miR-454\* | 79053 | ALG8 | asparagine-linked glycosylation 8, alpha-1,3-glucosyltransferase homolog (S. cerevisiae) | 1 | | 700 | hsa-miR-1233 | 11183 | MAP4K5 | mitogen-activated protein kinase kinase kinase kinase 5 | 1 | | 701 | hsa-miR-569 | 11183 | MAP4K5 | mitogen-activated protein kinase kinase kinase kinase 5 | 1 | | 702 | hsa-miR-766 | 11183 | MAP4K5 | mitogen-activated protein kinase kinase kinase kinase 5 | 1 | | 705 | hsa-miR-1233 | 9057 | SLC7A6 | solute carrier family 7 (cationic amino acid transporter, y+ system), member 6 | 1 | | 706 | hsa-miR-569 | 9057 | SLC7A6 | solute carrier family 7 (cationic amino acid transporter, y+ system), member 6 | 1 | | 707 | hsa-miR-571 | 9057 | SLC7A6 | solute carrier family 7 (cationic amino acid transporter, y+ system), member 6 | 1 | | 708 | hsa-miR-766 | 9057 | SLC7A6 | solute carrier family 7 (cationic amino acid transporter, y+ system), member 6 | 1 | | 709 | hsa-miR-874 | 9057 | SLC7A6 | solute carrier family 7 (cationic amino acid transporter, y+ system), member 6 | 1 | | 710 | hsa-miR-623 | 4123 | MAN2C1 | mannosidase, alpha, class 2C, member 1 | 1 | | 711 | hsa-miR-627 | 4123 | MAN2C1 | mannosidase, alpha, class 2C, member 1 | 1 | | 712 | hsa-miR-1233 | 22909 | MTMR15 | myotubularin related protein 15 | 1 | | 713 | hsa-miR-874 | 22909 | MTMR15 | myotubularin related protein 15 | 1 | | 714 | hsa-miR-1233 | 596 | BCL2 | B-cell CLL/lymphoma 2 | 1 | | 715 | hsa-miR-569 | 596 | BCL2 | B-cell CLL/lymphoma 2 | 1 | | 716 | hsa-miR-766 | 596 | BCL2 | B-cell CLL/lymphoma 2 | 1 | | 717 | hsa-miR-874 | 596 | BCL2 | B-cell CLL/lymphoma 2 | 1 | | 718 | hsa-miR-454\* | 5311 | PKD2 | polycystic kidney disease 2 (autosomal dominant) | 1 | | 720 | hsa-miR-1233 | 10127 | ZNF263 | zinc finger protein 263 | 1 | | 721 | hsa-miR-766 | 10127 | ZNF263 | zinc finger protein 263 | 1 | | 722 | hsa-miR-591 | 51096 | UTP18 | UTP18, small subunit (SSU) processome component, homolog (yeast) | 1 | | 723 | hsa-miR-623 | 3707 | ITPKB | inositol 1,4,5-trisphosphate 3-kinase B | 1 | | 724 | hsa-miR-593\* | 23082 | PPRC1 | peroxisome proliferator-activated receptor gamma, coactivator-related 1 | 1 | | 725 | hsa-miR-766 | 23082 | PPRC1 | peroxisome proliferator-activated receptor gamma, coactivator-related 1 | 1 | | 727 | hsa-mir-3130-1 | 5914 | RARA | retinoic acid receptor, alpha | 1 | | 728 | hsa-mir-3130-2 | 5914 | RARA | retinoic acid receptor, alpha | 1 | | 729 | hsa-mir-3130-3 | 5914 | RARA | retinoic acid receptor, alpha | 1 | | 730 | hsa-miR-95 | 5914 | RARA | retinoic acid receptor, alpha | 1 | | 732 | hsa-miR-569 | 4548 | MTR | 5-methyltetrahydrofolate-homocysteine methyltransferase | 1 | | 733 | hsa-miR-571 | 4548 | MTR | 5-methyltetrahydrofolate-homocysteine methyltransferase | 1 | | 735 | hsa-miR-766 | 4548 | MTR | 5-methyltetrahydrofolate-homocysteine methyltransferase | 1 | | 737 | hsa-miR-591 | 10165 | SLC25A13 | solute carrier family 25, member 13 (citrin) | 1 | | 738 | hsa-miR-604 | 3732 | CD82 | CD82 molecule | 1 | | 739 | hsa-miR-635 | 3732 | CD82 | CD82 molecule | 1 | | 740 | hsa-miR-938 | 3732 | CD82 | CD82 molecule | 1 | | 742 | hsa-miR-1233 | 3109 | HLA-DMB | major histocompatibility complex, class II, DM beta | 1 | | 743 | hsa-miR-617 | 3109 | HLA-DMB | major histocompatibility complex, class II, DM beta | 1 | | 744 | hsa-miR-766 | 3109 | HLA-DMB | major histocompatibility complex, class II, DM beta | 1 | | 745 | hsa-miR-874 | 3109 | HLA-DMB | major histocompatibility complex, class II, DM beta | 1 | | 746 | hsa-miR-1233 | 22880 | MORC2 | MORC family CW-type zinc finger 2 | 1 | | 748 | hsa-miR-874 | 22880 | MORC2 | MORC family CW-type zinc finger 2 | 1 | | 750 | hsa-miR-627 | 6901 | TAZ | tafazzin | 1 | | 752 | hsa-miR-671-5p | 6901 | TAZ | tafazzin | 1 | | 753 | hsa-miR-571 | 9338 | TCEAL1 | transcription elongation factor A (SII)-like 1 | 1 | | 754 | hsa-mir-10a | 1203 | CLN5 | ceroid-lipofuscinosis, neuronal 5 | 1 | | 755 | hsa-miR-576-5p | 1203 | CLN5 | ceroid-lipofuscinosis, neuronal 5 | 1 | | 756 | hsa-mir-10a | 9819 | TSC22D2 | TSC22 domain family, member 2 | 1 | | 757 | hsa-miR-934 | 9819 | TSC22D2 | TSC22 domain family, member 2 | 1 | | 758 | hsa-miR-591 | 51634 | RBMX2 | RNA binding motif protein, X-linked 2 | 1 | | 759 | hsa-miR-623 | 1938 | EEF2 | eukaryotic translation elongation factor 2 | 1 | | 760 | hsa-miR-593\* | 4242 | MFNG | MFNG O-fucosylpeptide 3-beta-N-acetylglucosaminyltransferase | 1 | | 761 | hsa-miR-623 | 4242 | MFNG | MFNG O-fucosylpeptide 3-beta-N-acetylglucosaminyltransferase | 1 | | 762 | hsa-miR-604 | 22904 | SBNO2 | strawberry notch homolog 2 (Drosophila) | 1 | | 764 | hsa-miR-765 | 22904 | SBNO2 | strawberry notch homolog 2 (Drosophila) | 1 | | 765 | hsa-miR-938 | 22904 | SBNO2 | strawberry notch homolog 2 (Drosophila) | 1 | | 766 | hsa-miR-1233 | 10208 | USPL1 | ubiquitin specific peptidase like 1 | 1 | | 767 | hsa-miR-569 | 10208 | USPL1 | ubiquitin specific peptidase like 1 | 1 | | 768 | hsa-miR-571 | 10208 | USPL1 | ubiquitin specific peptidase like 1 | 1 | | 769 | hsa-miR-766 | 10208 | USPL1 | ubiquitin specific peptidase like 1 | 1 | | 770 | hsa-miR-874 | 10208 | USPL1 | ubiquitin specific peptidase like 1 | 1 | | 771 | hsa-miR-635 | 5130 | PCYT1A | phosphate cytidylyltransferase 1, choline, alpha | 1 | | 772 | hsa-miR-635 | 10981 | RAB32 | RAB32, member RAS oncogene family | 1 | | 773 | hsa-miR-643 | 2207 | FCER1G | Fc fragment of IgE, high affinity I, receptor for; gamma polypeptide | 1 | | 775 | hsa-miR-1233 | 9166 | EBAG9 | estrogen receptor binding site associated, antigen, 9 | 1 | | 776 | hsa-miR-571 | 9166 | EBAG9 | estrogen receptor binding site associated, antigen, 9 | 1 | | 777 | hsa-miR-591 | 9166 | EBAG9 | estrogen receptor binding site associated, antigen, 9 | 1 | | 778 | hsa-miR-874 | 9166 | EBAG9 | estrogen receptor binding site associated, antigen, 9 | 1 | | 779 | hsa-miR-569 | 10667 | FARS2 | phenylalanyl-tRNA synthetase 2, mitochondrial | 1 | | 780 | hsa-miR-766 | 10667 | FARS2 | phenylalanyl-tRNA synthetase 2, mitochondrial | 1 | | 781 | hsa-mir-10a | 10772 | SFRS13A | splicing factor, arginine/serine-rich 13A | 1 | | 782 | hsa-miR-571 | 10772 | SFRS13A | splicing factor, arginine/serine-rich 13A | 1 | | 783 | hsa-miR-576-5p | 10772 | SFRS13A | splicing factor, arginine/serine-rich 13A | 1 | | 784 | hsa-miR-591 | 10772 | SFRS13A | splicing factor, arginine/serine-rich 13A | 1 | | 786 | hsa-miR-623 | 6183 | MRPS12 | mitochondrial ribosomal protein S12 | 1 | | 787 | hsa-miR-623 | 11186 | RASSF1 | Ras association (RalGDS/AF-6) domain family member 1 | 1 | | 788 | hsa-miR-1233 | 7188 | TRAF5 | TNF receptor-associated factor 5 | 1 | | 789 | hsa-miR-569 | 7188 | TRAF5 | TNF receptor-associated factor 5 | 1 | | 790 | hsa-miR-571 | 7188 | TRAF5 | TNF receptor-associated factor 5 | 1 | | 791 | hsa-miR-766 | 7188 | TRAF5 | TNF receptor-associated factor 5 | 1 | | 792 | hsa-miR-874 | 7188 | TRAF5 | TNF receptor-associated factor 5 | 1 | | 795 | hsa-miR-766 | 2976 | GTF3C2 | general transcription factor IIIC, polypeptide 2, beta 110kDa | 1 | | 796 | hsa-miR-1233 | 9730 | VPRBP | Vpr (HIV-1) binding protein | 1 | | 797 | hsa-miR-766 | 9730 | VPRBP | Vpr (HIV-1) binding protein | 1 | | 798 | hsa-miR-591 | 7699 | ZNF140 | zinc finger protein 140 | 1 | | 799 | hsa-miR-1233 | 9760 | TOX | thymocyte selection-associated high mobility group box | 1 | | 800 | hsa-miR-569 | 9760 | TOX | thymocyte selection-associated high mobility group box | 1 | | 801 | hsa-miR-571 | 9760 | TOX | thymocyte selection-associated high mobility group box | 1 | | 802 | hsa-miR-766 | 9760 | TOX | thymocyte selection-associated high mobility group box | 1 | | 803 | hsa-miR-874 | 9760 | TOX | thymocyte selection-associated high mobility group box | 1 | | 804 | hsa-miR-766 | 9284 | NPIP | nuclear pore complex interacting protein | 1 | | 805 | hsa-miR-635 | 5509 | PPP1R3D | protein phosphatase 1, regulatory (inhibitor) subunit 3D | 1 | | 806 | hsa-miR-770-5p | 354 | KLK3 | kallikrein-related peptidase 3 | 1 | | 807 | hsa-miR-95 | 354 | KLK3 | kallikrein-related peptidase 3 | 1 | | 808 | hsa-miR-591 | 9016 | SLC25A14 | solute carrier family 25 (mitochondrial carrier, brain), member 14 | 1 | | 809 | hsa-miR-1233 | 100 | ADA | adenosine deaminase | 1 | | 810 | hsa-miR-569 | 100 | ADA | adenosine deaminase | 1 | | 812 | hsa-miR-874 | 100 | ADA | adenosine deaminase | 1 | | 813 | hsa-miR-623 | 8405 | SPOP | speckle-type POZ protein | 1 | | 814 | hsa-mir-10a | 905 | CCNT2 | cyclin T2 | 1 | | 815 | hsa-mir-1224 | 905 | CCNT2 | cyclin T2 | 1 | | 816 | hsa-miR-1224-5p | 905 | CCNT2 | cyclin T2 | 1 | | 818 | hsa-miR-934 | 905 | CCNT2 | cyclin T2 | 1 | | 819 | hsa-miR-623 | 26100 | WIPI2 | WD repeat domain, phosphoinositide interacting 2 | 1 | | 820 | hsa-miR-627 | 26100 | WIPI2 | WD repeat domain, phosphoinositide interacting 2 | 1 | | 821 | hsa-miR-671-5p | 26100 | WIPI2 | WD repeat domain, phosphoinositide interacting 2 | 1 | | 822 | hsa-mir-10a | 1060 | CENPC1 | centromere protein C 1 | 1 | | 823 | hsa-miR-571 | 1060 | CENPC1 | centromere protein C 1 | 1 | | 824 | hsa-miR-591 | 1060 | CENPC1 | centromere protein C 1 | 1 | | 825 | hsa-mir-10a | 7270 | TTF1 | transcription termination factor, RNA polymerase I | 1 | | 826 | hsa-miR-1233 | 3590 | IL11RA | interleukin 11 receptor, alpha | 1 | | 827 | hsa-miR-569 | 3590 | IL11RA | interleukin 11 receptor, alpha | 1 | | 828 | hsa-miR-766 | 3590 | IL11RA | interleukin 11 receptor, alpha | 1 | | 829 | hsa-miR-874 | 3590 | IL11RA | interleukin 11 receptor, alpha | 1 | | 830 | hsa-miR-1233 | 4118 | MAL | mal, T-cell differentiation protein | 1 | | 831 | hsa-miR-569 | 4118 | MAL | mal, T-cell differentiation protein | 1 | | 832 | hsa-miR-766 | 4118 | MAL | mal, T-cell differentiation protein | 1 | | 833 | hsa-miR-874 | 4118 | MAL | mal, T-cell differentiation protein | 1 | | 834 | hsa-mir-10a | 7181 | NR2C1 | nuclear receptor subfamily 2, group C, member 1 | 1 | | 835 | hsa-miR-1233 | 7181 | NR2C1 | nuclear receptor subfamily 2, group C, member 1 | 1 | | 837 | hsa-miR-591 | 7181 | NR2C1 | nuclear receptor subfamily 2, group C, member 1 | 1 | | 838 | hsa-miR-766 | 7181 | NR2C1 | nuclear receptor subfamily 2, group C, member 1 | 1 | | 839 | hsa-miR-874 | 7181 | NR2C1 | nuclear receptor subfamily 2, group C, member 1 | 1 | | 840 | hsa-miR-934 | 7181 | NR2C1 | nuclear receptor subfamily 2, group C, member 1 | 1 | | 841 | hsa-miR-1233 | 5734 | PTGER4 | prostaglandin E receptor 4 (subtype EP4) | 1 | | 842 | hsa-miR-874 | 5734 | PTGER4 | prostaglandin E receptor 4 (subtype EP4) | 1 | | 843 | hsa-miR-766 | 399 | RHOH | ras homolog gene family, member H | 1 | | 844 | hsa-miR-1233 | 5001 | ORC5L | origin recognition complex, subunit 5-like (yeast) | 1 | | 845 | hsa-miR-571 | 5001 | ORC5L | origin recognition complex, subunit 5-like (yeast) | 1 | | 846 | hsa-miR-591 | 5001 | ORC5L | origin recognition complex, subunit 5-like (yeast) | 1 | | 847 | hsa-miR-766 | 5001 | ORC5L | origin recognition complex, subunit 5-like (yeast) | 1 | | 848 | hsa-mir-3130-1 | 2705 | GJB1 | gap junction protein, beta 1, 32kDa | 1 | | 849 | hsa-mir-3130-2 | 2705 | GJB1 | gap junction protein, beta 1, 32kDa | 1 | | 850 | hsa-mir-3130-3 | 2705 | GJB1 | gap junction protein, beta 1, 32kDa | 1 | | 851 | hsa-mir-10a | 9949 | AMMECR1 | Alport syndrome, mental retardation, midface hypoplasia and elliptocytosis chromosomal region gene 1 | 1 | | 852 | hsa-miR-627 | 11129 | SFRS16 | splicing factor, arginine/serine-rich 16 | 1 | | 854 | hsa-miR-765 | 6777 | STAT5B | signal transducer and activator of transcription 5B | 1 | | 855 | hsa-miR-766 | 5393 | EXOSC9 | exosome component 9 | 1 | | 856 | hsa-miR-874 | 5393 | EXOSC9 | exosome component 9 | 1 | | 857 | hsa-miR-1233 | 7553 | ZNF7 | zinc finger protein 7 | 1 | | 858 | hsa-miR-571 | 7553 | ZNF7 | zinc finger protein 7 | 1 | | 859 | hsa-miR-593\* | 51172 | NAGPA | N-acetylglucosamine-1-phosphodiester alpha-N-acetylglucosaminidase | 1 | | 860 | hsa-miR-571 | 26747 | NUFIP1 | nuclear fragile X mental retardation protein interacting protein 1 | 1 | | 861 | hsa-miR-874 | 26747 | NUFIP1 | nuclear fragile X mental retardation protein interacting protein 1 | 1 | | 862 | hsa-miR-765 | 215 | ABCD1 | ATP-binding cassette, sub-family D (ALD), member 1 | 1 | | 866 | hsa-miR-1233 | 5775 | PTPN4 | protein tyrosine phosphatase, non-receptor type 4 (megakaryocyte) | 1 | | 867 | hsa-miR-569 | 5775 | PTPN4 | protein tyrosine phosphatase, non-receptor type 4 (megakaryocyte) | 1 | | 868 | hsa-miR-766 | 5775 | PTPN4 | protein tyrosine phosphatase, non-receptor type 4 (megakaryocyte) | 1 | | 869 | hsa-miR-874 | 5775 | PTPN4 | protein tyrosine phosphatase, non-receptor type 4 (megakaryocyte) | 1 | | 870 | hsa-miR-188-5p | 4090 | SMAD5 | SMAD family member 5 | 1 | | 872 | hsa-miR-635 | 9744 | ACAP1 | ArfGAP with coiled-coil, ankyrin repeat and PH domains 1 | 1 | | 876 | hsa-miR-627 | 9744 | ACAP1 | ArfGAP with coiled-coil, ankyrin repeat and PH domains 1 | 1 | | 877 | hsa-miR-1233 | 80184 | CEP290 | centrosomal protein 290kDa | 1 | | 878 | hsa-miR-571 | 80184 | CEP290 | centrosomal protein 290kDa | 1 | | 879 | hsa-miR-591 | 80184 | CEP290 | centrosomal protein 290kDa | 1 | | 881 | hsa-miR-1233 | 6932 | TCF7 | transcription factor 7 (T-cell specific, HMG-box) | 1 | | 882 | hsa-miR-874 | 6932 | TCF7 | transcription factor 7 (T-cell specific, HMG-box) | 1 | | 883 | hsa-miR-10a | 10297 | APC2 | adenomatosis polyposis coli 2 | 1 | | 884 | hsa-miR-770-5p | 10297 | APC2 | adenomatosis polyposis coli 2 | 1 | | 885 | hsa-miR-1233 | 9841 | ZBTB24 | zinc finger and BTB domain containing 24 | 1 | | 886 | hsa-miR-571 | 9841 | ZBTB24 | zinc finger and BTB domain containing 24 | 1 | | 887 | hsa-miR-591 | 9841 | ZBTB24 | zinc finger and BTB domain containing 24 | 1 | | 888 | hsa-miR-874 | 9841 | ZBTB24 | zinc finger and BTB domain containing 24 | 1 | | 889 | hsa-miR-770-5p | 3216 | HOXB6 | homeobox B6 | 1 | | 890 | hsa-miR-95 | 3216 | HOXB6 | homeobox B6 | 1 | | 891 | hsa-miR-95 | 5645 | PRSS2 | protease, serine, 2 (trypsin 2) | 1 | | 892 | hsa-miR-1233 | 8434 | RECK | reversion-inducing-cysteine-rich protein with kazal motifs | 1 | | 893 | hsa-miR-571 | 8434 | RECK | reversion-inducing-cysteine-rich protein with kazal motifs | 1 | | 894 | hsa-miR-874 | 8434 | RECK | reversion-inducing-cysteine-rich protein with kazal motifs | 1 | | 895 | hsa-miR-766 | 1605 | DAG1 | dystroglycan 1 (dystrophin-associated glycoprotein 1) | 1 | | 896 | hsa-miR-1233 | 1880 | GPR183 | G protein-coupled receptor 183 | 1 | | 897 | hsa-miR-874 | 1880 | GPR183 | G protein-coupled receptor 183 | 1 | | 898 | hsa-miR-1233 | 10520 | ZNF211 | zinc finger protein 211 | 1 | | 899 | hsa-miR-569 | 10520 | ZNF211 | zinc finger protein 211 | 1 | | 900 | hsa-miR-571 | 10520 | ZNF211 | zinc finger protein 211 | 1 | | 901 | hsa-miR-766 | 10520 | ZNF211 | zinc finger protein 211 | 1 | | 902 | hsa-miR-1233 | 3001 | GZMA | granzyme A (granzyme 1, cytotoxic T-lymphocyte-associated serine esterase 3) | 1 | | 903 | hsa-miR-569 | 3001 | GZMA | granzyme A (granzyme 1, cytotoxic T-lymphocyte-associated serine esterase 3) | 1 | | 904 | hsa-miR-766 | 3001 | GZMA | granzyme A (granzyme 1, cytotoxic T-lymphocyte-associated serine esterase 3) | 1 | | 905 | hsa-miR-1233 | 50628 | GEMIN4 | gem (nuclear organelle) associated protein 4 | 1 | | 906 | hsa-miR-571 | 50628 | GEMIN4 | gem (nuclear organelle) associated protein 4 | 1 | | 907 | hsa-miR-766 | 50628 | GEMIN4 | gem (nuclear organelle) associated protein 4 | 1 | | 908 | hsa-miR-874 | 50628 | GEMIN4 | gem (nuclear organelle) associated protein 4 | 1 | | 909 | hsa-miR-635 | 7464 | CORO2A | coronin, actin binding protein, 2A | 1 | | 910 | hsa-miR-635 | 671 | BPI | bactericidal/permeability-increasing protein | 1 | | 911 | hsa-miR-604 | 11057 | ABHD2 | abhydrolase domain containing 2 | 1 | | 912 | hsa-miR-938 | 11057 | ABHD2 | abhydrolase domain containing 2 | 1 | | 913 | hsa-miR-576-5p | 22834 | ZNF652 | zinc finger protein 652 | 1 | | 914 | hsa-miR-770-5p | 3215 | HOXB5 | homeobox B5 | 1 | | 915 | hsa-miR-95 | 3215 | HOXB5 | homeobox B5 | 1 | | 919 | hsa-miR-635 | 978 | CDA | cytidine deaminase | 1 | | 920 | hsa-miR-1233 | 9734 | HDAC9 | histone deacetylase 9 | 1 | | 921 | hsa-mir-10a | 23545 | ATP6V0A2 | ATPase, H+ transporting, lysosomal V0 subunit a2 | 1 | | 922 | hsa-mir-199a-2 | 23545 | ATP6V0A2 | ATPase, H+ transporting, lysosomal V0 subunit a2 | 1 | | 923 | hsa-mir-214 | 23545 | ATP6V0A2 | ATPase, H+ transporting, lysosomal V0 subunit a2 | 1 | | 924 | hsa-mir-1224 | 23545 | ATP6V0A2 | ATPase, H+ transporting, lysosomal V0 subunit a2 | 1 | | 925 | hsa-miR-1224-5p | 23545 | ATP6V0A2 | ATPase, H+ transporting, lysosomal V0 subunit a2 | 1 | | 926 | hsa-miR-199a-5p | 23545 | ATP6V0A2 | ATPase, H+ transporting, lysosomal V0 subunit a2 | 1 | | 927 | hsa-miR-576-5p | 23545 | ATP6V0A2 | ATPase, H+ transporting, lysosomal V0 subunit a2 | 1 | | 928 | hsa-miR-934 | 23545 | ATP6V0A2 | ATPase, H+ transporting, lysosomal V0 subunit a2 | 1 | | 929 | hsa-miR-770-5p | 1271 | CNTFR | ciliary neurotrophic factor receptor | 1 | | 930 | hsa-miR-95 | 1271 | CNTFR | ciliary neurotrophic factor receptor | 1 | | 931 | hsa-miR-571 | 11062 | DUS4L | dihydrouridine synthase 4-like (S. cerevisiae) | 1 | | 932 | hsa-miR-591 | 11062 | DUS4L | dihydrouridine synthase 4-like (S. cerevisiae) | 1 | | 933 | hsa-miR-1233 | 8631 | SKAP1 | src kinase associated phosphoprotein 1 | 1 | | 934 | hsa-miR-569 | 8631 | SKAP1 | src kinase associated phosphoprotein 1 | 1 | | 935 | hsa-miR-766 | 8631 | SKAP1 | src kinase associated phosphoprotein 1 | 1 | | 936 | hsa-miR-874 | 8631 | SKAP1 | src kinase associated phosphoprotein 1 | 1 | | 937 | hsa-miR-95 | 8839 | WISP2 | WNT1 inducible signaling pathway protein 2 | 1 | | 938 | hsa-miR-1233 | 11232 | POLG2 | polymerase (DNA directed), gamma 2, accessory subunit | 1 | | 939 | hsa-miR-571 | 11232 | POLG2 | polymerase (DNA directed), gamma 2, accessory subunit | 1 | | 940 | hsa-miR-874 | 11232 | POLG2 | polymerase (DNA directed), gamma 2, accessory subunit | 1 | | 941 | hsa-miR-1233 | 914 | CD2 | CD2 molecule | 1 | | 942 | hsa-miR-569 | 914 | CD2 | CD2 molecule | 1 | | 943 | hsa-miR-766 | 914 | CD2 | CD2 molecule | 1 | | 944 | hsa-miR-874 | 914 | CD2 | CD2 molecule | 1 | | 945 | hsa-mir-423 | 1384 | CRAT | carnitine acetyltransferase | 1 | | 946 | hsa-miR-10a | 5781 | PTPN11 | protein tyrosine phosphatase, non-receptor type 11 | 1 | | 947 | hsa-miR-770-5p | 5781 | PTPN11 | protein tyrosine phosphatase, non-receptor type 11 | 1 | | 948 | hsa-miR-95 | 5781 | PTPN11 | protein tyrosine phosphatase, non-receptor type 11 | 1 | | 951 | hsa-mir-10a | 11168 | PSIP1 | PC4 and SFRS1 interacting protein 1 | 1 | | 955 | hsa-mir-423 | 23759 | PPIL2 | peptidylprolyl isomerase (cyclophilin)-like 2 | 1 | | 956 | hsa-miR-10a | 152 | ADRA2C | adrenergic, alpha-2C-, receptor | 1 | | 957 | hsa-miR-770-5p | 152 | ADRA2C | adrenergic, alpha-2C-, receptor | 1 | | 958 | hsa-miR-95 | 152 | ADRA2C | adrenergic, alpha-2C-, receptor | 1 | | 959 | hsa-miR-766 | 939 | CD27 | CD27 molecule | 1 | | 960 | hsa-miR-126\* | 9032 | TM4SF5 | transmembrane 4 L six family member 5 | 1 | | 964 | hsa-miR-638 | 5210 | PFKFB4 | 6-phosphofructo-2-kinase/fructose-2,6-biphosphatase 4 | 1 | | 965 | hsa-miR-10a | 8581 | LY6D | lymphocyte antigen 6 complex, locus D | 1 | | 966 | hsa-miR-770-5p | 8581 | LY6D | lymphocyte antigen 6 complex, locus D | 1 | | 967 | hsa-miR-95 | 8581 | LY6D | lymphocyte antigen 6 complex, locus D | 1 | | 968 | hsa-miR-10a | 4440 | MSI1 | musashi homolog 1 (Drosophila) | 1 | | 969 | hsa-miR-770-5p | 4440 | MSI1 | musashi homolog 1 (Drosophila) | 1 | | 970 | hsa-miR-95 | 4440 | MSI1 | musashi homolog 1 (Drosophila) | 1 | | 971 | hsa-miR-1233 | 1236 | CCR7 | chemokine (C-C motif) receptor 7 | 1 | | 972 | hsa-miR-765 | 9021 | SOCS3 | suppressor of cytokine signaling 3 | 1 | | 973 | hsa-miR-617 | 6375 | XCL1 | chemokine (C motif) ligand 1 | 1 | | 974 | hsa-miR-770-5p | 9745 | ZNF536 | zinc finger protein 536 | 1 | | 975 | hsa-miR-95 | 9745 | ZNF536 | zinc finger protein 536 | 1 | | 976 | hsa-miR-593\* | 3276 | PRMT1 | protein arginine methyltransferase 1 | 1 | | 977 | hsa-miR-623 | 3276 | PRMT1 | protein arginine methyltransferase 1 | 1 | | 978 | hsa-mir-3130-1 | 10202 | DHRS2 | dehydrogenase/reductase (SDR family) member 2 | 1 | | 979 | hsa-mir-3130-2 | 10202 | DHRS2 | dehydrogenase/reductase (SDR family) member 2 | 1 | | 980 | hsa-mir-3130-3 | 10202 | DHRS2 | dehydrogenase/reductase (SDR family) member 2 | 1 | | 981 | hsa-miR-770-5p | 4858 | NOVA2 | neuro-oncological ventral antigen 2 | 1 | | 982 | hsa-miR-95 | 4858 | NOVA2 | neuro-oncological ventral antigen 2 | 1 | | 983 | hsa-miR-571 | 2272 | FHIT | fragile histidine triad gene | 1 | | 984 | hsa-mir-10a | 25988 | HINFP | histone H4 transcription factor | 1 | | 985 | hsa-miR-934 | 25988 | HINFP | histone H4 transcription factor | 1 | | 986 | hsa-miR-1233 | 940 | CD28 | CD28 molecule | 1 | | 987 | hsa-miR-569 | 940 | CD28 | CD28 molecule | 1 | | 988 | hsa-miR-571 | 940 | CD28 | CD28 molecule | 1 | | 989 | hsa-miR-766 | 940 | CD28 | CD28 molecule | 1 | | 990 | hsa-miR-874 | 940 | CD28 | CD28 molecule | 1 | | 991 | hsa-miR-571 | 9631 | NUP155 | nucleoporin 155kDa | 1 | | 992 | hsa-miR-591 | 9631 | NUP155 | nucleoporin 155kDa | 1 | | 993 | hsa-miR-766 | 51230 | PHF20 | PHD finger protein 20 | 1 | | 994 | hsa-miR-635 | 8809 | IL18R1 | interleukin 18 receptor 1 | 1 | | 995 | hsa-miR-604 | 7030 | TFE3 | transcription factor binding to IGHM enhancer 3 | 1 | | 996 | hsa-miR-638 | 7030 | TFE3 | transcription factor binding to IGHM enhancer 3 | 1 | | 997 | hsa-miR-765 | 7030 | TFE3 | transcription factor binding to IGHM enhancer 3 | 1 | | 998 | hsa-miR-938 | 7030 | TFE3 | transcription factor binding to IGHM enhancer 3 | 1 | | 999 | hsa-miR-1233 | 3003 | GZMK | granzyme K (granzyme 3; tryptase II) | 1 | | 1000 | hsa-miR-569 | 3003 | GZMK | granzyme K (granzyme 3; tryptase II) | 1 | | 1001 | hsa-miR-766 | 3003 | GZMK | granzyme K (granzyme 3; tryptase II) | 1 | | 1002 | hsa-miR-10a | 3881 | KRT31 | keratin 31 | 1 | | 1003 | hsa-miR-770-5p | 3881 | KRT31 | keratin 31 | 1 | | 1004 | hsa-miR-95 | 3881 | KRT31 | keratin 31 | 1 | | 1005 | hsa-miR-643 | 3240 | HP | haptoglobin | 1 | | 1006 | hsa-miR-770-5p | 79774 | GRTP1 | growth hormone regulated TBC protein 1 | 1 | | 1007 | hsa-miR-95 | 79774 | GRTP1 | growth hormone regulated TBC protein 1 | 1 | | 1008 | hsa-mir-3130-1 | 1137 | CHRNA4 | cholinergic receptor, nicotinic, alpha 4 | 1 | | 1009 | hsa-mir-3130-2 | 1137 | CHRNA4 | cholinergic receptor, nicotinic, alpha 4 | 1 | | 1010 | hsa-mir-3130-3 | 1137 | CHRNA4 | cholinergic receptor, nicotinic, alpha 4 | 1 | | 1011 | hsa-miR-10a | 1137 | CHRNA4 | cholinergic receptor, nicotinic, alpha 4 | 1 | | 1012 | hsa-miR-770-5p | 1137 | CHRNA4 | cholinergic receptor, nicotinic, alpha 4 | 1 | | 1013 | hsa-miR-95 | 1137 | CHRNA4 | cholinergic receptor, nicotinic, alpha 4 | 1 | | 1014 | hsa-miR-1233 | 1677 | DFFB | DNA fragmentation factor, 40kDa, beta polypeptide (caspase-activated DNase) | 1 | | 1015 | hsa-miR-766 | 1677 | DFFB | DNA fragmentation factor, 40kDa, beta polypeptide (caspase-activated DNase) | 1 | | 1016 | hsa-miR-766 | 10225 | CD96 | CD96 molecule | 1 | | 1017 | hsa-miR-576-5p | 23443 | SLC35A3 | solute carrier family 35 (UDP-N-acetylglucosamine (UDP-GlcNAc) transporter), member A3 | 1 | | 1018 | hsa-mir-423 | 3338 | DNAJC4 | DnaJ (Hsp40) homolog, subfamily C, member 4 | 1 | | 1019 | hsa-mir-3130-1 | 3338 | DNAJC4 | DnaJ (Hsp40) homolog, subfamily C, member 4 | 1 | | 1020 | hsa-mir-3130-2 | 3338 | DNAJC4 | DnaJ (Hsp40) homolog, subfamily C, member 4 | 1 | | 1021 | hsa-mir-3130-3 | 3338 | DNAJC4 | DnaJ (Hsp40) homolog, subfamily C, member 4 | 1 | | 1022 | hsa-miR-10a | 3338 | DNAJC4 | DnaJ (Hsp40) homolog, subfamily C, member 4 | 1 | | 1023 | hsa-miR-770-5p | 3338 | DNAJC4 | DnaJ (Hsp40) homolog, subfamily C, member 4 | 1 | | 1024 | hsa-miR-95 | 3338 | DNAJC4 | DnaJ (Hsp40) homolog, subfamily C, member 4 | 1 | | 1025 | hsa-miR-10a | 51716 | CES4 | carboxylesterase 4, pseudogene | 1 | | 1026 | hsa-miR-770-5p | 51716 | CES4 | carboxylesterase 4, pseudogene | 1 | | 1027 | hsa-miR-95 | 51716 | CES4 | carboxylesterase 4, pseudogene | 1 | | 1028 | hsa-mir-885 | 6885 | MAP3K7 | mitogen-activated protein kinase kinase kinase 7 | 1 | | 1029 | hsa-miR-934 | 6885 | MAP3K7 | mitogen-activated protein kinase kinase kinase 7 | 1 | | 1030 | hsa-miR-765 | 7706 | TRIM25 | tripartite motif-containing 25 | 1 | | 1031 | hsa-miR-1233 | 7700 | ZNF141 | zinc finger protein 141 | 1 | | 1032 | hsa-miR-571 | 7700 | ZNF141 | zinc finger protein 141 | 1 | | 1033 | hsa-miR-874 | 7700 | ZNF141 | zinc finger protein 141 | 1 | | 1034 | hsa-mir-10a | 65110 | UPF3A | UPF3 regulator of nonsense transcripts homolog A (yeast) | 1 | | 1035 | hsa-mir-1224 | 65110 | UPF3A | UPF3 regulator of nonsense transcripts homolog A (yeast) | 1 | | 1036 | hsa-mir-885 | 65110 | UPF3A | UPF3 regulator of nonsense transcripts homolog A (yeast) | 1 | | 1037 | hsa-miR-1224-5p | 65110 | UPF3A | UPF3 regulator of nonsense transcripts homolog A (yeast) | 1 | | 1039 | hsa-miR-569 | 65110 | UPF3A | UPF3 regulator of nonsense transcripts homolog A (yeast) | 1 | | 1041 | hsa-miR-766 | 65110 | UPF3A | UPF3 regulator of nonsense transcripts homolog A (yeast) | 1 | | 1042 | hsa-miR-934 | 65110 | UPF3A | UPF3 regulator of nonsense transcripts homolog A (yeast) | 1 | | 1043 | hsa-miR-10a | 9394 | HS6ST1 | heparan sulfate 6-O-sulfotransferase 1 | 1 | | 1044 | hsa-miR-770-5p | 9394 | HS6ST1 | heparan sulfate 6-O-sulfotransferase 1 | 1 | | 1045 | hsa-miR-95 | 9394 | HS6ST1 | heparan sulfate 6-O-sulfotransferase 1 | 1 | | 1046 | hsa-mir-3130-1 | 4325 | MMP16 | matrix metallopeptidase 16 (membrane-inserted) | 1 | | 1047 | hsa-mir-3130-2 | 4325 | MMP16 | matrix metallopeptidase 16 (membrane-inserted) | 1 | | 1048 | hsa-mir-3130-3 | 4325 | MMP16 | matrix metallopeptidase 16 (membrane-inserted) | 1 | | 1049 | hsa-miR-10a | 4325 | MMP16 | matrix metallopeptidase 16 (membrane-inserted) | 1 | | 1050 | hsa-miR-770-5p | 4325 | MMP16 | matrix metallopeptidase 16 (membrane-inserted) | 1 | | 1051 | hsa-miR-95 | 4325 | MMP16 | matrix metallopeptidase 16 (membrane-inserted) | 1 | | 1052 | hsa-miR-770-5p | 4796 | NFKBIL2 | nuclear factor of kappa light polypeptide gene enhancer in B-cells inhibitor-like 2 | 1 | | 1053 | hsa-miR-635 | 4317 | MMP8 | matrix metallopeptidase 8 (neutrophil collagenase) | 1 | | 1054 | hsa-miR-643 | 4317 | MMP8 | matrix metallopeptidase 8 (neutrophil collagenase) | 1 | | 1055 | hsa-miR-126\* | 3166 | HMX1 | H6 family homeobox 1 | 1 | | 1056 | hsa-miR-10a | 954 | ENTPD2 | ectonucleoside triphosphate diphosphohydrolase 2 | 1 | | 1057 | hsa-miR-770-5p | 954 | ENTPD2 | ectonucleoside triphosphate diphosphohydrolase 2 | 1 | | 1058 | hsa-miR-95 | 954 | ENTPD2 | ectonucleoside triphosphate diphosphohydrolase 2 | 1 | | 1059 | hsa-miR-591 | 7696 | ZNF137 | zinc finger protein 137 | 1 | | 1060 | hsa-mir-10a | 5884 | RAD17 | RAD17 homolog (S. pombe) | 1 | | 1061 | hsa-miR-454\* | 5884 | RAD17 | RAD17 homolog (S. pombe) | 1 | | 1062 | hsa-miR-571 | 5884 | RAD17 | RAD17 homolog (S. pombe) | 1 | | 1063 | hsa-miR-591 | 5884 | RAD17 | RAD17 homolog (S. pombe) | 1 | | 1064 | hsa-miR-10a | 27296 | TP53TG5 | TP53 target 5 | 1 | | 1065 | hsa-miR-770-5p | 27296 | TP53TG5 | TP53 target 5 | 1 | | 1066 | hsa-miR-95 | 27296 | TP53TG5 | TP53 target 5 | 1 | | 1068 | hsa-miR-766 | 9533 | POLR1C | polymerase (RNA) I polypeptide C, 30kDa | 1 | | 1069 | hsa-miR-627 | 4791 | NFKB2 | nuclear factor of kappa light polypeptide gene enhancer in B-cells 2 (p49/p100) | 1 | | 1070 | hsa-miR-571 | 225 | ABCD2 | ATP-binding cassette, sub-family D (ALD), member 2 | 1 | | 1073 | hsa-miR-623 | 114049 | WBSCR22 | Williams Beuren syndrome chromosome region 22 | 1 | | 1074 | hsa-miR-766 | 114049 | WBSCR22 | Williams Beuren syndrome chromosome region 22 | 1 | | 1075 | hsa-miR-95 | 51 | ACOX1 | acyl-Coenzyme A oxidase 1, palmitoyl | 1 | | 1076 | hsa-miR-135a | 5606 | MAP2K3 | mitogen-activated protein kinase kinase 3 | 1 | | 1079 | hsa-miR-765 | 5606 | MAP2K3 | mitogen-activated protein kinase kinase 3 | 1 | | 1082 | hsa-mir-505 | 9048 | ARTN | artemin | 1 | | 1086 | hsa-miR-617 | 2833 | CXCR3 | chemokine (C-X-C motif) receptor 3 | 1 | | 1087 | hsa-miR-1233 | 54900 | LAX1 | lymphocyte transmembrane adaptor 1 | 1 | | 1088 | hsa-miR-569 | 54900 | LAX1 | lymphocyte transmembrane adaptor 1 | 1 | | 1089 | hsa-miR-766 | 54900 | LAX1 | lymphocyte transmembrane adaptor 1 | 1 | | 1090 | hsa-miR-604 | 80256 | KIAA1539 | KIAA1539 | 1 | | 1093 | hsa-miR-938 | 80256 | KIAA1539 | KIAA1539 | 1 | | 1094 | hsa-miR-770-5p | 10458 | BAIAP2 | BAI1-associated protein 2 | 1 | | 1095 | hsa-miR-95 | 10458 | BAIAP2 | BAI1-associated protein 2 | 1 | | 1096 | hsa-miR-1233 | 11126 | CD160 | CD160 molecule | 1 | | 1097 | hsa-miR-569 | 11126 | CD160 | CD160 molecule | 1 | | 1098 | hsa-miR-617 | 11126 | CD160 | CD160 molecule | 1 | | 1099 | hsa-miR-766 | 11126 | CD160 | CD160 molecule | 1 | | 1100 | hsa-miR-874 | 11126 | CD160 | CD160 molecule | 1 | | 1101 | hsa-miR-1233 | 4931 | NVL | nuclear VCP-like | 1 | | 1102 | hsa-miR-569 | 4931 | NVL | nuclear VCP-like | 1 | | 1103 | hsa-miR-571 | 4931 | NVL | nuclear VCP-like | 1 | | 1104 | hsa-miR-766 | 4931 | NVL | nuclear VCP-like | 1 | | 1105 | hsa-miR-766 | 2734 | GLG1 | golgi glycoprotein 1 | 1 | | 1106 | hsa-mir-10a | 10605 | PAIP1 | poly(A) binding protein interacting protein 1 | 1 | | 1108 | hsa-miR-571 | 10605 | PAIP1 | poly(A) binding protein interacting protein 1 | 1 | | 1113 | hsa-miR-766 | 9188 | DDX21 | DEAD (Asp-Glu-Ala-Asp) box polypeptide 21 | 1 | | 1114 | hsa-miR-10a | 6585 | SLIT1 | slit homolog 1 (Drosophila) | 1 | | 1115 | hsa-miR-770-5p | 6585 | SLIT1 | slit homolog 1 (Drosophila) | 1 | | 1116 | hsa-miR-95 | 6585 | SLIT1 | slit homolog 1 (Drosophila) | 1 | | 1118 | hsa-miR-10a | 8911 | CACNA1I | calcium channel, voltage-dependent, T type, alpha 1I subunit | 1 | | 1119 | hsa-miR-770-5p | 8911 | CACNA1I | calcium channel, voltage-dependent, T type, alpha 1I subunit | 1 | | 1120 | hsa-miR-95 | 8911 | CACNA1I | calcium channel, voltage-dependent, T type, alpha 1I subunit | 1 | | 1122 | hsa-miR-770-5p | 5024 | P2RX3 | purinergic receptor P2X, ligand-gated ion channel, 3 | 1 | | 1123 | hsa-miR-95 | 5024 | P2RX3 | purinergic receptor P2X, ligand-gated ion channel, 3 | 1 | | 1124 | hsa-miR-1233 | 10111 | RAD50 | RAD50 homolog (S. cerevisiae) | 1 | | 1125 | hsa-miR-571 | 10111 | RAD50 | RAD50 homolog (S. cerevisiae) | 1 | | 1126 | hsa-miR-591 | 10111 | RAD50 | RAD50 homolog (S. cerevisiae) | 1 | | 1127 | hsa-miR-765 | 6282 | S100A11 | S100 calcium binding protein A11 | 1 | | 1128 | hsa-mir-505 | 6623 | SNCG | synuclein, gamma (breast cancer-specific protein 1) | 1 | | 1129 | hsa-mir-3130-1 | 6623 | SNCG | synuclein, gamma (breast cancer-specific protein 1) | 1 | | 1130 | hsa-mir-3130-2 | 6623 | SNCG | synuclein, gamma (breast cancer-specific protein 1) | 1 | | 1131 | hsa-mir-3130-3 | 6623 | SNCG | synuclein, gamma (breast cancer-specific protein 1) | 1 | | 1132 | hsa-miR-10a | 6623 | SNCG | synuclein, gamma (breast cancer-specific protein 1) | 1 | | 1133 | hsa-miR-128 | 6623 | SNCG | synuclein, gamma (breast cancer-specific protein 1) | 1 | | 1134 | hsa-miR-770-5p | 6623 | SNCG | synuclein, gamma (breast cancer-specific protein 1) | 1 | | 1135 | hsa-miR-95 | 6623 | SNCG | synuclein, gamma (breast cancer-specific protein 1) | 1 | | 1136 | hsa-miR-636 | 10075 | HUWE1 | HECT, UBA and WWE domain containing 1 | 1 | | 1137 | hsa-miR-766 | 10075 | HUWE1 | HECT, UBA and WWE domain containing 1 | 1 | | 1138 | hsa-miR-636 | 1642 | DDB1 | damage-specific DNA binding protein 1, 127kDa | 1 | | 1139 | hsa-miR-623 | 6208 | RPS14 | ribosomal protein S14 | 1 | | 1140 | hsa-miR-623 | 10801 | SEPT9 | septin 9 | 1 | | 1141 | hsa-miR-766 | 23741 | EID1 | EP300 interacting inhibitor of differentiation 1 | 1 | | 1143 | hsa-miR-635 | 334 | APLP2 | amyloid beta (A4) precursor-like protein 2 | 1 | | 1146 | hsa-miR-765 | 23385 | NCSTN | nicastrin | 1 | | 1147 | hsa-miR-1233 | 7329 | UBE2I | ubiquitin-conjugating enzyme E2I (UBC9 homolog, yeast) | 1 | | 1148 | hsa-miR-571 | 7329 | UBE2I | ubiquitin-conjugating enzyme E2I (UBC9 homolog, yeast) | 1 | | 1149 | hsa-miR-874 | 7329 | UBE2I | ubiquitin-conjugating enzyme E2I (UBC9 homolog, yeast) | 1 | | 1150 | hsa-miR-623 | 10236 | HNRNPR | heterogeneous nuclear ribonucleoprotein R | 1 | | 1151 | hsa-miR-766 | 10236 | HNRNPR | heterogeneous nuclear ribonucleoprotein R | 1 | | 1156 | hsa-miR-1233 | 5717 | PSMD11 | proteasome (prosome, macropain) 26S subunit, non-ATPase, 11 | 1 | | 1157 | hsa-miR-766 | 5717 | PSMD11 | proteasome (prosome, macropain) 26S subunit, non-ATPase, 11 | 1 | | 1158 | hsa-miR-1233 | 6431 | SFRS6 | splicing factor, arginine/serine-rich 6 | 1 | | 1159 | hsa-miR-569 | 6431 | SFRS6 | splicing factor, arginine/serine-rich 6 | 1 | | 1160 | hsa-miR-571 | 6431 | SFRS6 | splicing factor, arginine/serine-rich 6 | 1 | | 1161 | hsa-miR-766 | 6431 | SFRS6 | splicing factor, arginine/serine-rich 6 | 1 | | 1162 | hsa-miR-874 | 6431 | SFRS6 | splicing factor, arginine/serine-rich 6 | 1 | | 1164 | hsa-miR-671-5p | 1107 | CHD3 | chromodomain helicase DNA binding protein 3 | 1 | | 1165 | hsa-miR-1233 | 7818 | DAP3 | death associated protein 3 | 1 | | 1166 | hsa-miR-569 | 7818 | DAP3 | death associated protein 3 | 1 | | 1167 | hsa-miR-571 | 7818 | DAP3 | death associated protein 3 | 1 | | 1168 | hsa-miR-766 | 7818 | DAP3 | death associated protein 3 | 1 | | 1169 | hsa-miR-1233 | 25814 | ATXN10 | ataxin 10 | 1 | | 1170 | hsa-miR-766 | 25814 | ATXN10 | ataxin 10 | 1 | | 1172 | hsa-miR-593\* | 9361 | LONP1 | lon peptidase 1, mitochondrial | 1 | | 1173 | hsa-miR-623 | 9361 | LONP1 | lon peptidase 1, mitochondrial | 1 | | 1174 | hsa-miR-766 | 9361 | LONP1 | lon peptidase 1, mitochondrial | 1 | | 1175 | hsa-miR-765 | 65018 | PINK1 | PTEN induced putative kinase 1 | 1 | | 1179 | hsa-miR-569 | 9987 | HNRPDL | heterogeneous nuclear ribonucleoprotein D-like | 1 | | 1180 | hsa-miR-571 | 9987 | HNRPDL | heterogeneous nuclear ribonucleoprotein D-like | 1 | | 1183 | hsa-miR-569 | 5860 | QDPR | quinoid dihydropteridine reductase | 1 | | 1184 | hsa-miR-766 | 5860 | QDPR | quinoid dihydropteridine reductase | 1 | | 1185 | hsa-mir-10a | 8575 | PRKRA | protein kinase, interferon-inducible double stranded RNA dependent activator | 1 | | 1186 | hsa-miR-190 | 8575 | PRKRA | protein kinase, interferon-inducible double stranded RNA dependent activator | 1 | | 1188 | hsa-miR-627 | 79143 | MBOAT7 | membrane bound O-acyltransferase domain containing 7 | 1 | | 1189 | hsa-miR-638 | 79143 | MBOAT7 | membrane bound O-acyltransferase domain containing 7 | 1 | | 1190 | hsa-miR-635 | 688 | KLF5 | Kruppel-like factor 5 (intestinal) | 1 | | 1191 | hsa-miR-1233 | 8473 | OGT | O-linked N-acetylglucosamine (GlcNAc) transferase (UDP-N-acetylglucosamine:polypeptide-N-acetylglucosaminyl transferase) | 1 | | 1192 | hsa-miR-874 | 8473 | OGT | O-linked N-acetylglucosamine (GlcNAc) transferase (UDP-N-acetylglucosamine:polypeptide-N-acetylglucosaminyl transferase) | 1 | | 1193 | hsa-miR-591 | 9126 | SMC3 | structural maintenance of chromosomes 3 | 1 | | 1194 | hsa-miR-766 | 56339 | METTL3 | methyltransferase like 3 | 1 | | 1195 | hsa-miR-635 | 2745 | GLRX | glutaredoxin (thioltransferase) | 1 | | 1196 | hsa-miR-1233 | 9533 | POLR1C | polymerase (RNA) I polypeptide C, 30kDa | 1 | | 1197 | hsa-miR-571 | 9533 | POLR1C | polymerase (RNA) I polypeptide C, 30kDa | 1 | | 1198 | hsa-miR-874 | 9533 | POLR1C | polymerase (RNA) I polypeptide C, 30kDa | 1 | | 1199 | hsa-mir-28 | 6004 | RGS16 | regulator of G-protein signaling 16 | 1 | | 1200 | hsa-miR-28-5p | 6004 | RGS16 | regulator of G-protein signaling 16 | 1 | | 1201 | hsa-miR-636 | 3184 | HNRNPD | heterogeneous nuclear ribonucleoprotein D (AU-rich element RNA binding protein 1, 37kDa) | 1 | | 1202 | hsa-miR-571 | 11168 | PSIP1 | PC4 and SFRS1 interacting protein 1 | 1 | | 1203 | hsa-miR-874 | 11168 | PSIP1 | PC4 and SFRS1 interacting protein 1 | 1 | | 1204 | hsa-miR-454\* | 6675 | UAP1 | UDP-N-acteylglucosamine pyrophosphorylase 1 | 1 | | 1205 | hsa-miR-1233 | 9044 | BTAF1 | BTAF1 RNA polymerase II, B-TFIID transcription factor-associated, 170kDa (Mot1 homolog, S. cerevisiae) | 1 | | 1206 | hsa-miR-569 | 9044 | BTAF1 | BTAF1 RNA polymerase II, B-TFIID transcription factor-associated, 170kDa (Mot1 homolog, S. cerevisiae) | 1 | | 1207 | hsa-miR-571 | 9044 | BTAF1 | BTAF1 RNA polymerase II, B-TFIID transcription factor-associated, 170kDa (Mot1 homolog, S. cerevisiae) | 1 | | 1208 | hsa-miR-766 | 9044 | BTAF1 | BTAF1 RNA polymerase II, B-TFIID transcription factor-associated, 170kDa (Mot1 homolog, S. cerevisiae) | 1 | | 1209 | hsa-miR-874 | 9044 | BTAF1 | BTAF1 RNA polymerase II, B-TFIID transcription factor-associated, 170kDa (Mot1 homolog, S. cerevisiae) | 1 | | 1210 | hsa-miR-1233 | 23598 | PATZ1 | POZ (BTB) and AT hook containing zinc finger 1 | 1 | | 1211 | hsa-miR-569 | 23598 | PATZ1 | POZ (BTB) and AT hook containing zinc finger 1 | 1 | | 1212 | hsa-miR-766 | 23598 | PATZ1 | POZ (BTB) and AT hook containing zinc finger 1 | 1 | | 1213 | hsa-miR-874 | 23598 | PATZ1 | POZ (BTB) and AT hook containing zinc finger 1 | 1 | | 1214 | hsa-miR-766 | 5631 | PRPS1 | phosphoribosyl pyrophosphate synthetase 1 | 1 | | 1215 | hsa-miR-593\* | 57418 | WDR18 | WD repeat domain 18 | 1 | | 1216 | hsa-miR-765 | 8569 | MKNK1 | MAP kinase interacting serine/threonine kinase 1 | 1 | | 1217 | hsa-miR-569 | 58473 | PLEKHB1 | pleckstrin homology domain containing, family B (evectins) member 1 | 1 | | 1218 | hsa-miR-766 | 58473 | PLEKHB1 | pleckstrin homology domain containing, family B (evectins) member 1 | 1 | | 1219 | hsa-mir-10a | 11236 | RNF139 | ring finger protein 139 | 1 | | 1220 | hsa-miR-1233 | 23404 | EXOSC2 | exosome component 2 | 1 | | 1221 | hsa-miR-571 | 23404 | EXOSC2 | exosome component 2 | 1 | | 1222 | hsa-miR-874 | 23404 | EXOSC2 | exosome component 2 | 1 | | 1223 | hsa-mir-423 | 3479 | IGF1 | insulin-like growth factor 1 (somatomedin C) | 1 | | 1227 | hsa-miR-576-5p | 8726 | EED | embryonic ectoderm development | 1 | | 1228 | hsa-miR-766 | 64087 | MCCC2 | methylcrotonoyl-Coenzyme A carboxylase 2 (beta) | 1 | | 1234 | hsa-miR-591 | 23379 | KIAA0947 | KIAA0947 | 1 | | 1235 | hsa-miR-627 | 11068 | CYB561D2 | cytochrome b-561 domain containing 2 | 1 | | 1236 | hsa-miR-671-5p | 11068 | CYB561D2 | cytochrome b-561 domain containing 2 | 1 | | 1237 | hsa-mir-628 | 11119 | BTN3A1 | butyrophilin, subfamily 3, member A1 | 1 | | 1238 | hsa-miR-1233 | 11119 | BTN3A1 | butyrophilin, subfamily 3, member A1 | 1 | | 1239 | hsa-miR-628-5p | 11119 | BTN3A1 | butyrophilin, subfamily 3, member A1 | 1 | | 1240 | hsa-miR-766 | 11119 | BTN3A1 | butyrophilin, subfamily 3, member A1 | 1 | | 1241 | hsa-miR-874 | 11119 | BTN3A1 | butyrophilin, subfamily 3, member A1 | 1 | | 1242 | hsa-miR-10a | 7262 | PHLDA2 | pleckstrin homology-like domain, family A, member 2 | 1 | | 1243 | hsa-miR-770-5p | 7262 | PHLDA2 | pleckstrin homology-like domain, family A, member 2 | 1 | | 1244 | hsa-miR-95 | 7262 | PHLDA2 | pleckstrin homology-like domain, family A, member 2 | 1 | | 1245 | hsa-miR-1233 | 5727 | PTCH1 | patched homolog 1 (Drosophila) | 1 | | 1246 | hsa-miR-569 | 5727 | PTCH1 | patched homolog 1 (Drosophila) | 1 | | 1247 | hsa-miR-571 | 5727 | PTCH1 | patched homolog 1 (Drosophila) | 1 | | 1248 | hsa-miR-766 | 5727 | PTCH1 | patched homolog 1 (Drosophila) | 1 | | 1249 | hsa-miR-874 | 5727 | PTCH1 | patched homolog 1 (Drosophila) | 1 | | 1250 | hsa-miR-770-5p | 22927 | HABP4 | hyaluronan binding protein 4 | 1 | | 1251 | hsa-miR-95 | 22927 | HABP4 | hyaluronan binding protein 4 | 1 | | 1252 | hsa-miR-10a | 9351 | SLC9A3R2 | solute carrier family 9 (sodium/hydrogen exchanger), member 3 regulator 2 | 1 | | 1253 | hsa-miR-770-5p | 9351 | SLC9A3R2 | solute carrier family 9 (sodium/hydrogen exchanger), member 3 regulator 2 | 1 | | 1254 | hsa-miR-95 | 9351 | SLC9A3R2 | solute carrier family 9 (sodium/hydrogen exchanger), member 3 regulator 2 | 1 | | 1256 | hsa-miR-766 | 54674 | LRRN3 | leucine rich repeat neuronal 3 | 1 | | 1258 | hsa-mir-10a | 23443 | SLC35A3 | solute carrier family 35 (UDP-N-acetylglucosamine (UDP-GlcNAc) transporter), member A3 | 1 | | 1259 | hsa-mir-1224 | 23443 | SLC35A3 | solute carrier family 35 (UDP-N-acetylglucosamine (UDP-GlcNAc) transporter), member A3 | 1 | | 1260 | hsa-miR-1224-5p | 23443 | SLC35A3 | solute carrier family 35 (UDP-N-acetylglucosamine (UDP-GlcNAc) transporter), member A3 | 1 | | 1261 | hsa-miR-571 | 23443 | SLC35A3 | solute carrier family 35 (UDP-N-acetylglucosamine (UDP-GlcNAc) transporter), member A3 | 1 | | 1262 | hsa-miR-571 | 9497 | SLC4A7 | solute carrier family 4, sodium bicarbonate cotransporter, member 7 | 1 | | 1264 | hsa-miR-190 | 545 | ATR | ataxia telangiectasia and Rad3 related | 1 | | 1269 | hsa-miR-591 | 545 | ATR | ataxia telangiectasia and Rad3 related | 1 | | 1270 | hsa-miR-604 | 4778 | NFE2 | nuclear factor (erythroid-derived 2), 45kDa | 1 | | 1271 | hsa-miR-635 | 4778 | NFE2 | nuclear factor (erythroid-derived 2), 45kDa | 1 | | 1272 | hsa-miR-638 | 4778 | NFE2 | nuclear factor (erythroid-derived 2), 45kDa | 1 | | 1273 | hsa-miR-765 | 4778 | NFE2 | nuclear factor (erythroid-derived 2), 45kDa | 1 | | 1274 | hsa-miR-938 | 4778 | NFE2 | nuclear factor (erythroid-derived 2), 45kDa | 1 | | 1275 | hsa-miR-1233 | 10795 | ZNF268 | zinc finger protein 268 | 1 | | 1276 | hsa-miR-571 | 10795 | ZNF268 | zinc finger protein 268 | 1 | | 1277 | hsa-miR-1233 | 5243 | ABCB1 | ATP-binding cassette, sub-family B (MDR/TAP), member 1 | 1 | | 1278 | hsa-miR-569 | 5243 | ABCB1 | ATP-binding cassette, sub-family B (MDR/TAP), member 1 | 1 | | 1279 | hsa-miR-571 | 5243 | ABCB1 | ATP-binding cassette, sub-family B (MDR/TAP), member 1 | 1 | | 1280 | hsa-miR-766 | 5243 | ABCB1 | ATP-binding cassette, sub-family B (MDR/TAP), member 1 | 1 | | 1282 | hsa-mir-3130-1 | 10411 | RAPGEF3 | Rap guanine nucleotide exchange factor (GEF) 3 | 1 | | 1283 | hsa-mir-3130-2 | 10411 | RAPGEF3 | Rap guanine nucleotide exchange factor (GEF) 3 | 1 | | 1284 | hsa-mir-3130-3 | 10411 | RAPGEF3 | Rap guanine nucleotide exchange factor (GEF) 3 | 1 | | 1285 | hsa-miR-10a | 10411 | RAPGEF3 | Rap guanine nucleotide exchange factor (GEF) 3 | 1 | | 1286 | hsa-miR-770-5p | 10411 | RAPGEF3 | Rap guanine nucleotide exchange factor (GEF) 3 | 1 | | 1287 | hsa-miR-95 | 10411 | RAPGEF3 | Rap guanine nucleotide exchange factor (GEF) 3 | 1 | | 1288 | hsa-mir-10a | 79441 | HAUS3 | HAUS augmin-like complex, subunit 3 | 1 | | 1289 | hsa-miR-1233 | 79441 | HAUS3 | HAUS augmin-like complex, subunit 3 | 1 | | 1290 | hsa-miR-571 | 79441 | HAUS3 | HAUS augmin-like complex, subunit 3 | 1 | | 1291 | hsa-miR-576-5p | 79441 | HAUS3 | HAUS augmin-like complex, subunit 3 | 1 | | 1292 | hsa-miR-591 | 79441 | HAUS3 | HAUS augmin-like complex, subunit 3 | 1 | | 1293 | hsa-miR-638 | 5603 | MAPK13 | mitogen-activated protein kinase 13 | 1 | | 1294 | hsa-miR-1233 | 4068 | SH2D1A | SH2 domain protein 1A | 1 | | 1295 | hsa-miR-569 | 4068 | SH2D1A | SH2 domain protein 1A | 1 | | 1296 | hsa-miR-571 | 4068 | SH2D1A | SH2 domain protein 1A | 1 | | 1297 | hsa-miR-766 | 4068 | SH2D1A | SH2 domain protein 1A | 1 | | 1298 | hsa-miR-874 | 4068 | SH2D1A | SH2 domain protein 1A | 1 | | 1299 | hsa-miR-635 | 4200 | ME2 | malic enzyme 2, NAD(+)-dependent, mitochondrial | 1 | | 1300 | hsa-miR-569 | 3002 | GZMB | granzyme B (granzyme 2, cytotoxic T-lymphocyte-associated serine esterase 1) | 1 | | 1301 | hsa-miR-766 | 3002 | GZMB | granzyme B (granzyme 2, cytotoxic T-lymphocyte-associated serine esterase 1) | 1 | | 1302 | hsa-miR-604 | 5795 | PTPRJ | protein tyrosine phosphatase, receptor type, J | 1 | | 1303 | hsa-miR-938 | 5795 | PTPRJ | protein tyrosine phosphatase, receptor type, J | 1 | | 1305 | hsa-miR-10a | 782 | CACNB1 | calcium channel, voltage-dependent, beta 1 subunit | 1 | | 1306 | hsa-miR-770-5p | 782 | CACNB1 | calcium channel, voltage-dependent, beta 1 subunit | 1 | | 1307 | hsa-miR-95 | 782 | CACNB1 | calcium channel, voltage-dependent, beta 1 subunit | 1 | | 1308 | hsa-miR-635 | 10745 | PHTF1 | putative homeodomain transcription factor 1 | 1 | | 1309 | hsa-miR-636 | 158 | ADSL | adenylosuccinate lyase | 1 | | 1310 | hsa-miR-766 | 158 | ADSL | adenylosuccinate lyase | 1 | | 1311 | hsa-miR-934 | 158 | ADSL | adenylosuccinate lyase | 1 | | 1312 | hsa-mir-3130-1 | 3754 | KCNF1 | potassium voltage-gated channel, subfamily F, member 1 | 1 | | 1313 | hsa-mir-3130-2 | 3754 | KCNF1 | potassium voltage-gated channel, subfamily F, member 1 | 1 | | 1314 | hsa-mir-3130-3 | 3754 | KCNF1 | potassium voltage-gated channel, subfamily F, member 1 | 1 | | 1315 | hsa-miR-10a | 3754 | KCNF1 | potassium voltage-gated channel, subfamily F, member 1 | 1 | | 1316 | hsa-miR-770-5p | 3754 | KCNF1 | potassium voltage-gated channel, subfamily F, member 1 | 1 | | 1317 | hsa-miR-95 | 3754 | KCNF1 | potassium voltage-gated channel, subfamily F, member 1 | 1 | | 1318 | hsa-miR-10a | 9356 | SLC22A6 | solute carrier family 22 (organic anion transporter), member 6 | 1 | | 1319 | hsa-miR-770-5p | 9356 | SLC22A6 | solute carrier family 22 (organic anion transporter), member 6 | 1 | | 1320 | hsa-miR-95 | 9356 | SLC22A6 | solute carrier family 22 (organic anion transporter), member 6 | 1 | | 1321 | hsa-miR-10a | 887 | CCKBR | cholecystokinin B receptor | 1 | | 1323 | hsa-mir-3130-1 | 5330 | PLCB2 | phospholipase C, beta 2 | 1 | | 1324 | hsa-mir-3130-2 | 5330 | PLCB2 | phospholipase C, beta 2 | 1 | | 1325 | hsa-mir-3130-3 | 5330 | PLCB2 | phospholipase C, beta 2 | 1 | | 1326 | hsa-miR-10a | 5330 | PLCB2 | phospholipase C, beta 2 | 1 | | 1327 | hsa-miR-128 | 5330 | PLCB2 | phospholipase C, beta 2 | 1 | | 1328 | hsa-miR-770-5p | 5330 | PLCB2 | phospholipase C, beta 2 | 1 | | 1329 | hsa-miR-95 | 5330 | PLCB2 | phospholipase C, beta 2 | 1 | | 1330 | hsa-miR-635 | 5023 | P2RX1 | purinergic receptor P2X, ligand-gated ion channel, 1 | 1 | | 1331 | hsa-mir-3130-1 | 9362 | CPNE6 | copine VI (neuronal) | 1 | | 1332 | hsa-mir-3130-2 | 9362 | CPNE6 | copine VI (neuronal) | 1 | | 1333 | hsa-mir-3130-3 | 9362 | CPNE6 | copine VI (neuronal) | 1 | | 1334 | hsa-miR-770-5p | 9362 | CPNE6 | copine VI (neuronal) | 1 | | 1335 | hsa-mir-3130-1 | 8538 | BARX2 | BARX homeobox 2 | 1 | | 1336 | hsa-mir-3130-2 | 8538 | BARX2 | BARX homeobox 2 | 1 | | 1337 | hsa-mir-3130-3 | 8538 | BARX2 | BARX homeobox 2 | 1 | | 1338 | hsa-miR-10a | 8538 | BARX2 | BARX homeobox 2 | 1 | | 1339 | hsa-miR-770-5p | 8538 | BARX2 | BARX homeobox 2 | 1 | | 1340 | hsa-miR-95 | 8538 | BARX2 | BARX homeobox 2 | 1 | | 1341 | hsa-miR-1233 | 440270 | GOLGA8B | golgin A8 family, member B | 1 | | 1342 | hsa-miR-569 | 440270 | GOLGA8B | golgin A8 family, member B | 1 | | 1343 | hsa-miR-766 | 440270 | GOLGA8B | golgin A8 family, member B | 1 | | 1344 | hsa-mir-3130-1 | 9468 | PCYT1B | phosphate cytidylyltransferase 1, choline, beta | 1 | | 1345 | hsa-mir-3130-2 | 9468 | PCYT1B | phosphate cytidylyltransferase 1, choline, beta | 1 | | 1346 | hsa-mir-3130-3 | 9468 | PCYT1B | phosphate cytidylyltransferase 1, choline, beta | 1 | | 1347 | hsa-miR-10a | 9468 | PCYT1B | phosphate cytidylyltransferase 1, choline, beta | 1 | | 1348 | hsa-miR-128 | 9468 | PCYT1B | phosphate cytidylyltransferase 1, choline, beta | 1 | | 1349 | hsa-miR-770-5p | 9468 | PCYT1B | phosphate cytidylyltransferase 1, choline, beta | 1 | | 1350 | hsa-miR-95 | 9468 | PCYT1B | phosphate cytidylyltransferase 1, choline, beta | 1 | | 1351 | hsa-miR-604 | 2529 | FUT7 | fucosyltransferase 7 (alpha (1,3) fucosyltransferase) | 1 | | 1352 | hsa-miR-635 | 2529 | FUT7 | fucosyltransferase 7 (alpha (1,3) fucosyltransferase) | 1 | | 1353 | hsa-miR-638 | 2529 | FUT7 | fucosyltransferase 7 (alpha (1,3) fucosyltransferase) | 1 | | 1354 | hsa-miR-938 | 2529 | FUT7 | fucosyltransferase 7 (alpha (1,3) fucosyltransferase) | 1 | | 1355 | hsa-miR-1233 | 330 | BIRC3 | baculoviral IAP repeat-containing 3 | 1 | | 1356 | hsa-miR-874 | 330 | BIRC3 | baculoviral IAP repeat-containing 3 | 1 | | 1357 | hsa-miR-10a | 2637 | GBX2 | gastrulation brain homeobox 2 | 1 | | 1358 | hsa-miR-770-5p | 2637 | GBX2 | gastrulation brain homeobox 2 | 1 | | 1359 | hsa-miR-95 | 2637 | GBX2 | gastrulation brain homeobox 2 | 1 | | 1360 | hsa-miR-627 | 7841 | MOGS | mannosyl-oligosaccharide glucosidase | 1 | | 1361 | hsa-miR-671-5p | 7841 | MOGS | mannosyl-oligosaccharide glucosidase | 1 | | 1362 | hsa-mir-423 | 6442 | SGCA | sarcoglycan, alpha (50kDa dystrophin-associated glycoprotein) | 1 | | 1363 | hsa-mir-505 | 6442 | SGCA | sarcoglycan, alpha (50kDa dystrophin-associated glycoprotein) | 1 | | 1364 | hsa-mir-3130-1 | 6442 | SGCA | sarcoglycan, alpha (50kDa dystrophin-associated glycoprotein) | 1 | | 1365 | hsa-mir-3130-2 | 6442 | SGCA | sarcoglycan, alpha (50kDa dystrophin-associated glycoprotein) | 1 | | 1366 | hsa-mir-3130-3 | 6442 | SGCA | sarcoglycan, alpha (50kDa dystrophin-associated glycoprotein) | 1 | | 1367 | hsa-miR-10a | 6442 | SGCA | sarcoglycan, alpha (50kDa dystrophin-associated glycoprotein) | 1 | | 1368 | hsa-miR-770-5p | 6442 | SGCA | sarcoglycan, alpha (50kDa dystrophin-associated glycoprotein) | 1 | | 1369 | hsa-miR-95 | 6442 | SGCA | sarcoglycan, alpha (50kDa dystrophin-associated glycoprotein) | 1 | | 1371 | hsa-miR-1233 | 4281 | MID1 | midline 1 (Opitz/BBB syndrome) | 1 | | 1372 | hsa-miR-874 | 4281 | MID1 | midline 1 (Opitz/BBB syndrome) | 1 | | 1373 | hsa-miR-604 | 11237 | RNF24 | ring finger protein 24 | 1 | | 1374 | hsa-miR-938 | 11237 | RNF24 | ring finger protein 24 | 1 | | 1375 | hsa-mir-3130-1 | 4771 | NF2 | neurofibromin 2 (merlin) | 1 | | 1376 | hsa-mir-3130-2 | 4771 | NF2 | neurofibromin 2 (merlin) | 1 | | 1377 | hsa-mir-3130-3 | 4771 | NF2 | neurofibromin 2 (merlin) | 1 | | 1378 | hsa-miR-10a | 4771 | NF2 | neurofibromin 2 (merlin) | 1 | | 1379 | hsa-miR-770-5p | 4771 | NF2 | neurofibromin 2 (merlin) | 1 | | 1380 | hsa-miR-95 | 4771 | NF2 | neurofibromin 2 (merlin) | 1 | | 1381 | hsa-miR-126\* | 861 | RUNX1 | runt-related transcription factor 1 | 1 | | 1384 | hsa-mir-10a | 27072 | VPS41 | vacuolar protein sorting 41 homolog (S. cerevisiae) | 1 | | 1385 | hsa-mir-199a-2 | 27072 | VPS41 | vacuolar protein sorting 41 homolog (S. cerevisiae) | 1 | | 1386 | hsa-mir-214 | 27072 | VPS41 | vacuolar protein sorting 41 homolog (S. cerevisiae) | 1 | | 1387 | hsa-miR-199a-5p | 27072 | VPS41 | vacuolar protein sorting 41 homolog (S. cerevisiae) | 1 | | 1389 | hsa-miR-934 | 27072 | VPS41 | vacuolar protein sorting 41 homolog (S. cerevisiae) | 1 | | 1397 | hsa-miR-571 | 8611 | PPAP2A | phosphatidic acid phosphatase type 2A | 1 | | 1398 | hsa-mir-3130-1 | 9783 | RIMS3 | regulating synaptic membrane exocytosis 3 | 1 | | 1399 | hsa-mir-3130-2 | 9783 | RIMS3 | regulating synaptic membrane exocytosis 3 | 1 | | 1400 | hsa-mir-3130-3 | 9783 | RIMS3 | regulating synaptic membrane exocytosis 3 | 1 | | 1401 | hsa-miR-770-5p | 23650 | TRIM29 | tripartite motif-containing 29 | 1 | | 1402 | hsa-miR-95 | 23650 | TRIM29 | tripartite motif-containing 29 | 1 | | 1403 | hsa-miR-576-5p | 4690 | NCK1 | NCK adaptor protein 1 | 1 | | 1404 | hsa-mir-505 | 3111 | HLA-DOA | major histocompatibility complex, class II, DO alpha | 1 | | 1405 | hsa-mir-3130-1 | 3111 | HLA-DOA | major histocompatibility complex, class II, DO alpha | 1 | | 1406 | hsa-mir-3130-2 | 3111 | HLA-DOA | major histocompatibility complex, class II, DO alpha | 1 | | 1407 | hsa-mir-3130-3 | 3111 | HLA-DOA | major histocompatibility complex, class II, DO alpha | 1 | | 1408 | hsa-miR-10a | 3111 | HLA-DOA | major histocompatibility complex, class II, DO alpha | 1 | | 1409 | hsa-miR-128 | 3111 | HLA-DOA | major histocompatibility complex, class II, DO alpha | 1 | | 1410 | hsa-miR-770-5p | 3111 | HLA-DOA | major histocompatibility complex, class II, DO alpha | 1 | | 1411 | hsa-miR-95 | 3111 | HLA-DOA | major histocompatibility complex, class II, DO alpha | 1 | | 1412 | hsa-miR-623 | 5976 | UPF1 | UPF1 regulator of nonsense transcripts homolog (yeast) | 1 | | 1413 | hsa-miR-627 | 5976 | UPF1 | UPF1 regulator of nonsense transcripts homolog (yeast) | 1 | | 1414 | hsa-miR-671-5p | 5976 | UPF1 | UPF1 regulator of nonsense transcripts homolog (yeast) | 1 | | 1415 | hsa-mir-3130-1 | 8646 | CHRD | chordin | 1 | | 1416 | hsa-mir-3130-2 | 8646 | CHRD | chordin | 1 | | 1417 | hsa-mir-3130-3 | 8646 | CHRD | chordin | 1 | | 1418 | hsa-miR-10a | 8646 | CHRD | chordin | 1 | | 1419 | hsa-miR-770-5p | 8646 | CHRD | chordin | 1 | | 1420 | hsa-miR-95 | 8646 | CHRD | chordin | 1 | | 1421 | hsa-miR-627 | 4802 | NFYC | nuclear transcription factor Y, gamma | 1 | | 1422 | hsa-miR-638 | 4802 | NFYC | nuclear transcription factor Y, gamma | 1 | | 1423 | hsa-miR-671-5p | 4802 | NFYC | nuclear transcription factor Y, gamma | 1 | | 1424 | hsa-mir-505 | 8021 | NUP214 | nucleoporin 214kDa | 1 | | 1425 | hsa-miR-770-5p | 8021 | NUP214 | nucleoporin 214kDa | 1 | | 1426 | hsa-miR-95 | 8021 | NUP214 | nucleoporin 214kDa | 1 | | 1427 | hsa-miR-10a | 5046 | PCSK6 | proprotein convertase subtilisin/kexin type 6 | 1 | | 1428 | hsa-miR-770-5p | 5046 | PCSK6 | proprotein convertase subtilisin/kexin type 6 | 1 | | 1429 | hsa-miR-95 | 5046 | PCSK6 | proprotein convertase subtilisin/kexin type 6 | 1 | | 1430 | hsa-miR-10a | 6899 | TBX1 | T-box 1 | 1 | | 1431 | hsa-miR-770-5p | 6899 | TBX1 | T-box 1 | 1 | | 1432 | hsa-miR-95 | 6899 | TBX1 | T-box 1 | 1 | | 1435 | hsa-miR-770-5p | 3077 | HFE | hemochromatosis | 1 | | 1437 | hsa-miR-635 | 7850 | IL1R2 | interleukin 1 receptor, type II | 1 | | 1444 | hsa-miR-604 | 23569 | PADI4 | peptidyl arginine deiminase, type IV | 1 | | 1446 | hsa-miR-938 | 23569 | PADI4 | peptidyl arginine deiminase, type IV | 1 | | 1447 | hsa-miR-770-5p | 5600 | MAPK11 | mitogen-activated protein kinase 11 | 1 | | 1448 | hsa-miR-95 | 5600 | MAPK11 | mitogen-activated protein kinase 11 | 1 | | 1449 | hsa-miR-10a | 3479 | IGF1 | insulin-like growth factor 1 (somatomedin C) | 1 | | 1450 | hsa-miR-770-5p | 3479 | IGF1 | insulin-like growth factor 1 (somatomedin C) | 1 | | 1451 | hsa-miR-95 | 3479 | IGF1 | insulin-like growth factor 1 (somatomedin C) | 1 | | 1452 | hsa-miR-1233 | 84525 | HOPX | HOP homeobox | 1 | | 1453 | hsa-miR-569 | 84525 | HOPX | HOP homeobox | 1 | | 1454 | hsa-miR-766 | 84525 | HOPX | HOP homeobox | 1 | | 1455 | hsa-miR-10a | 9001 | HAP1 | huntingtin-associated protein 1 | 1 | | 1457 | hsa-miR-591 | 10128 | LRPPRC | leucine-rich PPR-motif containing | 1 | | 1458 | hsa-miR-604 | 377 | ARF3 | ADP-ribosylation factor 3 | 1 | | 1459 | hsa-miR-638 | 377 | ARF3 | ADP-ribosylation factor 3 | 1 | | 1460 | hsa-miR-765 | 377 | ARF3 | ADP-ribosylation factor 3 | 1 | | 1461 | hsa-miR-938 | 377 | ARF3 | ADP-ribosylation factor 3 | 1 | | 1462 | hsa-miR-623 | 6122 | RPL3 | ribosomal protein L3 | 1 | | 1463 | hsa-miR-10a | 10214 | SSX3 | synovial sarcoma, X breakpoint 3 | 1 | | 1465 | hsa-miR-569 | 29969 | MDFIC | MyoD family inhibitor domain containing | 1 | | 1466 | hsa-miR-766 | 29969 | MDFIC | MyoD family inhibitor domain containing | 1 | | 1467 | hsa-miR-874 | 29969 | MDFIC | MyoD family inhibitor domain containing | 1 | | 1468 | hsa-miR-1233 | 83988 | NCALD | neurocalcin delta | 1 | | 1469 | hsa-miR-569 | 83988 | NCALD | neurocalcin delta | 1 | | 1470 | hsa-miR-617 | 83988 | NCALD | neurocalcin delta | 1 | | 1471 | hsa-miR-10a | 6194 | RPS6 | ribosomal protein S6 | 1 | | 1472 | hsa-miR-770-5p | 10580 | SORBS1 | sorbin and SH3 domain containing 1 | 1 | | 1473 | hsa-miR-95 | 10580 | SORBS1 | sorbin and SH3 domain containing 1 | 1 | | 1474 | hsa-miR-569 | 2205 | FCER1A | Fc fragment of IgE, high affinity I, receptor for; alpha polypeptide | 1 | | 1475 | hsa-miR-766 | 2205 | FCER1A | Fc fragment of IgE, high affinity I, receptor for; alpha polypeptide | 1 | | 1476 | hsa-miR-874 | 2205 | FCER1A | Fc fragment of IgE, high affinity I, receptor for; alpha polypeptide | 1 | | 1477 | hsa-miR-604 | 9341 | VAMP3 | vesicle-associated membrane protein 3 (cellubrevin) | 1 | | 1478 | hsa-miR-938 | 9341 | VAMP3 | vesicle-associated membrane protein 3 (cellubrevin) | 1 | | 1479 | hsa-miR-593\* | 374291 | NDUFS7 | NADH dehydrogenase (ubiquinone) Fe-S protein 7, 20kDa (NADH-coenzyme Q reductase) | 1 | | 1480 | hsa-miR-623 | 374291 | NDUFS7 | NADH dehydrogenase (ubiquinone) Fe-S protein 7, 20kDa (NADH-coenzyme Q reductase) | 1 | | 1483 | hsa-miR-770-5p | 629 | CFB | complement factor B | 1 | | 1484 | hsa-miR-604 | 4627 | MYH9 | myosin, heavy chain 9, non-muscle | 1 | | 1485 | hsa-miR-635 | 4627 | MYH9 | myosin, heavy chain 9, non-muscle | 1 | | 1486 | hsa-miR-638 | 4627 | MYH9 | myosin, heavy chain 9, non-muscle | 1 | | 1487 | hsa-miR-938 | 4627 | MYH9 | myosin, heavy chain 9, non-muscle | 1 | | 1489 | hsa-miR-874 | 220988 | HNRNPA3 | heterogeneous nuclear ribonucleoprotein A3 | 1 | | 1490 | hsa-miR-766 | 220988 | HNRNPA3 | heterogeneous nuclear ribonucleoprotein A3 | 1 | | 1492 | hsa-miR-1233 | 9221 | NOLC1 | nucleolar and coiled-body phosphoprotein 1 | 1 | | 1493 | hsa-miR-569 | 9221 | NOLC1 | nucleolar and coiled-body phosphoprotein 1 | 1 | | 1494 | hsa-miR-571 | 9221 | NOLC1 | nucleolar and coiled-body phosphoprotein 1 | 1 | | 1495 | hsa-miR-766 | 9221 | NOLC1 | nucleolar and coiled-body phosphoprotein 1 | 1 | | 1496 | hsa-miR-874 | 9221 | NOLC1 | nucleolar and coiled-body phosphoprotein 1 | 1 | | 1497 | hsa-miR-571 | 7155 | TOP2B | topoisomerase (DNA) II beta 180kDa | 1 | | 1498 | hsa-miR-638 | 26099 | C1orf144 | chromosome 1 open reading frame 144 | 1 | | 1499 | hsa-miR-576-5p | 58517 | RBM25 | RNA binding motif protein 25 | 1 | | 1500 | hsa-miR-1233 | 6122 | RPL3 | ribosomal protein L3 | 1 | | 1501 | hsa-miR-1233 | 8565 | YARS | tyrosyl-tRNA synthetase | 1 | | 1502 | hsa-miR-766 | 8565 | YARS | tyrosyl-tRNA synthetase | 1 | | 1503 | hsa-miR-617 | 9289 | GPR56 | G protein-coupled receptor 56 | 1 | | 1504 | hsa-mir-135b | 23126 | POGZ | pogo transposable element with ZNF domain | 1 | | 1505 | hsa-miR-934 | 23126 | POGZ | pogo transposable element with ZNF domain | 1 | | 1506 | hsa-miR-627 | 23141 | ANKLE2 | ankyrin repeat and LEM domain containing 2 | 1 | | 1507 | hsa-miR-671-5p | 23141 | ANKLE2 | ankyrin repeat and LEM domain containing 2 | 1 | | 1508 | hsa-mir-10a | 23360 | FNBP4 | formin binding protein 4 | 1 | | 1510 | hsa-miR-190 | 23360 | FNBP4 | formin binding protein 4 | 1 | | 1511 | hsa-miR-569 | 23360 | FNBP4 | formin binding protein 4 | 1 | | 1512 | hsa-miR-571 | 23360 | FNBP4 | formin binding protein 4 | 1 | | 1514 | hsa-miR-934 | 23360 | FNBP4 | formin binding protein 4 | 1 | | 1518 | hsa-miR-1233 | 5295 | PIK3R1 | phosphoinositide-3-kinase, regulatory subunit 1 (alpha) | 1 | | 1519 | hsa-miR-766 | 5295 | PIK3R1 | phosphoinositide-3-kinase, regulatory subunit 1 (alpha) | 1 | | 1520 | hsa-miR-874 | 5295 | PIK3R1 | phosphoinositide-3-kinase, regulatory subunit 1 (alpha) | 1 | | 1522 | hsa-miR-569 | 23175 | LPIN1 | lipin 1 | 1 | | 1526 | hsa-miR-454\* | 6541 | SLC7A1 | solute carrier family 7 (cationic amino acid transporter, y+ system), member 1 | 1 | | 1527 | hsa-mir-149 | 375056 | MIA3 | melanoma inhibitory activity family, member 3 | 1 | | 1528 | hsa-miR-591 | 375056 | MIA3 | melanoma inhibitory activity family, member 3 | 1 | | 1529 | hsa-miR-454\* | 64764 | CREB3L2 | cAMP responsive element binding protein 3-like 2 | 1 | | 1531 | hsa-mir-135b | 51029 | PPPDE1 | PPPDE peptidase domain containing 1 | 1 | | 1532 | hsa-miR-454\* | 51029 | PPPDE1 | PPPDE peptidase domain containing 1 | 1 | | 1533 | hsa-miR-1233 | 57634 | EP400 | E1A binding protein p400 | 1 | | 1534 | hsa-miR-569 | 57634 | EP400 | E1A binding protein p400 | 1 | | 1535 | hsa-miR-766 | 57634 | EP400 | E1A binding protein p400 | 1 | | 1536 | hsa-miR-1233 | 23358 | USP24 | ubiquitin specific peptidase 24 | 1 | | 1537 | hsa-miR-190 | 23358 | USP24 | ubiquitin specific peptidase 24 | 1 | | 1538 | hsa-miR-571 | 23358 | USP24 | ubiquitin specific peptidase 24 | 1 | | 1539 | hsa-miR-766 | 23358 | USP24 | ubiquitin specific peptidase 24 | 1 | | 1540 | hsa-miR-934 | 23358 | USP24 | ubiquitin specific peptidase 24 | 1 | | 1541 | hsa-miR-1233 | 23065 | KIAA0090 | KIAA0090 | 1 | | 1542 | hsa-miR-591 | 51603 | METTL13 | methyltransferase like 13 | 1 | | 1543 | hsa-miR-593\* | 22984 | PDCD11 | programmed cell death 11 | 1 | | 1545 | hsa-mir-199a-2 | 51592 | TRIM33 | tripartite motif-containing 33 | 1 | | 1546 | hsa-mir-214 | 51592 | TRIM33 | tripartite motif-containing 33 | 1 | | 1547 | hsa-miR-199a-5p | 51592 | TRIM33 | tripartite motif-containing 33 | 1 | | 1548 | hsa-miR-591 | 51592 | TRIM33 | tripartite motif-containing 33 | 1 | | 1549 | hsa-miR-454\* | 26128 | KIAA1279 | KIAA1279 | 1 | | 1550 | hsa-miR-569 | 2534 | FYN | FYN oncogene related to SRC, FGR, YES | 1 | | 1551 | hsa-miR-766 | 6597 | SMARCA4 | SWI/SNF related, matrix associated, actin dependent regulator of chromatin, subfamily a, member 4 | 1 | | 1552 | hsa-miR-1233 | 5151 | PDE8A | phosphodiesterase 8A | 1 | | 1553 | hsa-miR-591 | 5151 | PDE8A | phosphodiesterase 8A | 1 | | 1554 | hsa-miR-1233 | 7572 | ZNF24 | zinc finger protein 24 | 1 | | 1555 | hsa-miR-571 | 7572 | ZNF24 | zinc finger protein 24 | 1 | | 1556 | hsa-miR-766 | 7572 | ZNF24 | zinc finger protein 24 | 1 | | 1557 | hsa-miR-593\* | 80308 | FLAD1 | FAD1 flavin adenine dinucleotide synthetase homolog (S. cerevisiae) | 1 | | 1558 | hsa-miR-623 | 80308 | FLAD1 | FAD1 flavin adenine dinucleotide synthetase homolog (S. cerevisiae) | 1 | | 1559 | hsa-miR-1233 | 9326 | ZNHIT3 | zinc finger, HIT type 3 | 1 | | 1560 | hsa-miR-190 | 9326 | ZNHIT3 | zinc finger, HIT type 3 | 1 | | 1561 | hsa-miR-569 | 9326 | ZNHIT3 | zinc finger, HIT type 3 | 1 | | 1562 | hsa-miR-766 | 9326 | ZNHIT3 | zinc finger, HIT type 3 | 1 | | 1563 | hsa-miR-638 | 6777 | STAT5B | signal transducer and activator of transcription 5B | 1 | | 1565 | hsa-miR-617 | 27250 | PDCD4 | programmed cell death 4 (neoplastic transformation inhibitor) | 1 | | 1567 | hsa-mir-10a | 80205 | CHD9 | chromodomain helicase DNA binding protein 9 | 1 | | 1568 | hsa-miR-576-5p | 80205 | CHD9 | chromodomain helicase DNA binding protein 9 | 1 | | 1569 | hsa-miR-591 | 80205 | CHD9 | chromodomain helicase DNA binding protein 9 | 1 | | 1570 | hsa-miR-766 | 80205 | CHD9 | chromodomain helicase DNA binding protein 9 | 1 | | 1571 | hsa-miR-934 | 80205 | CHD9 | chromodomain helicase DNA binding protein 9 | 1 | | 1572 | hsa-miR-1233 | 201562 | PTPLB | protein tyrosine phosphatase-like (proline instead of catalytic arginine), member b | 1 | | 1573 | hsa-miR-571 | 201562 | PTPLB | protein tyrosine phosphatase-like (proline instead of catalytic arginine), member b | 1 | | 1574 | hsa-miR-591 | 201562 | PTPLB | protein tyrosine phosphatase-like (proline instead of catalytic arginine), member b | 1 | | 1575 | hsa-miR-766 | 201562 | PTPLB | protein tyrosine phosphatase-like (proline instead of catalytic arginine), member b | 1 | | 1578 | hsa-miR-1233 | 23173 | METAP1 | methionyl aminopeptidase 1 | 1 | | 1579 | hsa-miR-766 | 23173 | METAP1 | methionyl aminopeptidase 1 | 1 | | 1580 | hsa-miR-569 | 23195 | MDN1 | MDN1, midasin homolog (yeast) | 1 | | 1581 | hsa-miR-766 | 23195 | MDN1 | MDN1, midasin homolog (yeast) | 1 | | 1582 | hsa-miR-571 | 157922 | CAMSAP1 | calmodulin regulated spectrin-associated protein 1 | 1 | | 1583 | hsa-miR-591 | 157922 | CAMSAP1 | calmodulin regulated spectrin-associated protein 1 | 1 | | 1584 | hsa-miR-766 | 9711 | KIAA0226 | KIAA0226 | 1 | | 1585 | hsa-miR-576-5p | 30849 | PIK3R4 | phosphoinositide-3-kinase, regulatory subunit 4 | 1 | | 1586 | hsa-miR-569 | 27246 | RNF115 | ring finger protein 115 | 1 | | 1587 | hsa-miR-766 | 27246 | RNF115 | ring finger protein 115 | 1 | | 1592 | hsa-miR-576-5p | 23041 | MON2 | MON2 homolog (S. cerevisiae) | 1 | | 1593 | hsa-miR-591 | 23041 | MON2 | MON2 homolog (S. cerevisiae) | 1 | | 1594 | hsa-mir-10a | 81875 | ISG20L2 | interferon stimulated exonuclease gene 20kDa-like 2 | 1 | | 1595 | hsa-miR-576-5p | 81875 | ISG20L2 | interferon stimulated exonuclease gene 20kDa-like 2 | 1 | | 1596 | hsa-miR-569 | 9804 | TOMM20 | translocase of outer mitochondrial membrane 20 homolog (yeast) | 1 | | 1597 | hsa-miR-636 | 56252 | YLPM1 | YLP motif containing 1 | 1 | | 1599 | hsa-miR-934 | 56252 | YLPM1 | YLP motif containing 1 | 1 | | 1600 | hsa-miR-1233 | 57037 | ANKMY2 | ankyrin repeat and MYND domain containing 2 | 1 | | 1601 | hsa-miR-591 | 57037 | ANKMY2 | ankyrin repeat and MYND domain containing 2 | 1 | | 1602 | hsa-mir-10a | 23382 | AHCYL2 | adenosylhomocysteinase-like 2 | 1 | | 1603 | hsa-miR-591 | 23382 | AHCYL2 | adenosylhomocysteinase-like 2 | 1 | | 1605 | hsa-miR-571 | 8939 | FUBP3 | far upstream element (FUSE) binding protein 3 | 1 | | 1606 | hsa-miR-576-5p | 8939 | FUBP3 | far upstream element (FUSE) binding protein 3 | 1 | | 1607 | hsa-miR-591 | 8939 | FUBP3 | far upstream element (FUSE) binding protein 3 | 1 | | 1608 | hsa-miR-766 | 57470 | LRRC47 | leucine rich repeat containing 47 | 1 | | 1610 | hsa-miR-571 | 56950 | SMYD2 | SET and MYND domain containing 2 | 1 | | 1613 | hsa-miR-569 | 56950 | SMYD2 | SET and MYND domain containing 2 | 1 | | 1614 | hsa-mir-423 | 1291 | COL6A1 | collagen, type VI, alpha 1 | 1 | | 1615 | hsa-miR-10a | 1291 | COL6A1 | collagen, type VI, alpha 1 | 1 | | 1618 | hsa-miR-1233 | 23269 | MGA | MAX gene associated | 1 | | 1620 | hsa-miR-623 | 541578 | CXorf40B | chromosome X open reading frame 40B | 1 | | 1621 | hsa-miR-1233 | 23119 | HIC2 | hypermethylated in cancer 2 | 1 | | 1622 | hsa-miR-571 | 23119 | HIC2 | hypermethylated in cancer 2 | 1 | | 1623 | hsa-miR-591 | 23119 | HIC2 | hypermethylated in cancer 2 | 1 | | 1624 | hsa-miR-766 | 23119 | HIC2 | hypermethylated in cancer 2 | 1 | | 1625 | hsa-miR-188-5p | 9747 | FAM115A | family with sequence similarity 115, member A | 1 | | 1628 | hsa-miR-1233 | 9736 | USP34 | ubiquitin specific peptidase 34 | 1 | | 1629 | hsa-mir-3130-1 | 9479 | MAPK8IP1 | mitogen-activated protein kinase 8 interacting protein 1 | 1 | | 1630 | hsa-mir-3130-2 | 9479 | MAPK8IP1 | mitogen-activated protein kinase 8 interacting protein 1 | 1 | | 1631 | hsa-mir-3130-3 | 9479 | MAPK8IP1 | mitogen-activated protein kinase 8 interacting protein 1 | 1 | | 1632 | hsa-miR-10a | 9479 | MAPK8IP1 | mitogen-activated protein kinase 8 interacting protein 1 | 1 | | 1633 | hsa-mir-149 | 7110 | TMF1 | TATA element modulatory factor 1 | 1 | | 1635 | hsa-miR-1233 | 84942 | WDR73 | WD repeat domain 73 | 1 | | 1636 | hsa-miR-766 | 84942 | WDR73 | WD repeat domain 73 | 1 | | 1637 | hsa-miR-1233 | 23370 | ARHGEF18 | Rho/Rac guanine nucleotide exchange factor (GEF) 18 | 1 | | 1638 | hsa-miR-766 | 23370 | ARHGEF18 | Rho/Rac guanine nucleotide exchange factor (GEF) 18 | 1 | | 1639 | hsa-miR-874 | 23370 | ARHGEF18 | Rho/Rac guanine nucleotide exchange factor (GEF) 18 | 1 | | 1640 | hsa-miR-623 | 513 | ATP5D | ATP synthase, H+ transporting, mitochondrial F1 complex, delta subunit | 1 | | 1641 | hsa-miR-627 | 513 | ATP5D | ATP synthase, H+ transporting, mitochondrial F1 complex, delta subunit | 1 | | 1642 | hsa-miR-671-5p | 513 | ATP5D | ATP synthase, H+ transporting, mitochondrial F1 complex, delta subunit | 1 | | 1643 | hsa-miR-627 | 489 | ATP2A3 | ATPase, Ca++ transporting, ubiquitous | 1 | | 1644 | hsa-miR-638 | 489 | ATP2A3 | ATPase, Ca++ transporting, ubiquitous | 1 | | 1645 | hsa-miR-593\* | 9862 | MED24 | mediator complex subunit 24 | 1 | | 1646 | hsa-miR-1233 | 57493 | HEG1 | HEG homolog 1 (zebrafish) | 1 | | 1647 | hsa-miR-190 | 57493 | HEG1 | HEG homolog 1 (zebrafish) | 1 | | 1648 | hsa-miR-569 | 57493 | HEG1 | HEG homolog 1 (zebrafish) | 1 | | 1649 | hsa-miR-571 | 57493 | HEG1 | HEG homolog 1 (zebrafish) | 1 | | 1650 | hsa-miR-766 | 57493 | HEG1 | HEG homolog 1 (zebrafish) | 1 | | 1652 | hsa-miR-576-5p | 5286 | PIK3C2A | phosphoinositide-3-kinase, class 2, alpha polypeptide | 1 | | 1653 | hsa-miR-591 | 5286 | PIK3C2A | phosphoinositide-3-kinase, class 2, alpha polypeptide | 1 | | 1654 | hsa-miR-1233 | 100272216 | LOC100272216 | hypothetical LOC100272216 | 1 | | 1655 | hsa-miR-766 | 100272216 | LOC100272216 | hypothetical LOC100272216 | 1 | | 1656 | hsa-miR-571 | 23508 | TTC9 | tetratricopeptide repeat domain 9 | 1 | | 1657 | hsa-miR-591 | 10302 | SNAPC5 | small nuclear RNA activating complex, polypeptide 5, 19kDa | 1 | | 1658 | hsa-mir-10a | 57205 | ATP10D | ATPase, class V, type 10D | 1 | | 1659 | hsa-mir-10a | 10464 | PIBF1 | progesterone immunomodulatory binding factor 1 | 1 | | 1660 | hsa-miR-571 | 10464 | PIBF1 | progesterone immunomodulatory binding factor 1 | 1 | | 1661 | hsa-miR-576-5p | 10464 | PIBF1 | progesterone immunomodulatory binding factor 1 | 1 | | 1662 | hsa-miR-1233 | 3858 | KRT10 | keratin 10 | 1 | | 1663 | hsa-miR-1233 | 5198 | PFAS | phosphoribosylformylglycinamidine synthase | 1 | | 1664 | hsa-miR-571 | 5198 | PFAS | phosphoribosylformylglycinamidine synthase | 1 | | 1665 | hsa-miR-591 | 5198 | PFAS | phosphoribosylformylglycinamidine synthase | 1 | | 1666 | hsa-miR-874 | 5198 | PFAS | phosphoribosylformylglycinamidine synthase | 1 | | 1667 | hsa-mir-10a | 23116 | FAM179B | family with sequence similarity 179, member B | 1 | | 1668 | hsa-miR-1233 | 23116 | FAM179B | family with sequence similarity 179, member B | 1 | | 1669 | hsa-miR-571 | 23116 | FAM179B | family with sequence similarity 179, member B | 1 | | 1670 | hsa-miR-591 | 23116 | FAM179B | family with sequence similarity 179, member B | 1 | | 1671 | hsa-miR-591 | 10196 | PRMT3 | protein arginine methyltransferase 3 | 1 | | 1672 | hsa-mir-10a | 221443 | C6orf130 | chromosome 6 open reading frame 130 | 1 | | 1673 | hsa-mir-199a-2 | 221443 | C6orf130 | chromosome 6 open reading frame 130 | 1 | | 1674 | hsa-mir-214 | 221443 | C6orf130 | chromosome 6 open reading frame 130 | 1 | | 1676 | hsa-miR-199a-5p | 221443 | C6orf130 | chromosome 6 open reading frame 130 | 1 | | 1678 | hsa-miR-766 | 221443 | C6orf130 | chromosome 6 open reading frame 130 | 1 | | 1679 | hsa-miR-934 | 221443 | C6orf130 | chromosome 6 open reading frame 130 | 1 | | 1680 | hsa-miR-591 | 4750 | NEK1 | NIMA (never in mitosis gene a)-related kinase 1 | 1 | | 1681 | hsa-mir-423 | 4776 | NFATC4 | nuclear factor of activated T-cells, cytoplasmic, calcineurin-dependent 4 | 1 | | 1682 | hsa-mir-3130-1 | 4776 | NFATC4 | nuclear factor of activated T-cells, cytoplasmic, calcineurin-dependent 4 | 1 | | 1683 | hsa-mir-3130-2 | 4776 | NFATC4 | nuclear factor of activated T-cells, cytoplasmic, calcineurin-dependent 4 | 1 | | 1684 | hsa-mir-3130-3 | 4776 | NFATC4 | nuclear factor of activated T-cells, cytoplasmic, calcineurin-dependent 4 | 1 | | 1685 | hsa-miR-10a | 4776 | NFATC4 | nuclear factor of activated T-cells, cytoplasmic, calcineurin-dependent 4 | 1 | | 1686 | hsa-miR-770-5p | 4776 | NFATC4 | nuclear factor of activated T-cells, cytoplasmic, calcineurin-dependent 4 | 1 | | 1687 | hsa-miR-95 | 4776 | NFATC4 | nuclear factor of activated T-cells, cytoplasmic, calcineurin-dependent 4 | 1 | | 1690 | hsa-miR-1233 | 6642 | SNX1 | sorting nexin 1 | 1 | | 1691 | hsa-miR-766 | 6642 | SNX1 | sorting nexin 1 | 1 | | 1695 | hsa-miR-126\* | 25803 | SPDEF | SAM pointed domain containing ets transcription factor | 1 | | 1696 | hsa-miR-636 | 5510 | PPP1R7 | protein phosphatase 1, regulatory (inhibitor) subunit 7 | 1 | | 1697 | hsa-miR-1233 | 23398 | PPWD1 | peptidylprolyl isomerase domain and WD repeat containing 1 | 1 | | 1698 | hsa-miR-571 | 23398 | PPWD1 | peptidylprolyl isomerase domain and WD repeat containing 1 | 1 | | 1700 | hsa-miR-623 | 7329 | UBE2I | ubiquitin-conjugating enzyme E2I (UBC9 homolog, yeast) | 1 | | 1701 | hsa-miR-1233 | 9045 | RPL14 | ribosomal protein L14 | 1 | | 1702 | hsa-miR-617 | 9045 | RPL14 | ribosomal protein L14 | 1 | | 1703 | hsa-miR-874 | 9045 | RPL14 | ribosomal protein L14 | 1 | | 1704 | hsa-miR-1233 | 10772 | SFRS13A | splicing factor, arginine/serine-rich 13A | 1 | | 1705 | hsa-miR-569 | 10772 | SFRS13A | splicing factor, arginine/serine-rich 13A | 1 | | 1706 | hsa-miR-454\* | 23155 | CLCC1 | chloride channel CLIC-like 1 | 1 | | 1707 | hsa-miR-591 | 23155 | CLCC1 | chloride channel CLIC-like 1 | 1 | | 1710 | hsa-miR-1233 | 5378 | PMS1 | PMS1 postmeiotic segregation increased 1 (S. cerevisiae) | 1 | | 1711 | hsa-miR-571 | 5378 | PMS1 | PMS1 postmeiotic segregation increased 1 (S. cerevisiae) | 1 | | 1712 | hsa-miR-591 | 5378 | PMS1 | PMS1 postmeiotic segregation increased 1 (S. cerevisiae) | 1 | | 1713 | hsa-miR-874 | 5378 | PMS1 | PMS1 postmeiotic segregation increased 1 (S. cerevisiae) | 1 | | 1714 | hsa-miR-591 | 91298 | C12orf29 | chromosome 12 open reading frame 29 | 1 | | 1715 | hsa-miR-1233 | 150759 | LOC150759 | hypothetical protein LOC150759 | 1 | | 1716 | hsa-miR-766 | 150759 | LOC150759 | hypothetical protein LOC150759 | 1 | | 1717 | hsa-miR-770-5p | 399904 | LOC399904 | hypothetical LOC399904 | 1 | | 1718 | hsa-miR-591 | 905 | CCNT2 | cyclin T2 | 1 | | 1719 | hsa-miR-770-5p | 23254 | RP1-21O18.1 | kazrin | 1 | | 1720 | hsa-miR-95 | 23254 | RP1-21O18.1 | kazrin | 1 | | 1721 | hsa-miR-1233 | 25983 | NGDN | neuroguidin, EIF4E binding protein | 1 | | 1722 | hsa-miR-569 | 25983 | NGDN | neuroguidin, EIF4E binding protein | 1 | | 1723 | hsa-miR-766 | 25983 | NGDN | neuroguidin, EIF4E binding protein | 1 | | 1724 | hsa-miR-1233 | 3633 | INPP5B | inositol polyphosphate-5-phosphatase, 75kDa | 1 | | 1725 | hsa-miR-766 | 3633 | INPP5B | inositol polyphosphate-5-phosphatase, 75kDa | 1 | | 1726 | hsa-miR-1233 | 6964 | TRD@ | T cell receptor delta locus | 1 | | 1729 | hsa-miR-617 | 6964 | TRD@ | T cell receptor delta locus | 1 | | 1730 | hsa-miR-766 | 6964 | TRD@ | T cell receptor delta locus | 1 | | 1732 | hsa-miR-770-5p | 2898 | GRIK2 | glutamate receptor, ionotropic, kainate 2 | 1 | | 1733 | hsa-miR-591 | 120526 | DNAJC24 | DnaJ (Hsp40) homolog, subfamily C, member 24 | 1 | | 1734 | hsa-miR-1233 | 4603 | MYBL1 | v-myb myeloblastosis viral oncogene homolog (avian)-like 1 | 1 | | 1735 | hsa-miR-569 | 4603 | MYBL1 | v-myb myeloblastosis viral oncogene homolog (avian)-like 1 | 1 | | 1736 | hsa-miR-766 | 4603 | MYBL1 | v-myb myeloblastosis viral oncogene homolog (avian)-like 1 | 1 | | 1737 | hsa-miR-874 | 4603 | MYBL1 | v-myb myeloblastosis viral oncogene homolog (avian)-like 1 | 1 | | 1738 | hsa-miR-10a | 1312 | COMT | catechol-O-methyltransferase | 1 | | 1739 | hsa-mir-628 | 7535 | ZAP70 | zeta-chain (TCR) associated protein kinase 70kDa | 1 | | 1740 | hsa-miR-1233 | 7535 | ZAP70 | zeta-chain (TCR) associated protein kinase 70kDa | 1 | | 1741 | hsa-miR-617 | 7535 | ZAP70 | zeta-chain (TCR) associated protein kinase 70kDa | 1 | | 1742 | hsa-miR-628-5p | 7535 | ZAP70 | zeta-chain (TCR) associated protein kinase 70kDa | 1 | | 1743 | hsa-mir-10a | 11019 | LIAS | lipoic acid synthetase | 1 | | 1744 | hsa-miR-1233 | 11019 | LIAS | lipoic acid synthetase | 1 | | 1745 | hsa-miR-190 | 11019 | LIAS | lipoic acid synthetase | 1 | | 1746 | hsa-miR-569 | 11019 | LIAS | lipoic acid synthetase | 1 | | 1747 | hsa-miR-571 | 11019 | LIAS | lipoic acid synthetase | 1 | | 1748 | hsa-miR-766 | 11019 | LIAS | lipoic acid synthetase | 1 | | 1749 | hsa-miR-1233 | 6742 | SSBP1 | single-stranded DNA binding protein 1 | 1 | | 1750 | hsa-miR-766 | 6742 | SSBP1 | single-stranded DNA binding protein 1 | 1 | | 1752 | hsa-miR-874 | 10147 | SFRS14 | splicing factor, arginine/serine-rich 14 | 1 | | 1753 | hsa-miR-874 | 6432 | SFRS7 | splicing factor, arginine/serine-rich 7, 35kDa | 1 | | 1754 | hsa-mir-10a | 51668 | HSPB11 | heat shock protein family B (small), member 11 | 1 | | 1755 | hsa-miR-1233 | 51668 | HSPB11 | heat shock protein family B (small), member 11 | 1 | | 1756 | hsa-miR-571 | 51668 | HSPB11 | heat shock protein family B (small), member 11 | 1 | | 1757 | hsa-miR-766 | 51668 | HSPB11 | heat shock protein family B (small), member 11 | 1 | | 1759 | hsa-miR-571 | 57134 | MAN1C1 | mannosidase, alpha, class 1C, member 1 | 1 | | 1761 | hsa-miR-770-5p | 29775 | CARD10 | caspase recruitment domain family, member 10 | 1 | | 1762 | hsa-miR-95 | 29775 | CARD10 | caspase recruitment domain family, member 10 | 1 | | 1763 | hsa-miR-770-5p | 283212 | KLHL35 | kelch-like 35 (Drosophila) | 1 | | 1764 | hsa-miR-617 | 6136 | RPL12 | ribosomal protein L12 | 1 | | 1769 | hsa-miR-591 | 65110 | UPF3A | UPF3 regulator of nonsense transcripts homolog A (yeast) | 1 | | 1770 | hsa-miR-874 | 65110 | UPF3A | UPF3 regulator of nonsense transcripts homolog A (yeast) | 1 | | 1775 | hsa-miR-770-5p | 25803 | SPDEF | SAM pointed domain containing ets transcription factor | 1 | | 1776 | hsa-miR-95 | 25803 | SPDEF | SAM pointed domain containing ets transcription factor | 1 | | 1777 | hsa-miR-571 | 6019 | RLN2 | relaxin 2 | 1 | | 1778 | hsa-miR-874 | 6019 | RLN2 | relaxin 2 | 1 | | 1779 | hsa-mir-3130-1 | 79166 | LILRP2 | leukocyte immunoglobulin-like receptor pseudogene 2 | 1 | | 1780 | hsa-mir-3130-2 | 79166 | LILRP2 | leukocyte immunoglobulin-like receptor pseudogene 2 | 1 | | 1781 | hsa-mir-3130-3 | 79166 | LILRP2 | leukocyte immunoglobulin-like receptor pseudogene 2 | 1 | | 1782 | hsa-miR-643 | 79166 | LILRP2 | leukocyte immunoglobulin-like receptor pseudogene 2 | 1 | | 1783 | hsa-miR-604 | 6093 | ROCK1 | Rho-associated, coiled-coil containing protein kinase 1 | 1 | | 1784 | hsa-miR-938 | 6093 | ROCK1 | Rho-associated, coiled-coil containing protein kinase 1 | 1 | | 1785 | hsa-miR-623 | 2734 | GLG1 | golgi glycoprotein 1 | 1 | | 1786 | hsa-mir-10a | 90333 | ZNF468 | zinc finger protein 468 | 1 | | 1787 | hsa-miR-576-5p | 90333 | ZNF468 | zinc finger protein 468 | 1 | | 1788 | hsa-miR-591 | 90333 | ZNF468 | zinc finger protein 468 | 1 | | 1793 | hsa-miR-604 | 63940 | GPSM3 | G-protein signaling modulator 3 (AGS3-like, C. elegans) | 1 | | 1794 | hsa-miR-638 | 63940 | GPSM3 | G-protein signaling modulator 3 (AGS3-like, C. elegans) | 1 | | 1795 | hsa-miR-938 | 63940 | GPSM3 | G-protein signaling modulator 3 (AGS3-like, C. elegans) | 1 | | 1796 | hsa-miR-1233 | 7110 | TMF1 | TATA element modulatory factor 1 | 1 | | 1797 | hsa-miR-591 | 7110 | TMF1 | TATA element modulatory factor 1 | 1 | | 1798 | hsa-miR-874 | 7110 | TMF1 | TATA element modulatory factor 1 | 1 | | 1799 | hsa-mir-10a | 22872 | SEC31A | SEC31 homolog A (S. cerevisiae) | 1 | | 1800 | hsa-miR-1233 | 22872 | SEC31A | SEC31 homolog A (S. cerevisiae) | 1 | | 1801 | hsa-miR-190 | 22872 | SEC31A | SEC31 homolog A (S. cerevisiae) | 1 | | 1802 | hsa-miR-571 | 22872 | SEC31A | SEC31 homolog A (S. cerevisiae) | 1 | | 1803 | hsa-miR-766 | 22872 | SEC31A | SEC31 homolog A (S. cerevisiae) | 1 | | 1804 | hsa-miR-874 | 22872 | SEC31A | SEC31 homolog A (S. cerevisiae) | 1 | | 1805 | hsa-miR-1233 | 667 | DST | dystonin | 1 | | 1806 | hsa-miR-571 | 667 | DST | dystonin | 1 | | 1807 | hsa-miR-95 | 22997 | IGSF9B | immunoglobulin superfamily, member 9B | 1 | | 1808 | hsa-mir-10a | 10480 | EIF3M | eukaryotic translation initiation factor 3, subunit M | 1 | | 1809 | hsa-miR-1233 | 10480 | EIF3M | eukaryotic translation initiation factor 3, subunit M | 1 | | 1810 | hsa-miR-571 | 10480 | EIF3M | eukaryotic translation initiation factor 3, subunit M | 1 | | 1811 | hsa-miR-766 | 10480 | EIF3M | eukaryotic translation initiation factor 3, subunit M | 1 | | 1812 | hsa-mir-3130-1 | 800 | CALD1 | caldesmon 1 | 1 | | 1813 | hsa-mir-3130-2 | 800 | CALD1 | caldesmon 1 | 1 | | 1814 | hsa-mir-3130-3 | 800 | CALD1 | caldesmon 1 | 1 | | 1815 | hsa-miR-10a | 800 | CALD1 | caldesmon 1 | 1 | | 1816 | hsa-miR-770-5p | 800 | CALD1 | caldesmon 1 | 1 | | 1817 | hsa-miR-95 | 800 | CALD1 | caldesmon 1 | 1 | | 1818 | hsa-mir-3130-1 | 26220 | DGCR5 | DiGeorge syndrome critical region gene 5 (non-protein coding) | 1 | | 1819 | hsa-mir-3130-2 | 26220 | DGCR5 | DiGeorge syndrome critical region gene 5 (non-protein coding) | 1 | | 1820 | hsa-mir-3130-3 | 26220 | DGCR5 | DiGeorge syndrome critical region gene 5 (non-protein coding) | 1 | | 1821 | hsa-miR-770-5p | 26220 | DGCR5 | DiGeorge syndrome critical region gene 5 (non-protein coding) | 1 | | 1822 | hsa-miR-95 | 26220 | DGCR5 | DiGeorge syndrome critical region gene 5 (non-protein coding) | 1 | | 1823 | hsa-miR-635 | 10474 | TADA3 | transcriptional adaptor 3 | 1 | | 1824 | hsa-miR-638 | 10474 | TADA3 | transcriptional adaptor 3 | 1 | | 1828 | hsa-miR-1233 | 926 | CD8B | CD8b molecule | 1 | | 1829 | hsa-miR-766 | 926 | CD8B | CD8b molecule | 1 | | 1830 | hsa-miR-874 | 926 | CD8B | CD8b molecule | 1 | | 1831 | hsa-miR-1233 | 643376 | BTBD18 | BTB (POZ) domain containing 18 | 1 | | 1832 | hsa-miR-10a | 728882 | FAM182B | family with sequence similarity 182, member B | 1 | | 1833 | hsa-miR-635 | 5606 | MAP2K3 | mitogen-activated protein kinase kinase 3 | 1 | | 1835 | hsa-miR-454\* | 51668 | HSPB11 | heat shock protein family B (small), member 11 | 1 | | 1836 | hsa-miR-874 | 55661 | DDX27 | DEAD (Asp-Glu-Ala-Asp) box polypeptide 27 | 1 | | 1837 | hsa-miR-623 | 6597 | SMARCA4 | SWI/SNF related, matrix associated, actin dependent regulator of chromatin, subfamily a, member 4 | 1 | | 1838 | hsa-miR-627 | 6597 | SMARCA4 | SWI/SNF related, matrix associated, actin dependent regulator of chromatin, subfamily a, member 4 | 1 | | 1839 | hsa-miR-638 | 6597 | SMARCA4 | SWI/SNF related, matrix associated, actin dependent regulator of chromatin, subfamily a, member 4 | 1 | | 1840 | hsa-miR-671-5p | 6597 | SMARCA4 | SWI/SNF related, matrix associated, actin dependent regulator of chromatin, subfamily a, member 4 | 1 | | 1841 | hsa-miR-10a | 4661 | MYT1 | myelin transcription factor 1 | 1 | | 1842 | hsa-miR-770-5p | 4661 | MYT1 | myelin transcription factor 1 | 1 | | 1843 | hsa-miR-95 | 4661 | MYT1 | myelin transcription factor 1 | 1 | | 1844 | hsa-mir-10a | 23047 | PDS5B | PDS5, regulator of cohesion maintenance, homolog B (S. cerevisiae) | 1 | | 1845 | hsa-miR-576-5p | 23047 | PDS5B | PDS5, regulator of cohesion maintenance, homolog B (S. cerevisiae) | 1 | | 1846 | hsa-miR-10a | 492 | ATP2B3 | ATPase, Ca++ transporting, plasma membrane 3 | 1 | | 1847 | hsa-miR-770-5p | 492 | ATP2B3 | ATPase, Ca++ transporting, plasma membrane 3 | 1 | | 1848 | hsa-miR-95 | 492 | ATP2B3 | ATPase, Ca++ transporting, plasma membrane 3 | 1 | | 1849 | hsa-miR-623 | 2534 | FYN | FYN oncogene related to SRC, FGR, YES | 1 | | 1850 | hsa-miR-1233 | 387535 | HCRP1 | hepatocellular carcinoma-related HCRP1 | 1 | | 1851 | hsa-miR-569 | 387535 | HCRP1 | hepatocellular carcinoma-related HCRP1 | 1 | | 1852 | hsa-miR-766 | 387535 | HCRP1 | hepatocellular carcinoma-related HCRP1 | 1 | | 1853 | hsa-mir-10a | 55729 | ATF7IP | activating transcription factor 7 interacting protein | 1 | | 1854 | hsa-mir-1224 | 55729 | ATF7IP | activating transcription factor 7 interacting protein | 1 | | 1855 | hsa-miR-1224-5p | 55729 | ATF7IP | activating transcription factor 7 interacting protein | 1 | | 1856 | hsa-miR-635 | 3557 | IL1RN | interleukin 1 receptor antagonist | 1 | | 1857 | hsa-miR-638 | 3557 | IL1RN | interleukin 1 receptor antagonist | 1 | | 1858 | hsa-miR-604 | 10565 | ARFGEF1 | ADP-ribosylation factor guanine nucleotide-exchange factor 1(brefeldin A-inhibited) | 1 | | 1859 | hsa-miR-938 | 10565 | ARFGEF1 | ADP-ribosylation factor guanine nucleotide-exchange factor 1(brefeldin A-inhibited) | 1 | | 1860 | hsa-mir-423 | 1211 | CLTA | clathrin, light chain (Lca) | 1 | | 1861 | hsa-miR-10a | 1211 | CLTA | clathrin, light chain (Lca) | 1 | | 1862 | hsa-miR-95 | 1211 | CLTA | clathrin, light chain (Lca) | 1 | | 1863 | hsa-miR-1233 | 6936 | C2orf3 | chromosome 2 open reading frame 3 | 1 | | 1864 | hsa-miR-571 | 6936 | C2orf3 | chromosome 2 open reading frame 3 | 1 | | 1865 | hsa-miR-874 | 6936 | C2orf3 | chromosome 2 open reading frame 3 | 1 | | 1866 | hsa-miR-874 | 442216 | RPS17P5 | ribosomal protein S17 pseudogene 5 | 1 | | 1867 | hsa-miR-627 | 80344 | DCAF11 | DDB1 and CUL4 associated factor 11 | 1 | | 1868 | hsa-miR-638 | 80344 | DCAF11 | DDB1 and CUL4 associated factor 11 | 1 | | 1873 | hsa-miR-10a | 11155 | LDB3 | LIM domain binding 3 | 1 | | 1874 | hsa-miR-770-5p | 11155 | LDB3 | LIM domain binding 3 | 1 | | 1875 | hsa-miR-95 | 11155 | LDB3 | LIM domain binding 3 | 1 | | 1876 | hsa-miR-635 | 10367 | CBARA1 | calcium binding atopy-related autoantigen 1 | 1 | | 1877 | hsa-miR-765 | 10367 | CBARA1 | calcium binding atopy-related autoantigen 1 | 1 | | 1878 | hsa-mir-3130-1 | 23119 | HIC2 | hypermethylated in cancer 2 | 1 | | 1879 | hsa-mir-3130-2 | 23119 | HIC2 | hypermethylated in cancer 2 | 1 | | 1880 | hsa-mir-3130-3 | 23119 | HIC2 | hypermethylated in cancer 2 | 1 | | 1881 | hsa-miR-10a | 23119 | HIC2 | hypermethylated in cancer 2 | 1 | | 1882 | hsa-miR-770-5p | 23119 | HIC2 | hypermethylated in cancer 2 | 1 | | 1883 | hsa-miR-95 | 23119 | HIC2 | hypermethylated in cancer 2 | 1 | | 1884 | hsa-miR-635 | 965 | CD58 | CD58 molecule | 1 | | 1889 | hsa-miR-10a | 2778 | GNAS | GNAS complex locus | 1 | | 1890 | hsa-miR-770-5p | 2778 | GNAS | GNAS complex locus | 1 | | 1891 | hsa-miR-95 | 2778 | GNAS | GNAS complex locus | 1 | | 1892 | hsa-mir-3130-1 | 22996 | TTC39A | tetratricopeptide repeat domain 39A | 1 | | 1893 | hsa-mir-3130-2 | 22996 | TTC39A | tetratricopeptide repeat domain 39A | 1 | | 1894 | hsa-mir-3130-3 | 22996 | TTC39A | tetratricopeptide repeat domain 39A | 1 | | 1895 | hsa-miR-10a | 22996 | TTC39A | tetratricopeptide repeat domain 39A | 1 | | 1896 | hsa-miR-770-5p | 22996 | TTC39A | tetratricopeptide repeat domain 39A | 1 | | 1897 | hsa-miR-95 | 22996 | TTC39A | tetratricopeptide repeat domain 39A | 1 | | 1898 | hsa-miR-874 | 50852 | TRAT1 | T cell receptor associated transmembrane adaptor 1 | 1 | | 1899 | hsa-mir-3130-1 | 2099 | ESR1 | estrogen receptor 1 | 1 | | 1900 | hsa-mir-3130-2 | 2099 | ESR1 | estrogen receptor 1 | 1 | | 1901 | hsa-mir-3130-3 | 2099 | ESR1 | estrogen receptor 1 | 1 | | 1902 | hsa-miR-10a | 2099 | ESR1 | estrogen receptor 1 | 1 | | 1903 | hsa-miR-770-5p | 2099 | ESR1 | estrogen receptor 1 | 1 | | 1904 | hsa-miR-95 | 2099 | ESR1 | estrogen receptor 1 | 1 | | 1905 | hsa-miR-627 | 8241 | RBM10 | RNA binding motif protein 10 | 1 | | 1906 | hsa-miR-671-5p | 8241 | RBM10 | RNA binding motif protein 10 | 1 | | 1907 | hsa-miR-617 | 6173 | RPL36A | ribosomal protein L36a | 1 | | 1908 | hsa-mir-423 | 1294 | COL7A1 | collagen, type VII, alpha 1 | 1 | | 1909 | hsa-mir-3130-1 | 1294 | COL7A1 | collagen, type VII, alpha 1 | 1 | | 1910 | hsa-mir-3130-2 | 1294 | COL7A1 | collagen, type VII, alpha 1 | 1 | | 1911 | hsa-mir-3130-3 | 1294 | COL7A1 | collagen, type VII, alpha 1 | 1 | | 1912 | hsa-miR-10a | 3850 | KRT3 | keratin 3 | 1 | | 1913 | hsa-miR-770-5p | 3850 | KRT3 | keratin 3 | 1 | | 1914 | hsa-miR-95 | 3850 | KRT3 | keratin 3 | 1 | | 1915 | hsa-miR-95 | 9436 | NCR2 | natural cytotoxicity triggering receptor 2 | 1 | | 1916 | hsa-miR-571 | 171392 | ZNF675 | zinc finger protein 675 | 1 | | 1917 | hsa-mir-3130-1 | 1368 | CPM | carboxypeptidase M | 1 | | 1918 | hsa-mir-3130-2 | 1368 | CPM | carboxypeptidase M | 1 | | 1919 | hsa-mir-3130-3 | 1368 | CPM | carboxypeptidase M | 1 | | 1920 | hsa-miR-10a | 1368 | CPM | carboxypeptidase M | 1 | | 1921 | hsa-miR-770-5p | 1368 | CPM | carboxypeptidase M | 1 | | 1922 | hsa-miR-95 | 1368 | CPM | carboxypeptidase M | 1 | | 1923 | hsa-miR-95 | 7047 | TGM4 | transglutaminase 4 (prostate) | 1 | | 1924 | hsa-miR-190 | 29969 | MDFIC | MyoD family inhibitor domain containing | 1 | | 1925 | hsa-miR-571 | 29969 | MDFIC | MyoD family inhibitor domain containing | 1 | | 1929 | hsa-miR-16 | 7010 | TEK | TEK tyrosine kinase, endothelial | 1 | | 1930 | hsa-miR-1233 | 55748 | CNDP2 | CNDP dipeptidase 2 (metallopeptidase M20 family) | 1 | | 1931 | hsa-miR-874 | 55748 | CNDP2 | CNDP dipeptidase 2 (metallopeptidase M20 family) | 1 | | 1932 | hsa-miR-765 | 64397 | ZFP106 | zinc finger protein 106 homolog (mouse) | 1 | | 1936 | hsa-miR-1233 | 29997 | GLTSCR2 | glioma tumor suppressor candidate region gene 2 | 1 | | 1937 | hsa-miR-617 | 29997 | GLTSCR2 | glioma tumor suppressor candidate region gene 2 | 1 | | 1938 | hsa-miR-874 | 29997 | GLTSCR2 | glioma tumor suppressor candidate region gene 2 | 1 | | 1939 | hsa-miR-636 | 11198 | SUPT16H | suppressor of Ty 16 homolog (S. cerevisiae) | 1 | | 1940 | hsa-miR-766 | 11198 | SUPT16H | suppressor of Ty 16 homolog (S. cerevisiae) | 1 | | 1942 | hsa-miR-593\* | 5859 | QARS | glutaminyl-tRNA synthetase | 1 | | 1943 | hsa-miR-766 | 5859 | QARS | glutaminyl-tRNA synthetase | 1 | | 1945 | hsa-miR-1233 | 55226 | NAT10 | N-acetyltransferase 10 (GCN5-related) | 1 | | 1946 | hsa-miR-766 | 55226 | NAT10 | N-acetyltransferase 10 (GCN5-related) | 1 | | 1947 | hsa-mir-10a | 55037 | PTCD3 | Pentatricopeptide repeat domain 3 | 1 | | 1956 | hsa-miR-593\* | 10055 | SAE1 | SUMO1 activating enzyme subunit 1 | 1 | | 1957 | hsa-miR-766 | 10055 | SAE1 | SUMO1 activating enzyme subunit 1 | 1 | | 1958 | hsa-miR-623 | 51070 | NOSIP | nitric oxide synthase interacting protein | 1 | | 1959 | hsa-miR-1233 | 29105 | C16orf80 | chromosome 16 open reading frame 80 | 1 | | 1960 | hsa-miR-569 | 29105 | C16orf80 | chromosome 16 open reading frame 80 | 1 | | 1961 | hsa-miR-571 | 29105 | C16orf80 | chromosome 16 open reading frame 80 | 1 | | 1962 | hsa-miR-766 | 29105 | C16orf80 | chromosome 16 open reading frame 80 | 1 | | 1963 | hsa-miR-1233 | 54977 | SLC25A38 | solute carrier family 25, member 38 | 1 | | 1964 | hsa-miR-569 | 54977 | SLC25A38 | solute carrier family 25, member 38 | 1 | | 1965 | hsa-miR-766 | 54977 | SLC25A38 | solute carrier family 25, member 38 | 1 | | 1966 | hsa-miR-454\* | 54927 | CHCHD3 | coiled-coil-helix-coiled-coil-helix domain containing 3 | 1 | | 1967 | hsa-miR-765 | 51734 | SEPX1 | selenoprotein X, 1 | 1 | | 1968 | hsa-miR-635 | 58472 | SQRDL | sulfide quinone reductase-like (yeast) | 1 | | 1969 | hsa-miR-623 | 51116 | MRPS2 | mitochondrial ribosomal protein S2 | 1 | | 1970 | hsa-miR-1233 | 7570 | ZNF22 | zinc finger protein 22 (KOX 15) | 1 | | 1971 | hsa-miR-569 | 7570 | ZNF22 | zinc finger protein 22 (KOX 15) | 1 | | 1972 | hsa-miR-571 | 7570 | ZNF22 | zinc finger protein 22 (KOX 15) | 1 | | 1973 | hsa-miR-766 | 7570 | ZNF22 | zinc finger protein 22 (KOX 15) | 1 | | 1974 | hsa-miR-454\* | 51138 | COPS4 | COP9 constitutive photomorphogenic homolog subunit 4 (Arabidopsis) | 1 | | 1975 | hsa-miR-591 | 51138 | COPS4 | COP9 constitutive photomorphogenic homolog subunit 4 (Arabidopsis) | 1 | | 1977 | hsa-miR-454\* | 23609 | MKRN2 | makorin ring finger protein 2 | 1 | | 1978 | hsa-miR-591 | 23609 | MKRN2 | makorin ring finger protein 2 | 1 | | 1979 | hsa-miR-1233 | 55114 | ARHGAP17 | Rho GTPase activating protein 17 | 1 | | 1980 | hsa-miR-766 | 55114 | ARHGAP17 | Rho GTPase activating protein 17 | 1 | | 1981 | hsa-miR-1233 | 55326 | AGPAT5 | 1-acylglycerol-3-phosphate O-acyltransferase 5 (lysophosphatidic acid acyltransferase, epsilon) | 1 | | 1982 | hsa-miR-571 | 54881 | TEX10 | testis expressed 10 | 1 | | 1983 | hsa-miR-591 | 54881 | TEX10 | testis expressed 10 | 1 | | 1985 | hsa-miR-576-5p | 55696 | RBM22 | RNA binding motif protein 22 | 1 | | 1986 | hsa-miR-591 | 26060 | APPL1 | adaptor protein, phosphotyrosine interaction, PH domain and leucine zipper containing 1 | 1 | | 1987 | hsa-miR-766 | 64769 | MEAF6 | MYST/Esa1-associated factor 6 | 1 | | 1988 | hsa-miR-1233 | 64110 | MAGEF1 | melanoma antigen family F, 1 | 1 | | 1989 | hsa-miR-571 | 64110 | MAGEF1 | melanoma antigen family F, 1 | 1 | | 1990 | hsa-miR-766 | 65265 | C8orf33 | chromosome 8 open reading frame 33 | 1 | | 1991 | hsa-miR-623 | 51177 | PLEKHO1 | pleckstrin homology domain containing, family O member 1 | 1 | | 1992 | hsa-miR-576-5p | 23683 | PRKD3 | protein kinase D3 | 1 | | 1993 | hsa-miR-1233 | 23560 | GTPBP4 | GTP binding protein 4 | 1 | | 1994 | hsa-miR-571 | 23560 | GTPBP4 | GTP binding protein 4 | 1 | | 1995 | hsa-miR-1233 | 53371 | NUP54 | nucleoporin 54kDa | 1 | | 1996 | hsa-miR-571 | 53371 | NUP54 | nucleoporin 54kDa | 1 | | 1997 | hsa-miR-591 | 53371 | NUP54 | nucleoporin 54kDa | 1 | | 1998 | hsa-miR-874 | 53371 | NUP54 | nucleoporin 54kDa | 1 | | 2000 | hsa-miR-571 | 79048 | SECISBP2 | SECIS binding protein 2 | 1 | | 2001 | hsa-miR-1233 | 29102 | RNASEN | ribonuclease type III, nuclear | 1 | | 2002 | hsa-miR-571 | 29102 | RNASEN | ribonuclease type III, nuclear | 1 | | 2003 | hsa-miR-591 | 29102 | RNASEN | ribonuclease type III, nuclear | 1 | | 2004 | hsa-miR-874 | 29102 | RNASEN | ribonuclease type III, nuclear | 1 | | 2005 | hsa-miR-569 | 55316 | RSAD1 | radical S-adenosyl methionine domain containing 1 | 1 | | 2006 | hsa-miR-766 | 55316 | RSAD1 | radical S-adenosyl methionine domain containing 1 | 1 | | 2007 | hsa-miR-571 | 8491 | MAP4K3 | mitogen-activated protein kinase kinase kinase kinase 3 | 1 | | 2008 | hsa-miR-591 | 8491 | MAP4K3 | mitogen-activated protein kinase kinase kinase kinase 3 | 1 | | 2009 | hsa-mir-10a | 54906 | C10orf18 | chromosome 10 open reading frame 18 | 1 | | 2011 | hsa-miR-571 | 54906 | C10orf18 | chromosome 10 open reading frame 18 | 1 | | 2013 | hsa-miR-635 | 54930 | HAUS4 | HAUS augmin-like complex, subunit 4 | 1 | | 2014 | hsa-miR-638 | 79641 | ROGDI | rogdi homolog (Drosophila) | 1 | | 2015 | hsa-miR-571 | 51562 | MBIP | MAP3K12 binding inhibitory protein 1 | 1 | | 2016 | hsa-miR-591 | 51562 | MBIP | MAP3K12 binding inhibitory protein 1 | 1 | | 2017 | hsa-miR-1233 | 64062 | RBM26 | RNA binding motif protein 26 | 1 | | 2018 | hsa-miR-874 | 64062 | RBM26 | RNA binding motif protein 26 | 1 | | 2019 | hsa-miR-1233 | 54585 | LZTFL1 | leucine zipper transcription factor-like 1 | 1 | | 2021 | hsa-miR-874 | 54585 | LZTFL1 | leucine zipper transcription factor-like 1 | 1 | | 2022 | hsa-miR-1233 | 55325 | UFSP2 | UFM1-specific peptidase 2 | 1 | | 2023 | hsa-miR-571 | 55325 | UFSP2 | UFM1-specific peptidase 2 | 1 | | 2024 | hsa-miR-766 | 55325 | UFSP2 | UFM1-specific peptidase 2 | 1 | | 2025 | hsa-miR-874 | 55325 | UFSP2 | UFM1-specific peptidase 2 | 1 | | 2026 | hsa-miR-635 | 79887 | PLBD1 | phospholipase B domain containing 1 | 1 | | 2027 | hsa-miR-1233 | 65981 | CAPRIN2 | caprin family member 2 | 1 | | 2028 | hsa-miR-569 | 65981 | CAPRIN2 | caprin family member 2 | 1 | | 2029 | hsa-miR-571 | 65981 | CAPRIN2 | caprin family member 2 | 1 | | 2030 | hsa-miR-766 | 65981 | CAPRIN2 | caprin family member 2 | 1 | | 2031 | hsa-miR-874 | 65981 | CAPRIN2 | caprin family member 2 | 1 | | 2032 | hsa-miR-766 | 54919 | HEATR2 | HEAT repeat containing 2 | 1 | | 2036 | hsa-miR-1233 | 50650 | ARHGEF3 | Rho guanine nucleotide exchange factor (GEF) 3 | 1 | | 2037 | hsa-miR-569 | 50650 | ARHGEF3 | Rho guanine nucleotide exchange factor (GEF) 3 | 1 | | 2038 | hsa-miR-571 | 50650 | ARHGEF3 | Rho guanine nucleotide exchange factor (GEF) 3 | 1 | | 2039 | hsa-miR-766 | 50650 | ARHGEF3 | Rho guanine nucleotide exchange factor (GEF) 3 | 1 | | 2040 | hsa-miR-874 | 50650 | ARHGEF3 | Rho guanine nucleotide exchange factor (GEF) 3 | 1 | | 2041 | hsa-miR-638 | 29095 | ORMDL2 | ORM1-like 2 (S. cerevisiae) | 1 | | 2042 | hsa-miR-766 | 56954 | NIT2 | nitrilase family, member 2 | 1 | | 2043 | hsa-miR-1233 | 57128 | LYRM4 | LYR motif containing 4 | 1 | | 2044 | hsa-miR-571 | 57128 | LYRM4 | LYR motif containing 4 | 1 | | 2045 | hsa-miR-766 | 57128 | LYRM4 | LYR motif containing 4 | 1 | | 2046 | hsa-miR-874 | 57128 | LYRM4 | LYR motif containing 4 | 1 | | 2048 | hsa-miR-571 | 55750 | AGK | acylglycerol kinase | 1 | | 2049 | hsa-miR-591 | 55750 | AGK | acylglycerol kinase | 1 | | 2050 | hsa-miR-874 | 55750 | AGK | acylglycerol kinase | 1 | | 2052 | hsa-miR-454\* | 55364 | IMPACT | Impact homolog (mouse) | 1 | | 2053 | hsa-miR-591 | 55364 | IMPACT | Impact homolog (mouse) | 1 | | 2054 | hsa-miR-571 | 10166 | SLC25A15 | solute carrier family 25 (mitochondrial carrier; ornithine transporter) member 15 | 1 | | 2055 | hsa-miR-1233 | 64219 | PJA1 | praja ring finger 1 | 1 | | 2056 | hsa-miR-569 | 64219 | PJA1 | praja ring finger 1 | 1 | | 2057 | hsa-miR-571 | 64219 | PJA1 | praja ring finger 1 | 1 | | 2058 | hsa-miR-766 | 64219 | PJA1 | praja ring finger 1 | 1 | | 2059 | hsa-miR-874 | 64219 | PJA1 | praja ring finger 1 | 1 | | 2060 | hsa-mir-10a | 80006 | C5orf44 | chromosome 5 open reading frame 44 | 1 | | 2061 | hsa-miR-591 | 80006 | C5orf44 | chromosome 5 open reading frame 44 | 1 | | 2062 | hsa-miR-770-5p | 56667 | MUC13 | mucin 13, cell surface associated | 1 | | 2063 | hsa-miR-95 | 56667 | MUC13 | mucin 13, cell surface associated | 1 | | 2065 | hsa-miR-1233 | 9451 | EIF2AK3 | eukaryotic translation initiation factor 2-alpha kinase 3 | 1 | | 2066 | hsa-mir-10a | 54953 | C1orf27 | chromosome 1 open reading frame 27 | 1 | | 2067 | hsa-miR-934 | 54953 | C1orf27 | chromosome 1 open reading frame 27 | 1 | | 2068 | hsa-miR-638 | 55954 | ZMAT5 | zinc finger, matrin type 5 | 1 | | 2069 | hsa-miR-874 | 79707 | NOL9 | nucleolar protein 9 | 1 | | 2070 | hsa-miR-638 | 80149 | ZC3H12A | zinc finger CCCH-type containing 12A | 1 | | 2071 | hsa-miR-635 | 64757 | MOSC1 | MOCO sulphurase C-terminal domain containing 1 | 1 | | 2072 | hsa-miR-635 | 54997 | TESC | tescalcin | 1 | | 2073 | hsa-mir-10a | 60487 | TRMT11 | tRNA methyltransferase 11 homolog (S. cerevisiae) | 1 | | 2074 | hsa-miR-1233 | 60487 | TRMT11 | tRNA methyltransferase 11 homolog (S. cerevisiae) | 1 | | 2075 | hsa-miR-569 | 60487 | TRMT11 | tRNA methyltransferase 11 homolog (S. cerevisiae) | 1 | | 2076 | hsa-miR-571 | 60487 | TRMT11 | tRNA methyltransferase 11 homolog (S. cerevisiae) | 1 | | 2077 | hsa-miR-766 | 60487 | TRMT11 | tRNA methyltransferase 11 homolog (S. cerevisiae) | 1 | | 2078 | hsa-miR-874 | 60487 | TRMT11 | tRNA methyltransferase 11 homolog (S. cerevisiae) | 1 | | 2079 | hsa-miR-1233 | 64318 | NOC3L | nucleolar complex associated 3 homolog (S. cerevisiae) | 1 | | 2080 | hsa-miR-571 | 64318 | NOC3L | nucleolar complex associated 3 homolog (S. cerevisiae) | 1 | | 2081 | hsa-miR-766 | 64318 | NOC3L | nucleolar complex associated 3 homolog (S. cerevisiae) | 1 | | 2082 | hsa-miR-874 | 64318 | NOC3L | nucleolar complex associated 3 homolog (S. cerevisiae) | 1 | | 2083 | hsa-miR-635 | 55129 | ANO10 | anoctamin 10 | 1 | | 2084 | hsa-miR-569 | 57134 | MAN1C1 | mannosidase, alpha, class 1C, member 1 | 1 | | 2085 | hsa-miR-766 | 57134 | MAN1C1 | mannosidase, alpha, class 1C, member 1 | 1 | | 2086 | hsa-mir-10a | 79752 | ZFAND1 | zinc finger, AN1-type domain 1 | 1 | | 2087 | hsa-miR-1233 | 79752 | ZFAND1 | zinc finger, AN1-type domain 1 | 1 | | 2088 | hsa-miR-569 | 79752 | ZFAND1 | zinc finger, AN1-type domain 1 | 1 | | 2089 | hsa-miR-571 | 79752 | ZFAND1 | zinc finger, AN1-type domain 1 | 1 | | 2090 | hsa-miR-591 | 79752 | ZFAND1 | zinc finger, AN1-type domain 1 | 1 | | 2091 | hsa-miR-627 | 59307 | SIGIRR | single immunoglobulin and toll-interleukin 1 receptor (TIR) domain | 1 | | 2092 | hsa-mir-10a | 55602 | CDKN2AIP | CDKN2A interacting protein | 1 | | 2093 | hsa-miR-591 | 29080 | CCDC59 | coiled-coil domain containing 59 | 1 | | 2094 | hsa-mir-135b | 54925 | ZNF434 | zinc finger protein 434 | 1 | | 2095 | hsa-miR-1233 | 55278 | QRSL1 | glutaminyl-tRNA synthase (glutamine-hydrolyzing)-like 1 | 1 | | 2096 | hsa-mir-10a | 9317 | PTER | phosphotriesterase related | 1 | | 2097 | hsa-miR-1233 | 9317 | PTER | phosphotriesterase related | 1 | | 2098 | hsa-miR-188-5p | 9317 | PTER | phosphotriesterase related | 1 | | 2099 | hsa-miR-190 | 9317 | PTER | phosphotriesterase related | 1 | | 2100 | hsa-miR-569 | 9317 | PTER | phosphotriesterase related | 1 | | 2101 | hsa-miR-571 | 9317 | PTER | phosphotriesterase related | 1 | | 2102 | hsa-miR-766 | 9317 | PTER | phosphotriesterase related | 1 | | 2105 | hsa-miR-1233 | 64425 | POLR1E | polymerase (RNA) I polypeptide E, 53kDa | 1 | | 2106 | hsa-miR-571 | 64425 | POLR1E | polymerase (RNA) I polypeptide E, 53kDa | 1 | | 2107 | hsa-miR-766 | 64425 | POLR1E | polymerase (RNA) I polypeptide E, 53kDa | 1 | | 2108 | hsa-miR-874 | 64425 | POLR1E | polymerase (RNA) I polypeptide E, 53kDa | 1 | | 2109 | hsa-miR-591 | 64417 | C5orf28 | chromosome 5 open reading frame 28 | 1 | | 2110 | hsa-mir-10a | 51388 | NIP7 | nuclear import 7 homolog (S. cerevisiae) | 1 | | 2111 | hsa-miR-934 | 51388 | NIP7 | nuclear import 7 homolog (S. cerevisiae) | 1 | | 2112 | hsa-miR-591 | 51018 | RRP15 | ribosomal RNA processing 15 homolog (S. cerevisiae) | 1 | | 2113 | hsa-miR-1233 | 79582 | SPAG16 | sperm associated antigen 16 | 1 | | 2114 | hsa-miR-571 | 79582 | SPAG16 | sperm associated antigen 16 | 1 | | 2115 | hsa-miR-874 | 79582 | SPAG16 | sperm associated antigen 16 | 1 | | 2116 | hsa-miR-454\* | 54433 | GAR1 | GAR1 ribonucleoprotein homolog (yeast) | 1 | | 2117 | hsa-miR-1233 | 80173 | IFT74 | intraflagellar transport 74 homolog (Chlamydomonas) | 1 | | 2118 | hsa-miR-571 | 80173 | IFT74 | intraflagellar transport 74 homolog (Chlamydomonas) | 1 | | 2119 | hsa-miR-874 | 80173 | IFT74 | intraflagellar transport 74 homolog (Chlamydomonas) | 1 | | 2120 | hsa-miR-766 | 29928 | TIMM22 | translocase of inner mitochondrial membrane 22 homolog (yeast) | 1 | | 2121 | hsa-miR-1233 | 51182 | HSPA14 | heat shock 70kDa protein 14 | 1 | | 2122 | hsa-miR-591 | 51182 | HSPA14 | heat shock 70kDa protein 14 | 1 | | 2123 | hsa-miR-569 | 55876 | GSDMB | gasdermin B | 1 | | 2124 | hsa-miR-766 | 55876 | GSDMB | gasdermin B | 1 | | 2125 | hsa-miR-874 | 55876 | GSDMB | gasdermin B | 1 | | 2126 | hsa-miR-571 | 80007 | C10orf88 | chromosome 10 open reading frame 88 | 1 | | 2127 | hsa-miR-591 | 80007 | C10orf88 | chromosome 10 open reading frame 88 | 1 | | 2128 | hsa-miR-770-5p | 55753 | OGDHL | oxoglutarate dehydrogenase-like | 1 | | 2129 | hsa-miR-95 | 55753 | OGDHL | oxoglutarate dehydrogenase-like | 1 | | 2133 | hsa-miR-874 | 64783 | RBM15 | RNA binding motif protein 15 | 1 | | 2134 | hsa-miR-591 | 11201 | POLI | polymerase (DNA directed) iota | 1 | | 2135 | hsa-miR-636 | 51374 | C2orf28 | chromosome 2 open reading frame 28 | 1 | | 2136 | hsa-miR-591 | 60560 | NAA35 | N(alpha)-acetyltransferase 35, NatC auxiliary subunit | 1 | | 2137 | hsa-miR-1233 | 51001 | MTERFD1 | MTERF domain containing 1 | 1 | | 2138 | hsa-miR-571 | 51001 | MTERFD1 | MTERF domain containing 1 | 1 | | 2139 | hsa-miR-591 | 51001 | MTERFD1 | MTERF domain containing 1 | 1 | | 2140 | hsa-miR-874 | 51001 | MTERFD1 | MTERF domain containing 1 | 1 | | 2141 | hsa-miR-671-5p | 54344 | DPM3 | dolichyl-phosphate mannosyltransferase polypeptide 3 | 1 | | 2142 | hsa-miR-635 | 9489 | PGS1 | phosphatidylglycerophosphate synthase 1 | 1 | | 2143 | hsa-mir-10a | 55128 | TRIM68 | tripartite motif-containing 68 | 1 | | 2144 | hsa-miR-1233 | 55128 | TRIM68 | tripartite motif-containing 68 | 1 | | 2145 | hsa-miR-569 | 55128 | TRIM68 | tripartite motif-containing 68 | 1 | | 2146 | hsa-miR-571 | 55128 | TRIM68 | tripartite motif-containing 68 | 1 | | 2147 | hsa-miR-766 | 55128 | TRIM68 | tripartite motif-containing 68 | 1 | | 2148 | hsa-mir-628 | 8718 | TNFRSF25 | tumor necrosis factor receptor superfamily, member 25 | 1 | | 2149 | hsa-miR-617 | 8718 | TNFRSF25 | tumor necrosis factor receptor superfamily, member 25 | 1 | | 2150 | hsa-miR-628-5p | 8718 | TNFRSF25 | tumor necrosis factor receptor superfamily, member 25 | 1 | | 2151 | hsa-miR-95 | 51705 | EMCN | endomucin | 1 | | 2152 | hsa-miR-454\* | 55703 | POLR3B | polymerase (RNA) III (DNA directed) polypeptide B | 1 | | 2153 | hsa-miR-591 | 55703 | POLR3B | polymerase (RNA) III (DNA directed) polypeptide B | 1 | | 2154 | hsa-miR-567 | 79669 | C3orf52 | chromosome 3 open reading frame 52 | 1 | | 2155 | hsa-miR-1233 | 54093 | SETD4 | SET domain containing 4 | 1 | | 2156 | hsa-miR-569 | 54093 | SETD4 | SET domain containing 4 | 1 | | 2157 | hsa-miR-766 | 54093 | SETD4 | SET domain containing 4 | 1 | | 2158 | hsa-miR-454\* | 29915 | HCFC2 | host cell factor C2 | 1 | | 2159 | hsa-miR-591 | 29915 | HCFC2 | host cell factor C2 | 1 | | 2160 | hsa-miR-10a | 55714 | ODZ3 | odz, odd Oz/ten-m homolog 3 (Drosophila) | 1 | | 2161 | hsa-miR-770-5p | 55714 | ODZ3 | odz, odd Oz/ten-m homolog 3 (Drosophila) | 1 | | 2162 | hsa-miR-95 | 55714 | ODZ3 | odz, odd Oz/ten-m homolog 3 (Drosophila) | 1 | | 2163 | hsa-miR-591 | 54554 | WDR5B | WD repeat domain 5B | 1 | | 2165 | hsa-miR-591 | 7769 | ZNF226 | zinc finger protein 226 | 1 | | 2166 | hsa-miR-638 | 51548 | SIRT6 | sirtuin (silent mating type information regulation 2 homolog) 6 (S. cerevisiae) | 1 | | 2167 | hsa-miR-766 | 79970 | ZNF767 | zinc finger family member 767 | 1 | | 2168 | hsa-miR-874 | 79810 | PTCD2 | pentatricopeptide repeat domain 2 | 1 | | 2169 | hsa-miR-635 | 57126 | CD177 | CD177 molecule | 1 | | 2170 | hsa-miR-643 | 57126 | CD177 | CD177 molecule | 1 | | 2171 | hsa-miR-10a | 3069 | HDLBP | high density lipoprotein binding protein | 1 | | 2172 | hsa-miR-770-5p | 3069 | HDLBP | high density lipoprotein binding protein | 1 | | 2173 | hsa-miR-95 | 3069 | HDLBP | high density lipoprotein binding protein | 1 | | 2174 | hsa-miR-1233 | 79037 | PVRIG | poliovirus receptor related immunoglobulin domain containing | 1 | | 2175 | hsa-miR-617 | 79037 | PVRIG | poliovirus receptor related immunoglobulin domain containing | 1 | | 2176 | hsa-miR-766 | 79037 | PVRIG | poliovirus receptor related immunoglobulin domain containing | 1 | | 2177 | hsa-miR-1233 | 51275 | C12orf47 | chromosome 12 open reading frame 47 | 1 | | 2178 | hsa-miR-569 | 51275 | C12orf47 | chromosome 12 open reading frame 47 | 1 | | 2179 | hsa-miR-1233 | 7561 | ZNF14 | zinc finger protein 14 | 1 | | 2180 | hsa-miR-571 | 7561 | ZNF14 | zinc finger protein 14 | 1 | | 2181 | hsa-miR-874 | 7561 | ZNF14 | zinc finger protein 14 | 1 | | 2187 | hsa-miR-10a | 5169 | ENPP3 | ectonucleotide pyrophosphatase/phosphodiesterase 3 | 1 | | 2188 | hsa-miR-770-5p | 5169 | ENPP3 | ectonucleotide pyrophosphatase/phosphodiesterase 3 | 1 | | 2189 | hsa-miR-95 | 5169 | ENPP3 | ectonucleotide pyrophosphatase/phosphodiesterase 3 | 1 | | 2190 | hsa-miR-604 | 54518 | APBB1IP | amyloid beta (A4) precursor protein-binding, family B, member 1 interacting protein | 1 | | 2191 | hsa-miR-635 | 54518 | APBB1IP | amyloid beta (A4) precursor protein-binding, family B, member 1 interacting protein | 1 | | 2192 | hsa-miR-638 | 54518 | APBB1IP | amyloid beta (A4) precursor protein-binding, family B, member 1 interacting protein | 1 | | 2193 | hsa-miR-938 | 54518 | APBB1IP | amyloid beta (A4) precursor protein-binding, family B, member 1 interacting protein | 1 | | 2194 | hsa-mir-10a | 79872 | CBLL1 | Cas-Br-M (murine) ecotropic retroviral transforming sequence-like 1 | 1 | | 2203 | hsa-miR-770-5p | 79919 | C2orf54 | chromosome 2 open reading frame 54 | 1 | | 2204 | hsa-miR-95 | 79919 | C2orf54 | chromosome 2 open reading frame 54 | 1 | | 2205 | hsa-mir-186 | 25803 | SPDEF | SAM pointed domain containing ets transcription factor | 1 | | 2206 | hsa-miR-186 | 25803 | SPDEF | SAM pointed domain containing ets transcription factor | 1 | | 2207 | hsa-miR-635 | 387893 | SETD8 | SET domain containing (lysine methyltransferase) 8 | 1 | | 2208 | hsa-miR-604 | 55002 | TMCO3 | transmembrane and coiled-coil domains 3 | 1 | | 2210 | hsa-miR-938 | 55002 | TMCO3 | transmembrane and coiled-coil domains 3 | 1 | | 2211 | hsa-miR-604 | 63916 | ELMO2 | engulfment and cell motility 2 | 1 | | 2213 | hsa-miR-938 | 63916 | ELMO2 | engulfment and cell motility 2 | 1 | | 2214 | hsa-mir-135b | 79595 | SAP130 | Sin3A-associated protein, 130kDa | 1 | | 2215 | hsa-miR-591 | 79595 | SAP130 | Sin3A-associated protein, 130kDa | 1 | | 2216 | hsa-miR-874 | 57602 | USP36 | ubiquitin specific peptidase 36 | 1 | | 2217 | hsa-miR-770-5p | 7042 | TGFB2 | transforming growth factor, beta 2 | 1 | | 2218 | hsa-miR-95 | 7042 | TGFB2 | transforming growth factor, beta 2 | 1 | | 2219 | hsa-miR-10a | 79444 | BIRC7 | baculoviral IAP repeat-containing 7 | 1 | | 2220 | hsa-miR-770-5p | 79444 | BIRC7 | baculoviral IAP repeat-containing 7 | 1 | | 2221 | hsa-miR-95 | 79444 | BIRC7 | baculoviral IAP repeat-containing 7 | 1 | | 2222 | hsa-mir-423 | 80054 | LOC80054 | hypothetical LOC80054 | 1 | | 2223 | hsa-mir-3130-1 | 10880 | ACTL7B | actin-like 7B | 1 | | 2224 | hsa-mir-3130-2 | 10880 | ACTL7B | actin-like 7B | 1 | | 2225 | hsa-mir-3130-3 | 10880 | ACTL7B | actin-like 7B | 1 | | 2226 | hsa-miR-95 | 10880 | ACTL7B | actin-like 7B | 1 | | 2227 | hsa-miR-10a | 150160 | CCT8L2 | chaperonin containing TCP1, subunit 8 (theta)-like 2 | 1 | | 2228 | hsa-mir-3130-1 | 55237 | C14orf115 | chromosome 14 open reading frame 115 | 1 | | 2229 | hsa-mir-3130-2 | 55237 | C14orf115 | chromosome 14 open reading frame 115 | 1 | | 2230 | hsa-mir-3130-3 | 55237 | C14orf115 | chromosome 14 open reading frame 115 | 1 | | 2231 | hsa-miR-10a | 55237 | C14orf115 | chromosome 14 open reading frame 115 | 1 | | 2232 | hsa-miR-770-5p | 55237 | C14orf115 | chromosome 14 open reading frame 115 | 1 | | 2233 | hsa-miR-95 | 55237 | C14orf115 | chromosome 14 open reading frame 115 | 1 | | 2234 | hsa-miR-1233 | 54537 | FAM35A | family with sequence similarity 35, member A | 1 | | 2235 | hsa-miR-571 | 54537 | FAM35A | family with sequence similarity 35, member A | 1 | | 2236 | hsa-miR-591 | 54537 | FAM35A | family with sequence similarity 35, member A | 1 | | 2237 | hsa-miR-874 | 54537 | FAM35A | family with sequence similarity 35, member A | 1 | | 2238 | hsa-miR-10a | 51214 | IGF2AS | insulin-like growth factor 2 antisense | 1 | | 2239 | hsa-miR-635 | 56729 | RETN | resistin | 1 | | 2246 | hsa-mir-628 | 30009 | TBX21 | T-box 21 | 1 | | 2247 | hsa-miR-617 | 30009 | TBX21 | T-box 21 | 1 | | 2248 | hsa-miR-628-5p | 30009 | TBX21 | T-box 21 | 1 | | 2249 | hsa-mir-628 | 50854 | C6orf48 | chromosome 6 open reading frame 48 | 1 | | 2250 | hsa-miR-593\* | 50854 | C6orf48 | chromosome 6 open reading frame 48 | 1 | | 2251 | hsa-miR-623 | 50854 | C6orf48 | chromosome 6 open reading frame 48 | 1 | | 2252 | hsa-miR-628-5p | 50854 | C6orf48 | chromosome 6 open reading frame 48 | 1 | | 2253 | hsa-miR-766 | 50854 | C6orf48 | chromosome 6 open reading frame 48 | 1 | | 2254 | hsa-miR-10a | 55065 | GPR172B | G protein-coupled receptor 172B | 1 | | 2255 | hsa-miR-770-5p | 55065 | GPR172B | G protein-coupled receptor 172B | 1 | | 2256 | hsa-miR-95 | 55065 | GPR172B | G protein-coupled receptor 172B | 1 | | 2257 | hsa-mir-3130-1 | 10501 | SEMA6B | sema domain, transmembrane domain (TM), and cytoplasmic domain, (semaphorin) 6B | 1 | | 2258 | hsa-mir-3130-2 | 10501 | SEMA6B | sema domain, transmembrane domain (TM), and cytoplasmic domain, (semaphorin) 6B | 1 | | 2259 | hsa-mir-3130-3 | 10501 | SEMA6B | sema domain, transmembrane domain (TM), and cytoplasmic domain, (semaphorin) 6B | 1 | | 2260 | hsa-miR-10a | 10501 | SEMA6B | sema domain, transmembrane domain (TM), and cytoplasmic domain, (semaphorin) 6B | 1 | | 2261 | hsa-miR-770-5p | 10501 | SEMA6B | sema domain, transmembrane domain (TM), and cytoplasmic domain, (semaphorin) 6B | 1 | | 2262 | hsa-miR-95 | 10501 | SEMA6B | sema domain, transmembrane domain (TM), and cytoplasmic domain, (semaphorin) 6B | 1 | | 2263 | hsa-miR-623 | 55072 | RNF31 | ring finger protein 31 | 1 | | 2264 | hsa-miR-627 | 55072 | RNF31 | ring finger protein 31 | 1 | | 2265 | hsa-miR-638 | 55072 | RNF31 | ring finger protein 31 | 1 | | 2266 | hsa-miR-671-5p | 55072 | RNF31 | ring finger protein 31 | 1 | | 2267 | hsa-mir-3130-1 | 3274 | HRH2 | histamine receptor H2 | 1 | | 2268 | hsa-mir-3130-2 | 3274 | HRH2 | histamine receptor H2 | 1 | | 2269 | hsa-mir-3130-3 | 3274 | HRH2 | histamine receptor H2 | 1 | | 2270 | hsa-miR-770-5p | 266977 | GPR110 | G protein-coupled receptor 110 | 1 | | 2271 | hsa-miR-95 | 266977 | GPR110 | G protein-coupled receptor 110 | 1 | | 2272 | hsa-mir-10a | 60560 | NAA35 | N(alpha)-acetyltransferase 35, NatC auxiliary subunit | 1 | | 2273 | hsa-mir-885 | 60560 | NAA35 | N(alpha)-acetyltransferase 35, NatC auxiliary subunit | 1 | | 2274 | hsa-miR-766 | 60560 | NAA35 | N(alpha)-acetyltransferase 35, NatC auxiliary subunit | 1 | | 2275 | hsa-miR-934 | 60560 | NAA35 | N(alpha)-acetyltransferase 35, NatC auxiliary subunit | 1 | | 2276 | hsa-miR-635 | 55755 | CDK5RAP2 | CDK5 regulatory subunit associated protein 2 | 1 | | 2277 | hsa-miR-643 | 55755 | CDK5RAP2 | CDK5 regulatory subunit associated protein 2 | 1 | | 2278 | hsa-miR-638 | 26000 | TBC1D10B | TBC1 domain family, member 10B | 1 | | 2279 | hsa-miR-765 | 26000 | TBC1D10B | TBC1 domain family, member 10B | 1 | | 2280 | hsa-mir-423 | 81492 | RSPH6A | radial spoke head 6 homolog A (Chlamydomonas) | 1 | | 2281 | hsa-miR-638 | 81619 | TSPAN14 | tetraspanin 14 | 1 | | 2282 | hsa-miR-1233 | 81606 | LBH | limb bud and heart development homolog (mouse) | 1 | | 2283 | hsa-miR-569 | 81606 | LBH | limb bud and heart development homolog (mouse) | 1 | | 2284 | hsa-miR-766 | 81606 | LBH | limb bud and heart development homolog (mouse) | 1 | | 2285 | hsa-miR-874 | 81606 | LBH | limb bud and heart development homolog (mouse) | 1 | | 2286 | hsa-miR-635 | 81602 | CDADC1 | cytidine and dCMP deaminase domain containing 1 | 1 | | 2287 | hsa-mir-10a | 81034 | SLC25A32 | solute carrier family 25, member 32 | 1 | | 2288 | hsa-miR-454\* | 81034 | SLC25A32 | solute carrier family 25, member 32 | 1 | | 2289 | hsa-miR-591 | 81034 | SLC25A32 | solute carrier family 25, member 32 | 1 | | 2290 | hsa-miR-934 | 81034 | SLC25A32 | solute carrier family 25, member 32 | 1 | | 2291 | hsa-miR-635 | 81577 | GFOD2 | glucose-fructose oxidoreductase domain containing 2 | 1 | | 2292 | hsa-mir-3130-1 | 25959 | KANK2 | KN motif and ankyrin repeat domains 2 | 1 | | 2293 | hsa-mir-3130-2 | 25959 | KANK2 | KN motif and ankyrin repeat domains 2 | 1 | | 2294 | hsa-mir-3130-3 | 25959 | KANK2 | KN motif and ankyrin repeat domains 2 | 1 | | 2295 | hsa-miR-10a | 25959 | KANK2 | KN motif and ankyrin repeat domains 2 | 1 | | 2296 | hsa-miR-770-5p | 25959 | KANK2 | KN motif and ankyrin repeat domains 2 | 1 | | 2297 | hsa-miR-95 | 25959 | KANK2 | KN motif and ankyrin repeat domains 2 | 1 | | 2298 | hsa-miR-1233 | 79961 | DENND2D | DENN/MADD domain containing 2D | 1 | | 2299 | hsa-miR-569 | 79961 | DENND2D | DENN/MADD domain containing 2D | 1 | | 2300 | hsa-miR-766 | 79961 | DENND2D | DENN/MADD domain containing 2D | 1 | | 2301 | hsa-miR-874 | 79961 | DENND2D | DENN/MADD domain containing 2D | 1 | | 2302 | hsa-mir-423 | 9132 | KCNQ4 | potassium voltage-gated channel, KQT-like subfamily, member 4 | 1 | | 2303 | hsa-miR-10a | 9132 | KCNQ4 | potassium voltage-gated channel, KQT-like subfamily, member 4 | 1 | | 2304 | hsa-miR-770-5p | 9132 | KCNQ4 | potassium voltage-gated channel, KQT-like subfamily, member 4 | 1 | | 2305 | hsa-miR-95 | 9132 | KCNQ4 | potassium voltage-gated channel, KQT-like subfamily, member 4 | 1 | | 2306 | hsa-miR-766 | 55239 | OGFOD1 | 2-oxoglutarate and iron-dependent oxygenase domain containing 1 | 1 | | 2307 | hsa-mir-3130-1 | 51208 | CLDN18 | claudin 18 | 1 | | 2308 | hsa-mir-3130-2 | 51208 | CLDN18 | claudin 18 | 1 | | 2309 | hsa-mir-3130-3 | 51208 | CLDN18 | claudin 18 | 1 | | 2310 | hsa-miR-770-5p | 51208 | CLDN18 | claudin 18 | 1 | | 2311 | hsa-miR-95 | 51208 | CLDN18 | claudin 18 | 1 | | 2312 | hsa-miR-635 | 55825 | PECR | peroxisomal trans-2-enoyl-CoA reductase | 1 | | 2313 | hsa-miR-770-5p | 56979 | PRDM9 | PR domain containing 9 | 1 | | 2314 | hsa-miR-95 | 56979 | PRDM9 | PR domain containing 9 | 1 | | 2315 | hsa-miR-934 | 29919 | C18orf8 | chromosome 18 open reading frame 8 | 1 | | 2317 | hsa-mir-199a-2 | 79184 | BRCC3 | BRCA1/BRCA2-containing complex, subunit 3 | 1 | | 2318 | hsa-mir-214 | 79184 | BRCC3 | BRCA1/BRCA2-containing complex, subunit 3 | 1 | | 2319 | hsa-miR-199a-5p | 79184 | BRCC3 | BRCA1/BRCA2-containing complex, subunit 3 | 1 | | 2320 | hsa-miR-591 | 79184 | BRCC3 | BRCA1/BRCA2-containing complex, subunit 3 | 1 | | 2323 | hsa-miR-1233 | 23435 | TARDBP | TAR DNA binding protein | 1 | | 2324 | hsa-miR-569 | 23435 | TARDBP | TAR DNA binding protein | 1 | | 2325 | hsa-miR-766 | 23435 | TARDBP | TAR DNA binding protein | 1 | | 2326 | hsa-miR-874 | 23435 | TARDBP | TAR DNA binding protein | 1 | | 2327 | hsa-miR-623 | 81890 | QTRT1 | queuine tRNA-ribosyltransferase 1 | 1 | | 2328 | hsa-miR-10a | 5623 | PSPN | persephin | 1 | | 2329 | hsa-miR-770-5p | 5623 | PSPN | persephin | 1 | | 2330 | hsa-miR-95 | 5623 | PSPN | persephin | 1 | | 2331 | hsa-miR-10a | 56132 | PCDHB3 | protocadherin beta 3 | 1 | | 2332 | hsa-miR-770-5p | 56132 | PCDHB3 | protocadherin beta 3 | 1 | | 2333 | hsa-miR-770-5p | 3238 | HOXD12 | homeobox D12 | 1 | | 2334 | hsa-miR-95 | 3238 | HOXD12 | homeobox D12 | 1 | | 2335 | hsa-miR-10a | 81285 | OR51E2 | olfactory receptor, family 51, subfamily E, member 2 | 1 | | 2336 | hsa-miR-10a | 10741 | RBBP9 | retinoblastoma binding protein 9 | 1 | | 2337 | hsa-miR-770-5p | 10741 | RBBP9 | retinoblastoma binding protein 9 | 1 | | 2338 | hsa-miR-95 | 10741 | RBBP9 | retinoblastoma binding protein 9 | 1 | | 2339 | hsa-miR-635 | 51271 | UBAP1 | ubiquitin associated protein 1 | 1 | | 2340 | hsa-miR-765 | 51271 | UBAP1 | ubiquitin associated protein 1 | 1 | | 2341 | hsa-miR-591 | 51451 | LCMT1 | leucine carboxyl methyltransferase 1 | 1 | | 2342 | hsa-miR-627 | 63916 | ELMO2 | engulfment and cell motility 2 | 1 | | 2343 | hsa-miR-671-5p | 63916 | ELMO2 | engulfment and cell motility 2 | 1 | | 2345 | hsa-miR-1233 | 55341 | LSG1 | large subunit GTPase 1 homolog (S. cerevisiae) | 1 | | 2346 | hsa-miR-571 | 55341 | LSG1 | large subunit GTPase 1 homolog (S. cerevisiae) | 1 | | 2347 | hsa-miR-874 | 55341 | LSG1 | large subunit GTPase 1 homolog (S. cerevisiae) | 1 | | 2348 | hsa-miR-10a | 10025 | MED16 | mediator complex subunit 16 | 1 | | 2349 | hsa-miR-635 | 65010 | SLC26A6 | solute carrier family 26, member 6 | 1 | | 2350 | hsa-miR-765 | 65010 | SLC26A6 | solute carrier family 26, member 6 | 1 | | 2351 | hsa-miR-454\* | 4329 | ALDH6A1 | aldehyde dehydrogenase 6 family, member A1 | 1 | | 2352 | hsa-miR-591 | 4329 | ALDH6A1 | aldehyde dehydrogenase 6 family, member A1 | 1 | | 2353 | hsa-mir-10a | 55863 | TMEM126B | transmembrane protein 126B | 1 | | 2354 | hsa-miR-576-5p | 55863 | TMEM126B | transmembrane protein 126B | 1 | | 2355 | hsa-miR-1233 | 440515 | ZNF506 | zinc finger protein 506 | 1 | | 2356 | hsa-mir-423 | 83696 | TRAPPC9 | trafficking protein particle complex 9 | 1 | | 2357 | hsa-mir-3130-1 | 83696 | TRAPPC9 | trafficking protein particle complex 9 | 1 | | 2358 | hsa-mir-3130-2 | 83696 | TRAPPC9 | trafficking protein particle complex 9 | 1 | | 2359 | hsa-mir-3130-3 | 83696 | TRAPPC9 | trafficking protein particle complex 9 | 1 | | 2360 | hsa-miR-10a | 83696 | TRAPPC9 | trafficking protein particle complex 9 | 1 | | 2361 | hsa-miR-770-5p | 83696 | TRAPPC9 | trafficking protein particle complex 9 | 1 | | 2362 | hsa-miR-95 | 83696 | TRAPPC9 | trafficking protein particle complex 9 | 1 | | 2363 | hsa-miR-593\* | 55272 | IMP3 | IMP3, U3 small nucleolar ribonucleoprotein, homolog (yeast) | 1 | | 2364 | hsa-miR-623 | 55272 | IMP3 | IMP3, U3 small nucleolar ribonucleoprotein, homolog (yeast) | 1 | | 2365 | hsa-miR-638 | 10163 | WASF2 | WAS protein family, member 2 | 1 | | 2366 | hsa-miR-765 | 10163 | WASF2 | WAS protein family, member 2 | 1 | | 2367 | hsa-miR-766 | 6146 | RPL22 | ribosomal protein L22 | 1 | | 2368 | hsa-miR-623 | 10238 | DCAF7 | DDB1 and CUL4 associated factor 7 | 1 | | 2369 | hsa-miR-635 | 54434 | SSH1 | slingshot homolog 1 (Drosophila) | 1 | | 2370 | hsa-miR-765 | 54434 | SSH1 | slingshot homolog 1 (Drosophila) | 1 | | 2371 | hsa-miR-627 | 80305 | TRABD | TraB domain containing | 1 | | 2373 | hsa-miR-765 | 80305 | TRABD | TraB domain containing | 1 | | 2374 | hsa-miR-1233 | 90806 | ANGEL2 | angel homolog 2 (Drosophila) | 1 | | 2375 | hsa-miR-571 | 90806 | ANGEL2 | angel homolog 2 (Drosophila) | 1 | | 2377 | hsa-mir-10a | 90806 | ANGEL2 | angel homolog 2 (Drosophila) | 1 | | 2378 | hsa-miR-766 | 5911 | RAP2A | RAP2A, member of RAS oncogene family | 1 | | 2380 | hsa-miR-770-5p | 27147 | DENND2A | DENN/MADD domain containing 2A | 1 | | 2381 | hsa-miR-95 | 27147 | DENND2A | DENN/MADD domain containing 2A | 1 | | 2382 | hsa-miR-571 | 84851 | TRIM52 | tripartite motif-containing 52 | 1 | | 2383 | hsa-miR-576-5p | 84851 | TRIM52 | tripartite motif-containing 52 | 1 | | 2384 | hsa-miR-190 | 3178 | HNRNPA1 | heterogeneous nuclear ribonucleoprotein A1 | 1 | | 2385 | hsa-miR-454\* | 4869 | NPM1 | nucleophosmin (nucleolar phosphoprotein B23, numatrin) | 1 | | 2386 | hsa-miR-1233 | 3653 | IPW | imprinted in Prader-Willi syndrome (non-protein coding) | 1 | | 2387 | hsa-miR-874 | 3653 | IPW | imprinted in Prader-Willi syndrome (non-protein coding) | 1 | | 2389 | hsa-mir-10a | 339448 | C1orf174 | chromosome 1 open reading frame 174 | 1 | | 2390 | hsa-miR-1233 | 339448 | C1orf174 | chromosome 1 open reading frame 174 | 1 | | 2391 | hsa-miR-571 | 339448 | C1orf174 | chromosome 1 open reading frame 174 | 1 | | 2392 | hsa-miR-591 | 339448 | C1orf174 | chromosome 1 open reading frame 174 | 1 | | 2393 | hsa-miR-874 | 339448 | C1orf174 | chromosome 1 open reading frame 174 | 1 | | 2394 | hsa-mir-3130-1 | 752014 | CEMP1 | cementum protein 1 | 1 | | 2395 | hsa-mir-3130-2 | 752014 | CEMP1 | cementum protein 1 | 1 | | 2396 | hsa-mir-3130-3 | 752014 | CEMP1 | cementum protein 1 | 1 | | 2397 | hsa-miR-10a | 752014 | CEMP1 | cementum protein 1 | 1 | | 2398 | hsa-miR-770-5p | 752014 | CEMP1 | cementum protein 1 | 1 | | 2399 | hsa-miR-95 | 752014 | CEMP1 | cementum protein 1 | 1 | | 2400 | hsa-miR-1233 | 10399 | GNB2L1 | guanine nucleotide binding protein (G protein), beta polypeptide 2-like 1 | 1 | | 2401 | hsa-miR-766 | 10399 | GNB2L1 | guanine nucleotide binding protein (G protein), beta polypeptide 2-like 1 | 1 | | 2402 | hsa-miR-591 | 3178 | HNRNPA1 | heterogeneous nuclear ribonucleoprotein A1 | 1 | | 2403 | hsa-miR-10a | 2036 | EPB41L1 | erythrocyte membrane protein band 4.1-like 1 | 1 | | 2404 | hsa-miR-770-5p | 2036 | EPB41L1 | erythrocyte membrane protein band 4.1-like 1 | 1 | | 2405 | hsa-miR-95 | 2036 | EPB41L1 | erythrocyte membrane protein band 4.1-like 1 | 1 | | 2406 | hsa-miR-1233 | 347902 | AMIGO2 | adhesion molecule with Ig-like domain 2 | 1 | | 2407 | hsa-miR-569 | 347902 | AMIGO2 | adhesion molecule with Ig-like domain 2 | 1 | | 2408 | hsa-miR-571 | 347902 | AMIGO2 | adhesion molecule with Ig-like domain 2 | 1 | | 2409 | hsa-miR-766 | 347902 | AMIGO2 | adhesion molecule with Ig-like domain 2 | 1 | | 2410 | hsa-miR-874 | 347902 | AMIGO2 | adhesion molecule with Ig-like domain 2 | 1 | | 2412 | hsa-miR-569 | 55750 | AGK | acylglycerol kinase | 1 | | 2413 | hsa-miR-766 | 55750 | AGK | acylglycerol kinase | 1 | | 2414 | hsa-miR-766 | 84861 | KLHL22 | kelch-like 22 (Drosophila) | 1 | | 2415 | hsa-mir-10a | 9994 | CASP8AP2 | caspase 8 associated protein 2 | 1 | | 2416 | hsa-mir-199a-2 | 9994 | CASP8AP2 | caspase 8 associated protein 2 | 1 | | 2417 | hsa-mir-214 | 9994 | CASP8AP2 | caspase 8 associated protein 2 | 1 | | 2418 | hsa-mir-149 | 9994 | CASP8AP2 | caspase 8 associated protein 2 | 1 | | 2419 | hsa-miR-199a-5p | 9994 | CASP8AP2 | caspase 8 associated protein 2 | 1 | | 2420 | hsa-miR-576-5p | 9994 | CASP8AP2 | caspase 8 associated protein 2 | 1 | | 2421 | hsa-miR-934 | 9994 | CASP8AP2 | caspase 8 associated protein 2 | 1 | | 2422 | hsa-mir-10a | 54700 | RRN3 | RRN3 RNA polymerase I transcription factor homolog (S. cerevisiae) | 1 | | 2423 | hsa-miR-1233 | 54700 | RRN3 | RRN3 RNA polymerase I transcription factor homolog (S. cerevisiae) | 1 | | 2424 | hsa-miR-190 | 54700 | RRN3 | RRN3 RNA polymerase I transcription factor homolog (S. cerevisiae) | 1 | | 2425 | hsa-miR-571 | 54700 | RRN3 | RRN3 RNA polymerase I transcription factor homolog (S. cerevisiae) | 1 | | 2426 | hsa-miR-766 | 54700 | RRN3 | RRN3 RNA polymerase I transcription factor homolog (S. cerevisiae) | 1 | | 2427 | hsa-miR-874 | 54700 | RRN3 | RRN3 RNA polymerase I transcription factor homolog (S. cerevisiae) | 1 | | 2428 | hsa-mir-10a | 65084 | TMEM135 | transmembrane protein 135 | 1 | | 2429 | hsa-miR-576-5p | 65084 | TMEM135 | transmembrane protein 135 | 1 | | 2430 | hsa-miR-591 | 65084 | TMEM135 | transmembrane protein 135 | 1 | | 2431 | hsa-miR-635 | 10938 | EHD1 | EH-domain containing 1 | 1 | | 2432 | hsa-miR-1233 | 10016 | PDCD6 | programmed cell death 6 | 1 | | 2433 | hsa-miR-576-5p | 51322 | WAC | WW domain containing adaptor with coiled-coil | 1 | | 2434 | hsa-miR-766 | 27131 | SNX5 | sorting nexin 5 | 1 | | 2435 | hsa-miR-135a | 28988 | DBNL | drebrin-like | 1 | | 2436 | hsa-miR-638 | 28988 | DBNL | drebrin-like | 1 | | 2437 | hsa-miR-765 | 28988 | DBNL | drebrin-like | 1 | | 2438 | hsa-miR-454\* | 10927 | SPIN1 | spindlin 1 | 1 | | 2439 | hsa-miR-569 | 51727 | CMPK1 | cytidine monophosphate (UMP-CMP) kinase 1, cytosolic | 1 | | 2440 | hsa-miR-766 | 51727 | CMPK1 | cytidine monophosphate (UMP-CMP) kinase 1, cytosolic | 1 | | 2441 | hsa-miR-591 | 26985 | AP3M1 | adaptor-related protein complex 3, mu 1 subunit | 1 | | 2442 | hsa-mir-10a | 26060 | APPL1 | adaptor protein, phosphotyrosine interaction, PH domain and leucine zipper containing 1 | 1 | | 2443 | hsa-mir-885 | 26060 | APPL1 | adaptor protein, phosphotyrosine interaction, PH domain and leucine zipper containing 1 | 1 | | 2444 | hsa-miR-934 | 26060 | APPL1 | adaptor protein, phosphotyrosine interaction, PH domain and leucine zipper containing 1 | 1 | | 2445 | hsa-mir-3130-1 | 54982 | CLN6 | ceroid-lipofuscinosis, neuronal 6, late infantile, variant | 1 | | 2446 | hsa-mir-3130-2 | 54982 | CLN6 | ceroid-lipofuscinosis, neuronal 6, late infantile, variant | 1 | | 2447 | hsa-mir-3130-3 | 54982 | CLN6 | ceroid-lipofuscinosis, neuronal 6, late infantile, variant | 1 | | 2448 | hsa-mir-10a | 79139 | DERL1 | Der1-like domain family, member 1 | 1 | | 2449 | hsa-miR-454\* | 79139 | DERL1 | Der1-like domain family, member 1 | 1 | | 2450 | hsa-mir-10a | 51026 | GOLT1B | golgi transport 1 homolog B (S. cerevisiae) | 1 | | 2451 | hsa-miR-454\* | 51026 | GOLT1B | golgi transport 1 homolog B (S. cerevisiae) | 1 | | 2452 | hsa-miR-576-5p | 51026 | GOLT1B | golgi transport 1 homolog B (S. cerevisiae) | 1 | | 2453 | hsa-miR-591 | 51026 | GOLT1B | golgi transport 1 homolog B (S. cerevisiae) | 1 | | 2454 | hsa-miR-95 | 65083 | NOL6 | nucleolar protein family 6 (RNA-associated) | 1 | | 2455 | hsa-miR-10a | 55366 | LGR4 | leucine-rich repeat-containing G protein-coupled receptor 4 | 1 | | 2456 | hsa-miR-770-5p | 55366 | LGR4 | leucine-rich repeat-containing G protein-coupled receptor 4 | 1 | | 2457 | hsa-miR-95 | 55366 | LGR4 | leucine-rich repeat-containing G protein-coupled receptor 4 | 1 | | 2458 | hsa-miR-1236 | 51013 | EXOSC1 | exosome component 1 | 1 | | 2459 | hsa-miR-454\* | 55300 | PI4K2B | phosphatidylinositol 4-kinase type 2 beta | 1 | | 2460 | hsa-miR-770-5p | 125058 | TBC1D16 | TBC1 domain family, member 16 | 1 | | 2461 | hsa-miR-95 | 125058 | TBC1D16 | TBC1 domain family, member 16 | 1 | | 2462 | hsa-miR-1233 | 64328 | XPO4 | exportin 4 | 1 | | 2463 | hsa-miR-571 | 64328 | XPO4 | exportin 4 | 1 | | 2464 | hsa-miR-591 | 64328 | XPO4 | exportin 4 | 1 | | 2465 | hsa-miR-766 | 64328 | XPO4 | exportin 4 | 1 | | 2466 | hsa-miR-874 | 64328 | XPO4 | exportin 4 | 1 | | 2467 | hsa-miR-1233 | 8313 | AXIN2 | axin 2 | 1 | | 2468 | hsa-miR-571 | 8313 | AXIN2 | axin 2 | 1 | | 2469 | hsa-miR-874 | 8313 | AXIN2 | axin 2 | 1 | | 2470 | hsa-miR-569 | 55014 | STX17 | syntaxin 17 | 1 | | 2471 | hsa-miR-766 | 55014 | STX17 | syntaxin 17 | 1 | | 2472 | hsa-miR-635 | 56034 | PDGFC | platelet derived growth factor C | 1 | | 2473 | hsa-miR-591 | 51095 | TRNT1 | tRNA nucleotidyl transferase, CCA-adding, 1 | 1 | | 2474 | hsa-mir-3130-1 | 64093 | SMOC1 | SPARC related modular calcium binding 1 | 1 | | 2475 | hsa-mir-3130-2 | 64093 | SMOC1 | SPARC related modular calcium binding 1 | 1 | | 2476 | hsa-mir-3130-3 | 64093 | SMOC1 | SPARC related modular calcium binding 1 | 1 | | 2477 | hsa-miR-10a | 64093 | SMOC1 | SPARC related modular calcium binding 1 | 1 | | 2478 | hsa-miR-95 | 64093 | SMOC1 | SPARC related modular calcium binding 1 | 1 | | 2479 | hsa-miR-571 | 64940 | STAG3L4 | stromal antigen 3-like 4 | 1 | | 2480 | hsa-mir-149 | 51633 | OTUD6B | OTU domain containing 6B | 1 | | 2481 | hsa-miR-591 | 51633 | OTUD6B | OTU domain containing 6B | 1 | | 2482 | hsa-miR-454\* | 63979 | FIGNL1 | fidgetin-like 1 | 1 | | 2483 | hsa-miR-591 | 63979 | FIGNL1 | fidgetin-like 1 | 1 | | 2484 | hsa-miR-766 | 56919 | DHX33 | DEAH (Asp-Glu-Ala-His) box polypeptide 33 | 1 | | 2485 | hsa-miR-766 | 53335 | BCL11A | B-cell CLL/lymphoma 11A (zinc finger protein) | 1 | | 2486 | hsa-miR-1233 | 9840 | KIAA0748 | KIAA0748 | 1 | | 2487 | hsa-miR-569 | 9840 | KIAA0748 | KIAA0748 | 1 | | 2488 | hsa-miR-571 | 9840 | KIAA0748 | KIAA0748 | 1 | | 2489 | hsa-miR-766 | 9840 | KIAA0748 | KIAA0748 | 1 | | 2490 | hsa-miR-874 | 9840 | KIAA0748 | KIAA0748 | 1 | | 2492 | hsa-miR-569 | 23731 | C9orf5 | chromosome 9 open reading frame 5 | 1 | | 2494 | hsa-miR-934 | 23731 | C9orf5 | chromosome 9 open reading frame 5 | 1 | | 2495 | hsa-miR-454\* | 23731 | C9orf5 | chromosome 9 open reading frame 5 | 1 | | 2496 | hsa-miR-636 | 54940 | OCIAD1 | OCIA domain containing 1 | 1 | | 2497 | hsa-miR-1233 | 28987 | NOB1 | NIN1/RPN12 binding protein 1 homolog (S. cerevisiae) | 1 | | 2498 | hsa-miR-766 | 28987 | NOB1 | NIN1/RPN12 binding protein 1 homolog (S. cerevisiae) | 1 | | 2499 | hsa-miR-874 | 28987 | NOB1 | NIN1/RPN12 binding protein 1 homolog (S. cerevisiae) | 1 | | 2500 | hsa-miR-636 | 51234 | TMEM85 | transmembrane protein 85 | 1 | | 2501 | hsa-miR-604 | 23582 | CCNDBP1 | cyclin D-type binding-protein 1 | 1 | | 2502 | hsa-miR-635 | 23582 | CCNDBP1 | cyclin D-type binding-protein 1 | 1 | | 2503 | hsa-miR-938 | 23582 | CCNDBP1 | cyclin D-type binding-protein 1 | 1 | | 2504 | hsa-mir-10a | 55591 | VEZT | vezatin, adherens junctions transmembrane protein | 1 | | 2506 | hsa-miR-454\* | 55591 | VEZT | vezatin, adherens junctions transmembrane protein | 1 | | 2510 | hsa-miR-638 | 81603 | TRIM8 | tripartite motif-containing 8 | 1 | | 2511 | hsa-miR-623 | 57407 | NMRAL1 | NmrA-like family domain containing 1 | 1 | | 2512 | hsa-miR-623 | 94103 | ORMDL3 | ORM1-like 3 (S. cerevisiae) | 1 | | 2513 | hsa-miR-1233 | 54931 | RG9MTD1 | RNA (guanine-9-) methyltransferase domain containing 1 | 1 | | 2514 | hsa-miR-591 | 54931 | RG9MTD1 | RNA (guanine-9-) methyltransferase domain containing 1 | 1 | | 2515 | hsa-miR-591 | 51496 | CTDSPL2 | CTD (carboxy-terminal domain, RNA polymerase II, polypeptide A) small phosphatase like 2 | 1 | | 2517 | hsa-miR-571 | 10194 | TSHZ1 | teashirt zinc finger homeobox 1 | 1 | | 2518 | hsa-miR-874 | 10194 | TSHZ1 | teashirt zinc finger homeobox 1 | 1 | | 2519 | hsa-miR-569 | 10194 | TSHZ1 | teashirt zinc finger homeobox 1 | 1 | | 2520 | hsa-miR-766 | 10194 | TSHZ1 | teashirt zinc finger homeobox 1 | 1 | | 2521 | hsa-miR-934 | 10194 | TSHZ1 | teashirt zinc finger homeobox 1 | 1 | | 2522 | hsa-miR-1233 | 55692 | LUC7L | LUC7-like (S. cerevisiae) | 1 | | 2523 | hsa-miR-571 | 55692 | LUC7L | LUC7-like (S. cerevisiae) | 1 | | 2524 | hsa-miR-766 | 55692 | LUC7L | LUC7-like (S. cerevisiae) | 1 | | 2525 | hsa-miR-874 | 55692 | LUC7L | LUC7-like (S. cerevisiae) | 1 | | 2526 | hsa-miR-10a | 84266 | ALKBH7 | alkB, alkylation repair homolog 7 (E. coli) | 1 | | 2527 | hsa-miR-770-5p | 84266 | ALKBH7 | alkB, alkylation repair homolog 7 (E. coli) | 1 | | 2529 | hsa-miR-627 | 84266 | ALKBH7 | alkB, alkylation repair homolog 7 (E. coli) | 1 | | 2531 | hsa-miR-10a | 51129 | ANGPTL4 | angiopoietin-like 4 | 1 | | 2532 | hsa-miR-770-5p | 51129 | ANGPTL4 | angiopoietin-like 4 | 1 | | 2533 | hsa-miR-95 | 51129 | ANGPTL4 | angiopoietin-like 4 | 1 | | 2534 | hsa-miR-591 | 51249 | TMEM69 | transmembrane protein 69 | 1 | | 2535 | hsa-miR-604 | 25798 | BRI3 | brain protein I3 | 1 | | 2536 | hsa-miR-938 | 25798 | BRI3 | brain protein I3 | 1 | | 2539 | hsa-miR-766 | 84172 | POLR1B | polymerase (RNA) I polypeptide B, 128kDa | 1 | | 2541 | hsa-miR-766 | 55646 | LYAR | Ly1 antibody reactive homolog (mouse) | 1 | | 2542 | hsa-mir-10a | 83548 | COG3 | component of oligomeric golgi complex 3 | 1 | | 2543 | hsa-miR-604 | 83862 | TMEM120A | transmembrane protein 120A | 1 | | 2544 | hsa-miR-635 | 83862 | TMEM120A | transmembrane protein 120A | 1 | | 2545 | hsa-miR-638 | 83862 | TMEM120A | transmembrane protein 120A | 1 | | 2546 | hsa-miR-938 | 83862 | TMEM120A | transmembrane protein 120A | 1 | | 2547 | hsa-miR-591 | 90592 | ZNF700 | zinc finger protein 700 | 1 | | 2548 | hsa-miR-635 | 83853 | ROPN1L | ropporin 1-like | 1 | | 2549 | hsa-miR-454\* | 9406 | ZRANB2 | zinc finger, RAN-binding domain containing 2 | 1 | | 2550 | hsa-miR-591 | 9406 | ZRANB2 | zinc finger, RAN-binding domain containing 2 | 1 | | 2551 | hsa-miR-638 | 29933 | GPR132 | G protein-coupled receptor 132 | 1 | | 2553 | hsa-miR-569 | 83636 | C19orf12 | chromosome 19 open reading frame 12 | 1 | | 2554 | hsa-miR-571 | 83636 | C19orf12 | chromosome 19 open reading frame 12 | 1 | | 2556 | hsa-miR-874 | 83636 | C19orf12 | chromosome 19 open reading frame 12 | 1 | | 2557 | hsa-miR-635 | 29097 | CNIH4 | cornichon homolog 4 (Drosophila) | 1 | | 2558 | hsa-miR-1233 | 56477 | CCL28 | chemokine (C-C motif) ligand 28 | 1 | | 2559 | hsa-miR-766 | 56477 | CCL28 | chemokine (C-C motif) ligand 28 | 1 | | 2560 | hsa-miR-874 | 56477 | CCL28 | chemokine (C-C motif) ligand 28 | 1 | | 2561 | hsa-miR-10a | 84070 | FAM186B | family with sequence similarity 186, member B | 1 | | 2562 | hsa-miR-623 | 55929 | DMAP1 | DNA methyltransferase 1 associated protein 1 | 1 | | 2563 | hsa-miR-770-5p | 149986 | LSM14B | LSM14B, SCD6 homolog B (S. cerevisiae) | 1 | | 2564 | hsa-miR-95 | 149986 | LSM14B | LSM14B, SCD6 homolog B (S. cerevisiae) | 1 | | 2565 | hsa-miR-95 | 27123 | DKK2 | dickkopf homolog 2 (Xenopus laevis) | 1 | | 2566 | hsa-mir-3130-1 | 116519 | APOA5 | apolipoprotein A-V | 1 | | 2567 | hsa-mir-3130-2 | 116519 | APOA5 | apolipoprotein A-V | 1 | | 2568 | hsa-mir-3130-3 | 116519 | APOA5 | apolipoprotein A-V | 1 | | 2569 | hsa-miR-766 | 79048 | SECISBP2 | SECIS binding protein 2 | 1 | | 2570 | hsa-miR-770-5p | 7455 | ZAN | zonadhesin | 1 | | 2571 | hsa-miR-95 | 7455 | ZAN | zonadhesin | 1 | | 2572 | hsa-miR-10a | 57731 | SPTBN4 | spectrin, beta, non-erythrocytic 4 | 1 | | 2573 | hsa-miR-770-5p | 57731 | SPTBN4 | spectrin, beta, non-erythrocytic 4 | 1 | | 2574 | hsa-miR-95 | 57731 | SPTBN4 | spectrin, beta, non-erythrocytic 4 | 1 | | 2575 | hsa-mir-423 | 10841 | FTCD | formiminotransferase cyclodeaminase | 1 | | 2576 | hsa-miR-604 | 51719 | CAB39 | calcium binding protein 39 | 1 | | 2577 | hsa-miR-635 | 51719 | CAB39 | calcium binding protein 39 | 1 | | 2578 | hsa-miR-938 | 51719 | CAB39 | calcium binding protein 39 | 1 | | 2579 | hsa-miR-1233 | 51523 | CXXC5 | CXXC finger 5 | 1 | | 2580 | hsa-miR-766 | 51523 | CXXC5 | CXXC finger 5 | 1 | | 2581 | hsa-miR-1233 | 84820 | POLR2J4 | polymerase (RNA) II (DNA directed) polypeptide J4, pseudogene | 1 | | 2582 | hsa-miR-10a | 84552 | PARD6G | par-6 partitioning defective 6 homolog gamma (C. elegans) | 1 | | 2583 | hsa-miR-770-5p | 84552 | PARD6G | par-6 partitioning defective 6 homolog gamma (C. elegans) | 1 | | 2584 | hsa-miR-95 | 84552 | PARD6G | par-6 partitioning defective 6 homolog gamma (C. elegans) | 1 | | 2585 | hsa-miR-95 | 4773 | NFATC2 | nuclear factor of activated T-cells, cytoplasmic, calcineurin-dependent 2 | 1 | | 2586 | hsa-miR-766 | 6730 | SRP68 | signal recognition particle 68kDa | 1 | | 2587 | hsa-miR-454\* | 84992 | PIGY | phosphatidylinositol glycan anchor biosynthesis, class Y | 1 | | 2588 | hsa-mir-10a | 54467 | ANKIB1 | ankyrin repeat and IBR domain containing 1 | 1 | | 2589 | hsa-mir-135b | 54467 | ANKIB1 | ankyrin repeat and IBR domain containing 1 | 1 | | 2590 | hsa-miR-454\* | 54467 | ANKIB1 | ankyrin repeat and IBR domain containing 1 | 1 | | 2591 | hsa-miR-934 | 54467 | ANKIB1 | ankyrin repeat and IBR domain containing 1 | 1 | | 2592 | hsa-miR-1233 | 54467 | ANKIB1 | ankyrin repeat and IBR domain containing 1 | 1 | | 2593 | hsa-miR-766 | 54467 | ANKIB1 | ankyrin repeat and IBR domain containing 1 | 1 | | 2594 | hsa-miR-576-5p | 27327 | TNRC6A | trinucleotide repeat containing 6A | 1 | | 2595 | hsa-miR-874 | 56990 | CDC42SE2 | CDC42 small effector 2 | 1 | | 2597 | hsa-miR-571 | 84365 | MKI67IP | MKI67 (FHA domain) interacting nucleolar phosphoprotein | 1 | | 2599 | hsa-miR-569 | 84365 | MKI67IP | MKI67 (FHA domain) interacting nucleolar phosphoprotein | 1 | | 2600 | hsa-miR-874 | 84365 | MKI67IP | MKI67 (FHA domain) interacting nucleolar phosphoprotein | 1 | | 2601 | hsa-miR-627 | 54921 | CHTF8 | CTF8, chromosome transmission fidelity factor 8 homolog (S. cerevisiae) | 1 | | 2602 | hsa-miR-671-5p | 54921 | CHTF8 | CTF8, chromosome transmission fidelity factor 8 homolog (S. cerevisiae) | 1 | | 2603 | hsa-miR-604 | 415116 | PIM3 | pim-3 oncogene | 1 | | 2604 | hsa-miR-638 | 415116 | PIM3 | pim-3 oncogene | 1 | | 2605 | hsa-miR-938 | 415116 | PIM3 | pim-3 oncogene | 1 | | 2606 | hsa-mir-10a | 92912 | UBE2Q2 | ubiquitin-conjugating enzyme E2Q family member 2 | 1 | | 2607 | hsa-miR-1233 | 92912 | UBE2Q2 | ubiquitin-conjugating enzyme E2Q family member 2 | 1 | | 2608 | hsa-miR-571 | 92912 | UBE2Q2 | ubiquitin-conjugating enzyme E2Q family member 2 | 1 | | 2609 | hsa-miR-766 | 92912 | UBE2Q2 | ubiquitin-conjugating enzyme E2Q family member 2 | 1 | | 2610 | hsa-miR-604 | 7920 | BAT5 | HLA-B associated transcript 5 | 1 | | 2611 | hsa-miR-635 | 7920 | BAT5 | HLA-B associated transcript 5 | 1 | | 2612 | hsa-miR-638 | 7920 | BAT5 | HLA-B associated transcript 5 | 1 | | 2613 | hsa-miR-765 | 7920 | BAT5 | HLA-B associated transcript 5 | 1 | | 2614 | hsa-miR-938 | 7920 | BAT5 | HLA-B associated transcript 5 | 1 | | 2615 | hsa-miR-604 | 7706 | TRIM25 | tripartite motif-containing 25 | 1 | | 2616 | hsa-miR-635 | 7706 | TRIM25 | tripartite motif-containing 25 | 1 | | 2617 | hsa-miR-938 | 7706 | TRIM25 | tripartite motif-containing 25 | 1 | | 2619 | hsa-miR-635 | 57655 | GRAMD1A | GRAM domain containing 1A | 1 | | 2620 | hsa-miR-765 | 57655 | GRAMD1A | GRAM domain containing 1A | 1 | | 2622 | hsa-miR-627 | 25921 | ZDHHC5 | zinc finger, DHHC-type containing 5 | 1 | | 2623 | hsa-miR-638 | 25921 | ZDHHC5 | zinc finger, DHHC-type containing 5 | 1 | | 2624 | hsa-miR-766 | 57291 | KIAA0114 | KIAA0114 | 1 | | 2625 | hsa-mir-1224 | 134553 | C5orf24 | chromosome 5 open reading frame 24 | 1 | | 2626 | hsa-miR-1224-5p | 134553 | C5orf24 | chromosome 5 open reading frame 24 | 1 | | 2627 | hsa-miR-1233 | 64968 | MRPS6 | mitochondrial ribosomal protein S6 | 1 | | 2628 | hsa-miR-569 | 64968 | MRPS6 | mitochondrial ribosomal protein S6 | 1 | | 2629 | hsa-miR-766 | 64968 | MRPS6 | mitochondrial ribosomal protein S6 | 1 | | 2630 | hsa-miR-604 | 91663 | MYADM | myeloid-associated differentiation marker | 1 | | 2631 | hsa-miR-638 | 91663 | MYADM | myeloid-associated differentiation marker | 1 | | 2632 | hsa-miR-765 | 91663 | MYADM | myeloid-associated differentiation marker | 1 | | 2633 | hsa-miR-938 | 91663 | MYADM | myeloid-associated differentiation marker | 1 | | 2634 | hsa-miR-571 | 1968 | EIF2S3 | eukaryotic translation initiation factor 2, subunit 3 gamma, 52kDa | 1 | | 2635 | hsa-miR-874 | 1968 | EIF2S3 | eukaryotic translation initiation factor 2, subunit 3 gamma, 52kDa | 1 | | 2636 | hsa-miR-766 | 84317 | CCDC115 | coiled-coil domain containing 115 | 1 | | 2637 | hsa-miR-671-5p | 91012 | LASS5 | LAG1 homolog, ceramide synthase 5 | 1 | | 2638 | hsa-miR-591 | 64426 | SUDS3 | suppressor of defective silencing 3 homolog (S. cerevisiae) | 1 | | 2639 | hsa-miR-627 | 84335 | AKT1S1 | AKT1 substrate 1 (proline-rich) | 1 | | 2640 | hsa-miR-638 | 84335 | AKT1S1 | AKT1 substrate 1 (proline-rich) | 1 | | 2644 | hsa-mir-135b | 3069 | HDLBP | high density lipoprotein binding protein | 1 | | 2645 | hsa-mir-10a | 389203 | C4orf52 | chromosome 4 open reading frame 52 | 1 | | 2646 | hsa-miR-591 | 389203 | C4orf52 | chromosome 4 open reading frame 52 | 1 | | 2647 | hsa-miR-627 | 6482 | ST3GAL1 | ST3 beta-galactoside alpha-2,3-sialyltransferase 1 | 1 | | 2648 | hsa-miR-671-5p | 6482 | ST3GAL1 | ST3 beta-galactoside alpha-2,3-sialyltransferase 1 | 1 | | 2649 | hsa-miR-1236 | 94107 | TMEM203 | transmembrane protein 203 | 1 | | 2650 | hsa-miR-636 | 94107 | TMEM203 | transmembrane protein 203 | 1 | | 2651 | hsa-miR-766 | 94107 | TMEM203 | transmembrane protein 203 | 1 | | 2653 | hsa-miR-454\* | 123811 | C16orf63 | chromosome 16 open reading frame 63 | 1 | | 2654 | hsa-miR-591 | 123811 | C16orf63 | chromosome 16 open reading frame 63 | 1 | | 2655 | hsa-miR-1233 | 7402 | UTRN | utrophin | 1 | | 2656 | hsa-miR-766 | 7402 | UTRN | utrophin | 1 | | 2657 | hsa-miR-874 | 7402 | UTRN | utrophin | 1 | | 2658 | hsa-miR-1233 | 55239 | OGFOD1 | 2-oxoglutarate and iron-dependent oxygenase domain containing 1 | 1 | | 2659 | hsa-miR-591 | 55239 | OGFOD1 | 2-oxoglutarate and iron-dependent oxygenase domain containing 1 | 1 | | 2660 | hsa-miR-874 | 55239 | OGFOD1 | 2-oxoglutarate and iron-dependent oxygenase domain containing 1 | 1 | | 2661 | hsa-miR-571 | 57585 | CRAMP1L | Crm, cramped-like (Drosophila) | 1 | | 2662 | hsa-miR-454\* | 757 | TMEM50B | transmembrane protein 50B | 1 | | 2663 | hsa-miR-591 | 757 | TMEM50B | transmembrane protein 50B | 1 | | 2664 | hsa-miR-934 | 757 | TMEM50B | transmembrane protein 50B | 1 | | 2665 | hsa-miR-593\* | 64928 | MRPL14 | mitochondrial ribosomal protein L14 | 1 | | 2666 | hsa-miR-591 | 4090 | SMAD5 | SMAD family member 5 | 1 | | 2667 | hsa-miR-591 | 54545 | MTMR12 | myotubularin related protein 12 | 1 | | 2668 | hsa-miR-1233 | 5565 | PRKAB2 | protein kinase, AMP-activated, beta 2 non-catalytic subunit | 1 | | 2669 | hsa-miR-571 | 5565 | PRKAB2 | protein kinase, AMP-activated, beta 2 non-catalytic subunit | 1 | | 2670 | hsa-miR-591 | 5565 | PRKAB2 | protein kinase, AMP-activated, beta 2 non-catalytic subunit | 1 | | 2671 | hsa-miR-623 | 90809 | TMEM55B | transmembrane protein 55B | 1 | | 2672 | hsa-miR-627 | 90809 | TMEM55B | transmembrane protein 55B | 1 | | 2673 | hsa-miR-671-5p | 90809 | TMEM55B | transmembrane protein 55B | 1 | | 2674 | hsa-miR-454\* | 115106 | HAUS1 | HAUS augmin-like complex, subunit 1 | 1 | | 2675 | hsa-miR-591 | 115106 | HAUS1 | HAUS augmin-like complex, subunit 1 | 1 | | 2676 | hsa-miR-766 | 84844 | PHF5A | PHD finger protein 5A | 1 | | 2677 | hsa-miR-591 | 54994 | C20orf11 | chromosome 20 open reading frame 11 | 1 | | 2679 | hsa-mir-10a | 10838 | ZNF275 | zinc finger protein 275 | 1 | | 2680 | hsa-miR-1233 | 10838 | ZNF275 | zinc finger protein 275 | 1 | | 2684 | hsa-miR-874 | 5928 | RBBP4 | retinoblastoma binding protein 4 | 1 | | 2685 | hsa-miR-1233 | 84881 | RPUSD4 | RNA pseudouridylate synthase domain containing 4 | 1 | | 2686 | hsa-miR-874 | 84881 | RPUSD4 | RNA pseudouridylate synthase domain containing 4 | 1 | | 2687 | hsa-miR-627 | 221927 | C7orf27 | chromosome 7 open reading frame 27 | 1 | | 2688 | hsa-miR-638 | 221927 | C7orf27 | chromosome 7 open reading frame 27 | 1 | | 2689 | hsa-miR-671-5p | 221927 | C7orf27 | chromosome 7 open reading frame 27 | 1 | | 2690 | hsa-miR-591 | 254048 | UBN2 | ubinuclein 2 | 1 | | 2691 | hsa-mir-10a | 54014 | BRWD1 | bromodomain and WD repeat domain containing 1 | 1 | | 2692 | hsa-miR-1233 | 85013 | TMEM128 | transmembrane protein 128 | 1 | | 2693 | hsa-miR-571 | 85013 | TMEM128 | transmembrane protein 128 | 1 | | 2694 | hsa-miR-591 | 85013 | TMEM128 | transmembrane protein 128 | 1 | | 2695 | hsa-miR-766 | 85013 | TMEM128 | transmembrane protein 128 | 1 | | 2696 | hsa-mir-10a | 129401 | NUP35 | nucleoporin 35kDa | 1 | | 2697 | hsa-miR-1233 | 129401 | NUP35 | nucleoporin 35kDa | 1 | | 2698 | hsa-miR-571 | 129401 | NUP35 | nucleoporin 35kDa | 1 | | 2699 | hsa-miR-766 | 129401 | NUP35 | nucleoporin 35kDa | 1 | | 2700 | hsa-miR-874 | 129401 | NUP35 | nucleoporin 35kDa | 1 | | 2701 | hsa-mir-135b | 57614 | KIAA1468 | KIAA1468 | 1 | | 2702 | hsa-miR-591 | 51434 | ANAPC7 | anaphase promoting complex subunit 7 | 1 | | 2703 | hsa-miR-874 | 85379 | KIAA1671 | KIAA1671 | 1 | | 2704 | hsa-miR-454\* | 10431 | TIMM23 | translocase of inner mitochondrial membrane 23 homolog (yeast) | 1 | | 2705 | hsa-miR-591 | 10431 | TIMM23 | translocase of inner mitochondrial membrane 23 homolog (yeast) | 1 | | 2706 | hsa-miR-1233 | 22821 | RASA3 | RAS p21 protein activator 3 | 1 | | 2707 | hsa-miR-569 | 22821 | RASA3 | RAS p21 protein activator 3 | 1 | | 2708 | hsa-miR-766 | 22821 | RASA3 | RAS p21 protein activator 3 | 1 | | 2709 | hsa-miR-874 | 22821 | RASA3 | RAS p21 protein activator 3 | 1 | | 2710 | hsa-miR-766 | 26260 | FBXO25 | F-box protein 25 | 1 | | 2712 | hsa-miR-591 | 58487 | CREBZF | CREB/ATF bZIP transcription factor | 1 | | 2713 | hsa-miR-1233 | 115426 | UHRF2 | ubiquitin-like with PHD and ring finger domains 2 | 1 | | 2714 | hsa-miR-569 | 115426 | UHRF2 | ubiquitin-like with PHD and ring finger domains 2 | 1 | | 2715 | hsa-miR-766 | 115426 | UHRF2 | ubiquitin-like with PHD and ring finger domains 2 | 1 | | 2716 | hsa-miR-454\* | 122060 | SLAIN1 | SLAIN motif family, member 1 | 1 | | 2717 | hsa-miR-591 | 122060 | SLAIN1 | SLAIN motif family, member 1 | 1 | | 2718 | hsa-miR-454\* | 25879 | DCAF13 | DDB1 and CUL4 associated factor 13 | 1 | | 2720 | hsa-miR-1233 | 57666 | FBRSL1 | fibrosin-like 1 | 1 | | 2721 | hsa-miR-569 | 57666 | FBRSL1 | fibrosin-like 1 | 1 | | 2722 | hsa-miR-571 | 57666 | FBRSL1 | fibrosin-like 1 | 1 | | 2723 | hsa-miR-766 | 57666 | FBRSL1 | fibrosin-like 1 | 1 | | 2724 | hsa-miR-874 | 57666 | FBRSL1 | fibrosin-like 1 | 1 | | 2725 | hsa-miR-1233 | 90799 | CCDC45 | coiled-coil domain containing 45 | 1 | | 2726 | hsa-miR-571 | 90799 | CCDC45 | coiled-coil domain containing 45 | 1 | | 2727 | hsa-miR-874 | 90799 | CCDC45 | coiled-coil domain containing 45 | 1 | | 2728 | hsa-miR-617 | 93058 | COQ10A | coenzyme Q10 homolog A (S. cerevisiae) | 1 | | 2729 | hsa-miR-874 | 93058 | COQ10A | coenzyme Q10 homolog A (S. cerevisiae) | 1 | | 2730 | hsa-miR-1233 | 84946 | LTV1 | LTV1 homolog (S. cerevisiae) | 1 | | 2731 | hsa-miR-766 | 84946 | LTV1 | LTV1 homolog (S. cerevisiae) | 1 | | 2732 | hsa-miR-623 | 85378 | TUBGCP6 | tubulin, gamma complex associated protein 6 | 1 | | 2733 | hsa-mir-10a | 57511 | COG6 | component of oligomeric golgi complex 6 | 1 | | 2734 | hsa-mir-199a-2 | 57511 | COG6 | component of oligomeric golgi complex 6 | 1 | | 2735 | hsa-mir-214 | 57511 | COG6 | component of oligomeric golgi complex 6 | 1 | | 2736 | hsa-miR-199a-5p | 57511 | COG6 | component of oligomeric golgi complex 6 | 1 | | 2737 | hsa-miR-576-5p | 57511 | COG6 | component of oligomeric golgi complex 6 | 1 | | 2738 | hsa-miR-591 | 57511 | COG6 | component of oligomeric golgi complex 6 | 1 | | 2739 | hsa-miR-1233 | 284702 | NCRNA00201 | non-protein coding RNA 201 | 1 | | 2740 | hsa-miR-766 | 284702 | NCRNA00201 | non-protein coding RNA 201 | 1 | | 2741 | hsa-mir-10a | 3192 | HNRNPU | heterogeneous nuclear ribonucleoprotein U (scaffold attachment factor A) | 1 | | 2742 | hsa-miR-576-5p | 3192 | HNRNPU | heterogeneous nuclear ribonucleoprotein U (scaffold attachment factor A) | 1 | | 2743 | hsa-miR-593\* | 125988 | C19orf70 | chromosome 19 open reading frame 70 | 1 | | 2744 | hsa-miR-623 | 125988 | C19orf70 | chromosome 19 open reading frame 70 | 1 | | 2745 | hsa-miR-1233 | 157638 | FAM84B | family with sequence similarity 84, member B | 1 | | 2746 | hsa-miR-571 | 157638 | FAM84B | family with sequence similarity 84, member B | 1 | | 2747 | hsa-miR-874 | 157638 | FAM84B | family with sequence similarity 84, member B | 1 | | 2748 | hsa-miR-591 | 9931 | HELZ | helicase with zinc finger | 1 | | 2749 | hsa-mir-10a | 81617 | CAB39L | calcium binding protein 39-like | 1 | | 2750 | hsa-miR-874 | 55605 | KIF21A | kinesin family member 21A | 1 | | 2751 | hsa-miR-1233 | 56160 | NDNL2 | necdin-like 2 | 1 | | 2752 | hsa-miR-569 | 56160 | NDNL2 | necdin-like 2 | 1 | | 2753 | hsa-miR-571 | 56160 | NDNL2 | necdin-like 2 | 1 | | 2754 | hsa-miR-766 | 56160 | NDNL2 | necdin-like 2 | 1 | | 2755 | hsa-miR-1233 | 36 | ACADSB | acyl-Coenzyme A dehydrogenase, short/branched chain | 1 | | 2756 | hsa-miR-569 | 36 | ACADSB | acyl-Coenzyme A dehydrogenase, short/branched chain | 1 | | 2757 | hsa-miR-571 | 36 | ACADSB | acyl-Coenzyme A dehydrogenase, short/branched chain | 1 | | 2758 | hsa-miR-591 | 36 | ACADSB | acyl-Coenzyme A dehydrogenase, short/branched chain | 1 | | 2759 | hsa-miR-874 | 36 | ACADSB | acyl-Coenzyme A dehydrogenase, short/branched chain | 1 | | 2762 | hsa-miR-591 | 222236 | NAPEPLD | N-acyl phosphatidylethanolamine phospholipase D | 1 | | 2763 | hsa-miR-591 | 10818 | FRS2 | fibroblast growth factor receptor substrate 2 | 1 | | 2765 | hsa-mir-10a | 219854 | TMEM218 | transmembrane protein 218 | 1 | | 2766 | hsa-mir-199a-2 | 219854 | TMEM218 | transmembrane protein 218 | 1 | | 2767 | hsa-mir-214 | 219854 | TMEM218 | transmembrane protein 218 | 1 | | 2768 | hsa-miR-199a-5p | 219854 | TMEM218 | transmembrane protein 218 | 1 | | 2769 | hsa-miR-934 | 219854 | TMEM218 | transmembrane protein 218 | 1 | | 2770 | hsa-mir-1224 | 5286 | PIK3C2A | phosphoinositide-3-kinase, class 2, alpha polypeptide | 1 | | 2771 | hsa-mir-885 | 5286 | PIK3C2A | phosphoinositide-3-kinase, class 2, alpha polypeptide | 1 | | 2772 | hsa-miR-1224-5p | 5286 | PIK3C2A | phosphoinositide-3-kinase, class 2, alpha polypeptide | 1 | | 2773 | hsa-miR-934 | 5286 | PIK3C2A | phosphoinositide-3-kinase, class 2, alpha polypeptide | 1 | | 2774 | hsa-miR-576-5p | 25909 | AHCTF1 | AT hook containing transcription factor 1 | 1 | | 2775 | hsa-miR-454\* | 9946 | CRYZL1 | crystallin, zeta (quinone reductase)-like 1 | 1 | | 2776 | hsa-miR-591 | 9946 | CRYZL1 | crystallin, zeta (quinone reductase)-like 1 | 1 | | 2777 | hsa-miR-454\* | 10059 | DNM1L | dynamin 1-like | 1 | | 2778 | hsa-miR-591 | 10059 | DNM1L | dynamin 1-like | 1 | | 2779 | hsa-miR-454\* | 283489 | ZNF828 | zinc finger protein 828 | 1 | | 2780 | hsa-mir-10a | 139596 | UPRT | uracil phosphoribosyltransferase (FUR1) homolog (S. cerevisiae) | 1 | | 2781 | hsa-mir-1224 | 139596 | UPRT | uracil phosphoribosyltransferase (FUR1) homolog (S. cerevisiae) | 1 | | 2782 | hsa-mir-885 | 139596 | UPRT | uracil phosphoribosyltransferase (FUR1) homolog (S. cerevisiae) | 1 | | 2783 | hsa-miR-1224-5p | 139596 | UPRT | uracil phosphoribosyltransferase (FUR1) homolog (S. cerevisiae) | 1 | | 2784 | hsa-miR-1233 | 139596 | UPRT | uracil phosphoribosyltransferase (FUR1) homolog (S. cerevisiae) | 1 | | 2785 | hsa-miR-766 | 139596 | UPRT | uracil phosphoribosyltransferase (FUR1) homolog (S. cerevisiae) | 1 | | 2786 | hsa-miR-934 | 139596 | UPRT | uracil phosphoribosyltransferase (FUR1) homolog (S. cerevisiae) | 1 | | 2787 | hsa-mir-423 | 161198 | CLEC14A | C-type lectin domain family 14, member A | 1 | | 2788 | hsa-mir-3130-1 | 161198 | CLEC14A | C-type lectin domain family 14, member A | 1 | | 2789 | hsa-mir-3130-2 | 161198 | CLEC14A | C-type lectin domain family 14, member A | 1 | | 2790 | hsa-mir-3130-3 | 161198 | CLEC14A | C-type lectin domain family 14, member A | 1 | | 2791 | hsa-miR-10a | 161198 | CLEC14A | C-type lectin domain family 14, member A | 1 | | 2792 | hsa-miR-770-5p | 161198 | CLEC14A | C-type lectin domain family 14, member A | 1 | | 2793 | hsa-miR-95 | 161198 | CLEC14A | C-type lectin domain family 14, member A | 1 | | 2794 | hsa-miR-1233 | 57587 | KIAA1430 | KIAA1430 | 1 | | 2795 | hsa-miR-571 | 57587 | KIAA1430 | KIAA1430 | 1 | | 2796 | hsa-miR-874 | 57587 | KIAA1430 | KIAA1430 | 1 | | 2797 | hsa-miR-591 | 79832 | QSER1 | glutamine and serine rich 1 | 1 | | 2798 | hsa-miR-604 | 9489 | PGS1 | phosphatidylglycerophosphate synthase 1 | 1 | | 2799 | hsa-miR-638 | 9489 | PGS1 | phosphatidylglycerophosphate synthase 1 | 1 | | 2800 | hsa-miR-938 | 9489 | PGS1 | phosphatidylglycerophosphate synthase 1 | 1 | | 2801 | hsa-miR-1233 | 11098 | PRSS23 | protease, serine, 23 | 1 | | 2802 | hsa-miR-571 | 11098 | PRSS23 | protease, serine, 23 | 1 | | 2803 | hsa-miR-874 | 11098 | PRSS23 | protease, serine, 23 | 1 | | 2804 | hsa-miR-591 | 219541 | MED19 | mediator complex subunit 19 | 1 | | 2805 | hsa-miR-1233 | 84333 | PCGF5 | polycomb group ring finger 5 | 1 | | 2806 | hsa-mir-10a | 56987 | BBX | bobby sox homolog (Drosophila) | 1 | | 2807 | hsa-miR-571 | 92344 | GORAB | golgin, RAB6-interacting | 1 | | 2808 | hsa-miR-591 | 92344 | GORAB | golgin, RAB6-interacting | 1 | | 2809 | hsa-miR-1233 | 142940 | TRUB1 | TruB pseudouridine (psi) synthase homolog 1 (E. coli) | 1 | | 2810 | hsa-miR-571 | 142940 | TRUB1 | TruB pseudouridine (psi) synthase homolog 1 (E. coli) | 1 | | 2811 | hsa-miR-591 | 142940 | TRUB1 | TruB pseudouridine (psi) synthase homolog 1 (E. coli) | 1 | | 2812 | hsa-miR-766 | 142940 | TRUB1 | TruB pseudouridine (psi) synthase homolog 1 (E. coli) | 1 | | 2813 | hsa-mir-10a | 1122 | CHML | choroideremia-like (Rab escort protein 2) | 1 | | 2814 | hsa-miR-1233 | 1122 | CHML | choroideremia-like (Rab escort protein 2) | 1 | | 2815 | hsa-miR-1233 | 133746 | JMY | junction mediating and regulatory protein, p53 cofactor | 1 | | 2816 | hsa-miR-569 | 133746 | JMY | junction mediating and regulatory protein, p53 cofactor | 1 | | 2818 | hsa-miR-591 | 133746 | JMY | junction mediating and regulatory protein, p53 cofactor | 1 | | 2819 | hsa-miR-766 | 133746 | JMY | junction mediating and regulatory protein, p53 cofactor | 1 | | 2820 | hsa-miR-874 | 133746 | JMY | junction mediating and regulatory protein, p53 cofactor | 1 | | 2821 | hsa-mir-10a | 414327 | PS1TP4 | HBV preS1-transactivated protein 4 | 1 | | 2822 | hsa-mir-199a-2 | 414327 | PS1TP4 | HBV preS1-transactivated protein 4 | 1 | | 2823 | hsa-mir-214 | 414327 | PS1TP4 | HBV preS1-transactivated protein 4 | 1 | | 2824 | hsa-miR-199a-5p | 414327 | PS1TP4 | HBV preS1-transactivated protein 4 | 1 | | 2825 | hsa-miR-576-5p | 414327 | PS1TP4 | HBV preS1-transactivated protein 4 | 1 | | 2826 | hsa-miR-934 | 414327 | PS1TP4 | HBV preS1-transactivated protein 4 | 1 | | 2827 | hsa-miR-1233 | 120534 | C11orf46 | chromosome 11 open reading frame 46 | 1 | | 2828 | hsa-miR-571 | 120534 | C11orf46 | chromosome 11 open reading frame 46 | 1 | | 2829 | hsa-miR-874 | 120534 | C11orf46 | chromosome 11 open reading frame 46 | 1 | | 2830 | hsa-miR-1233 | 6920 | TCEA3 | transcription elongation factor A (SII), 3 | 1 | | 2831 | hsa-miR-874 | 6920 | TCEA3 | transcription elongation factor A (SII), 3 | 1 | | 2832 | hsa-miR-591 | 118924 | C10orf4 | chromosome 10 open reading frame 4 | 1 | | 2835 | hsa-miR-571 | 84186 | ZCCHC7 | zinc finger, CCHC domain containing 7 | 1 | | 2836 | hsa-miR-591 | 84186 | ZCCHC7 | zinc finger, CCHC domain containing 7 | 1 | | 2837 | hsa-mir-10a | 55183 | RIF1 | RAP1 interacting factor homolog (yeast) | 1 | | 2838 | hsa-miR-576-5p | 55183 | RIF1 | RAP1 interacting factor homolog (yeast) | 1 | | 2839 | hsa-miR-1233 | 80012 | PHC3 | polyhomeotic homolog 3 (Drosophila) | 1 | | 2840 | hsa-miR-571 | 80012 | PHC3 | polyhomeotic homolog 3 (Drosophila) | 1 | | 2841 | hsa-miR-591 | 80012 | PHC3 | polyhomeotic homolog 3 (Drosophila) | 1 | | 2842 | hsa-miR-874 | 80012 | PHC3 | polyhomeotic homolog 3 (Drosophila) | 1 | | 2843 | hsa-miR-591 | 83892 | KCTD10 | potassium channel tetramerisation domain containing 10 | 1 | | 2845 | hsa-miR-766 | 388228 | SBK1 | SH3-binding domain kinase 1 | 1 | | 2846 | hsa-mir-628 | 388228 | SBK1 | SH3-binding domain kinase 1 | 1 | | 2847 | hsa-miR-628-5p | 388228 | SBK1 | SH3-binding domain kinase 1 | 1 | | 2849 | hsa-miR-571 | 30837 | SOCS7 | suppressor of cytokine signaling 7 | 1 | | 2851 | hsa-miR-1233 | 285761 | DCBLD1 | discoidin, CUB and LCCL domain containing 1 | 1 | | 2852 | hsa-miR-569 | 285761 | DCBLD1 | discoidin, CUB and LCCL domain containing 1 | 1 | | 2853 | hsa-miR-571 | 285761 | DCBLD1 | discoidin, CUB and LCCL domain containing 1 | 1 | | 2854 | hsa-miR-766 | 285761 | DCBLD1 | discoidin, CUB and LCCL domain containing 1 | 1 | | 2855 | hsa-miR-874 | 285761 | DCBLD1 | discoidin, CUB and LCCL domain containing 1 | 1 | | 2856 | hsa-miR-1233 | 201161 | CENPV | centromere protein V | 1 | | 2857 | hsa-miR-571 | 201161 | CENPV | centromere protein V | 1 | | 2858 | hsa-miR-874 | 201161 | CENPV | centromere protein V | 1 | | 2859 | hsa-miR-766 | 84826 | SFT2D3 | SFT2 domain containing 3 | 1 | | 2860 | hsa-mir-505 | 83933 | HDAC10 | histone deacetylase 10 | 1 | | 2861 | hsa-mir-576 | 83933 | HDAC10 | histone deacetylase 10 | 1 | | 2862 | hsa-miR-591 | 55102 | ATG2B | ATG2 autophagy related 2 homolog B (S. cerevisiae) | 1 | | 2863 | hsa-miR-591 | 10741 | RBBP9 | retinoblastoma binding protein 9 | 1 | | 2864 | hsa-miR-766 | 124997 | WDR81 | WD repeat domain 81 | 1 | | 2865 | hsa-miR-1233 | 64965 | MRPS9 | mitochondrial ribosomal protein S9 | 1 | | 2866 | hsa-miR-571 | 64965 | MRPS9 | mitochondrial ribosomal protein S9 | 1 | | 2867 | hsa-miR-766 | 64965 | MRPS9 | mitochondrial ribosomal protein S9 | 1 | | 2868 | hsa-miR-874 | 64965 | MRPS9 | mitochondrial ribosomal protein S9 | 1 | | 2869 | hsa-miR-1233 | 221830 | TWISTNB | TWIST neighbor | 1 | | 2870 | hsa-miR-571 | 221830 | TWISTNB | TWIST neighbor | 1 | | 2871 | hsa-miR-591 | 221830 | TWISTNB | TWIST neighbor | 1 | | 2872 | hsa-miR-874 | 221830 | TWISTNB | TWIST neighbor | 1 | | 2873 | hsa-miR-1233 | 57456 | KIAA1143 | KIAA1143 | 1 | | 2874 | hsa-miR-571 | 57456 | KIAA1143 | KIAA1143 | 1 | | 2875 | hsa-miR-591 | 57456 | KIAA1143 | KIAA1143 | 1 | | 2876 | hsa-miR-874 | 57456 | KIAA1143 | KIAA1143 | 1 | | 2877 | hsa-mir-10a | 114799 | ESCO1 | establishment of cohesion 1 homolog 1 (S. cerevisiae) | 1 | | 2878 | hsa-miR-1233 | 114799 | ESCO1 | establishment of cohesion 1 homolog 1 (S. cerevisiae) | 1 | | 2879 | hsa-miR-571 | 114799 | ESCO1 | establishment of cohesion 1 homolog 1 (S. cerevisiae) | 1 | | 2880 | hsa-miR-591 | 114799 | ESCO1 | establishment of cohesion 1 homolog 1 (S. cerevisiae) | 1 | | 2881 | hsa-mir-10a | 283464 | GXYLT1 | glucoside xylosyltransferase 1 | 1 | | 2882 | hsa-miR-1233 | 3111 | HLA-DOA | major histocompatibility complex, class II, DO alpha | 1 | | 2883 | hsa-miR-766 | 3111 | HLA-DOA | major histocompatibility complex, class II, DO alpha | 1 | | 2884 | hsa-miR-1233 | 84329 | HVCN1 | hydrogen voltage-gated channel 1 | 1 | | 2885 | hsa-miR-766 | 84329 | HVCN1 | hydrogen voltage-gated channel 1 | 1 | | 2886 | hsa-miR-1233 | 134266 | GRPEL2 | GrpE-like 2, mitochondrial (E. coli) | 1 | | 2887 | hsa-miR-571 | 134266 | GRPEL2 | GrpE-like 2, mitochondrial (E. coli) | 1 | | 2888 | hsa-miR-591 | 23443 | SLC35A3 | solute carrier family 35 (UDP-N-acetylglucosamine (UDP-GlcNAc) transporter), member A3 | 1 | | 2889 | hsa-miR-591 | 29945 | ANAPC4 | anaphase promoting complex subunit 4 | 1 | | 2890 | hsa-miR-591 | 400657 | LOC400657 | hypothetical LOC400657 | 1 | | 2891 | hsa-miR-1233 | 11052 | CPSF6 | cleavage and polyadenylation specific factor 6, 68kDa | 1 | | 2892 | hsa-miR-571 | 11052 | CPSF6 | cleavage and polyadenylation specific factor 6, 68kDa | 1 | | 2893 | hsa-miR-874 | 11052 | CPSF6 | cleavage and polyadenylation specific factor 6, 68kDa | 1 | | 2894 | hsa-miR-1233 | 4773 | NFATC2 | nuclear factor of activated T-cells, cytoplasmic, calcineurin-dependent 2 | 1 | | 2895 | hsa-miR-569 | 4773 | NFATC2 | nuclear factor of activated T-cells, cytoplasmic, calcineurin-dependent 2 | 1 | | 2896 | hsa-miR-766 | 4773 | NFATC2 | nuclear factor of activated T-cells, cytoplasmic, calcineurin-dependent 2 | 1 | | 2897 | hsa-miR-874 | 4773 | NFATC2 | nuclear factor of activated T-cells, cytoplasmic, calcineurin-dependent 2 | 1 | | 2898 | hsa-miR-571 | 80155 | NAA15 | N(alpha)-acetyltransferase 15, NatA auxiliary subunit | 1 | | 2899 | hsa-miR-591 | 80155 | NAA15 | N(alpha)-acetyltransferase 15, NatA auxiliary subunit | 1 | | 2900 | hsa-miR-571 | 3631 | INPP4A | inositol polyphosphate-4-phosphatase, type I, 107kDa | 1 | | 2901 | hsa-miR-591 | 3631 | INPP4A | inositol polyphosphate-4-phosphatase, type I, 107kDa | 1 | | 2902 | hsa-miR-766 | 57602 | USP36 | ubiquitin specific peptidase 36 | 1 | | 2903 | hsa-miR-571 | 51123 | ZNF706 | zinc finger protein 706 | 1 | | 2904 | hsa-miR-591 | 51123 | ZNF706 | zinc finger protein 706 | 1 | | 2905 | hsa-mir-10a | 201973 | CCDC111 | coiled-coil domain containing 111 | 1 | | 2906 | hsa-miR-591 | 201973 | CCDC111 | coiled-coil domain containing 111 | 1 | | 2907 | hsa-miR-1233 | 112487 | C14orf126 | chromosome 14 open reading frame 126 | 1 | | 2908 | hsa-miR-591 | 112487 | C14orf126 | chromosome 14 open reading frame 126 | 1 | | 2909 | hsa-miR-874 | 112487 | C14orf126 | chromosome 14 open reading frame 126 | 1 | | 2910 | hsa-miR-593\* | 64975 | MRPL41 | mitochondrial ribosomal protein L41 | 1 | | 2911 | hsa-miR-623 | 64975 | MRPL41 | mitochondrial ribosomal protein L41 | 1 | | 2912 | hsa-miR-1233 | 57713 | SFMBT2 | Scm-like with four mbt domains 2 | 1 | | 2913 | hsa-miR-766 | 57713 | SFMBT2 | Scm-like with four mbt domains 2 | 1 | | 2914 | hsa-mir-628 | 51466 | EVL | Enah/Vasp-like | 1 | | 2915 | hsa-miR-1233 | 51466 | EVL | Enah/Vasp-like | 1 | | 2916 | hsa-miR-569 | 51466 | EVL | Enah/Vasp-like | 1 | | 2917 | hsa-miR-628-5p | 51466 | EVL | Enah/Vasp-like | 1 | | 2918 | hsa-mir-10a | 84668 | FAM126A | family with sequence similarity 126, member A | 1 | | 2919 | hsa-miR-591 | 84668 | FAM126A | family with sequence similarity 126, member A | 1 | | 2920 | hsa-mir-10a | 84725 | PLEKHA8 | pleckstrin homology domain containing, family A (phosphoinositide binding specific) member 8 | 1 | | 2921 | hsa-mir-3130-1 | 57799 | RAB40C | RAB40C, member RAS oncogene family | 1 | | 2922 | hsa-mir-3130-2 | 57799 | RAB40C | RAB40C, member RAS oncogene family | 1 | | 2923 | hsa-mir-3130-3 | 57799 | RAB40C | RAB40C, member RAS oncogene family | 1 | | 2924 | hsa-miR-10a | 57799 | RAB40C | RAB40C, member RAS oncogene family | 1 | | 2925 | hsa-miR-770-5p | 57799 | RAB40C | RAB40C, member RAS oncogene family | 1 | | 2926 | hsa-miR-95 | 57799 | RAB40C | RAB40C, member RAS oncogene family | 1 | | 2927 | hsa-miR-1233 | 56647 | BCCIP | BRCA2 and CDKN1A interacting protein | 1 | | 2928 | hsa-miR-874 | 56647 | BCCIP | BRCA2 and CDKN1A interacting protein | 1 | | 2932 | hsa-mir-10a | 84437 | KIAA1826 | KIAA1826 | 1 | | 2933 | hsa-miR-576-5p | 84437 | KIAA1826 | KIAA1826 | 1 | | 2934 | hsa-miR-591 | 84437 | KIAA1826 | KIAA1826 | 1 | | 2935 | hsa-miR-934 | 84437 | KIAA1826 | KIAA1826 | 1 | | 2936 | hsa-miR-766 | 115509 | ZNF689 | zinc finger protein 689 | 1 | | 2937 | hsa-miR-623 | 1731 | SEPT1 | septin 1 | 1 | | 2938 | hsa-miR-627 | 1731 | SEPT1 | septin 1 | 1 | | 2939 | hsa-miR-591 | 729614 | hCG\_2008140 | hypothetical LOC729614 | 1 | | 2940 | hsa-miR-1233 | 9255 | AIMP1 | aminoacyl tRNA synthetase complex-interacting multifunctional protein 1 | 1 | | 2941 | hsa-miR-571 | 9255 | AIMP1 | aminoacyl tRNA synthetase complex-interacting multifunctional protein 1 | 1 | | 2942 | hsa-miR-1233 | 85315 | PAQR8 | progestin and adipoQ receptor family member VIII | 1 | | 2943 | hsa-miR-569 | 85315 | PAQR8 | progestin and adipoQ receptor family member VIII | 1 | | 2944 | hsa-miR-571 | 85315 | PAQR8 | progestin and adipoQ receptor family member VIII | 1 | | 2945 | hsa-miR-591 | 85315 | PAQR8 | progestin and adipoQ receptor family member VIII | 1 | | 2946 | hsa-miR-766 | 85315 | PAQR8 | progestin and adipoQ receptor family member VIII | 1 | | 2947 | hsa-mir-3130-1 | 11076 | TPPP | tubulin polymerization promoting protein | 1 | | 2948 | hsa-mir-3130-2 | 11076 | TPPP | tubulin polymerization promoting protein | 1 | | 2949 | hsa-mir-3130-3 | 11076 | TPPP | tubulin polymerization promoting protein | 1 | | 2950 | hsa-miR-10a | 11076 | TPPP | tubulin polymerization promoting protein | 1 | | 2951 | hsa-miR-770-5p | 11076 | TPPP | tubulin polymerization promoting protein | 1 | | 2952 | hsa-miR-95 | 11076 | TPPP | tubulin polymerization promoting protein | 1 | | 2953 | hsa-miR-10a | 100113384 | SNORD123 | small nucleolar RNA, C/D box 123 | 1 | | 2954 | hsa-miR-1233 | 92106 | OXNAD1 | oxidoreductase NAD-binding domain containing 1 | 1 | | 2955 | hsa-miR-569 | 92106 | OXNAD1 | oxidoreductase NAD-binding domain containing 1 | 1 | | 2956 | hsa-miR-571 | 92106 | OXNAD1 | oxidoreductase NAD-binding domain containing 1 | 1 | | 2957 | hsa-miR-766 | 92106 | OXNAD1 | oxidoreductase NAD-binding domain containing 1 | 1 | | 2958 | hsa-miR-874 | 92106 | OXNAD1 | oxidoreductase NAD-binding domain containing 1 | 1 | | 2959 | hsa-miR-1233 | 1915 | EEF1A1 | eukaryotic translation elongation factor 1 alpha 1 | 1 | | 2960 | hsa-miR-571 | 1915 | EEF1A1 | eukaryotic translation elongation factor 1 alpha 1 | 1 | | 2961 | hsa-miR-874 | 1915 | EEF1A1 | eukaryotic translation elongation factor 1 alpha 1 | 1 | | 2962 | hsa-miR-10a | 55684 | C9orf86 | chromosome 9 open reading frame 86 | 1 | | 2963 | hsa-miR-770-5p | 55684 | C9orf86 | chromosome 9 open reading frame 86 | 1 | | 2964 | hsa-miR-95 | 55684 | C9orf86 | chromosome 9 open reading frame 86 | 1 | | 2965 | hsa-miR-1233 | 9141 | PDCD5 | programmed cell death 5 | 1 | | 2966 | hsa-miR-569 | 9141 | PDCD5 | programmed cell death 5 | 1 | | 2967 | hsa-miR-571 | 9141 | PDCD5 | programmed cell death 5 | 1 | | 2968 | hsa-miR-766 | 9141 | PDCD5 | programmed cell death 5 | 1 | | 2969 | hsa-miR-874 | 9141 | PDCD5 | programmed cell death 5 | 1 | | 2970 | hsa-miR-591 | 54906 | C10orf18 | chromosome 10 open reading frame 18 | 1 | | 2971 | hsa-miR-766 | 9382 | COG1 | component of oligomeric golgi complex 1 | 1 | | 2972 | hsa-miR-1233 | 643836 | ZFP62 | zinc finger protein 62 homolog (mouse) | 1 | | 2973 | hsa-miR-571 | 643836 | ZFP62 | zinc finger protein 62 homolog (mouse) | 1 | | 2974 | hsa-miR-591 | 643836 | ZFP62 | zinc finger protein 62 homolog (mouse) | 1 | | 2975 | hsa-miR-874 | 643836 | ZFP62 | zinc finger protein 62 homolog (mouse) | 1 | | 2976 | hsa-miR-591 | 376940 | ZC3H6 | zinc finger CCCH-type containing 6 | 1 | | 2977 | hsa-miR-1233 | 84294 | UTP23 | UTP23, small subunit (SSU) processome component, homolog (yeast) | 1 | | 2978 | hsa-miR-591 | 729570 | LOC729570 | hypothetical LOC729570 | 1 | | 2979 | hsa-miR-1233 | 6100 | RP9 | retinitis pigmentosa 9 (autosomal dominant) | 1 | | 2980 | hsa-miR-766 | 6100 | RP9 | retinitis pigmentosa 9 (autosomal dominant) | 1 | | 2981 | hsa-miR-1233 | 129293 | C2orf89 | chromosome 2 open reading frame 89 | 1 | | 2982 | hsa-miR-569 | 129293 | C2orf89 | chromosome 2 open reading frame 89 | 1 | | 2983 | hsa-miR-766 | 129293 | C2orf89 | chromosome 2 open reading frame 89 | 1 | | 2984 | hsa-miR-874 | 129293 | C2orf89 | chromosome 2 open reading frame 89 | 1 | | 2985 | hsa-mir-628 | 389289 | C5orf39 | chromosome 5 open reading frame 39 | 1 | | 2986 | hsa-miR-1233 | 389289 | C5orf39 | chromosome 5 open reading frame 39 | 1 | | 2987 | hsa-miR-569 | 389289 | C5orf39 | chromosome 5 open reading frame 39 | 1 | | 2988 | hsa-miR-628-5p | 389289 | C5orf39 | chromosome 5 open reading frame 39 | 1 | | 2989 | hsa-miR-766 | 389289 | C5orf39 | chromosome 5 open reading frame 39 | 1 | | 2990 | hsa-mir-10a | 619423 | FAM85A | family with sequence similarity 85, member A | 1 | | 2991 | hsa-miR-1233 | 619423 | FAM85A | family with sequence similarity 85, member A | 1 | | 2992 | hsa-miR-569 | 619423 | FAM85A | family with sequence similarity 85, member A | 1 | | 2993 | hsa-miR-766 | 619423 | FAM85A | family with sequence similarity 85, member A | 1 | | 2994 | hsa-miR-934 | 619423 | FAM85A | family with sequence similarity 85, member A | 1 | | 2999 | hsa-miR-874 | 114791 | TUBGCP5 | tubulin, gamma complex associated protein 5 | 1 | | 3000 | hsa-miR-623 | 284613 | CYB561D1 | cytochrome b-561 domain containing 1 | 1 | | 3001 | hsa-miR-1233 | 757 | TMEM50B | transmembrane protein 50B | 1 | | 3002 | hsa-mir-3130-1 | 81603 | TRIM8 | tripartite motif-containing 8 | 1 | | 3003 | hsa-mir-3130-2 | 81603 | TRIM8 | tripartite motif-containing 8 | 1 | | 3004 | hsa-mir-3130-3 | 81603 | TRIM8 | tripartite motif-containing 8 | 1 | | 3005 | hsa-miR-770-5p | 81603 | TRIM8 | tripartite motif-containing 8 | 1 | | 3006 | hsa-miR-95 | 81603 | TRIM8 | tripartite motif-containing 8 | 1 | | 3007 | hsa-miR-591 | 9879 | DDX46 | DEAD (Asp-Glu-Ala-Asp) box polypeptide 46 | 1 | | 3008 | hsa-miR-10a | 65083 | NOL6 | nucleolar protein family 6 (RNA-associated) | 1 | | 3009 | hsa-miR-1233 | 84245 | MRI1 | methylthioribose-1-phosphate isomerase homolog (S. cerevisiae) | 1 | | 3010 | hsa-miR-571 | 84245 | MRI1 | methylthioribose-1-phosphate isomerase homolog (S. cerevisiae) | 1 | | 3011 | hsa-miR-591 | 84245 | MRI1 | methylthioribose-1-phosphate isomerase homolog (S. cerevisiae) | 1 | | 3012 | hsa-miR-874 | 84245 | MRI1 | methylthioribose-1-phosphate isomerase homolog (S. cerevisiae) | 1 | | 3013 | hsa-miR-10a | 205428 | C3orf58 | chromosome 3 open reading frame 58 | 1 | | 3014 | hsa-miR-770-5p | 205428 | C3orf58 | chromosome 3 open reading frame 58 | 1 | | 3015 | hsa-miR-95 | 205428 | C3orf58 | chromosome 3 open reading frame 58 | 1 | | 3016 | hsa-miR-571 | 55014 | STX17 | syntaxin 17 | 1 | | 3017 | hsa-miR-190 | 29116 | MYLIP | myosin regulatory light chain interacting protein | 1 | | 3018 | hsa-miR-1233 | 162972 | ZNF550 | zinc finger protein 550 | 1 | | 3019 | hsa-miR-571 | 162972 | ZNF550 | zinc finger protein 550 | 1 | | 3020 | hsa-miR-874 | 162972 | ZNF550 | zinc finger protein 550 | 1 | | 3021 | hsa-miR-627 | 26090 | ABHD12 | abhydrolase domain containing 12 | 1 | | 3022 | hsa-miR-671-5p | 26090 | ABHD12 | abhydrolase domain containing 12 | 1 | | 3023 | hsa-miR-1233 | 2067 | ERCC1 | excision repair cross-complementing rodent repair deficiency, complementation group 1 (includes overlapping antisense sequence) | 1 | | 3024 | hsa-miR-571 | 2067 | ERCC1 | excision repair cross-complementing rodent repair deficiency, complementation group 1 (includes overlapping antisense sequence) | 1 | | 3025 | hsa-miR-766 | 2067 | ERCC1 | excision repair cross-complementing rodent repair deficiency, complementation group 1 (includes overlapping antisense sequence) | 1 | | 3026 | hsa-miR-874 | 2067 | ERCC1 | excision repair cross-complementing rodent repair deficiency, complementation group 1 (includes overlapping antisense sequence) | 1 | | 3027 | hsa-mir-149 | 148423 | C1orf52 | chromosome 1 open reading frame 52 | 1 | | 3028 | hsa-miR-576-5p | 148423 | C1orf52 | chromosome 1 open reading frame 52 | 1 | | 3029 | hsa-miR-591 | 148423 | C1orf52 | chromosome 1 open reading frame 52 | 1 | | 3030 | hsa-miR-770-5p | 54921 | CHTF8 | CTF8, chromosome transmission fidelity factor 8 homolog (S. cerevisiae) | 1 | | 3031 | hsa-miR-95 | 54921 | CHTF8 | CTF8, chromosome transmission fidelity factor 8 homolog (S. cerevisiae) | 1 | | 3032 | hsa-miR-576-5p | 219749 | ZNF25 | zinc finger protein 25 | 1 | | 3033 | hsa-miR-95 | 729234 | LOC729234 | fumarylacetoacetate hydrolase domain containing 2 pseudogene | 1 | | 3034 | hsa-miR-591 | 55728 | N4BP2 | NEDD4 binding protein 2 | 1 | | 3037 | hsa-miR-591 | 256471 | MFSD8 | major facilitator superfamily domain containing 8 | 1 | | 3038 | hsa-miR-766 | 256471 | MFSD8 | major facilitator superfamily domain containing 8 | 1 | | 3039 | hsa-miR-874 | 256471 | MFSD8 | major facilitator superfamily domain containing 8 | 1 | | 3040 | hsa-miR-571 | 91614 | DEPDC7 | DEP domain containing 7 | 1 | | 3041 | hsa-miR-571 | 147929 | ZNF565 | zinc finger protein 565 | 1 | | 3042 | hsa-miR-10a | 51704 | GPRC5B | G protein-coupled receptor, family C, group 5, member B | 1 | | 3043 | hsa-miR-770-5p | 51704 | GPRC5B | G protein-coupled receptor, family C, group 5, member B | 1 | | 3044 | hsa-miR-95 | 51704 | GPRC5B | G protein-coupled receptor, family C, group 5, member B | 1 | | 3046 | hsa-mir-10a | 55153 | SDAD1 | SDA1 domain containing 1 | 1 | | 3047 | hsa-miR-1233 | 55153 | SDAD1 | SDA1 domain containing 1 | 1 | | 3048 | hsa-miR-569 | 55153 | SDAD1 | SDA1 domain containing 1 | 1 | | 3049 | hsa-miR-571 | 55153 | SDAD1 | SDA1 domain containing 1 | 1 | | 3050 | hsa-miR-766 | 55153 | SDAD1 | SDA1 domain containing 1 | 1 | | 3051 | hsa-mir-16-2 | 729359 | PLIN4 | perilipin 4 | 1 | | 3052 | hsa-mir-155 | 729359 | PLIN4 | perilipin 4 | 1 | | 3053 | hsa-mir-3130-1 | 729359 | PLIN4 | perilipin 4 | 1 | | 3054 | hsa-mir-3130-2 | 729359 | PLIN4 | perilipin 4 | 1 | | 3055 | hsa-mir-3130-3 | 729359 | PLIN4 | perilipin 4 | 1 | | 3056 | hsa-miR-128 | 729359 | PLIN4 | perilipin 4 | 1 | | 3057 | hsa-miR-15b | 729359 | PLIN4 | perilipin 4 | 1 | | 3058 | hsa-miR-643 | 729359 | PLIN4 | perilipin 4 | 1 | | 3059 | hsa-miR-765 | 5210 | PFKFB4 | 6-phosphofructo-2-kinase/fructose-2,6-biphosphatase 4 | 1 | | 3060 | hsa-miR-635 | 128346 | C1orf162 | chromosome 1 open reading frame 162 | 1 | | 3062 | hsa-miR-1233 | 121551 | BTBD11 | BTB (POZ) domain containing 11 | 1 | | 3063 | hsa-miR-569 | 121551 | BTBD11 | BTB (POZ) domain containing 11 | 1 | | 3064 | hsa-miR-571 | 121551 | BTBD11 | BTB (POZ) domain containing 11 | 1 | | 3065 | hsa-miR-874 | 121551 | BTBD11 | BTB (POZ) domain containing 11 | 1 | | 3066 | hsa-miR-1233 | 57489 | ODF2L | outer dense fiber of sperm tails 2-like | 1 | | 3068 | hsa-miR-934 | 55037 | PTCD3 | Pentatricopeptide repeat domain 3 | 1 | | 3069 | hsa-miR-591 | 133686 | C5orf33 | chromosome 5 open reading frame 33 | 1 | | 3070 | hsa-miR-591 | 340277 | C7orf46 | chromosome 7 open reading frame 46 | 1 | | 3071 | hsa-miR-1233 | 9727 | RAB11FIP3 | RAB11 family interacting protein 3 (class II) | 1 | | 3072 | hsa-miR-766 | 9727 | RAB11FIP3 | RAB11 family interacting protein 3 (class II) | 1 | | 3073 | hsa-miR-874 | 9727 | RAB11FIP3 | RAB11 family interacting protein 3 (class II) | 1 | | 3074 | hsa-miR-1233 | 7587 | ZNF37A | zinc finger protein 37A | 1 | | 3075 | hsa-mir-3130-1 | 57537 | SORCS2 | sortilin-related VPS10 domain containing receptor 2 | 1 | | 3076 | hsa-mir-3130-2 | 57537 | SORCS2 | sortilin-related VPS10 domain containing receptor 2 | 1 | | 3077 | hsa-mir-3130-3 | 57537 | SORCS2 | sortilin-related VPS10 domain containing receptor 2 | 1 | | 3078 | hsa-miR-770-5p | 57537 | SORCS2 | sortilin-related VPS10 domain containing receptor 2 | 1 | | 3079 | hsa-miR-95 | 57537 | SORCS2 | sortilin-related VPS10 domain containing receptor 2 | 1 | | 3080 | hsa-miR-638 | 146880 | LOC146880 | hypothetical LOC146880 | 1 | | 3081 | hsa-miR-454\* | 375484 | C5orf25 | chromosome 5 open reading frame 25 | 1 | | 3082 | hsa-miR-591 | 375484 | C5orf25 | chromosome 5 open reading frame 25 | 1 | | 3083 | hsa-miR-10a | 153090 | DAB2IP | DAB2 interacting protein | 1 | | 3084 | hsa-miR-770-5p | 153090 | DAB2IP | DAB2 interacting protein | 1 | | 3085 | hsa-miR-95 | 153090 | DAB2IP | DAB2 interacting protein | 1 | | 3086 | hsa-miR-576-5p | 222194 | RSBN1L | round spermatid basic protein 1-like | 1 | | 3087 | hsa-miR-10a | 582 | BBS1 | Bardet-Biedl syndrome 1 | 1 | | 3088 | hsa-miR-1233 | 119504 | C10orf104 | chromosome 10 open reading frame 104 | 1 | | 3089 | hsa-miR-571 | 119504 | C10orf104 | chromosome 10 open reading frame 104 | 1 | | 3090 | hsa-miR-591 | 119504 | C10orf104 | chromosome 10 open reading frame 104 | 1 | | 3091 | hsa-miR-874 | 119504 | C10orf104 | chromosome 10 open reading frame 104 | 1 | | 3093 | hsa-mir-505 | 113220 | KIF12 | kinesin family member 12 | 1 | | 3094 | hsa-mir-3130-1 | 113220 | KIF12 | kinesin family member 12 | 1 | | 3095 | hsa-mir-3130-2 | 113220 | KIF12 | kinesin family member 12 | 1 | | 3096 | hsa-mir-3130-3 | 113220 | KIF12 | kinesin family member 12 | 1 | | 3097 | hsa-miR-10a | 113220 | KIF12 | kinesin family member 12 | 1 | | 3098 | hsa-miR-770-5p | 113220 | KIF12 | kinesin family member 12 | 1 | | 3099 | hsa-miR-95 | 113220 | KIF12 | kinesin family member 12 | 1 | | 3100 | hsa-miR-10a | 54756 | IL17RD | interleukin 17 receptor D | 1 | | 3101 | hsa-miR-571 | 64682 | ANAPC1 | anaphase promoting complex subunit 1 | 1 | | 3102 | hsa-miR-591 | 64682 | ANAPC1 | anaphase promoting complex subunit 1 | 1 | | 3103 | hsa-miR-95 | 3236 | HOXD10 | homeobox D10 | 1 | | 3104 | hsa-mir-10a | 5994 | RFXAP | regulatory factor X-associated protein | 1 | | 3105 | hsa-miR-454\* | 5994 | RFXAP | regulatory factor X-associated protein | 1 | | 3106 | hsa-miR-571 | 5994 | RFXAP | regulatory factor X-associated protein | 1 | | 3107 | hsa-miR-591 | 5994 | RFXAP | regulatory factor X-associated protein | 1 | | 3108 | hsa-miR-591 | 64062 | RBM26 | RNA binding motif protein 26 | 1 | | 3109 | hsa-miR-10a | 121260 | SLC15A4 | solute carrier family 15, member 4 | 1 | | 3110 | hsa-miR-770-5p | 121260 | SLC15A4 | solute carrier family 15, member 4 | 1 | | 3111 | hsa-miR-95 | 121260 | SLC15A4 | solute carrier family 15, member 4 | 1 | | 3112 | hsa-miR-10a | 65268 | WNK2 | WNK lysine deficient protein kinase 2 | 1 | | 3113 | hsa-miR-770-5p | 65268 | WNK2 | WNK lysine deficient protein kinase 2 | 1 | | 3114 | hsa-miR-95 | 65268 | WNK2 | WNK lysine deficient protein kinase 2 | 1 | | 3115 | hsa-miR-10a | 90668 | LRRC16B | leucine rich repeat containing 16B | 1 | | 3116 | hsa-miR-770-5p | 90668 | LRRC16B | leucine rich repeat containing 16B | 1 | | 3117 | hsa-miR-95 | 90668 | LRRC16B | leucine rich repeat containing 16B | 1 | | 3118 | hsa-miR-569 | 23545 | ATP6V0A2 | ATPase, H+ transporting, lysosomal V0 subunit a2 | 1 | | 3119 | hsa-miR-571 | 162967 | ZNF320 | zinc finger protein 320 | 1 | | 3120 | hsa-miR-591 | 162967 | ZNF320 | zinc finger protein 320 | 1 | | 3121 | hsa-miR-10a | 2350 | FOLR2 | folate receptor 2 (fetal) | 1 | | 3122 | hsa-miR-770-5p | 2350 | FOLR2 | folate receptor 2 (fetal) | 1 | | 3123 | hsa-miR-95 | 2350 | FOLR2 | folate receptor 2 (fetal) | 1 | | 3124 | hsa-miR-1233 | 55174 | INTS10 | integrator complex subunit 10 | 1 | | 3125 | hsa-miR-1233 | 5599 | MAPK8 | mitogen-activated protein kinase 8 | 1 | | 3126 | hsa-miR-874 | 5599 | MAPK8 | mitogen-activated protein kinase 8 | 1 | | 3127 | hsa-miR-571 | 6619 | SNAPC3 | small nuclear RNA activating complex, polypeptide 3, 50kDa | 1 | | 3129 | hsa-miR-1233 | 92345 | NAF1 | nuclear assembly factor 1 homolog (S. cerevisiae) | 1 | | 3130 | hsa-miR-571 | 92345 | NAF1 | nuclear assembly factor 1 homolog (S. cerevisiae) | 1 | | 3132 | hsa-miR-874 | 92345 | NAF1 | nuclear assembly factor 1 homolog (S. cerevisiae) | 1 | | 3133 | hsa-miR-571 | 147525 | C18orf18 | chromosome 18 open reading frame 18 | 1 | | 3134 | hsa-mir-199a-2 | 55599 | RNPC3 | RNA-binding region (RNP1, RRM) containing 3 | 1 | | 3135 | hsa-mir-214 | 55599 | RNPC3 | RNA-binding region (RNP1, RRM) containing 3 | 1 | | 3136 | hsa-miR-199a-5p | 55599 | RNPC3 | RNA-binding region (RNP1, RRM) containing 3 | 1 | | 3137 | hsa-miR-576-5p | 55599 | RNPC3 | RNA-binding region (RNP1, RRM) containing 3 | 1 | | 3138 | hsa-miR-770-5p | 93273 | LEMD1 | LEM domain containing 1 | 1 | | 3139 | hsa-miR-95 | 93273 | LEMD1 | LEM domain containing 1 | 1 | | 3140 | hsa-mir-3130-1 | 92369 | SPSB4 | splA/ryanodine receptor domain and SOCS box containing 4 | 1 | | 3141 | hsa-mir-3130-2 | 92369 | SPSB4 | splA/ryanodine receptor domain and SOCS box containing 4 | 1 | | 3142 | hsa-mir-3130-3 | 92369 | SPSB4 | splA/ryanodine receptor domain and SOCS box containing 4 | 1 | | 3143 | hsa-miR-766 | 11231 | SEC63 | SEC63 homolog (S. cerevisiae) | 1 | | 3144 | hsa-miR-617 | 221188 | GPR114 | G protein-coupled receptor 114 | 1 | | 3145 | hsa-miR-188-5p | 79832 | QSER1 | glutamine and serine rich 1 | 1 | | 3146 | hsa-miR-571 | 166815 | TIGD2 | tigger transposable element derived 2 | 1 | | 3147 | hsa-miR-591 | 166815 | TIGD2 | tigger transposable element derived 2 | 1 | | 3148 | hsa-miR-874 | 166815 | TIGD2 | tigger transposable element derived 2 | 1 | | 3149 | hsa-miR-10a | 1810 | DR1 | down-regulator of transcription 1, TBP-binding (negative cofactor 2) | 1 | | 3150 | hsa-miR-770-5p | 1810 | DR1 | down-regulator of transcription 1, TBP-binding (negative cofactor 2) | 1 | | 3151 | hsa-miR-95 | 1810 | DR1 | down-regulator of transcription 1, TBP-binding (negative cofactor 2) | 1 | | 3152 | hsa-miR-10a | 196740 | C10orf72 | chromosome 10 open reading frame 72 | 1 | | 3153 | hsa-miR-770-5p | 196740 | C10orf72 | chromosome 10 open reading frame 72 | 1 | | 3154 | hsa-miR-95 | 196740 | C10orf72 | chromosome 10 open reading frame 72 | 1 | | 3155 | hsa-miR-10a | 7920 | BAT5 | HLA-B associated transcript 5 | 1 | | 3156 | hsa-miR-770-5p | 7920 | BAT5 | HLA-B associated transcript 5 | 1 | | 3157 | hsa-miR-95 | 7920 | BAT5 | HLA-B associated transcript 5 | 1 | | 3158 | hsa-miR-1233 | 28985 | MCTS1 | malignant T cell amplified sequence 1 | 1 | | 3159 | hsa-miR-591 | 28985 | MCTS1 | malignant T cell amplified sequence 1 | 1 | | 3160 | hsa-miR-1233 | 283663 | LOC283663 | hypothetical LOC283663 | 1 | | 3161 | hsa-miR-1233 | 57121 | LPAR5 | lysophosphatidic acid receptor 5 | 1 | | 3162 | hsa-miR-1233 | 55119 | PRPF38B | PRP38 pre-mRNA processing factor 38 (yeast) domain containing B | 1 | | 3163 | hsa-miR-10a | 127294 | MYOM3 | myomesin family, member 3 | 1 | | 3165 | hsa-miR-766 | 730094 | C16orf52 | chromosome 16 open reading frame 52 | 1 | | 3166 | hsa-miR-934 | 730094 | C16orf52 | chromosome 16 open reading frame 52 | 1 | | 3167 | hsa-miR-1233 | 153364 | MBLAC2 | metallo-beta-lactamase domain containing 2 | 1 | | 3168 | hsa-miR-571 | 153364 | MBLAC2 | metallo-beta-lactamase domain containing 2 | 1 | | 3169 | hsa-miR-874 | 153364 | MBLAC2 | metallo-beta-lactamase domain containing 2 | 1 | | 3170 | hsa-miR-1233 | 10336 | PCGF3 | polycomb group ring finger 3 | 1 | | 3171 | hsa-miR-874 | 10336 | PCGF3 | polycomb group ring finger 3 | 1 | | 3172 | hsa-mir-423 | 57452 | GALNTL1 | UDP-N-acetyl-alpha-D-galactosamine:polypeptide N-acetylgalactosaminyltransferase-like 1 | 1 | | 3173 | hsa-miR-10a | 57452 | GALNTL1 | UDP-N-acetyl-alpha-D-galactosamine:polypeptide N-acetylgalactosaminyltransferase-like 1 | 1 | | 3174 | hsa-miR-770-5p | 57452 | GALNTL1 | UDP-N-acetyl-alpha-D-galactosamine:polypeptide N-acetylgalactosaminyltransferase-like 1 | 1 | | 3175 | hsa-miR-95 | 57452 | GALNTL1 | UDP-N-acetyl-alpha-D-galactosamine:polypeptide N-acetylgalactosaminyltransferase-like 1 | 1 | | 3176 | hsa-miR-591 | 9529 | BAG5 | BCL2-associated athanogene 5 | 1 | | 3177 | hsa-miR-10a | 94086 | HSPB9 | heat shock protein, alpha-crystallin-related, B9 | 1 | | 3178 | hsa-miR-770-5p | 94086 | HSPB9 | heat shock protein, alpha-crystallin-related, B9 | 1 | | 3179 | hsa-miR-95 | 94086 | HSPB9 | heat shock protein, alpha-crystallin-related, B9 | 1 | | 3180 | hsa-miR-874 | 57646 | USP28 | ubiquitin specific peptidase 28 | 1 | | 3181 | hsa-miR-770-5p | 201595 | STT3B | STT3, subunit of the oligosaccharyltransferase complex, homolog B (S. cerevisiae) | 1 | | 3182 | hsa-miR-95 | 201595 | STT3B | STT3, subunit of the oligosaccharyltransferase complex, homolog B (S. cerevisiae) | 1 | | 3183 | hsa-miR-874 | 119559 | SFXN4 | sideroflexin 4 | 1 | | 3184 | hsa-miR-10a | 3691 | ITGB4 | integrin, beta 4 | 1 | | 3185 | hsa-miR-591 | 730094 | C16orf52 | chromosome 16 open reading frame 52 | 1 | | 3188 | hsa-mir-3130-1 | 201140 | DHRS7C | dehydrogenase/reductase (SDR family) member 7C | 1 | | 3189 | hsa-mir-3130-2 | 201140 | DHRS7C | dehydrogenase/reductase (SDR family) member 7C | 1 | | 3190 | hsa-mir-3130-3 | 201140 | DHRS7C | dehydrogenase/reductase (SDR family) member 7C | 1 | | 3191 | hsa-miR-10a | 201140 | DHRS7C | dehydrogenase/reductase (SDR family) member 7C | 1 | | 3192 | hsa-miR-770-5p | 201140 | DHRS7C | dehydrogenase/reductase (SDR family) member 7C | 1 | | 3193 | hsa-miR-95 | 201140 | DHRS7C | dehydrogenase/reductase (SDR family) member 7C | 1 | | 3194 | hsa-miR-770-5p | 9099 | USP2 | ubiquitin specific peptidase 2 | 1 | | 3195 | hsa-miR-95 | 9099 | USP2 | ubiquitin specific peptidase 2 | 1 | | 3196 | hsa-mir-576 | 10361 | NPM2 | nucleophosmin/nucleoplasmin 2 | 1 | | 3197 | hsa-miR-10a | 122664 | TPPP2 | tubulin polymerization-promoting protein family member 2 | 1 | | 3198 | hsa-miR-770-5p | 122664 | TPPP2 | tubulin polymerization-promoting protein family member 2 | 1 | | 3199 | hsa-miR-95 | 122664 | TPPP2 | tubulin polymerization-promoting protein family member 2 | 1 | | 3200 | hsa-miR-874 | 54986 | ULK4 | unc-51-like kinase 4 (C. elegans) | 1 | | 3201 | hsa-miR-128 | 387644 | NCRNA00202 | non-protein coding RNA 202 | 1 | | 3202 | hsa-miR-770-5p | 23274 | CLEC16A | C-type lectin domain family 16, member A | 1 | | 3203 | hsa-miR-95 | 23274 | CLEC16A | C-type lectin domain family 16, member A | 1 | | 3204 | hsa-miR-1233 | 645744 | LOC645744 | similar to PCAF associated factor 65 beta | 1 | | 3205 | hsa-miR-569 | 645744 | LOC645744 | similar to PCAF associated factor 65 beta | 1 | | 3206 | hsa-miR-766 | 645744 | LOC645744 | similar to PCAF associated factor 65 beta | 1 | | 3207 | hsa-mir-3130-1 | 100288438 | LOC100288438 | hypothetical protein LOC100288438 | 1 | | 3208 | hsa-mir-3130-2 | 100288438 | LOC100288438 | hypothetical protein LOC100288438 | 1 | | 3209 | hsa-mir-3130-3 | 100288438 | LOC100288438 | hypothetical protein LOC100288438 | 1 | | 3210 | hsa-miR-10a | 100288438 | LOC100288438 | hypothetical protein LOC100288438 | 1 | | 3211 | hsa-miR-770-5p | 100288438 | LOC100288438 | hypothetical protein LOC100288438 | 1 | | 3212 | hsa-miR-95 | 100288438 | LOC100288438 | hypothetical protein LOC100288438 | 1 | | 3213 | hsa-miR-95 | 645524 | FLJ36840 | hypothetical LOC645524 | 1 | | 3214 | hsa-miR-10a | 149175 | MANEAL | mannosidase, endo-alpha-like | 1 | | 3215 | hsa-mir-3130-1 | 55072 | RNF31 | ring finger protein 31 | 1 | | 3216 | hsa-mir-3130-2 | 55072 | RNF31 | ring finger protein 31 | 1 | | 3217 | hsa-mir-3130-3 | 55072 | RNF31 | ring finger protein 31 | 1 | | 3218 | hsa-miR-10a | 55072 | RNF31 | ring finger protein 31 | 1 | | 3219 | hsa-miR-770-5p | 55072 | RNF31 | ring finger protein 31 | 1 | | 3220 | hsa-miR-95 | 55072 | RNF31 | ring finger protein 31 | 1 | | 3221 | hsa-mir-423 | 388503 | C3P1 | complement component 3 precursor pseudogene | 1 | | 3222 | hsa-mir-3130-1 | 388503 | C3P1 | complement component 3 precursor pseudogene | 1 | | 3223 | hsa-mir-3130-2 | 388503 | C3P1 | complement component 3 precursor pseudogene | 1 | | 3224 | hsa-mir-3130-3 | 388503 | C3P1 | complement component 3 precursor pseudogene | 1 | | 3225 | hsa-miR-10a | 388503 | C3P1 | complement component 3 precursor pseudogene | 1 | | 3226 | hsa-miR-770-5p | 828 | CAPS | calcyphosine | 1 | | 3227 | hsa-miR-190 | 10081 | PDCD7 | programmed cell death 7 | 1 | | 3228 | hsa-miR-10a | 155435 | RBM33 | RNA binding motif protein 33 | 1 | | 3229 | hsa-miR-1233 | 127281 | C1orf93 | chromosome 1 open reading frame 93 | 1 | | 3230 | hsa-miR-766 | 127281 | C1orf93 | chromosome 1 open reading frame 93 | 1 | | 3231 | hsa-miR-1233 | 57646 | USP28 | ubiquitin specific peptidase 28 | 1 | | 3232 | hsa-miR-766 | 57646 | USP28 | ubiquitin specific peptidase 28 | 1 | | 3233 | hsa-miR-576-5p | 54556 | ING3 | inhibitor of growth family, member 3 | 1 | | 3234 | hsa-miR-591 | 54556 | ING3 | inhibitor of growth family, member 3 | 1 | | 3235 | hsa-mir-1224 | 79184 | BRCC3 | BRCA1/BRCA2-containing complex, subunit 3 | 1 | | 3236 | hsa-mir-885 | 79184 | BRCC3 | BRCA1/BRCA2-containing complex, subunit 3 | 1 | | 3237 | hsa-miR-1224-5p | 79184 | BRCC3 | BRCA1/BRCA2-containing complex, subunit 3 | 1 | | 3238 | hsa-miR-218 | 79184 | BRCC3 | BRCA1/BRCA2-containing complex, subunit 3 | 1 | | 3239 | hsa-miR-571 | 79184 | BRCC3 | BRCA1/BRCA2-containing complex, subunit 3 | 1 | | 3240 | hsa-miR-10a | 441027 | TMEM150C | transmembrane protein 150C | 1 | | 3241 | hsa-miR-770-5p | 441027 | TMEM150C | transmembrane protein 150C | 1 | | 3242 | hsa-miR-95 | 441027 | TMEM150C | transmembrane protein 150C | 1 | | 3243 | hsa-miR-766 | 57711 | ZNF529 | zinc finger protein 529 | 1 | | 3244 | hsa-miR-576-5p | 2186 | BPTF | bromodomain PHD finger transcription factor | 1 | | 3245 | hsa-miR-1233 | 54014 | BRWD1 | bromodomain and WD repeat domain containing 1 | 1 | | 3246 | hsa-miR-591 | 54014 | BRWD1 | bromodomain and WD repeat domain containing 1 | 1 | | 3247 | hsa-miR-874 | 54014 | BRWD1 | bromodomain and WD repeat domain containing 1 | 1 | | 3248 | hsa-miR-591 | 4507 | MTAP | methylthioadenosine phosphorylase | 1 | | 3249 | hsa-miR-770-5p | 55349 | CHDH | choline dehydrogenase | 1 | | 3250 | hsa-miR-95 | 55349 | CHDH | choline dehydrogenase | 1 | | 3251 | hsa-miR-591 | 10236 | HNRNPR | heterogeneous nuclear ribonucleoprotein R | 1 | | 3252 | hsa-miR-874 | 10236 | HNRNPR | heterogeneous nuclear ribonucleoprotein R | 1 | | 3253 | hsa-miR-571 | 55326 | AGPAT5 | 1-acylglycerol-3-phosphate O-acyltransferase 5 (lysophosphatidic acid acyltransferase, epsilon) | 1 | | 3254 | hsa-miR-766 | 56987 | BBX | bobby sox homolog (Drosophila) | 1 | | 3255 | hsa-miR-188-5p | 283464 | GXYLT1 | glucoside xylosyltransferase 1 | 1 | | 3256 | hsa-miR-591 | 440944 | LOC440944 | hypothetical LOC440944 | 1 | | 3257 | hsa-miR-1233 | 100132352 | LOC100132352 | similar to hCG1989297 | 1 | | 3258 | hsa-miR-571 | 55745 | MUDENG | MU-2/AP1M2 domain containing, death-inducing | 1 | | 3259 | hsa-miR-591 | 55745 | MUDENG | MU-2/AP1M2 domain containing, death-inducing | 1 | | 3260 | hsa-mir-10a | 29945 | ANAPC4 | anaphase promoting complex subunit 4 | 1 | | 3261 | hsa-mir-199a-2 | 29945 | ANAPC4 | anaphase promoting complex subunit 4 | 1 | | 3262 | hsa-mir-214 | 29945 | ANAPC4 | anaphase promoting complex subunit 4 | 1 | | 3263 | hsa-miR-1233 | 29945 | ANAPC4 | anaphase promoting complex subunit 4 | 1 | | 3264 | hsa-miR-199a-5p | 29945 | ANAPC4 | anaphase promoting complex subunit 4 | 1 | | 3265 | hsa-miR-766 | 29945 | ANAPC4 | anaphase promoting complex subunit 4 | 1 | | 3266 | hsa-miR-576-5p | 11168 | PSIP1 | PC4 and SFRS1 interacting protein 1 | 1 | | 3267 | hsa-miR-95 | 57642 | COL20A1 | collagen, type XX, alpha 1 | 1 | | 3268 | hsa-miR-591 | 55958 | KLHL9 | kelch-like 9 (Drosophila) | 1 | | 3269 | hsa-miR-1233 | 55592 | GOLGA2B | golgin A2 family, member B | 1 | | 3270 | hsa-miR-571 | 55592 | GOLGA2B | golgin A2 family, member B | 1 | | 3271 | hsa-miR-766 | 55592 | GOLGA2B | golgin A2 family, member B | 1 | | 3272 | hsa-miR-874 | 55592 | GOLGA2B | golgin A2 family, member B | 1 | | 3273 | hsa-miR-1233 | 64087 | MCCC2 | methylcrotonoyl-Coenzyme A carboxylase 2 (beta) | 1 | | 3274 | hsa-miR-571 | 64087 | MCCC2 | methylcrotonoyl-Coenzyme A carboxylase 2 (beta) | 1 | | 3275 | hsa-miR-874 | 64087 | MCCC2 | methylcrotonoyl-Coenzyme A carboxylase 2 (beta) | 1 | | 3276 | hsa-miR-10a | 8991 | SELENBP1 | selenium binding protein 1 | 1 | | 3277 | hsa-miR-770-5p | 8991 | SELENBP1 | selenium binding protein 1 | 1 | | 3278 | hsa-miR-95 | 8991 | SELENBP1 | selenium binding protein 1 | 1 | | 3279 | hsa-miR-16 | 388662 | SLC6A17 | solute carrier family 6, member 17 | 1 | | 3280 | hsa-miR-591 | 84172 | POLR1B | polymerase (RNA) I polypeptide B, 128kDa | 1 | | 3281 | hsa-miR-10a | 64800 | EFCAB6 | EF-hand calcium binding domain 6 | 1 | | 3282 | hsa-miR-95 | 64800 | EFCAB6 | EF-hand calcium binding domain 6 | 1 | | 3283 | hsa-miR-770-5p | 60437 | CDH26 | cadherin 26 | 1 | | 3284 | hsa-miR-95 | 60437 | CDH26 | cadherin 26 | 1 | | 3285 | hsa-mir-423 | 53345 | TM6SF2 | transmembrane 6 superfamily member 2 | 1 | | 3286 | hsa-mir-3130-1 | 53345 | TM6SF2 | transmembrane 6 superfamily member 2 | 1 | | 3287 | hsa-mir-3130-2 | 53345 | TM6SF2 | transmembrane 6 superfamily member 2 | 1 | | 3288 | hsa-mir-3130-3 | 53345 | TM6SF2 | transmembrane 6 superfamily member 2 | 1 | | 3289 | hsa-miR-10a | 53345 | TM6SF2 | transmembrane 6 superfamily member 2 | 1 | | 3290 | hsa-miR-770-5p | 53345 | TM6SF2 | transmembrane 6 superfamily member 2 | 1 | | 3291 | hsa-miR-1233 | 7769 | ZNF226 | zinc finger protein 226 | 1 | | 3292 | hsa-miR-569 | 7769 | ZNF226 | zinc finger protein 226 | 1 | | 3293 | hsa-miR-766 | 7769 | ZNF226 | zinc finger protein 226 | 1 | | 3294 | hsa-miR-874 | 7769 | ZNF226 | zinc finger protein 226 | 1 | | 3295 | hsa-miR-190 | 29121 | CLEC2D | C-type lectin domain family 2, member D | 1 | | 3296 | hsa-mir-423 | 83959 | SLC4A11 | solute carrier family 4, sodium borate transporter, member 11 | 1 | | 3297 | hsa-mir-3130-1 | 83959 | SLC4A11 | solute carrier family 4, sodium borate transporter, member 11 | 1 | | 3298 | hsa-mir-3130-2 | 83959 | SLC4A11 | solute carrier family 4, sodium borate transporter, member 11 | 1 | | 3299 | hsa-mir-3130-3 | 83959 | SLC4A11 | solute carrier family 4, sodium borate transporter, member 11 | 1 | | 3300 | hsa-miR-10a | 83959 | SLC4A11 | solute carrier family 4, sodium borate transporter, member 11 | 1 | | 3301 | hsa-miR-770-5p | 83959 | SLC4A11 | solute carrier family 4, sodium borate transporter, member 11 | 1 | | 3302 | hsa-miR-95 | 83959 | SLC4A11 | solute carrier family 4, sodium borate transporter, member 11 | 1 | | 3303 | hsa-mir-3130-1 | 114800 | CCDC85A | coiled-coil domain containing 85A | 1 | | 3304 | hsa-mir-3130-2 | 114800 | CCDC85A | coiled-coil domain containing 85A | 1 | | 3305 | hsa-mir-3130-3 | 114800 | CCDC85A | coiled-coil domain containing 85A | 1 | | 3306 | hsa-mir-10a | 394 | ARHGAP5 | Rho GTPase activating protein 5 | 1 | | 3307 | hsa-miR-770-5p | 100133790 | LOC100133790 | intestinal mucin-like | 1 | | 3308 | hsa-miR-95 | 100133790 | LOC100133790 | intestinal mucin-like | 1 | | 3309 | hsa-miR-95 | 140880 | CST11 | cystatin 11 | 1 | | 3310 | hsa-miR-770-5p | 259291 | TAS2R45 | taste receptor, type 2, member 45 | 1 | | 3311 | hsa-miR-95 | 259291 | TAS2R45 | taste receptor, type 2, member 45 | 1 | | 3312 | hsa-mir-3130-1 | 56241 | SUSD2 | sushi domain containing 2 | 1 | | 3313 | hsa-mir-3130-2 | 56241 | SUSD2 | sushi domain containing 2 | 1 | | 3314 | hsa-mir-3130-3 | 56241 | SUSD2 | sushi domain containing 2 | 1 | | 3315 | hsa-miR-10a | 56241 | SUSD2 | sushi domain containing 2 | 1 | | 3316 | hsa-miR-770-5p | 56241 | SUSD2 | sushi domain containing 2 | 1 | | 3317 | hsa-miR-593\* | 29997 | GLTSCR2 | glioma tumor suppressor candidate region gene 2 | 1 | | 3318 | hsa-miR-95 | 9734 | HDAC9 | histone deacetylase 9 | 1 | | 3319 | hsa-mir-423 | 60506 | NYX | nyctalopin | 1 | | 3320 | hsa-mir-3130-1 | 60506 | NYX | nyctalopin | 1 | | 3321 | hsa-mir-3130-2 | 60506 | NYX | nyctalopin | 1 | | 3322 | hsa-mir-3130-3 | 60506 | NYX | nyctalopin | 1 | | 3323 | hsa-miR-10a | 60506 | NYX | nyctalopin | 1 | | 3324 | hsa-miR-770-5p | 60506 | NYX | nyctalopin | 1 | | 3325 | hsa-miR-10a | 140731 | ANKRD60 | ankyrin repeat domain 60 | 1 | | 3326 | hsa-miR-770-5p | 140731 | ANKRD60 | ankyrin repeat domain 60 | 1 | | 3327 | hsa-miR-95 | 57101 | ANO2 | anoctamin 2 | 1 | | 3328 | hsa-miR-10a | 54660 | PCDHB18 | protocadherin beta 18 pseudogene | 1 | | 3329 | hsa-miR-770-5p | 54660 | PCDHB18 | protocadherin beta 18 pseudogene | 1 | | 3330 | hsa-miR-95 | 54660 | PCDHB18 | protocadherin beta 18 pseudogene | 1 | | 3331 | hsa-miR-766 | 84532 | ACSS1 | acyl-CoA synthetase short-chain family member 1 | 1 | | 3332 | hsa-miR-95 | 66000 | TMEM108 | transmembrane protein 108 | 1 | | 3336 | hsa-miR-128 | 83999 | KREMEN1 | kringle containing transmembrane protein 1 | 1 | | 3337 | hsa-mir-3130-1 | 284187 | DKFZp761P0212 | hypothetical protein DKFZp761P0212 | 1 | | 3338 | hsa-mir-3130-2 | 284187 | DKFZp761P0212 | hypothetical protein DKFZp761P0212 | 1 | | 3339 | hsa-mir-3130-3 | 284187 | DKFZp761P0212 | hypothetical protein DKFZp761P0212 | 1 | | 3340 | hsa-miR-10a | 284187 | DKFZp761P0212 | hypothetical protein DKFZp761P0212 | 1 | | 3341 | hsa-miR-770-5p | 284187 | DKFZp761P0212 | hypothetical protein DKFZp761P0212 | 1 | | 3342 | hsa-miR-95 | 284187 | DKFZp761P0212 | hypothetical protein DKFZp761P0212 | 1 | | 3343 | hsa-miR-770-5p | 55354 | ORF1 | hypothetical protein, clone pT-Adv JuaX22 | 1 | | 3344 | hsa-miR-95 | 55354 | ORF1 | hypothetical protein, clone pT-Adv JuaX22 | 1 | | 3345 | hsa-miR-10a | 57224 | NHSL1 | NHS-like 1 | 1 | | 3346 | hsa-miR-770-5p | 57224 | NHSL1 | NHS-like 1 | 1 | | 3347 | hsa-miR-95 | 57224 | NHSL1 | NHS-like 1 | 1 | | 3348 | hsa-miR-770-5p | 56063 | C1orf91 | chromosome 1 open reading frame 91 | 1 | | 3349 | hsa-miR-95 | 56063 | C1orf91 | chromosome 1 open reading frame 91 | 1 | | 3350 | hsa-mir-423 | 93444 | LOC93444 | hypothetical protein LOC93444 | 1 | | 3351 | hsa-mir-3130-1 | 93444 | LOC93444 | hypothetical protein LOC93444 | 1 | | 3352 | hsa-mir-3130-2 | 93444 | LOC93444 | hypothetical protein LOC93444 | 1 | | 3353 | hsa-mir-3130-3 | 93444 | LOC93444 | hypothetical protein LOC93444 | 1 | | 3354 | hsa-miR-591 | 125228 | C18orf19 | chromosome 18 open reading frame 19 | 1 | | 3356 | hsa-miR-591 | 645212 | LOC645212 | hypothetical LOC645212 | 1 | | 3357 | hsa-miR-571 | 374986 | FAM73A | family with sequence similarity 73, member A | 1 | | 3358 | hsa-miR-591 | 374986 | FAM73A | family with sequence similarity 73, member A | 1 | | 3359 | hsa-miR-765 | 51043 | ZBTB7B | zinc finger and BTB domain containing 7B | 1 | | 3360 | hsa-miR-766 | 3707 | ITPKB | inositol 1,4,5-trisphosphate 3-kinase B | 1 | | 3361 | hsa-miR-576-5p | 10691 | GMEB1 | glucocorticoid modulatory element binding protein 1 | 1 | | 3362 | hsa-miR-591 | 10691 | GMEB1 | glucocorticoid modulatory element binding protein 1 | 1 | | 3363 | hsa-miR-1233 | 6428 | SFRS3 | splicing factor, arginine/serine-rich 3 | 1 | | 3364 | hsa-miR-1233 | 163087 | ZNF383 | zinc finger protein 383 | 1 | | 3365 | hsa-miR-571 | 163087 | ZNF383 | zinc finger protein 383 | 1 | | 3366 | hsa-miR-591 | 163087 | ZNF383 | zinc finger protein 383 | 1 | | 3367 | hsa-miR-874 | 163087 | ZNF383 | zinc finger protein 383 | 1 | | 3368 | hsa-miR-571 | 64794 | DDX31 | DEAD (Asp-Glu-Ala-Asp) box polypeptide 31 | 1 | | 3369 | hsa-miR-874 | 64794 | DDX31 | DEAD (Asp-Glu-Ala-Asp) box polypeptide 31 | 1 | | 3370 | hsa-miR-1233 | 84441 | MAML2 | mastermind-like 2 (Drosophila) | 1 | | 3371 | hsa-miR-766 | 84441 | MAML2 | mastermind-like 2 (Drosophila) | 1 | | 3372 | hsa-miR-874 | 84441 | MAML2 | mastermind-like 2 (Drosophila) | 1 | | 3373 | hsa-miR-874 | 29121 | CLEC2D | C-type lectin domain family 2, member D | 1 | | 3374 | hsa-miR-1233 | 3192 | HNRNPU | heterogeneous nuclear ribonucleoprotein U (scaffold attachment factor A) | 1 | | 3375 | hsa-miR-571 | 3192 | HNRNPU | heterogeneous nuclear ribonucleoprotein U (scaffold attachment factor A) | 1 | | 3376 | hsa-miR-874 | 394 | ARHGAP5 | Rho GTPase activating protein 5 | 1 | | 3379 | hsa-miR-591 | 163081 | ZNF567 | zinc finger protein 567 | 1 | | 3381 | hsa-mir-10a | 152815 | THAP6 | THAP domain containing 6 | 1 | | 3382 | hsa-miR-576-5p | 152815 | THAP6 | THAP domain containing 6 | 1 | | 3383 | hsa-miR-1233 | 124961 | ZFP3 | zinc finger protein 3 homolog (mouse) | 1 | | 3384 | hsa-miR-571 | 124961 | ZFP3 | zinc finger protein 3 homolog (mouse) | 1 | | 3385 | hsa-miR-591 | 124961 | ZFP3 | zinc finger protein 3 homolog (mouse) | 1 | | 3386 | hsa-miR-874 | 124961 | ZFP3 | zinc finger protein 3 homolog (mouse) | 1 | | 3387 | hsa-miR-571 | 100287482 | LOC100287482 | similar to hCG2038584 | 1 | | 3388 | hsa-miR-874 | 100287482 | LOC100287482 | similar to hCG2038584 | 1 | | 3389 | hsa-miR-95 | 162968 | ZNF497 | zinc finger protein 497 | 1 | | 3390 | hsa-miR-635 | 266747 | RGL4 | ral guanine nucleotide dissociation stimulator-like 4 | 1 | | 3391 | hsa-miR-874 | 54554 | WDR5B | WD repeat domain 5B | 1 | | 3392 | hsa-mir-423 | 4320 | MMP11 | matrix metallopeptidase 11 (stromelysin 3) | 1 | | 3393 | hsa-mir-3130-1 | 4320 | MMP11 | matrix metallopeptidase 11 (stromelysin 3) | 1 | | 3394 | hsa-mir-3130-2 | 4320 | MMP11 | matrix metallopeptidase 11 (stromelysin 3) | 1 | | 3395 | hsa-mir-3130-3 | 4320 | MMP11 | matrix metallopeptidase 11 (stromelysin 3) | 1 | | 3396 | hsa-miR-10a | 4320 | MMP11 | matrix metallopeptidase 11 (stromelysin 3) | 1 | | 3397 | hsa-miR-770-5p | 4320 | MMP11 | matrix metallopeptidase 11 (stromelysin 3) | 1 | | 3398 | hsa-miR-95 | 4320 | MMP11 | matrix metallopeptidase 11 (stromelysin 3) | 1 | | 3399 | hsa-miR-1233 | 7514 | XPO1 | exportin 1 (CRM1 homolog, yeast) | 1 | | 3400 | hsa-mir-423 | 254559 | LOC254559 | hypothetical LOC254559 | 1 | | 3401 | hsa-miR-10a | 254559 | LOC254559 | hypothetical LOC254559 | 1 | | 3402 | hsa-miR-770-5p | 254559 | LOC254559 | hypothetical LOC254559 | 1 | | 3403 | hsa-miR-95 | 254559 | LOC254559 | hypothetical LOC254559 | 1 | | 3404 | hsa-miR-1233 | 201475 | RAB12 | RAB12, member RAS oncogene family | 1 | | 3405 | hsa-miR-766 | 201475 | RAB12 | RAB12, member RAS oncogene family | 1 | | 3406 | hsa-miR-591 | 119392 | C10orf78 | chromosome 10 open reading frame 78 | 1 | | 3407 | hsa-miR-95 | 375775 | PNPLA7 | patatin-like phospholipase domain containing 7 | 1 | | 3408 | hsa-miR-1233 | 10075 | HUWE1 | HECT, UBA and WWE domain containing 1 | 1 | | 3409 | hsa-miR-591 | 132320 | SCLT1 | sodium channel and clathrin linker 1 | 1 | | 3410 | hsa-miR-10a | 359 | AQP2 | aquaporin 2 (collecting duct) | 1 | | 3411 | hsa-mir-3130-1 | 388780 | LOC388780 | hypothetical LOC388780 | 1 | | 3412 | hsa-mir-3130-2 | 388780 | LOC388780 | hypothetical LOC388780 | 1 | | 3413 | hsa-mir-3130-3 | 388780 | LOC388780 | hypothetical LOC388780 | 1 | | 3414 | hsa-miR-10a | 388780 | LOC388780 | hypothetical LOC388780 | 1 | | 3415 | hsa-miR-770-5p | 388780 | LOC388780 | hypothetical LOC388780 | 1 | | 3416 | hsa-miR-95 | 388780 | LOC388780 | hypothetical LOC388780 | 1 | | 3417 | hsa-miR-571 | 4012 | LNPEP | leucyl/cystinyl aminopeptidase | 1 | | 3418 | hsa-miR-10a | 4335 | MNT | MAX binding protein | 1 | | 3419 | hsa-miR-770-5p | 4335 | MNT | MAX binding protein | 1 | | 3420 | hsa-miR-95 | 4335 | MNT | MAX binding protein | 1 | | 3421 | hsa-miR-1233 | 154075 | SAMD3 | sterile alpha motif domain containing 3 | 1 | | 3422 | hsa-miR-569 | 154075 | SAMD3 | sterile alpha motif domain containing 3 | 1 | | 3423 | hsa-miR-766 | 154075 | SAMD3 | sterile alpha motif domain containing 3 | 1 | | 3424 | hsa-miR-874 | 154075 | SAMD3 | sterile alpha motif domain containing 3 | 1 | | 3425 | hsa-miR-874 | 60468 | BACH2 | BTB and CNC homology 1, basic leucine zipper transcription factor 2 | 1 | | 3429 | hsa-miR-1233 | 8575 | PRKRA | protein kinase, interferon-inducible double stranded RNA dependent activator | 1 | | 3430 | hsa-miR-569 | 8575 | PRKRA | protein kinase, interferon-inducible double stranded RNA dependent activator | 1 | | 3431 | hsa-miR-766 | 8575 | PRKRA | protein kinase, interferon-inducible double stranded RNA dependent activator | 1 | | 3432 | hsa-miR-874 | 8575 | PRKRA | protein kinase, interferon-inducible double stranded RNA dependent activator | 1 | | 3433 | hsa-miR-604 | 731789 | LOC731789 | hypothetical LOC731789 | 1 | | 3434 | hsa-miR-938 | 731789 | LOC731789 | hypothetical LOC731789 | 1 | | 3435 | hsa-miR-770-5p | 116179 | TGM7 | transglutaminase 7 | 1 | | 3436 | hsa-miR-554 | 1385 | CREB1 | cAMP responsive element binding protein 1 | 1 | | 3437 | hsa-mir-10a | 5928 | RBBP4 | retinoblastoma binding protein 4 | 1 | | 3439 | hsa-miR-10a | 2155 | F7 | coagulation factor VII (serum prothrombin conversion accelerator) | 1 | | 3440 | hsa-miR-770-5p | 2155 | F7 | coagulation factor VII (serum prothrombin conversion accelerator) | 1 | | 3441 | hsa-miR-95 | 2155 | F7 | coagulation factor VII (serum prothrombin conversion accelerator) | 1 | | 3442 | hsa-mir-3130-1 | 87769 | A2LD1 | AIG2-like domain 1 | 1 | | 3443 | hsa-mir-3130-2 | 87769 | A2LD1 | AIG2-like domain 1 | 1 | | 3444 | hsa-mir-3130-3 | 87769 | A2LD1 | AIG2-like domain 1 | 1 | | 3445 | hsa-miR-10a | 87769 | A2LD1 | AIG2-like domain 1 | 1 | | 3446 | hsa-miR-770-5p | 87769 | A2LD1 | AIG2-like domain 1 | 1 | | 3447 | hsa-miR-95 | 87769 | A2LD1 | AIG2-like domain 1 | 1 | | 3448 | hsa-mir-3130-1 | 440503 | PLIN5 | perilipin 5 | 1 | | 3449 | hsa-mir-3130-2 | 440503 | PLIN5 | perilipin 5 | 1 | | 3450 | hsa-mir-3130-3 | 440503 | PLIN5 | perilipin 5 | 1 | | 3451 | hsa-miR-10a | 440503 | PLIN5 | perilipin 5 | 1 | | 3452 | hsa-miR-770-5p | 440503 | PLIN5 | perilipin 5 | 1 | | 3453 | hsa-miR-95 | 440503 | PLIN5 | perilipin 5 | 1 | | 3454 | hsa-miR-591 | 728769 | LOC728769 | hypothetical protein LOC728769 | 1 | | 3455 | hsa-miR-95 | 729956 | SHISA7 | shisa homolog 7 (Xenopus laevis) | 1 | | 3456 | hsa-miR-1233 | 100130742 | LRRC69 | leucine rich repeat containing 69 | 1 | | 3457 | hsa-miR-874 | 100130742 | LRRC69 | leucine rich repeat containing 69 | 1 | | 3458 | hsa-miR-1233 | 201595 | STT3B | STT3, subunit of the oligosaccharyltransferase complex, homolog B (S. cerevisiae) | 1 | | 3459 | hsa-mir-3130-1 | 54815 | GATAD2A | GATA zinc finger domain containing 2A | 1 | | 3460 | hsa-mir-3130-2 | 54815 | GATAD2A | GATA zinc finger domain containing 2A | 1 | | 3461 | hsa-mir-3130-3 | 54815 | GATAD2A | GATA zinc finger domain containing 2A | 1 | | 3462 | hsa-miR-95 | 54815 | GATAD2A | GATA zinc finger domain containing 2A | 1 | | 3463 | hsa-mir-3130-1 | 284131 | FLJ35220 | hypothetical protein FLJ35220 | 1 | | 3464 | hsa-mir-3130-2 | 284131 | FLJ35220 | hypothetical protein FLJ35220 | 1 | | 3465 | hsa-mir-3130-3 | 284131 | FLJ35220 | hypothetical protein FLJ35220 | 1 | | 3466 | hsa-miR-128 | 284131 | FLJ35220 | hypothetical protein FLJ35220 | 1 | | 3467 | hsa-mir-423 | 124245 | ZC3H18 | zinc finger CCCH-type containing 18 | 1 | | 3468 | hsa-miR-10a | 124245 | ZC3H18 | zinc finger CCCH-type containing 18 | 1 | | 3469 | hsa-miR-1233 | 171425 | CLYBL | citrate lyase beta like | 1 | | 3470 | hsa-miR-571 | 171425 | CLYBL | citrate lyase beta like | 1 | | 3471 | hsa-miR-874 | 171425 | CLYBL | citrate lyase beta like | 1 | | 3472 | hsa-miR-571 | 440515 | ZNF506 | zinc finger protein 506 | 1 | | 3473 | hsa-miR-576-5p | 440515 | ZNF506 | zinc finger protein 506 | 1 | | 3474 | hsa-miR-569 | 286272 | LOC286272 | hypothetical protein LOC286272 | 1 | | 3475 | hsa-miR-766 | 286272 | LOC286272 | hypothetical protein LOC286272 | 1 | | 3476 | hsa-miR-571 | 134728 | IRAK1BP1 | interleukin-1 receptor-associated kinase 1 binding protein 1 | 1 | | 3477 | hsa-miR-1233 | 7699 | ZNF140 | zinc finger protein 140 | 1 | | 3478 | hsa-miR-571 | 7699 | ZNF140 | zinc finger protein 140 | 1 | | 3479 | hsa-miR-874 | 7699 | ZNF140 | zinc finger protein 140 | 1 | | 3480 | hsa-miR-571 | 89978 | ATPBD4 | ATP binding domain 4 | 1 | | 3481 | hsa-miR-591 | 89978 | ATPBD4 | ATP binding domain 4 | 1 | | 3482 | hsa-miR-1233 | 284161 | GDPD1 | glycerophosphodiester phosphodiesterase domain containing 1 | 1 | | 3483 | hsa-miR-571 | 284161 | GDPD1 | glycerophosphodiester phosphodiesterase domain containing 1 | 1 | | 3484 | hsa-miR-874 | 284161 | GDPD1 | glycerophosphodiester phosphodiesterase domain containing 1 | 1 | | 3485 | hsa-mir-10a | 222236 | NAPEPLD | N-acyl phosphatidylethanolamine phospholipase D | 1 | | 3486 | hsa-miR-188-5p | 222236 | NAPEPLD | N-acyl phosphatidylethanolamine phospholipase D | 1 | | 3487 | hsa-mir-10a | 119392 | C10orf78 | chromosome 10 open reading frame 78 | 1 | | 3488 | hsa-miR-874 | 221443 | C6orf130 | chromosome 6 open reading frame 130 | 1 | | 3489 | hsa-miR-569 | 2971 | GTF3A | general transcription factor IIIA | 1 | | 3490 | hsa-miR-766 | 2971 | GTF3A | general transcription factor IIIA | 1 | | 3491 | hsa-miR-591 | 10972 | TMED10 | transmembrane emp24-like trafficking protein 10 (yeast) | 1 | | 3492 | hsa-miR-591 | 51065 | RPS27L | ribosomal protein S27-like | 1 | | 3493 | hsa-miR-126\* | 113655 | MFSD3 | major facilitator superfamily domain containing 3 | 1 | | 3494 | hsa-miR-770-5p | 23023 | TMCC1 | transmembrane and coiled-coil domain family 1 | 1 | | 3495 | hsa-miR-95 | 23023 | TMCC1 | transmembrane and coiled-coil domain family 1 | 1 | | 3496 | hsa-miR-770-5p | 100303728 | LOC100303728 | hypothetical LOC100303728 | 1 | | 3497 | hsa-miR-1233 | 399753 | RP11-144G6.7 | hypothetical LOC399753 | 1 | | 3498 | hsa-miR-766 | 399753 | RP11-144G6.7 | hypothetical LOC399753 | 1 | | 3499 | hsa-mir-885 | 8939 | FUBP3 | far upstream element (FUSE) binding protein 3 | 1 | | 3500 | hsa-miR-10a | 286753 | TUSC5 | tumor suppressor candidate 5 | 1 | | 3501 | hsa-miR-770-5p | 286753 | TUSC5 | tumor suppressor candidate 5 | 1 | | 3502 | hsa-miR-95 | 286753 | TUSC5 | tumor suppressor candidate 5 | 1 | | 3503 | hsa-miR-95 | 145837 | LOC145837 | hypothetical LOC145837 | 1 | | 3504 | hsa-miR-10a | 100132074 | FOXO6 | forkhead box O6 | 1 | | 3505 | hsa-miR-770-5p | 100132074 | FOXO6 | forkhead box O6 | 1 | | 3506 | hsa-miR-95 | 100132074 | FOXO6 | forkhead box O6 | 1 | | 3507 | hsa-miR-1233 | 3716 | JAK1 | Janus kinase 1 | 1 | | 3508 | hsa-miR-569 | 3716 | JAK1 | Janus kinase 1 | 1 | | 3509 | hsa-miR-766 | 3716 | JAK1 | Janus kinase 1 | 1 | | 3510 | hsa-miR-10a | 404266 | hCG\_2042068 | hypothetical LOC404266 | 1 | | 3511 | hsa-miR-770-5p | 404266 | hCG\_2042068 | hypothetical LOC404266 | 1 | | 3512 | hsa-miR-95 | 404266 | hCG\_2042068 | hypothetical LOC404266 | 1 | | 3513 | hsa-miR-571 | 148254 | ZNF555 | zinc finger protein 555 | 1 | | 3514 | hsa-miR-95 | 5817 | PVR | poliovirus receptor | 1 | | 3515 | hsa-miR-770-5p | 114042 | C21orf89 | chromosome 21 open reading frame 89 | 1 | | 3516 | hsa-miR-1233 | 3708 | ITPR1 | inositol 1,4,5-triphosphate receptor, type 1 | 1 | | 3517 | hsa-miR-571 | 3708 | ITPR1 | inositol 1,4,5-triphosphate receptor, type 1 | 1 | | 3518 | hsa-miR-874 | 3708 | ITPR1 | inositol 1,4,5-triphosphate receptor, type 1 | 1 | | 3519 | hsa-miR-1233 | 84924 | ZNF566 | zinc finger protein 566 | 1 | | 3520 | hsa-miR-571 | 84924 | ZNF566 | zinc finger protein 566 | 1 | | 3521 | hsa-miR-766 | 84924 | ZNF566 | zinc finger protein 566 | 1 | | 3522 | hsa-miR-874 | 84924 | ZNF566 | zinc finger protein 566 | 1 | | 3523 | hsa-miR-95 | 359 | AQP2 | aquaporin 2 (collecting duct) | 1 | | 3524 | hsa-miR-10a | 79838 | TMC5 | transmembrane channel-like 5 | 1 | | 3525 | hsa-miR-770-5p | 79838 | TMC5 | transmembrane channel-like 5 | 1 | | 3526 | hsa-miR-95 | 79838 | TMC5 | transmembrane channel-like 5 | 1 | | 3527 | hsa-miR-874 | 149628 | PYHIN1 | pyrin and HIN domain family, member 1 | 1 | | 3528 | hsa-miR-10a | 7037 | TFRC | transferrin receptor (p90, CD71) | 1 | | 3529 | hsa-miR-770-5p | 7037 | TFRC | transferrin receptor (p90, CD71) | 1 | | 3530 | hsa-miR-604 | 833 | CARS | cysteinyl-tRNA synthetase | 1 | | 3531 | hsa-miR-938 | 833 | CARS | cysteinyl-tRNA synthetase | 1 | | 3532 | hsa-miR-128 | 125336 | LOXHD1 | lipoxygenase homology domains 1 | 1 | | 3533 | hsa-miR-95 | 125336 | LOXHD1 | lipoxygenase homology domains 1 | 1 | | 3534 | hsa-mir-3130-1 | 6101 | RP1 | retinitis pigmentosa 1 (autosomal dominant) | 1 | | 3535 | hsa-mir-3130-2 | 6101 | RP1 | retinitis pigmentosa 1 (autosomal dominant) | 1 | | 3536 | hsa-mir-3130-3 | 6101 | RP1 | retinitis pigmentosa 1 (autosomal dominant) | 1 | | 3537 | hsa-miR-770-5p | 6101 | RP1 | retinitis pigmentosa 1 (autosomal dominant) | 1 | | 3538 | hsa-miR-95 | 6101 | RP1 | retinitis pigmentosa 1 (autosomal dominant) | 1 | | 3539 | hsa-miR-576-5p | 286052 | LOC286052 | hypothetical protein LOC286052 | 1 | | 3540 | hsa-miR-591 | 286052 | LOC286052 | hypothetical protein LOC286052 | 1 | | 3541 | hsa-miR-10a | 142678 | MIB2 | mindbomb homolog 2 (Drosophila) | 1 | | 3542 | hsa-miR-770-5p | 142678 | MIB2 | mindbomb homolog 2 (Drosophila) | 1 | | 3543 | hsa-miR-95 | 142678 | MIB2 | mindbomb homolog 2 (Drosophila) | 1 | | 3544 | hsa-miR-10a | 389337 | FLJ41603 | FLJ41603 protein | 1 | | 3545 | hsa-miR-770-5p | 389337 | FLJ41603 | FLJ41603 protein | 1 | | 3546 | hsa-miR-95 | 389337 | FLJ41603 | FLJ41603 protein | 1 | | 3547 | hsa-miR-10a | 401237 | FLJ22536 | hypothetical locus LOC401237 | 1 | | 3548 | hsa-miR-770-5p | 401237 | FLJ22536 | hypothetical locus LOC401237 | 1 | | 3549 | hsa-miR-95 | 401237 | FLJ22536 | hypothetical locus LOC401237 | 1 | | 3550 | hsa-miR-571 | 126070 | ZNF440 | zinc finger protein 440 | 1 | | 3551 | hsa-miR-591 | 126070 | ZNF440 | zinc finger protein 440 | 1 | | 3552 | hsa-miR-638 | 84106 | PRAM1 | PML-RARA regulated adaptor molecule 1 | 1 | | 3553 | hsa-miR-571 | 8481 | OFD1 | oral-facial-digital syndrome 1 | 1 | | 3554 | hsa-miR-571 | 148203 | ZNF738 | zinc finger protein 738 | 1 | | 3555 | hsa-miR-591 | 10785 | WDR4 | WD repeat domain 4 | 1 | | 3556 | hsa-miR-1233 | 100233209 | LOC100233209 | hypothetical LOC100233209 | 1 | | 3557 | hsa-miR-10a | 162515 | SLC16A11 | solute carrier family 16, member 11 (monocarboxylic acid transporter 11) | 1 | | 3558 | hsa-miR-770-5p | 162515 | SLC16A11 | solute carrier family 16, member 11 (monocarboxylic acid transporter 11) | 1 | | 3559 | hsa-miR-95 | 162515 | SLC16A11 | solute carrier family 16, member 11 (monocarboxylic acid transporter 11) | 1 | | 3560 | hsa-miR-770-5p | 9744 | ACAP1 | ArfGAP with coiled-coil, ankyrin repeat and PH domains 1 | 1 | | 3561 | hsa-miR-95 | 9744 | ACAP1 | ArfGAP with coiled-coil, ankyrin repeat and PH domains 1 | 1 | | 3562 | hsa-miR-766 | 163081 | ZNF567 | zinc finger protein 567 | 1 | | 3563 | hsa-miR-571 | 171023 | ASXL1 | additional sex combs like 1 (Drosophila) | 1 | | 3564 | hsa-miR-874 | 171023 | ASXL1 | additional sex combs like 1 (Drosophila) | 1 | | 3565 | hsa-miR-1233 | 8662 | EIF3B | eukaryotic translation initiation factor 3, subunit B | 1 | | 3566 | hsa-miR-569 | 8662 | EIF3B | eukaryotic translation initiation factor 3, subunit B | 1 | | 3567 | hsa-miR-766 | 8662 | EIF3B | eukaryotic translation initiation factor 3, subunit B | 1 | | 3568 | hsa-miR-571 | 414777 | HCG18 | HLA complex group 18 | 1 | | 3569 | hsa-miR-571 | 51569 | UFM1 | ubiquitin-fold modifier 1 | 1 | | 3570 | hsa-miR-591 | 51569 | UFM1 | ubiquitin-fold modifier 1 | 1 | | 3571 | hsa-miR-1233 | 166785 | MMAA | methylmalonic aciduria (cobalamin deficiency) cblA type | 1 | | 3572 | hsa-miR-571 | 166785 | MMAA | methylmalonic aciduria (cobalamin deficiency) cblA type | 1 | | 3573 | hsa-miR-874 | 166785 | MMAA | methylmalonic aciduria (cobalamin deficiency) cblA type | 1 | | 3574 | hsa-miR-770-5p | 29995 | LMCD1 | LIM and cysteine-rich domains 1 | 1 | | 3575 | hsa-miR-95 | 29995 | LMCD1 | LIM and cysteine-rich domains 1 | 1 | | 3576 | hsa-miR-1233 | 91749 | KIAA1919 | KIAA1919 | 1 | | 3577 | hsa-miR-874 | 91749 | KIAA1919 | KIAA1919 | 1 | | 3578 | hsa-miR-770-5p | 111 | ADCY5 | adenylate cyclase 5 | 1 | | 3579 | hsa-miR-95 | 111 | ADCY5 | adenylate cyclase 5 | 1 | | 3580 | hsa-miR-591 | 56902 | PNO1 | partner of NOB1 homolog (S. cerevisiae) | 1 | | 3581 | hsa-miR-1233 | 91120 | ZNF682 | zinc finger protein 682 | 1 | | 3582 | hsa-miR-571 | 91120 | ZNF682 | zinc finger protein 682 | 1 | | 3583 | hsa-miR-874 | 91120 | ZNF682 | zinc finger protein 682 | 1 | | 3584 | hsa-mir-423 | 83999 | KREMEN1 | kringle containing transmembrane protein 1 | 1 | | 3585 | hsa-miR-10a | 83999 | KREMEN1 | kringle containing transmembrane protein 1 | 1 | | 3586 | hsa-miR-770-5p | 83999 | KREMEN1 | kringle containing transmembrane protein 1 | 1 | | 3587 | hsa-miR-95 | 83999 | KREMEN1 | kringle containing transmembrane protein 1 | 1 | | 3588 | hsa-miR-10a | 150572 | SMYD1 | SET and MYND domain containing 1 | 1 | | 3589 | hsa-mir-423 | 2738 | GLI4 | GLI family zinc finger 4 | 1 | | 3590 | hsa-mir-505 | 2738 | GLI4 | GLI family zinc finger 4 | 1 | | 3591 | hsa-mir-3130-1 | 2738 | GLI4 | GLI family zinc finger 4 | 1 | | 3592 | hsa-mir-3130-2 | 2738 | GLI4 | GLI family zinc finger 4 | 1 | | 3593 | hsa-mir-3130-3 | 2738 | GLI4 | GLI family zinc finger 4 | 1 | | 3594 | hsa-miR-770-5p | 2738 | GLI4 | GLI family zinc finger 4 | 1 | | 3595 | hsa-miR-95 | 2738 | GLI4 | GLI family zinc finger 4 | 1 | | 3596 | hsa-miR-604 | 150372 | NFAM1 | NFAT activating protein with ITAM motif 1 | 1 | | 3597 | hsa-miR-938 | 150372 | NFAM1 | NFAT activating protein with ITAM motif 1 | 1 | | 3598 | hsa-mir-3130-1 | 56950 | SMYD2 | SET and MYND domain containing 2 | 1 | | 3599 | hsa-mir-3130-2 | 56950 | SMYD2 | SET and MYND domain containing 2 | 1 | | 3600 | hsa-mir-3130-3 | 56950 | SMYD2 | SET and MYND domain containing 2 | 1 | | 3601 | hsa-miR-10a | 56950 | SMYD2 | SET and MYND domain containing 2 | 1 | | 3602 | hsa-miR-770-5p | 56950 | SMYD2 | SET and MYND domain containing 2 | 1 | | 3603 | hsa-miR-95 | 56950 | SMYD2 | SET and MYND domain containing 2 | 1 | | 3604 | hsa-mir-3130-1 | 25862 | USP49 | ubiquitin specific peptidase 49 | 1 | | 3605 | hsa-mir-3130-2 | 25862 | USP49 | ubiquitin specific peptidase 49 | 1 | | 3606 | hsa-mir-3130-3 | 25862 | USP49 | ubiquitin specific peptidase 49 | 1 | | 3607 | hsa-miR-10a | 25862 | USP49 | ubiquitin specific peptidase 49 | 1 | | 3608 | hsa-miR-770-5p | 25862 | USP49 | ubiquitin specific peptidase 49 | 1 | | 3609 | hsa-miR-1233 | 348035 | MGC40069 | hypothetical protein MGC40069 | 1 | | 3610 | hsa-miR-571 | 348035 | MGC40069 | hypothetical protein MGC40069 | 1 | | 3611 | hsa-miR-874 | 348035 | MGC40069 | hypothetical protein MGC40069 | 1 | | 3612 | hsa-miR-874 | 148103 | ZNF599 | zinc finger protein 599 | 1 | | 3613 | hsa-miR-571 | 7621 | ZNF70 | zinc finger protein 70 | 1 | | 3614 | hsa-miR-10a | 90249 | UNC5A | unc-5 homolog A (C. elegans) | 1 | | 3615 | hsa-miR-770-5p | 90249 | UNC5A | unc-5 homolog A (C. elegans) | 1 | | 3616 | hsa-miR-95 | 90249 | UNC5A | unc-5 homolog A (C. elegans) | 1 | | 3617 | hsa-miR-1233 | 340481 | ZDHHC21 | zinc finger, DHHC-type containing 21 | 1 | | 3618 | hsa-miR-766 | 340481 | ZDHHC21 | zinc finger, DHHC-type containing 21 | 1 | | 3619 | hsa-miR-10a | 401551 | WDR38 | WD repeat domain 38 | 1 | | 3620 | hsa-miR-10a | 200316 | APOBEC3F | apolipoprotein B mRNA editing enzyme, catalytic polypeptide-like 3F | 1 | | 3621 | hsa-miR-770-5p | 200316 | APOBEC3F | apolipoprotein B mRNA editing enzyme, catalytic polypeptide-like 3F | 1 | | 3622 | hsa-miR-95 | 200316 | APOBEC3F | apolipoprotein B mRNA editing enzyme, catalytic polypeptide-like 3F | 1 | | 3623 | hsa-mir-10a | 54813 | KLHL28 | kelch-like 28 (Drosophila) | 1 | | 3624 | hsa-miR-576-5p | 54813 | KLHL28 | kelch-like 28 (Drosophila) | 1 | | 3625 | hsa-miR-591 | 54813 | KLHL28 | kelch-like 28 (Drosophila) | 1 | | 3626 | hsa-miR-1233 | 152926 | PPM1K | protein phosphatase 1K (PP2C domain containing) | 1 | | 3627 | hsa-miR-571 | 152926 | PPM1K | protein phosphatase 1K (PP2C domain containing) | 1 | | 3628 | hsa-miR-874 | 152926 | PPM1K | protein phosphatase 1K (PP2C domain containing) | 1 | | 3629 | hsa-miR-10a | 7514 | XPO1 | exportin 1 (CRM1 homolog, yeast) | 1 | | 3630 | hsa-miR-10a | 29800 | ZDHHC1 | zinc finger, DHHC-type containing 1 | 1 | | 3631 | hsa-miR-770-5p | 29800 | ZDHHC1 | zinc finger, DHHC-type containing 1 | 1 | | 3632 | hsa-miR-95 | 29800 | ZDHHC1 | zinc finger, DHHC-type containing 1 | 1 | | 3633 | hsa-miR-770-5p | 375 | ARF1 | ADP-ribosylation factor 1 | 1 | | 3634 | hsa-miR-591 | 4801 | NFYB | nuclear transcription factor Y, beta | 1 | | 3635 | hsa-miR-770-5p | 55008 | HERC6 | hect domain and RLD 6 | 1 | | 3636 | hsa-miR-95 | 55008 | HERC6 | hect domain and RLD 6 | 1 | | 3637 | hsa-miR-627 | 8189 | SYMPK | symplekin | 1 | | 3638 | hsa-miR-638 | 8189 | SYMPK | symplekin | 1 | | 3639 | hsa-miR-671-5p | 8189 | SYMPK | symplekin | 1 | | 3640 | hsa-mir-423 | 5817 | PVR | poliovirus receptor | 1 | | 3641 | hsa-mir-3130-1 | 5817 | PVR | poliovirus receptor | 1 | | 3642 | hsa-mir-3130-2 | 5817 | PVR | poliovirus receptor | 1 | | 3643 | hsa-mir-3130-3 | 5817 | PVR | poliovirus receptor | 1 | | 3644 | hsa-mir-10a | 1477 | CSTF1 | cleavage stimulation factor, 3' pre-RNA, subunit 1, 50kDa | 1 | | 3645 | hsa-miR-576-5p | 1477 | CSTF1 | cleavage stimulation factor, 3' pre-RNA, subunit 1, 50kDa | 1 | | 3646 | hsa-miR-591 | 1477 | CSTF1 | cleavage stimulation factor, 3' pre-RNA, subunit 1, 50kDa | 1 | | 3647 | hsa-miR-638 | 10555 | AGPAT2 | 1-acylglycerol-3-phosphate O-acyltransferase 2 (lysophosphatidic acid acyltransferase, beta) | 1 | | 3648 | hsa-mir-423 | 9400 | RECQL5 | RecQ protein-like 5 | 1 | | 3649 | hsa-mir-576 | 9400 | RECQL5 | RecQ protein-like 5 | 1 | | 3650 | hsa-miR-10a | 9400 | RECQL5 | RecQ protein-like 5 | 1 | | 3651 | hsa-mir-28 | 23464 | GCAT | glycine C-acetyltransferase | 1 | | 3652 | hsa-mir-423 | 23464 | GCAT | glycine C-acetyltransferase | 1 | | 3653 | hsa-mir-576 | 23464 | GCAT | glycine C-acetyltransferase | 1 | | 3654 | hsa-miR-28-5p | 23464 | GCAT | glycine C-acetyltransferase | 1 | | 3655 | hsa-miR-604 | 527 | ATP6V0C | ATPase, H+ transporting, lysosomal 16kDa, V0 subunit c | 1 | | 3656 | hsa-miR-938 | 527 | ATP6V0C | ATPase, H+ transporting, lysosomal 16kDa, V0 subunit c | 1 | | 3657 | hsa-miR-571 | 9063 | PIAS2 | protein inhibitor of activated STAT, 2 | 1 | | 3658 | hsa-miR-571 | 27241 | BBS9 | Bardet-Biedl syndrome 9 | 1 | | 3659 | hsa-miR-1233 | 360 | AQP3 | aquaporin 3 (Gill blood group) | 1 | | 3660 | hsa-miR-569 | 360 | AQP3 | aquaporin 3 (Gill blood group) | 1 | | 3661 | hsa-mir-10a | 65125 | WNK1 | WNK lysine deficient protein kinase 1 | 1 | | 3662 | hsa-mir-1224 | 65125 | WNK1 | WNK lysine deficient protein kinase 1 | 1 | | 3663 | hsa-mir-885 | 65125 | WNK1 | WNK lysine deficient protein kinase 1 | 1 | | 3664 | hsa-miR-1224-5p | 65125 | WNK1 | WNK lysine deficient protein kinase 1 | 1 | | 3665 | hsa-miR-623 | 25970 | SH2B1 | SH2B adaptor protein 1 | 1 | | 3666 | hsa-miR-671-5p | 25970 | SH2B1 | SH2B adaptor protein 1 | 1 | | 3667 | hsa-miR-135a | 23770 | FKBP8 | FK506 binding protein 8, 38kDa | 1 | | 3668 | hsa-miR-638 | 23770 | FKBP8 | FK506 binding protein 8, 38kDa | 1 | | 3669 | hsa-miR-765 | 23770 | FKBP8 | FK506 binding protein 8, 38kDa | 1 | | 3670 | hsa-miR-571 | 10801 | SEPT9 | septin 9 | 1 | | 3671 | hsa-miR-617 | 10801 | SEPT9 | septin 9 | 1 | | 3672 | hsa-miR-874 | 10801 | SEPT9 | septin 9 | 1 | | 3673 | hsa-miR-576-5p | 57147 | SCYL3 | SCY1-like 3 (S. cerevisiae) | 1 | | 3674 | hsa-miR-591 | 57147 | SCYL3 | SCY1-like 3 (S. cerevisiae) | 1 | | 3675 | hsa-miR-571 | 26051 | PPP1R16B | protein phosphatase 1, regulatory (inhibitor) subunit 16B | 1 | | 3676 | hsa-miR-617 | 26051 | PPP1R16B | protein phosphatase 1, regulatory (inhibitor) subunit 16B | 1 | | 3677 | hsa-miR-593\* | 55049 | C19orf60 | chromosome 19 open reading frame 60 | 1 | | 3678 | hsa-miR-623 | 55049 | C19orf60 | chromosome 19 open reading frame 60 | 1 | | 3679 | hsa-miR-571 | 89781 | HPS4 | Hermansky-Pudlak syndrome 4 | 1 | | 3680 | hsa-miR-874 | 93210 | PGAP3 | post-GPI attachment to proteins 3 | 1 | | 3681 | hsa-miR-1233 | 57048 | PLSCR3 | phospholipid scramblase 3 | 1 | | 3682 | hsa-miR-874 | 57048 | PLSCR3 | phospholipid scramblase 3 | 1 | | 3683 | hsa-miR-571 | 92595 | ZNF764 | zinc finger protein 764 | 1 | | 3684 | hsa-miR-1233 | 1856 | DVL2 | dishevelled, dsh homolog 2 (Drosophila) | 1 | | 3685 | hsa-miR-766 | 1856 | DVL2 | dishevelled, dsh homolog 2 (Drosophila) | 1 | | 3686 | hsa-miR-638 | 5526 | PPP2R5B | protein phosphatase 2, regulatory subunit B', beta isoform | 1 | | 3687 | hsa-mir-10a | 388969 | C2orf68 | chromosome 2 open reading frame 68 | 1 | | 3688 | hsa-mir-1224 | 388969 | C2orf68 | chromosome 2 open reading frame 68 | 1 | | 3689 | hsa-miR-1224-5p | 388969 | C2orf68 | chromosome 2 open reading frame 68 | 1 | | 3690 | hsa-miR-571 | 388969 | C2orf68 | chromosome 2 open reading frame 68 | 1 | | 3691 | hsa-miR-576-5p | 388969 | C2orf68 | chromosome 2 open reading frame 68 | 1 | | 3692 | hsa-miR-1233 | 79874 | RABEP2 | rabaptin, RAB GTPase binding effector protein 2 | 1 | | 3693 | hsa-miR-766 | 79874 | RABEP2 | rabaptin, RAB GTPase binding effector protein 2 | 1 | |

---

Gene Ontology - Biological Process [Details: ]
[truncated: 28,835 more chars]
