# Supplementary material for: MMpred: functional miRNA – mRNA interaction analyses by miRNA expression prediction
Source: BMC Genomics. 2012 Nov 14;13:620. doi: 10.1186/1471-2164-13-620 (PMC3562514; doi:10.1186/1471-2164-13-620)
Supplement: Additional file 3 — Sample pipeline outputs in HTML format (compressed file). [file 1471-2164-13-620-S3.ZIP › Burn_early-late-control&chilren-adoult/BuntTtestSvsC_Thu-09-09-2010_05-03-05.html]

REPORT


## Report of miRNA-mRNA interactions for all arrays. [generated on 2010-09-09 05:03:06]

---

Statistical testing for messenger RNA arrays: 2356 genes found significantly up-/down-regulated. Details:

| |  | ArrayFile | FunctionalGroup.Characteristics..sampling.time.group. | FunctionalGroup.Characteristics..age.group. | | --- | --- | --- | --- | | 1 | GSM493655.CEL | Early | Ped | | 2 | GSM493656.CEL | Mid | Ped | | 3 | GSM493657.CEL | Early | Adult | | 4 | GSM493658.CEL | Mid | Adult | | 5 | GSM493659.CEL | Early | Ped | | 6 | GSM493660.CEL | Mid | Ped | | 7 | GSM493661.CEL | Early | Ped | | 8 | GSM493662.CEL | Mid | Ped | | 9 | GSM493663.CEL | Early | Ped | | 10 | GSM493664.CEL | Mid | Ped | | 11 | GSM493665.CEL | Early | Adult | | 12 | GSM493666.CEL | Mid | Adult | | 13 | GSM493667.CEL | Early | Adult | | 14 | GSM493668.CEL | Mid | Adult | | 15 | GSM493669.CEL | Early | Adult | | 16 | GSM493670.CEL | Mid | Adult | | 17 | GSM493671.CEL | Early | Adult | | 18 | GSM493672.CEL | Mid | Adult | | 19 | GSM493673.CEL | Early | Adult | | 20 | GSM493674.CEL | Mid | Adult | | 21 | GSM493675.CEL | Early | Adult | | 22 | GSM493676.CEL | Mid | Adult | | 23 | GSM493677.CEL | Early | Ped | | 24 | GSM493678.CEL | Mid | Ped | | 25 | GSM493679.CEL | Early | Ped | | 26 | GSM493680.CEL | Mid | Ped | | 27 | GSM493681.CEL | Early | Adult | | 28 | GSM493682.CEL | Mid | Adult | | 29 | GSM493683.CEL | Early | Ped | | 30 | GSM493684.CEL | Mid | Ped | | 31 | GSM493685.CEL | Early | Adult | | 32 | GSM493686.CEL | Mid | Adult | | 33 | GSM493687.CEL | Early | Ped | | 34 | GSM493688.CEL | Mid | Ped | | 35 | GSM493689.CEL | Early | Ped | | 36 | GSM493690.CEL | Mid | Ped | | 37 | GSM493691.CEL | Early | Adult | | 38 | GSM493692.CEL | Mid | Adult | | 39 | GSM493693.CEL | Early | Adult | | 40 | GSM493694.CEL | Mid | Adult | | 41 | GSM493695.CEL | Early | Adult | | 42 | GSM493696.CEL | Mid | Adult | | 43 | GSM493697.CEL | Early | Ped | | 44 | GSM493698.CEL | Mid | Ped | | 45 | GSM493699.CEL | Early | Adult | | 46 | GSM493700.CEL | Mid | Adult | | 47 | GSM493701.CEL | Early | Adult | | 48 | GSM493702.CEL | Mid | Adult | | 49 | GSM493703.CEL | Early | Adult | | 50 | GSM493704.CEL | Mid | Adult | | 51 | GSM493705.CEL | Early | Adult | | 52 | GSM493706.CEL | Mid | Adult | | 53 | GSM493707.CEL | Early | Ped | | 54 | GSM493708.CEL | Mid | Ped | | 55 | GSM493709.CEL | Early | Adult | | 56 | GSM493710.CEL | Mid | Adult | | 57 | GSM493711.CEL | Early | Ped | | 58 | GSM493712.CEL | Mid | Ped | | 59 | GSM493713.CEL | Early | Ped | | 60 | GSM493714.CEL | Mid | Ped | | 61 | GSM493715.CEL | Early | Ped | | 62 | GSM493716.CEL | Mid | Ped | | 63 | GSM493717.CEL | Early | Ped | | 64 | GSM493718.CEL | Mid | Ped | | 65 | GSM493719.CEL | Early | Adult | | 66 | GSM493720.CEL | Mid | Adult | | 67 | GSM493721.CEL | Early | Ped | | 68 | GSM493722.CEL | Mid | Ped | | 69 | GSM493723.CEL | Early | Ped | | 70 | GSM493724.CEL | Mid | Ped | | 71 | GSM493725.CEL | Early | Ped | | 72 | GSM493726.CEL | Mid | Ped | | 73 | GSM493727.CEL | Early | Adult | | 74 | GSM493728.CEL | Mid | Adult | | 75 | GSM493729.CEL | Early | Ped | | 76 | GSM493730.CEL | Mid | Ped | | 77 | GSM493731.CEL | Early | Ped | | 78 | GSM493732.CEL | Mid | Ped | | 79 | GSM493733.CEL | Early | Adult | | 80 | GSM493734.CEL | Mid | Adult | | 81 | GSM493735.CEL | Early | Adult | | 82 | GSM493736.CEL | Mid | Adult | | 83 | GSM493737.CEL | Early | Adult | | 84 | GSM493738.CEL | Mid | Adult | | 85 | GSM493739.CEL | Early | Adult | | 86 | GSM493740.CEL | Mid | Adult | | 87 | GSM493741.CEL | Early | Adult | | 88 | GSM493742.CEL | Mid | Adult | | 89 | GSM493743.CEL | Early | Ped | | 90 | GSM493744.CEL | Mid | Ped | | 91 | GSM493745.CEL | Early | Ped | | 92 | GSM493746.CEL | Mid | Ped | | 93 | GSM493747.CEL | Early | Ped | | 94 | GSM493748.CEL | Mid | Ped | | 95 | GSM493749.CEL | Early | Ped | | 96 | GSM493750.CEL | Mid | Ped | | 97 | GSM493751.CEL | Early | Adult | | 98 | GSM493752.CEL | Mid | Adult | | 99 | GSM493753.CEL | Early | Ped | | 100 | GSM493754.CEL | Mid | Ped | | 101 | GSM493755.CEL | Early | Adult | | 102 | GSM493756.CEL | Mid | Adult | | 103 | GSM493757.CEL | Early | Adult | | 104 | GSM493758.CEL | Mid | Adult | | 105 | GSM493759.CEL | Early | Adult | | 106 | GSM493760.CEL | Mid | Adult | | 107 | GSM493761.CEL | Early | Adult | | 108 | GSM493762.CEL | Mid | Adult | | 109 | GSM493763.CEL | Early | Adult | | 110 | GSM493764.CEL | Mid | Adult | | 111 | GSM493765.CEL | Early | Ped | | 112 | GSM493766.CEL | Mid | Ped | | 113 | GSM493767.CEL | Early | Adult | | 114 | GSM493768.CEL | Mid | Adult | | 115 | GSM493769.CEL | control | Ped | | 116 | GSM493770.CEL | control | Adult | | 117 | GSM493771.CEL | control | Adult | | 118 | GSM493772.CEL | control | Adult | | 119 | GSM493773.CEL | control | Ped | | 120 | GSM493774.CEL | control | Adult | | 121 | GSM493775.CEL | control | Adult | | 122 | GSM493776.CEL | control | Ped | | 123 | GSM493777.CEL | control | Adult | | 124 | GSM493778.CEL | control | Ped | | 125 | GSM493779.CEL | control | Ped | | 126 | GSM493780.CEL | control | Ped | | 127 | GSM493781.CEL | control | Ped | | 128 | GSM493782.CEL | control | Ped | | 129 | GSM493783.CEL | control | Adult | | 130 | GSM493784.CEL | control | Ped | | 131 | GSM493785.CEL | control | Adult | | 132 | GSM493786.CEL | control | Adult | | 133 | GSM493787.CEL | control | Ped | | 134 | GSM493788.CEL | control | Ped | | 135 | GSM493789.CEL | control | Ped | | 136 | GSM493790.CEL | control | Ped | | 137 | GSM493791.CEL | control | Adult | | 138 | GSM493792.CEL | control | Adult | | 139 | GSM493793.CEL | control | Ped | | 140 | GSM493794.CEL | control | Ped | | 141 | GSM493795.CEL | control | Adult | | 142 | GSM493796.CEL | control | Adult | | 143 | GSM493797.CEL | control | Adult | | 144 | GSM493798.CEL | control | Ped | | 145 | GSM493799.CEL | control | Ped | | 146 | GSM493800.CEL | control | Adult | | 147 | GSM493801.CEL | control | Adult | | 148 | GSM493802.CEL | control | Ped | | 149 | GSM493803.CEL | control | Adult | | 150 | GSM493804.CEL | control | Ped | | 151 | GSM493805.CEL | control | Ped | | 152 | GSM493806.CEL | control | Ped | | 153 | GSM493807.CEL | control | Ped | | 154 | GSM493808.CEL | control | Ped | | 155 | GSM493809.CEL | control | Adult | | 156 | GSM493810.CEL | control | Adult | | 157 | GSM493811.CEL | control | Adult | | 158 | GSM493812.CEL | control | Adult | | 159 | GSM493813.CEL | control | Ped | | 160 | GSM493814.CEL | control | Ped | | 161 | GSM493815.CEL | control | Ped | | 162 | GSM493816.CEL | control | Ped | | 163 | GSM493817.CEL | control | Ped | | 164 | GSM493818.CEL | control | Ped | | 165 | GSM493819.CEL | control | Adult | | 166 | GSM493820.CEL | control | Ped | | 167 | GSM493821.CEL | control | Adult | | 168 | GSM493822.CEL | control | Adult | | 169 | GSM493823.CEL | control | Ped | | 170 | GSM493824.CEL | control | Ped | | 171 | GSM493825.CEL | control | Ped | | 172 | GSM493826.CEL | control | Adult | | 173 | GSM493827.CEL | control | Adult | | 174 | GSM493828.CEL | control | Adult | | 175 | GSM493829.CEL | control | Ped | | 176 | GSM493830.CEL | control | Ped | | 177 | GSM493831.CEL | control | Adult | |

  

Principal Component Analyses:

Heatmap for top 50 geneses from statistical analyses (ordered by p-value):

Volcano plot with for auto cut-off calculation audit (cut-off shown with red line):

---

Statistical testing for microRNA prediction method I - scaling function: 35 genes found significantly up-/down-regulated. Details:

Heatmap for top 50 geneses from statistical analyses (ordered by p-value):

Volcano plot with for auto cut-off calculation audit (cut-off shown with red line):

---

Statistical testing for microRNA prediction method II - linear modelling: 44 genes found significantly up-/down-regulated. Details:

Principal Component Analysis:

Heatmap for top 50 geneses from statistical analyses (ordered by p-value):

Volcano plot with for auto cut-off calculation audit (cut-off shown with red line):

---

Mean anti-correlation detected between mRNA and miRNA = -0.284499. Details:

Histogram of most anti-correlated miRNA-mRNA pairs - potential miRNA-target interactions:

---

Total number of 59 miRNAs are predicted to have significantly up-/down-regulated targets. Expend:

| |  | microRNA | NoSuppresedGenes | | --- | --- | --- | | 1 | hsa-miR-608 | 343 | | 2 | hsa-miR-126\* | 308 | | 3 | hsa-miR-16 | 197 | | 4 | hsa-mir-424 | 161 | | 5 | hsa-miR-503 | 161 | | 6 | hsa-miR-190 | 156 | | 7 | hsa-mir-15a | 141 | | 8 | hsa-miR-617 | 135 | | 9 | hsa-mir-16-1 | 133 | | 10 | hsa-mir-628 | 132 | | 11 | hsa-mir-126 | 121 | | 12 | hsa-mir-556 | 104 | | 13 | hsa-miR-566 | 103 | | 14 | hsa-miR-623 | 98 | | 15 | hsa-miR-564 | 94 | | 16 | hsa-miR-1231 | 93 | | 17 | hsa-miR-586 | 87 | | 18 | hsa-miR-196b | 80 | | 19 | hsa-mir-218-2 | 70 | | 20 | hsa-miR-585 | 70 | | 21 | hsa-mir-196b | 69 | | 22 | hsa-mir-1915 | 57 | | 23 | hsa-miR-618 | 55 | | 24 | hsa-mir-490 | 45 | | 25 | hsa-miR-490-5p | 45 | | 26 | hsa-mir-30c-1 | 42 | | 27 | hsa-mir-30e | 42 | | 28 | hsa-miR-338-5p | 42 | | 29 | hsa-miR-657 | 42 | | 30 | hsa-miR-95 | 42 | | 31 | hsa-miR-634 | 41 | | 32 | hsa-miR-675 | 39 | | 33 | hsa-mir-339 | 37 | | 34 | hsa-miR-339-5p | 37 | | 35 | hsa-mir-454 | 33 | | 36 | hsa-mir-135b | 31 | | 37 | hsa-miR-454\* | 26 | | 38 | hsa-miR-574-5p | 25 | | 39 | hsa-mir-675 | 23 | | 40 | hsa-mir-574 | 18 | | 41 | hsa-miR-504 | 17 | | 42 | hsa-miR-548d-5p | 15 | | 43 | hsa-miR-30c | 14 | | 44 | hsa-miR-30e | 14 | | 45 | hsa-miR-616\* | 7 | | 46 | hsa-miR-650 | 5 | | 47 | hsa-miR-944 | 5 | | 48 | hsa-mir-106b | 4 | | 49 | hsa-mir-25 | 4 | | 50 | hsa-mir-93 | 4 | | 51 | hsa-mir-548d-1 | 3 | | 52 | hsa-miR-26a | 2 | | 53 | hsa-miR-455-5p | 2 | | 54 | hsa-miR-584 | 2 | | 55 | hsa-miR-603 | 2 | | 56 | hsa-mir-139 | 1 | | 57 | hsa-miR-139-5p | 1 | | 58 | hsa-mir-22 | 1 | | 59 | hsa-mir-3126 | 1 | |

---

Total number of 775 genes are predicted to be under differential miRNA repression. Expend:

| |  | GenSymbols | GeneName | NoTargetingMicroRNA | | --- | --- | --- | --- | | 1 | UBE2I | ubiquitin-conjugating enzyme E2I (UBC9 homolog, yeast) | 21 | | 2 | EIF3F | eukaryotic translation initiation factor 3, subunit F | 17 | | 3 | BTN2A2 | butyrophilin, subfamily 2, member A2 | 16 | | 4 | RPL14 | ribosomal protein L14 | 16 | | 5 | ZNF551 | zinc finger protein 551 | 16 | | 6 | CUL4A | cullin 4A | 15 | | 7 | EIF3D | eukaryotic translation initiation factor 3, subunit D | 15 | | 8 | RPS3A | ribosomal protein S3A | 15 | | 9 | CLEC2D | C-type lectin domain family 2, member D | 14 | | 10 | CSDE1 | cold shock domain containing E1, RNA-binding | 14 | | 11 | HSD17B7 | hydroxysteroid (17-beta) dehydrogenase 7 | 14 | | 12 | PPP3CC | protein phosphatase 3 (formerly 2B), catalytic subunit, gamma isoform | 14 | | 13 | RPL13 | ribosomal protein L13 | 14 | | 14 | RPL24 | ribosomal protein L24 | 14 | | 15 | RPL4 | ribosomal protein L4 | 14 | | 16 | TGIF2 | TGFB-induced factor homeobox 2 | 14 | | 17 | AGPAT5 | 1-acylglycerol-3-phosphate O-acyltransferase 5 (lysophosphatidic acid acyltransferase, epsilon) | 13 | | 18 | EHMT1 | euchromatic histone-lysine N-methyltransferase 1 | 13 | | 19 | EIF3K | eukaryotic translation initiation factor 3, subunit K | 13 | | 20 | LARS | leucyl-tRNA synthetase | 13 | | 21 | RPAIN | ribosomal protein L15 | 13 | | 22 | RPL15 | RPA interacting protein | 13 | | 23 | STRBP | spermatid perinuclear RNA binding protein | 13 | | 24 | ZCCHC7 | zinc finger protein 569 | 13 | | 25 | ZNF569 | zinc finger protein 805 | 13 | | 26 | ZNF805 | zinc finger, CCHC domain containing 7 | 13 | | 27 | ATXN10 | ataxin 10 | 12 | | 28 | CCDC50 | chemokine (C-C motif) receptor 6 | 12 | | 29 | CCR6 | coiled-coil domain containing 50 | 12 | | 30 | DDX31 | DEAD (Asp-Glu-Ala-Asp) box polypeptide 31 | 12 | | 31 | EIF3M | Enah/Vasp-like | 12 | | 32 | EVL | eukaryotic translation initiation factor 3, subunit M | 12 | | 33 | FBXO25 | F-box protein 25 | 12 | | 34 | HINT1 | glutaminyl-tRNA synthase (glutamine-hydrolyzing)-like 1 | 12 | | 35 | HLA-DRA | histidine triad nucleotide binding protein 1 | 12 | | 36 | ITPR1 | homolog of rat pragma of Rnd2 | 12 | | 37 | MBTD1 | inositol 1,4,5-triphosphate receptor, type 1 | 12 | | 38 | NBEAL1 | major histocompatibility complex, class II, DR alpha | 12 | | 39 | PRAGMIN | mbt domain containing 1 | 12 | | 40 | PTPMT1 | neurobeachin-like 1 | 12 | | 41 | QRSL1 | protein tyrosine phosphatase, mitochondrial 1 | 12 | | 42 | RPS6 | ribosomal protein S6 | 12 | | 43 | RPS7 | ribosomal protein S7 | 12 | | 44 | RUFY3 | RUN and FYVE domain containing 3 | 12 | | 45 | SNRPD3 | small nuclear ribonucleoprotein D3 polypeptide 18kDa | 12 | | 46 | TAF1A | TATA box binding protein (TBP)-associated factor, RNA polymerase I, A, 48kDa | 12 | | 47 | ZNF224 | zinc finger protein 224 | 12 | | 48 | BACH2 | B double prime 1, subunit of RNA polymerase III transcription initiation factor IIIB | 11 | | 49 | BDP1 | BTB and CNC homology 1, basic leucine zipper transcription factor 2 | 11 | | 50 | CRTC3 | CREB regulated transcription coactivator 3 | 11 | | 51 | EZH1 | enhancer of zeste homolog 1 (Drosophila) | 11 | | 52 | FAM165B | family with sequence similarity 165, member B | 11 | | 53 | GJC1 | gap junction protein, gamma 1, 45kDa | 11 | | 54 | GNB2L1 | guanine nucleotide binding protein (G protein), beta polypeptide 2-like 1 | 11 | | 55 | HERPUD1 | homocysteine-inducible, endoplasmic reticulum stress-inducible, ubiquitin-like domain member 1 | 11 | | 56 | MAGOH | mago-nashi homolog, proliferation-associated (Drosophila) | 11 | | 57 | RAB12 | RAB12, member RAS oncogene family | 11 | | 58 | RPL29 | ribosomal L1 domain containing 1 | 11 | | 59 | RPL35A | ribosomal protein L29 | 11 | | 60 | RPS3 | ribosomal protein L35a | 11 | | 61 | RSL1D1 | ribosomal protein S3 | 11 | | 62 | SART3 | squamous cell carcinoma antigen recognized by T cells 3 | 11 | | 63 | ATP5A1 | ATP synthase, H+ transporting, mitochondrial F1 complex, alpha subunit 1, cardiac muscle | 10 | | 64 | BBS4 | Bardet-Biedl syndrome 4 | 10 | | 65 | CCT3 | chaperonin containing TCP1, subunit 3 (gamma) | 10 | | 66 | CYP19A1 | cytochrome P450, family 19, subfamily A, polypeptide 1 | 10 | | 67 | CYP2R1 | cytochrome P450, family 2, subfamily R, polypeptide 1 | 10 | | 68 | EEF1A1 | eukaryotic translation elongation factor 1 alpha 1 | 10 | | 69 | EEF2 | eukaryotic translation elongation factor 2 | 10 | | 70 | EIF4A1 | eukaryotic translation initiation factor 4A1 | 10 | | 71 | FLJ12334 | GRAM domain containing 3 | 10 | | 72 | GRAMD3 | hypothetical gene supported by AK022396; AK097927 | 10 | | 73 | ILF3 | interleukin enhancer binding factor 3, 90kDa | 10 | | 74 | LOC494150 | matrin 3 | 10 | | 75 | MATR3 | platelet-activating factor acetylhydrolase 2, 40kDa | 10 | | 76 | PAFAH2 | pogo transposable element with ZNF domain | 10 | | 77 | POGZ | prohibitin pseudogene | 10 | | 78 | PSMB4 | proteasome (prosome, macropain) subunit, beta type, 4 | 10 | | 79 | RPL17 | ribosomal protein L17 | 10 | | 80 | RPL19 | ribosomal protein L19 | 10 | | 81 | RPL37 | ribosomal protein L37 | 10 | | 82 | SFRS2IP | splicing factor, arginine/serine-rich 2, interacting protein | 10 | | 83 | SMARCC2 | ST6 beta-galactosamide alpha-2,6-sialyltranferase 1 | 10 | | 84 | ST6GAL1 | stromal interaction molecule 2 | 10 | | 85 | STIM2 | suppressor of zeste 12 homolog (Drosophila) | 10 | | 86 | SUZ12 | SWI/SNF related, matrix associated, actin dependent regulator of chromatin, subfamily c, member 2 | 10 | | 87 | TDRD3 | tudor domain containing 3 | 10 | | 88 | WDR54 | WD repeat domain 54 | 10 | | 89 | ZFP82 | zinc finger protein 248 | 10 | | 90 | ZMYM6 | zinc finger protein 785 | 10 | | 91 | ZNF248 | zinc finger protein 82 homolog (mouse) | 10 | | 92 | ZNF785 | zinc finger, MYM-type 6 | 10 | | 93 | CIITA | class II, major histocompatibility complex, transactivator | 9 | | 94 | CYB561D2 | cytochrome b-561 domain containing 2 | 9 | | 95 | DCAF4 | DDB1 and CUL4 associated factor 4 | 9 | | 96 | DCLRE1C | DEAH (Asp-Glu-Ala-His) box polypeptide 36 | 9 | | 97 | DHX36 | DNA cross-link repair 1C (PSO2 homolog, S. cerevisiae) | 9 | | 98 | FAIM | Fas apoptotic inhibitory molecule | 9 | | 99 | KIAA1737 | hypothetical LOC147727 | 9 | | 100 | LOC147727 | hypothetical LOC642826 | 9 | | 101 | LOC642826 | KIAA1737 | 9 | | 102 | MRPL4 | mitochondrial ribosomal protein L4 | 9 | | 103 | MRPS26 | mitochondrial ribosomal protein S26 | 9 | | 104 | MYST4 | MYST histone acetyltransferase (monocytic leukemia) 4 | 9 | | 105 | PPIA | peptidylprolyl isomerase A (cyclophilin A) | 9 | | 106 | PRMT6 | protein arginine methyltransferase 6 | 9 | | 107 | RPL13A | ribosomal protein L13a | 9 | | 108 | RPL36 | ribosomal protein L36 | 9 | | 109 | SH2D1A | SH2 domain protein 1A | 9 | | 110 | SPN | sialophorin | 9 | | 111 | ACYP2 | acylphosphatase 2, muscle type | 8 | | 112 | C11orf57 | CD81 molecule | 8 | | 113 | C12orf65 | CD8b molecule | 8 | | 114 | C17orf42 | chromosome 11 open reading frame 57 | 8 | | 115 | CD81 | chromosome 12 open reading frame 65 | 8 | | 116 | CD8B | chromosome 17 open reading frame 42 | 8 | | 117 | DGKE | diacylglycerol kinase, epsilon 64kDa | 8 | | 118 | FLJ33630 | golgin A8 family, member A | 8 | | 119 | GOLGA8A | granzyme M (lymphocyte met-ase 1) | 8 | | 120 | GZMM | hypothetical LOC644873 | 8 | | 121 | HLA-DQB1 | interleukin 27 receptor, alpha | 8 | | 122 | IL27RA | LAS1-like (S. cerevisiae) | 8 | | 123 | LAS1L | major histocompatibility complex, class II, DQ beta 1 | 8 | | 124 | MCF2L | MCF.2 cell line derived transforming sequence-like | 8 | | 125 | PDE4DIP | phosphodiesterase 4D interacting protein | 8 | | 126 | SAPS2 | SAPS domain family, member 2 | 8 | | 127 | YSK4 | YSK4 Sps1/Ste20-related kinase homolog (S. cerevisiae) | 8 | | 128 | ZBTB40 | zinc finger and BTB domain containing 40 | 8 | | 129 | ZMYND11 | zinc finger and SCAN domain containing 2 | 8 | | 130 | ZNF350 | zinc finger protein 350 | 8 | | 131 | ZSCAN2 | zinc finger, MYND domain containing 11 | 8 | | 132 | AFF3 | advillin | 7 | | 133 | AVIL | AF4/FMR2 family, member 3 | 7 | | 134 | BHLHE41 | basic helix-loop-helix family, member e41 | 7 | | 135 | C1orf105 | chromosome 1 open reading frame 105 | 7 | | 136 | C8B | chromosome X open reading frame 21 | 7 | | 137 | CCDC21 | coiled-coil domain containing 21 | 7 | | 138 | CXorf21 | complement component 8, beta polypeptide | 7 | | 139 | CYP2C9 | cytochrome P450, family 2, subfamily C, polypeptide 9 | 7 | | 140 | ERG | general transcription factor IIH, polypeptide 3, 34kDa | 7 | | 141 | GTF2H3 | heat shock 22kDa protein 8 | 7 | | 142 | HSPB8 | interleukin 28A (interferon, lambda 2) | 7 | | 143 | IL28A | karyopherin alpha 5 (importin alpha 6) | 7 | | 144 | KIAA1009 | keratin associated protein 8-1 | 7 | | 145 | KIF3C | KIAA1009 | 7 | | 146 | KPNA5 | kinesin family member 3C | 7 | | 147 | KRTAP8-1 | microtubule associated monoxygenase, calponin and LIM domain containing 3 | 7 | | 148 | MICAL3 | neuronal calcium sensor 1 | 7 | | 149 | NCS1 | neurotrophic tyrosine kinase, receptor, type 3 | 7 | | 150 | NIPAL3 | NIPA-like domain containing 3 | 7 | | 151 | NTRK3 | olfactory receptor, family 5, subfamily AK, member 4 pseudogene | 7 | | 152 | OR5AK4P | pentatricopeptide repeat domain 1 | 7 | | 153 | PCGF3 | PHD finger protein 15 | 7 | | 154 | PDE5A | phosphodiesterase 5A, cGMP-specific | 7 | | 155 | PHF15 | phytanoyl-CoA 2-hydroxylase interacting protein-like | 7 | | 156 | PHYHIPL | polycomb group ring finger 3 | 7 | | 157 | PTCD1 | reticulon 4 interacting protein 1 | 7 | | 158 | RDH13 | retinol dehydrogenase 13 (all-trans/9-cis) | 7 | | 159 | RG9MTD3 | RNA (guanine-9-) methyltransferase domain containing 3 | 7 | | 160 | RTN4IP1 | runt-related transcription factor 2 | 7 | | 161 | RUNX2 | serpin peptidase inhibitor, clade B (ovalbumin), member 8 | 7 | | 162 | SDCBP2 | solute carrier family 11 (proton-coupled divalent metal ion transporters), member 1 | 7 | | 163 | SERPINB8 | solute carrier family 17 (sodium-dependent inorganic phosphate cotransporter), member 7 | 7 | | 164 | SLC11A1 | sortilin-related VPS10 domain containing receptor 1 | 7 | | 165 | SLC17A7 | synapse defective 1, Rho GTPase, homolog 1 (C. elegans) | 7 | | 166 | SORCS1 | syndecan binding protein (syntenin) 2 | 7 | | 167 | SYDE1 | transmembrane protein 37 | 7 | | 168 | TMEM37 | ubiquitin specific peptidase 6 (Tre-2 oncogene) | 7 | | 169 | USP6 | v-ets erythroblastosis virus E26 oncogene homolog (avian) | 7 | | 170 | WASL | Wiskott-Aldrich syndrome-like | 7 | | 171 | ZBED5 | zinc finger, BED-type containing 5 | 7 | | 172 | ZDHHC23 | zinc finger, DHHC-type containing 23 | 7 | | 173 | ACRV1 | acrosomal vesicle protein 1 | 6 | | 174 | ALDH1A1 | aldehyde dehydrogenase 1 family, member A1 | 6 | | 175 | ANKRD53 | ankyrin repeat domain 53 | 6 | | 176 | BANK1 | B-cell scaffold protein with ankyrin repeats 1 | 6 | | 177 | BEST2 | bassoon (presynaptic cytomatrix protein) | 6 | | 178 | BSN | bestrophin 2 | 6 | | 179 | BTNL9 | butyrophilin-like 9 | 6 | | 180 | C10orf114 | calcium regulated heat stable protein 1, 24kDa | 6 | | 181 | C10orf41 | ceroid-lipofuscinosis, neuronal 8 (epilepsy, progressive with mental retardation) | 6 | | 182 | C12orf39 | chromosome 10 open reading frame 114 | 6 | | 183 | C18orf22 | chromosome 10 open reading frame 41 | 6 | | 184 | C18orf55 | chromosome 12 open reading frame 39 | 6 | | 185 | C20orf117 | chromosome 18 open reading frame 22 | 6 | | 186 | C5orf24 | chromosome 18 open reading frame 55 | 6 | | 187 | CARHSP1 | chromosome 20 open reading frame 117 | 6 | | 188 | CLN8 | chromosome 5 open reading frame 24 | 6 | | 189 | CYLC1 | cylicin, basic protein of sperm head cytoskeleton 1 | 6 | | 190 | CYP2A13 | cytochrome P450, family 2, subfamily A, polypeptide 13 | 6 | | 191 | CYP3A4 | cytochrome P450, family 3, subfamily A, polypeptide 4 | 6 | | 192 | DAND5 | DAN domain family, member 5 | 6 | | 193 | EHD2 | EH-domain containing 2 | 6 | | 194 | ELL2 | elongation factor, RNA polymerase II, 2 | 6 | | 195 | ESCO1 | establishment of cohesion 1 homolog 1 (S. cerevisiae) | 6 | | 196 | FAM22B | F-box protein 43 | 6 | | 197 | FBXO43 | family with sequence similarity 22, member B | 6 | | 198 | FGF18 | fibroblast growth factor 18 | 6 | | 199 | GAD1 | G protein-coupled receptor 52 | 6 | | 200 | GNAO1 | glutamate decarboxylase 1 (brain, 67kDa) | 6 | | 201 | GOPC | golgi-associated PDZ and coiled-coil motif containing | 6 | | 202 | GPR52 | guanine nucleotide binding protein (G protein), alpha activating activity polypeptide O | 6 | | 203 | KLK4 | hypothetical LOC100128977 | 6 | | 204 | LOC100128977 | hypothetical LOC152217 | 6 | | 205 | LOC100130700 | hypothetical LOC340074 | 6 | | 206 | LOC152217 | hypothetical LOC642891 | 6 | | 207 | LOC340074 | kallikrein-related peptidase 4 | 6 | | 208 | LOC341912 | leucine zipper protein 4 | 6 | | 209 | LOC642891 | mesoderm posterior 1 homolog (mouse) | 6 | | 210 | LUZP4 | metastasis associated lung adenocarcinoma transcript 1 (non-protein coding) | 6 | | 211 | MALAT1 | microtubule-associated protein 1B | 6 | | 212 | MAP1B | mitochondrial ribosomal protein L40 | 6 | | 213 | MESP1 | mRNA turnover 4 homolog (S. cerevisiae) | 6 | | 214 | MRPL40 | myosin XVI | 6 | | 215 | MRTO4 | NEDD4 binding protein 1 | 6 | | 216 | MYO16 | nestin | 6 | | 217 | N4BP1 | netrin 1 | 6 | | 218 | NES | neurexophilin 3 | 6 | | 219 | NTN1 | nudix (nucleoside diphosphate linked moiety X)-type motif 16-like 1 | 6 | | 220 | NUDT16L1 | peptidoglycan recognition protein 4 | 6 | | 221 | NXPH3 | peptidyl arginine deiminase, type IV | 6 | | 222 | PABPC3 | phospholipase C, delta 3 | 6 | | 223 | PADI4 | poly(A) binding protein, cytoplasmic 3 | 6 | | 224 | PCDHB11 | pregnancy specific beta-1-glycoprotein 1 | 6 | | 225 | PGLYRP4 | protein phosphatase 1, regulatory (inhibitor) subunit 1B | 6 | | 226 | PLCD3 | protocadherin beta 11 | 6 | | 227 | PPP1R1B | PRR5-ARHGAP8 readthrough | 6 | | 228 | PRR5-ARHGAP8 | seizure related 6 homolog (mouse)-like 2 | 6 | | 229 | PSG1 | similar to developmental pluripotency associated 5; embryonal stem cell specific gene 1 | 6 | | 230 | SEZ6L2 | similar to hCG2038355 | 6 | | 231 | SNHG5 | small nucleolar RNA host gene 5 (non-protein coding) | 6 | | 232 | SYPL2 | synaptophysin-like 2 | 6 | | 233 | SYT12 | synaptotagmin XII | 6 | | 234 | TCEAL4 | transcription elongation factor A (SII)-like 4 | 6 | | 235 | TRIM15 | tripartite motif-containing 15 | 6 | | 236 | WISP1 | Wilms tumor upstream neighbor 1 | 6 | | 237 | WIT1 | wingless-type MMTV integration site family, member 6 | 6 | | 238 | WNT6 | WNT1 inducible signaling pathway protein 1 | 6 | | 239 | XDH | xanthine dehydrogenase | 6 | | 240 | ZNF611 | zinc finger protein 611 | 6 | | 241 | A1CF | adrenergic, beta-3-, receptor | 5 | | 242 | ACTR1B | APOBEC1 complementation factor | 5 | | 243 | ADC | arginine decarboxylase | 5 | | 244 | ADRB3 | ARP1 actin-related protein 1 homolog B, centractin beta (yeast) | 5 | | 245 | ARHGEF7 | ATPase, Ca++ transporting, plasma membrane 2 | 5 | | 246 | ATP2B2 | bridging integrator 1 | 5 | | 247 | B3GALT6 | calcium-sensing receptor | 5 | | 248 | BIN1 | cardiotrophin-like cytokine factor 1 | 5 | | 249 | C20orf141 | chromodomain helicase DNA binding protein 2 | 5 | | 250 | C2orf65 | chromosome 2 open reading frame 65 | 5 | | 251 | C6orf142 | chromosome 20 open reading frame 141 | 5 | | 252 | C8orf83 | chromosome 6 open reading frame 142 | 5 | | 253 | CASR | chromosome 8 open reading frame 83 | 5 | | 254 | CDK14 | collagen, type V, alpha 1 | 5 | | 255 | CHD2 | cyclin-dependent kinase 14 | 5 | | 256 | CLCF1 | DLX6 antisense RNA (non-protein coding) | 5 | | 257 | COL5A1 | E2F transcription factor 5, p130-binding | 5 | | 258 | DLX6AS | ectodysplasin A | 5 | | 259 | E2F5 | EDAR-associated death domain | 5 | | 260 | EDA | elaC homolog 2 (E. coli) | 5 | | 261 | EDARADD | endoplasmic reticulum protein 29 | 5 | | 262 | ELAC2 | four jointed box 1 (Drosophila) | 5 | | 263 | ERP29 | gap junction protein, beta 6, 30kDa | 5 | | 264 | FJX1 | glutamate receptor interacting protein 2 | 5 | | 265 | FLJ30375 | glutaredoxin-like protein YDR286C homolog | 5 | | 266 | FLJ38028 | glycerol-3-phosphate dehydrogenase 1 (soluble) | 5 | | 267 | FLJ44606 | H6 family homeobox 1 | 5 | | 268 | GJB6 | HBS1-like (S. cerevisiae) | 5 | | 269 | GPD1 | heparan sulfate (glucosamine) 3-O-sulfotransferase 4 | 5 | | 270 | GRIP2 | HMP19 protein | 5 | | 271 | HBS1L | homeobox A9 | 5 | | 272 | HMP19 | hypothetical gene supported by AK095347 | 5 | | 273 | HMX1 | hypothetical LOC100129098 | 5 | | 274 | HOXA9 | hypothetical LOC644192 | 5 | | 275 | HS3ST4 | hypothetical LOC646482 | 5 | | 276 | KIAA1244 | hypothetical protein LOC100286909 | 5 | | 277 | LARP1B | hypothetical protein LOC100287584 | 5 | | 278 | LEKR1 | hypothetical protein LOC152578 | 5 | | 279 | LLGL1 | hypothetical protein LOC253264 | 5 | | 280 | LOC100129098 | hypothetical protein LOC440982 | 5 | | 281 | LOC100133790 | hypothetical protein MGC12982 | 5 | | 282 | LOC100286909 | intestinal mucin-like | 5 | | 283 | LOC100287584 | KIAA1244 | 5 | | 284 | LOC152578 | La ribonucleoprotein domain family, member 1B | 5 | | 285 | LOC253264 | lethal giant larvae homolog 1 (Drosophila) | 5 | | 286 | LOC644192 | leucine, glutamate and lysine rich 1 | 5 | | 287 | LOC646482 | mannosidase, alpha, class 1B, member 1 | 5 | | 288 | MAN1B1 | maternally expressed 3 (non-protein coding) | 5 | | 289 | MEG3 | microsomal glutathione S-transferase 3 | 5 | | 290 | MGC12982 | myeloid leukemia factor 1 | 5 | | 291 | MGST3 | N-deacetylase/N-sulfotransferase (heparan glucosaminyl) 1 | 5 | | 292 | MLF1 | nascent polypeptide-associated complex alpha subunit | 5 | | 293 | NACA | neuregulin 2 | 5 | | 294 | NDST1 | neuroblastoma highly expressed 1 | 5 | | 295 | NHEG1 | olfactomedin-like 2A | 5 | | 296 | NRG2 | olfactory receptor, family 1, subfamily A, member 2 | 5 | | 297 | OLFML2A | optic atrophy 1 (autosomal dominant) | 5 | | 298 | OPA1 | period homolog 3 (Drosophila) | 5 | | 299 | OR1A2 | phosphoglucomutase 5 | 5 | | 300 | P2RX2 | platelet-derived growth factor beta polypeptide (simian sarcoma viral (v-sis) oncogene homolog) | 5 | | 301 | PDGFB | PR domain containing 2, with ZNF domain | 5 | | 302 | PER3 | protein phosphatase 1, regulatory (inhibitor) subunit 1C | 5 | | 303 | PGM5 | purinergic receptor P2X, ligand-gated ion channel, 2 | 5 | | 304 | PPP1R1C | RAB3A interacting protein (rabin3) | 5 | | 305 | PRDM2 | RAB8B, member RAS oncogene family | 5 | | 306 | RAB3IP | Rap guanine nucleotide exchange factor (GEF) 3 | 5 | | 307 | RAB8B | regulation of nuclear pre-mRNA domain containing 1A | 5 | | 308 | RAPGEF3 | rhabdomyosarcoma 2 associated transcript (non-protein coding) | 5 | | 309 | RMST | Rho guanine nucleotide exchange factor (GEF) 7 | 5 | | 310 | RPRD1A | serine/arginine repetitive matrix 4 | 5 | | 311 | SERPINA1 | serpin peptidase inhibitor, clade A (alpha-1 antiproteinase, antitrypsin), member 1 | 5 | | 312 | SFRS12 | SH3 domain containing, Ysc84-like 1 (S. cerevisiae) | 5 | | 313 | SH3YL1 | solute carrier family 12 (potassium/chloride transporters), member 7 | 5 | | 314 | SLC12A7 | solute carrier family 14 (urea transporter), member 2 | 5 | | 315 | SLC14A2 | solute carrier family 18 (vesicular monoamine), member 2 | 5 | | 316 | SLC18A2 | splicing factor, arginine/serine-rich 12 | 5 | | 317 | SRCIN1 | SRC kinase signaling inhibitor 1 | 5 | | 318 | SRRM4 | TEA domain family member 4 | 5 | | 319 | TEAD4 | transient receptor potential cation channel, subfamily M, member 6 | 5 | | 320 | TMC2 | transmembrane and coiled-coil domains 5A | 5 | | 321 | TMC7 | transmembrane channel-like 2 | 5 | | 322 | TMCO5A | transmembrane channel-like 7 | 5 | | 323 | TMEM160 | transmembrane protein 160 | 5 | | 324 | TRPM6 | tyrosine 3-monooxygenase/tryptophan 5-monooxygenase activation protein, eta polypeptide | 5 | | 325 | USP13 | ubiquitin specific peptidase 13 (isopeptidase T-3) | 5 | | 326 | WDR26 | UDP-Gal:betaGal beta 1,3-galactosyltransferase polypeptide 6 | 5 | | 327 | WWOX | WD repeat domain 26 | 5 | | 328 | YWHAH | WW domain containing oxidoreductase | 5 | | 329 | ZNF274 | zinc finger protein 274 | 5 | | 330 | ZNF316 | zinc finger protein 316 | 5 | | 331 | ABCC11 | adaptor-related protein complex 4, sigma 1 subunit | 4 | | 332 | ACAN | adenylate cyclase 4 | 4 | | 333 | ADCY4 | aggrecan | 4 | | 334 | AGAP1 | ankyrin repeat and SOCS box-containing 7 | 4 | | 335 | ANKRD43 | ankyrin repeat domain 43 | 4 | | 336 | AP4S1 | ArfGAP with GTPase domain, ankyrin repeat and PH domain 1 | 4 | | 337 | ASB7 | ATP-binding cassette, sub-family C (CFTR/MRP), member 11 | 4 | | 338 | BICD1 | bicaudal D homolog 1 (Drosophila) | 4 | | 339 | BRWD1 | bromodomain and WD repeat domain containing 1 | 4 | | 340 | C11orf85 | calcium channel, voltage-dependent, gamma subunit 8 | 4 | | 341 | C1orf91 | calcyphosine-like | 4 | | 342 | C1orf96 | cAMP responsive element binding protein 3-like 1 | 4 | | 343 | C6orf123 | cardiotrophin 1 | 4 | | 344 | C6orf153 | CD79a molecule, immunoglobulin-associated alpha | 4 | | 345 | C8orf46 | CD79b molecule, immunoglobulin-associated beta | 4 | | 346 | C9orf98 | choline dehydrogenase | 4 | | 347 | CACNG8 | chromosome 1 open reading frame 91 | 4 | | 348 | CAPSL | chromosome 1 open reading frame 96 | 4 | | 349 | CCDC61 | chromosome 11 open reading frame 85 | 4 | | 350 | CD79A | chromosome 6 open reading frame 123 | 4 | | 351 | CD79B | chromosome 6 open reading frame 153 | 4 | | 352 | CHDH | chromosome 8 open reading frame 46 | 4 | | 353 | CLRN1 | chromosome 9 open reading frame 98 | 4 | | 354 | CLU | chromosome X open reading frame 62 | 4 | | 355 | CREB3L1 | clarin 1 | 4 | | 356 | CTF1 | clusterin | 4 | | 357 | CXorf62 | coiled-coil domain containing 61 | 4 | | 358 | CYP2C8 | cytochrome P450, family 2, subfamily C, polypeptide 8 | 4 | | 359 | DES | desmin | 4 | | 360 | DNAJC17 | DnaJ (Hsp40) homolog, subfamily C, member 17 | 4 | | 361 | DNM2 | dual specificity phosphatase 21 | 4 | | 362 | DUSP21 | dynamin 2 | 4 | | 363 | EGFL7 | early growth response 4 | 4 | | 364 | EGR4 | EGF-like-domain, multiple 7 | 4 | | 365 | ENG | endoglin | 4 | | 366 | FAHD1 | FAD-dependent oxidoreductase domain containing 2 | 4 | | 367 | FAM124A | family with sequence similarity 124A | 4 | | 368 | FAM129C | family with sequence similarity 129, member C | 4 | | 369 | FAM170A | family with sequence similarity 170, member A | 4 | | 370 | FAM65A | family with sequence similarity 65, member A | 4 | | 371 | FCRLA | Fc receptor-like A | 4 | | 372 | FKBP3 | FK506 binding protein 3, 25kDa | 4 | | 373 | FOXP2 | forkhead box P2 | 4 | | 374 | FOXRED2 | fumarylacetoacetate hydrolase domain containing 1 | 4 | | 375 | GPR114 | G protein-coupled receptor 114 | 4 | | 376 | GPR26 | G protein-coupled receptor 26 | 4 | | 377 | HAUS5 | HAUS augmin-like complex, subunit 5 | 4 | | 378 | HOXD13 | homeobox D13 | 4 | | 379 | IGF2BP1 | hypothetical LOC145845 | 4 | | 380 | IL11 | hypothetical LOC415056 | 4 | | 381 | IL34 | hypothetical LOC651250 | 4 | | 382 | JMJD6 | hypothetical LOC729994 | 4 | | 383 | JUND | hypothetical locus LOC494558 | 4 | | 384 | KCNAB1 | hypothetical protein LOC100131864 | 4 | | 385 | KIAA0284 | hypothetical protein LOC283089 | 4 | | 386 | KLK2 | hypothetical protein LOC387763 | 4 | | 387 | LGI4 | hypothetical protein LOC644714 | 4 | | 388 | LHFP | insulin-like growth factor 2 mRNA binding protein 1 | 4 | | 389 | LIN7A | interleukin 11 | 4 | | 390 | LOC100131864 | interleukin 34 | 4 | | 391 | LOC145845 | jumonji domain containing 6 | 4 | | 392 | LOC283089 | jun D proto-oncogene | 4 | | 393 | LOC387763 | kallikrein-related peptidase 2 | 4 | | 394 | LOC415056 | KIAA0284 | 4 | | 395 | LOC494558 | leucine-rich repeat LGI family, member 4 | 4 | | 396 | LOC644714 | lin-7 homolog A (C. elegans) | 4 | | 397 | LOC651250 | lipoma HMGIC fusion partner | 4 | | 398 | LOC729994 | mediator complex subunit 26 | 4 | | 399 | MBOAT2 | membrane bound O-acyltransferase domain containing 2 | 4 | | 400 | MCM8 | minichromosome maintenance complex component 8 | 4 | | 401 | MED26 | NADPH oxidase, EF-hand calcium binding domain 5 | 4 | | 402 | MYCNOS | neuralized homolog 1B (Drosophila) | 4 | | 403 | NEURL1B | nicotinamide nucleotide adenylyltransferase 3 | 4 | | 404 | NMNAT3 | obscurin-like 1 | 4 | | 405 | NOX5 | olfactory receptor, family 10, subfamily A, member 5 | 4 | | 406 | OBSL1 | pancreatic and duodenal homeobox 1 | 4 | | 407 | OR10A5 | phosphatidylinositol transfer protein, alpha | 4 | | 408 | PCDHGA3 | placental growth factor | 4 | | 409 | PDX1 | pleckstrin homology domain containing, family A (phosphoinositide binding specific) member 4 | 4 | | 410 | PGF | potassium voltage-gated channel, shaker-related subfamily, beta member 1 | 4 | | 411 | PITPNA | proline rich 11 | 4 | | 412 | PLEKHA4 | prostaglandin reductase 1 | 4 | | 413 | PRR11 | protease, serine, 12 (neurotrypsin, motopsin) | 4 | | 414 | PRSS12 | protocadherin gamma subfamily A, 3 | 4 | | 415 | PTGR1 | ribosomal protein S6 kinase, 90kDa, polypeptide 6 | 4 | | 416 | RPS6KA6 | S-phase kinase-associated protein 1 | 4 | | 417 | SAP130 | Sin3A-associated protein, 130kDa | 4 | | 418 | SKP1 | SLIT and NTRK-like family, member 2 | 4 | | 419 | SLC10A2 | small nuclear RNA activating complex, polypeptide 5, 19kDa | 4 | | 420 | SLC16A2 | small nucleolar RNA host gene 10 (non-protein coding) | 4 | | 421 | SLC26A8 | solute carrier family 10 (sodium/bile acid cotransporter family), member 2 | 4 | | 422 | SLC36A4 | solute carrier family 16, member 2 (monocarboxylic acid transporter 8) | 4 | | 423 | SLITRK2 | solute carrier family 26, member 8 | 4 | | 424 | SNAPC5 | solute carrier family 36 (proton/amino acid symporter), member 4 | 4 | | 425 | SNHG10 | spinster homolog 2 (Drosophila) | 4 | | 426 | SOD1 | sulfite oxidase | 4 | | 427 | SPNS2 | superoxide dismutase 1, soluble | 4 | | 428 | SUOX | T-cell leukemia/lymphoma 1A | 4 | | 429 | TBCD | transmembrane protein 207 | 4 | | 430 | TCL1A | tubulin folding cofactor D | 4 | | 431 | TMEM207 | v-myc myelocytomatosis viral related oncogene, neuroblastoma derived (avian) opposite strand | 4 | | 432 | WDR51A | WD repeat domain 51A | 4 | | 433 | ZG16 | zinc finger protein 213 | 4 | | 434 | ZNF213 | zymogen granule protein 16 homolog (rat) | 4 | | 435 | ACACA | acetyl-Coenzyme A carboxylase alpha | 3 | | 436 | ACPT | acid phosphatase, testicular | 3 | | 437 | AK7 | activating transcription factor 7 | 3 | | 438 | ALDH1A3 | adaptor-related protein complex 1, gamma 1 subunit | 3 | | 439 | AP1G1 | additional sex combs like 3 (Drosophila) | 3 | | 440 | ART4 | adenylate kinase 7 | 3 | | 441 | ASB14 | ADP-ribosyltransferase 4 (Dombrock blood group) | 3 | | 442 | ASRGL1 | aldehyde dehydrogenase 1 family, member A3 | 3 | | 443 | ASXL3 | ankyrin repeat and SOCS box-containing 14 | 3 | | 444 | ATF7 | asparaginase like 1 | 3 | | 445 | ATP2C2 | ATPase, Ca++ transporting, type 2C, member 2 | 3 | | 446 | BRE | brain and reproductive organ-expressed (TNFRSF1A modulator) | 3 | | 447 | BRPF3 | bromodomain and PHD finger containing, 3 | 3 | | 448 | C10orf96 | cadherin 1, type 1, E-cadherin (epithelial) | 3 | | 449 | C11orf49 | calcium/calmodulin-dependent protein kinase II alpha | 3 | | 450 | C1orf100 | cancer/testis antigen 2 | 3 | | 451 | C4orf42 | Cas-Br-M (murine) ecotropic retroviral transforming sequence c | 3 | | 452 | C9orf25 | cat eye syndrome chromosome region, candidate 9 | 3 | | 453 | C9orf44 | chromosome 1 open reading frame 100 | 3 | | 454 | C9orf6 | chromosome 10 open reading frame 96 | 3 | | 455 | C9orf7 | chromosome 11 open reading frame 49 | 3 | | 456 | CAMK2A | chromosome 4 open reading frame 42 | 3 | | 457 | CBLC | chromosome 9 open reading frame 25 | 3 | | 458 | CCDC17 | chromosome 9 open reading frame 44 | 3 | | 459 | CCDC45 | chromosome 9 open reading frame 6 | 3 | | 460 | CCDC87 | chromosome 9 open reading frame 7 | 3 | | 461 | CCDC93 | ciliary neurotrophic factor | 3 | | 462 | CDH1 | coiled-coil domain containing 17 | 3 | | 463 | CECR9 | coiled-coil domain containing 45 | 3 | | 464 | CNTF | coiled-coil domain containing 87 | 3 | | 465 | CTAG2 | coiled-coil domain containing 93 | 3 | | 466 | DCAF13 | DDB1 and CUL4 associated factor 13 | 3 | | 467 | DDA1 | DDHD domain containing 1 | 3 | | 468 | DDHD1 | DEAD (Asp-Glu-Ala-Asp) box polypeptide 56 | 3 | | 469 | DDN | dendrin | 3 | | 470 | DDX56 | DET1 and DDB1 associated 1 | 3 | | 471 | DKK2 | dickkopf homolog 2 (Xenopus laevis) | 3 | | 472 | EFCAB1 | EF-hand calcium binding domain 1 | 3 | | 473 | ENAM | enamelin | 3 | | 474 | ERN1 | endoplasmic reticulum to nucleus signaling 1 | 3 | | 475 | EXD2 | exonuclease 3'-5' domain containing 2 | 3 | | 476 | FADS2 | family with sequence similarity 128, member B | 3 | | 477 | FAM128B | fatty acid desaturase 2 | 3 | | 478 | FER1L4 | feline leukemia virus subgroup C cellular receptor family, member 2 | 3 | | 479 | FIZ1 | fer-1-like 4 (C. elegans) | 3 | | 480 | FLJ39739 | FLT3-interacting zinc finger 1 | 3 | | 481 | FLJ42289 | forkhead box C1 | 3 | | 482 | FLVCR2 | fragile X mental retardation, autosomal homolog 2 | 3 | | 483 | FOXC1 | G protein-coupled receptor 176 | 3 | | 484 | FXR2 | gamma-aminobutyric acid (GABA) receptor, theta | 3 | | 485 | GABRQ | glutathione S-transferase mu 5 | 3 | | 486 | GPR176 | homeobox A10 | 3 | | 487 | GSTM5 | homeobox C11 | 3 | | 488 | HOXA10 | HtrA serine peptidase 3 | 3 | | 489 | HOXC11 | hypothetical FLJ39739 | 3 | | 490 | HTRA3 | hypothetical LOC100132319 | 3 | | 491 | IGDCC3 | hypothetical LOC388182 | 3 | | 492 | IGFBP5 | hypothetical LOC550113 | 3 | | 493 | IGSF10 | hypothetical protein LOC100129112 | 3 | | 494 | IPO9 | hypothetical protein LOC339988 | 3 | | 495 | IRF2BP2 | hypothetical protein LOC647309 | 3 | | 496 | IVD | immunoglobulin superfamily, DCC subclass, member 3 | 3 | | 497 | KIAA1755 | immunoglobulin superfamily, member 10 | 3 | | 498 | KLF14 | importin 9 | 3 | | 499 | KRT4 | insulin-like growth factor binding protein 5 | 3 | | 500 | LOC100129112 | interferon regulatory factor 2 binding protein 2 | 3 | | 501 | LOC100132319 | isovaleryl Coenzyme A dehydrogenase | 3 | | 502 | LOC339988 | keratin 4 | 3 | | 503 | LOC550113 | KIAA1755 | 3 | | 504 | LOC647309 | Kruppel-like factor 14 | 3 | | 505 | LRRFIP1 | leucine rich repeat (in FLII) interacting protein 1 | 3 | | 506 | LYPLAL1 | lysophospholipase-like 1 | 3 | | 507 | MAP7D3 | MAP7 domain containing 3 | 3 | | 508 | MCOLN3 | membrane protein, palmitoylated 3 (MAGUK p55 subfamily member 3) | 3 | | 509 | MPP3 | mitochondrial ribosomal protein L47 | 3 | | 510 | MRPL47 | mucin 5AC, oligomeric mucus/gel-forming | 3 | | 511 | MUC5AC | mucolipin 3 | 3 | | 512 | MYO5B | myosin VB | 3 | | 513 | MYOZ1 | myozenin 1 | 3 | | 514 | NEFH | natural killer-tumor recognition sequence | 3 | | 515 | NKTR | neurofilament, heavy polypeptide | 3 | | 516 | OR51B5 | olfactory receptor, family 51, subfamily B, member 5 | 3 | | 517 | PACSIN1 | p21 protein (Cdc42/Rac)-activated kinase 3 | 3 | | 518 | PAK3 | patched domain containing 2 | 3 | | 519 | PCBP3 | PERP, TP53 apoptosis effector | 3 | | 520 | PCDHB4 | phosphodiesterase 6H, cGMP-specific, cone, gamma | 3 | | 521 | PDE6H | pleckstrin homology domain containing, family G (with RhoGef domain) member 2 | 3 | | 522 | PERP | poly(rC) binding protein 3 | 3 | | 523 | PLEKHG2 | pregnancy specific beta-1-glycoprotein 9 | 3 | | 524 | PSG9 | protein kinase C and casein kinase substrate in neurons 1 | 3 | | 525 | PTCHD2 | protein tyrosine phosphatase, receptor type, C | 3 | | 526 | PTPRC | protocadherin beta 4 | 3 | | 527 | RDH5 | retinol dehydrogenase 5 (11-cis/9-cis) | 3 | | 528 | SCCPDH | saccharopine dehydrogenase (putative) | 3 | | 529 | SCN1B | shroom family member 4 | 3 | | 530 | SCUBE3 | signal peptide, CUB domain, EGF-like 3 | 3 | | 531 | SHROOM4 | SLAIN motif family, member 2 | 3 | | 532 | SLAIN2 | slingshot homolog 3 (Drosophila) | 3 | | 533 | SLC17A1 | sodium channel, voltage-gated, type I, beta | 3 | | 534 | SLC6A4 | solute carrier family 17 (sodium phosphate), member 1 | 3 | | 535 | SMC3 | solute carrier family 6 (neurotransmitter transporter, serotonin), member 4 | 3 | | 536 | SNCAIP | SON DNA binding protein | 3 | | 537 | SOHLH2 | spermatogenesis and oogenesis specific basic helix-loop-helix 2 | 3 | | 538 | SON | SRY (sex determining region Y)-box 15 | 3 | | 539 | SOX15 | ST7 overlapping transcript 2 (non-protein coding) | 3 | | 540 | SSH3 | stonin 2 | 3 | | 541 | ST7OT2 | structural maintenance of chromosomes 3 | 3 | | 542 | STON2 | synuclein, alpha interacting protein | 3 | | 543 | TAS2R7 | taste receptor, type 2, member 7 | 3 | | 544 | TBL1X | tenascin R (restrictin, janusin) | 3 | | 545 | TGFA | thymocyte selection-associated high mobility group box | 3 | | 546 | TGM4 | transducin (beta)-like 1X-linked | 3 | | 547 | TMEM185A | transforming growth factor, alpha | 3 | | 548 | TNK2 | transglutaminase 4 (prostate) | 3 | | 549 | TNR | transient receptor potential cation channel, subfamily V, member 5 | 3 | | 550 | TOX | transmembrane protein 185A | 3 | | 551 | TRIM3 | tripartite motif-containing 3 | 3 | | 552 | TRPV5 | tyrosine kinase, non-receptor, 2 | 3 | | 553 | UBXN6 | UBX domain protein 6 | 3 | | 554 | VDR | vitamin D (1,25- dihydroxyvitamin D3) receptor | 3 | | 555 | VWA3B | von Willebrand factor A domain containing 3B | 3 | | 556 | ZFYVE27 | zinc finger protein 284 | 3 | | 557 | ZNF284 | zinc finger protein 449 | 3 | | 558 | ZNF449 | zinc finger protein 618 | 3 | | 559 | ZNF618 | zinc finger, FYVE domain containing 27 | 3 | | 560 | AGXT2L2 | additional sex combs like 2 (Drosophila) | 2 | | 561 | ALX1 | alanine-glyoxylate aminotransferase 2-like 2 | 2 | | 562 | ASXL2 | ALX homeobox 1 | 2 | | 563 | ATXN8OS | ATXN8 opposite strand (non-protein coding) | 2 | | 564 | BACH1 | B-cell CLL/lymphoma 3 | 2 | | 565 | BAGE | B melanoma antigen | 2 | | 566 | BCL3 | BTB and CNC homology 1, basic leucine zipper transcription factor 1 | 2 | | 567 | C20orf199 | calcium binding and coiled-coil domain 2 | 2 | | 568 | C22orf30 | calcium channel, voltage-dependent, alpha 2/delta subunit 4 | 2 | | 569 | C5orf62 | caspase 10, apoptosis-related cysteine peptidase | 2 | | 570 | C9orf100 | CCR4-NOT transcription complex, subunit 4 | 2 | | 571 | CACNA2D4 | CD72 molecule | 2 | | 572 | CALCOCO2 | CDC-like kinase 4 | 2 | | 573 | CASP10 | chromosome 20 open reading frame 199 | 2 | | 574 | CCDC3 | chromosome 22 open reading frame 30 | 2 | | 575 | CCNK | chromosome 5 open reading frame 62 | 2 | | 576 | CD72 | chromosome 9 open reading frame 100 | 2 | | 577 | CLASP2 | coiled-coil domain containing 3 | 2 | | 578 | CLK4 | COMM domain containing 6 | 2 | | 579 | CNOT4 | complement component (3d/Epstein Barr virus) receptor 2 | 2 | | 580 | CNTD2 | CTD (carboxy-terminal domain, RNA polymerase II, polypeptide A) small phosphatase-like | 2 | | 581 | COMMD6 | cyclin G associated kinase | 2 | | 582 | CR2 | cyclin K | 2 | | 583 | CTDSPL | cyclin N-terminal domain containing 2 | 2 | | 584 | EPHB2 | cytoplasmic linker associated protein 2 | 2 | | 585 | EYA4 | EPH receptor B2 | 2 | | 586 | FAM131B | eyes absent homolog 4 (Drosophila) | 2 | | 587 | FANCI | family with sequence similarity 131, member B | 2 | | 588 | FOXA2 | family with sequence similarity 86, member A pseudogene | 2 | | 589 | GAK | Fanconi anemia, complementation group I | 2 | | 590 | GGTA1 | forkhead box A2 | 2 | | 591 | GPR173 | G protein-coupled receptor 173 | 2 | | 592 | GPRC5A | G protein-coupled receptor, family C, group 5, member A | 2 | | 593 | GRIN1 | glutamate receptor, ionotropic, N-methyl D-aspartate 1 | 2 | | 594 | GSK3A | glycogen synthase kinase 3 alpha | 2 | | 595 | H3F3B | glycoprotein, alpha-galactosyltransferase 1 | 2 | | 596 | HABP4 | H3 histone, family 3B (H3.3B) | 2 | | 597 | hCG\_1990547 | hect domain and RLD 2 pseudogene 3 | 2 | | 598 | HDGF | HECT domain containing 1 | 2 | | 599 | HECTD1 | hepatoma-derived growth factor (high-mobility group protein 1-like) | 2 | | 600 | HERC2P3 | histamine N-methyltransferase | 2 | | 601 | HNMT | hyaluronan binding protein 4 | 2 | | 602 | ID3 | hypothetical LOC148696 | 2 | | 603 | IGHM | hypothetical LOC25845 | 2 | | 604 | IQCF3 | hypothetical LOC497256 | 2 | | 605 | ITGB3 | hypothetical LOC731275 | 2 | | 606 | KANK1 | hypothetical protein LOC100131825 | 2 | | 607 | KDM5C | hypothetical protein LOC100287445 | 2 | | 608 | KRT33B | hypothetical protein LOC113230 | 2 | | 609 | LAMC2 | hypothetical protein LOC285556 | 2 | | 610 | LOC100131825 | hypothetical protein LOC93463 | 2 | | 611 | LOC100287081 | immunoglobulin heavy constant mu | 2 | | 612 | LOC100287445 | inhibitor of DNA binding 3, dominant negative helix-loop-helix protein | 2 | | 613 | LOC113230 | integrin, beta 3 (platelet glycoprotein IIIa, antigen CD61) | 2 | | 614 | LOC148696 | IQ motif containing F3 | 2 | | 615 | LOC25845 | keratin 33B | 2 | | 616 | LOC285556 | KN motif and ankyrin repeat domains 1 | 2 | | 617 | LOC440292 | laminin, gamma 2 | 2 | | 618 | LOC497256 | lysine (K)-specific demethylase 5C | 2 | | 619 | LOC731275 | membrane-associated ring finger (C3HC4) 7 | 2 | | 620 | LOC93463 | MLX interacting protein | 2 | | 621 | MARCH7 | monoacylglycerol O-acyltransferase 3 | 2 | | 622 | MLXIP | neuron navigator 1 | 2 | | 623 | MOGAT3 | neurotrophic tyrosine kinase, receptor, type 2 | 2 | | 624 | NAV1 | nuclear transcription factor Y, gamma | 2 | | 625 | NFYC | oligodendrocytic myelin paranodal and inner loop protein | 2 | | 626 | NTRK2 | one cut homeobox 2 | 2 | | 627 | ONECUT2 | pantothenate kinase 2 | 2 | | 628 | OPALIN | par-3 partitioning defective 3 homolog B (C. elegans) | 2 | | 629 | PANK2 | par-6 partitioning defective 6 homolog gamma (C. elegans) | 2 | | 630 | PARD3B | phosphoinositide-3-kinase, regulatory subunit 2 (beta) | 2 | | 631 | PARD6G | piwi-like 1 (Drosophila) | 2 | | 632 | PCDHGC4 | podoplanin | 2 | | 633 | PDPN | profilin family, member 4 | 2 | | 634 | PFN4 | protein kinase N2 | 2 | | 635 | PIK3R2 | protocadherin gamma subfamily C, 4 | 2 | | 636 | PIWIL1 | quaking homolog, KH domain RNA binding (mouse) | 2 | | 637 | PKN2 | RALY RNA binding protein-like | 2 | | 638 | QKI | RAS guanyl releasing protein 3 (calcium and DAG-regulated) | 2 | | 639 | RALYL | regenerating islet-derived 1 beta | 2 | | 640 | RASGRP3 | retinol dehydrogenase 12 (all-trans/9-cis/11-cis) | 2 | | 641 | RBM47 | ribosomal protein S2 pseudogene 45 | 2 | | 642 | RDH12 | ring finger protein 157 | 2 | | 643 | REG1B | RNA binding motif protein 47 | 2 | | 644 | RNF157 | SDA1 domain containing 1 | 2 | | 645 | RPS2P45 | serpin peptidase inhibitor, clade E (nexin, plasminogen activator inhibitor type 1), member 2 | 2 | | 646 | SDAD1 | SERTA domain containing 4 | 2 | | 647 | SERPINE2 | similar to COMM domain containing 4 | 2 | | 648 | SERTAD4 | similar to hCG1999172 | 2 | | 649 | SFRS17A | solute carrier family 25, member 35 | 2 | | 650 | SLC25A35 | somatostatin receptor 2 | 2 | | 651 | SPEF2 | spastic paraplegia 11 (autosomal recessive) | 2 | | 652 | SPG11 | sperm flagellar 2 | 2 | | 653 | SPHKAP | SPHK1 interactor, AKAP domain containing | 2 | | 654 | SSTR2 | splicing factor, arginine/serine-rich 17A | 2 | | 655 | ST14 | suppression of tumorigenicity 14 (colon carcinoma) | 2 | | 656 | ST7 | suppression of tumorigenicity 7 | 2 | | 657 | TECTB | tectorin beta | 2 | | 658 | TMEM75 | tetratricopeptide repeat domain 18 | 2 | | 659 | TMOD1 | tetratricopeptide repeat domain 7B | 2 | | 660 | TTC18 | transmembrane protein 75 | 2 | | 661 | TTC7B | tropomodulin 1 | 2 | | 662 | U2AF2 | U2 small nuclear RNA auxiliary factor 2 | 2 | | 663 | UBE2A | ubinuclein 2 | 2 | | 664 | UBN2 | ubiquitin-conjugating enzyme E2A (RAD6 homolog) | 2 | | 665 | VEGFA | vascular endothelial growth factor A | 2 | | 666 | ZBTB7B | zinc finger and BTB domain containing 7B | 2 | | 667 | ZNF180 | zinc finger protein 180 | 2 | | 668 | ZNF19 | zinc finger protein 19 | 2 | | 669 | ZNF556 | zinc finger protein 556 | 2 | | 670 | ABCC12 | 5'-nucleotidase domain containing 3 | 1 | | 671 | ABHD2 | abhydrolase domain containing 2 | 1 | | 672 | ABLIM3 | actin binding LIM protein family, member 3 | 1 | | 673 | ACTR3 | actin related protein 2/3 complex, subunit 5, 16kDa | 1 | | 674 | ADCYAP1 | activating transcription factor 6 | 1 | | 675 | ANXA10 | adenylate cyclase activating polypeptide 1 (pituitary) | 1 | | 676 | APH1B | annexin A10 | 1 | | 677 | AQP10 | anterior pharynx defective 1 homolog B (C. elegans) | 1 | | 678 | ARNT | aquaporin 10 | 1 | | 679 | ARPC5 | ARP3 actin-related protein 3 homolog (yeast) | 1 | | 680 | ATF6 | aryl hydrocarbon receptor nuclear translocator | 1 | | 681 | ATP1B3 | ATP-binding cassette, sub-family C (CFTR/MRP), member 12 | 1 | | 682 | BLK | ATPase, Na+/K+ transporting, beta 3 polypeptide | 1 | | 683 | BRD4 | B lymphoid tyrosine kinase | 1 | | 684 | C10orf118 | bromodomain containing 4 | 1 | | 685 | C13orf38 | Cas-Br-M (murine) ecotropic retroviral transforming sequence | 1 | | 686 | C20orf54 | CD300 molecule-like family member g | 1 | | 687 | C3orf75 | choline kinase alpha | 1 | | 688 | C9orf122 | chromodomain helicase DNA binding protein 7 | 1 | | 689 | C9orf72 | chromosome 10 open reading frame 118 | 1 | | 690 | CBL | chromosome 13 open reading frame 38 | 1 | | 691 | CBS | chromosome 20 open reading frame 54 | 1 | | 692 | CD300LG | chromosome 3 open reading frame 75 | 1 | | 693 | CHD7 | chromosome 9 open reading frame 122 | 1 | | 694 | CHKA | chromosome 9 open reading frame 72 | 1 | | 695 | DCN | cystathionine-beta-synthase | 1 | | 696 | DEFA6 | decorin | 1 | | 697 | DLGAP2 | defensin, alpha 6, Paneth cell-specific | 1 | | 698 | DLGAP4 | discs, large (Drosophila) homolog-associated protein 2 | 1 | | 699 | DNAH12 | discs, large (Drosophila) homolog-associated protein 4 | 1 | | 700 | DOK5 | docking protein 5 | 1 | | 701 | EIF2C4 | dynein, axonemal, heavy chain 12 | 1 | | 702 | EPB41L4B | erythrocyte membrane protein band 4.1 like 4B | 1 | | 703 | FAM125B | eukaryotic translation initiation factor 2C, 4 | 1 | | 704 | FAM73B | family with sequence similarity 125, member B | 1 | | 705 | FANCD2 | family with sequence similarity 73, member B | 1 | | 706 | FGD4 | Fanconi anemia, complementation group D2 | 1 | | 707 | FLRT3 | FERM domain containing 3 | 1 | | 708 | FRMD3 | fibronectin leucine rich transmembrane protein 3 | 1 | | 709 | GAD2 | FYVE, RhoGEF and PH domain containing 4 | 1 | | 710 | GALNTL4 | gap junction protein, alpha 4, 37kDa | 1 | | 711 | GJA4 | glutamate decarboxylase 2 (pancreatic islets and brain, 65kDa) | 1 | | 712 | GRIK2 | glutamate receptor, ionotropic, kainate 2 | 1 | | 713 | HBB | hect domain and RLD 6 | 1 | | 714 | HERC6 | hemoglobin, beta | 1 | | 715 | HTRA1 | HtrA serine peptidase 1 | 1 | | 716 | IGSF5 | hypothetical LOC338588 | 1 | | 717 | IL28RA | hypothetical LOC653110 | 1 | | 718 | INPP5B | hypothetical LOC728789 | 1 | | 719 | ITGB5 | hypothetical protein LOC100128687 | 1 | | 720 | KIAA0754 | hypothetical protein LOC100288447 | 1 | | 721 | KRTAP4-1 | immunoglobulin superfamily, member 5 | 1 | | 722 | LATS2 | inositol polyphosphate-5-phosphatase, 75kDa | 1 | | 723 | LOC100128687 | integrin, beta 5 | 1 | | 724 | LOC100288447 | interleukin 28 receptor, alpha (interferon, lambda receptor) | 1 | | 725 | LOC100289373 | keratin associated protein 4-1 | 1 | | 726 | LOC338588 | KIAA0754 | 1 | | 727 | LOC653110 | latent transforming growth factor beta binding protein 2 | 1 | | 728 | LOC728789 | LATS, large tumor suppressor, homolog 2 (Drosophila) | 1 | | 729 | LTBP2 | matrix-remodelling associated 5 | 1 | | 730 | MAGI1 | membrane associated guanylate kinase, WW and PDZ domain containing 1 | 1 | | 731 | MAP2K6 | mitogen-activated protein kinase kinase 6 | 1 | | 732 | MAP4K5 | mitogen-activated protein kinase kinase kinase kinase 5 | 1 | | 733 | MPDZ | multiple PDZ domain protein | 1 | | 734 | MXRA5 | Na+/K+ transporting ATPase interacting 1 | 1 | | 735 | NDUFA7 | NADH dehydrogenase (ubiquinone) 1 alpha subcomplex, 7, 14.5kDa | 1 | | 736 | NKAIN1 | nephroblastoma overexpressed gene | 1 | | 737 | NOS1 | nitric oxide synthase 1 (neuronal) | 1 | | 738 | NOV | paternally expressed 3 | 1 | | 739 | NT5DC3 | pecanex homolog (Drosophila) | 1 | | 740 | PAFAH1B2 | placenta-specific 9 | 1 | | 741 | PCNX | platelet-activating factor acetylhydrolase 1b, catalytic subunit 2 (30kDa) | 1 | | 742 | PDK3 | PTK6 protein tyrosine kinase 6 | 1 | | 743 | PEG3 | pyruvate dehydrogenase kinase, isozyme 3 | 1 | | 744 | PLAC9 | RAB39, member RAS oncogene family | 1 | | 745 | PTK6 | RB-associated KRAB zinc finger | 1 | | 746 | RAB39 | ring finger and FYVE-like domain containing 1 | 1 | | 747 | RBAK | sec1 family domain containing 2 | 1 | | 748 | RFFL | sema domain, transmembrane domain (TM), and cytoplasmic domain, (semaphorin) 6D | 1 | | 749 | SAMD4B | SFRS protein kinase 1 | 1 | | 750 | SCFD2 | SIK family kinase 3 | 1 | | 751 | SEMA6D | similar to hCG2041645 | 1 | | 752 | SIK3 | solute carrier family 1 (glutamate/neutral amino acid transporter), member 4 | 1 | | 753 | SLC1A4 | solute carrier family 4, anion exchanger, member 1 (erythrocyte membrane protein band 3, Diego blood group) | 1 | | 754 | SLC4A1 | solute carrier family 5 (choline transporter), member 7 | 1 | | 755 | SLC5A7 | solute carrier family 6 (neurotransmitter transporter, taurine), member 6 | 1 | | 756 | SLC6A6 | sorbin and SH3 domain containing 2 | 1 | | 757 | SNX19 | sorting nexin 19 | 1 | | 758 | SORBS2 | Sp1 transcription factor | 1 | | 759 | SP1 | Spi-B transcription factor (Spi-1/PU.1 related) | 1 | | 760 | SPIB | sterile alpha motif domain containing 4B | 1 | | 761 | SRPK1 | synaptojanin 1 | 1 | | 762 | SYCE2 | synaptonemal complex central element protein 2 | 1 | | 763 | SYNJ1 | tectonin beta-propeller repeat containing 2 | 1 | | 764 | TECPR2 | transmembrane protein 156 | 1 | | 765 | TMEM156 | UDP-N-acetyl-alpha-D-galactosamine:polypeptide N-acetylgalactosaminyltransferase-like 4 | 1 | | 766 | VAMP3 | vacuolar protein sorting 33 homolog A (S. cerevisiae) | 1 | | 767 | VPS33A | vesicle-associated membrane protein 3 (cellubrevin) | 1 | | 768 | ZC3H3 | zinc finger CCCH-type containing 3 | 1 | | 769 | ZFAND3 | zinc finger protein 319 | 1 | | 770 | ZFP41 | zinc finger protein 41 homolog (mouse) | 1 | | 771 | ZNF319 | zinc finger protein 462 | 1 | | 772 | ZNF462 | zinc finger protein 594 | 1 | | 773 | ZNF594 | zinc finger protein 74 | 1 | | 774 | ZNF74 | zinc finger protein 836 | 1 | | 775 | ZNF836 | zinc finger, AN1-type domain 3 | 1 | |

---

Total number of miRNA-mRNA 3682 interactions for given cut-off. Press for ALL:

| |  | miR | EntrezID | Gene | Name | Score | | --- | --- | --- | --- | --- | --- | | 1143 | hsa-mir-15a | 56 | ACRV1 | acrosomal vesicle protein 1 | 4 | | 1144 | hsa-mir-16-1 | 56 | ACRV1 | acrosomal vesicle protein 1 | 4 | | 1145 | hsa-miR-126\* | 56 | ACRV1 | acrosomal vesicle protein 1 | 4 | | 1146 | hsa-miR-16 | 56 | ACRV1 | acrosomal vesicle protein 1 | 4 | | 1147 | hsa-miR-608 | 56 | ACRV1 | acrosomal vesicle protein 1 | 4 | | 288 | hsa-mir-126 | 1915 | EEF1A1 | eukaryotic translation elongation factor 1 alpha 1 | 3 | | 289 | hsa-mir-424 | 1915 | EEF1A1 | eukaryotic translation elongation factor 1 alpha 1 | 3 | | 291 | hsa-miR-503 | 1915 | EEF1A1 | eukaryotic translation elongation factor 1 alpha 1 | 3 | | 340 | hsa-miR-338-5p | 55024 | BANK1 | B-cell scaffold protein with ankyrin repeats 1 | 3 | | 341 | hsa-miR-657 | 55024 | BANK1 | B-cell scaffold protein with ankyrin repeats 1 | 3 | | 490 | hsa-mir-424 | 6137 | RPL13 | ribosomal protein L13 | 3 | | 491 | hsa-mir-628 | 6137 | RPL13 | ribosomal protein L13 | 3 | | 492 | hsa-miR-190 | 6137 | RPL13 | ribosomal protein L13 | 3 | | 493 | hsa-miR-503 | 6137 | RPL13 | ribosomal protein L13 | 3 | | 495 | hsa-miR-617 | 6137 | RPL13 | ribosomal protein L13 | 3 | | 1566 | hsa-mir-424 | 4068 | SH2D1A | SH2 domain protein 1A | 3 | | 1567 | hsa-mir-628 | 4068 | SH2D1A | SH2 domain protein 1A | 3 | | 1569 | hsa-miR-503 | 4068 | SH2D1A | SH2 domain protein 1A | 3 | | 1570 | hsa-miR-504 | 4068 | SH2D1A | SH2 domain protein 1A | 3 | | 1571 | hsa-miR-586 | 4068 | SH2D1A | SH2 domain protein 1A | 3 | | 1572 | hsa-miR-617 | 4068 | SH2D1A | SH2 domain protein 1A | 3 | | 1836 | hsa-miR-126\* | 4916 | NTRK3 | neurotrophic tyrosine kinase, receptor, type 3 | 3 | | 1837 | hsa-miR-608 | 4916 | NTRK3 | neurotrophic tyrosine kinase, receptor, type 3 | 3 | | 2405 | hsa-miR-338-5p | 55342 | STRBP | spermatid perinuclear RNA binding protein | 3 | | 2406 | hsa-miR-657 | 55342 | STRBP | spermatid perinuclear RNA binding protein | 3 | | 132 | hsa-mir-218-2 | 146540 | ZNF785 | zinc finger protein 785 | 2 | | 133 | hsa-mir-126 | 146540 | ZNF785 | zinc finger protein 785 | 2 | | 134 | hsa-mir-424 | 146540 | ZNF785 | zinc finger protein 785 | 2 | | 135 | hsa-miR-190 | 146540 | ZNF785 | zinc finger protein 785 | 2 | | 136 | hsa-miR-503 | 146540 | ZNF785 | zinc finger protein 785 | 2 | | 137 | hsa-miR-585 | 146540 | ZNF785 | zinc finger protein 785 | 2 | | 181 | hsa-miR-1231 | 151242 | PPP1R1C | protein phosphatase 1, regulatory (inhibitor) subunit 1C | 2 | | 219 | hsa-miR-623 | 973 | CD79A | CD79a molecule, immunoglobulin-associated alpha | 2 | | 264 | hsa-mir-424 | 84268 | RPAIN | RPA interacting protein | 2 | | 266 | hsa-mir-628 | 84268 | RPAIN | RPA interacting protein | 2 | | 267 | hsa-miR-190 | 84268 | RPAIN | RPA interacting protein | 2 | | 268 | hsa-miR-503 | 84268 | RPAIN | RPA interacting protein | 2 | | 270 | hsa-miR-586 | 84268 | RPAIN | RPA interacting protein | 2 | | 271 | hsa-miR-617 | 84268 | RPAIN | RPA interacting protein | 2 | | 290 | hsa-mir-628 | 1915 | EEF1A1 | eukaryotic translation elongation factor 1 alpha 1 | 2 | | 313 | hsa-mir-126 | 7329 | UBE2I | ubiquitin-conjugating enzyme E2I (UBC9 homolog, yeast) | 2 | | 315 | hsa-mir-424 | 7329 | UBE2I | ubiquitin-conjugating enzyme E2I (UBC9 homolog, yeast) | 2 | | 318 | hsa-mir-628 | 7329 | UBE2I | ubiquitin-conjugating enzyme E2I (UBC9 homolog, yeast) | 2 | | 319 | hsa-miR-190 | 7329 | UBE2I | ubiquitin-conjugating enzyme E2I (UBC9 homolog, yeast) | 2 | | 322 | hsa-miR-503 | 7329 | UBE2I | ubiquitin-conjugating enzyme E2I (UBC9 homolog, yeast) | 2 | | 324 | hsa-miR-617 | 7329 | UBE2I | ubiquitin-conjugating enzyme E2I (UBC9 homolog, yeast) | 2 | | 339 | hsa-miR-190 | 55024 | BANK1 | B-cell scaffold protein with ankyrin repeats 1 | 2 | | 385 | hsa-miR-608 | 4842 | NOS1 | nitric oxide synthase 1 (neuronal) | 2 | | 404 | hsa-miR-1231 | 286144 | C8orf83 | chromosome 8 open reading frame 83 | 2 | | 434 | hsa-miR-586 | 339988 | LOC339988 | hypothetical protein LOC339988 | 2 | | 435 | hsa-miR-617 | 339988 | LOC339988 | hypothetical protein LOC339988 | 2 | | 494 | hsa-miR-586 | 6137 | RPL13 | ribosomal protein L13 | 2 | | 541 | hsa-miR-126\* | 140890 | SFRS12 | splicing factor, arginine/serine-rich 12 | 2 | | 542 | hsa-miR-608 | 140890 | SFRS12 | splicing factor, arginine/serine-rich 12 | 2 | | 547 | hsa-mir-196b | 6693 | SPN | sialophorin | 2 | | 549 | hsa-mir-628 | 6693 | SPN | sialophorin | 2 | | 550 | hsa-miR-196b | 6693 | SPN | sialophorin | 2 | | 704 | hsa-mir-424 | 6167 | RPL37 | ribosomal protein L37 | 2 | | 705 | hsa-mir-556 | 6167 | RPL37 | ribosomal protein L37 | 2 | | 707 | hsa-miR-190 | 6167 | RPL37 | ribosomal protein L37 | 2 | | 708 | hsa-miR-503 | 6167 | RPL37 | ribosomal protein L37 | 2 | | 723 | hsa-mir-424 | 6189 | RPS3A | ribosomal protein S3A | 2 | | 725 | hsa-mir-628 | 6189 | RPS3A | ribosomal protein S3A | 2 | | 727 | hsa-miR-190 | 6189 | RPS3A | ribosomal protein S3A | 2 | | 730 | hsa-miR-503 | 6189 | RPS3A | ribosomal protein S3A | 2 | | 794 | hsa-mir-196b | 1973 | EIF4A1 | eukaryotic translation initiation factor 4A1 | 2 | | 795 | hsa-mir-424 | 1973 | EIF4A1 | eukaryotic translation initiation factor 4A1 | 2 | | 796 | hsa-mir-490 | 1973 | EIF4A1 | eukaryotic translation initiation factor 4A1 | 2 | | 797 | hsa-mir-628 | 1973 | EIF4A1 | eukaryotic translation initiation factor 4A1 | 2 | | 798 | hsa-miR-196b | 1973 | EIF4A1 | eukaryotic translation initiation factor 4A1 | 2 | | 799 | hsa-miR-490-5p | 1973 | EIF4A1 | eukaryotic translation initiation factor 4A1 | 2 | | 800 | hsa-miR-503 | 1973 | EIF4A1 | eukaryotic translation initiation factor 4A1 | 2 | | 801 | hsa-miR-623 | 1973 | EIF4A1 | eukaryotic translation initiation factor 4A1 | 2 | | 918 | hsa-mir-339 | 3797 | KIF3C | kinesin family member 3C | 2 | | 921 | hsa-miR-339-5p | 3797 | KIF3C | kinesin family member 3C | 2 | | 922 | hsa-miR-564 | 3797 | KIF3C | kinesin family member 3C | 2 | | 923 | hsa-miR-618 | 3797 | KIF3C | kinesin family member 3C | 2 | | 924 | hsa-miR-634 | 3797 | KIF3C | kinesin family member 3C | 2 | | 1296 | hsa-miR-566 | 56 | ACRV1 | acrosomal vesicle protein 1 | 2 | | 1366 | hsa-mir-15a | 10052 | GJC1 | gap junction protein, gamma 1, 45kDa | 2 | | 1367 | hsa-mir-16-1 | 10052 | GJC1 | gap junction protein, gamma 1, 45kDa | 2 | | 1372 | hsa-miR-16 | 10052 | GJC1 | gap junction protein, gamma 1, 45kDa | 2 | | 1374 | hsa-miR-608 | 10052 | GJC1 | gap junction protein, gamma 1, 45kDa | 2 | | 1423 | hsa-mir-196b | 3122 | HLA-DRA | major histocompatibility complex, class II, DR alpha | 2 | | 1424 | hsa-mir-424 | 3122 | HLA-DRA | major histocompatibility complex, class II, DR alpha | 2 | | 1425 | hsa-mir-490 | 3122 | HLA-DRA | major histocompatibility complex, class II, DR alpha | 2 | | 1426 | hsa-mir-628 | 3122 | HLA-DRA | major histocompatibility complex, class II, DR alpha | 2 | | 1428 | hsa-miR-190 | 3122 | HLA-DRA | major histocompatibility complex, class II, DR alpha | 2 | | 1429 | hsa-miR-196b | 3122 | HLA-DRA | major histocompatibility complex, class II, DR alpha | 2 | | 1430 | hsa-miR-490-5p | 3122 | HLA-DRA | major histocompatibility complex, class II, DR alpha | 2 | | 1431 | hsa-miR-503 | 3122 | HLA-DRA | major histocompatibility complex, class II, DR alpha | 2 | | 1432 | hsa-miR-617 | 3122 | HLA-DRA | major histocompatibility complex, class II, DR alpha | 2 | | 1433 | hsa-miR-623 | 3122 | HLA-DRA | major histocompatibility complex, class II, DR alpha | 2 | | 1434 | hsa-mir-196b | 6137 | RPL13 | ribosomal protein L13 | 2 | | 1435 | hsa-miR-196b | 6137 | RPL13 | ribosomal protein L13 | 2 | | 1437 | hsa-miR-623 | 6137 | RPL13 | ribosomal protein L13 | 2 | | 1501 | hsa-mir-628 | 8115 | TCL1A | T-cell leukemia/lymphoma 1A | 2 | | 1519 | hsa-mir-15a | 89870 | TRIM15 | tripartite motif-containing 15 | 2 | | 1520 | hsa-mir-16-1 | 89870 | TRIM15 | tripartite motif-containing 15 | 2 | | 1521 | hsa-miR-126\* | 89870 | TRIM15 | tripartite motif-containing 15 | 2 | | 1522 | hsa-miR-16 | 89870 | TRIM15 | tripartite motif-containing 15 | 2 | | 1523 | hsa-miR-566 | 89870 | TRIM15 | tripartite motif-containing 15 | 2 | | 1524 | hsa-miR-608 | 89870 | TRIM15 | tripartite motif-containing 15 | 2 | | 1559 | hsa-miR-1231 | 843 | CASP10 | caspase 10, apoptosis-related cysteine peptidase | 2 | | 1568 | hsa-miR-190 | 4068 | SH2D1A | SH2 domain protein 1A | 2 | | 1601 | hsa-miR-574-5p | 9577 | BRE | brain and reproductive organ-expressed (TNFRSF1A modulator) | 2 | | 1602 | hsa-miR-634 | 9577 | BRE | brain and reproductive organ-expressed (TNFRSF1A modulator) | 2 | | 1734 | hsa-mir-196b | 1915 | EEF1A1 | eukaryotic translation elongation factor 1 alpha 1 | 2 | | 1735 | hsa-mir-1915 | 1915 | EEF1A1 | eukaryotic translation elongation factor 1 alpha 1 | 2 | | 1736 | hsa-miR-190 | 1915 | EEF1A1 | eukaryotic translation elongation factor 1 alpha 1 | 2 | | 1737 | hsa-miR-196b | 1915 | EEF1A1 | eukaryotic translation elongation factor 1 alpha 1 | 2 | | 1738 | hsa-miR-623 | 1915 | EEF1A1 | eukaryotic translation elongation factor 1 alpha 1 | 2 | | 1746 | hsa-miR-126\* | 10217 | CTDSPL | CTD (carboxy-terminal domain, RNA polymerase II, polypeptide A) small phosphatase-like | 2 | | 1843 | hsa-miR-16 | 4916 | NTRK3 | neurotrophic tyrosine kinase, receptor, type 3 | 2 | | 1891 | hsa-miR-1231 | 1559 | CYP2C9 | cytochrome P450, family 2, subfamily C, polypeptide 9 | 2 | | 1892 | hsa-miR-126\* | 1559 | CYP2C9 | cytochrome P450, family 2, subfamily C, polypeptide 9 | 2 | | 1895 | hsa-miR-608 | 1559 | CYP2C9 | cytochrome P450, family 2, subfamily C, polypeptide 9 | 2 | | 1906 | hsa-mir-424 | 60436 | TGIF2 | TGFB-induced factor homeobox 2 | 2 | | 1907 | hsa-mir-628 | 60436 | TGIF2 | TGFB-induced factor homeobox 2 | 2 | | 1908 | hsa-miR-190 | 60436 | TGIF2 | TGFB-induced factor homeobox 2 | 2 | | 1910 | hsa-miR-503 | 60436 | TGIF2 | TGFB-induced factor homeobox 2 | 2 | | 1912 | hsa-miR-586 | 60436 | TGIF2 | TGFB-induced factor homeobox 2 | 2 | | 1913 | hsa-miR-617 | 60436 | TGIF2 | TGFB-induced factor homeobox 2 | 2 | | 1914 | hsa-miR-623 | 60436 | TGIF2 | TGFB-induced factor homeobox 2 | 2 | | 2001 | hsa-mir-424 | 51520 | LARS | leucyl-tRNA synthetase | 2 | | 2003 | hsa-mir-628 | 51520 | LARS | leucyl-tRNA synthetase | 2 | | 2005 | hsa-miR-190 | 51520 | LARS | leucyl-tRNA synthetase | 2 | | 2007 | hsa-miR-503 | 51520 | LARS | leucyl-tRNA synthetase | 2 | | 2009 | hsa-miR-586 | 51520 | LARS | leucyl-tRNA synthetase | 2 | | 2010 | hsa-miR-617 | 51520 | LARS | leucyl-tRNA synthetase | 2 | | 2055 | hsa-mir-556 | 55278 | QRSL1 | glutaminyl-tRNA synthase (glutamine-hydrolyzing)-like 1 | 2 | | 2271 | hsa-miR-564 | 140803 | TRPM6 | transient receptor potential cation channel, subfamily M, member 6 | 2 | | 2272 | hsa-miR-618 | 140803 | TRPM6 | transient receptor potential cation channel, subfamily M, member 6 | 2 | | 2306 | hsa-mir-15a | 7475 | WNT6 | wingless-type MMTV integration site family, member 6 | 2 | | 2307 | hsa-mir-16-1 | 7475 | WNT6 | wingless-type MMTV integration site family, member 6 | 2 | | 2308 | hsa-miR-126\* | 7475 | WNT6 | wingless-type MMTV integration site family, member 6 | 2 | | 2309 | hsa-miR-16 | 7475 | WNT6 | wingless-type MMTV integration site family, member 6 | 2 | | 2311 | hsa-miR-608 | 7475 | WNT6 | wingless-type MMTV integration site family, member 6 | 2 | | 2321 | hsa-miR-618 | 9683 | N4BP1 | NEDD4 binding protein 1 | 2 | | 2404 | hsa-mir-126 | 55342 | STRBP | spermatid perinuclear RNA binding protein | 2 | | 2471 | hsa-mir-126 | 55814 | BDP1 | B double prime 1, subunit of RNA polymerase III transcription initiation factor IIIB | 2 | | 2472 | hsa-mir-30c-1 | 55814 | BDP1 | B double prime 1, subunit of RNA polymerase III transcription initiation factor IIIB | 2 | | 2473 | hsa-mir-30e | 55814 | BDP1 | B double prime 1, subunit of RNA polymerase III transcription initiation factor IIIB | 2 | | 2474 | hsa-mir-424 | 55814 | BDP1 | B double prime 1, subunit of RNA polymerase III transcription initiation factor IIIB | 2 | | 2477 | hsa-miR-503 | 55814 | BDP1 | B double prime 1, subunit of RNA polymerase III transcription initiation factor IIIB | 2 | | 2492 | hsa-mir-196b | 378938 | MALAT1 | metastasis associated lung adenocarcinoma transcript 1 (non-protein coding) | 2 | | 2494 | hsa-mir-574 | 378938 | MALAT1 | metastasis associated lung adenocarcinoma transcript 1 (non-protein coding) | 2 | | 2496 | hsa-miR-196b | 378938 | MALAT1 | metastasis associated lung adenocarcinoma transcript 1 (non-protein coding) | 2 | | 2670 | hsa-mir-126 | 152137 | CCDC50 | coiled-coil domain containing 50 | 2 | | 2671 | hsa-mir-424 | 152137 | CCDC50 | coiled-coil domain containing 50 | 2 | | 2672 | hsa-mir-556 | 152137 | CCDC50 | coiled-coil domain containing 50 | 2 | | 2674 | hsa-miR-190 | 152137 | CCDC50 | coiled-coil domain containing 50 | 2 | | 2676 | hsa-miR-503 | 152137 | CCDC50 | coiled-coil domain containing 50 | 2 | | 2891 | hsa-mir-30c-1 | 55342 | STRBP | spermatid perinuclear RNA binding protein | 2 | | 2892 | hsa-mir-30e | 55342 | STRBP | spermatid perinuclear RNA binding protein | 2 | | 2895 | hsa-miR-190 | 55342 | STRBP | spermatid perinuclear RNA binding protein | 2 | | 3083 | hsa-miR-586 | 22927 | HABP4 | hyaluronan binding protein 4 | 2 | | 3084 | hsa-miR-617 | 22927 | HABP4 | hyaluronan binding protein 4 | 2 | | 3163 | hsa-miR-608 | 145845 | LOC145845 | hypothetical LOC145845 | 2 | | 1 | hsa-mir-15a | 113026 | PLCD3 | phospholipase C, delta 3 | 1 | | 2 | hsa-mir-16-1 | 113026 | PLCD3 | phospholipase C, delta 3 | 1 | | 3 | hsa-miR-1231 | 113026 | PLCD3 | phospholipase C, delta 3 | 1 | | 4 | hsa-miR-126\* | 113026 | PLCD3 | phospholipase C, delta 3 | 1 | | 5 | hsa-miR-16 | 113026 | PLCD3 | phospholipase C, delta 3 | 1 | | 6 | hsa-miR-608 | 113026 | PLCD3 | phospholipase C, delta 3 | 1 | | 7 | hsa-mir-126 | 5788 | PTPRC | protein tyrosine phosphatase, receptor type, C | 1 | | 8 | hsa-mir-424 | 5788 | PTPRC | protein tyrosine phosphatase, receptor type, C | 1 | | 9 | hsa-miR-503 | 5788 | PTPRC | protein tyrosine phosphatase, receptor type, C | 1 | | 10 | hsa-miR-608 | 146894 | CD300LG | CD300 molecule-like family member g | 1 | | 11 | hsa-miR-126\* | 85439 | STON2 | stonin 2 | 1 | | 12 | hsa-miR-16 | 85439 | STON2 | stonin 2 | 1 | | 13 | hsa-miR-608 | 85439 | STON2 | stonin 2 | 1 | | 14 | hsa-miR-608 | 94160 | ABCC12 | ATP-binding cassette, sub-family C (CFTR/MRP), member 12 | 1 | | 15 | hsa-miR-126\* | 23210 | JMJD6 | jumonji domain containing 6 | 1 | | 16 | hsa-miR-16 | 23210 | JMJD6 | jumonji domain containing 6 | 1 | | 17 | hsa-miR-564 | 23210 | JMJD6 | jumonji domain containing 6 | 1 | | 18 | hsa-miR-608 | 23210 | JMJD6 | jumonji domain containing 6 | 1 | | 19 | hsa-miR-618 | 117584 | RFFL | ring finger and FYVE-like domain containing 1 | 1 | | 20 | hsa-miR-126\* | 6521 | SLC4A1 | solute carrier family 4, anion exchanger, member 1 (erythrocyte membrane protein band 3, Diego blood group) | 1 | | 21 | hsa-miR-586 | 79925 | SPEF2 | sperm flagellar 2 | 1 | | 22 | hsa-miR-617 | 79925 | SPEF2 | sperm flagellar 2 | 1 | | 23 | hsa-miR-1231 | 136259 | KLF14 | Kruppel-like factor 14 | 1 | | 24 | hsa-miR-126\* | 136259 | KLF14 | Kruppel-like factor 14 | 1 | | 25 | hsa-miR-634 | 136259 | KLF14 | Kruppel-like factor 14 | 1 | | 26 | hsa-mir-15a | 282616 | IL28A | interleukin 28A (interferon, lambda 2) | 1 | | 27 | hsa-mir-16-1 | 282616 | IL28A | interleukin 28A (interferon, lambda 2) | 1 | | 28 | hsa-miR-1231 | 282616 | IL28A | interleukin 28A (interferon, lambda 2) | 1 | | 29 | hsa-miR-126\* | 282616 | IL28A | interleukin 28A (interferon, lambda 2) | 1 | | 30 | hsa-miR-16 | 282616 | IL28A | interleukin 28A (interferon, lambda 2) | 1 | | 31 | hsa-miR-566 | 282616 | IL28A | interleukin 28A (interferon, lambda 2) | 1 | | 32 | hsa-miR-608 | 282616 | IL28A | interleukin 28A (interferon, lambda 2) | 1 | | 33 | hsa-mir-15a | 117532 | TMC2 | transmembrane channel-like 2 | 1 | | 34 | hsa-mir-16-1 | 117532 | TMC2 | transmembrane channel-like 2 | 1 | | 35 | hsa-miR-126\* | 117532 | TMC2 | transmembrane channel-like 2 | 1 | | 36 | hsa-miR-16 | 117532 | TMC2 | transmembrane channel-like 2 | 1 | | 37 | hsa-miR-608 | 117532 | TMC2 | transmembrane channel-like 2 | 1 | | 38 | hsa-mir-218-2 | 134553 | C5orf24 | chromosome 5 open reading frame 24 | 1 | | 39 | hsa-mir-126 | 134553 | C5orf24 | chromosome 5 open reading frame 24 | 1 | | 40 | hsa-mir-556 | 134553 | C5orf24 | chromosome 5 open reading frame 24 | 1 | | 41 | hsa-mir-1915 | 134553 | C5orf24 | chromosome 5 open reading frame 24 | 1 | | 42 | hsa-miR-190 | 134553 | C5orf24 | chromosome 5 open reading frame 24 | 1 | | 43 | hsa-miR-585 | 134553 | C5orf24 | chromosome 5 open reading frame 24 | 1 | | 44 | hsa-miR-608 | 5753 | PTK6 | PTK6 protein tyrosine kinase 6 | 1 | | 45 | hsa-miR-190 | 57786 | RBAK | RB-associated KRAB zinc finger | 1 | | 46 | hsa-miR-574-5p | 203228 | C9orf72 | chromosome 9 open reading frame 72 | 1 | | 47 | hsa-mir-15a | 153579 | BTNL9 | butyrophilin-like 9 | 1 | | 48 | hsa-mir-16-1 | 153579 | BTNL9 | butyrophilin-like 9 | 1 | | 49 | hsa-miR-126\* | 153579 | BTNL9 | butyrophilin-like 9 | 1 | | 50 | hsa-miR-16 | 153579 | BTNL9 | butyrophilin-like 9 | 1 | | 51 | hsa-miR-564 | 153579 | BTNL9 | butyrophilin-like 9 | 1 | | 52 | hsa-miR-608 | 153579 | BTNL9 | butyrophilin-like 9 | 1 | | 53 | hsa-mir-15a | 6571 | SLC18A2 | solute carrier family 18 (vesicular monoamine), member 2 | 1 | | 54 | hsa-mir-16-1 | 6571 | SLC18A2 | solute carrier family 18 (vesicular monoamine), member 2 | 1 | | 55 | hsa-miR-126\* | 6571 | SLC18A2 | solute carrier family 18 (vesicular monoamine), member 2 | 1 | | 56 | hsa-miR-16 | 6571 | SLC18A2 | solute carrier family 18 (vesicular monoamine), member 2 | 1 | | 57 | hsa-miR-608 | 6571 | SLC18A2 | solute carrier family 18 (vesicular monoamine), member 2 | 1 | | 58 | hsa-mir-15a | 128178 | EDARADD | EDAR-associated death domain | 1 | | 59 | hsa-mir-16-1 | 128178 | EDARADD | EDAR-associated death domain | 1 | | 60 | hsa-miR-126\* | 128178 | EDARADD | EDAR-associated death domain | 1 | | 61 | hsa-miR-16 | 128178 | EDARADD | EDAR-associated death domain | 1 | | 62 | hsa-miR-608 | 128178 | EDARADD | EDAR-associated death domain | 1 | | 63 | hsa-mir-15a | 142686 | ASB14 | ankyrin repeat and SOCS box-containing 14 | 1 | | 64 | hsa-mir-16-1 | 142686 | ASB14 | ankyrin repeat and SOCS box-containing 14 | 1 | | 65 | hsa-miR-608 | 142686 | ASB14 | ankyrin repeat and SOCS box-containing 14 | 1 | | 66 | hsa-miR-126\* | 439943 | CXorf62 | chromosome X open reading frame 62 | 1 | | 67 | hsa-miR-16 | 439943 | CXorf62 | chromosome X open reading frame 62 | 1 | | 68 | hsa-miR-564 | 439943 | CXorf62 | chromosome X open reading frame 62 | 1 | | 69 | hsa-miR-608 | 439943 | CXorf62 | chromosome X open reading frame 62 | 1 | | 70 | hsa-mir-15a | 145942 | TMCO5A | transmembrane and coiled-coil domains 5A | 1 | | 71 | hsa-mir-16-1 | 145942 | TMCO5A | transmembrane and coiled-coil domains 5A | 1 | | 72 | hsa-miR-1231 | 145942 | TMCO5A | transmembrane and coiled-coil domains 5A | 1 | | 73 | hsa-miR-126\* | 145942 | TMCO5A | transmembrane and coiled-coil domains 5A | 1 | | 74 | hsa-miR-608 | 145942 | TMCO5A | transmembrane and coiled-coil domains 5A | 1 | | 75 | hsa-miR-126\* | 6975 | TECTB | tectorin beta | 1 | | 76 | hsa-miR-608 | 6975 | TECTB | tectorin beta | 1 | | 77 | hsa-mir-218-2 | 148266 | ZNF569 | zinc finger protein 569 | 1 | | 78 | hsa-mir-126 | 148266 | ZNF569 | zinc finger protein 569 | 1 | | 79 | hsa-mir-30c-1 | 148266 | ZNF569 | zinc finger protein 569 | 1 | | 80 | hsa-mir-30e | 148266 | ZNF569 | zinc finger protein 569 | 1 | | 81 | hsa-mir-424 | 148266 | ZNF569 | zinc finger protein 569 | 1 | | 82 | hsa-mir-556 | 148266 | ZNF569 | zinc finger protein 569 | 1 | | 83 | hsa-miR-190 | 148266 | ZNF569 | zinc finger protein 569 | 1 | | 84 | hsa-miR-338-5p | 148266 | ZNF569 | zinc finger protein 569 | 1 | | 85 | hsa-miR-503 | 148266 | ZNF569 | zinc finger protein 569 | 1 | | 86 | hsa-miR-585 | 148266 | ZNF569 | zinc finger protein 569 | 1 | | 87 | hsa-miR-586 | 148266 | ZNF569 | zinc finger protein 569 | 1 | | 88 | hsa-miR-617 | 148266 | ZNF569 | zinc finger protein 569 | 1 | | 89 | hsa-miR-657 | 148266 | ZNF569 | zinc finger protein 569 | 1 | | 90 | hsa-mir-15a | 55132 | LARP1B | La ribonucleoprotein domain family, member 1B | 1 | | 91 | hsa-mir-16-1 | 55132 | LARP1B | La ribonucleoprotein domain family, member 1B | 1 | | 92 | hsa-miR-126\* | 55132 | LARP1B | La ribonucleoprotein domain family, member 1B | 1 | | 93 | hsa-miR-16 | 55132 | LARP1B | La ribonucleoprotein domain family, member 1B | 1 | | 94 | hsa-miR-608 | 55132 | LARP1B | La ribonucleoprotein domain family, member 1B | 1 | | 95 | hsa-mir-126 | 65065 | NBEAL1 | neurobeachin-like 1 | 1 | | 96 | hsa-mir-196b | 65065 | NBEAL1 | neurobeachin-like 1 | 1 | | 97 | hsa-mir-424 | 65065 | NBEAL1 | neurobeachin-like 1 | 1 | | 98 | hsa-mir-490 | 65065 | NBEAL1 | neurobeachin-like 1 | 1 | | 99 | hsa-mir-556 | 65065 | NBEAL1 | neurobeachin-like 1 | 1 | | 100 | hsa-mir-628 | 65065 | NBEAL1 | neurobeachin-like 1 | 1 | | 101 | hsa-miR-190 | 65065 | NBEAL1 | neurobeachin-like 1 | 1 | | 102 | hsa-miR-196b | 65065 | NBEAL1 | neurobeachin-like 1 | 1 | | 103 | hsa-miR-490-5p | 65065 | NBEAL1 | neurobeachin-like 1 | 1 | | 104 | hsa-miR-503 | 65065 | NBEAL1 | neurobeachin-like 1 | 1 | | 105 | hsa-miR-617 | 65065 | NBEAL1 | neurobeachin-like 1 | 1 | | 106 | hsa-miR-623 | 65065 | NBEAL1 | neurobeachin-like 1 | 1 | | 107 | hsa-mir-15a | 26751 | SH3YL1 | SH3 domain containing, Ysc84-like 1 (S. cerevisiae) | 1 | | 108 | hsa-mir-16-1 | 26751 | SH3YL1 | SH3 domain containing, Ysc84-like 1 (S. cerevisiae) | 1 | | 109 | hsa-miR-126\* | 26751 | SH3YL1 | SH3 domain containing, Ysc84-like 1 (S. cerevisiae) | 1 | | 110 | hsa-miR-16 | 26751 | SH3YL1 | SH3 domain containing, Ysc84-like 1 (S. cerevisiae) | 1 | | 111 | hsa-miR-608 | 26751 | SH3YL1 | SH3 domain containing, Ysc84-like 1 (S. cerevisiae) | 1 | | 112 | hsa-miR-16 | 3176 | HNMT | histamine N-methyltransferase | 1 | | 113 | hsa-miR-608 | 3176 | HNMT | histamine N-methyltransferase | 1 | | 114 | hsa-miR-126\* | 113451 | ADC | arginine decarboxylase | 1 | | 115 | hsa-miR-16 | 113451 | ADC | arginine decarboxylase | 1 | | 116 | hsa-miR-564 | 113451 | ADC | arginine decarboxylase | 1 | | 117 | hsa-miR-566 | 113451 | ADC | arginine decarboxylase | 1 | | 118 | hsa-miR-608 | 113451 | ADC | arginine decarboxylase | 1 | | 119 | hsa-mir-15a | 5271 | SERPINB8 | serpin peptidase inhibitor, clade B (ovalbumin), member 8 | 1 | | 120 | hsa-mir-16-1 | 5271 | SERPINB8 | serpin peptidase inhibitor, clade B (ovalbumin), member 8 | 1 | | 121 | hsa-miR-1231 | 5271 | SERPINB8 | serpin peptidase inhibitor, clade B (ovalbumin), member 8 | 1 | | 122 | hsa-miR-126\* | 5271 | SERPINB8 | serpin peptidase inhibitor, clade B (ovalbumin), member 8 | 1 | | 123 | hsa-miR-16 | 5271 | SERPINB8 | serpin peptidase inhibitor, clade B (ovalbumin), member 8 | 1 | | 124 | hsa-miR-574-5p | 5271 | SERPINB8 | serpin peptidase inhibitor, clade B (ovalbumin), member 8 | 1 | | 125 | hsa-miR-608 | 5271 | SERPINB8 | serpin peptidase inhibitor, clade B (ovalbumin), member 8 | 1 | | 126 | hsa-miR-608 | 100288447 | LOC100288447 | hypothetical protein LOC100288447 | 1 | | 127 | hsa-mir-15a | 80725 | SRCIN1 | SRC kinase signaling inhibitor 1 | 1 | | 128 | hsa-mir-16-1 | 80725 | SRCIN1 | SRC kinase signaling inhibitor 1 | 1 | | 129 | hsa-miR-126\* | 80725 | SRCIN1 | SRC kinase signaling inhibitor 1 | 1 | | 130 | hsa-miR-16 | 80725 | SRCIN1 | SRC kinase signaling inhibitor 1 | 1 | | 131 | hsa-miR-608 | 80725 | SRCIN1 | SRC kinase signaling inhibitor 1 | 1 | | 138 | hsa-mir-556 | 146540 | ZNF785 | zinc finger protein 785 | 1 | | 139 | hsa-miR-338-5p | 146540 | ZNF785 | zinc finger protein 785 | 1 | | 140 | hsa-miR-617 | 146540 | ZNF785 | zinc finger protein 785 | 1 | | 141 | hsa-miR-657 | 146540 | ZNF785 | zinc finger protein 785 | 1 | | 142 | hsa-miR-608 | 54734 | RAB39 | RAB39, member RAS oncogene family | 1 | | 143 | hsa-miR-608 | 55816 | DOK5 | docking protein 5 | 1 | | 144 | hsa-mir-339 | 85320 | ABCC11 | ATP-binding cassette, sub-family C (CFTR/MRP), member 11 | 1 | | 145 | hsa-miR-339-5p | 85320 | ABCC11 | ATP-binding cassette, sub-family C (CFTR/MRP), member 11 | 1 | | 146 | hsa-miR-564 | 85320 | ABCC11 | ATP-binding cassette, sub-family C (CFTR/MRP), member 11 | 1 | | 147 | hsa-miR-608 | 85320 | ABCC11 | ATP-binding cassette, sub-family C (CFTR/MRP), member 11 | 1 | | 148 | hsa-mir-15a | 176 | ACAN | aggrecan | 1 | | 149 | hsa-mir-16-1 | 176 | ACAN | aggrecan | 1 | | 150 | hsa-miR-126\* | 176 | ACAN | aggrecan | 1 | | 151 | hsa-miR-608 | 176 | ACAN | aggrecan | 1 | | 152 | hsa-miR-566 | 399979 | SNX19 | sorting nexin 19 | 1 | | 153 | hsa-mir-15a | 26238 | C6orf123 | chromosome 6 open reading frame 123 | 1 | | 154 | hsa-mir-16-1 | 26238 | C6orf123 | chromosome 6 open reading frame 123 | 1 | | 155 | hsa-miR-126\* | 26238 | C6orf123 | chromosome 6 open reading frame 123 | 1 | | 156 | hsa-miR-608 | 26238 | C6orf123 | chromosome 6 open reading frame 123 | 1 | | 157 | hsa-mir-339 | 117583 | PARD3B | par-3 partitioning defective 3 homolog B (C. elegans) | 1 | | 158 | hsa-miR-339-5p | 117583 | PARD3B | par-3 partitioning defective 3 homolog B (C. elegans) | 1 | | 159 | hsa-mir-15a | 8863 | PER3 | period homolog 3 (Drosophila) | 1 | | 160 | hsa-mir-16-1 | 8863 | PER3 | period homolog 3 (Drosophila) | 1 | | 161 | hsa-miR-126\* | 8863 | PER3 | period homolog 3 (Drosophila) | 1 | | 162 | hsa-miR-16 | 8863 | PER3 | period homolog 3 (Drosophila) | 1 | | 163 | hsa-miR-608 | 8863 | PER3 | period homolog 3 (Drosophila) | 1 | | 164 | hsa-mir-15a | 553158 | PRR5-ARHGAP8 | PRR5-ARHGAP8 readthrough | 1 | | 165 | hsa-mir-16-1 | 553158 | PRR5-ARHGAP8 | PRR5-ARHGAP8 readthrough | 1 | | 166 | hsa-miR-126\* | 553158 | PRR5-ARHGAP8 | PRR5-ARHGAP8 readthrough | 1 | | 167 | hsa-miR-16 | 553158 | PRR5-ARHGAP8 | PRR5-ARHGAP8 readthrough | 1 | | 168 | hsa-miR-564 | 553158 | PRR5-ARHGAP8 | PRR5-ARHGAP8 readthrough | 1 | | 169 | hsa-miR-608 | 553158 | PRR5-ARHGAP8 | PRR5-ARHGAP8 readthrough | 1 | | 170 | hsa-miR-126\* | 55252 | ASXL2 | additional sex combs like 2 (Drosophila) | 1 | | 171 | hsa-miR-566 | 55252 | ASXL2 | additional sex combs like 2 (Drosophila) | 1 | | 172 | hsa-miR-126\* | 89872 | AQP10 | aquaporin 10 | 1 | | 173 | hsa-miR-1231 | 54937 | SOHLH2 | spermatogenesis and oogenesis specific basic helix-loop-helix 2 | 1 | | 174 | hsa-miR-126\* | 54937 | SOHLH2 | spermatogenesis and oogenesis specific basic helix-loop-helix 2 | 1 | | 175 | hsa-miR-608 | 54937 | SOHLH2 | spermatogenesis and oogenesis specific basic helix-loop-helix 2 | 1 | | 176 | hsa-miR-1231 | 374355 | C10orf96 | chromosome 10 open reading frame 96 | 1 | | 177 | hsa-miR-126\* | 374355 | C10orf96 | chromosome 10 open reading frame 96 | 1 | | 178 | hsa-miR-608 | 374355 | C10orf96 | chromosome 10 open reading frame 96 | 1 | | 179 | hsa-mir-15a | 151242 | PPP1R1C | protein phosphatase 1, regulatory (inhibitor) subunit 1C | 1 | | 180 | hsa-mir-16-1 | 151242 | PPP1R1C | protein phosphatase 1, regulatory (inhibitor) subunit 1C | 1 | | 182 | hsa-miR-126\* | 151242 | PPP1R1C | protein phosphatase 1, regulatory (inhibitor) subunit 1C | 1 | | 183 | hsa-miR-608 | 151242 | PPP1R1C | protein phosphatase 1, regulatory (inhibitor) subunit 1C | 1 | | 184 | hsa-mir-218-2 | 84186 | ZCCHC7 | zinc finger, CCHC domain containing 7 | 1 | | 185 | hsa-mir-126 | 84186 | ZCCHC7 | zinc finger, CCHC domain containing 7 | 1 | | 186 | hsa-mir-196b | 84186 | ZCCHC7 | zinc finger, CCHC domain containing 7 | 1 | | 187 | hsa-mir-424 | 84186 | ZCCHC7 | zinc finger, CCHC domain containing 7 | 1 | | 188 | hsa-mir-490 | 84186 | ZCCHC7 | zinc finger, CCHC domain containing 7 | 1 | | 189 | hsa-mir-556 | 84186 | ZCCHC7 | zinc finger, CCHC domain containing 7 | 1 | | 190 | hsa-mir-1915 | 84186 | ZCCHC7 | zinc finger, CCHC domain containing 7 | 1 | | 191 | hsa-miR-190 | 84186 | ZCCHC7 | zinc finger, CCHC domain containing 7 | 1 | | 192 | hsa-miR-196b | 84186 | ZCCHC7 | zinc finger, CCHC domain containing 7 | 1 | | 193 | hsa-miR-490-5p | 84186 | ZCCHC7 | zinc finger, CCHC domain containing 7 | 1 | | 194 | hsa-miR-503 | 84186 | ZCCHC7 | zinc finger, CCHC domain containing 7 | 1 | | 195 | hsa-miR-585 | 84186 | ZCCHC7 | zinc finger, CCHC domain containing 7 | 1 | | 196 | hsa-miR-623 | 84186 | ZCCHC7 | zinc finger, CCHC domain containing 7 | 1 | | 197 | hsa-miR-126\* | 574 | BAGE | B melanoma antigen | 1 | | 198 | hsa-miR-566 | 574 | BAGE | B melanoma antigen | 1 | | 199 | hsa-mir-15a | 144124 | OR10A5 | olfactory receptor, family 10, subfamily A, member 5 | 1 | | 200 | hsa-mir-16-1 | 144124 | OR10A5 | olfactory receptor, family 10, subfamily A, member 5 | 1 | | 201 | hsa-miR-16 | 144124 | OR10A5 | olfactory receptor, family 10, subfamily A, member 5 | 1 | | 202 | hsa-miR-608 | 144124 | OR10A5 | olfactory receptor, family 10, subfamily A, member 5 | 1 | | 203 | hsa-mir-218-2 | 84816 | RTN4IP1 | reticulon 4 interacting protein 1 | 1 | | 204 | hsa-mir-126 | 84816 | RTN4IP1 | reticulon 4 interacting protein 1 | 1 | | 205 | hsa-mir-424 | 84816 | RTN4IP1 | reticulon 4 interacting protein 1 | 1 | | 206 | hsa-mir-556 | 84816 | RTN4IP1 | reticulon 4 interacting protein 1 | 1 | | 207 | hsa-miR-190 | 84816 | RTN4IP1 | reticulon 4 interacting protein 1 | 1 | | 208 | hsa-miR-503 | 84816 | RTN4IP1 | reticulon 4 interacting protein 1 | 1 | | 209 | hsa-miR-585 | 84816 | RTN4IP1 | reticulon 4 interacting protein 1 | 1 | | 210 | hsa-mir-339 | 7401 | CLRN1 | clarin 1 | 1 | | 211 | hsa-miR-1231 | 7401 | CLRN1 | clarin 1 | 1 | | 212 | hsa-miR-339-5p | 7401 | CLRN1 | clarin 1 | 1 | | 213 | hsa-miR-608 | 7401 | CLRN1 | clarin 1 | 1 | | 214 | hsa-mir-454 | 974 | CD79B | CD79b molecule, immunoglobulin-associated beta | 1 | | 215 | hsa-miR-675 | 974 | CD79B | CD79b molecule, immunoglobulin-associated beta | 1 | | 216 | hsa-miR-95 | 974 | CD79B | CD79b molecule, immunoglobulin-associated beta | 1 | | 217 | hsa-mir-196b | 973 | CD79A | CD79a molecule, immunoglobulin-associated alpha | 1 | | 218 | hsa-miR-196b | 973 | CD79A | CD79a molecule, immunoglobulin-associated alpha | 1 | | 220 | hsa-miR-650 | 973 | CD79A | CD79a molecule, immunoglobulin-associated alpha | 1 | | 221 | hsa-mir-218-2 | 284406 | ZFP82 | zinc finger protein 82 homolog (mouse) | 1 | | 222 | hsa-mir-126 | 284406 | ZFP82 | zinc finger protein 82 homolog (mouse) | 1 | | 223 | hsa-mir-424 | 284406 | ZFP82 | zinc finger protein 82 homolog (mouse) | 1 | | 224 | hsa-mir-556 | 284406 | ZFP82 | zinc finger protein 82 homolog (mouse) | 1 | | 225 | hsa-mir-628 | 284406 | ZFP82 | zinc finger protein 82 homolog (mouse) | 1 | | 226 | hsa-miR-190 | 284406 | ZFP82 | zinc finger protein 82 homolog (mouse) | 1 | | 227 | hsa-miR-503 | 284406 | ZFP82 | zinc finger protein 82 homolog (mouse) | 1 | | 228 | hsa-miR-585 | 284406 | ZFP82 | zinc finger protein 82 homolog (mouse) | 1 | | 229 | hsa-miR-586 | 284406 | ZFP82 | zinc finger protein 82 homolog (mouse) | 1 | | 230 | hsa-miR-617 | 284406 | ZFP82 | zinc finger protein 82 homolog (mouse) | 1 | | 231 | hsa-mir-454 | 10092 | ARPC5 | actin related protein 2/3 complex, subunit 5, 16kDa | 1 | | 232 | hsa-mir-15a | 55218 | EXD2 | exonuclease 3'-5' domain containing 2 | 1 | | 233 | hsa-mir-16-1 | 55218 | EXD2 | exonuclease 3'-5' domain containing 2 | 1 | | 234 | hsa-miR-608 | 55218 | EXD2 | exonuclease 3'-5' domain containing 2 | 1 | | 235 | hsa-mir-15a | 399726 | C10orf114 | chromosome 10 open reading frame 114 | 1 | | 236 | hsa-mir-16-1 | 399726 | C10orf114 | chromosome 10 open reading frame 114 | 1 | | 237 | hsa-miR-126\* | 399726 | C10orf114 | chromosome 10 open reading frame 114 | 1 | | 238 | hsa-miR-16 | 399726 | C10orf114 | chromosome 10 open reading frame 114 | 1 | | 239 | hsa-miR-566 | 399726 | C10orf114 | chromosome 10 open reading frame 114 | 1 | | 240 | hsa-miR-608 | 399726 | C10orf114 | chromosome 10 open reading frame 114 | 1 | | 241 | hsa-mir-126 | 3094 | HINT1 | histidine triad nucleotide binding protein 1 | 1 | | 242 | hsa-mir-30c-1 | 3094 | HINT1 | histidine triad nucleotide binding protein 1 | 1 | | 243 | hsa-mir-30e | 3094 | HINT1 | histidine triad nucleotide binding protein 1 | 1 | | 244 | hsa-mir-424 | 3094 | HINT1 | histidine triad nucleotide binding protein 1 | 1 | | 245 | hsa-mir-556 | 3094 | HINT1 | histidine triad nucleotide binding protein 1 | 1 | | 246 | hsa-mir-628 | 3094 | HINT1 | histidine triad nucleotide binding protein 1 | 1 | | 247 | hsa-miR-190 | 3094 | HINT1 | histidine triad nucleotide binding protein 1 | 1 | | 248 | hsa-miR-338-5p | 3094 | HINT1 | histidine triad nucleotide binding protein 1 | 1 | | 249 | hsa-miR-503 | 3094 | HINT1 | histidine triad nucleotide binding protein 1 | 1 | | 250 | hsa-miR-586 | 3094 | HINT1 | histidine triad nucleotide binding protein 1 | 1 | | 251 | hsa-miR-617 | 3094 | HINT1 | histidine triad nucleotide binding protein 1 | 1 | | 252 | hsa-miR-657 | 3094 | HINT1 | histidine triad nucleotide binding protein 1 | 1 | | 253 | hsa-mir-135b | 388685 | FLJ39739 | hypothetical FLJ39739 | 1 | | 254 | hsa-mir-675 | 388685 | FLJ39739 | hypothetical FLJ39739 | 1 | | 255 | hsa-miR-454\* | 388685 | FLJ39739 | hypothetical FLJ39739 | 1 | | 256 | hsa-miR-1231 | 54328 | GPR173 | G protein-coupled receptor 173 | 1 | | 257 | hsa-miR-608 | 54328 | GPR173 | G protein-coupled receptor 173 | 1 | | 258 | hsa-mir-30c-1 | 636 | BICD1 | bicaudal D homolog 1 (Drosophila) | 1 | | 259 | hsa-mir-30e | 636 | BICD1 | bicaudal D homolog 1 (Drosophila) | 1 | | 260 | hsa-miR-30c | 636 | BICD1 | bicaudal D homolog 1 (Drosophila) | 1 | | 261 | hsa-miR-30e | 636 | BICD1 | bicaudal D homolog 1 (Drosophila) | 1 | | 262 | hsa-mir-218-2 | 84268 | RPAIN | RPA interacting protein | 1 | | 263 | hsa-mir-126 | 84268 | RPAIN | RPA interacting protein | 1 | | 265 | hsa-mir-556 | 84268 | RPAIN | RPA interacting protein | 1 | | 269 | hsa-miR-585 | 84268 | RPAIN | RPA interacting protein | 1 | | 272 | hsa-miR-1231 | 415056 | LOC415056 | hypothetical LOC415056 | 1 | | 273 | hsa-miR-126\* | 415056 | LOC415056 | hypothetical LOC415056 | 1 | | 274 | hsa-miR-574-5p | 415056 | LOC415056 | hypothetical LOC415056 | 1 | | 275 | hsa-miR-608 | 415056 | LOC415056 | hypothetical LOC415056 | 1 | | 276 | hsa-mir-15a | 114815 | SORCS1 | sortilin-related VPS10 domain containing receptor 1 | 1 | | 277 | hsa-mir-16-1 | 114815 | SORCS1 | sortilin-related VPS10 domain containing receptor 1 | 1 | | 278 | hsa-mir-25 | 114815 | SORCS1 | sortilin-related VPS10 domain containing receptor 1 | 1 | | 279 | hsa-mir-93 | 114815 | SORCS1 | sortilin-related VPS10 domain containing receptor 1 | 1 | | 280 | hsa-mir-106b | 114815 | SORCS1 | sortilin-related VPS10 domain containing receptor 1 | 1 | | 281 | hsa-miR-16 | 114815 | SORCS1 | sortilin-related VPS10 domain containing receptor 1 | 1 | | 282 | hsa-miR-608 | 114815 | SORCS1 | sortilin-related VPS10 domain containing receptor 1 | 1 | | 283 | hsa-miR-1231 | 550113 | LOC550113 | hypothetical LOC550113 | 1 | | 284 | hsa-miR-126\* | 550113 | LOC550113 | hypothetical LOC550113 | 1 | | 285 | hsa-miR-608 | 550113 | LOC550113 | hypothetical LOC550113 | 1 | | 286 | hsa-miR-548d-5p | 158228 | C9orf122 | chromosome 9 open reading frame 122 | 1 | | 287 | hsa-miR-608 | 100289373 | LOC100289373 | similar to hCG2041645 | 1 | | 292 | hsa-miR-617 | 1915 | EEF1A1 | eukaryotic translation elongation factor 1 alpha 1 | 1 | | 293 | hsa-mir-15a | 440982 | FLJ30375 | hypothetical protein LOC440982 | 1 | | 294 | hsa-mir-16-1 | 440982 | FLJ30375 | hypothetical protein LOC440982 | 1 | | 295 | hsa-miR-126\* | 440982 | FLJ30375 | hypothetical protein LOC440982 | 1 | | 296 | hsa-miR-16 | 440982 | FLJ30375 | hypothetical protein LOC440982 | 1 | | 297 | hsa-miR-608 | 440982 | FLJ30375 | hypothetical protein LOC440982 | 1 | | 298 | hsa-miR-126\* | 55283 | MCOLN3 | mucolipin 3 | 1 | | 299 | hsa-miR-16 | 55283 | MCOLN3 | mucolipin 3 | 1 | | 300 | hsa-miR-608 | 55283 | MCOLN3 | mucolipin 3 | 1 | | 301 | hsa-miR-126\* | 375189 | PFN4 | profilin family, member 4 | 1 | | 302 | hsa-miR-16 | 375189 | PFN4 | profilin family, member 4 | 1 | | 303 | hsa-mir-339 | 158067 | C9orf98 | chromosome 9 open reading frame 98 | 1 | | 304 | hsa-miR-1231 | 158067 | C9orf98 | chromosome 9 open reading frame 98 | 1 | | 305 | hsa-miR-339-5p | 158067 | C9orf98 | chromosome 9 open reading frame 98 | 1 | | 306 | hsa-miR-608 | 158067 | C9orf98 | chromosome 9 open reading frame 98 | 1 | | 307 | hsa-mir-126 | 64844 | MARCH7 | membrane-associated ring finger (C3HC4) 7 | 1 | | 308 | hsa-miR-190 | 64844 | MARCH7 | membrane-associated ring finger (C3HC4) 7 | 1 | | 309 | hsa-mir-196b | 4820 | NKTR | natural killer-tumor recognition sequence | 1 | | 310 | hsa-mir-628 | 4820 | NKTR | natural killer-tumor recognition sequence | 1 | | 311 | hsa-miR-196b | 4820 | NKTR | natural killer-tumor recognition sequence | 1 | | 312 | hsa-mir-218-2 | 7329 | UBE2I | ubiquitin-conjugating enzyme E2I (UBC9 homolog, yeast) | 1 | | 314 | hsa-mir-196b | 7329 | UBE2I | ubiquitin-conjugating enzyme E2I (UBC9 homolog, yeast) | 1 | | 316 | hsa-mir-490 | 7329 | UBE2I | ubiquitin-conjugating enzyme E2I (UBC9 homolog, yeast) | 1 | | 317 | hsa-mir-556 | 7329 | UBE2I | ubiquitin-conjugating enzyme E2I (UBC9 homolog, yeast) | 1 | | 320 | hsa-miR-196b | 7329 | UBE2I | ubiquitin-conjugating enzyme E2I (UBC9 homolog, yeast) | 1 | | 321 | hsa-miR-490-5p | 7329 | UBE2I | ubiquitin-conjugating enzyme E2I (UBC9 homolog, yeast) | 1 | | 323 | hsa-miR-585 | 7329 | UBE2I | ubiquitin-conjugating enzyme E2I (UBC9 homolog, yeast) | 1 | | 325 | hsa-miR-623 | 7329 | UBE2I | ubiquitin-conjugating enzyme E2I (UBC9 homolog, yeast) | 1 | | 326 | hsa-mir-126 | 9782 | MATR3 | matrin 3 | 1 | | 327 | hsa-mir-424 | 9782 | MATR3 | matrin 3 | 1 | | 328 | hsa-mir-490 | 9782 | MATR3 | matrin 3 | 1 | | 329 | hsa-mir-1915 | 9782 | MATR3 | matrin 3 | 1 | | 330 | hsa-mir-3126 | 9782 | MATR3 | matrin 3 | 1 | | 331 | hsa-miR-190 | 9782 | MATR3 | matrin 3 | 1 | | 332 | hsa-miR-490-5p | 9782 | MATR3 | matrin 3 | 1 | | 333 | hsa-miR-503 | 9782 | MATR3 | matrin 3 | 1 | | 334 | hsa-miR-623 | 9782 | MATR3 | matrin 3 | 1 | | 335 | hsa-miR-944 | 9782 | MATR3 | matrin 3 | 1 | | 336 | hsa-miR-126\* | 113230 | LOC113230 | hypothetical protein LOC113230 | 1 | | 337 | hsa-miR-608 | 113230 | LOC113230 | hypothetical protein LOC113230 | 1 | | 338 | hsa-miR-608 | 100128687 | LOC100128687 | hypothetical protein LOC100128687 | 1 | | 342 | hsa-mir-196b | 84268 | RPAIN | RPA interacting protein | 1 | | 343 | hsa-miR-196b | 84268 | RPAIN | RPA interacting protein | 1 | | 344 | hsa-miR-623 | 84268 | RPAIN | RPA interacting protein | 1 | | 345 | hsa-mir-15a | 389170 | LEKR1 | leucine, glutamate and lysine rich 1 | 1 | | 346 | hsa-mir-16-1 | 389170 | LEKR1 | leucine, glutamate and lysine rich 1 | 1 | | 347 | hsa-miR-126\* | 389170 | LEKR1 | leucine, glutamate and lysine rich 1 | 1 | | 348 | hsa-miR-16 | 389170 | LEKR1 | leucine, glutamate and lysine rich 1 | 1 | | 349 | hsa-miR-608 | 389170 | LEKR1 | leucine, glutamate and lysine rich 1 | 1 | | 350 | hsa-miR-126\* | 4802 | NFYC | nuclear transcription factor Y, gamma | 1 | | 351 | hsa-miR-566 | 4802 | NFYC | nuclear transcription factor Y, gamma | 1 | | 352 | hsa-miR-608 | 9223 | MAGI1 | membrane associated guanylate kinase, WW and PDZ domain containing 1 | 1 | | 353 | hsa-miR-608 | 388182 | FLJ42289 | hypothetical LOC388182 | 1 | | 354 | hsa-miR-618 | 388182 | FLJ42289 | hypothetical LOC388182 | 1 | | 355 | hsa-miR-634 | 388182 | FLJ42289 | hypothetical LOC388182 | 1 | | 356 | hsa-mir-454 | 55349 | CHDH | choline dehydrogenase | 1 | | 357 | hsa-miR-564 | 55349 | CHDH | choline dehydrogenase | 1 | | 358 | hsa-miR-675 | 55349 | CHDH | choline dehydrogenase | 1 | | 359 | hsa-miR-95 | 55349 | CHDH | choline dehydrogenase | 1 | | 360 | hsa-mir-424 | 9441 | MED26 | mediator complex subunit 26 | 1 | | 361 | hsa-mir-628 | 9441 | MED26 | mediator complex subunit 26 | 1 | | 362 | hsa-miR-190 | 9441 | MED26 | mediator complex subunit 26 | 1 | | 363 | hsa-miR-503 | 9441 | MED26 | mediator complex subunit 26 | 1 | | 364 | hsa-miR-126\* | 641384 | TMEM75 | transmembrane protein 75 | 1 | | 365 | hsa-miR-566 | 641384 | TMEM75 | transmembrane protein 75 | 1 | | 366 | hsa-mir-126 | 55216 | C11orf57 | chromosome 11 open reading frame 57 | 1 | | 367 | hsa-mir-30c-1 | 55216 | C11orf57 | chromosome 11 open reading frame 57 | 1 | | 368 | hsa-mir-30e | 55216 | C11orf57 | chromosome 11 open reading frame 57 | 1 | | 369 | hsa-mir-424 | 55216 | C11orf57 | chromosome 11 open reading frame 57 | 1 | | 370 | hsa-miR-190 | 55216 | C11orf57 | chromosome 11 open reading frame 57 | 1 | | 371 | hsa-miR-338-5p | 55216 | C11orf57 | chromosome 11 open reading frame 57 | 1 | | 372 | hsa-miR-503 | 55216 | C11orf57 | chromosome 11 open reading frame 57 | 1 | | 373 | hsa-miR-657 | 55216 | C11orf57 | chromosome 11 open reading frame 57 | 1 | | 374 | hsa-mir-15a | 1588 | CYP19A1 | cytochrome P450, family 19, subfamily A, polypeptide 1 | 1 | | 375 | hsa-mir-16-1 | 1588 | CYP19A1 | cytochrome P450, family 19, subfamily A, polypeptide 1 | 1 | | 376 | hsa-mir-339 | 1588 | CYP19A1 | cytochrome P450, family 19, subfamily A, polypeptide 1 | 1 | | 377 | hsa-miR-16 | 1588 | CYP19A1 | cytochrome P450, family 19, subfamily A, polypeptide 1 | 1 | | 378 | hsa-miR-339-5p | 1588 | CYP19A1 | cytochrome P450, family 19, subfamily A, polypeptide 1 | 1 | | 379 | hsa-miR-564 | 1588 | CYP19A1 | cytochrome P450, family 19, subfamily A, polypeptide 1 | 1 | | 380 | hsa-miR-608 | 1588 | CYP19A1 | cytochrome P450, family 19, subfamily A, polypeptide 1 | 1 | | 381 | hsa-miR-675 | 497256 | LOC497256 | hypothetical LOC497256 | 1 | | 382 | hsa-miR-95 | 497256 | LOC497256 | hypothetical LOC497256 | 1 | | 383 | hsa-miR-608 | 338588 | LOC338588 | hypothetical LOC338588 | 1 | | 384 | hsa-miR-126\* | 728789 | LOC728789 | hypothetical LOC728789 | 1 | | 386 | hsa-mir-15a | 100287584 | LOC100287584 | hypothetical protein LOC100287584 | 1 | | 387 | hsa-mir-16-1 | 100287584 | LOC100287584 | hypothetical protein LOC100287584 | 1 | | 388 | hsa-miR-1231 | 100287584 | LOC100287584 | hypothetical protein LOC100287584 | 1 | | 389 | hsa-miR-608 | 100287584 | LOC100287584 | hypothetical protein LOC100287584 | 1 | | 390 | hsa-miR-634 | 100287584 | LOC100287584 | hypothetical protein LOC100287584 | 1 | | 391 | hsa-mir-454 | 85449 | KIAA1755 | KIAA1755 | 1 | | 392 | hsa-miR-675 | 85449 | KIAA1755 | KIAA1755 | 1 | | 393 | hsa-miR-95 | 85449 | KIAA1755 | KIAA1755 | 1 | | 394 | hsa-mir-218-2 | 6601 | SMARCC2 | SWI/SNF related, matrix associated, actin dependent regulator of chromatin, subfamily c, member 2 | 1 | | 395 | hsa-mir-126 | 6601 | SMARCC2 | SWI/SNF related, matrix associated, actin dependent regulator of chromatin, subfamily c, member 2 | 1 | | 396 | hsa-mir-424 | 6601 | SMARCC2 | SWI/SNF related, matrix associated, actin dependent regulator of chromatin, subfamily c, member 2 | 1 | | 397 | hsa-mir-490 | 6601 | SMARCC2 | SWI/SNF related, matrix associated, actin dependent regulator of chromatin, subfamily c, member 2 | 1 | | 398 | hsa-mir-556 | 6601 | SMARCC2 | SWI/SNF related, matrix associated, actin dependent regulator of chromatin, subfamily c, member 2 | 1 | | 399 | hsa-mir-1915 | 6601 | SMARCC2 | SWI/SNF related, matrix associated, actin dependent regulator of chromatin, subfamily c, member 2 | 1 | | 400 | hsa-miR-190 | 6601 | SMARCC2 | SWI/SNF related, matrix associated, actin dependent regulator of chromatin, subfamily c, member 2 | 1 | | 401 | hsa-miR-490-5p | 6601 | SMARCC2 | SWI/SNF related, matrix associated, actin dependent regulator of chromatin, subfamily c, member 2 | 1 | | 402 | hsa-miR-503 | 6601 | SMARCC2 | SWI/SNF related, matrix associated, actin dependent regulator of chromatin, subfamily c, member 2 | 1 | | 403 | hsa-miR-585 | 6601 | SMARCC2 | SWI/SNF related, matrix associated, actin dependent regulator of chromatin, subfamily c, member 2 | 1 | | 405 | hsa-miR-608 | 286144 | C8orf83 | chromosome 8 open reading frame 83 | 1 | | 406 | hsa-mir-15a | 8654 | PDE5A | phosphodiesterase 5A, cGMP-specific | 1 | | 407 | hsa-mir-16-1 | 8654 | PDE5A | phosphodiesterase 5A, cGMP-specific | 1 | | 408 | hsa-miR-1231 | 8654 | PDE5A | phosphodiesterase 5A, cGMP-specific | 1 | | 409 | hsa-miR-126\* | 8654 | PDE5A | phosphodiesterase 5A, cGMP-specific | 1 | | 410 | hsa-miR-16 | 8654 | PDE5A | phosphodiesterase 5A, cGMP-specific | 1 | | 411 | hsa-miR-564 | 8654 | PDE5A | phosphodiesterase 5A, cGMP-specific | 1 | | 412 | hsa-miR-608 | 8654 | PDE5A | phosphodiesterase 5A, cGMP-specific | 1 | | 413 | hsa-mir-218-2 | 23522 | MYST4 | MYST histone acetyltransferase (monocytic leukemia) 4 | 1 | | 414 | hsa-mir-126 | 23522 | MYST4 | MYST histone acetyltransferase (monocytic leukemia) 4 | 1 | | 415 | hsa-mir-424 | 23522 | MYST4 | MYST histone acetyltransferase (monocytic leukemia) 4 | 1 | | 416 | hsa-mir-556 | 23522 | MYST4 | MYST histone acetyltransferase (monocytic leukemia) 4 | 1 | | 417 | hsa-miR-190 | 23522 | MYST4 | MYST histone acetyltransferase (monocytic leukemia) 4 | 1 | | 418 | hsa-miR-338-5p | 23522 | MYST4 | MYST histone acetyltransferase (monocytic leukemia) 4 | 1 | | 419 | hsa-miR-503 | 23522 | MYST4 | MYST histone acetyltransferase (monocytic leukemia) 4 | 1 | | 420 | hsa-miR-585 | 23522 | MYST4 | MYST histone acetyltransferase (monocytic leukemia) 4 | 1 | | 421 | hsa-miR-657 | 23522 | MYST4 | MYST histone acetyltransferase (monocytic leukemia) 4 | 1 | | 422 | hsa-mir-126 | 8874 | ARHGEF7 | Rho guanine nucleotide exchange factor (GEF) 7 | 1 | | 423 | hsa-mir-424 | 8874 | ARHGEF7 | Rho guanine nucleotide exchange factor (GEF) 7 | 1 | | 424 | hsa-mir-556 | 8874 | ARHGEF7 | Rho guanine nucleotide exchange factor (GEF) 7 | 1 | | 425 | hsa-miR-190 | 8874 | ARHGEF7 | Rho guanine nucleotide exchange factor (GEF) 7 | 1 | | 426 | hsa-miR-503 | 8874 | ARHGEF7 | Rho guanine nucleotide exchange factor (GEF) 7 | 1 | | 427 | hsa-mir-15a | 340074 | LOC340074 | hypothetical LOC340074 | 1 | | 428 | hsa-mir-16-1 | 340074 | LOC340074 | hypothetical LOC340074 | 1 | | 429 | hsa-miR-1231 | 340074 | LOC340074 | hypothetical LOC340074 | 1 | | 430 | hsa-miR-126\* | 340074 | LOC340074 | hypothetical LOC340074 | 1 | | 431 | hsa-miR-16 | 340074 | LOC340074 | hypothetical LOC340074 | 1 | | 432 | hsa-miR-608 | 340074 | LOC340074 | hypothetical LOC340074 | 1 | | 433 | hsa-mir-628 | 339988 | LOC339988 | hypothetical protein LOC339988 | 1 | | 436 | hsa-mir-339 | 199699 | DAND5 | DAN domain family, member 5 | 1 | | 437 | hsa-miR-126\* | 199699 | DAND5 | DAN domain family, member 5 | 1 | | 438 | hsa-miR-16 | 199699 | DAND5 | DAN domain family, member 5 | 1 | | 439 | hsa-miR-339-5p | 199699 | DAND5 | DAN domain family, member 5 | 1 | | 440 | hsa-miR-564 | 199699 | DAND5 | DAN domain family, member 5 | 1 | | 441 | hsa-miR-608 | 199699 | DAND5 | DAN domain family, member 5 | 1 | | 442 | hsa-miR-608 | 55008 | HERC6 | hect domain and RLD 6 | 1 | | 443 | hsa-mir-454 | 340069 | FAM170A | family with sequence similarity 170, member A | 1 | | 444 | hsa-miR-16 | 340069 | FAM170A | family with sequence similarity 170, member A | 1 | | 445 | hsa-miR-675 | 340069 | FAM170A | family with sequence similarity 170, member A | 1 | | 446 | hsa-miR-95 | 340069 | FAM170A | family with sequence similarity 170, member A | 1 | | 447 | hsa-miR-126\* | 3043 | HBB | hemoglobin, beta | 1 | | 448 | hsa-miR-608 | 201625 | DNAH12 | dynein, axonemal, heavy chain 12 | 1 | | 449 | hsa-mir-126 | 3633 | INPP5B | inositol polyphosphate-5-phosphatase, 75kDa | 1 | | 450 | hsa-miR-608 | 2898 | GRIK2 | glutamate receptor, ionotropic, kainate 2 | 1 | | 451 | hsa-mir-15a | 23263 | MCF2L | MCF.2 cell line derived transforming sequence-like | 1 | | 452 | hsa-mir-16-1 | 23263 | MCF2L | MCF.2 cell line derived transforming sequence-like | 1 | | 453 | hsa-miR-1231 | 23263 | MCF2L | MCF.2 cell line derived transforming sequence-like | 1 | | 454 | hsa-miR-126\* | 23263 | MCF2L | MCF.2 cell line derived transforming sequence-like | 1 | | 455 | hsa-miR-16 | 23263 | MCF2L | MCF.2 cell line derived transforming sequence-like | 1 | | 456 | hsa-miR-566 | 23263 | MCF2L | MCF.2 cell line derived transforming sequence-like | 1 | | 457 | hsa-miR-574-5p | 23263 | MCF2L | MCF.2 cell line derived transforming sequence-like | 1 | | 458 | hsa-miR-608 | 23263 | MCF2L | MCF.2 cell line derived transforming sequence-like | 1 | | 459 | hsa-mir-15a | 283089 | LOC283089 | hypothetical protein LOC283089 | 1 | | 460 | hsa-mir-16-1 | 283089 | LOC283089 | hypothetical protein LOC283089 | 1 | | 461 | hsa-miR-126\* | 283089 | LOC283089 | hypothetical protein LOC283089 | 1 | | 462 | hsa-miR-608 | 283089 | LOC283089 | hypothetical protein LOC283089 | 1 | | 463 | hsa-mir-16-1 | 200403 | VWA3B | von Willebrand factor A domain containing 3B | 1 | | 464 | hsa-miR-16 | 200403 | VWA3B | von Willebrand factor A domain containing 3B | 1 | | 465 | hsa-miR-608 | 200403 | VWA3B | von Willebrand factor A domain containing 3B | 1 | | 466 | hsa-miR-1231 | 100131864 | LOC100131864 | hypothetical protein LOC100131864 | 1 | | 467 | hsa-miR-126\* | 100131864 | LOC100131864 | hypothetical protein LOC100131864 | 1 | | 468 | hsa-miR-564 | 100131864 | LOC100131864 | hypothetical protein LOC100131864 | 1 | | 469 | hsa-miR-608 | 100131864 | LOC100131864 | hypothetical protein LOC100131864 | 1 | | 470 | hsa-mir-454 | 93654 | ST7OT2 | ST7 overlapping transcript 2 (non-protein coding) | 1 | | 471 | hsa-miR-675 | 93654 | ST7OT2 | ST7 overlapping transcript 2 (non-protein coding) | 1 | | 472 | hsa-miR-95 | 93654 | ST7OT2 | ST7 overlapping transcript 2 (non-protein coding) | 1 | | 473 | hsa-mir-454 | 93986 | FOXP2 | forkhead box P2 | 1 | | 474 | hsa-miR-16 | 93986 | FOXP2 | forkhead box P2 | 1 | | 475 | hsa-miR-675 | 93986 | FOXP2 | forkhead box P2 | 1 | | 476 | hsa-miR-95 | 93986 | FOXP2 | forkhead box P2 | 1 | | 477 | hsa-mir-15a | 152578 | LOC152578 | hypothetical protein LOC152578 | 1 | | 478 | hsa-mir-16-1 | 152578 | LOC152578 | hypothetical protein LOC152578 | 1 | | 479 | hsa-miR-126\* | 152578 | LOC152578 | hypothetical protein LOC152578 | 1 | | 480 | hsa-miR-16 | 152578 | LOC152578 | hypothetical protein LOC152578 | 1 | | 481 | hsa-miR-608 | 152578 | LOC152578 | hypothetical protein LOC152578 | 1 | | 482 | hsa-mir-15a | 337879 | KRTAP8-1 | keratin associated protein 8-1 | 1 | | 483 | hsa-mir-16-1 | 337879 | KRTAP8-1 | keratin associated protein 8-1 | 1 | | 484 | hsa-mir-339 | 337879 | KRTAP8-1 | keratin associated protein 8-1 | 1 | | 485 | hsa-miR-126\* | 337879 | KRTAP8-1 | keratin associated protein 8-1 | 1 | | 486 | hsa-miR-16 | 337879 | KRTAP8-1 | keratin associated protein 8-1 | 1 | | 487 | hsa-miR-339-5p | 337879 | KRTAP8-1 | keratin associated protein 8-1 | 1 | | 488 | hsa-miR-608 | 337879 | KRTAP8-1 | keratin associated protein 8-1 | 1 | | 489 | hsa-mir-126 | 6137 | RPL13 | ribosomal protein L13 | 1 | | 496 | hsa-miR-126\* | 27154 | BRPF3 | bromodomain and PHD finger containing, 3 | 1 | | 497 | hsa-miR-566 | 27154 | BRPF3 | bromodomain and PHD finger containing, 3 | 1 | | 498 | hsa-miR-608 | 27154 | BRPF3 | bromodomain and PHD finger containing, 3 | 1 | | 499 | hsa-mir-15a | 23589 | CARHSP1 | calcium regulated heat stable protein 1, 24kDa | 1 | | 500 | hsa-mir-16-1 | 23589 | CARHSP1 | calcium regulated heat stable protein 1, 24kDa | 1 | | 501 | hsa-miR-126\* | 23589 | CARHSP1 | calcium regulated heat stable protein 1, 24kDa | 1 | | 502 | hsa-miR-16 | 23589 | CARHSP1 | calcium regulated heat stable protein 1, 24kDa | 1 | | 503 | hsa-miR-566 | 23589 | CARHSP1 | calcium regulated heat stable protein 1, 24kDa | 1 | | 504 | hsa-miR-608 | 23589 | CARHSP1 | calcium regulated heat stable protein 1, 24kDa | 1 | | 505 | hsa-mir-218-2 | 23512 | SUZ12 | suppressor of zeste 12 homolog (Drosophila) | 1 | | 506 | hsa-mir-126 | 23512 | SUZ12 | suppressor of zeste 12 homolog (Drosophila) | 1 | | 507 | hsa-mir-424 | 23512 | SUZ12 | suppressor of zeste 12 homolog (Drosophila) | 1 | | 508 | hsa-mir-490 | 23512 | SUZ12 | suppressor of zeste 12 homolog (Drosophila) | 1 | | 509 | hsa-mir-556 | 23512 | SUZ12 | suppressor of zeste 12 homolog (Drosophila) | 1 | | 510 | hsa-mir-1915 | 23512 | SUZ12 | suppressor of zeste 12 homolog (Drosophila) | 1 | | 511 | hsa-miR-190 | 23512 | SUZ12 | suppressor of zeste 12 homolog (Drosophila) | 1 | | 512 | hsa-miR-490-5p | 23512 | SUZ12 | suppressor of zeste 12 homolog (Drosophila) | 1 | | 513 | hsa-miR-503 | 23512 | SUZ12 | suppressor of zeste 12 homolog (Drosophila) | 1 | | 514 | hsa-miR-585 | 23512 | SUZ12 | suppressor of zeste 12 homolog (Drosophila) | 1 | | 515 | hsa-mir-339 | 651250 | LOC651250 | hypothetical LOC651250 | 1 | | 516 | hsa-miR-339-5p | 651250 | LOC651250 | hypothetical LOC651250 | 1 | | 517 | hsa-miR-564 | 651250 | LOC651250 | hypothetical LOC651250 | 1 | | 518 | hsa-miR-608 | 651250 | LOC651250 | hypothetical LOC651250 | 1 | | 519 | hsa-mir-15a | 219525 | OR5AK4P | olfactory receptor, family 5, subfamily AK, member 4 pseudogene | 1 | | 520 | hsa-mir-16-1 | 219525 | OR5AK4P | olfactory receptor, family 5, subfamily AK, member 4 pseudogene | 1 | | 521 | hsa-miR-1231 | 219525 | OR5AK4P | olfactory receptor, family 5, subfamily AK, member 4 pseudogene | 1 | | 522 | hsa-miR-126\* | 219525 | OR5AK4P | olfactory receptor, family 5, subfamily AK, member 4 pseudogene | 1 | | 523 | hsa-miR-16 | 219525 | OR5AK4P | olfactory receptor, family 5, subfamily AK, member 4 pseudogene | 1 | | 524 | hsa-miR-566 | 219525 | OR5AK4P | olfactory receptor, family 5, subfamily AK, member 4 pseudogene | 1 | | 525 | hsa-miR-608 | 219525 | OR5AK4P | olfactory receptor, family 5, subfamily AK, member 4 pseudogene | 1 | | 526 | hsa-mir-454 | 30847 | CECR9 | cat eye syndrome chromosome region, candidate 9 | 1 | | 527 | hsa-miR-675 | 30847 | CECR9 | cat eye syndrome chromosome region, candidate 9 | 1 | | 528 | hsa-miR-95 | 30847 | CECR9 | cat eye syndrome chromosome region, candidate 9 | 1 | | 529 | hsa-miR-608 | 2572 | GAD2 | glutamate decarboxylase 2 (pancreatic islets and brain, 65kDa) | 1 | | 530 | hsa-mir-454 | 387763 | LOC387763 | hypothetical protein LOC387763 | 1 | | 531 | hsa-miR-16 | 387763 | LOC387763 | hypothetical protein LOC387763 | 1 | | 532 | hsa-miR-675 | 387763 | LOC387763 | hypothetical protein LOC387763 | 1 | | 533 | hsa-miR-95 | 387763 | LOC387763 | hypothetical protein LOC387763 | 1 | | 534 | hsa-mir-126 | 10677 | AVIL | advillin | 1 | | 535 | hsa-mir-424 | 10677 | AVIL | advillin | 1 | | 536 | hsa-mir-490 | 10677 | AVIL | advillin | 1 | | 537 | hsa-mir-628 | 10677 | AVIL | advillin | 1 | | 538 | hsa-miR-190 | 10677 | AVIL | advillin | 1 | | 539 | hsa-miR-490-5p | 10677 | AVIL | advillin | 1 | | 540 | hsa-miR-503 | 10677 | AVIL | advillin | 1 | | 543 | hsa-miR-574-5p | 2177 | FANCD2 | Fanconi anemia, complementation group D2 | 1 | | 544 | hsa-mir-15a | 79645 | EFCAB1 | EF-hand calcium binding domain 1 | 1 | | 545 | hsa-mir-16-1 | 79645 | EFCAB1 | EF-hand calcium binding domain 1 | 1 | | 546 | hsa-miR-608 | 79645 | EFCAB1 | EF-hand calcium binding domain 1 | 1 | | 548 | hsa-mir-574 | 6693 | SPN | sialophorin | 1 | | 551 | hsa-miR-504 | 6693 | SPN | sialophorin | 1 | | 552 | hsa-miR-623 | 6693 | SPN | sialophorin | 1 | | 553 | hsa-mir-15a | 100294720 | NHEG1 | neuroblastoma highly expressed 1 | 1 | | 554 | hsa-mir-16-1 | 100294720 | NHEG1 | neuroblastoma highly expressed 1 | 1 | | 555 | hsa-miR-126\* | 100294720 | NHEG1 | neuroblastoma highly expressed 1 | 1 | | 556 | hsa-miR-16 | 100294720 | NHEG1 | neuroblastoma highly expressed 1 | 1 | | 557 | hsa-miR-608 | 100294720 | NHEG1 | neuroblastoma highly expressed 1 | 1 | | 558 | hsa-miR-454\* | 162962 | ZNF836 | zinc finger protein 836 | 1 | | 559 | hsa-mir-15a | 100129098 | LOC100129098 | hypothetical LOC100129098 | 1 | | 560 | hsa-mir-16-1 | 100129098 | LOC100129098 | hypothetical LOC100129098 | 1 | | 561 | hsa-miR-126\* | 100129098 | LOC100129098 | hypothetical LOC100129098 | 1 | | 562 | hsa-miR-16 | 100129098 | LOC100129098 | hypothetical LOC100129098 | 1 | | 563 | hsa-miR-608 | 100129098 | LOC100129098 | hypothetical LOC100129098 | 1 | | 564 | hsa-mir-15a | 646482 | LOC646482 | hypothetical LOC646482 | 1 | | 565 | hsa-mir-16-1 | 646482 | LOC646482 | hypothetical LOC646482 | 1 | | 566 | hsa-miR-126\* | 646482 | LOC646482 | hypothetical LOC646482 | 1 | | 567 | hsa-miR-16 | 646482 | LOC646482 | hypothetical LOC646482 | 1 | | 568 | hsa-miR-608 | 646482 | LOC646482 | hypothetical LOC646482 | 1 | | 569 | hsa-mir-15a | 647309 | LOC647309 | hypothetical protein LOC647309 | 1 | | 570 | hsa-mir-16-1 | 647309 | LOC647309 | hypothetical protein LOC647309 | 1 | | 571 | hsa-miR-608 | 647309 | LOC647309 | hypothetical protein LOC647309 | 1 | | 572 | hsa-mir-15a | 114804 | RNF157 | ring finger protein 157 | 1 | | 573 | hsa-miR-608 | 114804 | RNF157 | ring finger protein 157 | 1 | | 574 | hsa-miR-608 | 653110 | LOC653110 | hypothetical LOC653110 | 1 | | 575 | hsa-mir-218-2 | 9701 | SAPS2 | SAPS domain family, member 2 | 1 | | 576 | hsa-mir-126 | 9701 | SAPS2 | SAPS domain family, member 2 | 1 | | 577 | hsa-mir-424 | 9701 | SAPS2 | SAPS domain family, member 2 | 1 | | 578 | hsa-mir-556 | 9701 | SAPS2 | SAPS domain family, member 2 | 1 | | 579 | hsa-mir-1915 | 9701 | SAPS2 | SAPS domain family, member 2 | 1 | | 580 | hsa-miR-190 | 9701 | SAPS2 | SAPS domain family, member 2 | 1 | | 581 | hsa-miR-503 | 9701 | SAPS2 | SAPS domain family, member 2 | 1 | | 582 | hsa-miR-585 | 9701 | SAPS2 | SAPS domain family, member 2 | 1 | | 583 | hsa-miR-1231 | 6907 | TBL1X | transducin (beta)-like 1X-linked | 1 | | 584 | hsa-miR-126\* | 6907 | TBL1X | transducin (beta)-like 1X-linked | 1 | | 585 | hsa-miR-608 | 6907 | TBL1X | transducin (beta)-like 1X-linked | 1 | | 586 | hsa-mir-218-2 | 9169 | SFRS2IP | splicing factor, arginine/serine-rich 2, interacting protein | 1 | | 587 | hsa-mir-126 | 9169 | SFRS2IP | splicing factor, arginine/serine-rich 2, interacting protein | 1 | | 588 | hsa-mir-424 | 9169 | SFRS2IP | splicing factor, arginine/serine-rich 2, interacting protein | 1 | | 589 | hsa-mir-490 | 9169 | SFRS2IP | splicing factor, arginine/serine-rich 2, interacting protein | 1 | | 590 | hsa-mir-556 | 9169 | SFRS2IP | splicing factor, arginine/serine-rich 2, interacting protein | 1 | | 591 | hsa-mir-1915 | 9169 | SFRS2IP | splicing factor, arginine/serine-rich 2, interacting protein | 1 | | 592 | hsa-miR-190 | 9169 | SFRS2IP | splicing factor, arginine/serine-rich 2, interacting protein | 1 | | 593 | hsa-miR-490-5p | 9169 | SFRS2IP | splicing factor, arginine/serine-rich 2, interacting protein | 1 | | 594 | hsa-miR-503 | 9169 | SFRS2IP | splicing factor, arginine/serine-rich 2, interacting protein | 1 | | 595 | hsa-miR-585 | 9169 | SFRS2IP | splicing factor, arginine/serine-rich 2, interacting protein | 1 | | 596 | hsa-mir-218-2 | 8664 | EIF3D | eukaryotic translation initiation factor 3, subunit D | 1 | | 597 | hsa-mir-126 | 8664 | EIF3D | eukaryotic translation initiation factor 3, subunit D | 1 | | 598 | hsa-mir-196b | 8664 | EIF3D | eukaryotic translation initiation factor 3, subunit D | 1 | | 599 | hsa-mir-424 | 8664 | EIF3D | eukaryotic translation initiation factor 3, subunit D | 1 | | 600 | hsa-mir-490 | 8664 | EIF3D | eukaryotic translation initiation factor 3, subunit D | 1 | | 601 | hsa-mir-556 | 8664 | EIF3D | eukaryotic translation initiation factor 3, subunit D | 1 | | 602 | hsa-mir-628 | 8664 | EIF3D | eukaryotic translation initiation factor 3, subunit D | 1 | | 603 | hsa-mir-1915 | 8664 | EIF3D | eukaryotic translation initiation factor 3, subunit D | 1 | | 604 | hsa-miR-190 | 8664 | EIF3D | eukaryotic translation initiation factor 3, subunit D | 1 | | 605 | hsa-miR-196b | 8664 | EIF3D | eukaryotic translation initiation factor 3, subunit D | 1 | | 606 | hsa-miR-490-5p | 8664 | EIF3D | eukaryotic translation initiation factor 3, subunit D | 1 | | 607 | hsa-miR-503 | 8664 | EIF3D | eukaryotic translation initiation factor 3, subunit D | 1 | | 608 | hsa-miR-585 | 8664 | EIF3D | eukaryotic translation initiation factor 3, subunit D | 1 | | 609 | hsa-miR-617 | 8664 | EIF3D | eukaryotic translation initiation factor 3, subunit D | 1 | | 610 | hsa-miR-623 | 8664 | EIF3D | eukaryotic translation initiation factor 3, subunit D | 1 | | 611 | hsa-mir-196b | 6152 | RPL24 | ribosomal protein L24 | 1 | | 612 | hsa-mir-424 | 6152 | RPL24 | ribosomal protein L24 | 1 | | 613 | hsa-mir-490 | 6152 | RPL24 | ribosomal protein L24 | 1 | | 614 | hsa-mir-574 | 6152 | RPL24 | ribosomal protein L24 | 1 | | 615 | hsa-mir-628 | 6152 | RPL24 | ribosomal protein L24 | 1 | | 616 | hsa-miR-190 | 6152 | RPL24 | ribosomal protein L24 | 1 | | 617 | hsa-miR-196b | 6152 | RPL24 | ribosomal protein L24 | 1 | | 618 | hsa-miR-490-5p | 6152 | RPL24 | ribosomal protein L24 | 1 | | 619 | hsa-miR-503 | 6152 | RPL24 | ribosomal protein L24 | 1 | | 620 | hsa-miR-504 | 6152 | RPL24 | ribosomal protein L24 | 1 | | 621 | hsa-miR-586 | 6152 | RPL24 | ribosomal protein L24 | 1 | | 622 | hsa-miR-617 | 6152 | RPL24 | ribosomal protein L24 | 1 | | 623 | hsa-miR-623 | 6152 | RPL24 | ribosomal protein L24 | 1 | | 624 | hsa-miR-944 | 6152 | RPL24 | ribosomal protein L24 | 1 | | 625 | hsa-mir-218-2 | 8665 | EIF3F | eukaryotic translation initiation factor 3, subunit F | 1 | | 626 | hsa-mir-126 | 8665 | EIF3F | eukaryotic translation initiation factor 3, subunit F | 1 | | 627 | hsa-mir-424 | 8665 | EIF3F | eukaryotic translation initiation factor 3, subunit F | 1 | | 628 | hsa-mir-490 | 8665 | EIF3F | eukaryotic translation initiation factor 3, subunit F | 1 | | 629 | hsa-mir-556 | 8665 | EIF3F | eukaryotic translation initiation factor 3, subunit F | 1 | | 630 | hsa-mir-628 | 8665 | EIF3F | eukaryotic translation initiation factor 3, subunit F | 1 | | 631 | hsa-mir-1915 | 8665 | EIF3F | eukaryotic translation initiation factor 3, subunit F | 1 | | 632 | hsa-miR-190 | 8665 | EIF3F | eukaryotic translation initiation factor 3, subunit F | 1 | | 633 | hsa-miR-196b | 8665 | EIF3F | eukaryotic translation initiation factor 3, subunit F | 1 | | 634 | hsa-miR-338-5p | 8665 | EIF3F | eukaryotic translation initiation factor 3, subunit F | 1 | | 635 | hsa-miR-490-5p | 8665 | EIF3F | eukaryotic translation initiation factor 3, subunit F | 1 | | 636 | hsa-miR-503 | 8665 | EIF3F | eukaryotic translation initiation factor 3, subunit F | 1 | | 637 | hsa-miR-585 | 8665 | EIF3F | eukaryotic translation initiation factor 3, subunit F | 1 | | 638 | hsa-miR-586 | 8665 | EIF3F | eukaryotic translation initiation factor 3, subunit F | 1 | | 639 | hsa-miR-617 | 8665 | EIF3F | eukaryotic translation initiation factor 3, subunit F | 1 | | 640 | hsa-miR-623 | 8665 | EIF3F | eukaryotic translation initiation factor 3, subunit F | 1 | | 641 | hsa-miR-657 | 8665 | EIF3F | eukaryotic translation initiation factor 3, subunit F | 1 | | 642 | hsa-mir-196b | 6143 | RPL19 | ribosomal protein L19 | 1 | | 643 | hsa-mir-424 | 6143 | RPL19 | ribosomal protein L19 | 1 | | 644 | hsa-mir-628 | 6143 | RPL19 | ribosomal protein L19 | 1 | | 645 | hsa-miR-190 | 6143 | RPL19 | ribosomal protein L19 | 1 | | 646 | hsa-miR-196b | 6143 | RPL19 | ribosomal protein L19 | 1 | | 647 | hsa-miR-503 | 6143 | RPL19 | ribosomal protein L19 | 1 | | 648 | hsa-miR-504 | 6143 | RPL19 | ribosomal protein L19 | 1 | | 649 | hsa-miR-586 | 6143 | RPL19 | ribosomal protein L19 | 1 | | 650 | hsa-miR-617 | 6143 | RPL19 | ribosomal protein L19 | 1 | | 651 | hsa-miR-623 | 6143 | RPL19 | ribosomal protein L19 | 1 | | 652 | hsa-mir-196b | 6139 | RPL17 | ribosomal protein L17 | 1 | | 653 | hsa-mir-424 | 6139 | RPL17 | ribosomal protein L17 | 1 | | 654 | hsa-mir-628 | 6139 | RPL17 | ribosomal protein L17 | 1 | | 655 | hsa-mir-1915 | 6139 | RPL17 | ribosomal protein L17 | 1 | | 656 | hsa-miR-190 | 6139 | RPL17 | ribosomal protein L17 | 1 | | 657 | hsa-miR-196b | 6139 | RPL17 | ribosomal protein L17 | 1 | | 658 | hsa-miR-503 | 6139 | RPL17 | ribosomal protein L17 | 1 | | 659 | hsa-miR-586 | 6139 | RPL17 | ribosomal protein L17 | 1 | | 660 | hsa-miR-617 | 6139 | RPL17 | ribosomal protein L17 | 1 | | 661 | hsa-miR-623 | 6139 | RPL17 | ribosomal protein L17 | 1 | | 662 | hsa-mir-126 | 9045 | RPL14 | ribosomal protein L14 | 1 | | 663 | hsa-mir-196b | 9045 | RPL14 | ribosomal protein L14 | 1 | | 664 | hsa-mir-424 | 9045 | RPL14 | ribosomal protein L14 | 1 | | 665 | hsa-mir-490 | 9045 | RPL14 | ribosomal protein L14 | 1 | | 666 | hsa-mir-574 | 9045 | RPL14 | ribosomal protein L14 | 1 | | 667 | hsa-mir-628 | 9045 | RPL14 | ribosomal protein L14 | 1 | | 668 | hsa-mir-1915 | 9045 | RPL14 | ribosomal protein L14 | 1 | | 669 | hsa-miR-190 | 9045 | RPL14 | ribosomal protein L14 | 1 | | 670 | hsa-miR-196b | 9045 | RPL14 | ribosomal protein L14 | 1 | | 671 | hsa-miR-490-5p | 9045 | RPL14 | ribosomal protein L14 | 1 | | 672 | hsa-miR-503 | 9045 | RPL14 | ribosomal protein L14 | 1 | | 673 | hsa-miR-504 | 9045 | RPL14 | ribosomal protein L14 | 1 | | 674 | hsa-miR-586 | 9045 | RPL14 | ribosomal protein L14 | 1 | | 675 | hsa-miR-617 | 9045 | RPL14 | ribosomal protein L14 | 1 | | 676 | hsa-miR-623 | 9045 | RPL14 | ribosomal protein L14 | 1 | | 677 | hsa-miR-944 | 9045 | RPL14 | ribosomal protein L14 | 1 | | 678 | hsa-mir-126 | 6201 | RPS7 | ribosomal protein S7 | 1 | | 679 | hsa-mir-196b | 6201 | RPS7 | ribosomal protein S7 | 1 | | 680 | hsa-mir-424 | 6201 | RPS7 | ribosomal protein S7 | 1 | | 681 | hsa-mir-490 | 6201 | RPS7 | ribosomal protein S7 | 1 | | 682 | hsa-mir-574 | 6201 | RPS7 | ribosomal protein S7 | 1 | | 683 | hsa-mir-628 | 6201 | RPS7 | ribosomal protein S7 | 1 | | 684 | hsa-mir-1915 | 6201 | RPS7 | ribosomal protein S7 | 1 | | 685 | hsa-miR-190 | 6201 | RPS7 | ribosomal protein S7 | 1 | | 686 | hsa-miR-196b | 6201 | RPS7 | ribosomal protein S7 | 1 | | 687 | hsa-miR-490-5p | 6201 | RPS7 | ribosomal protein S7 | 1 | | 688 | hsa-miR-503 | 6201 | RPS7 | ribosomal protein S7 | 1 | | 689 | hsa-miR-623 | 6201 | RPS7 | ribosomal protein S7 | 1 | | 690 | hsa-mir-126 | 6124 | RPL4 | ribosomal protein L4 | 1 | | 691 | hsa-mir-196b | 6124 | RPL4 | ribosomal protein L4 | 1 | | 692 | hsa-mir-424 | 6124 | RPL4 | ribosomal protein L4 | 1 | | 693 | hsa-mir-556 | 6124 | RPL4 | ribosomal protein L4 | 1 | | 694 | hsa-mir-628 | 6124 | RPL4 | ribosomal protein L4 | 1 | | 695 | hsa-mir-1915 | 6124 | RPL4 | ribosomal protein L4 | 1 | | 696 | hsa-miR-190 | 6124 | RPL4 | ribosomal protein L4 | 1 | | 697 | hsa-miR-196b | 6124 | RPL4 | ribosomal protein L4 | 1 | | 698 | hsa-miR-338-5p | 6124 | RPL4 | ribosomal protein L4 | 1 | | 699 | hsa-miR-503 | 6124 | RPL4 | ribosomal protein L4 | 1 | | 700 | hsa-miR-586 | 6124 | RPL4 | ribosomal protein L4 | 1 | | 701 | hsa-miR-617 | 6124 | RPL4 | ribosomal protein L4 | 1 | | 702 | hsa-miR-623 | 6124 | RPL4 | ribosomal protein L4 | 1 | | 703 | hsa-miR-657 | 6124 | RPL4 | ribosomal protein L4 | 1 | | 706 | hsa-mir-628 | 6167 | RPL37 | ribosomal protein L37 | 1 | | 709 | hsa-miR-586 | 6167 | RPL37 | ribosomal protein L37 | 1 | | 710 | hsa-miR-617 | 6167 | RPL37 | ribosomal protein L37 | 1 | | 711 | hsa-mir-196b | 1938 | EEF2 | eukaryotic translation elongation factor 2 | 1 | | 712 | hsa-mir-424 | 1938 | EEF2 | eukaryotic translation elongation factor 2 | 1 | | 713 | hsa-mir-628 | 1938 | EEF2 | eukaryotic translation elongation factor 2 | 1 | | 714 | hsa-mir-1915 | 1938 | EEF2 | eukaryotic translation elongation factor 2 | 1 | | 715 | hsa-miR-190 | 1938 | EEF2 | eukaryotic translation elongation factor 2 | 1 | | 716 | hsa-miR-196b | 1938 | EEF2 | eukaryotic translation elongation factor 2 | 1 | | 717 | hsa-miR-503 | 1938 | EEF2 | eukaryotic translation elongation factor 2 | 1 | | 718 | hsa-miR-586 | 1938 | EEF2 | eukaryotic translation elongation factor 2 | 1 | | 719 | hsa-miR-617 | 1938 | EEF2 | eukaryotic translation elongation factor 2 | 1 | | 720 | hsa-miR-623 | 1938 | EEF2 | eukaryotic translation elongation factor 2 | 1 | | 721 | hsa-mir-126 | 6189 | RPS3A | ribosomal protein S3A | 1 | | 722 | hsa-mir-196b | 6189 | RPS3A | ribosomal protein S3A | 1 | | 724 | hsa-mir-490 | 6189 | RPS3A | ribosomal protein S3A | 1 | | 726 | hsa-mir-1915 | 6189 | RPS3A | ribosomal protein S3A | 1 | | 728 | hsa-miR-196b | 6189 | RPS3A | ribosomal protein S3A | 1 | | 729 | hsa-miR-490-5p | 6189 | RPS3A | ribosomal protein S3A | 1 | | 731 | hsa-miR-623 | 6189 | RPS3A | ribosomal protein S3A | 1 | | 732 | hsa-mir-628 | 6647 | SOD1 | superoxide dismutase 1, soluble | 1 | | 733 | hsa-miR-586 | 6647 | SOD1 | superoxide dismutase 1, soluble | 1 | | 734 | hsa-miR-617 | 6647 | SOD1 | superoxide dismutase 1, soluble | 1 | | 735 | hsa-miR-623 | 6647 | SOD1 | superoxide dismutase 1, soluble | 1 | | 736 | hsa-mir-196b | 10399 | GNB2L1 | guanine nucleotide binding protein (G protein), beta polypeptide 2-like 1 | 1 | | 737 | hsa-mir-424 | 10399 | GNB2L1 | guanine nucleotide binding protein (G protein), beta polypeptide 2-like 1 | 1 | | 738 | hsa-mir-490 | 10399 | GNB2L1 | guanine nucleotide binding protein (G protein), beta polypeptide 2-like 1 | 1 | | 739 | hsa-mir-574 | 10399 | GNB2L1 | guanine nucleotide binding protein (G protein), beta polypeptide 2-like 1 | 1 | | 740 | hsa-mir-628 | 10399 | GNB2L1 | guanine nucleotide binding protein (G protein), beta polypeptide 2-like 1 | 1 | | 741 | hsa-mir-1915 | 10399 | GNB2L1 | guanine nucleotide binding protein (G protein), beta polypeptide 2-like 1 | 1 | | 742 | hsa-miR-190 | 10399 | GNB2L1 | guanine nucleotide binding protein (G protein), beta polypeptide 2-like 1 | 1 | | 743 | hsa-miR-196b | 10399 | GNB2L1 | guanine nucleotide binding protein (G protein), beta polypeptide 2-like 1 | 1 | | 744 | hsa-miR-490-5p | 10399 | GNB2L1 | guanine nucleotide binding protein (G protein), beta polypeptide 2-like 1 | 1 | | 745 | hsa-miR-503 | 10399 | GNB2L1 | guanine nucleotide binding protein (G protein), beta polypeptide 2-like 1 | 1 | | 746 | hsa-miR-623 | 10399 | GNB2L1 | guanine nucleotide binding protein (G protein), beta polypeptide 2-like 1 | 1 | | 747 | hsa-mir-196b | 975 | CD81 | CD81 molecule | 1 | | 748 | hsa-mir-424 | 975 | CD81 | CD81 molecule | 1 | | 749 | hsa-mir-628 | 975 | CD81 | CD81 molecule | 1 | | 750 | hsa-miR-190 | 975 | CD81 | CD81 molecule | 1 | | 751 | hsa-miR-196b | 975 | CD81 | CD81 molecule | 1 | | 752 | hsa-miR-503 | 975 | CD81 | CD81 molecule | 1 | | 753 | hsa-miR-617 | 975 | CD81 | CD81 molecule | 1 | | 754 | hsa-miR-623 | 975 | CD81 | CD81 molecule | 1 | | 755 | hsa-mir-196b | 23521 | RPL13A | ribosomal protein L13a | 1 | | 756 | hsa-mir-424 | 23521 | RPL13A | ribosomal protein L13a | 1 | | 757 | hsa-mir-628 | 23521 | RPL13A | ribosomal protein L13a | 1 | | 758 | hsa-miR-190 | 23521 | RPL13A | ribosomal protein L13a | 1 | | 759 | hsa-miR-196b | 23521 | RPL13A | ribosomal protein L13a | 1 | | 760 | hsa-miR-503 | 23521 | RPL13A | ribosomal protein L13a | 1 | | 761 | hsa-miR-586 | 23521 | RPL13A | ribosomal protein L13a | 1 | | 762 | hsa-miR-617 | 23521 | RPL13A | ribosomal protein L13a | 1 | | 763 | hsa-miR-623 | 23521 | RPL13A | ribosomal protein L13a | 1 | | 764 | hsa-mir-135b | 6500 | SKP1 | S-phase kinase-associated protein 1 | 1 | | 765 | hsa-mir-556 | 6500 | SKP1 | S-phase kinase-associated protein 1 | 1 | | 766 | hsa-mir-675 | 6500 | SKP1 | S-phase kinase-associated protein 1 | 1 | | 767 | hsa-miR-454\* | 6500 | SKP1 | S-phase kinase-associated protein 1 | 1 | | 768 | hsa-mir-218-2 | 7203 | CCT3 | chaperonin containing TCP1, subunit 3 (gamma) | 1 | | 769 | hsa-mir-196b | 7203 | CCT3 | chaperonin containing TCP1, subunit 3 (gamma) | 1 | | 770 | hsa-mir-424 | 7203 | CCT3 | chaperonin containing TCP1, subunit 3 (gamma) | 1 | | 771 | hsa-mir-556 | 7203 | CCT3 | chaperonin containing TCP1, subunit 3 (gamma) | 1 | | 772 | hsa-mir-628 | 7203 | CCT3 | chaperonin containing TCP1, subunit 3 (gamma) | 1 | | 773 | hsa-miR-190 | 7203 | CCT3 | chaperonin containing TCP1, subunit 3 (gamma) | 1 | | 774 | hsa-miR-196b | 7203 | CCT3 | chaperonin containing TCP1, subunit 3 (gamma) | 1 | | 775 | hsa-miR-503 | 7203 | CCT3 | chaperonin containing TCP1, subunit 3 (gamma) | 1 | | 776 | hsa-miR-585 | 7203 | CCT3 | chaperonin containing TCP1, subunit 3 (gamma) | 1 | | 777 | hsa-miR-623 | 7203 | CCT3 | chaperonin containing TCP1, subunit 3 (gamma) | 1 | | 778 | hsa-miR-618 | 10096 | ACTR3 | ARP3 actin-related protein 3 homolog (yeast) | 1 | | 779 | hsa-miR-634 | 3693 | ITGB5 | integrin, beta 5 | 1 | | 780 | hsa-miR-126\* | 999 | CDH1 | cadherin 1, type 1, E-cadherin (epithelial) | 1 | | 781 | hsa-miR-16 | 999 | CDH1 | cadherin 1, type 1, E-cadherin (epithelial) | 1 | | 782 | hsa-miR-608 | 999 | CDH1 | cadherin 1, type 1, E-cadherin (epithelial) | 1 | | 783 | hsa-miR-16 | 5654 | HTRA1 | HtrA serine peptidase 1 | 1 | | 784 | hsa-mir-424 | 5306 | PITPNA | phosphatidylinositol transfer protein, alpha | 1 | | 785 | hsa-mir-556 | 5306 | PITPNA | phosphatidylinositol transfer protein, alpha | 1 | | 786 | hsa-miR-190 | 5306 | PITPNA | phosphatidylinositol transfer protein, alpha | 1 | | 787 | hsa-miR-503 | 5306 | PITPNA | phosphatidylinositol transfer protein, alpha | 1 | | 788 | hsa-mir-196b | 10961 | ERP29 | endoplasmic reticulum protein 29 | 1 | | 789 | hsa-mir-574 | 10961 | ERP29 | endoplasmic reticulum protein 29 | 1 | | 790 | hsa-miR-196b | 10961 | ERP29 | endoplasmic reticulum protein 29 | 1 | | 791 | hsa-miR-623 | 10961 | ERP29 | endoplasmic reticulum protein 29 | 1 | | 792 | hsa-miR-650 | 10961 | ERP29 | endoplasmic reticulum protein 29 | 1 | | 793 | hsa-miR-618 | 9341 | VAMP3 | vesicle-associated membrane protein 3 (cellubrevin) | 1 | | 802 | hsa-mir-628 | 60528 | ELAC2 | elaC homolog 2 (E. coli) | 1 | | 803 | hsa-miR-196b | 60528 | ELAC2 | elaC homolog 2 (E. coli) | 1 | | 804 | hsa-miR-586 | 60528 | ELAC2 | elaC homolog 2 (E. coli) | 1 | | 805 | hsa-miR-617 | 60528 | ELAC2 | elaC homolog 2 (E. coli) | 1 | | 806 | hsa-miR-623 | 60528 | ELAC2 | elaC homolog 2 (E. coli) | 1 | | 807 | hsa-miR-1231 | 51097 | SCCPDH | saccharopine dehydrogenase (putative) | 1 | | 808 | hsa-miR-574-5p | 51097 | SCCPDH | saccharopine dehydrogenase (putative) | 1 | | 809 | hsa-miR-634 | 51097 | SCCPDH | saccharopine dehydrogenase (putative) | 1 | | 810 | hsa-miR-608 | 1634 | DCN | decorin | 1 | | 811 | hsa-miR-1231 | 7319 | UBE2A | ubiquitin-conjugating enzyme E2A (RAD6 homolog) | 1 | | 812 | hsa-miR-618 | 7319 | UBE2A | ubiquitin-conjugating enzyme E2A (RAD6 homolog) | 1 | | 813 | hsa-mir-126 | 6480 | ST6GAL1 | ST6 beta-galactosamide alpha-2,6-sialyltranferase 1 | 1 | | 814 | hsa-mir-424 | 6480 | ST6GAL1 | ST6 beta-galactosamide alpha-2,6-sialyltranferase 1 | 1 | | 815 | hsa-mir-556 | 6480 | ST6GAL1 | ST6 beta-galactosamide alpha-2,6-sialyltranferase 1 | 1 | | 816 | hsa-mir-628 | 6480 | ST6GAL1 | ST6 beta-galactosamide alpha-2,6-sialyltranferase 1 | 1 | | 817 | hsa-miR-190 | 6480 | ST6GAL1 | ST6 beta-galactosamide alpha-2,6-sialyltranferase 1 | 1 | | 818 | hsa-miR-338-5p | 6480 | ST6GAL1 | ST6 beta-galactosamide alpha-2,6-sialyltranferase 1 | 1 | | 819 | hsa-miR-503 | 6480 | ST6GAL1 | ST6 beta-galactosamide alpha-2,6-sialyltranferase 1 | 1 | | 820 | hsa-miR-586 | 6480 | ST6GAL1 | ST6 beta-galactosamide alpha-2,6-sialyltranferase 1 | 1 | | 821 | hsa-miR-617 | 6480 | ST6GAL1 | ST6 beta-galactosamide alpha-2,6-sialyltranferase 1 | 1 | | 822 | hsa-miR-657 | 6480 | ST6GAL1 | ST6 beta-galactosamide alpha-2,6-sialyltranferase 1 | 1 | | 823 | hsa-mir-196b | 10120 | ACTR1B | ARP1 actin-related protein 1 homolog B, centractin beta (yeast) | 1 | | 824 | hsa-mir-628 | 10120 | ACTR1B | ARP1 actin-related protein 1 homolog B, centractin beta (yeast) | 1 | | 825 | hsa-miR-196b | 10120 | ACTR1B | ARP1 actin-related protein 1 homolog B, centractin beta (yeast) | 1 | | 826 | hsa-miR-623 | 10120 | ACTR1B | ARP1 actin-related protein 1 homolog B, centractin beta (yeast) | 1 | | 827 | hsa-miR-650 | 10120 | ACTR1B | ARP1 actin-related protein 1 homolog B, centractin beta (yeast) | 1 | | 828 | hsa-mir-218-2 | 10771 | ZMYND11 | zinc finger, MYND domain containing 11 | 1 | | 829 | hsa-mir-126 | 10771 | ZMYND11 | zinc finger, MYND domain containing 11 | 1 | | 830 | hsa-mir-424 | 10771 | ZMYND11 | zinc finger, MYND domain containing 11 | 1 | | 831 | hsa-mir-556 | 10771 | ZMYND11 | zinc finger, MYND domain containing 11 | 1 | | 832 | hsa-mir-1915 | 10771 | ZMYND11 | zinc finger, MYND domain containing 11 | 1 | | 833 | hsa-miR-190 | 10771 | ZMYND11 | zinc finger, MYND domain containing 11 | 1 | | 834 | hsa-miR-503 | 10771 | ZMYND11 | zinc finger, MYND domain containing 11 | 1 | | 835 | hsa-miR-585 | 10771 | ZMYND11 | zinc finger, MYND domain containing 11 | 1 | | 836 | hsa-miR-618 | 6732 | SRPK1 | SFRS protein kinase 1 | 1 | | 837 | hsa-miR-126\* | 2931 | GSK3A | glycogen synthase kinase 3 alpha | 1 | | 838 | hsa-miR-566 | 2931 | GSK3A | glycogen synthase kinase 3 alpha | 1 | | 839 | hsa-mir-548d-1 | 1674 | DES | desmin | 1 | | 840 | hsa-mir-454 | 1674 | DES | desmin | 1 | | 841 | hsa-miR-675 | 1674 | DES | desmin | 1 | | 842 | hsa-miR-95 | 1674 | DES | desmin | 1 | | 843 | hsa-mir-218-2 | 10480 | EIF3M | eukaryotic translation initiation factor 3, subunit M | 1 | | 844 | hsa-mir-126 | 10480 | EIF3M | eukaryotic translation initiation factor 3, subunit M | 1 | | 845 | hsa-mir-30c-1 | 10480 | EIF3M | eukaryotic translation initiation factor 3, subunit M | 1 | | 846 | hsa-mir-30e | 10480 | EIF3M | eukaryotic translation initiation factor 3, subunit M | 1 | | 847 | hsa-mir-424 | 10480 | EIF3M | eukaryotic translation initiation factor 3, subunit M | 1 | | 848 | hsa-mir-556 | 10480 | EIF3M | eukaryotic translation initiation factor 3, subunit M | 1 | | 849 | hsa-miR-190 | 10480 | EIF3M | eukaryotic translation initiation factor 3, subunit M | 1 | | 850 | hsa-miR-338-5p | 10480 | EIF3M | eukaryotic translation initiation factor 3, subunit M | 1 | | 851 | hsa-miR-503 | 10480 | EIF3M | eukaryotic translation initiation factor 3, subunit M | 1 | | 852 | hsa-miR-585 | 10480 | EIF3M | eukaryotic translation initiation factor 3, subunit M | 1 | | 853 | hsa-miR-586 | 10480 | EIF3M | eukaryotic translation initiation factor 3, subunit M | 1 | | 854 | hsa-miR-657 | 10480 | EIF3M | eukaryotic translation initiation factor 3, subunit M | 1 | | 855 | hsa-mir-196b | 5692 | PSMB4 | proteasome (prosome, macropain) subunit, beta type, 4 | 1 | | 856 | hsa-mir-424 | 5692 | PSMB4 | proteasome (prosome, macropain) subunit, beta type, 4 | 1 | | 857 | hsa-mir-490 | 5692 | PSMB4 | proteasome (prosome, macropain) subunit, beta type, 4 | 1 | | 858 | hsa-mir-628 | 5692 | PSMB4 | proteasome (prosome, macropain) subunit, beta type, 4 | 1 | | 859 | hsa-mir-1915 | 5692 | PSMB4 | proteasome (prosome, macropain) subunit, beta type, 4 | 1 | | 860 | hsa-miR-190 | 5692 | PSMB4 | proteasome (prosome, macropain) subunit, beta type, 4 | 1 | | 861 | hsa-miR-196b | 5692 | PSMB4 | proteasome (prosome, macropain) subunit, beta type, 4 | 1 | | 862 | hsa-miR-490-5p | 5692 | PSMB4 | proteasome (prosome, macropain) subunit, beta type, 4 | 1 | | 863 | hsa-miR-503 | 5692 | PSMB4 | proteasome (prosome, macropain) subunit, beta type, 4 | 1 | | 864 | hsa-miR-623 | 5692 | PSMB4 | proteasome (prosome, macropain) subunit, beta type, 4 | 1 | | 865 | hsa-miR-126\* | 3918 | LAMC2 | laminin, gamma 2 | 1 | | 866 | hsa-miR-608 | 3918 | LAMC2 | laminin, gamma 2 | 1 | | 867 | hsa-miR-126\* | 2580 | GAK | cyclin G associated kinase | 1 | | 868 | hsa-miR-608 | 2580 | GAK | cyclin G associated kinase | 1 | | 869 | hsa-mir-135b | 79921 | TCEAL4 | transcription elongation factor A (SII)-like 4 | 1 | | 870 | hsa-mir-556 | 79921 | TCEAL4 | transcription elongation factor A (SII)-like 4 | 1 | | 871 | hsa-mir-675 | 79921 | TCEAL4 | transcription elongation factor A (SII)-like 4 | 1 | | 872 | hsa-miR-190 | 79921 | TCEAL4 | transcription elongation factor A (SII)-like 4 | 1 | | 873 | hsa-miR-454\* | 79921 | TCEAL4 | transcription elongation factor A (SII)-like 4 | 1 | | 874 | hsa-miR-548d-5p | 79921 | TCEAL4 | transcription elongation factor A (SII)-like 4 | 1 | | 875 | hsa-miR-126\* | 8242 | KDM5C | lysine (K)-specific demethylase 5C | 1 | | 876 | hsa-miR-566 | 8242 | KDM5C | lysine (K)-specific demethylase 5C | 1 | | 877 | hsa-mir-218-2 | 6634 | SNRPD3 | small nuclear ribonucleoprotein D3 polypeptide 18kDa | 1 | | 878 | hsa-mir-126 | 6634 | SNRPD3 | small nuclear ribonucleoprotein D3 polypeptide 18kDa | 1 | | 879 | hsa-mir-424 | 6634 | SNRPD3 | small nuclear ribonucleoprotein D3 polypeptide 18kDa | 1 | | 880 | hsa-mir-490 | 6634 | SNRPD3 | small nuclear ribonucleoprotein D3 polypeptide 18kDa | 1 | | 881 | hsa-mir-556 | 6634 | SNRPD3 | small nuclear ribonucleoprotein D3 polypeptide 18kDa | 1 | | 882 | hsa-mir-1915 | 6634 | SNRPD3 | small nuclear ribonucleoprotein D3 polypeptide 18kDa | 1 | | 883 | hsa-miR-190 | 6634 | SNRPD3 | small nuclear ribonucleoprotein D3 polypeptide 18kDa | 1 | | 884 | hsa-miR-490-5p | 6634 | SNRPD3 | small nuclear ribonucleoprotein D3 polypeptide 18kDa | 1 | | 885 | hsa-miR-503 | 6634 | SNRPD3 | small nuclear ribonucleoprotein D3 polypeptide 18kDa | 1 | | 886 | hsa-miR-585 | 6634 | SNRPD3 | small nuclear ribonucleoprotein D3 polypeptide 18kDa | 1 | | 887 | hsa-miR-586 | 6634 | SNRPD3 | small nuclear ribonucleoprotein D3 polypeptide 18kDa | 1 | | 888 | hsa-miR-623 | 6634 | SNRPD3 | small nuclear ribonucleoprotein D3 polypeptide 18kDa | 1 | | 889 | hsa-miR-126\* | 22839 | DLGAP4 | discs, large (Drosophila) homolog-associated protein 4 | 1 | | 890 | hsa-miR-126\* | 3340 | NDST1 | N-deacetylase/N-sulfotransferase (heparan glucosaminyl) 1 | 1 | | 891 | hsa-miR-16 | 3340 | NDST1 | N-deacetylase/N-sulfotransferase (heparan glucosaminyl) 1 | 1 | | 892 | hsa-miR-564 | 3340 | NDST1 | N-deacetylase/N-sulfotransferase (heparan glucosaminyl) 1 | 1 | | 893 | hsa-miR-566 | 3340 | NDST1 | N-deacetylase/N-sulfotransferase (heparan glucosaminyl) 1 | 1 | | 894 | hsa-miR-608 | 3340 | NDST1 | N-deacetylase/N-sulfotransferase (heparan glucosaminyl) 1 | 1 | | 895 | hsa-miR-623 | 4701 | NDUFA7 | NADH dehydrogenase (ubiquinone) 1 alpha subcomplex, 7, 14.5kDa | 1 | | 896 | hsa-miR-1231 | 9052 | GPRC5A | G protein-coupled receptor, family C, group 5, member A | 1 | | 897 | hsa-miR-608 | 9052 | GPRC5A | G protein-coupled receptor, family C, group 5, member A | 1 | | 898 | hsa-mir-424 | 64976 | MRPL40 | mitochondrial ribosomal protein L40 | 1 | | 899 | hsa-mir-628 | 64976 | MRPL40 | mitochondrial ribosomal protein L40 | 1 | | 900 | hsa-miR-190 | 64976 | MRPL40 | mitochondrial ribosomal protein L40 | 1 | | 901 | hsa-miR-503 | 64976 | MRPL40 | mitochondrial ribosomal protein L40 | 1 | | 902 | hsa-miR-586 | 64976 | MRPL40 | mitochondrial ribosomal protein L40 | 1 | | 903 | hsa-miR-617 | 64976 | MRPL40 | mitochondrial ribosomal protein L40 | 1 | | 904 | hsa-miR-1231 | 220 | ALDH1A3 | aldehyde dehydrogenase 1 family, member A3 | 1 | | 905 | hsa-miR-126\* | 220 | ALDH1A3 | aldehyde dehydrogenase 1 family, member A3 | 1 | | 906 | hsa-miR-608 | 220 | ALDH1A3 | aldehyde dehydrogenase 1 family, member A3 | 1 | | 907 | hsa-mir-135b | 79096 | C11orf49 | chromosome 11 open reading frame 49 | 1 | | 908 | hsa-mir-675 | 79096 | C11orf49 | chromosome 11 open reading frame 49 | 1 | | 909 | hsa-miR-454\* | 79096 | C11orf49 | chromosome 11 open reading frame 49 | 1 | | 910 | hsa-mir-15a | 1289 | COL5A1 | collagen, type V, alpha 1 | 1 | | 911 | hsa-mir-16-1 | 1289 | COL5A1 | collagen, type V, alpha 1 | 1 | | 912 | hsa-miR-126\* | 1289 | COL5A1 | collagen, type V, alpha 1 | 1 | | 913 | hsa-miR-16 | 1289 | COL5A1 | collagen, type V, alpha 1 | 1 | | 914 | hsa-miR-608 | 1289 | COL5A1 | collagen, type V, alpha 1 | 1 | | 915 | hsa-miR-574-5p | 164 | AP1G1 | adaptor-related protein complex 1, gamma 1 subunit | 1 | | 916 | hsa-miR-618 | 164 | AP1G1 | adaptor-related protein complex 1, gamma 1 subunit | 1 | | 917 | hsa-miR-634 | 164 | AP1G1 | adaptor-related protein complex 1, gamma 1 subunit | 1 | | 919 | hsa-miR-126\* | 3797 | KIF3C | kinesin family member 3C | 1 | | 920 | hsa-miR-16 | 3797 | KIF3C | kinesin family member 3C | 1 | | 925 | hsa-miR-126\* | 3488 | IGFBP5 | insulin-like growth factor binding protein 5 | 1 | | 926 | hsa-miR-566 | 3488 | IGFBP5 | insulin-like growth factor binding protein 5 | 1 | | 927 | hsa-miR-608 | 3488 | IGFBP5 | insulin-like growth factor binding protein 5 | 1 | | 928 | hsa-miR-675 | 7111 | TMOD1 | tropomodulin 1 | 1 | | 929 | hsa-miR-95 | 7111 | TMOD1 | tropomodulin 1 | 1 | | 930 | hsa-mir-628 | 3712 | IVD | isovaleryl Coenzyme A dehydrogenase | 1 | | 931 | hsa-miR-617 | 3712 | IVD | isovaleryl Coenzyme A dehydrogenase | 1 | | 932 | hsa-miR-623 | 3712 | IVD | isovaleryl Coenzyme A dehydrogenase | 1 | | 933 | hsa-mir-424 | 3727 | JUND | jun D proto-oncogene | 1 | | 934 | hsa-mir-628 | 3727 | JUND | jun D proto-oncogene | 1 | | 935 | hsa-miR-503 | 3727 | JUND | jun D proto-oncogene | 1 | | 936 | hsa-miR-623 | 3727 | JUND | jun D proto-oncogene | 1 | | 937 | hsa-miR-126\* | 10188 | TNK2 | tyrosine kinase, non-receptor, 2 | 1 | | 938 | hsa-miR-16 | 10188 | TNK2 | tyrosine kinase, non-receptor, 2 | 1 | | 939 | hsa-miR-566 | 10188 | TNK2 | tyrosine kinase, non-receptor, 2 | 1 | | 940 | hsa-miR-618 | 22926 | ATF6 | activating transcription factor 6 | 1 | | 941 | hsa-mir-424 | 9923 | ZBTB40 | zinc finger and BTB domain containing 40 | 1 | | 942 | hsa-mir-556 | 9923 | ZBTB40 | zinc finger and BTB domain containing 40 | 1 | | 943 | hsa-mir-628 | 9923 | ZBTB40 | zinc finger and BTB domain containing 40 | 1 | | 944 | hsa-miR-190 | 9923 | ZBTB40 | zinc finger and BTB domain containing 40 | 1 | | 945 | hsa-miR-503 | 9923 | ZBTB40 | zinc finger and BTB domain containing 40 | 1 | | 946 | hsa-miR-586 | 9923 | ZBTB40 | zinc finger and BTB domain containing 40 | 1 | | 947 | hsa-miR-617 | 9923 | ZBTB40 | zinc finger and BTB domain containing 40 | 1 | | 948 | hsa-miR-623 | 9923 | ZBTB40 | zinc finger and BTB domain containing 40 | 1 | | 949 | hsa-mir-424 | 116987 | AGAP1 | ArfGAP with GTPase domain, ankyrin repeat and PH domain 1 | 1 | | 950 | hsa-mir-628 | 116987 | AGAP1 | ArfGAP with GTPase domain, ankyrin repeat and PH domain 1 | 1 | | 951 | hsa-miR-503 | 116987 | AGAP1 | ArfGAP with GTPase domain, ankyrin repeat and PH domain 1 | 1 | | 952 | hsa-miR-617 | 116987 | AGAP1 | ArfGAP with GTPase domain, ankyrin repeat and PH domain 1 | 1 | | 953 | hsa-miR-1231 | 6821 | SUOX | sulfite oxidase | 1 | | 954 | hsa-miR-454\* | 6821 | SUOX | sulfite oxidase | 1 | | 955 | hsa-miR-574-5p | 6821 | SUOX | sulfite oxidase | 1 | | 956 | hsa-miR-634 | 6821 | SUOX | sulfite oxidase | 1 | | 957 | hsa-mir-135b | 23387 | SIK3 | SIK family kinase 3 | 1 | | 958 | hsa-mir-15a | 57030 | SLC17A7 | solute carrier family 17 (sodium-dependent inorganic phosphate cotransporter), member 7 | 1 | | 959 | hsa-mir-16-1 | 57030 | SLC17A7 | solute carrier family 17 (sodium-dependent inorganic phosphate cotransporter), member 7 | 1 | | 960 | hsa-miR-1231 | 57030 | SLC17A7 | solute carrier family 17 (sodium-dependent inorganic phosphate cotransporter), member 7 | 1 | | 961 | hsa-miR-126\* | 57030 | SLC17A7 | solute carrier family 17 (sodium-dependent inorganic phosphate cotransporter), member 7 | 1 | | 962 | hsa-miR-16 | 57030 | SLC17A7 | solute carrier family 17 (sodium-dependent inorganic phosphate cotransporter), member 7 | 1 | | 963 | hsa-miR-574-5p | 57030 | SLC17A7 | solute carrier family 17 (sodium-dependent inorganic phosphate cotransporter), member 7 | 1 | | 964 | hsa-miR-608 | 57030 | SLC17A7 | solute carrier family 17 (sodium-dependent inorganic phosphate cotransporter), member 7 | 1 | | 965 | hsa-miR-95 | 1119 | CHKA | choline kinase alpha | 1 | | 966 | hsa-miR-618 | 9895 | TECPR2 | tectonin beta-propeller repeat containing 2 | 1 | | 967 | hsa-miR-126\* | 6567 | SLC16A2 | solute carrier family 16, member 2 (monocarboxylic acid transporter 8) | 1 | | 968 | hsa-miR-564 | 6567 | SLC16A2 | solute carrier family 16, member 2 (monocarboxylic acid transporter 8) | 1 | | 969 | hsa-miR-566 | 6567 | SLC16A2 | solute carrier family 16, member 2 (monocarboxylic acid transporter 8) | 1 | | 970 | hsa-miR-608 | 6567 | SLC16A2 | solute carrier family 16, member 2 (monocarboxylic acid transporter 8) | 1 | | 971 | hsa-miR-616\* | 4856 | NOV | nephroblastoma overexpressed gene | 1 | | 972 | hsa-mir-628 | 9760 | TOX | thymocyte selection-associated high mobility group box | 1 | | 973 | hsa-miR-586 | 9760 | TOX | thymocyte selection-associated high mobility group box | 1 | | 974 | hsa-miR-617 | 9760 | TOX | thymocyte selection-associated high mobility group box | 1 | | 975 | hsa-mir-15a | 8927 | BSN | bassoon (presynaptic cytomatrix protein) | 1 | | 976 | hsa-mir-16-1 | 8927 | BSN | bassoon (presynaptic cytomatrix protein) | 1 | | 977 | hsa-miR-126\* | 8927 | BSN | bassoon (presynaptic cytomatrix protein) | 1 | | 978 | hsa-miR-16 | 8927 | BSN | bassoon (presynaptic cytomatrix protein) | 1 | | 979 | hsa-miR-566 | 8927 | BSN | bassoon (presynaptic cytomatrix protein) | 1 | | 980 | hsa-miR-608 | 8927 | BSN | bassoon (presynaptic cytomatrix protein) | 1 | | 981 | hsa-mir-339 | 5218 | CDK14 | cyclin-dependent kinase 14 | 1 | | 982 | hsa-miR-339-5p | 5218 | CDK14 | cyclin-dependent kinase 14 | 1 | | 983 | hsa-miR-564 | 5218 | CDK14 | cyclin-dependent kinase 14 | 1 | | 984 | hsa-miR-618 | 5218 | CDK14 | cyclin-dependent kinase 14 | 1 | | 985 | hsa-miR-634 | 5218 | CDK14 | cyclin-dependent kinase 14 | 1 | | 986 | hsa-miR-126\* | 3690 | ITGB3 | integrin, beta 3 (platelet glycoprotein IIIa, antigen CD61) | 1 | | 987 | hsa-miR-566 | 3690 | ITGB3 | integrin, beta 3 (platelet glycoprotein IIIa, antigen CD61) | 1 | | 988 | hsa-mir-15a | 491 | ATP2B2 | ATPase, Ca++ transporting, plasma membrane 2 | 1 | | 989 | hsa-miR-1231 | 491 | ATP2B2 | ATPase, Ca++ transporting, plasma membrane 2 | 1 | | 990 | hsa-miR-126\* | 491 | ATP2B2 | ATPase, Ca++ transporting, plasma membrane 2 | 1 | | 991 | hsa-miR-16 | 491 | ATP2B2 | ATPase, Ca++ transporting, plasma membrane 2 | 1 | | 992 | hsa-miR-608 | 491 | ATP2B2 | ATPase, Ca++ transporting, plasma membrane 2 | 1 | | 993 | hsa-miR-1231 | 4291 | MLF1 | myeloid leukemia factor 1 | 1 | | 994 | hsa-miR-126\* | 4291 | MLF1 | myeloid leukemia factor 1 | 1 | | 995 | hsa-miR-16 | 4291 | MLF1 | myeloid leukemia factor 1 | 1 | | 996 | hsa-miR-566 | 4291 | MLF1 | myeloid leukemia factor 1 | 1 | | 997 | hsa-miR-608 | 4291 | MLF1 | myeloid leukemia factor 1 | 1 | | 998 | hsa-miR-126\* | 2701 | GJA4 | gap junction protein, alpha 4, 37kDa | 1 | | 999 | hsa-miR-126\* | 602 | BCL3 | B-cell CLL/lymphoma 3 | 1 | | 1000 | hsa-miR-566 | 602 | BCL3 | B-cell CLL/lymphoma 3 | 1 | | 1001 | hsa-miR-126\* | 10612 | TRIM3 | tripartite motif-containing 3 | 1 | | 1002 | hsa-miR-566 | 10612 | TRIM3 | tripartite motif-containing 3 | 1 | | 1003 | hsa-miR-608 | 10612 | TRIM3 | tripartite motif-containing 3 | 1 | | 1004 | hsa-mir-135b | 10782 | ZNF274 | zinc finger protein 274 | 1 | | 1005 | hsa-mir-556 | 10782 | ZNF274 | zinc finger protein 274 | 1 | | 1006 | hsa-mir-675 | 10782 | ZNF274 | zinc finger protein 274 | 1 | | 1007 | hsa-miR-190 | 10782 | ZNF274 | zinc finger protein 274 | 1 | | 1008 | hsa-miR-454\* | 10782 | ZNF274 | zinc finger protein 274 | 1 | | 1009 | hsa-miR-608 | 8777 | MPDZ | multiple PDZ domain protein | 1 | | 1010 | hsa-mir-424 | 4261 | CIITA | class II, major histocompatibility complex, transactivator | 1 | | 1011 | hsa-mir-556 | 4261 | CIITA | class II, major histocompatibility complex, transactivator | 1 | | 1012 | hsa-mir-628 | 4261 | CIITA | class II, major histocompatibility complex, transactivator | 1 | | 1013 | hsa-miR-190 | 4261 | CIITA | class II, major histocompatibility complex, transactivator | 1 | | 1014 | hsa-miR-338-5p | 4261 | CIITA | class II, major histocompatibility complex, transactivator | 1 | | 1015 | hsa-miR-503 | 4261 | CIITA | class II, major histocompatibility complex, transactivator | 1 | | 1016 | hsa-miR-586 | 4261 | CIITA | class II, major histocompatibility complex, transactivator | 1 | | 1017 | hsa-miR-617 | 4261 | CIITA | class II, major histocompatibility complex, transactivator | 1 | | 1018 | hsa-miR-657 | 4261 | CIITA | class II, major histocompatibility complex, transactivator | 1 | | 1019 | hsa-mir-218-2 | 5051 | PAFAH2 | platelet-activating factor acetylhydrolase 2, 40kDa | 1 | | 1020 | hsa-mir-126 | 5051 | PAFAH2 | platelet-activating factor acetylhydrolase 2, 40kDa | 1 | | 1021 | hsa-mir-424 | 5051 | PAFAH2 | platelet-activating factor acetylhydrolase 2, 40kDa | 1 | | 1022 | hsa-mir-556 | 5051 | PAFAH2 | platelet-activating factor acetylhydrolase 2, 40kDa | 1 | | 1023 | hsa-mir-628 | 5051 | PAFAH2 | platelet-activating factor acetylhydrolase 2, 40kDa | 1 | | 1024 | hsa-miR-190 | 5051 | PAFAH2 | platelet-activating factor acetylhydrolase 2, 40kDa | 1 | | 1025 | hsa-miR-503 | 5051 | PAFAH2 | platelet-activating factor acetylhydrolase 2, 40kDa | 1 | | 1026 | hsa-miR-585 | 5051 | PAFAH2 | platelet-activating factor acetylhydrolase 2, 40kDa | 1 | | 1027 | hsa-miR-617 | 5051 | PAFAH2 | platelet-activating factor acetylhydrolase 2, 40kDa | 1 | | 1028 | hsa-miR-623 | 5051 | PAFAH2 | platelet-activating factor acetylhydrolase 2, 40kDa | 1 | | 1029 | hsa-mir-628 | 974 | CD79B | CD79b molecule, immunoglobulin-associated beta | 1 | | 1030 | hsa-mir-218-2 | 10385 | BTN2A2 | butyrophilin, subfamily 2, member A2 | 1 | | 1031 | hsa-mir-126 | 10385 | BTN2A2 | butyrophilin, subfamily 2, member A2 | 1 | | 1032 | hsa-mir-196b | 10385 | BTN2A2 | butyrophilin, subfamily 2, member A2 | 1 | | 1033 | hsa-mir-424 | 10385 | BTN2A2 | butyrophilin, subfamily 2, member A2 | 1 | | 1034 | hsa-mir-490 | 10385 | BTN2A2 | butyrophilin, subfamily 2, member A2 | 1 | | 1035 | hsa-mir-556 | 10385 | BTN2A2 | butyrophilin, subfamily 2, member A2 | 1 | | 1036 | hsa-mir-628 | 10385 | BTN2A2 | butyrophilin, subfamily 2, member A2 | 1 | | 1037 | hsa-mir-1915 | 10385 | BTN2A2 | butyrophilin, subfamily 2, member A2 | 1 | | 1038 | hsa-miR-190 | 10385 | BTN2A2 | butyrophilin, subfamily 2, member A2 | 1 | | 1039 | hsa-miR-196b | 10385 | BTN2A2 | butyrophilin, subfamily 2, member A2 | 1 | | 1040 | hsa-miR-490-5p | 10385 | BTN2A2 | butyrophilin, subfamily 2, member A2 | 1 | | 1041 | hsa-miR-503 | 10385 | BTN2A2 | butyrophilin, subfamily 2, member A2 | 1 | | 1042 | hsa-miR-585 | 10385 | BTN2A2 | butyrophilin, subfamily 2, member A2 | 1 | | 1043 | hsa-miR-586 | 10385 | BTN2A2 | butyrophilin, subfamily 2, member A2 | 1 | | 1044 | hsa-miR-617 | 10385 | BTN2A2 | butyrophilin, subfamily 2, member A2 | 1 | | 1045 | hsa-miR-623 | 10385 | BTN2A2 | butyrophilin, subfamily 2, member A2 | 1 | | 1046 | hsa-miR-126\* | 9715 | FAM131B | family with sequence similarity 131, member B | 1 | | 1047 | hsa-miR-16 | 9715 | FAM131B | family with sequence similarity 131, member B | 1 | | 1048 | hsa-miR-126\* | 6324 | SCN1B | sodium channel, voltage-gated, type I, beta | 1 | | 1049 | hsa-miR-574-5p | 6324 | SCN1B | sodium channel, voltage-gated, type I, beta | 1 | | 1050 | hsa-miR-608 | 6324 | SCN1B | sodium channel, voltage-gated, type I, beta | 1 | | 1051 | hsa-mir-15a | 8492 | PRSS12 | protease, serine, 12 (neurotrypsin, motopsin) | 1 | | 1052 | hsa-mir-16-1 | 8492 | PRSS12 | protease, serine, 12 (neurotrypsin, motopsin) | 1 | | 1053 | hsa-miR-126\* | 8492 | PRSS12 | protease, serine, 12 (neurotrypsin, motopsin) | 1 | | 1054 | hsa-miR-608 | 8492 | PRSS12 | protease, serine, 12 (neurotrypsin, motopsin) | 1 | | 1055 | hsa-mir-556 | 1380 | CR2 | complement component (3d/Epstein Barr virus) receptor 2 | 1 | | 1056 | hsa-miR-190 | 1380 | CR2 | complement component (3d/Epstein Barr virus) receptor 2 | 1 | | 1057 | hsa-miR-618 | 5608 | MAP2K6 | mitogen-activated protein kinase kinase 6 | 1 | | 1058 | hsa-miR-126\* | 22885 | ABLIM3 | actin binding LIM protein family, member 3 | 1 | | 1059 | hsa-miR-1231 | 2949 | GSTM5 | glutathione S-transferase mu 5 | 1 | | 1060 | hsa-miR-126\* | 2949 | GSTM5 | glutathione S-transferase mu 5 | 1 | | 1061 | hsa-miR-608 | 2949 | GSTM5 | glutathione S-transferase mu 5 | 1 | | 1062 | hsa-mir-15a | 8976 | WASL | Wiskott-Aldrich syndrome-like | 1 | | 1063 | hsa-mir-16-1 | 8976 | WASL | Wiskott-Aldrich syndrome-like | 1 | | 1064 | hsa-miR-126\* | 8976 | WASL | Wiskott-Aldrich syndrome-like | 1 | | 1065 | hsa-miR-16 | 8976 | WASL | Wiskott-Aldrich syndrome-like | 1 | | 1066 | hsa-miR-564 | 8976 | WASL | Wiskott-Aldrich syndrome-like | 1 | | 1067 | hsa-miR-566 | 8976 | WASL | Wiskott-Aldrich syndrome-like | 1 | | 1068 | hsa-miR-608 | 8976 | WASL | Wiskott-Aldrich syndrome-like | 1 | | 1069 | hsa-miR-126\* | 51043 | ZBTB7B | zinc finger and BTB domain containing 7B | 1 | | 1070 | hsa-miR-566 | 51043 | ZBTB7B | zinc finger and BTB domain containing 7B | 1 | | 1071 | hsa-mir-628 | 7625 | ZNF74 | zinc finger protein 74 | 1 | | 1072 | hsa-miR-126\* | 5968 | REG1B | regenerating islet-derived 1 beta | 1 | | 1073 | hsa-miR-608 | 5968 | REG1B | regenerating islet-derived 1 beta | 1 | | 1074 | hsa-miR-126\* | 2902 | GRIN1 | glutamate receptor, ionotropic, N-methyl D-aspartate 1 | 1 | | 1075 | hsa-miR-608 | 2902 | GRIN1 | glutamate receptor, ionotropic, N-methyl D-aspartate 1 | 1 | | 1076 | hsa-miR-618 | 6533 | SLC6A6 | solute carrier family 6 (neurotransmitter transporter, taurine), member 6 | 1 | | 1077 | hsa-mir-196b | 9466 | IL27RA | interleukin 27 receptor, alpha | 1 | | 1078 | hsa-mir-424 | 9466 | IL27RA | interleukin 27 receptor, alpha | 1 | | 1079 | hsa-mir-628 | 9466 | IL27RA | interleukin 27 receptor, alpha | 1 | | 1080 | hsa-miR-190 | 9466 | IL27RA | interleukin 27 receptor, alpha | 1 | | 1081 | hsa-miR-196b | 9466 | IL27RA | interleukin 27 receptor, alpha | 1 | | 1082 | hsa-miR-503 | 9466 | IL27RA | interleukin 27 receptor, alpha | 1 | | 1083 | hsa-miR-617 | 9466 | IL27RA | interleukin 27 receptor, alpha | 1 | | 1084 | hsa-miR-623 | 9466 | IL27RA | interleukin 27 receptor, alpha | 1 | | 1085 | hsa-mir-218-2 | 22832 | KIAA1009 | KIAA1009 | 1 | | 1086 | hsa-mir-126 | 22832 | KIAA1009 | KIAA1009 | 1 | | 1087 | hsa-mir-30c-1 | 22832 | KIAA1009 | KIAA1009 | 1 | | 1088 | hsa-mir-30e | 22832 | KIAA1009 | KIAA1009 | 1 | | 1089 | hsa-mir-556 | 22832 | KIAA1009 | KIAA1009 | 1 | | 1090 | hsa-miR-190 | 22832 | KIAA1009 | KIAA1009 | 1 | | 1091 | hsa-miR-585 | 22832 | KIAA1009 | KIAA1009 | 1 | | 1092 | hsa-miR-126\* | 9914 | ATP2C2 | ATPase, Ca++ transporting, type 2C, member 2 | 1 | | 1093 | hsa-miR-564 | 9914 | ATP2C2 | ATPase, Ca++ transporting, type 2C, member 2 | 1 | | 1094 | hsa-miR-618 | 9914 | ATP2C2 | ATPase, Ca++ transporting, type 2C, member 2 | 1 | | 1095 | hsa-mir-424 | 6693 | SPN | sialophorin | 1 | | 1096 | hsa-miR-503 | 6693 | SPN | sialophorin | 1 | | 1097 | hsa-miR-617 | 6693 | SPN | sialophorin | 1 | | 1098 | hsa-miR-1231 | 4356 | MPP3 | membrane protein, palmitoylated 3 (MAGUK p55 subfamily member 3) | 1 | | 1099 | hsa-miR-126\* | 4356 | MPP3 | membrane protein, palmitoylated 3 (MAGUK p55 subfamily member 3) | 1 | | 1100 | hsa-miR-608 | 4356 | MPP3 | membrane protein, palmitoylated 3 (MAGUK p55 subfamily member 3) | 1 | | 1101 | hsa-miR-126\* | 1896 | EDA | ectodysplasin A | 1 | | 1102 | hsa-miR-16 | 1896 | EDA | ectodysplasin A | 1 | | 1103 | hsa-miR-564 | 1896 | EDA | ectodysplasin A | 1 | | 1104 | hsa-miR-566 | 1896 | EDA | ectodysplasin A | 1 | | 1105 | hsa-miR-608 | 1896 | EDA | ectodysplasin A | 1 | | 1106 | hsa-mir-126 | 3841 | KPNA5 | karyopherin alpha 5 (importin alpha 6) | 1 | | 1107 | hsa-mir-424 | 3841 | KPNA5 | karyopherin alpha 5 (importin alpha 6) | 1 | | 1108 | hsa-mir-628 | 3841 | KPNA5 | karyopherin alpha 5 (importin alpha 6) | 1 | | 1109 | hsa-miR-190 | 3841 | KPNA5 | karyopherin alpha 5 (importin alpha 6) | 1 | | 1110 | hsa-miR-503 | 3841 | KPNA5 | karyopherin alpha 5 (importin alpha 6) | 1 | | 1111 | hsa-miR-586 | 3841 | KPNA5 | karyopherin alpha 5 (importin alpha 6) | 1 | | 1112 | hsa-miR-617 | 3841 | KPNA5 | karyopherin alpha 5 (importin alpha 6) | 1 | | 1113 | hsa-miR-623 | 640 | BLK | B lymphoid tyrosine kinase | 1 | | 1114 | hsa-miR-608 | 116 | ADCYAP1 | adenylate cyclase activating polypeptide 1 (pituitary) | 1 | | 1115 | hsa-mir-339 | 8825 | LIN7A | lin-7 homolog A (C. elegans) | 1 | | 1116 | hsa-miR-339-5p | 8825 | LIN7A | lin-7 homolog A (C. elegans) | 1 | | 1117 | hsa-miR-564 | 8825 | LIN7A | lin-7 homolog A (C. elegans) | 1 | | 1118 | hsa-miR-618 | 8825 | LIN7A | lin-7 homolog A (C. elegans) | 1 | | 1119 | hsa-mir-218-2 | 9015 | TAF1A | TATA box binding protein (TBP)-associated factor, RNA polymerase I, A, 48kDa | 1 | | 1120 | hsa-mir-126 | 9015 | TAF1A | TATA box binding protein (TBP)-associated factor, RNA polymerase I, A, 48kDa | 1 | | 1121 | hsa-mir-30c-1 | 9015 | TAF1A | TATA box binding protein (TBP)-associated factor, RNA polymerase I, A, 48kDa | 1 | | 1122 | hsa-mir-30e | 9015 | TAF1A | TATA box binding protein (TBP)-associated factor, RNA polymerase I, A, 48kDa | 1 | | 1123 | hsa-mir-424 | 9015 | TAF1A | TATA box binding protein (TBP)-associated factor, RNA polymerase I, A, 48kDa | 1 | | 1124 | hsa-mir-556 | 9015 | TAF1A | TATA box binding protein (TBP)-associated factor, RNA polymerase I, A, 48kDa | 1 | | 1125 | hsa-mir-1915 | 9015 | TAF1A | TATA box binding protein (TBP)-associated factor, RNA polymerase I, A, 48kDa | 1 | | 1126 | hsa-miR-190 | 9015 | TAF1A | TATA box binding protein (TBP)-associated factor, RNA polymerase I, A, 48kDa | 1 | | 1127 | hsa-miR-338-5p | 9015 | TAF1A | TATA box binding protein (TBP)-associated factor, RNA polymerase I, A, 48kDa | 1 | | 1128 | hsa-miR-503 | 9015 | TAF1A | TATA box binding protein (TBP)-associated factor, RNA polymerase I, A, 48kDa | 1 | | 1129 | hsa-miR-585 | 9015 | TAF1A | TATA box binding protein (TBP)-associated factor, RNA polymerase I, A, 48kDa | 1 | | 1130 | hsa-miR-657 | 9015 | TAF1A | TATA box binding protein (TBP)-associated factor, RNA polymerase I, A, 48kDa | 1 | | 1131 | hsa-mir-15a | 2571 | GAD1 | glutamate decarboxylase 1 (brain, 67kDa) | 1 | | 1132 | hsa-mir-16-1 | 2571 | GAD1 | glutamate decarboxylase 1 (brain, 67kDa) | 1 | | 1133 | hsa-miR-126\* | 2571 | GAD1 | glutamate decarboxylase 1 (brain, 67kDa) | 1 | | 1134 | hsa-miR-16 | 2571 | GAD1 | glutamate decarboxylase 1 (brain, 67kDa) | 1 | | 1135 | hsa-miR-564 | 2571 | GAD1 | glutamate decarboxylase 1 (brain, 67kDa) | 1 | | 1136 | hsa-miR-608 | 2571 | GAD1 | glutamate decarboxylase 1 (brain, 67kDa) | 1 | | 1137 | hsa-miR-1231 | 11016 | ATF7 | activating transcription factor 7 | 1 | | 1138 | hsa-miR-126\* | 11016 | ATF7 | activating transcription factor 7 | 1 | | 1139 | hsa-miR-608 | 11016 | ATF7 | activating transcription factor 7 | 1 | | 1140 | hsa-mir-15a | 3227 | HOXC11 | homeobox C11 | 1 | | 1141 | hsa-miR-126\* | 3227 | HOXC11 | homeobox C11 | 1 | | 1142 | hsa-miR-608 | 3227 | HOXC11 | homeobox C11 | 1 | | 1148 | hsa-mir-15a | 155 | ADRB3 | adrenergic, beta-3-, receptor | 1 | | 1149 | hsa-mir-16-1 | 155 | ADRB3 | adrenergic, beta-3-, receptor | 1 | | 1150 | hsa-miR-126\* | 155 | ADRB3 | adrenergic, beta-3-, receptor | 1 | | 1151 | hsa-miR-16 | 155 | ADRB3 | adrenergic, beta-3-, receptor | 1 | | 1152 | hsa-miR-608 | 155 | ADRB3 | adrenergic, beta-3-, receptor | 1 | | 1153 | hsa-miR-126\* | 1489 | CTF1 | cardiotrophin 1 | 1 | | 1154 | hsa-miR-564 | 1489 | CTF1 | cardiotrophin 1 | 1 | | 1155 | hsa-miR-566 | 1489 | CTF1 | cardiotrophin 1 | 1 | | 1156 | hsa-miR-608 | 1489 | CTF1 | cardiotrophin 1 | 1 | | 1157 | hsa-mir-218-2 | 98 | ACYP2 | acylphosphatase 2, muscle type | 1 | | 1158 | hsa-mir-126 | 98 | ACYP2 | acylphosphatase 2, muscle type | 1 | | 1159 | hsa-mir-424 | 98 | ACYP2 | acylphosphatase 2, muscle type | 1 | | 1160 | hsa-mir-556 | 98 | ACYP2 | acylphosphatase 2, muscle type | 1 | | 1161 | hsa-miR-190 | 98 | ACYP2 | acylphosphatase 2, muscle type | 1 | | 1162 | hsa-miR-503 | 98 | ACYP2 | acylphosphatase 2, muscle type | 1 | | 1163 | hsa-miR-585 | 98 | ACYP2 | acylphosphatase 2, muscle type | 1 | | 1164 | hsa-miR-586 | 98 | ACYP2 | acylphosphatase 2, muscle type | 1 | | 1165 | hsa-miR-126\* | 8092 | ALX1 | ALX homeobox 1 | 1 | | 1166 | hsa-miR-608 | 8092 | ALX1 | ALX homeobox 1 | 1 | | 1167 | hsa-miR-126\* | 5149 | PDE6H | phosphodiesterase 6H, cGMP-specific, cone, gamma | 1 | | 1168 | hsa-miR-548d-5p | 5149 | PDE6H | phosphodiesterase 6H, cGMP-specific, cone, gamma | 1 | | 1169 | hsa-miR-608 | 5149 | PDE6H | phosphodiesterase 6H, cGMP-specific, cone, gamma | 1 | | 1170 | hsa-miR-126\* | 6568 | SLC17A1 | solute carrier family 17 (sodium phosphate), member 1 | 1 | | 1171 | hsa-miR-564 | 6568 | SLC17A1 | solute carrier family 17 (sodium phosphate), member 1 | 1 | | 1172 | hsa-miR-608 | 6568 | SLC17A1 | solute carrier family 17 (sodium phosphate), member 1 | 1 | | 1173 | hsa-miR-126\* | 3589 | IL11 | interleukin 11 | 1 | | 1174 | hsa-miR-16 | 3589 | IL11 | interleukin 11 | 1 | | 1175 | hsa-miR-566 | 3589 | IL11 | interleukin 11 | 1 | | 1176 | hsa-miR-608 | 3589 | IL11 | interleukin 11 | 1 | | 1177 | hsa-mir-15a | 51352 | WIT1 | Wilms tumor upstream neighbor 1 | 1 | | 1178 | hsa-mir-16-1 | 51352 | WIT1 | Wilms tumor upstream neighbor 1 | 1 | | 1179 | hsa-miR-126\* | 51352 | WIT1 | Wilms tumor upstream neighbor 1 | 1 | | 1180 | hsa-miR-16 | 51352 | WIT1 | Wilms tumor upstream neighbor 1 | 1 | | 1181 | hsa-miR-574-5p | 51352 | WIT1 | Wilms tumor upstream neighbor 1 | 1 | | 1182 | hsa-miR-608 | 51352 | WIT1 | Wilms tumor upstream neighbor 1 | 1 | | 1183 | hsa-mir-15a | 732 | C8B | complement component 8, beta polypeptide | 1 | | 1184 | hsa-mir-16-1 | 732 | C8B | complement component 8, beta polypeptide | 1 | | 1185 | hsa-miR-126\* | 732 | C8B | complement component 8, beta polypeptide | 1 | | 1186 | hsa-miR-16 | 732 | C8B | complement component 8, beta polypeptide | 1 | | 1187 | hsa-miR-564 | 732 | C8B | complement component 8, beta polypeptide | 1 | | 1188 | hsa-miR-566 | 732 | C8B | complement component 8, beta polypeptide | 1 | | 1189 | hsa-miR-608 | 732 | C8B | complement component 8, beta polypeptide | 1 | | 1190 | hsa-mir-126 | 1235 | CCR6 | chemokine (C-C motif) receptor 6 | 1 | | 1191 | hsa-mir-30c-1 | 1235 | CCR6 | chemokine (C-C motif) receptor 6 | 1 | | 1192 | hsa-mir-30e | 1235 | CCR6 | chemokine (C-C motif) receptor 6 | 1 | | 1193 | hsa-mir-424 | 1235 | CCR6 | chemokine (C-C motif) receptor 6 | 1 | | 1194 | hsa-mir-556 | 1235 | CCR6 | chemokine (C-C motif) receptor 6 | 1 | | 1195 | hsa-mir-628 | 1235 | CCR6 | chemokine (C-C motif) receptor 6 | 1 | | 1196 | hsa-miR-190 | 1235 | CCR6 | chemokine (C-C motif) receptor 6 | 1 | | 1197 | hsa-miR-338-5p | 1235 | CCR6 | chemokine (C-C motif) receptor 6 | 1 | | 1198 | hsa-miR-503 | 1235 | CCR6 | chemokine (C-C motif) receptor 6 | 1 | | 1199 | hsa-miR-586 | 1235 | CCR6 | chemokine (C-C motif) receptor 6 | 1 | | 1200 | hsa-miR-617 | 1235 | CCR6 | chemokine (C-C motif) receptor 6 | 1 | | 1201 | hsa-miR-657 | 1235 | CCR6 | chemokine (C-C motif) receptor 6 | 1 | | 1202 | hsa-miR-126\* | 7143 | TNR | tenascin R (restrictin, janusin) | 1 | | 1203 | hsa-miR-16 | 7143 | TNR | tenascin R (restrictin, janusin) | 1 | | 1204 | hsa-miR-608 | 7143 | TNR | tenascin R (restrictin, janusin) | 1 | | 1205 | hsa-mir-218-2 | 5533 | PPP3CC | protein phosphatase 3 (formerly 2B), catalytic subunit, gamma isoform | 1 | | 1206 | hsa-mir-126 | 5533 | PPP3CC | protein phosphatase 3 (formerly 2B), catalytic subunit, gamma isoform | 1 | | 1207 | hsa-mir-30c-1 | 5533 | PPP3CC | protein phosphatase 3 (formerly 2B), catalytic subunit, gamma isoform | 1 | | 1208 | hsa-mir-30e | 5533 | PPP3CC | protein phosphatase 3 (formerly 2B), catalytic subunit, gamma isoform | 1 | | 1209 | hsa-mir-424 | 5533 | PPP3CC | protein phosphatase 3 (formerly 2B), catalytic subunit, gamma isoform | 1 | | 1210 | hsa-mir-556 | 5533 | PPP3CC | protein phosphatase 3 (formerly 2B), catalytic subunit, gamma isoform | 1 | | 1211 | hsa-mir-628 | 5533 | PPP3CC | protein phosphatase 3 (formerly 2B), catalytic subunit, gamma isoform | 1 | | 1212 | hsa-miR-190 | 5533 | PPP3CC | protein phosphatase 3 (formerly 2B), catalytic subunit, gamma isoform | 1 | | 1213 | hsa-miR-338-5p | 5533 | PPP3CC | protein phosphatase 3 (formerly 2B), catalytic subunit, gamma isoform | 1 | | 1214 | hsa-miR-503 | 5533 | PPP3CC | protein phosphatase 3 (formerly 2B), catalytic subunit, gamma isoform | 1 | | 1215 | hsa-miR-585 | 5533 | PPP3CC | protein phosphatase 3 (formerly 2B), catalytic subunit, gamma isoform | 1 | | 1216 | hsa-miR-586 | 5533 | PPP3CC | protein phosphatase 3 (formerly 2B), catalytic subunit, gamma isoform | 1 | | 1217 | hsa-miR-617 | 5533 | PPP3CC | protein phosphatase 3 (formerly 2B), catalytic subunit, gamma isoform | 1 | | 1218 | hsa-miR-657 | 5533 | PPP3CC | protein phosphatase 3 (formerly 2B), catalytic subunit, gamma isoform | 1 | | 1219 | hsa-miR-126\* | 6555 | SLC10A2 | solute carrier family 10 (sodium/bile acid cotransporter family), member 2 | 1 | | 1220 | hsa-miR-16 | 6555 | SLC10A2 | solute carrier family 10 (sodium/bile acid cotransporter family), member 2 | 1 | | 1221 | hsa-miR-564 | 6555 | SLC10A2 | solute carrier family 10 (sodium/bile acid cotransporter family), member 2 | 1 | | 1222 | hsa-miR-608 | 6555 | SLC10A2 | solute carrier family 10 (sodium/bile acid cotransporter family), member 2 | 1 | | 1223 | hsa-miR-126\* | 5296 | PIK3R2 | phosphoinositide-3-kinase, regulatory subunit 2 (beta) | 1 | | 1224 | hsa-miR-566 | 5296 | PIK3R2 | phosphoinositide-3-kinase, regulatory subunit 2 (beta) | 1 | | 1225 | hsa-mir-139 | 54799 | MBTD1 | mbt domain containing 1 | 1 | | 1226 | hsa-mir-218-2 | 54799 | MBTD1 | mbt domain containing 1 | 1 | | 1227 | hsa-mir-126 | 54799 | MBTD1 | mbt domain containing 1 | 1 | | 1228 | hsa-mir-424 | 54799 | MBTD1 | mbt domain containing 1 | 1 | | 1229 | hsa-mir-490 | 54799 | MBTD1 | mbt domain containing 1 | 1 | | 1230 | hsa-mir-556 | 54799 | MBTD1 | mbt domain containing 1 | 1 | | 1231 | hsa-mir-1915 | 54799 | MBTD1 | mbt domain containing 1 | 1 | | 1232 | hsa-miR-139-5p | 54799 | MBTD1 | mbt domain containing 1 | 1 | | 1233 | hsa-miR-190 | 54799 | MBTD1 | mbt domain containing 1 | 1 | | 1234 | hsa-miR-490-5p | 54799 | MBTD1 | mbt domain containing 1 | 1 | | 1235 | hsa-miR-503 | 54799 | MBTD1 | mbt domain containing 1 | 1 | | 1236 | hsa-miR-585 | 54799 | MBTD1 | mbt domain containing 1 | 1 | | 1237 | hsa-mir-339 | 420 | ART4 | ADP-ribosyltransferase 4 (Dombrock blood group) | 1 | | 1238 | hsa-miR-339-5p | 420 | ART4 | ADP-ribosyltransferase 4 (Dombrock blood group) | 1 | | 1239 | hsa-miR-608 | 420 | ART4 | ADP-ribosyltransferase 4 (Dombrock blood group) | 1 | | 1240 | hsa-miR-126\* | 3166 | HMX1 | H6 family homeobox 1 | 1 | | 1241 | hsa-miR-16 | 3166 | HMX1 | H6 family homeobox 1 | 1 | | 1242 | hsa-miR-564 | 3166 | HMX1 | H6 family homeobox 1 | 1 | | 1243 | hsa-miR-566 | 3166 | HMX1 | H6 family homeobox 1 | 1 | | 1244 | hsa-miR-608 | 3166 | HMX1 | H6 family homeobox 1 | 1 | | 1245 | hsa-miR-126\* | 3239 | HOXD13 | homeobox D13 | 1 | | 1246 | hsa-miR-16 | 3239 | HOXD13 | homeobox D13 | 1 | | 1247 | hsa-miR-566 | 3239 | HOXD13 | homeobox D13 | 1 | | 1248 | hsa-miR-608 | 3239 | HOXD13 | homeobox D13 | 1 | | 1249 | hsa-mir-15a | 54831 | BEST2 | bestrophin 2 | 1 | | 1250 | hsa-mir-16-1 | 54831 | BEST2 | bestrophin 2 | 1 | | 1251 | hsa-miR-1231 | 54831 | BEST2 | bestrophin 2 | 1 | | 1252 | hsa-miR-126\* | 54831 | BEST2 | bestrophin 2 | 1 | | 1253 | hsa-miR-16 | 54831 | BEST2 | bestrophin 2 | 1 | | 1254 | hsa-miR-608 | 54831 | BEST2 | bestrophin 2 | 1 | | 1255 | hsa-mir-196b | 3004 | GZMM | granzyme M (lymphocyte met-ase 1) | 1 | | 1256 | hsa-mir-424 | 3004 | GZMM | granzyme M (lymphocyte met-ase 1) | 1 | | 1257 | hsa-mir-628 | 3004 | GZMM | granzyme M (lymphocyte met-ase 1) | 1 | | 1258 | hsa-miR-196b | 3004 | GZMM | granzyme M (lymphocyte met-ase 1) | 1 | | 1259 | hsa-miR-503 | 3004 | GZMM | granzyme M (lymphocyte met-ase 1) | 1 | | 1260 | hsa-miR-504 | 3004 | GZMM | granzyme M (lymphocyte met-ase 1) | 1 | | 1261 | hsa-miR-617 | 3004 | GZMM | granzyme M (lymphocyte met-ase 1) | 1 | | 1262 | hsa-miR-623 | 3004 | GZMM | granzyme M (lymphocyte met-ase 1) | 1 | | 1263 | hsa-mir-126 | 80052 | RPS2P45 | ribosomal protein S2 pseudogene 45 | 1 | | 1264 | hsa-miR-190 | 80052 | RPS2P45 | ribosomal protein S2 pseudogene 45 | 1 | | 1265 | hsa-mir-15a | 6532 | SLC6A4 | solute carrier family 6 (neurotransmitter transporter, serotonin), member 4 | 1 | | 1266 | hsa-mir-16-1 | 6532 | SLC6A4 | solute carrier family 6 (neurotransmitter transporter, serotonin), member 4 | 1 | | 1267 | hsa-miR-608 | 6532 | SLC6A4 | solute carrier family 6 (neurotransmitter transporter, serotonin), member 4 | 1 | | 1268 | hsa-miR-1231 | 7982 | ST7 | suppression of tumorigenicity 7 | 1 | | 1269 | hsa-miR-634 | 7982 | ST7 | suppression of tumorigenicity 7 | 1 | | 1270 | hsa-miR-618 | 8867 | SYNJ1 | synaptojanin 1 | 1 | | 1271 | hsa-mir-218-2 | 140710 | C20orf117 | chromosome 20 open reading frame 117 | 1 | | 1272 | hsa-mir-126 | 140710 | C20orf117 | chromosome 20 open reading frame 117 | 1 | | 1273 | hsa-mir-424 | 140710 | C20orf117 | chromosome 20 open reading frame 117 | 1 | | 1274 | hsa-miR-190 | 140710 | C20orf117 | chromosome 20 open reading frame 117 | 1 | | 1275 | hsa-miR-503 | 140710 | C20orf117 | chromosome 20 open reading frame 117 | 1 | | 1276 | hsa-miR-585 | 140710 | C20orf117 | chromosome 20 open reading frame 117 | 1 | | 1277 | hsa-miR-126\* | 1961 | EGR4 | early growth response 4 | 1 | | 1278 | hsa-miR-16 | 1961 | EGR4 | early growth response 4 | 1 | | 1279 | hsa-miR-564 | 1961 | EGR4 | early growth response 4 | 1 | | 1280 | hsa-miR-608 | 1961 | EGR4 | early growth response 4 | 1 | | 1281 | hsa-mir-126 | 120227 | CYP2R1 | cytochrome P450, family 2, subfamily R, polypeptide 1 | 1 | | 1282 | hsa-mir-196b | 120227 | CYP2R1 | cytochrome P450, family 2, subfamily R, polypeptide 1 | 1 | | 1283 | hsa-mir-424 | 120227 | CYP2R1 | cytochrome P450, family 2, subfamily R, polypeptide 1 | 1 | | 1284 | hsa-mir-628 | 120227 | CYP2R1 | cytochrome P450, family 2, subfamily R, polypeptide 1 | 1 | | 1285 | hsa-miR-190 | 120227 | CYP2R1 | cytochrome P450, family 2, subfamily R, polypeptide 1 | 1 | | 1286 | hsa-miR-196b | 120227 | CYP2R1 | cytochrome P450, family 2, subfamily R, polypeptide 1 | 1 | | 1287 | hsa-miR-503 | 120227 | CYP2R1 | cytochrome P450, family 2, subfamily R, polypeptide 1 | 1 | | 1288 | hsa-miR-586 | 120227 | CYP2R1 | cytochrome P450, family 2, subfamily R, polypeptide 1 | 1 | | 1289 | hsa-miR-617 | 120227 | CYP2R1 | cytochrome P450, family 2, subfamily R, polypeptide 1 | 1 | | 1290 | hsa-miR-623 | 120227 | CYP2R1 | cytochrome P450, family 2, subfamily R, polypeptide 1 | 1 | | 1291 | hsa-miR-126\* | 3884 | KRT33B | keratin 33B | 1 | | 1292 | hsa-miR-608 | 3884 | KRT33B | keratin 33B | 1 | | 1293 | hsa-miR-126\* | 1671 | DEFA6 | defensin, alpha 6, Paneth cell-specific | 1 | | 1294 | hsa-mir-628 | 3399 | ID3 | inhibitor of DNA binding 3, dominant negative helix-loop-helix protein | 1 | | 1295 | hsa-miR-617 | 3399 | ID3 | inhibitor of DNA binding 3, dominant negative helix-loop-helix protein | 1 | | 1297 | hsa-mir-196b | 926 | CD8B | CD8b molecule | 1 | | 1298 | hsa-mir-424 | 926 | CD8B | CD8b molecule | 1 | | 1299 | hsa-mir-628 | 926 | CD8B | CD8b molecule | 1 | | 1300 | hsa-miR-196b | 926 | CD8B | CD8b molecule | 1 | | 1301 | hsa-miR-503 | 926 | CD8B | CD8b molecule | 1 | | 1302 | hsa-miR-586 | 926 | CD8B | CD8b molecule | 1 | | 1303 | hsa-miR-617 | 926 | CD8B | CD8b molecule | 1 | | 1304 | hsa-miR-623 | 926 | CD8B | CD8b molecule | 1 | | 1305 | hsa-mir-15a | 9542 | NRG2 | neuregulin 2 | 1 | | 1306 | hsa-mir-16-1 | 9542 | NRG2 | neuregulin 2 | 1 | | 1307 | hsa-miR-126\* | 9542 | NRG2 | neuregulin 2 | 1 | | 1308 | hsa-miR-16 | 9542 | NRG2 | neuregulin 2 | 1 | | 1309 | hsa-miR-608 | 9542 | NRG2 | neuregulin 2 | 1 | | 1310 | hsa-mir-126 | 81550 | TDRD3 | tudor domain containing 3 | 1 | | 1311 | hsa-mir-424 | 81550 | TDRD3 | tudor domain containing 3 | 1 | | 1312 | hsa-mir-556 | 81550 | TDRD3 | tudor domain containing 3 | 1 | | 1313 | hsa-mir-628 | 81550 | TDRD3 | tudor domain containing 3 | 1 | | 1314 | hsa-miR-190 | 81550 | TDRD3 | tudor domain containing 3 | 1 | | 1315 | hsa-miR-338-5p | 81550 | TDRD3 | tudor domain containing 3 | 1 | | 1316 | hsa-miR-503 | 81550 | TDRD3 | tudor domain containing 3 | 1 | | 1317 | hsa-miR-586 | 81550 | TDRD3 | tudor domain containing 3 | 1 | | 1318 | hsa-miR-617 | 81550 | TDRD3 | tudor domain containing 3 | 1 | | 1319 | hsa-miR-657 | 81550 | TDRD3 | tudor domain containing 3 | 1 | | 1320 | hsa-mir-126 | 5042 | PABPC3 | poly(A) binding protein, cytoplasmic 3 | 1 | | 1321 | hsa-mir-424 | 5042 | PABPC3 | poly(A) binding protein, cytoplasmic 3 | 1 | | 1322 | hsa-mir-1915 | 5042 | PABPC3 | poly(A) binding protein, cytoplasmic 3 | 1 | | 1323 | hsa-miR-338-5p | 5042 | PABPC3 | poly(A) binding protein, cytoplasmic 3 | 1 | | 1324 | hsa-miR-503 | 5042 | PABPC3 | poly(A) binding protein, cytoplasmic 3 | 1 | | 1325 | hsa-miR-657 | 5042 | PABPC3 | poly(A) binding protein, cytoplasmic 3 | 1 | | 1326 | hsa-mir-196b | 81887 | LAS1L | LAS1-like (S. cerevisiae) | 1 | | 1327 | hsa-mir-424 | 81887 | LAS1L | LAS1-like (S. cerevisiae) | 1 | | 1328 | hsa-mir-628 | 81887 | LAS1L | LAS1-like (S. cerevisiae) | 1 | | 1329 | hsa-miR-196b | 81887 | LAS1L | LAS1-like (S. cerevisiae) | 1 | | 1330 | hsa-miR-503 | 81887 | LAS1L | LAS1-like (S. cerevisiae) | 1 | | 1331 | hsa-miR-586 | 81887 | LAS1L | LAS1-like (S. cerevisiae) | 1 | | 1332 | hsa-miR-617 | 81887 | LAS1L | LAS1-like (S. cerevisiae) | 1 | | 1333 | hsa-miR-623 | 81887 | LAS1L | LAS1-like (S. cerevisiae) | 1 | | 1334 | hsa-mir-126 | 81856 | ZNF611 | zinc finger protein 611 | 1 | | 1335 | hsa-mir-424 | 81856 | ZNF611 | zinc finger protein 611 | 1 | | 1336 | hsa-mir-1915 | 81856 | ZNF611 | zinc finger protein 611 | 1 | | 1337 | hsa-miR-190 | 81856 | ZNF611 | zinc finger protein 611 | 1 | | 1338 | hsa-miR-503 | 81856 | ZNF611 | zinc finger protein 611 | 1 | | 1339 | hsa-miR-584 | 81856 | ZNF611 | zinc finger protein 611 | 1 | | 1340 | hsa-mir-15a | 5669 | PSG1 | pregnancy specific beta-1-glycoprotein 1 | 1 | | 1341 | hsa-mir-16-1 | 5669 | PSG1 | pregnancy specific beta-1-glycoprotein 1 | 1 | | 1342 | hsa-miR-1231 | 5669 | PSG1 | pregnancy specific beta-1-glycoprotein 1 | 1 | | 1343 | hsa-miR-126\* | 5669 | PSG1 | pregnancy specific beta-1-glycoprotein 1 | 1 | | 1344 | hsa-miR-16 | 5669 | PSG1 | pregnancy specific beta-1-glycoprotein 1 | 1 | | 1345 | hsa-miR-608 | 5669 | PSG1 | pregnancy specific beta-1-glycoprotein 1 | 1 | | 1346 | hsa-miR-126\* | 56302 | TRPV5 | transient receptor potential cation channel, subfamily V, member 5 | 1 | | 1347 | hsa-miR-566 | 56302 | TRPV5 | transient receptor potential cation channel, subfamily V, member 5 | 1 | | 1348 | hsa-miR-608 | 56302 | TRPV5 | transient receptor potential cation channel, subfamily V, member 5 | 1 | | 1349 | hsa-mir-15a | 1553 | CYP2A13 | cytochrome P450, family 2, subfamily A, polypeptide 13 | 1 | | 1350 | hsa-mir-16-1 | 1553 | CYP2A13 | cytochrome P450, family 2, subfamily A, polypeptide 13 | 1 | | 1351 | hsa-miR-1231 | 1553 | CYP2A13 | cytochrome P450, family 2, subfamily A, polypeptide 13 | 1 | | 1352 | hsa-miR-126\* | 1553 | CYP2A13 | cytochrome P450, family 2, subfamily A, polypeptide 13 | 1 | | 1353 | hsa-miR-16 | 1553 | CYP2A13 | cytochrome P450, family 2, subfamily A, polypeptide 13 | 1 | | 1354 | hsa-miR-608 | 1553 | CYP2A13 | cytochrome P450, family 2, subfamily A, polypeptide 13 | 1 | | 1355 | hsa-mir-15a | 1576 | CYP3A4 | cytochrome P450, family 3, subfamily A, polypeptide 4 | 1 | | 1356 | hsa-mir-16-1 | 1576 | CYP3A4 | cytochrome P450, family 3, subfamily A, polypeptide 4 | 1 | | 1357 | hsa-miR-126\* | 1576 | CYP3A4 | cytochrome P450, family 3, subfamily A, polypeptide 4 | 1 | | 1358 | hsa-miR-16 | 1576 | CYP3A4 | cytochrome P450, family 3, subfamily A, polypeptide 4 | 1 | | 1359 | hsa-miR-564 | 1576 | CYP3A4 | cytochrome P450, family 3, subfamily A, polypeptide 4 | 1 | | 1360 | hsa-miR-608 | 1576 | CYP3A4 | cytochrome P450, family 3, subfamily A, polypeptide 4 | 1 | | 1361 | hsa-mir-15a | 8170 | SLC14A2 | solute carrier family 14 (urea transporter), member 2 | 1 | | 1362 | hsa-mir-16-1 | 8170 | SLC14A2 | solute carrier family 14 (urea transporter), member 2 | 1 | | 1363 | hsa-miR-126\* | 8170 | SLC14A2 | solute carrier family 14 (urea transporter), member 2 | 1 | | 1364 | hsa-miR-16 | 8170 | SLC14A2 | solute carrier family 14 (urea transporter), member 2 | 1 | | 1365 | hsa-miR-608 | 8170 | SLC14A2 | solute carrier family 14 (urea transporter), member 2 | 1 | | 1368 | hsa-mir-25 | 10052 | GJC1 | gap junction protein, gamma 1, 45kDa | 1 | | 1369 | hsa-mir-93 | 10052 | GJC1 | gap junction protein, gamma 1, 45kDa | 1 | | 1370 | hsa-mir-106b | 10052 | GJC1 | gap junction protein, gamma 1, 45kDa | 1 | | 1371 | hsa-mir-339 | 10052 | GJC1 | gap junction protein, gamma 1, 45kDa | 1 | | 1373 | hsa-miR-339-5p | 10052 | GJC1 | gap junction protein, gamma 1, 45kDa | 1 | | 1375 | hsa-miR-126\* | 5239 | PGM5 | phosphoglucomutase 5 | 1 | | 1376 | hsa-miR-16 | 5239 | PGM5 | phosphoglucomutase 5 | 1 | | 1377 | hsa-miR-564 | 5239 | PGM5 | phosphoglucomutase 5 | 1 | | 1378 | hsa-miR-566 | 5239 | PGM5 | phosphoglucomutase 5 | 1 | | 1379 | hsa-miR-608 | 5239 | PGM5 | phosphoglucomutase 5 | 1 | | 1380 | hsa-mir-15a | 56125 | PCDHB11 | protocadherin beta 11 | 1 | | 1381 | hsa-mir-16-1 | 56125 | PCDHB11 | protocadherin beta 11 | 1 | | 1382 | hsa-miR-126\* | 56125 | PCDHB11 | protocadherin beta 11 | 1 | | 1383 | hsa-miR-16 | 56125 | PCDHB11 | protocadherin beta 11 | 1 | | 1384 | hsa-miR-564 | 56125 | PCDHB11 | protocadherin beta 11 | 1 | | 1385 | hsa-miR-608 | 56125 | PCDHB11 | protocadherin beta 11 | 1 | | 1386 | hsa-mir-424 | 4666 | NACA | nascent polypeptide-associated complex alpha subunit | 1 | | 1387 | hsa-mir-628 | 4666 | NACA | nascent polypeptide-associated complex alpha subunit | 1 | | 1388 | hsa-miR-503 | 4666 | NACA | nascent polypeptide-associated complex alpha subunit | 1 | | 1389 | hsa-miR-586 | 4666 | NACA | nascent polypeptide-associated complex alpha subunit | 1 | | 1390 | hsa-miR-617 | 4666 | NACA | nascent polypeptide-associated complex alpha subunit | 1 | | 1391 | hsa-mir-196b | 6188 | RPS3 | ribosomal protein S3 | 1 | | 1392 | hsa-mir-424 | 6188 | RPS3 | ribosomal protein S3 | 1 | | 1393 | hsa-mir-490 | 6188 | RPS3 | ribosomal protein S3 | 1 | | 1394 | hsa-mir-628 | 6188 | RPS3 | ribosomal protein S3 | 1 | | 1395 | hsa-mir-1915 | 6188 | RPS3 | ribosomal protein S3 | 1 | | 1396 | hsa-miR-190 | 6188 | RPS3 | ribosomal protein S3 | 1 | | 1397 | hsa-miR-196b | 6188 | RPS3 | ribosomal protein S3 | 1 | | 1398 | hsa-miR-490-5p | 6188 | RPS3 | ribosomal protein S3 | 1 | | 1399 | hsa-miR-503 | 6188 | RPS3 | ribosomal protein S3 | 1 | | 1400 | hsa-miR-617 | 6188 | RPS3 | ribosomal protein S3 | 1 | | 1401 | hsa-miR-623 | 6188 | RPS3 | ribosomal protein S3 | 1 | | 1402 | hsa-mir-30c-1 | 23015 | GOLGA8A | golgin A8 family, member A | 1 | | 1403 | hsa-mir-30e | 23015 | GOLGA8A | golgin A8 family, member A | 1 | | 1404 | hsa-mir-574 | 23015 | GOLGA8A | golgin A8 family, member A | 1 | | 1405 | hsa-mir-628 | 23015 | GOLGA8A | golgin A8 family, member A | 1 | | 1406 | hsa-miR-30c | 23015 | GOLGA8A | golgin A8 family, member A | 1 | | 1407 | hsa-miR-30e | 23015 | GOLGA8A | golgin A8 family, member A | 1 | | 1408 | hsa-miR-586 | 23015 | GOLGA8A | golgin A8 family, member A | 1 | | 1409 | hsa-miR-617 | 23015 | GOLGA8A | golgin A8 family, member A | 1 | | 1410 | hsa-mir-126 | 25814 | ATXN10 | ataxin 10 | 1 | | 1411 | hsa-mir-30c-1 | 25814 | ATXN10 | ataxin 10 | 1 | | 1412 | hsa-mir-30e | 25814 | ATXN10 | ataxin 10 | 1 | | 1413 | hsa-mir-424 | 25814 | ATXN10 | ataxin 10 | 1 | | 1414 | hsa-mir-628 | 25814 | ATXN10 | ataxin 10 | 1 | | 1415 | hsa-miR-30c | 25814 | ATXN10 | ataxin 10 | 1 | | 1416 | hsa-miR-30e | 25814 | ATXN10 | ataxin 10 | 1 | | 1417 | hsa-miR-338-5p | 25814 | ATXN10 | ataxin 10 | 1 | | 1418 | hsa-miR-503 | 25814 | ATXN10 | ataxin 10 | 1 | | 1419 | hsa-miR-586 | 25814 | ATXN10 | ataxin 10 | 1 | | 1420 | hsa-miR-617 | 25814 | ATXN10 | ataxin 10 | 1 | | 1421 | hsa-miR-657 | 25814 | ATXN10 | ataxin 10 | 1 | | 1422 | hsa-mir-126 | 3122 | HLA-DRA | major histocompatibility complex, class II, DR alpha | 1 | | 1427 | hsa-mir-1915 | 3122 | HLA-DRA | major histocompatibility complex, class II, DR alpha | 1 | | 1436 | hsa-miR-504 | 6137 | RPL13 | ribosomal protein L13 | 1 | | 1438 | hsa-mir-196b | 3609 | ILF3 | interleukin enhancer binding factor 3, 90kDa | 1 | | 1439 | hsa-mir-424 | 3609 | ILF3 | interleukin enhancer binding factor 3, 90kDa | 1 | | 1440 | hsa-mir-490 | 3609 | ILF3 | interleukin enhancer binding factor 3, 90kDa | 1 | | 1441 | hsa-mir-628 | 3609 | ILF3 | interleukin enhancer binding factor 3, 90kDa | 1 | | 1442 | hsa-miR-190 | 3609 | ILF3 | interleukin enhancer binding factor 3, 90kDa | 1 | | 1443 | hsa-miR-196b | 3609 | ILF3 | interleukin enhancer binding factor 3, 90kDa | 1 | | 1444 | hsa-miR-490-5p | 3609 | ILF3 | interleukin enhancer binding factor 3, 90kDa | 1 | | 1445 | hsa-miR-503 | 3609 | ILF3 | interleukin enhancer binding factor 3, 90kDa | 1 | | 1446 | hsa-miR-617 | 3609 | ILF3 | interleukin enhancer binding factor 3, 90kDa | 1 | | 1447 | hsa-miR-623 | 3609 | ILF3 | interleukin enhancer binding factor 3, 90kDa | 1 | | 1448 | hsa-mir-218-2 | 9733 | SART3 | squamous cell carcinoma antigen recognized by T cells 3 | 1 | | 1449 | hsa-mir-126 | 9733 | SART3 | squamous cell carcinoma antigen recognized by T cells 3 | 1 | | 1450 | hsa-mir-424 | 9733 | SART3 | squamous cell carcinoma antigen recognized by T cells 3 | 1 | | 1451 | hsa-mir-490 | 9733 | SART3 | squamous cell carcinoma antigen recognized by T cells 3 | 1 | | 1452 | hsa-mir-556 | 9733 | SART3 | squamous cell carcinoma antigen recognized by T cells 3 | 1 | | 1453 | hsa-mir-1915 | 9733 | SART3 | squamous cell carcinoma antigen recognized by T cells 3 | 1 | | 1454 | hsa-miR-190 | 9733 | SART3 | squamous cell carcinoma antigen recognized by T cells 3 | 1 | | 1455 | hsa-miR-490-5p | 9733 | SART3 | squamous cell carcinoma antigen recognized by T cells 3 | 1 | | 1456 | hsa-miR-503 | 9733 | SART3 | squamous cell carcinoma antigen recognized by T cells 3 | 1 | | 1457 | hsa-miR-585 | 9733 | SART3 | squamous cell carcinoma antigen recognized by T cells 3 | 1 | | 1458 | hsa-miR-623 | 9733 | SART3 | squamous cell carcinoma antigen recognized by T cells 3 | 1 | | 1459 | hsa-mir-126 | 6194 | RPS6 | ribosomal protein S6 | 1 | | 1460 | hsa-mir-196b | 6194 | RPS6 | ribosomal protein S6 | 1 | | 1461 | hsa-mir-424 | 6194 | RPS6 | ribosomal protein S6 | 1 | | 1462 | hsa-mir-490 | 6194 | RPS6 | ribosomal protein S6 | 1 | | 1463 | hsa-mir-628 | 6194 | RPS6 | ribosomal protein S6 | 1 | | 1464 | hsa-mir-1915 | 6194 | RPS6 | ribosomal protein S6 | 1 | | 1465 | hsa-miR-190 | 6194 | RPS6 | ribosomal protein S6 | 1 | | 1466 | hsa-miR-196b | 6194 | RPS6 | ribosomal protein S6 | 1 | | 1467 | hsa-miR-490-5p | 6194 | RPS6 | ribosomal protein S6 | 1 | | 1468 | hsa-miR-503 | 6194 | RPS6 | ribosomal protein S6 | 1 | | 1469 | hsa-miR-617 | 6194 | RPS6 | ribosomal protein S6 | 1 | | 1470 | hsa-miR-623 | 6194 | RPS6 | ribosomal protein S6 | 1 | | 1471 | hsa-mir-126 | 9126 | SMC3 | structural maintenance of chromosomes 3 | 1 | | 1472 | hsa-mir-30c-1 | 9126 | SMC3 | structural maintenance of chromosomes 3 | 1 | | 1473 | hsa-mir-30e | 9126 | SMC3 | structural maintenance of chromosomes 3 | 1 | | 1474 | hsa-mir-218-2 | 10767 | HBS1L | HBS1-like (S. cerevisiae) | 1 | | 1475 | hsa-mir-126 | 10767 | HBS1L | HBS1-like (S. cerevisiae) | 1 | | 1476 | hsa-mir-556 | 10767 | HBS1L | HBS1-like (S. cerevisiae) | 1 | | 1477 | hsa-miR-190 | 10767 | HBS1L | HBS1-like (S. cerevisiae) | 1 | | 1478 | hsa-miR-585 | 10767 | HBS1L | HBS1-like (S. cerevisiae) | 1 | | 1479 | hsa-miR-1231 | 5678 | PSG9 | pregnancy specific beta-1-glycoprotein 9 | 1 | | 1480 | hsa-miR-126\* | 5678 | PSG9 | pregnancy specific beta-1-glycoprotein 9 | 1 | | 1481 | hsa-miR-608 | 5678 | PSG9 | pregnancy specific beta-1-glycoprotein 9 | 1 | | 1482 | hsa-miR-608 | 25878 | MXRA5 | matrix-remodelling associated 5 | 1 | | 1483 | hsa-miR-126\* | 6509 | SLC1A4 | solute carrier family 1 (glutamate/neutral amino acid transporter), member 4 | 1 | | 1484 | hsa-mir-196b | 3119 | HLA-DQB1 | major histocompatibility complex, class II, DQ beta 1 | 1 | | 1485 | hsa-mir-424 | 3119 | HLA-DQB1 | major histocompatibility complex, class II, DQ beta 1 | 1 | | 1486 | hsa-mir-628 | 3119 | HLA-DQB1 | major histocompatibility complex, class II, DQ beta 1 | 1 | | 1487 | hsa-miR-190 | 3119 | HLA-DQB1 | major histocompatibility complex, class II, DQ beta 1 | 1 | | 1488 | hsa-miR-196b | 3119 | HLA-DQB1 | major histocompatibility complex, class II, DQ beta 1 | 1 | | 1489 | hsa-miR-503 | 3119 | HLA-DQB1 | major histocompatibility complex, class II, DQ beta 1 | 1 | | 1490 | hsa-miR-617 | 3119 | HLA-DQB1 | major histocompatibility complex, class II, DQ beta 1 | 1 | | 1491 | hsa-miR-623 | 3119 | HLA-DQB1 | major histocompatibility complex, class II, DQ beta 1 | 1 | | 1492 | hsa-mir-454 | 3817 | KLK2 | kallikrein-related peptidase 2 | 1 | | 1493 | hsa-miR-16 | 3817 | KLK2 | kallikrein-related peptidase 2 | 1 | | 1494 | hsa-miR-675 | 3817 | KLK2 | kallikrein-related peptidase 2 | 1 | | 1495 | hsa-miR-95 | 3817 | KLK2 | kallikrein-related peptidase 2 | 1 | | 1496 | hsa-mir-339 | 3205 | HOXA9 | homeobox A9 | 1 | | 1497 | hsa-miR-1231 | 3205 | HOXA9 | homeobox A9 | 1 | | 1498 | hsa-miR-339-5p | 3205 | HOXA9 | homeobox A9 | 1 | | 1499 | hsa-miR-574-5p | 3205 | HOXA9 | homeobox A9 | 1 | | 1500 | hsa-miR-634 | 3205 | HOXA9 | homeobox A9 | 1 | | 1502 | hsa-miR-190 | 8115 | TCL1A | T-cell leukemia/lymphoma 1A | 1 | | 1503 | hsa-mir-218-2 | 4116 | MAGOH | mago-nashi homolog, proliferation-associated (Drosophila) | 1 | | 1504 | hsa-mir-126 | 4116 | MAGOH | mago-nashi homolog, proliferation-associated (Drosophila) | 1 | | 1505 | hsa-mir-424 | 4116 | MAGOH | mago-nashi homolog, proliferation-associated (Drosophila) | 1 | | 1506 | hsa-mir-490 | 4116 | MAGOH | mago-nashi homolog, proliferation-associated (Drosophila) | 1 | | 1507 | hsa-mir-556 | 4116 | MAGOH | mago-nashi homolog, proliferation-associated (Drosophila) | 1 | | 1508 | hsa-mir-1915 | 4116 | MAGOH | mago-nashi homolog, proliferation-associated (Drosophila) | 1 | | 1509 | hsa-miR-190 | 4116 | MAGOH | mago-nashi homolog, proliferation-associated (Drosophila) | 1 | | 1510 | hsa-miR-490-5p | 4116 | MAGOH | mago-nashi homolog, proliferation-associated (Drosophila) | 1 | | 1511 | hsa-miR-503 | 4116 | MAGOH | mago-nashi homolog, proliferation-associated (Drosophila) | 1 | | 1512 | hsa-miR-585 | 4116 | MAGOH | mago-nashi homolog, proliferation-associated (Drosophila) | 1 | | 1513 | hsa-miR-617 | 4116 | MAGOH | mago-nashi homolog, proliferation-associated (Drosophila) | 1 | | 1514 | hsa-miR-126\* | 5959 | RDH5 | retinol dehydrogenase 5 (11-cis/9-cis) | 1 | | 1515 | hsa-miR-564 | 5959 | RDH5 | retinol dehydrogenase 5 (11-cis/9-cis) | 1 | | 1516 | hsa-miR-608 | 5959 | RDH5 | retinol dehydrogenase 5 (11-cis/9-cis) | 1 | | 1517 | hsa-miR-608 | 11199 | ANXA10 | annexin A10 | 1 | | 1518 | hsa-miR-618 | 5049 | PAFAH1B2 | platelet-activating factor acetylhydrolase 1b, catalytic subunit 2 (30kDa) | 1 | | 1525 | hsa-miR-675 | 4850 | CNOT4 | CCR4-NOT transcription complex, subunit 4 | 1 | | 1526 | hsa-miR-95 | 4850 | CNOT4 | CCR4-NOT transcription complex, subunit 4 | 1 | | 1527 | hsa-miR-608 | 9228 | DLGAP2 | discs, large (Drosophila) homolog-associated protein 2 | 1 | | 1528 | hsa-mir-126 | 22902 | RUFY3 | RUN and FYVE domain containing 3 | 1 | | 1529 | hsa-mir-30c-1 | 22902 | RUFY3 | RUN and FYVE domain containing 3 | 1 | | 1530 | hsa-mir-30e | 22902 | RUFY3 | RUN and FYVE domain containing 3 | 1 | | 1531 | hsa-mir-424 | 22902 | RUFY3 | RUN and FYVE domain containing 3 | 1 | | 1532 | hsa-mir-556 | 22902 | RUFY3 | RUN and FYVE domain containing 3 | 1 | | 1533 | hsa-mir-628 | 22902 | RUFY3 | RUN and FYVE domain containing 3 | 1 | | 1534 | hsa-miR-190 | 22902 | RUFY3 | RUN and FYVE domain containing 3 | 1 | | 1535 | hsa-miR-338-5p | 22902 | RUFY3 | RUN and FYVE domain containing 3 | 1 | | 1536 | hsa-miR-503 | 22902 | RUFY3 | RUN and FYVE domain containing 3 | 1 | | 1537 | hsa-miR-586 | 22902 | RUFY3 | RUN and FYVE domain containing 3 | 1 | | 1538 | hsa-miR-617 | 22902 | RUFY3 | RUN and FYVE domain containing 3 | 1 | | 1539 | hsa-miR-657 | 22902 | RUFY3 | RUN and FYVE domain containing 3 | 1 | | 1540 | hsa-mir-218-2 | 11154 | AP4S1 | adaptor-related protein complex 4, sigma 1 subunit | 1 | | 1541 | hsa-mir-126 | 11154 | AP4S1 | adaptor-related protein complex 4, sigma 1 subunit | 1 | | 1542 | hsa-miR-190 | 11154 | AP4S1 | adaptor-related protein complex 4, sigma 1 subunit | 1 | | 1543 | hsa-miR-585 | 11154 | AP4S1 | adaptor-related protein complex 4, sigma 1 subunit | 1 | | 1544 | hsa-mir-15a | 7498 | XDH | xanthine dehydrogenase | 1 | | 1545 | hsa-mir-16-1 | 7498 | XDH | xanthine dehydrogenase | 1 | | 1546 | hsa-miR-1231 | 7498 | XDH | xanthine dehydrogenase | 1 | | 1547 | hsa-miR-126\* | 7498 | XDH | xanthine dehydrogenase | 1 | | 1548 | hsa-miR-16 | 7498 | XDH | xanthine dehydrogenase | 1 | | 1549 | hsa-miR-608 | 7498 | XDH | xanthine dehydrogenase | 1 | | 1550 | hsa-miR-454\* | 7422 | VEGFA | vascular endothelial growth factor A | 1 | | 1551 | hsa-miR-548d-5p | 7422 | VEGFA | vascular endothelial growth factor A | 1 | | 1552 | hsa-miR-1231 | 846 | CASR | calcium-sensing receptor | 1 | | 1553 | hsa-miR-126\* | 846 | CASR | calcium-sensing receptor | 1 | | 1554 | hsa-miR-16 | 846 | CASR | calcium-sensing receptor | 1 | | 1555 | hsa-miR-564 | 846 | CASR | calcium-sensing receptor | 1 | | 1556 | hsa-miR-608 | 846 | CASR | calcium-sensing receptor | 1 | | 1557 | hsa-miR-126\* | 2048 | EPHB2 | EPH receptor B2 | 1 | | 1558 | hsa-miR-608 | 2048 | EPHB2 | EPH receptor B2 | 1 | | 1560 | hsa-miR-608 | 843 | CASP10 | caspase 10, apoptosis-related cysteine peptidase | 1 | | 1561 | hsa-miR-126\* | 3651 | PDX1 | pancreatic and duodenal homeobox 1 | 1 | | 1562 | hsa-miR-16 | 3651 | PDX1 | pancreatic and duodenal homeobox 1 | 1 | | 1563 | hsa-miR-566 | 3651 | PDX1 | pancreatic and duodenal homeobox 1 | 1 | | 1564 | hsa-miR-608 | 3651 | PDX1 | pancreatic and duodenal homeobox 1 | 1 | | 1565 | hsa-miR-617 | 11183 | MAP4K5 | mitogen-activated protein kinase kinase kinase kinase 5 | 1 | | 1573 | hsa-mir-196b | 4068 | SH2D1A | SH2 domain protein 1A | 1 | | 1574 | hsa-miR-196b | 4068 | SH2D1A | SH2 domain protein 1A | 1 | | 1575 | hsa-miR-126\* | 7039 | TGFA | transforming growth factor, alpha | 1 | | 1576 | hsa-miR-566 | 7039 | TGFA | transforming growth factor, alpha | 1 | | 1577 | hsa-miR-618 | 7039 | TGFA | transforming growth factor, alpha | 1 | | 1578 | hsa-mir-218-2 | 2145 | EZH1 | enhancer of zeste homolog 1 (Drosophila) | 1 | | 1579 | hsa-mir-126 | 2145 | EZH1 | enhancer of zeste homolog 1 (Drosophila) | 1 | | 1580 | hsa-mir-424 | 2145 | EZH1 | enhancer of zeste homolog 1 (Drosophila) | 1 | | 1581 | hsa-mir-490 | 2145 | EZH1 | enhancer of zeste homolog 1 (Drosophila) | 1 | | 1582 | hsa-mir-556 | 2145 | EZH1 | enhancer of zeste homolog 1 (Drosophila) | 1 | | 1583 | hsa-mir-1915 | 2145 | EZH1 | enhancer of zeste homolog 1 (Drosophila) | 1 | | 1584 | hsa-miR-190 | 2145 | EZH1 | enhancer of zeste homolog 1 (Drosophila) | 1 | | 1585 | hsa-miR-455-5p | 2145 | EZH1 | enhancer of zeste homolog 1 (Drosophila) | 1 | | 1586 | hsa-miR-490-5p | 2145 | EZH1 | enhancer of zeste homolog 1 (Drosophila) | 1 | | 1587 | hsa-miR-503 | 2145 | EZH1 | enhancer of zeste homolog 1 (Drosophila) | 1 | | 1588 | hsa-miR-585 | 2145 | EZH1 | enhancer of zeste homolog 1 (Drosophila) | 1 | | 1589 | hsa-mir-15a | 8840 | WISP1 | WNT1 inducible signaling pathway protein 1 | 1 | | 1590 | hsa-mir-16-1 | 8840 | WISP1 | WNT1 inducible signaling pathway protein 1 | 1 | | 1591 | hsa-miR-126\* | 8840 | WISP1 | WNT1 inducible signaling pathway protein 1 | 1 | | 1592 | hsa-miR-16 | 8840 | WISP1 | WNT1 inducible signaling pathway protein 1 | 1 | | 1593 | hsa-miR-566 | 8840 | WISP1 | WNT1 inducible signaling pathway protein 1 | 1 | | 1594 | hsa-miR-608 | 8840 | WISP1 | WNT1 inducible signaling pathway protein 1 | 1 | | 1595 | hsa-mir-339 | 23569 | PADI4 | peptidyl arginine deiminase, type IV | 1 | | 1596 | hsa-miR-126\* | 23569 | PADI4 | peptidyl arginine deiminase, type IV | 1 | | 1597 | hsa-miR-339-5p | 23569 | PADI4 | peptidyl arginine deiminase, type IV | 1 | | 1598 | hsa-miR-564 | 23569 | PADI4 | peptidyl arginine deiminase, type IV | 1 | | 1599 | hsa-miR-618 | 23569 | PADI4 | peptidyl arginine deiminase, type IV | 1 | | 1600 | hsa-miR-634 | 23569 | PADI4 | peptidyl arginine deiminase, type IV | 1 | | 1603 | hsa-mir-15a | 2078 | ERG | v-ets erythroblastosis virus E26 oncogene homolog (avian) | 1 | | 1604 | hsa-mir-16-1 | 2078 | ERG | v-ets erythroblastosis virus E26 oncogene homolog (avian) | 1 | | 1605 | hsa-mir-339 | 2078 | ERG | v-ets erythroblastosis virus E26 oncogene homolog (avian) | 1 | | 1606 | hsa-miR-126\* | 2078 | ERG | v-ets erythroblastosis virus E26 oncogene homolog (avian) | 1 | | 1607 | hsa-miR-339-5p | 2078 | ERG | v-ets erythroblastosis virus E26 oncogene homolog (avian) | 1 | | 1608 | hsa-miR-564 | 2078 | ERG | v-ets erythroblastosis virus E26 oncogene homolog (avian) | 1 | | 1609 | hsa-miR-608 | 2078 | ERG | v-ets erythroblastosis virus E26 oncogene homolog (avian) | 1 | | 1610 | hsa-mir-218-2 | 90233 | ZNF551 | zinc finger protein 551 | 1 | | 1611 | hsa-mir-126 | 90233 | ZNF551 | zinc finger protein 551 | 1 | | 1612 | hsa-mir-196b | 90233 | ZNF551 | zinc finger protein 551 | 1 | | 1613 | hsa-mir-424 | 90233 | ZNF551 | zinc finger protein 551 | 1 | | 1614 | hsa-mir-490 | 90233 | ZNF551 | zinc finger protein 551 | 1 | | 1615 | hsa-mir-556 | 90233 | ZNF551 | zinc finger protein 551 | 1 | | 1616 | hsa-mir-628 | 90233 | ZNF551 | zinc finger protein 551 | 1 | | 1617 | hsa-mir-1915 | 90233 | ZNF551 | zinc finger protein 551 | 1 | | 1618 | hsa-miR-190 | 90233 | ZNF551 | zinc finger protein 551 | 1 | | 1619 | hsa-miR-196b | 90233 | ZNF551 | zinc finger protein 551 | 1 | | 1620 | hsa-miR-490-5p | 90233 | ZNF551 | zinc finger protein 551 | 1 | | 1621 | hsa-miR-503 | 90233 | ZNF551 | zinc finger protein 551 | 1 | | 1622 | hsa-miR-585 | 90233 | ZNF551 | zinc finger protein 551 | 1 | | 1623 | hsa-miR-586 | 90233 | ZNF551 | zinc finger protein 551 | 1 | | 1624 | hsa-miR-617 | 90233 | ZNF551 | zinc finger protein 551 | 1 | | 1625 | hsa-miR-623 | 90233 | ZNF551 | zinc finger protein 551 | 1 | | 1626 | hsa-mir-574 | 1973 | EIF4A1 | eukaryotic translation initiation factor 4A1 | 1 | | 1627 | hsa-mir-1915 | 1973 | EIF4A1 | eukaryotic translation initiation factor 4A1 | 1 | | 1628 | hsa-miR-126\* | 22877 | MLXIP | MLX interacting protein | 1 | | 1629 | hsa-miR-566 | 22877 | MLXIP | MLX interacting protein | 1 | | 1630 | hsa-mir-16-1 | 56112 | PCDHGA3 | protocadherin gamma subfamily A, 3 | 1 | | 1631 | hsa-miR-126\* | 56112 | PCDHGA3 | protocadherin gamma subfamily A, 3 | 1 | | 1632 | hsa-miR-16 | 56112 | PCDHGA3 | protocadherin gamma subfamily A, 3 | 1 | | 1633 | hsa-miR-608 | 56112 | PCDHGA3 | protocadherin gamma subfamily A, 3 | 1 | | 1634 | hsa-mir-135b | 3021 | H3F3B | H3 histone, family 3B (H3.3B) | 1 | | 1635 | hsa-miR-616\* | 3021 | H3F3B | H3 histone, family 3B (H3.3B) | 1 | | 1636 | hsa-mir-218-2 | 26156 | RSL1D1 | ribosomal L1 domain containing 1 | 1 | | 1637 | hsa-mir-424 | 26156 | RSL1D1 | ribosomal L1 domain containing 1 | 1 | | 1638 | hsa-mir-556 | 26156 | RSL1D1 | ribosomal L1 domain containing 1 | 1 | | 1639 | hsa-mir-628 | 26156 | RSL1D1 | ribosomal L1 domain containing 1 | 1 | | 1640 | hsa-mir-675 | 26156 | RSL1D1 | ribosomal L1 domain containing 1 | 1 | | 1641 | hsa-miR-190 | 26156 | RSL1D1 | ribosomal L1 domain containing 1 | 1 | | 1642 | hsa-miR-503 | 26156 | RSL1D1 | ribosomal L1 domain containing 1 | 1 | | 1643 | hsa-miR-585 | 26156 | RSL1D1 | ribosomal L1 domain containing 1 | 1 | | 1644 | hsa-miR-586 | 26156 | RSL1D1 | ribosomal L1 domain containing 1 | 1 | | 1645 | hsa-miR-617 | 26156 | RSL1D1 | ribosomal L1 domain containing 1 | 1 | | 1646 | hsa-miR-623 | 26156 | RSL1D1 | ribosomal L1 domain containing 1 | 1 | | 1647 | hsa-mir-490 | 6137 | RPL13 | ribosomal protein L13 | 1 | | 1648 | hsa-mir-574 | 6137 | RPL13 | ribosomal protein L13 | 1 | | 1649 | hsa-miR-490-5p | 6137 | RPL13 | ribosomal protein L13 | 1 | | 1650 | hsa-mir-135b | 4976 | OPA1 | optic atrophy 1 (autosomal dominant) | 1 | | 1651 | hsa-mir-556 | 4976 | OPA1 | optic atrophy 1 (autosomal dominant) | 1 | | 1652 | hsa-mir-675 | 4976 | OPA1 | optic atrophy 1 (autosomal dominant) | 1 | | 1653 | hsa-miR-190 | 4976 | OPA1 | optic atrophy 1 (autosomal dominant) | 1 | | 1654 | hsa-miR-454\* | 4976 | OPA1 | optic atrophy 1 (autosomal dominant) | 1 | | 1655 | hsa-mir-126 | 216 | ALDH1A1 | aldehyde dehydrogenase 1 family, member A1 | 1 | | 1656 | hsa-mir-424 | 216 | ALDH1A1 | aldehyde dehydrogenase 1 family, member A1 | 1 | | 1657 | hsa-mir-628 | 216 | ALDH1A1 | aldehyde dehydrogenase 1 family, member A1 | 1 | | 1658 | hsa-miR-190 | 216 | ALDH1A1 | aldehyde dehydrogenase 1 family, member A1 | 1 | | 1659 | hsa-miR-503 | 216 | ALDH1A1 | aldehyde dehydrogenase 1 family, member A1 | 1 | | 1660 | hsa-miR-617 | 216 | ALDH1A1 | aldehyde dehydrogenase 1 family, member A1 | 1 | | 1661 | hsa-miR-1231 | 9577 | BRE | brain and reproductive organ-expressed (TNFRSF1A modulator) | 1 | | 1662 | hsa-mir-196b | 27335 | EIF3K | eukaryotic translation initiation factor 3, subunit K | 1 | | 1663 | hsa-mir-424 | 27335 | EIF3K | eukaryotic translation initiation factor 3, subunit K | 1 | | 1664 | hsa-mir-490 | 27335 | EIF3K | eukaryotic translation initiation factor 3, subunit K | 1 | | 1665 | hsa-mir-556 | 27335 | EIF3K | eukaryotic translation initiation factor 3, subunit K | 1 | | 1666 | hsa-mir-628 | 27335 | EIF3K | eukaryotic translation initiation factor 3, subunit K | 1 | | 1667 | hsa-miR-190 | 27335 | EIF3K | eukaryotic translation initiation factor 3, subunit K | 1 | | 1668 | hsa-miR-196b | 27335 | EIF3K | eukaryotic translation initiation factor 3, subunit K | 1 | | 1669 | hsa-miR-490-5p | 27335 | EIF3K | eukaryotic translation initiation factor 3, subunit K | 1 | | 1670 | hsa-miR-503 | 27335 | EIF3K | eukaryotic translation initiation factor 3, subunit K | 1 | | 1671 | hsa-miR-504 | 27335 | EIF3K | eukaryotic translation initiation factor 3, subunit K | 1 | | 1672 | hsa-miR-586 | 27335 | EIF3K | eukaryotic translation initiation factor 3, subunit K | 1 | | 1673 | hsa-miR-617 | 27335 | EIF3K | eukaryotic translation initiation factor 3, subunit K | 1 | | 1674 | hsa-miR-623 | 27335 | EIF3K | eukaryotic translation initiation factor 3, subunit K | 1 | | 1675 | hsa-mir-218-2 | 585 | BBS4 | Bardet-Biedl syndrome 4 | 1 | | 1676 | hsa-mir-126 | 585 | BBS4 | Bardet-Biedl syndrome 4 | 1 | | 1677 | hsa-mir-424 | 585 | BBS4 | Bardet-Biedl syndrome 4 | 1 | | 1678 | hsa-mir-556 | 585 | BBS4 | Bardet-Biedl syndrome 4 | 1 | | 1679 | hsa-mir-628 | 585 | BBS4 | Bardet-Biedl syndrome 4 | 1 | | 1680 | hsa-miR-190 | 585 | BBS4 | Bardet-Biedl syndrome 4 | 1 | | 1681 | hsa-miR-196b | 585 | BBS4 | Bardet-Biedl syndrome 4 | 1 | | 1682 | hsa-miR-503 | 585 | BBS4 | Bardet-Biedl syndrome 4 | 1 | | 1683 | hsa-miR-585 | 585 | BBS4 | Bardet-Biedl syndrome 4 | 1 | | 1684 | hsa-miR-617 | 585 | BBS4 | Bardet-Biedl syndrome 4 | 1 | | 1685 | hsa-miR-618 | 875 | CBS | cystathionine-beta-synthase | 1 | | 1686 | hsa-mir-196b | 3507 | IGHM | immunoglobulin heavy constant mu | 1 | | 1687 | hsa-miR-196b | 3507 | IGHM | immunoglobulin heavy constant mu | 1 | | 1688 | hsa-mir-628 | 80097 | FAM128B | family with sequence similarity 128, member B | 1 | | 1689 | hsa-miR-623 | 80097 | FAM128B | family with sequence similarity 128, member B | 1 | | 1690 | hsa-miR-650 | 80097 | FAM128B | family with sequence similarity 128, member B | 1 | | 1691 | hsa-miR-454\* | 23189 | KANK1 | KN motif and ankyrin repeat domains 1 | 1 | | 1692 | hsa-miR-548d-5p | 23189 | KANK1 | KN motif and ankyrin repeat domains 1 | 1 | | 1693 | hsa-mir-15a | 169611 | OLFML2A | olfactomedin-like 2A | 1 | | 1694 | hsa-mir-16-1 | 169611 | OLFML2A | olfactomedin-like 2A | 1 | | 1695 | hsa-miR-1231 | 169611 | OLFML2A | olfactomedin-like 2A | 1 | | 1696 | hsa-miR-16 | 169611 | OLFML2A | olfactomedin-like 2A | 1 | | 1697 | hsa-miR-608 | 169611 | OLFML2A | olfactomedin-like 2A | 1 | | 1698 | hsa-miR-126\* | 815 | CAMK2A | calcium/calmodulin-dependent protein kinase II alpha | 1 | | 1699 | hsa-miR-16 | 815 | CAMK2A | calcium/calmodulin-dependent protein kinase II alpha | 1 | | 1700 | hsa-miR-608 | 815 | CAMK2A | calcium/calmodulin-dependent protein kinase II alpha | 1 | | 1701 | hsa-mir-454 | 3206 | HOXA10 | homeobox A10 | 1 | | 1702 | hsa-miR-675 | 3206 | HOXA10 | homeobox A10 | 1 | | 1703 | hsa-miR-95 | 3206 | HOXA10 | homeobox A10 | 1 | | 1704 | hsa-miR-618 | 22990 | PCNX | pecanex homolog (Drosophila) | 1 | | 1705 | hsa-mir-135b | 10302 | SNAPC5 | small nuclear RNA activating complex, polypeptide 5, 19kDa | 1 | | 1706 | hsa-mir-556 | 10302 | SNAPC5 | small nuclear RNA activating complex, polypeptide 5, 19kDa | 1 | | 1707 | hsa-mir-675 | 10302 | SNAPC5 | small nuclear RNA activating complex, polypeptide 5, 19kDa | 1 | | 1708 | hsa-miR-454\* | 10302 | SNAPC5 | small nuclear RNA activating complex, polypeptide 5, 19kDa | 1 | | 1709 | hsa-miR-1231 | 3851 | KRT4 | keratin 4 | 1 | | 1710 | hsa-miR-126\* | 3851 | KRT4 | keratin 4 | 1 | | 1711 | hsa-miR-608 | 3851 | KRT4 | keratin 4 | 1 | | 1712 | hsa-mir-454 | 283638 | KIAA0284 | KIAA0284 | 1 | | 1713 | hsa-miR-16 | 283638 | KIAA0284 | KIAA0284 | 1 | | 1714 | hsa-miR-675 | 283638 | KIAA0284 | KIAA0284 | 1 | | 1715 | hsa-miR-95 | 283638 | KIAA0284 | KIAA0284 | 1 | | 1716 | hsa-miR-1231 | 2296 | FOXC1 | forkhead box C1 | 1 | | 1717 | hsa-miR-126\* | 2296 | FOXC1 | forkhead box C1 | 1 | | 1718 | hsa-miR-608 | 2296 | FOXC1 | forkhead box C1 | 1 | | 1719 | hsa-mir-30c-1 | 57209 | ZNF248 | zinc finger protein 248 | 1 | | 1720 | hsa-mir-30e | 57209 | ZNF248 | zinc finger protein 248 | 1 | | 1721 | hsa-mir-196b | 57209 | ZNF248 | zinc finger protein 248 | 1 | | 1722 | hsa-mir-424 | 57209 | ZNF248 | zinc finger protein 248 | 1 | | 1723 | hsa-mir-574 | 57209 | ZNF248 | zinc finger protein 248 | 1 | | 1724 | hsa-mir-628 | 57209 | ZNF248 | zinc finger protein 248 | 1 | | 1725 | hsa-miR-196b | 57209 | ZNF248 | zinc finger protein 248 | 1 | | 1726 | hsa-miR-503 | 57209 | ZNF248 | zinc finger protein 248 | 1 | | 1727 | hsa-miR-586 | 57209 | ZNF248 | zinc finger protein 248 | 1 | | 1728 | hsa-miR-617 | 57209 | ZNF248 | zinc finger protein 248 | 1 | | 1729 | hsa-mir-339 | 129642 | MBOAT2 | membrane bound O-acyltransferase domain containing 2 | 1 | | 1730 | hsa-miR-339-5p | 129642 | MBOAT2 | membrane bound O-acyltransferase domain containing 2 | 1 | | 1731 | hsa-miR-564 | 129642 | MBOAT2 | membrane bound O-acyltransferase domain containing 2 | 1 | | 1732 | hsa-miR-618 | 129642 | MBOAT2 | membrane bound O-acyltransferase domain containing 2 | 1 | | 1733 | hsa-miR-566 | 23144 | ZC3H3 | zinc finger CCCH-type containing 3 | 1 | | 1739 | hsa-miR-126\* | 90993 | CREB3L1 | cAMP responsive element binding protein 3-like 1 | 1 | | 1740 | hsa-miR-16 | 90993 | CREB3L1 | cAMP responsive element binding protein 3-like 1 | 1 | | 1741 | hsa-miR-564 | 90993 | CREB3L1 | cAMP responsive element binding protein 3-like 1 | 1 | | 1742 | hsa-miR-608 | 90993 | CREB3L1 | cAMP responsive element binding protein 3-like 1 | 1 | | 1743 | hsa-mir-135b | 6651 | SON | SON DNA binding protein | 1 | | 1744 | hsa-mir-675 | 6651 | SON | SON DNA binding protein | 1 | | 1745 | hsa-miR-454\* | 6651 | SON | SON DNA binding protein | 1 | | 1747 | hsa-mir-196b | 6165 | RPL35A | ribosomal protein L35a | 1 | | 1748 | hsa-mir-424 | 6165 | RPL35A | ribosomal protein L35a | 1 | | 1749 | hsa-mir-556 | 6165 | RPL35A | ribosomal protein L35a | 1 | | 1750 | hsa-mir-628 | 6165 | RPL35A | ribosomal protein L35a | 1 | | 1751 | hsa-miR-190 | 6165 | RPL35A | ribosomal protein L35a | 1 | | 1752 | hsa-miR-196b | 6165 | RPL35A | ribosomal protein L35a | 1 | | 1753 | hsa-miR-503 | 6165 | RPL35A | ribosomal protein L35a | 1 | | 1754 | hsa-miR-504 | 6165 | RPL35A | ribosomal protein L35a | 1 | | 1755 | hsa-miR-586 | 6165 | RPL35A | ribosomal protein L35a | 1 | | 1756 | hsa-miR-617 | 6165 | RPL35A | ribosomal protein L35a | 1 | | 1757 | hsa-miR-623 | 6165 | RPL35A | ribosomal protein L35a | 1 | | 1758 | hsa-miR-1231 | 7421 | VDR | vitamin D (1,25- dihydroxyvitamin D3) receptor | 1 | | 1759 | hsa-miR-126\* | 7421 | VDR | vitamin D (1,25- dihydroxyvitamin D3) receptor | 1 | | 1760 | hsa-miR-608 | 7421 | VDR | vitamin D (1,25- dihydroxyvitamin D3) receptor | 1 | | 1761 | hsa-miR-126\* | 2819 | GPD1 | glycerol-3-phosphate dehydrogenase 1 (soluble) | 1 | | 1762 | hsa-miR-16 | 2819 | GPD1 | glycerol-3-phosphate dehydrogenase 1 (soluble) | 1 | | 1763 | hsa-miR-564 | 2819 | GPD1 | glycerol-3-phosphate dehydrogenase 1 (soluble) | 1 | | 1764 | hsa-miR-566 | 2819 | GPD1 | glycerol-3-phosphate dehydrogenase 1 (soluble) | 1 | | 1765 | hsa-miR-608 | 2819 | GPD1 | glycerol-3-phosphate dehydrogenase 1 (soluble) | 1 | | 1766 | hsa-mir-218-2 | 498 | ATP5A1 | ATP synthase, H+ transporting, mitochondrial F1 complex, alpha subunit 1, cardiac muscle | 1 | | 1767 | hsa-mir-126 | 498 | ATP5A1 | ATP synthase, H+ transporting, mitochondrial F1 complex, alpha subunit 1, cardiac muscle | 1 | | 1768 | hsa-mir-424 | 498 | ATP5A1 | ATP synthase, H+ transporting, mitochondrial F1 complex, alpha subunit 1, cardiac muscle | 1 | | 1769 | hsa-mir-556 | 498 | ATP5A1 | ATP synthase, H+ transporting, mitochondrial F1 complex, alpha subunit 1, cardiac muscle | 1 | | 1770 | hsa-mir-1915 | 498 | ATP5A1 | ATP synthase, H+ transporting, mitochondrial F1 complex, alpha subunit 1, cardiac muscle | 1 | | 1771 | hsa-miR-190 | 498 | ATP5A1 | ATP synthase, H+ transporting, mitochondrial F1 complex, alpha subunit 1, cardiac muscle | 1 | | 1772 | hsa-miR-503 | 498 | ATP5A1 | ATP synthase, H+ transporting, mitochondrial F1 complex, alpha subunit 1, cardiac muscle | 1 | | 1773 | hsa-miR-585 | 498 | ATP5A1 | ATP synthase, H+ transporting, mitochondrial F1 complex, alpha subunit 1, cardiac muscle | 1 | | 1774 | hsa-miR-617 | 498 | ATP5A1 | ATP synthase, H+ transporting, mitochondrial F1 complex, alpha subunit 1, cardiac muscle | 1 | | 1775 | hsa-miR-623 | 498 | ATP5A1 | ATP synthase, H+ transporting, mitochondrial F1 complex, alpha subunit 1, cardiac muscle | 1 | | 1776 | hsa-miR-608 | 138046 | RALYL | RALY RNA binding protein-like | 1 | | 1777 | hsa-miR-634 | 138046 | RALYL | RALY RNA binding protein-like | 1 | | 1778 | hsa-mir-218-2 | 9659 | PDE4DIP | phosphodiesterase 4D interacting protein | 1 | | 1779 | hsa-mir-424 | 9659 | PDE4DIP | phosphodiesterase 4D interacting protein | 1 | | 1780 | hsa-mir-556 | 9659 | PDE4DIP | phosphodiesterase 4D interacting protein | 1 | | 1781 | hsa-mir-628 | 9659 | PDE4DIP | phosphodiesterase 4D interacting protein | 1 | | 1782 | hsa-miR-190 | 9659 | PDE4DIP | phosphodiesterase 4D interacting protein | 1 | | 1783 | hsa-miR-503 | 9659 | PDE4DIP | phosphodiesterase 4D interacting protein | 1 | | 1784 | hsa-miR-585 | 9659 | PDE4DIP | phosphodiesterase 4D interacting protein | 1 | | 1785 | hsa-miR-623 | 9659 | PDE4DIP | phosphodiesterase 4D interacting protein | 1 | | 1786 | hsa-miR-126\* | 653808 | ZG16 | zymogen granule protein 16 homolog (rat) | 1 | | 1787 | hsa-miR-16 | 653808 | ZG16 | zymogen granule protein 16 homolog (rat) | 1 | | 1788 | hsa-miR-564 | 653808 | ZG16 | zymogen granule protein 16 homolog (rat) | 1 | | 1789 | hsa-miR-608 | 653808 | ZG16 | zymogen granule protein 16 homolog (rat) | 1 | | 1790 | hsa-miR-126\* | 11338 | U2AF2 | U2 small nuclear RNA auxiliary factor 2 | 1 | | 1791 | hsa-miR-608 | 11338 | U2AF2 | U2 small nuclear RNA auxiliary factor 2 | 1 | | 1792 | hsa-miR-608 | 10217 | CTDSPL | CTD (carboxy-terminal domain, RNA polymerase II, polypeptide A) small phosphatase-like | 1 | | 1793 | hsa-mir-15a | 92346 | C1orf105 | chromosome 1 open reading frame 105 | 1 | | 1794 | hsa-mir-16-1 | 92346 | C1orf105 | chromosome 1 open reading frame 105 | 1 | | 1795 | hsa-miR-1231 | 92346 | C1orf105 | chromosome 1 open reading frame 105 | 1 | | 1796 | hsa-miR-126\* | 92346 | C1orf105 | chromosome 1 open reading frame 105 | 1 | | 1797 | hsa-miR-16 | 92346 | C1orf105 | chromosome 1 open reading frame 105 | 1 | | 1798 | hsa-miR-564 | 92346 | C1orf105 | chromosome 1 open reading frame 105 | 1 | | 1799 | hsa-miR-608 | 92346 | C1orf105 | chromosome 1 open reading frame 105 | 1 | | 1800 | hsa-miR-16 | 31 | ACACA | acetyl-Coenzyme A carboxylase alpha | 1 | | 1801 | hsa-miR-675 | 31 | ACACA | acetyl-Coenzyme A carboxylase alpha | 1 | | 1802 | hsa-miR-95 | 31 | ACACA | acetyl-Coenzyme A carboxylase alpha | 1 | | 1803 | hsa-mir-339 | 22936 | ELL2 | elongation factor, RNA polymerase II, 2 | 1 | | 1804 | hsa-miR-339-5p | 22936 | ELL2 | elongation factor, RNA polymerase II, 2 | 1 | | 1805 | hsa-miR-564 | 22936 | ELL2 | elongation factor, RNA polymerase II, 2 | 1 | | 1806 | hsa-miR-608 | 22936 | ELL2 | elongation factor, RNA polymerase II, 2 | 1 | | 1807 | hsa-miR-618 | 22936 | ELL2 | elongation factor, RNA polymerase II, 2 | 1 | | 1808 | hsa-miR-634 | 22936 | ELL2 | elongation factor, RNA polymerase II, 2 | 1 | | 1809 | hsa-mir-15a | 4131 | MAP1B | microtubule-associated protein 1B | 1 | | 1810 | hsa-mir-16-1 | 4131 | MAP1B | microtubule-associated protein 1B | 1 | | 1811 | hsa-miR-126\* | 4131 | MAP1B | microtubule-associated protein 1B | 1 | | 1812 | hsa-miR-16 | 4131 | MAP1B | microtubule-associated protein 1B | 1 | | 1813 | hsa-miR-564 | 4131 | MAP1B | microtubule-associated protein 1B | 1 | | 1814 | hsa-miR-608 | 4131 | MAP1B | microtubule-associated protein 1B | 1 | | 1815 | hsa-mir-22 | 57185 | NIPAL3 | NIPA-like domain containing 3 | 1 | | 1816 | hsa-mir-196b | 57185 | NIPAL3 | NIPA-like domain containing 3 | 1 | | 1817 | hsa-mir-574 | 57185 | NIPAL3 | NIPA-like domain containing 3 | 1 | | 1818 | hsa-mir-628 | 57185 | NIPAL3 | NIPA-like domain containing 3 | 1 | | 1819 | hsa-miR-196b | 57185 | NIPAL3 | NIPA-like domain containing 3 | 1 | | 1820 | hsa-miR-617 | 57185 | NIPAL3 | NIPA-like domain containing 3 | 1 | | 1821 | hsa-miR-623 | 57185 | NIPAL3 | NIPA-like domain containing 3 | 1 | | 1822 | hsa-miR-1231 | 5063 | PAK3 | p21 protein (Cdc42/Rac)-activated kinase 3 | 1 | | 1823 | hsa-miR-126\* | 5063 | PAK3 | p21 protein (Cdc42/Rac)-activated kinase 3 | 1 | | 1824 | hsa-miR-608 | 5063 | PAK3 | p21 protein (Cdc42/Rac)-activated kinase 3 | 1 | | 1825 | hsa-mir-196b | 274 | BIN1 | bridging integrator 1 | 1 | | 1826 | hsa-mir-628 | 274 | BIN1 | bridging integrator 1 | 1 | | 1827 | hsa-miR-196b | 274 | BIN1 | bridging integrator 1 | 1 | | 1828 | hsa-miR-617 | 274 | BIN1 | bridging integrator 1 | 1 | | 1829 | hsa-miR-623 | 274 | BIN1 | bridging integrator 1 | 1 | | 1830 | hsa-miR-618 | 6667 | SP1 | Sp1 transcription factor | 1 | | 1831 | hsa-miR-126\* | 23109 | DDN | dendrin | 1 | | 1832 | hsa-miR-16 | 23109 | DDN | dendrin | 1 | | 1833 | hsa-miR-608 | 23109 | DDN | dendrin | 1 | | 1834 | hsa-miR-126\* | 9271 | PIWIL1 | piwi-like 1 (Drosophila) | 1 | | 1835 | hsa-miR-608 | 9271 | PIWIL1 | piwi-like 1 (Drosophila) | 1 | | 1838 | hsa-mir-15a | 10411 | RAPGEF3 | Rap guanine nucleotide exchange factor (GEF) 3 | 1 | | 1839 | hsa-mir-16-1 | 10411 | RAPGEF3 | Rap guanine nucleotide exchange factor (GEF) 3 | 1 | | 1840 | hsa-miR-126\* | 10411 | RAPGEF3 | Rap guanine nucleotide exchange factor (GEF) 3 | 1 | | 1841 | hsa-miR-16 | 10411 | RAPGEF3 | Rap guanine nucleotide exchange factor (GEF) 3 | 1 | | 1842 | hsa-miR-608 | 10411 | RAPGEF3 | Rap guanine nucleotide exchange factor (GEF) 3 | 1 | | 1844 | hsa-mir-15a | 23026 | MYO16 | myosin XVI | 1 | | 1845 | hsa-mir-16-1 | 23026 | MYO16 | myosin XVI | 1 | | 1846 | hsa-miR-1231 | 23026 | MYO16 | myosin XVI | 1 | | 1847 | hsa-miR-126\* | 23026 | MYO16 | myosin XVI | 1 | | 1848 | hsa-miR-16 | 23026 | MYO16 | myosin XVI | 1 | | 1849 | hsa-miR-608 | 23026 | MYO16 | myosin XVI | 1 | | 1850 | hsa-mir-126 | 5228 | PGF | placental growth factor | 1 | | 1851 | hsa-mir-424 | 5228 | PGF | placental growth factor | 1 | | 1852 | hsa-mir-1915 | 5228 | PGF | placental growth factor | 1 | | 1853 | hsa-miR-503 | 5228 | PGF | placental growth factor | 1 | | 1854 | hsa-mir-218-2 | 23126 | POGZ | pogo transposable element with ZNF domain | 1 | | 1855 | hsa-mir-126 | 23126 | POGZ | pogo transposable element with ZNF domain | 1 | | 1856 | hsa-mir-424 | 23126 | POGZ | pogo transposable element with ZNF domain | 1 | | 1857 | hsa-mir-490 | 23126 | POGZ | pogo transposable element with ZNF domain | 1 | | 1858 | hsa-mir-556 | 23126 | POGZ | pogo transposable element with ZNF domain | 1 | | 1859 | hsa-mir-1915 | 23126 | POGZ | pogo transposable element with ZNF domain | 1 | | 1860 | hsa-miR-190 | 23126 | POGZ | pogo transposable element with ZNF domain | 1 | | 1861 | hsa-miR-490-5p | 23126 | POGZ | pogo transposable element with ZNF domain | 1 | | 1862 | hsa-miR-503 | 23126 | POGZ | pogo transposable element with ZNF domain | 1 | | 1863 | hsa-miR-585 | 23126 | POGZ | pogo transposable element with ZNF domain | 1 | | 1864 | hsa-miR-126\* | 30848 | CTAG2 | cancer/testis antigen 2 | 1 | | 1865 | hsa-miR-16 | 30848 | CTAG2 | cancer/testis antigen 2 | 1 | | 1866 | hsa-miR-608 | 30848 | CTAG2 | cancer/testis antigen 2 | 1 | | 1867 | hsa-miR-126\* | 283755 | HERC2P3 | hect domain and RLD 2 pseudogene 3 | 1 | | 1868 | hsa-miR-608 | 283755 | HERC2P3 | hect domain and RLD 2 pseudogene 3 | 1 | | 1869 | hsa-miR-126\* | 118491 | TTC18 | tetratricopeptide repeat domain 18 | 1 | | 1870 | hsa-miR-608 | 118491 | TTC18 | tetratricopeptide repeat domain 18 | 1 | | 1871 | hsa-mir-15a | 91683 | SYT12 | synaptotagmin XII | 1 | | 1872 | hsa-mir-16-1 | 91683 | SYT12 | synaptotagmin XII | 1 | | 1873 | hsa-miR-126\* | 91683 | SYT12 | synaptotagmin XII | 1 | | 1874 | hsa-miR-16 | 91683 | SYT12 | synaptotagmin XII | 1 | | 1875 | hsa-miR-566 | 91683 | SYT12 | synaptotagmin XII | 1 | | 1876 | hsa-miR-608 | 91683 | SYT12 | synaptotagmin XII | 1 | | 1877 | hsa-mir-15a | 2775 | GNAO1 | guanine nucleotide binding protein (G protein), alpha activating activity polypeptide O | 1 | | 1878 | hsa-mir-16-1 | 2775 | GNAO1 | guanine nucleotide binding protein (G protein), alpha activating activity polypeptide O | 1 | | 1879 | hsa-miR-126\* | 2775 | GNAO1 | guanine nucleotide binding protein (G protein), alpha activating activity polypeptide O | 1 | | 1880 | hsa-miR-16 | 2775 | GNAO1 | guanine nucleotide binding protein (G protein), alpha activating activity polypeptide O | 1 | | 1881 | hsa-miR-603 | 2775 | GNAO1 | guanine nucleotide binding protein (G protein), alpha activating activity polypeptide O | 1 | | 1882 | hsa-miR-608 | 2775 | GNAO1 | guanine nucleotide binding protein (G protein), alpha activating activity polypeptide O | 1 | | 1883 | hsa-miR-190 | 971 | CD72 | CD72 molecule | 1 | | 1884 | hsa-miR-623 | 971 | CD72 | CD72 molecule | 1 | | 1885 | hsa-miR-126\* | 1785 | DNM2 | dynamin 2 | 1 | | 1886 | hsa-miR-16 | 1785 | DNM2 | dynamin 2 | 1 | | 1887 | hsa-miR-564 | 1785 | DNM2 | dynamin 2 | 1 | | 1888 | hsa-miR-608 | 1785 | DNM2 | dynamin 2 | 1 | | 1889 | hsa-mir-15a | 1559 | CYP2C9 | cytochrome P450, family 2, subfamily C, polypeptide 9 | 1 | | 1890 | hsa-mir-16-1 | 1559 | CYP2C9 | cytochrome P450, family 2, subfamily C, polypeptide 9 | 1 | | 1893 | hsa-miR-16 | 1559 | CYP2C9 | cytochrome P450, family 2, subfamily C, polypeptide 9 | 1 | | 1894 | hsa-miR-566 | 1559 | CYP2C9 | cytochrome P450, family 2, subfamily C, polypeptide 9 | 1 | | 1896 | hsa-mir-15a | 5155 | PDGFB | platelet-derived growth factor beta polypeptide (simian sarcoma viral (v-sis) oncogene homolog) | 1 | | 1897 | hsa-miR-126\* | 5155 | PDGFB | platelet-derived growth factor beta polypeptide (simian sarcoma viral (v-sis) oncogene homolog) | 1 | | 1898 | hsa-miR-16 | 5155 | PDGFB | platelet-derived growth factor beta polypeptide (simian sarcoma viral (v-sis) oncogene homolog) | 1 | | 1899 | hsa-miR-566 | 5155 | PDGFB | platelet-derived growth factor beta polypeptide (simian sarcoma viral (v-sis) oncogene homolog) | 1 | | 1900 | hsa-miR-608 | 5155 | PDGFB | platelet-derived growth factor beta polypeptide (simian sarcoma viral (v-sis) oncogene homolog) | 1 | | 1901 | hsa-mir-15a | 10408 | MYCNOS | v-myc myelocytomatosis viral related oncogene, neuroblastoma derived (avian) opposite strand | 1 | | 1902 | hsa-miR-126\* | 10408 | MYCNOS | v-myc myelocytomatosis viral related oncogene, neuroblastoma derived (avian) opposite strand | 1 | | 1903 | hsa-miR-16 | 10408 | MYCNOS | v-myc myelocytomatosis viral related oncogene, neuroblastoma derived (avian) opposite strand | 1 | | 1904 | hsa-miR-608 | 10408 | MYCNOS | v-myc myelocytomatosis viral related oncogene, neuroblastoma derived (avian) opposite strand | 1 | | 1905 | hsa-mir-196b | 60436 | TGIF2 | TGFB-induced factor homeobox 2 | 1 | | 1909 | hsa-miR-196b | 60436 | TGIF2 | TGFB-induced factor homeobox 2 | 1 | | 1911 | hsa-miR-504 | 60436 | TGIF2 | TGFB-induced factor homeobox 2 | 1 | | 1915 | hsa-miR-1231 | 6315 | ATXN8OS | ATXN8 opposite strand (non-protein coding) | 1 | | 1916 | hsa-miR-126\* | 6315 | ATXN8OS | ATXN8 opposite strand (non-protein coding) | 1 | | 1917 | hsa-mir-339 | 7799 | PRDM2 | PR domain containing 2, with ZNF domain | 1 | | 1918 | hsa-miR-126\* | 7799 | PRDM2 | PR domain containing 2, with ZNF domain | 1 | | 1919 | hsa-miR-339-5p | 7799 | PRDM2 | PR domain containing 2, with ZNF domain | 1 | | 1920 | hsa-miR-564 | 7799 | PRDM2 | PR domain containing 2, with ZNF domain | 1 | | 1921 | hsa-miR-608 | 7799 | PRDM2 | PR domain containing 2, with ZNF domain | 1 | | 1922 | hsa-miR-126\* | 80852 | GRIP2 | glutamate receptor interacting protein 2 | 1 | | 1923 | hsa-miR-16 | 80852 | GRIP2 | glutamate receptor interacting protein 2 | 1 | | 1924 | hsa-miR-564 | 80852 | GRIP2 | glutamate receptor interacting protein 2 | 1 | | 1925 | hsa-miR-566 | 80852 | GRIP2 | glutamate receptor interacting protein 2 | 1 | | 1926 | hsa-miR-608 | 80852 | GRIP2 | glutamate receptor interacting protein 2 | 1 | | 1927 | hsa-miR-126\* | 3068 | HDGF | hepatoma-derived growth factor (high-mobility group protein 1-like) | 1 | | 1928 | hsa-miR-634 | 3068 | HDGF | hepatoma-derived growth factor (high-mobility group protein 1-like) | 1 | | 1929 | hsa-mir-30c-1 | 253143 | C22orf30 | chromosome 22 open reading frame 30 | 1 | | 1930 | hsa-mir-30e | 253143 | C22orf30 | chromosome 22 open reading frame 30 | 1 | | 1931 | hsa-mir-196b | 6159 | RPL29 | ribosomal protein L29 | 1 | | 1932 | hsa-mir-424 | 6159 | RPL29 | ribosomal protein L29 | 1 | | 1933 | hsa-mir-490 | 6159 | RPL29 | ribosomal protein L29 | 1 | | 1934 | hsa-mir-574 | 6159 | RPL29 | ribosomal protein L29 | 1 | | 1935 | hsa-mir-628 | 6159 | RPL29 | ribosomal protein L29 | 1 | | 1936 | hsa-miR-190 | 6159 | RPL29 | ribosomal protein L29 | 1 | | 1937 | hsa-miR-196b | 6159 | RPL29 | ribosomal protein L29 | 1 | | 1938 | hsa-miR-490-5p | 6159 | RPL29 | ribosomal protein L29 | 1 | | 1939 | hsa-miR-503 | 6159 | RPL29 | ribosomal protein L29 | 1 | | 1940 | hsa-miR-617 | 6159 | RPL29 | ribosomal protein L29 | 1 | | 1941 | hsa-miR-623 | 6159 | RPL29 | ribosomal protein L29 | 1 | | 1942 | hsa-mir-15a | 1538 | CYLC1 | cylicin, basic protein of sperm head cytoskeleton 1 | 1 | | 1943 | hsa-mir-16-1 | 1538 | CYLC1 | cylicin, basic protein of sperm head cytoskeleton 1 | 1 | | 1944 | hsa-miR-126\* | 1538 | CYLC1 | cylicin, basic protein of sperm head cytoskeleton 1 | 1 | | 1945 | hsa-miR-16 | 1538 | CYLC1 | cylicin, basic protein of sperm head cytoskeleton 1 | 1 | | 1946 | hsa-miR-564 | 1538 | CYLC1 | cylicin, basic protein of sperm head cytoskeleton 1 | 1 | | 1947 | hsa-miR-608 | 1538 | CYLC1 | cylicin, basic protein of sperm head cytoskeleton 1 | 1 | | 1948 | hsa-miR-338-5p | 6189 | RPS3A | ribosomal protein S3A | 1 | | 1949 | hsa-miR-586 | 6189 | RPS3A | ribosomal protein S3A | 1 | | 1950 | hsa-miR-617 | 6189 | RPS3A | ribosomal protein S3A | 1 | | 1951 | hsa-miR-657 | 6189 | RPS3A | ribosomal protein S3A | 1 | | 1952 | hsa-miR-126\* | 6768 | ST14 | suppression of tumorigenicity 14 (colon carcinoma) | 1 | | 1953 | hsa-miR-566 | 6768 | ST14 | suppression of tumorigenicity 14 (colon carcinoma) | 1 | | 1954 | hsa-mir-15a | 4916 | NTRK3 | neurotrophic tyrosine kinase, receptor, type 3 | 1 | | 1955 | hsa-mir-16-1 | 4916 | NTRK3 | neurotrophic tyrosine kinase, receptor, type 3 | 1 | | 1956 | hsa-miR-564 | 4916 | NTRK3 | neurotrophic tyrosine kinase, receptor, type 3 | 1 | | 1957 | hsa-miR-566 | 4916 | NTRK3 | neurotrophic tyrosine kinase, receptor, type 3 | 1 | | 1958 | hsa-miR-126\* | 6665 | SOX15 | SRY (sex determining region Y)-box 15 | 1 | | 1959 | hsa-miR-566 | 6665 | SOX15 | SRY (sex determining region Y)-box 15 | 1 | | 1960 | hsa-miR-608 | 6665 | SOX15 | SRY (sex determining region Y)-box 15 | 1 | | 1961 | hsa-mir-218-2 | 9709 | HERPUD1 | homocysteine-inducible, endoplasmic reticulum stress-inducible, ubiquitin-like domain member 1 | 1 | | 1962 | hsa-mir-126 | 9709 | HERPUD1 | homocysteine-inducible, endoplasmic reticulum stress-inducible, ubiquitin-like domain member 1 | 1 | | 1963 | hsa-mir-424 | 9709 | HERPUD1 | homocysteine-inducible, endoplasmic reticulum stress-inducible, ubiquitin-like domain member 1 | 1 | | 1964 | hsa-mir-490 | 9709 | HERPUD1 | homocysteine-inducible, endoplasmic reticulum stress-inducible, ubiquitin-like domain member 1 | 1 | | 1965 | hsa-mir-556 | 9709 | HERPUD1 | homocysteine-inducible, endoplasmic reticulum stress-inducible, ubiquitin-like domain member 1 | 1 | | 1966 | hsa-mir-1915 | 9709 | HERPUD1 | homocysteine-inducible, endoplasmic reticulum stress-inducible, ubiquitin-like domain member 1 | 1 | | 1967 | hsa-miR-190 | 9709 | HERPUD1 | homocysteine-inducible, endoplasmic reticulum stress-inducible, ubiquitin-like domain member 1 | 1 | | 1968 | hsa-miR-490-5p | 9709 | HERPUD1 | homocysteine-inducible, endoplasmic reticulum stress-inducible, ubiquitin-like domain member 1 | 1 | | 1969 | hsa-miR-503 | 9709 | HERPUD1 | homocysteine-inducible, endoplasmic reticulum stress-inducible, ubiquitin-like domain member 1 | 1 | | 1970 | hsa-miR-585 | 9709 | HERPUD1 | homocysteine-inducible, endoplasmic reticulum stress-inducible, ubiquitin-like domain member 1 | 1 | | 1971 | hsa-miR-623 | 9709 | HERPUD1 | homocysteine-inducible, endoplasmic reticulum stress-inducible, ubiquitin-like domain member 1 | 1 | | 1972 | hsa-mir-454 | 4586 | MUC5AC | mucin 5AC, oligomeric mucus/gel-forming | 1 | | 1973 | hsa-miR-675 | 4586 | MUC5AC | mucin 5AC, oligomeric mucus/gel-forming | 1 | | 1974 | hsa-miR-95 | 4586 | MUC5AC | mucin 5AC, oligomeric mucus/gel-forming | 1 | | 1975 | hsa-mir-126 | 6138 | RPL15 | ribosomal protein L15 | 1 | | 1976 | hsa-mir-196b | 6138 | RPL15 | ribosomal protein L15 | 1 | | 1977 | hsa-mir-424 | 6138 | RPL15 | ribosomal protein L15 | 1 | | 1978 | hsa-mir-490 | 6138 | RPL15 | ribosomal protein L15 | 1 | | 1979 | hsa-mir-628 | 6138 | RPL15 | ribosomal protein L15 | 1 | | 1980 | hsa-mir-1915 | 6138 | RPL15 | ribosomal protein L15 | 1 | | 1981 | hsa-miR-190 | 6138 | RPL15 | ribosomal protein L15 | 1 | | 1982 | hsa-miR-196b | 6138 | RPL15 | ribosomal protein L15 | 1 | | 1983 | hsa-miR-490-5p | 6138 | RPL15 | ribosomal protein L15 | 1 | | 1984 | hsa-miR-503 | 6138 | RPL15 | ribosomal protein L15 | 1 | | 1985 | hsa-miR-617 | 6138 | RPL15 | ribosomal protein L15 | 1 | | 1986 | hsa-miR-623 | 6138 | RPL15 | ribosomal protein L15 | 1 | | 1987 | hsa-miR-944 | 6138 | RPL15 | ribosomal protein L15 | 1 | | 1988 | hsa-miR-126\* | 100131825 | LOC100131825 | hypothetical protein LOC100131825 | 1 | | 1989 | hsa-miR-608 | 100131825 | LOC100131825 | hypothetical protein LOC100131825 | 1 | | 1990 | hsa-miR-1231 | 6752 | SSTR2 | somatostatin receptor 2 | 1 | | 1991 | hsa-miR-608 | 6752 | SSTR2 | somatostatin receptor 2 | 1 | | 1992 | hsa-mir-454 | 7047 | TGM4 | transglutaminase 4 (prostate) | 1 | | 1993 | hsa-miR-675 | 7047 | TGM4 | transglutaminase 4 (prostate) | 1 | | 1994 | hsa-miR-95 | 7047 | TGM4 | transglutaminase 4 (prostate) | 1 | | 1995 | hsa-mir-126 | 5478 | PPIA | peptidylprolyl isomerase A (cyclophilin A) | 1 | | 1996 | hsa-mir-424 | 5478 | PPIA | peptidylprolyl isomerase A (cyclophilin A) | 1 | | 1997 | hsa-miR-503 | 5478 | PPIA | peptidylprolyl isomerase A (cyclophilin A) | 1 | | 1998 | hsa-miR-617 | 5478 | PPIA | peptidylprolyl isomerase A (cyclophilin A) | 1 | | 1999 | hsa-mir-218-2 | 51520 | LARS | leucyl-tRNA synthetase | 1 | | 2000 | hsa-mir-126 | 51520 | LARS | leucyl-tRNA synthetase | 1 | | 2002 | hsa-mir-556 | 51520 | LARS | leucyl-tRNA synthetase | 1 | | 2004 | hsa-mir-1915 | 51520 | LARS | leucyl-tRNA synthetase | 1 | | 2006 | hsa-miR-338-5p | 51520 | LARS | leucyl-tRNA synthetase | 1 | | 2008 | hsa-miR-585 | 51520 | LARS | leucyl-tRNA synthetase | 1 | | 2011 | hsa-miR-657 | 51520 | LARS | leucyl-tRNA synthetase | 1 | | 2012 | hsa-mir-424 | 10723 | SLC12A7 | solute carrier family 12 (potassium/chloride transporters), member 7 | 1 | | 2013 | hsa-mir-628 | 10723 | SLC12A7 | solute carrier family 12 (potassium/chloride transporters), member 7 | 1 | | 2014 | hsa-miR-503 | 10723 | SLC12A7 | solute carrier family 12 (potassium/chloride transporters), member 7 | 1 | | 2015 | hsa-miR-617 | 10723 | SLC12A7 | solute carrier family 12 (potassium/chloride transporters), member 7 | 1 | | 2016 | hsa-miR-623 | 10723 | SLC12A7 | solute carrier family 12 (potassium/chloride transporters), member 7 | 1 | | 2017 | hsa-miR-618 | 405 | ARNT | aryl hydrocarbon receptor nuclear translocator | 1 | | 2018 | hsa-mir-15a | 51617 | HMP19 | HMP19 protein | 1 | | 2019 | hsa-mir-16-1 | 51617 | HMP19 | HMP19 protein | 1 | | 2020 | hsa-miR-126\* | 51617 | HMP19 | HMP19 protein | 1 | | 2021 | hsa-miR-16 | 51617 | HMP19 | HMP19 protein | 1 | | 2022 | hsa-miR-608 | 51617 | HMP19 | HMP19 protein | 1 | | 2023 | hsa-mir-15a | 10763 | NES | nestin | 1 | | 2024 | hsa-mir-16-1 | 10763 | NES | nestin | 1 | | 2025 | hsa-miR-126\* | 10763 | NES | nestin | 1 | | 2026 | hsa-miR-16 | 10763 | NES | nestin | 1 | | 2027 | hsa-miR-566 | 10763 | NES | nestin | 1 | | 2028 | hsa-miR-608 | 10763 | NES | nestin | 1 | | 2029 | hsa-mir-218-2 | 65983 | GRAMD3 | GRAM domain containing 3 | 1 | | 2030 | hsa-mir-126 | 65983 | GRAMD3 | GRAM domain containing 3 | 1 | | 2031 | hsa-mir-424 | 65983 | GRAMD3 | GRAM domain containing 3 | 1 | | 2032 | hsa-mir-556 | 65983 | GRAMD3 | GRAM domain containing 3 | 1 | | 2033 | hsa-mir-628 | 65983 | GRAMD3 | GRAM domain containing 3 | 1 | | 2034 | hsa-miR-190 | 65983 | GRAMD3 | GRAM domain containing 3 | 1 | | 2035 | hsa-miR-503 | 65983 | GRAMD3 | GRAM domain containing 3 | 1 | | 2036 | hsa-miR-585 | 65983 | GRAMD3 | GRAM domain containing 3 | 1 | | 2037 | hsa-miR-586 | 65983 | GRAMD3 | GRAM domain containing 3 | 1 | | 2038 | hsa-miR-617 | 65983 | GRAMD3 | GRAM domain containing 3 | 1 | | 2039 | hsa-mir-218-2 | 60436 | TGIF2 | TGFB-induced factor homeobox 2 | 1 | | 2040 | hsa-mir-126 | 60436 | TGIF2 | TGFB-induced factor homeobox 2 | 1 | | 2041 | hsa-mir-556 | 60436 | TGIF2 | TGFB-induced factor homeobox 2 | 1 | | 2042 | hsa-miR-585 | 60436 | TGIF2 | TGFB-induced factor homeobox 2 | 1 | | 2043 | hsa-miR-618 | 51559 | NT5DC3 | 5'-nucleotidase domain containing 3 | 1 | | 2044 | hsa-miR-126\* | 51162 | EGFL7 | EGF-like-domain, multiple 7 | 1 | | 2045 | hsa-miR-16 | 51162 | EGFL7 | EGF-like-domain, multiple 7 | 1 | | 2046 | hsa-miR-566 | 51162 | EGFL7 | EGF-like-domain, multiple 7 | 1 | | 2047 | hsa-miR-608 | 51162 | EGFL7 | EGF-like-domain, multiple 7 | 1 | | 2048 | hsa-miR-618 | 55636 | CHD7 | chromodomain helicase DNA binding protein 7 | 1 | | 2049 | hsa-mir-135b | 80150 | ASRGL1 | asparaginase like 1 | 1 | | 2050 | hsa-miR-454\* | 80150 | ASRGL1 | asparaginase like 1 | 1 | | 2051 | hsa-miR-548d-5p | 80150 | ASRGL1 | asparaginase like 1 | 1 | | 2052 | hsa-mir-218-2 | 55278 | QRSL1 | glutaminyl-tRNA synthase (glutamine-hydrolyzing)-like 1 | 1 | | 2053 | hsa-mir-126 | 55278 | QRSL1 | glutaminyl-tRNA synthase (glutamine-hydrolyzing)-like 1 | 1 | | 2054 | hsa-mir-424 | 55278 | QRSL1 | glutaminyl-tRNA synthase (glutamine-hydrolyzing)-like 1 | 1 | | 2056 | hsa-mir-628 | 55278 | QRSL1 | glutaminyl-tRNA synthase (glutamine-hydrolyzing)-like 1 | 1 | | 2057 | hsa-miR-190 | 55278 | QRSL1 | glutaminyl-tRNA synthase (glutamine-hydrolyzing)-like 1 | 1 | | 2058 | hsa-miR-338-5p | 55278 | QRSL1 | glutaminyl-tRNA synthase (glutamine-hydrolyzing)-like 1 | 1 | | 2059 | hsa-miR-503 | 55278 | QRSL1 | glutaminyl-tRNA synthase (glutamine-hydrolyzing)-like 1 | 1 | | 2060 | hsa-miR-585 | 55278 | QRSL1 | glutaminyl-tRNA synthase (glutamine-hydrolyzing)-like 1 | 1 | | 2061 | hsa-miR-586 | 55278 | QRSL1 | glutaminyl-tRNA synthase (glutamine-hydrolyzing)-like 1 | 1 | | 2062 | hsa-miR-617 | 55278 | QRSL1 | glutaminyl-tRNA synthase (glutamine-hydrolyzing)-like 1 | 1 | | 2063 | hsa-miR-657 | 55278 | QRSL1 | glutaminyl-tRNA synthase (glutamine-hydrolyzing)-like 1 | 1 | | 2064 | hsa-miR-574-5p | 54942 | C9orf6 | chromosome 9 open reading frame 6 | 1 | | 2065 | hsa-miR-618 | 54942 | C9orf6 | chromosome 9 open reading frame 6 | 1 | | 2066 | hsa-miR-634 | 54942 | C9orf6 | chromosome 9 open reading frame 6 | 1 | | 2067 | hsa-miR-126\* | 57664 | PLEKHA4 | pleckstrin homology domain containing, family A (phosphoinositide binding specific) member 4 | 1 | | 2068 | hsa-miR-16 | 57664 | PLEKHA4 | pleckstrin homology domain containing, family A (phosphoinositide binding specific) member 4 | 1 | | 2069 | hsa-miR-566 | 57664 | PLEKHA4 | pleckstrin homology domain containing, family A (phosphoinositide binding specific) member 4 | 1 | | 2070 | hsa-miR-608 | 57664 | PLEKHA4 | pleckstrin homology domain containing, family A (phosphoinositide binding specific) member 4 | 1 | | 2071 | hsa-mir-135b | 51741 | WWOX | WW domain containing oxidoreductase | 1 | | 2072 | hsa-mir-556 | 51741 | WWOX | WW domain containing oxidoreductase | 1 | | 2073 | hsa-mir-675 | 51741 | WWOX | WW domain containing oxidoreductase | 1 | | 2074 | hsa-miR-454\* | 51741 | WWOX | WW domain containing oxidoreductase | 1 | | 2075 | hsa-miR-548d-5p | 51741 | WWOX | WW domain containing oxidoreductase | 1 | | 2076 | hsa-miR-618 | 192670 | EIF2C4 | eukaryotic translation initiation factor 2C, 4 | 1 | | 2077 | hsa-mir-339 | 51762 | RAB8B | RAB8B, member RAS oncogene family | 1 | | 2078 | hsa-miR-339-5p | 51762 | RAB8B | RAB8B, member RAS oncogene family | 1 | | 2079 | hsa-miR-564 | 51762 | RAB8B | RAB8B, member RAS oncogene family | 1 | | 2080 | hsa-miR-618 | 51762 | RAB8B | RAB8B, member RAS oncogene family | 1 | | 2081 | hsa-miR-634 | 51762 | RAB8B | RAB8B, member RAS oncogene family | 1 | | 2082 | hsa-mir-196b | 54958 | TMEM160 | transmembrane protein 160 | 1 | | 2083 | hsa-mir-628 | 54958 | TMEM160 | transmembrane protein 160 | 1 | | 2084 | hsa-miR-196b | 54958 | TMEM160 | transmembrane protein 160 | 1 | | 2085 | hsa-miR-623 | 54958 | TMEM160 | transmembrane protein 160 | 1 | | 2086 | hsa-miR-650 | 54958 | TMEM160 | transmembrane protein 160 | 1 | | 2087 | hsa-mir-196b | 2055 | CLN8 | ceroid-lipofuscinosis, neuronal 8 (epilepsy, progressive with mental retardation) | 1 | | 2088 | hsa-mir-424 | 2055 | CLN8 | ceroid-lipofuscinosis, neuronal 8 (epilepsy, progressive with mental retardation) | 1 | | 2089 | hsa-mir-628 | 2055 | CLN8 | ceroid-lipofuscinosis, neuronal 8 (epilepsy, progressive with mental retardation) | 1 | | 2090 | hsa-miR-196b | 2055 | CLN8 | ceroid-lipofuscinosis, neuronal 8 (epilepsy, progressive with mental retardation) | 1 | | 2091 | hsa-miR-503 | 2055 | CLN8 | ceroid-lipofuscinosis, neuronal 8 (epilepsy, progressive with mental retardation) | 1 | | 2092 | hsa-miR-623 | 2055 | CLN8 | ceroid-lipofuscinosis, neuronal 8 (epilepsy, progressive with mental retardation) | 1 | | 2093 | hsa-mir-126 | 55771 | PRR11 | proline rich 11 | 1 | | 2094 | hsa-mir-30c-1 | 55771 | PRR11 | proline rich 11 | 1 | | 2095 | hsa-mir-30e | 55771 | PRR11 | proline rich 11 | 1 | | 2096 | hsa-mir-1915 | 55771 | PRR11 | proline rich 11 | 1 | | 2097 | hsa-mir-196b | 79863 | C18orf22 | chromosome 18 open reading frame 22 | 1 | | 2098 | hsa-mir-574 | 79863 | C18orf22 | chromosome 18 open reading frame 22 | 1 | | 2099 | hsa-mir-628 | 79863 | C18orf22 | chromosome 18 open reading frame 22 | 1 | | 2100 | hsa-miR-196b | 79863 | C18orf22 | chromosome 18 open reading frame 22 | 1 | | 2101 | hsa-miR-586 | 79863 | C18orf22 | chromosome 18 open reading frame 22 | 1 | | 2102 | hsa-miR-617 | 79863 | C18orf22 | chromosome 18 open reading frame 22 | 1 | | 2103 | hsa-miR-126\* | 79570 | NKAIN1 | Na+/K+ transporting ATPase interacting 1 | 1 | | 2104 | hsa-miR-126\* | 58529 | MYOZ1 | myozenin 1 | 1 | | 2105 | hsa-miR-16 | 58529 | MYOZ1 | myozenin 1 | 1 | | 2106 | hsa-miR-608 | 58529 | MYOZ1 | myozenin 1 | 1 | | 2107 | hsa-mir-15a | 24147 | FJX1 | four jointed box 1 (Drosophila) | 1 | | 2108 | hsa-miR-126\* | 24147 | FJX1 | four jointed box 1 (Drosophila) | 1 | | 2109 | hsa-miR-16 | 24147 | FJX1 | four jointed box 1 (Drosophila) | 1 | | 2110 | hsa-miR-566 | 24147 | FJX1 | four jointed box 1 (Drosophila) | 1 | | 2111 | hsa-miR-608 | 24147 | FJX1 | four jointed box 1 (Drosophila) | 1 | | 2112 | hsa-mir-126 | 55024 | BANK1 | B-cell scaffold protein with ankyrin repeats 1 | 1 | | 2113 | hsa-mir-30c-1 | 55024 | BANK1 | B-cell scaffold protein with ankyrin repeats 1 | 1 | | 2114 | hsa-mir-30e | 55024 | BANK1 | B-cell scaffold protein with ankyrin repeats 1 | 1 | | 2115 | hsa-mir-126 | 64421 | DCLRE1C | DNA cross-link repair 1C (PSO2 homolog, S. cerevisiae) | 1 | | 2116 | hsa-mir-30c-1 | 64421 | DCLRE1C | DNA cross-link repair 1C (PSO2 homolog, S. cerevisiae) | 1 | | 2117 | hsa-mir-30e | 64421 | DCLRE1C | DNA cross-link repair 1C (PSO2 homolog, S. cerevisiae) | 1 | | 2118 | hsa-mir-424 | 64421 | DCLRE1C | DNA cross-link repair 1C (PSO2 homolog, S. cerevisiae) | 1 | | 2119 | hsa-mir-1915 | 64421 | DCLRE1C | DNA cross-link repair 1C (PSO2 homolog, S. cerevisiae) | 1 | | 2120 | hsa-miR-190 | 64421 | DCLRE1C | DNA cross-link repair 1C (PSO2 homolog, S. cerevisiae) | 1 | | 2121 | hsa-miR-338-5p | 64421 | DCLRE1C | DNA cross-link repair 1C (PSO2 homolog, S. cerevisiae) | 1 | | 2122 | hsa-miR-503 | 64421 | DCLRE1C | DNA cross-link repair 1C (PSO2 homolog, S. cerevisiae) | 1 | | 2123 | hsa-miR-657 | 64421 | DCLRE1C | DNA cross-link repair 1C (PSO2 homolog, S. cerevisiae) | 1 | | 2124 | hsa-mir-196b | 25873 | RPL36 | ribosomal protein L36 | 1 | | 2125 | hsa-mir-424 | 25873 | RPL36 | ribosomal protein L36 | 1 | | 2126 | hsa-mir-628 | 25873 | RPL36 | ribosomal protein L36 | 1 | | 2127 | hsa-miR-196b | 25873 | RPL36 | ribosomal protein L36 | 1 | | 2128 | hsa-miR-503 | 25873 | RPL36 | ribosomal protein L36 | 1 | | 2129 | hsa-miR-504 | 25873 | RPL36 | ribosomal protein L36 | 1 | | 2130 | hsa-miR-586 | 25873 | RPL36 | ribosomal protein L36 | 1 | | 2131 | hsa-miR-617 | 25873 | RPL36 | ribosomal protein L36 | 1 | | 2132 | hsa-miR-623 | 25873 | RPL36 | ribosomal protein L36 | 1 | | 2133 | hsa-miR-1231 | 54520 | CCDC93 | coiled-coil domain containing 93 | 1 | | 2134 | hsa-miR-618 | 54520 | CCDC93 | coiled-coil domain containing 93 | 1 | | 2135 | hsa-miR-634 | 54520 | CCDC93 | coiled-coil domain containing 93 | 1 | | 2136 | hsa-mir-218-2 | 55192 | DNAJC17 | DnaJ (Hsp40) homolog, subfamily C, member 17 | 1 | | 2137 | hsa-miR-190 | 55192 | DNAJC17 | DnaJ (Hsp40) homolog, subfamily C, member 17 | 1 | | 2138 | hsa-miR-585 | 55192 | DNAJC17 | DnaJ (Hsp40) homolog, subfamily C, member 17 | 1 | | 2139 | hsa-miR-623 | 55192 | DNAJC17 | DnaJ (Hsp40) homolog, subfamily C, member 17 | 1 | | 2140 | hsa-miR-126\* | 55231 | CCDC87 | coiled-coil domain containing 87 | 1 | | 2141 | hsa-miR-564 | 55231 | CCDC87 | coiled-coil domain containing 87 | 1 | | 2142 | hsa-miR-608 | 55231 | CCDC87 | coiled-coil domain containing 87 | 1 | | 2143 | hsa-mir-15a | 1558 | CYP2C8 | cytochrome P450, family 2, subfamily C, polypeptide 8 | 1 | | 2144 | hsa-mir-16-1 | 1558 | CYP2C8 | cytochrome P450, family 2, subfamily C, polypeptide 8 | 1 | | 2145 | hsa-miR-1231 | 1558 | CYP2C8 | cytochrome P450, family 2, subfamily C, polypeptide 8 | 1 | | 2146 | hsa-miR-608 | 1558 | CYP2C8 | cytochrome P450, family 2, subfamily C, polypeptide 8 | 1 | | 2147 | hsa-mir-218-2 | 9204 | ZMYM6 | zinc finger, MYM-type 6 | 1 | | 2148 | hsa-mir-126 | 9204 | ZMYM6 | zinc finger, MYM-type 6 | 1 | | 2149 | hsa-mir-30c-1 | 9204 | ZMYM6 | zinc finger, MYM-type 6 | 1 | | 2150 | hsa-mir-30e | 9204 | ZMYM6 | zinc finger, MYM-type 6 | 1 | | 2151 | hsa-mir-424 | 9204 | ZMYM6 | zinc finger, MYM-type 6 | 1 | | 2152 | hsa-mir-556 | 9204 | ZMYM6 | zinc finger, MYM-type 6 | 1 | | 2153 | hsa-mir-1915 | 9204 | ZMYM6 | zinc finger, MYM-type 6 | 1 | | 2154 | hsa-miR-190 | 9204 | ZMYM6 | zinc finger, MYM-type 6 | 1 | | 2155 | hsa-miR-503 | 9204 | ZMYM6 | zinc finger, MYM-type 6 | 1 | | 2156 | hsa-miR-585 | 9204 | ZMYM6 | zinc finger, MYM-type 6 | 1 | | 2157 | hsa-miR-1231 | 140460 | ASB7 | ankyrin repeat and SOCS box-containing 7 | 1 | | 2158 | hsa-miR-574-5p | 140460 | ASB7 | ankyrin repeat and SOCS box-containing 7 | 1 | | 2159 | hsa-miR-618 | 140460 | ASB7 | ankyrin repeat and SOCS box-containing 7 | 1 | | 2160 | hsa-miR-634 | 140460 | ASB7 | ankyrin repeat and SOCS box-containing 7 | 1 | | 2161 | hsa-miR-1231 | 79905 | TMC7 | transmembrane channel-like 7 | 1 | | 2162 | hsa-miR-126\* | 79905 | TMC7 | transmembrane channel-like 7 | 1 | | 2163 | hsa-miR-16 | 79905 | TMC7 | transmembrane channel-like 7 | 1 | | 2164 | hsa-miR-564 | 79905 | TMC7 | transmembrane channel-like 7 | 1 | | 2165 | hsa-miR-608 | 79905 | TMC7 | transmembrane channel-like 7 | 1 | | 2166 | hsa-mir-218-2 | 51478 | HSD17B7 | hydroxysteroid (17-beta) dehydrogenase 7 | 1 | | 2167 | hsa-mir-126 | 51478 | HSD17B7 | hydroxysteroid (17-beta) dehydrogenase 7 | 1 | | 2168 | hsa-mir-30c-1 | 51478 | HSD17B7 | hydroxysteroid (17-beta) dehydrogenase 7 | 1 | | 2169 | hsa-mir-30e | 51478 | HSD17B7 | hydroxysteroid (17-beta) dehydrogenase 7 | 1 | | 2170 | hsa-mir-424 | 51478 | HSD17B7 | hydroxysteroid (17-beta) dehydrogenase 7 | 1 | | 2171 | hsa-mir-490 | 51478 | HSD17B7 | hydroxysteroid (17-beta) dehydrogenase 7 | 1 | | 2172 | hsa-mir-556 | 51478 | HSD17B7 | hydroxysteroid (17-beta) dehydrogenase 7 | 1 | | 2173 | hsa-mir-1915 | 51478 | HSD17B7 | hydroxysteroid (17-beta) dehydrogenase 7 | 1 | | 2174 | hsa-miR-190 | 51478 | HSD17B7 | hydroxysteroid (17-beta) dehydrogenase 7 | 1 | | 2175 | hsa-miR-338-5p | 51478 | HSD17B7 | hydroxysteroid (17-beta) dehydrogenase 7 | 1 | | 2176 | hsa-miR-490-5p | 51478 | HSD17B7 | hydroxysteroid (17-beta) dehydrogenase 7 | 1 | | 2177 | hsa-miR-503 | 51478 | HSD17B7 | hydroxysteroid (17-beta) dehydrogenase 7 | 1 | | 2178 | hsa-miR-585 | 51478 | HSD17B7 | hydroxysteroid (17-beta) dehydrogenase 7 | 1 | | 2179 | hsa-miR-657 | 51478 | HSD17B7 | hydroxysteroid (17-beta) dehydrogenase 7 | 1 | | 2180 | hsa-mir-218-2 | 29121 | CLEC2D | C-type lectin domain family 2, member D | 1 | | 2181 | hsa-mir-126 | 29121 | CLEC2D | C-type lectin domain family 2, member D | 1 | | 2182 | hsa-mir-424 | 29121 | CLEC2D | C-type lectin domain family 2, member D | 1 | | 2183 | hsa-mir-490 | 29121 | CLEC2D | C-type lectin domain family 2, member D | 1 | | 2184 | hsa-mir-556 | 29121 | CLEC2D | C-type lectin domain family 2, member D | 1 | | 2185 | hsa-mir-628 | 29121 | CLEC2D | C-type lectin domain family 2, member D | 1 | | 2186 | hsa-miR-190 | 29121 | CLEC2D | C-type lectin domain family 2, member D | 1 | | 2187 | hsa-miR-196b | 29121 | CLEC2D | C-type lectin domain family 2, member D | 1 | | 2188 | hsa-miR-490-5p | 29121 | CLEC2D | C-type lectin domain family 2, member D | 1 | | 2189 | hsa-miR-503 | 29121 | CLEC2D | C-type lectin domain family 2, member D | 1 | | 2190 | hsa-miR-585 | 29121 | CLEC2D | C-type lectin domain family 2, member D | 1 | | 2191 | hsa-miR-586 | 29121 | CLEC2D | C-type lectin domain family 2, member D | 1 | | 2192 | hsa-miR-617 | 29121 | CLEC2D | C-type lectin domain family 2, member D | 1 | | 2193 | hsa-miR-623 | 29121 | CLEC2D | C-type lectin domain family 2, member D | 1 | | 2194 | hsa-mir-126 | 80231 | CXorf21 | chromosome X open reading frame 21 | 1 | | 2195 | hsa-mir-424 | 80231 | CXorf21 | chromosome X open reading frame 21 | 1 | | 2196 | hsa-mir-490 | 80231 | CXorf21 | chromosome X open reading frame 21 | 1 | | 2197 | hsa-mir-1915 | 80231 | CXorf21 | chromosome X open reading frame 21 | 1 | | 2198 | hsa-miR-190 | 80231 | CXorf21 | chromosome X open reading frame 21 | 1 | | 2199 | hsa-miR-490-5p | 80231 | CXorf21 | chromosome X open reading frame 21 | 1 | | 2200 | hsa-miR-503 | 80231 | CXorf21 | chromosome X open reading frame 21 | 1 | | 2201 | hsa-miR-1231 | 79935 | CNTD2 | cyclin N-terminal domain containing 2 | 1 | | 2202 | hsa-miR-608 | 79935 | CNTD2 | cyclin N-terminal domain containing 2 | 1 | | 2203 | hsa-mir-135b | 79595 | SAP130 | Sin3A-associated protein, 130kDa | 1 | | 2204 | hsa-mir-675 | 79595 | SAP130 | Sin3A-associated protein, 130kDa | 1 | | 2205 | hsa-miR-454\* | 79595 | SAP130 | Sin3A-associated protein, 130kDa | 1 | | 2206 | hsa-miR-548d-5p | 79595 | SAP130 | Sin3A-associated protein, 130kDa | 1 | | 2207 | hsa-miR-126\* | 63904 | DUSP21 | dual specificity phosphatase 21 | 1 | | 2208 | hsa-miR-16 | 63904 | DUSP21 | dual specificity phosphatase 21 | 1 | | 2209 | hsa-miR-564 | 63904 | DUSP21 | dual specificity phosphatase 21 | 1 | | 2210 | hsa-miR-608 | 63904 | DUSP21 | dual specificity phosphatase 21 | 1 | | 2211 | hsa-miR-608 | 54566 | EPB41L4B | erythrocyte membrane protein band 4.1 like 4B | 1 | | 2212 | hsa-miR-608 | 80031 | SEMA6D | sema domain, transmembrane domain (TM), and cytoplasmic domain, (semaphorin) 6D | 1 | | 2213 | hsa-miR-126\* | 79400 | NOX5 | NADPH oxidase, EF-hand calcium binding domain 5 | 1 | | 2214 | hsa-miR-16 | 79400 | NOX5 | NADPH oxidase, EF-hand calcium binding domain 5 | 1 | | 2215 | hsa-miR-566 | 79400 | NOX5 | NADPH oxidase, EF-hand calcium binding domain 5 | 1 | | 2216 | hsa-miR-608 | 79400 | NOX5 | NADPH oxidase, EF-hand calcium binding domain 5 | 1 | | 2217 | hsa-mir-218-2 | 55179 | FAIM | Fas apoptotic inhibitory molecule | 1 | | 2218 | hsa-mir-126 | 55179 | FAIM | Fas apoptotic inhibitory molecule | 1 | | 2219 | hsa-mir-424 | 55179 | FAIM | Fas apoptotic inhibitory molecule | 1 | | 2220 | hsa-mir-556 | 55179 | FAIM | Fas apoptotic inhibitory molecule | 1 | | 2221 | hsa-miR-190 | 55179 | FAIM | Fas apoptotic inhibitory molecule | 1 | | 2222 | hsa-miR-503 | 55179 | FAIM | Fas apoptotic inhibitory molecule | 1 | | 2223 | hsa-miR-585 | 55179 | FAIM | Fas apoptotic inhibitory molecule | 1 | | 2224 | hsa-miR-586 | 55179 | FAIM | Fas apoptotic inhibitory molecule | 1 | | 2225 | hsa-miR-617 | 55179 | FAIM | Fas apoptotic inhibitory molecule | 1 | | 2226 | hsa-mir-15a | 51213 | LUZP4 | leucine zipper protein 4 | 1 | | 2227 | hsa-mir-16-1 | 51213 | LUZP4 | leucine zipper protein 4 | 1 | | 2228 | hsa-miR-126\* | 51213 | LUZP4 | leucine zipper protein 4 | 1 | | 2229 | hsa-miR-16 | 51213 | LUZP4 | leucine zipper protein 4 | 1 | | 2230 | hsa-miR-566 | 51213 | LUZP4 | leucine zipper protein 4 | 1 | | 2231 | hsa-miR-608 | 51213 | LUZP4 | leucine zipper protein 4 | 1 | | 2232 | hsa-mir-424 | 51154 | MRTO4 | mRNA turnover 4 homolog (S. cerevisiae) | 1 | | 2233 | hsa-mir-628 | 51154 | MRTO4 | mRNA turnover 4 homolog (S. cerevisiae) | 1 | | 2234 | hsa-miR-503 | 51154 | MRTO4 | mRNA turnover 4 homolog (S. cerevisiae) | 1 | | 2235 | hsa-miR-586 | 51154 | MRTO4 | mRNA turnover 4 homolog (S. cerevisiae) | 1 | | 2236 | hsa-miR-617 | 51154 | MRTO4 | mRNA turnover 4 homolog (S. cerevisiae) | 1 | | 2237 | hsa-miR-623 | 51154 | MRTO4 | mRNA turnover 4 homolog (S. cerevisiae) | 1 | | 2238 | hsa-miR-126\* | 80032 | ZNF556 | zinc finger protein 556 | 1 | | 2239 | hsa-miR-16 | 80032 | ZNF556 | zinc finger protein 556 | 1 | | 2240 | hsa-miR-608 | 60482 | SLC5A7 | solute carrier family 5 (choline transporter), member 7 | 1 | | 2241 | hsa-mir-15a | 27330 | RPS6KA6 | ribosomal protein S6 kinase, 90kDa, polypeptide 6 | 1 | | 2242 | hsa-miR-1231 | 27330 | RPS6KA6 | ribosomal protein S6 kinase, 90kDa, polypeptide 6 | 1 | | 2243 | hsa-miR-126\* | 27330 | RPS6KA6 | ribosomal protein S6 kinase, 90kDa, polypeptide 6 | 1 | | 2244 | hsa-miR-608 | 27330 | RPS6KA6 | ribosomal protein S6 kinase, 90kDa, polypeptide 6 | 1 | | 2245 | hsa-miR-30c | 25879 | DCAF13 | DDB1 and CUL4 associated factor 13 | 1 | | 2246 | hsa-miR-30e | 25879 | DCAF13 | DDB1 and CUL4 associated factor 13 | 1 | | 2247 | hsa-miR-617 | 25879 | DCAF13 | DDB1 and CUL4 associated factor 13 | 1 | | 2248 | hsa-miR-608 | 8470 | SORBS2 | sorbin and SH3 domain containing 2 | 1 | | 2249 | hsa-miR-126\* | 55879 | GABRQ | gamma-aminobutyric acid (GABA) receptor, theta | 1 | | 2250 | hsa-miR-16 | 55879 | GABRQ | gamma-aminobutyric acid (GABA) receptor, theta | 1 | | 2251 | hsa-miR-608 | 55879 | GABRQ | gamma-aminobutyric acid (GABA) receptor, theta | 1 | | 2252 | hsa-mir-15a | 57115 | PGLYRP4 | peptidoglycan recognition protein 4 | 1 | | 2253 | hsa-mir-16-1 | 57115 | PGLYRP4 | peptidoglycan recognition protein 4 | 1 | | 2254 | hsa-mir-339 | 57115 | PGLYRP4 | peptidoglycan recognition protein 4 | 1 | | 2255 | hsa-miR-16 | 57115 | PGLYRP4 | peptidoglycan recognition protein 4 | 1 | | 2256 | hsa-miR-339-5p | 57115 | PGLYRP4 | peptidoglycan recognition protein 4 | 1 | | 2257 | hsa-miR-608 | 57115 | PGLYRP4 | peptidoglycan recognition protein 4 | 1 | | 2258 | hsa-mir-15a | 29974 | A1CF | APOBEC1 complementation factor | 1 | | 2259 | hsa-mir-16-1 | 29974 | A1CF | APOBEC1 complementation factor | 1 | | 2260 | hsa-miR-126\* | 29974 | A1CF | APOBEC1 complementation factor | 1 | | 2261 | hsa-miR-16 | 29974 | A1CF | APOBEC1 complementation factor | 1 | | 2262 | hsa-miR-608 | 29974 | A1CF | APOBEC1 complementation factor | 1 | | 2263 | hsa-mir-15a | 80763 | C12orf39 | chromosome 12 open reading frame 39 | 1 | | 2264 | hsa-mir-16-1 | 80763 | C12orf39 | chromosome 12 open reading frame 39 | 1 | | 2265 | hsa-miR-126\* | 80763 | C12orf39 | chromosome 12 open reading frame 39 | 1 | | 2266 | hsa-miR-16 | 80763 | C12orf39 | chromosome 12 open reading frame 39 | 1 | | 2267 | hsa-miR-566 | 80763 | C12orf39 | chromosome 12 open reading frame 39 | 1 | | 2268 | hsa-miR-608 | 80763 | C12orf39 | chromosome 12 open reading frame 39 | 1 | | 2269 | hsa-miR-1231 | 140803 | TRPM6 | transient receptor potential cation channel, subfamily M, member 6 | 1 | | 2270 | hsa-miR-126\* | 140803 | TRPM6 | transient receptor potential cation channel, subfamily M, member 6 | 1 | | 2273 | hsa-miR-634 | 140803 | TRPM6 | transient receptor potential cation channel, subfamily M, member 6 | 1 | | 2274 | hsa-mir-15a | 80122 | YSK4 | YSK4 Sps1/Ste20-related kinase homolog (S. cerevisiae) | 1 | | 2275 | hsa-mir-16-1 | 80122 | YSK4 | YSK4 Sps1/Ste20-related kinase homolog (S. cerevisiae) | 1 | | 2276 | hsa-miR-1231 | 80122 | YSK4 | YSK4 Sps1/Ste20-related kinase homolog (S. cerevisiae) | 1 | | 2277 | hsa-miR-126\* | 80122 | YSK4 | YSK4 Sps1/Ste20-related kinase homolog (S. cerevisiae) | 1 | | 2278 | hsa-miR-16 | 80122 | YSK4 | YSK4 Sps1/Ste20-related kinase homolog (S. cerevisiae) | 1 | | 2279 | hsa-miR-564 | 80122 | YSK4 | YSK4 Sps1/Ste20-related kinase homolog (S. cerevisiae) | 1 | | 2280 | hsa-miR-566 | 80122 | YSK4 | YSK4 Sps1/Ste20-related kinase homolog (S. cerevisiae) | 1 | | 2281 | hsa-miR-608 | 80122 | YSK4 | YSK4 Sps1/Ste20-related kinase homolog (S. cerevisiae) | 1 | | 2282 | hsa-miR-126\* | 860 | RUNX2 | runt-related transcription factor 2 | 1 | | 2283 | hsa-miR-16 | 860 | RUNX2 | runt-related transcription factor 2 | 1 | | 2284 | hsa-miR-564 | 860 | RUNX2 | runt-related transcription factor 2 | 1 | | 2285 | hsa-miR-566 | 860 | RUNX2 | runt-related transcription factor 2 | 1 | | 2286 | hsa-miR-608 | 860 | RUNX2 | runt-related transcription factor 2 | 1 | | 2287 | hsa-mir-15a | 9293 | GPR52 | G protein-coupled receptor 52 | 1 | | 2288 | hsa-mir-16-1 | 9293 | GPR52 | G protein-coupled receptor 52 | 1 | | 2289 | hsa-miR-126\* | 9293 | GPR52 | G protein-coupled receptor 52 | 1 | | 2290 | hsa-miR-16 | 9293 | GPR52 | G protein-coupled receptor 52 | 1 | | 2291 | hsa-miR-564 | 9293 | GPR52 | G protein-coupled receptor 52 | 1 | | 2292 | hsa-miR-608 | 9293 | GPR52 | G protein-coupled receptor 52 | 1 | | 2293 | hsa-miR-126\* | 50837 | TAS2R7 | taste receptor, type 2, member 7 | 1 | | 2294 | hsa-miR-16 | 50837 | TAS2R7 | taste receptor, type 2, member 7 | 1 | | 2295 | hsa-miR-608 | 50837 | TAS2R7 | taste receptor, type 2, member 7 | 1 | | 2296 | hsa-mir-15a | 26189 | OR1A2 | olfactory receptor, family 1, subfamily A, member 2 | 1 | | 2297 | hsa-mir-16-1 | 26189 | OR1A2 | olfactory receptor, family 1, subfamily A, member 2 | 1 | | 2298 | hsa-miR-126\* | 26189 | OR1A2 | olfactory receptor, family 1, subfamily A, member 2 | 1 | | 2299 | hsa-miR-16 | 26189 | OR1A2 | olfactory receptor, family 1, subfamily A, member 2 | 1 | | 2300 | hsa-miR-608 | 26189 | OR1A2 | olfactory receptor, family 1, subfamily A, member 2 | 1 | | 2301 | hsa-mir-218-2 | 1875 | E2F5 | E2F transcription factor 5, p130-binding | 1 | | 2302 | hsa-mir-126 | 1875 | E2F5 | E2F transcription factor 5, p130-binding | 1 | | 2303 | hsa-mir-556 | 1875 | E2F5 | E2F transcription factor 5, p130-binding | 1 | | 2304 | hsa-miR-190 | 1875 | E2F5 | E2F transcription factor 5, p130-binding | 1 | | 2305 | hsa-miR-585 | 1875 | E2F5 | E2F transcription factor 5, p130-binding | 1 | | 2310 | hsa-miR-566 | 7475 | WNT6 | wingless-type MMTV integration site family, member 6 | 1 | | 2312 | hsa-mir-15a | 26353 | HSPB8 | heat shock 22kDa protein 8 | 1 | | 2313 | hsa-mir-16-1 | 26353 | HSPB8 | heat shock 22kDa protein 8 | 1 | | 2314 | hsa-miR-126\* | 26353 | HSPB8 | heat shock 22kDa protein 8 | 1 | | 2315 | hsa-miR-16 | 26353 | HSPB8 | heat shock 22kDa protein 8 | 1 | | 2316 | hsa-miR-564 | 26353 | HSPB8 | heat shock 22kDa protein 8 | 1 | | 2317 | hsa-miR-566 | 26353 | HSPB8 | heat shock 22kDa protein 8 | 1 | | 2318 | hsa-miR-608 | 26353 | HSPB8 | heat shock 22kDa protein 8 | 1 | | 2319 | hsa-miR-126\* | 4915 | NTRK2 | neurotrophic tyrosine kinase, receptor, type 2 | 1 | | 2320 | hsa-miR-608 | 4915 | NTRK2 | neurotrophic tyrosine kinase, receptor, type 2 | 1 | | 2322 | hsa-mir-15a | 30846 | EHD2 | EH-domain containing 2 | 1 | | 2323 | hsa-mir-16-1 | 30846 | EHD2 | EH-domain containing 2 | 1 | | 2324 | hsa-miR-126\* | 30846 | EHD2 | EH-domain containing 2 | 1 | | 2325 | hsa-miR-16 | 30846 | EHD2 | EH-domain containing 2 | 1 | | 2326 | hsa-miR-564 | 30846 | EHD2 | EH-domain containing 2 | 1 | | 2327 | hsa-miR-608 | 30846 | EHD2 | EH-domain containing 2 | 1 | | 2328 | hsa-miR-618 | 5165 | PDK3 | pyruvate dehydrogenase kinase, isozyme 3 | 1 | | 2329 | hsa-mir-15a | 11248 | NXPH3 | neurexophilin 3 | 1 | | 2330 | hsa-mir-16-1 | 11248 | NXPH3 | neurexophilin 3 | 1 | | 2331 | hsa-miR-126\* | 11248 | NXPH3 | neurexophilin 3 | 1 | | 2332 | hsa-miR-16 | 11248 | NXPH3 | neurexophilin 3 | 1 | | 2333 | hsa-miR-564 | 11248 | NXPH3 | neurexophilin 3 | 1 | | 2334 | hsa-miR-608 | 11248 | NXPH3 | neurexophilin 3 | 1 | | 2335 | hsa-mir-339 | 1191 | CLU | clusterin | 1 | | 2336 | hsa-miR-339-5p | 1191 | CLU | clusterin | 1 | | 2337 | hsa-miR-618 | 1191 | CLU | clusterin | 1 | | 2338 | hsa-miR-634 | 1191 | CLU | clusterin | 1 | | 2339 | hsa-miR-1231 | 9513 | FXR2 | fragile X mental retardation, autosomal homolog 2 | 1 | | 2340 | hsa-miR-126\* | 9513 | FXR2 | fragile X mental retardation, autosomal homolog 2 | 1 | | 2341 | hsa-miR-608 | 9513 | FXR2 | fragile X mental retardation, autosomal homolog 2 | 1 | | 2342 | hsa-mir-126 | 2967 | GTF2H3 | general transcription factor IIH, polypeptide 3, 34kDa | 1 | | 2343 | hsa-mir-30c-1 | 2967 | GTF2H3 | general transcription factor IIH, polypeptide 3, 34kDa | 1 | | 2344 | hsa-mir-30e | 2967 | GTF2H3 | general transcription factor IIH, polypeptide 3, 34kDa | 1 | | 2345 | hsa-mir-424 | 2967 | GTF2H3 | general transcription factor IIH, polypeptide 3, 34kDa | 1 | | 2346 | hsa-mir-1915 | 2967 | GTF2H3 | general transcription factor IIH, polypeptide 3, 34kDa | 1 | | 2347 | hsa-miR-503 | 2967 | GTF2H3 | general transcription factor IIH, polypeptide 3, 34kDa | 1 | | 2348 | hsa-miR-584 | 2967 | GTF2H3 | general transcription factor IIH, polypeptide 3, 34kDa | 1 | | 2349 | hsa-miR-1231 | 80307 | FER1L4 | fer-1-like 4 (C. elegans) | 1 | | 2350 | hsa-miR-126\* | 80307 | FER1L4 | fer-1-like 4 (C. elegans) | 1 | | 2351 | hsa-miR-608 | 80307 | FER1L4 | fer-1-like 4 (C. elegans) | 1 | | 2352 | hsa-miR-126\* | 2287 | FKBP3 | FK506 binding protein 3, 25kDa | 1 | | 2353 | hsa-miR-16 | 2287 | FKBP3 | FK506 binding protein 3, 25kDa | 1 | | 2354 | hsa-miR-566 | 2287 | FKBP3 | FK506 binding protein 3, 25kDa | 1 | | 2355 | hsa-miR-608 | 2287 | FKBP3 | FK506 binding protein 3, 25kDa | 1 | | 2356 | hsa-miR-618 | 60685 | ZFAND3 | zinc finger, AN1-type domain 3 | 1 | | 2357 | hsa-mir-15a | 23413 | NCS1 | neuronal calcium sensor 1 | 1 | | 2358 | hsa-mir-16-1 | 23413 | NCS1 | neuronal calcium sensor 1 | 1 | | 2359 | hsa-miR-126\* | 23413 | NCS1 | neuronal calcium sensor 1 | 1 | | 2360 | hsa-miR-16 | 23413 | NCS1 | neuronal calcium sensor 1 | 1 | | 2361 | hsa-miR-564 | 23413 | NCS1 | neuronal calcium sensor 1 | 1 | | 2362 | hsa-miR-566 | 23413 | NCS1 | neuronal calcium sensor 1 | 1 | | 2363 | hsa-miR-608 | 23413 | NCS1 | neuronal calcium sensor 1 | 1 | | 2364 | hsa-mir-126 | 26024 | PTCD1 | pentatricopeptide repeat domain 1 | 1 | | 2365 | hsa-mir-30c-1 | 26024 | PTCD1 | pentatricopeptide repeat domain 1 | 1 | | 2366 | hsa-mir-30e | 26024 | PTCD1 | pentatricopeptide repeat domain 1 | 1 | | 2367 | hsa-mir-424 | 26024 | PTCD1 | pentatricopeptide repeat domain 1 | 1 | | 2368 | hsa-miR-338-5p | 26024 | PTCD1 | pentatricopeptide repeat domain 1 | 1 | | 2369 | hsa-miR-503 | 26024 | PTCD1 | pentatricopeptide repeat domain 1 | 1 | | 2370 | hsa-miR-657 | 26024 | PTCD1 | pentatricopeptide repeat domain 1 | 1 | | 2371 | hsa-miR-608 | 23767 | FLRT3 | fibronectin leucine rich transmembrane protein 3 | 1 | | 2372 | hsa-miR-1231 | 55640 | FLVCR2 | feline leukemia virus subgroup C cellular receptor family, member 2 | 1 | | 2373 | hsa-miR-618 | 55640 | FLVCR2 | feline leukemia virus subgroup C cellular receptor family, member 2 | 1 | | 2374 | hsa-miR-634 | 55640 | FLVCR2 | feline leukemia virus subgroup C cellular receptor family, member 2 | 1 | | 2375 | hsa-mir-218-2 | 7812 | CSDE1 | cold shock domain containing E1, RNA-binding | 1 | | 2376 | hsa-mir-126 | 7812 | CSDE1 | cold shock domain containing E1, RNA-binding | 1 | | 2377 | hsa-mir-196b | 7812 | CSDE1 | cold shock domain containing E1, RNA-binding | 1 | | 2378 | hsa-mir-424 | 7812 | CSDE1 | cold shock domain containing E1, RNA-binding | 1 | | 2379 | hsa-mir-490 | 7812 | CSDE1 | cold shock domain containing E1, RNA-binding | 1 | | 2380 | hsa-mir-556 | 7812 | CSDE1 | cold shock domain containing E1, RNA-binding | 1 | | 2381 | hsa-mir-1915 | 7812 | CSDE1 | cold shock domain containing E1, RNA-binding | 1 | | 2382 | hsa-miR-190 | 7812 | CSDE1 | cold shock domain containing E1, RNA-binding | 1 | | 2383 | hsa-miR-196b | 7812 | CSDE1 | cold shock domain containing E1, RNA-binding | 1 | | 2384 | hsa-miR-455-5p | 7812 | CSDE1 | cold shock domain containing E1, RNA-binding | 1 | | 2385 | hsa-miR-490-5p | 7812 | CSDE1 | cold shock domain containing E1, RNA-binding | 1 | | 2386 | hsa-miR-503 | 7812 | CSDE1 | cold shock domain containing E1, RNA-binding | 1 | | 2387 | hsa-miR-585 | 7812 | CSDE1 | cold shock domain containing E1, RNA-binding | 1 | | 2388 | hsa-miR-623 | 7812 | CSDE1 | cold shock domain containing E1, RNA-binding | 1 | | 2389 | hsa-mir-126 | 170506 | DHX36 | DEAH (Asp-Glu-Ala-His) box polypeptide 36 | 1 | | 2390 | hsa-mir-424 | 170506 | DHX36 | DEAH (Asp-Glu-Ala-His) box polypeptide 36 | 1 | | 2391 | hsa-mir-490 | 170506 | DHX36 | DEAH (Asp-Glu-Ala-His) box polypeptide 36 | 1 | | 2392 | hsa-mir-628 | 170506 | DHX36 | DEAH (Asp-Glu-Ala-His) box polypeptide 36 | 1 | | 2393 | hsa-miR-190 | 170506 | DHX36 | DEAH (Asp-Glu-Ala-His) box polypeptide 36 | 1 | | 2394 | hsa-miR-196b | 170506 | DHX36 | DEAH (Asp-Glu-Ala-His) box polypeptide 36 | 1 | | 2395 | hsa-miR-490-5p | 170506 | DHX36 | DEAH (Asp-Glu-Ala-His) box polypeptide 36 | 1 | | 2396 | hsa-miR-503 | 170506 | DHX36 | DEAH (Asp-Glu-Ala-His) box polypeptide 36 | 1 | | 2397 | hsa-miR-623 | 170506 | DHX36 | DEAH (Asp-Glu-Ala-His) box polypeptide 36 | 1 | | 2398 | hsa-mir-126 | 29090 | C18orf55 | chromosome 18 open reading frame 55 | 1 | | 2399 | hsa-mir-30c-1 | 29090 | C18orf55 | chromosome 18 open reading frame 55 | 1 | | 2400 | hsa-mir-30e | 29090 | C18orf55 | chromosome 18 open reading frame 55 | 1 | | 2401 | hsa-mir-424 | 29090 | C18orf55 | chromosome 18 open reading frame 55 | 1 | | 2402 | hsa-mir-556 | 29090 | C18orf55 | chromosome 18 open reading frame 55 | 1 | | 2403 | hsa-miR-503 | 29090 | C18orf55 | chromosome 18 open reading frame 55 | 1 | | 2407 | hsa-mir-218-2 | 55170 | PRMT6 | protein arginine methyltransferase 6 | 1 | | 2408 | hsa-mir-126 | 55170 | PRMT6 | protein arginine methyltransferase 6 | 1 | | 2409 | hsa-mir-424 | 55170 | PRMT6 | protein arginine methyltransferase 6 | 1 | | 2410 | hsa-mir-556 | 55170 | PRMT6 | protein arginine methyltransferase 6 | 1 | | 2411 | hsa-miR-190 | 55170 | PRMT6 | protein arginine methyltransferase 6 | 1 | | 2412 | hsa-miR-503 | 55170 | PRMT6 | protein arginine methyltransferase 6 | 1 | | 2413 | hsa-miR-585 | 55170 | PRMT6 | protein arginine methyltransferase 6 | 1 | | 2414 | hsa-miR-617 | 55170 | PRMT6 | protein arginine methyltransferase 6 | 1 | | 2415 | hsa-miR-623 | 55170 | PRMT6 | protein arginine methyltransferase 6 | 1 | | 2416 | hsa-miR-618 | 85027 | C5orf62 | chromosome 5 open reading frame 62 | 1 | | 2417 | hsa-miR-634 | 85027 | C5orf62 | chromosome 5 open reading frame 62 | 1 | | 2418 | hsa-miR-1231 | 83643 | CCDC3 | coiled-coil domain containing 3 | 1 | | 2419 | hsa-miR-634 | 83643 | CCDC3 | coiled-coil domain containing 3 | 1 | | 2420 | hsa-mir-15a | 26470 | SEZ6L2 | seizure related 6 homolog (mouse)-like 2 | 1 | | 2421 | hsa-mir-16-1 | 26470 | SEZ6L2 | seizure related 6 homolog (mouse)-like 2 | 1 | | 2422 | hsa-miR-126\* | 26470 | SEZ6L2 | seizure related 6 homolog (mouse)-like 2 | 1 | | 2423 | hsa-miR-16 | 26470 | SEZ6L2 | seizure related 6 homolog (mouse)-like 2 | 1 | | 2424 | hsa-miR-566 | 26470 | SEZ6L2 | seizure related 6 homolog (mouse)-like 2 | 1 | | 2425 | hsa-miR-608 | 26470 | SEZ6L2 | seizure related 6 homolog (mouse)-like 2 | 1 | | 2426 | hsa-mir-424 | 91574 | C12orf65 | chromosome 12 open reading frame 65 | 1 | | 2427 | hsa-mir-628 | 91574 | C12orf65 | chromosome 12 open reading frame 65 | 1 | | 2428 | hsa-mir-675 | 91574 | C12orf65 | chromosome 12 open reading frame 65 | 1 | | 2429 | hsa-miR-190 | 91574 | C12orf65 | chromosome 12 open reading frame 65 | 1 | | 2430 | hsa-miR-503 | 91574 | C12orf65 | chromosome 12 open reading frame 65 | 1 | | 2431 | hsa-miR-586 | 91574 | C12orf65 | chromosome 12 open reading frame 65 | 1 | | 2432 | hsa-miR-617 | 91574 | C12orf65 | chromosome 12 open reading frame 65 | 1 | | 2433 | hsa-miR-623 | 91574 | C12orf65 | chromosome 12 open reading frame 65 | 1 | | 2434 | hsa-mir-15a | 57129 | MRPL47 | mitochondrial ribosomal protein L47 | 1 | | 2435 | hsa-mir-16-1 | 57129 | MRPL47 | mitochondrial ribosomal protein L47 | 1 | | 2436 | hsa-miR-608 | 57129 | MRPL47 | mitochondrial ribosomal protein L47 | 1 | | 2437 | hsa-miR-126\* | 23624 | CBLC | Cas-Br-M (murine) ecotropic retroviral transforming sequence c | 1 | | 2438 | hsa-miR-16 | 23624 | CBLC | Cas-Br-M (murine) ecotropic retroviral transforming sequence c | 1 | | 2439 | hsa-miR-608 | 23624 | CBLC | Cas-Br-M (murine) ecotropic retroviral transforming sequence c | 1 | | 2440 | hsa-miR-574-5p | 4053 | LTBP2 | latent transforming growth factor beta binding protein 2 | 1 | | 2441 | hsa-mir-126 | 51073 | MRPL4 | mitochondrial ribosomal protein L4 | 1 | | 2442 | hsa-mir-196b | 51073 | MRPL4 | mitochondrial ribosomal protein L4 | 1 | | 2443 | hsa-mir-424 | 51073 | MRPL4 | mitochondrial ribosomal protein L4 | 1 | | 2444 | hsa-mir-628 | 51073 | MRPL4 | mitochondrial ribosomal protein L4 | 1 | | 2445 | hsa-miR-190 | 51073 | MRPL4 | mitochondrial ribosomal protein L4 | 1 | | 2446 | hsa-miR-196b | 51073 | MRPL4 | mitochondrial ribosomal protein L4 | 1 | | 2447 | hsa-miR-503 | 51073 | MRPL4 | mitochondrial ribosomal protein L4 | 1 | | 2448 | hsa-miR-617 | 51073 | MRPL4 | mitochondrial ribosomal protein L4 | 1 | | 2449 | hsa-miR-623 | 51073 | MRPL4 | mitochondrial ribosomal protein L4 | 1 | | 2450 | hsa-miR-1231 | 55215 | FANCI | Fanconi anemia, complementation group I | 1 | | 2451 | hsa-miR-634 | 55215 | FANCI | Fanconi anemia, complementation group I | 1 | | 2452 | hsa-mir-30c-1 | 90799 | CCDC45 | coiled-coil domain containing 45 | 1 | | 2453 | hsa-mir-30e | 90799 | CCDC45 | coiled-coil domain containing 45 | 1 | | 2454 | hsa-miR-617 | 90799 | CCDC45 | coiled-coil domain containing 45 | 1 | | 2455 | hsa-miR-126\* | 10117 | ENAM | enamelin | 1 | | 2456 | hsa-miR-16 | 10117 | ENAM | enamelin | 1 | | 2457 | hsa-miR-608 | 10117 | ENAM | enamelin | 1 | | 2458 | hsa-mir-30c-1 | 55153 | SDAD1 | SDA1 domain containing 1 | 1 | | 2459 | hsa-mir-30e | 55153 | SDAD1 | SDA1 domain containing 1 | 1 | | 2460 | hsa-mir-15a | 22953 | P2RX2 | purinergic receptor P2X, ligand-gated ion channel, 2 | 1 | | 2461 | hsa-mir-16-1 | 22953 | P2RX2 | purinergic receptor P2X, ligand-gated ion channel, 2 | 1 | | 2462 | hsa-miR-126\* | 22953 | P2RX2 | purinergic receptor P2X, ligand-gated ion channel, 2 | 1 | | 2463 | hsa-miR-16 | 22953 | P2RX2 | purinergic receptor P2X, ligand-gated ion channel, 2 | 1 | | 2464 | hsa-miR-608 | 22953 | P2RX2 | purinergic receptor P2X, ligand-gated ion channel, 2 | 1 | | 2465 | hsa-mir-339 | 286144 | C8orf83 | chromosome 8 open reading frame 83 | 1 | | 2466 | hsa-miR-339-5p | 286144 | C8orf83 | chromosome 8 open reading frame 83 | 1 | | 2467 | hsa-miR-634 | 286144 | C8orf83 | chromosome 8 open reading frame 83 | 1 | | 2468 | hsa-mir-454 | 27123 | DKK2 | dickkopf homolog 2 (Xenopus laevis) | 1 | | 2469 | hsa-miR-675 | 27123 | DKK2 | dickkopf homolog 2 (Xenopus laevis) | 1 | | 2470 | hsa-miR-95 | 27123 | DKK2 | dickkopf homolog 2 (Xenopus laevis) | 1 | | 2475 | hsa-miR-30c | 55814 | BDP1 | B double prime 1, subunit of RNA polymerase III transcription initiation factor IIIB | 1 | | 2476 | hsa-miR-30e | 55814 | BDP1 | B double prime 1, subunit of RNA polymerase III transcription initiation factor IIIB | 1 | | 2478 | hsa-miR-126\* | 84793 | MGC12982 | hypothetical protein MGC12982 | 1 | | 2479 | hsa-miR-16 | 84793 | MGC12982 | hypothetical protein MGC12982 | 1 | | 2480 | hsa-miR-564 | 84793 | MGC12982 | hypothetical protein MGC12982 | 1 | | 2481 | hsa-miR-566 | 84793 | MGC12982 | hypothetical protein MGC12982 | 1 | | 2482 | hsa-miR-608 | 84793 | MGC12982 | hypothetical protein MGC12982 | 1 | | 2483 | hsa-mir-424 | 84309 | NUDT16L1 | nudix (nucleoside diphosphate linked moiety X)-type motif 16-like 1 | 1 | | 2484 | hsa-mir-628 | 84309 | NUDT16L1 | nudix (nucleoside diphosphate linked moiety X)-type motif 16-like 1 | 1 | | 2485 | hsa-miR-196b | 84309 | NUDT16L1 | nudix (nucleoside diphosphate linked moiety X)-type motif 16-like 1 | 1 | | 2486 | hsa-miR-503 | 84309 | NUDT16L1 | nudix (nucleoside diphosphate linked moiety X)-type motif 16-like 1 | 1 | | 2487 | hsa-miR-617 | 84309 | NUDT16L1 | nudix (nucleoside diphosphate linked moiety X)-type motif 16-like 1 | 1 | | 2488 | hsa-miR-623 | 84309 | NUDT16L1 | nudix (nucleoside diphosphate linked moiety X)-type motif 16-like 1 | 1 | | 2489 | hsa-miR-126\* | 100132319 | LOC100132319 | hypothetical LOC100132319 | 1 | | 2490 | hsa-miR-566 | 100132319 | LOC100132319 | hypothetical LOC100132319 | 1 | | 2491 | hsa-miR-608 | 100132319 | LOC100132319 | hypothetical LOC100132319 | 1 | | 2493 | hsa-mir-424 | 378938 | MALAT1 | metastasis associated lung adenocarcinoma transcript 1 (non-protein coding) | 1 | | 2495 | hsa-mir-1915 | 378938 | MALAT1 | metastasis associated lung adenocarcinoma transcript 1 (non-protein coding) | 1 | | 2497 | hsa-miR-503 | 378938 | MALAT1 | metastasis associated lung adenocarcinoma transcript 1 (non-protein coding) | 1 | | 2498 | hsa-mir-218-2 | 6167 | RPL37 | ribosomal protein L37 | 1 | | 2499 | hsa-mir-126 | 6167 | RPL37 | ribosomal protein L37 | 1 | | 2500 | hsa-miR-585 | 6167 | RPL37 | ribosomal protein L37 | 1 | | 2501 | hsa-miR-1231 | 89796 | NAV1 | neuron navigator 1 | 1 | | 2502 | hsa-miR-634 | 89796 | NAV1 | neuron navigator 1 | 1 | | 2503 | hsa-miR-574-5p | 57606 | SLAIN2 | SLAIN motif family, member 2 | 1 | | 2504 | hsa-miR-618 | 57606 | SLAIN2 | SLAIN motif family, member 2 | 1 | | 2505 | hsa-miR-634 | 57606 | SLAIN2 | SLAIN motif family, member 2 | 1 | | 2506 | hsa-mir-339 | 80232 | WDR26 | WD repeat domain 26 | 1 | | 2507 | hsa-miR-339-5p | 80232 | WDR26 | WD repeat domain 26 | 1 | | 2508 | hsa-miR-564 | 80232 | WDR26 | WD repeat domain 26 | 1 | | 2509 | hsa-miR-618 | 80232 | WDR26 | WD repeat domain 26 | 1 | | 2510 | hsa-miR-634 | 80232 | WDR26 | WD repeat domain 26 | 1 | | 2511 | hsa-miR-126\* | 203259 | C9orf25 | chromosome 9 open reading frame 25 | 1 | | 2512 | hsa-miR-16 | 203259 | C9orf25 | chromosome 9 open reading frame 25 | 1 | | 2513 | hsa-miR-566 | 203259 | C9orf25 | chromosome 9 open reading frame 25 | 1 | | 2514 | hsa-mir-424 | 387066 | SNHG5 | small nucleolar RNA host gene 5 (non-protein coding) | 1 | | 2515 | hsa-mir-628 | 387066 | SNHG5 | small nucleolar RNA host gene 5 (non-protein coding) | 1 | | 2516 | hsa-miR-190 | 387066 | SNHG5 | small nucleolar RNA host gene 5 (non-protein coding) | 1 | | 2517 | hsa-miR-503 | 387066 | SNHG5 | small nucleolar RNA host gene 5 (non-protein coding) | 1 | | 2518 | hsa-miR-586 | 387066 | SNHG5 | small nucleolar RNA host gene 5 (non-protein coding) | 1 | | 2519 | hsa-miR-617 | 387066 | SNHG5 | small nucleolar RNA host gene 5 (non-protein coding) | 1 | | 2520 | hsa-miR-1231 | 84152 | PPP1R1B | protein phosphatase 1, regulatory (inhibitor) subunit 1B | 1 | | 2521 | hsa-miR-126\* | 84152 | PPP1R1B | protein phosphatase 1, regulatory (inhibitor) subunit 1B | 1 | | 2522 | hsa-miR-16 | 84152 | PPP1R1B | protein phosphatase 1, regulatory (inhibitor) subunit 1B | 1 | | 2523 | hsa-miR-564 | 84152 | PPP1R1B | protein phosphatase 1, regulatory (inhibitor) subunit 1B | 1 | | 2524 | hsa-miR-566 | 84152 | PPP1R1B | protein phosphatase 1, regulatory (inhibitor) subunit 1B | 1 | | 2525 | hsa-miR-608 | 84152 | PPP1R1B | protein phosphatase 1, regulatory (inhibitor) subunit 1B | 1 | | 2526 | hsa-mir-196b | 64949 | MRPS26 | mitochondrial ribosomal protein S26 | 1 | | 2527 | hsa-mir-424 | 64949 | MRPS26 | mitochondrial ribosomal protein S26 | 1 | | 2528 | hsa-mir-628 | 64949 | MRPS26 | mitochondrial ribosomal protein S26 | 1 | | 2529 | hsa-miR-196b | 64949 | MRPS26 | mitochondrial ribosomal protein S26 | 1 | | 2530 | hsa-miR-503 | 64949 | MRPS26 | mitochondrial ribosomal protein S26 | 1 | | 2531 | hsa-miR-504 | 64949 | MRPS26 | mitochondrial ribosomal protein S26 | 1 | | 2532 | hsa-miR-586 | 64949 | MRPS26 | mitochondrial ribosomal protein S26 | 1 | | 2533 | hsa-miR-617 | 64949 | MRPS26 | mitochondrial ribosomal protein S26 | 1 | | 2534 | hsa-miR-623 | 64949 | MRPS26 | mitochondrial ribosomal protein S26 | 1 | | 2535 | hsa-mir-135b | 118813 | ZFYVE27 | zinc finger, FYVE domain containing 27 | 1 | | 2536 | hsa-mir-675 | 118813 | ZFYVE27 | zinc finger, FYVE domain containing 27 | 1 | | 2537 | hsa-miR-623 | 118813 | ZFYVE27 | zinc finger, FYVE domain containing 27 | 1 | | 2538 | hsa-miR-618 | 867 | CBL | Cas-Br-M (murine) ecotropic retroviral transforming sequence | 1 | | 2539 | hsa-mir-218-2 | 57620 | STIM2 | stromal interaction molecule 2 | 1 | | 2540 | hsa-mir-126 | 57620 | STIM2 | stromal interaction molecule 2 | 1 | | 2541 | hsa-mir-424 | 57620 | STIM2 | stromal interaction molecule 2 | 1 | | 2542 | hsa-mir-556 | 57620 | STIM2 | stromal interaction molecule 2 | 1 | | 2543 | hsa-mir-628 | 57620 | STIM2 | stromal interaction molecule 2 | 1 | | 2544 | hsa-mir-1915 | 57620 | STIM2 | stromal interaction molecule 2 | 1 | | 2545 | hsa-miR-190 | 57620 | STIM2 | stromal interaction molecule 2 | 1 | | 2546 | hsa-miR-503 | 57620 | STIM2 | stromal interaction molecule 2 | 1 | | 2547 | hsa-miR-585 | 57620 | STIM2 | stromal interaction molecule 2 | 1 | | 2548 | hsa-miR-617 | 57620 | STIM2 | stromal interaction molecule 2 | 1 | | 2549 | hsa-mir-15a | 4645 | MYO5B | myosin VB | 1 | | 2550 | hsa-mir-16-1 | 4645 | MYO5B | myosin VB | 1 | | 2551 | hsa-miR-608 | 4645 | MYO5B | myosin VB | 1 | | 2552 | hsa-miR-586 | 170622 | COMMD6 | COMM domain containing 6 | 1 | | 2553 | hsa-miR-617 | 170622 | COMMD6 | COMM domain containing 6 | 1 | | 2554 | hsa-mir-15a | 54492 | NEURL1B | neuralized homolog 1B (Drosophila) | 1 | | 2555 | hsa-mir-16-1 | 54492 | NEURL1B | neuralized homolog 1B (Drosophila) | 1 | | 2556 | hsa-miR-126\* | 54492 | NEURL1B | neuralized homolog 1B (Drosophila) | 1 | | 2557 | hsa-miR-634 | 54492 | NEURL1B | neuralized homolog 1B (Drosophila) | 1 | | 2558 | hsa-mir-628 | 25845 | LOC25845 | hypothetical LOC25845 | 1 | | 2559 | hsa-miR-617 | 25845 | LOC25845 | hypothetical LOC25845 | 1 | | 2560 | hsa-mir-218-2 | 79813 | EHMT1 | euchromatic histone-lysine N-methyltransferase 1 | 1 | | 2561 | hsa-mir-126 | 79813 | EHMT1 | euchromatic histone-lysine N-methyltransferase 1 | 1 | | 2562 | hsa-mir-196b | 79813 | EHMT1 | euchromatic histone-lysine N-methyltransferase 1 | 1 | | 2563 | hsa-mir-424 | 79813 | EHMT1 | euchromatic histone-lysine N-methyltransferase 1 | 1 | | 2564 | hsa-mir-490 | 79813 | EHMT1 | euchromatic histone-lysine N-methyltransferase 1 | 1 | | 2565 | hsa-mir-556 | 79813 | EHMT1 | euchromatic histone-lysine N-methyltransferase 1 | 1 | | 2566 | hsa-mir-1915 | 79813 | EHMT1 | euchromatic histone-lysine N-methyltransferase 1 | 1 | | 2567 | hsa-miR-190 | 79813 | EHMT1 | euchromatic histone-lysine N-methyltransferase 1 | 1 | | 2568 | hsa-miR-196b | 79813 | EHMT1 | euchromatic histone-lysine N-methyltransferase 1 | 1 | | 2569 | hsa-miR-490-5p | 79813 | EHMT1 | euchromatic histone-lysine N-methyltransferase 1 | 1 | | 2570 | hsa-miR-503 | 79813 | EHMT1 | euchromatic histone-lysine N-methyltransferase 1 | 1 | | 2571 | hsa-miR-585 | 79813 | EHMT1 | euchromatic histone-lysine N-methyltransferase 1 | 1 | | 2572 | hsa-miR-623 | 79813 | EHMT1 | euchromatic histone-lysine N-methyltransferase 1 | 1 | | 2573 | hsa-mir-424 | 112724 | RDH13 | retinol dehydrogenase 13 (all-trans/9-cis) | 1 | | 2574 | hsa-mir-628 | 112724 | RDH13 | retinol dehydrogenase 13 (all-trans/9-cis) | 1 | | 2575 | hsa-miR-190 | 112724 | RDH13 | retinol dehydrogenase 13 (all-trans/9-cis) | 1 | | 2576 | hsa-miR-503 | 112724 | RDH13 | retinol dehydrogenase 13 (all-trans/9-cis) | 1 | | 2577 | hsa-miR-586 | 112724 | RDH13 | retinol dehydrogenase 13 (all-trans/9-cis) | 1 | | 2578 | hsa-miR-617 | 112724 | RDH13 | retinol dehydrogenase 13 (all-trans/9-cis) | 1 | | 2579 | hsa-miR-623 | 112724 | RDH13 | retinol dehydrogenase 13 (all-trans/9-cis) | 1 | | 2580 | hsa-mir-218-2 | 26260 | FBXO25 | F-box protein 25 | 1 | | 2581 | hsa-mir-126 | 26260 | FBXO25 | F-box protein 25 | 1 | | 2582 | hsa-mir-424 | 26260 | FBXO25 | F-box protein 25 | 1 | | 2583 | hsa-mir-556 | 26260 | FBXO25 | F-box protein 25 | 1 | | 2584 | hsa-mir-628 | 26260 | FBXO25 | F-box protein 25 | 1 | | 2585 | hsa-mir-1915 | 26260 | FBXO25 | F-box protein 25 | 1 | | 2586 | hsa-miR-190 | 26260 | FBXO25 | F-box protein 25 | 1 | | 2587 | hsa-miR-503 | 26260 | FBXO25 | F-box protein 25 | 1 | | 2588 | hsa-miR-585 | 26260 | FBXO25 | F-box protein 25 | 1 | | 2589 | hsa-miR-586 | 26260 | FBXO25 | F-box protein 25 | 1 | | 2590 | hsa-miR-617 | 26260 | FBXO25 | F-box protein 25 | 1 | | 2591 | hsa-miR-623 | 26260 | FBXO25 | F-box protein 25 | 1 | | 2592 | hsa-mir-218-2 | 85457 | KIAA1737 | KIAA1737 | 1 | | 2593 | hsa-mir-126 | 85457 | KIAA1737 | KIAA1737 | 1 | | 2594 | hsa-mir-424 | 85457 | KIAA1737 | KIAA1737 | 1 | | 2595 | hsa-mir-556 | 85457 | KIAA1737 | KIAA1737 | 1 | | 2596 | hsa-mir-628 | 85457 | KIAA1737 | KIAA1737 | 1 | | 2597 | hsa-miR-190 | 85457 | KIAA1737 | KIAA1737 | 1 | | 2598 | hsa-miR-503 | 85457 | KIAA1737 | KIAA1737 | 1 | | 2599 | hsa-miR-585 | 85457 | KIAA1737 | KIAA1737 | 1 | | 2600 | hsa-miR-617 | 85457 | KIAA1737 | KIAA1737 | 1 | | 2601 | hsa-mir-424 | 152217 | LOC152217 | hypothetical LOC152217 | 1 | | 2602 | hsa-mir-628 | 152217 | LOC152217 | hypothetical LOC152217 | 1 | | 2603 | hsa-miR-503 | 152217 | LOC152217 | hypothetical LOC152217 | 1 | | 2604 | hsa-miR-586 | 152217 | LOC152217 | hypothetical LOC152217 | 1 | | 2605 | hsa-miR-617 | 152217 | LOC152217 | hypothetical LOC152217 | 1 | | 2606 | hsa-miR-623 | 152217 | LOC152217 | hypothetical LOC152217 | 1 | | 2607 | hsa-miR-1231 | 124976 | SPNS2 | spinster homolog 2 (Drosophila) | 1 | | 2608 | hsa-miR-126\* | 124976 | SPNS2 | spinster homolog 2 (Drosophila) | 1 | | 2609 | hsa-miR-618 | 124976 | SPNS2 | spinster homolog 2 (Drosophila) | 1 | | 2610 | hsa-miR-634 | 124976 | SPNS2 | spinster homolog 2 (Drosophila) | 1 | | 2611 | hsa-mir-196b | 126792 | B3GALT6 | UDP-Gal:betaGal beta 1,3-galactosyltransferase polypeptide 6 | 1 | | 2612 | hsa-mir-628 | 126792 | B3GALT6 | UDP-Gal:betaGal beta 1,3-galactosyltransferase polypeptide 6 | 1 | | 2613 | hsa-miR-196b | 126792 | B3GALT6 | UDP-Gal:betaGal beta 1,3-galactosyltransferase polypeptide 6 | 1 | | 2614 | hsa-miR-617 | 126792 | B3GALT6 | UDP-Gal:betaGal beta 1,3-galactosyltransferase polypeptide 6 | 1 | | 2615 | hsa-miR-623 | 126792 | B3GALT6 | UDP-Gal:betaGal beta 1,3-galactosyltransferase polypeptide 6 | 1 | | 2616 | hsa-mir-135b | 88745 | C6orf153 | chromosome 6 open reading frame 153 | 1 | | 2617 | hsa-mir-675 | 88745 | C6orf153 | chromosome 6 open reading frame 153 | 1 | | 2618 | hsa-miR-454\* | 88745 | C6orf153 | chromosome 6 open reading frame 153 | 1 | | 2619 | hsa-miR-548d-5p | 88745 | C6orf153 | chromosome 6 open reading frame 153 | 1 | | 2620 | hsa-mir-135b | 8812 | CCNK | cyclin K | 1 | | 2621 | hsa-miR-616\* | 8812 | CCNK | cyclin K | 1 | | 2622 | hsa-mir-126 | 84058 | WDR54 | WD repeat domain 54 | 1 | | 2623 | hsa-mir-424 | 84058 | WDR54 | WD repeat domain 54 | 1 | | 2624 | hsa-mir-556 | 84058 | WDR54 | WD repeat domain 54 | 1 | | 2625 | hsa-mir-628 | 84058 | WDR54 | WD repeat domain 54 | 1 | | 2626 | hsa-miR-190 | 84058 | WDR54 | WD repeat domain 54 | 1 | | 2627 | hsa-miR-338-5p | 84058 | WDR54 | WD repeat domain 54 | 1 | | 2628 | hsa-miR-503 | 84058 | WDR54 | WD repeat domain 54 | 1 | | 2629 | hsa-miR-586 | 84058 | WDR54 | WD repeat domain 54 | 1 | | 2630 | hsa-miR-617 | 84058 | WDR54 | WD repeat domain 54 | 1 | | 2631 | hsa-miR-657 | 84058 | WDR54 | WD repeat domain 54 | 1 | | 2632 | hsa-mir-218-2 | 114971 | PTPMT1 | protein tyrosine phosphatase, mitochondrial 1 | 1 | | 2633 | hsa-mir-126 | 114971 | PTPMT1 | protein tyrosine phosphatase, mitochondrial 1 | 1 | | 2634 | hsa-mir-196b | 114971 | PTPMT1 | protein tyrosine phosphatase, mitochondrial 1 | 1 | | 2635 | hsa-mir-424 | 114971 | PTPMT1 | protein tyrosine phosphatase, mitochondrial 1 | 1 | | 2636 | hsa-mir-490 | 114971 | PTPMT1 | protein tyrosine phosphatase, mitochondrial 1 | 1 | | 2637 | hsa-mir-628 | 114971 | PTPMT1 | protein tyrosine phosphatase, mitochondrial 1 | 1 | | 2638 | hsa-miR-190 | 114971 | PTPMT1 | protein tyrosine phosphatase, mitochondrial 1 | 1 | | 2639 | hsa-miR-196b | 114971 | PTPMT1 | protein tyrosine phosphatase, mitochondrial 1 | 1 | | 2640 | hsa-miR-490-5p | 114971 | PTPMT1 | protein tyrosine phosphatase, mitochondrial 1 | 1 | | 2641 | hsa-miR-503 | 114971 | PTPMT1 | protein tyrosine phosphatase, mitochondrial 1 | 1 | | 2642 | hsa-miR-585 | 114971 | PTPMT1 | protein tyrosine phosphatase, mitochondrial 1 | 1 | | 2643 | hsa-miR-623 | 114971 | PTPMT1 | protein tyrosine phosphatase, mitochondrial 1 | 1 | | 2644 | hsa-mir-135b | 126731 | C1orf96 | chromosome 1 open reading frame 96 | 1 | | 2645 | hsa-mir-556 | 126731 | C1orf96 | chromosome 1 open reading frame 96 | 1 | | 2646 | hsa-miR-190 | 126731 | C1orf96 | chromosome 1 open reading frame 96 | 1 | | 2647 | hsa-miR-454\* | 126731 | C1orf96 | chromosome 1 open reading frame 96 | 1 | | 2648 | hsa-miR-126\* | 64857 | PLEKHG2 | pleckstrin homology domain containing, family G (with RhoGef domain) member 2 | 1 | | 2649 | hsa-miR-566 | 64857 | PLEKHG2 | pleckstrin homology domain containing, family G (with RhoGef domain) member 2 | 1 | | 2650 | hsa-miR-608 | 64857 | PLEKHG2 | pleckstrin homology domain containing, family G (with RhoGef domain) member 2 | 1 | | 2651 | hsa-miR-616\* | 23476 | BRD4 | bromodomain containing 4 | 1 | | 2652 | hsa-miR-548d-5p | 145567 | TTC7B | tetratricopeptide repeat domain 7B | 1 | | 2653 | hsa-miR-634 | 145567 | TTC7B | tetratricopeptide repeat domain 7B | 1 | | 2654 | hsa-miR-1231 | 25886 | WDR51A | WD repeat domain 51A | 1 | | 2655 | hsa-miR-126\* | 25886 | WDR51A | WD repeat domain 51A | 1 | | 2656 | hsa-miR-618 | 25886 | WDR51A | WD repeat domain 51A | 1 | | 2657 | hsa-miR-634 | 25886 | WDR51A | WD repeat domain 51A | 1 | | 2658 | hsa-mir-675 | 84895 | FAM73B | family with sequence similarity 73, member B | 1 | | 2659 | hsa-mir-135b | 83464 | APH1B | anterior pharynx defective 1 homolog B (C. elegans) | 1 | | 2660 | hsa-miR-608 | 58499 | ZNF462 | zinc finger protein 462 | 1 | | 2661 | hsa-mir-25 | 84457 | PHYHIPL | phytanoyl-CoA 2-hydroxylase interacting protein-like | 1 | | 2662 | hsa-mir-93 | 84457 | PHYHIPL | phytanoyl-CoA 2-hydroxylase interacting protein-like | 1 | | 2663 | hsa-mir-106b | 84457 | PHYHIPL | phytanoyl-CoA 2-hydroxylase interacting protein-like | 1 | | 2664 | hsa-mir-339 | 84457 | PHYHIPL | phytanoyl-CoA 2-hydroxylase interacting protein-like | 1 | | 2665 | hsa-miR-339-5p | 84457 | PHYHIPL | phytanoyl-CoA 2-hydroxylase interacting protein-like | 1 | | 2666 | hsa-miR-574-5p | 84457 | PHYHIPL | phytanoyl-CoA 2-hydroxylase interacting protein-like | 1 | | 2667 | hsa-miR-608 | 84457 | PHYHIPL | phytanoyl-CoA 2-hydroxylase interacting protein-like | 1 | | 2668 | hsa-miR-1231 | 10630 | PDPN | podoplanin | 1 | | 2669 | hsa-miR-608 | 10630 | PDPN | podoplanin | 1 | | 2673 | hsa-mir-628 | 152137 | CCDC50 | coiled-coil domain containing 50 | 1 | | 2675 | hsa-miR-338-5p | 152137 | CCDC50 | coiled-coil domain containing 50 | 1 | | 2677 | hsa-miR-586 | 152137 | CCDC50 | coiled-coil domain containing 50 | 1 | | 2678 | hsa-miR-617 | 152137 | CCDC50 | coiled-coil domain containing 50 | 1 | | 2679 | hsa-miR-657 | 152137 | CCDC50 | coiled-coil domain containing 50 | 1 | | 2680 | hsa-mir-196b | 81889 | FAHD1 | fumarylacetoacetate hydrolase domain containing 1 | 1 | | 2681 | hsa-mir-424 | 81889 | FAHD1 | fumarylacetoacetate hydrolase domain containing 1 | 1 | | 2682 | hsa-miR-196b | 81889 | FAHD1 | fumarylacetoacetate hydrolase domain containing 1 | 1 | | 2683 | hsa-miR-503 | 81889 | FAHD1 | fumarylacetoacetate hydrolase domain containing 1 | 1 | | 2684 | hsa-mir-339 | 1270 | CNTF | ciliary neurotrophic factor | 1 | | 2685 | hsa-miR-339-5p | 1270 | CNTF | ciliary neurotrophic factor | 1 | | 2686 | hsa-miR-564 | 1270 | CNTF | ciliary neurotrophic factor | 1 | | 2687 | hsa-mir-30c-1 | 8975 | USP13 | ubiquitin specific peptidase 13 (isopeptidase T-3) | 1 | | 2688 | hsa-mir-30e | 8975 | USP13 | ubiquitin specific peptidase 13 (isopeptidase T-3) | 1 | | 2689 | hsa-miR-30c | 8975 | USP13 | ubiquitin specific peptidase 13 (isopeptidase T-3) | 1 | | 2690 | hsa-miR-30e | 8975 | USP13 | ubiquitin specific peptidase 13 (isopeptidase T-3) | 1 | | 2691 | hsa-miR-617 | 8975 | USP13 | ubiquitin specific peptidase 13 (isopeptidase T-3) | 1 | | 2692 | hsa-mir-126 | 254887 | ZDHHC23 | zinc finger, DHHC-type containing 23 | 1 | | 2693 | hsa-mir-424 | 254887 | ZDHHC23 | zinc finger, DHHC-type containing 23 | 1 | | 2694 | hsa-mir-556 | 254887 | ZDHHC23 | zinc finger, DHHC-type containing 23 | 1 | | 2695 | hsa-miR-190 | 254887 | ZDHHC23 | zinc finger, DHHC-type containing 23 | 1 | | 2696 | hsa-miR-503 | 254887 | ZDHHC23 | zinc finger, DHHC-type containing 23 | 1 | | 2697 | hsa-miR-586 | 254887 | ZDHHC23 | zinc finger, DHHC-type containing 23 | 1 | | 2698 | hsa-miR-617 | 254887 | ZDHHC23 | zinc finger, DHHC-type containing 23 | 1 | | 2699 | hsa-miR-454\* | 152579 | SCFD2 | sec1 family domain containing 2 | 1 | | 2700 | hsa-mir-126 | 26094 | DCAF4 | DDB1 and CUL4 associated factor 4 | 1 | | 2701 | hsa-mir-424 | 26094 | DCAF4 | DDB1 and CUL4 associated factor 4 | 1 | | 2702 | hsa-mir-556 | 26094 | DCAF4 | DDB1 and CUL4 associated factor 4 | 1 | | 2703 | hsa-mir-628 | 26094 | DCAF4 | DDB1 and CUL4 associated factor 4 | 1 | | 2704 | hsa-miR-190 | 26094 | DCAF4 | DDB1 and CUL4 associated factor 4 | 1 | | 2705 | hsa-miR-503 | 26094 | DCAF4 | DDB1 and CUL4 associated factor 4 | 1 | | 2706 | hsa-miR-586 | 26094 | DCAF4 | DDB1 and CUL4 associated factor 4 | 1 | | 2707 | hsa-miR-617 | 26094 | DCAF4 | DDB1 and CUL4 associated factor 4 | 1 | | 2708 | hsa-miR-623 | 26094 | DCAF4 | DDB1 and CUL4 associated factor 4 | 1 | | 2709 | hsa-miR-126\* | 84922 | FIZ1 | FLT3-interacting zinc finger 1 | 1 | | 2710 | hsa-miR-566 | 84922 | FIZ1 | FLT3-interacting zinc finger 1 | 1 | | 2711 | hsa-miR-608 | 84922 | FIZ1 | FLT3-interacting zinc finger 1 | 1 | | 2712 | hsa-miR-618 | 26524 | LATS2 | LATS, large tumor suppressor, homolog 2 (Drosophila) | 1 | | 2713 | hsa-mir-628 | 29993 | PACSIN1 | protein kinase C and casein kinase substrate in neurons 1 | 1 | | 2714 | hsa-miR-586 | 29993 | PACSIN1 | protein kinase C and casein kinase substrate in neurons 1 | 1 | | 2715 | hsa-miR-617 | 29993 | PACSIN1 | protein kinase C and casein kinase substrate in neurons 1 | 1 | | 2716 | hsa-mir-126 | 60468 | BACH2 | BTB and CNC homology 1, basic leucine zipper transcription factor 2 | 1 | | 2717 | hsa-mir-424 | 60468 | BACH2 | BTB and CNC homology 1, basic leucine zipper transcription factor 2 | 1 | | 2718 | hsa-mir-556 | 60468 | BACH2 | BTB and CNC homology 1, basic leucine zipper transcription factor 2 | 1 | | 2719 | hsa-mir-628 | 60468 | BACH2 | BTB and CNC homology 1, basic leucine zipper transcription factor 2 | 1 | | 2720 | hsa-miR-190 | 60468 | BACH2 | BTB and CNC homology 1, basic leucine zipper transcription factor 2 | 1 | | 2721 | hsa-miR-196b | 60468 | BACH2 | BTB and CNC homology 1, basic leucine zipper transcription factor 2 | 1 | | 2722 | hsa-miR-338-5p | 60468 | BACH2 | BTB and CNC homology 1, basic leucine zipper transcription factor 2 | 1 | | 2723 | hsa-miR-503 | 60468 | BACH2 | BTB and CNC homology 1, basic leucine zipper transcription factor 2 | 1 | | 2724 | hsa-miR-586 | 60468 | BACH2 | BTB and CNC homology 1, basic leucine zipper transcription factor 2 | 1 | | 2725 | hsa-miR-617 | 60468 | BACH2 | BTB and CNC homology 1, basic leucine zipper transcription factor 2 | 1 | | 2726 | hsa-miR-657 | 60468 | BACH2 | BTB and CNC homology 1, basic leucine zipper transcription factor 2 | 1 | | 2727 | hsa-mir-15a | 140738 | TMEM37 | transmembrane protein 37 | 1 | | 2728 | hsa-mir-16-1 | 140738 | TMEM37 | transmembrane protein 37 | 1 | | 2729 | hsa-miR-126\* | 140738 | TMEM37 | transmembrane protein 37 | 1 | | 2730 | hsa-miR-16 | 140738 | TMEM37 | transmembrane protein 37 | 1 | | 2731 | hsa-miR-564 | 140738 | TMEM37 | transmembrane protein 37 | 1 | | 2732 | hsa-miR-566 | 140738 | TMEM37 | transmembrane protein 37 | 1 | | 2733 | hsa-miR-608 | 140738 | TMEM37 | transmembrane protein 37 | 1 | | 2734 | hsa-mir-126 | 3899 | AFF3 | AF4/FMR2 family, member 3 | 1 | | 2735 | hsa-mir-424 | 3899 | AFF3 | AF4/FMR2 family, member 3 | 1 | | 2736 | hsa-mir-556 | 3899 | AFF3 | AF4/FMR2 family, member 3 | 1 | | 2737 | hsa-miR-190 | 3899 | AFF3 | AF4/FMR2 family, member 3 | 1 | | 2738 | hsa-miR-338-5p | 3899 | AFF3 | AF4/FMR2 family, member 3 | 1 | | 2739 | hsa-miR-503 | 3899 | AFF3 | AF4/FMR2 family, member 3 | 1 | | 2740 | hsa-miR-657 | 3899 | AFF3 | AF4/FMR2 family, member 3 | 1 | | 2741 | hsa-miR-126\* | 7760 | ZNF213 | zinc finger protein 213 | 1 | | 2742 | hsa-miR-16 | 7760 | ZNF213 | zinc finger protein 213 | 1 | | 2743 | hsa-miR-566 | 7760 | ZNF213 | zinc finger protein 213 | 1 | | 2744 | hsa-miR-608 | 7760 | ZNF213 | zinc finger protein 213 | 1 | | 2745 | hsa-mir-424 | 57120 | GOPC | golgi-associated PDZ and coiled-coil motif containing | 1 | | 2746 | hsa-mir-556 | 57120 | GOPC | golgi-associated PDZ and coiled-coil motif containing | 1 | | 2747 | hsa-mir-628 | 57120 | GOPC | golgi-associated PDZ and coiled-coil motif containing | 1 | | 2748 | hsa-miR-503 | 57120 | GOPC | golgi-associated PDZ and coiled-coil motif containing | 1 | | 2749 | hsa-miR-617 | 57120 | GOPC | golgi-associated PDZ and coiled-coil motif containing | 1 | | 2750 | hsa-miR-623 | 57120 | GOPC | golgi-associated PDZ and coiled-coil motif containing | 1 | | 2751 | hsa-mir-126 | 51466 | EVL | Enah/Vasp-like | 1 | | 2752 | hsa-mir-196b | 51466 | EVL | Enah/Vasp-like | 1 | | 2753 | hsa-mir-424 | 51466 | EVL | Enah/Vasp-like | 1 | | 2754 | hsa-mir-556 | 51466 | EVL | Enah/Vasp-like | 1 | | 2755 | hsa-mir-628 | 51466 | EVL | Enah/Vasp-like | 1 | | 2756 | hsa-miR-190 | 51466 | EVL | Enah/Vasp-like | 1 | | 2757 | hsa-miR-196b | 51466 | EVL | Enah/Vasp-like | 1 | | 2758 | hsa-miR-503 | 51466 | EVL | Enah/Vasp-like | 1 | | 2759 | hsa-miR-586 | 51466 | EVL | Enah/Vasp-like | 1 | | 2760 | hsa-miR-617 | 51466 | EVL | Enah/Vasp-like | 1 | | 2761 | hsa-miR-623 | 51466 | EVL | Enah/Vasp-like | 1 | | 2762 | hsa-miR-944 | 51466 | EVL | Enah/Vasp-like | 1 | | 2763 | hsa-mir-15a | 10642 | IGF2BP1 | insulin-like growth factor 2 mRNA binding protein 1 | 1 | | 2764 | hsa-mir-16-1 | 10642 | IGF2BP1 | insulin-like growth factor 2 mRNA binding protein 1 | 1 | | 2765 | hsa-miR-126\* | 10642 | IGF2BP1 | insulin-like growth factor 2 mRNA binding protein 1 | 1 | | 2766 | hsa-miR-608 | 10642 | IGF2BP1 | insulin-like growth factor 2 mRNA binding protein 1 | 1 | | 2767 | hsa-miR-126\* | 359948 | IRF2BP2 | interferon regulatory factor 2 binding protein 2 | 1 | | 2768 | hsa-miR-566 | 359948 | IRF2BP2 | interferon regulatory factor 2 binding protein 2 | 1 | | 2769 | hsa-miR-608 | 359948 | IRF2BP2 | interferon regulatory factor 2 binding protein 2 | 1 | | 2770 | hsa-miR-126\* | 79016 | DDA1 | DET1 and DDB1 associated 1 | 1 | | 2771 | hsa-miR-566 | 79016 | DDA1 | DET1 and DDB1 associated 1 | 1 | | 2772 | hsa-miR-608 | 79016 | DDA1 | DET1 and DDB1 associated 1 | 1 | | 2773 | hsa-miR-126\* | 5270 | SERPINE2 | serpin peptidase inhibitor, clade E (nexin, plasminogen activator inhibitor type 1), member 2 | 1 | | 2774 | hsa-miR-608 | 5270 | SERPINE2 | serpin peptidase inhibitor, clade E (nexin, plasminogen activator inhibitor type 1), member 2 | 1 | | 2775 | hsa-mir-548d-1 | 23363 | OBSL1 | obscurin-like 1 | 1 | | 2776 | hsa-mir-454 | 23363 | OBSL1 | obscurin-like 1 | 1 | | 2777 | hsa-miR-675 | 23363 | OBSL1 | obscurin-like 1 | 1 | | 2778 | hsa-miR-95 | 23363 | OBSL1 | obscurin-like 1 | 1 | | 2779 | hsa-mir-218-2 | 64793 | CCDC21 | coiled-coil domain containing 21 | 1 | | 2780 | hsa-mir-490 | 64793 | CCDC21 | coiled-coil domain containing 21 | 1 | | 2781 | hsa-mir-1915 | 64793 | CCDC21 | coiled-coil domain containing 21 | 1 | | 2782 | hsa-miR-190 | 64793 | CCDC21 | coiled-coil domain containing 21 | 1 | | 2783 | hsa-miR-490-5p | 64793 | CCDC21 | coiled-coil domain containing 21 | 1 | | 2784 | hsa-miR-585 | 64793 | CCDC21 | coiled-coil domain containing 21 | 1 | | 2785 | hsa-miR-623 | 64793 | CCDC21 | coiled-coil domain containing 21 | 1 | | 2786 | hsa-mir-454 | 11245 | GPR176 | G protein-coupled receptor 176 | 1 | | 2787 | hsa-miR-675 | 11245 | GPR176 | G protein-coupled receptor 176 | 1 | | 2788 | hsa-miR-95 | 11245 | GPR176 | G protein-coupled receptor 176 | 1 | | 2789 | hsa-miR-617 | 286128 | ZFP41 | zinc finger protein 41 homolog (mouse) | 1 | | 2790 | hsa-mir-15a | 57221 | KIAA1244 | KIAA1244 | 1 | | 2791 | hsa-mir-16-1 | 57221 | KIAA1244 | KIAA1244 | 1 | | 2792 | hsa-miR-1231 | 57221 | KIAA1244 | KIAA1244 | 1 | | 2793 | hsa-miR-126\* | 57221 | KIAA1244 | KIAA1244 | 1 | | 2794 | hsa-miR-608 | 57221 | KIAA1244 | KIAA1244 | 1 | | 2795 | hsa-mir-135b | 93589 | CACNA2D4 | calcium channel, voltage-dependent, alpha 2/delta subunit 4 | 1 | | 2796 | hsa-mir-675 | 93589 | CACNA2D4 | calcium channel, voltage-dependent, alpha 2/delta subunit 4 | 1 | | 2797 | hsa-mir-15a | 9951 | HS3ST4 | heparan sulfate (glucosamine) 3-O-sulfotransferase 4 | 1 | | 2798 | hsa-mir-16-1 | 9951 | HS3ST4 | heparan sulfate (glucosamine) 3-O-sulfotransferase 4 | 1 | | 2799 | hsa-miR-126\* | 9951 | HS3ST4 | heparan sulfate (glucosamine) 3-O-sulfotransferase 4 | 1 | | 2800 | hsa-miR-16 | 9951 | HS3ST4 | heparan sulfate (glucosamine) 3-O-sulfotransferase 4 | 1 | | 2801 | hsa-miR-608 | 9951 | HS3ST4 | heparan sulfate (glucosamine) 3-O-sulfotransferase 4 | 1 | | 2802 | hsa-mir-628 | 100287081 | LOC100287081 | similar to hCG1999172 | 1 | | 2803 | hsa-miR-617 | 100287081 | LOC100287081 | similar to hCG1999172 | 1 | | 2804 | hsa-mir-126 | 54065 | FAM165B | family with sequence similarity 165, member B | 1 | | 2805 | hsa-mir-30c-1 | 54065 | FAM165B | family with sequence similarity 165, member B | 1 | | 2806 | hsa-mir-30e | 54065 | FAM165B | family with sequence similarity 165, member B | 1 | | 2807 | hsa-mir-424 | 54065 | FAM165B | family with sequence similarity 165, member B | 1 | | 2808 | hsa-mir-628 | 54065 | FAM165B | family with sequence similarity 165, member B | 1 | | 2809 | hsa-miR-26a | 54065 | FAM165B | family with sequence similarity 165, member B | 1 | | 2810 | hsa-miR-30c | 54065 | FAM165B | family with sequence similarity 165, member B | 1 | | 2811 | hsa-miR-30e | 54065 | FAM165B | family with sequence similarity 165, member B | 1 | | 2812 | hsa-miR-503 | 54065 | FAM165B | family with sequence similarity 165, member B | 1 | | 2813 | hsa-miR-586 | 54065 | FAM165B | family with sequence similarity 165, member B | 1 | | 2814 | hsa-miR-617 | 54065 | FAM165B | family with sequence similarity 165, member B | 1 | | 2815 | hsa-mir-454 | 80700 | UBXN6 | UBX domain protein 6 | 1 | | 2816 | hsa-miR-675 | 80700 | UBXN6 | UBX domain protein 6 | 1 | | 2817 | hsa-miR-95 | 80700 | UBXN6 | UBX domain protein 6 | 1 | | 2818 | hsa-miR-454\* | 2681 | GGTA1 | glycoprotein, alpha-galactosyltransferase 1 | 1 | | 2819 | hsa-miR-548d-5p | 2681 | GGTA1 | glycoprotein, alpha-galactosyltransferase 1 | 1 | | 2820 | hsa-mir-15a | 222663 | SCUBE3 | signal peptide, CUB domain, EGF-like 3 | 1 | | 2821 | hsa-mir-16-1 | 222663 | SCUBE3 | signal peptide, CUB domain, EGF-like 3 | 1 | | 2822 | hsa-miR-608 | 222663 | SCUBE3 | signal peptide, CUB domain, EGF-like 3 | 1 | | 2823 | hsa-mir-15a | 117177 | RAB3IP | RAB3A interacting protein (rabin3) | 1 | | 2824 | hsa-mir-339 | 117177 | RAB3IP | RAB3A interacting protein (rabin3) | 1 | | 2825 | hsa-miR-339-5p | 117177 | RAB3IP | RAB3A interacting protein (rabin3) | 1 | | 2826 | hsa-miR-564 | 117177 | RAB3IP | RAB3A interacting protein (rabin3) | 1 | | 2827 | hsa-miR-608 | 117177 | RAB3IP | RAB3A interacting protein (rabin3) | 1 | | 2828 | hsa-miR-126\* | 8227 | SFRS17A | splicing factor, arginine/serine-rich 17A | 1 | | 2829 | hsa-miR-566 | 8227 | SFRS17A | splicing factor, arginine/serine-rich 17A | 1 | | 2830 | hsa-miR-618 | 57567 | ZNF319 | zinc finger protein 319 | 1 | | 2831 | hsa-mir-454 | 729440 | CCDC61 | coiled-coil domain containing 61 | 1 | | 2832 | hsa-miR-16 | 729440 | CCDC61 | coiled-coil domain containing 61 | 1 | | 2833 | hsa-miR-675 | 729440 | CCDC61 | coiled-coil domain containing 61 | 1 | | 2834 | hsa-miR-95 | 729440 | CCDC61 | coiled-coil domain containing 61 | 1 | | 2835 | hsa-miR-618 | 11057 | ABHD2 | abhydrolase domain containing 2 | 1 | | 2836 | hsa-miR-574-5p | 80309 | SPHKAP | SPHK1 interactor, AKAP domain containing | 1 | | 2837 | hsa-miR-608 | 80309 | SPHKAP | SPHK1 interactor, AKAP domain containing | 1 | | 2838 | hsa-mir-135b | 100286909 | LOC100286909 | hypothetical protein LOC100286909 | 1 | | 2839 | hsa-mir-556 | 100286909 | LOC100286909 | hypothetical protein LOC100286909 | 1 | | 2840 | hsa-mir-628 | 100286909 | LOC100286909 | hypothetical protein LOC100286909 | 1 | | 2841 | hsa-miR-190 | 100286909 | LOC100286909 | hypothetical protein LOC100286909 | 1 | | 2842 | hsa-miR-616\* | 100286909 | LOC100286909 | hypothetical protein LOC100286909 | 1 | | 2843 | hsa-mir-135b | 55197 | RPRD1A | regulation of nuclear pre-mRNA domain containing 1A | 1 | | 2844 | hsa-mir-556 | 55197 | RPRD1A | regulation of nuclear pre-mRNA domain containing 1A | 1 | | 2845 | hsa-mir-675 | 55197 | RPRD1A | regulation of nuclear pre-mRNA domain containing 1A | 1 | | 2846 | hsa-miR-190 | 55197 | RPRD1A | regulation of nuclear pre-mRNA domain containing 1A | 1 | | 2847 | hsa-miR-454\* | 55197 | RPRD1A | regulation of nuclear pre-mRNA domain containing 1A | 1 | | 2848 | hsa-miR-126\* | 94031 | HTRA3 | HtrA serine peptidase 3 | 1 | | 2849 | hsa-miR-608 | 94031 | HTRA3 | HtrA serine peptidase 3 | 1 | | 2850 | hsa-miR-634 | 94031 | HTRA3 | HtrA serine peptidase 3 | 1 | | 2851 | hsa-mir-454 | 2022 | ENG | endoglin | 1 | | 2852 | hsa-miR-16 | 2022 | ENG | endoglin | 1 | | 2853 | hsa-miR-675 | 2022 | ENG | endoglin | 1 | | 2854 | hsa-miR-95 | 2022 | ENG | endoglin | 1 | | 2855 | hsa-miR-126\* | 254778 | C8orf46 | chromosome 8 open reading frame 46 | 1 | | 2856 | hsa-miR-16 | 254778 | C8orf46 | chromosome 8 open reading frame 46 | 1 | | 2857 | hsa-miR-566 | 254778 | C8orf46 | chromosome 8 open reading frame 46 | 1 | | 2858 | hsa-miR-608 | 254778 | C8orf46 | chromosome 8 open reading frame 46 | 1 | | 2859 | hsa-miR-1231 | 399512 | SLC25A35 | solute carrier family 25, member 35 | 1 | | 2860 | hsa-miR-574-5p | 399512 | SLC25A35 | solute carrier family 25, member 35 | 1 | | 2861 | hsa-miR-126\* | 80025 | PANK2 | pantothenate kinase 2 | 1 | | 2862 | hsa-miR-608 | 80025 | PANK2 | pantothenate kinase 2 | 1 | | 2863 | hsa-mir-135b | 203523 | ZNF449 | zinc finger protein 449 | 1 | | 2864 | hsa-miR-454\* | 203523 | ZNF449 | zinc finger protein 449 | 1 | | 2865 | hsa-miR-548d-5p | 203523 | ZNF449 | zinc finger protein 449 | 1 | | 2866 | hsa-mir-135b | 1106 | CHD2 | chromodomain helicase DNA binding protein 2 | 1 | | 2867 | hsa-mir-556 | 1106 | CHD2 | chromodomain helicase DNA binding protein 2 | 1 | | 2868 | hsa-mir-675 | 1106 | CHD2 | chromodomain helicase DNA binding protein 2 | 1 | | 2869 | hsa-miR-454\* | 1106 | CHD2 | chromodomain helicase DNA binding protein 2 | 1 | | 2870 | hsa-miR-548d-5p | 1106 | CHD2 | chromodomain helicase DNA binding protein 2 | 1 | | 2871 | hsa-mir-339 | 55897 | MESP1 | mesoderm posterior 1 homolog (mouse) | 1 | | 2872 | hsa-miR-126\* | 55897 | MESP1 | mesoderm posterior 1 homolog (mouse) | 1 | | 2873 | hsa-miR-16 | 55897 | MESP1 | mesoderm posterior 1 homolog (mouse) | 1 | | 2874 | hsa-miR-339-5p | 55897 | MESP1 | mesoderm posterior 1 homolog (mouse) | 1 | | 2875 | hsa-miR-564 | 55897 | MESP1 | mesoderm posterior 1 homolog (mouse) | 1 | | 2876 | hsa-miR-608 | 55897 | MESP1 | mesoderm posterior 1 homolog (mouse) | 1 | | 2877 | hsa-mir-454 | 283596 | SNHG10 | small nucleolar RNA host gene 10 (non-protein coding) | 1 | | 2878 | hsa-miR-16 | 283596 | SNHG10 | small nucleolar RNA host gene 10 (non-protein coding) | 1 | | 2879 | hsa-miR-675 | 283596 | SNHG10 | small nucleolar RNA host gene 10 (non-protein coding) | 1 | | 2880 | hsa-miR-95 | 283596 | SNHG10 | small nucleolar RNA host gene 10 (non-protein coding) | 1 | | 2881 | hsa-mir-126 | 55088 | C10orf118 | chromosome 10 open reading frame 118 | 1 | | 2882 | hsa-mir-126 | 10336 | PCGF3 | polycomb group ring finger 3 | 1 | | 2883 | hsa-mir-30c-1 | 10336 | PCGF3 | polycomb group ring finger 3 | 1 | | 2884 | hsa-mir-30e | 10336 | PCGF3 | polycomb group ring finger 3 | 1 | | 2885 | hsa-mir-556 | 10336 | PCGF3 | polycomb group ring finger 3 | 1 | | 2886 | hsa-miR-190 | 10336 | PCGF3 | polycomb group ring finger 3 | 1 | | 2887 | hsa-miR-338-5p | 10336 | PCGF3 | polycomb group ring finger 3 | 1 | | 2888 | hsa-miR-657 | 10336 | PCGF3 | polycomb group ring finger 3 | 1 | | 2889 | hsa-miR-566 | 54502 | RBM47 | RNA binding motif protein 47 | 1 | | 2890 | hsa-miR-618 | 54502 | RBM47 | RNA binding motif protein 47 | 1 | | 2893 | hsa-mir-424 | 55342 | STRBP | spermatid perinuclear RNA binding protein | 1 | | 2894 | hsa-mir-556 | 55342 | STRBP | spermatid perinuclear RNA binding protein | 1 | | 2896 | hsa-miR-30c | 55342 | STRBP | spermatid perinuclear RNA binding protein | 1 | | 2897 | hsa-miR-30e | 55342 | STRBP | spermatid perinuclear RNA binding protein | 1 | | 2898 | hsa-miR-503 | 55342 | STRBP | spermatid perinuclear RNA binding protein | 1 | | 2899 | hsa-miR-586 | 55342 | STRBP | spermatid perinuclear RNA binding protein | 1 | | 2900 | hsa-miR-617 | 55342 | STRBP | spermatid perinuclear RNA binding protein | 1 | | 2901 | hsa-mir-196b | 11068 | CYB561D2 | cytochrome b-561 domain containing 2 | 1 | | 2902 | hsa-mir-424 | 11068 | CYB561D2 | cytochrome b-561 domain containing 2 | 1 | | 2903 | hsa-mir-628 | 11068 | CYB561D2 | cytochrome b-561 domain containing 2 | 1 | | 2904 | hsa-miR-196b | 11068 | CYB561D2 | cytochrome b-561 domain containing 2 | 1 | | 2905 | hsa-miR-503 | 11068 | CYB561D2 | cytochrome b-561 domain containing 2 | 1 | | 2906 | hsa-miR-504 | 11068 | CYB561D2 | cytochrome b-561 domain containing 2 | 1 | | 2907 | hsa-miR-586 | 11068 | CYB561D2 | cytochrome b-561 domain containing 2 | 1 | | 2908 | hsa-miR-617 | 11068 | CYB561D2 | cytochrome b-561 domain containing 2 | 1 | | 2909 | hsa-miR-623 | 11068 | CYB561D2 | cytochrome b-561 domain containing 2 | 1 | | 2910 | hsa-miR-126\* | 56256 | SERTAD4 | SERTA domain containing 4 | 1 | | 2911 | hsa-miR-608 | 56256 | SERTAD4 | SERTA domain containing 4 | 1 | | 2912 | hsa-miR-126\* | 483 | ATP1B3 | ATPase, Na+/K+ transporting, beta 3 polypeptide | 1 | | 2913 | hsa-miR-126\* | 6904 | TBCD | tubulin folding cofactor D | 1 | | 2914 | hsa-miR-16 | 6904 | TBCD | tubulin folding cofactor D | 1 | | 2915 | hsa-miR-564 | 6904 | TBCD | tubulin folding cofactor D | 1 | | 2916 | hsa-miR-608 | 6904 | TBCD | tubulin folding cofactor D | 1 | | 2917 | hsa-mir-15a | 196475 | RMST | rhabdomyosarcoma 2 associated transcript (non-protein coding) | 1 | | 2918 | hsa-mir-16-1 | 196475 | RMST | rhabdomyosarcoma 2 associated transcript (non-protein coding) | 1 | | 2919 | hsa-miR-126\* | 196475 | RMST | rhabdomyosarcoma 2 associated transcript (non-protein coding) | 1 | | 2920 | hsa-miR-16 | 196475 | RMST | rhabdomyosarcoma 2 associated transcript (non-protein coding) | 1 | | 2921 | hsa-miR-608 | 196475 | RMST | rhabdomyosarcoma 2 associated transcript (non-protein coding) | 1 | | 2922 | hsa-miR-623 | 54859 | C3orf75 | chromosome 3 open reading frame 75 | 1 | | 2923 | hsa-mir-135b | 55095 | SAMD4B | sterile alpha motif domain containing 4B | 1 | | 2924 | hsa-miR-548d-5p | 257019 | FRMD3 | FERM domain containing 3 | 1 | | 2925 | hsa-mir-135b | 441951 | C20orf199 | chromosome 20 open reading frame 199 | 1 | | 2926 | hsa-miR-616\* | 441951 | C20orf199 | chromosome 20 open reading frame 199 | 1 | | 2927 | hsa-mir-628 | 221188 | GPR114 | G protein-coupled receptor 114 | 1 | | 2928 | hsa-miR-504 | 221188 | GPR114 | G protein-coupled receptor 114 | 1 | | 2929 | hsa-miR-586 | 221188 | GPR114 | G protein-coupled receptor 114 | 1 | | 2930 | hsa-miR-617 | 221188 | GPR114 | G protein-coupled receptor 114 | 1 | | 2931 | hsa-mir-15a | 84530 | SRRM4 | serine/arginine repetitive matrix 4 | 1 | | 2932 | hsa-mir-16-1 | 84530 | SRRM4 | serine/arginine repetitive matrix 4 | 1 | | 2933 | hsa-miR-126\* | 84530 | SRRM4 | serine/arginine repetitive matrix 4 | 1 | | 2934 | hsa-miR-16 | 84530 | SRRM4 | serine/arginine repetitive matrix 4 | 1 | | 2935 | hsa-miR-608 | 84530 | SRRM4 | serine/arginine repetitive matrix 4 | 1 | | 2936 | hsa-miR-30c | 7733 | ZNF180 | zinc finger protein 180 | 1 | | 2937 | hsa-miR-30e | 7733 | ZNF180 | zinc finger protein 180 | 1 | | 2938 | hsa-miR-126\* | 5178 | PEG3 | paternally expressed 3 | 1 | | 2939 | hsa-mir-135b | 127018 | LYPLAL1 | lysophospholipase-like 1 | 1 | | 2940 | hsa-mir-675 | 127018 | LYPLAL1 | lysophospholipase-like 1 | 1 | | 2941 | hsa-miR-454\* | 127018 | LYPLAL1 | lysophospholipase-like 1 | 1 | | 2942 | hsa-mir-15a | 134548 | ANKRD43 | ankyrin repeat domain 43 | 1 | | 2943 | hsa-mir-16-1 | 134548 | ANKRD43 | ankyrin repeat domain 43 | 1 | | 2944 | hsa-miR-16 | 134548 | ANKRD43 | ankyrin repeat domain 43 | 1 | | 2945 | hsa-miR-608 | 134548 | ANKRD43 | ankyrin repeat domain 43 | 1 | | 2946 | hsa-miR-126\* | 219348 | PLAC9 | placenta-specific 9 | 1 | | 2947 | hsa-miR-126\* | 9480 | ONECUT2 | one cut homeobox 2 | 1 | | 2948 | hsa-miR-608 | 9480 | ONECUT2 | one cut homeobox 2 | 1 | | 2949 | hsa-mir-339 | 5265 | SERPINA1 | serpin peptidase inhibitor, clade A (alpha-1 antiproteinase, antitrypsin), member 1 | 1 | | 2950 | hsa-miR-339-5p | 5265 | SERPINA1 | serpin peptidase inhibitor, clade A (alpha-1 antiproteinase, antitrypsin), member 1 | 1 | | 2951 | hsa-miR-574-5p | 5265 | SERPINA1 | serpin peptidase inhibitor, clade A (alpha-1 antiproteinase, antitrypsin), member 1 | 1 | | 2952 | hsa-miR-618 | 5265 | SERPINA1 | serpin peptidase inhibitor, clade A (alpha-1 antiproteinase, antitrypsin), member 1 | 1 | | 2953 | hsa-miR-634 | 5265 | SERPINA1 | serpin peptidase inhibitor, clade A (alpha-1 antiproteinase, antitrypsin), member 1 | 1 | | 2954 | hsa-mir-15a | 6556 | SLC11A1 | solute carrier family 11 (proton-coupled divalent metal ion transporters), member 1 | 1 | | 2955 | hsa-mir-16-1 | 6556 | SLC11A1 | solute carrier family 11 (proton-coupled divalent metal ion transporters), member 1 | 1 | | 2956 | hsa-miR-126\* | 6556 | SLC11A1 | solute carrier family 11 (proton-coupled divalent metal ion transporters), member 1 | 1 | | 2957 | hsa-miR-16 | 6556 | SLC11A1 | solute carrier family 11 (proton-coupled divalent metal ion transporters), member 1 | 1 | | 2958 | hsa-miR-564 | 6556 | SLC11A1 | solute carrier family 11 (proton-coupled divalent metal ion transporters), member 1 | 1 | | 2959 | hsa-miR-566 | 6556 | SLC11A1 | solute carrier family 11 (proton-coupled divalent metal ion transporters), member 1 | 1 | | 2960 | hsa-miR-608 | 6556 | SLC11A1 | solute carrier family 11 (proton-coupled divalent metal ion transporters), member 1 | 1 | | 2961 | hsa-mir-30c-1 | 5586 | PKN2 | protein kinase N2 | 1 | | 2962 | hsa-mir-30e | 5586 | PKN2 | protein kinase N2 | 1 | | 2963 | hsa-miR-1231 | 54039 | PCBP3 | poly(rC) binding protein 3 | 1 | | 2964 | hsa-miR-126\* | 54039 | PCBP3 | poly(rC) binding protein 3 | 1 | | 2965 | hsa-miR-608 | 54039 | PCBP3 | poly(rC) binding protein 3 | 1 | | 2966 | hsa-mir-15a | 284612 | SYPL2 | synaptophysin-like 2 | 1 | | 2967 | hsa-mir-16-1 | 284612 | SYPL2 | synaptophysin-like 2 | 1 | | 2968 | hsa-miR-1231 | 284612 | SYPL2 | synaptophysin-like 2 | 1 | | 2969 | hsa-miR-126\* | 284612 | SYPL2 | synaptophysin-like 2 | 1 | | 2970 | hsa-miR-16 | 284612 | SYPL2 | synaptophysin-like 2 | 1 | | 2971 | hsa-miR-608 | 284612 | SYPL2 | synaptophysin-like 2 | 1 | | 2972 | hsa-miR-126\* | 285313 | IGSF10 | immunoglobulin superfamily, member 10 | 1 | | 2973 | hsa-miR-16 | 285313 | IGSF10 | immunoglobulin superfamily, member 10 | 1 | | 2974 | hsa-miR-608 | 285313 | IGSF10 | immunoglobulin superfamily, member 10 | 1 | | 2975 | hsa-miR-126\* | 196883 | ADCY4 | adenylate cyclase 4 | 1 | | 2976 | hsa-miR-564 | 196883 | ADCY4 | adenylate cyclase 4 | 1 | | 2977 | hsa-miR-566 | 196883 | ADCY4 | adenylate cyclase 4 | 1 | | 2978 | hsa-miR-608 | 196883 | ADCY4 | adenylate cyclase 4 | 1 | | 2979 | hsa-miR-1231 | 79567 | FAM65A | family with sequence similarity 65, member A | 1 | | 2980 | hsa-miR-126\* | 79567 | FAM65A | family with sequence similarity 65, member A | 1 | | 2981 | hsa-miR-566 | 79567 | FAM65A | family with sequence similarity 65, member A | 1 | | 2982 | hsa-miR-608 | 79567 | FAM65A | family with sequence similarity 65, member A | 1 | | 2983 | hsa-mir-339 | 285987 | DLX6AS | DLX6 antisense RNA (non-protein coding) | 1 | | 2984 | hsa-miR-126\* | 285987 | DLX6AS | DLX6 antisense RNA (non-protein coding) | 1 | | 2985 | hsa-miR-339-5p | 285987 | DLX6AS | DLX6 antisense RNA (non-protein coding) | 1 | | 2986 | hsa-miR-564 | 285987 | DLX6AS | DLX6 antisense RNA (non-protein coding) | 1 | | 2987 | hsa-miR-608 | 285987 | DLX6AS | DLX6 antisense RNA (non-protein coding) | 1 | | 2988 | hsa-mir-424 | 199786 | FAM129C | family with sequence similarity 129, member C | 1 | | 2989 | hsa-mir-556 | 199786 | FAM129C | family with sequence similarity 129, member C | 1 | | 2990 | hsa-miR-190 | 199786 | FAM129C | family with sequence similarity 129, member C | 1 | | 2991 | hsa-miR-503 | 199786 | FAM129C | family with sequence similarity 129, member C | 1 | | 2992 | hsa-mir-30c-1 | 54993 | ZSCAN2 | zinc finger and SCAN domain containing 2 | 1 | | 2993 | hsa-mir-30e | 54993 | ZSCAN2 | zinc finger and SCAN domain containing 2 | 1 | | 2994 | hsa-mir-424 | 54993 | ZSCAN2 | zinc finger and SCAN domain containing 2 | 1 | | 2995 | hsa-mir-628 | 54993 | ZSCAN2 | zinc finger and SCAN domain containing 2 | 1 | | 2996 | hsa-miR-190 | 54993 | ZSCAN2 | zinc finger and SCAN domain containing 2 | 1 | | 2997 | hsa-miR-503 | 54993 | ZSCAN2 | zinc finger and SCAN domain containing 2 | 1 | | 2998 | hsa-miR-586 | 54993 | ZSCAN2 | zinc finger and SCAN domain containing 2 | 1 | | 2999 | hsa-miR-617 | 54993 | ZSCAN2 | zinc finger and SCAN domain containing 2 | 1 | | 3000 | hsa-miR-126\* | 283129 | C11orf85 | chromosome 11 open reading frame 85 | 1 | | 3001 | hsa-miR-16 | 283129 | C11orf85 | chromosome 11 open reading frame 85 | 1 | | 3002 | hsa-miR-566 | 283129 | C11orf85 | chromosome 11 open reading frame 85 | 1 | | 3003 | hsa-miR-608 | 283129 | C11orf85 | chromosome 11 open reading frame 85 | 1 | | 3004 | hsa-mir-15a | 79365 | BHLHE41 | basic helix-loop-helix family, member e41 | 1 | | 3005 | hsa-mir-16-1 | 79365 | BHLHE41 | basic helix-loop-helix family, member e41 | 1 | | 3006 | hsa-miR-1231 | 79365 | BHLHE41 | basic helix-loop-helix family, member e41 | 1 | | 3007 | hsa-miR-126\* | 79365 | BHLHE41 | basic helix-loop-helix family, member e41 | 1 | | 3008 | hsa-miR-16 | 79365 | BHLHE41 | basic helix-loop-helix family, member e41 | 1 | | 3009 | hsa-miR-566 | 79365 | BHLHE41 | basic helix-loop-helix family, member e41 | 1 | | 3010 | hsa-miR-608 | 79365 | BHLHE41 | basic helix-loop-helix family, member e41 | 1 | | 3011 | hsa-miR-16 | 84548 | TMEM185A | transmembrane protein 185A | 1 | | 3012 | hsa-miR-564 | 84548 | TMEM185A | transmembrane protein 185A | 1 | | 3013 | hsa-miR-608 | 84548 | TMEM185A | transmembrane protein 185A | 1 | | 3014 | hsa-miR-126\* | 128653 | C20orf141 | chromosome 20 open reading frame 141 | 1 | | 3015 | hsa-miR-16 | 128653 | C20orf141 | chromosome 20 open reading frame 141 | 1 | | 3016 | hsa-miR-564 | 128653 | C20orf141 | chromosome 20 open reading frame 141 | 1 | | 3017 | hsa-miR-566 | 128653 | C20orf141 | chromosome 20 open reading frame 141 | 1 | | 3018 | hsa-miR-608 | 128653 | C20orf141 | chromosome 20 open reading frame 141 | 1 | | 3019 | hsa-mir-15a | 8817 | FGF18 | fibroblast growth factor 18 | 1 | | 3020 | hsa-mir-16-1 | 8817 | FGF18 | fibroblast growth factor 18 | 1 | | 3021 | hsa-miR-1231 | 8817 | FGF18 | fibroblast growth factor 18 | 1 | | 3022 | hsa-miR-126\* | 8817 | FGF18 | fibroblast growth factor 18 | 1 | | 3023 | hsa-miR-574-5p | 8817 | FGF18 | fibroblast growth factor 18 | 1 | | 3024 | hsa-miR-608 | 8817 | FGF18 | fibroblast growth factor 18 | 1 | | 3025 | hsa-mir-339 | 10186 | LHFP | lipoma HMGIC fusion partner | 1 | | 3026 | hsa-miR-126\* | 10186 | LHFP | lipoma HMGIC fusion partner | 1 | | 3027 | hsa-miR-339-5p | 10186 | LHFP | lipoma HMGIC fusion partner | 1 | | 3028 | hsa-miR-608 | 10186 | LHFP | lipoma HMGIC fusion partner | 1 | | 3029 | hsa-miR-126\* | 7881 | KCNAB1 | potassium voltage-gated channel, shaker-related subfamily, beta member 1 | 1 | | 3030 | hsa-miR-16 | 7881 | KCNAB1 | potassium voltage-gated channel, shaker-related subfamily, beta member 1 | 1 | | 3031 | hsa-miR-566 | 7881 | KCNAB1 | potassium voltage-gated channel, shaker-related subfamily, beta member 1 | 1 | | 3032 | hsa-miR-608 | 7881 | KCNAB1 | potassium voltage-gated channel, shaker-related subfamily, beta member 1 | 1 | | 3033 | hsa-mir-15a | 57553 | MICAL3 | microtubule associated monoxygenase, calponin and LIM domain containing 3 | 1 | | 3034 | hsa-mir-16-1 | 57553 | MICAL3 | microtubule associated monoxygenase, calponin and LIM domain containing 3 | 1 | | 3035 | hsa-miR-1231 | 57553 | MICAL3 | microtubule associated monoxygenase, calponin and LIM domain containing 3 | 1 | | 3036 | hsa-miR-126\* | 57553 | MICAL3 | microtubule associated monoxygenase, calponin and LIM domain containing 3 | 1 | | 3037 | hsa-miR-16 | 57553 | MICAL3 | microtubule associated monoxygenase, calponin and LIM domain containing 3 | 1 | | 3038 | hsa-miR-566 | 57553 | MICAL3 | microtubule associated monoxygenase, calponin and LIM domain containing 3 | 1 | | 3039 | hsa-miR-608 | 57553 | MICAL3 | microtubule associated monoxygenase, calponin and LIM domain containing 3 | 1 | | 3040 | hsa-miR-1231 | 56131 | PCDHB4 | protocadherin beta 4 | 1 | | 3041 | hsa-miR-126\* | 56131 | PCDHB4 | protocadherin beta 4 | 1 | | 3042 | hsa-miR-608 | 56131 | PCDHB4 | protocadherin beta 4 | 1 | | 3043 | hsa-miR-16 | 56098 | PCDHGC4 | protocadherin gamma subfamily C, 4 | 1 | | 3044 | hsa-miR-608 | 56098 | PCDHGC4 | protocadherin gamma subfamily C, 4 | 1 | | 3045 | hsa-mir-339 | 10804 | GJB6 | gap junction protein, beta 6, 30kDa | 1 | | 3046 | hsa-miR-339-5p | 10804 | GJB6 | gap junction protein, beta 6, 30kDa | 1 | | 3047 | hsa-miR-564 | 10804 | GJB6 | gap junction protein, beta 6, 30kDa | 1 | | 3048 | hsa-miR-618 | 10804 | GJB6 | gap junction protein, beta 6, 30kDa | 1 | | 3049 | hsa-miR-634 | 10804 | GJB6 | gap junction protein, beta 6, 30kDa | 1 | | 3050 | hsa-miR-1231 | 84904 | C9orf100 | chromosome 9 open reading frame 100 | 1 | | 3051 | hsa-miR-608 | 84904 | C9orf100 | chromosome 9 open reading frame 100 | 1 | | 3052 | hsa-miR-190 | 55814 | BDP1 | B double prime 1, subunit of RNA polymerase III transcription initiation factor IIIB | 1 | | 3053 | hsa-miR-338-5p | 55814 | BDP1 | B double prime 1, subunit of RNA polymerase III transcription initiation factor IIIB | 1 | | 3054 | hsa-miR-617 | 55814 | BDP1 | B double prime 1, subunit of RNA polymerase III transcription initiation factor IIIB | 1 | | 3055 | hsa-miR-657 | 55814 | BDP1 | B double prime 1, subunit of RNA polymerase III transcription initiation factor IIIB | 1 | | 3056 | hsa-mir-218-2 | 55326 | AGPAT5 | 1-acylglycerol-3-phosphate O-acyltransferase 5 (lysophosphatidic acid acyltransferase, epsilon) | 1 | | 3057 | hsa-mir-126 | 55326 | AGPAT5 | 1-acylglycerol-3-phosphate O-acyltransferase 5 (lysophosphatidic acid acyltransferase, epsilon) | 1 | | 3058 | hsa-mir-30c-1 | 55326 | AGPAT5 | 1-acylglycerol-3-phosphate O-acyltransferase 5 (lysophosphatidic acid acyltransferase, epsilon) | 1 | | 3059 | hsa-mir-30e | 55326 | AGPAT5 | 1-acylglycerol-3-phosphate O-acyltransferase 5 (lysophosphatidic acid acyltransferase, epsilon) | 1 | | 3060 | hsa-mir-424 | 55326 | AGPAT5 | 1-acylglycerol-3-phosphate O-acyltransferase 5 (lysophosphatidic acid acyltransferase, epsilon) | 1 | | 3061 | hsa-mir-556 | 55326 | AGPAT5 | 1-acylglycerol-3-phosphate O-acyltransferase 5 (lysophosphatidic acid acyltransferase, epsilon) | 1 | | 3062 | hsa-miR-190 | 55326 | AGPAT5 | 1-acylglycerol-3-phosphate O-acyltransferase 5 (lysophosphatidic acid acyltransferase, epsilon) | 1 | | 3063 | hsa-miR-338-5p | 55326 | AGPAT5 | 1-acylglycerol-3-phosphate O-acyltransferase 5 (lysophosphatidic acid acyltransferase, epsilon) | 1 | | 3064 | hsa-miR-503 | 55326 | AGPAT5 | 1-acylglycerol-3-phosphate O-acyltransferase 5 (lysophosphatidic acid acyltransferase, epsilon) | 1 | | 3065 | hsa-miR-585 | 55326 | AGPAT5 | 1-acylglycerol-3-phosphate O-acyltransferase 5 (lysophosphatidic acid acyltransferase, epsilon) | 1 | | 3066 | hsa-miR-586 | 55326 | AGPAT5 | 1-acylglycerol-3-phosphate O-acyltransferase 5 (lysophosphatidic acid acyltransferase, epsilon) | 1 | | 3067 | hsa-miR-617 | 55326 | AGPAT5 | 1-acylglycerol-3-phosphate O-acyltransferase 5 (lysophosphatidic acid acyltransferase, epsilon) | 1 | | 3068 | hsa-miR-657 | 55326 | AGPAT5 | 1-acylglycerol-3-phosphate O-acyltransferase 5 (lysophosphatidic acid acyltransferase, epsilon) | 1 | | 3069 | hsa-miR-126\* | 9543 | IGDCC3 | immunoglobulin superfamily, DCC subclass, member 3 | 1 | | 3070 | hsa-miR-564 | 9543 | IGDCC3 | immunoglobulin superfamily, DCC subclass, member 3 | 1 | | 3071 | hsa-miR-608 | 9543 | IGDCC3 | immunoglobulin superfamily, DCC subclass, member 3 | 1 | | 3072 | hsa-miR-126\* | 84552 | PARD6G | par-6 partitioning defective 6 homolog gamma (C. elegans) | 1 | | 3073 | hsa-miR-608 | 84552 | PARD6G | par-6 partitioning defective 6 homolog gamma (C. elegans) | 1 | | 3074 | hsa-mir-556 | 860 | RUNX2 | runt-related transcription factor 2 | 1 | | 3075 | hsa-miR-190 | 860 | RUNX2 | runt-related transcription factor 2 | 1 | | 3076 | hsa-mir-196b | 23338 | PHF15 | PHD finger protein 15 | 1 | | 3077 | hsa-mir-574 | 23338 | PHF15 | PHD finger protein 15 | 1 | | 3078 | hsa-mir-628 | 23338 | PHF15 | PHD finger protein 15 | 1 | | 3079 | hsa-miR-196b | 23338 | PHF15 | PHD finger protein 15 | 1 | | 3080 | hsa-miR-504 | 23338 | PHF15 | PHD finger protein 15 | 1 | | 3081 | hsa-miR-617 | 23338 | PHF15 | PHD finger protein 15 | 1 | | 3082 | hsa-miR-623 | 23338 | PHF15 | PHD finger protein 15 | 1 | | 3085 | hsa-mir-126 | 7767 | ZNF224 | zinc finger protein 224 | 1 | | 3086 | hsa-mir-30c-1 | 7767 | ZNF224 | zinc finger protein 224 | 1 | | 3087 | hsa-mir-30e | 7767 | ZNF224 | zinc finger protein 224 | 1 | | 3088 | hsa-mir-424 | 7767 | ZNF224 | zinc finger protein 224 | 1 | | 3089 | hsa-mir-556 | 7767 | ZNF224 | zinc finger protein 224 | 1 | | 3090 | hsa-miR-190 | 7767 | ZNF224 | zinc finger protein 224 | 1 | | 3091 | hsa-miR-30c | 7767 | ZNF224 | zinc finger protein 224 | 1 | | 3092 | hsa-miR-30e | 7767 | ZNF224 | zinc finger protein 224 | 1 | | 3093 | hsa-miR-338-5p | 7767 | ZNF224 | zinc finger protein 224 | 1 | | 3094 | hsa-miR-503 | 7767 | ZNF224 | zinc finger protein 224 | 1 | | 3095 | hsa-miR-617 | 7767 | ZNF224 | zinc finger protein 224 | 1 | | 3096 | hsa-miR-657 | 7767 | ZNF224 | zinc finger protein 224 | 1 | | 3097 | hsa-miR-126\* | 148696 | LOC148696 | hypothetical LOC148696 | 1 | | 3098 | hsa-miR-608 | 148696 | LOC148696 | hypothetical LOC148696 | 1 | | 3099 | hsa-mir-218-2 | 8451 | CUL4A | cullin 4A | 1 | | 3100 | hsa-mir-126 | 8451 | CUL4A | cullin 4A | 1 | | 3101 | hsa-mir-196b | 8451 | CUL4A | cullin 4A | 1 | | 3102 | hsa-mir-424 | 8451 | CUL4A | cullin 4A | 1 | | 3103 | hsa-mir-490 | 8451 | CUL4A | cullin 4A | 1 | | 3104 | hsa-mir-556 | 8451 | CUL4A | cullin 4A | 1 | | 3105 | hsa-mir-628 | 8451 | CUL4A | cullin 4A | 1 | | 3106 | hsa-mir-1915 | 8451 | CUL4A | cullin 4A | 1 | | 3107 | hsa-miR-190 | 8451 | CUL4A | cullin 4A | 1 | | 3108 | hsa-miR-196b | 8451 | CUL4A | cullin 4A | 1 | | 3109 | hsa-miR-490-5p | 8451 | CUL4A | cullin 4A | 1 | | 3110 | hsa-miR-503 | 8451 | CUL4A | cullin 4A | 1 | | 3111 | hsa-miR-585 | 8451 | CUL4A | cullin 4A | 1 | | 3112 | hsa-miR-617 | 8451 | CUL4A | cullin 4A | 1 | | 3113 | hsa-miR-623 | 8451 | CUL4A | cullin 4A | 1 | | 3114 | hsa-mir-218-2 | 400946 | FLJ12334 | hypothetical gene supported by AK022396; AK097927 | 1 | | 3115 | hsa-mir-126 | 400946 | FLJ12334 | hypothetical gene supported by AK022396; AK097927 | 1 | | 3116 | hsa-mir-424 | 400946 | FLJ12334 | hypothetical gene supported by AK022396; AK097927 | 1 | | 3117 | hsa-mir-556 | 400946 | FLJ12334 | hypothetical gene supported by AK022396; AK097927 | 1 | | 3118 | hsa-mir-628 | 400946 | FLJ12334 | hypothetical gene supported by AK022396; AK097927 | 1 | | 3119 | hsa-miR-190 | 400946 | FLJ12334 | hypothetical gene supported by AK022396; AK097927 | 1 | | 3120 | hsa-miR-503 | 400946 | FLJ12334 | hypothetical gene supported by AK022396; AK097927 | 1 | | 3121 | hsa-miR-585 | 400946 | FLJ12334 | hypothetical gene supported by AK022396; AK097927 | 1 | | 3122 | hsa-miR-586 | 400946 | FLJ12334 | hypothetical gene supported by AK022396; AK097927 | 1 | | 3123 | hsa-miR-617 | 400946 | FLJ12334 | hypothetical gene supported by AK022396; AK097927 | 1 | | 3124 | hsa-mir-15a | 253264 | LOC253264 | hypothetical protein LOC253264 | 1 | | 3125 | hsa-mir-16-1 | 253264 | LOC253264 | hypothetical protein LOC253264 | 1 | | 3126 | hsa-miR-126\* | 253264 | LOC253264 | hypothetical protein LOC253264 | 1 | | 3127 | hsa-miR-16 | 253264 | LOC253264 | hypothetical protein LOC253264 | 1 | | 3128 | hsa-miR-608 | 253264 | LOC253264 | hypothetical protein LOC253264 | 1 | | 3129 | hsa-miR-617 | 6689 | SPIB | Spi-B transcription factor (Spi-1/PU.1 related) | 1 | | 3130 | hsa-mir-15a | 644192 | LOC644192 | hypothetical LOC644192 | 1 | | 3131 | hsa-mir-16-1 | 644192 | LOC644192 | hypothetical LOC644192 | 1 | | 3132 | hsa-miR-126\* | 644192 | LOC644192 | hypothetical LOC644192 | 1 | | 3133 | hsa-miR-16 | 644192 | LOC644192 | hypothetical LOC644192 | 1 | | 3134 | hsa-miR-608 | 644192 | LOC644192 | hypothetical LOC644192 | 1 | | 3135 | hsa-mir-218-2 | 64784 | CRTC3 | CREB regulated transcription coactivator 3 | 1 | | 3136 | hsa-mir-126 | 64784 | CRTC3 | CREB regulated transcription coactivator 3 | 1 | | 3137 | hsa-mir-424 | 64784 | CRTC3 | CREB regulated transcription coactivator 3 | 1 | | 3138 | hsa-mir-490 | 64784 | CRTC3 | CREB regulated transcription coactivator 3 | 1 | | 3139 | hsa-mir-556 | 64784 | CRTC3 | CREB regulated transcription coactivator 3 | 1 | | 3140 | hsa-mir-1915 | 64784 | CRTC3 | CREB regulated transcription coactivator 3 | 1 | | 3141 | hsa-miR-190 | 64784 | CRTC3 | CREB regulated transcription coactivator 3 | 1 | | 3142 | hsa-miR-490-5p | 64784 | CRTC3 | CREB regulated transcription coactivator 3 | 1 | | 3143 | hsa-miR-503 | 64784 | CRTC3 | CREB regulated transcription coactivator 3 | 1 | | 3144 | hsa-miR-585 | 64784 | CRTC3 | CREB regulated transcription coactivator 3 | 1 | | 3145 | hsa-miR-623 | 64784 | CRTC3 | CREB regulated transcription coactivator 3 | 1 | | 3146 | hsa-miR-126\* | 84631 | SLITRK2 | SLIT and NTRK-like family, member 2 | 1 | | 3147 | hsa-miR-16 | 84631 | SLITRK2 | SLIT and NTRK-like family, member 2 | 1 | | 3148 | hsa-miR-564 | 84631 | SLITRK2 | SLIT and NTRK-like family, member 2 | 1 | | 3149 | hsa-miR-608 | 84631 | SLITRK2 | SLIT and NTRK-like family, member 2 | 1 | | 3150 | hsa-mir-218-2 | 59348 | ZNF350 | zinc finger protein 350 | 1 | | 3151 | hsa-mir-126 | 59348 | ZNF350 | zinc finger protein 350 | 1 | | 3152 | hsa-mir-424 | 59348 | ZNF350 | zinc finger protein 350 | 1 | | 3153 | hsa-mir-556 | 59348 | ZNF350 | zinc finger protein 350 | 1 | | 3154 | hsa-mir-1915 | 59348 | ZNF350 | zinc finger protein 350 | 1 | | 3155 | hsa-miR-190 | 59348 | ZNF350 | zinc finger protein 350 | 1 | | 3156 | hsa-miR-503 | 59348 | ZNF350 | zinc finger protein 350 | 1 | | 3157 | hsa-miR-585 | 59348 | ZNF350 | zinc finger protein 350 | 1 | | 3158 | hsa-miR-126\* | 374378 | GALNTL4 | UDP-N-acetyl-alpha-D-galactosamine:polypeptide N-acetylgalactosaminyltransferase-like 4 | 1 | | 3159 | hsa-miR-126\* | 80020 | FOXRED2 | FAD-dependent oxidoreductase domain containing 2 | 1 | | 3160 | hsa-miR-16 | 80020 | FOXRED2 | FAD-dependent oxidoreductase domain containing 2 | 1 | | 3161 | hsa-miR-566 | 80020 | FOXRED2 | FAD-dependent oxidoreductase domain containing 2 | 1 | | 3162 | hsa-miR-608 | 80020 | FOXRED2 | FAD-dependent oxidoreductase domain containing 2 | 1 | | 3164 | hsa-mir-30c-1 | 7329 | UBE2I | ubiquitin-conjugating enzyme E2I (UBC9 homolog, yeast) | 1 | | 3165 | hsa-mir-30e | 7329 | UBE2I | ubiquitin-conjugating enzyme E2I (UBC9 homolog, yeast) | 1 | | 3166 | hsa-miR-30c | 7329 | UBE2I | ubiquitin-conjugating enzyme E2I (UBC9 homolog, yeast) | 1 | | 3167 | hsa-miR-30e | 7329 | UBE2I | ubiquitin-conjugating enzyme E2I (UBC9 homolog, yeast) | 1 | | 3168 | hsa-miR-338-5p | 7329 | UBE2I | ubiquitin-conjugating enzyme E2I (UBC9 homolog, yeast) | 1 | | 3169 | hsa-miR-586 | 7329 | UBE2I | ubiquitin-conjugating enzyme E2I (UBC9 homolog, yeast) | 1 | | 3170 | hsa-miR-657 | 7329 | UBE2I | ubiquitin-conjugating enzyme E2I (UBC9 homolog, yeast) | 1 | | 3171 | hsa-mir-15a | 145845 | LOC145845 | hypothetical LOC145845 | 1 | | 3172 | hsa-mir-16-1 | 145845 | LOC145845 | hypothetical LOC145845 | 1 | | 3173 | hsa-miR-16 | 145845 | LOC145845 | hypothetical LOC145845 | 1 | | 3174 | hsa-miR-608 | 113278 | C20orf54 | chromosome 20 open reading frame 54 | 1 | | 3175 | hsa-miR-608 | 728591 | C13orf38 | chromosome 13 open reading frame 38 | 1 | | 3176 | hsa-miR-126\* | 158314 | C9orf44 | chromosome 9 open reading frame 44 | 1 | | 3177 | hsa-miR-564 | 158314 | C9orf44 | chromosome 9 open reading frame 44 | 1 | | 3178 | hsa-miR-603 | 158314 | C9orf44 | chromosome 9 open reading frame 44 | 1 | | 3179 | hsa-mir-339 | 80816 | ASXL3 | additional sex combs like 3 (Drosophila) | 1 | | 3180 | hsa-miR-339-5p | 80816 | ASXL3 | additional sex combs like 3 (Drosophila) | 1 | | 3181 | hsa-miR-608 | 80816 | ASXL3 | additional sex combs like 3 (Drosophila) | 1 | | 3182 | hsa-miR-126\* | 644714 | LOC644714 | hypothetical protein LOC644714 | 1 | | 3183 | hsa-miR-16 | 644714 | LOC644714 | hypothetical protein LOC644714 | 1 | | 3184 | hsa-miR-566 | 644714 | LOC644714 | hypothetical protein LOC644714 | 1 | | 3185 | hsa-miR-608 | 644714 | LOC644714 | hypothetical protein LOC644714 | 1 | | 3186 | hsa-mir-454 | 84515 | MCM8 | minichromosome maintenance complex component 8 | 1 | | 3187 | hsa-miR-16 | 84515 | MCM8 | minichromosome maintenance complex component 8 | 1 | | 3188 | hsa-miR-675 | 84515 | MCM8 | minichromosome maintenance complex component 8 | 1 | | 3189 | hsa-miR-95 | 84515 | MCM8 | minichromosome maintenance complex component 8 | 1 | | 3190 | hsa-mir-15a | 27111 | SDCBP2 | syndecan binding protein (syntenin) 2 | 1 | | 3191 | hsa-mir-16-1 | 27111 | SDCBP2 | syndecan binding protein (syntenin) 2 | 1 | | 3192 | hsa-miR-1231 | 27111 | SDCBP2 | syndecan binding protein (syntenin) 2 | 1 | | 3193 | hsa-miR-126\* | 27111 | SDCBP2 | syndecan binding protein (syntenin) 2 | 1 | | 3194 | hsa-miR-16 | 27111 | SDCBP2 | syndecan binding protein (syntenin) 2 | 1 | | 3195 | hsa-miR-566 | 27111 | SDCBP2 | syndecan binding protein (syntenin) 2 | 1 | | 3196 | hsa-miR-608 | 27111 | SDCBP2 | syndecan binding protein (syntenin) 2 | 1 | | 3197 | hsa-mir-15a | 9622 | KLK4 | kallikrein-related peptidase 4 | 1 | | 3198 | hsa-mir-16-1 | 9622 | KLK4 | kallikrein-related peptidase 4 | 1 | | 3199 | hsa-miR-1231 | 9622 | KLK4 | kallikrein-related peptidase 4 | 1 | | 3200 | hsa-miR-126\* | 9622 | KLK4 | kallikrein-related peptidase 4 | 1 | | 3201 | hsa-miR-16 | 9622 | KLK4 | kallikrein-related peptidase 4 | 1 | | 3202 | hsa-miR-608 | 9622 | KLK4 | kallikrein-related peptidase 4 | 1 | | 3203 | hsa-mir-548d-1 | 100133790 | LOC100133790 | intestinal mucin-like | 1 | | 3204 | hsa-mir-454 | 100133790 | LOC100133790 | intestinal mucin-like | 1 | | 3205 | hsa-miR-16 | 100133790 | LOC100133790 | intestinal mucin-like | 1 | | 3206 | hsa-miR-675 | 100133790 | LOC100133790 | intestinal mucin-like | 1 | | 3207 | hsa-miR-95 | 100133790 | LOC100133790 | intestinal mucin-like | 1 | | 3208 | hsa-miR-126\* | 57540 | PTCHD2 | patched domain containing 2 | 1 | | 3209 | hsa-miR-16 | 57540 | PTCHD2 | patched domain containing 2 | 1 | | 3210 | hsa-miR-608 | 57540 | PTCHD2 | patched domain containing 2 | 1 | | 3211 | hsa-mir-15a | 341912 | LOC341912 | similar to developmental pluripotency associated 5; embryonal stem cell specific gene 1 | 1 | | 3212 | hsa-mir-16-1 | 341912 | LOC341912 | similar to developmental pluripotency associated 5; embryonal stem cell specific gene 1 | 1 | | 3213 | hsa-miR-126\* | 341912 | LOC341912 | similar to developmental pluripotency associated 5; embryonal stem cell specific gene 1 | 1 | | 3214 | hsa-miR-16 | 341912 | LOC341912 | similar to developmental pluripotency associated 5; embryonal stem cell specific gene 1 | 1 | | 3215 | hsa-miR-566 | 341912 | LOC341912 | similar to developmental pluripotency associated 5; embryonal stem cell specific gene 1 | 1 | | 3216 | hsa-miR-608 | 341912 | LOC341912 | similar to developmental pluripotency associated 5; embryonal stem cell specific gene 1 | 1 | | 3217 | hsa-miR-1231 | 130951 | C2orf65 | chromosome 2 open reading frame 65 | 1 | | 3218 | hsa-miR-126\* | 130951 | C2orf65 | chromosome 2 open reading frame 65 | 1 | | 3219 | hsa-miR-564 | 130951 | C2orf65 | chromosome 2 open reading frame 65 | 1 | | 3220 | hsa-miR-566 | 130951 | C2orf65 | chromosome 2 open reading frame 65 | 1 | | 3221 | hsa-miR-608 | 130951 | C2orf65 | chromosome 2 open reading frame 65 | 1 | | 3222 | hsa-miR-126\* | 440292 | LOC440292 | similar to COMM domain containing 4 | 1 | | 3223 | hsa-miR-608 | 440292 | LOC440292 | similar to COMM domain containing 4 | 1 | | 3224 | hsa-miR-608 | 85285 | KRTAP4-1 | keratin associated protein 4-1 | 1 | | 3225 | hsa-miR-566 | 731275 | LOC731275 | hypothetical LOC731275 | 1 | | 3226 | hsa-miR-618 | 731275 | LOC731275 | hypothetical LOC731275 | 1 | | 3227 | hsa-mir-15a | 729262 | FAM22B | family with sequence similarity 22, member B | 1 | | 3228 | hsa-miR-1231 | 729262 | FAM22B | family with sequence similarity 22, member B | 1 | | 3229 | hsa-miR-126\* | 729262 | FAM22B | family with sequence similarity 22, member B | 1 | | 3230 | hsa-miR-16 | 729262 | FAM22B | family with sequence similarity 22, member B | 1 | | 3231 | hsa-miR-566 | 729262 | FAM22B | family with sequence similarity 22, member B | 1 | | 3232 | hsa-miR-608 | 729262 | FAM22B | family with sequence similarity 22, member B | 1 | | 3233 | hsa-mir-454 | 59283 | CACNG8 | calcium channel, voltage-dependent, gamma subunit 8 | 1 | | 3234 | hsa-miR-16 | 59283 | CACNG8 | calcium channel, voltage-dependent, gamma subunit 8 | 1 | | 3235 | hsa-miR-675 | 59283 | CACNG8 | calcium channel, voltage-dependent, gamma subunit 8 | 1 | | 3236 | hsa-miR-95 | 59283 | CACNG8 | calcium channel, voltage-dependent, gamma subunit 8 | 1 | | 3237 | hsa-miR-1231 | 282763 | OR51B5 | olfactory receptor, family 51, subfamily B, member 5 | 1 | | 3238 | hsa-miR-574-5p | 282763 | OR51B5 | olfactory receptor, family 51, subfamily B, member 5 | 1 | | 3239 | hsa-miR-608 | 282763 | OR51B5 | olfactory receptor, family 51, subfamily B, member 5 | 1 | | 3240 | hsa-miR-126\* | 346606 | MOGAT3 | monoacylglycerol O-acyltransferase 3 | 1 | | 3241 | hsa-miR-608 | 346606 | MOGAT3 | monoacylglycerol O-acyltransferase 3 | 1 | | 3242 | hsa-miR-675 | 93463 | LOC93463 | hypothetical protein LOC93463 | 1 | | 3243 | hsa-miR-95 | 93463 | LOC93463 | hypothetical protein LOC93463 | 1 | | 3244 | hsa-mir-454 | 56063 | C1orf91 | chromosome 1 open reading frame 91 | 1 | | 3245 | hsa-miR-16 | 56063 | C1orf91 | chromosome 1 open reading frame 91 | 1 | | 3246 | hsa-miR-675 | 56063 | C1orf91 | chromosome 1 open reading frame 91 | 1 | | 3247 | hsa-miR-95 | 56063 | C1orf91 | chromosome 1 open reading frame 91 | 1 | | 3248 | hsa-miR-574-5p | 571 | BACH1 | BTB and CNC homology 1, basic leucine zipper transcription factor 1 | 1 | | 3249 | hsa-miR-608 | 571 | BACH1 | BTB and CNC homology 1, basic leucine zipper transcription factor 1 | 1 | | 3250 | hsa-mir-574 | 7567 | ZNF19 | zinc finger protein 19 | 1 | | 3251 | hsa-miR-196b | 7567 | ZNF19 | zinc finger protein 19 | 1 | | 3252 | hsa-mir-339 | 120103 | SLC36A4 | solute carrier family 36 (proton/amino acid symporter), member 4 | 1 | | 3253 | hsa-miR-339-5p | 120103 | SLC36A4 | solute carrier family 36 (proton/amino acid symporter), member 4 | 1 | | 3254 | hsa-miR-574-5p | 120103 | SLC36A4 | solute carrier family 36 (proton/amino acid symporter), member 4 | 1 | | 3255 | hsa-miR-634 | 120103 | SLC36A4 | solute carrier family 36 (proton/amino acid symporter), member 4 | 1 | | 3256 | hsa-mir-196b | 147727 | LOC147727 | hypothetical LOC147727 | 1 | | 3257 | hsa-mir-424 | 147727 | LOC147727 | hypothetical LOC147727 | 1 | | 3258 | hsa-mir-628 | 147727 | LOC147727 | hypothetical LOC147727 | 1 | | 3259 | hsa-miR-190 | 147727 | LOC147727 | hypothetical LOC147727 | 1 | | 3260 | hsa-miR-196b | 147727 | LOC147727 | hypothetical LOC147727 | 1 | | 3261 | hsa-miR-503 | 147727 | LOC147727 | hypothetical LOC147727 | 1 | | 3262 | hsa-miR-586 | 147727 | LOC147727 | hypothetical LOC147727 | 1 | | 3263 | hsa-miR-617 | 147727 | LOC147727 | hypothetical LOC147727 | 1 | | 3264 | hsa-miR-623 | 147727 | LOC147727 | hypothetical LOC147727 | 1 | | 3265 | hsa-mir-135b | 10241 | CALCOCO2 | calcium binding and coiled-coil domain 2 | 1 | | 3266 | hsa-miR-454\* | 10241 | CALCOCO2 | calcium binding and coiled-coil domain 2 | 1 | | 3267 | hsa-mir-15a | 55384 | MEG3 | maternally expressed 3 (non-protein coding) | 1 | | 3268 | hsa-mir-16-1 | 55384 | MEG3 | maternally expressed 3 (non-protein coding) | 1 | | 3269 | hsa-miR-126\* | 55384 | MEG3 | maternally expressed 3 (non-protein coding) | 1 | | 3270 | hsa-miR-16 | 55384 | MEG3 | maternally expressed 3 (non-protein coding) | 1 | | 3271 | hsa-miR-608 | 55384 | MEG3 | maternally expressed 3 (non-protein coding) | 1 | | 3272 | hsa-mir-126 | 157285 | PRAGMIN | homolog of rat pragma of Rnd2 | 1 | | 3273 | hsa-mir-30c-1 | 157285 | PRAGMIN | homolog of rat pragma of Rnd2 | 1 | | 3274 | hsa-mir-30e | 157285 | PRAGMIN | homolog of rat pragma of Rnd2 | 1 | | 3275 | hsa-mir-424 | 157285 | PRAGMIN | homolog of rat pragma of Rnd2 | 1 | | 3276 | hsa-mir-556 | 157285 | PRAGMIN | homolog of rat pragma of Rnd2 | 1 | | 3277 | hsa-mir-628 | 157285 | PRAGMIN | homolog of rat pragma of Rnd2 | 1 | | 3278 | hsa-miR-190 | 157285 | PRAGMIN | homolog of rat pragma of Rnd2 | 1 | | 3279 | hsa-miR-338-5p | 157285 | PRAGMIN | homolog of rat pragma of Rnd2 | 1 | | 3280 | hsa-miR-503 | 157285 | PRAGMIN | homolog of rat pragma of Rnd2 | 1 | | 3281 | hsa-miR-586 | 157285 | PRAGMIN | homolog of rat pragma of Rnd2 | 1 | | 3282 | hsa-miR-617 | 157285 | PRAGMIN | homolog of rat pragma of Rnd2 | 1 | | 3283 | hsa-miR-657 | 157285 | PRAGMIN | homolog of rat pragma of Rnd2 | 1 | | 3284 | hsa-mir-126 | 114799 | ESCO1 | establishment of cohesion 1 homolog 1 (S. cerevisiae) | 1 | | 3285 | hsa-mir-30c-1 | 114799 | ESCO1 | establishment of cohesion 1 homolog 1 (S. cerevisiae) | 1 | | 3286 | hsa-mir-30e | 114799 | ESCO1 | establishment of cohesion 1 homolog 1 (S. cerevisiae) | 1 | | 3287 | hsa-miR-30c | 114799 | ESCO1 | establishment of cohesion 1 homolog 1 (S. cerevisiae) | 1 | | 3288 | hsa-miR-30e | 114799 | ESCO1 | establishment of cohesion 1 homolog 1 (S. cerevisiae) | 1 | | 3289 | hsa-miR-617 | 114799 | ESCO1 | establishment of cohesion 1 homolog 1 (S. cerevisiae) | 1 | | 3290 | hsa-mir-15a | 90523 | C6orf142 | chromosome 6 open reading frame 142 | 1 | | 3291 | hsa-mir-16-1 | 90523 | C6orf142 | chromosome 6 open reading frame 142 | 1 | | 3292 | hsa-miR-126\* | 90523 | C6orf142 | chromosome 6 open reading frame 142 | 1 | | 3293 | hsa-miR-16 | 90523 | C6orf142 | chromosome 6 open reading frame 142 | 1 | | 3294 | hsa-miR-608 | 90523 | C6orf142 | chromosome 6 open reading frame 142 | 1 | | 3295 | hsa-mir-218-2 | 84824 | FCRLA | Fc receptor-like A | 1 | | 3296 | hsa-mir-556 | 84824 | FCRLA | Fc receptor-like A | 1 | | 3297 | hsa-miR-190 | 84824 | FCRLA | Fc receptor-like A | 1 | | 3298 | hsa-miR-585 | 84824 | FCRLA | Fc receptor-like A | 1 | | 3299 | hsa-mir-126 | 64794 | DDX31 | DEAD (Asp-Glu-Ala-Asp) box polypeptide 31 | 1 | | 3300 | hsa-mir-30c-1 | 64794 | DDX31 | DEAD (Asp-Glu-Ala-Asp) box polypeptide 31 | 1 | | 3301 | hsa-mir-30e | 64794 | DDX31 | DEAD (Asp-Glu-Ala-Asp) box polypeptide 31 | 1 | | 3302 | hsa-mir-424 | 64794 | DDX31 | DEAD (Asp-Glu-Ala-Asp) box polypeptide 31 | 1 | | 3303 | hsa-mir-556 | 64794 | DDX31 | DEAD (Asp-Glu-Ala-Asp) box polypeptide 31 | 1 | | 3304 | hsa-mir-628 | 64794 | DDX31 | DEAD (Asp-Glu-Ala-Asp) box polypeptide 31 | 1 | | 3305 | hsa-miR-190 | 64794 | DDX31 | DEAD (Asp-Glu-Ala-Asp) box polypeptide 31 | 1 | | 3306 | hsa-miR-338-5p | 64794 | DDX31 | DEAD (Asp-Glu-Ala-Asp) box polypeptide 31 | 1 | | 3307 | hsa-miR-503 | 64794 | DDX31 | DEAD (Asp-Glu-Ala-Asp) box polypeptide 31 | 1 | | 3308 | hsa-miR-586 | 64794 | DDX31 | DEAD (Asp-Glu-Ala-Asp) box polypeptide 31 | 1 | | 3309 | hsa-miR-617 | 64794 | DDX31 | DEAD (Asp-Glu-Ala-Asp) box polypeptide 31 | 1 | | 3310 | hsa-miR-657 | 64794 | DDX31 | DEAD (Asp-Glu-Ala-Asp) box polypeptide 31 | 1 | | 3311 | hsa-mir-15a | 23529 | CLCF1 | cardiotrophin-like cytokine factor 1 | 1 | | 3312 | hsa-mir-16-1 | 23529 | CLCF1 | cardiotrophin-like cytokine factor 1 | 1 | | 3313 | hsa-miR-126\* | 23529 | CLCF1 | cardiotrophin-like cytokine factor 1 | 1 | | 3314 | hsa-miR-16 | 23529 | CLCF1 | cardiotrophin-like cytokine factor 1 | 1 | | 3315 | hsa-miR-608 | 23529 | CLCF1 | cardiotrophin-like cytokine factor 1 | 1 | | 3316 | hsa-mir-30c-1 | 644873 | FLJ33630 | hypothetical LOC644873 | 1 | | 3317 | hsa-mir-30e | 644873 | FLJ33630 | hypothetical LOC644873 | 1 | | 3318 | hsa-miR-30c | 644873 | FLJ33630 | hypothetical LOC644873 | 1 | | 3319 | hsa-miR-30e | 644873 | FLJ33630 | hypothetical LOC644873 | 1 | | 3320 | hsa-miR-338-5p | 644873 | FLJ33630 | hypothetical LOC644873 | 1 | | 3321 | hsa-miR-586 | 644873 | FLJ33630 | hypothetical LOC644873 | 1 | | 3322 | hsa-miR-617 | 644873 | FLJ33630 | hypothetical LOC644873 | 1 | | 3323 | hsa-miR-657 | 644873 | FLJ33630 | hypothetical LOC644873 | 1 | | 3324 | hsa-miR-126\* | 85007 | AGXT2L2 | alanine-glyoxylate aminotransferase 2-like 2 | 1 | | 3325 | hsa-miR-566 | 85007 | AGXT2L2 | alanine-glyoxylate aminotransferase 2-like 2 | 1 | | 3326 | hsa-mir-556 | 84622 | ZNF594 | zinc finger protein 594 | 1 | | 3327 | hsa-mir-15a | 5478 | PPIA | peptidylprolyl isomerase A (cyclophilin A) | 1 | | 3328 | hsa-mir-16-1 | 5478 | PPIA | peptidylprolyl isomerase A (cyclophilin A) | 1 | | 3329 | hsa-miR-126\* | 5478 | PPIA | peptidylprolyl isomerase A (cyclophilin A) | 1 | | 3330 | hsa-miR-16 | 5478 | PPIA | peptidylprolyl isomerase A (cyclophilin A) | 1 | | 3331 | hsa-miR-608 | 5478 | PPIA | peptidylprolyl isomerase A (cyclophilin A) | 1 | | 3332 | hsa-mir-135b | 2081 | ERN1 | endoplasmic reticulum to nucleus signaling 1 | 1 | | 3333 | hsa-mir-675 | 2081 | ERN1 | endoplasmic reticulum to nucleus signaling 1 | 1 | | 3334 | hsa-miR-616\* | 2081 | ERN1 | endoplasmic reticulum to nucleus signaling 1 | 1 | | 3335 | hsa-mir-15a | 100131017 | ZNF316 | zinc finger protein 316 | 1 | | 3336 | hsa-mir-16-1 | 100131017 | ZNF316 | zinc finger protein 316 | 1 | | 3337 | hsa-miR-126\* | 100131017 | ZNF316 | zinc finger protein 316 | 1 | | 3338 | hsa-miR-16 | 100131017 | ZNF316 | zinc finger protein 316 | 1 | | 3339 | hsa-miR-608 | 100131017 | ZNF316 | zinc finger protein 316 | 1 | | 3340 | hsa-mir-218-2 | 201475 | RAB12 | RAB12, member RAS oncogene family | 1 | | 3341 | hsa-mir-126 | 201475 | RAB12 | RAB12, member RAS oncogene family | 1 | | 3342 | hsa-mir-424 | 201475 | RAB12 | RAB12, member RAS oncogene family | 1 | | 3343 | hsa-mir-556 | 201475 | RAB12 | RAB12, member RAS oncogene family | 1 | | 3344 | hsa-mir-1915 | 201475 | RAB12 | RAB12, member RAS oncogene family | 1 | | 3345 | hsa-miR-190 | 201475 | RAB12 | RAB12, member RAS oncogene family | 1 | | 3346 | hsa-miR-338-5p | 201475 | RAB12 | RAB12, member RAS oncogene family | 1 | | 3347 | hsa-miR-503 | 201475 | RAB12 | RAB12, member RAS oncogene family | 1 | | 3348 | hsa-miR-585 | 201475 | RAB12 | RAB12, member RAS oncogene family | 1 | | 3349 | hsa-miR-617 | 201475 | RAB12 | RAB12, member RAS oncogene family | 1 | | 3350 | hsa-miR-657 | 201475 | RAB12 | RAB12, member RAS oncogene family | 1 | | 3351 | hsa-miR-126\* | 494558 | LOC494558 | hypothetical locus LOC494558 | 1 | | 3352 | hsa-miR-16 | 494558 | LOC494558 | hypothetical locus LOC494558 | 1 | | 3353 | hsa-miR-564 | 494558 | LOC494558 | hypothetical locus LOC494558 | 1 | | 3354 | hsa-miR-608 | 494558 | LOC494558 | hypothetical locus LOC494558 | 1 | | 3355 | hsa-miR-126\* | 133690 | CAPSL | calcyphosine-like | 1 | | 3356 | hsa-miR-16 | 133690 | CAPSL | calcyphosine-like | 1 | | 3357 | hsa-miR-564 | 133690 | CAPSL | calcyphosine-like | 1 | | 3358 | hsa-miR-608 | 133690 | CAPSL | calcyphosine-like | 1 | | 3359 | hsa-mir-135b | 9444 | QKI | quaking homolog, KH domain RNA binding (mouse) | 1 | | 3360 | hsa-miR-190 | 9444 | QKI | quaking homolog, KH domain RNA binding (mouse) | 1 | | 3361 | hsa-mir-25 | 149483 | CCDC17 | coiled-coil domain containing 17 | 1 | | 3362 | hsa-mir-93 | 149483 | CCDC17 | coiled-coil domain containing 17 | 1 | | 3363 | hsa-mir-106b | 149483 | CCDC17 | coiled-coil domain containing 17 | 1 | | 3364 | hsa-miR-126\* | 285556 | LOC285556 | hypothetical protein LOC285556 | 1 | | 3365 | hsa-miR-608 | 285556 | LOC285556 | hypothetical protein LOC285556 | 1 | | 3366 | hsa-mir-15a | 7533 | YWHAH | tyrosine 3-monooxygenase/tryptophan 5-monooxygenase activation protein, eta polypeptide | 1 | | 3367 | hsa-mir-16-1 | 7533 | YWHAH | tyrosine 3-monooxygenase/tryptophan 5-monooxygenase activation protein, eta polypeptide | 1 | | 3368 | hsa-miR-1231 | 7533 | YWHAH | tyrosine 3-monooxygenase/tryptophan 5-monooxygenase activation protein, eta polypeptide | 1 | | 3369 | hsa-miR-608 | 7533 | YWHAH | tyrosine 3-monooxygenase/tryptophan 5-monooxygenase activation protein, eta polypeptide | 1 | | 3370 | hsa-miR-634 | 7533 | YWHAH | tyrosine 3-monooxygenase/tryptophan 5-monooxygenase activation protein, eta polypeptide | 1 | | 3371 | hsa-mir-574 | 3996 | LLGL1 | lethal giant larvae homolog 1 (Drosophila) | 1 | | 3372 | hsa-mir-628 | 3996 | LLGL1 | lethal giant larvae homolog 1 (Drosophila) | 1 | | 3373 | hsa-miR-196b | 3996 | LLGL1 | lethal giant larvae homolog 1 (Drosophila) | 1 | | 3374 | hsa-miR-617 | 3996 | LLGL1 | lethal giant larvae homolog 1 (Drosophila) | 1 | | 3375 | hsa-miR-95 | 3996 | LLGL1 | lethal giant larvae homolog 1 (Drosophila) | 1 | | 3376 | hsa-mir-15a | 100128977 | LOC100128977 | hypothetical LOC100128977 | 1 | | 3377 | hsa-mir-16-1 | 100128977 | LOC100128977 | hypothetical LOC100128977 | 1 | | 3378 | hsa-miR-1231 | 100128977 | LOC100128977 | hypothetical LOC100128977 | 1 | | 3379 | hsa-miR-126\* | 100128977 | LOC100128977 | hypothetical LOC100128977 | 1 | | 3380 | hsa-miR-16 | 100128977 | LOC100128977 | hypothetical LOC100128977 | 1 | | 3381 | hsa-miR-608 | 100128977 | LOC100128977 | hypothetical LOC100128977 | 1 | | 3382 | hsa-mir-218-2 | 152137 | CCDC50 | coiled-coil domain containing 50 | 1 | | 3383 | hsa-miR-585 | 152137 | CCDC50 | coiled-coil domain containing 50 | 1 | | 3384 | hsa-mir-15a | 286151 | FBXO43 | F-box protein 43 | 1 | | 3385 | hsa-mir-16-1 | 286151 | FBXO43 | F-box protein 43 | 1 | | 3386 | hsa-miR-126\* | 286151 | FBXO43 | F-box protein 43 | 1 | | 3387 | hsa-miR-16 | 286151 | FBXO43 | F-box protein 43 | 1 | | 3388 | hsa-miR-564 | 286151 | FBXO43 | F-box protein 43 | 1 | | 3389 | hsa-miR-608 | 286151 | FBXO43 | F-box protein 43 | 1 | | 3390 | hsa-miR-675 | 401067 | IQCF3 | IQ motif containing F3 | 1 | | 3391 | hsa-miR-95 | 401067 | IQCF3 | IQ motif containing F3 | 1 | | 3392 | hsa-mir-15a | 100131213 | C10orf41 | chromosome 10 open reading frame 41 | 1 | | 3393 | hsa-mir-16-1 | 100131213 | C10orf41 | chromosome 10 open reading frame 41 | 1 | | 3394 | hsa-miR-1231 | 100131213 | C10orf41 | chromosome 10 open reading frame 41 | 1 | | 3395 | hsa-miR-126\* | 100131213 | C10orf41 | chromosome 10 open reading frame 41 | 1 | | 3396 | hsa-miR-566 | 100131213 | C10orf41 | chromosome 10 open reading frame 41 | 1 | | 3397 | hsa-miR-608 | 100131213 | C10orf41 | chromosome 10 open reading frame 41 | 1 | | 3398 | hsa-miR-126\* | 146433 | IL34 | interleukin 34 | 1 | | 3399 | hsa-miR-16 | 146433 | IL34 | interleukin 34 | 1 | | 3400 | hsa-miR-566 | 146433 | IL34 | interleukin 34 | 1 | | 3401 | hsa-miR-608 | 146433 | IL34 | interleukin 34 | 1 | | 3402 | hsa-miR-608 | 256126 | SYCE2 | synaptonemal complex central element protein 2 | 1 | | 3403 | hsa-mir-339 | 116369 | SLC26A8 | solute carrier family 26, member 8 | 1 | | 3404 | hsa-miR-339-5p | 116369 | SLC26A8 | solute carrier family 26, member 8 | 1 | | 3405 | hsa-miR-564 | 116369 | SLC26A8 | solute carrier family 26, member 8 | 1 | | 3406 | hsa-miR-618 | 116369 | SLC26A8 | solute carrier family 26, member 8 | 1 | | 3407 | hsa-mir-15a | 200159 | C1orf100 | chromosome 1 open reading frame 100 | 1 | | 3408 | hsa-miR-126\* | 200159 | C1orf100 | chromosome 1 open reading frame 100 | 1 | | 3409 | hsa-miR-608 | 200159 | C1orf100 | chromosome 1 open reading frame 100 | 1 | | 3410 | hsa-miR-126\* | 286042 | hCG\_1990547 | family with sequence similarity 86, member A pseudogene | 1 | | 3411 | hsa-miR-564 | 286042 | hCG\_1990547 | family with sequence similarity 86, member A pseudogene | 1 | | 3412 | hsa-miR-126\* | 131920 | TMEM207 | transmembrane protein 207 | 1 | | 3413 | hsa-miR-16 | 131920 | TMEM207 | transmembrane protein 207 | 1 | | 3414 | hsa-miR-566 | 131920 | TMEM207 | transmembrane protein 207 | 1 | | 3415 | hsa-miR-608 | 131920 | TMEM207 | transmembrane protein 207 | 1 | | 3416 | hsa-miR-126\* | 92070 | C4orf42 | chromosome 4 open reading frame 42 | 1 | | 3417 | hsa-miR-566 | 92070 | C4orf42 | chromosome 4 open reading frame 42 | 1 | | 3418 | hsa-miR-608 | 92070 | C4orf42 | chromosome 4 open reading frame 42 | 1 | | 3419 | hsa-mir-15a | 100130700 | LOC100130700 | similar to hCG2038355 | 1 | | 3420 | hsa-mir-16-1 | 100130700 | LOC100130700 | similar to hCG2038355 | 1 | | 3421 | hsa-miR-1231 | 100130700 | LOC100130700 | similar to hCG2038355 | 1 | | 3422 | hsa-miR-126\* | 100130700 | LOC100130700 | similar to hCG2038355 | 1 | | 3423 | hsa-miR-16 | 100130700 | LOC100130700 | similar to hCG2038355 | 1 | | 3424 | hsa-miR-608 | 100130700 | LOC100130700 | similar to hCG2038355 | 1 | | 3425 | hsa-mir-15a | 9627 | SNCAIP | synuclein, alpha interacting protein | 1 | | 3426 | hsa-mir-16-1 | 9627 | SNCAIP | synuclein, alpha interacting protein | 1 | | 3427 | hsa-miR-608 | 9627 | SNCAIP | synuclein, alpha interacting protein | 1 | | 3428 | hsa-miR-1231 | 79649 | MAP7D3 | MAP7 domain containing 3 | 1 | | 3429 | hsa-miR-126\* | 79649 | MAP7D3 | MAP7 domain containing 3 | 1 | | 3430 | hsa-miR-608 | 79649 | MAP7D3 | MAP7 domain containing 3 | 1 | | 3431 | hsa-mir-15a | 9423 | NTN1 | netrin 1 | 1 | | 3432 | hsa-mir-16-1 | 9423 | NTN1 | netrin 1 | 1 | | 3433 | hsa-miR-126\* | 9423 | NTN1 | netrin 1 | 1 | | 3434 | hsa-miR-16 | 9423 | NTN1 | netrin 1 | 1 | | 3435 | hsa-miR-564 | 9423 | NTN1 | netrin 1 | 1 | | 3436 | hsa-miR-608 | 9423 | NTN1 | netrin 1 | 1 | | 3437 | hsa-mir-15a | 729994 | LOC729994 | hypothetical LOC729994 | 1 | | 3438 | hsa-mir-16-1 | 729994 | LOC729994 | hypothetical LOC729994 | 1 | | 3439 | hsa-miR-126\* | 729994 | LOC729994 | hypothetical LOC729994 | 1 | | 3440 | hsa-miR-608 | 729994 | LOC729994 | hypothetical LOC729994 | 1 | | 3441 | hsa-mir-454 | 9208 | LRRFIP1 | leucine rich repeat (in FLII) interacting protein 1 | 1 | | 3442 | hsa-miR-675 | 9208 | LRRFIP1 | leucine rich repeat (in FLII) interacting protein 1 | 1 | | 3443 | hsa-miR-95 | 9208 | LRRFIP1 | leucine rich repeat (in FLII) interacting protein 1 | 1 | | 3444 | hsa-miR-126\* | 23122 | CLASP2 | cytoplasmic linker associated protein 2 | 1 | | 3445 | hsa-miR-608 | 23122 | CLASP2 | cytoplasmic linker associated protein 2 | 1 | | 3446 | hsa-mir-126 | 79736 | C17orf42 | chromosome 17 open reading frame 42 | 1 | | 3447 | hsa-mir-30c-1 | 79736 | C17orf42 | chromosome 17 open reading frame 42 | 1 | | 3448 | hsa-mir-30e | 79736 | C17orf42 | chromosome 17 open reading frame 42 | 1 | | 3449 | hsa-miR-26a | 79736 | C17orf42 | chromosome 17 open reading frame 42 | 1 | | 3450 | hsa-miR-30c | 79736 | C17orf42 | chromosome 17 open reading frame 42 | 1 | | 3451 | hsa-miR-30e | 79736 | C17orf42 | chromosome 17 open reading frame 42 | 1 | | 3452 | hsa-miR-338-5p | 79736 | C17orf42 | chromosome 17 open reading frame 42 | 1 | | 3453 | hsa-miR-657 | 79736 | C17orf42 | chromosome 17 open reading frame 42 | 1 | | 3454 | hsa-mir-628 | 254048 | UBN2 | ubinuclein 2 | 1 | | 3455 | hsa-miR-504 | 254048 | UBN2 | ubinuclein 2 | 1 | | 3456 | hsa-mir-218-2 | 390980 | ZNF805 | zinc finger protein 805 | 1 | | 3457 | hsa-mir-126 | 390980 | ZNF805 | zinc finger protein 805 | 1 | | 3458 | hsa-mir-196b | 390980 | ZNF805 | zinc finger protein 805 | 1 | | 3459 | hsa-mir-424 | 390980 | ZNF805 | zinc finger protein 805 | 1 | | 3460 | hsa-mir-556 | 390980 | ZNF805 | zinc finger protein 805 | 1 | | 3461 | hsa-mir-628 | 390980 | ZNF805 | zinc finger protein 805 | 1 | | 3462 | hsa-mir-1915 | 390980 | ZNF805 | zinc finger protein 805 | 1 | | 3463 | hsa-miR-190 | 390980 | ZNF805 | zinc finger protein 805 | 1 | | 3464 | hsa-miR-196b | 390980 | ZNF805 | zinc finger protein 805 | 1 | | 3465 | hsa-miR-503 | 390980 | ZNF805 | zinc finger protein 805 | 1 | | 3466 | hsa-miR-585 | 390980 | ZNF805 | zinc finger protein 805 | 1 | | 3467 | hsa-miR-617 | 390980 | ZNF805 | zinc finger protein 805 | 1 | | 3468 | hsa-miR-623 | 390980 | ZNF805 | zinc finger protein 805 | 1 | | 3469 | hsa-mir-628 | 114991 | ZNF618 | zinc finger protein 618 | 1 | | 3470 | hsa-miR-586 | 114991 | ZNF618 | zinc finger protein 618 | 1 | | 3471 | hsa-miR-617 | 114991 | ZNF618 | zinc finger protein 618 | 1 | | 3472 | hsa-mir-218-2 | 8526 | DGKE | diacylglycerol kinase, epsilon 64kDa | 1 | | 3473 | hsa-mir-424 | 8526 | DGKE | diacylglycerol kinase, epsilon 64kDa | 1 | | 3474 | hsa-mir-556 | 8526 | DGKE | diacylglycerol kinase, epsilon 64kDa | 1 | | 3475 | hsa-mir-628 | 8526 | DGKE | diacylglycerol kinase, epsilon 64kDa | 1 | | 3476 | hsa-miR-190 | 8526 | DGKE | diacylglycerol kinase, epsilon 64kDa | 1 | | 3477 | hsa-miR-503 | 8526 | DGKE | diacylglycerol kinase, epsilon 64kDa | 1 | | 3478 | hsa-miR-585 | 8526 | DGKE | diacylglycerol kinase, epsilon 64kDa | 1 | | 3479 | hsa-miR-623 | 8526 | DGKE | diacylglycerol kinase, epsilon 64kDa | 1 | | 3480 | hsa-mir-424 | 54606 | DDX56 | DEAD (Asp-Glu-Ala-Asp) box polypeptide 56 | 1 | | 3481 | hsa-miR-196b | 54606 | DDX56 | DEAD (Asp-Glu-Ala-Asp) box polypeptide 56 | 1 | | 3482 | hsa-miR-503 | 54606 | DDX56 | DEAD (Asp-Glu-Ala-Asp) box polypeptide 56 | 1 | | 3483 | hsa-mir-218-2 | 494150 | LOC494150 | prohibitin pseudogene | 1 | | 3484 | hsa-mir-196b | 494150 | LOC494150 | prohibitin pseudogene | 1 | | 3485 | hsa-mir-424 | 494150 | LOC494150 | prohibitin pseudogene | 1 | | 3486 | hsa-mir-490 | 494150 | LOC494150 | prohibitin pseudogene | 1 | | 3487 | hsa-miR-190 | 494150 | LOC494150 | prohibitin pseudogene | 1 | | 3488 | hsa-miR-196b | 494150 | LOC494150 | prohibitin pseudogene | 1 | | 3489 | hsa-miR-490-5p | 494150 | LOC494150 | prohibitin pseudogene | 1 | | 3490 | hsa-miR-503 | 494150 | LOC494150 | prohibitin pseudogene | 1 | | 3491 | hsa-miR-585 | 494150 | LOC494150 | prohibitin pseudogene | 1 | | 3492 | hsa-miR-623 | 494150 | LOC494150 | prohibitin pseudogene | 1 | | 3493 | hsa-miR-1231 | 2070 | EYA4 | eyes absent homolog 4 (Drosophila) | 1 | | 3494 | hsa-miR-608 | 2070 | EYA4 | eyes absent homolog 4 (Drosophila) | 1 | | 3495 | hsa-miR-608 | 643314 | KIAA0754 | KIAA0754 | 1 | | 3496 | hsa-mir-454 | 1588 | CYP19A1 | cytochrome P450, family 19, subfamily A, polypeptide 1 | 1 | | 3497 | hsa-miR-675 | 1588 | CYP19A1 | cytochrome P450, family 19, subfamily A, polypeptide 1 | 1 | | 3498 | hsa-miR-95 | 1588 | CYP19A1 | cytochrome P450, family 19, subfamily A, polypeptide 1 | 1 | | 3499 | hsa-mir-135b | 342909 | ZNF284 | zinc finger protein 284 | 1 | | 3500 | hsa-mir-675 | 342909 | ZNF284 | zinc finger protein 284 | 1 | | 3501 | hsa-miR-454\* | 342909 | ZNF284 | zinc finger protein 284 | 1 | | 3502 | hsa-miR-126\* | 93377 | OPALIN | oligodendrocytic myelin paranodal and inner loop protein | 1 | | 3503 | hsa-miR-608 | 93377 | OPALIN | oligodendrocytic myelin paranodal and inner loop protein | 1 | | 3504 | hsa-mir-126 | 3708 | ITPR1 | inositol 1,4,5-triphosphate receptor, type 1 | 1 | | 3505 | hsa-mir-30c-1 | 3708 | ITPR1 | inositol 1,4,5-triphosphate receptor, type 1 | 1 | | 3506 | hsa-mir-30e | 3708 | ITPR1 | inositol 1,4,5-triphosphate receptor, type 1 | 1 | | 3507 | hsa-mir-424 | 3708 | ITPR1 | inositol 1,4,5-triphosphate receptor, type 1 | 1 | | 3508 | hsa-mir-556 | 3708 | ITPR1 | inositol 1,4,5-triphosphate receptor, type 1 | 1 | | 3509 | hsa-mir-628 | 3708 | ITPR1 | inositol 1,4,5-triphosphate receptor, type 1 | 1 | | 3510 | hsa-miR-190 | 3708 | ITPR1 | inositol 1,4,5-triphosphate receptor, type 1 | 1 | | 3511 | hsa-miR-338-5p | 3708 | ITPR1 | inositol 1,4,5-triphosphate receptor, type 1 | 1 | | 3512 | hsa-miR-503 | 3708 | ITPR1 | inositol 1,4,5-triphosphate receptor, type 1 | 1 | | 3513 | hsa-miR-586 | 3708 | ITPR1 | inositol 1,4,5-triphosphate receptor, type 1 | 1 | | 3514 | hsa-miR-617 | 3708 | ITPR1 | inositol 1,4,5-triphosphate receptor, type 1 | 1 | | 3515 | hsa-miR-657 | 3708 | ITPR1 | inositol 1,4,5-triphosphate receptor, type 1 | 1 | | 3516 | hsa-mir-218-2 | 158234 | RG9MTD3 | RNA (guanine-9-) methyltransferase domain containing 3 | 1 | | 3517 | hsa-mir-126 | 158234 | RG9MTD3 | RNA (guanine-9-) methyltransferase domain containing 3 | 1 | | 3518 | hsa-mir-424 | 158234 | RG9MTD3 | RNA (guanine-9-) methyltransferase domain containing 3 | 1 | | 3519 | hsa-mir-1915 | 158234 | RG9MTD3 | RNA (guanine-9-) methyltransferase domain containing 3 | 1 | | 3520 | hsa-miR-190 | 158234 | RG9MTD3 | RNA (guanine-9-) methyltransferase domain containing 3 | 1 | | 3521 | hsa-miR-503 | 158234 | RG9MTD3 | RNA (guanine-9-) methyltransferase domain containing 3 | 1 | | 3522 | hsa-miR-585 | 158234 | RG9MTD3 | RNA (guanine-9-) methyltransferase domain containing 3 | 1 | | 3523 | hsa-mir-196b | 642826 | LOC642826 | hypothetical LOC642826 | 1 | | 3524 | hsa-mir-424 | 642826 | LOC642826 | hypothetical LOC642826 | 1 | | 3525 | hsa-mir-628 | 642826 | LOC642826 | hypothetical LOC642826 | 1 | | 3526 | hsa-miR-190 | 642826 | LOC642826 | hypothetical LOC642826 | 1 | | 3527 | hsa-miR-196b | 642826 | LOC642826 | hypothetical LOC642826 | 1 | | 3528 | hsa-miR-503 | 642826 | LOC642826 | hypothetical LOC642826 | 1 | | 3529 | hsa-miR-504 | 642826 | LOC642826 | hypothetical LOC642826 | 1 | | 3530 | hsa-miR-586 | 642826 | LOC642826 | hypothetical LOC642826 | 1 | | 3531 | hsa-miR-617 | 642826 | LOC642826 | hypothetical LOC642826 | 1 | | 3532 | hsa-mir-15a | 58486 | ZBED5 | zinc finger, BED-type containing 5 | 1 | | 3533 | hsa-mir-16-1 | 58486 | ZBED5 | zinc finger, BED-type containing 5 | 1 | | 3534 | hsa-miR-126\* | 58486 | ZBED5 | zinc finger, BED-type containing 5 | 1 | | 3535 | hsa-miR-16 | 58486 | ZBED5 | zinc finger, BED-type containing 5 | 1 | | 3536 | hsa-miR-564 | 58486 | ZBED5 | zinc finger, BED-type containing 5 | 1 | | 3537 | hsa-miR-566 | 58486 | ZBED5 | zinc finger, BED-type containing 5 | 1 | | 3538 | hsa-miR-608 | 58486 | ZBED5 | zinc finger, BED-type containing 5 | 1 | | 3539 | hsa-miR-126\* | 64065 | PERP | PERP, TP53 apoptosis effector | 1 | | 3540 | hsa-miR-564 | 64065 | PERP | PERP, TP53 apoptosis effector | 1 | | 3541 | hsa-miR-608 | 64065 | PERP | PERP, TP53 apoptosis effector | 1 | | 3542 | hsa-mir-15a | 220108 | FAM124A | family with sequence similarity 124A | 1 | | 3543 | hsa-mir-16-1 | 220108 | FAM124A | family with sequence similarity 124A | 1 | | 3544 | hsa-miR-126\* | 220108 | FAM124A | family with sequence similarity 124A | 1 | | 3545 | hsa-miR-608 | 220108 | FAM124A | family with sequence similarity 124A | 1 | | 3546 | hsa-mir-454 | 100129112 | LOC100129112 | hypothetical protein LOC100129112 | 1 | | 3547 | hsa-miR-675 | 100129112 | LOC100129112 | hypothetical protein LOC100129112 | 1 | | 3548 | hsa-miR-95 | 100129112 | LOC100129112 | hypothetical protein LOC100129112 | 1 | | 3549 | hsa-miR-126\* | 122481 | AK7 | adenylate kinase 7 | 1 | | 3550 | hsa-miR-16 | 122481 | AK7 | adenylate kinase 7 | 1 | | 3551 | hsa-miR-608 | 122481 | AK7 | adenylate kinase 7 | 1 | | 3552 | hsa-mir-424 | 57396 | CLK4 | CDC-like kinase 4 | 1 | | 3553 | hsa-miR-503 | 57396 | CLK4 | CDC-like kinase 4 | 1 | | 3554 | hsa-mir-454 | 93650 | ACPT | acid phosphatase, testicular | 1 | | 3555 | hsa-miR-675 | 93650 | ACPT | acid phosphatase, testicular | 1 | | 3556 | hsa-miR-95 | 93650 | ACPT | acid phosphatase, testicular | 1 | | 3557 | hsa-miR-586 | 80008 | TMEM156 | transmembrane protein 156 | 1 | | 3558 | hsa-miR-564 | 80208 | SPG11 | spastic paraplegia 11 (autosomal recessive) | 1 | | 3559 | hsa-miR-608 | 80208 | SPG11 | spastic paraplegia 11 (autosomal recessive) | 1 | | 3560 | hsa-mir-126 | 25831 | HECTD1 | HECT domain containing 1 | 1 | | 3561 | hsa-miR-190 | 25831 | HECTD1 | HECT domain containing 1 | 1 | | 3562 | hsa-mir-15a | 140890 | SFRS12 | splicing factor, arginine/serine-rich 12 | 1 | | 3563 | hsa-mir-16-1 | 140890 | SFRS12 | splicing factor, arginine/serine-rich 12 | 1 | | 3564 | hsa-miR-16 | 140890 | SFRS12 | splicing factor, arginine/serine-rich 12 | 1 | | 3565 | hsa-mir-15a | 9098 | USP6 | ubiquitin specific peptidase 6 (Tre-2 oncogene) | 1 | | 3566 | hsa-mir-16-1 | 9098 | USP6 | ubiquitin specific peptidase 6 (Tre-2 oncogene) | 1 | | 3567 | hsa-miR-126\* | 9098 | USP6 | ubiquitin specific peptidase 6 (Tre-2 oncogene) | 1 | | 3568 | hsa-miR-16 | 9098 | USP6 | ubiquitin specific peptidase 6 (Tre-2 oncogene) | 1 | | 3569 | hsa-miR-564 | 9098 | USP6 | ubiquitin specific peptidase 6 (Tre-2 oncogene) | 1 | | 3570 | hsa-miR-566 | 9098 | USP6 | ubiquitin specific peptidase 6 (Tre-2 oncogene) | 1 | | 3571 | hsa-miR-608 | 9098 | USP6 | ubiquitin specific peptidase 6 (Tre-2 oncogene) | 1 | | 3572 | hsa-miR-618 | 121512 | FGD4 | FYVE, RhoGEF and PH domain containing 4 | 1 | | 3573 | hsa-miR-126\* | 163175 | LGI4 | leucine-rich repeat LGI family, member 4 | 1 | | 3574 | hsa-miR-16 | 163175 | LGI4 | leucine-rich repeat LGI family, member 4 | 1 | | 3575 | hsa-miR-566 | 163175 | LGI4 | leucine-rich repeat LGI family, member 4 | 1 | | 3576 | hsa-miR-608 | 163175 | LGI4 | leucine-rich repeat LGI family, member 4 | 1 | | 3577 | hsa-mir-15a | 400643 | FLJ38028 | hypothetical gene supported by AK095347 | 1 | | 3578 | hsa-mir-16-1 | 400643 | FLJ38028 | hypothetical gene supported by AK095347 | 1 | | 3579 | hsa-miR-126\* | 400643 | FLJ38028 | hypothetical gene supported by AK095347 | 1 | | 3580 | hsa-miR-16 | 400643 | FLJ38028 | hypothetical gene supported by AK095347 | 1 | | 3581 | hsa-miR-608 | 400643 | FLJ38028 | hypothetical gene supported by AK095347 | 1 | | 3582 | hsa-mir-339 | 22949 | PTGR1 | prostaglandin reductase 1 | 1 | | 3583 | hsa-miR-339-5p | 22949 | PTGR1 | prostaglandin reductase 1 | 1 | | 3584 | hsa-miR-618 | 22949 | PTGR1 | prostaglandin reductase 1 | 1 | | 3585 | hsa-miR-634 | 22949 | PTGR1 | prostaglandin reductase 1 | 1 | | 3586 | hsa-miR-126\* | 145226 | RDH12 | retinol dehydrogenase 12 (all-trans/9-cis/11-cis) | 1 | | 3587 | hsa-miR-608 | 145226 | RDH12 | retinol dehydrogenase 12 (all-trans/9-cis/11-cis) | 1 | | 3588 | hsa-miR-608 | 150084 | IGSF5 | immunoglobulin superfamily, member 5 | 1 | | 3589 | hsa-mir-15a | 642891 | LOC642891 | hypothetical LOC642891 | 1 | | 3590 | hsa-mir-16-1 | 642891 | LOC642891 | hypothetical LOC642891 | 1 | | 3591 | hsa-miR-126\* | 642891 | LOC642891 | hypothetical LOC642891 | 1 | | 3592 | hsa-miR-16 | 642891 | LOC642891 | hypothetical LOC642891 | 1 | | 3593 | hsa-miR-564 | 642891 | LOC642891 | hypothetical LOC642891 | 1 | | 3594 | hsa-miR-608 | 642891 | LOC642891 | hypothetical LOC642891 | 1 | | 3595 | hsa-miR-1231 | 10052 | GJC1 | gap junction protein, gamma 1, 45kDa | 1 | | 3596 | hsa-miR-126\* | 10052 | GJC1 | gap junction protein, gamma 1, 45kDa | 1 | | 3597 | hsa-miR-126\* | 349565 | NMNAT3 | nicotinamide nucleotide adenylyltransferase 3 | 1 | | 3598 | hsa-miR-564 | 349565 | NMNAT3 | nicotinamide nucleotide adenylyltransferase 3 | 1 | | 3599 | hsa-miR-566 | 349565 | NMNAT3 | nicotinamide nucleotide adenylyltransferase 3 | 1 | | 3600 | hsa-miR-608 | 349565 | NMNAT3 | nicotinamide nucleotide adenylyltransferase 3 | 1 | | 3601 | hsa-miR-126\* | 100287445 | LOC100287445 | hypothetical protein LOC100287445 | 1 | | 3602 | hsa-miR-608 | 100287445 | LOC100287445 | hypothetical protein LOC100287445 | 1 | | 3603 | hsa-miR-126\* | 9415 | FADS2 | fatty acid desaturase 2 | 1 | | 3604 | hsa-miR-16 | 9415 | FADS2 | fatty acid desaturase 2 | 1 | | 3605 | hsa-miR-608 | 9415 | FADS2 | fatty acid desaturase 2 | 1 | | 3606 | hsa-mir-339 | 4259 | MGST3 | microsomal glutathione S-transferase 3 | 1 | | 3607 | hsa-miR-126\* | 4259 | MGST3 | microsomal glutathione S-transferase 3 | 1 | | 3608 | hsa-miR-339-5p | 4259 | MGST3 | microsomal glutathione S-transferase 3 | 1 | | 3609 | hsa-miR-564 | 4259 | MGST3 | microsomal glutathione S-transferase 3 | 1 | | 3610 | hsa-miR-608 | 4259 | MGST3 | microsomal glutathione S-transferase 3 | 1 | | 3611 | hsa-mir-675 | 80821 | DDHD1 | DDHD domain containing 1 | 1 | | 3612 | hsa-miR-586 | 80821 | DDHD1 | DDHD domain containing 1 | 1 | | 3613 | hsa-miR-617 | 80821 | DDHD1 | DDHD domain containing 1 | 1 | | 3614 | hsa-miR-190 | 163702 | IL28RA | interleukin 28 receptor, alpha (interferon, lambda receptor) | 1 | | 3615 | hsa-miR-95 | 65082 | VPS33A | vacuolar protein sorting 33 homolog A (S. cerevisiae) | 1 | | 3616 | hsa-mir-15a | 79998 | ANKRD53 | ankyrin repeat domain 53 | 1 | | 3617 | hsa-mir-16-1 | 79998 | ANKRD53 | ankyrin repeat domain 53 | 1 | | 3618 | hsa-miR-126\* | 79998 | ANKRD53 | ankyrin repeat domain 53 | 1 | | 3619 | hsa-miR-16 | 79998 | ANKRD53 | ankyrin repeat domain 53 | 1 | | 3620 | hsa-miR-566 | 79998 | ANKRD53 | ankyrin repeat domain 53 | 1 | | 3621 | hsa-miR-608 | 79998 | ANKRD53 | ankyrin repeat domain 53 | 1 | | 3622 | hsa-miR-126\* | 25780 | RASGRP3 | RAS guanyl releasing protein 3 (calcium and DAG-regulated) | 1 | | 3623 | hsa-miR-608 | 25780 | RASGRP3 | RAS guanyl releasing protein 3 (calcium and DAG-regulated) | 1 | | 3624 | hsa-mir-454 | 2849 | GPR26 | G protein-coupled receptor 26 | 1 | | 3625 | hsa-miR-16 | 2849 | GPR26 | G protein-coupled receptor 26 | 1 | | 3626 | hsa-miR-675 | 2849 | GPR26 | G protein-coupled receptor 26 | 1 | | 3627 | hsa-miR-95 | 2849 | GPR26 | G protein-coupled receptor 26 | 1 | | 3628 | hsa-mir-30c-1 | 54014 | BRWD1 | bromodomain and WD repeat domain containing 1 | 1 | | 3629 | hsa-mir-30e | 54014 | BRWD1 | bromodomain and WD repeat domain containing 1 | 1 | | 3630 | hsa-mir-628 | 54014 | BRWD1 | bromodomain and WD repeat domain containing 1 | 1 | | 3631 | hsa-miR-617 | 54014 | BRWD1 | bromodomain and WD repeat domain containing 1 | 1 | | 3632 | hsa-mir-424 | 55705 | IPO9 | importin 9 | 1 | | 3633 | hsa-miR-503 | 55705 | IPO9 | importin 9 | 1 | | 3634 | hsa-miR-617 | 55705 | IPO9 | importin 9 | 1 | | 3635 | hsa-mir-628 | 401207 | FLJ44606 | glutaredoxin-like protein YDR286C homolog | 1 | | 3636 | hsa-miR-338-5p | 401207 | FLJ44606 | glutaredoxin-like protein YDR286C homolog | 1 | | 3637 | hsa-miR-586 | 401207 | FLJ44606 | glutaredoxin-like protein YDR286C homolog | 1 | | 3638 | hsa-miR-617 | 401207 | FLJ44606 | glutaredoxin-like protein YDR286C homolog | 1 | | 3639 | hsa-miR-657 | 401207 | FLJ44606 | glutaredoxin-like protein YDR286C homolog | 1 | | 3640 | hsa-miR-126\* | 57477 | SHROOM4 | shroom family member 4 | 1 | | 3641 | hsa-miR-564 | 57477 | SHROOM4 | shroom family member 4 | 1 | | 3642 | hsa-miR-608 | 57477 | SHROOM4 | shroom family member 4 | 1 | | 3643 | hsa-miR-190 | 4744 | NEFH | neurofilament, heavy polypeptide | 1 | | 3644 | hsa-miR-338-5p | 4744 | NEFH | neurofilament, heavy polypeptide | 1 | | 3645 | hsa-miR-657 | 4744 | NEFH | neurofilament, heavy polypeptide | 1 | | 3646 | hsa-mir-135b | 23354 | HAUS5 | HAUS augmin-like complex, subunit 5 | 1 | | 3647 | hsa-mir-675 | 23354 | HAUS5 | HAUS augmin-like complex, subunit 5 | 1 | | 3648 | hsa-miR-454\* | 23354 | HAUS5 | HAUS augmin-like complex, subunit 5 | 1 | | 3649 | hsa-miR-548d-5p | 23354 | HAUS5 | HAUS augmin-like complex, subunit 5 | 1 | | 3650 | hsa-miR-196b | 8115 | TCL1A | T-cell leukemia/lymphoma 1A | 1 | | 3651 | hsa-miR-623 | 8115 | TCL1A | T-cell leukemia/lymphoma 1A | 1 | | 3652 | hsa-miR-675 | 3170 | FOXA2 | forkhead box A2 | 1 | | 3653 | hsa-miR-95 | 3170 | FOXA2 | forkhead box A2 | 1 | | 3654 | hsa-mir-454 | 7004 | TEAD4 | TEA domain family member 4 | 1 | | 3655 | hsa-miR-16 | 7004 | TEAD4 | TEA domain family member 4 | 1 | | 3656 | hsa-miR-564 | 7004 | TEAD4 | TEA domain family member 4 | 1 | | 3657 | hsa-miR-675 | 7004 | TEAD4 | TEA domain family member 4 | 1 | | 3658 | hsa-miR-95 | 7004 | TEAD4 | TEA domain family member 4 | 1 | | 3659 | hsa-mir-15a | 85360 | SYDE1 | synapse defective 1, Rho GTPase, homolog 1 (C. elegans) | 1 | | 3660 | hsa-mir-16-1 | 85360 | SYDE1 | synapse defective 1, Rho GTPase, homolog 1 (C. elegans) | 1 | | 3661 | hsa-miR-126\* | 85360 | SYDE1 | synapse defective 1, Rho GTPase, homolog 1 (C. elegans) | 1 | | 3662 | hsa-miR-16 | 85360 | SYDE1 | synapse defective 1, Rho GTPase, homolog 1 (C. elegans) | 1 | | 3663 | hsa-miR-564 | 85360 | SYDE1 | synapse defective 1, Rho GTPase, homolog 1 (C. elegans) | 1 | | 3664 | hsa-miR-566 | 85360 | SYDE1 | synapse defective 1, Rho GTPase, homolog 1 (C. elegans) | 1 | | 3665 | hsa-miR-608 | 85360 | SYDE1 | synapse defective 1, Rho GTPase, homolog 1 (C. elegans) | 1 | | 3666 | hsa-miR-1231 | 9683 | N4BP1 | NEDD4 binding protein 1 | 1 | | 3667 | hsa-miR-126\* | 9683 | N4BP1 | NEDD4 binding protein 1 | 1 | | 3668 | hsa-miR-564 | 9683 | N4BP1 | NEDD4 binding protein 1 | 1 | | 3669 | hsa-miR-566 | 9683 | N4BP1 | NEDD4 binding protein 1 | 1 | | 3670 | hsa-miR-608 | 9683 | N4BP1 | NEDD4 binding protein 1 | 1 | | 3671 | hsa-miR-126\* | 54961 | SSH3 | slingshot homolog 3 (Drosophila) | 1 | | 3672 | hsa-miR-564 | 54961 | SSH3 | slingshot homolog 3 (Drosophila) | 1 | | 3673 | hsa-miR-618 | 54961 | SSH3 | slingshot homolog 3 (Drosophila) | 1 | | 3674 | hsa-miR-634 | 89853 | FAM125B | family with sequence similarity 125, member B | 1 | | 3675 | hsa-mir-454 | 11094 | C9orf7 | chromosome 9 open reading frame 7 | 1 | | 3676 | hsa-miR-675 | 11094 | C9orf7 | chromosome 9 open reading frame 7 | 1 | | 3677 | hsa-miR-95 | 11094 | C9orf7 | chromosome 9 open reading frame 7 | 1 | | 3678 | hsa-mir-196b | 11253 | MAN1B1 | mannosidase, alpha, class 1B, member 1 | 1 | | 3679 | hsa-mir-574 | 11253 | MAN1B1 | mannosidase, alpha, class 1B, member 1 | 1 | | 3680 | hsa-mir-628 | 11253 | MAN1B1 | mannosidase, alpha, class 1B, member 1 | 1 | | 3681 | hsa-miR-196b | 11253 | MAN1B1 | mannosidase, alpha, class 1B, member 1 | 1 | | 3682 | hsa-miR-623 | 11253 | MAN1B1 | mannosidase, alpha, class 1B, member 1 | 1 | |

---

Gene Ontology - Biological Process [Details: ]

| |  | genes in Category | percent in the observed List | percent in the genome | fold of overrepresents | odds ratio | p value | | --- | --- | --- | --- | --- | --- | --- | | translational elongation | 18 | 0.0325 | 0.00745 | 4.4 | 5.2 | 1.3e-07 | | translation | 38 | 0.0687 | 0.02890 | 2.4 | 2.6 | 6.8e-07 | | neurotransmitter biosynthetic process | 4 | 0.0072 | 0.00056 | 12.9 | 24.9 | 1.4e-04 | | cellular protein metabolic process | 134 | 0.2423 | 0.18142 | 1.3 | 1.5 | 1.5e-04 | | cellular macromolecule metabolic process | 265 | 0.4792 | 0.40707 | 1.2 | 1.4 | 2.7e-04 | | gene expression | 180 | 0.3255 | 0.26158 | 1.2 | 1.4 | 3.8e-04 | | eye development | 14 | 0.0253 | 0.00900 | 2.8 | 3.1 | 4.5e-04 | | response to protozoan | 3 | 0.0054 | 0.00035 | 15.4 | 37.3 | 5.5e-04 | | macromolecule biosynthetic process | 171 | 0.3092 | 0.24851 | 1.2 | 1.4 | 5.7e-04 | | ribosomal large subunit biogenesis | 4 | 0.0072 | 0.00077 | 9.4 | 14.2 | 6.0e-04 | | divalent metal ion transport | 17 | 0.0307 | 0.01259 | 2.4 | 2.6 | 6.2e-04 | | primary metabolic process | 331 | 0.5986 | 0.53154 | 1.1 | 1.3 | 7.1e-04 | | biosynthetic process | 203 | 0.3671 | 0.30455 | 1.2 | 1.3 | 7.7e-04 | | protein metabolic process | 149 | 0.2694 | 0.21335 | 1.3 | 1.4 | 8.1e-04 | | macromolecule metabolic process | 283 | 0.5118 | 0.44575 | 1.1 | 1.3 | 8.7e-04 | | cellular macromolecule biosynthetic process | 167 | 0.3020 | 0.24408 | 1.2 | 1.4 | 9.1e-04 | | negative regulation of calcium ion transport | 4 | 0.0072 | 0.00091 | 7.9 | 11.1 | 1.2e-03 | | cellular biosynthetic process | 197 | 0.3562 | 0.29688 | 1.2 | 1.3 | 1.2e-03 | | nitrogen compound metabolic process | 206 | 0.3725 | 0.31257 | 1.2 | 1.3 | 1.3e-03 | | retina development in camera-type eye | 6 | 0.0108 | 0.00225 | 4.8 | 5.8 | 1.3e-03 | | cellular developmental process | 95 | 0.1718 | 0.12763 | 1.3 | 1.4 | 1.4e-03 | | regulation of primary metabolic process | 159 | 0.2875 | 0.23275 | 1.2 | 1.3 | 1.4e-03 | | calcium ion transport | 16 | 0.0289 | 0.01238 | 2.3 | 2.5 | 1.4e-03 | | camera-type eye development | 11 | 0.0199 | 0.00689 | 2.9 | 3.2 | 1.5e-03 | | anatomical structure morphogenesis | 70 | 0.1266 | 0.08874 | 1.4 | 1.5 | 1.5e-03 | | regulation of neuron projection development | 9 | 0.0163 | 0.00492 | 3.3 | 3.7 | 1.5e-03 | | glutamate decarboxylation to succinate | 2 | 0.0036 | 0.00014 | 25.7 | Inf | 1.5e-03 | | positive regulation of T-helper 1 type immune response | 2 | 0.0036 | 0.00014 | 25.7 | Inf | 1.5e-03 | | positive regulation of vascular endothelial growth factor receptor signaling pathway | 3 | 0.0054 | 0.00049 | 11.0 | 18.6 | 1.8e-03 | | endocrine pancreas development | 4 | 0.0072 | 0.00105 | 6.9 | 9.0 | 2.2e-03 | | regulation of nucleobase, nucleoside, nucleotide and nucleic acid metabolic process | 137 | 0.2477 | 0.19844 | 1.2 | 1.3 | 2.2e-03 | | regulation of nitrogen compound metabolic process | 138 | 0.2495 | 0.20020 | 1.2 | 1.3 | 2.3e-03 | | regulation of cell projection organization | 10 | 0.0181 | 0.00626 | 2.9 | 3.2 | 2.3e-03 | | cell differentiation | 89 | 0.1609 | 0.12046 | 1.3 | 1.4 | 2.4e-03 | | regulation of cellular metabolic process | 164 | 0.2966 | 0.24457 | 1.2 | 1.3 | 2.6e-03 | | neurofilament cytoskeleton organization | 3 | 0.0054 | 0.00056 | 9.6 | 14.9 | 2.8e-03 | | striatum development | 3 | 0.0054 | 0.00056 | 9.6 | 14.9 | 2.8e-03 | | nervous system development | 59 | 0.1067 | 0.07412 | 1.4 | 1.5 | 2.8e-03 | | metabolic process | 356 | 0.6438 | 0.58786 | 1.1 | 1.3 | 3.5e-03 | | di-, tri-valent inorganic cation transport | 17 | 0.0307 | 0.01477 | 2.1 | 2.2 | 3.5e-03 | | cellular metabolic process | 317 | 0.5732 | 0.51635 | 1.1 | 1.3 | 3.5e-03 | | T-helper 1 type immune response | 4 | 0.0072 | 0.00120 | 6.1 | 7.7 | 3.6e-03 | | regulation of macromolecule metabolic process | 154 | 0.2785 | 0.23022 | 1.2 | 1.3 | 4.0e-03 | | positive regulation of axon extension | 3 | 0.0054 | 0.00063 | 8.6 | 12.4 | 4.1e-03 | | regulation of vascular endothelial growth factor receptor signaling pathway | 3 | 0.0054 | 0.00063 | 8.6 | 12.4 | 4.1e-03 | | positive regulation of axon regeneration | 2 | 0.0036 | 0.00021 | 17.1 | 49.6 | 4.4e-03 | | convergent extension | 2 | 0.0036 | 0.00021 | 17.1 | 49.6 | 4.4e-03 | | positive regulation of neuron projection development | 2 | 0.0036 | 0.00021 | 17.1 | 49.6 | 4.4e-03 | | positive regulation of neuron projection regeneration | 2 | 0.0036 | 0.00021 | 17.1 | 49.6 | 4.4e-03 | | regulation of biosynthetic process | 141 | 0.2550 | 0.20885 | 1.2 | 1.3 | 4.4e-03 | | regulation of neurotransmitter levels | 9 | 0.0163 | 0.00577 | 2.8 | 3.1 | 4.5e-03 | | nucleobase, nucleoside, nucleotide and nucleic acid metabolic process | 186 | 0.3363 | 0.28570 | 1.2 | 1.3 | 4.6e-03 | | regulation of metabolic process | 168 | 0.3038 | 0.25561 | 1.2 | 1.3 | 5.2e-03 | | sensory organ development | 17 | 0.0307 | 0.01540 | 2.0 | 2.1 | 5.3e-03 | | transmembrane receptor protein tyrosine kinase signaling pathway | 20 | 0.0362 | 0.01934 | 1.9 | 2.0 | 5.5e-03 | | glutamine family amino acid catabolic process | 4 | 0.0072 | 0.00134 | 5.4 | 6.6 | 5.5e-03 | | negative regulation of ion transport | 4 | 0.0072 | 0.00134 | 5.4 | 6.6 | 5.5e-03 | | positive regulation of interferon-gamma production | 3 | 0.0054 | 0.00070 | 7.7 | 10.6 | 5.7e-03 | | negative regulation of adaptive immune response | 3 | 0.0054 | 0.00070 | 7.7 | 10.6 | 5.7e-03 | | negative regulation of adaptive immune response based on somatic recombination of immune receptors built from immunoglobulin superfamily domains | 3 | 0.0054 | 0.00070 | 7.7 | 10.6 | 5.7e-03 | | subpallium development | 3 | 0.0054 | 0.00070 | 7.7 | 10.6 | 5.7e-03 | | regulation of macromolecule biosynthetic process | 134 | 0.2423 | 0.19893 | 1.2 | 1.3 | 6.2e-03 | | regulation of cellular biosynthetic process | 139 | 0.2514 | 0.20758 | 1.2 | 1.3 | 6.4e-03 | | vascular endothelial growth factor receptor signaling pathway | 4 | 0.0072 | 0.00141 | 5.1 | 6.2 | 6.7e-03 | | regulation of transcription | 124 | 0.2242 | 0.18269 | 1.2 | 1.3 | 6.8e-03 | | response to toxin | 7 | 0.0127 | 0.00408 | 3.1 | 3.4 | 7.0e-03 | | developmental process | 149 | 0.2694 | 0.22551 | 1.2 | 1.3 | 7.6e-03 | | response to interferon-gamma | 3 | 0.0054 | 0.00077 | 7.0 | 9.3 | 7.6e-03 | | positive regulation of endothelial cell proliferation | 4 | 0.0072 | 0.00148 | 4.9 | 5.9 | 8.0e-03 | | multicellular organismal development | 135 | 0.2441 | 0.20245 | 1.2 | 1.3 | 8.4e-03 | | defense response to protozoan | 2 | 0.0036 | 0.00028 | 12.9 | 24.8 | 8.6e-03 | | positive regulation of cell projection organization | 6 | 0.0108 | 0.00330 | 3.3 | 3.6 | 9.3e-03 | | photoreceptor cell maintenance | 4 | 0.0072 | 0.00155 | 4.7 | 5.5 | 9.5e-03 | | neurotransmitter metabolic process | 4 | 0.0072 | 0.00155 | 4.7 | 5.5 | 9.5e-03 | | regulation of gene expression | 134 | 0.2423 | 0.20153 | 1.2 | 1.3 | 9.6e-03 | | positive regulation of biological process | 97 | 0.1754 | 0.13993 | 1.3 | 1.3 | 9.9e-03 | |

---

Gene Ontology - Biological Process, level II [Details: ]

| |  | genes in Category | percent in the observed List | percent in the genome | fold of overrepresents | odds ratio | p value | | --- | --- | --- | --- | --- | --- | --- | | translational elongation | 18 | 0.0327 | 0.00745 | 4.4 | 5.2 | 1.2e-07 | | translation | 38 | 0.0690 | 0.02890 | 2.4 | 2.6 | 6.2e-07 | | cellular protein metabolic process | 134 | 0.2432 | 0.18142 | 1.3 | 1.5 | 1.3e-04 | | neurotransmitter biosynthetic process | 4 | 0.0073 | 0.00056 | 12.9 | 25.0 | 1.4e-04 | | cellular macromolecule metabolic process | 265 | 0.4809 | 0.40707 | 1.2 | 1.4 | 2.1e-04 | | gene expression | 180 | 0.3267 | 0.26158 | 1.2 | 1.4 | 3.1e-04 | | eye development | 14 | 0.0254 | 0.00900 | 2.8 | 3.1 | 4.4e-04 | | macromolecule biosynthetic process | 171 | 0.3103 | 0.24851 | 1.2 | 1.4 | 4.8e-04 | | primary metabolic process | 331 | 0.6007 | 0.53154 | 1.1 | 1.3 | 5.0e-04 | | response to protozoan | 3 | 0.0054 | 0.00035 | 15.5 | 37.4 | 5.5e-04 | | ribosomal large subunit biogenesis | 4 | 0.0073 | 0.00077 | 9.4 | 14.3 | 5.9e-04 | | divalent metal ion transport | 17 | 0.0309 | 0.01259 | 2.5 | 2.7 | 6.0e-04 | | biosynthetic process | 203 | 0.3684 | 0.30455 | 1.2 | 1.3 | 6.3e-04 | | macromolecule metabolic process | 283 | 0.5136 | 0.44575 | 1.2 | 1.3 | 6.5e-04 | | protein metabolic process | 149 | 0.2704 | 0.21335 | 1.3 | 1.4 | 6.8e-04 | | cellular macromolecule biosynthetic process | 167 | 0.3031 | 0.24408 | 1.2 | 1.4 | 7.6e-04 | | cellular biosynthetic process | 197 | 0.3575 | 0.29688 | 1.2 | 1.3 | 1.0e-03 | | nitrogen compound metabolic process | 206 | 0.3739 | 0.31257 | 1.2 | 1.3 | 1.0e-03 | | regulation of primary metabolic process | 159 | 0.2886 | 0.23275 | 1.2 | 1.4 | 1.2e-03 | | negative regulation of calcium ion transport | 4 | 0.0073 | 0.00091 | 7.9 | 11.1 | 1.2e-03 | | cellular developmental process | 95 | 0.1724 | 0.12763 | 1.4 | 1.4 | 1.2e-03 | | retina development in camera-type eye | 6 | 0.0109 | 0.00225 | 4.8 | 5.8 | 1.3e-03 | | anatomical structure morphogenesis | 70 | 0.1270 | 0.08874 | 1.4 | 1.5 | 1.3e-03 | | calcium ion transport | 16 | 0.0290 | 0.01238 | 2.3 | 2.5 | 1.4e-03 | | camera-type eye development | 11 | 0.0200 | 0.00689 | 2.9 | 3.2 | 1.4e-03 | | regulation of neuron projection development | 9 | 0.0163 | 0.00492 | 3.3 | 3.7 | 1.5e-03 | | glutamate decarboxylation to succinate | 2 | 0.0036 | 0.00014 | 25.8 | Inf | 1.5e-03 | | positive regulation of T-helper 1 type immune response | 2 | 0.0036 | 0.00014 | 25.8 | Inf | 1.5e-03 | | positive regulation of vascular endothelial growth factor receptor signaling pathway | 3 | 0.0054 | 0.00049 | 11.1 | 18.7 | 1.8e-03 | | regulation of nucleobase, nucleoside, nucleotide and nucleic acid metabolic process | 137 | 0.2486 | 0.19844 | 1.3 | 1.4 | 1.9e-03 | | regulation of nitrogen compound metabolic process | 138 | 0.2505 | 0.20020 | 1.3 | 1.4 | 2.0e-03 | | endocrine pancreas development | 4 | 0.0073 | 0.00105 | 6.9 | 9.1 | 2.2e-03 | | regulation of cellular metabolic process | 164 | 0.2976 | 0.24457 | 1.2 | 1.3 | 2.2e-03 | | cell differentiation | 89 | 0.1615 | 0.12046 | 1.3 | 1.4 | 2.2e-03 | | regulation of cell projection organization | 10 | 0.0181 | 0.00626 | 2.9 | 3.2 | 2.3e-03 | | nervous system development | 59 | 0.1071 | 0.07412 | 1.4 | 1.5 | 2.6e-03 | | cellular metabolic process | 317 | 0.5753 | 0.51635 | 1.1 | 1.3 | 2.7e-03 | | neurofilament cytoskeleton organization | 3 | 0.0054 | 0.00056 | 9.7 | 15.0 | 2.8e-03 | | striatum development | 3 | 0.0054 | 0.00056 | 9.7 | 15.0 | 2.8e-03 | | di-, tri-valent inorganic cation transport | 17 | 0.0309 | 0.01477 | 2.1 | 2.2 | 3.4e-03 | | regulation of macromolecule metabolic process | 154 | 0.2795 | 0.23022 | 1.2 | 1.3 | 3.5e-03 | | T-helper 1 type immune response | 4 | 0.0073 | 0.00120 | 6.1 | 7.7 | 3.5e-03 | | nucleobase, nucleoside, nucleotide and nucleic acid metabolic process | 186 | 0.3376 | 0.28570 | 1.2 | 1.3 | 3.9e-03 | | regulation of biosynthetic process | 141 | 0.2559 | 0.20885 | 1.2 | 1.3 | 3.9e-03 | | positive regulation of axon extension | 3 | 0.0054 | 0.00063 | 8.6 | 12.5 | 4.1e-03 | | regulation of vascular endothelial growth factor receptor signaling pathway | 3 | 0.0054 | 0.00063 | 8.6 | 12.5 | 4.1e-03 | | regulation of neurotransmitter levels | 9 | 0.0163 | 0.00577 | 2.8 | 3.1 | 4.4e-03 | | positive regulation of axon regeneration | 2 | 0.0036 | 0.00021 | 17.2 | 49.8 | 4.4e-03 | | convergent extension | 2 | 0.0036 | 0.00021 | 17.2 | 49.8 | 4.4e-03 | | positive regulation of neuron projection development | 2 | 0.0036 | 0.00021 | 17.2 | 49.8 | 4.4e-03 | | positive regulation of neuron projection regeneration | 2 | 0.0036 | 0.00021 | 17.2 | 49.8 | 4.4e-03 | | regulation of metabolic process | 168 | 0.3049 | 0.25561 | 1.2 | 1.3 | 4.5e-03 | | sensory organ development | 17 | 0.0309 | 0.01540 | 2.0 | 2.1 | 5.1e-03 | | transmembrane receptor protein tyrosine kinase signaling pathway | 20 | 0.0363 | 0.01934 | 1.9 | 2.0 | 5.3e-03 | | regulation of macromolecule biosynthetic process | 134 | 0.2432 | 0.19893 | 1.2 | 1.3 | 5.4e-03 | | glutamine family amino acid catabolic process | 4 | 0.0073 | 0.00134 | 5.4 | 6.7 | 5.4e-03 | | negative regulation of ion transport | 4 | 0.0073 | 0.00134 | 5.4 | 6.7 | 5.4e-03 | | regulation of cellular biosynthetic process | 139 | 0.2523 | 0.20758 | 1.2 | 1.3 | 5.6e-03 | | positive regulation of interferon-gamma production | 3 | 0.0054 | 0.00070 | 7.7 | 10.7 | 5.7e-03 | | negative regulation of adaptive immune response | 3 | 0.0054 | 0.00070 | 7.7 | 10.7 | 5.7e-03 | | negative regulation of adaptive immune response based on somatic recombination of immune receptors built from immunoglobulin superfamily domains | 3 | 0.0054 | 0.00070 | 7.7 | 10.7 | 5.7e-03 | | subpallium development | 3 | 0.0054 | 0.00070 | 7.7 | 10.7 | 5.7e-03 | | regulation of transcription | 124 | 0.2250 | 0.18269 | 1.2 | 1.3 | 6.0e-03 | | vascular endothelial growth factor receptor signaling pathway | 4 | 0.0073 | 0.00141 | 5.2 | 6.2 | 6.6e-03 | | response to toxin | 7 | 0.0127 | 0.00408 | 3.1 | 3.4 | 6.9e-03 | | multicellular organismal development | 135 | 0.2450 | 0.20245 | 1.2 | 1.3 | 7.4e-03 | | response to interferon-gamma | 3 | 0.0054 | 0.00077 | 7.0 | 9.3 | 7.6e-03 | | positive regulation of endothelial cell proliferation | 4 | 0.0073 | 0.00148 | 4.9 | 5.9 | 7.9e-03 | | regulation of gene expression | 134 | 0.2432 | 0.20153 | 1.2 | 1.3 | 8.5e-03 | | defense response to protozoan | 2 | 0.0036 | 0.00028 | 12.9 | 24.9 | 8.5e-03 | | positive regulation of cell projection organization | 6 | 0.0109 | 0.00330 | 3.3 | 3.7 | 9.2e-03 | | photoreceptor cell maintenance | 4 | 0.0073 | 0.00155 | 4.7 | 5.5 | 9.4e-03 | | neurotransmitter metabolic process | 4 | 0.0073 | 0.00155 | 4.7 | 5.5 | 9.4e-03 | | positive regulation of transcription from RNA polymerase II promoter | 23 | 0.0417 | 0.02461 | 1.7 | 1.8 | 9.7e-03 | |

---

KEGG pathways [Details: ]

| |  | genes in Category | percent in the observed List | percent in the genome | fold of overrepresents | odds ratio | p value | | --- | --- | --- | --- | --- | --- | --- | | Ribosome | 16 | 0.082 | 0.0174 | 4.7 | 6.0 | 1.5e-07 | | B cell receptor signaling pathway | 8 | 0.041 | 0.0148 | 2.8 | 3.1 | 7.6e-03 | | Primary immunodeficiency | 5 | 0.026 | 0.0069 | 3.7 | 4.3 | 1.0e-02 | | Retinol metabolism | 7 | 0.036 | 0.0127 | 2.9 | 3.2 | 1.1e-02 | | Amyotrophic lateral sclerosis (ALS) | 6 | 0.031 | 0.0105 | 3.0 | 3.3 | 1.5e-02 | | Renal cell carcinoma | 7 | 0.036 | 0.0138 | 2.6 | 2.9 | 1.7e-02 | | Drug metabolism - cytochrome P450 | 7 | 0.036 | 0.0142 | 2.5 | 2.8 | 2.0e-02 | | Caffeine metabolism | 2 | 0.010 | 0.0014 | 7.4 | 10.1 | 2.7e-02 | | Oocyte meiosis | 9 | 0.046 | 0.0225 | 2.1 | 2.2 | 3.0e-02 | | mTOR signaling pathway | 5 | 0.026 | 0.0103 | 2.5 | 2.7 | 4.8e-02 | | ErbB signaling pathway | 7 | 0.036 | 0.0172 | 2.1 | 2.2 | 4.8e-02 | | Metabolism of xenobiotics by cytochrome P450 | 6 | 0.031 | 0.0138 | 2.2 | 2.4 | 5.1e-02 | | Long-term potentiation | 6 | 0.031 | 0.0138 | 2.2 | 2.4 | 5.1e-02 | | Taurine and hypotaurine metabolism | 2 | 0.010 | 0.0020 | 5.2 | 6.3 | 5.4e-02 | | Phosphatidylinositol signaling system | 6 | 0.031 | 0.0150 | 2.1 | 2.2 | 7.0e-02 | | Circadian rhythm - mammal | 2 | 0.010 | 0.0026 | 4.0 | 4.6 | 8.7e-02 | | Type I diabetes mellitus | 4 | 0.021 | 0.0087 | 2.4 | 2.5 | 8.7e-02 | | Linoleic acid metabolism | 3 | 0.015 | 0.0057 | 2.7 | 2.9 | 9.8e-02 | |

---

Disease Ontology Lite terms [Details: ]

| |  | genes in Category | percent in the observed List | percent in the genome | fold of overrepresents | odds ratio | p value | | --- | --- | --- | --- | --- | --- | --- | | Anxiety disorder | 2 | 0.0122 | 0.00074 | 16.5 | 48.0 | 0.0048 | | Generalized anxiety disorder | 3 | 0.0183 | 0.00296 | 6.2 | 8.0 | 0.0109 | | Down syndrome | 8 | 0.0488 | 0.01901 | 2.6 | 2.8 | 0.0119 | | Bipolar disorder | 8 | 0.0488 | 0.01925 | 2.5 | 2.8 | 0.0128 | | Dental enamel hypoplasia | 2 | 0.0122 | 0.00148 | 8.2 | 12.0 | 0.0219 | | Uterine disease | 2 | 0.0122 | 0.00173 | 7.1 | 9.6 | 0.0299 | | Atherosclerosis | 14 | 0.0854 | 0.05060 | 1.7 | 1.8 | 0.0364 | | Stomach disease | 2 | 0.0122 | 0.00197 | 6.2 | 8.0 | 0.0388 | | Uterine fibroids | 2 | 0.0122 | 0.00197 | 6.2 | 8.0 | 0.0388 | | Female reproductive cancer | 2 | 0.0122 | 0.00222 | 5.5 | 6.8 | 0.0486 | | Spondylarthropathies | 2 | 0.0122 | 0.00222 | 5.5 | 6.8 | 0.0486 | | Capillaries disease | 2 | 0.0122 | 0.00247 | 4.9 | 6.0 | 0.0592 | | Movement disorder | 3 | 0.0183 | 0.00568 | 3.2 | 3.6 | 0.0637 | | Aplastic anemia | 3 | 0.0183 | 0.00568 | 3.2 | 3.6 | 0.0637 | | Pre-Eclampsia | 6 | 0.0366 | 0.01777 | 2.1 | 2.2 | 0.0697 | | Uterine cancer | 1 | 0.0061 | 0.00049 | 12.4 | 23.8 | 0.0793 | | Von Hippel-Lindau syndrome | 1 | 0.0061 | 0.00049 | 12.4 | 23.8 | 0.0793 | | Pancreatitis | 4 | 0.0244 | 0.01012 | 2.4 | 2.6 | 0.0822 | | Spinocerebellar ataxias | 2 | 0.0122 | 0.00296 | 4.1 | 4.8 | 0.0824 | | Alzheimer's disease | 12 | 0.0732 | 0.04764 | 1.5 | 1.6 | 0.0893 | | Mental retardation | 5 | 0.0305 | 0.01481 | 2.1 | 2.2 | 0.0938 | |

---

USER DEFINED THERMS: inflammation, apoptosis, necrosis, bunt, burn [Details: ]

| |  | genes in Category | percent in the observed List | percent in the genome | fold of overrepresents | odds ratio | p value | | --- | --- | --- | --- | --- | --- | --- | | apoptosis | 108 | 0.8372 | 0.05777 | 14 | 87 | 5.0e-112 | | inflammation | 34 | 0.2636 | 0.01712 | 15 | 21 | 1.5e-30 | | necrosis | 19 | 0.1473 | 0.01113 | 13 | 16 | 4.5e-16 | | burn | 1 | 0.0078 | 0.00031 | 25 | 27 | 3.9e-02 | |

---
