# Supplementary material for: MMpred: functional miRNA – mRNA interaction analyses by miRNA expression prediction
Source: BMC Genomics. 2012 Nov 14;13:620. doi: 10.1186/1471-2164-13-620 (PMC3562514; doi:10.1186/1471-2164-13-620)
Supplement: Additional file 3 — Sample pipeline outputs in HTML format (compressed file). [file 1471-2164-13-620-S3.ZIP › Burn_early-late-control/BuntTtestSvsC_Thu-09-09-2010_03-26-46.html]

REPORT


## Report of miRNA-mRNA interactions for all arrays. [generated on 2010-09-09 03:26:46]

---

Statistical testing for messenger RNA arrays: 32141 genes found significantly up-/down-regulated. Details:

| |  | ArrayFile | FunctionalGroup | | --- | --- | --- | | 1 | GSM493655.CEL | burn | | 2 | GSM493656.CEL | burn | | 3 | GSM493657.CEL | burn | | 4 | GSM493658.CEL | burn | | 5 | GSM493659.CEL | burn | | 6 | GSM493660.CEL | burn | | 7 | GSM493661.CEL | burn | | 8 | GSM493662.CEL | burn | | 9 | GSM493663.CEL | burn | | 10 | GSM493664.CEL | burn | | 11 | GSM493665.CEL | burn | | 12 | GSM493666.CEL | burn | | 13 | GSM493667.CEL | burn | | 14 | GSM493668.CEL | burn | | 15 | GSM493669.CEL | burn | | 16 | GSM493670.CEL | burn | | 17 | GSM493671.CEL | burn | | 18 | GSM493672.CEL | burn | | 19 | GSM493673.CEL | burn | | 20 | GSM493674.CEL | burn | | 21 | GSM493675.CEL | burn | | 22 | GSM493676.CEL | burn | | 23 | GSM493677.CEL | burn | | 24 | GSM493678.CEL | burn | | 25 | GSM493679.CEL | burn | | 26 | GSM493680.CEL | burn | | 27 | GSM493681.CEL | burn | | 28 | GSM493682.CEL | burn | | 29 | GSM493683.CEL | burn | | 30 | GSM493684.CEL | burn | | 31 | GSM493685.CEL | burn | | 32 | GSM493686.CEL | burn | | 33 | GSM493687.CEL | burn | | 34 | GSM493688.CEL | burn | | 35 | GSM493689.CEL | burn | | 36 | GSM493690.CEL | burn | | 37 | GSM493691.CEL | burn | | 38 | GSM493692.CEL | burn | | 39 | GSM493693.CEL | burn | | 40 | GSM493694.CEL | burn | | 41 | GSM493695.CEL | burn | | 42 | GSM493696.CEL | burn | | 43 | GSM493697.CEL | burn | | 44 | GSM493698.CEL | burn | | 45 | GSM493699.CEL | burn | | 46 | GSM493700.CEL | burn | | 47 | GSM493701.CEL | burn | | 48 | GSM493702.CEL | burn | | 49 | GSM493703.CEL | burn | | 50 | GSM493704.CEL | burn | | 51 | GSM493705.CEL | burn | | 52 | GSM493706.CEL | burn | | 53 | GSM493707.CEL | burn | | 54 | GSM493708.CEL | burn | | 55 | GSM493709.CEL | burn | | 56 | GSM493710.CEL | burn | | 57 | GSM493711.CEL | burn | | 58 | GSM493712.CEL | burn | | 59 | GSM493713.CEL | burn | | 60 | GSM493714.CEL | burn | | 61 | GSM493715.CEL | burn | | 62 | GSM493716.CEL | burn | | 63 | GSM493717.CEL | burn | | 64 | GSM493718.CEL | burn | | 65 | GSM493719.CEL | burn | | 66 | GSM493720.CEL | burn | | 67 | GSM493721.CEL | burn | | 68 | GSM493722.CEL | burn | | 69 | GSM493723.CEL | burn | | 70 | GSM493724.CEL | burn | | 71 | GSM493725.CEL | burn | | 72 | GSM493726.CEL | burn | | 73 | GSM493727.CEL | burn | | 74 | GSM493728.CEL | burn | | 75 | GSM493729.CEL | burn | | 76 | GSM493730.CEL | burn | | 77 | GSM493731.CEL | burn | | 78 | GSM493732.CEL | burn | | 79 | GSM493733.CEL | burn | | 80 | GSM493734.CEL | burn | | 81 | GSM493735.CEL | burn | | 82 | GSM493736.CEL | burn | | 83 | GSM493737.CEL | burn | | 84 | GSM493738.CEL | burn | | 85 | GSM493739.CEL | burn | | 86 | GSM493740.CEL | burn | | 87 | GSM493741.CEL | burn | | 88 | GSM493742.CEL | burn | | 89 | GSM493743.CEL | burn | | 90 | GSM493744.CEL | burn | | 91 | GSM493745.CEL | burn | | 92 | GSM493746.CEL | burn | | 93 | GSM493747.CEL | burn | | 94 | GSM493748.CEL | burn | | 95 | GSM493749.CEL | burn | | 96 | GSM493750.CEL | burn | | 97 | GSM493751.CEL | burn | | 98 | GSM493752.CEL | burn | | 99 | GSM493753.CEL | burn | | 100 | GSM493754.CEL | burn | | 101 | GSM493755.CEL | burn | | 102 | GSM493756.CEL | burn | | 103 | GSM493757.CEL | burn | | 104 | GSM493758.CEL | burn | | 105 | GSM493759.CEL | burn | | 106 | GSM493760.CEL | burn | | 107 | GSM493761.CEL | burn | | 108 | GSM493762.CEL | burn | | 109 | GSM493763.CEL | burn | | 110 | GSM493764.CEL | burn | | 111 | GSM493765.CEL | burn | | 112 | GSM493766.CEL | burn | | 113 | GSM493767.CEL | burn | | 114 | GSM493768.CEL | burn | | 115 | GSM493769.CEL | control | | 116 | GSM493770.CEL | control | | 117 | GSM493771.CEL | control | | 118 | GSM493772.CEL | control | | 119 | GSM493773.CEL | control | | 120 | GSM493774.CEL | control | | 121 | GSM493775.CEL | control | | 122 | GSM493776.CEL | control | | 123 | GSM493777.CEL | control | | 124 | GSM493778.CEL | control | | 125 | GSM493779.CEL | control | | 126 | GSM493780.CEL | control | | 127 | GSM493781.CEL | control | | 128 | GSM493782.CEL | control | | 129 | GSM493783.CEL | control | | 130 | GSM493784.CEL | control | | 131 | GSM493785.CEL | control | | 132 | GSM493786.CEL | control | | 133 | GSM493787.CEL | control | | 134 | GSM493788.CEL | control | | 135 | GSM493789.CEL | control | | 136 | GSM493790.CEL | control | | 137 | GSM493791.CEL | control | | 138 | GSM493792.CEL | control | | 139 | GSM493793.CEL | control | | 140 | GSM493794.CEL | control | | 141 | GSM493795.CEL | control | | 142 | GSM493796.CEL | control | | 143 | GSM493797.CEL | control | | 144 | GSM493798.CEL | control | | 145 | GSM493799.CEL | control | | 146 | GSM493800.CEL | control | | 147 | GSM493801.CEL | control | | 148 | GSM493802.CEL | control | | 149 | GSM493803.CEL | control | | 150 | GSM493804.CEL | control | | 151 | GSM493805.CEL | control | | 152 | GSM493806.CEL | control | | 153 | GSM493807.CEL | control | | 154 | GSM493808.CEL | control | | 155 | GSM493809.CEL | control | | 156 | GSM493810.CEL | control | | 157 | GSM493811.CEL | control | | 158 | GSM493812.CEL | control | | 159 | GSM493813.CEL | control | | 160 | GSM493814.CEL | control | | 161 | GSM493815.CEL | control | | 162 | GSM493816.CEL | control | | 163 | GSM493817.CEL | control | | 164 | GSM493818.CEL | control | | 165 | GSM493819.CEL | control | | 166 | GSM493820.CEL | control | | 167 | GSM493821.CEL | control | | 168 | GSM493822.CEL | control | | 169 | GSM493823.CEL | control | | 170 | GSM493824.CEL | control | | 171 | GSM493825.CEL | control | | 172 | GSM493826.CEL | control | | 173 | GSM493827.CEL | control | | 174 | GSM493828.CEL | control | | 175 | GSM493829.CEL | control | | 176 | GSM493830.CEL | control | | 177 | GSM493831.CEL | control | |

  

Principal Component Analyses:

Heatmap for top 50 geneses from statistical analyses (ordered by p-value):

Volcano plot with for auto cut-off calculation audit (cut-off shown with red line):

---

Statistical testing for microRNA prediction method I - scaling function: 105 genes found significantly up-/down-regulated. Details:

Heatmap for top 50 geneses from statistical analyses (ordered by p-value):

Volcano plot with for auto cut-off calculation audit (cut-off shown with red line):

---

Statistical testing for microRNA prediction method II - linear modelling: 156 genes found significantly up-/down-regulated. Details:

Principal Component Analysis:

Heatmap for top 50 geneses from statistical analyses (ordered by p-value):

Volcano plot with for auto cut-off calculation audit (cut-off shown with red line):

---

Mean anti-correlation detected between mRNA and miRNA = -0.318962. Details:

Histogram of most anti-correlated miRNA-mRNA pairs - potential miRNA-target interactions:

---

Total number of 63 miRNAs are predicted to have significantly up-/down-regulated targets. Expend:

| |  | microRNA | NoSuppresedGenes | | --- | --- | --- | | 1 | hsa-miR-1233 | 355 | | 2 | hsa-miR-95 | 291 | | 3 | hsa-miR-766 | 275 | | 4 | hsa-miR-571 | 259 | | 5 | hsa-miR-874 | 239 | | 6 | hsa-miR-10a | 214 | | 7 | hsa-miR-558 | 195 | | 8 | hsa-miR-423-5p | 187 | | 9 | hsa-mir-10a | 131 | | 10 | hsa-miR-877 | 127 | | 11 | hsa-miR-569 | 119 | | 12 | hsa-miR-555 | 93 | | 13 | hsa-miR-623 | 91 | | 14 | hsa-miR-618 | 87 | | 15 | hsa-mir-3130-1 | 84 | | 16 | hsa-mir-3130-2 | 84 | | 17 | hsa-mir-3130-3 | 84 | | 18 | hsa-mir-3130-4 | 84 | | 19 | hsa-miR-576-5p | 82 | | 20 | hsa-miR-454\* | 49 | | 21 | hsa-miR-938 | 49 | | 22 | hsa-miR-593\* | 35 | | 23 | hsa-miR-580 | 31 | | 24 | hsa-miR-499-5p | 28 | | 25 | hsa-miR-608 | 28 | | 26 | hsa-mir-505 | 16 | | 27 | hsa-miR-505\* | 16 | | 28 | hsa-miR-128 | 14 | | 29 | hsa-mir-199a-2 | 13 | | 30 | hsa-mir-214 | 13 | | 31 | hsa-mir-149 | 11 | | 32 | hsa-miR-149 | 11 | | 33 | hsa-miR-1224-5p | 10 | | 34 | hsa-mir-885 | 10 | | 35 | hsa-miR-885-5p | 10 | | 36 | hsa-miR-503 | 7 | | 37 | hsa-mir-628 | 7 | | 38 | hsa-miR-628-5p | 7 | | 39 | hsa-miR-643 | 7 | | 40 | hsa-miR-135a | 6 | | 41 | hsa-mir-135b | 5 | | 42 | hsa-mir-576 | 5 | | 43 | hsa-miR-126\* | 4 | | 44 | hsa-miR-1236 | 3 | | 45 | hsa-mir-499 | 3 | | 46 | hsa-miR-586 | 3 | | 47 | hsa-miR-1231 | 1 | | 48 | hsa-mir-155 | 1 | | 49 | hsa-mir-15b | 1 | | 50 | hsa-mir-16-2 | 1 | | 51 | hsa-mir-186 | 1 | | 52 | hsa-miR-186 | 1 | | 53 | hsa-miR-301a | 1 | | 54 | hsa-mir-342 | 1 | | 55 | hsa-miR-449b | 1 | | 56 | hsa-mir-454 | 1 | | 57 | hsa-miR-554 | 1 | | 58 | hsa-mir-556 | 1 | | 59 | hsa-miR-556-5p | 1 | | 60 | hsa-miR-567 | 1 | | 61 | hsa-miR-619 | 1 | | 62 | hsa-miR-626 | 1 | | 63 | hsa-miR-675 | 1 | |

---

Total number of 1351 genes are predicted to be under differential miRNA repression. Expend:

| |  | GenSymbols | GeneName | NoTargetingMicroRNA | | --- | --- | --- | --- | | 1 | ATP6V0A2 | ATPase, H+ transporting, lysosomal V0 subunit a2 | 11 | | 2 | C20orf181 | chromosome 20 open reading frame 181 | 11 | | 3 | CAND1 | cullin-associated and neddylation-dissociated 1 | 11 | | 4 | RALGAPA1 | Ral GTPase activating protein, alpha subunit 1 (catalytic) | 11 | | 5 | SMYD2 | SET and MYND domain containing 2 | 11 | | 6 | CREBZF | CREB/ATF bZIP transcription factor | 10 | | 7 | LRCH3 | leucine-rich repeats and calponin homology (CH) domain containing 3 | 10 | | 8 | LRTM2 | leucine-rich repeats and transmembrane domains 2 | 10 | | 9 | SNCG | synuclein, gamma (breast cancer-specific protein 1) | 10 | | 10 | C6orf130 | chromosome 6 open reading frame 130 | 9 | | 11 | KIF1C | kinesin family member 1C | 9 | | 12 | OPRL1 | opiate receptor-like 1 | 9 | | 13 | PLIN4 | PC4 and SFRS1 interacting protein 1 | 9 | | 14 | PSIP1 | perilipin 4 | 9 | | 15 | WIZ | widely interspaced zinc finger motifs | 9 | | 16 | ABLIM2 | actin binding LIM protein family, member 2 | 8 | | 17 | CAMK2B | calcium/calmodulin-dependent protein kinase II beta | 8 | | 18 | FUBP1 | far upstream element (FUSE) binding protein 1 | 8 | | 19 | GLI4 | GLI family zinc finger 4 | 8 | | 20 | NELF | nasal embryonic LHRH factor | 8 | | 21 | NF2 | neurofibromin 2 (merlin) | 8 | | 22 | PALM3 | Paralemmin-3 | 8 | | 23 | SLURP1 | secreted LY6/PLAUR domain containing 1 | 8 | | 24 | SYNRG | synergin, gamma | 8 | | 25 | TRNP1 | tetratricopeptide repeat domain 37 | 8 | | 26 | TTC37 | TMF1-regulated nuclear protein 1 | 8 | | 27 | TXK | TXK tyrosine kinase | 8 | | 28 | ZNF227 | zinc finger protein 227 | 8 | | 29 | ADAMTSL3 | ADAMTS-like 3 | 7 | | 30 | C1orf95 | CCR4-NOT transcription complex, subunit 7 | 7 | | 31 | C2orf64 | chemokine (C-C motif) ligand 24 | 7 | | 32 | C5orf28 | chromosome 1 open reading frame 95 | 7 | | 33 | CCDC57 | chromosome 2 open reading frame 64 | 7 | | 34 | CCL24 | chromosome 5 open reading frame 28 | 7 | | 35 | CLASP2 | coiled-coil domain containing 57 | 7 | | 36 | CNOT7 | cytoplasmic linker associated protein 2 | 7 | | 37 | ESR1 | estrogen receptor 1 | 7 | | 38 | HPS6 | Hermansky-Pudlak syndrome 6 | 7 | | 39 | IPO5 | importin 5 | 7 | | 40 | LAMA3 | laminin, alpha 3 | 7 | | 41 | MFGE8 | milk fat globule-EGF factor 8 protein | 7 | | 42 | MORN1 | MORN repeat containing 1 | 7 | | 43 | OR7E104P | olfactory receptor, family 7, subfamily E, member 104 pseudogene | 7 | | 44 | PCYT1B | Pentatricopeptide repeat domain 3 | 7 | | 45 | PTCD3 | phosphate cytidylyltransferase 1, choline, beta | 7 | | 46 | RAD1 | RAD1 homolog (S. pombe) | 7 | | 47 | REXO1 | REX1, RNA exonuclease 1 homolog (S. cerevisiae) | 7 | | 48 | RPL13 | ribosomal protein L13 | 7 | | 49 | SFRS13A | solute carrier family 22 (organic anion transporter), member 7 | 7 | | 50 | SLC22A7 | splicing factor, arginine/serine-rich 13A | 7 | | 51 | TM9SF3 | transmembrane 9 superfamily member 3 | 7 | | 52 | WFDC2 | WAP four-disulfide core domain 2 | 7 | | 53 | ZNF567 | zinc finger protein 567 | 7 | | 54 | ZNF639 | zinc finger protein 639 | 7 | | 55 | AKAP7 | A kinase (PRKA) anchor protein 7 | 6 | | 56 | AKNA | angel homolog 2 (Drosophila) | 6 | | 57 | ANGEL2 | ankylosis, progressive homolog (mouse) | 6 | | 58 | ANKH | AT-hook transcription factor | 6 | | 59 | BCAM | basal cell adhesion molecule (Lutheran blood group) | 6 | | 60 | BRWD1 | bromodomain and WD repeat domain containing 1 | 6 | | 61 | C19orf12 | caldesmon 1 | 6 | | 62 | C6orf48 | cholinergic receptor, nicotinic, beta 2 (neuronal) | 6 | | 63 | CALD1 | chromosome 19 open reading frame 12 | 6 | | 64 | CCDC66 | chromosome 6 open reading frame 48 | 6 | | 65 | CCNT2 | coiled-coil domain containing 66 | 6 | | 66 | CHIC1 | cyclin T2 | 6 | | 67 | CHRNB2 | cysteine-rich hydrophobic domain 1 | 6 | | 68 | DDX18 | DEAD (Asp-Glu-Ala-Asp) box polypeptide 18 | 6 | | 69 | DENND4C | DENN/MADD domain containing 4C | 6 | | 70 | DKFZp761P0212 | dual-specificity tyrosine-(Y)-phosphorylation regulated kinase 2 | 6 | | 71 | DNAH7 | dynein, axonemal, heavy chain 7 | 6 | | 72 | DYRK2 | eukaryotic translation initiation factor 3, subunit M | 6 | | 73 | EIF3M | FAST kinase domains 2 | 6 | | 74 | FASTKD2 | G2/M-phase specific E3 ubiquitin ligase | 6 | | 75 | G2E3 | HAUS augmin-like complex, subunit 6 | 6 | | 76 | HAUS6 | heparin-binding EGF-like growth factor | 6 | | 77 | HBEGF | heterogeneous nuclear ribonucleoprotein A1 | 6 | | 78 | HNRNPA1 | heterogeneous nuclear ribonucleoprotein D-like | 6 | | 79 | HNRPDL | hypothetical LOC100133461 | 6 | | 80 | IGF1 | hypothetical protein DKFZp761P0212 | 6 | | 81 | KIF3A | insulin-like growth factor 1 (somatomedin C) | 6 | | 82 | LCORL | kinesin family member 3A | 6 | | 83 | LOC100133461 | ligand dependent nuclear receptor corepressor-like | 6 | | 84 | LOC100293596 | matrix metallopeptidase 11 (stromelysin 3) | 6 | | 85 | MAPK8IP3 | mitogen-activated protein kinase 8 interacting protein 3 | 6 | | 86 | MGC34796 | monocyte to macrophage differentiation-associated 2 | 6 | | 87 | MMD2 | natural cytotoxicity triggering receptor 2 | 6 | | 88 | MMP11 | NLR family, CARD domain containing 3 | 6 | | 89 | NCR2 | nuclear protein, transcriptional regulator, 1 | 6 | | 90 | NLRC3 | nucleoporin 133kDa | 6 | | 91 | NUP133 | OCIA domain containing 2 | 6 | | 92 | NUPR1 | PAP associated domain containing 5 | 6 | | 93 | OCIAD2 | podocan | 6 | | 94 | PAPD5 | prominin 2 | 6 | | 95 | PODN | PWWP domain containing 2A | 6 | | 96 | PROM2 | Rap guanine nucleotide exchange factor (GEF) 3 | 6 | | 97 | PWWP2A | regulatory factor X, 5 (influences HLA class II expression) | 6 | | 98 | RAPGEF3 | retinoic acid receptor, alpha | 6 | | 99 | RARA | ribonuclease H1 | 6 | | 100 | RFX5 | ring finger protein 125 | 6 | | 101 | RNASEH1 | ring finger protein 219 | 6 | | 102 | RNF125 | sex comb on midleg-like 4 (Drosophila) | 6 | | 103 | RNF219 | similar to mucin | 6 | | 104 | SAMM50 | small nuclear ribonucleoprotein polypeptide A' | 6 | | 105 | SCML4 | small nucleolar RNA host gene 12 (non-protein coding) | 6 | | 106 | SFRS12 | small nucleolar RNA host gene 8 (non-protein coding) | 6 | | 107 | SLC26A2 | solute carrier family 26 (sulfate transporter), member 2 | 6 | | 108 | SLC4A9 | solute carrier family 4, sodium bicarbonate cotransporter, member 9 | 6 | | 109 | SMARCA4 | sorting and assembly machinery component 50 homolog (S. cerevisiae) | 6 | | 110 | SMOC1 | SPARC related modular calcium binding 1 | 6 | | 111 | SNHG12 | spastic paraplegia 7 (pure and complicated autosomal recessive) | 6 | | 112 | SNHG8 | splicing factor, arginine/serine-rich 12 | 6 | | 113 | SNRPA1 | SPR pseudogene | 6 | | 114 | SPG7 | SWI/SNF related, matrix associated, actin dependent regulator of chromatin, subfamily a, member 4 | 6 | | 115 | TBC1D29 | tankyrase, TRF1-interacting ankyrin-related ADP-ribose polymerase | 6 | | 116 | TBXA2R | TBC1 domain family, member 29 | 6 | | 117 | TMPRSS6 | thromboxane A2 receptor | 6 | | 118 | TNKS | trafficking protein particle complex 9 | 6 | | 119 | TPPP | transmembrane protease, serine 6 | 6 | | 120 | TRAPPC9 | tubulin polymerization promoting protein | 6 | | 121 | WDR67 | WD repeat domain 67 | 6 | | 122 | WDR75 | WD repeat domain 75 | 6 | | 123 | ZCCHC11 | zinc finger protein 167 | 6 | | 124 | ZFAND1 | zinc finger protein 195 | 6 | | 125 | ZFP90 | zinc finger protein 226 | 6 | | 126 | ZNF167 | zinc finger protein 432 | 6 | | 127 | ZNF195 | zinc finger protein 45 | 6 | | 128 | ZNF226 | zinc finger protein 90 homolog (mouse) | 6 | | 129 | ZNF432 | zinc finger, AN1-type domain 1 | 6 | | 130 | ZNF45 | zinc finger, CCHC domain containing 11 | 6 | | 131 | ABCB1 | 2-aminoethanethiol (cysteamine) dioxygenase | 5 | | 132 | ACADSB | 3-hydroxybutyrate dehydrogenase, type 2 | 5 | | 133 | ADO | acyl-Coenzyme A dehydrogenase, short/branched chain | 5 | | 134 | ADSL | adenylosuccinate lyase | 5 | | 135 | BBS2 | ATP-binding cassette, sub-family B (MDR/TAP), member 1 | 5 | | 136 | BDH2 | Bardet-Biedl syndrome 2 | 5 | | 137 | BTF3L4 | basic transcription factor 3-like 4 | 5 | | 138 | C10orf18 | caprin family member 2 | 5 | | 139 | C16orf80 | casein kinase 1, gamma 3 | 5 | | 140 | C3orf17 | CD47 molecule | 5 | | 141 | CAPRIN2 | CD6 molecule | 5 | | 142 | CCDC55 | CD96 molecule | 5 | | 143 | CD47 | chromodomain protein, Y-like 2 | 5 | | 144 | CD6 | chromosome 10 open reading frame 18 | 5 | | 145 | CD96 | chromosome 16 open reading frame 80 | 5 | | 146 | CDYL2 | chromosome 3 open reading frame 17 | 5 | | 147 | COG6 | coiled-coil domain containing 55 | 5 | | 148 | CSNK1G3 | component of oligomeric golgi complex 6 | 5 | | 149 | CWF19L2 | CWF19-like 2, cell cycle control (S. pombe) | 5 | | 150 | DBF4B | DBF4 homolog B (S. cerevisiae) | 5 | | 151 | DCAF16 | DDB1 and CUL4 associated factor 16 | 5 | | 152 | DCAF17 | DDB1 and CUL4 associated factor 17 | 5 | | 153 | DCBLD1 | DEAD (Asp-Glu-Ala-As) box polypeptide 19A | 5 | | 154 | DDX19A | DEAH (Asp-Glu-Ala-His) box polypeptide 30 | 5 | | 155 | DHX30 | dedicator of cytokinesis 9 | 5 | | 156 | DNAJC21 | discoidin, CUB and LCCL domain containing 1 | 5 | | 157 | DNMT1 | DNA (cytosine-5-)-methyltransferase 1 | 5 | | 158 | DOCK9 | DnaJ (Hsp40) homolog, subfamily C, member 21 | 5 | | 159 | EIF5B | elongation protein 2 homolog (S. cerevisiae) | 5 | | 160 | ELP2 | enolase superfamily member 1 | 5 | | 161 | ENOSF1 | eukaryotic translation initiation factor 5B | 5 | | 162 | FAM169A | family with sequence similarity 169, member A | 5 | | 163 | FAM85A | family with sequence similarity 85, member A | 5 | | 164 | FOXO1 | forkhead box O1 | 5 | | 165 | GOLGA8A | G protein-coupled receptor associated sorting protein 1 | 5 | | 166 | GPRASP1 | golgin A8 family, member A | 5 | | 167 | HNRNPA3 | heterogeneous nuclear ribonucleoprotein A3 | 5 | | 168 | HNRNPR | heterogeneous nuclear ribonucleoprotein R | 5 | | 169 | HTATSF1 | HIV-1 Tat specific factor 1 | 5 | | 170 | IDH3B | hypothetical LOC550643 | 5 | | 171 | KIAA1683 | hypothetical protein LOC100132987 | 5 | | 172 | KIF25 | hypothetical protein LOC100291944 | 5 | | 173 | KPNA5 | hypothetical protein LOC647107 | 5 | | 174 | LBH | isocitrate dehydrogenase 3 (NAD+) beta | 5 | | 175 | LDHB | karyopherin alpha 5 (importin alpha 6) | 5 | | 176 | LOC100132987 | KIAA1683 | 5 | | 177 | LOC100291944 | kinesin family member 25 | 5 | | 178 | LOC550643 | lactate dehydrogenase B | 5 | | 179 | LOC646808 | limb bud and heart development homolog (mouse) | 5 | | 180 | LOC647107 | MDN1, midasin homolog (yeast) | 5 | | 181 | MAPK8IP1 | mitogen-activated protein kinase 8 interacting protein 1 | 5 | | 182 | MDN1 | MTERF domain containing 2 | 5 | | 183 | MTERFD2 | N(alpha)-acetyltransferase 35, NatC auxiliary subunit | 5 | | 184 | NAA35 | neurotrimin | 5 | | 185 | NCBP2 | nuclear cap binding protein subunit 2, 20kDa | 5 | | 186 | NTM | nucleoporin 35kDa | 5 | | 187 | NUP35 | phosphoinositide-3-kinase, class 2, beta polypeptide | 5 | | 188 | PATZ1 | pleckstrin homology domain containing, family A (phosphoinositide binding specific) member 1 | 5 | | 189 | PDCD5 | polyhomeotic homolog 3 (Drosophila) | 5 | | 190 | PHC3 | polymerase (RNA) I polypeptide C, 30kDa | 5 | | 191 | PIK3C2B | POZ (BTB) and AT hook containing zinc finger 1 | 5 | | 192 | PLEKHA1 | programmed cell death 5 | 5 | | 193 | POLR1C | proteasome (prosome, macropain) activator subunit 4 | 5 | | 194 | PRKCQ | proteasome (prosome, macropain) assembly chaperone 4 | 5 | | 195 | PSME4 | protein kinase C, theta | 5 | | 196 | PSMG4 | RB-associated KRAB zinc finger | 5 | | 197 | RBAK | regulatory factor X, 7 | 5 | | 198 | RFX7 | SET nuclear oncogene | 5 | | 199 | SACS | Sfi1 homolog, spindle assembly associated (yeast) | 5 | | 200 | SET | similar to L antigen family, member 3 | 5 | | 201 | SFI1 | solute carrier family 22 (organic anion transporter), member 8 | 5 | | 202 | SFRS15 | solute carrier family 7 (cationic amino acid transporter, y+ system), member 6 | 5 | | 203 | SLC22A8 | spastic ataxia of Charlevoix-Saguenay (sacsin) | 5 | | 204 | SLC7A6 | splicing factor, arginine/serine-rich 15 | 5 | | 205 | SUPV3L1 | suppressor of var1, 3-like 1 (S. cerevisiae) | 5 | | 206 | TARSL2 | T cell receptor delta locus | 5 | | 207 | THADA | thioredoxin domain containing 16 | 5 | | 208 | TM6SF2 | threonyl-tRNA synthetase-like 2 | 5 | | 209 | TMEM106B | thyroid adenoma associated | 5 | | 210 | TNFRSF25 | translocase of outer mitochondrial membrane 70 homolog A (S. cerevisiae) | 5 | | 211 | TOMM70A | transmembrane 6 superfamily member 2 | 5 | | 212 | TRD@ | transmembrane protein 106B | 5 | | 213 | TRIM8 | tripartite motif-containing 8 | 5 | | 214 | TXNDC16 | tumor necrosis factor receptor superfamily, member 25 | 5 | | 215 | UBE2I | ubiquitin-conjugating enzyme E2I (UBC9 homolog, yeast) | 5 | | 216 | USPL1 | ubiquitin specific peptidase like 1 | 5 | | 217 | ZBTB10 | zinc finger and BTB domain containing 10 | 5 | | 218 | ZNF512 | zinc finger protein 512 | 5 | | 219 | ZNF544 | zinc finger protein 544 | 5 | | 220 | ZNF600 | zinc finger protein 600 | 5 | | 221 | ZNF827 | zinc finger protein 827 | 5 | | 222 | ZNF83 | zinc finger protein 83 | 5 | | 223 | AASDH | 2-hydroxyacyl-CoA lyase 1 | 4 | | 224 | ABLIM1 | 2-oxoglutarate and iron-dependent oxygenase domain containing 1 | 4 | | 225 | ADARB1 | A kinase (PRKA) anchor protein 8 | 4 | | 226 | ADNP | actin binding LIM protein 1 | 4 | | 227 | AGAP4 | activity-dependent neuroprotector homeobox | 4 | | 228 | AIMP1 | adenosine deaminase, RNA-specific, B1 (RED1 homolog rat) | 4 | | 229 | AKAP8 | aminoacyl tRNA synthetase complex-interacting multifunctional protein 1 | 4 | | 230 | ANKRD10 | aminoadipate-semialdehyde dehydrogenase | 4 | | 231 | ARHGAP5 | ankyrin repeat domain 10 | 4 | | 232 | ATP11B | ArfGAP with GTPase domain, ankyrin repeat and PH domain 4 | 4 | | 233 | C12orf23 | ATPase, class VI, type 11B | 4 | | 234 | C14orf64 | C2 calcium-dependent domain containing 2 | 4 | | 235 | C2CD2 | cAMP responsive element binding protein 3-like 3 | 4 | | 236 | C8orf54 | CD3g molecule, gamma (CD3-TCR complex) | 4 | | 237 | C9orf41 | CD79b molecule, immunoglobulin-associated beta | 4 | | 238 | C9orf5 | centrosomal protein 290kDa | 4 | | 239 | CCDC50 | chloride channel Kb | 4 | | 240 | CCND2 | choroideremia-like (Rab escort protein 2) | 4 | | 241 | CCNL2 | chromosome 12 open reading frame 23 | 4 | | 242 | CD3G | chromosome 14 open reading frame 64 | 4 | | 243 | CD79B | chromosome 8 open reading frame 54 | 4 | | 244 | CEP290 | chromosome 9 open reading frame 41 | 4 | | 245 | CHML | chromosome 9 open reading frame 5 | 4 | | 246 | CLCNKB | CKLF-like MARVEL transmembrane domain containing 4 | 4 | | 247 | CMTM4 | coiled-coil domain containing 50 | 4 | | 248 | COL9A1 | collagen, type IX, alpha 1 | 4 | | 249 | COX18 | COX18 cytochrome c oxidase assembly homolog (S. cerevisiae) | 4 | | 250 | COX7C | cullin 2 | 4 | | 251 | CREB3L3 | cyclin D2 | 4 | | 252 | CUL2 | cyclin L2 | 4 | | 253 | DGCR7 | cytochrome c oxidase subunit VIIc | 4 | | 254 | DHX40 | DEAH (Asp-Glu-Ala-His) box polypeptide 40 | 4 | | 255 | ECM1 | DiGeorge syndrome critical region gene 7 | 4 | | 256 | EEF1A1 | eomesodermin homolog (Xenopus laevis) | 4 | | 257 | EOMES | eukaryotic translation elongation factor 1 alpha 1 | 4 | | 258 | ERCC5 | Ewing tumor-associated antigen 1 | 4 | | 259 | ESYT2 | excision repair cross-complementing rodent repair deficiency, complementation group 5 | 4 | | 260 | ETAA1 | extended synaptotagmin-like protein 2 | 4 | | 261 | FAM179B | extracellular matrix protein 1 | 4 | | 262 | FAM76B | family with sequence similarity 179, member B | 4 | | 263 | FOLR2 | family with sequence similarity 76, member B | 4 | | 264 | FTSJD1 | folate receptor 2 (fetal) | 4 | | 265 | FUNDC2 | fracture callus 1 homolog (rat) | 4 | | 266 | FXC1 | FtsJ methyltransferase domain containing 1 | 4 | | 267 | GALNT12 | FUN14 domain containing 2 | 4 | | 268 | GIMAP1 | G-rich RNA sequence binding factor 1 | 4 | | 269 | GOLT1B | glycoprotein hormone beta 5 | 4 | | 270 | GORASP2 | golgi reassembly stacking protein 2, 55kDa | 4 | | 271 | GPHB5 | golgi transport 1 homolog B (S. cerevisiae) | 4 | | 272 | GRSF1 | GTP binding protein 1 | 4 | | 273 | GTPBP1 | GTPase, IMAP family member 1 | 4 | | 274 | HACL1 | HEG homolog 1 (zebrafish) | 4 | | 275 | HDAC10 | helicase, POLQ-like | 4 | | 276 | HEG1 | histamine receptor H2 | 4 | | 277 | HELQ | histone deacetylase 10 | 4 | | 278 | HRASLS2 | HRAS-like suppressor 2 | 4 | | 279 | HRH2 | IKAROS family zinc finger 5 (Pegasus) | 4 | | 280 | IKZF5 | InaD-like (Drosophila) | 4 | | 281 | IL11RA | integral membrane protein 2A | 4 | | 282 | ILF3 | interleukin 11 receptor, alpha | 4 | | 283 | INADL | interleukin enhancer binding factor 3, 90kDa | 4 | | 284 | ITM2A | KIAA1826 | 4 | | 285 | KIAA1826 | killer cell lectin-like receptor subfamily D, member 1 | 4 | | 286 | KLRD1 | Leo1, Paf1/RNA polymerase II complex component, homolog (S. cerevisiae) | 4 | | 287 | LEO1 | lon peptidase 1, mitochondrial | 4 | | 288 | LONP1 | LUC7-like (S. cerevisiae) | 4 | | 289 | LUC7L | lymphocyte antigen 6 complex, locus D | 4 | | 290 | LY6D | mediator complex subunit 6 | 4 | | 291 | MAGEH1 | melanoma antigen family H, 1 | 4 | | 292 | MBLAC2 | membrane-bound transcription factor peptidase, site 1 | 4 | | 293 | MBTPS1 | mesoderm induction early response 1, family member 3 | 4 | | 294 | MED6 | metallo-beta-lactamase domain containing 2 | 4 | | 295 | MIB1 | mindbomb homolog 1 (Drosophila) | 4 | | 296 | MIER3 | mitochondrial ribosomal protein S18B | 4 | | 297 | MRPS18B | mitochondrial translation optimization 1 homolog (S. cerevisiae) | 4 | | 298 | MSI2 | musashi homolog 2 (Drosophila) | 4 | | 299 | MTO1 | NGNL6975 | 4 | | 300 | NFIX | nuclear factor I/X (CCAAT-binding transcription factor) | 4 | | 301 | NUP214 | nuclear VCP-like | 4 | | 302 | NUP43 | nucleoporin 214kDa | 4 | | 303 | NUP54 | nucleoporin 43kDa | 4 | | 304 | NVL | nucleoporin 54kDa | 4 | | 305 | OFD1 | oral-facial-digital syndrome 1 | 4 | | 306 | OGFOD1 | OTU domain containing 4 | 4 | | 307 | OTUD4 | OTU domain containing 6B | 4 | | 308 | OTUD6B | pappalysin 2 | 4 | | 309 | P2RY10 | peptidylprolyl isomerase (cyclophilin)-like 4 | 4 | | 310 | PAPPA2 | peroxisomal biogenesis factor 3 | 4 | | 311 | PCMTD2 | postmeiotic segregation increased 2-like 1 pseudogene | 4 | | 312 | PEX3 | protein-kinase, interferon-inducible double stranded RNA dependent inhibitor, repressor of (P58 repressor) | 4 | | 313 | PMS2L1 | protein-L-isoaspartate (D-aspartate) O-methyltransferase domain containing 2 | 4 | | 314 | PPIL4 | protein phosphatase 1, regulatory (inhibitor) subunit 3E | 4 | | 315 | PPP1R3E | PRP4 pre-mRNA processing factor 4 homolog B (yeast) | 4 | | 316 | PRKRIR | purinergic receptor P2Y, G-protein coupled, 10 | 4 | | 317 | PRPF4B | RAD50 interactor 1 | 4 | | 318 | RANGRF | RAN guanine nucleotide release factor | 4 | | 319 | RCC2 | regulator of chromosome condensation 2 | 4 | | 320 | RDH14 | retinol dehydrogenase 14 (all-trans/9-cis/11-cis) | 4 | | 321 | RINT1 | Rho GTPase activating protein 5 | 4 | | 322 | RNASEN | ribonuclease type III, nuclear | 4 | | 323 | RPL7A | ribosomal protein L7a | 4 | | 324 | RRP15 | ribosomal RNA processing 15 homolog (S. cerevisiae) | 4 | | 325 | SCML1 | sex comb on midleg-like 1 (Drosophila) | 4 | | 326 | SFPQ | SMEK homolog 2, suppressor of mek1 (Dictyostelium) | 4 | | 327 | SFRS18 | solute carrier family 30 (zinc transporter), member 7 | 4 | | 328 | SLC30A7 | splA/ryanodine receptor domain and SOCS box containing 4 | 4 | | 329 | SMEK2 | splicing factor proline/glutamine-rich (polypyrimidine tract binding protein associated) | 4 | | 330 | SPSB4 | splicing factor, arginine/serine-rich 18 | 4 | | 331 | SYTL2 | synaptotagmin-like 2 | 4 | | 332 | TARDBP | TAR DNA binding protein | 4 | | 333 | TASP1 | taspase, threonine aspartase, 1 | 4 | | 334 | TBRG1 | tetratricopeptide repeat domain 27 | 4 | | 335 | TGFBR3 | thiosulfate sulfurtransferase (rhodanese)-like domain containing 2 | 4 | | 336 | TMEM105 | transforming growth factor beta regulator 1 | 4 | | 337 | TMEM204 | transforming growth factor, beta receptor III | 4 | | 338 | TOMM22 | translocase of outer mitochondrial membrane 22 homolog (yeast) | 4 | | 339 | TSTD2 | transmembrane protein 105 | 4 | | 340 | TTC27 | transmembrane protein 204 | 4 | | 341 | UBASH3A | ubiquitin-fold modifier 1 | 4 | | 342 | UFM1 | ubiquitin associated and SH3 domain containing, A | 4 | | 343 | UNQ6975 | ubiquitin specific peptidase 16 | 4 | | 344 | USP16 | ubiquitin specific peptidase 24 | 4 | | 345 | USP24 | ubiquitin specific peptidase 28 | 4 | | 346 | USP28 | ubiquitin specific peptidase 8 | 4 | | 347 | USP8 | UDP-N-acetyl-alpha-D-galactosamine:polypeptide N-acetylgalactosaminyltransferase 12 (GalNAc-T12) | 4 | | 348 | WBP11 | WNK lysine deficient protein kinase 1 | 4 | | 349 | WNK1 | WW domain binding protein 11 | 4 | | 350 | ZBTB25 | zinc finger and BTB domain containing 25 | 4 | | 351 | ZC3H8 | zinc finger CCCH-type containing 8 | 4 | | 352 | ZFP62 | zinc finger family member 673 | 4 | | 353 | ZNF224 | zinc finger family member 783 | 4 | | 354 | ZNF302 | zinc finger protein 224 | 4 | | 355 | ZNF304 | zinc finger protein 302 | 4 | | 356 | ZNF430 | zinc finger protein 304 | 4 | | 357 | ZNF550 | zinc finger protein 430 | 4 | | 358 | ZNF57 | zinc finger protein 550 | 4 | | 359 | ZNF673 | zinc finger protein 57 | 4 | | 360 | ZNF783 | zinc finger protein 62 homolog (mouse) | 4 | | 361 | ZNF84 | zinc finger protein 84 | 4 | | 362 | ZXDB | zinc finger, X-linked, duplicated B | 4 | | 363 | ADAMTSL1 | 3-ketodihydrosphingosine reductase | 3 | | 364 | AKR7A2 | 3-oxoacid CoA transferase 1 | 3 | | 365 | ALDH5A1 | ADAMTS-like 1 | 3 | | 366 | ANAPC10 | aldehyde dehydrogenase 5 family, member A1 | 3 | | 367 | ANKMY2 | aldo-keto reductase family 7, member A2 (aflatoxin aldehyde reductase) | 3 | | 368 | APOBEC3F | anaphase promoting complex subunit 10 | 3 | | 369 | ARHGEF9 | ankyrin repeat and MYND domain containing 2 | 3 | | 370 | ARID2 | apolipoprotein B mRNA editing enzyme, catalytic polypeptide-like 3F | 3 | | 371 | ARMCX5 | armadillo repeat containing, X-linked 5 | 3 | | 372 | ATP5D | asparaginyl-tRNA synthetase | 3 | | 373 | ATP8B1 | AT rich interactive domain 2 (ARID, RFX-like) | 3 | | 374 | ATPAF1 | ATP synthase mitochondrial F1 complex assembly factor 1 | 3 | | 375 | AXIN2 | ATP synthase, H+ transporting, mitochondrial F1 complex, delta subunit | 3 | | 376 | B9D1 | ATPase, class I, type 8B, member 1 | 3 | | 377 | BACH2 | axin 2 | 3 | | 378 | BAG5 | B9 protein domain 1 | 3 | | 379 | BCCIP | BCL2-associated athanogene 5 | 3 | | 380 | C10orf72 | BRCA2 and CDKN1A interacting protein | 3 | | 381 | C12orf24 | BTB and CNC homology 1, basic leucine zipper transcription factor 2 | 3 | | 382 | C12orf47 | calcium/calmodulin-dependent serine protein kinase (MAGUK family) | 3 | | 383 | C12orf57 | calpain 7 | 3 | | 384 | C14orf126 | cAMP responsive element binding protein 1 | 3 | | 385 | C16orf81 | CD5 molecule | 3 | | 386 | C19orf70 | CD52 molecule | 3 | | 387 | C3orf37 | Cdc42 guanine nucleotide exchange factor (GEF) 9 | 3 | | 388 | C9orf82 | centrosomal protein 164kDa | 3 | | 389 | CAPN7 | chemokine (C-C motif) ligand 28 | 3 | | 390 | CASK | chemokine (C-X-C motif) receptor 5 | 3 | | 391 | CCDC25 | chromosome 10 open reading frame 72 | 3 | | 392 | CCDC59 | chromosome 12 open reading frame 24 | 3 | | 393 | CCDC84 | chromosome 12 open reading frame 47 | 3 | | 394 | CCL28 | chromosome 12 open reading frame 57 | 3 | | 395 | CD5 | chromosome 14 open reading frame 126 | 3 | | 396 | CD52 | chromosome 16 open reading frame 81 | 3 | | 397 | CEP164 | chromosome 19 open reading frame 70 | 3 | | 398 | CLTA | chromosome 3 open reading frame 37 | 3 | | 399 | CREB1 | chromosome 9 open reading frame 82 | 3 | | 400 | CUTA | clathrin, light chain (Lca) | 3 | | 401 | CXCR5 | coiled-coil domain containing 25 | 3 | | 402 | CYP2U1 | coiled-coil domain containing 59 | 3 | | 403 | DCAF8 | coiled-coil domain containing 84 | 3 | | 404 | DDX10 | cutA divalent cation tolerance homolog (E. coli) | 3 | | 405 | DEGS2 | cytochrome P450, family 2, subfamily U, polypeptide 1 | 3 | | 406 | DMP1 | DDB1 and CUL4 associated factor 8 | 3 | | 407 | DOPEY1 | DEAD (Asp-Glu-Ala-Asp) box polypeptide 10 | 3 | | 408 | EHMT2 | degenerative spermatocyte homolog 2, lipid desaturase (Drosophila) | 3 | | 409 | EIF2A | dentin matrix acidic phosphoprotein 1 | 3 | | 410 | EIF3L | dopey family member 1 | 3 | | 411 | FAM108B1 | euchromatic histone-lysine N-methyltransferase 2 | 3 | | 412 | FAM38A | eukaryotic translation initiation factor 2A, 65kDa | 3 | | 413 | FAM3D | eukaryotic translation initiation factor 3, subunit L | 3 | | 414 | FAM76A | F-box protein 4 | 3 | | 415 | FARS2 | FAD1 flavin adenine dinucleotide synthetase homolog (S. cerevisiae) | 3 | | 416 | FBXO4 | family with sequence similarity 108, member B1 | 3 | | 417 | FLAD1 | family with sequence similarity 3, member D | 3 | | 418 | FNIP2 | family with sequence similarity 38, member A | 3 | | 419 | FNTA | family with sequence similarity 76, member A | 3 | | 420 | FYCO1 | farnesyltransferase, CAAX box, alpha | 3 | | 421 | GDPD1 | folliculin interacting protein 2 | 3 | | 422 | GGPS1 | FYVE and coiled-coil domain containing 1 | 3 | | 423 | GIMAP5 | G1 to S phase transition 2 | 3 | | 424 | GLMN | geranylgeranyl diphosphate synthase 1 | 3 | | 425 | GLP1R | glomulin, FKBP associated protein | 3 | | 426 | GMEB1 | glucagon-like peptide 1 receptor | 3 | | 427 | GSPT2 | glucocorticoid modulatory element binding protein 1 | 3 | | 428 | H2AFV | glycerophosphodiester phosphodiesterase domain containing 1 | 3 | | 429 | HCRP1 | GTPase, IMAP family member 5 | 3 | | 430 | HELZ | H2A histone family, member V | 3 | | 431 | HOPX | HECT, UBA and WWE domain containing 1 | 3 | | 432 | HOXB3 | helicase with zinc finger | 3 | | 433 | HP1BP3 | hepatocellular carcinoma-related HCRP1 | 3 | | 434 | HUWE1 | heterochromatin protein 1, binding protein 3 | 3 | | 435 | ILKAP | homeobox B3 | 3 | | 436 | IMP3 | HOP homeobox | 3 | | 437 | ITGB4 | hypothetical protein LOC286114 | 3 | | 438 | ITPKB | IMP3, U3 small nucleolar ribonucleoprotein, homolog (yeast) | 3 | | 439 | ITPR1 | inositol 1,4,5-triphosphate receptor, type 1 | 3 | | 440 | JAK1 | inositol 1,4,5-trisphosphate 3-kinase B | 3 | | 441 | KDSR | integrin-linked kinase-associated serine/threonine phosphatase 2C | 3 | | 442 | KIAA0226 | integrin, beta 4 | 3 | | 443 | KLHL7 | Janus kinase 1 | 3 | | 444 | LOC286114 | kelch-like 7 (Drosophila) | 3 | | 445 | MAF | KIAA0226 | 3 | | 446 | MAN2A1 | mannosidase, alpha, class 2A, member 1 | 3 | | 447 | MAPK1 | mediator complex subunit 31 | 3 | | 448 | MED31 | metastasis associated 1 | 3 | | 449 | MIOS | methylmalonic aciduria (cobalamin deficiency) cblA type | 3 | | 450 | MIS12 | MIS12, MIND kinetochore complex component, homolog (S. pombe) | 3 | | 451 | MMAA | missing oocyte, meiosis regulator, homolog (Drosophila) | 3 | | 452 | MRPL41 | mitochondrial ribosomal protein L41 | 3 | | 453 | MTA1 | mitogen-activated protein kinase 1 | 3 | | 454 | MYO7A | myosin VIIA | 3 | | 455 | NARS | N-acetyltransferase 10 (GCN5-related) | 3 | | 456 | NAT10 | neuroguidin, EIF4E binding protein | 3 | | 457 | NGDN | NIN1/RPN12 binding protein 1 homolog (S. cerevisiae) | 3 | | 458 | NKX6-1 | NK6 homeobox 1 | 3 | | 459 | NMD3 | NMD3 homolog (S. cerevisiae) | 3 | | 460 | NME3 | non-metastatic cells 3, protein expressed in | 3 | | 461 | NOB1 | NOP58 ribonucleoprotein homolog (yeast) | 3 | | 462 | NOP58 | NSA2 ribosome biogenesis homolog (S. cerevisiae) | 3 | | 463 | NPM1 | nuclear fragile X mental retardation protein interacting protein 1 | 3 | | 464 | NSA2 | nucleophosmin (nucleolar phosphoprotein B23, numatrin) | 3 | | 465 | NUDCD3 | NudC domain containing 3 | 3 | | 466 | NUFIP1 | peptidylprolyl isomerase E (cyclophilin E) | 3 | | 467 | OXCT1 | phenylalanyl-tRNA synthetase 2, mitochondrial | 3 | | 468 | PAAF1 | phosphatidylinositol glycan anchor biosynthesis, class G | 3 | | 469 | PCSK7 | phosphohistidine phosphatase 1 | 3 | | 470 | PCYOX1 | phosphoinositide-3-kinase, regulatory subunit 4 | 3 | | 471 | PHPT1 | polo-like kinase 1 substrate 1 | 3 | | 472 | PIGG | PPPDE peptidase domain containing 1 | 3 | | 473 | PIK3R4 | prenylcysteine oxidase 1 | 3 | | 474 | PLK1S1 | proprotein convertase subtilisin/kexin type 7 | 3 | | 475 | PPIE | protease, serine, 23 | 3 | | 476 | PPP2R2C | proteasomal ATPase-associated factor 1 | 3 | | 477 | PPPDE1 | protein phosphatase 2 (formerly 2A), regulatory subunit B, gamma isoform | 3 | | 478 | PRPF19 | PRP19/PSO4 pre-mRNA processing factor 19 homolog (S. cerevisiae) | 3 | | 479 | PRSS23 | PYD (pyrin domain) containing 1 | 3 | | 480 | PYDC1 | rabaptin, RAB GTPase binding effector protein 2 | 3 | | 481 | RABEP2 | RAP1 interacting factor homolog (yeast) | 3 | | 482 | RBM18 | ras homolog gene family, member Q | 3 | | 483 | RBM4 | ribosomal protein L24 | 3 | | 484 | RHOQ | ribosomal protein L37 | 3 | | 485 | RIF1 | ribosomal protein SA | 3 | | 486 | RPL24 | RNA binding motif protein 18 | 3 | | 487 | RPL37 | RNA binding motif protein 4 | 3 | | 488 | RPSA | sarcoglycan, delta (35kDa dystrophin-associated glycoprotein) | 3 | | 489 | SCOC | short coiled-coil protein | 3 | | 490 | SENP6 | signal transducer and activator of transcription 4 | 3 | | 491 | SFRS3 | single stranded DNA binding protein 4 | 3 | | 492 | SGCD | solute carrier family 25 (mitochondrial carrier; adenine nucleotide translocator), member 6 | 3 | | 493 | SLC25A6 | solute carrier family 39 (zinc transporter), member 6 | 3 | | 494 | SLC39A6 | spermatogenesis associated 5 | 3 | | 495 | SMARCC1 | splicing factor, arginine/serine-rich 3 | 3 | | 496 | SPATA5 | ST6 beta-galactosamide alpha-2,6-sialyltranferase 1 | 3 | | 497 | SSBP4 | SUMO1/sentrin specific peptidase 6 | 3 | | 498 | ST6GAL1 | SWI/SNF related, matrix associated, actin dependent regulator of chromatin, subfamily c, member 1 | 3 | | 499 | STAT4 | TAF9B RNA polymerase II, TATA box binding protein (TBP)-associated factor, 31kDa | 3 | | 500 | TAF9B | tectonic family member 3 | 3 | | 501 | TCTN3 | tenascin XB | 3 | | 502 | THOC1 | THO complex 1 | 3 | | 503 | TLE6 | TRAF3 interacting protein 3 | 3 | | 504 | TMEM135 | transducin-like enhancer of split 6 (E(sp1) homolog, Drosophila) | 3 | | 505 | TMEM147 | transmembrane protein 135 | 3 | | 506 | TMEM209 | transmembrane protein 147 | 3 | | 507 | TNXB | transmembrane protein 209 | 3 | | 508 | TRAF3IP3 | ubiquitin-like modifier activating enzyme 2 | 3 | | 509 | UBA2 | ubiquitin family domain containing 1 | 3 | | 510 | UBFD1 | ubiquitin protein ligase E3 component n-recognin 5 | 3 | | 511 | UBR5 | UTP15, U3 small nucleolar ribonucleoprotein, homolog (S. cerevisiae) | 3 | | 512 | UTP15 | UTP3, small subunit (SSU) processome component, homolog (S. cerevisiae) | 3 | | 513 | UTP3 | v-maf musculoaponeurotic fibrosarcoma oncogene homolog (avian) | 3 | | 514 | VAX2 | ventral anterior homeobox 2 | 3 | | 515 | VEZT | vezatin, adherens junctions transmembrane protein | 3 | | 516 | WDR11 | WD repeat domain 11 | 3 | | 517 | ZNF141 | zinc finger protein 141 | 3 | | 518 | ZNF182 | zinc finger protein 182 | 3 | | 519 | ZNF268 | zinc finger protein 268 | 3 | | 520 | ZNF277 | zinc finger protein 277 | 3 | | 521 | ZNF280D | zinc finger protein 280D | 3 | | 522 | ZNF345 | zinc finger protein 345 | 3 | | 523 | ZNF512B | zinc finger protein 512B | 3 | | 524 | ZNF571 | zinc finger protein 571 | 3 | | 525 | ABCB6 | 1-acylglycerol-3-phosphate O-acyltransferase 5 (lysophosphatidic acid acyltransferase, epsilon) | 2 | | 526 | ACAP1 | actinin, alpha 1 | 2 | | 527 | ACTN1 | adenylosuccinate synthase like 1 | 2 | | 528 | ADRA2B | adrenergic, alpha-2B-, receptor | 2 | | 529 | ADSSL1 | aldo-keto reductase family 1, member E2 | 2 | | 530 | AGGF1 | Alport syndrome, mental retardation, midface hypoplasia and elliptocytosis chromosomal region gene 1 | 2 | | 531 | AGPAT5 | anaphase promoting complex subunit 1 | 2 | | 532 | AIDA | angiogenic factor with G patch and FHA domains 1 | 2 | | 533 | AKR1E2 | anoctamin 1, calcium activated chloride channel | 2 | | 534 | ALG10B | APEX nuclease (multifunctional DNA repair enzyme) 1 | 2 | | 535 | AMMECR1 | ArfGAP with coiled-coil, ankyrin repeat and PH domains 1 | 2 | | 536 | ANAPC1 | asparagine-linked glycosylation 10, alpha-1,2-glucosyltransferase homolog B (yeast) | 2 | | 537 | ANO1 | ATP-binding cassette, sub-family B (MDR/TAP), member 6 | 2 | | 538 | APEX1 | ATPase, Ca++ transporting, plasma membrane 2 | 2 | | 539 | ATP2B2 | ATPase, Ca++ transporting, plasma membrane 3 | 2 | | 540 | ATP2B3 | axin interactor, dorsalization associated | 2 | | 541 | B3GNTL1 | B-cell CLL/lymphoma 9-like | 2 | | 542 | BCL9L | B double prime 1, subunit of RNA polymerase III transcription initiation factor IIIB | 2 | | 543 | BDP1 | baculoviral IAP repeat-containing 7 | 2 | | 544 | BIRC7 | bladder cancer associated protein | 2 | | 545 | BLCAP | butyrophilin, subfamily 3, member A2 | 2 | | 546 | BTN3A2 | cancer susceptibility candidate 2 | 2 | | 547 | C10orf116 | carcinoembryonic antigen-related cell adhesion molecule 1 (biliary glycoprotein) | 2 | | 548 | C10orf32 | CCR4-NOT transcription complex, subunit 6-like | 2 | | 549 | C10orf78 | CD27 molecule | 2 | | 550 | C11orf57 | CD84 molecule | 2 | | 551 | C11orf92 | CDK5 regulatory subunit associated protein 3 | 2 | | 552 | C12orf26 | centrosomal protein 350kDa | 2 | | 553 | C14orf180 | chromodomain helicase DNA binding protein 2 | 2 | | 554 | C14orf73 | chromosome 1 open reading frame 109 | 2 | | 555 | C16orf87 | chromosome 1 open reading frame 228 | 2 | | 556 | C19orf62 | chromosome 10 open reading frame 116 | 2 | | 557 | C1orf109 | chromosome 10 open reading frame 32 | 2 | | 558 | C1orf228 | chromosome 10 open reading frame 78 | 2 | | 559 | C21orf59 | chromosome 11 open reading frame 57 | 2 | | 560 | C3orf15 | chromosome 11 open reading frame 92 | 2 | | 561 | C3orf58 | chromosome 12 open reading frame 26 | 2 | | 562 | C6orf136 | chromosome 14 open reading frame 180 | 2 | | 563 | C9orf79 | chromosome 14 open reading frame 73 | 2 | | 564 | CASC2 | chromosome 16 open reading frame 87 | 2 | | 565 | CCDC27 | chromosome 19 open reading frame 62 | 2 | | 566 | CCDC41 | chromosome 21 open reading frame 59 | 2 | | 567 | CCDC61 | chromosome 3 open reading frame 15 | 2 | | 568 | CCNJ | chromosome 3 open reading frame 58 | 2 | | 569 | CD27 | chromosome 6 open reading frame 136 | 2 | | 570 | CD84 | chromosome 9 open reading frame 79 | 2 | | 571 | CDK12 | ClpP caseinolytic peptidase, ATP-dependent, proteolytic subunit homolog (E. coli) | 2 | | 572 | CDK5RAP3 | coiled-coil domain containing 27 | 2 | | 573 | CEACAM1 | coiled-coil domain containing 41 | 2 | | 574 | CEP350 | coiled-coil domain containing 61 | 2 | | 575 | CHD2 | collagen, type VI, alpha 1 | 2 | | 576 | CLPP | CSE1 chromosome segregation 1-like (yeast) | 2 | | 577 | CNGB1 | cyclic nucleotide gated channel beta 1 | 2 | | 578 | CNOT6L | cyclin-dependent kinase 12 | 2 | | 579 | COL6A1 | cyclin J | 2 | | 580 | COX4I1 | cytochrome c oxidase subunit IV isoform 1 | 2 | | 581 | CSE1L | cytochrome c, somatic | 2 | | 582 | CYCS | cytochrome P450, family 4, subfamily F, polypeptide 8 | 2 | | 583 | CYP4F8 | cytohesin 3 | 2 | | 584 | CYTH3 | DAB2 interacting protein | 2 | | 585 | DAB2IP | dapper, antagonist of beta-catenin, homolog 3 (Xenopus laevis) | 2 | | 586 | DACT3 | DEAD (Asp-Glu-Ala-Asp) box polypeptide 28 | 2 | | 587 | DDX28 | deleted in lung and esophageal cancer 1 | 2 | | 588 | DENR | density-regulated protein | 2 | | 589 | DLAT | dihydrolipoamide S-acetyltransferase | 2 | | 590 | DLEC1 | dual-specificity tyrosine-(Y)-phosphorylation regulated kinase 1A | 2 | | 591 | DNM1L | dual specificity phosphatase 7 | 2 | | 592 | DUSP7 | dynamin 1-like | 2 | | 593 | DYRK1A | EF-hand calcium binding domain 4B | 2 | | 594 | EFCAB4B | EPS8-like 1 | 2 | | 595 | EPRS | exosome component 9 | 2 | | 596 | EPS8L1 | exportin 1 (CRM1 homolog, yeast) | 2 | | 597 | EXOSC9 | family with sequence similarity 113, member B | 2 | | 598 | FABP6 | family with sequence similarity 162, member A | 2 | | 599 | FAHD1 | family with sequence similarity 50, member A | 2 | | 600 | FAM113B | family with sequence similarity 83, member H | 2 | | 601 | FAM162A | fat mass and obesity associated | 2 | | 602 | FAM50A | fatty acid binding protein 6, ileal | 2 | | 603 | FAM83H | FERM domain containing 8 | 2 | | 604 | FLJ31356 | fibroblast growth factor receptor substrate 2 | 2 | | 605 | FLJ39609 | forty-two-three domain containing 1 | 2 | | 606 | FRMD8 | FtsJ homolog 1 (E. coli) | 2 | | 607 | FRS2 | fucosyltransferase 5 (alpha (1,3) fucosyltransferase) | 2 | | 608 | FTO | fumarylacetoacetate hydrolase domain containing 1 | 2 | | 609 | FTSJ1 | G patch domain containing 8 | 2 | | 610 | FUT5 | GA binding protein transcription factor, alpha subunit 60kDa | 2 | | 611 | FYTTD1 | GCN1 general control of amino-acid synthesis 1-like 1 (yeast) | 2 | | 612 | GABPA | general transcription factor IIA, 1, 19/37kDa | 2 | | 613 | GANAB | glucosidase, alpha; neutral AB | 2 | | 614 | GCN1L1 | glucoside xylosyltransferase 1 | 2 | | 615 | GLG1 | glutamate receptor, ionotropic, N-methyl D-aspartate 1 | 2 | | 616 | GLO1 | glutamyl-prolyl-tRNA synthetase | 2 | | 617 | GPATCH8 | glyoxalase I | 2 | | 618 | GRIN1 | golgi glycoprotein 1 | 2 | | 619 | GTF2A1 | heat shock protein, alpha-crystallin-related, B9 | 2 | | 620 | GXYLT1 | high mobility group AT-hook 2 | 2 | | 621 | hCG\_2038428 | homolog of rat pragma of Rnd2 | 2 | | 622 | HMGA2 | hypothetical LOC285830 | 2 | | 623 | HSPB9 | hypothetical LOC728701 | 2 | | 624 | IGH@ | hypothetical protein FLJ31356 | 2 | | 625 | IPCEF1 | hypothetical protein LOC100128198 | 2 | | 626 | IREB2 | hypothetical protein LOC144776 | 2 | | 627 | ITGA9 | hypothetical protein LOC150759 | 2 | | 628 | IWS1 | hypothetical protein LOC254128 | 2 | | 629 | KALRN | immunoglobulin heavy locus | 2 | | 630 | KCNG2 | integrin, alpha 9 | 2 | | 631 | KIAA0090 | interaction protein for cytohesin exchange factors 1 | 2 | | 632 | KIAA0776 | iron-responsive element binding protein 2 | 2 | | 633 | KLK15 | IWS1 homolog (S. cerevisiae) | 2 | | 634 | KRT31 | kalirin, RhoGEF kinase | 2 | | 635 | KTN1 | kallikrein-related peptidase 15 | 2 | | 636 | LARGE | keratin 31 | 2 | | 637 | LOC100128198 | KIAA0090 | 2 | | 638 | LOC100129196 | KIAA0776 | 2 | | 639 | LOC100132352 | kinectin 1 (kinesin receptor) | 2 | | 640 | LOC100133315 | latent transforming growth factor beta binding protein 3 | 2 | | 641 | LOC100240726 | like-glycosyltransferase | 2 | | 642 | LOC100287166 | LSM4 homolog, U6 small nuclear RNA associated (S. cerevisiae) | 2 | | 643 | LOC150759 | makorin ring finger protein 1 pseudogene | 2 | | 644 | LOC203510 | matrix metallopeptidase 9 (gelatinase B, 92kDa gelatinase, 92kDa type IV collagenase) | 2 | | 645 | LOC254128 | mediator complex subunit 17 | 2 | | 646 | LOC728701 | Meis homeobox 3 | 2 | | 647 | LSM4 | methyltransferase like 14 | 2 | | 648 | LTBP3 | mindbomb homolog 2 (Drosophila) | 2 | | 649 | MAP2K3 | minichromosome maintenance complex component 3 associated protein | 2 | | 650 | MAP3K7 | mitochondrial ribosomal protein L14 | 2 | | 651 | MCM3AP | mitochondrial ribosomal protein S2 | 2 | | 652 | MCOLN2 | mitochondrial ribosomal protein S24 | 2 | | 653 | MED17 | mitochondrial ribosomal protein S26 | 2 | | 654 | MEIS3 | mitogen-activated protein kinase kinase 3 | 2 | | 655 | METTL14 | mitogen-activated protein kinase kinase kinase 7 | 2 | | 656 | MIB2 | mucin 4, cell surface associated | 2 | | 657 | MLL4 | mucolipin 2 | 2 | | 658 | MMP9 | myeloid/lymphoid or mixed-lineage leukemia 4 | 2 | | 659 | MRPL14 | myosin, heavy chain 13, skeletal muscle | 2 | | 660 | MRPS2 | myotubularin related protein 3 | 2 | | 661 | MRPS24 | N(alpha)-acetyltransferase 16, NatA auxiliary subunit | 2 | | 662 | MRPS26 | nestin | 2 | | 663 | MTMR3 | neugrin, neurite outgrowth associated | 2 | | 664 | MUC4 | neural cell adhesion molecule 1 | 2 | | 665 | MYH13 | neutral sphingomyelinase (N-SMase) activation associated factor | 2 | | 666 | NAA16 | nicotinamide nucleotide transhydrogenase | 2 | | 667 | NCAM1 | non-protein coding RNA 81 | 2 | | 668 | NCOR2 | nuclear fragile X mental retardation protein interacting protein 2 | 2 | | 669 | NCRNA00081 | nuclear pore complex interacting protein | 2 | | 670 | NES | nuclear receptor co-repressor 2 | 2 | | 671 | NGRN | nucleolar protein 8 | 2 | | 672 | NNT | olfactory receptor, family 1, subfamily J, member 4 | 2 | | 673 | NOL8 | otopetrin 2 | 2 | | 674 | NPIP | outer dense fiber of sperm tails 3B | 2 | | 675 | NSMAF | oxidation resistance 1 | 2 | | 676 | NUFIP2 | PAX interacting (with transcription-activation domain) protein 1 | 2 | | 677 | ODF3B | PDZ and LIM domain 7 (enigma) | 2 | | 678 | OR1J4 | peroxisomal biogenesis factor 13 | 2 | | 679 | OTOP2 | phosphatidylglycerophosphate synthase 1 | 2 | | 680 | OXR1 | phosphatidylinositol glycan anchor biosynthesis, class L | 2 | | 681 | PABPN1 | phosphodiesterase 12 | 2 | | 682 | PAPOLA | phosphoinositide-3-kinase adaptor protein 1 | 2 | | 683 | PAPOLG | phospholipid scramblase 3 | 2 | | 684 | PAXIP1 | platelet-derived growth factor receptor, alpha polypeptide | 2 | | 685 | PCDHB13 | pogo transposable element with KRAB domain | 2 | | 686 | PCGF5 | poly(A) binding protein, nuclear 1 | 2 | | 687 | PDE12 | poly(A) polymerase alpha | 2 | | 688 | PDGFRA | poly(A) polymerase gamma | 2 | | 689 | PDLIM7 | polycomb group ring finger 5 | 2 | | 690 | PEX13 | polymerase (RNA) II (DNA directed) polypeptide I, 14.5kDa | 2 | | 691 | PGS1 | polymerase (RNA) III (DNA directed) polypeptide C (62kD) | 2 | | 692 | PIGL | postmeiotic segregation increased 2-like 5 | 2 | | 693 | PIK3AP1 | potassium voltage-gated channel, subfamily G, member 2 | 2 | | 694 | PKIA | POU class 3 homeobox 2 | 2 | | 695 | PLSCR3 | proteasome (prosome, macropain) 26S subunit, non-ATPase, 11 | 2 | | 696 | PMS2L5 | proteasome (prosome, macropain) subunit, beta type, 1 | 2 | | 697 | POGK | protein arginine methyltransferase 3 | 2 | | 698 | POLR2I | protein kinase (cAMP-dependent, catalytic) inhibitor alpha | 2 | | 699 | POLR3C | protein kinase D3 | 2 | | 700 | POU3F2 | protein phosphatase 1, regulatory (inhibitor) subunit 3B | 2 | | 701 | PPM1G | protein phosphatase 1, regulatory (inhibitor) subunit 8 | 2 | | 702 | PPP1R3B | protein phosphatase 1G (formerly 2C), magnesium-dependent, gamma isoform | 2 | | 703 | PPP1R8 | protein tyrosine phosphatase, non-receptor type 11 | 2 | | 704 | PRAGMIN | protocadherin beta 13 | 2 | | 705 | PRKD3 | PRP31 pre-mRNA processing factor 31 homolog (S. cerevisiae) | 2 | | 706 | PRMT3 | RAB12, member RAS oncogene family | 2 | | 707 | PRPF31 | RAB31, member RAS oncogene family | 2 | | 708 | PSMB1 | RAB40B, member RAS oncogene family | 2 | | 709 | PSMD11 | RAN binding protein 6 | 2 | | 710 | PTPN11 | Rap guanine nucleotide exchange factor (GEF) 6 | 2 | | 711 | RAB12 | Ras and Rab interactor 3 | 2 | | 712 | RAB31 | Ras association (RalGDS/AF-6) domain family member 1 | 2 | | 713 | RAB40B | RecQ protein-like 5 | 2 | | 714 | RALB | REST corepressor 3 | 2 | | 715 | RANBP6 | ribosomal protein L10 | 2 | | 716 | RAPGEF6 | ribosomal protein L36 | 2 | | 717 | RASSF1 | ribosomal protein S6 | 2 | | 718 | RBM17 | ribosomal protein S7 | 2 | | 719 | RCOR3 | ribosomal protein S9 | 2 | | 720 | RECQL5 | ring finger protein 115 | 2 | | 721 | RIN3 | RIO kinase 2 (yeast) | 2 | | 722 | RIOK2 | RNA binding motif protein 17 | 2 | | 723 | RNF115 | RNA binding protein S1, serine-rich domain | 2 | | 724 | RNPS1 | scavenger receptor cysteine-rich glycoprotein | 2 | | 725 | RP3-377H14.5 | SEC24 family, member B (S. cerevisiae) | 2 | | 726 | RPL10 | secreted frizzled-related protein 5 | 2 | | 727 | RPL36 | selenium binding protein 1 | 2 | | 728 | RPS6 | seven in absentia homolog 1 (Drosophila) | 2 | | 729 | RPS7 | signal-regulatory protein alpha | 2 | | 730 | RPS9 | similar to hCG1644442 | 2 | | 731 | SDC3 | similar to hCG1989297 | 2 | | 732 | SEC24B | similar to hCG1995469 | 2 | | 733 | SELENBP1 | similar to hCG2019076 | 2 | | 734 | SFRP5 | similar to hCG2033298 | 2 | | 735 | SFTA1P | SIN3 homolog A, transcription regulator (yeast) | 2 | | 736 | SIAH1 | single-stranded DNA binding protein 1 | 2 | | 737 | SIN3A | Sjogren syndrome antigen B (autoantigen La) | 2 | | 738 | SIRPA | slingshot homolog 1 (Drosophila) | 2 | | 739 | SLC15A4 | slit homolog 1 (Drosophila) | 2 | | 740 | SLC22A6 | slowmo homolog 2 (Drosophila) | 2 | | 741 | SLC28A1 | small nuclear ribonucleoprotein D2 polypeptide 16.5kDa | 2 | | 742 | SLC34A1 | small nuclear ribonucleoprotein polypeptide A | 2 | | 743 | SLIT1 | solute carrier family 15, member 4 | 2 | | 744 | SLMO2 | solute carrier family 22 (organic anion transporter), member 6 | 2 | | 745 | SMARCAD1 | solute carrier family 28 (sodium-coupled nucleoside transporter), member 1 | 2 | | 746 | SNRPA | solute carrier family 34 (sodium phosphate), member 1 | 2 | | 747 | SNRPD2 | sorting nexin 4 | 2 | | 748 | SNX4 | sorting nexin 5 | 2 | | 749 | SNX5 | spectrin, beta, non-erythrocytic 4 | 2 | | 750 | SPAG9 | sperm associated antigen 9 | 2 | | 751 | SPATA3 | spermatogenesis associated 3 | 2 | | 752 | SPTBN4 | stromal antigen 3-like 4 | 2 | | 753 | SSB | STT3, subunit of the oligosaccharyltransferase complex, homolog B (S. cerevisiae) | 2 | | 754 | SSBP1 | surfactant associated 1 (pseudogene) | 2 | | 755 | SSC5D | SWI/SNF-related, matrix-associated actin-dependent regulator of chromatin, subfamily a, containing DEAD/H box 1 | 2 | | 756 | SSH1 | syndecan 3 | 2 | | 757 | STAG3L4 | T-box 1 | 2 | | 758 | STT3B | TAF2 RNA polymerase II, TATA box binding protein (TBP)-associated factor, 150kDa | 2 | | 759 | TAF2 | tetratricopeptide repeat domain 23 | 2 | | 760 | TBX1 | tetratricopeptide repeat domain 38 | 2 | | 761 | TFAM | threonine synthase-like 2 (S. cerevisiae) | 2 | | 762 | THNSL2 | TIMP metallopeptidase inhibitor 2 | 2 | | 763 | TIMP2 | TNFRSF1A-associated via death domain | 2 | | 764 | TMEM126B | transcription factor A, mitochondrial | 2 | | 765 | TMEM150C | transcriptional regulating factor 1 | 2 | | 766 | TMEM176B | transient receptor potential cation channel, subfamily C, member 2-like | 2 | | 767 | TMEM184C | translin | 2 | | 768 | TRADD | transmembrane protein 126B | 2 | | 769 | TRERF1 | transmembrane protein 150C | 2 | | 770 | TRIM23 | transmembrane protein 176B | 2 | | 771 | TRIM24 | transmembrane protein 184C | 2 | | 772 | TSC22D2 | tripartite motif-containing 23 | 2 | | 773 | TSN | tripartite motif-containing 24 | 2 | | 774 | TTC23 | TSC22 domain family, member 2 | 2 | | 775 | TTC38 | tubulin, gamma complex associated protein 6 | 2 | | 776 | TUBGCP6 | ubiquitin-like modifier activating enzyme 7 | 2 | | 777 | UBA7 | ubiquitously-expressed transcript | 2 | | 778 | UXT | UDP-GlcNAc:betaGal beta-1,3-N-acetylglucosaminyltransferase-like 1 | 2 | | 779 | VPRBP | v-ral simian leukemia viral oncogene homolog B (ras related; GTP binding protein) | 2 | | 780 | WDR19 | Vpr (HIV-1) binding protein | 2 | | 781 | WDR73 | WD repeat domain 19 | 2 | | 782 | WWC1 | WD repeat domain 73 | 2 | | 783 | XPO1 | WW and C2 domain containing 1 | 2 | | 784 | XYLT1 | xylosyltransferase I | 2 | | 785 | YTHDC1 | YTH domain containing 1 | 2 | | 786 | ZC3H13 | zinc finger CCCH-type containing 13 | 2 | | 787 | ZDHHC1 | zinc finger protein 180 | 2 | | 788 | ZDHHC19 | zinc finger protein 264 | 2 | | 789 | ZMYM4 | zinc finger protein 347 | 2 | | 790 | ZNF180 | zinc finger protein 518B | 2 | | 791 | ZNF264 | zinc finger protein 599 | 2 | | 792 | ZNF347 | zinc finger protein 627 | 2 | | 793 | ZNF518B | zinc finger protein 652 | 2 | | 794 | ZNF599 | zinc finger protein 711 | 2 | | 795 | ZNF627 | zinc finger protein 814 | 2 | | 796 | ZNF652 | zinc finger protein 823 | 2 | | 797 | ZNF711 | zinc finger, DHHC-type containing 1 | 2 | | 798 | ZNF814 | zinc finger, DHHC-type containing 19 | 2 | | 799 | ZNF823 | zinc finger, MYM-type 4 | 2 | | 800 | ABHD12 | abhydrolase domain containing 12 | 1 | | 801 | ABHD15 | abhydrolase domain containing 15 | 1 | | 802 | ACAD11 | acidic (leucine-rich) nuclear phosphoprotein 32 family, member B | 1 | | 803 | ACBD6 | actin-like 6A | 1 | | 804 | ACTL6A | actin related protein 2/3 complex, subunit 3, 21kDa | 1 | | 805 | ACTR1A | acyl-Coenzyme A binding domain containing 6 | 1 | | 806 | ADAD2 | acyl-Coenzyme A dehydrogenase family, member 11 | 1 | | 807 | ADAM9 | ADAM metallopeptidase domain 9 (meltrin gamma) | 1 | | 808 | ADAMTS8 | ADAM metallopeptidase with thrombospondin type 1 motif, 8 | 1 | | 809 | ADCY1 | adaptor-related protein complex 1, sigma 2 subunit | 1 | | 810 | ADCY5 | adaptor-related protein complex 4, epsilon 1 subunit | 1 | | 811 | AGAP3 | additional sex combs like 2 (Drosophila) | 1 | | 812 | AGFG1 | adenomatosis polyposis coli 2 | 1 | | 813 | AKIRIN2 | adenosine deaminase domain containing 2 | 1 | | 814 | AKT1 | adenylate cyclase 1 (brain) | 1 | | 815 | ALPL | adenylate cyclase 5 | 1 | | 816 | ANGPT2 | ADP-ribosylation factor-like 8B | 1 | | 817 | ANGPTL2 | akirin 2 | 1 | | 818 | ANKHD1 | alkaline phosphatase, liver/bone/kidney | 1 | | 819 | ANKRD22 | amyloid beta (A4) precursor protein-binding, family A, member 2 | 1 | | 820 | ANKRD49 | angiopoietin-like 2 | 1 | | 821 | ANP32B | angiopoietin 2 | 1 | | 822 | ANXA3 | ankyrin repeat and KH domain containing 1 | 1 | | 823 | AP1S2 | ankyrin repeat and SOCS box-containing 11 | 1 | | 824 | AP4E1 | ankyrin repeat domain 22 | 1 | | 825 | APBA2 | ankyrin repeat domain 49 | 1 | | 826 | APC2 | annexin A3 | 1 | | 827 | APOC2 | apolipoprotein C-II | 1 | | 828 | AQP9 | aquaporin 9 | 1 | | 829 | ARHGAP23 | ArfGAP with FG repeats 1 | 1 | | 830 | ARHGDIA | ArfGAP with GTPase domain, ankyrin repeat and PH domain 3 | 1 | | 831 | ARL8B | ArfGAP with SH3 domain, ankyrin repeat and PH domain 1 | 1 | | 832 | ARPC3 | ARP1 actin-related protein 1 homolog A, centractin alpha (yeast) | 1 | | 833 | ASAP1 | ash1 (absent, small, or homeotic)-like (Drosophila) | 1 | | 834 | ASB11 | asteroid homolog 1 (Drosophila) | 1 | | 835 | ASH1L | ATP synthase, H+ transporting, mitochondrial F1 complex, gamma polypeptide 1 | 1 | | 836 | ASTE1 | ATPase, class VI, type 11A | 1 | | 837 | ASXL2 | ATPase, class VI, type 11C | 1 | | 838 | ATP11A | ATPase, H+ transporting, lysosomal 16kDa, V0 subunit c | 1 | | 839 | ATP11C | B-cell CLL/lymphoma 7C | 1 | | 840 | ATP5C1 | BCL2-interacting killer (apoptosis-inducing) | 1 | | 841 | ATP6V0C | BCL2-related ovarian killer | 1 | | 842 | B3GALT6 | BCL2/adenovirus E1B 19kDa interacting protein 2 | 1 | | 843 | BCAR1 | BMX non-receptor tyrosine kinase | 1 | | 844 | BCL7C | bone morphogenetic protein 8a | 1 | | 845 | BIK | breast cancer anti-estrogen resistance 1 | 1 | | 846 | BMP8A | BTB (POZ) domain containing 18 | 1 | | 847 | BMX | C-type lectin-like 1 | 1 | | 848 | BNIP2 | C-type lectin domain family 4, member D | 1 | | 849 | BOK | cadherin-related family member 5 | 1 | | 850 | BTBD18 | cadherin 6, type 2, K-cadherin (fetal kidney) | 1 | | 851 | C11orf54 | calcium binding protein 2 | 1 | | 852 | C11orf94 | calcium regulated heat stable protein 1, 24kDa | 1 | | 853 | C14orf174 | calcium/calmodulin-dependent protein kinase I | 1 | | 854 | C15orf17 | carbamoyl-phosphate synthetase 2, aspartate transcarbamylase, and dihydroorotase | 1 | | 855 | C15orf57 | castor zinc finger 1 | 1 | | 856 | C15orf59 | CD82 molecule | 1 | | 857 | C16orf13 | CDKN2A interacting protein | 1 | | 858 | C17orf101 | cell cycle progression 1 | 1 | | 859 | C17orf28 | cell division cycle 20 homolog B (S. cerevisiae) | 1 | | 860 | C17orf81 | chemokine (C-C motif) ligand 5 | 1 | | 861 | C18orf18 | chemokine (C-X-C motif) receptor 1 | 1 | | 862 | C19orf53 | chromodomain helicase DNA binding protein 5 | 1 | | 863 | C19orf59 | chromosome 1 open reading frame 105 | 1 | | 864 | C1orf105 | chromosome 1 open reading frame 200 | 1 | | 865 | C1orf200 | chromosome 1 open reading frame 50 | 1 | | 866 | C1orf50 | chromosome 1 open reading frame 53 | 1 | | 867 | C1orf53 | chromosome 1 open reading frame 65 | 1 | | 868 | C1orf65 | chromosome 11 open reading frame 54 | 1 | | 869 | C1RL | chromosome 11 open reading frame 94 | 1 | | 870 | C2orf19 | chromosome 14 open reading frame 174 | 1 | | 871 | C2orf54 | chromosome 15 open reading frame 17 | 1 | | 872 | C3orf52 | chromosome 15 open reading frame 57 | 1 | | 873 | C3orf63 | chromosome 15 open reading frame 59 | 1 | | 874 | C4BPB | chromosome 16 open reading frame 13 | 1 | | 875 | C4orf29 | chromosome 17 open reading frame 101 | 1 | | 876 | C4orf38 | chromosome 17 open reading frame 28 | 1 | | 877 | C5orf13 | chromosome 17 open reading frame 81 | 1 | | 878 | C5orf33 | chromosome 18 open reading frame 18 | 1 | | 879 | C6orf153 | chromosome 19 open reading frame 53 | 1 | | 880 | C8orf33 | chromosome 19 open reading frame 59 | 1 | | 881 | C9orf123 | chromosome 2 open reading frame 19 | 1 | | 882 | C9orf85 | chromosome 2 open reading frame 54 | 1 | | 883 | CABP2 | chromosome 3 open reading frame 52 | 1 | | 884 | CAD | chromosome 3 open reading frame 63 | 1 | | 885 | CAMK1 | chromosome 4 open reading frame 29 | 1 | | 886 | CARHSP1 | chromosome 4 open reading frame 38 | 1 | | 887 | CASZ1 | chromosome 5 open reading frame 13 | 1 | | 888 | CBFB | chromosome 5 open reading frame 33 | 1 | | 889 | CCDC116 | chromosome 6 open reading frame 153 | 1 | | 890 | CCDC43 | chromosome 8 open reading frame 33 | 1 | | 891 | CCDC47 | chromosome 9 open reading frame 123 | 1 | | 892 | CCL5 | chromosome 9 open reading frame 85 | 1 | | 893 | CCPG1 | chromosome X open reading frame 26 | 1 | | 894 | CD82 | chromosome X open reading frame 57 | 1 | | 895 | CDC20B | ciliary rootlet coiled-coil, rootletin-like 1 | 1 | | 896 | CDH6 | cofactor of BRCA1 | 1 | | 897 | CDHR5 | coiled-coil domain containing 116 | 1 | | 898 | CDKN2AIP | coiled-coil domain containing 43 | 1 | | 899 | CFDP1 | coiled-coil domain containing 47 | 1 | | 900 | CHD5 | complement component 1, r subcomponent-like | 1 | | 901 | CKAP4 | complement component 4 binding protein, beta | 1 | | 902 | CLEC4D | component of oligomeric golgi complex 5 | 1 | | 903 | CLECL1 | contactin associated protein-like 4 | 1 | | 904 | CMC1 | COP9 constitutive photomorphogenic homolog subunit 2 (Arabidopsis) | 1 | | 905 | CNGB3 | core-binding factor, beta subunit | 1 | | 906 | CNIH4 | cornichon homolog 4 (Drosophila) | 1 | | 907 | CNTNAP4 | COX assembly mitochondrial protein homolog (S. cerevisiae) | 1 | | 908 | COBRA1 | craniofacial development protein 1 | 1 | | 909 | COG5 | CXXC finger 1 (PHD domain) | 1 | | 910 | COPS2 | cyclic nucleotide gated channel beta 3 | 1 | | 911 | CROCCL1 | cystatin C | 1 | | 912 | CST3 | cytochrome P450, family 2, subfamily A, polypeptide 7 pseudogene 1 | 1 | | 913 | CXCR1 | cytohesin 1 | 1 | | 914 | CXorf26 | cytoskeleton-associated protein 4 | 1 | | 915 | CXorf57 | DBF4 homolog (S. cerevisiae) | 1 | | 916 | CXXC1 | DDB1 and CUL4 associated factor 7 | 1 | | 917 | CYP2A7P1 | DEAD (Asp-Glu-Ala-Asp) box polypeptide 23 | 1 | | 918 | CYTH1 | DEAH (Asp-Glu-Ala-His) box polypeptide 15 | 1 | | 919 | DBF4 | DEAH (Asp-Glu-Ala-His) box polypeptide 36 | 1 | | 920 | DCAF7 | DENN/MADD domain containing 4A | 1 | | 921 | DDX23 | DEP domain containing 7 | 1 | | 922 | DENND4A | dermatan sulfate epimerase | 1 | | 923 | DEPDC7 | developmental pluripotency associated 4 | 1 | | 924 | DHX15 | diablo homolog (Drosophila) | 1 | | 925 | DHX36 | dishevelled, dsh homolog 3 (Drosophila) | 1 | | 926 | DIABLO | disrupted in renal carcinoma 2 | 1 | | 927 | DIRC2 | distal-less homeobox 2 | 1 | | 928 | DKFZp761E198 | DKFZp761E198 protein | 1 | | 929 | DLX2 | DNA-damage regulated autophagy modulator 1 | 1 | | 930 | DNAJC11 | DnaJ (Hsp40) homolog, subfamily C, member 11 | 1 | | 931 | DNAJC16 | DnaJ (Hsp40) homolog, subfamily C, member 16 | 1 | | 932 | DNAJC19 | DnaJ (Hsp40) homolog, subfamily C, member 19 | 1 | | 933 | DNAJC8 | DnaJ (Hsp40) homolog, subfamily C, member 8 | 1 | | 934 | DPPA4 | dpy-19-like 3 (C. elegans) | 1 | | 935 | DPY19L3 | E2F-associated phosphoprotein | 1 | | 936 | DRAM1 | early B-cell factor 3 | 1 | | 937 | DSE | embryonic ectoderm development | 1 | | 938 | DVL3 | EMG1 nucleolar protein homolog (S. cerevisiae) | 1 | | 939 | EAPP | EMI domain containing 1 | 1 | | 940 | EBF3 | endomucin | 1 | | 941 | ECHDC1 | endoplasmic reticulum protein 29 | 1 | | 942 | EED | enolase-phosphatase 1 | 1 | | 943 | EEF1G | enoyl Coenzyme A hydratase domain containing 1 | 1 | | 944 | EEF2 | ERGIC and golgi 3 | 1 | | 945 | EIF4A1 | ERO1-like (S. cerevisiae) | 1 | | 946 | EMCN | erythrocyte membrane protein band 4.1 like 4B | 1 | | 947 | EMG1 | eukaryotic translation elongation factor 1 gamma | 1 | | 948 | EMID1 | eukaryotic translation elongation factor 2 | 1 | | 949 | ENOPH1 | eukaryotic translation initiation factor 4A1 | 1 | | 950 | ENPEP | exosome component 1 | 1 | | 951 | EPB41L4B | F-box protein 40 | 1 | | 952 | ERGIC3 | F-box protein 42 | 1 | | 953 | ERO1L | F-box protein 9 | 1 | | 954 | ERP29 | FAD-dependent oxidoreductase domain containing 2 | 1 | | 955 | EXOSC1 | family with sequence similarity 108, member A1 | 1 | | 956 | FAM108A1 | family with sequence similarity 133, member B | 1 | | 957 | FAM133B | family with sequence similarity 160, member A2 | 1 | | 958 | FAM160A2 | family with sequence similarity 176, member B | 1 | | 959 | FAM176B | family with sequence similarity 186, member B | 1 | | 960 | FAM186B | family with sequence similarity 69, member A | 1 | | 961 | FAM69A | Fanconi anemia, complementation group A | 1 | | 962 | FANCA | farnesyl diphosphate synthase (farnesyl pyrophosphate synthetase, dimethylallyltranstransferase, geranyltranstransferase) | 1 | | 963 | FAR1 | fatty acyl CoA reductase 1 | 1 | | 964 | FBXO40 | Fc fragment of IgE, high affinity I, receptor for; gamma polypeptide | 1 | | 965 | FBXO42 | fem-1 homolog c (C. elegans) | 1 | | 966 | FBXO9 | fibroblast growth factor 20 | 1 | | 967 | FCER1G | fibronectin type III domain containing 3B | 1 | | 968 | FDPS | FK506 binding protein 5 | 1 | | 969 | FEM1C | formyl peptide receptor 2 | 1 | | 970 | FGF20 | FOS-like antigen 2 | 1 | | 971 | FKBP5 | G protein-coupled receptor 171 | 1 | | 972 | FLJ32065 | G protein-coupled receptor 84 | 1 | | 973 | FLJ36840 | G protein-coupled receptor kinase interacting ArfGAP 2 | 1 | | 974 | FNDC3B | G protein-coupled receptor, family C, group 5, member C | 1 | | 975 | FOSL2 | germinal center expressed transcript 2 | 1 | | 976 | FOXRED2 | glutamate-cysteine ligase, modifier subunit | 1 | | 977 | FPR2 | glutamyl aminopeptidase (aminopeptidase A) | 1 | | 978 | GADD45A | glutathione S-transferase kappa 1 | 1 | | 979 | GALNT14 | glycerol-3-phosphate dehydrogenase 1-like | 1 | | 980 | GCET2 | glycerol kinase 3 pseudogene | 1 | | 981 | GCLM | glycine cleavage system protein H (aminomethyl carrier) | 1 | | 982 | GCSH | glycogen synthase kinase 3 alpha | 1 | | 983 | GHRH | golgi integral membrane protein 4 | 1 | | 984 | GIT2 | golgin A3 | 1 | | 985 | GK3P | growth arrest and DNA-damage-inducible, alpha | 1 | | 986 | GNAI3 | growth hormone releasing hormone | 1 | | 987 | GOLGA3 | GTP binding protein 6 (putative) | 1 | | 988 | GOLIM4 | guanine nucleotide binding protein (G protein), alpha inhibiting activity polypeptide 3 | 1 | | 989 | GPD1L | hairless homolog (mouse) | 1 | | 990 | GPR171 | HAUS augmin-like complex, subunit 2 | 1 | | 991 | GPR84 | HEAT repeat containing 2 | 1 | | 992 | GPRC5C | HEAT repeat containing 5B | 1 | | 993 | GSK3A | heat shock 70kDa protein 1A | 1 | | 994 | GSTK1 | heat shock protein, alpha-crystallin-related, B6 | 1 | | 995 | GTPBP6 | hect domain and RLD 6 | 1 | | 996 | HAP1 | HECT domain containing 1 | 1 | | 997 | HAUS2 | heme oxygenase (decycling) 2 | 1 | | 998 | HCK | hemopoietic cell kinase | 1 | | 999 | HDAC3 | hepatocellular carcinoma-associated gene TD26 | 1 | | 1000 | HEATR2 | Hermansky-Pudlak syndrome 5 | 1 | | 1001 | HEATR5B | heterogeneous nuclear ribonucleoprotein C (C1/C2) | 1 | | 1002 | HECTD1 | heterogeneous nuclear ribonucleoprotein M | 1 | | 1003 | HERC6 | high-mobility group box 2 | 1 | | 1004 | HINFP | histocompatibility (minor) HB-1 | 1 | | 1005 | HIST1H1A | histone cluster 1, H1a | 1 | | 1006 | HLA-E | histone deacetylase 3 | 1 | | 1007 | HMGB2 | histone H4 transcription factor | 1 | | 1008 | HMHB1 | homeobox C12 | 1 | | 1009 | HMOX2 | homeobox D12 | 1 | | 1010 | HNRNPC | huntingtin-associated protein 1 | 1 | | 1011 | HNRNPM | hypothetical LOC100129098 | 1 | | 1012 | HOXC12 | hypothetical LOC100233209 | 1 | | 1013 | HOXD12 | hypothetical LOC145837 | 1 | | 1014 | HPS5 | hypothetical LOC150527 | 1 | | 1015 | HR | hypothetical LOC158376 | 1 | | 1016 | HSPA1A | hypothetical LOC284900 | 1 | | 1017 | HSPB6 | hypothetical LOC400657 | 1 | | 1018 | IDUA | hypothetical LOC440173 | 1 | | 1019 | IL18R1 | hypothetical LOC440900 | 1 | | 1020 | IL1R1 | hypothetical LOC645212 | 1 | | 1021 | IL1R2 | hypothetical LOC645524 | 1 | | 1022 | IL21R | hypothetical protein FLJ32065 | 1 | | 1023 | IMPAD1 | hypothetical protein LOC100129129 | 1 | | 1024 | ING5 | hypothetical protein LOC100130344 | 1 | | 1025 | INTS9 | hypothetical protein LOC100288432 | 1 | | 1026 | IQCC | hypothetical protein LOC100293830 | 1 | | 1027 | IRG1 | hypothetical protein LOC149086 | 1 | | 1028 | ITGAM | hypothetical protein LOC283688 | 1 | | 1029 | ITGB1BP3 | hypothetical protein LOC283875 | 1 | | 1030 | ITPA | hypothetical protein LOC284669 | 1 | | 1031 | JARID2 | hypothetical protein LOC285463 | 1 | | 1032 | KCNJ12 | hypothetical protein LOC285708 | 1 | | 1033 | KCNQ1 | hypothetical protein LOC728114 | 1 | | 1034 | KCNS2 | hypothetical protein MGC10814 | 1 | | 1035 | KCNT1 | hypothetical protein, clone pT-Adv JuaX22 | 1 | | 1036 | KCP | iduronidase, alpha-L- | 1 | | 1037 | KDM1A | immunoresponsive 1 homolog (mouse) | 1 | | 1038 | KIAA0317 | inhibitor of growth family, member 5 | 1 | | 1039 | KIAA0528 | inosine triphosphatase (nucleoside triphosphate pyrophosphatase) | 1 | | 1040 | KIAA1377 | inositol monophosphatase domain containing 1 | 1 | | 1041 | KIF21A | integrator complex subunit 9 | 1 | | 1042 | KLF5 | integrin beta 1 binding protein 3 | 1 | | 1043 | KLK12 | integrin, alpha M (complement component 3 receptor 3 subunit) | 1 | | 1044 | KLK4 | interleukin 1 receptor, type I | 1 | | 1045 | KRT19P2 | interleukin 1 receptor, type II | 1 | | 1046 | KSR1 | interleukin 18 receptor 1 | 1 | | 1047 | LAGE3 | interleukin 21 receptor | 1 | | 1048 | LAMB1 | intestinal mucin-like | 1 | | 1049 | LARP4 | IQ motif containing C | 1 | | 1050 | LASP1 | jumonji, AT rich interactive domain 2 | 1 | | 1051 | LAT2 | kallikrein-related peptidase 12 | 1 | | 1052 | LIN7C | kallikrein-related peptidase 4 | 1 | | 1053 | LMO7 | kazrin | 1 | | 1054 | LOC100129098 | keratin 19 pseudogene 2 | 1 | | 1055 | LOC100129129 | KIAA0317 | 1 | | 1056 | LOC100129633 | KIAA0528 | 1 | | 1057 | LOC100130344 | KIAA1377 | 1 | | 1058 | LOC100132741 | kielin/chordin-like protein | 1 | | 1059 | LOC100133790 | kinase suppressor of ras 1 | 1 | | 1060 | LOC100233209 | kinesin family member 21A | 1 | | 1061 | LOC100288432 | kinesin family member 27 pseudogene | 1 | | 1062 | LOC100289833 | Kruppel-like factor 5 (intestinal) | 1 | | 1063 | LOC100293830 | L antigen family, member 3 | 1 | | 1064 | LOC145837 | La ribonucleoprotein domain family, member 4 | 1 | | 1065 | LOC149086 | laminin, beta 1 | 1 | | 1066 | LOC150527 | leucine-rich alpha-2-glycoprotein 1 | 1 | | 1067 | LOC158376 | leucine-rich repeats and calponin homology (CH) domain containing 4 | 1 | | 1068 | LOC283688 | leucine rich repeat and fibronectin type III domain containing 2 | 1 | | 1069 | LOC283875 | leucine rich repeat containing 58 | 1 | | 1070 | LOC284669 | LIM and SH3 protein 1 | 1 | | 1071 | LOC284900 | LIM domain 7 | 1 | | 1072 | LOC285463 | lin-7 homolog C (C. elegans) | 1 | | 1073 | LOC285708 | linker for activation of T cells family, member 2 | 1 | | 1074 | LOC388630 | LON peptidase N-terminal domain and ring finger 1 | 1 | | 1075 | LOC389765 | LSM14A, SCD6 homolog A (S. cerevisiae) | 1 | | 1076 | LOC400657 | lymphotoxin beta (TNF superfamily, member 3) | 1 | | 1077 | LOC440173 | lysine (K)-specific demethylase 1A | 1 | | 1078 | LOC440900 | major facilitator superfamily domain containing 3 | 1 | | 1079 | LOC55908 | major histocompatibility complex, class I, E | 1 | | 1080 | LOC645212 | malignant T cell amplified sequence 1 | 1 | | 1081 | LOC728114 | MAP kinase interacting serine/threonine kinase 1 | 1 | | 1082 | LONRF1 | MAX dimerization protein 1 | 1 | | 1083 | LRCH4 | mbt domain containing 1 | 1 | | 1084 | LRFN2 | McKusick-Kaufman syndrome | 1 | | 1085 | LRG1 | mediator complex subunit 29 | 1 | | 1086 | LRRC58 | membrane bound O-acyltransferase domain containing 2 | 1 | | 1087 | LSM14A | metallo-beta-lactamase domain containing 1 | 1 | | 1088 | LTB | methionine sulfoxide reductase A | 1 | | 1089 | MAFG | methyltransferase like 13 | 1 | | 1090 | MARCKS | methyltransferase like 3 | 1 | | 1091 | MBLAC1 | methyltransferase like 9 | 1 | | 1092 | MBOAT2 | migration and invasion inhibitory protein | 1 | | 1093 | MBTD1 | mitochondrial carrier homolog 2 (C. elegans) | 1 | | 1094 | MCTS1 | mitochondrial ribosomal protein 63 | 1 | | 1095 | MEAF6 | mitochondrial ribosomal protein L11 | 1 | | 1096 | MED29 | mitochondrial ribosomal protein L15 | 1 | | 1097 | METTL13 | mitochondrial ribosomal protein L17 | 1 | | 1098 | METTL3 | mitochondrial ribosomal protein L19 | 1 | | 1099 | METTL9 | mitochondrial ribosomal protein S10 | 1 | | 1100 | MFSD3 | mitochondrial ribosomal protein S7 | 1 | | 1101 | MGC10814 | MOB1, Mps One Binder kinase activator-like 1B (yeast) | 1 | | 1102 | MIIP | motile sperm domain containing 2 | 1 | | 1103 | MKKS | mutL homolog 1, colon cancer, nonpolyposis type 2 (E. coli) | 1 | | 1104 | MKNK1 | myosin, light chain 3, alkali; ventricular, skeletal, slow | 1 | | 1105 | MLH1 | myristoylated alanine-rich protein kinase C substrate | 1 | | 1106 | MOBKL1B | MYST/Esa1-associated factor 6 | 1 | | 1107 | MOSPD2 | N-acetyltransferase 8 (GCN5-related, putative) | 1 | | 1108 | MRP63 | N-ethylmaleimide-sensitive factor attachment protein, beta | 1 | | 1109 | MRPL11 | N(alpha)-acetyltransferase 20, NatB catalytic subunit | 1 | | 1110 | MRPL15 | N(alpha)-acetyltransferase 40, NatD catalytic subunit, homolog (S. cerevisiae) | 1 | | 1111 | MRPL17 | NADH dehydrogenase (ubiquinone) 1 beta subcomplex, 3, 12kDa | 1 | | 1112 | MRPL19 | NECAP endocytosis associated 2 | 1 | | 1113 | MRPS10 | neurexophilin 3 | 1 | | 1114 | MRPS7 | neuron navigator 1 | 1 | | 1115 | MSRA | neuronal PAS domain protein 1 | 1 | | 1116 | MTCH2 | nicotinamide N-methyltransferase | 1 | | 1117 | MXD1 | nitrilase family, member 2 | 1 | | 1118 | MYL3 | NK1 homeobox 1 | 1 | | 1119 | NAA20 | NLR family, apoptosis inhibitory protein | 1 | | 1120 | NAA40 | NLR family, pyrin domain containing 12 | 1 | | 1121 | NAIP | non-POU domain containing, octamer-binding | 1 | | 1122 | NAP1L3 | non-protein coding RNA 202 | 1 | | 1123 | NAPB | non-protein coding RNA 213 | 1 | | 1124 | NAT8 | non-protein coding RNA 94 | 1 | | 1125 | NAV1 | non-SMC condensin II complex, subunit H2 | 1 | | 1126 | NCAPH2 | nucleoporin 155kDa | 1 | | 1127 | NCRNA00094 | nucleosome assembly protein 1-like 3 | 1 | | 1128 | NCRNA00202 | Obg-like ATPase 1 | 1 | | 1129 | NCRNA00213 | obscurin-like 1 | 1 | | 1130 | NDUFB3 | olfactory receptor, family 1, subfamily F, member 1 | 1 | | 1131 | NECAP2 | olfactory receptor, family 10, subfamily D, member 1 pseudogene | 1 | | 1132 | NIT2 | olfactory receptor, family 2, subfamily H, member 2 | 1 | | 1133 | NKX1-1 | olfactory receptor, family 2, subfamily L, member 2 | 1 | | 1134 | NLRP12 | ornithine aminotransferase | 1 | | 1135 | NNMT | outer dense fiber of sperm tails 2 | 1 | | 1136 | NONO | oxoglutarate dehydrogenase-like | 1 | | 1137 | NPAS1 | pantothenate kinase 4 | 1 | | 1138 | NUP155 | paralemmin | 1 | | 1139 | NXPH3 | patatin-like phospholipase domain containing 1 | 1 | | 1140 | OAT | patatin-like phospholipase domain containing 5 | 1 | | 1141 | OBSL1 | paxillin | 1 | | 1142 | ODF2 | PBX/knotted 1 homeobox 2 | 1 | | 1143 | OGDHL | pentatricopeptide repeat domain 2 | 1 | | 1144 | OLA1 | peptidase (mitochondrial processing) alpha | 1 | | 1145 | OR10D1P | peptidase M20 domain containing 2 | 1 | | 1146 | OR1F1 | peptidyl arginine deiminase, type IV | 1 | | 1147 | OR2H2 | peptidylprolyl isomerase (cyclophilin)-like 5 | 1 | | 1148 | OR2L2 | peptidylprolyl isomerase G (cyclophilin G) | 1 | | 1149 | ORF1 | pericentrin | 1 | | 1150 | PADI4 | PHD finger protein 17 | 1 | | 1151 | PALM | PHD finger protein 21A | 1 | | 1152 | PANK4 | phenylethanolamine N-methyltransferase | 1 | | 1153 | PATE1 | phosphatidylinositol glycan anchor biosynthesis, class U | 1 | | 1154 | PATL1 | phosphatidylinositol transfer protein, beta | 1 | | 1155 | PBRM1 | phosphogluconate dehydrogenase | 1 | | 1156 | PBXIP1 | phospholipase B domain containing 1 | 1 | | 1157 | PCNT | PIF1 5'-to-3' DNA helicase homolog (S. cerevisiae) | 1 | | 1158 | PCSK5 | pim-3 oncogene | 1 | | 1159 | PDK3 | pleiomorphic adenoma gene-like 1 | 1 | | 1160 | PDP1 | polybromo 1 | 1 | | 1161 | PFDN6 | polycystic kidney disease 2 (autosomal dominant) | 1 | | 1162 | PGD | polymerase (DNA directed), epsilon 3 (p17 subunit) | 1 | | 1163 | PHF17 | polymerase (RNA) II (DNA directed) polypeptide K, 7.0kDa | 1 | | 1164 | PHF21A | potassium channel, subfamily T, member 1 | 1 | | 1165 | PIAS2 | potassium inwardly-rectifying channel, subfamily J, member 12 | 1 | | 1166 | PIF1 | potassium voltage-gated channel, delayed-rectifier, subfamily S, member 2 | 1 | | 1167 | PIGU | potassium voltage-gated channel, KQT-like subfamily, member 1 | 1 | | 1168 | PIM3 | PQ loop repeat containing 3 | 1 | | 1169 | PITPNB | pre-B-cell leukemia homeobox interacting protein 1 | 1 | | 1170 | PKD2 | pre T-cell antigen receptor alpha | 1 | | 1171 | PKNOX2 | prefoldin subunit 6 | 1 | | 1172 | PLAGL1 | pregnancy specific beta-1-glycoprotein 1 | 1 | | 1173 | PLBD1 | primase, DNA, polypeptide 1 (49kDa) | 1 | | 1174 | PM20D2 | proline-serine-threonine phosphatase interacting protein 2 | 1 | | 1175 | PMPCA | prolyl endopeptidase | 1 | | 1176 | PNMT | proprotein convertase subtilisin/kexin type 5 | 1 | | 1177 | PNPLA1 | prostate and testis expressed 1 | 1 | | 1178 | PNPLA5 | protamine 3 | 1 | | 1179 | POLE3 | protein arginine methyltransferase 2 | 1 | | 1180 | POLR2K | protein arginine methyltransferase 6 | 1 | | 1181 | PP14571 | protein associated with topoisomerase II homolog 1 (yeast) | 1 | | 1182 | PPIG | protein inhibitor of activated STAT, 2 | 1 | | 1183 | PPIL5 | protein kinase, cAMP-dependent, regulatory, type I, alpha (tissue specific extinguisher 1) | 1 | | 1184 | PPP4R2 | protein phosphatase 4, regulatory subunit 2 | 1 | | 1185 | PPP4R4 | protein phosphatase 4, regulatory subunit 4 | 1 | | 1186 | PQLC3 | prothymosin, alpha | 1 | | 1187 | PREP | pyruvate dehydrogenase kinase, isozyme 3 | 1 | | 1188 | PRIM1 | pyruvate dehyrogenase phosphatase catalytic subunit 1 | 1 | | 1189 | PRKAR1A | RAB18, member RAS oncogene family | 1 | | 1190 | PRM3 | RAB4A, member RAS oncogene family | 1 | | 1191 | PRMT2 | RAD23 homolog B (S. cerevisiae) | 1 | | 1192 | PRMT6 | RAD9 homolog A (S. pombe) | 1 | | 1193 | PSG1 | ral guanine nucleotide dissociation stimulator-like 4 | 1 | | 1194 | PSTPIP2 | RAP2A, member of RAS oncogene family | 1 | | 1195 | PTCD2 | Ras-like without CAAX 1 | 1 | | 1196 | PTCRA | Ras-related associated with diabetes | 1 | | 1197 | PTMA | RD RNA binding protein | 1 | | 1198 | PXN | regulator of chromosome condensation (RCC1) and BTB (POZ) domain containing protein 1 | 1 | | 1199 | RAB18 | regulator of G-protein signaling 14 | 1 | | 1200 | RAB4A | Rho GDP dissociation inhibitor (GDI) alpha | 1 | | 1201 | RAD23B | Rho GTPase activating protein 23 | 1 | | 1202 | RAD9A | ribonucleotide reductase M1 | 1 | | 1203 | RAP2A | ribonucleotide reductase M2 B (TP53 inducible) | 1 | | 1204 | RBM3 | ribosomal protein L28 | 1 | | 1205 | RBM33 | ribosomal protein L31 | 1 | | 1206 | RBM5 | ribosomal protein S19 | 1 | | 1207 | RCBTB1 | ribosomal protein S2 | 1 | | 1208 | RDBP | ribosomal protein S6 kinase, 90kDa, polypeptide 5 | 1 | | 1209 | RGL4 | ribosomal protein S8 | 1 | | 1210 | RGS14 | ribosomal RNA processing 8, methyltransferase, homolog (yeast) | 1 | | 1211 | RIT1 | ring finger protein 146 | 1 | | 1212 | RNF146 | ring finger protein 187 | 1 | | 1213 | RNF187 | RNA (guanine-7-) methyltransferase | 1 | | 1214 | RNMT | RNA binding motif (RNP1, RRM) protein 3 | 1 | | 1215 | RP1-21O18.1 | RNA binding motif protein 33 | 1 | | 1216 | RPAP3 | RNA binding motif protein 5 | 1 | | 1217 | RPL28 | RNA polymerase II associated protein 3 | 1 | | 1218 | RPL31 | rotatin | 1 | | 1219 | RPS19 | runt-related transcription factor 2 | 1 | | 1220 | RPS2 | S-phase kinase-associated protein 2 (p45) | 1 | | 1221 | RPS6KA5 | SAPS domain family, member 2 | 1 | | 1222 | RPS8 | sec1 family domain containing 2 | 1 | | 1223 | RRAD | SEC14-like 2 (S. cerevisiae) | 1 | | 1224 | RRM1 | sema domain, immunoglobulin domain (Ig), short basic domain, secreted, (semaphorin) 3F | 1 | | 1225 | RRM2B | sema domain, immunoglobulin domain (Ig), transmembrane domain (TM) and short cytoplasmic domain, (semaphorin) 4G | 1 | | 1226 | RRP8 | septin 7 | 1 | | 1227 | RTTN | serine/threonine kinase 25 (STE20 homolog, yeast) | 1 | | 1228 | RUNX2 | serine/threonine kinase 35 | 1 | | 1229 | SAPS2 | serpin peptidase inhibitor, clade B (ovalbumin), member 1 | 1 | | 1230 | SBNO2 | SERTA domain containing 2 | 1 | | 1231 | SCFD2 | serum response factor binding protein 1 | 1 | | 1232 | SDF2 | SET and MYND domain containing 1 | 1 | | 1233 | SEC14L2 | SFRS protein kinase 2 | 1 | | 1234 | SEMA3F | SFT2 domain containing 1 | 1 | | 1235 | SEMA4G | SFT2 domain containing 3 | 1 | | 1236 | SEPT7 | SH3-binding domain protein 5-like | 1 | | 1237 | SERPINB1 | shroom family member 1 | 1 | | 1238 | SERTAD2 | sialic acid binding Ig-like lectin 9 | 1 | | 1239 | SF3B3 | signal-induced proliferation-associated 1 like 2 | 1 | | 1240 | SFT2D1 | signal recognition particle 68kDa | 1 | | 1241 | SFT2D3 | signal sequence receptor, beta (translocon-associated protein beta) | 1 | | 1242 | SH3BP5L | similar to hCG1651427 | 1 | | 1243 | SHROOM1 | similar to hCG1655084 | 1 | | 1244 | SIGLEC9 | similar to hCG1777210 | 1 | | 1245 | SIPA1L2 | similar to hCG2038441 | 1 | | 1246 | SKP2 | Sjogren syndrome/scleroderma autoantigen 1 | 1 | | 1247 | SLAIN1 | SLAIN motif family, member 1 | 1 | | 1248 | SLC13A3 | SMAD family member 5 | 1 | | 1249 | SLC18A3 | small nuclear RNA activating complex, polypeptide 3, 50kDa | 1 | | 1250 | SLC25A30 | solute carrier family 13 (sodium-dependent dicarboxylate transporter), member 3 | 1 | | 1251 | SLC26A8 | solute carrier family 18 (vesicular acetylcholine), member 3 | 1 | | 1252 | SLC36A1 | solute carrier family 25, member 30 | 1 | | 1253 | SLC37A3 | solute carrier family 26, member 8 | 1 | | 1254 | SLC39A5 | solute carrier family 36 (proton/amino acid symporter), member 1 | 1 | | 1255 | SMAD5 | solute carrier family 37 (glycerol-3-phosphate transporter), member 3 | 1 | | 1256 | SMC1A | solute carrier family 39 (metal ion transporter), member 5 | 1 | | 1257 | SMYD1 | sorbin and SH3 domain containing 1 | 1 | | 1258 | SNAPC3 | SP140 nuclear body protein-like | 1 | | 1259 | SORBS1 | spectrin, beta, non-erythrocytic 2 | 1 | | 1260 | SP140L | splicing factor 3b, subunit 3, 130kDa | 1 | | 1261 | SPTBN2 | strawberry notch homolog 2 (Drosophila) | 1 | | 1262 | SQRDL | stromal cell-derived factor 2 | 1 | | 1263 | SRC | structural maintenance of chromosomes 1A | 1 | | 1264 | SRFBP1 | sulfide quinone reductase-like (yeast) | 1 | | 1265 | SRP68 | suppressor of Ty 3 homolog (S. cerevisiae) | 1 | | 1266 | SRPK2 | synapsin II | 1 | | 1267 | SS18 | synovial sarcoma translocation, chromosome 18 | 1 | | 1268 | SSR2 | syntaxin 11 | 1 | | 1269 | SSSCA1 | syntaxin 16 | 1 | | 1270 | STK25 | T cell receptor associated transmembrane adaptor 1 | 1 | | 1271 | STK35 | tafazzin | 1 | | 1272 | STX11 | TATA box binding protein (TBP)-associated factor, RNA polymerase I, A, 48kDa | 1 | | 1273 | STX16 | TatD DNase domain containing 3 | 1 | | 1274 | SUPT3H | TBC1 domain family, member 14 | 1 | | 1275 | SYN2 | TBC1 domain family, member 16 | 1 | | 1276 | TAF1A | teashirt zinc finger homeobox 3 | 1 | | 1277 | TATDN3 | testis expressed 10 | 1 | | 1278 | TAZ | testis expressed 13A | 1 | | 1279 | TBC1D14 | tetraspanin 3 | 1 | | 1280 | TBC1D16 | tetratricopeptide repeat domain 17 | 1 | | 1281 | TBCC | tetratricopeptide repeat domain 9 | 1 | | 1282 | TDRD9 | THAP domain containing 9 | 1 | | 1283 | TEX10 | thyroid hormone receptor interactor 11 | 1 | | 1284 | TEX13A | toll interacting protein | 1 | | 1285 | TFB2M | transcription factor B2, mitochondrial | 1 | | 1286 | TGFB2 | transcription termination factor, RNA polymerase I | 1 | | 1287 | THAP9 | transforming growth factor, beta 2 | 1 | | 1288 | TIMM9 | transient receptor potential cation channel, subfamily M, member 7 | 1 | | 1289 | TM4SF1 | translocase of inner mitochondrial membrane 9 homolog (yeast) | 1 | | 1290 | TMEM14B | transmembrane 4 L six family member 1 | 1 | | 1291 | TMEM57 | transmembrane protein 14B | 1 | | 1292 | TMEM66 | transmembrane protein 57 | 1 | | 1293 | TMEM69 | transmembrane protein 66 | 1 | | 1294 | TNPO1 | transmembrane protein 69 | 1 | | 1295 | TOLLIP | transportin 1 | 1 | | 1296 | TP53I11 | tripartite motif-containing 54 | 1 | | 1297 | TRAT1 | tripartite motif-containing 56 | 1 | | 1298 | TRIM54 | tubulin folding cofactor C | 1 | | 1299 | TRIM56 | tubulin, gamma complex associated protein 5 | 1 | | 1300 | TRIP11 | tudor domain containing 9 | 1 | | 1301 | TRPM7 | tumor protein p53 inducible protein 11 | 1 | | 1302 | TSHZ3 | ubiquilin 4 | 1 | | 1303 | TSPAN3 | ubiquitin specific peptidase 46 | 1 | | 1304 | TTC17 | UDP-Gal:betaGal beta 1,3-galactosyltransferase polypeptide 6 | 1 | | 1305 | TTC9 | UDP-N-acetyl-alpha-D-galactosamine:polypeptide N-acetylgalactosaminyltransferase 14 (GalNAc-T14) | 1 | | 1306 | TTF1 | UPF0632 protein A | 1 | | 1307 | TUBGCP5 | URB2 ribosome biogenesis 2 homolog (S. cerevisiae) | 1 | | 1308 | UBQLN4 | v-akt murine thymoma viral oncogene homolog 1 | 1 | | 1309 | URB2 | v-maf musculoaponeurotic fibrosarcoma oncogene homolog G (avian) | 1 | | 1310 | USP46 | v-src sarcoma (Schmidt-Ruppin A-2) viral oncogene homolog (avian) | 1 | | 1311 | VAMP1 | vacuolar protein sorting 13 homolog D (S. cerevisiae) | 1 | | 1312 | VEZF1 | vacuolar protein sorting 45 homolog (S. cerevisiae) | 1 | | 1313 | VPS13D | vascular endothelial zinc finger 1 | 1 | | 1314 | VPS45 | vesicle-associated membrane protein 1 (synaptobrevin 1) | 1 | | 1315 | WDR38 | WAP four-disulfide core domain 10A | 1 | | 1316 | WDR5B | WAP, follistatin/kazal, immunoglobulin, kunitz and netrin domain containing 1 | 1 | | 1317 | WDR7 | WD repeat domain 38 | 1 | | 1318 | WDR74 | WD repeat domain 5B | 1 | | 1319 | WFDC10A | WD repeat domain 7 | 1 | | 1320 | WFIKKN1 | WD repeat domain 74 | 1 | | 1321 | WISP2 | wingless-type MMTV integration site family, member 3 | 1 | | 1322 | WNT3 | wingless-type MMTV integration site family, member 5B | 1 | | 1323 | WNT5B | WNT1 inducible signaling pathway protein 2 | 1 | | 1324 | YBX1 | Y box binding protein 1 | 1 | | 1325 | YME1L1 | yippee-like 2 (Drosophila) | 1 | | 1326 | YPEL2 | YME1-like 1 (S. cerevisiae) | 1 | | 1327 | YTHDF1 | YTH domain family, member 1 | 1 | | 1328 | ZAN | zinc finger and BTB domain containing 11 | 1 | | 1329 | ZBTB11 | zinc finger and BTB domain containing 40 | 1 | | 1330 | ZBTB40 | zinc finger CCCH-type containing 6 | 1 | | 1331 | ZC3H6 | zinc finger protein 106 homolog (mouse) | 1 | | 1332 | ZDHHC20 | zinc finger protein 205 | 1 | | 1333 | ZFP106 | zinc finger protein 207 | 1 | | 1334 | ZFYVE28 | zinc finger protein 219 | 1 | | 1335 | ZHX1 | zinc finger protein 223 | 1 | | 1336 | ZNF205 | zinc finger protein 234 | 1 | | 1337 | ZNF207 | zinc finger protein 33B | 1 | | 1338 | ZNF219 | zinc finger protein 434 | 1 | | 1339 | ZNF223 | zinc finger protein 440 | 1 | | 1340 | ZNF234 | zinc finger protein 449 | 1 | | 1341 | ZNF33B | zinc finger protein 497 | 1 | | 1342 | ZNF434 | zinc finger protein 532 | 1 | | 1343 | ZNF440 | zinc finger protein 540 | 1 | | 1344 | ZNF449 | zinc finger protein 555 | 1 | | 1345 | ZNF497 | zinc finger protein 564 | 1 | | 1346 | ZNF532 | zinc finger protein 589 | 1 | | 1347 | ZNF540 | zinc finger protein 853 | 1 | | 1348 | ZNF555 | zinc finger, DHHC-type containing 20 | 1 | | 1349 | ZNF564 | zinc finger, FYVE domain containing 28 | 1 | | 1350 | ZNF589 | zinc fingers and homeoboxes 1 | 1 | | 1351 | ZNF853 | zonadhesin | 1 | |

---

Total number of miRNA-mRNA 3499 interactions for given cut-off. Press for ALL:

| |  | miR | EntrezID | Gene | Name | Score | | --- | --- | --- | --- | --- | --- | | 315 | hsa-miR-766 | 6418 | SET | SET nuclear oncogene | 5 | | 67 | hsa-miR-618 | 23200 | ATP11B | ATPase, class VI, type 11B | 4 | | 299 | hsa-miR-874 | 3178 | HNRNPA1 | heterogeneous nuclear ribonucleoprotein A1 | 4 | | 560 | hsa-miR-1233 | 8445 | DYRK2 | dual-specificity tyrosine-(Y)-phosphorylation regulated kinase 2 | 4 | | 568 | hsa-miR-1233 | 58487 | CREBZF | CREB/ATF bZIP transcription factor | 4 | | 891 | hsa-miR-618 | 634 | CEACAM1 | carcinoembryonic antigen-related cell adhesion molecule 1 (biliary glycoprotein) | 4 | | 1540 | hsa-miR-766 | 5379 | PMS2L1 | postmeiotic segregation increased 2-like 1 pseudogene | 4 | | 1684 | hsa-miR-766 | 6130 | RPL7A | ribosomal protein L7a | 4 | | 1705 | hsa-miR-1233 | 55037 | PTCD3 | Pentatricopeptide repeat domain 3 | 4 | | 1709 | hsa-miR-874 | 55037 | PTCD3 | Pentatricopeptide repeat domain 3 | 4 | | 1836 | hsa-miR-618 | 29097 | CNIH4 | cornichon homolog 4 (Drosophila) | 4 | | 51 | hsa-miR-766 | 51275 | C12orf47 | chromosome 12 open reading frame 47 | 3 | | 115 | hsa-miR-1233 | 256380 | SCML4 | sex comb on midleg-like 4 (Drosophila) | 3 | | 117 | hsa-miR-874 | 256380 | SCML4 | sex comb on midleg-like 4 (Drosophila) | 3 | | 133 | hsa-miR-1233 | 64417 | C5orf28 | chromosome 5 open reading frame 28 | 3 | | 137 | hsa-miR-874 | 64417 | C5orf28 | chromosome 5 open reading frame 28 | 3 | | 173 | hsa-miR-423-5p | 84859 | LRCH3 | leucine-rich repeats and calponin homology (CH) domain containing 3 | 3 | | 325 | hsa-miR-623 | 6134 | RPL10 | ribosomal protein L10 | 3 | | 350 | hsa-miR-1233 | 9669 | EIF5B | eukaryotic translation initiation factor 5B | 3 | | 392 | hsa-miR-877 | 3420 | IDH3B | isocitrate dehydrogenase 3 (NAD+) beta | 3 | | 407 | hsa-miR-623 | 8106 | PABPN1 | poly(A) binding protein, nuclear 1 | 3 | | 437 | hsa-miR-1233 | 9987 | HNRPDL | heterogeneous nuclear ribonucleoprotein D-like | 3 | | 450 | hsa-miR-623 | 6687 | SPG7 | spastic paraplegia 7 (pure and complicated autosomal recessive) | 3 | | 451 | hsa-miR-877 | 6687 | SPG7 | spastic paraplegia 7 (pure and complicated autosomal recessive) | 3 | | 470 | hsa-miR-938 | 8897 | MTMR3 | myotubularin related protein 3 | 3 | | 563 | hsa-miR-766 | 8445 | DYRK2 | dual-specificity tyrosine-(Y)-phosphorylation regulated kinase 2 | 3 | | 564 | hsa-miR-874 | 8445 | DYRK2 | dual-specificity tyrosine-(Y)-phosphorylation regulated kinase 2 | 3 | | 595 | hsa-miR-623 | 6187 | RPS2 | ribosomal protein S2 | 3 | | 681 | hsa-miR-874 | 55556 | ENOSF1 | enolase superfamily member 1 | 3 | | 975 | hsa-miR-1233 | 3824 | KLRD1 | killer cell lectin-like receptor subfamily D, member 1 | 3 | | 1080 | hsa-miR-877 | 3609 | ILF3 | interleukin enhancer binding factor 3, 90kDa | 3 | | 1301 | hsa-miR-423-5p | 23122 | CLASP2 | cytoplasmic linker associated protein 2 | 3 | | 1522 | hsa-miR-1233 | 3178 | HNRNPA1 | heterogeneous nuclear ribonucleoprotein A1 | 3 | | 1525 | hsa-miR-766 | 3178 | HNRNPA1 | heterogeneous nuclear ribonucleoprotein A1 | 3 | | 1541 | hsa-miR-877 | 5379 | PMS2L1 | postmeiotic segregation increased 2-like 1 pseudogene | 3 | | 1574 | hsa-miR-571 | 54014 | BRWD1 | bromodomain and WD repeat domain containing 1 | 3 | | 1601 | hsa-miR-1233 | 55340 | GIMAP5 | GTPase, IMAP family member 5 | 3 | | 1685 | hsa-miR-874 | 6130 | RPL7A | ribosomal protein L7a | 3 | | 1708 | hsa-miR-766 | 55037 | PTCD3 | Pentatricopeptide repeat domain 3 | 3 | | 1714 | hsa-miR-571 | 394 | ARHGAP5 | Rho GTPase activating protein 5 | 3 | | 1783 | hsa-miR-1233 | 10600 | USP16 | ubiquitin specific peptidase 16 | 3 | | 1785 | hsa-miR-766 | 10600 | USP16 | ubiquitin specific peptidase 16 | 3 | | 1795 | hsa-miR-1233 | 55900 | ZNF302 | zinc finger protein 302 | 3 | | 1796 | hsa-miR-558 | 55900 | ZNF302 | zinc finger protein 302 | 3 | | 1797 | hsa-miR-571 | 55900 | ZNF302 | zinc finger protein 302 | 3 | | 1915 | hsa-miR-571 | 65986 | ZBTB10 | zinc finger and BTB domain containing 10 | 3 | | 1974 | hsa-miR-1233 | 56172 | ANKH | ankylosis, progressive homolog (mouse) | 3 | | 1975 | hsa-miR-571 | 56172 | ANKH | ankylosis, progressive homolog (mouse) | 3 | | 1976 | hsa-miR-874 | 56172 | ANKH | ankylosis, progressive homolog (mouse) | 3 | | 2022 | hsa-miR-1233 | 26355 | FAM162A | family with sequence similarity 162, member A | 3 | | 2031 | hsa-miR-618 | 9236 | CCPG1 | cell cycle progression 1 | 3 | | 2035 | hsa-miR-1233 | 60468 | BACH2 | BTB and CNC homology 1, basic leucine zipper transcription factor 2 | 3 | | 2126 | hsa-miR-1233 | 11276 | SYNRG | synergin, gamma | 3 | | 2129 | hsa-miR-571 | 11276 | SYNRG | synergin, gamma | 3 | | 2131 | hsa-miR-874 | 11276 | SYNRG | synergin, gamma | 3 | | 2292 | hsa-mir-149 | 55632 | G2E3 | G2/M-phase specific E3 ubiquitin ligase | 3 | | 2293 | hsa-miR-149 | 55632 | G2E3 | G2/M-phase specific E3 ubiquitin ligase | 3 | | 2351 | hsa-miR-95 | 57731 | SPTBN4 | spectrin, beta, non-erythrocytic 4 | 3 | | 2457 | hsa-miR-766 | 124540 | MSI2 | musashi homolog 2 (Drosophila) | 3 | | 2630 | hsa-miR-1233 | 152137 | CCDC50 | coiled-coil domain containing 50 | 3 | | 2631 | hsa-miR-571 | 152137 | CCDC50 | coiled-coil domain containing 50 | 3 | | 2645 | hsa-miR-1233 | 152485 | ZNF827 | zinc finger protein 827 | 3 | | 3190 | hsa-miR-1233 | 163081 | ZNF567 | zinc finger protein 567 | 3 | | 1 | hsa-miR-623 | 7318 | UBA7 | ubiquitin-like modifier activating enzyme 7 | 2 | | 2 | hsa-mir-3130-1 | 10406 | WFDC2 | WAP four-disulfide core domain 2 | 2 | | 3 | hsa-mir-3130-2 | 10406 | WFDC2 | WAP four-disulfide core domain 2 | 2 | | 4 | hsa-mir-3130-3 | 10406 | WFDC2 | WAP four-disulfide core domain 2 | 2 | | 5 | hsa-mir-3130-4 | 10406 | WFDC2 | WAP four-disulfide core domain 2 | 2 | | 9 | hsa-miR-618 | 5594 | MAPK1 | mitogen-activated protein kinase 1 | 2 | | 12 | hsa-miR-1233 | 170575 | GIMAP1 | GTPase, IMAP family member 1 | 2 | | 17 | hsa-miR-618 | 338339 | CLEC4D | C-type lectin domain family 4, member D | 2 | | 22 | hsa-miR-423-5p | 196528 | ARID2 | AT rich interactive domain 2 (ARID, RFX-like) | 2 | | 31 | hsa-miR-95 | 283860 | C16orf81 | chromosome 16 open reading frame 81 | 2 | | 38 | hsa-miR-571 | 112487 | C14orf126 | chromosome 14 open reading frame 126 | 2 | | 42 | hsa-miR-1233 | 27334 | P2RY10 | purinergic receptor P2Y, G-protein coupled, 10 | 2 | | 43 | hsa-miR-569 | 27334 | P2RY10 | purinergic receptor P2Y, G-protein coupled, 10 | 2 | | 44 | hsa-miR-766 | 27334 | P2RY10 | purinergic receptor P2Y, G-protein coupled, 10 | 2 | | 80 | hsa-miR-938 | 91662 | NLRP12 | NLR family, pyrin domain containing 12 | 2 | | 82 | hsa-miR-1233 | 10225 | CD96 | CD96 molecule | 2 | | 83 | hsa-miR-569 | 10225 | CD96 | CD96 molecule | 2 | | 84 | hsa-miR-571 | 10225 | CD96 | CD96 molecule | 2 | | 85 | hsa-miR-874 | 10225 | CD96 | CD96 molecule | 2 | | 87 | hsa-miR-423-5p | 11179 | ZNF277 | zinc finger protein 277 | 2 | | 88 | hsa-miR-576-5p | 11179 | ZNF277 | zinc finger protein 277 | 2 | | 91 | hsa-miR-618 | 8754 | ADAM9 | ADAM metallopeptidase domain 9 (meltrin gamma) | 2 | | 104 | hsa-miR-95 | 974 | CD79B | CD79b molecule, immunoglobulin-associated beta | 2 | | 109 | hsa-mir-10a | 55216 | C11orf57 | chromosome 11 open reading frame 57 | 2 | | 116 | hsa-miR-766 | 256380 | SCML4 | sex comb on midleg-like 4 (Drosophila) | 2 | | 135 | hsa-miR-571 | 64417 | C5orf28 | chromosome 5 open reading frame 28 | 2 | | 138 | hsa-miR-938 | 249 | ALPL | alkaline phosphatase, liver/bone/kidney | 2 | | 139 | hsa-miR-1233 | 130916 | MTERFD2 | MTERF domain containing 2 | 2 | | 142 | hsa-miR-571 | 130916 | MTERFD2 | MTERF domain containing 2 | 2 | | 147 | hsa-miR-576-5p | 6733 | SRPK2 | SFRS protein kinase 2 | 2 | | 149 | hsa-miR-95 | 400258 | C14orf180 | chromosome 14 open reading frame 180 | 2 | | 172 | hsa-mir-885 | 84859 | LRCH3 | leucine-rich repeats and calponin homology (CH) domain containing 3 | 2 | | 174 | hsa-miR-885-5p | 84859 | LRCH3 | leucine-rich repeats and calponin homology (CH) domain containing 3 | 2 | | 215 | hsa-miR-95 | 89876 | C3orf15 | chromosome 3 open reading frame 15 | 2 | | 241 | hsa-miR-571 | 1122 | CHML | choroideremia-like (Rab escort protein 2) | 2 | | 247 | hsa-miR-1233 | 923 | CD6 | CD6 molecule | 2 | | 250 | hsa-miR-766 | 923 | CD6 | CD6 molecule | 2 | | 251 | hsa-miR-874 | 923 | CD6 | CD6 molecule | 2 | | 257 | hsa-miR-10a | 81137 | OR7E104P | olfactory receptor, family 7, subfamily E, member 104 pseudogene | 2 | | 271 | hsa-miR-95 | 26246 | OR2L2 | olfactory receptor, family 2, subfamily L, member 2 | 2 | | 275 | hsa-mir-3130-1 | 4240 | MFGE8 | milk fat globule-EGF factor 8 protein | 2 | | 276 | hsa-mir-3130-2 | 4240 | MFGE8 | milk fat globule-EGF factor 8 protein | 2 | | 277 | hsa-mir-3130-3 | 4240 | MFGE8 | milk fat globule-EGF factor 8 protein | 2 | | 278 | hsa-mir-3130-4 | 4240 | MFGE8 | milk fat globule-EGF factor 8 protein | 2 | | 280 | hsa-miR-95 | 4240 | MFGE8 | milk fat globule-EGF factor 8 protein | 2 | | 289 | hsa-miR-1233 | 23195 | MDN1 | MDN1, midasin homolog (yeast) | 2 | | 290 | hsa-miR-571 | 23195 | MDN1 | MDN1, midasin homolog (yeast) | 2 | | 291 | hsa-miR-874 | 23195 | MDN1 | MDN1, midasin homolog (yeast) | 2 | | 298 | hsa-miR-558 | 3178 | HNRNPA1 | heterogeneous nuclear ribonucleoprotein A1 | 2 | | 316 | hsa-miR-1233 | 6418 | SET | SET nuclear oncogene | 2 | | 317 | hsa-miR-569 | 6418 | SET | SET nuclear oncogene | 2 | | 318 | hsa-miR-874 | 6418 | SET | SET nuclear oncogene | 2 | | 330 | hsa-miR-1233 | 2058 | EPRS | glutamyl-prolyl-tRNA synthetase | 2 | | 331 | hsa-miR-766 | 2058 | EPRS | glutamyl-prolyl-tRNA synthetase | 2 | | 343 | hsa-miR-1233 | 3983 | ABLIM1 | actin binding LIM protein 1 | 2 | | 345 | hsa-miR-766 | 3983 | ABLIM1 | actin binding LIM protein 1 | 2 | | 346 | hsa-miR-874 | 3983 | ABLIM1 | actin binding LIM protein 1 | 2 | | 349 | hsa-miR-618 | 10970 | CKAP4 | cytoskeleton-associated protein 4 | 2 | | 351 | hsa-miR-874 | 9669 | EIF5B | eukaryotic translation initiation factor 5B | 2 | | 353 | hsa-miR-766 | 9669 | EIF5B | eukaryotic translation initiation factor 5B | 2 | | 355 | hsa-miR-1233 | 3945 | LDHB | lactate dehydrogenase B | 2 | | 357 | hsa-miR-586 | 3945 | LDHB | lactate dehydrogenase B | 2 | | 358 | hsa-miR-766 | 3945 | LDHB | lactate dehydrogenase B | 2 | | 359 | hsa-miR-874 | 3945 | LDHB | lactate dehydrogenase B | 2 | | 363 | hsa-miR-877 | 6599 | SMARCC1 | SWI/SNF related, matrix associated, actin dependent regulator of chromatin, subfamily c, member 1 | 2 | | 365 | hsa-miR-938 | 5829 | PXN | paxillin | 2 | | 366 | hsa-miR-454\* | 1434 | CSE1L | CSE1 chromosome segregation 1-like (yeast) | 2 | | 371 | hsa-miR-135a | 396 | ARHGDIA | Rho GDP dissociation inhibitor (GDI) alpha | 2 | | 387 | hsa-miR-623 | 9416 | DDX23 | DEAD (Asp-Glu-Ala-Asp) box polypeptide 23 | 2 | | 406 | hsa-miR-623 | 1973 | EIF4A1 | eukaryotic translation initiation factor 4A1 | 2 | | 431 | hsa-miR-555 | 23394 | ADNP | activity-dependent neuroprotector homeobox | 2 | | 439 | hsa-miR-874 | 9987 | HNRPDL | heterogeneous nuclear ribonucleoprotein D-like | 2 | | 453 | hsa-miR-555 | 8899 | PRPF4B | PRP4 pre-mRNA processing factor 4 homolog B (yeast) | 2 | | 458 | hsa-miR-1233 | 158 | ADSL | adenylosuccinate lyase | 2 | | 469 | hsa-miR-618 | 8897 | MTMR3 | myotubularin related protein 3 | 2 | | 489 | hsa-miR-423-5p | 2531 | KDSR | 3-ketodihydrosphingosine reductase | 2 | | 499 | hsa-miR-423-5p | 9255 | AIMP1 | aminoacyl tRNA synthetase complex-interacting multifunctional protein 1 | 2 | | 519 | hsa-miR-623 | 6223 | RPS19 | ribosomal protein S19 | 2 | | 533 | hsa-miR-1233 | 9452 | ITM2A | integral membrane protein 2A | 2 | | 534 | hsa-miR-766 | 9452 | ITM2A | integral membrane protein 2A | 2 | | 550 | hsa-miR-938 | 140885 | SIRPA | signal-regulatory protein alpha | 2 | | 561 | hsa-miR-569 | 8445 | DYRK2 | dual-specificity tyrosine-(Y)-phosphorylation regulated kinase 2 | 2 | | 562 | hsa-miR-571 | 8445 | DYRK2 | dual-specificity tyrosine-(Y)-phosphorylation regulated kinase 2 | 2 | | 565 | hsa-miR-558 | 8445 | DYRK2 | dual-specificity tyrosine-(Y)-phosphorylation regulated kinase 2 | 2 | | 570 | hsa-miR-766 | 58487 | CREBZF | CREB/ATF bZIP transcription factor | 2 | | 573 | hsa-miR-571 | 58487 | CREBZF | CREB/ATF bZIP transcription factor | 2 | | 576 | hsa-mir-10a | 9529 | BAG5 | BCL2-associated athanogene 5 | 2 | | 577 | hsa-miR-423-5p | 9529 | BAG5 | BCL2-associated athanogene 5 | 2 | | 587 | hsa-miR-423-5p | 8453 | CUL2 | cullin 2 | 2 | | 588 | hsa-miR-576-5p | 8453 | CUL2 | cullin 2 | 2 | | 598 | hsa-miR-618 | 7077 | TIMP2 | TIMP metallopeptidase inhibitor 2 | 2 | | 614 | hsa-mir-10a | 23473 | CAPN7 | calpain 7 | 2 | | 615 | hsa-miR-423-5p | 23473 | CAPN7 | calpain 7 | 2 | | 623 | hsa-miR-1233 | 8481 | OFD1 | oral-facial-digital syndrome 1 | 2 | | 625 | hsa-miR-874 | 8481 | OFD1 | oral-facial-digital syndrome 1 | 2 | | 651 | hsa-miR-877 | 55794 | DDX28 | DEAD (Asp-Glu-Ala-Asp) box polypeptide 28 | 2 | | 662 | hsa-miR-95 | 4320 | MMP11 | matrix metallopeptidase 11 (stromelysin 3) | 2 | | 665 | hsa-miR-593\* | 513 | ATP5D | ATP synthase, H+ transporting, mitochondrial F1 complex, delta subunit | 2 | | 670 | hsa-miR-1233 | 8504 | PEX3 | peroxisomal biogenesis factor 3 | 2 | | 672 | hsa-miR-571 | 8504 | PEX3 | peroxisomal biogenesis factor 3 | 2 | | 680 | hsa-miR-877 | 5550 | PREP | prolyl endopeptidase | 2 | | 682 | hsa-miR-1233 | 55556 | ENOSF1 | enolase superfamily member 1 | 2 | | 684 | hsa-miR-571 | 55556 | ENOSF1 | enolase superfamily member 1 | 2 | | 707 | hsa-miR-576-5p | 1385 | CREB1 | cAMP responsive element binding protein 1 | 2 | | 712 | hsa-miR-877 | 22907 | DHX30 | DEAH (Asp-Glu-Ala-His) box polypeptide 30 | 2 | | 720 | hsa-miR-1233 | 7637 | ZNF84 | zinc finger protein 84 | 2 | | 722 | hsa-miR-571 | 7637 | ZNF84 | zinc finger protein 84 | 2 | | 723 | hsa-miR-874 | 7637 | ZNF84 | zinc finger protein 84 | 2 | | 727 | hsa-miR-571 | 5810 | RAD1 | RAD1 homolog (S. pombe) | 2 | | 748 | hsa-miR-576-5p | 905 | CCNT2 | cyclin T2 | 2 | | 749 | hsa-mir-628 | 1043 | CD52 | CD52 molecule | 2 | | 750 | hsa-miR-623 | 1043 | CD52 | CD52 molecule | 2 | | 751 | hsa-miR-628-5p | 1043 | CD52 | CD52 molecule | 2 | | 752 | hsa-miR-1233 | 7049 | TGFBR3 | transforming growth factor, beta receptor III | 2 | | 753 | hsa-miR-569 | 7049 | TGFBR3 | transforming growth factor, beta receptor III | 2 | | 754 | hsa-miR-766 | 7049 | TGFBR3 | transforming growth factor, beta receptor III | 2 | | 755 | hsa-miR-874 | 7049 | TGFBR3 | transforming growth factor, beta receptor III | 2 | | 773 | hsa-miR-618 | 4671 | NAIP | NLR family, apoptosis inhibitory protein | 2 | | 779 | hsa-miR-423-5p | 9949 | AMMECR1 | Alport syndrome, mental retardation, midface hypoplasia and elliptocytosis chromosomal region gene 1 | 2 | | 795 | hsa-miR-938 | 9744 | ACAP1 | ArfGAP with coiled-coil, ankyrin repeat and PH domains 1 | 2 | | 800 | hsa-miR-874 | 80184 | CEP290 | centrosomal protein 290kDa | 2 | | 809 | hsa-miR-618 | 7850 | IL1R2 | interleukin 1 receptor, type II | 2 | | 842 | hsa-miR-1233 | 80342 | TRAF3IP3 | TRAF3 interacting protein 3 | 2 | | 843 | hsa-miR-766 | 80342 | TRAF3IP3 | TRAF3 interacting protein 3 | 2 | | 844 | hsa-miR-874 | 80342 | TRAF3IP3 | TRAF3 interacting protein 3 | 2 | | 850 | hsa-miR-1233 | 11168 | PSIP1 | PC4 and SFRS1 interacting protein 1 | 2 | | 851 | hsa-miR-569 | 11168 | PSIP1 | PC4 and SFRS1 interacting protein 1 | 2 | | 852 | hsa-miR-766 | 11168 | PSIP1 | PC4 and SFRS1 interacting protein 1 | 2 | | 853 | hsa-miR-1233 | 22868 | FASTKD2 | FAST kinase domains 2 | 2 | | 854 | hsa-miR-571 | 22868 | FASTKD2 | FAST kinase domains 2 | 2 | | 855 | hsa-miR-874 | 22868 | FASTKD2 | FAST kinase domains 2 | 2 | | 862 | hsa-miR-1233 | 6627 | SNRPA1 | small nuclear ribonucleoprotein polypeptide A' | 2 | | 873 | hsa-miR-618 | 5165 | PDK3 | pyruvate dehydrogenase kinase, isozyme 3 | 2 | | 874 | hsa-miR-1233 | 4094 | MAF | v-maf musculoaponeurotic fibrosarcoma oncogene homolog (avian) | 2 | | 875 | hsa-miR-874 | 4094 | MAF | v-maf musculoaponeurotic fibrosarcoma oncogene homolog (avian) | 2 | | 925 | hsa-miR-423-5p | 54468 | MIOS | missing oocyte, meiosis regulator, homolog (Drosophila) | 2 | | 948 | hsa-mir-10a | 55832 | CAND1 | cullin-associated and neddylation-dissociated 1 | 2 | | 950 | hsa-miR-1233 | 55832 | CAND1 | cullin-associated and neddylation-dissociated 1 | 2 | | 951 | hsa-miR-423-5p | 55832 | CAND1 | cullin-associated and neddylation-dissociated 1 | 2 | | 953 | hsa-miR-571 | 55832 | CAND1 | cullin-associated and neddylation-dissociated 1 | 2 | | 968 | hsa-miR-938 | 5606 | MAP2K3 | mitogen-activated protein kinase kinase 3 | 2 | | 969 | hsa-miR-95 | 3834 | KIF25 | kinesin family member 25 | 2 | | 976 | hsa-miR-569 | 3824 | KLRD1 | killer cell lectin-like receptor subfamily D, member 1 | 2 | | 977 | hsa-miR-766 | 3824 | KLRD1 | killer cell lectin-like receptor subfamily D, member 1 | 2 | | 980 | hsa-miR-454\* | 26003 | GORASP2 | golgi reassembly stacking protein 2, 55kDa | 2 | | 1014 | hsa-miR-10a | 2740 | GLP1R | glucagon-like peptide 1 receptor | 2 | | 1034 | hsa-miR-10a | 7148 | TNXB | tenascin XB | 2 | | 1037 | hsa-miR-938 | 87 | ACTN1 | actinin, alpha 1 | 2 | | 1046 | hsa-miR-1233 | 10236 | HNRNPR | heterogeneous nuclear ribonucleoprotein R | 2 | | 1062 | hsa-miR-423-5p | 51366 | UBR5 | ubiquitin protein ligase E3 component n-recognin 5 | 2 | | 1064 | hsa-miR-576-5p | 51366 | UBR5 | ubiquitin protein ligase E3 component n-recognin 5 | 2 | | 1067 | hsa-miR-1233 | 8886 | DDX18 | DEAD (Asp-Glu-Ala-Asp) box polypeptide 18 | 2 | | 1068 | hsa-miR-766 | 8886 | DDX18 | DEAD (Asp-Glu-Ala-Asp) box polypeptide 18 | 2 | | 1079 | hsa-miR-766 | 3609 | ILF3 | interleukin enhancer binding factor 3, 90kDa | 2 | | 1090 | hsa-miR-766 | 9987 | HNRPDL | heterogeneous nuclear ribonucleoprotein D-like | 2 | | 1120 | hsa-miR-423-5p | 11168 | PSIP1 | PC4 and SFRS1 interacting protein 1 | 2 | | 1142 | hsa-mir-3130-1 | 3479 | IGF1 | insulin-like growth factor 1 (somatomedin C) | 2 | | 1143 | hsa-mir-3130-2 | 3479 | IGF1 | insulin-like growth factor 1 (somatomedin C) | 2 | | 1144 | hsa-mir-3130-3 | 3479 | IGF1 | insulin-like growth factor 1 (somatomedin C) | 2 | | 1145 | hsa-mir-3130-4 | 3479 | IGF1 | insulin-like growth factor 1 (somatomedin C) | 2 | | 1154 | hsa-miR-766 | 11118 | BTN3A2 | butyrophilin, subfamily 3, member A2 | 2 | | 1164 | hsa-miR-874 | 5243 | ABCB1 | ATP-binding cassette, sub-family B (MDR/TAP), member 1 | 2 | | 1167 | hsa-miR-623 | 3420 | IDH3B | isocitrate dehydrogenase 3 (NAD+) beta | 2 | | 1171 | hsa-miR-1233 | 5588 | PRKCQ | protein kinase C, theta | 2 | | 1172 | hsa-miR-569 | 5588 | PRKCQ | protein kinase C, theta | 2 | | 1173 | hsa-miR-571 | 5588 | PRKCQ | protein kinase C, theta | 2 | | 1174 | hsa-miR-766 | 5588 | PRKCQ | protein kinase C, theta | 2 | | 1175 | hsa-miR-874 | 5588 | PRKCQ | protein kinase C, theta | 2 | | 1182 | hsa-miR-766 | 10772 | SFRS13A | splicing factor, arginine/serine-rich 13A | 2 | | 1219 | hsa-miR-938 | 2358 | FPR2 | formyl peptide receptor 2 | 2 | | 1222 | hsa-miR-1233 | 8718 | TNFRSF25 | tumor necrosis factor receptor superfamily, member 25 | 2 | | 1223 | hsa-miR-766 | 8718 | TNFRSF25 | tumor necrosis factor receptor superfamily, member 25 | 2 | | 1254 | hsa-miR-95 | 3691 | ITGB4 | integrin, beta 4 | 2 | | 1259 | hsa-miR-1233 | 220988 | HNRNPA3 | heterogeneous nuclear ribonucleoprotein A3 | 2 | | 1266 | hsa-miR-1233 | 3843 | IPO5 | importin 5 | 2 | | 1268 | hsa-miR-766 | 3843 | IPO5 | importin 5 | 2 | | 1294 | hsa-miR-618 | 1992 | SERPINB1 | serpin peptidase inhibitor, clade B (ovalbumin), member 1 | 2 | | 1297 | hsa-mir-10a | 23122 | CLASP2 | cytoplasmic linker associated protein 2 | 2 | | 1303 | hsa-miR-571 | 23122 | CLASP2 | cytoplasmic linker associated protein 2 | 2 | | 1306 | hsa-mir-10a | 51029 | PPPDE1 | PPPDE peptidase domain containing 1 | 2 | | 1314 | hsa-miR-423-5p | 23091 | ZC3H13 | zinc finger CCCH-type containing 13 | 2 | | 1315 | hsa-miR-576-5p | 23091 | ZC3H13 | zinc finger CCCH-type containing 13 | 2 | | 1321 | hsa-miR-766 | 23131 | GPATCH8 | G patch domain containing 8 | 2 | | 1327 | hsa-miR-1233 | 23348 | DOCK9 | dedicator of cytokinesis 9 | 2 | | 1329 | hsa-miR-571 | 23348 | DOCK9 | dedicator of cytokinesis 9 | 2 | | 1331 | hsa-miR-874 | 23348 | DOCK9 | dedicator of cytokinesis 9 | 2 | | 1335 | hsa-miR-454\* | 1737 | DLAT | dihydrolipoamide S-acetyltransferase | 2 | | 1337 | hsa-miR-423-5p | 23376 | KIAA0776 | KIAA0776 | 2 | | 1365 | hsa-miR-1233 | 8880 | FUBP1 | far upstream element (FUSE) binding protein 1 | 2 | | 1367 | hsa-miR-571 | 8880 | FUBP1 | far upstream element (FUSE) binding protein 1 | 2 | | 1368 | hsa-miR-766 | 8880 | FUBP1 | far upstream element (FUSE) binding protein 1 | 2 | | 1380 | hsa-miR-1233 | 56950 | SMYD2 | SET and MYND domain containing 2 | 2 | | 1382 | hsa-miR-766 | 56950 | SMYD2 | SET and MYND domain containing 2 | 2 | | 1383 | hsa-miR-874 | 56950 | SMYD2 | SET and MYND domain containing 2 | 2 | | 1385 | hsa-miR-1233 | 6137 | RPL13 | ribosomal protein L13 | 2 | | 1386 | hsa-miR-874 | 6137 | RPL13 | ribosomal protein L13 | 2 | | 1388 | hsa-miR-95 | 1291 | COL6A1 | collagen, type VI, alpha 1 | 2 | | 1404 | hsa-miR-1233 | 253959 | RALGAPA1 | Ral GTPase activating protein, alpha subunit 1 (catalytic) | 2 | | 1407 | hsa-miR-571 | 253959 | RALGAPA1 | Ral GTPase activating protein, alpha subunit 1 (catalytic) | 2 | | 1434 | hsa-miR-618 | 129642 | MBOAT2 | membrane bound O-acyltransferase domain containing 2 | 2 | | 1444 | hsa-miR-1233 | 221443 | C6orf130 | chromosome 6 open reading frame 130 | 2 | | 1446 | hsa-miR-571 | 221443 | C6orf130 | chromosome 6 open reading frame 130 | 2 | | 1452 | hsa-miR-1233 | 11127 | KIF3A | kinesin family member 3A | 2 | | 1453 | hsa-miR-569 | 11127 | KIF3A | kinesin family member 3A | 2 | | 1454 | hsa-miR-571 | 11127 | KIF3A | kinesin family member 3A | 2 | | 1455 | hsa-miR-766 | 11127 | KIF3A | kinesin family member 3A | 2 | | 1456 | hsa-miR-874 | 11127 | KIF3A | kinesin family member 3A | 2 | | 1458 | hsa-miR-1233 | 388650 | FAM69A | family with sequence similarity 69, member A | 2 | | 1478 | hsa-miR-569 | 6964 | TRD@ | T cell receptor delta locus | 2 | | 1479 | hsa-miR-571 | 6964 | TRD@ | T cell receptor delta locus | 2 | | 1481 | hsa-miR-874 | 6964 | TRD@ | T cell receptor delta locus | 2 | | 1493 | hsa-miR-1233 | 26049 | FAM169A | family with sequence similarity 169, member A | 2 | | 1494 | hsa-miR-571 | 26049 | FAM169A | family with sequence similarity 169, member A | 2 | | 1495 | hsa-miR-874 | 26049 | FAM169A | family with sequence similarity 169, member A | 2 | | 1497 | hsa-miR-1233 | 6421 | SFPQ | splicing factor proline/glutamine-rich (polypyrimidine tract binding protein associated) | 2 | | 1523 | hsa-miR-569 | 3178 | HNRNPA1 | heterogeneous nuclear ribonucleoprotein A1 | 2 | | 1524 | hsa-miR-571 | 3178 | HNRNPA1 | heterogeneous nuclear ribonucleoprotein A1 | 2 | | 1528 | hsa-miR-623 | 6203 | RPS9 | ribosomal protein S9 | 2 | | 1536 | hsa-miR-766 | 6687 | SPG7 | spastic paraplegia 7 (pure and complicated autosomal recessive) | 2 | | 1538 | hsa-miR-1233 | 5379 | PMS2L1 | postmeiotic segregation increased 2-like 1 pseudogene | 2 | | 1553 | hsa-miR-1233 | 10207 | INADL | InaD-like (Drosophila) | 2 | | 1554 | hsa-miR-571 | 10207 | INADL | InaD-like (Drosophila) | 2 | | 1555 | hsa-miR-874 | 10207 | INADL | InaD-like (Drosophila) | 2 | | 1557 | hsa-miR-877 | 6597 | SMARCA4 | SWI/SNF related, matrix associated, actin dependent regulator of chromatin, subfamily a, member 4 | 2 | | 1565 | hsa-miR-1233 | 51018 | RRP15 | ribosomal RNA processing 15 homolog (S. cerevisiae) | 2 | | 1566 | hsa-miR-571 | 51018 | RRP15 | ribosomal RNA processing 15 homolog (S. cerevisiae) | 2 | | 1567 | hsa-miR-874 | 51018 | RRP15 | ribosomal RNA processing 15 homolog (S. cerevisiae) | 2 | | 1602 | hsa-miR-874 | 55340 | GIMAP5 | GTPase, IMAP family member 5 | 2 | | 1616 | hsa-mir-3130-1 | 23162 | MAPK8IP3 | mitogen-activated protein kinase 8 interacting protein 3 | 2 | | 1617 | hsa-mir-3130-2 | 23162 | MAPK8IP3 | mitogen-activated protein kinase 8 interacting protein 3 | 2 | | 1618 | hsa-mir-3130-3 | 23162 | MAPK8IP3 | mitogen-activated protein kinase 8 interacting protein 3 | 2 | | 1619 | hsa-mir-3130-4 | 23162 | MAPK8IP3 | mitogen-activated protein kinase 8 interacting protein 3 | 2 | | 1620 | hsa-miR-95 | 23162 | MAPK8IP3 | mitogen-activated protein kinase 8 interacting protein 3 | 2 | | 1641 | hsa-miR-766 | 6627 | SNRPA1 | small nuclear ribonucleoprotein polypeptide A' | 2 | | 1645 | hsa-mir-3130-1 | 9436 | NCR2 | natural cytotoxicity triggering receptor 2 | 2 | | 1646 | hsa-mir-3130-2 | 9436 | NCR2 | natural cytotoxicity triggering receptor 2 | 2 | | 1647 | hsa-mir-3130-3 | 9436 | NCR2 | natural cytotoxicity triggering receptor 2 | 2 | | 1648 | hsa-mir-3130-4 | 9436 | NCR2 | natural cytotoxicity triggering receptor 2 | 2 | | 1649 | hsa-miR-10a | 9436 | NCR2 | natural cytotoxicity triggering receptor 2 | 2 | | 1675 | hsa-miR-1233 | 23545 | ATP6V0A2 | ATPase, H+ transporting, lysosomal V0 subunit a2 | 2 | | 1676 | hsa-miR-571 | 23545 | ATP6V0A2 | ATPase, H+ transporting, lysosomal V0 subunit a2 | 2 | | 1677 | hsa-miR-874 | 23545 | ATP6V0A2 | ATPase, H+ transporting, lysosomal V0 subunit a2 | 2 | | 1683 | hsa-miR-1233 | 6130 | RPL7A | ribosomal protein L7a | 2 | | 1689 | hsa-miR-618 | 11031 | RAB31 | RAB31, member RAS oncogene family | 2 | | 1699 | hsa-miR-576-5p | 51012 | SLMO2 | slowmo homolog 2 (Drosophila) | 2 | | 1700 | hsa-miR-618 | 51108 | METTL9 | methyltransferase like 9 | 2 | | 1706 | hsa-miR-569 | 55037 | PTCD3 | Pentatricopeptide repeat domain 3 | 2 | | 1707 | hsa-miR-571 | 55037 | PTCD3 | Pentatricopeptide repeat domain 3 | 2 | | 1713 | hsa-miR-1233 | 394 | ARHGAP5 | Rho GTPase activating protein 5 | 2 | | 1725 | hsa-miR-1233 | 51569 | UFM1 | ubiquitin-fold modifier 1 | 2 | | 1727 | hsa-miR-1233 | 55717 | WDR11 | WD repeat domain 11 | 2 | | 1729 | hsa-miR-618 | 3267 | AGFG1 | ArfGAP with FG repeats 1 | 2 | | 1738 | hsa-miR-1233 | 55246 | CCDC25 | coiled-coil domain containing 25 | 2 | | 1739 | hsa-miR-571 | 55246 | CCDC25 | coiled-coil domain containing 25 | 2 | | 1773 | hsa-miR-423-5p | 55216 | C11orf57 | chromosome 11 open reading frame 57 | 2 | | 1776 | hsa-miR-1233 | 54906 | C10orf18 | chromosome 10 open reading frame 18 | 2 | | 1777 | hsa-miR-423-5p | 54906 | C10orf18 | chromosome 10 open reading frame 18 | 2 | | 1779 | hsa-miR-576-5p | 54906 | C10orf18 | chromosome 10 open reading frame 18 | 2 | | 1780 | hsa-miR-576-5p | 55758 | RCOR3 | REST corepressor 3 | 2 | | 1784 | hsa-miR-423-5p | 10600 | USP16 | ubiquitin specific peptidase 16 | 2 | | 1786 | hsa-miR-1233 | 64864 | RFX7 | regulatory factor X, 7 | 2 | | 1787 | hsa-miR-571 | 64864 | RFX7 | regulatory factor X, 7 | 2 | | 1798 | hsa-miR-874 | 55900 | ZNF302 | zinc finger protein 302 | 2 | | 1804 | hsa-miR-571 | 246243 | RNASEH1 | ribonuclease H1 | 2 | | 1807 | hsa-miR-618 | 30001 | ERO1L | ERO1-like (S. cerevisiae) | 2 | | 1818 | hsa-miR-1233 | 64682 | ANAPC1 | anaphase promoting complex subunit 1 | 2 | | 1835 | hsa-miR-576-5p | 25821 | MTO1 | mitochondrial translation optimization 1 homolog (S. cerevisiae) | 2 | | 1850 | hsa-miR-571 | 6322 | SCML1 | sex comb on midleg-like 1 (Drosophila) | 2 | | 1851 | hsa-miR-874 | 6322 | SCML1 | sex comb on midleg-like 1 (Drosophila) | 2 | | 1852 | hsa-miR-766 | 55340 | GIMAP5 | GTPase, IMAP family member 5 | 2 | | 1854 | hsa-miR-938 | 2355 | FOSL2 | FOS-like antigen 2 | 2 | | 1881 | hsa-miR-1233 | 59338 | PLEKHA1 | pleckstrin homology domain containing, family A (phosphoinositide binding specific) member 1 | 2 | | 1883 | hsa-miR-571 | 59338 | PLEKHA1 | pleckstrin homology domain containing, family A (phosphoinositide binding specific) member 1 | 2 | | 1884 | hsa-miR-766 | 59338 | PLEKHA1 | pleckstrin homology domain containing, family A (phosphoinositide binding specific) member 1 | 2 | | 1885 | hsa-miR-874 | 59338 | PLEKHA1 | pleckstrin homology domain containing, family A (phosphoinositide binding specific) member 1 | 2 | | 1905 | hsa-miR-558 | 79886 | C9orf82 | chromosome 9 open reading frame 82 | 2 | | 1944 | hsa-miR-571 | 7769 | ZNF226 | zinc finger protein 226 | 2 | | 1948 | hsa-miR-766 | 54876 | DCAF16 | DDB1 and CUL4 associated factor 16 | 2 | | 1980 | hsa-miR-766 | 63892 | THADA | thyroid adenoma associated | 2 | | 1999 | hsa-miR-10a | 10864 | SLC22A7 | solute carrier family 22 (organic anion transporter), member 7 | 2 | | 2001 | hsa-miR-95 | 10864 | SLC22A7 | solute carrier family 22 (organic anion transporter), member 7 | 2 | | 2036 | hsa-miR-766 | 60468 | BACH2 | BTB and CNC homology 1, basic leucine zipper transcription factor 2 | 2 | | 2084 | hsa-miR-1233 | 55769 | ZNF83 | zinc finger protein 83 | 2 | | 2085 | hsa-miR-569 | 55769 | ZNF83 | zinc finger protein 83 | 2 | | 2086 | hsa-miR-571 | 55769 | ZNF83 | zinc finger protein 83 | 2 | | 2087 | hsa-miR-766 | 55769 | ZNF83 | zinc finger protein 83 | 2 | | 2088 | hsa-miR-874 | 55769 | ZNF83 | zinc finger protein 83 | 2 | | 2127 | hsa-miR-558 | 11276 | SYNRG | synergin, gamma | 2 | | 2128 | hsa-miR-569 | 11276 | SYNRG | synergin, gamma | 2 | | 2130 | hsa-miR-766 | 11276 | SYNRG | synergin, gamma | 2 | | 2152 | hsa-miR-623 | 10471 | PFDN6 | prefoldin subunit 6 | 2 | | 2167 | hsa-miR-1233 | 57466 | SFRS15 | splicing factor, arginine/serine-rich 15 | 2 | | 2168 | hsa-miR-423-5p | 57466 | SFRS15 | splicing factor, arginine/serine-rich 15 | 2 | | 2183 | hsa-miR-558 | 51068 | NMD3 | NMD3 homolog (S. cerevisiae) | 2 | | 2191 | hsa-miR-423-5p | 51193 | ZNF639 | zinc finger protein 639 | 2 | | 2207 | hsa-miR-618 | 64778 | FNDC3B | fibronectin type III domain containing 3B | 2 | | 2236 | hsa-miR-1233 | 23731 | C9orf5 | chromosome 9 open reading frame 5 | 2 | | 2238 | hsa-miR-766 | 23731 | C9orf5 | chromosome 9 open reading frame 5 | 2 | | 2253 | hsa-miR-571 | 55591 | VEZT | vezatin, adherens junctions transmembrane protein | 2 | | 2255 | hsa-miR-569 | 56172 | ANKH | ankylosis, progressive homolog (mouse) | 2 | | 2256 | hsa-miR-766 | 56172 | ANKH | ankylosis, progressive homolog (mouse) | 2 | | 2273 | hsa-miR-593\* | 170463 | SSBP4 | single stranded DNA binding protein 4 | 2 | | 2274 | hsa-miR-766 | 170463 | SSBP4 | single stranded DNA binding protein 4 | 2 | | 2275 | hsa-miR-877 | 170463 | SSBP4 | single stranded DNA binding protein 4 | 2 | | 2294 | hsa-miR-454\* | 55632 | G2E3 | G2/M-phase specific E3 ubiquitin ligase | 2 | | 2295 | hsa-miR-555 | 55632 | G2E3 | G2/M-phase specific E3 ubiquitin ligase | 2 | | 2310 | hsa-mir-10a | 60592 | SCOC | short coiled-coil protein | 2 | | 2312 | hsa-miR-618 | 57616 | TSHZ3 | teashirt zinc finger homeobox 3 | 2 | | 2332 | hsa-miR-1233 | 83636 | C19orf12 | chromosome 19 open reading frame 12 | 2 | | 2336 | hsa-miR-766 | 83636 | C19orf12 | chromosome 19 open reading frame 12 | 2 | | 2350 | hsa-miR-10a | 57731 | SPTBN4 | spectrin, beta, non-erythrocytic 4 | 2 | | 2352 | hsa-miR-95 | 7455 | ZAN | zonadhesin | 2 | | 2355 | hsa-mir-10a | 25821 | MTO1 | mitochondrial translation optimization 1 homolog (S. cerevisiae) | 2 | | 2378 | hsa-miR-423-5p | 57534 | MIB1 | mindbomb homolog 1 (Drosophila) | 2 | | 2379 | hsa-miR-576-5p | 57534 | MIB1 | mindbomb homolog 1 (Drosophila) | 2 | | 2438 | hsa-miR-618 | 57568 | SIPA1L2 | signal-induced proliferation-associated 1 like 2 | 2 | | 2453 | hsa-miR-555 | 4090 | SMAD5 | SMAD family member 5 | 2 | | 2459 | hsa-miR-1233 | 124540 | MSI2 | musashi homolog 2 (Drosophila) | 2 | | 2464 | hsa-miR-571 | 25871 | C3orf17 | chromosome 3 open reading frame 17 | 2 | | 2481 | hsa-mir-10a | 493753 | C2orf64 | chromosome 2 open reading frame 64 | 2 | | 2500 | hsa-miR-1233 | 54843 | SYTL2 | synaptotagmin-like 2 | 2 | | 2501 | hsa-miR-569 | 54843 | SYTL2 | synaptotagmin-like 2 | 2 | | 2502 | hsa-miR-766 | 54843 | SYTL2 | synaptotagmin-like 2 | 2 | | 2503 | hsa-miR-874 | 54843 | SYTL2 | synaptotagmin-like 2 | 2 | | 2505 | hsa-miR-555 | 1385 | CREB1 | cAMP responsive element binding protein 1 | 2 | | 2508 | hsa-miR-874 | 58487 | CREBZF | CREB/ATF bZIP transcription factor | 2 | | 2539 | hsa-miR-423-5p | 91408 | BTF3L4 | basic transcription factor 3-like 4 | 2 | | 2542 | hsa-miR-576-5p | 91408 | BTF3L4 | basic transcription factor 3-like 4 | 2 | | 2547 | hsa-miR-766 | 961 | CD47 | CD47 molecule | 2 | | 2560 | hsa-miR-1233 | 146198 | ZFP90 | zinc finger protein 90 homolog (mouse) | 2 | | 2561 | hsa-miR-571 | 146198 | ZFP90 | zinc finger protein 90 homolog (mouse) | 2 | | 2562 | hsa-miR-766 | 146198 | ZFP90 | zinc finger protein 90 homolog (mouse) | 2 | | 2563 | hsa-miR-1233 | 246175 | CNOT6L | CCR4-NOT transcription complex, subunit 6-like | 2 | | 2564 | hsa-miR-766 | 246175 | CNOT6L | CCR4-NOT transcription complex, subunit 6-like | 2 | | 2567 | hsa-miR-423-5p | 148867 | SLC30A7 | solute carrier family 30 (zinc transporter), member 7 | 2 | | 2568 | hsa-miR-555 | 148867 | SLC30A7 | solute carrier family 30 (zinc transporter), member 7 | 2 | | 2577 | hsa-miR-1233 | 84897 | TBRG1 | transforming growth factor beta regulator 1 | 2 | | 2596 | hsa-miR-423-5p | 55183 | RIF1 | RAP1 interacting factor homolog (yeast) | 2 | | 2632 | hsa-miR-766 | 152137 | CCDC50 | coiled-coil domain containing 50 | 2 | | 2633 | hsa-miR-874 | 152137 | CCDC50 | coiled-coil domain containing 50 | 2 | | 2646 | hsa-miR-766 | 152485 | ZNF827 | zinc finger protein 827 | 2 | | 2680 | hsa-miR-1233 | 90673 | PPP1R3E | protein phosphatase 1, regulatory (inhibitor) subunit 3E | 2 | | 2681 | hsa-miR-766 | 90673 | PPP1R3E | protein phosphatase 1, regulatory (inhibitor) subunit 3E | 2 | | 2693 | hsa-miR-877 | 221545 | C6orf136 | chromosome 6 open reading frame 136 | 2 | | 2698 | hsa-miR-423-5p | 57721 | METTL14 | methyltransferase like 14 | 2 | | 2746 | hsa-miR-1233 | 3841 | KPNA5 | karyopherin alpha 5 (importin alpha 6) | 2 | | 2747 | hsa-miR-571 | 3841 | KPNA5 | karyopherin alpha 5 (importin alpha 6) | 2 | | 2748 | hsa-miR-766 | 3841 | KPNA5 | karyopherin alpha 5 (importin alpha 6) | 2 | | 2749 | hsa-miR-874 | 3841 | KPNA5 | karyopherin alpha 5 (importin alpha 6) | 2 | | 2764 | hsa-miR-95 | 5522 | PPP2R2C | protein phosphatase 2 (formerly 2A), regulatory subunit B, gamma isoform | 2 | | 2766 | hsa-miR-423-5p | 132949 | AASDH | aminoadipate-semialdehyde dehydrogenase | 2 | | 2866 | hsa-miR-571 | 143884 | CWF19L2 | CWF19-like 2, cell cycle control (S. pombe) | 2 | | 2907 | hsa-miR-571 | 54205 | CYCS | cytochrome c, somatic | 2 | | 2996 | hsa-miR-423-5p | 134218 | DNAJC21 | DnaJ (Hsp40) homolog, subfamily C, member 21 | 2 | | 3106 | hsa-miR-10a | 57455 | REXO1 | REX1, RNA exonuclease 1 homolog (S. cerevisiae) | 2 | | 3194 | hsa-miR-571 | 163081 | ZNF567 | zinc finger protein 567 | 2 | | 3195 | hsa-miR-874 | 163081 | ZNF567 | zinc finger protein 567 | 2 | | 3212 | hsa-mir-10a | 254251 | LCORL | ligand dependent nuclear receptor corepressor-like | 2 | | 3213 | hsa-mir-149 | 254251 | LCORL | ligand dependent nuclear receptor corepressor-like | 2 | | 3214 | hsa-miR-149 | 254251 | LCORL | ligand dependent nuclear receptor corepressor-like | 2 | | 3215 | hsa-miR-423-5p | 254251 | LCORL | ligand dependent nuclear receptor corepressor-like | 2 | | 3216 | hsa-miR-576-5p | 254251 | LCORL | ligand dependent nuclear receptor corepressor-like | 2 | | 3265 | hsa-miR-1233 | 11146 | GLMN | glomulin, FKBP associated protein | 2 | | 3266 | hsa-miR-571 | 11146 | GLMN | glomulin, FKBP associated protein | 2 | | 3267 | hsa-miR-874 | 11146 | GLMN | glomulin, FKBP associated protein | 2 | | 3293 | hsa-miR-95 | 339541 | C1orf228 | chromosome 1 open reading frame 228 | 2 | | 3295 | hsa-miR-618 | 118932 | ANKRD22 | ankyrin repeat domain 22 | 2 | | 6 | hsa-miR-10a | 10406 | WFDC2 | WAP four-disulfide core domain 2 | 1 | | 7 | hsa-miR-580 | 10406 | WFDC2 | WAP four-disulfide core domain 2 | 1 | | 8 | hsa-miR-95 | 10406 | WFDC2 | WAP four-disulfide core domain 2 | 1 | | 10 | hsa-miR-10a | 283375 | SLC39A5 | solute carrier family 39 (metal ion transporter), member 5 | 1 | | 11 | hsa-miR-623 | 170575 | GIMAP1 | GTPase, IMAP family member 1 | 1 | | 13 | hsa-miR-503 | 170575 | GIMAP1 | GTPase, IMAP family member 1 | 1 | | 14 | hsa-miR-766 | 170575 | GIMAP1 | GTPase, IMAP family member 1 | 1 | | 15 | hsa-miR-95 | 164127 | C1orf65 | chromosome 1 open reading frame 65 | 1 | | 16 | hsa-miR-938 | 79660 | PPP1R3B | protein phosphatase 1, regulatory (inhibitor) subunit 3B | 1 | | 18 | hsa-miR-10a | 117166 | WFIKKN1 | WAP, follistatin/kazal, immunoglobulin, kunitz and netrin domain containing 1 | 1 | | 19 | hsa-miR-10a | 148870 | CCDC27 | coiled-coil domain containing 27 | 1 | | 20 | hsa-miR-95 | 148870 | CCDC27 | coiled-coil domain containing 27 | 1 | | 21 | hsa-miR-10a | 140456 | ASB11 | ankyrin repeat and SOCS box-containing 11 | 1 | | 23 | hsa-miR-618 | 285848 | PNPLA1 | patatin-like phospholipase domain containing 1 | 1 | | 24 | hsa-miR-95 | 85445 | CNTNAP4 | contactin associated protein-like 4 | 1 | | 25 | hsa-mir-3130-1 | 439941 | C8orf54 | chromosome 8 open reading frame 54 | 1 | | 26 | hsa-mir-3130-2 | 439941 | C8orf54 | chromosome 8 open reading frame 54 | 1 | | 27 | hsa-mir-3130-3 | 439941 | C8orf54 | chromosome 8 open reading frame 54 | 1 | | 28 | hsa-mir-3130-4 | 439941 | C8orf54 | chromosome 8 open reading frame 54 | 1 | | 29 | hsa-mir-454 | 283860 | C16orf81 | chromosome 16 open reading frame 81 | 1 | | 30 | hsa-miR-301a | 283860 | C16orf81 | chromosome 16 open reading frame 81 | 1 | | 32 | hsa-miR-608 | 3228 | HOXC12 | homeobox C12 | 1 | | 33 | hsa-mir-3130-1 | 124359 | CDYL2 | chromodomain protein, Y-like 2 | 1 | | 34 | hsa-mir-3130-2 | 124359 | CDYL2 | chromodomain protein, Y-like 2 | 1 | | 35 | hsa-mir-3130-3 | 124359 | CDYL2 | chromodomain protein, Y-like 2 | 1 | | 36 | hsa-mir-3130-4 | 124359 | CDYL2 | chromodomain protein, Y-like 2 | 1 | | 37 | hsa-miR-643 | 124359 | CDYL2 | chromodomain protein, Y-like 2 | 1 | | 39 | hsa-mir-505 | 79816 | TLE6 | transducin-like enhancer of split 6 (E(sp1) homolog, Drosophila) | 1 | | 40 | hsa-miR-505\* | 79816 | TLE6 | transducin-like enhancer of split 6 (E(sp1) homolog, Drosophila) | 1 | | 41 | hsa-miR-95 | 79816 | TLE6 | transducin-like enhancer of split 6 (E(sp1) homolog, Drosophila) | 1 | | 45 | hsa-mir-3130-1 | 122876 | GPHB5 | glycoprotein hormone beta 5 | 1 | | 46 | hsa-mir-3130-2 | 122876 | GPHB5 | glycoprotein hormone beta 5 | 1 | | 47 | hsa-mir-3130-3 | 122876 | GPHB5 | glycoprotein hormone beta 5 | 1 | | 48 | hsa-mir-3130-4 | 122876 | GPHB5 | glycoprotein hormone beta 5 | 1 | | 49 | hsa-miR-571 | 163050 | ZNF564 | zinc finger protein 564 | 1 | | 50 | hsa-miR-1233 | 126792 | B3GALT6 | UDP-Gal:betaGal beta 1,3-galactosyltransferase polypeptide 6 | 1 | | 52 | hsa-miR-938 | 51317 | PHF21A | PHD finger protein 21A | 1 | | 53 | hsa-mir-576 | 255374 | MBLAC1 | metallo-beta-lactamase domain containing 1 | 1 | | 54 | hsa-miR-10a | 3788 | KCNS2 | potassium voltage-gated channel, delayed-rectifier, subfamily S, member 2 | 1 | | 55 | hsa-mir-10a | 50809 | HP1BP3 | heterochromatin protein 1, binding protein 3 | 1 | | 56 | hsa-miR-423-5p | 50809 | HP1BP3 | heterochromatin protein 1, binding protein 3 | 1 | | 57 | hsa-miR-423-5p | 10260 | DENND4A | DENN/MADD domain containing 4A | 1 | | 58 | hsa-miR-623 | 84326 | C16orf13 | chromosome 16 open reading frame 13 | 1 | | 59 | hsa-miR-938 | 118788 | PIK3AP1 | phosphoinositide-3-kinase adaptor protein 1 | 1 | | 60 | hsa-mir-505 | 284001 | CCDC57 | coiled-coil domain containing 57 | 1 | | 61 | hsa-mir-3130-1 | 284001 | CCDC57 | coiled-coil domain containing 57 | 1 | | 62 | hsa-mir-3130-2 | 284001 | CCDC57 | coiled-coil domain containing 57 | 1 | | 63 | hsa-mir-3130-3 | 284001 | CCDC57 | coiled-coil domain containing 57 | 1 | | 64 | hsa-mir-3130-4 | 284001 | CCDC57 | coiled-coil domain containing 57 | 1 | | 65 | hsa-miR-505\* | 284001 | CCDC57 | coiled-coil domain containing 57 | 1 | | 66 | hsa-miR-580 | 284001 | CCDC57 | coiled-coil domain containing 57 | 1 | | 68 | hsa-miR-10a | 9711 | KIAA0226 | KIAA0226 | 1 | | 69 | hsa-miR-1233 | 126295 | ZNF57 | zinc finger protein 57 | 1 | | 70 | hsa-miR-558 | 126295 | ZNF57 | zinc finger protein 57 | 1 | | 71 | hsa-miR-571 | 126295 | ZNF57 | zinc finger protein 57 | 1 | | 72 | hsa-miR-874 | 126295 | ZNF57 | zinc finger protein 57 | 1 | | 73 | hsa-mir-3130-1 | 146223 | CMTM4 | CKLF-like MARVEL transmembrane domain containing 4 | 1 | | 74 | hsa-mir-3130-2 | 146223 | CMTM4 | CKLF-like MARVEL transmembrane domain containing 4 | 1 | | 75 | hsa-mir-3130-3 | 146223 | CMTM4 | CKLF-like MARVEL transmembrane domain containing 4 | 1 | | 76 | hsa-mir-3130-4 | 146223 | CMTM4 | CKLF-like MARVEL transmembrane domain containing 4 | 1 | | 77 | hsa-miR-608 | 394261 | C2orf19 | chromosome 2 open reading frame 19 | 1 | | 78 | hsa-miR-10a | 9672 | SDC3 | syndecan 3 | 1 | | 79 | hsa-miR-95 | 9672 | SDC3 | syndecan 3 | 1 | | 81 | hsa-miR-95 | 9870 | KIAA0317 | KIAA0317 | 1 | | 86 | hsa-mir-10a | 11179 | ZNF277 | zinc finger protein 277 | 1 | | 89 | hsa-miR-10a | 55107 | ANO1 | anoctamin 1, calcium activated chloride channel | 1 | | 90 | hsa-miR-95 | 55107 | ANO1 | anoctamin 1, calcium activated chloride channel | 1 | | 92 | hsa-miR-10a | 3680 | ITGA9 | integrin, alpha 9 | 1 | | 93 | hsa-miR-95 | 3680 | ITGA9 | integrin, alpha 9 | 1 | | 94 | hsa-mir-3130-1 | 80174 | DBF4B | DBF4 homolog B (S. cerevisiae) | 1 | | 95 | hsa-mir-3130-2 | 80174 | DBF4B | DBF4 homolog B (S. cerevisiae) | 1 | | 96 | hsa-mir-3130-3 | 80174 | DBF4B | DBF4 homolog B (S. cerevisiae) | 1 | | 97 | hsa-mir-3130-4 | 80174 | DBF4B | DBF4 homolog B (S. cerevisiae) | 1 | | 98 | hsa-miR-10a | 80174 | DBF4B | DBF4 homolog B (S. cerevisiae) | 1 | | 99 | hsa-miR-95 | 80119 | PIF1 | PIF1 5'-to-3' DNA helicase homolog (S. cerevisiae) | 1 | | 100 | hsa-miR-619 | 54434 | SSH1 | slingshot homolog 1 (Drosophila) | 1 | | 101 | hsa-miR-10a | 3492 | IGH@ | immunoglobulin heavy locus | 1 | | 102 | hsa-miR-95 | 3492 | IGH@ | immunoglobulin heavy locus | 1 | | 103 | hsa-miR-10a | 974 | CD79B | CD79b molecule, immunoglobulin-associated beta | 1 | | 105 | hsa-miR-128 | 974 | CD79B | CD79b molecule, immunoglobulin-associated beta | 1 | | 106 | hsa-miR-580 | 974 | CD79B | CD79b molecule, immunoglobulin-associated beta | 1 | | 107 | hsa-miR-623 | 6352 | CCL5 | chemokine (C-C motif) ligand 5 | 1 | | 108 | hsa-miR-10a | 164592 | CCDC116 | coiled-coil domain containing 116 | 1 | | 110 | hsa-miR-10a | 100130417 | FLJ39609 | similar to hCG1995469 | 1 | | 111 | hsa-miR-95 | 100130417 | FLJ39609 | similar to hCG1995469 | 1 | | 112 | hsa-miR-10a | 3842 | TNPO1 | transportin 1 | 1 | | 113 | hsa-miR-766 | 23065 | KIAA0090 | KIAA0090 | 1 | | 114 | hsa-miR-874 | 100132741 | LOC100132741 | similar to hCG1655084 | 1 | | 118 | hsa-miR-558 | 256380 | SCML4 | sex comb on midleg-like 4 (Drosophila) | 1 | | 119 | hsa-miR-571 | 256380 | SCML4 | sex comb on midleg-like 4 (Drosophila) | 1 | | 120 | hsa-miR-10a | 255082 | CASC2 | cancer susceptibility candidate 2 | 1 | | 121 | hsa-miR-95 | 255082 | CASC2 | cancer susceptibility candidate 2 | 1 | | 122 | hsa-miR-95 | 9815 | GIT2 | G protein-coupled receptor kinase interacting ArfGAP 2 | 1 | | 123 | hsa-miR-608 | 158376 | LOC158376 | hypothetical LOC158376 | 1 | | 124 | hsa-miR-1233 | 100129196 | LOC100129196 | similar to hCG2033298 | 1 | | 125 | hsa-miR-874 | 100129196 | LOC100129196 | similar to hCG2033298 | 1 | | 126 | hsa-miR-95 | 161394 | C14orf174 | chromosome 14 open reading frame 174 | 1 | | 127 | hsa-mir-3130-1 | 100132987 | LOC100132987 | hypothetical protein LOC100132987 | 1 | | 128 | hsa-mir-3130-2 | 100132987 | LOC100132987 | hypothetical protein LOC100132987 | 1 | | 129 | hsa-mir-3130-3 | 100132987 | LOC100132987 | hypothetical protein LOC100132987 | 1 | | 130 | hsa-mir-3130-4 | 100132987 | LOC100132987 | hypothetical protein LOC100132987 | 1 | | 131 | hsa-miR-95 | 100132987 | LOC100132987 | hypothetical protein LOC100132987 | 1 | | 132 | hsa-mir-10a | 64417 | C5orf28 | chromosome 5 open reading frame 28 | 1 | | 134 | hsa-miR-423-5p | 64417 | C5orf28 | chromosome 5 open reading frame 28 | 1 | | 136 | hsa-miR-766 | 64417 | C5orf28 | chromosome 5 open reading frame 28 | 1 | | 140 | hsa-miR-558 | 130916 | MTERFD2 | MTERF domain containing 2 | 1 | | 141 | hsa-miR-569 | 130916 | MTERFD2 | MTERF domain containing 2 | 1 | | 143 | hsa-miR-766 | 130916 | MTERFD2 | MTERF domain containing 2 | 1 | | 144 | hsa-miR-95 | 51614 | ERGIC3 | ERGIC and golgi 3 | 1 | | 145 | hsa-miR-95 | 6714 | SRC | v-src sarcoma (Schmidt-Ruppin A-2) viral oncogene homolog (avian) | 1 | | 146 | hsa-miR-938 | 8675 | STX16 | syntaxin 16 | 1 | | 148 | hsa-miR-95 | 148103 | ZNF599 | zinc finger protein 599 | 1 | | 150 | hsa-miR-10a | 400258 | C14orf180 | chromosome 14 open reading frame 180 | 1 | | 151 | hsa-miR-95 | 388722 | C1orf53 | chromosome 1 open reading frame 53 | 1 | | 152 | hsa-mir-505 | 654429 | LRTM2 | leucine-rich repeats and transmembrane domains 2 | 1 | | 153 | hsa-mir-3130-1 | 654429 | LRTM2 | leucine-rich repeats and transmembrane domains 2 | 1 | | 154 | hsa-mir-3130-2 | 654429 | LRTM2 | leucine-rich repeats and transmembrane domains 2 | 1 | | 155 | hsa-mir-3130-3 | 654429 | LRTM2 | leucine-rich repeats and transmembrane domains 2 | 1 | | 156 | hsa-mir-3130-4 | 654429 | LRTM2 | leucine-rich repeats and transmembrane domains 2 | 1 | | 157 | hsa-miR-10a | 654429 | LRTM2 | leucine-rich repeats and transmembrane domains 2 | 1 | | 158 | hsa-miR-128 | 654429 | LRTM2 | leucine-rich repeats and transmembrane domains 2 | 1 | | 159 | hsa-miR-505\* | 654429 | LRTM2 | leucine-rich repeats and transmembrane domains 2 | 1 | | 160 | hsa-miR-580 | 654429 | LRTM2 | leucine-rich repeats and transmembrane domains 2 | 1 | | 161 | hsa-miR-95 | 654429 | LRTM2 | leucine-rich repeats and transmembrane domains 2 | 1 | | 162 | hsa-miR-571 | 7582 | ZNF33B | zinc finger protein 33B | 1 | | 163 | hsa-miR-95 | 8091 | HMGA2 | high mobility group AT-hook 2 | 1 | | 164 | hsa-miR-10a | 29940 | DSE | dermatan sulfate epimerase | 1 | | 165 | hsa-miR-1233 | 22897 | CEP164 | centrosomal protein 164kDa | 1 | | 166 | hsa-miR-571 | 22897 | CEP164 | centrosomal protein 164kDa | 1 | | 167 | hsa-miR-766 | 22897 | CEP164 | centrosomal protein 164kDa | 1 | | 168 | hsa-miR-1233 | 388011 | C14orf64 | chromosome 14 open reading frame 64 | 1 | | 169 | hsa-miR-571 | 388011 | C14orf64 | chromosome 14 open reading frame 64 | 1 | | 170 | hsa-miR-766 | 388011 | C14orf64 | chromosome 14 open reading frame 64 | 1 | | 171 | hsa-miR-874 | 388011 | C14orf64 | chromosome 14 open reading frame 64 | 1 | | 175 | hsa-miR-95 | 283688 | LOC283688 | hypothetical protein LOC283688 | 1 | | 176 | hsa-mir-576 | 644997 | C1orf200 | chromosome 1 open reading frame 200 | 1 | | 177 | hsa-mir-3130-1 | 57188 | ADAMTSL3 | ADAMTS-like 3 | 1 | | 178 | hsa-mir-3130-2 | 57188 | ADAMTSL3 | ADAMTS-like 3 | 1 | | 179 | hsa-mir-3130-3 | 57188 | ADAMTSL3 | ADAMTS-like 3 | 1 | | 180 | hsa-mir-3130-4 | 57188 | ADAMTSL3 | ADAMTS-like 3 | 1 | | 181 | hsa-miR-10a | 57188 | ADAMTSL3 | ADAMTS-like 3 | 1 | | 182 | hsa-miR-580 | 57188 | ADAMTSL3 | ADAMTS-like 3 | 1 | | 183 | hsa-miR-95 | 57188 | ADAMTSL3 | ADAMTS-like 3 | 1 | | 184 | hsa-miR-10a | 100129129 | LOC100129129 | hypothetical protein LOC100129129 | 1 | | 185 | hsa-miR-10a | 8091 | HMGA2 | high mobility group AT-hook 2 | 1 | | 186 | hsa-miR-10a | 83592 | AKR1E2 | aldo-keto reductase family 1, member E2 | 1 | | 187 | hsa-miR-95 | 83592 | AKR1E2 | aldo-keto reductase family 1, member E2 | 1 | | 188 | hsa-mir-3130-1 | 3909 | LAMA3 | laminin, alpha 3 | 1 | | 189 | hsa-mir-3130-2 | 3909 | LAMA3 | laminin, alpha 3 | 1 | | 190 | hsa-mir-3130-3 | 3909 | LAMA3 | laminin, alpha 3 | 1 | | 191 | hsa-mir-3130-4 | 3909 | LAMA3 | laminin, alpha 3 | 1 | | 192 | hsa-miR-10a | 3909 | LAMA3 | laminin, alpha 3 | 1 | | 193 | hsa-miR-499-5p | 3909 | LAMA3 | laminin, alpha 3 | 1 | | 194 | hsa-miR-95 | 3909 | LAMA3 | laminin, alpha 3 | 1 | | 195 | hsa-miR-608 | 56172 | ANKH | ankylosis, progressive homolog (mouse) | 1 | | 196 | hsa-miR-571 | 1211 | CLTA | clathrin, light chain (Lca) | 1 | | 197 | hsa-miR-10a | 100128198 | LOC100128198 | hypothetical protein LOC100128198 | 1 | | 198 | hsa-miR-95 | 100128198 | LOC100128198 | hypothetical protein LOC100128198 | 1 | | 199 | hsa-miR-95 | 150527 | LOC150527 | hypothetical LOC150527 | 1 | | 200 | hsa-miR-10a | 84766 | EFCAB4B | EF-hand calcium binding domain 4B | 1 | | 201 | hsa-miR-95 | 84766 | EFCAB4B | EF-hand calcium binding domain 4B | 1 | | 202 | hsa-mir-3130-1 | 100133461 | LOC100133461 | hypothetical LOC100133461 | 1 | | 203 | hsa-mir-3130-2 | 100133461 | LOC100133461 | hypothetical LOC100133461 | 1 | | 204 | hsa-mir-3130-3 | 100133461 | LOC100133461 | hypothetical LOC100133461 | 1 | | 205 | hsa-mir-3130-4 | 100133461 | LOC100133461 | hypothetical LOC100133461 | 1 | | 206 | hsa-miR-10a | 100133461 | LOC100133461 | hypothetical LOC100133461 | 1 | | 207 | hsa-miR-95 | 100133461 | LOC100133461 | hypothetical LOC100133461 | 1 | | 208 | hsa-miR-10a | 403150 | FLJ31356 | hypothetical protein FLJ31356 | 1 | | 209 | hsa-miR-95 | 403150 | FLJ31356 | hypothetical protein FLJ31356 | 1 | | 210 | hsa-miR-10a | 284669 | LOC284669 | hypothetical protein LOC284669 | 1 | | 211 | hsa-mir-3130-1 | 284186 | TMEM105 | transmembrane protein 105 | 1 | | 212 | hsa-mir-3130-2 | 284186 | TMEM105 | transmembrane protein 105 | 1 | | 213 | hsa-mir-3130-3 | 284186 | TMEM105 | transmembrane protein 105 | 1 | | 214 | hsa-mir-3130-4 | 284186 | TMEM105 | transmembrane protein 105 | 1 | | 216 | hsa-miR-95 | 160365 | CLECL1 | C-type lectin-like 1 | 1 | | 217 | hsa-mir-3130-1 | 400952 | UNQ6975 | NGNL6975 | 1 | | 218 | hsa-mir-3130-2 | 400952 | UNQ6975 | NGNL6975 | 1 | | 219 | hsa-mir-3130-3 | 400952 | UNQ6975 | NGNL6975 | 1 | | 220 | hsa-mir-3130-4 | 400952 | UNQ6975 | NGNL6975 | 1 | | 221 | hsa-miR-10a | 286114 | LOC286114 | hypothetical protein LOC286114 | 1 | | 222 | hsa-miR-499-5p | 286114 | LOC286114 | hypothetical protein LOC286114 | 1 | | 223 | hsa-miR-95 | 286114 | LOC286114 | hypothetical protein LOC286114 | 1 | | 224 | hsa-miR-95 | 285463 | LOC285463 | hypothetical protein LOC285463 | 1 | | 225 | hsa-miR-10a | 286234 | C9orf79 | chromosome 9 open reading frame 79 | 1 | | 226 | hsa-miR-95 | 286234 | C9orf79 | chromosome 9 open reading frame 79 | 1 | | 227 | hsa-mir-342 | 23200 | ATP11B | ATPase, class VI, type 11B | 1 | | 228 | hsa-miR-626 | 23200 | ATP11B | ATPase, class VI, type 11B | 1 | | 229 | hsa-miR-643 | 23200 | ATP11B | ATPase, class VI, type 11B | 1 | | 230 | hsa-miR-608 | 100316868 | NCRNA00213 | non-protein coding RNA 213 | 1 | | 231 | hsa-miR-95 | 283875 | LOC283875 | hypothetical protein LOC283875 | 1 | | 232 | hsa-miR-608 | 285708 | LOC285708 | hypothetical protein LOC285708 | 1 | | 233 | hsa-miR-10a | 144776 | hCG\_2038428 | hypothetical protein LOC144776 | 1 | | 234 | hsa-miR-95 | 144776 | hCG\_2038428 | hypothetical protein LOC144776 | 1 | | 235 | hsa-miR-449b | 166979 | CDC20B | cell division cycle 20 homolog B (S. cerevisiae) | 1 | | 236 | hsa-miR-608 | 54729 | NKX1-1 | NK1 homeobox 1 | 1 | | 237 | hsa-miR-1233 | 119016 | AGAP4 | ArfGAP with GTPase domain, ankyrin repeat and PH domain 4 | 1 | | 238 | hsa-miR-571 | 119016 | AGAP4 | ArfGAP with GTPase domain, ankyrin repeat and PH domain 4 | 1 | | 239 | hsa-miR-766 | 119016 | AGAP4 | ArfGAP with GTPase domain, ankyrin repeat and PH domain 4 | 1 | | 240 | hsa-miR-874 | 119016 | AGAP4 | ArfGAP with GTPase domain, ankyrin repeat and PH domain 4 | 1 | | 242 | hsa-mir-3130-1 | 266621 | DGCR7 | DiGeorge syndrome critical region gene 7 | 1 | | 243 | hsa-mir-3130-2 | 266621 | DGCR7 | DiGeorge syndrome critical region gene 7 | 1 | | 244 | hsa-mir-3130-3 | 266621 | DGCR7 | DiGeorge syndrome critical region gene 7 | 1 | | 245 | hsa-mir-3130-4 | 266621 | DGCR7 | DiGeorge syndrome critical region gene 7 | 1 | | 246 | hsa-miR-608 | 23589 | CARHSP1 | calcium regulated heat stable protein 1, 24kDa | 1 | | 248 | hsa-miR-569 | 923 | CD6 | CD6 molecule | 1 | | 249 | hsa-miR-571 | 923 | CD6 | CD6 molecule | 1 | | 252 | hsa-miR-10a | 149086 | LOC149086 | hypothetical protein LOC149086 | 1 | | 253 | hsa-mir-3130-1 | 81137 | OR7E104P | olfactory receptor, family 7, subfamily E, member 104 pseudogene | 1 | | 254 | hsa-mir-3130-2 | 81137 | OR7E104P | olfactory receptor, family 7, subfamily E, member 104 pseudogene | 1 | | 255 | hsa-mir-3130-3 | 81137 | OR7E104P | olfactory receptor, family 7, subfamily E, member 104 pseudogene | 1 | | 256 | hsa-mir-3130-4 | 81137 | OR7E104P | olfactory receptor, family 7, subfamily E, member 104 pseudogene | 1 | | 258 | hsa-miR-499-5p | 81137 | OR7E104P | olfactory receptor, family 7, subfamily E, member 104 pseudogene | 1 | | 259 | hsa-miR-95 | 81137 | OR7E104P | olfactory receptor, family 7, subfamily E, member 104 pseudogene | 1 | | 260 | hsa-mir-505 | 100128998 | C20orf181 | chromosome 20 open reading frame 181 | 1 | | 261 | hsa-mir-3130-1 | 100128998 | C20orf181 | chromosome 20 open reading frame 181 | 1 | | 262 | hsa-mir-3130-2 | 100128998 | C20orf181 | chromosome 20 open reading frame 181 | 1 | | 263 | hsa-mir-3130-3 | 100128998 | C20orf181 | chromosome 20 open reading frame 181 | 1 | | 264 | hsa-mir-3130-4 | 100128998 | C20orf181 | chromosome 20 open reading frame 181 | 1 | | 265 | hsa-miR-10a | 100128998 | C20orf181 | chromosome 20 open reading frame 181 | 1 | | 266 | hsa-miR-128 | 100128998 | C20orf181 | chromosome 20 open reading frame 181 | 1 | | 267 | hsa-miR-499-5p | 100128998 | C20orf181 | chromosome 20 open reading frame 181 | 1 | | 268 | hsa-miR-505\* | 100128998 | C20orf181 | chromosome 20 open reading frame 181 | 1 | | 269 | hsa-miR-580 | 100128998 | C20orf181 | chromosome 20 open reading frame 181 | 1 | | 270 | hsa-miR-95 | 100128998 | C20orf181 | chromosome 20 open reading frame 181 | 1 | | 272 | hsa-miR-95 | 26541 | OR10D1P | olfactory receptor, family 10, subfamily D, member 1 pseudogene | 1 | | 273 | hsa-miR-10a | 26219 | OR1J4 | olfactory receptor, family 1, subfamily J, member 4 | 1 | | 274 | hsa-miR-95 | 26219 | OR1J4 | olfactory receptor, family 1, subfamily J, member 4 | 1 | | 279 | hsa-miR-10a | 4240 | MFGE8 | milk fat globule-EGF factor 8 protein | 1 | | 281 | hsa-miR-10a | 6597 | SMARCA4 | SWI/SNF related, matrix associated, actin dependent regulator of chromatin, subfamily a, member 4 | 1 | | 282 | hsa-miR-95 | 6597 | SMARCA4 | SWI/SNF related, matrix associated, actin dependent regulator of chromatin, subfamily a, member 4 | 1 | | 283 | hsa-miR-569 | 256380 | SCML4 | sex comb on midleg-like 4 (Drosophila) | 1 | | 284 | hsa-miR-608 | 100129098 | LOC100129098 | hypothetical LOC100129098 | 1 | | 285 | hsa-miR-874 | 57732 | ZFYVE28 | zinc finger, FYVE domain containing 28 | 1 | | 286 | hsa-miR-10a | 56917 | MEIS3 | Meis homeobox 3 | 1 | | 287 | hsa-miR-95 | 56917 | MEIS3 | Meis homeobox 3 | 1 | | 288 | hsa-miR-95 | 57582 | KCNT1 | potassium channel, subfamily T, member 1 | 1 | | 292 | hsa-miR-95 | 728114 | LOC728114 | hypothetical protein LOC728114 | 1 | | 293 | hsa-miR-95 | 100289833 | LOC100289833 | similar to hCG2038441 | 1 | | 294 | hsa-miR-623 | 8717 | TRADD | TNFRSF1A-associated via death domain | 1 | | 295 | hsa-miR-877 | 8717 | TRADD | TNFRSF1A-associated via death domain | 1 | | 296 | hsa-miR-623 | 6158 | RPL28 | ribosomal protein L28 | 1 | | 297 | hsa-miR-623 | 6152 | RPL24 | ribosomal protein L24 | 1 | | 300 | hsa-miR-1233 | 4677 | NARS | asparaginyl-tRNA synthetase | 1 | | 301 | hsa-miR-558 | 4677 | NARS | asparaginyl-tRNA synthetase | 1 | | 302 | hsa-miR-874 | 4677 | NARS | asparaginyl-tRNA synthetase | 1 | | 303 | hsa-miR-593\* | 4841 | NONO | non-POU domain containing, octamer-binding | 1 | | 304 | hsa-miR-593\* | 10921 | RNPS1 | RNA binding protein S1, serine-rich domain | 1 | | 305 | hsa-miR-877 | 10921 | RNPS1 | RNA binding protein S1, serine-rich domain | 1 | | 306 | hsa-miR-1233 | 4869 | NPM1 | nucleophosmin (nucleolar phosphoprotein B23, numatrin) | 1 | | 307 | hsa-miR-874 | 4869 | NPM1 | nucleophosmin (nucleolar phosphoprotein B23, numatrin) | 1 | | 308 | hsa-miR-623 | 4670 | HNRNPM | heterogeneous nuclear ribonucleoprotein M | 1 | | 309 | hsa-miR-503 | 6194 | RPS6 | ribosomal protein S6 | 1 | | 310 | hsa-miR-1233 | 2339 | FNTA | farnesyltransferase, CAAX box, alpha | 1 | | 311 | hsa-miR-558 | 2339 | FNTA | farnesyltransferase, CAAX box, alpha | 1 | | 312 | hsa-miR-874 | 2339 | FNTA | farnesyltransferase, CAAX box, alpha | 1 | | 313 | hsa-miR-938 | 5573 | PRKAR1A | protein kinase, cAMP-dependent, regulatory, type I, alpha (tissue specific extinguisher 1) | 1 | | 314 | hsa-miR-938 | 3927 | LASP1 | LIM and SH3 protein 1 | 1 | | 319 | hsa-miR-623 | 6746 | SSR2 | signal sequence receptor, beta (translocon-associated protein beta) | 1 | | 320 | hsa-miR-1233 | 2739 | GLO1 | glyoxalase I | 1 | | 321 | hsa-miR-766 | 2739 | GLO1 | glyoxalase I | 1 | | 322 | hsa-miR-454\* | 23450 | SF3B3 | splicing factor 3b, subunit 3, 130kDa | 1 | | 323 | hsa-miR-623 | 1937 | EEF1G | eukaryotic translation elongation factor 1 gamma | 1 | | 324 | hsa-miR-938 | 10121 | ACTR1A | ARP1 actin-related protein 1 homolog A, centractin alpha (yeast) | 1 | | 326 | hsa-miR-623 | 5757 | PTMA | prothymosin, alpha | 1 | | 327 | hsa-miR-618 | 3303 | HSPA1A | heat shock 70kDa protein 1A | 1 | | 328 | hsa-miR-593\* | 6633 | SNRPD2 | small nuclear ribonucleoprotein D2 polypeptide 16.5kDa | 1 | | 329 | hsa-miR-877 | 6633 | SNRPD2 | small nuclear ribonucleoprotein D2 polypeptide 16.5kDa | 1 | | 332 | hsa-miR-503 | 51669 | TMEM66 | transmembrane protein 66 | 1 | | 333 | hsa-miR-503 | 6202 | RPS8 | ribosomal protein S8 | 1 | | 334 | hsa-miR-623 | 5689 | PSMB1 | proteasome (prosome, macropain) subunit, beta type, 1 | 1 | | 335 | hsa-miR-623 | 3133 | HLA-E | major histocompatibility complex, class I, E | 1 | | 336 | hsa-miR-623 | 5496 | PPM1G | protein phosphatase 1G (formerly 2C), magnesium-dependent, gamma isoform | 1 | | 337 | hsa-miR-877 | 5496 | PPM1G | protein phosphatase 1G (formerly 2C), magnesium-dependent, gamma isoform | 1 | | 338 | hsa-miR-558 | 3895 | KTN1 | kinectin 1 (kinesin receptor) | 1 | | 339 | hsa-miR-1233 | 894 | CCND2 | cyclin D2 | 1 | | 340 | hsa-miR-569 | 894 | CCND2 | cyclin D2 | 1 | | 341 | hsa-miR-766 | 894 | CCND2 | cyclin D2 | 1 | | 342 | hsa-miR-877 | 894 | CCND2 | cyclin D2 | 1 | | 344 | hsa-miR-569 | 3983 | ABLIM1 | actin binding LIM protein 1 | 1 | | 347 | hsa-miR-766 | 10099 | TSPAN3 | tetraspanin 3 | 1 | | 348 | hsa-miR-623 | 5936 | RBM4 | RNA binding motif protein 4 | 1 | | 352 | hsa-miR-569 | 9669 | EIF5B | eukaryotic translation initiation factor 5B | 1 | | 354 | hsa-miR-877 | 9669 | EIF5B | eukaryotic translation initiation factor 5B | 1 | | 356 | hsa-miR-569 | 3945 | LDHB | lactate dehydrogenase B | 1 | | 360 | hsa-miR-766 | 10904 | BLCAP | bladder cancer associated protein | 1 | | 361 | hsa-miR-877 | 10904 | BLCAP | bladder cancer associated protein | 1 | | 362 | hsa-miR-766 | 6599 | SMARCC1 | SWI/SNF related, matrix associated, actin dependent regulator of chromatin, subfamily c, member 1 | 1 | | 364 | hsa-miR-623 | 6599 | SMARCC1 | SWI/SNF related, matrix associated, actin dependent regulator of chromatin, subfamily c, member 1 | 1 | | 367 | hsa-miR-766 | 1434 | CSE1L | CSE1 chromosome segregation 1-like (yeast) | 1 | | 368 | hsa-miR-938 | 5226 | PGD | phosphogluconate dehydrogenase | 1 | | 369 | hsa-miR-1233 | 6741 | SSB | Sjogren syndrome antigen B (autoantigen La) | 1 | | 370 | hsa-miR-766 | 6741 | SSB | Sjogren syndrome antigen B (autoantigen La) | 1 | | 372 | hsa-miR-1233 | 10054 | UBA2 | ubiquitin-like modifier activating enzyme 2 | 1 | | 373 | hsa-miR-558 | 10054 | UBA2 | ubiquitin-like modifier activating enzyme 2 | 1 | | 374 | hsa-miR-571 | 10054 | UBA2 | ubiquitin-like modifier activating enzyme 2 | 1 | | 375 | hsa-miR-618 | 2773 | GNAI3 | guanine nucleotide binding protein (G protein), alpha inhibiting activity polypeptide 3 | 1 | | 376 | hsa-miR-623 | 10961 | ERP29 | endoplasmic reticulum protein 29 | 1 | | 377 | hsa-miR-618 | 5887 | RAD23B | RAD23 homolog B (S. cerevisiae) | 1 | | 378 | hsa-miR-1233 | 23386 | NUDCD3 | NudC domain containing 3 | 1 | | 379 | hsa-miR-569 | 23386 | NUDCD3 | NudC domain containing 3 | 1 | | 380 | hsa-miR-766 | 23386 | NUDCD3 | NudC domain containing 3 | 1 | | 381 | hsa-miR-618 | 55233 | MOBKL1B | MOB1, Mps One Binder kinase activator-like 1B (yeast) | 1 | | 382 | hsa-miR-558 | 10541 | ANP32B | acidic (leucine-rich) nuclear phosphoprotein 32 family, member B | 1 | | 383 | hsa-miR-877 | 10494 | STK25 | serine/threonine kinase 25 (STE20 homolog, yeast) | 1 | | 384 | hsa-miR-623 | 1471 | CST3 | cystatin C | 1 | | 385 | hsa-miR-558 | 1665 | DHX15 | DEAH (Asp-Glu-Ala-His) box polypeptide 15 | 1 | | 386 | hsa-miR-623 | 10181 | RBM5 | RNA binding motif protein 5 | 1 | | 388 | hsa-miR-454\* | 6240 | RRM1 | ribonucleotide reductase M1 | 1 | | 389 | hsa-miR-555 | 2926 | GRSF1 | G-rich RNA sequence binding factor 1 | 1 | | 390 | hsa-miR-558 | 2926 | GRSF1 | G-rich RNA sequence binding factor 1 | 1 | | 391 | hsa-miR-571 | 2926 | GRSF1 | G-rich RNA sequence binding factor 1 | 1 | | 393 | hsa-miR-558 | 7247 | TSN | translin | 1 | | 394 | hsa-miR-454\* | 7247 | TSN | translin | 1 | | 395 | hsa-miR-1233 | 22916 | NCBP2 | nuclear cap binding protein subunit 2, 20kDa | 1 | | 396 | hsa-miR-569 | 22916 | NCBP2 | nuclear cap binding protein subunit 2, 20kDa | 1 | | 397 | hsa-miR-571 | 22916 | NCBP2 | nuclear cap binding protein subunit 2, 20kDa | 1 | | 398 | hsa-miR-766 | 22916 | NCBP2 | nuclear cap binding protein subunit 2, 20kDa | 1 | | 399 | hsa-miR-874 | 22916 | NCBP2 | nuclear cap binding protein subunit 2, 20kDa | 1 | | 400 | hsa-miR-1233 | 9868 | TOMM70A | translocase of outer mitochondrial membrane 70 homolog A (S. cerevisiae) | 1 | | 401 | hsa-miR-558 | 9868 | TOMM70A | translocase of outer mitochondrial membrane 70 homolog A (S. cerevisiae) | 1 | | 402 | hsa-miR-571 | 9868 | TOMM70A | translocase of outer mitochondrial membrane 70 homolog A (S. cerevisiae) | 1 | | 403 | hsa-miR-766 | 9868 | TOMM70A | translocase of outer mitochondrial membrane 70 homolog A (S. cerevisiae) | 1 | | 404 | hsa-miR-874 | 9868 | TOMM70A | translocase of outer mitochondrial membrane 70 homolog A (S. cerevisiae) | 1 | | 405 | hsa-miR-766 | 2926 | GRSF1 | G-rich RNA sequence binding factor 1 | 1 | | 408 | hsa-miR-571 | 25813 | SAMM50 | sorting and assembly machinery component 50 homolog (S. cerevisiae) | 1 | | 409 | hsa-miR-593\* | 25813 | SAMM50 | sorting and assembly machinery component 50 homolog (S. cerevisiae) | 1 | | 410 | hsa-miR-623 | 25813 | SAMM50 | sorting and assembly machinery component 50 homolog (S. cerevisiae) | 1 | | 411 | hsa-miR-766 | 25813 | SAMM50 | sorting and assembly machinery component 50 homolog (S. cerevisiae) | 1 | | 412 | hsa-miR-877 | 25813 | SAMM50 | sorting and assembly machinery component 50 homolog (S. cerevisiae) | 1 | | 413 | hsa-miR-454\* | 8243 | SMC1A | structural maintenance of chromosomes 1A | 1 | | 414 | hsa-miR-618 | 4942 | OAT | ornithine aminotransferase | 1 | | 415 | hsa-miR-593\* | 8720 | MBTPS1 | membrane-bound transcription factor peptidase, site 1 | 1 | | 416 | hsa-miR-623 | 8720 | MBTPS1 | membrane-bound transcription factor peptidase, site 1 | 1 | | 417 | hsa-miR-766 | 8720 | MBTPS1 | membrane-bound transcription factor peptidase, site 1 | 1 | | 418 | hsa-miR-877 | 8720 | MBTPS1 | membrane-bound transcription factor peptidase, site 1 | 1 | | 419 | hsa-miR-938 | 4082 | MARCKS | myristoylated alanine-rich protein kinase C substrate | 1 | | 420 | hsa-mir-10a | 56941 | C3orf37 | chromosome 3 open reading frame 37 | 1 | | 421 | hsa-miR-1233 | 56941 | C3orf37 | chromosome 3 open reading frame 37 | 1 | | 422 | hsa-miR-766 | 56941 | C3orf37 | chromosome 3 open reading frame 37 | 1 | | 423 | hsa-miR-1233 | 1786 | DNMT1 | DNA (cytosine-5-)-methyltransferase 1 | 1 | | 424 | hsa-miR-569 | 1786 | DNMT1 | DNA (cytosine-5-)-methyltransferase 1 | 1 | | 425 | hsa-miR-766 | 1786 | DNMT1 | DNA (cytosine-5-)-methyltransferase 1 | 1 | | 426 | hsa-miR-874 | 1786 | DNMT1 | DNA (cytosine-5-)-methyltransferase 1 | 1 | | 427 | hsa-miR-877 | 1786 | DNMT1 | DNA (cytosine-5-)-methyltransferase 1 | 1 | | 428 | hsa-miR-593\* | 6626 | SNRPA | small nuclear ribonucleoprotein polypeptide A | 1 | | 429 | hsa-miR-877 | 6626 | SNRPA | small nuclear ribonucleoprotein polypeptide A | 1 | | 430 | hsa-miR-423-5p | 23394 | ADNP | activity-dependent neuroprotector homeobox | 1 | | 432 | hsa-miR-576-5p | 23394 | ADNP | activity-dependent neuroprotector homeobox | 1 | | 433 | hsa-miR-95 | 1857 | DVL3 | dishevelled, dsh homolog 3 (Drosophila) | 1 | | 434 | hsa-miR-1233 | 10412 | NSA2 | NSA2 ribosome biogenesis homolog (S. cerevisiae) | 1 | | 435 | hsa-miR-766 | 10412 | NSA2 | NSA2 ribosome biogenesis homolog (S. cerevisiae) | 1 | | 436 | hsa-miR-874 | 10412 | NSA2 | NSA2 ribosome biogenesis homolog (S. cerevisiae) | 1 | | 438 | hsa-miR-558 | 9987 | HNRPDL | heterogeneous nuclear ribonucleoprotein D-like | 1 | | 440 | hsa-miR-1233 | 6480 | ST6GAL1 | ST6 beta-galactosamide alpha-2,6-sialyltranferase 1 | 1 | | 441 | hsa-miR-766 | 6480 | ST6GAL1 | ST6 beta-galactosamide alpha-2,6-sialyltranferase 1 | 1 | | 442 | hsa-miR-874 | 6480 | ST6GAL1 | ST6 beta-galactosamide alpha-2,6-sialyltranferase 1 | 1 | | 443 | hsa-miR-454\* | 9202 | ZMYM4 | zinc finger, MYM-type 4 | 1 | | 444 | hsa-miR-558 | 9202 | ZMYM4 | zinc finger, MYM-type 4 | 1 | | 445 | hsa-miR-555 | 25800 | SLC39A6 | solute carrier family 39 (zinc transporter), member 6 | 1 | | 446 | hsa-miR-558 | 25800 | SLC39A6 | solute carrier family 39 (zinc transporter), member 6 | 1 | | 447 | hsa-miR-571 | 25800 | SLC39A6 | solute carrier family 39 (zinc transporter), member 6 | 1 | | 448 | hsa-miR-618 | 5899 | RALB | v-ral simian leukemia viral oncogene homolog B (ras related; GTP binding protein) | 1 | | 449 | hsa-miR-938 | 5899 | RALB | v-ral simian leukemia viral oncogene homolog B (ras related; GTP binding protein) | 1 | | 452 | hsa-miR-877 | 2802 | GOLGA3 | golgin A3 | 1 | | 454 | hsa-miR-558 | 8899 | PRPF4B | PRP4 pre-mRNA processing factor 4 homolog B (yeast) | 1 | | 455 | hsa-miR-1233 | 8899 | PRPF4B | PRP4 pre-mRNA processing factor 4 homolog B (yeast) | 1 | | 456 | hsa-miR-623 | 8574 | AKR7A2 | aldo-keto reductase family 7, member A2 (aflatoxin aldehyde reductase) | 1 | | 457 | hsa-miR-877 | 8574 | AKR7A2 | aldo-keto reductase family 7, member A2 (aflatoxin aldehyde reductase) | 1 | | 459 | hsa-miR-558 | 158 | ADSL | adenylosuccinate lyase | 1 | | 460 | hsa-miR-571 | 158 | ADSL | adenylosuccinate lyase | 1 | | 461 | hsa-miR-874 | 158 | ADSL | adenylosuccinate lyase | 1 | | 462 | hsa-miR-555 | 7716 | VEZF1 | vascular endothelial zinc finger 1 | 1 | | 463 | hsa-mir-10a | 55746 | NUP133 | nucleoporin 133kDa | 1 | | 464 | hsa-miR-1233 | 55746 | NUP133 | nucleoporin 133kDa | 1 | | 465 | hsa-miR-423-5p | 55746 | NUP133 | nucleoporin 133kDa | 1 | | 466 | hsa-miR-555 | 55746 | NUP133 | nucleoporin 133kDa | 1 | | 467 | hsa-miR-558 | 55746 | NUP133 | nucleoporin 133kDa | 1 | | 468 | hsa-miR-571 | 55746 | NUP133 | nucleoporin 133kDa | 1 | | 471 | hsa-miR-126\* | 2931 | GSK3A | glycogen synthase kinase 3 alpha | 1 | | 472 | hsa-miR-558 | 10480 | EIF3M | eukaryotic translation initiation factor 3, subunit M | 1 | | 473 | hsa-miR-95 | 4837 | NNMT | nicotinamide N-methyltransferase | 1 | | 474 | hsa-miR-766 | 50717 | DCAF8 | DDB1 and CUL4 associated factor 8 | 1 | | 475 | hsa-miR-877 | 50717 | DCAF8 | DDB1 and CUL4 associated factor 8 | 1 | | 476 | hsa-miR-555 | 9453 | GGPS1 | geranylgeranyl diphosphate synthase 1 | 1 | | 477 | hsa-miR-576-5p | 9453 | GGPS1 | geranylgeranyl diphosphate synthase 1 | 1 | | 478 | hsa-miR-423-5p | 9453 | GGPS1 | geranylgeranyl diphosphate synthase 1 | 1 | | 479 | hsa-miR-593\* | 10919 | EHMT2 | euchromatic histone-lysine N-methyltransferase 2 | 1 | | 480 | hsa-miR-877 | 10919 | EHMT2 | euchromatic histone-lysine N-methyltransferase 2 | 1 | | 481 | hsa-miR-423-5p | 865 | CBFB | core-binding factor, beta subunit | 1 | | 482 | hsa-miR-593\* | 26121 | PRPF31 | PRP31 pre-mRNA processing factor 31 homolog (S. cerevisiae) | 1 | | 483 | hsa-miR-877 | 26121 | PRPF31 | PRP31 pre-mRNA processing factor 31 homolog (S. cerevisiae) | 1 | | 484 | hsa-miR-1233 | 2073 | ERCC5 | excision repair cross-complementing rodent repair deficiency, complementation group 5 | 1 | | 485 | hsa-miR-558 | 2073 | ERCC5 | excision repair cross-complementing rodent repair deficiency, complementation group 5 | 1 | | 486 | hsa-miR-571 | 2073 | ERCC5 | excision repair cross-complementing rodent repair deficiency, complementation group 5 | 1 | | 487 | hsa-miR-874 | 2073 | ERCC5 | excision repair cross-complementing rodent repair deficiency, complementation group 5 | 1 | | 488 | hsa-mir-10a | 2531 | KDSR | 3-ketodihydrosphingosine reductase | 1 | | 490 | hsa-miR-766 | 2531 | KDSR | 3-ketodihydrosphingosine reductase | 1 | | 491 | hsa-miR-576-5p | 9318 | COPS2 | COP9 constitutive photomorphogenic homolog subunit 2 (Arabidopsis) | 1 | | 492 | hsa-miR-593\* | 10430 | TMEM147 | transmembrane protein 147 | 1 | | 493 | hsa-miR-623 | 10430 | TMEM147 | transmembrane protein 147 | 1 | | 494 | hsa-miR-877 | 10430 | TMEM147 | transmembrane protein 147 | 1 | | 495 | hsa-miR-766 | 10450 | PPIE | peptidylprolyl isomerase E (cyclophilin E) | 1 | | 496 | hsa-miR-877 | 6903 | TBCC | tubulin folding cofactor C | 1 | | 497 | hsa-miR-454\* | 4292 | MLH1 | mutL homolog 1, colon cancer, nonpolyposis type 2 (E. coli) | 1 | | 498 | hsa-miR-558 | 23760 | PITPNB | phosphatidylinositol transfer protein, beta | 1 | | 500 | hsa-miR-558 | 9255 | AIMP1 | aminoacyl tRNA synthetase complex-interacting multifunctional protein 1 | 1 | | 501 | hsa-mir-10a | 8658 | TNKS | tankyrase, TRF1-interacting ankyrin-related ADP-ribose polymerase | 1 | | 502 | hsa-miR-1233 | 8658 | TNKS | tankyrase, TRF1-interacting ankyrin-related ADP-ribose polymerase | 1 | | 503 | hsa-miR-423-5p | 8658 | TNKS | tankyrase, TRF1-interacting ankyrin-related ADP-ribose polymerase | 1 | | 504 | hsa-miR-571 | 8658 | TNKS | tankyrase, TRF1-interacting ankyrin-related ADP-ribose polymerase | 1 | | 505 | hsa-miR-766 | 8658 | TNKS | tankyrase, TRF1-interacting ankyrin-related ADP-ribose polymerase | 1 | | 506 | hsa-miR-874 | 8658 | TNKS | tankyrase, TRF1-interacting ankyrin-related ADP-ribose polymerase | 1 | | 507 | hsa-miR-1233 | 55308 | DDX19A | DEAD (Asp-Glu-Ala-As) box polypeptide 19A | 1 | | 508 | hsa-miR-569 | 55308 | DDX19A | DEAD (Asp-Glu-Ala-As) box polypeptide 19A | 1 | | 509 | hsa-miR-571 | 55308 | DDX19A | DEAD (Asp-Glu-Ala-As) box polypeptide 19A | 1 | | 510 | hsa-miR-766 | 55308 | DDX19A | DEAD (Asp-Glu-Ala-As) box polypeptide 19A | 1 | | 511 | hsa-miR-877 | 55308 | DDX19A | DEAD (Asp-Glu-Ala-As) box polypeptide 19A | 1 | | 512 | hsa-mir-10a | 27336 | HTATSF1 | HIV-1 Tat specific factor 1 | 1 | | 513 | hsa-miR-1233 | 27336 | HTATSF1 | HIV-1 Tat specific factor 1 | 1 | | 514 | hsa-miR-423-5p | 27336 | HTATSF1 | HIV-1 Tat specific factor 1 | 1 | | 515 | hsa-miR-571 | 27336 | HTATSF1 | HIV-1 Tat specific factor 1 | 1 | | 516 | hsa-miR-766 | 27336 | HTATSF1 | HIV-1 Tat specific factor 1 | 1 | | 517 | hsa-miR-423-5p | 55837 | EAPP | E2F-associated phosphoprotein | 1 | | 518 | hsa-miR-454\* | 5440 | POLR2K | polymerase (RNA) II (DNA directed) polypeptide K, 7.0kDa | 1 | | 520 | hsa-miR-423-5p | 9792 | SERTAD2 | SERTA domain containing 2 | 1 | | 521 | hsa-miR-454\* | 86 | ACTL6A | actin-like 6A | 1 | | 522 | hsa-miR-1233 | 8731 | RNMT | RNA (guanine-7-) methyltransferase | 1 | | 523 | hsa-miR-593\* | 1327 | COX4I1 | cytochrome c oxidase subunit IV isoform 1 | 1 | | 524 | hsa-miR-623 | 1327 | COX4I1 | cytochrome c oxidase subunit IV isoform 1 | 1 | | 525 | hsa-miR-877 | 790 | CAD | carbamoyl-phosphate synthetase 2, aspartate transcarbamylase, and dihydroorotase | 1 | | 526 | hsa-miR-1233 | 2308 | FOXO1 | forkhead box O1 | 1 | | 527 | hsa-miR-555 | 2308 | FOXO1 | forkhead box O1 | 1 | | 528 | hsa-miR-558 | 2308 | FOXO1 | forkhead box O1 | 1 | | 529 | hsa-miR-571 | 2308 | FOXO1 | forkhead box O1 | 1 | | 530 | hsa-miR-874 | 2308 | FOXO1 | forkhead box O1 | 1 | | 531 | hsa-miR-623 | 25804 | LSM4 | LSM4 homolog, U6 small nuclear RNA associated (S. cerevisiae) | 1 | | 532 | hsa-miR-877 | 25804 | LSM4 | LSM4 homolog, U6 small nuclear RNA associated (S. cerevisiae) | 1 | | 535 | hsa-miR-874 | 9452 | ITM2A | integral membrane protein 2A | 1 | | 536 | hsa-miR-569 | 9452 | ITM2A | integral membrane protein 2A | 1 | | 537 | hsa-miR-877 | 25920 | COBRA1 | cofactor of BRCA1 | 1 | | 538 | hsa-miR-593\* | 9780 | FAM38A | family with sequence similarity 38, member A | 1 | | 539 | hsa-miR-623 | 9780 | FAM38A | family with sequence similarity 38, member A | 1 | | 540 | hsa-miR-877 | 9780 | FAM38A | family with sequence similarity 38, member A | 1 | | 541 | hsa-miR-1233 | 5019 | OXCT1 | 3-oxoacid CoA transferase 1 | 1 | | 542 | hsa-miR-569 | 5019 | OXCT1 | 3-oxoacid CoA transferase 1 | 1 | | 543 | hsa-miR-766 | 5019 | OXCT1 | 3-oxoacid CoA transferase 1 | 1 | | 544 | hsa-miR-766 | 9701 | SAPS2 | SAPS domain family, member 2 | 1 | | 545 | hsa-miR-423-5p | 10427 | SEC24B | SEC24 family, member B (S. cerevisiae) | 1 | | 546 | hsa-miR-576-5p | 10427 | SEC24B | SEC24 family, member B (S. cerevisiae) | 1 | | 547 | hsa-miR-623 | 8192 | CLPP | ClpP caseinolytic peptidase, ATP-dependent, proteolytic subunit homolog (E. coli) | 1 | | 548 | hsa-miR-877 | 8192 | CLPP | ClpP caseinolytic peptidase, ATP-dependent, proteolytic subunit homolog (E. coli) | 1 | | 549 | hsa-miR-623 | 9267 | CYTH1 | cytohesin 1 | 1 | | 551 | hsa-miR-618 | 140885 | SIRPA | signal-regulatory protein alpha | 1 | | 552 | hsa-miR-623 | 6428 | SFRS3 | splicing factor, arginine/serine-rich 3 | 1 | | 553 | hsa-miR-618 | 3554 | IL1R1 | interleukin 1 receptor, type I | 1 | | 554 | hsa-miR-1233 | 5993 | RFX5 | regulatory factor X, 5 (influences HLA class II expression) | 1 | | 555 | hsa-miR-569 | 5993 | RFX5 | regulatory factor X, 5 (influences HLA class II expression) | 1 | | 556 | hsa-miR-571 | 5993 | RFX5 | regulatory factor X, 5 (influences HLA class II expression) | 1 | | 557 | hsa-miR-766 | 5993 | RFX5 | regulatory factor X, 5 (influences HLA class II expression) | 1 | | 558 | hsa-miR-874 | 5993 | RFX5 | regulatory factor X, 5 (influences HLA class II expression) | 1 | | 559 | hsa-miR-877 | 5993 | RFX5 | regulatory factor X, 5 (influences HLA class II expression) | 1 | | 566 | hsa-miR-10a | 58487 | CREBZF | CREB/ATF bZIP transcription factor | 1 | | 567 | hsa-miR-95 | 58487 | CREBZF | CREB/ATF bZIP transcription factor | 1 | | 569 | hsa-miR-569 | 58487 | CREBZF | CREB/ATF bZIP transcription factor | 1 | | 571 | hsa-mir-10a | 58487 | CREBZF | CREB/ATF bZIP transcription factor | 1 | | 572 | hsa-miR-423-5p | 58487 | CREBZF | CREB/ATF bZIP transcription factor | 1 | | 574 | hsa-miR-555 | 6477 | SIAH1 | seven in absentia homolog 1 (Drosophila) | 1 | | 575 | hsa-miR-576-5p | 6477 | SIAH1 | seven in absentia homolog 1 (Drosophila) | 1 | | 578 | hsa-miR-766 | 9529 | BAG5 | BCL2-associated athanogene 5 | 1 | | 579 | hsa-mir-10a | 9652 | TTC37 | tetratricopeptide repeat domain 37 | 1 | | 580 | hsa-miR-1233 | 9652 | TTC37 | tetratricopeptide repeat domain 37 | 1 | | 581 | hsa-miR-569 | 9652 | TTC37 | tetratricopeptide repeat domain 37 | 1 | | 582 | hsa-miR-571 | 9652 | TTC37 | tetratricopeptide repeat domain 37 | 1 | | 583 | hsa-miR-766 | 9652 | TTC37 | tetratricopeptide repeat domain 37 | 1 | | 584 | hsa-miR-423-5p | 9652 | TTC37 | tetratricopeptide repeat domain 37 | 1 | | 585 | hsa-miR-555 | 9652 | TTC37 | tetratricopeptide repeat domain 37 | 1 | | 586 | hsa-miR-558 | 9652 | TTC37 | tetratricopeptide repeat domain 37 | 1 | | 589 | hsa-mir-199a-2 | 8453 | CUL2 | cullin 2 | 1 | | 590 | hsa-mir-214 | 8453 | CUL2 | cullin 2 | 1 | | 591 | hsa-miR-618 | 6388 | SDF2 | stromal cell-derived factor 2 | 1 | | 592 | hsa-miR-593\* | 27339 | PRPF19 | PRP19/PSO4 pre-mRNA processing factor 19 homolog (S. cerevisiae) | 1 | | 593 | hsa-miR-623 | 27339 | PRPF19 | PRP19/PSO4 pre-mRNA processing factor 19 homolog (S. cerevisiae) | 1 | | 594 | hsa-miR-877 | 27339 | PRPF19 | PRP19/PSO4 pre-mRNA processing factor 19 homolog (S. cerevisiae) | 1 | | 596 | hsa-miR-877 | 10534 | SSSCA1 | Sjogren syndrome/scleroderma autoantigen 1 | 1 | | 597 | hsa-miR-877 | 9159 | PCSK7 | proprotein convertase subtilisin/kexin type 7 | 1 | | 599 | hsa-miR-877 | 23378 | RRP8 | ribosomal RNA processing 8, methyltransferase, homolog (yeast) | 1 | | 600 | hsa-miR-555 | 7019 | TFAM | transcription factor A, mitochondrial | 1 | | 601 | hsa-miR-576-5p | 7019 | TFAM | transcription factor A, mitochondrial | 1 | | 602 | hsa-miR-10a | 10058 | ABCB6 | ATP-binding cassette, sub-family B (MDR/TAP), member 6 | 1 | | 603 | hsa-miR-95 | 10058 | ABCB6 | ATP-binding cassette, sub-family B (MDR/TAP), member 6 | 1 | | 604 | hsa-mir-10a | 266655 | NCRNA00094 | non-protein coding RNA 94 | 1 | | 605 | hsa-miR-623 | 9130 | FAM50A | family with sequence similarity 50, member A | 1 | | 606 | hsa-miR-877 | 9130 | FAM50A | family with sequence similarity 50, member A | 1 | | 607 | hsa-miR-1233 | 23229 | ARHGEF9 | Cdc42 guanine nucleotide exchange factor (GEF) 9 | 1 | | 608 | hsa-miR-571 | 23229 | ARHGEF9 | Cdc42 guanine nucleotide exchange factor (GEF) 9 | 1 | | 609 | hsa-miR-766 | 23229 | ARHGEF9 | Cdc42 guanine nucleotide exchange factor (GEF) 9 | 1 | | 610 | hsa-miR-877 | 7318 | UBA7 | ubiquitin-like modifier activating enzyme 7 | 1 | | 611 | hsa-miR-555 | 3720 | JARID2 | jumonji, AT rich interactive domain 2 | 1 | | 612 | hsa-miR-558 | 8905 | AP1S2 | adaptor-related protein complex 1, sigma 2 subunit | 1 | | 613 | hsa-miR-877 | 8225 | GTPBP6 | GTP binding protein 6 (putative) | 1 | | 616 | hsa-miR-1224-5p | 23473 | CAPN7 | calpain 7 | 1 | | 617 | hsa-miR-618 | 4709 | NDUFB3 | NADH dehydrogenase (ubiquinone) 1 beta subcomplex, 3, 12kDa | 1 | | 618 | hsa-miR-618 | 9537 | TP53I11 | tumor protein p53 inducible protein 11 | 1 | | 619 | hsa-miR-1233 | 54726 | OTUD4 | OTU domain containing 4 | 1 | | 620 | hsa-miR-558 | 54726 | OTUD4 | OTU domain containing 4 | 1 | | 621 | hsa-miR-571 | 54726 | OTUD4 | OTU domain containing 4 | 1 | | 622 | hsa-miR-766 | 54726 | OTUD4 | OTU domain containing 4 | 1 | | 624 | hsa-miR-558 | 8481 | OFD1 | oral-facial-digital syndrome 1 | 1 | | 626 | hsa-miR-10a | 10974 | C10orf116 | chromosome 10 open reading frame 116 | 1 | | 627 | hsa-miR-95 | 10974 | C10orf116 | chromosome 10 open reading frame 116 | 1 | | 628 | hsa-miR-1233 | 9057 | SLC7A6 | solute carrier family 7 (cationic amino acid transporter, y+ system), member 6 | 1 | | 629 | hsa-miR-569 | 9057 | SLC7A6 | solute carrier family 7 (cationic amino acid transporter, y+ system), member 6 | 1 | | 630 | hsa-miR-571 | 9057 | SLC7A6 | solute carrier family 7 (cationic amino acid transporter, y+ system), member 6 | 1 | | 631 | hsa-miR-766 | 9057 | SLC7A6 | solute carrier family 7 (cationic amino acid transporter, y+ system), member 6 | 1 | | 632 | hsa-miR-874 | 9057 | SLC7A6 | solute carrier family 7 (cationic amino acid transporter, y+ system), member 6 | 1 | | 633 | hsa-miR-766 | 5867 | RAB4A | RAB4A, member RAS oncogene family | 1 | | 634 | hsa-miR-1233 | 7915 | ALDH5A1 | aldehyde dehydrogenase 5 family, member A1 | 1 | | 635 | hsa-miR-571 | 7915 | ALDH5A1 | aldehyde dehydrogenase 5 family, member A1 | 1 | | 636 | hsa-miR-874 | 7915 | ALDH5A1 | aldehyde dehydrogenase 5 family, member A1 | 1 | | 637 | hsa-miR-454\* | 6502 | SKP2 | S-phase kinase-associated protein 2 (p45) | 1 | | 638 | hsa-miR-454\* | 10466 | COG5 | component of oligomeric golgi complex 5 | 1 | | 639 | hsa-miR-766 | 5116 | PCNT | pericentrin | 1 | | 640 | hsa-miR-454\* | 5311 | PKD2 | polycystic kidney disease 2 (autosomal dominant) | 1 | | 641 | hsa-miR-623 | 3707 | ITPKB | inositol 1,4,5-trisphosphate 3-kinase B | 1 | | 642 | hsa-miR-877 | 3707 | ITPKB | inositol 1,4,5-trisphosphate 3-kinase B | 1 | | 643 | hsa-miR-618 | 1647 | GADD45A | growth arrest and DNA-damage-inducible, alpha | 1 | | 644 | hsa-mir-3130-1 | 5914 | RARA | retinoic acid receptor, alpha | 1 | | 645 | hsa-mir-3130-2 | 5914 | RARA | retinoic acid receptor, alpha | 1 | | 646 | hsa-mir-3130-3 | 5914 | RARA | retinoic acid receptor, alpha | 1 | | 647 | hsa-mir-3130-4 | 5914 | RARA | retinoic acid receptor, alpha | 1 | | 648 | hsa-miR-580 | 5914 | RARA | retinoic acid receptor, alpha | 1 | | 649 | hsa-miR-95 | 5914 | RARA | retinoic acid receptor, alpha | 1 | | 650 | hsa-miR-766 | 55794 | DDX28 | DEAD (Asp-Glu-Ala-Asp) box polypeptide 28 | 1 | | 652 | hsa-miR-1233 | 10270 | AKAP8 | A kinase (PRKA) anchor protein 8 | 1 | | 653 | hsa-miR-558 | 10270 | AKAP8 | A kinase (PRKA) anchor protein 8 | 1 | | 654 | hsa-miR-571 | 10270 | AKAP8 | A kinase (PRKA) anchor protein 8 | 1 | | 655 | hsa-miR-766 | 10270 | AKAP8 | A kinase (PRKA) anchor protein 8 | 1 | | 656 | hsa-miR-95 | 5064 | PALM | paralemmin | 1 | | 657 | hsa-miR-1233 | 104 | ADARB1 | adenosine deaminase, RNA-specific, B1 (RED1 homolog rat) | 1 | | 658 | hsa-miR-569 | 104 | ADARB1 | adenosine deaminase, RNA-specific, B1 (RED1 homolog rat) | 1 | | 659 | hsa-miR-766 | 104 | ADARB1 | adenosine deaminase, RNA-specific, B1 (RED1 homolog rat) | 1 | | 660 | hsa-miR-874 | 104 | ADARB1 | adenosine deaminase, RNA-specific, B1 (RED1 homolog rat) | 1 | | 661 | hsa-miR-571 | 64854 | USP46 | ubiquitin specific peptidase 46 | 1 | | 663 | hsa-miR-938 | 3732 | CD82 | CD82 molecule | 1 | | 664 | hsa-miR-618 | 2730 | GCLM | glutamate-cysteine ligase, modifier subunit | 1 | | 666 | hsa-miR-618 | 4318 | MMP9 | matrix metallopeptidase 9 (gelatinase B, 92kDa gelatinase, 92kDa type IV collagenase) | 1 | | 667 | hsa-miR-643 | 4318 | MMP9 | matrix metallopeptidase 9 (gelatinase B, 92kDa gelatinase, 92kDa type IV collagenase) | 1 | | 668 | hsa-miR-877 | 55756 | INTS9 | integrator complex subunit 9 | 1 | | 669 | hsa-miR-1233 | 9923 | ZBTB40 | zinc finger and BTB domain containing 40 | 1 | | 671 | hsa-miR-558 | 8504 | PEX3 | peroxisomal biogenesis factor 3 | 1 | | 673 | hsa-miR-874 | 8504 | PEX3 | peroxisomal biogenesis factor 3 | 1 | | 674 | hsa-miR-1233 | 9984 | THOC1 | THO complex 1 | 1 | | 675 | hsa-miR-558 | 9984 | THOC1 | THO complex 1 | 1 | | 676 | hsa-miR-874 | 9984 | THOC1 | THO complex 1 | 1 | | 677 | hsa-mir-10a | 9819 | TSC22D2 | TSC22 domain family, member 2 | 1 | | 678 | hsa-miR-423-5p | 9819 | TSC22D2 | TSC22 domain family, member 2 | 1 | | 679 | hsa-miR-623 | 1938 | EEF2 | eukaryotic translation elongation factor 2 | 1 | | 683 | hsa-miR-569 | 55556 | ENOSF1 | enolase superfamily member 1 | 1 | | 685 | hsa-miR-766 | 55556 | ENOSF1 | enolase superfamily member 1 | 1 | | 686 | hsa-miR-938 | 22904 | SBNO2 | strawberry notch homolog 2 (Drosophila) | 1 | | 687 | hsa-miR-1233 | 10208 | USPL1 | ubiquitin specific peptidase like 1 | 1 | | 688 | hsa-miR-569 | 10208 | USPL1 | ubiquitin specific peptidase like 1 | 1 | | 689 | hsa-miR-571 | 10208 | USPL1 | ubiquitin specific peptidase like 1 | 1 | | 690 | hsa-miR-766 | 10208 | USPL1 | ubiquitin specific peptidase like 1 | 1 | | 691 | hsa-miR-874 | 10208 | USPL1 | ubiquitin specific peptidase like 1 | 1 | | 692 | hsa-miR-643 | 2207 | FCER1G | Fc fragment of IgE, high affinity I, receptor for; gamma polypeptide | 1 | | 693 | hsa-miR-1233 | 7748 | ZNF195 | zinc finger protein 195 | 1 | | 694 | hsa-miR-423-5p | 7748 | ZNF195 | zinc finger protein 195 | 1 | | 695 | hsa-miR-569 | 7748 | ZNF195 | zinc finger protein 195 | 1 | | 696 | hsa-miR-571 | 7748 | ZNF195 | zinc finger protein 195 | 1 | | 697 | hsa-miR-766 | 7748 | ZNF195 | zinc finger protein 195 | 1 | | 698 | hsa-miR-874 | 7748 | ZNF195 | zinc finger protein 195 | 1 | | 699 | hsa-miR-454\* | 10926 | DBF4 | DBF4 homolog (S. cerevisiae) | 1 | | 700 | hsa-miR-569 | 10667 | FARS2 | phenylalanyl-tRNA synthetase 2, mitochondrial | 1 | | 701 | hsa-miR-766 | 10667 | FARS2 | phenylalanyl-tRNA synthetase 2, mitochondrial | 1 | | 702 | hsa-miR-877 | 10667 | FARS2 | phenylalanyl-tRNA synthetase 2, mitochondrial | 1 | | 703 | hsa-mir-10a | 10772 | SFRS13A | splicing factor, arginine/serine-rich 13A | 1 | | 704 | hsa-miR-423-5p | 10772 | SFRS13A | splicing factor, arginine/serine-rich 13A | 1 | | 705 | hsa-miR-571 | 10772 | SFRS13A | splicing factor, arginine/serine-rich 13A | 1 | | 706 | hsa-miR-576-5p | 10772 | SFRS13A | splicing factor, arginine/serine-rich 13A | 1 | | 708 | hsa-miR-608 | 27333 | GOLIM4 | golgi integral membrane protein 4 | 1 | | 709 | hsa-miR-623 | 11186 | RASSF1 | Ras association (RalGDS/AF-6) domain family member 1 | 1 | | 710 | hsa-miR-877 | 11186 | RASSF1 | Ras association (RalGDS/AF-6) domain family member 1 | 1 | | 711 | hsa-miR-623 | 22907 | DHX30 | DEAH (Asp-Glu-Ala-His) box polypeptide 30 | 1 | | 713 | hsa-miR-1233 | 9730 | VPRBP | Vpr (HIV-1) binding protein | 1 | | 714 | hsa-miR-766 | 9730 | VPRBP | Vpr (HIV-1) binding protein | 1 | | 715 | hsa-miR-558 | 78988 | MRP63 | mitochondrial ribosomal protein 63 | 1 | | 716 | hsa-miR-10a | 8805 | TRIM24 | tripartite motif-containing 24 | 1 | | 717 | hsa-miR-95 | 8805 | TRIM24 | tripartite motif-containing 24 | 1 | | 718 | hsa-miR-623 | 8536 | CAMK1 | calcium/calmodulin-dependent protein kinase I | 1 | | 719 | hsa-miR-95 | 9215 | LARGE | like-glycosyltransferase | 1 | | 721 | hsa-miR-558 | 7637 | ZNF84 | zinc finger protein 84 | 1 | | 724 | hsa-mir-10a | 5810 | RAD1 | RAD1 homolog (S. pombe) | 1 | | 725 | hsa-miR-1233 | 5810 | RAD1 | RAD1 homolog (S. pombe) | 1 | | 726 | hsa-miR-423-5p | 5810 | RAD1 | RAD1 homolog (S. pombe) | 1 | | 728 | hsa-miR-766 | 5810 | RAD1 | RAD1 homolog (S. pombe) | 1 | | 729 | hsa-miR-1233 | 5287 | PIK3C2B | phosphoinositide-3-kinase, class 2, beta polypeptide | 1 | | 730 | hsa-miR-569 | 5287 | PIK3C2B | phosphoinositide-3-kinase, class 2, beta polypeptide | 1 | | 731 | hsa-miR-571 | 5287 | PIK3C2B | phosphoinositide-3-kinase, class 2, beta polypeptide | 1 | | 732 | hsa-miR-766 | 5287 | PIK3C2B | phosphoinositide-3-kinase, class 2, beta polypeptide | 1 | | 733 | hsa-miR-874 | 5287 | PIK3C2B | phosphoinositide-3-kinase, class 2, beta polypeptide | 1 | | 734 | hsa-miR-95 | 3784 | KCNQ1 | potassium voltage-gated channel, KQT-like subfamily, member 1 | 1 | | 735 | hsa-miR-1233 | 29902 | C12orf24 | chromosome 12 open reading frame 24 | 1 | | 736 | hsa-miR-571 | 29902 | C12orf24 | chromosome 12 open reading frame 24 | 1 | | 737 | hsa-miR-874 | 29902 | C12orf24 | chromosome 12 open reading frame 24 | 1 | | 738 | hsa-miR-766 | 9284 | NPIP | nuclear pore complex interacting protein | 1 | | 739 | hsa-miR-877 | 9284 | NPIP | nuclear pore complex interacting protein | 1 | | 740 | hsa-miR-95 | 23541 | SEC14L2 | SEC14-like 2 (S. cerevisiae) | 1 | | 741 | hsa-miR-423-5p | 11234 | HPS5 | Hermansky-Pudlak syndrome 5 | 1 | | 742 | hsa-miR-571 | 10966 | RAB40B | RAB40B, member RAS oncogene family | 1 | | 743 | hsa-miR-874 | 10966 | RAB40B | RAB40B, member RAS oncogene family | 1 | | 744 | hsa-miR-1233 | 9252 | RPS6KA5 | ribosomal protein S6 kinase, 90kDa, polypeptide 5 | 1 | | 745 | hsa-mir-10a | 905 | CCNT2 | cyclin T2 | 1 | | 746 | hsa-miR-1224-5p | 905 | CCNT2 | cyclin T2 | 1 | | 747 | hsa-miR-423-5p | 905 | CCNT2 | cyclin T2 | 1 | | 756 | hsa-miR-423-5p | 373 | TRIM23 | tripartite motif-containing 23 | 1 | | 757 | hsa-miR-576-5p | 373 | TRIM23 | tripartite motif-containing 23 | 1 | | 758 | hsa-miR-571 | 4675 | NAP1L3 | nucleosome assembly protein 1-like 3 | 1 | | 759 | hsa-mir-10a | 7270 | TTF1 | transcription termination factor, RNA polymerase I | 1 | | 760 | hsa-miR-1233 | 3590 | IL11RA | interleukin 11 receptor, alpha | 1 | | 761 | hsa-miR-569 | 3590 | IL11RA | interleukin 11 receptor, alpha | 1 | | 762 | hsa-miR-766 | 3590 | IL11RA | interleukin 11 receptor, alpha | 1 | | 763 | hsa-miR-874 | 3590 | IL11RA | interleukin 11 receptor, alpha | 1 | | 764 | hsa-miR-1233 | 9737 | GPRASP1 | G protein-coupled receptor associated sorting protein 1 | 1 | | 765 | hsa-miR-569 | 9737 | GPRASP1 | G protein-coupled receptor associated sorting protein 1 | 1 | | 766 | hsa-miR-571 | 9737 | GPRASP1 | G protein-coupled receptor associated sorting protein 1 | 1 | | 767 | hsa-miR-766 | 9737 | GPRASP1 | G protein-coupled receptor associated sorting protein 1 | 1 | | 768 | hsa-miR-874 | 9737 | GPRASP1 | G protein-coupled receptor associated sorting protein 1 | 1 | | 769 | hsa-miR-95 | 6236 | RRAD | Ras-related associated with diabetes | 1 | | 770 | hsa-miR-877 | 5883 | RAD9A | RAD9 homolog A (S. pombe) | 1 | | 771 | hsa-miR-95 | 2028 | ENPEP | glutamyl aminopeptidase (aminopeptidase A) | 1 | | 772 | hsa-miR-576-5p | 27107 | ZBTB11 | zinc finger and BTB domain containing 11 | 1 | | 774 | hsa-miR-593\* | 4832 | NME3 | non-metastatic cells 3, protein expressed in | 1 | | 775 | hsa-miR-623 | 4832 | NME3 | non-metastatic cells 3, protein expressed in | 1 | | 776 | hsa-miR-877 | 4832 | NME3 | non-metastatic cells 3, protein expressed in | 1 | | 777 | hsa-miR-618 | 4097 | MAFG | v-maf musculoaponeurotic fibrosarcoma oncogene homolog G (avian) | 1 | | 778 | hsa-mir-10a | 9949 | AMMECR1 | Alport syndrome, mental retardation, midface hypoplasia and elliptocytosis chromosomal region gene 1 | 1 | | 780 | hsa-miR-1233 | 1662 | DDX10 | DEAD (Asp-Glu-Ala-Asp) box polypeptide 10 | 1 | | 781 | hsa-miR-766 | 1662 | DDX10 | DEAD (Asp-Glu-Ala-Asp) box polypeptide 10 | 1 | | 782 | hsa-miR-874 | 1662 | DDX10 | DEAD (Asp-Glu-Ala-Asp) box polypeptide 10 | 1 | | 783 | hsa-miR-454\* | 5557 | PRIM1 | primase, DNA, polypeptide 1 (49kDa) | 1 | | 784 | hsa-miR-766 | 5393 | EXOSC9 | exosome component 9 | 1 | | 785 | hsa-miR-874 | 5393 | EXOSC9 | exosome component 9 | 1 | | 786 | hsa-mir-10a | 1836 | SLC26A2 | solute carrier family 26 (sulfate transporter), member 2 | 1 | | 787 | hsa-mir-885 | 1836 | SLC26A2 | solute carrier family 26 (sulfate transporter), member 2 | 1 | | 788 | hsa-miR-423-5p | 1836 | SLC26A2 | solute carrier family 26 (sulfate transporter), member 2 | 1 | | 789 | hsa-miR-766 | 1836 | SLC26A2 | solute carrier family 26 (sulfate transporter), member 2 | 1 | | 790 | hsa-miR-885-5p | 1836 | SLC26A2 | solute carrier family 26 (sulfate transporter), member 2 | 1 | | 791 | hsa-miR-558 | 26747 | NUFIP1 | nuclear fragile X mental retardation protein interacting protein 1 | 1 | | 792 | hsa-miR-571 | 26747 | NUFIP1 | nuclear fragile X mental retardation protein interacting protein 1 | 1 | | 793 | hsa-miR-874 | 26747 | NUFIP1 | nuclear fragile X mental retardation protein interacting protein 1 | 1 | | 794 | hsa-miR-95 | 6712 | SPTBN2 | spectrin, beta, non-erythrocytic 2 | 1 | | 796 | hsa-miR-10a | 64849 | SLC13A3 | solute carrier family 13 (sodium-dependent dicarboxylate transporter), member 3 | 1 | | 797 | hsa-miR-1233 | 80184 | CEP290 | centrosomal protein 290kDa | 1 | | 798 | hsa-miR-558 | 80184 | CEP290 | centrosomal protein 290kDa | 1 | | 799 | hsa-miR-571 | 80184 | CEP290 | centrosomal protein 290kDa | 1 | | 801 | hsa-mir-3130-1 | 9567 | GTPBP1 | GTP binding protein 1 | 1 | | 802 | hsa-mir-3130-2 | 9567 | GTPBP1 | GTP binding protein 1 | 1 | | 803 | hsa-mir-3130-3 | 9567 | GTPBP1 | GTP binding protein 1 | 1 | | 804 | hsa-mir-3130-4 | 9567 | GTPBP1 | GTP binding protein 1 | 1 | | 805 | hsa-miR-874 | 9816 | URB2 | URB2 ribosome biogenesis 2 homolog (S. cerevisiae) | 1 | | 806 | hsa-miR-10a | 10297 | APC2 | adenomatosis polyposis coli 2 | 1 | | 807 | hsa-miR-1233 | 24140 | FTSJ1 | FtsJ homolog 1 (E. coli) | 1 | | 808 | hsa-miR-766 | 24140 | FTSJ1 | FtsJ homolog 1 (E. coli) | 1 | | 810 | hsa-miR-1233 | 23708 | GSPT2 | G1 to S phase transition 2 | 1 | | 811 | hsa-miR-571 | 23708 | GSPT2 | G1 to S phase transition 2 | 1 | | 812 | hsa-miR-874 | 23708 | GSPT2 | G1 to S phase transition 2 | 1 | | 813 | hsa-miR-766 | 22826 | DNAJC8 | DnaJ (Hsp40) homolog, subfamily C, member 8 | 1 | | 814 | hsa-miR-618 | 366 | AQP9 | aquaporin 9 | 1 | | 815 | hsa-miR-95 | 4634 | MYL3 | myosin, light chain 3, alkali; ventricular, skeletal, slow | 1 | | 816 | hsa-miR-423-5p | 22834 | ZNF652 | zinc finger protein 652 | 1 | | 817 | hsa-miR-576-5p | 22834 | ZNF652 | zinc finger protein 652 | 1 | | 818 | hsa-miR-10a | 27077 | B9D1 | B9 protein domain 1 | 1 | | 819 | hsa-miR-499-5p | 27077 | B9D1 | B9 protein domain 1 | 1 | | 820 | hsa-miR-95 | 27077 | B9D1 | B9 protein domain 1 | 1 | | 821 | hsa-mir-10a | 55667 | DENND4C | DENN/MADD domain containing 4C | 1 | | 822 | hsa-mir-885 | 55667 | DENND4C | DENN/MADD domain containing 4C | 1 | | 823 | hsa-miR-1224-5p | 55667 | DENND4C | DENN/MADD domain containing 4C | 1 | | 824 | hsa-miR-423-5p | 55667 | DENND4C | DENN/MADD domain containing 4C | 1 | | 825 | hsa-miR-766 | 55667 | DENND4C | DENN/MADD domain containing 4C | 1 | | 826 | hsa-miR-885-5p | 55667 | DENND4C | DENN/MADD domain containing 4C | 1 | | 827 | hsa-mir-10a | 23545 | ATP6V0A2 | ATPase, H+ transporting, lysosomal V0 subunit a2 | 1 | | 828 | hsa-mir-199a-2 | 23545 | ATP6V0A2 | ATPase, H+ transporting, lysosomal V0 subunit a2 | 1 | | 829 | hsa-mir-214 | 23545 | ATP6V0A2 | ATPase, H+ transporting, lysosomal V0 subunit a2 | 1 | | 830 | hsa-miR-1224-5p | 23545 | ATP6V0A2 | ATPase, H+ transporting, lysosomal V0 subunit a2 | 1 | | 831 | hsa-miR-423-5p | 23545 | ATP6V0A2 | ATPase, H+ transporting, lysosomal V0 subunit a2 | 1 | | 832 | hsa-miR-576-5p | 23545 | ATP6V0A2 | ATPase, H+ transporting, lysosomal V0 subunit a2 | 1 | | 833 | hsa-miR-1233 | 9465 | AKAP7 | A kinase (PRKA) anchor protein 7 | 1 | | 834 | hsa-miR-558 | 9465 | AKAP7 | A kinase (PRKA) anchor protein 7 | 1 | | 835 | hsa-miR-569 | 9465 | AKAP7 | A kinase (PRKA) anchor protein 7 | 1 | | 836 | hsa-miR-571 | 9465 | AKAP7 | A kinase (PRKA) anchor protein 7 | 1 | | 837 | hsa-miR-766 | 9465 | AKAP7 | A kinase (PRKA) anchor protein 7 | 1 | | 838 | hsa-miR-874 | 9465 | AKAP7 | A kinase (PRKA) anchor protein 7 | 1 | | 839 | hsa-miR-618 | 638 | BIK | BCL2-interacting killer (apoptosis-inducing) | 1 | | 840 | hsa-miR-618 | 3684 | ITGAM | integrin, alpha M (complement component 3 receptor 3 subunit) | 1 | | 841 | hsa-miR-95 | 8839 | WISP2 | WNT1 inducible signaling pathway protein 2 | 1 | | 845 | hsa-miR-10a | 5781 | PTPN11 | protein tyrosine phosphatase, non-receptor type 11 | 1 | | 846 | hsa-miR-95 | 5781 | PTPN11 | protein tyrosine phosphatase, non-receptor type 11 | 1 | | 847 | hsa-mir-10a | 9422 | ZNF264 | zinc finger protein 264 | 1 | | 848 | hsa-miR-423-5p | 9422 | ZNF264 | zinc finger protein 264 | 1 | | 849 | hsa-mir-10a | 11168 | PSIP1 | PC4 and SFRS1 interacting protein 1 | 1 | | 856 | hsa-mir-3130-1 | 1188 | CLCNKB | chloride channel Kb | 1 | | 857 | hsa-mir-3130-2 | 1188 | CLCNKB | chloride channel Kb | 1 | | 858 | hsa-mir-3130-3 | 1188 | CLCNKB | chloride channel Kb | 1 | | 859 | hsa-mir-3130-4 | 1188 | CLCNKB | chloride channel Kb | 1 | | 860 | hsa-miR-766 | 8832 | CD84 | CD84 molecule | 1 | | 861 | hsa-miR-877 | 8832 | CD84 | CD84 molecule | 1 | | 863 | hsa-miR-558 | 6627 | SNRPA1 | small nuclear ribonucleoprotein polypeptide A' | 1 | | 864 | hsa-miR-1233 | 6775 | STAT4 | signal transducer and activator of transcription 4 | 1 | | 865 | hsa-miR-569 | 6775 | STAT4 | signal transducer and activator of transcription 4 | 1 | | 866 | hsa-miR-766 | 6775 | STAT4 | signal transducer and activator of transcription 4 | 1 | | 867 | hsa-miR-766 | 939 | CD27 | CD27 molecule | 1 | | 868 | hsa-miR-877 | 939 | CD27 | CD27 molecule | 1 | | 869 | hsa-miR-10a | 8581 | LY6D | lymphocyte antigen 6 complex, locus D | 1 | | 870 | hsa-miR-499-5p | 8581 | LY6D | lymphocyte antigen 6 complex, locus D | 1 | | 871 | hsa-miR-580 | 8581 | LY6D | lymphocyte antigen 6 complex, locus D | 1 | | 872 | hsa-miR-95 | 8581 | LY6D | lymphocyte antigen 6 complex, locus D | 1 | | 876 | hsa-miR-95 | 7755 | ZNF205 | zinc finger protein 205 | 1 | | 877 | hsa-miR-618 | 660 | BMX | BMX non-receptor tyrosine kinase | 1 | | 878 | hsa-mir-10a | 25988 | HINFP | histone H4 transcription factor | 1 | | 879 | hsa-miR-586 | 8464 | SUPT3H | suppressor of Ty 3 homolog (S. cerevisiae) | 1 | | 880 | hsa-miR-571 | 9631 | NUP155 | nucleoporin 155kDa | 1 | | 881 | hsa-miR-503 | 1915 | EEF1A1 | eukaryotic translation elongation factor 1 alpha 1 | 1 | | 882 | hsa-mir-505 | 4987 | OPRL1 | opiate receptor-like 1 | 1 | | 883 | hsa-mir-3130-1 | 4987 | OPRL1 | opiate receptor-like 1 | 1 | | 884 | hsa-mir-3130-2 | 4987 | OPRL1 | opiate receptor-like 1 | 1 | | 885 | hsa-mir-3130-3 | 4987 | OPRL1 | opiate receptor-like 1 | 1 | | 886 | hsa-mir-3130-4 | 4987 | OPRL1 | opiate receptor-like 1 | 1 | | 887 | hsa-miR-128 | 4987 | OPRL1 | opiate receptor-like 1 | 1 | | 888 | hsa-miR-505\* | 4987 | OPRL1 | opiate receptor-like 1 | 1 | | 889 | hsa-miR-580 | 4987 | OPRL1 | opiate receptor-like 1 | 1 | | 890 | hsa-miR-95 | 4987 | OPRL1 | opiate receptor-like 1 | 1 | | 892 | hsa-miR-643 | 634 | CEACAM1 | carcinoembryonic antigen-related cell adhesion molecule 1 (biliary glycoprotein) | 1 | | 893 | hsa-miR-1233 | 55634 | ZNF673 | zinc finger family member 673 | 1 | | 894 | hsa-miR-558 | 55634 | ZNF673 | zinc finger family member 673 | 1 | | 895 | hsa-miR-571 | 55634 | ZNF673 | zinc finger family member 673 | 1 | | 896 | hsa-miR-874 | 55634 | ZNF673 | zinc finger family member 673 | 1 | | 897 | hsa-miR-571 | 9015 | TAF1A | TATA box binding protein (TBP)-associated factor, RNA polymerase I, A, 48kDa | 1 | | 898 | hsa-miR-618 | 8809 | IL18R1 | interleukin 18 receptor 1 | 1 | | 899 | hsa-miR-1233 | 51276 | ZNF571 | zinc finger protein 571 | 1 | | 900 | hsa-miR-571 | 51276 | ZNF571 | zinc finger protein 571 | 1 | | 901 | hsa-miR-874 | 51276 | ZNF571 | zinc finger protein 571 | 1 | | 902 | hsa-miR-95 | 55721 | IQCC | IQ motif containing C | 1 | | 903 | hsa-miR-10a | 3881 | KRT31 | keratin 31 | 1 | | 904 | hsa-miR-95 | 3881 | KRT31 | keratin 31 | 1 | | 905 | hsa-miR-10a | 4861 | NPAS1 | neuronal PAS domain protein 1 | 1 | | 906 | hsa-miR-766 | 10225 | CD96 | CD96 molecule | 1 | | 907 | hsa-miR-95 | 5409 | PNMT | phenylethanolamine N-methyltransferase | 1 | | 908 | hsa-miR-1233 | 917 | CD3G | CD3g molecule, gamma (CD3-TCR complex) | 1 | | 909 | hsa-miR-569 | 917 | CD3G | CD3g molecule, gamma (CD3-TCR complex) | 1 | | 910 | hsa-miR-766 | 917 | CD3G | CD3g molecule, gamma (CD3-TCR complex) | 1 | | 911 | hsa-miR-874 | 917 | CD3G | CD3g molecule, gamma (CD3-TCR complex) | 1 | | 912 | hsa-mir-10a | 7294 | TXK | TXK tyrosine kinase | 1 | | 913 | hsa-miR-1233 | 7294 | TXK | TXK tyrosine kinase | 1 | | 914 | hsa-miR-423-5p | 7294 | TXK | TXK tyrosine kinase | 1 | | 915 | hsa-miR-503 | 7294 | TXK | TXK tyrosine kinase | 1 | | 916 | hsa-miR-569 | 7294 | TXK | TXK tyrosine kinase | 1 | | 917 | hsa-miR-571 | 7294 | TXK | TXK tyrosine kinase | 1 | | 918 | hsa-miR-766 | 7294 | TXK | TXK tyrosine kinase | 1 | | 919 | hsa-miR-874 | 7294 | TXK | TXK tyrosine kinase | 1 | | 920 | hsa-miR-423-5p | 80264 | ZNF430 | zinc finger protein 430 | 1 | | 921 | hsa-miR-766 | 80264 | ZNF430 | zinc finger protein 430 | 1 | | 922 | hsa-mir-885 | 6885 | MAP3K7 | mitogen-activated protein kinase kinase kinase 7 | 1 | | 923 | hsa-miR-885-5p | 6885 | MAP3K7 | mitogen-activated protein kinase kinase kinase 7 | 1 | | 924 | hsa-mir-10a | 54468 | MIOS | missing oocyte, meiosis regulator, homolog (Drosophila) | 1 | | 926 | hsa-miR-938 | 4084 | MXD1 | MAX dimerization protein 1 | 1 | | 927 | hsa-miR-1233 | 7700 | ZNF141 | zinc finger protein 141 | 1 | | 928 | hsa-miR-571 | 7700 | ZNF141 | zinc finger protein 141 | 1 | | 929 | hsa-miR-874 | 7700 | ZNF141 | zinc finger protein 141 | 1 | | 930 | hsa-miR-555 | 5325 | PLAGL1 | pleiomorphic adenoma gene-like 1 | 1 | | 931 | hsa-mir-10a | 10001 | MED6 | mediator complex subunit 6 | 1 | | 932 | hsa-miR-1233 | 10001 | MED6 | mediator complex subunit 6 | 1 | | 933 | hsa-miR-423-5p | 10001 | MED6 | mediator complex subunit 6 | 1 | | 934 | hsa-miR-571 | 10001 | MED6 | mediator complex subunit 6 | 1 | | 935 | hsa-miR-10a | 5454 | POU3F2 | POU class 3 homeobox 2 | 1 | | 936 | hsa-miR-95 | 5454 | POU3F2 | POU class 3 homeobox 2 | 1 | | 937 | hsa-miR-938 | 3577 | CXCR1 | chemokine (C-X-C motif) receptor 1 | 1 | | 938 | hsa-miR-95 | 6843 | VAMP1 | vesicle-associated membrane protein 1 (synaptobrevin 1) | 1 | | 939 | hsa-miR-874 | 7766 | ZNF223 | zinc finger protein 223 | 1 | | 940 | hsa-miR-10a | 6572 | SLC18A3 | solute carrier family 18 (vesicular acetylcholine), member 3 | 1 | | 941 | hsa-miR-623 | 207 | AKT1 | v-akt murine thymoma viral oncogene homolog 1 | 1 | | 942 | hsa-miR-1233 | 25850 | ZNF345 | zinc finger protein 345 | 1 | | 943 | hsa-miR-571 | 25850 | ZNF345 | zinc finger protein 345 | 1 | | 944 | hsa-miR-874 | 25850 | ZNF345 | zinc finger protein 345 | 1 | | 945 | hsa-miR-623 | 4050 | LTB | lymphotoxin beta (TNF superfamily, member 3) | 1 | | 946 | hsa-miR-10a | 6425 | SFRP5 | secreted frizzled-related protein 5 | 1 | | 947 | hsa-miR-95 | 6425 | SFRP5 | secreted frizzled-related protein 5 | 1 | | 949 | hsa-mir-885 | 55832 | CAND1 | cullin-associated and neddylation-dissociated 1 | 1 | | 952 | hsa-miR-569 | 55832 | CAND1 | cullin-associated and neddylation-dissociated 1 | 1 | | 954 | hsa-miR-766 | 55832 | CAND1 | cullin-associated and neddylation-dissociated 1 | 1 | | 955 | hsa-miR-885-5p | 55832 | CAND1 | cullin-associated and neddylation-dissociated 1 | 1 | | 956 | hsa-miR-766 | 9533 | POLR1C | polymerase (RNA) I polypeptide C, 30kDa | 1 | | 957 | hsa-miR-877 | 9533 | POLR1C | polymerase (RNA) I polypeptide C, 30kDa | 1 | | 958 | hsa-mir-3130-1 | 6915 | TBXA2R | thromboxane A2 receptor | 1 | | 959 | hsa-mir-3130-2 | 6915 | TBXA2R | thromboxane A2 receptor | 1 | | 960 | hsa-mir-3130-3 | 6915 | TBXA2R | thromboxane A2 receptor | 1 | | 961 | hsa-mir-3130-4 | 6915 | TBXA2R | thromboxane A2 receptor | 1 | | 962 | hsa-miR-10a | 6915 | TBXA2R | thromboxane A2 receptor | 1 | | 963 | hsa-miR-95 | 6915 | TBXA2R | thromboxane A2 receptor | 1 | | 964 | hsa-miR-10a | 9154 | SLC28A1 | solute carrier family 28 (sodium-coupled nucleoside transporter), member 1 | 1 | | 965 | hsa-miR-95 | 9154 | SLC28A1 | solute carrier family 28 (sodium-coupled nucleoside transporter), member 1 | 1 | | 966 | hsa-miR-1233 | 29909 | GPR171 | G protein-coupled receptor 171 | 1 | | 967 | hsa-miR-135a | 5606 | MAP2K3 | mitogen-activated protein kinase kinase 3 | 1 | | 970 | hsa-miR-580 | 51475 | CABP2 | calcium binding protein 2 | 1 | | 971 | hsa-miR-1233 | 57343 | ZNF304 | zinc finger protein 304 | 1 | | 972 | hsa-miR-571 | 57343 | ZNF304 | zinc finger protein 304 | 1 | | 973 | hsa-miR-766 | 57343 | ZNF304 | zinc finger protein 304 | 1 | | 974 | hsa-miR-874 | 57343 | ZNF304 | zinc finger protein 304 | 1 | | 978 | hsa-miR-874 | 3824 | KLRD1 | killer cell lectin-like receptor subfamily D, member 1 | 1 | | 979 | hsa-mir-10a | 26003 | GORASP2 | golgi reassembly stacking protein 2, 55kDa | 1 | | 981 | hsa-mir-10a | 5511 | PPP1R8 | protein phosphatase 1, regulatory (inhibitor) subunit 8 | 1 | | 982 | hsa-miR-766 | 5511 | PPP1R8 | protein phosphatase 1, regulatory (inhibitor) subunit 8 | 1 | | 983 | hsa-mir-10a | 10393 | ANAPC10 | anaphase promoting complex subunit 10 | 1 | | 984 | hsa-miR-1233 | 10393 | ANAPC10 | anaphase promoting complex subunit 10 | 1 | | 985 | hsa-miR-571 | 10393 | ANAPC10 | anaphase promoting complex subunit 10 | 1 | | 986 | hsa-miR-1233 | 4931 | NVL | nuclear VCP-like | 1 | | 987 | hsa-miR-569 | 4931 | NVL | nuclear VCP-like | 1 | | 988 | hsa-miR-571 | 4931 | NVL | nuclear VCP-like | 1 | | 989 | hsa-miR-766 | 4931 | NVL | nuclear VCP-like | 1 | | 990 | hsa-miR-10a | 9940 | DLEC1 | deleted in lung and esophageal cancer 1 | 1 | | 991 | hsa-miR-95 | 9940 | DLEC1 | deleted in lung and esophageal cancer 1 | 1 | | 992 | hsa-miR-766 | 2734 | GLG1 | golgi glycoprotein 1 | 1 | | 993 | hsa-miR-95 | 11311 | VPS45 | vacuolar protein sorting 45 homolog (S. cerevisiae) | 1 | | 994 | hsa-mir-3130-1 | 26083 | TBC1D29 | TBC1 domain family, member 29 | 1 | | 995 | hsa-mir-3130-2 | 26083 | TBC1D29 | TBC1 domain family, member 29 | 1 | | 996 | hsa-mir-3130-3 | 26083 | TBC1D29 | TBC1 domain family, member 29 | 1 | | 997 | hsa-mir-3130-4 | 26083 | TBC1D29 | TBC1 domain family, member 29 | 1 | | 998 | hsa-miR-10a | 26083 | TBC1D29 | TBC1 domain family, member 29 | 1 | | 999 | hsa-miR-95 | 26083 | TBC1D29 | TBC1 domain family, member 29 | 1 | | 1000 | hsa-miR-938 | 3055 | HCK | hemopoietic cell kinase | 1 | | 1001 | hsa-mir-3130-1 | 3834 | KIF25 | kinesin family member 25 | 1 | | 1002 | hsa-mir-3130-2 | 3834 | KIF25 | kinesin family member 25 | 1 | | 1003 | hsa-mir-3130-3 | 3834 | KIF25 | kinesin family member 25 | 1 | | 1004 | hsa-mir-3130-4 | 3834 | KIF25 | kinesin family member 25 | 1 | | 1005 | hsa-miR-95 | 6569 | SLC34A1 | solute carrier family 34 (sodium phosphate), member 1 | 1 | | 1006 | hsa-miR-10a | 8735 | MYH13 | myosin, heavy chain 13, skeletal muscle | 1 | | 1007 | hsa-miR-95 | 8735 | MYH13 | myosin, heavy chain 13, skeletal muscle | 1 | | 1008 | hsa-miR-95 | 725 | C4BPB | complement component 4 binding protein, beta | 1 | | 1009 | hsa-miR-10a | 6585 | SLIT1 | slit homolog 1 (Drosophila) | 1 | | 1010 | hsa-miR-95 | 6585 | SLIT1 | slit homolog 1 (Drosophila) | 1 | | 1011 | hsa-miR-608 | 5669 | PSG1 | pregnancy specific beta-1-glycoprotein 1 | 1 | | 1012 | hsa-miR-95 | 57824 | HMHB1 | histocompatibility (minor) HB-1 | 1 | | 1013 | hsa-miR-938 | 5594 | MAPK1 | mitogen-activated protein kinase 1 | 1 | | 1015 | hsa-miR-128 | 2740 | GLP1R | glucagon-like peptide 1 receptor | 1 | | 1016 | hsa-miR-95 | 2740 | GLP1R | glucagon-like peptide 1 receptor | 1 | | 1017 | hsa-miR-95 | 3024 | HIST1H1A | histone cluster 1, H1a | 1 | | 1018 | hsa-miR-10a | 151 | ADRA2B | adrenergic, alpha-2B-, receptor | 1 | | 1019 | hsa-miR-95 | 151 | ADRA2B | adrenergic, alpha-2B-, receptor | 1 | | 1020 | hsa-miR-10a | 26251 | KCNG2 | potassium voltage-gated channel, subfamily G, member 2 | 1 | | 1021 | hsa-miR-95 | 26251 | KCNG2 | potassium voltage-gated channel, subfamily G, member 2 | 1 | | 1022 | hsa-mir-505 | 6623 | SNCG | synuclein, gamma (breast cancer-specific protein 1) | 1 | | 1023 | hsa-mir-3130-1 | 6623 | SNCG | synuclein, gamma (breast cancer-specific protein 1) | 1 | | 1024 | hsa-mir-3130-2 | 6623 | SNCG | synuclein, gamma (breast cancer-specific protein 1) | 1 | | 1025 | hsa-mir-3130-3 | 6623 | SNCG | synuclein, gamma (breast cancer-specific protein 1) | 1 | | 1026 | hsa-mir-3130-4 | 6623 | SNCG | synuclein, gamma (breast cancer-specific protein 1) | 1 | | 1027 | hsa-miR-10a | 6623 | SNCG | synuclein, gamma (breast cancer-specific protein 1) | 1 | | 1028 | hsa-miR-128 | 6623 | SNCG | synuclein, gamma (breast cancer-specific protein 1) | 1 | | 1029 | hsa-miR-505\* | 6623 | SNCG | synuclein, gamma (breast cancer-specific protein 1) | 1 | | 1030 | hsa-miR-580 | 6623 | SNCG | synuclein, gamma (breast cancer-specific protein 1) | 1 | | 1031 | hsa-miR-95 | 6623 | SNCG | synuclein, gamma (breast cancer-specific protein 1) | 1 | | 1032 | hsa-miR-766 | 10075 | HUWE1 | HECT, UBA and WWE domain containing 1 | 1 | | 1033 | hsa-miR-877 | 10075 | HUWE1 | HECT, UBA and WWE domain containing 1 | 1 | | 1035 | hsa-miR-623 | 4904 | YBX1 | Y box binding protein 1 | 1 | | 1036 | hsa-miR-618 | 87 | ACTN1 | actinin, alpha 1 | 1 | | 1038 | hsa-miR-423-5p | 6428 | SFRS3 | splicing factor, arginine/serine-rich 3 | 1 | | 1039 | hsa-miR-938 | 10094 | ARPC3 | actin related protein 2/3 complex, subunit 3, 21kDa | 1 | | 1040 | hsa-miR-1233 | 7329 | UBE2I | ubiquitin-conjugating enzyme E2I (UBC9 homolog, yeast) | 1 | | 1041 | hsa-miR-558 | 7329 | UBE2I | ubiquitin-conjugating enzyme E2I (UBC9 homolog, yeast) | 1 | | 1042 | hsa-miR-571 | 7329 | UBE2I | ubiquitin-conjugating enzyme E2I (UBC9 homolog, yeast) | 1 | | 1043 | hsa-miR-874 | 7329 | UBE2I | ubiquitin-conjugating enzyme E2I (UBC9 homolog, yeast) | 1 | | 1044 | hsa-miR-623 | 10236 | HNRNPR | heterogeneous nuclear ribonucleoprotein R | 1 | | 1045 | hsa-miR-766 | 10236 | HNRNPR | heterogeneous nuclear ribonucleoprotein R | 1 | | 1047 | hsa-miR-1233 | 5717 | PSMD11 | proteasome (prosome, macropain) 26S subunit, non-ATPase, 11 | 1 | | 1048 | hsa-miR-766 | 5717 | PSMD11 | proteasome (prosome, macropain) 26S subunit, non-ATPase, 11 | 1 | | 1049 | hsa-miR-423-5p | 6597 | SMARCA4 | SWI/SNF related, matrix associated, actin dependent regulator of chromatin, subfamily a, member 4 | 1 | | 1050 | hsa-miR-1233 | 23015 | GOLGA8A | golgin A8 family, member A | 1 | | 1051 | hsa-miR-569 | 23015 | GOLGA8A | golgin A8 family, member A | 1 | | 1052 | hsa-miR-571 | 23015 | GOLGA8A | golgin A8 family, member A | 1 | | 1053 | hsa-miR-766 | 23015 | GOLGA8A | golgin A8 family, member A | 1 | | 1054 | hsa-miR-874 | 23015 | GOLGA8A | golgin A8 family, member A | 1 | | 1055 | hsa-miR-618 | 3148 | HMGB2 | high-mobility group box 2 | 1 | | 1056 | hsa-mir-10a | 54107 | POLE3 | polymerase (DNA directed), epsilon 3 (p17 subunit) | 1 | | 1057 | hsa-miR-558 | 55832 | CAND1 | cullin-associated and neddylation-dissociated 1 | 1 | | 1058 | hsa-miR-454\* | 55832 | CAND1 | cullin-associated and neddylation-dissociated 1 | 1 | | 1059 | hsa-miR-576-5p | 55832 | CAND1 | cullin-associated and neddylation-dissociated 1 | 1 | | 1060 | hsa-miR-877 | 26003 | GORASP2 | golgi reassembly stacking protein 2, 55kDa | 1 | | 1061 | hsa-miR-558 | 26003 | GORASP2 | golgi reassembly stacking protein 2, 55kDa | 1 | | 1063 | hsa-miR-555 | 51366 | UBR5 | ubiquitin protein ligase E3 component n-recognin 5 | 1 | | 1065 | hsa-miR-10a | 9612 | NCOR2 | nuclear receptor co-repressor 2 | 1 | | 1066 | hsa-miR-95 | 9612 | NCOR2 | nuclear receptor co-repressor 2 | 1 | | 1069 | hsa-miR-877 | 8886 | DDX18 | DEAD (Asp-Glu-Ala-Asp) box polypeptide 18 | 1 | | 1070 | hsa-mir-10a | 8886 | DDX18 | DEAD (Asp-Glu-Ala-Asp) box polypeptide 18 | 1 | | 1071 | hsa-miR-423-5p | 8886 | DDX18 | DEAD (Asp-Glu-Ala-Asp) box polypeptide 18 | 1 | | 1072 | hsa-miR-569 | 8886 | DDX18 | DEAD (Asp-Glu-Ala-Asp) box polypeptide 18 | 1 | | 1073 | hsa-miR-454\* | 54205 | CYCS | cytochrome c, somatic | 1 | | 1074 | hsa-miR-593\* | 28973 | MRPS18B | mitochondrial ribosomal protein S18B | 1 | | 1075 | hsa-miR-623 | 28973 | MRPS18B | mitochondrial ribosomal protein S18B | 1 | | 1076 | hsa-miR-766 | 28973 | MRPS18B | mitochondrial ribosomal protein S18B | 1 | | 1077 | hsa-miR-877 | 28973 | MRPS18B | mitochondrial ribosomal protein S18B | 1 | | 1078 | hsa-miR-593\* | 6137 | RPL13 | ribosomal protein L13 | 1 | | 1081 | hsa-miR-423-5p | 9360 | PPIG | peptidylprolyl isomerase G (cyclophilin G) | 1 | | 1082 | hsa-miR-593\* | 9361 | LONP1 | lon peptidase 1, mitochondrial | 1 | | 1083 | hsa-miR-623 | 9361 | LONP1 | lon peptidase 1, mitochondrial | 1 | | 1084 | hsa-miR-766 | 9361 | LONP1 | lon peptidase 1, mitochondrial | 1 | | 1085 | hsa-miR-877 | 9361 | LONP1 | lon peptidase 1, mitochondrial | 1 | | 1086 | hsa-miR-555 | 1859 | DYRK1A | dual-specificity tyrosine-(Y)-phosphorylation regulated kinase 1A | 1 | | 1087 | hsa-miR-576-5p | 1859 | DYRK1A | dual-specificity tyrosine-(Y)-phosphorylation regulated kinase 1A | 1 | | 1088 | hsa-miR-569 | 9987 | HNRPDL | heterogeneous nuclear ribonucleoprotein D-like | 1 | | 1089 | hsa-miR-571 | 9987 | HNRPDL | heterogeneous nuclear ribonucleoprotein D-like | 1 | | 1091 | hsa-miR-877 | 3704 | ITPA | inosine triphosphatase (nucleoside triphosphate pyrophosphatase) | 1 | | 1092 | hsa-miR-618 | 688 | KLF5 | Kruppel-like factor 5 (intestinal) | 1 | | 1093 | hsa-miR-1236 | 7936 | RDBP | RD RNA binding protein | 1 | | 1094 | hsa-mir-3130-1 | 26471 | NUPR1 | nuclear protein, transcriptional regulator, 1 | 1 | | 1095 | hsa-mir-3130-2 | 26471 | NUPR1 | nuclear protein, transcriptional regulator, 1 | 1 | | 1096 | hsa-mir-3130-3 | 26471 | NUPR1 | nuclear protein, transcriptional regulator, 1 | 1 | | 1097 | hsa-mir-3130-4 | 26471 | NUPR1 | nuclear protein, transcriptional regulator, 1 | 1 | | 1098 | hsa-miR-10a | 26471 | NUPR1 | nuclear protein, transcriptional regulator, 1 | 1 | | 1099 | hsa-miR-95 | 26471 | NUPR1 | nuclear protein, transcriptional regulator, 1 | 1 | | 1100 | hsa-miR-766 | 10436 | EMG1 | EMG1 nucleolar protein homolog (S. cerevisiae) | 1 | | 1101 | hsa-mir-3130-1 | 10749 | KIF1C | kinesin family member 1C | 1 | | 1102 | hsa-mir-3130-2 | 10749 | KIF1C | kinesin family member 1C | 1 | | 1103 | hsa-mir-3130-3 | 10749 | KIF1C | kinesin family member 1C | 1 | | 1104 | hsa-mir-3130-4 | 10749 | KIF1C | kinesin family member 1C | 1 | | 1105 | hsa-miR-10a | 10749 | KIF1C | kinesin family member 1C | 1 | | 1106 | hsa-miR-128 | 10749 | KIF1C | kinesin family member 1C | 1 | | 1107 | hsa-miR-499-5p | 10749 | KIF1C | kinesin family member 1C | 1 | | 1108 | hsa-miR-580 | 10749 | KIF1C | kinesin family member 1C | 1 | | 1109 | hsa-miR-95 | 10749 | KIF1C | kinesin family member 1C | 1 | | 1110 | hsa-miR-766 | 56339 | METTL3 | methyltransferase like 3 | 1 | | 1111 | hsa-miR-558 | 23272 | C3orf63 | chromosome 3 open reading frame 63 | 1 | | 1112 | hsa-miR-618 | 663 | BNIP2 | BCL2/adenovirus E1B 19kDa interacting protein 2 | 1 | | 1113 | hsa-miR-1233 | 9533 | POLR1C | polymerase (RNA) I polypeptide C, 30kDa | 1 | | 1114 | hsa-miR-571 | 9533 | POLR1C | polymerase (RNA) I polypeptide C, 30kDa | 1 | | 1115 | hsa-miR-874 | 9533 | POLR1C | polymerase (RNA) I polypeptide C, 30kDa | 1 | | 1116 | hsa-mir-10a | 5612 | PRKRIR | protein-kinase, interferon-inducible double stranded RNA dependent inhibitor, repressor of (P58 repressor) | 1 | | 1117 | hsa-miR-1233 | 5612 | PRKRIR | protein-kinase, interferon-inducible double stranded RNA dependent inhibitor, repressor of (P58 repressor) | 1 | | 1118 | hsa-miR-423-5p | 5612 | PRKRIR | protein-kinase, interferon-inducible double stranded RNA dependent inhibitor, repressor of (P58 repressor) | 1 | | 1119 | hsa-miR-766 | 5612 | PRKRIR | protein-kinase, interferon-inducible double stranded RNA dependent inhibitor, repressor of (P58 repressor) | 1 | | 1121 | hsa-miR-571 | 11168 | PSIP1 | PC4 and SFRS1 interacting protein 1 | 1 | | 1122 | hsa-miR-874 | 11168 | PSIP1 | PC4 and SFRS1 interacting protein 1 | 1 | | 1123 | hsa-miR-766 | 4094 | MAF | v-maf musculoaponeurotic fibrosarcoma oncogene homolog (avian) | 1 | | 1124 | hsa-mir-3130-1 | 1893 | ECM1 | extracellular matrix protein 1 | 1 | | 1125 | hsa-mir-3130-2 | 1893 | ECM1 | extracellular matrix protein 1 | 1 | | 1126 | hsa-mir-3130-3 | 1893 | ECM1 | extracellular matrix protein 1 | 1 | | 1127 | hsa-mir-3130-4 | 1893 | ECM1 | extracellular matrix protein 1 | 1 | | 1128 | hsa-miR-618 | 306 | ANXA3 | annexin A3 | 1 | | 1129 | hsa-miR-1233 | 10623 | POLR3C | polymerase (RNA) III (DNA directed) polypeptide C (62kD) | 1 | | 1130 | hsa-miR-766 | 10623 | POLR3C | polymerase (RNA) III (DNA directed) polypeptide C (62kD) | 1 | | 1131 | hsa-miR-1233 | 23598 | PATZ1 | POZ (BTB) and AT hook containing zinc finger 1 | 1 | | 1132 | hsa-miR-569 | 23598 | PATZ1 | POZ (BTB) and AT hook containing zinc finger 1 | 1 | | 1133 | hsa-miR-766 | 23598 | PATZ1 | POZ (BTB) and AT hook containing zinc finger 1 | 1 | | 1134 | hsa-miR-874 | 23598 | PATZ1 | POZ (BTB) and AT hook containing zinc finger 1 | 1 | | 1135 | hsa-miR-877 | 23598 | PATZ1 | POZ (BTB) and AT hook containing zinc finger 1 | 1 | | 1136 | hsa-miR-618 | 8569 | MKNK1 | MAP kinase interacting serine/threonine kinase 1 | 1 | | 1137 | hsa-miR-1233 | 57050 | UTP3 | UTP3, small subunit (SSU) processome component, homolog (S. cerevisiae) | 1 | | 1138 | hsa-miR-571 | 57050 | UTP3 | UTP3, small subunit (SSU) processome component, homolog (S. cerevisiae) | 1 | | 1139 | hsa-miR-766 | 57050 | UTP3 | UTP3, small subunit (SSU) processome component, homolog (S. cerevisiae) | 1 | | 1140 | hsa-miR-555 | 6873 | TAF2 | TAF2 RNA polymerase II, TATA box binding protein (TBP)-associated factor, 150kDa | 1 | | 1141 | hsa-miR-558 | 6873 | TAF2 | TAF2 RNA polymerase II, TATA box binding protein (TBP)-associated factor, 150kDa | 1 | | 1146 | hsa-miR-576-5p | 8726 | EED | embryonic ectoderm development | 1 | | 1147 | hsa-miR-558 | 79068 | FTO | fat mass and obesity associated | 1 | | 1148 | hsa-miR-874 | 79068 | FTO | fat mass and obesity associated | 1 | | 1149 | hsa-miR-95 | 6405 | SEMA3F | sema domain, immunoglobulin domain (Ig), short basic domain, secreted, (semaphorin) 3F | 1 | | 1150 | hsa-mir-505 | 4784 | NFIX | nuclear factor I/X (CCAAT-binding transcription factor) | 1 | | 1151 | hsa-miR-505\* | 4784 | NFIX | nuclear factor I/X (CCAAT-binding transcription factor) | 1 | | 1152 | hsa-miR-580 | 4784 | NFIX | nuclear factor I/X (CCAAT-binding transcription factor) | 1 | | 1153 | hsa-miR-95 | 4784 | NFIX | nuclear factor I/X (CCAAT-binding transcription factor) | 1 | | 1155 | hsa-miR-1233 | 321 | APBA2 | amyloid beta (A4) precursor protein-binding, family A, member 2 | 1 | | 1156 | hsa-miR-938 | 6016 | RIT1 | Ras-like without CAAX 1 | 1 | | 1157 | hsa-miR-1233 | 10795 | ZNF268 | zinc finger protein 268 | 1 | | 1158 | hsa-miR-423-5p | 10795 | ZNF268 | zinc finger protein 268 | 1 | | 1159 | hsa-miR-571 | 10795 | ZNF268 | zinc finger protein 268 | 1 | | 1160 | hsa-miR-1233 | 5243 | ABCB1 | ATP-binding cassette, sub-family B (MDR/TAP), member 1 | 1 | | 1161 | hsa-miR-569 | 5243 | ABCB1 | ATP-binding cassette, sub-family B (MDR/TAP), member 1 | 1 | | 1162 | hsa-miR-571 | 5243 | ABCB1 | ATP-binding cassette, sub-family B (MDR/TAP), member 1 | 1 | | 1163 | hsa-miR-766 | 5243 | ABCB1 | ATP-binding cassette, sub-family B (MDR/TAP), member 1 | 1 | | 1165 | hsa-miR-1236 | 3420 | IDH3B | isocitrate dehydrogenase 3 (NAD+) beta | 1 | | 1166 | hsa-miR-593\* | 3420 | IDH3B | isocitrate dehydrogenase 3 (NAD+) beta | 1 | | 1168 | hsa-miR-766 | 3420 | IDH3B | isocitrate dehydrogenase 3 (NAD+) beta | 1 | | 1169 | hsa-miR-766 | 328 | APEX1 | APEX nuclease (multifunctional DNA repair enzyme) 1 | 1 | | 1170 | hsa-miR-877 | 328 | APEX1 | APEX nuclease (multifunctional DNA repair enzyme) 1 | 1 | | 1176 | hsa-mir-3130-1 | 10411 | RAPGEF3 | Rap guanine nucleotide exchange factor (GEF) 3 | 1 | | 1177 | hsa-mir-3130-2 | 10411 | RAPGEF3 | Rap guanine nucleotide exchange factor (GEF) 3 | 1 | | 1178 | hsa-mir-3130-3 | 10411 | RAPGEF3 | Rap guanine nucleotide exchange factor (GEF) 3 | 1 | | 1179 | hsa-mir-3130-4 | 10411 | RAPGEF3 | Rap guanine nucleotide exchange factor (GEF) 3 | 1 | | 1180 | hsa-miR-10a | 10411 | RAPGEF3 | Rap guanine nucleotide exchange factor (GEF) 3 | 1 | | 1181 | hsa-miR-95 | 10411 | RAPGEF3 | Rap guanine nucleotide exchange factor (GEF) 3 | 1 | | 1183 | hsa-miR-938 | 8676 | STX11 | syntaxin 11 | 1 | | 1184 | hsa-miR-454\* | 5810 | RAD1 | RAD1 homolog (S. pombe) | 1 | | 1185 | hsa-miR-618 | 6854 | SYN2 | synapsin II | 1 | | 1186 | hsa-miR-766 | 158 | ADSL | adenylosuccinate lyase | 1 | | 1187 | hsa-miR-95 | 9027 | NAT8 | N-acetyltransferase 8 (GCN5-related, putative) | 1 | | 1188 | hsa-miR-10a | 6444 | SGCD | sarcoglycan, delta (35kDa dystrophin-associated glycoprotein) | 1 | | 1189 | hsa-miR-499-5p | 6444 | SGCD | sarcoglycan, delta (35kDa dystrophin-associated glycoprotein) | 1 | | 1190 | hsa-miR-95 | 6444 | SGCD | sarcoglycan, delta (35kDa dystrophin-associated glycoprotein) | 1 | | 1191 | hsa-miR-10a | 9356 | SLC22A6 | solute carrier family 22 (organic anion transporter), member 6 | 1 | | 1192 | hsa-miR-95 | 9356 | SLC22A6 | solute carrier family 22 (organic anion transporter), member 6 | 1 | | 1193 | hsa-miR-10a | 2172 | FABP6 | fatty acid binding protein 6, ileal | 1 | | 1194 | hsa-miR-95 | 2172 | FABP6 | fatty acid binding protein 6, ileal | 1 | | 1195 | hsa-mir-3130-1 | 9468 | PCYT1B | phosphate cytidylyltransferase 1, choline, beta | 1 | | 1196 | hsa-mir-3130-2 | 9468 | PCYT1B | phosphate cytidylyltransferase 1, choline, beta | 1 | | 1197 | hsa-mir-3130-3 | 9468 | PCYT1B | phosphate cytidylyltransferase 1, choline, beta | 1 | | 1198 | hsa-mir-3130-4 | 9468 | PCYT1B | phosphate cytidylyltransferase 1, choline, beta | 1 | | 1199 | hsa-miR-10a | 9468 | PCYT1B | phosphate cytidylyltransferase 1, choline, beta | 1 | | 1200 | hsa-miR-128 | 9468 | PCYT1B | phosphate cytidylyltransferase 1, choline, beta | 1 | | 1201 | hsa-miR-95 | 9468 | PCYT1B | phosphate cytidylyltransferase 1, choline, beta | 1 | | 1202 | hsa-miR-623 | 10450 | PPIE | peptidylprolyl isomerase E (cyclophilin E) | 1 | | 1203 | hsa-miR-877 | 10450 | PPIE | peptidylprolyl isomerase E (cyclophilin E) | 1 | | 1204 | hsa-miR-938 | 27180 | SIGLEC9 | sialic acid binding Ig-like lectin 9 | 1 | | 1205 | hsa-miR-10a | 11283 | CYP4F8 | cytochrome P450, family 4, subfamily F, polypeptide 8 | 1 | | 1206 | hsa-miR-95 | 11283 | CYP4F8 | cytochrome P450, family 4, subfamily F, polypeptide 8 | 1 | | 1207 | hsa-miR-95 | 1004 | CDH6 | cadherin 6, type 2, K-cadherin (fetal kidney) | 1 | | 1208 | hsa-miR-580 | 4240 | MFGE8 | milk fat globule-EGF factor 8 protein | 1 | | 1209 | hsa-miR-618 | 26268 | FBXO9 | F-box protein 9 | 1 | | 1210 | hsa-miR-423-5p | 10428 | CFDP1 | craniofacial development protein 1 | 1 | | 1211 | hsa-mir-3130-1 | 4771 | NF2 | neurofibromin 2 (merlin) | 1 | | 1212 | hsa-mir-3130-2 | 4771 | NF2 | neurofibromin 2 (merlin) | 1 | | 1213 | hsa-mir-3130-3 | 4771 | NF2 | neurofibromin 2 (merlin) | 1 | | 1214 | hsa-mir-3130-4 | 4771 | NF2 | neurofibromin 2 (merlin) | 1 | | 1215 | hsa-miR-10a | 4771 | NF2 | neurofibromin 2 (merlin) | 1 | | 1216 | hsa-miR-95 | 4771 | NF2 | neurofibromin 2 (merlin) | 1 | | 1217 | hsa-miR-10a | 1258 | CNGB1 | cyclic nucleotide gated channel beta 1 | 1 | | 1218 | hsa-miR-95 | 1258 | CNGB1 | cyclic nucleotide gated channel beta 1 | 1 | | 1220 | hsa-miR-10a | 2902 | GRIN1 | glutamate receptor, ionotropic, N-methyl D-aspartate 1 | 1 | | 1221 | hsa-miR-95 | 2902 | GRIN1 | glutamate receptor, ionotropic, N-methyl D-aspartate 1 | 1 | | 1224 | hsa-miR-1233 | 84809 | CROCCL1 | ciliary rootlet coiled-coil, rootletin-like 1 | 1 | | 1225 | hsa-miR-10a | 4647 | MYO7A | myosin VIIA | 1 | | 1226 | hsa-miR-580 | 4647 | MYO7A | myosin VIIA | 1 | | 1227 | hsa-miR-95 | 4647 | MYO7A | myosin VIIA | 1 | | 1228 | hsa-miR-95 | 285 | ANGPT2 | angiopoietin 2 | 1 | | 1229 | hsa-miR-10a | 2527 | FUT5 | fucosyltransferase 5 (alpha (1,3) fucosyltransferase) | 1 | | 1230 | hsa-miR-95 | 2527 | FUT5 | fucosyltransferase 5 (alpha (1,3) fucosyltransferase) | 1 | | 1231 | hsa-mir-505 | 8021 | NUP214 | nucleoporin 214kDa | 1 | | 1232 | hsa-miR-505\* | 8021 | NUP214 | nucleoporin 214kDa | 1 | | 1233 | hsa-miR-580 | 8021 | NUP214 | nucleoporin 214kDa | 1 | | 1234 | hsa-miR-95 | 8021 | NUP214 | nucleoporin 214kDa | 1 | | 1235 | hsa-miR-10a | 6899 | TBX1 | T-box 1 | 1 | | 1236 | hsa-miR-95 | 6899 | TBX1 | T-box 1 | 1 | | 1237 | hsa-miR-938 | 23569 | PADI4 | peptidyl arginine deiminase, type IV | 1 | | 1238 | hsa-miR-10a | 3479 | IGF1 | insulin-like growth factor 1 (somatomedin C) | 1 | | 1239 | hsa-miR-95 | 3479 | IGF1 | insulin-like growth factor 1 (somatomedin C) | 1 | | 1240 | hsa-miR-608 | 491 | ATP2B2 | ATPase, Ca++ transporting, plasma membrane 2 | 1 | | 1241 | hsa-miR-1233 | 84525 | HOPX | HOP homeobox | 1 | | 1242 | hsa-miR-569 | 84525 | HOPX | HOP homeobox | 1 | | 1243 | hsa-miR-766 | 84525 | HOPX | HOP homeobox | 1 | | 1244 | hsa-miR-10a | 9001 | HAP1 | huntingtin-associated protein 1 | 1 | | 1245 | hsa-miR-95 | 3912 | LAMB1 | laminin, beta 1 | 1 | | 1246 | hsa-miR-10a | 6194 | RPS6 | ribosomal protein S6 | 1 | | 1247 | hsa-miR-95 | 10580 | SORBS1 | sorbin and SH3 domain containing 1 | 1 | | 1248 | hsa-miR-558 | 54468 | MIOS | missing oocyte, meiosis regulator, homolog (Drosophila) | 1 | | 1249 | hsa-miR-623 | 9112 | MTA1 | metastasis associated 1 | 1 | | 1250 | hsa-miR-766 | 9112 | MTA1 | metastasis associated 1 | 1 | | 1251 | hsa-miR-877 | 9112 | MTA1 | metastasis associated 1 | 1 | | 1252 | hsa-miR-95 | 171558 | PTCRA | pre T-cell antigen receptor alpha | 1 | | 1253 | hsa-miR-499-5p | 3691 | ITGB4 | integrin, beta 4 | 1 | | 1255 | hsa-mir-3130-1 | 60676 | PAPPA2 | pappalysin 2 | 1 | | 1256 | hsa-mir-3130-2 | 60676 | PAPPA2 | pappalysin 2 | 1 | | 1257 | hsa-mir-3130-3 | 60676 | PAPPA2 | pappalysin 2 | 1 | | 1258 | hsa-mir-3130-4 | 60676 | PAPPA2 | pappalysin 2 | 1 | | 1260 | hsa-miR-558 | 220988 | HNRNPA3 | heterogeneous nuclear ribonucleoprotein A3 | 1 | | 1261 | hsa-miR-874 | 220988 | HNRNPA3 | heterogeneous nuclear ribonucleoprotein A3 | 1 | | 1262 | hsa-miR-766 | 220988 | HNRNPA3 | heterogeneous nuclear ribonucleoprotein A3 | 1 | | 1263 | hsa-miR-423-5p | 220988 | HNRNPA3 | heterogeneous nuclear ribonucleoprotein A3 | 1 | | 1264 | hsa-mir-10a | 3843 | IPO5 | importin 5 | 1 | | 1265 | hsa-miR-423-5p | 3843 | IPO5 | importin 5 | 1 | | 1267 | hsa-miR-571 | 3843 | IPO5 | importin 5 | 1 | | 1269 | hsa-miR-569 | 3843 | IPO5 | importin 5 | 1 | | 1270 | hsa-miR-874 | 3843 | IPO5 | importin 5 | 1 | | 1271 | hsa-miR-623 | 293 | SLC25A6 | solute carrier family 25 (mitochondrial carrier; adenine nucleotide translocator), member 6 | 1 | | 1272 | hsa-miR-877 | 23203 | PMPCA | peptidase (mitochondrial processing) alpha | 1 | | 1273 | hsa-miR-1233 | 26123 | TCTN3 | tectonic family member 3 | 1 | | 1274 | hsa-miR-569 | 26123 | TCTN3 | tectonic family member 3 | 1 | | 1275 | hsa-miR-766 | 26123 | TCTN3 | tectonic family member 3 | 1 | | 1276 | hsa-mir-149 | 23433 | RHOQ | ras homolog gene family, member Q | 1 | | 1277 | hsa-miR-149 | 23433 | RHOQ | ras homolog gene family, member Q | 1 | | 1278 | hsa-miR-555 | 23433 | RHOQ | ras homolog gene family, member Q | 1 | | 1279 | hsa-miR-766 | 10985 | GCN1L1 | GCN1 general control of amino-acid synthesis 1-like 1 (yeast) | 1 | | 1280 | hsa-miR-1233 | 25957 | SFRS18 | splicing factor, arginine/serine-rich 18 | 1 | | 1281 | hsa-miR-555 | 25957 | SFRS18 | splicing factor, arginine/serine-rich 18 | 1 | | 1282 | hsa-miR-558 | 25957 | SFRS18 | splicing factor, arginine/serine-rich 18 | 1 | | 1283 | hsa-miR-874 | 25957 | SFRS18 | splicing factor, arginine/serine-rich 18 | 1 | | 1284 | hsa-miR-623 | 6137 | RPL13 | ribosomal protein L13 | 1 | | 1285 | hsa-miR-423-5p | 94239 | H2AFV | H2A histone family, member V | 1 | | 1286 | hsa-miR-555 | 94239 | H2AFV | H2A histone family, member V | 1 | | 1287 | hsa-miR-558 | 94239 | H2AFV | H2A histone family, member V | 1 | | 1288 | hsa-miR-766 | 23198 | PSME4 | proteasome (prosome, macropain) activator subunit 4 | 1 | | 1289 | hsa-miR-1233 | 23198 | PSME4 | proteasome (prosome, macropain) activator subunit 4 | 1 | | 1290 | hsa-miR-558 | 23198 | PSME4 | proteasome (prosome, macropain) activator subunit 4 | 1 | | 1291 | hsa-miR-571 | 23198 | PSME4 | proteasome (prosome, macropain) activator subunit 4 | 1 | | 1292 | hsa-miR-874 | 23198 | PSME4 | proteasome (prosome, macropain) activator subunit 4 | 1 | | 1293 | hsa-miR-623 | 57326 | PBXIP1 | pre-B-cell leukemia homeobox interacting protein 1 | 1 | | 1295 | hsa-miR-766 | 8888 | MCM3AP | minichromosome maintenance complex component 3 associated protein | 1 | | 1296 | hsa-miR-877 | 8888 | MCM3AP | minichromosome maintenance complex component 3 associated protein | 1 | | 1298 | hsa-mir-199a-2 | 23122 | CLASP2 | cytoplasmic linker associated protein 2 | 1 | | 1299 | hsa-mir-214 | 23122 | CLASP2 | cytoplasmic linker associated protein 2 | 1 | | 1300 | hsa-mir-499 | 23122 | CLASP2 | cytoplasmic linker associated protein 2 | 1 | | 1302 | hsa-miR-576-5p | 23122 | CLASP2 | cytoplasmic linker associated protein 2 | 1 | | 1304 | hsa-miR-766 | 55187 | VPS13D | vacuolar protein sorting 13 homolog D (S. cerevisiae) | 1 | | 1305 | hsa-miR-766 | 23028 | KDM1A | lysine (K)-specific demethylase 1A | 1 | | 1307 | hsa-mir-135b | 51029 | PPPDE1 | PPPDE peptidase domain containing 1 | 1 | | 1308 | hsa-miR-454\* | 51029 | PPPDE1 | PPPDE peptidase domain containing 1 | 1 | | 1309 | hsa-miR-1233 | 23358 | USP24 | ubiquitin specific peptidase 24 | 1 | | 1310 | hsa-miR-423-5p | 23358 | USP24 | ubiquitin specific peptidase 24 | 1 | | 1311 | hsa-miR-571 | 23358 | USP24 | ubiquitin specific peptidase 24 | 1 | | 1312 | hsa-miR-766 | 23358 | USP24 | ubiquitin specific peptidase 24 | 1 | | 1313 | hsa-miR-1233 | 23065 | KIAA0090 | KIAA0090 | 1 | | 1316 | hsa-miR-558 | 51603 | METTL13 | methyltransferase like 13 | 1 | | 1317 | hsa-miR-1233 | 55251 | PCMTD2 | protein-L-isoaspartate (D-aspartate) O-methyltransferase domain containing 2 | 1 | | 1318 | hsa-miR-423-5p | 55251 | PCMTD2 | protein-L-isoaspartate (D-aspartate) O-methyltransferase domain containing 2 | 1 | | 1319 | hsa-miR-576-5p | 55251 | PCMTD2 | protein-L-isoaspartate (D-aspartate) O-methyltransferase domain containing 2 | 1 | | 1320 | hsa-miR-1233 | 91746 | YTHDC1 | YTH domain containing 1 | 1 | | 1322 | hsa-miR-423-5p | 23131 | GPATCH8 | G patch domain containing 8 | 1 | | 1323 | hsa-miR-558 | 84890 | ADO | 2-aminoethanethiol (cysteamine) dioxygenase | 1 | | 1324 | hsa-miR-571 | 84890 | ADO | 2-aminoethanethiol (cysteamine) dioxygenase | 1 | | 1325 | hsa-miR-454\* | 23171 | GPD1L | glycerol-3-phosphate dehydrogenase 1-like | 1 | | 1326 | hsa-miR-766 | 6597 | SMARCA4 | SWI/SNF related, matrix associated, actin dependent regulator of chromatin, subfamily a, member 4 | 1 | | 1328 | hsa-miR-569 | 23348 | DOCK9 | dedicator of cytokinesis 9 | 1 | | 1330 | hsa-miR-766 | 23348 | DOCK9 | dedicator of cytokinesis 9 | 1 | | 1332 | hsa-miR-593\* | 80308 | FLAD1 | FAD1 flavin adenine dinucleotide synthetase homolog (S. cerevisiae) | 1 | | 1333 | hsa-miR-623 | 80308 | FLAD1 | FAD1 flavin adenine dinucleotide synthetase homolog (S. cerevisiae) | 1 | | 1334 | hsa-miR-877 | 80308 | FLAD1 | FAD1 flavin adenine dinucleotide synthetase homolog (S. cerevisiae) | 1 | | 1336 | hsa-miR-569 | 11118 | BTN3A2 | butyrophilin, subfamily 3, member A2 | 1 | | 1338 | hsa-miR-558 | 23376 | KIAA0776 | KIAA0776 | 1 | | 1339 | hsa-miR-423-5p | 8723 | SNX4 | sorting nexin 4 | 1 | | 1340 | hsa-miR-454\* | 8723 | SNX4 | sorting nexin 4 | 1 | | 1341 | hsa-miR-569 | 23195 | MDN1 | MDN1, midasin homolog (yeast) | 1 | | 1342 | hsa-miR-766 | 23195 | MDN1 | MDN1, midasin homolog (yeast) | 1 | | 1343 | hsa-mir-10a | 113251 | LARP4 | La ribonucleoprotein domain family, member 4 | 1 | | 1344 | hsa-mir-10a | 140890 | SFRS12 | splicing factor, arginine/serine-rich 12 | 1 | | 1345 | hsa-mir-199a-2 | 140890 | SFRS12 | splicing factor, arginine/serine-rich 12 | 1 | | 1346 | hsa-mir-214 | 140890 | SFRS12 | splicing factor, arginine/serine-rich 12 | 1 | | 1347 | hsa-mir-499 | 140890 | SFRS12 | splicing factor, arginine/serine-rich 12 | 1 | | 1348 | hsa-miR-423-5p | 140890 | SFRS12 | splicing factor, arginine/serine-rich 12 | 1 | | 1349 | hsa-miR-576-5p | 140890 | SFRS12 | splicing factor, arginine/serine-rich 12 | 1 | | 1350 | hsa-miR-766 | 9711 | KIAA0226 | KIAA0226 | 1 | | 1351 | hsa-miR-877 | 9711 | KIAA0226 | KIAA0226 | 1 | | 1352 | hsa-miR-555 | 30849 | PIK3R4 | phosphoinositide-3-kinase, regulatory subunit 4 | 1 | | 1353 | hsa-miR-558 | 30849 | PIK3R4 | phosphoinositide-3-kinase, regulatory subunit 4 | 1 | | 1354 | hsa-miR-576-5p | 30849 | PIK3R4 | phosphoinositide-3-kinase, regulatory subunit 4 | 1 | | 1355 | hsa-miR-569 | 27246 | RNF115 | ring finger protein 115 | 1 | | 1356 | hsa-miR-766 | 27246 | RNF115 | ring finger protein 115 | 1 | | 1357 | hsa-miR-1233 | 57037 | ANKMY2 | ankyrin repeat and MYND domain containing 2 | 1 | | 1358 | hsa-miR-423-5p | 57037 | ANKMY2 | ankyrin repeat and MYND domain containing 2 | 1 | | 1359 | hsa-miR-558 | 57037 | ANKMY2 | ankyrin repeat and MYND domain containing 2 | 1 | | 1360 | hsa-mir-10a | 22976 | PAXIP1 | PAX interacting (with transcription-activation domain) protein 1 | 1 | | 1361 | hsa-miR-454\* | 22976 | PAXIP1 | PAX interacting (with transcription-activation domain) protein 1 | 1 | | 1362 | hsa-miR-593\* | 293 | SLC25A6 | solute carrier family 25 (mitochondrial carrier; adenine nucleotide translocator), member 6 | 1 | | 1363 | hsa-miR-877 | 293 | SLC25A6 | solute carrier family 25 (mitochondrial carrier; adenine nucleotide translocator), member 6 | 1 | | 1364 | hsa-mir-10a | 8880 | FUBP1 | far upstream element (FUSE) binding protein 1 | 1 | | 1366 | hsa-miR-423-5p | 8880 | FUBP1 | far upstream element (FUSE) binding protein 1 | 1 | | 1369 | hsa-miR-1233 | 25966 | C2CD2 | C2 calcium-dependent domain containing 2 | 1 | | 1370 | hsa-miR-569 | 25966 | C2CD2 | C2 calcium-dependent domain containing 2 | 1 | | 1371 | hsa-miR-571 | 25966 | C2CD2 | C2 calcium-dependent domain containing 2 | 1 | | 1372 | hsa-miR-766 | 25966 | C2CD2 | C2 calcium-dependent domain containing 2 | 1 | | 1373 | hsa-miR-454\* | 23335 | WDR7 | WD repeat domain 7 | 1 | | 1374 | hsa-miR-1233 | 6832 | SUPV3L1 | suppressor of var1, 3-like 1 (S. cerevisiae) | 1 | | 1375 | hsa-miR-569 | 6832 | SUPV3L1 | suppressor of var1, 3-like 1 (S. cerevisiae) | 1 | | 1376 | hsa-miR-571 | 6832 | SUPV3L1 | suppressor of var1, 3-like 1 (S. cerevisiae) | 1 | | 1377 | hsa-miR-766 | 6832 | SUPV3L1 | suppressor of var1, 3-like 1 (S. cerevisiae) | 1 | | 1378 | hsa-miR-874 | 6832 | SUPV3L1 | suppressor of var1, 3-like 1 (S. cerevisiae) | 1 | | 1379 | hsa-mir-135b | 23341 | DNAJC16 | DnaJ (Hsp40) homolog, subfamily C, member 16 | 1 | | 1381 | hsa-miR-571 | 56950 | SMYD2 | SET and MYND domain containing 2 | 1 | | 1384 | hsa-miR-569 | 56950 | SMYD2 | SET and MYND domain containing 2 | 1 | | 1387 | hsa-miR-10a | 1291 | COL6A1 | collagen, type VI, alpha 1 | 1 | | 1389 | hsa-miR-558 | 9847 | KIAA0528 | KIAA0528 | 1 | | 1390 | hsa-miR-766 | 5438 | POLR2I | polymerase (RNA) II (DNA directed) polypeptide I, 14.5kDa | 1 | | 1391 | hsa-miR-877 | 5438 | POLR2I | polymerase (RNA) II (DNA directed) polypeptide I, 14.5kDa | 1 | | 1392 | hsa-mir-3130-1 | 9479 | MAPK8IP1 | mitogen-activated protein kinase 8 interacting protein 1 | 1 | | 1393 | hsa-mir-3130-2 | 9479 | MAPK8IP1 | mitogen-activated protein kinase 8 interacting protein 1 | 1 | | 1394 | hsa-mir-3130-3 | 9479 | MAPK8IP1 | mitogen-activated protein kinase 8 interacting protein 1 | 1 | | 1395 | hsa-mir-3130-4 | 9479 | MAPK8IP1 | mitogen-activated protein kinase 8 interacting protein 1 | 1 | | 1396 | hsa-miR-10a | 9479 | MAPK8IP1 | mitogen-activated protein kinase 8 interacting protein 1 | 1 | | 1397 | hsa-miR-423-5p | 26953 | RANBP6 | RAN binding protein 6 | 1 | | 1398 | hsa-miR-558 | 26953 | RANBP6 | RAN binding protein 6 | 1 | | 1399 | hsa-miR-1233 | 84942 | WDR73 | WD repeat domain 73 | 1 | | 1400 | hsa-miR-766 | 84942 | WDR73 | WD repeat domain 73 | 1 | | 1401 | hsa-miR-623 | 513 | ATP5D | ATP synthase, H+ transporting, mitochondrial F1 complex, delta subunit | 1 | | 1402 | hsa-miR-877 | 513 | ATP5D | ATP synthase, H+ transporting, mitochondrial F1 complex, delta subunit | 1 | | 1403 | hsa-miR-877 | 8106 | PABPN1 | poly(A) binding protein, nuclear 1 | 1 | | 1405 | hsa-miR-555 | 253959 | RALGAPA1 | Ral GTPase activating protein, alpha subunit 1 (catalytic) | 1 | | 1406 | hsa-miR-558 | 253959 | RALGAPA1 | Ral GTPase activating protein, alpha subunit 1 (catalytic) | 1 | | 1408 | hsa-miR-874 | 253959 | RALGAPA1 | Ral GTPase activating protein, alpha subunit 1 (catalytic) | 1 | | 1409 | hsa-miR-1233 | 57493 | HEG1 | HEG homolog 1 (zebrafish) | 1 | | 1410 | hsa-miR-569 | 57493 | HEG1 | HEG homolog 1 (zebrafish) | 1 | | 1411 | hsa-miR-571 | 57493 | HEG1 | HEG homolog 1 (zebrafish) | 1 | | 1412 | hsa-miR-766 | 57493 | HEG1 | HEG homolog 1 (zebrafish) | 1 | | 1413 | hsa-miR-618 | 206358 | SLC36A1 | solute carrier family 36 (proton/amino acid symporter), member 1 | 1 | | 1414 | hsa-miR-454\* | 2653 | GCSH | glycine cleavage system protein H (aminomethyl carrier) | 1 | | 1415 | hsa-miR-558 | 1737 | DLAT | dihydrolipoamide S-acetyltransferase | 1 | | 1416 | hsa-miR-558 | 989 | SEPT7 | septin 7 | 1 | | 1417 | hsa-miR-1233 | 158427 | TSTD2 | thiosulfate sulfurtransferase (rhodanese)-like domain containing 2 | 1 | | 1418 | hsa-miR-569 | 158427 | TSTD2 | thiosulfate sulfurtransferase (rhodanese)-like domain containing 2 | 1 | | 1419 | hsa-miR-766 | 158427 | TSTD2 | thiosulfate sulfurtransferase (rhodanese)-like domain containing 2 | 1 | | 1420 | hsa-miR-877 | 158427 | TSTD2 | thiosulfate sulfurtransferase (rhodanese)-like domain containing 2 | 1 | | 1421 | hsa-miR-555 | 9857 | CEP350 | centrosomal protein 350kDa | 1 | | 1422 | hsa-miR-576-5p | 9857 | CEP350 | centrosomal protein 350kDa | 1 | | 1423 | hsa-miR-571 | 23508 | TTC9 | tetratricopeptide repeat domain 9 | 1 | | 1424 | hsa-miR-555 | 92482 | NCRNA00081 | non-protein coding RNA 81 | 1 | | 1425 | hsa-miR-576-5p | 92482 | NCRNA00081 | non-protein coding RNA 81 | 1 | | 1426 | hsa-miR-1233 | 26278 | SACS | spastic ataxia of Charlevoix-Saguenay (sacsin) | 1 | | 1427 | hsa-miR-569 | 26278 | SACS | spastic ataxia of Charlevoix-Saguenay (sacsin) | 1 | | 1428 | hsa-miR-571 | 26278 | SACS | spastic ataxia of Charlevoix-Saguenay (sacsin) | 1 | | 1429 | hsa-miR-766 | 26278 | SACS | spastic ataxia of Charlevoix-Saguenay (sacsin) | 1 | | 1430 | hsa-miR-874 | 26278 | SACS | spastic ataxia of Charlevoix-Saguenay (sacsin) | 1 | | 1431 | hsa-miR-1233 | 23033 | DOPEY1 | dopey family member 1 | 1 | | 1432 | hsa-miR-571 | 23033 | DOPEY1 | dopey family member 1 | 1 | | 1433 | hsa-miR-558 | 23033 | DOPEY1 | dopey family member 1 | 1 | | 1435 | hsa-mir-10a | 23116 | FAM179B | family with sequence similarity 179, member B | 1 | | 1436 | hsa-miR-1233 | 23116 | FAM179B | family with sequence similarity 179, member B | 1 | | 1437 | hsa-miR-423-5p | 23116 | FAM179B | family with sequence similarity 179, member B | 1 | | 1438 | hsa-miR-571 | 23116 | FAM179B | family with sequence similarity 179, member B | 1 | | 1439 | hsa-miR-423-5p | 10196 | PRMT3 | protein arginine methyltransferase 3 | 1 | | 1440 | hsa-miR-558 | 10196 | PRMT3 | protein arginine methyltransferase 3 | 1 | | 1441 | hsa-mir-10a | 221443 | C6orf130 | chromosome 6 open reading frame 130 | 1 | | 1442 | hsa-mir-199a-2 | 221443 | C6orf130 | chromosome 6 open reading frame 130 | 1 | | 1443 | hsa-mir-214 | 221443 | C6orf130 | chromosome 6 open reading frame 130 | 1 | | 1445 | hsa-miR-423-5p | 221443 | C6orf130 | chromosome 6 open reading frame 130 | 1 | | 1447 | hsa-miR-766 | 221443 | C6orf130 | chromosome 6 open reading frame 130 | 1 | | 1448 | hsa-miR-618 | 56929 | FEM1C | fem-1 homolog c (C. elegans) | 1 | | 1449 | hsa-miR-623 | 7329 | UBE2I | ubiquitin-conjugating enzyme E2I (UBC9 homolog, yeast) | 1 | | 1450 | hsa-miR-1233 | 10772 | SFRS13A | splicing factor, arginine/serine-rich 13A | 1 | | 1451 | hsa-miR-569 | 10772 | SFRS13A | splicing factor, arginine/serine-rich 13A | 1 | | 1457 | hsa-miR-874 | 5125 | PCSK5 | proprotein convertase subtilisin/kexin type 5 | 1 | | 1459 | hsa-miR-1233 | 150759 | LOC150759 | hypothetical protein LOC150759 | 1 | | 1460 | hsa-miR-766 | 150759 | LOC150759 | hypothetical protein LOC150759 | 1 | | 1461 | hsa-miR-1233 | 5936 | RBM4 | RNA binding motif protein 4 | 1 | | 1462 | hsa-miR-874 | 5936 | RBM4 | RNA binding motif protein 4 | 1 | | 1463 | hsa-miR-95 | 3425 | IDUA | iduronidase, alpha-L- | 1 | | 1464 | hsa-miR-555 | 64131 | XYLT1 | xylosyltransferase I | 1 | | 1465 | hsa-miR-558 | 64131 | XYLT1 | xylosyltransferase I | 1 | | 1466 | hsa-miR-555 | 905 | CCNT2 | cyclin T2 | 1 | | 1467 | hsa-miR-558 | 905 | CCNT2 | cyclin T2 | 1 | | 1468 | hsa-miR-95 | 23254 | RP1-21O18.1 | kazrin | 1 | | 1469 | hsa-miR-10a | 8844 | KSR1 | kinase suppressor of ras 1 | 1 | | 1470 | hsa-miR-608 | 129080 | EMID1 | EMI domain containing 1 | 1 | | 1471 | hsa-miR-1233 | 25983 | NGDN | neuroguidin, EIF4E binding protein | 1 | | 1472 | hsa-miR-569 | 25983 | NGDN | neuroguidin, EIF4E binding protein | 1 | | 1473 | hsa-miR-766 | 25983 | NGDN | neuroguidin, EIF4E binding protein | 1 | | 1474 | hsa-miR-1233 | 3921 | RPSA | ribosomal protein SA | 1 | | 1475 | hsa-miR-766 | 3921 | RPSA | ribosomal protein SA | 1 | | 1476 | hsa-miR-874 | 3921 | RPSA | ribosomal protein SA | 1 | | 1477 | hsa-miR-1233 | 6964 | TRD@ | T cell receptor delta locus | 1 | | 1480 | hsa-miR-766 | 6964 | TRD@ | T cell receptor delta locus | 1 | | 1482 | hsa-miR-1233 | 1350 | COX7C | cytochrome c oxidase subunit VIIc | 1 | | 1483 | hsa-miR-569 | 1350 | COX7C | cytochrome c oxidase subunit VIIc | 1 | | 1484 | hsa-miR-571 | 1350 | COX7C | cytochrome c oxidase subunit VIIc | 1 | | 1485 | hsa-miR-766 | 1350 | COX7C | cytochrome c oxidase subunit VIIc | 1 | | 1486 | hsa-miR-1233 | 1849 | DUSP7 | dual specificity phosphatase 7 | 1 | | 1487 | hsa-miR-766 | 1849 | DUSP7 | dual specificity phosphatase 7 | 1 | | 1488 | hsa-miR-558 | 961 | CD47 | CD47 molecule | 1 | | 1489 | hsa-miR-1233 | 5383 | PMS2L5 | postmeiotic segregation increased 2-like 5 | 1 | | 1490 | hsa-miR-766 | 5383 | PMS2L5 | postmeiotic segregation increased 2-like 5 | 1 | | 1491 | hsa-miR-1233 | 6201 | RPS7 | ribosomal protein S7 | 1 | | 1492 | hsa-miR-874 | 6201 | RPS7 | ribosomal protein S7 | 1 | | 1496 | hsa-miR-95 | 26038 | CHD5 | chromodomain helicase DNA binding protein 5 | 1 | | 1498 | hsa-miR-558 | 6421 | SFPQ | splicing factor proline/glutamine-rich (polypyrimidine tract binding protein associated) | 1 | | 1499 | hsa-miR-1233 | 6742 | SSBP1 | single-stranded DNA binding protein 1 | 1 | | 1500 | hsa-miR-766 | 6742 | SSBP1 | single-stranded DNA binding protein 1 | 1 | | 1501 | hsa-miR-1233 | 93594 | WDR67 | WD repeat domain 67 | 1 | | 1502 | hsa-miR-558 | 93594 | WDR67 | WD repeat domain 67 | 1 | | 1503 | hsa-miR-569 | 93594 | WDR67 | WD repeat domain 67 | 1 | | 1504 | hsa-miR-571 | 93594 | WDR67 | WD repeat domain 67 | 1 | | 1505 | hsa-miR-766 | 93594 | WDR67 | WD repeat domain 67 | 1 | | 1506 | hsa-miR-874 | 93594 | WDR67 | WD repeat domain 67 | 1 | | 1507 | hsa-miR-558 | 8880 | FUBP1 | far upstream element (FUSE) binding protein 1 | 1 | | 1508 | hsa-miR-569 | 8880 | FUBP1 | far upstream element (FUSE) binding protein 1 | 1 | | 1509 | hsa-miR-874 | 8880 | FUBP1 | far upstream element (FUSE) binding protein 1 | 1 | | 1510 | hsa-miR-938 | 9260 | PDLIM7 | PDZ and LIM domain 7 (enigma) | 1 | | 1511 | hsa-miR-95 | 9260 | PDLIM7 | PDZ and LIM domain 7 (enigma) | 1 | | 1512 | hsa-miR-1233 | 509 | ATP5C1 | ATP synthase, H+ transporting, mitochondrial F1 complex, gamma polypeptide 1 | 1 | | 1513 | hsa-miR-1233 | 6152 | RPL24 | ribosomal protein L24 | 1 | | 1514 | hsa-miR-874 | 6152 | RPL24 | ribosomal protein L24 | 1 | | 1515 | hsa-mir-3130-1 | 56171 | DNAH7 | dynein, axonemal, heavy chain 7 | 1 | | 1516 | hsa-mir-3130-2 | 56171 | DNAH7 | dynein, axonemal, heavy chain 7 | 1 | | 1517 | hsa-mir-3130-3 | 56171 | DNAH7 | dynein, axonemal, heavy chain 7 | 1 | | 1518 | hsa-mir-3130-4 | 56171 | DNAH7 | dynein, axonemal, heavy chain 7 | 1 | | 1519 | hsa-miR-10a | 56171 | DNAH7 | dynein, axonemal, heavy chain 7 | 1 | | 1520 | hsa-miR-95 | 56171 | DNAH7 | dynein, axonemal, heavy chain 7 | 1 | | 1521 | hsa-miR-558 | 8574 | AKR7A2 | aldo-keto reductase family 7, member A2 (aflatoxin aldehyde reductase) | 1 | | 1526 | hsa-miR-558 | 5689 | PSMB1 | proteasome (prosome, macropain) subunit, beta type, 1 | 1 | | 1527 | hsa-miR-593\* | 6203 | RPS9 | ribosomal protein S9 | 1 | | 1529 | hsa-mir-628 | 6137 | RPL13 | ribosomal protein L13 | 1 | | 1530 | hsa-miR-628-5p | 6137 | RPL13 | ribosomal protein L13 | 1 | | 1531 | hsa-miR-608 | 92346 | C1orf105 | chromosome 1 open reading frame 105 | 1 | | 1532 | hsa-miR-1233 | 7597 | ZBTB25 | zinc finger and BTB domain containing 25 | 1 | | 1533 | hsa-miR-571 | 7597 | ZBTB25 | zinc finger and BTB domain containing 25 | 1 | | 1534 | hsa-miR-766 | 7597 | ZBTB25 | zinc finger and BTB domain containing 25 | 1 | | 1535 | hsa-miR-874 | 7597 | ZBTB25 | zinc finger and BTB domain containing 25 | 1 | | 1537 | hsa-miR-10a | 2691 | GHRH | growth hormone releasing hormone | 1 | | 1539 | hsa-miR-569 | 5379 | PMS2L1 | postmeiotic segregation increased 2-like 1 pseudogene | 1 | | 1542 | hsa-mir-3130-1 | 57152 | SLURP1 | secreted LY6/PLAUR domain containing 1 | 1 | | 1543 | hsa-mir-3130-2 | 57152 | SLURP1 | secreted LY6/PLAUR domain containing 1 | 1 | | 1544 | hsa-mir-3130-3 | 57152 | SLURP1 | secreted LY6/PLAUR domain containing 1 | 1 | | 1545 | hsa-mir-3130-4 | 57152 | SLURP1 | secreted LY6/PLAUR domain containing 1 | 1 | | 1546 | hsa-miR-10a | 57152 | SLURP1 | secreted LY6/PLAUR domain containing 1 | 1 | | 1547 | hsa-miR-499-5p | 57152 | SLURP1 | secreted LY6/PLAUR domain containing 1 | 1 | | 1548 | hsa-miR-580 | 57152 | SLURP1 | secreted LY6/PLAUR domain containing 1 | 1 | | 1549 | hsa-miR-95 | 57152 | SLURP1 | secreted LY6/PLAUR domain containing 1 | 1 | | 1550 | hsa-miR-874 | 27334 | P2RY10 | purinergic receptor P2Y, G-protein coupled, 10 | 1 | | 1551 | hsa-miR-623 | 23193 | GANAB | glucosidase, alpha; neutral AB | 1 | | 1552 | hsa-miR-877 | 23193 | GANAB | glucosidase, alpha; neutral AB | 1 | | 1556 | hsa-miR-423-5p | 3895 | KTN1 | kinectin 1 (kinesin receptor) | 1 | | 1558 | hsa-miR-623 | 2734 | GLG1 | golgi glycoprotein 1 | 1 | | 1559 | hsa-miR-555 | 26034 | IPCEF1 | interaction protein for cytohesin exchange factors 1 | 1 | | 1560 | hsa-miR-558 | 26034 | IPCEF1 | interaction protein for cytohesin exchange factors 1 | 1 | | 1561 | hsa-mir-10a | 84859 | LRCH3 | leucine-rich repeats and calponin homology (CH) domain containing 3 | 1 | | 1562 | hsa-mir-199a-2 | 84859 | LRCH3 | leucine-rich repeats and calponin homology (CH) domain containing 3 | 1 | | 1563 | hsa-mir-214 | 84859 | LRCH3 | leucine-rich repeats and calponin homology (CH) domain containing 3 | 1 | | 1564 | hsa-mir-499 | 84859 | LRCH3 | leucine-rich repeats and calponin homology (CH) domain containing 3 | 1 | | 1568 | hsa-miR-608 | 126393 | HSPB6 | heat shock protein, alpha-crystallin-related, B6 | 1 | | 1569 | hsa-miR-1233 | 26054 | SENP6 | SUMO1/sentrin specific peptidase 6 | 1 | | 1570 | hsa-miR-558 | 26054 | SENP6 | SUMO1/sentrin specific peptidase 6 | 1 | | 1571 | hsa-miR-571 | 26054 | SENP6 | SUMO1/sentrin specific peptidase 6 | 1 | | 1572 | hsa-miR-1233 | 93349 | SP140L | SP140 nuclear body protein-like | 1 | | 1573 | hsa-miR-423-5p | 91746 | YTHDC1 | YTH domain containing 1 | 1 | | 1575 | hsa-mir-10a | 253959 | RALGAPA1 | Ral GTPase activating protein, alpha subunit 1 (catalytic) | 1 | | 1576 | hsa-mir-885 | 253959 | RALGAPA1 | Ral GTPase activating protein, alpha subunit 1 (catalytic) | 1 | | 1577 | hsa-miR-1224-5p | 253959 | RALGAPA1 | Ral GTPase activating protein, alpha subunit 1 (catalytic) | 1 | | 1578 | hsa-miR-423-5p | 253959 | RALGAPA1 | Ral GTPase activating protein, alpha subunit 1 (catalytic) | 1 | | 1579 | hsa-miR-766 | 253959 | RALGAPA1 | Ral GTPase activating protein, alpha subunit 1 (catalytic) | 1 | | 1580 | hsa-miR-885-5p | 253959 | RALGAPA1 | Ral GTPase activating protein, alpha subunit 1 (catalytic) | 1 | | 1581 | hsa-miR-95 | 100130449 | PP14571 | similar to hCG1777210 | 1 | | 1582 | hsa-miR-10a | 4684 | NCAM1 | neural cell adhesion molecule 1 | 1 | | 1583 | hsa-miR-95 | 4684 | NCAM1 | neural cell adhesion molecule 1 | 1 | | 1584 | hsa-miR-10a | 146712 | B3GNTL1 | UDP-GlcNAc:betaGal beta-1,3-N-acetylglucosaminyltransferase-like 1 | 1 | | 1585 | hsa-miR-95 | 146712 | B3GNTL1 | UDP-GlcNAc:betaGal beta-1,3-N-acetylglucosaminyltransferase-like 1 | 1 | | 1586 | hsa-mir-10a | 10480 | EIF3M | eukaryotic translation initiation factor 3, subunit M | 1 | | 1587 | hsa-miR-1233 | 10480 | EIF3M | eukaryotic translation initiation factor 3, subunit M | 1 | | 1588 | hsa-miR-423-5p | 10480 | EIF3M | eukaryotic translation initiation factor 3, subunit M | 1 | | 1589 | hsa-miR-571 | 10480 | EIF3M | eukaryotic translation initiation factor 3, subunit M | 1 | | 1590 | hsa-miR-766 | 10480 | EIF3M | eukaryotic translation initiation factor 3, subunit M | 1 | | 1591 | hsa-mir-3130-1 | 800 | CALD1 | caldesmon 1 | 1 | | 1592 | hsa-mir-3130-2 | 800 | CALD1 | caldesmon 1 | 1 | | 1593 | hsa-mir-3130-3 | 800 | CALD1 | caldesmon 1 | 1 | | 1594 | hsa-mir-3130-4 | 800 | CALD1 | caldesmon 1 | 1 | | 1595 | hsa-miR-10a | 800 | CALD1 | caldesmon 1 | 1 | | 1596 | hsa-miR-95 | 800 | CALD1 | caldesmon 1 | 1 | | 1597 | hsa-miR-10a | 5156 | PDGFRA | platelet-derived growth factor receptor, alpha polypeptide | 1 | | 1598 | hsa-miR-95 | 5156 | PDGFRA | platelet-derived growth factor receptor, alpha polypeptide | 1 | | 1599 | hsa-miR-10a | 107 | ADCY1 | adenylate cyclase 1 (brain) | 1 | | 1600 | hsa-miR-1233 | 643376 | BTBD18 | BTB (POZ) domain containing 18 | 1 | | 1603 | hsa-miR-10a | 9215 | LARGE | like-glycosyltransferase | 1 | | 1604 | hsa-miR-10a | 1746 | DLX2 | distal-less homeobox 2 | 1 | | 1605 | hsa-miR-623 | 6597 | SMARCA4 | SWI/SNF related, matrix associated, actin dependent regulator of chromatin, subfamily a, member 4 | 1 | | 1606 | hsa-mir-3130-1 | 100293596 | LOC100293596 | similar to mucin | 1 | | 1607 | hsa-mir-3130-2 | 100293596 | LOC100293596 | similar to mucin | 1 | | 1608 | hsa-mir-3130-3 | 100293596 | LOC100293596 | similar to mucin | 1 | | 1609 | hsa-mir-3130-4 | 100293596 | LOC100293596 | similar to mucin | 1 | | 1610 | hsa-miR-10a | 100293596 | LOC100293596 | similar to mucin | 1 | | 1611 | hsa-miR-95 | 100293596 | LOC100293596 | similar to mucin | 1 | | 1612 | hsa-miR-877 | 55735 | DNAJC11 | DnaJ (Hsp40) homolog, subfamily C, member 11 | 1 | | 1613 | hsa-miR-10a | 492 | ATP2B3 | ATPase, Ca++ transporting, plasma membrane 3 | 1 | | 1614 | hsa-miR-95 | 492 | ATP2B3 | ATPase, Ca++ transporting, plasma membrane 3 | 1 | | 1615 | hsa-miR-938 | 2713 | GK3P | glycerol kinase 3 pseudogene | 1 | | 1621 | hsa-miR-10a | 23162 | MAPK8IP3 | mitogen-activated protein kinase 8 interacting protein 3 | 1 | | 1622 | hsa-miR-1233 | 387535 | HCRP1 | hepatocellular carcinoma-related HCRP1 | 1 | | 1623 | hsa-miR-569 | 387535 | HCRP1 | hepatocellular carcinoma-related HCRP1 | 1 | | 1624 | hsa-miR-766 | 387535 | HCRP1 | hepatocellular carcinoma-related HCRP1 | 1 | | 1625 | hsa-miR-623 | 10985 | GCN1L1 | GCN1 general control of amino-acid synthesis 1-like 1 (yeast) | 1 | | 1626 | hsa-miR-10a | 1211 | CLTA | clathrin, light chain (Lca) | 1 | | 1627 | hsa-miR-95 | 1211 | CLTA | clathrin, light chain (Lca) | 1 | | 1628 | hsa-miR-877 | 8841 | HDAC3 | histone deacetylase 3 | 1 | | 1629 | hsa-miR-95 | 1550 | CYP2A7P1 | cytochrome P450, family 2, subfamily A, polypeptide 7 pseudogene 1 | 1 | | 1630 | hsa-miR-10a | 55020 | TTC38 | tetratricopeptide repeat domain 38 | 1 | | 1631 | hsa-miR-95 | 55020 | TTC38 | tetratricopeptide repeat domain 38 | 1 | | 1632 | hsa-miR-938 | 23250 | ATP11A | ATPase, class VI, type 11A | 1 | | 1633 | hsa-miR-95 | 6760 | SS18 | synovial sarcoma translocation, chromosome 18 | 1 | | 1634 | hsa-miR-10a | 643 | CXCR5 | chemokine (C-X-C motif) receptor 5 | 1 | | 1635 | hsa-miR-580 | 643 | CXCR5 | chemokine (C-X-C motif) receptor 5 | 1 | | 1636 | hsa-miR-95 | 643 | CXCR5 | chemokine (C-X-C motif) receptor 5 | 1 | | 1637 | hsa-mir-3130-1 | 54979 | HRASLS2 | HRAS-like suppressor 2 | 1 | | 1638 | hsa-mir-3130-2 | 54979 | HRASLS2 | HRAS-like suppressor 2 | 1 | | 1639 | hsa-mir-3130-3 | 54979 | HRASLS2 | HRAS-like suppressor 2 | 1 | | 1640 | hsa-mir-3130-4 | 54979 | HRASLS2 | HRAS-like suppressor 2 | 1 | | 1642 | hsa-mir-10a | 22868 | FASTKD2 | FAST kinase domains 2 | 1 | | 1643 | hsa-miR-569 | 22868 | FASTKD2 | FAST kinase domains 2 | 1 | | 1644 | hsa-miR-766 | 22868 | FASTKD2 | FAST kinase domains 2 | 1 | | 1650 | hsa-miR-10a | 1758 | DMP1 | dentin matrix acidic phosphoprotein 1 | 1 | | 1651 | hsa-miR-499-5p | 1758 | DMP1 | dentin matrix acidic phosphoprotein 1 | 1 | | 1652 | hsa-miR-95 | 1758 | DMP1 | dentin matrix acidic phosphoprotein 1 | 1 | | 1653 | hsa-miR-10a | 9757 | MLL4 | myeloid/lymphoid or mixed-lineage leukemia 4 | 1 | | 1654 | hsa-miR-95 | 9757 | MLL4 | myeloid/lymphoid or mixed-lineage leukemia 4 | 1 | | 1655 | hsa-miR-10a | 7932 | OR2H2 | olfactory receptor, family 2, subfamily H, member 2 | 1 | | 1656 | hsa-miR-10a | 100240726 | LOC100240726 | makorin ring finger protein 1 pseudogene | 1 | | 1657 | hsa-miR-95 | 100240726 | LOC100240726 | makorin ring finger protein 1 pseudogene | 1 | | 1658 | hsa-miR-874 | 50852 | TRAT1 | T cell receptor associated transmembrane adaptor 1 | 1 | | 1659 | hsa-miR-608 | 4771 | NF2 | neurofibromin 2 (merlin) | 1 | | 1660 | hsa-mir-3130-1 | 2099 | ESR1 | estrogen receptor 1 | 1 | | 1661 | hsa-mir-3130-2 | 2099 | ESR1 | estrogen receptor 1 | 1 | | 1662 | hsa-mir-3130-3 | 2099 | ESR1 | estrogen receptor 1 | 1 | | 1663 | hsa-mir-3130-4 | 2099 | ESR1 | estrogen receptor 1 | 1 | | 1664 | hsa-miR-10a | 2099 | ESR1 | estrogen receptor 1 | 1 | | 1665 | hsa-miR-499-5p | 2099 | ESR1 | estrogen receptor 1 | 1 | | 1666 | hsa-miR-95 | 2099 | ESR1 | estrogen receptor 1 | 1 | | 1667 | hsa-miR-95 | 2224 | FDPS | farnesyl diphosphate synthase (farnesyl pyrophosphate synthetase, dimethylallyltranstransferase, geranyltranstransferase) | 1 | | 1668 | hsa-miR-95 | 160313 | KRT19P2 | keratin 19 pseudogene 2 | 1 | | 1669 | hsa-miR-499-5p | 7148 | TNXB | tenascin XB | 1 | | 1670 | hsa-miR-95 | 7148 | TNXB | tenascin XB | 1 | | 1671 | hsa-miR-10a | 203510 | LOC203510 | similar to hCG1644442 | 1 | | 1672 | hsa-miR-95 | 203510 | LOC203510 | similar to hCG1644442 | 1 | | 1673 | hsa-miR-95 | 9436 | NCR2 | natural cytotoxicity triggering receptor 2 | 1 | | 1674 | hsa-miR-10a | 6569 | SLC34A1 | solute carrier family 34 (sodium phosphate), member 1 | 1 | | 1678 | hsa-miR-1233 | 51386 | EIF3L | eukaryotic translation initiation factor 3, subunit L | 1 | | 1679 | hsa-miR-558 | 51386 | EIF3L | eukaryotic translation initiation factor 3, subunit L | 1 | | 1680 | hsa-miR-874 | 51386 | EIF3L | eukaryotic translation initiation factor 3, subunit L | 1 | | 1681 | hsa-miR-1233 | 51335 | NGRN | neugrin, neurite outgrowth associated | 1 | | 1682 | hsa-miR-766 | 51335 | NGRN | neugrin, neurite outgrowth associated | 1 | | 1686 | hsa-miR-623 | 373156 | GSTK1 | glutathione S-transferase kappa 1 | 1 | | 1687 | hsa-miR-555 | 56889 | TM9SF3 | transmembrane 9 superfamily member 3 | 1 | | 1688 | hsa-miR-558 | 56889 | TM9SF3 | transmembrane 9 superfamily member 3 | 1 | | 1690 | hsa-miR-938 | 11031 | RAB31 | RAB31, member RAS oncogene family | 1 | | 1691 | hsa-miR-618 | 64397 | ZFP106 | zinc finger protein 106 homolog (mouse) | 1 | | 1692 | hsa-miR-593\* | 3609 | ILF3 | interleukin enhancer binding factor 3, 90kDa | 1 | | 1693 | hsa-miR-623 | 3609 | ILF3 | interleukin enhancer binding factor 3, 90kDa | 1 | | 1694 | hsa-mir-10a | 51729 | WBP11 | WW domain binding protein 11 | 1 | | 1695 | hsa-miR-1233 | 51729 | WBP11 | WW domain binding protein 11 | 1 | | 1696 | hsa-miR-423-5p | 51729 | WBP11 | WW domain binding protein 11 | 1 | | 1697 | hsa-miR-766 | 51729 | WBP11 | WW domain binding protein 11 | 1 | | 1698 | hsa-miR-423-5p | 51012 | SLMO2 | slowmo homolog 2 (Drosophila) | 1 | | 1701 | hsa-miR-1233 | 55226 | NAT10 | N-acetyltransferase 10 (GCN5-related) | 1 | | 1702 | hsa-miR-766 | 55226 | NAT10 | N-acetyltransferase 10 (GCN5-related) | 1 | | 1703 | hsa-miR-877 | 55226 | NAT10 | N-acetyltransferase 10 (GCN5-related) | 1 | | 1704 | hsa-mir-10a | 55037 | PTCD3 | Pentatricopeptide repeat domain 3 | 1 | | 1710 | hsa-miR-593\* | 28974 | C19orf53 | chromosome 19 open reading frame 53 | 1 | | 1711 | hsa-miR-135a | 54472 | TOLLIP | toll interacting protein | 1 | | 1712 | hsa-miR-877 | 51081 | MRPS7 | mitochondrial ribosomal protein S7 | 1 | | 1715 | hsa-miR-1233 | 58478 | ENOPH1 | enolase-phosphatase 1 | 1 | | 1716 | hsa-miR-1233 | 29105 | C16orf80 | chromosome 16 open reading frame 80 | 1 | | 1717 | hsa-miR-569 | 29105 | C16orf80 | chromosome 16 open reading frame 80 | 1 | | 1718 | hsa-miR-571 | 29105 | C16orf80 | chromosome 16 open reading frame 80 | 1 | | 1719 | hsa-miR-766 | 29105 | C16orf80 | chromosome 16 open reading frame 80 | 1 | | 1720 | hsa-miR-877 | 29105 | C16orf80 | chromosome 16 open reading frame 80 | 1 | | 1721 | hsa-miR-618 | 58472 | SQRDL | sulfide quinone reductase-like (yeast) | 1 | | 1722 | hsa-miR-623 | 51116 | MRPS2 | mitochondrial ribosomal protein S2 | 1 | | 1723 | hsa-miR-877 | 51116 | MRPS2 | mitochondrial ribosomal protein S2 | 1 | | 1724 | hsa-miR-766 | 29088 | MRPL15 | mitochondrial ribosomal protein L15 | 1 | | 1726 | hsa-miR-423-5p | 51569 | UFM1 | ubiquitin-fold modifier 1 | 1 | | 1728 | hsa-miR-558 | 55717 | WDR11 | WD repeat domain 11 | 1 | | 1730 | hsa-mir-10a | 55608 | ANKRD10 | ankyrin repeat domain 10 | 1 | | 1731 | hsa-miR-423-5p | 55608 | ANKRD10 | ankyrin repeat domain 10 | 1 | | 1732 | hsa-miR-1233 | 55326 | AGPAT5 | 1-acylglycerol-3-phosphate O-acyltransferase 5 (lysophosphatidic acid acyltransferase, epsilon) | 1 | | 1733 | hsa-miR-571 | 54881 | TEX10 | testis expressed 10 | 1 | | 1734 | hsa-miR-454\* | 55173 | MRPS10 | mitochondrial ribosomal protein S10 | 1 | | 1735 | hsa-miR-623 | 3163 | HMOX2 | heme oxygenase (decycling) 2 | 1 | | 1736 | hsa-miR-1233 | 56683 | C21orf59 | chromosome 21 open reading frame 59 | 1 | | 1737 | hsa-miR-766 | 56683 | C21orf59 | chromosome 21 open reading frame 59 | 1 | | 1740 | hsa-miR-874 | 55246 | CCDC25 | coiled-coil domain containing 25 | 1 | | 1741 | hsa-mir-10a | 8195 | MKKS | McKusick-Kaufman syndrome | 1 | | 1742 | hsa-miR-766 | 64769 | MEAF6 | MYST/Esa1-associated factor 6 | 1 | | 1743 | hsa-miR-766 | 65265 | C8orf33 | chromosome 8 open reading frame 33 | 1 | | 1744 | hsa-mir-10a | 55074 | OXR1 | oxidation resistance 1 | 1 | | 1745 | hsa-miR-423-5p | 55074 | OXR1 | oxidation resistance 1 | 1 | | 1746 | hsa-miR-1233 | 79443 | FYCO1 | FYVE and coiled-coil domain containing 1 | 1 | | 1747 | hsa-miR-571 | 79443 | FYCO1 | FYVE and coiled-coil domain containing 1 | 1 | | 1748 | hsa-miR-874 | 79443 | FYCO1 | FYVE and coiled-coil domain containing 1 | 1 | | 1749 | hsa-miR-423-5p | 23683 | PRKD3 | protein kinase D3 | 1 | | 1750 | hsa-miR-576-5p | 23683 | PRKD3 | protein kinase D3 | 1 | | 1751 | hsa-miR-1233 | 55035 | NOL8 | nucleolar protein 8 | 1 | | 1752 | hsa-miR-766 | 55035 | NOL8 | nucleolar protein 8 | 1 | | 1753 | hsa-miR-1233 | 29883 | CNOT7 | CCR4-NOT transcription complex, subunit 7 | 1 | | 1754 | hsa-miR-766 | 29883 | CNOT7 | CCR4-NOT transcription complex, subunit 7 | 1 | | 1755 | hsa-miR-1233 | 53371 | NUP54 | nucleoporin 54kDa | 1 | | 1756 | hsa-miR-558 | 53371 | NUP54 | nucleoporin 54kDa | 1 | | 1757 | hsa-miR-571 | 53371 | NUP54 | nucleoporin 54kDa | 1 | | 1758 | hsa-miR-874 | 53371 | NUP54 | nucleoporin 54kDa | 1 | | 1759 | hsa-miR-1233 | 29102 | RNASEN | ribonuclease type III, nuclear | 1 | | 1760 | hsa-miR-558 | 29102 | RNASEN | ribonuclease type III, nuclear | 1 | | 1761 | hsa-miR-571 | 29102 | RNASEN | ribonuclease type III, nuclear | 1 | | 1762 | hsa-miR-874 | 29102 | RNASEN | ribonuclease type III, nuclear | 1 | | 1763 | hsa-miR-766 | 54704 | PDP1 | pyruvate dehyrogenase phosphatase catalytic subunit 1 | 1 | | 1764 | hsa-mir-199a-2 | 79665 | DHX40 | DEAH (Asp-Glu-Ala-His) box polypeptide 40 | 1 | | 1765 | hsa-mir-214 | 79665 | DHX40 | DEAH (Asp-Glu-Ala-His) box polypeptide 40 | 1 | | 1766 | hsa-miR-423-5p | 79665 | DHX40 | DEAH (Asp-Glu-Ala-His) box polypeptide 40 | 1 | | 1767 | hsa-miR-576-5p | 79665 | DHX40 | DEAH (Asp-Glu-Ala-His) box polypeptide 40 | 1 | | 1768 | hsa-miR-1233 | 56898 | BDH2 | 3-hydroxybutyrate dehydrogenase, type 2 | 1 | | 1769 | hsa-miR-569 | 56898 | BDH2 | 3-hydroxybutyrate dehydrogenase, type 2 | 1 | | 1770 | hsa-miR-571 | 56898 | BDH2 | 3-hydroxybutyrate dehydrogenase, type 2 | 1 | | 1771 | hsa-miR-766 | 56898 | BDH2 | 3-hydroxybutyrate dehydrogenase, type 2 | 1 | | 1772 | hsa-miR-874 | 56898 | BDH2 | 3-hydroxybutyrate dehydrogenase, type 2 | 1 | | 1774 | hsa-miR-558 | 26520 | TIMM9 | translocase of inner mitochondrial membrane 9 homolog (yeast) | 1 | | 1775 | hsa-mir-10a | 54906 | C10orf18 | chromosome 10 open reading frame 18 | 1 | | 1778 | hsa-miR-571 | 54906 | C10orf18 | chromosome 10 open reading frame 18 | 1 | | 1781 | hsa-miR-576-5p | 55213 | RCBTB1 | regulator of chromosome condensation (RCC1) and BTB (POZ) domain containing protein 1 | 1 | | 1782 | hsa-mir-10a | 10600 | USP16 | ubiquitin specific peptidase 16 | 1 | | 1788 | hsa-miR-618 | 79887 | PLBD1 | phospholipase B domain containing 1 | 1 | | 1789 | hsa-miR-1233 | 65981 | CAPRIN2 | caprin family member 2 | 1 | | 1790 | hsa-miR-569 | 65981 | CAPRIN2 | caprin family member 2 | 1 | | 1791 | hsa-miR-571 | 65981 | CAPRIN2 | caprin family member 2 | 1 | | 1792 | hsa-miR-766 | 65981 | CAPRIN2 | caprin family member 2 | 1 | | 1793 | hsa-miR-874 | 65981 | CAPRIN2 | caprin family member 2 | 1 | | 1794 | hsa-miR-766 | 54919 | HEATR2 | HEAT repeat containing 2 | 1 | | 1799 | hsa-miR-593\* | 8409 | UXT | ubiquitously-expressed transcript | 1 | | 1800 | hsa-miR-877 | 8409 | UXT | ubiquitously-expressed transcript | 1 | | 1801 | hsa-mir-10a | 246243 | RNASEH1 | ribonuclease H1 | 1 | | 1802 | hsa-miR-1233 | 246243 | RNASEH1 | ribonuclease H1 | 1 | | 1803 | hsa-miR-423-5p | 246243 | RNASEH1 | ribonuclease H1 | 1 | | 1805 | hsa-miR-766 | 246243 | RNASEH1 | ribonuclease H1 | 1 | | 1806 | hsa-miR-874 | 246243 | RNASEH1 | ribonuclease H1 | 1 | | 1808 | hsa-miR-593\* | 29098 | RANGRF | RAN guanine nucleotide release factor | 1 | | 1809 | hsa-miR-623 | 29098 | RANGRF | RAN guanine nucleotide release factor | 1 | | 1810 | hsa-miR-766 | 29098 | RANGRF | RAN guanine nucleotide release factor | 1 | | 1811 | hsa-miR-877 | 29098 | RANGRF | RAN guanine nucleotide release factor | 1 | | 1812 | hsa-miR-423-5p | 55781 | RIOK2 | RIO kinase 2 (yeast) | 1 | | 1813 | hsa-miR-766 | 56954 | NIT2 | nitrilase family, member 2 | 1 | | 1814 | hsa-miR-1233 | 28986 | MAGEH1 | melanoma antigen family H, 1 | 1 | | 1815 | hsa-miR-558 | 28986 | MAGEH1 | melanoma antigen family H, 1 | 1 | | 1816 | hsa-miR-571 | 28986 | MAGEH1 | melanoma antigen family H, 1 | 1 | | 1817 | hsa-miR-874 | 28986 | MAGEH1 | melanoma antigen family H, 1 | 1 | | 1819 | hsa-miR-1233 | 60561 | RINT1 | RAD50 interactor 1 | 1 | | 1820 | hsa-miR-423-5p | 60561 | RINT1 | RAD50 interactor 1 | 1 | | 1821 | hsa-miR-558 | 60561 | RINT1 | RAD50 interactor 1 | 1 | | 1822 | hsa-miR-571 | 60561 | RINT1 | RAD50 interactor 1 | 1 | | 1823 | hsa-mir-10a | 54801 | HAUS6 | HAUS augmin-like complex, subunit 6 | 1 | | 1824 | hsa-mir-885 | 54801 | HAUS6 | HAUS augmin-like complex, subunit 6 | 1 | | 1825 | hsa-miR-423-5p | 54801 | HAUS6 | HAUS augmin-like complex, subunit 6 | 1 | | 1826 | hsa-miR-885-5p | 54801 | HAUS6 | HAUS augmin-like complex, subunit 6 | 1 | | 1827 | hsa-miR-558 | 64216 | TFB2M | transcription factor B2, mitochondrial | 1 | | 1828 | hsa-miR-618 | 55332 | DRAM1 | DNA-damage regulated autophagy modulator 1 | 1 | | 1829 | hsa-miR-1233 | 55622 | TTC27 | tetratricopeptide repeat domain 27 | 1 | | 1830 | hsa-miR-558 | 55622 | TTC27 | tetratricopeptide repeat domain 27 | 1 | | 1831 | hsa-miR-571 | 55622 | TTC27 | tetratricopeptide repeat domain 27 | 1 | | 1832 | hsa-miR-874 | 55622 | TTC27 | tetratricopeptide repeat domain 27 | 1 | | 1833 | hsa-miR-1233 | 54955 | C1orf109 | chromosome 1 open reading frame 109 | 1 | | 1834 | hsa-miR-571 | 54955 | C1orf109 | chromosome 1 open reading frame 109 | 1 | | 1837 | hsa-miR-877 | 79829 | NAA40 | N(alpha)-acetyltransferase 40, NatD catalytic subunit, homolog (S. cerevisiae) | 1 | | 1838 | hsa-miR-1233 | 27300 | ZNF544 | zinc finger protein 544 | 1 | | 1839 | hsa-miR-569 | 27300 | ZNF544 | zinc finger protein 544 | 1 | | 1840 | hsa-miR-571 | 27300 | ZNF544 | zinc finger protein 544 | 1 | | 1841 | hsa-miR-766 | 27300 | ZNF544 | zinc finger protein 544 | 1 | | 1842 | hsa-miR-874 | 27300 | ZNF544 | zinc finger protein 544 | 1 | | 1843 | hsa-miR-623 | 80279 | CDK5RAP3 | CDK5 regulatory subunit associated protein 3 | 1 | | 1844 | hsa-miR-877 | 80279 | CDK5RAP3 | CDK5 regulatory subunit associated protein 3 | 1 | | 1845 | hsa-miR-877 | 55229 | PANK4 | pantothenate kinase 4 | 1 | | 1846 | hsa-miR-10a | 54869 | EPS8L1 | EPS8-like 1 | 1 | | 1847 | hsa-miR-95 | 54869 | EPS8L1 | EPS8-like 1 | 1 | | 1848 | hsa-miR-1233 | 6322 | SCML1 | sex comb on midleg-like 1 (Drosophila) | 1 | | 1849 | hsa-miR-423-5p | 6322 | SCML1 | sex comb on midleg-like 1 (Drosophila) | 1 | | 1853 | hsa-mir-10a | 79657 | RPAP3 | RNA polymerase II associated protein 3 | 1 | | 1855 | hsa-miR-1233 | 79695 | GALNT12 | UDP-N-acetyl-alpha-D-galactosamine:polypeptide N-acetylgalactosaminyltransferase 12 (GalNAc-T12) | 1 | | 1856 | hsa-miR-569 | 79695 | GALNT12 | UDP-N-acetyl-alpha-D-galactosamine:polypeptide N-acetylgalactosaminyltransferase 12 (GalNAc-T12) | 1 | | 1857 | hsa-miR-571 | 79695 | GALNT12 | UDP-N-acetyl-alpha-D-galactosamine:polypeptide N-acetylgalactosaminyltransferase 12 (GalNAc-T12) | 1 | | 1858 | hsa-miR-874 | 79695 | GALNT12 | UDP-N-acetyl-alpha-D-galactosamine:polypeptide N-acetylgalactosaminyltransferase 12 (GalNAc-T12) | 1 | | 1859 | hsa-miR-877 | 4771 | NF2 | neurofibromin 2 (merlin) | 1 | | 1860 | hsa-mir-10a | 79752 | ZFAND1 | zinc finger, AN1-type domain 1 | 1 | | 1861 | hsa-miR-1233 | 79752 | ZFAND1 | zinc finger, AN1-type domain 1 | 1 | | 1862 | hsa-miR-423-5p | 79752 | ZFAND1 | zinc finger, AN1-type domain 1 | 1 | | 1863 | hsa-miR-558 | 79752 | ZFAND1 | zinc finger, AN1-type domain 1 | 1 | | 1864 | hsa-miR-569 | 79752 | ZFAND1 | zinc finger, AN1-type domain 1 | 1 | | 1865 | hsa-miR-571 | 79752 | ZFAND1 | zinc finger, AN1-type domain 1 | 1 | | 1866 | hsa-mir-10a | 55602 | CDKN2AIP | CDKN2A interacting protein | 1 | | 1867 | hsa-miR-423-5p | 54664 | TMEM106B | transmembrane protein 106B | 1 | | 1868 | hsa-miR-576-5p | 54664 | TMEM106B | transmembrane protein 106B | 1 | | 1869 | hsa-miR-423-5p | 29080 | CCDC59 | coiled-coil domain containing 59 | 1 | | 1870 | hsa-miR-555 | 29080 | CCDC59 | coiled-coil domain containing 59 | 1 | | 1871 | hsa-miR-558 | 29080 | CCDC59 | coiled-coil domain containing 59 | 1 | | 1872 | hsa-mir-135b | 54925 | ZNF434 | zinc finger protein 434 | 1 | | 1873 | hsa-miR-1233 | 80227 | PAAF1 | proteasomal ATPase-associated factor 1 | 1 | | 1874 | hsa-miR-571 | 80227 | PAAF1 | proteasomal ATPase-associated factor 1 | 1 | | 1875 | hsa-miR-766 | 80227 | PAAF1 | proteasomal ATPase-associated factor 1 | 1 | | 1876 | hsa-miR-618 | 51279 | C1RL | complement component 1, r subcomponent-like | 1 | | 1877 | hsa-miR-1233 | 348995 | NUP43 | nucleoporin 43kDa | 1 | | 1878 | hsa-miR-558 | 348995 | NUP43 | nucleoporin 43kDa | 1 | | 1879 | hsa-miR-571 | 348995 | NUP43 | nucleoporin 43kDa | 1 | | 1880 | hsa-miR-874 | 348995 | NUP43 | nucleoporin 43kDa | 1 | | 1882 | hsa-miR-569 | 59338 | PLEKHA1 | pleckstrin homology domain containing, family A (phosphoinositide binding specific) member 1 | 1 | | 1886 | hsa-miR-558 | 64417 | C5orf28 | chromosome 5 open reading frame 28 | 1 | | 1887 | hsa-miR-558 | 51018 | RRP15 | ribosomal RNA processing 15 homolog (S. cerevisiae) | 1 | | 1888 | hsa-miR-10a | 55258 | THNSL2 | threonine synthase-like 2 (S. cerevisiae) | 1 | | 1889 | hsa-miR-95 | 55258 | THNSL2 | threonine synthase-like 2 (S. cerevisiae) | 1 | | 1890 | hsa-miR-608 | 63876 | PKNOX2 | PBX/knotted 1 homeobox 2 | 1 | | 1891 | hsa-miR-877 | 8270 | LAGE3 | L antigen family, member 3 | 1 | | 1892 | hsa-mir-135b | 54851 | ANKRD49 | ankyrin repeat domain 49 | 1 | | 1893 | hsa-miR-623 | 9274 | BCL7C | B-cell CLL/lymphoma 7C | 1 | | 1894 | hsa-miR-555 | 55751 | TMEM184C | transmembrane protein 184C | 1 | | 1895 | hsa-miR-558 | 55751 | TMEM184C | transmembrane protein 184C | 1 | | 1896 | hsa-miR-877 | 65003 | MRPL11 | mitochondrial ribosomal protein L11 | 1 | | 1897 | hsa-miR-608 | 57715 | SEMA4G | sema domain, immunoglobulin domain (Ig), transmembrane domain (TM) and short cytoplasmic domain, (semaphorin) 4G | 1 | | 1898 | hsa-miR-1233 | 54465 | ETAA1 | Ewing tumor-associated antigen 1 | 1 | | 1899 | hsa-miR-558 | 54465 | ETAA1 | Ewing tumor-associated antigen 1 | 1 | | 1900 | hsa-miR-571 | 54465 | ETAA1 | Ewing tumor-associated antigen 1 | 1 | | 1901 | hsa-miR-874 | 54465 | ETAA1 | Ewing tumor-associated antigen 1 | 1 | | 1902 | hsa-miR-618 | 79623 | GALNT14 | UDP-N-acetyl-alpha-D-galactosamine:polypeptide N-acetylgalactosaminyltransferase 14 (GalNAc-T14) | 1 | | 1903 | hsa-miR-423-5p | 79886 | C9orf82 | chromosome 9 open reading frame 82 | 1 | | 1904 | hsa-miR-454\* | 79886 | C9orf82 | chromosome 9 open reading frame 82 | 1 | | 1906 | hsa-miR-95 | 55753 | OGDHL | oxoglutarate dehydrogenase-like | 1 | | 1907 | hsa-miR-618 | 4482 | MSRA | methionine sulfoxide reductase A | 1 | | 1908 | hsa-miR-558 | 29789 | OLA1 | Obg-like ATPase 1 | 1 | | 1909 | hsa-mir-10a | 79596 | RNF219 | ring finger protein 219 | 1 | | 1910 | hsa-mir-199a-2 | 79596 | RNF219 | ring finger protein 219 | 1 | | 1911 | hsa-mir-214 | 79596 | RNF219 | ring finger protein 219 | 1 | | 1912 | hsa-miR-423-5p | 79596 | RNF219 | ring finger protein 219 | 1 | | 1913 | hsa-miR-571 | 79596 | RNF219 | ring finger protein 219 | 1 | | 1914 | hsa-miR-576-5p | 79596 | RNF219 | ring finger protein 219 | 1 | | 1916 | hsa-miR-580 | 51222 | ZNF219 | zinc finger protein 219 | 1 | | 1917 | hsa-miR-1233 | 79652 | TMEM204 | transmembrane protein 204 | 1 | | 1918 | hsa-miR-569 | 79652 | TMEM204 | transmembrane protein 204 | 1 | | 1919 | hsa-miR-766 | 79652 | TMEM204 | transmembrane protein 204 | 1 | | 1920 | hsa-miR-877 | 79652 | TMEM204 | transmembrane protein 204 | 1 | | 1921 | hsa-miR-1233 | 64860 | ARMCX5 | armadillo repeat containing, X-linked 5 | 1 | | 1922 | hsa-miR-558 | 64860 | ARMCX5 | armadillo repeat containing, X-linked 5 | 1 | | 1923 | hsa-miR-571 | 64860 | ARMCX5 | armadillo repeat containing, X-linked 5 | 1 | | 1924 | hsa-miR-877 | 56616 | DIABLO | diablo homolog (Drosophila) | 1 | | 1925 | hsa-miR-874 | 55086 | CXorf57 | chromosome X open reading frame 57 | 1 | | 1926 | hsa-miR-555 | 79612 | NAA16 | N(alpha)-acetyltransferase 16, NatA auxiliary subunit | 1 | | 1927 | hsa-miR-558 | 79612 | NAA16 | N(alpha)-acetyltransferase 16, NatA auxiliary subunit | 1 | | 1928 | hsa-miR-618 | 9489 | PGS1 | phosphatidylglycerophosphate synthase 1 | 1 | | 1929 | hsa-mir-628 | 8718 | TNFRSF25 | tumor necrosis factor receptor superfamily, member 25 | 1 | | 1930 | hsa-miR-628-5p | 8718 | TNFRSF25 | tumor necrosis factor receptor superfamily, member 25 | 1 | | 1931 | hsa-miR-877 | 8718 | TNFRSF25 | tumor necrosis factor receptor superfamily, member 25 | 1 | | 1932 | hsa-miR-95 | 51705 | EMCN | endomucin | 1 | | 1933 | hsa-miR-1233 | 55617 | TASP1 | taspase, threonine aspartase, 1 | 1 | | 1934 | hsa-miR-558 | 55617 | TASP1 | taspase, threonine aspartase, 1 | 1 | | 1935 | hsa-miR-571 | 55617 | TASP1 | taspase, threonine aspartase, 1 | 1 | | 1936 | hsa-miR-874 | 55617 | TASP1 | taspase, threonine aspartase, 1 | 1 | | 1937 | hsa-miR-135a | 79890 | RIN3 | Ras and Rab interactor 3 | 1 | | 1938 | hsa-miR-938 | 79890 | RIN3 | Ras and Rab interactor 3 | 1 | | 1939 | hsa-miR-567 | 79669 | C3orf52 | chromosome 3 open reading frame 52 | 1 | | 1940 | hsa-miR-555 | 7733 | ZNF180 | zinc finger protein 180 | 1 | | 1941 | hsa-miR-571 | 7733 | ZNF180 | zinc finger protein 180 | 1 | | 1942 | hsa-miR-10a | 23452 | ANGPTL2 | angiopoietin-like 2 | 1 | | 1943 | hsa-miR-558 | 7769 | ZNF226 | zinc finger protein 226 | 1 | | 1945 | hsa-mir-10a | 51134 | CCDC41 | coiled-coil domain containing 41 | 1 | | 1946 | hsa-miR-423-5p | 51134 | CCDC41 | coiled-coil domain containing 41 | 1 | | 1947 | hsa-miR-874 | 79810 | PTCD2 | pentatricopeptide repeat domain 2 | 1 | | 1949 | hsa-mir-628 | 25873 | RPL36 | ribosomal protein L36 | 1 | | 1950 | hsa-miR-628-5p | 25873 | RPL36 | ribosomal protein L36 | 1 | | 1951 | hsa-miR-95 | 53841 | CDHR5 | cadherin-related family member 5 | 1 | | 1952 | hsa-miR-1233 | 51275 | C12orf47 | chromosome 12 open reading frame 47 | 1 | | 1953 | hsa-miR-569 | 51275 | C12orf47 | chromosome 12 open reading frame 47 | 1 | | 1954 | hsa-miR-1233 | 9668 | ZNF432 | zinc finger protein 432 | 1 | | 1955 | hsa-miR-423-5p | 9668 | ZNF432 | zinc finger protein 432 | 1 | | 1956 | hsa-miR-555 | 9668 | ZNF432 | zinc finger protein 432 | 1 | | 1957 | hsa-miR-558 | 9668 | ZNF432 | zinc finger protein 432 | 1 | | 1958 | hsa-miR-571 | 9668 | ZNF432 | zinc finger protein 432 | 1 | | 1959 | hsa-miR-874 | 9668 | ZNF432 | zinc finger protein 432 | 1 | | 1960 | hsa-mir-3130-1 | 79906 | MORN1 | MORN repeat containing 1 | 1 | | 1961 | hsa-mir-3130-2 | 79906 | MORN1 | MORN repeat containing 1 | 1 | | 1962 | hsa-mir-3130-3 | 79906 | MORN1 | MORN repeat containing 1 | 1 | | 1963 | hsa-mir-3130-4 | 79906 | MORN1 | MORN repeat containing 1 | 1 | | 1964 | hsa-miR-10a | 79906 | MORN1 | MORN repeat containing 1 | 1 | | 1965 | hsa-miR-95 | 79906 | MORN1 | MORN repeat containing 1 | 1 | | 1966 | hsa-miR-623 | 4054 | LTBP3 | latent transforming growth factor beta binding protein 3 | 1 | | 1967 | hsa-miR-877 | 4054 | LTBP3 | latent transforming growth factor beta binding protein 3 | 1 | | 1968 | hsa-miR-618 | 9050 | PSTPIP2 | proline-serine-threonine phosphatase interacting protein 2 | 1 | | 1969 | hsa-miR-423-5p | 55857 | PLK1S1 | polo-like kinase 1 substrate 1 | 1 | | 1970 | hsa-miR-877 | 51385 | ZNF589 | zinc finger protein 589 | 1 | | 1971 | hsa-miR-423-5p | 80167 | C4orf29 | chromosome 4 open reading frame 29 | 1 | | 1972 | hsa-miR-95 | 54897 | CASZ1 | castor zinc finger 1 | 1 | | 1973 | hsa-miR-571 | 7767 | ZNF224 | zinc finger protein 224 | 1 | | 1977 | hsa-miR-95 | 79919 | C2orf54 | chromosome 2 open reading frame 54 | 1 | | 1978 | hsa-miR-95 | 55806 | HR | hairless homolog (mouse) | 1 | | 1979 | hsa-miR-608 | 353500 | BMP8A | bone morphogenetic protein 8a | 1 | | 1981 | hsa-miR-877 | 63892 | THADA | thyroid adenoma associated | 1 | | 1982 | hsa-miR-608 | 54714 | CNGB3 | cyclic nucleotide gated channel beta 3 | 1 | | 1983 | hsa-miR-95 | 26281 | FGF20 | fibroblast growth factor 20 | 1 | | 1984 | hsa-miR-95 | 7042 | TGFB2 | transforming growth factor, beta 2 | 1 | | 1985 | hsa-miR-1233 | 53347 | UBASH3A | ubiquitin associated and SH3 domain containing, A | 1 | | 1986 | hsa-miR-569 | 53347 | UBASH3A | ubiquitin associated and SH3 domain containing, A | 1 | | 1987 | hsa-miR-766 | 53347 | UBASH3A | ubiquitin associated and SH3 domain containing, A | 1 | | 1988 | hsa-miR-874 | 53347 | UBASH3A | ubiquitin associated and SH3 domain containing, A | 1 | | 1989 | hsa-miR-95 | 55908 | LOC55908 | hepatocellular carcinoma-associated gene TD26 | 1 | | 1990 | hsa-miR-10a | 25806 | VAX2 | ventral anterior homeobox 2 | 1 | | 1991 | hsa-miR-499-5p | 25806 | VAX2 | ventral anterior homeobox 2 | 1 | | 1992 | hsa-miR-95 | 25806 | VAX2 | ventral anterior homeobox 2 | 1 | | 1993 | hsa-miR-10a | 79444 | BIRC7 | baculoviral IAP repeat-containing 7 | 1 | | 1994 | hsa-miR-95 | 79444 | BIRC7 | baculoviral IAP repeat-containing 7 | 1 | | 1995 | hsa-mir-3130-1 | 10864 | SLC22A7 | solute carrier family 22 (organic anion transporter), member 7 | 1 | | 1996 | hsa-mir-3130-2 | 10864 | SLC22A7 | solute carrier family 22 (organic anion transporter), member 7 | 1 | | 1997 | hsa-mir-3130-3 | 10864 | SLC22A7 | solute carrier family 22 (organic anion transporter), member 7 | 1 | | 1998 | hsa-mir-3130-4 | 10864 | SLC22A7 | solute carrier family 22 (organic anion transporter), member 7 | 1 | | 2000 | hsa-miR-499-5p | 10864 | SLC22A7 | solute carrier family 22 (organic anion transporter), member 7 | 1 | | 2002 | hsa-miR-95 | 57718 | PPP4R4 | protein phosphatase 4, regulatory subunit 4 | 1 | | 2003 | hsa-miR-10a | 11095 | ADAMTS8 | ADAM metallopeptidase with thrombospondin type 1 motif, 8 | 1 | | 2004 | hsa-miR-623 | 55707 | NECAP2 | NECAP endocytosis associated 2 | 1 | | 2005 | hsa-mir-628 | 50854 | C6orf48 | chromosome 6 open reading frame 48 | 1 | | 2006 | hsa-miR-593\* | 50854 | C6orf48 | chromosome 6 open reading frame 48 | 1 | | 2007 | hsa-miR-623 | 50854 | C6orf48 | chromosome 6 open reading frame 48 | 1 | | 2008 | hsa-miR-628-5p | 50854 | C6orf48 | chromosome 6 open reading frame 48 | 1 | | 2009 | hsa-miR-766 | 50854 | C6orf48 | chromosome 6 open reading frame 48 | 1 | | 2010 | hsa-miR-877 | 50854 | C6orf48 | chromosome 6 open reading frame 48 | 1 | | 2011 | hsa-mir-3130-1 | 3274 | HRH2 | histamine receptor H2 | 1 | | 2012 | hsa-mir-3130-2 | 3274 | HRH2 | histamine receptor H2 | 1 | | 2013 | hsa-mir-3130-3 | 3274 | HRH2 | histamine receptor H2 | 1 | | 2014 | hsa-mir-3130-4 | 3274 | HRH2 | histamine receptor H2 | 1 | | 2015 | hsa-miR-1233 | 57728 | WDR19 | WD repeat domain 19 | 1 | | 2016 | hsa-miR-571 | 57728 | WDR19 | WD repeat domain 19 | 1 | | 2017 | hsa-mir-10a | 60560 | NAA35 | N(alpha)-acetyltransferase 35, NatC auxiliary subunit | 1 | | 2018 | hsa-mir-885 | 60560 | NAA35 | N(alpha)-acetyltransferase 35, NatC auxiliary subunit | 1 | | 2019 | hsa-miR-423-5p | 60560 | NAA35 | N(alpha)-acetyltransferase 35, NatC auxiliary subunit | 1 | | 2020 | hsa-miR-766 | 60560 | NAA35 | N(alpha)-acetyltransferase 35, NatC auxiliary subunit | 1 | | 2021 | hsa-miR-885-5p | 60560 | NAA35 | N(alpha)-acetyltransferase 35, NatC auxiliary subunit | 1 | | 2023 | hsa-miR-1233 | 81606 | LBH | limb bud and heart development homolog (mouse) | 1 | | 2024 | hsa-miR-569 | 81606 | LBH | limb bud and heart development homolog (mouse) | 1 | | 2025 | hsa-miR-766 | 81606 | LBH | limb bud and heart development homolog (mouse) | 1 | | 2026 | hsa-miR-874 | 81606 | LBH | limb bud and heart development homolog (mouse) | 1 | | 2027 | hsa-miR-877 | 81606 | LBH | limb bud and heart development homolog (mouse) | 1 | | 2028 | hsa-miR-10a | 27231 | ITGB1BP3 | integrin beta 1 binding protein 3 | 1 | | 2029 | hsa-miR-766 | 55239 | OGFOD1 | 2-oxoglutarate and iron-dependent oxygenase domain containing 1 | 1 | | 2030 | hsa-miR-1233 | 28990 | ASTE1 | asteroid homolog 1 (Drosophila) | 1 | | 2032 | hsa-mir-10a | 54816 | ZNF280D | zinc finger protein 280D | 1 | | 2033 | hsa-miR-423-5p | 54816 | ZNF280D | zinc finger protein 280D | 1 | | 2034 | hsa-miR-576-5p | 54816 | ZNF280D | zinc finger protein 280D | 1 | | 2037 | hsa-miR-1233 | 23435 | TARDBP | TAR DNA binding protein | 1 | | 2038 | hsa-miR-569 | 23435 | TARDBP | TAR DNA binding protein | 1 | | 2039 | hsa-miR-766 | 23435 | TARDBP | TAR DNA binding protein | 1 | | 2040 | hsa-miR-874 | 23435 | TARDBP | TAR DNA binding protein | 1 | | 2041 | hsa-miR-623 | 81926 | FAM108A1 | family with sequence similarity 108, member A1 | 1 | | 2042 | hsa-mir-505 | 9376 | SLC22A8 | solute carrier family 22 (organic anion transporter), member 8 | 1 | | 2043 | hsa-miR-10a | 9376 | SLC22A8 | solute carrier family 22 (organic anion transporter), member 8 | 1 | | 2044 | hsa-miR-505\* | 9376 | SLC22A8 | solute carrier family 22 (organic anion transporter), member 8 | 1 | | 2045 | hsa-miR-580 | 9376 | SLC22A8 | solute carrier family 22 (organic anion transporter), member 8 | 1 | | 2046 | hsa-miR-95 | 9376 | SLC22A8 | solute carrier family 22 (organic anion transporter), member 8 | 1 | | 2047 | hsa-miR-95 | 84991 | RBM17 | RNA binding motif protein 17 | 1 | | 2048 | hsa-miR-10a | 4825 | NKX6-1 | NK6 homeobox 1 | 1 | | 2049 | hsa-miR-499-5p | 4825 | NKX6-1 | NK6 homeobox 1 | 1 | | 2050 | hsa-miR-95 | 4825 | NKX6-1 | NK6 homeobox 1 | 1 | | 2051 | hsa-miR-95 | 4992 | OR1F1 | olfactory receptor, family 1, subfamily F, member 1 | 1 | | 2052 | hsa-miR-95 | 3238 | HOXD12 | homeobox D12 | 1 | | 2053 | hsa-miR-938 | 81847 | RNF146 | ring finger protein 146 | 1 | | 2054 | hsa-miR-10a | 56123 | PCDHB13 | protocadherin beta 13 | 1 | | 2055 | hsa-miR-95 | 56123 | PCDHB13 | protocadherin beta 13 | 1 | | 2056 | hsa-miR-877 | 81853 | TMEM14B | transmembrane protein 14B | 1 | | 2057 | hsa-miR-10a | 666 | BOK | BCL2-related ovarian killer | 1 | | 2058 | hsa-mir-3130-1 | 6369 | CCL24 | chemokine (C-C motif) ligand 24 | 1 | | 2059 | hsa-mir-3130-2 | 6369 | CCL24 | chemokine (C-C motif) ligand 24 | 1 | | 2060 | hsa-mir-3130-3 | 6369 | CCL24 | chemokine (C-C motif) ligand 24 | 1 | | 2061 | hsa-mir-3130-4 | 6369 | CCL24 | chemokine (C-C motif) ligand 24 | 1 | | 2062 | hsa-miR-10a | 6369 | CCL24 | chemokine (C-C motif) ligand 24 | 1 | | 2063 | hsa-miR-128 | 6369 | CCL24 | chemokine (C-C motif) ligand 24 | 1 | | 2064 | hsa-miR-95 | 6369 | CCL24 | chemokine (C-C motif) ligand 24 | 1 | | 2065 | hsa-miR-1233 | 51596 | CUTA | cutA divalent cation tolerance homolog (E. coli) | 1 | | 2066 | hsa-miR-766 | 51596 | CUTA | cutA divalent cation tolerance homolog (E. coli) | 1 | | 2067 | hsa-miR-874 | 51596 | CUTA | cutA divalent cation tolerance homolog (E. coli) | 1 | | 2068 | hsa-miR-555 | 8562 | DENR | density-regulated protein | 1 | | 2069 | hsa-miR-558 | 8562 | DENR | density-regulated protein | 1 | | 2070 | hsa-mir-10a | 9440 | MED17 | mediator complex subunit 17 | 1 | | 2071 | hsa-miR-1224-5p | 9440 | MED17 | mediator complex subunit 17 | 1 | | 2072 | hsa-miR-1233 | 80895 | ILKAP | integrin-linked kinase-associated serine/threonine phosphatase 2C | 1 | | 2073 | hsa-miR-569 | 80895 | ILKAP | integrin-linked kinase-associated serine/threonine phosphatase 2C | 1 | | 2074 | hsa-miR-766 | 80895 | ILKAP | integrin-linked kinase-associated serine/threonine phosphatase 2C | 1 | | 2075 | hsa-mir-10a | 79003 | MIS12 | MIS12, MIND kinetochore complex component, homolog (S. pombe) | 1 | | 2076 | hsa-miR-1233 | 79003 | MIS12 | MIS12, MIND kinetochore complex component, homolog (S. pombe) | 1 | | 2077 | hsa-miR-571 | 79003 | MIS12 | MIS12, MIND kinetochore complex component, homolog (S. pombe) | 1 | | 2078 | hsa-miR-558 | 55327 | LIN7C | lin-7 homolog C (C. elegans) | 1 | | 2079 | hsa-miR-938 | 7462 | LAT2 | linker for activation of T cells family, member 2 | 1 | | 2080 | hsa-miR-558 | 6160 | RPL31 | ribosomal protein L31 | 1 | | 2081 | hsa-mir-10a | 55863 | TMEM126B | transmembrane protein 126B | 1 | | 2082 | hsa-miR-576-5p | 55863 | TMEM126B | transmembrane protein 126B | 1 | | 2083 | hsa-miR-10a | 29781 | NCAPH2 | non-SMC condensin II complex, subunit H2 | 1 | | 2089 | hsa-miR-877 | 50615 | IL21R | interleukin 21 receptor | 1 | | 2090 | hsa-mir-3130-1 | 83696 | TRAPPC9 | trafficking protein particle complex 9 | 1 | | 2091 | hsa-mir-3130-2 | 83696 | TRAPPC9 | trafficking protein particle complex 9 | 1 | | 2092 | hsa-mir-3130-3 | 83696 | TRAPPC9 | trafficking protein particle complex 9 | 1 | | 2093 | hsa-mir-3130-4 | 83696 | TRAPPC9 | trafficking protein particle complex 9 | 1 | | 2094 | hsa-miR-10a | 83696 | TRAPPC9 | trafficking protein particle complex 9 | 1 | | 2095 | hsa-miR-95 | 83696 | TRAPPC9 | trafficking protein particle complex 9 | 1 | | 2096 | hsa-miR-593\* | 55272 | IMP3 | IMP3, U3 small nucleolar ribonucleoprotein, homolog (yeast) | 1 | | 2097 | hsa-miR-623 | 55272 | IMP3 | IMP3, U3 small nucleolar ribonucleoprotein, homolog (yeast) | 1 | | 2098 | hsa-miR-877 | 55272 | IMP3 | IMP3, U3 small nucleolar ribonucleoprotein, homolog (yeast) | 1 | | 2099 | hsa-miR-623 | 29086 | C19orf62 | chromosome 19 open reading frame 62 | 1 | | 2100 | hsa-miR-877 | 29086 | C19orf62 | chromosome 19 open reading frame 62 | 1 | | 2101 | hsa-miR-877 | 54663 | WDR74 | WD repeat domain 74 | 1 | | 2102 | hsa-miR-423-5p | 54915 | YTHDF1 | YTH domain family, member 1 | 1 | | 2103 | hsa-miR-623 | 10238 | DCAF7 | DDB1 and CUL4 associated factor 7 | 1 | | 2104 | hsa-miR-618 | 54434 | SSH1 | slingshot homolog 1 (Drosophila) | 1 | | 2105 | hsa-miR-766 | 6421 | SFPQ | splicing factor proline/glutamine-rich (polypyrimidine tract binding protein associated) | 1 | | 2106 | hsa-miR-877 | 6421 | SFPQ | splicing factor proline/glutamine-rich (polypyrimidine tract binding protein associated) | 1 | | 2107 | hsa-mir-186 | 58525 | WIZ | widely interspaced zinc finger motifs | 1 | | 2108 | hsa-mir-3130-1 | 58525 | WIZ | widely interspaced zinc finger motifs | 1 | | 2109 | hsa-mir-3130-2 | 58525 | WIZ | widely interspaced zinc finger motifs | 1 | | 2110 | hsa-mir-3130-3 | 58525 | WIZ | widely interspaced zinc finger motifs | 1 | | 2111 | hsa-mir-3130-4 | 58525 | WIZ | widely interspaced zinc finger motifs | 1 | | 2112 | hsa-miR-10a | 58525 | WIZ | widely interspaced zinc finger motifs | 1 | | 2113 | hsa-miR-128 | 58525 | WIZ | widely interspaced zinc finger motifs | 1 | | 2114 | hsa-miR-186 | 58525 | WIZ | widely interspaced zinc finger motifs | 1 | | 2115 | hsa-miR-95 | 58525 | WIZ | widely interspaced zinc finger motifs | 1 | | 2116 | hsa-miR-1233 | 90806 | ANGEL2 | angel homolog 2 (Drosophila) | 1 | | 2117 | hsa-miR-423-5p | 90806 | ANGEL2 | angel homolog 2 (Drosophila) | 1 | | 2118 | hsa-miR-555 | 90806 | ANGEL2 | angel homolog 2 (Drosophila) | 1 | | 2119 | hsa-miR-558 | 90806 | ANGEL2 | angel homolog 2 (Drosophila) | 1 | | 2120 | hsa-miR-571 | 90806 | ANGEL2 | angel homolog 2 (Drosophila) | 1 | | 2121 | hsa-mir-10a | 90806 | ANGEL2 | angel homolog 2 (Drosophila) | 1 | | 2122 | hsa-miR-766 | 5911 | RAP2A | RAP2A, member of RAS oncogene family | 1 | | 2123 | hsa-mir-628 | 155060 | ZNF783 | zinc finger family member 783 | 1 | | 2124 | hsa-miR-628-5p | 155060 | ZNF783 | zinc finger family member 783 | 1 | | 2125 | hsa-miR-454\* | 4869 | NPM1 | nucleophosmin (nucleolar phosphoprotein B23, numatrin) | 1 | | 2132 | hsa-miR-126\* | 4034 | LRCH4 | leucine-rich repeats and calponin homology (CH) domain containing 4 | 1 | | 2133 | hsa-miR-877 | 6134 | RPL10 | ribosomal protein L10 | 1 | | 2134 | hsa-mir-3130-1 | 1297 | COL9A1 | collagen, type IX, alpha 1 | 1 | | 2135 | hsa-mir-3130-2 | 1297 | COL9A1 | collagen, type IX, alpha 1 | 1 | | 2136 | hsa-mir-3130-3 | 1297 | COL9A1 | collagen, type IX, alpha 1 | 1 | | 2137 | hsa-mir-3130-4 | 1297 | COL9A1 | collagen, type IX, alpha 1 | 1 | | 2138 | hsa-miR-555 | 25821 | MTO1 | mitochondrial translation optimization 1 homolog (S. cerevisiae) | 1 | | 2139 | hsa-miR-558 | 25821 | MTO1 | mitochondrial translation optimization 1 homolog (S. cerevisiae) | 1 | | 2140 | hsa-mir-3130-1 | 50863 | NTM | neurotrimin | 1 | | 2141 | hsa-mir-3130-2 | 50863 | NTM | neurotrimin | 1 | | 2142 | hsa-mir-3130-3 | 50863 | NTM | neurotrimin | 1 | | 2143 | hsa-mir-3130-4 | 50863 | NTM | neurotrimin | 1 | | 2144 | hsa-miR-95 | 50863 | NTM | neurotrimin | 1 | | 2145 | hsa-miR-1233 | 5935 | RBM3 | RNA binding motif (RNP1, RRM) protein 3 | 1 | | 2146 | hsa-mir-10a | 7596 | ZNF45 | zinc finger protein 45 | 1 | | 2147 | hsa-miR-1233 | 7596 | ZNF45 | zinc finger protein 45 | 1 | | 2148 | hsa-miR-423-5p | 7596 | ZNF45 | zinc finger protein 45 | 1 | | 2149 | hsa-miR-555 | 7596 | ZNF45 | zinc finger protein 45 | 1 | | 2150 | hsa-miR-558 | 7596 | ZNF45 | zinc finger protein 45 | 1 | | 2151 | hsa-miR-571 | 7596 | ZNF45 | zinc finger protein 45 | 1 | | 2153 | hsa-miR-558 | 26065 | LSM14A | LSM14A, SCD6 homolog A (S. cerevisiae) | 1 | | 2154 | hsa-miR-1233 | 57665 | RDH14 | retinol dehydrogenase 14 (all-trans/9-cis/11-cis) | 1 | | 2155 | hsa-miR-558 | 57665 | RDH14 | retinol dehydrogenase 14 (all-trans/9-cis/11-cis) | 1 | | 2156 | hsa-miR-571 | 57665 | RDH14 | retinol dehydrogenase 14 (all-trans/9-cis/11-cis) | 1 | | 2157 | hsa-miR-874 | 57665 | RDH14 | retinol dehydrogenase 14 (all-trans/9-cis/11-cis) | 1 | | 2158 | hsa-mir-10a | 65084 | TMEM135 | transmembrane protein 135 | 1 | | 2159 | hsa-miR-423-5p | 65084 | TMEM135 | transmembrane protein 135 | 1 | | 2160 | hsa-miR-576-5p | 65084 | TMEM135 | transmembrane protein 135 | 1 | | 2161 | hsa-miR-877 | 63875 | MRPL17 | mitochondrial ribosomal protein L17 | 1 | | 2162 | hsa-miR-555 | 64895 | PAPOLG | poly(A) polymerase gamma | 1 | | 2163 | hsa-miR-558 | 64895 | PAPOLG | poly(A) polymerase gamma | 1 | | 2164 | hsa-miR-1233 | 285830 | RP3-377H14.5 | hypothetical LOC285830 | 1 | | 2165 | hsa-miR-874 | 285830 | RP3-377H14.5 | hypothetical LOC285830 | 1 | | 2166 | hsa-mir-10a | 57466 | SFRS15 | splicing factor, arginine/serine-rich 15 | 1 | | 2169 | hsa-miR-571 | 57466 | SFRS15 | splicing factor, arginine/serine-rich 15 | 1 | | 2170 | hsa-mir-10a | 23788 | MTCH2 | mitochondrial carrier homolog 2 (C. elegans) | 1 | | 2171 | hsa-miR-766 | 27131 | SNX5 | sorting nexin 5 | 1 | | 2172 | hsa-miR-618 | 57003 | CCDC47 | coiled-coil domain containing 47 | 1 | | 2173 | hsa-miR-618 | 55207 | ARL8B | ADP-ribosylation factor-like 8B | 1 | | 2174 | hsa-miR-593\* | 56993 | TOMM22 | translocase of outer mitochondrial membrane 22 homolog (yeast) | 1 | | 2175 | hsa-miR-623 | 56993 | TOMM22 | translocase of outer mitochondrial membrane 22 homolog (yeast) | 1 | | 2176 | hsa-miR-766 | 56993 | TOMM22 | translocase of outer mitochondrial membrane 22 homolog (yeast) | 1 | | 2177 | hsa-miR-877 | 56993 | TOMM22 | translocase of outer mitochondrial membrane 22 homolog (yeast) | 1 | | 2178 | hsa-miR-1233 | 26515 | FXC1 | fracture callus 1 homolog (rat) | 1 | | 2179 | hsa-miR-558 | 26515 | FXC1 | fracture callus 1 homolog (rat) | 1 | | 2180 | hsa-miR-571 | 26515 | FXC1 | fracture callus 1 homolog (rat) | 1 | | 2181 | hsa-miR-874 | 26515 | FXC1 | fracture callus 1 homolog (rat) | 1 | | 2182 | hsa-miR-454\* | 51068 | NMD3 | NMD3 homolog (S. cerevisiae) | 1 | | 2184 | hsa-mir-10a | 51026 | GOLT1B | golgi transport 1 homolog B (S. cerevisiae) | 1 | | 2185 | hsa-miR-423-5p | 51026 | GOLT1B | golgi transport 1 homolog B (S. cerevisiae) | 1 | | 2186 | hsa-miR-454\* | 51026 | GOLT1B | golgi transport 1 homolog B (S. cerevisiae) | 1 | | 2187 | hsa-miR-576-5p | 51026 | GOLT1B | golgi transport 1 homolog B (S. cerevisiae) | 1 | | 2188 | hsa-miR-423-5p | 55758 | RCOR3 | REST corepressor 3 | 1 | | 2189 | hsa-miR-1236 | 51013 | EXOSC1 | exosome component 1 | 1 | | 2190 | hsa-mir-10a | 51193 | ZNF639 | zinc finger protein 639 | 1 | | 2192 | hsa-miR-571 | 51193 | ZNF639 | zinc finger protein 639 | 1 | | 2193 | hsa-mir-149 | 51193 | ZNF639 | zinc finger protein 639 | 1 | | 2194 | hsa-miR-149 | 51193 | ZNF639 | zinc finger protein 639 | 1 | | 2195 | hsa-miR-454\* | 51193 | ZNF639 | zinc finger protein 639 | 1 | | 2196 | hsa-miR-558 | 51193 | ZNF639 | zinc finger protein 639 | 1 | | 2197 | hsa-miR-423-5p | 64864 | RFX7 | regulatory factor X, 7 | 1 | | 2198 | hsa-miR-558 | 64864 | RFX7 | regulatory factor X, 7 | 1 | | 2199 | hsa-miR-874 | 64864 | RFX7 | regulatory factor X, 7 | 1 | | 2200 | hsa-miR-95 | 125058 | TBC1D16 | TBC1 domain family, member 16 | 1 | | 2201 | hsa-miR-423-5p | 55109 | AGGF1 | angiogenic factor with G patch and FHA domains 1 | 1 | | 2202 | hsa-miR-576-5p | 55109 | AGGF1 | angiogenic factor with G patch and FHA domains 1 | 1 | | 2203 | hsa-miR-618 | 79660 | PPP1R3B | protein phosphatase 1, regulatory (inhibitor) subunit 3B | 1 | | 2204 | hsa-miR-571 | 55781 | RIOK2 | RIO kinase 2 (yeast) | 1 | | 2205 | hsa-miR-454\* | 54801 | HAUS6 | HAUS augmin-like complex, subunit 6 | 1 | | 2206 | hsa-miR-558 | 54801 | HAUS6 | HAUS augmin-like complex, subunit 6 | 1 | | 2208 | hsa-miR-1233 | 8313 | AXIN2 | axin 2 | 1 | | 2209 | hsa-miR-571 | 8313 | AXIN2 | axin 2 | 1 | | 2210 | hsa-miR-874 | 8313 | AXIN2 | axin 2 | 1 | | 2211 | hsa-mir-10a | 11276 | SYNRG | synergin, gamma | 1 | | 2212 | hsa-miR-423-5p | 11276 | SYNRG | synergin, gamma | 1 | | 2213 | hsa-mir-3130-1 | 64093 | SMOC1 | SPARC related modular calcium binding 1 | 1 | | 2214 | hsa-mir-3130-2 | 64093 | SMOC1 | SPARC related modular calcium binding 1 | 1 | | 2215 | hsa-mir-3130-3 | 64093 | SMOC1 | SPARC related modular calcium binding 1 | 1 | | 2216 | hsa-mir-3130-4 | 64093 | SMOC1 | SPARC related modular calcium binding 1 | 1 | | 2217 | hsa-miR-10a | 64093 | SMOC1 | SPARC related modular calcium binding 1 | 1 | | 2218 | hsa-miR-95 | 64093 | SMOC1 | SPARC related modular calcium binding 1 | 1 | | 2219 | hsa-miR-766 | 54664 | TMEM106B | transmembrane protein 106B | 1 | | 2220 | hsa-miR-558 | 64940 | STAG3L4 | stromal antigen 3-like 4 | 1 | | 2221 | hsa-miR-571 | 64940 | STAG3L4 | stromal antigen 3-like 4 | 1 | | 2222 | hsa-mir-10a | 55783 | FTSJD1 | FtsJ methyltransferase domain containing 1 | 1 | | 2223 | hsa-miR-423-5p | 55783 | FTSJD1 | FtsJ methyltransferase domain containing 1 | 1 | | 2224 | hsa-miR-555 | 55783 | FTSJD1 | FtsJ methyltransferase domain containing 1 | 1 | | 2225 | hsa-miR-558 | 55783 | FTSJD1 | FtsJ methyltransferase domain containing 1 | 1 | | 2226 | hsa-mir-149 | 51633 | OTUD6B | OTU domain containing 6B | 1 | | 2227 | hsa-miR-149 | 51633 | OTUD6B | OTU domain containing 6B | 1 | | 2228 | hsa-miR-555 | 51633 | OTUD6B | OTU domain containing 6B | 1 | | 2229 | hsa-miR-558 | 51633 | OTUD6B | OTU domain containing 6B | 1 | | 2230 | hsa-miR-608 | 79906 | MORN1 | MORN repeat containing 1 | 1 | | 2231 | hsa-miR-95 | 51725 | FBXO40 | F-box protein 40 | 1 | | 2232 | hsa-miR-1233 | 81669 | CCNL2 | cyclin L2 | 1 | | 2233 | hsa-miR-569 | 81669 | CCNL2 | cyclin L2 | 1 | | 2234 | hsa-miR-766 | 81669 | CCNL2 | cyclin L2 | 1 | | 2235 | hsa-miR-874 | 81669 | CCNL2 | cyclin L2 | 1 | | 2237 | hsa-miR-569 | 23731 | C9orf5 | chromosome 9 open reading frame 5 | 1 | | 2239 | hsa-miR-454\* | 23731 | C9orf5 | chromosome 9 open reading frame 5 | 1 | | 2240 | hsa-miR-1233 | 83939 | EIF2A | eukaryotic translation initiation factor 2A, 65kDa | 1 | | 2241 | hsa-miR-558 | 83939 | EIF2A | eukaryotic translation initiation factor 2A, 65kDa | 1 | | 2242 | hsa-miR-874 | 83939 | EIF2A | eukaryotic translation initiation factor 2A, 65kDa | 1 | | 2243 | hsa-miR-1233 | 28987 | NOB1 | NIN1/RPN12 binding protein 1 homolog (S. cerevisiae) | 1 | | 2244 | hsa-miR-766 | 28987 | NOB1 | NIN1/RPN12 binding protein 1 homolog (S. cerevisiae) | 1 | | 2245 | hsa-miR-874 | 28987 | NOB1 | NIN1/RPN12 binding protein 1 homolog (S. cerevisiae) | 1 | | 2246 | hsa-miR-766 | 51126 | NAA20 | N(alpha)-acetyltransferase 20, NatB catalytic subunit | 1 | | 2247 | hsa-miR-1233 | 65991 | FUNDC2 | FUN14 domain containing 2 | 1 | | 2248 | hsa-miR-569 | 65991 | FUNDC2 | FUN14 domain containing 2 | 1 | | 2249 | hsa-miR-766 | 65991 | FUNDC2 | FUN14 domain containing 2 | 1 | | 2250 | hsa-miR-877 | 65991 | FUNDC2 | FUN14 domain containing 2 | 1 | | 2251 | hsa-miR-558 | 55862 | ECHDC1 | enoyl Coenzyme A hydratase domain containing 1 | 1 | | 2252 | hsa-mir-10a | 55591 | VEZT | vezatin, adherens junctions transmembrane protein | 1 | | 2254 | hsa-miR-454\* | 55591 | VEZT | vezatin, adherens junctions transmembrane protein | 1 | | 2257 | hsa-miR-1233 | 51602 | NOP58 | NOP58 ribonucleoprotein homolog (yeast) | 1 | | 2258 | hsa-miR-558 | 51602 | NOP58 | NOP58 ribonucleoprotein homolog (yeast) | 1 | | 2259 | hsa-miR-874 | 51602 | NOP58 | NOP58 ribonucleoprotein homolog (yeast) | 1 | | 2260 | hsa-miR-10a | 9564 | BCAR1 | breast cancer anti-estrogen resistance 1 | 1 | | 2261 | hsa-miR-423-5p | 170506 | DHX36 | DEAH (Asp-Glu-Ala-His) box polypeptide 36 | 1 | | 2262 | hsa-miR-618 | 55122 | AKIRIN2 | akirin 2 | 1 | | 2263 | hsa-miR-1233 | 56916 | SMARCAD1 | SWI/SNF-related, matrix-associated actin-dependent regulator of chromatin, subfamily a, containing DEAD/H box 1 | 1 | | 2264 | hsa-miR-874 | 56916 | SMARCAD1 | SWI/SNF-related, matrix-associated actin-dependent regulator of chromatin, subfamily a, containing DEAD/H box 1 | 1 | | 2265 | hsa-miR-593\* | 29085 | PHPT1 | phosphohistidine phosphatase 1 | 1 | | 2266 | hsa-miR-623 | 29085 | PHPT1 | phosphohistidine phosphatase 1 | 1 | | 2267 | hsa-miR-877 | 29085 | PHPT1 | phosphohistidine phosphatase 1 | 1 | | 2268 | hsa-miR-1233 | 26061 | HACL1 | 2-hydroxyacyl-CoA lyase 1 | 1 | | 2269 | hsa-miR-558 | 26061 | HACL1 | 2-hydroxyacyl-CoA lyase 1 | 1 | | 2270 | hsa-miR-571 | 26061 | HACL1 | 2-hydroxyacyl-CoA lyase 1 | 1 | | 2271 | hsa-miR-874 | 26061 | HACL1 | 2-hydroxyacyl-CoA lyase 1 | 1 | | 2272 | hsa-miR-423-5p | 11244 | ZHX1 | zinc fingers and homeoboxes 1 | 1 | | 2276 | hsa-miR-1233 | 583 | BBS2 | Bardet-Biedl syndrome 2 | 1 | | 2277 | hsa-miR-423-5p | 583 | BBS2 | Bardet-Biedl syndrome 2 | 1 | | 2278 | hsa-miR-571 | 583 | BBS2 | Bardet-Biedl syndrome 2 | 1 | | 2279 | hsa-miR-766 | 583 | BBS2 | Bardet-Biedl syndrome 2 | 1 | | 2280 | hsa-miR-874 | 583 | BBS2 | Bardet-Biedl syndrome 2 | 1 | | 2281 | hsa-mir-10a | 84081 | CCDC55 | coiled-coil domain containing 55 | 1 | | 2282 | hsa-mir-199a-2 | 84081 | CCDC55 | coiled-coil domain containing 55 | 1 | | 2283 | hsa-mir-214 | 84081 | CCDC55 | coiled-coil domain containing 55 | 1 | | 2284 | hsa-miR-423-5p | 84081 | CCDC55 | coiled-coil domain containing 55 | 1 | | 2285 | hsa-miR-576-5p | 84081 | CCDC55 | coiled-coil domain containing 55 | 1 | | 2286 | hsa-miR-423-5p | 55193 | PBRM1 | polybromo 1 | 1 | | 2287 | hsa-mir-149 | 55975 | KLHL7 | kelch-like 7 (Drosophila) | 1 | | 2288 | hsa-miR-149 | 55975 | KLHL7 | kelch-like 7 (Drosophila) | 1 | | 2289 | hsa-miR-423-5p | 55975 | KLHL7 | kelch-like 7 (Drosophila) | 1 | | 2290 | hsa-miR-1233 | 55608 | ANKRD10 | ankyrin repeat domain 10 | 1 | | 2291 | hsa-miR-558 | 55608 | ANKRD10 | ankyrin repeat domain 10 | 1 | | 2296 | hsa-miR-423-5p | 55632 | G2E3 | G2/M-phase specific E3 ubiquitin ligase | 1 | | 2297 | hsa-miR-576-5p | 55632 | G2E3 | G2/M-phase specific E3 ubiquitin ligase | 1 | | 2298 | hsa-miR-938 | 80851 | SH3BP5L | SH3-binding domain protein 5-like | 1 | | 2299 | hsa-miR-555 | 28970 | C11orf54 | chromosome 11 open reading frame 54 | 1 | | 2300 | hsa-miR-766 | 55170 | PRMT6 | protein arginine methyltransferase 6 | 1 | | 2301 | hsa-miR-877 | 23587 | C17orf81 | chromosome 17 open reading frame 81 | 1 | | 2302 | hsa-miR-454\* | 51260 | CXorf26 | chromosome X open reading frame 26 | 1 | | 2303 | hsa-miR-1233 | 55692 | LUC7L | LUC7-like (S. cerevisiae) | 1 | | 2304 | hsa-miR-571 | 55692 | LUC7L | LUC7-like (S. cerevisiae) | 1 | | 2305 | hsa-miR-766 | 55692 | LUC7L | LUC7-like (S. cerevisiae) | 1 | | 2306 | hsa-miR-874 | 55692 | LUC7L | LUC7-like (S. cerevisiae) | 1 | | 2307 | hsa-miR-618 | 84255 | SLC37A3 | solute carrier family 37 (glycerol-3-phosphate transporter), member 3 | 1 | | 2308 | hsa-miR-766 | 54822 | TRPM7 | transient receptor potential cation channel, subfamily M, member 7 | 1 | | 2309 | hsa-miR-555 | 51249 | TMEM69 | transmembrane protein 69 | 1 | | 2311 | hsa-miR-555 | 50484 | RRM2B | ribonucleotide reductase M2 B (TP53 inducible) | 1 | | 2313 | hsa-miR-10a | 54566 | EPB41L4B | erythrocyte membrane protein band 4.1 like 4B | 1 | | 2314 | hsa-miR-766 | 201283 | FLJ32065 | hypothetical protein FLJ32065 | 1 | | 2315 | hsa-miR-1233 | 26272 | FBXO4 | F-box protein 4 | 1 | | 2316 | hsa-miR-571 | 26272 | FBXO4 | F-box protein 4 | 1 | | 2317 | hsa-miR-874 | 26272 | FBXO4 | F-box protein 4 | 1 | | 2318 | hsa-miR-1233 | 84524 | ZC3H8 | zinc finger CCCH-type containing 8 | 1 | | 2319 | hsa-miR-571 | 84524 | ZC3H8 | zinc finger CCCH-type containing 8 | 1 | | 2320 | hsa-miR-766 | 84524 | ZC3H8 | zinc finger CCCH-type containing 8 | 1 | | 2321 | hsa-miR-874 | 84524 | ZC3H8 | zinc finger CCCH-type containing 8 | 1 | | 2322 | hsa-miR-95 | 81029 | WNT5B | wingless-type MMTV integration site family, member 5B | 1 | | 2323 | hsa-miR-938 | 84067 | FAM160A2 | family with sequence similarity 160, member A2 | 1 | | 2324 | hsa-miR-766 | 10207 | INADL | InaD-like (Drosophila) | 1 | | 2325 | hsa-miR-618 | 53831 | GPR84 | G protein-coupled receptor 84 | 1 | | 2326 | hsa-miR-1233 | 85028 | SNHG12 | small nucleolar RNA host gene 12 (non-protein coding) | 1 | | 2327 | hsa-miR-558 | 85028 | SNHG12 | small nucleolar RNA host gene 12 (non-protein coding) | 1 | | 2328 | hsa-miR-571 | 85028 | SNHG12 | small nucleolar RNA host gene 12 (non-protein coding) | 1 | | 2329 | hsa-miR-766 | 85028 | SNHG12 | small nucleolar RNA host gene 12 (non-protein coding) | 1 | | 2330 | hsa-miR-874 | 85028 | SNHG12 | small nucleolar RNA host gene 12 (non-protein coding) | 1 | | 2331 | hsa-miR-623 | 85028 | SNHG12 | small nucleolar RNA host gene 12 (non-protein coding) | 1 | | 2333 | hsa-miR-558 | 83636 | C19orf12 | chromosome 19 open reading frame 12 | 1 | | 2334 | hsa-miR-569 | 83636 | C19orf12 | chromosome 19 open reading frame 12 | 1 | | 2335 | hsa-miR-571 | 83636 | C19orf12 | chromosome 19 open reading frame 12 | 1 | | 2337 | hsa-miR-874 | 83636 | C19orf12 | chromosome 19 open reading frame 12 | 1 | | 2338 | hsa-miR-1233 | 56477 | CCL28 | chemokine (C-C motif) ligand 28 | 1 | | 2339 | hsa-miR-766 | 56477 | CCL28 | chemokine (C-C motif) ligand 28 | 1 | | 2340 | hsa-miR-874 | 56477 | CCL28 | chemokine (C-C motif) ligand 28 | 1 | | 2341 | hsa-miR-10a | 84070 | FAM186B | family with sequence similarity 186, member B | 1 | | 2342 | hsa-mir-3130-1 | 83697 | SLC4A9 | solute carrier family 4, sodium bicarbonate cotransporter, member 9 | 1 | | 2343 | hsa-mir-3130-2 | 83697 | SLC4A9 | solute carrier family 4, sodium bicarbonate cotransporter, member 9 | 1 | | 2344 | hsa-mir-3130-3 | 83697 | SLC4A9 | solute carrier family 4, sodium bicarbonate cotransporter, member 9 | 1 | | 2345 | hsa-mir-3130-4 | 83697 | SLC4A9 | solute carrier family 4, sodium bicarbonate cotransporter, member 9 | 1 | | 2346 | hsa-miR-10a | 83697 | SLC4A9 | solute carrier family 4, sodium bicarbonate cotransporter, member 9 | 1 | | 2347 | hsa-miR-95 | 83697 | SLC4A9 | solute carrier family 4, sodium bicarbonate cotransporter, member 9 | 1 | | 2348 | hsa-miR-608 | 9622 | KLK4 | kallikrein-related peptidase 4 | 1 | | 2349 | hsa-miR-95 | 84757 | MGC10814 | hypothetical protein MGC10814 | 1 | | 2353 | hsa-miR-874 | 26355 | FAM162A | family with sequence similarity 162, member A | 1 | | 2354 | hsa-miR-618 | 22931 | RAB18 | RAB18, member RAS oncogene family | 1 | | 2356 | hsa-miR-423-5p | 25831 | HECTD1 | HECT domain containing 1 | 1 | | 2357 | hsa-miR-423-5p | 199692 | ZNF627 | zinc finger protein 627 | 1 | | 2358 | hsa-miR-576-5p | 199692 | ZNF627 | zinc finger protein 627 | 1 | | 2359 | hsa-miR-874 | 56893 | UBQLN4 | ubiquilin 4 | 1 | | 2360 | hsa-miR-938 | 7077 | TIMP2 | TIMP metallopeptidase inhibitor 2 | 1 | | 2361 | hsa-miR-593\* | 55920 | RCC2 | regulator of chromosome condensation 2 | 1 | | 2362 | hsa-miR-623 | 55920 | RCC2 | regulator of chromosome condensation 2 | 1 | | 2363 | hsa-miR-766 | 55920 | RCC2 | regulator of chromosome condensation 2 | 1 | | 2364 | hsa-miR-877 | 55920 | RCC2 | regulator of chromosome condensation 2 | 1 | | 2365 | hsa-miR-558 | 50809 | HP1BP3 | heterochromatin protein 1, binding protein 3 | 1 | | 2366 | hsa-miR-766 | 6730 | SRP68 | signal recognition particle 68kDa | 1 | | 2367 | hsa-miR-938 | 57533 | TBC1D14 | TBC1 domain family, member 14 | 1 | | 2368 | hsa-mir-10a | 84248 | FYTTD1 | forty-two-three domain containing 1 | 1 | | 2369 | hsa-miR-423-5p | 84248 | FYTTD1 | forty-two-three domain containing 1 | 1 | | 2370 | hsa-miR-1233 | 57488 | ESYT2 | extended synaptotagmin-like protein 2 | 1 | | 2371 | hsa-miR-569 | 57488 | ESYT2 | extended synaptotagmin-like protein 2 | 1 | | 2372 | hsa-miR-766 | 57488 | ESYT2 | extended synaptotagmin-like protein 2 | 1 | | 2373 | hsa-miR-877 | 57488 | ESYT2 | extended synaptotagmin-like protein 2 | 1 | | 2374 | hsa-miR-623 | 113246 | C12orf57 | chromosome 12 open reading frame 57 | 1 | | 2375 | hsa-miR-766 | 113246 | C12orf57 | chromosome 12 open reading frame 57 | 1 | | 2376 | hsa-miR-877 | 113246 | C12orf57 | chromosome 12 open reading frame 57 | 1 | | 2377 | hsa-mir-10a | 57534 | MIB1 | mindbomb homolog 1 (Drosophila) | 1 | | 2380 | hsa-miR-1233 | 84128 | WDR75 | WD repeat domain 75 | 1 | | 2381 | hsa-miR-558 | 84128 | WDR75 | WD repeat domain 75 | 1 | | 2382 | hsa-miR-569 | 84128 | WDR75 | WD repeat domain 75 | 1 | | 2383 | hsa-miR-571 | 84128 | WDR75 | WD repeat domain 75 | 1 | | 2384 | hsa-miR-766 | 84128 | WDR75 | WD repeat domain 75 | 1 | | 2385 | hsa-miR-874 | 84128 | WDR75 | WD repeat domain 75 | 1 | | 2386 | hsa-miR-555 | 57534 | MIB1 | mindbomb homolog 1 (Drosophila) | 1 | | 2387 | hsa-mir-10a | 64756 | ATPAF1 | ATP synthase mitochondrial F1 complex assembly factor 1 | 1 | | 2388 | hsa-miR-423-5p | 64756 | ATPAF1 | ATP synthase mitochondrial F1 complex assembly factor 1 | 1 | | 2389 | hsa-miR-571 | 64756 | ATPAF1 | ATP synthase mitochondrial F1 complex assembly factor 1 | 1 | | 2390 | hsa-miR-938 | 415116 | PIM3 | pim-3 oncogene | 1 | | 2391 | hsa-mir-135b | 54928 | IMPAD1 | inositol monophosphatase domain containing 1 | 1 | | 2392 | hsa-miR-1233 | 56889 | TM9SF3 | transmembrane 9 superfamily member 3 | 1 | | 2393 | hsa-miR-423-5p | 56889 | TM9SF3 | transmembrane 9 superfamily member 3 | 1 | | 2394 | hsa-miR-571 | 56889 | TM9SF3 | transmembrane 9 superfamily member 3 | 1 | | 2395 | hsa-miR-766 | 56889 | TM9SF3 | transmembrane 9 superfamily member 3 | 1 | | 2396 | hsa-miR-874 | 56889 | TM9SF3 | transmembrane 9 superfamily member 3 | 1 | | 2397 | hsa-miR-1233 | 90488 | C12orf23 | chromosome 12 open reading frame 23 | 1 | | 2398 | hsa-miR-569 | 90488 | C12orf23 | chromosome 12 open reading frame 23 | 1 | | 2399 | hsa-miR-571 | 90488 | C12orf23 | chromosome 12 open reading frame 23 | 1 | | 2400 | hsa-miR-874 | 90488 | C12orf23 | chromosome 12 open reading frame 23 | 1 | | 2401 | hsa-miR-1233 | 6167 | RPL37 | ribosomal protein L37 | 1 | | 2402 | hsa-miR-571 | 6167 | RPL37 | ribosomal protein L37 | 1 | | 2403 | hsa-miR-874 | 6167 | RPL37 | ribosomal protein L37 | 1 | | 2404 | hsa-miR-423-5p | 55677 | IWS1 | IWS1 homolog (S. cerevisiae) | 1 | | 2405 | hsa-miR-576-5p | 55677 | IWS1 | IWS1 homolog (S. cerevisiae) | 1 | | 2406 | hsa-miR-1231 | 89796 | NAV1 | neuron navigator 1 | 1 | | 2407 | hsa-miR-454\* | 84991 | RBM17 | RNA binding motif protein 17 | 1 | | 2408 | hsa-miR-423-5p | 60592 | SCOC | short coiled-coil protein | 1 | | 2409 | hsa-miR-576-5p | 60592 | SCOC | short coiled-coil protein | 1 | | 2410 | hsa-miR-618 | 50807 | ASAP1 | ArfGAP with SH3 domain, ankyrin repeat and PH domain 1 | 1 | | 2411 | hsa-miR-95 | 57184 | C15orf17 | chromosome 15 open reading frame 17 | 1 | | 2412 | hsa-miR-454\* | 55761 | TTC17 | tetratricopeptide repeat domain 17 | 1 | | 2413 | hsa-miR-618 | 2289 | FKBP5 | FK506 binding protein 5 | 1 | | 2414 | hsa-miR-571 | 90871 | C9orf123 | chromosome 9 open reading frame 123 | 1 | | 2415 | hsa-miR-618 | 84188 | FAR1 | fatty acyl CoA reductase 1 | 1 | | 2416 | hsa-miR-569 | 56061 | UBFD1 | ubiquitin family domain containing 1 | 1 | | 2417 | hsa-miR-766 | 56061 | UBFD1 | ubiquitin family domain containing 1 | 1 | | 2418 | hsa-miR-877 | 56061 | UBFD1 | ubiquitin family domain containing 1 | 1 | | 2419 | hsa-miR-503 | 6130 | RPL7A | ribosomal protein L7a | 1 | | 2420 | hsa-miR-555 | 57532 | NUFIP2 | nuclear fragile X mental retardation protein interacting protein 2 | 1 | | 2421 | hsa-miR-576-5p | 57532 | NUFIP2 | nuclear fragile X mental retardation protein interacting protein 2 | 1 | | 2422 | hsa-miR-593\* | 64951 | MRPS24 | mitochondrial ribosomal protein S24 | 1 | | 2423 | hsa-miR-877 | 64951 | MRPS24 | mitochondrial ribosomal protein S24 | 1 | | 2424 | hsa-miR-454\* | 1836 | SLC26A2 | solute carrier family 26 (sulfate transporter), member 2 | 1 | | 2425 | hsa-miR-571 | 55205 | ZNF532 | zinc finger protein 532 | 1 | | 2426 | hsa-miR-571 | 550643 | LOC550643 | hypothetical LOC550643 | 1 | | 2427 | hsa-miR-874 | 550643 | LOC550643 | hypothetical LOC550643 | 1 | | 2428 | hsa-miR-1233 | 550643 | LOC550643 | hypothetical LOC550643 | 1 | | 2429 | hsa-miR-569 | 550643 | LOC550643 | hypothetical LOC550643 | 1 | | 2430 | hsa-miR-766 | 550643 | LOC550643 | hypothetical LOC550643 | 1 | | 2431 | hsa-mir-10a | 84450 | ZNF512 | zinc finger protein 512 | 1 | | 2432 | hsa-mir-199a-2 | 84450 | ZNF512 | zinc finger protein 512 | 1 | | 2433 | hsa-mir-214 | 84450 | ZNF512 | zinc finger protein 512 | 1 | | 2434 | hsa-miR-1233 | 84450 | ZNF512 | zinc finger protein 512 | 1 | | 2435 | hsa-miR-423-5p | 84450 | ZNF512 | zinc finger protein 512 | 1 | | 2436 | hsa-miR-558 | 29883 | CNOT7 | CCR4-NOT transcription complex, subunit 7 | 1 | | 2437 | hsa-miR-874 | 29883 | CNOT7 | CCR4-NOT transcription complex, subunit 7 | 1 | | 2439 | hsa-miR-1233 | 55239 | OGFOD1 | 2-oxoglutarate and iron-dependent oxygenase domain containing 1 | 1 | | 2440 | hsa-miR-558 | 55239 | OGFOD1 | 2-oxoglutarate and iron-dependent oxygenase domain containing 1 | 1 | | 2441 | hsa-miR-874 | 55239 | OGFOD1 | 2-oxoglutarate and iron-dependent oxygenase domain containing 1 | 1 | | 2442 | hsa-mir-10a | 63908 | NAPB | N-ethylmaleimide-sensitive factor attachment protein, beta | 1 | | 2443 | hsa-mir-628 | 64949 | MRPS26 | mitochondrial ribosomal protein S26 | 1 | | 2444 | hsa-miR-628-5p | 64949 | MRPS26 | mitochondrial ribosomal protein S26 | 1 | | 2445 | hsa-miR-593\* | 64928 | MRPL14 | mitochondrial ribosomal protein L14 | 1 | | 2446 | hsa-miR-877 | 64928 | MRPL14 | mitochondrial ribosomal protein L14 | 1 | | 2447 | hsa-miR-1233 | 100093630 | SNHG8 | small nucleolar RNA host gene 8 (non-protein coding) | 1 | | 2448 | hsa-miR-569 | 100093630 | SNHG8 | small nucleolar RNA host gene 8 (non-protein coding) | 1 | | 2449 | hsa-miR-571 | 100093630 | SNHG8 | small nucleolar RNA host gene 8 (non-protein coding) | 1 | | 2450 | hsa-miR-766 | 100093630 | SNHG8 | small nucleolar RNA host gene 8 (non-protein coding) | 1 | | 2451 | hsa-miR-874 | 100093630 | SNHG8 | small nucleolar RNA host gene 8 (non-protein coding) | 1 | | 2452 | hsa-miR-877 | 100093630 | SNHG8 | small nucleolar RNA host gene 8 (non-protein coding) | 1 | | 2454 | hsa-mir-10a | 92400 | RBM18 | RNA binding motif protein 18 | 1 | | 2455 | hsa-miR-555 | 92400 | RBM18 | RNA binding motif protein 18 | 1 | | 2456 | hsa-miR-576-5p | 92400 | RBM18 | RNA binding motif protein 18 | 1 | | 2458 | hsa-miR-877 | 124540 | MSI2 | musashi homolog 2 (Drosophila) | 1 | | 2460 | hsa-mir-149 | 51449 | PCYOX1 | prenylcysteine oxidase 1 | 1 | | 2461 | hsa-miR-149 | 51449 | PCYOX1 | prenylcysteine oxidase 1 | 1 | | 2462 | hsa-miR-555 | 51449 | PCYOX1 | prenylcysteine oxidase 1 | 1 | | 2463 | hsa-mir-10a | 25871 | C3orf17 | chromosome 3 open reading frame 17 | 1 | | 2465 | hsa-miR-1233 | 25871 | C3orf17 | chromosome 3 open reading frame 17 | 1 | | 2466 | hsa-miR-555 | 25871 | C3orf17 | chromosome 3 open reading frame 17 | 1 | | 2467 | hsa-miR-558 | 25871 | C3orf17 | chromosome 3 open reading frame 17 | 1 | | 2468 | hsa-miR-1233 | 132299 | OCIAD2 | OCIA domain containing 2 | 1 | | 2469 | hsa-miR-569 | 132299 | OCIAD2 | OCIA domain containing 2 | 1 | | 2470 | hsa-miR-571 | 132299 | OCIAD2 | OCIA domain containing 2 | 1 | | 2471 | hsa-miR-586 | 132299 | OCIAD2 | OCIA domain containing 2 | 1 | | 2472 | hsa-miR-766 | 132299 | OCIAD2 | OCIA domain containing 2 | 1 | | 2473 | hsa-miR-874 | 132299 | OCIAD2 | OCIA domain containing 2 | 1 | | 2474 | hsa-miR-571 | 84320 | ACBD6 | acyl-Coenzyme A binding domain containing 6 | 1 | | 2475 | hsa-miR-423-5p | 119032 | C10orf32 | chromosome 10 open reading frame 32 | 1 | | 2476 | hsa-miR-576-5p | 119032 | C10orf32 | chromosome 10 open reading frame 32 | 1 | | 2477 | hsa-miR-555 | 9043 | SPAG9 | sperm associated antigen 9 | 1 | | 2478 | hsa-miR-576-5p | 9043 | SPAG9 | sperm associated antigen 9 | 1 | | 2479 | hsa-miR-571 | 131118 | DNAJC19 | DnaJ (Hsp40) homolog, subfamily C, member 19 | 1 | | 2480 | hsa-mir-10a | 90416 | C15orf57 | chromosome 15 open reading frame 57 | 1 | | 2482 | hsa-mir-199a-2 | 493753 | C2orf64 | chromosome 2 open reading frame 64 | 1 | | 2483 | hsa-mir-214 | 493753 | C2orf64 | chromosome 2 open reading frame 64 | 1 | | 2484 | hsa-miR-1233 | 493753 | C2orf64 | chromosome 2 open reading frame 64 | 1 | | 2485 | hsa-miR-423-5p | 493753 | C2orf64 | chromosome 2 open reading frame 64 | 1 | | 2486 | hsa-miR-571 | 493753 | C2orf64 | chromosome 2 open reading frame 64 | 1 | | 2487 | hsa-miR-766 | 493753 | C2orf64 | chromosome 2 open reading frame 64 | 1 | | 2488 | hsa-miR-555 | 135293 | PM20D2 | peptidase M20 domain containing 2 | 1 | | 2489 | hsa-miR-423-5p | 2957 | GTF2A1 | general transcription factor IIA, 1, 19/37kDa | 1 | | 2490 | hsa-miR-576-5p | 2957 | GTF2A1 | general transcription factor IIA, 1, 19/37kDa | 1 | | 2491 | hsa-mir-10a | 54014 | BRWD1 | bromodomain and WD repeat domain containing 1 | 1 | | 2492 | hsa-mir-10a | 129401 | NUP35 | nucleoporin 35kDa | 1 | | 2493 | hsa-miR-1233 | 129401 | NUP35 | nucleoporin 35kDa | 1 | | 2494 | hsa-miR-571 | 129401 | NUP35 | nucleoporin 35kDa | 1 | | 2495 | hsa-miR-766 | 129401 | NUP35 | nucleoporin 35kDa | 1 | | 2496 | hsa-miR-874 | 129401 | NUP35 | nucleoporin 35kDa | 1 | | 2497 | hsa-miR-454\* | 116064 | LRRC58 | leucine rich repeat containing 58 | 1 | | 2498 | hsa-miR-555 | 196528 | ARID2 | AT rich interactive domain 2 (ARID, RFX-like) | 1 | | 2499 | hsa-miR-576-5p | 196528 | ARID2 | AT rich interactive domain 2 (ARID, RFX-like) | 1 | | 2504 | hsa-miR-618 | 151987 | PPP4R2 | protein phosphatase 4, regulatory subunit 2 | 1 | | 2506 | hsa-miR-558 | 84129 | ACAD11 | acyl-Coenzyme A dehydrogenase family, member 11 | 1 | | 2507 | hsa-miR-1233 | 130814 | PQLC3 | PQ loop repeat containing 3 | 1 | | 2509 | hsa-miR-558 | 58487 | CREBZF | CREB/ATF bZIP transcription factor | 1 | | 2510 | hsa-miR-423-5p | 124808 | CCDC43 | coiled-coil domain containing 43 | 1 | | 2511 | hsa-miR-877 | 4957 | ODF2 | outer dense fiber of sperm tails 2 | 1 | | 2512 | hsa-miR-454\* | 122060 | SLAIN1 | SLAIN motif family, member 1 | 1 | | 2513 | hsa-miR-618 | 147991 | DPY19L3 | dpy-19-like 3 (C. elegans) | 1 | | 2514 | hsa-miR-555 | 140901 | STK35 | serine/threonine kinase 35 | 1 | | 2515 | hsa-miR-423-5p | 51755 | CDK12 | cyclin-dependent kinase 12 | 1 | | 2516 | hsa-miR-576-5p | 51755 | CDK12 | cyclin-dependent kinase 12 | 1 | | 2517 | hsa-miR-623 | 55588 | MED29 | mediator complex subunit 29 | 1 | | 2518 | hsa-miR-623 | 85378 | TUBGCP6 | tubulin, gamma complex associated protein 6 | 1 | | 2519 | hsa-miR-877 | 85378 | TUBGCP6 | tubulin, gamma complex associated protein 6 | 1 | | 2520 | hsa-mir-10a | 57511 | COG6 | component of oligomeric golgi complex 6 | 1 | | 2521 | hsa-mir-199a-2 | 57511 | COG6 | component of oligomeric golgi complex 6 | 1 | | 2522 | hsa-mir-214 | 57511 | COG6 | component of oligomeric golgi complex 6 | 1 | | 2523 | hsa-miR-423-5p | 57511 | COG6 | component of oligomeric golgi complex 6 | 1 | | 2524 | hsa-miR-576-5p | 57511 | COG6 | component of oligomeric golgi complex 6 | 1 | | 2525 | hsa-miR-555 | 79960 | PHF17 | PHD finger protein 17 | 1 | | 2526 | hsa-miR-593\* | 125988 | C19orf70 | chromosome 19 open reading frame 70 | 1 | | 2527 | hsa-miR-623 | 125988 | C19orf70 | chromosome 19 open reading frame 70 | 1 | | 2528 | hsa-miR-877 | 125988 | C19orf70 | chromosome 19 open reading frame 70 | 1 | | 2529 | hsa-miR-938 | 113402 | SFT2D1 | SFT2 domain containing 1 | 1 | | 2530 | hsa-miR-423-5p | 3658 | IREB2 | iron-responsive element binding protein 2 | 1 | | 2531 | hsa-miR-576-5p | 3658 | IREB2 | iron-responsive element binding protein 2 | 1 | | 2532 | hsa-miR-877 | 128869 | PIGU | phosphatidylinositol glycan anchor biosynthesis, class U | 1 | | 2533 | hsa-miR-423-5p | 9931 | HELZ | helicase with zinc finger | 1 | | 2534 | hsa-miR-555 | 9931 | HELZ | helicase with zinc finger | 1 | | 2535 | hsa-miR-558 | 9931 | HELZ | helicase with zinc finger | 1 | | 2536 | hsa-mir-10a | 57600 | FNIP2 | folliculin interacting protein 2 | 1 | | 2537 | hsa-miR-555 | 57600 | FNIP2 | folliculin interacting protein 2 | 1 | | 2538 | hsa-miR-423-5p | 57600 | FNIP2 | folliculin interacting protein 2 | 1 | | 2540 | hsa-miR-555 | 91408 | BTF3L4 | basic transcription factor 3-like 4 | 1 | | 2541 | hsa-miR-558 | 91408 | BTF3L4 | basic transcription factor 3-like 4 | 1 | | 2543 | hsa-miR-10a | 283987 | C17orf28 | chromosome 17 open reading frame 28 | 1 | | 2544 | hsa-miR-874 | 55605 | KIF21A | kinesin family member 21A | 1 | | 2545 | hsa-miR-1233 | 961 | CD47 | CD47 molecule | 1 | | 2546 | hsa-miR-571 | 961 | CD47 | CD47 molecule | 1 | | 2548 | hsa-miR-874 | 961 | CD47 | CD47 molecule | 1 | | 2549 | hsa-miR-618 | 84925 | DIRC2 | disrupted in renal carcinoma 2 | 1 | | 2550 | hsa-miR-1233 | 36 | ACADSB | acyl-Coenzyme A dehydrogenase, short/branched chain | 1 | | 2551 | hsa-miR-558 | 36 | ACADSB | acyl-Coenzyme A dehydrogenase, short/branched chain | 1 | | 2552 | hsa-miR-569 | 36 | ACADSB | acyl-Coenzyme A dehydrogenase, short/branched chain | 1 | | 2553 | hsa-miR-571 | 36 | ACADSB | acyl-Coenzyme A dehydrogenase, short/branched chain | 1 | | 2554 | hsa-miR-874 | 36 | ACADSB | acyl-Coenzyme A dehydrogenase, short/branched chain | 1 | | 2555 | hsa-miR-1233 | 51616 | TAF9B | TAF9B RNA polymerase II, TATA box binding protein (TBP)-associated factor, 31kDa | 1 | | 2556 | hsa-miR-571 | 51616 | TAF9B | TAF9B RNA polymerase II, TATA box binding protein (TBP)-associated factor, 31kDa | 1 | | 2557 | hsa-miR-874 | 51616 | TAF9B | TAF9B RNA polymerase II, TATA box binding protein (TBP)-associated factor, 31kDa | 1 | | 2558 | hsa-miR-555 | 10818 | FRS2 | fibroblast growth factor receptor substrate 2 | 1 | | 2559 | hsa-miR-558 | 10818 | FRS2 | fibroblast growth factor receptor substrate 2 | 1 | | 2565 | hsa-miR-454\* | 10059 | DNM1L | dynamin 1-like | 1 | | 2566 | hsa-miR-558 | 10059 | DNM1L | dynamin 1-like | 1 | | 2569 | hsa-miR-555 | 57223 | SMEK2 | SMEK homolog 2, suppressor of mek1 (Dictyostelium) | 1 | | 2570 | hsa-miR-558 | 57223 | SMEK2 | SMEK homolog 2, suppressor of mek1 (Dictyostelium) | 1 | | 2571 | hsa-miR-938 | 9489 | PGS1 | phosphatidylglycerophosphate synthase 1 | 1 | | 2572 | hsa-miR-1233 | 11098 | PRSS23 | protease, serine, 23 | 1 | | 2573 | hsa-miR-571 | 11098 | PRSS23 | protease, serine, 23 | 1 | | 2574 | hsa-miR-874 | 11098 | PRSS23 | protease, serine, 23 | 1 | | 2575 | hsa-miR-423-5p | 55814 | BDP1 | B double prime 1, subunit of RNA polymerase III transcription initiation factor IIIB | 1 | | 2576 | hsa-miR-558 | 55814 | BDP1 | B double prime 1, subunit of RNA polymerase III transcription initiation factor IIIB | 1 | | 2578 | hsa-miR-423-5p | 84897 | TBRG1 | transforming growth factor beta regulator 1 | 1 | | 2579 | hsa-miR-1233 | 84333 | PCGF5 | polycomb group ring finger 5 | 1 | | 2580 | hsa-miR-558 | 84333 | PCGF5 | polycomb group ring finger 5 | 1 | | 2581 | hsa-miR-423-5p | 257415 | FAM133B | family with sequence similarity 133, member B | 1 | | 2582 | hsa-mir-10a | 1122 | CHML | choroideremia-like (Rab escort protein 2) | 1 | | 2583 | hsa-miR-1233 | 1122 | CHML | choroideremia-like (Rab escort protein 2) | 1 | | 2584 | hsa-miR-423-5p | 1122 | CHML | choroideremia-like (Rab escort protein 2) | 1 | | 2585 | hsa-miR-1233 | 113612 | CYP2U1 | cytochrome P450, family 2, subfamily U, polypeptide 1 | 1 | | 2586 | hsa-miR-571 | 113612 | CYP2U1 | cytochrome P450, family 2, subfamily U, polypeptide 1 | 1 | | 2587 | hsa-miR-874 | 113612 | CYP2U1 | cytochrome P450, family 2, subfamily U, polypeptide 1 | 1 | | 2588 | hsa-miR-558 | 23394 | ADNP | activity-dependent neuroprotector homeobox | 1 | | 2589 | hsa-miR-555 | 55870 | ASH1L | ash1 (absent, small, or homeotic)-like (Drosophila) | 1 | | 2590 | hsa-miR-618 | 118788 | PIK3AP1 | phosphoinositide-3-kinase adaptor protein 1 | 1 | | 2591 | hsa-mir-10a | 85313 | PPIL4 | peptidylprolyl isomerase (cyclophilin)-like 4 | 1 | | 2592 | hsa-miR-1224-5p | 85313 | PPIL4 | peptidylprolyl isomerase (cyclophilin)-like 4 | 1 | | 2593 | hsa-miR-423-5p | 85313 | PPIL4 | peptidylprolyl isomerase (cyclophilin)-like 4 | 1 | | 2594 | hsa-miR-576-5p | 85313 | PPIL4 | peptidylprolyl isomerase (cyclophilin)-like 4 | 1 | | 2595 | hsa-mir-10a | 55183 | RIF1 | RAP1 interacting factor homolog (yeast) | 1 | | 2597 | hsa-miR-576-5p | 55183 | RIF1 | RAP1 interacting factor homolog (yeast) | 1 | | 2598 | hsa-miR-1233 | 80012 | PHC3 | polyhomeotic homolog 3 (Drosophila) | 1 | | 2599 | hsa-miR-555 | 80012 | PHC3 | polyhomeotic homolog 3 (Drosophila) | 1 | | 2600 | hsa-miR-558 | 80012 | PHC3 | polyhomeotic homolog 3 (Drosophila) | 1 | | 2601 | hsa-miR-571 | 80012 | PHC3 | polyhomeotic homolog 3 (Drosophila) | 1 | | 2602 | hsa-miR-874 | 80012 | PHC3 | polyhomeotic homolog 3 (Drosophila) | 1 | | 2603 | hsa-mir-3130-1 | 127435 | PODN | podocan | 1 | | 2604 | hsa-mir-3130-2 | 127435 | PODN | podocan | 1 | | 2605 | hsa-mir-3130-3 | 127435 | PODN | podocan | 1 | | 2606 | hsa-mir-3130-4 | 127435 | PODN | podocan | 1 | | 2607 | hsa-miR-10a | 127435 | PODN | podocan | 1 | | 2608 | hsa-miR-95 | 127435 | PODN | podocan | 1 | | 2609 | hsa-mir-149 | 54664 | TMEM106B | transmembrane protein 106B | 1 | | 2610 | hsa-miR-149 | 54664 | TMEM106B | transmembrane protein 106B | 1 | | 2611 | hsa-miR-558 | 4124 | MAN2A1 | mannosidase, alpha, class 2A, member 1 | 1 | | 2612 | hsa-mir-10a | 148867 | SLC30A7 | solute carrier family 30 (zinc transporter), member 7 | 1 | | 2613 | hsa-miR-576-5p | 148867 | SLC30A7 | solute carrier family 30 (zinc transporter), member 7 | 1 | | 2614 | hsa-mir-10a | 388272 | C16orf87 | chromosome 16 open reading frame 87 | 1 | | 2615 | hsa-miR-423-5p | 388272 | C16orf87 | chromosome 16 open reading frame 87 | 1 | | 2616 | hsa-miR-1233 | 285761 | DCBLD1 | discoidin, CUB and LCCL domain containing 1 | 1 | | 2617 | hsa-miR-569 | 285761 | DCBLD1 | discoidin, CUB and LCCL domain containing 1 | 1 | | 2618 | hsa-miR-571 | 285761 | DCBLD1 | discoidin, CUB and LCCL domain containing 1 | 1 | | 2619 | hsa-miR-766 | 285761 | DCBLD1 | discoidin, CUB and LCCL domain containing 1 | 1 | | 2620 | hsa-miR-874 | 285761 | DCBLD1 | discoidin, CUB and LCCL domain containing 1 | 1 | | 2621 | hsa-miR-766 | 84826 | SFT2D3 | SFT2 domain containing 3 | 1 | | 2622 | hsa-mir-505 | 83933 | HDAC10 | histone deacetylase 10 | 1 | | 2623 | hsa-mir-576 | 83933 | HDAC10 | histone deacetylase 10 | 1 | | 2624 | hsa-miR-505\* | 83933 | HDAC10 | histone deacetylase 10 | 1 | | 2625 | hsa-miR-580 | 83933 | HDAC10 | histone deacetylase 10 | 1 | | 2626 | hsa-mir-10a | 64376 | IKZF5 | IKAROS family zinc finger 5 (Pegasus) | 1 | | 2627 | hsa-miR-423-5p | 64376 | IKZF5 | IKAROS family zinc finger 5 (Pegasus) | 1 | | 2628 | hsa-miR-555 | 64376 | IKZF5 | IKAROS family zinc finger 5 (Pegasus) | 1 | | 2629 | hsa-miR-576-5p | 64376 | IKZF5 | IKAROS family zinc finger 5 (Pegasus) | 1 | | 2634 | hsa-miR-423-5p | 114825 | PWWP2A | PWWP domain containing 2A | 1 | | 2635 | hsa-miR-576-5p | 114825 | PWWP2A | PWWP domain containing 2A | 1 | | 2636 | hsa-mir-10a | 57544 | TXNDC16 | thioredoxin domain containing 16 | 1 | | 2637 | hsa-miR-1233 | 57544 | TXNDC16 | thioredoxin domain containing 16 | 1 | | 2638 | hsa-miR-569 | 57544 | TXNDC16 | thioredoxin domain containing 16 | 1 | | 2639 | hsa-miR-571 | 57544 | TXNDC16 | thioredoxin domain containing 16 | 1 | | 2640 | hsa-miR-766 | 57544 | TXNDC16 | thioredoxin domain containing 16 | 1 | | 2641 | hsa-miR-423-5p | 143684 | FAM76B | family with sequence similarity 76, member B | 1 | | 2642 | hsa-miR-555 | 143684 | FAM76B | family with sequence similarity 76, member B | 1 | | 2643 | hsa-miR-558 | 143684 | FAM76B | family with sequence similarity 76, member B | 1 | | 2644 | hsa-miR-576-5p | 143684 | FAM76B | family with sequence similarity 76, member B | 1 | | 2647 | hsa-miR-555 | 253512 | SLC25A30 | solute carrier family 25, member 30 | 1 | | 2648 | hsa-miR-555 | 286410 | ATP11C | ATPase, class VI, type 11C | 1 | | 2649 | hsa-miR-877 | 116236 | ABHD15 | abhydrolase domain containing 15 | 1 | | 2650 | hsa-miR-576-5p | 54799 | MBTD1 | mbt domain containing 1 | 1 | | 2651 | hsa-miR-1233 | 64853 | AIDA | axin interactor, dorsalization associated | 1 | | 2652 | hsa-miR-558 | 64853 | AIDA | axin interactor, dorsalization associated | 1 | | 2653 | hsa-mir-10a | 64282 | PAPD5 | PAP associated domain containing 5 | 1 | | 2654 | hsa-miR-423-5p | 64282 | PAPD5 | PAP associated domain containing 5 | 1 | | 2655 | hsa-miR-571 | 5569 | PKIA | protein kinase (cAMP-dependent, catalytic) inhibitor alpha | 1 | | 2656 | hsa-miR-874 | 5569 | PKIA | protein kinase (cAMP-dependent, catalytic) inhibitor alpha | 1 | | 2657 | hsa-mir-10a | 283464 | GXYLT1 | glucoside xylosyltransferase 1 | 1 | | 2658 | hsa-miR-423-5p | 283464 | GXYLT1 | glucoside xylosyltransferase 1 | 1 | | 2659 | hsa-miR-423-5p | 85460 | ZNF518B | zinc finger protein 518B | 1 | | 2660 | hsa-miR-576-5p | 85460 | ZNF518B | zinc finger protein 518B | 1 | | 2661 | hsa-miR-555 | 400657 | LOC400657 | hypothetical LOC400657 | 1 | | 2662 | hsa-mir-10a | 91408 | BTF3L4 | basic transcription factor 3-like 4 | 1 | | 2663 | hsa-miR-874 | 25914 | RTTN | rotatin | 1 | | 2664 | hsa-miR-423-5p | 3183 | HNRNPC | heterogeneous nuclear ribonucleoprotein C (C1/C2) | 1 | | 2665 | hsa-miR-1233 | 8573 | CASK | calcium/calmodulin-dependent serine protein kinase (MAGUK family) | 1 | | 2666 | hsa-miR-571 | 8573 | CASK | calcium/calmodulin-dependent serine protein kinase (MAGUK family) | 1 | | 2667 | hsa-miR-874 | 8573 | CASK | calcium/calmodulin-dependent serine protein kinase (MAGUK family) | 1 | | 2668 | hsa-miR-1233 | 112487 | C14orf126 | chromosome 14 open reading frame 126 | 1 | | 2669 | hsa-miR-874 | 112487 | C14orf126 | chromosome 14 open reading frame 126 | 1 | | 2670 | hsa-miR-593\* | 64975 | MRPL41 | mitochondrial ribosomal protein L41 | 1 | | 2671 | hsa-miR-623 | 64975 | MRPL41 | mitochondrial ribosomal protein L41 | 1 | | 2672 | hsa-miR-877 | 64975 | MRPL41 | mitochondrial ribosomal protein L41 | 1 | | 2673 | hsa-miR-1233 | 338657 | CCDC84 | coiled-coil domain containing 84 | 1 | | 2674 | hsa-miR-558 | 338657 | CCDC84 | coiled-coil domain containing 84 | 1 | | 2675 | hsa-miR-874 | 338657 | CCDC84 | coiled-coil domain containing 84 | 1 | | 2676 | hsa-miR-95 | 253738 | EBF3 | early B-cell factor 3 | 1 | | 2677 | hsa-miR-1233 | 56647 | BCCIP | BRCA2 and CDKN1A interacting protein | 1 | | 2678 | hsa-miR-558 | 56647 | BCCIP | BRCA2 and CDKN1A interacting protein | 1 | | 2679 | hsa-miR-874 | 56647 | BCCIP | BRCA2 and CDKN1A interacting protein | 1 | | 2682 | hsa-miR-874 | 90673 | PPP1R3E | protein phosphatase 1, regulatory (inhibitor) subunit 3E | 1 | | 2683 | hsa-mir-10a | 84437 | KIAA1826 | KIAA1826 | 1 | | 2684 | hsa-miR-423-5p | 84437 | KIAA1826 | KIAA1826 | 1 | | 2685 | hsa-miR-555 | 84437 | KIAA1826 | KIAA1826 | 1 | | 2686 | hsa-miR-576-5p | 84437 | KIAA1826 | KIAA1826 | 1 | | 2687 | hsa-miR-423-5p | 2551 | GABPA | GA binding protein transcription factor, alpha subunit 60kDa | 1 | | 2688 | hsa-miR-576-5p | 2551 | GABPA | GA binding protein transcription factor, alpha subunit 60kDa | 1 | | 2689 | hsa-miR-1233 | 285521 | COX18 | COX18 cytochrome c oxidase assembly homolog (S. cerevisiae) | 1 | | 2690 | hsa-miR-423-5p | 285521 | COX18 | COX18 cytochrome c oxidase assembly homolog (S. cerevisiae) | 1 | | 2691 | hsa-miR-571 | 285521 | COX18 | COX18 cytochrome c oxidase assembly homolog (S. cerevisiae) | 1 | | 2692 | hsa-miR-766 | 285521 | COX18 | COX18 cytochrome c oxidase assembly homolog (S. cerevisiae) | 1 | | 2694 | hsa-miR-766 | 221545 | C6orf136 | chromosome 6 open reading frame 136 | 1 | | 2695 | hsa-miR-555 | 51104 | FAM108B1 | family with sequence similarity 108, member B1 | 1 | | 2696 | hsa-miR-558 | 51104 | FAM108B1 | family with sequence similarity 108, member B1 | 1 | | 2697 | hsa-miR-95 | 23363 | OBSL1 | obscurin-like 1 | 1 | | 2699 | hsa-miR-1233 | 9255 | AIMP1 | aminoacyl tRNA synthetase complex-interacting multifunctional protein 1 | 1 | | 2700 | hsa-miR-571 | 9255 | AIMP1 | aminoacyl tRNA synthetase complex-interacting multifunctional protein 1 | 1 | | 2701 | hsa-miR-1233 | 123283 | TARSL2 | threonyl-tRNA synthetase-like 2 | 1 | | 2702 | hsa-miR-569 | 123283 | TARSL2 | threonyl-tRNA synthetase-like 2 | 1 | | 2703 | hsa-miR-571 | 123283 | TARSL2 | threonyl-tRNA synthetase-like 2 | 1 | | 2704 | hsa-miR-766 | 123283 | TARSL2 | threonyl-tRNA synthetase-like 2 | 1 | | 2705 | hsa-miR-874 | 123283 | TARSL2 | threonyl-tRNA synthetase-like 2 | 1 | | 2706 | hsa-mir-3130-1 | 11076 | TPPP | tubulin polymerization promoting protein | 1 | | 2707 | hsa-mir-3130-2 | 11076 | TPPP | tubulin polymerization promoting protein | 1 | | 2708 | hsa-mir-3130-3 | 11076 | TPPP | tubulin polymerization promoting protein | 1 | | 2709 | hsa-mir-3130-4 | 11076 | TPPP | tubulin polymerization promoting protein | 1 | | 2710 | hsa-miR-10a | 11076 | TPPP | tubulin polymerization promoting protein | 1 | | 2711 | hsa-miR-95 | 11076 | TPPP | tubulin polymerization promoting protein | 1 | | 2712 | hsa-miR-10a | 131177 | FAM3D | family with sequence similarity 3, member D | 1 | | 2713 | hsa-miR-499-5p | 131177 | FAM3D | family with sequence similarity 3, member D | 1 | | 2714 | hsa-miR-95 | 131177 | FAM3D | family with sequence similarity 3, member D | 1 | | 2715 | hsa-mir-10a | 7770 | ZNF227 | zinc finger protein 227 | 1 | | 2716 | hsa-mir-556 | 7770 | ZNF227 | zinc finger protein 227 | 1 | | 2717 | hsa-miR-1224-5p | 7770 | ZNF227 | zinc finger protein 227 | 1 | | 2718 | hsa-miR-1233 | 7770 | ZNF227 | zinc finger protein 227 | 1 | | 2719 | hsa-miR-423-5p | 7770 | ZNF227 | zinc finger protein 227 | 1 | | 2720 | hsa-miR-556-5p | 7770 | ZNF227 | zinc finger protein 227 | 1 | | 2721 | hsa-miR-571 | 7770 | ZNF227 | zinc finger protein 227 | 1 | | 2722 | hsa-miR-766 | 7770 | ZNF227 | zinc finger protein 227 | 1 | | 2723 | hsa-miR-1233 | 1915 | EEF1A1 | eukaryotic translation elongation factor 1 alpha 1 | 1 | | 2724 | hsa-miR-571 | 1915 | EEF1A1 | eukaryotic translation elongation factor 1 alpha 1 | 1 | | 2725 | hsa-miR-874 | 1915 | EEF1A1 | eukaryotic translation elongation factor 1 alpha 1 | 1 | | 2726 | hsa-miR-1233 | 9141 | PDCD5 | programmed cell death 5 | 1 | | 2727 | hsa-miR-569 | 9141 | PDCD5 | programmed cell death 5 | 1 | | 2728 | hsa-miR-571 | 9141 | PDCD5 | programmed cell death 5 | 1 | | 2729 | hsa-miR-766 | 9141 | PDCD5 | programmed cell death 5 | 1 | | 2730 | hsa-miR-874 | 9141 | PDCD5 | programmed cell death 5 | 1 | | 2731 | hsa-mir-149 | 1456 | CSNK1G3 | casein kinase 1, gamma 3 | 1 | | 2732 | hsa-miR-149 | 1456 | CSNK1G3 | casein kinase 1, gamma 3 | 1 | | 2733 | hsa-miR-423-5p | 1456 | CSNK1G3 | casein kinase 1, gamma 3 | 1 | | 2734 | hsa-miR-555 | 1456 | CSNK1G3 | casein kinase 1, gamma 3 | 1 | | 2735 | hsa-miR-576-5p | 1456 | CSNK1G3 | casein kinase 1, gamma 3 | 1 | | 2736 | hsa-miR-1233 | 643836 | ZFP62 | zinc finger protein 62 homolog (mouse) | 1 | | 2737 | hsa-miR-558 | 643836 | ZFP62 | zinc finger protein 62 homolog (mouse) | 1 | | 2738 | hsa-miR-571 | 643836 | ZFP62 | zinc finger protein 62 homolog (mouse) | 1 | | 2739 | hsa-miR-874 | 643836 | ZFP62 | zinc finger protein 62 homolog (mouse) | 1 | | 2740 | hsa-miR-555 | 376940 | ZC3H6 | zinc finger CCCH-type containing 6 | 1 | | 2741 | hsa-mir-10a | 619423 | FAM85A | family with sequence similarity 85, member A | 1 | | 2742 | hsa-miR-1233 | 619423 | FAM85A | family with sequence similarity 85, member A | 1 | | 2743 | hsa-miR-423-5p | 619423 | FAM85A | family with sequence similarity 85, member A | 1 | | 2744 | hsa-miR-569 | 619423 | FAM85A | family with sequence similarity 85, member A | 1 | | 2745 | hsa-miR-766 | 619423 | FAM85A | family with sequence similarity 85, member A | 1 | | 2750 | hsa-miR-558 | 81889 | FAHD1 | fumarylacetoacetate hydrolase domain containing 1 | 1 | | 2751 | hsa-miR-576-5p | 81889 | FAHD1 | fumarylacetoacetate hydrolase domain containing 1 | 1 | | 2752 | hsa-miR-555 | 83786 | FRMD8 | FERM domain containing 8 | 1 | | 2753 | hsa-miR-576-5p | 83786 | FRMD8 | FERM domain containing 8 | 1 | | 2754 | hsa-miR-874 | 114791 | TUBGCP5 | tubulin, gamma complex associated protein 5 | 1 | | 2755 | hsa-miR-1233 | 158586 | ZXDB | zinc finger, X-linked, duplicated B | 1 | | 2756 | hsa-miR-558 | 158586 | ZXDB | zinc finger, X-linked, duplicated B | 1 | | 2757 | hsa-miR-571 | 158586 | ZXDB | zinc finger, X-linked, duplicated B | 1 | | 2758 | hsa-miR-874 | 158586 | ZXDB | zinc finger, X-linked, duplicated B | 1 | | 2759 | hsa-mir-3130-1 | 81603 | TRIM8 | tripartite motif-containing 8 | 1 | | 2760 | hsa-mir-3130-2 | 81603 | TRIM8 | tripartite motif-containing 8 | 1 | | 2761 | hsa-mir-3130-3 | 81603 | TRIM8 | tripartite motif-containing 8 | 1 | | 2762 | hsa-mir-3130-4 | 81603 | TRIM8 | tripartite motif-containing 8 | 1 | | 2763 | hsa-miR-95 | 81603 | TRIM8 | tripartite motif-containing 8 | 1 | | 2765 | hsa-mir-10a | 132949 | AASDH | aminoadipate-semialdehyde dehydrogenase | 1 | | 2767 | hsa-miR-569 | 152485 | ZNF827 | zinc finger protein 827 | 1 | | 2768 | hsa-miR-571 | 152485 | ZNF827 | zinc finger protein 827 | 1 | | 2769 | hsa-miR-874 | 152485 | ZNF827 | zinc finger protein 827 | 1 | | 2770 | hsa-miR-1233 | 84135 | UTP15 | UTP15, U3 small nucleolar ribonucleoprotein, homolog (S. cerevisiae) | 1 | | 2771 | hsa-miR-571 | 84135 | UTP15 | UTP15, U3 small nucleolar ribonucleoprotein, homolog (S. cerevisiae) | 1 | | 2772 | hsa-miR-874 | 84135 | UTP15 | UTP15, U3 small nucleolar ribonucleoprotein, homolog (S. cerevisiae) | 1 | | 2773 | hsa-miR-766 | 283149 | BCL9L | B-cell CLL/lymphoma 9-like | 1 | | 2774 | hsa-miR-877 | 283149 | BCL9L | B-cell CLL/lymphoma 9-like | 1 | | 2775 | hsa-miR-10a | 205428 | C3orf58 | chromosome 3 open reading frame 58 | 1 | | 2776 | hsa-miR-95 | 205428 | C3orf58 | chromosome 3 open reading frame 58 | 1 | | 2777 | hsa-miR-1233 | 162972 | ZNF550 | zinc finger protein 550 | 1 | | 2778 | hsa-miR-558 | 162972 | ZNF550 | zinc finger protein 550 | 1 | | 2779 | hsa-miR-571 | 162972 | ZNF550 | zinc finger protein 550 | 1 | | 2780 | hsa-miR-874 | 162972 | ZNF550 | zinc finger protein 550 | 1 | | 2781 | hsa-mir-10a | 54876 | DCAF16 | DDB1 and CUL4 associated factor 16 | 1 | | 2782 | hsa-miR-1233 | 54876 | DCAF16 | DDB1 and CUL4 associated factor 16 | 1 | | 2783 | hsa-miR-423-5p | 54876 | DCAF16 | DDB1 and CUL4 associated factor 16 | 1 | | 2784 | hsa-miR-571 | 54876 | DCAF16 | DDB1 and CUL4 associated factor 16 | 1 | | 2785 | hsa-mir-10a | 285331 | CCDC66 | coiled-coil domain containing 66 | 1 | | 2786 | hsa-miR-1233 | 285331 | CCDC66 | coiled-coil domain containing 66 | 1 | | 2787 | hsa-miR-423-5p | 285331 | CCDC66 | coiled-coil domain containing 66 | 1 | | 2788 | hsa-miR-571 | 285331 | CCDC66 | coiled-coil domain containing 66 | 1 | | 2789 | hsa-miR-576-5p | 285331 | CCDC66 | coiled-coil domain containing 66 | 1 | | 2790 | hsa-miR-874 | 285331 | CCDC66 | coiled-coil domain containing 66 | 1 | | 2791 | hsa-miR-877 | 26090 | ABHD12 | abhydrolase domain containing 12 | 1 | | 2792 | hsa-miR-10a | 5522 | PPP2R2C | protein phosphatase 2 (formerly 2A), regulatory subunit B, gamma isoform | 1 | | 2793 | hsa-miR-499-5p | 5522 | PPP2R2C | protein phosphatase 2 (formerly 2A), regulatory subunit B, gamma isoform | 1 | | 2794 | hsa-miR-555 | 7756 | ZNF207 | zinc finger protein 207 | 1 | | 2795 | hsa-miR-454\* | 23431 | AP4E1 | adaptor-related protein complex 4, epsilon 1 subunit | 1 | | 2796 | hsa-miR-10a | 11248 | NXPH3 | neurexophilin 3 | 1 | | 2797 | hsa-miR-1233 | 389362 | PSMG4 | proteasome (prosome, macropain) assembly chaperone 4 | 1 | | 2798 | hsa-miR-558 | 389362 | PSMG4 | proteasome (prosome, macropain) assembly chaperone 4 | 1 | | 2799 | hsa-miR-571 | 389362 | PSMG4 | proteasome (prosome, macropain) assembly chaperone 4 | 1 | | 2800 | hsa-miR-874 | 389362 | PSMG4 | proteasome (prosome, macropain) assembly chaperone 4 | 1 | | 2801 | hsa-miR-10a | 54753 | ZNF853 | zinc finger protein 853 | 1 | | 2802 | hsa-miR-558 | 152100 | CMC1 | COX assembly mitochondrial protein homolog (S. cerevisiae) | 1 | | 2803 | hsa-miR-618 | 122402 | TDRD9 | tudor domain containing 9 | 1 | | 2804 | hsa-miR-571 | 84289 | ING5 | inhibitor of growth family, member 5 | 1 | | 2805 | hsa-miR-558 | 55857 | PLK1S1 | polo-like kinase 1 substrate 1 | 1 | | 2806 | hsa-miR-571 | 55857 | PLK1S1 | polo-like kinase 1 substrate 1 | 1 | | 2807 | hsa-miR-571 | 91614 | DEPDC7 | DEP domain containing 7 | 1 | | 2808 | hsa-miR-1233 | 91523 | FAM113B | family with sequence similarity 113, member B | 1 | | 2809 | hsa-miR-766 | 91523 | FAM113B | family with sequence similarity 113, member B | 1 | | 2810 | hsa-miR-1233 | 138199 | C9orf41 | chromosome 9 open reading frame 41 | 1 | | 2811 | hsa-miR-558 | 138199 | C9orf41 | chromosome 9 open reading frame 41 | 1 | | 2812 | hsa-miR-571 | 138199 | C9orf41 | chromosome 9 open reading frame 41 | 1 | | 2813 | hsa-miR-874 | 138199 | C9orf41 | chromosome 9 open reading frame 41 | 1 | | 2814 | hsa-mir-149 | 114825 | PWWP2A | PWWP domain containing 2A | 1 | | 2815 | hsa-miR-149 | 114825 | PWWP2A | PWWP domain containing 2A | 1 | | 2816 | hsa-miR-555 | 114825 | PWWP2A | PWWP domain containing 2A | 1 | | 2817 | hsa-miR-558 | 114825 | PWWP2A | PWWP domain containing 2A | 1 | | 2818 | hsa-miR-1233 | 53344 | CHIC1 | cysteine-rich hydrophobic domain 1 | 1 | | 2819 | hsa-miR-558 | 53344 | CHIC1 | cysteine-rich hydrophobic domain 1 | 1 | | 2820 | hsa-miR-569 | 53344 | CHIC1 | cysteine-rich hydrophobic domain 1 | 1 | | 2821 | hsa-miR-571 | 53344 | CHIC1 | cysteine-rich hydrophobic domain 1 | 1 | | 2822 | hsa-miR-766 | 53344 | CHIC1 | cysteine-rich hydrophobic domain 1 | 1 | | 2823 | hsa-miR-874 | 53344 | CHIC1 | cysteine-rich hydrophobic domain 1 | 1 | | 2824 | hsa-mir-16-2 | 729359 | PLIN4 | perilipin 4 | 1 | | 2825 | hsa-mir-15b | 729359 | PLIN4 | perilipin 4 | 1 | | 2826 | hsa-mir-155 | 729359 | PLIN4 | perilipin 4 | 1 | | 2827 | hsa-mir-3130-1 | 729359 | PLIN4 | perilipin 4 | 1 | | 2828 | hsa-mir-3130-2 | 729359 | PLIN4 | perilipin 4 | 1 | | 2829 | hsa-mir-3130-3 | 729359 | PLIN4 | perilipin 4 | 1 | | 2830 | hsa-mir-3130-4 | 729359 | PLIN4 | perilipin 4 | 1 | | 2831 | hsa-miR-128 | 729359 | PLIN4 | perilipin 4 | 1 | | 2832 | hsa-miR-643 | 729359 | PLIN4 | perilipin 4 | 1 | | 2833 | hsa-miR-95 | 729440 | CCDC61 | coiled-coil domain containing 61 | 1 | | 2834 | hsa-miR-558 | 5810 | RAD1 | RAD1 homolog (S. pombe) | 1 | | 2835 | hsa-miR-1233 | 65986 | ZBTB10 | zinc finger and BTB domain containing 10 | 1 | | 2836 | hsa-miR-558 | 65986 | ZBTB10 | zinc finger and BTB domain containing 10 | 1 | | 2837 | hsa-miR-874 | 65986 | ZBTB10 | zinc finger and BTB domain containing 10 | 1 | | 2838 | hsa-miR-1233 | 10914 | PAPOLA | poly(A) polymerase alpha | 1 | | 2839 | hsa-miR-423-5p | 10914 | PAPOLA | poly(A) polymerase alpha | 1 | | 2840 | hsa-miR-1233 | 57786 | RBAK | RB-associated KRAB zinc finger | 1 | | 2841 | hsa-miR-423-5p | 57786 | RBAK | RB-associated KRAB zinc finger | 1 | | 2842 | hsa-miR-555 | 57786 | RBAK | RB-associated KRAB zinc finger | 1 | | 2843 | hsa-miR-558 | 57786 | RBAK | RB-associated KRAB zinc finger | 1 | | 2844 | hsa-miR-571 | 57786 | RBAK | RB-associated KRAB zinc finger | 1 | | 2845 | hsa-miR-423-5p | 55037 | PTCD3 | Pentatricopeptide repeat domain 3 | 1 | | 2846 | hsa-miR-558 | 133686 | C5orf33 | chromosome 5 open reading frame 33 | 1 | | 2847 | hsa-miR-1233 | 199870 | FAM76A | family with sequence similarity 76, member A | 1 | | 2848 | hsa-miR-558 | 199870 | FAM76A | family with sequence similarity 76, member A | 1 | | 2849 | hsa-miR-571 | 199870 | FAM76A | family with sequence similarity 76, member A | 1 | | 2850 | hsa-miR-618 | 116844 | LRG1 | leucine-rich alpha-2-glycoprotein 1 | 1 | | 2851 | hsa-miR-558 | 11127 | KIF3A | kinesin family member 3A | 1 | | 2852 | hsa-miR-555 | 3275 | PRMT2 | protein arginine methyltransferase 2 | 1 | | 2853 | hsa-mir-10a | 113510 | HELQ | helicase, POLQ-like | 1 | | 2854 | hsa-miR-1233 | 113510 | HELQ | helicase, POLQ-like | 1 | | 2855 | hsa-miR-423-5p | 113510 | HELQ | helicase, POLQ-like | 1 | | 2856 | hsa-miR-571 | 113510 | HELQ | helicase, POLQ-like | 1 | | 2857 | hsa-miR-454\* | 55142 | HAUS2 | HAUS augmin-like complex, subunit 2 | 1 | | 2858 | hsa-miR-558 | 128387 | TATDN3 | TatD DNase domain containing 3 | 1 | | 2859 | hsa-miR-95 | 51104 | FAM108B1 | family with sequence similarity 108, member B1 | 1 | | 2860 | hsa-miR-10a | 729440 | CCDC61 | coiled-coil domain containing 61 | 1 | | 2861 | hsa-miR-558 | 3213 | HOXB3 | homeobox B3 | 1 | | 2862 | hsa-miR-571 | 3213 | HOXB3 | homeobox B3 | 1 | | 2863 | hsa-miR-874 | 3213 | HOXB3 | homeobox B3 | 1 | | 2864 | hsa-mir-10a | 143884 | CWF19L2 | CWF19-like 2, cell cycle control (S. pombe) | 1 | | 2865 | hsa-miR-423-5p | 143884 | CWF19L2 | CWF19-like 2, cell cycle control (S. pombe) | 1 | | 2867 | hsa-miR-576-5p | 143884 | CWF19L2 | CWF19-like 2, cell cycle control (S. pombe) | 1 | | 2868 | hsa-miR-558 | 144245 | ALG10B | asparagine-linked glycosylation 10, alpha-1,2-glucosyltransferase homolog B (yeast) | 1 | | 2869 | hsa-miR-571 | 144245 | ALG10B | asparagine-linked glycosylation 10, alpha-1,2-glucosyltransferase homolog B (yeast) | 1 | | 2870 | hsa-miR-10a | 153090 | DAB2IP | DAB2 interacting protein | 1 | | 2871 | hsa-miR-95 | 153090 | DAB2IP | DAB2 interacting protein | 1 | | 2872 | hsa-miR-423-5p | 166968 | MIER3 | mesoderm induction early response 1, family member 3 | 1 | | 2873 | hsa-miR-555 | 166968 | MIER3 | mesoderm induction early response 1, family member 3 | 1 | | 2874 | hsa-miR-558 | 166968 | MIER3 | mesoderm induction early response 1, family member 3 | 1 | | 2875 | hsa-miR-576-5p | 166968 | MIER3 | mesoderm induction early response 1, family member 3 | 1 | | 2876 | hsa-miR-571 | 7552 | ZNF711 | zinc finger protein 711 | 1 | | 2877 | hsa-miR-874 | 7552 | ZNF711 | zinc finger protein 711 | 1 | | 2878 | hsa-miR-555 | 1106 | CHD2 | chromodomain helicase DNA binding protein 2 | 1 | | 2879 | hsa-miR-558 | 1106 | CHD2 | chromodomain helicase DNA binding protein 2 | 1 | | 2880 | hsa-miR-877 | 90673 | PPP1R3E | protein phosphatase 1, regulatory (inhibitor) subunit 3E | 1 | | 2881 | hsa-miR-555 | 84190 | C12orf26 | chromosome 12 open reading frame 26 | 1 | | 2882 | hsa-miR-558 | 84190 | C12orf26 | chromosome 12 open reading frame 26 | 1 | | 2883 | hsa-mir-149 | 64282 | PAPD5 | PAP associated domain containing 5 | 1 | | 2884 | hsa-miR-149 | 64282 | PAPD5 | PAP associated domain containing 5 | 1 | | 2885 | hsa-miR-555 | 64282 | PAPD5 | PAP associated domain containing 5 | 1 | | 2886 | hsa-miR-558 | 64282 | PAPD5 | PAP associated domain containing 5 | 1 | | 2887 | hsa-miR-623 | 388403 | YPEL2 | yippee-like 2 (Drosophila) | 1 | | 2888 | hsa-miR-1233 | 166378 | SPATA5 | spermatogenesis associated 5 | 1 | | 2889 | hsa-miR-571 | 166378 | SPATA5 | spermatogenesis associated 5 | 1 | | 2890 | hsa-miR-874 | 166378 | SPATA5 | spermatogenesis associated 5 | 1 | | 2891 | hsa-miR-555 | 54619 | CCNJ | cyclin J | 1 | | 2892 | hsa-miR-558 | 54619 | CCNJ | cyclin J | 1 | | 2893 | hsa-miR-608 | 149603 | RNF187 | ring finger protein 187 | 1 | | 2894 | hsa-miR-1233 | 23318 | ZCCHC11 | zinc finger, CCHC domain containing 11 | 1 | | 2895 | hsa-miR-423-5p | 23318 | ZCCHC11 | zinc finger, CCHC domain containing 11 | 1 | | 2896 | hsa-miR-555 | 23318 | ZCCHC11 | zinc finger, CCHC domain containing 11 | 1 | | 2897 | hsa-miR-558 | 23318 | ZCCHC11 | zinc finger, CCHC domain containing 11 | 1 | | 2898 | hsa-miR-571 | 23318 | ZCCHC11 | zinc finger, CCHC domain containing 11 | 1 | | 2899 | hsa-miR-874 | 23318 | ZCCHC11 | zinc finger, CCHC domain containing 11 | 1 | | 2900 | hsa-miR-571 | 64682 | ANAPC1 | anaphase promoting complex subunit 1 | 1 | | 2901 | hsa-miR-558 | 3841 | KPNA5 | karyopherin alpha 5 (importin alpha 6) | 1 | | 2902 | hsa-miR-10a | 10763 | NES | nestin | 1 | | 2903 | hsa-miR-95 | 10763 | NES | nestin | 1 | | 2904 | hsa-miR-10a | 84890 | ADO | 2-aminoethanethiol (cysteamine) dioxygenase | 1 | | 2905 | hsa-miR-499-5p | 84890 | ADO | 2-aminoethanethiol (cysteamine) dioxygenase | 1 | | 2906 | hsa-miR-95 | 84890 | ADO | 2-aminoethanethiol (cysteamine) dioxygenase | 1 | | 2908 | hsa-miR-423-5p | 54882 | ANKHD1 | ankyrin repeat and KH domain containing 1 | 1 | | 2909 | hsa-mir-199a-2 | 9101 | USP8 | ubiquitin specific peptidase 8 | 1 | | 2910 | hsa-mir-214 | 9101 | USP8 | ubiquitin specific peptidase 8 | 1 | | 2911 | hsa-miR-423-5p | 9101 | USP8 | ubiquitin specific peptidase 8 | 1 | | 2912 | hsa-miR-576-5p | 9101 | USP8 | ubiquitin specific peptidase 8 | 1 | | 2913 | hsa-miR-10a | 121260 | SLC15A4 | solute carrier family 15, member 4 | 1 | | 2914 | hsa-miR-95 | 121260 | SLC15A4 | solute carrier family 15, member 4 | 1 | | 2915 | hsa-miR-558 | 23545 | ATP6V0A2 | ATPase, H+ transporting, lysosomal V0 subunit a2 | 1 | | 2916 | hsa-miR-569 | 23545 | ATP6V0A2 | ATPase, H+ transporting, lysosomal V0 subunit a2 | 1 | | 2917 | hsa-miR-571 | 6137 | RPL13 | ribosomal protein L13 | 1 | | 2918 | hsa-miR-10a | 2350 | FOLR2 | folate receptor 2 (fetal) | 1 | | 2919 | hsa-miR-499-5p | 2350 | FOLR2 | folate receptor 2 (fetal) | 1 | | 2920 | hsa-miR-580 | 2350 | FOLR2 | folate receptor 2 (fetal) | 1 | | 2921 | hsa-miR-95 | 2350 | FOLR2 | folate receptor 2 (fetal) | 1 | | 2922 | hsa-miR-766 | 55717 | WDR11 | WD repeat domain 11 | 1 | | 2923 | hsa-miR-571 | 6619 | SNAPC3 | small nuclear RNA activating complex, polypeptide 3, 50kDa | 1 | | 2924 | hsa-miR-571 | 55552 | ZNF823 | zinc finger protein 823 | 1 | | 2925 | hsa-miR-874 | 55552 | ZNF823 | zinc finger protein 823 | 1 | | 2926 | hsa-miR-10a | 147906 | DACT3 | dapper, antagonist of beta-catenin, homolog 3 (Xenopus laevis) | 1 | | 2927 | hsa-miR-95 | 147906 | DACT3 | dapper, antagonist of beta-catenin, homolog 3 (Xenopus laevis) | 1 | | 2928 | hsa-miR-10a | 440173 | LOC440173 | hypothetical LOC440173 | 1 | | 2929 | hsa-miR-571 | 147525 | C18orf18 | chromosome 18 open reading frame 18 | 1 | | 2930 | hsa-miR-95 | 5594 | MAPK1 | mitogen-activated protein kinase 1 | 1 | | 2931 | hsa-mir-3130-1 | 92369 | SPSB4 | splA/ryanodine receptor domain and SOCS box containing 4 | 1 | | 2932 | hsa-mir-3130-2 | 92369 | SPSB4 | splA/ryanodine receptor domain and SOCS box containing 4 | 1 | | 2933 | hsa-mir-3130-3 | 92369 | SPSB4 | splA/ryanodine receptor domain and SOCS box containing 4 | 1 | | 2934 | hsa-mir-3130-4 | 92369 | SPSB4 | splA/ryanodine receptor domain and SOCS box containing 4 | 1 | | 2935 | hsa-miR-558 | 27131 | SNX5 | sorting nexin 5 | 1 | | 2936 | hsa-miR-10a | 55194 | FAM176B | family with sequence similarity 176, member B | 1 | | 2937 | hsa-miR-555 | 51735 | RAPGEF6 | Rap guanine nucleotide exchange factor (GEF) 6 | 1 | | 2938 | hsa-miR-558 | 51735 | RAPGEF6 | Rap guanine nucleotide exchange factor (GEF) 6 | 1 | | 2939 | hsa-miR-10a | 196740 | C10orf72 | chromosome 10 open reading frame 72 | 1 | | 2940 | hsa-miR-499-5p | 196740 | C10orf72 | chromosome 10 open reading frame 72 | 1 | | 2941 | hsa-miR-95 | 196740 | C10orf72 | chromosome 10 open reading frame 72 | 1 | | 2942 | hsa-miR-1233 | 255231 | MCOLN2 | mucolipin 2 | 1 | | 2943 | hsa-miR-874 | 255231 | MCOLN2 | mucolipin 2 | 1 | | 2944 | hsa-miR-571 | 79725 | THAP9 | THAP domain containing 9 | 1 | | 2945 | hsa-mir-3130-1 | 26012 | NELF | nasal embryonic LHRH factor | 1 | | 2946 | hsa-mir-3130-2 | 26012 | NELF | nasal embryonic LHRH factor | 1 | | 2947 | hsa-mir-3130-3 | 26012 | NELF | nasal embryonic LHRH factor | 1 | | 2948 | hsa-mir-3130-4 | 26012 | NELF | nasal embryonic LHRH factor | 1 | | 2949 | hsa-miR-10a | 26012 | NELF | nasal embryonic LHRH factor | 1 | | 2950 | hsa-miR-499-5p | 26012 | NELF | nasal embryonic LHRH factor | 1 | | 2951 | hsa-miR-580 | 26012 | NELF | nasal embryonic LHRH factor | 1 | | 2952 | hsa-miR-95 | 26012 | NELF | nasal embryonic LHRH factor | 1 | | 2953 | hsa-miR-10a | 57636 | ARHGAP23 | Rho GTPase activating protein 23 | 1 | | 2954 | hsa-miR-10a | 284297 | SSC5D | scavenger receptor cysteine-rich glycoprotein | 1 | | 2955 | hsa-miR-95 | 284297 | SSC5D | scavenger receptor cysteine-rich glycoprotein | 1 | | 2956 | hsa-miR-1233 | 28985 | MCTS1 | malignant T cell amplified sequence 1 | 1 | | 2957 | hsa-miR-1233 | 153364 | MBLAC2 | metallo-beta-lactamase domain containing 2 | 1 | | 2958 | hsa-miR-558 | 153364 | MBLAC2 | metallo-beta-lactamase domain containing 2 | 1 | | 2959 | hsa-miR-571 | 153364 | MBLAC2 | metallo-beta-lactamase domain containing 2 | 1 | | 2960 | hsa-miR-874 | 153364 | MBLAC2 | metallo-beta-lactamase domain containing 2 | 1 | | 2961 | hsa-miR-766 | 84897 | TBRG1 | transforming growth factor beta regulator 1 | 1 | | 2962 | hsa-miR-874 | 84897 | TBRG1 | transforming growth factor beta regulator 1 | 1 | | 2963 | hsa-mir-3130-1 | 79803 | HPS6 | Hermansky-Pudlak syndrome 6 | 1 | | 2964 | hsa-mir-3130-2 | 79803 | HPS6 | Hermansky-Pudlak syndrome 6 | 1 | | 2965 | hsa-mir-3130-3 | 79803 | HPS6 | Hermansky-Pudlak syndrome 6 | 1 | | 2966 | hsa-mir-3130-4 | 79803 | HPS6 | Hermansky-Pudlak syndrome 6 | 1 | | 2967 | hsa-miR-10a | 79803 | HPS6 | Hermansky-Pudlak syndrome 6 | 1 | | 2968 | hsa-miR-499-5p | 79803 | HPS6 | Hermansky-Pudlak syndrome 6 | 1 | | 2969 | hsa-miR-95 | 79803 | HPS6 | Hermansky-Pudlak syndrome 6 | 1 | | 2970 | hsa-miR-95 | 25813 | SAMM50 | sorting and assembly machinery component 50 homolog (S. cerevisiae) | 1 | | 2971 | hsa-miR-10a | 388135 | C15orf59 | chromosome 15 open reading frame 59 | 1 | | 2972 | hsa-miR-1233 | 921 | CD5 | CD5 molecule | 1 | | 2973 | hsa-miR-569 | 921 | CD5 | CD5 molecule | 1 | | 2974 | hsa-miR-766 | 921 | CD5 | CD5 molecule | 1 | | 2975 | hsa-miR-10a | 94086 | HSPB9 | heat shock protein, alpha-crystallin-related, B9 | 1 | | 2976 | hsa-miR-95 | 94086 | HSPB9 | heat shock protein, alpha-crystallin-related, B9 | 1 | | 2977 | hsa-miR-10a | 152641 | C4orf38 | chromosome 4 open reading frame 38 | 1 | | 2978 | hsa-miR-558 | 57646 | USP28 | ubiquitin specific peptidase 28 | 1 | | 2979 | hsa-miR-874 | 57646 | USP28 | ubiquitin specific peptidase 28 | 1 | | 2980 | hsa-miR-95 | 201595 | STT3B | STT3, subunit of the oligosaccharyltransferase complex, homolog B (S. cerevisiae) | 1 | | 2981 | hsa-miR-618 | 100288432 | LOC100288432 | hypothetical protein LOC100288432 | 1 | | 2982 | hsa-miR-10a | 91828 | C14orf73 | chromosome 14 open reading frame 73 | 1 | | 2983 | hsa-miR-95 | 91828 | C14orf73 | chromosome 14 open reading frame 73 | 1 | | 2984 | hsa-miR-10a | 3691 | ITGB4 | integrin, beta 4 | 1 | | 2985 | hsa-miR-555 | 389765 | LOC389765 | kinesin family member 27 pseudogene | 1 | | 2986 | hsa-mir-3130-1 | 221938 | MMD2 | monocyte to macrophage differentiation-associated 2 | 1 | | 2987 | hsa-mir-3130-2 | 221938 | MMD2 | monocyte to macrophage differentiation-associated 2 | 1 | | 2988 | hsa-mir-3130-3 | 221938 | MMD2 | monocyte to macrophage differentiation-associated 2 | 1 | | 2989 | hsa-mir-3130-4 | 221938 | MMD2 | monocyte to macrophage differentiation-associated 2 | 1 | | 2990 | hsa-miR-10a | 221938 | MMD2 | monocyte to macrophage differentiation-associated 2 | 1 | | 2991 | hsa-miR-95 | 221938 | MMD2 | monocyte to macrophage differentiation-associated 2 | 1 | | 2992 | hsa-miR-454\* | 22907 | DHX30 | DEAH (Asp-Glu-Ala-His) box polypeptide 30 | 1 | | 2993 | hsa-miR-1233 | 6687 | SPG7 | spastic paraplegia 7 (pure and complicated autosomal recessive) | 1 | | 2994 | hsa-miR-571 | 6687 | SPG7 | spastic paraplegia 7 (pure and complicated autosomal recessive) | 1 | | 2995 | hsa-miR-874 | 6687 | SPG7 | spastic paraplegia 7 (pure and complicated autosomal recessive) | 1 | | 2997 | hsa-miR-571 | 134218 | DNAJC21 | DnaJ (Hsp40) homolog, subfamily C, member 21 | 1 | | 2998 | hsa-miR-95 | 55890 | GPRC5C | G protein-coupled receptor, family C, group 5, member C | 1 | | 2999 | hsa-miR-10a | 399948 | C11orf92 | chromosome 11 open reading frame 92 | 1 | | 3000 | hsa-miR-95 | 399948 | C11orf92 | chromosome 11 open reading frame 92 | 1 | | 3001 | hsa-mir-505 | 342979 | PALM3 | Paralemmin-3 | 1 | | 3002 | hsa-mir-3130-1 | 342979 | PALM3 | Paralemmin-3 | 1 | | 3003 | hsa-mir-3130-2 | 342979 | PALM3 | Paralemmin-3 | 1 | | 3004 | hsa-mir-3130-3 | 342979 | PALM3 | Paralemmin-3 | 1 | | 3005 | hsa-mir-3130-4 | 342979 | PALM3 | Paralemmin-3 | 1 | | 3006 | hsa-miR-10a | 342979 | PALM3 | Paralemmin-3 | 1 | | 3007 | hsa-miR-505\* | 342979 | PALM3 | Paralemmin-3 | 1 | | 3008 | hsa-miR-95 | 342979 | PALM3 | Paralemmin-3 | 1 | | 3009 | hsa-miR-618 | 131540 | ZDHHC19 | zinc finger, DHHC-type containing 19 | 1 | | 3010 | hsa-miR-643 | 131540 | ZDHHC19 | zinc finger, DHHC-type containing 19 | 1 | | 3011 | hsa-miR-128 | 387644 | NCRNA00202 | non-protein coding RNA 202 | 1 | | 3012 | hsa-miR-608 | 100129633 | LOC100129633 | similar to hCG1651427 | 1 | | 3013 | hsa-miR-95 | 645524 | FLJ36840 | hypothetical LOC645524 | 1 | | 3014 | hsa-miR-10a | 344 | APOC2 | apolipoprotein C-II | 1 | | 3015 | hsa-mir-505 | 54872 | PIGG | phosphatidylinositol glycan anchor biosynthesis, class G | 1 | | 3016 | hsa-miR-505\* | 54872 | PIGG | phosphatidylinositol glycan anchor biosynthesis, class G | 1 | | 3017 | hsa-miR-95 | 54872 | PIGG | phosphatidylinositol glycan anchor biosynthesis, class G | 1 | | 3018 | hsa-miR-1233 | 55250 | ELP2 | elongation protein 2 homolog (S. cerevisiae) | 1 | | 3019 | hsa-miR-569 | 55250 | ELP2 | elongation protein 2 homolog (S. cerevisiae) | 1 | | 3020 | hsa-miR-571 | 55250 | ELP2 | elongation protein 2 homolog (S. cerevisiae) | 1 | | 3021 | hsa-miR-766 | 55250 | ELP2 | elongation protein 2 homolog (S. cerevisiae) | 1 | | 3022 | hsa-miR-874 | 55250 | ELP2 | elongation protein 2 homolog (S. cerevisiae) | 1 | | 3023 | hsa-miR-95 | 7473 | WNT3 | wingless-type MMTV integration site family, member 3 | 1 | | 3024 | hsa-miR-95 | 58531 | PRM3 | protamine 3 | 1 | | 3025 | hsa-miR-1233 | 8320 | EOMES | eomesodermin homolog (Xenopus laevis) | 1 | | 3026 | hsa-miR-569 | 8320 | EOMES | eomesodermin homolog (Xenopus laevis) | 1 | | 3027 | hsa-miR-766 | 8320 | EOMES | eomesodermin homolog (Xenopus laevis) | 1 | | 3028 | hsa-miR-874 | 8320 | EOMES | eomesodermin homolog (Xenopus laevis) | 1 | | 3029 | hsa-miR-10a | 155435 | RBM33 | RNA binding motif protein 33 | 1 | | 3030 | hsa-miR-1233 | 57646 | USP28 | ubiquitin specific peptidase 28 | 1 | | 3031 | hsa-miR-766 | 57646 | USP28 | ubiquitin specific peptidase 28 | 1 | | 3032 | hsa-miR-454\* | 201626 | PDE12 | phosphodiesterase 12 | 1 | | 3033 | hsa-miR-558 | 201626 | PDE12 | phosphodiesterase 12 | 1 | | 3034 | hsa-miR-938 | 91056 | DKFZp761E198 | DKFZp761E198 protein | 1 | | 3035 | hsa-miR-571 | 51068 | NMD3 | NMD3 homolog (S. cerevisiae) | 1 | | 3036 | hsa-miR-623 | 81844 | TRIM56 | tripartite motif-containing 56 | 1 | | 3037 | hsa-mir-10a | 80067 | DCAF17 | DDB1 and CUL4 associated factor 17 | 1 | | 3038 | hsa-miR-1233 | 80067 | DCAF17 | DDB1 and CUL4 associated factor 17 | 1 | | 3039 | hsa-miR-569 | 80067 | DCAF17 | DDB1 and CUL4 associated factor 17 | 1 | | 3040 | hsa-miR-571 | 80067 | DCAF17 | DDB1 and CUL4 associated factor 17 | 1 | | 3041 | hsa-miR-766 | 80067 | DCAF17 | DDB1 and CUL4 associated factor 17 | 1 | | 3042 | hsa-miR-10a | 441027 | TMEM150C | transmembrane protein 150C | 1 | | 3043 | hsa-miR-95 | 441027 | TMEM150C | transmembrane protein 150C | 1 | | 3044 | hsa-miR-1233 | 54014 | BRWD1 | bromodomain and WD repeat domain containing 1 | 1 | | 3045 | hsa-miR-555 | 54014 | BRWD1 | bromodomain and WD repeat domain containing 1 | 1 | | 3046 | hsa-miR-558 | 54014 | BRWD1 | bromodomain and WD repeat domain containing 1 | 1 | | 3047 | hsa-miR-874 | 54014 | BRWD1 | bromodomain and WD repeat domain containing 1 | 1 | | 3048 | hsa-miR-558 | 10236 | HNRNPR | heterogeneous nuclear ribonucleoprotein R | 1 | | 3049 | hsa-miR-874 | 10236 | HNRNPR | heterogeneous nuclear ribonucleoprotein R | 1 | | 3050 | hsa-miR-571 | 55326 | AGPAT5 | 1-acylglycerol-3-phosphate O-acyltransferase 5 (lysophosphatidic acid acyltransferase, epsilon) | 1 | | 3051 | hsa-miR-1233 | 9801 | MRPL19 | mitochondrial ribosomal protein L19 | 1 | | 3052 | hsa-mir-3130-1 | 375057 | C1orf95 | chromosome 1 open reading frame 95 | 1 | | 3053 | hsa-mir-3130-2 | 375057 | C1orf95 | chromosome 1 open reading frame 95 | 1 | | 3054 | hsa-mir-3130-3 | 375057 | C1orf95 | chromosome 1 open reading frame 95 | 1 | | 3055 | hsa-mir-3130-4 | 375057 | C1orf95 | chromosome 1 open reading frame 95 | 1 | | 3056 | hsa-miR-10a | 375057 | C1orf95 | chromosome 1 open reading frame 95 | 1 | | 3057 | hsa-miR-499-5p | 375057 | C1orf95 | chromosome 1 open reading frame 95 | 1 | | 3058 | hsa-miR-95 | 375057 | C1orf95 | chromosome 1 open reading frame 95 | 1 | | 3059 | hsa-miR-1233 | 100132352 | LOC100132352 | similar to hCG1989297 | 1 | | 3060 | hsa-miR-558 | 100132352 | LOC100132352 | similar to hCG1989297 | 1 | | 3061 | hsa-miR-423-5p | 10730 | YME1L1 | YME1-like 1 (S. cerevisiae) | 1 | | 3062 | hsa-miR-555 | 860 | RUNX2 | runt-related transcription factor 2 | 1 | | 3063 | hsa-miR-1233 | 9487 | PIGL | phosphatidylinositol glycan anchor biosynthesis, class L | 1 | | 3064 | hsa-miR-874 | 9487 | PIGL | phosphatidylinositol glycan anchor biosynthesis, class L | 1 | | 3065 | hsa-miR-95 | 3768 | KCNJ12 | potassium inwardly-rectifying channel, subfamily J, member 12 | 1 | | 3066 | hsa-mir-3130-1 | 80726 | KIAA1683 | KIAA1683 | 1 | | 3067 | hsa-mir-3130-2 | 80726 | KIAA1683 | KIAA1683 | 1 | | 3068 | hsa-mir-3130-3 | 80726 | KIAA1683 | KIAA1683 | 1 | | 3069 | hsa-mir-3130-4 | 80726 | KIAA1683 | KIAA1683 | 1 | | 3070 | hsa-miR-10a | 80726 | KIAA1683 | KIAA1683 | 1 | | 3071 | hsa-miR-1233 | 9159 | PCSK7 | proprotein convertase subtilisin/kexin type 7 | 1 | | 3072 | hsa-miR-766 | 9159 | PCSK7 | proprotein convertase subtilisin/kexin type 7 | 1 | | 3073 | hsa-miR-1233 | 100133315 | LOC100133315 | transient receptor potential cation channel, subfamily C, member 2-like | 1 | | 3074 | hsa-miR-874 | 100133315 | LOC100133315 | transient receptor potential cation channel, subfamily C, member 2-like | 1 | | 3075 | hsa-miR-555 | 11168 | PSIP1 | PC4 and SFRS1 interacting protein 1 | 1 | | 3076 | hsa-miR-576-5p | 11168 | PSIP1 | PC4 and SFRS1 interacting protein 1 | 1 | | 3077 | hsa-miR-95 | 57497 | LRFN2 | leucine rich repeat and fibronectin type III domain containing 2 | 1 | | 3078 | hsa-miR-675 | 8997 | KALRN | kalirin, RhoGEF kinase | 1 | | 3079 | hsa-miR-95 | 8997 | KALRN | kalirin, RhoGEF kinase | 1 | | 3080 | hsa-mir-10a | 29883 | CNOT7 | CCR4-NOT transcription complex, subunit 7 | 1 | | 3081 | hsa-miR-423-5p | 29883 | CNOT7 | CCR4-NOT transcription complex, subunit 7 | 1 | | 3082 | hsa-miR-571 | 29883 | CNOT7 | CCR4-NOT transcription complex, subunit 7 | 1 | | 3083 | hsa-miR-126\* | 80020 | FOXRED2 | FAD-dependent oxidoreductase domain containing 2 | 1 | | 3084 | hsa-miR-10a | 8991 | SELENBP1 | selenium binding protein 1 | 1 | | 3085 | hsa-miR-95 | 8991 | SELENBP1 | selenium binding protein 1 | 1 | | 3086 | hsa-mir-3130-1 | 53345 | TM6SF2 | transmembrane 6 superfamily member 2 | 1 | | 3087 | hsa-mir-3130-2 | 53345 | TM6SF2 | transmembrane 6 superfamily member 2 | 1 | | 3088 | hsa-mir-3130-3 | 53345 | TM6SF2 | transmembrane 6 superfamily member 2 | 1 | | 3089 | hsa-mir-3130-4 | 53345 | TM6SF2 | transmembrane 6 superfamily member 2 | 1 | | 3090 | hsa-miR-10a | 53345 | TM6SF2 | transmembrane 6 superfamily member 2 | 1 | | 3091 | hsa-miR-95 | 389362 | PSMG4 | proteasome (prosome, macropain) assembly chaperone 4 | 1 | | 3092 | hsa-miR-1233 | 7769 | ZNF226 | zinc finger protein 226 | 1 | | 3093 | hsa-miR-569 | 7769 | ZNF226 | zinc finger protein 226 | 1 | | 3094 | hsa-miR-766 | 7769 | ZNF226 | zinc finger protein 226 | 1 | | 3095 | hsa-miR-874 | 7769 | ZNF226 | zinc finger protein 226 | 1 | | 3096 | hsa-miR-95 | 43849 | KLK12 | kallikrein-related peptidase 12 | 1 | | 3097 | hsa-miR-580 | 150379 | PNPLA5 | patatin-like phospholipase domain containing 5 | 1 | | 3098 | hsa-miR-423-5p | 50717 | DCAF8 | DDB1 and CUL4 associated factor 8 | 1 | | 3099 | hsa-miR-766 | 54497 | HEATR5B | HEAT repeat containing 5B | 1 | | 3100 | hsa-miR-95 | 57159 | TRIM54 | tripartite motif-containing 54 | 1 | | 3101 | hsa-miR-423-5p | 57223 | SMEK2 | SMEK homolog 2, suppressor of mek1 (Dictyostelium) | 1 | | 3102 | hsa-miR-576-5p | 57223 | SMEK2 | SMEK homolog 2, suppressor of mek1 (Dictyostelium) | 1 | | 3103 | hsa-mir-10a | 394 | ARHGAP5 | Rho GTPase activating protein 5 | 1 | | 3104 | hsa-miR-95 | 100133790 | LOC100133790 | intestinal mucin-like | 1 | | 3105 | hsa-miR-95 | 140832 | WFDC10A | WAP four-disulfide core domain 10A | 1 | | 3107 | hsa-miR-10a | 100287166 | LOC100287166 | similar to hCG2019076 | 1 | | 3108 | hsa-miR-95 | 100287166 | LOC100287166 | similar to hCG2019076 | 1 | | 3109 | hsa-miR-10a | 92736 | OTOP2 | otopetrin 2 | 1 | | 3110 | hsa-miR-95 | 92736 | OTOP2 | otopetrin 2 | 1 | | 3111 | hsa-mir-3130-1 | 55888 | ZNF167 | zinc finger protein 167 | 1 | | 3112 | hsa-mir-3130-2 | 55888 | ZNF167 | zinc finger protein 167 | 1 | | 3113 | hsa-mir-3130-3 | 55888 | ZNF167 | zinc finger protein 167 | 1 | | 3114 | hsa-mir-3130-4 | 55888 | ZNF167 | zinc finger protein 167 | 1 | | 3115 | hsa-miR-10a | 55888 | ZNF167 | zinc finger protein 167 | 1 | | 3116 | hsa-miR-95 | 55888 | ZNF167 | zinc finger protein 167 | 1 | | 3117 | hsa-mir-505 | 84699 | CREB3L3 | cAMP responsive element binding protein 3-like 3 | 1 | | 3118 | hsa-miR-505\* | 84699 | CREB3L3 | cAMP responsive element binding protein 3-like 3 | 1 | | 3119 | hsa-miR-580 | 84699 | CREB3L3 | cAMP responsive element binding protein 3-like 3 | 1 | | 3120 | hsa-miR-95 | 84699 | CREB3L3 | cAMP responsive element binding protein 3-like 3 | 1 | | 3121 | hsa-mir-3130-1 | 164656 | TMPRSS6 | transmembrane protease, serine 6 | 1 | | 3122 | hsa-mir-3130-2 | 164656 | TMPRSS6 | transmembrane protease, serine 6 | 1 | | 3123 | hsa-mir-3130-3 | 164656 | TMPRSS6 | transmembrane protease, serine 6 | 1 | | 3124 | hsa-mir-3130-4 | 164656 | TMPRSS6 | transmembrane protease, serine 6 | 1 | | 3125 | hsa-miR-128 | 164656 | TMPRSS6 | transmembrane protease, serine 6 | 1 | | 3126 | hsa-miR-95 | 164656 | TMPRSS6 | transmembrane protease, serine 6 | 1 | | 3127 | hsa-miR-10a | 728701 | LOC728701 | hypothetical LOC728701 | 1 | | 3128 | hsa-miR-95 | 728701 | LOC728701 | hypothetical LOC728701 | 1 | | 3129 | hsa-mir-3130-1 | 100291944 | LOC100291944 | hypothetical protein LOC100291944 | 1 | | 3130 | hsa-mir-3130-2 | 100291944 | LOC100291944 | hypothetical protein LOC100291944 | 1 | | 3131 | hsa-mir-3130-3 | 100291944 | LOC100291944 | hypothetical protein LOC100291944 | 1 | | 3132 | hsa-mir-3130-4 | 100291944 | LOC100291944 | hypothetical protein LOC100291944 | 1 | | 3133 | hsa-miR-10a | 100291944 | LOC100291944 | hypothetical protein LOC100291944 | 1 | | 3134 | hsa-miR-10a | 55554 | KLK15 | kallikrein-related peptidase 15 | 1 | | 3135 | hsa-miR-95 | 55554 | KLK15 | kallikrein-related peptidase 15 | 1 | | 3136 | hsa-miR-10a | 491 | ATP2B2 | ATPase, Ca++ transporting, plasma membrane 2 | 1 | | 3137 | hsa-mir-3130-1 | 284187 | DKFZp761P0212 | hypothetical protein DKFZp761P0212 | 1 | | 3138 | hsa-mir-3130-2 | 284187 | DKFZp761P0212 | hypothetical protein DKFZp761P0212 | 1 | | 3139 | hsa-mir-3130-3 | 284187 | DKFZp761P0212 | hypothetical protein DKFZp761P0212 | 1 | | 3140 | hsa-mir-3130-4 | 284187 | DKFZp761P0212 | hypothetical protein DKFZp761P0212 | 1 | | 3141 | hsa-miR-10a | 284187 | DKFZp761P0212 | hypothetical protein DKFZp761P0212 | 1 | | 3142 | hsa-miR-95 | 284187 | DKFZp761P0212 | hypothetical protein DKFZp761P0212 | 1 | | 3143 | hsa-miR-95 | 55354 | ORF1 | hypothetical protein, clone pT-Adv JuaX22 | 1 | | 3144 | hsa-miR-558 | 134218 | DNAJC21 | DnaJ (Hsp40) homolog, subfamily C, member 21 | 1 | | 3145 | hsa-miR-569 | 26049 | FAM169A | family with sequence similarity 169, member A | 1 | | 3146 | hsa-miR-766 | 26049 | FAM169A | family with sequence similarity 169, member A | 1 | | 3147 | hsa-miR-10a | 4585 | MUC4 | mucin 4, cell surface associated | 1 | | 3148 | hsa-miR-95 | 4585 | MUC4 | mucin 4, cell surface associated | 1 | | 3149 | hsa-miR-1233 | 157285 | PRAGMIN | homolog of rat pragma of Rnd2 | 1 | | 3150 | hsa-miR-874 | 157285 | PRAGMIN | homolog of rat pragma of Rnd2 | 1 | | 3151 | hsa-miR-555 | 5194 | PEX13 | peroxisomal biogenesis factor 13 | 1 | | 3152 | hsa-miR-576-5p | 5194 | PEX13 | peroxisomal biogenesis factor 13 | 1 | | 3153 | hsa-mir-10a | 123169 | LEO1 | Leo1, Paf1/RNA polymerase II complex component, homolog (S. cerevisiae) | 1 | | 3154 | hsa-miR-1233 | 123169 | LEO1 | Leo1, Paf1/RNA polymerase II complex component, homolog (S. cerevisiae) | 1 | | 3155 | hsa-miR-571 | 123169 | LEO1 | Leo1, Paf1/RNA polymerase II complex component, homolog (S. cerevisiae) | 1 | | 3156 | hsa-miR-766 | 123169 | LEO1 | Leo1, Paf1/RNA polymerase II complex component, homolog (S. cerevisiae) | 1 | | 3157 | hsa-mir-10a | 4124 | MAN2A1 | mannosidase, alpha, class 2A, member 1 | 1 | | 3158 | hsa-miR-576-5p | 4124 | MAN2A1 | mannosidase, alpha, class 2A, member 1 | 1 | | 3159 | hsa-miR-454\* | 122769 | PPIL5 | peptidylprolyl isomerase (cyclophilin)-like 5 | 1 | | 3160 | hsa-miR-423-5p | 645212 | LOC645212 | hypothetical LOC645212 | 1 | | 3161 | hsa-mir-10a | 254128 | LOC254128 | hypothetical protein LOC254128 | 1 | | 3162 | hsa-miR-423-5p | 254128 | LOC254128 | hypothetical protein LOC254128 | 1 | | 3163 | hsa-miR-10a | 134549 | SHROOM1 | shroom family member 1 | 1 | | 3164 | hsa-mir-10a | 84928 | TMEM209 | transmembrane protein 209 | 1 | | 3165 | hsa-miR-423-5p | 84928 | TMEM209 | transmembrane protein 209 | 1 | | 3166 | hsa-miR-454\* | 84928 | TMEM209 | transmembrane protein 209 | 1 | | 3167 | hsa-miR-1233 | 54941 | RNF125 | ring finger protein 125 | 1 | | 3168 | hsa-miR-558 | 54941 | RNF125 | ring finger protein 125 | 1 | | 3169 | hsa-miR-569 | 54941 | RNF125 | ring finger protein 125 | 1 | | 3170 | hsa-miR-571 | 54941 | RNF125 | ring finger protein 125 | 1 | | 3171 | hsa-miR-766 | 54941 | RNF125 | ring finger protein 125 | 1 | | 3172 | hsa-miR-874 | 54941 | RNF125 | ring finger protein 125 | 1 | | 3173 | hsa-miR-766 | 3707 | ITPKB | inositol 1,4,5-trisphosphate 3-kinase B | 1 | | 3174 | hsa-miR-571 | 88745 | C6orf153 | chromosome 6 open reading frame 153 | 1 | | 3175 | hsa-miR-555 | 10691 | GMEB1 | glucocorticoid modulatory element binding protein 1 | 1 | | 3176 | hsa-miR-558 | 10691 | GMEB1 | glucocorticoid modulatory element binding protein 1 | 1 | | 3177 | hsa-miR-576-5p | 10691 | GMEB1 | glucocorticoid modulatory element binding protein 1 | 1 | | 3178 | hsa-miR-938 | 219988 | PATL1 | protein associated with topoisomerase II homolog 1 (yeast) | 1 | | 3179 | hsa-miR-1233 | 257144 | GCET2 | germinal center expressed transcript 2 | 1 | | 3180 | hsa-miR-1233 | 6428 | SFRS3 | splicing factor, arginine/serine-rich 3 | 1 | | 3181 | hsa-miR-1233 | 84859 | LRCH3 | leucine-rich repeats and calponin homology (CH) domain containing 3 | 1 | | 3182 | hsa-miR-558 | 84859 | LRCH3 | leucine-rich repeats and calponin homology (CH) domain containing 3 | 1 | | 3183 | hsa-miR-571 | 84859 | LRCH3 | leucine-rich repeats and calponin homology (CH) domain containing 3 | 1 | | 3184 | hsa-miR-1233 | 132949 | AASDH | aminoadipate-semialdehyde dehydrogenase | 1 | | 3185 | hsa-miR-766 | 132949 | AASDH | aminoadipate-semialdehyde dehydrogenase | 1 | | 3186 | hsa-miR-95 | 65986 | ZBTB10 | zinc finger and BTB domain containing 10 | 1 | | 3187 | hsa-miR-454\* | 57721 | METTL14 | methyltransferase like 14 | 1 | | 3188 | hsa-miR-618 | 199675 | C19orf59 | chromosome 19 open reading frame 59 | 1 | | 3189 | hsa-miR-874 | 394 | ARHGAP5 | Rho GTPase activating protein 5 | 1 | | 3191 | hsa-miR-423-5p | 163081 | ZNF567 | zinc finger protein 567 | 1 | | 3192 | hsa-miR-555 | 163081 | ZNF567 | zinc finger protein 567 | 1 | | 3193 | hsa-miR-558 | 163081 | ZNF567 | zinc finger protein 567 | 1 | | 3196 | hsa-mir-10a | 146198 | ZFP90 | zinc finger protein 90 homolog (mouse) | 1 | | 3197 | hsa-miR-423-5p | 146198 | ZFP90 | zinc finger protein 90 homolog (mouse) | 1 | | 3198 | hsa-miR-569 | 146198 | ZFP90 | zinc finger protein 90 homolog (mouse) | 1 | | 3199 | hsa-miR-95 | 162968 | ZNF497 | zinc finger protein 497 | 1 | | 3200 | hsa-mir-10a | 7569 | ZNF182 | zinc finger protein 182 | 1 | | 3201 | hsa-miR-423-5p | 7569 | ZNF182 | zinc finger protein 182 | 1 | | 3202 | hsa-miR-571 | 7569 | ZNF182 | zinc finger protein 182 | 1 | | 3203 | hsa-miR-618 | 266747 | RGL4 | ral guanine nucleotide dissociation stimulator-like 4 | 1 | | 3204 | hsa-miR-874 | 54554 | WDR5B | WD repeat domain 5B | 1 | | 3205 | hsa-mir-3130-1 | 4320 | MMP11 | matrix metallopeptidase 11 (stromelysin 3) | 1 | | 3206 | hsa-mir-3130-2 | 4320 | MMP11 | matrix metallopeptidase 11 (stromelysin 3) | 1 | | 3207 | hsa-mir-3130-3 | 4320 | MMP11 | matrix metallopeptidase 11 (stromelysin 3) | 1 | | 3208 | hsa-mir-3130-4 | 4320 | MMP11 | matrix metallopeptidase 11 (stromelysin 3) | 1 | | 3209 | hsa-miR-10a | 4320 | MMP11 | matrix metallopeptidase 11 (stromelysin 3) | 1 | | 3210 | hsa-miR-1233 | 7514 | XPO1 | exportin 1 (CRM1 homolog, yeast) | 1 | | 3211 | hsa-miR-874 | 57562 | KIAA1377 | KIAA1377 | 1 | | 3217 | hsa-miR-1233 | 201475 | RAB12 | RAB12, member RAS oncogene family | 1 | | 3218 | hsa-miR-766 | 201475 | RAB12 | RAB12, member RAS oncogene family | 1 | | 3219 | hsa-miR-558 | 119392 | C10orf78 | chromosome 10 open reading frame 78 | 1 | | 3220 | hsa-mir-3130-1 | 388610 | TRNP1 | TMF1-regulated nuclear protein 1 | 1 | | 3221 | hsa-mir-3130-2 | 388610 | TRNP1 | TMF1-regulated nuclear protein 1 | 1 | | 3222 | hsa-mir-3130-3 | 388610 | TRNP1 | TMF1-regulated nuclear protein 1 | 1 | | 3223 | hsa-mir-3130-4 | 388610 | TRNP1 | TMF1-regulated nuclear protein 1 | 1 | | 3224 | hsa-miR-10a | 388610 | TRNP1 | TMF1-regulated nuclear protein 1 | 1 | | 3225 | hsa-miR-499-5p | 388610 | TRNP1 | TMF1-regulated nuclear protein 1 | 1 | | 3226 | hsa-miR-580 | 388610 | TRNP1 | TMF1-regulated nuclear protein 1 | 1 | | 3227 | hsa-miR-95 | 388610 | TRNP1 | TMF1-regulated nuclear protein 1 | 1 | | 3228 | hsa-mir-3130-1 | 84448 | ABLIM2 | actin binding LIM protein family, member 2 | 1 | | 3229 | hsa-mir-3130-2 | 84448 | ABLIM2 | actin binding LIM protein family, member 2 | 1 | | 3230 | hsa-mir-3130-3 | 84448 | ABLIM2 | actin binding LIM protein family, member 2 | 1 | | 3231 | hsa-mir-3130-4 | 84448 | ABLIM2 | actin binding LIM protein family, member 2 | 1 | | 3232 | hsa-miR-10a | 84448 | ABLIM2 | actin binding LIM protein family, member 2 | 1 | | 3233 | hsa-miR-128 | 84448 | ABLIM2 | actin binding LIM protein family, member 2 | 1 | | 3234 | hsa-miR-580 | 84448 | ABLIM2 | actin binding LIM protein family, member 2 | 1 | | 3235 | hsa-miR-95 | 84448 | ABLIM2 | actin binding LIM protein family, member 2 | 1 | | 3236 | hsa-miR-10a | 9265 | CYTH3 | cytohesin 3 | 1 | | 3237 | hsa-miR-95 | 9265 | CYTH3 | cytohesin 3 | 1 | | 3238 | hsa-miR-423-5p | 9321 | TRIP11 | thyroid hormone receptor interactor 11 | 1 | | 3239 | hsa-miR-10a | 89876 | C3orf15 | chromosome 3 open reading frame 15 | 1 | | 3240 | hsa-mir-10a | 51003 | MED31 | mediator complex subunit 31 | 1 | | 3241 | hsa-miR-1224-5p | 51003 | MED31 | mediator complex subunit 31 | 1 | | 3242 | hsa-miR-423-5p | 51003 | MED31 | mediator complex subunit 31 | 1 | | 3243 | hsa-miR-1233 | 10075 | HUWE1 | HECT, UBA and WWE domain containing 1 | 1 | | 3244 | hsa-miR-1233 | 197358 | NLRC3 | NLR family, CARD domain containing 3 | 1 | | 3245 | hsa-miR-558 | 197358 | NLRC3 | NLR family, CARD domain containing 3 | 1 | | 3246 | hsa-miR-569 | 197358 | NLRC3 | NLR family, CARD domain containing 3 | 1 | | 3247 | hsa-miR-571 | 197358 | NLRC3 | NLR family, CARD domain containing 3 | 1 | | 3248 | hsa-miR-766 | 197358 | NLRC3 | NLR family, CARD domain containing 3 | 1 | | 3249 | hsa-miR-874 | 197358 | NLRC3 | NLR family, CARD domain containing 3 | 1 | | 3250 | hsa-miR-10a | 28959 | TMEM176B | transmembrane protein 176B | 1 | | 3251 | hsa-miR-95 | 28959 | TMEM176B | transmembrane protein 176B | 1 | | 3252 | hsa-miR-95 | 161931 | ADAD2 | adenosine deaminase domain containing 2 | 1 | | 3253 | hsa-miR-10a | 123099 | DEGS2 | degenerative spermatocyte homolog 2, lipid desaturase (Drosophila) | 1 | | 3254 | hsa-miR-499-5p | 123099 | DEGS2 | degenerative spermatocyte homolog 2, lipid desaturase (Drosophila) | 1 | | 3255 | hsa-miR-95 | 123099 | DEGS2 | degenerative spermatocyte homolog 2, lipid desaturase (Drosophila) | 1 | | 3256 | hsa-miR-95 | 91694 | LONRF1 | LON peptidase N-terminal domain and ring finger 1 | 1 | | 3257 | hsa-mir-3130-1 | 646808 | LOC646808 | similar to L antigen family, member 3 | 1 | | 3258 | hsa-mir-3130-2 | 646808 | LOC646808 | similar to L antigen family, member 3 | 1 | | 3259 | hsa-mir-3130-3 | 646808 | LOC646808 | similar to L antigen family, member 3 | 1 | | 3260 | hsa-mir-3130-4 | 646808 | LOC646808 | similar to L antigen family, member 3 | 1 | | 3261 | hsa-miR-10a | 646808 | LOC646808 | similar to L antigen family, member 3 | 1 | | 3262 | hsa-miR-874 | 60468 | BACH2 | BTB and CNC homology 1, basic leucine zipper transcription factor 2 | 1 | | 3263 | hsa-miR-571 | 152579 | SCFD2 | sec1 family domain containing 2 | 1 | | 3264 | hsa-miR-95 | 56157 | TEX13A | testis expressed 13A | 1 | | 3268 | hsa-miR-608 | 2175 | FANCA | Fanconi anemia, complementation group A | 1 | | 3269 | hsa-miR-1233 | 143884 | CWF19L2 | CWF19-like 2, cell cycle control (S. pombe) | 1 | | 3270 | hsa-miR-608 | 8899 | PRPF4B | PRP4 pre-mRNA processing factor 4 homolog B (yeast) | 1 | | 3271 | hsa-miR-874 | 55251 | PCMTD2 | protein-L-isoaspartate (D-aspartate) O-methyltransferase domain containing 2 | 1 | | 3272 | hsa-miR-554 | 1385 | CREB1 | cAMP responsive element binding protein 1 | 1 | | 3273 | hsa-miR-618 | 116369 | SLC26A8 | solute carrier family 26, member 8 | 1 | | 3274 | hsa-mir-3130-1 | 80709 | AKNA | AT-hook transcription factor | 1 | | 3275 | hsa-mir-3130-2 | 80709 | AKNA | AT-hook transcription factor | 1 | | 3276 | hsa-mir-3130-3 | 80709 | AKNA | AT-hook transcription factor | 1 | | 3277 | hsa-mir-3130-4 | 80709 | AKNA | AT-hook transcription factor | 1 | | 3278 | hsa-miR-10a | 80709 | AKNA | AT-hook transcription factor | 1 | | 3279 | hsa-miR-95 | 80709 | AKNA | AT-hook transcription factor | 1 | | 3280 | hsa-miR-558 | 25942 | SIN3A | SIN3 homolog A, transcription regulator (yeast) | 1 | | 3281 | hsa-miR-571 | 25942 | SIN3A | SIN3 homolog A, transcription regulator (yeast) | 1 | | 3282 | hsa-miR-1233 | 5205 | ATP8B1 | ATPase, class I, type 8B, member 1 | 1 | | 3283 | hsa-miR-558 | 5205 | ATP8B1 | ATPase, class I, type 8B, member 1 | 1 | | 3284 | hsa-miR-874 | 5205 | ATP8B1 | ATPase, class I, type 8B, member 1 | 1 | | 3285 | hsa-miR-95 | 4071 | TM4SF1 | transmembrane 4 L six family member 1 | 1 | | 3286 | hsa-miR-580 | 122622 | ADSSL1 | adenylosuccinate synthase like 1 | 1 | | 3287 | hsa-miR-95 | 122622 | ADSSL1 | adenylosuccinate synthase like 1 | 1 | | 3288 | hsa-miR-1233 | 201595 | STT3B | STT3, subunit of the oligosaccharyltransferase complex, homolog B (S. cerevisiae) | 1 | | 3289 | hsa-miR-10a | 440836 | ODF3B | outer dense fiber of sperm tails 3B | 1 | | 3290 | hsa-miR-95 | 440836 | ODF3B | outer dense fiber of sperm tails 3B | 1 | | 3291 | hsa-mir-10a | 134218 | DNAJC21 | DnaJ (Hsp40) homolog, subfamily C, member 21 | 1 | | 3292 | hsa-miR-576-5p | 134218 | DNAJC21 | DnaJ (Hsp40) homolog, subfamily C, member 21 | 1 | | 3294 | hsa-miR-95 | 9315 | C5orf13 | chromosome 5 open reading frame 13 | 1 | | 3296 | hsa-miR-874 | 163255 | ZNF540 | zinc finger protein 540 | 1 | | 3297 | hsa-miR-1233 | 55809 | TRERF1 | transcriptional regulating factor 1 | 1 | | 3298 | hsa-miR-874 | 55809 | TRERF1 | transcriptional regulating factor 1 | 1 | | 3299 | hsa-mir-10a | 23530 | NNT | nicotinamide nucleotide transhydrogenase | 1 | | 3300 | hsa-miR-423-5p | 23530 | NNT | nicotinamide nucleotide transhydrogenase | 1 | | 3301 | hsa-miR-555 | 80264 | ZNF430 | zinc finger protein 430 | 1 | | 3302 | hsa-miR-576-5p | 80264 | ZNF430 | zinc finger protein 430 | 1 | | 3303 | hsa-miR-1233 | 284161 | GDPD1 | glycerophosphodiester phosphodiesterase domain containing 1 | 1 | | 3304 | hsa-miR-571 | 284161 | GDPD1 | glycerophosphodiester phosphodiesterase domain containing 1 | 1 | | 3305 | hsa-miR-874 | 284161 | GDPD1 | glycerophosphodiester phosphodiesterase domain containing 1 | 1 | | 3306 | hsa-mir-10a | 119392 | C10orf78 | chromosome 10 open reading frame 78 | 1 | | 3307 | hsa-miR-571 | 84671 | ZNF347 | zinc finger protein 347 | 1 | | 3308 | hsa-miR-874 | 84671 | ZNF347 | zinc finger protein 347 | 1 | | 3309 | hsa-miR-558 | 221443 | C6orf130 | chromosome 6 open reading frame 130 | 1 | | 3310 | hsa-miR-874 | 221443 | C6orf130 | chromosome 6 open reading frame 130 | 1 | | 3311 | hsa-miR-558 | 138241 | C9orf85 | chromosome 9 open reading frame 85 | 1 | | 3312 | hsa-miR-126\* | 113655 | MFSD3 | major facilitator superfamily domain containing 3 | 1 | | 3313 | hsa-miR-95 | 116988 | AGAP3 | ArfGAP with GTPase domain, ankyrin repeat and PH domain 3 | 1 | | 3314 | hsa-miR-874 | 124540 | MSI2 | musashi homolog 2 (Drosophila) | 1 | | 3315 | hsa-mir-10a | 57645 | POGK | pogo transposable element with KRAB domain | 1 | | 3316 | hsa-miR-423-5p | 57645 | POGK | pogo transposable element with KRAB domain | 1 | | 3317 | hsa-mir-3130-1 | 150696 | PROM2 | prominin 2 | 1 | | 3318 | hsa-mir-3130-2 | 150696 | PROM2 | prominin 2 | 1 | | 3319 | hsa-mir-3130-3 | 150696 | PROM2 | prominin 2 | 1 | | 3320 | hsa-mir-3130-4 | 150696 | PROM2 | prominin 2 | 1 | | 3321 | hsa-miR-10a | 150696 | PROM2 | prominin 2 | 1 | | 3322 | hsa-miR-95 | 150696 | PROM2 | prominin 2 | 1 | | 3323 | hsa-miR-95 | 145837 | LOC145837 | hypothetical LOC145837 | 1 | | 3324 | hsa-miR-1233 | 3716 | JAK1 | Janus kinase 1 | 1 | | 3325 | hsa-miR-569 | 3716 | JAK1 | Janus kinase 1 | 1 | | 3326 | hsa-miR-766 | 3716 | JAK1 | Janus kinase 1 | 1 | | 3327 | hsa-miR-10a | 22907 | DHX30 | DEAH (Asp-Glu-Ala-His) box polypeptide 30 | 1 | | 3328 | hsa-miR-95 | 22907 | DHX30 | DEAH (Asp-Glu-Ala-His) box polypeptide 30 | 1 | | 3329 | hsa-miR-571 | 148254 | ZNF555 | zinc finger protein 555 | 1 | | 3330 | hsa-miR-10a | 339541 | C1orf228 | chromosome 1 open reading frame 228 | 1 | | 3331 | hsa-miR-608 | 100293830 | LOC100293830 | hypothetical protein LOC100293830 | 1 | | 3332 | hsa-mir-505 | 92949 | ADAMTSL1 | ADAMTS-like 1 | 1 | | 3333 | hsa-miR-505\* | 92949 | ADAMTSL1 | ADAMTS-like 1 | 1 | | 3334 | hsa-miR-95 | 92949 | ADAMTSL1 | ADAMTS-like 1 | 1 | | 3335 | hsa-miR-95 | 143678 | C11orf94 | chromosome 11 open reading frame 94 | 1 | | 3336 | hsa-miR-1233 | 3708 | ITPR1 | inositol 1,4,5-triphosphate receptor, type 1 | 1 | | 3337 | hsa-miR-571 | 3708 | ITPR1 | inositol 1,4,5-triphosphate receptor, type 1 | 1 | | 3338 | hsa-miR-874 | 3708 | ITPR1 | inositol 1,4,5-triphosphate receptor, type 1 | 1 | | 3339 | hsa-miR-10a | 8439 | NSMAF | neutral sphingomyelinase (N-SMase) activation associated factor | 1 | | 3340 | hsa-miR-95 | 8439 | NSMAF | neutral sphingomyelinase (N-SMase) activation associated factor | 1 | | 3341 | hsa-miR-423-5p | 55252 | ASXL2 | additional sex combs like 2 (Drosophila) | 1 | | 3342 | hsa-miR-608 | 730249 | IRG1 | immunoresponsive 1 homolog (mouse) | 1 | | 3343 | hsa-miR-10a | 130560 | SPATA3 | spermatogenesis associated 3 | 1 | | 3344 | hsa-miR-95 | 130560 | SPATA3 | spermatogenesis associated 3 | 1 | | 3345 | hsa-mir-3130-1 | 647107 | LOC647107 | hypothetical protein LOC647107 | 1 | | 3346 | hsa-mir-3130-2 | 647107 | LOC647107 | hypothetical protein LOC647107 | 1 | | 3347 | hsa-mir-3130-3 | 647107 | LOC647107 | hypothetical protein LOC647107 | 1 | | 3348 | hsa-mir-3130-4 | 647107 | LOC647107 | hypothetical protein LOC647107 | 1 | | 3349 | hsa-miR-95 | 647107 | LOC647107 | hypothetical protein LOC647107 | 1 | | 3350 | hsa-miR-555 | 254251 | LCORL | ligand dependent nuclear receptor corepressor-like | 1 | | 3351 | hsa-mir-3130-1 | 414927 | MGC34796 | SPR pseudogene | 1 | | 3352 | hsa-mir-3130-2 | 414927 | MGC34796 | SPR pseudogene | 1 | | 3353 | hsa-mir-3130-3 | 414927 | MGC34796 | SPR pseudogene | 1 | | 3354 | hsa-mir-3130-4 | 414927 | MGC34796 | SPR pseudogene | 1 | | 3355 | hsa-miR-10a | 414927 | MGC34796 | SPR pseudogene | 1 | | 3356 | hsa-miR-95 | 414927 | MGC34796 | SPR pseudogene | 1 | | 3357 | hsa-miR-10a | 64927 | TTC23 | tetratricopeptide repeat domain 23 | 1 | | 3358 | hsa-miR-95 | 64927 | TTC23 | tetratricopeptide repeat domain 23 | 1 | | 3359 | hsa-miR-95 | 203523 | ZNF449 | zinc finger protein 449 | 1 | | 3360 | hsa-miR-95 | 55211 | DPPA4 | developmental pluripotency associated 4 | 1 | | 3361 | hsa-mir-10a | 55219 | TMEM57 | transmembrane protein 57 | 1 | | 3362 | hsa-miR-10a | 142678 | MIB2 | mindbomb homolog 2 (Drosophila) | 1 | | 3363 | hsa-miR-95 | 142678 | MIB2 | mindbomb homolog 2 (Drosophila) | 1 | | 3364 | hsa-mir-3130-1 | 1141 | CHRNB2 | cholinergic receptor, nicotinic, beta 2 (neuronal) | 1 | | 3365 | hsa-mir-3130-2 | 1141 | CHRNB2 | cholinergic receptor, nicotinic, beta 2 (neuronal) | 1 | | 3366 | hsa-mir-3130-3 | 1141 | CHRNB2 | cholinergic receptor, nicotinic, beta 2 (neuronal) | 1 | | 3367 | hsa-mir-3130-4 | 1141 | CHRNB2 | cholinergic receptor, nicotinic, beta 2 (neuronal) | 1 | | 3368 | hsa-miR-10a | 1141 | CHRNB2 | cholinergic receptor, nicotinic, beta 2 (neuronal) | 1 | | 3369 | hsa-miR-95 | 1141 | CHRNB2 | cholinergic receptor, nicotinic, beta 2 (neuronal) | 1 | | 3370 | hsa-miR-10a | 286077 | FAM83H | family with sequence similarity 83, member H | 1 | | 3371 | hsa-miR-95 | 286077 | FAM83H | family with sequence similarity 83, member H | 1 | | 3372 | hsa-miR-10a | 388630 | LOC388630 | UPF0632 protein A | 1 | | 3373 | hsa-miR-571 | 126070 | ZNF440 | zinc finger protein 440 | 1 | | 3374 | hsa-miR-571 | 153443 | SRFBP1 | serum response factor binding protein 1 | 1 | | 3375 | hsa-miR-571 | 8481 | OFD1 | oral-facial-digital syndrome 1 | 1 | | 3376 | hsa-miR-1233 | 730051 | ZNF814 | zinc finger protein 814 | 1 | | 3377 | hsa-miR-571 | 730051 | ZNF814 | zinc finger protein 814 | 1 | | 3378 | hsa-miR-1233 | 100233209 | LOC100233209 | hypothetical LOC100233209 | 1 | | 3379 | hsa-miR-95 | 9744 | ACAP1 | ArfGAP with coiled-coil, ankyrin repeat and PH domains 1 | 1 | | 3380 | hsa-miR-569 | 6627 | SNRPA1 | small nuclear ribonucleoprotein polypeptide A' | 1 | | 3381 | hsa-miR-571 | 6627 | SNRPA1 | small nuclear ribonucleoprotein polypeptide A' | 1 | | 3382 | hsa-miR-874 | 6627 | SNRPA1 | small nuclear ribonucleoprotein polypeptide A' | 1 | | 3383 | hsa-miR-766 | 163081 | ZNF567 | zinc finger protein 567 | 1 | | 3384 | hsa-miR-1233 | 162966 | ZNF600 | zinc finger protein 600 | 1 | | 3385 | hsa-miR-558 | 162966 | ZNF600 | zinc finger protein 600 | 1 | | 3386 | hsa-miR-569 | 162966 | ZNF600 | zinc finger protein 600 | 1 | | 3387 | hsa-miR-571 | 162966 | ZNF600 | zinc finger protein 600 | 1 | | 3388 | hsa-miR-874 | 162966 | ZNF600 | zinc finger protein 600 | 1 | | 3389 | hsa-miR-558 | 51569 | UFM1 | ubiquitin-fold modifier 1 | 1 | | 3390 | hsa-miR-571 | 51569 | UFM1 | ubiquitin-fold modifier 1 | 1 | | 3391 | hsa-miR-874 | 4008 | LMO7 | LIM domain 7 | 1 | | 3392 | hsa-miR-1233 | 166785 | MMAA | methylmalonic aciduria (cobalamin deficiency) cblA type | 1 | | 3393 | hsa-miR-571 | 166785 | MMAA | methylmalonic aciduria (cobalamin deficiency) cblA type | 1 | | 3394 | hsa-miR-874 | 166785 | MMAA | methylmalonic aciduria (cobalamin deficiency) cblA type | 1 | | 3395 | hsa-miR-95 | 375616 | KCP | kielin/chordin-like protein | 1 | | 3396 | hsa-miR-95 | 111 | ADCY5 | adenylate cyclase 5 | 1 | | 3397 | hsa-miR-571 | 10780 | ZNF234 | zinc finger protein 234 | 1 | | 3398 | hsa-miR-10a | 150572 | SMYD1 | SET and MYND domain containing 1 | 1 | | 3399 | hsa-mir-505 | 2738 | GLI4 | GLI family zinc finger 4 | 1 | | 3400 | hsa-mir-3130-1 | 2738 | GLI4 | GLI family zinc finger 4 | 1 | | 3401 | hsa-mir-3130-2 | 2738 | GLI4 | GLI family zinc finger 4 | 1 | | 3402 | hsa-mir-3130-3 | 2738 | GLI4 | GLI family zinc finger 4 | 1 | | 3403 | hsa-mir-3130-4 | 2738 | GLI4 | GLI family zinc finger 4 | 1 | | 3404 | hsa-miR-505\* | 2738 | GLI4 | GLI family zinc finger 4 | 1 | | 3405 | hsa-miR-580 | 2738 | GLI4 | GLI family zinc finger 4 | 1 | | 3406 | hsa-miR-95 | 2738 | GLI4 | GLI family zinc finger 4 | 1 | | 3407 | hsa-mir-3130-1 | 56950 | SMYD2 | SET and MYND domain containing 2 | 1 | | 3408 | hsa-mir-3130-2 | 56950 | SMYD2 | SET and MYND domain containing 2 | 1 | | 3409 | hsa-mir-3130-3 | 56950 | SMYD2 | SET and MYND domain containing 2 | 1 | | 3410 | hsa-mir-3130-4 | 56950 | SMYD2 | SET and MYND domain containing 2 | 1 | | 3411 | hsa-miR-10a | 56950 | SMYD2 | SET and MYND domain containing 2 | 1 | | 3412 | hsa-miR-95 | 56950 | SMYD2 | SET and MYND domain containing 2 | 1 | | 3413 | hsa-miR-10a | 23286 | WWC1 | WW and C2 domain containing 1 | 1 | | 3414 | hsa-miR-95 | 23286 | WWC1 | WW and C2 domain containing 1 | 1 | | 3415 | hsa-miR-95 | 160065 | PATE1 | prostate and testis expressed 1 | 1 | | 3416 | hsa-miR-10a | 260434 | PYDC1 | PYD (pyrin domain) containing 1 | 1 | | 3417 | hsa-miR-580 | 260434 | PYDC1 | PYD (pyrin domain) containing 1 | 1 | | 3418 | hsa-miR-95 | 260434 | PYDC1 | PYD (pyrin domain) containing 1 | 1 | | 3419 | hsa-miR-874 | 148103 | ZNF599 | zinc finger protein 599 | 1 | | 3420 | hsa-miR-766 | 57466 | SFRS15 | splicing factor, arginine/serine-rich 15 | 1 | | 3421 | hsa-miR-618 | 253832 | ZDHHC20 | zinc finger, DHHC-type containing 20 | 1 | | 3422 | hsa-miR-95 | 440900 | LOC440900 | hypothetical LOC440900 | 1 | | 3423 | hsa-miR-10a | 207107 | SFTA1P | surfactant associated 1 (pseudogene) | 1 | | 3424 | hsa-miR-95 | 207107 | SFTA1P | surfactant associated 1 (pseudogene) | 1 | | 3425 | hsa-miR-10a | 401551 | WDR38 | WD repeat domain 38 | 1 | | 3426 | hsa-miR-10a | 200316 | APOBEC3F | apolipoprotein B mRNA editing enzyme, catalytic polypeptide-like 3F | 1 | | 3427 | hsa-miR-499-5p | 200316 | APOBEC3F | apolipoprotein B mRNA editing enzyme, catalytic polypeptide-like 3F | 1 | | 3428 | hsa-miR-95 | 200316 | APOBEC3F | apolipoprotein B mRNA editing enzyme, catalytic polypeptide-like 3F | 1 | | 3429 | hsa-miR-95 | 10919 | EHMT2 | euchromatic histone-lysine N-methyltransferase 2 | 1 | | 3430 | hsa-miR-10a | 7514 | XPO1 | exportin 1 (CRM1 homolog, yeast) | 1 | | 3431 | hsa-miR-558 | 284900 | LOC284900 | hypothetical LOC284900 | 1 | | 3432 | hsa-mir-3130-1 | 57455 | REXO1 | REX1, RNA exonuclease 1 homolog (S. cerevisiae) | 1 | | 3433 | hsa-mir-3130-2 | 57455 | REXO1 | REX1, RNA exonuclease 1 homolog (S. cerevisiae) | 1 | | 3434 | hsa-mir-3130-3 | 57455 | REXO1 | REX1, RNA exonuclease 1 homolog (S. cerevisiae) | 1 | | 3435 | hsa-mir-3130-4 | 57455 | REXO1 | REX1, RNA exonuclease 1 homolog (S. cerevisiae) | 1 | | 3436 | hsa-miR-499-5p | 57455 | REXO1 | REX1, RNA exonuclease 1 homolog (S. cerevisiae) | 1 | | 3437 | hsa-miR-95 | 57455 | REXO1 | REX1, RNA exonuclease 1 homolog (S. cerevisiae) | 1 | | 3438 | hsa-mir-10a | 7767 | ZNF224 | zinc finger protein 224 | 1 | | 3439 | hsa-mir-885 | 7767 | ZNF224 | zinc finger protein 224 | 1 | | 3440 | hsa-miR-885-5p | 7767 | ZNF224 | zinc finger protein 224 | 1 | | 3441 | hsa-miR-10a | 29800 | ZDHHC1 | zinc finger, DHHC-type containing 1 | 1 | | 3442 | hsa-miR-95 | 29800 | ZDHHC1 | zinc finger, DHHC-type containing 1 | 1 | | 3443 | hsa-miR-95 | 55008 | HERC6 | hect domain and RLD 6 | 1 | | 3444 | hsa-miR-95 | 100130344 | LOC100130344 | hypothetical protein LOC100130344 | 1 | | 3445 | hsa-mir-3130-1 | 1839 | HBEGF | heparin-binding EGF-like growth factor | 1 | | 3446 | hsa-mir-3130-2 | 1839 | HBEGF | heparin-binding EGF-like growth factor | 1 | | 3447 | hsa-mir-3130-3 | 1839 | HBEGF | heparin-binding EGF-like growth factor | 1 | | 3448 | hsa-mir-3130-4 | 1839 | HBEGF | heparin-binding EGF-like growth factor | 1 | | 3449 | hsa-miR-10a | 1839 | HBEGF | heparin-binding EGF-like growth factor | 1 | | 3450 | hsa-miR-95 | 1839 | HBEGF | heparin-binding EGF-like growth factor | 1 | | 3451 | hsa-mir-576 | 9400 | RECQL5 | RecQ protein-like 5 | 1 | | 3452 | hsa-miR-10a | 9400 | RECQL5 | RecQ protein-like 5 | 1 | | 3453 | hsa-mir-505 | 816 | CAMK2B | calcium/calmodulin-dependent protein kinase II beta | 1 | | 3454 | hsa-mir-3130-1 | 816 | CAMK2B | calcium/calmodulin-dependent protein kinase II beta | 1 | | 3455 | hsa-mir-3130-2 | 816 | CAMK2B | calcium/calmodulin-dependent protein kinase II beta | 1 | | 3456 | hsa-mir-3130-3 | 816 | CAMK2B | calcium/calmodulin-dependent protein kinase II beta | 1 | | 3457 | hsa-mir-3130-4 | 816 | CAMK2B | calcium/calmodulin-dependent protein kinase II beta | 1 | | 3458 | hsa-miR-505\* | 816 | CAMK2B | calcium/calmodulin-dependent protein kinase II beta | 1 | | 3459 | hsa-miR-580 | 816 | CAMK2B | calcium/calmodulin-dependent protein kinase II beta | 1 | | 3460 | hsa-miR-95 | 816 | CAMK2B | calcium/calmodulin-dependent protein kinase II beta | 1 | | 3461 | hsa-miR-1233 | 9814 | SFI1 | Sfi1 homolog, spindle assembly associated (yeast) | 1 | | 3462 | hsa-miR-569 | 9814 | SFI1 | Sfi1 homolog, spindle assembly associated (yeast) | 1 | | 3463 | hsa-miR-766 | 9814 | SFI1 | Sfi1 homolog, spindle assembly associated (yeast) | 1 | | 3464 | hsa-miR-874 | 9814 | SFI1 | Sfi1 homolog, spindle assembly associated (yeast) | 1 | | 3465 | hsa-miR-877 | 9814 | SFI1 | Sfi1 homolog, spindle assembly associated (yeast) | 1 | | 3466 | hsa-miR-938 | 527 | ATP6V0C | ATPase, H+ transporting, lysosomal 16kDa, V0 subunit c | 1 | | 3467 | hsa-miR-135a | 6901 | TAZ | tafazzin | 1 | | 3468 | hsa-miR-571 | 9063 | PIAS2 | protein inhibitor of activated STAT, 2 | 1 | | 3469 | hsa-miR-938 | 10636 | RGS14 | regulator of G-protein signaling 14 | 1 | | 3470 | hsa-mir-10a | 65125 | WNK1 | WNK lysine deficient protein kinase 1 | 1 | | 3471 | hsa-mir-885 | 65125 | WNK1 | WNK lysine deficient protein kinase 1 | 1 | | 3472 | hsa-miR-1224-5p | 65125 | WNK1 | WNK lysine deficient protein kinase 1 | 1 | | 3473 | hsa-miR-885-5p | 65125 | WNK1 | WNK lysine deficient protein kinase 1 | 1 | | 3474 | hsa-mir-3130-1 | 4059 | BCAM | basal cell adhesion molecule (Lutheran blood group) | 1 | | 3475 | hsa-mir-3130-2 | 4059 | BCAM | basal cell adhesion molecule (Lutheran blood group) | 1 | | 3476 | hsa-mir-3130-3 | 4059 | BCAM | basal cell adhesion molecule (Lutheran blood group) | 1 | | 3477 | hsa-mir-3130-4 | 4059 | BCAM | basal cell adhesion molecule (Lutheran blood group) | 1 | | 3478 | hsa-miR-10a | 4059 | BCAM | basal cell adhesion molecule (Lutheran blood group) | 1 | | 3479 | hsa-miR-95 | 4059 | BCAM | basal cell adhesion molecule (Lutheran blood group) | 1 | | 3480 | hsa-miR-571 | 6418 | SET | SET nuclear oncogene | 1 | | 3481 | hsa-miR-423-5p | 54455 | FBXO42 | F-box protein 42 | 1 | | 3482 | hsa-miR-877 | 30827 | CXXC1 | CXXC finger 1 (PHD domain) | 1 | | 3483 | hsa-miR-135a | 60672 | MIIP | migration and invasion inhibitory protein | 1 | | 3484 | hsa-miR-571 | 79701 | C17orf101 | chromosome 17 open reading frame 101 | 1 | | 3485 | hsa-mir-10a | 63892 | THADA | thyroid adenoma associated | 1 | | 3486 | hsa-miR-1233 | 63892 | THADA | thyroid adenoma associated | 1 | | 3487 | hsa-miR-423-5p | 63892 | THADA | thyroid adenoma associated | 1 | | 3488 | hsa-miR-1233 | 57473 | ZNF512B | zinc finger protein 512B | 1 | | 3489 | hsa-miR-766 | 57473 | ZNF512B | zinc finger protein 512B | 1 | | 3490 | hsa-miR-874 | 57473 | ZNF512B | zinc finger protein 512B | 1 | | 3491 | hsa-miR-1233 | 57048 | PLSCR3 | phospholipid scramblase 3 | 1 | | 3492 | hsa-miR-874 | 57048 | PLSCR3 | phospholipid scramblase 3 | 1 | | 3493 | hsa-miR-558 | 79078 | C1orf50 | chromosome 1 open reading frame 50 | 1 | | 3494 | hsa-miR-618 | 158747 | MOSPD2 | motile sperm domain containing 2 | 1 | | 3495 | hsa-miR-1233 | 79874 | RABEP2 | rabaptin, RAB GTPase binding effector protein 2 | 1 | | 3496 | hsa-miR-766 | 79874 | RABEP2 | rabaptin, RAB GTPase binding effector protein 2 | 1 | | 3497 | hsa-mir-576 | 79874 | RABEP2 | rabaptin, RAB GTPase binding effector protein 2 | 1 | | 3498 | hsa-miR-1233 | 155060 | ZNF783 | zinc finger family member 783 | 1 | | 3499 | hsa-miR-874 | 155060 | ZNF783 | zinc finger family member 783 | 1 | |

---

Gene Ontology - Biological Process [Details: ]

| |  | genes in Category | percent in the observed List | percent in the genome | fold of overrepresents | odds ratio | p value | | --- | --- | --- | --- | --- | --- | --- | | cellular macromolecule metabolic process | 506 | 0.5169 | 0.40707 | 1.3 | 1.6 | 4.4e-13 | | gene expression | 354 | 0.3616 | 0.26158 | 1.4 | 1.7 | 5.6e-13 | | macromolecule metabolic process | 540 | 0.5516 | 0.44575 | 1.2 | 1.6 | 3.8e-12 | | nucleobase, nucleoside, nucleotide and nucleic acid metabolic process | 371 | 0.3790 | 0.28570 | 1.3 | 1.6 | 4.2e-11 | | macromolecule biosynthetic process | 330 | 0.3371 | 0.24851 | 1.4 | 1.6 | 7.3e-11 | | cellular macromolecule biosynthetic process | 324 | 0.3309 | 0.24408 | 1.4 | 1.6 | 1.3e-10 | | nitrogen compound metabolic process | 393 | 0.4014 | 0.31257 | 1.3 | 1.5 | 6.8e-10 | | RNA metabolic process | 244 | 0.2492 | 0.17769 | 1.4 | 1.6 | 3.6e-09 | | cellular biosynthetic process | 370 | 0.3779 | 0.29688 | 1.3 | 1.5 | 1.1e-08 | | cellular metabolic process | 588 | 0.6006 | 0.51635 | 1.2 | 1.4 | 2.4e-08 | | primary metabolic process | 602 | 0.6149 | 0.53154 | 1.2 | 1.4 | 3.1e-08 | | biosynthetic process | 372 | 0.3800 | 0.30455 | 1.2 | 1.4 | 1.1e-07 | | RNA processing | 73 | 0.0746 | 0.04036 | 1.8 | 2.0 | 2.1e-07 | | metabolic process | 649 | 0.6629 | 0.58786 | 1.1 | 1.4 | 3.5e-07 | | regulation of macromolecule biosynthetic process | 253 | 0.2584 | 0.19893 | 1.3 | 1.4 | 1.6e-06 | | transcription | 242 | 0.2472 | 0.18902 | 1.3 | 1.4 | 1.8e-06 | | regulation of nitrogen compound metabolic process | 253 | 0.2584 | 0.20020 | 1.3 | 1.4 | 2.7e-06 | | regulation of biosynthetic process | 262 | 0.2676 | 0.20885 | 1.3 | 1.4 | 3.0e-06 | | regulation of cellular biosynthetic process | 260 | 0.2656 | 0.20758 | 1.3 | 1.4 | 3.8e-06 | | regulation of macromolecule metabolic process | 284 | 0.2901 | 0.23022 | 1.3 | 1.4 | 4.0e-06 | | regulation of gene expression | 253 | 0.2584 | 0.20153 | 1.3 | 1.4 | 4.6e-06 | | mRNA processing | 44 | 0.0449 | 0.02208 | 2.0 | 2.3 | 5.0e-06 | | regulation of nucleobase, nucleoside, nucleotide and nucleic acid metabolic process | 249 | 0.2543 | 0.19844 | 1.3 | 1.4 | 5.9e-06 | | RNA splicing | 42 | 0.0429 | 0.02095 | 2.0 | 2.3 | 7.0e-06 | | regulation of transcription | 231 | 0.2360 | 0.18269 | 1.3 | 1.4 | 8.6e-06 | | regulation of cellular metabolic process | 294 | 0.3003 | 0.24457 | 1.2 | 1.4 | 2.2e-05 | | regulation of metabolic process | 304 | 0.3105 | 0.25561 | 1.2 | 1.3 | 3.6e-05 | | regulation of primary metabolic process | 280 | 0.2860 | 0.23275 | 1.2 | 1.4 | 3.7e-05 | | ribonucleoprotein complex biogenesis | 29 | 0.0296 | 0.01350 | 2.2 | 2.4 | 5.1e-05 | | mRNA metabolic process | 47 | 0.0480 | 0.02651 | 1.8 | 2.0 | 5.3e-05 | | translation | 50 | 0.0511 | 0.02890 | 1.8 | 1.9 | 5.8e-05 | | organelle organization | 133 | 0.1359 | 0.09950 | 1.4 | 1.5 | 9.7e-05 | | nuclear transport | 29 | 0.0296 | 0.01399 | 2.1 | 2.3 | 9.8e-05 | | mitochondrial membrane organization | 9 | 0.0092 | 0.00211 | 4.4 | 5.8 | 1.3e-04 | | mRNA transport | 17 | 0.0174 | 0.00640 | 2.7 | 3.1 | 1.4e-04 | | transcription from RNA polymerase I promoter | 5 | 0.0051 | 0.00063 | 8.1 | 17.0 | 1.5e-04 | | nucleobase, nucleoside, nucleotide and nucleic acid transport | 20 | 0.0204 | 0.00837 | 2.4 | 2.8 | 1.7e-04 | | nucleic acid transport | 18 | 0.0184 | 0.00724 | 2.5 | 2.9 | 2.1e-04 | | RNA transport | 18 | 0.0184 | 0.00724 | 2.5 | 2.9 | 2.1e-04 | | establishment of RNA localization | 18 | 0.0184 | 0.00724 | 2.5 | 2.9 | 2.1e-04 | | nucleocytoplasmic transport | 28 | 0.0286 | 0.01392 | 2.1 | 2.3 | 2.2e-04 | | mitochondrion organization | 21 | 0.0215 | 0.00928 | 2.3 | 2.6 | 2.6e-04 | | translational elongation | 18 | 0.0184 | 0.00745 | 2.5 | 2.8 | 3.1e-04 | | RNA localization | 18 | 0.0184 | 0.00745 | 2.5 | 2.8 | 3.1e-04 | | RNA biosynthetic process | 166 | 0.1696 | 0.13248 | 1.3 | 1.4 | 3.4e-04 | | transcription, DNA-dependent | 165 | 0.1685 | 0.13220 | 1.3 | 1.4 | 4.3e-04 | | cellular component biogenesis | 102 | 0.1042 | 0.07651 | 1.4 | 1.4 | 7.3e-04 | | ribosomal small subunit biogenesis | 5 | 0.0051 | 0.00084 | 6.1 | 9.7 | 8.1e-04 | | cellular process | 872 | 0.8907 | 0.85718 | 1.0 | 1.4 | 8.1e-04 | | RNA splicing, via transesterification reactions | 17 | 0.0174 | 0.00752 | 2.3 | 2.6 | 9.8e-04 | | nuclear mRNA splicing, via spliceosome | 16 | 0.0163 | 0.00696 | 2.3 | 2.6 | 1.1e-03 | | RNA splicing, via transesterification reactions with bulged adenosine as nucleophile | 16 | 0.0163 | 0.00696 | 2.3 | 2.6 | 1.1e-03 | | ribosome biogenesis | 19 | 0.0194 | 0.00907 | 2.1 | 2.4 | 1.3e-03 | | regulation of RNA metabolic process | 155 | 0.1583 | 0.12664 | 1.3 | 1.3 | 1.5e-03 | | cellular protein metabolic process | 213 | 0.2176 | 0.18142 | 1.2 | 1.3 | 1.6e-03 | | ribonucleoprotein complex assembly | 13 | 0.0133 | 0.00527 | 2.5 | 2.9 | 1.7e-03 | | chromatin organization | 43 | 0.0439 | 0.02778 | 1.6 | 1.7 | 1.9e-03 | | chromatin modification | 34 | 0.0347 | 0.02060 | 1.7 | 1.8 | 1.9e-03 | | regulation of transcription, DNA-dependent | 150 | 0.1532 | 0.12320 | 1.2 | 1.3 | 2.3e-03 | | nuclear export | 12 | 0.0123 | 0.00485 | 2.5 | 2.9 | 2.4e-03 | | cellular component organization | 216 | 0.2206 | 0.18592 | 1.2 | 1.3 | 2.6e-03 | | RNA export from nucleus | 9 | 0.0092 | 0.00309 | 3.0 | 3.5 | 2.7e-03 | | positive regulation of keratinocyte migration | 3 | 0.0031 | 0.00035 | 8.7 | 20.3 | 2.9e-03 | | regulation of keratinocyte migration | 3 | 0.0031 | 0.00035 | 8.7 | 20.3 | 2.9e-03 | | establishment of protein localization | 81 | 0.0827 | 0.06132 | 1.3 | 1.4 | 3.3e-03 | | RNA methylation | 4 | 0.0041 | 0.00070 | 5.8 | 9.1 | 3.3e-03 | | rRNA metabolic process | 15 | 0.0153 | 0.00703 | 2.2 | 2.4 | 3.4e-03 | | chromosome organization | 51 | 0.0521 | 0.03572 | 1.5 | 1.5 | 4.2e-03 | | protein localization | 90 | 0.0919 | 0.07004 | 1.3 | 1.4 | 4.3e-03 | | microtubule-based process | 30 | 0.0306 | 0.01856 | 1.7 | 1.8 | 4.7e-03 | | positive regulation of membrane potential | 2 | 0.0020 | 0.00014 | 14.5 | Inf | 4.7e-03 | | pyrimidine transport | 2 | 0.0020 | 0.00014 | 14.5 | Inf | 4.7e-03 | | negative regulation of JNK cascade | 4 | 0.0041 | 0.00077 | 5.3 | 7.8 | 5.0e-03 | | negative regulation of stress-activated protein kinase signaling pathway | 4 | 0.0041 | 0.00077 | 5.3 | 7.8 | 5.0e-03 | | protein import | 20 | 0.0204 | 0.01097 | 1.9 | 2.0 | 5.2e-03 | | negative regulation of interferon-gamma production | 3 | 0.0031 | 0.00042 | 7.3 | 13.6 | 5.6e-03 | | multicellular organism growth | 11 | 0.0112 | 0.00471 | 2.4 | 2.7 | 5.7e-03 | | rRNA processing | 14 | 0.0143 | 0.00675 | 2.1 | 2.3 | 5.9e-03 | | spindle assembly | 5 | 0.0051 | 0.00127 | 4.0 | 5.2 | 6.2e-03 | | negative regulation of MAPKKK cascade | 5 | 0.0051 | 0.00127 | 4.0 | 5.2 | 6.2e-03 | | organelle assembly | 9 | 0.0092 | 0.00352 | 2.6 | 3.0 | 6.5e-03 | | protein transport | 78 | 0.0797 | 0.06054 | 1.3 | 1.4 | 7.2e-03 | | protein metabolic process | 240 | 0.2451 | 0.21335 | 1.1 | 1.2 | 7.2e-03 | | microtubule cytoskeleton organization | 20 | 0.0204 | 0.01132 | 1.8 | 1.9 | 7.4e-03 | | establishment of protein localization in plasma membrane | 3 | 0.0031 | 0.00049 | 6.2 | 10.2 | 9.2e-03 | | regulation of establishment of protein localization in plasma membrane | 3 | 0.0031 | 0.00049 | 6.2 | 10.2 | 9.2e-03 | | establishment of protein localization in membrane | 3 | 0.0031 | 0.00049 | 6.2 | 10.2 | 9.2e-03 | |

---

Gene Ontology - Biological Process, level II [Details: ]

| |  | genes in Category | percent in the observed List | percent in the genome | fold of overrepresents | odds ratio | p value | | --- | --- | --- | --- | --- | --- | --- | | cellular macromolecule metabolic process | 506 | 0.5211 | 0.40707 | 1.3 | 1.6 | 7.2e-14 | | gene expression | 354 | 0.3646 | 0.26158 | 1.4 | 1.7 | 1.5e-13 | | macromolecule metabolic process | 540 | 0.5561 | 0.44575 | 1.2 | 1.6 | 5.8e-13 | | nucleobase, nucleoside, nucleotide and nucleic acid metabolic process | 371 | 0.3821 | 0.28570 | 1.3 | 1.6 | 1.2e-11 | | macromolecule biosynthetic process | 330 | 0.3399 | 0.24851 | 1.4 | 1.6 | 2.4e-11 | | cellular macromolecule biosynthetic process | 324 | 0.3337 | 0.24408 | 1.4 | 1.6 | 4.4e-11 | | nitrogen compound metabolic process | 393 | 0.4047 | 0.31257 | 1.3 | 1.5 | 2.0e-10 | | RNA metabolic process | 244 | 0.2513 | 0.17769 | 1.4 | 1.6 | 1.6e-09 | | cellular biosynthetic process | 370 | 0.3811 | 0.29688 | 1.3 | 1.5 | 3.6e-09 | | cellular metabolic process | 588 | 0.6056 | 0.51635 | 1.2 | 1.5 | 4.4e-09 | | primary metabolic process | 602 | 0.6200 | 0.53154 | 1.2 | 1.5 | 5.3e-09 | | biosynthetic process | 372 | 0.3831 | 0.30455 | 1.3 | 1.5 | 3.9e-08 | | RNA processing | 73 | 0.0752 | 0.04036 | 1.9 | 2.1 | 1.6e-07 | | regulation of macromolecule biosynthetic process | 253 | 0.2606 | 0.19893 | 1.3 | 1.5 | 8.1e-07 | | transcription | 242 | 0.2492 | 0.18902 | 1.3 | 1.5 | 9.3e-07 | | regulation of nitrogen compound metabolic process | 253 | 0.2606 | 0.20020 | 1.3 | 1.4 | 1.4e-06 | | regulation of biosynthetic process | 262 | 0.2698 | 0.20885 | 1.3 | 1.4 | 1.5e-06 | | regulation of macromolecule metabolic process | 284 | 0.2925 | 0.23022 | 1.3 | 1.4 | 1.9e-06 | | regulation of cellular biosynthetic process | 260 | 0.2678 | 0.20758 | 1.3 | 1.4 | 1.9e-06 | | regulation of gene expression | 253 | 0.2606 | 0.20153 | 1.3 | 1.4 | 2.4e-06 | | regulation of nucleobase, nucleoside, nucleotide and nucleic acid metabolic process | 249 | 0.2564 | 0.19844 | 1.3 | 1.4 | 3.1e-06 | | mRNA processing | 44 | 0.0453 | 0.02208 | 2.1 | 2.3 | 4.0e-06 | | regulation of transcription | 231 | 0.2379 | 0.18269 | 1.3 | 1.4 | 4.6e-06 | | RNA splicing | 42 | 0.0433 | 0.02095 | 2.1 | 2.3 | 5.7e-06 | | regulation of cellular metabolic process | 294 | 0.3028 | 0.24457 | 1.2 | 1.4 | 1.1e-05 | | regulation of metabolic process | 304 | 0.3131 | 0.25561 | 1.2 | 1.4 | 1.8e-05 | | regulation of primary metabolic process | 280 | 0.2884 | 0.23275 | 1.2 | 1.4 | 1.9e-05 | | mRNA metabolic process | 47 | 0.0484 | 0.02651 | 1.8 | 2.0 | 4.3e-05 | | ribonucleoprotein complex biogenesis | 29 | 0.0299 | 0.01350 | 2.2 | 2.5 | 4.4e-05 | | translation | 50 | 0.0515 | 0.02890 | 1.8 | 1.9 | 4.7e-05 | | organelle organization | 133 | 0.1370 | 0.09950 | 1.4 | 1.5 | 6.6e-05 | | nuclear transport | 29 | 0.0299 | 0.01399 | 2.1 | 2.4 | 8.5e-05 | | mitochondrial membrane organization | 9 | 0.0093 | 0.00211 | 4.4 | 5.9 | 1.2e-04 | | mRNA transport | 17 | 0.0175 | 0.00640 | 2.7 | 3.2 | 1.2e-04 | | transcription from RNA polymerase I promoter | 5 | 0.0051 | 0.00063 | 8.1 | 17.1 | 1.5e-04 | | nucleobase, nucleoside, nucleotide and nucleic acid transport | 20 | 0.0206 | 0.00837 | 2.5 | 2.8 | 1.5e-04 | | nucleocytoplasmic transport | 28 | 0.0288 | 0.01392 | 2.1 | 2.3 | 1.9e-04 | | nucleic acid transport | 18 | 0.0185 | 0.00724 | 2.6 | 2.9 | 1.9e-04 | | RNA transport | 18 | 0.0185 | 0.00724 | 2.6 | 2.9 | 1.9e-04 | | establishment of RNA localization | 18 | 0.0185 | 0.00724 | 2.6 | 2.9 | 1.9e-04 | | RNA biosynthetic process | 166 | 0.1710 | 0.13248 | 1.3 | 1.4 | 2.3e-04 | | mitochondrion organization | 21 | 0.0216 | 0.00928 | 2.3 | 2.6 | 2.3e-04 | | translational elongation | 18 | 0.0185 | 0.00745 | 2.5 | 2.8 | 2.8e-04 | | RNA localization | 18 | 0.0185 | 0.00745 | 2.5 | 2.8 | 2.8e-04 | | transcription, DNA-dependent | 165 | 0.1699 | 0.13220 | 1.3 | 1.4 | 2.9e-04 | | ribosomal small subunit biogenesis | 5 | 0.0051 | 0.00084 | 6.1 | 9.8 | 7.8e-04 | | RNA splicing, via transesterification reactions | 17 | 0.0175 | 0.00752 | 2.3 | 2.6 | 9.0e-04 | | nuclear mRNA splicing, via spliceosome | 16 | 0.0165 | 0.00696 | 2.4 | 2.7 | 1.0e-03 | | RNA splicing, via transesterification reactions with bulged adenosine as nucleophile | 16 | 0.0165 | 0.00696 | 2.4 | 2.7 | 1.0e-03 | | cellular protein metabolic process | 213 | 0.2194 | 0.18142 | 1.2 | 1.3 | 1.1e-03 | | regulation of RNA metabolic process | 155 | 0.1596 | 0.12664 | 1.3 | 1.3 | 1.1e-03 | | ribosome biogenesis | 19 | 0.0196 | 0.00907 | 2.2 | 2.4 | 1.2e-03 | | ribonucleoprotein complex assembly | 13 | 0.0134 | 0.00527 | 2.5 | 2.9 | 1.6e-03 | | chromatin organization | 43 | 0.0443 | 0.02778 | 1.6 | 1.7 | 1.6e-03 | | regulation of transcription, DNA-dependent | 150 | 0.1545 | 0.12320 | 1.3 | 1.3 | 1.6e-03 | | chromatin modification | 34 | 0.0350 | 0.02060 | 1.7 | 1.8 | 1.7e-03 | | nuclear export | 12 | 0.0124 | 0.00485 | 2.5 | 2.9 | 2.3e-03 | | RNA export from nucleus | 9 | 0.0093 | 0.00309 | 3.0 | 3.5 | 2.5e-03 | | establishment of protein localization | 81 | 0.0834 | 0.06132 | 1.4 | 1.4 | 2.6e-03 | | positive regulation of keratinocyte migration | 3 | 0.0031 | 0.00035 | 8.8 | 20.5 | 2.9e-03 | | regulation of keratinocyte migration | 3 | 0.0031 | 0.00035 | 8.8 | 20.5 | 2.9e-03 | | rRNA metabolic process | 15 | 0.0154 | 0.00703 | 2.2 | 2.4 | 3.1e-03 | | RNA methylation | 4 | 0.0041 | 0.00070 | 5.9 | 9.1 | 3.3e-03 | | protein localization | 90 | 0.0927 | 0.07004 | 1.3 | 1.4 | 3.4e-03 | | chromosome organization | 51 | 0.0525 | 0.03572 | 1.5 | 1.6 | 3.5e-03 | | microtubule-based process | 30 | 0.0309 | 0.01856 | 1.7 | 1.8 | 4.1e-03 | | positive regulation of membrane potential | 2 | 0.0021 | 0.00014 | 14.6 | Inf | 4.7e-03 | | pyrimidine transport | 2 | 0.0021 | 0.00014 | 14.6 | Inf | 4.7e-03 | | protein import | 20 | 0.0206 | 0.01097 | 1.9 | 2.0 | 4.7e-03 | | protein metabolic process | 240 | 0.2472 | 0.21335 | 1.2 | 1.2 | 4.8e-03 | | negative regulation of JNK cascade | 4 | 0.0041 | 0.00077 | 5.3 | 7.8 | 4.8e-03 | | negative regulation of stress-activated protein kinase signaling pathway | 4 | 0.0041 | 0.00077 | 5.3 | 7.8 | 4.8e-03 | | multicellular organism growth | 11 | 0.0113 | 0.00471 | 2.4 | 2.7 | 5.4e-03 | | negative regulation of interferon-gamma production | 3 | 0.0031 | 0.00042 | 7.3 | 13.7 | 5.4e-03 | | rRNA processing | 14 | 0.0144 | 0.00675 | 2.1 | 2.3 | 5.5e-03 | | protein transport | 78 | 0.0803 | 0.06054 | 1.3 | 1.4 | 5.9e-03 | | spindle assembly | 5 | 0.0051 | 0.00127 | 4.1 | 5.3 | 6.0e-03 | | negative regulation of MAPKKK cascade | 5 | 0.0051 | 0.00127 | 4.1 | 5.3 | 6.0e-03 | | organelle assembly | 9 | 0.0093 | 0.00352 | 2.6 | 3.0 | 6.2e-03 | | microtubule cytoskeleton organization | 20 | 0.0206 | 0.01132 | 1.8 | 2.0 | 6.7e-03 | | establishment of protein localization in plasma membrane | 3 | 0.0031 | 0.00049 | 6.3 | 10.3 | 9.0e-03 | | regulation of establishment of protein localization in plasma membrane | 3 | 0.0031 | 0.00049 | 6.3 | 10.3 | 9.0e-03 | | establishment of protein localization in membrane | 3 | 0.0031 | 0.00049 | 6.3 | 10.3 | 9.0e-03 | | cellular component assembly | 85 | 0.0875 | 0.06807 | 1.3 | 1.3 | 9.2e-03 | | DNA metabolic process | 53 | 0.0546 | 0.03945 | 1.4 | 1.4 | 9.9e-03 | |

---

KEGG pathways [Details: ]

| |  | genes in Category | percent in the observed List | percent in the genome | fold of overrepresents | odds ratio | p value | | --- | --- | --- | --- | --- | --- | --- | | Spliceosome | 21 | 0.068 | 0.0253 | 2.7 | 3.1 | 2.8e-05 | | Ribosome | 15 | 0.048 | 0.0174 | 2.8 | 3.2 | 2.5e-04 | | RNA degradation | 9 | 0.029 | 0.0117 | 2.5 | 2.8 | 9.2e-03 | | Pyrimidine metabolism | 11 | 0.035 | 0.0194 | 1.8 | 2.0 | 3.7e-02 | | Huntington's disease | 17 | 0.055 | 0.0366 | 1.5 | 1.6 | 6.1e-02 | | Colorectal cancer | 9 | 0.029 | 0.0166 | 1.7 | 1.9 | 7.1e-02 | | Ubiquitin mediated proteolysis | 13 | 0.042 | 0.0273 | 1.5 | 1.6 | 8.1e-02 | | Melanogenesis | 10 | 0.032 | 0.0202 | 1.6 | 1.7 | 9.5e-02 | | RNA polymerase | 4 | 0.013 | 0.0057 | 2.2 | 2.5 | 9.9e-02 | |

---

Disease Ontology Lite terms [Details: ]

| |  | genes in Category | percent in the observed List | percent in the genome | fold of overrepresents | odds ratio | p value | | --- | --- | --- | --- | --- | --- | --- | | Solid tumor | 4 | 0.016 | 0.00296 | 5.4 | 7.7 | 0.0048 | | Carcinoma | 7 | 0.028 | 0.00864 | 3.2 | 3.9 | 0.0048 | | HTLV-I infection | 3 | 0.012 | 0.00222 | 5.4 | 7.6 | 0.0149 | | Renal tubular acidosis | 3 | 0.012 | 0.00222 | 5.4 | 7.6 | 0.0149 | | Brain tumor | 17 | 0.068 | 0.03876 | 1.7 | 1.9 | 0.0161 | | Lipoidosis | 2 | 0.008 | 0.00099 | 8.1 | 15.3 | 0.0211 | | Skin cancer | 4 | 0.016 | 0.00469 | 3.4 | 4.1 | 0.0267 | | Leukemia | 28 | 0.112 | 0.07801 | 1.4 | 1.5 | 0.0316 | | Esotropia | 3 | 0.012 | 0.00296 | 4.0 | 5.1 | 0.0341 | | Cockayne syndrome | 2 | 0.008 | 0.00148 | 5.4 | 7.6 | 0.0486 | | Dental enamel hypoplasia | 2 | 0.008 | 0.00148 | 5.4 | 7.6 | 0.0486 | | Cancer | 56 | 0.223 | 0.18168 | 1.2 | 1.3 | 0.0497 | | Gastrointestinal infection | 1 | 0.004 | 0.00025 | 16.1 | Inf | 0.0620 | | Tropical spastic paraparesis | 2 | 0.008 | 0.00173 | 4.6 | 6.1 | 0.0653 | | Bardet-Biedl syndrome | 2 | 0.008 | 0.00173 | 4.6 | 6.1 | 0.0653 | | Synovitis | 2 | 0.008 | 0.00197 | 4.0 | 5.1 | 0.0836 | | CNS metastases | 2 | 0.008 | 0.00197 | 4.0 | 5.1 | 0.0836 | | Hepatitis B | 3 | 0.012 | 0.00420 | 2.8 | 3.3 | 0.0839 | | Lupus vulgaris | 4 | 0.016 | 0.00691 | 2.3 | 2.5 | 0.0915 | | Vitiligo | 3 | 0.012 | 0.00444 | 2.7 | 3.1 | 0.0963 | | Osteoporosis | 5 | 0.020 | 0.00987 | 2.0 | 2.2 | 0.0984 | |

---

USER DEFINED THERMS: inflammation, apoptosis, necrosis, bunt, burn [Details: ]

| |  | genes in Category | percent in the observed List | percent in the genome | fold of overrepresents | odds ratio | p value | | --- | --- | --- | --- | --- | --- | --- | | apoptosis | 180 | 0.8824 | 0.05777 | 15 | 131 | 9.7e-196 | | inflammation | 37 | 0.1814 | 0.01712 | 11 | 13 | 8.2e-27 | | necrosis | 27 | 0.1324 | 0.01113 | 12 | 14 | 5.1e-21 | | burn | 2 | 0.0098 | 0.00031 | 32 | 37 | 1.8e-03 | |

---
