# Supplementary figures and images for: MMpred: functional miRNA – mRNA interaction analyses by miRNA expression prediction (part 2 of 3)
Source: BMC Genomics. 2012 Nov 14;13:620. doi: 10.1186/1471-2164-13-620 (PMC3562514; doi:10.1186/1471-2164-13-620)

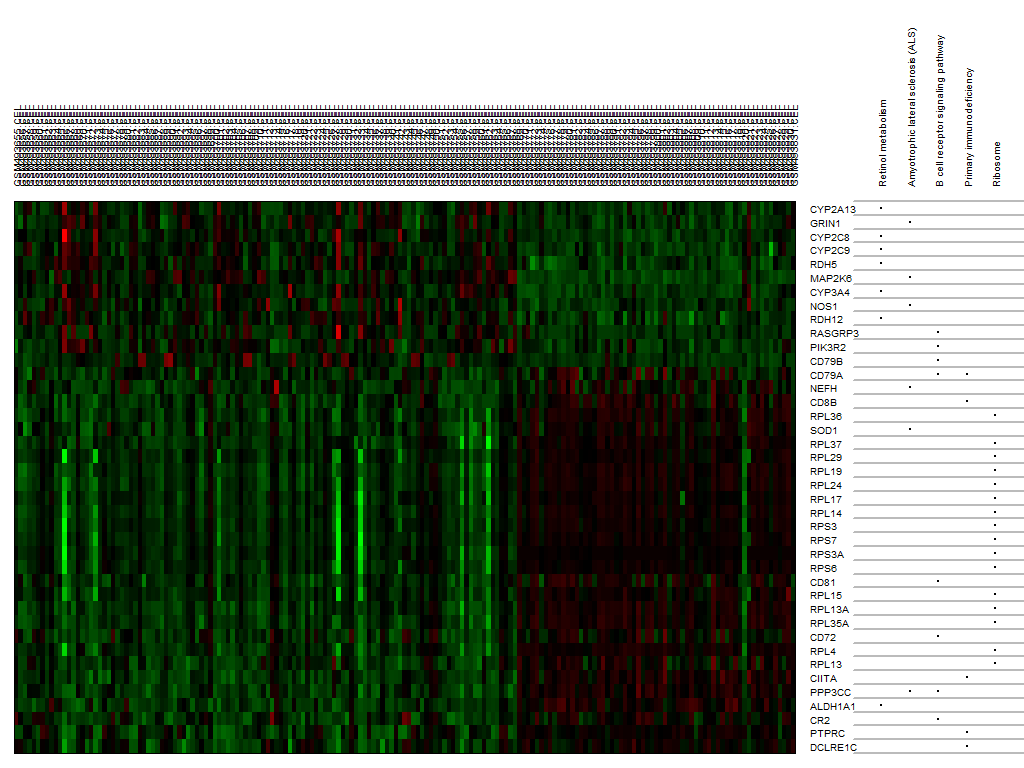

Supplement: Additional file 3 — Sample pipeline outputs in HTML format (compressed file). [file 1471-2164-13-620-S3.ZIP › Burn_early-late-control&chilren-adoult/GRAPH_Sep09_055645.png]

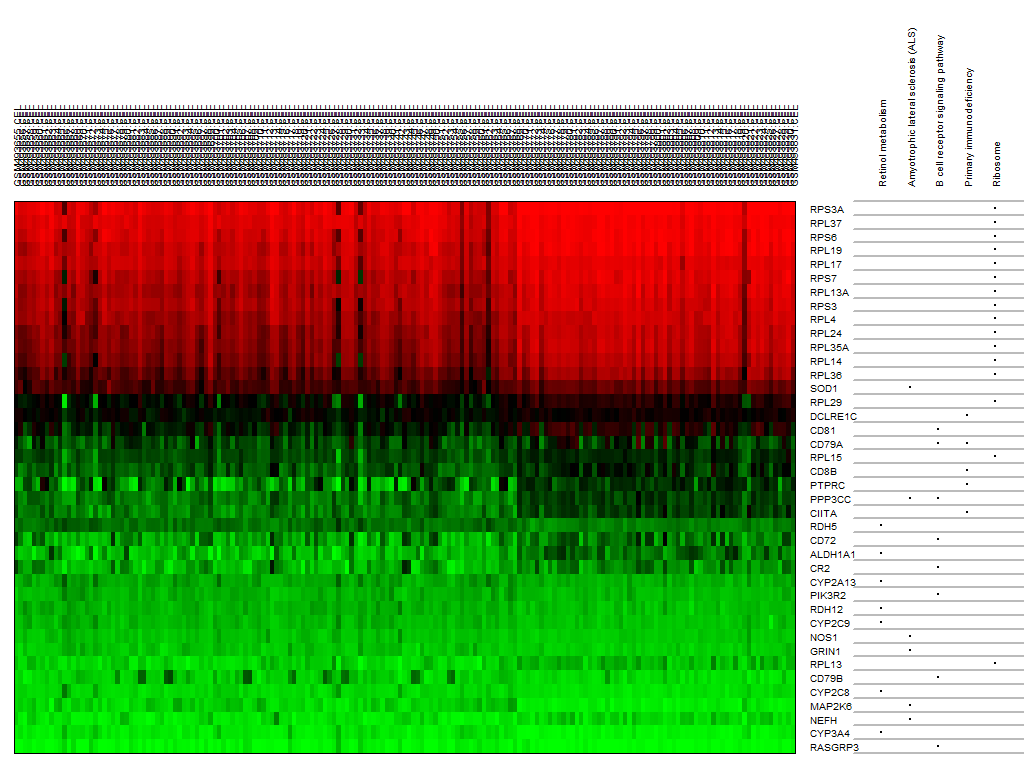

Supplement: Additional file 3 — Sample pipeline outputs in HTML format (compressed file). [file 1471-2164-13-620-S3.ZIP › Burn_early-late-control&chilren-adoult/GRAPH_Sep09_055652.png]

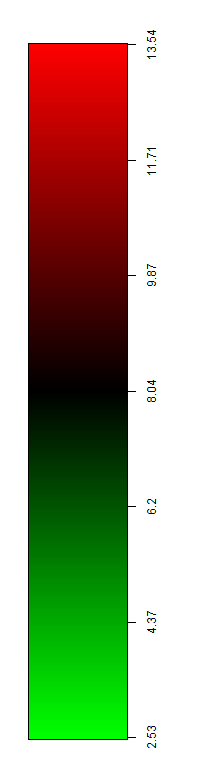

Supplement: Additional file 3 — Sample pipeline outputs in HTML format (compressed file). [file 1471-2164-13-620-S3.ZIP › Burn_early-late-control&chilren-adoult/GRAPH_Sep09_055657.png]

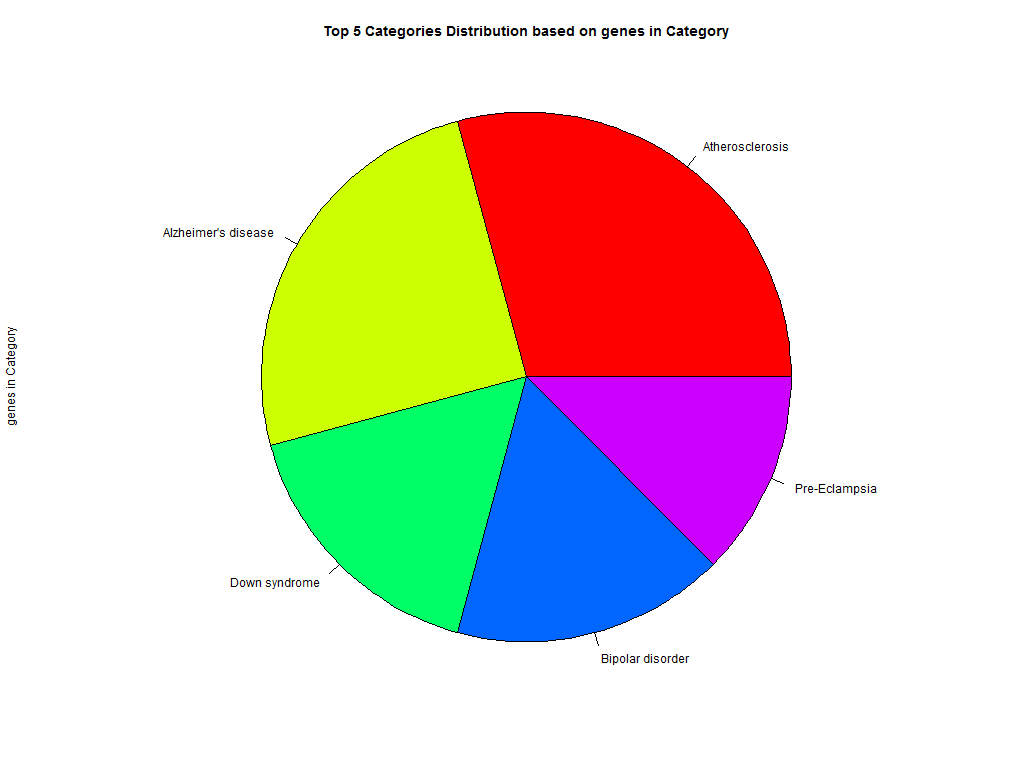

Supplement: Additional file 3 — Sample pipeline outputs in HTML format (compressed file). [file 1471-2164-13-620-S3.ZIP › Burn_early-late-control&chilren-adoult/GRAPH_Sep09_055714.png]

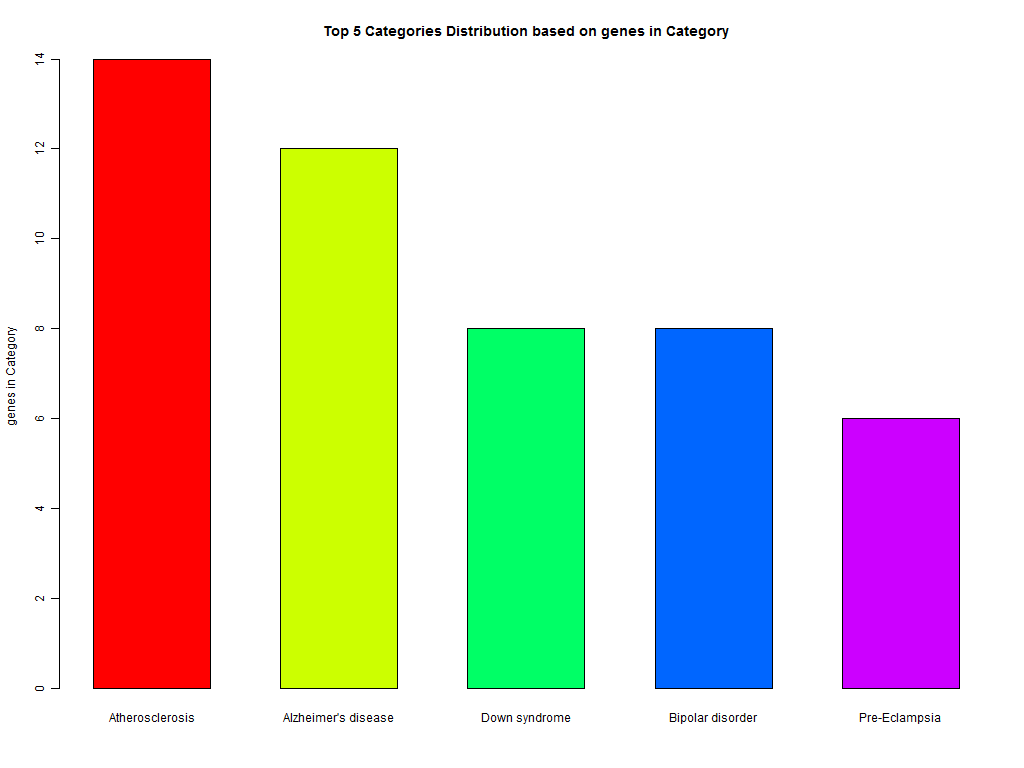

Supplement: Additional file 3 — Sample pipeline outputs in HTML format (compressed file). [file 1471-2164-13-620-S3.ZIP › Burn_early-late-control&chilren-adoult/GRAPH_Sep09_055719.png]

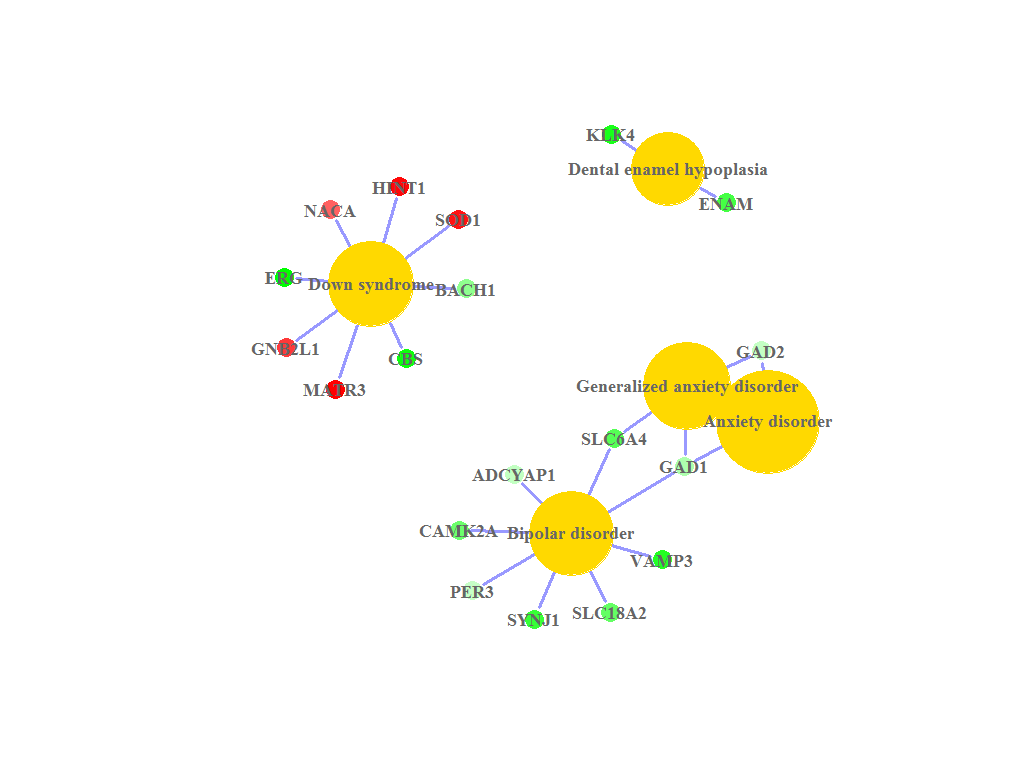

Supplement: Additional file 3 — Sample pipeline outputs in HTML format (compressed file). [file 1471-2164-13-620-S3.ZIP › Burn_early-late-control&chilren-adoult/GRAPH_Sep09_055724.png]

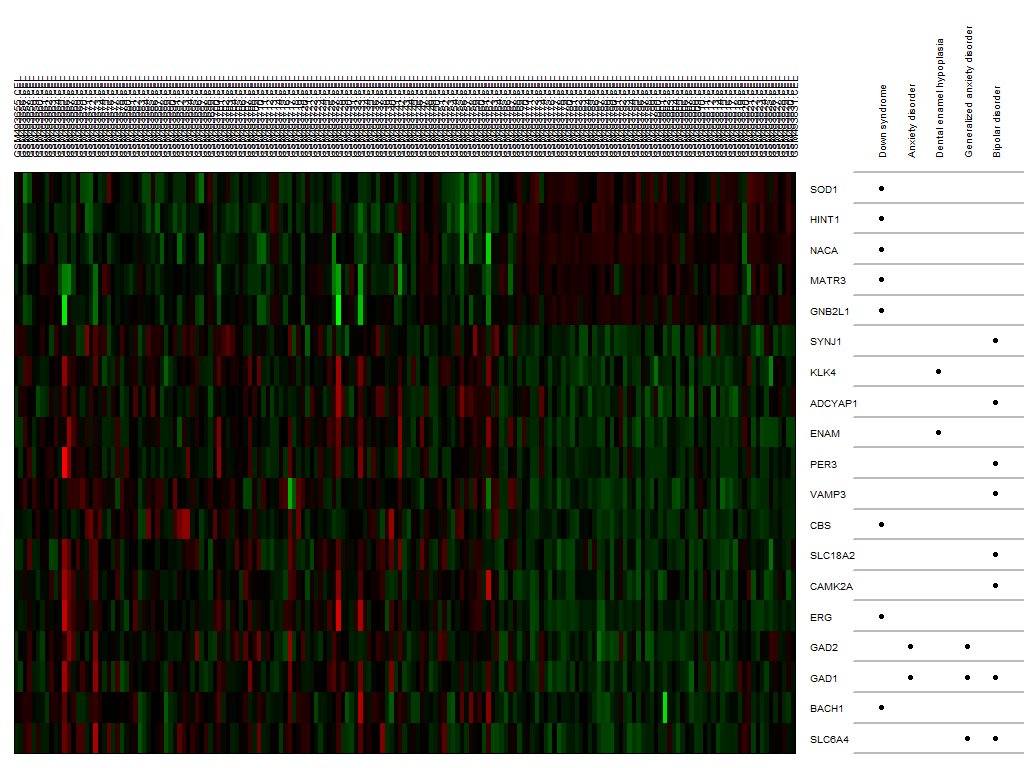

Supplement: Additional file 3 — Sample pipeline outputs in HTML format (compressed file). [file 1471-2164-13-620-S3.ZIP › Burn_early-late-control&chilren-adoult/GRAPH_Sep09_055730.png]

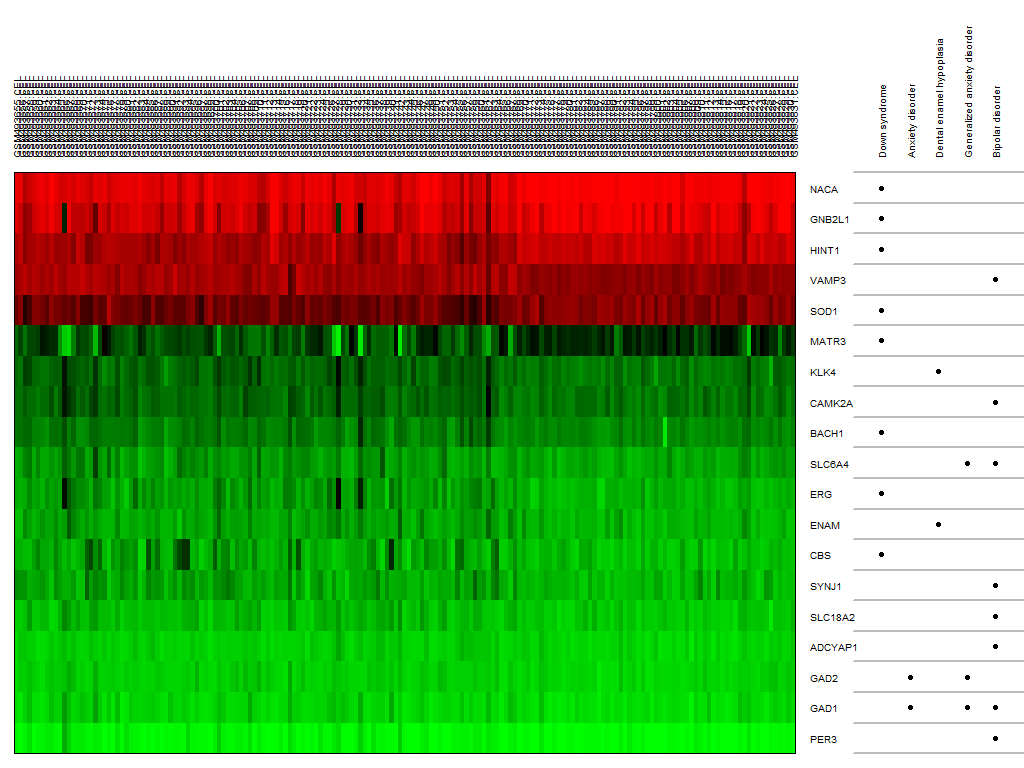

Supplement: Additional file 3 — Sample pipeline outputs in HTML format (compressed file). [file 1471-2164-13-620-S3.ZIP › Burn_early-late-control&chilren-adoult/GRAPH_Sep09_055737.png]

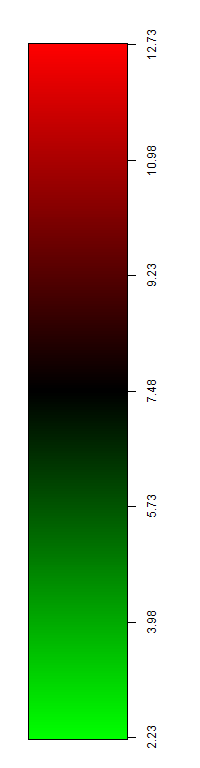

Supplement: Additional file 3 — Sample pipeline outputs in HTML format (compressed file). [file 1471-2164-13-620-S3.ZIP › Burn_early-late-control&chilren-adoult/GRAPH_Sep09_055742.png]

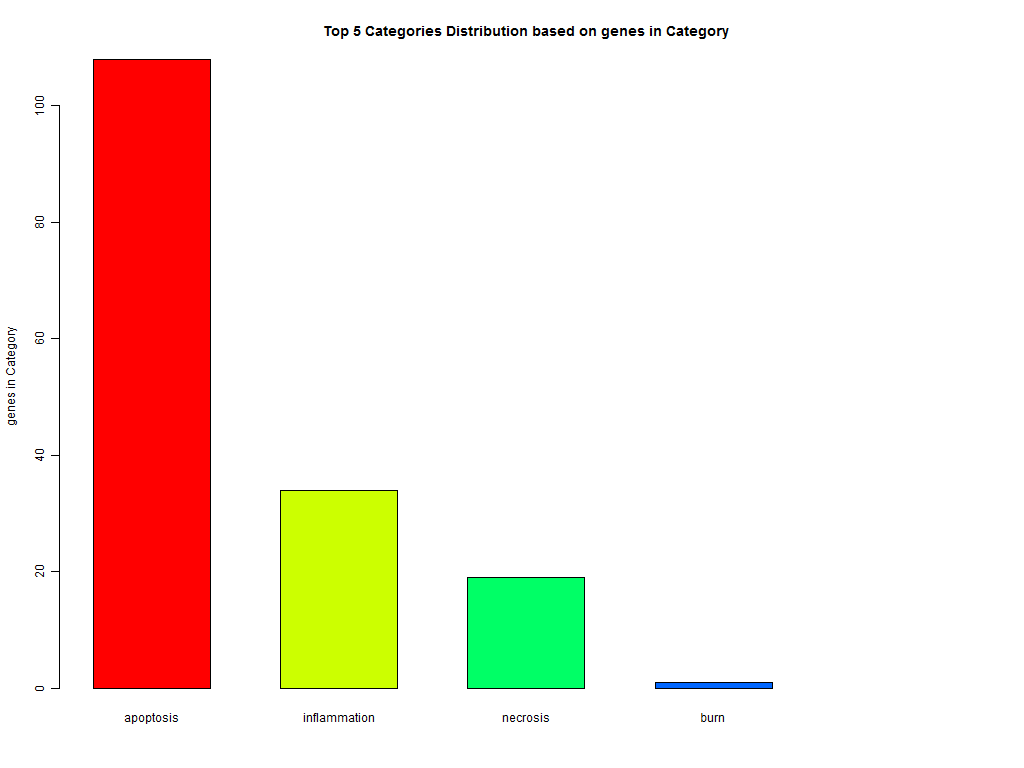

Supplement: Additional file 3 — Sample pipeline outputs in HTML format (compressed file). [file 1471-2164-13-620-S3.ZIP › Burn_early-late-control&chilren-adoult/GRAPH_Sep09_055820.png]

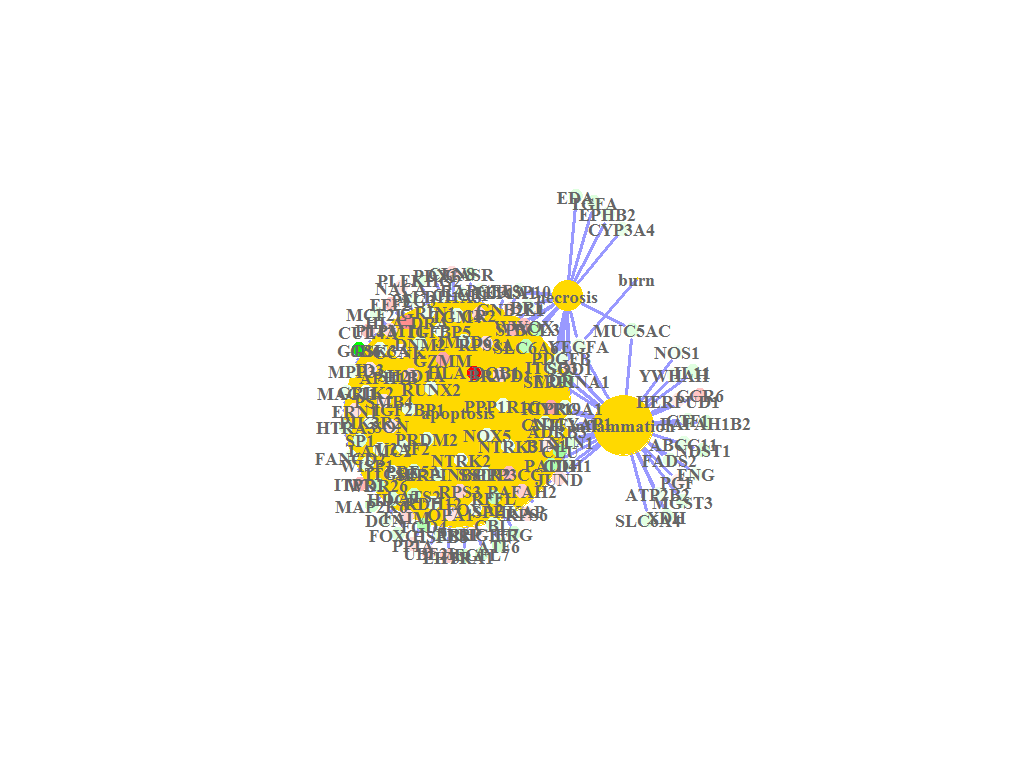

Supplement: Additional file 3 — Sample pipeline outputs in HTML format (compressed file). [file 1471-2164-13-620-S3.ZIP › Burn_early-late-control&chilren-adoult/GRAPH_Sep09_055828.png]

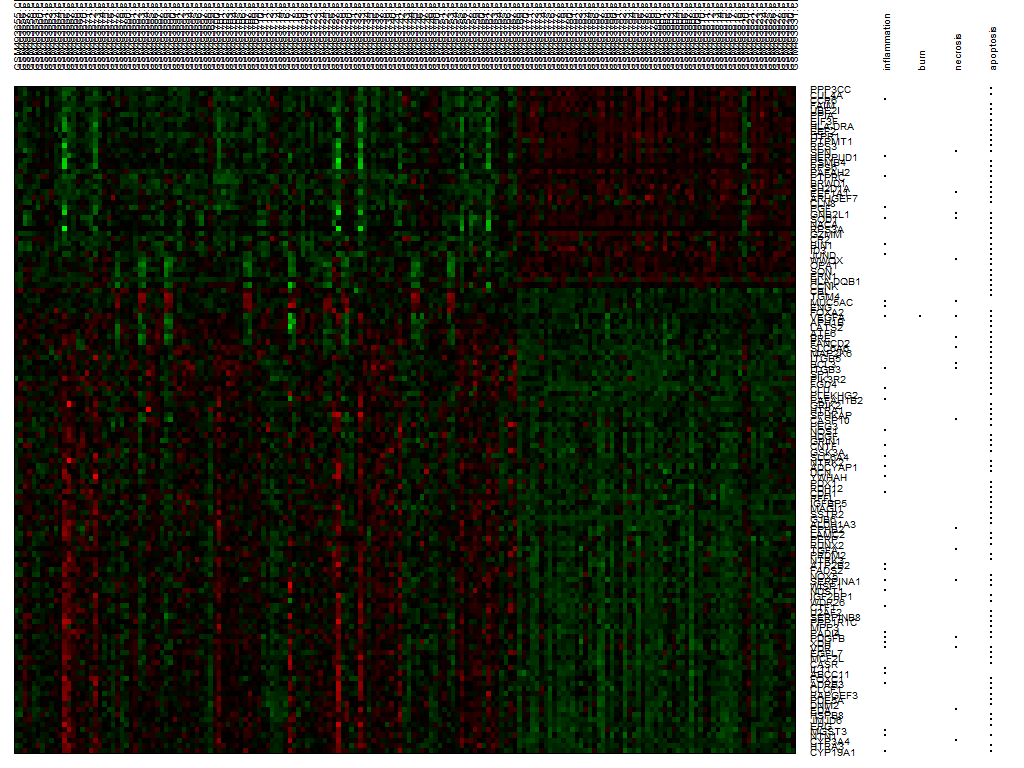

Supplement: Additional file 3 — Sample pipeline outputs in HTML format (compressed file). [file 1471-2164-13-620-S3.ZIP › Burn_early-late-control&chilren-adoult/GRAPH_Sep09_055833.png]

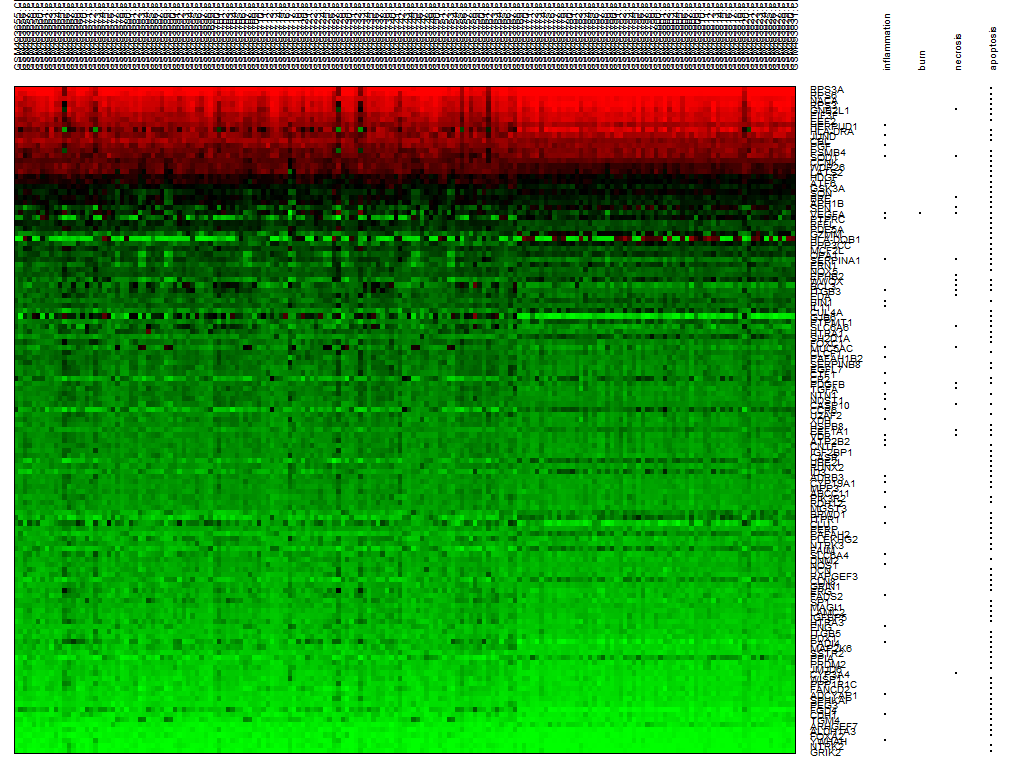

Supplement: Additional file 3 — Sample pipeline outputs in HTML format (compressed file). [file 1471-2164-13-620-S3.ZIP › Burn_early-late-control&chilren-adoult/GRAPH_Sep09_055841.png]

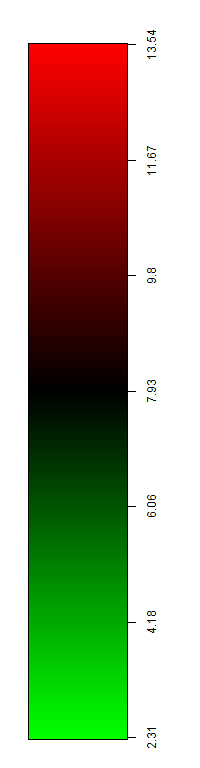

Supplement: Additional file 3 — Sample pipeline outputs in HTML format (compressed file). [file 1471-2164-13-620-S3.ZIP › Burn_early-late-control&chilren-adoult/GRAPH_Sep09_055846.png]

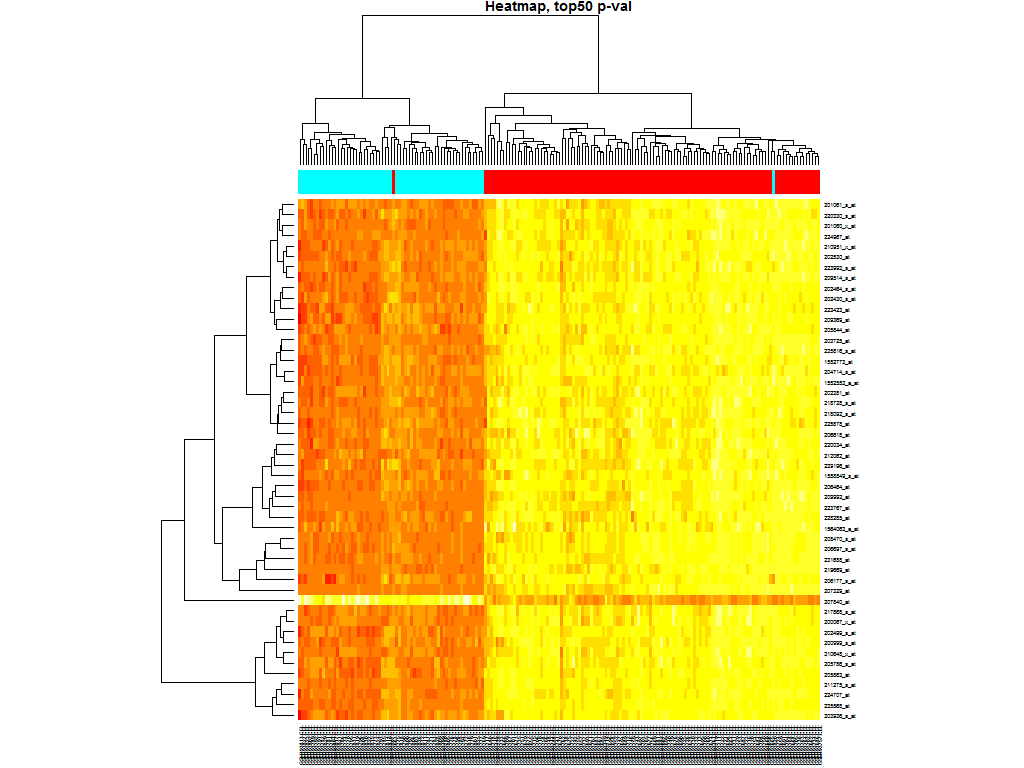

Supplement: Additional file 3 — Sample pipeline outputs in HTML format (compressed file). [file 1471-2164-13-620-S3.ZIP › Burn_early-late-control/GRAPH_Sep09_040313.png]

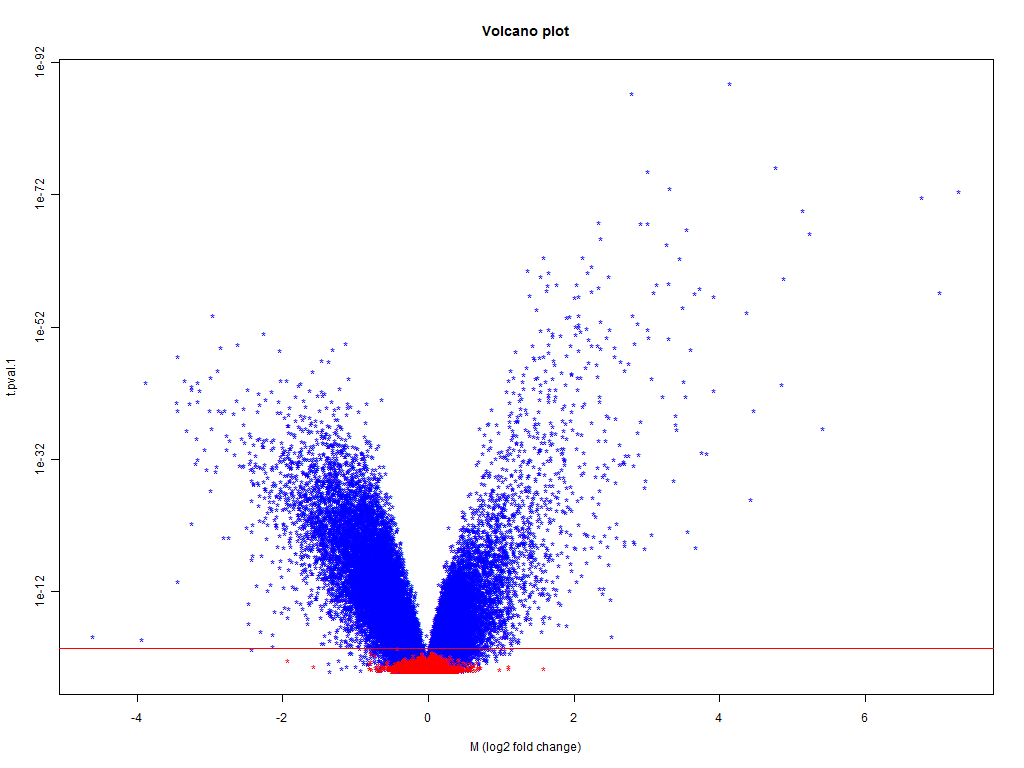

Supplement: Additional file 3 — Sample pipeline outputs in HTML format (compressed file). [file 1471-2164-13-620-S3.ZIP › Burn_early-late-control/GRAPH_Sep09_040315.png]

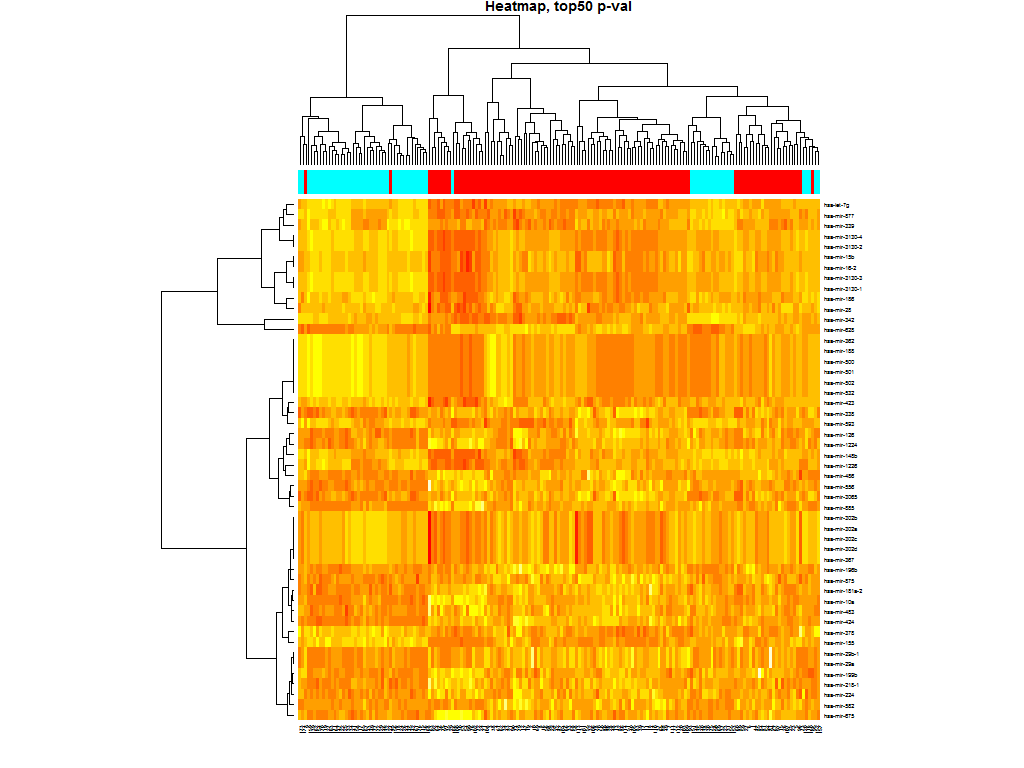

Supplement: Additional file 3 — Sample pipeline outputs in HTML format (compressed file). [file 1471-2164-13-620-S3.ZIP › Burn_early-late-control/GRAPH_Sep09_044532.png]

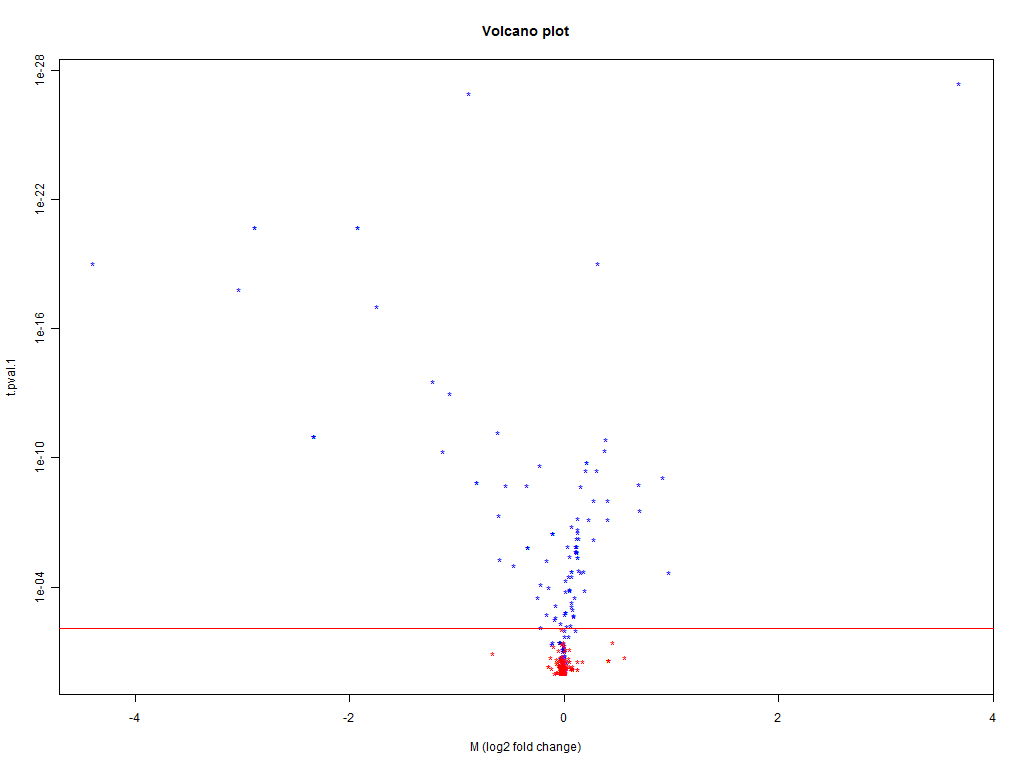

Supplement: Additional file 3 — Sample pipeline outputs in HTML format (compressed file). [file 1471-2164-13-620-S3.ZIP › Burn_early-late-control/GRAPH_Sep09_044535.png]

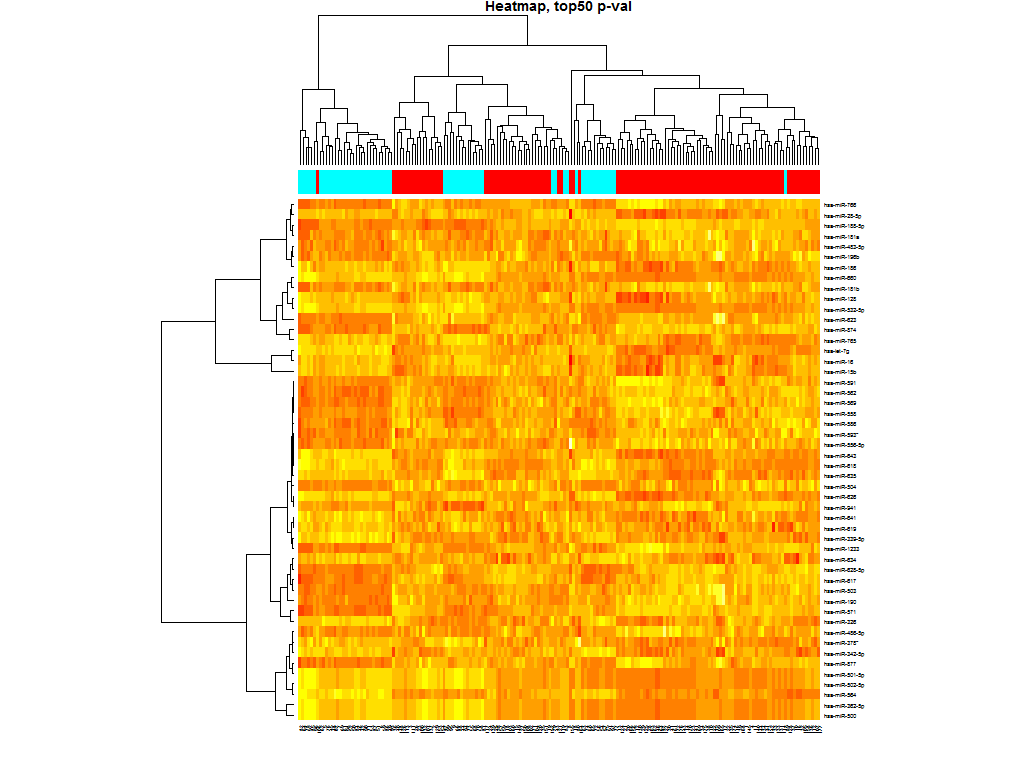

Supplement: Additional file 3 — Sample pipeline outputs in HTML format (compressed file). [file 1471-2164-13-620-S3.ZIP › Burn_early-late-control/GRAPH_Sep09_044610.png]

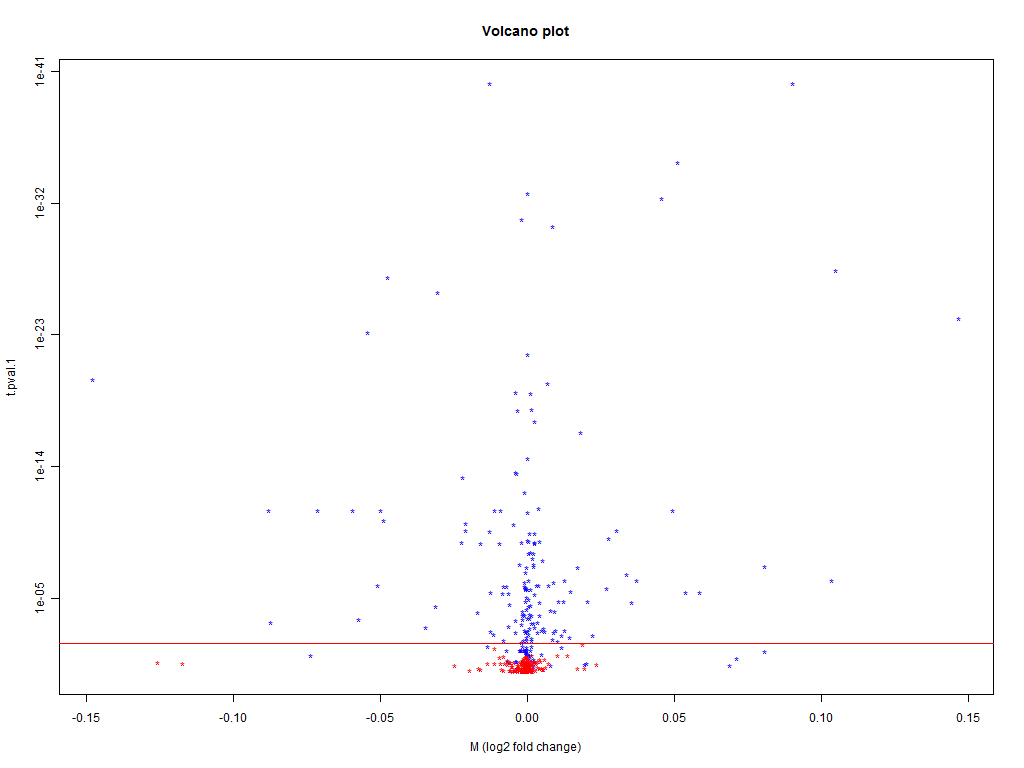

Supplement: Additional file 3 — Sample pipeline outputs in HTML format (compressed file). [file 1471-2164-13-620-S3.ZIP › Burn_early-late-control/GRAPH_Sep09_044613.png]

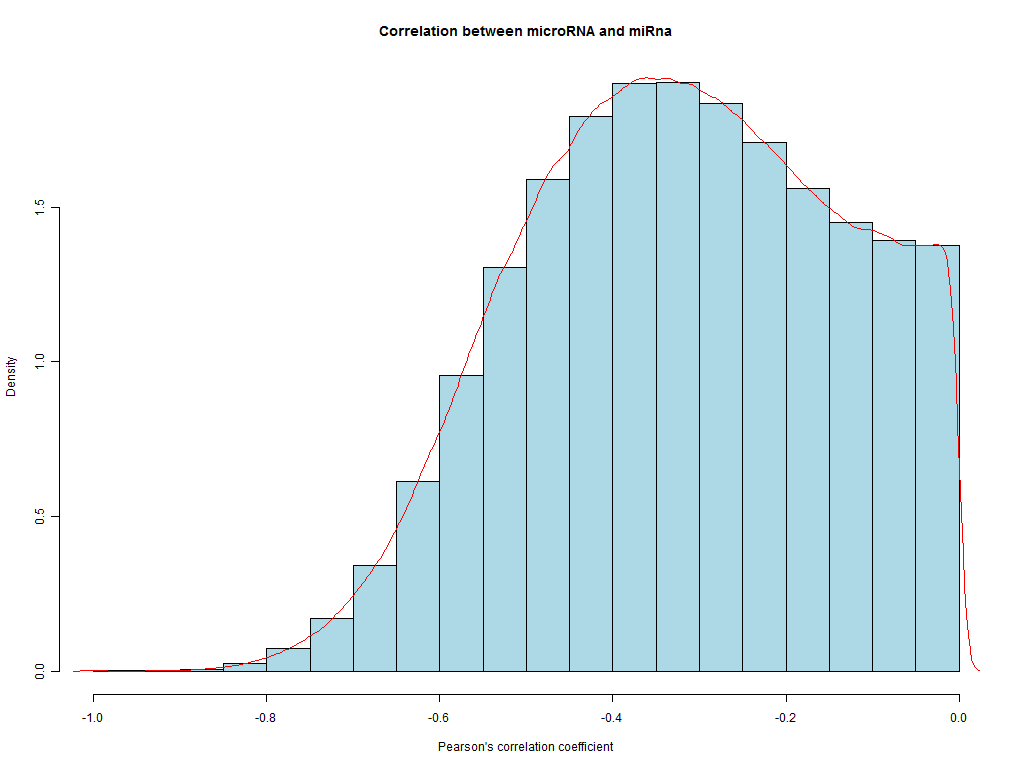

Supplement: Additional file 3 — Sample pipeline outputs in HTML format (compressed file). [file 1471-2164-13-620-S3.ZIP › Burn_early-late-control/GRAPH_Sep09_044633.png]

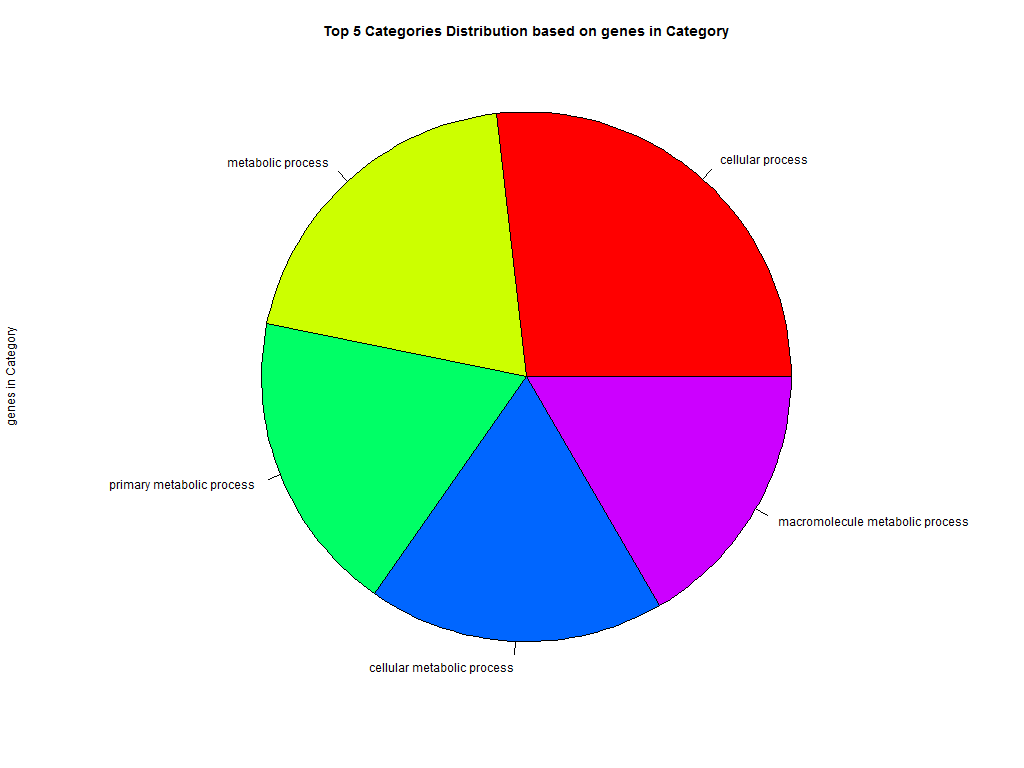

Supplement: Additional file 3 — Sample pipeline outputs in HTML format (compressed file). [file 1471-2164-13-620-S3.ZIP › Burn_early-late-control/GRAPH_Sep09_044913.png]

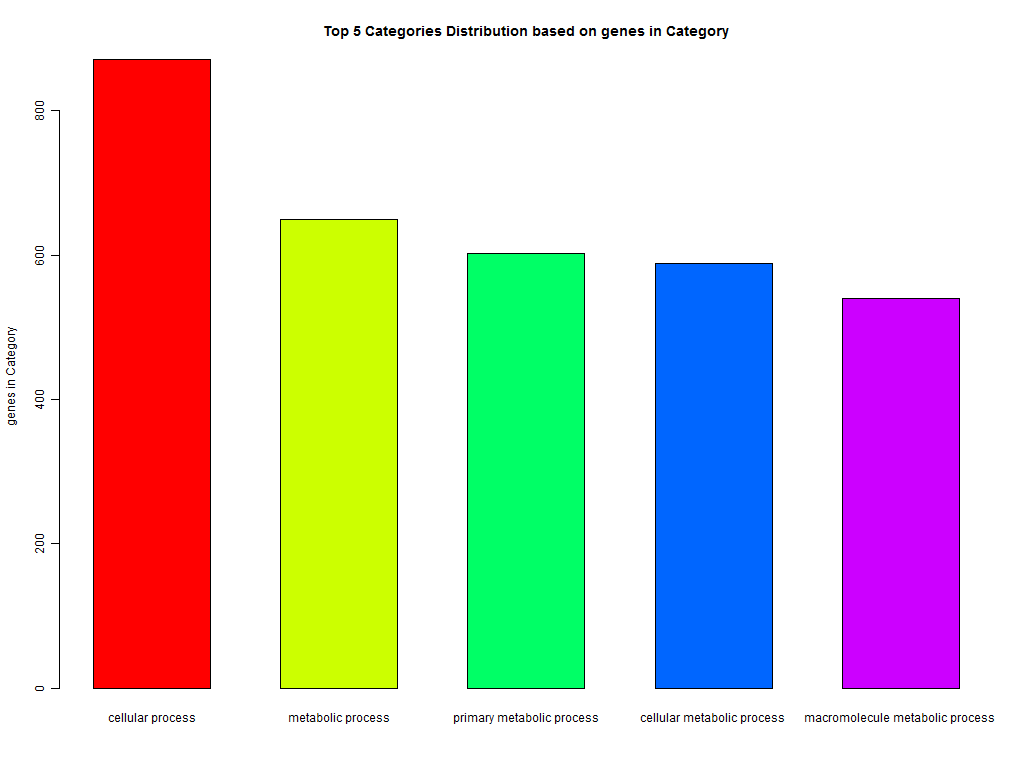

Supplement: Additional file 3 — Sample pipeline outputs in HTML format (compressed file). [file 1471-2164-13-620-S3.ZIP › Burn_early-late-control/GRAPH_Sep09_044919.png]

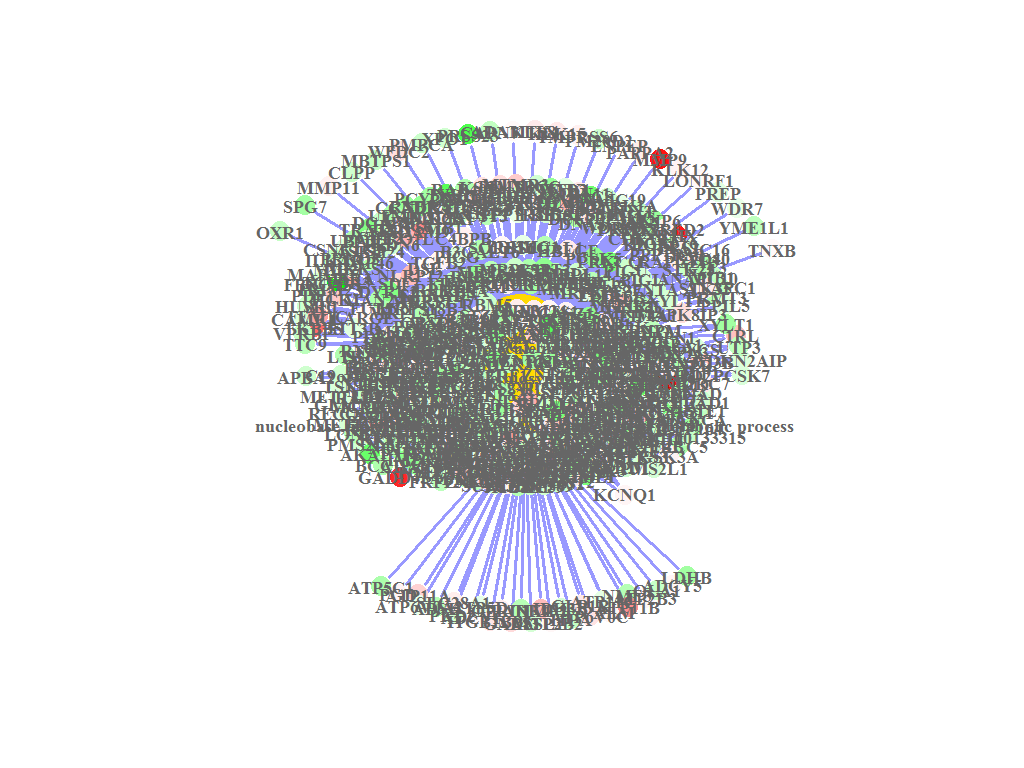

Supplement: Additional file 3 — Sample pipeline outputs in HTML format (compressed file). [file 1471-2164-13-620-S3.ZIP › Burn_early-late-control/GRAPH_Sep09_044934.png]

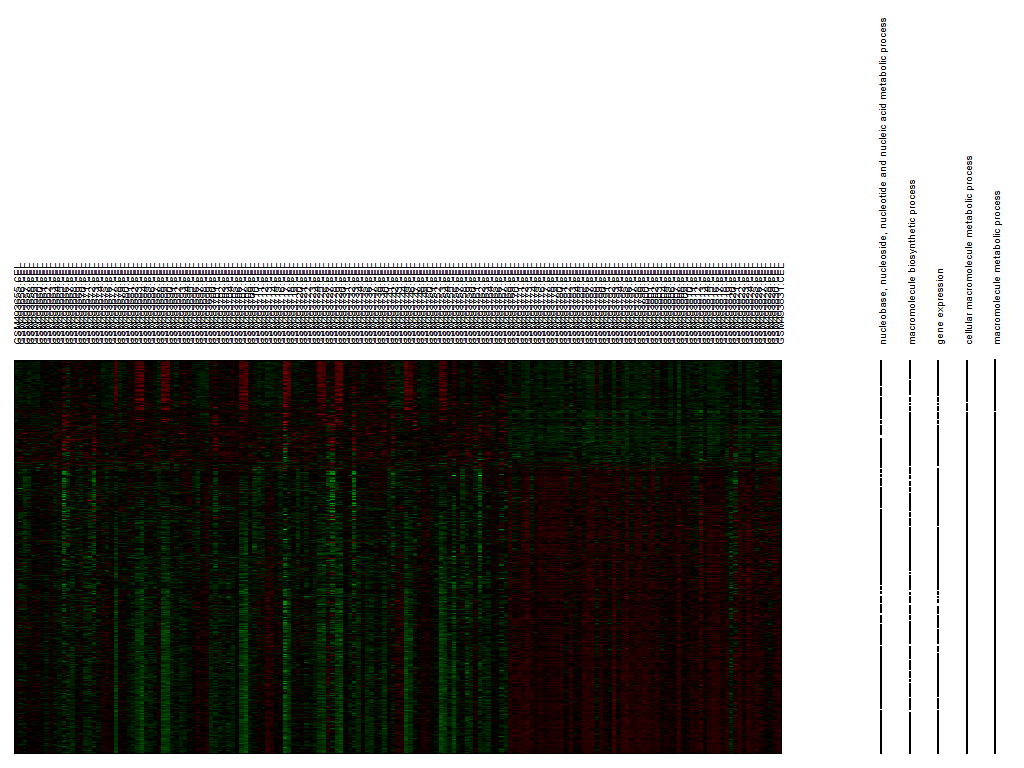

Supplement: Additional file 3 — Sample pipeline outputs in HTML format (compressed file). [file 1471-2164-13-620-S3.ZIP › Burn_early-late-control/GRAPH_Sep09_044944.png]

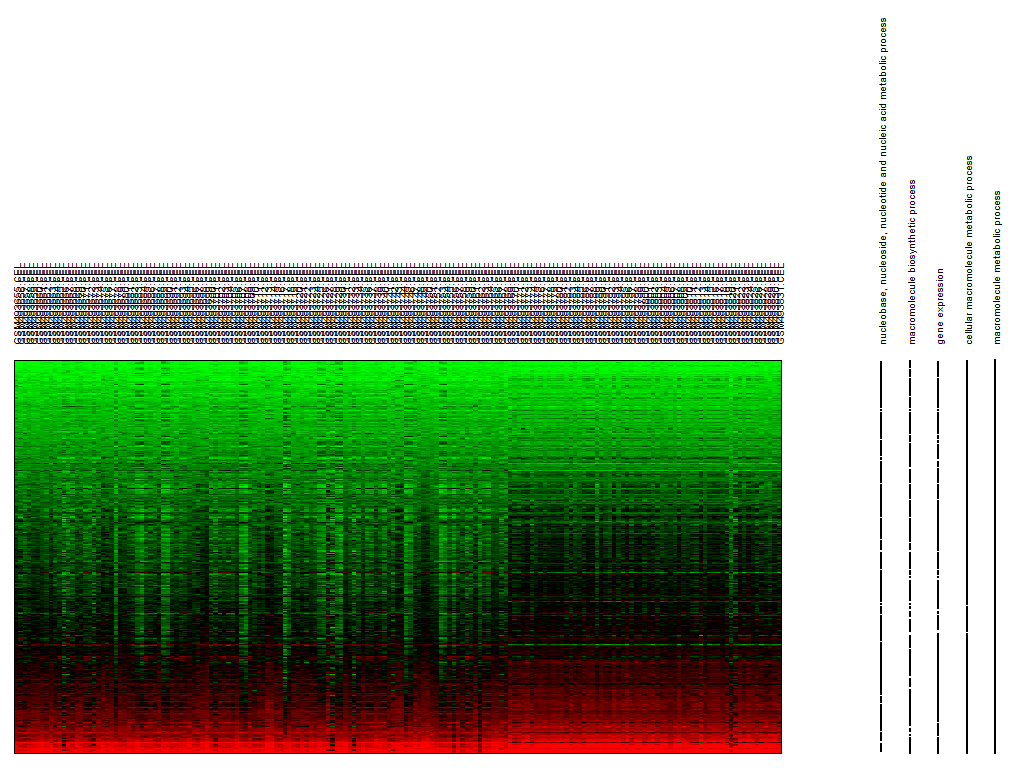

Supplement: Additional file 3 — Sample pipeline outputs in HTML format (compressed file). [file 1471-2164-13-620-S3.ZIP › Burn_early-late-control/GRAPH_Sep09_045000.png]

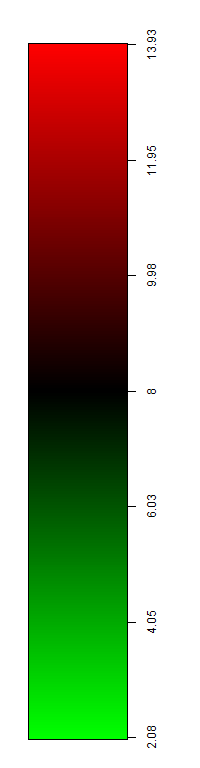

Supplement: Additional file 3 — Sample pipeline outputs in HTML format (compressed file). [file 1471-2164-13-620-S3.ZIP › Burn_early-late-control/GRAPH_Sep09_045021.png]

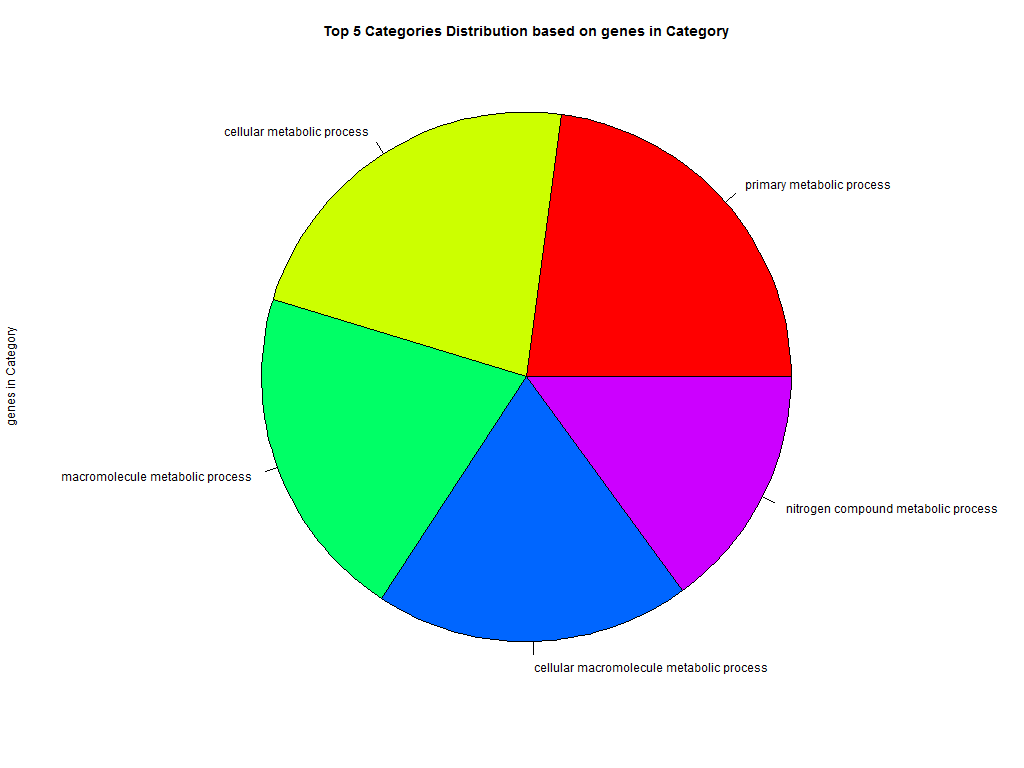

Supplement: Additional file 3 — Sample pipeline outputs in HTML format (compressed file). [file 1471-2164-13-620-S3.ZIP › Burn_early-late-control/GRAPH_Sep09_045321.png]

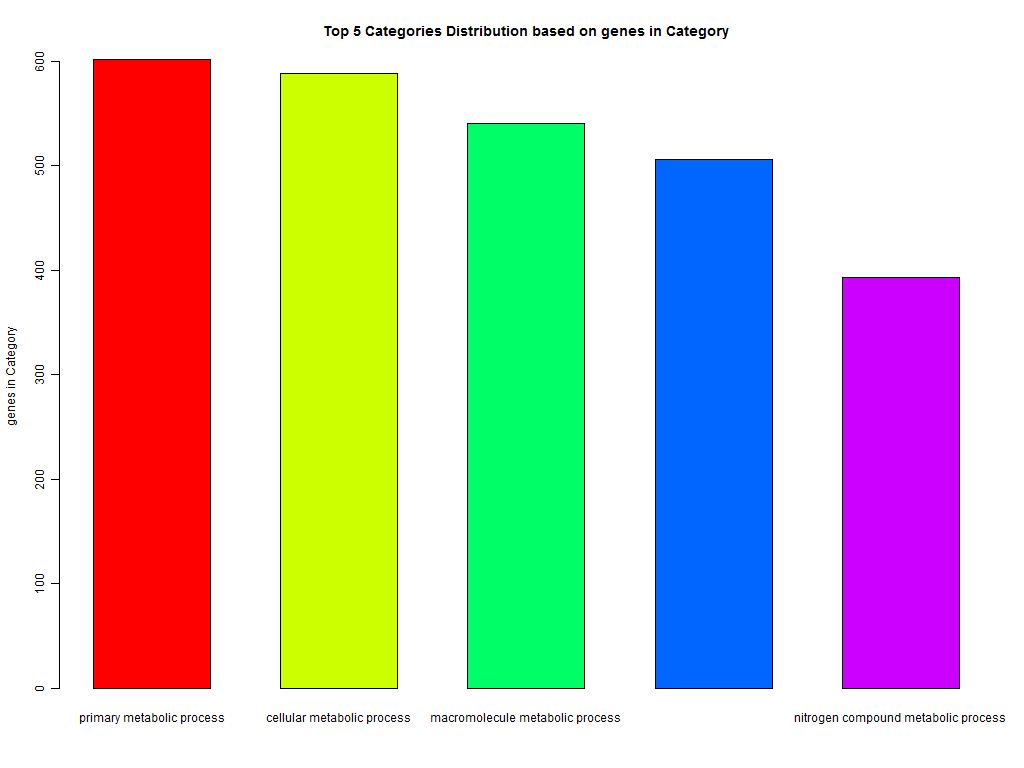

Supplement: Additional file 3 — Sample pipeline outputs in HTML format (compressed file). [file 1471-2164-13-620-S3.ZIP › Burn_early-late-control/GRAPH_Sep09_045327.png]

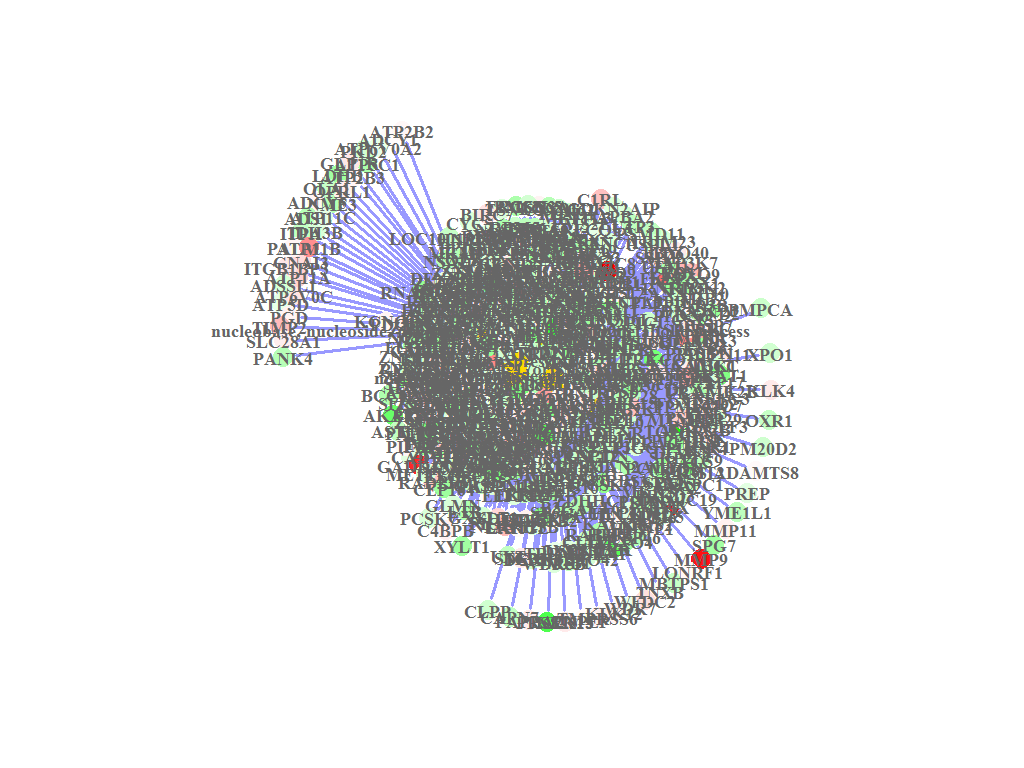

Supplement: Additional file 3 — Sample pipeline outputs in HTML format (compressed file). [file 1471-2164-13-620-S3.ZIP › Burn_early-late-control/GRAPH_Sep09_045351.png]

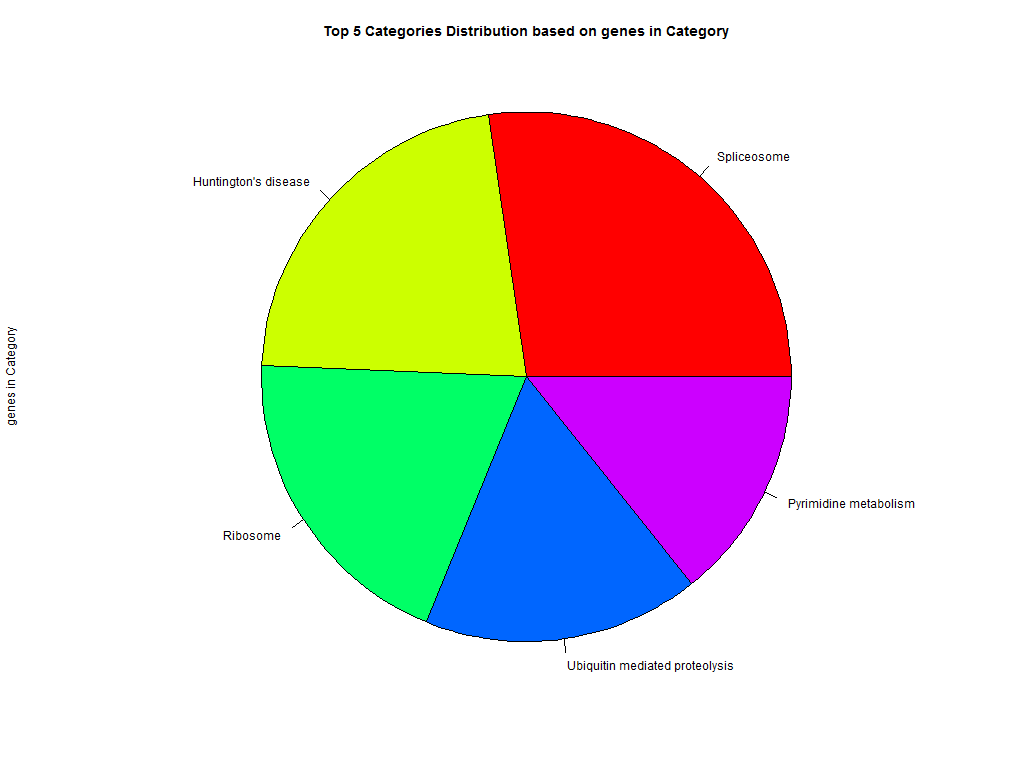

Supplement: Additional file 3 — Sample pipeline outputs in HTML format (compressed file). [file 1471-2164-13-620-S3.ZIP › Burn_early-late-control/GRAPH_Sep09_045454.png]

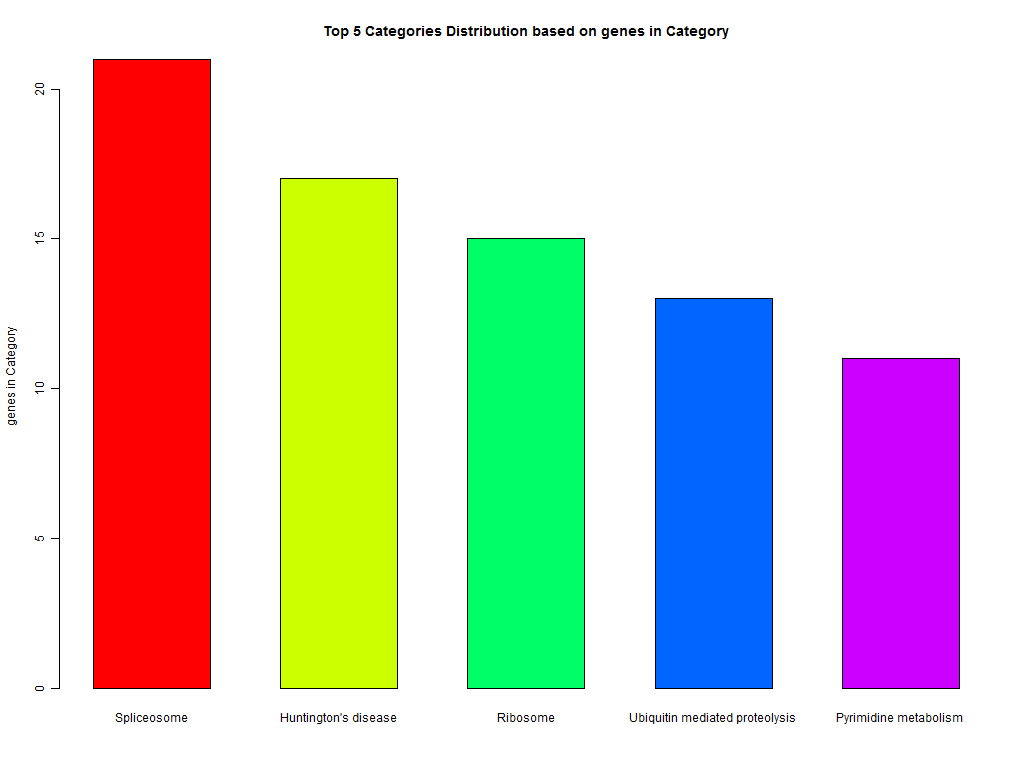

Supplement: Additional file 3 — Sample pipeline outputs in HTML format (compressed file). [file 1471-2164-13-620-S3.ZIP › Burn_early-late-control/GRAPH_Sep09_045500.png]

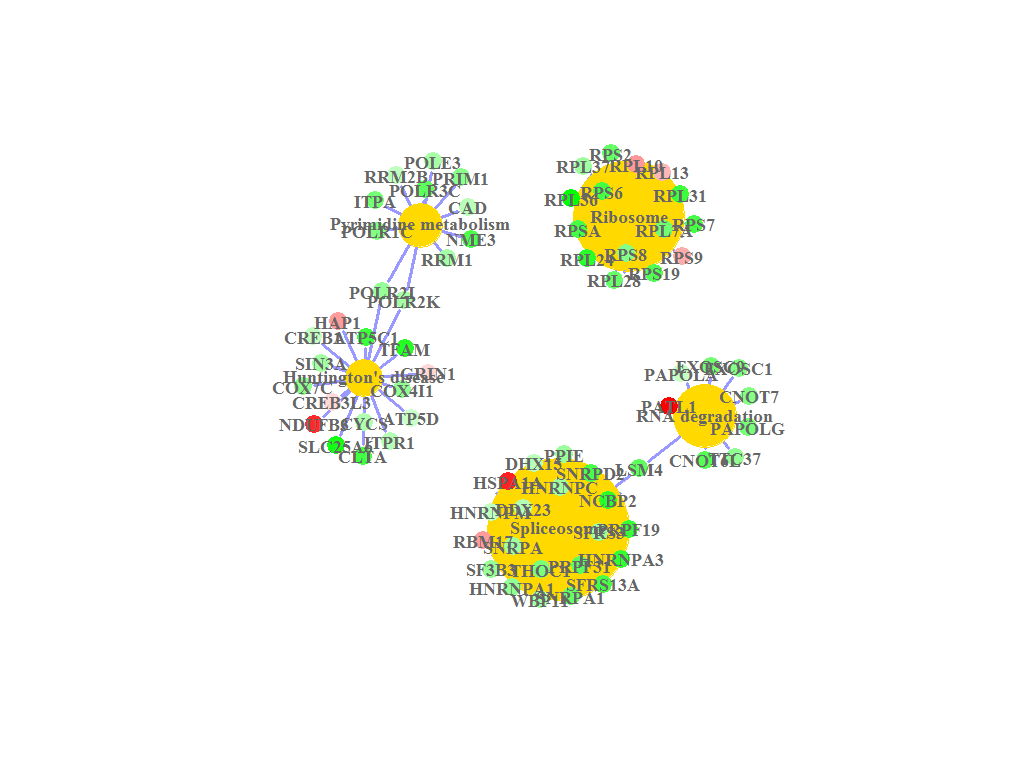

Supplement: Additional file 3 — Sample pipeline outputs in HTML format (compressed file). [file 1471-2164-13-620-S3.ZIP › Burn_early-late-control/GRAPH_Sep09_045505.png]

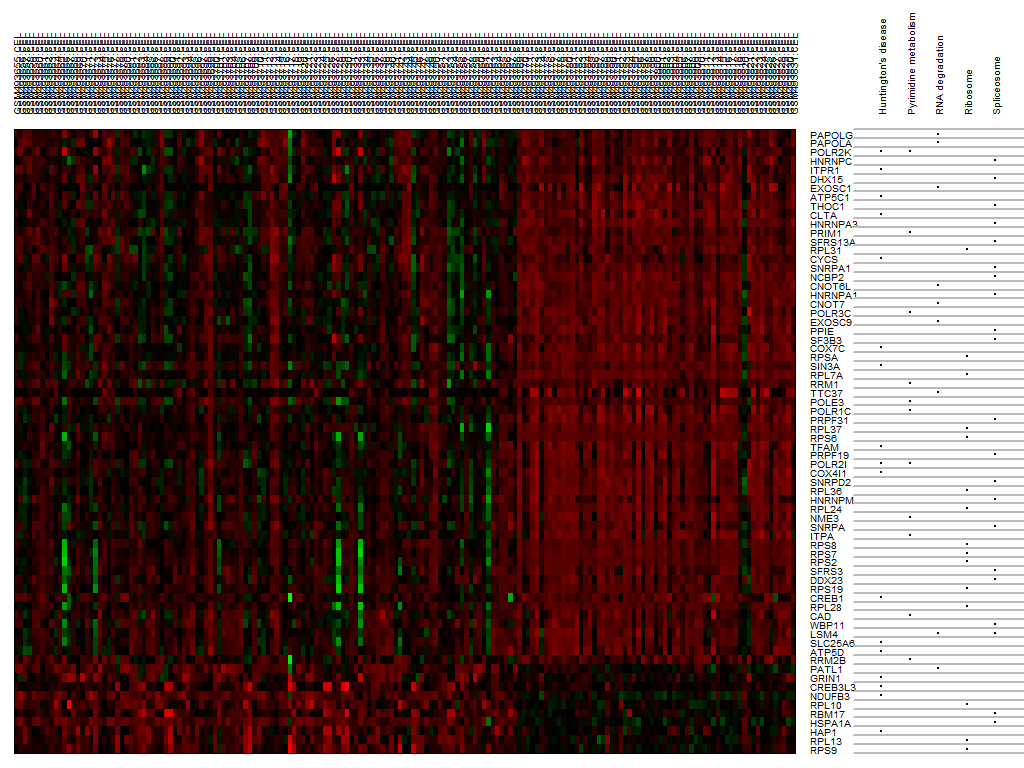

Supplement: Additional file 3 — Sample pipeline outputs in HTML format (compressed file). [file 1471-2164-13-620-S3.ZIP › Burn_early-late-control/GRAPH_Sep09_045511.png]

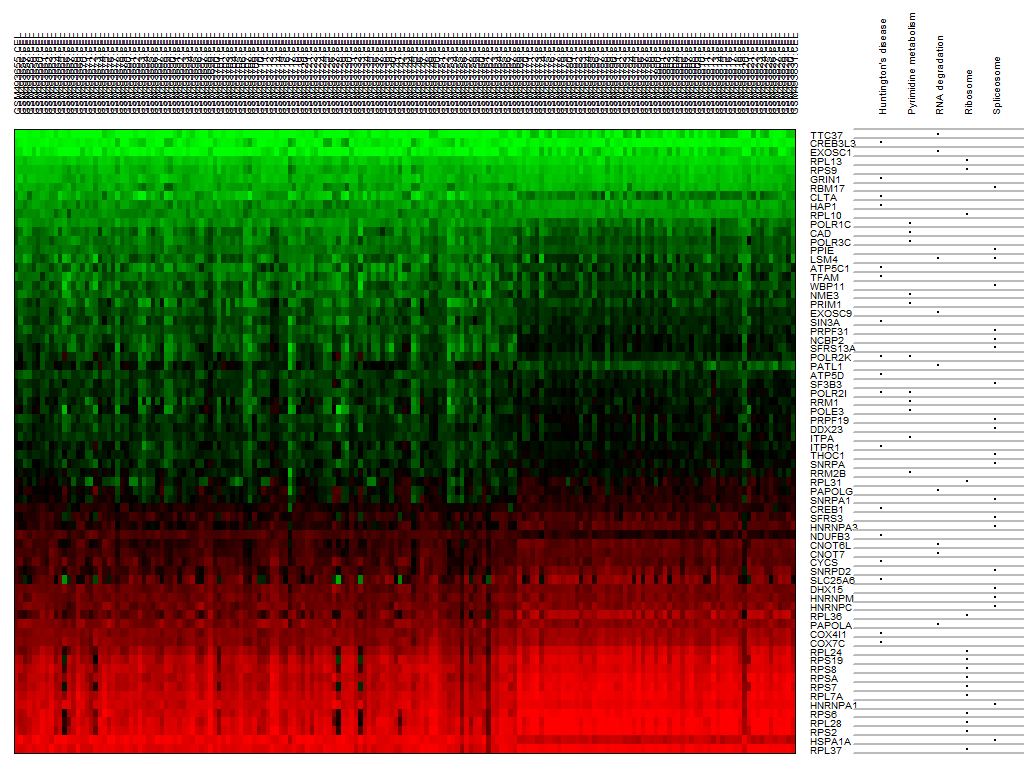

Supplement: Additional file 3 — Sample pipeline outputs in HTML format (compressed file). [file 1471-2164-13-620-S3.ZIP › Burn_early-late-control/GRAPH_Sep09_045519.png]

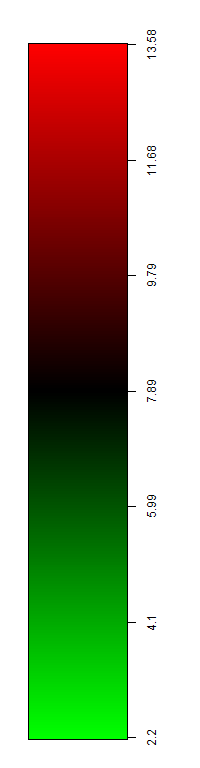

Supplement: Additional file 3 — Sample pipeline outputs in HTML format (compressed file). [file 1471-2164-13-620-S3.ZIP › Burn_early-late-control/GRAPH_Sep09_045525.png]

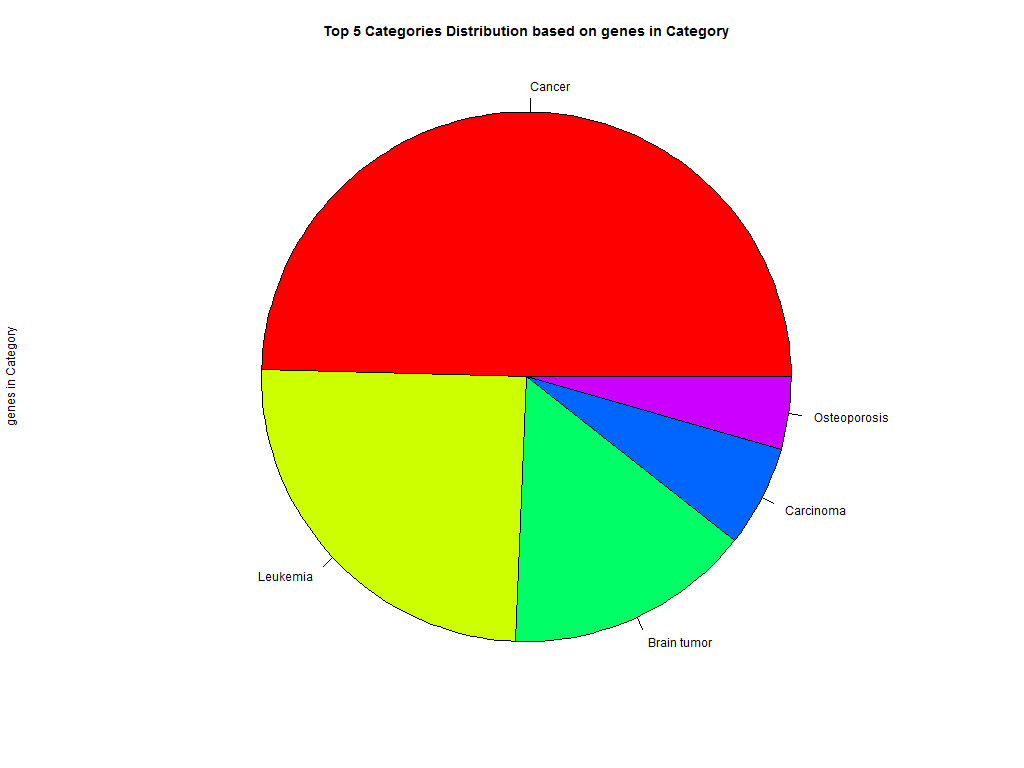

Supplement: Additional file 3 — Sample pipeline outputs in HTML format (compressed file). [file 1471-2164-13-620-S3.ZIP › Burn_early-late-control/GRAPH_Sep09_045544.png]

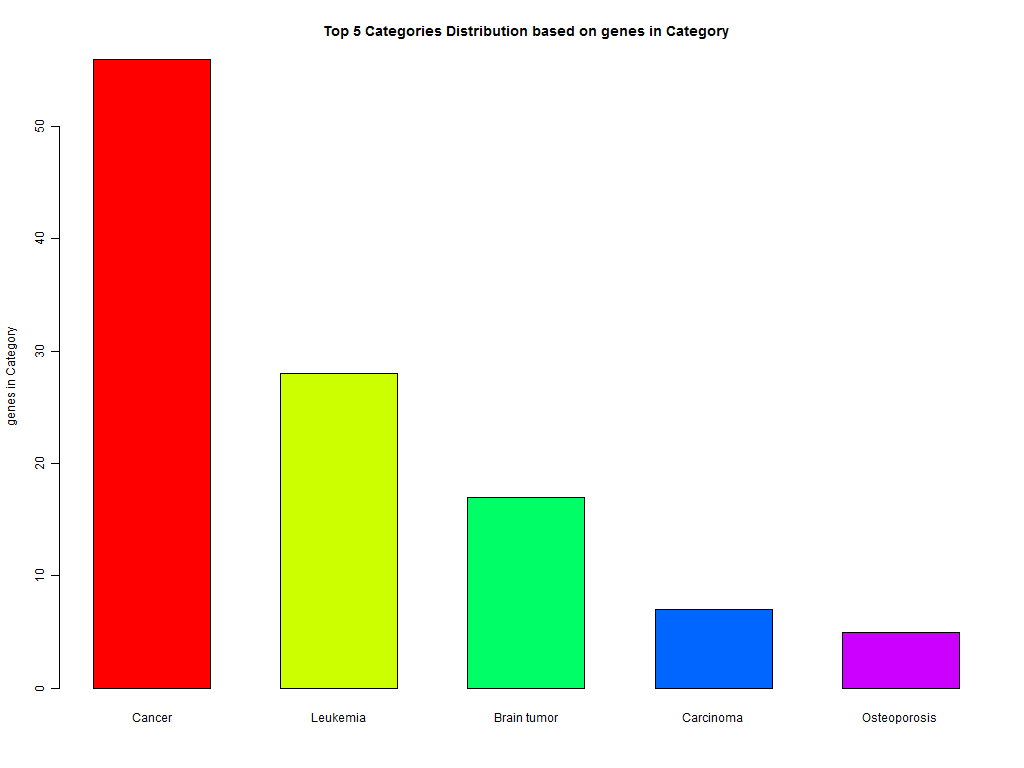

Supplement: Additional file 3 — Sample pipeline outputs in HTML format (compressed file). [file 1471-2164-13-620-S3.ZIP › Burn_early-late-control/GRAPH_Sep09_045549.png]

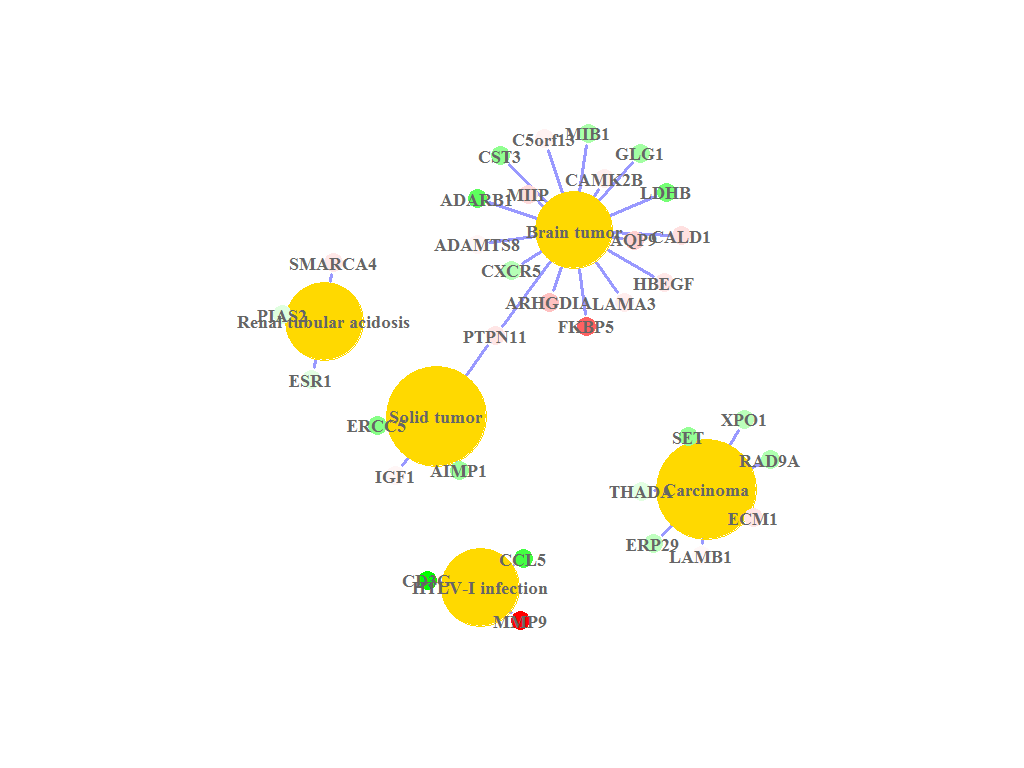

Supplement: Additional file 3 — Sample pipeline outputs in HTML format (compressed file). [file 1471-2164-13-620-S3.ZIP › Burn_early-late-control/GRAPH_Sep09_045555.png]

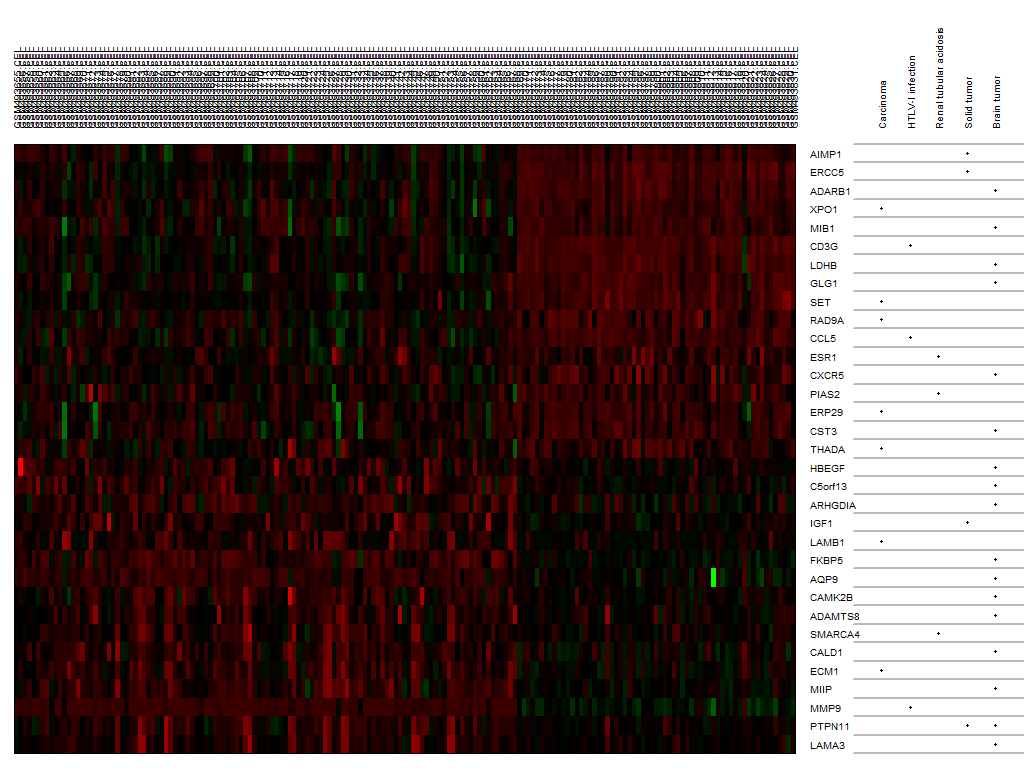

Supplement: Additional file 3 — Sample pipeline outputs in HTML format (compressed file). [file 1471-2164-13-620-S3.ZIP › Burn_early-late-control/GRAPH_Sep09_045601.png]

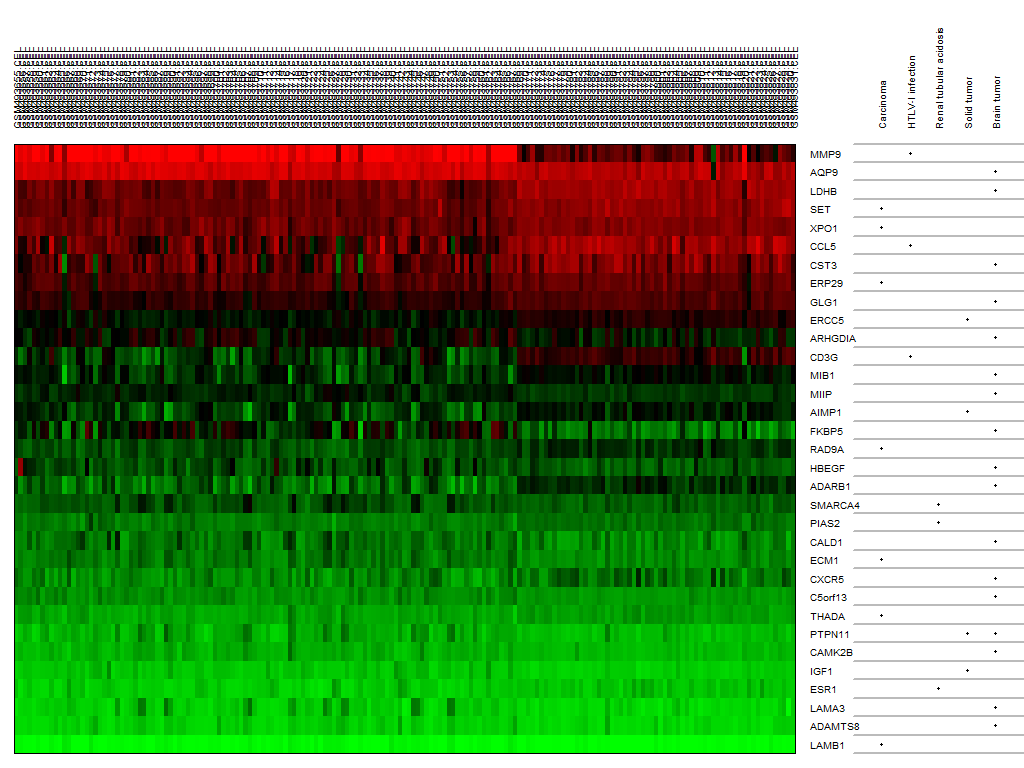

Supplement: Additional file 3 — Sample pipeline outputs in HTML format (compressed file). [file 1471-2164-13-620-S3.ZIP › Burn_early-late-control/GRAPH_Sep09_045608.png]

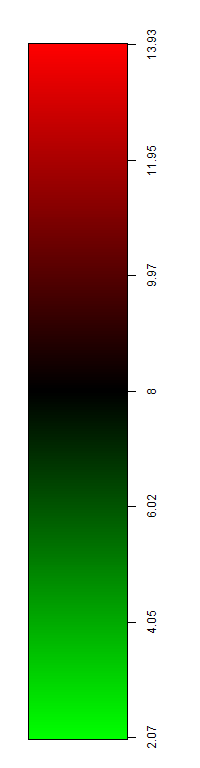

Supplement: Additional file 3 — Sample pipeline outputs in HTML format (compressed file). [file 1471-2164-13-620-S3.ZIP › Burn_early-late-control/GRAPH_Sep09_045614.png]

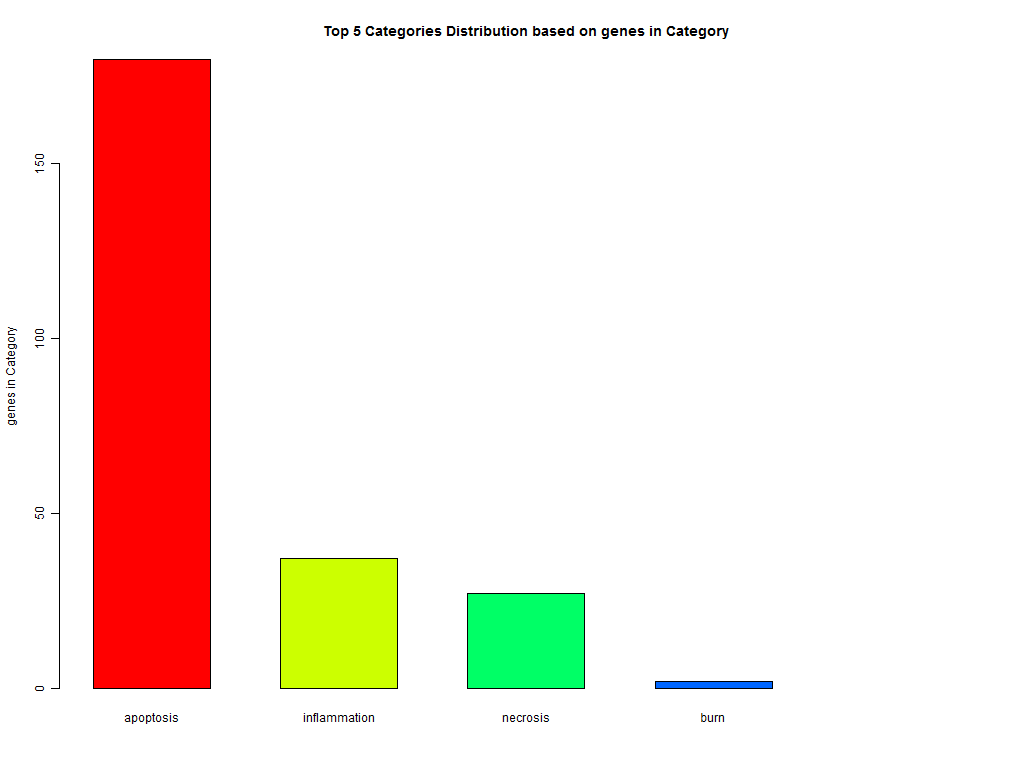

Supplement: Additional file 3 — Sample pipeline outputs in HTML format (compressed file). [file 1471-2164-13-620-S3.ZIP › Burn_early-late-control/GRAPH_Sep09_045654.png]

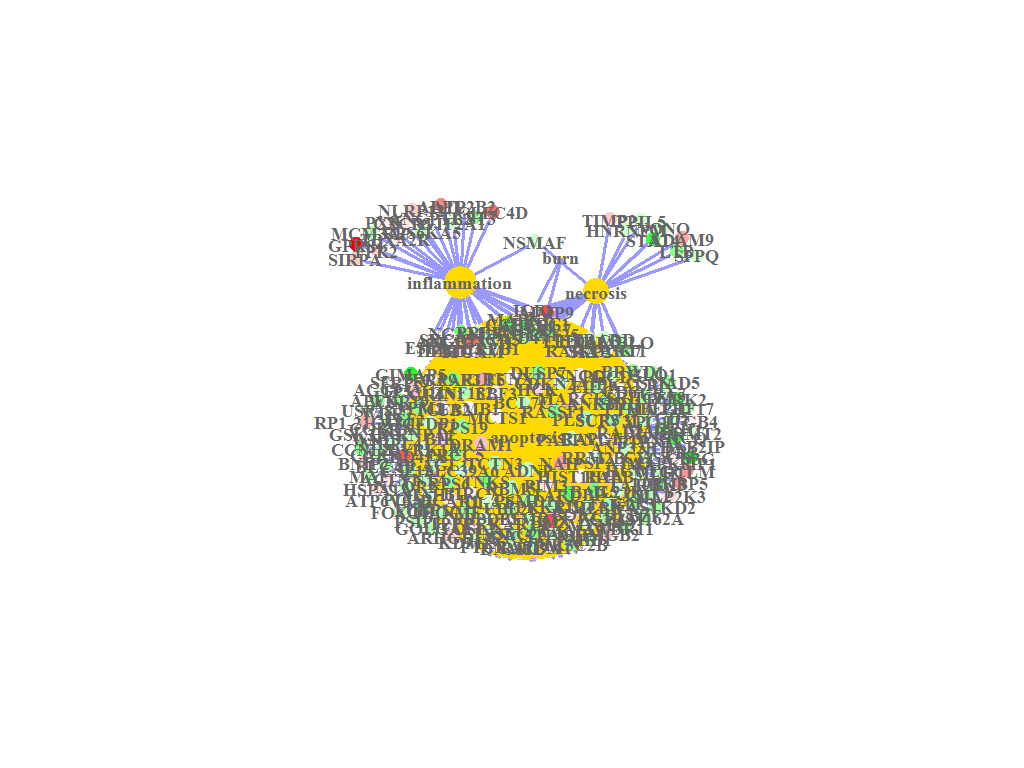

Supplement: Additional file 3 — Sample pipeline outputs in HTML format (compressed file). [file 1471-2164-13-620-S3.ZIP › Burn_early-late-control/GRAPH_Sep09_045705.png]

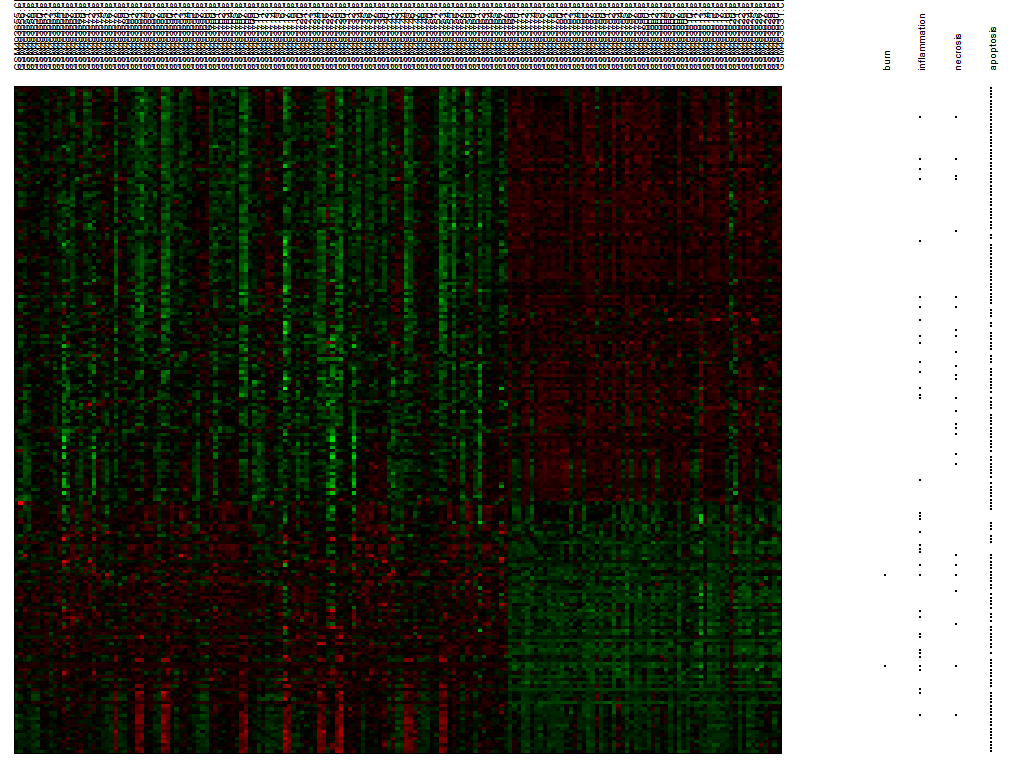

Supplement: Additional file 3 — Sample pipeline outputs in HTML format (compressed file). [file 1471-2164-13-620-S3.ZIP › Burn_early-late-control/GRAPH_Sep09_045712.png]

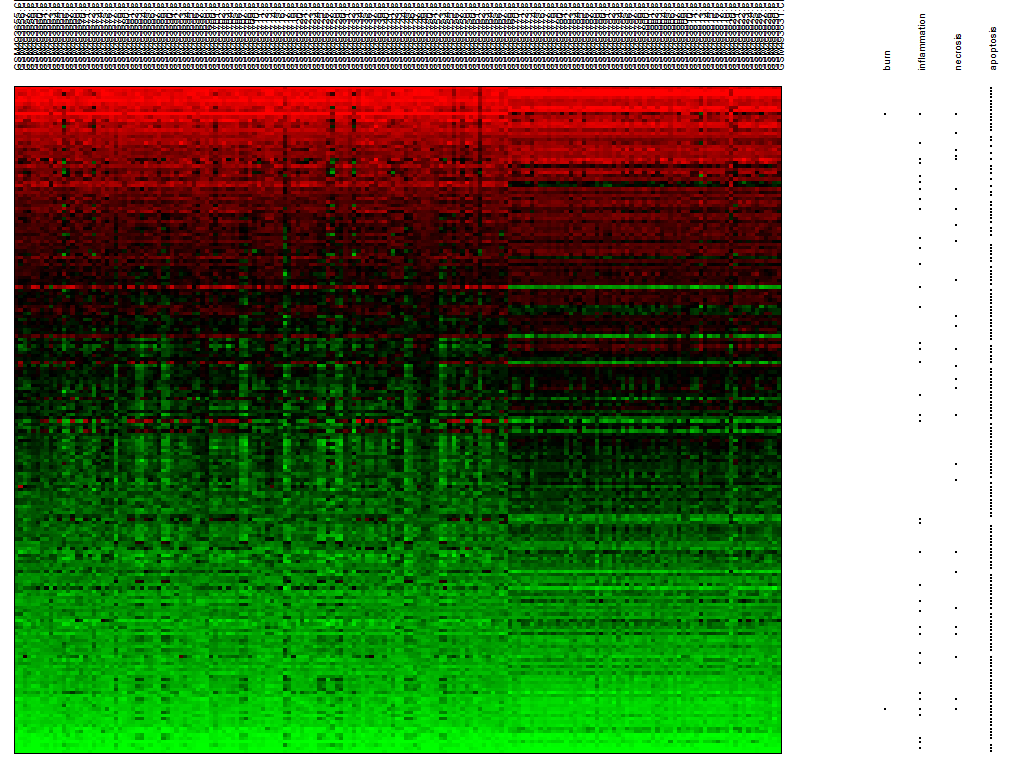

Supplement: Additional file 3 — Sample pipeline outputs in HTML format (compressed file). [file 1471-2164-13-620-S3.ZIP › Burn_early-late-control/GRAPH_Sep09_045720.png]

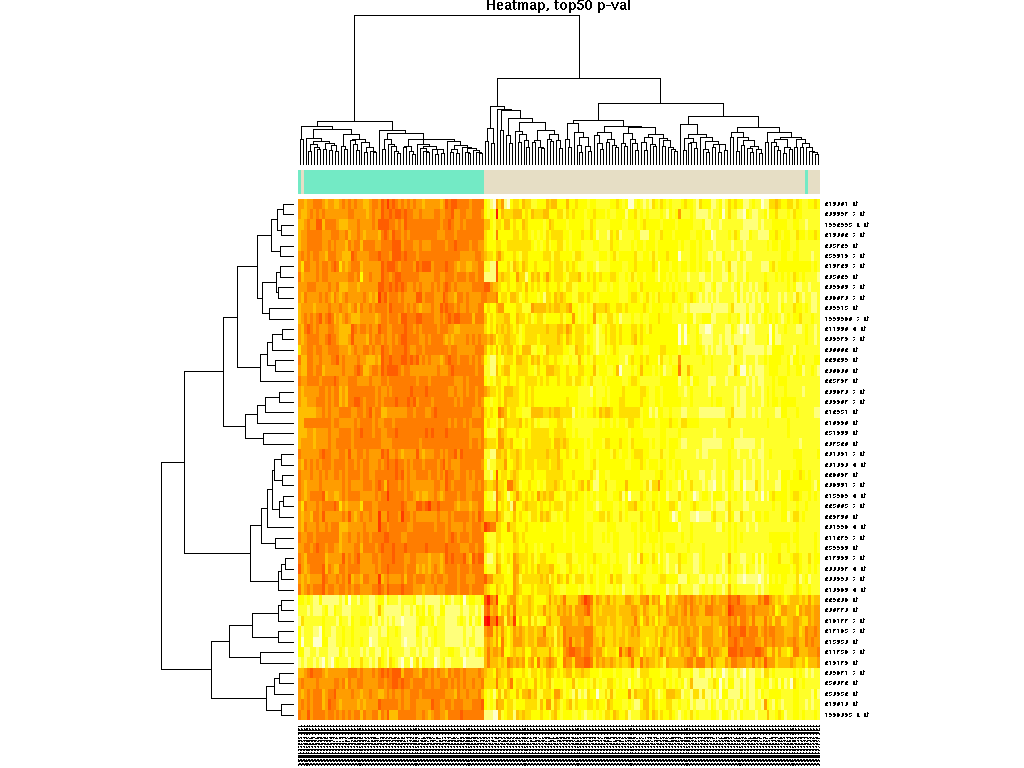

Supplement: Additional file 3 — Sample pipeline outputs in HTML format (compressed file). [file 1471-2164-13-620-S3.ZIP › TL4+LPS_activated/GRAPH_Jul21_190709.png]

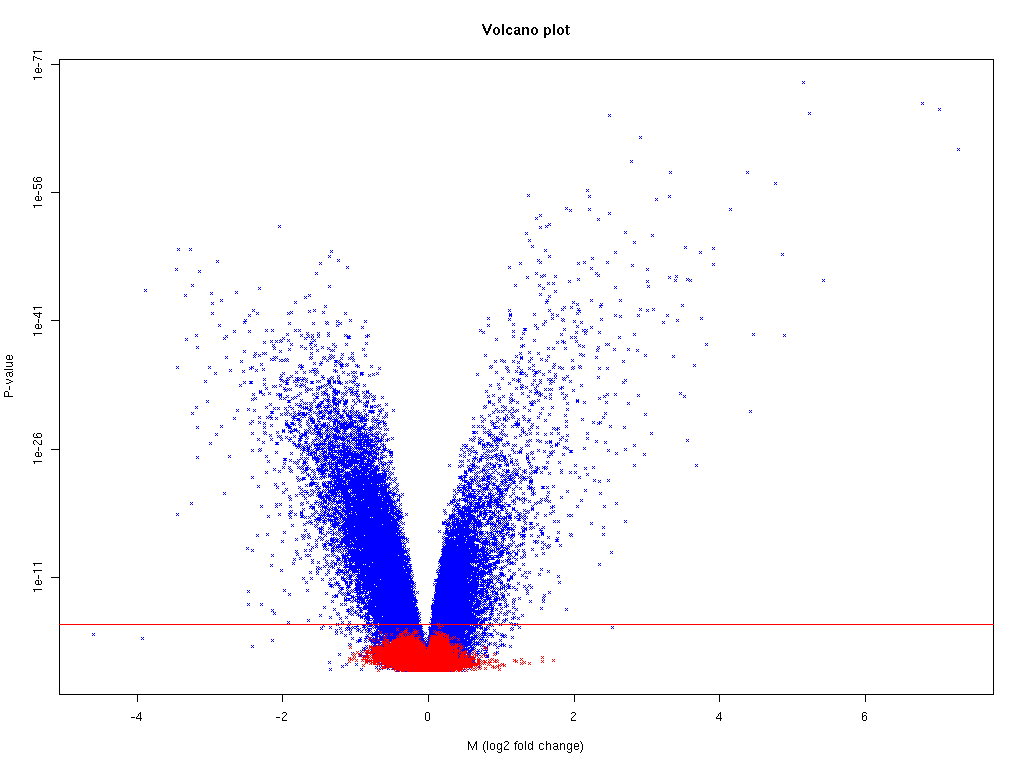

Supplement: Additional file 3 — Sample pipeline outputs in HTML format (compressed file). [file 1471-2164-13-620-S3.ZIP › TL4+LPS_activated/GRAPH_Jul21_190712.png]

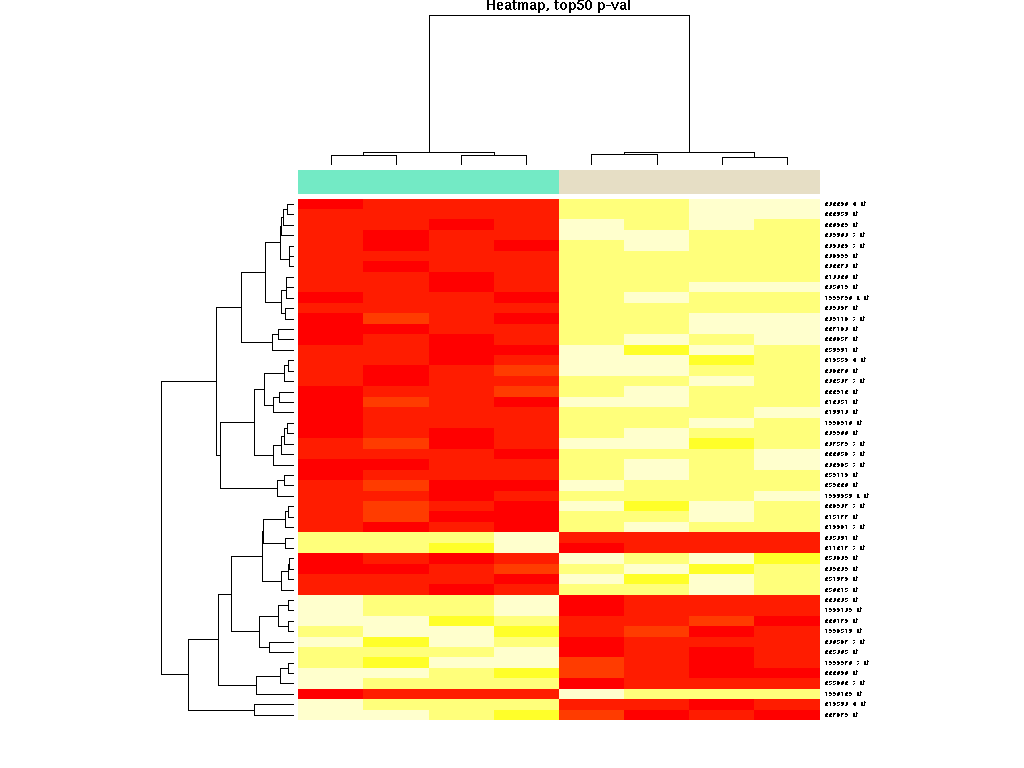

Supplement: Additional file 3 — Sample pipeline outputs in HTML format (compressed file). [file 1471-2164-13-620-S3.ZIP › TL4+LPS_activated/GRAPH_Jul21_190716.png]

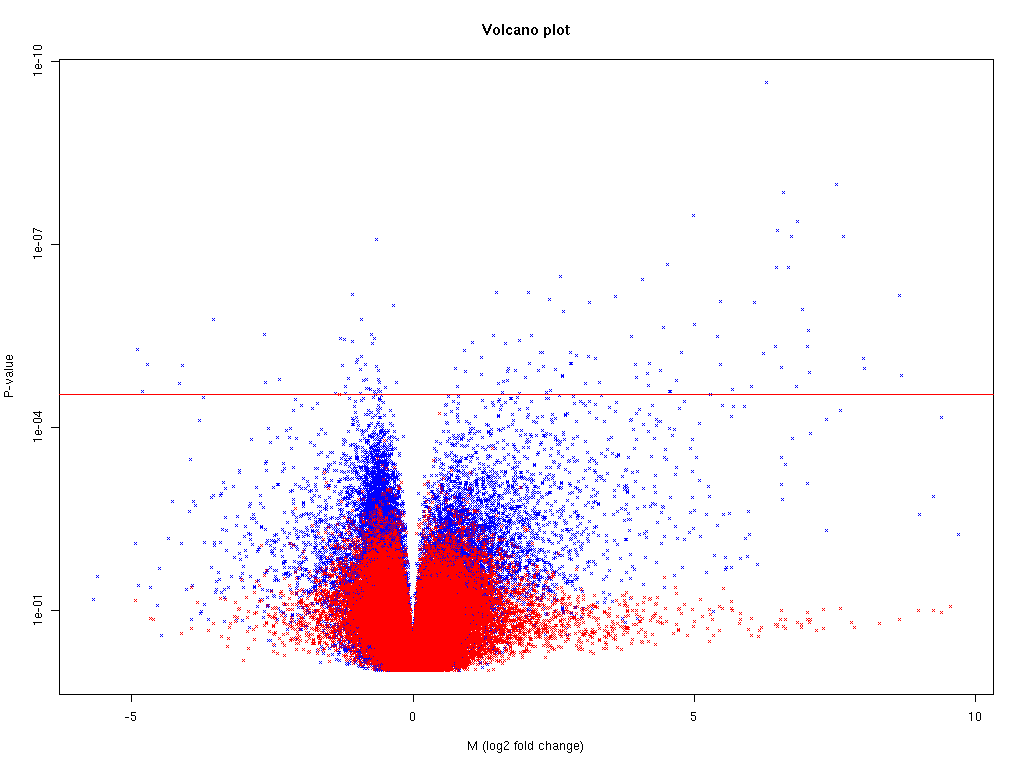

Supplement: Additional file 3 — Sample pipeline outputs in HTML format (compressed file). [file 1471-2164-13-620-S3.ZIP › TL4+LPS_activated/GRAPH_Jul21_190718.png]

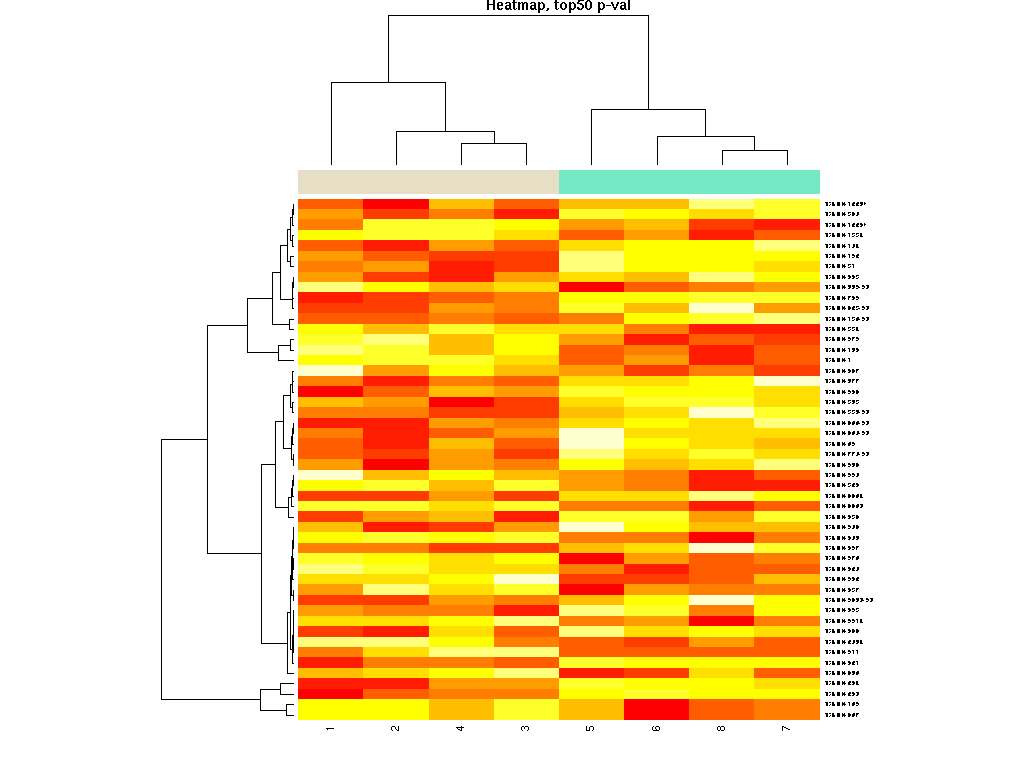

Supplement: Additional file 3 — Sample pipeline outputs in HTML format (compressed file). [file 1471-2164-13-620-S3.ZIP › TL4+LPS_activated/GRAPH_Jul21_191006.png]

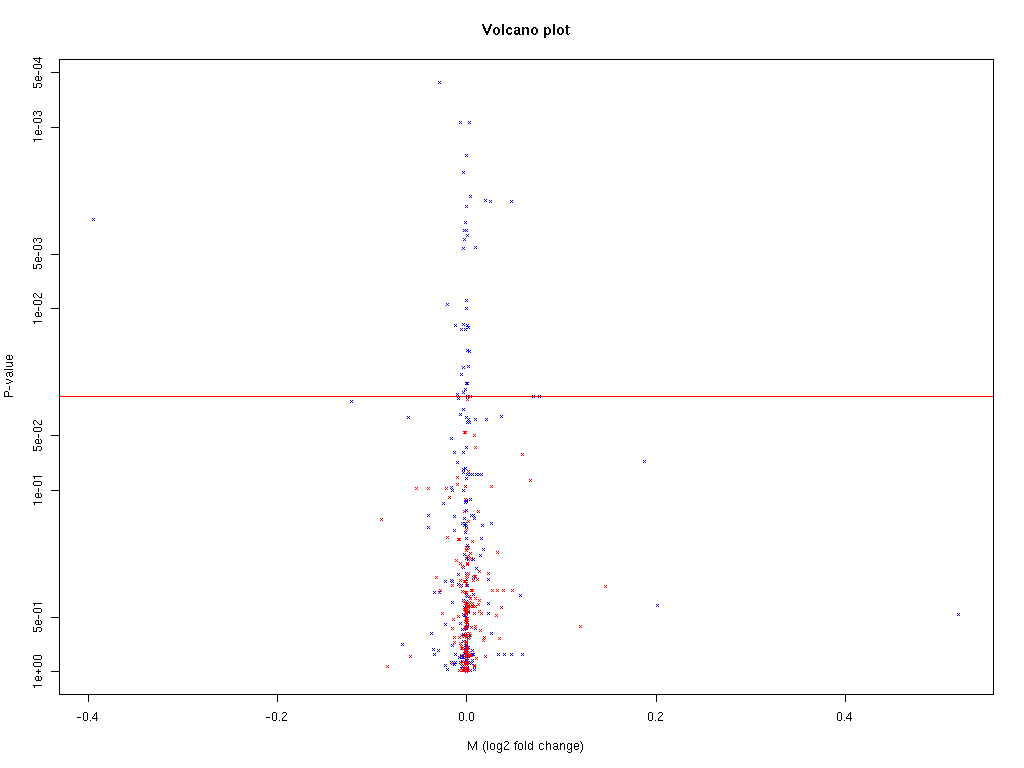

Supplement: Additional file 3 — Sample pipeline outputs in HTML format (compressed file). [file 1471-2164-13-620-S3.ZIP › TL4+LPS_activated/GRAPH_Jul21_191008.png]

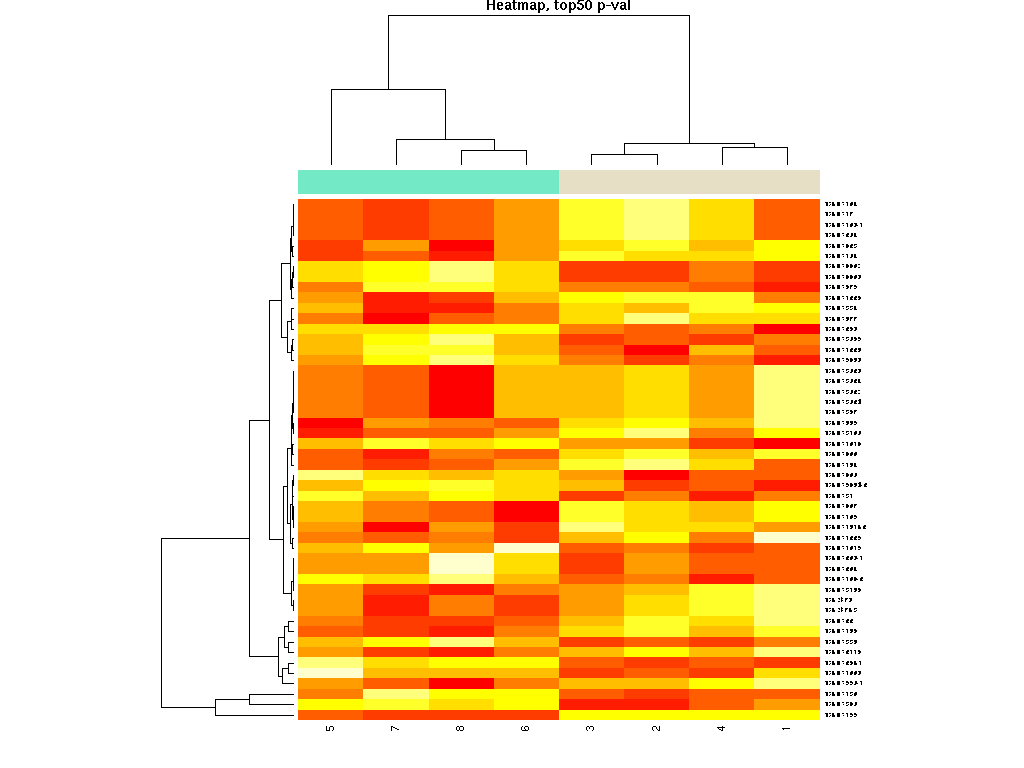

Supplement: Additional file 3 — Sample pipeline outputs in HTML format (compressed file). [file 1471-2164-13-620-S3.ZIP › TL4+LPS_activated/GRAPH_Jul21_191011.png]

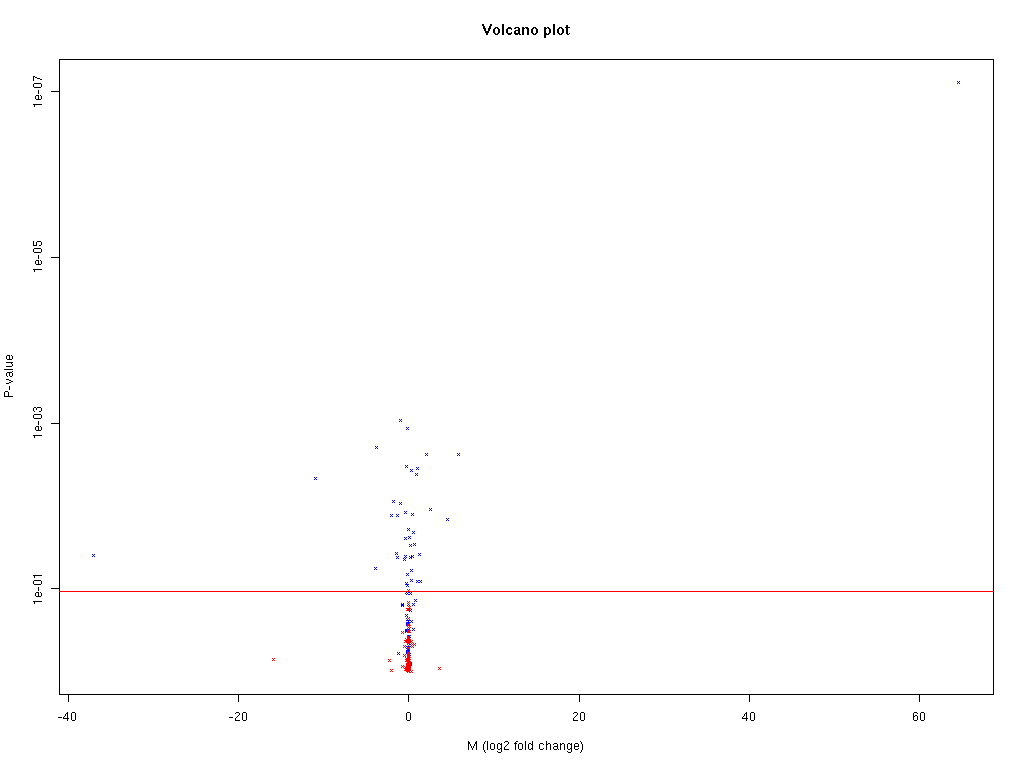

Supplement: Additional file 3 — Sample pipeline outputs in HTML format (compressed file). [file 1471-2164-13-620-S3.ZIP › TL4+LPS_activated/GRAPH_Jul21_191013.png]

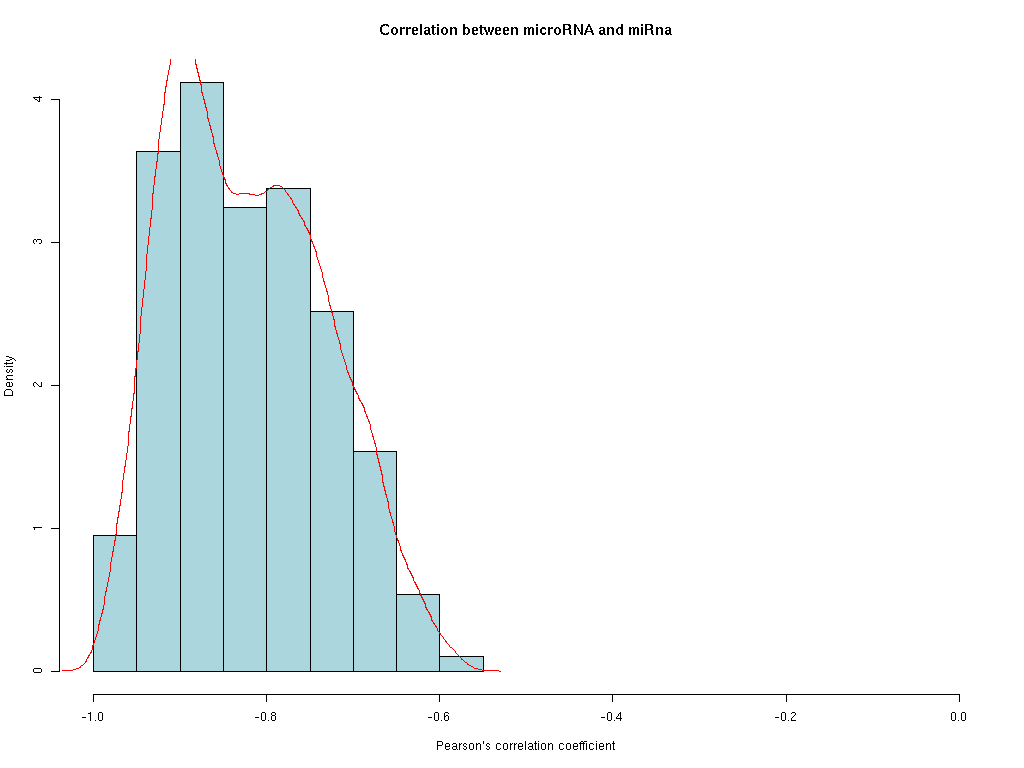

Supplement: Additional file 3 — Sample pipeline outputs in HTML format (compressed file). [file 1471-2164-13-620-S3.ZIP › TL4+LPS_activated/GRAPH_Jul21_191016.png]

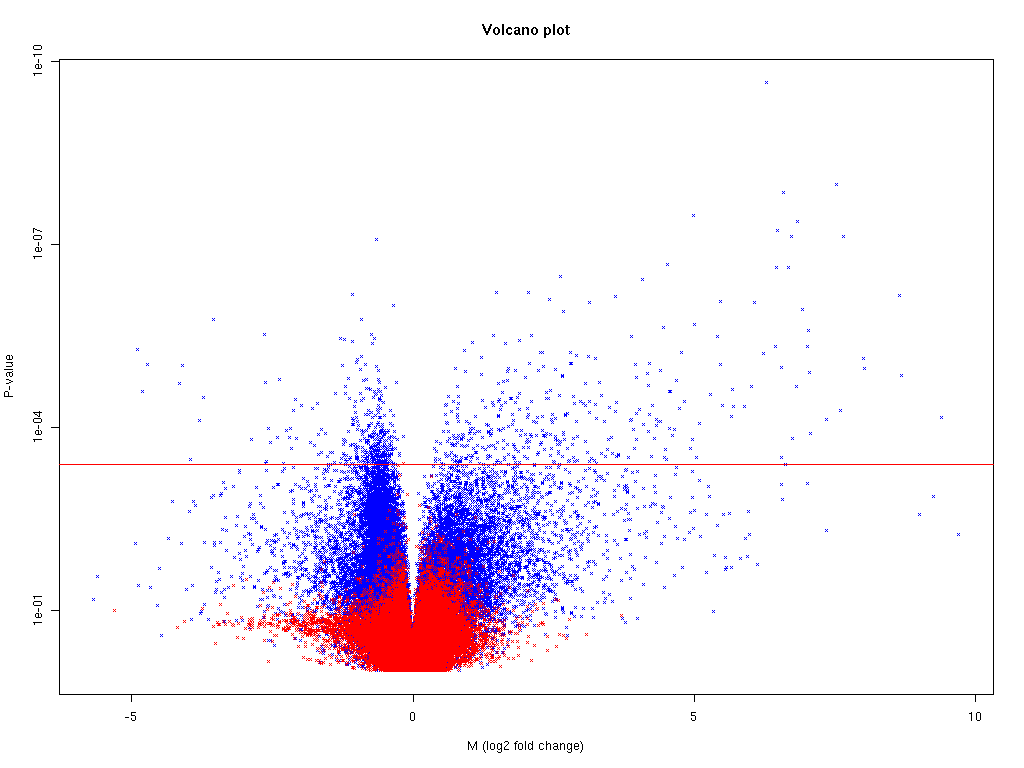

Supplement: Additional file 3 — Sample pipeline outputs in HTML format (compressed file). [file 1471-2164-13-620-S3.ZIP › TL4+LPS_activated/GRAPH_Jul21_191027.png]

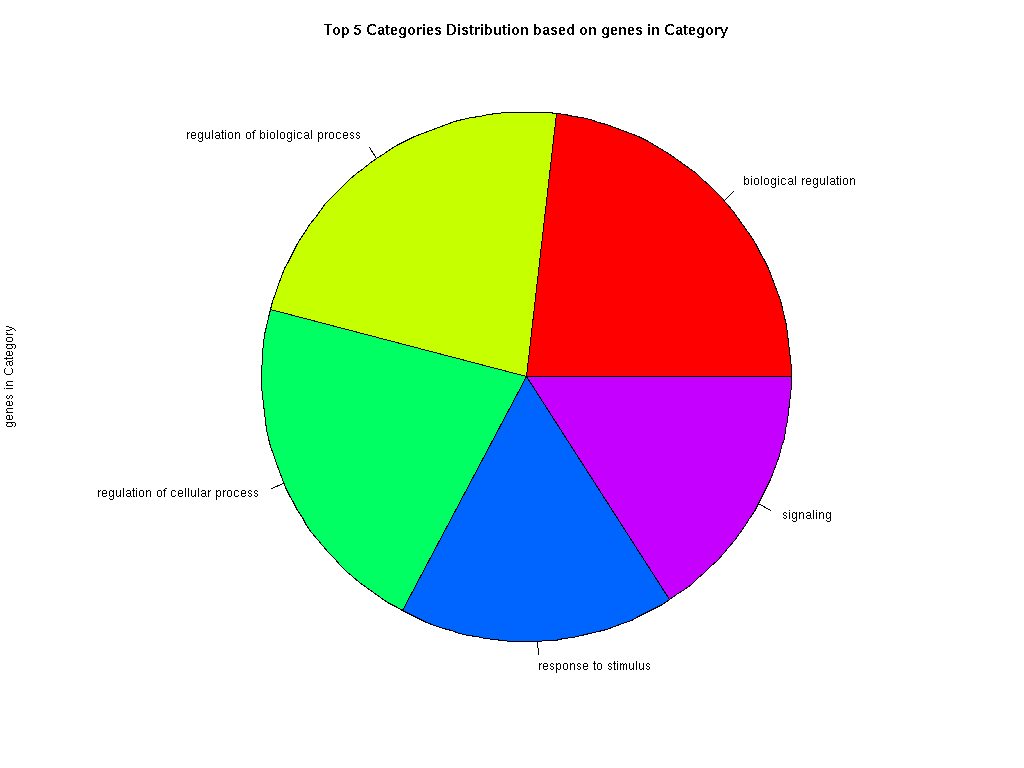

Supplement: Additional file 3 — Sample pipeline outputs in HTML format (compressed file). [file 1471-2164-13-620-S3.ZIP › TL4+LPS_activated/GRAPH_Jul21_191205.png]

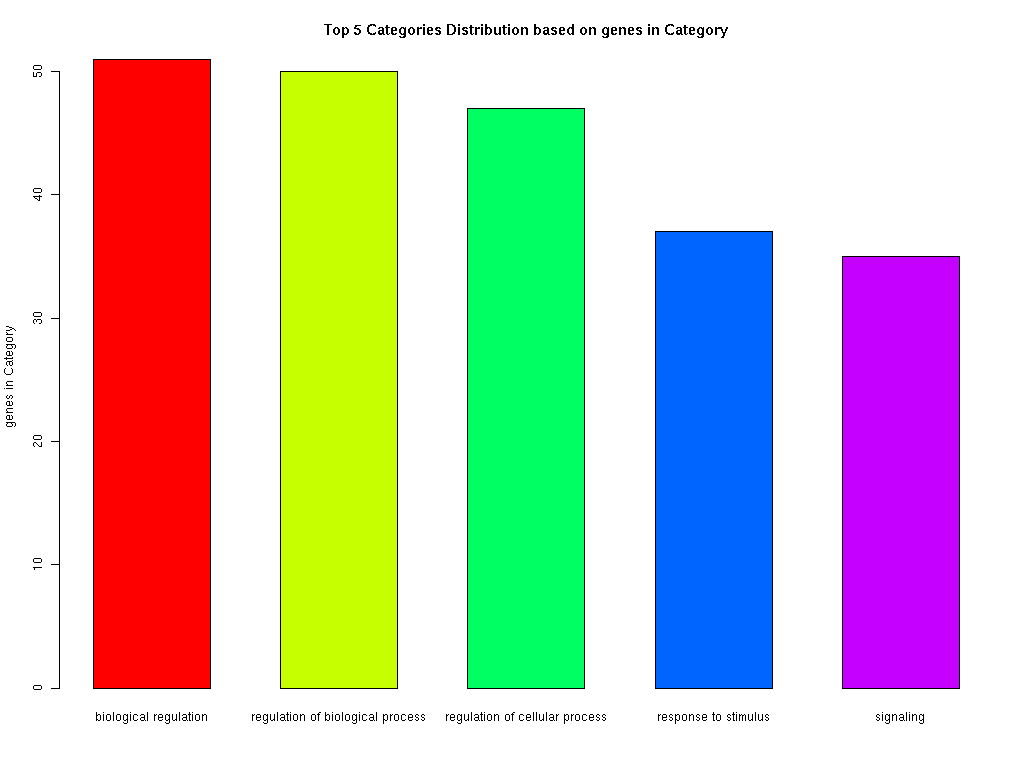

Supplement: Additional file 3 — Sample pipeline outputs in HTML format (compressed file). [file 1471-2164-13-620-S3.ZIP › TL4+LPS_activated/GRAPH_Jul21_191207.png]

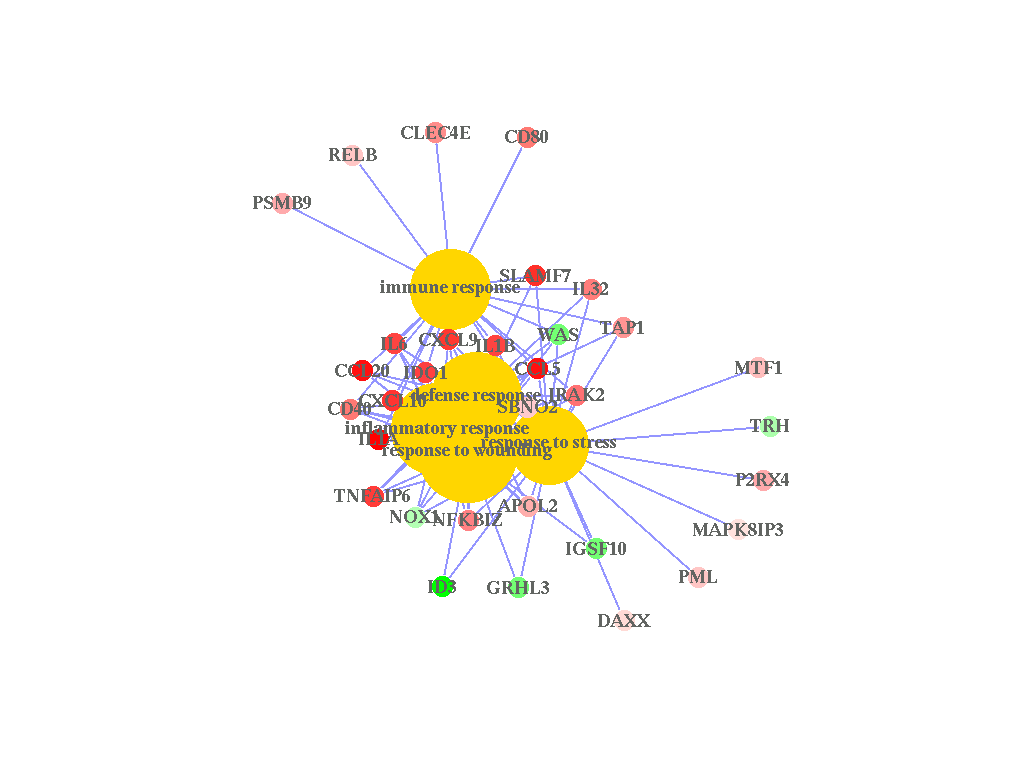

Supplement: Additional file 3 — Sample pipeline outputs in HTML format (compressed file). [file 1471-2164-13-620-S3.ZIP › TL4+LPS_activated/GRAPH_Jul21_191208.png]

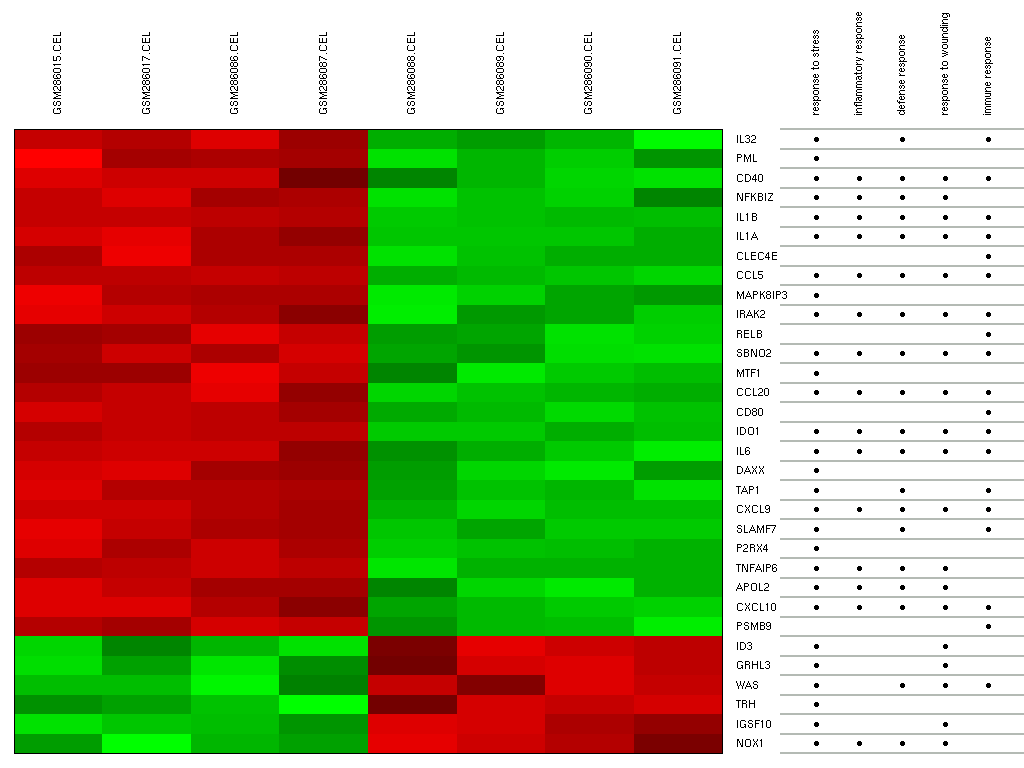

Supplement: Additional file 3 — Sample pipeline outputs in HTML format (compressed file). [file 1471-2164-13-620-S3.ZIP › TL4+LPS_activated/GRAPH_Jul21_191210.png]

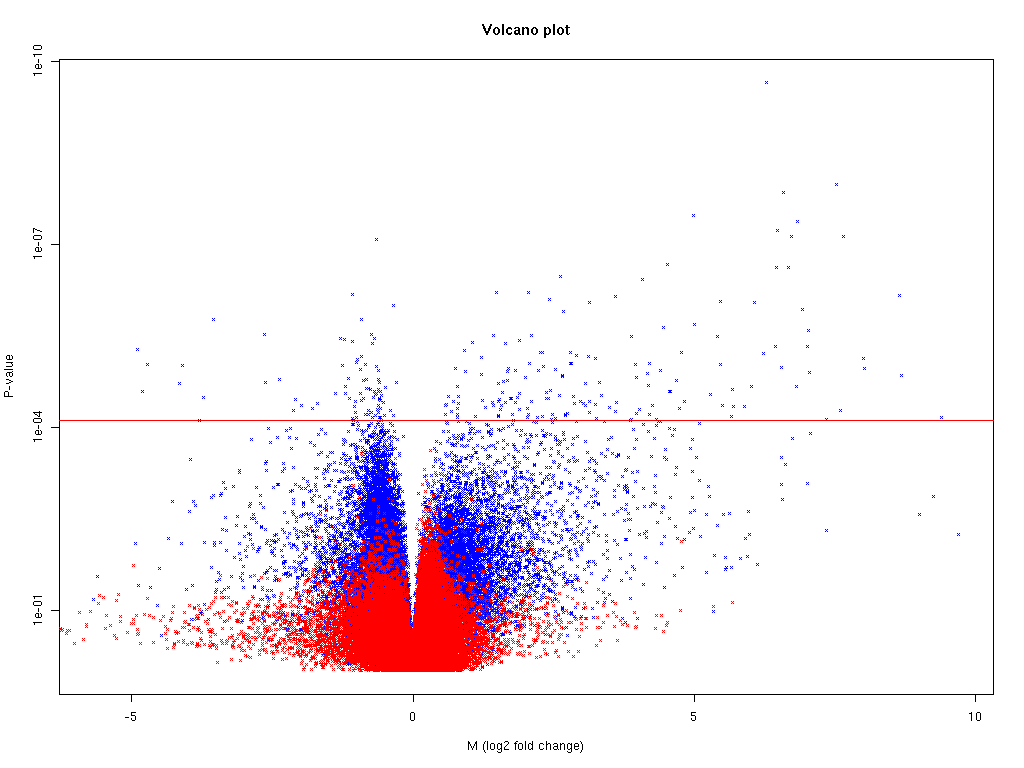

Supplement: Additional file 3 — Sample pipeline outputs in HTML format (compressed file). [file 1471-2164-13-620-S3.ZIP › TL4+LPS_activated/GRAPH_Jul21_191327.png]

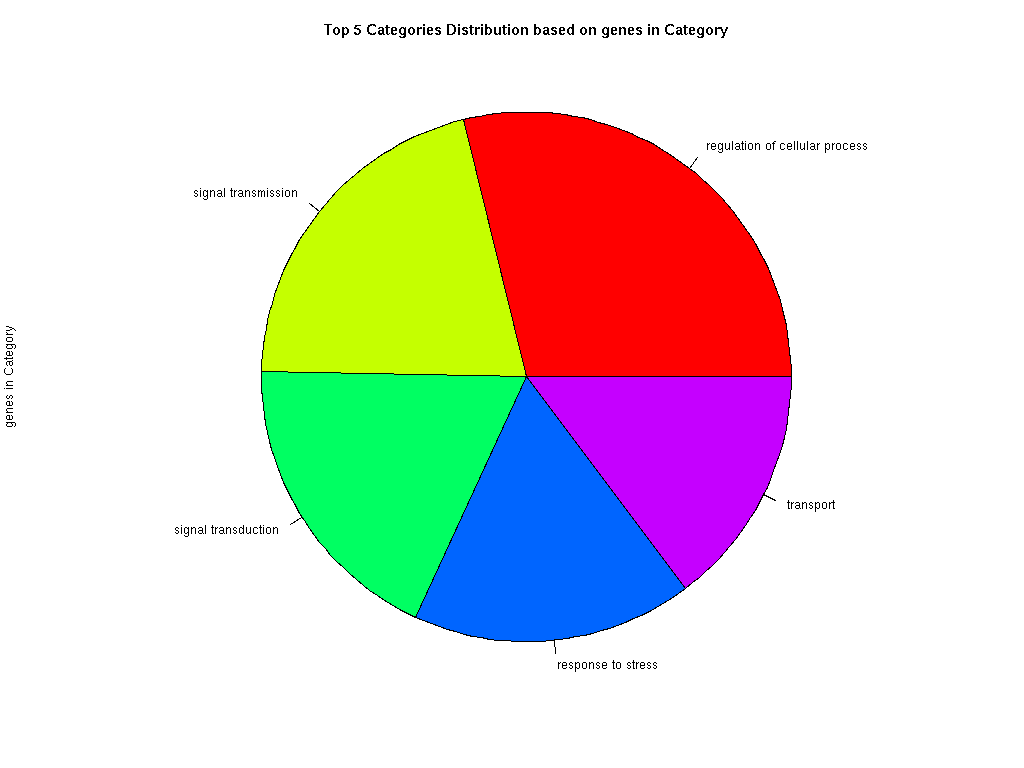

Supplement: Additional file 3 — Sample pipeline outputs in HTML format (compressed file). [file 1471-2164-13-620-S3.ZIP › TL4+LPS_activated/GRAPH_Jul21_191331.png]

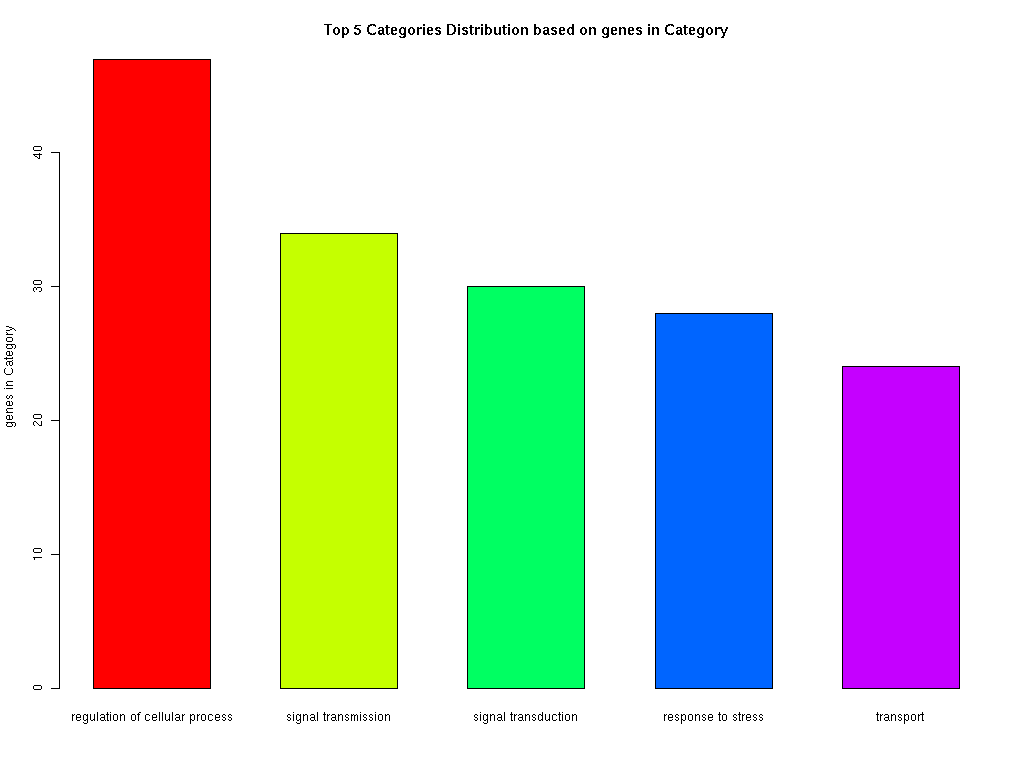

Supplement: Additional file 3 — Sample pipeline outputs in HTML format (compressed file). [file 1471-2164-13-620-S3.ZIP › TL4+LPS_activated/GRAPH_Jul21_191332.png]

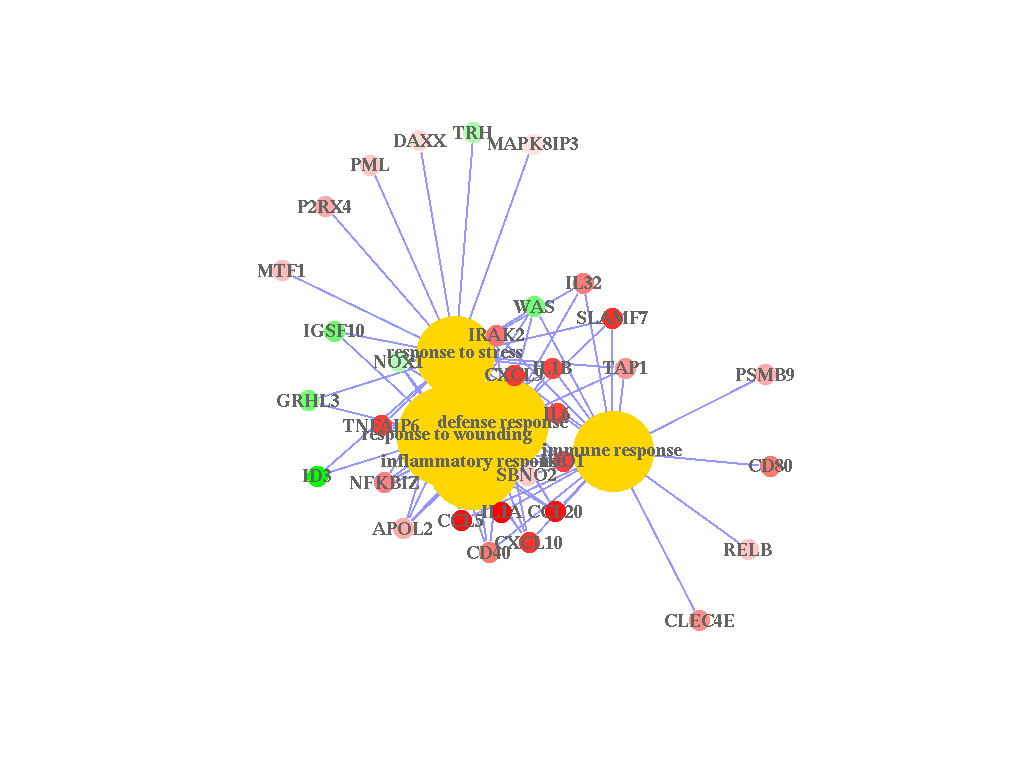

Supplement: Additional file 3 — Sample pipeline outputs in HTML format (compressed file). [file 1471-2164-13-620-S3.ZIP › TL4+LPS_activated/GRAPH_Jul21_191334.png]

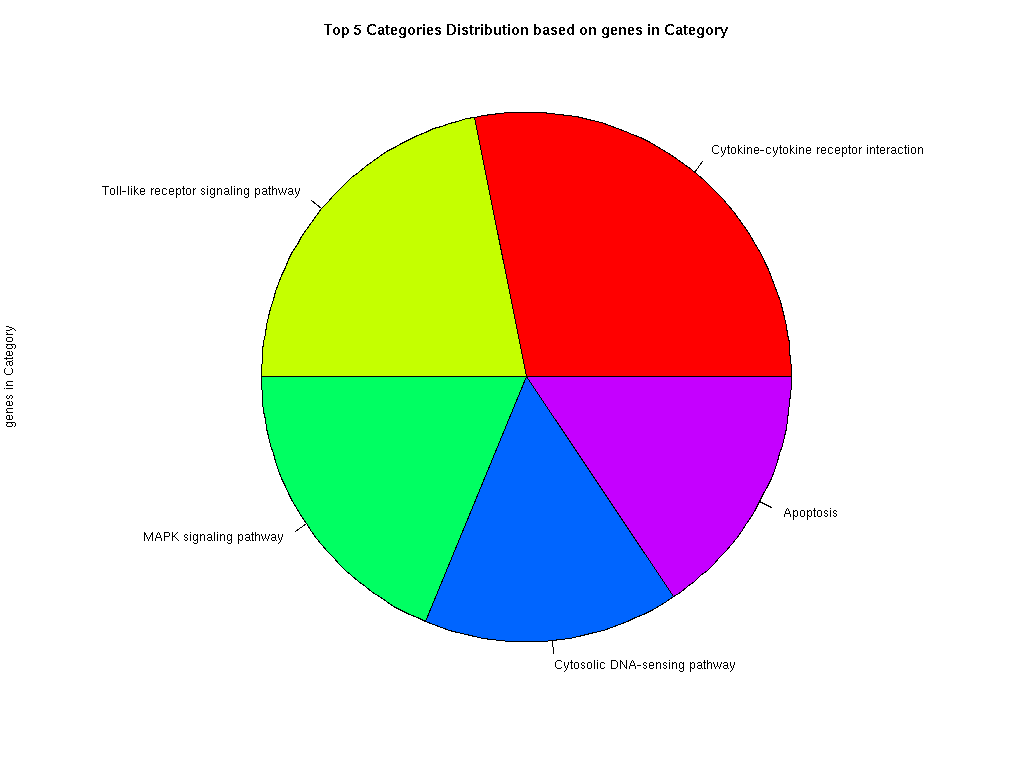

Supplement: Additional file 3 — Sample pipeline outputs in HTML format (compressed file). [file 1471-2164-13-620-S3.ZIP › TL4+LPS_activated/GRAPH_Jul21_191339.png]

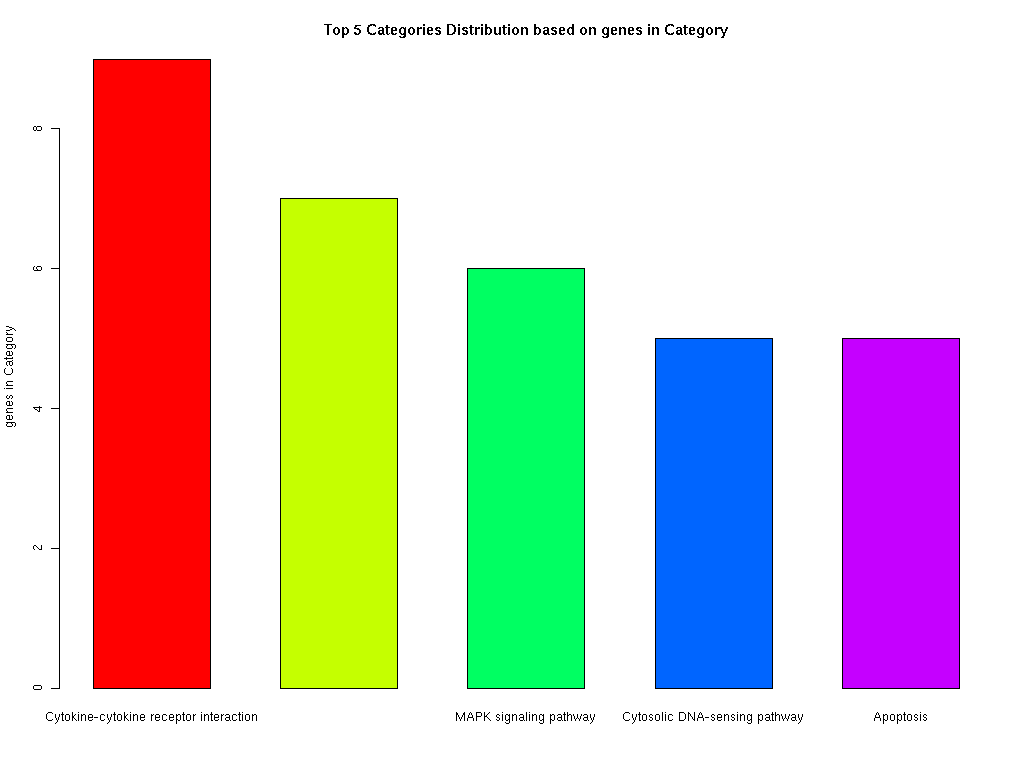

Supplement: Additional file 3 — Sample pipeline outputs in HTML format (compressed file). [file 1471-2164-13-620-S3.ZIP › TL4+LPS_activated/GRAPH_Jul21_191340.png]

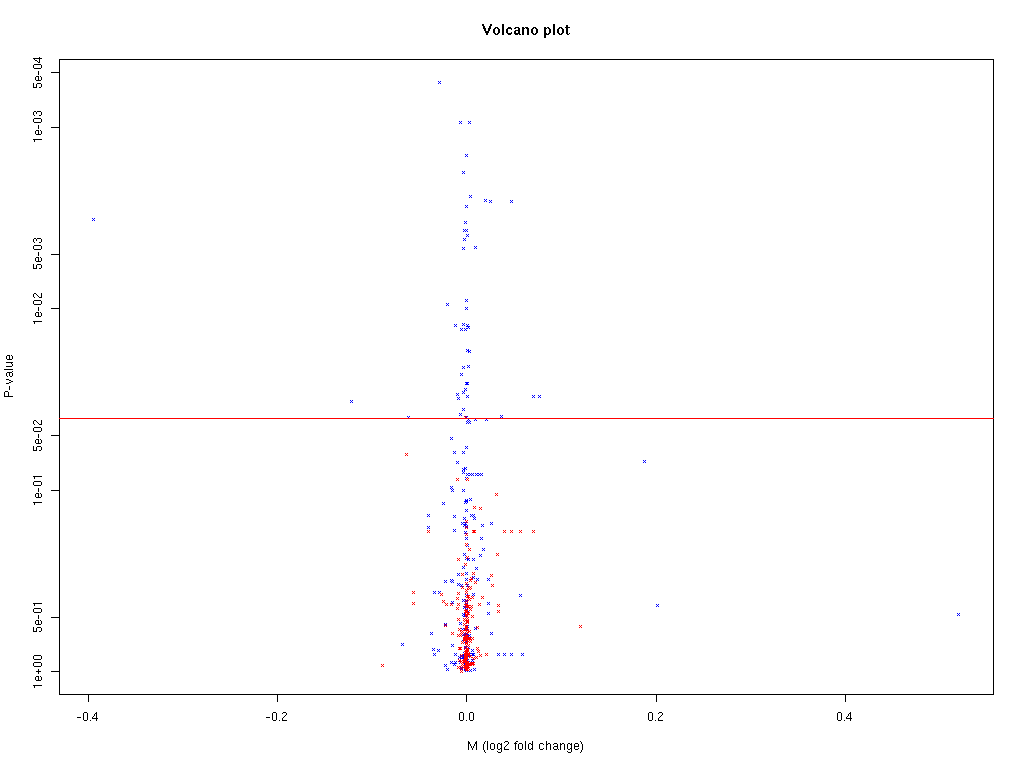

Supplement: Additional file 3 — Sample pipeline outputs in HTML format (compressed file). [file 1471-2164-13-620-S3.ZIP › TL4+LPS_activated/GRAPH_Jul21_191341.png]

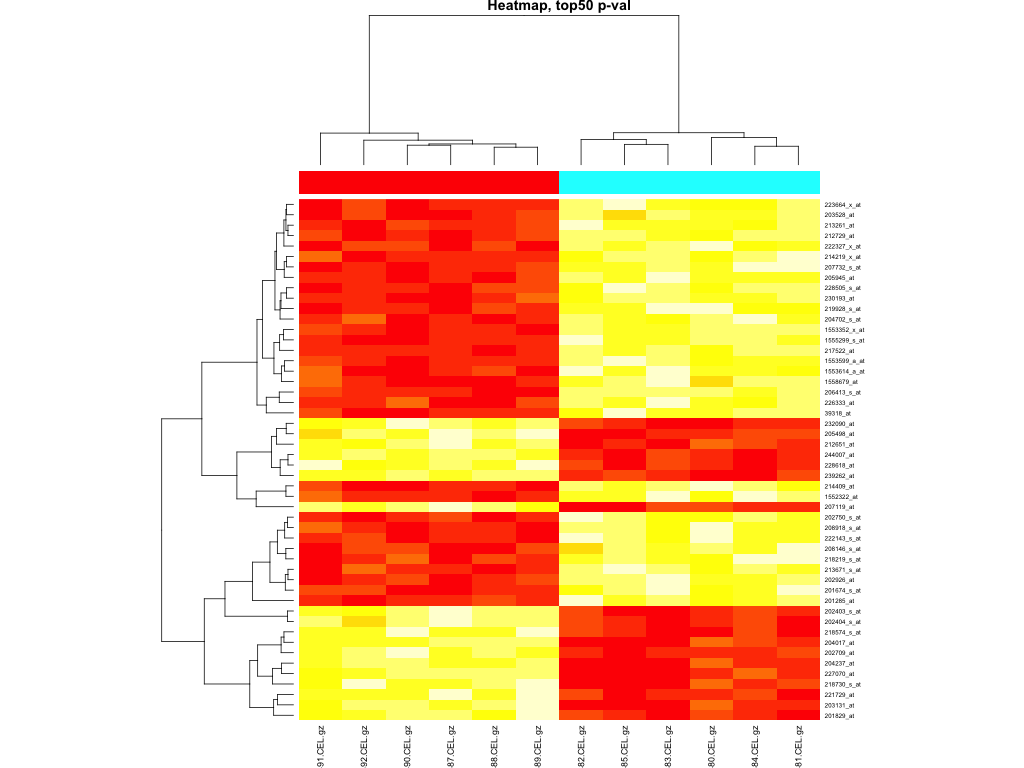

Supplement: Additional file 9 — Examples of MMpred predictions supported by experimental data and mapping against current databases. [file 1471-2164-13-620-S9.ZIP › Additional file 11 - Examples of MMpred predictions supported by experimental data and mapping against current databases/GSE19350/GRAPH_Sep 2_124114.png]

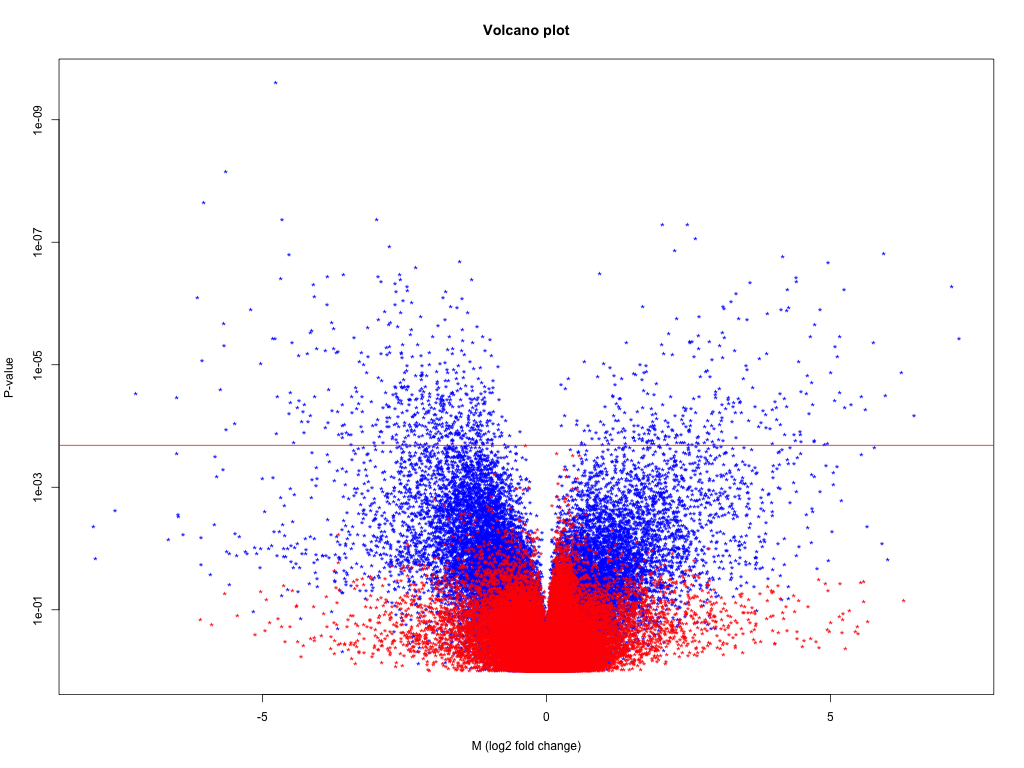

Supplement: Additional file 9 — Examples of MMpred predictions supported by experimental data and mapping against current databases. [file 1471-2164-13-620-S9.ZIP › Additional file 11 - Examples of MMpred predictions supported by experimental data and mapping against current databases/GSE19350/GRAPH_Sep 2_124115.png]

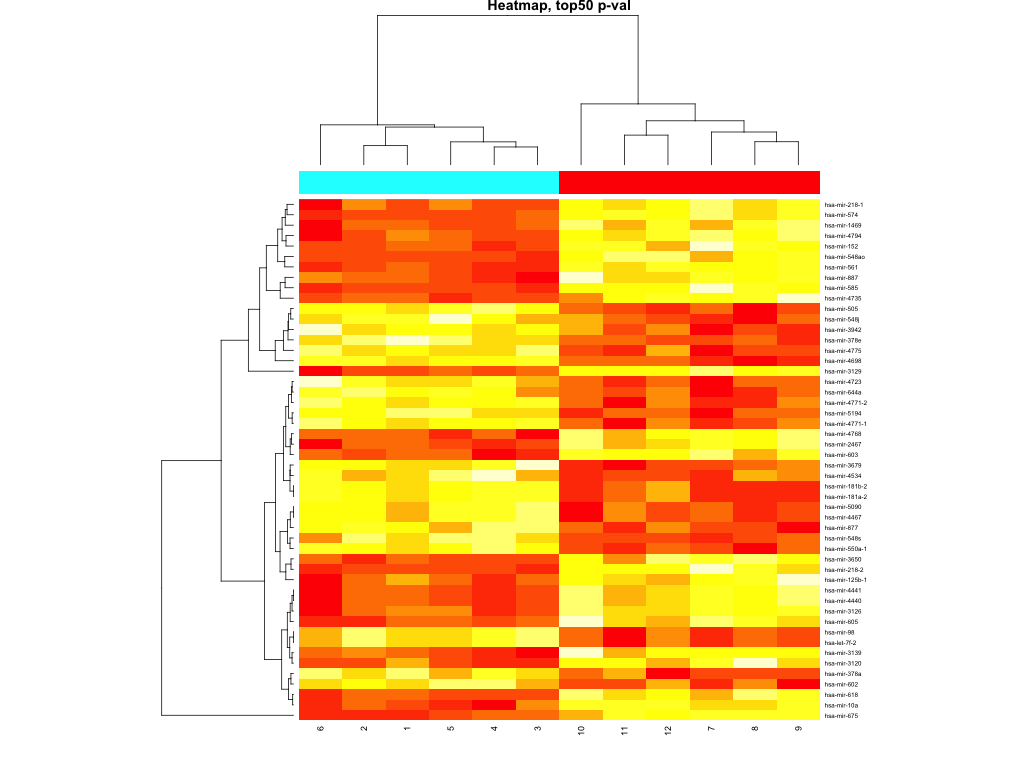

Supplement: Additional file 9 — Examples of MMpred predictions supported by experimental data and mapping against current databases. [file 1471-2164-13-620-S9.ZIP › Additional file 11 - Examples of MMpred predictions supported by experimental data and mapping against current databases/GSE19350/GRAPH_Sep 2_124443.png]

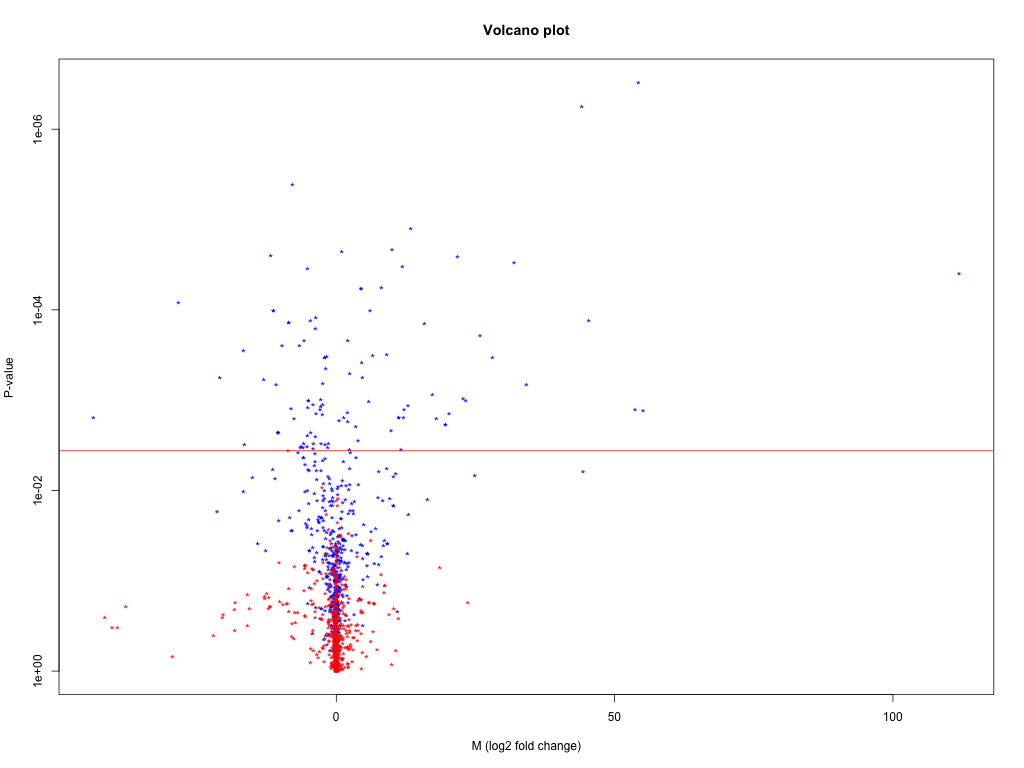

Supplement: Additional file 9 — Examples of MMpred predictions supported by experimental data and mapping against current databases. [file 1471-2164-13-620-S9.ZIP › Additional file 11 - Examples of MMpred predictions supported by experimental data and mapping against current databases/GSE19350/GRAPH_Sep 2_124444.png]

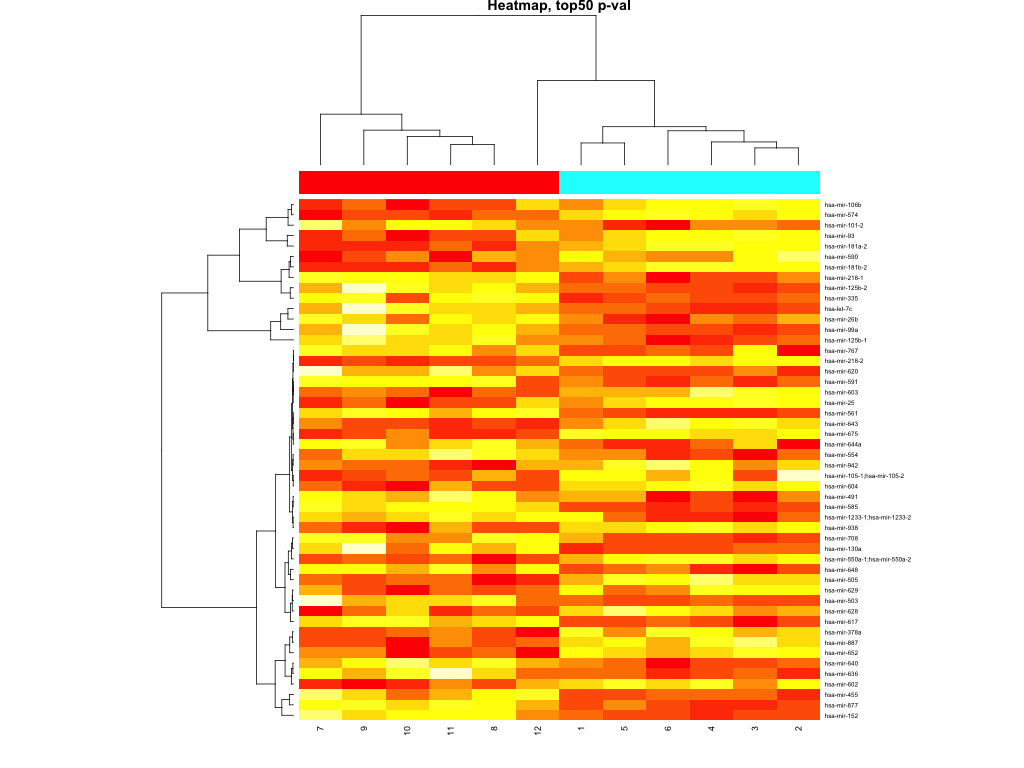

Supplement: Additional file 9 — Examples of MMpred predictions supported by experimental data and mapping against current databases. [file 1471-2164-13-620-S9.ZIP › Additional file 11 - Examples of MMpred predictions supported by experimental data and mapping against current databases/GSE19350/GRAPH_Sep 2_124447.png]

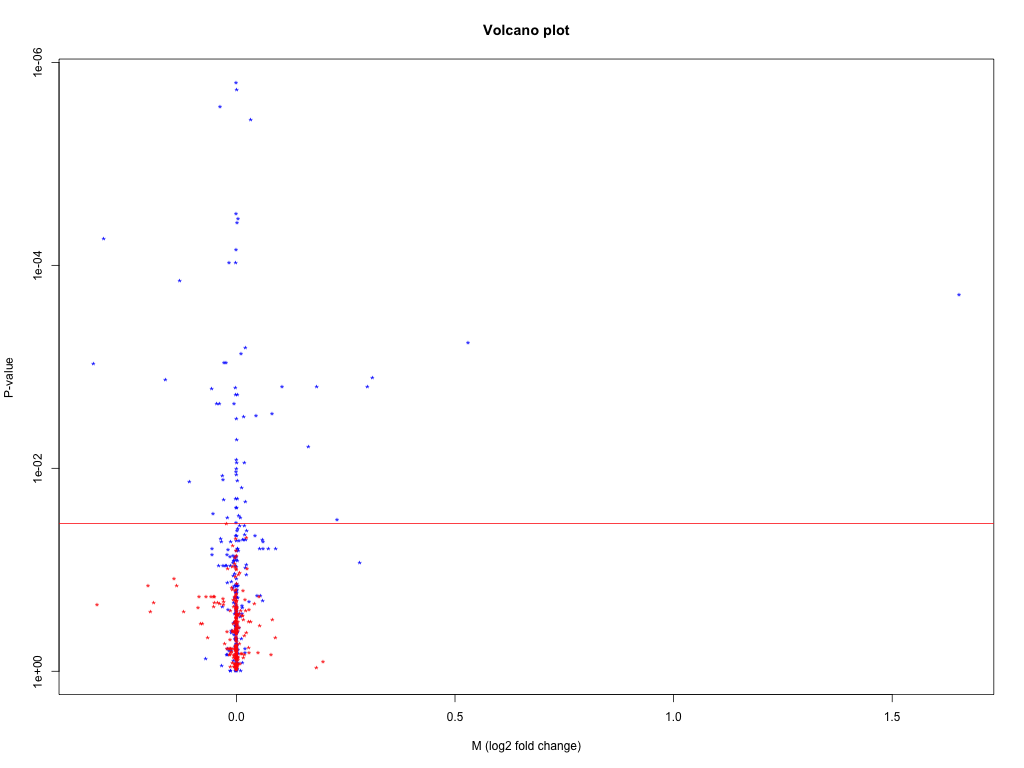

Supplement: Additional file 9 — Examples of MMpred predictions supported by experimental data and mapping against current databases. [file 1471-2164-13-620-S9.ZIP › Additional file 11 - Examples of MMpred predictions supported by experimental data and mapping against current databases/GSE19350/GRAPH_Sep 2_124448.png]

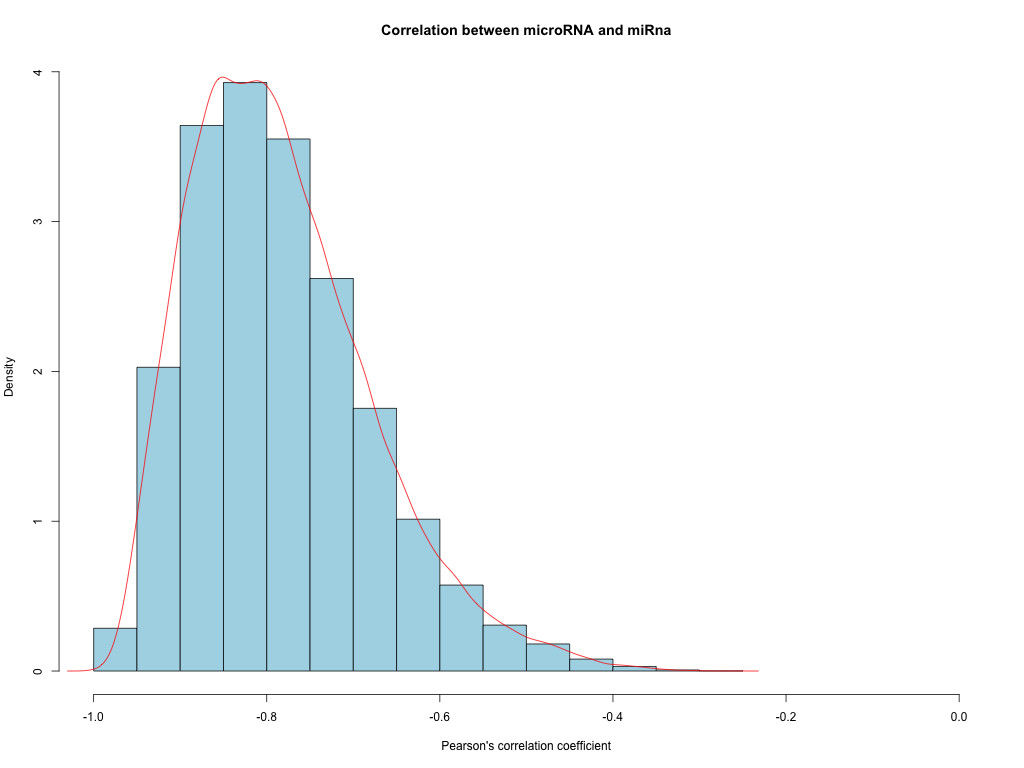

Supplement: Additional file 9 — Examples of MMpred predictions supported by experimental data and mapping against current databases. [file 1471-2164-13-620-S9.ZIP › Additional file 11 - Examples of MMpred predictions supported by experimental data and mapping against current databases/GSE19350/GRAPH_Sep 2_124450.png]

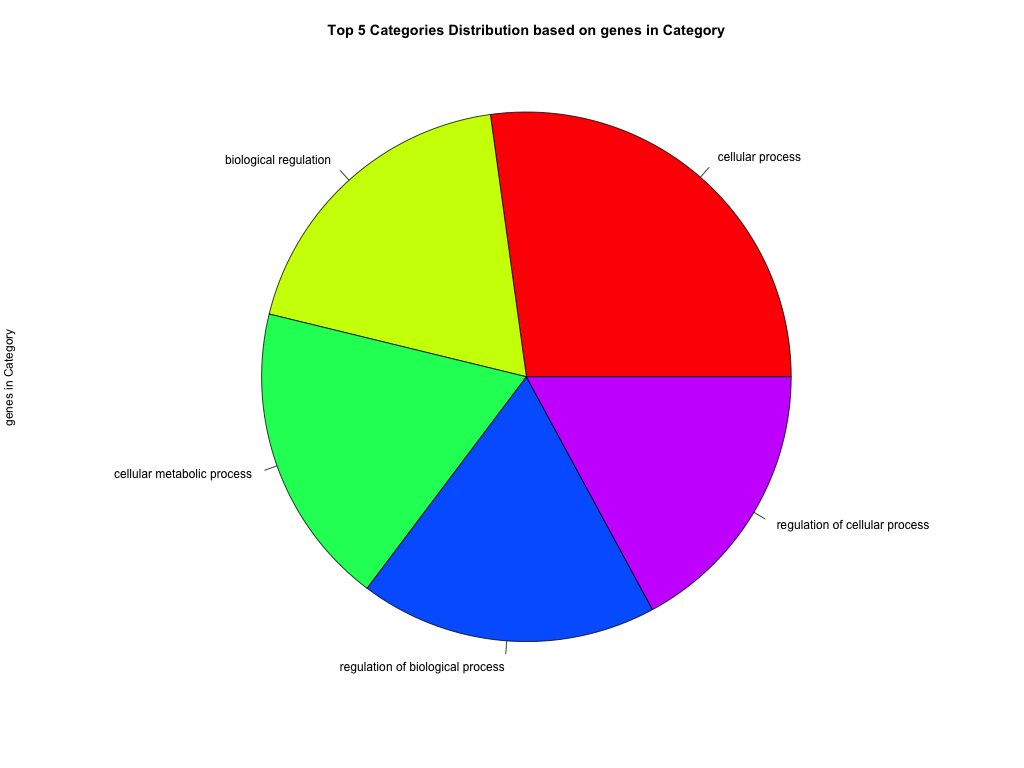

Supplement: Additional file 9 — Examples of MMpred predictions supported by experimental data and mapping against current databases. [file 1471-2164-13-620-S9.ZIP › Additional file 11 - Examples of MMpred predictions supported by experimental data and mapping against current databases/GSE19350/GRAPH_Sep 2_125535.png]

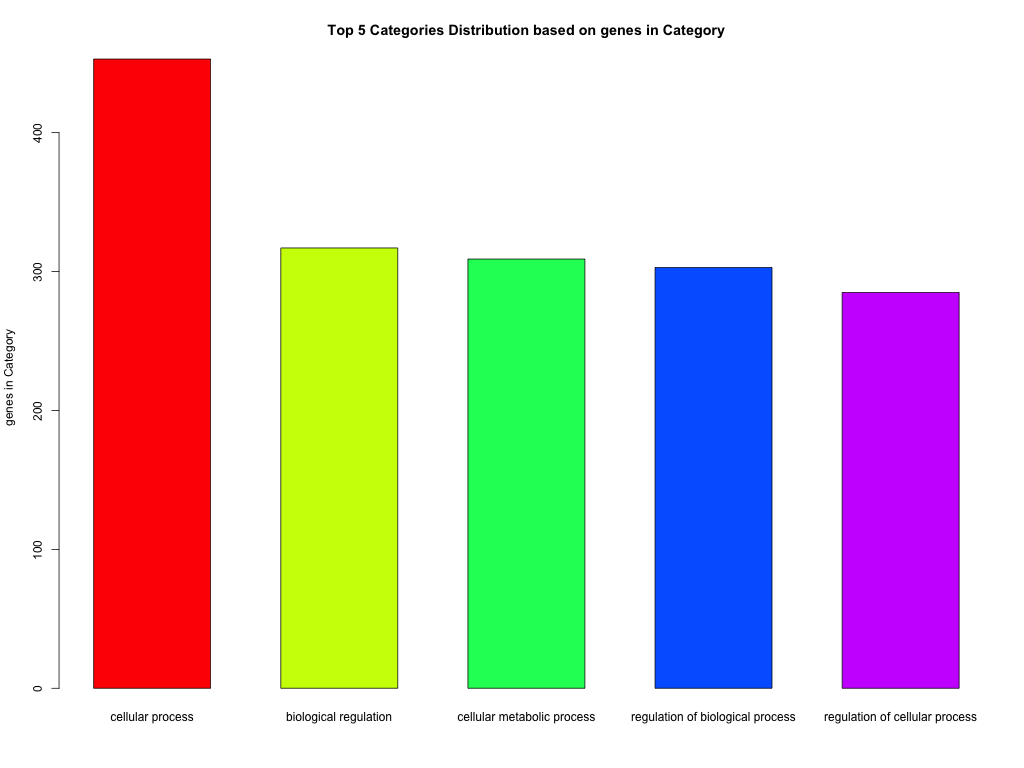

Supplement: Additional file 9 — Examples of MMpred predictions supported by experimental data and mapping against current databases. [file 1471-2164-13-620-S9.ZIP › Additional file 11 - Examples of MMpred predictions supported by experimental data and mapping against current databases/GSE19350/GRAPH_Sep 2_125537.png]

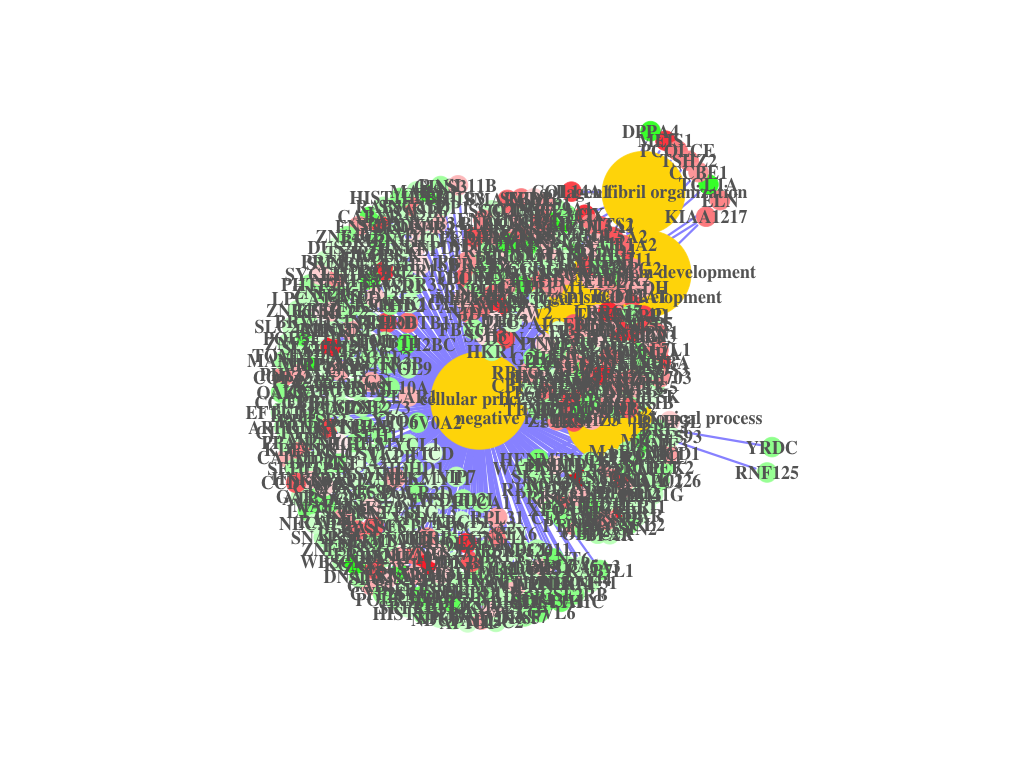

Supplement: Additional file 9 — Examples of MMpred predictions supported by experimental data and mapping against current databases. [file 1471-2164-13-620-S9.ZIP › Additional file 11 - Examples of MMpred predictions supported by experimental data and mapping against current databases/GSE19350/GRAPH_Sep 2_125540.png]

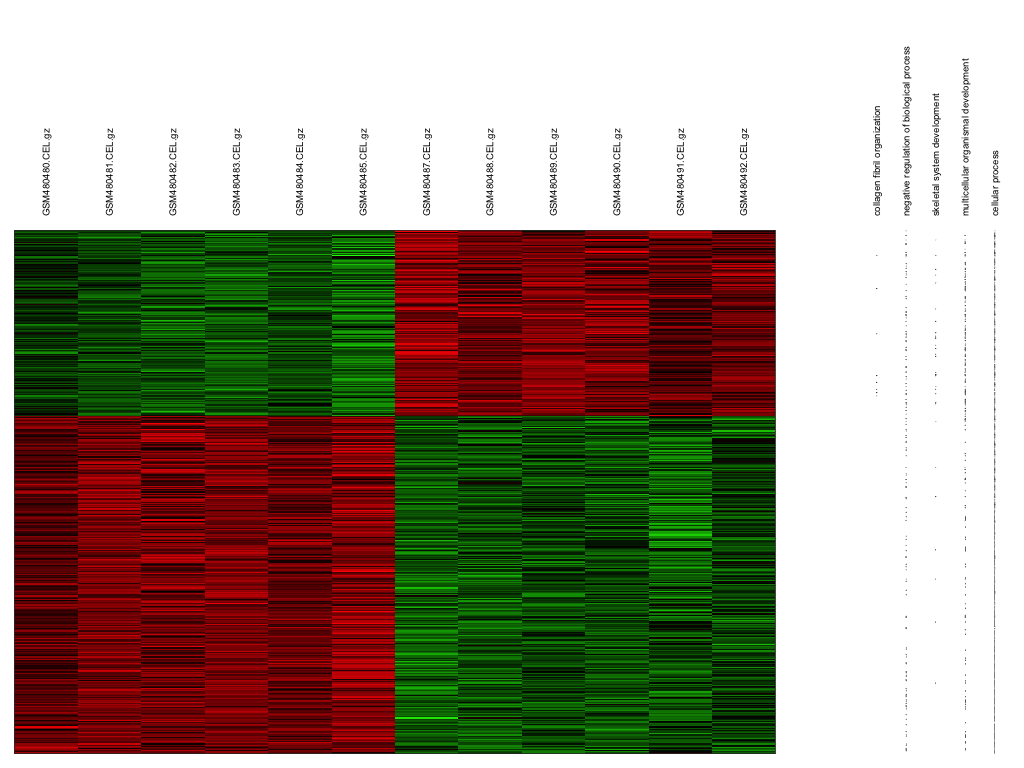

Supplement: Additional file 9 — Examples of MMpred predictions supported by experimental data and mapping against current databases. [file 1471-2164-13-620-S9.ZIP › Additional file 11 - Examples of MMpred predictions supported by experimental data and mapping against current databases/GSE19350/GRAPH_Sep 2_125546.png]

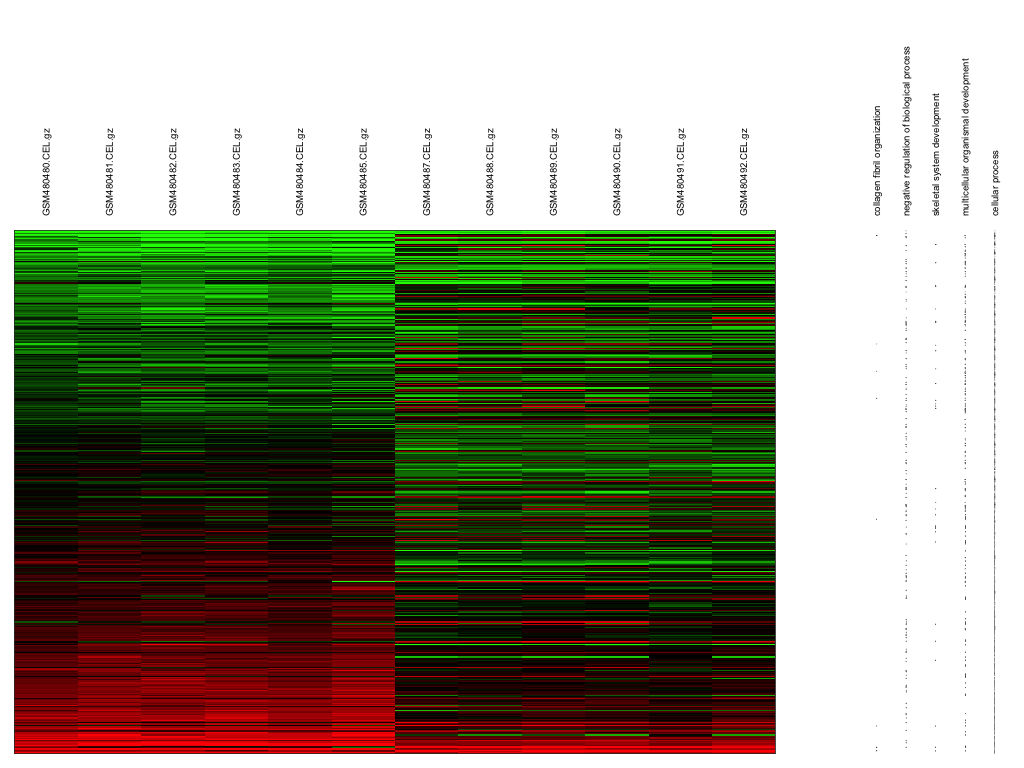

Supplement: Additional file 9 — Examples of MMpred predictions supported by experimental data and mapping against current databases. [file 1471-2164-13-620-S9.ZIP › Additional file 11 - Examples of MMpred predictions supported by experimental data and mapping against current databases/GSE19350/GRAPH_Sep 2_125549.png]

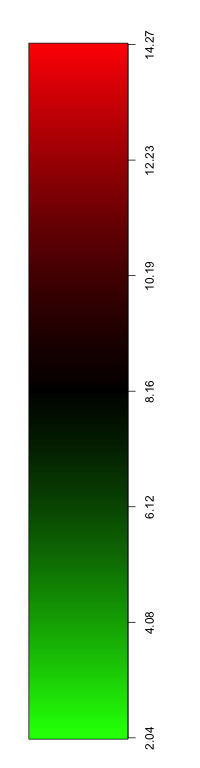

Supplement: Additional file 9 — Examples of MMpred predictions supported by experimental data and mapping against current databases. [file 1471-2164-13-620-S9.ZIP › Additional file 11 - Examples of MMpred predictions supported by experimental data and mapping against current databases/GSE19350/GRAPH_Sep 2_125550.png]

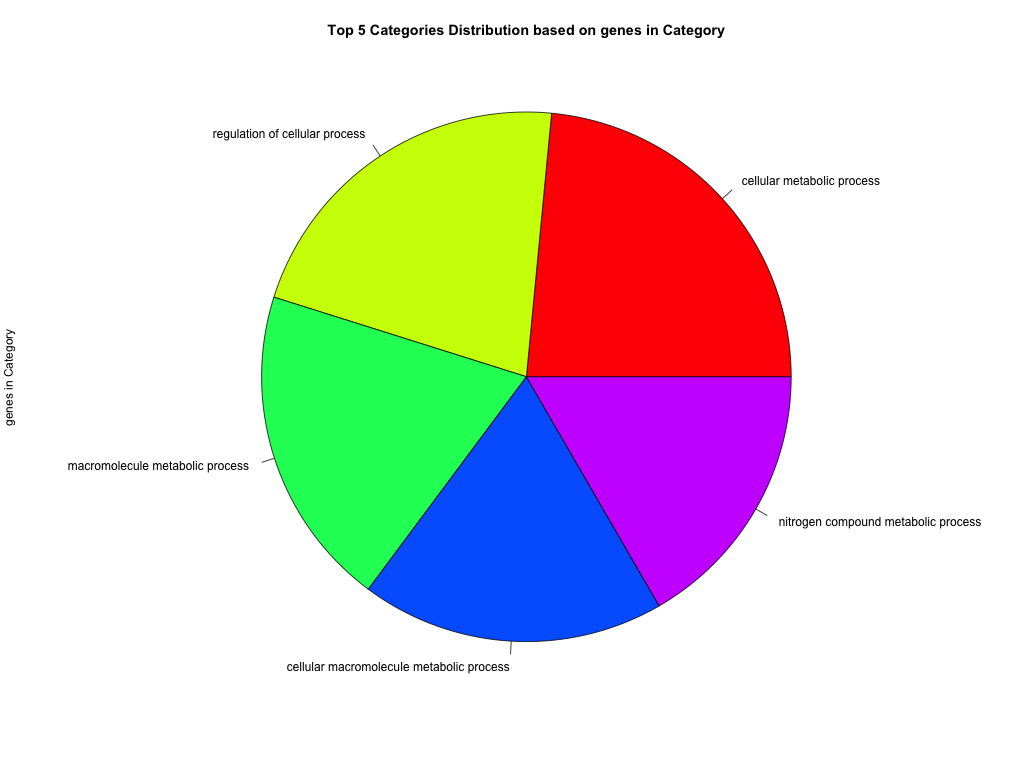

Supplement: Additional file 9 — Examples of MMpred predictions supported by experimental data and mapping against current databases. [file 1471-2164-13-620-S9.ZIP › Additional file 11 - Examples of MMpred predictions supported by experimental data and mapping against current databases/GSE19350/GRAPH_Sep 2_125648.png]

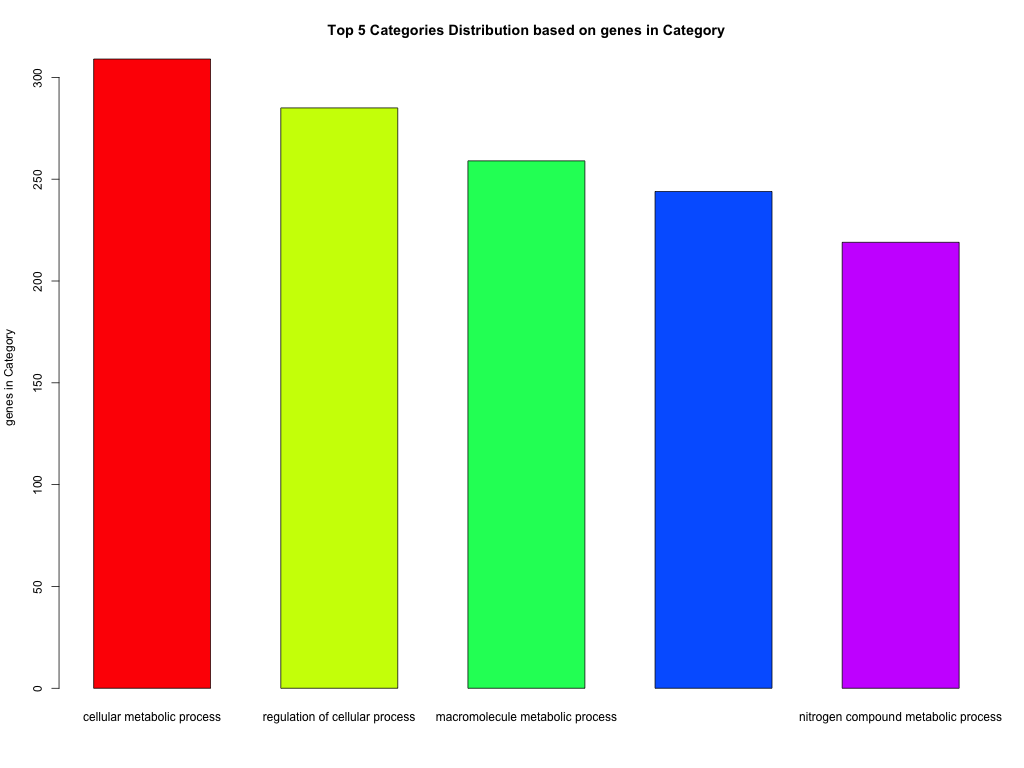

Supplement: Additional file 9 — Examples of MMpred predictions supported by experimental data and mapping against current databases. [file 1471-2164-13-620-S9.ZIP › Additional file 11 - Examples of MMpred predictions supported by experimental data and mapping against current databases/GSE19350/GRAPH_Sep 2_125649.png]

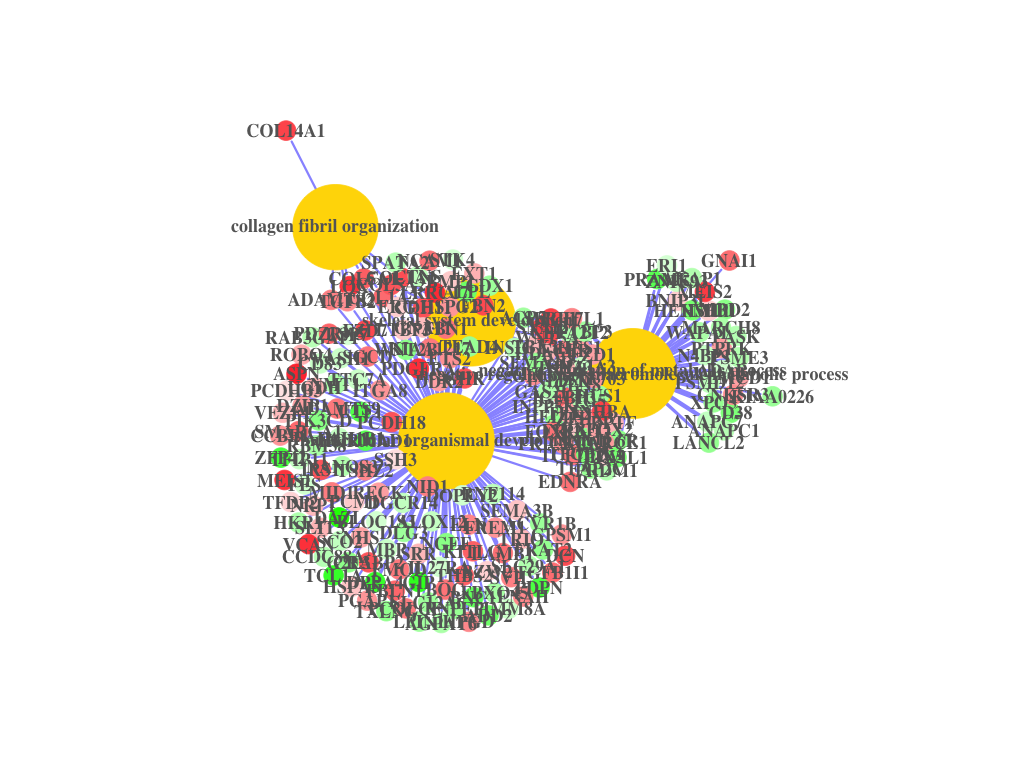

Supplement: Additional file 9 — Examples of MMpred predictions supported by experimental data and mapping against current databases. [file 1471-2164-13-620-S9.ZIP › Additional file 11 - Examples of MMpred predictions supported by experimental data and mapping against current databases/GSE19350/GRAPH_Sep 2_125650.png]

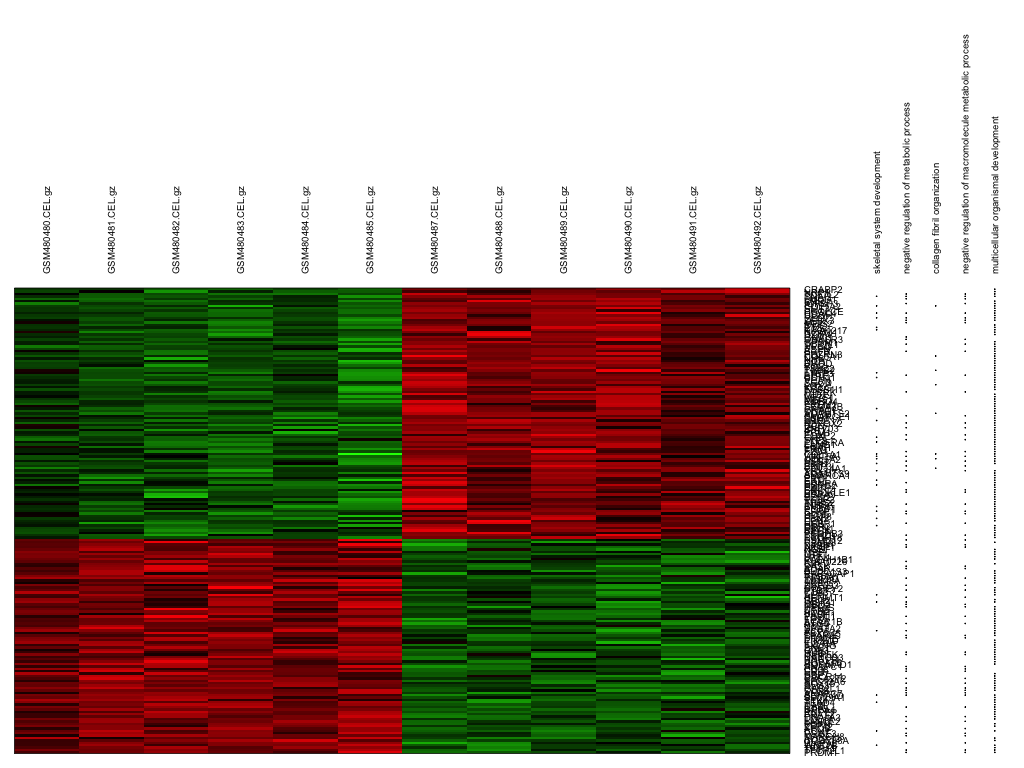

Supplement: Additional file 9 — Examples of MMpred predictions supported by experimental data and mapping against current databases. [file 1471-2164-13-620-S9.ZIP › Additional file 11 - Examples of MMpred predictions supported by experimental data and mapping against current databases/GSE19350/GRAPH_Sep 2_125652.png]

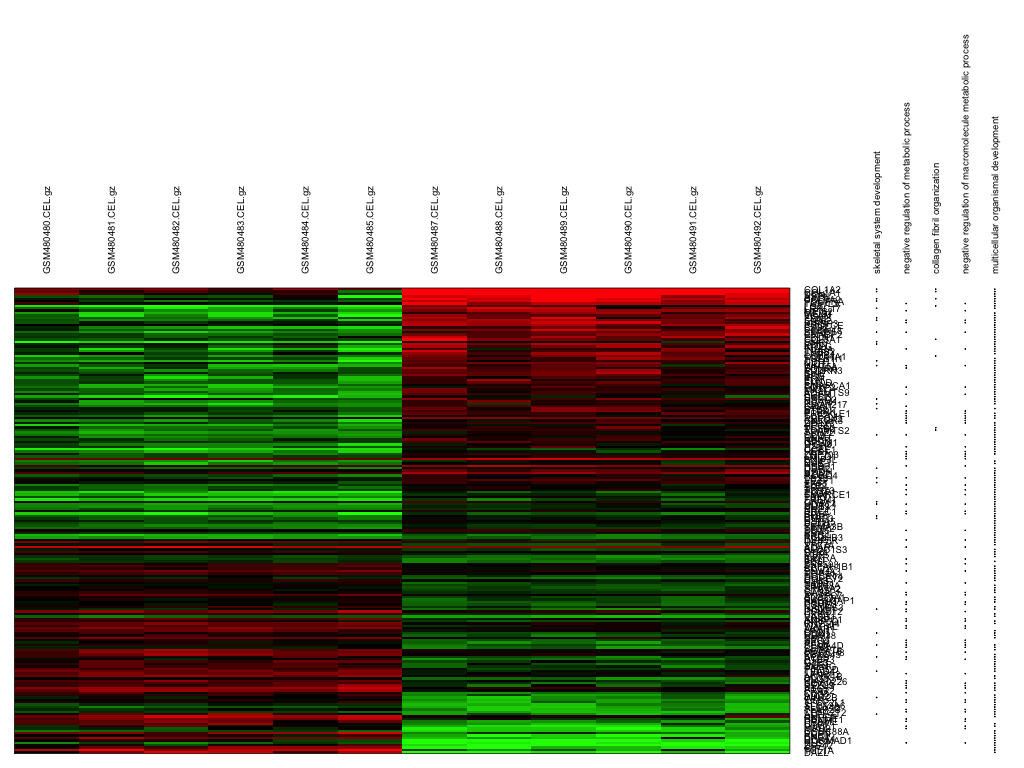

Supplement: Additional file 9 — Examples of MMpred predictions supported by experimental data and mapping against current databases. [file 1471-2164-13-620-S9.ZIP › Additional file 11 - Examples of MMpred predictions supported by experimental data and mapping against current databases/GSE19350/GRAPH_Sep 2_125654.png]

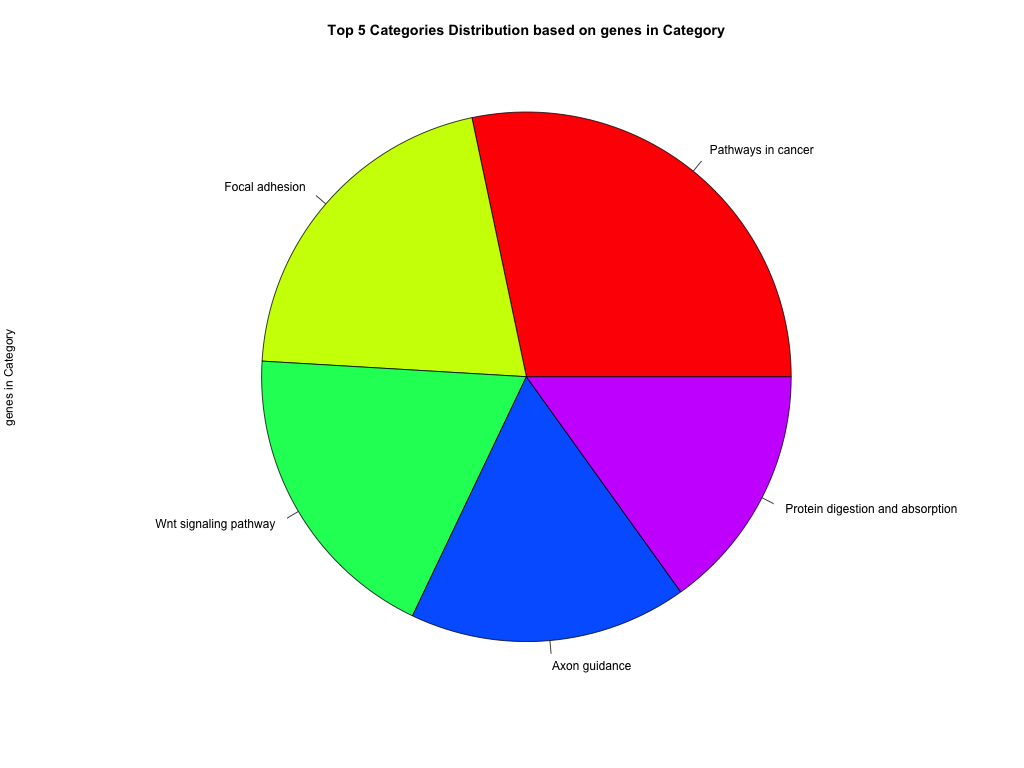

Supplement: Additional file 9 — Examples of MMpred predictions supported by experimental data and mapping against current databases. [file 1471-2164-13-620-S9.ZIP › Additional file 11 - Examples of MMpred predictions supported by experimental data and mapping against current databases/GSE19350/GRAPH_Sep 2_125657.png]

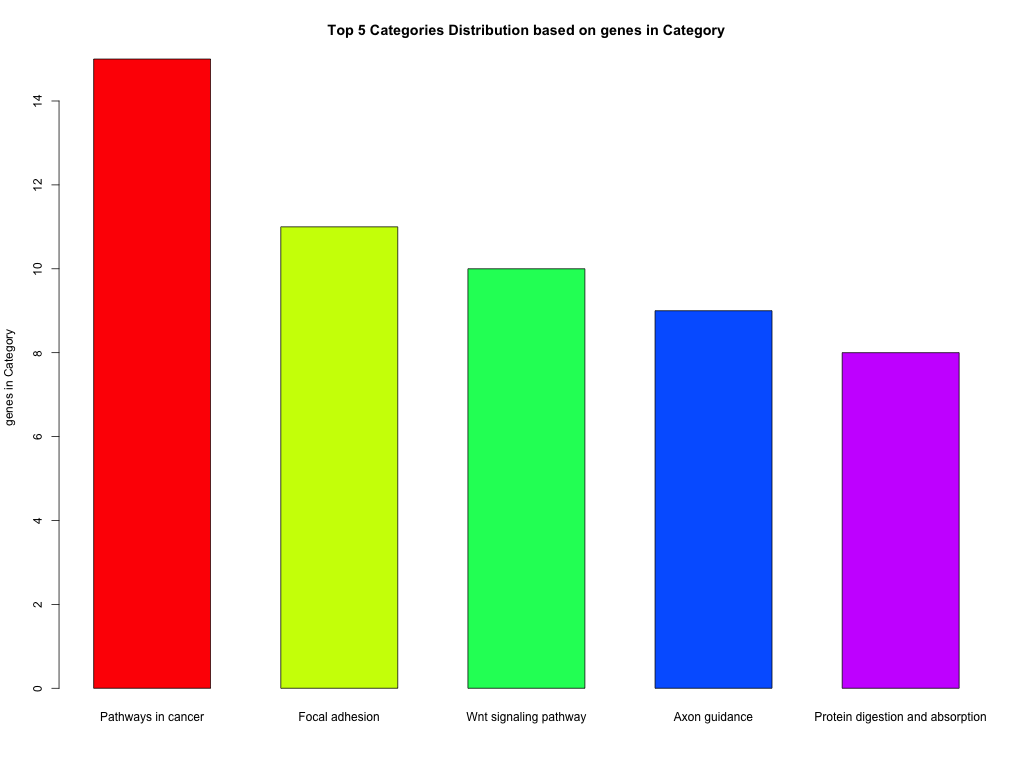

Supplement: Additional file 9 — Examples of MMpred predictions supported by experimental data and mapping against current databases. [file 1471-2164-13-620-S9.ZIP › Additional file 11 - Examples of MMpred predictions supported by experimental data and mapping against current databases/GSE19350/GRAPH_Sep 2_125658.png]

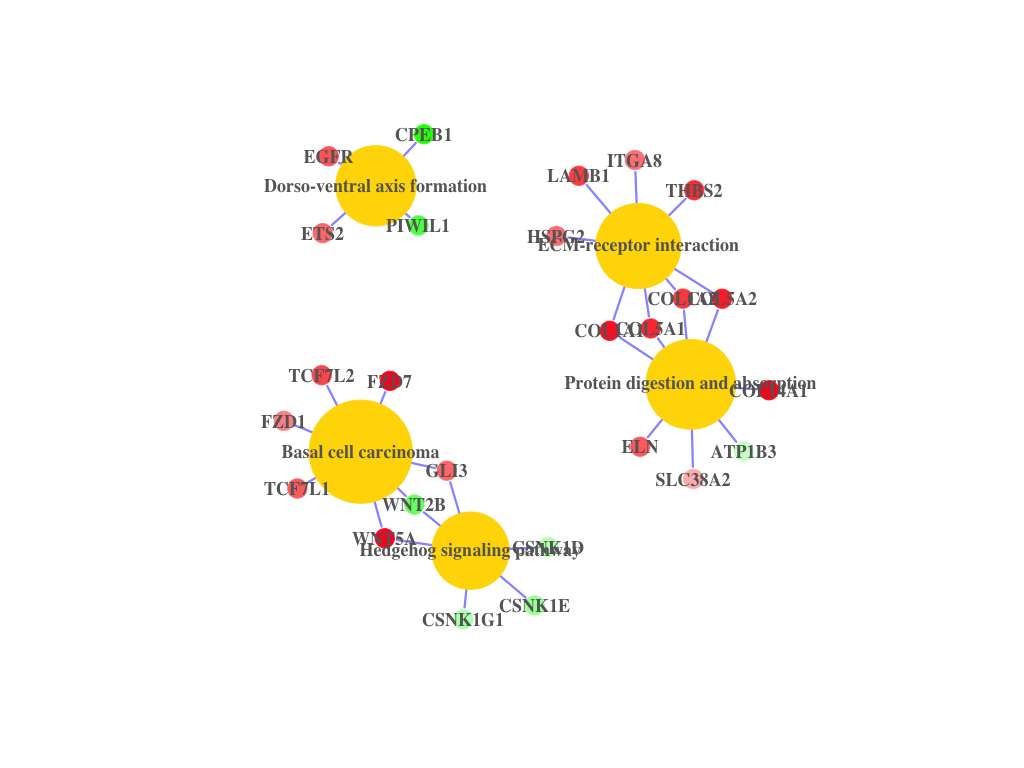

Supplement: Additional file 9 — Examples of MMpred predictions supported by experimental data and mapping against current databases. [file 1471-2164-13-620-S9.ZIP › Additional file 11 - Examples of MMpred predictions supported by experimental data and mapping against current databases/GSE19350/GRAPH_Sep 2_125700.png]

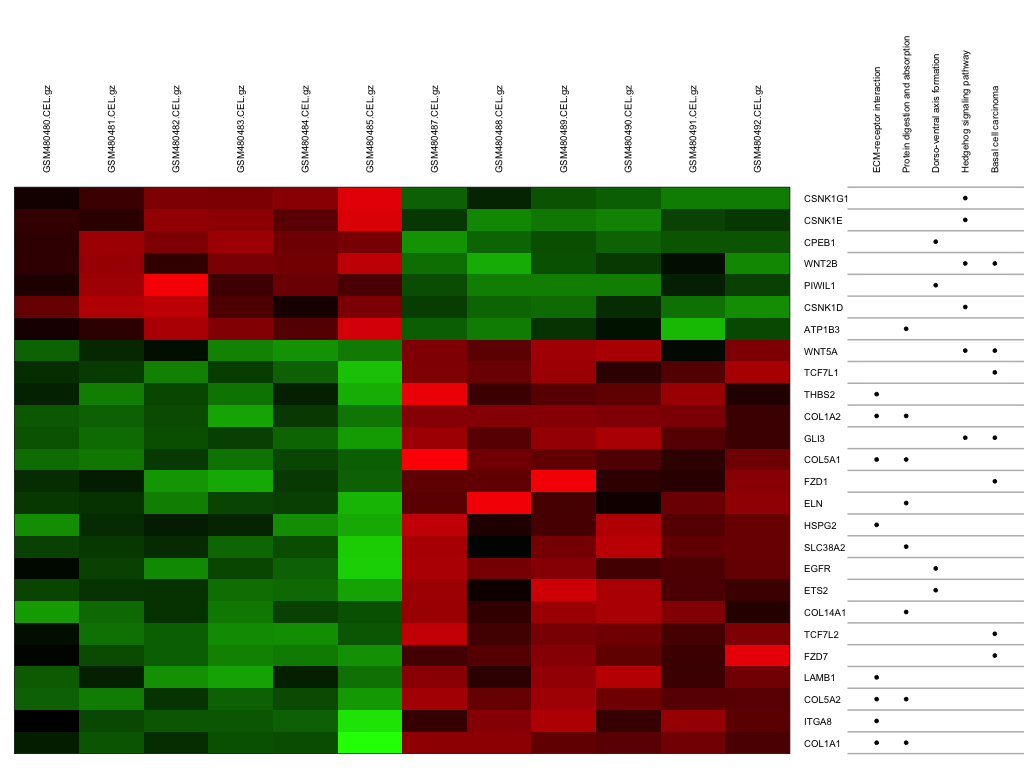

Supplement: Additional file 9 — Examples of MMpred predictions supported by experimental data and mapping against current databases. [file 1471-2164-13-620-S9.ZIP › Additional file 11 - Examples of MMpred predictions supported by experimental data and mapping against current databases/GSE19350/GRAPH_Sep 2_125701.png]

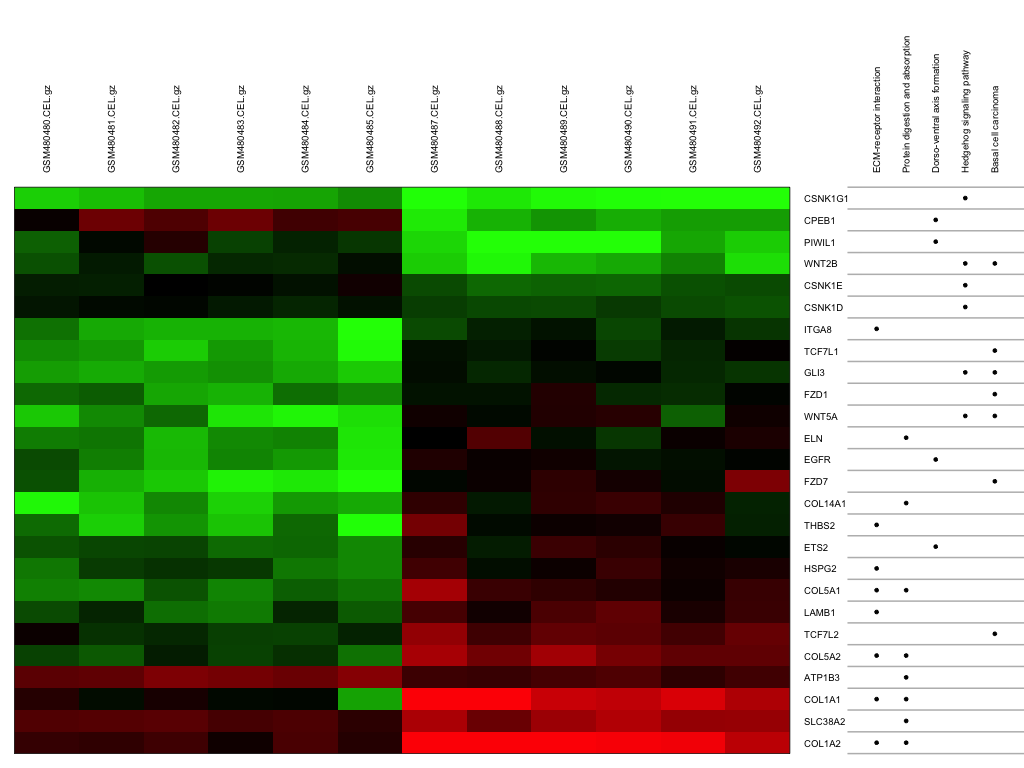

Supplement: Additional file 9 — Examples of MMpred predictions supported by experimental data and mapping against current databases. [file 1471-2164-13-620-S9.ZIP › Additional file 11 - Examples of MMpred predictions supported by experimental data and mapping against current databases/GSE19350/GRAPH_Sep 2_125702.png]

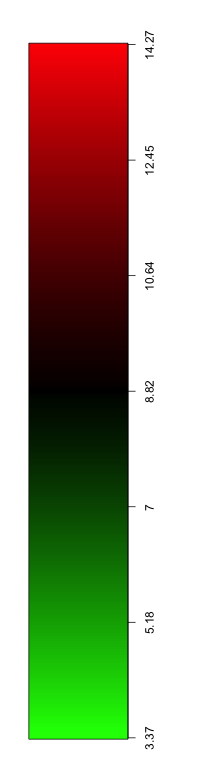

Supplement: Additional file 9 — Examples of MMpred predictions supported by experimental data and mapping against current databases. [file 1471-2164-13-620-S9.ZIP › Additional file 11 - Examples of MMpred predictions supported by experimental data and mapping against current databases/GSE19350/GRAPH_Sep 2_125703.png]

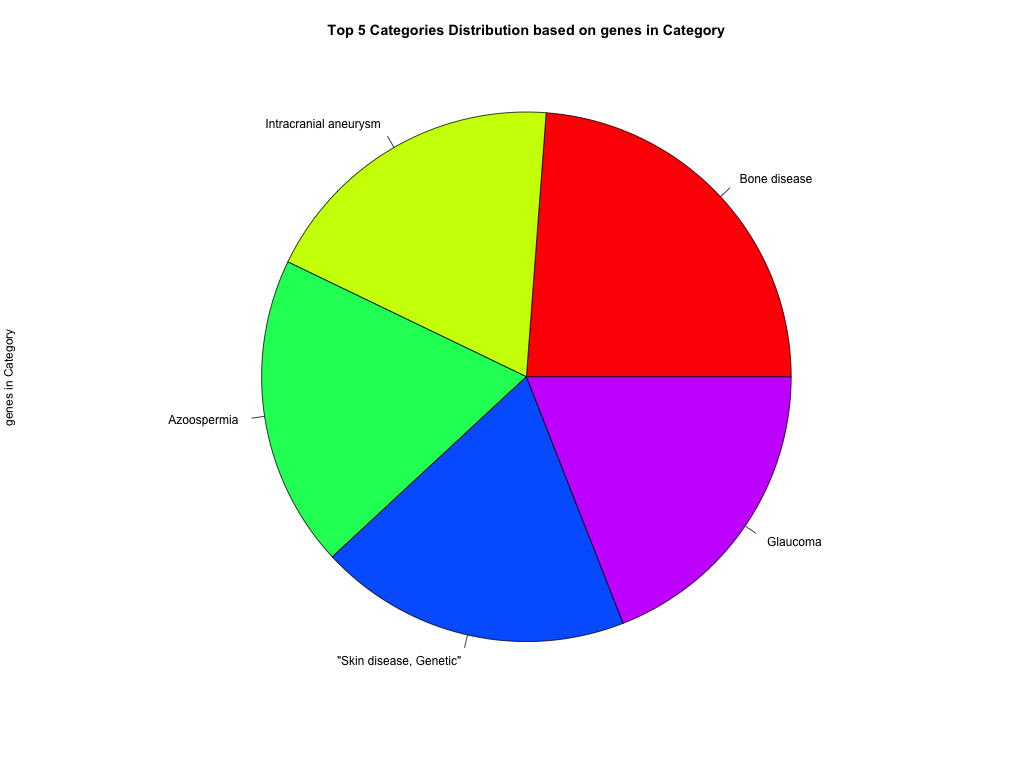

Supplement: Additional file 9 — Examples of MMpred predictions supported by experimental data and mapping against current databases. [file 1471-2164-13-620-S9.ZIP › Additional file 11 - Examples of MMpred predictions supported by experimental data and mapping against current databases/GSE19350/GRAPH_Sep 2_125706.png]

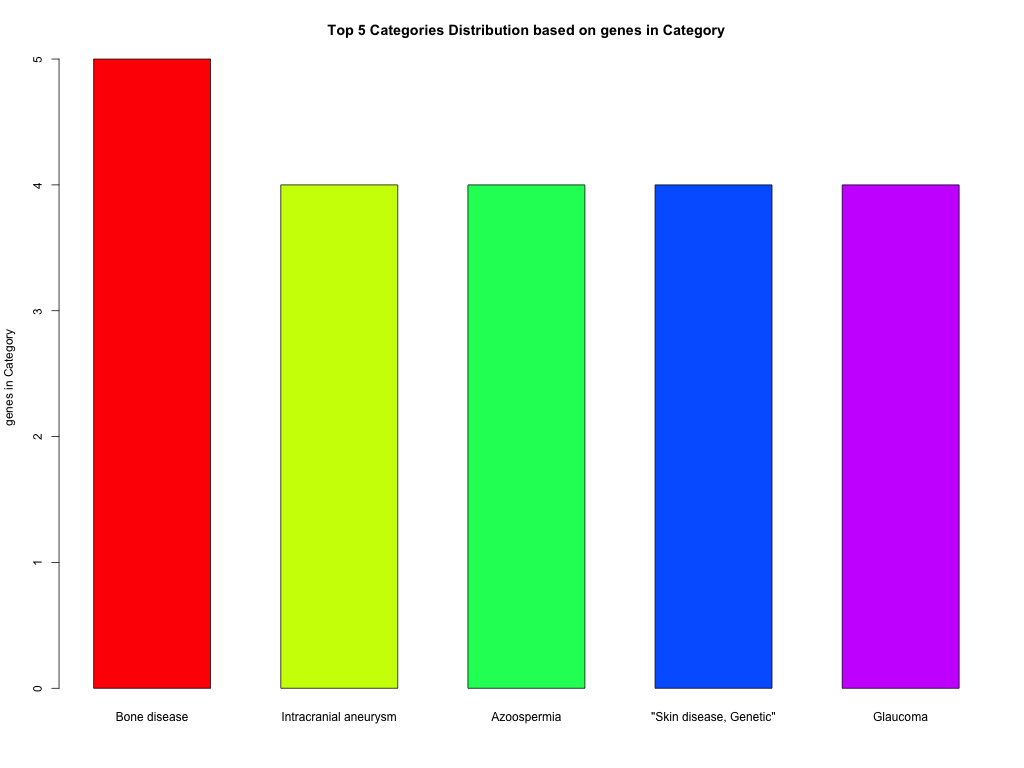

Supplement: Additional file 9 — Examples of MMpred predictions supported by experimental data and mapping against current databases. [file 1471-2164-13-620-S9.ZIP › Additional file 11 - Examples of MMpred predictions supported by experimental data and mapping against current databases/GSE19350/GRAPH_Sep 2_125707.png]

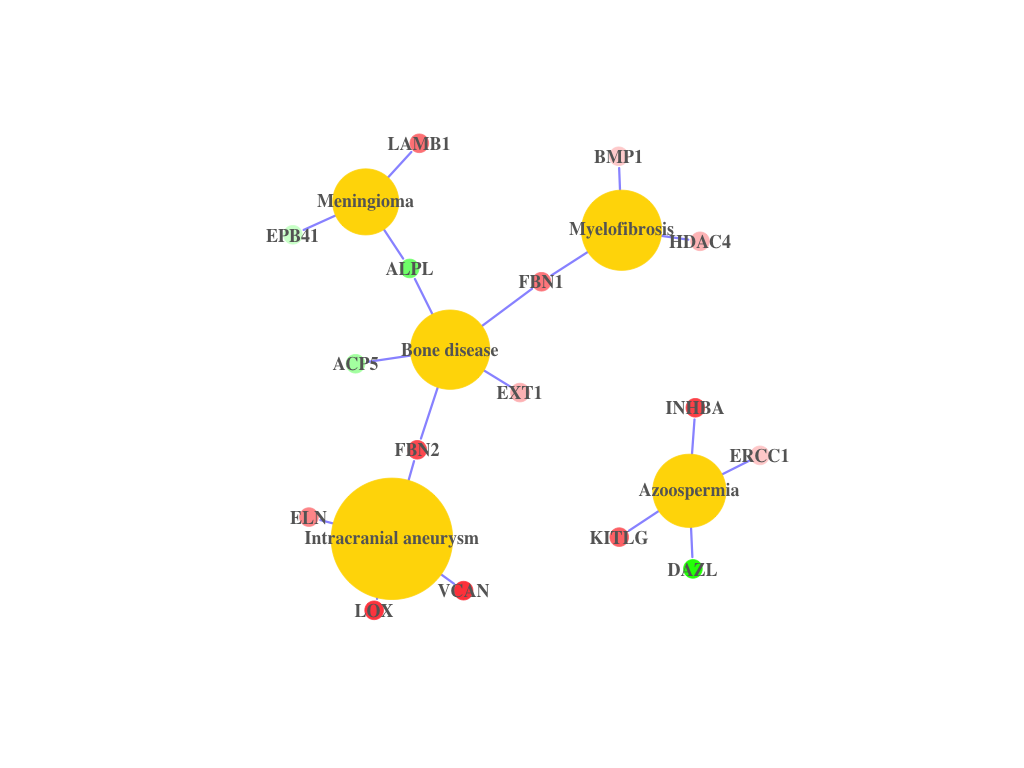

Supplement: Additional file 9 — Examples of MMpred predictions supported by experimental data and mapping against current databases. [file 1471-2164-13-620-S9.ZIP › Additional file 11 - Examples of MMpred predictions supported by experimental data and mapping against current databases/GSE19350/GRAPH_Sep 2_125708.png]

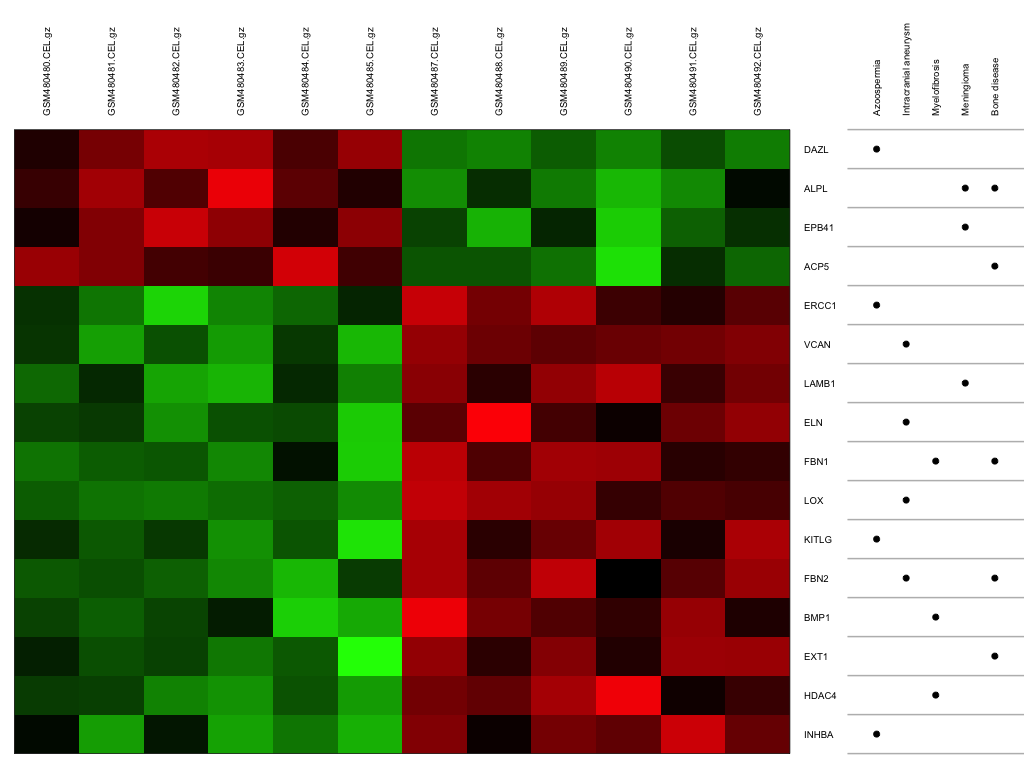

Supplement: Additional file 9 — Examples of MMpred predictions supported by experimental data and mapping against current databases. [file 1471-2164-13-620-S9.ZIP › Additional file 11 - Examples of MMpred predictions supported by experimental data and mapping against current databases/GSE19350/GRAPH_Sep 2_125709.png]

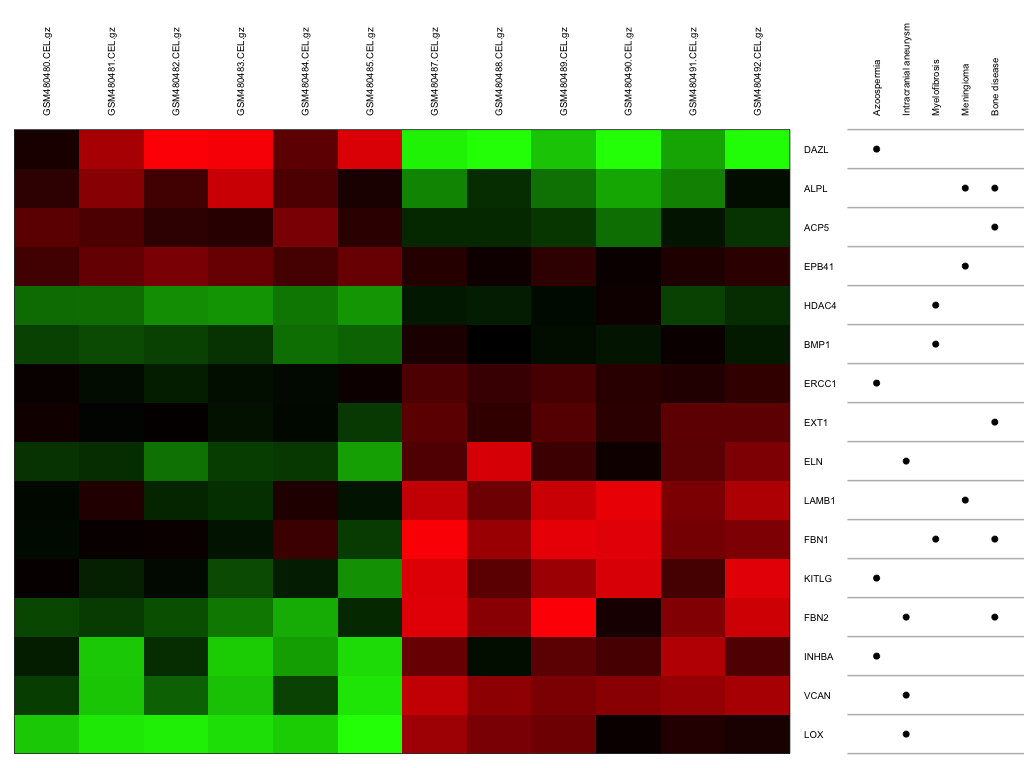

Supplement: Additional file 9 — Examples of MMpred predictions supported by experimental data and mapping against current databases. [file 1471-2164-13-620-S9.ZIP › Additional file 11 - Examples of MMpred predictions supported by experimental data and mapping against current databases/GSE19350/GRAPH_Sep 2_125710.png]

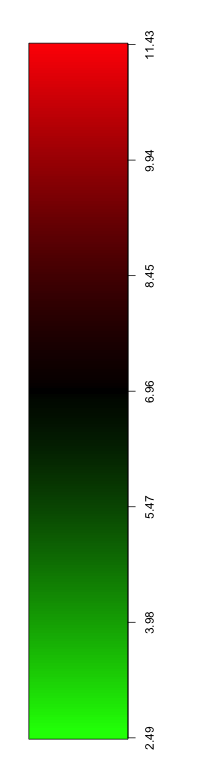

Supplement: Additional file 9 — Examples of MMpred predictions supported by experimental data and mapping against current databases. [file 1471-2164-13-620-S9.ZIP › Additional file 11 - Examples of MMpred predictions supported by experimental data and mapping against current databases/GSE19350/GRAPH_Sep 2_125711.png]

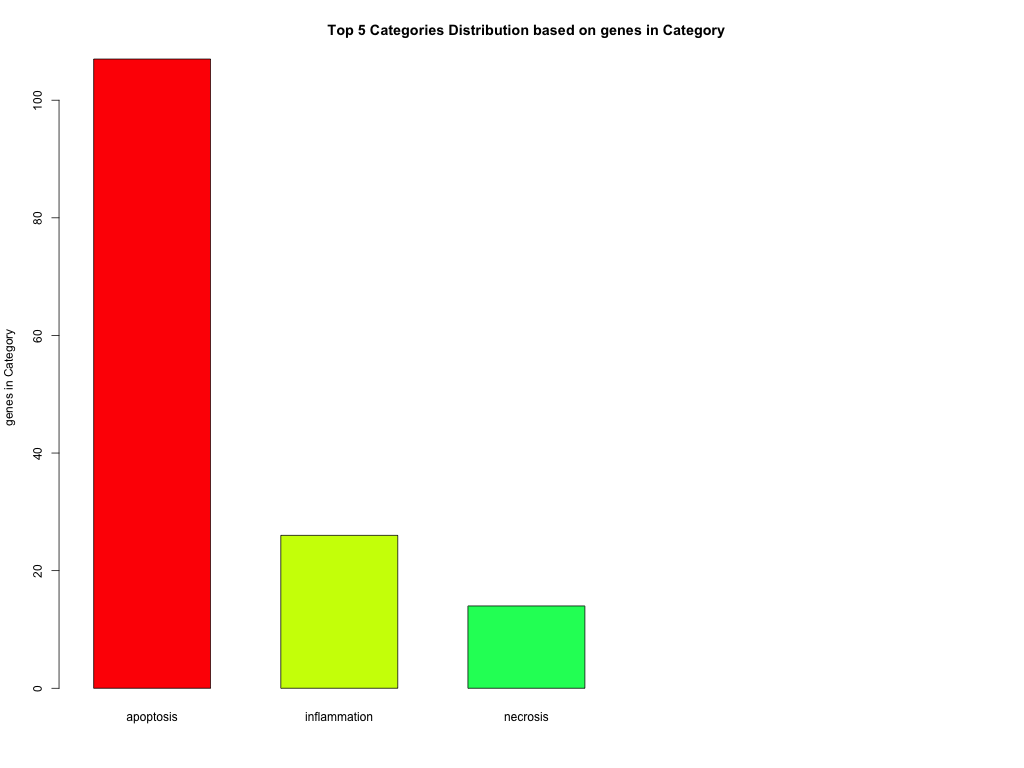

Supplement: Additional file 9 — Examples of MMpred predictions supported by experimental data and mapping against current databases. [file 1471-2164-13-620-S9.ZIP › Additional file 11 - Examples of MMpred predictions supported by experimental data and mapping against current databases/GSE19350/GRAPH_Sep 2_125714.png]

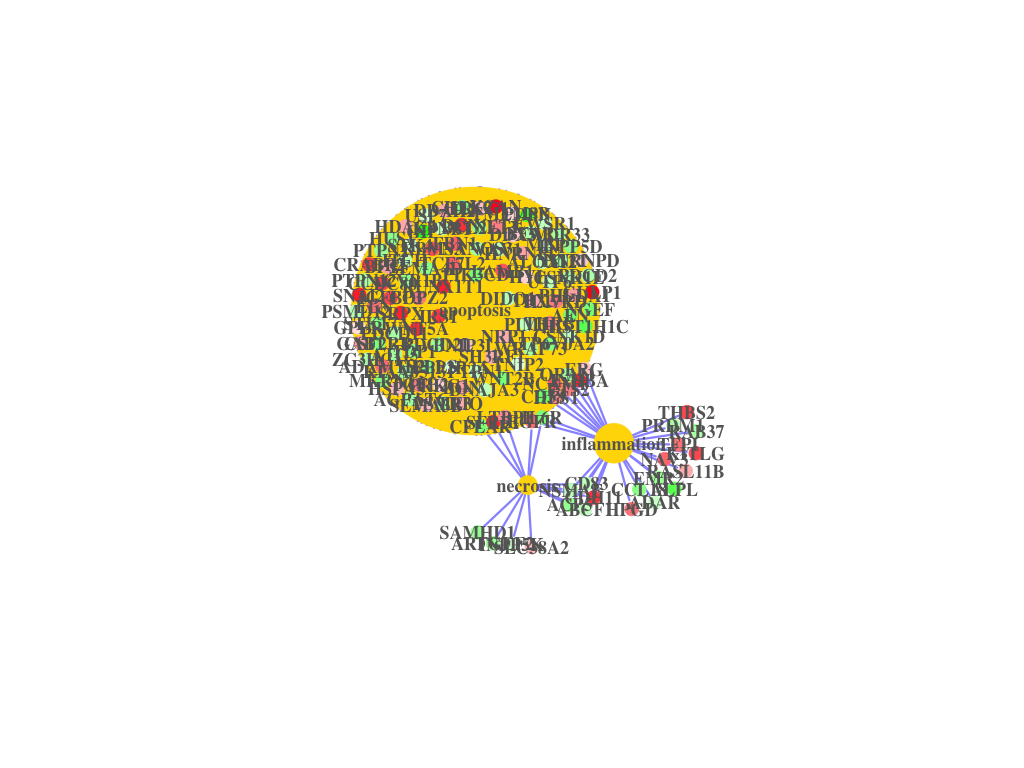

Supplement: Additional file 9 — Examples of MMpred predictions supported by experimental data and mapping against current databases. [file 1471-2164-13-620-S9.ZIP › Additional file 11 - Examples of MMpred predictions supported by experimental data and mapping against current databases/GSE19350/GRAPH_Sep 2_125715.png]

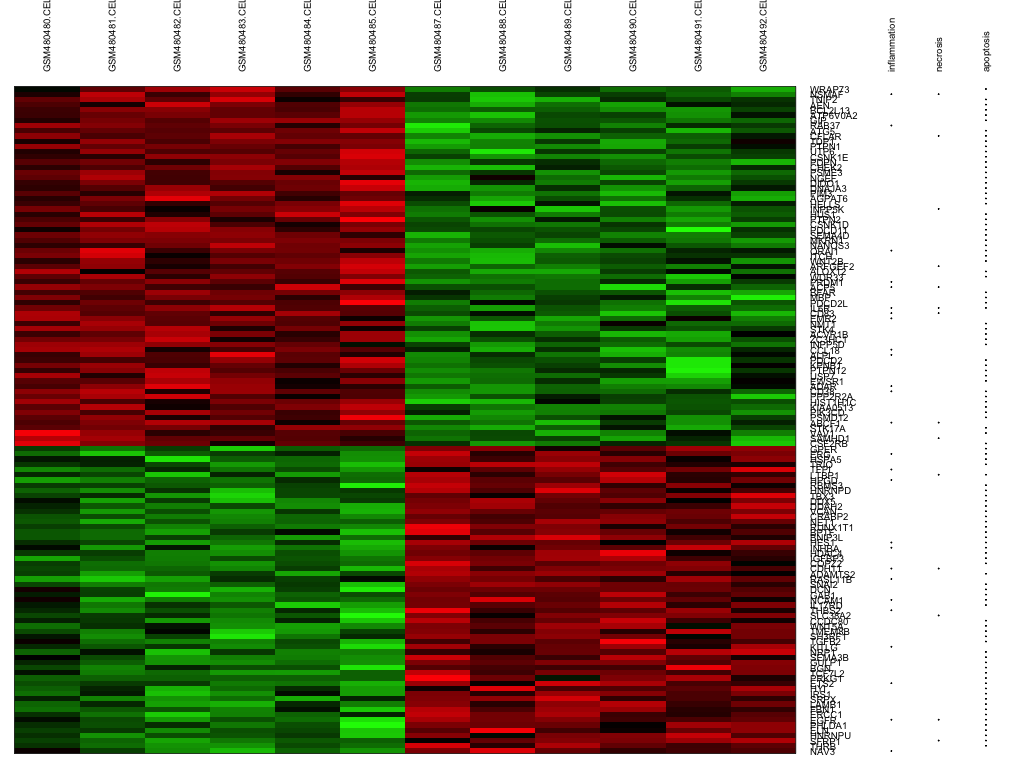

Supplement: Additional file 9 — Examples of MMpred predictions supported by experimental data and mapping against current databases. [file 1471-2164-13-620-S9.ZIP › Additional file 11 - Examples of MMpred predictions supported by experimental data and mapping against current databases/GSE19350/GRAPH_Sep 2_125717.png]
