# Supplementary figures and images for: MMpred: functional miRNA – mRNA interaction analyses by miRNA expression prediction (part 1 of 3)
Source: BMC Genomics. 2012 Nov 14;13:620. doi: 10.1186/1471-2164-13-620 (PMC3562514; doi:10.1186/1471-2164-13-620)

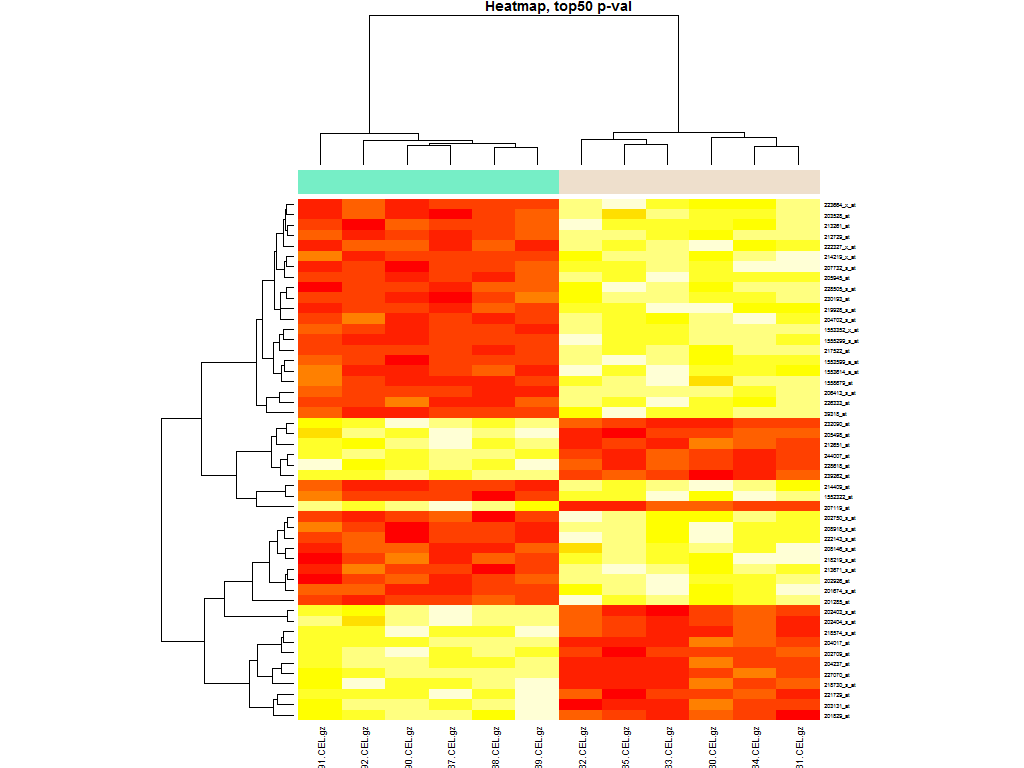

Supplement: Additional file 3 — Sample pipeline outputs in HTML format (compressed file). [file 1471-2164-13-620-S3.ZIP › BrainCancer12arraysPaired/FULL_pliki/GRAPH_Jul21_024756.png]

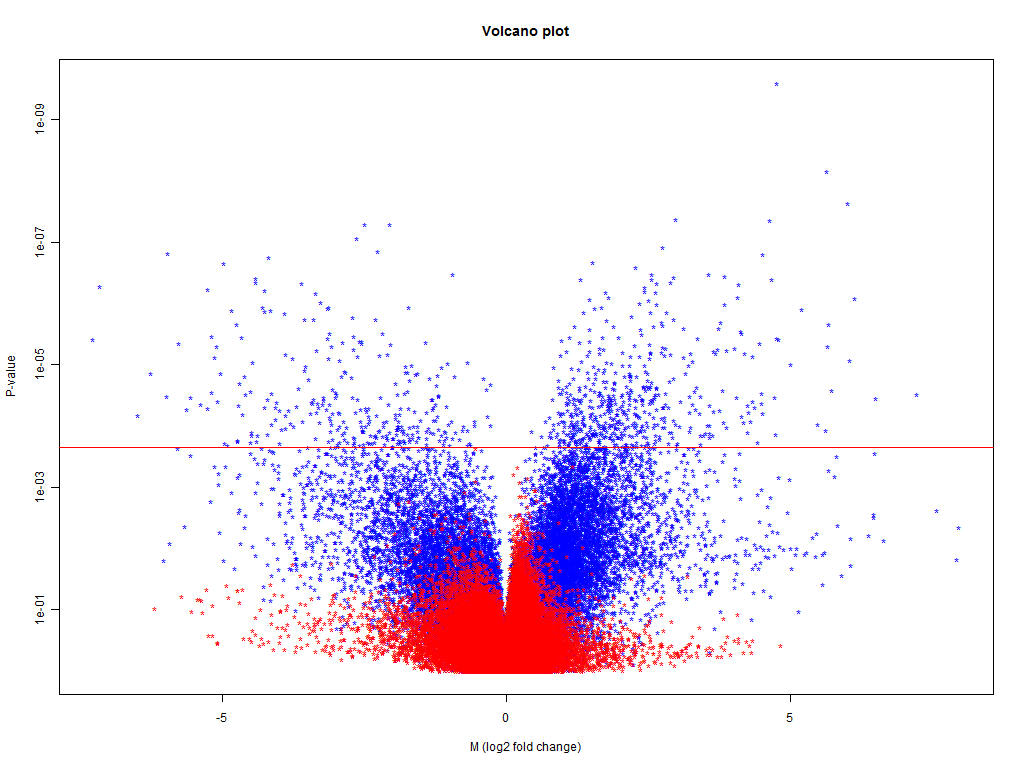

Supplement: Additional file 3 — Sample pipeline outputs in HTML format (compressed file). [file 1471-2164-13-620-S3.ZIP › BrainCancer12arraysPaired/FULL_pliki/GRAPH_Jul21_024757.png]

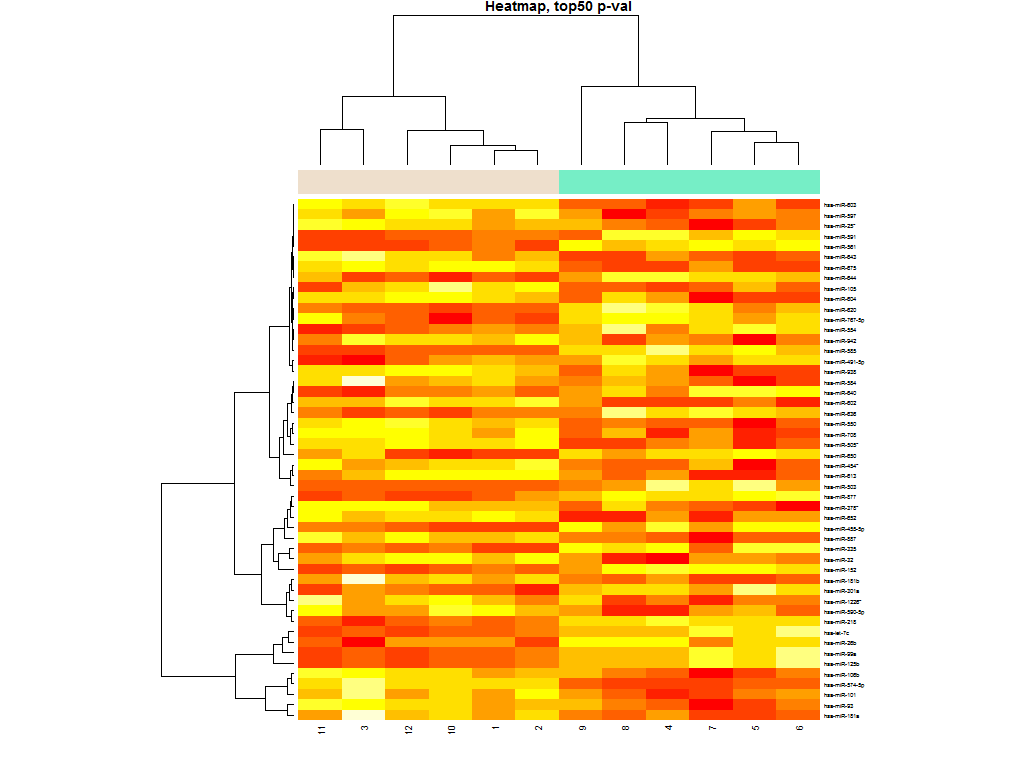

Supplement: Additional file 3 — Sample pipeline outputs in HTML format (compressed file). [file 1471-2164-13-620-S3.ZIP › BrainCancer12arraysPaired/FULL_pliki/GRAPH_Jul21_025007.png]

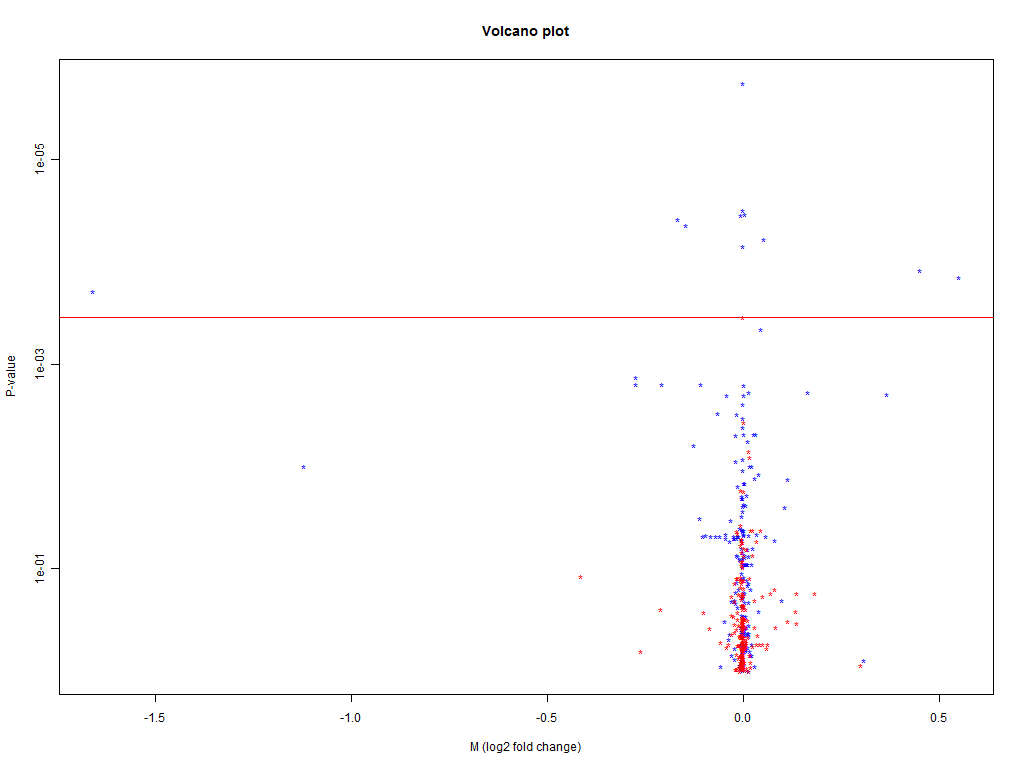

Supplement: Additional file 3 — Sample pipeline outputs in HTML format (compressed file). [file 1471-2164-13-620-S3.ZIP › BrainCancer12arraysPaired/FULL_pliki/GRAPH_Jul21_025008.png]

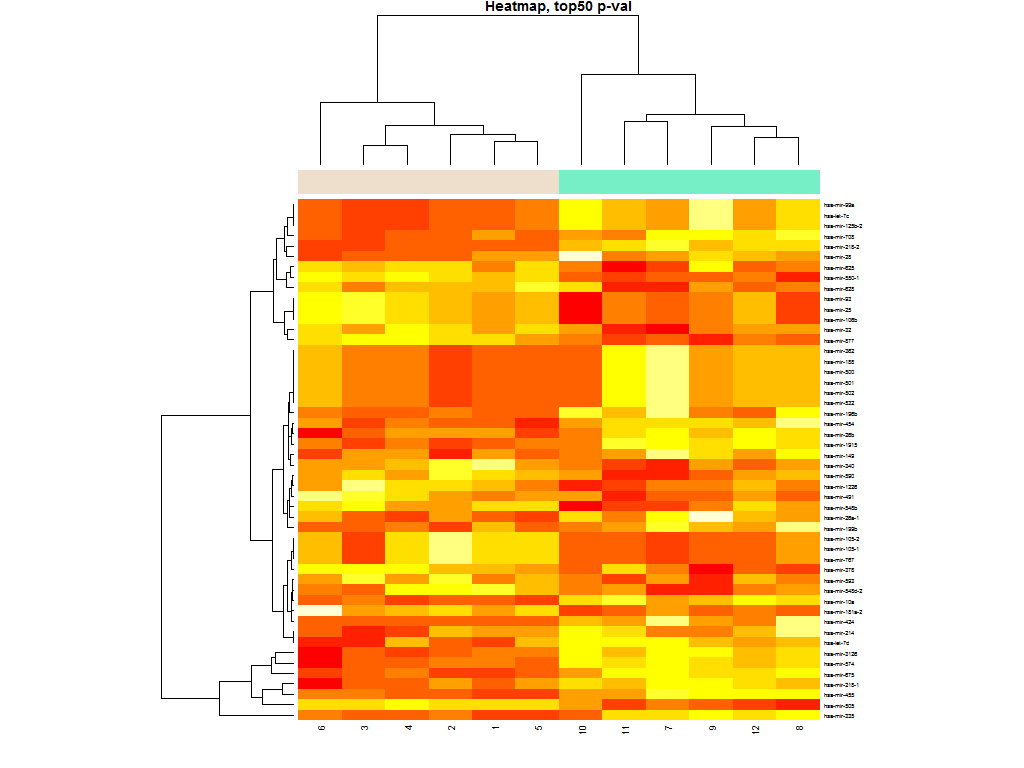

Supplement: Additional file 3 — Sample pipeline outputs in HTML format (compressed file). [file 1471-2164-13-620-S3.ZIP › BrainCancer12arraysPaired/FULL_pliki/GRAPH_Jul21_025010.png]

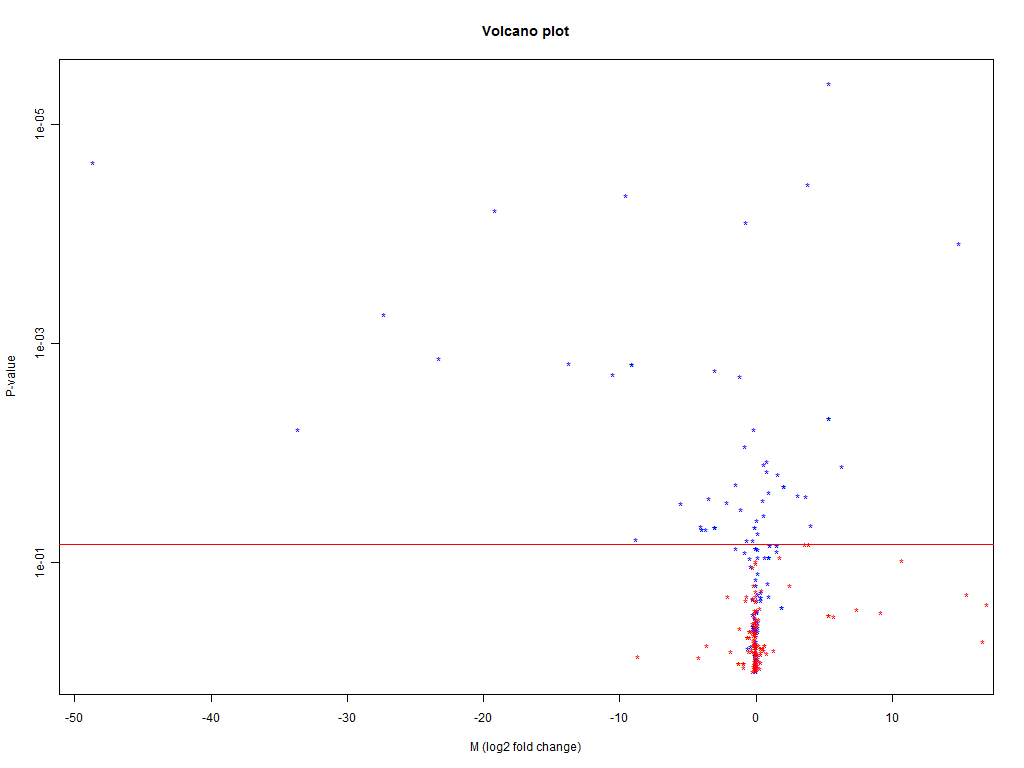

Supplement: Additional file 3 — Sample pipeline outputs in HTML format (compressed file). [file 1471-2164-13-620-S3.ZIP › BrainCancer12arraysPaired/FULL_pliki/GRAPH_Jul21_025012.png]

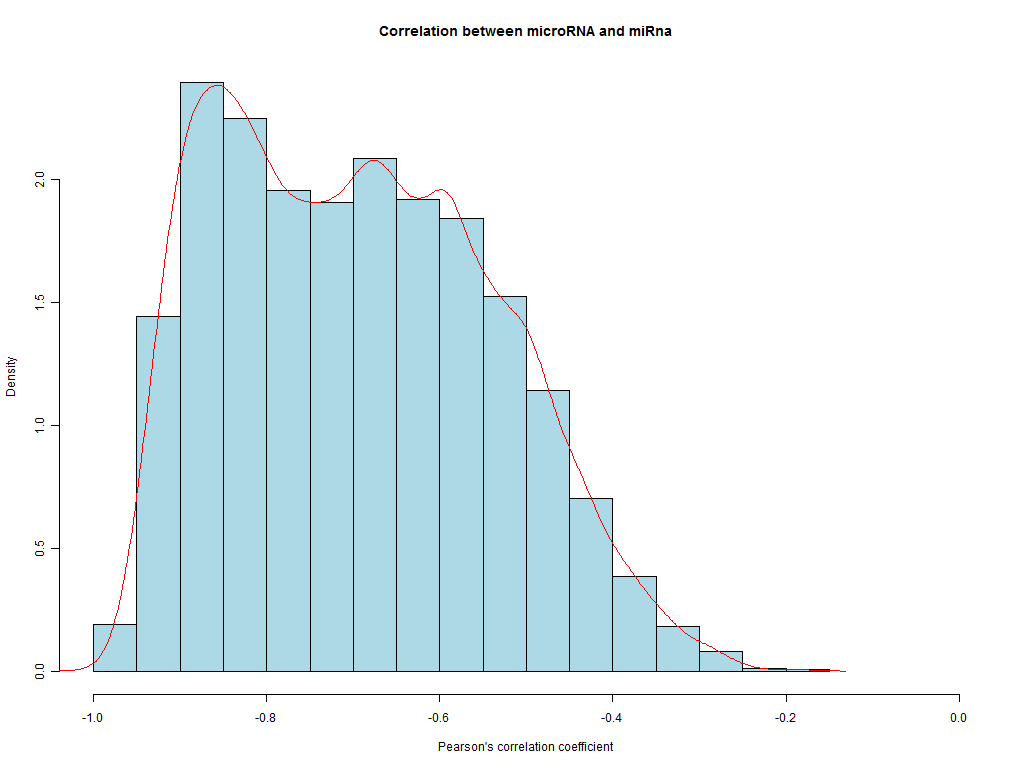

Supplement: Additional file 3 — Sample pipeline outputs in HTML format (compressed file). [file 1471-2164-13-620-S3.ZIP › BrainCancer12arraysPaired/FULL_pliki/GRAPH_Jul21_025014.png]

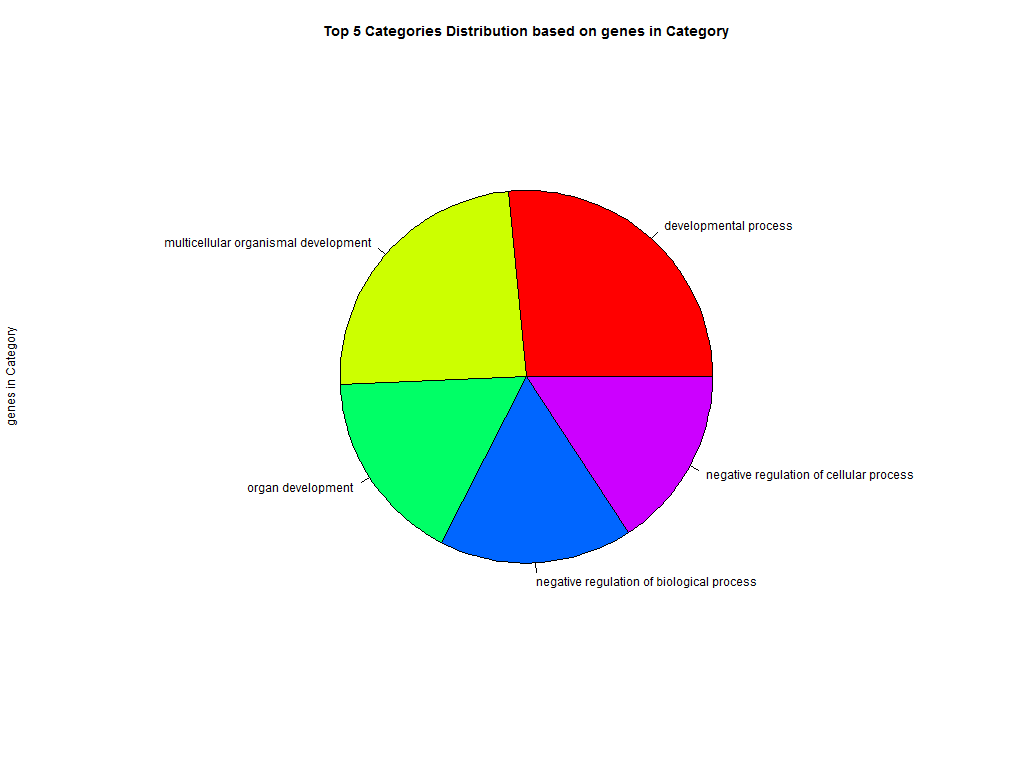

Supplement: Additional file 3 — Sample pipeline outputs in HTML format (compressed file). [file 1471-2164-13-620-S3.ZIP › BrainCancer12arraysPaired/FULL_pliki/GRAPH_Jul21_025207.png]

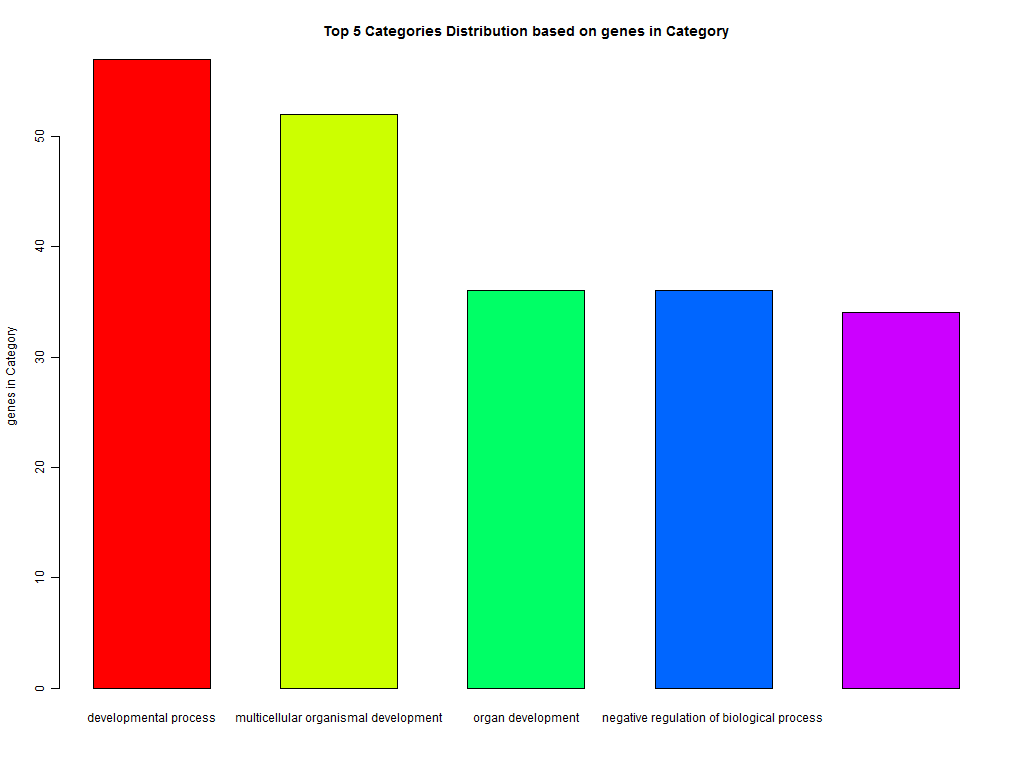

Supplement: Additional file 3 — Sample pipeline outputs in HTML format (compressed file). [file 1471-2164-13-620-S3.ZIP › BrainCancer12arraysPaired/FULL_pliki/GRAPH_Jul21_025213.png]

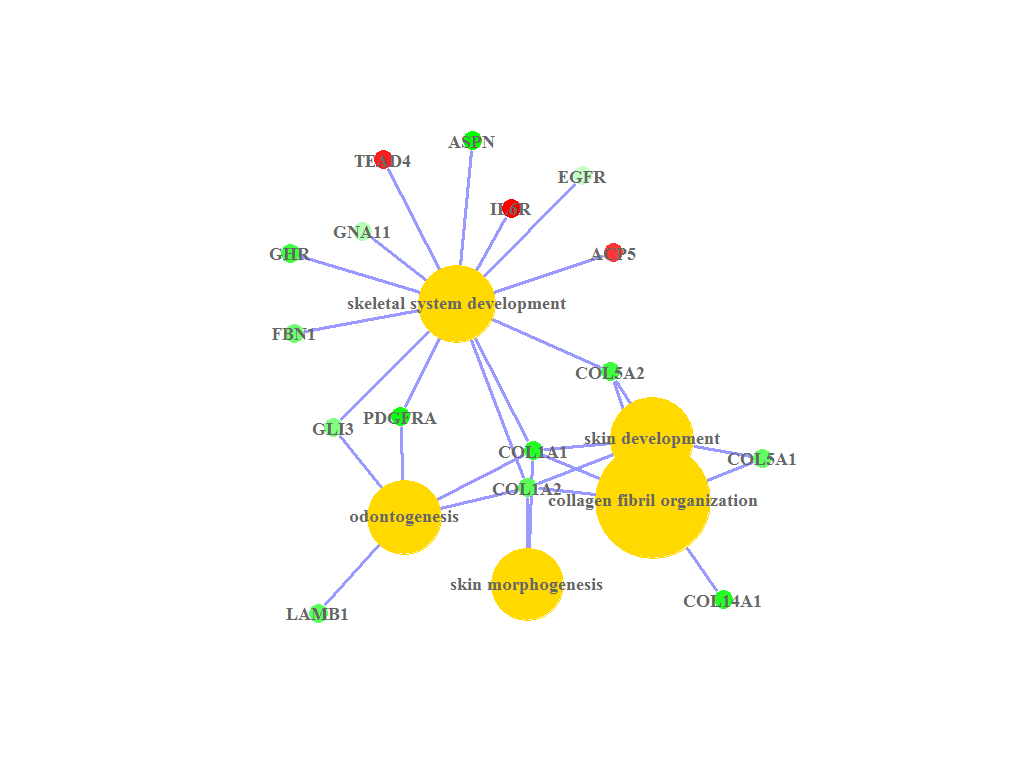

Supplement: Additional file 3 — Sample pipeline outputs in HTML format (compressed file). [file 1471-2164-13-620-S3.ZIP › BrainCancer12arraysPaired/FULL_pliki/GRAPH_Jul21_025219.png]

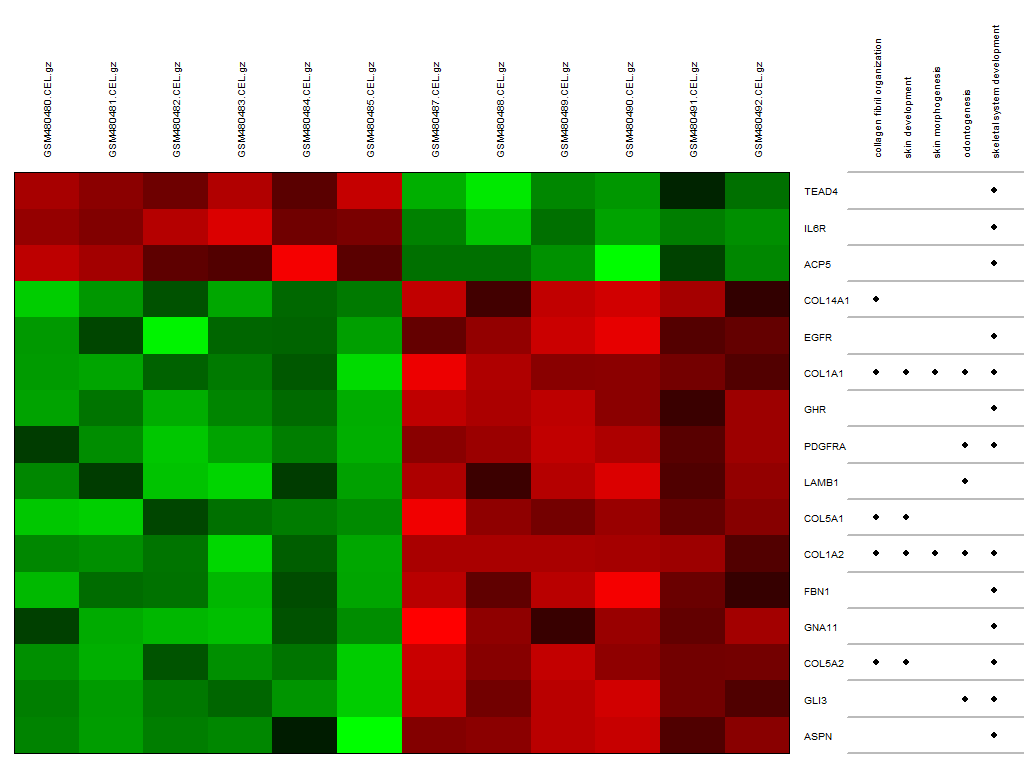

Supplement: Additional file 3 — Sample pipeline outputs in HTML format (compressed file). [file 1471-2164-13-620-S3.ZIP › BrainCancer12arraysPaired/FULL_pliki/GRAPH_Jul21_025224.png]

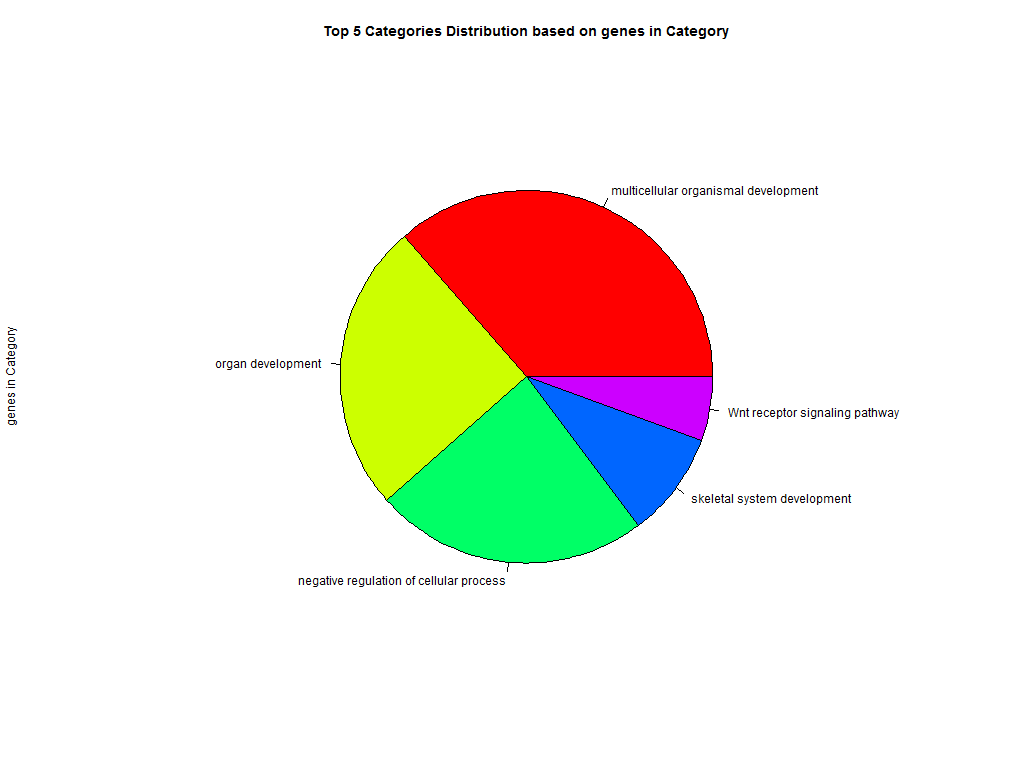

Supplement: Additional file 3 — Sample pipeline outputs in HTML format (compressed file). [file 1471-2164-13-620-S3.ZIP › BrainCancer12arraysPaired/FULL_pliki/GRAPH_Jul21_025318.png]

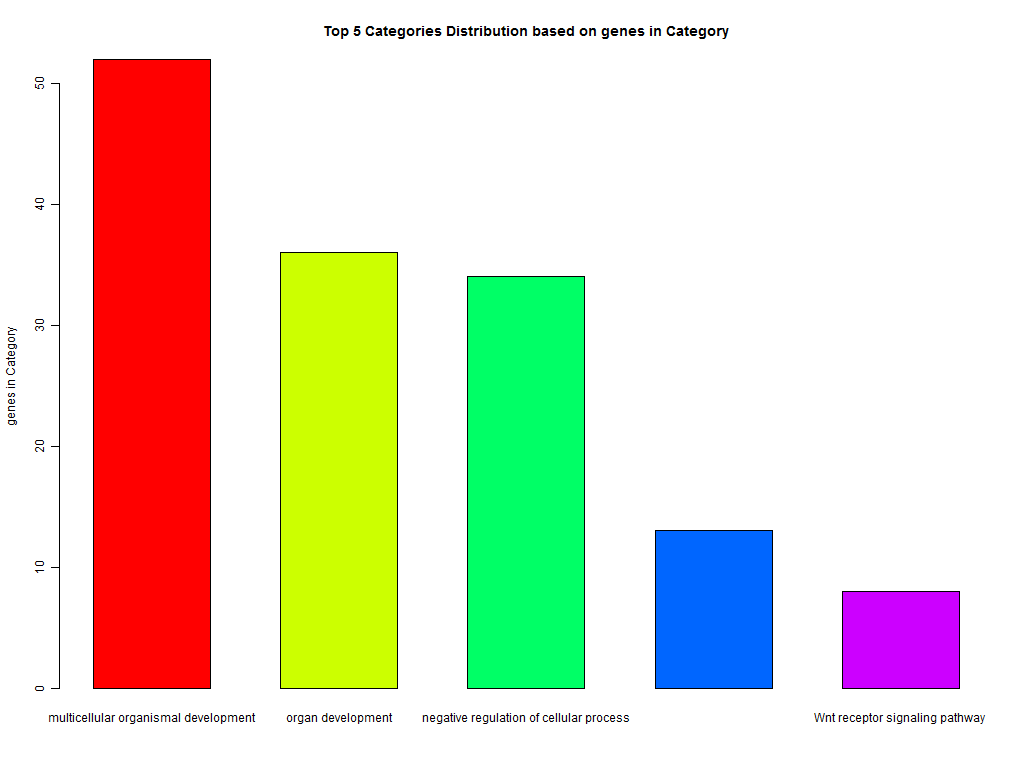

Supplement: Additional file 3 — Sample pipeline outputs in HTML format (compressed file). [file 1471-2164-13-620-S3.ZIP › BrainCancer12arraysPaired/FULL_pliki/GRAPH_Jul21_025325.png]

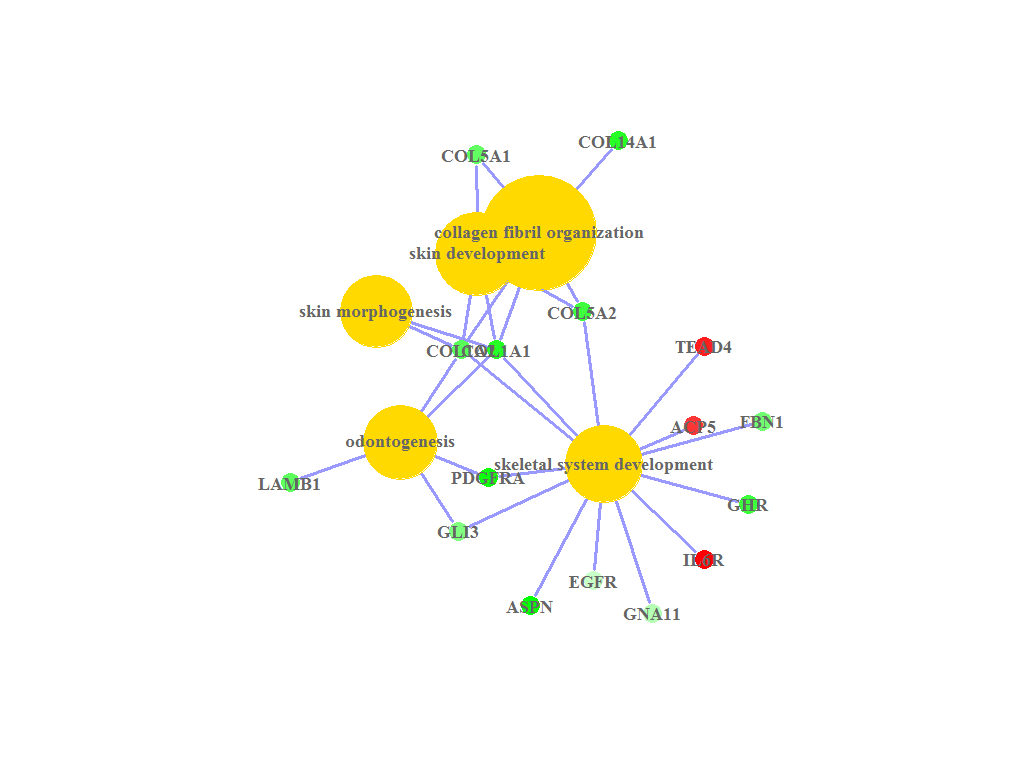

Supplement: Additional file 3 — Sample pipeline outputs in HTML format (compressed file). [file 1471-2164-13-620-S3.ZIP › BrainCancer12arraysPaired/FULL_pliki/GRAPH_Jul21_025340.png]

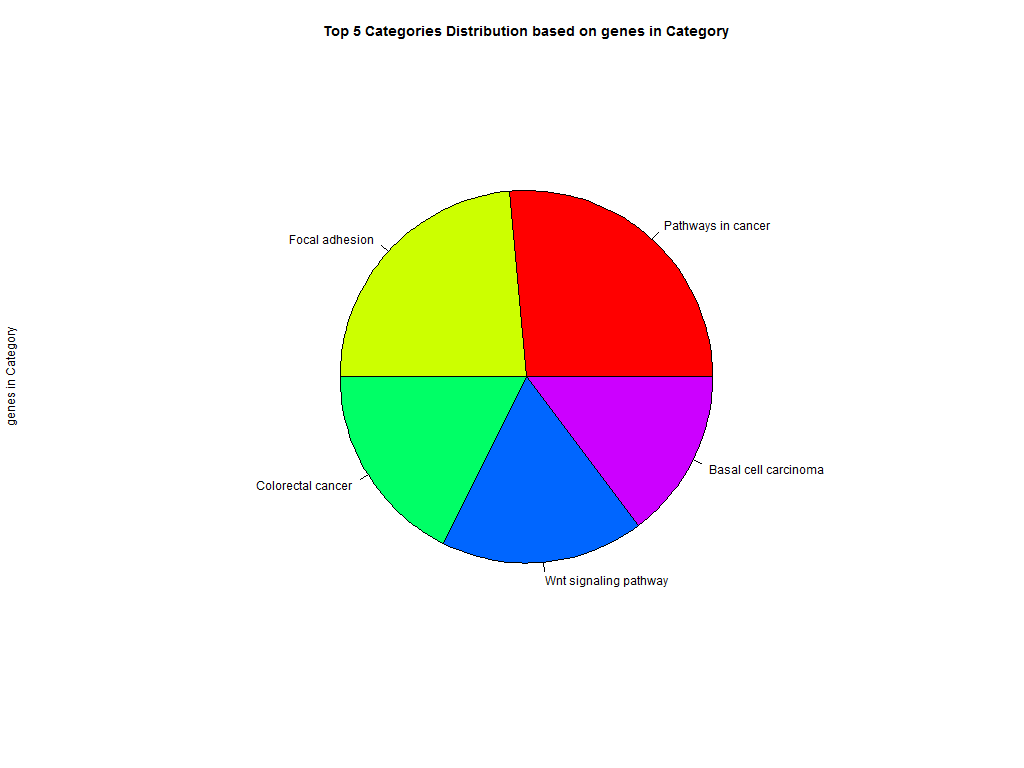

Supplement: Additional file 3 — Sample pipeline outputs in HTML format (compressed file). [file 1471-2164-13-620-S3.ZIP › BrainCancer12arraysPaired/FULL_pliki/GRAPH_Jul21_025407.png]

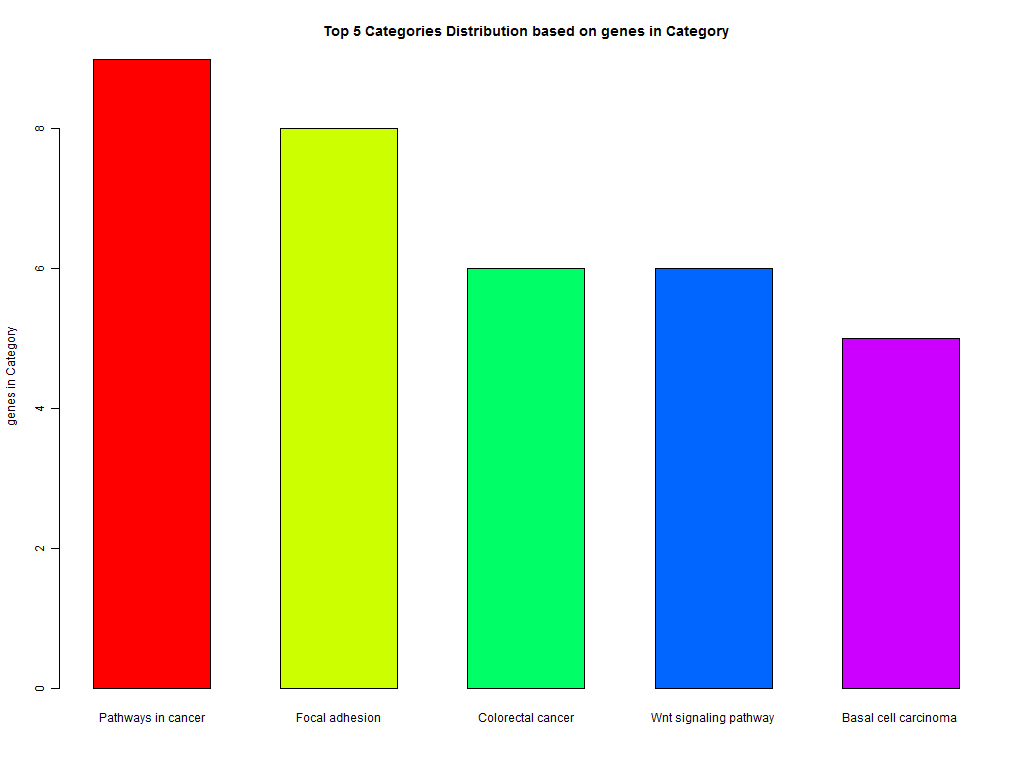

Supplement: Additional file 3 — Sample pipeline outputs in HTML format (compressed file). [file 1471-2164-13-620-S3.ZIP › BrainCancer12arraysPaired/FULL_pliki/GRAPH_Jul21_025413.png]

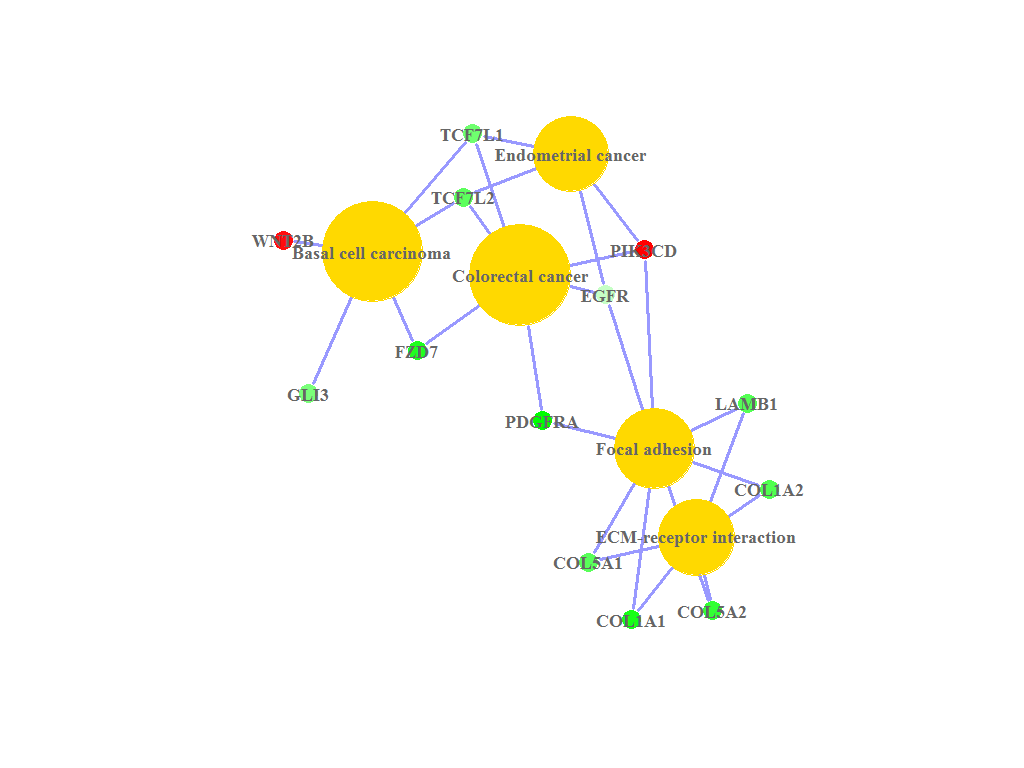

Supplement: Additional file 3 — Sample pipeline outputs in HTML format (compressed file). [file 1471-2164-13-620-S3.ZIP › BrainCancer12arraysPaired/FULL_pliki/GRAPH_Jul21_025419.png]

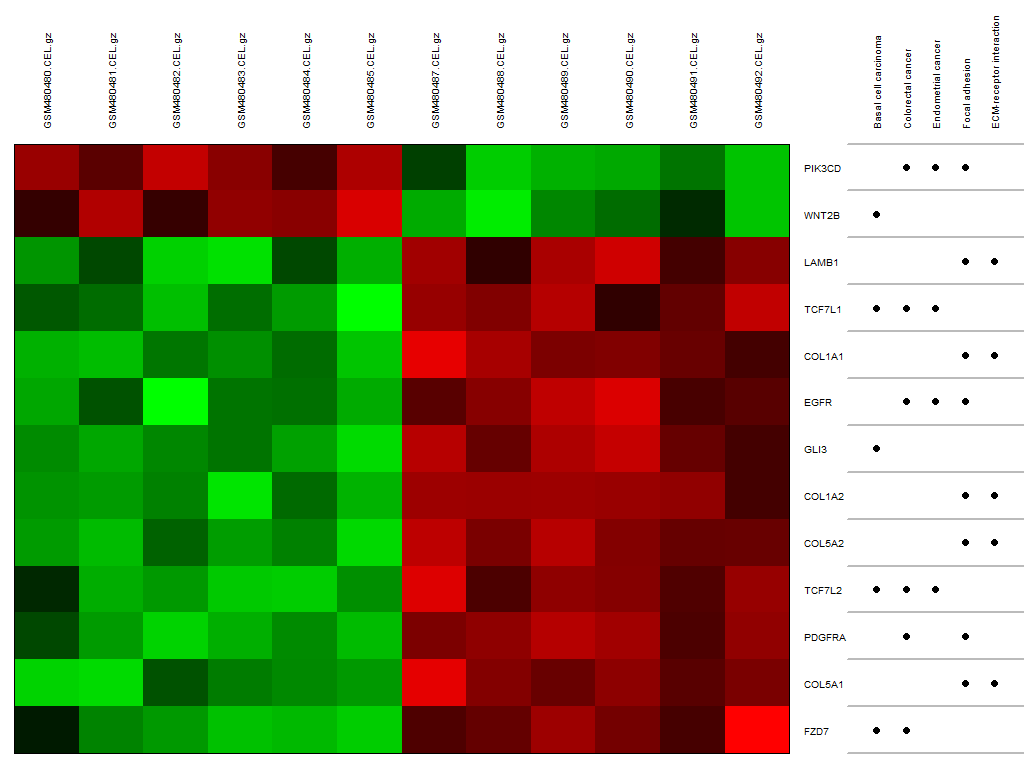

Supplement: Additional file 3 — Sample pipeline outputs in HTML format (compressed file). [file 1471-2164-13-620-S3.ZIP › BrainCancer12arraysPaired/FULL_pliki/GRAPH_Jul21_025425.png]

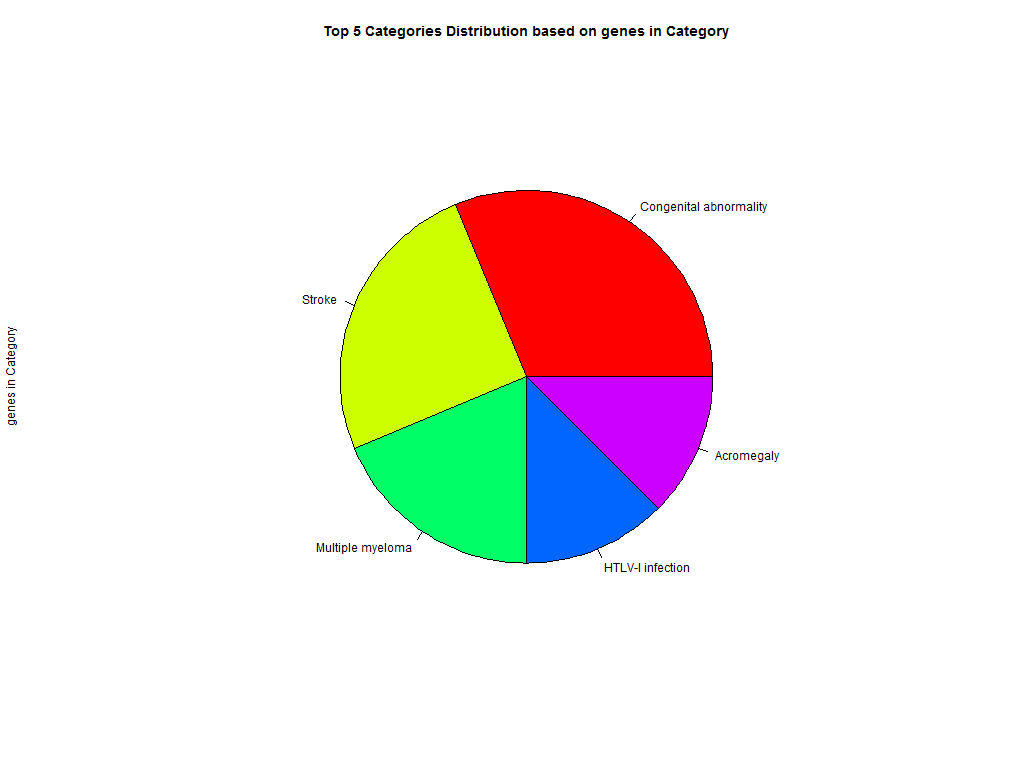

Supplement: Additional file 3 — Sample pipeline outputs in HTML format (compressed file). [file 1471-2164-13-620-S3.ZIP › BrainCancer12arraysPaired/FULL_pliki/GRAPH_Jul21_025440.png]

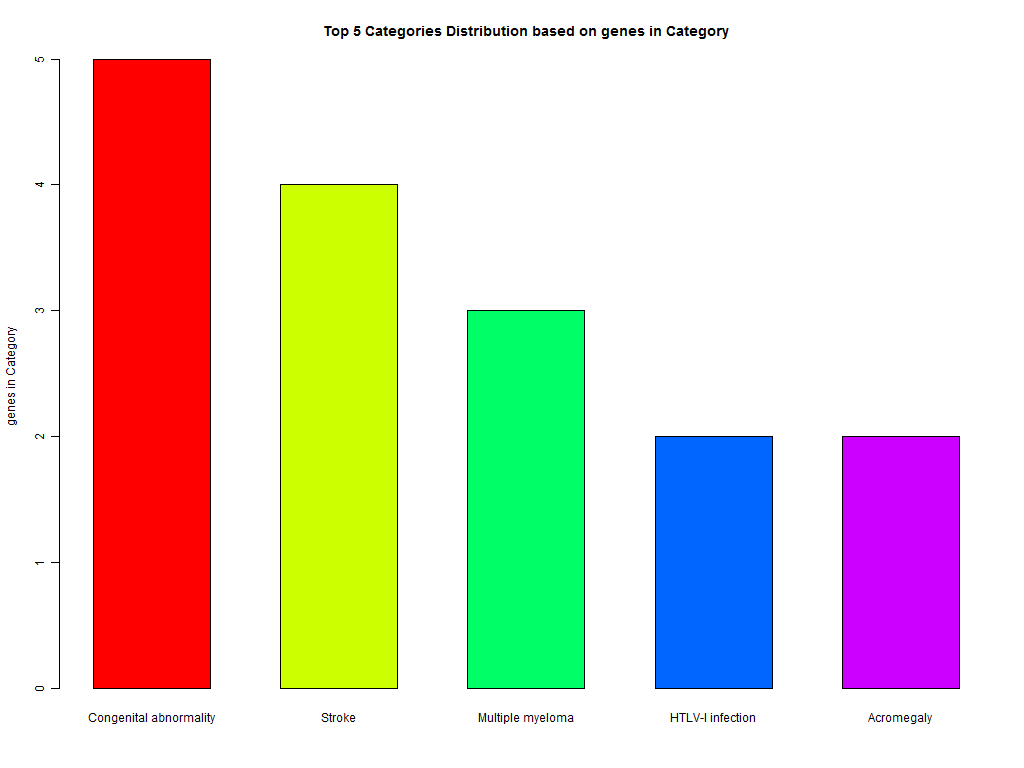

Supplement: Additional file 3 — Sample pipeline outputs in HTML format (compressed file). [file 1471-2164-13-620-S3.ZIP › BrainCancer12arraysPaired/FULL_pliki/GRAPH_Jul21_025446.png]

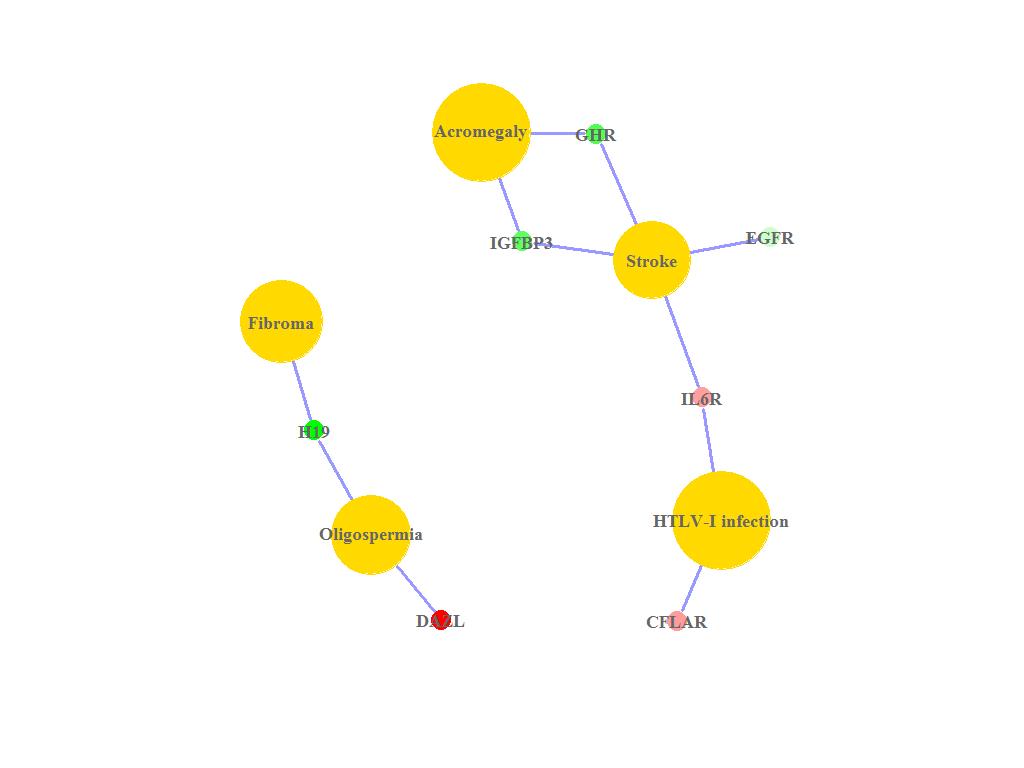

Supplement: Additional file 3 — Sample pipeline outputs in HTML format (compressed file). [file 1471-2164-13-620-S3.ZIP › BrainCancer12arraysPaired/FULL_pliki/GRAPH_Jul21_025452.png]

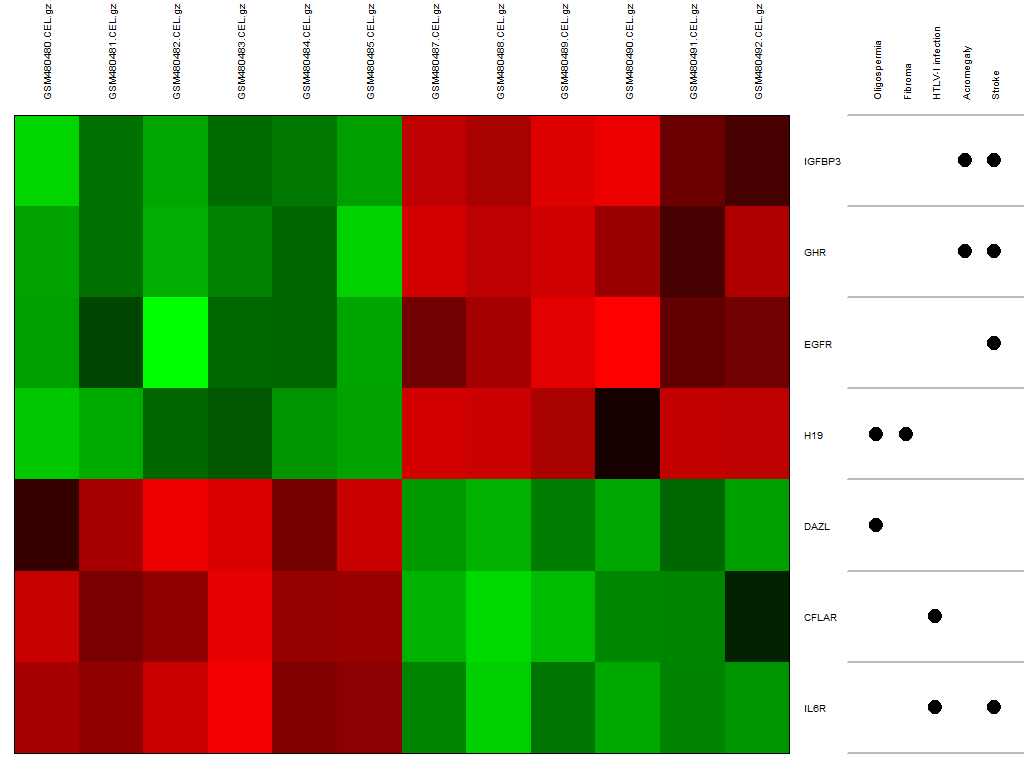

Supplement: Additional file 3 — Sample pipeline outputs in HTML format (compressed file). [file 1471-2164-13-620-S3.ZIP › BrainCancer12arraysPaired/FULL_pliki/GRAPH_Jul21_025458.png]

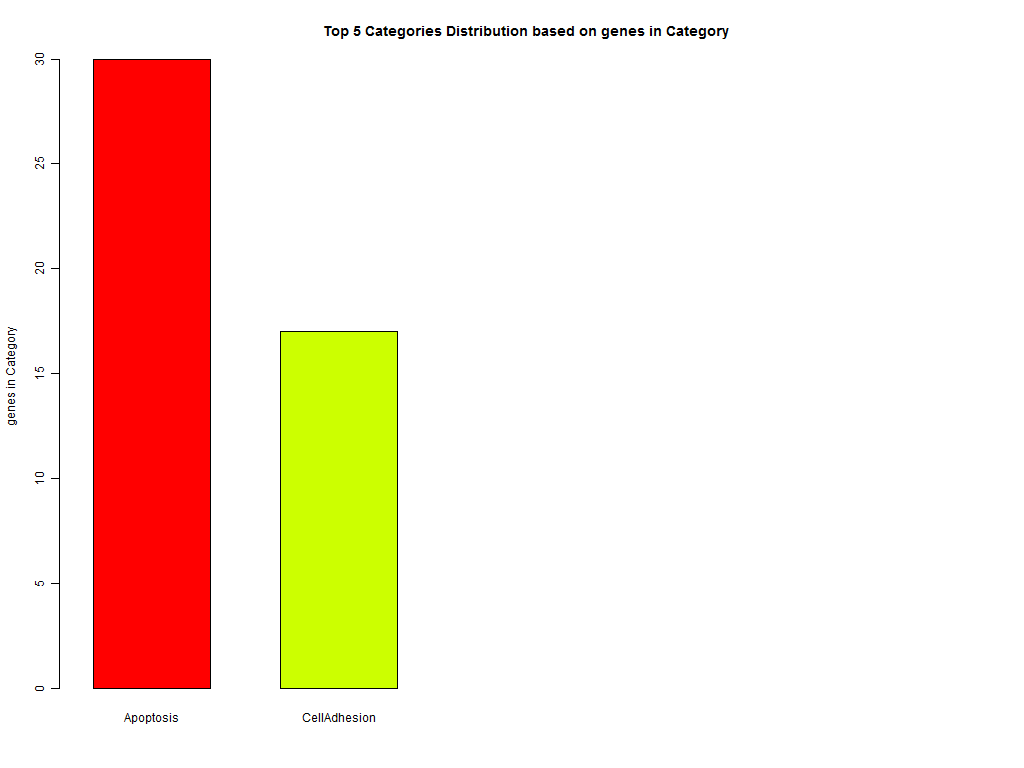

Supplement: Additional file 3 — Sample pipeline outputs in HTML format (compressed file). [file 1471-2164-13-620-S3.ZIP › BrainCancer12arraysPaired/FULL_pliki/GRAPH_Jul21_025516.png]

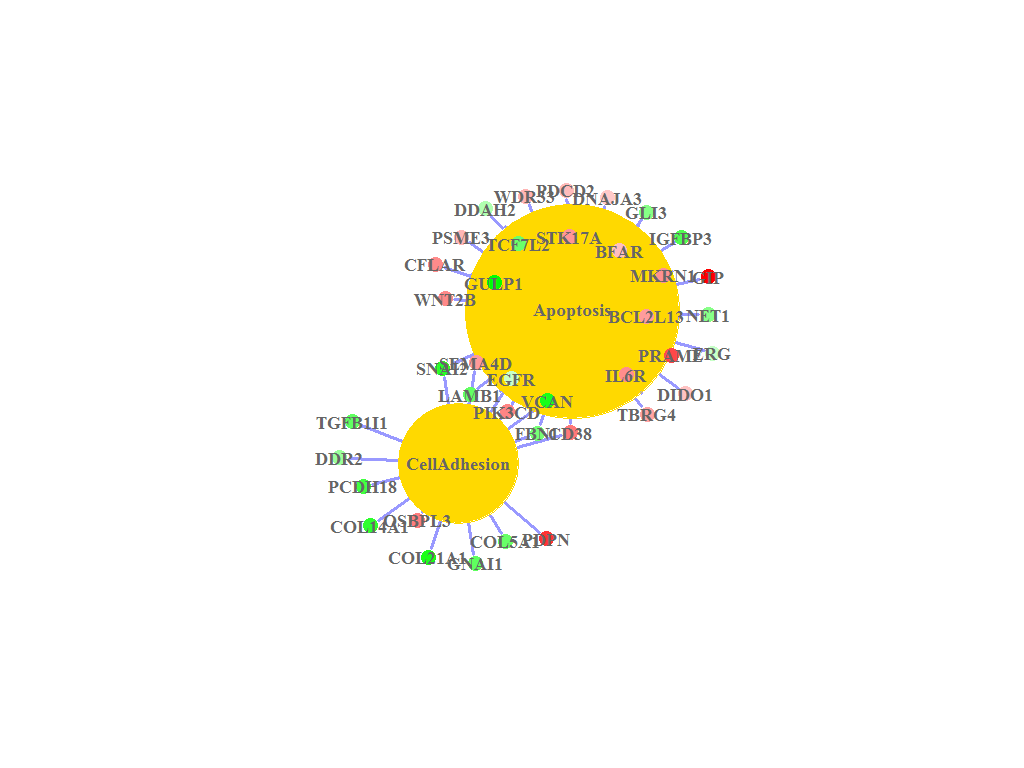

Supplement: Additional file 3 — Sample pipeline outputs in HTML format (compressed file). [file 1471-2164-13-620-S3.ZIP › BrainCancer12arraysPaired/FULL_pliki/GRAPH_Jul21_025522.png]

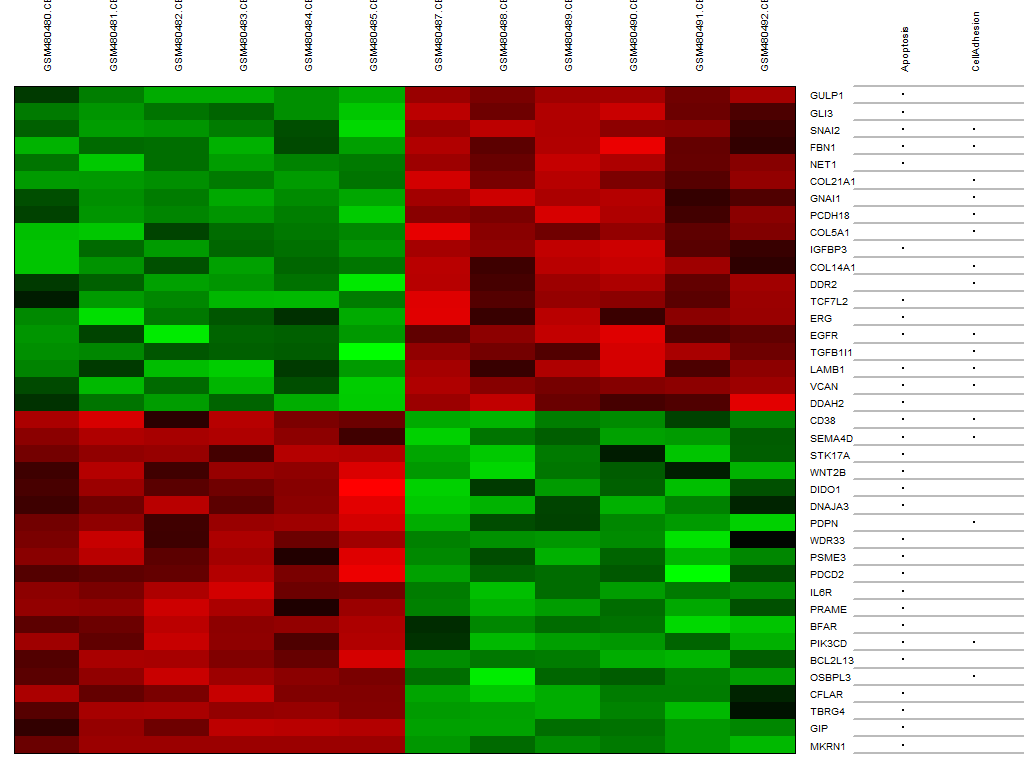

Supplement: Additional file 3 — Sample pipeline outputs in HTML format (compressed file). [file 1471-2164-13-620-S3.ZIP › BrainCancer12arraysPaired/FULL_pliki/GRAPH_Jul21_025528.png]

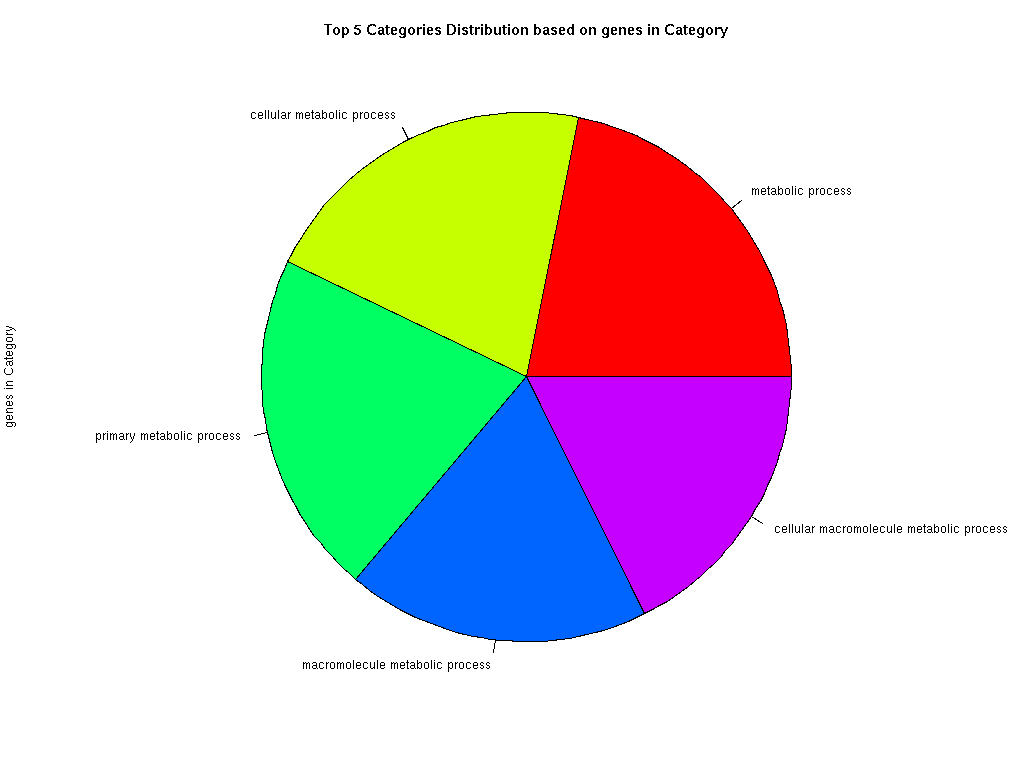

Supplement: Additional file 3 — Sample pipeline outputs in HTML format (compressed file). [file 1471-2164-13-620-S3.ZIP › Bunt182-anova1w2g/GRAPH_Jul23_185154.png]

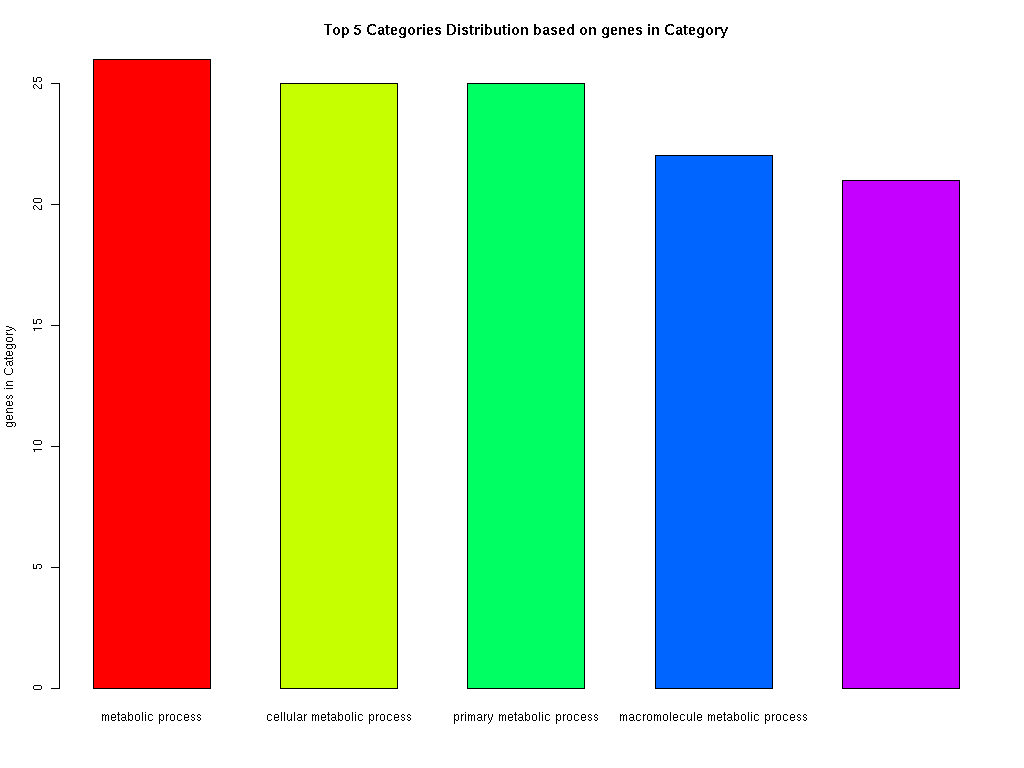

Supplement: Additional file 3 — Sample pipeline outputs in HTML format (compressed file). [file 1471-2164-13-620-S3.ZIP › Bunt182-anova1w2g/GRAPH_Jul23_185156.png]

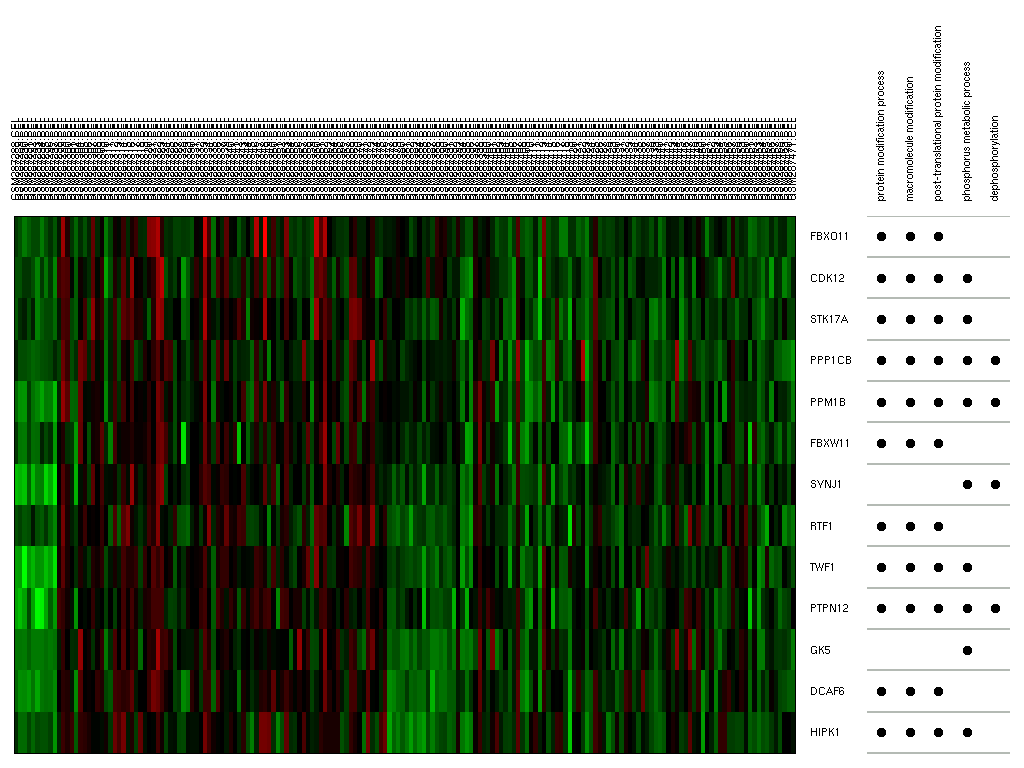

Supplement: Additional file 3 — Sample pipeline outputs in HTML format (compressed file). [file 1471-2164-13-620-S3.ZIP › Bunt182-anova1w2g/GRAPH_Jul23_185159.png]

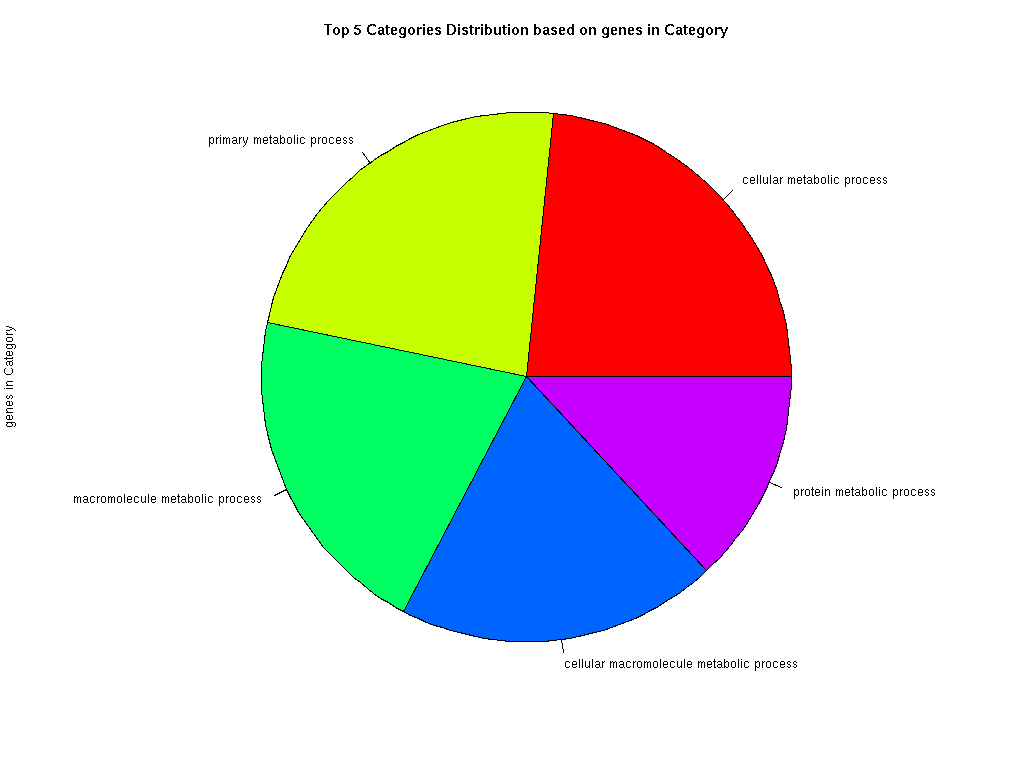

Supplement: Additional file 3 — Sample pipeline outputs in HTML format (compressed file). [file 1471-2164-13-620-S3.ZIP › Bunt182-anova1w2g/GRAPH_Jul23_185238.png]

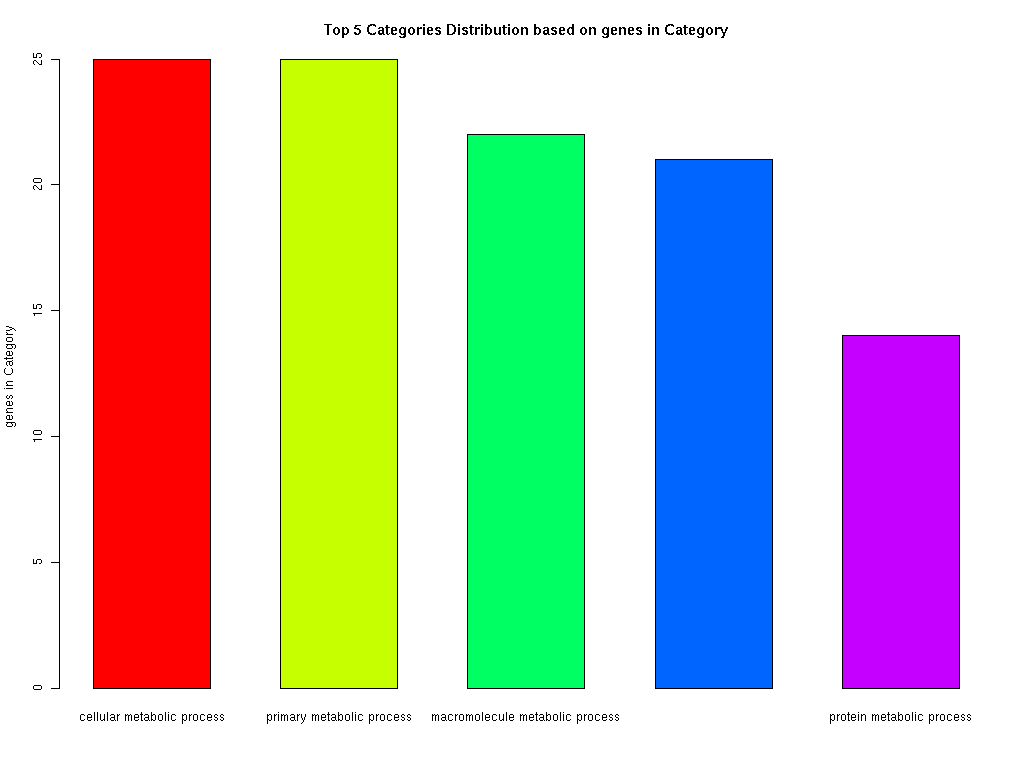

Supplement: Additional file 3 — Sample pipeline outputs in HTML format (compressed file). [file 1471-2164-13-620-S3.ZIP › Bunt182-anova1w2g/GRAPH_Jul23_185240.png]

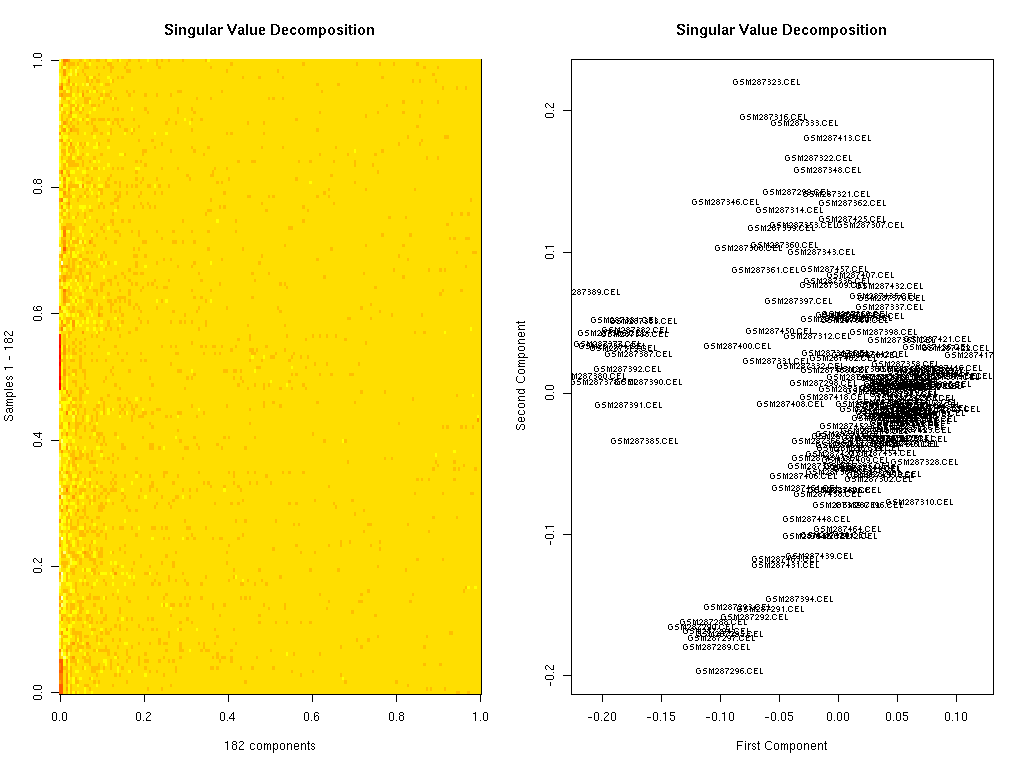

Supplement: Additional file 3 — Sample pipeline outputs in HTML format (compressed file). [file 1471-2164-13-620-S3.ZIP › Bunt182-anova1w2g/GRAPH_Jul26_211457.png]

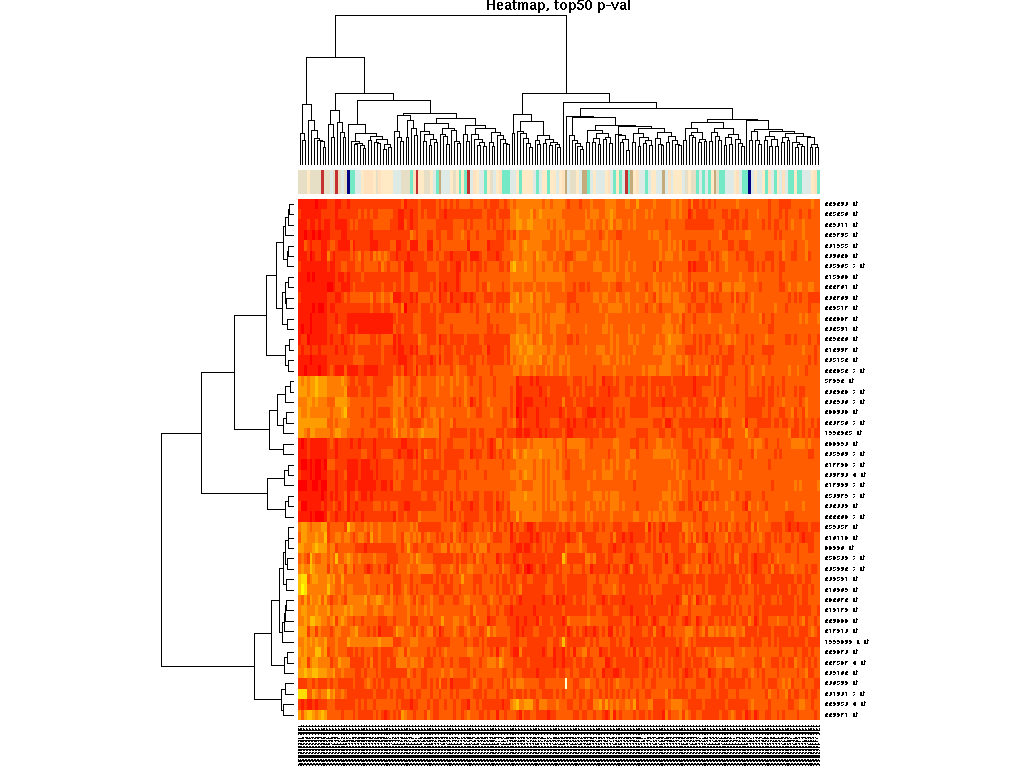

Supplement: Additional file 3 — Sample pipeline outputs in HTML format (compressed file). [file 1471-2164-13-620-S3.ZIP › Bunt182-anova1w2g/GRAPH_Jul26_211500.png]

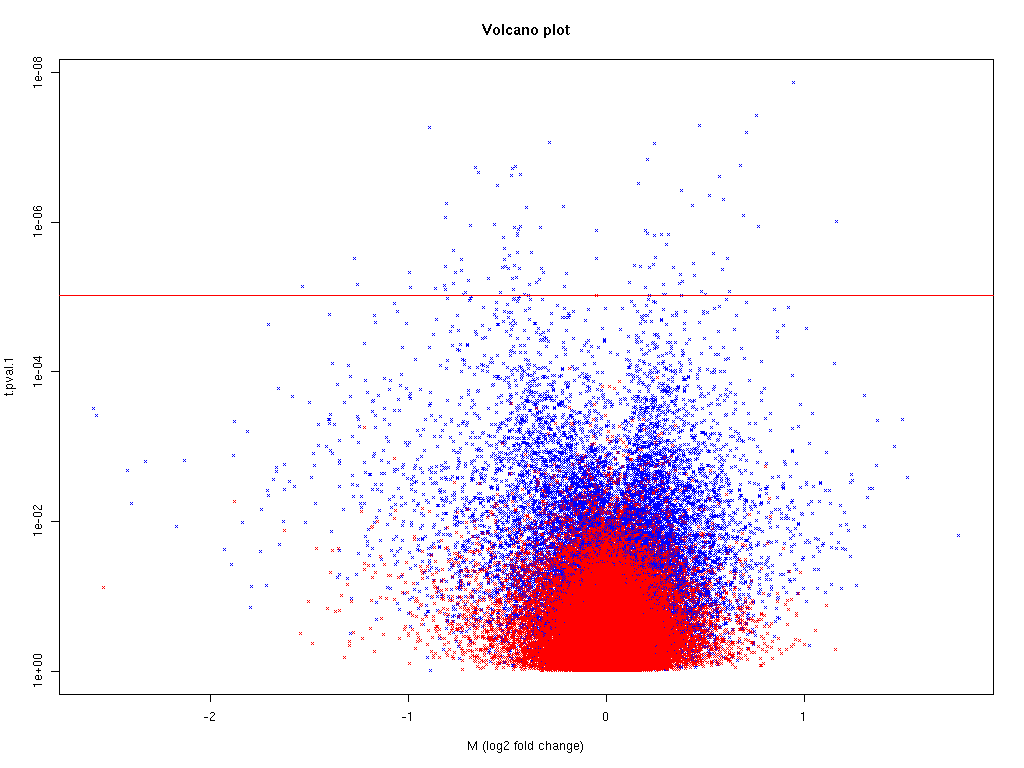

Supplement: Additional file 3 — Sample pipeline outputs in HTML format (compressed file). [file 1471-2164-13-620-S3.ZIP › Bunt182-anova1w2g/GRAPH_Jul26_211502.png]

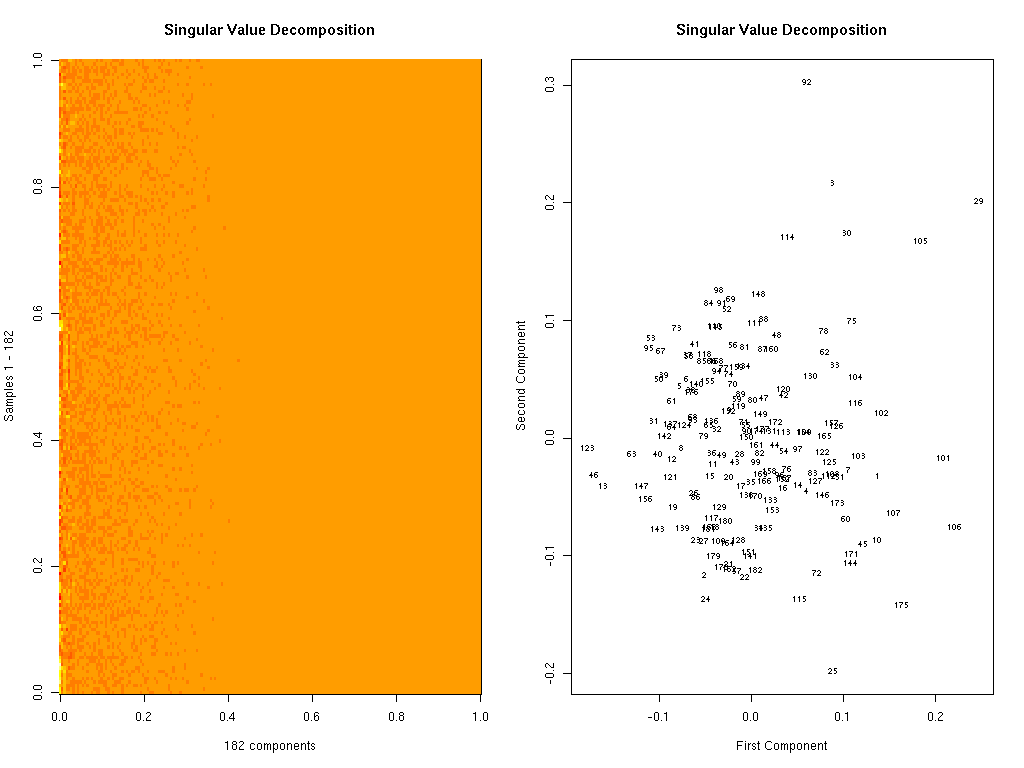

Supplement: Additional file 3 — Sample pipeline outputs in HTML format (compressed file). [file 1471-2164-13-620-S3.ZIP › Bunt182-anova1w2g/GRAPH_Jul26_224606.png]

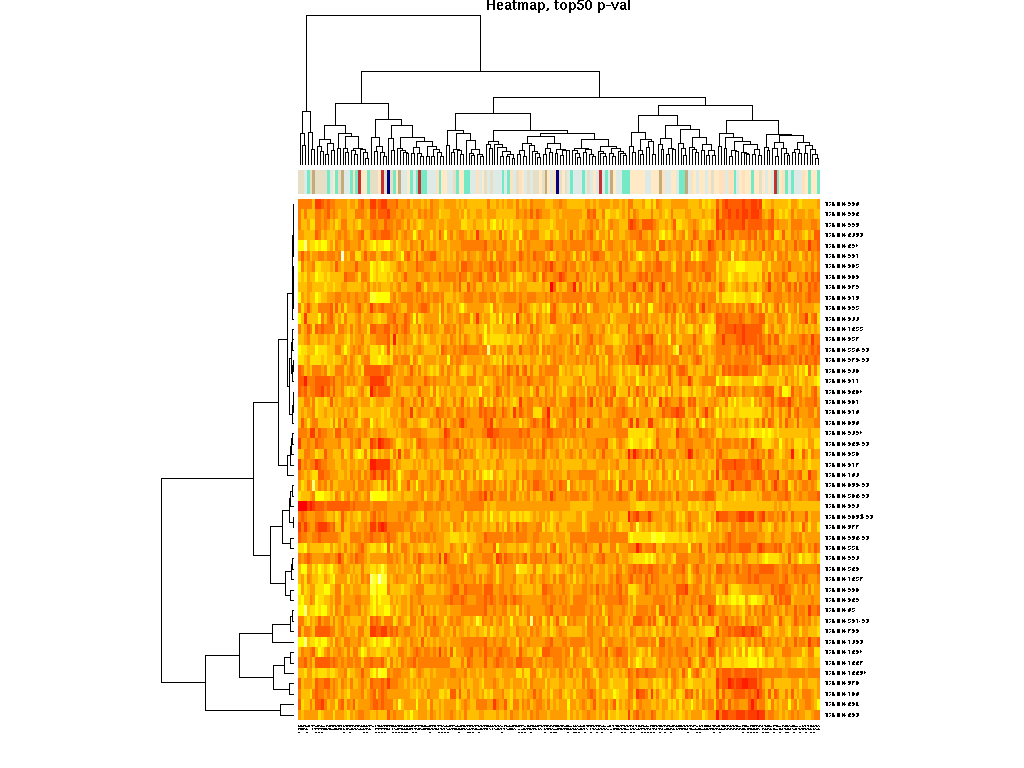

Supplement: Additional file 3 — Sample pipeline outputs in HTML format (compressed file). [file 1471-2164-13-620-S3.ZIP › Bunt182-anova1w2g/GRAPH_Jul26_224608.png]

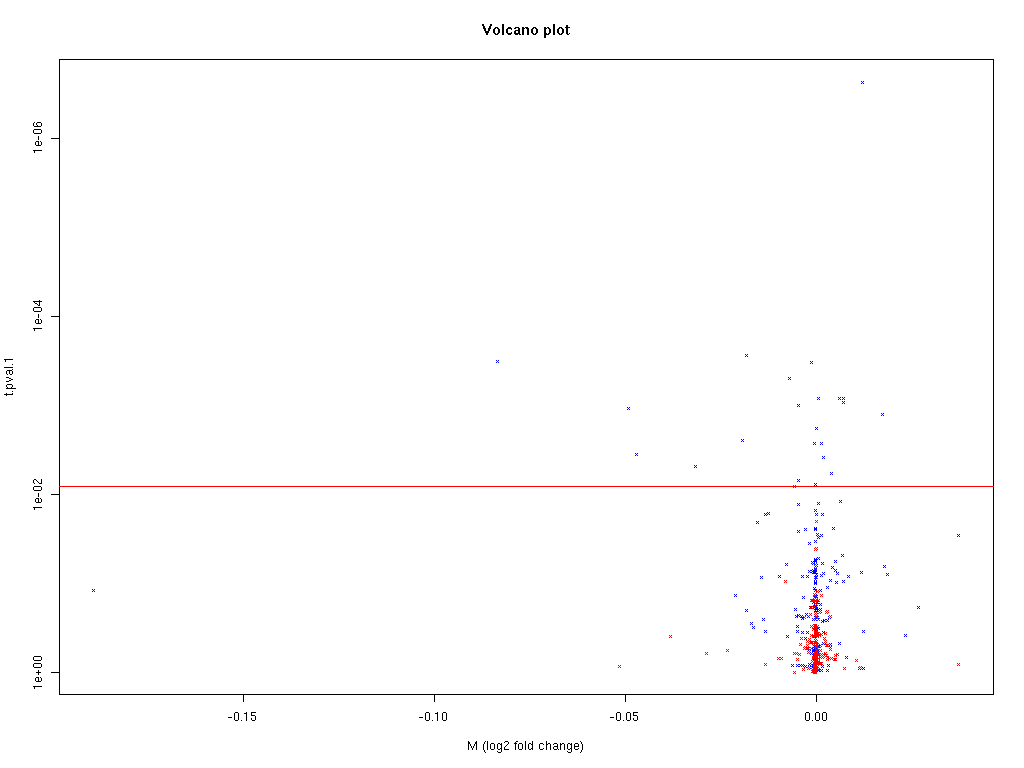

Supplement: Additional file 3 — Sample pipeline outputs in HTML format (compressed file). [file 1471-2164-13-620-S3.ZIP › Bunt182-anova1w2g/GRAPH_Jul26_224610.png]

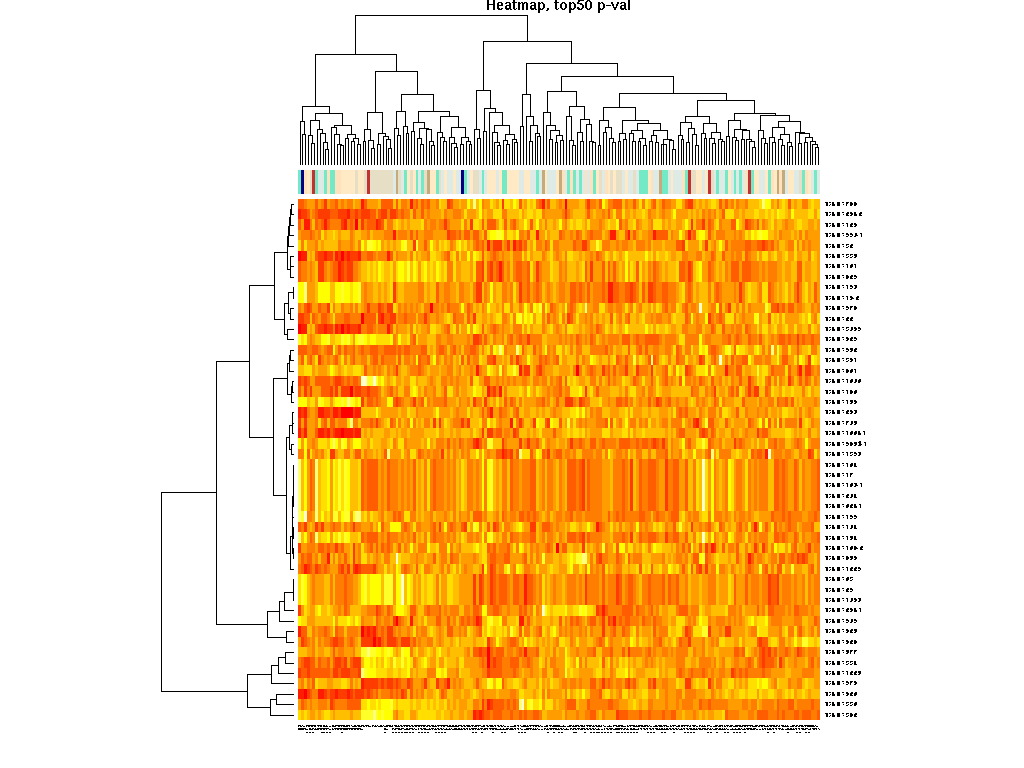

Supplement: Additional file 3 — Sample pipeline outputs in HTML format (compressed file). [file 1471-2164-13-620-S3.ZIP › Bunt182-anova1w2g/GRAPH_Jul26_224626.png]

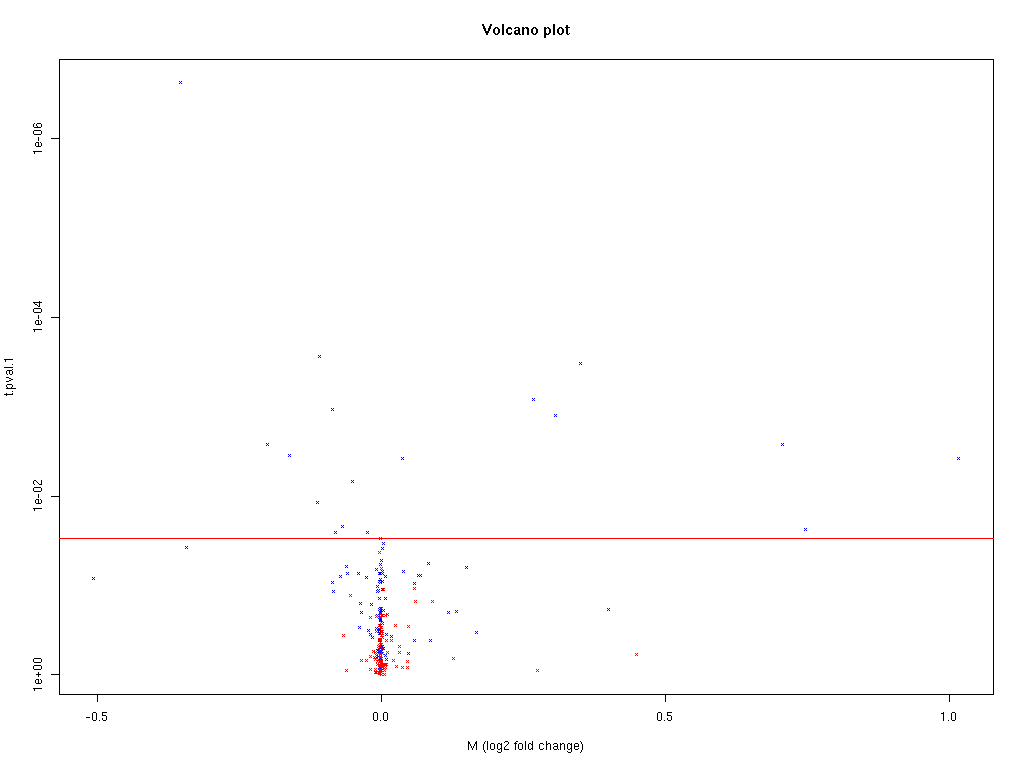

Supplement: Additional file 3 — Sample pipeline outputs in HTML format (compressed file). [file 1471-2164-13-620-S3.ZIP › Bunt182-anova1w2g/GRAPH_Jul26_224629.png]

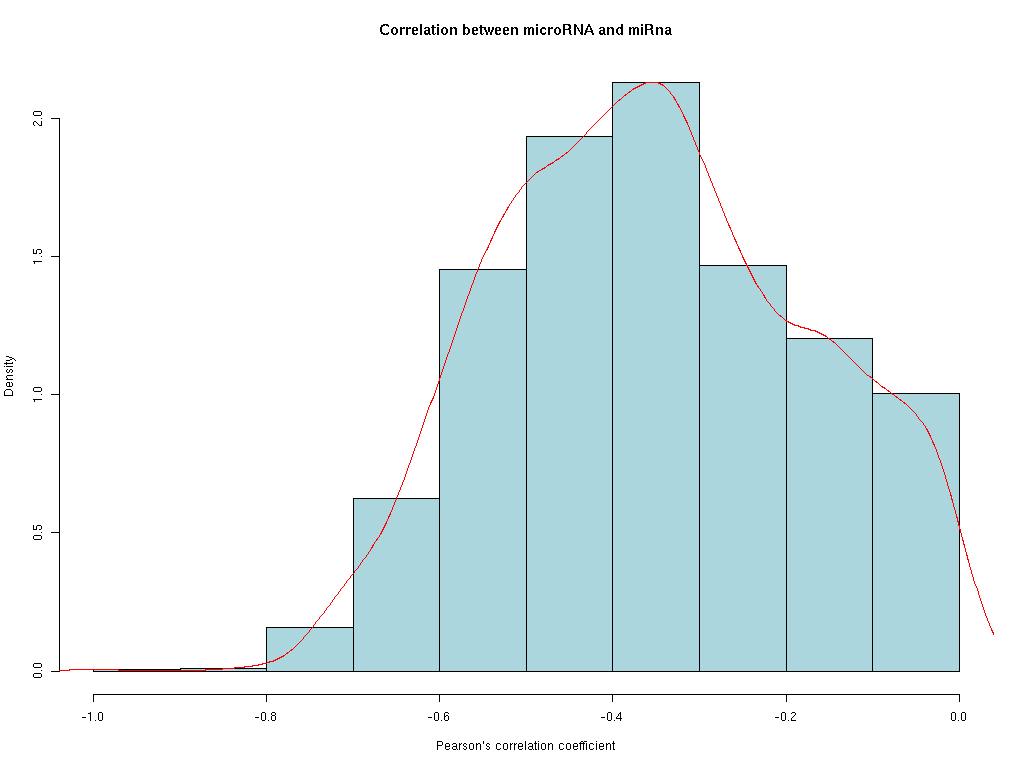

Supplement: Additional file 3 — Sample pipeline outputs in HTML format (compressed file). [file 1471-2164-13-620-S3.ZIP › Bunt182-anova1w2g/GRAPH_Jul26_224633.png]

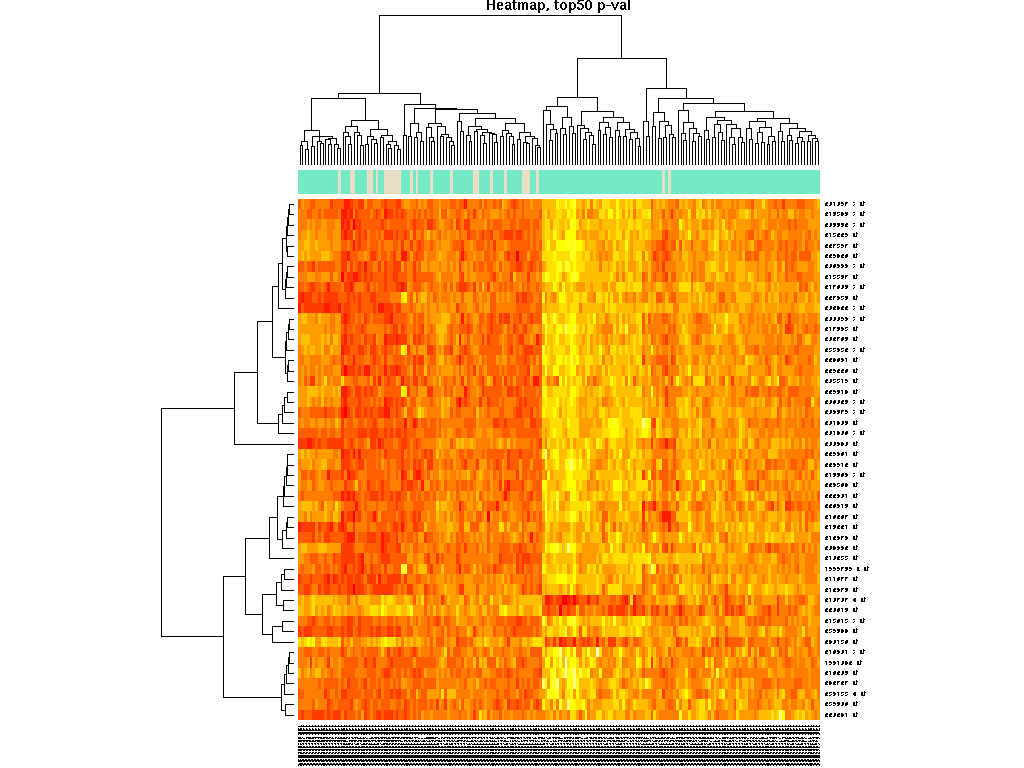

Supplement: Additional file 3 — Sample pipeline outputs in HTML format (compressed file). [file 1471-2164-13-620-S3.ZIP › Bunt182-ttest0.8/GRAPH_Jul23_135711.png]

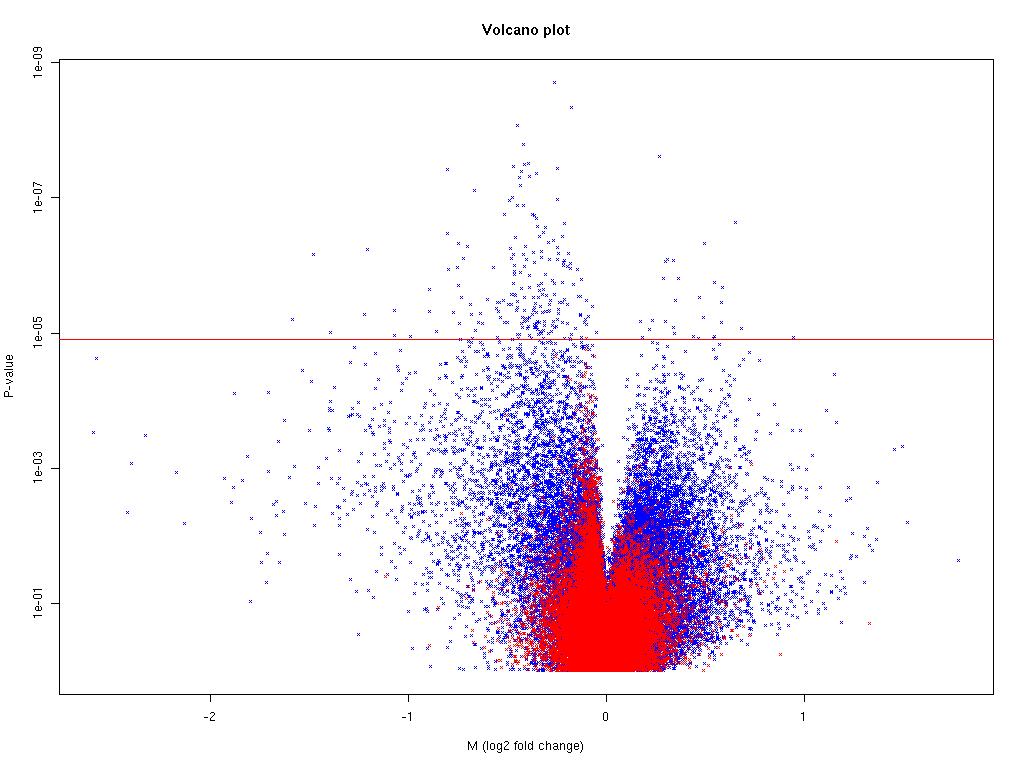

Supplement: Additional file 3 — Sample pipeline outputs in HTML format (compressed file). [file 1471-2164-13-620-S3.ZIP › Bunt182-ttest0.8/GRAPH_Jul23_135713.png]

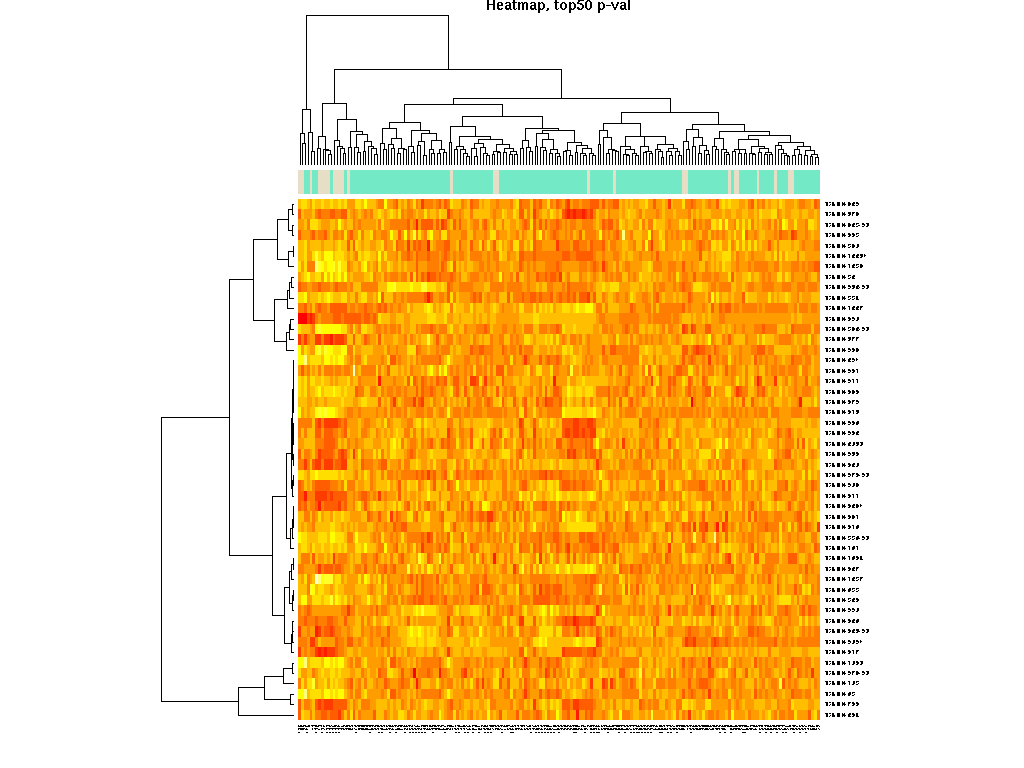

Supplement: Additional file 3 — Sample pipeline outputs in HTML format (compressed file). [file 1471-2164-13-620-S3.ZIP › Bunt182-ttest0.8/GRAPH_Jul23_153222.png]

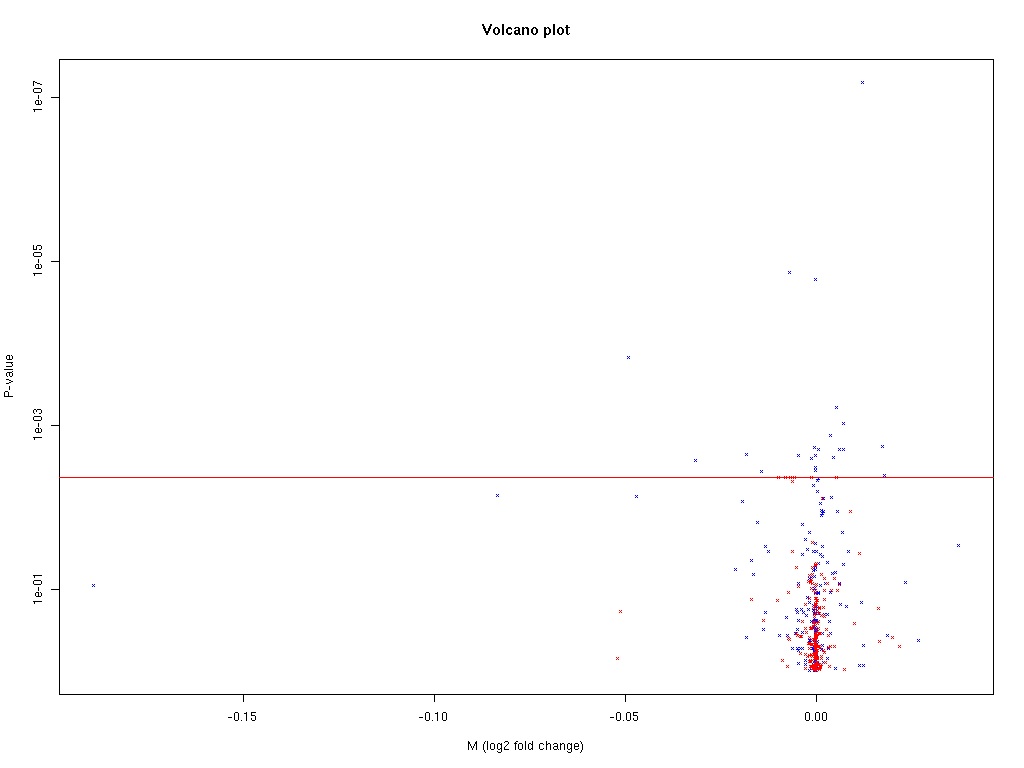

Supplement: Additional file 3 — Sample pipeline outputs in HTML format (compressed file). [file 1471-2164-13-620-S3.ZIP › Bunt182-ttest0.8/GRAPH_Jul23_153224.png]

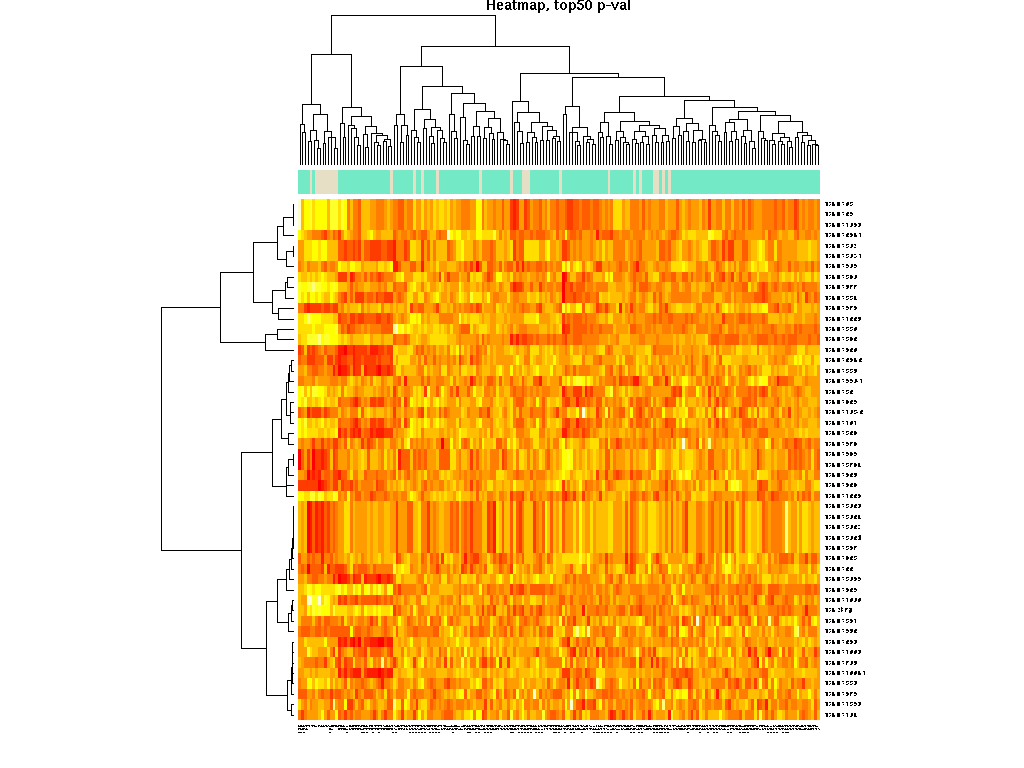

Supplement: Additional file 3 — Sample pipeline outputs in HTML format (compressed file). [file 1471-2164-13-620-S3.ZIP › Bunt182-ttest0.8/GRAPH_Jul23_153233.png]

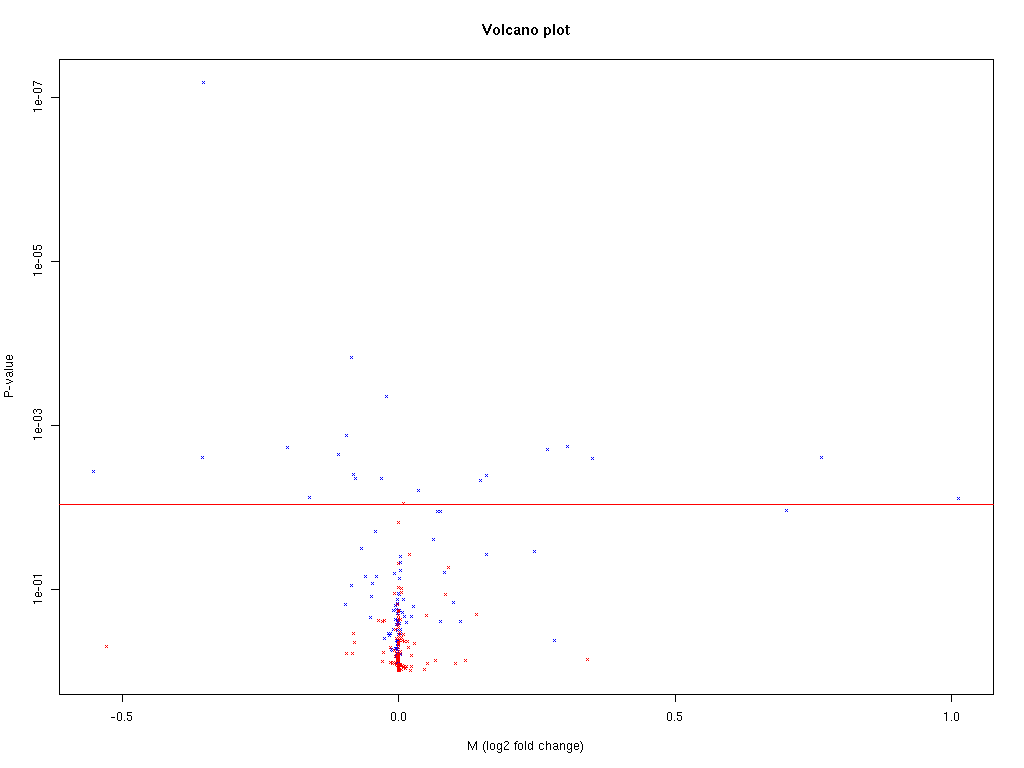

Supplement: Additional file 3 — Sample pipeline outputs in HTML format (compressed file). [file 1471-2164-13-620-S3.ZIP › Bunt182-ttest0.8/GRAPH_Jul23_153235.png]

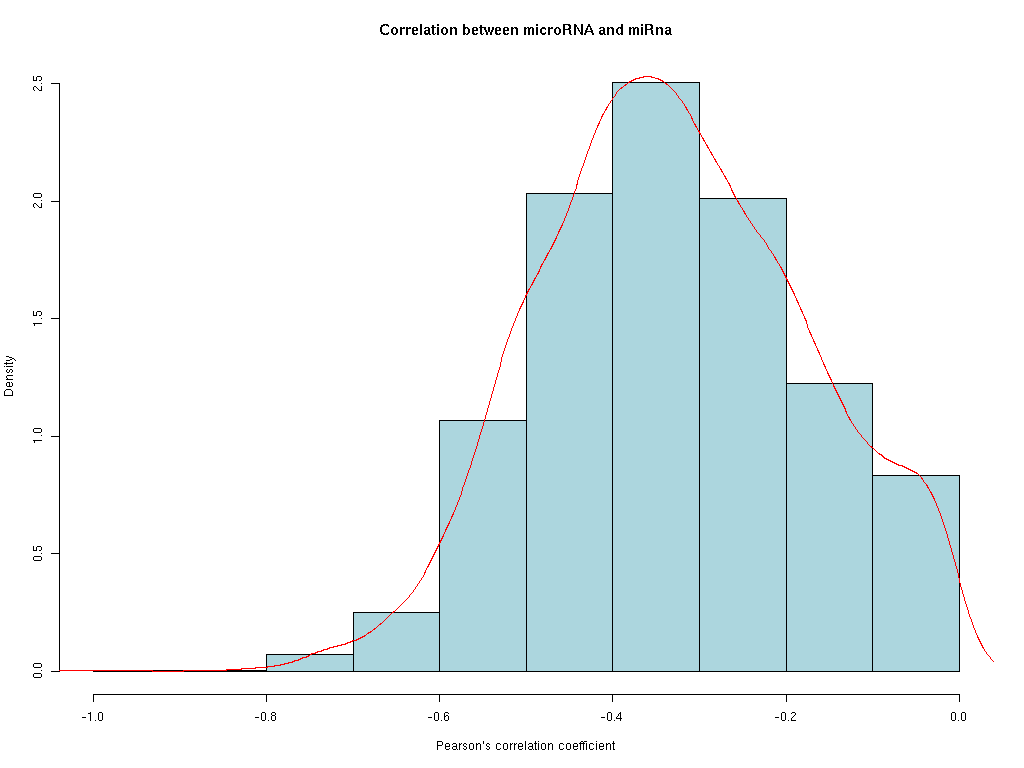

Supplement: Additional file 3 — Sample pipeline outputs in HTML format (compressed file). [file 1471-2164-13-620-S3.ZIP › Bunt182-ttest0.8/GRAPH_Jul23_153239.png]

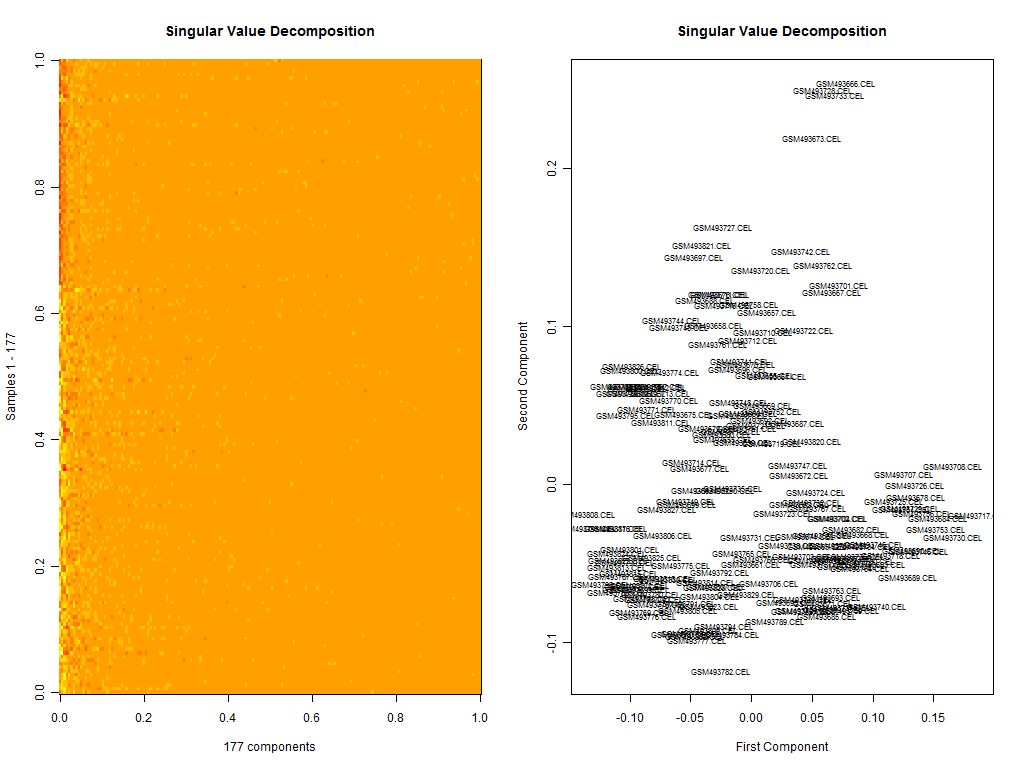

Supplement: Additional file 3 — Sample pipeline outputs in HTML format (compressed file). [file 1471-2164-13-620-S3.ZIP › Burn_dead-alive-control/GRAPH_Sep09_025044.png]

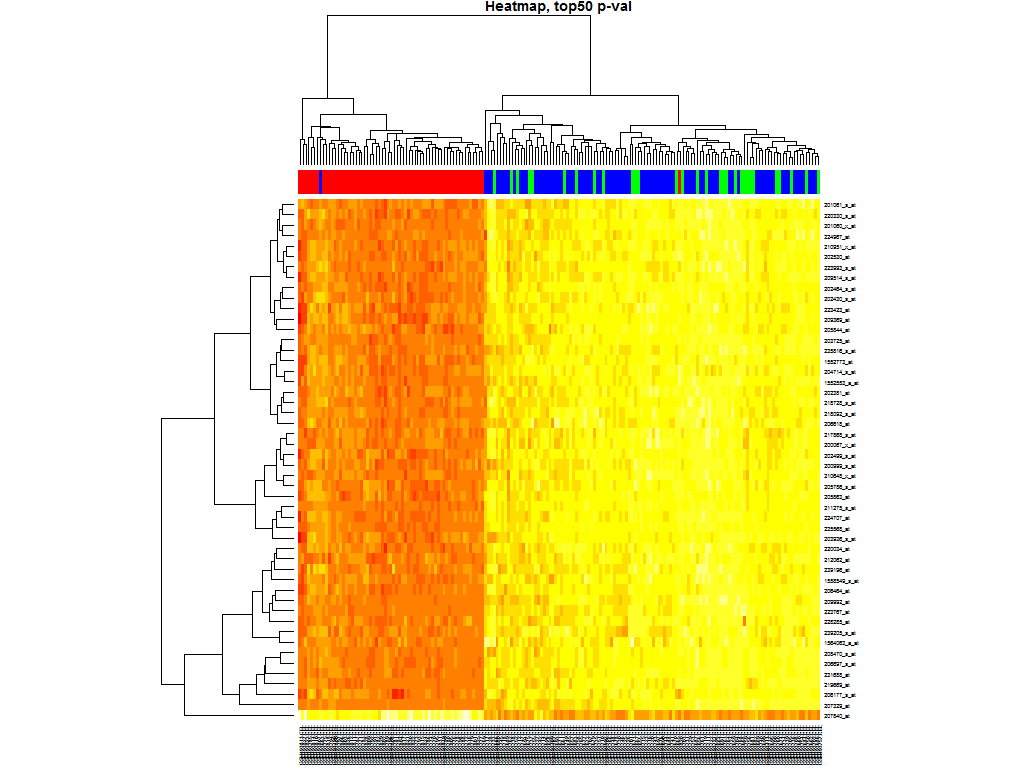

Supplement: Additional file 3 — Sample pipeline outputs in HTML format (compressed file). [file 1471-2164-13-620-S3.ZIP › Burn_dead-alive-control/GRAPH_Sep09_025046.png]

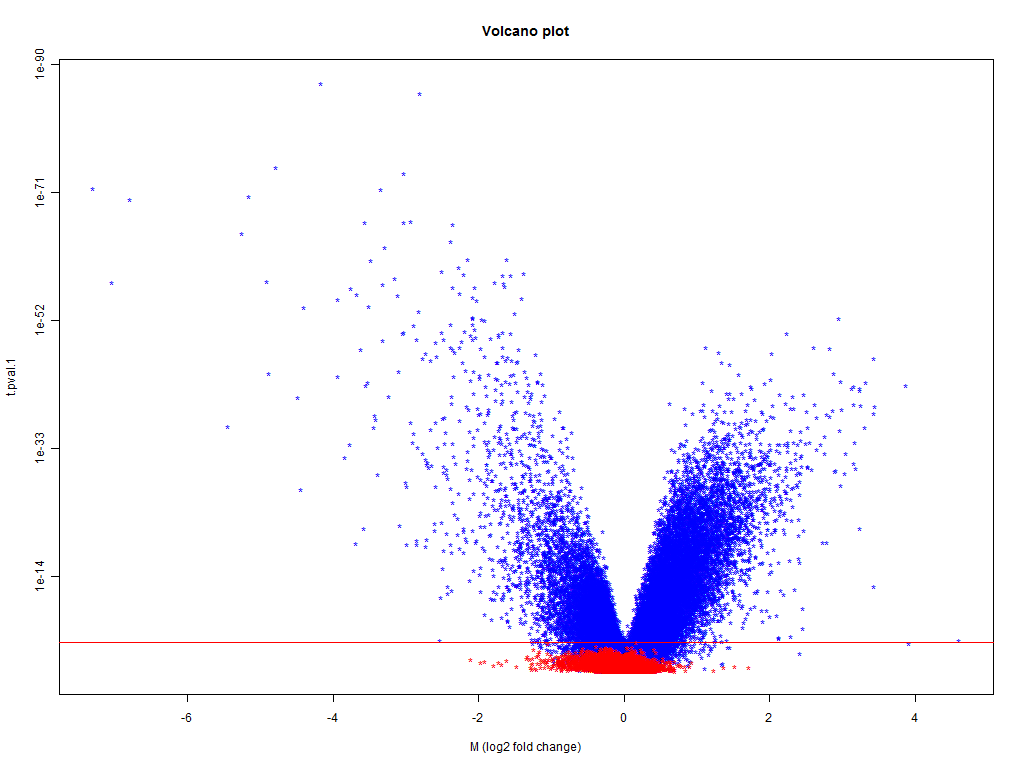

Supplement: Additional file 3 — Sample pipeline outputs in HTML format (compressed file). [file 1471-2164-13-620-S3.ZIP › Burn_dead-alive-control/GRAPH_Sep09_025048.png]

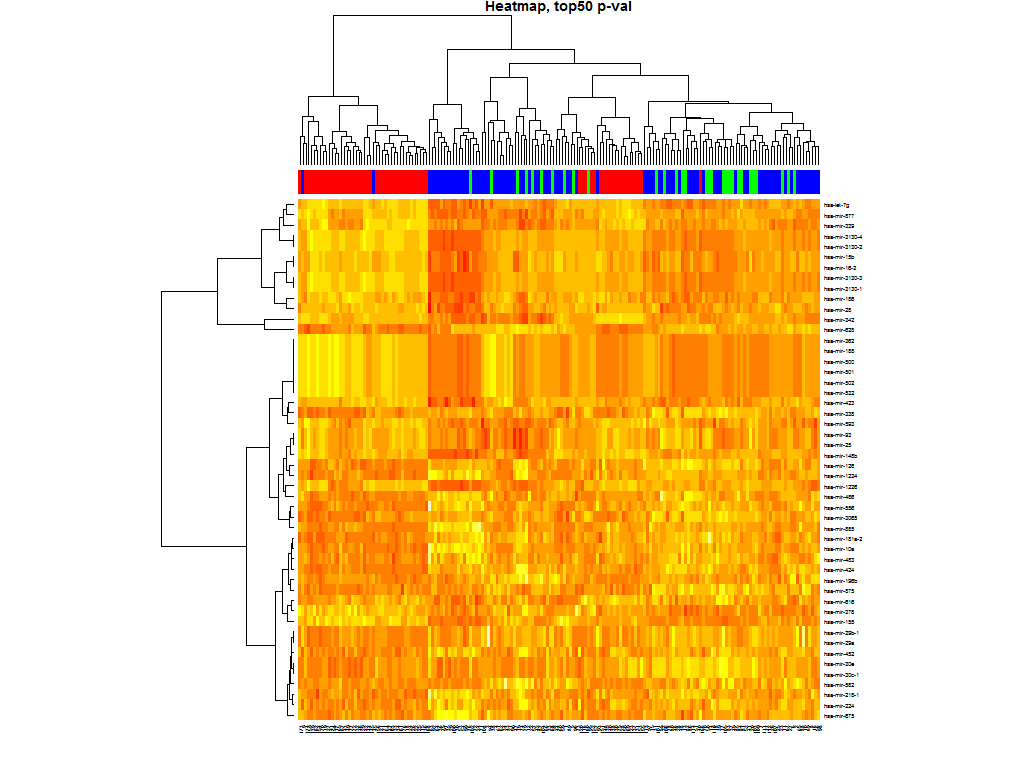

Supplement: Additional file 3 — Sample pipeline outputs in HTML format (compressed file). [file 1471-2164-13-620-S3.ZIP › Burn_dead-alive-control/GRAPH_Sep09_031710.png]

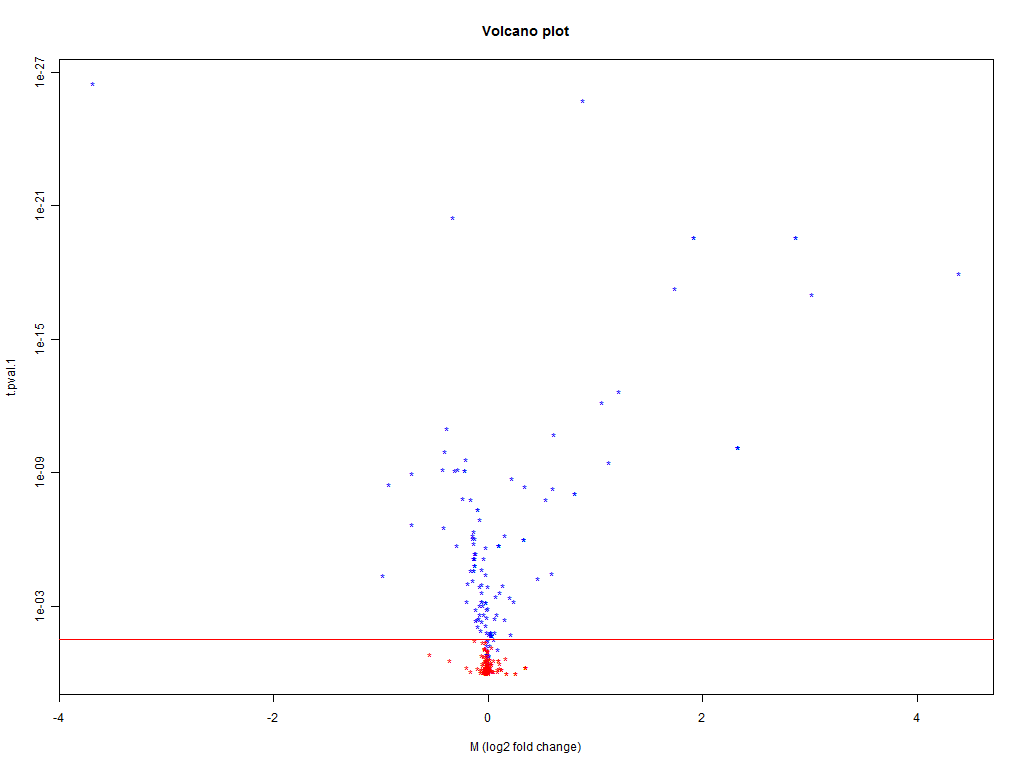

Supplement: Additional file 3 — Sample pipeline outputs in HTML format (compressed file). [file 1471-2164-13-620-S3.ZIP › Burn_dead-alive-control/GRAPH_Sep09_031711.png]

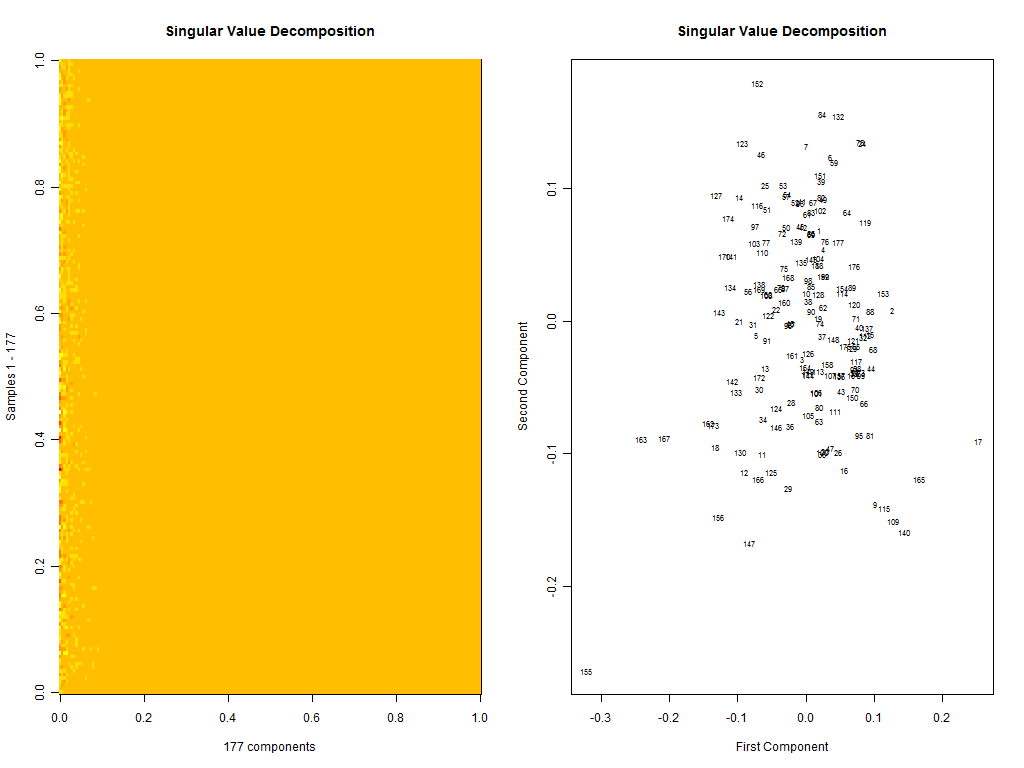

Supplement: Additional file 3 — Sample pipeline outputs in HTML format (compressed file). [file 1471-2164-13-620-S3.ZIP › Burn_dead-alive-control/GRAPH_Sep09_031719.png]

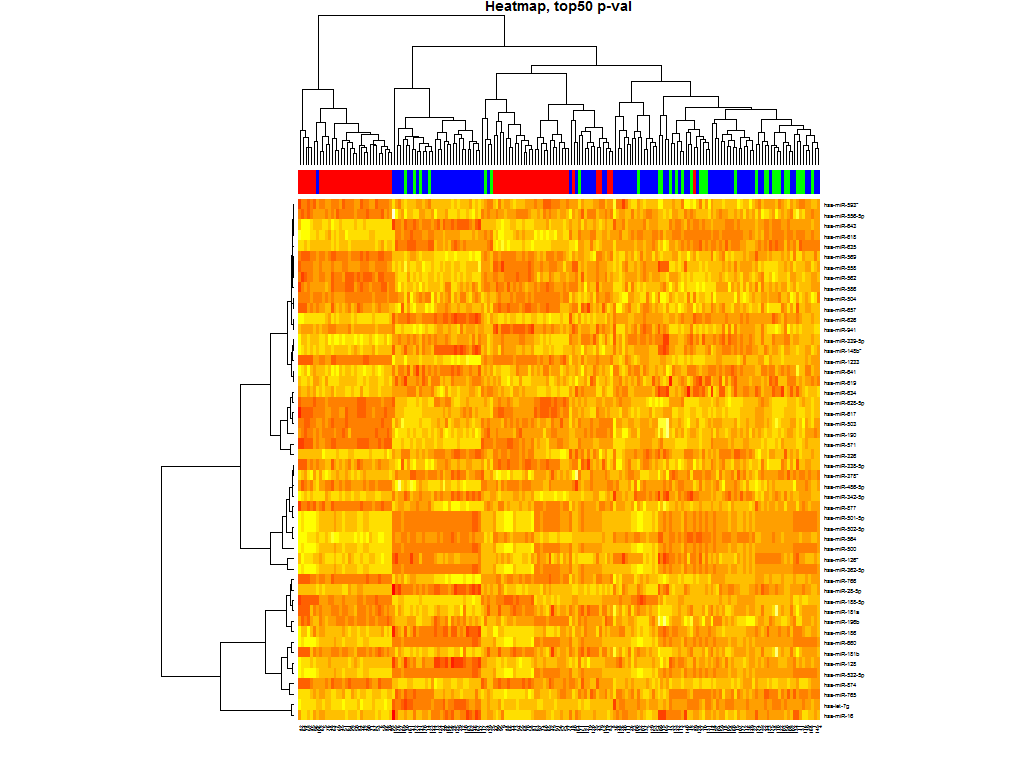

Supplement: Additional file 3 — Sample pipeline outputs in HTML format (compressed file). [file 1471-2164-13-620-S3.ZIP › Burn_dead-alive-control/GRAPH_Sep09_031721.png]

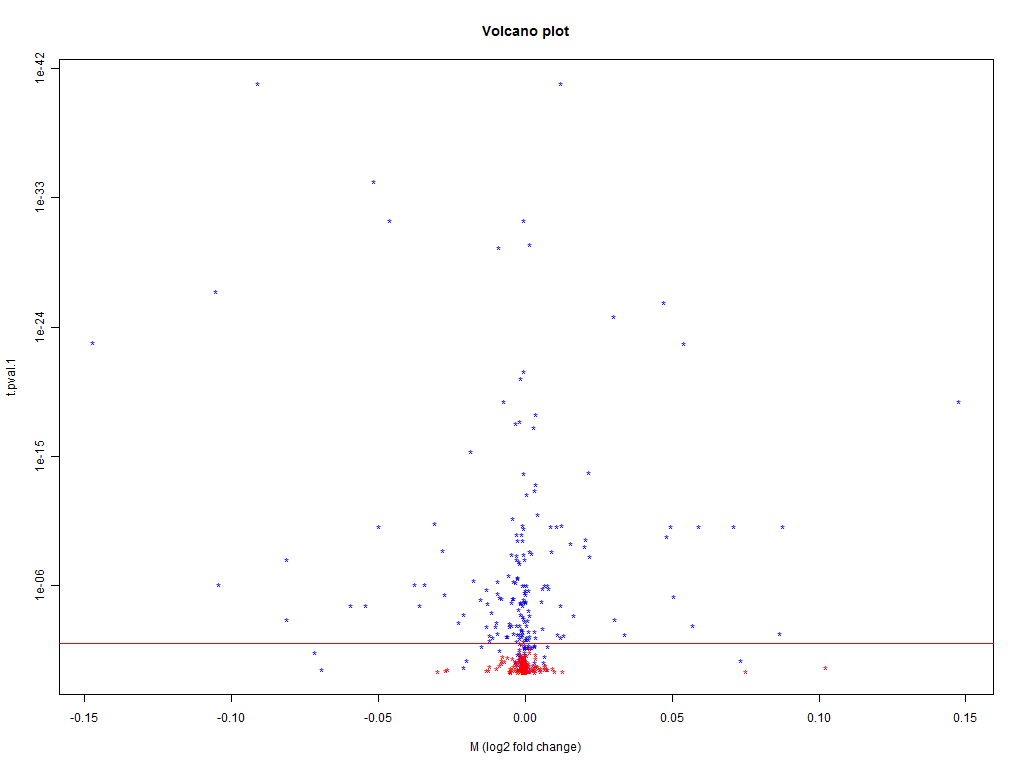

Supplement: Additional file 3 — Sample pipeline outputs in HTML format (compressed file). [file 1471-2164-13-620-S3.ZIP › Burn_dead-alive-control/GRAPH_Sep09_031722.png]

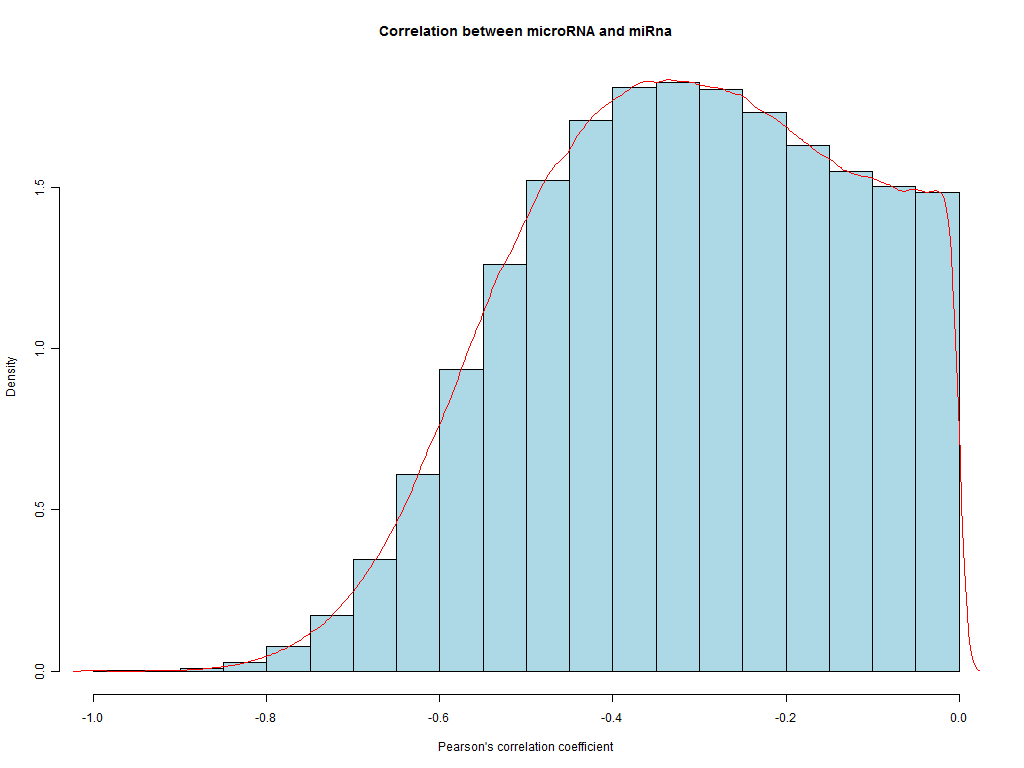

Supplement: Additional file 3 — Sample pipeline outputs in HTML format (compressed file). [file 1471-2164-13-620-S3.ZIP › Burn_dead-alive-control/GRAPH_Sep09_031742.png]

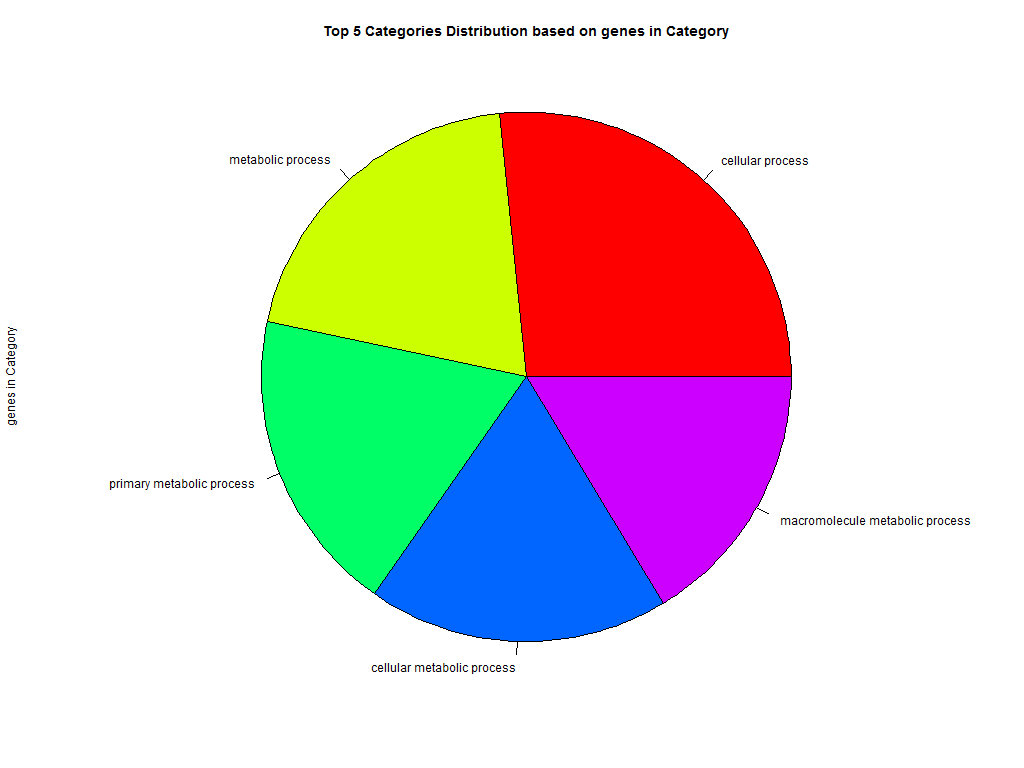

Supplement: Additional file 3 — Sample pipeline outputs in HTML format (compressed file). [file 1471-2164-13-620-S3.ZIP › Burn_dead-alive-control/GRAPH_Sep09_031947.png]

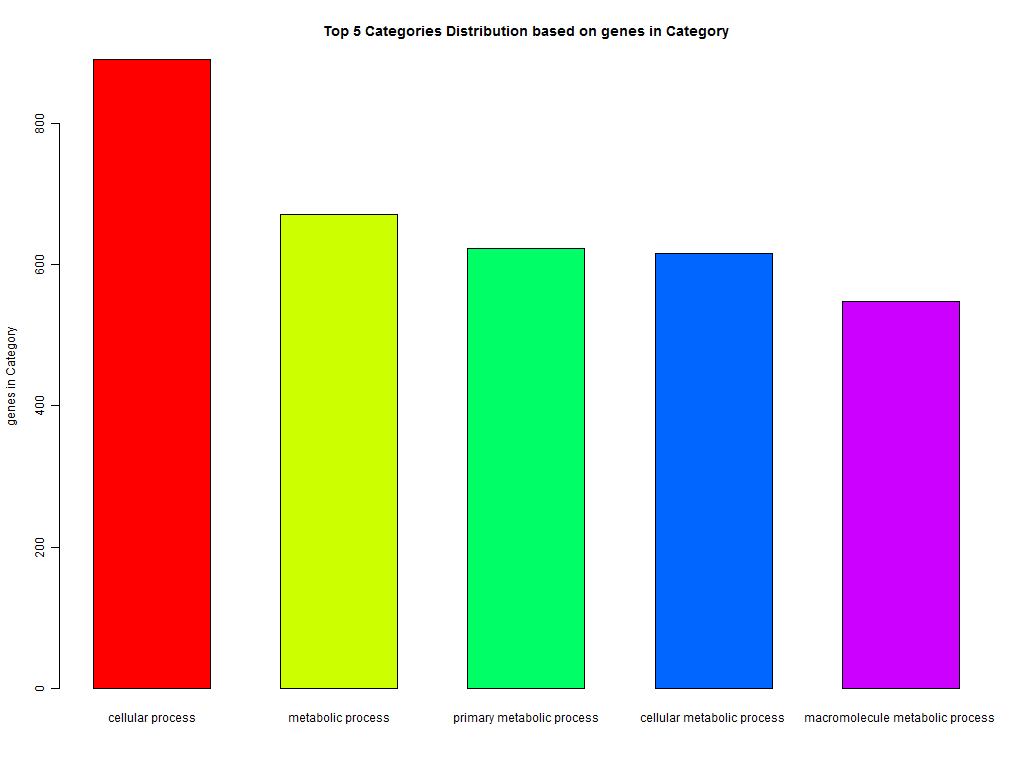

Supplement: Additional file 3 — Sample pipeline outputs in HTML format (compressed file). [file 1471-2164-13-620-S3.ZIP › Burn_dead-alive-control/GRAPH_Sep09_031953.png]

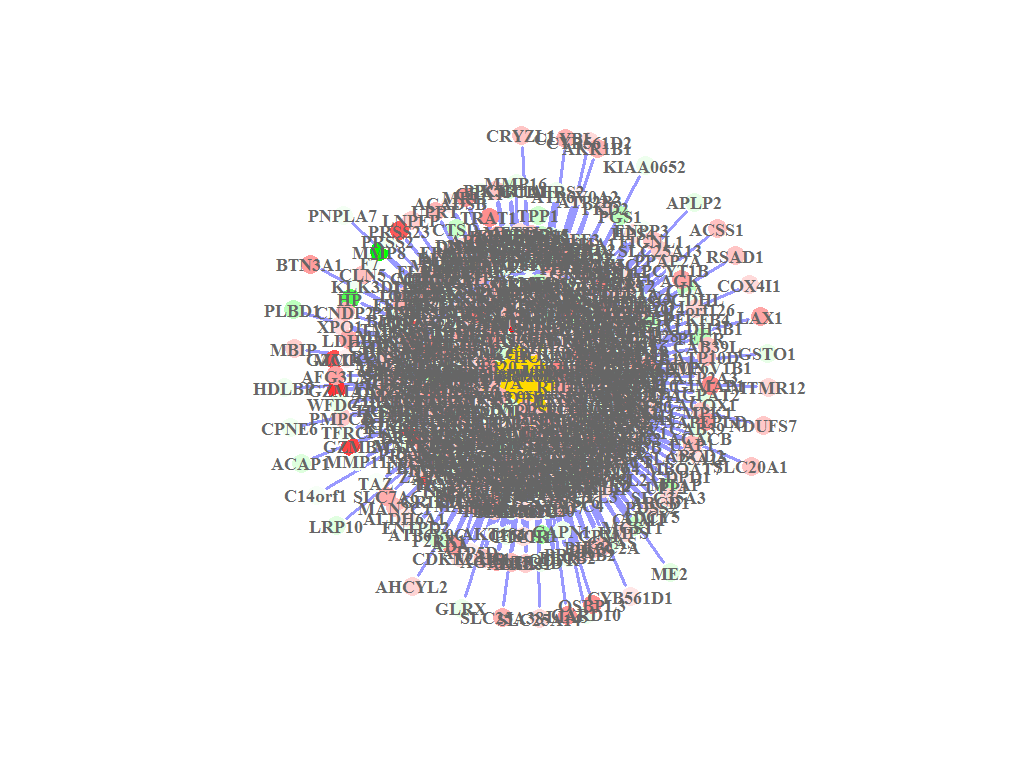

Supplement: Additional file 3 — Sample pipeline outputs in HTML format (compressed file). [file 1471-2164-13-620-S3.ZIP › Burn_dead-alive-control/GRAPH_Sep09_032031.png]

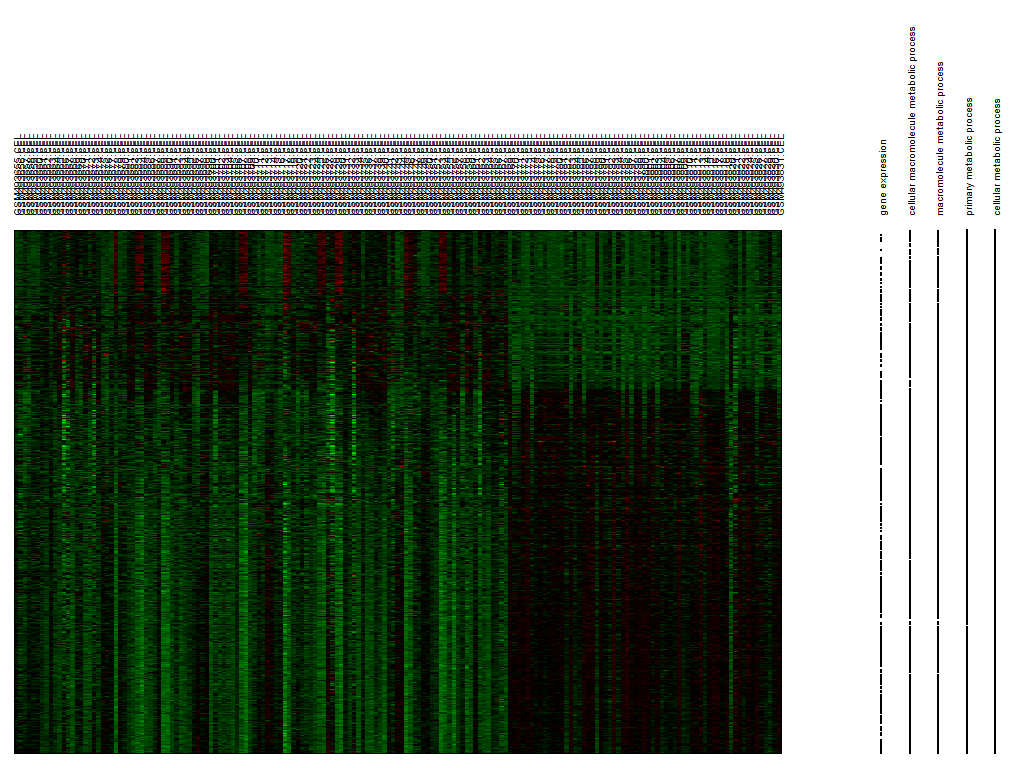

Supplement: Additional file 3 — Sample pipeline outputs in HTML format (compressed file). [file 1471-2164-13-620-S3.ZIP › Burn_dead-alive-control/GRAPH_Sep09_032046.png]

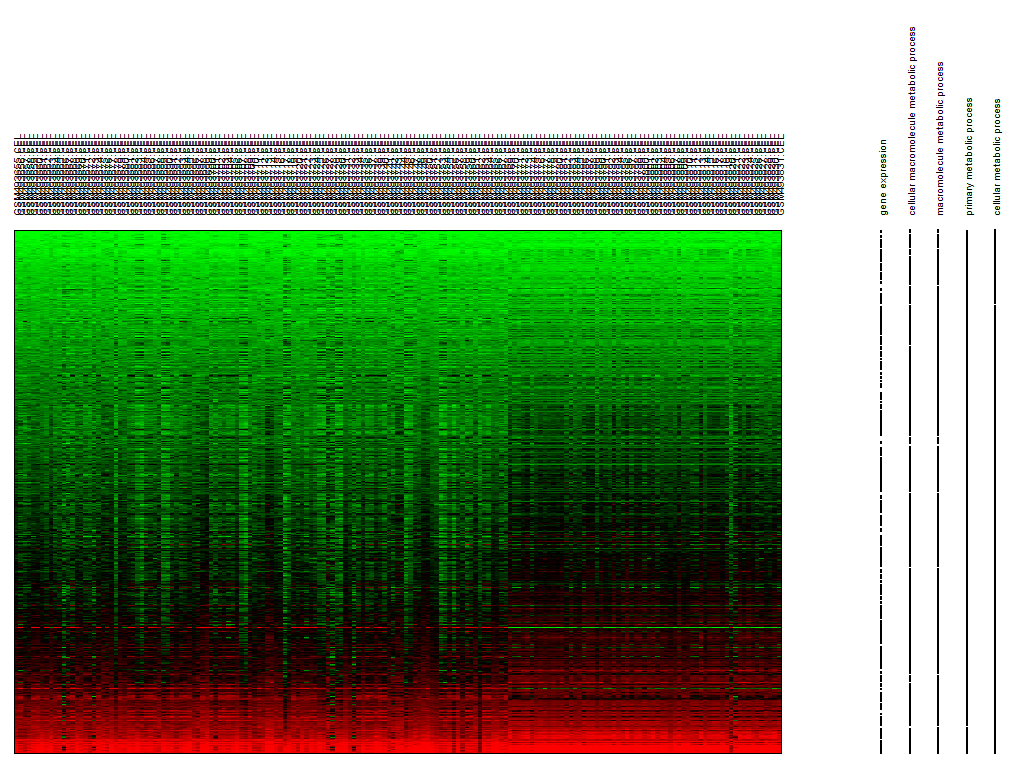

Supplement: Additional file 3 — Sample pipeline outputs in HTML format (compressed file). [file 1471-2164-13-620-S3.ZIP › Burn_dead-alive-control/GRAPH_Sep09_032100.png]

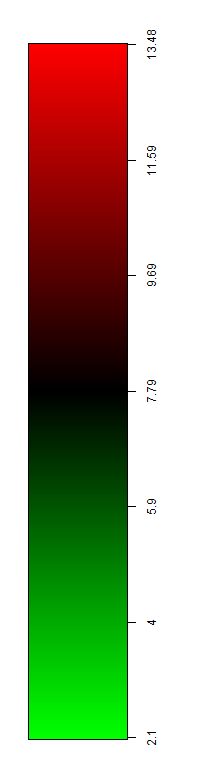

Supplement: Additional file 3 — Sample pipeline outputs in HTML format (compressed file). [file 1471-2164-13-620-S3.ZIP › Burn_dead-alive-control/GRAPH_Sep09_032106.png]

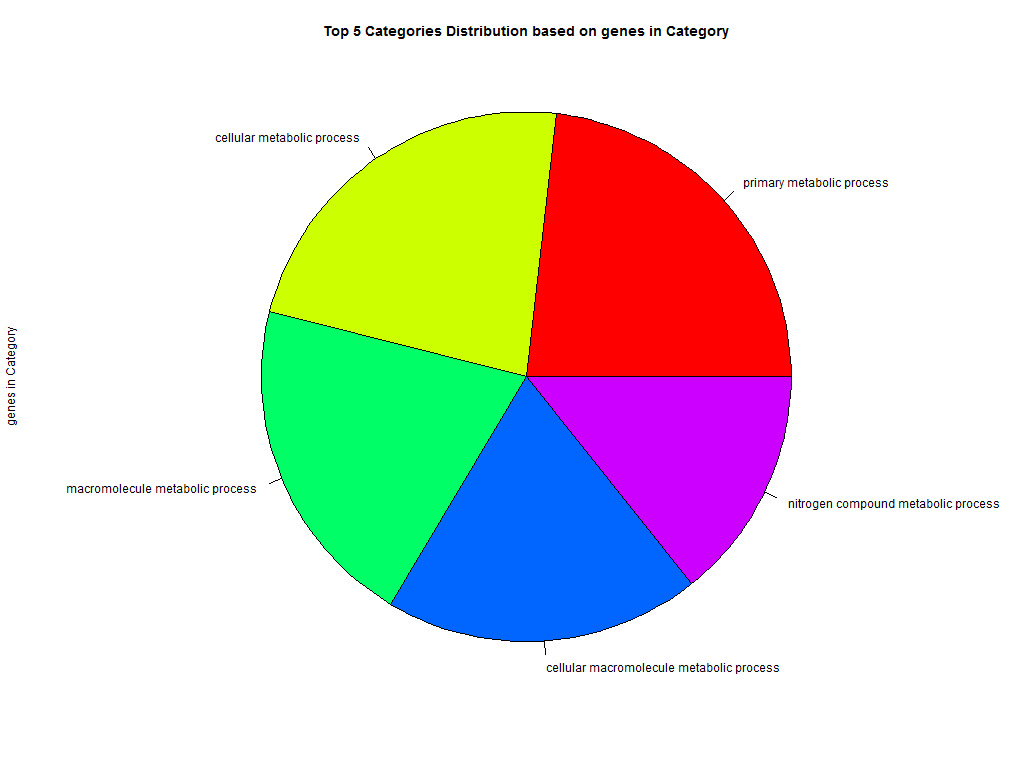

Supplement: Additional file 3 — Sample pipeline outputs in HTML format (compressed file). [file 1471-2164-13-620-S3.ZIP › Burn_dead-alive-control/GRAPH_Sep09_032226.png]

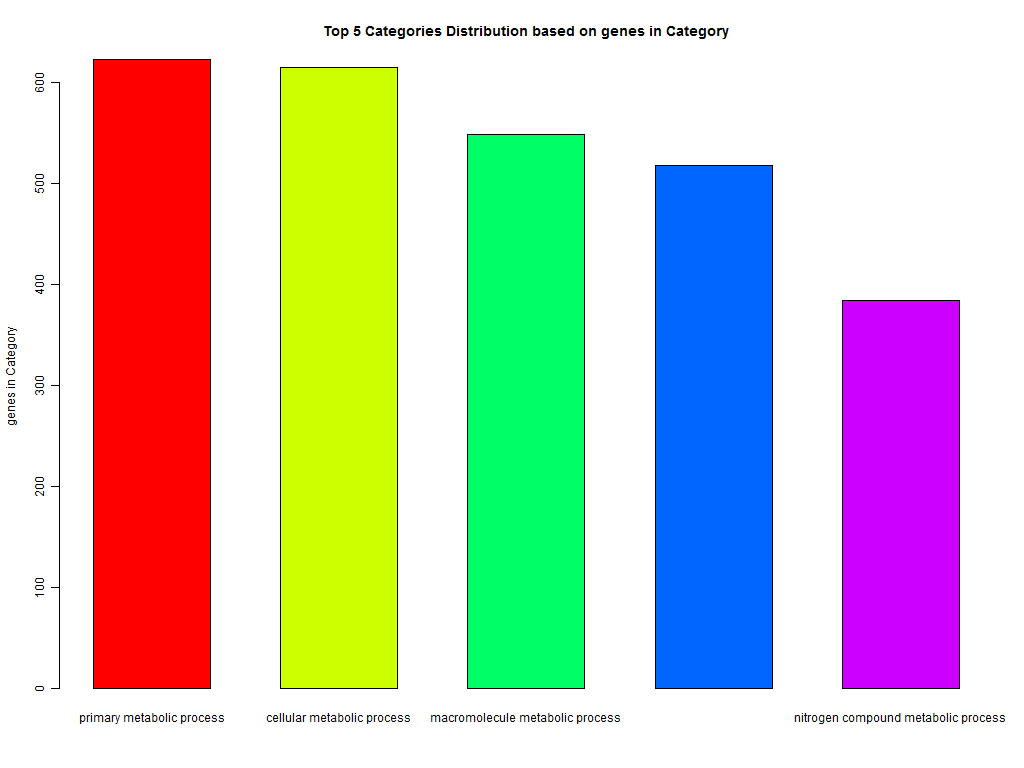

Supplement: Additional file 3 — Sample pipeline outputs in HTML format (compressed file). [file 1471-2164-13-620-S3.ZIP › Burn_dead-alive-control/GRAPH_Sep09_032232.png]

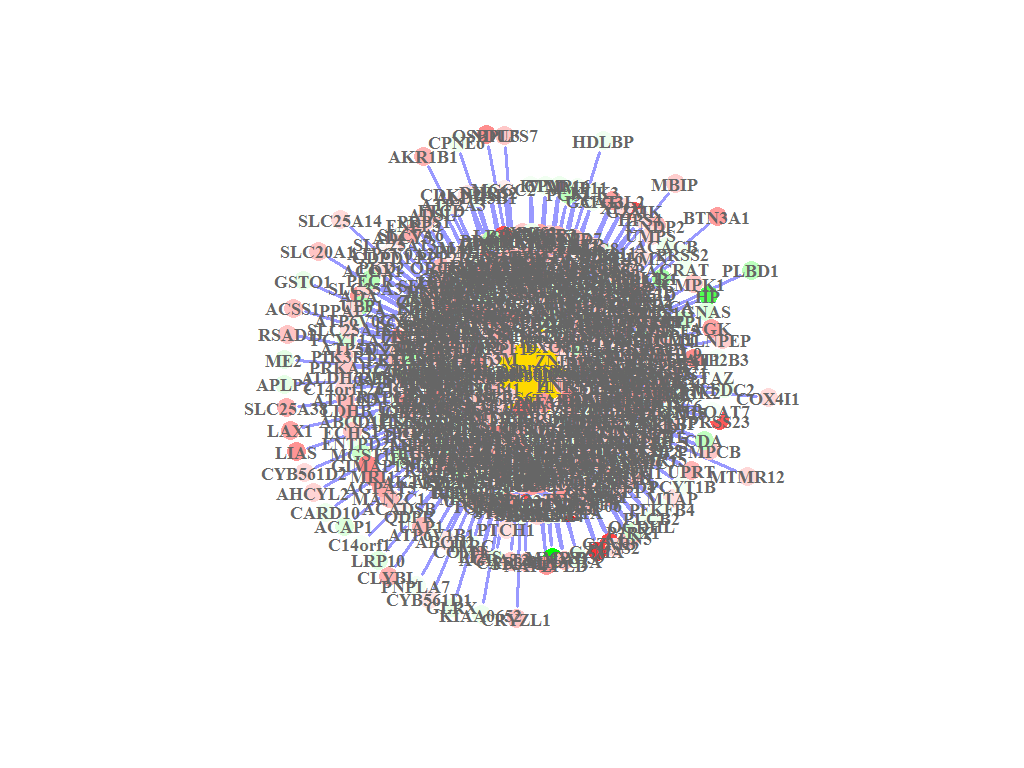

Supplement: Additional file 3 — Sample pipeline outputs in HTML format (compressed file). [file 1471-2164-13-620-S3.ZIP › Burn_dead-alive-control/GRAPH_Sep09_032249.png]

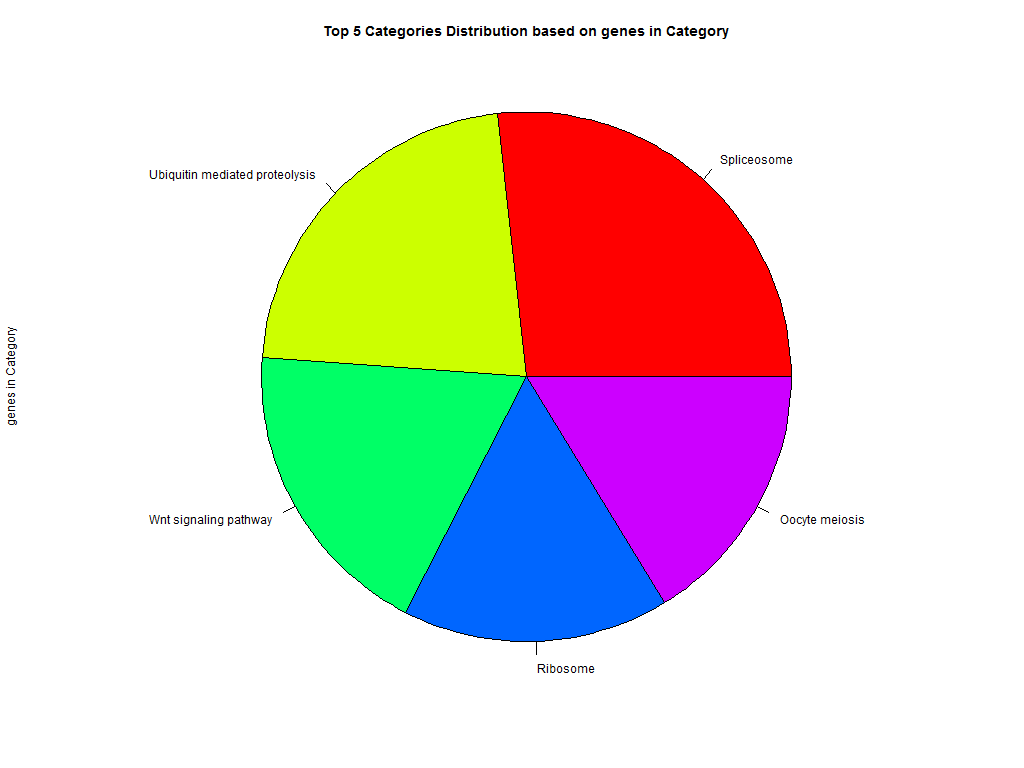

Supplement: Additional file 3 — Sample pipeline outputs in HTML format (compressed file). [file 1471-2164-13-620-S3.ZIP › Burn_dead-alive-control/GRAPH_Sep09_032342.png]

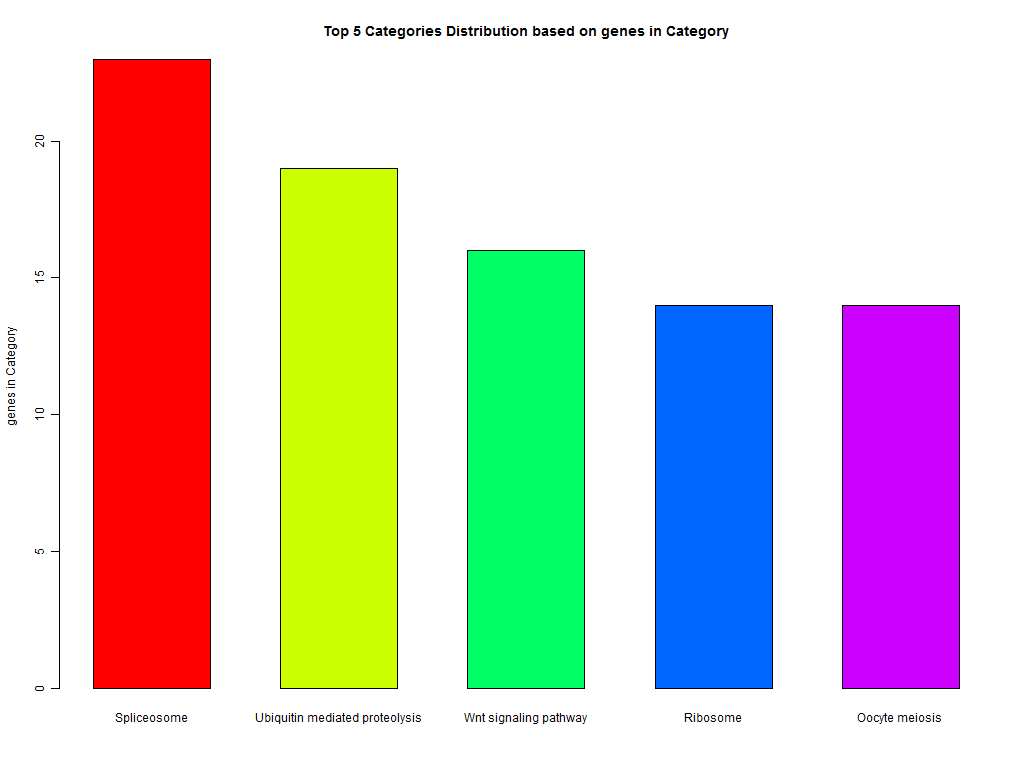

Supplement: Additional file 3 — Sample pipeline outputs in HTML format (compressed file). [file 1471-2164-13-620-S3.ZIP › Burn_dead-alive-control/GRAPH_Sep09_032348.png]

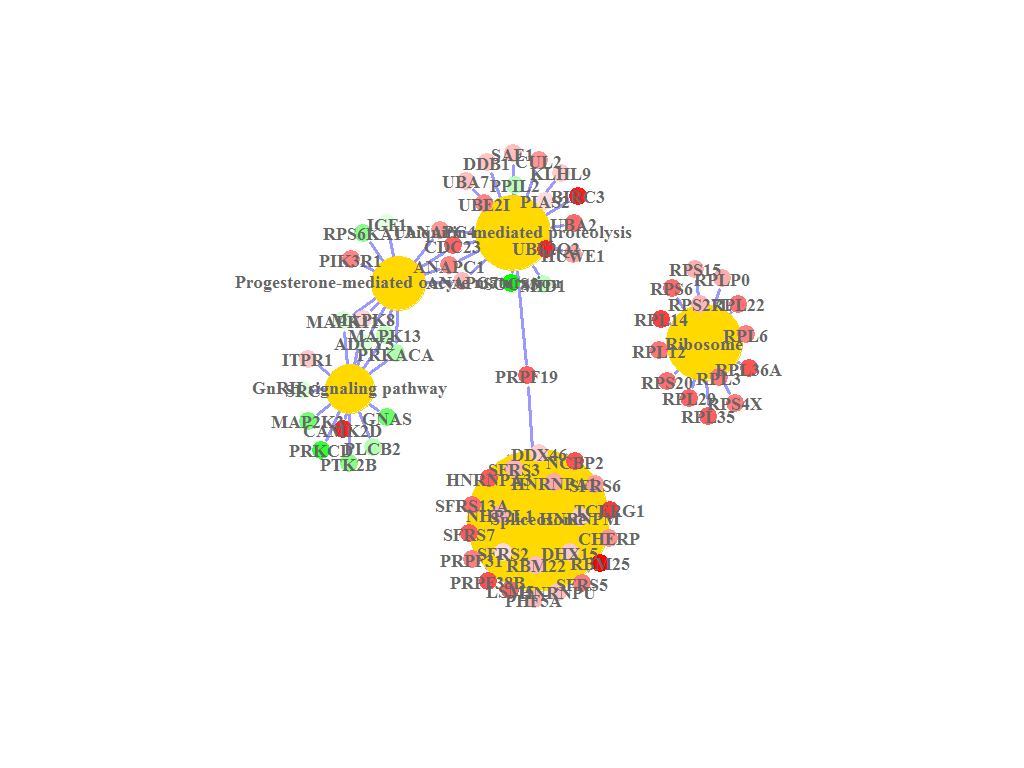

Supplement: Additional file 3 — Sample pipeline outputs in HTML format (compressed file). [file 1471-2164-13-620-S3.ZIP › Burn_dead-alive-control/GRAPH_Sep09_032401.png]

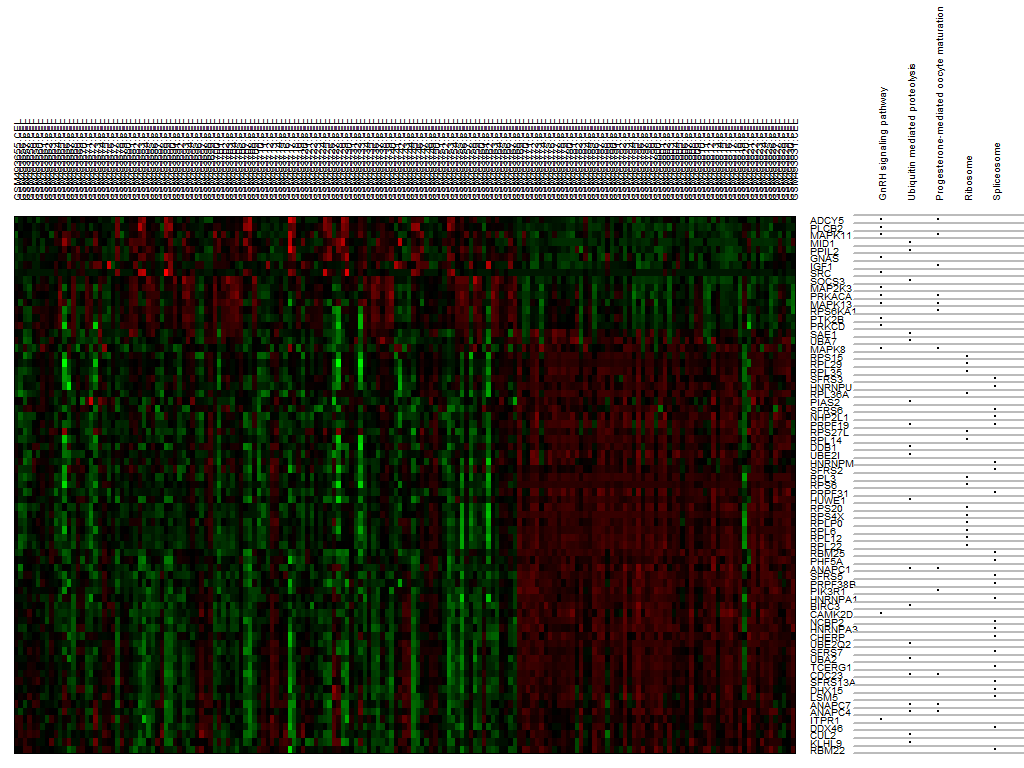

Supplement: Additional file 3 — Sample pipeline outputs in HTML format (compressed file). [file 1471-2164-13-620-S3.ZIP › Burn_dead-alive-control/GRAPH_Sep09_032418.png]

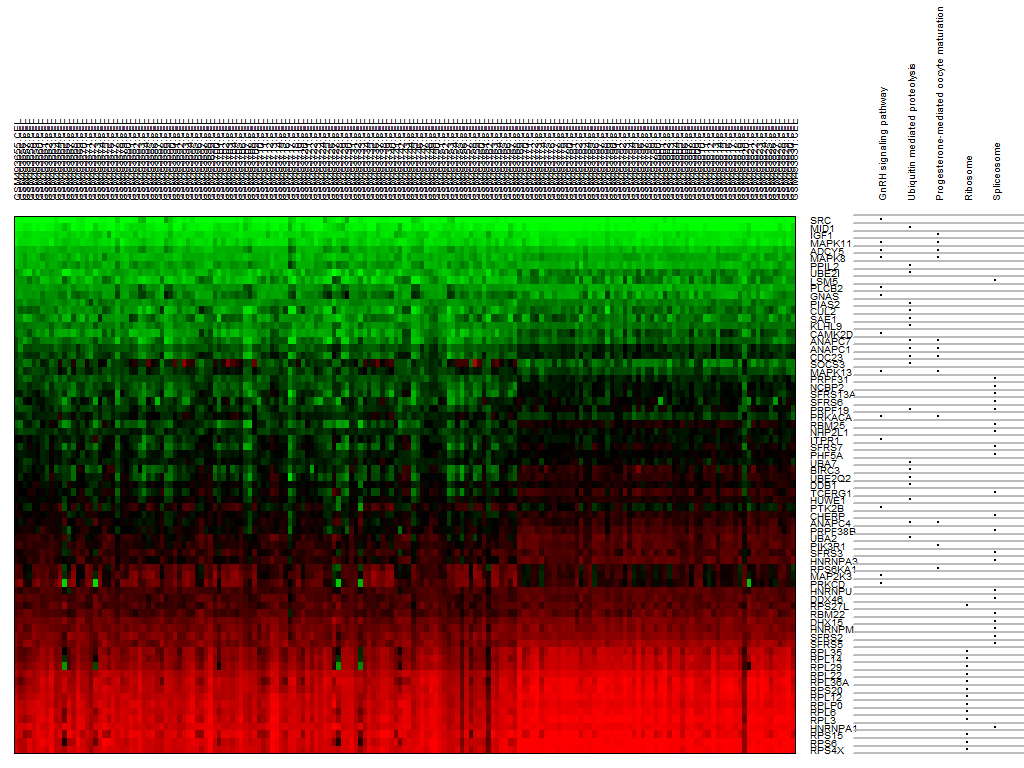

Supplement: Additional file 3 — Sample pipeline outputs in HTML format (compressed file). [file 1471-2164-13-620-S3.ZIP › Burn_dead-alive-control/GRAPH_Sep09_032429.png]

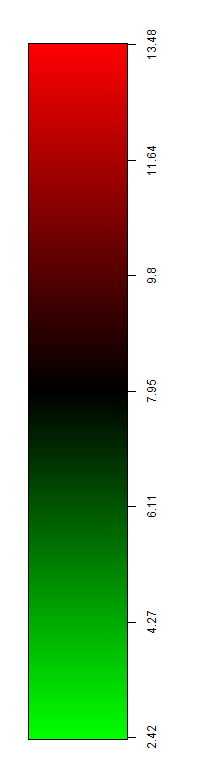

Supplement: Additional file 3 — Sample pipeline outputs in HTML format (compressed file). [file 1471-2164-13-620-S3.ZIP › Burn_dead-alive-control/GRAPH_Sep09_032435.png]

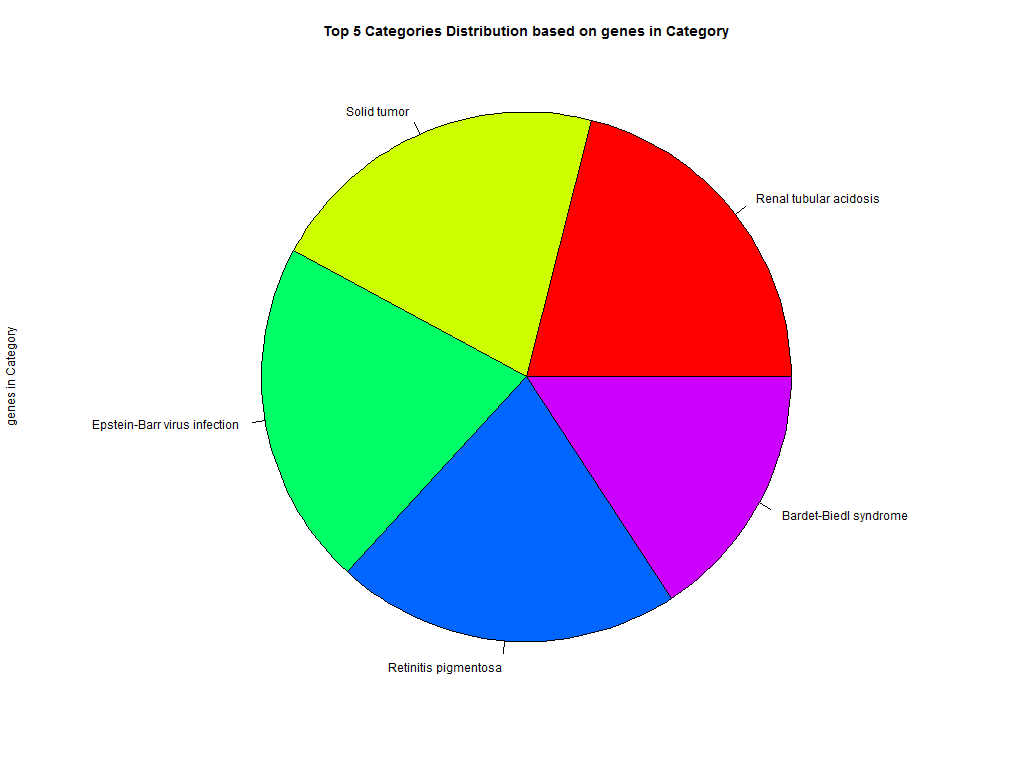

Supplement: Additional file 3 — Sample pipeline outputs in HTML format (compressed file). [file 1471-2164-13-620-S3.ZIP › Burn_dead-alive-control/GRAPH_Sep09_032454.png]

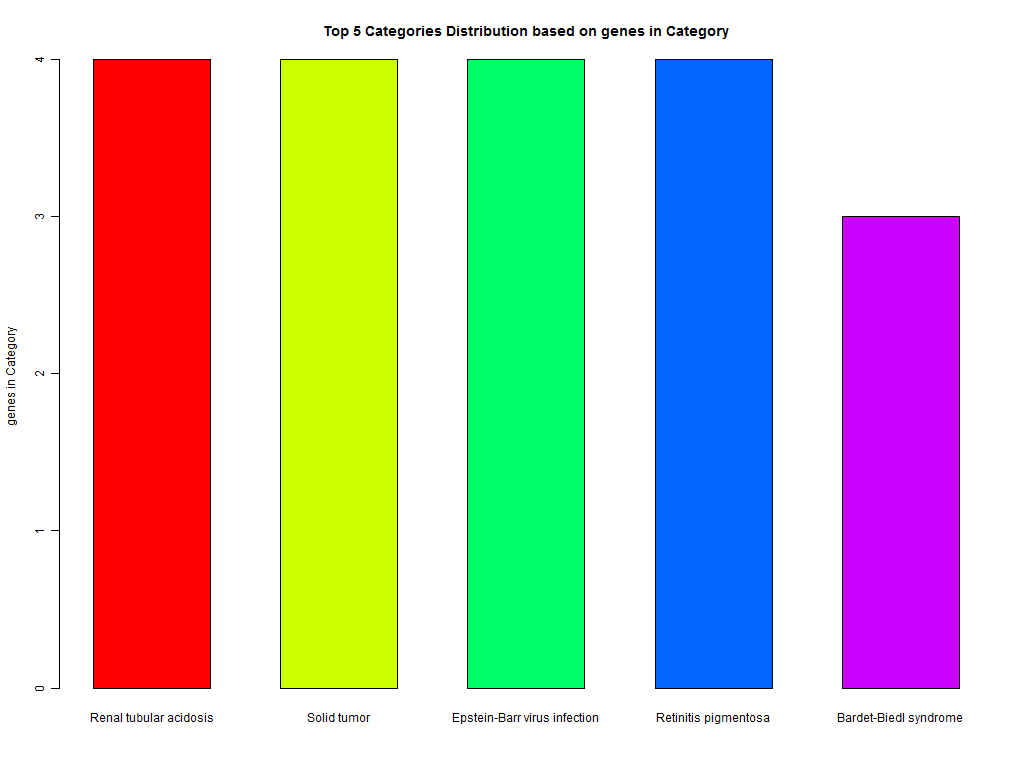

Supplement: Additional file 3 — Sample pipeline outputs in HTML format (compressed file). [file 1471-2164-13-620-S3.ZIP › Burn_dead-alive-control/GRAPH_Sep09_032500.png]

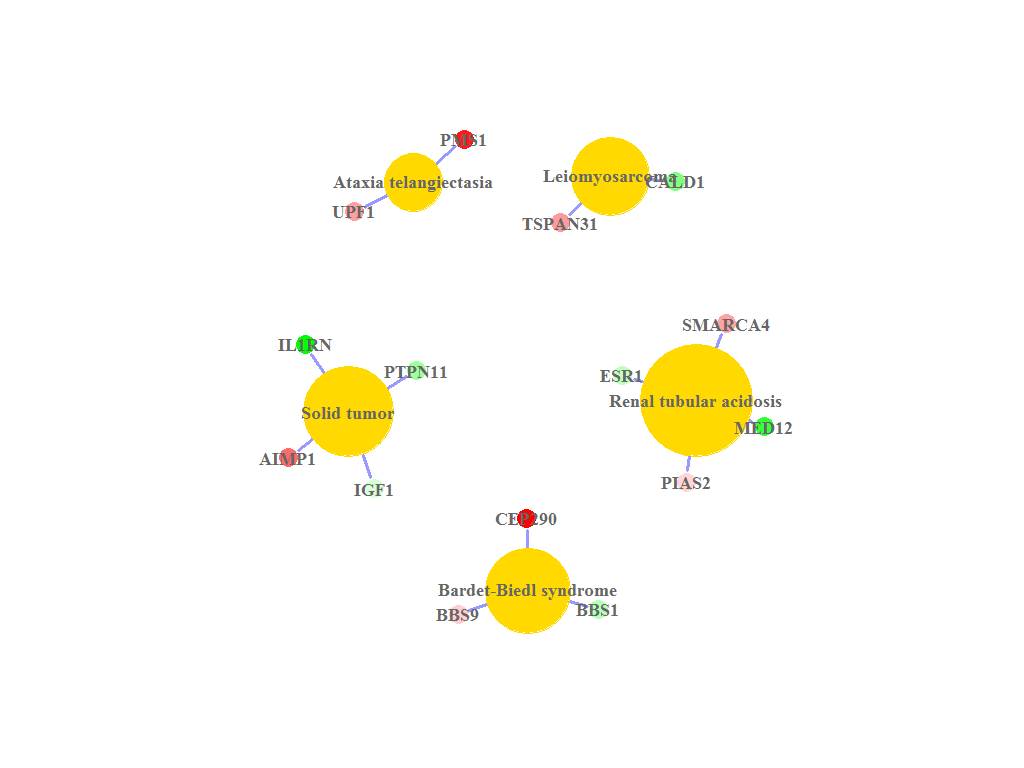

Supplement: Additional file 3 — Sample pipeline outputs in HTML format (compressed file). [file 1471-2164-13-620-S3.ZIP › Burn_dead-alive-control/GRAPH_Sep09_032506.png]

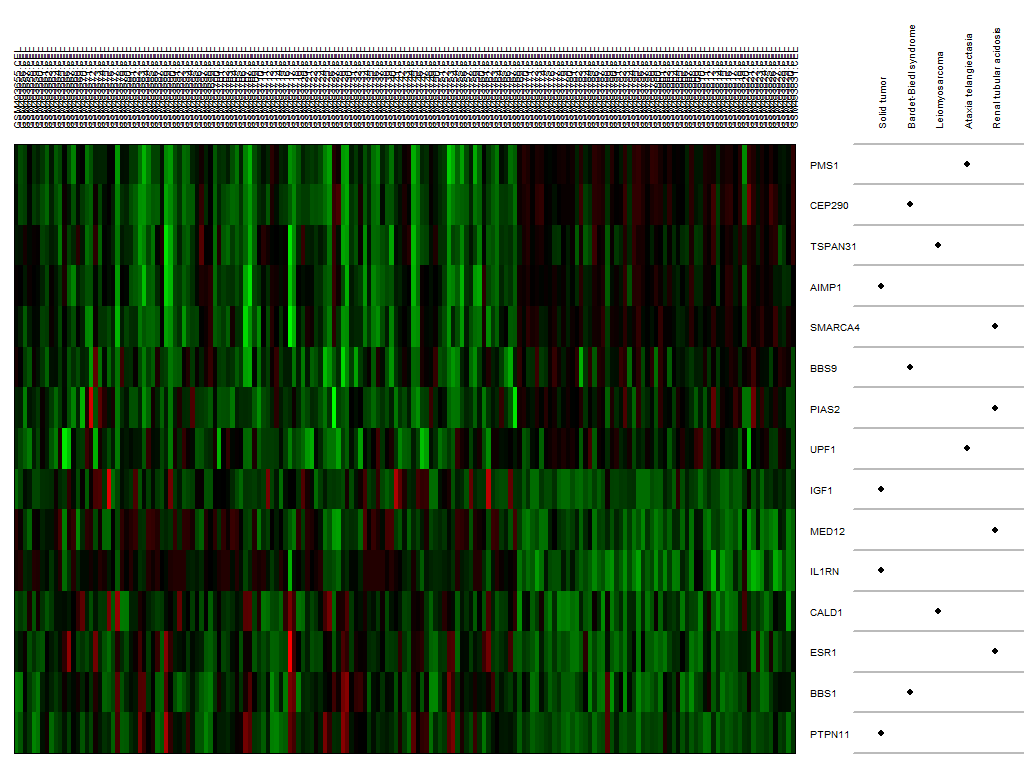

Supplement: Additional file 3 — Sample pipeline outputs in HTML format (compressed file). [file 1471-2164-13-620-S3.ZIP › Burn_dead-alive-control/GRAPH_Sep09_032512.png]

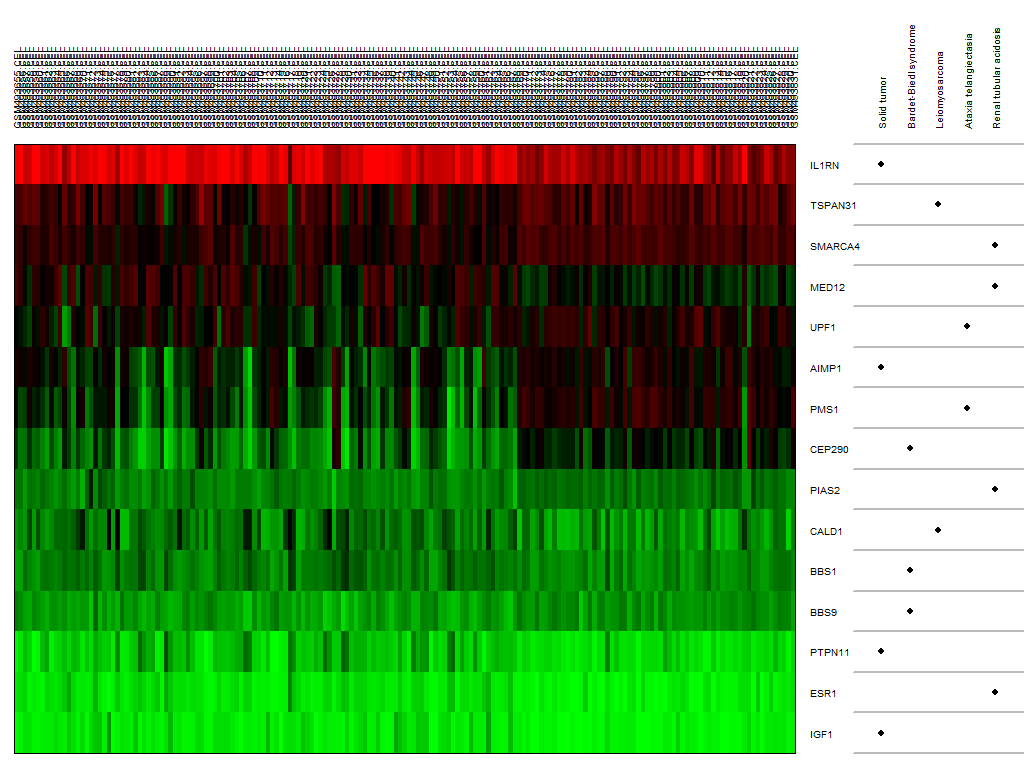

Supplement: Additional file 3 — Sample pipeline outputs in HTML format (compressed file). [file 1471-2164-13-620-S3.ZIP › Burn_dead-alive-control/GRAPH_Sep09_032519.png]

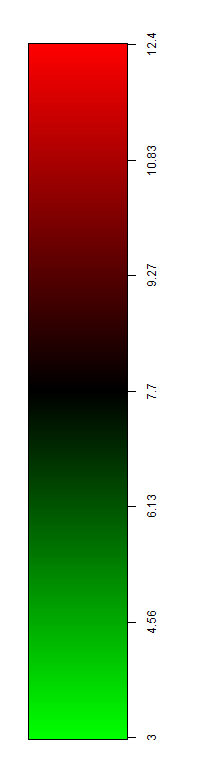

Supplement: Additional file 3 — Sample pipeline outputs in HTML format (compressed file). [file 1471-2164-13-620-S3.ZIP › Burn_dead-alive-control/GRAPH_Sep09_032525.png]

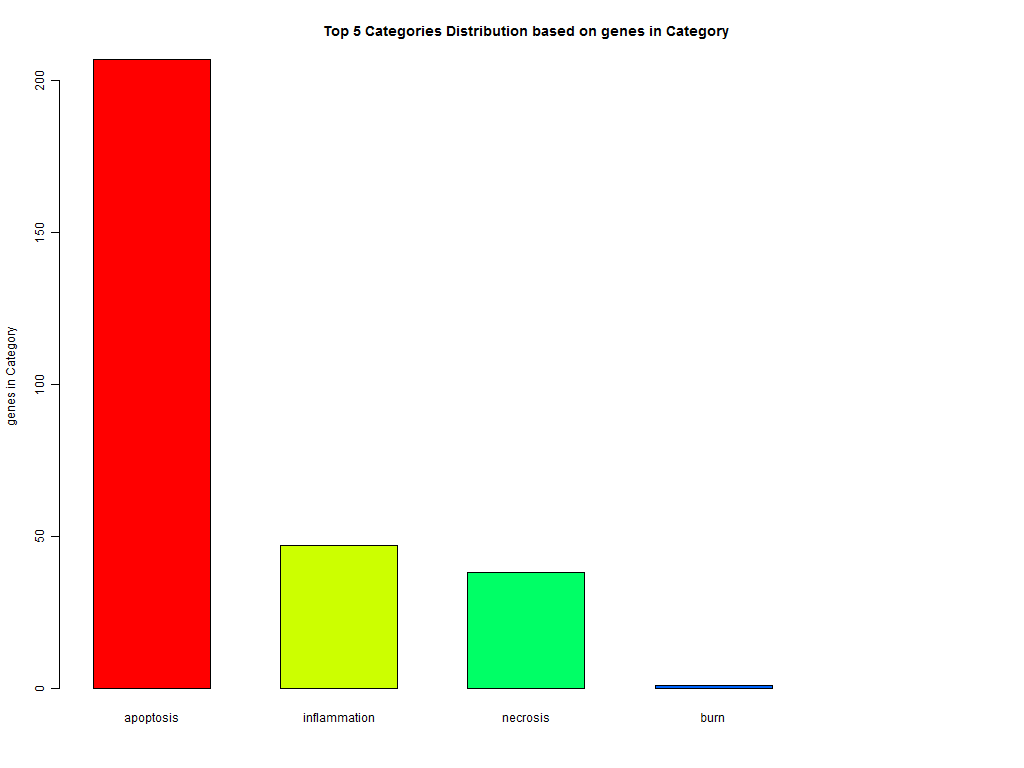

Supplement: Additional file 3 — Sample pipeline outputs in HTML format (compressed file). [file 1471-2164-13-620-S3.ZIP › Burn_dead-alive-control/GRAPH_Sep09_032546.png]

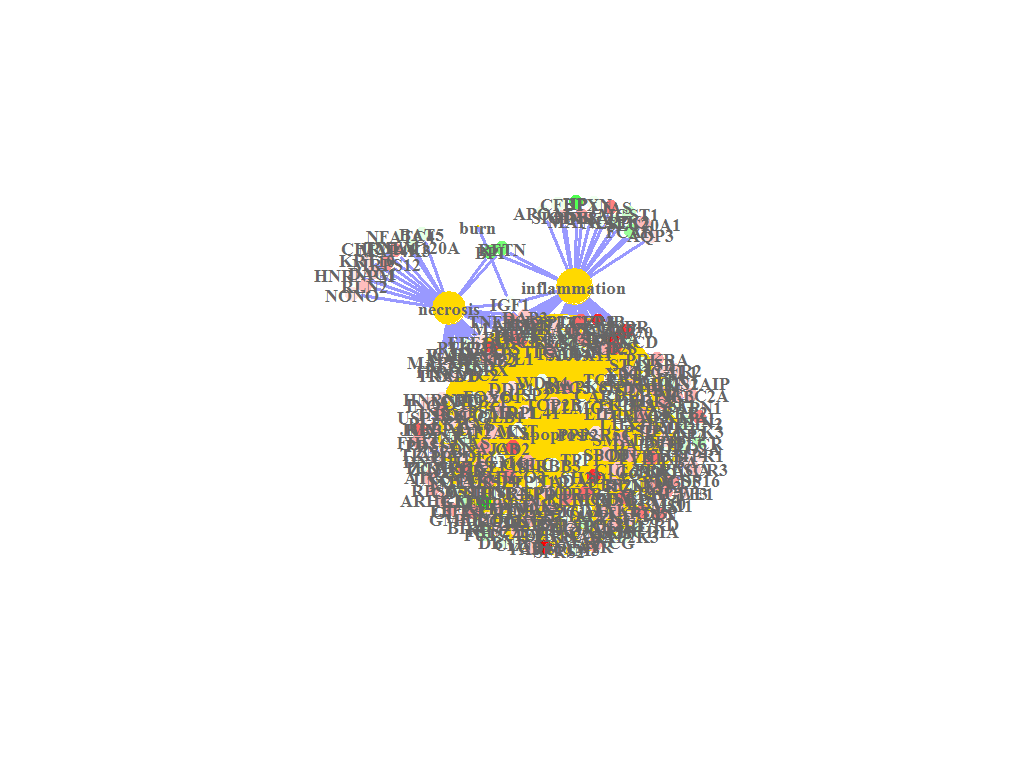

Supplement: Additional file 3 — Sample pipeline outputs in HTML format (compressed file). [file 1471-2164-13-620-S3.ZIP › Burn_dead-alive-control/GRAPH_Sep09_032553.png]

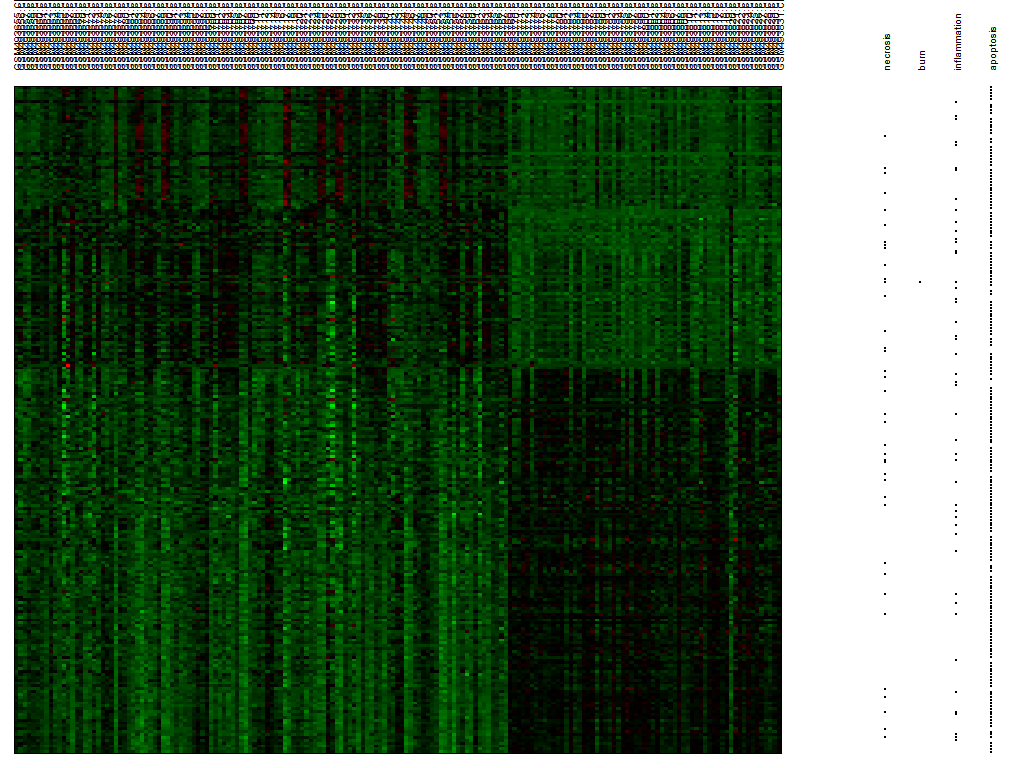

Supplement: Additional file 3 — Sample pipeline outputs in HTML format (compressed file). [file 1471-2164-13-620-S3.ZIP › Burn_dead-alive-control/GRAPH_Sep09_032600.png]

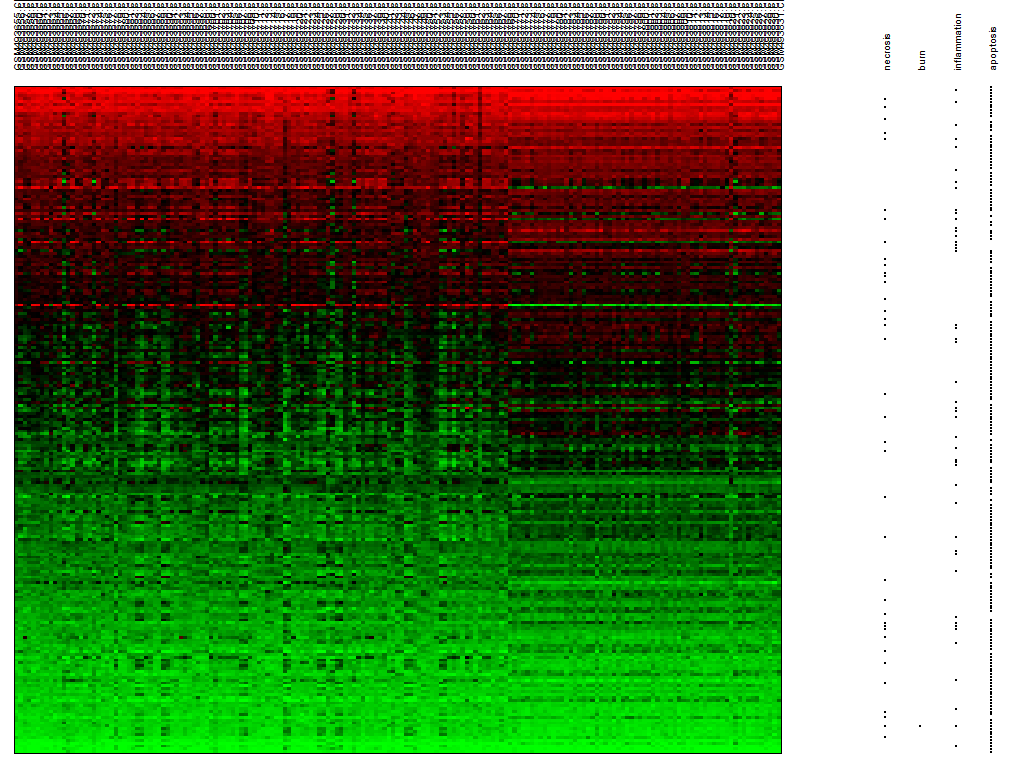

Supplement: Additional file 3 — Sample pipeline outputs in HTML format (compressed file). [file 1471-2164-13-620-S3.ZIP › Burn_dead-alive-control/GRAPH_Sep09_032623.png]

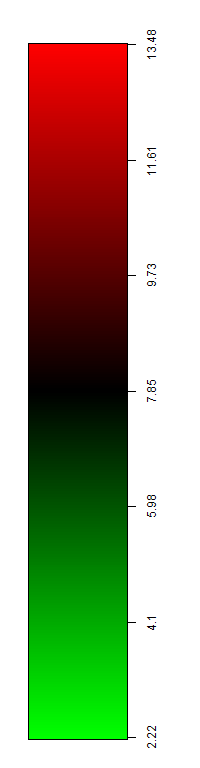

Supplement: Additional file 3 — Sample pipeline outputs in HTML format (compressed file). [file 1471-2164-13-620-S3.ZIP › Burn_dead-alive-control/GRAPH_Sep09_032631.png]

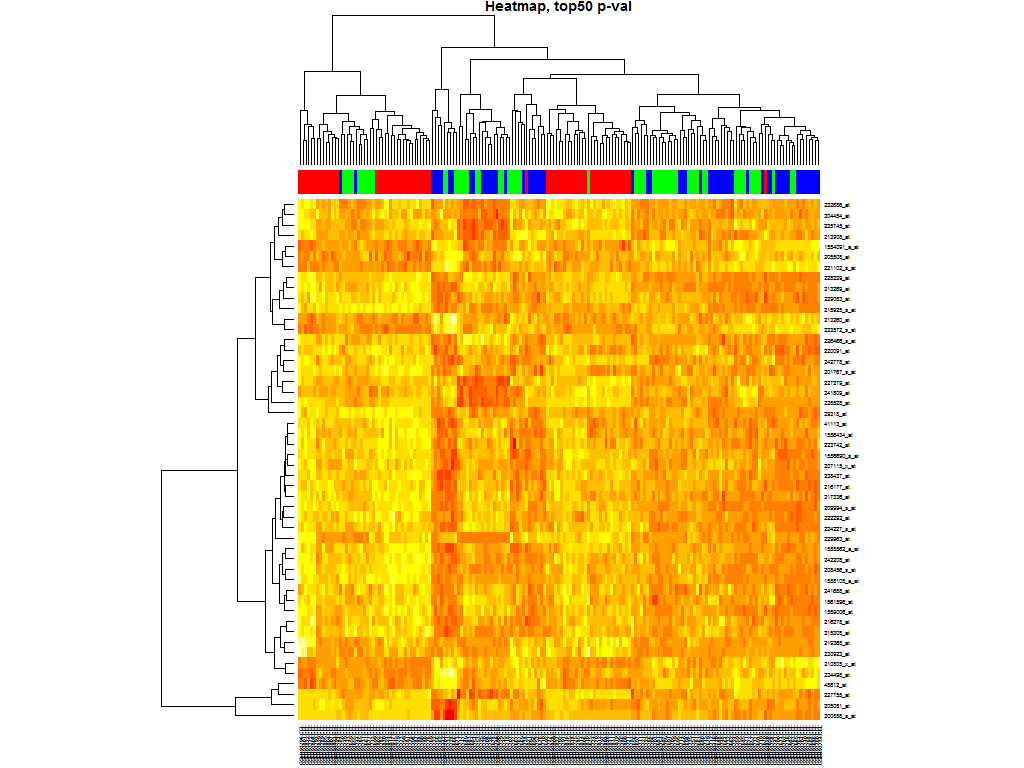

Supplement: Additional file 3 — Sample pipeline outputs in HTML format (compressed file). [file 1471-2164-13-620-S3.ZIP › Burn_early-late-control&chilren-adoult/GRAPH_Sep09_052708.png]

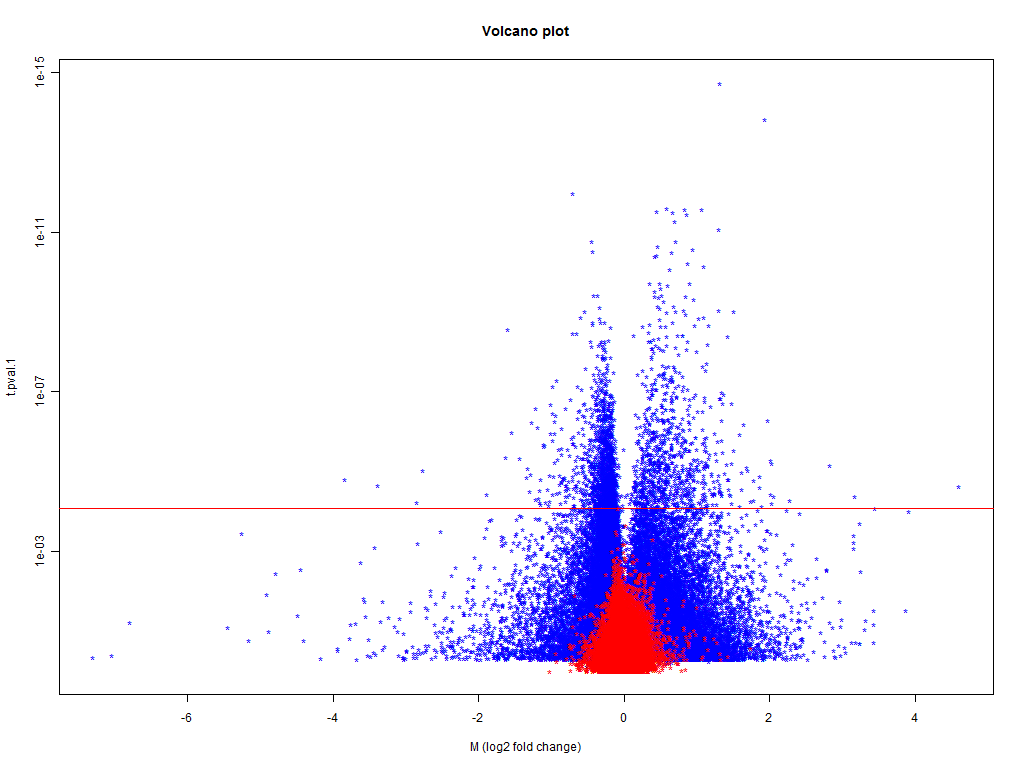

Supplement: Additional file 3 — Sample pipeline outputs in HTML format (compressed file). [file 1471-2164-13-620-S3.ZIP › Burn_early-late-control&chilren-adoult/GRAPH_Sep09_052710.png]

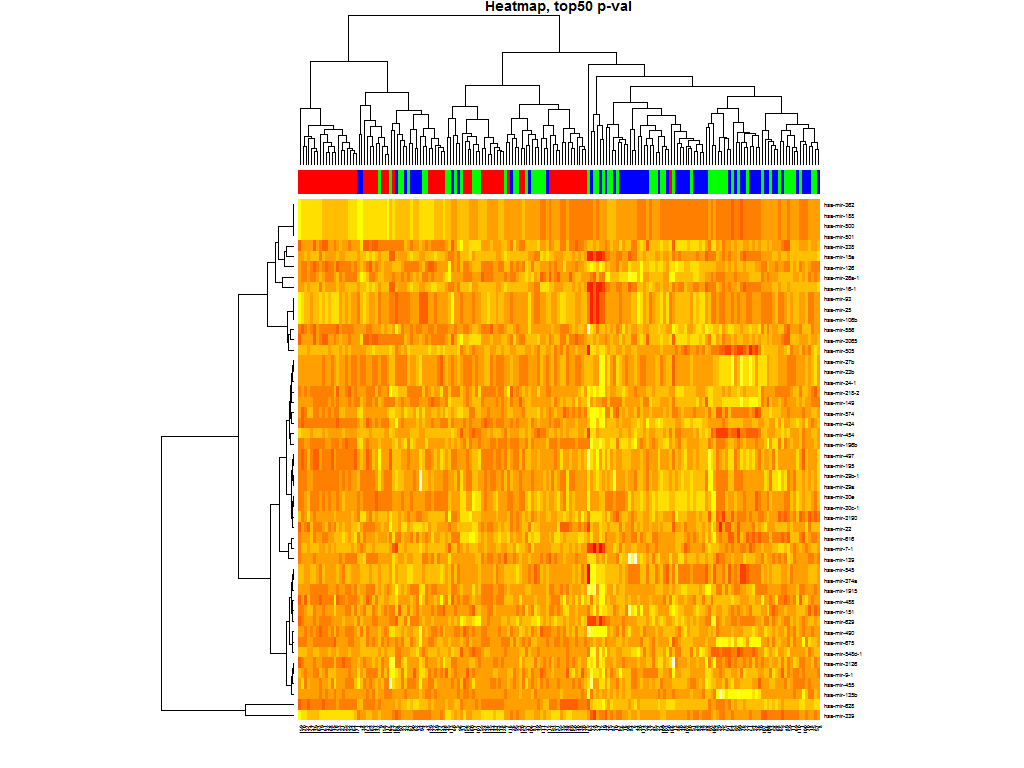

Supplement: Additional file 3 — Sample pipeline outputs in HTML format (compressed file). [file 1471-2164-13-620-S3.ZIP › Burn_early-late-control&chilren-adoult/GRAPH_Sep09_055144.png]

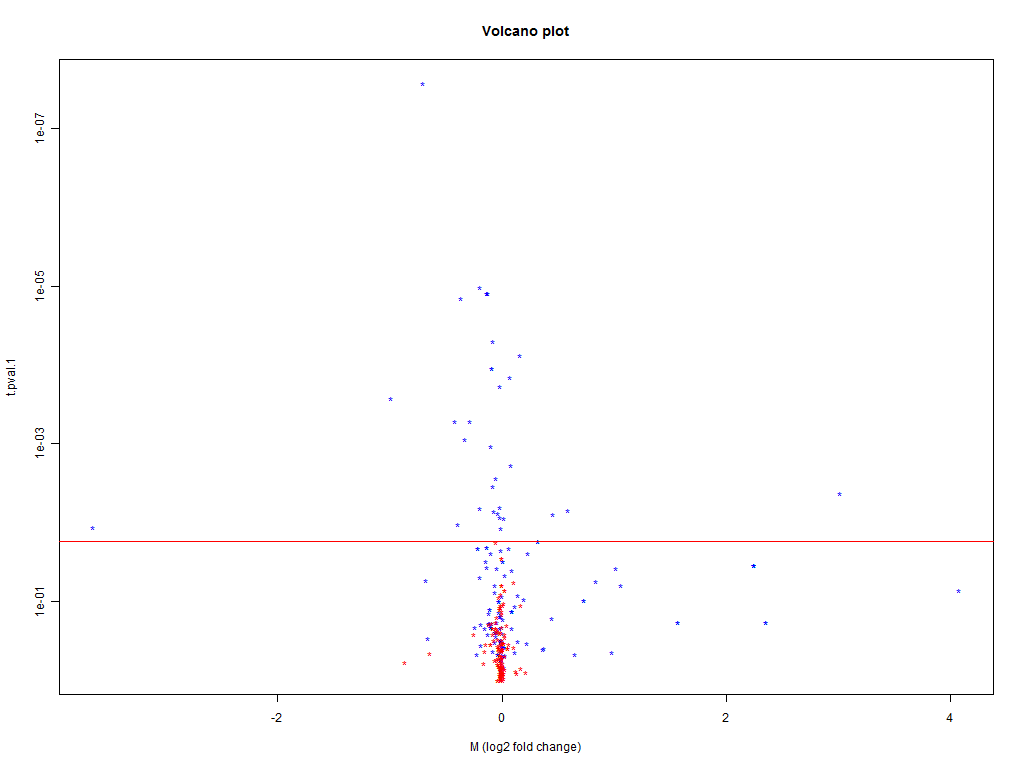

Supplement: Additional file 3 — Sample pipeline outputs in HTML format (compressed file). [file 1471-2164-13-620-S3.ZIP › Burn_early-late-control&chilren-adoult/GRAPH_Sep09_055147.png]

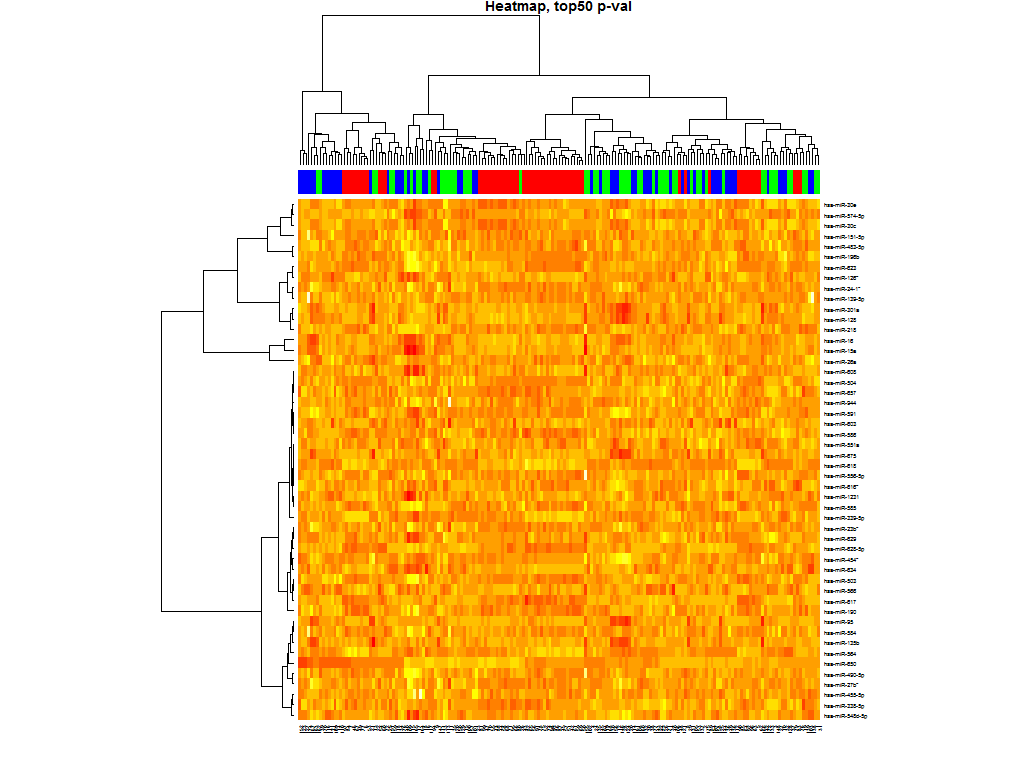

Supplement: Additional file 3 — Sample pipeline outputs in HTML format (compressed file). [file 1471-2164-13-620-S3.ZIP › Burn_early-late-control&chilren-adoult/GRAPH_Sep09_055204.png]

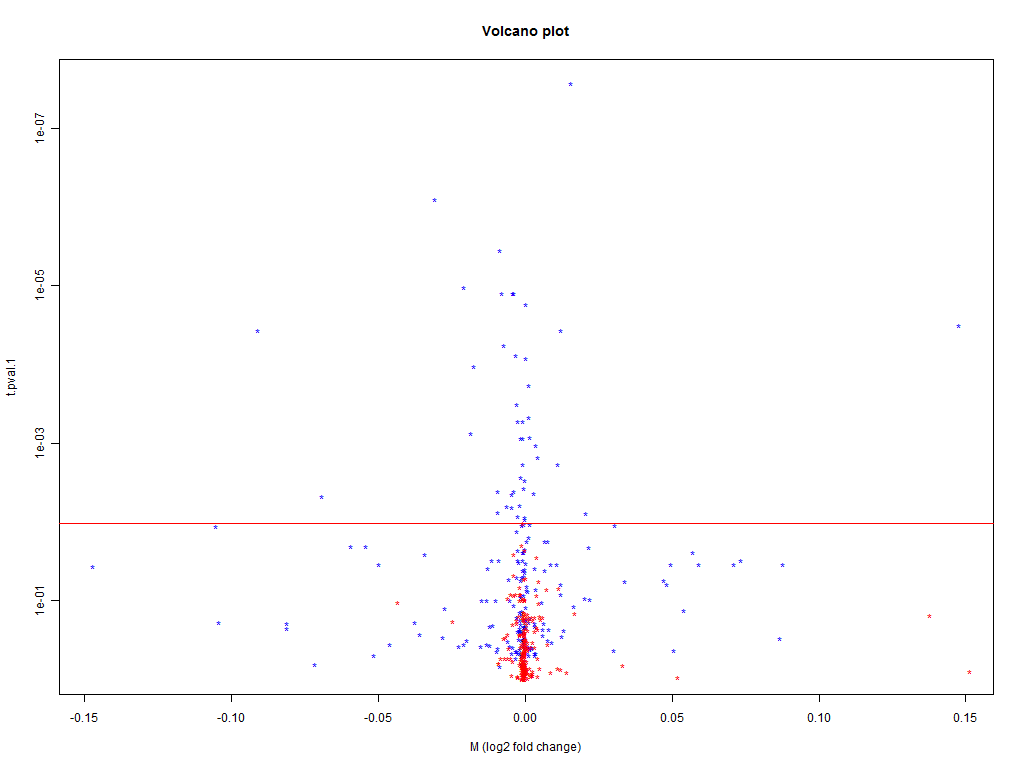

Supplement: Additional file 3 — Sample pipeline outputs in HTML format (compressed file). [file 1471-2164-13-620-S3.ZIP › Burn_early-late-control&chilren-adoult/GRAPH_Sep09_055205.png]

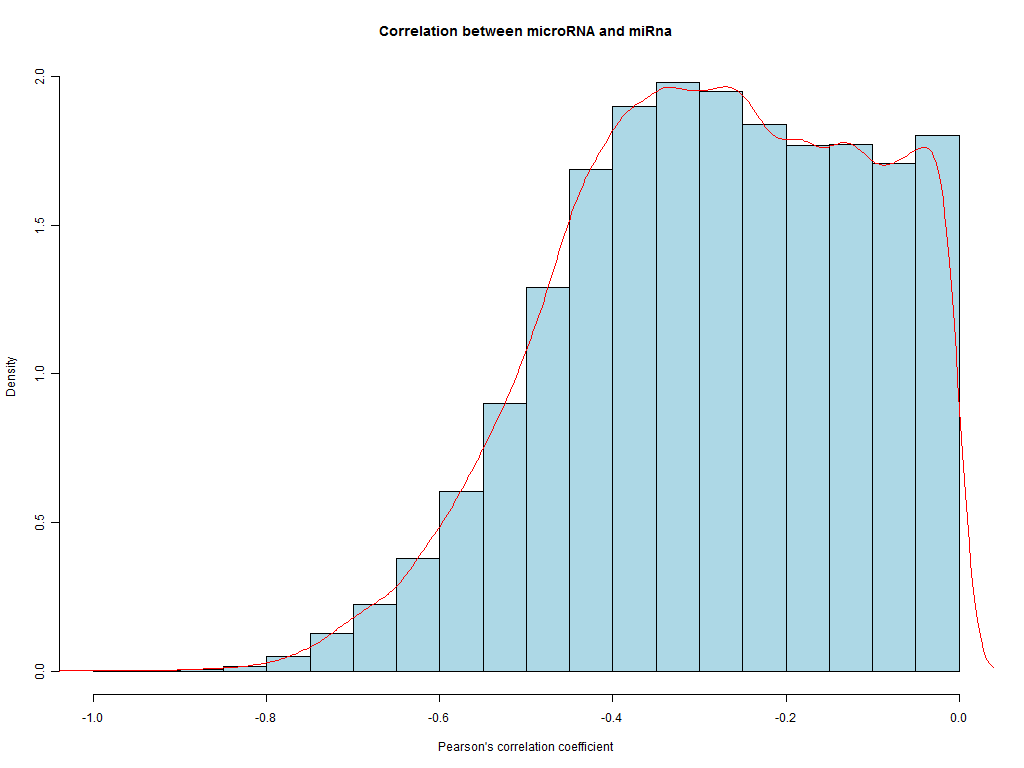

Supplement: Additional file 3 — Sample pipeline outputs in HTML format (compressed file). [file 1471-2164-13-620-S3.ZIP › Burn_early-late-control&chilren-adoult/GRAPH_Sep09_055207.png]

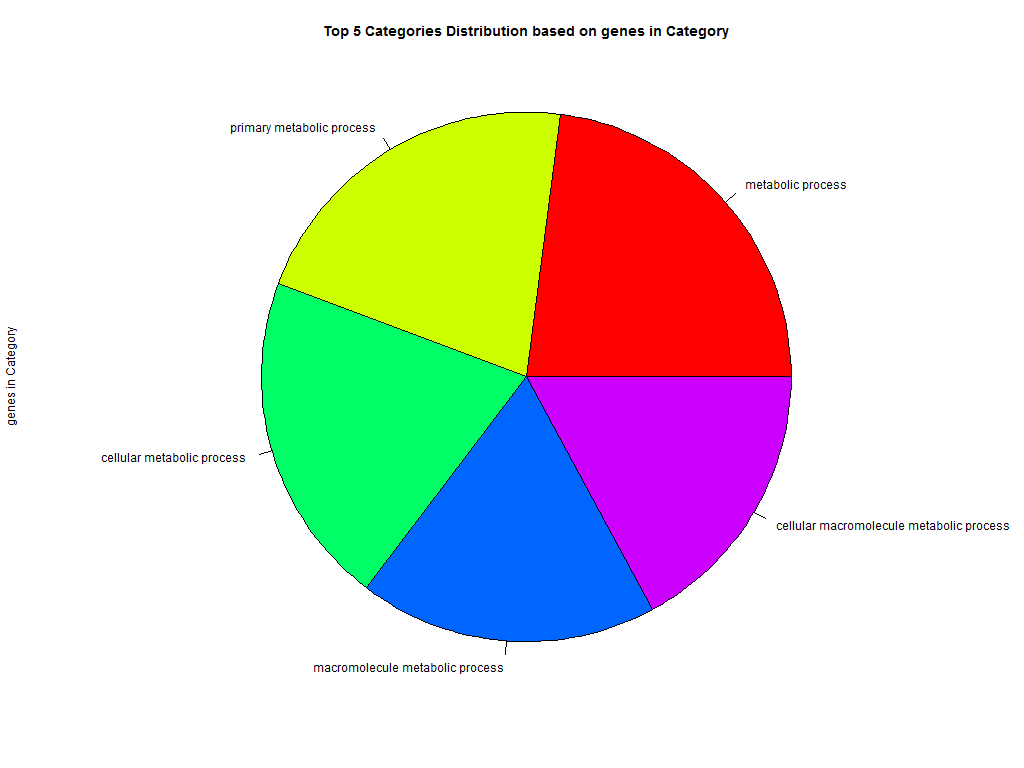

Supplement: Additional file 3 — Sample pipeline outputs in HTML format (compressed file). [file 1471-2164-13-620-S3.ZIP › Burn_early-late-control&chilren-adoult/GRAPH_Sep09_055344.png]

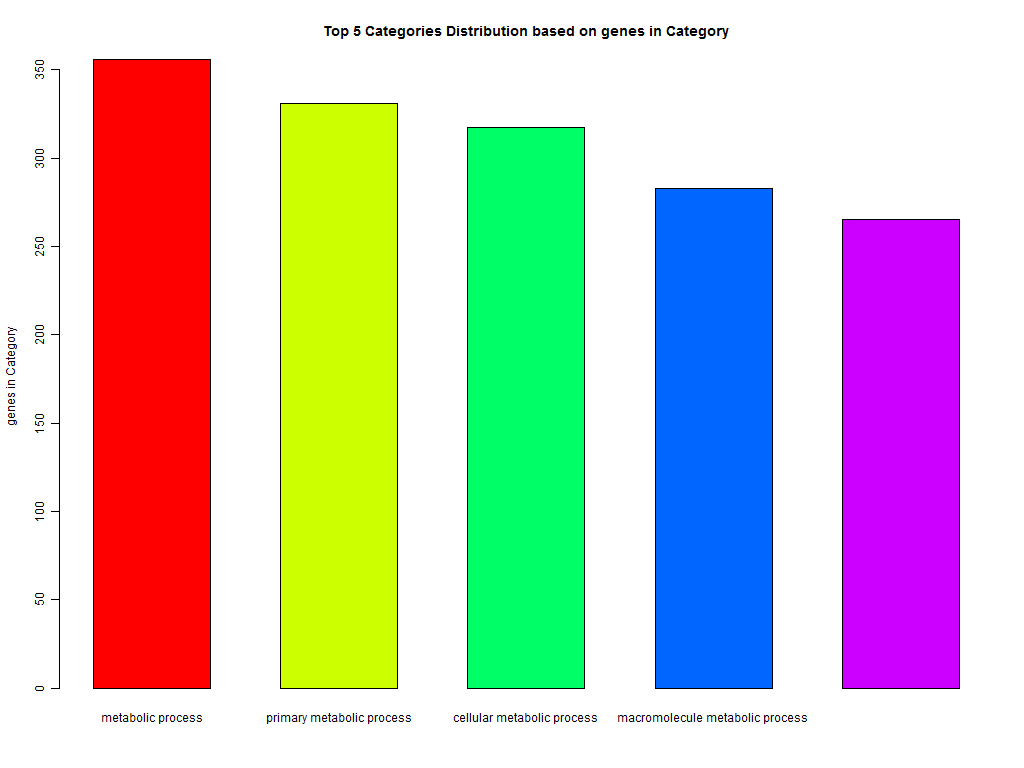

Supplement: Additional file 3 — Sample pipeline outputs in HTML format (compressed file). [file 1471-2164-13-620-S3.ZIP › Burn_early-late-control&chilren-adoult/GRAPH_Sep09_055349.png]

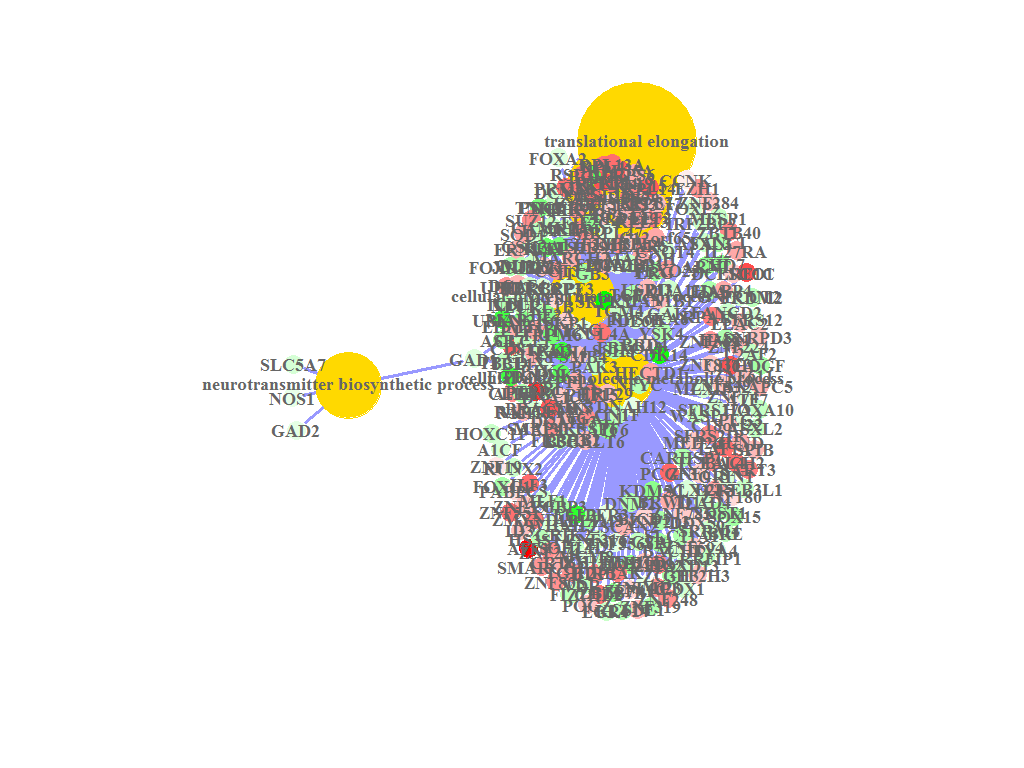

Supplement: Additional file 3 — Sample pipeline outputs in HTML format (compressed file). [file 1471-2164-13-620-S3.ZIP › Burn_early-late-control&chilren-adoult/GRAPH_Sep09_055356.png]

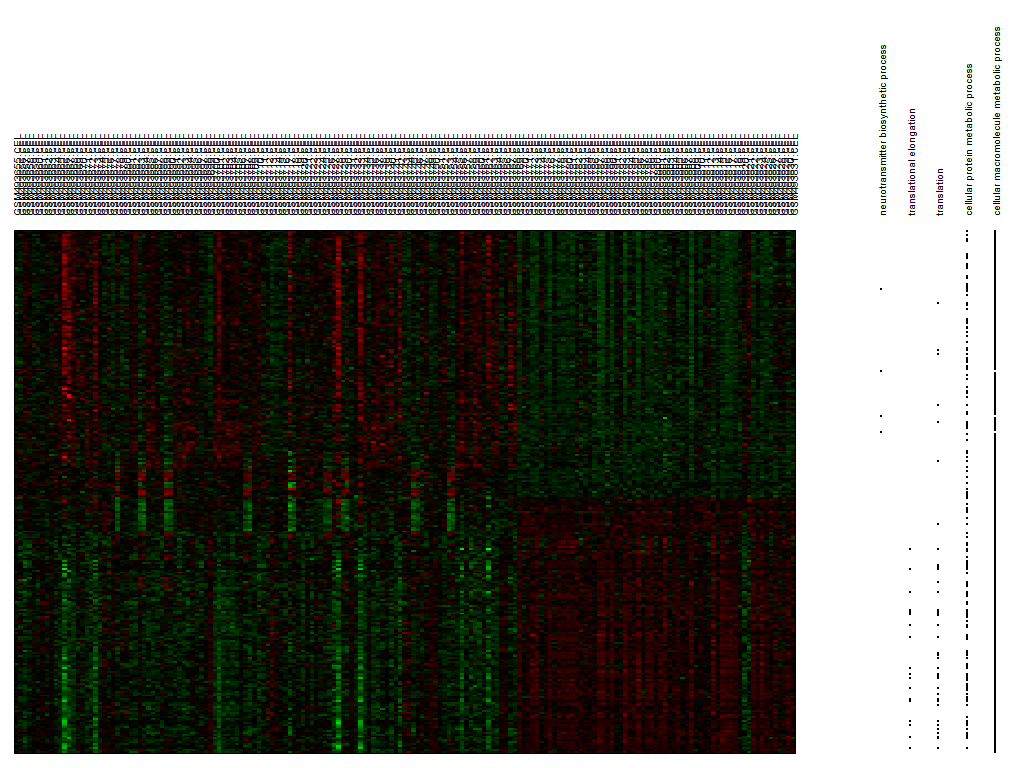

Supplement: Additional file 3 — Sample pipeline outputs in HTML format (compressed file). [file 1471-2164-13-620-S3.ZIP › Burn_early-late-control&chilren-adoult/GRAPH_Sep09_055403.png]

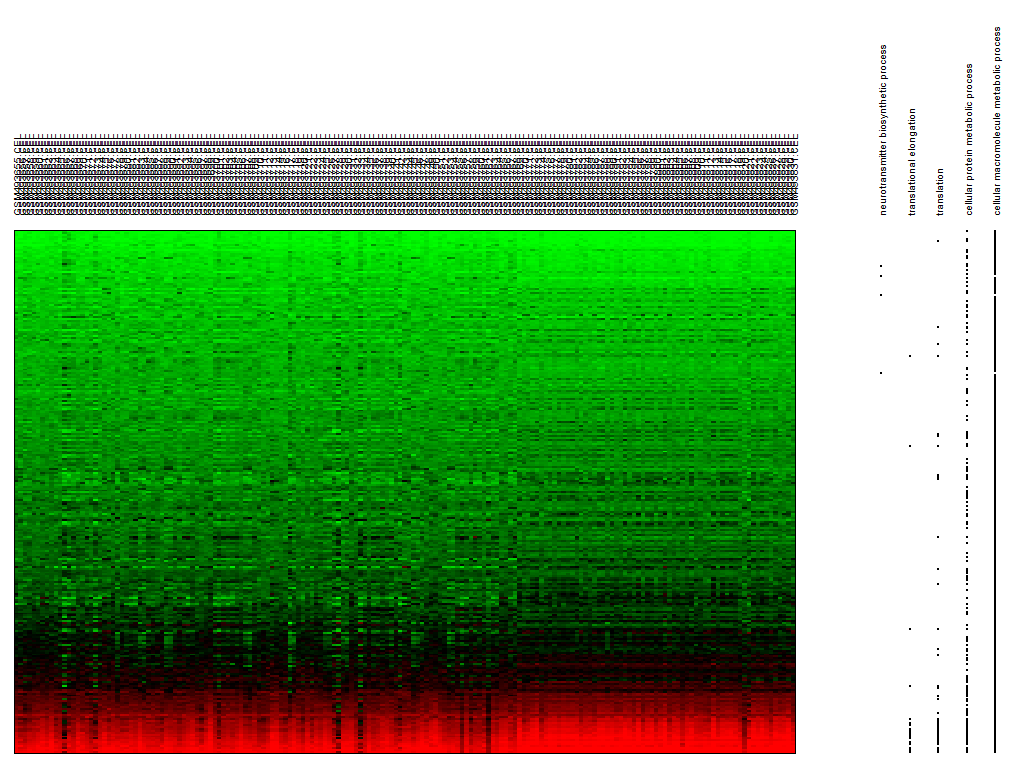

Supplement: Additional file 3 — Sample pipeline outputs in HTML format (compressed file). [file 1471-2164-13-620-S3.ZIP › Burn_early-late-control&chilren-adoult/GRAPH_Sep09_055411.png]

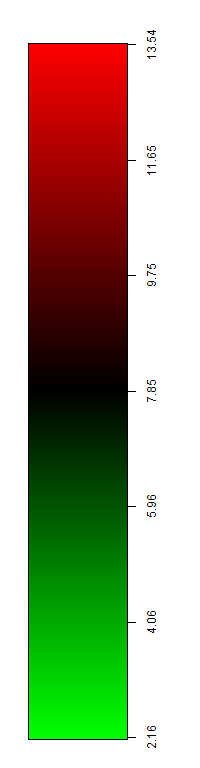

Supplement: Additional file 3 — Sample pipeline outputs in HTML format (compressed file). [file 1471-2164-13-620-S3.ZIP › Burn_early-late-control&chilren-adoult/GRAPH_Sep09_055416.png]

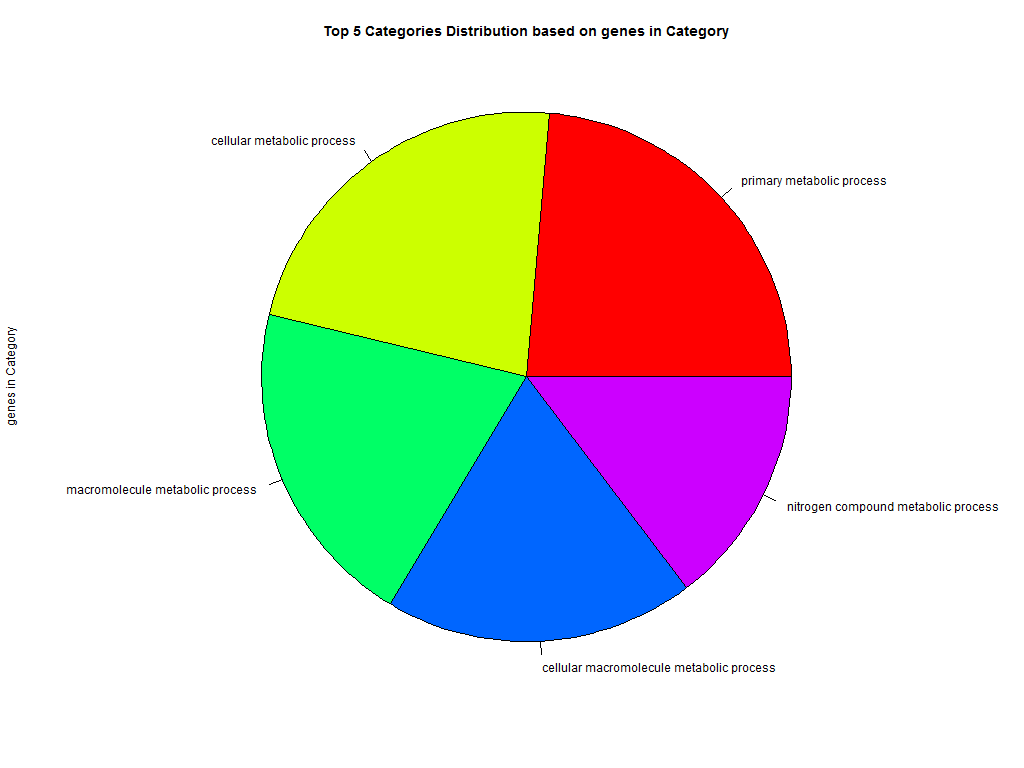

Supplement: Additional file 3 — Sample pipeline outputs in HTML format (compressed file). [file 1471-2164-13-620-S3.ZIP › Burn_early-late-control&chilren-adoult/GRAPH_Sep09_055540.png]

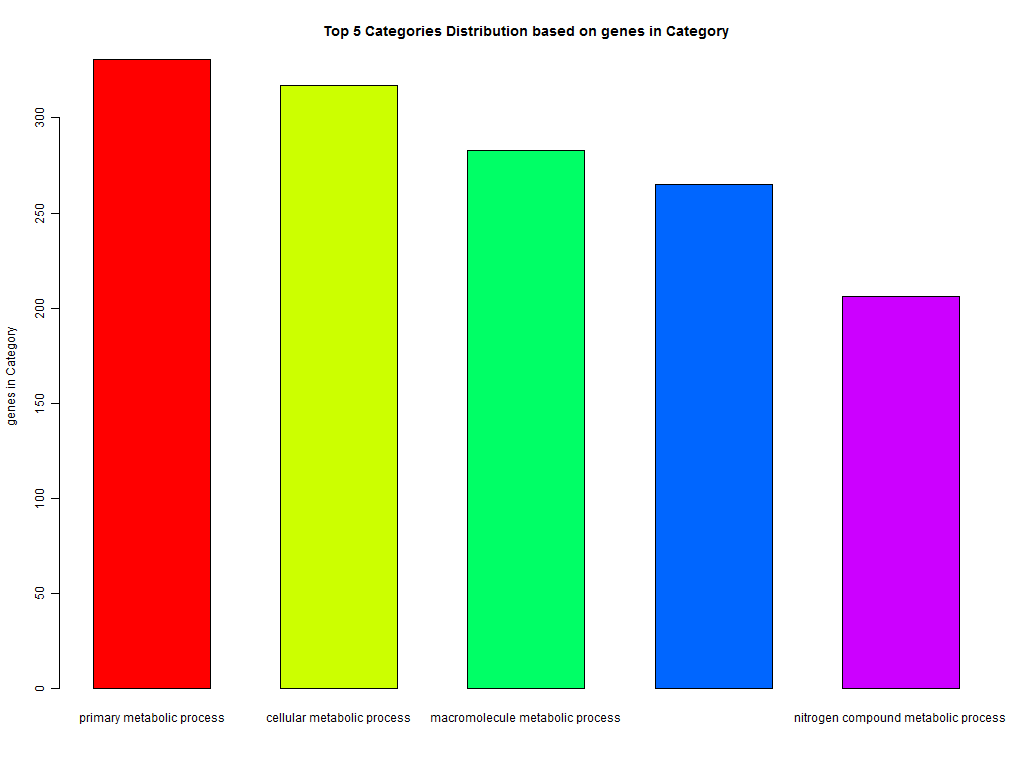

Supplement: Additional file 3 — Sample pipeline outputs in HTML format (compressed file). [file 1471-2164-13-620-S3.ZIP › Burn_early-late-control&chilren-adoult/GRAPH_Sep09_055545.png]

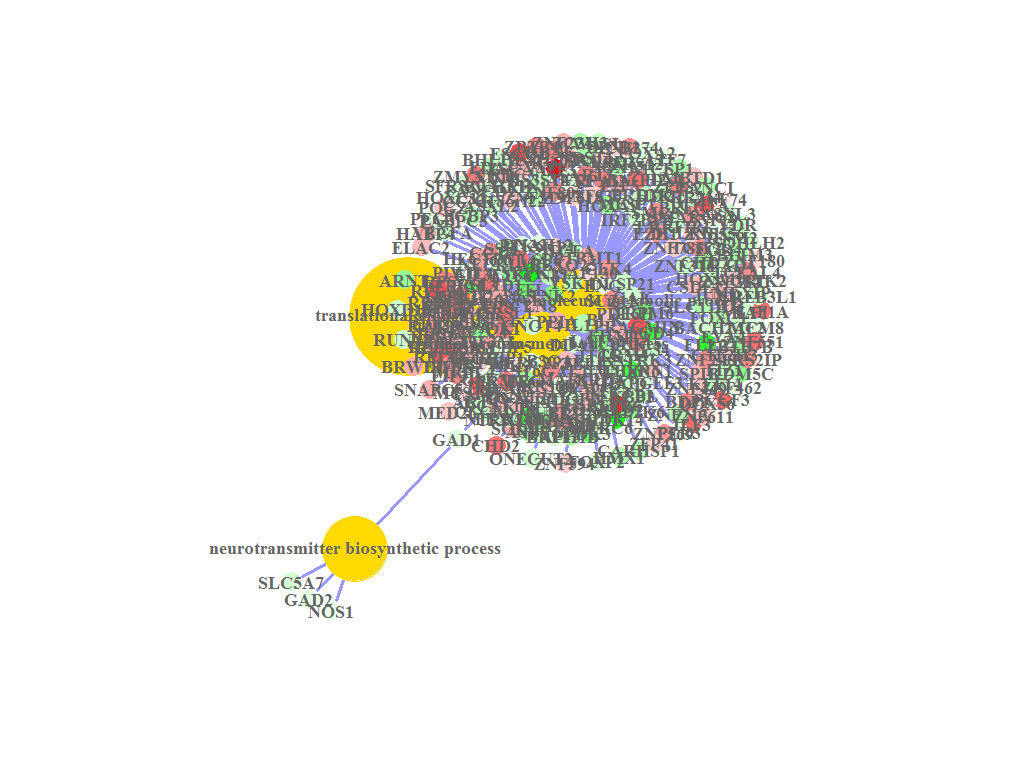

Supplement: Additional file 3 — Sample pipeline outputs in HTML format (compressed file). [file 1471-2164-13-620-S3.ZIP › Burn_early-late-control&chilren-adoult/GRAPH_Sep09_055552.png]

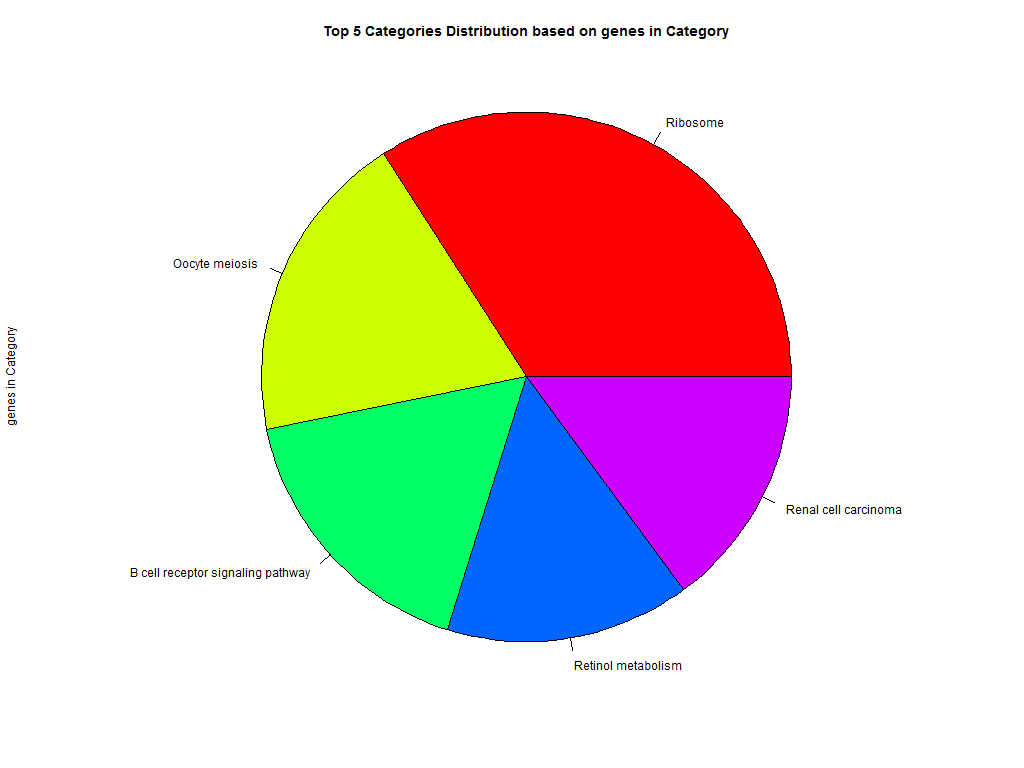

Supplement: Additional file 3 — Sample pipeline outputs in HTML format (compressed file). [file 1471-2164-13-620-S3.ZIP › Burn_early-late-control&chilren-adoult/GRAPH_Sep09_055629.png]

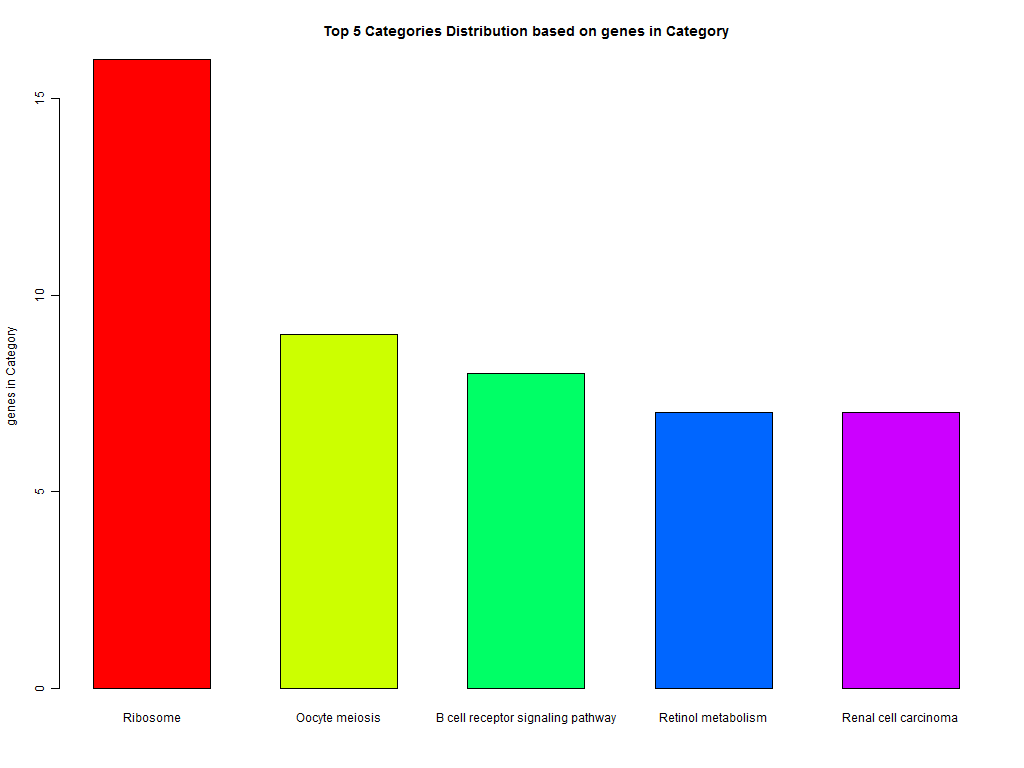

Supplement: Additional file 3 — Sample pipeline outputs in HTML format (compressed file). [file 1471-2164-13-620-S3.ZIP › Burn_early-late-control&chilren-adoult/GRAPH_Sep09_055634.png]

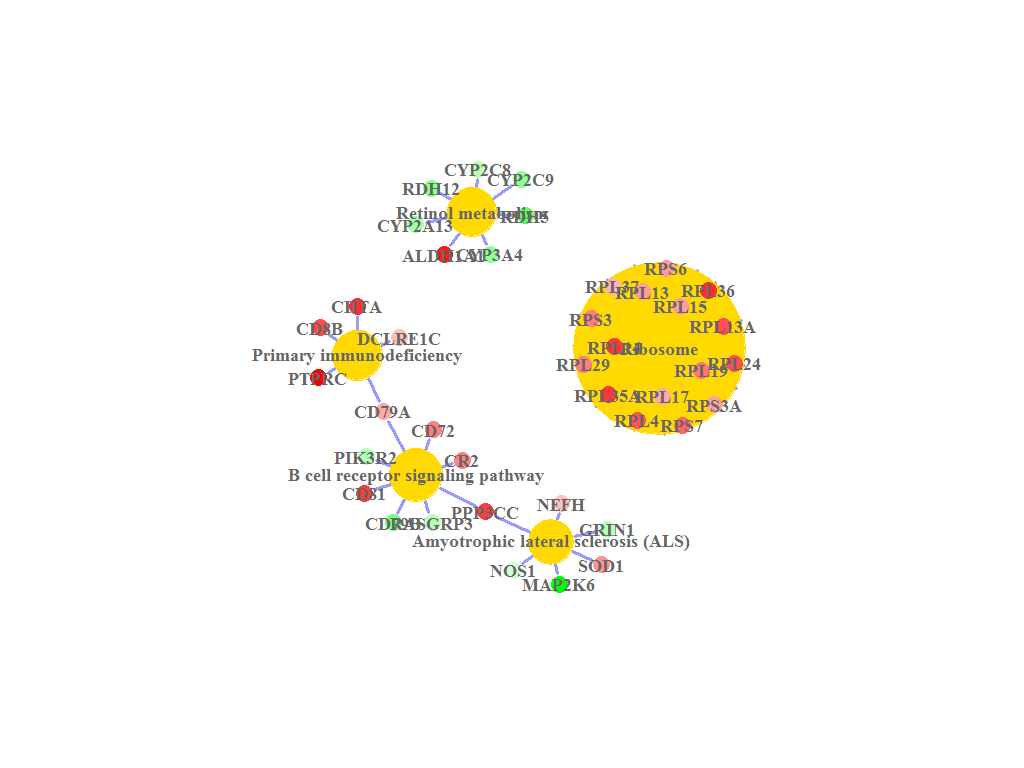

Supplement: Additional file 3 — Sample pipeline outputs in HTML format (compressed file). [file 1471-2164-13-620-S3.ZIP › Burn_early-late-control&chilren-adoult/GRAPH_Sep09_055639.png]
