# Supplementary material for: MMpred: functional miRNA – mRNA interaction analyses by miRNA expression prediction
Source: BMC Genomics. 2012 Nov 14;13:620. doi: 10.1186/1471-2164-13-620 (PMC3562514; doi:10.1186/1471-2164-13-620)
Supplement: Additional file 4 — Overview of the pipeline outputs (raw MMpred output for both case studies). [file 1471-2164-13-620-S4.pdf]

Additional file 6 - Overview of the pipeline output

The output presented in this chapter is standard pipe run (using ANOVA) on the mRNA expression data driven from "*Array-based bioinformatic analysis on pediatric primary central nervous system germ cell tumors"* dataset. This dataset was chosen for illustrating the standard output of the pipeline due to its simplicity and robustness. The user is presented with a HTML report in which particular sections can be expanded and collapsed for better clarity of viewing the results. Examples of such reports are supplied in Additional Materials. The chosen elements from the report have been accompanied by comments and some additional information.

1.1.1 Expression data pre-processing and statistical analyses

The first step of the pipeline produces various outputs, which permit the assay of the experimental design and the quality of the input data to be determined. The design matrix is first presented to the user (**Table 0.1**). This table enables the user to verify the parameters, in addition to giving the opportunity to familiarize oneself with the experimental design.

Table 0.1 The matrix explaining the design of experiment.

	ArrayFile	FunctionalGroup
1	1	Group 1
2	2	Group 1
3	3	Group 1
4	4	Group 1
5	5	Group 1
6	6	Group 1
7	7	Group 2
8	8	Group 2
9	9	Group 2
10	10	Group 2
11	11	Group 2
12	12	Group 2

The user is next presented with Principal Components Analysis (PCA) plots (**Figure 0.1**). This plot consists of 2 subplots – the heatmap visualizing the captured variance for each component (**Figure 0.1A**) and dot-plot showing first component plotted against second (**Figure 0.1B**). PCA plots are intended to quickly assess the general quality of the data by seeing if samples group together in the way determined by the design matrix/vector. Usually the dataset characterised with high variance in the first few components and showing good clustering on the scatter-plot are much more likely to produce robust miRNA expression prediction and accurate prediction about miRNA-mRNA interactions in the dataset.

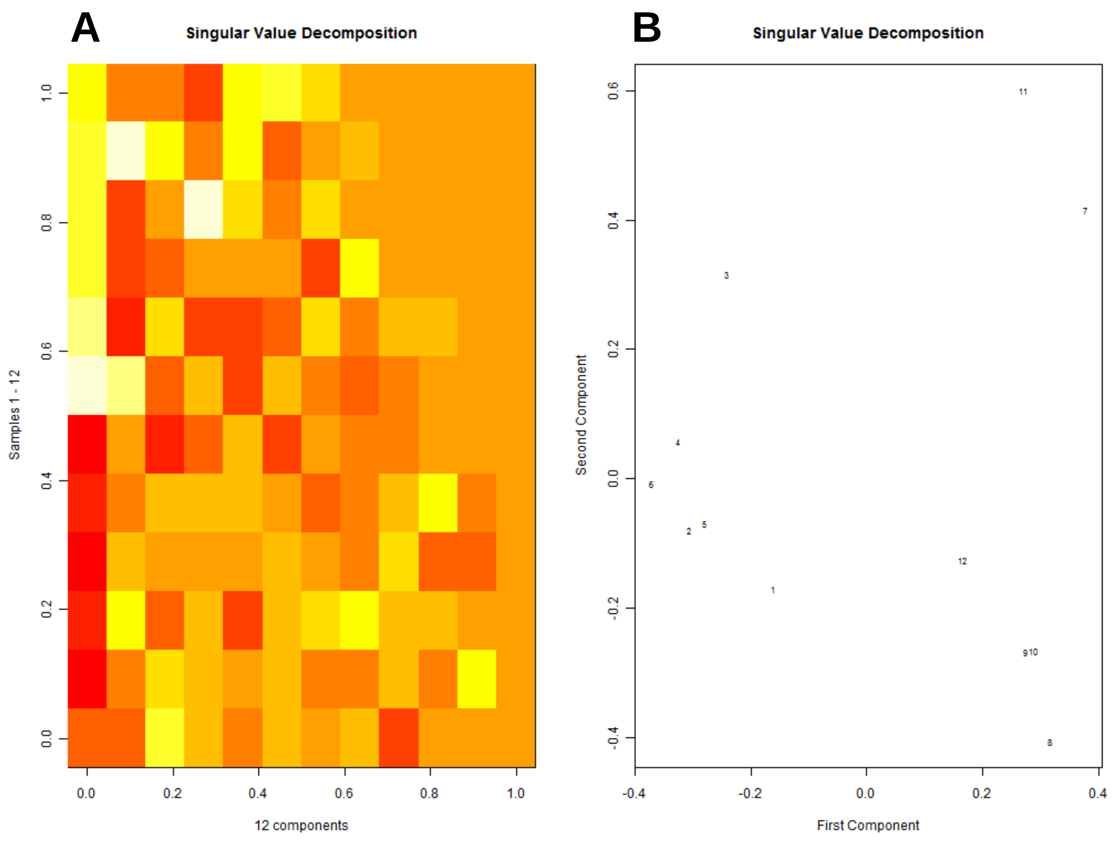

Figure 0.1 Principal Component Analyses obtained for predictor I pseudo-expression matrix. The plot consists of heatmap visualizing the captured variance for each component (subplot A) and scatter-plot showing first component plotted against second (subplot B).

The expression and clustering of the top 50 genes ranked by p-values is presented graphically as a heatmap (**Figure 0.2**). This figure permits the assessment of the output of the statistical testing (t-test or ANOVA). In the case of a simple experimental design (sample vs. control or group 1 vs. group 2) perfect clustering is expected. When the experimental design is more complicated (in which few functional groups are tested by ANOVA) some samples may not be perfectly clustered, yet generally good clustering is also expected. The poor clustering between experiment groups may indicate a wrong design of experiment or very bad quality of data. In such cases the prediction and functional analyses returned by the pipeline cannot be trusted – using a different data set or changing the experimental design matrix is suggested. Alternatively the pipeline can be modified to use non-parametric methods of testing the statistical significance of genes,

for example Rank Product method for identifying differentially expressed genes (implemented in BioConductor RankProd Package).

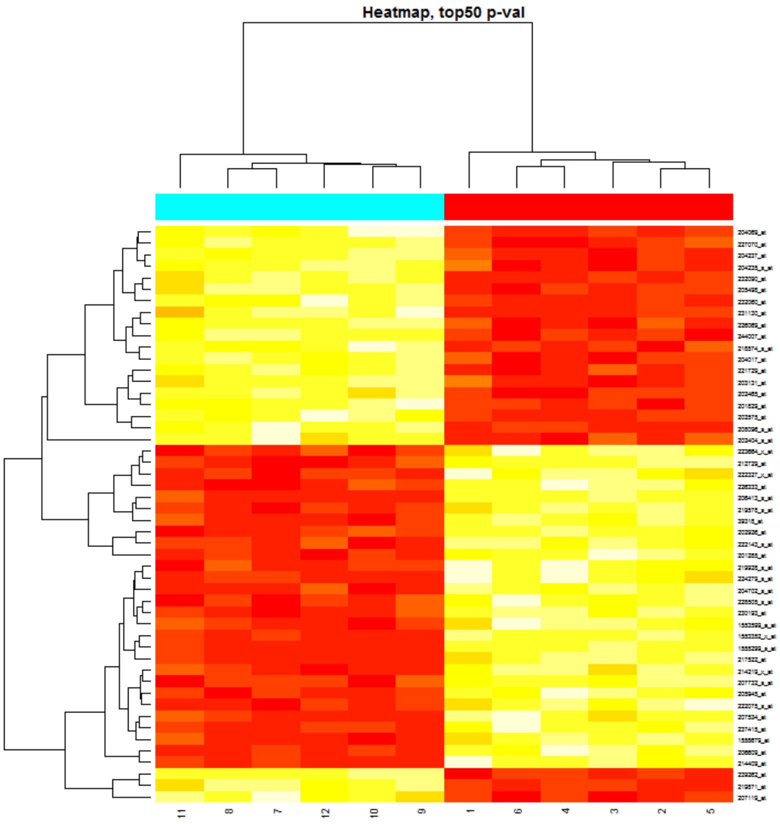

Figure 0.2 The heatmap and hierarchical clustering plot featuring the top 50 differentially expressed mRNAs (ordered by increasing p-value, obtained from ANOVA). Red colour determines low, yellow intermediate and white high expression index value

The final step of pre-processing uses permutated expression matrix statistical testing in order to determine the p-value cut-off. The results are visualised as a volcano plot presenting experimental fold change – p-value ratio values as blue and randomised values as red points (**Figure 0.3**). The red, horizontal line indicates p-value cut-off (auto-determined). This plot permits the determination of the number of genes that were recognized as significantly over- or under-expressed. In case of robust datasets (high variance captured by the first three PCA components, good hierarchical clustering) the cut-off boundary (p-value of most significant gene determined by statistics from randomized expression matrix) is usually much lower than the p-values of most significant experimental genes – the big group of genes are used in further analyses.

However, if the user decides to employ a dataset characterised by poor clustering and relatively high p-values of the most significant genes the automatically determined cut-off may be too strict. This problem can be worked around by modifying the pipeline to use a pre-determined, hard-coded cut-off value. Nevertheless, when the statistical analyses generate ambiguous output the consistency of the final output cannot be guaranteed.

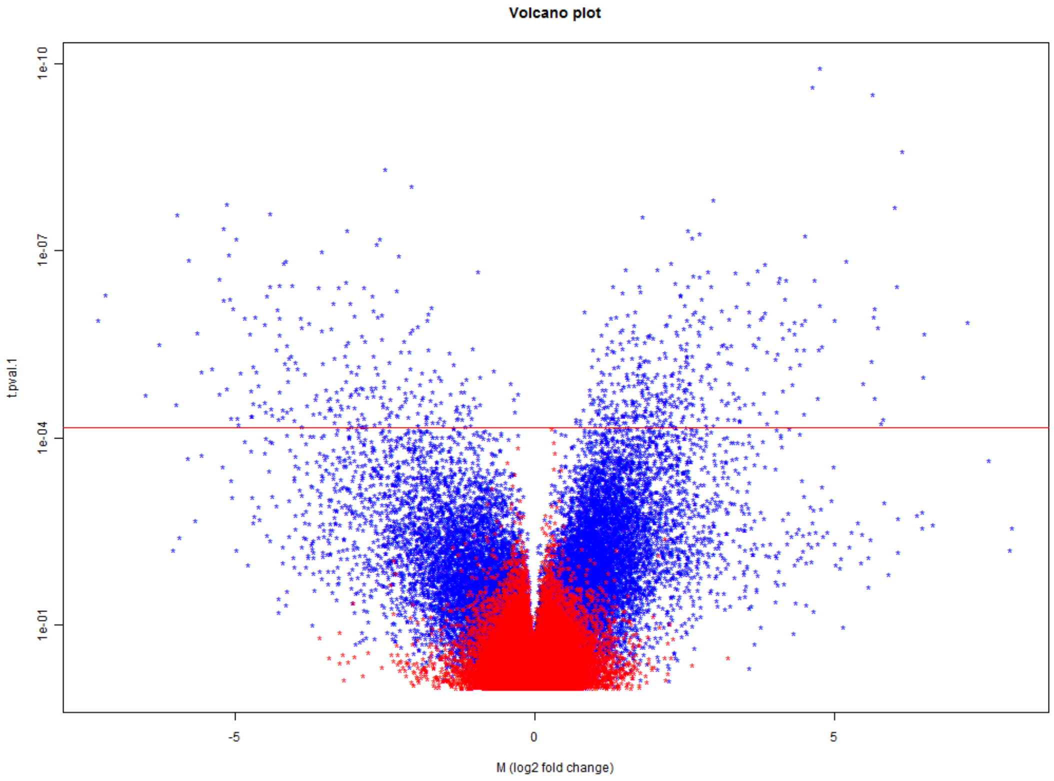

Figure 0.3 The volcano plot created using the input mRNA expression matrix. The fold change is marked on the X-axis and p-value on the Y-axis. The blue points indicate actual fold change – p-value ratio, while randomised values are marked as red points.

1.1.2 Mapping

Although the mapping is a very important component of the pipeline it is not directly displayed to the user in the HTML report. The mapping is usually read from a binary file in order to speed-up the pipeline run, but it can be also recalculated if there is a need to do so. Since the mapping is based on certain, curated information (driven from Ensembl and miRBase) and the different methods of mapping have been validated multiple times,

it was decided not to confuse the user with unnecessary information. The graphical ideogram of the host genes overlaps with miRNAs enriched with numbers of overlaps on specific features (sense/anti-sense strands, exon, intron 3’ and 5’UTR) is presented on **Figure 0.4**.

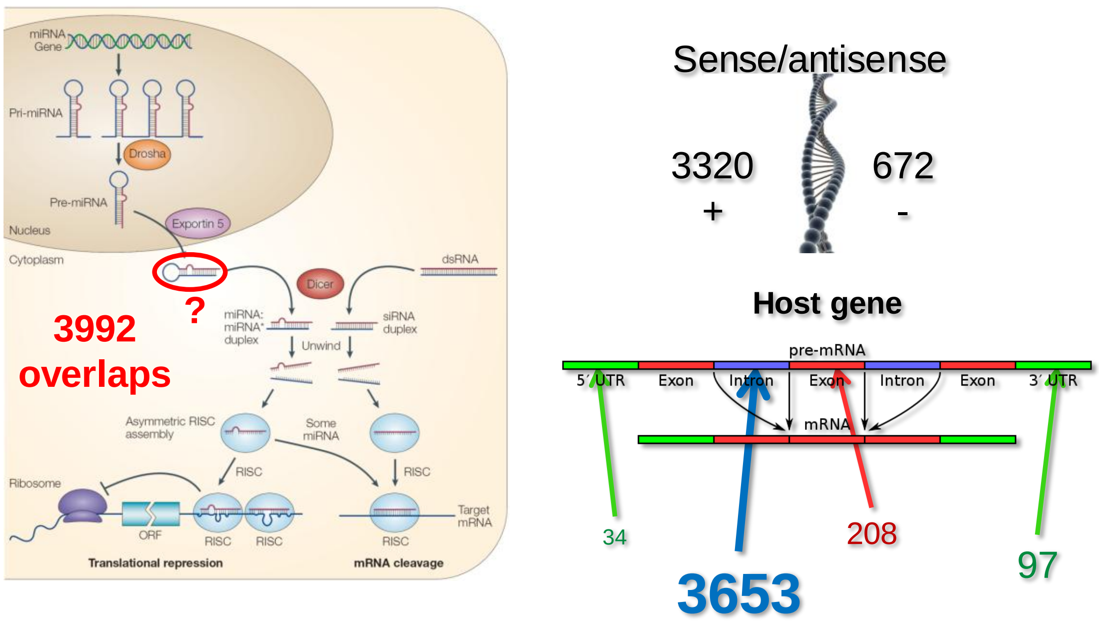

Figure 0.4 The simple graphical presentation of mapping results – the numbers indicates the number of overlaps between pre-miRNA and coding genes.

1.1.3 Predictor I: Scaling function

Although the direct output of the predictors, which is miRNA pseudo-expression matrix, is not presented directly to the user the plots summarizing the statistical analyses of the data, as well as the lists of the most significantly up- and down- regulated genes are included in the HTML report.

Similar to the statistical pre-processing of the mRNA expression input, the Principal Component Analyses plot produced using the scaling function predictor output is being generated (**Figure 0.5**). The robust predictor output is indicated by high variance of the few first components and good clustering on scatter-plot.

A B

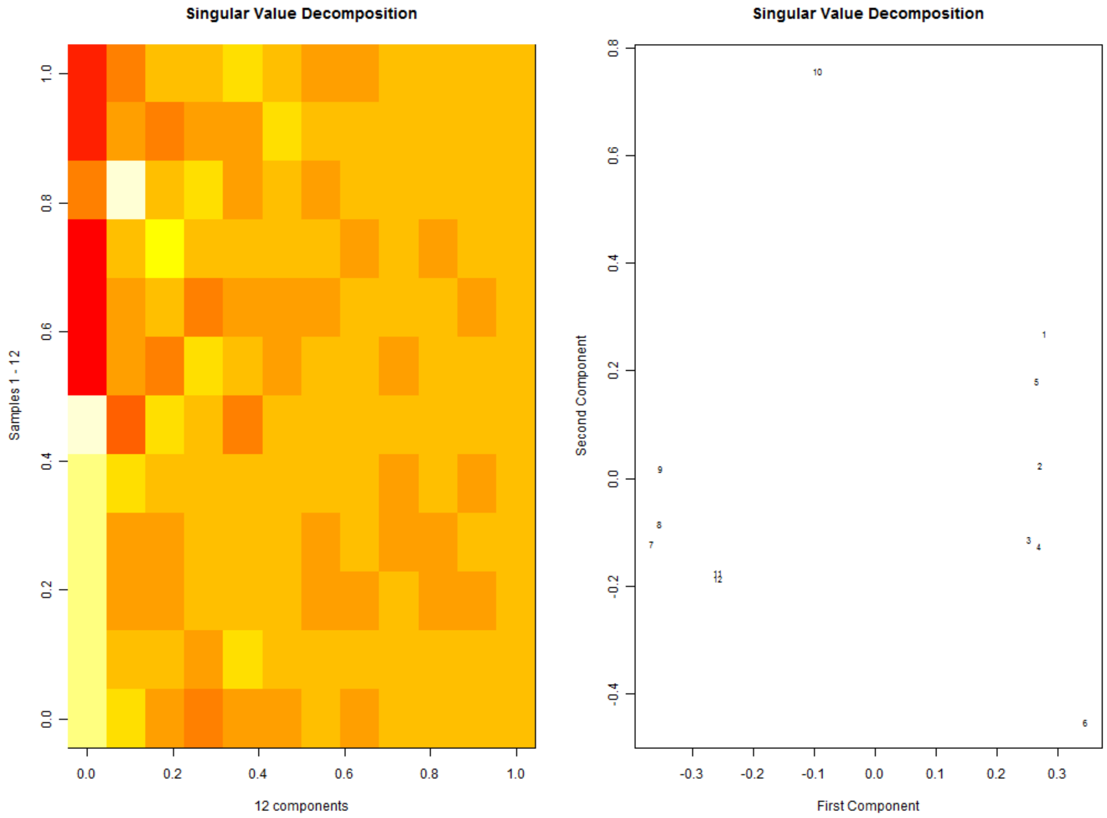

Figure 0.5 The Principal Component Analyses obtained from the predictor I pseudo-expression matrix. The plot consists of: heatmap visualizing the captured variance for each component (A) and scatter plot showing first component plotted against second (B). Numbers on the scatter-plot represent different microarrays (as in **Table 0.1**)

Furthermore the heatmap and cluster plot summarizing the top 50 differentially expressed miRNAs is also produced (**Figure 0.6**). In case of good quality prediction the similar clustering to the one achieved by the statistical testing input mRNA expression matrix is expected.

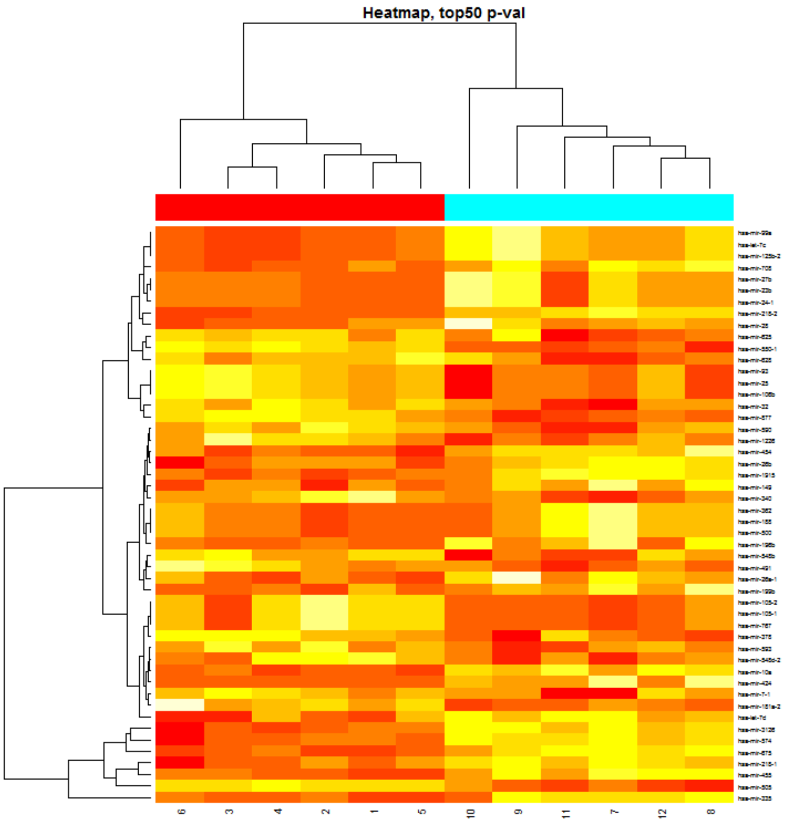

Figure 0.6 The heatmap and hierarchical clustering plot featuring the top 50 differentially expressed miRNAs produced by predictor I (ordered by increasing, p-value obtained from ANOVA).

Finally, the most p-value cut-off is determined by performing the same statistics on the permutated miRNA pseudo-expression matrix. This step is visualized in the volcano plot featuring the cut-off as a horizontal red line (**Figure 0.7**). It should be noted that this volcano plot is significantly different from the one produced using the experimental mRNA matrix. The values below the red line usually are characterized by very low (close to 0) fold change and form a vertical line instead of standard volcano-like structure. For a robust predictor output this value should be observed below the cut-off line only because it indicates the predictor errors (which should be filtered out by statistics). Above the cut-off line the usual volcano-like structure should be observed. Further, the fold change values might be much higher than is normally obtained from experimental expression matrixes. This fact should not concern the user, since in the case of miRNA pseudo-expression the fold-change is calculated for visualization purposes only and have no impact on final output.

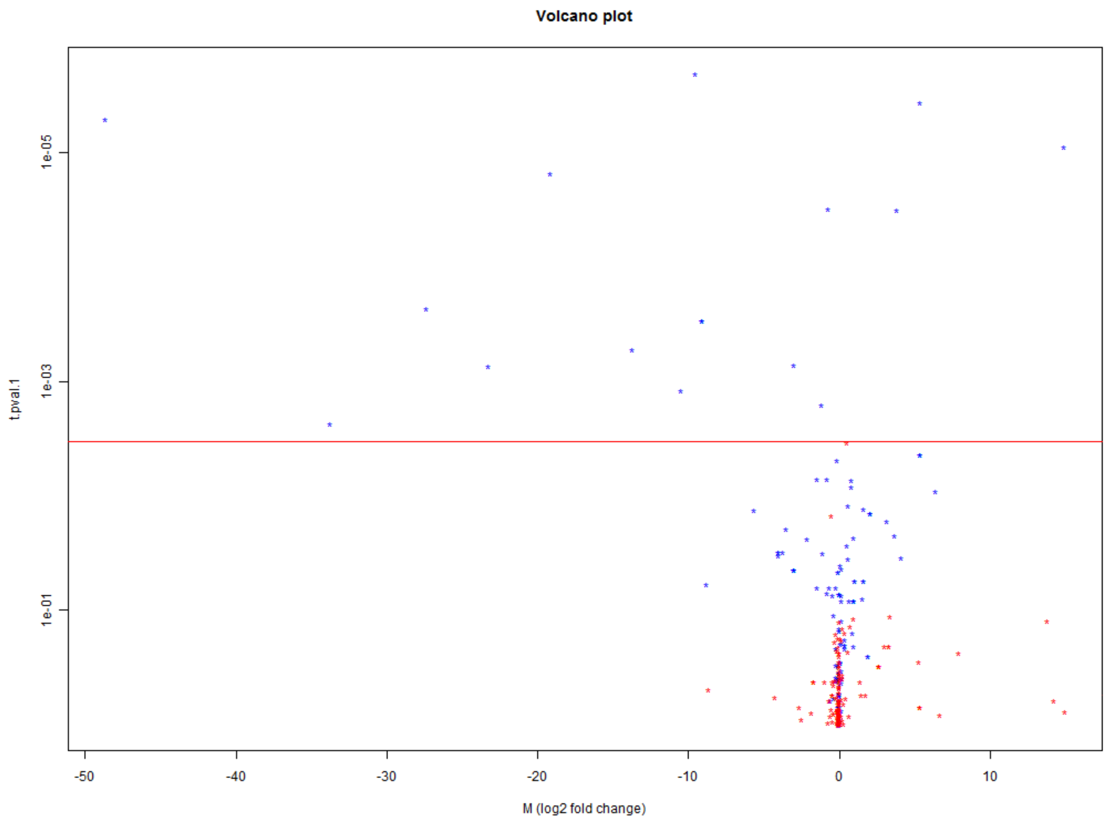

Figure 0.7 The volcano plot created using the miRNA pseudo-expression matrix obtained from predictor I. The fold change is marked on the X-axis and p-value on the Y-

axis. The blue points indicate actual fold change – p-value ratio, while randomised values are marked as red points.

1.1.4 Predictor II: Linear model

The output summarizing the prediction of the linear model based predictor is very similar to the scaling function based one. Although the prediction method is different the pseudo-expression matrix returned by this method has the same structure, so its robustness can be assessed in the same way. The corresponding plots and differentially regulated miRNA list should be interpreted in a similar way as the ones produced using the output of predictor I; therefore only the significant differences will be covered in this section.

The PCA plot should not differ much from the one obtained from the predictor I. If significant differences occur the user might consider using the output of only one predictor for further analyses.

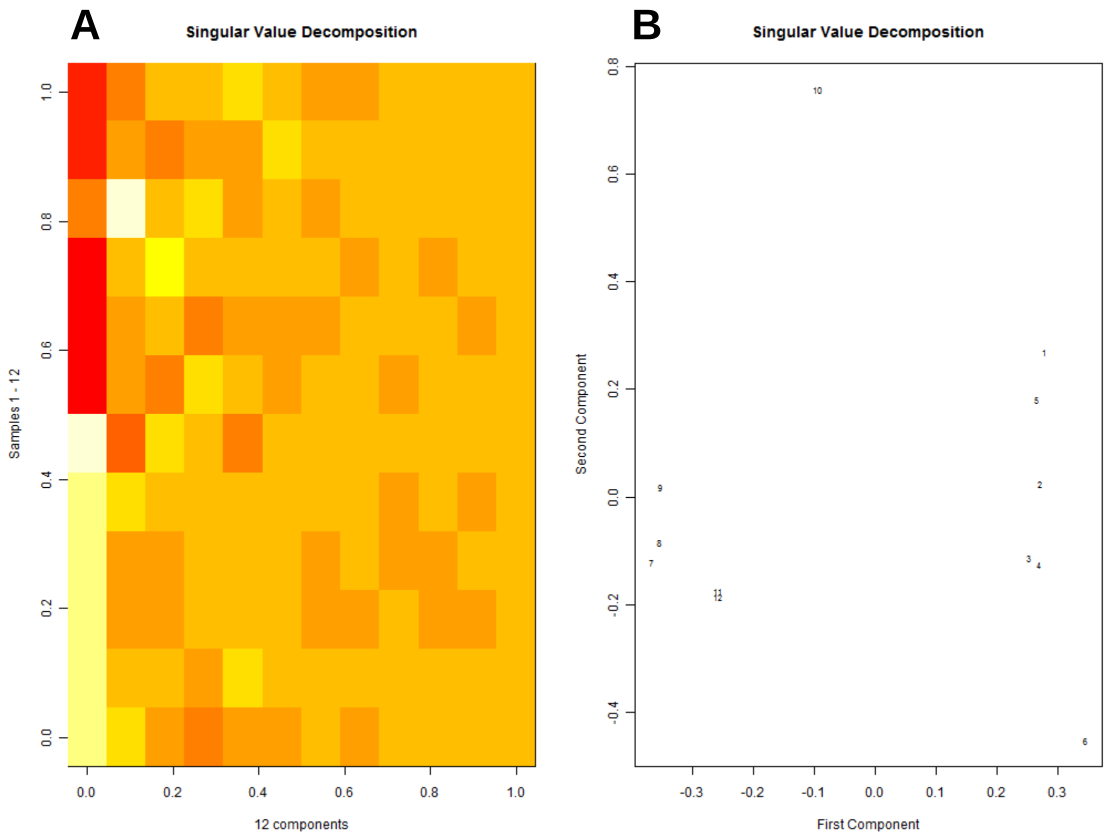

Figure 0.8 The Principal Component Analyses plot obtained from the predictor II pseudo-expression matrix. The plot consists of: heatmap visualizing the captured

variance for each component (A) and scatter-plot showing the first component plotted against second (B).

The heatmap may look slightly different (usually different miRNAs are ranked as the top 50), but the clustering pattern should be conserved (**Figure 0.9**). If the clustering achieved by predictors differs it may suggest that the output of the better performing predictor should be used for further analyses. These differences should be consistent with differences visualised in the PCA plot. Furthermore, similar to the previous example, the volcano plot presenting the p-value cut-off is being presented to the user (**Figure 0.10**).

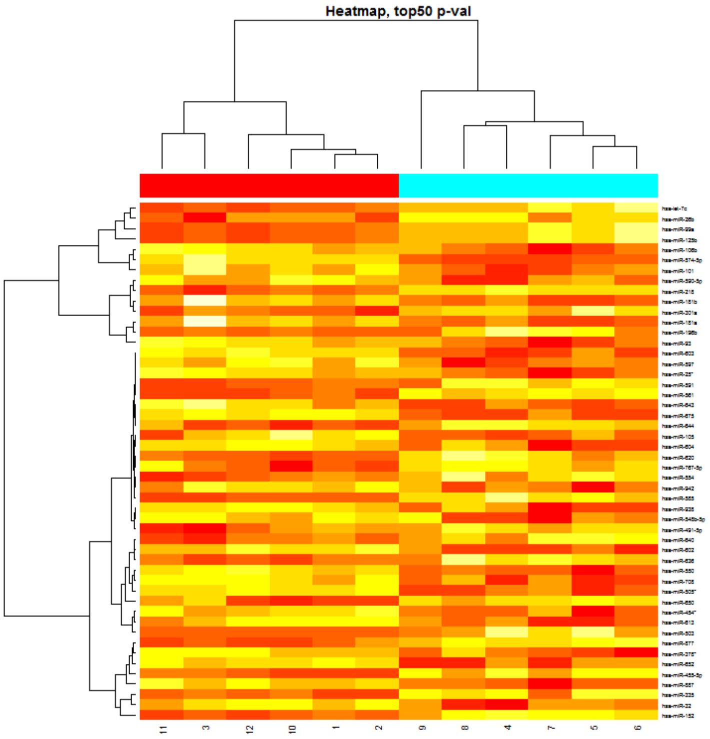

Figure 0.9 The heatmap and hierarchical clustering plot featuring the top 50 differentially expressed miRNAs produced by predictor II (ordered by increasing p-value, obtained from ANOVA).

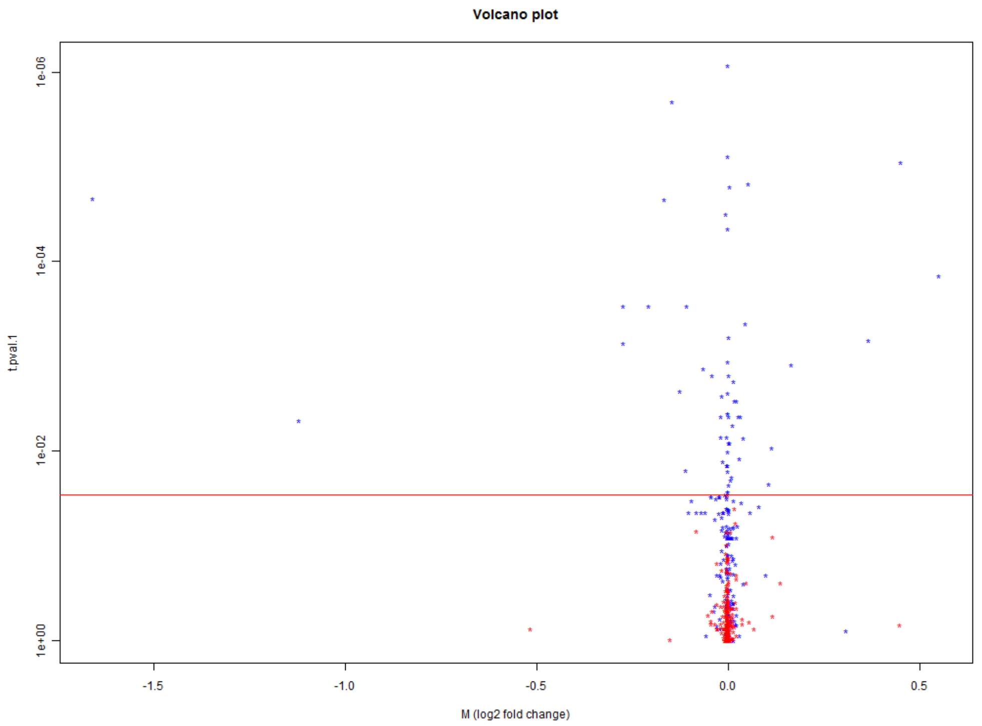

Figure 0.10 The volcano plot created using the miRNA pseudo-expression matrix obtained from predictor II. The fold change is marked on the X-axis and p-value on the Y-axis. The blue points indicate actual fold change – p-value ratio, while randomised values are marked as red points.

1.1.5 Statistical processing and consensus between predictions

This step produces a unified list of significantly up- and down-regulated miRNAs preceded by statistical testing. The plots generated after this step have been presented in the appropriate sections corresponding to the predictors. Since the output of this step is hard to verify and usually not important to a user those lists are not directly presented in the HTML report. However, the number of miRNA found significant by each predictor is displayed to a user in purpose of quality control. The results obtained for the analysed dataset are:

- Statistical testing for microRNA prediction method I - scaling function: 17 genes found significantly up/down-regulated
- Statistical testing for microRNA prediction method II - linear modelling: 57 genes found significantly up/down-regulated

1.1.6 Correlation analyzes

The output from this component allows the user to assess the quality of anti-correlation based miRNA-targets predictions. The correlation matrix itself is not presented to the user; depending on the quality of the data it may be very big. The histogram and distribution function summarizing all negative values from the matrix is presented instead (**Figure 0.11**).

This plot allows the user to audit the correlation cut-off value. The default cut-off is set to -0.8 and has been determined to be good for most robust datasets. In some cases this value may be too low – the low number of miRNA-mRNA interactions is returned, which results in uncertain output of hypergeometric overrepresentation testing. On the other hand in case of some datasets producing very strong statistics this boundary may be too high and should be lowered by the user to achieve more specific outputs.

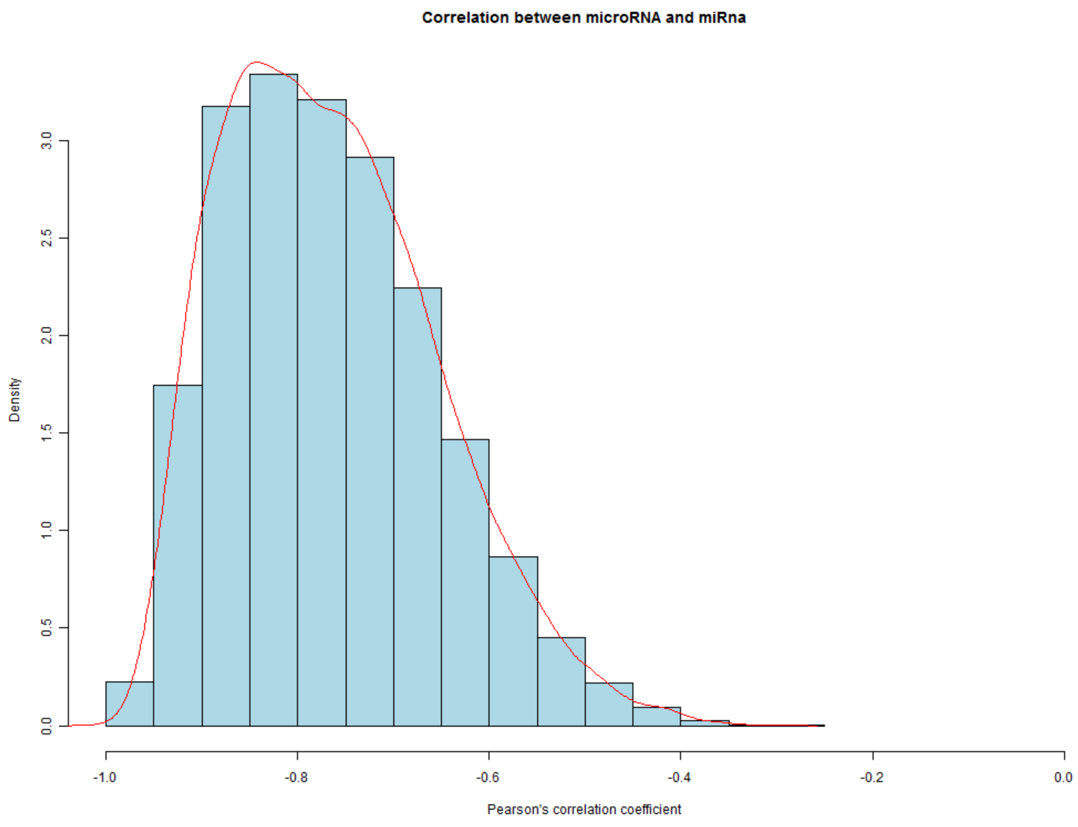

Figure 0.11 The histograms (blue bar-plot) and probability density function (red curve) summarizing all anti-correlated (negative correlation values) miRNA-mRNA interaction

(indicating putative targets). The X-axis shows Pearson's product correlation coefficient value, Y – the density of probability.

Subsequently the three table listing regulatory microRNAs, regulated genes and all interaction are presented in the HTML report. Each table is hidden by default in the collapsible section distinguished by the number of entities included in that table. The results for the analysed dataset are:

- Total number of 67 miRNAs is predicted to have significantly down-regulated targets.
- Total number of 500 genes is predicted to be under miRNA repression.
- Total number of 7662 miRNA-mRNA interactions has been detected for the given cut-off.

Regulatory microRNAs that were indentified to actively suppress messenger RNA transcripts are presented in the first table (**Table 0.2**). Besides miRBase identifiers the table summarizes the number of targets that were connected with the particular miRNA. This table is usually the shortest one - robust output is indicated by a much greater number of active miRNAs than the genes they are suppressing. This relationship is connected with the biological background of miRNA activity – the single miRNA is able to control up to a thousand targets, so observing much more regulated mRNA sequences than its regulators is expected.

Table 0.2 The summary of microRNAs that have been identified to significantly control the expression of messenger RNA transcripts. "microRNA" column lists the miRBase identifiers of miRNAs while "NoSuppressedGenes" indicates the number of putative targets regulated by each miRNA. The rows are sorted by the decreasing number of targets.

	microRNA	NoSuppresedGenes
1	hsa-mir-218-2	328
2	hsa-miR-585	328
3	hsa-miR-561	327
4	hsa-mir-675	316
5	hsa-miR-218	314
6	hsa-miR-152	313

7	hsa-mir-574	313
8	hsa-miR-877	297
9	hsa-mir-3126	266
10	hsa-mir-218-1	209
...	...	...
67	hsa-miR-326	1

The structure of the second table is very similar to the first one. This table summarises the genes that are suspected to be significantly regulated by miRNAs – for each gene the HNGC gene symbol, brief description and the number of putative controlling miRNA is presented (**Table 0.3**). Usually there is a group of miRNA controlling each gene; however there tends to be much fewer miRNAs controlling the single gene than genes controlled by single miRNA. Analysing this table the postulated many-to-many relationship nature of miRNA gene suppressing becomes visible.

Table 0.3 The summary of mRNA transcripts being under putative control of miRNA. The “GeneSymbol” column lists the HNGC gene identifiers, “GenesName” provides brief description of source gene and ”NoTargetingMicroRNA” provides the number of miRNAs that were found to control each gene. The rows are sorted by the decreasing number of controlling miRNAs.

	GenSymbols	GeneName	NoTargetingMicroRNA
1	MARS	methionyl-tRNA synthetase	26
2	NBAS	neuroblastoma amplified sequence	26
3	FAM122C	exportin 5	24
4	IL6R	family with sequence similarity 122C	24
5	KLRG2	interleukin 6 receptor	24
6	OSBPL3	killer cell lectin-like receptor subfamily G, member 2	24
7	PI4K2A	oxysterol binding protein-like 3	24
8	PPTC7	phosphatidylinositol 4-kinase type 2 alpha	24
9	RNF125	PTC7 protein phosphatase homolog (S. cerevisiae)	24
10	TFIP11	ring finger protein 125	24
...	...	...	...
500	PTGFRN	prostaglandin F2 receptor negative regulator	5

The final table summarizes all the miRNA-target interactions identified by the pipeline (**Table 0.4**). This output may be very long and hard to overlook. However, it is the most important source pipeline output that can be used in further analyses. Saved as Comma Separated Values (CSV) files, the table can be imported into various networks analyses software (for example Cytoscape) in order to visualise the results and perform additional analyses (for example assay of connected sub-networks, network clusters, etc.). This table contains all the information that can be found in the previous two tables in addition to Entrez gene id and significance score of the interaction. The score is simply the sum of the “not unique” interaction (Affymetrix IDs connected to mature miRNA IDs, see

Table 0.4 The summary of all unique interactions identified below given correlation cut-off. The columns lists miRBase IDs, Entrez Gene IDs, HNGC gene IDs, brief gene descriptions and significance score. The rows are sorted by the decreasing score values.

	miR	EntrezID	Gene	Name	Score
2358	hsa-let-7c	11184	MAP4K1	mitogen-activated protein kinase 1	6
1	hsa-let-7c	159091	FAM122C	family with sequence similarity 122C	4
69	hsa-let-7c	138009	DCAF4L2	DDB1 and CUL4 associated factor 4-like 2	4
87	hsa-let-7c	30816	ERVWE1	endogenous retroviral family W, env(C7), member 1	4
258	hsa-let-7c	132625	ZFP42	zinc finger protein 42 homolog (mouse)	4
322	hsa-let-7c	84690	SPATA22	spermatogenesis associated 22	4
588	hsa-let-7c	4141	MARS	methionyl-tRNA synthetase	4
739	hsa-mir-505	1634	DCN	decorin	4
740	hsa-mir-550-1	1634	DCN	decorin	4
741	hsa-mir-877	1634	DCN	decorin	4
...	...	...	...	...	...
7662	hsa-miR-578	26119	LDLRAP1	low density lipoprotein receptor adaptor protein 1	1

1.1.7 Final analyses – GO, KEGG, DOLight and user defined terms overrepresentation testing

This final output allows the user to assay functional dependencies between miRNA and the genes controlled by them. These analyses utilize the list of regulated genes only – genes have well established ontology and functional description. Such ontology for miRNA is being built, and relatively small amount of laboratory confirmed targets and an uncertain final set of miRNAs indicates that no established ontology will be built for miRNA in the foreseeable future.

This output is divided in to five sections, each summarizing the overrepresentation testing of different terms set:

- Gene Ontology for Biological Process - level I

- Gene Ontology for Biological Process - level II
- KEGG pathways
- Disease Ontology Light
- User defined Entrez terms

Each section contains a similar set of elements summarizing the different aspects of hypergeometric overrepresentation testing:

- The table presenting overrepresented terms, as well as statistics produced during overrepresentation testing (**Table 0.5** and **Table 0.6**)
- Pie-chart presenting the percentage contribution of top five terms (sorted by p-value) - (**Figure 0.12**)
- Bar-chat presenting the number of genes overrepresented in the top five categories (**Figure 0.13** and **Figure 0.16**)
- Concept-gene network presenting the connection between genes and terms (**Figure 0.14** and **Figure 0.17**)
- Heatmap of the genes most contributing to the top five categories enriched with terms cross tabulation (**Figure 0.15** and **Figure 0.18**)

Since this output schema recurrently repeats for different categories and many examples of the output are being shown in case studies only the output for Gene Ontology level I and user determined terms are presented here with intention to familiarize the reader with the output structure rather than analysing specific ontology.

Gene Ontology level I:

Table 0.5: The summary table of Gene Ontology – Biological Process level I overrepresentation testing. The columns indicates the category name, the number of genes that falls into each category, percentages of genes observed in the list and whole genome, fold change between those values, odds ratio and p-value returned by hypergeometric test. The list is sorted by statistical significance (p-value)

	genes in Category	percent in the observed List	percent in the genome	fold of overrepresents	odds ratio	p value
collagen fibril organization	7	0.0186	0.00183	10.2	13.8	3.6e-06
multicellular organismal development	109	0.2899	0.20245	1.4	1.6	2.6e-05
developmental process	118	0.3138	0.22551	1.4	1.6	3.9e-05
organ development	73	0.1941	0.12397	1.6	1.7	5.5e-05
extracellular matrix organization	11	0.0293	0.00696	4.2	4.7	6.0e-05
skeletal system development	22	0.0585	0.02503	2.3	2.5	2.1e-04
skin development	5	0.0133	0.00190	7.0	8.5	6.3e-04
negative regulation of biological process	70	0.1862	0.12798	1.5	1.6	7.1e-04
anatomical structure development	92	0.2447	0.18149	1.3	1.5	1.1e-03
negative regulation of cellular process	64	0.1702	0.11687	1.5	1.6	1.2e-03
…	…	…	…	…	…	…
embryonic digestive tract morphogenesis	2	0.0053	0.00042	12.6	18.5	9.7e-03

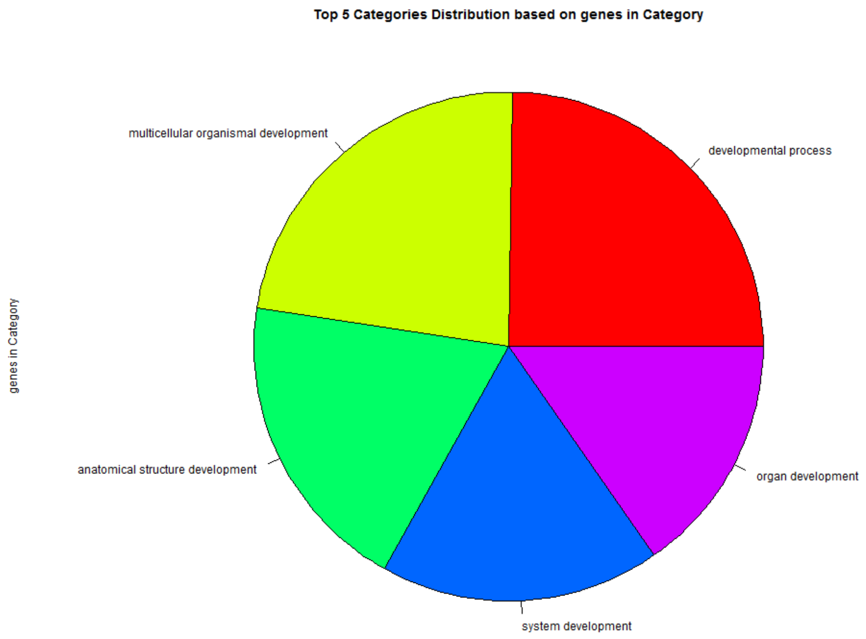

Figure 0.12 Pie-chart presenting the percentage contribution of the top 5 (sorted by p-value) Gene Ontology – Biological Process level I terms.

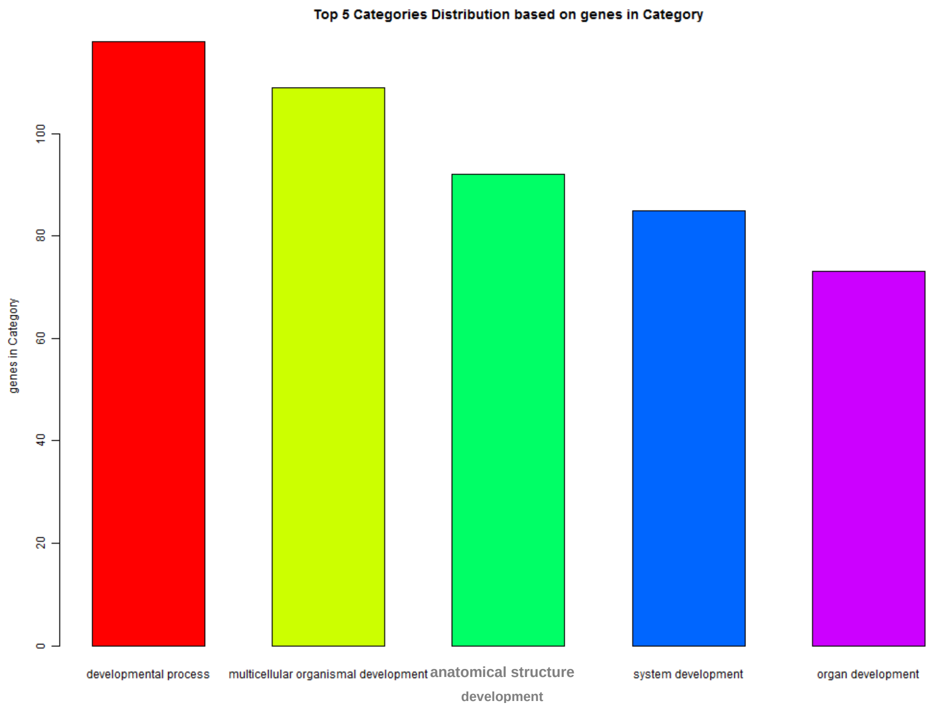

Figure 0.13 Bar chart presenting the number of genes overrepresented in the top 5 categories of Gene Ontology – Biological Process level I.

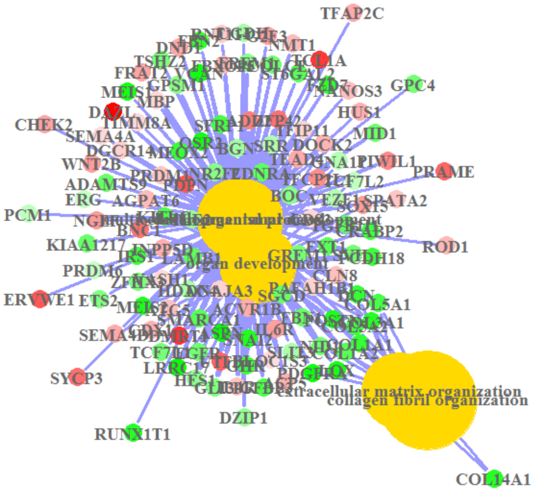

Figure 0.14 Concept-gene network presenting connection between genes and terms overrepresented in Gene Ontology – Biological Process level I.

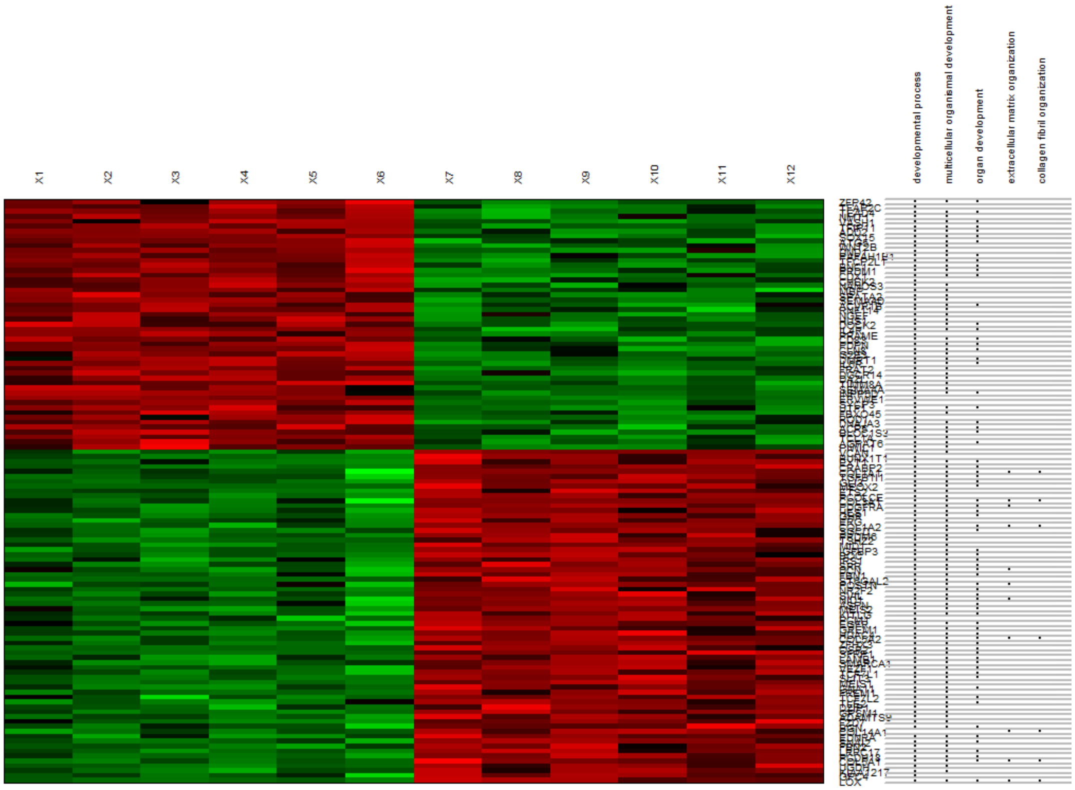

Figure 0.15 Heatmap of the genes most contributing to top 5 categories enriched with Gene Ontology – Biological Process level I terms cross tabulation. The green indicates low while red indicates high expressions (z-score standardized).

User determined Entrez terms

This section has been presented because of the little differences between other functional analyses sections. Only the terms provided by the user are being tested – so all of them are listed in the summary table. The number of rows of the table is equal to the terms listed by the user. Since the parameter defining the terms is not mandatory this section would not be produced if the *"TERMS"* parameter is omitted. Also the pie-chart is not produced in this section – the pie-chart requires at least 5 categories to be available and in many cases the user may be interested in the lower number of them, so producing percentage representation is pointless. The categories in this section may be any biological term supported by the Entrez Gene database. In the given example the overrepresentation of "inflammation", "apoptosis" and "necrosis" have been tested.

Table 0.6: The summary of the user defined Entrez terms overrepresentation testing. The columns indicates the category name, the number of genes that falls into each category, percentages of genes observed in the list and whole genome, fold change between those values, odds ratio and p-value returned by hypergeometric test. The list is sorted by statistical significance (p-value).

	genes in Category	percent in the observed List	percent in the genome	fold of overrepresents	odds ratio	p value
apoptosis	68	0.840	0.058	15	88	4.8e-71
inflammation	18	0.222	0.017	13	17	2.2e-15
necrosis	8	0.099	0.011	9	10	3.4e-06

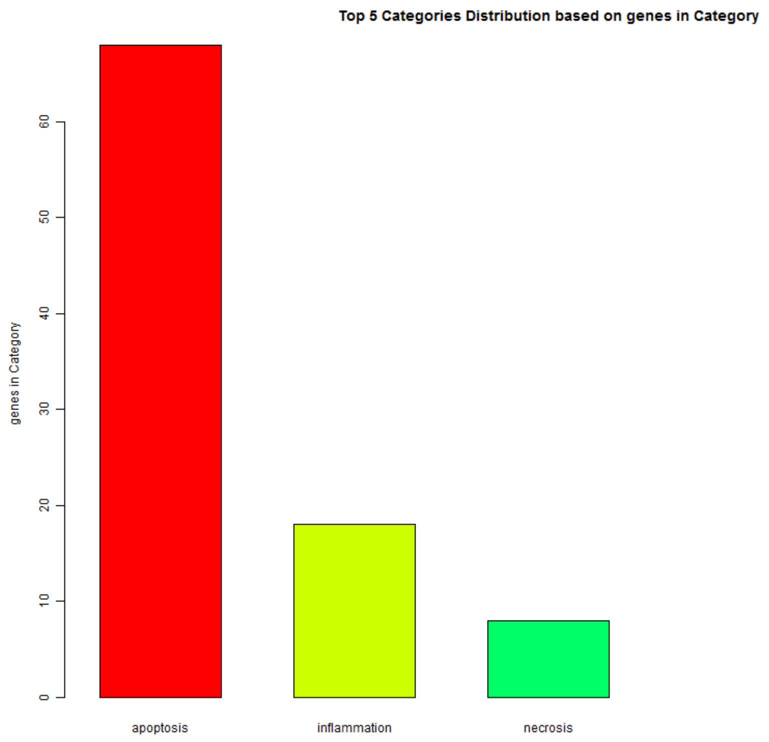

Figure 0.16 Bar chart presenting the number of genes overrepresented in user defined Entrez terms.

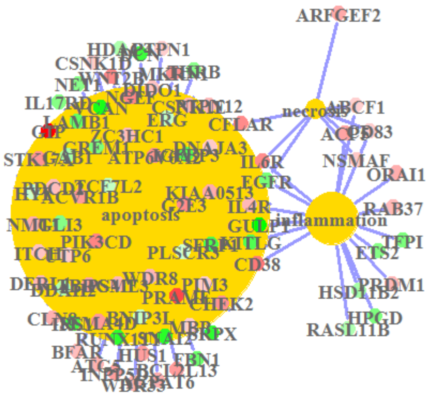

Figure 0.17 Concept-gene network presenting connection between genes and terms overrepresented in user defined Entrez terms

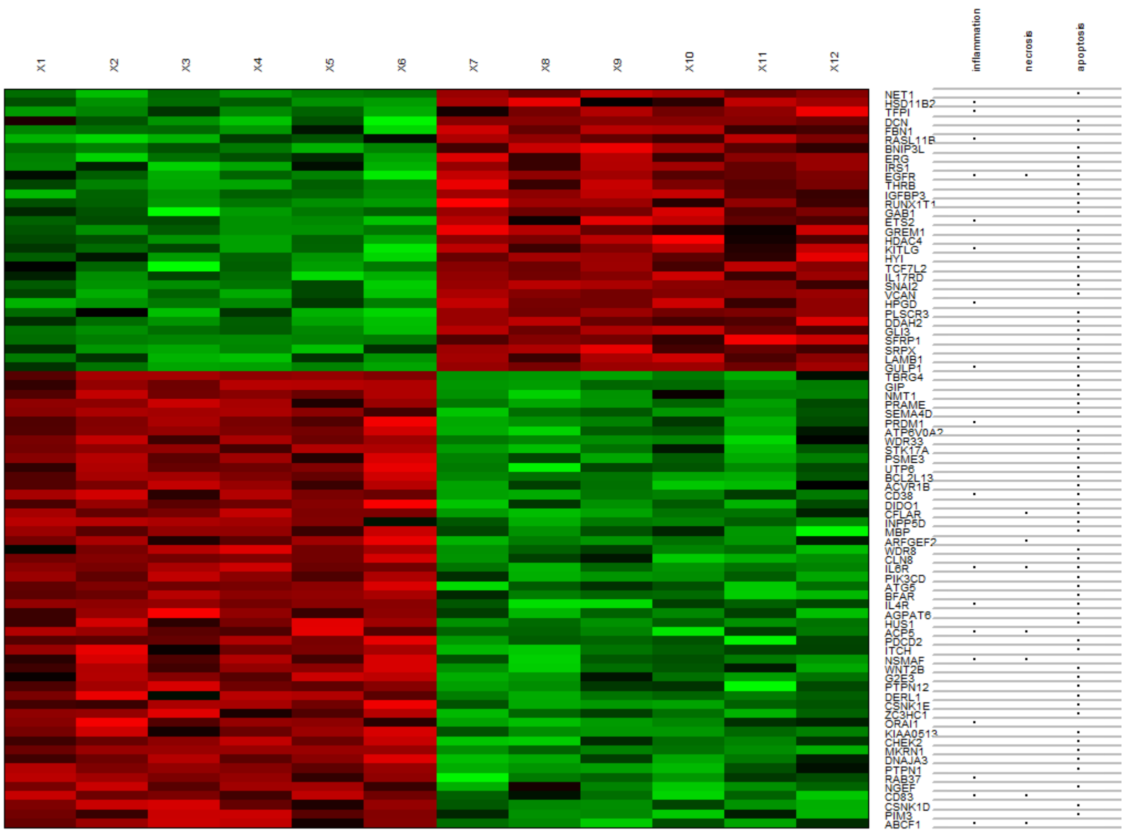

Figure 0.18 Heatmap of the genes most contributing to top 5 categories enriched with user defined Entrez terms cross tabulation. The green indicates low while red indicates high expressions (z-score standardized).
