# Supplementary material for: MMpred: functional miRNA – mRNA interaction analyses by miRNA expression prediction
Source: BMC Genomics. 2012 Nov 14;13:620. doi: 10.1186/1471-2164-13-620 (PMC3562514; doi:10.1186/1471-2164-13-620)
Supplement: Additional file 5 — The short description of analysed case studies. [file 1471-2164-13-620-S5.pdf]

Additional file 10 – The short description of analysed case studies.

Case Study I: Toll-like 4 receptor activated by Lipopolysaccharide (LPS)
Toll-like receptor (TLR) 4 activation of inflammatory networks in the presence of interferon (IFN)-g induces cytokine secretion is considered a canonical inflammatory pathway. Given the small size and simple design of the *"Dendritic cells activated with LPS IFNg over 48 hours"* dataset (8 microarrays, GSE11327, [1]) makes it an ideal to illustrate the workflow and output of the pipeline. The sample/control (*i.e.* dendritic cells activated with LPS/unstimulated dendritic cells) design of the experiment facilitates the use of simple t-test statistics. Standard quality assessment tools indicated that the dataset was of high quality and applying a 10^{-4} p-value cut-off resulted in a list of 244 genes that were significantly differentially expressed. The relevant expression values generated perfect hierarchical clustering within design groups. Consequently the predictors performed well. After statistical filtering 44 and 29 predicted miRNAs were found to be differentially expressed by the scaling function and the linear model predictors respectively. Applying correlation based filtering identified 64 miRNAs that are predicted to regulate 179 genes via 4080 interactions. Functional analyses of this predicted regulatory network revealed overrepresentation of numerous GO and KEGG terms that are characteristic of bacterial infection induced inflammation. The most significant GO terms included "response to external stimuli", "response to stress immune process", "defence response" and various inflammatory signalling pathways. The predicted KEGG pathways reported a number of genes involved in Toll-like receptor singling pathway, B cell receptor signalling pathway, apoptosis, MAPK signalling pathway and cytokine-cytokine receptor interaction. Further analysis of the predicted

gene-to-gene interaction concept networks identified a rich connectivity between the predicted genes and these GO terms and KEGG pathways. The top ten overrepresented Disease Ontology Light terms also indicates a strong association with inflammatory conditions. Analysis of the predicted expression pattern indicated that the predicted miRNA were involved in down-regulation of the inflammatory response, cellular differentiation processes and cell metabolic activity. These predicted expression patterns suggest that, following LPS activated inflammation, miRNA regulatory processes blocks cellular differentiation, active metabolism and the repress the inflammatory process itself, thereby providing negative feedback control that may prevent transition to a chronic inflammatory state. Such conclusions are consistent with the postulated role of microRNA in inflammation [1–6].

Case Study II: Comparison of miRNA regulation in human severe blunt trauma and severe burn injury

The second case study involved comparative analyses of a much larger and complex microarray experiment: That is *"Transcription profiling of human severe blunt trauma patients to predict outcome"* (184 Affymetrix HG-U 133 Plus 2.0 arrays (GSE11375, [7]), and "*A large-scale clinical study of gene expression response to severe burn injury"* (177 Affymetrix HG-U 133 Plus 2.0 arrays (GSE19743, [8]).

The primary objective was the investigation of possible miRNA regulation of the inflammatory response in burn and non-penetrative damages. It has been reported that the most important cause of mortality following severe blunt and burn traumas is multiple organ failure. Over-activation of innate immunity system can lead to severe inflammation and loss of function of multiple organs, which ultimately result in patient death. It is

possible that modelling peripheral blood leukocyte gene expression could be used to profile patient survival.

Our findings (see Table 2) indicated a crucial role for miRNA regulation of inflammatory process in both datasets as well as important differences between these two types of trauma. A predicted commonality between the case studies is that of posttraumatic miRNA repression lifting on genes involved with metabolic activity of leucocytes, metabolism of macro compounds (*i.e.* nucleic acids, proteins) and inflammatory specific pathways. In contrast, the macromolecule biosynthetic process is predicted as activated in burn injury while remaining constant during blunt trauma. MicroRNA regulation of cellular biosynthetic process, translational elongation genes suppression, signalling pathways genes over-expression and suppression of "Ribosome" KEGG pathway's genes are predicted in the burn damage dataset. There is no equivalent evidence of those processes occurring in the blunt trauma dataset. A strong overrepresentation of apoptosis connected genes and a marginal overrepresentation of inflammatory pathway genes was detected in both datasets. Also of interest is that overrepresentation of necrosis genes were predicted as blunt specific. The predictions made using this dataset are summarized in **Table 1**, while further discussion of both case studies can be found in the **Supplementary materials**.

Table 1 - The summary of cooperative blunt damage and burn injury datasets analyses.

Description	Blunt damage	Burn injury
**Increases metabolic activity of leucocytes**	YES	YES
**Increased metabolism of macro compounds (nucleic acids, proteins)**	YES	YES
**Activation of inflammatory pathways**	YES	YES
**Activated macromolecule modification pathways**	YES	NO
**Activated macromolecule biosynthetic process**	NO	YES
**Very specific regulation of cellular biosynthetic process, possibly involving miRNA**	NOT DETECTED	YES
**Translation and translational elongation genes suppression, possibly by miRNA**	NOT DETECTED	YES
**Signalling pathways genes over-expression**	NOT DETECTED	YES
**Suppression of “Ribosome” KEGG pathway’s genes**	NOT DETECTED	YES
**Very strong overrepresentation of genes connected with “Apoptosis”**	YES	YES
**Some overrepresentation of genes connected with “Inflammation”**	YES	YES
**Some overrepresentation of genes connected with “Necrosis”**	YES	NO

References

1. Dohnal AM, Luger R, Paul P, Fuchs D, Felzmann T: **CD40 ligation restores type 1 polarizing capacity in TLR4 activated dendritic cells that have ceased interleukin-12 expression.** *Journal of cellular and molecular medicine* 2008.

2. Tang B, Xiao B, Liu Z, Li N, Zhu E-D, Li B-S, Xie Q-H, Zhuang Y, Zou Q-M, Mao X-H: **Identification of MyD88 as a novel target of miR-155, involved in negative regulation of Helicobacter pylori-induced inflammation.** *FEBS letters* 2010, **584**:1481-6.

3. Perry MM, Moschos SA, Williams AE, Shepherd NJ, Larner-Svensson HM, Lindsay MA: **Rapid changes in microRNA-146a expression negatively regulate the IL-1beta-induced inflammatory response in human lung alveolar epithelial cells.** *Journal of immunology (Baltimore, Md. : 1950)* 2008, **180**:5689-98.

4. Sonkoly E, Pivarcsi A: **microRNAs in inflammation.** *International reviews of immunology* 2009, **28**:535-61.

5. Pauley KM, Cha S, Chan EKL: **MicroRNA in autoimmunity and autoimmune diseases.** *Journal of autoimmunity* 2009, **32**:189-94.

6. Moschos S a, Williams AE, Perry MM, Birrell M a, Belvisi MG, Lindsay M a: **Expression profiling in vivo demonstrates rapid changes in lung microRNA levels following lipopolysaccharide-induced inflammation but not in the anti-inflammatory action of glucocorticoids.** *BMC genomics* 2007, **8**:240.

7. Warren HS, Elson CM, Hayden DL, Schoenfeld DA, Cobb JP, Maier RV, Moldawer LL, Moore EE, Harbrecht BG, Pelak K, Cuschieri J, Herndon DN, Jeschke MG, Finnerty CC, Brownstein BH, Hennessy L, Mason PH, Tompkins RG: **A genomic score prognostic of outcome in trauma patients.** *Molecular medicine (Cambridge, Mass.)* , **15**:220-7.

8. Zhou B, Xu W, Herndon D, Tompkins R, Davis R, Xiao W, Wong WH, Toner M, Warren HS, Schoenfeld DA, Rahme L, McDonald-Smith GP, Hayden D, Mason P, Fagan S, Yu Y-M, Cobb JP, Remick DG, Mannick JA, Lederer JA, Gamelli RL, Silver GM, West MA, Shapiro MB, Smith R, Camp DG, Qian W, Storey J, Mindrinos M, Tibshirani R, Lowry S, Calvano S, Chaudry I, Cohen M, Moore EE, Johnson J, Moldawer LL, Baker HV, Efron PA, Balis UGJ, Billiar TR, Ochoa JB, Sperry JL, Miller-Graziano CL, De AK, Bankey PE, Finnerty CC, Jeschke MG, Minei JP, Arnoldo BD, Hunt JL, Horton J, Brownstein B, Freeman B, Maier RV, Nathens AB, Cuschieri J, Gibran N, Klein M, O'Keefe G: **Analysis of factorial time-course microarrays with application to a clinical study of burn injury.** *Proceedings of the National Academy of Sciences of the United States of America* 2010, **107**:9923-8.
