# Supplementary material for: MMpred: functional miRNA – mRNA interaction analyses by miRNA expression prediction
Source: BMC Genomics. 2012 Nov 14;13:620. doi: 10.1186/1471-2164-13-620 (PMC3562514; doi:10.1186/1471-2164-13-620)
Supplement: Additional file 6 — Detailed report on case study I: Toll-like 4 receptor activated by Lipopolysaccharide (LPS). [file 1471-2164-13-620-S6.pdf]

Additional file 4 – Detailed report on case study I: Toll-like 4 receptor activated by Lipopolysaccharide (LPS)

Toll-like receptor (TLR) 4 activation of inflammation in the presence of interferon (IFN)-g induces cytokine secretion, triggered by microbial lipopolysaccharide (LPS) is one of the canonical inflammatory pathways.

A.1 Design of the experiment

The datasets analysed in this case study is a simple sample/control plus time series experiment. The targeted cell culture was dendritic cells (DCs) in the differentiation process.

During the experiment monocyte derived DCs were matured for 6 hours with 30 ng/ml lipopolysaccharide and 1.000 U/ml IFN-g. RNA from that culture was isolated after 6, 12, 24, and 48 hours. Global gene expression has been measured using the Affymetrix HG-U133 plus 2.0 arrays. The summary of the samples are presented in **Table 0.1**.

The dataset has been chosen for its simplicity – there are only 8 arrays assaying very well described inflammatory pathway. The second reason for the author's interest in this particular dataset is that differentiating cells are subjected to an inflammatory inducer. MicroRNA regulation is known to be specifically active in differentiating cells, so some significant disturbances in microRNA and its targets are expected to be observed.

Table 0.1 The experimental design of "Transcription profiling of human dendritic cells activated with LPS IFNg over 48 hours" experiment.

ArrayExpress ID	Array Data File	Description
**GSE11327GSM286015**	GSM286015.CEL	DCs from a healthy donor activated for 6 hours with LPS IFNg analyzed after 6 hours
**GSE11327GSM286017**	GSM286017.CEL	DCs from a healthy donor activated for 6 hours with LPS IFNg analyzed after 12 hours
**GSE11327GSM286086**	GSM286086.CEL	DCs from a healthy donor activated for 6 hours with LPS IFNg analyzed after 24 hours
**GSE11327GSM286087**	GSM286087.CEL	DCs from a healthy donor activated for 6 hours with LPS IFNg analyzed after 48 hours
**GSE11327GSM286088**	GSM286088.CEL	DCs from a healthy donor, unstimulated control analyzed after 6h hours
**GSE11327GSM286089**	GSM286089.CEL	DCs from a healthy donor, unstimulated control analyzed after 12 hours
**GSE11327GSM286090**	GSM286090.CEL	DCs from a healthy donor, unstimulated control analyzed after 24 hours
**GSE11327GSM286091**	GSM286091.CEL	DCs from a healthy donor, unstimulated control analyzed after 48 hours

A.2 Assessing the quality of data and accuracy of prediction

For the purpose of making this case study as simple as possible only the sample/control design has been used for analyses (t-test). The output of statistical pre-processing revealed perfect clustering among the groups (**Figure 0.1**) and relatively low p-values for such small dataset.

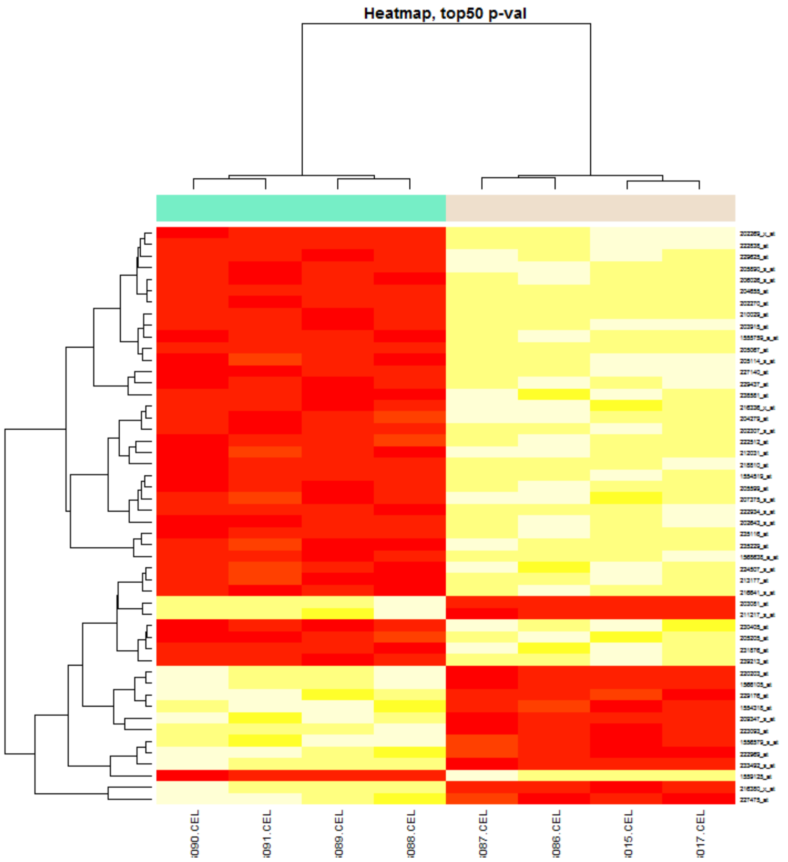

Figure 0.1 The heatmap and hierarchical clustering plot featuring top 50 differentially expressed genes (ordered by increasing, p-value obtained from t-test).

The 244 genes have been found significantly up- or down-regulated and have been used in the correlation matrix construction (**Figure 0.2**).

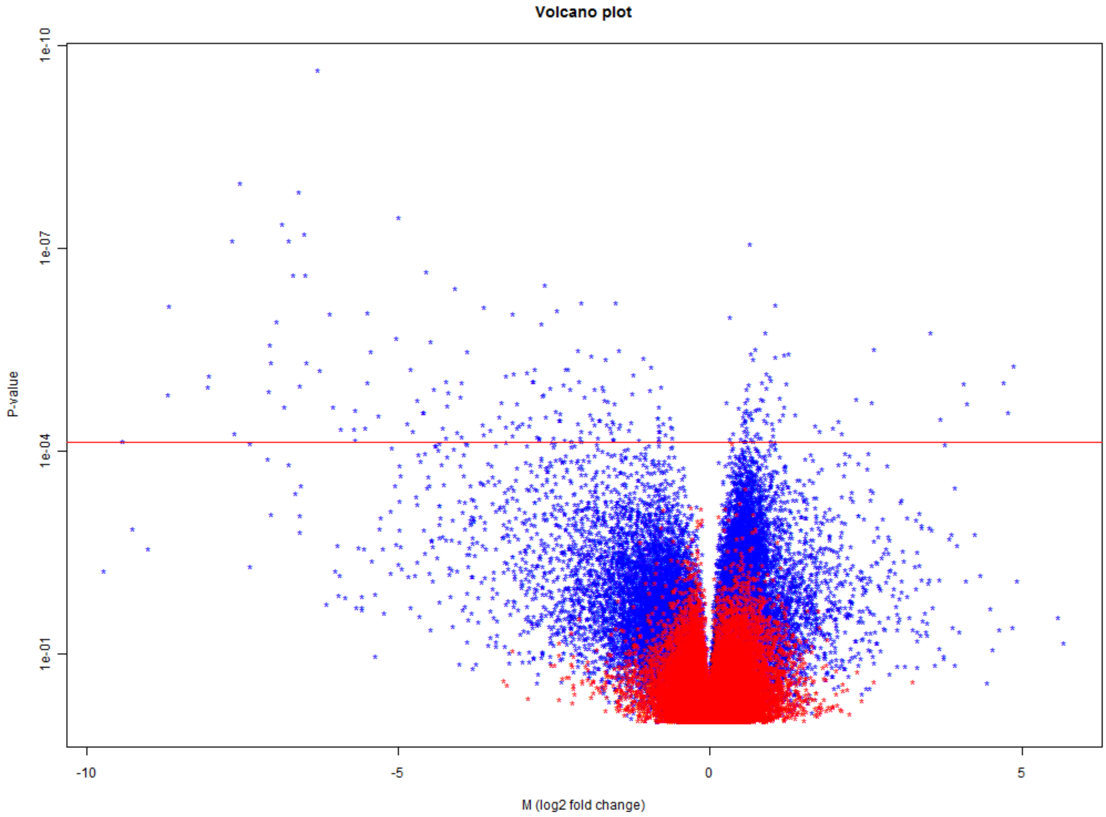

Figure 0.2 The volcano plot for "Transcription profiling of human dendritic cells activated with LPS IFNg over 48 hours" experiment. The fold change is marked on the X-axis and p-value on the Y-axis. The blue points indicate actual fold change – p-value ratio, while randomised values are marked as red points.

The statistical testing performed on miRNA pseudo-expression matrixes (output of both predictors) is consistent with results achieved for mRNA expression data. In both cases the perfect clustering is conserved (**Figure 0.3** and **Figure 0.4**); also p-values indicate robust prediction.

After statistical analyses 44 microRNA predicted by method I - scaling function and 29 predicted by method II - linear modelling: have been found significantly up/down-regulated

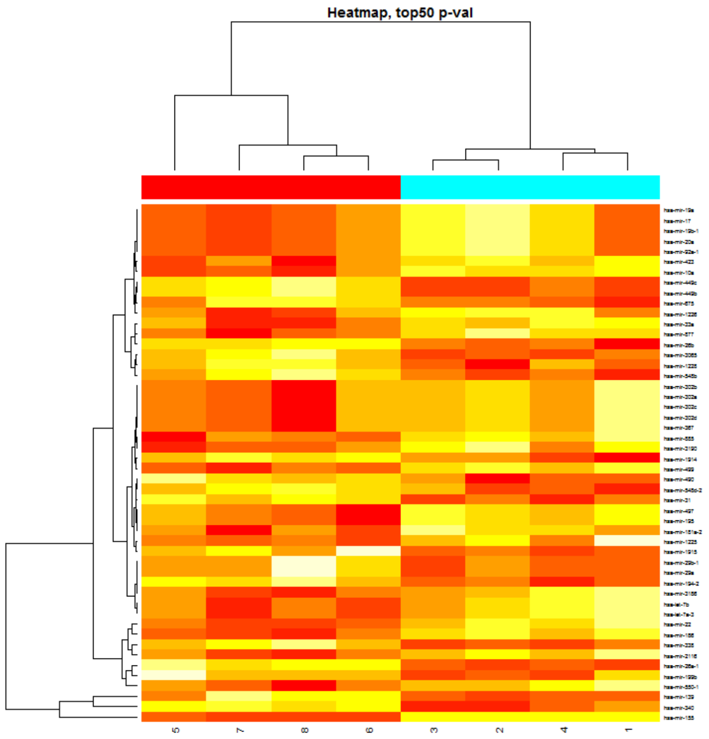

Figure 0.3 The heatmap and hierarchical clustering plot featuring top 50 differentially expressed miRNAs predicted by scaling function (ordered by increasing p-value, obtained from ANOVA).

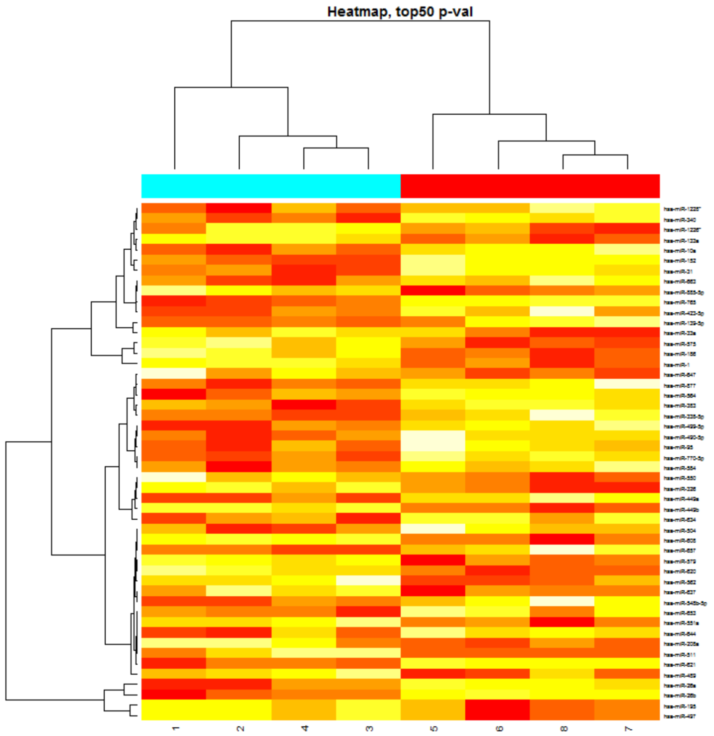

Figure 0.4 The heatmap and hierarchical clustering plot featuring top 50 differentially expressed miRNAs predicted by linear model (ordered by increasing p-value, obtained from ANOVA).

In this case the selection performed by statistical testing was very strict – only 15 miRNA were used for building the correlation matrix. The analyses of the negative correlation histogram shows that the cut-off boundary have been chosen at the right level – many interactions are characterised by very low correlation, the majority is below the default -0.8 correlation cut-off (**Figure 0.5**).

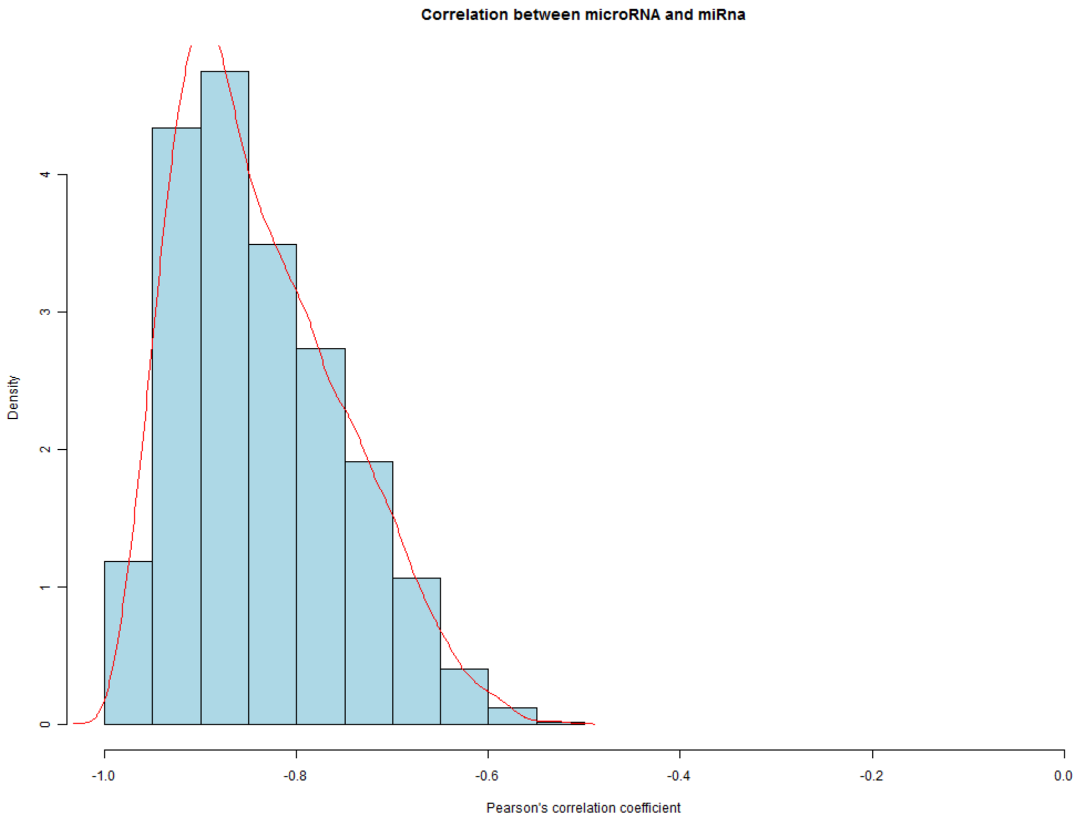

Figure 0.5 The histogram (blue bar-plot) and probability density function (red curve) summarizing all anti-correlated miRNA-mRNA interaction predicted for *"Transcription profiling of human dendritic cells activated with LPS IFNg over 48 hours*" dataset. The X-axis shows Pearson's product correlation coefficient value, Y-axis – the density of probability.

Finally, after performing correlation based filtering 64 miRNAs have been found to regulate 179 genes through 4080 interactions.

A.3 Functional analyses

The output of functional analyses is very consistent with both inflammatory pathways and well-recognised fields of miRNA regulation activity. The categories of Gene Ontology - Biological Process that include most genes are:

- biological regulation
- response to stimulus
- signalling
- signal transmission
- signalling process
- signal transduction
- response to stress
- cell communication
- positive regulation of biological process
- immune system process

Each one of those categories can be easily related to miRNA activity. Also the number of genes supporting most significant categories is very high; 3 top rated contain over 100 genes out of 235 found to be under miRNA control (**Figure 0.6**).

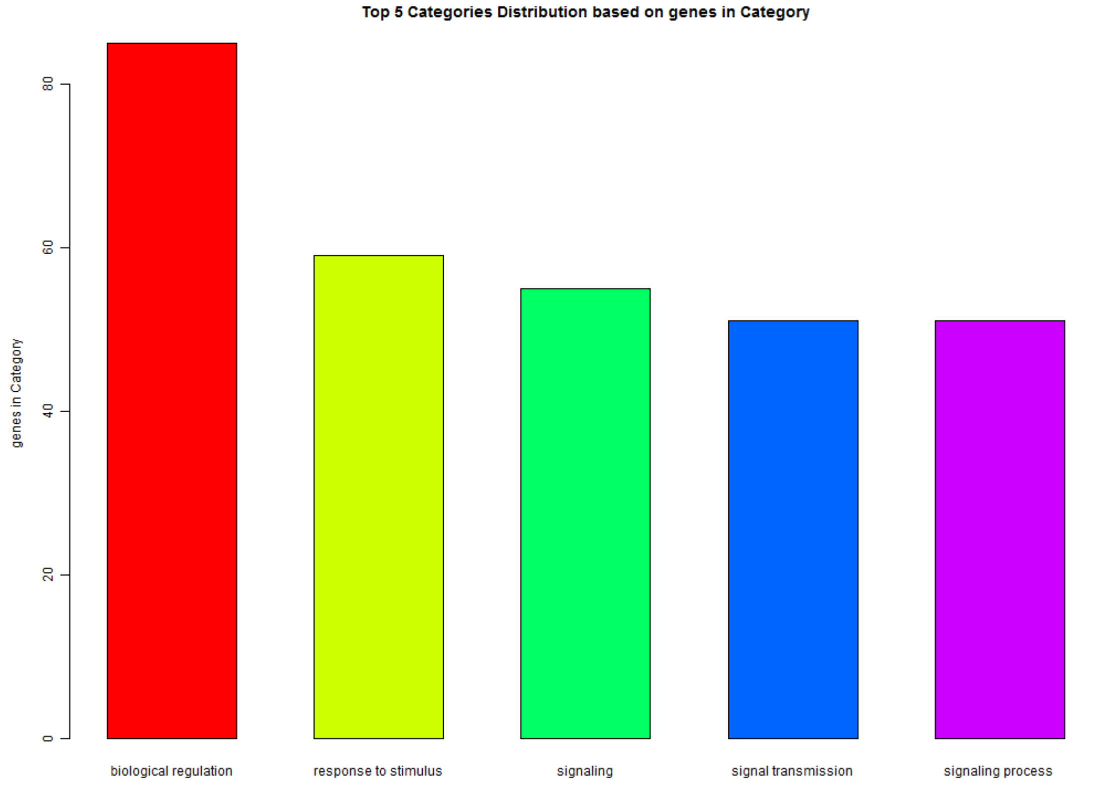

Figure 0.6 Bar chart presenting the number of genes in 5 most overrepresented categories of Gene Ontology – Biological Process level I for "Transcription profiling of human dendritic cells activated with LPS IFNg over 48 hours" dataset

Also very similar terms are found in level 2 Ontology. The 10 most overrepresented categories are:

- signal transmission
- signal transduction
- response to stress
- cell communication
- organ development
- positive regulation of cellular process
- immune response
- regulation of biological quality
- defence response
- response to external stimulus

The expression of most genes in LPS threaded group (arrays 1-4, marked in red) is much lower than expressions of genes in the control group (arrays 5-8, marked in green). This heatmap (**Figure 0.7**) indicates that after LPS treatment the transcription of certain miRNAs have increased targeting genes connected with cell differentiation process and cell metabolic activity (biological regulation, regulation of biological process, regulation of cellular process).

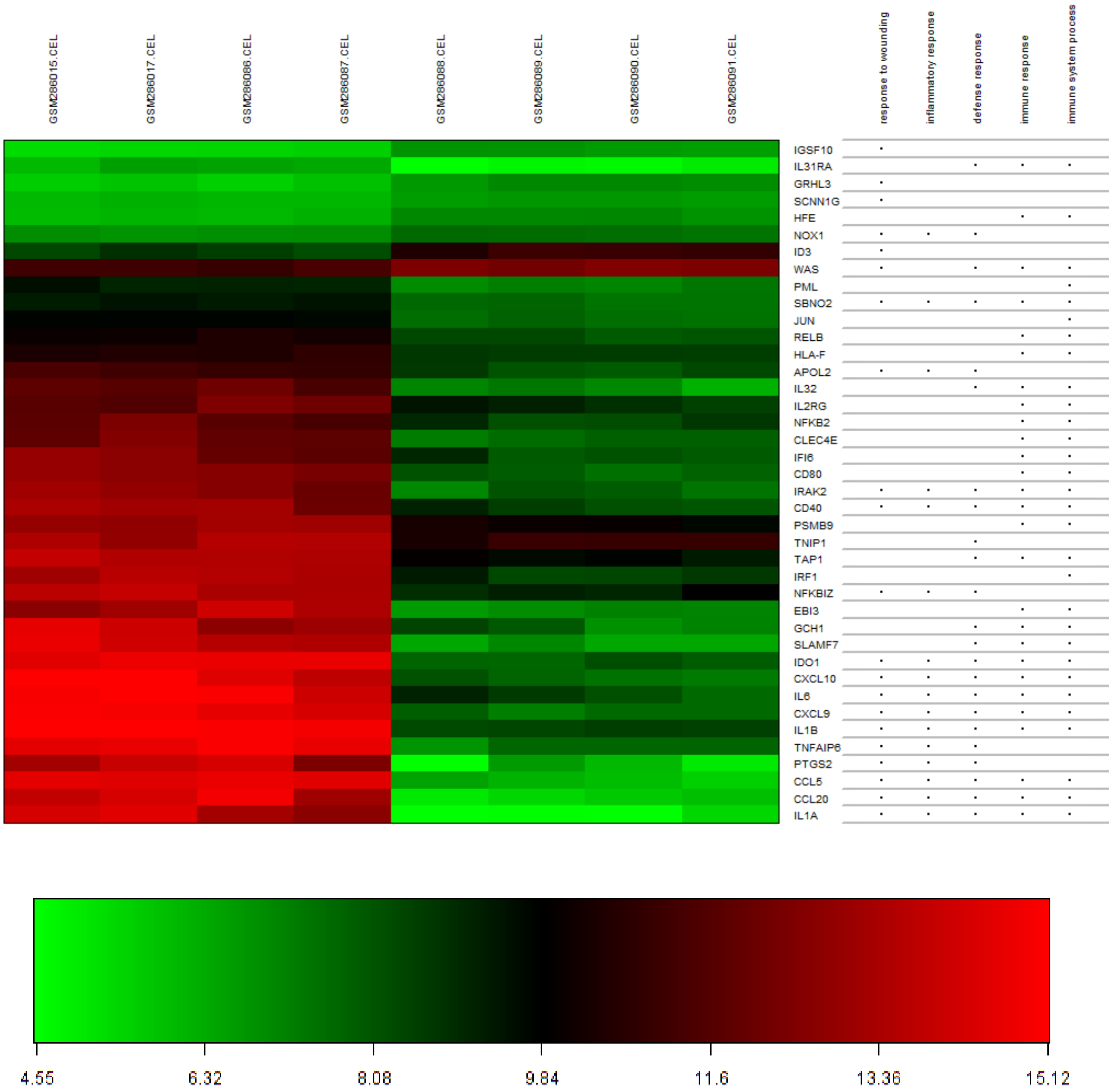

Figure 0.7 Heatmap of the genes most contributing to top 5 categories enriched with Gene Ontology – Biological Process level I terms cross tabulation. The green indicates low while red indicates high expression index (visualised on the colour bar below the heatmap).

Also the KEGG pathways are indicating the processes that should be under miRNA control in inflammatory state. Ten of the most overrepresented categories are known inflammatory activating pathways:

- Cytokine-cytokine receptor interaction
- MAPK signaling pathway
- Toll-like receptor signaling pathway
- Graft-versus-host disease
- Cytosolic DNA-sensing pathway
- B cell receptor signaling pathway
- Apoptosis
- Cell adhesion molecules (CAMs)
- Prion diseases
- Type I diabetes mellitus

The concept-gene network indicates good connectivity between different overrepresented KEGG pathways through many common genes. This suggests coherent regulation directed to promote inflammatory response.

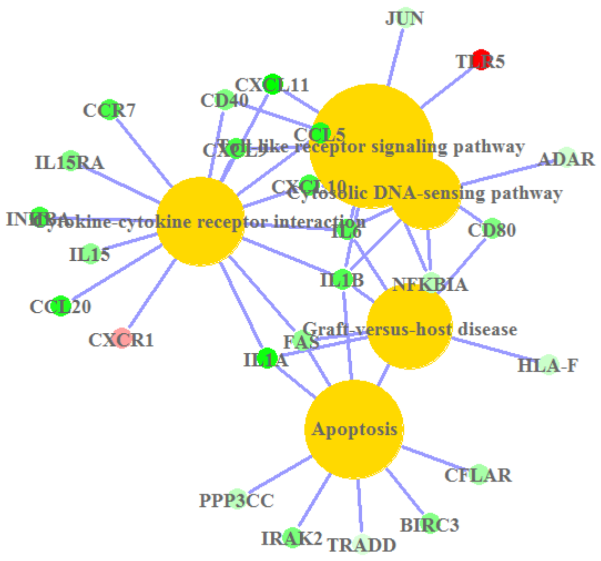

Figure 0.8 Concept-gene network presenting connection between genes and terms overrepresented in KEGG pathways.

The analyses of heatmap summarizing the most contributing genes for the top five KEGG categories (Cytokine-cytokine receptor interaction, Toll-like receptor signaling pathway, MAPK signaling pathway, Graft-versus-host disease and Cytosolic DNA-sensing pathway) suggest that in this case the miRNA works as an inflammatory repressing mechanism.

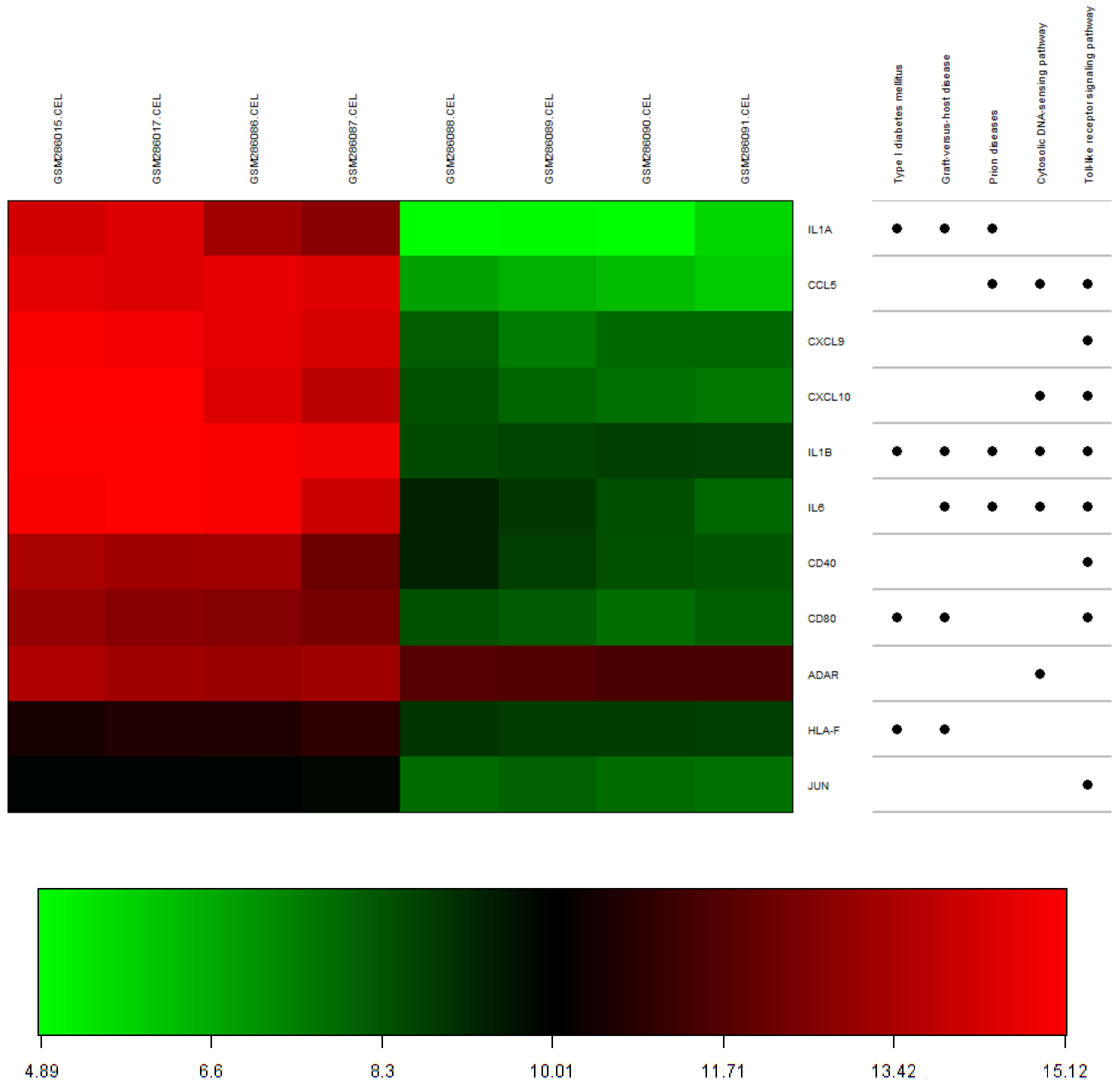

Figure 0.9 Heatmap of the genes most contributing to top 5 KEGG categories enriched cross tabulation. The green indicates low while red indicates high expression index (visualised on the colour bar below the heatmap).

Also the Disease Oncology Light terms are consistent with GO and KEGG pathways:

- Rheumatoid arthritis
- Embryoma
- Leukemia
- Breast cancer
- Atherosclerosis
- Hepatitis C
- Ulcerative colitis

- Dermatitis
- Melanoma
- Asthma

It should be noted, that although none of those diseases is directly caused by bacterial infection carrying LPS stimuli, all ten diseases are connected to inflammation. The expression pattern is very similar to the ones observed for GO and KEGG, so it would not be presented here.

Finally the user determined Entrez terms overrepresentation assay results indicates high (for apoptosis) to moderate (for necrosis and inflammation) overrepresentation in putative miRNA targets (**Table 0.2**).

Table 0.2: The summary table of user defined Entrez terms overrepresentation testing sorted by statistical significance (p-value).

	genes in Category	percent in the observed List	percent in the genome	fold of overrepresents	odds ratio	p value
apoptosis	43	0.74	0.058	13	48	5.1e-41
inflammation	29	0.50	0.017	29	60	6.6e-36
necrosis	23	0.40	0.011	36	61	4.0e-30

The heatmap presents expression change of most contributing genes for each category.

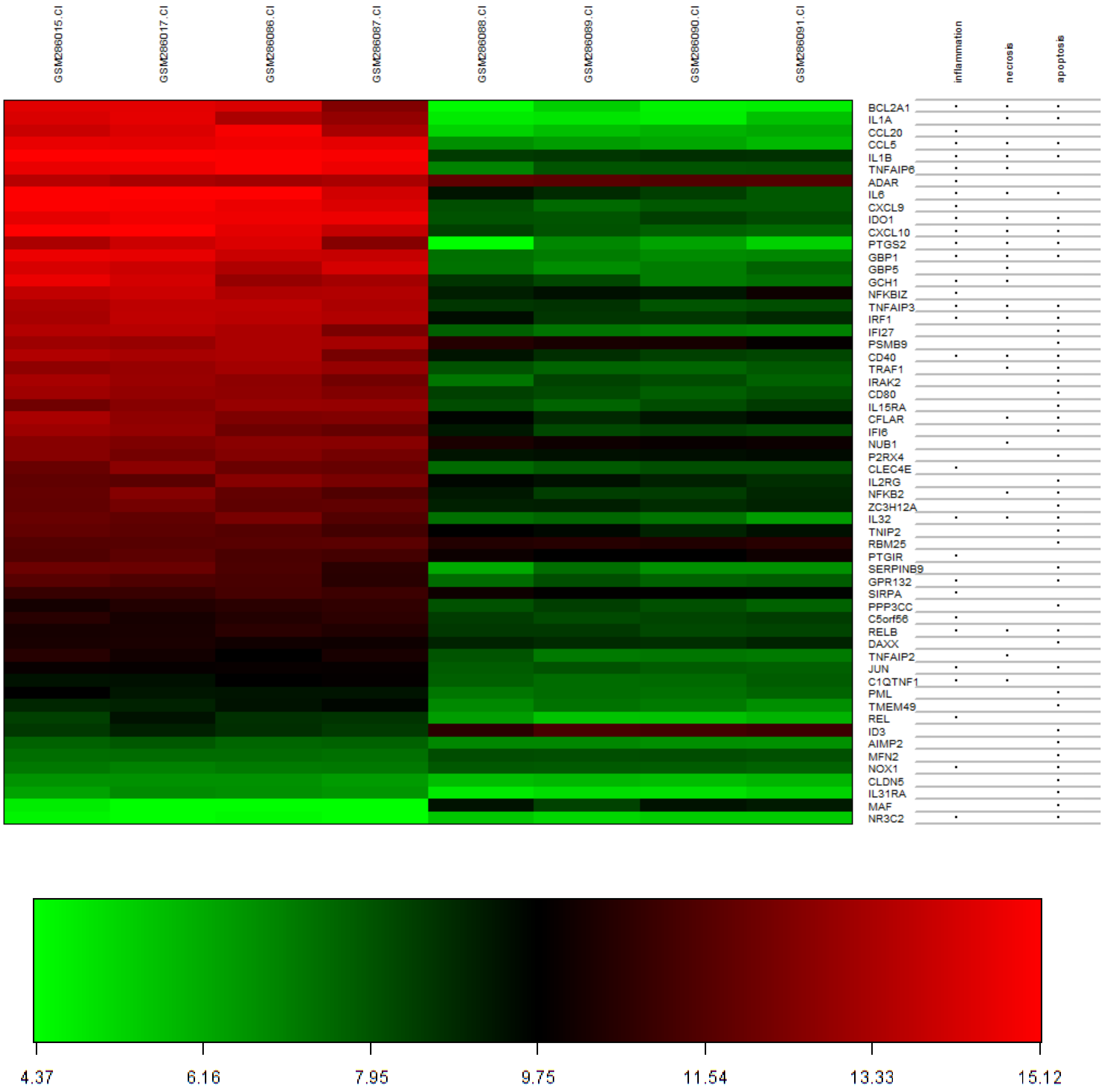

Figure 0.10 Heatmap of the genes most contributing to user defined terms enriched cross tabulation. The green indicates low while red indicates high expression index (visualised on the colour bar below the heatmap).
