# Supplementary material for: MMpred: functional miRNA – mRNA interaction analyses by miRNA expression prediction
Source: BMC Genomics. 2012 Nov 14;13:620. doi: 10.1186/1471-2164-13-620 (PMC3562514; doi:10.1186/1471-2164-13-620)
Supplement: Additional file 7 — Detailed report on case study II: Comparison of miRNA regulation in human severe blunt trauma and severe burn injury. [file 1471-2164-13-620-S7.pdf]

Additional file 5 – Detailed report on case study II: Comparison of miRNA regulation in human severe blunt trauma and severe burn injury

In presented case study two relatively big datasets have been analysed:

- *"Transcription profiling of human severe blunt trauma patients to predict outcome"* 184 Affymetrix HG-U 133 Plus 2.0 arrays (E-GEOD-11375)
- *"A large-scale clinical study of gene expression response to severe burn injury"* - 177 Affymetrix HG-U 133 Plus 2.0 arrays (E-GEOD-19743)

The main purpose of this work was to determine the differences in miRNA regulation of inflammatory response between burn and non-penetrative damages.

1.1.1 Design of the experiments

The main purpose of profiling human gene expression in blunt damages was the assumption that peripheral blood leukocyte gene expression could determine the patient survival chance. The most important mortality reason in the case of severe blunt trauma is multiple organ failure. The over-activation of innate immunity system may lead to massive inflammation resulting in loss of function of many organs ultimately causing the patient's death.

RNA has been isolated from whole blood leukocytes obtained within 12 hours of hospital admission. The whole experimental design containing all patients' background information forms a very long table, so only a summary has been presented here (**Table 0.1**).

Table 0.1 Summary of the *"Transcription profiling of human severe blunt trauma patients to predict outcome"* dataset (E-GEOD-11375) [1].

**Baseline**	
Age, mean (SD), y	34.0 (11.2)
Over 40 y old, N (%)	51 (32%)
Male, N (%)	101 (64%)
No comorbidities, N (%)	45 (28%)
Hypotension, medicated, N (%)	11 (7%)
Smoker, N (%)	63 (40%)
Chronic alcohol abuse, N (%)	24 (15%)
**Injury severity**	
Admitted on ventilator, N (%)	88 (56%)
APACHE score, mean (SD)	27.3 (6.0)
Injury Severity Score, mean (SD)	31.1 (13.5)
Maximum anatomic injury score, mean (SD)	4.0 (0.9)
Blood transfused, 0–12 h, mean (SD), mL	2460 (2050)
Worst base deficit, 0–12 h, mean (SD)	−10.0 (4.6)
**Outcome**	
Days in ICU, mean (SD)	13.2 (11.5)
Days on ventilator, mean (SD)	9.7 (8.5)
Hospital length of stay, mean (SD)	24.8 (18.3)
Maximum Marshall score, mean (SD)	5.5 (2.8)
Maximum Denver score, mean (SD)	2.1 (1.9)
MODS (Marshall criterion: Marshall score ≥ 6), N (%)	67 (42%)
MODS (Denver criterion: Denver score ≥ 4), N (%)	24 (15%)
Complications, N (%)	82 (52%)
Nosocomial infections, N (%)	87 (55%)
Death, N (%)	7 (4%)

The burn injury experiment has been designed to understand the age-dependent response to heat related inflammation stimuli. Blood samples were collected at different times after severe burn injury. Depending on the time of collection the samples were divided into two groups - early stage for <11 days and middle stage for 11-49 days after injury. The dataset contains 114 array experiments for 57 patients - 2 time points per patient. The control group contained 63 unaffected, healthy individuals.

Table 0.2 Summary of the *"A large-scale clinical study of gene expression response to severe burn injury"* dataset (E-GEOD-19743) [2].

	**Adults**	**Children**
Sample size	31	26
Age (years)	40 ± 12	4 ± 3
Gender (F/M)	7/24	6/20
TBSA (%)	65 ± 18	68 ± 14
Inhalation injury (yes/no)	19/12	18/8
Length of hospital stay (days)	70 ± 70	50 ± 30
Length of hospital stay/TBSA	0.9 ± 0.9	0.7 ± 0.4
Survival (yes/no)	20/11	24/2

1.1.2 Assessing the quality of data and accuracy of prediction

For both datasets both the t-test and ANOVA analyses have been performed.

The blunt dataset expression seems to be ambiguous – no strong signal determining the experimental groups have been detected. The first attempt utilized one-way ANOVA with multiple level factor indicating therapeutic outcomes of each patient plus control. The possible outcomes, depending on severity of damage (featured on the heatmap colour bar (**Figure 0.1**) with different colours), were:

- Sending home
- Sending home with services
- Sending to skilled nursing facility
- Sending to inpatient rehabilitation facility
- Sending to another acute care facility
- Other treatment
- Death
- Control group

The heatmap revealed externally poor clustering (**Figure 0.1**); the volcano plot also revealed that random experiment design gives similar level of most p-values to actual experimental data (**Figure 0.2**).

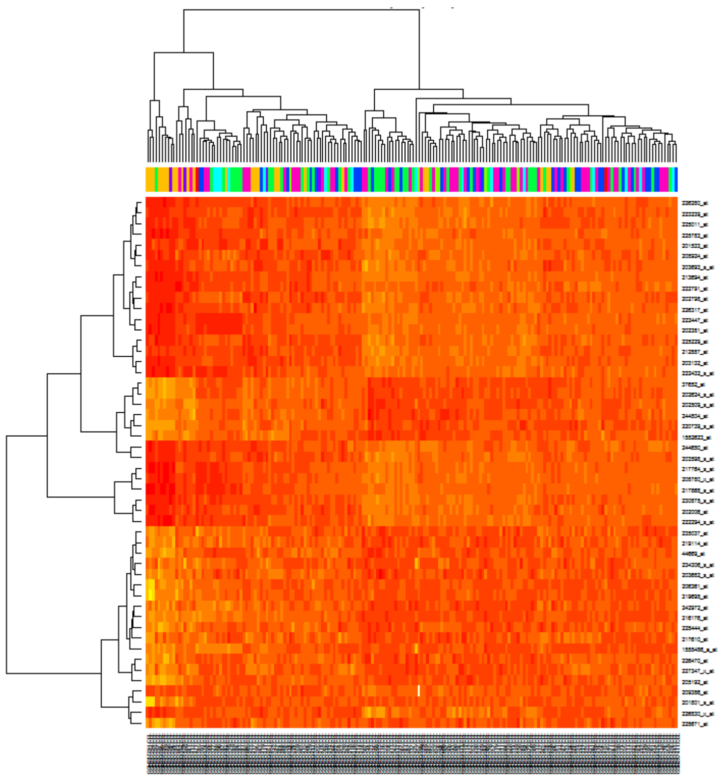

Figure 0.1 The heatmap and hierarchical clustering plot featuring very bad clustering based on the top 50 differentially expressed genes (ordered by increasing, p-value obtained from the t-test).

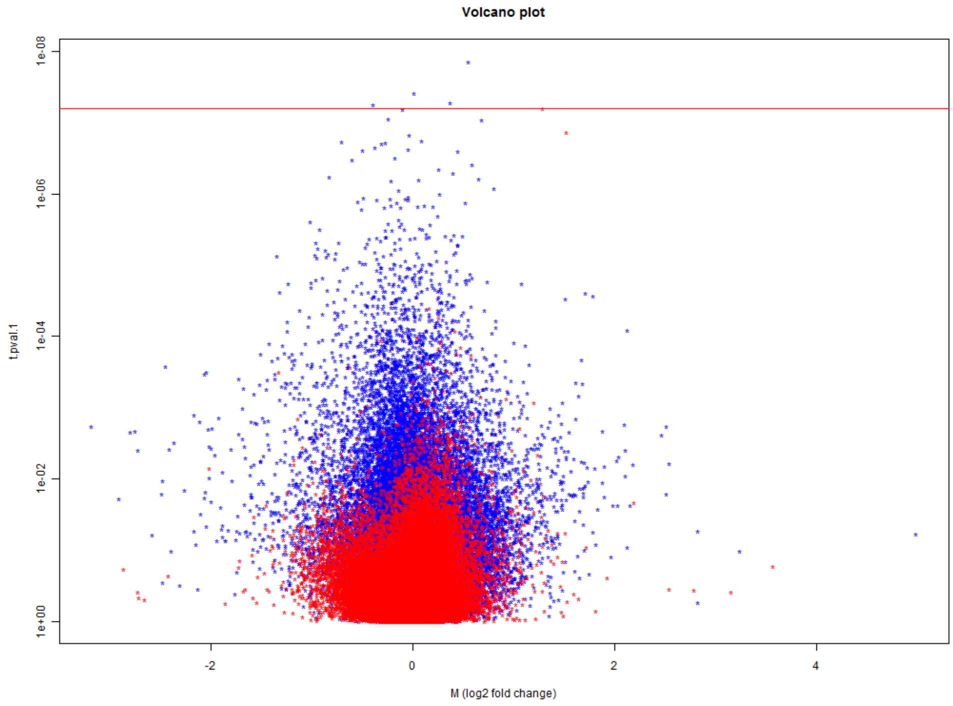

Figure 0.2 The volcano plot for blunt damage experiment. The fold change is marked on the X-axis and p-value on the Y-axis. The plot shows that actual experimental data (blue points) are only a little better than randomised values are (red points).

Better results were achieved using simpler, sample vs. control experimental design. The array experiments clusters into two groups: in one the control (unaffected individuals) are strongly overrepresented (**Figure 0.3**). However, the clustering is still far from perfect, clearly clustering into two groups, which do not overlap with the experimental design, indicates that there is some factor indicating much stronger expression signal than blunt damage itself.

The p-values achieved for experimental design were significantly lower than the randomized ones. The volcano plot has characteristic shape indicating rising absolute value of fold change with lowering p-value (**Figure 0.4**).

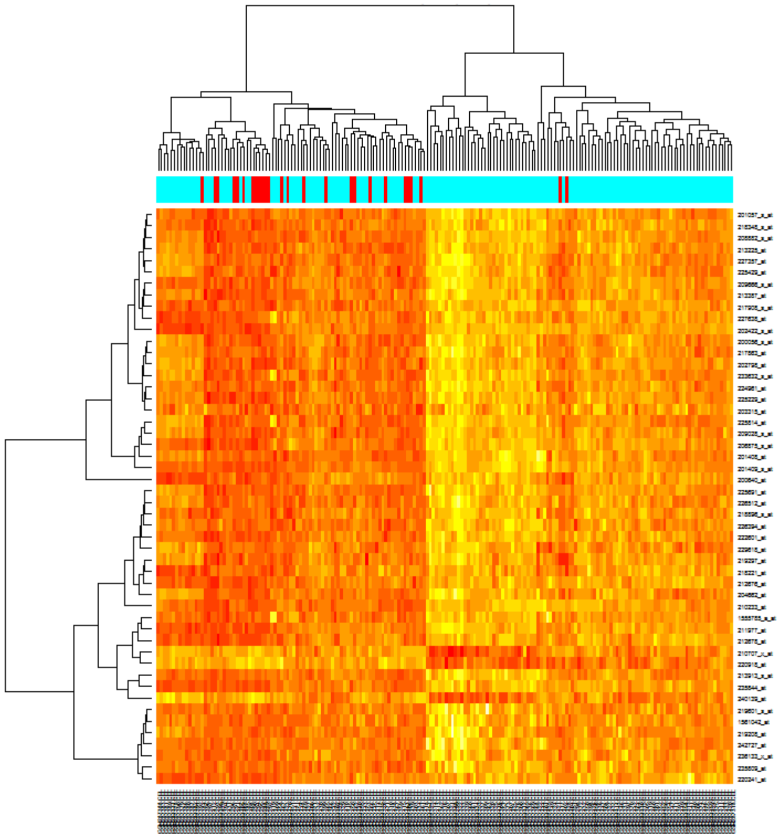

Figure 0.3 The heatmap and hierarchical clustering plot featuring imperfect clustering of sample (red) vs. control (cyan) on the top 50 differentially expressed genes (ordered by increasing, p-value obtained from the t-test).

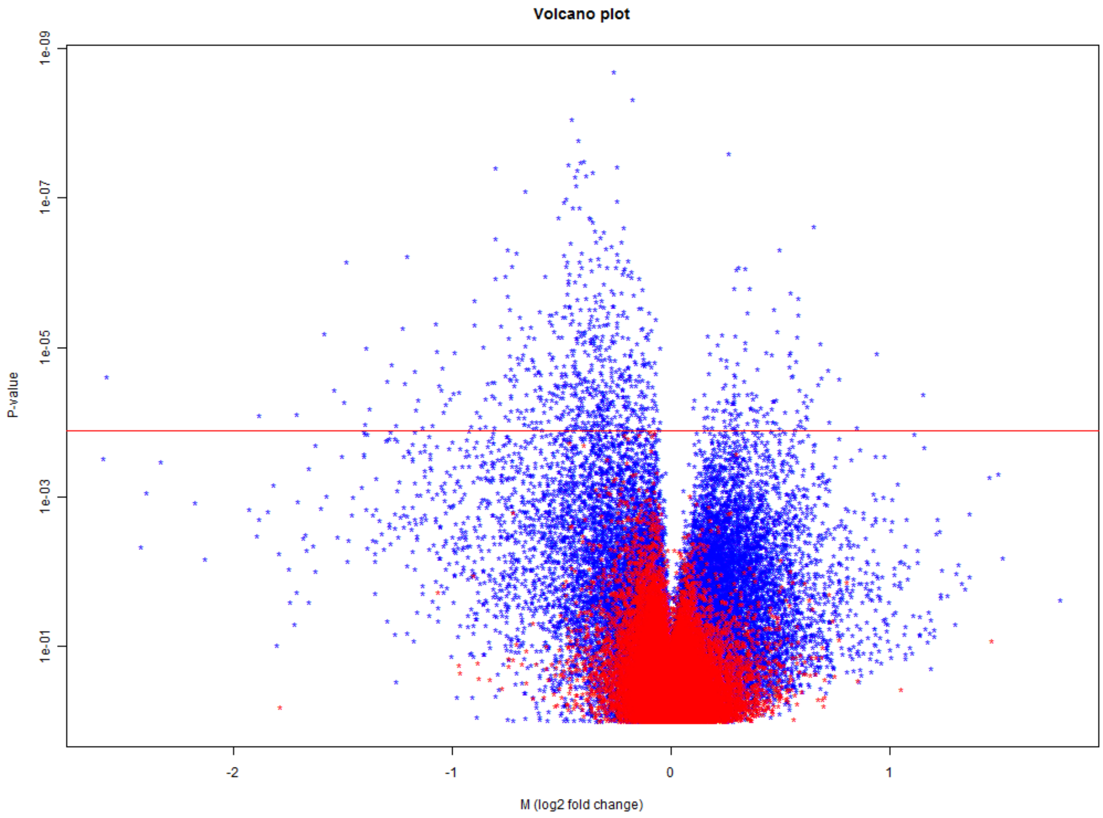

Figure 0.4 The volcano plot for blunt damage experiment. The fold change is marked on the X-axis and p-value on the Y-axis. In this case actual experimental data (blue points) are characterised with significantly lower p-values than randomized ones (red points).

Experiments with more complex designs (multi-way ANOVA, ANOVA with other factors) produced much ambiguous and confusing result than t-test sample/control design – this design have been decided to be utilized as a baseline for the pipeline.

The final mean correlation achieved in the correlation table was relatively high (**Figure 0.5**). The correlation cut-off has to be raised to -0.4 in order to obtain sufficient group of genes for overrepresentation testing. Finally 2105 interaction between miRNA and its targets have been identified. 27 miRNAs are controlling 376 putative target gens.

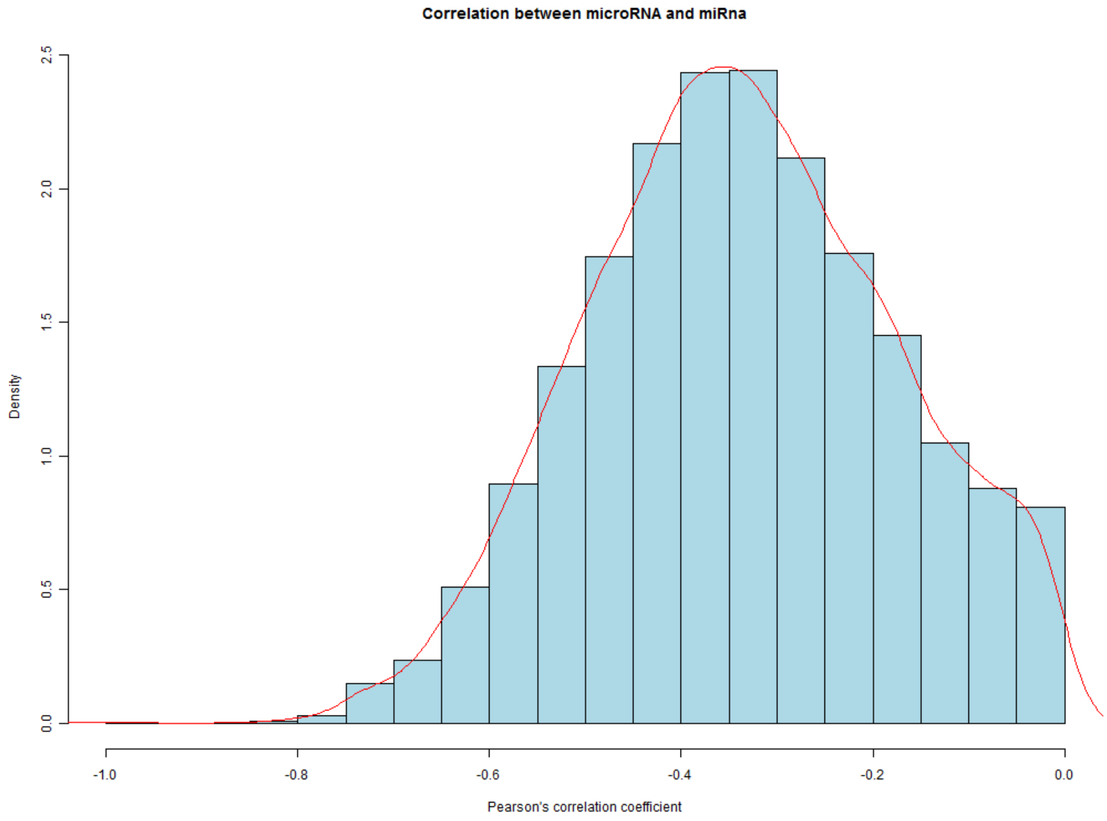

Figure 0.5 The histogram (blue bar-plot) and probability density function (red curve) summarizing all anti-correlated miRNA-mRNA interaction predicted for blunt damage dataset. The X-axis shows Pearson's product correlation coefficient value, Y-axis – the density of probability.

Much more consistent results have been achieved for the burn injury dataset. It seems that signal from burn damage must be much stronger and more specific than blunt damage one. The first design utilised the original experimental design with small modification. The two-way ANOVA had been performed. The first factor indicates time after treatment addition (as design in section 1.1.1) and control group. The second factor determines if patient or control individual is infant or adult. Utilizing this design brought the same clustering (**Figure 0.6**) and very good p-value level, much lower than in the case of permutated design (**Figure 0.7**).

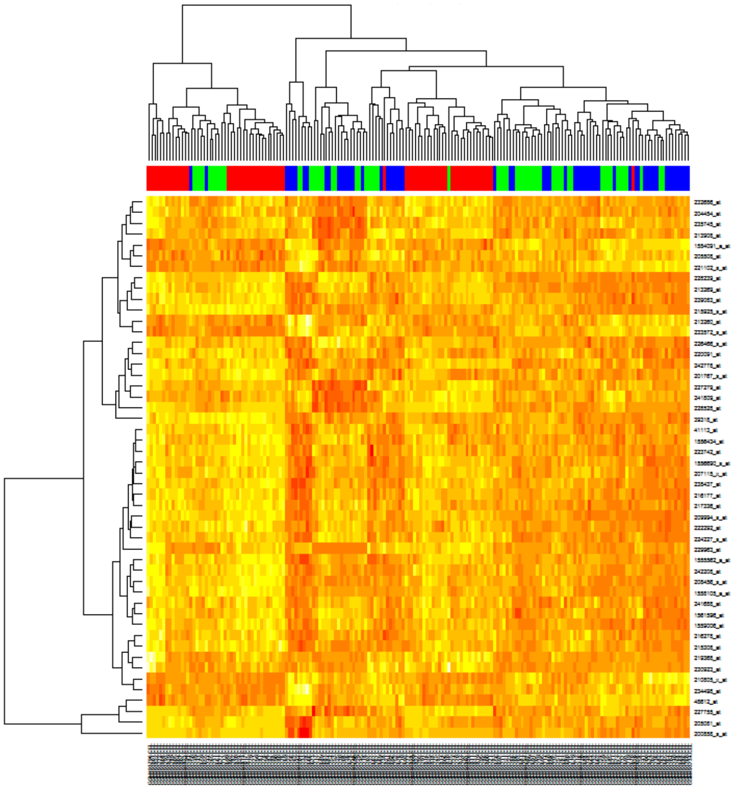

Figure 0.6 The heatmap and hierarchical clustering plot featuring some clustering of early (green), medium time taken samples control (blue) and control (red) on top 50 differentially expressed genes (ordered by increasing, p-value obtained from t-test).

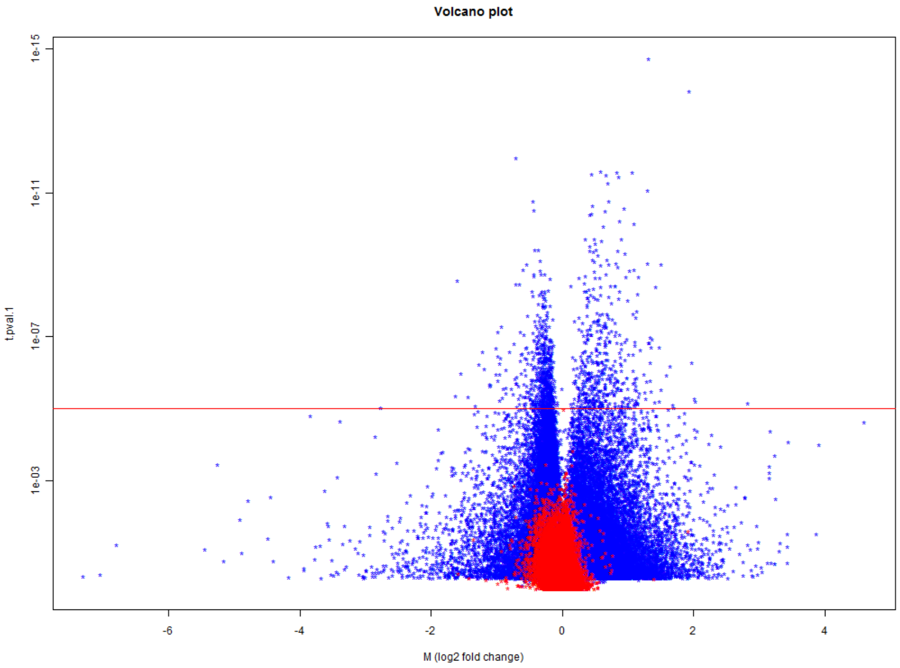

Figure 0.7 The volcano plot for burn injury experiment. The fold change is marked on the X-axis and p-value on the Y-axis. Actual experimental data (blue points) are characterised with significantly lower p-values than randomized ones (red points).

The interesting fact is that the clustering based on predicted miRNA activity may be considered better than the one based on the experimental data. Although the results obtained for each predictors differs for both scaling function predictor (**Figure 0.8**) and linear model predictor (**Figure 0.9**) good clustering had been achieved. Particularly the clustering of the control group (red) seems much better for predicted miRNA based results.

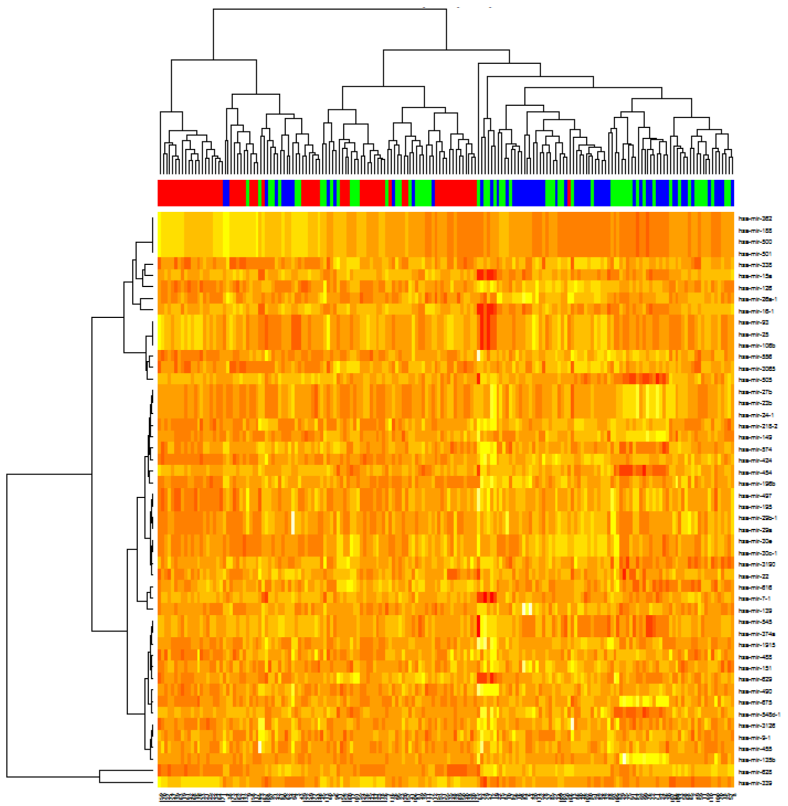

Figure 0.8 The heatmap and hierarchical clustering plot featuring good clustering of early (green), medium time taken samples control (blue) and control (red) on top 50 predicted by scaling function, most active miRNAs (ordered by increasing, p-value obtained from multi-way ANOVA).

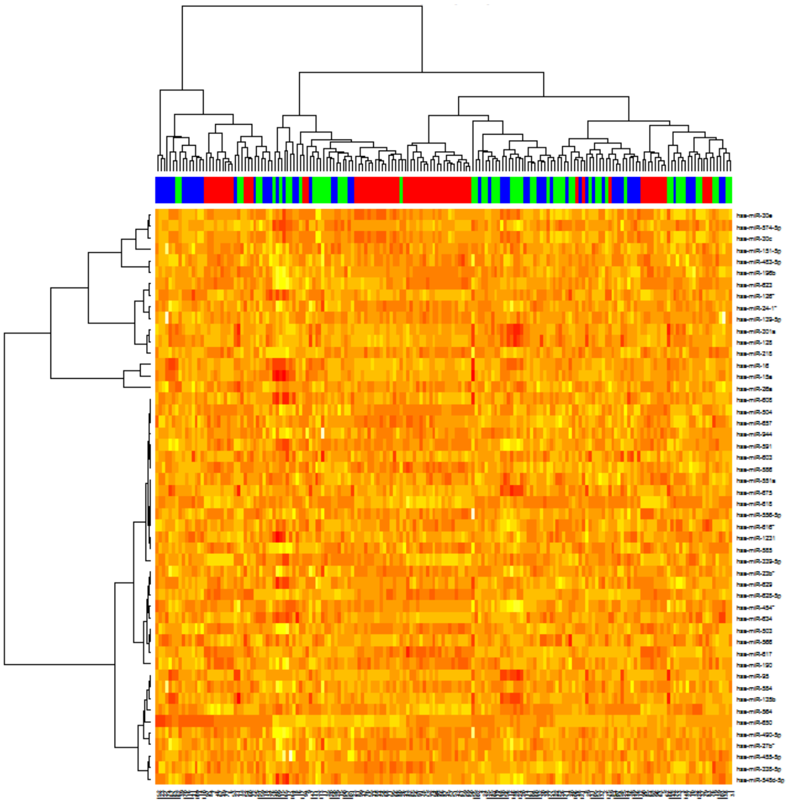

Figure 0.9 The heatmap and hierarchical clustering plot featuring good clustering of early (green), medium time taken samples control (blue) and control (red) on top 50 predicted by linear modelling, most active miRNAs (ordered by increasing, p-value obtained from multi-way ANOVA).

The second experimental design producing interesting, consistent results were one-way ANOVA assaying of gene expression differences between patients that recovered from the burn injury, the ones that passed away and the control group of individuals.

This design produces near-perfect (only one individual misclassified) clustering in sample injured patients and some clustering between recovered and dead patients **(Figure 0.10**). The p-values in that case reached extremely low level (some values reaching 10^{-90}, most of the genes below 10^{-10} level); the top randomised values were detected in 80 fold lower level (**Figure 0.11**). These strong statistics brings hope for robust miRNA activity prediction.

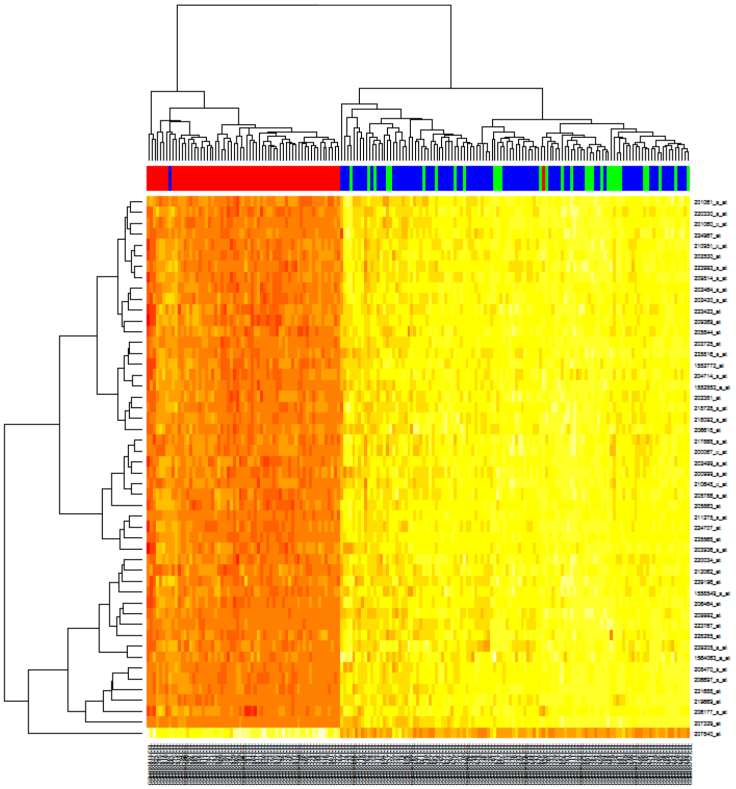

Figure 0.10 The heatmap and hierarchical clustering plot featuring near perfect clustering of control group (red) and some clustering of dead (green), and recovered (blue) patient based on the top 50 differentially expressed genes (ordered by increasing, p-value obtained from the t-test).

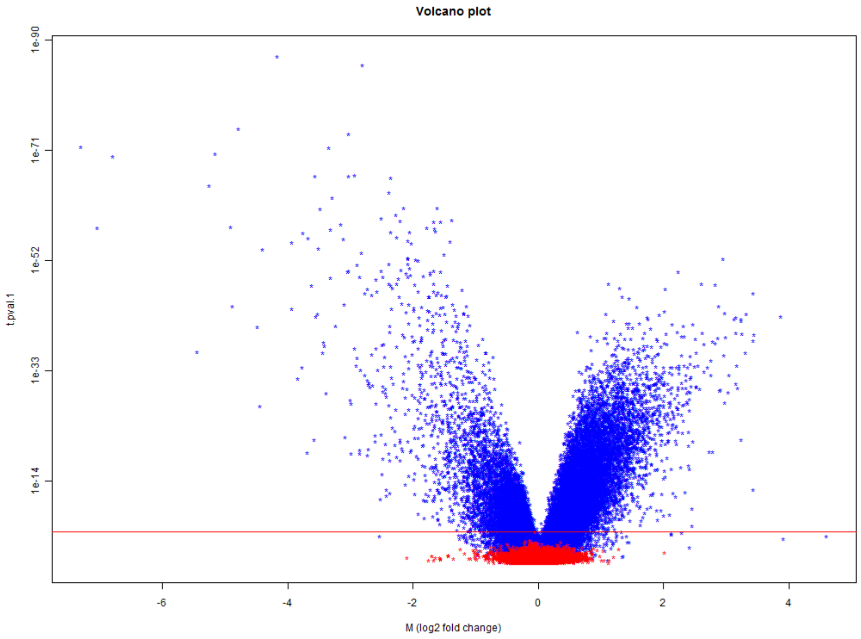

Figure 0.11 The volcano plot for burn injury experiment (recovered, dead and control design). The fold change is marked on the X-axis and p-value on the Y-axis. Actual experimental data (blue points) are characterised with extremely low p-values comparing to randomized design (red points).

Generally good clustering is conserved after prediction. However, the near-perfect clustering of the unaffected individuals is lost. For that price we can observe a little better clustering in recovered and dead patients groups (**Figure 0.12** and **Figure 0.13**).

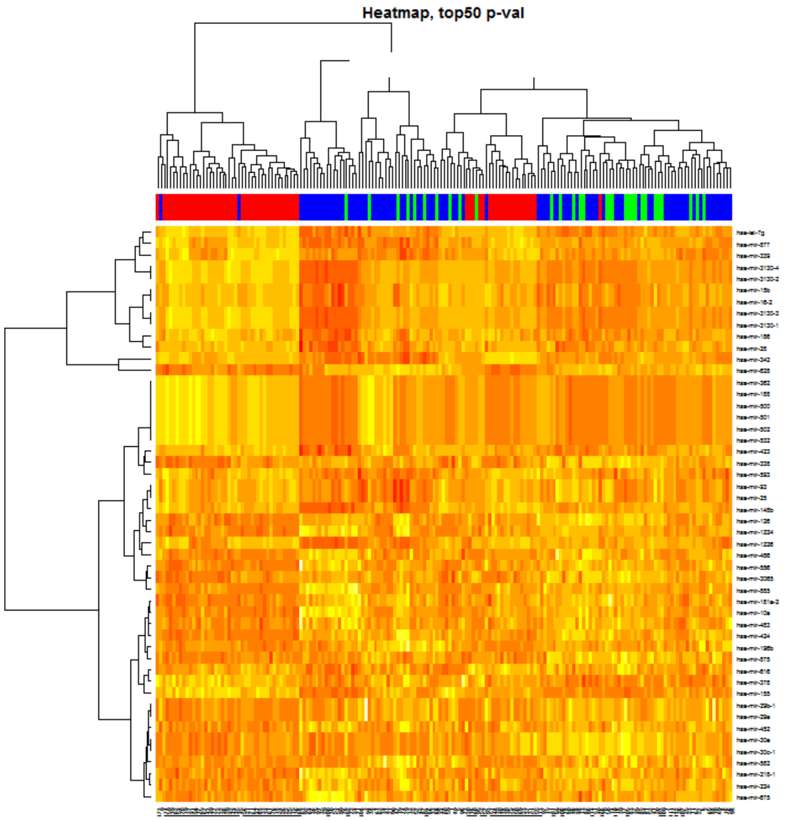

Figure 0.12 The heatmap and hierarchical clustering plot featuring good clustering of control group (red) and some clustering of dead (green), and recovered (blue) patients. Based on the top 50 predicted by scaling function, most active miRNAs (ordered by increasing, p-value from one-way ANOVA).

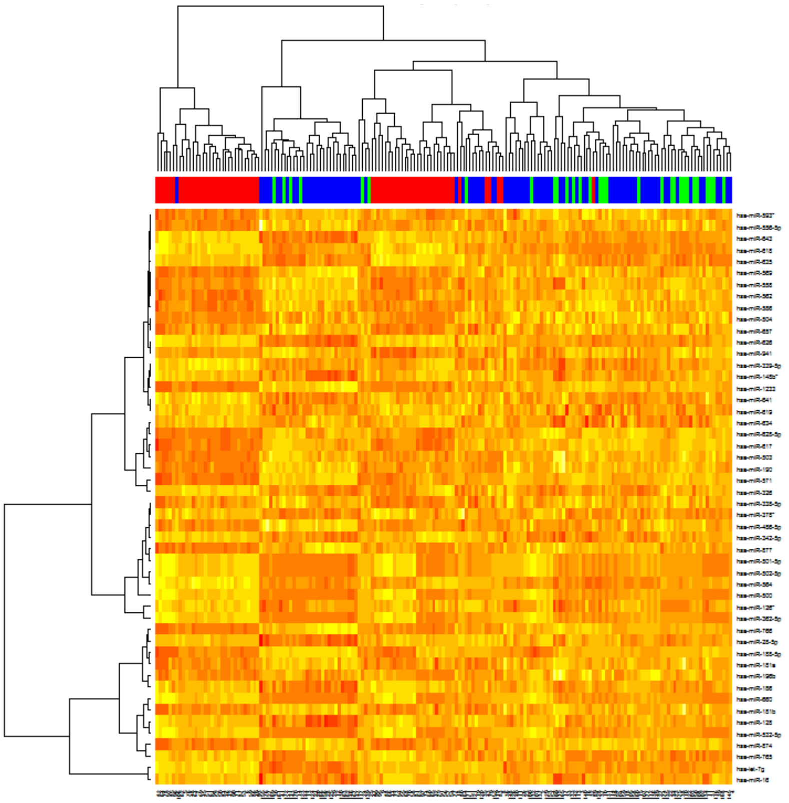

Figure 0.13 The heatmap and hierarchical clustering plot featuring good clustering of control group (red) and some clustering of dead (green), and recovered (blue) patients. Based on the top 50 predicted by scaling function, most active miRNAs (ordered by increasing, p-value from one-way ANOVA).

1.1.3 Functional analyses

Since only the t-test sample/control design returned reliable results in the case of blunt damage dataset the functional prediction output was much smaller than in case of the burn injury dataset. Hoverer, in both datasets some interesting insights may be formed basing on miRNA activity predictions.

The ten most numerous Gene Ontology categories for blunt damage dataset are:

- cellular process
- primary metabolic process
- cellular metabolic process
- macromolecule metabolic process
- cellular macromolecule metabolic process
- protein metabolic process
- cellular protein metabolic process
- catabolic process
- macromolecule modification
- protein modification process

Very many genes fall into overrepresented categories. All the top five categories (sorted by hypergeometric test p-value) contain more than 100 genes (**Figure 0.14**). For example 277 out of 376 total genes found to be under significant genes fall into the "cellular process" category - this number represents a vast majority of all genes falling into this category in whole human genome. On the other hand the "cellular process" is not specific so it can be interpreted in different ways. Analysing more specific overrepresented categories reveals that most of those genes are connected with metabolism or catabolism.

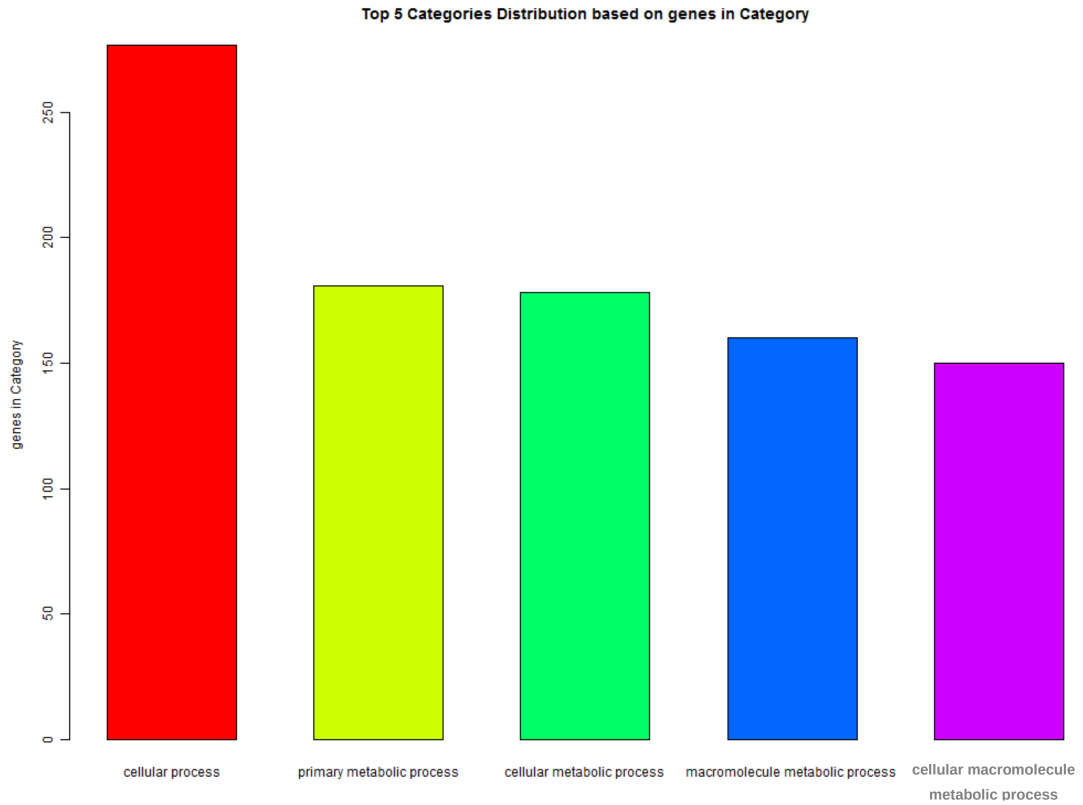

Figure 0.14 Bar chart presenting the number of genes in the 5 most overrepresented categories of Gene Ontology – Biological Process level I for blunt damage dataset. All the categories are connected with cellular metabolic process.

The heatmap shows that all those genes are slightly over-expressed in the sample group. It may indicate inflammatory processes happening on the whole organism level (**Figure 0.15**).

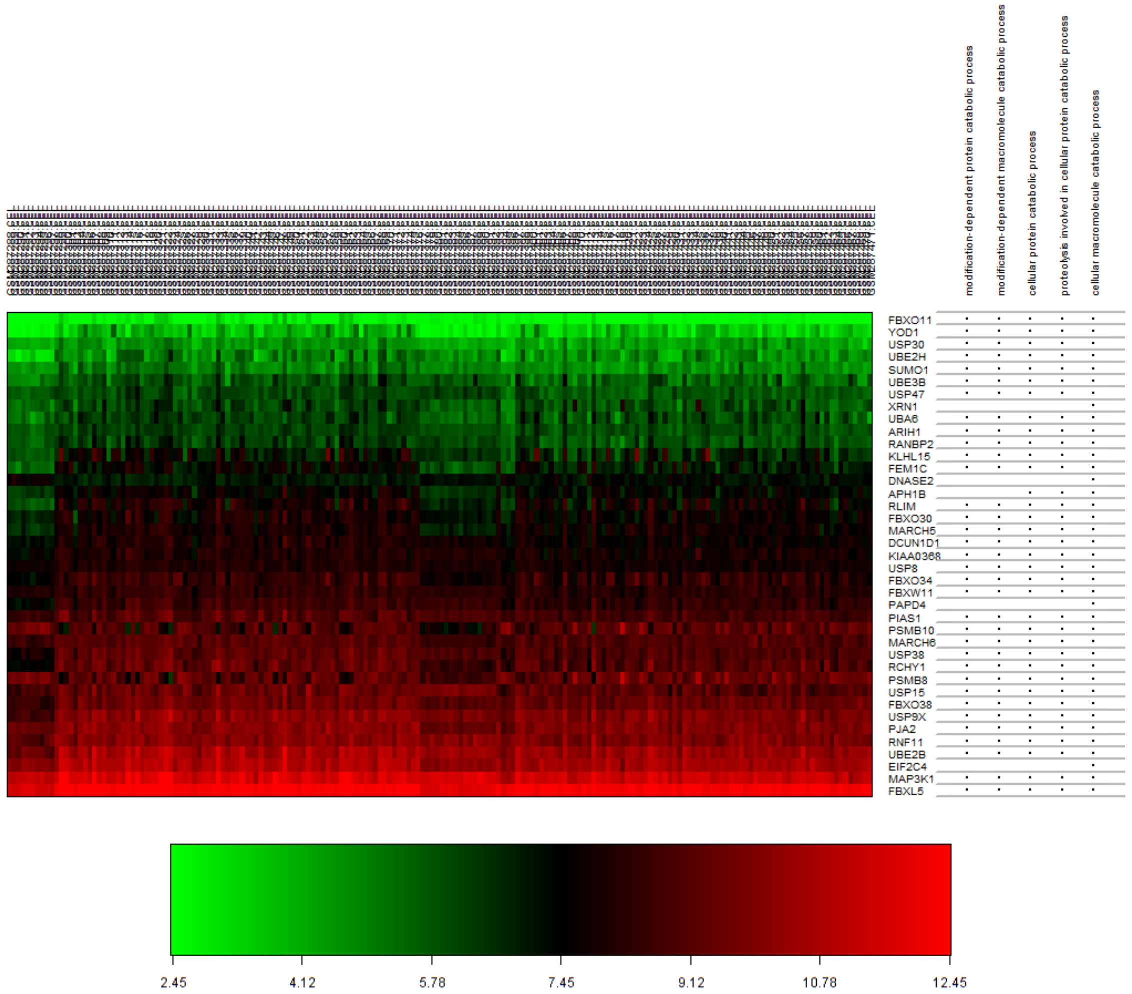

Figure 0.15 Heatmap of the genes most contributing to top 5 Gene Ontology – Biological Process level I terms enriched with cross tabulation. The green indicates low while red indicates high expression index (visualised on the colour bar below the heatmap). Please note two characteristic clusters of lower expression (at right side and in the middle of the heatmap) representing the control (unaffected) group.

The output of the KEGG categories was not as consistent and numerous as the GO terms, but still supports the abundant similar processes, in particular inflammation. The top 10 overrepresented categories are:

- Pathways in cancer
- Cytokine-cytokine receptor interaction
- Ubiquitin mediated proteolysis
- Endocytosis

- Insulin signaling pathway
- Pancreatic cancer
- Small cell lung cancer
- Prostate cancer
- SNARE interactions in vesicular transport
- Fatty acid metabolism

The heatmap for KEGG categories presents similar expression pattern as in the case of GO supporting genes (**Figure 0.16**). The gene concept network shows very poor connectivity between the groups connected with different terms (**Figure 0.17**).

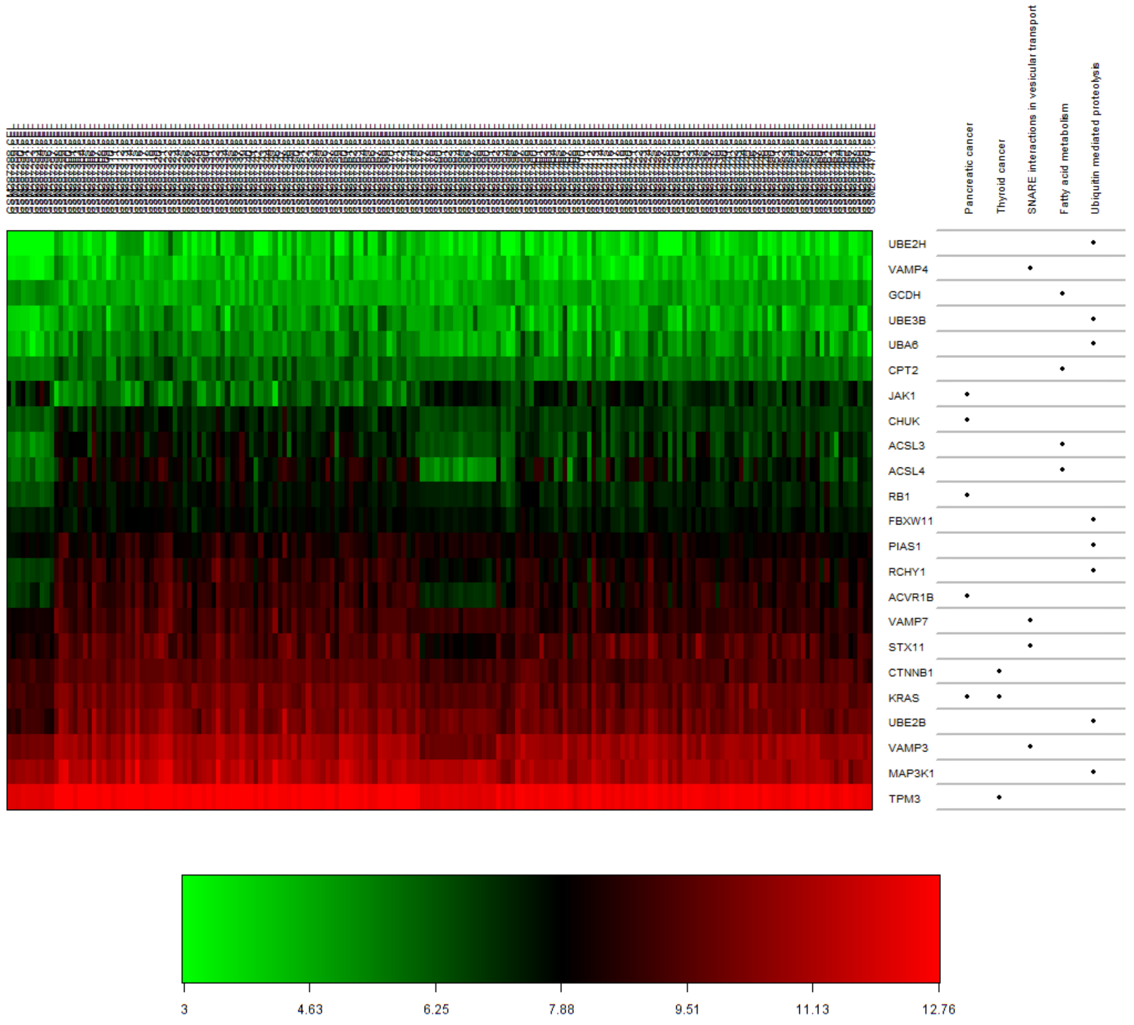

Figure 0.16 Heatmap of the genes most contributing to the top 5 KEGG categories enriched with cross tabulation. The green indicates low while red indicates high

expression index (visualised on the colour bar below the heatmap). Please note two characteristic clusters of lower expression (at right side and in the middle of the heatmap) representing control (unaffected) group.

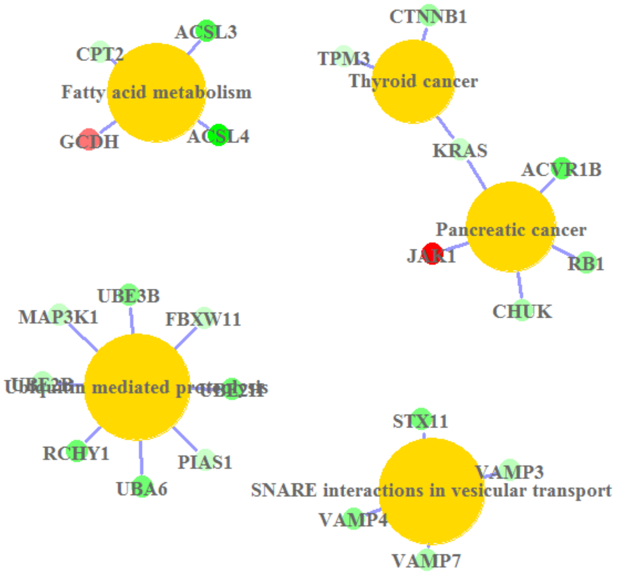

Figure 0.17 Concept-gene network presenting connection between genes and terms overrepresented in KEGG pathways.

The ten most overrepresented terms in Disease Ontology light are:

- Embryoma
- Congenital abnormality
- Alopecia
- Immunologic deficiency syndrome
- Leukoencephalopathy
- Periodontitis
- Depression
- "Myopathies, Nemaline"
- Neurofibromatosis

- "Purpura, Thrombocytopenic, Idiopathic"

Again, the expression pattern for genes supporting DOLight is similar to GO and KEGG – two clusters of lower gene expression corresponding to unaffected control individuals. The diseases are not consistent with each other and the categories are supported by relatively low number of genes (less than 10 in each category). The only positive thing supporting the previous output is that all these diseases are connected to inflammation.

Finally the user determined terms "Entrez" identified strong overrepresentation of genes connected with apoptosis (63 genes) and significant shift in expression of those genes (expression pattern is consistent with the GO and KEGG output). Inflammation (11 genes) and necrosis (10 genes) connected genes are present, but not strongly overrepresented.

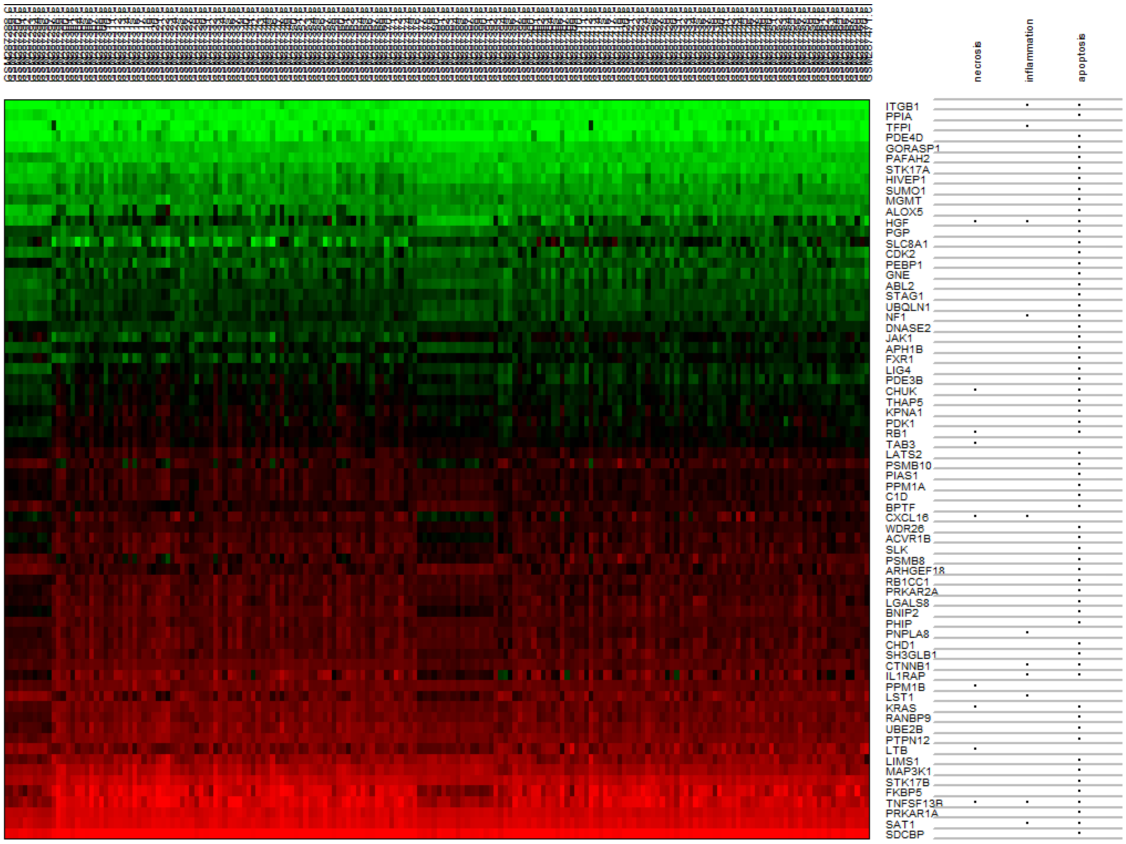

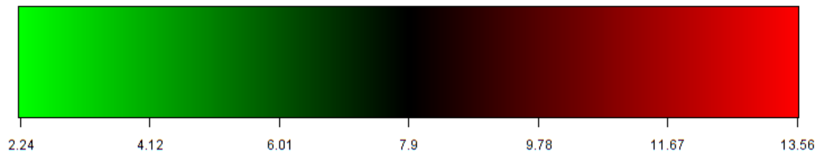

Figure 0.18 Heatmap of the genes most contributing to user determined Entrez terms enriched with cross tabulation. The green indicates low while red indicates high expression index (visualised on the colour bar below the heatmap). Two characteristic clusters of lower expression (at right side and in the middle of the heatmap) reveals similar expression pattern to KEGG and GO.

Since many more experiments were possible to conduct with high confidence on burn damage dataset the functional output of those analyses becomes externally complicated to present in this document. However, many consistencies between experiments have been found and this output report will focus on similarities between different experimental designs.

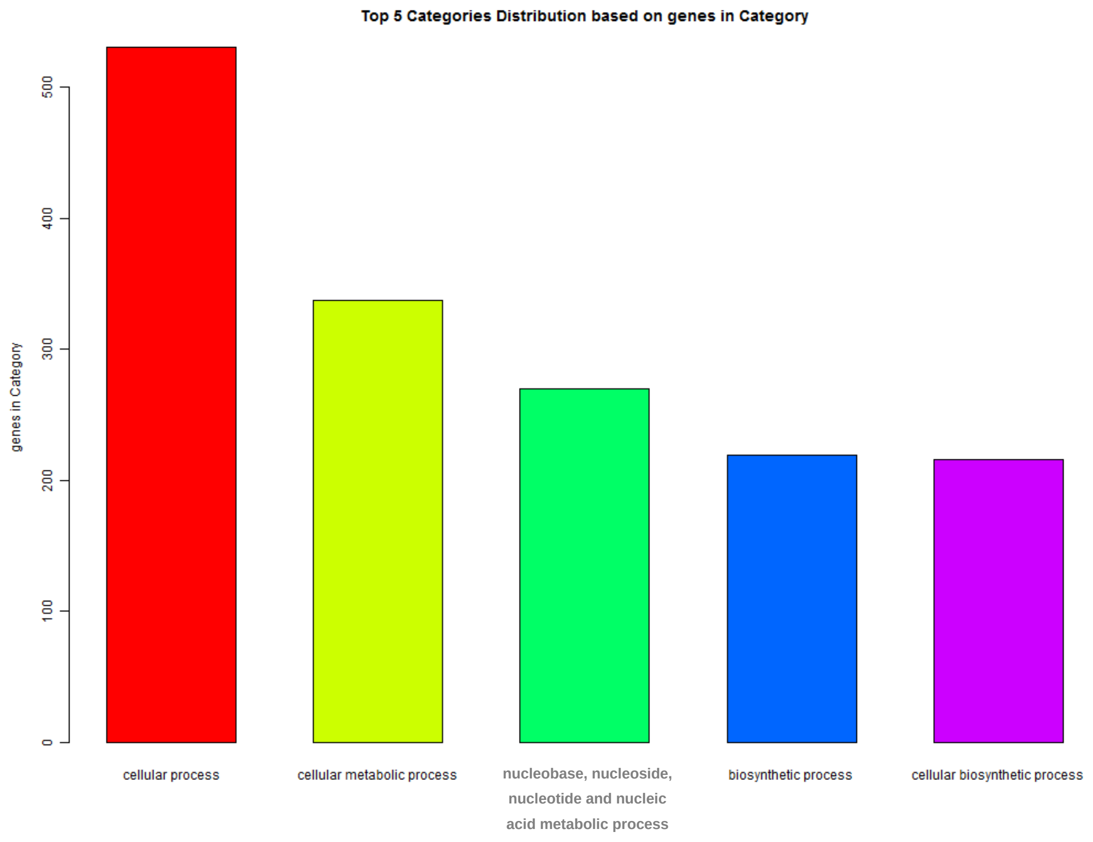

Figure 0.19 Bar chart presenting the number of genes in 5 most overrepresented categories of Gene Ontology – Biological Process level I for burn injury dataset. All the categories are connected with cellular metabolic process.

The overview of most overrepresented Gene Ontology terms for two-way ANOVA experimental design (including time after accident and age of the patient) reveals high consistency with the blunt damage dataset. Most of the top ten categories are directly connected with metabolic activity:

- cellular process
- cellular metabolic process
- cellular macromolecule metabolic process
- biosynthetic process
- cellular biosynthetic process
- nucleobase, nucleoside, nucleotide and nucleic acid metabolic process
- gene expression
- regulation of metabolic process
- macromolecule biosynthetic process
- regulation of cellular metabolic process
- regulation of primary metabolic process

The top five overrepresented categories are supported by at least 200 genes (more than 500 for "Cellular process") (**Figure 0.19**). The general expression of those genes is similar to the blunt damage dataset. However, the heatmap presenting more specific GO categories reveals significant differences between control and affected group (**Figure 0.20**). Those expression differences are very small, which makes them hard to notice on the heatmap presenting direct values. For that reason also the z-score standardized heatmap have been provided (**Figure 0.21**).

The GO terms obtained from level II ontology, as well as terms obtained using different experimental designs are consistent with two-way ANOVA output.

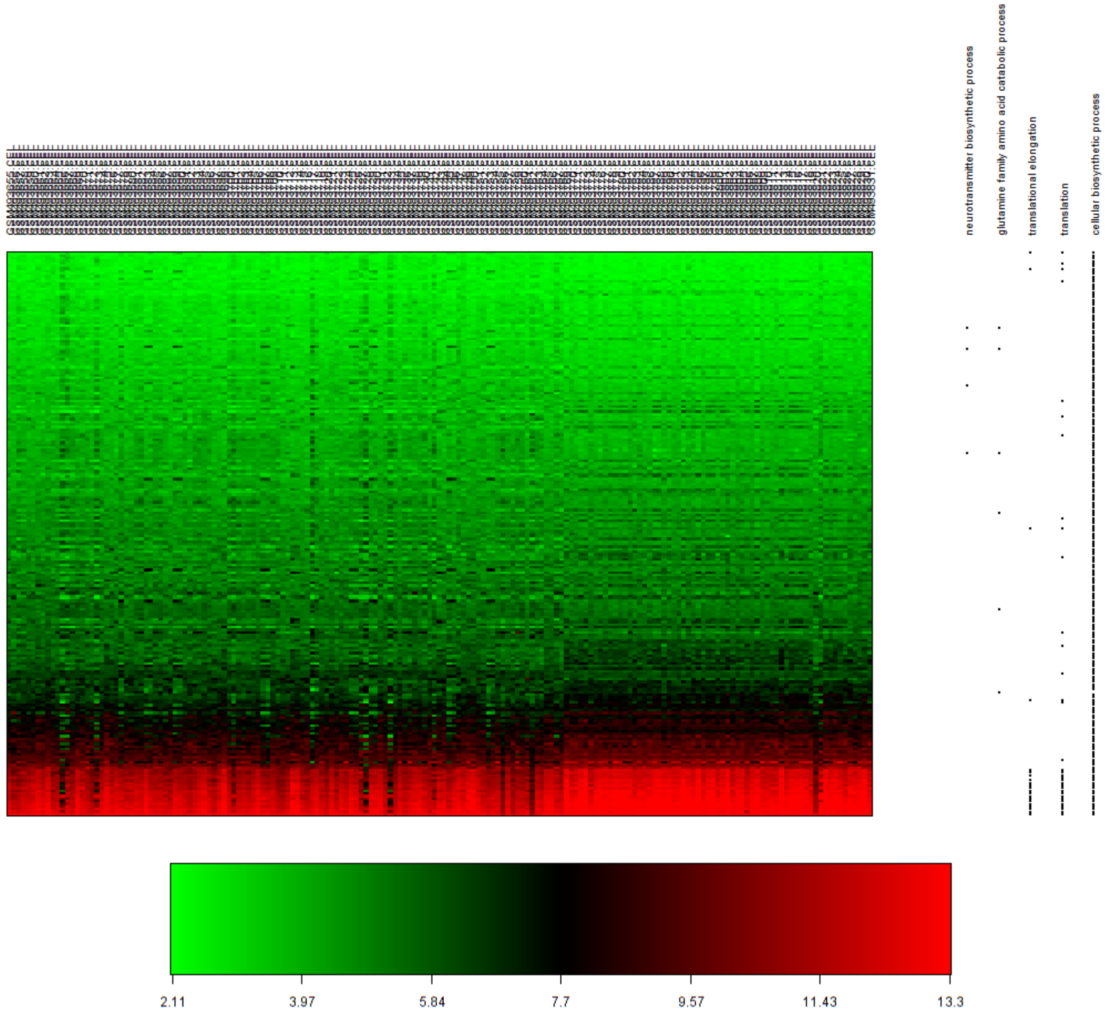

Figure 0.20 Heatmap presenting the genes most contributing to top 5 overrepresented categories of Gene Ontology – Biological Process level I enriched with cross tabulation. The overrepresentation has been measured by the p-value of hypergeometric test. The green indicates low while red indicates high expression index (visualised on the colour bar below the heatmap).

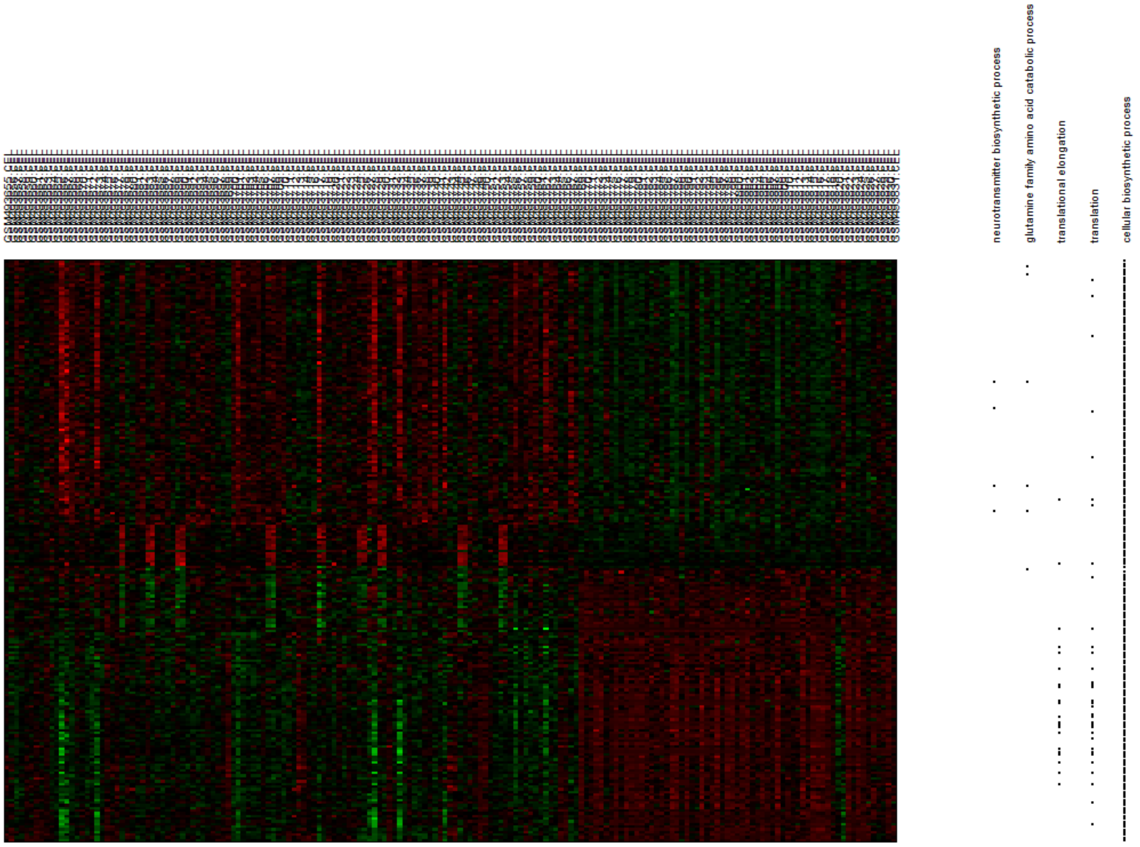

Figure 0.21 Heatmap presenting the same genes as **Figure 0.20**. The expression values have been standardized using z-score technique for better visualization and functional group separation. The green indicates low while red indicates high expressions. Please note characteristic clusters corresponding to affected patients (the left side and the middle part of the heatmap) and control unaffected control group (the right side).

The KEGG pathways found to be overrepresented in genes subjected to putative miRNA regulation, obtained using two-way ANOVA design are:

- Ribosome
- Calcium signaling pathway
- Cell adhesion molecules (CAMs)
- Phosphatidylinositol signaling system
- Drug metabolism - cytochrome P450
- Metabolism of xenobiotics by cytochrome P450
- Type I diabetes mellitus
- Asthma
- Primary immunodeficiency

- Allograft rejection

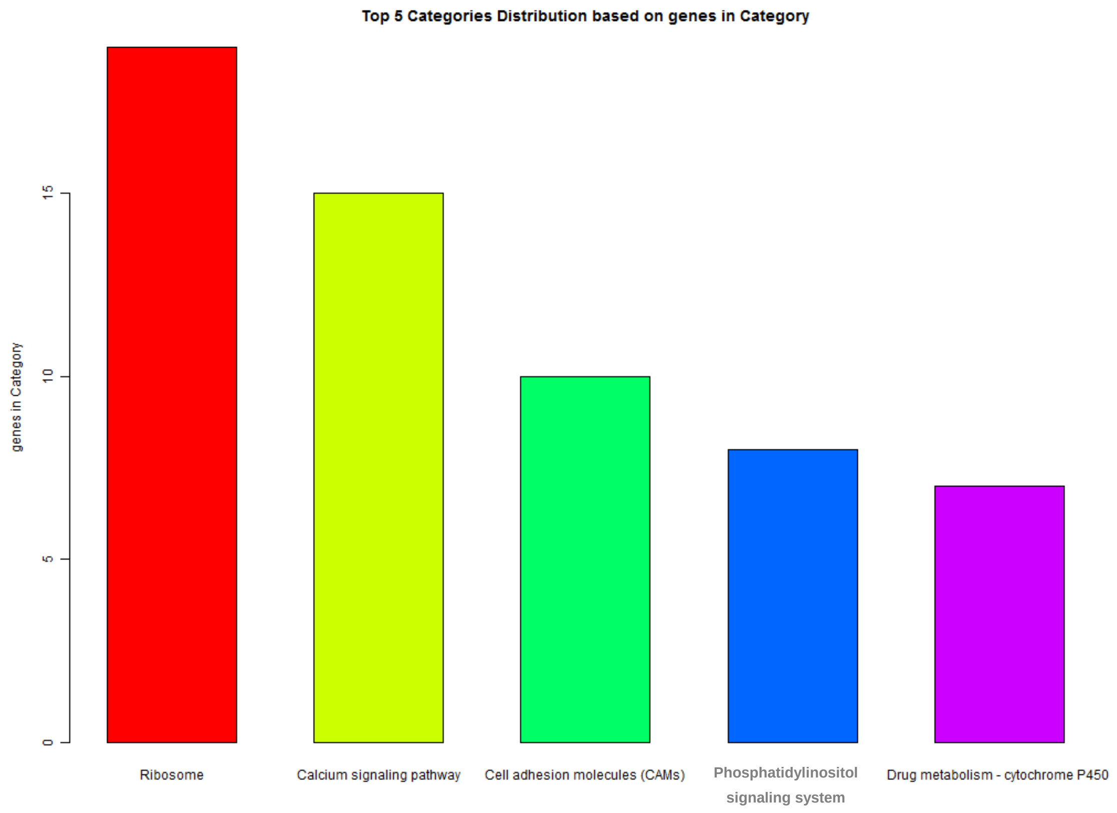

Figure 0.22 Bar chart presenting the number of genes in the 5 most overrepresented categories of the KEGG pathways for burn injury dataset. All the categories are connected with cellular metabolic process.

It should be noted, that those terms are supported by very few genes (**Figure 0.22**) and they are only partly consistent with other experimental designs (survival, sample/control t-test, etc.). On the other hand those categories are very specific (containing small number of genes), so p-values obtained by hypergeometric test are relatively small. Other KEGG categories commonly present in other designs are:

- MAPK signaling pathway
- Lipoic acid metabolism
- RNA degradation
- Leukocyte transendothelial migration
- T cell receptor signalling pathway
- Ubiquitin mediated proteolysis

- Focal adhesion

The expression profile is similar to the profile obtained for genes supporting overrepresented GO categories (**Figure 0.23**). Again, the differences in expression between the groups are very small, so z-score standardization has to be performed to achieve human readable output (**Figure 0.24**)

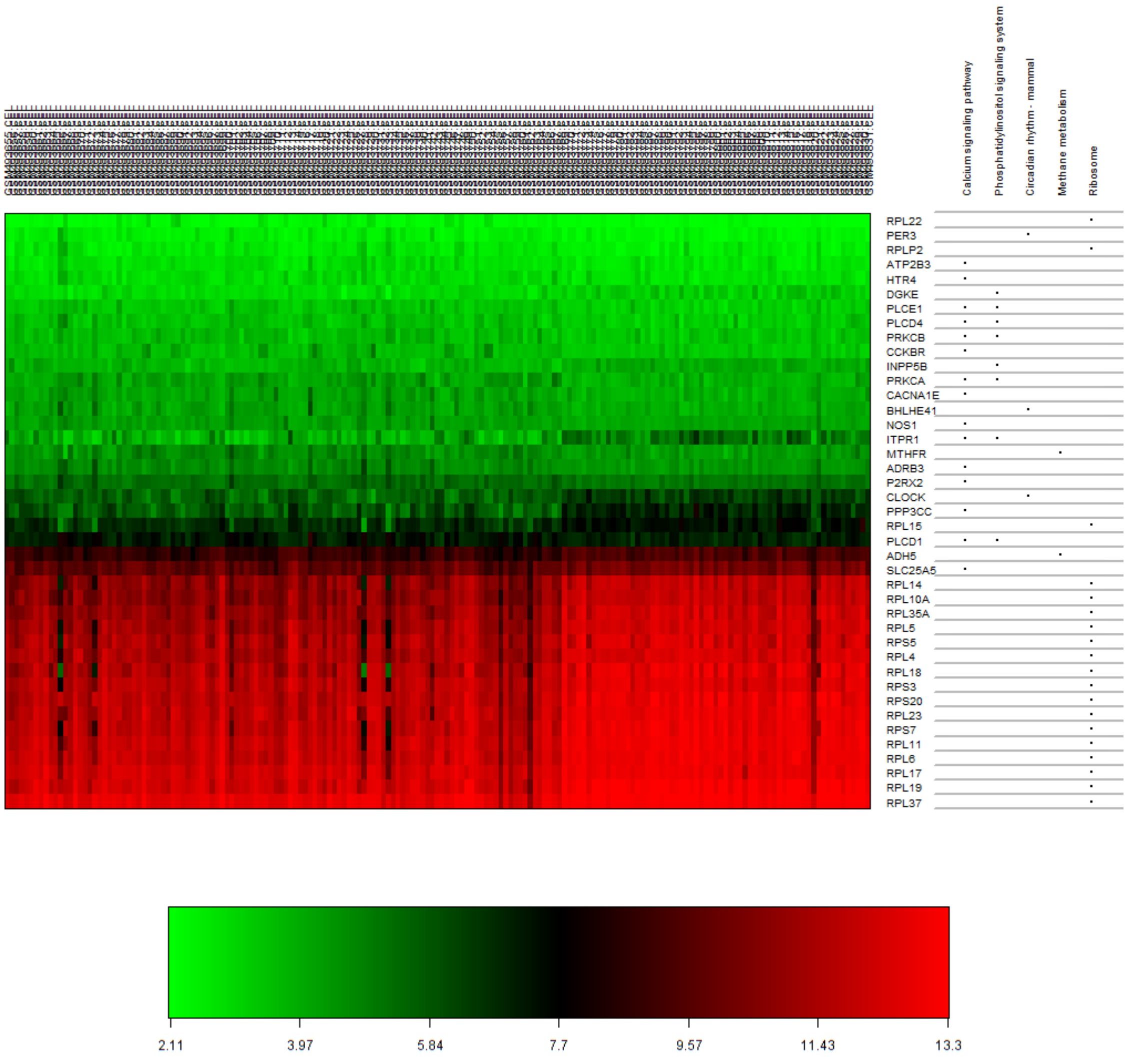

Figure 0.23 Heatmap presenting the genes most contributing to top 5 overrepresented categories KEGG pathways enriched with cross tabulation. The overrepresentation has been measured by p-value of hypergeometric test. The green indicates low while red indicates high expression index (visualised on the colour bar below the heatmap).

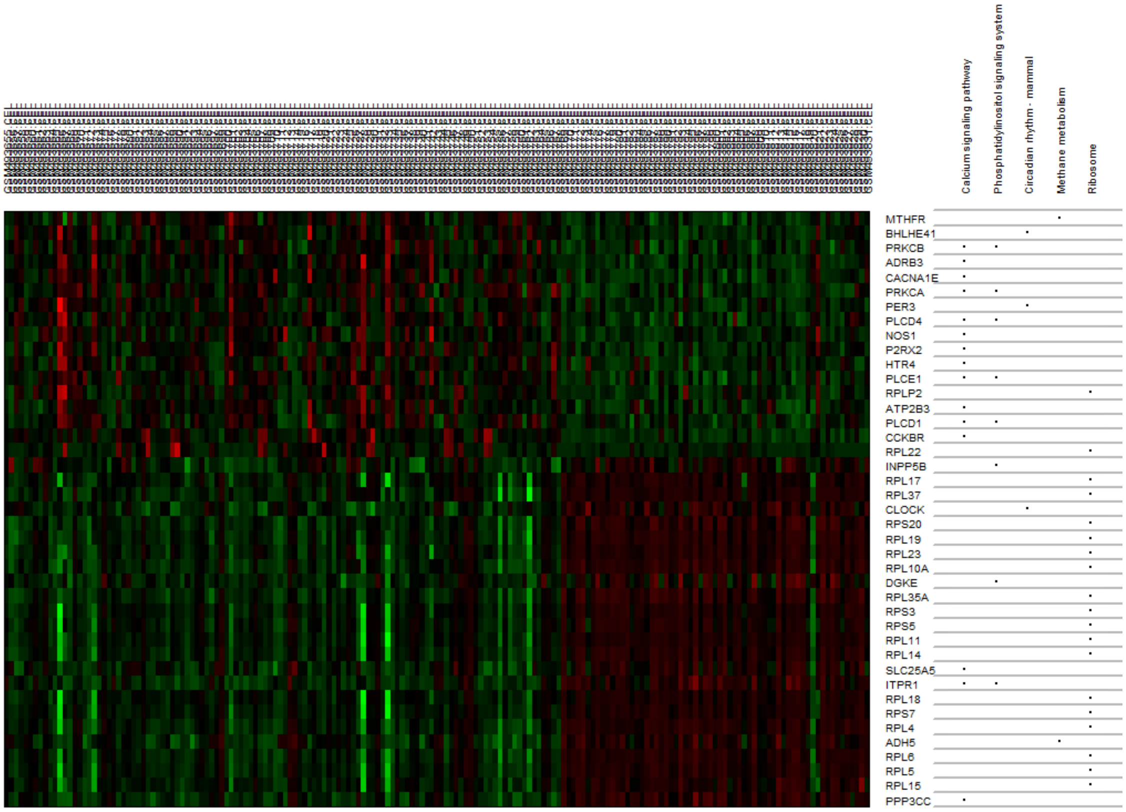

Figure 0.24 Heatmap presenting the same genes as **Figure 0.23**. The expression values have been standardized using z-score technique for better visualization and functional group separation. The green indicates low while red indicates high expression. Please note characteristic clusters corresponding to affected patients (the left site and the middle part of the heatmap) and control unaffected control group (the right side).

The top overrepresented Disease Ontology light terms involve:

- Bipolar disorder
- Heart failure
- Mental retardation
- Sickle cell disease
- Hemorrhagic disorder
- Endometrium cancer
- Generalized anxiety disorder
- Cleft palate
- Thrombocytopenia
- Thrombophilia

Similar to the previous examples many of these diseases can be connected to inflammatory states. The interesting discoveries are many mental health-related diseases. This can be explained as observed previously for GO terms and KEGG pathways overrepresentation of neurotransmitters connected genes. These categories may also come with signal determining infant patients from adult ones (see experimental design).

From user defined Entrez terms the “Apoptosis” seems to be very strongly overrepresented. The inflammatory gene seems to be fairy overrepresented, while necrosis is almost not present. The detailed output of hypergeometric test is presented in **Table 0.3**. The gene expression does not form any specific pattern – the genes supporting each category seems to be equally distributed in over- and under-expressed in the sample group (**Figure 0.25**)

Table 0.3 The summary of hypergeometric test for user provided Entrez terms for burn injury dataset (two-way ANOVA design).

	genes in Category	percent in the observed List	percent in the genome	fold of overrepresents	odds ratio	p value
apoptosis	115	0.852	0.058	14.8	98.1	4.2e-121
inflammation	33	0.244	0.017	14.3	19.4	1.6e-28
necrosis	13	0.096	0.011	8.7	9.7	4.2e-09

Please note low values of p-value obtained from hypergeometric test (**Table 0.3**). The reason for that is as many as 115 found apoptotic genes are 85% of the genes considered to be significantly overrepresented. The genes falling into “apoptosis” category are only 5.8% of all annotated genes. The chance of shuffling about 134 genes from all annotated and getting 115 falling into the same category, which is as small as ~5% of all genes is actually very small.

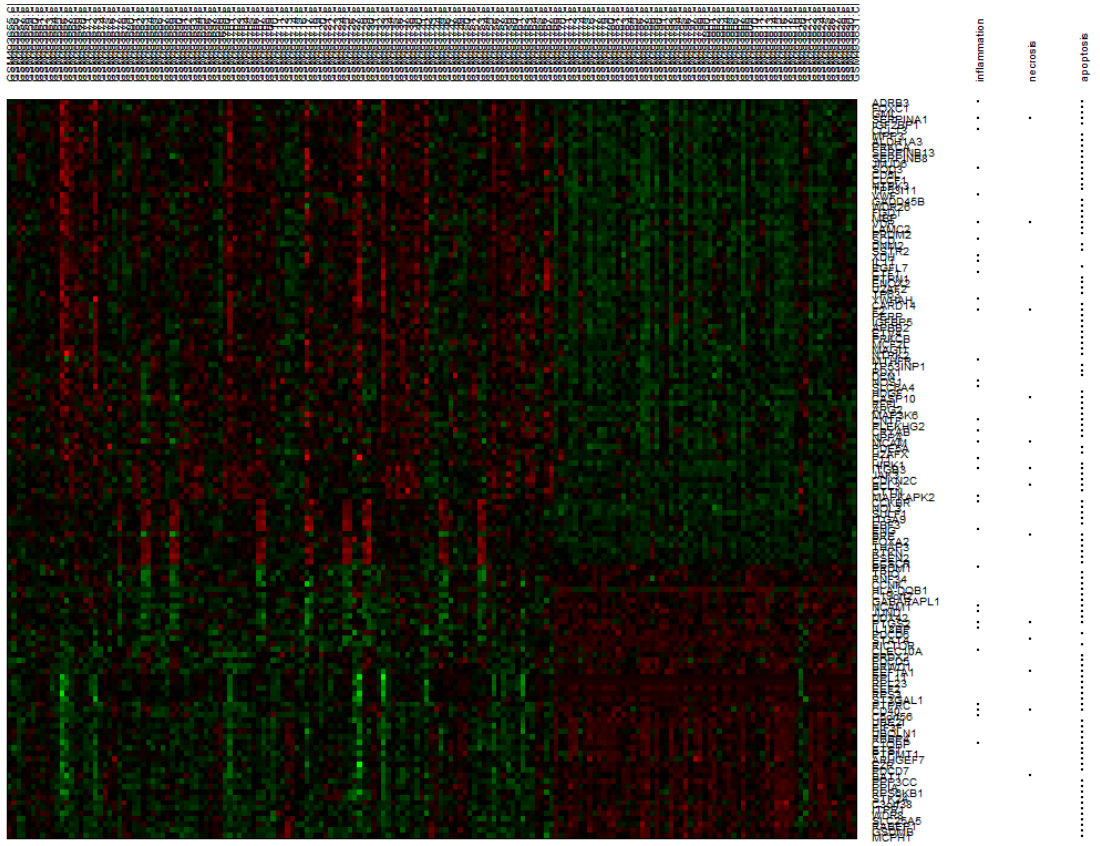

Figure 0.25 Heatmap summarizing the gene expression of genes supporting user provided Entrez terms for burn injury dataset (two-way ANOVA design). The expression values have been standardized using z-score technique for better visualization and functional group separation. The green indicates low while red indicates high expression. Please note characteristic clusters corresponding to affected patients (the left side and the middle part of the heatmap) and control unaffected control group (the right side). The genes for each category are equally distributed between over- and under-expressed.

Discussion

Comparing to the blunt damage dataset, the general quality of the signal was much better in burn injury datasets. This observation is supported both by clustering within the design groups and level of achieved p-value (**Figure 0.6, Figure 0.7, Figure 0.10** and **Figure 0.11**). Consequently, the functional analyses of burn injury datasets are simpler, because many different experimental designs produced robust, consistent predictor output (**Figure 0.8, Figure 0.9, Figure 0.12** and **Figure 0.13**).

The analyses of the most numerous Gene Ontology categories represented on the gene list reveal very strong consistency with blunt damages dataset. Most of the top overrepresented terms are associated with macromolecules (in particular the nucleotide and nucleic acid metabolic process) and general cellular metabolic process, as well as the regulation of metabolism. This similarity was expected – both blunt damage and burn injury causes massive tissue damage leading to increased necrosis and apoptosis. Also the inflammatory pathway must be activated to protect organism from infections and promote damaged tissues recovery. The anti-infection inflammatory function is even more important in the case of burn, since this is penetrative damage, in many cases destroying the organism’s natural protective barrier from viruses, bacteria and noxious stimuli. Consequently, the energy demands of immune system should be even higher than in case of blunt damage.

The second group of overrepresented GO categories are general and macromolecule biosynthetic process. The presence of these terms indicates the tissue riper process happening on the whole organism level (the RNA samples were taken from human blood). The appearance of these terms is the first significant difference between datasets, resulting from the specifity of tissue damage. Blunt damage causes physical damage to a tissue structure resulting in destroying the integrity of cells causing necrosis or apoptosis. However, the force of damage is usually strong enough to disrupt the structure of the macromolecules (proteins, nucleic acids, etc), so they can be recycled to form a structure of new cells. For that reason the terms and pathways connected with macromolecule modification are found to be overrepresented in this dataset. On the other hand the heat

energy causing burn damages can easily destroy the structure of organic macromolecules, for example by denaturation of proteins. The molecules subjected to high temperature lose its biological function and cannot be reused in the damaged tissue repair process. Consequently, the overrepresentation of macromolecule biosynthetic process terms is observed in burn injury dataset. The alternative explanation of this phenomenon is connected with the time of taking the samples. In the case of the blunt dataset the samples were taken as soon as possible after hospital admission, while samples from burn injury patients were taken in two time periods (in early and middle stage of the therapy). It is possible that advanced tissue repair mechanisms utilizing macromolecule biosynthetic process were not active in the early phase after accident and for that reason have been identified only in the burn dataset.

The top categories are supported by more than two hundred genes (**Figure 0.19**), and are indentified by low p-values obtained from hypergeometric test, which indicates high confidence of this functional analyses. The general expression profile is similar to the one driven from blunt damage datasets – the genes putatively repressed by miRNA connected with both immune system cellular metabolic process and biosynthetic process are over-expressed in burn affected group (**Figure 0.20**). Unfortunately, the poor clustering within time serious groups makes it impossible to predict how the expression of that genes are changing between early and the middle stage of hospital treatment. This indicates similar, characteristic for inflammatory states miRNA expression control – the immune system's cells metabolic genes, which normally are under miRNA suppression becomes active during inflammation due to the lower miRNA expression. Also the cellular macromolecule biosynthesis, normally active during the development process, is known to be under miRNA expression in mature, basal state tissues.

Further interesting differences between datasets are revealed by examining the top GO terms sorted by p-value obtained from hypergeometric test rather than the number of genes falling into each category. Those terms are more specific and all of them are connected with cellular biosynthetic process. The expression profile of those genes, visualised on the heatmap (**Figure 0.21**) shows that most of the genes falling into "translation" and "translational elongation" are under-expressed while all the genes

supporting terms like “neurotransmitter biosynthetic process” and “glutamine family amino acid catabolic process” are over-expressed in sample (burn injury affected) group. This may indicate very specific gene regulation during burn damage induced inflammatory process and subsequent repair process, which may suggest miRNA regulation involvement. Additional factor supporting advanced miRNA regulation comes with very small differences between expression indexes in that group – the differences become visible only after z-score standardization of gene expression (please compare **Figure 0.20** with **Figure 0.21**).

The output generated by overrepresentation testing for KEGG pathways seems to be consistent with GO terms sorted by strict hypergeometric testing. The identified KEGG pathways using two-way ANOVA are quite specific and supported by 10 – 20 genes only. Other experimental design ware able to return more general pathways like “MAPK signaling pathway”, “Leukocyte transendothelial migration” and “T cell receptor signaling pathway” supporting the assumption of inflammatory process happening on the organism level. Other terms including “Lipoic acid metabolism”, “RNA degradation” and “Ubiquitin mediated proteolysis” supports the hypothesis of metabolic activation of immune system cells and macromolecule metabolism.

The expression profile of specific KEGG pathways genes is very consistent with the one obtained for GO terms (**Figure 0.23** and **Figure 0.24**). The “Ribosome” pathway genes, strongly connected with translation are explicitly down-regulated in the sample group. On the other hand signalling pathways genes connected with “Calcium signalling pathway” and “Phosphatidylinositol signaling system” are strongly up-regulated. This may indicate ongoing inflammatory state and involvement of miRNA repression on translational processes.

Similarly to blunt damage dataset the strong overrepresentation of apoptosis connected genes have been detected for overrepresentation testing of user provided Entrez terms. Also the inflammatory genes have been found to be under putative miRNA control in the burn injury dataset. The important difference is much lower overrepresentation of necrotic process genes comparing to blunt datasets. It seems that in case of burn damage

the preferred method of cleaning survived cells in damaged areas is apoptosis. This may seem right, since most of the compounds in burned areas are not reusable, so the big deficit of macromolecules should accrue. In these circumstances “programmed cell death” is the most efficient way to reuse the resources of survived, but damaged cells to rebuild missing tissues. No significant expression pattern has been detected for those genes (**Figure 0.25**). The overview of comparative blunt damage and burn injury datasets analyses have been summarised in **Table 0.4**.

Table 0.4 The summary of cooperative blunt damage and burn injury datasets analyses.

Description	Blunt damage	Burn injury
Increases metabolic activity of leucocytes	YES	YES
Increased metabolism of macro compounds (nucleic acids, proteins)	YES	YES
Activation of inflammatory pathways	YES	YES
Activated macromolecule modification pathways	YES	NO
Activated macromolecule biosynthetic process	NO	YES
Very specific regulation of cellular biosynthetic process, possibly involving miRNA	NOT DETECTED	YES
Translation and translational elongation genes suppression, possibly by miRNA	NOT DETECTED	YES
Signalling pathways genes over-expression	NOT DETECTED	YES
Suppression of “Ribosome” KEGG pathway’s genes	NOT DETECTED	YES
Very strong overrepresentation of genes connected with “Apoptosis”	YES	YES
Some overrepresentation of genes connected with “Inflammation”	YES	YES
Some overrepresentation of genes connected with “Necrosis”	YES	NO

References

1. Warren HS, Elson CM, Hayden DL, et al.: **A genomic score prognostic of outcome in trauma patients.** *Molecular medicine (Cambridge, Mass.)* , **15**:220-7.

2. Zhou B, Xu W, Herndon D, et al.: **Analysis of factorial time-course microarrays with application to a clinical study of burn injury.** *Proceedings of the National Academy of Sciences of the United States of America* 2010, **107**:9923-8.
