# Supplementary material for: MMpred: functional miRNA – mRNA interaction analyses by miRNA expression prediction
Source: BMC Genomics. 2012 Nov 14;13:620. doi: 10.1186/1471-2164-13-620 (PMC3562514; doi:10.1186/1471-2164-13-620)
Supplement: Additional file 8 — Systematic validation of target prediction by the similarity of binding free energy distribution with miRecords. [file 1471-2164-13-620-S8.pdf]

Additional file 9 – Systematic validation of target prediction by the similarity of binding free energy distribution with miRecords.

The attempt to validate the pipeline using both experimentally validated (from miRecords, version 3) and predicted (TargetScan 6.0) targets have been made. Most of prediction methods (including TargetScan) give multiple miRNA predicted to target specific gene. For TargetScan 6.0 the genes can be regulated by up to 1059 miRNAs (188 on average, **Figure 1**). On the other hand experimentally validated targets databases cover only small fraction of all possible interaction.

Our method aims to produce case specific regulatory clusters of miRNA-targets interactions. The number of predicted targets for each gene is relatively small, so overlap with TargetScan predictions is insignificant. Also due to small number of miRNAs predicted for each gene the chance of hitting one of the validated miRecords targets is fairly small. Additionally, the MMpred model is suitable only for intronic miRNAs (the limitation of miRNA expression predictors).

For his reasons a systematic validation of case specific regulatory clusters using different prediction methods or large sets of published data is not feasible. Therefore, we have implemented alternative systematic validation method based on the binding energy between the mature miRNA and 3' UTR region of the gene. The method is modified "energy walk" procedure described in the paper by Ritchie *et al.* [1], which utilizes the impact of binding energy in proper miRNA-target pairing [2, 3].

The procedure utilizes the Vienna RNA Package version 1.8.5 to calculate minimum free energy of miRNA binding. The 3'UTR sequences are scanned using sliding window of 25bp and 5bp step. Since RNAfold algorithm allows only the calculation of free energy for single stranded RNA molecule, the scanned 25bp fragments of 3'UTR mRNA have been joined with mature miRNA sequence using 8bp artificial inter-linker sequence containing 'X' bases that cannot be paired (as described by Enright *et al.* [4]). The region of lowest free energy is considered to be the optimum binding site.

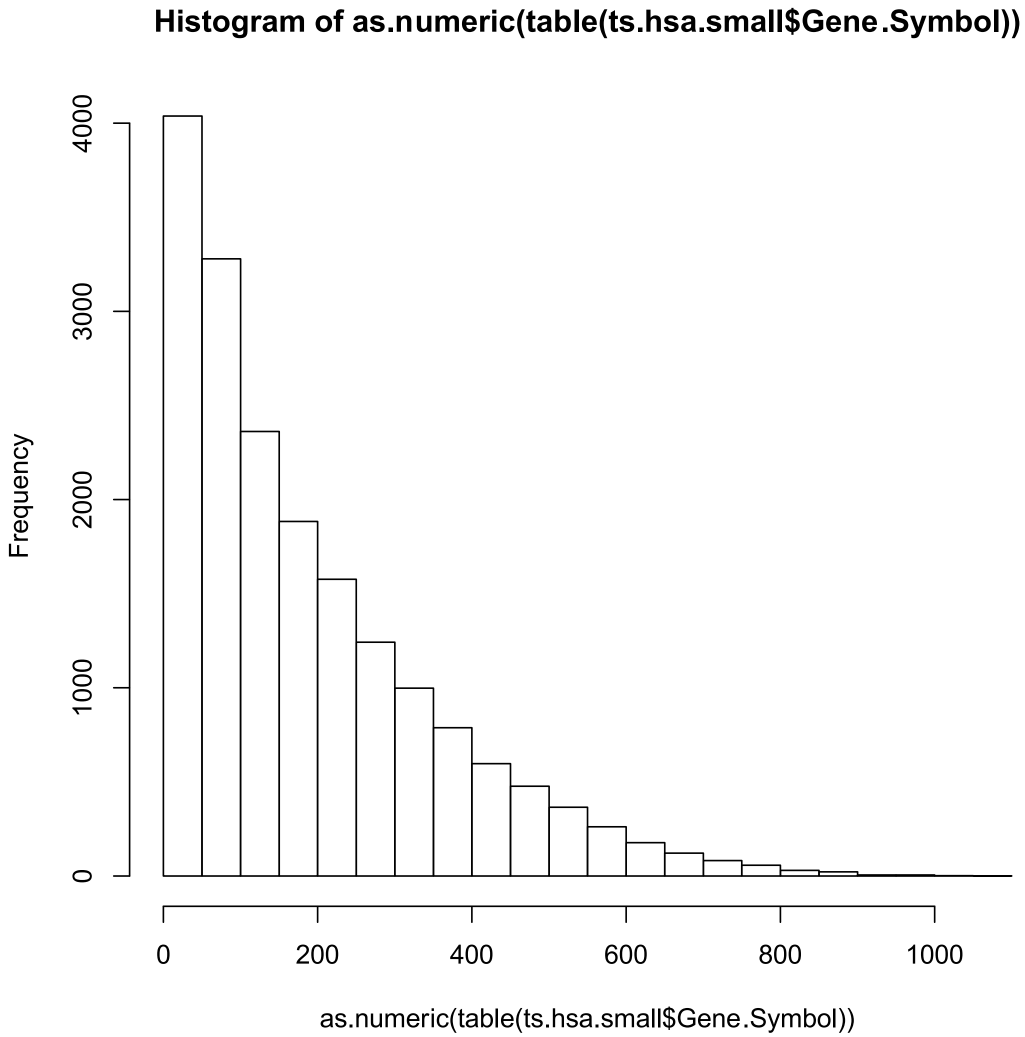

Figure 1 The histogram presenting the numbers of miRNAs predicted to target each of genes gathered in TargetScan 6.0 database. Each gene is targeted by 188 miRNAs on average.

The validation has been implemented in R language. Mature miRNA sequences have been obtained from miRBase version 17.0 using miRbase.db R library. 3'UTR sequences have been downloaded from Ensembl via biomaRt R interface. For genes with multiple 5'UTR transcripts the longest isoform was selected to ensure the sampling of all possible binding locations. Genes with 3'UTRs shorter that 100bp were discarded from analysis. The free energy calculations have been executed using GeneRfold R interface for Vienna RNA library. The miRecords (version 3, *mirecords.biolead.org/download.php*) have been used as comprehensive collection of validated targets.

All possible miRNA-mRNA interactions from miRecords were sampled in order to calibrate the free energy based validation. To confirm the significance of the method, two random sets of free binding energies were calculated: by permutation of genes name vector (using the same set of 3'UTR sequences) and by substituting the original set of 3'UTR with random gene sequences. The results are shown on **Figure 2**.

The study of lowest binding energy distributions revealed that using fixed free energy cut-off (-20 Kcal, Ritchie *et al*) would discard most of validated targets. For this reason we have decided to compare the distributions of minimal energy among the miRNA-target pairs rather than the number of high energy binding sites like in original procedure.

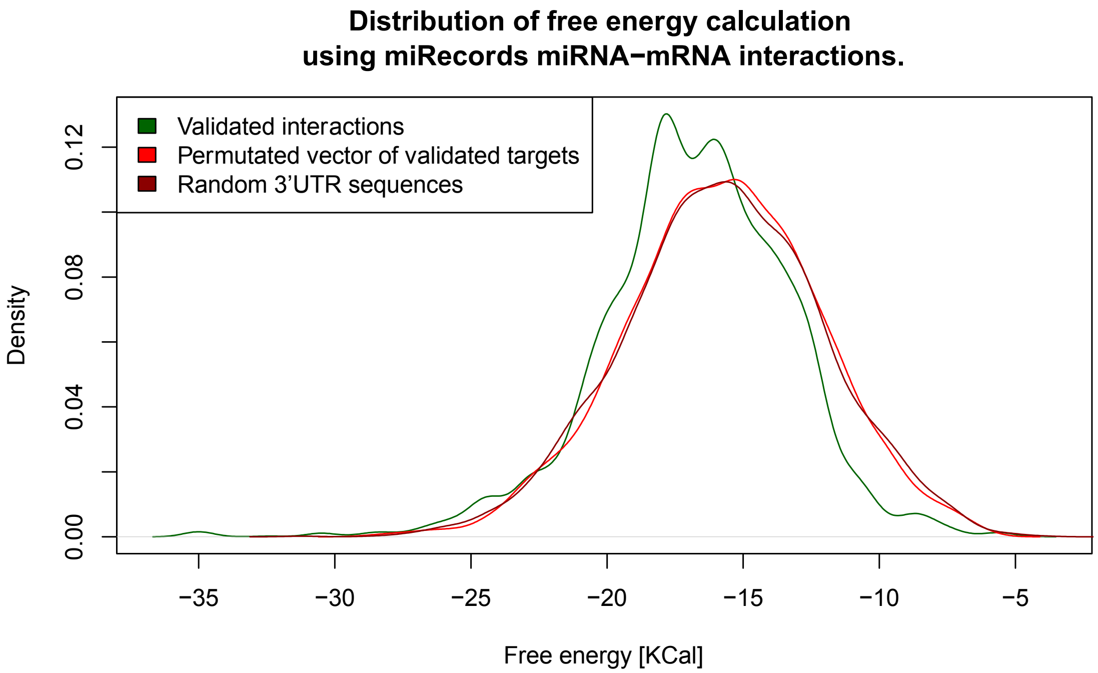

Figure 2 The distribution of calculated free energies in validated targets set driven from miRecords. The calculation has been obtained by sampling 3240 sequences of 3'UTR human target genes for optimal miRNA binding free energy. The randomized sampling contains the same number of free energy calculations.

To further assay the significance between actual and randomized energy calculation the Welch Two Sample t-test has been performed. The null hypothesis (true difference in means between actual and randomized data is equal to 0) has been rejected with p-value < 2.2e-16 for both randomizations. It should be noted that the randomized samples have the same mean with p-value = 0.9776.

Using the same methodology the predictions based on miRNA-mRNA expression anti-correlation has been performed (as described in the manuscript). To validate both experimentally measured values and the predictor output, the paired microarray dataset "*Comparative genomics matches mutations and cells to generate faithful ependymoma models*" (GSE21687) has been used. This is newly obtained dataset, used exclusively in cover letter to address referees comments.

At first, measured miRNA expression matrix has been correlated against mRNA expression matrix. Then the correlations have been filtered using *GetHT* function from MMpred pipeline with correlation cut-off equal -0.6. The predicted interactions have been run through Vienna RAN package free energy calculation similarly to validated miRecords interactions. Two randomized energy calculations have been prepared: using permutated vector of predicted targets (**Figure 3**) and the permutated target sequence (not shown on the figure)**.** The mean of predicted targets is significantly different from the randomized values (p-values equal 2.925e-11 and 5.663e-07, Welch Two Sample t-test). Furthermore, the distribution is similar to validated targets (p-value = 0.3748).

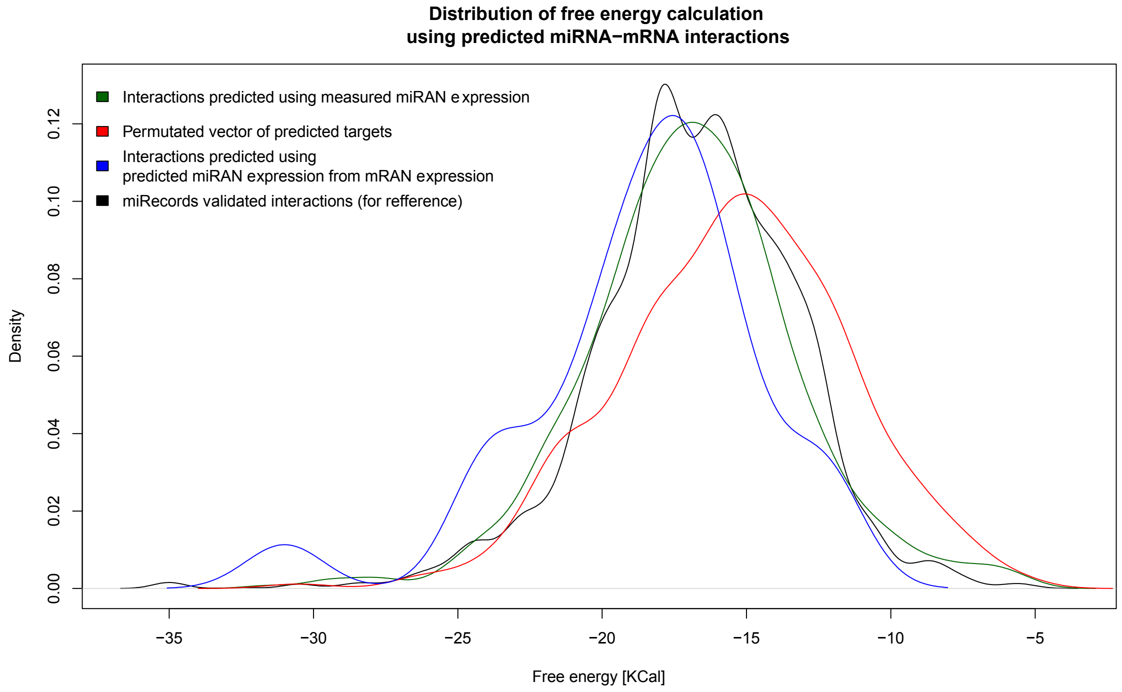

Figure 3 The distribution of calculated free energies in expression anti-correlation based target predictions. The calculations have been obtained by sampling sequences of 697 3'UTR human target genes candidates for experimental miRNA expression dataset and 26 for predictor drive miRNA expression (full MMpred pipeline). The randomized sampling contains 697 free energy calculations.

Finally, to assess both predictive power of miRNA expression predictor and targets predictive capabilities, the full MMpred pipeline has been run on mRNA expression data only, repeating the same free energy calculation procedure. The distribution is shown on **Figure 3** in blue. The distribution is similar to both miRecords and experimental data driven distributions (p-values equals 0.03952 and 0.05833) and dissimilar to randomized ones (see **Table 1** for all p-value comparisons). It can be seen that free energy is even more shifted towards more favourable (lower) values, than in case of validated targets. This is due to much smaller number of predicted interactions (only 26) comparing to validated set (3240) and experimentally measured expression driven one (697).

	Validated miRecords targets	Randomized miRecords 1	Randomized miRecords 2	Measured expression predicted	Measured expression randomized 1	Measured expression randomized 2	mRNAonly expression predicted
Validated miRecords targets		6.01E-31	9.96E-31	0.374818132	1.33E-13	1.45E-08	0.039524689
Randomized miRecords 1	6.01E-31		0.977604912	7.58E-14	0.133514965	0.168947724	0.001974776
Randomized miRecords 2	9.96E-31	0.977604912		8.77E-14	0.129887014	0.174625096	0.001990673
Measured expression predicted	0.374818132	7.58E-14	8.77E-14		2.92E-11	5.66E-07	0.058329623
Measured expression randomized 1	1.33E-13	0.133514965	0.129887014	2.92E-11		0.026457267	0.000946147
Measured expression randomized 2	1.45E-08	0.168947724	0.174625096	5.66E-07	0.026457267		0.003859031
mRNAonly expression predicted	0.039524689	0.001974776	0.001990673	0.058329623	0.000946147	0.003859031	

Table 1 P-values obtained from Welch Two Sample t-test. The cases where the null hypothesis has been rejected are marked in red (p-value cut-off equals 0.01), otherwise marked in green.

References:

1. Ritchie W, Rajasekhar M, Flamant S, Rasko JEJ: **Conserved expression patterns predict microRNA targets.** *PLoS computational biology* 2009, **5**:e1000513.

2. Kertesz M, Iovino N, Unnerstall U, Gaul U, Segal E: **The role of site accessibility in microRNA target recognition.** *Nature genetics* 2007, **39**:1278-84.

3. Long D, Lee R, Williams P, Chan CY, Ambros V, Ding Y: **Potent effect of target structure on microRNA function.** *Nature structural & molecular biology* 2007, **14**:287-94.

4. Enright AJ, John B, Gaul U, Tuschl T, Sander C, Marks DS: **MicroRNA targets in Drosophila.** *Genome biology* 2003, **5**:R1.
