# Supplementary material for: MMpred: functional miRNA – mRNA interaction analyses by miRNA expression prediction
Source: BMC Genomics. 2012 Nov 14;13:620. doi: 10.1186/1471-2164-13-620 (PMC3562514; doi:10.1186/1471-2164-13-620)
Supplement: Additional file 9 — Examples of MMpred predictions supported by experimental data and mapping against current databases. [file 1471-2164-13-620-S9.ZIP › Additional file 11 - Examples of MMpred predictions supported by experimental data and mapping against current databases/Additional file 11 - Examples of MMpred predictions.pdf]

Additional file 11 – Examples of MMpred predictions supported by experimental data and mapping against current databases.

The presented examples are produced using the updated mapping. We re-mapped the miRNAs both to their host genes, and to the transcripts represented on mRNA expression arrays, using the recent release of miRbase (version 18) and Ensemble Genes (version 68), respectively. The updated mapping covers a significantly larger pool of microRNAs with genomic context than previously reported. The number of microRNAs has now been increased to 1035 (of 1527 represented in miRbase), which corresponds to 975 mature 3p miRNAs and 385 mature 5p miRNAs. Furthermore, to avoid any potential naming conflicts arising from changing the *"-/*"* to *"3p/5p"* naming convention, etc. in later versions of miRbase, our new mapping utilises the mature sequence accession number rather than the mature sequence ID to both build the linear model and validate the scaling function model.

Moreover, with updated mapping we significantly increased the pool of supported microarray platforms – in addition to previously supported *affy_hg_u133_plus_2* and *affy_huex_1_0_st_v2* the following platforms were included: *affy_hc_g110, affy_hg_focus, affy_hg_u133a_2, affy_hg_u133a, affy_hg_u133b, affy_hg_u95av2, affy_hg_u95b, affy_hg_u95c, affy_hg_u95d, affy_hg_u95e, affy_hg_u95a, affy_hugenefl, affy_hugene_1_0_st_v1, affy_u133_x3p*. The new mapping data and updated MMpred scripts are available as R package.

Increasing the coverage of predicted miRNAs has significantly enhanced the model prediction power. For example, when compared to the data as presented by Wang et al. our method predicted all 6 targets of hsa-mir-218. Considering 6 MMpred predictions overlapping and 0 not overlapping with published data, relative to 302 further MMpred predictions and 21668 other possible predictions (based on 21976 protein coding genes represented in HGU-133plus2 microarray; source: Ensembl67) the Fisher's exact test p-value decreased from 8.52e-11 to 6.900841e-12. Furthermore, MMpred correctly predicted targets for a further two miRNAs indicated by Wang et al. - hsa-mir-335 and hsa-mir-503. Although Wang et al. did not indicate these miRNAs as intragenic, miRbase reports their overlap with MEST and AC004383.4 gene transcripts. Our method predicted one of 4 hsa-miR-335 targets (p-value=0.0084), and three of 9 targets (p-value=2.080172e-05) for hsa-miR-503. Furthermore, 6 of 7 significant KEGG pathways reported by Wang et al. on figure 3 were identified by MMpred (compared to 4 in previous example). Additionally, the MMpred predicted 2 signaling pathways that fall into the missing "Cell communication" KEGG category. So indirectly all the pathways from the publication were reproduced using MMpred.

The additional evidence of MMpred's performance and usability comes from the similar analyses we performed on new paired expression dataset [GSE26158] cited in Ghisi et al. (Blood, 2011)[1]. The MMpred running with default settings (correlation cutoff for target prediction = -0.8, p-values cut-offs auto determined by permutation) detected 10 out of 12 functional annotations (GO terms) indicated in Table 2, cluster 1. Since this publication gives extended spreadsheet of target predictions by miRanda and TargetScan algorithms instead of the compact miRNA target list (table S2, *http://bloodjournal.hematologylibrary.org/content/suppl/2011/04/18/blood-2010-12-326629.DC1/TableS2.xls*), we took a different approach to validate the pipeline's predictions. The spreadsheet created by Ghisi et al. contains predictions observed on transcription level marked by colours (upregulated miRNAs and genes highlighted in red, downregulated ones highlighted in green). Instead of using experimental miRNA expressions we predicted (using MMpred) the expression of 57 to be significantly different between functional groups. Five of resulting miRNAs were present in the spreadsheet. Further we correlated the predicted miRNA expression values with all 54675 probesets represented on HG-U133plus2 array. Finally we have calculated the median correlation of predicted miRNAs with both predicted and experimentally observed targets. The results are shown in **Table 1**.

miRNA ID	All predicted targets	Targets observed to be differentially expressed	Median correlation for all predicted targets	Median correlation for differentially expressed targets
hsa-miR-101	362	5	-0.2000	-0.9379
hsa-miR-151-5p	146	4	0.0113	-0.9806
hsa-miR-342-3p	351	7	-0.988	-0.8965
hsa-miR-342-5p	276	4	-0.0988	-0.9279
hsa-miR-128	450	9	0.0581	-0.9655

Table 1 The result of correlation test based on overlap between MMpred prediction and target genes of differentially expressed miRNAs identified by Ghisi et al. in tale S2.

References:

1. Ghisi M, Corradin A, Basso K, et al.: **Modulation of microRNA expression in human T-cell development: targeting of NOTCH3 by miR-150.** *Blood* 2011, **117**:7053–62.

2. Wang Y-P, Li K-B: **Correlation of expression profiles between microRNAs and mRNA targets using NCI-60 data.** *BMC genomics* 2009, **10**:218.
