# Supplementary material for: MMpred: functional miRNA – mRNA interaction analyses by miRNA expression prediction
Source: BMC Genomics. 2012 Nov 14;13:620. doi: 10.1186/1471-2164-13-620 (PMC3562514; doi:10.1186/1471-2164-13-620)
Supplement: Additional file 9 — Examples of MMpred predictions supported by experimental data and mapping against current databases. [file 1471-2164-13-620-S9.ZIP › Additional file 11 - Examples of MMpred predictions supported by experimental data and mapping against current databases/GSE19350/REPORT_Sun-02-09-2012_12-39-47.html]

REPORT


## Report of miRNA-mRNA interactions for all arrays. [generated on 2012-09-02 12:39:47]

---

Statistical testing for messenger RNA arrays: 812 genes found significantly up-/down-regulated. Details:

| |  | ArrayFile | FunctionalGroup | | --- | --- | --- | | 1 | GSM480480.CEL.gz | T | | 2 | GSM480481.CEL.gz | T | | 3 | GSM480482.CEL.gz | T | | 4 | GSM480483.CEL.gz | T | | 5 | GSM480484.CEL.gz | T | | 6 | GSM480485.CEL.gz | T | | 7 | GSM480487.CEL.gz | C | | 8 | GSM480488.CEL.gz | C | | 9 | GSM480489.CEL.gz | C | | 10 | GSM480490.CEL.gz | C | | 11 | GSM480491.CEL.gz | C | | 12 | GSM480492.CEL.gz | C | |

  

Principal Component Analyses:

Heatmap for top 50 geneses from statistical analyses (ordered by p-value):

Volcano plot with for auto cut-off calculation audit (cut-off shown with red line):

---

Statistical testing for microRNA prediction method I - scaling function: 102 genes found significantly up-/down-regulated. Details:

Principal Component Analyses:

Heatmap for top 50 geneses from statistical analyses (ordered by p-value):

Volcano plot with for auto cut-off calculation audit (cut-off shown with red line):

---

Statistical testing for microRNA prediction method II - linear modelling: 60 genes found significantly up-/down-regulated. Details:

Principal Component Analysis:

Heatmap for top 50 geneses from statistical analyses (ordered by p-value):

Volcano plot with for auto cut-off calculation audit (cut-off shown with red line):

---

Mean anti-correlation detected between mRNA and miRNA = -0.783731. Details:

Histogram of most anti-correlated miRNA-mRNA pairs - potential miRNA-target interactions:

---

Total number of 120 miRNAs are predicted to have significantly up-/down-regulated targets. Expend:

| |  | microRNA | NoSuppresedGenes | | --- | --- | --- | | 1 | hsa-mir-218-2 | 635 | | 2 | hsa-mir-574 | 628 | | 3 | hsa-mir-675 | 562 | | 4 | hsa-mir-877 | 537 | | 5 | hsa-mir-585 | 419 | | 6 | hsa-mir-3129 | 417 | | 7 | hsa-mir-561 | 412 | | 8 | hsa-mir-887 | 409 | | 9 | hsa-mir-938 | 398 | | 10 | hsa-mir-4794 | 391 | | 11 | hsa-mir-603 | 385 | | 12 | hsa-mir-604 | 380 | | 13 | hsa-mir-548ao | 364 | | 14 | hsa-mir-1245a | 362 | | 15 | hsa-mir-3606 | 361 | | 16 | hsa-mir-4441 | 360 | | 17 | hsa-mir-10a | 355 | | 18 | hsa-mir-2467 | 350 | | 19 | hsa-mir-3126 | 333 | | 20 | hsa-mir-618 | 329 | | 21 | hsa-mir-640 | 326 | | 22 | hsa-mir-605 | 316 | | 23 | hsa-mir-218-1 | 308 | | 24 | hsa-mir-3120 | 299 | | 25 | hsa-mir-4768 | 283 | | 26 | hsa-mir-620 | 283 | | 27 | hsa-mir-3650 | 280 | | 28 | hsa-mir-4735 | 279 | | 29 | hsa-mir-636 | 273 | | 30 | hsa-mir-617 | 263 | | 31 | hsa-mir-1271 | 254 | | 32 | hsa-mir-125b-1 | 247 | | 33 | hsa-mir-1469 | 241 | | 34 | hsa-mir-125b-2 | 237 | | 35 | hsa-mir-99a | 237 | | 36 | hsa-mir-4636 | 219 | | 37 | hsa-mir-550a-1 | 212 | | 38 | hsa-let-7c | 210 | | 39 | hsa-mir-4698 | 203 | | 40 | hsa-mir-4731 | 203 | | 41 | hsa-mir-181a-2 | 199 | | 42 | hsa-mir-181b-2 | 199 | | 43 | hsa-mir-505 | 183 | | 44 | hsa-mir-644a | 180 | | 45 | hsa-mir-455 | 178 | | 46 | hsa-mir-1915 | 177 | | 47 | hsa-mir-3139 | 172 | | 48 | hsa-mir-4467 | 172 | | 49 | hsa-mir-5090 | 172 | | 50 | hsa-mir-4723 | 168 | | 51 | hsa-mir-4771-1 | 163 | | 52 | hsa-mir-4771-2 | 162 | | 53 | hsa-mir-5579 | 162 | | 54 | hsa-mir-708 | 162 | | 55 | hsa-mir-4486 | 160 | | 56 | hsa-mir-548s | 144 | | 57 | hsa-mir-3170 | 142 | | 58 | hsa-mir-554 | 142 | | 59 | hsa-mir-591 | 142 | | 60 | hsa-mir-4519 | 141 | | 61 | hsa-mir-648 | 135 | | 62 | hsa-mir-378e | 129 | | 63 | hsa-mir-3942 | 129 | | 64 | hsa-mir-4775 | 125 | | 65 | hsa-mir-643 | 125 | | 66 | hsa-mir-3679 | 124 | | 67 | hsa-mir-602 | 124 | | 68 | hsa-mir-4517 | 123 | | 69 | hsa-mir-5194 | 121 | | 70 | hsa-mir-663b | 120 | | 71 | hsa-mir-548o-2 | 118 | | 72 | hsa-mir-378a | 110 | | 73 | hsa-let-7f-2 | 105 | | 74 | hsa-mir-548j | 105 | | 75 | hsa-mir-98 | 105 | | 76 | hsa-mir-106b | 104 | | 77 | hsa-mir-25 | 104 | | 78 | hsa-mir-93 | 104 | | 79 | hsa-mir-503 | 102 | | 80 | hsa-mir-4534 | 94 | | 81 | hsa-mir-613 | 93 | | 82 | hsa-mir-1233-1;hsa-mir-1233-2 | 90 | | 83 | hsa-mir-548n | 89 | | 84 | hsa-mir-23c | 80 | | 85 | hsa-mir-2909 | 76 | | 86 | hsa-mir-2355 | 75 | | 87 | hsa-mir-4498 | 75 | | 88 | hsa-mir-4695 | 70 | | 89 | hsa-mir-652 | 69 | | 90 | hsa-mir-1290 | 68 | | 91 | hsa-mir-491 | 68 | | 92 | hsa-mir-101-2 | 64 | | 93 | hsa-mir-1284 | 62 | | 94 | hsa-mir-3978 | 60 | | 95 | hsa-mir-548aa-2 | 58 | | 96 | hsa-mir-26b | 57 | | 97 | hsa-mir-4646 | 56 | | 98 | hsa-mir-4728 | 55 | | 99 | hsa-mir-4709 | 50 | | 100 | hsa-mir-335 | 46 | | 101 | hsa-mir-4668 | 46 | | 102 | hsa-mir-629 | 42 | | 103 | hsa-mir-4774 | 39 | | 104 | hsa-mir-3609 | 33 | | 105 | hsa-mir-3657 | 30 | | 106 | hsa-mir-4772 | 29 | | 107 | hsa-mir-130a | 25 | | 108 | hsa-mir-628 | 18 | | 109 | hsa-mir-1976 | 17 | | 110 | hsa-mir-4685 | 15 | | 111 | hsa-mir-767 | 13 | | 112 | hsa-mir-944 | 11 | | 113 | hsa-mir-196b | 9 | | 114 | hsa-mir-105-1;hsa-mir-105-2 | 6 | | 115 | hsa-mir-942 | 5 | | 116 | hsa-mir-590 | 4 | | 117 | hsa-mir-548b | 3 | | 118 | hsa-mir-107 | 2 | | 119 | hsa-mir-26a-1 | 2 | | 120 | hsa-mir-625 | 1 | |

---

Total number of 638 genes are predicted to be under differential miRNA repression. Expend:

| |  | GenSymbols | GeneName | NoTargetingMicroRNA | | --- | --- | --- | --- | | 1 | BPTF | bromodomain PHD finger transcription factor | 52 | | 2 | PRICKLE1 | prickle homolog 1 (Drosophila) | 52 | | 3 | AKAP1 | A kinase (PRKA) anchor protein 1 | 51 | | 4 | FAM122C | exportin 5 | 51 | | 5 | IL6R | family with sequence similarity 122C | 51 | | 6 | MARS | interleukin 6 receptor | 51 | | 7 | OSBPL3 | methionyl-tRNA synthetase | 51 | | 8 | RNF125 | oxysterol binding protein-like 3 | 51 | | 9 | SLIT3 | ring finger protein 125, E3 ubiquitin protein ligase | 51 | | 10 | XPO5 | slit homolog 3 (Drosophila) | 51 | | 11 | CDH11 | KIAA0226 | 50 | | 12 | CPEB1 | cadherin 11, type 2, OB-cadherin (osteoblast) | 50 | | 13 | KIAA0226 | cytoplasmic polyadenylation element binding protein 1 | 50 | | 14 | VCAN | versican | 50 | | 15 | CFLAR | CASP8 and FADD-like apoptosis regulator | 49 | | 16 | COL1A2 | collagen, type I, alpha 2 | 49 | | 17 | KLRG2 | killer cell lectin-like receptor subfamily G, member 2 | 49 | | 18 | LUZP4 | leucine zipper protein 4 | 49 | | 19 | NFE2L3 | nuclear factor (erythroid-derived 2)-like 3 | 49 | | 20 | SPATA22 | spermatogenesis associated 22 | 49 | | 21 | TCL1B | T-cell leukemia/lymphoma 1B | 48 | | 22 | TDRD12 | TEA domain family member 4 | 48 | | 23 | TEAD4 | transketolase | 48 | | 24 | TFIP11 | tudor domain containing 12 | 48 | | 25 | TKT | tuftelin interacting protein 11 | 48 | | 26 | YRDC | yrdC domain containing (E. coli) | 48 | | 27 | DNAJA3 | DnaJ (Hsp40) homolog, subfamily A, member 3 | 47 | | 28 | ERVW-1 | endogenous retrovirus group W, member 1 | 47 | | 29 | GAS2L1 | growth arrest-specific 2 like 1 | 47 | | 30 | INPP5F | inositol polyphosphate-5-phosphatase F | 47 | | 31 | MID1 | midline 1 (Opitz/BBB syndrome) | 47 | | 32 | PI4K2A | phosphatidylinositol 4-kinase type 2 alpha | 47 | | 33 | POLRMT | polymerase (RNA) mitochondrial (DNA directed) | 47 | | 34 | TBRG4 | transcription factor CP2-like 1 | 47 | | 35 | TFCP2L1 | transforming growth factor beta regulator 4 | 47 | | 36 | ZNF200 | zinc finger protein 200 | 47 | | 37 | C17orf63 | HUS1 checkpoint homolog (S. pombe) | 46 | | 38 | DLG3 | KDEL (Lys-Asp-Glu-Leu) endoplasmic reticulum protein retention receptor 3 | 46 | | 39 | FAM113B | N(alpha)-acetyltransferase 11, NatA catalytic subunit | 46 | | 40 | FRAT2 | NAD kinase | 46 | | 41 | GIP | chromosome 17 open reading frame 63 | 46 | | 42 | HUS1 | discs, large homolog 3 (Drosophila) | 46 | | 43 | KCNV2 | family with sequence similarity 113, member B | 46 | | 44 | KDELR3 | frequently rearranged in advanced T-cell lymphomas 2 | 46 | | 45 | MKRN1 | gastric inhibitory polypeptide | 46 | | 46 | NAA11 | makorin ring finger protein 1 | 46 | | 47 | NADK | neuroepithelial cell transforming 1 | 46 | | 48 | NET1 | nucleolar protein 9 | 46 | | 49 | NOL9 | potassium channel, subfamily V, member 2 | 46 | | 50 | PRAME | preferentially expressed antigen in melanoma | 46 | | 51 | RNF114 | ring finger protein 114 | 46 | | 52 | SLC12A6 | solute carrier family 12 (potassium/chloride transporters), member 6 | 46 | | 53 | SLC25A16 | solute carrier family 25 (mitochondrial carrier; Graves disease autoantigen), member 16 | 46 | | 54 | ANAPC7 | BCL2-like 13 (apoptosis facilitator) | 45 | | 55 | BCL2L13 | DDB1 and CUL4 associated factor 4-like 2 | 45 | | 56 | CCT6A | DDHD domain containing 1 | 45 | | 57 | CSNK1E | GULP, engulfment adaptor PTB domain containing 1 | 45 | | 58 | DAZL | LIM and cysteine-rich domains 1 | 45 | | 59 | DCAF4L2 | Meis homeobox 1 | 45 | | 60 | DDHD1 | PR domain containing 1, with ZNF domain | 45 | | 61 | DUS2L | Rho-related BTB domain containing 1 | 45 | | 62 | FAM46B | anaphase promoting complex subunit 7 | 45 | | 63 | GULP1 | casein kinase 1, epsilon | 45 | | 64 | LMCD1 | chaperonin containing TCP1, subunit 6A (zeta 1) | 45 | | 65 | MAGEC1 | deleted in azoospermia-like | 45 | | 66 | MAP4K1 | dihydrouridine synthase 2-like, SMM1 homolog (S. cerevisiae) | 45 | | 67 | MEIS1 | family with sequence similarity 46, member B | 45 | | 68 | MYCL1 | melanoma antigen family C, 1 | 45 | | 69 | OAZ3 | mitogen-activated protein kinase kinase kinase kinase 1 | 45 | | 70 | PRDM1 | ornithine decarboxylase antizyme 3 | 45 | | 71 | RHOBTB1 | serine/threonine kinase 31 | 45 | | 72 | SLC43A1 | solute carrier family 43, member 1 | 45 | | 73 | STK31 | transmembrane protein 170A | 45 | | 74 | TDRKH | tudor and KH domain containing | 45 | | 75 | TMEM170A | uromodulin-like 1 | 45 | | 76 | UMODL1 | v-myc myelocytomatosis viral oncogene homolog 1, lung carcinoma derived (avian) | 45 | | 77 | ADD2 | B-box and SPRY domain containing | 44 | | 78 | BSPRY | ELOVL fatty acid elongase 6 | 44 | | 79 | C12orf49 | RAB43, member RAS oncogene family | 44 | | 80 | C15orf41 | RRS1 ribosome biogenesis regulator homolog (S. cerevisiae) | 44 | | 81 | C20orf112 | Scm-like with four mbt domains 1 | 44 | | 82 | CIRH1A | T-cell leukemia/lymphoma 1A | 44 | | 83 | ELOVL6 | Ts translation elongation factor, mitochondrial | 44 | | 84 | GALNT6 | UDP-N-acetyl-alpha-D-galactosamine:polypeptide N-acetylgalactosaminyltransferase 6 (GalNAc-T6) | 44 | | 85 | LDLRAP1 | adducin 2 (beta) | 44 | | 86 | LOX | chromosome 12 open reading frame 49 | 44 | | 87 | MTHFD2 | chromosome 15 open reading frame 41 | 44 | | 88 | PRKG1 | chromosome 20 open reading frame 112 | 44 | | 89 | RAB43 | cirrhosis, autosomal recessive 1A (cirhin) | 44 | | 90 | RRP12 | low density lipoprotein receptor adaptor protein 1 | 44 | | 91 | RRS1 | lysyl oxidase | 44 | | 92 | SFMBT1 | methylenetetrahydrofolate dehydrogenase (NADP+ dependent) 2, methenyltetrahydrofolate cyclohydrolase | 44 | | 93 | SYCP3 | protein kinase, cGMP-dependent, type I | 44 | | 94 | TCL1A | ribosomal RNA processing 12 homolog (S. cerevisiae) | 44 | | 95 | TSFM | synaptonemal complex protein 3 | 44 | | 96 | UBAP2 | ubiquitin associated protein 2 | 44 | | 97 | ZFYVE1 | zinc and ring finger 2 | 44 | | 98 | ZNF462 | zinc finger protein 462 | 44 | | 99 | ZNF526 | zinc finger protein 526 | 44 | | 100 | ZNRF2 | zinc finger, FYVE domain containing 1 | 44 | | 101 | ATG13 | ATG13 autophagy related 13 homolog (S. cerevisiae) | 43 | | 102 | BYSL | DEAD (Asp-Glu-Ala-Asp) box polypeptide 43 | 43 | | 103 | C12orf44 | POU class 5 homeobox 1 pseudogene 3 | 43 | | 104 | C17orf51 | RFPL3 antisense RNA 1 (non-protein coding) | 43 | | 105 | CABYR | RNA pseudouridylate synthase domain containing 3 | 43 | | 106 | COL1A1 | bystin-like | 43 | | 107 | COL5A2 | calcium binding tyrosine-(Y)-phosphorylation regulated | 43 | | 108 | DCN | chromosome 12 open reading frame 44 | 43 | | 109 | DDX43 | chromosome 17 open reading frame 51 | 43 | | 110 | EDNRA | collagen, type I, alpha 1 | 43 | | 111 | FLJ13197 | collagen, type V, alpha 2 | 43 | | 112 | GLT8D2 | decorin | 43 | | 113 | INTS9 | endothelin receptor type A | 43 | | 114 | MMP12 | glycosyltransferase 8 domain containing 2 | 43 | | 115 | PDCD2 | integrator complex subunit 9 | 43 | | 116 | POU5F1P3 | matrix metallopeptidase 12 (macrophage elastase) | 43 | | 117 | PTPN1 | programmed cell death 2 | 43 | | 118 | RFPL3-AS1 | protein tyrosine phosphatase, non-receptor type 1 | 43 | | 119 | RFWD3 | ring finger and WD repeat domain 3 | 43 | | 120 | RPUSD3 | runt-related transcription factor 1; translocated to, 1 (cyclin D-related) | 43 | | 121 | RUNX1T1 | transcription factor AP-2 gamma (activating enhancer binding protein 2 gamma) | 43 | | 122 | TFAP2C | uncharacterized FLJ13197 | 43 | | 123 | AMOTL1 | ATPase, Ca++ transporting, ubiquitous | 42 | | 124 | ATP2A3 | ATPase, H+ transporting, lysosomal V0 subunit a2 | 42 | | 125 | ATP6V0A2 | DiGeorge syndrome critical region gene 14 | 42 | | 126 | BNC1 | GLI family zinc finger 3 | 42 | | 127 | COL21A1 | POU class 5 homeobox 1 pseudogene 4 | 42 | | 128 | COL5A1 | POU class 5 homeobox 1B | 42 | | 129 | DGCR14 | SPANXA2 overlapping transcript 1 (non-protein coding) | 42 | | 130 | DPPA4 | angiomotin like 1 | 42 | | 131 | FES | basonuclin 1 | 42 | | 132 | FLRT2 | collagen, type V, alpha 1 | 42 | | 133 | GLI3 | collagen, type XXI, alpha 1 | 42 | | 134 | LOC100128178 | developmental pluripotency associated 4 | 42 | | 135 | MTMR14 | feline sarcoma oncogene | 42 | | 136 | OR7E156P | fibronectin leucine rich transmembrane protein 2 | 42 | | 137 | POU5F1B | myotubularin related protein 14 | 42 | | 138 | POU5F1P4 | olfactory receptor, family 7, subfamily E, member 156 pseudogene | 42 | | 139 | PPAN | peter pan homolog (Drosophila) | 42 | | 140 | SNAI2 | snail homolog 2 (Drosophila) | 42 | | 141 | SPANXA2-OT1 | teashirt zinc finger homeobox 2 | 42 | | 142 | THRB | thyroid hormone receptor, beta | 42 | | 143 | TSHZ2 | uncharacterized LOC100128178 | 42 | | 144 | ZFHX3 | zinc finger homeobox 3 | 42 | | 145 | AVL9 | AVL9 homolog (S. cerevisiase) | 41 | | 146 | C1orf135 | G patch domain containing 3 | 41 | | 147 | CDX1 | HEAT repeat containing 7A | 41 | | 148 | CXorf67 | RAS-like, family 10, member A | 41 | | 149 | DACT3 | V-set and immunoglobulin domain containing 10 | 41 | | 150 | GARS | caudal type homeobox 1 | 41 | | 151 | GCAT | chromosome 1 open reading frame 135 | 41 | | 152 | GPATCH3 | chromosome X open reading frame 67 | 41 | | 153 | GPX8 | dapper, antagonist of beta-catenin, homolog 3 (Xenopus laevis) | 41 | | 154 | HEATR7A | glutathione peroxidase 8 (putative) | 41 | | 155 | MAGEB1 | glycine C-acetyltransferase | 41 | | 156 | MXRA5 | glycyl-tRNA synthetase | 41 | | 157 | NCAM1 | matrix-remodelling associated 5 | 41 | | 158 | PCOLCE | melanoma antigen family B, 1 | 41 | | 159 | PIK3CD | neural cell adhesion molecule 1 | 41 | | 160 | RASL10A | phosphoinositide-3-kinase, catalytic, delta polypeptide | 41 | | 161 | SGCD | procollagen C-endopeptidase enhancer | 41 | | 162 | SLC38A7 | sarcoglycan, delta (35kDa dystrophin-associated glycoprotein) | 41 | | 163 | VSIG10 | solute carrier family 38, member 7 | 41 | | 164 | C7orf26 | LanC lantibiotic synthetase component C-like 2 (bacterial) | 40 | | 165 | COL14A1 | SRY (sex determining region Y)-box 15 | 40 | | 166 | COPZ2 | WD repeat containing, antisense to TP73 | 40 | | 167 | FBN1 | chromosome 7 open reading frame 26 | 40 | | 168 | LANCL2 | coatomer protein complex, subunit zeta 2 | 40 | | 169 | NUP50 | collagen, type XIV, alpha 1 | 40 | | 170 | SLC7A6 | fibrillin 1 | 40 | | 171 | SOX15 | nucleoporin 50kDa | 40 | | 172 | TRAPPC10 | solute carrier family 7 (amino acid transporter light chain, y+L system), member 6 | 40 | | 173 | WRAP73 | trafficking protein particle complex 10 | 40 | | 174 | AEN | 1-acylglycerol-3-phosphate O-acyltransferase 6 (lysophosphatidic acid acyltransferase, zeta) | 39 | | 175 | AGPAT6 | DENN/MADD domain containing 2A | 39 | | 176 | CCDC86 | RNA pseudouridylate synthase domain containing 2 | 39 | | 177 | CCDC88A | apoptosis enhancing nuclease | 39 | | 178 | CHEK2 | arginyl aminopeptidase (aminopeptidase B) | 39 | | 179 | DENND2A | checkpoint kinase 2 | 39 | | 180 | ERG | coiled-coil domain containing 86 | 39 | | 181 | GNA11 | coiled-coil domain containing 88A | 39 | | 182 | NANOS3 | exportin 4 | 39 | | 183 | NBAS | guanine nucleotide binding protein (G protein), alpha 11 (Gq class) | 39 | | 184 | PCM1 | nanos homolog 3 (Drosophila) | 39 | | 185 | POLR2D | neuroblastoma amplified sequence | 39 | | 186 | RCC2 | pericentriolar material 1 | 39 | | 187 | RNPEP | polymerase (RNA) II (DNA directed) polypeptide D | 39 | | 188 | RPUSD2 | regulator of chromosome condensation 2 | 39 | | 189 | SEMA3B | sema domain, immunoglobulin domain (Ig), short basic domain, secreted, (semaphorin) 3B | 39 | | 190 | SEMA4D | sema domain, immunoglobulin domain (Ig), transmembrane domain (TM) and short cytoplasmic domain, (semaphorin) 4D | 39 | | 191 | SLC23A2 | solute carrier family 23 (nucleobase transporters), member 2 | 39 | | 192 | SLC45A4 | solute carrier family 45, member 4 | 39 | | 193 | TCF7L2 | transcription factor 7-like 2 (T-cell specific, HMG-box) | 39 | | 194 | UPP1 | uridine phosphorylase 1 | 39 | | 195 | WNT2B | v-ets erythroblastosis virus E26 oncogene homolog (avian) | 39 | | 196 | XPO4 | wingless-type MMTV integration site family, member 2B | 39 | | 197 | ZDHHC23 | zinc finger, DHHC-type containing 23 | 39 | | 198 | ACVR1B | RNA binding motif protein, X-linked-like 2 | 38 | | 199 | ADTRP | RNA binding protein, fox-1 homolog (C. elegans) 2 | 38 | | 200 | EGFR | Ras association (RalGDS/AF-6) domain family (N-terminal) member 8 | 38 | | 201 | EPB41 | WD repeat domain 66 | 38 | | 202 | FAM162B | Williams Beuren syndrome chromosome region 22 | 38 | | 203 | GNAI1 | activin A receptor, type IB | 38 | | 204 | LTBP3 | androgen-dependent TFPI-regulating protein | 38 | | 205 | OTOGL | epidermal growth factor receptor | 38 | | 206 | PCDH18 | erythrocyte membrane protein band 4.1 (elliptocytosis 1, RH-linked) | 38 | | 207 | POLR1A | family with sequence similarity 162, member B | 38 | | 208 | RASSF8 | guanine nucleotide binding protein (G protein), alpha inhibiting activity polypeptide 1 | 38 | | 209 | RBFOX2 | latent transforming growth factor beta binding protein 3 | 38 | | 210 | RBMXL2 | otogelin-like | 38 | | 211 | ROR1 | polymerase (RNA) I polypeptide A, 194kDa | 38 | | 212 | SPSB2 | protocadherin 18 | 38 | | 213 | STX6 | receptor tyrosine kinase-like orphan receptor 1 | 38 | | 214 | SURF6 | splA/ryanodine receptor domain and SOCS box containing 2 | 38 | | 215 | TRANK1 | surfeit 6 | 38 | | 216 | WBSCR22 | syntaxin 6 | 38 | | 217 | WDR66 | tetratricopeptide repeat and ankyrin repeat containing 1 | 38 | | 218 | ABCF1 | ATP-binding cassette, sub-family F (GCN20), member 1 | 37 | | 219 | ASPHD2 | CD38 molecule | 37 | | 220 | CD38 | G patch domain containing 4 | 37 | | 221 | CYP2S1 | IMP4, U3 small nucleolar ribonucleoprotein, homolog (yeast) | 37 | | 222 | FMOD | PTC7 protein phosphatase homolog (S. cerevisiae) | 37 | | 223 | GPATCH4 | StAR-related lipid transfer (START) domain containing 13 | 37 | | 224 | HPGD | UbiA prenyltransferase domain containing 1 | 37 | | 225 | IGFBP3 | aspartate beta-hydroxylase domain containing 2 | 37 | | 226 | IMP4 | cytochrome P450, family 2, subfamily S, polypeptide 1 | 37 | | 227 | KLHL18 | fibromodulin | 37 | | 228 | LRWD1 | hydroxyprostaglandin dehydrogenase 15-(NAD) | 37 | | 229 | NSMAF | insulin-like growth factor binding protein 3 | 37 | | 230 | PDGFRA | kelch-like 18 (Drosophila) | 37 | | 231 | PLAC9 | leucine-rich repeats and WD repeat domain containing 1 | 37 | | 232 | PPTC7 | neutral sphingomyelinase (N-SMase) activation associated factor | 37 | | 233 | SNCAIP | placenta-specific 9 | 37 | | 234 | STARD13 | platelet-derived growth factor receptor, alpha polypeptide | 37 | | 235 | TDP1 | synuclein, alpha interacting protein | 37 | | 236 | TGFB1I1 | taxilin gamma | 37 | | 237 | TUBBP5 | transforming growth factor beta 1 induced transcript 1 | 37 | | 238 | TXLNG | tubulin, beta pseudogene 5 | 37 | | 239 | UBIAD1 | tyrosyl-DNA phosphodiesterase 1 | 37 | | 240 | ZNF273 | zinc finger protein 273 | 37 | | 241 | BEND3 | BEN domain containing 3 | 36 | | 242 | BRF2 | BRF2, subunit of RNA polymerase III transcription initiation factor, BRF1-like | 36 | | 243 | C6orf136 | Ewing sarcoma breakpoint region 1 | 36 | | 244 | EWSR1 | KIAA0513 | 36 | | 245 | KIAA0513 | SET binding protein 1 | 36 | | 246 | MBD2 | UTP18 small subunit (SSU) processome component homolog (yeast) | 36 | | 247 | PIWIL1 | chromosome 6 open reading frame 136 | 36 | | 248 | SETBP1 | methyl-CpG binding domain protein 2 | 36 | | 249 | SLC4A8 | piwi-like 1 (Drosophila) | 36 | | 250 | TMEM117 | solute carrier family 4, sodium bicarbonate cotransporter, member 8 | 36 | | 251 | TOR3A | torsin family 3, member A | 36 | | 252 | UBE2O | transmembrane protein 117 | 36 | | 253 | UTP18 | ubiquitin-conjugating enzyme E2O | 36 | | 254 | VASH1 | vasohibin 1 | 36 | | 255 | ABCF2 | ADAM metallopeptidase with thrombospondin type 1 motif, 2 | 35 | | 256 | ADAMTS2 | ADP-ribosylation factor guanine nucleotide-exchange factor 2 (brefeldin A-inhibited) | 35 | | 257 | ARFGEF2 | ATP-binding cassette, sub-family F (GCN20), member 2 | 35 | | 258 | BFAR | CGG triplet repeat binding protein 1 | 35 | | 259 | CACHD1 | DDB1 and CUL4 associated factor 10 | 35 | | 260 | CGGBP1 | EGF containing fibulin-like extracellular matrix protein 2 | 35 | | 261 | CTNS | F-box protein 45 | 35 | | 262 | DCAF10 | GINS complex subunit 3 (Psf3 homolog) | 35 | | 263 | EFEMP2 | MDN1, midasin homolog (yeast) | 35 | | 264 | EFTUD2 | RNA binding motif protein 38 | 35 | | 265 | EIF4ENIF1 | UTP6, small subunit (SSU) processome component, homolog (yeast) | 35 | | 266 | FBXO45 | bifunctional apoptosis regulator | 35 | | 267 | GINS3 | cache domain containing 1 | 35 | | 268 | MAGEB2 | cystinosin, lysosomal cystine transporter | 35 | | 269 | MDN1 | elongation factor Tu GTP binding domain containing 2 | 35 | | 270 | PEAR1 | eukaryotic translation initiation factor 4E nuclear import factor 1 | 35 | | 271 | PIM3 | melanoma antigen family B, 2 | 35 | | 272 | PSME3 | pim-3 oncogene | 35 | | 273 | RBM38 | platelet endothelial aggregation receptor 1 | 35 | | 274 | SESN2 | proteasome (prosome, macropain) activator subunit 3 (PA28 gamma; Ki) | 35 | | 275 | SURF1 | sestrin 2 | 35 | | 276 | TCP11L1 | surfeit 1 | 35 | | 277 | THBS2 | t-complex 11 (mouse)-like 1 | 35 | | 278 | UTP6 | thrombospondin 2 | 35 | | 279 | WAPAL | wings apart-like homolog (Drosophila) | 35 | | 280 | ZNF341 | zinc finger protein 341 | 35 | | 281 | ZNF814 | zinc finger protein 814 | 35 | | 282 | ALOX12 | Boc homolog (mouse) | 34 | | 283 | ASB6 | CTD (carboxy-terminal domain, RNA polymerase II, polypeptide A) phosphatase, subunit 1 | 34 | | 284 | BATF3 | FK506 binding protein 7 | 34 | | 285 | BICD1 | G protein-coupled estrogen receptor 1 | 34 | | 286 | BOC | HORMA domain containing 1 | 34 | | 287 | CCDC163P | KIAA1217 | 34 | | 288 | CSTF2 | Meis homeobox 2 | 34 | | 289 | CTDP1 | PDZ domain containing ring finger 3 | 34 | | 290 | DOPEY2 | Rab geranylgeranyltransferase, alpha subunit | 34 | | 291 | EXT1 | Ras homolog enriched in brain like 1 | 34 | | 292 | FAM114A1 | WD repeat domain 85 | 34 | | 293 | FAM214B | ankyrin repeat and SOCS box containing 6 | 34 | | 294 | FBLN1 | arachidonate 12-lipoxygenase | 34 | | 295 | FKBP7 | basic leucine zipper transcription factor, ATF-like 3 | 34 | | 296 | GNG11 | bicaudal D homolog 1 (Drosophila) | 34 | | 297 | GPER | cleavage stimulation factor, 3' pre-RNA, subunit 2, 64kDa | 34 | | 298 | HORMAD1 | coiled-coil domain containing 163, pseudogene | 34 | | 299 | KIAA1217 | dopey family member 2 | 34 | | 300 | LOXL4 | exostosin 1 | 34 | | 301 | MEIS2 | family with sequence similarity 114, member A1 | 34 | | 302 | PDZRN3 | family with sequence similarity 214, member B | 34 | | 303 | RABGGTA | fibulin 1 | 34 | | 304 | RGS16 | guanine nucleotide binding protein (G protein), gamma 11 | 34 | | 305 | RHEBL1 | lysyl oxidase-like 4 | 34 | | 306 | SLC2A11 | regulator of G-protein signaling 16 | 34 | | 307 | WDR85 | solute carrier family 2 (facilitated glucose transporter), member 11 | 34 | | 308 | ASPN | G2/M-phase specific E3 ubiquitin protein ligase | 33 | | 309 | BRWD3 | N-myristoyltransferase 1 | 33 | | 310 | C5orf24 | PAS domain containing serine/threonine kinase | 33 | | 311 | CRABP2 | RAB4B, member RAS oncogene family | 33 | | 312 | CSNK1G1 | asporin | 33 | | 313 | CXorf38 | bromodomain and WD repeat domain containing 3 | 33 | | 314 | DDR2 | casein kinase 1, gamma 1 | 33 | | 315 | G2E3 | cellular retinoic acid binding protein 2 | 33 | | 316 | KPNA2 | chromosome 5 open reading frame 24 | 33 | | 317 | LY6K | chromosome X open reading frame 38 | 33 | | 318 | MED13L | discoidin domain receptor tyrosine kinase 2 | 33 | | 319 | NIP7 | karyopherin alpha 2 (RAG cohort 1, importin alpha 1) | 33 | | 320 | NMT1 | lymphocyte antigen 6 complex, locus K | 33 | | 321 | PASK | mediator complex subunit 13-like | 33 | | 322 | PKMYT1 | nuclear import 7 homolog (S. cerevisiae) | 33 | | 323 | PPM1G | protein kinase, membrane associated tyrosine/threonine 1 | 33 | | 324 | RAB4B | protein phosphatase, Mg2+/Mn2+ dependent, 1G | 33 | | 325 | STK17A | serine/threonine kinase 17a | 33 | | 326 | UTF1 | undifferentiated embryonic cell transcription factor 1 | 33 | | 327 | ZFP42 | zinc finger protein 42 homolog (mouse) | 33 | | 328 | ZNF655 | zinc finger protein 655 | 33 | | 329 | ANAPC1 | G-protein signaling modulator 1 | 32 | | 330 | ANKRD55 | NADH dehydrogenase (ubiquinone) 1, alpha/beta subcomplex, 1, 8kDa | 32 | | 331 | CSNK1D | VAMP (vesicle-associated membrane protein)-associated protein B and C | 32 | | 332 | GPC4 | anaphase promoting complex subunit 1 | 32 | | 333 | GPSM1 | ankyrin repeat domain 55 | 32 | | 334 | KLHL22 | casein kinase 1, delta | 32 | | 335 | LOC100505728 | glypican 4 | 32 | | 336 | LRRC59 | kelch-like 22 (Drosophila) | 32 | | 337 | NAV3 | leucine rich repeat containing 59 | 32 | | 338 | NDUFAB1 | neuron navigator 3 | 32 | | 339 | OBFC2A | oligonucleotide/oligosaccharide-binding fold containing 2A | 32 | | 340 | OGN | osteoglycin | 32 | | 341 | PNO1 | partner of NOB1 homolog (S. cerevisiae) | 32 | | 342 | PTBP3 | polypyrimidine tract binding protein 3 | 32 | | 343 | RNASE1 | ribonuclease, RNase A family, 1 (pancreatic) | 32 | | 344 | SLC45A3 | signal recognition particle receptor, B subunit | 32 | | 345 | SRPRB | solute carrier family 45, member 3 | 32 | | 346 | TTC7A | tetratricopeptide repeat domain 7A | 32 | | 347 | VAPB | uncharacterized LOC100505728 | 32 | | 348 | ZNF492 | zinc finger protein 492 | 32 | | 349 | ZNF503 | zinc finger protein 503 | 32 | | 350 | ADPGK | 5-hydroxytryptamine (serotonin) receptor 7 pseudogene 1 | 31 | | 351 | ARFGEF1 | ADP-dependent glucokinase | 31 | | 352 | ATP1B3 | ADP-ribosylation factor guanine nucleotide-exchange factor 1 (brefeldin A-inhibited) | 31 | | 353 | CCDC149 | ATPase, Na+/K+ transporting, beta 3 polypeptide | 31 | | 354 | CPVL | DEAD (Asp-Glu-Ala-Asp) box polypeptide 55 | 31 | | 355 | CYP2R1 | DnaJ (Hsp40) homolog, subfamily A, member 2 | 31 | | 356 | DDX55 | H19, imprinted maternally expressed transcript (non-protein coding) | 31 | | 357 | DNAJA2 | H2B histone family, member X, pseudogene | 31 | | 358 | GHR | LAS1-like (S. cerevisiae) | 31 | | 359 | H19 | RAB37, member RAS oncogene family | 31 | | 360 | H2BFXP | carboxypeptidase, vitellogenic-like | 31 | | 361 | HIST1H2BK | coiled-coil domain containing 149 | 31 | | 362 | HTR7P1 | cytochrome P450, family 2, subfamily R, polypeptide 1 | 31 | | 363 | LAS1L | growth hormone receptor | 31 | | 364 | LRRC17 | histone cluster 1, H2bk | 31 | | 365 | MAN1C1 | leucine rich repeat containing 17 | 31 | | 366 | PHTF2 | mannosidase, alpha, class 1C, member 1 | 31 | | 367 | RAB37 | putative homeodomain transcription factor 2 | 31 | | 368 | RPRML | reprimo-like | 31 | | 369 | SLC29A1 | solute carrier family 29 (nucleoside transporters), member 1 | 31 | | 370 | ZNF394 | zinc finger protein 394 | 31 | | 371 | ABCF3 | 5'-nucleotidase, cytosolic II | 30 | | 372 | ADAR | ATP-binding cassette, sub-family F (GCN20), member 3 | 30 | | 373 | CBFA2T2 | CNKSR family member 3 | 30 | | 374 | CNKSR3 | ORAI calcium release-activated calcium modulator 1 | 30 | | 375 | EMR2 | RNA binding motif protein 48 | 30 | | 376 | FOXP2 | RNA binding motif, single stranded interacting protein 3 | 30 | | 377 | FUCA1 | SWI5-dependent recombination repair 1 | 30 | | 378 | HDAC4 | WD repeat domain 33 | 30 | | 379 | HNRNPU | adenosine deaminase, RNA-specific | 30 | | 380 | INSIG1 | core-binding factor, runt domain, alpha subunit 2; translocated to, 2 | 30 | | 381 | LAMB1 | egf-like module containing, mucin-like, hormone receptor-like 2 | 30 | | 382 | LOC100129195 | exportin 6 | 30 | | 383 | LOC100505549 | forkhead box P2 | 30 | | 384 | MAGED2 | fucosidase, alpha-L- 1, tissue | 30 | | 385 | MTFR1 | heterogeneous nuclear ribonucleoprotein U (scaffold attachment factor A) | 30 | | 386 | NID2 | histone deacetylase 4 | 30 | | 387 | NIPA2 | insulin induced gene 1 | 30 | | 388 | NT5C2 | laminin, beta 1 | 30 | | 389 | ORAI1 | melanoma antigen family D, 2 | 30 | | 390 | PLBD1 | mitochondrial fission regulator 1 | 30 | | 391 | PSMD12 | nidogen 2 (osteonidogen) | 30 | | 392 | RBM48 | non imprinted in Prader-Willi/Angelman syndrome 2 | 30 | | 393 | RBMS3 | phospholipase B domain containing 1 | 30 | | 394 | SFR1 | proteasome (prosome, macropain) 26S subunit, non-ATPase, 12 | 30 | | 395 | THSD7A | thrombospondin, type I, domain containing 7A | 30 | | 396 | TMC7 | transmembrane channel-like 7 | 30 | | 397 | WDR33 | uncharacterized LOC100129195 | 30 | | 398 | XPO6 | uncharacterized LOC100505549 | 30 | | 399 | ZNF593 | zinc finger protein 593 | 30 | | 400 | AKAP3 | A kinase (PRKA) anchor protein 3 | 29 | | 401 | C12orf23 | F-box protein 28 | 29 | | 402 | C1orf38 | GRB2-associated binding protein 1 | 29 | | 403 | CES5A | SWI/SNF related, matrix associated, actin dependent regulator of chromatin, subfamily a, member 1 | 29 | | 404 | DIDO1 | carboxylesterase 5A | 29 | | 405 | FAM182B | chromosome 1 open reading frame 38 | 29 | | 406 | FBXO28 | chromosome 12 open reading frame 23 | 29 | | 407 | GAB1 | death inducer-obliterator 1 | 29 | | 408 | HSBP1 | family with sequence similarity 182, member B | 29 | | 409 | MCART1 | heat shock factor binding protein 1 | 29 | | 410 | PCCB | mitochondrial carrier triple repeat 1 | 29 | | 411 | PLEKHB2 | peroxiredoxin 4 | 29 | | 412 | PRDX4 | pleckstrin homology domain containing, family B (evectins) member 2 | 29 | | 413 | SMARCA1 | propionyl CoA carboxylase, beta polypeptide | 29 | | 414 | TIMM8A | teashirt zinc finger homeobox 3 | 29 | | 415 | TSHZ3 | translocase of inner mitochondrial membrane 8 homolog A (yeast) | 29 | | 416 | ZC3HAV1L | zinc finger CCCH-type, antiviral 1-like | 29 | | 417 | ZNF142 | zinc finger protein 142 | 29 | | 418 | ARPC1B | ATG5 autophagy related 5 homolog (S. cerevisiae) | 28 | | 419 | ATG5 | BCL2/adenovirus E1B 19kDa interacting protein 3-like | 28 | | 420 | BLOC1S3 | KDEL (Lys-Asp-Glu-Leu) endoplasmic reticulum protein retention receptor 2 | 28 | | 421 | BNIP3L | MHC class I polypeptide-related sequence B | 28 | | 422 | C8orf76 | NEDD4 binding protein 1 | 28 | | 423 | CCBE1 | SCO cytochrome oxidase deficient homolog 2 (yeast) | 28 | | 424 | CCDC80 | SWIM-type zinc finger 7 associated protein 1 | 28 | | 425 | ETS2 | UDP-glucose 6-dehydrogenase | 28 | | 426 | FAM124B | actin related protein 2/3 complex, subunit 1B, 41kDa | 28 | | 427 | HDHD1 | biogenesis of lysosomal organelles complex-1, subunit 3 | 28 | | 428 | HELLS | chromosome 8 open reading frame 76 | 28 | | 429 | HNRNPD | coiled-coil domain containing 80 | 28 | | 430 | IL17RD | collagen and calcium binding EGF domains 1 | 28 | | 431 | INHBA | family with sequence similarity 124B | 28 | | 432 | ITCH | haloacid dehalogenase-like hydrolase domain containing 1 | 28 | | 433 | KDELR2 | helicase, lymphoid-specific | 28 | | 434 | LOC100505876 | heterogeneous nuclear ribonucleoprotein D (AU-rich element RNA binding protein 1, 37kDa) | 28 | | 435 | MICB | inhibin, beta A | 28 | | 436 | N4BP1 | interleukin 17 receptor D | 28 | | 437 | PTPN2 | itchy E3 ubiquitin protein ligase | 28 | | 438 | SCO2 | protein tyrosine phosphatase, non-receptor type 2 | 28 | | 439 | SRPX | serine racemase | 28 | | 440 | SRR | supervillin | 28 | | 441 | SVIL | sushi-repeat containing protein, X-linked | 28 | | 442 | SWSAP1 | transmembrane protein 51 | 28 | | 443 | TMEM51 | uncharacterized LOC100505876 | 28 | | 444 | UGDH | v-ets erythroblastosis virus E26 oncogene homolog 2 (avian) | 28 | | 445 | ZNF296 | zinc finger protein 296 | 28 | | 446 | ZNF703 | zinc finger protein 703 | 28 | | 447 | ALPL | HKR1, GLI-Kruppel zinc finger family member | 27 | | 448 | AP1G1 | adaptor-related protein complex 1, gamma 1 subunit | 27 | | 449 | BCHE | alkaline phosphatase, liver/bone/kidney | 27 | | 450 | BMP1 | bone morphogenetic protein 1 | 27 | | 451 | CLN8 | butyrylcholinesterase | 27 | | 452 | DCTN5 | ceroid-lipofuscinosis, neuronal 8 (epilepsy, progressive with mental retardation) | 27 | | 453 | ERCC1 | dynactin 5 (p25) | 27 | | 454 | HKR1 | excision repair cross-complementing rodent repair deficiency, complementation group 1 (includes overlapping antisense sequence) | 27 | | 455 | LOC100506451 | polyribonucleotide nucleotidyltransferase 1 | 27 | | 456 | PACSIN2 | post-GPI attachment to proteins 1 | 27 | | 457 | PGAP1 | protein kinase C and casein kinase substrate in neurons 2 | 27 | | 458 | PNPT1 | uncharacterized LOC100506451 | 27 | | 459 | ZNF318 | zinc finger protein 318 | 27 | | 460 | ZNF579 | zinc finger protein 579 | 27 | | 461 | ZNF629 | zinc finger protein 629 | 27 | | 462 | ADAMTS9 | ADAM metallopeptidase with thrombospondin type 1 motif, 9 | 26 | | 463 | ATP11C | ATPase, class VI, type 11C | 26 | | 464 | CLPTM1L | CLPTM1-like | 26 | | 465 | CMBL | KIAA1704 | 26 | | 466 | EFS | carboxymethylenebutenolidase homolog (Pseudomonas) | 26 | | 467 | INPP5D | embryonal Fyn-associated substrate | 26 | | 468 | KIAA1704 | inositol polyphosphate-5-phosphatase, 145kDa | 26 | | 469 | LOC100505759 | lysophosphatidylcholine acyltransferase 4 | 26 | | 470 | LPCAT4 | mucolipin 2 | 26 | | 471 | MCOLN2 | prostaglandin F2 receptor negative regulator | 26 | | 472 | PTGFRN | transcription factor 7-like 1 (T-cell specific, HMG-box) | 26 | | 473 | TCF7L1 | transforming growth factor, beta 2 | 26 | | 474 | TGFB2 | transmembrane protein 199 | 26 | | 475 | TMEM199 | transmembrane protein 8B | 26 | | 476 | TMEM8B | triple functional domain (PTPRF interacting) | 26 | | 477 | TRIO | uncharacterized LOC100505759 | 26 | | 478 | ACP5 | ARP3 actin-related protein 3 homolog B (yeast) | 25 | | 479 | ACTR3B | DEAD (Asp-Glu-Ala-Asp) box polypeptide 23 | 25 | | 480 | C12orf66 | HEN1 methyltransferase homolog 1 (Arabidopsis) | 25 | | 481 | CALD1 | La ribonucleoprotein domain family, member 1B | 25 | | 482 | DDAH2 | SEH1-like (S. cerevisiae) | 25 | | 483 | DDX23 | acid phosphatase 5, tartrate resistant | 25 | | 484 | HENMT1 | caldesmon 1 | 25 | | 485 | HIST1H2BC | chromosome 12 open reading frame 66 | 25 | | 486 | ITGA8 | dimethylarginine dimethylaminohydrolase 2 | 25 | | 487 | LARP1B | histone cluster 1, H2bc | 25 | | 488 | MARCH8 | integrin, alpha 8 | 25 | | 489 | MFAP4 | membrane-associated ring finger (C3HC4) 8, E3 ubiquitin protein ligase | 25 | | 490 | PDCD11 | microfibrillar-associated protein 4 | 25 | | 491 | PDPN | phosphorylase kinase, alpha 1 (muscle) | 25 | | 492 | PHKA1 | podoplanin | 25 | | 493 | PTPN12 | programmed cell death 11 | 25 | | 494 | PURG | protein tyrosine phosphatase, non-receptor type 12 | 25 | | 495 | RHOD | purine-rich element binding protein G | 25 | | 496 | ROBO4 | ras homolog family member D | 25 | | 497 | SEH1L | roundabout, axon guidance receptor, homolog 4 (Drosophila) | 25 | | 498 | SMAGP | small cell adhesion glycoprotein | 25 | | 499 | SPATA2 | spermatogenesis associated 2 | 25 | | 500 | TCP1 | t-complex 1 | 25 | | 501 | ZC3HC1 | zinc finger, C3HC-type containing 1 | 25 | | 502 | CHTF8 | CTF8, chromosome transmission fidelity factor 8 homolog (S. cerevisiae) | 24 | | 503 | CKAP4 | DNA-damage-inducible transcript 4-like | 24 | | 504 | DDIT4L | FIC domain containing | 24 | | 505 | FICD | TAF4b RNA polymerase II, TATA box binding protein (TBP)-associated factor, 105kDa | 24 | | 506 | HIST1H2BF | TNFAIP3 interacting protein 2 | 24 | | 507 | IRS1 | WD repeat domain 86 | 24 | | 508 | L3MBTL2 | cytoskeleton-associated protein 4 | 24 | | 509 | RNF31 | histone cluster 1, H2bf | 24 | | 510 | TAF4B | insulin receptor substrate 1 | 24 | | 511 | TNIP2 | l(3)mbt-like 2 (Drosophila) | 24 | | 512 | WDR86 | ring finger protein 31 | 24 | | 513 | ACPL2 | 2-oxoglutarate and iron-dependent oxygenase domain containing 1 | 23 | | 514 | APEX2 | APEX nuclease (apurinic/apyrimidinic endonuclease) 2 | 23 | | 515 | CCDC117 | DAZ interacting protein 1 | 23 | | 516 | DZIP1 | RAB7, member RAS oncogene family-like 1 | 23 | | 517 | IL27RA | SAM domain and HD domain 1 | 23 | | 518 | MRPS18B | acid phosphatase-like 2 | 23 | | 519 | OGFOD1 | coiled-coil domain containing 117 | 23 | | 520 | PREB | interleukin 27 receptor, alpha | 23 | | 521 | RAB7L1 | mitochondrial ribosomal protein S18B | 23 | | 522 | RMND1 | prolactin regulatory element binding | 23 | | 523 | SAMHD1 | required for meiotic nuclear division 1 homolog (S. cerevisiae) | 23 | | 524 | SFRP1 | secreted frizzled-related protein 1 | 23 | | 525 | SLC38A2 | solute carrier family 38, member 2 | 23 | | 526 | TFPI | tissue factor pathway inhibitor (lipoprotein-associated coagulation inhibitor) | 23 | | 527 | VAV1 | vav 1 guanine nucleotide exchange factor | 23 | | 528 | BGN | F-box and leucine-rich repeat protein 7 | 22 | | 529 | C1orf85 | FRAS1 related extracellular matrix 1 | 22 | | 530 | CREBL2 | KIT ligand | 22 | | 531 | FBXL7 | PQ loop repeat containing 3 | 22 | | 532 | FIGN | RAS-like, family 11, member B | 22 | | 533 | FREM1 | biglycan | 22 | | 534 | FZD7 | cAMP responsive element binding protein-like 2 | 22 | | 535 | HYI | chromosome 1 open reading frame 85 | 22 | | 536 | INPP5K | fidgetin | 22 | | 537 | KITLG | frizzled family receptor 7 | 22 | | 538 | MRPS5 | hydroxypyruvate isomerase (putative) | 22 | | 539 | PHLDA1 | inositol polyphosphate-5-phosphatase K | 22 | | 540 | PQLC3 | mitochondrial ribosomal protein S5 | 22 | | 541 | PTPRD | pleckstrin homology-like domain, family A, member 1 | 22 | | 542 | PTPRK | protein tyrosine phosphatase, receptor type, D | 22 | | 543 | RASL11B | protein tyrosine phosphatase, receptor type, K | 22 | | 544 | RECK | reversion-inducing-cysteine-rich protein with kazal motifs | 22 | | 545 | RNF138 | ring finger protein 138, E3 ubiquitin protein ligase | 22 | | 546 | SYCE1L | synaptonemal complex central element protein 1-like | 22 | | 547 | TFDP2 | transcription factor Dp-2 (E2F dimerization partner 2) | 22 | | 548 | TLE2 | transducin-like enhancer of split 2 (E(sp1) homolog, Drosophila) | 22 | | 549 | ZNF445 | zinc finger protein 445 | 22 | | 550 | ERI1 | HOXB cluster antisense RNA 3 (non-protein coding) | 21 | | 551 | HES1 | NECAP endocytosis associated 2 | 21 | | 552 | HIST1H1C | SWI/SNF related, matrix associated, actin dependent regulator of chromatin, subfamily e, member 1 | 21 | | 553 | HNRNPR | exoribonuclease 1 | 21 | | 554 | HOXB-AS3 | hairy and enhancer of split 1, (Drosophila) | 21 | | 555 | IGSF5 | heterogeneous nuclear ribonucleoprotein R | 21 | | 556 | KPNB1 | histone cluster 1, H1c | 21 | | 557 | LPIN1 | immunoglobulin superfamily, member 5 | 21 | | 558 | LTBP1 | karyopherin (importin) beta 1 | 21 | | 559 | MBP | latent transforming growth factor beta binding protein 1 | 21 | | 560 | NECAP2 | lipin 1 | 21 | | 561 | NGEF | myelin basic protein | 21 | | 562 | NID1 | neuronal guanine nucleotide exchange factor | 21 | | 563 | PAFAH1B1 | nidogen 1 | 21 | | 564 | SMARCE1 | platelet-activating factor acetylhydrolase 1b, regulatory subunit 1 (45kDa) | 21 | | 565 | STOM | stomatin | 21 | | 566 | WNT5A | wingless-type MMTV integration site family, member 5A | 21 | | 567 | ZNF827 | zinc finger protein 827 | 21 | | 568 | CCDC3 | CD83 molecule | 20 | | 569 | CD83 | N-acetylated alpha-linked acidic dipeptidase 2 | 20 | | 570 | COQ10B | RNA methyltransferase like 1 | 20 | | 571 | ELN | SEC14-like 3 (S. cerevisiae) | 20 | | 572 | ENAH | coenzyme Q10 homolog B (S. cerevisiae) | 20 | | 573 | LURAP1L | coiled-coil domain containing 3 | 20 | | 574 | METTL8 | elastin | 20 | | 575 | NAALAD2 | enabled homolog (Drosophila) | 20 | | 576 | NUP62 | leucine rich adaptor protein 1-like | 20 | | 577 | PDCD2L | methyltransferase like 8 | 20 | | 578 | RNMTL1 | nucleoporin 62kDa | 20 | | 579 | RPL31 | programmed cell death 2-like | 20 | | 580 | SEC14L3 | ribosomal protein L31 | 20 | | 581 | SEPT6 | septin 6 | 20 | | 582 | SSH3 | serine/threonine kinase 4 | 20 | | 583 | STK4 | slingshot homolog 3 (Drosophila) | 20 | | 584 | USP7 | ubiquitin specific peptidase 7 (herpes virus-associated) | 20 | | 585 | VEZF1 | vascular endothelial zinc finger 1 | 20 | | 586 | C1orf198 | DEAD (Asp-Glu-Ala-Asp) box helicase 5 | 19 | | 587 | DDX5 | K(lysine) acetyltransferase 7 | 19 | | 588 | KAT7 | Nance-Horan syndrome (congenital cataracts and dental anomalies) | 19 | | 589 | LOC100134937 | SH3 domain containing ring finger 1 | 19 | | 590 | NARF | chromosome 1 open reading frame 198 | 19 | | 591 | NHS | nuclear prelamin A recognition factor | 19 | | 592 | PABPC4L | poly(A) binding protein, cytoplasmic 4-like | 19 | | 593 | SH3RF1 | synaptosomal-associated protein, 29kDa | 19 | | 594 | SNAP29 | uncharacterized LOC100134937 | 19 | | 595 | BZW2 | DCP2 decapping enzyme homolog (S. cerevisiae) | 18 | | 596 | DCP2 | RAB3 GTPase activating protein subunit 1 (catalytic) | 18 | | 597 | FBN2 | basic leucine zipper and W2 domains 2 | 18 | | 598 | FZD1 | exportin 7 | 18 | | 599 | HIST1H2BI | fibrillin 2 | 18 | | 600 | KBTBD8 | frizzled family receptor 1 | 18 | | 601 | LOC389831 | histone cluster 1, H2bi | 18 | | 602 | LY75 | kelch repeat and BTB (POZ) domain containing 8 | 18 | | 603 | RAB3GAP1 | lymphocyte antigen 75 | 18 | | 604 | TM6SF2 | translocase of outer mitochondrial membrane 70 homolog A (S. cerevisiae) | 18 | | 605 | TOMM70A | transmembrane 6 superfamily member 2 | 18 | | 606 | XPO7 | uncharacterized LOC389831 | 18 | | 607 | ZNF2 | zinc finger protein 2 | 18 | | 608 | ZNF589 | zinc finger protein 589 | 18 | | 609 | CBLL1 | Cbl proto-oncogene, E3 ubiquitin protein ligase-like 1 | 17 | | 610 | CCL18 | RAB34, member RAS oncogene family | 17 | | 611 | ENY2 | chemokine (C-C motif) ligand 18 (pulmonary and activation-regulated) | 17 | | 612 | HIST1H2BD | enhancer of yellow 2 homolog (Drosophila) | 17 | | 613 | MXRA8 | histone cluster 1, H2bd | 17 | | 614 | RAB34 | matrix-remodelling associated 8 | 17 | | 615 | RNF19B | ring finger protein 19B | 17 | | 616 | TMLHE | trimethyllysine hydroxylase, epsilon | 17 | | 617 | ZBED3 | zinc finger, BED-type containing 3 | 17 | | 618 | FMN1 | LIM domain kinase 2 | 16 | | 619 | HIST1H2BE | T-box 3 | 16 | | 620 | HSPG2 | formin 1 | 16 | | 621 | LIMK2 | heparan sulfate proteoglycan 2 | 16 | | 622 | PCDHB3 | histone cluster 1, H2be | 16 | | 623 | PPP2R2A | protein phosphatase 2, regulatory subunit B, alpha | 16 | | 624 | TBX3 | protocadherin beta 3 | 16 | | 625 | SYNRG | synergin, gamma | 15 | | 626 | USP14 | ubiquitin specific peptidase 14 (tRNA-guanine transglycosylase) | 15 | | 627 | FAM115A | family with sequence similarity 115, member A | 14 | | 628 | HSPA5 | heat shock 70kDa protein 5 (glucose-regulated protein, 78kDa) | 14 | | 629 | SKAP2 | src kinase associated phosphoprotein 2 | 14 | | 630 | AS3MT | arsenic (+3 oxidation state) methyltransferase | 13 | | 631 | CSF2RB | colony stimulating factor 2 receptor, beta, low-affinity (granulocyte-macrophage) | 13 | | 632 | SDF2L1 | stromal cell-derived factor 2-like 1 | 13 | | 633 | MAST3 | microtubule associated serine/threonine kinase 3 | 12 | | 634 | CRLF3 | cytokine receptor-like factor 3 | 11 | | 635 | NRP1 | SRR1 domain containing | 10 | | 636 | SRRD | neuropilin 1 | 10 | | 637 | C15orf61 | chromosome 15 open reading frame 61 | 9 | | 638 | DCHS1 | dachsous 1 (Drosophila) | 8 | |

---

Total number of miRNA-mRNA 20897 interactions for given cut-off. Press for ALL:

| |  | miR | EntrezID | Gene | Name | Score | | --- | --- | --- | --- | --- | --- | | 2109 | hsa-mir-25 | 1634 | DCN | decorin | 8 | | 2110 | hsa-mir-93 | 1634 | DCN | decorin | 8 | | 2111 | hsa-mir-181a-2 | 1634 | DCN | decorin | 8 | | 2112 | hsa-mir-181b-2 | 1634 | DCN | decorin | 8 | | 2113 | hsa-mir-106b | 1634 | DCN | decorin | 8 | | 2114 | hsa-mir-505 | 1634 | DCN | decorin | 8 | | 2120 | hsa-mir-643 | 1634 | DCN | decorin | 8 | | 2117 | hsa-mir-602 | 1634 | DCN | decorin | 7 | | 2202 | hsa-mir-25 | 1956 | EGFR | epidermal growth factor receptor | 6 | | 2203 | hsa-mir-93 | 1956 | EGFR | epidermal growth factor receptor | 6 | | 2204 | hsa-mir-181a-2 | 1956 | EGFR | epidermal growth factor receptor | 6 | | 2205 | hsa-mir-181b-2 | 1956 | EGFR | epidermal growth factor receptor | 6 | | 2206 | hsa-mir-106b | 1956 | EGFR | epidermal growth factor receptor | 6 | | 2210 | hsa-mir-643 | 1956 | EGFR | epidermal growth factor receptor | 6 | | 3443 | hsa-mir-181a-2 | 1289 | COL5A1 | collagen, type V, alpha 1 | 6 | | 3444 | hsa-mir-181b-2 | 1289 | COL5A1 | collagen, type V, alpha 1 | 6 | | 3445 | hsa-mir-378a | 1289 | COL5A1 | collagen, type V, alpha 1 | 6 | | 3446 | hsa-mir-505 | 1289 | COL5A1 | collagen, type V, alpha 1 | 6 | | 3448 | hsa-mir-602 | 1289 | COL5A1 | collagen, type V, alpha 1 | 6 | | 5125 | hsa-mir-181a-2 | 1462 | VCAN | versican | 6 | | 5126 | hsa-mir-181b-2 | 1462 | VCAN | versican | 6 | | 5128 | hsa-mir-378a | 1462 | VCAN | versican | 6 | | 5129 | hsa-mir-505 | 1462 | VCAN | versican | 6 | | 5134 | hsa-mir-643 | 1462 | VCAN | versican | 6 | | 5676 | hsa-let-7c | 119 | ADD2 | adducin 2 (beta) | 6 | | 5677 | hsa-mir-99a | 119 | ADD2 | adducin 2 (beta) | 6 | | 5679 | hsa-mir-218-1 | 119 | ADD2 | adducin 2 (beta) | 6 | | 5681 | hsa-mir-125b-1 | 119 | ADD2 | adducin 2 (beta) | 6 | | 5682 | hsa-mir-125b-2 | 119 | ADD2 | adducin 2 (beta) | 6 | | 5683 | hsa-mir-561 | 119 | ADD2 | adducin 2 (beta) | 6 | | 5685 | hsa-mir-585 | 119 | ADD2 | adducin 2 (beta) | 6 | | 6291 | hsa-let-7c | 11184 | MAP4K1 | mitogen-activated protein kinase kinase kinase kinase 1 | 6 | | 6292 | hsa-mir-99a | 11184 | MAP4K1 | mitogen-activated protein kinase kinase kinase kinase 1 | 6 | | 6294 | hsa-mir-218-1 | 11184 | MAP4K1 | mitogen-activated protein kinase kinase kinase kinase 1 | 6 | | 6296 | hsa-mir-125b-1 | 11184 | MAP4K1 | mitogen-activated protein kinase kinase kinase kinase 1 | 6 | | 6297 | hsa-mir-125b-2 | 11184 | MAP4K1 | mitogen-activated protein kinase kinase kinase kinase 1 | 6 | | 6298 | hsa-mir-455 | 11184 | MAP4K1 | mitogen-activated protein kinase kinase kinase kinase 1 | 6 | | 6299 | hsa-mir-561 | 11184 | MAP4K1 | mitogen-activated protein kinase kinase kinase kinase 1 | 6 | | 6301 | hsa-mir-585 | 11184 | MAP4K1 | mitogen-activated protein kinase kinase kinase kinase 1 | 6 | | 6308 | hsa-mir-708 | 11184 | MAP4K1 | mitogen-activated protein kinase kinase kinase kinase 1 | 6 | | 7049 | hsa-mir-561 | 1741 | DLG3 | discs, large homolog 3 (Drosophila) | 6 | | 7051 | hsa-mir-585 | 1741 | DLG3 | discs, large homolog 3 (Drosophila) | 6 | | 9495 | hsa-mir-181a-2 | 6934 | TCF7L2 | transcription factor 7-like 2 (T-cell specific, HMG-box) | 6 | | 9496 | hsa-mir-181b-2 | 6934 | TCF7L2 | transcription factor 7-like 2 (T-cell specific, HMG-box) | 6 | | 9498 | hsa-mir-505 | 6934 | TCF7L2 | transcription factor 7-like 2 (T-cell specific, HMG-box) | 6 | | 11561 | hsa-mir-561 | 55731 | C17orf63 | chromosome 17 open reading frame 63 | 6 | | 11563 | hsa-mir-585 | 55731 | C17orf63 | chromosome 17 open reading frame 63 | 6 | | 12646 | hsa-mir-218-1 | 29842 | TFCP2L1 | transcription factor CP2-like 1 | 6 | | 12649 | hsa-mir-561 | 29842 | TFCP2L1 | transcription factor CP2-like 1 | 6 | | 12651 | hsa-mir-585 | 29842 | TFCP2L1 | transcription factor CP2-like 1 | 6 | | 12688 | hsa-let-7c | 53916 | RAB4B | RAB4B, member RAS oncogene family | 6 | | 12689 | hsa-mir-99a | 53916 | RAB4B | RAB4B, member RAS oncogene family | 6 | | 12692 | hsa-mir-125b-2 | 53916 | RAB4B | RAB4B, member RAS oncogene family | 6 | | 12693 | hsa-mir-561 | 53916 | RAB4B | RAB4B, member RAS oncogene family | 6 | | 12695 | hsa-mir-585 | 53916 | RAB4B | RAB4B, member RAS oncogene family | 6 | | 14316 | hsa-mir-99a | 57510 | XPO5 | exportin 5 | 6 | | 14318 | hsa-mir-218-1 | 57510 | XPO5 | exportin 5 | 6 | | 14320 | hsa-mir-125b-1 | 57510 | XPO5 | exportin 5 | 6 | | 14321 | hsa-mir-125b-2 | 57510 | XPO5 | exportin 5 | 6 | | 14322 | hsa-mir-561 | 57510 | XPO5 | exportin 5 | 6 | | 14324 | hsa-mir-585 | 57510 | XPO5 | exportin 5 | 6 | | 16512 | hsa-mir-181a-2 | 144165 | PRICKLE1 | prickle homolog 1 (Drosophila) | 6 | | 16513 | hsa-mir-181b-2 | 144165 | PRICKLE1 | prickle homolog 1 (Drosophila) | 6 | | 16518 | hsa-mir-643 | 144165 | PRICKLE1 | prickle homolog 1 (Drosophila) | 6 | | 2207 | hsa-mir-505 | 1956 | EGFR | epidermal growth factor receptor | 5 | | 12682 | hsa-mir-99a | 29842 | TFCP2L1 | transcription factor CP2-like 1 | 5 | | 12686 | hsa-mir-125b-2 | 29842 | TFCP2L1 | transcription factor CP2-like 1 | 5 | | 14329 | hsa-mir-620 | 57510 | XPO5 | exportin 5 | 5 | | 16515 | hsa-mir-505 | 144165 | PRICKLE1 | prickle homolog 1 (Drosophila) | 5 | | 1 | hsa-let-7c | 159091 | FAM122C | family with sequence similarity 122C | 4 | | 2 | hsa-mir-99a | 159091 | FAM122C | family with sequence similarity 122C | 4 | | 6 | hsa-mir-125b-2 | 159091 | FAM122C | family with sequence similarity 122C | 4 | | 7 | hsa-mir-561 | 159091 | FAM122C | family with sequence similarity 122C | 4 | | 9 | hsa-mir-585 | 159091 | FAM122C | family with sequence similarity 122C | 4 | | 100 | hsa-mir-561 | 10228 | STX6 | syntaxin 6 | 4 | | 102 | hsa-mir-585 | 10228 | STX6 | syntaxin 6 | 4 | | 172 | hsa-let-7c | 138009 | DCAF4L2 | DDB1 and CUL4 associated factor 4-like 2 | 4 | | 173 | hsa-mir-99a | 138009 | DCAF4L2 | DDB1 and CUL4 associated factor 4-like 2 | 4 | | 175 | hsa-mir-218-1 | 138009 | DCAF4L2 | DDB1 and CUL4 associated factor 4-like 2 | 4 | | 177 | hsa-mir-125b-1 | 138009 | DCAF4L2 | DDB1 and CUL4 associated factor 4-like 2 | 4 | | 178 | hsa-mir-125b-2 | 138009 | DCAF4L2 | DDB1 and CUL4 associated factor 4-like 2 | 4 | | 179 | hsa-mir-561 | 138009 | DCAF4L2 | DDB1 and CUL4 associated factor 4-like 2 | 4 | | 181 | hsa-mir-585 | 138009 | DCAF4L2 | DDB1 and CUL4 associated factor 4-like 2 | 4 | | 189 | hsa-mir-708 | 138009 | DCAF4L2 | DDB1 and CUL4 associated factor 4-like 2 | 4 | | 217 | hsa-let-7c | 30816 | ERVW-1 | endogenous retrovirus group W, member 1 | 4 | | 218 | hsa-mir-99a | 30816 | ERVW-1 | endogenous retrovirus group W, member 1 | 4 | | 220 | hsa-mir-218-1 | 30816 | ERVW-1 | endogenous retrovirus group W, member 1 | 4 | | 223 | hsa-mir-125b-2 | 30816 | ERVW-1 | endogenous retrovirus group W, member 1 | 4 | | 224 | hsa-mir-455 | 30816 | ERVW-1 | endogenous retrovirus group W, member 1 | 4 | | 225 | hsa-mir-561 | 30816 | ERVW-1 | endogenous retrovirus group W, member 1 | 4 | | 227 | hsa-mir-585 | 30816 | ERVW-1 | endogenous retrovirus group W, member 1 | 4 | | 236 | hsa-mir-708 | 30816 | ERVW-1 | endogenous retrovirus group W, member 1 | 4 | | 483 | hsa-mir-218-1 | 9093 | DNAJA3 | DnaJ (Hsp40) homolog, subfamily A, member 3 | 4 | | 485 | hsa-mir-125b-1 | 9093 | DNAJA3 | DnaJ (Hsp40) homolog, subfamily A, member 3 | 4 | | 486 | hsa-mir-561 | 9093 | DNAJA3 | DnaJ (Hsp40) homolog, subfamily A, member 3 | 4 | | 488 | hsa-mir-585 | 9093 | DNAJA3 | DnaJ (Hsp40) homolog, subfamily A, member 3 | 4 | | 573 | hsa-mir-218-1 | 9498 | SLC4A8 | solute carrier family 4, sodium bicarbonate cotransporter, member 8 | 4 | | 575 | hsa-mir-125b-1 | 9498 | SLC4A8 | solute carrier family 4, sodium bicarbonate cotransporter, member 8 | 4 | | 577 | hsa-mir-561 | 9498 | SLC4A8 | solute carrier family 4, sodium bicarbonate cotransporter, member 8 | 4 | | 579 | hsa-mir-585 | 9498 | SLC4A8 | solute carrier family 4, sodium bicarbonate cotransporter, member 8 | 4 | | 583 | hsa-mir-620 | 9498 | SLC4A8 | solute carrier family 4, sodium bicarbonate cotransporter, member 8 | 4 | | 805 | hsa-mir-378a | 83871 | RAB34 | RAB34, member RAS oncogene family | 4 | | 806 | hsa-mir-505 | 83871 | RAB34 | RAB34, member RAS oncogene family | 4 | | 870 | hsa-mir-455 | 55787 | TXLNG | taxilin gamma | 4 | | 871 | hsa-mir-561 | 55787 | TXLNG | taxilin gamma | 4 | | 873 | hsa-mir-585 | 55787 | TXLNG | taxilin gamma | 4 | | 889 | hsa-let-7c | 84690 | SPATA22 | spermatogenesis associated 22 | 4 | | 890 | hsa-mir-99a | 84690 | SPATA22 | spermatogenesis associated 22 | 4 | | 892 | hsa-mir-218-1 | 84690 | SPATA22 | spermatogenesis associated 22 | 4 | | 894 | hsa-mir-125b-1 | 84690 | SPATA22 | spermatogenesis associated 22 | 4 | | 895 | hsa-mir-125b-2 | 84690 | SPATA22 | spermatogenesis associated 22 | 4 | | 896 | hsa-mir-561 | 84690 | SPATA22 | spermatogenesis associated 22 | 4 | | 898 | hsa-mir-585 | 84690 | SPATA22 | spermatogenesis associated 22 | 4 | | 906 | hsa-mir-708 | 84690 | SPATA22 | spermatogenesis associated 22 | 4 | | 1234 | hsa-mir-218-1 | 55905 | RNF114 | ring finger protein 114 | 4 | | 1236 | hsa-mir-561 | 55905 | RNF114 | ring finger protein 114 | 4 | | 1238 | hsa-mir-585 | 55905 | RNF114 | ring finger protein 114 | 4 | | 1653 | hsa-let-7c | 4141 | MARS | methionyl-tRNA synthetase | 4 | | 1654 | hsa-mir-99a | 4141 | MARS | methionyl-tRNA synthetase | 4 | | 1656 | hsa-mir-218-1 | 4141 | MARS | methionyl-tRNA synthetase | 4 | | 1658 | hsa-mir-125b-1 | 4141 | MARS | methionyl-tRNA synthetase | 4 | | 1659 | hsa-mir-125b-2 | 4141 | MARS | methionyl-tRNA synthetase | 4 | | 1661 | hsa-mir-561 | 4141 | MARS | methionyl-tRNA synthetase | 4 | | 1663 | hsa-mir-585 | 4141 | MARS | methionyl-tRNA synthetase | 4 | | 1718 | hsa-mir-181a-2 | 3912 | LAMB1 | laminin, beta 1 | 4 | | 1719 | hsa-mir-181b-2 | 3912 | LAMB1 | laminin, beta 1 | 4 | | 1721 | hsa-mir-378a | 3912 | LAMB1 | laminin, beta 1 | 4 | | 1883 | hsa-mir-561 | 8165 | AKAP1 | A kinase (PRKA) anchor protein 1 | 4 | | 1885 | hsa-mir-585 | 8165 | AKAP1 | A kinase (PRKA) anchor protein 1 | 4 | | 1891 | hsa-mir-620 | 8165 | AKAP1 | A kinase (PRKA) anchor protein 1 | 4 | | 2116 | hsa-mir-550a-1 | 1634 | DCN | decorin | 4 | | 2118 | hsa-mir-636 | 1634 | DCN | decorin | 4 | | 2119 | hsa-mir-640 | 1634 | DCN | decorin | 4 | | 2122 | hsa-mir-877 | 1634 | DCN | decorin | 4 | | 2125 | hsa-mir-3679 | 1634 | DCN | decorin | 4 | | 2127 | hsa-mir-548o-2 | 1634 | DCN | decorin | 4 | | 2128 | hsa-mir-4467 | 1634 | DCN | decorin | 4 | | 2129 | hsa-mir-4498 | 1634 | DCN | decorin | 4 | | 2130 | hsa-mir-4517 | 1634 | DCN | decorin | 4 | | 2132 | hsa-mir-4668 | 1634 | DCN | decorin | 4 | | 2133 | hsa-mir-4698 | 1634 | DCN | decorin | 4 | | 2134 | hsa-mir-4723 | 1634 | DCN | decorin | 4 | | 2135 | hsa-mir-4771-1 | 1634 | DCN | decorin | 4 | | 2136 | hsa-mir-4771-2 | 1634 | DCN | decorin | 4 | | 2137 | hsa-mir-4775 | 1634 | DCN | decorin | 4 | | 2138 | hsa-mir-5090 | 1634 | DCN | decorin | 4 | | 2139 | hsa-mir-604 | 1634 | DCN | decorin | 4 | | 2141 | hsa-mir-218-2 | 1634 | DCN | decorin | 4 | | 2142 | hsa-mir-574 | 1634 | DCN | decorin | 4 | | 2143 | hsa-mir-887 | 1634 | DCN | decorin | 4 | | 2144 | hsa-mir-938 | 1634 | DCN | decorin | 4 | | 2262 | hsa-mir-378a | 2131 | EXT1 | exostosin 1 | 4 | | 2263 | hsa-mir-505 | 2131 | EXT1 | exostosin 1 | 4 | | 2402 | hsa-mir-181a-2 | 23564 | DDAH2 | dimethylarginine dimethylaminohydrolase 2 | 4 | | 2403 | hsa-mir-181b-2 | 23564 | DDAH2 | dimethylarginine dimethylaminohydrolase 2 | 4 | | 2404 | hsa-mir-378a | 23564 | DDAH2 | dimethylarginine dimethylaminohydrolase 2 | 4 | | 2405 | hsa-mir-505 | 23564 | DDAH2 | dimethylarginine dimethylaminohydrolase 2 | 4 | | 2407 | hsa-mir-643 | 23564 | DDAH2 | dimethylarginine dimethylaminohydrolase 2 | 4 | | 2426 | hsa-mir-25 | 1277 | COL1A1 | collagen, type I, alpha 1 | 4 | | 2427 | hsa-mir-93 | 1277 | COL1A1 | collagen, type I, alpha 1 | 4 | | 2428 | hsa-mir-181a-2 | 1277 | COL1A1 | collagen, type I, alpha 1 | 4 | | 2429 | hsa-mir-181b-2 | 1277 | COL1A1 | collagen, type I, alpha 1 | 4 | | 2430 | hsa-mir-106b | 1277 | COL1A1 | collagen, type I, alpha 1 | 4 | | 2432 | hsa-mir-505 | 1277 | COL1A1 | collagen, type I, alpha 1 | 4 | | 2546 | hsa-mir-25 | 1278 | COL1A2 | collagen, type I, alpha 2 | 4 | | 2547 | hsa-mir-93 | 1278 | COL1A2 | collagen, type I, alpha 2 | 4 | | 2549 | hsa-mir-181a-2 | 1278 | COL1A2 | collagen, type I, alpha 2 | 4 | | 2550 | hsa-mir-181b-2 | 1278 | COL1A2 | collagen, type I, alpha 2 | 4 | | 2551 | hsa-mir-106b | 1278 | COL1A2 | collagen, type I, alpha 2 | 4 | | 2553 | hsa-mir-505 | 1278 | COL1A2 | collagen, type I, alpha 2 | 4 | | 2555 | hsa-mir-602 | 1278 | COL1A2 | collagen, type I, alpha 2 | 4 | | 2917 | hsa-let-7c | 24144 | TFIP11 | tuftelin interacting protein 11 | 4 | | 2918 | hsa-mir-99a | 24144 | TFIP11 | tuftelin interacting protein 11 | 4 | | 2923 | hsa-mir-125b-2 | 24144 | TFIP11 | tuftelin interacting protein 11 | 4 | | 2924 | hsa-mir-561 | 24144 | TFIP11 | tuftelin interacting protein 11 | 4 | | 2926 | hsa-mir-585 | 24144 | TFIP11 | tuftelin interacting protein 11 | 4 | | 2965 | hsa-mir-25 | 2200 | FBN1 | fibrillin 1 | 4 | | 2966 | hsa-mir-93 | 2200 | FBN1 | fibrillin 1 | 4 | | 2967 | hsa-mir-181a-2 | 2200 | FBN1 | fibrillin 1 | 4 | | 2968 | hsa-mir-181b-2 | 2200 | FBN1 | fibrillin 1 | 4 | | 2969 | hsa-mir-106b | 2200 | FBN1 | fibrillin 1 | 4 | | 3118 | hsa-mir-181a-2 | 2192 | FBLN1 | fibulin 1 | 4 | | 3119 | hsa-mir-181b-2 | 2192 | FBLN1 | fibulin 1 | 4 | | 3344 | hsa-mir-585 | 9650 | MTFR1 | mitochondrial fission regulator 1 | 4 | | 3347 | hsa-mir-620 | 9650 | MTFR1 | mitochondrial fission regulator 1 | 4 | | 3410 | hsa-mir-218-1 | 8439 | NSMAF | neutral sphingomyelinase (N-SMase) activation associated factor | 4 | | 3413 | hsa-mir-561 | 8439 | NSMAF | neutral sphingomyelinase (N-SMase) activation associated factor | 4 | | 3415 | hsa-mir-585 | 8439 | NSMAF | neutral sphingomyelinase (N-SMase) activation associated factor | 4 | | 3530 | hsa-let-7c | 9776 | ATG13 | ATG13 autophagy related 13 homolog (S. cerevisiae) | 4 | | 3531 | hsa-mir-99a | 9776 | ATG13 | ATG13 autophagy related 13 homolog (S. cerevisiae) | 4 | | 3534 | hsa-mir-125b-1 | 9776 | ATG13 | ATG13 autophagy related 13 homolog (S. cerevisiae) | 4 | | 3535 | hsa-mir-125b-2 | 9776 | ATG13 | ATG13 autophagy related 13 homolog (S. cerevisiae) | 4 | | 3536 | hsa-mir-561 | 9776 | ATG13 | ATG13 autophagy related 13 homolog (S. cerevisiae) | 4 | | 3538 | hsa-mir-585 | 9776 | ATG13 | ATG13 autophagy related 13 homolog (S. cerevisiae) | 4 | | 3565 | hsa-mir-181a-2 | 3280 | HES1 | hairy and enhancer of split 1, (Drosophila) | 4 | | 3566 | hsa-mir-181b-2 | 3280 | HES1 | hairy and enhancer of split 1, (Drosophila) | 4 | | 3570 | hsa-mir-643 | 3280 | HES1 | hairy and enhancer of split 1, (Drosophila) | 4 | | 3686 | hsa-let-7c | 9057 | SLC7A6 | solute carrier family 7 (amino acid transporter light chain, y+L system), member 6 | 4 | | 3687 | hsa-mir-99a | 9057 | SLC7A6 | solute carrier family 7 (amino acid transporter light chain, y+L system), member 6 | 4 | | 3690 | hsa-mir-125b-1 | 9057 | SLC7A6 | solute carrier family 7 (amino acid transporter light chain, y+L system), member 6 | 4 | | 3691 | hsa-mir-125b-2 | 9057 | SLC7A6 | solute carrier family 7 (amino acid transporter light chain, y+L system), member 6 | 4 | | 3692 | hsa-mir-561 | 9057 | SLC7A6 | solute carrier family 7 (amino acid transporter light chain, y+L system), member 6 | 4 | | 3694 | hsa-mir-585 | 9057 | SLC7A6 | solute carrier family 7 (amino acid transporter light chain, y+L system), member 6 | 4 | | 3770 | hsa-mir-25 | 4281 | MID1 | midline 1 (Opitz/BBB syndrome) | 4 | | 3771 | hsa-mir-93 | 4281 | MID1 | midline 1 (Opitz/BBB syndrome) | 4 | | 3773 | hsa-mir-181a-2 | 4281 | MID1 | midline 1 (Opitz/BBB syndrome) | 4 | | 3774 | hsa-mir-181b-2 | 4281 | MID1 | midline 1 (Opitz/BBB syndrome) | 4 | | 3775 | hsa-mir-106b | 4281 | MID1 | midline 1 (Opitz/BBB syndrome) | 4 | | 3776 | hsa-mir-505 | 4281 | MID1 | midline 1 (Opitz/BBB syndrome) | 4 | | 3780 | hsa-mir-643 | 4281 | MID1 | midline 1 (Opitz/BBB syndrome) | 4 | | 3988 | hsa-mir-25 | 6586 | SLIT3 | slit homolog 3 (Drosophila) | 4 | | 3989 | hsa-mir-93 | 6586 | SLIT3 | slit homolog 3 (Drosophila) | 4 | | 3991 | hsa-mir-181a-2 | 6586 | SLIT3 | slit homolog 3 (Drosophila) | 4 | | 3992 | hsa-mir-181b-2 | 6586 | SLIT3 | slit homolog 3 (Drosophila) | 4 | | 3993 | hsa-mir-106b | 6586 | SLIT3 | slit homolog 3 (Drosophila) | 4 | | 3994 | hsa-mir-505 | 6586 | SLIT3 | slit homolog 3 (Drosophila) | 4 | | 4038 | hsa-mir-181a-2 | 6594 | SMARCA1 | SWI/SNF related, matrix associated, actin dependent regulator of chromatin, subfamily a, member 1 | 4 | | 4039 | hsa-mir-181b-2 | 6594 | SMARCA1 | SWI/SNF related, matrix associated, actin dependent regulator of chromatin, subfamily a, member 1 | 4 | | 4040 | hsa-mir-505 | 6594 | SMARCA1 | SWI/SNF related, matrix associated, actin dependent regulator of chromatin, subfamily a, member 1 | 4 | | 4043 | hsa-mir-643 | 6594 | SMARCA1 | SWI/SNF related, matrix associated, actin dependent regulator of chromatin, subfamily a, member 1 | 4 | | 4044 | hsa-mir-652 | 6594 | SMARCA1 | SWI/SNF related, matrix associated, actin dependent regulator of chromatin, subfamily a, member 1 | 4 | | 4263 | hsa-mir-218-1 | 5134 | PDCD2 | programmed cell death 2 | 4 | | 4265 | hsa-mir-125b-1 | 5134 | PDCD2 | programmed cell death 2 | 4 | | 4266 | hsa-mir-561 | 5134 | PDCD2 | programmed cell death 2 | 4 | | 4268 | hsa-mir-585 | 5134 | PDCD2 | programmed cell death 2 | 4 | | 4480 | hsa-mir-25 | 51454 | GULP1 | GULP, engulfment adaptor PTB domain containing 1 | 4 | | 4481 | hsa-mir-93 | 51454 | GULP1 | GULP, engulfment adaptor PTB domain containing 1 | 4 | | 4483 | hsa-mir-181a-2 | 51454 | GULP1 | GULP, engulfment adaptor PTB domain containing 1 | 4 | | 4484 | hsa-mir-181b-2 | 51454 | GULP1 | GULP, engulfment adaptor PTB domain containing 1 | 4 | | 4485 | hsa-mir-106b | 51454 | GULP1 | GULP, engulfment adaptor PTB domain containing 1 | 4 | | 4487 | hsa-mir-505 | 51454 | GULP1 | GULP, engulfment adaptor PTB domain containing 1 | 4 | | 4491 | hsa-mir-643 | 51454 | GULP1 | GULP, engulfment adaptor PTB domain containing 1 | 4 | | 4493 | hsa-mir-652 | 51454 | GULP1 | GULP, engulfment adaptor PTB domain containing 1 | 4 | | 4529 | hsa-mir-125b-1 | 79071 | ELOVL6 | ELOVL fatty acid elongase 6 | 4 | | 4531 | hsa-mir-561 | 79071 | ELOVL6 | ELOVL fatty acid elongase 6 | 4 | | 4533 | hsa-mir-585 | 79071 | ELOVL6 | ELOVL fatty acid elongase 6 | 4 | | 4599 | hsa-let-7c | 7004 | TEAD4 | TEA domain family member 4 | 4 | | 4600 | hsa-mir-99a | 7004 | TEAD4 | TEA domain family member 4 | 4 | | 4602 | hsa-mir-218-1 | 7004 | TEAD4 | TEA domain family member 4 | 4 | | 4604 | hsa-mir-125b-1 | 7004 | TEAD4 | TEA domain family member 4 | 4 | | 4605 | hsa-mir-125b-2 | 7004 | TEAD4 | TEA domain family member 4 | 4 | | 4606 | hsa-mir-561 | 7004 | TEAD4 | TEA domain family member 4 | 4 | | 4608 | hsa-mir-585 | 7004 | TEAD4 | TEA domain family member 4 | 4 | | 4616 | hsa-mir-708 | 7004 | TEAD4 | TEA domain family member 4 | 4 | | 4720 | hsa-let-7c | 8220 | DGCR14 | DiGeorge syndrome critical region gene 14 | 4 | | 4721 | hsa-mir-99a | 8220 | DGCR14 | DiGeorge syndrome critical region gene 14 | 4 | | 4724 | hsa-mir-125b-2 | 8220 | DGCR14 | DiGeorge syndrome critical region gene 14 | 4 | | 4725 | hsa-mir-561 | 8220 | DGCR14 | DiGeorge syndrome critical region gene 14 | 4 | | 4727 | hsa-mir-585 | 8220 | DGCR14 | DiGeorge syndrome critical region gene 14 | 4 | | 4790 | hsa-mir-181a-2 | 10278 | EFS | embryonal Fyn-associated substrate | 4 | | 4791 | hsa-mir-181b-2 | 10278 | EFS | embryonal Fyn-associated substrate | 4 | | 4795 | hsa-mir-643 | 10278 | EFS | embryonal Fyn-associated substrate | 4 | | 4957 | hsa-mir-181a-2 | 1909 | EDNRA | endothelin receptor type A | 4 | | 4958 | hsa-mir-181b-2 | 1909 | EDNRA | endothelin receptor type A | 4 | | 4959 | hsa-mir-505 | 1909 | EDNRA | endothelin receptor type A | 4 | | 4961 | hsa-mir-602 | 1909 | EDNRA | endothelin receptor type A | 4 | | 5220 | hsa-let-7c | 9603 | NFE2L3 | nuclear factor (erythroid-derived 2)-like 3 | 4 | | 5221 | hsa-mir-99a | 9603 | NFE2L3 | nuclear factor (erythroid-derived 2)-like 3 | 4 | | 5223 | hsa-mir-218-1 | 9603 | NFE2L3 | nuclear factor (erythroid-derived 2)-like 3 | 4 | | 5226 | hsa-mir-125b-2 | 9603 | NFE2L3 | nuclear factor (erythroid-derived 2)-like 3 | 4 | | 5227 | hsa-mir-561 | 9603 | NFE2L3 | nuclear factor (erythroid-derived 2)-like 3 | 4 | | 5229 | hsa-mir-585 | 9603 | NFE2L3 | nuclear factor (erythroid-derived 2)-like 3 | 4 | | 5296 | hsa-mir-218-1 | 10630 | PDPN | podoplanin | 4 | | 5299 | hsa-mir-561 | 10630 | PDPN | podoplanin | 4 | | 5301 | hsa-mir-585 | 10630 | PDPN | podoplanin | 4 | | 5305 | hsa-mir-620 | 10630 | PDPN | podoplanin | 4 | | 5322 | hsa-mir-455 | 3364 | HUS1 | HUS1 checkpoint homolog (S. pombe) | 4 | | 5323 | hsa-mir-561 | 3364 | HUS1 | HUS1 checkpoint homolog (S. pombe) | 4 | | 5325 | hsa-mir-585 | 3364 | HUS1 | HUS1 checkpoint homolog (S. pombe) | 4 | | 5518 | hsa-let-7c | 23464 | GCAT | glycine C-acetyltransferase | 4 | | 5519 | hsa-mir-99a | 23464 | GCAT | glycine C-acetyltransferase | 4 | | 5521 | hsa-mir-218-1 | 23464 | GCAT | glycine C-acetyltransferase | 4 | | 5523 | hsa-mir-125b-1 | 23464 | GCAT | glycine C-acetyltransferase | 4 | | 5524 | hsa-mir-125b-2 | 23464 | GCAT | glycine C-acetyltransferase | 4 | | 5525 | hsa-mir-561 | 23464 | GCAT | glycine C-acetyltransferase | 4 | | 5527 | hsa-mir-585 | 23464 | GCAT | glycine C-acetyltransferase | 4 | | 5927 | hsa-mir-181a-2 | 862 | RUNX1T1 | runt-related transcription factor 1; translocated to, 1 (cyclin D-related) | 4 | | 5928 | hsa-mir-181b-2 | 862 | RUNX1T1 | runt-related transcription factor 1; translocated to, 1 (cyclin D-related) | 4 | | 5930 | hsa-mir-505 | 862 | RUNX1T1 | runt-related transcription factor 1; translocated to, 1 (cyclin D-related) | 4 | | 5932 | hsa-mir-602 | 862 | RUNX1T1 | runt-related transcription factor 1; translocated to, 1 (cyclin D-related) | 4 | | 6119 | hsa-mir-218-1 | 3570 | IL6R | interleukin 6 receptor | 4 | | 6123 | hsa-mir-561 | 3570 | IL6R | interleukin 6 receptor | 4 | | 6125 | hsa-mir-585 | 3570 | IL6R | interleukin 6 receptor | 4 | | 6132 | hsa-mir-708 | 3570 | IL6R | interleukin 6 receptor | 4 | | 6778 | hsa-mir-218-1 | 7752 | ZNF200 | zinc finger protein 200 | 4 | | 6782 | hsa-mir-561 | 7752 | ZNF200 | zinc finger protein 200 | 4 | | 6784 | hsa-mir-585 | 7752 | ZNF200 | zinc finger protein 200 | 4 | | 6877 | hsa-mir-218-1 | 489 | ATP2A3 | ATPase, Ca++ transporting, ubiquitous | 4 | | 6880 | hsa-mir-561 | 489 | ATP2A3 | ATPase, Ca++ transporting, ubiquitous | 4 | | 6882 | hsa-mir-585 | 489 | ATP2A3 | ATPase, Ca++ transporting, ubiquitous | 4 | | 6957 | hsa-mir-455 | 9473 | C1orf38 | chromosome 1 open reading frame 38 | 4 | | 6958 | hsa-mir-561 | 9473 | C1orf38 | chromosome 1 open reading frame 38 | 4 | | 6960 | hsa-mir-585 | 9473 | C1orf38 | chromosome 1 open reading frame 38 | 4 | | 7044 | hsa-let-7c | 1741 | DLG3 | discs, large homolog 3 (Drosophila) | 4 | | 7045 | hsa-mir-99a | 1741 | DLG3 | discs, large homolog 3 (Drosophila) | 4 | | 7048 | hsa-mir-125b-2 | 1741 | DLG3 | discs, large homolog 3 (Drosophila) | 4 | | 7057 | hsa-mir-620 | 1741 | DLG3 | discs, large homolog 3 (Drosophila) | 4 | | 7084 | hsa-mir-561 | 54941 | RNF125 | ring finger protein 125, E3 ubiquitin protein ligase | 4 | | 7086 | hsa-mir-585 | 54941 | RNF125 | ring finger protein 125, E3 ubiquitin protein ligase | 4 | | 7093 | hsa-mir-708 | 54941 | RNF125 | ring finger protein 125, E3 ubiquitin protein ligase | 4 | | 7136 | hsa-let-7c | 80256 | FAM214B | family with sequence similarity 214, member B | 4 | | 7137 | hsa-mir-99a | 80256 | FAM214B | family with sequence similarity 214, member B | 4 | | 7140 | hsa-mir-125b-2 | 80256 | FAM214B | family with sequence similarity 214, member B | 4 | | 7142 | hsa-mir-561 | 80256 | FAM214B | family with sequence similarity 214, member B | 4 | | 7144 | hsa-mir-585 | 80256 | FAM214B | family with sequence similarity 214, member B | 4 | | 7148 | hsa-mir-620 | 80256 | FAM214B | family with sequence similarity 214, member B | 4 | | 7172 | hsa-mir-561 | 120227 | CYP2R1 | cytochrome P450, family 2, subfamily R, polypeptide 1 | 4 | | 7174 | hsa-mir-585 | 120227 | CYP2R1 | cytochrome P450, family 2, subfamily R, polypeptide 1 | 4 | | 7668 | hsa-let-7c | 65220 | NADK | NAD kinase | 4 | | 7669 | hsa-mir-99a | 65220 | NADK | NAD kinase | 4 | | 7674 | hsa-mir-125b-2 | 65220 | NADK | NAD kinase | 4 | | 7677 | hsa-mir-585 | 65220 | NADK | NAD kinase | 4 | | 8109 | hsa-mir-561 | 26031 | OSBPL3 | oxysterol binding protein-like 3 | 4 | | 8111 | hsa-mir-585 | 26031 | OSBPL3 | oxysterol binding protein-like 3 | 4 | | 8118 | hsa-mir-708 | 26031 | OSBPL3 | oxysterol binding protein-like 3 | 4 | | 8215 | hsa-let-7c | 10634 | GAS2L1 | growth arrest-specific 2 like 1 | 4 | | 8216 | hsa-mir-99a | 10634 | GAS2L1 | growth arrest-specific 2 like 1 | 4 | | 8220 | hsa-mir-125b-2 | 10634 | GAS2L1 | growth arrest-specific 2 like 1 | 4 | | 8222 | hsa-mir-561 | 10634 | GAS2L1 | growth arrest-specific 2 like 1 | 4 | | 8224 | hsa-mir-585 | 10634 | GAS2L1 | growth arrest-specific 2 like 1 | 4 | | 8353 | hsa-mir-218-1 | 8034 | SLC25A16 | solute carrier family 25 (mitochondrial carrier; Graves disease autoantigen), member 16 | 4 | | 8355 | hsa-mir-125b-1 | 8034 | SLC25A16 | solute carrier family 25 (mitochondrial carrier; Graves disease autoantigen), member 16 | 4 | | 8356 | hsa-mir-561 | 8034 | SLC25A16 | solute carrier family 25 (mitochondrial carrier; Graves disease autoantigen), member 16 | 4 | | 8358 | hsa-mir-585 | 8034 | SLC25A16 | solute carrier family 25 (mitochondrial carrier; Graves disease autoantigen), member 16 | 4 | | 8363 | hsa-mir-708 | 8034 | SLC25A16 | solute carrier family 25 (mitochondrial carrier; Graves disease autoantigen), member 16 | 4 | | 8415 | hsa-mir-585 | 8837 | CFLAR | CASP8 and FADD-like apoptosis regulator | 4 | | 8453 | hsa-let-7c | 8115 | TCL1A | T-cell leukemia/lymphoma 1A | 4 | | 8454 | hsa-mir-99a | 8115 | TCL1A | T-cell leukemia/lymphoma 1A | 4 | | 8456 | hsa-mir-218-1 | 8115 | TCL1A | T-cell leukemia/lymphoma 1A | 4 | | 8459 | hsa-mir-125b-2 | 8115 | TCL1A | T-cell leukemia/lymphoma 1A | 4 | | 8460 | hsa-mir-561 | 8115 | TCL1A | T-cell leukemia/lymphoma 1A | 4 | | 8462 | hsa-mir-585 | 8115 | TCL1A | T-cell leukemia/lymphoma 1A | 4 | | 8469 | hsa-mir-708 | 8115 | TCL1A | T-cell leukemia/lymphoma 1A | 4 | | 8774 | hsa-mir-378a | 7035 | TFPI | tissue factor pathway inhibitor (lipoprotein-associated coagulation inhibitor) | 4 | | 8775 | hsa-mir-505 | 7035 | TFPI | tissue factor pathway inhibitor (lipoprotein-associated coagulation inhibitor) | 4 | | 8777 | hsa-mir-643 | 7035 | TFPI | tissue factor pathway inhibitor (lipoprotein-associated coagulation inhibitor) | 4 | | 8851 | hsa-mir-181a-2 | 2078 | ERG | v-ets erythroblastosis virus E26 oncogene homolog (avian) | 4 | | 8852 | hsa-mir-181b-2 | 2078 | ERG | v-ets erythroblastosis virus E26 oncogene homolog (avian) | 4 | | 9429 | hsa-mir-708 | 1741 | DLG3 | discs, large homolog 3 (Drosophila) | 4 | | 9442 | hsa-mir-218-1 | 9711 | KIAA0226 | KIAA0226 | 4 | | 9444 | hsa-mir-561 | 9711 | KIAA0226 | KIAA0226 | 4 | | 9446 | hsa-mir-585 | 9711 | KIAA0226 | KIAA0226 | 4 | | 10983 | hsa-mir-643 | 6934 | TCF7L2 | transcription factor 7-like 2 (T-cell specific, HMG-box) | 4 | | 11315 | hsa-mir-561 | 58477 | SRPRB | signal recognition particle receptor, B subunit | 4 | | 11317 | hsa-mir-585 | 58477 | SRPRB | signal recognition particle receptor, B subunit | 4 | | 11449 | hsa-mir-218-1 | 10762 | NUP50 | nucleoporin 50kDa | 4 | | 11451 | hsa-mir-455 | 10762 | NUP50 | nucleoporin 50kDa | 4 | | 11452 | hsa-mir-561 | 10762 | NUP50 | nucleoporin 50kDa | 4 | | 11454 | hsa-mir-585 | 10762 | NUP50 | nucleoporin 50kDa | 4 | | 11457 | hsa-mir-620 | 10762 | NUP50 | nucleoporin 50kDa | 4 | | 11524 | hsa-mir-218-1 | 64222 | TOR3A | torsin family 3, member A | 4 | | 11527 | hsa-mir-561 | 64222 | TOR3A | torsin family 3, member A | 4 | | 11529 | hsa-mir-585 | 64222 | TOR3A | torsin family 3, member A | 4 | | 11554 | hsa-let-7c | 55731 | C17orf63 | chromosome 17 open reading frame 63 | 4 | | 11555 | hsa-mir-99a | 55731 | C17orf63 | chromosome 17 open reading frame 63 | 4 | | 11557 | hsa-mir-218-1 | 55731 | C17orf63 | chromosome 17 open reading frame 63 | 4 | | 11560 | hsa-mir-125b-2 | 55731 | C17orf63 | chromosome 17 open reading frame 63 | 4 | | 11791 | hsa-let-7c | 79693 | YRDC | yrdC domain containing (E. coli) | 4 | | 11792 | hsa-mir-99a | 79693 | YRDC | yrdC domain containing (E. coli) | 4 | | 11794 | hsa-mir-218-1 | 79693 | YRDC | yrdC domain containing (E. coli) | 4 | | 11796 | hsa-mir-125b-1 | 79693 | YRDC | yrdC domain containing (E. coli) | 4 | | 11797 | hsa-mir-125b-2 | 79693 | YRDC | yrdC domain containing (E. coli) | 4 | | 11798 | hsa-mir-561 | 79693 | YRDC | yrdC domain containing (E. coli) | 4 | | 11800 | hsa-mir-585 | 79693 | YRDC | yrdC domain containing (E. coli) | 4 | | 11853 | hsa-mir-455 | 8934 | RAB7L1 | RAB7, member RAS oncogene family-like 1 | 4 | | 11854 | hsa-mir-561 | 8934 | RAB7L1 | RAB7, member RAS oncogene family-like 1 | 4 | | 11856 | hsa-mir-585 | 8934 | RAB7L1 | RAB7, member RAS oncogene family-like 1 | 4 | | 11858 | hsa-mir-620 | 8934 | RAB7L1 | RAB7, member RAS oncogene family-like 1 | 4 | | 11910 | hsa-mir-218-1 | 64785 | GINS3 | GINS complex subunit 3 (Psf3 homolog) | 4 | | 11914 | hsa-mir-561 | 64785 | GINS3 | GINS complex subunit 3 (Psf3 homolog) | 4 | | 11916 | hsa-mir-585 | 64785 | GINS3 | GINS complex subunit 3 (Psf3 homolog) | 4 | | 11942 | hsa-let-7c | 55238 | SLC38A7 | solute carrier family 38, member 7 | 4 | | 11943 | hsa-mir-99a | 55238 | SLC38A7 | solute carrier family 38, member 7 | 4 | | 11945 | hsa-mir-218-1 | 55238 | SLC38A7 | solute carrier family 38, member 7 | 4 | | 11947 | hsa-mir-125b-1 | 55238 | SLC38A7 | solute carrier family 38, member 7 | 4 | | 11948 | hsa-mir-125b-2 | 55238 | SLC38A7 | solute carrier family 38, member 7 | 4 | | 11949 | hsa-mir-561 | 55238 | SLC38A7 | solute carrier family 38, member 7 | 4 | | 11951 | hsa-mir-585 | 55238 | SLC38A7 | solute carrier family 38, member 7 | 4 | | 11976 | hsa-mir-181a-2 | 4969 | OGN | osteoglycin | 4 | | 11977 | hsa-mir-181b-2 | 4969 | OGN | osteoglycin | 4 | | 11978 | hsa-mir-378a | 4969 | OGN | osteoglycin | 4 | | 11979 | hsa-mir-505 | 4969 | OGN | osteoglycin | 4 | | 11981 | hsa-mir-602 | 4969 | OGN | osteoglycin | 4 | | 11983 | hsa-mir-643 | 4969 | OGN | osteoglycin | 4 | | 12326 | hsa-let-7c | 55833 | UBAP2 | ubiquitin associated protein 2 | 4 | | 12327 | hsa-mir-99a | 55833 | UBAP2 | ubiquitin associated protein 2 | 4 | | 12330 | hsa-mir-125b-1 | 55833 | UBAP2 | ubiquitin associated protein 2 | 4 | | 12331 | hsa-mir-125b-2 | 55833 | UBAP2 | ubiquitin associated protein 2 | 4 | | 12332 | hsa-mir-561 | 55833 | UBAP2 | ubiquitin associated protein 2 | 4 | | 12334 | hsa-mir-585 | 55833 | UBAP2 | ubiquitin associated protein 2 | 4 | | 12559 | hsa-let-7c | 64506 | CPEB1 | cytoplasmic polyadenylation element binding protein 1 | 4 | | 12560 | hsa-mir-99a | 64506 | CPEB1 | cytoplasmic polyadenylation element binding protein 1 | 4 | | 12564 | hsa-mir-125b-1 | 64506 | CPEB1 | cytoplasmic polyadenylation element binding protein 1 | 4 | | 12565 | hsa-mir-125b-2 | 64506 | CPEB1 | cytoplasmic polyadenylation element binding protein 1 | 4 | | 12567 | hsa-mir-561 | 64506 | CPEB1 | cytoplasmic polyadenylation element binding protein 1 | 4 | | 12569 | hsa-mir-585 | 64506 | CPEB1 | cytoplasmic polyadenylation element binding protein 1 | 4 | | 12577 | hsa-mir-708 | 64506 | CPEB1 | cytoplasmic polyadenylation element binding protein 1 | 4 | | 12648 | hsa-mir-125b-1 | 29842 | TFCP2L1 | transcription factor CP2-like 1 | 4 | | 12658 | hsa-mir-708 | 29842 | TFCP2L1 | transcription factor CP2-like 1 | 4 | | 12699 | hsa-mir-620 | 53916 | RAB4B | RAB4B, member RAS oncogene family | 4 | | 12796 | hsa-let-7c | 26256 | CABYR | calcium binding tyrosine-(Y)-phosphorylation regulated | 4 | | 12799 | hsa-mir-218-1 | 26256 | CABYR | calcium binding tyrosine-(Y)-phosphorylation regulated | 4 | | 12803 | hsa-mir-561 | 26256 | CABYR | calcium binding tyrosine-(Y)-phosphorylation regulated | 4 | | 12805 | hsa-mir-585 | 26256 | CABYR | calcium binding tyrosine-(Y)-phosphorylation regulated | 4 | | 12832 | hsa-let-7c | 11226 | GALNT6 | UDP-N-acetyl-alpha-D-galactosamine:polypeptide N-acetylgalactosaminyltransferase 6 (GalNAc-T6) | 4 | | 12833 | hsa-mir-99a | 11226 | GALNT6 | UDP-N-acetyl-alpha-D-galactosamine:polypeptide N-acetylgalactosaminyltransferase 6 (GalNAc-T6) | 4 | | 12836 | hsa-mir-125b-2 | 11226 | GALNT6 | UDP-N-acetyl-alpha-D-galactosamine:polypeptide N-acetylgalactosaminyltransferase 6 (GalNAc-T6) | 4 | | 12837 | hsa-mir-561 | 11226 | GALNT6 | UDP-N-acetyl-alpha-D-galactosamine:polypeptide N-acetylgalactosaminyltransferase 6 (GalNAc-T6) | 4 | | 12839 | hsa-mir-585 | 11226 | GALNT6 | UDP-N-acetyl-alpha-D-galactosamine:polypeptide N-acetylgalactosaminyltransferase 6 (GalNAc-T6) | 4 | | 13052 | hsa-mir-505 | 80055 | PGAP1 | post-GPI attachment to proteins 1 | 4 | | 13177 | hsa-mir-218-1 | 9990 | SLC12A6 | solute carrier family 12 (potassium/chloride transporters), member 6 | 4 | | 13179 | hsa-mir-125b-1 | 9990 | SLC12A6 | solute carrier family 12 (potassium/chloride transporters), member 6 | 4 | | 13180 | hsa-mir-455 | 9990 | SLC12A6 | solute carrier family 12 (potassium/chloride transporters), member 6 | 4 | | 13181 | hsa-mir-561 | 9990 | SLC12A6 | solute carrier family 12 (potassium/chloride transporters), member 6 | 4 | | 13183 | hsa-mir-585 | 9990 | SLC12A6 | solute carrier family 12 (potassium/chloride transporters), member 6 | 4 | | 13189 | hsa-mir-708 | 9990 | SLC12A6 | solute carrier family 12 (potassium/chloride transporters), member 6 | 4 | | 13363 | hsa-mir-378a | 81888 | HYI | hydroxypyruvate isomerase (putative) | 4 | | 13364 | hsa-mir-505 | 81888 | HYI | hydroxypyruvate isomerase (putative) | 4 | | 13368 | hsa-mir-643 | 81888 | HYI | hydroxypyruvate isomerase (putative) | 4 | | 13550 | hsa-mir-181a-2 | 1290 | COL5A2 | collagen, type V, alpha 2 | 4 | | 13551 | hsa-mir-181b-2 | 1290 | COL5A2 | collagen, type V, alpha 2 | 4 | | 13553 | hsa-mir-378a | 1290 | COL5A2 | collagen, type V, alpha 2 | 4 | | 13554 | hsa-mir-505 | 1290 | COL5A2 | collagen, type V, alpha 2 | 4 | | 13556 | hsa-mir-602 | 1290 | COL5A2 | collagen, type V, alpha 2 | 4 | | 13558 | hsa-mir-643 | 1290 | COL5A2 | collagen, type V, alpha 2 | 4 | | 13590 | hsa-mir-99a | 26119 | LDLRAP1 | low density lipoprotein receptor adaptor protein 1 | 4 | | 13592 | hsa-mir-218-1 | 26119 | LDLRAP1 | low density lipoprotein receptor adaptor protein 1 | 4 | | 13594 | hsa-mir-125b-1 | 26119 | LDLRAP1 | low density lipoprotein receptor adaptor protein 1 | 4 | | 13595 | hsa-mir-125b-2 | 26119 | LDLRAP1 | low density lipoprotein receptor adaptor protein 1 | 4 | | 13596 | hsa-mir-561 | 26119 | LDLRAP1 | low density lipoprotein receptor adaptor protein 1 | 4 | | 13598 | hsa-mir-585 | 26119 | LDLRAP1 | low density lipoprotein receptor adaptor protein 1 | 4 | | 13604 | hsa-mir-708 | 26119 | LDLRAP1 | low density lipoprotein receptor adaptor protein 1 | 4 | | 13665 | hsa-mir-181a-2 | 27147 | DENND2A | DENN/MADD domain containing 2A | 4 | | 13666 | hsa-mir-181b-2 | 27147 | DENND2A | DENN/MADD domain containing 2A | 4 | | 13667 | hsa-mir-505 | 27147 | DENND2A | DENN/MADD domain containing 2A | 4 | | 13669 | hsa-mir-602 | 27147 | DENND2A | DENN/MADD domain containing 2A | 4 | | 14315 | hsa-let-7c | 57510 | XPO5 | exportin 5 | 4 | | 14502 | hsa-mir-561 | 55632 | G2E3 | G2/M-phase specific E3 ubiquitin protein ligase | 4 | | 14504 | hsa-mir-585 | 55632 | G2E3 | G2/M-phase specific E3 ubiquitin protein ligase | 4 | | 14508 | hsa-mir-620 | 55632 | G2E3 | G2/M-phase specific E3 ubiquitin protein ligase | 4 | | 15211 | hsa-mir-181a-2 | 283120 | H19 | H19, imprinted maternally expressed transcript (non-protein coding) | 4 | | 15212 | hsa-mir-181b-2 | 283120 | H19 | H19, imprinted maternally expressed transcript (non-protein coding) | 4 | | 15213 | hsa-mir-378a | 283120 | H19 | H19, imprinted maternally expressed transcript (non-protein coding) | 4 | | 15214 | hsa-mir-505 | 283120 | H19 | H19, imprinted maternally expressed transcript (non-protein coding) | 4 | | 15668 | hsa-mir-561 | 160760 | PPTC7 | PTC7 protein phosphatase homolog (S. cerevisiae) | 4 | | 15670 | hsa-mir-585 | 160760 | PPTC7 | PTC7 protein phosphatase homolog (S. cerevisiae) | 4 | | 15673 | hsa-mir-620 | 160760 | PPTC7 | PTC7 protein phosphatase homolog (S. cerevisiae) | 4 | | 16004 | hsa-mir-561 | 57210 | SLC45A4 | solute carrier family 45, member 4 | 4 | | 16006 | hsa-mir-585 | 57210 | SLC45A4 | solute carrier family 45, member 4 | 4 | | 16342 | hsa-mir-561 | 80821 | DDHD1 | DDHD domain containing 1 | 4 | | 16344 | hsa-mir-585 | 80821 | DDHD1 | DDHD domain containing 1 | 4 | | 16546 | hsa-mir-652 | 144165 | PRICKLE1 | prickle homolog 1 (Drosophila) | 4 | | 16557 | hsa-mir-25 | 463 | ZFHX3 | zinc finger homeobox 3 | 4 | | 16558 | hsa-mir-93 | 463 | ZFHX3 | zinc finger homeobox 3 | 4 | | 16559 | hsa-mir-181a-2 | 463 | ZFHX3 | zinc finger homeobox 3 | 4 | | 16560 | hsa-mir-181b-2 | 463 | ZFHX3 | zinc finger homeobox 3 | 4 | | 16561 | hsa-mir-106b | 463 | ZFHX3 | zinc finger homeobox 3 | 4 | | 16563 | hsa-mir-505 | 463 | ZFHX3 | zinc finger homeobox 3 | 4 | | 16569 | hsa-mir-643 | 463 | ZFHX3 | zinc finger homeobox 3 | 4 | | 16714 | hsa-mir-181a-2 | 79269 | DCAF10 | DDB1 and CUL4 associated factor 10 | 4 | | 16715 | hsa-mir-181b-2 | 79269 | DCAF10 | DDB1 and CUL4 associated factor 10 | 4 | | 16716 | hsa-mir-378a | 79269 | DCAF10 | DDB1 and CUL4 associated factor 10 | 4 | | 16717 | hsa-mir-505 | 79269 | DCAF10 | DDB1 and CUL4 associated factor 10 | 4 | | 16721 | hsa-mir-643 | 79269 | DCAF10 | DDB1 and CUL4 associated factor 10 | 4 | | 16791 | hsa-mir-181a-2 | 58499 | ZNF462 | zinc finger protein 462 | 4 | | 16792 | hsa-mir-181b-2 | 58499 | ZNF462 | zinc finger protein 462 | 4 | | 16793 | hsa-mir-505 | 58499 | ZNF462 | zinc finger protein 462 | 4 | | 17045 | hsa-let-7c | 57168 | ASPHD2 | aspartate beta-hydroxylase domain containing 2 | 4 | | 17046 | hsa-mir-99a | 57168 | ASPHD2 | aspartate beta-hydroxylase domain containing 2 | 4 | | 17049 | hsa-mir-125b-2 | 57168 | ASPHD2 | aspartate beta-hydroxylase domain containing 2 | 4 | | 17051 | hsa-mir-561 | 57168 | ASPHD2 | aspartate beta-hydroxylase domain containing 2 | 4 | | 17053 | hsa-mir-585 | 57168 | ASPHD2 | aspartate beta-hydroxylase domain containing 2 | 4 | | 17058 | hsa-mir-620 | 57168 | ASPHD2 | aspartate beta-hydroxylase domain containing 2 | 4 | | 17598 | hsa-mir-561 | 124491 | TMEM170A | transmembrane protein 170A | 4 | | 17600 | hsa-mir-585 | 124491 | TMEM170A | transmembrane protein 170A | 4 | | 17604 | hsa-mir-620 | 124491 | TMEM170A | transmembrane protein 170A | 4 | | 17629 | hsa-let-7c | 29842 | TFCP2L1 | transcription factor CP2-like 1 | 4 | | 19014 | hsa-mir-218-1 | 22876 | INPP5F | inositol polyphosphate-5-phosphatase F | 4 | | 19016 | hsa-mir-125b-1 | 22876 | INPP5F | inositol polyphosphate-5-phosphatase F | 4 | | 19017 | hsa-mir-561 | 22876 | INPP5F | inositol polyphosphate-5-phosphatase F | 4 | | 19019 | hsa-mir-585 | 22876 | INPP5F | inositol polyphosphate-5-phosphatase F | 4 | | 19026 | hsa-mir-708 | 22876 | INPP5F | inositol polyphosphate-5-phosphatase F | 4 | | 19730 | hsa-mir-181a-2 | 93986 | FOXP2 | forkhead box P2 | 4 | | 19731 | hsa-mir-181b-2 | 93986 | FOXP2 | forkhead box P2 | 4 | | 19732 | hsa-mir-505 | 93986 | FOXP2 | forkhead box P2 | 4 | | 19734 | hsa-mir-602 | 93986 | FOXP2 | forkhead box P2 | 4 | | 19802 | hsa-mir-181a-2 | 128553 | TSHZ2 | teashirt zinc finger homeobox 2 | 4 | | 19803 | hsa-mir-181b-2 | 128553 | TSHZ2 | teashirt zinc finger homeobox 2 | 4 | | 19804 | hsa-mir-505 | 128553 | TSHZ2 | teashirt zinc finger homeobox 2 | 4 | | 19972 | hsa-mir-25 | 1009 | CDH11 | cadherin 11, type 2, OB-cadherin (osteoblast) | 4 | | 19973 | hsa-mir-93 | 1009 | CDH11 | cadherin 11, type 2, OB-cadherin (osteoblast) | 4 | | 19975 | hsa-mir-181a-2 | 1009 | CDH11 | cadherin 11, type 2, OB-cadherin (osteoblast) | 4 | | 19976 | hsa-mir-181b-2 | 1009 | CDH11 | cadherin 11, type 2, OB-cadherin (osteoblast) | 4 | | 19977 | hsa-mir-106b | 1009 | CDH11 | cadherin 11, type 2, OB-cadherin (osteoblast) | 4 | | 19979 | hsa-mir-505 | 1009 | CDH11 | cadherin 11, type 2, OB-cadherin (osteoblast) | 4 | | 19981 | hsa-mir-602 | 1009 | CDH11 | cadherin 11, type 2, OB-cadherin (osteoblast) | 4 | | 19984 | hsa-mir-643 | 1009 | CDH11 | cadherin 11, type 2, OB-cadherin (osteoblast) | 4 | | 5 | hsa-mir-125b-1 | 159091 | FAM122C | family with sequence similarity 122C | 3 | | 98 | hsa-mir-125b-1 | 10228 | STX6 | syntaxin 6 | 3 | | 222 | hsa-mir-125b-1 | 30816 | ERVW-1 | endogenous retrovirus group W, member 1 | 3 | | 492 | hsa-mir-620 | 9093 | DNAJA3 | DnaJ (Hsp40) homolog, subfamily A, member 3 | 3 | | 875 | hsa-mir-620 | 55787 | TXLNG | taxilin gamma | 3 | | 1241 | hsa-mir-620 | 55905 | RNF114 | ring finger protein 114 | 3 | | 1669 | hsa-mir-620 | 4141 | MARS | methionyl-tRNA synthetase | 3 | | 1722 | hsa-mir-505 | 3912 | LAMB1 | laminin, beta 1 | 3 | | 1726 | hsa-mir-652 | 3912 | LAMB1 | laminin, beta 1 | 3 | | 1876 | hsa-mir-99a | 8165 | AKAP1 | A kinase (PRKA) anchor protein 1 | 3 | | 1881 | hsa-mir-125b-2 | 8165 | AKAP1 | A kinase (PRKA) anchor protein 1 | 3 | | 2126 | hsa-mir-3942 | 1634 | DCN | decorin | 3 | | 2131 | hsa-mir-4534 | 1634 | DCN | decorin | 3 | | 2208 | hsa-mir-550a-1 | 1956 | EGFR | epidermal growth factor receptor | 3 | | 2209 | hsa-mir-640 | 1956 | EGFR | epidermal growth factor receptor | 3 | | 2212 | hsa-mir-877 | 1956 | EGFR | epidermal growth factor receptor | 3 | | 2218 | hsa-mir-4467 | 1956 | EGFR | epidermal growth factor receptor | 3 | | 2220 | hsa-mir-4517 | 1956 | EGFR | epidermal growth factor receptor | 3 | | 2221 | hsa-mir-4698 | 1956 | EGFR | epidermal growth factor receptor | 3 | | 2223 | hsa-mir-4771-1 | 1956 | EGFR | epidermal growth factor receptor | 3 | | 2224 | hsa-mir-4771-2 | 1956 | EGFR | epidermal growth factor receptor | 3 | | 2226 | hsa-mir-5090 | 1956 | EGFR | epidermal growth factor receptor | 3 | | 2227 | hsa-mir-604 | 1956 | EGFR | epidermal growth factor receptor | 3 | | 2229 | hsa-mir-218-2 | 1956 | EGFR | epidermal growth factor receptor | 3 | | 2230 | hsa-mir-574 | 1956 | EGFR | epidermal growth factor receptor | 3 | | 2231 | hsa-mir-887 | 1956 | EGFR | epidermal growth factor receptor | 3 | | 2232 | hsa-mir-938 | 1956 | EGFR | epidermal growth factor receptor | 3 | | 2431 | hsa-mir-378a | 1277 | COL1A1 | collagen, type I, alpha 1 | 3 | | 2453 | hsa-mir-602 | 1277 | COL1A1 | collagen, type I, alpha 1 | 3 | | 2970 | hsa-mir-378a | 2200 | FBN1 | fibrillin 1 | 3 | | 2987 | hsa-mir-602 | 2200 | FBN1 | fibrillin 1 | 3 | | 3342 | hsa-mir-455 | 9650 | MTFR1 | mitochondrial fission regulator 1 | 3 | | 3441 | hsa-let-7f-2 | 1289 | COL5A1 | collagen, type V, alpha 1 | 3 | | 3442 | hsa-mir-98 | 1289 | COL5A1 | collagen, type V, alpha 1 | 3 | | 3447 | hsa-mir-550a-1 | 1289 | COL5A1 | collagen, type V, alpha 1 | 3 | | 3449 | hsa-mir-644a | 1289 | COL5A1 | collagen, type V, alpha 1 | 3 | | 3450 | hsa-mir-877 | 1289 | COL5A1 | collagen, type V, alpha 1 | 3 | | 3451 | hsa-mir-548j | 1289 | COL5A1 | collagen, type V, alpha 1 | 3 | | 3452 | hsa-mir-548n | 1289 | COL5A1 | collagen, type V, alpha 1 | 3 | | 3453 | hsa-mir-1976 | 1289 | COL5A1 | collagen, type V, alpha 1 | 3 | | 3454 | hsa-mir-2909 | 1289 | COL5A1 | collagen, type V, alpha 1 | 3 | | 3455 | hsa-mir-548s | 1289 | COL5A1 | collagen, type V, alpha 1 | 3 | | 3456 | hsa-mir-2355 | 1289 | COL5A1 | collagen, type V, alpha 1 | 3 | | 3457 | hsa-mir-3942 | 1289 | COL5A1 | collagen, type V, alpha 1 | 3 | | 3458 | hsa-mir-548aa-2 | 1289 | COL5A1 | collagen, type V, alpha 1 | 3 | | 3459 | hsa-mir-378e | 1289 | COL5A1 | collagen, type V, alpha 1 | 3 | | 3460 | hsa-mir-4467 | 1289 | COL5A1 | collagen, type V, alpha 1 | 3 | | 3462 | hsa-mir-4698 | 1289 | COL5A1 | collagen, type V, alpha 1 | 3 | | 3463 | hsa-mir-4709 | 1289 | COL5A1 | collagen, type V, alpha 1 | 3 | | 3464 | hsa-mir-4723 | 1289 | COL5A1 | collagen, type V, alpha 1 | 3 | | 3465 | hsa-mir-4771-1 | 1289 | COL5A1 | collagen, type V, alpha 1 | 3 | | 3466 | hsa-mir-4771-2 | 1289 | COL5A1 | collagen, type V, alpha 1 | 3 | | 3468 | hsa-mir-5090 | 1289 | COL5A1 | collagen, type V, alpha 1 | 3 | | 3469 | hsa-mir-5194 | 1289 | COL5A1 | collagen, type V, alpha 1 | 3 | | 3470 | hsa-mir-604 | 1289 | COL5A1 | collagen, type V, alpha 1 | 3 | | 3471 | hsa-mir-675 | 1289 | COL5A1 | collagen, type V, alpha 1 | 3 | | 3472 | hsa-mir-218-2 | 1289 | COL5A1 | collagen, type V, alpha 1 | 3 | | 3473 | hsa-mir-574 | 1289 | COL5A1 | collagen, type V, alpha 1 | 3 | | 3474 | hsa-mir-887 | 1289 | COL5A1 | collagen, type V, alpha 1 | 3 | | 3475 | hsa-mir-938 | 1289 | COL5A1 | collagen, type V, alpha 1 | 3 | | 3555 | hsa-mir-218-1 | 9776 | ATG13 | ATG13 autophagy related 13 homolog (S. cerevisiae) | 3 | | 3568 | hsa-mir-505 | 3280 | HES1 | hairy and enhancer of split 1, (Drosophila) | 3 | | 3778 | hsa-mir-602 | 4281 | MID1 | midline 1 (Opitz/BBB syndrome) | 3 | | 4271 | hsa-mir-620 | 5134 | PDCD2 | programmed cell death 2 | 3 | | 4486 | hsa-mir-378a | 51454 | GULP1 | GULP, engulfment adaptor PTB domain containing 1 | 3 | | 4489 | hsa-mir-602 | 51454 | GULP1 | GULP, engulfment adaptor PTB domain containing 1 | 3 | | 4525 | hsa-mir-99a | 79071 | ELOVL6 | ELOVL fatty acid elongase 6 | 3 | | 4527 | hsa-mir-218-1 | 79071 | ELOVL6 | ELOVL fatty acid elongase 6 | 3 | | 4530 | hsa-mir-125b-2 | 79071 | ELOVL6 | ELOVL fatty acid elongase 6 | 3 | | 4539 | hsa-mir-620 | 79071 | ELOVL6 | ELOVL fatty acid elongase 6 | 3 | | 4731 | hsa-mir-620 | 8220 | DGCR14 | DiGeorge syndrome critical region gene 14 | 3 | | 4792 | hsa-mir-378a | 10278 | EFS | embryonal Fyn-associated substrate | 3 | | 4793 | hsa-mir-505 | 10278 | EFS | embryonal Fyn-associated substrate | 3 | | 5121 | hsa-let-7f-2 | 1462 | VCAN | versican | 3 | | 5124 | hsa-mir-98 | 1462 | VCAN | versican | 3 | | 5130 | hsa-mir-550a-1 | 1462 | VCAN | versican | 3 | | 5131 | hsa-mir-602 | 1462 | VCAN | versican | 3 | | 5135 | hsa-mir-644a | 1462 | VCAN | versican | 3 | | 5137 | hsa-mir-877 | 1462 | VCAN | versican | 3 | | 5138 | hsa-mir-548j | 1462 | VCAN | versican | 3 | | 5139 | hsa-mir-548n | 1462 | VCAN | versican | 3 | | 5140 | hsa-mir-2909 | 1462 | VCAN | versican | 3 | | 5141 | hsa-mir-548s | 1462 | VCAN | versican | 3 | | 5142 | hsa-mir-2355 | 1462 | VCAN | versican | 3 | | 5143 | hsa-mir-23c | 1462 | VCAN | versican | 3 | | 5144 | hsa-mir-3679 | 1462 | VCAN | versican | 3 | | 5145 | hsa-mir-3942 | 1462 | VCAN | versican | 3 | | 5146 | hsa-mir-548o-2 | 1462 | VCAN | versican | 3 | | 5147 | hsa-mir-378e | 1462 | VCAN | versican | 3 | | 5148 | hsa-mir-4467 | 1462 | VCAN | versican | 3 | | 5149 | hsa-mir-4498 | 1462 | VCAN | versican | 3 | | 5150 | hsa-mir-4517 | 1462 | VCAN | versican | 3 | | 5154 | hsa-mir-4698 | 1462 | VCAN | versican | 3 | | 5156 | hsa-mir-4723 | 1462 | VCAN | versican | 3 | | 5157 | hsa-mir-4771-1 | 1462 | VCAN | versican | 3 | | 5158 | hsa-mir-4771-2 | 1462 | VCAN | versican | 3 | | 5160 | hsa-mir-4775 | 1462 | VCAN | versican | 3 | | 5161 | hsa-mir-5090 | 1462 | VCAN | versican | 3 | | 5164 | hsa-mir-604 | 1462 | VCAN | versican | 3 | | 5165 | hsa-mir-675 | 1462 | VCAN | versican | 3 | | 5166 | hsa-mir-218-2 | 1462 | VCAN | versican | 3 | | 5167 | hsa-mir-574 | 1462 | VCAN | versican | 3 | | 5168 | hsa-mir-887 | 1462 | VCAN | versican | 3 | | 5169 | hsa-mir-938 | 1462 | VCAN | versican | 3 | | 5298 | hsa-mir-455 | 10630 | PDPN | podoplanin | 3 | | 5531 | hsa-mir-620 | 23464 | GCAT | glycine C-acetyltransferase | 3 | | 5678 | hsa-mir-10a | 119 | ADD2 | adducin 2 (beta) | 3 | | 5680 | hsa-mir-218-2 | 119 | ADD2 | adducin 2 (beta) | 3 | | 5684 | hsa-mir-574 | 119 | ADD2 | adducin 2 (beta) | 3 | | 5686 | hsa-mir-603 | 119 | ADD2 | adducin 2 (beta) | 3 | | 5687 | hsa-mir-604 | 119 | ADD2 | adducin 2 (beta) | 3 | | 5688 | hsa-mir-605 | 119 | ADD2 | adducin 2 (beta) | 3 | | 5689 | hsa-mir-618 | 119 | ADD2 | adducin 2 (beta) | 3 | | 5691 | hsa-mir-675 | 119 | ADD2 | adducin 2 (beta) | 3 | | 5692 | hsa-mir-887 | 119 | ADD2 | adducin 2 (beta) | 3 | | 5693 | hsa-mir-938 | 119 | ADD2 | adducin 2 (beta) | 3 | | 5694 | hsa-mir-1245a | 119 | ADD2 | adducin 2 (beta) | 3 | | 5695 | hsa-mir-1284 | 119 | ADD2 | adducin 2 (beta) | 3 | | 5696 | hsa-mir-1469 | 119 | ADD2 | adducin 2 (beta) | 3 | | 5697 | hsa-mir-3126 | 119 | ADD2 | adducin 2 (beta) | 3 | | 5698 | hsa-mir-3129 | 119 | ADD2 | adducin 2 (beta) | 3 | | 5699 | hsa-mir-3139 | 119 | ADD2 | adducin 2 (beta) | 3 | | 5701 | hsa-mir-3606 | 119 | ADD2 | adducin 2 (beta) | 3 | | 5702 | hsa-mir-3650 | 119 | ADD2 | adducin 2 (beta) | 3 | | 5703 | hsa-mir-4441 | 119 | ADD2 | adducin 2 (beta) | 3 | | 5705 | hsa-mir-4636 | 119 | ADD2 | adducin 2 (beta) | 3 | | 5706 | hsa-mir-4731 | 119 | ADD2 | adducin 2 (beta) | 3 | | 5707 | hsa-mir-4735 | 119 | ADD2 | adducin 2 (beta) | 3 | | 5708 | hsa-mir-4768 | 119 | ADD2 | adducin 2 (beta) | 3 | | 5709 | hsa-mir-2467 | 119 | ADD2 | adducin 2 (beta) | 3 | | 5710 | hsa-mir-4794 | 119 | ADD2 | adducin 2 (beta) | 3 | | 5711 | hsa-mir-548ao | 119 | ADD2 | adducin 2 (beta) | 3 | | 5712 | hsa-mir-617 | 119 | ADD2 | adducin 2 (beta) | 3 | | 5713 | hsa-mir-640 | 119 | ADD2 | adducin 2 (beta) | 3 | | 5715 | hsa-mir-877 | 119 | ADD2 | adducin 2 (beta) | 3 | | 5924 | hsa-mir-25 | 862 | RUNX1T1 | runt-related transcription factor 1; translocated to, 1 (cyclin D-related) | 3 | | 5925 | hsa-mir-93 | 862 | RUNX1T1 | runt-related transcription factor 1; translocated to, 1 (cyclin D-related) | 3 | | 5929 | hsa-mir-106b | 862 | RUNX1T1 | runt-related transcription factor 1; translocated to, 1 (cyclin D-related) | 3 | | 6117 | hsa-mir-99a | 3570 | IL6R | interleukin 6 receptor | 3 | | 6121 | hsa-mir-125b-1 | 3570 | IL6R | interleukin 6 receptor | 3 | | 6122 | hsa-mir-125b-2 | 3570 | IL6R | interleukin 6 receptor | 3 | | 6293 | hsa-mir-10a | 11184 | MAP4K1 | mitogen-activated protein kinase kinase kinase kinase 1 | 3 | | 6295 | hsa-mir-218-2 | 11184 | MAP4K1 | mitogen-activated protein kinase kinase kinase kinase 1 | 3 | | 6300 | hsa-mir-574 | 11184 | MAP4K1 | mitogen-activated protein kinase kinase kinase kinase 1 | 3 | | 6302 | hsa-mir-603 | 11184 | MAP4K1 | mitogen-activated protein kinase kinase kinase kinase 1 | 3 | | 6303 | hsa-mir-604 | 11184 | MAP4K1 | mitogen-activated protein kinase kinase kinase kinase 1 | 3 | | 6304 | hsa-mir-605 | 11184 | MAP4K1 | mitogen-activated protein kinase kinase kinase kinase 1 | 3 | | 6305 | hsa-mir-618 | 11184 | MAP4K1 | mitogen-activated protein kinase kinase kinase kinase 1 | 3 | | 6307 | hsa-mir-675 | 11184 | MAP4K1 | mitogen-activated protein kinase kinase kinase kinase 1 | 3 | | 6309 | hsa-mir-887 | 11184 | MAP4K1 | mitogen-activated protein kinase kinase kinase kinase 1 | 3 | | 6310 | hsa-mir-938 | 11184 | MAP4K1 | mitogen-activated protein kinase kinase kinase kinase 1 | 3 | | 6311 | hsa-mir-1245a | 11184 | MAP4K1 | mitogen-activated protein kinase kinase kinase kinase 1 | 3 | | 6312 | hsa-mir-1469 | 11184 | MAP4K1 | mitogen-activated protein kinase kinase kinase kinase 1 | 3 | | 6314 | hsa-mir-3126 | 11184 | MAP4K1 | mitogen-activated protein kinase kinase kinase kinase 1 | 3 | | 6315 | hsa-mir-3129 | 11184 | MAP4K1 | mitogen-activated protein kinase kinase kinase kinase 1 | 3 | | 6316 | hsa-mir-3139 | 11184 | MAP4K1 | mitogen-activated protein kinase kinase kinase kinase 1 | 3 | | 6317 | hsa-mir-3170 | 11184 | MAP4K1 | mitogen-activated protein kinase kinase kinase kinase 1 | 3 | | 6318 | hsa-mir-3606 | 11184 | MAP4K1 | mitogen-activated protein kinase kinase kinase kinase 1 | 3 | | 6319 | hsa-mir-3650 | 11184 | MAP4K1 | mitogen-activated protein kinase kinase kinase kinase 1 | 3 | | 6320 | hsa-mir-4441 | 11184 | MAP4K1 | mitogen-activated protein kinase kinase kinase kinase 1 | 3 | | 6321 | hsa-mir-4486 | 11184 | MAP4K1 | mitogen-activated protein kinase kinase kinase kinase 1 | 3 | | 6322 | hsa-mir-4636 | 11184 | MAP4K1 | mitogen-activated protein kinase kinase kinase kinase 1 | 3 | | 6323 | hsa-mir-4731 | 11184 | MAP4K1 | mitogen-activated protein kinase kinase kinase kinase 1 | 3 | | 6324 | hsa-mir-4735 | 11184 | MAP4K1 | mitogen-activated protein kinase kinase kinase kinase 1 | 3 | | 6325 | hsa-mir-4768 | 11184 | MAP4K1 | mitogen-activated protein kinase kinase kinase kinase 1 | 3 | | 6326 | hsa-mir-2467 | 11184 | MAP4K1 | mitogen-activated protein kinase kinase kinase kinase 1 | 3 | | 6327 | hsa-mir-4794 | 11184 | MAP4K1 | mitogen-activated protein kinase kinase kinase kinase 1 | 3 | | 6328 | hsa-mir-548ao | 11184 | MAP4K1 | mitogen-activated protein kinase kinase kinase kinase 1 | 3 | | 6329 | hsa-mir-5579 | 11184 | MAP4K1 | mitogen-activated protein kinase kinase kinase kinase 1 | 3 | | 6330 | hsa-mir-617 | 11184 | MAP4K1 | mitogen-activated protein kinase kinase kinase kinase 1 | 3 | | 6331 | hsa-mir-640 | 11184 | MAP4K1 | mitogen-activated protein kinase kinase kinase kinase 1 | 3 | | 6332 | hsa-mir-877 | 11184 | MAP4K1 | mitogen-activated protein kinase kinase kinase kinase 1 | 3 | | 6780 | hsa-mir-125b-1 | 7752 | ZNF200 | zinc finger protein 200 | 3 | | 6781 | hsa-mir-455 | 7752 | ZNF200 | zinc finger protein 200 | 3 | | 6787 | hsa-mir-620 | 7752 | ZNF200 | zinc finger protein 200 | 3 | | 7047 | hsa-mir-218-2 | 1741 | DLG3 | discs, large homolog 3 (Drosophila) | 3 | | 7052 | hsa-mir-603 | 1741 | DLG3 | discs, large homolog 3 (Drosophila) | 3 | | 7053 | hsa-mir-604 | 1741 | DLG3 | discs, large homolog 3 (Drosophila) | 3 | | 7054 | hsa-mir-605 | 1741 | DLG3 | discs, large homolog 3 (Drosophila) | 3 | | 7056 | hsa-mir-618 | 1741 | DLG3 | discs, large homolog 3 (Drosophila) | 3 | | 7059 | hsa-mir-887 | 1741 | DLG3 | discs, large homolog 3 (Drosophila) | 3 | | 7060 | hsa-mir-938 | 1741 | DLG3 | discs, large homolog 3 (Drosophila) | 3 | | 7062 | hsa-mir-1469 | 1741 | DLG3 | discs, large homolog 3 (Drosophila) | 3 | | 7063 | hsa-mir-3120 | 1741 | DLG3 | discs, large homolog 3 (Drosophila) | 3 | | 7064 | hsa-mir-3126 | 1741 | DLG3 | discs, large homolog 3 (Drosophila) | 3 | | 7065 | hsa-mir-3129 | 1741 | DLG3 | discs, large homolog 3 (Drosophila) | 3 | | 7066 | hsa-mir-3139 | 1741 | DLG3 | discs, large homolog 3 (Drosophila) | 3 | | 7068 | hsa-mir-3650 | 1741 | DLG3 | discs, large homolog 3 (Drosophila) | 3 | | 7069 | hsa-mir-4441 | 1741 | DLG3 | discs, large homolog 3 (Drosophila) | 3 | | 7070 | hsa-mir-4768 | 1741 | DLG3 | discs, large homolog 3 (Drosophila) | 3 | | 7071 | hsa-mir-2467 | 1741 | DLG3 | discs, large homolog 3 (Drosophila) | 3 | | 7072 | hsa-mir-4794 | 1741 | DLG3 | discs, large homolog 3 (Drosophila) | 3 | | 7073 | hsa-mir-548ao | 1741 | DLG3 | discs, large homolog 3 (Drosophila) | 3 | | 7074 | hsa-mir-617 | 1741 | DLG3 | discs, large homolog 3 (Drosophila) | 3 | | 7076 | hsa-mir-640 | 1741 | DLG3 | discs, large homolog 3 (Drosophila) | 3 | | 7082 | hsa-mir-125b-1 | 54941 | RNF125 | ring finger protein 125, E3 ubiquitin protein ligase | 3 | | 7141 | hsa-mir-455 | 80256 | FAM214B | family with sequence similarity 214, member B | 3 | | 7675 | hsa-mir-561 | 65220 | NADK | NAD kinase | 3 | | 8103 | hsa-mir-99a | 26031 | OSBPL3 | oxysterol binding protein-like 3 | 3 | | 8107 | hsa-mir-125b-2 | 26031 | OSBPL3 | oxysterol binding protein-like 3 | 3 | | 8108 | hsa-mir-455 | 26031 | OSBPL3 | oxysterol binding protein-like 3 | 3 | | 8227 | hsa-mir-605 | 10634 | GAS2L1 | growth arrest-specific 2 like 1 | 3 | | 8458 | hsa-mir-125b-1 | 8115 | TCL1A | T-cell leukemia/lymphoma 1A | 3 | | 8853 | hsa-mir-378a | 2078 | ERG | v-ets erythroblastosis virus E26 oncogene homolog (avian) | 3 | | 9449 | hsa-mir-620 | 9711 | KIAA0226 | KIAA0226 | 3 | | 9499 | hsa-mir-550a-1 | 6934 | TCF7L2 | transcription factor 7-like 2 (T-cell specific, HMG-box) | 3 | | 9506 | hsa-mir-548s | 6934 | TCF7L2 | transcription factor 7-like 2 (T-cell specific, HMG-box) | 3 | | 9507 | hsa-mir-2355 | 6934 | TCF7L2 | transcription factor 7-like 2 (T-cell specific, HMG-box) | 3 | | 9512 | hsa-mir-4467 | 6934 | TCF7L2 | transcription factor 7-like 2 (T-cell specific, HMG-box) | 3 | | 9514 | hsa-mir-4698 | 6934 | TCF7L2 | transcription factor 7-like 2 (T-cell specific, HMG-box) | 3 | | 9518 | hsa-mir-5090 | 6934 | TCF7L2 | transcription factor 7-like 2 (T-cell specific, HMG-box) | 3 | | 9520 | hsa-mir-604 | 6934 | TCF7L2 | transcription factor 7-like 2 (T-cell specific, HMG-box) | 3 | | 9522 | hsa-mir-218-2 | 6934 | TCF7L2 | transcription factor 7-like 2 (T-cell specific, HMG-box) | 3 | | 9523 | hsa-mir-574 | 6934 | TCF7L2 | transcription factor 7-like 2 (T-cell specific, HMG-box) | 3 | | 9524 | hsa-mir-887 | 6934 | TCF7L2 | transcription factor 7-like 2 (T-cell specific, HMG-box) | 3 | | 9525 | hsa-mir-938 | 6934 | TCF7L2 | transcription factor 7-like 2 (T-cell specific, HMG-box) | 3 | | 10982 | hsa-mir-378a | 6934 | TCF7L2 | transcription factor 7-like 2 (T-cell specific, HMG-box) | 3 | | 11318 | hsa-mir-620 | 58477 | SRPRB | signal recognition particle receptor, B subunit | 3 | | 11532 | hsa-mir-620 | 64222 | TOR3A | torsin family 3, member A | 3 | | 11558 | hsa-mir-218-2 | 55731 | C17orf63 | chromosome 17 open reading frame 63 | 3 | | 11559 | hsa-mir-125b-1 | 55731 | C17orf63 | chromosome 17 open reading frame 63 | 3 | | 11562 | hsa-mir-574 | 55731 | C17orf63 | chromosome 17 open reading frame 63 | 3 | | 11564 | hsa-mir-603 | 55731 | C17orf63 | chromosome 17 open reading frame 63 | 3 | | 11565 | hsa-mir-604 | 55731 | C17orf63 | chromosome 17 open reading frame 63 | 3 | | 11566 | hsa-mir-605 | 55731 | C17orf63 | chromosome 17 open reading frame 63 | 3 | | 11568 | hsa-mir-620 | 55731 | C17orf63 | chromosome 17 open reading frame 63 | 3 | | 11572 | hsa-mir-887 | 55731 | C17orf63 | chromosome 17 open reading frame 63 | 3 | | 11573 | hsa-mir-938 | 55731 | C17orf63 | chromosome 17 open reading frame 63 | 3 | | 11574 | hsa-mir-1245a | 55731 | C17orf63 | chromosome 17 open reading frame 63 | 3 | | 11577 | hsa-mir-3126 | 55731 | C17orf63 | chromosome 17 open reading frame 63 | 3 | | 11578 | hsa-mir-3129 | 55731 | C17orf63 | chromosome 17 open reading frame 63 | 3 | | 11581 | hsa-mir-3606 | 55731 | C17orf63 | chromosome 17 open reading frame 63 | 3 | | 11582 | hsa-mir-3650 | 55731 | C17orf63 | chromosome 17 open reading frame 63 | 3 | | 11583 | hsa-mir-4441 | 55731 | C17orf63 | chromosome 17 open reading frame 63 | 3 | | 11586 | hsa-mir-4731 | 55731 | C17orf63 | chromosome 17 open reading frame 63 | 3 | | 11588 | hsa-mir-4768 | 55731 | C17orf63 | chromosome 17 open reading frame 63 | 3 | | 11589 | hsa-mir-2467 | 55731 | C17orf63 | chromosome 17 open reading frame 63 | 3 | | 11590 | hsa-mir-4794 | 55731 | C17orf63 | chromosome 17 open reading frame 63 | 3 | | 11596 | hsa-mir-877 | 55731 | C17orf63 | chromosome 17 open reading frame 63 | 3 | | 11805 | hsa-mir-620 | 79693 | YRDC | yrdC domain containing (E. coli) | 3 | | 11920 | hsa-mir-620 | 64785 | GINS3 | GINS complex subunit 3 (Psf3 homolog) | 3 | | 12562 | hsa-mir-218-1 | 64506 | CPEB1 | cytoplasmic polyadenylation element binding protein 1 | 3 | | 12645 | hsa-mir-10a | 29842 | TFCP2L1 | transcription factor CP2-like 1 | 3 | | 12647 | hsa-mir-218-2 | 29842 | TFCP2L1 | transcription factor CP2-like 1 | 3 | | 12650 | hsa-mir-574 | 29842 | TFCP2L1 | transcription factor CP2-like 1 | 3 | | 12652 | hsa-mir-603 | 29842 | TFCP2L1 | transcription factor CP2-like 1 | 3 | | 12653 | hsa-mir-604 | 29842 | TFCP2L1 | transcription factor CP2-like 1 | 3 | | 12654 | hsa-mir-605 | 29842 | TFCP2L1 | transcription factor CP2-like 1 | 3 | | 12655 | hsa-mir-618 | 29842 | TFCP2L1 | transcription factor CP2-like 1 | 3 | | 12656 | hsa-mir-1271 | 29842 | TFCP2L1 | transcription factor CP2-like 1 | 3 | | 12657 | hsa-mir-675 | 29842 | TFCP2L1 | transcription factor CP2-like 1 | 3 | | 12659 | hsa-mir-887 | 29842 | TFCP2L1 | transcription factor CP2-like 1 | 3 | | 12660 | hsa-mir-938 | 29842 | TFCP2L1 | transcription factor CP2-like 1 | 3 | | 12662 | hsa-mir-1245a | 29842 | TFCP2L1 | transcription factor CP2-like 1 | 3 | | 12663 | hsa-mir-1469 | 29842 | TFCP2L1 | transcription factor CP2-like 1 | 3 | | 12664 | hsa-mir-3120 | 29842 | TFCP2L1 | transcription factor CP2-like 1 | 3 | | 12665 | hsa-mir-3126 | 29842 | TFCP2L1 | transcription factor CP2-like 1 | 3 | | 12666 | hsa-mir-3129 | 29842 | TFCP2L1 | transcription factor CP2-like 1 | 3 | | 12667 | hsa-mir-3139 | 29842 | TFCP2L1 | transcription factor CP2-like 1 | 3 | | 12668 | hsa-mir-3170 | 29842 | TFCP2L1 | transcription factor CP2-like 1 | 3 | | 12669 | hsa-mir-3606 | 29842 | TFCP2L1 | transcription factor CP2-like 1 | 3 | | 12670 | hsa-mir-3650 | 29842 | TFCP2L1 | transcription factor CP2-like 1 | 3 | | 12671 | hsa-mir-4441 | 29842 | TFCP2L1 | transcription factor CP2-like 1 | 3 | | 12673 | hsa-mir-4519 | 29842 | TFCP2L1 | transcription factor CP2-like 1 | 3 | | 12674 | hsa-mir-4636 | 29842 | TFCP2L1 | transcription factor CP2-like 1 | 3 | | 12675 | hsa-mir-4731 | 29842 | TFCP2L1 | transcription factor CP2-like 1 | 3 | | 12677 | hsa-mir-4768 | 29842 | TFCP2L1 | transcription factor CP2-like 1 | 3 | | 12678 | hsa-mir-2467 | 29842 | TFCP2L1 | transcription factor CP2-like 1 | 3 | | 12679 | hsa-mir-4794 | 29842 | TFCP2L1 | transcription factor CP2-like 1 | 3 | | 12680 | hsa-mir-548ao | 29842 | TFCP2L1 | transcription factor CP2-like 1 | 3 | | 12684 | hsa-mir-617 | 29842 | TFCP2L1 | transcription factor CP2-like 1 | 3 | | 12685 | hsa-mir-640 | 29842 | TFCP2L1 | transcription factor CP2-like 1 | 3 | | 12687 | hsa-mir-877 | 29842 | TFCP2L1 | transcription factor CP2-like 1 | 3 | | 12690 | hsa-mir-10a | 53916 | RAB4B | RAB4B, member RAS oncogene family | 3 | | 12691 | hsa-mir-218-2 | 53916 | RAB4B | RAB4B, member RAS oncogene family | 3 | | 12694 | hsa-mir-574 | 53916 | RAB4B | RAB4B, member RAS oncogene family | 3 | | 12696 | hsa-mir-603 | 53916 | RAB4B | RAB4B, member RAS oncogene family | 3 | | 12697 | hsa-mir-604 | 53916 | RAB4B | RAB4B, member RAS oncogene family | 3 | | 12701 | hsa-mir-938 | 53916 | RAB4B | RAB4B, member RAS oncogene family | 3 | | 12702 | hsa-mir-3129 | 53916 | RAB4B | RAB4B, member RAS oncogene family | 3 | | 12703 | hsa-mir-4441 | 53916 | RAB4B | RAB4B, member RAS oncogene family | 3 | | 12705 | hsa-mir-617 | 53916 | RAB4B | RAB4B, member RAS oncogene family | 3 | | 12708 | hsa-mir-767 | 53916 | RAB4B | RAB4B, member RAS oncogene family | 3 | | 12797 | hsa-mir-99a | 26256 | CABYR | calcium binding tyrosine-(Y)-phosphorylation regulated | 3 | | 12801 | hsa-mir-125b-2 | 26256 | CABYR | calcium binding tyrosine-(Y)-phosphorylation regulated | 3 | | 12809 | hsa-mir-620 | 26256 | CABYR | calcium binding tyrosine-(Y)-phosphorylation regulated | 3 | | 12864 | hsa-mir-218-1 | 11226 | GALNT6 | UDP-N-acetyl-alpha-D-galactosamine:polypeptide N-acetylgalactosaminyltransferase 6 (GalNAc-T6) | 3 | | 13212 | hsa-mir-99a | 9990 | SLC12A6 | solute carrier family 12 (potassium/chloride transporters), member 6 | 3 | | 13215 | hsa-mir-125b-2 | 9990 | SLC12A6 | solute carrier family 12 (potassium/chloride transporters), member 6 | 3 | | 13361 | hsa-mir-181a-2 | 81888 | HYI | hydroxypyruvate isomerase (putative) | 3 | | 13362 | hsa-mir-181b-2 | 81888 | HYI | hydroxypyruvate isomerase (putative) | 3 | | 14317 | hsa-mir-10a | 57510 | XPO5 | exportin 5 | 3 | | 14319 | hsa-mir-218-2 | 57510 | XPO5 | exportin 5 | 3 | | 14323 | hsa-mir-574 | 57510 | XPO5 | exportin 5 | 3 | | 14325 | hsa-mir-603 | 57510 | XPO5 | exportin 5 | 3 | | 14327 | hsa-mir-605 | 57510 | XPO5 | exportin 5 | 3 | | 14330 | hsa-mir-1271 | 57510 | XPO5 | exportin 5 | 3 | | 14331 | hsa-mir-675 | 57510 | XPO5 | exportin 5 | 3 | | 14333 | hsa-mir-887 | 57510 | XPO5 | exportin 5 | 3 | | 14335 | hsa-mir-1245a | 57510 | XPO5 | exportin 5 | 3 | | 14338 | hsa-mir-3120 | 57510 | XPO5 | exportin 5 | 3 | | 14339 | hsa-mir-3126 | 57510 | XPO5 | exportin 5 | 3 | | 14340 | hsa-mir-3129 | 57510 | XPO5 | exportin 5 | 3 | | 14342 | hsa-mir-3606 | 57510 | XPO5 | exportin 5 | 3 | | 14344 | hsa-mir-4441 | 57510 | XPO5 | exportin 5 | 3 | | 14347 | hsa-mir-4636 | 57510 | XPO5 | exportin 5 | 3 | | 14348 | hsa-mir-4731 | 57510 | XPO5 | exportin 5 | 3 | | 14350 | hsa-mir-4768 | 57510 | XPO5 | exportin 5 | 3 | | 14351 | hsa-mir-2467 | 57510 | XPO5 | exportin 5 | 3 | | 14352 | hsa-mir-4794 | 57510 | XPO5 | exportin 5 | 3 | | 14353 | hsa-mir-548ao | 57510 | XPO5 | exportin 5 | 3 | | 14355 | hsa-mir-491 | 57510 | XPO5 | exportin 5 | 3 | | 14357 | hsa-mir-591 | 57510 | XPO5 | exportin 5 | 3 | | 14359 | hsa-mir-636 | 57510 | XPO5 | exportin 5 | 3 | | 14360 | hsa-mir-640 | 57510 | XPO5 | exportin 5 | 3 | | 14362 | hsa-mir-877 | 57510 | XPO5 | exportin 5 | 3 | | 16510 | hsa-let-7f-2 | 144165 | PRICKLE1 | prickle homolog 1 (Drosophila) | 3 | | 16511 | hsa-mir-98 | 144165 | PRICKLE1 | prickle homolog 1 (Drosophila) | 3 | | 16517 | hsa-mir-550a-1 | 144165 | PRICKLE1 | prickle homolog 1 (Drosophila) | 3 | | 16519 | hsa-mir-644a | 144165 | PRICKLE1 | prickle homolog 1 (Drosophila) | 3 | | 16520 | hsa-mir-877 | 144165 | PRICKLE1 | prickle homolog 1 (Drosophila) | 3 | | 16523 | hsa-mir-548s | 144165 | PRICKLE1 | prickle homolog 1 (Drosophila) | 3 | | 16525 | hsa-mir-3679 | 144165 | PRICKLE1 | prickle homolog 1 (Drosophila) | 3 | | 16526 | hsa-mir-3942 | 144165 | PRICKLE1 | prickle homolog 1 (Drosophila) | 3 | | 16527 | hsa-mir-548o-2 | 144165 | PRICKLE1 | prickle homolog 1 (Drosophila) | 3 | | 16528 | hsa-mir-378e | 144165 | PRICKLE1 | prickle homolog 1 (Drosophila) | 3 | | 16529 | hsa-mir-4517 | 144165 | PRICKLE1 | prickle homolog 1 (Drosophila) | 3 | | 16531 | hsa-mir-4698 | 144165 | PRICKLE1 | prickle homolog 1 (Drosophila) | 3 | | 16533 | hsa-mir-4723 | 144165 | PRICKLE1 | prickle homolog 1 (Drosophila) | 3 | | 16534 | hsa-mir-4771-1 | 144165 | PRICKLE1 | prickle homolog 1 (Drosophila) | 3 | | 16535 | hsa-mir-4771-2 | 144165 | PRICKLE1 | prickle homolog 1 (Drosophila) | 3 | | 16536 | hsa-mir-4775 | 144165 | PRICKLE1 | prickle homolog 1 (Drosophila) | 3 | | 16537 | hsa-mir-675 | 144165 | PRICKLE1 | prickle homolog 1 (Drosophila) | 3 | | 16538 | hsa-mir-218-2 | 144165 | PRICKLE1 | prickle homolog 1 (Drosophila) | 3 | | 16539 | hsa-mir-574 | 144165 | PRICKLE1 | prickle homolog 1 (Drosophila) | 3 | | 16544 | hsa-mir-602 | 144165 | PRICKLE1 | prickle homolog 1 (Drosophila) | 3 | | 16728 | hsa-mir-93 | 79269 | DCAF10 | DDB1 and CUL4 associated factor 10 | 3 | | 16729 | hsa-mir-106b | 79269 | DCAF10 | DDB1 and CUL4 associated factor 10 | 3 | | 16731 | hsa-mir-652 | 79269 | DCAF10 | DDB1 and CUL4 associated factor 10 | 3 | | 16732 | hsa-mir-25 | 79269 | DCAF10 | DDB1 and CUL4 associated factor 10 | 3 | | 17618 | hsa-mir-218-1 | 124491 | TMEM170A | transmembrane protein 170A | 3 | | 19807 | hsa-mir-602 | 128553 | TSHZ2 | teashirt zinc finger homeobox 2 | 3 | | 3 | hsa-mir-10a | 159091 | FAM122C | family with sequence similarity 122C | 2 | | 4 | hsa-mir-218-2 | 159091 | FAM122C | family with sequence similarity 122C | 2 | | 8 | hsa-mir-574 | 159091 | FAM122C | family with sequence similarity 122C | 2 | | 10 | hsa-mir-603 | 159091 | FAM122C | family with sequence similarity 122C | 2 | | 12 | hsa-mir-605 | 159091 | FAM122C | family with sequence similarity 122C | 2 | | 14 | hsa-mir-618 | 159091 | FAM122C | family with sequence similarity 122C | 2 | | 15 | hsa-mir-1271 | 159091 | FAM122C | family with sequence similarity 122C | 2 | | 16 | hsa-mir-675 | 159091 | FAM122C | family with sequence similarity 122C | 2 | | 17 | hsa-mir-708 | 159091 | FAM122C | family with sequence similarity 122C | 2 | | 18 | hsa-mir-887 | 159091 | FAM122C | family with sequence similarity 122C | 2 | | 20 | hsa-mir-1245a | 159091 | FAM122C | family with sequence similarity 122C | 2 | | 22 | hsa-mir-3120 | 159091 | FAM122C | family with sequence similarity 122C | 2 | | 23 | hsa-mir-3126 | 159091 | FAM122C | family with sequence similarity 122C | 2 | | 24 | hsa-mir-3129 | 159091 | FAM122C | family with sequence similarity 122C | 2 | | 27 | hsa-mir-3606 | 159091 | FAM122C | family with sequence similarity 122C | 2 | | 28 | hsa-mir-3650 | 159091 | FAM122C | family with sequence similarity 122C | 2 | | 29 | hsa-mir-4441 | 159091 | FAM122C | family with sequence similarity 122C | 2 | | 32 | hsa-mir-4731 | 159091 | FAM122C | family with sequence similarity 122C | 2 | | 33 | hsa-mir-4735 | 159091 | FAM122C | family with sequence similarity 122C | 2 | | 34 | hsa-mir-4768 | 159091 | FAM122C | family with sequence similarity 122C | 2 | | 35 | hsa-mir-2467 | 159091 | FAM122C | family with sequence similarity 122C | 2 | | 36 | hsa-mir-4794 | 159091 | FAM122C | family with sequence similarity 122C | 2 | | 37 | hsa-mir-548ao | 159091 | FAM122C | family with sequence similarity 122C | 2 | | 39 | hsa-mir-218-1 | 159091 | FAM122C | family with sequence similarity 122C | 2 | | 40 | hsa-mir-617 | 159091 | FAM122C | family with sequence similarity 122C | 2 | | 41 | hsa-mir-640 | 159091 | FAM122C | family with sequence similarity 122C | 2 | | 42 | hsa-mir-877 | 159091 | FAM122C | family with sequence similarity 122C | 2 | | 44 | hsa-mir-620 | 159091 | FAM122C | family with sequence similarity 122C | 2 | | 52 | hsa-let-7c | 646 | BNC1 | basonuclin 1 | 2 | | 53 | hsa-mir-99a | 646 | BNC1 | basonuclin 1 | 2 | | 55 | hsa-mir-218-1 | 646 | BNC1 | basonuclin 1 | 2 | | 57 | hsa-mir-125b-1 | 646 | BNC1 | basonuclin 1 | 2 | | 58 | hsa-mir-125b-2 | 646 | BNC1 | basonuclin 1 | 2 | | 59 | hsa-mir-561 | 646 | BNC1 | basonuclin 1 | 2 | | 61 | hsa-mir-585 | 646 | BNC1 | basonuclin 1 | 2 | | 94 | hsa-let-7c | 10228 | STX6 | syntaxin 6 | 2 | | 95 | hsa-mir-99a | 10228 | STX6 | syntaxin 6 | 2 | | 96 | hsa-mir-10a | 10228 | STX6 | syntaxin 6 | 2 | | 97 | hsa-mir-218-2 | 10228 | STX6 | syntaxin 6 | 2 | | 99 | hsa-mir-125b-2 | 10228 | STX6 | syntaxin 6 | 2 | | 101 | hsa-mir-574 | 10228 | STX6 | syntaxin 6 | 2 | | 103 | hsa-mir-603 | 10228 | STX6 | syntaxin 6 | 2 | | 105 | hsa-mir-605 | 10228 | STX6 | syntaxin 6 | 2 | | 106 | hsa-mir-613 | 10228 | STX6 | syntaxin 6 | 2 | | 107 | hsa-mir-618 | 10228 | STX6 | syntaxin 6 | 2 | | 110 | hsa-mir-1245a | 10228 | STX6 | syntaxin 6 | 2 | | 112 | hsa-mir-3120 | 10228 | STX6 | syntaxin 6 | 2 | | 113 | hsa-mir-3126 | 10228 | STX6 | syntaxin 6 | 2 | | 114 | hsa-mir-3129 | 10228 | STX6 | syntaxin 6 | 2 | | 115 | hsa-mir-3606 | 10228 | STX6 | syntaxin 6 | 2 | | 117 | hsa-mir-4441 | 10228 | STX6 | syntaxin 6 | 2 | | 122 | hsa-mir-2467 | 10228 | STX6 | syntaxin 6 | 2 | | 123 | hsa-mir-4794 | 10228 | STX6 | syntaxin 6 | 2 | | 125 | hsa-mir-636 | 10228 | STX6 | syntaxin 6 | 2 | | 126 | hsa-mir-640 | 10228 | STX6 | syntaxin 6 | 2 | | 127 | hsa-let-7c | 89766 | UMODL1 | uromodulin-like 1 | 2 | | 128 | hsa-mir-99a | 89766 | UMODL1 | uromodulin-like 1 | 2 | | 130 | hsa-mir-218-1 | 89766 | UMODL1 | uromodulin-like 1 | 2 | | 132 | hsa-mir-125b-1 | 89766 | UMODL1 | uromodulin-like 1 | 2 | | 133 | hsa-mir-125b-2 | 89766 | UMODL1 | uromodulin-like 1 | 2 | | 134 | hsa-mir-561 | 89766 | UMODL1 | uromodulin-like 1 | 2 | | 136 | hsa-mir-585 | 89766 | UMODL1 | uromodulin-like 1 | 2 | | 144 | hsa-mir-708 | 89766 | UMODL1 | uromodulin-like 1 | 2 | | 174 | hsa-mir-10a | 138009 | DCAF4L2 | DDB1 and CUL4 associated factor 4-like 2 | 2 | | 176 | hsa-mir-218-2 | 138009 | DCAF4L2 | DDB1 and CUL4 associated factor 4-like 2 | 2 | | 180 | hsa-mir-574 | 138009 | DCAF4L2 | DDB1 and CUL4 associated factor 4-like 2 | 2 | | 182 | hsa-mir-603 | 138009 | DCAF4L2 | DDB1 and CUL4 associated factor 4-like 2 | 2 | | 183 | hsa-mir-604 | 138009 | DCAF4L2 | DDB1 and CUL4 associated factor 4-like 2 | 2 | | 184 | hsa-mir-605 | 138009 | DCAF4L2 | DDB1 and CUL4 associated factor 4-like 2 | 2 | | 185 | hsa-mir-618 | 138009 | DCAF4L2 | DDB1 and CUL4 associated factor 4-like 2 | 2 | | 186 | hsa-mir-620 | 138009 | DCAF4L2 | DDB1 and CUL4 associated factor 4-like 2 | 2 | | 187 | hsa-mir-1271 | 138009 | DCAF4L2 | DDB1 and CUL4 associated factor 4-like 2 | 2 | | 188 | hsa-mir-675 | 138009 | DCAF4L2 | DDB1 and CUL4 associated factor 4-like 2 | 2 | | 190 | hsa-mir-887 | 138009 | DCAF4L2 | DDB1 and CUL4 associated factor 4-like 2 | 2 | | 191 | hsa-mir-938 | 138009 | DCAF4L2 | DDB1 and CUL4 associated factor 4-like 2 | 2 | | 192 | hsa-mir-1245a | 138009 | DCAF4L2 | DDB1 and CUL4 associated factor 4-like 2 | 2 | | 193 | hsa-mir-1469 | 138009 | DCAF4L2 | DDB1 and CUL4 associated factor 4-like 2 | 2 | | 194 | hsa-mir-3120 | 138009 | DCAF4L2 | DDB1 and CUL4 associated factor 4-like 2 | 2 | | 195 | hsa-mir-3126 | 138009 | DCAF4L2 | DDB1 and CUL4 associated factor 4-like 2 | 2 | | 196 | hsa-mir-3129 | 138009 | DCAF4L2 | DDB1 and CUL4 associated factor 4-like 2 | 2 | | 197 | hsa-mir-3170 | 138009 | DCAF4L2 | DDB1 and CUL4 associated factor 4-like 2 | 2 | | 198 | hsa-mir-3606 | 138009 | DCAF4L2 | DDB1 and CUL4 associated factor 4-like 2 | 2 | | 199 | hsa-mir-3650 | 138009 | DCAF4L2 | DDB1 and CUL4 associated factor 4-like 2 | 2 | | 200 | hsa-mir-4441 | 138009 | DCAF4L2 | DDB1 and CUL4 associated factor 4-like 2 | 2 | | 201 | hsa-mir-4486 | 138009 | DCAF4L2 | DDB1 and CUL4 associated factor 4-like 2 | 2 | | 202 | hsa-mir-4636 | 138009 | DCAF4L2 | DDB1 and CUL4 associated factor 4-like 2 | 2 | | 203 | hsa-mir-4731 | 138009 | DCAF4L2 | DDB1 and CUL4 associated factor 4-like 2 | 2 | | 204 | hsa-mir-4735 | 138009 | DCAF4L2 | DDB1 and CUL4 associated factor 4-like 2 | 2 | | 205 | hsa-mir-4768 | 138009 | DCAF4L2 | DDB1 and CUL4 associated factor 4-like 2 | 2 | | 206 | hsa-mir-2467 | 138009 | DCAF4L2 | DDB1 and CUL4 associated factor 4-like 2 | 2 | | 207 | hsa-mir-4794 | 138009 | DCAF4L2 | DDB1 and CUL4 associated factor 4-like 2 | 2 | | 208 | hsa-mir-548ao | 138009 | DCAF4L2 | DDB1 and CUL4 associated factor 4-like 2 | 2 | | 209 | hsa-mir-5579 | 138009 | DCAF4L2 | DDB1 and CUL4 associated factor 4-like 2 | 2 | | 210 | hsa-mir-617 | 138009 | DCAF4L2 | DDB1 and CUL4 associated factor 4-like 2 | 2 | | 212 | hsa-mir-640 | 138009 | DCAF4L2 | DDB1 and CUL4 associated factor 4-like 2 | 2 | | 213 | hsa-mir-877 | 138009 | DCAF4L2 | DDB1 and CUL4 associated factor 4-like 2 | 2 | | 219 | hsa-mir-10a | 30816 | ERVW-1 | endogenous retrovirus group W, member 1 | 2 | | 221 | hsa-mir-218-2 | 30816 | ERVW-1 | endogenous retrovirus group W, member 1 | 2 | | 226 | hsa-mir-574 | 30816 | ERVW-1 | endogenous retrovirus group W, member 1 | 2 | | 228 | hsa-mir-603 | 30816 | ERVW-1 | endogenous retrovirus group W, member 1 | 2 | | 229 | hsa-mir-604 | 30816 | ERVW-1 | endogenous retrovirus group W, member 1 | 2 | | 230 | hsa-mir-605 | 30816 | ERVW-1 | endogenous retrovirus group W, member 1 | 2 | | 232 | hsa-mir-618 | 30816 | ERVW-1 | endogenous retrovirus group W, member 1 | 2 | | 233 | hsa-mir-620 | 30816 | ERVW-1 | endogenous retrovirus group W, member 1 | 2 | | 234 | hsa-mir-1271 | 30816 | ERVW-1 | endogenous retrovirus group W, member 1 | 2 | | 235 | hsa-mir-675 | 30816 | ERVW-1 | endogenous retrovirus group W, member 1 | 2 | | 237 | hsa-mir-887 | 30816 | ERVW-1 | endogenous retrovirus group W, member 1 | 2 | | 238 | hsa-mir-938 | 30816 | ERVW-1 | endogenous retrovirus group W, member 1 | 2 | | 239 | hsa-mir-1245a | 30816 | ERVW-1 | endogenous retrovirus group W, member 1 | 2 | | 240 | hsa-mir-1469 | 30816 | ERVW-1 | endogenous retrovirus group W, member 1 | 2 | | 241 | hsa-mir-1915 | 30816 | ERVW-1 | endogenous retrovirus group W, member 1 | 2 | | 242 | hsa-mir-3120 | 30816 | ERVW-1 | endogenous retrovirus group W, member 1 | 2 | | 243 | hsa-mir-3126 | 30816 | ERVW-1 | endogenous retrovirus group W, member 1 | 2 | | 244 | hsa-mir-3129 | 30816 | ERVW-1 | endogenous retrovirus group W, member 1 | 2 | | 245 | hsa-mir-3139 | 30816 | ERVW-1 | endogenous retrovirus group W, member 1 | 2 | | 246 | hsa-mir-3606 | 30816 | ERVW-1 | endogenous retrovirus group W, member 1 | 2 | | 247 | hsa-mir-3650 | 30816 | ERVW-1 | endogenous retrovirus group W, member 1 | 2 | | 248 | hsa-mir-4441 | 30816 | ERVW-1 | endogenous retrovirus group W, member 1 | 2 | | 249 | hsa-mir-4486 | 30816 | ERVW-1 | endogenous retrovirus group W, member 1 | 2 | | 250 | hsa-mir-4636 | 30816 | ERVW-1 | endogenous retrovirus group W, member 1 | 2 | | 251 | hsa-mir-4728 | 30816 | ERVW-1 | endogenous retrovirus group W, member 1 | 2 | | 252 | hsa-mir-4731 | 30816 | ERVW-1 | endogenous retrovirus group W, member 1 | 2 | | 253 | hsa-mir-4735 | 30816 | ERVW-1 | endogenous retrovirus group W, member 1 | 2 | | 254 | hsa-mir-4768 | 30816 | ERVW-1 | endogenous retrovirus group W, member 1 | 2 | | 255 | hsa-mir-2467 | 30816 | ERVW-1 | endogenous retrovirus group W, member 1 | 2 | | 256 | hsa-mir-4794 | 30816 | ERVW-1 | endogenous retrovirus group W, member 1 | 2 | | 257 | hsa-mir-548ao | 30816 | ERVW-1 | endogenous retrovirus group W, member 1 | 2 | | 258 | hsa-mir-5579 | 30816 | ERVW-1 | endogenous retrovirus group W, member 1 | 2 | | 259 | hsa-mir-617 | 30816 | ERVW-1 | endogenous retrovirus group W, member 1 | 2 | | 260 | hsa-mir-640 | 30816 | ERVW-1 | endogenous retrovirus group W, member 1 | 2 | | 261 | hsa-mir-648 | 30816 | ERVW-1 | endogenous retrovirus group W, member 1 | 2 | | 262 | hsa-mir-877 | 30816 | ERVW-1 | endogenous retrovirus group W, member 1 | 2 | | 265 | hsa-mir-181a-2 | 221223 | CES5A | carboxylesterase 5A | 2 | | 266 | hsa-mir-181b-2 | 221223 | CES5A | carboxylesterase 5A | 2 | | 292 | hsa-let-7c | 50511 | SYCP3 | synaptonemal complex protein 3 | 2 | | 293 | hsa-mir-99a | 50511 | SYCP3 | synaptonemal complex protein 3 | 2 | | 295 | hsa-mir-218-1 | 50511 | SYCP3 | synaptonemal complex protein 3 | 2 | | 297 | hsa-mir-125b-1 | 50511 | SYCP3 | synaptonemal complex protein 3 | 2 | | 298 | hsa-mir-125b-2 | 50511 | SYCP3 | synaptonemal complex protein 3 | 2 | | 299 | hsa-mir-561 | 50511 | SYCP3 | synaptonemal complex protein 3 | 2 | | 301 | hsa-mir-585 | 50511 | SYCP3 | synaptonemal complex protein 3 | 2 | | 309 | hsa-mir-708 | 50511 | SYCP3 | synaptonemal complex protein 3 | 2 | | 337 | hsa-mir-218-1 | 121268 | RHEBL1 | Ras homolog enriched in brain like 1 | 2 | | 339 | hsa-mir-125b-1 | 121268 | RHEBL1 | Ras homolog enriched in brain like 1 | 2 | | 340 | hsa-mir-561 | 121268 | RHEBL1 | Ras homolog enriched in brain like 1 | 2 | | 342 | hsa-mir-585 | 121268 | RHEBL1 | Ras homolog enriched in brain like 1 | 2 | | 346 | hsa-mir-620 | 121268 | RHEBL1 | Ras homolog enriched in brain like 1 | 2 | | 370 | hsa-let-7c | 283310 | OTOGL | otogelin-like | 2 | | 371 | hsa-mir-99a | 283310 | OTOGL | otogelin-like | 2 | | 373 | hsa-mir-218-1 | 283310 | OTOGL | otogelin-like | 2 | | 375 | hsa-mir-125b-1 | 283310 | OTOGL | otogelin-like | 2 | | 376 | hsa-mir-125b-2 | 283310 | OTOGL | otogelin-like | 2 | | 377 | hsa-mir-561 | 283310 | OTOGL | otogelin-like | 2 | | 379 | hsa-mir-585 | 283310 | OTOGL | otogelin-like | 2 | | 384 | hsa-mir-620 | 283310 | OTOGL | otogelin-like | 2 | | 409 | hsa-mir-218-1 | 54621 | VSIG10 | V-set and immunoglobulin domain containing 10 | 2 | | 411 | hsa-mir-125b-1 | 54621 | VSIG10 | V-set and immunoglobulin domain containing 10 | 2 | | 412 | hsa-mir-561 | 54621 | VSIG10 | V-set and immunoglobulin domain containing 10 | 2 | | 414 | hsa-mir-585 | 54621 | VSIG10 | V-set and immunoglobulin domain containing 10 | 2 | | 421 | hsa-mir-708 | 54621 | VSIG10 | V-set and immunoglobulin domain containing 10 | 2 | | 450 | hsa-mir-218-1 | 636 | BICD1 | bicaudal D homolog 1 (Drosophila) | 2 | | 452 | hsa-mir-125b-1 | 636 | BICD1 | bicaudal D homolog 1 (Drosophila) | 2 | | 453 | hsa-mir-561 | 636 | BICD1 | bicaudal D homolog 1 (Drosophila) | 2 | | 455 | hsa-mir-585 | 636 | BICD1 | bicaudal D homolog 1 (Drosophila) | 2 | | 462 | hsa-mir-708 | 636 | BICD1 | bicaudal D homolog 1 (Drosophila) | 2 | | 484 | hsa-mir-218-2 | 9093 | DNAJA3 | DnaJ (Hsp40) homolog, subfamily A, member 3 | 2 | | 487 | hsa-mir-574 | 9093 | DNAJA3 | DnaJ (Hsp40) homolog, subfamily A, member 3 | 2 | | 489 | hsa-mir-603 | 9093 | DNAJA3 | DnaJ (Hsp40) homolog, subfamily A, member 3 | 2 | | 490 | hsa-mir-605 | 9093 | DNAJA3 | DnaJ (Hsp40) homolog, subfamily A, member 3 | 2 | | 491 | hsa-mir-618 | 9093 | DNAJA3 | DnaJ (Hsp40) homolog, subfamily A, member 3 | 2 | | 493 | hsa-mir-1271 | 9093 | DNAJA3 | DnaJ (Hsp40) homolog, subfamily A, member 3 | 2 | | 494 | hsa-mir-675 | 9093 | DNAJA3 | DnaJ (Hsp40) homolog, subfamily A, member 3 | 2 | | 495 | hsa-mir-708 | 9093 | DNAJA3 | DnaJ (Hsp40) homolog, subfamily A, member 3 | 2 | | 496 | hsa-mir-887 | 9093 | DNAJA3 | DnaJ (Hsp40) homolog, subfamily A, member 3 | 2 | | 498 | hsa-mir-663b | 9093 | DNAJA3 | DnaJ (Hsp40) homolog, subfamily A, member 3 | 2 | | 499 | hsa-mir-1245a | 9093 | DNAJA3 | DnaJ (Hsp40) homolog, subfamily A, member 3 | 2 | | 500 | hsa-mir-1469 | 9093 | DNAJA3 | DnaJ (Hsp40) homolog, subfamily A, member 3 | 2 | | 501 | hsa-mir-3120 | 9093 | DNAJA3 | DnaJ (Hsp40) homolog, subfamily A, member 3 | 2 | | 502 | hsa-mir-3126 | 9093 | DNAJA3 | DnaJ (Hsp40) homolog, subfamily A, member 3 | 2 | | 503 | hsa-mir-3129 | 9093 | DNAJA3 | DnaJ (Hsp40) homolog, subfamily A, member 3 | 2 | | 506 | hsa-mir-3606 | 9093 | DNAJA3 | DnaJ (Hsp40) homolog, subfamily A, member 3 | 2 | | 508 | hsa-mir-4441 | 9093 | DNAJA3 | DnaJ (Hsp40) homolog, subfamily A, member 3 | 2 | | 510 | hsa-mir-4731 | 9093 | DNAJA3 | DnaJ (Hsp40) homolog, subfamily A, member 3 | 2 | | 511 | hsa-mir-4768 | 9093 | DNAJA3 | DnaJ (Hsp40) homolog, subfamily A, member 3 | 2 | | 512 | hsa-mir-2467 | 9093 | DNAJA3 | DnaJ (Hsp40) homolog, subfamily A, member 3 | 2 | | 513 | hsa-mir-4794 | 9093 | DNAJA3 | DnaJ (Hsp40) homolog, subfamily A, member 3 | 2 | | 514 | hsa-mir-548ao | 9093 | DNAJA3 | DnaJ (Hsp40) homolog, subfamily A, member 3 | 2 | | 516 | hsa-mir-99a | 9093 | DNAJA3 | DnaJ (Hsp40) homolog, subfamily A, member 3 | 2 | | 517 | hsa-mir-101-2 | 9093 | DNAJA3 | DnaJ (Hsp40) homolog, subfamily A, member 3 | 2 | | 519 | hsa-mir-503 | 9093 | DNAJA3 | DnaJ (Hsp40) homolog, subfamily A, member 3 | 2 | | 520 | hsa-mir-591 | 9093 | DNAJA3 | DnaJ (Hsp40) homolog, subfamily A, member 3 | 2 | | 521 | hsa-mir-617 | 9093 | DNAJA3 | DnaJ (Hsp40) homolog, subfamily A, member 3 | 2 | | 522 | hsa-mir-636 | 9093 | DNAJA3 | DnaJ (Hsp40) homolog, subfamily A, member 3 | 2 | | 523 | hsa-mir-640 | 9093 | DNAJA3 | DnaJ (Hsp40) homolog, subfamily A, member 3 | 2 | | 524 | hsa-mir-125b-2 | 9093 | DNAJA3 | DnaJ (Hsp40) homolog, subfamily A, member 3 | 2 | | 525 | hsa-mir-877 | 9093 | DNAJA3 | DnaJ (Hsp40) homolog, subfamily A, member 3 | 2 | | 526 | hsa-let-7c | 79707 | NOL9 | nucleolar protein 9 | 2 | | 527 | hsa-mir-99a | 79707 | NOL9 | nucleolar protein 9 | 2 | | 529 | hsa-mir-218-1 | 79707 | NOL9 | nucleolar protein 9 | 2 | | 531 | hsa-mir-125b-1 | 79707 | NOL9 | nucleolar protein 9 | 2 | | 532 | hsa-mir-125b-2 | 79707 | NOL9 | nucleolar protein 9 | 2 | | 533 | hsa-mir-561 | 79707 | NOL9 | nucleolar protein 9 | 2 | | 535 | hsa-mir-585 | 79707 | NOL9 | nucleolar protein 9 | 2 | | 542 | hsa-mir-708 | 79707 | NOL9 | nucleolar protein 9 | 2 | | 572 | hsa-mir-10a | 9498 | SLC4A8 | solute carrier family 4, sodium bicarbonate cotransporter, member 8 | 2 | | 574 | hsa-mir-218-2 | 9498 | SLC4A8 | solute carrier family 4, sodium bicarbonate cotransporter, member 8 | 2 | | 578 | hsa-mir-574 | 9498 | SLC4A8 | solute carrier family 4, sodium bicarbonate cotransporter, member 8 | 2 | | 580 | hsa-mir-603 | 9498 | SLC4A8 | solute carrier family 4, sodium bicarbonate cotransporter, member 8 | 2 | | 581 | hsa-mir-604 | 9498 | SLC4A8 | solute carrier family 4, sodium bicarbonate cotransporter, member 8 | 2 | | 582 | hsa-mir-605 | 9498 | SLC4A8 | solute carrier family 4, sodium bicarbonate cotransporter, member 8 | 2 | | 585 | hsa-mir-675 | 9498 | SLC4A8 | solute carrier family 4, sodium bicarbonate cotransporter, member 8 | 2 | | 586 | hsa-mir-938 | 9498 | SLC4A8 | solute carrier family 4, sodium bicarbonate cotransporter, member 8 | 2 | | 587 | hsa-mir-1245a | 9498 | SLC4A8 | solute carrier family 4, sodium bicarbonate cotransporter, member 8 | 2 | | 588 | hsa-mir-3126 | 9498 | SLC4A8 | solute carrier family 4, sodium bicarbonate cotransporter, member 8 | 2 | | 589 | hsa-mir-3129 | 9498 | SLC4A8 | solute carrier family 4, sodium bicarbonate cotransporter, member 8 | 2 | | 590 | hsa-mir-3606 | 9498 | SLC4A8 | solute carrier family 4, sodium bicarbonate cotransporter, member 8 | 2 | | 591 | hsa-mir-4441 | 9498 | SLC4A8 | solute carrier family 4, sodium bicarbonate cotransporter, member 8 | 2 | | 593 | hsa-mir-4636 | 9498 | SLC4A8 | solute carrier family 4, sodium bicarbonate cotransporter, member 8 | 2 | | 594 | hsa-mir-4735 | 9498 | SLC4A8 | solute carrier family 4, sodium bicarbonate cotransporter, member 8 | 2 | | 595 | hsa-mir-4768 | 9498 | SLC4A8 | solute carrier family 4, sodium bicarbonate cotransporter, member 8 | 2 | | 596 | hsa-mir-2467 | 9498 | SLC4A8 | solute carrier family 4, sodium bicarbonate cotransporter, member 8 | 2 | | 597 | hsa-mir-4794 | 9498 | SLC4A8 | solute carrier family 4, sodium bicarbonate cotransporter, member 8 | 2 | | 598 | hsa-let-7c | 9764 | KIAA0513 | KIAA0513 | 2 | | 599 | hsa-mir-99a | 9764 | KIAA0513 | KIAA0513 | 2 | | 601 | hsa-mir-218-1 | 9764 | KIAA0513 | KIAA0513 | 2 | | 603 | hsa-mir-125b-1 | 9764 | KIAA0513 | KIAA0513 | 2 | | 604 | hsa-mir-125b-2 | 9764 | KIAA0513 | KIAA0513 | 2 | | 605 | hsa-mir-561 | 9764 | KIAA0513 | KIAA0513 | 2 | | 607 | hsa-mir-585 | 9764 | KIAA0513 | KIAA0513 | 2 | | 637 | hsa-mir-652 | 10003 | NAALAD2 | N-acetylated alpha-linked acidic dipeptidase 2 | 2 | | 654 | hsa-let-7c | 388394 | RPRML | reprimo-like | 2 | | 655 | hsa-mir-99a | 388394 | RPRML | reprimo-like | 2 | | 657 | hsa-mir-218-1 | 388394 | RPRML | reprimo-like | 2 | | 659 | hsa-mir-125b-2 | 388394 | RPRML | reprimo-like | 2 | | 661 | hsa-mir-561 | 388394 | RPRML | reprimo-like | 2 | | 663 | hsa-mir-585 | 388394 | RPRML | reprimo-like | 2 | | 666 | hsa-mir-620 | 388394 | RPRML | reprimo-like | 2 | | 687 | hsa-mir-561 | 79828 | METTL8 | methyltransferase like 8 | 2 | | 689 | hsa-mir-585 | 79828 | METTL8 | methyltransferase like 8 | 2 | | 705 | hsa-let-7c | 132625 | ZFP42 | zinc finger protein 42 homolog (mouse) | 2 | | 706 | hsa-mir-99a | 132625 | ZFP42 | zinc finger protein 42 homolog (mouse) | 2 | | 708 | hsa-mir-218-1 | 132625 | ZFP42 | zinc finger protein 42 homolog (mouse) | 2 | | 710 | hsa-mir-125b-1 | 132625 | ZFP42 | zinc finger protein 42 homolog (mouse) | 2 | | 711 | hsa-mir-125b-2 | 132625 | ZFP42 | zinc finger protein 42 homolog (mouse) | 2 | | 712 | hsa-mir-561 | 132625 | ZFP42 | zinc finger protein 42 homolog (mouse) | 2 | | 714 | hsa-mir-585 | 132625 | ZFP42 | zinc finger protein 42 homolog (mouse) | 2 | | 739 | hsa-let-7c | 340602 | CXorf67 | chromosome X open reading frame 67 | 2 | | 740 | hsa-mir-99a | 340602 | CXorf67 | chromosome X open reading frame 67 | 2 | | 742 | hsa-mir-218-1 | 340602 | CXorf67 | chromosome X open reading frame 67 | 2 | | 744 | hsa-mir-125b-1 | 340602 | CXorf67 | chromosome X open reading frame 67 | 2 | | 745 | hsa-mir-125b-2 | 340602 | CXorf67 | chromosome X open reading frame 67 | 2 | | 746 | hsa-mir-561 | 340602 | CXorf67 | chromosome X open reading frame 67 | 2 | | 748 | hsa-mir-585 | 340602 | CXorf67 | chromosome X open reading frame 67 | 2 | | 756 | hsa-mir-708 | 340602 | CXorf67 | chromosome X open reading frame 67 | 2 | | 781 | hsa-mir-218-1 | 57180 | ACTR3B | ARP3 actin-related protein 3 homolog B (yeast) | 2 | | 783 | hsa-mir-561 | 57180 | ACTR3B | ARP3 actin-related protein 3 homolog B (yeast) | 2 | | 785 | hsa-mir-585 | 57180 | ACTR3B | ARP3 actin-related protein 3 homolog B (yeast) | 2 | | 788 | hsa-mir-620 | 57180 | ACTR3B | ARP3 actin-related protein 3 homolog B (yeast) | 2 | | 807 | hsa-mir-877 | 83871 | RAB34 | RAB34, member RAS oncogene family | 2 | | 808 | hsa-mir-548s | 83871 | RAB34 | RAB34, member RAS oncogene family | 2 | | 809 | hsa-mir-378e | 83871 | RAB34 | RAB34, member RAS oncogene family | 2 | | 810 | hsa-mir-4517 | 83871 | RAB34 | RAB34, member RAS oncogene family | 2 | | 811 | hsa-mir-4698 | 83871 | RAB34 | RAB34, member RAS oncogene family | 2 | | 812 | hsa-mir-4772 | 83871 | RAB34 | RAB34, member RAS oncogene family | 2 | | 813 | hsa-mir-675 | 83871 | RAB34 | RAB34, member RAS oncogene family | 2 | | 814 | hsa-mir-887 | 83871 | RAB34 | RAB34, member RAS oncogene family | 2 | | 815 | hsa-let-7c | 83746 | L3MBTL2 | l(3)mbt-like 2 (Drosophila) | 2 | | 816 | hsa-mir-99a | 83746 | L3MBTL2 | l(3)mbt-like 2 (Drosophila) | 2 | | 819 | hsa-mir-125b-2 | 83746 | L3MBTL2 | l(3)mbt-like 2 (Drosophila) | 2 | | 821 | hsa-mir-561 | 83746 | L3MBTL2 | l(3)mbt-like 2 (Drosophila) | 2 | | 823 | hsa-mir-585 | 83746 | L3MBTL2 | l(3)mbt-like 2 (Drosophila) | 2 | | 826 | hsa-mir-620 | 83746 | L3MBTL2 | l(3)mbt-like 2 (Drosophila) | 2 | | 842 | hsa-mir-561 | 23219 | FBXO28 | F-box protein 28 | 2 | | 844 | hsa-mir-585 | 23219 | FBXO28 | F-box protein 28 | 2 | | 847 | hsa-mir-620 | 23219 | FBXO28 | F-box protein 28 | 2 | | 868 | hsa-mir-10a | 55787 | TXLNG | taxilin gamma | 2 | | 869 | hsa-mir-218-2 | 55787 | TXLNG | taxilin gamma | 2 | | 872 | hsa-mir-574 | 55787 | TXLNG | taxilin gamma | 2 | | 874 | hsa-mir-603 | 55787 | TXLNG | taxilin gamma | 2 | | 876 | hsa-mir-675 | 55787 | TXLNG | taxilin gamma | 2 | | 877 | hsa-mir-1245a | 55787 | TXLNG | taxilin gamma | 2 | | 878 | hsa-mir-1915 | 55787 | TXLNG | taxilin gamma | 2 | | 879 | hsa-mir-3120 | 55787 | TXLNG | taxilin gamma | 2 | | 880 | hsa-mir-3129 | 55787 | TXLNG | taxilin gamma | 2 | | 881 | hsa-mir-3606 | 55787 | TXLNG | taxilin gamma | 2 | | 882 | hsa-mir-4735 | 55787 | TXLNG | taxilin gamma | 2 | | 883 | hsa-mir-4794 | 55787 | TXLNG | taxilin gamma | 2 | | 884 | hsa-mir-548ao | 55787 | TXLNG | taxilin gamma | 2 | | 887 | hsa-mir-877 | 55787 | TXLNG | taxilin gamma | 2 | | 888 | hsa-mir-1233-1;hsa-mir-1233-2 | 55787 | TXLNG | taxilin gamma | 2 | | 891 | hsa-mir-10a | 84690 | SPATA22 | spermatogenesis associated 22 | 2 | | 893 | hsa-mir-218-2 | 84690 | SPATA22 | spermatogenesis associated 22 | 2 | | 897 | hsa-mir-574 | 84690 | SPATA22 | spermatogenesis associated 22 | 2 | | 899 | hsa-mir-603 | 84690 | SPATA22 | spermatogenesis associated 22 | 2 | | 900 | hsa-mir-604 | 84690 | SPATA22 | spermatogenesis associated 22 | 2 | | 901 | hsa-mir-605 | 84690 | SPATA22 | spermatogenesis associated 22 | 2 | | 902 | hsa-mir-618 | 84690 | SPATA22 | spermatogenesis associated 22 | 2 | | 903 | hsa-mir-620 | 84690 | SPATA22 | spermatogenesis associated 22 | 2 | | 904 | hsa-mir-1271 | 84690 | SPATA22 | spermatogenesis associated 22 | 2 | | 905 | hsa-mir-675 | 84690 | SPATA22 | spermatogenesis associated 22 | 2 | | 907 | hsa-mir-887 | 84690 | SPATA22 | spermatogenesis associated 22 | 2 | | 908 | hsa-mir-938 | 84690 | SPATA22 | spermatogenesis associated 22 | 2 | | 909 | hsa-mir-1245a | 84690 | SPATA22 | spermatogenesis associated 22 | 2 | | 911 | hsa-mir-1469 | 84690 | SPATA22 | spermatogenesis associated 22 | 2 | | 912 | hsa-mir-1915 | 84690 | SPATA22 | spermatogenesis associated 22 | 2 | | 913 | hsa-mir-3120 | 84690 | SPATA22 | spermatogenesis associated 22 | 2 | | 914 | hsa-mir-3126 | 84690 | SPATA22 | spermatogenesis associated 22 | 2 | | 915 | hsa-mir-3129 | 84690 | SPATA22 | spermatogenesis associated 22 | 2 | | 916 | hsa-mir-3139 | 84690 | SPATA22 | spermatogenesis associated 22 | 2 | | 917 | hsa-mir-3170 | 84690 | SPATA22 | spermatogenesis associated 22 | 2 | | 918 | hsa-mir-3606 | 84690 | SPATA22 | spermatogenesis associated 22 | 2 | | 919 | hsa-mir-3650 | 84690 | SPATA22 | spermatogenesis associated 22 | 2 | | 920 | hsa-mir-4441 | 84690 | SPATA22 | spermatogenesis associated 22 | 2 | | 921 | hsa-mir-4519 | 84690 | SPATA22 | spermatogenesis associated 22 | 2 | | 922 | hsa-mir-4636 | 84690 | SPATA22 | spermatogenesis associated 22 | 2 | | 923 | hsa-mir-4731 | 84690 | SPATA22 | spermatogenesis associated 22 | 2 | | 924 | hsa-mir-4735 | 84690 | SPATA22 | spermatogenesis associated 22 | 2 | | 925 | hsa-mir-4768 | 84690 | SPATA22 | spermatogenesis associated 22 | 2 | | 926 | hsa-mir-2467 | 84690 | SPATA22 | spermatogenesis associated 22 | 2 | | 927 | hsa-mir-4794 | 84690 | SPATA22 | spermatogenesis associated 22 | 2 | | 928 | hsa-mir-548ao | 84690 | SPATA22 | spermatogenesis associated 22 | 2 | | 929 | hsa-mir-5579 | 84690 | SPATA22 | spermatogenesis associated 22 | 2 | | 931 | hsa-mir-591 | 84690 | SPATA22 | spermatogenesis associated 22 | 2 | | 932 | hsa-mir-617 | 84690 | SPATA22 | spermatogenesis associated 22 | 2 | | 933 | hsa-mir-640 | 84690 | SPATA22 | spermatogenesis associated 22 | 2 | | 934 | hsa-mir-648 | 84690 | SPATA22 | spermatogenesis associated 22 | 2 | | 935 | hsa-mir-877 | 84690 | SPATA22 | spermatogenesis associated 22 | 2 | | 936 | hsa-mir-1233-1;hsa-mir-1233-2 | 84690 | SPATA22 | spermatogenesis associated 22 | 2 | | 937 | hsa-let-7c | 140688 | C20orf112 | chromosome 20 open reading frame 112 | 2 | | 938 | hsa-mir-99a | 140688 | C20orf112 | chromosome 20 open reading frame 112 | 2 | | 940 | hsa-mir-218-1 | 140688 | C20orf112 | chromosome 20 open reading frame 112 | 2 | | 942 | hsa-mir-125b-1 | 140688 | C20orf112 | chromosome 20 open reading frame 112 | 2 | | 943 | hsa-mir-125b-2 | 140688 | C20orf112 | chromosome 20 open reading frame 112 | 2 | | 945 | hsa-mir-561 | 140688 | C20orf112 | chromosome 20 open reading frame 112 | 2 | | 947 | hsa-mir-585 | 140688 | C20orf112 | chromosome 20 open reading frame 112 | 2 | | 956 | hsa-mir-708 | 140688 | C20orf112 | chromosome 20 open reading frame 112 | 2 | | 983 | hsa-mir-181a-2 | 112770 | C1orf85 | chromosome 1 open reading frame 85 | 2 | | 984 | hsa-mir-181b-2 | 112770 | C1orf85 | chromosome 1 open reading frame 85 | 2 | | 985 | hsa-mir-378a | 112770 | C1orf85 | chromosome 1 open reading frame 85 | 2 | | 1003 | hsa-let-7c | 126661 | CCDC163P | coiled-coil domain containing 163, pseudogene | 2 | | 1004 | hsa-mir-99a | 126661 | CCDC163P | coiled-coil domain containing 163, pseudogene | 2 | | 1006 | hsa-mir-218-1 | 126661 | CCDC163P | coiled-coil domain containing 163, pseudogene | 2 | | 1008 | hsa-mir-125b-1 | 126661 | CCDC163P | coiled-coil domain containing 163, pseudogene | 2 | | 1009 | hsa-mir-125b-2 | 126661 | CCDC163P | coiled-coil domain containing 163, pseudogene | 2 | | 1010 | hsa-mir-561 | 126661 | CCDC163P | coiled-coil domain containing 163, pseudogene | 2 | | 1012 | hsa-mir-585 | 126661 | CCDC163P | coiled-coil domain containing 163, pseudogene | 2 | | 1042 | hsa-mir-643 | 100130958 | SYCE1L | synaptonemal complex central element protein 1-like | 2 | | 1060 | hsa-mir-218-1 | 100505876 | LOC100505876 | uncharacterized LOC100505876 | 2 | | 1062 | hsa-mir-125b-1 | 100505876 | LOC100505876 | uncharacterized LOC100505876 | 2 | | 1063 | hsa-mir-561 | 100505876 | LOC100505876 | uncharacterized LOC100505876 | 2 | | 1065 | hsa-mir-585 | 100505876 | LOC100505876 | uncharacterized LOC100505876 | 2 | | 1068 | hsa-mir-620 | 100505876 | LOC100505876 | uncharacterized LOC100505876 | 2 | | 1088 | hsa-mir-218-1 | 619455 | SPANXA2-OT1 | SPANXA2 overlapping transcript 1 (non-protein coding) | 2 | | 1091 | hsa-mir-561 | 619455 | SPANXA2-OT1 | SPANXA2 overlapping transcript 1 (non-protein coding) | 2 | | 1093 | hsa-mir-585 | 619455 | SPANXA2-OT1 | SPANXA2 overlapping transcript 1 (non-protein coding) | 2 | | 1101 | hsa-mir-708 | 619455 | SPANXA2-OT1 | SPANXA2 overlapping transcript 1 (non-protein coding) | 2 | | 1129 | hsa-let-7c | 23 | ABCF1 | ATP-binding cassette, sub-family F (GCN20), member 1 | 2 | | 1130 | hsa-mir-99a | 23 | ABCF1 | ATP-binding cassette, sub-family F (GCN20), member 1 | 2 | | 1132 | hsa-mir-218-1 | 23 | ABCF1 | ATP-binding cassette, sub-family F (GCN20), member 1 | 2 | | 1134 | hsa-mir-125b-1 | 23 | ABCF1 | ATP-binding cassette, sub-family F (GCN20), member 1 | 2 | | 1135 | hsa-mir-125b-2 | 23 | ABCF1 | ATP-binding cassette, sub-family F (GCN20), member 1 | 2 | | 1136 | hsa-mir-561 | 23 | ABCF1 | ATP-binding cassette, sub-family F (GCN20), member 1 | 2 | | 1138 | hsa-mir-585 | 23 | ABCF1 | ATP-binding cassette, sub-family F (GCN20), member 1 | 2 | | 1168 | hsa-mir-455 | 11143 | KAT7 | K(lysine) acetyltransferase 7 | 2 | | 1170 | hsa-mir-585 | 11143 | KAT7 | K(lysine) acetyltransferase 7 | 2 | | 1172 | hsa-mir-620 | 11143 | KAT7 | K(lysine) acetyltransferase 7 | 2 | | 1185 | hsa-mir-181a-2 | 11014 | KDELR2 | KDEL (Lys-Asp-Glu-Leu) endoplasmic reticulum protein retention receptor 2 | 2 | | 1186 | hsa-mir-181b-2 | 11014 | KDELR2 | KDEL (Lys-Asp-Glu-Leu) endoplasmic reticulum protein retention receptor 2 | 2 | | 1190 | hsa-mir-602 | 11014 | KDELR2 | KDEL (Lys-Asp-Glu-Leu) endoplasmic reticulum protein retention receptor 2 | 2 | | 1214 | hsa-mir-218-1 | 5048 | PAFAH1B1 | platelet-activating factor acetylhydrolase 1b, regulatory subunit 1 (45kDa) | 2 | | 1216 | hsa-mir-455 | 5048 | PAFAH1B1 | platelet-activating factor acetylhydrolase 1b, regulatory subunit 1 (45kDa) | 2 | | 1217 | hsa-mir-561 | 5048 | PAFAH1B1 | platelet-activating factor acetylhydrolase 1b, regulatory subunit 1 (45kDa) | 2 | | 1219 | hsa-mir-585 | 5048 | PAFAH1B1 | platelet-activating factor acetylhydrolase 1b, regulatory subunit 1 (45kDa) | 2 | | 1222 | hsa-mir-620 | 5048 | PAFAH1B1 | platelet-activating factor acetylhydrolase 1b, regulatory subunit 1 (45kDa) | 2 | | 1235 | hsa-mir-218-2 | 55905 | RNF114 | ring finger protein 114 | 2 | | 1237 | hsa-mir-574 | 55905 | RNF114 | ring finger protein 114 | 2 | | 1239 | hsa-mir-603 | 55905 | RNF114 | ring finger protein 114 | 2 | | 1240 | hsa-mir-605 | 55905 | RNF114 | ring finger protein 114 | 2 | | 1242 | hsa-mir-1271 | 55905 | RNF114 | ring finger protein 114 | 2 | | 1243 | hsa-mir-675 | 55905 | RNF114 | ring finger protein 114 | 2 | | 1244 | hsa-mir-663b | 55905 | RNF114 | ring finger protein 114 | 2 | | 1245 | hsa-mir-1245a | 55905 | RNF114 | ring finger protein 114 | 2 | | 1247 | hsa-mir-3120 | 55905 | RNF114 | ring finger protein 114 | 2 | | 1248 | hsa-mir-3126 | 55905 | RNF114 | ring finger protein 114 | 2 | | 1249 | hsa-mir-3129 | 55905 | RNF114 | ring finger protein 114 | 2 | | 1250 | hsa-mir-3606 | 55905 | RNF114 | ring finger protein 114 | 2 | | 1251 | hsa-mir-4441 | 55905 | RNF114 | ring finger protein 114 | 2 | | 1252 | hsa-mir-4794 | 55905 | RNF114 | ring finger protein 114 | 2 | | 1253 | hsa-mir-548ao | 55905 | RNF114 | ring finger protein 114 | 2 | | 1255 | hsa-mir-491 | 55905 | RNF114 | ring finger protein 114 | 2 | | 1257 | hsa-mir-554 | 55905 | RNF114 | ring finger protein 114 | 2 | | 1258 | hsa-mir-591 | 55905 | RNF114 | ring finger protein 114 | 2 | | 1259 | hsa-mir-636 | 55905 | RNF114 | ring finger protein 114 | 2 | | 1260 | hsa-mir-648 | 55905 | RNF114 | ring finger protein 114 | 2 | | 1261 | hsa-mir-877 | 55905 | RNF114 | ring finger protein 114 | 2 | | 1262 | hsa-mir-1233-1;hsa-mir-1233-2 | 55905 | RNF114 | ring finger protein 114 | 2 | | 1263 | hsa-let-7c | 5496 | PPM1G | protein phosphatase, Mg2+/Mn2+ dependent, 1G | 2 | | 1264 | hsa-mir-99a | 5496 | PPM1G | protein phosphatase, Mg2+/Mn2+ dependent, 1G | 2 | | 1266 | hsa-mir-125b-2 | 5496 | PPM1G | protein phosphatase, Mg2+/Mn2+ dependent, 1G | 2 | | 1268 | hsa-mir-561 | 5496 | PPM1G | protein phosphatase, Mg2+/Mn2+ dependent, 1G | 2 | | 1270 | hsa-mir-585 | 5496 | PPM1G | protein phosphatase, Mg2+/Mn2+ dependent, 1G | 2 | | 1273 | hsa-mir-620 | 5496 | PPM1G | protein phosphatase, Mg2+/Mn2+ dependent, 1G | 2 | | 1298 | hsa-mir-181a-2 | 3281 | HSBP1 | heat shock factor binding protein 1 | 2 | | 1299 | hsa-mir-181b-2 | 3281 | HSBP1 | heat shock factor binding protein 1 | 2 | | 1300 | hsa-mir-505 | 3281 | HSBP1 | heat shock factor binding protein 1 | 2 | | 1325 | hsa-mir-25 | 6160 | RPL31 | ribosomal protein L31 | 2 | | 1326 | hsa-mir-93 | 6160 | RPL31 | ribosomal protein L31 | 2 | | 1327 | hsa-mir-181a-2 | 6160 | RPL31 | ribosomal protein L31 | 2 | | 1328 | hsa-mir-181b-2 | 6160 | RPL31 | ribosomal protein L31 | 2 | | 1329 | hsa-mir-106b | 6160 | RPL31 | ribosomal protein L31 | 2 | | 1330 | hsa-mir-505 | 6160 | RPL31 | ribosomal protein L31 | 2 | | 1334 | hsa-mir-643 | 6160 | RPL31 | ribosomal protein L31 | 2 | | 1345 | hsa-let-7c | 10197 | PSME3 | proteasome (prosome, macropain) activator subunit 3 (PA28 gamma; Ki) | 2 | | 1346 | hsa-mir-99a | 10197 | PSME3 | proteasome (prosome, macropain) activator subunit 3 (PA28 gamma; Ki) | 2 | | 1348 | hsa-mir-218-1 | 10197 | PSME3 | proteasome (prosome, macropain) activator subunit 3 (PA28 gamma; Ki) | 2 | | 1350 | hsa-mir-125b-1 | 10197 | PSME3 | proteasome (prosome, macropain) activator subunit 3 (PA28 gamma; Ki) | 2 | | 1351 | hsa-mir-125b-2 | 10197 | PSME3 | proteasome (prosome, macropain) activator subunit 3 (PA28 gamma; Ki) | 2 | | 1353 | hsa-mir-561 | 10197 | PSME3 | proteasome (prosome, macropain) activator subunit 3 (PA28 gamma; Ki) | 2 | | 1355 | hsa-mir-585 | 10197 | PSME3 | proteasome (prosome, macropain) activator subunit 3 (PA28 gamma; Ki) | 2 | | 1360 | hsa-mir-620 | 10197 | PSME3 | proteasome (prosome, macropain) activator subunit 3 (PA28 gamma; Ki) | 2 | | 1380 | hsa-mir-378a | 10970 | CKAP4 | cytoskeleton-associated protein 4 | 2 | | 1406 | hsa-mir-455 | 2040 | STOM | stomatin | 2 | | 1407 | hsa-mir-561 | 2040 | STOM | stomatin | 2 | | 1409 | hsa-mir-585 | 2040 | STOM | stomatin | 2 | | 1410 | hsa-mir-620 | 2040 | STOM | stomatin | 2 | | 1412 | hsa-mir-708 | 2040 | STOM | stomatin | 2 | | 1426 | hsa-mir-218-1 | 4836 | NMT1 | N-myristoyltransferase 1 | 2 | | 1428 | hsa-mir-125b-1 | 4836 | NMT1 | N-myristoyltransferase 1 | 2 | | 1429 | hsa-mir-455 | 4836 | NMT1 | N-myristoyltransferase 1 | 2 | | 1430 | hsa-mir-561 | 4836 | NMT1 | N-myristoyltransferase 1 | 2 | | 1432 | hsa-mir-585 | 4836 | NMT1 | N-myristoyltransferase 1 | 2 | | 1438 | hsa-mir-708 | 4836 | NMT1 | N-myristoyltransferase 1 | 2 | | 1458 | hsa-mir-505 | 633 | BGN | biglycan | 2 | | 1461 | hsa-mir-643 | 633 | BGN | biglycan | 2 | | 1480 | hsa-let-7c | 23608 | MKRN1 | makorin ring finger protein 1 | 2 | | 1481 | hsa-mir-99a | 23608 | MKRN1 | makorin ring finger protein 1 | 2 | | 1483 | hsa-mir-218-1 | 23608 | MKRN1 | makorin ring finger protein 1 | 2 | | 1486 | hsa-mir-125b-2 | 23608 | MKRN1 | makorin ring finger protein 1 | 2 | | 1487 | hsa-mir-455 | 23608 | MKRN1 | makorin ring finger protein 1 | 2 | | 1488 | hsa-mir-561 | 23608 | MKRN1 | makorin ring finger protein 1 | 2 | | 1490 | hsa-mir-585 | 23608 | MKRN1 | makorin ring finger protein 1 | 2 | | 1495 | hsa-mir-620 | 23608 | MKRN1 | makorin ring finger protein 1 | 2 | | 1498 | hsa-mir-708 | 23608 | MKRN1 | makorin ring finger protein 1 | 2 | | 1526 | hsa-let-7c | 908 | CCT6A | chaperonin containing TCP1, subunit 6A (zeta 1) | 2 | | 1527 | hsa-mir-99a | 908 | CCT6A | chaperonin containing TCP1, subunit 6A (zeta 1) | 2 | | 1529 | hsa-mir-218-1 | 908 | CCT6A | chaperonin containing TCP1, subunit 6A (zeta 1) | 2 | | 1531 | hsa-mir-125b-1 | 908 | CCT6A | chaperonin containing TCP1, subunit 6A (zeta 1) | 2 | | 1532 | hsa-mir-125b-2 | 908 | CCT6A | chaperonin containing TCP1, subunit 6A (zeta 1) | 2 | | 1533 | hsa-mir-455 | 908 | CCT6A | chaperonin containing TCP1, subunit 6A (zeta 1) | 2 | | 1534 | hsa-mir-561 | 908 | CCT6A | chaperonin containing TCP1, subunit 6A (zeta 1) | 2 | | 1536 | hsa-mir-585 | 908 | CCT6A | chaperonin containing TCP1, subunit 6A (zeta 1) | 2 | | 1540 | hsa-mir-620 | 908 | CCT6A | chaperonin containing TCP1, subunit 6A (zeta 1) | 2 | | 1543 | hsa-mir-708 | 908 | CCT6A | chaperonin containing TCP1, subunit 6A (zeta 1) | 2 | | 1571 | hsa-mir-25 | 2114 | ETS2 | v-ets erythroblastosis virus E26 oncogene homolog 2 (avian) | 2 | | 1572 | hsa-mir-93 | 2114 | ETS2 | v-ets erythroblastosis virus E26 oncogene homolog 2 (avian) | 2 | | 1573 | hsa-mir-181a-2 | 2114 | ETS2 | v-ets erythroblastosis virus E26 oncogene homolog 2 (avian) | 2 | | 1574 | hsa-mir-181b-2 | 2114 | ETS2 | v-ets erythroblastosis virus E26 oncogene homolog 2 (avian) | 2 | | 1575 | hsa-mir-106b | 2114 | ETS2 | v-ets erythroblastosis virus E26 oncogene homolog 2 (avian) | 2 | | 1577 | hsa-mir-602 | 2114 | ETS2 | v-ets erythroblastosis virus E26 oncogene homolog 2 (avian) | 2 | | 1600 | hsa-mir-218-1 | 55041 | PLEKHB2 | pleckstrin homology domain containing, family B (evectins) member 2 | 2 | | 1603 | hsa-mir-561 | 55041 | PLEKHB2 | pleckstrin homology domain containing, family B (evectins) member 2 | 2 | | 1605 | hsa-mir-585 | 55041 | PLEKHB2 | pleckstrin homology domain containing, family B (evectins) member 2 | 2 | | 1607 | hsa-mir-620 | 55041 | PLEKHB2 | pleckstrin homology domain containing, family B (evectins) member 2 | 2 | | 1628 | hsa-let-7c | 9416 | DDX23 | DEAD (Asp-Glu-Ala-Asp) box polypeptide 23 | 2 | | 1629 | hsa-mir-99a | 9416 | DDX23 | DEAD (Asp-Glu-Ala-Asp) box polypeptide 23 | 2 | | 1631 | hsa-mir-125b-2 | 9416 | DDX23 | DEAD (Asp-Glu-Ala-Asp) box polypeptide 23 | 2 | | 1632 | hsa-mir-561 | 9416 | DDX23 | DEAD (Asp-Glu-Ala-Asp) box polypeptide 23 | 2 | | 1634 | hsa-mir-585 | 9416 | DDX23 | DEAD (Asp-Glu-Ala-Asp) box polypeptide 23 | 2 | | 1655 | hsa-mir-10a | 4141 | MARS | methionyl-tRNA synthetase | 2 | | 1657 | hsa-mir-218-2 | 4141 | MARS | methionyl-tRNA synthetase | 2 | | 1662 | hsa-mir-574 | 4141 | MARS | methionyl-tRNA synthetase | 2 | | 1664 | hsa-mir-603 | 4141 | MARS | methionyl-tRNA synthetase | 2 | | 1665 | hsa-mir-604 | 4141 | MARS | methionyl-tRNA synthetase | 2 | | 1666 | hsa-mir-605 | 4141 | MARS | methionyl-tRNA synthetase | 2 | | 1667 | hsa-mir-613 | 4141 | MARS | methionyl-tRNA synthetase | 2 | | 1668 | hsa-mir-618 | 4141 | MARS | methionyl-tRNA synthetase | 2 | | 1670 | hsa-mir-1271 | 4141 | MARS | methionyl-tRNA synthetase | 2 | | 1671 | hsa-mir-675 | 4141 | MARS | methionyl-tRNA synthetase | 2 | | 1672 | hsa-mir-887 | 4141 | MARS | methionyl-tRNA synthetase | 2 | | 1673 | hsa-mir-938 | 4141 | MARS | methionyl-tRNA synthetase | 2 | | 1674 | hsa-mir-663b | 4141 | MARS | methionyl-tRNA synthetase | 2 | | 1675 | hsa-mir-1245a | 4141 | MARS | methionyl-tRNA synthetase | 2 | | 1676 | hsa-mir-1469 | 4141 | MARS | methionyl-tRNA synthetase | 2 | | 1677 | hsa-mir-3120 | 4141 | MARS | methionyl-tRNA synthetase | 2 | | 1678 | hsa-mir-3126 | 4141 | MARS | methionyl-tRNA synthetase | 2 | | 1679 | hsa-mir-3129 | 4141 | MARS | methionyl-tRNA synthetase | 2 | | 1680 | hsa-mir-3139 | 4141 | MARS | methionyl-tRNA synthetase | 2 | | 1681 | hsa-mir-3606 | 4141 | MARS | methionyl-tRNA synthetase | 2 | | 1682 | hsa-mir-3650 | 4141 | MARS | methionyl-tRNA synthetase | 2 | | 1683 | hsa-mir-4441 | 4141 | MARS | methionyl-tRNA synthetase | 2 | | 1684 | hsa-mir-4731 | 4141 | MARS | methionyl-tRNA synthetase | 2 | | 1685 | hsa-mir-4735 | 4141 | MARS | methionyl-tRNA synthetase | 2 | | 1686 | hsa-mir-4768 | 4141 | MARS | methionyl-tRNA synthetase | 2 | | 1687 | hsa-mir-2467 | 4141 | MARS | methionyl-tRNA synthetase | 2 | | 1688 | hsa-mir-4794 | 4141 | MARS | methionyl-tRNA synthetase | 2 | | 1689 | hsa-mir-548ao | 4141 | MARS | methionyl-tRNA synthetase | 2 | | 1690 | hsa-mir-591 | 4141 | MARS | methionyl-tRNA synthetase | 2 | | 1691 | hsa-mir-617 | 4141 | MARS | methionyl-tRNA synthetase | 2 | | 1692 | hsa-mir-636 | 4141 | MARS | methionyl-tRNA synthetase | 2 | | 1693 | hsa-mir-640 | 4141 | MARS | methionyl-tRNA synthetase | 2 | | 1695 | hsa-mir-877 | 4141 | MARS | methionyl-tRNA synthetase | 2 | | 1699 | hsa-mir-585 | 7874 | USP7 | ubiquitin specific peptidase 7 (herpes virus-associated) | 2 | | 1716 | hsa-mir-25 | 3912 | LAMB1 | laminin, beta 1 | 2 | | 1717 | hsa-mir-93 | 3912 | LAMB1 | laminin, beta 1 | 2 | | 1720 | hsa-mir-106b | 3912 | LAMB1 | laminin, beta 1 | 2 | | 1723 | hsa-mir-550a-1 | 3912 | LAMB1 | laminin, beta 1 | 2 | | 1725 | hsa-mir-643 | 3912 | LAMB1 | laminin, beta 1 | 2 | | 1727 | hsa-mir-877 | 3912 | LAMB1 | laminin, beta 1 | 2 | | 1729 | hsa-mir-4467 | 3912 | LAMB1 | laminin, beta 1 | 2 | | 1730 | hsa-mir-4517 | 3912 | LAMB1 | laminin, beta 1 | 2 | | 1731 | hsa-mir-4534 | 3912 | LAMB1 | laminin, beta 1 | 2 | | 1733 | hsa-mir-4668 | 3912 | LAMB1 | laminin, beta 1 | 2 | | 1734 | hsa-mir-4698 | 3912 | LAMB1 | laminin, beta 1 | 2 | | 1737 | hsa-mir-5090 | 3912 | LAMB1 | laminin, beta 1 | 2 | | 1739 | hsa-mir-604 | 3912 | LAMB1 | laminin, beta 1 | 2 | | 1740 | hsa-mir-218-2 | 3912 | LAMB1 | laminin, beta 1 | 2 | | 1741 | hsa-mir-574 | 3912 | LAMB1 | laminin, beta 1 | 2 | | 1742 | hsa-mir-887 | 3912 | LAMB1 | laminin, beta 1 | 2 | | 1743 | hsa-mir-938 | 3912 | LAMB1 | laminin, beta 1 | 2 | | 1746 | hsa-mir-561 | 9868 | TOMM70A | translocase of outer mitochondrial membrane 70 homolog A (S. cerevisiae) | 2 | | 1748 | hsa-mir-585 | 9868 | TOMM70A | translocase of outer mitochondrial membrane 70 homolog A (S. cerevisiae) | 2 | | 1749 | hsa-mir-620 | 9868 | TOMM70A | translocase of outer mitochondrial membrane 70 homolog A (S. cerevisiae) | 2 | | 1762 | hsa-mir-25 | 800 | CALD1 | caldesmon 1 | 2 | | 1763 | hsa-mir-93 | 800 | CALD1 | caldesmon 1 | 2 | | 1764 | hsa-mir-181a-2 | 800 | CALD1 | caldesmon 1 | 2 | | 1765 | hsa-mir-181b-2 | 800 | CALD1 | caldesmon 1 | 2 | | 1766 | hsa-mir-106b | 800 | CALD1 | caldesmon 1 | 2 | | 1770 | hsa-mir-643 | 800 | CALD1 | caldesmon 1 | 2 | | 1771 | hsa-mir-652 | 800 | CALD1 | caldesmon 1 | 2 | | 1787 | hsa-let-7c | 3638 | INSIG1 | insulin induced gene 1 | 2 | | 1788 | hsa-mir-99a | 3638 | INSIG1 | insulin induced gene 1 | 2 | | 1790 | hsa-mir-218-1 | 3638 | INSIG1 | insulin induced gene 1 | 2 | | 1793 | hsa-mir-125b-2 | 3638 | INSIG1 | insulin induced gene 1 | 2 | | 1794 | hsa-mir-561 | 3638 | INSIG1 | insulin induced gene 1 | 2 | | 1796 | hsa-mir-585 | 3638 | INSIG1 | insulin induced gene 1 | 2 | | 1819 | hsa-mir-561 | 11252 | PACSIN2 | protein kinase C and casein kinase substrate in neurons 2 | 2 | | 1821 | hsa-mir-585 | 11252 | PACSIN2 | protein kinase C and casein kinase substrate in neurons 2 | 2 | | 1864 | hsa-mir-585 | 9097 | USP14 | ubiquitin specific peptidase 14 (tRNA-guanine transglycosylase) | 2 | | 1865 | hsa-mir-620 | 9097 | USP14 | ubiquitin specific peptidase 14 (tRNA-guanine transglycosylase) | 2 | | 1875 | hsa-let-7c | 8165 | AKAP1 | A kinase (PRKA) anchor protein 1 | 2 | | 1877 | hsa-mir-10a | 8165 | AKAP1 | A kinase (PRKA) anchor protein 1 | 2 | | 1878 | hsa-mir-218-1 | 8165 | AKAP1 | A kinase (PRKA) anchor protein 1 | 2 | | 1879 | hsa-mir-218-2 | 8165 | AKAP1 | A kinase (PRKA) anchor protein 1 | 2 | | 1880 | hsa-mir-125b-1 | 8165 | AKAP1 | A kinase (PRKA) anchor protein 1 | 2 | | 1882 | hsa-mir-455 | 8165 | AKAP1 | A kinase (PRKA) anchor protein 1 | 2 | | 1884 | hsa-mir-574 | 8165 | AKAP1 | A kinase (PRKA) anchor protein 1 | 2 | | 1886 | hsa-mir-603 | 8165 | AKAP1 | A kinase (PRKA) anchor protein 1 | 2 | | 1893 | hsa-mir-675 | 8165 | AKAP1 | A kinase (PRKA) anchor protein 1 | 2 | | 1894 | hsa-mir-708 | 8165 | AKAP1 | A kinase (PRKA) anchor protein 1 | 2 | | 1897 | hsa-mir-1245a | 8165 | AKAP1 | A kinase (PRKA) anchor protein 1 | 2 | | 1899 | hsa-mir-3120 | 8165 | AKAP1 | A kinase (PRKA) anchor protein 1 | 2 | | 1901 | hsa-mir-3129 | 8165 | AKAP1 | A kinase (PRKA) anchor protein 1 | 2 | | 1904 | hsa-mir-3606 | 8165 | AKAP1 | A kinase (PRKA) anchor protein 1 | 2 | | 1906 | hsa-mir-4441 | 8165 | AKAP1 | A kinase (PRKA) anchor protein 1 | 2 | | 1910 | hsa-mir-4735 | 8165 | AKAP1 | A kinase (PRKA) anchor protein 1 | 2 | | 1913 | hsa-mir-4794 | 8165 | AKAP1 | A kinase (PRKA) anchor protein 1 | 2 | | 1914 | hsa-mir-548ao | 8165 | AKAP1 | A kinase (PRKA) anchor protein 1 | 2 | | 1919 | hsa-mir-636 | 8165 | AKAP1 | A kinase (PRKA) anchor protein 1 | 2 | | 1921 | hsa-mir-877 | 8165 | AKAP1 | A kinase (PRKA) anchor protein 1 | 2 | | 1927 | hsa-mir-218-1 | 10797 | MTHFD2 | methylenetetrahydrofolate dehydrogenase (NADP+ dependent) 2, methenyltetrahydrofolate cyclohydrolase | 2 | | 1929 | hsa-mir-125b-1 | 10797 | MTHFD2 | methylenetetrahydrofolate dehydrogenase (NADP+ dependent) 2, methenyltetrahydrofolate cyclohydrolase | 2 | | 1930 | hsa-mir-455 | 10797 | MTHFD2 | methylenetetrahydrofolate dehydrogenase (NADP+ dependent) 2, methenyltetrahydrofolate cyclohydrolase | 2 | | 1931 | hsa-mir-561 | 10797 | MTHFD2 | methylenetetrahydrofolate dehydrogenase (NADP+ dependent) 2, methenyltetrahydrofolate cyclohydrolase | 2 | | 1933 | hsa-mir-585 | 10797 | MTHFD2 | methylenetetrahydrofolate dehydrogenase (NADP+ dependent) 2, methenyltetrahydrofolate cyclohydrolase | 2 | | 1938 | hsa-mir-620 | 10797 | MTHFD2 | methylenetetrahydrofolate dehydrogenase (NADP+ dependent) 2, methenyltetrahydrofolate cyclohydrolase | 2 | | 1941 | hsa-mir-708 | 10797 | MTHFD2 | methylenetetrahydrofolate dehydrogenase (NADP+ dependent) 2, methenyltetrahydrofolate cyclohydrolase | 2 | | 1971 | hsa-mir-25 | 6035 | RNASE1 | ribonuclease, RNase A family, 1 (pancreatic) | 2 | | 1972 | hsa-mir-93 | 6035 | RNASE1 | ribonuclease, RNase A family, 1 (pancreatic) | 2 | | 1974 | hsa-mir-181a-2 | 6035 | RNASE1 | ribonuclease, RNase A family, 1 (pancreatic) | 2 | | 1975 | hsa-mir-181b-2 | 6035 | RNASE1 | ribonuclease, RNase A family, 1 (pancreatic) | 2 | | 1976 | hsa-mir-106b | 6035 | RNASE1 | ribonuclease, RNase A family, 1 (pancreatic) | 2 | | 1980 | hsa-mir-629 | 6035 | RNASE1 | ribonuclease, RNase A family, 1 (pancreatic) | 2 | | 1983 | hsa-mir-652 | 6035 | RNASE1 | ribonuclease, RNase A family, 1 (pancreatic) | 2 | | 2002 | hsa-let-7c | 103 | ADAR | adenosine deaminase, RNA-specific | 2 | | 2003 | hsa-mir-99a | 103 | ADAR | adenosine deaminase, RNA-specific | 2 | | 2005 | hsa-mir-125b-2 | 103 | ADAR | adenosine deaminase, RNA-specific | 2 | | 2006 | hsa-mir-561 | 103 | ADAR | adenosine deaminase, RNA-specific | 2 | | 2008 | hsa-mir-585 | 103 | ADAR | adenosine deaminase, RNA-specific | 2 | | 2032 | hsa-let-7c | 2030 | SLC29A1 | solute carrier family 29 (nucleoside transporters), member 1 | 2 | | 2033 | hsa-mir-99a | 2030 | SLC29A1 | solute carrier family 29 (nucleoside transporters), member 1 | 2 | | 2035 | hsa-mir-218-1 | 2030 | SLC29A1 | solute carrier family 29 (nucleoside transporters), member 1 | 2 | | 2037 | hsa-mir-125b-2 | 2030 | SLC29A1 | solute carrier family 29 (nucleoside transporters), member 1 | 2 | | 2038 | hsa-mir-561 | 2030 | SLC29A1 | solute carrier family 29 (nucleoside transporters), member 1 | 2 | | 2040 | hsa-mir-585 | 2030 | SLC29A1 | solute carrier family 29 (nucleoside transporters), member 1 | 2 | | 2043 | hsa-mir-620 | 2030 | SLC29A1 | solute carrier family 29 (nucleoside transporters), member 1 | 2 | | 2064 | hsa-mir-25 | 10276 | NET1 | neuroepithelial cell transforming 1 | 2 | | 2065 | hsa-mir-93 | 10276 | NET1 | neuroepithelial cell transforming 1 | 2 | | 2067 | hsa-mir-181a-2 | 10276 | NET1 | neuroepithelial cell transforming 1 | 2 | | 2068 | hsa-mir-181b-2 | 10276 | NET1 | neuroepithelial cell transforming 1 | 2 | | 2069 | hsa-mir-106b | 10276 | NET1 | neuroepithelial cell transforming 1 | 2 | | 2070 | hsa-mir-378a | 10276 | NET1 | neuroepithelial cell transforming 1 | 2 | | 2071 | hsa-mir-505 | 10276 | NET1 | neuroepithelial cell transforming 1 | 2 | | 2073 | hsa-mir-602 | 10276 | NET1 | neuroepithelial cell transforming 1 | 2 | | 2076 | hsa-mir-652 | 10276 | NET1 | neuroepithelial cell transforming 1 | 2 | | 2115 | hsa-mir-554 | 1634 | DCN | decorin | 2 | | 2121 | hsa-mir-644a | 1634 | DCN | decorin | 2 | | 2123 | hsa-mir-548n | 1634 | DCN | decorin | 2 | | 2124 | hsa-mir-23c | 1634 | DCN | decorin | 2 | | 2140 | hsa-mir-675 | 1634 | DCN | decorin | 2 | | 2147 | hsa-mir-455 | 10549 | PRDX4 | peroxiredoxin 4 | 2 | | 2148 | hsa-mir-561 | 10549 | PRDX4 | peroxiredoxin 4 | 2 | | 2150 | hsa-mir-585 | 10549 | PRDX4 | peroxiredoxin 4 | 2 | | 2153 | hsa-mir-620 | 10549 | PRDX4 | peroxiredoxin 4 | 2 | | 2174 | hsa-let-7c | 10095 | ARPC1B | actin related protein 2/3 complex, subunit 1B, 41kDa | 2 | | 2175 | hsa-mir-99a | 10095 | ARPC1B | actin related protein 2/3 complex, subunit 1B, 41kDa | 2 | | 2177 | hsa-mir-125b-2 | 10095 | ARPC1B | actin related protein 2/3 complex, subunit 1B, 41kDa | 2 | | 2178 | hsa-mir-455 | 10095 | ARPC1B | actin related protein 2/3 complex, subunit 1B, 41kDa | 2 | | 2179 | hsa-mir-561 | 10095 | ARPC1B | actin related protein 2/3 complex, subunit 1B, 41kDa | 2 | | 2180 | hsa-mir-585 | 10095 | ARPC1B | actin related protein 2/3 complex, subunit 1B, 41kDa | 2 | | 2185 | hsa-mir-708 | 10095 | ARPC1B | actin related protein 2/3 complex, subunit 1B, 41kDa | 2 | | 2215 | hsa-mir-3679 | 1956 | EGFR | epidermal growth factor receptor | 2 | | 2217 | hsa-mir-548o-2 | 1956 | EGFR | epidermal growth factor receptor | 2 | | 2219 | hsa-mir-4498 | 1956 | EGFR | epidermal growth factor receptor | 2 | | 2225 | hsa-mir-4775 | 1956 | EGFR | epidermal growth factor receptor | 2 | | 2228 | hsa-mir-675 | 1956 | EGFR | epidermal growth factor receptor | 2 | | 2234 | hsa-mir-652 | 1956 | EGFR | epidermal growth factor receptor | 2 | | 2239 | hsa-mir-218-1 | 1389 | CREBL2 | cAMP responsive element binding protein-like 2 | 2 | | 2241 | hsa-mir-455 | 1389 | CREBL2 | cAMP responsive element binding protein-like 2 | 2 | | 2242 | hsa-mir-561 | 1389 | CREBL2 | cAMP responsive element binding protein-like 2 | 2 | | 2244 | hsa-mir-585 | 1389 | CREBL2 | cAMP responsive element binding protein-like 2 | 2 | | 2247 | hsa-mir-620 | 1389 | CREBL2 | cAMP responsive element binding protein-like 2 | 2 | | 2260 | hsa-mir-181a-2 | 2131 | EXT1 | exostosin 1 | 2 | | 2261 | hsa-mir-181b-2 | 2131 | EXT1 | exostosin 1 | 2 | | 2264 | hsa-mir-550a-1 | 2131 | EXT1 | exostosin 1 | 2 | | 2265 | hsa-mir-643 | 2131 | EXT1 | exostosin 1 | 2 | | 2266 | hsa-mir-644a | 2131 | EXT1 | exostosin 1 | 2 | | 2267 | hsa-mir-877 | 2131 | EXT1 | exostosin 1 | 2 | | 2268 | hsa-mir-548n | 2131 | EXT1 | exostosin 1 | 2 | | 2269 | hsa-mir-2355 | 2131 | EXT1 | exostosin 1 | 2 | | 2270 | hsa-mir-23c | 2131 | EXT1 | exostosin 1 | 2 | | 2271 | hsa-mir-3942 | 2131 | EXT1 | exostosin 1 | 2 | | 2273 | hsa-mir-4534 | 2131 | EXT1 | exostosin 1 | 2 | | 2274 | hsa-mir-4698 | 2131 | EXT1 | exostosin 1 | 2 | | 2275 | hsa-mir-4723 | 2131 | EXT1 | exostosin 1 | 2 | | 2276 | hsa-mir-4771-1 | 2131 | EXT1 | exostosin 1 | 2 | | 2277 | hsa-mir-4771-2 | 2131 | EXT1 | exostosin 1 | 2 | | 2278 | hsa-mir-675 | 2131 | EXT1 | exostosin 1 | 2 | | 2279 | hsa-mir-218-2 | 2131 | EXT1 | exostosin 1 | 2 | | 2280 | hsa-mir-574 | 2131 | EXT1 | exostosin 1 | 2 | | 2282 | hsa-mir-561 | 5782 | PTPN12 | protein tyrosine phosphatase, non-receptor type 12 | 2 | | 2284 | hsa-mir-585 | 5782 | PTPN12 | protein tyrosine phosphatase, non-receptor type 12 | 2 | | 2286 | hsa-mir-620 | 5782 | PTPN12 | protein tyrosine phosphatase, non-receptor type 12 | 2 | | 2306 | hsa-mir-181a-2 | 4811 | NID1 | nidogen 1 | 2 | | 2307 | hsa-mir-181b-2 | 4811 | NID1 | nidogen 1 | 2 | | 2308 | hsa-mir-378a | 4811 | NID1 | nidogen 1 | 2 | | 2309 | hsa-mir-505 | 4811 | NID1 | nidogen 1 | 2 | | 2311 | hsa-mir-643 | 4811 | NID1 | nidogen 1 | 2 | | 2327 | hsa-mir-505 | 6422 | SFRP1 | secreted frizzled-related protein 1 | 2 | | 2331 | hsa-mir-643 | 6422 | SFRP1 | secreted frizzled-related protein 1 | 2 | | 2332 | hsa-mir-652 | 6422 | SFRP1 | secreted frizzled-related protein 1 | 2 | | 2351 | hsa-mir-218-1 | 4706 | NDUFAB1 | NADH dehydrogenase (ubiquinone) 1, alpha/beta subcomplex, 1, 8kDa | 2 | | 2355 | hsa-mir-561 | 4706 | NDUFAB1 | NADH dehydrogenase (ubiquinone) 1, alpha/beta subcomplex, 1, 8kDa | 2 | | 2357 | hsa-mir-585 | 4706 | NDUFAB1 | NADH dehydrogenase (ubiquinone) 1, alpha/beta subcomplex, 1, 8kDa | 2 | | 2362 | hsa-mir-620 | 4706 | NDUFAB1 | NADH dehydrogenase (ubiquinone) 1, alpha/beta subcomplex, 1, 8kDa | 2 | | 2382 | hsa-mir-181a-2 | 7716 | VEZF1 | vascular endothelial zinc finger 1 | 2 | | 2383 | hsa-mir-181b-2 | 7716 | VEZF1 | vascular endothelial zinc finger 1 | 2 | | 2385 | hsa-mir-505 | 7716 | VEZF1 | vascular endothelial zinc finger 1 | 2 | | 2388 | hsa-mir-643 | 7716 | VEZF1 | vascular endothelial zinc finger 1 | 2 | | 2406 | hsa-mir-550a-1 | 23564 | DDAH2 | dimethylarginine dimethylaminohydrolase 2 | 2 | | 2409 | hsa-mir-548j | 23564 | DDAH2 | dimethylarginine dimethylaminohydrolase 2 | 2 | | 2410 | hsa-mir-548s | 23564 | DDAH2 | dimethylarginine dimethylaminohydrolase 2 | 2 | | 2411 | hsa-mir-2355 | 23564 | DDAH2 | dimethylarginine dimethylaminohydrolase 2 | 2 | | 2412 | hsa-mir-3942 | 23564 | DDAH2 | dimethylarginine dimethylaminohydrolase 2 | 2 | | 2414 | hsa-mir-4467 | 23564 | DDAH2 | dimethylarginine dimethylaminohydrolase 2 | 2 | | 2416 | hsa-mir-4698 | 23564 | DDAH2 | dimethylarginine dimethylaminohydrolase 2 | 2 | | 2418 | hsa-mir-4771-1 | 23564 | DDAH2 | dimethylarginine dimethylaminohydrolase 2 | 2 | | 2419 | hsa-mir-4771-2 | 23564 | DDAH2 | dimethylarginine dimethylaminohydrolase 2 | 2 | | 2420 | hsa-mir-5090 | 23564 | DDAH2 | dimethylarginine dimethylaminohydrolase 2 | 2 | | 2421 | hsa-mir-604 | 23564 | DDAH2 | dimethylarginine dimethylaminohydrolase 2 | 2 | | 2422 | hsa-mir-675 | 23564 | DDAH2 | dimethylarginine dimethylaminohydrolase 2 | 2 | | 2423 | hsa-mir-218-2 | 23564 | DDAH2 | dimethylarginine dimethylaminohydrolase 2 | 2 | | 2424 | hsa-mir-574 | 23564 | DDAH2 | dimethylarginine dimethylaminohydrolase 2 | 2 | | 2425 | hsa-mir-938 | 23564 | DDAH2 | dimethylarginine dimethylaminohydrolase 2 | 2 | | 2433 | hsa-mir-550a-1 | 1277 | COL1A1 | collagen, type I, alpha 1 | 2 | | 2434 | hsa-mir-636 | 1277 | COL1A1 | collagen, type I, alpha 1 | 2 | | 2436 | hsa-mir-643 | 1277 | COL1A1 | collagen, type I, alpha 1 | 2 | | 2437 | hsa-mir-644a | 1277 | COL1A1 | collagen, type I, alpha 1 | 2 | | 2438 | hsa-mir-877 | 1277 | COL1A1 | collagen, type I, alpha 1 | 2 | | 2439 | hsa-mir-548j | 1277 | COL1A1 | collagen, type I, alpha 1 | 2 | | 2440 | hsa-mir-548n | 1277 | COL1A1 | collagen, type I, alpha 1 | 2 | | 2441 | hsa-mir-3942 | 1277 | COL1A1 | collagen, type I, alpha 1 | 2 | | 2442 | hsa-mir-548o-2 | 1277 | COL1A1 | collagen, type I, alpha 1 | 2 | | 2443 | hsa-mir-4467 | 1277 | COL1A1 | collagen, type I, alpha 1 | 2 | | 2445 | hsa-mir-4698 | 1277 | COL1A1 | collagen, type I, alpha 1 | 2 | | 2446 | hsa-mir-4709 | 1277 | COL1A1 | collagen, type I, alpha 1 | 2 | | 2447 | hsa-mir-4723 | 1277 | COL1A1 | collagen, type I, alpha 1 | 2 | | 2448 | hsa-mir-4771-1 | 1277 | COL1A1 | collagen, type I, alpha 1 | 2 | | 2449 | hsa-mir-4771-2 | 1277 | COL1A1 | collagen, type I, alpha 1 | 2 | | 2450 | hsa-mir-4774 | 1277 | COL1A1 | collagen, type I, alpha 1 | 2 | | 2451 | hsa-mir-4775 | 1277 | COL1A1 | collagen, type I, alpha 1 | 2 | | 2452 | hsa-mir-5090 | 1277 | COL1A1 | collagen, type I, alpha 1 | 2 | | 2454 | hsa-mir-604 | 1277 | COL1A1 | collagen, type I, alpha 1 | 2 | | 2455 | hsa-mir-675 | 1277 | COL1A1 | collagen, type I, alpha 1 | 2 | | 2456 | hsa-mir-218-2 | 1277 | COL1A1 | collagen, type I, alpha 1 | 2 | | 2457 | hsa-mir-574 | 1277 | COL1A1 | collagen, type I, alpha 1 | 2 | | 2458 | hsa-mir-938 | 1277 | COL1A1 | collagen, type I, alpha 1 | 2 | | 2470 | hsa-mir-455 | 5520 | PPP2R2A | protein phosphatase 2, regulatory subunit B, alpha | 2 | | 2471 | hsa-mir-561 | 5520 | PPP2R2A | protein phosphatase 2, regulatory subunit B, alpha | 2 | | 2473 | hsa-mir-585 | 5520 | PPP2R2A | protein phosphatase 2, regulatory subunit B, alpha | 2 | | 2486 | hsa-mir-218-1 | 5718 | PSMD12 | proteasome (prosome, macropain) 26S subunit, non-ATPase, 12 | 2 | | 2488 | hsa-mir-125b-1 | 5718 | PSMD12 | proteasome (prosome, macropain) 26S subunit, non-ATPase, 12 | 2 | | 2489 | hsa-mir-455 | 5718 | PSMD12 | proteasome (prosome, macropain) 26S subunit, non-ATPase, 12 | 2 | | 2490 | hsa-mir-561 | 5718 | PSMD12 | proteasome (prosome, macropain) 26S subunit, non-ATPase, 12 | 2 | | 2492 | hsa-mir-585 | 5718 | PSMD12 | proteasome (prosome, macropain) 26S subunit, non-ATPase, 12 | 2 | | 2494 | hsa-mir-620 | 5718 | PSMD12 | proteasome (prosome, macropain) 26S subunit, non-ATPase, 12 | 2 | | 2516 | hsa-mir-218-1 | 55324 | ABCF3 | ATP-binding cassette, sub-family F (GCN20), member 3 | 2 | | 2518 | hsa-mir-561 | 55324 | ABCF3 | ATP-binding cassette, sub-family F (GCN20), member 3 | 2 | | 2520 | hsa-mir-585 | 55324 | ABCF3 | ATP-binding cassette, sub-family F (GCN20), member 3 | 2 | | 2523 | hsa-mir-620 | 55324 | ABCF3 | ATP-binding cassette, sub-family F (GCN20), member 3 | 2 | | 2545 | hsa-let-7f-2 | 1278 | COL1A2 | collagen, type I, alpha 2 | 2 | | 2548 | hsa-mir-98 | 1278 | COL1A2 | collagen, type I, alpha 2 | 2 | | 2552 | hsa-mir-378a | 1278 | COL1A2 | collagen, type I, alpha 2 | 2 | | 2554 | hsa-mir-550a-1 | 1278 | COL1A2 | collagen, type I, alpha 2 | 2 | | 2556 | hsa-mir-636 | 1278 | COL1A2 | collagen, type I, alpha 2 | 2 | | 2557 | hsa-mir-644a | 1278 | COL1A2 | collagen, type I, alpha 2 | 2 | | 2558 | hsa-mir-877 | 1278 | COL1A2 | collagen, type I, alpha 2 | 2 | | 2559 | hsa-mir-548j | 1278 | COL1A2 | collagen, type I, alpha 2 | 2 | | 2560 | hsa-mir-1290 | 1278 | COL1A2 | collagen, type I, alpha 2 | 2 | | 2561 | hsa-mir-548n | 1278 | COL1A2 | collagen, type I, alpha 2 | 2 | | 2562 | hsa-mir-2909 | 1278 | COL1A2 | collagen, type I, alpha 2 | 2 | | 2563 | hsa-mir-548s | 1278 | COL1A2 | collagen, type I, alpha 2 | 2 | | 2564 | hsa-mir-3609 | 1278 | COL1A2 | collagen, type I, alpha 2 | 2 | | 2565 | hsa-mir-23c | 1278 | COL1A2 | collagen, type I, alpha 2 | 2 | | 2566 | hsa-mir-3679 | 1278 | COL1A2 | collagen, type I, alpha 2 | 2 | | 2567 | hsa-mir-3942 | 1278 | COL1A2 | collagen, type I, alpha 2 | 2 | | 2568 | hsa-mir-548aa-2 | 1278 | COL1A2 | collagen, type I, alpha 2 | 2 | | 2569 | hsa-mir-548o-2 | 1278 | COL1A2 | collagen, type I, alpha 2 | 2 | | 2570 | hsa-mir-378e | 1278 | COL1A2 | collagen, type I, alpha 2 | 2 | | 2571 | hsa-mir-4467 | 1278 | COL1A2 | collagen, type I, alpha 2 | 2 | | 2572 | hsa-mir-4498 | 1278 | COL1A2 | collagen, type I, alpha 2 | 2 | | 2574 | hsa-mir-4534 | 1278 | COL1A2 | collagen, type I, alpha 2 | 2 | | 2575 | hsa-mir-4646 | 1278 | COL1A2 | collagen, type I, alpha 2 | 2 | | 2576 | hsa-mir-4668 | 1278 | COL1A2 | collagen, type I, alpha 2 | 2 | | 2577 | hsa-mir-4695 | 1278 | COL1A2 | collagen, type I, alpha 2 | 2 | | 2578 | hsa-mir-4698 | 1278 | COL1A2 | collagen, type I, alpha 2 | 2 | | 2579 | hsa-mir-4709 | 1278 | COL1A2 | collagen, type I, alpha 2 | 2 | | 2580 | hsa-mir-4723 | 1278 | COL1A2 | collagen, type I, alpha 2 | 2 | | 2581 | hsa-mir-4771-1 | 1278 | COL1A2 | collagen, type I, alpha 2 | 2 | | 2582 | hsa-mir-4771-2 | 1278 | COL1A2 | collagen, type I, alpha 2 | 2 | | 2583 | hsa-mir-4774 | 1278 | COL1A2 | collagen, type I, alpha 2 | 2 | | 2584 | hsa-mir-4775 | 1278 | COL1A2 | collagen, type I, alpha 2 | 2 | | 2585 | hsa-mir-5090 | 1278 | COL1A2 | collagen, type I, alpha 2 | 2 | | 2586 | hsa-mir-5194 | 1278 | COL1A2 | collagen, type I, alpha 2 | 2 | | 2587 | hsa-mir-604 | 1278 | COL1A2 | collagen, type I, alpha 2 | 2 | | 2588 | hsa-mir-675 | 1278 | COL1A2 | collagen, type I, alpha 2 | 2 | | 2589 | hsa-mir-218-2 | 1278 | COL1A2 | collagen, type I, alpha 2 | 2 | | 2590 | hsa-mir-574 | 1278 | COL1A2 | collagen, type I, alpha 2 | 2 | | 2591 | hsa-mir-887 | 1278 | COL1A2 | collagen, type I, alpha 2 | 2 | | 2592 | hsa-mir-938 | 1278 | COL1A2 | collagen, type I, alpha 2 | 2 | | 2596 | hsa-mir-181a-2 | 5118 | PCOLCE | procollagen C-endopeptidase enhancer | 2 | | 2597 | hsa-mir-181b-2 | 5118 | PCOLCE | procollagen C-endopeptidase enhancer | 2 | | 2598 | hsa-mir-378a | 5118 | PCOLCE | procollagen C-endopeptidase enhancer | 2 | | 2599 | hsa-mir-505 | 5118 | PCOLCE | procollagen C-endopeptidase enhancer | 2 | | 2601 | hsa-mir-602 | 5118 | PCOLCE | procollagen C-endopeptidase enhancer | 2 | | 2602 | hsa-mir-643 | 5118 | PCOLCE | procollagen C-endopeptidase enhancer | 2 | | 2636 | hsa-mir-218-1 | 9474 | ATG5 | ATG5 autophagy related 5 homolog (S. cerevisiae) | 2 | | 2638 | hsa-mir-455 | 9474 | ATG5 | ATG5 autophagy related 5 homolog (S. cerevisiae) | 2 | | 2639 | hsa-mir-561 | 9474 | ATG5 | ATG5 autophagy related 5 homolog (S. cerevisiae) | 2 | | 2641 | hsa-mir-585 | 9474 | ATG5 | ATG5 autophagy related 5 homolog (S. cerevisiae) | 2 | | 2644 | hsa-mir-620 | 9474 | ATG5 | ATG5 autophagy related 5 homolog (S. cerevisiae) | 2 | | 2663 | hsa-mir-218-1 | 9217 | VAPB | VAMP (vesicle-associated membrane protein)-associated protein B and C | 2 | | 2665 | hsa-mir-125b-1 | 9217 | VAPB | VAMP (vesicle-associated membrane protein)-associated protein B and C | 2 | | 2666 | hsa-mir-561 | 9217 | VAPB | VAMP (vesicle-associated membrane protein)-associated protein B and C | 2 | | 2668 | hsa-mir-585 | 9217 | VAPB | VAMP (vesicle-associated membrane protein)-associated protein B and C | 2 | | 2672 | hsa-mir-620 | 9217 | VAPB | VAMP (vesicle-associated membrane protein)-associated protein B and C | 2 | | 2695 | hsa-mir-25 | 6840 | SVIL | supervillin | 2 | | 2696 | hsa-mir-93 | 6840 | SVIL | supervillin | 2 | | 2697 | hsa-mir-181a-2 | 6840 | SVIL | supervillin | 2 | | 2698 | hsa-mir-181b-2 | 6840 | SVIL | supervillin | 2 | | 2699 | hsa-mir-106b | 6840 | SVIL | supervillin | 2 | | 2701 | hsa-mir-505 | 6840 | SVIL | supervillin | 2 | | 2703 | hsa-mir-602 | 6840 | SVIL | supervillin | 2 | | 2704 | hsa-mir-629 | 6840 | SVIL | supervillin | 2 | | 2725 | hsa-mir-181a-2 | 1382 | CRABP2 | cellular retinoic acid binding protein 2 | 2 | | 2726 | hsa-mir-181b-2 | 1382 | CRABP2 | cellular retinoic acid binding protein 2 | 2 | | 2727 | hsa-mir-378a | 1382 | CRABP2 | cellular retinoic acid binding protein 2 | 2 | | 2728 | hsa-mir-505 | 1382 | CRABP2 | cellular retinoic acid binding protein 2 | 2 | | 2730 | hsa-mir-643 | 1382 | CRABP2 | cellular retinoic acid binding protein 2 | 2 | | 2731 | hsa-mir-652 | 1382 | CRABP2 | cellular retinoic acid binding protein 2 | 2 | | 2757 | hsa-mir-218-1 | 9263 | STK17A | serine/threonine kinase 17a | 2 | | 2759 | hsa-mir-455 | 9263 | STK17A | serine/threonine kinase 17a | 2 | | 2760 | hsa-mir-561 | 9263 | STK17A | serine/threonine kinase 17a | 2 | | 2762 | hsa-mir-585 | 9263 | STK17A | serine/threonine kinase 17a | 2 | | 2764 | hsa-mir-620 | 9263 | STK17A | serine/threonine kinase 17a | 2 | | 2767 | hsa-mir-708 | 9263 | STK17A | serine/threonine kinase 17a | 2 | | 2789 | hsa-mir-181a-2 | 649 | BMP1 | bone morphogenetic protein 1 | 2 | | 2790 | hsa-mir-181b-2 | 649 | BMP1 | bone morphogenetic protein 1 | 2 | | 2816 | hsa-mir-25 | 2331 | FMOD | fibromodulin | 2 | | 2817 | hsa-mir-93 | 2331 | FMOD | fibromodulin | 2 | | 2818 | hsa-mir-181a-2 | 2331 | FMOD | fibromodulin | 2 | | 2819 | hsa-mir-181b-2 | 2331 | FMOD | fibromodulin | 2 | | 2820 | hsa-mir-106b | 2331 | FMOD | fibromodulin | 2 | | 2821 | hsa-mir-378a | 2331 | FMOD | fibromodulin | 2 | | 2822 | hsa-mir-505 | 2331 | FMOD | fibromodulin | 2 | | 2826 | hsa-mir-643 | 2331 | FMOD | fibromodulin | 2 | | 2828 | hsa-mir-652 | 2331 | FMOD | fibromodulin | 2 | | 2853 | hsa-let-7c | 5770 | PTPN1 | protein tyrosine phosphatase, non-receptor type 1 | 2 | | 2854 | hsa-mir-99a | 5770 | PTPN1 | protein tyrosine phosphatase, non-receptor type 1 | 2 | | 2856 | hsa-mir-218-1 | 5770 | PTPN1 | protein tyrosine phosphatase, non-receptor type 1 | 2 | | 2858 | hsa-mir-125b-1 | 5770 | PTPN1 | protein tyrosine phosphatase, non-receptor type 1 | 2 | | 2859 | hsa-mir-125b-2 | 5770 | PTPN1 | protein tyrosine phosphatase, non-receptor type 1 | 2 | | 2860 | hsa-mir-561 | 5770 | PTPN1 | protein tyrosine phosphatase, non-receptor type 1 | 2 | | 2862 | hsa-mir-585 | 5770 | PTPN1 | protein tyrosine phosphatase, non-receptor type 1 | 2 | | 2896 | hsa-mir-25 | 4052 | LTBP1 | latent transforming growth factor beta binding protein 1 | 2 | | 2897 | hsa-mir-93 | 4052 | LTBP1 | latent transforming growth factor beta binding protein 1 | 2 | | 2898 | hsa-mir-181a-2 | 4052 | LTBP1 | latent transforming growth factor beta binding protein 1 | 2 | | 2899 | hsa-mir-181b-2 | 4052 | LTBP1 | latent transforming growth factor beta binding protein 1 | 2 | | 2900 | hsa-mir-106b | 4052 | LTBP1 | latent transforming growth factor beta binding protein 1 | 2 | | 2919 | hsa-mir-10a | 24144 | TFIP11 | tuftelin interacting protein 11 | 2 | | 2920 | hsa-mir-218-1 | 24144 | TFIP11 | tuftelin interacting protein 11 | 2 | | 2921 | hsa-mir-218-2 | 24144 | TFIP11 | tuftelin interacting protein 11 | 2 | | 2925 | hsa-mir-574 | 24144 | TFIP11 | tuftelin interacting protein 11 | 2 | | 2927 | hsa-mir-603 | 24144 | TFIP11 | tuftelin interacting protein 11 | 2 | | 2930 | hsa-mir-618 | 24144 | TFIP11 | tuftelin interacting protein 11 | 2 | | 2932 | hsa-mir-675 | 24144 | TFIP11 | tuftelin interacting protein 11 | 2 | | 2933 | hsa-mir-708 | 24144 | TFIP11 | tuftelin interacting protein 11 | 2 | | 2934 | hsa-mir-887 | 24144 | TFIP11 | tuftelin interacting protein 11 | 2 | | 2936 | hsa-mir-1245a | 24144 | TFIP11 | tuftelin interacting protein 11 | 2 | | 2937 | hsa-mir-1469 | 24144 | TFIP11 | tuftelin interacting protein 11 | 2 | | 2938 | hsa-mir-1915 | 24144 | TFIP11 | tuftelin interacting protein 11 | 2 | | 2939 | hsa-mir-3120 | 24144 | TFIP11 | tuftelin interacting protein 11 | 2 | | 2940 | hsa-mir-3126 | 24144 | TFIP11 | tuftelin interacting protein 11 | 2 | | 2941 | hsa-mir-3129 | 24144 | TFIP11 | tuftelin interacting protein 11 | 2 | | 2944 | hsa-mir-3606 | 24144 | TFIP11 | tuftelin interacting protein 11 | 2 | | 2945 | hsa-mir-3650 | 24144 | TFIP11 | tuftelin interacting protein 11 | 2 | | 2946 | hsa-mir-4441 | 24144 | TFIP11 | tuftelin interacting protein 11 | 2 | | 2951 | hsa-mir-4735 | 24144 | TFIP11 | tuftelin interacting protein 11 | 2 | | 2952 | hsa-mir-4768 | 24144 | TFIP11 | tuftelin interacting protein 11 | 2 | | 2953 | hsa-mir-2467 | 24144 | TFIP11 | tuftelin interacting protein 11 | 2 | | 2954 | hsa-mir-4794 | 24144 | TFIP11 | tuftelin interacting protein 11 | 2 | | 2955 | hsa-mir-548ao | 24144 | TFIP11 | tuftelin interacting protein 11 | 2 | | 2957 | hsa-mir-554 | 24144 | TFIP11 | tuftelin interacting protein 11 | 2 | | 2958 | hsa-mir-617 | 24144 | TFIP11 | tuftelin interacting protein 11 | 2 | | 2959 | hsa-mir-640 | 24144 | TFIP11 | tuftelin interacting protein 11 | 2 | | 2960 | hsa-mir-877 | 24144 | TFIP11 | tuftelin interacting protein 11 | 2 | | 2961 | hsa-mir-620 | 24144 | TFIP11 | tuftelin interacting protein 11 | 2 | | 2971 | hsa-mir-550a-1 | 2200 | FBN1 | fibrillin 1 | 2 | | 2972 | hsa-mir-644a | 2200 | FBN1 | fibrillin 1 | 2 | | 2973 | hsa-mir-877 | 2200 | FBN1 | fibrillin 1 | 2 | | 2974 | hsa-mir-23c | 2200 | FBN1 | fibrillin 1 | 2 | | 2975 | hsa-mir-3942 | 2200 | FBN1 | fibrillin 1 | 2 | | 2976 | hsa-mir-548aa-2 | 2200 | FBN1 | fibrillin 1 | 2 | | 2977 | hsa-mir-378e | 2200 | FBN1 | fibrillin 1 | 2 | | 2978 | hsa-mir-4467 | 2200 | FBN1 | fibrillin 1 | 2 | | 2980 | hsa-mir-4698 | 2200 | FBN1 | fibrillin 1 | 2 | | 2981 | hsa-mir-4723 | 2200 | FBN1 | fibrillin 1 | 2 | | 2982 | hsa-mir-4771-1 | 2200 | FBN1 | fibrillin 1 | 2 | | 2983 | hsa-mir-4771-2 | 2200 | FBN1 | fibrillin 1 | 2 | | 2984 | hsa-mir-4775 | 2200 | FBN1 | fibrillin 1 | 2 | | 2985 | hsa-mir-5090 | 2200 | FBN1 | fibrillin 1 | 2 | | 2986 | hsa-mir-5194 | 2200 | FBN1 | fibrillin 1 | 2 | | 2988 | hsa-mir-604 | 2200 | FBN1 | fibrillin 1 | 2 | | 2989 | hsa-mir-675 | 2200 | FBN1 | fibrillin 1 | 2 | | 2990 | hsa-mir-218-2 | 2200 | FBN1 | fibrillin 1 | 2 | | 2991 | hsa-mir-574 | 2200 | FBN1 | fibrillin 1 | 2 | | 2992 | hsa-mir-887 | 2200 | FBN1 | fibrillin 1 | 2 | | 2993 | hsa-mir-938 | 2200 | FBN1 | fibrillin 1 | 2 | | 2995 | hsa-mir-218-1 | 51763 | INPP5K | inositol polyphosphate-5-phosphatase K | 2 | | 2998 | hsa-mir-561 | 51763 | INPP5K | inositol polyphosphate-5-phosphatase K | 2 | | 3000 | hsa-mir-585 | 51763 | INPP5K | inositol polyphosphate-5-phosphatase K | 2 | | 3002 | hsa-mir-620 | 51763 | INPP5K | inositol polyphosphate-5-phosphatase K | 2 | | 3016 | hsa-let-7c | 2517 | FUCA1 | fucosidase, alpha-L- 1, tissue | 2 | | 3017 | hsa-mir-99a | 2517 | FUCA1 | fucosidase, alpha-L- 1, tissue | 2 | | 3020 | hsa-mir-125b-2 | 2517 | FUCA1 | fucosidase, alpha-L- 1, tissue | 2 | | 3021 | hsa-mir-455 | 2517 | FUCA1 | fucosidase, alpha-L- 1, tissue | 2 | | 3022 | hsa-mir-561 | 2517 | FUCA1 | fucosidase, alpha-L- 1, tissue | 2 | | 3024 | hsa-mir-585 | 2517 | FUCA1 | fucosidase, alpha-L- 1, tissue | 2 | | 3028 | hsa-mir-620 | 2517 | FUCA1 | fucosidase, alpha-L- 1, tissue | 2 | | 3046 | hsa-let-7c | 51594 | NBAS | neuroblastoma amplified sequence | 2 | | 3047 | hsa-mir-99a | 51594 | NBAS | neuroblastoma amplified sequence | 2 | | 3049 | hsa-mir-218-1 | 51594 | NBAS | neuroblastoma amplified sequence | 2 | | 3052 | hsa-mir-125b-2 | 51594 | NBAS | neuroblastoma amplified sequence | 2 | | 3053 | hsa-mir-455 | 51594 | NBAS | neuroblastoma amplified sequence | 2 | | 3054 | hsa-mir-561 | 51594 | NBAS | neuroblastoma amplified sequence | 2 | | 3056 | hsa-mir-585 | 51594 | NBAS | neuroblastoma amplified sequence | 2 | | 3060 | hsa-mir-620 | 51594 | NBAS | neuroblastoma amplified sequence | 2 | | 3087 | hsa-mir-455 | 10565 | ARFGEF1 | ADP-ribosylation factor guanine nucleotide-exchange factor 1 (brefeldin A-inhibited) | 2 | | 3088 | hsa-mir-561 | 10565 | ARFGEF1 | ADP-ribosylation factor guanine nucleotide-exchange factor 1 (brefeldin A-inhibited) | 2 | | 3090 | hsa-mir-585 | 10565 | ARFGEF1 | ADP-ribosylation factor guanine nucleotide-exchange factor 1 (brefeldin A-inhibited) | 2 | | 3096 | hsa-mir-708 | 10565 | ARFGEF1 | ADP-ribosylation factor guanine nucleotide-exchange factor 1 (brefeldin A-inhibited) | 2 | | 3116 | hsa-let-7f-2 | 2192 | FBLN1 | fibulin 1 | 2 | | 3117 | hsa-mir-98 | 2192 | FBLN1 | fibulin 1 | 2 | | 3120 | hsa-mir-550a-1 | 2192 | FBLN1 | fibulin 1 | 2 | | 3121 | hsa-mir-602 | 2192 | FBLN1 | fibulin 1 | 2 | | 3123 | hsa-mir-877 | 2192 | FBLN1 | fibulin 1 | 2 | | 3124 | hsa-mir-548j | 2192 | FBLN1 | fibulin 1 | 2 | | 3125 | hsa-mir-1290 | 2192 | FBLN1 | fibulin 1 | 2 | | 3128 | hsa-mir-548s | 2192 | FBLN1 | fibulin 1 | 2 | | 3129 | hsa-mir-3609 | 2192 | FBLN1 | fibulin 1 | 2 | | 3132 | hsa-mir-548o-2 | 2192 | FBLN1 | fibulin 1 | 2 | | 3133 | hsa-mir-378e | 2192 | FBLN1 | fibulin 1 | 2 | | 3135 | hsa-mir-4695 | 2192 | FBLN1 | fibulin 1 | 2 | | 3136 | hsa-mir-4698 | 2192 | FBLN1 | fibulin 1 | 2 | | 3141 | hsa-mir-5194 | 2192 | FBLN1 | fibulin 1 | 2 | | 3143 | hsa-mir-675 | 2192 | FBLN1 | fibulin 1 | 2 | | 3144 | hsa-mir-218-2 | 2192 | FBLN1 | fibulin 1 | 2 | | 3145 | hsa-mir-574 | 2192 | FBLN1 | fibulin 1 | 2 | | 3146 | hsa-mir-887 | 2192 | FBLN1 | fibulin 1 | 2 | | 3150 | hsa-mir-181a-2 | 5796 | PTPRK | protein tyrosine phosphatase, receptor type, K | 2 | | 3151 | hsa-mir-181b-2 | 5796 | PTPRK | protein tyrosine phosphatase, receptor type, K | 2 | | 3152 | hsa-mir-378a | 5796 | PTPRK | protein tyrosine phosphatase, receptor type, K | 2 | | 3153 | hsa-mir-505 | 5796 | PTPRK | protein tyrosine phosphatase, receptor type, K | 2 | | 3156 | hsa-mir-643 | 5796 | PTPRK | protein tyrosine phosphatase, receptor type, K | 2 | | 3173 | hsa-mir-25 | 7869 | SEMA3B | sema domain, immunoglobulin domain (Ig), short basic domain, secreted, (semaphorin) 3B | 2 | | 3174 | hsa-mir-93 | 7869 | SEMA3B | sema domain, immunoglobulin domain (Ig), short basic domain, secreted, (semaphorin) 3B | 2 | | 3176 | hsa-mir-181a-2 | 7869 | SEMA3B | sema domain, immunoglobulin domain (Ig), short basic domain, secreted, (semaphorin) 3B | 2 | | 3177 | hsa-mir-181b-2 | 7869 | SEMA3B | sema domain, immunoglobulin domain (Ig), short basic domain, secreted, (semaphorin) 3B | 2 | | 3178 | hsa-mir-106b | 7869 | SEMA3B | sema domain, immunoglobulin domain (Ig), short basic domain, secreted, (semaphorin) 3B | 2 | | 3180 | hsa-mir-602 | 7869 | SEMA3B | sema domain, immunoglobulin domain (Ig), short basic domain, secreted, (semaphorin) 3B | 2 | | 3183 | hsa-mir-643 | 7869 | SEMA3B | sema domain, immunoglobulin domain (Ig), short basic domain, secreted, (semaphorin) 3B | 2 | | 3212 | hsa-mir-25 | 7058 | THBS2 | thrombospondin 2 | 2 | | 3213 | hsa-mir-93 | 7058 | THBS2 | thrombospondin 2 | 2 | | 3215 | hsa-mir-181a-2 | 7058 | THBS2 | thrombospondin 2 | 2 | | 3216 | hsa-mir-181b-2 | 7058 | THBS2 | thrombospondin 2 | 2 | | 3217 | hsa-mir-106b | 7058 | THBS2 | thrombospondin 2 | 2 | | 3246 | hsa-let-7c | 79080 | CCDC86 | coiled-coil domain containing 86 | 2 | | 3247 | hsa-mir-99a | 79080 | CCDC86 | coiled-coil domain containing 86 | 2 | | 3249 | hsa-mir-218-1 | 79080 | CCDC86 | coiled-coil domain containing 86 | 2 | | 3251 | hsa-mir-125b-1 | 79080 | CCDC86 | coiled-coil domain containing 86 | 2 | | 3252 | hsa-mir-125b-2 | 79080 | CCDC86 | coiled-coil domain containing 86 | 2 | | 3253 | hsa-mir-561 | 79080 | CCDC86 | coiled-coil domain containing 86 | 2 | | 3255 | hsa-mir-585 | 79080 | CCDC86 | coiled-coil domain containing 86 | 2 | | 3286 | hsa-mir-25 | 5156 | PDGFRA | platelet-derived growth factor receptor, alpha polypeptide | 2 | | 3287 | hsa-mir-93 | 5156 | PDGFRA | platelet-derived growth factor receptor, alpha polypeptide | 2 | | 3289 | hsa-mir-181a-2 | 5156 | PDGFRA | platelet-derived growth factor receptor, alpha polypeptide | 2 | | 3290 | hsa-mir-181b-2 | 5156 | PDGFRA | platelet-derived growth factor receptor, alpha polypeptide | 2 | | 3291 | hsa-mir-106b | 5156 | PDGFRA | platelet-derived growth factor receptor, alpha polypeptide | 2 | | 3293 | hsa-mir-505 | 5156 | PDGFRA | platelet-derived growth factor receptor, alpha polypeptide | 2 | | 3298 | hsa-mir-643 | 5156 | PDGFRA | platelet-derived growth factor receptor, alpha polypeptide | 2 | | 3299 | hsa-mir-652 | 5156 | PDGFRA | platelet-derived growth factor receptor, alpha polypeptide | 2 | | 3322 | hsa-mir-181a-2 | 2201 | FBN2 | fibrillin 2 | 2 | | 3323 | hsa-mir-181b-2 | 2201 | FBN2 | fibrillin 2 | 2 | | 3324 | hsa-mir-505 | 2201 | FBN2 | fibrillin 2 | 2 | | 3340 | hsa-mir-10a | 9650 | MTFR1 | mitochondrial fission regulator 1 | 2 | | 3341 | hsa-mir-218-2 | 9650 | MTFR1 | mitochondrial fission regulator 1 | 2 | | 3343 | hsa-mir-574 | 9650 | MTFR1 | mitochondrial fission regulator 1 | 2 | | 3345 | hsa-mir-603 | 9650 | MTFR1 | mitochondrial fission regulator 1 | 2 | | 3346 | hsa-mir-605 | 9650 | MTFR1 | mitochondrial fission regulator 1 | 2 | | 3348 | hsa-mir-675 | 9650 | MTFR1 | mitochondrial fission regulator 1 | 2 | | 3349 | hsa-mir-1245a | 9650 | MTFR1 | mitochondrial fission regulator 1 | 2 | | 3350 | hsa-mir-1915 | 9650 | MTFR1 | mitochondrial fission regulator 1 | 2 | | 3351 | hsa-mir-3120 | 9650 | MTFR1 | mitochondrial fission regulator 1 | 2 | | 3352 | hsa-mir-3129 | 9650 | MTFR1 | mitochondrial fission regulator 1 | 2 | | 3353 | hsa-mir-3606 | 9650 | MTFR1 | mitochondrial fission regulator 1 | 2 | | 3354 | hsa-mir-4794 | 9650 | MTFR1 | mitochondrial fission regulator 1 | 2 | | 3355 | hsa-mir-548ao | 9650 | MTFR1 | mitochondrial fission regulator 1 | 2 | | 3356 | hsa-mir-636 | 9650 | MTFR1 | mitochondrial fission regulator 1 | 2 | | 3358 | hsa-mir-877 | 9650 | MTFR1 | mitochondrial fission regulator 1 | 2 | | 3359 | hsa-mir-561 | 9650 | MTFR1 | mitochondrial fission regulator 1 | 2 | | 3363 | hsa-mir-708 | 9650 | MTFR1 | mitochondrial fission regulator 1 | 2 | | 3370 | hsa-mir-218-1 | 7378 | UPP1 | uridine phosphorylase 1 | 2 | | 3373 | hsa-mir-455 | 7378 | UPP1 | uridine phosphorylase 1 | 2 | | 3374 | hsa-mir-561 | 7378 | UPP1 | uridine phosphorylase 1 | 2 | | 3376 | hsa-mir-585 | 7378 | UPP1 | uridine phosphorylase 1 | 2 | | 3383 | hsa-mir-708 | 7378 | UPP1 | uridine phosphorylase 1 | 2 | | 3409 | hsa-mir-10a | 8439 | NSMAF | neutral sphingomyelinase (N-SMase) activation associated factor | 2 | | 3411 | hsa-mir-218-2 | 8439 | NSMAF | neutral sphingomyelinase (N-SMase) activation associated factor | 2 | | 3412 | hsa-mir-125b-1 | 8439 | NSMAF | neutral sphingomyelinase (N-SMase) activation associated factor | 2 | | 3414 | hsa-mir-574 | 8439 | NSMAF | neutral sphingomyelinase (N-SMase) activation associated factor | 2 | | 3417 | hsa-mir-605 | 8439 | NSMAF | neutral sphingomyelinase (N-SMase) activation associated factor | 2 | | 3419 | hsa-mir-675 | 8439 | NSMAF | neutral sphingomyelinase (N-SMase) activation associated factor | 2 | | 3420 | hsa-mir-708 | 8439 | NSMAF | neutral sphingomyelinase (N-SMase) activation associated factor | 2 | | 3422 | hsa-mir-1245a | 8439 | NSMAF | neutral sphingomyelinase (N-SMase) activation associated factor | 2 | | 3424 | hsa-mir-3120 | 8439 | NSMAF | neutral sphingomyelinase (N-SMase) activation associated factor | 2 | | 3425 | hsa-mir-3126 | 8439 | NSMAF | neutral sphingomyelinase (N-SMase) activation associated factor | 2 | | 3426 | hsa-mir-3129 | 8439 | NSMAF | neutral sphingomyelinase (N-SMase) activation associated factor | 2 | | 3427 | hsa-mir-3606 | 8439 | NSMAF | neutral sphingomyelinase (N-SMase) activation associated factor | 2 | | 3428 | hsa-mir-3650 | 8439 | NSMAF | neutral sphingomyelinase (N-SMase) activation associated factor | 2 | | 3429 | hsa-mir-4441 | 8439 | NSMAF | neutral sphingomyelinase (N-SMase) activation associated factor | 2 | | 3435 | hsa-mir-4794 | 8439 | NSMAF | neutral sphingomyelinase (N-SMase) activation associated factor | 2 | | 3436 | hsa-mir-548ao | 8439 | NSMAF | neutral sphingomyelinase (N-SMase) activation associated factor | 2 | | 3440 | hsa-mir-877 | 8439 | NSMAF | neutral sphingomyelinase (N-SMase) activation associated factor | 2 | | 3467 | hsa-mir-4774 | 1289 | COL5A1 | collagen, type V, alpha 1 | 2 | | 3476 | hsa-let-7c | 3635 | INPP5D | inositol polyphosphate-5-phosphatase, 145kDa | 2 | | 3477 | hsa-mir-99a | 3635 | INPP5D | inositol polyphosphate-5-phosphatase, 145kDa | 2 | | 3480 | hsa-mir-125b-2 | 3635 | INPP5D | inositol polyphosphate-5-phosphatase, 145kDa | 2 | | 3482 | hsa-mir-561 | 3635 | INPP5D | inositol polyphosphate-5-phosphatase, 145kDa | 2 | | 3484 | hsa-mir-585 | 3635 | INPP5D | inositol polyphosphate-5-phosphatase, 145kDa | 2 | | 3502 | hsa-mir-181a-2 | 7358 | UGDH | UDP-glucose 6-dehydrogenase | 2 | | 3503 | hsa-mir-181b-2 | 7358 | UGDH | UDP-glucose 6-dehydrogenase | 2 | | 3504 | hsa-mir-378a | 7358 | UGDH | UDP-glucose 6-dehydrogenase | 2 | | 3505 | hsa-mir-505 | 7358 | UGDH | UDP-glucose 6-dehydrogenase | 2 | | 3508 | hsa-mir-652 | 7358 | UGDH | UDP-glucose 6-dehydrogenase | 2 | | 3533 | hsa-mir-218-2 | 9776 | ATG13 | ATG13 autophagy related 13 homolog (S. cerevisiae) | 2 | | 3537 | hsa-mir-574 | 9776 | ATG13 | ATG13 autophagy related 13 homolog (S. cerevisiae) | 2 | | 3539 | hsa-mir-603 | 9776 | ATG13 | ATG13 autophagy related 13 homolog (S. cerevisiae) | 2 | | 3540 | hsa-mir-605 | 9776 | ATG13 | ATG13 autophagy related 13 homolog (S. cerevisiae) | 2 | | 3541 | hsa-mir-618 | 9776 | ATG13 | ATG13 autophagy related 13 homolog (S. cerevisiae) | 2 | | 3543 | hsa-mir-887 | 9776 | ATG13 | ATG13 autophagy related 13 homolog (S. cerevisiae) | 2 | | 3544 | hsa-mir-1245a | 9776 | ATG13 | ATG13 autophagy related 13 homolog (S. cerevisiae) | 2 | | 3545 | hsa-mir-1284 | 9776 | ATG13 | ATG13 autophagy related 13 homolog (S. cerevisiae) | 2 | | 3546 | hsa-mir-3126 | 9776 | ATG13 | ATG13 autophagy related 13 homolog (S. cerevisiae) | 2 | | 3547 | hsa-mir-3129 | 9776 | ATG13 | ATG13 autophagy related 13 homolog (S. cerevisiae) | 2 | | 3548 | hsa-mir-3606 | 9776 | ATG13 | ATG13 autophagy related 13 homolog (S. cerevisiae) | 2 | | 3549 | hsa-mir-4441 | 9776 | ATG13 | ATG13 autophagy related 13 homolog (S. cerevisiae) | 2 | | 3550 | hsa-mir-4731 | 9776 | ATG13 | ATG13 autophagy related 13 homolog (S. cerevisiae) | 2 | | 3551 | hsa-mir-4768 | 9776 | ATG13 | ATG13 autophagy related 13 homolog (S. cerevisiae) | 2 | | 3552 | hsa-mir-2467 | 9776 | ATG13 | ATG13 autophagy related 13 homolog (S. cerevisiae) | 2 | | 3553 | hsa-mir-4794 | 9776 | ATG13 | ATG13 autophagy related 13 homolog (S. cerevisiae) | 2 | | 3554 | hsa-mir-548ao | 9776 | ATG13 | ATG13 autophagy related 13 homolog (S. cerevisiae) | 2 | | 3557 | hsa-mir-591 | 9776 | ATG13 | ATG13 autophagy related 13 homolog (S. cerevisiae) | 2 | | 3558 | hsa-mir-617 | 9776 | ATG13 | ATG13 autophagy related 13 homolog (S. cerevisiae) | 2 | | 3559 | hsa-mir-636 | 9776 | ATG13 | ATG13 autophagy related 13 homolog (S. cerevisiae) | 2 | | 3560 | hsa-mir-640 | 9776 | ATG13 | ATG13 autophagy related 13 homolog (S. cerevisiae) | 2 | | 3562 | hsa-mir-877 | 9776 | ATG13 | ATG13 autophagy related 13 homolog (S. cerevisiae) | 2 | | 3567 | hsa-mir-378a | 3280 | HES1 | hairy and enhancer of split 1, (Drosophila) | 2 | | 3569 | hsa-mir-550a-1 | 3280 | HES1 | hairy and enhancer of split 1, (Drosophila) | 2 | | 3571 | hsa-mir-877 | 3280 | HES1 | hairy and enhancer of split 1, (Drosophila) | 2 | | 3573 | hsa-mir-3679 | 3280 | HES1 | hairy and enhancer of split 1, (Drosophila) | 2 | | 3574 | hsa-mir-3942 | 3280 | HES1 | hairy and enhancer of split 1, (Drosophila) | 2 | | 3575 | hsa-mir-548o-2 | 3280 | HES1 | hairy and enhancer of split 1, (Drosophila) | 2 | | 3576 | hsa-mir-4517 | 3280 | HES1 | hairy and enhancer of split 1, (Drosophila) | 2 | | 3577 | hsa-mir-4698 | 3280 | HES1 | hairy and enhancer of split 1, (Drosophila) | 2 | | 3578 | hsa-mir-4723 | 3280 | HES1 | hairy and enhancer of split 1, (Drosophila) | 2 | | 3579 | hsa-mir-4771-1 | 3280 | HES1 | hairy and enhancer of split 1, (Drosophila) | 2 | | 3580 | hsa-mir-4771-2 | 3280 | HES1 | hairy and enhancer of split 1, (Drosophila) | 2 | | 3581 | hsa-mir-4775 | 3280 | HES1 | hairy and enhancer of split 1, (Drosophila) | 2 | | 3582 | hsa-mir-675 | 3280 | HES1 | hairy and enhancer of split 1, (Drosophila) | 2 | | 3583 | hsa-mir-218-2 | 3280 | HES1 | hairy and enhancer of split 1, (Drosophila) | 2 | | 3584 | hsa-mir-574 | 3280 | HES1 | hairy and enhancer of split 1, (Drosophila) | 2 | | 3586 | hsa-let-7c | 24149 | ZNF318 | zinc finger protein 318 | 2 | | 3587 | hsa-mir-99a | 24149 | ZNF318 | zinc finger protein 318 | 2 | | 3589 | hsa-mir-125b-2 | 24149 | ZNF318 | zinc finger protein 318 | 2 | | 3590 | hsa-mir-561 | 24149 | ZNF318 | zinc finger protein 318 | 2 | | 3592 | hsa-mir-585 | 24149 | ZNF318 | zinc finger protein 318 | 2 | | 3596 | hsa-mir-708 | 24149 | ZNF318 | zinc finger protein 318 | 2 | | 3613 | hsa-let-7c | 10507 | SEMA4D | sema domain, immunoglobulin domain (Ig), transmembrane domain (TM) and short cytoplasmic domain, (semaphorin) 4D | 2 | | 3614 | hsa-mir-99a | 10507 | SEMA4D | sema domain, immunoglobulin domain (Ig), transmembrane domain (TM) and short cytoplasmic domain, (semaphorin) 4D | 2 | | 3617 | hsa-mir-125b-2 | 10507 | SEMA4D | sema domain, immunoglobulin domain (Ig), transmembrane domain (TM) and short cytoplasmic domain, (semaphorin) 4D | 2 | | 3618 | hsa-mir-455 | 10507 | SEMA4D | sema domain, immunoglobulin domain (Ig), transmembrane domain (TM) and short cytoplasmic domain, (semaphorin) 4D | 2 | | 3619 | hsa-mir-561 | 10507 | SEMA4D | sema domain, immunoglobulin domain (Ig), transmembrane domain (TM) and short cytoplasmic domain, (semaphorin) 4D | 2 | | 3621 | hsa-mir-585 | 10507 | SEMA4D | sema domain, immunoglobulin domain (Ig), transmembrane domain (TM) and short cytoplasmic domain, (semaphorin) 4D | 2 | | 3626 | hsa-mir-620 | 10507 | SEMA4D | sema domain, immunoglobulin domain (Ig), transmembrane domain (TM) and short cytoplasmic domain, (semaphorin) 4D | 2 | | 3652 | hsa-let-7c | 5875 | RABGGTA | Rab geranylgeranyltransferase, alpha subunit | 2 | | 3653 | hsa-mir-99a | 5875 | RABGGTA | Rab geranylgeranyltransferase, alpha subunit | 2 | | 3655 | hsa-mir-218-1 | 5875 | RABGGTA | Rab geranylgeranyltransferase, alpha subunit | 2 | | 3658 | hsa-mir-125b-2 | 5875 | RABGGTA | Rab geranylgeranyltransferase, alpha subunit | 2 | | 3659 | hsa-mir-561 | 5875 | RABGGTA | Rab geranylgeranyltransferase, alpha subunit | 2 | | 3661 | hsa-mir-585 | 5875 | RABGGTA | Rab geranylgeranyltransferase, alpha subunit | 2 | | 3666 | hsa-mir-620 | 5875 | RABGGTA | Rab geranylgeranyltransferase, alpha subunit | 2 | | 3688 | hsa-mir-10a | 9057 | SLC7A6 | solute carrier family 7 (amino acid transporter light chain, y+L system), member 6 | 2 | | 3689 | hsa-mir-218-2 | 9057 | SLC7A6 | solute carrier family 7 (amino acid transporter light chain, y+L system), member 6 | 2 | | 3693 | hsa-mir-574 | 9057 | SLC7A6 | solute carrier family 7 (amino acid transporter light chain, y+L system), member 6 | 2 | | 3697 | hsa-mir-605 | 9057 | SLC7A6 | solute carrier family 7 (amino acid transporter light chain, y+L system), member 6 | 2 | | 3698 | hsa-mir-618 | 9057 | SLC7A6 | solute carrier family 7 (amino acid transporter light chain, y+L system), member 6 | 2 | | 3699 | hsa-mir-708 | 9057 | SLC7A6 | solute carrier family 7 (amino acid transporter light chain, y+L system), member 6 | 2 | | 3700 | hsa-mir-887 | 9057 | SLC7A6 | solute carrier family 7 (amino acid transporter light chain, y+L system), member 6 | 2 | | 3701 | hsa-mir-938 | 9057 | SLC7A6 | solute carrier family 7 (amino acid transporter light chain, y+L system), member 6 | 2 | | 3702 | hsa-mir-1245a | 9057 | SLC7A6 | solute carrier family 7 (amino acid transporter light chain, y+L system), member 6 | 2 | | 3703 | hsa-mir-1469 | 9057 | SLC7A6 | solute carrier family 7 (amino acid transporter light chain, y+L system), member 6 | 2 | | 3704 | hsa-mir-3120 | 9057 | SLC7A6 | solute carrier family 7 (amino acid transporter light chain, y+L system), member 6 | 2 | | 3705 | hsa-mir-3126 | 9057 | SLC7A6 | solute carrier family 7 (amino acid transporter light chain, y+L system), member 6 | 2 | | 3706 | hsa-mir-3129 | 9057 | SLC7A6 | solute carrier family 7 (amino acid transporter light chain, y+L system), member 6 | 2 | | 3707 | hsa-mir-3139 | 9057 | SLC7A6 | solute carrier family 7 (amino acid transporter light chain, y+L system), member 6 | 2 | | 3708 | hsa-mir-3606 | 9057 | SLC7A6 | solute carrier family 7 (amino acid transporter light chain, y+L system), member 6 | 2 | | 3709 | hsa-mir-3650 | 9057 | SLC7A6 | solute carrier family 7 (amino acid transporter light chain, y+L system), member 6 | 2 | | 3710 | hsa-mir-4441 | 9057 | SLC7A6 | solute carrier family 7 (amino acid transporter light chain, y+L system), member 6 | 2 | | 3713 | hsa-mir-4731 | 9057 | SLC7A6 | solute carrier family 7 (amino acid transporter light chain, y+L system), member 6 | 2 | | 3714 | hsa-mir-4768 | 9057 | SLC7A6 | solute carrier family 7 (amino acid transporter light chain, y+L system), member 6 | 2 | | 3715 | hsa-mir-2467 | 9057 | SLC7A6 | solute carrier family 7 (amino acid transporter light chain, y+L system), member 6 | 2 | | 3716 | hsa-mir-4794 | 9057 | SLC7A6 | solute carrier family 7 (amino acid transporter light chain, y+L system), member 6 | 2 | | 3717 | hsa-mir-548ao | 9057 | SLC7A6 | solute carrier family 7 (amino acid transporter light chain, y+L system), member 6 | 2 | | 3719 | hsa-mir-617 | 9057 | SLC7A6 | solute carrier family 7 (amino acid transporter light chain, y+L system), member 6 | 2 | | 3720 | hsa-mir-640 | 9057 | SLC7A6 | solute carrier family 7 (amino acid transporter light chain, y+L system), member 6 | 2 | | 3721 | hsa-mir-877 | 9057 | SLC7A6 | solute carrier family 7 (amino acid transporter light chain, y+L system), member 6 | 2 | | 3727 | hsa-mir-218-1 | 705 | BYSL | bystin-like | 2 | | 3729 | hsa-mir-125b-1 | 705 | BYSL | bystin-like | 2 | | 3730 | hsa-mir-561 | 705 | BYSL | bystin-like | 2 | | 3732 | hsa-mir-585 | 705 | BYSL | bystin-like | 2 | | 3737 | hsa-mir-620 | 705 | BYSL | bystin-like | 2 | | 3740 | hsa-mir-708 | 705 | BYSL | bystin-like | 2 | | 3769 | hsa-let-7f-2 | 4281 | MID1 | midline 1 (Opitz/BBB syndrome) | 2 | | 3772 | hsa-mir-98 | 4281 | MID1 | midline 1 (Opitz/BBB syndrome) | 2 | | 3777 | hsa-mir-550a-1 | 4281 | MID1 | midline 1 (Opitz/BBB syndrome) | 2 | | 3779 | hsa-mir-636 | 4281 | MID1 | midline 1 (Opitz/BBB syndrome) | 2 | | 3781 | hsa-mir-644a | 4281 | MID1 | midline 1 (Opitz/BBB syndrome) | 2 | | 3782 | hsa-mir-877 | 4281 | MID1 | midline 1 (Opitz/BBB syndrome) | 2 | | 3784 | hsa-mir-548n | 4281 | MID1 | midline 1 (Opitz/BBB syndrome) | 2 | | 3786 | hsa-mir-548s | 4281 | MID1 | midline 1 (Opitz/BBB syndrome) | 2 | | 3788 | hsa-mir-23c | 4281 | MID1 | midline 1 (Opitz/BBB syndrome) | 2 | | 3789 | hsa-mir-3679 | 4281 | MID1 | midline 1 (Opitz/BBB syndrome) | 2 | | 3790 | hsa-mir-3942 | 4281 | MID1 | midline 1 (Opitz/BBB syndrome) | 2 | | 3791 | hsa-mir-548o-2 | 4281 | MID1 | midline 1 (Opitz/BBB syndrome) | 2 | | 3792 | hsa-mir-4467 | 4281 | MID1 | midline 1 (Opitz/BBB syndrome) | 2 | | 3793 | hsa-mir-4498 | 4281 | MID1 | midline 1 (Opitz/BBB syndrome) | 2 | | 3794 | hsa-mir-4517 | 4281 | MID1 | midline 1 (Opitz/BBB syndrome) | 2 | | 3795 | hsa-mir-4534 | 4281 | MID1 | midline 1 (Opitz/BBB syndrome) | 2 | | 3796 | hsa-mir-4646 | 4281 | MID1 | midline 1 (Opitz/BBB syndrome) | 2 | | 3798 | hsa-mir-4698 | 4281 | MID1 | midline 1 (Opitz/BBB syndrome) | 2 | | 3800 | hsa-mir-4723 | 4281 | MID1 | midline 1 (Opitz/BBB syndrome) | 2 | | 3801 | hsa-mir-4771-1 | 4281 | MID1 | midline 1 (Opitz/BBB syndrome) | 2 | | 3802 | hsa-mir-4771-2 | 4281 | MID1 | midline 1 (Opitz/BBB syndrome) | 2 | | 3804 | hsa-mir-4775 | 4281 | MID1 | midline 1 (Opitz/BBB syndrome) | 2 | | 3805 | hsa-mir-5090 | 4281 | MID1 | midline 1 (Opitz/BBB syndrome) | 2 | | 3807 | hsa-mir-603 | 4281 | MID1 | midline 1 (Opitz/BBB syndrome) | 2 | | 3808 | hsa-mir-604 | 4281 | MID1 | midline 1 (Opitz/BBB syndrome) | 2 | | 3809 | hsa-mir-675 | 4281 | MID1 | midline 1 (Opitz/BBB syndrome) | 2 | | 3810 | hsa-mir-218-2 | 4281 | MID1 | midline 1 (Opitz/BBB syndrome) | 2 | | 3811 | hsa-mir-574 | 4281 | MID1 | midline 1 (Opitz/BBB syndrome) | 2 | | 3812 | hsa-mir-887 | 4281 | MID1 | midline 1 (Opitz/BBB syndrome) | 2 | | 3813 | hsa-mir-938 | 4281 | MID1 | midline 1 (Opitz/BBB syndrome) | 2 | | 3817 | hsa-mir-218-1 | 5433 | POLR2D | polymerase (RNA) II (DNA directed) polypeptide D | 2 | | 3820 | hsa-mir-561 | 5433 | POLR2D | polymerase (RNA) II (DNA directed) polypeptide D | 2 | | 3822 | hsa-mir-585 | 5433 | POLR2D | polymerase (RNA) II (DNA directed) polypeptide D | 2 | | 3827 | hsa-mir-620 | 5433 | POLR2D | polymerase (RNA) II (DNA directed) polypeptide D | 2 | | 3855 | hsa-mir-181a-2 | 8324 | FZD7 | frizzled family receptor 7 | 2 | | 3856 | hsa-mir-181b-2 | 8324 | FZD7 | frizzled family receptor 7 | 2 | | 3857 | hsa-mir-378a | 8324 | FZD7 | frizzled family receptor 7 | 2 | | 3858 | hsa-mir-505 | 8324 | FZD7 | frizzled family receptor 7 | 2 | | 3860 | hsa-mir-643 | 8324 | FZD7 | frizzled family receptor 7 | 2 | | 3879 | hsa-mir-181a-2 | 2067 | ERCC1 | excision repair cross-complementing rodent repair deficiency, complementation group 1 (includes overlapping antisense sequence) | 2 | | 3880 | hsa-mir-181b-2 | 2067 | ERCC1 | excision repair cross-complementing rodent repair deficiency, complementation group 1 (includes overlapping antisense sequence) | 2 | | 3881 | hsa-mir-505 | 2067 | ERCC1 | excision repair cross-complementing rodent repair deficiency, complementation group 1 (includes overlapping antisense sequence) | 2 | | 3905 | hsa-mir-218-1 | 51096 | UTP18 | UTP18 small subunit (SSU) processome component homolog (yeast) | 2 | | 3907 | hsa-mir-125b-1 | 51096 | UTP18 | UTP18 small subunit (SSU) processome component homolog (yeast) | 2 | | 3908 | hsa-mir-561 | 51096 | UTP18 | UTP18 small subunit (SSU) processome component homolog (yeast) | 2 | | 3910 | hsa-mir-585 | 51096 | UTP18 | UTP18 small subunit (SSU) processome component homolog (yeast) | 2 | | 3915 | hsa-mir-620 | 51096 | UTP18 | UTP18 small subunit (SSU) processome component homolog (yeast) | 2 | | 3940 | hsa-let-7c | 5442 | POLRMT | polymerase (RNA) mitochondrial (DNA directed) | 2 | | 3941 | hsa-mir-99a | 5442 | POLRMT | polymerase (RNA) mitochondrial (DNA directed) | 2 | | 3943 | hsa-mir-218-1 | 5442 | POLRMT | polymerase (RNA) mitochondrial (DNA directed) | 2 | | 3945 | hsa-mir-125b-1 | 5442 | POLRMT | polymerase (RNA) mitochondrial (DNA directed) | 2 | | 3946 | hsa-mir-125b-2 | 5442 | POLRMT | polymerase (RNA) mitochondrial (DNA directed) | 2 | | 3947 | hsa-mir-561 | 5442 | POLRMT | polymerase (RNA) mitochondrial (DNA directed) | 2 | | 3949 | hsa-mir-585 | 5442 | POLRMT | polymerase (RNA) mitochondrial (DNA directed) | 2 | | 3957 | hsa-mir-708 | 5442 | POLRMT | polymerase (RNA) mitochondrial (DNA directed) | 2 | | 3987 | hsa-let-7f-2 | 6586 | SLIT3 | slit homolog 3 (Drosophila) | 2 | | 3990 | hsa-mir-98 | 6586 | SLIT3 | slit homolog 3 (Drosophila) | 2 | | 3995 | hsa-mir-550a-1 | 6586 | SLIT3 | slit homolog 3 (Drosophila) | 2 | | 3996 | hsa-mir-602 | 6586 | SLIT3 | slit homolog 3 (Drosophila) | 2 | | 3999 | hsa-mir-644a | 6586 | SLIT3 | slit homolog 3 (Drosophila) | 2 | | 4000 | hsa-mir-652 | 6586 | SLIT3 | slit homolog 3 (Drosophila) | 2 | | 4001 | hsa-mir-877 | 6586 | SLIT3 | slit homolog 3 (Drosophila) | 2 | | 4003 | hsa-mir-548n | 6586 | SLIT3 | slit homolog 3 (Drosophila) | 2 | | 4005 | hsa-mir-548s | 6586 | SLIT3 | slit homolog 3 (Drosophila) | 2 | | 4009 | hsa-mir-3679 | 6586 | SLIT3 | slit homolog 3 (Drosophila) | 2 | | 4010 | hsa-mir-548o-2 | 6586 | SLIT3 | slit homolog 3 (Drosophila) | 2 | | 4011 | hsa-mir-378e | 6586 | SLIT3 | slit homolog 3 (Drosophila) | 2 | | 4012 | hsa-mir-4467 | 6586 | SLIT3 | slit homolog 3 (Drosophila) | 2 | | 4014 | hsa-mir-4534 | 6586 | SLIT3 | slit homolog 3 (Drosophila) | 2 | | 4017 | hsa-mir-4698 | 6586 | SLIT3 | slit homolog 3 (Drosophila) | 2 | | 4018 | hsa-mir-4723 | 6586 | SLIT3 | slit homolog 3 (Drosophila) | 2 | | 4019 | hsa-mir-4771-1 | 6586 | SLIT3 | slit homolog 3 (Drosophila) | 2 | | 4020 | hsa-mir-4771-2 | 6586 | SLIT3 | slit homolog 3 (Drosophila) | 2 | | 4021 | hsa-mir-4775 | 6586 | SLIT3 | slit homolog 3 (Drosophila) | 2 | | 4022 | hsa-mir-5090 | 6586 | SLIT3 | slit homolog 3 (Drosophila) | 2 | | 4023 | hsa-mir-5194 | 6586 | SLIT3 | slit homolog 3 (Drosophila) | 2 | | 4025 | hsa-mir-675 | 6586 | SLIT3 | slit homolog 3 (Drosophila) | 2 | | 4026 | hsa-mir-218-2 | 6586 | SLIT3 | slit homolog 3 (Drosophila) | 2 | | 4027 | hsa-mir-574 | 6586 | SLIT3 | slit homolog 3 (Drosophila) | 2 | | 4029 | hsa-mir-887 | 6586 | SLIT3 | slit homolog 3 (Drosophila) | 2 | | 4031 | hsa-mir-378a | 6586 | SLIT3 | slit homolog 3 (Drosophila) | 2 | | 4041 | hsa-mir-550a-1 | 6594 | SMARCA1 | SWI/SNF related, matrix associated, actin dependent regulator of chromatin, subfamily a, member 1 | 2 | | 4042 | hsa-mir-629 | 6594 | SMARCA1 | SWI/SNF related, matrix associated, actin dependent regulator of chromatin, subfamily a, member 1 | 2 | | 4045 | hsa-mir-877 | 6594 | SMARCA1 | SWI/SNF related, matrix associated, actin dependent regulator of chromatin, subfamily a, member 1 | 2 | | 4046 | hsa-mir-548s | 6594 | SMARCA1 | SWI/SNF related, matrix associated, actin dependent regulator of chromatin, subfamily a, member 1 | 2 | | 4047 | hsa-mir-3679 | 6594 | SMARCA1 | SWI/SNF related, matrix associated, actin dependent regulator of chromatin, subfamily a, member 1 | 2 | | 4048 | hsa-mir-4467 | 6594 | SMARCA1 | SWI/SNF related, matrix associated, actin dependent regulator of chromatin, subfamily a, member 1 | 2 | | 4049 | hsa-mir-4517 | 6594 | SMARCA1 | SWI/SNF related, matrix associated, actin dependent regulator of chromatin, subfamily a, member 1 | 2 | | 4050 | hsa-mir-3978 | 6594 | SMARCA1 | SWI/SNF related, matrix associated, actin dependent regulator of chromatin, subfamily a, member 1 | 2 | | 4051 | hsa-mir-4698 | 6594 | SMARCA1 | SWI/SNF related, matrix associated, actin dependent regulator of chromatin, subfamily a, member 1 | 2 | | 4052 | hsa-mir-5090 | 6594 | SMARCA1 | SWI/SNF related, matrix associated, actin dependent regulator of chromatin, subfamily a, member 1 | 2 | | 4053 | hsa-mir-604 | 6594 | SMARCA1 | SWI/SNF related, matrix associated, actin dependent regulator of chromatin, subfamily a, member 1 | 2 | | 4054 | hsa-mir-218-2 | 6594 | SMARCA1 | SWI/SNF related, matrix associated, actin dependent regulator of chromatin, subfamily a, member 1 | 2 | | 4055 | hsa-mir-574 | 6594 | SMARCA1 | SWI/SNF related, matrix associated, actin dependent regulator of chromatin, subfamily a, member 1 | 2 | | 4056 | hsa-mir-887 | 6594 | SMARCA1 | SWI/SNF related, matrix associated, actin dependent regulator of chromatin, subfamily a, member 1 | 2 | | 4057 | hsa-mir-938 | 6594 | SMARCA1 | SWI/SNF related, matrix associated, actin dependent regulator of chromatin, subfamily a, member 1 | 2 | | 4067 | hsa-let-7c | 5293 | PIK3CD | phosphoinositide-3-kinase, catalytic, delta polypeptide | 2 | | 4068 | hsa-mir-99a | 5293 | PIK3CD | phosphoinositide-3-kinase, catalytic, delta polypeptide | 2 | | 4070 | hsa-mir-218-1 | 5293 | PIK3CD | phosphoinositide-3-kinase, catalytic, delta polypeptide | 2 | | 4072 | hsa-mir-125b-1 | 5293 | PIK3CD | phosphoinositide-3-kinase, catalytic, delta polypeptide | 2 | | 4073 | hsa-mir-125b-2 | 5293 | PIK3CD | phosphoinositide-3-kinase, catalytic, delta polypeptide | 2 | | 4074 | hsa-mir-561 | 5293 | PIK3CD | phosphoinositide-3-kinase, catalytic, delta polypeptide | 2 | | 4076 | hsa-mir-585 | 5293 | PIK3CD | phosphoinositide-3-kinase, catalytic, delta polypeptide | 2 | | 4109 | hsa-mir-25 | 3248 | HPGD | hydroxyprostaglandin dehydrogenase 15-(NAD) | 2 | | 4110 | hsa-mir-93 | 3248 | HPGD | hydroxyprostaglandin dehydrogenase 15-(NAD) | 2 | | 4112 | hsa-mir-181a-2 | 3248 | HPGD | hydroxyprostaglandin dehydrogenase 15-(NAD) | 2 | | 4113 | hsa-mir-181b-2 | 3248 | HPGD | hydroxyprostaglandin dehydrogenase 15-(NAD) | 2 | | 4114 | hsa-mir-106b | 3248 | HPGD | hydroxyprostaglandin dehydrogenase 15-(NAD) | 2 | | 4115 | hsa-mir-378a | 3248 | HPGD | hydroxyprostaglandin dehydrogenase 15-(NAD) | 2 | | 4116 | hsa-mir-505 | 3248 | HPGD | hydroxyprostaglandin dehydrogenase 15-(NAD) | 2 | | 4120 | hsa-mir-652 | 3248 | HPGD | hydroxyprostaglandin dehydrogenase 15-(NAD) | 2 | | 4145 | hsa-let-7c | 55756 | INTS9 | integrator complex subunit 9 | 2 | | 4146 | hsa-mir-99a | 55756 | INTS9 | integrator complex subunit 9 | 2 | | 4147 | hsa-mir-218-1 | 55756 | INTS9 | integrator complex subunit 9 | 2 | | 4149 | hsa-mir-125b-1 | 55756 | INTS9 | integrator complex subunit 9 | 2 | | 4150 | hsa-mir-125b-2 | 55756 | INTS9 | integrator complex subunit 9 | 2 | | 4151 | hsa-mir-561 | 55756 | INTS9 | integrator complex subunit 9 | 2 | | 4153 | hsa-mir-585 | 55756 | INTS9 | integrator complex subunit 9 | 2 | | 4160 | hsa-mir-708 | 55756 | INTS9 | integrator complex subunit 9 | 2 | | 4189 | hsa-mir-218-1 | 8226 | HDHD1 | haloacid dehalogenase-like hydrolase domain containing 1 | 2 | | 4192 | hsa-mir-561 | 8226 | HDHD1 | haloacid dehalogenase-like hydrolase domain containing 1 | 2 | | 4194 | hsa-mir-585 | 8226 | HDHD1 | haloacid dehalogenase-like hydrolase domain containing 1 | 2 | | 4197 | hsa-mir-620 | 8226 | HDHD1 | haloacid dehalogenase-like hydrolase domain containing 1 | 2 | | 4217 | hsa-mir-25 | 11015 | KDELR3 | KDEL (Lys-Asp-Glu-Leu) endoplasmic reticulum protein retention receptor 3 | 2 | | 4218 | hsa-mir-93 | 11015 | KDELR3 | KDEL (Lys-Asp-Glu-Leu) endoplasmic reticulum protein retention receptor 3 | 2 | | 4220 | hsa-mir-181a-2 | 11015 | KDELR3 | KDEL (Lys-Asp-Glu-Leu) endoplasmic reticulum protein retention receptor 3 | 2 | | 4221 | hsa-mir-181b-2 | 11015 | KDELR3 | KDEL (Lys-Asp-Glu-Leu) endoplasmic reticulum protein retention receptor 3 | 2 | | 4222 | hsa-mir-106b | 11015 | KDELR3 | KDEL (Lys-Asp-Glu-Leu) endoplasmic reticulum protein retention receptor 3 | 2 | | 4224 | hsa-mir-505 | 11015 | KDELR3 | KDEL (Lys-Asp-Glu-Leu) endoplasmic reticulum protein retention receptor 3 | 2 | | 4230 | hsa-mir-643 | 11015 | KDELR3 | KDEL (Lys-Asp-Glu-Leu) endoplasmic reticulum protein retention receptor 3 | 2 | | 4262 | hsa-mir-10a | 5134 | PDCD2 | programmed cell death 2 | 2 | | 4264 | hsa-mir-218-2 | 5134 | PDCD2 | programmed cell death 2 | 2 | | 4267 | hsa-mir-574 | 5134 | PDCD2 | programmed cell death 2 | 2 | | 4269 | hsa-mir-603 | 5134 | PDCD2 | programmed cell death 2 | 2 | | 4272 | hsa-mir-1271 | 5134 | PDCD2 | programmed cell death 2 | 2 | | 4273 | hsa-mir-675 | 5134 | PDCD2 | programmed cell death 2 | 2 | | 4274 | hsa-mir-1245a | 5134 | PDCD2 | programmed cell death 2 | 2 | | 4275 | hsa-mir-1915 | 5134 | PDCD2 | programmed cell death 2 | 2 | | 4276 | hsa-mir-3120 | 5134 | PDCD2 | programmed cell death 2 | 2 | | 4277 | hsa-mir-3126 | 5134 | PDCD2 | programmed cell death 2 | 2 | | 4278 | hsa-mir-3129 | 5134 | PDCD2 | programmed cell death 2 | 2 | | 4279 | hsa-mir-3606 | 5134 | PDCD2 | programmed cell death 2 | 2 | | 4280 | hsa-mir-4441 | 5134 | PDCD2 | programmed cell death 2 | 2 | | 4281 | hsa-mir-4735 | 5134 | PDCD2 | programmed cell death 2 | 2 | | 4282 | hsa-mir-2467 | 5134 | PDCD2 | programmed cell death 2 | 2 | | 4283 | hsa-mir-4794 | 5134 | PDCD2 | programmed cell death 2 | 2 | | 4284 | hsa-mir-548ao | 5134 | PDCD2 | programmed cell death 2 | 2 | | 4285 | hsa-mir-26b | 5134 | PDCD2 | programmed cell death 2 | 2 | | 4292 | hsa-mir-877 | 5134 | PDCD2 | programmed cell death 2 | 2 | | 4293 | hsa-mir-1233-1;hsa-mir-1233-2 | 5134 | PDCD2 | programmed cell death 2 | 2 | | 4295 | hsa-mir-25 | 4211 | MEIS1 | Meis homeobox 1 | 2 | | 4296 | hsa-mir-93 | 4211 | MEIS1 | Meis homeobox 1 | 2 | | 4298 | hsa-mir-181a-2 | 4211 | MEIS1 | Meis homeobox 1 | 2 | | 4299 | hsa-mir-181b-2 | 4211 | MEIS1 | Meis homeobox 1 | 2 | | 4300 | hsa-mir-106b | 4211 | MEIS1 | Meis homeobox 1 | 2 | | 4302 | hsa-mir-505 | 4211 | MEIS1 | Meis homeobox 1 | 2 | | 4304 | hsa-mir-602 | 4211 | MEIS1 | Meis homeobox 1 | 2 | | 4307 | hsa-mir-643 | 4211 | MEIS1 | Meis homeobox 1 | 2 | | 4309 | hsa-mir-652 | 4211 | MEIS1 | Meis homeobox 1 | 2 | | 4339 | hsa-let-7c | 23532 | PRAME | preferentially expressed antigen in melanoma | 2 | | 4340 | hsa-mir-99a | 23532 | PRAME | preferentially expressed antigen in melanoma | 2 | | 4342 | hsa-mir-218-1 | 23532 | PRAME | preferentially expressed antigen in melanoma | 2 | | 4344 | hsa-mir-125b-1 | 23532 | PRAME | preferentially expressed antigen in melanoma | 2 | | 4345 | hsa-mir-125b-2 | 23532 | PRAME | preferentially expressed antigen in melanoma | 2 | | 4346 | hsa-mir-561 | 23532 | PRAME | preferentially expressed antigen in melanoma | 2 | | 4348 | hsa-mir-585 | 23532 | PRAME | preferentially expressed antigen in melanoma | 2 | | 4387 | hsa-mir-181a-2 | 22795 | NID2 | nidogen 2 (osteonidogen) | 2 | | 4388 | hsa-mir-181b-2 | 22795 | NID2 | nidogen 2 (osteonidogen) | 2 | | 4389 | hsa-mir-505 | 22795 | NID2 | nidogen 2 (osteonidogen) | 2 | | 4391 | hsa-mir-602 | 22795 | NID2 | nidogen 2 (osteonidogen) | 2 | | 4416 | hsa-mir-25 | 2791 | GNG11 | guanine nucleotide binding protein (G protein), gamma 11 | 2 | | 4417 | hsa-mir-93 | 2791 | GNG11 | guanine nucleotide binding protein (G protein), gamma 11 | 2 | | 4419 | hsa-mir-181a-2 | 2791 | GNG11 | guanine nucleotide binding protein (G protein), gamma 11 | 2 | | 4420 | hsa-mir-181b-2 | 2791 | GNG11 | guanine nucleotide binding protein (G protein), gamma 11 | 2 | | 4421 | hsa-mir-106b | 2791 | GNG11 | guanine nucleotide binding protein (G protein), gamma 11 | 2 | | 4423 | hsa-mir-505 | 2791 | GNG11 | guanine nucleotide binding protein (G protein), gamma 11 | 2 | | 4427 | hsa-mir-643 | 2791 | GNG11 | guanine nucleotide binding protein (G protein), gamma 11 | 2 | | 4449 | hsa-let-7c | 51042 | ZNF593 | zinc finger protein 593 | 2 | | 4450 | hsa-mir-99a | 51042 | ZNF593 | zinc finger protein 593 | 2 | | 4452 | hsa-mir-218-1 | 51042 | ZNF593 | zinc finger protein 593 | 2 | | 4454 | hsa-mir-125b-1 | 51042 | ZNF593 | zinc finger protein 593 | 2 | | 4455 | hsa-mir-125b-2 | 51042 | ZNF593 | zinc finger protein 593 | 2 | | 4456 | hsa-mir-561 | 51042 | ZNF593 | zinc finger protein 593 | 2 | | 4458 | hsa-mir-585 | 51042 | ZNF593 | zinc finger protein 593 | 2 | | 4479 | hsa-let-7f-2 | 51454 | GULP1 | GULP, engulfment adaptor PTB domain containing 1 | 2 | | 4482 | hsa-mir-98 | 51454 | GULP1 | GULP, engulfment adaptor PTB domain containing 1 | 2 | | 4488 | hsa-mir-550a-1 | 51454 | GULP1 | GULP, engulfment adaptor PTB domain containing 1 | 2 | | 4490 | hsa-mir-640 | 51454 | GULP1 | GULP, engulfment adaptor PTB domain containing 1 | 2 | | 4492 | hsa-mir-644a | 51454 | GULP1 | GULP, engulfment adaptor PTB domain containing 1 | 2 | | 4494 | hsa-mir-877 | 51454 | GULP1 | GULP, engulfment adaptor PTB domain containing 1 | 2 | | 4495 | hsa-mir-548s | 51454 | GULP1 | GULP, engulfment adaptor PTB domain containing 1 | 2 | | 4497 | hsa-mir-23c | 51454 | GULP1 | GULP, engulfment adaptor PTB domain containing 1 | 2 | | 4498 | hsa-mir-3679 | 51454 | GULP1 | GULP, engulfment adaptor PTB domain containing 1 | 2 | | 4499 | hsa-mir-3942 | 51454 | GULP1 | GULP, engulfment adaptor PTB domain containing 1 | 2 | | 4500 | hsa-mir-548o-2 | 51454 | GULP1 | GULP, engulfment adaptor PTB domain containing 1 | 2 | | 4501 | hsa-mir-378e | 51454 | GULP1 | GULP, engulfment adaptor PTB domain containing 1 | 2 | | 4502 | hsa-mir-4467 | 51454 | GULP1 | GULP, engulfment adaptor PTB domain containing 1 | 2 | | 4503 | hsa-mir-4498 | 51454 | GULP1 | GULP, engulfment adaptor PTB domain containing 1 | 2 | | 4504 | hsa-mir-4517 | 51454 | GULP1 | GULP, engulfment adaptor PTB domain containing 1 | 2 | | 4505 | hsa-mir-4534 | 51454 | GULP1 | GULP, engulfment adaptor PTB domain containing 1 | 2 | | 4506 | hsa-mir-3978 | 51454 | GULP1 | GULP, engulfment adaptor PTB domain containing 1 | 2 | | 4507 | hsa-mir-4668 | 51454 | GULP1 | GULP, engulfment adaptor PTB domain containing 1 | 2 | | 4508 | hsa-mir-4698 | 51454 | GULP1 | GULP, engulfment adaptor PTB domain containing 1 | 2 | | 4509 | hsa-mir-4723 | 51454 | GULP1 | GULP, engulfment adaptor PTB domain containing 1 | 2 | | 4510 | hsa-mir-4771-1 | 51454 | GULP1 | GULP, engulfment adaptor PTB domain containing 1 | 2 | | 4511 | hsa-mir-4771-2 | 51454 | GULP1 | GULP, engulfment adaptor PTB domain containing 1 | 2 | | 4512 | hsa-mir-4775 | 51454 | GULP1 | GULP, engulfment adaptor PTB domain containing 1 | 2 | | 4513 | hsa-mir-5090 | 51454 | GULP1 | GULP, engulfment adaptor PTB domain containing 1 | 2 | | 4514 | hsa-mir-5194 | 51454 | GULP1 | GULP, engulfment adaptor PTB domain containing 1 | 2 | | 4515 | hsa-mir-603 | 51454 | GULP1 | GULP, engulfment adaptor PTB domain containing 1 | 2 | | 4516 | hsa-mir-604 | 51454 | GULP1 | GULP, engulfment adaptor PTB domain containing 1 | 2 | | 4517 | hsa-mir-675 | 51454 | GULP1 | GULP, engulfment adaptor PTB domain containing 1 | 2 | | 4518 | hsa-mir-218-2 | 51454 | GULP1 | GULP, engulfment adaptor PTB domain containing 1 | 2 | | 4519 | hsa-mir-574 | 51454 | GULP1 | GULP, engulfment adaptor PTB domain containing 1 | 2 | | 4520 | hsa-mir-887 | 51454 | GULP1 | GULP, engulfment adaptor PTB domain containing 1 | 2 | | 4521 | hsa-mir-938 | 51454 | GULP1 | GULP, engulfment adaptor PTB domain containing 1 | 2 | | 4524 | hsa-let-7c | 79071 | ELOVL6 | ELOVL fatty acid elongase 6 | 2 | | 4526 | hsa-mir-10a | 79071 | ELOVL6 | ELOVL fatty acid elongase 6 | 2 | | 4528 | hsa-mir-218-2 | 79071 | ELOVL6 | ELOVL fatty acid elongase 6 | 2 | | 4532 | hsa-mir-574 | 79071 | ELOVL6 | ELOVL fatty acid elongase 6 | 2 | | 4534 | hsa-mir-603 | 79071 | ELOVL6 | ELOVL fatty acid elongase 6 | 2 | | 4536 | hsa-mir-605 | 79071 | ELOVL6 | ELOVL fatty acid elongase 6 | 2 | | 4540 | hsa-mir-1271 | 79071 | ELOVL6 | ELOVL fatty acid elongase 6 | 2 | | 4541 | hsa-mir-675 | 79071 | ELOVL6 | ELOVL fatty acid elongase 6 | 2 | | 4544 | hsa-mir-663b | 79071 | ELOVL6 | ELOVL fatty acid elongase 6 | 2 | | 4545 | hsa-mir-1245a | 79071 | ELOVL6 | ELOVL fatty acid elongase 6 | 2 | | 4547 | hsa-mir-3120 | 79071 | ELOVL6 | ELOVL fatty acid elongase 6 | 2 | | 4548 | hsa-mir-3126 | 79071 | ELOVL6 | ELOVL fatty acid elongase 6 | 2 | | 4549 | hsa-mir-3129 | 79071 | ELOVL6 | ELOVL fatty acid elongase 6 | 2 | | 4550 | hsa-mir-3606 | 79071 | ELOVL6 | ELOVL fatty acid elongase 6 | 2 | | 4552 | hsa-mir-4441 | 79071 | ELOVL6 | ELOVL fatty acid elongase 6 | 2 | | 4555 | hsa-mir-4735 | 79071 | ELOVL6 | ELOVL fatty acid elongase 6 | 2 | | 4557 | hsa-mir-2467 | 79071 | ELOVL6 | ELOVL fatty acid elongase 6 | 2 | | 4558 | hsa-mir-4794 | 79071 | ELOVL6 | ELOVL fatty acid elongase 6 | 2 | | 4559 | hsa-mir-548ao | 79071 | ELOVL6 | ELOVL fatty acid elongase 6 | 2 | | 4561 | hsa-mir-591 | 79071 | ELOVL6 | ELOVL fatty acid elongase 6 | 2 | | 4563 | hsa-mir-636 | 79071 | ELOVL6 | ELOVL fatty acid elongase 6 | 2 | | 4564 | hsa-mir-640 | 79071 | ELOVL6 | ELOVL fatty acid elongase 6 | 2 | | 4565 | hsa-mir-877 | 79071 | ELOVL6 | ELOVL fatty acid elongase 6 | 2 | | 4566 | hsa-let-7c | 9088 | PKMYT1 | protein kinase, membrane associated tyrosine/threonine 1 | 2 | | 4567 | hsa-mir-99a | 9088 | PKMYT1 | protein kinase, membrane associated tyrosine/threonine 1 | 2 | | 4570 | hsa-mir-125b-1 | 9088 | PKMYT1 | protein kinase, membrane associated tyrosine/threonine 1 | 2 | | 4571 | hsa-mir-125b-2 | 9088 | PKMYT1 | protein kinase, membrane associated tyrosine/threonine 1 | 2 | | 4572 | hsa-mir-561 | 9088 | PKMYT1 | protein kinase, membrane associated tyrosine/threonine 1 | 2 | | 4574 | hsa-mir-585 | 9088 | PKMYT1 | protein kinase, membrane associated tyrosine/threonine 1 | 2 | | 4601 | hsa-mir-10a | 7004 | TEAD4 | TEA domain family member 4 | 2 | | 4603 | hsa-mir-218-2 | 7004 | TEAD4 | TEA domain family member 4 | 2 | | 4607 | hsa-mir-574 | 7004 | TEAD4 | TEA domain family member 4 | 2 | | 4609 | hsa-mir-603 | 7004 | TEAD4 | TEA domain family member 4 | 2 | | 4610 | hsa-mir-604 | 7004 | TEAD4 | TEA domain family member 4 | 2 | | 4611 | hsa-mir-605 | 7004 | TEAD4 | TEA domain family member 4 | 2 | | 4613 | hsa-mir-618 | 7004 | TEAD4 | TEA domain family member 4 | 2 | | 4614 | hsa-mir-1271 | 7004 | TEAD4 | TEA domain family member 4 | 2 | | 4615 | hsa-mir-675 | 7004 | TEAD4 | TEA domain family member 4 | 2 | | 4617 | hsa-mir-887 | 7004 | TEAD4 | TEA domain family member 4 | 2 | | 4618 | hsa-mir-938 | 7004 | TEAD4 | TEA domain family member 4 | 2 | | 4619 | hsa-mir-1245a | 7004 | TEAD4 | TEA domain family member 4 | 2 | | 4620 | hsa-mir-1469 | 7004 | TEAD4 | TEA domain family member 4 | 2 | | 4621 | hsa-mir-3120 | 7004 | TEAD4 | TEA domain family member 4 | 2 | | 4622 | hsa-mir-3126 | 7004 | TEAD4 | TEA domain family member 4 | 2 | | 4623 | hsa-mir-3129 | 7004 | TEAD4 | TEA domain family member 4 | 2 | | 4624 | hsa-mir-3139 | 7004 | TEAD4 | TEA domain family member 4 | 2 | | 4625 | hsa-mir-3170 | 7004 | TEAD4 | TEA domain family member 4 | 2 | | 4626 | hsa-mir-3606 | 7004 | TEAD4 | TEA domain family member 4 | 2 | | 4627 | hsa-mir-3650 | 7004 | TEAD4 | TEA domain family member 4 | 2 | | 4628 | hsa-mir-4441 | 7004 | TEAD4 | TEA domain family member 4 | 2 | | 4629 | hsa-mir-4486 | 7004 | TEAD4 | TEA domain family member 4 | 2 | | 4630 | hsa-mir-4636 | 7004 | TEAD4 | TEA domain family member 4 | 2 | | 4631 | hsa-mir-4731 | 7004 | TEAD4 | TEA domain family member 4 | 2 | | 4633 | hsa-mir-4768 | 7004 | TEAD4 | TEA domain family member 4 | 2 | | 4634 | hsa-mir-2467 | 7004 | TEAD4 | TEA domain family member 4 | 2 | | 4635 | hsa-mir-4794 | 7004 | TEAD4 | TEA domain family member 4 | 2 | | 4637 | hsa-mir-5579 | 7004 | TEAD4 | TEA domain family member 4 | 2 | | 4638 | hsa-mir-503 | 7004 | TEAD4 | TEA domain family member 4 | 2 | | 4639 | hsa-mir-455 | 7004 | TEAD4 | TEA domain family member 4 | 2 | | 4640 | hsa-mir-617 | 7004 | TEAD4 | TEA domain family member 4 | 2 | | 4641 | hsa-mir-640 | 7004 | TEAD4 | TEA domain family member 4 | 2 | | 4642 | hsa-mir-877 | 7004 | TEAD4 | TEA domain family member 4 | 2 | | 4644 | hsa-mir-218-1 | 6834 | SURF1 | surfeit 1 | 2 | | 4646 | hsa-mir-125b-1 | 6834 | SURF1 | surfeit 1 | 2 | | 4647 | hsa-mir-561 | 6834 | SURF1 | surfeit 1 | 2 | | 4649 | hsa-mir-585 | 6834 | SURF1 | surfeit 1 | 2 | | 4679 | hsa-mir-25 | 23768 | FLRT2 | fibronectin leucine rich transmembrane protein 2 | 2 | | 4680 | hsa-mir-93 | 23768 | FLRT2 | fibronectin leucine rich transmembrane protein 2 | 2 | | 4682 | hsa-mir-181a-2 | 23768 | FLRT2 | fibronectin leucine rich transmembrane protein 2 | 2 | | 4683 | hsa-mir-181b-2 | 23768 | FLRT2 | fibronectin leucine rich transmembrane protein 2 | 2 | | 4684 | hsa-mir-106b | 23768 | FLRT2 | fibronectin leucine rich transmembrane protein 2 | 2 | | 4685 | hsa-mir-505 | 23768 | FLRT2 | fibronectin leucine rich transmembrane protein 2 | 2 | | 4687 | hsa-mir-602 | 23768 | FLRT2 | fibronectin leucine rich transmembrane protein 2 | 2 | | 4689 | hsa-mir-643 | 23768 | FLRT2 | fibronectin leucine rich transmembrane protein 2 | 2 | | 4722 | hsa-mir-10a | 8220 | DGCR14 | DiGeorge syndrome critical region gene 14 | 2 | | 4723 | hsa-mir-218-2 | 8220 | DGCR14 | DiGeorge syndrome critical region gene 14 | 2 | | 4726 | hsa-mir-574 | 8220 | DGCR14 | DiGeorge syndrome critical region gene 14 | 2 | | 4728 | hsa-mir-603 | 8220 | DGCR14 | DiGeorge syndrome critical region gene 14 | 2 | | 4729 | hsa-mir-604 | 8220 | DGCR14 | DiGeorge syndrome critical region gene 14 | 2 | | 4730 | hsa-mir-605 | 8220 | DGCR14 | DiGeorge syndrome critical region gene 14 | 2 | | 4732 | hsa-mir-887 | 8220 | DGCR14 | DiGeorge syndrome critical region gene 14 | 2 | | 4733 | hsa-mir-938 | 8220 | DGCR14 | DiGeorge syndrome critical region gene 14 | 2 | | 4734 | hsa-mir-3126 | 8220 | DGCR14 | DiGeorge syndrome critical region gene 14 | 2 | | 4735 | hsa-mir-3129 | 8220 | DGCR14 | DiGeorge syndrome critical region gene 14 | 2 | | 4736 | hsa-mir-4441 | 8220 | DGCR14 | DiGeorge syndrome critical region gene 14 | 2 | | 4737 | hsa-mir-4768 | 8220 | DGCR14 | DiGeorge syndrome critical region gene 14 | 2 | | 4738 | hsa-mir-2467 | 8220 | DGCR14 | DiGeorge syndrome critical region gene 14 | 2 | | 4739 | hsa-mir-4794 | 8220 | DGCR14 | DiGeorge syndrome critical region gene 14 | 2 | | 4740 | hsa-mir-548ao | 8220 | DGCR14 | DiGeorge syndrome critical region gene 14 | 2 | | 4741 | hsa-mir-617 | 8220 | DGCR14 | DiGeorge syndrome critical region gene 14 | 2 | | 4744 | hsa-mir-877 | 8220 | DGCR14 | DiGeorge syndrome critical region gene 14 | 2 | | 4745 | hsa-let-7c | 8501 | SLC43A1 | solute carrier family 43, member 1 | 2 | | 4746 | hsa-mir-99a | 8501 | SLC43A1 | solute carrier family 43, member 1 | 2 | | 4749 | hsa-mir-125b-1 | 8501 | SLC43A1 | solute carrier family 43, member 1 | 2 | | 4750 | hsa-mir-125b-2 | 8501 | SLC43A1 | solute carrier family 43, member 1 | 2 | | 4751 | hsa-mir-561 | 8501 | SLC43A1 | solute carrier family 43, member 1 | 2 | | 4753 | hsa-mir-585 | 8501 | SLC43A1 | solute carrier family 43, member 1 | 2 | | 4760 | hsa-mir-708 | 8501 | SLC43A1 | solute carrier family 43, member 1 | 2 | | 4794 | hsa-mir-550a-1 | 10278 | EFS | embryonal Fyn-associated substrate | 2 | | 4796 | hsa-mir-644a | 10278 | EFS | embryonal Fyn-associated substrate | 2 | | 4797 | hsa-mir-877 | 10278 | EFS | embryonal Fyn-associated substrate | 2 | | 4798 | hsa-mir-548j | 10278 | EFS | embryonal Fyn-associated substrate | 2 | | 4799 | hsa-mir-548n | 10278 | EFS | embryonal Fyn-associated substrate | 2 | | 4800 | hsa-mir-2355 | 10278 | EFS | embryonal Fyn-associated substrate | 2 | | 4801 | hsa-mir-3942 | 10278 | EFS | embryonal Fyn-associated substrate | 2 | | 4802 | hsa-mir-548o-2 | 10278 | EFS | embryonal Fyn-associated substrate | 2 | | 4803 | hsa-mir-378e | 10278 | EFS | embryonal Fyn-associated substrate | 2 | | 4804 | hsa-mir-4698 | 10278 | EFS | embryonal Fyn-associated substrate | 2 | | 4805 | hsa-mir-4723 | 10278 | EFS | embryonal Fyn-associated substrate | 2 | | 4806 | hsa-mir-4771-1 | 10278 | EFS | embryonal Fyn-associated substrate | 2 | | 4807 | hsa-mir-4771-2 | 10278 | EFS | embryonal Fyn-associated substrate | 2 | | 4808 | hsa-mir-4775 | 10278 | EFS | embryonal Fyn-associated substrate | 2 | | 4810 | hsa-mir-675 | 10278 | EFS | embryonal Fyn-associated substrate | 2 | | 4811 | hsa-mir-218-2 | 10278 | EFS | embryonal Fyn-associated substrate | 2 | | 4812 | hsa-mir-574 | 10278 | EFS | embryonal Fyn-associated substrate | 2 | | 4813 | hsa-let-7c | 27301 | APEX2 | APEX nuclease (apurinic/apyrimidinic endonuclease) 2 | 2 | | 4814 | hsa-mir-99a | 27301 | APEX2 | APEX nuclease (apurinic/apyrimidinic endonuclease) 2 | 2 | | 4817 | hsa-mir-125b-2 | 27301 | APEX2 | APEX nuclease (apurinic/apyrimidinic endonuclease) 2 | 2 | | 4819 | hsa-mir-561 | 27301 | APEX2 | APEX nuclease (apurinic/apyrimidinic endonuclease) 2 | 2 | | 4821 | hsa-mir-585 | 27301 | APEX2 | APEX nuclease (apurinic/apyrimidinic endonuclease) 2 | 2 | | 4824 | hsa-mir-620 | 27301 | APEX2 | APEX nuclease (apurinic/apyrimidinic endonuclease) 2 | 2 | | 4836 | hsa-mir-181a-2 | 7089 | TLE2 | transducin-like enhancer of split 2 (E(sp1) homolog, Drosophila) | 2 | | 4837 | hsa-mir-181b-2 | 7089 | TLE2 | transducin-like enhancer of split 2 (E(sp1) homolog, Drosophila) | 2 | | 4838 | hsa-mir-505 | 7089 | TLE2 | transducin-like enhancer of split 2 (E(sp1) homolog, Drosophila) | 2 | | 4858 | hsa-let-7c | 9825 | SPATA2 | spermatogenesis associated 2 | 2 | | 4859 | hsa-mir-99a | 9825 | SPATA2 | spermatogenesis associated 2 | 2 | | 4862 | hsa-mir-125b-2 | 9825 | SPATA2 | spermatogenesis associated 2 | 2 | | 4864 | hsa-mir-561 | 9825 | SPATA2 | spermatogenesis associated 2 | 2 | | 4866 | hsa-mir-585 | 9825 | SPATA2 | spermatogenesis associated 2 | 2 | | 4869 | hsa-mir-620 | 9825 | SPATA2 | spermatogenesis associated 2 | 2 | | 4885 | hsa-mir-561 | 9308 | CD83 | CD83 molecule | 2 | | 4887 | hsa-mir-585 | 9308 | CD83 | CD83 molecule | 2 | | 4903 | hsa-mir-181a-2 | 8321 | FZD1 | frizzled family receptor 1 | 2 | | 4904 | hsa-mir-181b-2 | 8321 | FZD1 | frizzled family receptor 1 | 2 | | 4905 | hsa-mir-378a | 8321 | FZD1 | frizzled family receptor 1 | 2 | | 4906 | hsa-mir-505 | 8321 | FZD1 | frizzled family receptor 1 | 2 | | 4908 | hsa-mir-643 | 8321 | FZD1 | frizzled family receptor 1 | 2 | | 4922 | hsa-mir-218-1 | 1478 | CSTF2 | cleavage stimulation factor, 3' pre-RNA, subunit 2, 64kDa | 2 | | 4925 | hsa-mir-455 | 1478 | CSTF2 | cleavage stimulation factor, 3' pre-RNA, subunit 2, 64kDa | 2 | | 4926 | hsa-mir-561 | 1478 | CSTF2 | cleavage stimulation factor, 3' pre-RNA, subunit 2, 64kDa | 2 | | 4928 | hsa-mir-585 | 1478 | CSTF2 | cleavage stimulation factor, 3' pre-RNA, subunit 2, 64kDa | 2 | | 4932 | hsa-mir-620 | 1478 | CSTF2 | cleavage stimulation factor, 3' pre-RNA, subunit 2, 64kDa | 2 | | 4935 | hsa-mir-708 | 1478 | CSTF2 | cleavage stimulation factor, 3' pre-RNA, subunit 2, 64kDa | 2 | | 4955 | hsa-let-7f-2 | 1909 | EDNRA | endothelin receptor type A | 2 | | 4956 | hsa-mir-98 | 1909 | EDNRA | endothelin receptor type A | 2 | | 4960 | hsa-mir-550a-1 | 1909 | EDNRA | endothelin receptor type A | 2 | | 4962 | hsa-mir-644a | 1909 | EDNRA | endothelin receptor type A | 2 | | 4963 | hsa-mir-877 | 1909 | EDNRA | endothelin receptor type A | 2 | | 4964 | hsa-mir-548j | 1909 | EDNRA | endothelin receptor type A | 2 | | 4965 | hsa-mir-1290 | 1909 | EDNRA | endothelin receptor type A | 2 | | 4966 | hsa-mir-548n | 1909 | EDNRA | endothelin receptor type A | 2 | | 4967 | hsa-mir-2909 | 1909 | EDNRA | endothelin receptor type A | 2 | | 4968 | hsa-mir-548s | 1909 | EDNRA | endothelin receptor type A | 2 | | 4971 | hsa-mir-23c | 1909 | EDNRA | endothelin receptor type A | 2 | | 4974 | hsa-mir-378e | 1909 | EDNRA | endothelin receptor type A | 2 | | 4975 | hsa-mir-4534 | 1909 | EDNRA | endothelin receptor type A | 2 | | 4976 | hsa-mir-4695 | 1909 | EDNRA | endothelin receptor type A | 2 | | 4977 | hsa-mir-4698 | 1909 | EDNRA | endothelin receptor type A | 2 | | 4979 | hsa-mir-4723 | 1909 | EDNRA | endothelin receptor type A | 2 | | 4983 | hsa-mir-5194 | 1909 | EDNRA | endothelin receptor type A | 2 | | 4985 | hsa-mir-604 | 1909 | EDNRA | endothelin receptor type A | 2 | | 4987 | hsa-mir-218-2 | 1909 | EDNRA | endothelin receptor type A | 2 | | 4988 | hsa-mir-574 | 1909 | EDNRA | endothelin receptor type A | 2 | | 4989 | hsa-mir-938 | 1909 | EDNRA | endothelin receptor type A | 2 | | 4990 | hsa-mir-25 | 1909 | EDNRA | endothelin receptor type A | 2 | | 4991 | hsa-mir-93 | 1909 | EDNRA | endothelin receptor type A | 2 | | 4992 | hsa-mir-106b | 1909 | EDNRA | endothelin receptor type A | 2 | | 4999 | hsa-mir-218-1 | 7701 | ZNF142 | zinc finger protein 142 | 2 | | 5002 | hsa-mir-561 | 7701 | ZNF142 | zinc finger protein 142 | 2 | | 5004 | hsa-mir-585 | 7701 | ZNF142 | zinc finger protein 142 | 2 | | 5008 | hsa-mir-620 | 7701 | ZNF142 | zinc finger protein 142 | 2 | | 5027 | hsa-mir-181a-2 | 22873 | DZIP1 | DAZ interacting protein 1 | 2 | | 5028 | hsa-mir-181b-2 | 22873 | DZIP1 | DAZ interacting protein 1 | 2 | | 5030 | hsa-mir-505 | 22873 | DZIP1 | DAZ interacting protein 1 | 2 | | 5035 | hsa-mir-643 | 22873 | DZIP1 | DAZ interacting protein 1 | 2 | | 5050 | hsa-let-7c | 4321 | MMP12 | matrix metallopeptidase 12 (macrophage elastase) | 2 | | 5051 | hsa-mir-99a | 4321 | MMP12 | matrix metallopeptidase 12 (macrophage elastase) | 2 | | 5053 | hsa-mir-218-1 | 4321 | MMP12 | matrix metallopeptidase 12 (macrophage elastase) | 2 | | 5055 | hsa-mir-125b-1 | 4321 | MMP12 | matrix metallopeptidase 12 (macrophage elastase) | 2 | | 5056 | hsa-mir-125b-2 | 4321 | MMP12 | matrix metallopeptidase 12 (macrophage elastase) | 2 | | 5057 | hsa-mir-561 | 4321 | MMP12 | matrix metallopeptidase 12 (macrophage elastase) | 2 | | 5059 | hsa-mir-585 | 4321 | MMP12 | matrix metallopeptidase 12 (macrophage elastase) | 2 | | 5067 | hsa-mir-708 | 4321 | MMP12 | matrix metallopeptidase 12 (macrophage elastase) | 2 | | 5093 | hsa-mir-218-1 | 9683 | N4BP1 | NEDD4 binding protein 1 | 2 | | 5095 | hsa-mir-455 | 9683 | N4BP1 | NEDD4 binding protein 1 | 2 | | 5096 | hsa-mir-561 | 9683 | N4BP1 | NEDD4 binding protein 1 | 2 | | 5098 | hsa-mir-585 | 9683 | N4BP1 | NEDD4 binding protein 1 | 2 | | 5100 | hsa-mir-620 | 9683 | N4BP1 | NEDD4 binding protein 1 | 2 | | 5103 | hsa-mir-708 | 9683 | N4BP1 | NEDD4 binding protein 1 | 2 | | 5122 | hsa-mir-25 | 1462 | VCAN | versican | 2 | | 5123 | hsa-mir-93 | 1462 | VCAN | versican | 2 | | 5127 | hsa-mir-106b | 1462 | VCAN | versican | 2 | | 5132 | hsa-mir-636 | 1462 | VCAN | versican | 2 | | 5133 | hsa-mir-640 | 1462 | VCAN | versican | 2 | | 5151 | hsa-mir-4534 | 1462 | VCAN | versican | 2 | | 5153 | hsa-mir-4646 | 1462 | VCAN | versican | 2 | | 5155 | hsa-mir-4709 | 1462 | VCAN | versican | 2 | | 5159 | hsa-mir-4774 | 1462 | VCAN | versican | 2 | | 5163 | hsa-mir-603 | 1462 | VCAN | versican | 2 | | 5170 | hsa-mir-548aa-2 | 1462 | VCAN | versican | 2 | | 5171 | hsa-let-7c | 54 | ACP5 | acid phosphatase 5, tartrate resistant | 2 | | 5172 | hsa-mir-99a | 54 | ACP5 | acid phosphatase 5, tartrate resistant | 2 | | 5175 | hsa-mir-125b-2 | 54 | ACP5 | acid phosphatase 5, tartrate resistant | 2 | | 5176 | hsa-mir-561 | 54 | ACP5 | acid phosphatase 5, tartrate resistant | 2 | | 5178 | hsa-mir-585 | 54 | ACP5 | acid phosphatase 5, tartrate resistant | 2 | | 5196 | hsa-mir-181a-2 | 3667 | IRS1 | insulin receptor substrate 1 | 2 | | 5197 | hsa-mir-181b-2 | 3667 | IRS1 | insulin receptor substrate 1 | 2 | | 5198 | hsa-mir-378a | 3667 | IRS1 | insulin receptor substrate 1 | 2 | | 5200 | hsa-mir-643 | 3667 | IRS1 | insulin receptor substrate 1 | 2 | | 5222 | hsa-mir-10a | 9603 | NFE2L3 | nuclear factor (erythroid-derived 2)-like 3 | 2 | | 5224 | hsa-mir-218-2 | 9603 | NFE2L3 | nuclear factor (erythroid-derived 2)-like 3 | 2 | | 5228 | hsa-mir-574 | 9603 | NFE2L3 | nuclear factor (erythroid-derived 2)-like 3 | 2 | | 5230 | hsa-mir-603 | 9603 | NFE2L3 | nuclear factor (erythroid-derived 2)-like 3 | 2 | | 5231 | hsa-mir-604 | 9603 | NFE2L3 | nuclear factor (erythroid-derived 2)-like 3 | 2 | | 5234 | hsa-mir-618 | 9603 | NFE2L3 | nuclear factor (erythroid-derived 2)-like 3 | 2 | | 5236 | hsa-mir-1271 | 9603 | NFE2L3 | nuclear factor (erythroid-derived 2)-like 3 | 2 | | 5237 | hsa-mir-675 | 9603 | NFE2L3 | nuclear factor (erythroid-derived 2)-like 3 | 2 | | 5238 | hsa-mir-887 | 9603 | NFE2L3 | nuclear factor (erythroid-derived 2)-like 3 | 2 | | 5239 | hsa-mir-938 | 9603 | NFE2L3 | nuclear factor (erythroid-derived 2)-like 3 | 2 | | 5242 | hsa-mir-1469 | 9603 | NFE2L3 | nuclear factor (erythroid-derived 2)-like 3 | 2 | | 5243 | hsa-mir-3120 | 9603 | NFE2L3 | nuclear factor (erythroid-derived 2)-like 3 | 2 | | 5244 | hsa-mir-3126 | 9603 | NFE2L3 | nuclear factor (erythroid-derived 2)-like 3 | 2 | | 5245 | hsa-mir-3129 | 9603 | NFE2L3 | nuclear factor (erythroid-derived 2)-like 3 | 2 | | 5246 | hsa-mir-3139 | 9603 | NFE2L3 | nuclear factor (erythroid-derived 2)-like 3 | 2 | | 5248 | hsa-mir-3650 | 9603 | NFE2L3 | nuclear factor (erythroid-derived 2)-like 3 | 2 | | 5249 | hsa-mir-4441 | 9603 | NFE2L3 | nuclear factor (erythroid-derived 2)-like 3 | 2 | | 5250 | hsa-mir-4636 | 9603 | NFE2L3 | nuclear factor (erythroid-derived 2)-like 3 | 2 | | 5252 | hsa-mir-4735 | 9603 | NFE2L3 | nuclear factor (erythroid-derived 2)-like 3 | 2 | | 5253 | hsa-mir-4768 | 9603 | NFE2L3 | nuclear factor (erythroid-derived 2)-like 3 | 2 | | 5254 | hsa-mir-2467 | 9603 | NFE2L3 | nuclear factor (erythroid-derived 2)-like 3 | 2 | | 5255 | hsa-mir-4794 | 9603 | NFE2L3 | nuclear factor (erythroid-derived 2)-like 3 | 2 | | 5256 | hsa-mir-548ao | 9603 | NFE2L3 | nuclear factor (erythroid-derived 2)-like 3 | 2 | | 5259 | hsa-mir-617 | 9603 | NFE2L3 | nuclear factor (erythroid-derived 2)-like 3 | 2 | | 5261 | hsa-mir-640 | 9603 | NFE2L3 | nuclear factor (erythroid-derived 2)-like 3 | 2 | | 5262 | hsa-mir-877 | 9603 | NFE2L3 | nuclear factor (erythroid-derived 2)-like 3 | 2 | | 5263 | hsa-mir-181a-2 | 89795 | NAV3 | neuron navigator 3 | 2 | | 5264 | hsa-mir-181b-2 | 89795 | NAV3 | neuron navigator 3 | 2 | | 5265 | hsa-mir-505 | 89795 | NAV3 | neuron navigator 3 | 2 | | 5268 | hsa-mir-643 | 89795 | NAV3 | neuron navigator 3 | 2 | | 5295 | hsa-mir-10a | 10630 | PDPN | podoplanin | 2 | | 5297 | hsa-mir-218-2 | 10630 | PDPN | podoplanin | 2 | | 5300 | hsa-mir-574 | 10630 | PDPN | podoplanin | 2 | | 5302 | hsa-mir-603 | 10630 | PDPN | podoplanin | 2 | | 5303 | hsa-mir-605 | 10630 | PDPN | podoplanin | 2 | | 5306 | hsa-mir-675 | 10630 | PDPN | podoplanin | 2 | | 5308 | hsa-mir-1245a | 10630 | PDPN | podoplanin | 2 | | 5310 | hsa-mir-1915 | 10630 | PDPN | podoplanin | 2 | | 5311 | hsa-mir-3126 | 10630 | PDPN | podoplanin | 2 | | 5312 | hsa-mir-3129 | 10630 | PDPN | podoplanin | 2 | | 5313 | hsa-mir-3606 | 10630 | PDPN | podoplanin | 2 | | 5314 | hsa-mir-3650 | 10630 | PDPN | podoplanin | 2 | | 5315 | hsa-mir-4441 | 10630 | PDPN | podoplanin | 2 | | 5316 | hsa-mir-4735 | 10630 | PDPN | podoplanin | 2 | | 5317 | hsa-mir-2467 | 10630 | PDPN | podoplanin | 2 | | 5318 | hsa-mir-4794 | 10630 | PDPN | podoplanin | 2 | | 5319 | hsa-mir-548ao | 10630 | PDPN | podoplanin | 2 | | 5321 | hsa-mir-218-2 | 3364 | HUS1 | HUS1 checkpoint homolog (S. pombe) | 2 | | 5324 | hsa-mir-574 | 3364 | HUS1 | HUS1 checkpoint homolog (S. pombe) | 2 | | 5326 | hsa-mir-603 | 3364 | HUS1 | HUS1 checkpoint homolog (S. pombe) | 2 | | 5327 | hsa-mir-605 | 3364 | HUS1 | HUS1 checkpoint homolog (S. pombe) | 2 | | 5328 | hsa-mir-620 | 3364 | HUS1 | HUS1 checkpoint homolog (S. pombe) | 2 | | 5329 | hsa-mir-675 | 3364 | HUS1 | HUS1 checkpoint homolog (S. pombe) | 2 | | 5330 | hsa-mir-1245a | 3364 | HUS1 | HUS1 checkpoint homolog (S. pombe) | 2 | | 5331 | hsa-mir-1915 | 3364 | HUS1 | HUS1 checkpoint homolog (S. pombe) | 2 | | 5332 | hsa-mir-3120 | 3364 | HUS1 | HUS1 checkpoint homolog (S. pombe) | 2 | | 5333 | hsa-mir-3126 | 3364 | HUS1 | HUS1 checkpoint homolog (S. pombe) | 2 | | 5334 | hsa-mir-3129 | 3364 | HUS1 | HUS1 checkpoint homolog (S. pombe) | 2 | | 5335 | hsa-mir-3606 | 3364 | HUS1 | HUS1 checkpoint homolog (S. pombe) | 2 | | 5336 | hsa-mir-3650 | 3364 | HUS1 | HUS1 checkpoint homolog (S. pombe) | 2 | | 5337 | hsa-mir-4441 | 3364 | HUS1 | HUS1 checkpoint homolog (S. pombe) | 2 | | 5338 | hsa-mir-4486 | 3364 | HUS1 | HUS1 checkpoint homolog (S. pombe) | 2 | | 5339 | hsa-mir-4735 | 3364 | HUS1 | HUS1 checkpoint homolog (S. pombe) | 2 | | 5340 | hsa-mir-4768 | 3364 | HUS1 | HUS1 checkpoint homolog (S. pombe) | 2 | | 5341 | hsa-mir-2467 | 3364 | HUS1 | HUS1 checkpoint homolog (S. pombe) | 2 | | 5342 | hsa-mir-4794 | 3364 | HUS1 | HUS1 checkpoint homolog (S. pombe) | 2 | | 5343 | hsa-mir-548ao | 3364 | HUS1 | HUS1 checkpoint homolog (S. pombe) | 2 | | 5347 | hsa-mir-877 | 3364 | HUS1 | HUS1 checkpoint homolog (S. pombe) | 2 | | 5348 | hsa-let-7c | 1497 | CTNS | cystinosin, lysosomal cystine transporter | 2 | | 5349 | hsa-mir-99a | 1497 | CTNS | cystinosin, lysosomal cystine transporter | 2 | | 5350 | hsa-mir-218-1 | 1497 | CTNS | cystinosin, lysosomal cystine transporter | 2 | | 5352 | hsa-mir-125b-2 | 1497 | CTNS | cystinosin, lysosomal cystine transporter | 2 | | 5353 | hsa-mir-561 | 1497 | CTNS | cystinosin, lysosomal cystine transporter | 2 | | 5355 | hsa-mir-585 | 1497 | CTNS | cystinosin, lysosomal cystine transporter | 2 | | 5383 | hsa-mir-218-1 | 5771 | PTPN2 | protein tyrosine phosphatase, non-receptor type 2 | 2 | | 5385 | hsa-mir-561 | 5771 | PTPN2 | protein tyrosine phosphatase, non-receptor type 2 | 2 | | 5387 | hsa-mir-585 | 5771 | PTPN2 | protein tyrosine phosphatase, non-receptor type 2 | 2 | | 5413 | hsa-mir-181a-2 | 8406 | SRPX | sushi-repeat containing protein, X-linked | 2 | | 5414 | hsa-mir-181b-2 | 8406 | SRPX | sushi-repeat containing protein, X-linked | 2 | | 5415 | hsa-mir-505 | 8406 | SRPX | sushi-repeat containing protein, X-linked | 2 | | 5417 | hsa-mir-602 | 8406 | SRPX | sushi-repeat containing protein, X-linked | 2 | | 5439 | hsa-mir-25 | 2239 | GPC4 | glypican 4 | 2 | | 5440 | hsa-mir-93 | 2239 | GPC4 | glypican 4 | 2 | | 5441 | hsa-mir-181a-2 | 2239 | GPC4 | glypican 4 | 2 | | 5442 | hsa-mir-181b-2 | 2239 | GPC4 | glypican 4 | 2 | | 5443 | hsa-mir-106b | 2239 | GPC4 | glypican 4 | 2 | | 5448 | hsa-mir-643 | 2239 | GPC4 | glypican 4 | 2 | | 5471 | hsa-let-7c | 9150 | CTDP1 | CTD (carboxy-terminal domain, RNA polymerase II, polypeptide A) phosphatase, subunit 1 | 2 | | 5472 | hsa-mir-99a | 9150 | CTDP1 | CTD (carboxy-terminal domain, RNA polymerase II, polypeptide A) phosphatase, subunit 1 | 2 | | 5474 | hsa-mir-218-1 | 9150 | CTDP1 | CTD (carboxy-terminal domain, RNA polymerase II, polypeptide A) phosphatase, subunit 1 | 2 | | 5476 | hsa-mir-125b-1 | 9150 | CTDP1 | CTD (carboxy-terminal domain, RNA polymerase II, polypeptide A) phosphatase, subunit 1 | 2 | | 5477 | hsa-mir-125b-2 | 9150 | CTDP1 | CTD (carboxy-terminal domain, RNA polymerase II, polypeptide A) phosphatase, subunit 1 | 2 | | 5479 | hsa-mir-561 | 9150 | CTDP1 | CTD (carboxy-terminal domain, RNA polymerase II, polypeptide A) phosphatase, subunit 1 | 2 | | 5481 | hsa-mir-585 | 9150 | CTDP1 | CTD (carboxy-terminal domain, RNA polymerase II, polypeptide A) phosphatase, subunit 1 | 2 | | 5506 | hsa-mir-455 | 1439 | CSF2RB | colony stimulating factor 2 receptor, beta, low-affinity (granulocyte-macrophage) | 2 | | 5507 | hsa-mir-561 | 1439 | CSF2RB | colony stimulating factor 2 receptor, beta, low-affinity (granulocyte-macrophage) | 2 | | 5508 | hsa-mir-585 | 1439 | CSF2RB | colony stimulating factor 2 receptor, beta, low-affinity (granulocyte-macrophage) | 2 | | 5520 | hsa-mir-10a | 23464 | GCAT | glycine C-acetyltransferase | 2 | | 5522 | hsa-mir-218-2 | 23464 | GCAT | glycine C-acetyltransferase | 2 | | 5526 | hsa-mir-574 | 23464 | GCAT | glycine C-acetyltransferase | 2 | | 5528 | hsa-mir-603 | 23464 | GCAT | glycine C-acetyltransferase | 2 | | 5529 | hsa-mir-605 | 23464 | GCAT | glycine C-acetyltransferase | 2 | | 5530 | hsa-mir-618 | 23464 | GCAT | glycine C-acetyltransferase | 2 | | 5532 | hsa-mir-1271 | 23464 | GCAT | glycine C-acetyltransferase | 2 | | 5533 | hsa-mir-675 | 23464 | GCAT | glycine C-acetyltransferase | 2 | | 5534 | hsa-mir-1245a | 23464 | GCAT | glycine C-acetyltransferase | 2 | | 5535 | hsa-mir-1284 | 23464 | GCAT | glycine C-acetyltransferase | 2 | | 5536 | hsa-mir-1469 | 23464 | GCAT | glycine C-acetyltransferase | 2 | | 5538 | hsa-mir-3126 | 23464 | GCAT | glycine C-acetyltransferase | 2 | | 5539 | hsa-mir-3129 | 23464 | GCAT | glycine C-acetyltransferase | 2 | | 5540 | hsa-mir-3606 | 23464 | GCAT | glycine C-acetyltransferase | 2 | | 5541 | hsa-mir-4441 | 23464 | GCAT | glycine C-acetyltransferase | 2 | | 5543 | hsa-mir-4735 | 23464 | GCAT | glycine C-acetyltransferase | 2 | | 5544 | hsa-mir-2467 | 23464 | GCAT | glycine C-acetyltransferase | 2 | | 5545 | hsa-mir-4794 | 23464 | GCAT | glycine C-acetyltransferase | 2 | | 5546 | hsa-mir-548ao | 23464 | GCAT | glycine C-acetyltransferase | 2 | | 5547 | hsa-mir-617 | 23464 | GCAT | glycine C-acetyltransferase | 2 | | 5548 | hsa-mir-636 | 23464 | GCAT | glycine C-acetyltransferase | 2 | | 5551 | hsa-mir-877 | 23464 | GCAT | glycine C-acetyltransferase | 2 | | 5552 | hsa-mir-25 | 4921 | DDR2 | discoidin domain receptor tyrosine kinase 2 | 2 | | 5553 | hsa-mir-93 | 4921 | DDR2 | discoidin domain receptor tyrosine kinase 2 | 2 | | 5554 | hsa-mir-181a-2 | 4921 | DDR2 | discoidin domain receptor tyrosine kinase 2 | 2 | | 5555 | hsa-mir-181b-2 | 4921 | DDR2 | discoidin domain receptor tyrosine kinase 2 | 2 | | 5556 | hsa-mir-106b | 4921 | DDR2 | discoidin domain receptor tyrosine kinase 2 | 2 | | 5557 | hsa-mir-378a | 4921 | DDR2 | discoidin domain receptor tyrosine kinase 2 | 2 | | 5558 | hsa-mir-505 | 4921 | DDR2 | discoidin domain receptor tyrosine kinase 2 | 2 | | 5562 | hsa-mir-643 | 4921 | DDR2 | discoidin domain receptor tyrosine kinase 2 | 2 | | 5586 | hsa-mir-218-1 | 1678 | TIMM8A | translocase of inner mitochondrial membrane 8 homolog A (yeast) | 2 | | 5589 | hsa-mir-561 | 1678 | TIMM8A | translocase of inner mitochondrial membrane 8 homolog A (yeast) | 2 | | 5591 | hsa-mir-585 | 1678 | TIMM8A | translocase of inner mitochondrial membrane 8 homolog A (yeast) | 2 | | 5616 | hsa-mir-455 | 9997 | SCO2 | SCO cytochrome oxidase deficient homolog 2 (yeast) | 2 | | 5617 | hsa-mir-561 | 9997 | SCO2 | SCO cytochrome oxidase deficient homolog 2 (yeast) | 2 | | 5619 | hsa-mir-585 | 9997 | SCO2 | SCO cytochrome oxidase deficient homolog 2 (yeast) | 2 | | 5642 | hsa-let-7c | 9980 | DOPEY2 | dopey family member 2 | 2 | | 5643 | hsa-mir-99a | 9980 | DOPEY2 | dopey family member 2 | 2 | | 5645 | hsa-mir-218-1 | 9980 | DOPEY2 | dopey family member 2 | 2 | | 5647 | hsa-mir-125b-1 | 9980 | DOPEY2 | dopey family member 2 | 2 | | 5648 | hsa-mir-125b-2 | 9980 | DOPEY2 | dopey family member 2 | 2 | | 5649 | hsa-mir-561 | 9980 | DOPEY2 | dopey family member 2 | 2 | | 5651 | hsa-mir-585 | 9980 | DOPEY2 | dopey family member 2 | 2 | | 5690 | hsa-mir-620 | 119 | ADD2 | adducin 2 (beta) | 2 | | 5700 | hsa-mir-3170 | 119 | ADD2 | adducin 2 (beta) | 2 | | 5714 | hsa-mir-648 | 119 | ADD2 | adducin 2 (beta) | 2 | | 5716 | hsa-let-7c | 7022 | TFAP2C | transcription factor AP-2 gamma (activating enhancer binding protein 2 gamma) | 2 | | 5717 | hsa-mir-99a | 7022 | TFAP2C | transcription factor AP-2 gamma (activating enhancer binding protein 2 gamma) | 2 | | 5719 | hsa-mir-218-1 | 7022 | TFAP2C | transcription factor AP-2 gamma (activating enhancer binding protein 2 gamma) | 2 | | 5721 | hsa-mir-125b-1 | 7022 | TFAP2C | transcription factor AP-2 gamma (activating enhancer binding protein 2 gamma) | 2 | | 5722 | hsa-mir-125b-2 | 7022 | TFAP2C | transcription factor AP-2 gamma (activating enhancer binding protein 2 gamma) | 2 | | 5723 | hsa-mir-561 | 7022 | TFAP2C | transcription factor AP-2 gamma (activating enhancer binding protein 2 gamma) | 2 | | 5725 | hsa-mir-585 | 7022 | TFAP2C | transcription factor AP-2 gamma (activating enhancer binding protein 2 gamma) | 2 | | 5732 | hsa-mir-708 | 7022 | TFAP2C | transcription factor AP-2 gamma (activating enhancer binding protein 2 gamma) | 2 | | 5761 | hsa-mir-181a-2 | 10234 | LRRC17 | leucine rich repeat containing 17 | 2 | | 5762 | hsa-mir-181b-2 | 10234 | LRRC17 | leucine rich repeat containing 17 | 2 | | 5764 | hsa-mir-505 | 10234 | LRRC17 | leucine rich repeat containing 17 | 2 | | 5766 | hsa-mir-643 | 10234 | LRRC17 | leucine rich repeat containing 17 | 2 | | 5791 | hsa-mir-25 | 8434 | RECK | reversion-inducing-cysteine-rich protein with kazal motifs | 2 | | 5792 | hsa-mir-93 | 8434 | RECK | reversion-inducing-cysteine-rich protein with kazal motifs | 2 | | 5794 | hsa-mir-181a-2 | 8434 | RECK | reversion-inducing-cysteine-rich protein with kazal motifs | 2 | | 5795 | hsa-mir-181b-2 | 8434 | RECK | reversion-inducing-cysteine-rich protein with kazal motifs | 2 | | 5796 | hsa-mir-106b | 8434 | RECK | reversion-inducing-cysteine-rich protein with kazal motifs | 2 | | 5812 | hsa-let-7c | 2242 | FES | feline sarcoma oncogene | 2 | | 5813 | hsa-mir-99a | 2242 | FES | feline sarcoma oncogene | 2 | | 5815 | hsa-mir-218-1 | 2242 | FES | feline sarcoma oncogene | 2 | | 5817 | hsa-mir-125b-1 | 2242 | FES | feline sarcoma oncogene | 2 | | 5818 | hsa-mir-125b-2 | 2242 | FES | feline sarcoma oncogene | 2 | | 5819 | hsa-mir-455 | 2242 | FES | feline sarcoma oncogene | 2 | | 5820 | hsa-mir-561 | 2242 | FES | feline sarcoma oncogene | 2 | | 5822 | hsa-mir-585 | 2242 | FES | feline sarcoma oncogene | 2 | | 5829 | hsa-mir-708 | 2242 | FES | feline sarcoma oncogene | 2 | | 5854 | hsa-mir-25 | 590 | BCHE | butyrylcholinesterase | 2 | | 5855 | hsa-mir-93 | 590 | BCHE | butyrylcholinesterase | 2 | | 5856 | hsa-mir-181a-2 | 590 | BCHE | butyrylcholinesterase | 2 | | 5857 | hsa-mir-181b-2 | 590 | BCHE | butyrylcholinesterase | 2 | | 5858 | hsa-mir-106b | 590 | BCHE | butyrylcholinesterase | 2 | | 5859 | hsa-mir-505 | 590 | BCHE | butyrylcholinesterase | 2 | | 5861 | hsa-mir-643 | 590 | BCHE | butyrylcholinesterase | 2 | | 5882 | hsa-mir-561 | 51379 | CRLF3 | cytokine receptor-like factor 3 | 2 | | 5883 | hsa-mir-585 | 51379 | CRLF3 | cytokine receptor-like factor 3 | 2 | | 5892 | hsa-mir-181a-2 | 2690 | GHR | growth hormone receptor | 2 | | 5893 | hsa-mir-181b-2 | 2690 | GHR | growth hormone receptor | 2 | | 5894 | hsa-mir-378a | 2690 | GHR | growth hormone receptor | 2 | | 5895 | hsa-mir-505 | 2690 | GHR | growth hormone receptor | 2 | | 5897 | hsa-mir-643 | 2690 | GHR | growth hormone receptor | 2 | | 5923 | hsa-let-7f-2 | 862 | RUNX1T1 | runt-related transcription factor 1; translocated to, 1 (cyclin D-related) | 2 | | 5926 | hsa-mir-98 | 862 | RUNX1T1 | runt-related transcription factor 1; translocated to, 1 (cyclin D-related) | 2 | | 5931 | hsa-mir-550a-1 | 862 | RUNX1T1 | runt-related transcription factor 1; translocated to, 1 (cyclin D-related) | 2 | | 5933 | hsa-mir-644a | 862 | RUNX1T1 | runt-related transcription factor 1; translocated to, 1 (cyclin D-related) | 2 | | 5934 | hsa-mir-877 | 862 | RUNX1T1 | runt-related transcription factor 1; translocated to, 1 (cyclin D-related) | 2 | | 5935 | hsa-mir-548j | 862 | RUNX1T1 | runt-related transcription factor 1; translocated to, 1 (cyclin D-related) | 2 | | 5936 | hsa-mir-1290 | 862 | RUNX1T1 | runt-related transcription factor 1; translocated to, 1 (cyclin D-related) | 2 | | 5937 | hsa-mir-548n | 862 | RUNX1T1 | runt-related transcription factor 1; translocated to, 1 (cyclin D-related) | 2 | | 5938 | hsa-mir-2909 | 862 | RUNX1T1 | runt-related transcription factor 1; translocated to, 1 (cyclin D-related) | 2 | | 5939 | hsa-mir-548s | 862 | RUNX1T1 | runt-related transcription factor 1; translocated to, 1 (cyclin D-related) | 2 | | 5942 | hsa-mir-3657 | 862 | RUNX1T1 | runt-related transcription factor 1; translocated to, 1 (cyclin D-related) | 2 | | 5944 | hsa-mir-3942 | 862 | RUNX1T1 | runt-related transcription factor 1; translocated to, 1 (cyclin D-related) | 2 | | 5945 | hsa-mir-548o-2 | 862 | RUNX1T1 | runt-related transcription factor 1; translocated to, 1 (cyclin D-related) | 2 | | 5946 | hsa-mir-378e | 862 | RUNX1T1 | runt-related transcription factor 1; translocated to, 1 (cyclin D-related) | 2 | | 5948 | hsa-mir-4695 | 862 | RUNX1T1 | runt-related transcription factor 1; translocated to, 1 (cyclin D-related) | 2 | | 5949 | hsa-mir-4698 | 862 | RUNX1T1 | runt-related transcription factor 1; translocated to, 1 (cyclin D-related) | 2 | | 5950 | hsa-mir-4709 | 862 | RUNX1T1 | runt-related transcription factor 1; translocated to, 1 (cyclin D-related) | 2 | | 5951 | hsa-mir-4723 | 862 | RUNX1T1 | runt-related transcription factor 1; translocated to, 1 (cyclin D-related) | 2 | | 5952 | hsa-mir-4771-1 | 862 | RUNX1T1 | runt-related transcription factor 1; translocated to, 1 (cyclin D-related) | 2 | | 5953 | hsa-mir-4771-2 | 862 | RUNX1T1 | runt-related transcription factor 1; translocated to, 1 (cyclin D-related) | 2 | | 5954 | hsa-mir-4775 | 862 | RUNX1T1 | runt-related transcription factor 1; translocated to, 1 (cyclin D-related) | 2 | | 5955 | hsa-mir-5194 | 862 | RUNX1T1 | runt-related transcription factor 1; translocated to, 1 (cyclin D-related) | 2 | | 5956 | hsa-mir-675 | 862 | RUNX1T1 | runt-related transcription factor 1; translocated to, 1 (cyclin D-related) | 2 | | 5957 | hsa-mir-218-2 | 862 | RUNX1T1 | runt-related transcription factor 1; translocated to, 1 (cyclin D-related) | 2 | | 5958 | hsa-mir-574 | 862 | RUNX1T1 | runt-related transcription factor 1; translocated to, 1 (cyclin D-related) | 2 | | 5960 | hsa-mir-887 | 862 | RUNX1T1 | runt-related transcription factor 1; translocated to, 1 (cyclin D-related) | 2 | | 5962 | hsa-mir-455 | 4065 | LY75 | lymphocyte antigen 75 | 2 | | 5963 | hsa-mir-561 | 4065 | LY75 | lymphocyte antigen 75 | 2 | | 5965 | hsa-mir-585 | 4065 | LY75 | lymphocyte antigen 75 | 2 | | 5968 | hsa-mir-708 | 4065 | LY75 | lymphocyte antigen 75 | 2 | | 5981 | hsa-mir-455 | 952 | CD38 | CD38 molecule | 2 | | 5982 | hsa-mir-561 | 952 | CD38 | CD38 molecule | 2 | | 5984 | hsa-mir-585 | 952 | CD38 | CD38 molecule | 2 | | 6016 | hsa-mir-218-1 | 23545 | ATP6V0A2 | ATPase, H+ transporting, lysosomal V0 subunit a2 | 2 | | 6018 | hsa-mir-125b-1 | 23545 | ATP6V0A2 | ATPase, H+ transporting, lysosomal V0 subunit a2 | 2 | | 6019 | hsa-mir-561 | 23545 | ATP6V0A2 | ATPase, H+ transporting, lysosomal V0 subunit a2 | 2 | | 6021 | hsa-mir-585 | 23545 | ATP6V0A2 | ATPase, H+ transporting, lysosomal V0 subunit a2 | 2 | | 6028 | hsa-mir-708 | 23545 | ATP6V0A2 | ATPase, H+ transporting, lysosomal V0 subunit a2 | 2 | | 6059 | hsa-mir-218-1 | 55346 | TCP11L1 | t-complex 11 (mouse)-like 1 | 2 | | 6061 | hsa-mir-125b-1 | 55346 | TCP11L1 | t-complex 11 (mouse)-like 1 | 2 | | 6062 | hsa-mir-455 | 55346 | TCP11L1 | t-complex 11 (mouse)-like 1 | 2 | | 6063 | hsa-mir-561 | 55346 | TCP11L1 | t-complex 11 (mouse)-like 1 | 2 | | 6065 | hsa-mir-585 | 55346 | TCP11L1 | t-complex 11 (mouse)-like 1 | 2 | | 6068 | hsa-mir-620 | 55346 | TCP11L1 | t-complex 11 (mouse)-like 1 | 2 | | 6071 | hsa-mir-708 | 55346 | TCP11L1 | t-complex 11 (mouse)-like 1 | 2 | | 6093 | hsa-let-7c | 9466 | IL27RA | interleukin 27 receptor, alpha | 2 | | 6094 | hsa-mir-99a | 9466 | IL27RA | interleukin 27 receptor, alpha | 2 | | 6096 | hsa-mir-125b-2 | 9466 | IL27RA | interleukin 27 receptor, alpha | 2 | | 6097 | hsa-mir-561 | 9466 | IL27RA | interleukin 27 receptor, alpha | 2 | | 6116 | hsa-let-7c | 3570 | IL6R | interleukin 6 receptor | 2 | | 6118 | hsa-mir-10a | 3570 | IL6R | interleukin 6 receptor | 2 | | 6120 | hsa-mir-218-2 | 3570 | IL6R | interleukin 6 receptor | 2 | | 6124 | hsa-mir-574 | 3570 | IL6R | interleukin 6 receptor | 2 | | 6126 | hsa-mir-603 | 3570 | IL6R | interleukin 6 receptor | 2 | | 6127 | hsa-mir-604 | 3570 | IL6R | interleukin 6 receptor | 2 | | 6128 | hsa-mir-605 | 3570 | IL6R | interleukin 6 receptor | 2 | | 6129 | hsa-mir-618 | 3570 | IL6R | interleukin 6 receptor | 2 | | 6130 | hsa-mir-1271 | 3570 | IL6R | interleukin 6 receptor | 2 | | 6131 | hsa-mir-675 | 3570 | IL6R | interleukin 6 receptor | 2 | | 6133 | hsa-mir-887 | 3570 | IL6R | interleukin 6 receptor | 2 | | 6134 | hsa-mir-938 | 3570 | IL6R | interleukin 6 receptor | 2 | | 6135 | hsa-mir-663b | 3570 | IL6R | interleukin 6 receptor | 2 | | 6136 | hsa-mir-1245a | 3570 | IL6R | interleukin 6 receptor | 2 | | 6138 | hsa-mir-3120 | 3570 | IL6R | interleukin 6 receptor | 2 | | 6139 | hsa-mir-3126 | 3570 | IL6R | interleukin 6 receptor | 2 | | 6140 | hsa-mir-3129 | 3570 | IL6R | interleukin 6 receptor | 2 | | 6141 | hsa-mir-3139 | 3570 | IL6R | interleukin 6 receptor | 2 | | 6142 | hsa-mir-3170 | 3570 | IL6R | interleukin 6 receptor | 2 | | 6143 | hsa-mir-3606 | 3570 | IL6R | interleukin 6 receptor | 2 | | 6144 | hsa-mir-3650 | 3570 | IL6R | interleukin 6 receptor | 2 | | 6145 | hsa-mir-4441 | 3570 | IL6R | interleukin 6 receptor | 2 | | 6146 | hsa-mir-4486 | 3570 | IL6R | interleukin 6 receptor | 2 | | 6147 | hsa-mir-4519 | 3570 | IL6R | interleukin 6 receptor | 2 | | 6148 | hsa-mir-4636 | 3570 | IL6R | interleukin 6 receptor | 2 | | 6150 | hsa-mir-4735 | 3570 | IL6R | interleukin 6 receptor | 2 | | 6151 | hsa-mir-4768 | 3570 | IL6R | interleukin 6 receptor | 2 | | 6152 | hsa-mir-2467 | 3570 | IL6R | interleukin 6 receptor | 2 | | 6153 | hsa-mir-4794 | 3570 | IL6R | interleukin 6 receptor | 2 | | 6154 | hsa-mir-548ao | 3570 | IL6R | interleukin 6 receptor | 2 | | 6155 | hsa-mir-5579 | 3570 | IL6R | interleukin 6 receptor | 2 | | 6157 | hsa-mir-617 | 3570 | IL6R | interleukin 6 receptor | 2 | | 6159 | hsa-mir-648 | 3570 | IL6R | interleukin 6 receptor | 2 | | 6160 | hsa-mir-877 | 3570 | IL6R | interleukin 6 receptor | 2 | | 6165 | hsa-let-7c | 6665 | SOX15 | SRY (sex determining region Y)-box 15 | 2 | | 6166 | hsa-mir-99a | 6665 | SOX15 | SRY (sex determining region Y)-box 15 | 2 | | 6168 | hsa-mir-218-1 | 6665 | SOX15 | SRY (sex determining region Y)-box 15 | 2 | | 6171 | hsa-mir-125b-2 | 6665 | SOX15 | SRY (sex determining region Y)-box 15 | 2 | | 6173 | hsa-mir-561 | 6665 | SOX15 | SRY (sex determining region Y)-box 15 | 2 | | 6175 | hsa-mir-585 | 6665 | SOX15 | SRY (sex determining region Y)-box 15 | 2 | | 6206 | hsa-mir-218-1 | 4113 | MAGEB2 | melanoma antigen family B, 2 | 2 | | 6208 | hsa-mir-455 | 4113 | MAGEB2 | melanoma antigen family B, 2 | 2 | | 6209 | hsa-mir-561 | 4113 | MAGEB2 | melanoma antigen family B, 2 | 2 | | 6211 | hsa-mir-585 | 4113 | MAGEB2 | melanoma antigen family B, 2 | 2 | | 6217 | hsa-mir-620 | 4113 | MAGEB2 | melanoma antigen family B, 2 | 2 | | 6240 | hsa-let-7c | 7409 | VAV1 | vav 1 guanine nucleotide exchange factor | 2 | | 6245 | hsa-mir-455 | 7409 | VAV1 | vav 1 guanine nucleotide exchange factor | 2 | | 6246 | hsa-mir-561 | 7409 | VAV1 | vav 1 guanine nucleotide exchange factor | 2 | | 6248 | hsa-mir-585 | 7409 | VAV1 | vav 1 guanine nucleotide exchange factor | 2 | | 6263 | hsa-mir-218-1 | 4277 | MICB | MHC class I polypeptide-related sequence B | 2 | | 6265 | hsa-mir-455 | 4277 | MICB | MHC class I polypeptide-related sequence B | 2 | | 6266 | hsa-mir-561 | 4277 | MICB | MHC class I polypeptide-related sequence B | 2 | | 6268 | hsa-mir-585 | 4277 | MICB | MHC class I polypeptide-related sequence B | 2 | | 6271 | hsa-mir-708 | 4277 | MICB | MHC class I polypeptide-related sequence B | 2 | | 6306 | hsa-mir-1271 | 11184 | MAP4K1 | mitogen-activated protein kinase kinase kinase kinase 1 | 2 | | 6313 | hsa-mir-3120 | 11184 | MAP4K1 | mitogen-activated protein kinase kinase kinase kinase 1 | 2 | | 6333 | hsa-let-7c | 9623 | TCL1B | T-cell leukemia/lymphoma 1B | 2 | | 6334 | hsa-mir-99a | 9623 | TCL1B | T-cell leukemia/lymphoma 1B | 2 | | 6336 | hsa-mir-218-1 | 9623 | TCL1B | T-cell leukemia/lymphoma 1B | 2 | | 6338 | hsa-mir-125b-1 | 9623 | TCL1B | T-cell leukemia/lymphoma 1B | 2 | | 6339 | hsa-mir-125b-2 | 9623 | TCL1B | T-cell leukemia/lymphoma 1B | 2 | | 6340 | hsa-mir-455 | 9623 | TCL1B | T-cell leukemia/lymphoma 1B | 2 | | 6341 | hsa-mir-561 | 9623 | TCL1B | T-cell leukemia/lymphoma 1B | 2 | | 6343 | hsa-mir-585 | 9623 | TCL1B | T-cell leukemia/lymphoma 1B | 2 | | 6351 | hsa-mir-708 | 9623 | TCL1B | T-cell leukemia/lymphoma 1B | 2 | | 6381 | hsa-let-7c | 1044 | CDX1 | caudal type homeobox 1 | 2 | | 6382 | hsa-mir-99a | 1044 | CDX1 | caudal type homeobox 1 | 2 | | 6384 | hsa-mir-218-1 | 1044 | CDX1 | caudal type homeobox 1 | 2 | | 6386 | hsa-mir-125b-1 | 1044 | CDX1 | caudal type homeobox 1 | 2 | | 6387 | hsa-mir-125b-2 | 1044 | CDX1 | caudal type homeobox 1 | 2 | | 6388 | hsa-mir-455 | 1044 | CDX1 | caudal type homeobox 1 | 2 | | 6389 | hsa-mir-561 | 1044 | CDX1 | caudal type homeobox 1 | 2 | | 6391 | hsa-mir-585 | 1044 | CDX1 | caudal type homeobox 1 | 2 | | 6398 | hsa-mir-708 | 1044 | CDX1 | caudal type homeobox 1 | 2 | | 6423 | hsa-mir-218-1 | 7482 | WNT2B | wingless-type MMTV integration site family, member 2B | 2 | | 6425 | hsa-mir-125b-1 | 7482 | WNT2B | wingless-type MMTV integration site family, member 2B | 2 | | 6426 | hsa-mir-455 | 7482 | WNT2B | wingless-type MMTV integration site family, member 2B | 2 | | 6427 | hsa-mir-561 | 7482 | WNT2B | wingless-type MMTV integration site family, member 2B | 2 | | 6429 | hsa-mir-585 | 7482 | WNT2B | wingless-type MMTV integration site family, member 2B | 2 | | 6436 | hsa-mir-708 | 7482 | WNT2B | wingless-type MMTV integration site family, member 2B | 2 | | 6461 | hsa-mir-25 | 30008 | EFEMP2 | EGF containing fibulin-like extracellular matrix protein 2 | 2 | | 6462 | hsa-mir-93 | 30008 | EFEMP2 | EGF containing fibulin-like extracellular matrix protein 2 | 2 | | 6463 | hsa-mir-181a-2 | 30008 | EFEMP2 | EGF containing fibulin-like extracellular matrix protein 2 | 2 | | 6464 | hsa-mir-181b-2 | 30008 | EFEMP2 | EGF containing fibulin-like extracellular matrix protein 2 | 2 | | 6465 | hsa-mir-106b | 30008 | EFEMP2 | EGF containing fibulin-like extracellular matrix protein 2 | 2 | | 6466 | hsa-mir-378a | 30008 | EFEMP2 | EGF containing fibulin-like extracellular matrix protein 2 | 2 | | 6467 | hsa-mir-505 | 30008 | EFEMP2 | EGF containing fibulin-like extracellular matrix protein 2 | 2 | | 6471 | hsa-mir-643 | 30008 | EFEMP2 | EGF containing fibulin-like extracellular matrix protein 2 | 2 | | 6496 | hsa-let-7c | 1618 | DAZL | deleted in azoospermia-like | 2 | | 6497 | hsa-mir-99a | 1618 | DAZL | deleted in azoospermia-like | 2 | | 6499 | hsa-mir-218-1 | 1618 | DAZL | deleted in azoospermia-like | 2 | | 6501 | hsa-mir-125b-1 | 1618 | DAZL | deleted in azoospermia-like | 2 | | 6502 | hsa-mir-125b-2 | 1618 | DAZL | deleted in azoospermia-like | 2 | | 6503 | hsa-mir-561 | 1618 | DAZL | deleted in azoospermia-like | 2 | | 6505 | hsa-mir-585 | 1618 | DAZL | deleted in azoospermia-like | 2 | | 6512 | hsa-mir-708 | 1618 | DAZL | deleted in azoospermia-like | 2 | | 6541 | hsa-let-7c | 9947 | MAGEC1 | melanoma antigen family C, 1 | 2 | | 6542 | hsa-mir-99a | 9947 | MAGEC1 | melanoma antigen family C, 1 | 2 | | 6545 | hsa-mir-125b-2 | 9947 | MAGEC1 | melanoma antigen family C, 1 | 2 | | 6546 | hsa-mir-455 | 9947 | MAGEC1 | melanoma antigen family C, 1 | 2 | | 6547 | hsa-mir-561 | 9947 | MAGEC1 | melanoma antigen family C, 1 | 2 | | 6549 | hsa-mir-585 | 9947 | MAGEC1 | melanoma antigen family C, 1 | 2 | | 6556 | hsa-mir-708 | 9947 | MAGEC1 | melanoma antigen family C, 1 | 2 | | 6586 | hsa-let-7c | 10633 | RASL10A | RAS-like, family 10, member A | 2 | | 6587 | hsa-mir-99a | 10633 | RASL10A | RAS-like, family 10, member A | 2 | | 6589 | hsa-mir-218-1 | 10633 | RASL10A | RAS-like, family 10, member A | 2 | | 6591 | hsa-mir-125b-1 | 10633 | RASL10A | RAS-like, family 10, member A | 2 | | 6592 | hsa-mir-125b-2 | 10633 | RASL10A | RAS-like, family 10, member A | 2 | | 6594 | hsa-mir-561 | 10633 | RASL10A | RAS-like, family 10, member A | 2 | | 6596 | hsa-mir-585 | 10633 | RASL10A | RAS-like, family 10, member A | 2 | | 6602 | hsa-mir-708 | 10633 | RASL10A | RAS-like, family 10, member A | 2 | | 6628 | hsa-mir-218-1 | 8545 | CGGBP1 | CGG triplet repeat binding protein 1 | 2 | | 6630 | hsa-mir-125b-1 | 8545 | CGGBP1 | CGG triplet repeat binding protein 1 | 2 | | 6631 | hsa-mir-561 | 8545 | CGGBP1 | CGG triplet repeat binding protein 1 | 2 | | 6633 | hsa-mir-585 | 8545 | CGGBP1 | CGG triplet repeat binding protein 1 | 2 | | 6638 | hsa-mir-620 | 8545 | CGGBP1 | CGG triplet repeat binding protein 1 | 2 | | 6663 | hsa-mir-25 | 5592 | PRKG1 | protein kinase, cGMP-dependent, type I | 2 | | 6664 | hsa-mir-93 | 5592 | PRKG1 | protein kinase, cGMP-dependent, type I | 2 | | 6666 | hsa-mir-181a-2 | 5592 | PRKG1 | protein kinase, cGMP-dependent, type I | 2 | | 6667 | hsa-mir-181b-2 | 5592 | PRKG1 | protein kinase, cGMP-dependent, type I | 2 | | 6668 | hsa-mir-106b | 5592 | PRKG1 | protein kinase, cGMP-dependent, type I | 2 | | 6669 | hsa-mir-505 | 5592 | PRKG1 | protein kinase, cGMP-dependent, type I | 2 | | 6673 | hsa-mir-643 | 5592 | PRKG1 | protein kinase, cGMP-dependent, type I | 2 | | 6675 | hsa-mir-652 | 5592 | PRKG1 | protein kinase, cGMP-dependent, type I | 2 | | 6706 | hsa-let-7c | 239 | ALOX12 | arachidonate 12-lipoxygenase | 2 | | 6707 | hsa-mir-99a | 239 | ALOX12 | arachidonate 12-lipoxygenase | 2 | | 6709 | hsa-mir-218-1 | 239 | ALOX12 | arachidonate 12-lipoxygenase | 2 | | 6711 | hsa-mir-125b-1 | 239 | ALOX12 | arachidonate 12-lipoxygenase | 2 | | 6712 | hsa-mir-125b-2 | 239 | ALOX12 | arachidonate 12-lipoxygenase | 2 | | 6713 | hsa-mir-561 | 239 | ALOX12 | arachidonate 12-lipoxygenase | 2 | | 6715 | hsa-mir-585 | 239 | ALOX12 | arachidonate 12-lipoxygenase | 2 | | 6720 | hsa-mir-708 | 239 | ALOX12 | arachidonate 12-lipoxygenase | 2 | | 6741 | hsa-mir-218-1 | 27288 | RBMXL2 | RNA binding motif protein, X-linked-like 2 | 2 | | 6743 | hsa-mir-125b-1 | 27288 | RBMXL2 | RNA binding motif protein, X-linked-like 2 | 2 | | 6744 | hsa-mir-561 | 27288 | RBMXL2 | RNA binding motif protein, X-linked-like 2 | 2 | | 6746 | hsa-mir-585 | 27288 | RBMXL2 | RNA binding motif protein, X-linked-like 2 | 2 | | 6753 | hsa-mir-708 | 27288 | RBMXL2 | RNA binding motif protein, X-linked-like 2 | 2 | | 6779 | hsa-mir-218-2 | 7752 | ZNF200 | zinc finger protein 200 | 2 | | 6783 | hsa-mir-574 | 7752 | ZNF200 | zinc finger protein 200 | 2 | | 6785 | hsa-mir-603 | 7752 | ZNF200 | zinc finger protein 200 | 2 | | 6786 | hsa-mir-605 | 7752 | ZNF200 | zinc finger protein 200 | 2 | | 6788 | hsa-mir-1271 | 7752 | ZNF200 | zinc finger protein 200 | 2 | | 6789 | hsa-mir-675 | 7752 | ZNF200 | zinc finger protein 200 | 2 | | 6791 | hsa-mir-1245a | 7752 | ZNF200 | zinc finger protein 200 | 2 | | 6792 | hsa-mir-1915 | 7752 | ZNF200 | zinc finger protein 200 | 2 | | 6793 | hsa-mir-3120 | 7752 | ZNF200 | zinc finger protein 200 | 2 | | 6794 | hsa-mir-3126 | 7752 | ZNF200 | zinc finger protein 200 | 2 | | 6795 | hsa-mir-3129 | 7752 | ZNF200 | zinc finger protein 200 | 2 | | 6796 | hsa-mir-3606 | 7752 | ZNF200 | zinc finger protein 200 | 2 | | 6797 | hsa-mir-4441 | 7752 | ZNF200 | zinc finger protein 200 | 2 | | 6798 | hsa-mir-4735 | 7752 | ZNF200 | zinc finger protein 200 | 2 | | 6799 | hsa-mir-4768 | 7752 | ZNF200 | zinc finger protein 200 | 2 | | 6800 | hsa-mir-2467 | 7752 | ZNF200 | zinc finger protein 200 | 2 | | 6801 | hsa-mir-4794 | 7752 | ZNF200 | zinc finger protein 200 | 2 | | 6802 | hsa-mir-548ao | 7752 | ZNF200 | zinc finger protein 200 | 2 | | 6806 | hsa-mir-503 | 7752 | ZNF200 | zinc finger protein 200 | 2 | | 6810 | hsa-mir-877 | 7752 | ZNF200 | zinc finger protein 200 | 2 | | 6813 | hsa-mir-455 | 10566 | AKAP3 | A kinase (PRKA) anchor protein 3 | 2 | | 6814 | hsa-mir-561 | 10566 | AKAP3 | A kinase (PRKA) anchor protein 3 | 2 | | 6816 | hsa-mir-585 | 10566 | AKAP3 | A kinase (PRKA) anchor protein 3 | 2 | | 6820 | hsa-mir-708 | 10566 | AKAP3 | A kinase (PRKA) anchor protein 3 | 2 | | 6841 | hsa-mir-25 | 4212 | MEIS2 | Meis homeobox 2 | 2 | | 6842 | hsa-mir-93 | 4212 | MEIS2 | Meis homeobox 2 | 2 | | 6844 | hsa-mir-181a-2 | 4212 | MEIS2 | Meis homeobox 2 | 2 | | 6845 | hsa-mir-181b-2 | 4212 | MEIS2 | Meis homeobox 2 | 2 | | 6846 | hsa-mir-106b | 4212 | MEIS2 | Meis homeobox 2 | 2 | | 6847 | hsa-mir-505 | 4212 | MEIS2 | Meis homeobox 2 | 2 | | 6851 | hsa-mir-643 | 4212 | MEIS2 | Meis homeobox 2 | 2 | | 6874 | hsa-let-7c | 489 | ATP2A3 | ATPase, Ca++ transporting, ubiquitous | 2 | | 6878 | hsa-mir-218-2 | 489 | ATP2A3 | ATPase, Ca++ transporting, ubiquitous | 2 | | 6881 | hsa-mir-574 | 489 | ATP2A3 | ATPase, Ca++ transporting, ubiquitous | 2 | | 6883 | hsa-mir-603 | 489 | ATP2A3 | ATPase, Ca++ transporting, ubiquitous | 2 | | 6885 | hsa-mir-605 | 489 | ATP2A3 | ATPase, Ca++ transporting, ubiquitous | 2 | | 6886 | hsa-mir-618 | 489 | ATP2A3 | ATPase, Ca++ transporting, ubiquitous | 2 | | 6887 | hsa-mir-620 | 489 | ATP2A3 | ATPase, Ca++ transporting, ubiquitous | 2 | | 6888 | hsa-mir-675 | 489 | ATP2A3 | ATPase, Ca++ transporting, ubiquitous | 2 | | 6889 | hsa-mir-887 | 489 | ATP2A3 | ATPase, Ca++ transporting, ubiquitous | 2 | | 6891 | hsa-mir-1245a | 489 | ATP2A3 | ATPase, Ca++ transporting, ubiquitous | 2 | | 6892 | hsa-mir-1915 | 489 | ATP2A3 | ATPase, Ca++ transporting, ubiquitous | 2 | | 6893 | hsa-mir-3120 | 489 | ATP2A3 | ATPase, Ca++ transporting, ubiquitous | 2 | | 6894 | hsa-mir-3126 | 489 | ATP2A3 | ATPase, Ca++ transporting, ubiquitous | 2 | | 6895 | hsa-mir-3129 | 489 | ATP2A3 | ATPase, Ca++ transporting, ubiquitous | 2 | | 6896 | hsa-mir-3139 | 489 | ATP2A3 | ATPase, Ca++ transporting, ubiquitous | 2 | | 6897 | hsa-mir-3170 | 489 | ATP2A3 | ATPase, Ca++ transporting, ubiquitous | 2 | | 6898 | hsa-mir-3606 | 489 | ATP2A3 | ATPase, Ca++ transporting, ubiquitous | 2 | | 6899 | hsa-mir-3650 | 489 | ATP2A3 | ATPase, Ca++ transporting, ubiquitous | 2 | | 6900 | hsa-mir-4441 | 489 | ATP2A3 | ATPase, Ca++ transporting, ubiquitous | 2 | | 6901 | hsa-mir-4519 | 489 | ATP2A3 | ATPase, Ca++ transporting, ubiquitous | 2 | | 6902 | hsa-mir-4735 | 489 | ATP2A3 | ATPase, Ca++ transporting, ubiquitous | 2 | | 6903 | hsa-mir-4768 | 489 | ATP2A3 | ATPase, Ca++ transporting, ubiquitous | 2 | | 6904 | hsa-mir-2467 | 489 | ATP2A3 | ATPase, Ca++ transporting, ubiquitous | 2 | | 6905 | hsa-mir-4794 | 489 | ATP2A3 | ATPase, Ca++ transporting, ubiquitous | 2 | | 6906 | hsa-mir-548ao | 489 | ATP2A3 | ATPase, Ca++ transporting, ubiquitous | 2 | | 6907 | hsa-mir-554 | 489 | ATP2A3 | ATPase, Ca++ transporting, ubiquitous | 2 | | 6908 | hsa-mir-617 | 489 | ATP2A3 | ATPase, Ca++ transporting, ubiquitous | 2 | | 6909 | hsa-mir-648 | 489 | ATP2A3 | ATPase, Ca++ transporting, ubiquitous | 2 | | 6910 | hsa-mir-877 | 489 | ATP2A3 | ATPase, Ca++ transporting, ubiquitous | 2 | | 6911 | hsa-mir-1233-1;hsa-mir-1233-2 | 489 | ATP2A3 | ATPase, Ca++ transporting, ubiquitous | 2 | | 6912 | hsa-let-7c | 4112 | MAGEB1 | melanoma antigen family B, 1 | 2 | | 6913 | hsa-mir-99a | 4112 | MAGEB1 | melanoma antigen family B, 1 | 2 | | 6915 | hsa-mir-218-1 | 4112 | MAGEB1 | melanoma antigen family B, 1 | 2 | | 6917 | hsa-mir-125b-1 | 4112 | MAGEB1 | melanoma antigen family B, 1 | 2 | | 6918 | hsa-mir-125b-2 | 4112 | MAGEB1 | melanoma antigen family B, 1 | 2 | | 6919 | hsa-mir-455 | 4112 | MAGEB1 | melanoma antigen family B, 1 | 2 | | 6920 | hsa-mir-561 | 4112 | MAGEB1 | melanoma antigen family B, 1 | 2 | | 6922 | hsa-mir-585 | 4112 | MAGEB1 | melanoma antigen family B, 1 | 2 | | 6928 | hsa-mir-620 | 4112 | MAGEB1 | melanoma antigen family B, 1 | 2 | | 6953 | hsa-let-7c | 9473 | C1orf38 | chromosome 1 open reading frame 38 | 2 | | 6955 | hsa-mir-218-2 | 9473 | C1orf38 | chromosome 1 open reading frame 38 | 2 | | 6959 | hsa-mir-574 | 9473 | C1orf38 | chromosome 1 open reading frame 38 | 2 | | 6961 | hsa-mir-603 | 9473 | C1orf38 | chromosome 1 open reading frame 38 | 2 | | 6964 | hsa-mir-675 | 9473 | C1orf38 | chromosome 1 open reading frame 38 | 2 | | 6965 | hsa-mir-887 | 9473 | C1orf38 | chromosome 1 open reading frame 38 | 2 | | 6967 | hsa-mir-3129 | 9473 | C1orf38 | chromosome 1 open reading frame 38 | 2 | | 6969 | hsa-mir-3650 | 9473 | C1orf38 | chromosome 1 open reading frame 38 | 2 | | 6972 | hsa-mir-4728 | 9473 | C1orf38 | chromosome 1 open reading frame 38 | 2 | | 6973 | hsa-mir-4768 | 9473 | C1orf38 | chromosome 1 open reading frame 38 | 2 | | 6975 | hsa-mir-617 | 9473 | C1orf38 | chromosome 1 open reading frame 38 | 2 | | 6976 | hsa-let-7c | 30817 | EMR2 | egf-like module containing, mucin-like, hormone receptor-like 2 | 2 | | 6977 | hsa-mir-99a | 30817 | EMR2 | egf-like module containing, mucin-like, hormone receptor-like 2 | 2 | | 6980 | hsa-mir-125b-2 | 30817 | EMR2 | egf-like module containing, mucin-like, hormone receptor-like 2 | 2 | | 6981 | hsa-mir-455 | 30817 | EMR2 | egf-like module containing, mucin-like, hormone receptor-like 2 | 2 | | 6982 | hsa-mir-561 | 30817 | EMR2 | egf-like module containing, mucin-like, hormone receptor-like 2 | 2 | | 6984 | hsa-mir-585 | 30817 | EMR2 | egf-like module containing, mucin-like, hormone receptor-like 2 | 2 | | 6988 | hsa-mir-708 | 30817 | EMR2 | egf-like module containing, mucin-like, hormone receptor-like 2 | 2 | | 7007 | hsa-mir-218-1 | 114049 | WBSCR22 | Williams Beuren syndrome chromosome region 22 | 2 | | 7009 | hsa-mir-125b-1 | 114049 | WBSCR22 | Williams Beuren syndrome chromosome region 22 | 2 | | 7010 | hsa-mir-561 | 114049 | WBSCR22 | Williams Beuren syndrome chromosome region 22 | 2 | | 7012 | hsa-mir-585 | 114049 | WBSCR22 | Williams Beuren syndrome chromosome region 22 | 2 | | 7016 | hsa-mir-620 | 114049 | WBSCR22 | Williams Beuren syndrome chromosome region 22 | 2 | | 7046 | hsa-mir-10a | 1741 | DLG3 | discs, large homolog 3 (Drosophila) | 2 | | 7050 | hsa-mir-574 | 1741 | DLG3 | discs, large homolog 3 (Drosophila) | 2 | | 7058 | hsa-mir-675 | 1741 | DLG3 | discs, large homolog 3 (Drosophila) | 2 | | 7061 | hsa-mir-1245a | 1741 | DLG3 | discs, large homolog 3 (Drosophila) | 2 | | 7067 | hsa-mir-3606 | 1741 | DLG3 | discs, large homolog 3 (Drosophila) | 2 | | 7077 | hsa-mir-877 | 1741 | DLG3 | discs, large homolog 3 (Drosophila) | 2 | | 7078 | hsa-let-7c | 54941 | RNF125 | ring finger protein 125, E3 ubiquitin protein ligase | 2 | | 7079 | hsa-mir-99a | 54941 | RNF125 | ring finger protein 125, E3 ubiquitin protein ligase | 2 | | 7080 | hsa-mir-10a | 54941 | RNF125 | ring finger protein 125, E3 ubiquitin protein ligase | 2 | | 7081 | hsa-mir-218-2 | 54941 | RNF125 | ring finger protein 125, E3 ubiquitin protein ligase | 2 | | 7083 | hsa-mir-125b-2 | 54941 | RNF125 | ring finger protein 125, E3 ubiquitin protein ligase | 2 | | 7085 | hsa-mir-574 | 54941 | RNF125 | ring finger protein 125, E3 ubiquitin protein ligase | 2 | | 7087 | hsa-mir-603 | 54941 | RNF125 | ring finger protein 125, E3 ubiquitin protein ligase | 2 | | 7091 | hsa-mir-618 | 54941 | RNF125 | ring finger protein 125, E3 ubiquitin protein ligase | 2 | | 7092 | hsa-mir-675 | 54941 | RNF125 | ring finger protein 125, E3 ubiquitin protein ligase | 2 | | 7096 | hsa-mir-1469 | 54941 | RNF125 | ring finger protein 125, E3 ubiquitin protein ligase | 2 | | 7097 | hsa-mir-3120 | 54941 | RNF125 | ring finger protein 125, E3 ubiquitin protein ligase | 2 | | 7098 | hsa-mir-3126 | 54941 | RNF125 | ring finger protein 125, E3 ubiquitin protein ligase | 2 | | 7099 | hsa-mir-3129 | 54941 | RNF125 | ring finger protein 125, E3 ubiquitin protein ligase | 2 | | 7101 | hsa-mir-3170 | 54941 | RNF125 | ring finger protein 125, E3 ubiquitin protein ligase | 2 | | 7102 | hsa-mir-3650 | 54941 | RNF125 | ring finger protein 125, E3 ubiquitin protein ligase | 2 | | 7103 | hsa-mir-4441 | 54941 | RNF125 | ring finger protein 125, E3 ubiquitin protein ligase | 2 | | 7104 | hsa-mir-4486 | 54941 | RNF125 | ring finger protein 125, E3 ubiquitin protein ligase | 2 | | 7105 | hsa-mir-4636 | 54941 | RNF125 | ring finger protein 125, E3 ubiquitin protein ligase | 2 | | 7107 | hsa-mir-4735 | 54941 | RNF125 | ring finger protein 125, E3 ubiquitin protein ligase | 2 | | 7108 | hsa-mir-4768 | 54941 | RNF125 | ring finger protein 125, E3 ubiquitin protein ligase | 2 | | 7109 | hsa-mir-2467 | 54941 | RNF125 | ring finger protein 125, E3 ubiquitin protein ligase | 2 | | 7110 | hsa-mir-4794 | 54941 | RNF125 | ring finger protein 125, E3 ubiquitin protein ligase | 2 | | 7111 | hsa-mir-548ao | 54941 | RNF125 | ring finger protein 125, E3 ubiquitin protein ligase | 2 | | 7112 | hsa-mir-5579 | 54941 | RNF125 | ring finger protein 125, E3 ubiquitin protein ligase | 2 | | 7113 | hsa-mir-617 | 54941 | RNF125 | ring finger protein 125, E3 ubiquitin protein ligase | 2 | | 7115 | hsa-mir-877 | 54941 | RNF125 | ring finger protein 125, E3 ubiquitin protein ligase | 2 | | 7120 | hsa-mir-585 | 23636 | NUP62 | nucleoporin 62kDa | 2 | | 7124 | hsa-mir-620 | 23636 | NUP62 | nucleoporin 62kDa | 2 | | 7138 | hsa-mir-10a | 80256 | FAM214B | family with sequence similarity 214, member B | 2 | | 7139 | hsa-mir-218-2 | 80256 | FAM214B | family with sequence similarity 214, member B | 2 | | 7143 | hsa-mir-574 | 80256 | FAM214B | family with sequence similarity 214, member B | 2 | | 7145 | hsa-mir-603 | 80256 | FAM214B | family with sequence similarity 214, member B | 2 | | 7146 | hsa-mir-604 | 80256 | FAM214B | family with sequence similarity 214, member B | 2 | | 7147 | hsa-mir-618 | 80256 | FAM214B | family with sequence similarity 214, member B | 2 | | 7149 | hsa-mir-675 | 80256 | FAM214B | family with sequence similarity 214, member B | 2 | | 7150 | hsa-mir-887 | 80256 | FAM214B | family with sequence similarity 214, member B | 2 | | 7151 | hsa-mir-938 | 80256 | FAM214B | family with sequence similarity 214, member B | 2 | | 7152 | hsa-mir-1245a | 80256 | FAM214B | family with sequence similarity 214, member B | 2 | | 7154 | hsa-mir-3126 | 80256 | FAM214B | family with sequence similarity 214, member B | 2 | | 7155 | hsa-mir-3129 | 80256 | FAM214B | family with sequence similarity 214, member B | 2 | | 7156 | hsa-mir-3606 | 80256 | FAM214B | family with sequence similarity 214, member B | 2 | | 7158 | hsa-mir-4441 | 80256 | FAM214B | family with sequence similarity 214, member B | 2 | | 7159 | hsa-mir-4735 | 80256 | FAM214B | family with sequence similarity 214, member B | 2 | | 7160 | hsa-mir-4768 | 80256 | FAM214B | family with sequence similarity 214, member B | 2 | | 7161 | hsa-mir-2467 | 80256 | FAM214B | family with sequence similarity 214, member B | 2 | | 7162 | hsa-mir-4794 | 80256 | FAM214B | family with sequence similarity 214, member B | 2 | | 7163 | hsa-mir-548ao | 80256 | FAM214B | family with sequence similarity 214, member B | 2 | | 7164 | hsa-mir-617 | 80256 | FAM214B | family with sequence similarity 214, member B | 2 | | 7165 | hsa-mir-648 | 80256 | FAM214B | family with sequence similarity 214, member B | 2 | | 7167 | hsa-mir-877 | 80256 | FAM214B | family with sequence similarity 214, member B | 2 | | 7168 | hsa-mir-10a | 120227 | CYP2R1 | cytochrome P450, family 2, subfamily R, polypeptide 1 | 2 | | 7169 | hsa-mir-218-1 | 120227 | CYP2R1 | cytochrome P450, family 2, subfamily R, polypeptide 1 | 2 | | 7170 | hsa-mir-218-2 | 120227 | CYP2R1 | cytochrome P450, family 2, subfamily R, polypeptide 1 | 2 | | 7173 | hsa-mir-574 | 120227 | CYP2R1 | cytochrome P450, family 2, subfamily R, polypeptide 1 | 2 | | 7177 | hsa-mir-605 | 120227 | CYP2R1 | cytochrome P450, family 2, subfamily R, polypeptide 1 | 2 | | 7178 | hsa-mir-618 | 120227 | CYP2R1 | cytochrome P450, family 2, subfamily R, polypeptide 1 | 2 | | 7179 | hsa-mir-620 | 120227 | CYP2R1 | cytochrome P450, family 2, subfamily R, polypeptide 1 | 2 | | 7180 | hsa-mir-675 | 120227 | CYP2R1 | cytochrome P450, family 2, subfamily R, polypeptide 1 | 2 | | 7182 | hsa-mir-1245a | 120227 | CYP2R1 | cytochrome P450, family 2, subfamily R, polypeptide 1 | 2 | | 7183 | hsa-mir-1469 | 120227 | CYP2R1 | cytochrome P450, family 2, subfamily R, polypeptide 1 | 2 | | 7184 | hsa-mir-3126 | 120227 | CYP2R1 | cytochrome P450, family 2, subfamily R, polypeptide 1 | 2 | | 7185 | hsa-mir-3129 | 120227 | CYP2R1 | cytochrome P450, family 2, subfamily R, polypeptide 1 | 2 | | 7186 | hsa-mir-3606 | 120227 | CYP2R1 | cytochrome P450, family 2, subfamily R, polypeptide 1 | 2 | | 7192 | hsa-mir-548ao | 120227 | CYP2R1 | cytochrome P450, family 2, subfamily R, polypeptide 1 | 2 | | 7194 | hsa-mir-636 | 120227 | CYP2R1 | cytochrome P450, family 2, subfamily R, polypeptide 1 | 2 | | 7195 | hsa-mir-640 | 120227 | CYP2R1 | cytochrome P450, family 2, subfamily R, polypeptide 1 | 2 | | 7197 | hsa-mir-218-1 | 2695 | GIP | gastric inhibitory polypeptide | 2 | | 7199 | hsa-mir-125b-1 | 2695 | GIP | gastric inhibitory polypeptide | 2 | | 7200 | hsa-mir-455 | 2695 | GIP | gastric inhibitory polypeptide | 2 | | 7201 | hsa-mir-561 | 2695 | GIP | gastric inhibitory polypeptide | 2 | | 7203 | hsa-mir-585 | 2695 | GIP | gastric inhibitory polypeptide | 2 | | 7208 | hsa-mir-620 | 2695 | GIP | gastric inhibitory polypeptide | 2 | | 7211 | hsa-mir-708 | 2695 | GIP | gastric inhibitory polypeptide | 2 | | 7242 | hsa-let-7c | 1453 | CSNK1D | casein kinase 1, delta | 2 | | 7243 | hsa-mir-99a | 1453 | CSNK1D | casein kinase 1, delta | 2 | | 7244 | hsa-mir-218-1 | 1453 | CSNK1D | casein kinase 1, delta | 2 | | 7246 | hsa-mir-125b-2 | 1453 | CSNK1D | casein kinase 1, delta | 2 | | 7248 | hsa-mir-561 | 1453 | CSNK1D | casein kinase 1, delta | 2 | | 7250 | hsa-mir-585 | 1453 | CSNK1D | casein kinase 1, delta | 2 | | 7276 | hsa-mir-181a-2 | 81578 | COL21A1 | collagen, type XXI, alpha 1 | 2 | | 7277 | hsa-mir-181b-2 | 81578 | COL21A1 | collagen, type XXI, alpha 1 | 2 | | 7278 | hsa-mir-378a | 81578 | COL21A1 | collagen, type XXI, alpha 1 | 2 | | 7279 | hsa-mir-505 | 81578 | COL21A1 | collagen, type XXI, alpha 1 | 2 | | 7281 | hsa-mir-602 | 81578 | COL21A1 | collagen, type XXI, alpha 1 | 2 | | 7283 | hsa-mir-652 | 81578 | COL21A1 | collagen, type XXI, alpha 1 | 2 | | 7318 | hsa-mir-125b-1 | 81887 | LAS1L | LAS1-like (S. cerevisiae) | 2 | | 7319 | hsa-mir-561 | 81887 | LAS1L | LAS1-like (S. cerevisiae) | 2 | | 7321 | hsa-mir-585 | 81887 | LAS1L | LAS1-like (S. cerevisiae) | 2 | | 7327 | hsa-mir-708 | 81887 | LAS1L | LAS1-like (S. cerevisiae) | 2 | | 7348 | hsa-mir-218-1 | 54504 | CPVL | carboxypeptidase, vitellogenic-like | 2 | | 7350 | hsa-mir-455 | 54504 | CPVL | carboxypeptidase, vitellogenic-like | 2 | | 7351 | hsa-mir-561 | 54504 | CPVL | carboxypeptidase, vitellogenic-like | 2 | | 7353 | hsa-mir-585 | 54504 | CPVL | carboxypeptidase, vitellogenic-like | 2 | | 7358 | hsa-mir-620 | 54504 | CPVL | carboxypeptidase, vitellogenic-like | 2 | | 7378 | hsa-let-7c | 7109 | TRAPPC10 | trafficking protein particle complex 10 | 2 | | 7379 | hsa-mir-99a | 7109 | TRAPPC10 | trafficking protein particle complex 10 | 2 | | 7382 | hsa-mir-125b-1 | 7109 | TRAPPC10 | trafficking protein particle complex 10 | 2 | | 7383 | hsa-mir-125b-2 | 7109 | TRAPPC10 | trafficking protein particle complex 10 | 2 | | 7384 | hsa-mir-561 | 7109 | TRAPPC10 | trafficking protein particle complex 10 | 2 | | 7386 | hsa-mir-585 | 7109 | TRAPPC10 | trafficking protein particle complex 10 | 2 | | 7391 | hsa-mir-708 | 7109 | TRAPPC10 | trafficking protein particle complex 10 | 2 | | 7418 | hsa-let-7c | 6051 | RNPEP | arginyl aminopeptidase (aminopeptidase B) | 2 | | 7419 | hsa-mir-99a | 6051 | RNPEP | arginyl aminopeptidase (aminopeptidase B) | 2 | | 7420 | hsa-mir-218-1 | 6051 | RNPEP | arginyl aminopeptidase (aminopeptidase B) | 2 | | 7422 | hsa-mir-125b-1 | 6051 | RNPEP | arginyl aminopeptidase (aminopeptidase B) | 2 | | 7423 | hsa-mir-125b-2 | 6051 | RNPEP | arginyl aminopeptidase (aminopeptidase B) | 2 | | 7424 | hsa-mir-561 | 6051 | RNPEP | arginyl aminopeptidase (aminopeptidase B) | 2 | | 7426 | hsa-mir-585 | 6051 | RNPEP | arginyl aminopeptidase (aminopeptidase B) | 2 | | 7457 | hsa-let-7c | 8433 | UTF1 | undifferentiated embryonic cell transcription factor 1 | 2 | | 7458 | hsa-mir-99a | 8433 | UTF1 | undifferentiated embryonic cell transcription factor 1 | 2 | | 7461 | hsa-mir-125b-2 | 8433 | UTF1 | undifferentiated embryonic cell transcription factor 1 | 2 | | 7462 | hsa-mir-561 | 8433 | UTF1 | undifferentiated embryonic cell transcription factor 1 | 2 | | 7464 | hsa-mir-585 | 8433 | UTF1 | undifferentiated embryonic cell transcription factor 1 | 2 | | 7470 | hsa-mir-708 | 8433 | UTF1 | undifferentiated embryonic cell transcription factor 1 | 2 | | 7491 | hsa-mir-218-1 | 8343 | HIST1H2BF | histone cluster 1, H2bf | 2 | | 7493 | hsa-mir-455 | 8343 | HIST1H2BF | histone cluster 1, H2bf | 2 | | 7496 | hsa-mir-585 | 8343 | HIST1H2BF | histone cluster 1, H2bf | 2 | | 7499 | hsa-mir-620 | 8343 | HIST1H2BF | histone cluster 1, H2bf | 2 | | 7516 | hsa-mir-455 | 8346 | HIST1H2BI | histone cluster 1, H2bi | 2 | | 7518 | hsa-mir-585 | 8346 | HIST1H2BI | histone cluster 1, H2bi | 2 | | 7520 | hsa-mir-620 | 8346 | HIST1H2BI | histone cluster 1, H2bi | 2 | | 7534 | hsa-mir-455 | 8344 | HIST1H2BE | histone cluster 1, H2be | 2 | | 7536 | hsa-mir-585 | 8344 | HIST1H2BE | histone cluster 1, H2be | 2 | | 7537 | hsa-mir-620 | 8344 | HIST1H2BE | histone cluster 1, H2be | 2 | | 7548 | hsa-let-7c | 2617 | GARS | glycyl-tRNA synthetase | 2 | | 7549 | hsa-mir-99a | 2617 | GARS | glycyl-tRNA synthetase | 2 | | 7551 | hsa-mir-218-1 | 2617 | GARS | glycyl-tRNA synthetase | 2 | | 7553 | hsa-mir-125b-1 | 2617 | GARS | glycyl-tRNA synthetase | 2 | | 7554 | hsa-mir-125b-2 | 2617 | GARS | glycyl-tRNA synthetase | 2 | | 7555 | hsa-mir-561 | 2617 | GARS | glycyl-tRNA synthetase | 2 | | 7557 | hsa-mir-585 | 2617 | GARS | glycyl-tRNA synthetase | 2 | | 7564 | hsa-mir-708 | 2617 | GARS | glycyl-tRNA synthetase | 2 | | 7590 | hsa-mir-218-1 | 6950 | TCP1 | t-complex 1 | 2 | | 7593 | hsa-mir-561 | 6950 | TCP1 | t-complex 1 | 2 | | 7595 | hsa-mir-585 | 6950 | TCP1 | t-complex 1 | 2 | | 7598 | hsa-mir-620 | 6950 | TCP1 | t-complex 1 | 2 | | 7615 | hsa-mir-218-1 | 483 | ATP1B3 | ATPase, Na+/K+ transporting, beta 3 polypeptide | 2 | | 7617 | hsa-mir-125b-1 | 483 | ATP1B3 | ATPase, Na+/K+ transporting, beta 3 polypeptide | 2 | | 7618 | hsa-mir-561 | 483 | ATP1B3 | ATPase, Na+/K+ transporting, beta 3 polypeptide | 2 | | 7620 | hsa-mir-585 | 483 | ATP1B3 | ATPase, Na+/K+ transporting, beta 3 polypeptide | 2 | | 7646 | hsa-mir-218-1 | 28973 | MRPS18B | mitochondrial ribosomal protein S18B | 2 | | 7648 | hsa-mir-561 | 28973 | MRPS18B | mitochondrial ribosomal protein S18B | 2 | | 7650 | hsa-mir-585 | 28973 | MRPS18B | mitochondrial ribosomal protein S18B | 2 | | 7653 | hsa-mir-620 | 28973 | MRPS18B | mitochondrial ribosomal protein S18B | 2 | | 7670 | hsa-mir-10a | 65220 | NADK | NAD kinase | 2 | | 7671 | hsa-mir-218-1 | 65220 | NADK | NAD kinase | 2 | | 7672 | hsa-mir-218-2 | 65220 | NADK | NAD kinase | 2 | | 7673 | hsa-mir-125b-1 | 65220 | NADK | NAD kinase | 2 | | 7676 | hsa-mir-574 | 65220 | NADK | NAD kinase | 2 | | 7678 | hsa-mir-603 | 65220 | NADK | NAD kinase | 2 | | 7680 | hsa-mir-605 | 65220 | NADK | NAD kinase | 2 | | 7683 | hsa-mir-675 | 65220 | NADK | NAD kinase | 2 | | 7684 | hsa-mir-887 | 65220 | NADK | NAD kinase | 2 | | 7691 | hsa-mir-3129 | 65220 | NADK | NAD kinase | 2 | | 7696 | hsa-mir-4441 | 65220 | NADK | NAD kinase | 2 | | 7702 | hsa-mir-4768 | 65220 | NADK | NAD kinase | 2 | | 7703 | hsa-mir-2467 | 65220 | NADK | NAD kinase | 2 | | 7705 | hsa-mir-548ao | 65220 | NADK | NAD kinase | 2 | | 7706 | hsa-mir-617 | 65220 | NADK | NAD kinase | 2 | | 7708 | hsa-mir-877 | 65220 | NADK | NAD kinase | 2 | | 7710 | hsa-mir-218-1 | 3837 | KPNB1 | karyopherin (importin) beta 1 | 2 | | 7713 | hsa-mir-585 | 3837 | KPNB1 | karyopherin (importin) beta 1 | 2 | | 7717 | hsa-mir-620 | 3837 | KPNB1 | karyopherin (importin) beta 1 | 2 | | 7730 | hsa-mir-181a-2 | 7204 | TRIO | triple functional domain (PTPRF interacting) | 2 | | 7731 | hsa-mir-181b-2 | 7204 | TRIO | triple functional domain (PTPRF interacting) | 2 | | 7732 | hsa-mir-505 | 7204 | TRIO | triple functional domain (PTPRF interacting) | 2 | | 7734 | hsa-mir-602 | 7204 | TRIO | triple functional domain (PTPRF interacting) | 2 | | 7764 | hsa-let-7c | 9139 | CBFA2T2 | core-binding factor, runt domain, alpha subunit 2; translocated to, 2 | 2 | | 7765 | hsa-mir-99a | 9139 | CBFA2T2 | core-binding factor, runt domain, alpha subunit 2; translocated to, 2 | 2 | | 7768 | hsa-mir-125b-2 | 9139 | CBFA2T2 | core-binding factor, runt domain, alpha subunit 2; translocated to, 2 | 2 | | 7770 | hsa-mir-561 | 9139 | CBFA2T2 | core-binding factor, runt domain, alpha subunit 2; translocated to, 2 | 2 | | 7772 | hsa-mir-585 | 9139 | CBFA2T2 | core-binding factor, runt domain, alpha subunit 2; translocated to, 2 | 2 | | 7796 | hsa-mir-561 | 22978 | NT5C2 | 5'-nucleotidase, cytosolic II | 2 | | 7798 | hsa-mir-585 | 22978 | NT5C2 | 5'-nucleotidase, cytosolic II | 2 | | 7802 | hsa-mir-620 | 22978 | NT5C2 | 5'-nucleotidase, cytosolic II | 2 | | 7825 | hsa-mir-218-1 | 10294 | DNAJA2 | DnaJ (Hsp40) homolog, subfamily A, member 2 | 2 | | 7827 | hsa-mir-455 | 10294 | DNAJA2 | DnaJ (Hsp40) homolog, subfamily A, member 2 | 2 | | 7828 | hsa-mir-561 | 10294 | DNAJA2 | DnaJ (Hsp40) homolog, subfamily A, member 2 | 2 | | 7830 | hsa-mir-585 | 10294 | DNAJA2 | DnaJ (Hsp40) homolog, subfamily A, member 2 | 2 | | 7833 | hsa-mir-620 | 10294 | DNAJA2 | DnaJ (Hsp40) homolog, subfamily A, member 2 | 2 | | 7857 | hsa-mir-455 | 84516 | DCTN5 | dynactin 5 (p25) | 2 | | 7858 | hsa-mir-561 | 84516 | DCTN5 | dynactin 5 (p25) | 2 | | 7860 | hsa-mir-585 | 84516 | DCTN5 | dynactin 5 (p25) | 2 | | 7862 | hsa-mir-620 | 84516 | DCTN5 | dynactin 5 (p25) | 2 | | 7865 | hsa-mir-708 | 84516 | DCTN5 | dynactin 5 (p25) | 2 | | 7882 | hsa-let-7c | 9962 | SLC23A2 | solute carrier family 23 (nucleobase transporters), member 2 | 2 | | 7883 | hsa-mir-99a | 9962 | SLC23A2 | solute carrier family 23 (nucleobase transporters), member 2 | 2 | | 7884 | hsa-mir-218-1 | 9962 | SLC23A2 | solute carrier family 23 (nucleobase transporters), member 2 | 2 | | 7886 | hsa-mir-125b-2 | 9962 | SLC23A2 | solute carrier family 23 (nucleobase transporters), member 2 | 2 | | 7888 | hsa-mir-561 | 9962 | SLC23A2 | solute carrier family 23 (nucleobase transporters), member 2 | 2 | | 7890 | hsa-mir-585 | 9962 | SLC23A2 | solute carrier family 23 (nucleobase transporters), member 2 | 2 | | 7896 | hsa-mir-708 | 9962 | SLC23A2 | solute carrier family 23 (nucleobase transporters), member 2 | 2 | | 7921 | hsa-let-7c | 10061 | ABCF2 | ATP-binding cassette, sub-family F (GCN20), member 2 | 2 | | 7922 | hsa-mir-99a | 10061 | ABCF2 | ATP-binding cassette, sub-family F (GCN20), member 2 | 2 | | 7924 | hsa-mir-125b-2 | 10061 | ABCF2 | ATP-binding cassette, sub-family F (GCN20), member 2 | 2 | | 7925 | hsa-mir-455 | 10061 | ABCF2 | ATP-binding cassette, sub-family F (GCN20), member 2 | 2 | | 7926 | hsa-mir-561 | 10061 | ABCF2 | ATP-binding cassette, sub-family F (GCN20), member 2 | 2 | | 7928 | hsa-mir-585 | 10061 | ABCF2 | ATP-binding cassette, sub-family F (GCN20), member 2 | 2 | | 7936 | hsa-mir-708 | 10061 | ABCF2 | ATP-binding cassette, sub-family F (GCN20), member 2 | 2 | | 7956 | hsa-mir-218-1 | 6004 | RGS16 | regulator of G-protein signaling 16 | 2 | | 7958 | hsa-mir-455 | 6004 | RGS16 | regulator of G-protein signaling 16 | 2 | | 7959 | hsa-mir-561 | 6004 | RGS16 | regulator of G-protein signaling 16 | 2 | | 7961 | hsa-mir-585 | 6004 | RGS16 | regulator of G-protein signaling 16 | 2 | | 7963 | hsa-mir-620 | 6004 | RGS16 | regulator of G-protein signaling 16 | 2 | | 7966 | hsa-mir-708 | 6004 | RGS16 | regulator of G-protein signaling 16 | 2 | | 7997 | hsa-mir-218-1 | 3006 | HIST1H1C | histone cluster 1, H1c | 2 | | 7999 | hsa-mir-455 | 3006 | HIST1H1C | histone cluster 1, H1c | 2 | | 8000 | hsa-mir-561 | 3006 | HIST1H1C | histone cluster 1, H1c | 2 | | 8002 | hsa-mir-585 | 3006 | HIST1H1C | histone cluster 1, H1c | 2 | | 8004 | hsa-mir-620 | 3006 | HIST1H1C | histone cluster 1, H1c | 2 | | 8017 | hsa-let-7c | 23212 | RRS1 | RRS1 ribosome biogenesis regulator homolog (S. cerevisiae) | 2 | | 8018 | hsa-mir-99a | 23212 | RRS1 | RRS1 ribosome biogenesis regulator homolog (S. cerevisiae) | 2 | | 8020 | hsa-mir-218-1 | 23212 | RRS1 | RRS1 ribosome biogenesis regulator homolog (S. cerevisiae) | 2 | | 8022 | hsa-mir-125b-1 | 23212 | RRS1 | RRS1 ribosome biogenesis regulator homolog (S. cerevisiae) | 2 | | 8023 | hsa-mir-125b-2 | 23212 | RRS1 | RRS1 ribosome biogenesis regulator homolog (S. cerevisiae) | 2 | | 8024 | hsa-mir-561 | 23212 | RRS1 | RRS1 ribosome biogenesis regulator homolog (S. cerevisiae) | 2 | | 8026 | hsa-mir-585 | 23212 | RRS1 | RRS1 ribosome biogenesis regulator homolog (S. cerevisiae) | 2 | | 8032 | hsa-mir-708 | 23212 | RRS1 | RRS1 ribosome biogenesis regulator homolog (S. cerevisiae) | 2 | | 8062 | hsa-mir-25 | 25878 | MXRA5 | matrix-remodelling associated 5 | 2 | | 8063 | hsa-mir-93 | 25878 | MXRA5 | matrix-remodelling associated 5 | 2 | | 8065 | hsa-mir-181a-2 | 25878 | MXRA5 | matrix-remodelling associated 5 | 2 | | 8066 | hsa-mir-181b-2 | 25878 | MXRA5 | matrix-remodelling associated 5 | 2 | | 8067 | hsa-mir-106b | 25878 | MXRA5 | matrix-remodelling associated 5 | 2 | | 8070 | hsa-mir-602 | 25878 | MXRA5 | matrix-remodelling associated 5 | 2 | | 8072 | hsa-mir-643 | 25878 | MXRA5 | matrix-remodelling associated 5 | 2 | | 8102 | hsa-let-7c | 26031 | OSBPL3 | oxysterol binding protein-like 3 | 2 | | 8105 | hsa-mir-218-2 | 26031 | OSBPL3 | oxysterol binding protein-like 3 | 2 | | 8110 | hsa-mir-574 | 26031 | OSBPL3 | oxysterol binding protein-like 3 | 2 | | 8112 | hsa-mir-603 | 26031 | OSBPL3 | oxysterol binding protein-like 3 | 2 | | 8115 | hsa-mir-618 | 26031 | OSBPL3 | oxysterol binding protein-like 3 | 2 | | 8116 | hsa-mir-1271 | 26031 | OSBPL3 | oxysterol binding protein-like 3 | 2 | | 8117 | hsa-mir-675 | 26031 | OSBPL3 | oxysterol binding protein-like 3 | 2 | | 8119 | hsa-mir-887 | 26031 | OSBPL3 | oxysterol binding protein-like 3 | 2 | | 8121 | hsa-mir-1245a | 26031 | OSBPL3 | oxysterol binding protein-like 3 | 2 | | 8123 | hsa-mir-1915 | 26031 | OSBPL3 | oxysterol binding protein-like 3 | 2 | | 8124 | hsa-mir-3120 | 26031 | OSBPL3 | oxysterol binding protein-like 3 | 2 | | 8126 | hsa-mir-3129 | 26031 | OSBPL3 | oxysterol binding protein-like 3 | 2 | | 8129 | hsa-mir-3606 | 26031 | OSBPL3 | oxysterol binding protein-like 3 | 2 | | 8130 | hsa-mir-3650 | 26031 | OSBPL3 | oxysterol binding protein-like 3 | 2 | | 8133 | hsa-mir-4519 | 26031 | OSBPL3 | oxysterol binding protein-like 3 | 2 | | 8136 | hsa-mir-4735 | 26031 | OSBPL3 | oxysterol binding protein-like 3 | 2 | | 8137 | hsa-mir-4768 | 26031 | OSBPL3 | oxysterol binding protein-like 3 | 2 | | 8139 | hsa-mir-4794 | 26031 | OSBPL3 | oxysterol binding protein-like 3 | 2 | | 8140 | hsa-mir-548ao | 26031 | OSBPL3 | oxysterol binding protein-like 3 | 2 | | 8141 | hsa-mir-5579 | 26031 | OSBPL3 | oxysterol binding protein-like 3 | 2 | | 8143 | hsa-mir-335 | 26031 | OSBPL3 | oxysterol binding protein-like 3 | 2 | | 8144 | hsa-mir-554 | 26031 | OSBPL3 | oxysterol binding protein-like 3 | 2 | | 8145 | hsa-mir-617 | 26031 | OSBPL3 | oxysterol binding protein-like 3 | 2 | | 8147 | hsa-mir-877 | 26031 | OSBPL3 | oxysterol binding protein-like 3 | 2 | | 8153 | hsa-mir-25 | 7041 | TGFB1I1 | transforming growth factor beta 1 induced transcript 1 | 2 | | 8154 | hsa-mir-93 | 7041 | TGFB1I1 | transforming growth factor beta 1 induced transcript 1 | 2 | | 8155 | hsa-mir-181a-2 | 7041 | TGFB1I1 | transforming growth factor beta 1 induced transcript 1 | 2 | | 8156 | hsa-mir-181b-2 | 7041 | TGFB1I1 | transforming growth factor beta 1 induced transcript 1 | 2 | | 8157 | hsa-mir-106b | 7041 | TGFB1I1 | transforming growth factor beta 1 induced transcript 1 | 2 | | 8159 | hsa-mir-505 | 7041 | TGFB1I1 | transforming growth factor beta 1 induced transcript 1 | 2 | | 8163 | hsa-mir-643 | 7041 | TGFB1I1 | transforming growth factor beta 1 induced transcript 1 | 2 | | 8165 | hsa-mir-652 | 7041 | TGFB1I1 | transforming growth factor beta 1 induced transcript 1 | 2 | | 8192 | hsa-mir-561 | 57228 | SMAGP | small cell adhesion glycoprotein | 2 | | 8194 | hsa-mir-585 | 57228 | SMAGP | small cell adhesion glycoprotein | 2 | | 8199 | hsa-mir-620 | 57228 | SMAGP | small cell adhesion glycoprotein | 2 | | 8218 | hsa-mir-218-1 | 10634 | GAS2L1 | growth arrest-specific 2 like 1 | 2 | | 8219 | hsa-mir-218-2 | 10634 | GAS2L1 | growth arrest-specific 2 like 1 | 2 | | 8223 | hsa-mir-574 | 10634 | GAS2L1 | growth arrest-specific 2 like 1 | 2 | | 8225 | hsa-mir-603 | 10634 | GAS2L1 | growth arrest-specific 2 like 1 | 2 | | 8226 | hsa-mir-604 | 10634 | GAS2L1 | growth arrest-specific 2 like 1 | 2 | | 8228 | hsa-mir-618 | 10634 | GAS2L1 | growth arrest-specific 2 like 1 | 2 | | 8229 | hsa-mir-620 | 10634 | GAS2L1 | growth arrest-specific 2 like 1 | 2 | | 8230 | hsa-mir-675 | 10634 | GAS2L1 | growth arrest-specific 2 like 1 | 2 | | 8231 | hsa-mir-887 | 10634 | GAS2L1 | growth arrest-specific 2 like 1 | 2 | | 8232 | hsa-mir-938 | 10634 | GAS2L1 | growth arrest-specific 2 like 1 | 2 | | 8234 | hsa-mir-1245a | 10634 | GAS2L1 | growth arrest-specific 2 like 1 | 2 | | 8235 | hsa-mir-3126 | 10634 | GAS2L1 | growth arrest-specific 2 like 1 | 2 | | 8236 | hsa-mir-3129 | 10634 | GAS2L1 | growth arrest-specific 2 like 1 | 2 | | 8237 | hsa-mir-3139 | 10634 | GAS2L1 | growth arrest-specific 2 like 1 | 2 | | 8238 | hsa-mir-3606 | 10634 | GAS2L1 | growth arrest-specific 2 like 1 | 2 | | 8239 | hsa-mir-3650 | 10634 | GAS2L1 | growth arrest-specific 2 like 1 | 2 | | 8240 | hsa-mir-4441 | 10634 | GAS2L1 | growth arrest-specific 2 like 1 | 2 | | 8241 | hsa-mir-4735 | 10634 | GAS2L1 | growth arrest-specific 2 like 1 | 2 | | 8242 | hsa-mir-4768 | 10634 | GAS2L1 | growth arrest-specific 2 like 1 | 2 | | 8243 | hsa-mir-2467 | 10634 | GAS2L1 | growth arrest-specific 2 like 1 | 2 | | 8244 | hsa-mir-4794 | 10634 | GAS2L1 | growth arrest-specific 2 like 1 | 2 | | 8245 | hsa-mir-548ao | 10634 | GAS2L1 | growth arrest-specific 2 like 1 | 2 | | 8246 | hsa-mir-617 | 10634 | GAS2L1 | growth arrest-specific 2 like 1 | 2 | | 8249 | hsa-mir-877 | 10634 | GAS2L1 | growth arrest-specific 2 like 1 | 2 | | 8251 | hsa-mir-218-1 | 85236 | HIST1H2BK | histone cluster 1, H2bk | 2 | | 8253 | hsa-mir-455 | 85236 | HIST1H2BK | histone cluster 1, H2bk | 2 | | 8254 | hsa-mir-561 | 85236 | HIST1H2BK | histone cluster 1, H2bk | 2 | | 8256 | hsa-mir-585 | 85236 | HIST1H2BK | histone cluster 1, H2bk | 2 | | 8260 | hsa-mir-620 | 85236 | HIST1H2BK | histone cluster 1, H2bk | 2 | | 8281 | hsa-let-7c | 23401 | FRAT2 | frequently rearranged in advanced T-cell lymphomas 2 | 2 | | 8282 | hsa-mir-99a | 23401 | FRAT2 | frequently rearranged in advanced T-cell lymphomas 2 | 2 | | 8284 | hsa-mir-218-1 | 23401 | FRAT2 | frequently rearranged in advanced T-cell lymphomas 2 | 2 | | 8286 | hsa-mir-125b-1 | 23401 | FRAT2 | frequently rearranged in advanced T-cell lymphomas 2 | 2 | | 8287 | hsa-mir-125b-2 | 23401 | FRAT2 | frequently rearranged in advanced T-cell lymphomas 2 | 2 | | 8288 | hsa-mir-561 | 23401 | FRAT2 | frequently rearranged in advanced T-cell lymphomas 2 | 2 | | 8290 | hsa-mir-585 | 23401 | FRAT2 | frequently rearranged in advanced T-cell lymphomas 2 | 2 | | 8295 | hsa-mir-620 | 23401 | FRAT2 | frequently rearranged in advanced T-cell lymphomas 2 | 2 | | 8298 | hsa-mir-708 | 23401 | FRAT2 | frequently rearranged in advanced T-cell lymphomas 2 | 2 | | 8327 | hsa-mir-181a-2 | 29984 | RHOD | ras homolog family member D | 2 | | 8328 | hsa-mir-181b-2 | 29984 | RHOD | ras homolog family member D | 2 | | 8329 | hsa-mir-378a | 29984 | RHOD | ras homolog family member D | 2 | | 8330 | hsa-mir-505 | 29984 | RHOD | ras homolog family member D | 2 | | 8332 | hsa-mir-643 | 29984 | RHOD | ras homolog family member D | 2 | | 8352 | hsa-mir-10a | 8034 | SLC25A16 | solute carrier family 25 (mitochondrial carrier; Graves disease autoantigen), member 16 | 2 | | 8354 | hsa-mir-218-2 | 8034 | SLC25A16 | solute carrier family 25 (mitochondrial carrier; Graves disease autoantigen), member 16 | 2 | | 8357 | hsa-mir-574 | 8034 | SLC25A16 | solute carrier family 25 (mitochondrial carrier; Graves disease autoantigen), member 16 | 2 | | 8359 | hsa-mir-605 | 8034 | SLC25A16 | solute carrier family 25 (mitochondrial carrier; Graves disease autoantigen), member 16 | 2 | | 8360 | hsa-mir-618 | 8034 | SLC25A16 | solute carrier family 25 (mitochondrial carrier; Graves disease autoantigen), member 16 | 2 | | 8361 | hsa-mir-1271 | 8034 | SLC25A16 | solute carrier family 25 (mitochondrial carrier; Graves disease autoantigen), member 16 | 2 | | 8362 | hsa-mir-675 | 8034 | SLC25A16 | solute carrier family 25 (mitochondrial carrier; Graves disease autoantigen), member 16 | 2 | | 8364 | hsa-mir-887 | 8034 | SLC25A16 | solute carrier family 25 (mitochondrial carrier; Graves disease autoantigen), member 16 | 2 | | 8365 | hsa-mir-1245a | 8034 | SLC25A16 | solute carrier family 25 (mitochondrial carrier; Graves disease autoantigen), member 16 | 2 | | 8366 | hsa-mir-1469 | 8034 | SLC25A16 | solute carrier family 25 (mitochondrial carrier; Graves disease autoantigen), member 16 | 2 | | 8367 | hsa-mir-3120 | 8034 | SLC25A16 | solute carrier family 25 (mitochondrial carrier; Graves disease autoantigen), member 16 | 2 | | 8368 | hsa-mir-3126 | 8034 | SLC25A16 | solute carrier family 25 (mitochondrial carrier; Graves disease autoantigen), member 16 | 2 | | 8369 | hsa-mir-3129 | 8034 | SLC25A16 | solute carrier family 25 (mitochondrial carrier; Graves disease autoantigen), member 16 | 2 | | 8370 | hsa-mir-3139 | 8034 | SLC25A16 | solute carrier family 25 (mitochondrial carrier; Graves disease autoantigen), member 16 | 2 | | 8371 | hsa-mir-3170 | 8034 | SLC25A16 | solute carrier family 25 (mitochondrial carrier; Graves disease autoantigen), member 16 | 2 | | 8372 | hsa-mir-3606 | 8034 | SLC25A16 | solute carrier family 25 (mitochondrial carrier; Graves disease autoantigen), member 16 | 2 | | 8373 | hsa-mir-3650 | 8034 | SLC25A16 | solute carrier family 25 (mitochondrial carrier; Graves disease autoantigen), member 16 | 2 | | 8374 | hsa-mir-4441 | 8034 | SLC25A16 | solute carrier family 25 (mitochondrial carrier; Graves disease autoantigen), member 16 | 2 | | 8375 | hsa-mir-4486 | 8034 | SLC25A16 | solute carrier family 25 (mitochondrial carrier; Graves disease autoantigen), member 16 | 2 | | 8376 | hsa-mir-4519 | 8034 | SLC25A16 | solute carrier family 25 (mitochondrial carrier; Graves disease autoantigen), member 16 | 2 | | 8377 | hsa-mir-4636 | 8034 | SLC25A16 | solute carrier family 25 (mitochondrial carrier; Graves disease autoantigen), member 16 | 2 | | 8378 | hsa-mir-4731 | 8034 | SLC25A16 | solute carrier family 25 (mitochondrial carrier; Graves disease autoantigen), member 16 | 2 | | 8379 | hsa-mir-4735 | 8034 | SLC25A16 | solute carrier family 25 (mitochondrial carrier; Graves disease autoantigen), member 16 | 2 | | 8380 | hsa-mir-4768 | 8034 | SLC25A16 | solute carrier family 25 (mitochondrial carrier; Graves disease autoantigen), member 16 | 2 | | 8381 | hsa-mir-2467 | 8034 | SLC25A16 | solute carrier family 25 (mitochondrial carrier; Graves disease autoantigen), member 16 | 2 | | 8382 | hsa-mir-4794 | 8034 | SLC25A16 | solute carrier family 25 (mitochondrial carrier; Graves disease autoantigen), member 16 | 2 | | 8383 | hsa-mir-548ao | 8034 | SLC25A16 | solute carrier family 25 (mitochondrial carrier; Graves disease autoantigen), member 16 | 2 | | 8384 | hsa-mir-5579 | 8034 | SLC25A16 | solute carrier family 25 (mitochondrial carrier; Graves disease autoantigen), member 16 | 2 | | 8386 | hsa-mir-640 | 8034 | SLC25A16 | solute carrier family 25 (mitochondrial carrier; Graves disease autoantigen), member 16 | 2 | | 8387 | hsa-mir-1233-1;hsa-mir-1233-2 | 8034 | SLC25A16 | solute carrier family 25 (mitochondrial carrier; Graves disease autoantigen), member 16 | 2 | | 8390 | hsa-mir-455 | 3017 | HIST1H2BD | histone cluster 1, H2bd | 2 | | 8391 | hsa-mir-561 | 3017 | HIST1H2BD | histone cluster 1, H2bd | 2 | | 8393 | hsa-mir-585 | 3017 | HIST1H2BD | histone cluster 1, H2bd | 2 | | 8405 | hsa-let-7c | 8837 | CFLAR | CASP8 and FADD-like apoptosis regulator | 2 | | 8406 | hsa-mir-99a | 8837 | CFLAR | CASP8 and FADD-like apoptosis regulator | 2 | | 8408 | hsa-mir-218-1 | 8837 | CFLAR | CASP8 and FADD-like apoptosis regulator | 2 | | 8409 | hsa-mir-218-2 | 8837 | CFLAR | CASP8 and FADD-like apoptosis regulator | 2 | | 8410 | hsa-mir-125b-1 | 8837 | CFLAR | CASP8 and FADD-like apoptosis regulator | 2 | | 8411 | hsa-mir-125b-2 | 8837 | CFLAR | CASP8 and FADD-like apoptosis regulator | 2 | | 8413 | hsa-mir-561 | 8837 | CFLAR | CASP8 and FADD-like apoptosis regulator | 2 | | 8414 | hsa-mir-574 | 8837 | CFLAR | CASP8 and FADD-like apoptosis regulator | 2 | | 8416 | hsa-mir-603 | 8837 | CFLAR | CASP8 and FADD-like apoptosis regulator | 2 | | 8421 | hsa-mir-620 | 8837 | CFLAR | CASP8 and FADD-like apoptosis regulator | 2 | | 8422 | hsa-mir-1271 | 8837 | CFLAR | CASP8 and FADD-like apoptosis regulator | 2 | | 8423 | hsa-mir-675 | 8837 | CFLAR | CASP8 and FADD-like apoptosis regulator | 2 | | 8424 | hsa-mir-708 | 8837 | CFLAR | CASP8 and FADD-like apoptosis regulator | 2 | | 8427 | hsa-mir-663b | 8837 | CFLAR | CASP8 and FADD-like apoptosis regulator | 2 | | 8432 | hsa-mir-3129 | 8837 | CFLAR | CASP8 and FADD-like apoptosis regulator | 2 | | 8444 | hsa-mir-548ao | 8837 | CFLAR | CASP8 and FADD-like apoptosis regulator | 2 | | 8448 | hsa-mir-591 | 8837 | CFLAR | CASP8 and FADD-like apoptosis regulator | 2 | | 8450 | hsa-mir-636 | 8837 | CFLAR | CASP8 and FADD-like apoptosis regulator | 2 | | 8452 | hsa-mir-877 | 8837 | CFLAR | CASP8 and FADD-like apoptosis regulator | 2 | | 8455 | hsa-mir-10a | 8115 | TCL1A | T-cell leukemia/lymphoma 1A | 2 | | 8457 | hsa-mir-218-2 | 8115 | TCL1A | T-cell leukemia/lymphoma 1A | 2 | | 8461 | hsa-mir-574 | 8115 | TCL1A | T-cell leukemia/lymphoma 1A | 2 | | 8463 | hsa-mir-603 | 8115 | TCL1A | T-cell leukemia/lymphoma 1A | 2 | | 8464 | hsa-mir-604 | 8115 | TCL1A | T-cell leukemia/lymphoma 1A | 2 | | 8465 | hsa-mir-605 | 8115 | TCL1A | T-cell leukemia/lymphoma 1A | 2 | | 8466 | hsa-mir-613 | 8115 | TCL1A | T-cell leukemia/lymphoma 1A | 2 | | 8467 | hsa-mir-618 | 8115 | TCL1A | T-cell leukemia/lymphoma 1A | 2 | | 8468 | hsa-mir-675 | 8115 | TCL1A | T-cell leukemia/lymphoma 1A | 2 | | 8470 | hsa-mir-887 | 8115 | TCL1A | T-cell leukemia/lymphoma 1A | 2 | | 8471 | hsa-mir-938 | 8115 | TCL1A | T-cell leukemia/lymphoma 1A | 2 | | 8472 | hsa-mir-1245a | 8115 | TCL1A | T-cell leukemia/lymphoma 1A | 2 | | 8473 | hsa-mir-1469 | 8115 | TCL1A | T-cell leukemia/lymphoma 1A | 2 | | 8474 | hsa-mir-3120 | 8115 | TCL1A | T-cell leukemia/lymphoma 1A | 2 | | 8475 | hsa-mir-3126 | 8115 | TCL1A | T-cell leukemia/lymphoma 1A | 2 | | 8476 | hsa-mir-3129 | 8115 | TCL1A | T-cell leukemia/lymphoma 1A | 2 | | 8477 | hsa-mir-3139 | 8115 | TCL1A | T-cell leukemia/lymphoma 1A | 2 | | 8478 | hsa-mir-3170 | 8115 | TCL1A | T-cell leukemia/lymphoma 1A | 2 | | 8479 | hsa-mir-3606 | 8115 | TCL1A | T-cell leukemia/lymphoma 1A | 2 | | 8480 | hsa-mir-3650 | 8115 | TCL1A | T-cell leukemia/lymphoma 1A | 2 | | 8481 | hsa-mir-4441 | 8115 | TCL1A | T-cell leukemia/lymphoma 1A | 2 | | 8482 | hsa-mir-4486 | 8115 | TCL1A | T-cell leukemia/lymphoma 1A | 2 | | 8483 | hsa-mir-4636 | 8115 | TCL1A | T-cell leukemia/lymphoma 1A | 2 | | 8484 | hsa-mir-4731 | 8115 | TCL1A | T-cell leukemia/lymphoma 1A | 2 | | 8485 | hsa-mir-4735 | 8115 | TCL1A | T-cell leukemia/lymphoma 1A | 2 | | 8486 | hsa-mir-4768 | 8115 | TCL1A | T-cell leukemia/lymphoma 1A | 2 | | 8487 | hsa-mir-2467 | 8115 | TCL1A | T-cell leukemia/lymphoma 1A | 2 | | 8488 | hsa-mir-4794 | 8115 | TCL1A | T-cell leukemia/lymphoma 1A | 2 | | 8489 | hsa-mir-548ao | 8115 | TCL1A | T-cell leukemia/lymphoma 1A | 2 | | 8490 | hsa-mir-5579 | 8115 | TCL1A | T-cell leukemia/lymphoma 1A | 2 | | 8491 | hsa-mir-617 | 8115 | TCL1A | T-cell leukemia/lymphoma 1A | 2 | | 8492 | hsa-mir-640 | 8115 | TCL1A | T-cell leukemia/lymphoma 1A | 2 | | 8493 | hsa-mir-877 | 8115 | TCL1A | T-cell leukemia/lymphoma 1A | 2 | | 8494 | hsa-mir-218-1 | 2130 | EWSR1 | Ewing sarcoma breakpoint region 1 | 2 | | 8497 | hsa-mir-561 | 2130 | EWSR1 | Ewing sarcoma breakpoint region 1 | 2 | | 8499 | hsa-mir-585 | 2130 | EWSR1 | Ewing sarcoma breakpoint region 1 | 2 | | 8532 | hsa-mir-561 | 51385 | ZNF589 | zinc finger protein 589 | 2 | | 8534 | hsa-mir-585 | 51385 | ZNF589 | zinc finger protein 589 | 2 | | 8538 | hsa-mir-620 | 51385 | ZNF589 | zinc finger protein 589 | 2 | | 8549 | hsa-mir-25 | 3486 | IGFBP3 | insulin-like growth factor binding protein 3 | 2 | | 8550 | hsa-mir-93 | 3486 | IGFBP3 | insulin-like growth factor binding protein 3 | 2 | | 8552 | hsa-mir-181a-2 | 3486 | IGFBP3 | insulin-like growth factor binding protein 3 | 2 | | 8553 | hsa-mir-181b-2 | 3486 | IGFBP3 | insulin-like growth factor binding protein 3 | 2 | | 8554 | hsa-mir-106b | 3486 | IGFBP3 | insulin-like growth factor binding protein 3 | 2 | | 8557 | hsa-mir-602 | 3486 | IGFBP3 | insulin-like growth factor binding protein 3 | 2 | | 8585 | hsa-let-7c | 642559 | POU5F1P3 | POU class 5 homeobox 1 pseudogene 3 | 2 | | 8586 | hsa-mir-99a | 642559 | POU5F1P3 | POU class 5 homeobox 1 pseudogene 3 | 2 | | 8588 | hsa-mir-218-1 | 642559 | POU5F1P3 | POU class 5 homeobox 1 pseudogene 3 | 2 | | 8590 | hsa-mir-125b-1 | 642559 | POU5F1P3 | POU class 5 homeobox 1 pseudogene 3 | 2 | | 8591 | hsa-mir-125b-2 | 642559 | POU5F1P3 | POU class 5 homeobox 1 pseudogene 3 | 2 | | 8592 | hsa-mir-561 | 642559 | POU5F1P3 | POU class 5 homeobox 1 pseudogene 3 | 2 | | 8594 | hsa-mir-585 | 642559 | POU5F1P3 | POU class 5 homeobox 1 pseudogene 3 | 2 | | 8601 | hsa-mir-708 | 642559 | POU5F1P3 | POU class 5 homeobox 1 pseudogene 3 | 2 | | 8629 | hsa-mir-218-1 | 11200 | CHEK2 | checkpoint kinase 2 | 2 | | 8631 | hsa-mir-125b-1 | 11200 | CHEK2 | checkpoint kinase 2 | 2 | | 8632 | hsa-mir-561 | 11200 | CHEK2 | checkpoint kinase 2 | 2 | | 8634 | hsa-mir-585 | 11200 | CHEK2 | checkpoint kinase 2 | 2 | | 8641 | hsa-mir-708 | 11200 | CHEK2 | checkpoint kinase 2 | 2 | | 8668 | hsa-mir-643 | 8829 | NRP1 | neuropilin 1 | 2 | | 8669 | hsa-mir-652 | 8829 | NRP1 | neuropilin 1 | 2 | | 8680 | hsa-mir-455 | 3985 | LIMK2 | LIM domain kinase 2 | 2 | | 8681 | hsa-mir-561 | 3985 | LIMK2 | LIM domain kinase 2 | 2 | | 8683 | hsa-mir-585 | 3985 | LIMK2 | LIM domain kinase 2 | 2 | | 8694 | hsa-let-7c | 84779 | NAA11 | N(alpha)-acetyltransferase 11, NatA catalytic subunit | 2 | | 8695 | hsa-mir-99a | 84779 | NAA11 | N(alpha)-acetyltransferase 11, NatA catalytic subunit | 2 | | 8697 | hsa-mir-218-1 | 84779 | NAA11 | N(alpha)-acetyltransferase 11, NatA catalytic subunit | 2 | | 8699 | hsa-mir-125b-1 | 84779 | NAA11 | N(alpha)-acetyltransferase 11, NatA catalytic subunit | 2 | | 8700 | hsa-mir-125b-2 | 84779 | NAA11 | N(alpha)-acetyltransferase 11, NatA catalytic subunit | 2 | | 8702 | hsa-mir-561 | 84779 | NAA11 | N(alpha)-acetyltransferase 11, NatA catalytic subunit | 2 | | 8704 | hsa-mir-585 | 84779 | NAA11 | N(alpha)-acetyltransferase 11, NatA catalytic subunit | 2 | | 8712 | hsa-mir-708 | 84779 | NAA11 | N(alpha)-acetyltransferase 11, NatA catalytic subunit | 2 | | 8742 | hsa-mir-181a-2 | 2852 | GPER | G protein-coupled estrogen receptor 1 | 2 | | 8743 | hsa-mir-181b-2 | 2852 | GPER | G protein-coupled estrogen receptor 1 | 2 | | 8745 | hsa-mir-505 | 2852 | GPER | G protein-coupled estrogen receptor 1 | 2 | | 8748 | hsa-mir-643 | 2852 | GPER | G protein-coupled estrogen receptor 1 | 2 | | 8750 | hsa-mir-652 | 2852 | GPER | G protein-coupled estrogen receptor 1 | 2 | | 8776 | hsa-mir-550a-1 | 7035 | TFPI | tissue factor pathway inhibitor (lipoprotein-associated coagulation inhibitor) | 2 | | 8778 | hsa-mir-652 | 7035 | TFPI | tissue factor pathway inhibitor (lipoprotein-associated coagulation inhibitor) | 2 | | 8781 | hsa-mir-378e | 7035 | TFPI | tissue factor pathway inhibitor (lipoprotein-associated coagulation inhibitor) | 2 | | 8782 | hsa-mir-4467 | 7035 | TFPI | tissue factor pathway inhibitor (lipoprotein-associated coagulation inhibitor) | 2 | | 8785 | hsa-mir-4685 | 7035 | TFPI | tissue factor pathway inhibitor (lipoprotein-associated coagulation inhibitor) | 2 | | 8786 | hsa-mir-4698 | 7035 | TFPI | tissue factor pathway inhibitor (lipoprotein-associated coagulation inhibitor) | 2 | | 8788 | hsa-mir-5090 | 7035 | TFPI | tissue factor pathway inhibitor (lipoprotein-associated coagulation inhibitor) | 2 | | 8789 | hsa-mir-604 | 7035 | TFPI | tissue factor pathway inhibitor (lipoprotein-associated coagulation inhibitor) | 2 | | 8790 | hsa-mir-675 | 7035 | TFPI | tissue factor pathway inhibitor (lipoprotein-associated coagulation inhibitor) | 2 | | 8791 | hsa-mir-218-2 | 7035 | TFPI | tissue factor pathway inhibitor (lipoprotein-associated coagulation inhibitor) | 2 | | 8792 | hsa-mir-574 | 7035 | TFPI | tissue factor pathway inhibitor (lipoprotein-associated coagulation inhibitor) | 2 | | 8793 | hsa-mir-938 | 7035 | TFPI | tissue factor pathway inhibitor (lipoprotein-associated coagulation inhibitor) | 2 | | 8805 | hsa-let-7c | 645682 | POU5F1P4 | POU class 5 homeobox 1 pseudogene 4 | 2 | | 8806 | hsa-mir-99a | 645682 | POU5F1P4 | POU class 5 homeobox 1 pseudogene 4 | 2 | | 8808 | hsa-mir-218-1 | 645682 | POU5F1P4 | POU class 5 homeobox 1 pseudogene 4 | 2 | | 8810 | hsa-mir-125b-1 | 645682 | POU5F1P4 | POU class 5 homeobox 1 pseudogene 4 | 2 | | 8811 | hsa-mir-125b-2 | 645682 | POU5F1P4 | POU class 5 homeobox 1 pseudogene 4 | 2 | | 8812 | hsa-mir-561 | 645682 | POU5F1P4 | POU class 5 homeobox 1 pseudogene 4 | 2 | | 8814 | hsa-mir-585 | 645682 | POU5F1P4 | POU class 5 homeobox 1 pseudogene 4 | 2 | | 8821 | hsa-mir-708 | 645682 | POU5F1P4 | POU class 5 homeobox 1 pseudogene 4 | 2 | | 8854 | hsa-mir-550a-1 | 2078 | ERG | v-ets erythroblastosis virus E26 oncogene homolog (avian) | 2 | | 8856 | hsa-mir-877 | 2078 | ERG | v-ets erythroblastosis virus E26 oncogene homolog (avian) | 2 | | 8861 | hsa-mir-3942 | 2078 | ERG | v-ets erythroblastosis virus E26 oncogene homolog (avian) | 2 | | 8866 | hsa-mir-4698 | 2078 | ERG | v-ets erythroblastosis virus E26 oncogene homolog (avian) | 2 | | 8870 | hsa-mir-4775 | 2078 | ERG | v-ets erythroblastosis virus E26 oncogene homolog (avian) | 2 | | 8871 | hsa-mir-5194 | 2078 | ERG | v-ets erythroblastosis virus E26 oncogene homolog (avian) | 2 | | 8874 | hsa-mir-218-2 | 2078 | ERG | v-ets erythroblastosis virus E26 oncogene homolog (avian) | 2 | | 8875 | hsa-mir-574 | 2078 | ERG | v-ets erythroblastosis virus E26 oncogene homolog (avian) | 2 | | 8878 | hsa-let-7c | 55905 | RNF114 | ring finger protein 114 | 2 | | 8879 | hsa-mir-99a | 55905 | RNF114 | ring finger protein 114 | 2 | | 8881 | hsa-mir-125b-1 | 55905 | RNF114 | ring finger protein 114 | 2 | | 8882 | hsa-mir-125b-2 | 55905 | RNF114 | ring finger protein 114 | 2 | | 8897 | hsa-mir-125b-1 | 3838 | KPNA2 | karyopherin alpha 2 (RAG cohort 1, importin alpha 1) | 2 | | 8898 | hsa-mir-561 | 3838 | KPNA2 | karyopherin alpha 2 (RAG cohort 1, importin alpha 1) | 2 | | 8900 | hsa-mir-585 | 3838 | KPNA2 | karyopherin alpha 2 (RAG cohort 1, importin alpha 1) | 2 | | 8931 | hsa-mir-455 | 81614 | NIPA2 | non imprinted in Prader-Willi/Angelman syndrome 2 | 2 | | 8932 | hsa-mir-561 | 81614 | NIPA2 | non imprinted in Prader-Willi/Angelman syndrome 2 | 2 | | 8934 | hsa-mir-585 | 81614 | NIPA2 | non imprinted in Prader-Willi/Angelman syndrome 2 | 2 | | 8937 | hsa-mir-620 | 81614 | NIPA2 | non imprinted in Prader-Willi/Angelman syndrome 2 | 2 | | 8940 | hsa-mir-708 | 81614 | NIPA2 | non imprinted in Prader-Willi/Angelman syndrome 2 | 2 | | 8961 | hsa-mir-455 | 23039 | XPO7 | exportin 7 | 2 | | 8964 | hsa-mir-585 | 23039 | XPO7 | exportin 7 | 2 | | 8966 | hsa-mir-620 | 23039 | XPO7 | exportin 7 | 2 | | 8977 | hsa-mir-25 | 23389 | MED13L | mediator complex subunit 13-like | 2 | | 8978 | hsa-mir-93 | 23389 | MED13L | mediator complex subunit 13-like | 2 | | 8979 | hsa-mir-181a-2 | 23389 | MED13L | mediator complex subunit 13-like | 2 | | 8980 | hsa-mir-181b-2 | 23389 | MED13L | mediator complex subunit 13-like | 2 | | 8981 | hsa-mir-106b | 23389 | MED13L | mediator complex subunit 13-like | 2 | | 8982 | hsa-mir-378a | 23389 | MED13L | mediator complex subunit 13-like | 2 | | 8983 | hsa-mir-505 | 23389 | MED13L | mediator complex subunit 13-like | 2 | | 8985 | hsa-mir-602 | 23389 | MED13L | mediator complex subunit 13-like | 2 | | 8986 | hsa-mir-643 | 23389 | MED13L | mediator complex subunit 13-like | 2 | | 9010 | hsa-mir-218-1 | 23063 | WAPAL | wings apart-like homolog (Drosophila) | 2 | | 9013 | hsa-mir-455 | 23063 | WAPAL | wings apart-like homolog (Drosophila) | 2 | | 9014 | hsa-mir-561 | 23063 | WAPAL | wings apart-like homolog (Drosophila) | 2 | | 9016 | hsa-mir-585 | 23063 | WAPAL | wings apart-like homolog (Drosophila) | 2 | | 9019 | hsa-mir-620 | 23063 | WAPAL | wings apart-like homolog (Drosophila) | 2 | | 9022 | hsa-mir-708 | 23063 | WAPAL | wings apart-like homolog (Drosophila) | 2 | | 9047 | hsa-mir-455 | 23175 | LPIN1 | lipin 1 | 2 | | 9048 | hsa-mir-561 | 23175 | LPIN1 | lipin 1 | 2 | | 9050 | hsa-mir-585 | 23175 | LPIN1 | lipin 1 | 2 | | 9052 | hsa-mir-620 | 23175 | LPIN1 | lipin 1 | 2 | | 9067 | hsa-mir-218-1 | 92856 | IMP4 | IMP4, U3 small nucleolar ribonucleoprotein, homolog (yeast) | 2 | | 9071 | hsa-mir-561 | 92856 | IMP4 | IMP4, U3 small nucleolar ribonucleoprotein, homolog (yeast) | 2 | | 9073 | hsa-mir-585 | 92856 | IMP4 | IMP4, U3 small nucleolar ribonucleoprotein, homolog (yeast) | 2 | | 9079 | hsa-mir-620 | 92856 | IMP4 | IMP4, U3 small nucleolar ribonucleoprotein, homolog (yeast) | 2 | | 9104 | hsa-mir-455 | 23157 | SEPT6 | septin 6 | 2 | | 9105 | hsa-mir-561 | 23157 | SEPT6 | septin 6 | 2 | | 9107 | hsa-mir-585 | 23157 | SEPT6 | septin 6 | 2 | | 9125 | hsa-mir-561 | 22984 | PDCD11 | programmed cell death 11 | 2 | | 9127 | hsa-mir-585 | 22984 | PDCD11 | programmed cell death 11 | 2 | | 9129 | hsa-mir-620 | 22984 | PDCD11 | programmed cell death 11 | 2 | | 9148 | hsa-let-7c | 55544 | RBM38 | RNA binding motif protein 38 | 2 | | 9149 | hsa-mir-99a | 55544 | RBM38 | RNA binding motif protein 38 | 2 | | 9151 | hsa-mir-125b-2 | 55544 | RBM38 | RNA binding motif protein 38 | 2 | | 9152 | hsa-mir-561 | 55544 | RBM38 | RNA binding motif protein 38 | 2 | | 9154 | hsa-mir-585 | 55544 | RBM38 | RNA binding motif protein 38 | 2 | | 9161 | hsa-mir-708 | 55544 | RBM38 | RNA binding motif protein 38 | 2 | | 9184 | hsa-mir-218-1 | 23080 | AVL9 | AVL9 homolog (S. cerevisiase) | 2 | | 9186 | hsa-mir-125b-1 | 23080 | AVL9 | AVL9 homolog (S. cerevisiase) | 2 | | 9187 | hsa-mir-561 | 23080 | AVL9 | AVL9 homolog (S. cerevisiase) | 2 | | 9189 | hsa-mir-585 | 23080 | AVL9 | AVL9 homolog (S. cerevisiase) | 2 | | 9197 | hsa-mir-708 | 23080 | AVL9 | AVL9 homolog (S. cerevisiase) | 2 | | 9226 | hsa-mir-4775 | 1289 | COL5A1 | collagen, type V, alpha 1 | 2 | | 9227 | hsa-mir-25 | 1289 | COL5A1 | collagen, type V, alpha 1 | 2 | | 9228 | hsa-mir-93 | 1289 | COL5A1 | collagen, type V, alpha 1 | 2 | | 9229 | hsa-mir-106b | 1289 | COL5A1 | collagen, type V, alpha 1 | 2 | | 9232 | hsa-mir-25 | 9886 | RHOBTB1 | Rho-related BTB domain containing 1 | 2 | | 9233 | hsa-mir-93 | 9886 | RHOBTB1 | Rho-related BTB domain containing 1 | 2 | | 9235 | hsa-mir-181a-2 | 9886 | RHOBTB1 | Rho-related BTB domain containing 1 | 2 | | 9236 | hsa-mir-181b-2 | 9886 | RHOBTB1 | Rho-related BTB domain containing 1 | 2 | | 9237 | hsa-mir-106b | 9886 | RHOBTB1 | Rho-related BTB domain containing 1 | 2 | | 9238 | hsa-mir-505 | 9886 | RHOBTB1 | Rho-related BTB domain containing 1 | 2 | | 9240 | hsa-mir-602 | 9886 | RHOBTB1 | Rho-related BTB domain containing 1 | 2 | | 9243 | hsa-mir-643 | 9886 | RHOBTB1 | Rho-related BTB domain containing 1 | 2 | | 9276 | hsa-let-7c | 10102 | TSFM | Ts translation elongation factor, mitochondrial | 2 | | 9277 | hsa-mir-99a | 10102 | TSFM | Ts translation elongation factor, mitochondrial | 2 | | 9279 | hsa-mir-218-1 | 10102 | TSFM | Ts translation elongation factor, mitochondrial | 2 | | 9281 | hsa-mir-125b-1 | 10102 | TSFM | Ts translation elongation factor, mitochondrial | 2 | | 9282 | hsa-mir-125b-2 | 10102 | TSFM | Ts translation elongation factor, mitochondrial | 2 | | 9283 | hsa-mir-561 | 10102 | TSFM | Ts translation elongation factor, mitochondrial | 2 | | 9285 | hsa-mir-585 | 10102 | TSFM | Ts translation elongation factor, mitochondrial | 2 | | 9290 | hsa-mir-620 | 10102 | TSFM | Ts translation elongation factor, mitochondrial | 2 | | 9293 | hsa-mir-708 | 10102 | TSFM | Ts translation elongation factor, mitochondrial | 2 | | 9320 | hsa-mir-181a-2 | 2006 | ELN | elastin | 2 | | 9321 | hsa-mir-181b-2 | 2006 | ELN | elastin | 2 | | 9322 | hsa-mir-378a | 2006 | ELN | elastin | 2 | | 9323 | hsa-mir-505 | 2006 | ELN | elastin | 2 | | 9327 | hsa-mir-643 | 2006 | ELN | elastin | 2 | | 9341 | hsa-mir-125b-1 | 23195 | MDN1 | MDN1, midasin homolog (yeast) | 2 | | 9342 | hsa-mir-561 | 23195 | MDN1 | MDN1, midasin homolog (yeast) | 2 | | 9344 | hsa-mir-585 | 23195 | MDN1 | MDN1, midasin homolog (yeast) | 2 | | 9348 | hsa-mir-708 | 23195 | MDN1 | MDN1, midasin homolog (yeast) | 2 | | 9376 | hsa-mir-218-1 | 5096 | PCCB | propionyl CoA carboxylase, beta polypeptide | 2 | | 9379 | hsa-mir-561 | 5096 | PCCB | propionyl CoA carboxylase, beta polypeptide | 2 | | 9381 | hsa-mir-585 | 5096 | PCCB | propionyl CoA carboxylase, beta polypeptide | 2 | | 9385 | hsa-mir-620 | 5096 | PCCB | propionyl CoA carboxylase, beta polypeptide | 2 | | 9404 | hsa-mir-181a-2 | 4239 | MFAP4 | microfibrillar-associated protein 4 | 2 | | 9405 | hsa-mir-181b-2 | 4239 | MFAP4 | microfibrillar-associated protein 4 | 2 | | 9406 | hsa-mir-378a | 4239 | MFAP4 | microfibrillar-associated protein 4 | 2 | | 9407 | hsa-mir-505 | 4239 | MFAP4 | microfibrillar-associated protein 4 | 2 | | 9409 | hsa-mir-643 | 4239 | MFAP4 | microfibrillar-associated protein 4 | 2 | | 9430 | hsa-mir-3170 | 1741 | DLG3 | discs, large homolog 3 (Drosophila) | 2 | | 9431 | hsa-mir-4486 | 1741 | DLG3 | discs, large homolog 3 (Drosophila) | 2 | | 9432 | hsa-mir-4636 | 1741 | DLG3 | discs, large homolog 3 (Drosophila) | 2 | | 9434 | hsa-mir-5579 | 1741 | DLG3 | discs, large homolog 3 (Drosophila) | 2 | | 9435 | hsa-mir-218-1 | 1741 | DLG3 | discs, large homolog 3 (Drosophila) | 2 | | 9437 | hsa-mir-455 | 1741 | DLG3 | discs, large homolog 3 (Drosophila) | 2 | | 9441 | hsa-mir-10a | 9711 | KIAA0226 | KIAA0226 | 2 | | 9443 | hsa-mir-218-2 | 9711 | KIAA0226 | KIAA0226 | 2 | | 9445 | hsa-mir-574 | 9711 | KIAA0226 | KIAA0226 | 2 | | 9447 | hsa-mir-603 | 9711 | KIAA0226 | KIAA0226 | 2 | | 9448 | hsa-mir-618 | 9711 | KIAA0226 | KIAA0226 | 2 | | 9450 | hsa-mir-675 | 9711 | KIAA0226 | KIAA0226 | 2 | | 9451 | hsa-mir-887 | 9711 | KIAA0226 | KIAA0226 | 2 | | 9452 | hsa-mir-1245a | 9711 | KIAA0226 | KIAA0226 | 2 | | 9454 | hsa-mir-1915 | 9711 | KIAA0226 | KIAA0226 | 2 | | 9455 | hsa-mir-3126 | 9711 | KIAA0226 | KIAA0226 | 2 | | 9456 | hsa-mir-3129 | 9711 | KIAA0226 | KIAA0226 | 2 | | 9458 | hsa-mir-3606 | 9711 | KIAA0226 | KIAA0226 | 2 | | 9459 | hsa-mir-3650 | 9711 | KIAA0226 | KIAA0226 | 2 | | 9460 | hsa-mir-4735 | 9711 | KIAA0226 | KIAA0226 | 2 | | 9461 | hsa-mir-4768 | 9711 | KIAA0226 | KIAA0226 | 2 | | 9462 | hsa-mir-4794 | 9711 | KIAA0226 | KIAA0226 | 2 | | 9463 | hsa-mir-548ao | 9711 | KIAA0226 | KIAA0226 | 2 | | 9465 | hsa-mir-617 | 9711 | KIAA0226 | KIAA0226 | 2 | | 9467 | hsa-mir-877 | 9711 | KIAA0226 | KIAA0226 | 2 | | 9469 | hsa-let-7c | 9711 | KIAA0226 | KIAA0226 | 2 | | 9470 | hsa-mir-99a | 9711 | KIAA0226 | KIAA0226 | 2 | | 9471 | hsa-mir-125b-1 | 9711 | KIAA0226 | KIAA0226 | 2 | | 9472 | hsa-mir-125b-2 | 9711 | KIAA0226 | KIAA0226 | 2 | | 9473 | hsa-mir-455 | 9711 | KIAA0226 | KIAA0226 | 2 | | 9477 | hsa-mir-708 | 9711 | KIAA0226 | KIAA0226 | 2 | | 9492 | hsa-mir-25 | 6934 | TCF7L2 | transcription factor 7-like 2 (T-cell specific, HMG-box) | 2 | | 9493 | hsa-mir-93 | 6934 | TCF7L2 | transcription factor 7-like 2 (T-cell specific, HMG-box) | 2 | | 9497 | hsa-mir-106b | 6934 | TCF7L2 | transcription factor 7-like 2 (T-cell specific, HMG-box) | 2 | | 9500 | hsa-mir-602 | 6934 | TCF7L2 | transcription factor 7-like 2 (T-cell specific, HMG-box) | 2 | | 9513 | hsa-mir-4534 | 6934 | TCF7L2 | transcription factor 7-like 2 (T-cell specific, HMG-box) | 2 | | 9527 | hsa-mir-25 | 7373 | COL14A1 | collagen, type XIV, alpha 1 | 2 | | 9528 | hsa-mir-93 | 7373 | COL14A1 | collagen, type XIV, alpha 1 | 2 | | 9530 | hsa-mir-181a-2 | 7373 | COL14A1 | collagen, type XIV, alpha 1 | 2 | | 9531 | hsa-mir-181b-2 | 7373 | COL14A1 | collagen, type XIV, alpha 1 | 2 | | 9532 | hsa-mir-106b | 7373 | COL14A1 | collagen, type XIV, alpha 1 | 2 | | 9534 | hsa-mir-602 | 7373 | COL14A1 | collagen, type XIV, alpha 1 | 2 | | 9567 | hsa-mir-218-1 | 23276 | KLHL18 | kelch-like 18 (Drosophila) | 2 | | 9569 | hsa-mir-125b-1 | 23276 | KLHL18 | kelch-like 18 (Drosophila) | 2 | | 9570 | hsa-mir-561 | 23276 | KLHL18 | kelch-like 18 (Drosophila) | 2 | | 9572 | hsa-mir-585 | 23276 | KLHL18 | kelch-like 18 (Drosophila) | 2 | | 9577 | hsa-mir-620 | 23276 | KLHL18 | kelch-like 18 (Drosophila) | 2 | | 9604 | hsa-mir-25 | 23024 | PDZRN3 | PDZ domain containing ring finger 3 | 2 | | 9605 | hsa-mir-93 | 23024 | PDZRN3 | PDZ domain containing ring finger 3 | 2 | | 9607 | hsa-mir-181a-2 | 23024 | PDZRN3 | PDZ domain containing ring finger 3 | 2 | | 9608 | hsa-mir-181b-2 | 23024 | PDZRN3 | PDZ domain containing ring finger 3 | 2 | | 9609 | hsa-mir-106b | 23024 | PDZRN3 | PDZ domain containing ring finger 3 | 2 | | 9610 | hsa-mir-505 | 23024 | PDZRN3 | PDZ domain containing ring finger 3 | 2 | | 9612 | hsa-mir-602 | 23024 | PDZRN3 | PDZ domain containing ring finger 3 | 2 | | 9615 | hsa-mir-652 | 23024 | PDZRN3 | PDZ domain containing ring finger 3 | 2 | | 9638 | hsa-mir-561 | 167227 | DCP2 | DCP2 decapping enzyme homolog (S. cerevisiae) | 2 | | 9639 | hsa-mir-585 | 167227 | DCP2 | DCP2 decapping enzyme homolog (S. cerevisiae) | 2 | | 9660 | hsa-mir-585 | 22930 | RAB3GAP1 | RAB3 GTPase activating protein subunit 1 (catalytic) | 2 | | 9663 | hsa-mir-620 | 22930 | RAB3GAP1 | RAB3 GTPase activating protein subunit 1 (catalytic) | 2 | | 9673 | hsa-mir-505 | 9747 | FAM115A | family with sequence similarity 115, member A | 2 | | 9675 | hsa-mir-602 | 9747 | FAM115A | family with sequence similarity 115, member A | 2 | | 9689 | hsa-mir-708 | 489 | ATP2A3 | ATPase, Ca++ transporting, ubiquitous | 2 | | 9692 | hsa-mir-218-1 | 127544 | RNF19B | ring finger protein 19B | 2 | | 9694 | hsa-mir-455 | 127544 | RNF19B | ring finger protein 19B | 2 | | 9697 | hsa-mir-585 | 127544 | RNF19B | ring finger protein 19B | 2 | | 9699 | hsa-mir-620 | 127544 | RNF19B | ring finger protein 19B | 2 | | 9709 | hsa-mir-218-1 | 23031 | MAST3 | microtubule associated serine/threonine kinase 3 | 2 | | 9711 | hsa-mir-561 | 23031 | MAST3 | microtubule associated serine/threonine kinase 3 | 2 | | 9713 | hsa-mir-585 | 23031 | MAST3 | microtubule associated serine/threonine kinase 3 | 2 | | 9723 | hsa-mir-561 | 254531 | LPCAT4 | lysophosphatidylcholine acyltransferase 4 | 2 | | 9725 | hsa-mir-585 | 254531 | LPCAT4 | lysophosphatidylcholine acyltransferase 4 | 2 | | 9729 | hsa-mir-620 | 254531 | LPCAT4 | lysophosphatidylcholine acyltransferase 4 | 2 | | 9746 | hsa-mir-25 | 90627 | STARD13 | StAR-related lipid transfer (START) domain containing 13 | 2 | | 9747 | hsa-mir-93 | 90627 | STARD13 | StAR-related lipid transfer (START) domain containing 13 | 2 | | 9748 | hsa-mir-181a-2 | 90627 | STARD13 | StAR-related lipid transfer (START) domain containing 13 | 2 | | 9749 | hsa-mir-181b-2 | 90627 | STARD13 | StAR-related lipid transfer (START) domain containing 13 | 2 | | 9750 | hsa-mir-106b | 90627 | STARD13 | StAR-related lipid transfer (START) domain containing 13 | 2 | | 9751 | hsa-mir-505 | 90627 | STARD13 | StAR-related lipid transfer (START) domain containing 13 | 2 | | 9753 | hsa-mir-602 | 90627 | STARD13 | StAR-related lipid transfer (START) domain containing 13 | 2 | | 9757 | hsa-mir-643 | 90627 | STARD13 | StAR-related lipid transfer (START) domain containing 13 | 2 | | 9784 | hsa-mir-25 | 6591 | SNAI2 | snail homolog 2 (Drosophila) | 2 | | 9785 | hsa-mir-93 | 6591 | SNAI2 | snail homolog 2 (Drosophila) | 2 | | 9787 | hsa-mir-181a-2 | 6591 | SNAI2 | snail homolog 2 (Drosophila) | 2 | | 9788 | hsa-mir-181b-2 | 6591 | SNAI2 | snail homolog 2 (Drosophila) | 2 | | 9789 | hsa-mir-106b | 6591 | SNAI2 | snail homolog 2 (Drosophila) | 2 | | 9790 | hsa-mir-505 | 6591 | SNAI2 | snail homolog 2 (Drosophila) | 2 | | 9795 | hsa-mir-643 | 6591 | SNAI2 | snail homolog 2 (Drosophila) | 2 | | 9825 | hsa-mir-181a-2 | 23361 | ZNF629 | zinc finger protein 629 | 2 | | 9826 | hsa-mir-181b-2 | 23361 | ZNF629 | zinc finger protein 629 | 2 | | 9827 | hsa-mir-378a | 23361 | ZNF629 | zinc finger protein 629 | 2 | | 9828 | hsa-mir-505 | 23361 | ZNF629 | zinc finger protein 629 | 2 | | 9830 | hsa-mir-643 | 23361 | ZNF629 | zinc finger protein 629 | 2 | | 9852 | hsa-let-7c | 91 | ACVR1B | activin A receptor, type IB | 2 | | 9853 | hsa-mir-99a | 91 | ACVR1B | activin A receptor, type IB | 2 | | 9855 | hsa-mir-218-1 | 91 | ACVR1B | activin A receptor, type IB | 2 | | 9857 | hsa-mir-125b-1 | 91 | ACVR1B | activin A receptor, type IB | 2 | | 9858 | hsa-mir-125b-2 | 91 | ACVR1B | activin A receptor, type IB | 2 | | 9859 | hsa-mir-561 | 91 | ACVR1B | activin A receptor, type IB | 2 | | 9861 | hsa-mir-585 | 91 | ACVR1B | activin A receptor, type IB | 2 | | 9865 | hsa-mir-620 | 91 | ACVR1B | activin A receptor, type IB | 2 | | 9890 | hsa-mir-25 | 23194 | FBXL7 | F-box and leucine-rich repeat protein 7 | 2 | | 9891 | hsa-mir-93 | 23194 | FBXL7 | F-box and leucine-rich repeat protein 7 | 2 | | 9892 | hsa-mir-181a-2 | 23194 | FBXL7 | F-box and leucine-rich repeat protein 7 | 2 | | 9893 | hsa-mir-181b-2 | 23194 | FBXL7 | F-box and leucine-rich repeat protein 7 | 2 | | 9894 | hsa-mir-106b | 23194 | FBXL7 | F-box and leucine-rich repeat protein 7 | 2 | | 9916 | hsa-mir-218-1 | 9881 | TRANK1 | tetratricopeptide repeat and ankyrin repeat containing 1 | 2 | | 9918 | hsa-mir-455 | 9881 | TRANK1 | tetratricopeptide repeat and ankyrin repeat containing 1 | 2 | | 9919 | hsa-mir-561 | 9881 | TRANK1 | tetratricopeptide repeat and ankyrin repeat containing 1 | 2 | | 9921 | hsa-mir-585 | 9881 | TRANK1 | tetratricopeptide repeat and ankyrin repeat containing 1 | 2 | | 9925 | hsa-mir-620 | 9881 | TRANK1 | tetratricopeptide repeat and ankyrin repeat containing 1 | 2 | | 9927 | hsa-mir-708 | 9881 | TRANK1 | tetratricopeptide repeat and ankyrin repeat containing 1 | 2 | | 9954 | hsa-mir-218-1 | 51460 | SFMBT1 | Scm-like with four mbt domains 1 | 2 | | 9956 | hsa-mir-125b-1 | 51460 | SFMBT1 | Scm-like with four mbt domains 1 | 2 | | 9957 | hsa-mir-561 | 51460 | SFMBT1 | Scm-like with four mbt domains 1 | 2 | | 9959 | hsa-mir-585 | 51460 | SFMBT1 | Scm-like with four mbt domains 1 | 2 | | 9965 | hsa-mir-708 | 51460 | SFMBT1 | Scm-like with four mbt domains 1 | 2 | | 9998 | hsa-mir-505 | 54587 | MXRA8 | matrix-remodelling associated 8 | 2 | | 10000 | hsa-mir-643 | 54587 | MXRA8 | matrix-remodelling associated 8 | 2 | | 10014 | hsa-mir-181a-2 | 7474 | WNT5A | wingless-type MMTV integration site family, member 5A | 2 | | 10015 | hsa-mir-181b-2 | 7474 | WNT5A | wingless-type MMTV integration site family, member 5A | 2 | | 10016 | hsa-mir-378a | 7474 | WNT5A | wingless-type MMTV integration site family, member 5A | 2 | | 10017 | hsa-mir-505 | 7474 | WNT5A | wingless-type MMTV integration site family, member 5A | 2 | | 10019 | hsa-mir-602 | 7474 | WNT5A | wingless-type MMTV integration site family, member 5A | 2 | | 10035 | hsa-mir-25 | 92689 | FAM114A1 | family with sequence similarity 114, member A1 | 2 | | 10036 | hsa-mir-93 | 92689 | FAM114A1 | family with sequence similarity 114, member A1 | 2 | | 10037 | hsa-mir-181a-2 | 92689 | FAM114A1 | family with sequence similarity 114, member A1 | 2 | | 10038 | hsa-mir-181b-2 | 92689 | FAM114A1 | family with sequence similarity 114, member A1 | 2 | | 10039 | hsa-mir-106b | 92689 | FAM114A1 | family with sequence similarity 114, member A1 | 2 | | 10041 | hsa-mir-505 | 92689 | FAM114A1 | family with sequence similarity 114, member A1 | 2 | | 10045 | hsa-mir-643 | 92689 | FAM114A1 | family with sequence similarity 114, member A1 | 2 | | 10069 | hsa-let-7c | 23178 | PASK | PAS domain containing serine/threonine kinase | 2 | | 10070 | hsa-mir-99a | 23178 | PASK | PAS domain containing serine/threonine kinase | 2 | | 10073 | hsa-mir-125b-2 | 23178 | PASK | PAS domain containing serine/threonine kinase | 2 | | 10074 | hsa-mir-561 | 23178 | PASK | PAS domain containing serine/threonine kinase | 2 | | 10076 | hsa-mir-585 | 23178 | PASK | PAS domain containing serine/threonine kinase | 2 | | 10081 | hsa-mir-708 | 23178 | PASK | PAS domain containing serine/threonine kinase | 2 | | 10102 | hsa-mir-25 | 2078 | ERG | v-ets erythroblastosis virus E26 oncogene homolog (avian) | 2 | | 10103 | hsa-mir-93 | 2078 | ERG | v-ets erythroblastosis virus E26 oncogene homolog (avian) | 2 | | 10104 | hsa-mir-106b | 2078 | ERG | v-ets erythroblastosis virus E26 oncogene homolog (avian) | 2 | | 10105 | hsa-mir-505 | 2078 | ERG | v-ets erythroblastosis virus E26 oncogene homolog (avian) | 2 | | 10115 | hsa-mir-25 | 6444 | SGCD | sarcoglycan, delta (35kDa dystrophin-associated glycoprotein) | 2 | | 10116 | hsa-mir-93 | 6444 | SGCD | sarcoglycan, delta (35kDa dystrophin-associated glycoprotein) | 2 | | 10118 | hsa-mir-181a-2 | 6444 | SGCD | sarcoglycan, delta (35kDa dystrophin-associated glycoprotein) | 2 | | 10119 | hsa-mir-181b-2 | 6444 | SGCD | sarcoglycan, delta (35kDa dystrophin-associated glycoprotein) | 2 | | 10120 | hsa-mir-106b | 6444 | SGCD | sarcoglycan, delta (35kDa dystrophin-associated glycoprotein) | 2 | | 10123 | hsa-mir-602 | 6444 | SGCD | sarcoglycan, delta (35kDa dystrophin-associated glycoprotein) | 2 | | 10156 | hsa-mir-620 | 65220 | NADK | NAD kinase | 2 | | 10163 | hsa-mir-585 | 402055 | SRRD | SRR1 domain containing | 2 | | 10165 | hsa-mir-620 | 402055 | SRRD | SRR1 domain containing | 2 | | 10172 | hsa-mir-181a-2 | 10916 | MAGED2 | melanoma antigen family D, 2 | 2 | | 10173 | hsa-mir-181b-2 | 10916 | MAGED2 | melanoma antigen family D, 2 | 2 | | 10174 | hsa-mir-505 | 10916 | MAGED2 | melanoma antigen family D, 2 | 2 | | 10200 | hsa-mir-708 | 4141 | MARS | methionyl-tRNA synthetase | 2 | | 10209 | hsa-mir-25 | 2767 | GNA11 | guanine nucleotide binding protein (G protein), alpha 11 (Gq class) | 2 | | 10210 | hsa-mir-93 | 2767 | GNA11 | guanine nucleotide binding protein (G protein), alpha 11 (Gq class) | 2 | | 10212 | hsa-mir-181a-2 | 2767 | GNA11 | guanine nucleotide binding protein (G protein), alpha 11 (Gq class) | 2 | | 10213 | hsa-mir-181b-2 | 2767 | GNA11 | guanine nucleotide binding protein (G protein), alpha 11 (Gq class) | 2 | | 10214 | hsa-mir-106b | 2767 | GNA11 | guanine nucleotide binding protein (G protein), alpha 11 (Gq class) | 2 | | 10216 | hsa-mir-505 | 2767 | GNA11 | guanine nucleotide binding protein (G protein), alpha 11 (Gq class) | 2 | | 10218 | hsa-mir-643 | 2767 | GNA11 | guanine nucleotide binding protein (G protein), alpha 11 (Gq class) | 2 | | 10220 | hsa-mir-652 | 2767 | GNA11 | guanine nucleotide binding protein (G protein), alpha 11 (Gq class) | 2 | | 10247 | hsa-mir-181a-2 | 5789 | PTPRD | protein tyrosine phosphatase, receptor type, D | 2 | | 10248 | hsa-mir-181b-2 | 5789 | PTPRD | protein tyrosine phosphatase, receptor type, D | 2 | | 10249 | hsa-mir-505 | 5789 | PTPRD | protein tyrosine phosphatase, receptor type, D | 2 | | 10252 | hsa-mir-643 | 5789 | PTPRD | protein tyrosine phosphatase, receptor type, D | 2 | | 10269 | hsa-let-7c | 8034 | SLC25A16 | solute carrier family 25 (mitochondrial carrier; Graves disease autoantigen), member 16 | 2 | | 10270 | hsa-mir-99a | 8034 | SLC25A16 | solute carrier family 25 (mitochondrial carrier; Graves disease autoantigen), member 16 | 2 | | 10271 | hsa-mir-125b-2 | 8034 | SLC25A16 | solute carrier family 25 (mitochondrial carrier; Graves disease autoantigen), member 16 | 2 | | 10279 | hsa-mir-620 | 11184 | MAP4K1 | mitogen-activated protein kinase kinase kinase kinase 1 | 2 | | 10282 | hsa-let-7c | 8932 | MBD2 | methyl-CpG binding domain protein 2 | 2 | | 10283 | hsa-mir-99a | 8932 | MBD2 | methyl-CpG binding domain protein 2 | 2 | | 10285 | hsa-mir-218-1 | 8932 | MBD2 | methyl-CpG binding domain protein 2 | 2 | | 10287 | hsa-mir-125b-1 | 8932 | MBD2 | methyl-CpG binding domain protein 2 | 2 | | 10288 | hsa-mir-125b-2 | 8932 | MBD2 | methyl-CpG binding domain protein 2 | 2 | | 10289 | hsa-mir-561 | 8932 | MBD2 | methyl-CpG binding domain protein 2 | 2 | | 10291 | hsa-mir-585 | 8932 | MBD2 | methyl-CpG binding domain protein 2 | 2 | | 10297 | hsa-mir-620 | 8932 | MBD2 | methyl-CpG binding domain protein 2 | 2 | | 10318 | hsa-let-7c | 10737 | RFPL3-AS1 | RFPL3 antisense RNA 1 (non-protein coding) | 2 | | 10319 | hsa-mir-99a | 10737 | RFPL3-AS1 | RFPL3 antisense RNA 1 (non-protein coding) | 2 | | 10321 | hsa-mir-218-1 | 10737 | RFPL3-AS1 | RFPL3 antisense RNA 1 (non-protein coding) | 2 | | 10324 | hsa-mir-125b-2 | 10737 | RFPL3-AS1 | RFPL3 antisense RNA 1 (non-protein coding) | 2 | | 10326 | hsa-mir-561 | 10737 | RFPL3-AS1 | RFPL3 antisense RNA 1 (non-protein coding) | 2 | | 10328 | hsa-mir-585 | 10737 | RFPL3-AS1 | RFPL3 antisense RNA 1 (non-protein coding) | 2 | | 10361 | hsa-mir-620 | 10228 | STX6 | syntaxin 6 | 2 | | 10367 | hsa-mir-218-1 | 8347 | HIST1H2BC | histone cluster 1, H2bc | 2 | | 10369 | hsa-mir-455 | 8347 | HIST1H2BC | histone cluster 1, H2bc | 2 | | 10370 | hsa-mir-561 | 8347 | HIST1H2BC | histone cluster 1, H2bc | 2 | | 10372 | hsa-mir-585 | 8347 | HIST1H2BC | histone cluster 1, H2bc | 2 | | 10377 | hsa-mir-620 | 8347 | HIST1H2BC | histone cluster 1, H2bc | 2 | | 10391 | hsa-let-7c | 5462 | POU5F1B | POU class 5 homeobox 1B | 2 | | 10392 | hsa-mir-99a | 5462 | POU5F1B | POU class 5 homeobox 1B | 2 | | 10394 | hsa-mir-218-1 | 5462 | POU5F1B | POU class 5 homeobox 1B | 2 | | 10396 | hsa-mir-125b-1 | 5462 | POU5F1B | POU class 5 homeobox 1B | 2 | | 10397 | hsa-mir-125b-2 | 5462 | POU5F1B | POU class 5 homeobox 1B | 2 | | 10398 | hsa-mir-561 | 5462 | POU5F1B | POU class 5 homeobox 1B | 2 | | 10400 | hsa-mir-585 | 5462 | POU5F1B | POU class 5 homeobox 1B | 2 | | 10407 | hsa-mir-708 | 5462 | POU5F1B | POU class 5 homeobox 1B | 2 | | 10434 | hsa-mir-218-1 | 9991 | PTBP3 | polypyrimidine tract binding protein 3 | 2 | | 10436 | hsa-mir-125b-1 | 9991 | PTBP3 | polypyrimidine tract binding protein 3 | 2 | | 10437 | hsa-mir-561 | 9991 | PTBP3 | polypyrimidine tract binding protein 3 | 2 | | 10439 | hsa-mir-585 | 9991 | PTBP3 | polypyrimidine tract binding protein 3 | 2 | | 10444 | hsa-mir-620 | 9991 | PTBP3 | polypyrimidine tract binding protein 3 | 2 | | 10467 | hsa-mir-708 | 7752 | ZNF200 | zinc finger protein 200 | 2 | | 10480 | hsa-mir-218-1 | 84124 | ZNF394 | zinc finger protein 394 | 2 | | 10482 | hsa-mir-561 | 84124 | ZNF394 | zinc finger protein 394 | 2 | | 10484 | hsa-mir-585 | 84124 | ZNF394 | zinc finger protein 394 | 2 | | 10510 | hsa-let-7c | 23214 | XPO6 | exportin 6 | 2 | | 10511 | hsa-mir-99a | 23214 | XPO6 | exportin 6 | 2 | | 10514 | hsa-mir-125b-2 | 23214 | XPO6 | exportin 6 | 2 | | 10515 | hsa-mir-561 | 23214 | XPO6 | exportin 6 | 2 | | 10517 | hsa-mir-585 | 23214 | XPO6 | exportin 6 | 2 | | 10540 | hsa-let-7c | 9271 | PIWIL1 | piwi-like 1 (Drosophila) | 2 | | 10541 | hsa-mir-99a | 9271 | PIWIL1 | piwi-like 1 (Drosophila) | 2 | | 10543 | hsa-mir-125b-1 | 9271 | PIWIL1 | piwi-like 1 (Drosophila) | 2 | | 10544 | hsa-mir-125b-2 | 9271 | PIWIL1 | piwi-like 1 (Drosophila) | 2 | | 10545 | hsa-mir-561 | 9271 | PIWIL1 | piwi-like 1 (Drosophila) | 2 | | 10547 | hsa-mir-585 | 9271 | PIWIL1 | piwi-like 1 (Drosophila) | 2 | | 10552 | hsa-mir-708 | 9271 | PIWIL1 | piwi-like 1 (Drosophila) | 2 | | 10576 | hsa-mir-25 | 221981 | THSD7A | thrombospondin, type I, domain containing 7A | 2 | | 10577 | hsa-mir-93 | 221981 | THSD7A | thrombospondin, type I, domain containing 7A | 2 | | 10578 | hsa-mir-181a-2 | 221981 | THSD7A | thrombospondin, type I, domain containing 7A | 2 | | 10579 | hsa-mir-181b-2 | 221981 | THSD7A | thrombospondin, type I, domain containing 7A | 2 | | 10580 | hsa-mir-106b | 221981 | THSD7A | thrombospondin, type I, domain containing 7A | 2 | | 10581 | hsa-mir-505 | 221981 | THSD7A | thrombospondin, type I, domain containing 7A | 2 | | 10584 | hsa-mir-643 | 221981 | THSD7A | thrombospondin, type I, domain containing 7A | 2 | | 10606 | hsa-let-7c | 55361 | PI4K2A | phosphatidylinositol 4-kinase type 2 alpha | 2 | | 10607 | hsa-mir-99a | 55361 | PI4K2A | phosphatidylinositol 4-kinase type 2 alpha | 2 | | 10609 | hsa-mir-218-1 | 55361 | PI4K2A | phosphatidylinositol 4-kinase type 2 alpha | 2 | | 10611 | hsa-mir-125b-1 | 55361 | PI4K2A | phosphatidylinositol 4-kinase type 2 alpha | 2 | | 10612 | hsa-mir-125b-2 | 55361 | PI4K2A | phosphatidylinositol 4-kinase type 2 alpha | 2 | | 10613 | hsa-mir-561 | 55361 | PI4K2A | phosphatidylinositol 4-kinase type 2 alpha | 2 | | 10615 | hsa-mir-585 | 55361 | PI4K2A | phosphatidylinositol 4-kinase type 2 alpha | 2 | | 10623 | hsa-mir-708 | 55361 | PI4K2A | phosphatidylinositol 4-kinase type 2 alpha | 2 | | 10653 | hsa-let-7c | 10793 | ZNF273 | zinc finger protein 273 | 2 | | 10654 | hsa-mir-99a | 10793 | ZNF273 | zinc finger protein 273 | 2 | | 10656 | hsa-mir-218-1 | 10793 | ZNF273 | zinc finger protein 273 | 2 | | 10658 | hsa-mir-125b-2 | 10793 | ZNF273 | zinc finger protein 273 | 2 | | 10659 | hsa-mir-561 | 10793 | ZNF273 | zinc finger protein 273 | 2 | | 10661 | hsa-mir-585 | 10793 | ZNF273 | zinc finger protein 273 | 2 | | 10691 | hsa-mir-218-1 | 57157 | PHTF2 | putative homeodomain transcription factor 2 | 2 | | 10694 | hsa-mir-455 | 57157 | PHTF2 | putative homeodomain transcription factor 2 | 2 | | 10695 | hsa-mir-561 | 57157 | PHTF2 | putative homeodomain transcription factor 2 | 2 | | 10697 | hsa-mir-585 | 57157 | PHTF2 | putative homeodomain transcription factor 2 | 2 | | 10700 | hsa-mir-620 | 57157 | PHTF2 | putative homeodomain transcription factor 2 | 2 | | 10703 | hsa-mir-708 | 57157 | PHTF2 | putative homeodomain transcription factor 2 | 2 | | 10721 | hsa-let-7c | 91646 | TDRD12 | tudor domain containing 12 | 2 | | 10722 | hsa-mir-99a | 91646 | TDRD12 | tudor domain containing 12 | 2 | | 10724 | hsa-mir-218-1 | 91646 | TDRD12 | tudor domain containing 12 | 2 | | 10726 | hsa-mir-125b-1 | 91646 | TDRD12 | tudor domain containing 12 | 2 | | 10727 | hsa-mir-125b-2 | 91646 | TDRD12 | tudor domain containing 12 | 2 | | 10728 | hsa-mir-561 | 91646 | TDRD12 | tudor domain containing 12 | 2 | | 10730 | hsa-mir-585 | 91646 | TDRD12 | tudor domain containing 12 | 2 | | 10738 | hsa-mir-708 | 91646 | TDRD12 | tudor domain containing 12 | 2 | | 10770 | hsa-mir-25 | 4015 | LOX | lysyl oxidase | 2 | | 10771 | hsa-mir-93 | 4015 | LOX | lysyl oxidase | 2 | | 10773 | hsa-mir-181a-2 | 4015 | LOX | lysyl oxidase | 2 | | 10774 | hsa-mir-181b-2 | 4015 | LOX | lysyl oxidase | 2 | | 10775 | hsa-mir-106b | 4015 | LOX | lysyl oxidase | 2 | | 10777 | hsa-mir-505 | 4015 | LOX | lysyl oxidase | 2 | | 10779 | hsa-mir-602 | 4015 | LOX | lysyl oxidase | 2 | | 10780 | hsa-mir-643 | 4015 | LOX | lysyl oxidase | 2 | | 10813 | hsa-let-7c | 728882 | FAM182B | family with sequence similarity 182, member B | 2 | | 10814 | hsa-mir-99a | 728882 | FAM182B | family with sequence similarity 182, member B | 2 | | 10816 | hsa-mir-218-1 | 728882 | FAM182B | family with sequence similarity 182, member B | 2 | | 10818 | hsa-mir-125b-1 | 728882 | FAM182B | family with sequence similarity 182, member B | 2 | | 10819 | hsa-mir-125b-2 | 728882 | FAM182B | family with sequence similarity 182, member B | 2 | | 10820 | hsa-mir-561 | 728882 | FAM182B | family with sequence similarity 182, member B | 2 | | 10822 | hsa-mir-585 | 728882 | FAM182B | family with sequence similarity 182, member B | 2 | | 10842 | hsa-let-7c | 4610 | MYCL1 | v-myc myelocytomatosis viral oncogene homolog 1, lung carcinoma derived (avian) | 2 | | 10843 | hsa-mir-99a | 4610 | MYCL1 | v-myc myelocytomatosis viral oncogene homolog 1, lung carcinoma derived (avian) | 2 | | 10845 | hsa-mir-218-1 | 4610 | MYCL1 | v-myc myelocytomatosis viral oncogene homolog 1, lung carcinoma derived (avian) | 2 | | 10847 | hsa-mir-125b-1 | 4610 | MYCL1 | v-myc myelocytomatosis viral oncogene homolog 1, lung carcinoma derived (avian) | 2 | | 10848 | hsa-mir-125b-2 | 4610 | MYCL1 | v-myc myelocytomatosis viral oncogene homolog 1, lung carcinoma derived (avian) | 2 | | 10849 | hsa-mir-561 | 4610 | MYCL1 | v-myc myelocytomatosis viral oncogene homolog 1, lung carcinoma derived (avian) | 2 | | 10851 | hsa-mir-585 | 4610 | MYCL1 | v-myc myelocytomatosis viral oncogene homolog 1, lung carcinoma derived (avian) | 2 | | 10858 | hsa-mir-708 | 4610 | MYCL1 | v-myc myelocytomatosis viral oncogene homolog 1, lung carcinoma derived (avian) | 2 | | 10888 | hsa-mir-218-1 | 57615 | ZNF492 | zinc finger protein 492 | 2 | | 10891 | hsa-mir-561 | 57615 | ZNF492 | zinc finger protein 492 | 2 | | 10893 | hsa-mir-585 | 57615 | ZNF492 | zinc finger protein 492 | 2 | | 10920 | hsa-let-7c | 249 | ALPL | alkaline phosphatase, liver/bone/kidney | 2 | | 10921 | hsa-mir-99a | 249 | ALPL | alkaline phosphatase, liver/bone/kidney | 2 | | 10924 | hsa-mir-125b-2 | 249 | ALPL | alkaline phosphatase, liver/bone/kidney | 2 | | 10925 | hsa-mir-561 | 249 | ALPL | alkaline phosphatase, liver/bone/kidney | 2 | | 10927 | hsa-mir-585 | 249 | ALPL | alkaline phosphatase, liver/bone/kidney | 2 | | 10932 | hsa-mir-620 | 249 | ALPL | alkaline phosphatase, liver/bone/kidney | 2 | | 10948 | hsa-mir-218-1 | 10564 | ARFGEF2 | ADP-ribosylation factor guanine nucleotide-exchange factor 2 (brefeldin A-inhibited) | 2 | | 10950 | hsa-mir-125b-1 | 10564 | ARFGEF2 | ADP-ribosylation factor guanine nucleotide-exchange factor 2 (brefeldin A-inhibited) | 2 | | 10951 | hsa-mir-561 | 10564 | ARFGEF2 | ADP-ribosylation factor guanine nucleotide-exchange factor 2 (brefeldin A-inhibited) | 2 | | 10953 | hsa-mir-585 | 10564 | ARFGEF2 | ADP-ribosylation factor guanine nucleotide-exchange factor 2 (brefeldin A-inhibited) | 2 | | 10958 | hsa-mir-620 | 10564 | ARFGEF2 | ADP-ribosylation factor guanine nucleotide-exchange factor 2 (brefeldin A-inhibited) | 2 | | 10984 | hsa-mir-4517 | 6934 | TCF7L2 | transcription factor 7-like 2 (T-cell specific, HMG-box) | 2 | | 10990 | hsa-mir-181a-2 | 23543 | RBFOX2 | RNA binding protein, fox-1 homolog (C. elegans) 2 | 2 | | 10991 | hsa-mir-181b-2 | 23543 | RBFOX2 | RNA binding protein, fox-1 homolog (C. elegans) 2 | 2 | | 10993 | hsa-mir-505 | 23543 | RBFOX2 | RNA binding protein, fox-1 homolog (C. elegans) 2 | 2 | | 10995 | hsa-mir-602 | 23543 | RBFOX2 | RNA binding protein, fox-1 homolog (C. elegans) 2 | 2 | | 11026 | hsa-mir-505 | 53345 | TM6SF2 | transmembrane 6 superfamily member 2 | 2 | | 11029 | hsa-mir-652 | 53345 | TM6SF2 | transmembrane 6 superfamily member 2 | 2 | | 11044 | hsa-mir-455 | 8935 | SKAP2 | src kinase associated phosphoprotein 2 | 2 | | 11045 | hsa-mir-561 | 8935 | SKAP2 | src kinase associated phosphoprotein 2 | 2 | | 11047 | hsa-mir-585 | 8935 | SKAP2 | src kinase associated phosphoprotein 2 | 2 | | 11050 | hsa-mir-620 | 8935 | SKAP2 | src kinase associated phosphoprotein 2 | 2 | | 11056 | hsa-let-7c | 23223 | RRP12 | ribosomal RNA processing 12 homolog (S. cerevisiae) | 2 | | 11057 | hsa-mir-99a | 23223 | RRP12 | ribosomal RNA processing 12 homolog (S. cerevisiae) | 2 | | 11058 | hsa-mir-218-1 | 23223 | RRP12 | ribosomal RNA processing 12 homolog (S. cerevisiae) | 2 | | 11060 | hsa-mir-125b-1 | 23223 | RRP12 | ribosomal RNA processing 12 homolog (S. cerevisiae) | 2 | | 11061 | hsa-mir-125b-2 | 23223 | RRP12 | ribosomal RNA processing 12 homolog (S. cerevisiae) | 2 | | 11062 | hsa-mir-561 | 23223 | RRP12 | ribosomal RNA processing 12 homolog (S. cerevisiae) | 2 | | 11064 | hsa-mir-585 | 23223 | RRP12 | ribosomal RNA processing 12 homolog (S. cerevisiae) | 2 | | 11071 | hsa-mir-708 | 23223 | RRP12 | ribosomal RNA processing 12 homolog (S. cerevisiae) | 2 | | 11102 | hsa-mir-455 | 83737 | ITCH | itchy E3 ubiquitin protein ligase | 2 | | 11103 | hsa-mir-561 | 83737 | ITCH | itchy E3 ubiquitin protein ligase | 2 | | 11105 | hsa-mir-585 | 83737 | ITCH | itchy E3 ubiquitin protein ligase | 2 | | 11129 | hsa-mir-218-1 | 639 | PRDM1 | PR domain containing 1, with ZNF domain | 2 | | 11131 | hsa-mir-125b-1 | 639 | PRDM1 | PR domain containing 1, with ZNF domain | 2 | | 11132 | hsa-mir-561 | 639 | PRDM1 | PR domain containing 1, with ZNF domain | 2 | | 11134 | hsa-mir-585 | 639 | PRDM1 | PR domain containing 1, with ZNF domain | 2 | | 11138 | hsa-mir-620 | 639 | PRDM1 | PR domain containing 1, with ZNF domain | 2 | | 11141 | hsa-mir-708 | 639 | PRDM1 | PR domain containing 1, with ZNF domain | 2 | | 11173 | hsa-let-7c | 169522 | KCNV2 | potassium channel, subfamily V, member 2 | 2 | | 11174 | hsa-mir-99a | 169522 | KCNV2 | potassium channel, subfamily V, member 2 | 2 | | 11176 | hsa-mir-218-1 | 169522 | KCNV2 | potassium channel, subfamily V, member 2 | 2 | | 11178 | hsa-mir-125b-1 | 169522 | KCNV2 | potassium channel, subfamily V, member 2 | 2 | | 11179 | hsa-mir-125b-2 | 169522 | KCNV2 | potassium channel, subfamily V, member 2 | 2 | | 11180 | hsa-mir-455 | 169522 | KCNV2 | potassium channel, subfamily V, member 2 | 2 | | 11181 | hsa-mir-561 | 169522 | KCNV2 | potassium channel, subfamily V, member 2 | 2 | | 11183 | hsa-mir-585 | 169522 | KCNV2 | potassium channel, subfamily V, member 2 | 2 | | 11191 | hsa-mir-708 | 169522 | KCNV2 | potassium channel, subfamily V, member 2 | 2 | | 11219 | hsa-mir-218-1 | 3364 | HUS1 | HUS1 checkpoint homolog (S. pombe) | 2 | | 11220 | hsa-mir-125b-1 | 3364 | HUS1 | HUS1 checkpoint homolog (S. pombe) | 2 | | 11224 | hsa-mir-708 | 3364 | HUS1 | HUS1 checkpoint homolog (S. pombe) | 2 | | 11241 | hsa-mir-643 | 28969 | BZW2 | basic leucine zipper and W2 domains 2 | 2 | | 11257 | hsa-mir-455 | 10113 | PREB | prolactin regulatory element binding | 2 | | 11258 | hsa-mir-561 | 10113 | PREB | prolactin regulatory element binding | 2 | | 11260 | hsa-mir-585 | 10113 | PREB | prolactin regulatory element binding | 2 | | 11262 | hsa-mir-620 | 10113 | PREB | prolactin regulatory element binding | 2 | | 11278 | hsa-let-7c | 51283 | BFAR | bifunctional apoptosis regulator | 2 | | 11279 | hsa-mir-99a | 51283 | BFAR | bifunctional apoptosis regulator | 2 | | 11281 | hsa-mir-218-1 | 51283 | BFAR | bifunctional apoptosis regulator | 2 | | 11283 | hsa-mir-125b-2 | 51283 | BFAR | bifunctional apoptosis regulator | 2 | | 11284 | hsa-mir-561 | 51283 | BFAR | bifunctional apoptosis regulator | 2 | | 11286 | hsa-mir-585 | 51283 | BFAR | bifunctional apoptosis regulator | 2 | | 11313 | hsa-mir-10a | 58477 | SRPRB | signal recognition particle receptor, B subunit | 2 | | 11314 | hsa-mir-218-2 | 58477 | SRPRB | signal recognition particle receptor, B subunit | 2 | | 11316 | hsa-mir-574 | 58477 | SRPRB | signal recognition particle receptor, B subunit | 2 | | 11319 | hsa-mir-1271 | 58477 | SRPRB | signal recognition particle receptor, B subunit | 2 | | 11320 | hsa-mir-675 | 58477 | SRPRB | signal recognition particle receptor, B subunit | 2 | | 11322 | hsa-mir-3120 | 58477 | SRPRB | signal recognition particle receptor, B subunit | 2 | | 11323 | hsa-mir-3129 | 58477 | SRPRB | signal recognition particle receptor, B subunit | 2 | | 11326 | hsa-mir-4794 | 58477 | SRPRB | signal recognition particle receptor, B subunit | 2 | | 11329 | hsa-let-7c | 63893 | UBE2O | ubiquitin-conjugating enzyme E2O | 2 | | 11330 | hsa-mir-99a | 63893 | UBE2O | ubiquitin-conjugating enzyme E2O | 2 | | 11332 | hsa-mir-218-1 | 63893 | UBE2O | ubiquitin-conjugating enzyme E2O | 2 | | 11334 | hsa-mir-125b-1 | 63893 | UBE2O | ubiquitin-conjugating enzyme E2O | 2 | | 11335 | hsa-mir-125b-2 | 63893 | UBE2O | ubiquitin-conjugating enzyme E2O | 2 | | 11336 | hsa-mir-561 | 63893 | UBE2O | ubiquitin-conjugating enzyme E2O | 2 | | 11338 | hsa-mir-585 | 63893 | UBE2O | ubiquitin-conjugating enzyme E2O | 2 | | 11365 | hsa-let-7c | 60673 | C12orf44 | chromosome 12 open reading frame 44 | 2 | | 11366 | hsa-mir-99a | 60673 | C12orf44 | chromosome 12 open reading frame 44 | 2 | | 11369 | hsa-mir-125b-1 | 60673 | C12orf44 | chromosome 12 open reading frame 44 | 2 | | 11370 | hsa-mir-125b-2 | 60673 | C12orf44 | chromosome 12 open reading frame 44 | 2 | | 11371 | hsa-mir-561 | 60673 | C12orf44 | chromosome 12 open reading frame 44 | 2 | | 11373 | hsa-mir-585 | 60673 | C12orf44 | chromosome 12 open reading frame 44 | 2 | | 11409 | hsa-mir-218-1 | 55915 | LANCL2 | LanC lantibiotic synthetase component C-like 2 (bacterial) | 2 | | 11412 | hsa-mir-455 | 55915 | LANCL2 | LanC lantibiotic synthetase component C-like 2 (bacterial) | 2 | | 11413 | hsa-mir-561 | 55915 | LANCL2 | LanC lantibiotic synthetase component C-like 2 (bacterial) | 2 | | 11415 | hsa-mir-585 | 55915 | LANCL2 | LanC lantibiotic synthetase component C-like 2 (bacterial) | 2 | | 11419 | hsa-mir-620 | 55915 | LANCL2 | LanC lantibiotic synthetase component C-like 2 (bacterial) | 2 | | 11422 | hsa-mir-708 | 55915 | LANCL2 | LanC lantibiotic synthetase component C-like 2 (bacterial) | 2 | | 11448 | hsa-mir-10a | 10762 | NUP50 | nucleoporin 50kDa | 2 | | 11450 | hsa-mir-218-2 | 10762 | NUP50 | nucleoporin 50kDa | 2 | | 11453 | hsa-mir-574 | 10762 | NUP50 | nucleoporin 50kDa | 2 | | 11455 | hsa-mir-603 | 10762 | NUP50 | nucleoporin 50kDa | 2 | | 11456 | hsa-mir-605 | 10762 | NUP50 | nucleoporin 50kDa | 2 | | 11458 | hsa-mir-1271 | 10762 | NUP50 | nucleoporin 50kDa | 2 | | 11459 | hsa-mir-675 | 10762 | NUP50 | nucleoporin 50kDa | 2 | | 11460 | hsa-mir-1245a | 10762 | NUP50 | nucleoporin 50kDa | 2 | | 11461 | hsa-mir-1915 | 10762 | NUP50 | nucleoporin 50kDa | 2 | | 11462 | hsa-mir-3120 | 10762 | NUP50 | nucleoporin 50kDa | 2 | | 11463 | hsa-mir-3129 | 10762 | NUP50 | nucleoporin 50kDa | 2 | | 11464 | hsa-mir-3606 | 10762 | NUP50 | nucleoporin 50kDa | 2 | | 11465 | hsa-mir-3650 | 10762 | NUP50 | nucleoporin 50kDa | 2 | | 11466 | hsa-mir-4441 | 10762 | NUP50 | nucleoporin 50kDa | 2 | | 11468 | hsa-mir-4636 | 10762 | NUP50 | nucleoporin 50kDa | 2 | | 11469 | hsa-mir-4735 | 10762 | NUP50 | nucleoporin 50kDa | 2 | | 11470 | hsa-mir-2467 | 10762 | NUP50 | nucleoporin 50kDa | 2 | | 11471 | hsa-mir-4794 | 10762 | NUP50 | nucleoporin 50kDa | 2 | | 11472 | hsa-mir-503 | 10762 | NUP50 | nucleoporin 50kDa | 2 | | 11475 | hsa-mir-455 | 9342 | SNAP29 | synaptosomal-associated protein, 29kDa | 2 | | 11476 | hsa-mir-561 | 9342 | SNAP29 | synaptosomal-associated protein, 29kDa | 2 | | 11478 | hsa-mir-585 | 9342 | SNAP29 | synaptosomal-associated protein, 29kDa | 2 | | 11480 | hsa-mir-620 | 9342 | SNAP29 | synaptosomal-associated protein, 29kDa | 2 | | 11493 | hsa-mir-218-1 | 79887 | PLBD1 | phospholipase B domain containing 1 | 2 | | 11495 | hsa-mir-455 | 79887 | PLBD1 | phospholipase B domain containing 1 | 2 | | 11496 | hsa-mir-561 | 79887 | PLBD1 | phospholipase B domain containing 1 | 2 | | 11498 | hsa-mir-585 | 79887 | PLBD1 | phospholipase B domain containing 1 | 2 | | 11522 | hsa-mir-99a | 64222 | TOR3A | torsin family 3, member A | 2 | | 11523 | hsa-mir-10a | 64222 | TOR3A | torsin family 3, member A | 2 | | 11525 | hsa-mir-218-2 | 64222 | TOR3A | torsin family 3, member A | 2 | | 11526 | hsa-mir-125b-2 | 64222 | TOR3A | torsin family 3, member A | 2 | | 11528 | hsa-mir-574 | 64222 | TOR3A | torsin family 3, member A | 2 | | 11530 | hsa-mir-603 | 64222 | TOR3A | torsin family 3, member A | 2 | | 11531 | hsa-mir-618 | 64222 | TOR3A | torsin family 3, member A | 2 | | 11533 | hsa-mir-1271 | 64222 | TOR3A | torsin family 3, member A | 2 | | 11534 | hsa-mir-675 | 64222 | TOR3A | torsin family 3, member A | 2 | | 11536 | hsa-mir-1245a | 64222 | TOR3A | torsin family 3, member A | 2 | | 11537 | hsa-mir-3120 | 64222 | TOR3A | torsin family 3, member A | 2 | | 11538 | hsa-mir-3126 | 64222 | TOR3A | torsin family 3, member A | 2 | | 11539 | hsa-mir-3129 | 64222 | TOR3A | torsin family 3, member A | 2 | | 11540 | hsa-mir-3606 | 64222 | TOR3A | torsin family 3, member A | 2 | | 11541 | hsa-mir-4441 | 64222 | TOR3A | torsin family 3, member A | 2 | | 11543 | hsa-mir-4768 | 64222 | TOR3A | torsin family 3, member A | 2 | | 11544 | hsa-mir-2467 | 64222 | TOR3A | torsin family 3, member A | 2 | | 11545 | hsa-mir-4794 | 64222 | TOR3A | torsin family 3, member A | 2 | | 11546 | hsa-mir-548ao | 64222 | TOR3A | torsin family 3, member A | 2 | | 11549 | hsa-mir-591 | 64222 | TOR3A | torsin family 3, member A | 2 | | 11550 | hsa-mir-617 | 64222 | TOR3A | torsin family 3, member A | 2 | | 11551 | hsa-mir-636 | 64222 | TOR3A | torsin family 3, member A | 2 | | 11552 | hsa-mir-640 | 64222 | TOR3A | torsin family 3, member A | 2 | | 11556 | hsa-mir-10a | 55731 | C17orf63 | chromosome 17 open reading frame 63 | 2 | | 11567 | hsa-mir-618 | 55731 | C17orf63 | chromosome 17 open reading frame 63 | 2 | | 11569 | hsa-mir-1271 | 55731 | C17orf63 | chromosome 17 open reading frame 63 | 2 | | 11570 | hsa-mir-675 | 55731 | C17orf63 | chromosome 17 open reading frame 63 | 2 | | 11571 | hsa-mir-708 | 55731 | C17orf63 | chromosome 17 open reading frame 63 | 2 | | 11575 | hsa-mir-1469 | 55731 | C17orf63 | chromosome 17 open reading frame 63 | 2 | | 11576 | hsa-mir-3120 | 55731 | C17orf63 | chromosome 17 open reading frame 63 | 2 | | 11579 | hsa-mir-3139 | 55731 | C17orf63 | chromosome 17 open reading frame 63 | 2 | | 11584 | hsa-mir-4486 | 55731 | C17orf63 | chromosome 17 open reading frame 63 | 2 | | 11585 | hsa-mir-4636 | 55731 | C17orf63 | chromosome 17 open reading frame 63 | 2 | | 11591 | hsa-mir-548ao | 55731 | C17orf63 | chromosome 17 open reading frame 63 | 2 | | 11593 | hsa-mir-617 | 55731 | C17orf63 | chromosome 17 open reading frame 63 | 2 | | 11594 | hsa-mir-636 | 55731 | C17orf63 | chromosome 17 open reading frame 63 | 2 | | 11599 | hsa-mir-125b-1 | 64328 | XPO4 | exportin 4 | 2 | | 11600 | hsa-mir-561 | 64328 | XPO4 | exportin 4 | 2 | | 11602 | hsa-mir-585 | 64328 | XPO4 | exportin 4 | 2 | | 11609 | hsa-mir-708 | 64328 | XPO4 | exportin 4 | 2 | | 11636 | hsa-let-7c | 55159 | RFWD3 | ring finger and WD repeat domain 3 | 2 | | 11637 | hsa-mir-99a | 55159 | RFWD3 | ring finger and WD repeat domain 3 | 2 | | 11639 | hsa-mir-218-1 | 55159 | RFWD3 | ring finger and WD repeat domain 3 | 2 | | 11641 | hsa-mir-125b-1 | 55159 | RFWD3 | ring finger and WD repeat domain 3 | 2 | | 11642 | hsa-mir-125b-2 | 55159 | RFWD3 | ring finger and WD repeat domain 3 | 2 | | 11643 | hsa-mir-561 | 55159 | RFWD3 | ring finger and WD repeat domain 3 | 2 | | 11645 | hsa-mir-585 | 55159 | RFWD3 | ring finger and WD repeat domain 3 | 2 | | 11652 | hsa-mir-708 | 55159 | RFWD3 | ring finger and WD repeat domain 3 | 2 | | 11680 | hsa-mir-25 | 29995 | LMCD1 | LIM and cysteine-rich domains 1 | 2 | | 11681 | hsa-mir-93 | 29995 | LMCD1 | LIM and cysteine-rich domains 1 | 2 | | 11683 | hsa-mir-181a-2 | 29995 | LMCD1 | LIM and cysteine-rich domains 1 | 2 | | 11684 | hsa-mir-181b-2 | 29995 | LMCD1 | LIM and cysteine-rich domains 1 | 2 | | 11685 | hsa-mir-106b | 29995 | LMCD1 | LIM and cysteine-rich domains 1 | 2 | | 11686 | hsa-mir-378a | 29995 | LMCD1 | LIM and cysteine-rich domains 1 | 2 | | 11687 | hsa-mir-505 | 29995 | LMCD1 | LIM and cysteine-rich domains 1 | 2 | | 11689 | hsa-mir-602 | 29995 | LMCD1 | LIM and cysteine-rich domains 1 | 2 | | 11690 | hsa-mir-629 | 29995 | LMCD1 | LIM and cysteine-rich domains 1 | 2 | | 11694 | hsa-mir-652 | 29995 | LMCD1 | LIM and cysteine-rich domains 1 | 2 | | 11724 | hsa-mir-218-1 | 64682 | ANAPC1 | anaphase promoting complex subunit 1 | 2 | | 11727 | hsa-mir-561 | 64682 | ANAPC1 | anaphase promoting complex subunit 1 | 2 | | 11729 | hsa-mir-585 | 64682 | ANAPC1 | anaphase promoting complex subunit 1 | 2 | | 11731 | hsa-mir-620 | 64682 | ANAPC1 | anaphase promoting complex subunit 1 | 2 | | 11757 | hsa-mir-218-1 | 56478 | EIF4ENIF1 | eukaryotic translation initiation factor 4E nuclear import factor 1 | 2 | | 11759 | hsa-mir-125b-1 | 56478 | EIF4ENIF1 | eukaryotic translation initiation factor 4E nuclear import factor 1 | 2 | | 11760 | hsa-mir-561 | 56478 | EIF4ENIF1 | eukaryotic translation initiation factor 4E nuclear import factor 1 | 2 | | 11762 | hsa-mir-585 | 56478 | EIF4ENIF1 | eukaryotic translation initiation factor 4E nuclear import factor 1 | 2 | | 11764 | hsa-mir-620 | 56478 | EIF4ENIF1 | eukaryotic translation initiation factor 4E nuclear import factor 1 | 2 | | 11793 | hsa-mir-10a | 79693 | YRDC | yrdC domain containing (E. coli) | 2 | | 11795 | hsa-mir-218-2 | 79693 | YRDC | yrdC domain containing (E. coli) | 2 | | 11799 | hsa-mir-574 | 79693 | YRDC | yrdC domain containing (E. coli) | 2 | | 11801 | hsa-mir-603 | 79693 | YRDC | yrdC domain containing (E. coli) | 2 | | 11802 | hsa-mir-604 | 79693 | YRDC | yrdC domain containing (E. coli) | 2 | | 11803 | hsa-mir-605 | 79693 | YRDC | yrdC domain containing (E. coli) | 2 | | 11804 | hsa-mir-618 | 79693 | YRDC | yrdC domain containing (E. coli) | 2 | | 11806 | hsa-mir-1271 | 79693 | YRDC | yrdC domain containing (E. coli) | 2 | | 11807 | hsa-mir-675 | 79693 | YRDC | yrdC domain containing (E. coli) | 2 | | 11808 | hsa-mir-887 | 79693 | YRDC | yrdC domain containing (E. coli) | 2 | | 11809 | hsa-mir-938 | 79693 | YRDC | yrdC domain containing (E. coli) | 2 | | 11810 | hsa-mir-1245a | 79693 | YRDC | yrdC domain containing (E. coli) | 2 | | 11812 | hsa-mir-1469 | 79693 | YRDC | yrdC domain containing (E. coli) | 2 | | 11813 | hsa-mir-3120 | 79693 | YRDC | yrdC domain containing (E. coli) | 2 | | 11814 | hsa-mir-3126 | 79693 | YRDC | yrdC domain containing (E. coli) | 2 | | 11815 | hsa-mir-3129 | 79693 | YRDC | yrdC domain containing (E. coli) | 2 | | 11818 | hsa-mir-3606 | 79693 | YRDC | yrdC domain containing (E. coli) | 2 | | 11820 | hsa-mir-4441 | 79693 | YRDC | yrdC domain containing (E. coli) | 2 | | 11822 | hsa-mir-4636 | 79693 | YRDC | yrdC domain containing (E. coli) | 2 | | 11823 | hsa-mir-4731 | 79693 | YRDC | yrdC domain containing (E. coli) | 2 | | 11825 | hsa-mir-4768 | 79693 | YRDC | yrdC domain containing (E. coli) | 2 | | 11826 | hsa-mir-2467 | 79693 | YRDC | yrdC domain containing (E. coli) | 2 | | 11827 | hsa-mir-4794 | 79693 | YRDC | yrdC domain containing (E. coli) | 2 | | 11828 | hsa-mir-548ao | 79693 | YRDC | yrdC domain containing (E. coli) | 2 | | 11833 | hsa-mir-636 | 79693 | YRDC | yrdC domain containing (E. coli) | 2 | | 11834 | hsa-mir-640 | 79693 | YRDC | yrdC domain containing (E. coli) | 2 | | 11836 | hsa-mir-877 | 79693 | YRDC | yrdC domain containing (E. coli) | 2 | | 11839 | hsa-mir-455 | 23753 | SDF2L1 | stromal cell-derived factor 2-like 1 | 2 | | 11841 | hsa-mir-585 | 23753 | SDF2L1 | stromal cell-derived factor 2-like 1 | 2 | | 11844 | hsa-mir-620 | 23753 | SDF2L1 | stromal cell-derived factor 2-like 1 | 2 | | 11852 | hsa-mir-218-2 | 8934 | RAB7L1 | RAB7, member RAS oncogene family-like 1 | 2 | | 11855 | hsa-mir-574 | 8934 | RAB7L1 | RAB7, member RAS oncogene family-like 1 | 2 | | 11857 | hsa-mir-603 | 8934 | RAB7L1 | RAB7, member RAS oncogene family-like 1 | 2 | | 11859 | hsa-mir-675 | 8934 | RAB7L1 | RAB7, member RAS oncogene family-like 1 | 2 | | 11861 | hsa-mir-1915 | 8934 | RAB7L1 | RAB7, member RAS oncogene family-like 1 | 2 | | 11863 | hsa-mir-3129 | 8934 | RAB7L1 | RAB7, member RAS oncogene family-like 1 | 2 | | 11864 | hsa-mir-3650 | 8934 | RAB7L1 | RAB7, member RAS oncogene family-like 1 | 2 | | 11865 | hsa-mir-4735 | 8934 | RAB7L1 | RAB7, member RAS oncogene family-like 1 | 2 | | 11866 | hsa-mir-4768 | 8934 | RAB7L1 | RAB7, member RAS oncogene family-like 1 | 2 | | 11867 | hsa-mir-548ao | 8934 | RAB7L1 | RAB7, member RAS oncogene family-like 1 | 2 | | 11868 | hsa-mir-335 | 8934 | RAB7L1 | RAB7, member RAS oncogene family-like 1 | 2 | | 11869 | hsa-mir-617 | 8934 | RAB7L1 | RAB7, member RAS oncogene family-like 1 | 2 | | 11870 | hsa-mir-648 | 8934 | RAB7L1 | RAB7, member RAS oncogene family-like 1 | 2 | | 11871 | hsa-mir-877 | 8934 | RAB7L1 | RAB7, member RAS oncogene family-like 1 | 2 | | 11875 | hsa-mir-218-1 | 55813 | UTP6 | UTP6, small subunit (SSU) processome component, homolog (yeast) | 2 | | 11877 | hsa-mir-125b-1 | 55813 | UTP6 | UTP6, small subunit (SSU) processome component, homolog (yeast) | 2 | | 11878 | hsa-mir-561 | 55813 | UTP6 | UTP6, small subunit (SSU) processome component, homolog (yeast) | 2 | | 11880 | hsa-mir-585 | 55813 | UTP6 | UTP6, small subunit (SSU) processome component, homolog (yeast) | 2 | | 11885 | hsa-mir-708 | 55813 | UTP6 | UTP6, small subunit (SSU) processome component, homolog (yeast) | 2 | | 11909 | hsa-mir-10a | 64785 | GINS3 | GINS complex subunit 3 (Psf3 homolog) | 2 | | 11911 | hsa-mir-218-2 | 64785 | GINS3 | GINS complex subunit 3 (Psf3 homolog) | 2 | | 11912 | hsa-mir-125b-1 | 64785 | GINS3 | GINS complex subunit 3 (Psf3 homolog) | 2 | | 11915 | hsa-mir-574 | 64785 | GINS3 | GINS complex subunit 3 (Psf3 homolog) | 2 | | 11918 | hsa-mir-605 | 64785 | GINS3 | GINS complex subunit 3 (Psf3 homolog) | 2 | | 11919 | hsa-mir-618 | 64785 | GINS3 | GINS complex subunit 3 (Psf3 homolog) | 2 | | 11921 | hsa-mir-1271 | 64785 | GINS3 | GINS complex subunit 3 (Psf3 homolog) | 2 | | 11922 | hsa-mir-675 | 64785 | GINS3 | GINS complex subunit 3 (Psf3 homolog) | 2 | | 11923 | hsa-mir-663b | 64785 | GINS3 | GINS complex subunit 3 (Psf3 homolog) | 2 | | 11924 | hsa-mir-1245a | 64785 | GINS3 | GINS complex subunit 3 (Psf3 homolog) | 2 | | 11925 | hsa-mir-1469 | 64785 | GINS3 | GINS complex subunit 3 (Psf3 homolog) | 2 | | 11927 | hsa-mir-3120 | 64785 | GINS3 | GINS complex subunit 3 (Psf3 homolog) | 2 | | 11928 | hsa-mir-3126 | 64785 | GINS3 | GINS complex subunit 3 (Psf3 homolog) | 2 | | 11929 | hsa-mir-3129 | 64785 | GINS3 | GINS complex subunit 3 (Psf3 homolog) | 2 | | 11930 | hsa-mir-3606 | 64785 | GINS3 | GINS complex subunit 3 (Psf3 homolog) | 2 | | 11931 | hsa-mir-4441 | 64785 | GINS3 | GINS complex subunit 3 (Psf3 homolog) | 2 | | 11933 | hsa-mir-4735 | 64785 | GINS3 | GINS complex subunit 3 (Psf3 homolog) | 2 | | 11934 | hsa-mir-2467 | 64785 | GINS3 | GINS complex subunit 3 (Psf3 homolog) | 2 | | 11935 | hsa-mir-4794 | 64785 | GINS3 | GINS complex subunit 3 (Psf3 homolog) | 2 | | 11936 | hsa-mir-548ao | 64785 | GINS3 | GINS complex subunit 3 (Psf3 homolog) | 2 | | 11938 | hsa-mir-591 | 64785 | GINS3 | GINS complex subunit 3 (Psf3 homolog) | 2 | | 11939 | hsa-mir-636 | 64785 | GINS3 | GINS complex subunit 3 (Psf3 homolog) | 2 | | 11940 | hsa-mir-640 | 64785 | GINS3 | GINS complex subunit 3 (Psf3 homolog) | 2 | | 11941 | hsa-mir-877 | 64785 | GINS3 | GINS complex subunit 3 (Psf3 homolog) | 2 | | 11944 | hsa-mir-10a | 55238 | SLC38A7 | solute carrier family 38, member 7 | 2 | | 11946 | hsa-mir-218-2 | 55238 | SLC38A7 | solute carrier family 38, member 7 | 2 | | 11950 | hsa-mir-574 | 55238 | SLC38A7 | solute carrier family 38, member 7 | 2 | | 11952 | hsa-mir-603 | 55238 | SLC38A7 | solute carrier family 38, member 7 | 2 | | 11953 | hsa-mir-604 | 55238 | SLC38A7 | solute carrier family 38, member 7 | 2 | | 11954 | hsa-mir-605 | 55238 | SLC38A7 | solute carrier family 38, member 7 | 2 | | 11955 | hsa-mir-618 | 55238 | SLC38A7 | solute carrier family 38, member 7 | 2 | | 11956 | hsa-mir-887 | 55238 | SLC38A7 | solute carrier family 38, member 7 | 2 | | 11957 | hsa-mir-938 | 55238 | SLC38A7 | solute carrier family 38, member 7 | 2 | | 11958 | hsa-mir-663b | 55238 | SLC38A7 | solute carrier family 38, member 7 | 2 | | 11959 | hsa-mir-1245a | 55238 | SLC38A7 | solute carrier family 38, member 7 | 2 | | 11960 | hsa-mir-1469 | 55238 | SLC38A7 | solute carrier family 38, member 7 | 2 | | 11961 | hsa-mir-3126 | 55238 | SLC38A7 | solute carrier family 38, member 7 | 2 | | 11962 | hsa-mir-3129 | 55238 | SLC38A7 | solute carrier family 38, member 7 | 2 | | 11963 | hsa-mir-3139 | 55238 | SLC38A7 | solute carrier family 38, member 7 | 2 | | 11964 | hsa-mir-3606 | 55238 | SLC38A7 | solute carrier family 38, member 7 | 2 | | 11965 | hsa-mir-4441 | 55238 | SLC38A7 | solute carrier family 38, member 7 | 2 | | 11966 | hsa-mir-4731 | 55238 | SLC38A7 | solute carrier family 38, member 7 | 2 | | 11967 | hsa-mir-4768 | 55238 | SLC38A7 | solute carrier family 38, member 7 | 2 | | 11968 | hsa-mir-2467 | 55238 | SLC38A7 | solute carrier family 38, member 7 | 2 | | 11969 | hsa-mir-4794 | 55238 | SLC38A7 | solute carrier family 38, member 7 | 2 | | 11970 | hsa-mir-591 | 55238 | SLC38A7 | solute carrier family 38, member 7 | 2 | | 11971 | hsa-mir-617 | 55238 | SLC38A7 | solute carrier family 38, member 7 | 2 | | 11972 | hsa-mir-636 | 55238 | SLC38A7 | solute carrier family 38, member 7 | 2 | | 11973 | hsa-mir-640 | 55238 | SLC38A7 | solute carrier family 38, member 7 | 2 | | 11974 | hsa-mir-877 | 55238 | SLC38A7 | solute carrier family 38, member 7 | 2 | | 11980 | hsa-mir-550a-1 | 4969 | OGN | osteoglycin | 2 | | 11982 | hsa-mir-640 | 4969 | OGN | osteoglycin | 2 | | 11984 | hsa-mir-877 | 4969 | OGN | osteoglycin | 2 | | 11985 | hsa-mir-548j | 4969 | OGN | osteoglycin | 2 | | 11986 | hsa-mir-548s | 4969 | OGN | osteoglycin | 2 | | 11987 | hsa-mir-2355 | 4969 | OGN | osteoglycin | 2 | | 11988 | hsa-mir-3679 | 4969 | OGN | osteoglycin | 2 | | 11989 | hsa-mir-3942 | 4969 | OGN | osteoglycin | 2 | | 11990 | hsa-mir-548o-2 | 4969 | OGN | osteoglycin | 2 | | 11991 | hsa-mir-378e | 4969 | OGN | osteoglycin | 2 | | 11992 | hsa-mir-4467 | 4969 | OGN | osteoglycin | 2 | | 11993 | hsa-mir-4517 | 4969 | OGN | osteoglycin | 2 | | 11994 | hsa-mir-4534 | 4969 | OGN | osteoglycin | 2 | | 11995 | hsa-mir-4698 | 4969 | OGN | osteoglycin | 2 | | 11996 | hsa-mir-4723 | 4969 | OGN | osteoglycin | 2 | | 11997 | hsa-mir-4771-1 | 4969 | OGN | osteoglycin | 2 | | 11998 | hsa-mir-4771-2 | 4969 | OGN | osteoglycin | 2 | | 11999 | hsa-mir-5090 | 4969 | OGN | osteoglycin | 2 | | 12000 | hsa-mir-604 | 4969 | OGN | osteoglycin | 2 | | 12001 | hsa-mir-675 | 4969 | OGN | osteoglycin | 2 | | 12002 | hsa-mir-218-2 | 4969 | OGN | osteoglycin | 2 | | 12003 | hsa-mir-574 | 4969 | OGN | osteoglycin | 2 | | 12004 | hsa-mir-887 | 4969 | OGN | osteoglycin | 2 | | 12005 | hsa-mir-938 | 4969 | OGN | osteoglycin | 2 | | 12007 | hsa-mir-218-1 | 51444 | RNF138 | ring finger protein 138, E3 ubiquitin protein ligase | 2 | | 12009 | hsa-mir-561 | 51444 | RNF138 | ring finger protein 138, E3 ubiquitin protein ligase | 2 | | 12011 | hsa-mir-585 | 51444 | RNF138 | ring finger protein 138, E3 ubiquitin protein ligase | 2 | | 12014 | hsa-mir-620 | 51444 | RNF138 | ring finger protein 138, E3 ubiquitin protein ligase | 2 | | 12032 | hsa-mir-643 | 55217 | TMLHE | trimethyllysine hydroxylase, epsilon | 2 | | 12033 | hsa-mir-652 | 55217 | TMLHE | trimethyllysine hydroxylase, epsilon | 2 | | 12046 | hsa-mir-218-1 | 55092 | TMEM51 | transmembrane protein 51 | 2 | | 12048 | hsa-mir-125b-1 | 55092 | TMEM51 | transmembrane protein 51 | 2 | | 12049 | hsa-mir-561 | 55092 | TMEM51 | transmembrane protein 51 | 2 | | 12051 | hsa-mir-585 | 55092 | TMEM51 | transmembrane protein 51 | 2 | | 12055 | hsa-mir-620 | 55092 | TMEM51 | transmembrane protein 51 | 2 | | 12073 | hsa-let-7c | 63906 | GPATCH3 | G patch domain containing 3 | 2 | | 12074 | hsa-mir-99a | 63906 | GPATCH3 | G patch domain containing 3 | 2 | | 12076 | hsa-mir-218-1 | 63906 | GPATCH3 | G patch domain containing 3 | 2 | | 12078 | hsa-mir-125b-1 | 63906 | GPATCH3 | G patch domain containing 3 | 2 | | 12079 | hsa-mir-125b-2 | 63906 | GPATCH3 | G patch domain containing 3 | 2 | | 12080 | hsa-mir-561 | 63906 | GPATCH3 | G patch domain containing 3 | 2 | | 12082 | hsa-mir-585 | 63906 | GPATCH3 | G patch domain containing 3 | 2 | | 12088 | hsa-mir-620 | 63906 | GPATCH3 | G patch domain containing 3 | 2 | | 12115 | hsa-mir-218-1 | 57134 | MAN1C1 | mannosidase, alpha, class 1C, member 1 | 2 | | 12117 | hsa-mir-125b-1 | 57134 | MAN1C1 | mannosidase, alpha, class 1C, member 1 | 2 | | 12119 | hsa-mir-561 | 57134 | MAN1C1 | mannosidase, alpha, class 1C, member 1 | 2 | | 12121 | hsa-mir-585 | 57134 | MAN1C1 | mannosidase, alpha, class 1C, member 1 | 2 | | 12124 | hsa-mir-620 | 57134 | MAN1C1 | mannosidase, alpha, class 1C, member 1 | 2 | | 12126 | hsa-mir-708 | 57134 | MAN1C1 | mannosidase, alpha, class 1C, member 1 | 2 | | 12145 | hsa-mir-218-1 | 55290 | BRF2 | BRF2, subunit of RNA polymerase III transcription initiation factor, BRF1-like | 2 | | 12147 | hsa-mir-455 | 55290 | BRF2 | BRF2, subunit of RNA polymerase III transcription initiation factor, BRF1-like | 2 | | 12148 | hsa-mir-561 | 55290 | BRF2 | BRF2, subunit of RNA polymerase III transcription initiation factor, BRF1-like | 2 | | 12150 | hsa-mir-585 | 55290 | BRF2 | BRF2, subunit of RNA polymerase III transcription initiation factor, BRF1-like | 2 | | 12156 | hsa-mir-708 | 55290 | BRF2 | BRF2, subunit of RNA polymerase III transcription initiation factor, BRF1-like | 2 | | 12182 | hsa-mir-218-1 | 55178 | RNMTL1 | RNA methyltransferase like 1 | 2 | | 12184 | hsa-mir-561 | 55178 | RNMTL1 | RNA methyltransferase like 1 | 2 | | 12186 | hsa-mir-585 | 55178 | RNMTL1 | RNA methyltransferase like 1 | 2 | | 12187 | hsa-mir-620 | 55178 | RNMTL1 | RNA methyltransferase like 1 | 2 | | 12201 | hsa-let-7c | 51388 | NIP7 | nuclear import 7 homolog (S. cerevisiae) | 2 | | 12202 | hsa-mir-99a | 51388 | NIP7 | nuclear import 7 homolog (S. cerevisiae) | 2 | | 12203 | hsa-mir-218-1 | 51388 | NIP7 | nuclear import 7 homolog (S. cerevisiae) | 2 | | 12205 | hsa-mir-125b-1 | 51388 | NIP7 | nuclear import 7 homolog (S. cerevisiae) | 2 | | 12206 | hsa-mir-125b-2 | 51388 | NIP7 | nuclear import 7 homolog (S. cerevisiae) | 2 | | 12207 | hsa-mir-561 | 51388 | NIP7 | nuclear import 7 homolog (S. cerevisiae) | 2 | | 12209 | hsa-mir-585 | 51388 | NIP7 | nuclear import 7 homolog (S. cerevisiae) | 2 | | 12234 | hsa-mir-25 | 54829 | ASPN | asporin | 2 | | 12235 | hsa-mir-93 | 54829 | ASPN | asporin | 2 | | 12236 | hsa-mir-181a-2 | 54829 | ASPN | asporin | 2 | | 12237 | hsa-mir-181b-2 | 54829 | ASPN | asporin | 2 | | 12238 | hsa-mir-106b | 54829 | ASPN | asporin | 2 | | 12239 | hsa-mir-378a | 54829 | ASPN | asporin | 2 | | 12240 | hsa-mir-505 | 54829 | ASPN | asporin | 2 | | 12244 | hsa-mir-643 | 54829 | ASPN | asporin | 2 | | 12267 | hsa-let-7c | 29914 | UBIAD1 | UbiA prenyltransferase domain containing 1 | 2 | | 12268 | hsa-mir-99a | 29914 | UBIAD1 | UbiA prenyltransferase domain containing 1 | 2 | | 12270 | hsa-mir-218-1 | 29914 | UBIAD1 | UbiA prenyltransferase domain containing 1 | 2 | | 12272 | hsa-mir-125b-1 | 29914 | UBIAD1 | UbiA prenyltransferase domain containing 1 | 2 | | 12273 | hsa-mir-125b-2 | 29914 | UBIAD1 | UbiA prenyltransferase domain containing 1 | 2 | | 12275 | hsa-mir-561 | 29914 | UBIAD1 | UbiA prenyltransferase domain containing 1 | 2 | | 12277 | hsa-mir-585 | 29914 | UBIAD1 | UbiA prenyltransferase domain containing 1 | 2 | | 12283 | hsa-mir-620 | 29914 | UBIAD1 | UbiA prenyltransferase domain containing 1 | 2 | | 12328 | hsa-mir-10a | 55833 | UBAP2 | ubiquitin associated protein 2 | 2 | | 12329 | hsa-mir-218-2 | 55833 | UBAP2 | ubiquitin associated protein 2 | 2 | | 12333 | hsa-mir-574 | 55833 | UBAP2 | ubiquitin associated protein 2 | 2 | | 12335 | hsa-mir-603 | 55833 | UBAP2 | ubiquitin associated protein 2 | 2 | | 12336 | hsa-mir-604 | 55833 | UBAP2 | ubiquitin associated protein 2 | 2 | | 12337 | hsa-mir-605 | 55833 | UBAP2 | ubiquitin associated protein 2 | 2 | | 12338 | hsa-mir-618 | 55833 | UBAP2 | ubiquitin associated protein 2 | 2 | | 12339 | hsa-mir-887 | 55833 | UBAP2 | ubiquitin associated protein 2 | 2 | | 12340 | hsa-mir-938 | 55833 | UBAP2 | ubiquitin associated protein 2 | 2 | | 12341 | hsa-mir-1245a | 55833 | UBAP2 | ubiquitin associated protein 2 | 2 | | 12342 | hsa-mir-1469 | 55833 | UBAP2 | ubiquitin associated protein 2 | 2 | | 12343 | hsa-mir-3120 | 55833 | UBAP2 | ubiquitin associated protein 2 | 2 | | 12344 | hsa-mir-3126 | 55833 | UBAP2 | ubiquitin associated protein 2 | 2 | | 12345 | hsa-mir-3129 | 55833 | UBAP2 | ubiquitin associated protein 2 | 2 | | 12346 | hsa-mir-3139 | 55833 | UBAP2 | ubiquitin associated protein 2 | 2 | | 12347 | hsa-mir-3170 | 55833 | UBAP2 | ubiquitin associated protein 2 | 2 | | 12348 | hsa-mir-3606 | 55833 | UBAP2 | ubiquitin associated protein 2 | 2 | | 12349 | hsa-mir-3650 | 55833 | UBAP2 | ubiquitin associated protein 2 | 2 | | 12350 | hsa-mir-4441 | 55833 | UBAP2 | ubiquitin associated protein 2 | 2 | | 12351 | hsa-mir-4636 | 55833 | UBAP2 | ubiquitin associated protein 2 | 2 | | 12352 | hsa-mir-4731 | 55833 | UBAP2 | ubiquitin associated protein 2 | 2 | | 12353 | hsa-mir-4735 | 55833 | UBAP2 | ubiquitin associated protein 2 | 2 | | 12354 | hsa-mir-4768 | 55833 | UBAP2 | ubiquitin associated protein 2 | 2 | | 12355 | hsa-mir-2467 | 55833 | UBAP2 | ubiquitin associated protein 2 | 2 | | 12356 | hsa-mir-4794 | 55833 | UBAP2 | ubiquitin associated protein 2 | 2 | | 12357 | hsa-mir-548ao | 55833 | UBAP2 | ubiquitin associated protein 2 | 2 | | 12358 | hsa-mir-218-1 | 55833 | UBAP2 | ubiquitin associated protein 2 | 2 | | 12359 | hsa-mir-554 | 55833 | UBAP2 | ubiquitin associated protein 2 | 2 | | 12360 | hsa-mir-617 | 55833 | UBAP2 | ubiquitin associated protein 2 | 2 | | 12361 | hsa-mir-640 | 55833 | UBAP2 | ubiquitin associated protein 2 | 2 | | 12362 | hsa-mir-877 | 55833 | UBAP2 | ubiquitin associated protein 2 | 2 | | 12363 | hsa-mir-25 | 63826 | SRR | serine racemase | 2 | | 12364 | hsa-mir-93 | 63826 | SRR | serine racemase | 2 | | 12365 | hsa-mir-181a-2 | 63826 | SRR | serine racemase | 2 | | 12366 | hsa-mir-181b-2 | 63826 | SRR | serine racemase | 2 | | 12367 | hsa-mir-106b | 63826 | SRR | serine racemase | 2 | | 12368 | hsa-mir-505 | 63826 | SRR | serine racemase | 2 | | 12373 | hsa-mir-643 | 63826 | SRR | serine racemase | 2 | | 12393 | hsa-mir-455 | 64859 | OBFC2A | oligonucleotide/oligosaccharide-binding fold containing 2A | 2 | | 12394 | hsa-mir-561 | 64859 | OBFC2A | oligonucleotide/oligosaccharide-binding fold containing 2A | 2 | | 12396 | hsa-mir-585 | 64859 | OBFC2A | oligonucleotide/oligosaccharide-binding fold containing 2A | 2 | | 12402 | hsa-mir-708 | 64859 | OBFC2A | oligonucleotide/oligosaccharide-binding fold containing 2A | 2 | | 12423 | hsa-let-7c | 64782 | AEN | apoptosis enhancing nuclease | 2 | | 12424 | hsa-mir-99a | 64782 | AEN | apoptosis enhancing nuclease | 2 | | 12425 | hsa-mir-218-1 | 64782 | AEN | apoptosis enhancing nuclease | 2 | | 12427 | hsa-mir-125b-1 | 64782 | AEN | apoptosis enhancing nuclease | 2 | | 12428 | hsa-mir-125b-2 | 64782 | AEN | apoptosis enhancing nuclease | 2 | | 12429 | hsa-mir-561 | 64782 | AEN | apoptosis enhancing nuclease | 2 | | 12431 | hsa-mir-585 | 64782 | AEN | apoptosis enhancing nuclease | 2 | | 12437 | hsa-mir-708 | 64782 | AEN | apoptosis enhancing nuclease | 2 | | 12462 | hsa-mir-218-1 | 80219 | COQ10B | coenzyme Q10 homolog B (S. cerevisiae) | 2 | | 12464 | hsa-mir-455 | 80219 | COQ10B | coenzyme Q10 homolog B (S. cerevisiae) | 2 | | 12465 | hsa-mir-561 | 80219 | COQ10B | coenzyme Q10 homolog B (S. cerevisiae) | 2 | | 12467 | hsa-mir-585 | 80219 | COQ10B | coenzyme Q10 homolog B (S. cerevisiae) | 2 | | 12468 | hsa-mir-620 | 80219 | COQ10B | coenzyme Q10 homolog B (S. cerevisiae) | 2 | | 12482 | hsa-mir-25 | 9627 | SNCAIP | synuclein, alpha interacting protein | 2 | | 12483 | hsa-mir-93 | 9627 | SNCAIP | synuclein, alpha interacting protein | 2 | | 12484 | hsa-mir-181a-2 | 9627 | SNCAIP | synuclein, alpha interacting protein | 2 | | 12485 | hsa-mir-181b-2 | 9627 | SNCAIP | synuclein, alpha interacting protein | 2 | | 12486 | hsa-mir-106b | 9627 | SNCAIP | synuclein, alpha interacting protein | 2 | | 12487 | hsa-mir-505 | 9627 | SNCAIP | synuclein, alpha interacting protein | 2 | | 12489 | hsa-mir-602 | 9627 | SNCAIP | synuclein, alpha interacting protein | 2 | | 12493 | hsa-mir-643 | 9627 | SNCAIP | synuclein, alpha interacting protein | 2 | | 12520 | hsa-mir-25 | 51226 | COPZ2 | coatomer protein complex, subunit zeta 2 | 2 | | 12521 | hsa-mir-93 | 51226 | COPZ2 | coatomer protein complex, subunit zeta 2 | 2 | | 12523 | hsa-mir-181a-2 | 51226 | COPZ2 | coatomer protein complex, subunit zeta 2 | 2 | | 12524 | hsa-mir-181b-2 | 51226 | COPZ2 | coatomer protein complex, subunit zeta 2 | 2 | | 12525 | hsa-mir-106b | 51226 | COPZ2 | coatomer protein complex, subunit zeta 2 | 2 | | 12528 | hsa-mir-602 | 51226 | COPZ2 | coatomer protein complex, subunit zeta 2 | 2 | | 12561 | hsa-mir-10a | 64506 | CPEB1 | cytoplasmic polyadenylation element binding protein 1 | 2 | | 12563 | hsa-mir-218-2 | 64506 | CPEB1 | cytoplasmic polyadenylation element binding protein 1 | 2 | | 12566 | hsa-mir-455 | 64506 | CPEB1 | cytoplasmic polyadenylation element binding protein 1 | 2 | | 12568 | hsa-mir-574 | 64506 | CPEB1 | cytoplasmic polyadenylation element binding protein 1 | 2 | | 12570 | hsa-mir-603 | 64506 | CPEB1 | cytoplasmic polyadenylation element binding protein 1 | 2 | | 12571 | hsa-mir-604 | 64506 | CPEB1 | cytoplasmic polyadenylation element binding protein 1 | 2 | | 12572 | hsa-mir-605 | 64506 | CPEB1 | cytoplasmic polyadenylation element binding protein 1 | 2 | | 12573 | hsa-mir-618 | 64506 | CPEB1 | cytoplasmic polyadenylation element binding protein 1 | 2 | | 12574 | hsa-mir-620 | 64506 | CPEB1 | cytoplasmic polyadenylation element binding protein 1 | 2 | | 12575 | hsa-mir-1271 | 64506 | CPEB1 | cytoplasmic polyadenylation element binding protein 1 | 2 | | 12576 | hsa-mir-675 | 64506 | CPEB1 | cytoplasmic polyadenylation element binding protein 1 | 2 | | 12578 | hsa-mir-887 | 64506 | CPEB1 | cytoplasmic polyadenylation element binding protein 1 | 2 | | 12579 | hsa-mir-938 | 64506 | CPEB1 | cytoplasmic polyadenylation element binding protein 1 | 2 | | 12580 | hsa-mir-1245a | 64506 | CPEB1 | cytoplasmic polyadenylation element binding protein 1 | 2 | | 12581 | hsa-mir-1469 | 64506 | CPEB1 | cytoplasmic polyadenylation element binding protein 1 | 2 | | 12583 | hsa-mir-3120 | 64506 | CPEB1 | cytoplasmic polyadenylation element binding protein 1 | 2 | | 12584 | hsa-mir-3126 | 64506 | CPEB1 | cytoplasmic polyadenylation element binding protein 1 | 2 | | 12585 | hsa-mir-3129 | 64506 | CPEB1 | cytoplasmic polyadenylation element binding protein 1 | 2 | | 12586 | hsa-mir-3139 | 64506 | CPEB1 | cytoplasmic polyadenylation element binding protein 1 | 2 | | 12587 | hsa-mir-3170 | 64506 | CPEB1 | cytoplasmic polyadenylation element binding protein 1 | 2 | | 12588 | hsa-mir-3606 | 64506 | CPEB1 | cytoplasmic polyadenylation element binding protein 1 | 2 | | 12589 | hsa-mir-3650 | 64506 | CPEB1 | cytoplasmic polyadenylation element binding protein 1 | 2 | | 12590 | hsa-mir-4441 | 64506 | CPEB1 | cytoplasmic polyadenylation element binding protein 1 | 2 | | 12591 | hsa-mir-4486 | 64506 | CPEB1 | cytoplasmic polyadenylation element binding protein 1 | 2 | | 12592 | hsa-mir-4636 | 64506 | CPEB1 | cytoplasmic polyadenylation element binding protein 1 | 2 | | 12593 | hsa-mir-4731 | 64506 | CPEB1 | cytoplasmic polyadenylation element binding protein 1 | 2 | | 12594 | hsa-mir-4735 | 64506 | CPEB1 | cytoplasmic polyadenylation element binding protein 1 | 2 | | 12595 | hsa-mir-4768 | 64506 | CPEB1 | cytoplasmic polyadenylation element binding protein 1 | 2 | | 12596 | hsa-mir-2467 | 64506 | CPEB1 | cytoplasmic polyadenylation element binding protein 1 | 2 | | 12597 | hsa-mir-4794 | 64506 | CPEB1 | cytoplasmic polyadenylation element binding protein 1 | 2 | | 12598 | hsa-mir-548ao | 64506 | CPEB1 | cytoplasmic polyadenylation element binding protein 1 | 2 | | 12599 | hsa-mir-5579 | 64506 | CPEB1 | cytoplasmic polyadenylation element binding protein 1 | 2 | | 12602 | hsa-mir-617 | 64506 | CPEB1 | cytoplasmic polyadenylation element binding protein 1 | 2 | | 12604 | hsa-mir-640 | 64506 | CPEB1 | cytoplasmic polyadenylation element binding protein 1 | 2 | | 12606 | hsa-mir-877 | 64506 | CPEB1 | cytoplasmic polyadenylation element binding protein 1 | 2 | | 12610 | hsa-mir-125b-1 | 55775 | TDP1 | tyrosyl-DNA phosphodiesterase 1 | 2 | | 12611 | hsa-mir-561 | 55775 | TDP1 | tyrosyl-DNA phosphodiesterase 1 | 2 | | 12613 | hsa-mir-585 | 55775 | TDP1 | tyrosyl-DNA phosphodiesterase 1 | 2 | | 12619 | hsa-mir-620 | 55775 | TDP1 | tyrosyl-DNA phosphodiesterase 1 | 2 | | 12661 | hsa-mir-663b | 29842 | TFCP2L1 | transcription factor CP2-like 1 | 2 | | 12672 | hsa-mir-4486 | 29842 | TFCP2L1 | transcription factor CP2-like 1 | 2 | | 12676 | hsa-mir-4735 | 29842 | TFCP2L1 | transcription factor CP2-like 1 | 2 | | 12681 | hsa-mir-5579 | 29842 | TFCP2L1 | transcription factor CP2-like 1 | 2 | | 12700 | hsa-mir-675 | 53916 | RAB4B | RAB4B, member RAS oncogene family | 2 | | 12704 | hsa-mir-548ao | 53916 | RAB4B | RAB4B, member RAS oncogene family | 2 | | 12709 | hsa-mir-877 | 53916 | RAB4B | RAB4B, member RAS oncogene family | 2 | | 12711 | hsa-mir-218-1 | 26502 | NARF | nuclear prelamin A recognition factor | 2 | | 12712 | hsa-mir-561 | 26502 | NARF | nuclear prelamin A recognition factor | 2 | | 12714 | hsa-mir-585 | 26502 | NARF | nuclear prelamin A recognition factor | 2 | | 12730 | hsa-mir-25 | 79667 | FLJ13197 | uncharacterized FLJ13197 | 2 | | 12731 | hsa-mir-93 | 79667 | FLJ13197 | uncharacterized FLJ13197 | 2 | | 12733 | hsa-mir-181a-2 | 79667 | FLJ13197 | uncharacterized FLJ13197 | 2 | | 12734 | hsa-mir-181b-2 | 79667 | FLJ13197 | uncharacterized FLJ13197 | 2 | | 12735 | hsa-mir-106b | 79667 | FLJ13197 | uncharacterized FLJ13197 | 2 | | 12736 | hsa-mir-378a | 79667 | FLJ13197 | uncharacterized FLJ13197 | 2 | | 12737 | hsa-mir-505 | 79667 | FLJ13197 | uncharacterized FLJ13197 | 2 | | 12739 | hsa-mir-602 | 79667 | FLJ13197 | uncharacterized FLJ13197 | 2 | | 12742 | hsa-mir-643 | 79667 | FLJ13197 | uncharacterized FLJ13197 | 2 | | 12772 | hsa-let-7c | 11153 | FICD | FIC domain containing | 2 | | 12773 | hsa-mir-99a | 11153 | FICD | FIC domain containing | 2 | | 12775 | hsa-mir-125b-2 | 11153 | FICD | FIC domain containing | 2 | | 12776 | hsa-mir-561 | 11153 | FICD | FIC domain containing | 2 | | 12798 | hsa-mir-10a | 26256 | CABYR | calcium binding tyrosine-(Y)-phosphorylation regulated | 2 | | 12800 | hsa-mir-218-2 | 26256 | CABYR | calcium binding tyrosine-(Y)-phosphorylation regulated | 2 | | 12804 | hsa-mir-574 | 26256 | CABYR | calcium binding tyrosine-(Y)-phosphorylation regulated | 2 | | 12806 | hsa-mir-603 | 26256 | CABYR | calcium binding tyrosine-(Y)-phosphorylation regulated | 2 | | 12807 | hsa-mir-605 | 26256 | CABYR | calcium binding tyrosine-(Y)-phosphorylation regulated | 2 | | 12808 | hsa-mir-618 | 26256 | CABYR | calcium binding tyrosine-(Y)-phosphorylation regulated | 2 | | 12810 | hsa-mir-1271 | 26256 | CABYR | calcium binding tyrosine-(Y)-phosphorylation regulated | 2 | | 12811 | hsa-mir-675 | 26256 | CABYR | calcium binding tyrosine-(Y)-phosphorylation regulated | 2 | | 12812 | hsa-mir-887 | 26256 | CABYR | calcium binding tyrosine-(Y)-phosphorylation regulated | 2 | | 12813 | hsa-mir-938 | 26256 | CABYR | calcium binding tyrosine-(Y)-phosphorylation regulated | 2 | | 12814 | hsa-mir-663b | 26256 | CABYR | calcium binding tyrosine-(Y)-phosphorylation regulated | 2 | | 12815 | hsa-mir-1245a | 26256 | CABYR | calcium binding tyrosine-(Y)-phosphorylation regulated | 2 | | 12817 | hsa-mir-3126 | 26256 | CABYR | calcium binding tyrosine-(Y)-phosphorylation regulated | 2 | | 12818 | hsa-mir-3129 | 26256 | CABYR | calcium binding tyrosine-(Y)-phosphorylation regulated | 2 | | 12819 | hsa-mir-3606 | 26256 | CABYR | calcium binding tyrosine-(Y)-phosphorylation regulated | 2 | | 12820 | hsa-mir-3650 | 26256 | CABYR | calcium binding tyrosine-(Y)-phosphorylation regulated | 2 | | 12821 | hsa-mir-4441 | 26256 | CABYR | calcium binding tyrosine-(Y)-phosphorylation regulated | 2 | | 12822 | hsa-mir-4728 | 26256 | CABYR | calcium binding tyrosine-(Y)-phosphorylation regulated | 2 | | 12823 | hsa-mir-4735 | 26256 | CABYR | calcium binding tyrosine-(Y)-phosphorylation regulated | 2 | | 12824 | hsa-mir-4768 | 26256 | CABYR | calcium binding tyrosine-(Y)-phosphorylation regulated | 2 | | 12825 | hsa-mir-2467 | 26256 | CABYR | calcium binding tyrosine-(Y)-phosphorylation regulated | 2 | | 12826 | hsa-mir-4794 | 26256 | CABYR | calcium binding tyrosine-(Y)-phosphorylation regulated | 2 | | 12827 | hsa-mir-548ao | 26256 | CABYR | calcium binding tyrosine-(Y)-phosphorylation regulated | 2 | | 12828 | hsa-mir-617 | 26256 | CABYR | calcium binding tyrosine-(Y)-phosphorylation regulated | 2 | | 12830 | hsa-mir-648 | 26256 | CABYR | calcium binding tyrosine-(Y)-phosphorylation regulated | 2 | | 12831 | hsa-mir-877 | 26256 | CABYR | calcium binding tyrosine-(Y)-phosphorylation regulated | 2 | | 12834 | hsa-mir-10a | 11226 | GALNT6 | UDP-N-acetyl-alpha-D-galactosamine:polypeptide N-acetylgalactosaminyltransferase 6 (GalNAc-T6) | 2 | | 12835 | hsa-mir-218-2 | 11226 | GALNT6 | UDP-N-acetyl-alpha-D-galactosamine:polypeptide N-acetylgalactosaminyltransferase 6 (GalNAc-T6) | 2 | | 12838 | hsa-mir-574 | 11226 | GALNT6 | UDP-N-acetyl-alpha-D-galactosamine:polypeptide N-acetylgalactosaminyltransferase 6 (GalNAc-T6) | 2 | | 12840 | hsa-mir-603 | 11226 | GALNT6 | UDP-N-acetyl-alpha-D-galactosamine:polypeptide N-acetylgalactosaminyltransferase 6 (GalNAc-T6) | 2 | | 12841 | hsa-mir-604 | 11226 | GALNT6 | UDP-N-acetyl-alpha-D-galactosamine:polypeptide N-acetylgalactosaminyltransferase 6 (GalNAc-T6) | 2 | | 12842 | hsa-mir-605 | 11226 | GALNT6 | UDP-N-acetyl-alpha-D-galactosamine:polypeptide N-acetylgalactosaminyltransferase 6 (GalNAc-T6) | 2 | | 12843 | hsa-mir-618 | 11226 | GALNT6 | UDP-N-acetyl-alpha-D-galactosamine:polypeptide N-acetylgalactosaminyltransferase 6 (GalNAc-T6) | 2 | | 12844 | hsa-mir-620 | 11226 | GALNT6 | UDP-N-acetyl-alpha-D-galactosamine:polypeptide N-acetylgalactosaminyltransferase 6 (GalNAc-T6) | 2 | | 12846 | hsa-mir-675 | 11226 | GALNT6 | UDP-N-acetyl-alpha-D-galactosamine:polypeptide N-acetylgalactosaminyltransferase 6 (GalNAc-T6) | 2 | | 12847 | hsa-mir-887 | 11226 | GALNT6 | UDP-N-acetyl-alpha-D-galactosamine:polypeptide N-acetylgalactosaminyltransferase 6 (GalNAc-T6) | 2 | | 12848 | hsa-mir-938 | 11226 | GALNT6 | UDP-N-acetyl-alpha-D-galactosamine:polypeptide N-acetylgalactosaminyltransferase 6 (GalNAc-T6) | 2 | | 12850 | hsa-mir-1245a | 11226 | GALNT6 | UDP-N-acetyl-alpha-D-galactosamine:polypeptide N-acetylgalactosaminyltransferase 6 (GalNAc-T6) | 2 | | 12851 | hsa-mir-3120 | 11226 | GALNT6 | UDP-N-acetyl-alpha-D-galactosamine:polypeptide N-acetylgalactosaminyltransferase 6 (GalNAc-T6) | 2 | | 12852 | hsa-mir-3126 | 11226 | GALNT6 | UDP-N-acetyl-alpha-D-galactosamine:polypeptide N-acetylgalactosaminyltransferase 6 (GalNAc-T6) | 2 | | 12853 | hsa-mir-3129 | 11226 | GALNT6 | UDP-N-acetyl-alpha-D-galactosamine:polypeptide N-acetylgalactosaminyltransferase 6 (GalNAc-T6) | 2 | | 12854 | hsa-mir-3139 | 11226 | GALNT6 | UDP-N-acetyl-alpha-D-galactosamine:polypeptide N-acetylgalactosaminyltransferase 6 (GalNAc-T6) | 2 | | 12855 | hsa-mir-3606 | 11226 | GALNT6 | UDP-N-acetyl-alpha-D-galactosamine:polypeptide N-acetylgalactosaminyltransferase 6 (GalNAc-T6) | 2 | | 12856 | hsa-mir-3650 | 11226 | GALNT6 | UDP-N-acetyl-alpha-D-galactosamine:polypeptide N-acetylgalactosaminyltransferase 6 (GalNAc-T6) | 2 | | 12857 | hsa-mir-4441 | 11226 | GALNT6 | UDP-N-acetyl-alpha-D-galactosamine:polypeptide N-acetylgalactosaminyltransferase 6 (GalNAc-T6) | 2 | | 12858 | hsa-mir-4731 | 11226 | GALNT6 | UDP-N-acetyl-alpha-D-galactosamine:polypeptide N-acetylgalactosaminyltransferase 6 (GalNAc-T6) | 2 | | 12860 | hsa-mir-4768 | 11226 | GALNT6 | UDP-N-acetyl-alpha-D-galactosamine:polypeptide N-acetylgalactosaminyltransferase 6 (GalNAc-T6) | 2 | | 12861 | hsa-mir-2467 | 11226 | GALNT6 | UDP-N-acetyl-alpha-D-galactosamine:polypeptide N-acetylgalactosaminyltransferase 6 (GalNAc-T6) | 2 | | 12862 | hsa-mir-4794 | 11226 | GALNT6 | UDP-N-acetyl-alpha-D-galactosamine:polypeptide N-acetylgalactosaminyltransferase 6 (GalNAc-T6) | 2 | | 12863 | hsa-mir-548ao | 11226 | GALNT6 | UDP-N-acetyl-alpha-D-galactosamine:polypeptide N-acetylgalactosaminyltransferase 6 (GalNAc-T6) | 2 | | 12865 | hsa-mir-554 | 11226 | GALNT6 | UDP-N-acetyl-alpha-D-galactosamine:polypeptide N-acetylgalactosaminyltransferase 6 (GalNAc-T6) | 2 | | 12867 | hsa-mir-617 | 11226 | GALNT6 | UDP-N-acetyl-alpha-D-galactosamine:polypeptide N-acetylgalactosaminyltransferase 6 (GalNAc-T6) | 2 | | 12868 | hsa-mir-636 | 11226 | GALNT6 | UDP-N-acetyl-alpha-D-galactosamine:polypeptide N-acetylgalactosaminyltransferase 6 (GalNAc-T6) | 2 | | 12869 | hsa-mir-640 | 11226 | GALNT6 | UDP-N-acetyl-alpha-D-galactosamine:polypeptide N-acetylgalactosaminyltransferase 6 (GalNAc-T6) | 2 | | 12870 | hsa-mir-648 | 11226 | GALNT6 | UDP-N-acetyl-alpha-D-galactosamine:polypeptide N-acetylgalactosaminyltransferase 6 (GalNAc-T6) | 2 | | 12871 | hsa-mir-877 | 11226 | GALNT6 | UDP-N-acetyl-alpha-D-galactosamine:polypeptide N-acetylgalactosaminyltransferase 6 (GalNAc-T6) | 2 | | 12872 | hsa-let-7c | 55510 | DDX43 | DEAD (Asp-Glu-Ala-Asp) box polypeptide 43 | 2 | | 12873 | hsa-mir-99a | 55510 | DDX43 | DEAD (Asp-Glu-Ala-Asp) box polypeptide 43 | 2 | | 12875 | hsa-mir-218-1 | 55510 | DDX43 | DEAD (Asp-Glu-Ala-Asp) box polypeptide 43 | 2 | | 12877 | hsa-mir-125b-1 | 55510 | DDX43 | DEAD (Asp-Glu-Ala-Asp) box polypeptide 43 | 2 | | 12878 | hsa-mir-125b-2 | 55510 | DDX43 | DEAD (Asp-Glu-Ala-Asp) box polypeptide 43 | 2 | | 12879 | hsa-mir-561 | 55510 | DDX43 | DEAD (Asp-Glu-Ala-Asp) box polypeptide 43 | 2 | | 12881 | hsa-mir-585 | 55510 | DDX43 | DEAD (Asp-Glu-Ala-Asp) box polypeptide 43 | 2 | | 12888 | hsa-mir-708 | 55510 | DDX43 | DEAD (Asp-Glu-Ala-Asp) box polypeptide 43 | 2 | | 12915 | hsa-let-7c | 79872 | CBLL1 | Cbl proto-oncogene, E3 ubiquitin protein ligase-like 1 | 2 | | 12916 | hsa-mir-99a | 79872 | CBLL1 | Cbl proto-oncogene, E3 ubiquitin protein ligase-like 1 | 2 | | 12919 | hsa-mir-125b-2 | 79872 | CBLL1 | Cbl proto-oncogene, E3 ubiquitin protein ligase-like 1 | 2 | | 12920 | hsa-mir-561 | 79872 | CBLL1 | Cbl proto-oncogene, E3 ubiquitin protein ligase-like 1 | 2 | | 12922 | hsa-mir-585 | 79872 | CBLL1 | Cbl proto-oncogene, E3 ubiquitin protein ligase-like 1 | 2 | | 12923 | hsa-mir-620 | 79872 | CBLL1 | Cbl proto-oncogene, E3 ubiquitin protein ligase-like 1 | 2 | | 12932 | hsa-let-7c | 79905 | TMC7 | transmembrane channel-like 7 | 2 | | 12933 | hsa-mir-99a | 79905 | TMC7 | transmembrane channel-like 7 | 2 | | 12935 | hsa-mir-218-1 | 79905 | TMC7 | transmembrane channel-like 7 | 2 | | 12937 | hsa-mir-125b-1 | 79905 | TMC7 | transmembrane channel-like 7 | 2 | | 12938 | hsa-mir-125b-2 | 79905 | TMC7 | transmembrane channel-like 7 | 2 | | 12939 | hsa-mir-561 | 79905 | TMC7 | transmembrane channel-like 7 | 2 | | 12941 | hsa-mir-585 | 79905 | TMC7 | transmembrane channel-like 7 | 2 | | 12963 | hsa-mir-218-1 | 79722 | ANKRD55 | ankyrin repeat domain 55 | 2 | | 12965 | hsa-mir-125b-1 | 79722 | ANKRD55 | ankyrin repeat domain 55 | 2 | | 12967 | hsa-mir-561 | 79722 | ANKRD55 | ankyrin repeat domain 55 | 2 | | 12969 | hsa-mir-585 | 79722 | ANKRD55 | ankyrin repeat domain 55 | 2 | | 12972 | hsa-mir-620 | 79722 | ANKRD55 | ankyrin repeat domain 55 | 2 | | 12975 | hsa-mir-708 | 79722 | ANKRD55 | ankyrin repeat domain 55 | 2 | | 12995 | hsa-mir-218-1 | 55005 | RMND1 | required for meiotic nuclear division 1 homolog (S. cerevisiae) | 2 | | 12997 | hsa-mir-561 | 55005 | RMND1 | required for meiotic nuclear division 1 homolog (S. cerevisiae) | 2 | | 12999 | hsa-mir-585 | 55005 | RMND1 | required for meiotic nuclear division 1 homolog (S. cerevisiae) | 2 | | 13002 | hsa-mir-620 | 55005 | RMND1 | required for meiotic nuclear division 1 homolog (S. cerevisiae) | 2 | | 13017 | hsa-let-7c | 55509 | BATF3 | basic leucine zipper transcription factor, ATF-like 3 | 2 | | 13018 | hsa-mir-99a | 55509 | BATF3 | basic leucine zipper transcription factor, ATF-like 3 | 2 | | 13021 | hsa-mir-125b-1 | 55509 | BATF3 | basic leucine zipper transcription factor, ATF-like 3 | 2 | | 13022 | hsa-mir-125b-2 | 55509 | BATF3 | basic leucine zipper transcription factor, ATF-like 3 | 2 | | 13023 | hsa-mir-561 | 55509 | BATF3 | basic leucine zipper transcription factor, ATF-like 3 | 2 | | 13025 | hsa-mir-585 | 55509 | BATF3 | basic leucine zipper transcription factor, ATF-like 3 | 2 | | 13051 | hsa-mir-378a | 80055 | PGAP1 | post-GPI attachment to proteins 1 | 2 | | 13055 | hsa-mir-644a | 80055 | PGAP1 | post-GPI attachment to proteins 1 | 2 | | 13056 | hsa-mir-548j | 80055 | PGAP1 | post-GPI attachment to proteins 1 | 2 | | 13058 | hsa-mir-2909 | 80055 | PGAP1 | post-GPI attachment to proteins 1 | 2 | | 13059 | hsa-mir-548s | 80055 | PGAP1 | post-GPI attachment to proteins 1 | 2 | | 13060 | hsa-mir-2355 | 80055 | PGAP1 | post-GPI attachment to proteins 1 | 2 | | 13064 | hsa-mir-378e | 80055 | PGAP1 | post-GPI attachment to proteins 1 | 2 | | 13066 | hsa-mir-4685 | 80055 | PGAP1 | post-GPI attachment to proteins 1 | 2 | | 13067 | hsa-mir-4698 | 80055 | PGAP1 | post-GPI attachment to proteins 1 | 2 | | 13074 | hsa-mir-675 | 80055 | PGAP1 | post-GPI attachment to proteins 1 | 2 | | 13075 | hsa-mir-218-2 | 80055 | PGAP1 | post-GPI attachment to proteins 1 | 2 | | 13079 | hsa-mir-218-1 | 79843 | FAM124B | family with sequence similarity 124B | 2 | | 13081 | hsa-mir-455 | 79843 | FAM124B | family with sequence similarity 124B | 2 | | 13082 | hsa-mir-561 | 79843 | FAM124B | family with sequence similarity 124B | 2 | | 13084 | hsa-mir-585 | 79843 | FAM124B | family with sequence similarity 124B | 2 | | 13106 | hsa-let-7c | 51213 | LUZP4 | leucine zipper protein 4 | 2 | | 13107 | hsa-mir-99a | 51213 | LUZP4 | leucine zipper protein 4 | 2 | | 13109 | hsa-mir-218-1 | 51213 | LUZP4 | leucine zipper protein 4 | 2 | | 13111 | hsa-mir-125b-1 | 51213 | LUZP4 | leucine zipper protein 4 | 2 | | 13112 | hsa-mir-125b-2 | 51213 | LUZP4 | leucine zipper protein 4 | 2 | | 13113 | hsa-mir-455 | 51213 | LUZP4 | leucine zipper protein 4 | 2 | | 13114 | hsa-mir-561 | 51213 | LUZP4 | leucine zipper protein 4 | 2 | | 13116 | hsa-mir-585 | 51213 | LUZP4 | leucine zipper protein 4 | 2 | | 13121 | hsa-mir-620 | 51213 | LUZP4 | leucine zipper protein 4 | 2 | | 13124 | hsa-mir-708 | 51213 | LUZP4 | leucine zipper protein 4 | 2 | | 13156 | hsa-mir-218-1 | 55707 | NECAP2 | NECAP endocytosis associated 2 | 2 | | 13158 | hsa-mir-455 | 55707 | NECAP2 | NECAP endocytosis associated 2 | 2 | | 13159 | hsa-mir-561 | 55707 | NECAP2 | NECAP endocytosis associated 2 | 2 | | 13161 | hsa-mir-585 | 55707 | NECAP2 | NECAP endocytosis associated 2 | 2 | | 13162 | hsa-mir-620 | 55707 | NECAP2 | NECAP endocytosis associated 2 | 2 | | 13176 | hsa-mir-10a | 9990 | SLC12A6 | solute carrier family 12 (potassium/chloride transporters), member 6 | 2 | | 13178 | hsa-mir-218-2 | 9990 | SLC12A6 | solute carrier family 12 (potassium/chloride transporters), member 6 | 2 | | 13182 | hsa-mir-574 | 9990 | SLC12A6 | solute carrier family 12 (potassium/chloride transporters), member 6 | 2 | | 13184 | hsa-mir-603 | 9990 | SLC12A6 | solute carrier family 12 (potassium/chloride transporters), member 6 | 2 | | 13185 | hsa-mir-604 | 9990 | SLC12A6 | solute carrier family 12 (potassium/chloride transporters), member 6 | 2 | | 13186 | hsa-mir-618 | 9990 | SLC12A6 | solute carrier family 12 (potassium/chloride transporters), member 6 | 2 | | 13187 | hsa-mir-1271 | 9990 | SLC12A6 | solute carrier family 12 (potassium/chloride transporters), member 6 | 2 | | 13188 | hsa-mir-675 | 9990 | SLC12A6 | solute carrier family 12 (potassium/chloride transporters), member 6 | 2 | | 13190 | hsa-mir-887 | 9990 | SLC12A6 | solute carrier family 12 (potassium/chloride transporters), member 6 | 2 | | 13191 | hsa-mir-938 | 9990 | SLC12A6 | solute carrier family 12 (potassium/chloride transporters), member 6 | 2 | | 13192 | hsa-mir-1245a | 9990 | SLC12A6 | solute carrier family 12 (potassium/chloride transporters), member 6 | 2 | | 13193 | hsa-mir-1469 | 9990 | SLC12A6 | solute carrier family 12 (potassium/chloride transporters), member 6 | 2 | | 13194 | hsa-mir-1915 | 9990 | SLC12A6 | solute carrier family 12 (potassium/chloride transporters), member 6 | 2 | | 13195 | hsa-mir-3120 | 9990 | SLC12A6 | solute carrier family 12 (potassium/chloride transporters), member 6 | 2 | | 13196 | hsa-mir-3126 | 9990 | SLC12A6 | solute carrier family 12 (potassium/chloride transporters), member 6 | 2 | | 13197 | hsa-mir-3129 | 9990 | SLC12A6 | solute carrier family 12 (potassium/chloride transporters), member 6 | 2 | | 13198 | hsa-mir-3170 | 9990 | SLC12A6 | solute carrier family 12 (potassium/chloride transporters), member 6 | 2 | | 13199 | hsa-mir-3606 | 9990 | SLC12A6 | solute carrier family 12 (potassium/chl
[truncated: 1,448,718 more chars]
