# Supplementary material for: MMpred: functional miRNA – mRNA interaction analyses by miRNA expression prediction
Source: BMC Genomics. 2012 Nov 14;13:620. doi: 10.1186/1471-2164-13-620 (PMC3562514; doi:10.1186/1471-2164-13-620)
Supplement: Additional file 9 — Examples of MMpred predictions supported by experimental data and mapping against current databases. [file 1471-2164-13-620-S9.ZIP › Additional file 11 - Examples of MMpred predictions supported by experimental data and mapping against current databases/GSE26158/REPORT_Mon-03-09-2012_13-00-39.html]

REPORT


## Report of miRNA-mRNA interactions for all arrays. [generated on 2012-09-03 13:00:39]

---

Statistical testing for messenger RNA arrays: 116 genes found significantly up-/down-regulated. Details:

| |  | ArrayFile | FunctionalGroup | | --- | --- | --- | | 1 | GSM642299\_DP\_15.CEL.gz | T | | 2 | GSM642300\_DP\_16.CEL.gz | T | | 3 | GSM642301\_DP\_17.CEL.gz | T | | 4 | GSM642302\_SP\_1.CEL.gz | C | | 5 | GSM642303\_SP\_2.CEL.gz | C | | 6 | GSM642304\_SP\_3.CEL.gz | C | |

  

Principal Component Analyses:

Heatmap for top 50 geneses from statistical analyses (ordered by p-value):

Volcano plot with for auto cut-off calculation audit (cut-off shown with red line):

---

Statistical testing for microRNA prediction method I - scaling function: 36 genes found significantly up-/down-regulated. Details:

Principal Component Analyses:

Heatmap for top 50 geneses from statistical analyses (ordered by p-value):

Volcano plot with for auto cut-off calculation audit (cut-off shown with red line):

---

Statistical testing for microRNA prediction method II - linear modelling: 36 genes found significantly up-/down-regulated. Details:

Principal Component Analysis:

Heatmap for top 50 geneses from statistical analyses (ordered by p-value):

Volcano plot with for auto cut-off calculation audit (cut-off shown with red line):

---

Mean anti-correlation detected between mRNA and miRNA = -0.975612. Details:

Histogram of most anti-correlated miRNA-mRNA pairs - potential miRNA-target interactions:

---

Total number of 57 miRNAs are predicted to have significantly up-/down-regulated targets. Expend:

| |  | microRNA | NoSuppresedGenes | | --- | --- | --- | | 1 | hsa-mir-101-2 | 94 | | 2 | hsa-mir-302b | 94 | | 3 | hsa-mir-302c | 94 | | 4 | hsa-mir-302d | 94 | | 5 | hsa-mir-367 | 94 | | 6 | hsa-mir-562 | 94 | | 7 | hsa-mir-604 | 94 | | 8 | hsa-mir-938 | 94 | | 9 | hsa-mir-128-1;hsa-mir-128-2 | 61 | | 10 | hsa-mir-15a | 61 | | 11 | hsa-mir-16-1 | 61 | | 12 | hsa-mir-301a | 61 | | 13 | hsa-mir-302a | 61 | | 14 | hsa-mir-3135a | 61 | | 15 | hsa-mir-3177 | 61 | | 16 | hsa-mir-33b | 61 | | 17 | hsa-mir-3655 | 61 | | 18 | hsa-mir-4526 | 61 | | 19 | hsa-mir-4635 | 61 | | 20 | hsa-mir-4651 | 61 | | 21 | hsa-mir-4744 | 61 | | 22 | hsa-mir-511-1;hsa-mir-511-2 | 61 | | 23 | hsa-mir-5190 | 61 | | 24 | hsa-mir-548d-1 | 61 | | 25 | hsa-mir-548h-1 | 61 | | 26 | hsa-mir-586 | 61 | | 27 | hsa-mir-593 | 61 | | 28 | hsa-mir-620 | 61 | | 29 | hsa-mir-942 | 61 | | 30 | hsa-let-7i | 33 | | 31 | hsa-mir-1236 | 33 | | 32 | hsa-mir-1284 | 33 | | 33 | hsa-mir-151a | 33 | | 34 | hsa-mir-3181 | 33 | | 35 | hsa-mir-326 | 33 | | 36 | hsa-mir-339 | 33 | | 37 | hsa-mir-340 | 33 | | 38 | hsa-mir-342 | 33 | | 39 | hsa-mir-3651 | 33 | | 40 | hsa-mir-454 | 33 | | 41 | hsa-mir-4632 | 33 | | 42 | hsa-mir-4648 | 33 | | 43 | hsa-mir-4726 | 33 | | 44 | hsa-mir-4785 | 33 | | 45 | hsa-mir-4794 | 33 | | 46 | hsa-mir-4802 | 33 | | 47 | hsa-mir-511-1 | 33 | | 48 | hsa-mir-511-2 | 33 | | 49 | hsa-mir-548b | 33 | | 50 | hsa-mir-548d-1;hsa-mir-548d-2 | 33 | | 51 | hsa-mir-567 | 33 | | 52 | hsa-mir-576 | 33 | | 53 | hsa-mir-579 | 33 | | 54 | hsa-mir-582 | 33 | | 55 | hsa-mir-608 | 33 | | 56 | hsa-mir-621 | 33 | | 57 | hsa-mir-627 | 33 | |

---

Total number of 94 genes are predicted to be under differential miRNA repression. Expend:

| |  | GenSymbols | GeneName | NoTargetingMicroRNA.Var1 | NoTargetingMicroRNA.Freq | | --- | --- | --- | --- | --- | | 1 | HLA-E | major histocompatibility complex, class I, E | ABCB1 | 29 | | 2 | IDH3A | isocitrate dehydrogenase 3 (NAD+) alpha | ACTN1 | 29 | | 3 | LAPTM4B | lysosomal protein transmembrane 4 beta | ADAM19 | 29 | | 4 | RHBDD1 | rhomboid domain containing 1 | ADAM23 | 29 | | 5 | GIMAP1 | GTPase, IMAP family member 1 | ANTXR2 | 29 | | 6 | CD200R1 | CD200 receptor 1 | APH1B | 36 | | 7 | SEPW1 | selenoprotein W, 1 | ARHGAP21 | 29 | | 8 | LINC00282 | long intergenic non-protein coding RNA 282 | BCL2 | 29 | | 9 | LOC400965 | uncharacterized LOC400965 | BCL2L1 | 36 | | 10 | ZNF519 | zinc finger protein 519 | BRP44L | 36 | | 11 | RPS19 | ribosomal protein S19 | C10orf54 | 29 | | 12 | DNAJB12 | DnaJ (Hsp40) homolog, subfamily B, member 12 | C11orf80 | 36 | | 13 | HK2 | hexokinase 2 | C14orf182 | 29 | | 14 | HDDC2 | HD domain containing 2 | C2orf89 | 29 | | 15 | SOCS2 | suppressor of cytokine signaling 2 | C7orf31 | 36 | | 16 | LSP1 | lymphocyte-specific protein 1 | CAMSAP2 | 29 | | 17 | BCL2 | B-cell CLL/lymphoma 2 | CARD11 | 29 | | 18 | WASF1 | WAS protein family, member 1 | CASK | 36 | | 19 | FCER1G | Fc fragment of IgE, high affinity I, receptor for; gamma polypeptide | CASP1 | 29 | | 20 | CD44 | CD44 molecule (Indian blood group) | CCDC71L | 36 | | 21 | SELL | selectin L | CD200R1 | 29 | | 22 | IARS | isoleucyl-tRNA synthetase | CD44 | 29 | | 23 | CD5 | CD5 molecule | CD5 | 29 | | 24 | GFI1 | growth factor independent 1 transcription repressor | CDC42 | 29 | | 25 | HTR2B | 5-hydroxytryptamine (serotonin) receptor 2B, G protein-coupled | CEP152 | 36 | | 26 | PRMT8 | protein arginine methyltransferase 8 | CHD1 | 36 | | 27 | ACTN1 | actinin, alpha 1 | CIT | 36 | | 28 | CDC42 | cell division cycle 42 (GTP binding protein, 25kDa) | CUX1 | 36 | | 29 | HLA-B | major histocompatibility complex, class I, B | CYLD | 29 | | 30 | WDR46 | WD repeat domain 46 | DNAJB12 | 36 | | 31 | POLR1C | polymerase (RNA) I polypeptide C, 30kDa | DNAJC9 | 36 | | 32 | ADAM19 | ADAM metallopeptidase domain 19 | EMB | 29 | | 33 | CASP1 | caspase 1, apoptosis-related cysteine peptidase | FCER1G | 29 | | 34 | ABCB1 | ATP-binding cassette, sub-family B (MDR/TAP), member 1 | FCRL3 | 29 | | 35 | SH2D1A | SH2 domain containing 1A | FXYD5 | 29 | | 36 | TRMT1 | TRM1 tRNA methyltransferase 1 homolog (S. cerevisiae) | GALNT10 | 29 | | 37 | NCK1 | NCK adaptor protein 1 | GFI1 | 36 | | 38 | CASK | calcium/calmodulin-dependent serine protein kinase (MAGUK family) | GIMAP1 | 29 | | 39 | MKI67 | antigen identified by monoclonal antibody Ki-67 | GLCCI1 | 36 | | 40 | BCL2L1 | BCL2-like 1 | GNG8 | 29 | | 41 | CIT | citron (rho-interacting, serine/threonine kinase 21) | HDDC2 | 29 | | 42 | DNAJC9 | DnaJ (Hsp40) homolog, subfamily C, member 9 | HDGF | 36 | | 43 | ADAM23 | ADAM metallopeptidase domain 23 | HGSNAT | 29 | | 44 | IFITM1 | interferon induced transmembrane protein 1 (9-27) | HIVEP3 | 29 | | 45 | MAN1C1 | mannosidase, alpha, class 1C, member 1 | HK2 | 29 | | 46 | CUX1 | cut-like homeobox 1 | HLA-B | 29 | | 47 | NR4A2 | nuclear receptor subfamily 4, group A, member 2 | HLA-E | 29 | | 48 | HDGF | hepatoma-derived growth factor | HTR2B | 29 | | 49 | CAMSAP2 | calmodulin regulated spectrin-associated protein family, member 2 | IARS | 29 | | 50 | POLR3E | polymerase (RNA) III (DNA directed) polypeptide E (80kD) | IDH3A | 36 | | 51 | HGSNAT | heparan-alpha-glucosaminide N-acetyltransferase | IFITM1 | 29 | | 52 | BRP44L | brain protein 44-like | IKZF4 | 29 | | 53 | PAK1IP1 | PAK1 interacting protein 1 | IRAK2 | 29 | | 54 | KLF2 | Kruppel-like factor 2 (lung) | IRF2BP2 | 29 | | 55 | TMEM156 | transmembrane protein 156 | KLF2 | 29 | | 56 | UPB1 | ureidopropionase, beta | KLHL5 | 29 | | 57 | TNIP3 | TNFAIP3 interacting protein 3 | LAPTM4B | 29 | | 58 | PLEKHA5 | pleckstrin homology domain containing, family A member 5 | LINC00282 | 29 | | 59 | TRAF3 | TNF receptor-associated factor 3 | LOC400965 | 36 | | 60 | CYLD | cylindromatosis (turban tumor syndrome) | LSP1 | 29 | | 61 | PDCD6 | programmed cell death 6 | MAN1C1 | 29 | | 62 | ORMDL1 | ORM1-like 1 (S. cerevisiae) | MKI67 | 36 | | 63 | NFKBIZ | nuclear factor of kappa light polypeptide gene enhancer in B-cells inhibitor, zeta | NCK1 | 36 | | 64 | CARD11 | caspase recruitment domain family, member 11 | NFATC3 | 36 | | 65 | FXYD5 | FXYD domain containing ion transport regulator 5 | NFKBIZ | 29 | | 66 | IRF2BP2 | interferon regulatory factor 2 binding protein 2 | NR4A2 | 29 | | 67 | ARHGAP21 | Rho GTPase activating protein 21 | ORMDL1 | 36 | | 68 | NFATC3 | nuclear factor of activated T-cells, cytoplasmic, calcineurin-dependent 3 | PAK1IP1 | 29 | | 69 | PPP1R16A | protein phosphatase 1, regulatory subunit 16A | PDCD6 | 36 | | 70 | C10orf54 | chromosome 10 open reading frame 54 | PLEKHA5 | 29 | | 71 | GLCCI1 | glucocorticoid induced transcript 1 | POLR1C | 29 | | 72 | KLHL5 | kelch-like 5 (Drosophila) | POLR3E | 29 | | 73 | APH1B | anterior pharynx defective 1 homolog B (C. elegans) | PPP1R16A | 36 | | 74 | CCDC71L | coiled-coil domain containing 71-like | PRMT8 | 29 | | 75 | IKZF4 | IKAROS family zinc finger 4 (Eos) | PTPRJ | 29 | | 76 | EMB | embigin | RASSF6 | 36 | | 77 | PTPRJ | protein tyrosine phosphatase, receptor type, J | RHBDD1 | 36 | | 78 | TAPT1 | transmembrane anterior posterior transformation 1 | RORC | 36 | | 79 | C2orf89 | chromosome 2 open reading frame 89 | RPS19 | 29 | | 80 | ANTXR2 | anthrax toxin receptor 2 | RUFY2 | 36 | | 81 | RORC | RAR-related orphan receptor C | SELL | 29 | | 82 | C7orf31 | chromosome 7 open reading frame 31 | SEPW1 | 29 | | 83 | GALNT10 | UDP-N-acetyl-alpha-D-galactosamine:polypeptide N-acetylgalactosaminyltransferase 10 (GalNAc-T10) | SH2D1A | 36 | | 84 | ZNRF1 | zinc and ring finger 1, E3 ubiquitin protein ligase | SOCS2 | 29 | | 85 | FCRL3 | Fc receptor-like 3 | TAPT1 | 36 | | 86 | IRAK2 | interleukin-1 receptor-associated kinase 2 | TMEM156 | 29 | | 87 | RUFY2 | RUN and FYVE domain containing 2 | TNIP3 | 29 | | 88 | GNG8 | guanine nucleotide binding protein (G protein), gamma 8 | TRAF3 | 29 | | 89 | HIVEP3 | human immunodeficiency virus type I enhancer binding protein 3 | TRMT1 | 29 | | 90 | RASSF6 | Ras association (RalGDS/AF-6) domain family member 6 | UPB1 | 36 | | 91 | CHD1 | chromodomain helicase DNA binding protein 1 | WASF1 | 29 | | 92 | C11orf80 | chromosome 11 open reading frame 80 | WDR46 | 29 | | 93 | CEP152 | centrosomal protein 152kDa | ZNF519 | 36 | | 94 | C14orf182 | chromosome 14 open reading frame 182 | ZNRF1 | 36 | |

---

Total number of miRNA-mRNA 2957 interactions for given cut-off. Press for ALL:

| |  | miR | EntrezID | Gene | Name | Score | | --- | --- | --- | --- | --- | --- | | 189 | hsa-mir-15a | 3133 | HLA-E | major histocompatibility complex, class I, E | 4 | | 190 | hsa-mir-16-1 | 3133 | HLA-E | major histocompatibility complex, class I, E | 4 | | 191 | hsa-mir-302a | 3133 | HLA-E | major histocompatibility complex, class I, E | 4 | | 219 | hsa-mir-340 | 3419 | IDH3A | isocitrate dehydrogenase 3 (NAD+) alpha | 4 | | 220 | hsa-mir-342 | 3419 | IDH3A | isocitrate dehydrogenase 3 (NAD+) alpha | 4 | | 221 | hsa-mir-326 | 3419 | IDH3A | isocitrate dehydrogenase 3 (NAD+) alpha | 4 | | 222 | hsa-mir-151a | 3419 | IDH3A | isocitrate dehydrogenase 3 (NAD+) alpha | 4 | | 790 | hsa-mir-15a | 55353 | LAPTM4B | lysosomal protein transmembrane 4 beta | 4 | | 791 | hsa-mir-16-1 | 55353 | LAPTM4B | lysosomal protein transmembrane 4 beta | 4 | | 792 | hsa-mir-302a | 55353 | LAPTM4B | lysosomal protein transmembrane 4 beta | 4 | | 2338 | hsa-mir-340 | 84236 | RHBDD1 | rhomboid domain containing 1 | 4 | | 2339 | hsa-mir-342 | 84236 | RHBDD1 | rhomboid domain containing 1 | 4 | | 2340 | hsa-mir-326 | 84236 | RHBDD1 | rhomboid domain containing 1 | 4 | | 2341 | hsa-mir-151a | 84236 | RHBDD1 | rhomboid domain containing 1 | 4 | | 1 | hsa-mir-15a | 170575 | GIMAP1 | GTPase, IMAP family member 1 | 2 | | 2 | hsa-mir-16-1 | 170575 | GIMAP1 | GTPase, IMAP family member 1 | 2 | | 3 | hsa-mir-302a | 170575 | GIMAP1 | GTPase, IMAP family member 1 | 2 | | 30 | hsa-mir-15a | 131450 | CD200R1 | CD200 receptor 1 | 2 | | 31 | hsa-mir-16-1 | 131450 | CD200R1 | CD200 receptor 1 | 2 | | 32 | hsa-mir-302a | 131450 | CD200R1 | CD200 receptor 1 | 2 | | 59 | hsa-mir-15a | 6415 | SEPW1 | selenoprotein W, 1 | 2 | | 60 | hsa-mir-16-1 | 6415 | SEPW1 | selenoprotein W, 1 | 2 | | 61 | hsa-mir-302a | 6415 | SEPW1 | selenoprotein W, 1 | 2 | | 88 | hsa-mir-15a | 283521 | LINC00282 | long intergenic non-protein coding RNA 282 | 2 | | 89 | hsa-mir-16-1 | 283521 | LINC00282 | long intergenic non-protein coding RNA 282 | 2 | | 90 | hsa-mir-302a | 283521 | LINC00282 | long intergenic non-protein coding RNA 282 | 2 | | 118 | hsa-mir-340 | 400965 | LOC400965 | uncharacterized LOC400965 | 2 | | 119 | hsa-mir-342 | 400965 | LOC400965 | uncharacterized LOC400965 | 2 | | 120 | hsa-mir-326 | 400965 | LOC400965 | uncharacterized LOC400965 | 2 | | 121 | hsa-mir-151a | 400965 | LOC400965 | uncharacterized LOC400965 | 2 | | 154 | hsa-mir-340 | 162655 | ZNF519 | zinc finger protein 519 | 2 | | 155 | hsa-mir-342 | 162655 | ZNF519 | zinc finger protein 519 | 2 | | 156 | hsa-mir-326 | 162655 | ZNF519 | zinc finger protein 519 | 2 | | 157 | hsa-mir-151a | 162655 | ZNF519 | zinc finger protein 519 | 2 | | 192 | hsa-mir-302b | 3133 | HLA-E | major histocompatibility complex, class I, E | 2 | | 193 | hsa-mir-302c | 3133 | HLA-E | major histocompatibility complex, class I, E | 2 | | 194 | hsa-mir-302d | 3133 | HLA-E | major histocompatibility complex, class I, E | 2 | | 195 | hsa-mir-367 | 3133 | HLA-E | major histocompatibility complex, class I, E | 2 | | 196 | hsa-mir-562 | 3133 | HLA-E | major histocompatibility complex, class I, E | 2 | | 197 | hsa-mir-604 | 3133 | HLA-E | major histocompatibility complex, class I, E | 2 | | 198 | hsa-mir-548d-1 | 3133 | HLA-E | major histocompatibility complex, class I, E | 2 | | 199 | hsa-mir-938 | 3133 | HLA-E | major histocompatibility complex, class I, E | 2 | | 200 | hsa-mir-548h-1 | 3133 | HLA-E | major histocompatibility complex, class I, E | 2 | | 201 | hsa-mir-3135a | 3133 | HLA-E | major histocompatibility complex, class I, E | 2 | | 202 | hsa-mir-3177 | 3133 | HLA-E | major histocompatibility complex, class I, E | 2 | | 203 | hsa-mir-3655 | 3133 | HLA-E | major histocompatibility complex, class I, E | 2 | | 204 | hsa-mir-4526 | 3133 | HLA-E | major histocompatibility complex, class I, E | 2 | | 205 | hsa-mir-4635 | 3133 | HLA-E | major histocompatibility complex, class I, E | 2 | | 206 | hsa-mir-4651 | 3133 | HLA-E | major histocompatibility complex, class I, E | 2 | | 207 | hsa-mir-4744 | 3133 | HLA-E | major histocompatibility complex, class I, E | 2 | | 208 | hsa-mir-5190 | 3133 | HLA-E | major histocompatibility complex, class I, E | 2 | | 209 | hsa-mir-101-2 | 3133 | HLA-E | major histocompatibility complex, class I, E | 2 | | 210 | hsa-mir-128-1;hsa-mir-128-2 | 3133 | HLA-E | major histocompatibility complex, class I, E | 2 | | 211 | hsa-mir-301a | 3133 | HLA-E | major histocompatibility complex, class I, E | 2 | | 212 | hsa-mir-511-1;hsa-mir-511-2 | 3133 | HLA-E | major histocompatibility complex, class I, E | 2 | | 213 | hsa-mir-586 | 3133 | HLA-E | major histocompatibility complex, class I, E | 2 | | 214 | hsa-mir-593 | 3133 | HLA-E | major histocompatibility complex, class I, E | 2 | | 215 | hsa-mir-620 | 3133 | HLA-E | major histocompatibility complex, class I, E | 2 | | 216 | hsa-mir-33b | 3133 | HLA-E | major histocompatibility complex, class I, E | 2 | | 217 | hsa-mir-942 | 3133 | HLA-E | major histocompatibility complex, class I, E | 2 | | 218 | hsa-mir-101-2 | 3419 | IDH3A | isocitrate dehydrogenase 3 (NAD+) alpha | 2 | | 223 | hsa-mir-511-1 | 3419 | IDH3A | isocitrate dehydrogenase 3 (NAD+) alpha | 2 | | 224 | hsa-mir-511-2 | 3419 | IDH3A | isocitrate dehydrogenase 3 (NAD+) alpha | 2 | | 225 | hsa-mir-1284 | 3419 | IDH3A | isocitrate dehydrogenase 3 (NAD+) alpha | 2 | | 226 | hsa-mir-3181 | 3419 | IDH3A | isocitrate dehydrogenase 3 (NAD+) alpha | 2 | | 227 | hsa-mir-3651 | 3419 | IDH3A | isocitrate dehydrogenase 3 (NAD+) alpha | 2 | | 228 | hsa-mir-4632 | 3419 | IDH3A | isocitrate dehydrogenase 3 (NAD+) alpha | 2 | | 229 | hsa-mir-4648 | 3419 | IDH3A | isocitrate dehydrogenase 3 (NAD+) alpha | 2 | | 230 | hsa-mir-4726 | 3419 | IDH3A | isocitrate dehydrogenase 3 (NAD+) alpha | 2 | | 231 | hsa-mir-4785 | 3419 | IDH3A | isocitrate dehydrogenase 3 (NAD+) alpha | 2 | | 232 | hsa-mir-4794 | 3419 | IDH3A | isocitrate dehydrogenase 3 (NAD+) alpha | 2 | | 233 | hsa-mir-4802 | 3419 | IDH3A | isocitrate dehydrogenase 3 (NAD+) alpha | 2 | | 234 | hsa-let-7i | 3419 | IDH3A | isocitrate dehydrogenase 3 (NAD+) alpha | 2 | | 235 | hsa-mir-302b | 3419 | IDH3A | isocitrate dehydrogenase 3 (NAD+) alpha | 2 | | 236 | hsa-mir-302c | 3419 | IDH3A | isocitrate dehydrogenase 3 (NAD+) alpha | 2 | | 237 | hsa-mir-339 | 3419 | IDH3A | isocitrate dehydrogenase 3 (NAD+) alpha | 2 | | 238 | hsa-mir-562 | 3419 | IDH3A | isocitrate dehydrogenase 3 (NAD+) alpha | 2 | | 239 | hsa-mir-567 | 3419 | IDH3A | isocitrate dehydrogenase 3 (NAD+) alpha | 2 | | 240 | hsa-mir-576 | 3419 | IDH3A | isocitrate dehydrogenase 3 (NAD+) alpha | 2 | | 241 | hsa-mir-579 | 3419 | IDH3A | isocitrate dehydrogenase 3 (NAD+) alpha | 2 | | 242 | hsa-mir-582 | 3419 | IDH3A | isocitrate dehydrogenase 3 (NAD+) alpha | 2 | | 243 | hsa-mir-604 | 3419 | IDH3A | isocitrate dehydrogenase 3 (NAD+) alpha | 2 | | 244 | hsa-mir-608 | 3419 | IDH3A | isocitrate dehydrogenase 3 (NAD+) alpha | 2 | | 245 | hsa-mir-621 | 3419 | IDH3A | isocitrate dehydrogenase 3 (NAD+) alpha | 2 | | 246 | hsa-mir-627 | 3419 | IDH3A | isocitrate dehydrogenase 3 (NAD+) alpha | 2 | | 247 | hsa-mir-454 | 3419 | IDH3A | isocitrate dehydrogenase 3 (NAD+) alpha | 2 | | 248 | hsa-mir-302d | 3419 | IDH3A | isocitrate dehydrogenase 3 (NAD+) alpha | 2 | | 249 | hsa-mir-367 | 3419 | IDH3A | isocitrate dehydrogenase 3 (NAD+) alpha | 2 | | 250 | hsa-mir-548b | 3419 | IDH3A | isocitrate dehydrogenase 3 (NAD+) alpha | 2 | | 251 | hsa-mir-548d-1;hsa-mir-548d-2 | 3419 | IDH3A | isocitrate dehydrogenase 3 (NAD+) alpha | 2 | | 252 | hsa-mir-938 | 3419 | IDH3A | isocitrate dehydrogenase 3 (NAD+) alpha | 2 | | 253 | hsa-mir-1236 | 3419 | IDH3A | isocitrate dehydrogenase 3 (NAD+) alpha | 2 | | 254 | hsa-mir-15a | 6223 | RPS19 | ribosomal protein S19 | 2 | | 255 | hsa-mir-16-1 | 6223 | RPS19 | ribosomal protein S19 | 2 | | 256 | hsa-mir-302a | 6223 | RPS19 | ribosomal protein S19 | 2 | | 284 | hsa-mir-340 | 54788 | DNAJB12 | DnaJ (Hsp40) homolog, subfamily B, member 12 | 2 | | 285 | hsa-mir-342 | 54788 | DNAJB12 | DnaJ (Hsp40) homolog, subfamily B, member 12 | 2 | | 286 | hsa-mir-326 | 54788 | DNAJB12 | DnaJ (Hsp40) homolog, subfamily B, member 12 | 2 | | 287 | hsa-mir-151a | 54788 | DNAJB12 | DnaJ (Hsp40) homolog, subfamily B, member 12 | 2 | | 319 | hsa-mir-15a | 3099 | HK2 | hexokinase 2 | 2 | | 320 | hsa-mir-16-1 | 3099 | HK2 | hexokinase 2 | 2 | | 321 | hsa-mir-302a | 3099 | HK2 | hexokinase 2 | 2 | | 348 | hsa-mir-15a | 51020 | HDDC2 | HD domain containing 2 | 2 | | 349 | hsa-mir-16-1 | 51020 | HDDC2 | HD domain containing 2 | 2 | | 350 | hsa-mir-302a | 51020 | HDDC2 | HD domain containing 2 | 2 | | 377 | hsa-mir-15a | 8835 | SOCS2 | suppressor of cytokine signaling 2 | 2 | | 378 | hsa-mir-16-1 | 8835 | SOCS2 | suppressor of cytokine signaling 2 | 2 | | 379 | hsa-mir-302a | 8835 | SOCS2 | suppressor of cytokine signaling 2 | 2 | | 406 | hsa-mir-15a | 4046 | LSP1 | lymphocyte-specific protein 1 | 2 | | 407 | hsa-mir-16-1 | 4046 | LSP1 | lymphocyte-specific protein 1 | 2 | | 408 | hsa-mir-302a | 4046 | LSP1 | lymphocyte-specific protein 1 | 2 | | 435 | hsa-mir-15a | 596 | BCL2 | B-cell CLL/lymphoma 2 | 2 | | 436 | hsa-mir-16-1 | 596 | BCL2 | B-cell CLL/lymphoma 2 | 2 | | 437 | hsa-mir-302a | 596 | BCL2 | B-cell CLL/lymphoma 2 | 2 | | 464 | hsa-mir-15a | 8936 | WASF1 | WAS protein family, member 1 | 2 | | 465 | hsa-mir-16-1 | 8936 | WASF1 | WAS protein family, member 1 | 2 | | 466 | hsa-mir-302a | 8936 | WASF1 | WAS protein family, member 1 | 2 | | 493 | hsa-mir-15a | 2207 | FCER1G | Fc fragment of IgE, high affinity I, receptor for; gamma polypeptide | 2 | | 494 | hsa-mir-16-1 | 2207 | FCER1G | Fc fragment of IgE, high affinity I, receptor for; gamma polypeptide | 2 | | 495 | hsa-mir-302a | 2207 | FCER1G | Fc fragment of IgE, high affinity I, receptor for; gamma polypeptide | 2 | | 522 | hsa-mir-15a | 960 | CD44 | CD44 molecule (Indian blood group) | 2 | | 523 | hsa-mir-16-1 | 960 | CD44 | CD44 molecule (Indian blood group) | 2 | | 524 | hsa-mir-302a | 960 | CD44 | CD44 molecule (Indian blood group) | 2 | | 551 | hsa-mir-15a | 6402 | SELL | selectin L | 2 | | 552 | hsa-mir-16-1 | 6402 | SELL | selectin L | 2 | | 553 | hsa-mir-302a | 6402 | SELL | selectin L | 2 | | 580 | hsa-mir-15a | 3376 | IARS | isoleucyl-tRNA synthetase | 2 | | 581 | hsa-mir-16-1 | 3376 | IARS | isoleucyl-tRNA synthetase | 2 | | 582 | hsa-mir-302a | 3376 | IARS | isoleucyl-tRNA synthetase | 2 | | 609 | hsa-mir-15a | 921 | CD5 | CD5 molecule | 2 | | 610 | hsa-mir-16-1 | 921 | CD5 | CD5 molecule | 2 | | 611 | hsa-mir-302a | 921 | CD5 | CD5 molecule | 2 | | 639 | hsa-mir-340 | 2672 | GFI1 | growth factor independent 1 transcription repressor | 2 | | 640 | hsa-mir-342 | 2672 | GFI1 | growth factor independent 1 transcription repressor | 2 | | 641 | hsa-mir-326 | 2672 | GFI1 | growth factor independent 1 transcription repressor | 2 | | 642 | hsa-mir-151a | 2672 | GFI1 | growth factor independent 1 transcription repressor | 2 | | 674 | hsa-mir-15a | 3357 | HTR2B | 5-hydroxytryptamine (serotonin) receptor 2B, G protein-coupled | 2 | | 675 | hsa-mir-16-1 | 3357 | HTR2B | 5-hydroxytryptamine (serotonin) receptor 2B, G protein-coupled | 2 | | 676 | hsa-mir-302a | 3357 | HTR2B | 5-hydroxytryptamine (serotonin) receptor 2B, G protein-coupled | 2 | | 703 | hsa-mir-15a | 56341 | PRMT8 | protein arginine methyltransferase 8 | 2 | | 704 | hsa-mir-16-1 | 56341 | PRMT8 | protein arginine methyltransferase 8 | 2 | | 705 | hsa-mir-302a | 56341 | PRMT8 | protein arginine methyltransferase 8 | 2 | | 732 | hsa-mir-15a | 87 | ACTN1 | actinin, alpha 1 | 2 | | 733 | hsa-mir-16-1 | 87 | ACTN1 | actinin, alpha 1 | 2 | | 734 | hsa-mir-302a | 87 | ACTN1 | actinin, alpha 1 | 2 | | 761 | hsa-mir-15a | 998 | CDC42 | cell division cycle 42 (GTP binding protein, 25kDa) | 2 | | 762 | hsa-mir-16-1 | 998 | CDC42 | cell division cycle 42 (GTP binding protein, 25kDa) | 2 | | 763 | hsa-mir-302a | 998 | CDC42 | cell division cycle 42 (GTP binding protein, 25kDa) | 2 | | 793 | hsa-mir-302b | 55353 | LAPTM4B | lysosomal protein transmembrane 4 beta | 2 | | 794 | hsa-mir-302c | 55353 | LAPTM4B | lysosomal protein transmembrane 4 beta | 2 | | 795 | hsa-mir-302d | 55353 | LAPTM4B | lysosomal protein transmembrane 4 beta | 2 | | 796 | hsa-mir-367 | 55353 | LAPTM4B | lysosomal protein transmembrane 4 beta | 2 | | 797 | hsa-mir-562 | 55353 | LAPTM4B | lysosomal protein transmembrane 4 beta | 2 | | 798 | hsa-mir-604 | 55353 | LAPTM4B | lysosomal protein transmembrane 4 beta | 2 | | 799 | hsa-mir-548d-1 | 55353 | LAPTM4B | lysosomal protein transmembrane 4 beta | 2 | | 800 | hsa-mir-938 | 55353 | LAPTM4B | lysosomal protein transmembrane 4 beta | 2 | | 801 | hsa-mir-548h-1 | 55353 | LAPTM4B | lysosomal protein transmembrane 4 beta | 2 | | 802 | hsa-mir-3135a | 55353 | LAPTM4B | lysosomal protein transmembrane 4 beta | 2 | | 803 | hsa-mir-3177 | 55353 | LAPTM4B | lysosomal protein transmembrane 4 beta | 2 | | 804 | hsa-mir-3655 | 55353 | LAPTM4B | lysosomal protein transmembrane 4 beta | 2 | | 805 | hsa-mir-4526 | 55353 | LAPTM4B | lysosomal protein transmembrane 4 beta | 2 | | 806 | hsa-mir-4635 | 55353 | LAPTM4B | lysosomal protein transmembrane 4 beta | 2 | | 807 | hsa-mir-4651 | 55353 | LAPTM4B | lysosomal protein transmembrane 4 beta | 2 | | 808 | hsa-mir-4744 | 55353 | LAPTM4B | lysosomal protein transmembrane 4 beta | 2 | | 809 | hsa-mir-5190 | 55353 | LAPTM4B | lysosomal protein transmembrane 4 beta | 2 | | 810 | hsa-mir-101-2 | 55353 | LAPTM4B | lysosomal protein transmembrane 4 beta | 2 | | 811 | hsa-mir-128-1;hsa-mir-128-2 | 55353 | LAPTM4B | lysosomal protein transmembrane 4 beta | 2 | | 812 | hsa-mir-301a | 55353 | LAPTM4B | lysosomal protein transmembrane 4 beta | 2 | | 813 | hsa-mir-511-1;hsa-mir-511-2 | 55353 | LAPTM4B | lysosomal protein transmembrane 4 beta | 2 | | 814 | hsa-mir-586 | 55353 | LAPTM4B | lysosomal protein transmembrane 4 beta | 2 | | 815 | hsa-mir-593 | 55353 | LAPTM4B | lysosomal protein transmembrane 4 beta | 2 | | 816 | hsa-mir-620 | 55353 | LAPTM4B | lysosomal protein transmembrane 4 beta | 2 | | 817 | hsa-mir-33b | 55353 | LAPTM4B | lysosomal protein transmembrane 4 beta | 2 | | 818 | hsa-mir-942 | 55353 | LAPTM4B | lysosomal protein transmembrane 4 beta | 2 | | 819 | hsa-mir-15a | 3106 | HLA-B | major histocompatibility complex, class I, B | 2 | | 820 | hsa-mir-16-1 | 3106 | HLA-B | major histocompatibility complex, class I, B | 2 | | 821 | hsa-mir-302a | 3106 | HLA-B | major histocompatibility complex, class I, B | 2 | | 848 | hsa-mir-15a | 9277 | WDR46 | WD repeat domain 46 | 2 | | 849 | hsa-mir-16-1 | 9277 | WDR46 | WD repeat domain 46 | 2 | | 850 | hsa-mir-302a | 9277 | WDR46 | WD repeat domain 46 | 2 | | 877 | hsa-mir-15a | 9533 | POLR1C | polymerase (RNA) I polypeptide C, 30kDa | 2 | | 878 | hsa-mir-16-1 | 9533 | POLR1C | polymerase (RNA) I polypeptide C, 30kDa | 2 | | 879 | hsa-mir-302a | 9533 | POLR1C | polymerase (RNA) I polypeptide C, 30kDa | 2 | | 906 | hsa-mir-15a | 8728 | ADAM19 | ADAM metallopeptidase domain 19 | 2 | | 907 | hsa-mir-16-1 | 8728 | ADAM19 | ADAM metallopeptidase domain 19 | 2 | | 908 | hsa-mir-302a | 8728 | ADAM19 | ADAM metallopeptidase domain 19 | 2 | | 935 | hsa-mir-15a | 834 | CASP1 | caspase 1, apoptosis-related cysteine peptidase | 2 | | 936 | hsa-mir-16-1 | 834 | CASP1 | caspase 1, apoptosis-related cysteine peptidase | 2 | | 937 | hsa-mir-302a | 834 | CASP1 | caspase 1, apoptosis-related cysteine peptidase | 2 | | 964 | hsa-mir-15a | 5243 | ABCB1 | ATP-binding cassette, sub-family B (MDR/TAP), member 1 | 2 | | 965 | hsa-mir-16-1 | 5243 | ABCB1 | ATP-binding cassette, sub-family B (MDR/TAP), member 1 | 2 | | 966 | hsa-mir-302a | 5243 | ABCB1 | ATP-binding cassette, sub-family B (MDR/TAP), member 1 | 2 | | 994 | hsa-mir-340 | 4068 | SH2D1A | SH2 domain containing 1A | 2 | | 995 | hsa-mir-342 | 4068 | SH2D1A | SH2 domain containing 1A | 2 | | 996 | hsa-mir-326 | 4068 | SH2D1A | SH2 domain containing 1A | 2 | | 997 | hsa-mir-151a | 4068 | SH2D1A | SH2 domain containing 1A | 2 | | 1029 | hsa-mir-15a | 55621 | TRMT1 | TRM1 tRNA methyltransferase 1 homolog (S. cerevisiae) | 2 | | 1030 | hsa-mir-16-1 | 55621 | TRMT1 | TRM1 tRNA methyltransferase 1 homolog (S. cerevisiae) | 2 | | 1031 | hsa-mir-302a | 55621 | TRMT1 | TRM1 tRNA methyltransferase 1 homolog (S. cerevisiae) | 2 | | 1059 | hsa-mir-340 | 4690 | NCK1 | NCK adaptor protein 1 | 2 | | 1060 | hsa-mir-342 | 4690 | NCK1 | NCK adaptor protein 1 | 2 | | 1061 | hsa-mir-326 | 4690 | NCK1 | NCK adaptor protein 1 | 2 | | 1062 | hsa-mir-151a | 4690 | NCK1 | NCK adaptor protein 1 | 2 | | 1095 | hsa-mir-340 | 8573 | CASK | calcium/calmodulin-dependent serine protein kinase (MAGUK family) | 2 | | 1096 | hsa-mir-342 | 8573 | CASK | calcium/calmodulin-dependent serine protein kinase (MAGUK family) | 2 | | 1097 | hsa-mir-326 | 8573 | CASK | calcium/calmodulin-dependent serine protein kinase (MAGUK family) | 2 | | 1098 | hsa-mir-151a | 8573 | CASK | calcium/calmodulin-dependent serine protein kinase (MAGUK family) | 2 | | 1131 | hsa-mir-340 | 4288 | MKI67 | antigen identified by monoclonal antibody Ki-67 | 2 | | 1132 | hsa-mir-342 | 4288 | MKI67 | antigen identified by monoclonal antibody Ki-67 | 2 | | 1133 | hsa-mir-326 | 4288 | MKI67 | antigen identified by monoclonal antibody Ki-67 | 2 | | 1134 | hsa-mir-151a | 4288 | MKI67 | antigen identified by monoclonal antibody Ki-67 | 2 | | 1167 | hsa-mir-340 | 598 | BCL2L1 | BCL2-like 1 | 2 | | 1168 | hsa-mir-342 | 598 | BCL2L1 | BCL2-like 1 | 2 | | 1169 | hsa-mir-326 | 598 | BCL2L1 | BCL2-like 1 | 2 | | 1170 | hsa-mir-151a | 598 | BCL2L1 | BCL2-like 1 | 2 | | 1203 | hsa-mir-340 | 11113 | CIT | citron (rho-interacting, serine/threonine kinase 21) | 2 | | 1204 | hsa-mir-342 | 11113 | CIT | citron (rho-interacting, serine/threonine kinase 21) | 2 | | 1205 | hsa-mir-326 | 11113 | CIT | citron (rho-interacting, serine/threonine kinase 21) | 2 | | 1206 | hsa-mir-151a | 11113 | CIT | citron (rho-interacting, serine/threonine kinase 21) | 2 | | 1239 | hsa-mir-340 | 23234 | DNAJC9 | DnaJ (Hsp40) homolog, subfamily C, member 9 | 2 | | 1240 | hsa-mir-342 | 23234 | DNAJC9 | DnaJ (Hsp40) homolog, subfamily C, member 9 | 2 | | 1241 | hsa-mir-326 | 23234 | DNAJC9 | DnaJ (Hsp40) homolog, subfamily C, member 9 | 2 | | 1242 | hsa-mir-151a | 23234 | DNAJC9 | DnaJ (Hsp40) homolog, subfamily C, member 9 | 2 | | 1274 | hsa-mir-15a | 8745 | ADAM23 | ADAM metallopeptidase domain 23 | 2 | | 1275 | hsa-mir-16-1 | 8745 | ADAM23 | ADAM metallopeptidase domain 23 | 2 | | 1276 | hsa-mir-302a | 8745 | ADAM23 | ADAM metallopeptidase domain 23 | 2 | | 1303 | hsa-mir-15a | 8519 | IFITM1 | interferon induced transmembrane protein 1 (9-27) | 2 | | 1304 | hsa-mir-16-1 | 8519 | IFITM1 | interferon induced transmembrane protein 1 (9-27) | 2 | | 1305 | hsa-mir-302a | 8519 | IFITM1 | interferon induced transmembrane protein 1 (9-27) | 2 | | 1332 | hsa-mir-15a | 57134 | MAN1C1 | mannosidase, alpha, class 1C, member 1 | 2 | | 1333 | hsa-mir-16-1 | 57134 | MAN1C1 | mannosidase, alpha, class 1C, member 1 | 2 | | 1334 | hsa-mir-302a | 57134 | MAN1C1 | mannosidase, alpha, class 1C, member 1 | 2 | | 1362 | hsa-mir-340 | 1523 | CUX1 | cut-like homeobox 1 | 2 | | 1363 | hsa-mir-342 | 1523 | CUX1 | cut-like homeobox 1 | 2 | | 1364 | hsa-mir-326 | 1523 | CUX1 | cut-like homeobox 1 | 2 | | 1365 | hsa-mir-151a | 1523 | CUX1 | cut-like homeobox 1 | 2 | | 1397 | hsa-mir-15a | 4929 | NR4A2 | nuclear receptor subfamily 4, group A, member 2 | 2 | | 1398 | hsa-mir-16-1 | 4929 | NR4A2 | nuclear receptor subfamily 4, group A, member 2 | 2 | | 1399 | hsa-mir-302a | 4929 | NR4A2 | nuclear receptor subfamily 4, group A, member 2 | 2 | | 1427 | hsa-mir-340 | 3068 | HDGF | hepatoma-derived growth factor | 2 | | 1428 | hsa-mir-342 | 3068 | HDGF | hepatoma-derived growth factor | 2 | | 1429 | hsa-mir-326 | 3068 | HDGF | hepatoma-derived growth factor | 2 | | 1430 | hsa-mir-151a | 3068 | HDGF | hepatoma-derived growth factor | 2 | | 1462 | hsa-mir-15a | 23271 | CAMSAP2 | calmodulin regulated spectrin-associated protein family, member 2 | 2 | | 1463 | hsa-mir-16-1 | 23271 | CAMSAP2 | calmodulin regulated spectrin-associated protein family, member 2 | 2 | | 1464 | hsa-mir-302a | 23271 | CAMSAP2 | calmodulin regulated spectrin-associated protein family, member 2 | 2 | | 1491 | hsa-mir-15a | 55718 | POLR3E | polymerase (RNA) III (DNA directed) polypeptide E (80kD) | 2 | | 1492 | hsa-mir-16-1 | 55718 | POLR3E | polymerase (RNA) III (DNA directed) polypeptide E (80kD) | 2 | | 1493 | hsa-mir-302a | 55718 | POLR3E | polymerase (RNA) III (DNA directed) polypeptide E (80kD) | 2 | | 1520 | hsa-mir-15a | 138050 | HGSNAT | heparan-alpha-glucosaminide N-acetyltransferase | 2 | | 1521 | hsa-mir-16-1 | 138050 | HGSNAT | heparan-alpha-glucosaminide N-acetyltransferase | 2 | | 1522 | hsa-mir-302a | 138050 | HGSNAT | heparan-alpha-glucosaminide N-acetyltransferase | 2 | | 1550 | hsa-mir-340 | 51660 | BRP44L | brain protein 44-like | 2 | | 1551 | hsa-mir-342 | 51660 | BRP44L | brain protein 44-like | 2 | | 1552 | hsa-mir-326 | 51660 | BRP44L | brain protein 44-like | 2 | | 1553 | hsa-mir-151a | 51660 | BRP44L | brain protein 44-like | 2 | | 1585 | hsa-mir-15a | 55003 | PAK1IP1 | PAK1 interacting protein 1 | 2 | | 1586 | hsa-mir-16-1 | 55003 | PAK1IP1 | PAK1 interacting protein 1 | 2 | | 1587 | hsa-mir-302a | 55003 | PAK1IP1 | PAK1 interacting protein 1 | 2 | | 1614 | hsa-mir-15a | 10365 | KLF2 | Kruppel-like factor 2 (lung) | 2 | | 1615 | hsa-mir-16-1 | 10365 | KLF2 | Kruppel-like factor 2 (lung) | 2 | | 1616 | hsa-mir-302a | 10365 | KLF2 | Kruppel-like factor 2 (lung) | 2 | | 1643 | hsa-mir-15a | 80008 | TMEM156 | transmembrane protein 156 | 2 | | 1644 | hsa-mir-16-1 | 80008 | TMEM156 | transmembrane protein 156 | 2 | | 1645 | hsa-mir-302a | 80008 | TMEM156 | transmembrane protein 156 | 2 | | 1673 | hsa-mir-340 | 51733 | UPB1 | ureidopropionase, beta | 2 | | 1674 | hsa-mir-342 | 51733 | UPB1 | ureidopropionase, beta | 2 | | 1675 | hsa-mir-326 | 51733 | UPB1 | ureidopropionase, beta | 2 | | 1676 | hsa-mir-151a | 51733 | UPB1 | ureidopropionase, beta | 2 | | 1708 | hsa-mir-15a | 79931 | TNIP3 | TNFAIP3 interacting protein 3 | 2 | | 1709 | hsa-mir-16-1 | 79931 | TNIP3 | TNFAIP3 interacting protein 3 | 2 | | 1710 | hsa-mir-302a | 79931 | TNIP3 | TNFAIP3 interacting protein 3 | 2 | | 1737 | hsa-mir-15a | 54477 | PLEKHA5 | pleckstrin homology domain containing, family A member 5 | 2 | | 1738 | hsa-mir-16-1 | 54477 | PLEKHA5 | pleckstrin homology domain containing, family A member 5 | 2 | | 1739 | hsa-mir-302a | 54477 | PLEKHA5 | pleckstrin homology domain containing, family A member 5 | 2 | | 1766 | hsa-mir-15a | 7187 | TRAF3 | TNF receptor-associated factor 3 | 2 | | 1767 | hsa-mir-16-1 | 7187 | TRAF3 | TNF receptor-associated factor 3 | 2 | | 1768 | hsa-mir-302a | 7187 | TRAF3 | TNF receptor-associated factor 3 | 2 | | 1795 | hsa-mir-15a | 1540 | CYLD | cylindromatosis (turban tumor syndrome) | 2 | | 1796 | hsa-mir-16-1 | 1540 | CYLD | cylindromatosis (turban tumor syndrome) | 2 | | 1797 | hsa-mir-302a | 1540 | CYLD | cylindromatosis (turban tumor syndrome) | 2 | | 1825 | hsa-mir-340 | 10016 | PDCD6 | programmed cell death 6 | 2 | | 1826 | hsa-mir-342 | 10016 | PDCD6 | programmed cell death 6 | 2 | | 1827 | hsa-mir-326 | 10016 | PDCD6 | programmed cell death 6 | 2 | | 1828 | hsa-mir-151a | 10016 | PDCD6 | programmed cell death 6 | 2 | | 1861 | hsa-mir-340 | 94101 | ORMDL1 | ORM1-like 1 (S. cerevisiae) | 2 | | 1862 | hsa-mir-342 | 94101 | ORMDL1 | ORM1-like 1 (S. cerevisiae) | 2 | | 1863 | hsa-mir-326 | 94101 | ORMDL1 | ORM1-like 1 (S. cerevisiae) | 2 | | 1864 | hsa-mir-151a | 94101 | ORMDL1 | ORM1-like 1 (S. cerevisiae) | 2 | | 1896 | hsa-mir-15a | 64332 | NFKBIZ | nuclear factor of kappa light polypeptide gene enhancer in B-cells inhibitor, zeta | 2 | | 1897 | hsa-mir-16-1 | 64332 | NFKBIZ | nuclear factor of kappa light polypeptide gene enhancer in B-cells inhibitor, zeta | 2 | | 1898 | hsa-mir-302a | 64332 | NFKBIZ | nuclear factor of kappa light polypeptide gene enhancer in B-cells inhibitor, zeta | 2 | | 1925 | hsa-mir-15a | 84433 | CARD11 | caspase recruitment domain family, member 11 | 2 | | 1926 | hsa-mir-16-1 | 84433 | CARD11 | caspase recruitment domain family, member 11 | 2 | | 1927 | hsa-mir-302a | 84433 | CARD11 | caspase recruitment domain family, member 11 | 2 | | 1954 | hsa-mir-15a | 53827 | FXYD5 | FXYD domain containing ion transport regulator 5 | 2 | | 1955 | hsa-mir-16-1 | 53827 | FXYD5 | FXYD domain containing ion transport regulator 5 | 2 | | 1956 | hsa-mir-302a | 53827 | FXYD5 | FXYD domain containing ion transport regulator 5 | 2 | | 1983 | hsa-mir-15a | 359948 | IRF2BP2 | interferon regulatory factor 2 binding protein 2 | 2 | | 1984 | hsa-mir-16-1 | 359948 | IRF2BP2 | interferon regulatory factor 2 binding protein 2 | 2 | | 1985 | hsa-mir-302a | 359948 | IRF2BP2 | interferon regulatory factor 2 binding protein 2 | 2 | | 2012 | hsa-mir-15a | 57584 | ARHGAP21 | Rho GTPase activating protein 21 | 2 | | 2013 | hsa-mir-16-1 | 57584 | ARHGAP21 | Rho GTPase activating protein 21 | 2 | | 2014 | hsa-mir-302a | 57584 | ARHGAP21 | Rho GTPase activating protein 21 | 2 | | 2042 | hsa-mir-340 | 4775 | NFATC3 | nuclear factor of activated T-cells, cytoplasmic, calcineurin-dependent 3 | 2 | | 2043 | hsa-mir-342 | 4775 | NFATC3 | nuclear factor of activated T-cells, cytoplasmic, calcineurin-dependent 3 | 2 | | 2044 | hsa-mir-326 | 4775 | NFATC3 | nuclear factor of activated T-cells, cytoplasmic, calcineurin-dependent 3 | 2 | | 2045 | hsa-mir-151a | 4775 | NFATC3 | nuclear factor of activated T-cells, cytoplasmic, calcineurin-dependent 3 | 2 | | 2078 | hsa-mir-340 | 84988 | PPP1R16A | protein phosphatase 1, regulatory subunit 16A | 2 | | 2079 | hsa-mir-342 | 84988 | PPP1R16A | protein phosphatase 1, regulatory subunit 16A | 2 | | 2080 | hsa-mir-326 | 84988 | PPP1R16A | protein phosphatase 1, regulatory subunit 16A | 2 | | 2081 | hsa-mir-151a | 84988 | PPP1R16A | protein phosphatase 1, regulatory subunit 16A | 2 | | 2113 | hsa-mir-15a | 64115 | C10orf54 | chromosome 10 open reading frame 54 | 2 | | 2114 | hsa-mir-16-1 | 64115 | C10orf54 | chromosome 10 open reading frame 54 | 2 | | 2115 | hsa-mir-302a | 64115 | C10orf54 | chromosome 10 open reading frame 54 | 2 | | 2143 | hsa-mir-340 | 113263 | GLCCI1 | glucocorticoid induced transcript 1 | 2 | | 2144 | hsa-mir-342 | 113263 | GLCCI1 | glucocorticoid induced transcript 1 | 2 | | 2145 | hsa-mir-326 | 113263 | GLCCI1 | glucocorticoid induced transcript 1 | 2 | | 2146 | hsa-mir-151a | 113263 | GLCCI1 | glucocorticoid induced transcript 1 | 2 | | 2178 | hsa-mir-15a | 51088 | KLHL5 | kelch-like 5 (Drosophila) | 2 | | 2179 | hsa-mir-16-1 | 51088 | KLHL5 | kelch-like 5 (Drosophila) | 2 | | 2180 | hsa-mir-302a | 51088 | KLHL5 | kelch-like 5 (Drosophila) | 2 | | 2208 | hsa-mir-340 | 83464 | APH1B | anterior pharynx defective 1 homolog B (C. elegans) | 2 | | 2209 | hsa-mir-342 | 83464 | APH1B | anterior pharynx defective 1 homolog B (C. elegans) | 2 | | 2210 | hsa-mir-326 | 83464 | APH1B | anterior pharynx defective 1 homolog B (C. elegans) | 2 | | 2211 | hsa-mir-151a | 83464 | APH1B | anterior pharynx defective 1 homolog B (C. elegans) | 2 | | 2244 | hsa-mir-340 | 168455 | CCDC71L | coiled-coil domain containing 71-like | 2 | | 2245 | hsa-mir-342 | 168455 | CCDC71L | coiled-coil domain containing 71-like | 2 | | 2246 | hsa-mir-326 | 168455 | CCDC71L | coiled-coil domain containing 71-like | 2 | | 2247 | hsa-mir-151a | 168455 | CCDC71L | coiled-coil domain containing 71-like | 2 | | 2279 | hsa-mir-15a | 64375 | IKZF4 | IKAROS family zinc finger 4 (Eos) | 2 | | 2280 | hsa-mir-16-1 | 64375 | IKZF4 | IKAROS family zinc finger 4 (Eos) | 2 | | 2281 | hsa-mir-302a | 64375 | IKZF4 | IKAROS family zinc finger 4 (Eos) | 2 | | 2308 | hsa-mir-15a | 133418 | EMB | embigin | 2 | | 2309 | hsa-mir-16-1 | 133418 | EMB | embigin | 2 | | 2310 | hsa-mir-302a | 133418 | EMB | embigin | 2 | | 2337 | hsa-mir-101-2 | 84236 | RHBDD1 | rhomboid domain containing 1 | 2 | | 2342 | hsa-mir-511-1 | 84236 | RHBDD1 | rhomboid domain containing 1 | 2 | | 2343 | hsa-mir-511-2 | 84236 | RHBDD1 | rhomboid domain containing 1 | 2 | | 2344 | hsa-mir-1284 | 84236 | RHBDD1 | rhomboid domain containing 1 | 2 | | 2345 | hsa-mir-3181 | 84236 | RHBDD1 | rhomboid domain containing 1 | 2 | | 2346 | hsa-mir-3651 | 84236 | RHBDD1 | rhomboid domain containing 1 | 2 | | 2347 | hsa-mir-4632 | 84236 | RHBDD1 | rhomboid domain containing 1 | 2 | | 2348 | hsa-mir-4648 | 84236 | RHBDD1 | rhomboid domain containing 1 | 2 | | 2349 | hsa-mir-4726 | 84236 | RHBDD1 | rhomboid domain containing 1 | 2 | | 2350 | hsa-mir-4785 | 84236 | RHBDD1 | rhomboid domain containing 1 | 2 | | 2351 | hsa-mir-4794 | 84236 | RHBDD1 | rhomboid domain containing 1 | 2 | | 2352 | hsa-mir-4802 | 84236 | RHBDD1 | rhomboid domain containing 1 | 2 | | 2353 | hsa-let-7i | 84236 | RHBDD1 | rhomboid domain containing 1 | 2 | | 2354 | hsa-mir-302b | 84236 | RHBDD1 | rhomboid domain containing 1 | 2 | | 2355 | hsa-mir-302c | 84236 | RHBDD1 | rhomboid domain containing 1 | 2 | | 2356 | hsa-mir-339 | 84236 | RHBDD1 | rhomboid domain containing 1 | 2 | | 2357 | hsa-mir-562 | 84236 | RHBDD1 | rhomboid domain containing 1 | 2 | | 2358 | hsa-mir-567 | 84236 | RHBDD1 | rhomboid domain containing 1 | 2 | | 2359 | hsa-mir-576 | 84236 | RHBDD1 | rhomboid domain containing 1 | 2 | | 2360 | hsa-mir-579 | 84236 | RHBDD1 | rhomboid domain containing 1 | 2 | | 2361 | hsa-mir-582 | 84236 | RHBDD1 | rhomboid domain containing 1 | 2 | | 2362 | hsa-mir-604 | 84236 | RHBDD1 | rhomboid domain containing 1 | 2 | | 2363 | hsa-mir-608 | 84236 | RHBDD1 | rhomboid domain containing 1 | 2 | | 2364 | hsa-mir-621 | 84236 | RHBDD1 | rhomboid domain containing 1 | 2 | | 2365 | hsa-mir-627 | 84236 | RHBDD1 | rhomboid domain containing 1 | 2 | | 2366 | hsa-mir-454 | 84236 | RHBDD1 | rhomboid domain containing 1 | 2 | | 2367 | hsa-mir-302d | 84236 | RHBDD1 | rhomboid domain containing 1 | 2 | | 2368 | hsa-mir-367 | 84236 | RHBDD1 | rhomboid domain containing 1 | 2 | | 2369 | hsa-mir-548b | 84236 | RHBDD1 | rhomboid domain containing 1 | 2 | | 2370 | hsa-mir-548d-1;hsa-mir-548d-2 | 84236 | RHBDD1 | rhomboid domain containing 1 | 2 | | 2371 | hsa-mir-938 | 84236 | RHBDD1 | rhomboid domain containing 1 | 2 | | 2372 | hsa-mir-1236 | 84236 | RHBDD1 | rhomboid domain containing 1 | 2 | | 2373 | hsa-mir-15a | 5795 | PTPRJ | protein tyrosine phosphatase, receptor type, J | 2 | | 2374 | hsa-mir-16-1 | 5795 | PTPRJ | protein tyrosine phosphatase, receptor type, J | 2 | | 2375 | hsa-mir-302a | 5795 | PTPRJ | protein tyrosine phosphatase, receptor type, J | 2 | | 2403 | hsa-mir-340 | 202018 | TAPT1 | transmembrane anterior posterior transformation 1 | 2 | | 2404 | hsa-mir-342 | 202018 | TAPT1 | transmembrane anterior posterior transformation 1 | 2 | | 2405 | hsa-mir-326 | 202018 | TAPT1 | transmembrane anterior posterior transformation 1 | 2 | | 2406 | hsa-mir-151a | 202018 | TAPT1 | transmembrane anterior posterior transformation 1 | 2 | | 2438 | hsa-mir-15a | 129293 | C2orf89 | chromosome 2 open reading frame 89 | 2 | | 2439 | hsa-mir-16-1 | 129293 | C2orf89 | chromosome 2 open reading frame 89 | 2 | | 2440 | hsa-mir-302a | 129293 | C2orf89 | chromosome 2 open reading frame 89 | 2 | | 2467 | hsa-mir-15a | 118429 | ANTXR2 | anthrax toxin receptor 2 | 2 | | 2468 | hsa-mir-16-1 | 118429 | ANTXR2 | anthrax toxin receptor 2 | 2 | | 2469 | hsa-mir-302a | 118429 | ANTXR2 | anthrax toxin receptor 2 | 2 | | 2497 | hsa-mir-340 | 6097 | RORC | RAR-related orphan receptor C | 2 | | 2498 | hsa-mir-342 | 6097 | RORC | RAR-related orphan receptor C | 2 | | 2499 | hsa-mir-326 | 6097 | RORC | RAR-related orphan receptor C | 2 | | 2500 | hsa-mir-151a | 6097 | RORC | RAR-related orphan receptor C | 2 | | 2533 | hsa-mir-340 | 136895 | C7orf31 | chromosome 7 open reading frame 31 | 2 | | 2534 | hsa-mir-342 | 136895 | C7orf31 | chromosome 7 open reading frame 31 | 2 | | 2535 | hsa-mir-326 | 136895 | C7orf31 | chromosome 7 open reading frame 31 | 2 | | 2536 | hsa-mir-151a | 136895 | C7orf31 | chromosome 7 open reading frame 31 | 2 | | 2568 | hsa-mir-15a | 55568 | GALNT10 | UDP-N-acetyl-alpha-D-galactosamine:polypeptide N-acetylgalactosaminyltransferase 10 (GalNAc-T10) | 2 | | 2569 | hsa-mir-16-1 | 55568 | GALNT10 | UDP-N-acetyl-alpha-D-galactosamine:polypeptide N-acetylgalactosaminyltransferase 10 (GalNAc-T10) | 2 | | 2570 | hsa-mir-302a | 55568 | GALNT10 | UDP-N-acetyl-alpha-D-galactosamine:polypeptide N-acetylgalactosaminyltransferase 10 (GalNAc-T10) | 2 | | 2598 | hsa-mir-340 | 84937 | ZNRF1 | zinc and ring finger 1, E3 ubiquitin protein ligase | 2 | | 2599 | hsa-mir-342 | 84937 | ZNRF1 | zinc and ring finger 1, E3 ubiquitin protein ligase | 2 | | 2600 | hsa-mir-326 | 84937 | ZNRF1 | zinc and ring finger 1, E3 ubiquitin protein ligase | 2 | | 2601 | hsa-mir-151a | 84937 | ZNRF1 | zinc and ring finger 1, E3 ubiquitin protein ligase | 2 | | 2633 | hsa-mir-15a | 115352 | FCRL3 | Fc receptor-like 3 | 2 | | 2634 | hsa-mir-16-1 | 115352 | FCRL3 | Fc receptor-like 3 | 2 | | 2635 | hsa-mir-302a | 115352 | FCRL3 | Fc receptor-like 3 | 2 | | 2662 | hsa-mir-15a | 3656 | IRAK2 | interleukin-1 receptor-associated kinase 2 | 2 | | 2663 | hsa-mir-16-1 | 3656 | IRAK2 | interleukin-1 receptor-associated kinase 2 | 2 | | 2664 | hsa-mir-302a | 3656 | IRAK2 | interleukin-1 receptor-associated kinase 2 | 2 | | 2692 | hsa-mir-340 | 55680 | RUFY2 | RUN and FYVE domain containing 2 | 2 | | 2693 | hsa-mir-342 | 55680 | RUFY2 | RUN and FYVE domain containing 2 | 2 | | 2694 | hsa-mir-326 | 55680 | RUFY2 | RUN and FYVE domain containing 2 | 2 | | 2695 | hsa-mir-151a | 55680 | RUFY2 | RUN and FYVE domain containing 2 | 2 | | 2727 | hsa-mir-15a | 94235 | GNG8 | guanine nucleotide binding protein (G protein), gamma 8 | 2 | | 2728 | hsa-mir-16-1 | 94235 | GNG8 | guanine nucleotide binding protein (G protein), gamma 8 | 2 | | 2729 | hsa-mir-302a | 94235 | GNG8 | guanine nucleotide binding protein (G protein), gamma 8 | 2 | | 2756 | hsa-mir-15a | 59269 | HIVEP3 | human immunodeficiency virus type I enhancer binding protein 3 | 2 | | 2757 | hsa-mir-16-1 | 59269 | HIVEP3 | human immunodeficiency virus type I enhancer binding protein 3 | 2 | | 2758 | hsa-mir-302a | 59269 | HIVEP3 | human immunodeficiency virus type I enhancer binding protein 3 | 2 | | 2786 | hsa-mir-340 | 166824 | RASSF6 | Ras association (RalGDS/AF-6) domain family member 6 | 2 | | 2787 | hsa-mir-342 | 166824 | RASSF6 | Ras association (RalGDS/AF-6) domain family member 6 | 2 | | 2788 | hsa-mir-326 | 166824 | RASSF6 | Ras association (RalGDS/AF-6) domain family member 6 | 2 | | 2789 | hsa-mir-151a | 166824 | RASSF6 | Ras association (RalGDS/AF-6) domain family member 6 | 2 | | 2822 | hsa-mir-340 | 1105 | CHD1 | chromodomain helicase DNA binding protein 1 | 2 | | 2823 | hsa-mir-342 | 1105 | CHD1 | chromodomain helicase DNA binding protein 1 | 2 | | 2824 | hsa-mir-326 | 1105 | CHD1 | chromodomain helicase DNA binding protein 1 | 2 | | 2825 | hsa-mir-151a | 1105 | CHD1 | chromodomain helicase DNA binding protein 1 | 2 | | 2858 | hsa-mir-340 | 79703 | C11orf80 | chromosome 11 open reading frame 80 | 2 | | 2859 | hsa-mir-342 | 79703 | C11orf80 | chromosome 11 open reading frame 80 | 2 | | 2860 | hsa-mir-326 | 79703 | C11orf80 | chromosome 11 open reading frame 80 | 2 | | 2861 | hsa-mir-151a | 79703 | C11orf80 | chromosome 11 open reading frame 80 | 2 | | 2894 | hsa-mir-340 | 22995 | CEP152 | centrosomal protein 152kDa | 2 | | 2895 | hsa-mir-342 | 22995 | CEP152 | centrosomal protein 152kDa | 2 | | 2896 | hsa-mir-326 | 22995 | CEP152 | centrosomal protein 152kDa | 2 | | 2897 | hsa-mir-151a | 22995 | CEP152 | centrosomal protein 152kDa | 2 | | 2929 | hsa-mir-15a | 283551 | C14orf182 | chromosome 14 open reading frame 182 | 2 | | 2930 | hsa-mir-16-1 | 283551 | C14orf182 | chromosome 14 open reading frame 182 | 2 | | 2931 | hsa-mir-302a | 283551 | C14orf182 | chromosome 14 open reading frame 182 | 2 | | 4 | hsa-mir-302b | 170575 | GIMAP1 | GTPase, IMAP family member 1 | 1 | | 5 | hsa-mir-302c | 170575 | GIMAP1 | GTPase, IMAP family member 1 | 1 | | 6 | hsa-mir-302d | 170575 | GIMAP1 | GTPase, IMAP family member 1 | 1 | | 7 | hsa-mir-367 | 170575 | GIMAP1 | GTPase, IMAP family member 1 | 1 | | 8 | hsa-mir-562 | 170575 | GIMAP1 | GTPase, IMAP family member 1 | 1 | | 9 | hsa-mir-604 | 170575 | GIMAP1 | GTPase, IMAP family member 1 | 1 | | 10 | hsa-mir-548d-1 | 170575 | GIMAP1 | GTPase, IMAP family member 1 | 1 | | 11 | hsa-mir-938 | 170575 | GIMAP1 | GTPase, IMAP family member 1 | 1 | | 12 | hsa-mir-548h-1 | 170575 | GIMAP1 | GTPase, IMAP family member 1 | 1 | | 13 | hsa-mir-3135a | 170575 | GIMAP1 | GTPase, IMAP family member 1 | 1 | | 14 | hsa-mir-3177 | 170575 | GIMAP1 | GTPase, IMAP family member 1 | 1 | | 15 | hsa-mir-3655 | 170575 | GIMAP1 | GTPase, IMAP family member 1 | 1 | | 16 | hsa-mir-4526 | 170575 | GIMAP1 | GTPase, IMAP family member 1 | 1 | | 17 | hsa-mir-4635 | 170575 | GIMAP1 | GTPase, IMAP family member 1 | 1 | | 18 | hsa-mir-4651 | 170575 | GIMAP1 | GTPase, IMAP family member 1 | 1 | | 19 | hsa-mir-4744 | 170575 | GIMAP1 | GTPase, IMAP family member 1 | 1 | | 20 | hsa-mir-5190 | 170575 | GIMAP1 | GTPase, IMAP family member 1 | 1 | | 21 | hsa-mir-101-2 | 170575 | GIMAP1 | GTPase, IMAP family member 1 | 1 | | 22 | hsa-mir-128-1;hsa-mir-128-2 | 170575 | GIMAP1 | GTPase, IMAP family member 1 | 1 | | 23 | hsa-mir-301a | 170575 | GIMAP1 | GTPase, IMAP family member 1 | 1 | | 24 | hsa-mir-511-1;hsa-mir-511-2 | 170575 | GIMAP1 | GTPase, IMAP family member 1 | 1 | | 25 | hsa-mir-586 | 170575 | GIMAP1 | GTPase, IMAP family member 1 | 1 | | 26 | hsa-mir-593 | 170575 | GIMAP1 | GTPase, IMAP family member 1 | 1 | | 27 | hsa-mir-620 | 170575 | GIMAP1 | GTPase, IMAP family member 1 | 1 | | 28 | hsa-mir-33b | 170575 | GIMAP1 | GTPase, IMAP family member 1 | 1 | | 29 | hsa-mir-942 | 170575 | GIMAP1 | GTPase, IMAP family member 1 | 1 | | 33 | hsa-mir-302b | 131450 | CD200R1 | CD200 receptor 1 | 1 | | 34 | hsa-mir-302c | 131450 | CD200R1 | CD200 receptor 1 | 1 | | 35 | hsa-mir-302d | 131450 | CD200R1 | CD200 receptor 1 | 1 | | 36 | hsa-mir-367 | 131450 | CD200R1 | CD200 receptor 1 | 1 | | 37 | hsa-mir-562 | 131450 | CD200R1 | CD200 receptor 1 | 1 | | 38 | hsa-mir-604 | 131450 | CD200R1 | CD200 receptor 1 | 1 | | 39 | hsa-mir-548d-1 | 131450 | CD200R1 | CD200 receptor 1 | 1 | | 40 | hsa-mir-938 | 131450 | CD200R1 | CD200 receptor 1 | 1 | | 41 | hsa-mir-548h-1 | 131450 | CD200R1 | CD200 receptor 1 | 1 | | 42 | hsa-mir-3135a | 131450 | CD200R1 | CD200 receptor 1 | 1 | | 43 | hsa-mir-3177 | 131450 | CD200R1 | CD200 receptor 1 | 1 | | 44 | hsa-mir-3655 | 131450 | CD200R1 | CD200 receptor 1 | 1 | | 45 | hsa-mir-4526 | 131450 | CD200R1 | CD200 receptor 1 | 1 | | 46 | hsa-mir-4635 | 131450 | CD200R1 | CD200 receptor 1 | 1 | | 47 | hsa-mir-4651 | 131450 | CD200R1 | CD200 receptor 1 | 1 | | 48 | hsa-mir-4744 | 131450 | CD200R1 | CD200 receptor 1 | 1 | | 49 | hsa-mir-5190 | 131450 | CD200R1 | CD200 receptor 1 | 1 | | 50 | hsa-mir-101-2 | 131450 | CD200R1 | CD200 receptor 1 | 1 | | 51 | hsa-mir-128-1;hsa-mir-128-2 | 131450 | CD200R1 | CD200 receptor 1 | 1 | | 52 | hsa-mir-301a | 131450 | CD200R1 | CD200 receptor 1 | 1 | | 53 | hsa-mir-511-1;hsa-mir-511-2 | 131450 | CD200R1 | CD200 receptor 1 | 1 | | 54 | hsa-mir-586 | 131450 | CD200R1 | CD200 receptor 1 | 1 | | 55 | hsa-mir-593 | 131450 | CD200R1 | CD200 receptor 1 | 1 | | 56 | hsa-mir-620 | 131450 | CD200R1 | CD200 receptor 1 | 1 | | 57 | hsa-mir-33b | 131450 | CD200R1 | CD200 receptor 1 | 1 | | 58 | hsa-mir-942 | 131450 | CD200R1 | CD200 receptor 1 | 1 | | 62 | hsa-mir-302b | 6415 | SEPW1 | selenoprotein W, 1 | 1 | | 63 | hsa-mir-302c | 6415 | SEPW1 | selenoprotein W, 1 | 1 | | 64 | hsa-mir-302d | 6415 | SEPW1 | selenoprotein W, 1 | 1 | | 65 | hsa-mir-367 | 6415 | SEPW1 | selenoprotein W, 1 | 1 | | 66 | hsa-mir-562 | 6415 | SEPW1 | selenoprotein W, 1 | 1 | | 67 | hsa-mir-604 | 6415 | SEPW1 | selenoprotein W, 1 | 1 | | 68 | hsa-mir-548d-1 | 6415 | SEPW1 | selenoprotein W, 1 | 1 | | 69 | hsa-mir-938 | 6415 | SEPW1 | selenoprotein W, 1 | 1 | | 70 | hsa-mir-548h-1 | 6415 | SEPW1 | selenoprotein W, 1 | 1 | | 71 | hsa-mir-3135a | 6415 | SEPW1 | selenoprotein W, 1 | 1 | | 72 | hsa-mir-3177 | 6415 | SEPW1 | selenoprotein W, 1 | 1 | | 73 | hsa-mir-3655 | 6415 | SEPW1 | selenoprotein W, 1 | 1 | | 74 | hsa-mir-4526 | 6415 | SEPW1 | selenoprotein W, 1 | 1 | | 75 | hsa-mir-4635 | 6415 | SEPW1 | selenoprotein W, 1 | 1 | | 76 | hsa-mir-4651 | 6415 | SEPW1 | selenoprotein W, 1 | 1 | | 77 | hsa-mir-4744 | 6415 | SEPW1 | selenoprotein W, 1 | 1 | | 78 | hsa-mir-5190 | 6415 | SEPW1 | selenoprotein W, 1 | 1 | | 79 | hsa-mir-101-2 | 6415 | SEPW1 | selenoprotein W, 1 | 1 | | 80 | hsa-mir-128-1;hsa-mir-128-2 | 6415 | SEPW1 | selenoprotein W, 1 | 1 | | 81 | hsa-mir-301a | 6415 | SEPW1 | selenoprotein W, 1 | 1 | | 82 | hsa-mir-511-1;hsa-mir-511-2 | 6415 | SEPW1 | selenoprotein W, 1 | 1 | | 83 | hsa-mir-586 | 6415 | SEPW1 | selenoprotein W, 1 | 1 | | 84 | hsa-mir-593 | 6415 | SEPW1 | selenoprotein W, 1 | 1 | | 85 | hsa-mir-620 | 6415 | SEPW1 | selenoprotein W, 1 | 1 | | 86 | hsa-mir-33b | 6415 | SEPW1 | selenoprotein W, 1 | 1 | | 87 | hsa-mir-942 | 6415 | SEPW1 | selenoprotein W, 1 | 1 | | 91 | hsa-mir-302b | 283521 | LINC00282 | long intergenic non-protein coding RNA 282 | 1 | | 92 | hsa-mir-302c | 283521 | LINC00282 | long intergenic non-protein coding RNA 282 | 1 | | 93 | hsa-mir-302d | 283521 | LINC00282 | long intergenic non-protein coding RNA 282 | 1 | | 94 | hsa-mir-367 | 283521 | LINC00282 | long intergenic non-protein coding RNA 282 | 1 | | 95 | hsa-mir-562 | 283521 | LINC00282 | long intergenic non-protein coding RNA 282 | 1 | | 96 | hsa-mir-604 | 283521 | LINC00282 | long intergenic non-protein coding RNA 282 | 1 | | 97 | hsa-mir-548d-1 | 283521 | LINC00282 | long intergenic non-protein coding RNA 282 | 1 | | 98 | hsa-mir-938 | 283521 | LINC00282 | long intergenic non-protein coding RNA 282 | 1 | | 99 | hsa-mir-548h-1 | 283521 | LINC00282 | long intergenic non-protein coding RNA 282 | 1 | | 100 | hsa-mir-3135a | 283521 | LINC00282 | long intergenic non-protein coding RNA 282 | 1 | | 101 | hsa-mir-3177 | 283521 | LINC00282 | long intergenic non-protein coding RNA 282 | 1 | | 102 | hsa-mir-3655 | 283521 | LINC00282 | long intergenic non-protein coding RNA 282 | 1 | | 103 | hsa-mir-4526 | 283521 | LINC00282 | long intergenic non-protein coding RNA 282 | 1 | | 104 | hsa-mir-4635 | 283521 | LINC00282 | long intergenic non-protein coding RNA 282 | 1 | | 105 | hsa-mir-4651 | 283521 | LINC00282 | long intergenic non-protein coding RNA 282 | 1 | | 106 | hsa-mir-4744 | 283521 | LINC00282 | long intergenic non-protein coding RNA 282 | 1 | | 107 | hsa-mir-5190 | 283521 | LINC00282 | long intergenic non-protein coding RNA 282 | 1 | | 108 | hsa-mir-101-2 | 283521 | LINC00282 | long intergenic non-protein coding RNA 282 | 1 | | 109 | hsa-mir-128-1;hsa-mir-128-2 | 283521 | LINC00282 | long intergenic non-protein coding RNA 282 | 1 | | 110 | hsa-mir-301a | 283521 | LINC00282 | long intergenic non-protein coding RNA 282 | 1 | | 111 | hsa-mir-511-1;hsa-mir-511-2 | 283521 | LINC00282 | long intergenic non-protein coding RNA 282 | 1 | | 112 | hsa-mir-586 | 283521 | LINC00282 | long intergenic non-protein coding RNA 282 | 1 | | 113 | hsa-mir-593 | 283521 | LINC00282 | long intergenic non-protein coding RNA 282 | 1 | | 114 | hsa-mir-620 | 283521 | LINC00282 | long intergenic non-protein coding RNA 282 | 1 | | 115 | hsa-mir-33b | 283521 | LINC00282 | long intergenic non-protein coding RNA 282 | 1 | | 116 | hsa-mir-942 | 283521 | LINC00282 | long intergenic non-protein coding RNA 282 | 1 | | 117 | hsa-mir-101-2 | 400965 | LOC400965 | uncharacterized LOC400965 | 1 | | 122 | hsa-mir-511-1 | 400965 | LOC400965 | uncharacterized LOC400965 | 1 | | 123 | hsa-mir-511-2 | 400965 | LOC400965 | uncharacterized LOC400965 | 1 | | 124 | hsa-mir-1284 | 400965 | LOC400965 | uncharacterized LOC400965 | 1 | | 125 | hsa-mir-3181 | 400965 | LOC400965 | uncharacterized LOC400965 | 1 | | 126 | hsa-mir-3651 | 400965 | LOC400965 | uncharacterized LOC400965 | 1 | | 127 | hsa-mir-4632 | 400965 | LOC400965 | uncharacterized LOC400965 | 1 | | 128 | hsa-mir-4648 | 400965 | LOC400965 | uncharacterized LOC400965 | 1 | | 129 | hsa-mir-4726 | 400965 | LOC400965 | uncharacterized LOC400965 | 1 | | 130 | hsa-mir-4785 | 400965 | LOC400965 | uncharacterized LOC400965 | 1 | | 131 | hsa-mir-4794 | 400965 | LOC400965 | uncharacterized LOC400965 | 1 | | 132 | hsa-mir-4802 | 400965 | LOC400965 | uncharacterized LOC400965 | 1 | | 133 | hsa-let-7i | 400965 | LOC400965 | uncharacterized LOC400965 | 1 | | 134 | hsa-mir-302b | 400965 | LOC400965 | uncharacterized LOC400965 | 1 | | 135 | hsa-mir-302c | 400965 | LOC400965 | uncharacterized LOC400965 | 1 | | 136 | hsa-mir-339 | 400965 | LOC400965 | uncharacterized LOC400965 | 1 | | 137 | hsa-mir-562 | 400965 | LOC400965 | uncharacterized LOC400965 | 1 | | 138 | hsa-mir-567 | 400965 | LOC400965 | uncharacterized LOC400965 | 1 | | 139 | hsa-mir-576 | 400965 | LOC400965 | uncharacterized LOC400965 | 1 | | 140 | hsa-mir-579 | 400965 | LOC400965 | uncharacterized LOC400965 | 1 | | 141 | hsa-mir-582 | 400965 | LOC400965 | uncharacterized LOC400965 | 1 | | 142 | hsa-mir-604 | 400965 | LOC400965 | uncharacterized LOC400965 | 1 | | 143 | hsa-mir-608 | 400965 | LOC400965 | uncharacterized LOC400965 | 1 | | 144 | hsa-mir-621 | 400965 | LOC400965 | uncharacterized LOC400965 | 1 | | 145 | hsa-mir-627 | 400965 | LOC400965 | uncharacterized LOC400965 | 1 | | 146 | hsa-mir-454 | 400965 | LOC400965 | uncharacterized LOC400965 | 1 | | 147 | hsa-mir-302d | 400965 | LOC400965 | uncharacterized LOC400965 | 1 | | 148 | hsa-mir-367 | 400965 | LOC400965 | uncharacterized LOC400965 | 1 | | 149 | hsa-mir-548b | 400965 | LOC400965 | uncharacterized LOC400965 | 1 | | 150 | hsa-mir-548d-1;hsa-mir-548d-2 | 400965 | LOC400965 | uncharacterized LOC400965 | 1 | | 151 | hsa-mir-938 | 400965 | LOC400965 | uncharacterized LOC400965 | 1 | | 152 | hsa-mir-1236 | 400965 | LOC400965 | uncharacterized LOC400965 | 1 | | 153 | hsa-mir-101-2 | 162655 | ZNF519 | zinc finger protein 519 | 1 | | 158 | hsa-mir-511-1 | 162655 | ZNF519 | zinc finger protein 519 | 1 | | 159 | hsa-mir-511-2 | 162655 | ZNF519 | zinc finger protein 519 | 1 | | 160 | hsa-mir-1284 | 162655 | ZNF519 | zinc finger protein 519 | 1 | | 161 | hsa-mir-3181 | 162655 | ZNF519 | zinc finger protein 519 | 1 | | 162 | hsa-mir-3651 | 162655 | ZNF519 | zinc finger protein 519 | 1 | | 163 | hsa-mir-4632 | 162655 | ZNF519 | zinc finger protein 519 | 1 | | 164 | hsa-mir-4648 | 162655 | ZNF519 | zinc finger protein 519 | 1 | | 165 | hsa-mir-4726 | 162655 | ZNF519 | zinc finger protein 519 | 1 | | 166 | hsa-mir-4785 | 162655 | ZNF519 | zinc finger protein 519 | 1 | | 167 | hsa-mir-4794 | 162655 | ZNF519 | zinc finger protein 519 | 1 | | 168 | hsa-mir-4802 | 162655 | ZNF519 | zinc finger protein 519 | 1 | | 169 | hsa-let-7i | 162655 | ZNF519 | zinc finger protein 519 | 1 | | 170 | hsa-mir-302b | 162655 | ZNF519 | zinc finger protein 519 | 1 | | 171 | hsa-mir-302c | 162655 | ZNF519 | zinc finger protein 519 | 1 | | 172 | hsa-mir-339 | 162655 | ZNF519 | zinc finger protein 519 | 1 | | 173 | hsa-mir-562 | 162655 | ZNF519 | zinc finger protein 519 | 1 | | 174 | hsa-mir-567 | 162655 | ZNF519 | zinc finger protein 519 | 1 | | 175 | hsa-mir-576 | 162655 | ZNF519 | zinc finger protein 519 | 1 | | 176 | hsa-mir-579 | 162655 | ZNF519 | zinc finger protein 519 | 1 | | 177 | hsa-mir-582 | 162655 | ZNF519 | zinc finger protein 519 | 1 | | 178 | hsa-mir-604 | 162655 | ZNF519 | zinc finger protein 519 | 1 | | 179 | hsa-mir-608 | 162655 | ZNF519 | zinc finger protein 519 | 1 | | 180 | hsa-mir-621 | 162655 | ZNF519 | zinc finger protein 519 | 1 | | 181 | hsa-mir-627 | 162655 | ZNF519 | zinc finger protein 519 | 1 | | 182 | hsa-mir-454 | 162655 | ZNF519 | zinc finger protein 519 | 1 | | 183 | hsa-mir-302d | 162655 | ZNF519 | zinc finger protein 519 | 1 | | 184 | hsa-mir-367 | 162655 | ZNF519 | zinc finger protein 519 | 1 | | 185 | hsa-mir-548b | 162655 | ZNF519 | zinc finger protein 519 | 1 | | 186 | hsa-mir-548d-1;hsa-mir-548d-2 | 162655 | ZNF519 | zinc finger protein 519 | 1 | | 187 | hsa-mir-938 | 162655 | ZNF519 | zinc finger protein 519 | 1 | | 188 | hsa-mir-1236 | 162655 | ZNF519 | zinc finger protein 519 | 1 | | 257 | hsa-mir-302b | 6223 | RPS19 | ribosomal protein S19 | 1 | | 258 | hsa-mir-302c | 6223 | RPS19 | ribosomal protein S19 | 1 | | 259 | hsa-mir-302d | 6223 | RPS19 | ribosomal protein S19 | 1 | | 260 | hsa-mir-367 | 6223 | RPS19 | ribosomal protein S19 | 1 | | 261 | hsa-mir-562 | 6223 | RPS19 | ribosomal protein S19 | 1 | | 262 | hsa-mir-604 | 6223 | RPS19 | ribosomal protein S19 | 1 | | 263 | hsa-mir-548d-1 | 6223 | RPS19 | ribosomal protein S19 | 1 | | 264 | hsa-mir-938 | 6223 | RPS19 | ribosomal protein S19 | 1 | | 265 | hsa-mir-548h-1 | 6223 | RPS19 | ribosomal protein S19 | 1 | | 266 | hsa-mir-3135a | 6223 | RPS19 | ribosomal protein S19 | 1 | | 267 | hsa-mir-3177 | 6223 | RPS19 | ribosomal protein S19 | 1 | | 268 | hsa-mir-3655 | 6223 | RPS19 | ribosomal protein S19 | 1 | | 269 | hsa-mir-4526 | 6223 | RPS19 | ribosomal protein S19 | 1 | | 270 | hsa-mir-4635 | 6223 | RPS19 | ribosomal protein S19 | 1 | | 271 | hsa-mir-4651 | 6223 | RPS19 | ribosomal protein S19 | 1 | | 272 | hsa-mir-4744 | 6223 | RPS19 | ribosomal protein S19 | 1 | | 273 | hsa-mir-5190 | 6223 | RPS19 | ribosomal protein S19 | 1 | | 274 | hsa-mir-101-2 | 6223 | RPS19 | ribosomal protein S19 | 1 | | 275 | hsa-mir-128-1;hsa-mir-128-2 | 6223 | RPS19 | ribosomal protein S19 | 1 | | 276 | hsa-mir-301a | 6223 | RPS19 | ribosomal protein S19 | 1 | | 277 | hsa-mir-511-1;hsa-mir-511-2 | 6223 | RPS19 | ribosomal protein S19 | 1 | | 278 | hsa-mir-586 | 6223 | RPS19 | ribosomal protein S19 | 1 | | 279 | hsa-mir-593 | 6223 | RPS19 | ribosomal protein S19 | 1 | | 280 | hsa-mir-620 | 6223 | RPS19 | ribosomal protein S19 | 1 | | 281 | hsa-mir-33b | 6223 | RPS19 | ribosomal protein S19 | 1 | | 282 | hsa-mir-942 | 6223 | RPS19 | ribosomal protein S19 | 1 | | 283 | hsa-mir-101-2 | 54788 | DNAJB12 | DnaJ (Hsp40) homolog, subfamily B, member 12 | 1 | | 288 | hsa-mir-511-1 | 54788 | DNAJB12 | DnaJ (Hsp40) homolog, subfamily B, member 12 | 1 | | 289 | hsa-mir-511-2 | 54788 | DNAJB12 | DnaJ (Hsp40) homolog, subfamily B, member 12 | 1 | | 290 | hsa-mir-1284 | 54788 | DNAJB12 | DnaJ (Hsp40) homolog, subfamily B, member 12 | 1 | | 291 | hsa-mir-3181 | 54788 | DNAJB12 | DnaJ (Hsp40) homolog, subfamily B, member 12 | 1 | | 292 | hsa-mir-3651 | 54788 | DNAJB12 | DnaJ (Hsp40) homolog, subfamily B, member 12 | 1 | | 293 | hsa-mir-4632 | 54788 | DNAJB12 | DnaJ (Hsp40) homolog, subfamily B, member 12 | 1 | | 294 | hsa-mir-4648 | 54788 | DNAJB12 | DnaJ (Hsp40) homolog, subfamily B, member 12 | 1 | | 295 | hsa-mir-4726 | 54788 | DNAJB12 | DnaJ (Hsp40) homolog, subfamily B, member 12 | 1 | | 296 | hsa-mir-4785 | 54788 | DNAJB12 | DnaJ (Hsp40) homolog, subfamily B, member 12 | 1 | | 297 | hsa-mir-4794 | 54788 | DNAJB12 | DnaJ (Hsp40) homolog, subfamily B, member 12 | 1 | | 298 | hsa-mir-4802 | 54788 | DNAJB12 | DnaJ (Hsp40) homolog, subfamily B, member 12 | 1 | | 299 | hsa-let-7i | 54788 | DNAJB12 | DnaJ (Hsp40) homolog, subfamily B, member 12 | 1 | | 300 | hsa-mir-302b | 54788 | DNAJB12 | DnaJ (Hsp40) homolog, subfamily B, member 12 | 1 | | 301 | hsa-mir-302c | 54788 | DNAJB12 | DnaJ (Hsp40) homolog, subfamily B, member 12 | 1 | | 302 | hsa-mir-339 | 54788 | DNAJB12 | DnaJ (Hsp40) homolog, subfamily B, member 12 | 1 | | 303 | hsa-mir-562 | 54788 | DNAJB12 | DnaJ (Hsp40) homolog, subfamily B, member 12 | 1 | | 304 | hsa-mir-567 | 54788 | DNAJB12 | DnaJ (Hsp40) homolog, subfamily B, member 12 | 1 | | 305 | hsa-mir-576 | 54788 | DNAJB12 | DnaJ (Hsp40) homolog, subfamily B, member 12 | 1 | | 306 | hsa-mir-579 | 54788 | DNAJB12 | DnaJ (Hsp40) homolog, subfamily B, member 12 | 1 | | 307 | hsa-mir-582 | 54788 | DNAJB12 | DnaJ (Hsp40) homolog, subfamily B, member 12 | 1 | | 308 | hsa-mir-604 | 54788 | DNAJB12 | DnaJ (Hsp40) homolog, subfamily B, member 12 | 1 | | 309 | hsa-mir-608 | 54788 | DNAJB12 | DnaJ (Hsp40) homolog, subfamily B, member 12 | 1 | | 310 | hsa-mir-621 | 54788 | DNAJB12 | DnaJ (Hsp40) homolog, subfamily B, member 12 | 1 | | 311 | hsa-mir-627 | 54788 | DNAJB12 | DnaJ (Hsp40) homolog, subfamily B, member 12 | 1 | | 312 | hsa-mir-454 | 54788 | DNAJB12 | DnaJ (Hsp40) homolog, subfamily B, member 12 | 1 | | 313 | hsa-mir-302d | 54788 | DNAJB12 | DnaJ (Hsp40) homolog, subfamily B, member 12 | 1 | | 314 | hsa-mir-367 | 54788 | DNAJB12 | DnaJ (Hsp40) homolog, subfamily B, member 12 | 1 | | 315 | hsa-mir-548b | 54788 | DNAJB12 | DnaJ (Hsp40) homolog, subfamily B, member 12 | 1 | | 316 | hsa-mir-548d-1;hsa-mir-548d-2 | 54788 | DNAJB12 | DnaJ (Hsp40) homolog, subfamily B, member 12 | 1 | | 317 | hsa-mir-938 | 54788 | DNAJB12 | DnaJ (Hsp40) homolog, subfamily B, member 12 | 1 | | 318 | hsa-mir-1236 | 54788 | DNAJB12 | DnaJ (Hsp40) homolog, subfamily B, member 12 | 1 | | 322 | hsa-mir-302b | 3099 | HK2 | hexokinase 2 | 1 | | 323 | hsa-mir-302c | 3099 | HK2 | hexokinase 2 | 1 | | 324 | hsa-mir-302d | 3099 | HK2 | hexokinase 2 | 1 | | 325 | hsa-mir-367 | 3099 | HK2 | hexokinase 2 | 1 | | 326 | hsa-mir-562 | 3099 | HK2 | hexokinase 2 | 1 | | 327 | hsa-mir-604 | 3099 | HK2 | hexokinase 2 | 1 | | 328 | hsa-mir-548d-1 | 3099 | HK2 | hexokinase 2 | 1 | | 329 | hsa-mir-938 | 3099 | HK2 | hexokinase 2 | 1 | | 330 | hsa-mir-548h-1 | 3099 | HK2 | hexokinase 2 | 1 | | 331 | hsa-mir-3135a | 3099 | HK2 | hexokinase 2 | 1 | | 332 | hsa-mir-3177 | 3099 | HK2 | hexokinase 2 | 1 | | 333 | hsa-mir-3655 | 3099 | HK2 | hexokinase 2 | 1 | | 334 | hsa-mir-4526 | 3099 | HK2 | hexokinase 2 | 1 | | 335 | hsa-mir-4635 | 3099 | HK2 | hexokinase 2 | 1 | | 336 | hsa-mir-4651 | 3099 | HK2 | hexokinase 2 | 1 | | 337 | hsa-mir-4744 | 3099 | HK2 | hexokinase 2 | 1 | | 338 | hsa-mir-5190 | 3099 | HK2 | hexokinase 2 | 1 | | 339 | hsa-mir-101-2 | 3099 | HK2 | hexokinase 2 | 1 | | 340 | hsa-mir-128-1;hsa-mir-128-2 | 3099 | HK2 | hexokinase 2 | 1 | | 341 | hsa-mir-301a | 3099 | HK2 | hexokinase 2 | 1 | | 342 | hsa-mir-511-1;hsa-mir-511-2 | 3099 | HK2 | hexokinase 2 | 1 | | 343 | hsa-mir-586 | 3099 | HK2 | hexokinase 2 | 1 | | 344 | hsa-mir-593 | 3099 | HK2 | hexokinase 2 | 1 | | 345 | hsa-mir-620 | 3099 | HK2 | hexokinase 2 | 1 | | 346 | hsa-mir-33b | 3099 | HK2 | hexokinase 2 | 1 | | 347 | hsa-mir-942 | 3099 | HK2 | hexokinase 2 | 1 | | 351 | hsa-mir-302b | 51020 | HDDC2 | HD domain containing 2 | 1 | | 352 | hsa-mir-302c | 51020 | HDDC2 | HD domain containing 2 | 1 | | 353 | hsa-mir-302d | 51020 | HDDC2 | HD domain containing 2 | 1 | | 354 | hsa-mir-367 | 51020 | HDDC2 | HD domain containing 2 | 1 | | 355 | hsa-mir-562 | 51020 | HDDC2 | HD domain containing 2 | 1 | | 356 | hsa-mir-604 | 51020 | HDDC2 | HD domain containing 2 | 1 | | 357 | hsa-mir-548d-1 | 51020 | HDDC2 | HD domain containing 2 | 1 | | 358 | hsa-mir-938 | 51020 | HDDC2 | HD domain containing 2 | 1 | | 359 | hsa-mir-548h-1 | 51020 | HDDC2 | HD domain containing 2 | 1 | | 360 | hsa-mir-3135a | 51020 | HDDC2 | HD domain containing 2 | 1 | | 361 | hsa-mir-3177 | 51020 | HDDC2 | HD domain containing 2 | 1 | | 362 | hsa-mir-3655 | 51020 | HDDC2 | HD domain containing 2 | 1 | | 363 | hsa-mir-4526 | 51020 | HDDC2 | HD domain containing 2 | 1 | | 364 | hsa-mir-4635 | 51020 | HDDC2 | HD domain containing 2 | 1 | | 365 | hsa-mir-4651 | 51020 | HDDC2 | HD domain containing 2 | 1 | | 366 | hsa-mir-4744 | 51020 | HDDC2 | HD domain containing 2 | 1 | | 367 | hsa-mir-5190 | 51020 | HDDC2 | HD domain containing 2 | 1 | | 368 | hsa-mir-101-2 | 51020 | HDDC2 | HD domain containing 2 | 1 | | 369 | hsa-mir-128-1;hsa-mir-128-2 | 51020 | HDDC2 | HD domain containing 2 | 1 | | 370 | hsa-mir-301a | 51020 | HDDC2 | HD domain containing 2 | 1 | | 371 | hsa-mir-511-1;hsa-mir-511-2 | 51020 | HDDC2 | HD domain containing 2 | 1 | | 372 | hsa-mir-586 | 51020 | HDDC2 | HD domain containing 2 | 1 | | 373 | hsa-mir-593 | 51020 | HDDC2 | HD domain containing 2 | 1 | | 374 | hsa-mir-620 | 51020 | HDDC2 | HD domain containing 2 | 1 | | 375 | hsa-mir-33b | 51020 | HDDC2 | HD domain containing 2 | 1 | | 376 | hsa-mir-942 | 51020 | HDDC2 | HD domain containing 2 | 1 | | 380 | hsa-mir-302b | 8835 | SOCS2 | suppressor of cytokine signaling 2 | 1 | | 381 | hsa-mir-302c | 8835 | SOCS2 | suppressor of cytokine signaling 2 | 1 | | 382 | hsa-mir-302d | 8835 | SOCS2 | suppressor of cytokine signaling 2 | 1 | | 383 | hsa-mir-367 | 8835 | SOCS2 | suppressor of cytokine signaling 2 | 1 | | 384 | hsa-mir-562 | 8835 | SOCS2 | suppressor of cytokine signaling 2 | 1 | | 385 | hsa-mir-604 | 8835 | SOCS2 | suppressor of cytokine signaling 2 | 1 | | 386 | hsa-mir-548d-1 | 8835 | SOCS2 | suppressor of cytokine signaling 2 | 1 | | 387 | hsa-mir-938 | 8835 | SOCS2 | suppressor of cytokine signaling 2 | 1 | | 388 | hsa-mir-548h-1 | 8835 | SOCS2 | suppressor of cytokine signaling 2 | 1 | | 389 | hsa-mir-3135a | 8835 | SOCS2 | suppressor of cytokine signaling 2 | 1 | | 390 | hsa-mir-3177 | 8835 | SOCS2 | suppressor of cytokine signaling 2 | 1 | | 391 | hsa-mir-3655 | 8835 | SOCS2 | suppressor of cytokine signaling 2 | 1 | | 392 | hsa-mir-4526 | 8835 | SOCS2 | suppressor of cytokine signaling 2 | 1 | | 393 | hsa-mir-4635 | 8835 | SOCS2 | suppressor of cytokine signaling 2 | 1 | | 394 | hsa-mir-4651 | 8835 | SOCS2 | suppressor of cytokine signaling 2 | 1 | | 395 | hsa-mir-4744 | 8835 | SOCS2 | suppressor of cytokine signaling 2 | 1 | | 396 | hsa-mir-5190 | 8835 | SOCS2 | suppressor of cytokine signaling 2 | 1 | | 397 | hsa-mir-101-2 | 8835 | SOCS2 | suppressor of cytokine signaling 2 | 1 | | 398 | hsa-mir-128-1;hsa-mir-128-2 | 8835 | SOCS2 | suppressor of cytokine signaling 2 | 1 | | 399 | hsa-mir-301a | 8835 | SOCS2 | suppressor of cytokine signaling 2 | 1 | | 400 | hsa-mir-511-1;hsa-mir-511-2 | 8835 | SOCS2 | suppressor of cytokine signaling 2 | 1 | | 401 | hsa-mir-586 | 8835 | SOCS2 | suppressor of cytokine signaling 2 | 1 | | 402 | hsa-mir-593 | 8835 | SOCS2 | suppressor of cytokine signaling 2 | 1 | | 403 | hsa-mir-620 | 8835 | SOCS2 | suppressor of cytokine signaling 2 | 1 | | 404 | hsa-mir-33b | 8835 | SOCS2 | suppressor of cytokine signaling 2 | 1 | | 405 | hsa-mir-942 | 8835 | SOCS2 | suppressor of cytokine signaling 2 | 1 | | 409 | hsa-mir-302b | 4046 | LSP1 | lymphocyte-specific protein 1 | 1 | | 410 | hsa-mir-302c | 4046 | LSP1 | lymphocyte-specific protein 1 | 1 | | 411 | hsa-mir-302d | 4046 | LSP1 | lymphocyte-specific protein 1 | 1 | | 412 | hsa-mir-367 | 4046 | LSP1 | lymphocyte-specific protein 1 | 1 | | 413 | hsa-mir-562 | 4046 | LSP1 | lymphocyte-specific protein 1 | 1 | | 414 | hsa-mir-604 | 4046 | LSP1 | lymphocyte-specific protein 1 | 1 | | 415 | hsa-mir-548d-1 | 4046 | LSP1 | lymphocyte-specific protein 1 | 1 | | 416 | hsa-mir-938 | 4046 | LSP1 | lymphocyte-specific protein 1 | 1 | | 417 | hsa-mir-548h-1 | 4046 | LSP1 | lymphocyte-specific protein 1 | 1 | | 418 | hsa-mir-3135a | 4046 | LSP1 | lymphocyte-specific protein 1 | 1 | | 419 | hsa-mir-3177 | 4046 | LSP1 | lymphocyte-specific protein 1 | 1 | | 420 | hsa-mir-3655 | 4046 | LSP1 | lymphocyte-specific protein 1 | 1 | | 421 | hsa-mir-4526 | 4046 | LSP1 | lymphocyte-specific protein 1 | 1 | | 422 | hsa-mir-4635 | 4046 | LSP1 | lymphocyte-specific protein 1 | 1 | | 423 | hsa-mir-4651 | 4046 | LSP1 | lymphocyte-specific protein 1 | 1 | | 424 | hsa-mir-4744 | 4046 | LSP1 | lymphocyte-specific protein 1 | 1 | | 425 | hsa-mir-5190 | 4046 | LSP1 | lymphocyte-specific protein 1 | 1 | | 426 | hsa-mir-101-2 | 4046 | LSP1 | lymphocyte-specific protein 1 | 1 | | 427 | hsa-mir-128-1;hsa-mir-128-2 | 4046 | LSP1 | lymphocyte-specific protein 1 | 1 | | 428 | hsa-mir-301a | 4046 | LSP1 | lymphocyte-specific protein 1 | 1 | | 429 | hsa-mir-511-1;hsa-mir-511-2 | 4046 | LSP1 | lymphocyte-specific protein 1 | 1 | | 430 | hsa-mir-586 | 4046 | LSP1 | lymphocyte-specific protein 1 | 1 | | 431 | hsa-mir-593 | 4046 | LSP1 | lymphocyte-specific protein 1 | 1 | | 432 | hsa-mir-620 | 4046 | LSP1 | lymphocyte-specific protein 1 | 1 | | 433 | hsa-mir-33b | 4046 | LSP1 | lymphocyte-specific protein 1 | 1 | | 434 | hsa-mir-942 | 4046 | LSP1 | lymphocyte-specific protein 1 | 1 | | 438 | hsa-mir-302b | 596 | BCL2 | B-cell CLL/lymphoma 2 | 1 | | 439 | hsa-mir-302c | 596 | BCL2 | B-cell CLL/lymphoma 2 | 1 | | 440 | hsa-mir-302d | 596 | BCL2 | B-cell CLL/lymphoma 2 | 1 | | 441 | hsa-mir-367 | 596 | BCL2 | B-cell CLL/lymphoma 2 | 1 | | 442 | hsa-mir-562 | 596 | BCL2 | B-cell CLL/lymphoma 2 | 1 | | 443 | hsa-mir-604 | 596 | BCL2 | B-cell CLL/lymphoma 2 | 1 | | 444 | hsa-mir-548d-1 | 596 | BCL2 | B-cell CLL/lymphoma 2 | 1 | | 445 | hsa-mir-938 | 596 | BCL2 | B-cell CLL/lymphoma 2 | 1 | | 446 | hsa-mir-548h-1 | 596 | BCL2 | B-cell CLL/lymphoma 2 | 1 | | 447 | hsa-mir-3135a | 596 | BCL2 | B-cell CLL/lymphoma 2 | 1 | | 448 | hsa-mir-3177 | 596 | BCL2 | B-cell CLL/lymphoma 2 | 1 | | 449 | hsa-mir-3655 | 596 | BCL2 | B-cell CLL/lymphoma 2 | 1 | | 450 | hsa-mir-4526 | 596 | BCL2 | B-cell CLL/lymphoma 2 | 1 | | 451 | hsa-mir-4635 | 596 | BCL2 | B-cell CLL/lymphoma 2 | 1 | | 452 | hsa-mir-4651 | 596 | BCL2 | B-cell CLL/lymphoma 2 | 1 | | 453 | hsa-mir-4744 | 596 | BCL2 | B-cell CLL/lymphoma 2 | 1 | | 454 | hsa-mir-5190 | 596 | BCL2 | B-cell CLL/lymphoma 2 | 1 | | 455 | hsa-mir-101-2 | 596 | BCL2 | B-cell CLL/lymphoma 2 | 1 | | 456 | hsa-mir-128-1;hsa-mir-128-2 | 596 | BCL2 | B-cell CLL/lymphoma 2 | 1 | | 457 | hsa-mir-301a | 596 | BCL2 | B-cell CLL/lymphoma 2 | 1 | | 458 | hsa-mir-511-1;hsa-mir-511-2 | 596 | BCL2 | B-cell CLL/lymphoma 2 | 1 | | 459 | hsa-mir-586 | 596 | BCL2 | B-cell CLL/lymphoma 2 | 1 | | 460 | hsa-mir-593 | 596 | BCL2 | B-cell CLL/lymphoma 2 | 1 | | 461 | hsa-mir-620 | 596 | BCL2 | B-cell CLL/lymphoma 2 | 1 | | 462 | hsa-mir-33b | 596 | BCL2 | B-cell CLL/lymphoma 2 | 1 | | 463 | hsa-mir-942 | 596 | BCL2 | B-cell CLL/lymphoma 2 | 1 | | 467 | hsa-mir-302b | 8936 | WASF1 | WAS protein family, member 1 | 1 | | 468 | hsa-mir-302c | 8936 | WASF1 | WAS protein family, member 1 | 1 | | 469 | hsa-mir-302d | 8936 | WASF1 | WAS protein family, member 1 | 1 | | 470 | hsa-mir-367 | 8936 | WASF1 | WAS protein family, member 1 | 1 | | 471 | hsa-mir-562 | 8936 | WASF1 | WAS protein family, member 1 | 1 | | 472 | hsa-mir-604 | 8936 | WASF1 | WAS protein family, member 1 | 1 | | 473 | hsa-mir-548d-1 | 8936 | WASF1 | WAS protein family, member 1 | 1 | | 474 | hsa-mir-938 | 8936 | WASF1 | WAS protein family, member 1 | 1 | | 475 | hsa-mir-548h-1 | 8936 | WASF1 | WAS protein family, member 1 | 1 | | 476 | hsa-mir-3135a | 8936 | WASF1 | WAS protein family, member 1 | 1 | | 477 | hsa-mir-3177 | 8936 | WASF1 | WAS protein family, member 1 | 1 | | 478 | hsa-mir-3655 | 8936 | WASF1 | WAS protein family, member 1 | 1 | | 479 | hsa-mir-4526 | 8936 | WASF1 | WAS protein family, member 1 | 1 | | 480 | hsa-mir-4635 | 8936 | WASF1 | WAS protein family, member 1 | 1 | | 481 | hsa-mir-4651 | 8936 | WASF1 | WAS protein family, member 1 | 1 | | 482 | hsa-mir-4744 | 8936 | WASF1 | WAS protein family, member 1 | 1 | | 483 | hsa-mir-5190 | 8936 | WASF1 | WAS protein family, member 1 | 1 | | 484 | hsa-mir-101-2 | 8936 | WASF1 | WAS protein family, member 1 | 1 | | 485 | hsa-mir-128-1;hsa-mir-128-2 | 8936 | WASF1 | WAS protein family, member 1 | 1 | | 486 | hsa-mir-301a | 8936 | WASF1 | WAS protein family, member 1 | 1 | | 487 | hsa-mir-511-1;hsa-mir-511-2 | 8936 | WASF1 | WAS protein family, member 1 | 1 | | 488 | hsa-mir-586 | 8936 | WASF1 | WAS protein family, member 1 | 1 | | 489 | hsa-mir-593 | 8936 | WASF1 | WAS protein family, member 1 | 1 | | 490 | hsa-mir-620 | 8936 | WASF1 | WAS protein family, member 1 | 1 | | 491 | hsa-mir-33b | 8936 | WASF1 | WAS protein family, member 1 | 1 | | 492 | hsa-mir-942 | 8936 | WASF1 | WAS protein family, member 1 | 1 | | 496 | hsa-mir-302b | 2207 | FCER1G | Fc fragment of IgE, high affinity I, receptor for; gamma polypeptide | 1 | | 497 | hsa-mir-302c | 2207 | FCER1G | Fc fragment of IgE, high affinity I, receptor for; gamma polypeptide | 1 | | 498 | hsa-mir-302d | 2207 | FCER1G | Fc fragment of IgE, high affinity I, receptor for; gamma polypeptide | 1 | | 499 | hsa-mir-367 | 2207 | FCER1G | Fc fragment of IgE, high affinity I, receptor for; gamma polypeptide | 1 | | 500 | hsa-mir-562 | 2207 | FCER1G | Fc fragment of IgE, high affinity I, receptor for; gamma polypeptide | 1 | | 501 | hsa-mir-604 | 2207 | FCER1G | Fc fragment of IgE, high affinity I, receptor for; gamma polypeptide | 1 | | 502 | hsa-mir-548d-1 | 2207 | FCER1G | Fc fragment of IgE, high affinity I, receptor for; gamma polypeptide | 1 | | 503 | hsa-mir-938 | 2207 | FCER1G | Fc fragment of IgE, high affinity I, receptor for; gamma polypeptide | 1 | | 504 | hsa-mir-548h-1 | 2207 | FCER1G | Fc fragment of IgE, high affinity I, receptor for; gamma polypeptide | 1 | | 505 | hsa-mir-3135a | 2207 | FCER1G | Fc fragment of IgE, high affinity I, receptor for; gamma polypeptide | 1 | | 506 | hsa-mir-3177 | 2207 | FCER1G | Fc fragment of IgE, high affinity I, receptor for; gamma polypeptide | 1 | | 507 | hsa-mir-3655 | 2207 | FCER1G | Fc fragment of IgE, high affinity I, receptor for; gamma polypeptide | 1 | | 508 | hsa-mir-4526 | 2207 | FCER1G | Fc fragment of IgE, high affinity I, receptor for; gamma polypeptide | 1 | | 509 | hsa-mir-4635 | 2207 | FCER1G | Fc fragment of IgE, high affinity I, receptor for; gamma polypeptide | 1 | | 510 | hsa-mir-4651 | 2207 | FCER1G | Fc fragment of IgE, high affinity I, receptor for; gamma polypeptide | 1 | | 511 | hsa-mir-4744 | 2207 | FCER1G | Fc fragment of IgE, high affinity I, receptor for; gamma polypeptide | 1 | | 512 | hsa-mir-5190 | 2207 | FCER1G | Fc fragment of IgE, high affinity I, receptor for; gamma polypeptide | 1 | | 513 | hsa-mir-101-2 | 2207 | FCER1G | Fc fragment of IgE, high affinity I, receptor for; gamma polypeptide | 1 | | 514 | hsa-mir-128-1;hsa-mir-128-2 | 2207 | FCER1G | Fc fragment of IgE, high affinity I, receptor for; gamma polypeptide | 1 | | 515 | hsa-mir-301a | 2207 | FCER1G | Fc fragment of IgE, high affinity I, receptor for; gamma polypeptide | 1 | | 516 | hsa-mir-511-1;hsa-mir-511-2 | 2207 | FCER1G | Fc fragment of IgE, high affinity I, receptor for; gamma polypeptide | 1 | | 517 | hsa-mir-586 | 2207 | FCER1G | Fc fragment of IgE, high affinity I, receptor for; gamma polypeptide | 1 | | 518 | hsa-mir-593 | 2207 | FCER1G | Fc fragment of IgE, high affinity I, receptor for; gamma polypeptide | 1 | | 519 | hsa-mir-620 | 2207 | FCER1G | Fc fragment of IgE, high affinity I, receptor for; gamma polypeptide | 1 | | 520 | hsa-mir-33b | 2207 | FCER1G | Fc fragment of IgE, high affinity I, receptor for; gamma polypeptide | 1 | | 521 | hsa-mir-942 | 2207 | FCER1G | Fc fragment of IgE, high affinity I, receptor for; gamma polypeptide | 1 | | 525 | hsa-mir-302b | 960 | CD44 | CD44 molecule (Indian blood group) | 1 | | 526 | hsa-mir-302c | 960 | CD44 | CD44 molecule (Indian blood group) | 1 | | 527 | hsa-mir-302d | 960 | CD44 | CD44 molecule (Indian blood group) | 1 | | 528 | hsa-mir-367 | 960 | CD44 | CD44 molecule (Indian blood group) | 1 | | 529 | hsa-mir-562 | 960 | CD44 | CD44 molecule (Indian blood group) | 1 | | 530 | hsa-mir-604 | 960 | CD44 | CD44 molecule (Indian blood group) | 1 | | 531 | hsa-mir-548d-1 | 960 | CD44 | CD44 molecule (Indian blood group) | 1 | | 532 | hsa-mir-938 | 960 | CD44 | CD44 molecule (Indian blood group) | 1 | | 533 | hsa-mir-548h-1 | 960 | CD44 | CD44 molecule (Indian blood group) | 1 | | 534 | hsa-mir-3135a | 960 | CD44 | CD44 molecule (Indian blood group) | 1 | | 535 | hsa-mir-3177 | 960 | CD44 | CD44 molecule (Indian blood group) | 1 | | 536 | hsa-mir-3655 | 960 | CD44 | CD44 molecule (Indian blood group) | 1 | | 537 | hsa-mir-4526 | 960 | CD44 | CD44 molecule (Indian blood group) | 1 | | 538 | hsa-mir-4635 | 960 | CD44 | CD44 molecule (Indian blood group) | 1 | | 539 | hsa-mir-4651 | 960 | CD44 | CD44 molecule (Indian blood group) | 1 | | 540 | hsa-mir-4744 | 960 | CD44 | CD44 molecule (Indian blood group) | 1 | | 541 | hsa-mir-5190 | 960 | CD44 | CD44 molecule (Indian blood group) | 1 | | 542 | hsa-mir-101-2 | 960 | CD44 | CD44 molecule (Indian blood group) | 1 | | 543 | hsa-mir-128-1;hsa-mir-128-2 | 960 | CD44 | CD44 molecule (Indian blood group) | 1 | | 544 | hsa-mir-301a | 960 | CD44 | CD44 molecule (Indian blood group) | 1 | | 545 | hsa-mir-511-1;hsa-mir-511-2 | 960 | CD44 | CD44 molecule (Indian blood group) | 1 | | 546 | hsa-mir-586 | 960 | CD44 | CD44 molecule (Indian blood group) | 1 | | 547 | hsa-mir-593 | 960 | CD44 | CD44 molecule (Indian blood group) | 1 | | 548 | hsa-mir-620 | 960 | CD44 | CD44 molecule (Indian blood group) | 1 | | 549 | hsa-mir-33b | 960 | CD44 | CD44 molecule (Indian blood group) | 1 | | 550 | hsa-mir-942 | 960 | CD44 | CD44 molecule (Indian blood group) | 1 | | 554 | hsa-mir-302b | 6402 | SELL | selectin L | 1 | | 555 | hsa-mir-302c | 6402 | SELL | selectin L | 1 | | 556 | hsa-mir-302d | 6402 | SELL | selectin L | 1 | | 557 | hsa-mir-367 | 6402 | SELL | selectin L | 1 | | 558 | hsa-mir-562 | 6402 | SELL | selectin L | 1 | | 559 | hsa-mir-604 | 6402 | SELL | selectin L | 1 | | 560 | hsa-mir-548d-1 | 6402 | SELL | selectin L | 1 | | 561 | hsa-mir-938 | 6402 | SELL | selectin L | 1 | | 562 | hsa-mir-548h-1 | 6402 | SELL | selectin L | 1 | | 563 | hsa-mir-3135a | 6402 | SELL | selectin L | 1 | | 564 | hsa-mir-3177 | 6402 | SELL | selectin L | 1 | | 565 | hsa-mir-3655 | 6402 | SELL | selectin L | 1 | | 566 | hsa-mir-4526 | 6402 | SELL | selectin L | 1 | | 567 | hsa-mir-4635 | 6402 | SELL | selectin L | 1 | | 568 | hsa-mir-4651 | 6402 | SELL | selectin L | 1 | | 569 | hsa-mir-4744 | 6402 | SELL | selectin L | 1 | | 570 | hsa-mir-5190 | 6402 | SELL | selectin L | 1 | | 571 | hsa-mir-101-2 | 6402 | SELL | selectin L | 1 | | 572 | hsa-mir-128-1;hsa-mir-128-2 | 6402 | SELL | selectin L | 1 | | 573 | hsa-mir-301a | 6402 | SELL | selectin L | 1 | | 574 | hsa-mir-511-1;hsa-mir-511-2 | 6402 | SELL | selectin L | 1 | | 575 | hsa-mir-586 | 6402 | SELL | selectin L | 1 | | 576 | hsa-mir-593 | 6402 | SELL | selectin L | 1 | | 577 | hsa-mir-620 | 6402 | SELL | selectin L | 1 | | 578 | hsa-mir-33b | 6402 | SELL | selectin L | 1 | | 579 | hsa-mir-942 | 6402 | SELL | selectin L | 1 | | 583 | hsa-mir-302b | 3376 | IARS | isoleucyl-tRNA synthetase | 1 | | 584 | hsa-mir-302c | 3376 | IARS | isoleucyl-tRNA synthetase | 1 | | 585 | hsa-mir-302d | 3376 | IARS | isoleucyl-tRNA synthetase | 1 | | 586 | hsa-mir-367 | 3376 | IARS | isoleucyl-tRNA synthetase | 1 | | 587 | hsa-mir-562 | 3376 | IARS | isoleucyl-tRNA synthetase | 1 | | 588 | hsa-mir-604 | 3376 | IARS | isoleucyl-tRNA synthetase | 1 | | 589 | hsa-mir-548d-1 | 3376 | IARS | isoleucyl-tRNA synthetase | 1 | | 590 | hsa-mir-938 | 3376 | IARS | isoleucyl-tRNA synthetase | 1 | | 591 | hsa-mir-548h-1 | 3376 | IARS | isoleucyl-tRNA synthetase | 1 | | 592 | hsa-mir-3135a | 3376 | IARS | isoleucyl-tRNA synthetase | 1 | | 593 | hsa-mir-3177 | 3376 | IARS | isoleucyl-tRNA synthetase | 1 | | 594 | hsa-mir-3655 | 3376 | IARS | isoleucyl-tRNA synthetase | 1 | | 595 | hsa-mir-4526 | 3376 | IARS | isoleucyl-tRNA synthetase | 1 | | 596 | hsa-mir-4635 | 3376 | IARS | isoleucyl-tRNA synthetase | 1 | | 597 | hsa-mir-4651 | 3376 | IARS | isoleucyl-tRNA synthetase | 1 | | 598 | hsa-mir-4744 | 3376 | IARS | isoleucyl-tRNA synthetase | 1 | | 599 | hsa-mir-5190 | 3376 | IARS | isoleucyl-tRNA synthetase | 1 | | 600 | hsa-mir-101-2 | 3376 | IARS | isoleucyl-tRNA synthetase | 1 | | 601 | hsa-mir-128-1;hsa-mir-128-2 | 3376 | IARS | isoleucyl-tRNA synthetase | 1 | | 602 | hsa-mir-301a | 3376 | IARS | isoleucyl-tRNA synthetase | 1 | | 603 | hsa-mir-511-1;hsa-mir-511-2 | 3376 | IARS | isoleucyl-tRNA synthetase | 1 | | 604 | hsa-mir-586 | 3376 | IARS | isoleucyl-tRNA synthetase | 1 | | 605 | hsa-mir-593 | 3376 | IARS | isoleucyl-tRNA synthetase | 1 | | 606 | hsa-mir-620 | 3376 | IARS | isoleucyl-tRNA synthetase | 1 | | 607 | hsa-mir-33b | 3376 | IARS | isoleucyl-tRNA synthetase | 1 | | 608 | hsa-mir-942 | 3376 | IARS | isoleucyl-tRNA synthetase | 1 | | 612 | hsa-mir-302b | 921 | CD5 | CD5 molecule | 1 | | 613 | hsa-mir-302c | 921 | CD5 | CD5 molecule | 1 | | 614 | hsa-mir-302d | 921 | CD5 | CD5 molecule | 1 | | 615 | hsa-mir-367 | 921 | CD5 | CD5 molecule | 1 | | 616 | hsa-mir-562 | 921 | CD5 | CD5 molecule | 1 | | 617 | hsa-mir-604 | 921 | CD5 | CD5 molecule | 1 | | 618 | hsa-mir-548d-1 | 921 | CD5 | CD5 molecule | 1 | | 619 | hsa-mir-938 | 921 | CD5 | CD5 molecule | 1 | | 620 | hsa-mir-548h-1 | 921 | CD5 | CD5 molecule | 1 | | 621 | hsa-mir-3135a | 921 | CD5 | CD5 molecule | 1 | | 622 | hsa-mir-3177 | 921 | CD5 | CD5 molecule | 1 | | 623 | hsa-mir-3655 | 921 | CD5 | CD5 molecule | 1 | | 624 | hsa-mir-4526 | 921 | CD5 | CD5 molecule | 1 | | 625 | hsa-mir-4635 | 921 | CD5 | CD5 molecule | 1 | | 626 | hsa-mir-4651 | 921 | CD5 | CD5 molecule | 1 | | 627 | hsa-mir-4744 | 921 | CD5 | CD5 molecule | 1 | | 628 | hsa-mir-5190 | 921 | CD5 | CD5 molecule | 1 | | 629 | hsa-mir-101-2 | 921 | CD5 | CD5 molecule | 1 | | 630 | hsa-mir-128-1;hsa-mir-128-2 | 921 | CD5 | CD5 molecule | 1 | | 631 | hsa-mir-301a | 921 | CD5 | CD5 molecule | 1 | | 632 | hsa-mir-511-1;hsa-mir-511-2 | 921 | CD5 | CD5 molecule | 1 | | 633 | hsa-mir-586 | 921 | CD5 | CD5 molecule | 1 | | 634 | hsa-mir-593 | 921 | CD5 | CD5 molecule | 1 | | 635 | hsa-mir-620 | 921 | CD5 | CD5 molecule | 1 | | 636 | hsa-mir-33b | 921 | CD5 | CD5 molecule | 1 | | 637 | hsa-mir-942 | 921 | CD5 | CD5 molecule | 1 | | 638 | hsa-mir-101-2 | 2672 | GFI1 | growth factor independent 1 transcription repressor | 1 | | 643 | hsa-mir-511-1 | 2672 | GFI1 | growth factor independent 1 transcription repressor | 1 | | 644 | hsa-mir-511-2 | 2672 | GFI1 | growth factor independent 1 transcription repressor | 1 | | 645 | hsa-mir-1284 | 2672 | GFI1 | growth factor independent 1 transcription repressor | 1 | | 646 | hsa-mir-3181 | 2672 | GFI1 | growth factor independent 1 transcription repressor | 1 | | 647 | hsa-mir-3651 | 2672 | GFI1 | growth factor independent 1 transcription repressor | 1 | | 648 | hsa-mir-4632 | 2672 | GFI1 | growth factor independent 1 transcription repressor | 1 | | 649 | hsa-mir-4648 | 2672 | GFI1 | growth factor independent 1 transcription repressor | 1 | | 650 | hsa-mir-4726 | 2672 | GFI1 | growth factor independent 1 transcription repressor | 1 | | 651 | hsa-mir-4785 | 2672 | GFI1 | growth factor independent 1 transcription repressor | 1 | | 652 | hsa-mir-4794 | 2672 | GFI1 | growth factor independent 1 transcription repressor | 1 | | 653 | hsa-mir-4802 | 2672 | GFI1 | growth factor independent 1 transcription repressor | 1 | | 654 | hsa-let-7i | 2672 | GFI1 | growth factor independent 1 transcription repressor | 1 | | 655 | hsa-mir-302b | 2672 | GFI1 | growth factor independent 1 transcription repressor | 1 | | 656 | hsa-mir-302c | 2672 | GFI1 | growth factor independent 1 transcription repressor | 1 | | 657 | hsa-mir-339 | 2672 | GFI1 | growth factor independent 1 transcription repressor | 1 | | 658 | hsa-mir-562 | 2672 | GFI1 | growth factor independent 1 transcription repressor | 1 | | 659 | hsa-mir-567 | 2672 | GFI1 | growth factor independent 1 transcription repressor | 1 | | 660 | hsa-mir-576 | 2672 | GFI1 | growth factor independent 1 transcription repressor | 1 | | 661 | hsa-mir-579 | 2672 | GFI1 | growth factor independent 1 transcription repressor | 1 | | 662 | hsa-mir-582 | 2672 | GFI1 | growth factor independent 1 transcription repressor | 1 | | 663 | hsa-mir-604 | 2672 | GFI1 | growth factor independent 1 transcription repressor | 1 | | 664 | hsa-mir-608 | 2672 | GFI1 | growth factor independent 1 transcription repressor | 1 | | 665 | hsa-mir-621 | 2672 | GFI1 | growth factor independent 1 transcription repressor | 1 | | 666 | hsa-mir-627 | 2672 | GFI1 | growth factor independent 1 transcription repressor | 1 | | 667 | hsa-mir-454 | 2672 | GFI1 | growth factor independent 1 transcription repressor | 1 | | 668 | hsa-mir-302d | 2672 | GFI1 | growth factor independent 1 transcription repressor | 1 | | 669 | hsa-mir-367 | 2672 | GFI1 | growth factor independent 1 transcription repressor | 1 | | 670 | hsa-mir-548b | 2672 | GFI1 | growth factor independent 1 transcription repressor | 1 | | 671 | hsa-mir-548d-1;hsa-mir-548d-2 | 2672 | GFI1 | growth factor independent 1 transcription repressor | 1 | | 672 | hsa-mir-938 | 2672 | GFI1 | growth factor independent 1 transcription repressor | 1 | | 673 | hsa-mir-1236 | 2672 | GFI1 | growth factor independent 1 transcription repressor | 1 | | 677 | hsa-mir-302b | 3357 | HTR2B | 5-hydroxytryptamine (serotonin) receptor 2B, G protein-coupled | 1 | | 678 | hsa-mir-302c | 3357 | HTR2B | 5-hydroxytryptamine (serotonin) receptor 2B, G protein-coupled | 1 | | 679 | hsa-mir-302d | 3357 | HTR2B | 5-hydroxytryptamine (serotonin) receptor 2B, G protein-coupled | 1 | | 680 | hsa-mir-367 | 3357 | HTR2B | 5-hydroxytryptamine (serotonin) receptor 2B, G protein-coupled | 1 | | 681 | hsa-mir-562 | 3357 | HTR2B | 5-hydroxytryptamine (serotonin) receptor 2B, G protein-coupled | 1 | | 682 | hsa-mir-604 | 3357 | HTR2B | 5-hydroxytryptamine (serotonin) receptor 2B, G protein-coupled | 1 | | 683 | hsa-mir-548d-1 | 3357 | HTR2B | 5-hydroxytryptamine (serotonin) receptor 2B, G protein-coupled | 1 | | 684 | hsa-mir-938 | 3357 | HTR2B | 5-hydroxytryptamine (serotonin) receptor 2B, G protein-coupled | 1 | | 685 | hsa-mir-548h-1 | 3357 | HTR2B | 5-hydroxytryptamine (serotonin) receptor 2B, G protein-coupled | 1 | | 686 | hsa-mir-3135a | 3357 | HTR2B | 5-hydroxytryptamine (serotonin) receptor 2B, G protein-coupled | 1 | | 687 | hsa-mir-3177 | 3357 | HTR2B | 5-hydroxytryptamine (serotonin) receptor 2B, G protein-coupled | 1 | | 688 | hsa-mir-3655 | 3357 | HTR2B | 5-hydroxytryptamine (serotonin) receptor 2B, G protein-coupled | 1 | | 689 | hsa-mir-4526 | 3357 | HTR2B | 5-hydroxytryptamine (serotonin) receptor 2B, G protein-coupled | 1 | | 690 | hsa-mir-4635 | 3357 | HTR2B | 5-hydroxytryptamine (serotonin) receptor 2B, G protein-coupled | 1 | | 691 | hsa-mir-4651 | 3357 | HTR2B | 5-hydroxytryptamine (serotonin) receptor 2B, G protein-coupled | 1 | | 692 | hsa-mir-4744 | 3357 | HTR2B | 5-hydroxytryptamine (serotonin) receptor 2B, G protein-coupled | 1 | | 693 | hsa-mir-5190 | 3357 | HTR2B | 5-hydroxytryptamine (serotonin) receptor 2B, G protein-coupled | 1 | | 694 | hsa-mir-101-2 | 3357 | HTR2B | 5-hydroxytryptamine (serotonin) receptor 2B, G protein-coupled | 1 | | 695 | hsa-mir-128-1;hsa-mir-128-2 | 3357 | HTR2B | 5-hydroxytryptamine (serotonin) receptor 2B, G protein-coupled | 1 | | 696 | hsa-mir-301a | 3357 | HTR2B | 5-hydroxytryptamine (serotonin) receptor 2B, G protein-coupled | 1 | | 697 | hsa-mir-511-1;hsa-mir-511-2 | 3357 | HTR2B | 5-hydroxytryptamine (serotonin) receptor 2B, G protein-coupled | 1 | | 698 | hsa-mir-586 | 3357 | HTR2B | 5-hydroxytryptamine (serotonin) receptor 2B, G protein-coupled | 1 | | 699 | hsa-mir-593 | 3357 | HTR2B | 5-hydroxytryptamine (serotonin) receptor 2B, G protein-coupled | 1 | | 700 | hsa-mir-620 | 3357 | HTR2B | 5-hydroxytryptamine (serotonin) receptor 2B, G protein-coupled | 1 | | 701 | hsa-mir-33b | 3357 | HTR2B | 5-hydroxytryptamine (serotonin) receptor 2B, G protein-coupled | 1 | | 702 | hsa-mir-942 | 3357 | HTR2B | 5-hydroxytryptamine (serotonin) receptor 2B, G protein-coupled | 1 | | 706 | hsa-mir-302b | 56341 | PRMT8 | protein arginine methyltransferase 8 | 1 | | 707 | hsa-mir-302c | 56341 | PRMT8 | protein arginine methyltransferase 8 | 1 | | 708 | hsa-mir-302d | 56341 | PRMT8 | protein arginine methyltransferase 8 | 1 | | 709 | hsa-mir-367 | 56341 | PRMT8 | protein arginine methyltransferase 8 | 1 | | 710 | hsa-mir-562 | 56341 | PRMT8 | protein arginine methyltransferase 8 | 1 | | 711 | hsa-mir-604 | 56341 | PRMT8 | protein arginine methyltransferase 8 | 1 | | 712 | hsa-mir-548d-1 | 56341 | PRMT8 | protein arginine methyltransferase 8 | 1 | | 713 | hsa-mir-938 | 56341 | PRMT8 | protein arginine methyltransferase 8 | 1 | | 714 | hsa-mir-548h-1 | 56341 | PRMT8 | protein arginine methyltransferase 8 | 1 | | 715 | hsa-mir-3135a | 56341 | PRMT8 | protein arginine methyltransferase 8 | 1 | | 716 | hsa-mir-3177 | 56341 | PRMT8 | protein arginine methyltransferase 8 | 1 | | 717 | hsa-mir-3655 | 56341 | PRMT8 | protein arginine methyltransferase 8 | 1 | | 718 | hsa-mir-4526 | 56341 | PRMT8 | protein arginine methyltransferase 8 | 1 | | 719 | hsa-mir-4635 | 56341 | PRMT8 | protein arginine methyltransferase 8 | 1 | | 720 | hsa-mir-4651 | 56341 | PRMT8 | protein arginine methyltransferase 8 | 1 | | 721 | hsa-mir-4744 | 56341 | PRMT8 | protein arginine methyltransferase 8 | 1 | | 722 | hsa-mir-5190 | 56341 | PRMT8 | protein arginine methyltransferase 8 | 1 | | 723 | hsa-mir-101-2 | 56341 | PRMT8 | protein arginine methyltransferase 8 | 1 | | 724 | hsa-mir-128-1;hsa-mir-128-2 | 56341 | PRMT8 | protein arginine methyltransferase 8 | 1 | | 725 | hsa-mir-301a | 56341 | PRMT8 | protein arginine methyltransferase 8 | 1 | | 726 | hsa-mir-511-1;hsa-mir-511-2 | 56341 | PRMT8 | protein arginine methyltransferase 8 | 1 | | 727 | hsa-mir-586 | 56341 | PRMT8 | protein arginine methyltransferase 8 | 1 | | 728 | hsa-mir-593 | 56341 | PRMT8 | protein arginine methyltransferase 8 | 1 | | 729 | hsa-mir-620 | 56341 | PRMT8 | protein arginine methyltransferase 8 | 1 | | 730 | hsa-mir-33b | 56341 | PRMT8 | protein arginine methyltransferase 8 | 1 | | 731 | hsa-mir-942 | 56341 | PRMT8 | protein arginine methyltransferase 8 | 1 | | 735 | hsa-mir-302b | 87 | ACTN1 | actinin, alpha 1 | 1 | | 736 | hsa-mir-302c | 87 | ACTN1 | actinin, alpha 1 | 1 | | 737 | hsa-mir-302d | 87 | ACTN1 | actinin, alpha 1 | 1 | | 738 | hsa-mir-367 | 87 | ACTN1 | actinin, alpha 1 | 1 | | 739 | hsa-mir-562 | 87 | ACTN1 | actinin, alpha 1 | 1 | | 740 | hsa-mir-604 | 87 | ACTN1 | actinin, alpha 1 | 1 | | 741 | hsa-mir-548d-1 | 87 | ACTN1 | actinin, alpha 1 | 1 | | 742 | hsa-mir-938 | 87 | ACTN1 | actinin, alpha 1 | 1 | | 743 | hsa-mir-548h-1 | 87 | ACTN1 | actinin, alpha 1 | 1 | | 744 | hsa-mir-3135a | 87 | ACTN1 | actinin, alpha 1 | 1 | | 745 | hsa-mir-3177 | 87 | ACTN1 | actinin, alpha 1 | 1 | | 746 | hsa-mir-3655 | 87 | ACTN1 | actinin, alpha 1 | 1 | | 747 | hsa-mir-4526 | 87 | ACTN1 | actinin, alpha 1 | 1 | | 748 | hsa-mir-4635 | 87 | ACTN1 | actinin, alpha 1 | 1 | | 749 | hsa-mir-4651 | 87 | ACTN1 | actinin, alpha 1 | 1 | | 750 | hsa-mir-4744 | 87 | ACTN1 | actinin, alpha 1 | 1 | | 751 | hsa-mir-5190 | 87 | ACTN1 | actinin, alpha 1 | 1 | | 752 | hsa-mir-101-2 | 87 | ACTN1 | actinin, alpha 1 | 1 | | 753 | hsa-mir-128-1;hsa-mir-128-2 | 87 | ACTN1 | actinin, alpha 1 | 1 | | 754 | hsa-mir-301a | 87 | ACTN1 | actinin, alpha 1 | 1 | | 755 | hsa-mir-511-1;hsa-mir-511-2 | 87 | ACTN1 | actinin, alpha 1 | 1 | | 756 | hsa-mir-586 | 87 | ACTN1 | actinin, alpha 1 | 1 | | 757 | hsa-mir-593 | 87 | ACTN1 | actinin, alpha 1 | 1 | | 758 | hsa-mir-620 | 87 | ACTN1 | actinin, alpha 1 | 1 | | 759 | hsa-mir-33b | 87 | ACTN1 | actinin, alpha 1 | 1 | | 760 | hsa-mir-942 | 87 | ACTN1 | actinin, alpha 1 | 1 | | 764 | hsa-mir-302b | 998 | CDC42 | cell division cycle 42 (GTP binding protein, 25kDa) | 1 | | 765 | hsa-mir-302c | 998 | CDC42 | cell division cycle 42 (GTP binding protein, 25kDa) | 1 | | 766 | hsa-mir-302d | 998 | CDC42 | cell division cycle 42 (GTP binding protein, 25kDa) | 1 | | 767 | hsa-mir-367 | 998 | CDC42 | cell division cycle 42 (GTP binding protein, 25kDa) | 1 | | 768 | hsa-mir-562 | 998 | CDC42 | cell division cycle 42 (GTP binding protein, 25kDa) | 1 | | 769 | hsa-mir-604 | 998 | CDC42 | cell division cycle 42 (GTP binding protein, 25kDa) | 1 | | 770 | hsa-mir-548d-1 | 998 | CDC42 | cell division cycle 42 (GTP binding protein, 25kDa) | 1 | | 771 | hsa-mir-938 | 998 | CDC42 | cell division cycle 42 (GTP binding protein, 25kDa) | 1 | | 772 | hsa-mir-548h-1 | 998 | CDC42 | cell division cycle 42 (GTP binding protein, 25kDa) | 1 | | 773 | hsa-mir-3135a | 998 | CDC42 | cell division cycle 42 (GTP binding protein, 25kDa) | 1 | | 774 | hsa-mir-3177 | 998 | CDC42 | cell division cycle 42 (GTP binding protein, 25kDa) | 1 | | 775 | hsa-mir-3655 | 998 | CDC42 | cell division cycle 42 (GTP binding protein, 25kDa) | 1 | | 776 | hsa-mir-4526 | 998 | CDC42 | cell division cycle 42 (GTP binding protein, 25kDa) | 1 | | 777 | hsa-mir-4635 | 998 | CDC42 | cell division cycle 42 (GTP binding protein, 25kDa) | 1 | | 778 | hsa-mir-4651 | 998 | CDC42 | cell division cycle 42 (GTP binding protein, 25kDa) | 1 | | 779 | hsa-mir-4744 | 998 | CDC42 | cell division cycle 42 (GTP binding protein, 25kDa) | 1 | | 780 | hsa-mir-5190 | 998 | CDC42 | cell division cycle 42 (GTP binding protein, 25kDa) | 1 | | 781 | hsa-mir-101-2 | 998 | CDC42 | cell division cycle 42 (GTP binding protein, 25kDa) | 1 | | 782 | hsa-mir-128-1;hsa-mir-128-2 | 998 | CDC42 | cell division cycle 42 (GTP binding protein, 25kDa) | 1 | | 783 | hsa-mir-301a | 998 | CDC42 | cell division cycle 42 (GTP binding protein, 25kDa) | 1 | | 784 | hsa-mir-511-1;hsa-mir-511-2 | 998 | CDC42 | cell division cycle 42 (GTP binding protein, 25kDa) | 1 | | 785 | hsa-mir-586 | 998 | CDC42 | cell division cycle 42 (GTP binding protein, 25kDa) | 1 | | 786 | hsa-mir-593 | 998 | CDC42 | cell division cycle 42 (GTP binding protein, 25kDa) | 1 | | 787 | hsa-mir-620 | 998 | CDC42 | cell division cycle 42 (GTP binding protein, 25kDa) | 1 | | 788 | hsa-mir-33b | 998 | CDC42 | cell division cycle 42 (GTP binding protein, 25kDa) | 1 | | 789 | hsa-mir-942 | 998 | CDC42 | cell division cycle 42 (GTP binding protein, 25kDa) | 1 | | 822 | hsa-mir-302b | 3106 | HLA-B | major histocompatibility complex, class I, B | 1 | | 823 | hsa-mir-302c | 3106 | HLA-B | major histocompatibility complex, class I, B | 1 | | 824 | hsa-mir-302d | 3106 | HLA-B | major histocompatibility complex, class I, B | 1 | | 825 | hsa-mir-367 | 3106 | HLA-B | major histocompatibility complex, class I, B | 1 | | 826 | hsa-mir-562 | 3106 | HLA-B | major histocompatibility complex, class I, B | 1 | | 827 | hsa-mir-604 | 3106 | HLA-B | major histocompatibility complex, class I, B | 1 | | 828 | hsa-mir-548d-1 | 3106 | HLA-B | major histocompatibility complex, class I, B | 1 | | 829 | hsa-mir-938 | 3106 | HLA-B | major histocompatibility complex, class I, B | 1 | | 830 | hsa-mir-548h-1 | 3106 | HLA-B | major histocompatibility complex, class I, B | 1 | | 831 | hsa-mir-3135a | 3106 | HLA-B | major histocompatibility complex, class I, B | 1 | | 832 | hsa-mir-3177 | 3106 | HLA-B | major histocompatibility complex, class I, B | 1 | | 833 | hsa-mir-3655 | 3106 | HLA-B | major histocompatibility complex, class I, B | 1 | | 834 | hsa-mir-4526 | 3106 | HLA-B | major histocompatibility complex, class I, B | 1 | | 835 | hsa-mir-4635 | 3106 | HLA-B | major histocompatibility complex, class I, B | 1 | | 836 | hsa-mir-4651 | 3106 | HLA-B | major histocompatibility complex, class I, B | 1 | | 837 | hsa-mir-4744 | 3106 | HLA-B | major histocompatibility complex, class I, B | 1 | | 838 | hsa-mir-5190 | 3106 | HLA-B | major histocompatibility complex, class I, B | 1 | | 839 | hsa-mir-101-2 | 3106 | HLA-B | major histocompatibility complex, class I, B | 1 | | 840 | hsa-mir-128-1;hsa-mir-128-2 | 3106 | HLA-B | major histocompatibility complex, class I, B | 1 | | 841 | hsa-mir-301a | 3106 | HLA-B | major histocompatibility complex, class I, B | 1 | | 842 | hsa-mir-511-1;hsa-mir-511-2 | 3106 | HLA-B | major histocompatibility complex, class I, B | 1 | | 843 | hsa-mir-586 | 3106 | HLA-B | major histocompatibility complex, class I, B | 1 | | 844 | hsa-mir-593 | 3106 | HLA-B | major histocompatibility complex, class I, B | 1 | | 845 | hsa-mir-620 | 3106 | HLA-B | major histocompatibility complex, class I, B | 1 | | 846 | hsa-mir-33b | 3106 | HLA-B | major histocompatibility complex, class I, B | 1 | | 847 | hsa-mir-942 | 3106 | HLA-B | major histocompatibility complex, class I, B | 1 | | 851 | hsa-mir-302b | 9277 | WDR46 | WD repeat domain 46 | 1 | | 852 | hsa-mir-302c | 9277 | WDR46 | WD repeat domain 46 | 1 | | 853 | hsa-mir-302d | 9277 | WDR46 | WD repeat domain 46 | 1 | | 854 | hsa-mir-367 | 9277 | WDR46 | WD repeat domain 46 | 1 | | 855 | hsa-mir-562 | 9277 | WDR46 | WD repeat domain 46 | 1 | | 856 | hsa-mir-604 | 9277 | WDR46 | WD repeat domain 46 | 1 | | 857 | hsa-mir-548d-1 | 9277 | WDR46 | WD repeat domain 46 | 1 | | 858 | hsa-mir-938 | 9277 | WDR46 | WD repeat domain 46 | 1 | | 859 | hsa-mir-548h-1 | 9277 | WDR46 | WD repeat domain 46 | 1 | | 860 | hsa-mir-3135a | 9277 | WDR46 | WD repeat domain 46 | 1 | | 861 | hsa-mir-3177 | 9277 | WDR46 | WD repeat domain 46 | 1 | | 862 | hsa-mir-3655 | 9277 | WDR46 | WD repeat domain 46 | 1 | | 863 | hsa-mir-4526 | 9277 | WDR46 | WD repeat domain 46 | 1 | | 864 | hsa-mir-4635 | 9277 | WDR46 | WD repeat domain 46 | 1 | | 865 | hsa-mir-4651 | 9277 | WDR46 | WD repeat domain 46 | 1 | | 866 | hsa-mir-4744 | 9277 | WDR46 | WD repeat domain 46 | 1 | | 867 | hsa-mir-5190 | 9277 | WDR46 | WD repeat domain 46 | 1 | | 868 | hsa-mir-101-2 | 9277 | WDR46 | WD repeat domain 46 | 1 | | 869 | hsa-mir-128-1;hsa-mir-128-2 | 9277 | WDR46 | WD repeat domain 46 | 1 | | 870 | hsa-mir-301a | 9277 | WDR46 | WD repeat domain 46 | 1 | | 871 | hsa-mir-511-1;hsa-mir-511-2 | 9277 | WDR46 | WD repeat domain 46 | 1 | | 872 | hsa-mir-586 | 9277 | WDR46 | WD repeat domain 46 | 1 | | 873 | hsa-mir-593 | 9277 | WDR46 | WD repeat domain 46 | 1 | | 874 | hsa-mir-620 | 9277 | WDR46 | WD repeat domain 46 | 1 | | 875 | hsa-mir-33b | 9277 | WDR46 | WD repeat domain 46 | 1 | | 876 | hsa-mir-942 | 9277 | WDR46 | WD repeat domain 46 | 1 | | 880 | hsa-mir-302b | 9533 | POLR1C | polymerase (RNA) I polypeptide C, 30kDa | 1 | | 881 | hsa-mir-302c | 9533 | POLR1C | polymerase (RNA) I polypeptide C, 30kDa | 1 | | 882 | hsa-mir-302d | 9533 | POLR1C | polymerase (RNA) I polypeptide C, 30kDa | 1 | | 883 | hsa-mir-367 | 9533 | POLR1C | polymerase (RNA) I polypeptide C, 30kDa | 1 | | 884 | hsa-mir-562 | 9533 | POLR1C | polymerase (RNA) I polypeptide C, 30kDa | 1 | | 885 | hsa-mir-604 | 9533 | POLR1C | polymerase (RNA) I polypeptide C, 30kDa | 1 | | 886 | hsa-mir-548d-1 | 9533 | POLR1C | polymerase (RNA) I polypeptide C, 30kDa | 1 | | 887 | hsa-mir-938 | 9533 | POLR1C | polymerase (RNA) I polypeptide C, 30kDa | 1 | | 888 | hsa-mir-548h-1 | 9533 | POLR1C | polymerase (RNA) I polypeptide C, 30kDa | 1 | | 889 | hsa-mir-3135a | 9533 | POLR1C | polymerase (RNA) I polypeptide C, 30kDa | 1 | | 890 | hsa-mir-3177 | 9533 | POLR1C | polymerase (RNA) I polypeptide C, 30kDa | 1 | | 891 | hsa-mir-3655 | 9533 | POLR1C | polymerase (RNA) I polypeptide C, 30kDa | 1 | | 892 | hsa-mir-4526 | 9533 | POLR1C | polymerase (RNA) I polypeptide C, 30kDa | 1 | | 893 | hsa-mir-4635 | 9533 | POLR1C | polymerase (RNA) I polypeptide C, 30kDa | 1 | | 894 | hsa-mir-4651 | 9533 | POLR1C | polymerase (RNA) I polypeptide C, 30kDa | 1 | | 895 | hsa-mir-4744 | 9533 | POLR1C | polymerase (RNA) I polypeptide C, 30kDa | 1 | | 896 | hsa-mir-5190 | 9533 | POLR1C | polymerase (RNA) I polypeptide C, 30kDa | 1 | | 897 | hsa-mir-101-2 | 9533 | POLR1C | polymerase (RNA) I polypeptide C, 30kDa | 1 | | 898 | hsa-mir-128-1;hsa-mir-128-2 | 9533 | POLR1C | polymerase (RNA) I polypeptide C, 30kDa | 1 | | 899 | hsa-mir-301a | 9533 | POLR1C | polymerase (RNA) I polypeptide C, 30kDa | 1 | | 900 | hsa-mir-511-1;hsa-mir-511-2 | 9533 | POLR1C | polymerase (RNA) I polypeptide C, 30kDa | 1 | | 901 | hsa-mir-586 | 9533 | POLR1C | polymerase (RNA) I polypeptide C, 30kDa | 1 | | 902 | hsa-mir-593 | 9533 | POLR1C | polymerase (RNA) I polypeptide C, 30kDa | 1 | | 903 | hsa-mir-620 | 9533 | POLR1C | polymerase (RNA) I polypeptide C, 30kDa | 1 | | 904 | hsa-mir-33b | 9533 | POLR1C | polymerase (RNA) I polypeptide C, 30kDa | 1 | | 905 | hsa-mir-942 | 9533 | POLR1C | polymerase (RNA) I polypeptide C, 30kDa | 1 | | 909 | hsa-mir-302b | 8728 | ADAM19 | ADAM metallopeptidase domain 19 | 1 | | 910 | hsa-mir-302c | 8728 | ADAM19 | ADAM metallopeptidase domain 19 | 1 | | 911 | hsa-mir-302d | 8728 | ADAM19 | ADAM metallopeptidase domain 19 | 1 | | 912 | hsa-mir-367 | 8728 | ADAM19 | ADAM metallopeptidase domain 19 | 1 | | 913 | hsa-mir-562 | 8728 | ADAM19 | ADAM metallopeptidase domain 19 | 1 | | 914 | hsa-mir-604 | 8728 | ADAM19 | ADAM metallopeptidase domain 19 | 1 | | 915 | hsa-mir-548d-1 | 8728 | ADAM19 | ADAM metallopeptidase domain 19 | 1 | | 916 | hsa-mir-938 | 8728 | ADAM19 | ADAM metallopeptidase domain 19 | 1 | | 917 | hsa-mir-548h-1 | 8728 | ADAM19 | ADAM metallopeptidase domain 19 | 1 | | 918 | hsa-mir-3135a | 8728 | ADAM19 | ADAM metallopeptidase domain 19 | 1 | | 919 | hsa-mir-3177 | 8728 | ADAM19 | ADAM metallopeptidase domain 19 | 1 | | 920 | hsa-mir-3655 | 8728 | ADAM19 | ADAM metallopeptidase domain 19 | 1 | | 921 | hsa-mir-4526 | 8728 | ADAM19 | ADAM metallopeptidase domain 19 | 1 | | 922 | hsa-mir-4635 | 8728 | ADAM19 | ADAM metallopeptidase domain 19 | 1 | | 923 | hsa-mir-4651 | 8728 | ADAM19 | ADAM metallopeptidase domain 19 | 1 | | 924 | hsa-mir-4744 | 8728 | ADAM19 | ADAM metallopeptidase domain 19 | 1 | | 925 | hsa-mir-5190 | 8728 | ADAM19 | ADAM metallopeptidase domain 19 | 1 | | 926 | hsa-mir-101-2 | 8728 | ADAM19 | ADAM metallopeptidase domain 19 | 1 | | 927 | hsa-mir-128-1;hsa-mir-128-2 | 8728 | ADAM19 | ADAM metallopeptidase domain 19 | 1 | | 928 | hsa-mir-301a | 8728 | ADAM19 | ADAM metallopeptidase domain 19 | 1 | | 929 | hsa-mir-511-1;hsa-mir-511-2 | 8728 | ADAM19 | ADAM metallopeptidase domain 19 | 1 | | 930 | hsa-mir-586 | 8728 | ADAM19 | ADAM metallopeptidase domain 19 | 1 | | 931 | hsa-mir-593 | 8728 | ADAM19 | ADAM metallopeptidase domain 19 | 1 | | 932 | hsa-mir-620 | 8728 | ADAM19 | ADAM metallopeptidase domain 19 | 1 | | 933 | hsa-mir-33b | 8728 | ADAM19 | ADAM metallopeptidase domain 19 | 1 | | 934 | hsa-mir-942 | 8728 | ADAM19 | ADAM metallopeptidase domain 19 | 1 | | 938 | hsa-mir-302b | 834 | CASP1 | caspase 1, apoptosis-related cysteine peptidase | 1 | | 939 | hsa-mir-302c | 834 | CASP1 | caspase 1, apoptosis-related cysteine peptidase | 1 | | 940 | hsa-mir-302d | 834 | CASP1 | caspase 1, apoptosis-related cysteine peptidase | 1 | | 941 | hsa-mir-367 | 834 | CASP1 | caspase 1, apoptosis-related cysteine peptidase | 1 | | 942 | hsa-mir-562 | 834 | CASP1 | caspase 1, apoptosis-related cysteine peptidase | 1 | | 943 | hsa-mir-604 | 834 | CASP1 | caspase 1, apoptosis-related cysteine peptidase | 1 | | 944 | hsa-mir-548d-1 | 834 | CASP1 | caspase 1, apoptosis-related cysteine peptidase | 1 | | 945 | hsa-mir-938 | 834 | CASP1 | caspase 1, apoptosis-related cysteine peptidase | 1 | | 946 | hsa-mir-548h-1 | 834 | CASP1 | caspase 1, apoptosis-related cysteine peptidase | 1 | | 947 | hsa-mir-3135a | 834 | CASP1 | caspase 1, apoptosis-related cysteine peptidase | 1 | | 948 | hsa-mir-3177 | 834 | CASP1 | caspase 1, apoptosis-related cysteine peptidase | 1 | | 949 | hsa-mir-3655 | 834 | CASP1 | caspase 1, apoptosis-related cysteine peptidase | 1 | | 950 | hsa-mir-4526 | 834 | CASP1 | caspase 1, apoptosis-related cysteine peptidase | 1 | | 951 | hsa-mir-4635 | 834 | CASP1 | caspase 1, apoptosis-related cysteine peptidase | 1 | | 952 | hsa-mir-4651 | 834 | CASP1 | caspase 1, apoptosis-related cysteine peptidase | 1 | | 953 | hsa-mir-4744 | 834 | CASP1 | caspase 1, apoptosis-related cysteine peptidase | 1 | | 954 | hsa-mir-5190 | 834 | CASP1 | caspase 1, apoptosis-related cysteine peptidase | 1 | | 955 | hsa-mir-101-2 | 834 | CASP1 | caspase 1, apoptosis-related cysteine peptidase | 1 | | 956 | hsa-mir-128-1;hsa-mir-128-2 | 834 | CASP1 | caspase 1, apoptosis-related cysteine peptidase | 1 | | 957 | hsa-mir-301a | 834 | CASP1 | caspase 1, apoptosis-related cysteine peptidase | 1 | | 958 | hsa-mir-511-1;hsa-mir-511-2 | 834 | CASP1 | caspase 1, apoptosis-related cysteine peptidase | 1 | | 959 | hsa-mir-586 | 834 | CASP1 | caspase 1, apoptosis-related cysteine peptidase | 1 | | 960 | hsa-mir-593 | 834 | CASP1 | caspase 1, apoptosis-related cysteine peptidase | 1 | | 961 | hsa-mir-620 | 834 | CASP1 | caspase 1, apoptosis-related cysteine peptidase | 1 | | 962 | hsa-mir-33b | 834 | CASP1 | caspase 1, apoptosis-related cysteine peptidase | 1 | | 963 | hsa-mir-942 | 834 | CASP1 | caspase 1, apoptosis-related cysteine peptidase | 1 | | 967 | hsa-mir-302b | 5243 | ABCB1 | ATP-binding cassette, sub-family B (MDR/TAP), member 1 | 1 | | 968 | hsa-mir-302c | 5243 | ABCB1 | ATP-binding cassette, sub-family B (MDR/TAP), member 1 | 1 | | 969 | hsa-mir-302d | 5243 | ABCB1 | ATP-binding cassette, sub-family B (MDR/TAP), member 1 | 1 | | 970 | hsa-mir-367 | 5243 | ABCB1 | ATP-binding cassette, sub-family B (MDR/TAP), member 1 | 1 | | 971 | hsa-mir-562 | 5243 | ABCB1 | ATP-binding cassette, sub-family B (MDR/TAP), member 1 | 1 | | 972 | hsa-mir-604 | 5243 | ABCB1 | ATP-binding cassette, sub-family B (MDR/TAP), member 1 | 1 | | 973 | hsa-mir-548d-1 | 5243 | ABCB1 | ATP-binding cassette, sub-family B (MDR/TAP), member 1 | 1 | | 974 | hsa-mir-938 | 5243 | ABCB1 | ATP-binding cassette, sub-family B (MDR/TAP), member 1 | 1 | | 975 | hsa-mir-548h-1 | 5243 | ABCB1 | ATP-binding cassette, sub-family B (MDR/TAP), member 1 | 1 | | 976 | hsa-mir-3135a | 5243 | ABCB1 | ATP-binding cassette, sub-family B (MDR/TAP), member 1 | 1 | | 977 | hsa-mir-3177 | 5243 | ABCB1 | ATP-binding cassette, sub-family B (MDR/TAP), member 1 | 1 | | 978 | hsa-mir-3655 | 5243 | ABCB1 | ATP-binding cassette, sub-family B (MDR/TAP), member 1 | 1 | | 979 | hsa-mir-4526 | 5243 | ABCB1 | ATP-binding cassette, sub-family B (MDR/TAP), member 1 | 1 | | 980 | hsa-mir-4635 | 5243 | ABCB1 | ATP-binding cassette, sub-family B (MDR/TAP), member 1 | 1 | | 981 | hsa-mir-4651 | 5243 | ABCB1 | ATP-binding cassette, sub-family B (MDR/TAP), member 1 | 1 | | 982 | hsa-mir-4744 | 5243 | ABCB1 | ATP-binding cassette, sub-family B (MDR/TAP), member 1 | 1 | | 983 | hsa-mir-5190 | 5243 | ABCB1 | ATP-binding cassette, sub-family B (MDR/TAP), member 1 | 1 | | 984 | hsa-mir-101-2 | 5243 | ABCB1 | ATP-binding cassette, sub-family B (MDR/TAP), member 1 | 1 | | 985 | hsa-mir-128-1;hsa-mir-128-2 | 5243 | ABCB1 | ATP-binding cassette, sub-family B (MDR/TAP), member 1 | 1 | | 986 | hsa-mir-301a | 5243 | ABCB1 | ATP-binding cassette, sub-family B (MDR/TAP), member 1 | 1 | | 987 | hsa-mir-511-1;hsa-mir-511-2 | 5243 | ABCB1 | ATP-binding cassette, sub-family B (MDR/TAP), member 1 | 1 | | 988 | hsa-mir-586 | 5243 | ABCB1 | ATP-binding cassette, sub-family B (MDR/TAP), member 1 | 1 | | 989 | hsa-mir-593 | 5243 | ABCB1 | ATP-binding cassette, sub-family B (MDR/TAP), member 1 | 1 | | 990 | hsa-mir-620 | 5243 | ABCB1 | ATP-binding cassette, sub-family B (MDR/TAP), member 1 | 1 | | 991 | hsa-mir-33b | 5243 | ABCB1 | ATP-binding cassette, sub-family B (MDR/TAP), member 1 | 1 | | 992 | hsa-mir-942 | 5243 | ABCB1 | ATP-binding cassette, sub-family B (MDR/TAP), member 1 | 1 | | 993 | hsa-mir-101-2 | 4068 | SH2D1A | SH2 domain containing 1A | 1 | | 998 | hsa-mir-511-1 | 4068 | SH2D1A | SH2 domain containing 1A | 1 | | 999 | hsa-mir-511-2 | 4068 | SH2D1A | SH2 domain containing 1A | 1 | | 1000 | hsa-mir-1284 | 4068 | SH2D1A | SH2 domain containing 1A | 1 | | 1001 | hsa-mir-3181 | 4068 | SH2D1A | SH2 domain containing 1A | 1 | | 1002 | hsa-mir-3651 | 4068 | SH2D1A | SH2 domain containing 1A | 1 | | 1003 | hsa-mir-4632 | 4068 | SH2D1A | SH2 domain containing 1A | 1 | | 1004 | hsa-mir-4648 | 4068 | SH2D1A | SH2 domain containing 1A | 1 | | 1005 | hsa-mir-4726 | 4068 | SH2D1A | SH2 domain containing 1A | 1 | | 1006 | hsa-mir-4785 | 4068 | SH2D1A | SH2 domain containing 1A | 1 | | 1007 | hsa-mir-4794 | 4068 | SH2D1A | SH2 domain containing 1A | 1 | | 1008 | hsa-mir-4802 | 4068 | SH2D1A | SH2 domain containing 1A | 1 | | 1009 | hsa-let-7i | 4068 | SH2D1A | SH2 domain containing 1A | 1 | | 1010 | hsa-mir-302b | 4068 | SH2D1A | SH2 domain containing 1A | 1 | | 1011 | hsa-mir-302c | 4068 | SH2D1A | SH2 domain containing 1A | 1 | | 1012 | hsa-mir-339 | 4068 | SH2D1A | SH2 domain containing 1A | 1 | | 1013 | hsa-mir-562 | 4068 | SH2D1A | SH2 domain containing 1A | 1 | | 1014 | hsa-mir-567 | 4068 | SH2D1A | SH2 domain containing 1A | 1 | | 1015 | hsa-mir-576 | 4068 | SH2D1A | SH2 domain containing 1A | 1 | | 1016 | hsa-mir-579 | 4068 | SH2D1A | SH2 domain containing 1A | 1 | | 1017 | hsa-mir-582 | 4068 | SH2D1A | SH2 domain containing 1A | 1 | | 1018 | hsa-mir-604 | 4068 | SH2D1A | SH2 domain containing 1A | 1 | | 1019 | hsa-mir-608 | 4068 | SH2D1A | SH2 domain containing 1A | 1 | | 1020 | hsa-mir-621 | 4068 | SH2D1A | SH2 domain containing 1A | 1 | | 1021 | hsa-mir-627 | 4068 | SH2D1A | SH2 domain containing 1A | 1 | | 1022 | hsa-mir-454 | 4068 | SH2D1A | SH2 domain containing 1A | 1 | | 1023 | hsa-mir-302d | 4068 | SH2D1A | SH2 domain containing 1A | 1 | | 1024 | hsa-mir-367 | 4068 | SH2D1A | SH2 domain containing 1A | 1 | | 1025 | hsa-mir-548b | 4068 | SH2D1A | SH2 domain containing 1A | 1 | | 1026 | hsa-mir-548d-1;hsa-mir-548d-2 | 4068 | SH2D1A | SH2 domain containing 1A | 1 | | 1027 | hsa-mir-938 | 4068 | SH2D1A | SH2 domain containing 1A | 1 | | 1028 | hsa-mir-1236 | 4068 | SH2D1A | SH2 domain containing 1A | 1 | | 1032 | hsa-mir-302b | 55621 | TRMT1 | TRM1 tRNA methyltransferase 1 homolog (S. cerevisiae) | 1 | | 1033 | hsa-mir-302c | 55621 | TRMT1 | TRM1 tRNA methyltransferase 1 homolog (S. cerevisiae) | 1 | | 1034 | hsa-mir-302d | 55621 | TRMT1 | TRM1 tRNA methyltransferase 1 homolog (S. cerevisiae) | 1 | | 1035 | hsa-mir-367 | 55621 | TRMT1 | TRM1 tRNA methyltransferase 1 homolog (S. cerevisiae) | 1 | | 1036 | hsa-mir-562 | 55621 | TRMT1 | TRM1 tRNA methyltransferase 1 homolog (S. cerevisiae) | 1 | | 1037 | hsa-mir-604 | 55621 | TRMT1 | TRM1 tRNA methyltransferase 1 homolog (S. cerevisiae) | 1 | | 1038 | hsa-mir-548d-1 | 55621 | TRMT1 | TRM1 tRNA methyltransferase 1 homolog (S. cerevisiae) | 1 | | 1039 | hsa-mir-938 | 55621 | TRMT1 | TRM1 tRNA methyltransferase 1 homolog (S. cerevisiae) | 1 | | 1040 | hsa-mir-548h-1 | 55621 | TRMT1 | TRM1 tRNA methyltransferase 1 homolog (S. cerevisiae) | 1 | | 1041 | hsa-mir-3135a | 55621 | TRMT1 | TRM1 tRNA methyltransferase 1 homolog (S. cerevisiae) | 1 | | 1042 | hsa-mir-3177 | 55621 | TRMT1 | TRM1 tRNA methyltransferase 1 homolog (S. cerevisiae) | 1 | | 1043 | hsa-mir-3655 | 55621 | TRMT1 | TRM1 tRNA methyltransferase 1 homolog (S. cerevisiae) | 1 | | 1044 | hsa-mir-4526 | 55621 | TRMT1 | TRM1 tRNA methyltransferase 1 homolog (S. cerevisiae) | 1 | | 1045 | hsa-mir-4635 | 55621 | TRMT1 | TRM1 tRNA methyltransferase 1 homolog (S. cerevisiae) | 1 | | 1046 | hsa-mir-4651 | 55621 | TRMT1 | TRM1 tRNA methyltransferase 1 homolog (S. cerevisiae) | 1 | | 1047 | hsa-mir-4744 | 55621 | TRMT1 | TRM1 tRNA methyltransferase 1 homolog (S. cerevisiae) | 1 | | 1048 | hsa-mir-5190 | 55621 | TRMT1 | TRM1 tRNA methyltransferase 1 homolog (S. cerevisiae) | 1 | | 1049 | hsa-mir-101-2 | 55621 | TRMT1 | TRM1 tRNA methyltransferase 1 homolog (S. cerevisiae) | 1 | | 1050 | hsa-mir-128-1;hsa-mir-128-2 | 55621 | TRMT1 | TRM1 tRNA methyltransferase 1 homolog (S. cerevisiae) | 1 | | 1051 | hsa-mir-301a | 55621 | TRMT1 | TRM1 tRNA methyltransferase 1 homolog (S. cerevisiae) | 1 | | 1052 | hsa-mir-511-1;hsa-mir-511-2 | 55621 | TRMT1 | TRM1 tRNA methyltransferase 1 homolog (S. cerevisiae) | 1 | | 1053 | hsa-mir-586 | 55621 | TRMT1 | TRM1 tRNA methyltransferase 1 homolog (S. cerevisiae) | 1 | | 1054 | hsa-mir-593 | 55621 | TRMT1 | TRM1 tRNA methyltransferase 1 homolog (S. cerevisiae) | 1 | | 1055 | hsa-mir-620 | 55621 | TRMT1 | TRM1 tRNA methyltransferase 1 homolog (S. cerevisiae) | 1 | | 1056 | hsa-mir-33b | 55621 | TRMT1 | TRM1 tRNA methyltransferase 1 homolog (S. cerevisiae) | 1 | | 1057 | hsa-mir-942 | 55621 | TRMT1 | TRM1 tRNA methyltransferase 1 homolog (S. cerevisiae) | 1 | | 1058 | hsa-mir-101-2 | 4690 | NCK1 | NCK adaptor protein 1 | 1 | | 1063 | hsa-mir-511-1 | 4690 | NCK1 | NCK adaptor protein 1 | 1 | | 1064 | hsa-mir-511-2 | 4690 | NCK1 | NCK adaptor protein 1 | 1 | | 1065 | hsa-mir-1284 | 4690 | NCK1 | NCK adaptor protein 1 | 1 | | 1066 | hsa-mir-3181 | 4690 | NCK1 | NCK adaptor protein 1 | 1 | | 1067 | hsa-mir-3651 | 4690 | NCK1 | NCK adaptor protein 1 | 1 | | 1068 | hsa-mir-4632 | 4690 | NCK1 | NCK adaptor protein 1 | 1 | | 1069 | hsa-mir-4648 | 4690 | NCK1 | NCK adaptor protein 1 | 1 | | 1070 | hsa-mir-4726 | 4690 | NCK1 | NCK adaptor protein 1 | 1 | | 1071 | hsa-mir-4785 | 4690 | NCK1 | NCK adaptor protein 1 | 1 | | 1072 | hsa-mir-4794 | 4690 | NCK1 | NCK adaptor protein 1 | 1 | | 1073 | hsa-mir-4802 | 4690 | NCK1 | NCK adaptor protein 1 | 1 | | 1074 | hsa-let-7i | 4690 | NCK1 | NCK adaptor protein 1 | 1 | | 1075 | hsa-mir-302b | 4690 | NCK1 | NCK adaptor protein 1 | 1 | | 1076 | hsa-mir-302c | 4690 | NCK1 | NCK adaptor protein 1 | 1 | | 1077 | hsa-mir-339 | 4690 | NCK1 | NCK adaptor protein 1 | 1 | | 1078 | hsa-mir-562 | 4690 | NCK1 | NCK adaptor protein 1 | 1 | | 1079 | hsa-mir-567 | 4690 | NCK1 | NCK adaptor protein 1 | 1 | | 1080 | hsa-mir-576 | 4690 | NCK1 | NCK adaptor protein 1 | 1 | | 1081 | hsa-mir-579 | 4690 | NCK1 | NCK adaptor protein 1 | 1 | | 1082 | hsa-mir-582 | 4690 | NCK1 | NCK adaptor protein 1 | 1 | | 1083 | hsa-mir-604 | 4690 | NCK1 | NCK adaptor protein 1 | 1 | | 1084 | hsa-mir-608 | 4690 | NCK1 | NCK adaptor protein 1 | 1 | | 1085 | hsa-mir-621 | 4690 | NCK1 | NCK adaptor protein 1 | 1 | | 1086 | hsa-mir-627 | 4690 | NCK1 | NCK adaptor protein 1 | 1 | | 1087 | hsa-mir-454 | 4690 | NCK1 | NCK adaptor protein 1 | 1 | | 1088 | hsa-mir-302d | 4690 | NCK1 | NCK adaptor protein 1 | 1 | | 1089 | hsa-mir-367 | 4690 | NCK1 | NCK adaptor protein 1 | 1 | | 1090 | hsa-mir-548b | 4690 | NCK1 | NCK adaptor protein 1 | 1 | | 1091 | hsa-mir-548d-1;hsa-mir-548d-2 | 4690 | NCK1 | NCK adaptor protein 1 | 1 | | 1092 | hsa-mir-938 | 4690 | NCK1 | NCK adaptor protein 1 | 1 | | 1093 | hsa-mir-1236 | 4690 | NCK1 | NCK adaptor protein 1 | 1 | | 1094 | hsa-mir-101-2 | 8573 | CASK | calcium/calmodulin-dependent serine protein kinase (MAGUK family) | 1 | | 1099 | hsa-mir-511-1 | 8573 | CASK | calcium/calmodulin-dependent serine protein kinase (MAGUK family) | 1 | | 1100 | hsa-mir-511-2 | 8573 | CASK | calcium/calmodulin-dependent serine protein kinase (MAGUK family) | 1 | | 1101 | hsa-mir-1284 | 8573 | CASK | calcium/calmodulin-dependent serine protein kinase (MAGUK family) | 1 | | 1102 | hsa-mir-3181 | 8573 | CASK | calcium/calmodulin-dependent serine protein kinase (MAGUK family) | 1 | | 1103 | hsa-mir-3651 | 8573 | CASK | calcium/calmodulin-dependent serine protein kinase (MAGUK family) | 1 | | 1104 | hsa-mir-4632 | 8573 | CASK | calcium/calmodulin-dependent serine protein kinase (MAGUK family) | 1 | | 1105 | hsa-mir-4648 | 8573 | CASK | calcium/calmodulin-dependent serine protein kinase (MAGUK family) | 1 | | 1106 | hsa-mir-4726 | 8573 | CASK | calcium/calmodulin-dependent serine protein kinase (MAGUK family) | 1 | | 1107 | hsa-mir-4785 | 8573 | CASK | calcium/calmodulin-dependent serine protein kinase (MAGUK family) | 1 | | 1108 | hsa-mir-4794 | 8573 | CASK | calcium/calmodulin-dependent serine protein kinase (MAGUK family) | 1 | | 1109 | hsa-mir-4802 | 8573 | CASK | calcium/calmodulin-dependent serine protein kinase (MAGUK family) | 1 | | 1110 | hsa-let-7i | 8573 | CASK | calcium/calmodulin-dependent serine protein kinase (MAGUK family) | 1 | | 1111 | hsa-mir-302b | 8573 | CASK | calcium/calmodulin-dependent serine protein kinase (MAGUK family) | 1 | | 1112 | hsa-mir-302c | 8573 | CASK | calcium/calmodulin-dependent serine protein kinase (MAGUK family) | 1 | | 1113 | hsa-mir-339 | 8573 | CASK | calcium/calmodulin-dependent serine protein kinase (MAGUK family) | 1 | | 1114 | hsa-mir-562 | 8573 | CASK | calcium/calmodulin-dependent serine protein kinase (MAGUK family) | 1 | | 1115 | hsa-mir-567 | 8573 | CASK | calcium/calmodulin-dependent serine protein kinase (MAGUK family) | 1 | | 1116 | hsa-mir-576 | 8573 | CASK | calcium/calmodulin-dependent serine protein kinase (MAGUK family) | 1 | | 1117 | hsa-mir-579 | 8573 | CASK | calcium/calmodulin-dependent serine protein kinase (MAGUK family) | 1 | | 1118 | hsa-mir-582 | 8573 | CASK | calcium/calmodulin-dependent serine protein kinase (MAGUK family) | 1 | | 1119 | hsa-mir-604 | 8573 | CASK | calcium/calmodulin-dependent serine protein kinase (MAGUK family) | 1 | | 1120 | hsa-mir-608 | 8573 | CASK | calcium/calmodulin-dependent serine protein kinase (MAGUK family) | 1 | | 1121 | hsa-mir-621 | 8573 | CASK | calcium/calmodulin-dependent serine protein kinase (MAGUK family) | 1 | | 1122 | hsa-mir-627 | 8573 | CASK | calcium/calmodulin-dependent serine protein kinase (MAGUK family) | 1 | | 1123 | hsa-mir-454 | 8573 | CASK | calcium/calmodulin-dependent serine protein kinase (MAGUK family) | 1 | | 1124 | hsa-mir-302d | 8573 | CASK | calcium/calmodulin-dependent serine protein kinase (MAGUK family) | 1 | | 1125 | hsa-mir-367 | 8573 | CASK | calcium/calmodulin-dependent serine protein kinase (MAGUK family) | 1 | | 1126 | hsa-mir-548b | 8573 | CASK | calcium/calmodulin-dependent serine protein kinase (MAGUK family) | 1 | | 1127 | hsa-mir-548d-1;hsa-mir-548d-2 | 8573 | CASK | calcium/calmodulin-dependent serine protein kinase (MAGUK family) | 1 | | 1128 | hsa-mir-938 | 8573 | CASK | calcium/calmodulin-dependent serine protein kinase (MAGUK family) | 1 | | 1129 | hsa-mir-1236 | 8573 | CASK | calcium/calmodulin-dependent serine protein kinase (MAGUK family) | 1 | | 1130 | hsa-mir-101-2 | 4288 | MKI67 | antigen identified by monoclonal antibody Ki-67 | 1 | | 1135 | hsa-mir-511-1 | 4288 | MKI67 | antigen identified by monoclonal antibody Ki-67 | 1 | | 1136 | hsa-mir-511-2 | 4288 | MKI67 | antigen identified by monoclonal antibody Ki-67 | 1 | | 1137 | hsa-mir-1284 | 4288 | MKI67 | antigen identified by monoclonal antibody Ki-67 | 1 | | 1138 | hsa-mir-3181 | 4288 | MKI67 | antigen identified by monoclonal antibody Ki-67 | 1 | | 1139 | hsa-mir-3651 | 4288 | MKI67 | antigen identified by monoclonal antibody Ki-67 | 1 | | 1140 | hsa-mir-4632 | 4288 | MKI67 | antigen identified by monoclonal antibody Ki-67 | 1 | | 1141 | hsa-mir-4648 | 4288 | MKI67 | antigen identified by monoclonal antibody Ki-67 | 1 | | 1142 | hsa-mir-4726 | 4288 | MKI67 | antigen identified by monoclonal antibody Ki-67 | 1 | | 1143 | hsa-mir-4785 | 4288 | MKI67 | antigen identified by monoclonal antibody Ki-67 | 1 | | 1144 | hsa-mir-4794 | 4288 | MKI67 | antigen identified by monoclonal antibody Ki-67 | 1 | | 1145 | hsa-mir-4802 | 4288 | MKI67 | antigen identified by monoclonal antibody Ki-67 | 1 | | 1146 | hsa-let-7i | 4288 | MKI67 | antigen identified by monoclonal antibody Ki-67 | 1 | | 1147 | hsa-mir-302b | 4288 | MKI67 | antigen identified by monoclonal antibody Ki-67 | 1 | | 1148 | hsa-mir-302c | 4288 | MKI67 | antigen identified by monoclonal antibody Ki-67 | 1 | | 1149 | hsa-mir-339 | 4288 | MKI67 | antigen identified by monoclonal antibody Ki-67 | 1 | | 1150 | hsa-mir-562 | 4288 | MKI67 | antigen identified by monoclonal antibody Ki-67 | 1 | | 1151 | hsa-mir-567 | 4288 | MKI67 | antigen identified by monoclonal antibody Ki-67 | 1 | | 1152 | hsa-mir-576 | 4288 | MKI67 | antigen identified by monoclonal antibody Ki-67 | 1 | | 1153 | hsa-mir-579 | 4288 | MKI67 | antigen identified by monoclonal antibody Ki-67 | 1 | | 1154 | hsa-mir-582 | 4288 | MKI67 | antigen identified by monoclonal antibody Ki-67 | 1 | | 1155 | hsa-mir-604 | 4288 | MKI67 | antigen identified by monoclonal antibody Ki-67 | 1 | | 1156 | hsa-mir-608 | 4288 | MKI67 | antigen identified by monoclonal antibody Ki-67 | 1 | | 1157 | hsa-mir-621 | 4288 | MKI67 | antigen identified by monoclonal antibody Ki-67 | 1 | | 1158 | hsa-mir-627 | 4288 | MKI67 | antigen identified by monoclonal antibody Ki-67 | 1 | | 1159 | hsa-mir-454 | 4288 | MKI67 | antigen identified by monoclonal antibody Ki-67 | 1 | | 1160 | hsa-mir-302d | 4288 | MKI67 | antigen identified by monoclonal antibody Ki-67 | 1 | | 1161 | hsa-mir-367 | 4288 | MKI67 | antigen identified by monoclonal antibody Ki-67 | 1 | | 1162 | hsa-mir-548b | 4288 | MKI67 | antigen identified by monoclonal antibody Ki-67 | 1 | | 1163 | hsa-mir-548d-1;hsa-mir-548d-2 | 4288 | MKI67 | antigen identified by monoclonal antibody Ki-67 | 1 | | 1164 | hsa-mir-938 | 4288 | MKI67 | antigen identified by monoclonal antibody Ki-67 | 1 | | 1165 | hsa-mir-1236 | 4288 | MKI67 | antigen identified by monoclonal antibody Ki-67 | 1 | | 1166 | hsa-mir-101-2 | 598 | BCL2L1 | BCL2-like 1 | 1 | | 1171 | hsa-mir-511-1 | 598 | BCL2L1 | BCL2-like 1 | 1 | | 1172 | hsa-mir-511-2 | 598 | BCL2L1 | BCL2-like 1 | 1 | | 1173 | hsa-mir-1284 | 598 | BCL2L1 | BCL2-like 1 | 1 | | 1174 | hsa-mir-3181 | 598 | BCL2L1 | BCL2-like 1 | 1 | | 1175 | hsa-mir-3651 | 598 | BCL2L1 | BCL2-like 1 | 1 | | 1176 | hsa-mir-4632 | 598 | BCL2L1 | BCL2-like 1 | 1 | | 1177 | hsa-mir-4648 | 598 | BCL2L1 | BCL2-like 1 | 1 | | 1178 | hsa-mir-4726 | 598 | BCL2L1 | BCL2-like 1 | 1 | | 1179 | hsa-mir-4785 | 598 | BCL2L1 | BCL2-like 1 | 1 | | 1180 | hsa-mir-4794 | 598 | BCL2L1 | BCL2-like 1 | 1 | | 1181 | hsa-mir-4802 | 598 | BCL2L1 | BCL2-like 1 | 1 | | 1182 | hsa-let-7i | 598 | BCL2L1 | BCL2-like 1 | 1 | | 1183 | hsa-mir-302b | 598 | BCL2L1 | BCL2-like 1 | 1 | | 1184 | hsa-mir-302c | 598 | BCL2L1 | BCL2-like 1 | 1 | | 1185 | hsa-mir-339 | 598 | BCL2L1 | BCL2-like 1 | 1 | | 1186 | hsa-mir-562 | 598 | BCL2L1 | BCL2-like 1 | 1 | | 1187 | hsa-mir-567 | 598 | BCL2L1 | BCL2-like 1 | 1 | | 1188 | hsa-mir-576 | 598 | BCL2L1 | BCL2-like 1 | 1 | | 1189 | hsa-mir-579 | 598 | BCL2L1 | BCL2-like 1 | 1 | | 1190 | hsa-mir-582 | 598 | BCL2L1 | BCL2-like 1 | 1 | | 1191 | hsa-mir-604 | 598 | BCL2L1 | BCL2-like 1 | 1 | | 1192 | hsa-mir-608 | 598 | BCL2L1 | BCL2-like 1 | 1 | | 1193 | hsa-mir-621 | 598 | BCL2L1 | BCL2-like 1 | 1 | | 1194 | hsa-mir-627 | 598 | BCL2L1 | BCL2-like 1 | 1 | | 1195 | hsa-mir-454 | 598 | BCL2L1 | BCL2-like 1 | 1 | | 1196 | hsa-mir-302d | 598 | BCL2L1 | BCL2-like 1 | 1 | | 1197 | hsa-mir-367 | 598 | BCL2L1 | BCL2-like 1 | 1 | | 1198 | hsa-mir-548b | 598 | BCL2L1 | BCL2-like 1 | 1 | | 1199 | hsa-mir-548d-1;hsa-mir-548d-2 | 598 | BCL2L1 | BCL2-like 1 | 1 | | 1200 | hsa-mir-938 | 598 | BCL2L1 | BCL2-like 1 | 1 | | 1201 | hsa-mir-1236 | 598 | BCL2L1 | BCL2-like 1 | 1 | | 1202 | hsa-mir-101-2 | 11113 | CIT | citron (rho-interacting, serine/threonine kinase 21) | 1 | | 1207 | hsa-mir-511-1 | 11113 | CIT | citron (rho-interacting, serine/threonine kinase 21) | 1 | | 1208 | hsa-mir-511-2 | 11113 | CIT | citron (rho-interacting, serine/threonine kinase 21) | 1 | | 1209 | hsa-mir-1284 | 11113 | CIT | citron (rho-interacting, serine/threonine kinase 21) | 1 | | 1210 | hsa-mir-3181 | 11113 | CIT | citron (rho-interacting, serine/threonine kinase 21) | 1 | | 1211 | hsa-mir-3651 | 11113 | CIT | citron (rho-interacting, serine/threonine kinase 21) | 1 | | 1212 | hsa-mir-4632 | 11113 | CIT | citron (rho-interacting, serine/threonine kinase 21) | 1 | | 1213 | hsa-mir-4648 | 11113 | CIT | citron (rho-interacting, serine/threonine kinase 21) | 1 | | 1214 | hsa-mir-4726 | 11113 | CIT | citron (rho-interacting, serine/threonine kinase 21) | 1 | | 1215 | hsa-mir-4785 | 11113 | CIT | citron (rho-interacting, serine/threonine kinase 21) | 1 | | 1216 | hsa-mir-4794 | 11113 | CIT | citron (rho-interacting, serine/threonine kinase 21) | 1 | | 1217 | hsa-mir-4802 | 11113 | CIT | citron (rho-interacting, serine/threonine kinase 21) | 1 | | 1218 | hsa-let-7i | 11113 | CIT | citron (rho-interacting, serine/threonine kinase 21) | 1 | | 1219 | hsa-mir-302b | 11113 | CIT | citron (rho-interacting, serine/threonine kinase 21) | 1 | | 1220 | hsa-mir-302c | 11113 | CIT | citron (rho-interacting, serine/threonine kinase 21) | 1 | | 1221 | hsa-mir-339 | 11113 | CIT | citron (rho-interacting, serine/threonine kinase 21) | 1 | | 1222 | hsa-mir-562 | 11113 | CIT | citron (rho-interacting, serine/threonine kinase 21) | 1 | | 1223 | hsa-mir-567 | 11113 | CIT | citron (rho-interacting, serine/threonine kinase 21) | 1 | | 1224 | hsa-mir-576 | 11113 | CIT | citron (rho-interacting, serine/threonine kinase 21) | 1 | | 1225 | hsa-mir-579 | 11113 | CIT | citron (rho-interacting, serine/threonine kinase 21) | 1 | | 1226 | hsa-mir-582 | 11113 | CIT | citron (rho-interacting, serine/threonine kinase 21) | 1 | | 1227 | hsa-mir-604 | 11113 | CIT | citron (rho-interacting, serine/threonine kinase 21) | 1 | | 1228 | hsa-mir-608 | 11113 | CIT | citron (rho-interacting, serine/threonine kinase 21) | 1 | | 1229 | hsa-mir-621 | 11113 | CIT | citron (rho-interacting, serine/threonine kinase 21) | 1 | | 1230 | hsa-mir-627 | 11113 | CIT | citron (rho-interacting, serine/threonine kinase 21) | 1 | | 1231 | hsa-mir-454 | 11113 | CIT | citron (rho-interacting, serine/threonine kinase 21) | 1 | | 1232 | hsa-mir-302d | 11113 | CIT | citron (rho-interacting, serine/threonine kinase 21) | 1 | | 1233 | hsa-mir-367 | 11113 | CIT | citron (rho-interacting, serine/threonine kinase 21) | 1 | | 1234 | hsa-mir-548b | 11113 | CIT | citron (rho-interacting, serine/threonine kinase 21) | 1 | | 1235 | hsa-mir-548d-1;hsa-mir-548d-2 | 11113 | CIT | citron (rho-interacting, serine/threonine kinase 21) | 1 | | 1236 | hsa-mir-938 | 11113 | CIT | citron (rho-interacting, serine/threonine kinase 21) | 1 | | 1237 | hsa-mir-1236 | 11113 | CIT | citron (rho-interacting, serine/threonine kinase 21) | 1 | | 1238 | hsa-mir-101-2 | 23234 | DNAJC9 | DnaJ (Hsp40) homolog, subfamily C, member 9 | 1 | | 1243 | hsa-mir-511-1 | 23234 | DNAJC9 | DnaJ (Hsp40) homolog, subfamily C, member 9 | 1 | | 1244 | hsa-mir-511-2 | 23234 | DNAJC9 | DnaJ (Hsp40) homolog, subfamily C, member 9 | 1 | | 1245 | hsa-mir-1284 | 23234 | DNAJC9 | DnaJ (Hsp40) homolog, subfamily C, member 9 | 1 | | 1246 | hsa-mir-3181 | 23234 | DNAJC9 | DnaJ (Hsp40) homolog, subfamily C, member 9 | 1 | | 1247 | hsa-mir-3651 | 23234 | DNAJC9 | DnaJ (Hsp40) homolog, subfamily C, member 9 | 1 | | 1248 | hsa-mir-4632 | 23234 | DNAJC9 | DnaJ (Hsp40) homolog, subfamily C, member 9 | 1 | | 1249 | hsa-mir-4648 | 23234 | DNAJC9 | DnaJ (Hsp40) homolog, subfamily C, member 9 | 1 | | 1250 | hsa-mir-4726 | 23234 | DNAJC9 | DnaJ (Hsp40) homolog, subfamily C, member 9 | 1 | | 1251 | hsa-mir-4785 | 23234 | DNAJC9 | DnaJ (Hsp40) homolog, subfamily C, member 9 | 1 | | 1252 | hsa-mir-4794 | 23234 | DNAJC9 | DnaJ (Hsp40) homolog, subfamily C, member 9 | 1 | | 1253 | hsa-mir-4802 | 23234 | DNAJC9 | DnaJ (Hsp40) homolog, subfamily C, member 9 | 1 | | 1254 | hsa-let-7i | 23234 | DNAJC9 | DnaJ (Hsp40) homolog, subfamily C, member 9 | 1 | | 1255 | hsa-mir-302b | 23234 | DNAJC9 | DnaJ (Hsp40) homolog, subfamily C, member 9 | 1 | | 1256 | hsa-mir-302c | 23234 | DNAJC9 | DnaJ (Hsp40) homolog, subfamily C, member 9 | 1 | | 1257 | hsa-mir-339 | 23234 | DNAJC9 | DnaJ (Hsp40) homolog, subfamily C, member 9 | 1 | | 1258 | hsa-mir-562 | 23234 | DNAJC9 | DnaJ (Hsp40) homolog, subfamily C, member 9 | 1 | | 1259 | hsa-mir-567 | 23234 | DNAJC9 | DnaJ (Hsp40) homolog, subfamily C, member 9 | 1 | | 1260 | hsa-mir-576 | 23234 | DNAJC9 | DnaJ (Hsp40) homolog, subfamily C, member 9 | 1 | | 1261 | hsa-mir-579 | 23234 | DNAJC9 | DnaJ (Hsp40) homolog, subfamily C, member 9 | 1 | | 1262 | hsa-mir-582 | 23234 | DNAJC9 | DnaJ (Hsp40) homolog, subfamily C, member 9 | 1 | | 1263 | hsa-mir-604 | 23234 | DNAJC9 | DnaJ (Hsp40) homolog, subfamily C, member 9 | 1 | | 1264 | hsa-mir-608 | 23234 | DNAJC9 | DnaJ (Hsp40) homolog, subfamily C, member 9 | 1 | | 1265 | hsa-mir-621 | 23234 | DNAJC9 | DnaJ (Hsp40) homolog, subfamily C, member 9 | 1 | | 1266 | hsa-mir-627 | 23234 | DNAJC9 | DnaJ (Hsp40) homolog, subfamily C, member 9 | 1 | | 1267 | hsa-mir-454 | 23234 | DNAJC9 | DnaJ (Hsp40) homolog, subfamily C, member 9 | 1 | | 1268 | hsa-mir-302d | 23234 | DNAJC9 | DnaJ (Hsp40) homolog, subfamily C, member 9 | 1 | | 1269 | hsa-mir-367 | 23234 | DNAJC9 | DnaJ (Hsp40) homolog, subfamily C, member 9 | 1 | | 1270 | hsa-mir-548b | 23234 | DNAJC9 | DnaJ (Hsp40) homolog, subfamily C, member 9 | 1 | | 1271 | hsa-mir-548d-1;hsa-mir-548d-2 | 23234 | DNAJC9 | DnaJ (Hsp40) homolog, subfamily C, member 9 | 1 | | 1272 | hsa-mir-938 | 23234 | DNAJC9 | DnaJ (Hsp40) homolog, subfamily C, member 9 | 1 | | 1273 | hsa-mir-1236 | 23234 | DNAJC9 | DnaJ (Hsp40) homolog, subfamily C, member 9 | 1 | | 1277 | hsa-mir-302b | 8745 | ADAM23 | ADAM metallopeptidase domain 23 | 1 | | 1278 | hsa-mir-302c | 8745 | ADAM23 | ADAM metallopeptidase domain 23 | 1 | | 1279 | hsa-mir-302d | 8745 | ADAM23 | ADAM metallopeptidase domain 23 | 1 | | 1280 | hsa-mir-367 | 8745 | ADAM23 | ADAM metallopeptidase domain 23 | 1 | | 1281 | hsa-mir-562 | 8745 | ADAM23 | ADAM metallopeptidase domain 23 | 1 | | 1282 | hsa-mir-604 | 8745 | ADAM23 | ADAM metallopeptidase domain 23 | 1 | | 1283 | hsa-mir-548d-1 | 8745 | ADAM23 | ADAM metallopeptidase domain 23 | 1 | | 1284 | hsa-mir-938 | 8745 | ADAM23 | ADAM metallopeptidase domain 23 | 1 | | 1285 | hsa-mir-548h-1 | 8745 | ADAM23 | ADAM metallopeptidase domain 23 | 1 | | 1286 | hsa-mir-3135a | 8745 | ADAM23 | ADAM metallopeptidase domain 23 | 1 | | 1287 | hsa-mir-3177 | 8745 | ADAM23 | ADAM metallopeptidase domain 23 | 1 | | 1288 | hsa-mir-3655 | 8745 | ADAM23 | ADAM metallopeptidase domain 23 | 1 | | 1289 | hsa-mir-4526 | 8745 | ADAM23 | ADAM metallopeptidase domain 23 | 1 | | 1290 | hsa-mir-4635 | 8745 | ADAM23 | ADAM metallopeptidase domain 23 | 1 | | 1291 | hsa-mir-4651 | 8745 | ADAM23 | ADAM metallopeptidase domain 23 | 1 | | 1292 | hsa-mir-4744 | 8745 | ADAM23 | ADAM metallopeptidase domain 23 | 1 | | 1293 | hsa-mir-5190 | 8745 | ADAM23 | ADAM metallopeptidase domain 23 | 1 | | 1294 | hsa-mir-101-2 | 8745 | ADAM23 | ADAM metallopeptidase domain 23 | 1 | | 1295 | hsa-mir-128-1;hsa-mir-128-2 | 8745 | ADAM23 | ADAM metallopeptidase domain 23 | 1 | | 1296 | hsa-mir-301a | 8745 | ADAM23 | ADAM metallopeptidase domain 23 | 1 | | 1297 | hsa-mir-511-1;hsa-mir-511-2 | 8745 | ADAM23 | ADAM metallopeptidase domain 23 | 1 | | 1298 | hsa-mir-586 | 8745 | ADAM23 | ADAM metallopeptidase domain 23 | 1 | | 1299 | hsa-mir-593 | 8745 | ADAM23 | ADAM metallopeptidase domain 23 | 1 | | 1300 | hsa-mir-620 | 8745 | ADAM23 | ADAM metallopeptidase domain 23 | 1 | | 1301 | hsa-mir-33b | 8745 | ADAM23 | ADAM metallopeptidase domain 23 | 1 | | 1302 | hsa-mir-942 | 8745 | ADAM23 | ADAM metallopeptidase domain 23 | 1 | | 1306 | hsa-mir-302b | 8519 | IFITM1 | interferon induced transmembrane protein 1 (9-27) | 1 | | 1307 | hsa-mir-302c | 8519 | IFITM1 | interferon induced transmembrane protein 1 (9-27) | 1 | | 1308 | hsa-mir-302d | 8519 | IFITM1 | interferon induced transmembrane protein 1 (9-27) | 1 | | 1309 | hsa-mir-367 | 8519 | IFITM1 | interferon induced transmembrane protein 1 (9-27) | 1 | | 1310 | hsa-mir-562 | 8519 | IFITM1 | interferon induced transmembrane protein 1 (9-27) | 1 | | 1311 | hsa-mir-604 | 8519 | IFITM1 | interferon induced transmembrane protein 1 (9-27) | 1 | | 1312 | hsa-mir-548d-1 | 8519 | IFITM1 | interferon induced transmembrane protein 1 (9-27) | 1 | | 1313 | hsa-mir-938 | 8519 | IFITM1 | interferon induced transmembrane protein 1 (9-27) | 1 | | 1314 | hsa-mir-548h-1 | 8519 | IFITM1 | interferon induced transmembrane protein 1 (9-27) | 1 | | 1315 | hsa-mir-3135a | 8519 | IFITM1 | interferon induced transmembrane protein 1 (9-27) | 1 | | 1316 | hsa-mir-3177 | 8519 | IFITM1 | interferon induced transmembrane protein 1 (9-27) | 1 | | 1317 | hsa-mir-3655 | 8519 | IFITM1 | interferon induced transmembrane protein 1 (9-27) | 1 | | 1318 | hsa-mir-4526 | 8519 | IFITM1 | interferon induced transmembrane protein 1 (9-27) | 1 | | 1319 | hsa-mir-4635 | 8519 | IFITM1 | interferon induced transmembrane protein 1 (9-27) | 1 | | 1320 | hsa-mir-4651 | 8519 | IFITM1 | interferon induced transmembrane protein 1 (9-27) | 1 | | 1321 | hsa-mir-4744 | 8519 | IFITM1 | interferon induced transmembrane protein 1 (9-27) | 1 | | 1322 | hsa-mir-5190 | 8519 | IFITM1 | interferon induced transmembrane protein 1 (9-27) | 1 | | 1323 | hsa-mir-101-2 | 8519 | IFITM1 | interferon induced transmembrane protein 1 (9-27) | 1 | | 1324 | hsa-mir-128-1;hsa-mir-128-2 | 8519 | IFITM1 | interferon induced transmembrane protein 1 (9-27) | 1 | | 1325 | hsa-mir-301a | 8519 | IFITM1 | interferon induced transmembrane protein 1 (9-27) | 1 | | 1326 | hsa-mir-511-1;hsa-mir-511-2 | 8519 | IFITM1 | interferon induced transmembrane protein 1 (9-27) | 1 | | 1327 | hsa-mir-586 | 8519 | IFITM1 | interferon induced transmembrane protein 1 (9-27) | 1 | | 1328 | hsa-mir-593 | 8519 | IFITM1 | interferon induced transmembrane protein 1 (9-27) | 1 | | 1329 | hsa-mir-620 | 8519 | IFITM1 | interferon induced transmembrane protein 1 (9-27) | 1 | | 1330 | hsa-mir-33b | 8519 | IFITM1 | interferon induced transmembrane protein 1 (9-27) | 1 | | 1331 | hsa-mir-942 | 8519 | IFITM1 | interferon induced transmembrane protein 1 (9-27) | 1 | | 1335 | hsa-mir-302b | 57134 | MAN1C1 | mannosidase, alpha, class 1C, member 1 | 1 | | 1336 | hsa-mir-302c | 57134 | MAN1C1 | mannosidase, alpha, class 1C, member 1 | 1 | | 1337 | hsa-mir-302d | 57134 | MAN1C1 | mannosidase, alpha, class 1C, member 1 | 1 | | 1338 | hsa-mir-367 | 57134 | MAN1C1 | mannosidase, alpha, class 1C, member 1 | 1 | | 1339 | hsa-mir-562 | 57134 | MAN1C1 | mannosidase, alpha, class 1C, member 1 | 1 | | 1340 | hsa-mir-604 | 57134 | MAN1C1 | mannosidase, alpha, class 1C, member 1 | 1 | | 1341 | hsa-mir-548d-1 | 57134 | MAN1C1 | mannosidase, alpha, class 1C, member 1 | 1 | | 1342 | hsa-mir-938 | 57134 | MAN1C1 | mannosidase, alpha, class 1C, member 1 | 1 | | 1343 | hsa-mir-548h-1 | 57134 | MAN1C1 | mannosidase, alpha, class 1C, member 1 | 1 | | 1344 | hsa-mir-3135a | 57134 | MAN1C1 | mannosidase, alpha, class 1C, member 1 | 1 | | 1345 | hsa-mir-3177 | 57134 | MAN1C1 | mannosidase, alpha, class 1C, member 1 | 1 | | 1346 | hsa-mir-3655 | 57134 | MAN1C1 | mannosidase, alpha, class 1C, member 1 | 1 | | 1347 | hsa-mir-4526 | 57134 | MAN1C1 | mannosidase, alpha, class 1C, member 1 | 1 | | 1348 | hsa-mir-4635 | 57134 | MAN1C1 | mannosidase, alpha, class 1C, member 1 | 1 | | 1349 | hsa-mir-4651 | 57134 | MAN1C1 | mannosidase, alpha, class 1C, member 1 | 1 | | 1350 | hsa-mir-4744 | 57134 | MAN1C1 | mannosidase, alpha, class 1C, member 1 | 1 | | 1351 | hsa-mir-5190 | 57134 | MAN1C1 | mannosidase, alpha, class 1C, member 1 | 1 | | 1352 | hsa-mir-101-2 | 57134 | MAN1C1 | mannosidase, alpha, class 1C, member 1 | 1 | | 1353 | hsa-mir-128-1;hsa-mir-128-2 | 57134 | MAN1C1 | mannosidase, alpha, class 1C, member 1 | 1 | | 1354 | hsa-mir-301a | 57134 | MAN1C1 | mannosidase, alpha, class 1C, member 1 | 1 | | 1355 | hsa-mir-511-1;hsa-mir-511-2 | 57134 | MAN1C1 | mannosidase, alpha, class 1C, member 1 | 1 | | 1356 | hsa-mir-586 | 57134 | MAN1C1 | mannosidase, alpha, class 1C, member 1 | 1 | | 1357 | hsa-mir-593 | 57134 | MAN1C1 | mannosidase, alpha, class 1C, member 1 | 1 | | 1358 | hsa-mir-620 | 57134 | MAN1C1 | mannosidase, alpha, class 1C, member 1 | 1 | | 1359 | hsa-mir-33b | 57134 | MAN1C1 | mannosidase, alpha, class 1C, member 1 | 1 | | 1360 | hsa-mir-942 | 57134 | MAN1C1 | mannosidase, alpha, class 1C, member 1 | 1 | | 1361 | hsa-mir-101-2 | 1523 | CUX1 | cut-like homeobox 1 | 1 | | 1366 | hsa-mir-511-1 | 1523 | CUX1 | cut-like homeobox 1 | 1 | | 1367 | hsa-mir-511-2 | 1523 | CUX1 | cut-like homeobox 1 | 1 | | 1368 | hsa-mir-1284 | 1523 | CUX1 | cut-like homeobox 1 | 1 | | 1369 | hsa-mir-3181 | 1523 | CUX1 | cut-like homeobox 1 | 1 | | 1370 | hsa-mir-3651 | 1523 | CUX1 | cut-like homeobox 1 | 1 | | 1371 | hsa-mir-4632 | 1523 | CUX1 | cut-like homeobox 1 | 1 | | 1372 | hsa-mir-4648 | 1523 | CUX1 | cut-like homeobox 1 | 1 | | 1373 | hsa-mir-4726 | 1523 | CUX1 | cut-like homeobox 1 | 1 | | 1374 | hsa-mir-4785 | 1523 | CUX1 | cut-like homeobox 1 | 1 | | 1375 | hsa-mir-4794 | 1523 | CUX1 | cut-like homeobox 1 | 1 | | 1376 | hsa-mir-4802 | 1523 | CUX1 | cut-like homeobox 1 | 1 | | 1377 | hsa-let-7i | 1523 | CUX1 | cut-like homeobox 1 | 1 | | 1378 | hsa-mir-302b | 1523 | CUX1 | cut-like homeobox 1 | 1 | | 1379 | hsa-mir-302c | 1523 | CUX1 | cut-like homeobox 1 | 1 | | 1380 | hsa-mir-339 | 1523 | CUX1 | cut-like homeobox 1 | 1 | | 1381 | hsa-mir-562 | 1523 | CUX1 | cut-like homeobox 1 | 1 | | 1382 | hsa-mir-567 | 1523 | CUX1 | cut-like homeobox 1 | 1 | | 1383 | hsa-mir-576 | 1523 | CUX1 | cut-like homeobox 1 | 1 | | 1384 | hsa-mir-579 | 1523 | CUX1 | cut-like homeobox 1 | 1 | | 1385 | hsa-mir-582 | 1523 | CUX1 | cut-like homeobox 1 | 1 | | 1386 | hsa-mir-604 | 1523 | CUX1 | cut-like homeobox 1 | 1 | | 1387 | hsa-mir-608 | 1523 | CUX1 | cut-like homeobox 1 | 1 | | 1388 | hsa-mir-621 | 1523 | CUX1 | cut-like homeobox 1 | 1 | | 1389 | hsa-mir-627 | 1523 | CUX1 | cut-like homeobox 1 | 1 | | 1390 | hsa-mir-454 | 1523 | CUX1 | cut-like homeobox 1 | 1 | | 1391 | hsa-mir-302d | 1523 | CUX1 | cut-like homeobox 1 | 1 | | 1392 | hsa-mir-367 | 1523 | CUX1 | cut-like homeobox 1 | 1 | | 1393 | hsa-mir-548b | 1523 | CUX1 | cut-like homeobox 1 | 1 | | 1394 | hsa-mir-548d-1;hsa-mir-548d-2 | 1523 | CUX1 | cut-like homeobox 1 | 1 | | 1395 | hsa-mir-938 | 1523 | CUX1 | cut-like homeobox 1 | 1 | | 1396 | hsa-mir-1236 | 1523 | CUX1 | cut-like homeobox 1 | 1 | | 1400 | hsa-mir-302b | 4929 | NR4A2 | nuclear receptor subfamily 4, group A, member 2 | 1 | | 1401 | hsa-mir-302c | 4929 | NR4A2 | nuclear receptor subfamily 4, group A, member 2 | 1 | | 1402 | hsa-mir-302d | 4929 | NR4A2 | nuclear receptor subfamily 4, group A, member 2 | 1 | | 1403 | hsa-mir-367 | 4929 | NR4A2 | nuclear receptor subfamily 4, group A, member 2 | 1 | | 1404 | hsa-mir-562 | 4929 | NR4A2 | nuclear receptor subfamily 4, group A, member 2 | 1 | | 1405 | hsa-mir-604 | 4929 | NR4A2 | nuclear receptor subfamily 4, group A, member 2 | 1 | | 1406 | hsa-mir-548d-1 | 4929 | NR4A2 | nuclear receptor subfamily 4, group A, member 2 | 1 | | 1407 | hsa-mir-938 | 4929 | NR4A2 | nuclear receptor subfamily 4, group A, member 2 | 1 | | 1408 | hsa-mir-548h-1 | 4929 | NR4A2 | nuclear receptor subfamily 4, group A, member 2 | 1 | | 1409 | hsa-mir-3135a | 4929 | NR4A2 | nuclear receptor subfamily 4, group A, member 2 | 1 | | 1410 | hsa-mir-3177 | 4929 | NR4A2 | nuclear receptor subfamily 4, group A, member 2 | 1 | | 1411 | hsa-mir-3655 | 4929 | NR4A2 | nuclear receptor subfamily 4, group A, member 2 | 1 | | 1412 | hsa-mir-4526 | 4929 | NR4A2 | nuclear receptor subfamily 4, group A, member 2 | 1 | | 1413 | hsa-mir-4635 | 4929 | NR4A2 | nuclear receptor subfamily 4, group A, member 2 | 1 | | 1414 | hsa-mir-4651 | 4929 | NR4A2 | nuclear receptor subfamily 4, group A, member 2 | 1 | | 1415 | hsa-mir-4744 | 4929 | NR4A2 | nuclear receptor subfamily 4, group A, member 2 | 1 | | 1416 | hsa-mir-5190 | 4929 | NR4A2 | nuclear receptor subfamily 4, group A, member 2 | 1 | | 1417 | hsa-mir-101-2 | 4929 | NR4A2 | nuclear receptor subfamily 4, group A, member 2 | 1 | | 1418 | hsa-mir-128-1;hsa-mir-128-2 | 4929 | NR4A2 | nuclear receptor subfamily 4, group A, member 2 | 1 | | 1419 | hsa-mir-301a | 4929 | NR4A2 | nuclear receptor subfamily 4, group A, member 2 | 1 | | 1420 | hsa-mir-511-1;hsa-mir-511-2 | 4929 | NR4A2 | nuclear receptor subfamily 4, group A, member 2 | 1 | | 1421 | hsa-mir-586 | 4929 | NR4A2 | nuclear receptor subfamily 4, group A, member 2 | 1 | | 1422 | hsa-mir-593 | 4929 | NR4A2 | nuclear receptor subfamily 4, group A, member 2 | 1 | | 1423 | hsa-mir-620 | 4929 | NR4A2 | nuclear receptor subfamily 4, group A, member 2 | 1 | | 1424 | hsa-mir-33b | 4929 | NR4A2 | nuclear receptor subfamily 4, group A, member 2 | 1 | | 1425 | hsa-mir-942 | 4929 | NR4A2 | nuclear receptor subfamily 4, group A, member 2 | 1 | | 1426 | hsa-mir-101-2 | 3068 | HDGF | hepatoma-derived growth factor | 1 | | 1431 | hsa-mir-511-1 | 3068 | HDGF | hepatoma-derived growth factor | 1 | | 1432 | hsa-mir-511-2 | 3068 | HDGF | hepatoma-derived growth factor | 1 | | 1433 | hsa-mir-1284 | 3068 | HDGF | hepatoma-derived growth factor | 1 | | 1434 | hsa-mir-3181 | 3068 | HDGF | hepatoma-derived growth factor | 1 | | 1435 | hsa-mir-3651 | 3068 | HDGF | hepatoma-derived growth factor | 1 | | 1436 | hsa-mir-4632 | 3068 | HDGF | hepatoma-derived growth factor | 1 | | 1437 | hsa-mir-4648 | 3068 | HDGF | hepatoma-derived growth factor | 1 | | 1438 | hsa-mir-4726 | 3068 | HDGF | hepatoma-derived growth factor | 1 | | 1439 | hsa-mir-4785 | 3068 | HDGF | hepatoma-derived growth factor | 1 | | 1440 | hsa-mir-4794 | 3068 | HDGF | hepatoma-derived growth factor | 1 | | 1441 | hsa-mir-4802 | 3068 | HDGF | hepatoma-derived growth factor | 1 | | 1442 | hsa-let-7i | 3068 | HDGF | hepatoma-derived growth factor | 1 | | 1443 | hsa-mir-302b | 3068 | HDGF | hepatoma-derived growth factor | 1 | | 1444 | hsa-mir-302c | 3068 | HDGF | hepatoma-derived growth factor | 1 | | 1445 | hsa-mir-339 | 3068 | HDGF | hepatoma-derived growth factor | 1 | | 1446 | hsa-mir-562 | 3068 | HDGF | hepatoma-derived growth factor | 1 | | 1447 | hsa-mir-567 | 3068 | HDGF | hepatoma-derived growth factor | 1 | | 1448 | hsa-mir-576 | 3068 | HDGF | hepatoma-derived growth factor | 1 | | 1449 | hsa-mir-579 | 3068 | HDGF | hepatoma-derived growth factor | 1 | | 1450 | hsa-mir-582 | 3068 | HDGF | hepatoma-derived growth factor | 1 | | 1451 | hsa-mir-604 | 3068 | HDGF | hepatoma-derived growth factor | 1 | | 1452 | hsa-mir-608 | 3068 | HDGF | hepatoma-derived growth factor | 1 | | 1453 | hsa-mir-621 | 3068 | HDGF | hepatoma-derived growth factor | 1 | | 1454 | hsa-mir-627 | 3068 | HDGF | hepatoma-derived growth factor | 1 | | 1455 | hsa-mir-454 | 3068 | HDGF | hepatoma-derived growth factor | 1 | | 1456 | hsa-mir-302d | 3068 | HDGF | hepatoma-derived growth factor | 1 | | 1457 | hsa-mir-367 | 3068 | HDGF | hepatoma-derived growth factor | 1 | | 1458 | hsa-mir-548b | 3068 | HDGF | hepatoma-derived growth factor | 1 | | 1459 | hsa-mir-548d-1;hsa-mir-548d-2 | 3068 | HDGF | hepatoma-derived growth factor | 1 | | 1460 | hsa-mir-938 | 3068 | HDGF | hepatoma-derived growth factor | 1 | | 1461 | hsa-mir-1236 | 3068 | HDGF | hepatoma-derived growth factor | 1 | | 1465 | hsa-mir-302b | 23271 | CAMSAP2 | calmodulin regulated spectrin-associated protein family, member 2 | 1 | | 1466 | hsa-mir-302c | 23271 | CAMSAP2 | calmodulin regulated spectrin-associated protein family, member 2 | 1 | | 1467 | hsa-mir-302d | 23271 | CAMSAP2 | calmodulin regulated spectrin-associated protein family, member 2 | 1 | | 1468 | hsa-mir-367 | 23271 | CAMSAP2 | calmodulin regulated spectrin-associated protein family, member 2 | 1 | | 1469 | hsa-mir-562 | 23271 | CAMSAP2 | calmodulin regulated spectrin-associated protein family, member 2 | 1 | | 1470 | hsa-mir-604 | 23271 | CAMSAP2 | calmodulin regulated spectrin-associated protein family, member 2 | 1 | | 1471 | hsa-mir-548d-1 | 23271 | CAMSAP2 | calmodulin regulated spectrin-associated protein family, member 2 | 1 | | 1472 | hsa-mir-938 | 23271 | CAMSAP2 | calmodulin regulated spectrin-associated protein family, member 2 | 1 | | 1473 | hsa-mir-548h-1 | 23271 | CAMSAP2 | calmodulin regulated spectrin-associated protein family, member 2 | 1 | | 1474 | hsa-mir-3135a | 23271 | CAMSAP2 | calmodulin regulated spectrin-associated protein family, member 2 | 1 | | 1475 | hsa-mir-3177 | 23271 | CAMSAP2 | calmodulin regulated spectrin-associated protein family, member 2 | 1 | | 1476 | hsa-mir-3655 | 23271 | CAMSAP2 | calmodulin regulated spectrin-associated protein family, member 2 | 1 | | 1477 | hsa-mir-4526 | 23271 | CAMSAP2 | calmodulin regulated spectrin-associated protein family, member 2 | 1 | | 1478 | hsa-mir-4635 | 23271 | CAMSAP2 | calmodulin regulated spectrin-associated protein family, member 2 | 1 | | 1479 | hsa-mir-4651 | 23271 | CAMSAP2 | calmodulin regulated spectrin-associated protein family, member 2 | 1 | | 1480 | hsa-mir-4744 | 23271 | CAMSAP2 | calmodulin regulated spectrin-associated protein family, member 2 | 1 | | 1481 | hsa-mir-5190 | 23271 | CAMSAP2 | calmodulin regulated spectrin-associated protein family, member 2 | 1 | | 1482 | hsa-mir-101-2 | 23271 | CAMSAP2 | calmodulin regulated spectrin-associated protein family, member 2 | 1 | | 1483 | hsa-mir-128-1;hsa-mir-128-2 | 23271 | CAMSAP2 | calmodulin regulated spectrin-associated protein family, member 2 | 1 | | 1484 | hsa-mir-301a | 23271 | CAMSAP2 | calmodulin regulated spectrin-associated protein family, member 2 | 1 | | 1485 | hsa-mir-511-1;hsa-mir-511-2 | 23271 | CAMSAP2 | calmodulin regulated spectrin-associated protein family, member 2 | 1 | | 1486 | hsa-mir-586 | 23271 | CAMSAP2 | calmodulin regulated spectrin-associated protein family, member 2 | 1 | | 1487 | hsa-mir-593 | 23271 | CAMSAP2 | calmodulin regulated spectrin-associated protein family, member 2 | 1 | | 1488 | hsa-mir-620 | 23271 | CAMSAP2 | calmodulin regulated spectrin-associated protein family, member 2 | 1 | | 1489 | hsa-mir-33b | 23271 | CAMSAP2 | calmodulin regulated spectrin-associated protein family, member 2 | 1 | | 1490 | hsa-mir-942 | 23271 | CAMSAP2 | calmodulin regulated spectrin-associated protein family, member 2 | 1 | | 1494 | hsa-mir-302b | 55718 | POLR3E | polymerase (RNA) III (DNA directed) polypeptide E (80kD) | 1 | | 1495 | hsa-mir-302c | 55718 | POLR3E | polymerase (RNA) III (DNA directed) polypeptide E (80kD) | 1 | | 1496 | hsa-mir-302d | 55718 | POLR3E | polymerase (RNA) III (DNA directed) polypeptide E (80kD) | 1 | | 1497 | hsa-mir-367 | 55718 | POLR3E | polymerase (RNA) III (DNA directed) polypeptide E (80kD) | 1 | | 1498 | hsa-mir-562 | 55718 | POLR3E | polymerase (RNA) III (DNA directed) polypeptide E (80kD) | 1 | | 1499 | hsa-mir-604 | 55718 | POLR3E | polymerase (RNA) III (DNA directed) polypeptide E (80kD) | 1 | | 1500 | hsa-mir-548d-1 | 55718 | POLR3E | polymerase (RNA) III (DNA directed) polypeptide E (80kD) | 1 | | 1501 | hsa-mir-938 | 55718 | POLR3E | polymerase (RNA) III (DNA directed) polypeptide E (80kD) | 1 | | 1502 | hsa-mir-548h-1 | 55718 | POLR3E | polymerase (RNA) III (DNA directed) polypeptide E (80kD) | 1 | | 1503 | hsa-mir-3135a | 55718 | POLR3E | polymerase (RNA) III (DNA directed) polypeptide E (80kD) | 1 | | 1504 | hsa-mir-3177 | 55718 | POLR3E | polymerase (RNA) III (DNA directed) polypeptide E (80kD) | 1 | | 1505 | hsa-mir-3655 | 55718 | POLR3E | polymerase (RNA) III (DNA directed) polypeptide E (80kD) | 1 | | 1506 | hsa-mir-4526 | 55718 | POLR3E | polymerase (RNA) III (DNA directed) polypeptide E (80kD) | 1 | | 1507 | hsa-mir-4635 | 55718 | POLR3E | polymerase (RNA) III (DNA directed) polypeptide E (80kD) | 1 | | 1508 | hsa-mir-4651 | 55718 | POLR3E | polymerase (RNA) III (DNA directed) polypeptide E (80kD) | 1 | | 1509 | hsa-mir-4744 | 55718 | POLR3E | polymerase (RNA) III (DNA directed) polypeptide E (80kD) | 1 | | 1510 | hsa-mir-5190 | 55718 | POLR3E | polymerase (RNA) III (DNA directed) polypeptide E (80kD) | 1 | | 1511 | hsa-mir-101-2 | 55718 | POLR3E | polymerase (RNA) III (DNA directed) polypeptide E (80kD) | 1 | | 1512 | hsa-mir-128-1;hsa-mir-128-2 | 55718 | POLR3E | polymerase (RNA) III (DNA directed) polypeptide E (80kD) | 1 | | 1513 | hsa-mir-301a | 55718 | POLR3E | polymerase (RNA) III (DNA directed) polypeptide E (80kD) | 1 | | 1514 | hsa-mir-511-1;hsa-mir-511-2 | 55718 | POLR3E | polymerase (RNA) III (DNA directed) polypeptide E (80kD) | 1 | | 1515 | hsa-mir-586 | 55718 | POLR3E | polymerase (RNA) III (DNA directed) polypeptide E (80kD) | 1 | | 1516 | hsa-mir-593 | 55718 | POLR3E | polymerase (RNA) III (DNA directed) polypeptide E (80kD) | 1 | | 1517 | hsa-mir-620 | 55718 | POLR3E | polymerase (RNA) III (DNA directed) polypeptide E (80kD) | 1 | | 1518 | hsa-mir-33b | 55718 | POLR3E | polymerase (RNA) III (DNA directed) polypeptide E (80kD) | 1 | | 1519 | hsa-mir-942 | 55718 | POLR3E | polymerase (RNA) III (DNA directed) polypeptide E (80kD) | 1 | | 1523 | hsa-mir-302b | 138050 | HGSNAT | heparan-alpha-glucosaminide N-acetyltransferase | 1 | | 1524 | hsa-mir-302c | 138050 | HGSNAT | heparan-alpha-glucosaminide N-acetyltransferase | 1 | | 1525 | hsa-mir-302d | 138050 | HGSNAT | heparan-alpha-glucosaminide N-acetyltransferase | 1 | | 1526 | hsa-mir-367 | 138050 | HGSNAT | heparan-alpha-glucosaminide N-acetyltransferase | 1 | | 1527 | hsa-mir-562 | 138050 | HGSNAT | heparan-alpha-glucosaminide N-acetyltransferase | 1 | | 1528 | hsa-mir-604 | 138050 | HGSNAT | heparan-alpha-glucosaminide N-acetyltransferase | 1 | | 1529 | hsa-mir-548d-1 | 138050 | HGSNAT | heparan-alpha-glucosaminide N-acetyltransferase | 1 | | 1530 | hsa-mir-938 | 138050 | HGSNAT | heparan-alpha-glucosaminide N-acetyltransferase | 1 | | 1531 | hsa-mir-548h-1 | 138050 | HGSNAT | heparan-alpha-glucosaminide N-acetyltransferase | 1 | | 1532 | hsa-mir-3135a | 138050 | HGSNAT | heparan-alpha-glucosaminide N-acetyltransferase | 1 | | 1533 | hsa-mir-3177 | 138050 | HGSNAT | heparan-alpha-glucosaminide N-acetyltransferase | 1 | | 1534 | hsa-mir-3655 | 138050 | HGSNAT | heparan-alpha-glucosaminide N-acetyltransferase | 1 | | 1535 | hsa-mir-4526 | 138050 | HGSNAT | heparan-alpha-glucosaminide N-acetyltransferase | 1 | | 1536 | hsa-mir-4635 | 138050 | HGSNAT | heparan-alpha-glucosaminide N-acetyltransferase | 1 | | 1537 | hsa-mir-4651 | 138050 | HGSNAT | heparan-alpha-glucosaminide N-acetyltransferase | 1 | | 1538 | hsa-mir-4744 | 138050 | HGSNAT | heparan-alpha-glucosaminide N-acetyltransferase | 1 | | 1539 | hsa-mir-5190 | 138050 | HGSNAT | heparan-alpha-glucosaminide N-acetyltransferase | 1 | | 1540 | hsa-mir-101-2 | 138050 | HGSNAT | heparan-alpha-glucosaminide N-acetyltransferase | 1 | | 1541 | hsa-mir-128-1;hsa-mir-128-2 | 138050 | HGSNAT | heparan-alpha-glucosaminide N-acetyltransferase | 1 | | 1542 | hsa-mir-301a | 138050 | HGSNAT | heparan-alpha-glucosaminide N-acetyltransferase | 1 | | 1543 | hsa-mir-511-1;hsa-mir-511-2 | 138050 | HGSNAT | heparan-alpha-glucosaminide N-acetyltransferase | 1 | | 1544 | hsa-mir-586 | 138050 | HGSNAT | heparan-alpha-glucosaminide N-acetyltransferase | 1 | | 1545 | hsa-mir-593 | 138050 | HGSNAT | heparan-alpha-glucosaminide N-acetyltransferase | 1 | | 1546 | hsa-mir-620 | 138050 | HGSNAT | heparan-alpha-glucosaminide N-acetyltransferase | 1 | | 1547 | hsa-mir-33b | 138050 | HGSNAT | heparan-alpha-glucosaminide N-acetyltransferase | 1 | | 1548 | hsa-mir-942 | 138050 | HGSNAT | heparan-alpha-glucosaminide N-acetyltransferase | 1 | | 1549 | hsa-mir-101-2 | 51660 | BRP44L | brain protein 44-like | 1 | | 1554 | hsa-mir-511-1 | 51660 | BRP44L | brain protein 44-like | 1 | | 1555 | hsa-mir-511-2 | 51660 | BRP44L | brain protein 44-like | 1 | | 1556 | hsa-mir-1284 | 51660 | BRP44L | brain protein 44-like | 1 | | 1557 | hsa-mir-3181 | 51660 | BRP44L | brain protein 44-like | 1 | | 1558 | hsa-mir-3651 | 51660 | BRP44L | brain protein 44-like | 1 | | 1559 | hsa-mir-4632 | 51660 | BRP44L | brain protein 44-like | 1 | | 1560 | hsa-mir-4648 | 51660 | BRP44L | brain protein 44-like | 1 | | 1561 | hsa-mir-4726 | 51660 | BRP44L | brain protein 44-like | 1 | | 1562 | hsa-mir-4785 | 51660 | BRP44L | brain protein 44-like | 1 | | 1563 | hsa-mir-4794 | 51660 | BRP44L | brain protein 44-like | 1 | | 1564 | hsa-mir-4802 | 51660 | BRP44L | brain protein 44-like | 1 | | 1565 | hsa-let-7i | 51660 | BRP44L | brain protein 44-like | 1 | | 1566 | hsa-mir-302b | 51660 | BRP44L | brain protein 44-like | 1 | | 1567 | hsa-mir-302c | 51660 | BRP44L | brain protein 44-like | 1 | | 1568 | hsa-mir-339 | 51660 | BRP44L | brain protein 44-like | 1 | | 1569 | hsa-mir-562 | 51660 | BRP44L | brain protein 44-like | 1 | | 1570 | hsa-mir-567 | 51660 | BRP44L | brain protein 44-like | 1 | | 1571 | hsa-mir-576 | 51660 | BRP44L | brain protein 44-like | 1 | | 1572 | hsa-mir-579 | 51660 | BRP44L | brain protein 44-like | 1 | | 1573 | hsa-mir-582 | 51660 | BRP44L | brain protein 44-like | 1 | | 1574 | hsa-mir-604 | 51660 | BRP44L | brain protein 44-like | 1 | | 1575 | hsa-mir-608 | 51660 | BRP44L | brain protein 44-like | 1 | | 1576 | hsa-mir-621 | 51660 | BRP44L | brain protein 44-like | 1 | | 1577 | hsa-mir-627 | 51660 | BRP44L | brain protein 44-like | 1 | | 1578 | hsa-mir-454 | 51660 | BRP44L | brain protein 44-like | 1 | | 1579 | hsa-mir-302d | 51660 | BRP44L | brain protein 44-like | 1 | | 1580 | hsa-mir-367 | 51660 | BRP44L | brain protein 44-like | 1 | | 1581 | hsa-mir-548b | 51660 | BRP44L | brain protein 44-like | 1 | | 1582 | hsa-mir-548d-1;hsa-mir-548d-2 | 51660 | BRP44L | brain protein 44-like | 1 | | 1583 | hsa-mir-938 | 51660 | BRP44L | brain protein 44-like | 1 | | 1584 | hsa-mir-1236 | 51660 | BRP44L | brain protein 44-like | 1 | | 1588 | hsa-mir-302b | 55003 | PAK1IP1 | PAK1 interacting protein 1 | 1 | | 1589 | hsa-mir-302c | 55003 | PAK1IP1 | PAK1 interacting protein 1 | 1 | | 1590 | hsa-mir-302d | 55003 | PAK1IP1 | PAK1 interacting protein 1 | 1 | | 1591 | hsa-mir-367 | 55003 | PAK1IP1 | PAK1 interacting protein 1 | 1 | | 1592 | hsa-mir-562 | 55003 | PAK1IP1 | PAK1 interacting protein 1 | 1 | | 1593 | hsa-mir-604 | 55003 | PAK1IP1 | PAK1 interacting protein 1 | 1 | | 1594 | hsa-mir-548d-1 | 55003 | PAK1IP1 | PAK1 interacting protein 1 | 1 | | 1595 | hsa-mir-938 | 55003 | PAK1IP1 | PAK1 interacting protein 1 | 1 | | 1596 | hsa-mir-548h-1 | 55003 | PAK1IP1 | PAK1 interacting protein 1 | 1 | | 1597 | hsa-mir-3135a | 55003 | PAK1IP1 | PAK1 interacting protein 1 | 1 | | 1598 | hsa-mir-3177 | 55003 | PAK1IP1 | PAK1 interacting protein 1 | 1 | | 1599 | hsa-mir-3655 | 55003 | PAK1IP1 | PAK1 interacting protein 1 | 1 | | 1600 | hsa-mir-4526 | 55003 | PAK1IP1 | PAK1 interacting protein 1 | 1 | | 1601 | hsa-mir-4635 | 55003 | PAK1IP1 | PAK1 interacting protein 1 | 1 | | 1602 | hsa-mir-4651 | 55003 | PAK1IP1 | PAK1 interacting protein 1 | 1 | | 1603 | hsa-mir-4744 | 55003 | PAK1IP1 | PAK1 interacting protein 1 | 1 | | 1604 | hsa-mir-5190 | 55003 | PAK1IP1 | PAK1 interacting protein 1 | 1 | | 1605 | hsa-mir-101-2 | 55003 | PAK1IP1 | PAK1 interacting protein 1 | 1 | | 1606 | hsa-mir-128-1;hsa-mir-128-2 | 55003 | PAK1IP1 | PAK1 interacting protein 1 | 1 | | 1607 | hsa-mir-301a | 55003 | PAK1IP1 | PAK1 interacting protein 1 | 1 | | 1608 | hsa-mir-511-1;hsa-mir-511-2 | 55003 | PAK1IP1 | PAK1 interacting protein 1 | 1 | | 1609 | hsa-mir-586 | 55003 | PAK1IP1 | PAK1 interacting protein 1 | 1 | | 1610 | hsa-mir-593 | 55003 | PAK1IP1 | PAK1 interacting protein 1 | 1 | | 1611 | hsa-mir-620 | 55003 | PAK1IP1 | PAK1 interacting protein 1 | 1 | | 1612 | hsa-mir-33b | 55003 | PAK1IP1 | PAK1 interacting protein 1 | 1 | | 1613 | hsa-mir-942 | 55003 | PAK1IP1 | PAK1 interacting protein 1 | 1 | | 1617 | hsa-mir-302b | 10365 | KLF2 | Kruppel-like factor 2 (lung) | 1 | | 1618 | hsa-mir-302c | 10365 | KLF2 | Kruppel-like factor 2 (lung) | 1 | | 1619 | hsa-mir-302d | 10365 | KLF2 | Kruppel-like factor 2 (lung) | 1 | | 1620 | hsa-mir-367 | 10365 | KLF2 | Kruppel-like factor 2 (lung) | 1 | | 1621 | hsa-mir-562 | 10365 | KLF2 | Kruppel-like factor 2 (lung) | 1 | | 1622 | hsa-mir-604 | 10365 | KLF2 | Kruppel-like factor 2 (lung) | 1 | | 1623 | hsa-mir-548d-1 | 10365 | KLF2 | Kruppel-like factor 2 (lung) | 1 | | 1624 | hsa-mir-938 | 10365 | KLF2 | Kruppel-like factor 2 (lung) | 1 | | 1625 | hsa-mir-548h-1 | 10365 | KLF2 | Kruppel-like factor 2 (lung) | 1 | | 1626 | hsa-mir-3135a | 10365 | KLF2 | Kruppel-like factor 2 (lung) | 1 | | 1627 | hsa-mir-3177 | 10365 | KLF2 | Kruppel-like factor 2 (lung) | 1 | | 1628 | hsa-mir-3655 | 10365 | KLF2 | Kruppel-like factor 2 (lung) | 1 | | 1629 | hsa-mir-4526 | 10365 | KLF2 | Kruppel-like factor 2 (lung) | 1 | | 1630 | hsa-mir-4635 | 10365 | KLF2 | Kruppel-like factor 2 (lung) | 1 | | 1631 | hsa-mir-4651 | 10365 | KLF2 | Kruppel-like factor 2 (lung) | 1 | | 1632 | hsa-mir-4744 | 10365 | KLF2 | Kruppel-like factor 2 (lung) | 1 | | 1633 | hsa-mir-5190 | 10365 | KLF2 | Kruppel-like factor 2 (lung) | 1 | | 1634 | hsa-mir-101-2 | 10365 | KLF2 | Kruppel-like factor 2 (lung) | 1 | | 1635 | hsa-mir-128-1;hsa-mir-128-2 | 10365 | KLF2 | Kruppel-like factor 2 (lung) | 1 | | 1636 | hsa-mir-301a | 10365 | KLF2 | Kruppel-like factor 2 (lung) | 1 | | 1637 | hsa-mir-511-1;hsa-mir-511-2 | 10365 | KLF2 | Kruppel-like factor 2 (lung) | 1 | | 1638 | hsa-mir-586 | 10365 | KLF2 | Kruppel-like factor 2 (lung) | 1 | | 1639 | hsa-mir-593 | 10365 | KLF2 | Kruppel-like factor 2 (lung) | 1 | | 1640 | hsa-mir-620 | 10365 | KLF2 | Kruppel-like factor 2 (lung) | 1 | | 1641 | hsa-mir-33b | 10365 | KLF2 | Kruppel-like factor 2 (lung) | 1 | | 1642 | hsa-mir-942 | 10365 | KLF2 | Kruppel-like factor 2 (lung) | 1 | | 1646 | hsa-mir-302b | 80008 | TMEM156 | transmembrane protein 156 | 1 | | 1647 | hsa-mir-302c | 80008 | TMEM156 | transmembrane protein 156 | 1 | | 1648 | hsa-mir-302d | 80008 | TMEM156 | transmembrane protein 156 | 1 | | 1649 | hsa-mir-367 | 80008 | TMEM156 | transmembrane protein 156 | 1 | | 1650 | hsa-mir-562 | 80008 | TMEM156 | transmembrane protein 156 | 1 | | 1651 | hsa-mir-604 | 80008 | TMEM156 | transmembrane protein 156 | 1 | | 1652 | hsa-mir-548d-1 | 80008 | TMEM156 | transmembrane protein 156 | 1 | | 1653 | hsa-mir-938 | 80008 | TMEM156 | transmembrane protein 156 | 1 | | 1654 | hsa-mir-548h-1 | 80008 | TMEM156 | transmembrane protein 156 | 1 | | 1655 | hsa-mir-3135a | 80008 | TMEM156 | transmembrane protein 156 | 1 | | 1656 | hsa-mir-3177 | 80008 | TMEM156 | transmembrane protein 156 | 1 | | 1657 | hsa-mir-3655 | 80008 | TMEM156 | transmembrane protein 156 | 1 | | 1658 | hsa-mir-4526 | 80008 | TMEM156 | transmembrane protein 156 | 1 | | 1659 | hsa-mir-4635 | 80008 | TMEM156 | transmembrane protein 156 | 1 | | 1660 | hsa-mir-4651 | 80008 | TMEM156 | transmembrane protein 156 | 1 | | 1661 | hsa-mir-4744 | 80008 | TMEM156 | transmembrane protein 156 | 1 | | 1662 | hsa-mir-5190 | 80008 | TMEM156 | transmembrane protein 156 | 1 | | 1663 | hsa-mir-101-2 | 80008 | TMEM156 | transmembrane protein 156 | 1 | | 1664 | hsa-mir-128-1;hsa-mir-128-2 | 80008 | TMEM156 | transmembrane protein 156 | 1 | | 1665 | hsa-mir-301a | 80008 | TMEM156 | transmembrane protein 156 | 1 | | 1666 | hsa-mir-511-1;hsa-mir-511-2 | 80008 | TMEM156 | transmembrane protein 156 | 1 | | 1667 | hsa-mir-586 | 80008 | TMEM156 | transmembrane protein 156 | 1 | | 1668 | hsa-mir-593 | 80008 | TMEM156 | transmembrane protein 156 | 1 | | 1669 | hsa-mir-620 | 80008 | TMEM156 | transmembrane protein 156 | 1 | | 1670 | hsa-mir-33b | 80008 | TMEM156 | transmembrane protein 156 | 1 | | 1671 | hsa-mir-942 | 80008 | TMEM156 | transmembrane protein 156 | 1 | | 1672 | hsa-mir-101-2 | 51733 | UPB1 | ureidopropionase, beta | 1 | | 1677 | hsa-mir-511-1 | 51733 | UPB1 | ureidopropionase, beta | 1 | | 1678 | hsa-mir-511-2 | 51733 | UPB1 | ureidopropionase, beta | 1 | | 1679 | hsa-mir-1284 | 51733 | UPB1 | ureidopropionase, beta | 1 | | 1680 | hsa-mir-3181 | 51733 | UPB1 | ureidopropionase, beta | 1 | | 1681 | hsa-mir-3651 | 51733 | UPB1 | ureidopropionase, beta | 1 | | 1682 | hsa-mir-4632 | 51733 | UPB1 | ureidopropionase, beta | 1 | | 1683 | hsa-mir-4648 | 51733 | UPB1 | ureidopropionase, beta | 1 | | 1684 | hsa-mir-4726 | 51733 | UPB1 | ureidopropionase, beta | 1 | | 1685 | hsa-mir-4785 | 51733 | UPB1 | ureidopropionase, beta | 1 | | 1686 | hsa-mir-4794 | 51733 | UPB1 | ureidopropionase, beta | 1 | | 1687 | hsa-mir-4802 | 51733 | UPB1 | ureidopropionase, beta | 1 | | 1688 | hsa-let-7i | 51733 | UPB1 | ureidopropionase, beta | 1 | | 1689 | hsa-mir-302b | 51733 | UPB1 | ureidopropionase, beta | 1 | | 1690 | hsa-mir-302c | 51733 | UPB1 | ureidopropionase, beta | 1 | | 1691 | hsa-mir-339 | 51733 | UPB1 | ureidopropionase, beta | 1 | | 1692 | hsa-mir-562 | 51733 | UPB1 | ureidopropionase, beta | 1 | | 1693 | hsa-mir-567 | 51733 | UPB1 | ureidopropionase, beta | 1 | | 1694 | hsa-mir-576 | 51733 | UPB1 | ureidopropionase, beta | 1 | | 1695 | hsa-mir-579 | 51733 | UPB1 | ureidopropionase, beta | 1 | | 1696 | hsa-mir-582 | 51733 | UPB1 | ureidopropionase, beta | 1 | | 1697 | hsa-mir-604 | 51733 | UPB1 | ureidopropionase, beta | 1 | | 1698 | hsa-mir-608 | 51733 | UPB1 | ureidopropionase, beta | 1 | | 1699 | hsa-mir-621 | 51733 | UPB1 | ureidopropionase, beta | 1 | | 1700 | hsa-mir-627 | 51733 | UPB1 | ureidopropionase, beta | 1 | | 1701 | hsa-mir-454 | 51733 | UPB1 | ureidopropionase, beta | 1 | | 1702 | hsa-mir-302d | 51733 | UPB1 | ureidopropionase, beta | 1 | | 1703 | hsa-mir-367 | 51733 | UPB1 | ureidopropionase, beta | 1 | | 1704 | hsa-mir-548b | 51733 | UPB1 | ureidopropionase, beta | 1 | | 1705 | hsa-mir-548d-1;hsa-mir-548d-2 | 51733 | UPB1 | ureidopropionase, beta | 1 | | 1706 | hsa-mir-938 | 51733 | UPB1 | ureidopropionase, beta | 1 | | 1707 | hsa-mir-1236 | 51733 | UPB1 | ureidopropionase, beta | 1 | | 1711 | hsa-mir-302b | 79931 | TNIP3 | TNFAIP3 interacting protein 3 | 1 | | 1712 | hsa-mir-302c | 79931 | TNIP3 | TNFAIP3 interacting protein 3 | 1 | | 1713 | hsa-mir-302d | 79931 | TNIP3 | TNFAIP3 interacting protein 3 | 1 | | 1714 | hsa-mir-367 | 79931 | TNIP3 | TNFAIP3 interacting protein 3 | 1 | | 1715 | hsa-mir-562 | 79931 | TNIP3 | TNFAIP3 interacting protein 3 | 1 | | 1716 | hsa-mir-604 | 79931 | TNIP3 | TNFAIP3 interacting protein 3 | 1 | | 1717 | hsa-mir-548d-1 | 79931 | TNIP3 | TNFAIP3 interacting protein 3 | 1 | | 1718 | hsa-mir-938 | 79931 | TNIP3 | TNFAIP3 interacting protein 3 | 1 | | 1719 | hsa-mir-548h-1 | 79931 | TNIP3 | TNFAIP3 interacting protein 3 | 1 | | 1720 | hsa-mir-3135a | 79931 | TNIP3 | TNFAIP3 interacting protein 3 | 1 | | 1721 | hsa-mir-3177 | 79931 | TNIP3 | TNFAIP3 interacting protein 3 | 1 | | 1722 | hsa-mir-3655 | 79931 | TNIP3 | TNFAIP3 interacting protein 3 | 1 | | 1723 | hsa-mir-4526 | 79931 | TNIP3 | TNFAIP3 interacting protein 3 | 1 | | 1724 | hsa-mir-4635 | 79931 | TNIP3 | TNFAIP3 interacting protein 3 | 1 | | 1725 | hsa-mir-4651 | 79931 | TNIP3 | TNFAIP3 interacting protein 3 | 1 | | 1726 | hsa-mir-4744 | 79931 | TNIP3 | TNFAIP3 interacting protein 3 | 1 | | 1727 | hsa-mir-5190 | 79931 | TNIP3 | TNFAIP3 interacting protein 3 | 1 | | 1728 | hsa-mir-101-2 | 79931 | TNIP3 | TNFAIP3 interacting protein 3 | 1 | | 1729 | hsa-mir-128-1;hsa-mir-128-2 | 79931 | TNIP3 | TNFAIP3 interacting protein 3 | 1 | | 1730 | hsa-mir-301a | 79931 | TNIP3 | TNFAIP3 interacting protein 3 | 1 | | 1731 | hsa-mir-511-1;hsa-mir-511-2 | 79931 | TNIP3 | TNFAIP3 interacting protein 3 | 1 | | 1732 | hsa-mir-586 | 79931 | TNIP3 | TNFAIP3 interacting protein 3 | 1 | | 1733 | hsa-mir-593 | 79931 | TNIP3 | TNFAIP3 interacting protein 3 | 1 | | 1734 | hsa-mir-620 | 79931 | TNIP3 | TNFAIP3 interacting protein 3 | 1 | | 1735 | hsa-mir-33b | 79931 | TNIP3 | TNFAIP3 interacting protein 3 | 1 | | 1736 | hsa-mir-942 | 79931 | TNIP3 | TNFAIP3 interacting protein 3 | 1 | | 1740 | hsa-mir-302b | 54477 | PLEKHA5 | pleckstrin homology domain containing, family A member 5 | 1 | | 1741 | hsa-mir-302c | 54477 | PLEKHA5 | pleckstrin homology domain containing, family A member 5 | 1 | | 1742 | hsa-mir-302d | 54477 | PLEKHA5 | pleckstrin homology domain containing, family A member 5 | 1 | | 1743 | hsa-mir-367 | 54477 | PLEKHA5 | pleckstrin homology domain containing, family A member 5 | 1 | | 1744 | hsa-mir-562 | 54477 | PLEKHA5 | pleckstrin homology domain containing, family A member 5 | 1 | | 1745 | hsa-mir-604 | 54477 | PLEKHA5 | pleckstrin homology domain containing, family A member 5 | 1 | | 1746 | hsa-mir-548d-1 | 54477 | PLEKHA5 | pleckstrin homology domain containing, family A member 5 | 1 | | 1747 | hsa-mir-938 | 54477 | PLEKHA5 | pleckstrin homology domain containing, family A member 5 | 1 | | 1748 | hsa-mir-548h-1 | 54477 | PLEKHA5 | pleckstrin homology domain containing, family A member 5 | 1 | | 1749 | hsa-mir-3135a | 54477 | PLEKHA5 | pleckstrin homology domain containing, family A member 5 | 1 | | 1750 | hsa-mir-3177 | 54477 | PLEKHA5 | pleckstrin homology domain containing, family A member 5 | 1 | | 1751 | hsa-mir-3655 | 54477 | PLEKHA5 | pleckstrin homology domain containing, family A member 5 | 1 | | 1752 | hsa-mir-4526 | 54477 | PLEKHA5 | pleckstrin homology domain containing, family A member 5 | 1 | | 1753 | hsa-mir-4635 | 54477 | PLEKHA5 | pleckstrin homology domain containing, family A member 5 | 1 | | 1754 | hsa-mir-4651 | 54477 | PLEKHA5 | pleckstrin homology domain containing, family A member 5 | 1 | | 1755 | hsa-mir-4744 | 54477 | PLEKHA5 | pleckstrin homology domain containing, family A member 5 | 1 | | 1756 | hsa-mir-5190 | 54477 | PLEKHA5 | pleckstrin homology domain containing, family A member 5 | 1 | | 1757 | hsa-mir-101-2 | 54477 | PLEKHA5 | pleckstrin homology domain containing, family A member 5 | 1 | | 1758 | hsa-mir-128-1;hsa-mir-128-2 | 54477 | PLEKHA5 | pleckstrin homology domain containing, family A member 5 | 1 | | 1759 | hsa-mir-301a | 54477 | PLEKHA5 | pleckstrin homology domain containing, family A member 5 | 1 | | 1760 | hsa-mir-511-1;hsa-mir-511-2 | 54477 | PLEKHA5 | pleckstrin homology domain containing, family A member 5 | 1 | | 1761 | hsa-mir-586 | 54477 | PLEKHA5 | pleckstrin homology domain containing, family A member 5 | 1 | | 1762 | hsa-mir-593 | 54477 | PLEKHA5 | pleckstrin homology domain containing, family A member 5 | 1 | | 1763 | hsa-mir-620 | 54477 | PLEKHA5 | pleckstrin homology domain containing, family A member 5 | 1 | | 1764 | hsa-mir-33b | 54477 | PLEKHA5 | pleckstrin homology domain containing, family A member 5 | 1 | | 1765 | hsa-mir-942 | 54477 | PLEKHA5 | pleckstrin homology domain containing, family A member 5 | 1 | | 1769 | hsa-mir-302b | 7187 | TRAF3 | TNF receptor-associated factor 3 | 1 | | 1770 | hsa-mir-302c | 7187 | TRAF3 | TNF receptor-associated factor 3 | 1 | | 1771 | hsa-mir-302d | 7187 | TRAF3 | TNF receptor-associated factor 3 | 1 | | 1772 | hsa-mir-367 | 7187 | TRAF3 | TNF receptor-associated factor 3 | 1 | | 1773 | hsa-mir-562 | 7187 | TRAF3 | TNF receptor-associated factor 3 | 1 | | 1774 | hsa-mir-604 | 7187 | TRAF3 | TNF receptor-associated factor 3 | 1 | | 1775 | hsa-mir-548d-1 | 7187 | TRAF3 | TNF receptor-associated factor 3 | 1 | | 1776 | hsa-mir-938 | 7187 | TRAF3 | TNF receptor-associated factor 3 | 1 | | 1777 | hsa-mir-548h-1 | 7187 | TRAF3 | TNF receptor-associated factor 3 | 1 | | 1778 | hsa-mir-3135a | 7187 | TRAF3 | TNF receptor-associated factor 3 | 1 | | 1779 | hsa-mir-3177 | 7187 | TRAF3 | TNF receptor-associated factor 3 | 1 | | 1780 | hsa-mir-3655 | 7187 | TRAF3 | TNF receptor-associated factor 3 | 1 | | 1781 | hsa-mir-4526 | 7187 | TRAF3 | TNF receptor-associated factor 3 | 1 | | 1782 | hsa-mir-4635 | 7187 | TRAF3 | TNF receptor-associated factor 3 | 1 | | 1783 | hsa-mir-4651 | 7187 | TRAF3 | TNF receptor-associated factor 3 | 1 | | 1784 | hsa-mir-4744 | 7187 | TRAF3 | TNF receptor-associated factor 3 | 1 | | 1785 | hsa-mir-5190 | 7187 | TRAF3 | TNF receptor-associated factor 3 | 1 | | 1786 | hsa-mir-101-2 | 7187 | TRAF3 | TNF receptor-associated factor 3 | 1 | | 1787 | hsa-mir-128-1;hsa-mir-128-2 | 7187 | TRAF3 | TNF receptor-associated factor 3 | 1 | | 1788 | hsa-mir-301a | 7187 | TRAF3 | TNF receptor-associated factor 3 | 1 | | 1789 | hsa-mir-511-1;hsa-mir-511-2 | 7187 | TRAF3 | TNF receptor-associated factor 3 | 1 | | 1790 | hsa-mir-586 | 7187 | TRAF3 | TNF receptor-associated factor 3 | 1 | | 1791 | hsa-mir-593 | 7187 | TRAF3 | TNF receptor-associated factor 3 | 1 | | 1792 | hsa-mir-620 | 7187 | TRAF3 | TNF receptor-associated factor 3 | 1 | | 1793 | hsa-mir-33b | 7187 | TRAF3 | TNF receptor-associated factor 3 | 1 | | 1794 | hsa-mir-942 | 7187 | TRAF3 | TNF receptor-associated factor 3 | 1 | | 1798 | hsa-mir-302b | 1540 | CYLD | cylindromatosis (turban tumor syndrome) | 1 | | 1799 | hsa-mir-302c | 1540 | CYLD | cylindromatosis (turban tumor syndrome) | 1 | | 1800 | hsa-mir-302d | 1540 | CYLD | cylindromatosis (turban tumor syndrome) | 1 | | 1801 | hsa-mir-367 | 1540 | CYLD | cylindromatosis (turban tumor syndrome) | 1 | | 1802 | hsa-mir-562 | 1540 | CYLD | cylindromatosis (turban tumor syndrome) | 1 | | 1803 | hsa-mir-604 | 1540 | CYLD | cylindromatosis (turban tumor syndrome) | 1 | | 1804 | hsa-mir-548d-1 | 1540 | CYLD | cylindromatosis (turban tumor syndrome) | 1 | | 1805 | hsa-mir-938 | 1540 | CYLD | cylindromatosis (turban tumor syndrome) | 1 | | 1806 | hsa-mir-548h-1 | 1540 | CYLD | cylindromatosis (turban tumor syndrome) | 1 | | 1807 | hsa-mir-3135a | 1540 | CYLD | cylindromatosis (turban tumor syndrome) | 1 | | 1808 | hsa-mir-3177 | 1540 | CYLD | cylindromatosis (turban tumor syndrome) | 1 | | 1809 | hsa-mir-3655 | 1540 | CYLD | cylindromatosis (turban tumor syndrome) | 1 | | 1810 | hsa-mir-4526 | 1540 | CYLD | cylindromatosis (turban tumor syndrome) | 1 | | 1811 | hsa-mir-4635 | 1540 | CYLD | cylindromatosis (turban tumor syndrome) | 1 | | 1812 | hsa-mir-4651 | 1540 | CYLD | cylindromatosis (turban tumor syndrome) | 1 | | 1813 | hsa-mir-4744 | 1540 | CYLD | cylindromatosis (turban tumor syndrome) | 1 | | 1814 | hsa-mir-5190 | 1540 | CYLD | cylindromatosis (turban tumor syndrome) | 1 | | 1815 | hsa-mir-101-2 | 1540 | CYLD | cylindromatosis (turban tumor syndrome) | 1 | | 1816 | hsa-mir-128-1;hsa-mir-128-2 | 1540 | CYLD | cylindromatosis (turban tumor syndrome) | 1 | | 1817 | hsa-mir-301a | 1540 | CYLD | cylindromatosis (turban tumor syndrome) | 1 | | 1818 | hsa-mir-511-1;hsa-mir-511-2 | 1540 | CYLD | cylindromatosis (turban tumor syndrome) | 1 | | 1819 | hsa-mir-586 | 1540 | CYLD | cylindromatosis (turban tumor syndrome) | 1 | | 1820 | hsa-mir-593 | 1540 | CYLD | cylindromatosis (turban tumor syndrome) | 1 | | 1821 | hsa-mir-620 | 1540 | CYLD | cylindromatosis (turban tumor syndrome) | 1 | | 1822 | hsa-mir-33b | 1540 | CYLD | cylindromatosis (turban tumor syndrome) | 1 | | 1823 | hsa-mir-942 | 1540 | CYLD | cylindromatosis (turban tumor syndrome) | 1 | | 1824 | hsa-mir-101-2 | 10016 | PDCD6 | programmed cell death 6 | 1 | | 1829 | hsa-mir-511-1 | 10016 | PDCD6 | programmed cell death 6 | 1 | | 1830 | hsa-mir-511-2 | 10016 | PDCD6 | programmed cell death 6 | 1 | | 1831 | hsa-mir-1284 | 10016 | PDCD6 | programmed cell death 6 | 1 | | 1832 | hsa-mir-3181 | 10016 | PDCD6 | programmed cell death 6 | 1 | | 1833 | hsa-mir-3651 | 10016 | PDCD6 | programmed cell death 6 | 1 | | 1834 | hsa-mir-4632 | 10016 | PDCD6 | programmed cell death 6 | 1 | | 1835 | hsa-mir-4648 | 10016 | PDCD6 | programmed cell death 6 | 1 | | 1836 | hsa-mir-4726 | 10016 | PDCD6 | programmed cell death 6 | 1 | | 1837 | hsa-mir-4785 | 10016 | PDCD6 | programmed cell death 6 | 1 | | 1838 | hsa-mir-4794 | 10016 | PDCD6 | programmed cell death 6 | 1 | | 1839 | hsa-mir-4802 | 10016 | PDCD6 | programmed cell death 6 | 1 | | 1840 | hsa-let-7i | 10016 | PDCD6 | programmed cell death 6 | 1 | | 1841 | hsa-mir-302b | 10016 | PDCD6 | programmed cell death 6 | 1 | | 1842 | hsa-mir-302c | 10016 | PDCD6 | programmed cell death 6 | 1 | | 1843 | hsa-mir-339 | 10016 | PDCD6 | programmed cell death 6 | 1 | | 1844 | hsa-mir-562 | 10016 | PDCD6 | programmed cell death 6 | 1 | | 1845 | hsa-mir-567 | 10016 | PDCD6 | programmed cell death 6 | 1 | | 1846 | hsa-mir-576 | 10016 | PDCD6 | programmed cell death 6 | 1 | | 1847 | hsa-mir-579 | 10016 | PDCD6 | programmed cell death 6 | 1 | | 1848 | hsa-mir-582 | 10016 | PDCD6 | programmed cell death 6 | 1 | | 1849 | hsa-mir-604 | 10016 | PDCD6 | programmed cell death 6 | 1 | | 1850 | hsa-mir-608 | 10016 | PDCD6 | programmed cell death 6 | 1 | | 1851 | hsa-mir-621 | 10016 | PDCD6 | programmed cell death 6 | 1 | | 1852 | hsa-mir-627 | 10016 | PDCD6 | programmed cell death 6 | 1 | | 1853 | hsa-mir-454 | 10016 | PDCD6 | programmed cell death 6 | 1 | | 1854 | hsa-mir-302d | 10016 | PDCD6 | programmed cell death 6 | 1 | | 1855 | hsa-mir-367 | 10016 | PDCD6 | programmed cell death 6 | 1 | | 1856 | hsa-mir-548b | 10016 | PDCD6 | programmed cell death 6 | 1 | | 1857 | hsa-mir-548d-1;hsa-mir-548d-2 | 10016 | PDCD6 | programmed cell death 6 | 1 | | 1858 | hsa-mir-938 | 10016 | PDCD6 | programmed cell death 6 | 1 | | 1859 | hsa-mir-1236 | 10016 | PDCD6 | programmed cell death 6 | 1 | | 1860 | hsa-mir-101-2 | 94101 | ORMDL1 | ORM1-like 1 (S. cerevisiae) | 1 | | 1865 | hsa-mir-511-1 | 94101 | ORMDL1 | ORM1-like 1 (S. cerevisiae) | 1 | | 1866 | hsa-mir-511-2 | 94101 | ORMDL1 | ORM1-like 1 (S. cerevisiae) | 1 | | 1867 | hsa-mir-1284 | 94101 | ORMDL1 | ORM1-like 1 (S. cerevisiae) | 1 | | 1868 | hsa-mir-3181 | 94101 | ORMDL1 | ORM1-like 1 (S. cerevisiae) | 1 | | 1869 | hsa-mir-3651 | 94101 | ORMDL1 | ORM1-like 1 (S. cerevisiae) | 1 | | 1870 | hsa-mir-4632 | 94101 | ORMDL1 | ORM1-like 1 (S. cerevisiae) | 1 | | 1871 | hsa-mir-4648 | 94101 | ORMDL1 | ORM1-like 1 (S. cerevisiae) | 1 | | 1872 | hsa-mir-4726 | 94101 | ORMDL1 | ORM1-like 1 (S. cerevisiae) | 1 | | 1873 | hsa-mir-4785 | 94101 | ORMDL1 | ORM1-like 1 (S. cerevisiae) | 1 | | 1874 | hsa-mir-4794 | 94101 | ORMDL1 | ORM1-like 1 (S. cerevisiae) | 1 | | 1875 | hsa-mir-4802 | 94101 | ORMDL1 | ORM1-like 1 (S. cerevisiae) | 1 | | 1876 | hsa-let-7i | 94101 | ORMDL1 | ORM1-like 1 (S. cerevisiae) | 1 | | 1877 | hsa-mir-302b | 94101 | ORMDL1 | ORM1-like 1 (S. cerevisiae) | 1 | | 1878 | hsa-mir-302c | 94101 | ORMDL1 | ORM1-like 1 (S. cerevisiae) | 1 | | 1879 | hsa-mir-339 | 94101 | ORMDL1 | ORM1-like 1 (S. cerevisiae) | 1 | | 1880 | hsa-mir-562 | 94101 | ORMDL1 | ORM1-like 1 (S. cerevisiae) | 1 | | 1881 | hsa-mir-567 | 94101 | ORMDL1 | ORM1-like 1 (S. cerevisiae) | 1 | | 1882 | hsa-mir-576 | 94101 | ORMDL1 | ORM1-like 1 (S. cerevisiae) | 1 | | 1883 | hsa-mir-579 | 94101 | ORMDL1 | ORM1-like 1 (S. cerevisiae) | 1 | | 1884 | hsa-mir-582 | 94101 | ORMDL1 | ORM1-like 1 (S. cerevisiae) | 1 | | 1885 | hsa-mir-604 | 94101 | ORMDL1 | ORM1-like 1 (S. cerevisiae) | 1 | | 1886 | hsa-mir-608 | 94101 | ORMDL1 | ORM1-like 1 (S. cerevisiae) | 1 | | 1887 | hsa-mir-621 | 94101 | ORMDL1 | ORM1-like 1 (S. cerevisiae) | 1 | | 1888 | hsa-mir-627 | 94101 | ORMDL1 | ORM1-like 1 (S. cerevisiae) | 1 | | 1889 | hsa-mir-454 | 94101 | ORMDL1 | ORM1-like 1 (S. cerevisiae) | 1 | | 1890 | hsa-mir-302d | 94101 | ORMDL1 | ORM1-like 1 (S. cerevisiae) | 1 | | 1891 | hsa-mir-367 | 94101 | ORMDL1 | ORM1-like 1 (S. cerevisiae) | 1 | | 1892 | hsa-mir-548b | 94101 | ORMDL1 | ORM1-like 1 (S. cerevisiae) | 1 | | 1893 | hsa-mir-548d-1;hsa-mir-548d-2 | 94101 | ORMDL1 | ORM1-like 1 (S. cerevisiae) | 1 | | 1894 | hsa-mir-938 | 94101 | ORMDL1 | ORM1-like 1 (S. cerevisiae) | 1 | | 1895 | hsa-mir-1236 | 94101 | ORMDL1 | ORM1-like 1 (S. cerevisiae) | 1 | | 1899 | hsa-mir-302b | 64332 | NFKBIZ | nuclear factor of kappa light polypeptide gene enhancer in B-cells inhibitor, zeta | 1 | | 1900 | hsa-mir-302c | 64332 | NFKBIZ | nuclear factor of kappa light polypeptide gene enhancer in B-cells inhibitor, zeta | 1 | | 1901 | hsa-mir-302d | 64332 | NFKBIZ | nuclear factor of kappa light polypeptide gene enhancer in B-cells inhibitor, zeta | 1 | | 1902 | hsa-mir-367 | 64332 | NFKBIZ | nuclear factor of kappa light polypeptide gene enhancer in B-cells inhibitor, zeta | 1 | | 1903 | hsa-mir-562 | 64332 | NFKBIZ | nuclear factor of kappa light polypeptide gene enhancer in B-cells inhibitor, zeta | 1 | | 1904 | hsa-mir-604 | 64332 | NFKBIZ | nuclear factor of kappa light polypeptide gene enhancer in B-cells inhibitor, zeta | 1 | | 1905 | hsa-mir-548d-1 | 64332 | NFKBIZ | nuclear factor of kappa light polypeptide gene enhancer in B-cells inhibitor, zeta | 1 | | 1906 | hsa-mir-938 | 64332 | NFKBIZ | nuclear factor of kappa light polypeptide gene enhancer in B-cells inhibitor, zeta | 1 | | 1907 | hsa-mir-548h-1 | 64332 | NFKBIZ | nuclear factor of kappa light polypeptide gene enhancer in B-cells inhibitor, zeta | 1 | | 1908 | hsa-mir-3135a | 64332 | NFKBIZ | nuclear factor of kappa light polypeptide gene enhancer in B-cells inhibitor, zeta | 1 | | 1909 | hsa-mir-3177 | 64332 | NFKBIZ | nuclear factor of kappa light polypeptide gene enhancer in B-cells inhibitor, zeta | 1 | | 1910 | hsa-mir-3655 | 64332 | NFKBIZ | nuclear factor of kappa light polypeptide gene enhancer in B-cells inhibitor, zeta | 1 | | 1911 | hsa-mir-4526 | 64332 | NFKBIZ | nuclear factor of kappa light polypeptide gene enhancer in B-cells inhibitor, zeta | 1 | | 1912 | hsa-mir-4635 | 64332 | NFKBIZ | nuclear factor of kappa light polypeptide gene enhancer in B-cells inhibitor, zeta | 1 | | 1913 | hsa-mir-4651 | 64332 | NFKBIZ | nuclear factor of kappa light polypeptide gene enhancer in B-cells inhibitor, zeta | 1 | | 1914 | hsa-mir-4744 | 64332 | NFKBIZ | nuclear factor of kappa light polypeptide gene enhancer in B-cells inhibitor, zeta | 1 | | 1915 | hsa-mir-5190 | 64332 | NFKBIZ | nuclear factor of kappa light polypeptide gene enhancer in B-cells inhibitor, zeta | 1 | | 1916 | hsa-mir-101-2 | 64332 | NFKBIZ | nuclear factor of kappa light polypeptide gene enhancer in B-cells inhibitor, zeta | 1 | | 1917 | hsa-mir-128-1;hsa-mir-128-2 | 64332 | NFKBIZ | nuclear factor of kappa light polypeptide gene enhancer in B-cells inhibitor, zeta | 1 | | 1918 | hsa-mir-301a | 64332 | NFKBIZ | nuclear factor of kappa light polypeptide gene enhancer in B-cells inhibitor, zeta | 1 | | 1919 | hsa-mir-511-1;hsa-mir-511-2 | 64332 | NFKBIZ | nuclear factor of kappa light polypeptide gene enhancer in B-cells inhibitor, zeta | 1 | | 1920 | hsa-mir-586 | 64332 | NFKBIZ | nuclear factor of kappa light polypeptide gene enhancer in B-cells inhibitor, zeta | 1 | | 1921 | hsa-mir-593 | 64332 | NFKBIZ | nuclear factor of kappa light polypeptide gene enhancer in B-cells inhibitor, zeta | 1 | | 1922 | hsa-mir-620 | 64332 | NFKBIZ | nuclear factor of kappa light polypeptide gene enhancer in B-cells inhibitor, zeta | 1 | | 1923 | hsa-mir-33b | 64332 | NFKBIZ | nuclear factor of kappa light polypeptide gene enhancer in B-cells inhibitor, zeta | 1 | | 1924 | hsa-mir-942 | 64332 | NFKBIZ | nuclear factor of kappa light polypeptide gene enhancer in B-cells inhibitor, zeta | 1 | | 1928 | hsa-mir-302b | 84433 | CARD11 | caspase recruitment domain family, member 11 | 1 | | 1929 | hsa-mir-302c | 84433 | CARD11 | caspase recruitment domain family, member 11 | 1 | | 1930 | hsa-mir-302d | 84433 | CARD11 | caspase recruitment domain family, member 11 | 1 | | 1931 | hsa-mir-367 | 84433 | CARD11 | caspase recruitment domain family, member 11 | 1 | | 1932 | hsa-mir-562 | 84433 | CARD11 | caspase recruitment domain family, member 11 | 1 | | 1933 | hsa-mir-604 | 84433 | CARD11 | caspase recruitment domain family, member 11 | 1 | | 1934 | hsa-mir-548d-1 | 84433 | CARD11 | caspase recruitment domain family, member 11 | 1 | | 1935 | hsa-mir-938 | 84433 | CARD11 | caspase recruitment domain family, member 11 | 1 | | 1936 | hsa-mir-548h-1 | 84433 | CARD11 | caspase recruitment domain family, member 11 | 1 | | 1937 | hsa-mir-3135a | 84433 | CARD11 | caspase recruitment domain family, member 11 | 1 | | 1938 | hsa-mir-3177 | 84433 | CARD11 | caspase recruitment domain family, member 11 | 1 | | 1939 | hsa-mir-3655 | 84433 | CARD11 | caspase recruitment domain family, member 11 | 1 | | 1940 | hsa-mir-4526 | 84433 | CARD11 | caspase recruitment domain family, member 11 | 1 | | 1941 | hsa-mir-4635 | 84433 | CARD11 | caspase recruitment domain family, member 11 | 1 | | 1942 | hsa-mir-4651 | 84433 | CARD11 | caspase recruitment domain family, member 11 | 1 | | 1943 | hsa-mir-4744 | 84433 | CARD11 | caspase recruitment domain family, member 11 | 1 | | 1944 | hsa-mir-5190 | 84433 | CARD11 | caspase recruitment domain family, member 11 | 1 | | 1945 | hsa-mir-101-2 | 84433 | CARD11 | caspase recruitment domain family, member 11 | 1 | | 1946 | hsa-mir-128-1;hsa-mir-128-2 | 84433 | CARD11 | caspase recruitment domain family, member 11 | 1 | | 1947 | hsa-mir-301a | 84433 | CARD11 | caspase recruitment domain family, member 11 | 1 | | 1948 | hsa-mir-511-1;hsa-mir-511-2 | 84433 | CARD11 | caspase recruitment domain family, member 11 | 1 | | 1949 | hsa-mir-586 | 84433 | CARD11 | caspase recruitment domain family, member 11 | 1 | | 1950 | hsa-mir-593 | 84433 | CARD11 | caspase recruitment domain family, member 11 | 1 | | 1951 | hsa-mir-620 | 84433 | CARD11 | caspase recruitment domain family, member 11 | 1 | | 1952 | hsa-mir-33b | 84433 | CARD11 | caspase recruitment domain family, member 11 | 1 | | 1953 | hsa-mir-942 | 84433 | CARD11 | caspase recruitment domain family, member 11 | 1 | | 1957 | hsa-mir-302b | 53827 | FXYD5 | FXYD domain containing ion transport regulator 5 | 1 | | 1958 | hsa-mir-302c | 53827 | FXYD5 | FXYD domain containing ion transport regulator 5 | 1 | | 1959 | hsa-mir-302d | 53827 | FXYD5 | FXYD domain containing ion transport regulator 5 | 1 | | 1960 | hsa-mir-367 | 53827 | FXYD5 | FXYD domain containing ion transport regulator 5 | 1 | | 1961 | hsa-mir-562 | 53827 | FXYD5 | FXYD domain containing ion transport regulator 5 | 1 | | 1962 | hsa-mir-604 | 53827 | FXYD5 | FXYD domain containing ion transport regulator 5 | 1 | | 1963 | hsa-mir-548d-1 | 53827 | FXYD5 | FXYD domain containing ion transport regulator 5 | 1 | | 1964 | hsa-mir-938 | 53827 | FXYD5 | FXYD domain containing ion transport regulator 5 | 1 | | 1965 | hsa-mir-548h-1 | 53827 | FXYD5 | FXYD domain containing ion transport regulator 5 | 1 | | 1966 | hsa-mir-3135a | 53827 | FXYD5 | FXYD domain containing ion transport regulator 5 | 1 | | 1967 | hsa-mir-3177 | 53827 | FXYD5 | FXYD domain containing ion transport regulator 5 | 1 | | 1968 | hsa-mir-3655 | 53827 | FXYD5 | FXYD domain containing ion transport regulator 5 | 1 | | 1969 | hsa-mir-4526 | 53827 | FXYD5 | FXYD domain containing ion transport regulator 5 | 1 | | 1970 | hsa-mir-4635 | 53827 | FXYD5 | FXYD domain containing ion transport regulator 5 | 1 | | 1971 | hsa-mir-4651 | 53827 | FXYD5 | FXYD domain containing ion transport regulator 5 | 1 | | 1972 | hsa-mir-4744 | 53827 | FXYD5 | FXYD domain containing ion transport regulator 5 | 1 | | 1973 | hsa-mir-5190 | 53827 | FXYD5 | FXYD domain containing ion transport regulator 5 | 1 | | 1974 | hsa-mir-101-2 | 53827 | FXYD5 | FXYD domain containing ion transport regulator 5 | 1 | | 1975 | hsa-mir-128-1;hsa-mir-128-2 | 53827 | FXYD5 | FXYD domain containing ion transport regulator 5 | 1 | | 1976 | hsa-mir-301a | 53827 | FXYD5 | FXYD domain containing ion transport regulator 5 | 1 | | 1977 | hsa-mir-511-1;hsa-mir-511-2 | 53827 | FXYD5 | FXYD domain containing ion transport regulator 5 | 1 | | 1978 | hsa-mir-586 | 53827 | FXYD5 | FXYD domain containing ion transport regulator 5 | 1 | | 1979 | hsa-mir-593 | 53827 | FXYD5 | FXYD domain containing ion transport regulator 5 | 1 | | 1980 | hsa-mir-620 | 53827 | FXYD5 | FXYD domain containing ion transport regulator 5 | 1 | | 1981 | hsa-mir-33b | 53827 | FXYD5 | FXYD domain containing ion transport regulator 5 | 1 | | 1982 | hsa-mir-942 | 53827 | FXYD5 | FXYD domain containing ion transport regulator 5 | 1 | | 1986 | hsa-mir-302b | 359948 | IRF2BP2 | interferon regulatory factor 2 binding protein 2 | 1 | | 1987 | hsa-mir-302c | 359948 | IRF2BP2 | interferon regulatory factor 2 binding protein 2 | 1 | | 1988 | hsa-mir-302d | 359948 | IRF2BP2 | interferon regulatory factor 2 binding protein 2 | 1 | | 1989 | hsa-mir-367 | 359948 | IRF2BP2 | interferon regulatory factor 2 binding protein 2 | 1 | | 1990 | hsa-mir-562 | 359948 | IRF2BP2 | interferon regulatory factor 2 binding protein 2 | 1 | | 1991 | hsa-mir-604 | 359948 | IRF2BP2 | interferon regulatory factor 2 binding protein 2 | 1 | | 1992 | hsa-mir-548d-1 | 359948 | IRF2BP2 | interferon regulatory factor 2 binding protein 2 | 1 | | 1993 | hsa-mir-938 | 359948 | IRF2BP2 | interferon regulatory factor 2 binding protein 2 | 1 | | 1994 | hsa-mir-548h-1 | 359948 | IRF2BP2 | interferon regulatory factor 2 binding protein 2 | 1 | | 1995 | hsa-mir-3135a | 359948 | IRF2BP2 | interferon regulatory factor 2 binding protein 2 | 1 | | 1996 | hsa-mir-3177 | 359948 | IRF2BP2 | interferon regulatory factor 2 binding protein 2 | 1 | | 1997 | hsa-mir-3655 | 359948 | IRF2BP2 | interferon regulatory factor 2 binding protein 2 | 1 | | 1998 | hsa-mir-4526 | 359948 | IRF2BP2 | interferon regulatory factor 2 binding protein 2 | 1 | | 1999 | hsa-mir-4635 | 359948 | IRF2BP2 | interferon regulatory factor 2 binding protein 2 | 1 | | 2000 | hsa-mir-4651 | 359948 | IRF2BP2 | interferon regulatory factor 2 binding protein 2 | 1 | | 2001 | hsa-mir-4744 | 359948 | IRF2BP2 | interferon regulatory factor 2 binding protein 2 | 1 | | 2002 | hsa-mir-5190 | 359948 | IRF2BP2 | interferon regulatory factor 2 binding protein 2 | 1 | | 2003 | hsa-mir-101-2 | 359948 | IRF2BP2 | interferon regulatory factor 2 binding protein 2 | 1 | | 2004 | hsa-mir-128-1;hsa-mir-128-2 | 359948 | IRF2BP2 | interferon regulatory factor 2 binding protein 2 | 1 | | 2005 | hsa-mir-301a | 359948 | IRF2BP2 | interferon regulatory factor 2 binding protein 2 | 1 | | 2006 | hsa-mir-511-1;hsa-mir-511-2 | 359948 | IRF2BP2 | interferon regulatory factor 2 binding protein 2 | 1 | | 2007 | hsa-mir-586 | 359948 | IRF2BP2 | interferon regulatory factor 2 binding protein 2 | 1 | | 2008 | hsa-mir-593 | 359948 | IRF2BP2 | interferon regulatory factor 2 binding protein 2 | 1 | | 2009 | hsa-mir-620 | 359948 | IRF2BP2 | interferon regulatory factor 2 binding protein 2 | 1 | | 2010 | hsa-mir-33b | 359948 | IRF2BP2 | interferon regulatory factor 2 binding protein 2 | 1 | | 2011 | hsa-mir-942 | 359948 | IRF2BP2 | interferon regulatory factor 2 binding protein 2 | 1 | | 2015 | hsa-mir-302b | 57584 | ARHGAP21 | Rho GTPase activating protein 21 | 1 | | 2016 | hsa-mir-302c | 57584 | ARHGAP21 | Rho GTPase activating protein 21 | 1 | | 2017 | hsa-mir-302d | 57584 | ARHGAP21 | Rho GTPase activating protein 21 | 1 | | 2018 | hsa-mir-367 | 57584 | ARHGAP21 | Rho GTPase activating protein 21 | 1 | | 2019 | hsa-mir-562 | 57584 | ARHGAP21 | Rho GTPase activating protein 21 | 1 | | 2020 | hsa-mir-604 | 57584 | ARHGAP21 | Rho GTPase activating protein 21 | 1 | | 2021 | hsa-mir-548d-1 | 57584 | ARHGAP21 | Rho GTPase activating protein 21 | 1 | | 2022 | hsa-mir-938 | 57584 | ARHGAP21 | Rho GTPase activating protein 21 | 1 | | 2023 | hsa-mir-548h-1 | 57584 | ARHGAP21 | Rho GTPase activating protein 21 | 1 | | 2024 | hsa-mir-3135a | 57584 | ARHGAP21 | Rho GTPase activating protein 21 | 1 | | 2025 | hsa-mir-3177 | 57584 | ARHGAP21 | Rho GTPase activating protein 21 | 1 | | 2026 | hsa-mir-3655 | 57584 | ARHGAP21 | Rho GTPase activating protein 21 | 1 | | 2027 | hsa-mir-4526 | 57584 | ARHGAP21 | Rho GTPase activating protein 21 | 1 | | 2028 | hsa-mir-4635 | 57584 | ARHGAP21 | Rho GTPase activating protein 21 | 1 | | 2029 | hsa-mir-4651 | 57584 | ARHGAP21 | Rho GTPase activating protein 21 | 1 | | 2030 | hsa-mir-4744 | 57584 | ARHGAP21 | Rho GTPase activating protein 21 | 1 | | 2031 | hsa-mir-5190 | 57584 | ARHGAP21 | Rho GTPase activating protein 21 | 1 | | 2032 | hsa-mir-101-2 | 57584 | ARHGAP21 | Rho GTPase activating protein 21 | 1 | | 2033 | hsa-mir-128-1;hsa-mir-128-2 | 57584 | ARHGAP21 | Rho GTPase activating protein 21 | 1 | | 2034 | hsa-mir-301a | 57584 | ARHGAP21 | Rho GTPase activating protein 21 | 1 | | 2035 | hsa-mir-511-1;hsa-mir-511-2 | 57584 | ARHGAP21 | Rho GTPase activating protein 21 | 1 | | 2036 | hsa-mir-586 | 57584 | ARHGAP21 | Rho GTPase activating protein 21 | 1 | | 2037 | hsa-mir-593 | 57584 | ARHGAP21 | Rho GTPase activating protein 21 | 1 | | 2038 | hsa-mir-620 | 57584 | ARHGAP21 | Rho GTPase activating protein 21 | 1 | | 2039 | hsa-mir-33b | 57584 | ARHGAP21 | Rho GTPase activating protein 21 | 1 | | 2040 | hsa-mir-942 | 57584 | ARHGAP21 | Rho GTPase activating protein 21 | 1 | | 2041 | hsa-mir-101-2 | 4775 | NFATC3 | nuclear factor of activated T-cells, cytoplasmic, calcineurin-dependent 3 | 1 | | 2046 | hsa-mir-511-1 | 4775 | NFATC3 | nuclear factor of activated T-cells, cytoplasmic, calcineurin-dependent 3 | 1 | | 2047 | hsa-mir-511-2 | 4775 | NFATC3 | nuclear factor of activated T-cells, cytoplasmic, calcineurin-dependent 3 | 1 | | 2048 | hsa-mir-1284 | 4775 | NFATC3 | nuclear factor of activated T-cells, cytoplasmic, calcineurin-dependent 3 | 1 | | 2049 | hsa-mir-3181 | 4775 | NFATC3 | nuclear factor of activated T-cells, cytoplasmic, calcineurin-dependent 3 | 1 | | 2050 | hsa-mir-3651 | 4775 | NFATC3 | nuclear factor of activated T-cells, cytoplasmic, calcineurin-dependent 3 | 1 | | 2051 | hsa-mir-4632 | 4775 | NFATC3 | nuclear factor of activated T-cells, cytoplasmic, calcineurin-dependent 3 | 1 | | 2052 | hsa-mir-4648 | 4775 | NFATC3 | nuclear factor of activated T-cells, cytoplasmic, calcineurin-dependent 3 | 1 | | 2053 | hsa-mir-4726 | 4775 | NFATC3 | nuclear factor of activated T-cells, cytoplasmic, calcineurin-dependent 3 | 1 | | 2054 | hsa-mir-4785 | 4775 | NFATC3 | nuclear factor of activated T-cells, cytoplasmic, calcineurin-dependent 3 | 1 | | 2055 | hsa-mir-4794 | 4775 | NFATC3 | nuclear factor of activated T-cells, cytoplasmic, calcineurin-dependent 3 | 1 | | 2056 | hsa-mir-4802 | 4775 | NFATC3 | nuclear factor of activated T-cells, cytoplasmic, calcineurin-dependent 3 | 1 | | 2057 | hsa-let-7i | 4775 | NFATC3 | nuclear factor of activated T-cells, cytoplasmic, calcineurin-dependent 3 | 1 | | 2058 | hsa-mir-302b | 4775 | NFATC3 | nuclear factor of activated T-cells, cytoplasmic, calcineurin-dependent 3 | 1 | | 2059 | hsa-mir-302c | 4775 | NFATC3 | nuclear factor of activated T-cells, cytoplasmic, calcineurin-dependent 3 | 1 | | 2060 | hsa-mir-339 | 4775 | NFATC3 | nuclear factor of activated T-cells, cytoplasmic, calcineurin-dependent 3 | 1 | | 2061 | hsa-mir-562 | 4775 | NFATC3 | nuclear factor of activated T-cells, cytoplasmic, calcineurin-dependent 3 | 1 | | 2062 | hsa-mir-567 | 4775 | NFATC3 | nuclear factor of activated T-cells, cytoplasmic, calcineurin-dependent 3 | 1 | | 2063 | hsa-mir-576 | 4775 | NFATC3 | nuclear factor of activated T-cells, cytoplasmic, calcineurin-dependent 3 | 1 | | 2064 | hsa-mir-579 | 4775 | NFATC3 | nuclear factor of activated T-cells, cytoplasmic, calcineurin-dependent 3 | 1 | | 2065 | hsa-mir-582 | 4775 | NFATC3 | nuclear factor of activated T-cells, cytoplasmic, calcineurin-dependent 3 | 1 | | 2066 | hsa-mir-604 | 4775 | NFATC3 | nuclear factor of activated T-cells, cytoplasmic, calcineurin-dependent 3 | 1 | | 2067 | hsa-mir-608 | 4775 | NFATC3 | nuclear factor of activated T-cells, cytoplasmic, calcineurin-dependent 3 | 1 | | 2068 | hsa-mir-621 | 4775 | NFATC3 | nuclear factor of activated T-cells, cytoplasmic, calcineurin-dependent 3 | 1 | | 2069 | hsa-mir-627 | 4775 | NFATC3 | nuclear factor of activated T-cells, cytoplasmic, calcineurin-dependent 3 | 1 | | 2070 | hsa-mir-454 | 4775 | NFATC3 | nuclear factor of activated T-cells, cytoplasmic, calcineurin-dependent 3 | 1 | | 2071 | hsa-mir-302d | 4775 | NFATC3 | nuclear factor of activated T-cells, cytoplasmic, calcineurin-dependent 3 | 1 | | 2072 | hsa-mir-367 | 4775 | NFATC3 | nuclear factor of activated T-cells, cytoplasmic, calcineurin-dependent 3 | 1 | | 2073 | hsa-mir-548b | 4775 | NFATC3 | nuclear factor of activated T-cells, cytoplasmic, calcineurin-dependent 3 | 1 | | 2074 | hsa-mir-548d-1;hsa-mir-548d-2 | 4775 | NFATC3 | nuclear factor of activated T-cells, cytoplasmic, calcineurin-dependent 3 | 1 | | 2075 | hsa-mir-938 | 4775 | NFATC3 | nuclear factor of activated T-cells, cytoplasmic, calcineurin-dependent 3 | 1 | | 2076 | hsa-mir-1236 | 4775 | NFATC3 | nuclear factor of activated T-cells, cytoplasmic, calcineurin-dependent 3 | 1 | | 2077 | hsa-mir-101-2 | 84988 | PPP1R16A | protein phosphatase 1, regulatory subunit 16A | 1 | | 2082 | hsa-mir-511-1 | 84988 | PPP1R16A | protein phosphatase 1, regulatory subunit 16A | 1 | | 2083 | hsa-mir-511-2 | 84988 | PPP1R16A | protein phosphatase 1, regulatory subunit 16A | 1 | | 2084 | hsa-mir-1284 | 84988 | PPP1R16A | protein phosphatase 1, regulatory subunit 16A | 1 | | 2085 | hsa-mir-3181 | 84988 | PPP1R16A | protein phosphatase 1, regulatory subunit 16A | 1 | | 2086 | hsa-mir-3651 | 84988 | PPP1R16A | protein phosphatase 1, regulatory subunit 16A | 1 | | 2087 | hsa-mir-4632 | 84988 | PPP1R16A | protein phosphatase 1, regulatory subunit 16A | 1 | | 2088 | hsa-mir-4648 | 84988 | PPP1R16A | protein phosphatase 1, regulatory subunit 16A | 1 | | 2089 | hsa-mir-4726 | 84988 | PPP1R16A | protein phosphatase 1, regulatory subunit 16A | 1 | | 2090 | hsa-mir-4785 | 84988 | PPP1R16A | protein phosphatase 1, regulatory subunit 16A | 1 | | 2091 | hsa-mir-4794 | 84988 | PPP1R16A | protein phosphatase 1, regulatory subunit 16A | 1 | | 2092 | hsa-mir-4802 | 84988 | PPP1R16A | protein phosphatase 1, regulatory subunit 16A | 1 | | 2093 | hsa-let-7i | 84988 | PPP1R16A | protein phosphatase 1, regulatory subunit 16A | 1 | | 2094 | hsa-mir-302b | 84988 | PPP1R16A | protein phosphatase 1, regulatory subunit 16A | 1 | | 2095 | hsa-mir-302c | 84988 | PPP1R16A | protein phosphatase 1, regulatory subunit 16A | 1 | | 2096 | hsa-mir-339 | 84988 | PPP1R16A | protein phosphatase 1, regulatory subunit 16A | 1 | | 2097 | hsa-mir-562 | 84988 | PPP1R16A | protein phosphatase 1, regulatory subunit 16A | 1 | | 2098 | hsa-mir-567 | 84988 | PPP1R16A | protein phosphatase 1, regulatory subunit 16A | 1 | | 2099 | hsa-mir-576 | 84988 | PPP1R16A | protein phosphatase 1, regulatory subunit 16A | 1 | | 2100 | hsa-mir-579 | 84988 | PPP1R16A | protein phosphatase 1, regulatory subunit 16A | 1 | | 2101 | hsa-mir-582 | 84988 | PPP1R16A | protein phosphatase 1, regulatory subunit 16A | 1 | | 2102 | hsa-mir-604 | 84988 | PPP1R16A | protein phosphatase 1, regulatory subunit 16A | 1 | | 2103 | hsa-mir-608 | 84988 | PPP1R16A | protein phosphatase 1, regulatory subunit 16A | 1 | | 2104 | hsa-mir-621 | 84988 | PPP1R16A | protein phosphatase 1, regulatory subunit 16A | 1 | | 2105 | hsa-mir-627 | 84988 | PPP1R16A | protein phosphatase 1, regulatory subunit 16A | 1 | | 2106 | hsa-mir-454 | 84988 | PPP1R16A | protein phosphatase 1, regulatory subunit 16A | 1 | | 2107 | hsa-mir-302d | 84988 | PPP1R16A | protein phosphatase 1, regulatory subunit 16A | 1 | | 2108 | hsa-mir-367 | 84988 | PPP1R16A | protein phosphatase 1, regulatory subunit 16A | 1 | | 2109 | hsa-mir-548b | 84988 | PPP1R16A | protein phosphatase 1, regulatory subunit 16A | 1 | | 2110 | hsa-mir-548d-1;hsa-mir-548d-2 | 84988 | PPP1R16A | protein phosphatase 1, regulatory subunit 16A | 1 | | 2111 | hsa-mir-938 | 84988 | PPP1R16A | protein phosphatase 1, regulatory subunit 16A | 1 | | 2112 | hsa-mir-1236 | 84988 | PPP1R16A | protein phosphatase 1, regulatory subunit 16A | 1 | | 2116 | hsa-mir-302b | 64115 | C10orf54 | chromosome 10 open reading frame 54 | 1 | | 2117 | hsa-mir-302c | 64115 | C10orf54 | chromosome 10 open reading frame 54 | 1 | | 2118 | hsa-mir-302d | 64115 | C10orf54 | chromosome 10 open reading frame 54 | 1 | | 2119 | hsa-mir-367 | 64115 | C10orf54 | chromosome 10 open reading frame 54 | 1 | | 2120 | hsa-mir-562 | 64115 | C10orf54 | chromosome 10 open reading frame 54 | 1 | | 2121 | hsa-mir-604 | 64115 | C10orf54 | chromosome 10 open reading frame 54 | 1 | | 2122 | hsa-mir-548d-1 | 64115 | C10orf54 | chromosome 10 open reading frame 54 | 1 | | 2123 | hsa-mir-938 | 64115 | C10orf54 | chromosome 10 open reading frame 54 | 1 | | 2124 | hsa-mir-548h-1 | 64115 | C10orf54 | chromosome 10 open reading frame 54 | 1 | | 2125 | hsa-mir-3135a | 64115 | C10orf54 | chromosome 10 open reading frame 54 | 1 | | 2126 | hsa-mir-3177 | 64115 | C10orf54 | chromosome 10 open reading frame 54 | 1 | | 2127 | hsa-mir-3655 | 64115 | C10orf54 | chromosome 10 open reading frame 54 | 1 | | 2128 | hsa-mir-4526 | 64115 | C10orf54 | chromosome 10 open reading frame 54 | 1 | | 2129 | hsa-mir-4635 | 64115 | C10orf54 | chromosome 10 open reading frame 54 | 1 | | 2130 | hsa-mir-4651 | 64115 | C10orf54 | chromosome 10 open reading frame 54 | 1 | | 2131 | hsa-mir-4744 | 64115 | C10orf54 | chromosome 10 open reading frame 54 | 1 | | 2132 | hsa-mir-5190 | 64115 | C10orf54 | chromosome 10 open reading frame 54 | 1 | | 2133 | hsa-mir-101-2 | 64115 | C10orf54 | chromosome 10 open reading frame 54 | 1 | | 2134 | hsa-mir-128-1;hsa-mir-128-2 | 64115 | C10orf54 | chromosome 10 open reading frame 54 | 1 | | 2135 | hsa-mir-301a | 64115 | C10orf54 | chromosome 10 open reading frame 54 | 1 | | 2136 | hsa-mir-511-1;hsa-mir-511-2 | 64115 | C10orf54 | chromosome 10 open reading frame 54 | 1 | | 2137 | hsa-mir-586 | 64115 | C10orf54 | chromosome 10 open reading frame 54 | 1 | | 2138 | hsa-mir-593 | 64115 | C10orf54 | chromosome 10 open reading frame 54 | 1 | | 2139 | hsa-mir-620 | 64115 | C10orf54 | chromosome 10 open reading frame 54 | 1 | | 2140 | hsa-mir-33b | 64115 | C10orf54 | chromosome 10 open reading frame 54 | 1 | | 2141 | hsa-mir-942 | 64115 | C10orf54 | chromosome 10 open reading frame 54 | 1 | | 2142 | hsa-mir-101-2 | 113263 | GLCCI1 | glucocorticoid induced transcript 1 | 1 | | 2147 | hsa-mir-511-1 | 113263 | GLCCI1 | glucocorticoid induced transcript 1 | 1 | | 2148 | hsa-mir-511-2 | 113263 | GLCCI1 | glucocorticoid induced transcript 1 | 1 | | 2149 | hsa-mir-1284 | 113263 | GLCCI1 | glucocorticoid induced transcript 1 | 1 | | 2150 | hsa-mir-3181 | 113263 | GLCCI1 | glucocorticoid induced transcript 1 | 1 | | 2151 | hsa-mir-3651 | 113263 | GLCCI1 | glucocorticoid induced transcript 1 | 1 | | 2152 | hsa-mir-4632 | 113263 | GLCCI1 | glucocorticoid induced transcript 1 | 1 | | 2153 | hsa-mir-4648 | 113263 | GLCCI1 | glucocorticoid induced transcript 1 | 1 | | 2154 | hsa-mir-4726 | 113263 | GLCCI1 | glucocorticoid induced transcript 1 | 1 | | 2155 | hsa-mir-4785 | 113263 | GLCCI1 | glucocorticoid induced transcript 1 | 1 | | 2156 | hsa-mir-4794 | 113263 | GLCCI1 | glucocorticoid induced transcript 1 | 1 | | 2157 | hsa-mir-4802 | 113263 | GLCCI1 | glucocorticoid induced transcript 1 | 1 | | 2158 | hsa-let-7i | 113263 | GLCCI1 | glucocorticoid induced transcript 1 | 1 | | 2159 | hsa-mir-302b | 113263 | GLCCI1 | glucocorticoid induced transcript 1 | 1 | | 2160 | hsa-mir-302c | 113263 | GLCCI1 | glucocorticoid induced transcript 1 | 1 | | 2161 | hsa-mir-339 | 113263 | GLCCI1 | glucocorticoid induced transcript 1 | 1 | | 2162 | hsa-mir-562 | 113263 | GLCCI1 | glucocorticoid induced transcript 1 | 1 | | 2163 | hsa-mir-567 | 113263 | GLCCI1 | glucocorticoid induced transcript 1 | 1 | | 2164 | hsa-mir-576 | 113263 | GLCCI1 | glucocorticoid induced transcript 1 | 1 | | 2165 | hsa-mir-579 | 113263 | GLCCI1 | glucocorticoid induced transcript 1 | 1 | | 2166 | hsa-mir-582 | 113263 | GLCCI1 | glucocorticoid induced transcript 1 | 1 | | 2167 | hsa-mir-604 | 113263 | GLCCI1 | glucocorticoid induced transcript 1 | 1 | | 2168 | hsa-mir-608 | 113263 | GLCCI1 | glucocorticoid induced transcript 1 | 1 | | 2169 | hsa-mir-621 | 113263 | GLCCI1 | glucocorticoid induced transcript 1 | 1 | | 2170 | hsa-mir-627 | 113263 | GLCCI1 | glucocorticoid induced transcript 1 | 1 | | 2171 | hsa-mir-454 | 113263 | GLCCI1 | glucocorticoid induced transcript 1 | 1 | | 2172 | hsa-mir-302d | 113263 | GLCCI1 | glucocorticoid induced transcript 1 | 1 | | 2173 | hsa-mir-367 | 113263 | GLCCI1 | glucocorticoid induced transcript 1 | 1 | | 2174 | hsa-mir-548b | 113263 | GLCCI1 | glucocorticoid induced transcript 1 | 1 | | 2175 | hsa-mir-548d-1;hsa-mir-548d-2 | 113263 | GLCCI1 | glucocorticoid induced transcript 1 | 1 | | 2176 | hsa-mir-938 | 113263 | GLCCI1 | glucocorticoid induced transcript 1 | 1 | | 2177 | hsa-mir-1236 | 113263 | GLCCI1 | glucocorticoid induced transcript 1 | 1 | | 2181 | hsa-mir-302b | 51088 | KLHL5 | kelch-like 5 (Drosophila) | 1 | | 2182 | hsa-mir-302c | 51088 | KLHL5 | kelch-like 5 (Drosophila) | 1 | | 2183 | hsa-mir-302d | 51088 | KLHL5 | kelch-like 5 (Drosophila) | 1 | | 2184 | hsa-mir-367 | 51088 | KLHL5 | kelch-like 5 (Drosophila) | 1 | | 2185 | hsa-mir-562 | 51088 | KLHL5 | kelch-like 5 (Drosophila) | 1 | | 2186 | hsa-mir-604 | 51088 | KLHL5 | kelch-like 5 (Drosophila) | 1 | | 2187 | hsa-mir-548d-1 | 51088 | KLHL5 | kelch-like 5 (Drosophila) | 1 | | 2188 | hsa-mir-938 | 51088 | KLHL5 | kelch-like 5 (Drosophila) | 1 | | 2189 | hsa-mir-548h-1 | 51088 | KLHL5 | kelch-like 5 (Drosophila) | 1 | | 2190 | hsa-mir-3135a | 51088 | KLHL5 | kelch-like 5 (Drosophila) | 1 | | 2191 | hsa-mir-3177 | 51088 | KLHL5 | kelch-like 5 (Drosophila) | 1 | | 2192 | hsa-mir-3655 | 51088 | KLHL5 | kelch-like 5 (Drosophila) | 1 | | 2193 | hsa-mir-4526 | 51088 | KLHL5 | kelch-like 5 (Drosophila) | 1 | | 2194 | hsa-mir-4635 | 51088 | KLHL5 | kelch-like 5 (Drosophila) | 1 | | 2195 | hsa-mir-4651 | 51088 | KLHL5 | kelch-like 5 (Drosophila) | 1 | | 2196 | hsa-mir-4744 | 51088 | KLHL5 | kelch-like 5 (Drosophila) | 1 | | 2197 | hsa-mir-5190 | 51088 | KLHL5 | kelch-like 5 (Drosophila) | 1 | | 2198 | hsa-mir-101-2 | 51088 | KLHL5 | kelch-like 5 (Drosophila) | 1 | | 2199 | hsa-mir-128-1;hsa-mir-128-2 | 51088 | KLHL5 | kelch-like 5 (Drosophila) | 1 | | 2200 | hsa-mir-301a | 51088 | KLHL5 | kelch-like 5 (Drosophila) | 1 | | 2201 | hsa-mir-511-1;hsa-mir-511-2 | 51088 | KLHL5 | kelch-like 5 (Drosophila) | 1 | | 2202 | hsa-mir-586 | 51088 | KLHL5 | kelch-like 5 (Drosophila) | 1 | | 2203 | hsa-mir-593 | 51088 | KLHL5 | kelch-like 5 (Drosophila) | 1 | | 2204 | hsa-mir-620 | 51088 | KLHL5 | kelch-like 5 (Drosophila) | 1 | | 2205 | hsa-mir-33b | 51088 | KLHL5 | kelch-like 5 (Drosophila) | 1 | | 2206 | hsa-mir-942 | 51088 | KLHL5 | kelch-like 5 (Drosophila) | 1 | | 2207 | hsa-mir-101-2 | 83464 | APH1B | anterior pharynx defective 1 homolog B (C. elegans) | 1 | | 2212 | hsa-mir-511-1 | 83464 | APH1B | anterior pharynx defective 1 homolog B (C. elegans) | 1 | | 2213 | hsa-mir-511-2 | 83464 | APH1B | anterior pharynx defective 1 homolog B (C. elegans) | 1 | | 2214 | hsa-mir-1284 | 83464 | APH1B | anterior pharynx defective 1 homolog B (C. elegans) | 1 | | 2215 | hsa-mir-3181 | 83464 | APH1B | anterior pharynx defective 1 homolog B (C. elegans) | 1 | | 2216 | hsa-mir-3651 | 83464 | APH1B | anterior pharynx defective 1 homolog B (C. elegans) | 1 | | 2217 | hsa-mir-4632 | 83464 | APH1B | anterior pharynx defective 1 homolog B (C. elegans) | 1 | | 2218 | hsa-mir-4648 | 83464 | APH1B | anterior pharynx defective 1 homolog B (C. elegans) | 1 | | 2219 | hsa-mir-4726 | 83464 | APH1B | anterior pharynx defective 1 homolog B (C. elegans) | 1 | | 2220 | hsa-mir-4785 | 83464 | APH1B | anterior pharynx defective 1 homolog B (C. elegans) | 1 | | 2221 | hsa-mir-4794 | 83464 | APH1B | anterior pharynx defective 1 homolog B (C. elegans) | 1 | | 2222 | hsa-mir-4802 | 83464 | APH1B | anterior pharynx defective 1 homolog B (C. elegans) | 1 | | 2223 | hsa-let-7i | 83464 | APH1B | anterior pharynx defective 1 homolog B (C. elegans) | 1 | | 2224 | hsa-mir-302b | 83464 | APH1B | anterior pharynx defective 1 homolog B (C. elegans) | 1 | | 2225 | hsa-mir-302c | 83464 | APH1B | anterior pharynx defective 1 homolog B (C. elegans) | 1 | | 2226 | hsa-mir-339 | 83464 | APH1B | anterior pharynx defective 1 homolog B (C. elegans) | 1 | | 2227 | hsa-mir-562 | 83464 | APH1B | anterior pharynx defective 1 homolog B (C. elegans) | 1 | | 2228 | hsa-mir-567 | 83464 | APH1B | anterior pharynx defective 1 homolog B (C. elegans) | 1 | | 2229 | hsa-mir-576 | 83464 | APH1B | anterior pharynx defective 1 homolog B (C. elegans) | 1 | | 2230 | hsa-mir-579 | 83464 | APH1B | anterior pharynx defective 1 homolog B (C. elegans) | 1 | | 2231 | hsa-mir-582 | 83464 | APH1B | anterior pharynx defective 1 homolog B (C. elegans) | 1 | | 2232 | hsa-mir-604 | 83464 | APH1B | anterior pharynx defective 1 homolog B (C. elegans) | 1 | | 2233 | hsa-mir-608 | 83464 | APH1B | anterior pharynx defective 1 homolog B (C. elegans) | 1 | | 2234 | hsa-mir-621 | 83464 | APH1B | anterior pharynx defective 1 homolog B (C. elegans) | 1 | | 2235 | hsa-mir-627 | 83464 | APH1B | anterior pharynx defective 1 homolog B (C. elegans) | 1 | | 2236 | hsa-mir-454 | 83464 | APH1B | anterior pharynx defective 1 homolog B (C. elegans) | 1 | | 2237 | hsa-mir-302d | 83464 | APH1B | anterior pharynx defective 1 homolog B (C. elegans) | 1 | | 2238 | hsa-mir-367 | 83464 | APH1B | anterior pharynx defective 1 homolog B (C. elegans) | 1 | | 2239 | hsa-mir-548b | 83464 | APH1B | anterior pharynx defective 1 homolog B (C. elegans) | 1 | | 2240 | hsa-mir-548d-1;hsa-mir-548d-2 | 83464 | APH1B | anterior pharynx defective 1 homolog B (C. elegans) | 1 | | 2241 | hsa-mir-938 | 83464 | APH1B | anterior pharynx defective 1 homolog B (C. elegans) | 1 | | 2242 | hsa-mir-1236 | 83464 | APH1B | anterior pharynx defective 1 homolog B (C. elegans) | 1 | | 2243 | hsa-mir-101-2 | 168455 | CCDC71L | coiled-coil domain containing 71-like | 1 | | 2248 | hsa-mir-511-1 | 168455 | CCDC71L | coiled-coil domain containing 71-like | 1 | | 2249 | hsa-mir-511-2 | 168455 | CCDC71L | coiled-coil domain containing 71-like | 1 | | 2250 | hsa-mir-1284 | 168455 | CCDC71L | coiled-coil domain containing 71-like | 1 | | 2251 | hsa-mir-3181 | 168455 | CCDC71L | coiled-coil domain containing 71-like | 1 | | 2252 | hsa-mir-3651 | 168455 | CCDC71L | coiled-coil domain containing 71-like | 1 | | 2253 | hsa-mir-4632 | 168455 | CCDC71L | coiled-coil domain containing 71-like | 1 | | 2254 | hsa-mir-4648 | 168455 | CCDC71L | coiled-coil domain containing 71-like | 1 | | 2255 | hsa-mir-4726 | 168455 | CCDC71L | coiled-coil domain containing 71-like | 1 | | 2256 | hsa-mir-4785 | 168455 | CCDC71L | coiled-coil domain containing 71-like | 1 | | 2257 | hsa-mir-4794 | 168455 | CCDC71L | coiled-coil domain containing 71-like | 1 | | 2258 | hsa-mir-4802 | 168455 | CCDC71L | coiled-coil domain containing 71-like | 1 | | 2259 | hsa-let-7i | 168455 | CCDC71L | coiled-coil domain containing 71-like | 1 | | 2260 | hsa-mir-302b | 168455 | CCDC71L | coiled-coil domain containing 71-like | 1 | | 2261 | hsa-mir-302c | 168455 | CCDC71L | coiled-coil domain containing 71-like | 1 | | 2262 | hsa-mir-339 | 168455 | CCDC71L | coiled-coil domain containing 71-like | 1 | | 2263 | hsa-mir-562 | 168455 | CCDC71L | coiled-coil domain containing 71-like | 1 | | 2264 | hsa-mir-567 | 168455 | CCDC71L | coiled-coil domain containing 71-like | 1 | | 2265 | hsa-mir-576 | 168455 | CCDC71L | coiled-coil domain containing 71-like | 1 | | 2266 | hsa-mir-579 | 168455 | CCDC71L | coiled-coil domain containing 71-like | 1 | | 2267 | hsa-mir-582 | 168455 | CCDC71L | coiled-coil domain containing 71-like | 1 | | 2268 | hsa-mir-604 | 168455 | CCDC71L | coiled-coil domain containing 71-like | 1 | | 2269 | hsa-mir-608 | 168455 | CCDC71L | coiled-coil domain containing 71-like | 1 | | 2270 | hsa-mir-621 | 168455 | CCDC71L | coiled-coil domain containing 71-like | 1 | | 2271 | hsa-mir-627 | 168455 | CCDC71L | coiled-coil domain containing 71-like | 1 | | 2272 | hsa-mir-454 | 168455 | CCDC71L | coiled-coil domain containing 71-like | 1 | | 2273 | hsa-mir-302d | 168455 | CCDC71L | coiled-coil domain containing 71-like | 1 | | 2274 | hsa-mir-367 | 168455 | CCDC71L | coiled-coil domain containing 71-like | 1 | | 2275 | hsa-mir-548b | 168455 | CCDC71L | coiled-coil domain containing 71-like | 1 | | 2276 | hsa-mir-548d-1;hsa-mir-548d-2 | 168455 | CCDC71L | coiled-coil domain containing 71-like | 1 | | 2277 | hsa-mir-938 | 168455 | CCDC71L | coiled-coil domain containing 71-like | 1 | | 2278 | hsa-mir-1236 | 168455 | CCDC71L | coiled-coil domain containing 71-like | 1 | | 2282 | hsa-mir-302b | 64375 | IKZF4 | IKAROS family zinc finger 4 (Eos) | 1 | | 2283 | hsa-mir-302c | 64375 | IKZF4 | IKAROS family zinc finger 4 (Eos) | 1 | | 2284 | hsa-mir-302d | 64375 | IKZF4 | IKAROS family zinc finger 4 (Eos) | 1 | | 2285 | hsa-mir-367 | 64375 | IKZF4 | IKAROS family zinc finger 4 (Eos) | 1 | | 2286 | hsa-mir-562 | 64375 | IKZF4 | IKAROS family zinc finger 4 (Eos) | 1 | | 2287 | hsa-mir-604 | 64375 | IKZF4 | IKAROS family zinc finger 4 (Eos) | 1 | | 2288 | hsa-mir-548d-1 | 64375 | IKZF4 | IKAROS family zinc finger 4 (Eos) | 1 | | 2289 | hsa-mir-938 | 64375 | IKZF4 | IKAROS family zinc finger 4 (Eos) | 1 | | 2290 | hsa-mir-548h-1 | 64375 | IKZF4 | IKAROS family zinc finger 4 (Eos) | 1 | | 2291 | hsa-mir-3135a | 64375 | IKZF4 | IKAROS family zinc finger 4 (Eos) | 1 | | 2292 | hsa-mir-3177 | 64375 | IKZF4 | IKAROS family zinc finger 4 (Eos) | 1 | | 2293 | hsa-mir-3655 | 64375 | IKZF4 | IKAROS family zinc finger 4 (Eos) | 1 | | 2294 | hsa-mir-4526 | 64375 | IKZF4 | IKAROS family zinc finger 4 (Eos) | 1 | | 2295 | hsa-mir-4635 | 64375 | IKZF4 | IKAROS family zinc finger 4 (Eos) | 1 | | 2296 | hsa-mir-4651 | 64375 | IKZF4 | IKAROS family zinc finger 4 (Eos) | 1 | | 2297 | hsa-mir-4744 | 64375 | IKZF4 | IKAROS family zinc finger 4 (Eos) | 1 | | 2298 | hsa-mir-5190 | 64375 | IKZF4 | IKAROS family zinc finger 4 (Eos) | 1 | | 2299 | hsa-mir-101-2 | 64375 | IKZF4 | IKAROS family zinc finger 4 (Eos) | 1 | | 2300 | hsa-mir-128-1;hsa-mir-128-2 | 64375 | IKZF4 | IKAROS family zinc finger 4 (Eos) | 1 | | 2301 | hsa-mir-301a | 64375 | IKZF4 | IKAROS family zinc finger 4 (Eos) | 1 | | 2302 | hsa-mir-511-1;hsa-mir-511-2 | 64375 | IKZF4 | IKAROS family zinc finger 4 (Eos) | 1 | | 2303 | hsa-mir-586 | 64375 | IKZF4 | IKAROS family zinc finger 4 (Eos) | 1 | | 2304 | hsa-mir-593 | 64375 | IKZF4 | IKAROS family zinc finger 4 (Eos) | 1 | | 2305 | hsa-mir-620 | 64375 | IKZF4 | IKAROS family zinc finger 4 (Eos) | 1 | | 2306 | hsa-mir-33b | 64375 | IKZF4 | IKAROS family zinc finger 4 (Eos) | 1 | | 2307 | hsa-mir-942 | 64375 | IKZF4 | IKAROS family zinc finger 4 (Eos) | 1 | | 2311 | hsa-mir-302b | 133418 | EMB | embigin | 1 | | 2312 | hsa-mir-302c | 133418 | EMB | embigin | 1 | | 2313 | hsa-mir-302d | 133418 | EMB | embigin | 1 | | 2314 | hsa-mir-367 | 133418 | EMB | embigin | 1 | | 2315 | hsa-mir-562 | 133418 | EMB | embigin | 1 | | 2316 | hsa-mir-604 | 133418 | EMB | embigin | 1 | | 2317 | hsa-mir-548d-1 | 133418 | EMB | embigin | 1 | | 2318 | hsa-mir-938 | 133418 | EMB | embigin | 1 | | 2319 | hsa-mir-548h-1 | 133418 | EMB | embigin | 1 | | 2320 | hsa-mir-3135a | 133418 | EMB | embigin | 1 | | 2321 | hsa-mir-3177 | 133418 | EMB | embigin | 1 | | 2322 | hsa-mir-3655 | 133418 | EMB | embigin | 1 | | 2323 | hsa-mir-4526 | 133418 | EMB | embigin | 1 | | 2324 | hsa-mir-4635 | 133418 | EMB | embigin | 1 | | 2325 | hsa-mir-4651 | 133418 | EMB | embigin | 1 | | 2326 | hsa-mir-4744 | 133418 | EMB | embigin | 1 | | 2327 | hsa-mir-5190 | 133418 | EMB | embigin | 1 | | 2328 | hsa-mir-101-2 | 133418 | EMB | embigin | 1 | | 2329 | hsa-mir-128-1;hsa-mir-128-2 | 133418 | EMB | embigin | 1 | | 2330 | hsa-mir-301a | 133418 | EMB | embigin | 1 | | 2331 | hsa-mir-511-1;hsa-mir-511-2 | 133418 | EMB | embigin | 1 | | 2332 | hsa-mir-586 | 133418 | EMB | embigin | 1 | | 2333 | hsa-mir-593 | 133418 | EMB | embigin | 1 | | 2334 | hsa-mir-620 | 133418 | EMB | embigin | 1 | | 2335 | hsa-mir-33b | 133418 | EMB | embigin | 1 | | 2336 | hsa-mir-942 | 133418 | EMB | embigin | 1 | | 2376 | hsa-mir-302b | 5795 | PTPRJ | protein tyrosine phosphatase, receptor type, J | 1 | | 2377 | hsa-mir-302c | 5795 | PTPRJ | protein tyrosine phosphatase, receptor type, J | 1 | | 2378 | hsa-mir-302d | 5795 | PTPRJ | protein tyrosine phosphatase, receptor type, J | 1 | | 2379 | hsa-mir-367 | 5795 | PTPRJ | protein tyrosine phosphatase, receptor type, J | 1 | | 2380 | hsa-mir-562 | 5795 | PTPRJ | protein tyrosine phosphatase, receptor type, J | 1 | | 2381 | hsa-mir-604 | 5795 | PTPRJ | protein tyrosine phosphatase, receptor type, J | 1 | | 2382 | hsa-mir-548d-1 | 5795 | PTPRJ | protein tyrosine phosphatase, receptor type, J | 1 | | 2383 | hsa-mir-938 | 5795 | PTPRJ | protein tyrosine phosphatase, receptor type, J | 1 | | 2384 | hsa-mir-548h-1 | 5795 | PTPRJ | protein tyrosine phosphatase, receptor type, J | 1 | | 2385 | hsa-mir-3135a | 5795 | PTPRJ | protein tyrosine phosphatase, receptor type, J | 1 | | 2386 | hsa-mir-3177 | 5795 | PTPRJ | protein tyrosine phosphatase, receptor type, J | 1 | | 2387 | hsa-mir-3655 | 5795 | PTPRJ | protein tyrosine phosphatase, receptor type, J | 1 | | 2388 | hsa-mir-4526 | 5795 | PTPRJ | protein tyrosine phosphatase, receptor type, J | 1 | | 2389 | hsa-mir-4635 | 5795 | PTPRJ | protein tyrosine phosphatase, receptor type, J | 1 | | 2390 | hsa-mir-4651 | 5795 | PTPRJ | protein tyrosine phosphatase, receptor type, J | 1 | | 2391 | hsa-mir-4744 | 5795 | PTPRJ | protein tyrosine phosphatase, receptor type, J | 1 | | 2392 | hsa-mir-5190 | 5795 | PTPRJ | protein tyrosine phosphatase, receptor type, J | 1 | | 2393 | hsa-mir-101-2 | 5795 | PTPRJ | protein tyrosine phosphatase, receptor type, J | 1 | | 2394 | hsa-mir-128-1;hsa-mir-128-2 | 5795 | PTPRJ | protein tyrosine phosphatase, receptor type, J | 1 | | 2395 | hsa-mir-301a | 5795 | PTPRJ | protein tyrosine phosphatase, receptor type, J | 1 | | 2396 | hsa-mir-511-1;hsa-mir-511-2 | 5795 | PTPRJ | protein tyrosine phosphatase, receptor type, J | 1 | | 2397 | hsa-mir-586 | 5795 | PTPRJ | protein tyrosine phosphatase, receptor type, J | 1 | | 2398 | hsa-mir-593 | 5795 | PTPRJ | protein tyrosine phosphatase, receptor type, J | 1 | | 2399 | hsa-mir-620 | 5795 | PTPRJ | protein tyrosine phosphatase, receptor type, J | 1 | | 2400 | hsa-mir-33b | 5795 | PTPRJ | protein tyrosine phosphatase, receptor type, J | 1 | | 2401 | hsa-mir-942 | 5795 | PTPRJ | protein tyrosine phosphatase, receptor type, J | 1 | | 2402 | hsa-mir-101-2 | 202018 | TAPT1 | transmembrane anterior posterior transformation 1 | 1 | | 2407 | hsa-mir-511-1 | 202018 | TAPT1 | transmembrane anterior posterior transformation 1 | 1 | | 2408 | hsa-mir-511-2 | 202018 | TAPT1 | transmembrane anterior posterior transformation 1 | 1 | | 2409 | hsa-mir-1284 | 202018 | TAPT1 | transmembrane anterior posterior transformation 1 | 1 | | 2410 | hsa-mir-3181 | 202018 | TAPT1 | transmembrane anterior posterior transformation 1 | 1 | | 2411 | hsa-mir-3651 | 202018 | TAPT1 | transmembrane anterior posterior transformation 1 | 1 | | 2412 | hsa-mir-4632 | 202018 | TAPT1 | transmembrane anterior posterior transformation 1 | 1 | | 2413 | hsa-mir-4648 | 202018 | TAPT1 | transmembrane anterior posterior transformation 1 | 1 | | 2414 | hsa-mir-4726 | 202018 | TAPT1 | transmembrane anterior posterior transformation 1 | 1 | | 2415 | hsa-mir-4785 | 202018 | TAPT1 | transmembrane anterior posterior transformation 1 | 1 | | 2416 | hsa-mir-4794 | 202018 | TAPT1 | transmembrane anterior posterior transformation 1 | 1 | | 2417 | hsa-mir-4802 | 202018 | TAPT1 | transmembrane anterior posterior transformation 1 | 1 | | 2418 | hsa-let-7i | 202018 | TAPT1 | transmembrane anterior posterior transformation 1 | 1 | | 2419 | hsa-mir-302b | 202018 | TAPT1 | transmembrane anterior posterior transformation 1 | 1 | | 2420 | hsa-mir-302c | 202018 | TAPT1 | transmembrane anterior posterior transformation 1 | 1 | | 2421 | hsa-mir-339 | 202018 | TAPT1 | transmembrane anterior posterior transformation 1 | 1 | | 2422 | hsa-mir-562 | 202018 | TAPT1 | transmembrane anterior posterior transformation 1 | 1 | | 2423 | hsa-mir-567 | 202018 | TAPT1 | transmembrane anterior posterior transformation 1 | 1 | | 2424 | hsa-mir-576 | 202018 | TAPT1 | transmembrane anterior posterior transformation 1 | 1 | | 2425 | hsa-mir-579 | 202018 | TAPT1 | transmembrane anterior posterior transformation 1 | 1 | | 2426 | hsa-mir-582 | 202018 | TAPT1 | transmembrane anterior posterior transformation 1 | 1 | | 2427 | hsa-mir-604 | 202018 | TAPT1 | transmembrane anterior posterior transformation 1 | 1 | | 2428 | hsa-mir-608 | 202018 | TAPT1 | transmembrane anterior posterior transformation 1 | 1 | | 2429 | hsa-mir-621 | 202018 | TAPT1 | transmembrane anterior posterior transformation 1 | 1 | | 2430 | hsa-mir-627 | 202018 | TAPT1 | transmembrane anterior posterior transformation 1 | 1 | | 2431 | hsa-mir-454 | 202018 | TAPT1 | transmembrane anterior posterior transformation 1 | 1 | | 2432 | hsa-mir-302d | 202018 | TAPT1 | transmembrane anterior posterior transformation 1 | 1 | | 2433 | hsa-mir-367 | 202018 | TAPT1 | transmembrane anterior posterior transformation 1 | 1 | | 2434 | hsa-mir-548b | 202018 | TAPT1 | transmembrane anterior posterior transformation 1 | 1 | | 2435 | hsa-mir-548d-1;hsa-mir-548d-2 | 202018 | TAPT1 | transmembrane anterior posterior transformation 1 | 1 | | 2436 | hsa-mir-938 | 202018 | TAPT1 | transmembrane anterior posterior transformation 1 | 1 | | 2437 | hsa-mir-1236 | 202018 | TAPT1 | transmembrane anterior posterior transformation 1 | 1 | | 2441 | hsa-mir-302b | 129293 | C2orf89 | chromosome 2 open reading frame 89 | 1 | | 2442 | hsa-mir-302c | 129293 | C2orf89 | chromosome 2 open reading frame 89 | 1 | | 2443 | hsa-mir-302d | 129293 | C2orf89 | chromosome 2 open reading frame 89 | 1 | | 2444 | hsa-mir-367 | 129293 | C2orf89 | chromosome 2 open reading frame 89 | 1 | | 2445 | hsa-mir-562 | 129293 | C2orf89 | chromosome 2 open reading frame 89 | 1 | | 2446 | hsa-mir-604 | 129293 | C2orf89 | chromosome 2 open reading frame 89 | 1 | | 2447 | hsa-mir-548d-1 | 129293 | C2orf89 | chromosome 2 open reading frame 89 | 1 | | 2448 | hsa-mir-938 | 129293 | C2orf89 | chromosome 2 open reading frame 89 | 1 | | 2449 | hsa-mir-548h-1 | 129293 | C2orf89 | chromosome 2 open reading frame 89 | 1 | | 2450 | hsa-mir-3135a | 129293 | C2orf89 | chromosome 2 open reading frame 89 | 1 | | 2451 | hsa-mir-3177 | 129293 | C2orf89 | chromosome 2 open reading frame 89 | 1 | | 2452 | hsa-mir-3655 | 129293 | C2orf89 | chromosome 2 open reading frame 89 | 1 | | 2453 | hsa-mir-4526 | 129293 | C2orf89 | chromosome 2 open reading frame 89 | 1 | | 2454 | hsa-mir-4635 | 129293 | C2orf89 | chromosome 2 open reading frame 89 | 1 | | 2455 | hsa-mir-4651 | 129293 | C2orf89 | chromosome 2 open reading frame 89 | 1 | | 2456 | hsa-mir-4744 | 129293 | C2orf89 | chromosome 2 open reading frame 89 | 1 | | 2457 | hsa-mir-5190 | 129293 | C2orf89 | chromosome 2 open reading frame 89 | 1 | | 2458 | hsa-mir-101-2 | 129293 | C2orf89 | chromosome 2 open reading frame 89 | 1 | | 2459 | hsa-mir-128-1;hsa-mir-128-2 | 129293 | C2orf89 | chromosome 2 open reading frame 89 | 1 | | 2460 | hsa-mir-301a | 129293 | C2orf89 | chromosome 2 open reading frame 89 | 1 | | 2461 | hsa-mir-511-1;hsa-mir-511-2 | 129293 | C2orf89 | chromosome 2 open reading frame 89 | 1 | | 2462 | hsa-mir-586 | 129293 | C2orf89 | chromosome 2 open reading frame 89 | 1 | | 2463 | hsa-mir-593 | 129293 | C2orf89 | chromosome 2 open reading frame 89 | 1 | | 2464 | hsa-mir-620 | 129293 | C2orf89 | chromosome 2 open reading frame 89 | 1 | | 2465 | hsa-mir-33b | 129293 | C2orf89 | chromosome 2 open reading frame 89 | 1 | | 2466 | hsa-mir-942 | 129293 | C2orf89 | chromosome 2 open reading frame 89 | 1 | | 2470 | hsa-mir-302b | 118429 | ANTXR2 | anthrax toxin receptor 2 | 1 | | 2471 | hsa-mir-302c | 118429 | ANTXR2 | anthrax toxin receptor 2 | 1 | | 2472 | hsa-mir-302d | 118429 | ANTXR2 | anthrax toxin receptor 2 | 1 | | 2473 | hsa-mir-367 | 118429 | ANTXR2 | anthrax toxin receptor 2 | 1 | | 2474 | hsa-mir-562 | 118429 | ANTXR2 | anthrax toxin receptor 2 | 1 | | 2475 | hsa-mir-604 | 118429 | ANTXR2 | anthrax toxin receptor 2 | 1 | | 2476 | hsa-mir-548d-1 | 118429 | ANTXR2 | anthrax toxin receptor 2 | 1 | | 2477 | hsa-mir-938 | 118429 | ANTXR2 | anthrax toxin receptor 2 | 1 | | 2478 | hsa-mir-548h-1 | 118429 | ANTXR2 | anthrax toxin receptor 2 | 1 | | 2479 | hsa-mir-3135a | 118429 | ANTXR2 | anthrax toxin receptor 2 | 1 | | 2480 | hsa-mir-3177 | 118429 | ANTXR2 | anthrax toxin receptor 2 | 1 | | 2481 | hsa-mir-3655 | 118429 | ANTXR2 | anthrax toxin receptor 2 | 1 | | 2482 | hsa-mir-4526 | 118429 | ANTXR2 | anthrax toxin receptor 2 | 1 | | 2483 | hsa-mir-4635 | 118429 | ANTXR2 | anthrax toxin receptor 2 | 1 | | 2484 | hsa-mir-4651 | 118429 | ANTXR2 | anthrax toxin receptor 2 | 1 | | 2485 | hsa-mir-4744 | 118429 | ANTXR2 | anthrax toxin receptor 2 | 1 | | 2486 | hsa-mir-5190 | 118429 | ANTXR2 | anthrax toxin receptor 2 | 1 | | 2487 | hsa-mir-101-2 | 118429 | ANTXR2 | anthrax toxin receptor 2 | 1 | | 2488 | hsa-mir-128-1;hsa-mir-128-2 | 118429 | ANTXR2 | anthrax toxin receptor 2 | 1 | | 2489 | hsa-mir-301a | 118429 | ANTXR2 | anthrax toxin receptor 2 | 1 | | 2490 | hsa-mir-511-1;hsa-mir-511-2 | 118429 | ANTXR2 | anthrax toxin receptor 2 | 1 | | 2491 | hsa-mir-586 | 118429 | ANTXR2 | anthrax toxin receptor 2 | 1 | | 2492 | hsa-mir-593 | 118429 | ANTXR2 | anthrax toxin receptor 2 | 1 | | 2493 | hsa-mir-620 | 118429 | ANTXR2 | anthrax toxin receptor 2 | 1 | | 2494 | hsa-mir-33b | 118429 | ANTXR2 | anthrax toxin receptor 2 | 1 | | 2495 | hsa-mir-942 | 118429 | ANTXR2 | anthrax toxin receptor 2 | 1 | | 2496 | hsa-mir-101-2 | 6097 | RORC | RAR-related orphan receptor C | 1 | | 2501 | hsa-mir-511-1 | 6097 | RORC | RAR-related orphan receptor C | 1 | | 2502 | hsa-mir-511-2 | 6097 | RORC | RAR-related orphan receptor C | 1 | | 2503 | hsa-mir-1284 | 6097 | RORC | RAR-related orphan receptor C | 1 | | 2504 | hsa-mir-3181 | 6097 | RORC | RAR-related orphan receptor C | 1 | | 2505 | hsa-mir-3651 | 6097 | RORC | RAR-related orphan receptor C | 1 | | 2506 | hsa-mir-4632 | 6097 | RORC | RAR-related orphan receptor C | 1 | | 2507 | hsa-mir-4648 | 6097 | RORC | RAR-related orphan receptor C | 1 | | 2508 | hsa-mir-4726 | 6097 | RORC | RAR-related orphan receptor C | 1 | | 2509 | hsa-mir-4785 | 6097 | RORC | RAR-related orphan receptor C | 1 | | 2510 | hsa-mir-4794 | 6097 | RORC | RAR-related orphan receptor C | 1 | | 2511 | hsa-mir-4802 | 6097 | RORC | RAR-related orphan receptor C | 1 | | 2512 | hsa-let-7i | 6097 | RORC | RAR-related orphan receptor C | 1 | | 2513 | hsa-mir-302b | 6097 | RORC | RAR-related orphan receptor C | 1 | | 2514 | hsa-mir-302c | 6097 | RORC | RAR-related orphan receptor C | 1 | | 2515 | hsa-mir-339 | 6097 | RORC | RAR-related orphan receptor C | 1 | | 2516 | hsa-mir-562 | 6097 | RORC | RAR-related orphan receptor C | 1 | | 2517 | hsa-mir-567 | 6097 | RORC | RAR-related orphan receptor C | 1 | | 2518 | hsa-mir-576 | 6097 | RORC | RAR-related orphan receptor C | 1 | | 2519 | hsa-mir-579 | 6097 | RORC | RAR-related orphan receptor C | 1 | | 2520 | hsa-mir-582 | 6097 | RORC | RAR-related orphan receptor C | 1 | | 2521 | hsa-mir-604 | 6097 | RORC | RAR-related orphan receptor C | 1 | | 2522 | hsa-mir-608 | 6097 | RORC | RAR-related orphan receptor C | 1 | | 2523 | hsa-mir-621 | 6097 | RORC | RAR-related orphan receptor C | 1 | | 2524 | hsa-mir-627 | 6097 | RORC | RAR-related orphan receptor C | 1 | | 2525 | hsa-mir-454 | 6097 | RORC | RAR-related orphan receptor C | 1 | | 2526 | hsa-mir-302d | 6097 | RORC | RAR-related orphan receptor C | 1 | | 2527 | hsa-mir-367 | 6097 | RORC | RAR-related orphan receptor C | 1 | | 2528 | hsa-mir-548b | 6097 | RORC | RAR-related orphan receptor C | 1 | | 2529 | hsa-mir-548d-1;hsa-mir-548d-2 | 6097 | RORC | RAR-related orphan receptor C | 1 | | 2530 | hsa-mir-938 | 6097 | RORC | RAR-related orphan receptor C | 1 | | 2531 | hsa-mir-1236 | 6097 | RORC | RAR-related orphan receptor C | 1 | | 2532 | hsa-mir-101-2 | 136895 | C7orf31 | chromosome 7 open reading frame 31 | 1 | | 2537 | hsa-mir-511-1 | 136895 | C7orf31 | chromosome 7 open reading frame 31 | 1 | | 2538 | hsa-mir-511-2 | 136895 | C7orf31 | chromosome 7 open reading frame 31 | 1 | | 2539 | hsa-mir-1284 | 136895 | C7orf31 | chromosome 7 open reading frame 31 | 1 | | 2540 | hsa-mir-3181 | 136895 | C7orf31 | chromosome 7 open reading frame 31 | 1 | | 2541 | hsa-mir-3651 | 136895 | C7orf31 | chromosome 7 open reading frame 31 | 1 | | 2542 | hsa-mir-4632 | 136895 | C7orf31 | chromosome 7 open reading frame 31 | 1 | | 2543 | hsa-mir-4648 | 136895 | C7orf31 | chromosome 7 open reading frame 31 | 1 | | 2544 | hsa-mir-4726 | 136895 | C7orf31 | chromosome 7 open reading frame 31 | 1 | | 2545 | hsa-mir-4785 | 136895 | C7orf31 | chromosome 7 open reading frame 31 | 1 | | 2546 | hsa-mir-4794 | 136895 | C7orf31 | chromosome 7 open reading frame 31 | 1 | | 2547 | hsa-mir-4802 | 136895 | C7orf31 | chromosome 7 open reading frame 31 | 1 | | 2548 | hsa-let-7i | 136895 | C7orf31 | chromosome 7 open reading frame 31 | 1 | | 2549 | hsa-mir-302b | 136895 | C7orf31 | chromosome 7 open reading frame 31 | 1 | | 2550 | hsa-mir-302c | 136895 | C7orf31 | chromosome 7 open reading frame 31 | 1 | | 2551 | hsa-mir-339 | 136895 | C7orf31 | chromosome 7 open reading frame 31 | 1 | | 2552 | hsa-mir-562 | 136895 | C7orf31 | chromosome 7 open reading frame 31 | 1 | | 2553 | hsa-mir-567 | 136895 | C7orf31 | chromosome 7 open reading frame 31 | 1 | | 2554 | hsa-mir-576 | 136895 | C7orf31 | chromosome 7 open reading frame 31 | 1 | | 2555 | hsa-mir-579 | 136895 | C7orf31 | chromosome 7 open reading frame 31 | 1 | | 2556 | hsa-mir-582 | 136895 | C7orf31 | chromosome 7 open reading frame 31 | 1 | | 2557 | hsa-mir-604 | 136895 | C7orf31 | chromosome 7 open reading frame 31 | 1 | | 2558 | hsa-mir-608 | 136895 | C7orf31 | chromosome 7 open reading frame 31 | 1 | | 2559 | hsa-mir-621 | 136895 | C7orf31 | chromosome 7 open reading frame 31 | 1 | | 2560 | hsa-mir-627 | 136895 | C7orf31 | chromosome 7 open reading frame 31 | 1 | | 2561 | hsa-mir-454 | 136895 | C7orf31 | chromosome 7 open reading frame 31 | 1 | | 2562 | hsa-mir-302d | 136895 | C7orf31 | chromosome 7 open reading frame 31 | 1 | | 2563 | hsa-mir-367 | 136895 | C7orf31 | chromosome 7 open reading frame 31 | 1 | | 2564 | hsa-mir-548b | 136895 | C7orf31 | chromosome 7 open reading frame 31 | 1 | | 2565 | hsa-mir-548d-1;hsa-mir-548d-2 | 136895 | C7orf31 | chromosome 7 open reading frame 31 | 1 | | 2566 | hsa-mir-938 | 136895 | C7orf31 | chromosome 7 open reading frame 31 | 1 | | 2567 | hsa-mir-1236 | 136895 | C7orf31 | chromosome 7 open reading frame 31 | 1 | | 2571 | hsa-mir-302b | 55568 | GALNT10 | UDP-N-acetyl-alpha-D-galactosamine:polypeptide N-acetylgalactosaminyltransferase 10 (GalNAc-T10) | 1 | | 2572 | hsa-mir-302c | 55568 | GALNT10 | UDP-N-acetyl-alpha-D-galactosamine:polypeptide N-acetylgalactosaminyltransferase 10 (GalNAc-T10) | 1 | | 2573 | hsa-mir-302d | 55568 | GALNT10 | UDP-N-acetyl-alpha-D-galactosamine:polypeptide N-acetylgalactosaminyltransferase 10 (GalNAc-T10) | 1 | | 2574 | hsa-mir-367 | 55568 | GALNT10 | UDP-N-acetyl-alpha-D-galactosamine:polypeptide N-acetylgalactosaminyltransferase 10 (GalNAc-T10) | 1 | | 2575 | hsa-mir-562 | 55568 | GALNT10 | UDP-N-acetyl-alpha-D-galactosamine:polypeptide N-acetylgalactosaminyltransferase 10 (GalNAc-T10) | 1 | | 2576 | hsa-mir-604 | 55568 | GALNT10 | UDP-N-acetyl-alpha-D-galactosamine:polypeptide N-acetylgalactosaminyltransferase 10 (GalNAc-T10) | 1 | | 2577 | hsa-mir-548d-1 | 55568 | GALNT10 | UDP-N-acetyl-alpha-D-galactosamine:polypeptide N-acetylgalactosaminyltransferase 10 (GalNAc-T10) | 1 | | 2578 | hsa-mir-938 | 55568 | GALNT10 | UDP-N-acetyl-alpha-D-galactosamine:polypeptide N-acetylgalactosaminyltransferase 10 (GalNAc-T10) | 1 | | 2579 | hsa-mir-548h-1 | 55568 | GALNT10 | UDP-N-acetyl-alpha-D-galactosamine:polypeptide N-acetylgalactosaminyltransferase 10 (GalNAc-T10) | 1 | | 2580 | hsa-mir-3135a | 55568 | GALNT10 | UDP-N-acetyl-alpha-D-galactosamine:polypeptide N-acetylgalactosaminyltransferase 10 (GalNAc-T10) | 1 | | 2581 | hsa-mir-3177 | 55568 | GALNT10 | UDP-N-acetyl-alpha-D-galactosamine:polypeptide N-acetylgalactosaminyltransferase 10 (GalNAc-T10) | 1 | | 2582 | hsa-mir-3655 | 55568 | GALNT10 | UDP-N-acetyl-alpha-D-galactosamine:polypeptide N-acetylgalactosaminyltransferase 10 (GalNAc-T10) | 1 | | 2583 | hsa-mir-4526 | 55568 | GALNT10 | UDP-N-acetyl-alpha-D-galactosamine:polypeptide N-acetylgalactosaminyltransferase 10 (GalNAc-T10) | 1 | | 2584 | hsa-mir-4635 | 55568 | GALNT10 | UDP-N-acetyl-alpha-D-galactosamine:polypeptide N-acetylgalactosaminyltransferase 10 (GalNAc-T10) | 1 | | 2585 | hsa-mir-4651 | 55568 | GALNT10 | UDP-N-acetyl-alpha-D-galactosamine:polypeptide N-acetylgalactosaminyltransferase 10 (GalNAc-T10) | 1 | | 2586 | hsa-mir-4744 | 55568 | GALNT10 | UDP-N-acetyl-alpha-D-galactosamine:polypeptide N-acetylgalactosaminyltransferase 10 (GalNAc-T10) | 1 | | 2587 | hsa-mir-5190 | 55568 | GALNT10 | UDP-N-acetyl-alpha-D-galactosamine:polypeptide N-acetylgalactosaminyltransferase 10 (GalNAc-T10) | 1 | | 2588 | hsa-mir-101-2 | 55568 | GALNT10 | UDP-N-acetyl-alpha-D-galactosamine:polypeptide N-acetylgalactosaminyltransferase 10 (GalNAc-T10) | 1 | | 2589 | hsa-mir-128-1;hsa-mir-128-2 | 55568 | GALNT10 | UDP-N-acetyl-alpha-D-galactosamine:polypeptide N-acetylgalactosaminyltransferase 10 (GalNAc-T10) | 1 | | 2590 | hsa-mir-301a | 55568 | GALNT10 | UDP-N-acetyl-alpha-D-galactosamine:polypeptide N-acetylgalactosaminyltransferase 10 (GalNAc-T10) | 1 | | 2591 | hsa-mir-511-1;hsa-mir-511-2 | 55568 | GALNT10 | UDP-N-acetyl-alpha-D-galactosamine:polypeptide N-acetylgalactosaminyltransferase 10 (GalNAc-T10) | 1 | | 2592 | hsa-mir-586 | 55568 | GALNT10 | UDP-N-acetyl-alpha-D-galactosamine:polypeptide N-acetylgalactosaminyltransferase 10 (GalNAc-T10) | 1 | | 2593 | hsa-mir-593 | 55568 | GALNT10 | UDP-N-acetyl-alpha-D-galactosamine:polypeptide N-acetylgalactosaminyltransferase 10 (GalNAc-T10) | 1 | | 2594 | hsa-mir-620 | 55568 | GALNT10 | UDP-N-acetyl-alpha-D-galactosamine:polypeptide N-acetylgalactosaminyltransferase 10 (GalNAc-T10) | 1 | | 2595 | hsa-mir-33b | 55568 | GALNT10 | UDP-N-acetyl-alpha-D-galactosamine:polypeptide N-acetylgalactosaminyltransferase 10 (GalNAc-T10) | 1 | | 2596 | hsa-mir-942 | 55568 | GALNT10 | UDP-N-acetyl-alpha-D-galactosamine:polypeptide N-acetylgalactosaminyltransferase 10 (GalNAc-T10) | 1 | | 2597 | hsa-mir-101-2 | 84937 | ZNRF1 | zinc and ring finger 1, E3 ubiquitin protein ligase | 1 | | 2602 | hsa-mir-511-1 | 84937 | ZNRF1 | zinc and ring finger 1, E3 ubiquitin protein ligase | 1 | | 2603 | hsa-mir-511-2 | 84937 | ZNRF1 | zinc and ring finger 1, E3 ubiquitin protein ligase | 1 | | 2604 | hsa-mir-1284 | 84937 | ZNRF1 | zinc and ring finger 1, E3 ubiquitin protein ligase | 1 | | 2605 | hsa-mir-3181 | 84937 | ZNRF1 | zinc and ring finger 1, E3 ubiquitin protein ligase | 1 | | 2606 | hsa-mir-3651 | 84937 | ZNRF1 | zinc and ring finger 1, E3 ubiquitin protein ligase | 1 | | 2607 | hsa-mir-4632 | 84937 | ZNRF1 | zinc and ring finger 1, E3 ubiquitin protein ligase | 1 | | 2608 | hsa-mir-4648 | 84937 | ZNRF1 | zinc and ring finger 1, E3 ubiquitin protein ligase | 1 | | 2609 | hsa-mir-4726 | 84937 | ZNRF1 | zinc and ring finger 1, E3 ubiquitin protein ligase | 1 | | 2610 | hsa-mir-4785 | 84937 | ZNRF1 | zinc and ring finger 1, E3 ubiquitin protein ligase | 1 | | 2611 | hsa-mir-4794 | 84937 | ZNRF1 | zinc and ring finger 1, E3 ubiquitin protein ligase | 1 | | 2612 | hsa-mir-4802 | 84937 | ZNRF1 | zinc and ring finger 1, E3 ubiquitin protein ligase | 1 | | 2613 | hsa-let-7i | 84937 | ZNRF1 | zinc and ring finger 1, E3 ubiquitin protein ligase | 1 | | 2614 | hsa-mir-302b | 84937 | ZNRF1 | zinc and ring finger 1, E3 ubiquitin protein ligase | 1 | | 2615 | hsa-mir-302c | 84937 | ZNRF1 | zinc and ring finger 1, E3 ubiquitin protein ligase | 1 | | 2616 | hsa-mir-339 | 84937 | ZNRF1 | zinc and ring finger 1, E3 ubiquitin protein ligase | 1 | | 2617 | hsa-mir-562 | 84937 | ZNRF1 | zinc and ring finger 1, E3 ubiquitin protein ligase | 1 | | 2618 | hsa-mir-567 | 84937 | ZNRF1 | zinc and ring finger 1, E3 ubiquitin protein ligase | 1 | | 2619 | hsa-mir-576 | 84937 | ZNRF1 | zinc and ring finger 1, E3 ubiquitin protein ligase | 1 | | 2620 | hsa-mir-579 | 84937 | ZNRF1 | zinc and ring finger 1, E3 ubiquitin protein ligase | 1 | | 2621 | hsa-mir-582 | 84937 | ZNRF1 | zinc and ring finger 1, E3 ubiquitin protein ligase | 1 | | 2622 | hsa-mir-604 | 84937 | ZNRF1 | zinc and ring finger 1, E3 ubiquitin protein ligase | 1 | | 2623 | hsa-mir-608 | 84937 | ZNRF1 | zinc and ring finger 1, E3 ubiquitin protein ligase | 1 | | 2624 | hsa-mir-621 | 84937 | ZNRF1 | zinc and ring finger 1, E3 ubiquitin protein ligase | 1 | | 2625 | hsa-mir-627 | 84937 | ZNRF1 | zinc and ring finger 1, E3 ubiquitin protein ligase | 1 | | 2626 | hsa-mir-454 | 84937 | ZNRF1 | zinc and ring finger 1, E3 ubiquitin protein ligase | 1 | | 2627 | hsa-mir-302d | 84937 | ZNRF1 | zinc and ring finger 1, E3 ubiquitin protein ligase | 1 | | 2628 | hsa-mir-367 | 84937 | ZNRF1 | zinc and ring finger 1, E3 ubiquitin protein ligase | 1 | | 2629 | hsa-mir-548b | 84937 | ZNRF1 | zinc and ring finger 1, E3 ubiquitin protein ligase | 1 | | 2630 | hsa-mir-548d-1;hsa-mir-548d-2 | 84937 | ZNRF1 | zinc and ring finger 1, E3 ubiquitin protein ligase | 1 | | 2631 | hsa-mir-938 | 84937 | ZNRF1 | zinc and ring finger 1, E3 ubiquitin protein ligase | 1 | | 2632 | hsa-mir-1236 | 84937 | ZNRF1 | zinc and ring finger 1, E3 ubiquitin protein ligase | 1 | | 2636 | hsa-mir-302b | 115352 | FCRL3 | Fc receptor-like 3 | 1 | | 2637 | hsa-mir-302c | 115352 | FCRL3 | Fc receptor-like 3 | 1 | | 2638 | hsa-mir-302d | 115352 | FCRL3 | Fc receptor-like 3 | 1 | | 2639 | hsa-mir-367 | 115352 | FCRL3 | Fc receptor-like 3 | 1 | | 2640 | hsa-mir-562 | 115352 | FCRL3 | Fc receptor-like 3 | 1 | | 2641 | hsa-mir-604 | 115352 | FCRL3 | Fc receptor-like 3 | 1 | | 2642 | hsa-mir-548d-1 | 115352 | FCRL3 | Fc receptor-like 3 | 1 | | 2643 | hsa-mir-938 | 115352 | FCRL3 | Fc receptor-like 3 | 1 | | 2644 | hsa-mir-548h-1 | 115352 | FCRL3 | Fc receptor-like 3 | 1 | | 2645 | hsa-mir-3135a | 115352 | FCRL3 | Fc receptor-like 3 | 1 | | 2646 | hsa-mir-3177 | 115352 | FCRL3 | Fc receptor-like 3 | 1 | | 2647 | hsa-mir-3655 | 115352 | FCRL3 | Fc receptor-like 3 | 1 | | 2648 | hsa-mir-4526 | 115352 | FCRL3 | Fc receptor-like 3 | 1 | | 2649 | hsa-mir-4635 | 115352 | FCRL3 | Fc receptor-like 3 | 1 | | 2650 | hsa-mir-4651 | 115352 | FCRL3 | Fc receptor-like 3 | 1 | | 2651 | hsa-mir-4744 | 115352 | FCRL3 | Fc receptor-like 3 | 1 | | 2652 | hsa-mir-5190 | 115352 | FCRL3 | Fc receptor-like 3 | 1 | | 2653 | hsa-mir-101-2 | 115352 | FCRL3 | Fc receptor-like 3 | 1 | | 2654 | hsa-mir-128-1;hsa-mir-128-2 | 115352 | FCRL3 | Fc receptor-like 3 | 1 | | 2655 | hsa-mir-301a | 115352 | FCRL3 | Fc receptor-like 3 | 1 | | 2656 | hsa-mir-511-1;hsa-mir-511-2 | 115352 | FCRL3 | Fc receptor-like 3 | 1 | | 2657 | hsa-mir-586 | 115352 | FCRL3 | Fc receptor-like 3 | 1 | | 2658 | hsa-mir-593 | 115352 | FCRL3 | Fc receptor-like 3 | 1 | | 2659 | hsa-mir-620 | 115352 | FCRL3 | Fc receptor-like 3 | 1 | | 2660 | hsa-mir-33b | 115352 | FCRL3 | Fc receptor-like 3 | 1 | | 2661 | hsa-mir-942 | 115352 | FCRL3 | Fc receptor-like 3 | 1 | | 2665 | hsa-mir-302b | 3656 | IRAK2 | interleukin-1 receptor-associated kinase 2 | 1 | | 2666 | hsa-mir-302c | 3656 | IRAK2 | interleukin-1 receptor-associated kinase 2 | 1 | | 2667 | hsa-mir-302d | 3656 | IRAK2 | interleukin-1 receptor-associated kinase 2 | 1 | | 2668 | hsa-mir-367 | 3656 | IRAK2 | interleukin-1 receptor-associated kinase 2 | 1 | | 2669 | hsa-mir-562 | 3656 | IRAK2 | interleukin-1 receptor-associated kinase 2 | 1 | | 2670 | hsa-mir-604 | 3656 | IRAK2 | interleukin-1 receptor-associated kinase 2 | 1 | | 2671 | hsa-mir-548d-1 | 3656 | IRAK2 | interleukin-1 receptor-associated kinase 2 | 1 | | 2672 | hsa-mir-938 | 3656 | IRAK2 | interleukin-1 receptor-associated kinase 2 | 1 | | 2673 | hsa-mir-548h-1 | 3656 | IRAK2 | interleukin-1 receptor-associated kinase 2 | 1 | | 2674 | hsa-mir-3135a | 3656 | IRAK2 | interleukin-1 receptor-associated kinase 2 | 1 | | 2675 | hsa-mir-3177 | 3656 | IRAK2 | interleukin-1 receptor-associated kinase 2 | 1 | | 2676 | hsa-mir-3655 | 3656 | IRAK2 | interleukin-1 receptor-associated kinase 2 | 1 | | 2677 | hsa-mir-4526 | 3656 | IRAK2 | interleukin-1 receptor-associated kinase 2 | 1 | | 2678 | hsa-mir-4635 | 3656 | IRAK2 | interleukin-1 receptor-associated kinase 2 | 1 | | 2679 | hsa-mir-4651 | 3656 | IRAK2 | interleukin-1 receptor-associated kinase 2 | 1 | | 2680 | hsa-mir-4744 | 3656 | IRAK2 | interleukin-1 receptor-associated kinase 2 | 1 | | 2681 | hsa-mir-5190 | 3656 | IRAK2 | interleukin-1 receptor-associated kinase 2 | 1 | | 2682 | hsa-mir-101-2 | 3656 | IRAK2 | interleukin-1 receptor-associated kinase 2 | 1 | | 2683 | hsa-mir-128-1;hsa-mir-128-2 | 3656 | IRAK2 | interleukin-1 receptor-associated kinase 2 | 1 | | 2684 | hsa-mir-301a | 3656 | IRAK2 | interleukin-1 receptor-associated kinase 2 | 1 | | 2685 | hsa-mir-511-1;hsa-mir-511-2 | 3656 | IRAK2 | interleukin-1 receptor-associated kinase 2 | 1 | | 2686 | hsa-mir-586 | 3656 | IRAK2 | interleukin-1 receptor-associated kinase 2 | 1 | | 2687 | hsa-mir-593 | 3656 | IRAK2 | interleukin-1 receptor-associated kinase 2 | 1 | | 2688 | hsa-mir-620 | 3656 | IRAK2 | interleukin-1 receptor-associated kinase 2 | 1 | | 2689 | hsa-mir-33b | 3656 | IRAK2 | interleukin-1 receptor-associated kinase 2 | 1 | | 2690 | hsa-mir-942 | 3656 | IRAK2 | interleukin-1 receptor-associated kinase 2 | 1 | | 2691 | hsa-mir-101-2 | 55680 | RUFY2 | RUN and FYVE domain containing 2 | 1 | | 2696 | hsa-mir-511-1 | 55680 | RUFY2 | RUN and FYVE domain containing 2 | 1 | | 2697 | hsa-mir-511-2 | 55680 | RUFY2 | RUN and FYVE domain containing 2 | 1 | | 2698 | hsa-mir-1284 | 55680 | RUFY2 | RUN and FYVE domain containing 2 | 1 | | 2699 | hsa-mir-3181 | 55680 | RUFY2 | RUN and FYVE domain containing 2 | 1 | | 2700 | hsa-mir-3651 | 55680 | RUFY2 | RUN and FYVE domain containing 2 | 1 | | 2701 | hsa-mir-4632 | 55680 | RUFY2 | RUN and FYVE domain containing 2 | 1 | | 2702 | hsa-mir-4648 | 55680 | RUFY2 | RUN and FYVE domain containing 2 | 1 | | 2703 | hsa-mir-4726 | 55680 | RUFY2 | RUN and FYVE domain containing 2 | 1 | | 2704 | hsa-mir-4785 | 55680 | RUFY2 | RUN and FYVE domain containing 2 | 1 | | 2705 | hsa-mir-4794 | 55680 | RUFY2 | RUN and FYVE domain containing 2 | 1 | | 2706 | hsa-mir-4802 | 55680 | RUFY2 | RUN and FYVE domain containing 2 | 1 | | 2707 | hsa-let-7i | 55680 | RUFY2 | RUN and FYVE domain containing 2 | 1 | | 2708 | hsa-mir-302b | 55680 | RUFY2 | RUN and FYVE domain containing 2 | 1 | | 2709 | hsa-mir-302c | 55680 | RUFY2 | RUN and FYVE domain containing 2 | 1 | | 2710 | hsa-mir-339 | 55680 | RUFY2 | RUN and FYVE domain containing 2 | 1 | | 2711 | hsa-mir-562 | 55680 | RUFY2 | RUN and FYVE domain containing 2 | 1 | | 2712 | hsa-mir-567 | 55680 | RUFY2 | RUN and FYVE domain containing 2 | 1 | | 2713 | hsa-mir-576 | 55680 | RUFY2 | RUN and FYVE domain containing 2 | 1 | | 2714 | hsa-mir-579 | 55680 | RUFY2 | RUN and FYVE domain containing 2 | 1 | | 2715 | hsa-mir-582 | 55680 | RUFY2 | RUN and FYVE domain containing 2 | 1 | | 2716 | hsa-mir-604 | 55680 | RUFY2 | RUN and FYVE domain containing 2 | 1 | | 2717 | hsa-mir-608 | 55680 | RUFY2 | RUN and FYVE domain containing 2 | 1 | | 2718 | hsa-mir-621 | 55680 | RUFY2 | RUN and FYVE domain containing 2 | 1 | | 2719 | hsa-mir-627 | 55680 | RUFY2 | RUN and FYVE domain containing 2 | 1 | | 2720 | hsa-mir-454 | 55680 | RUFY2 | RUN and FYVE domain containing 2 | 1 | | 2721 | hsa-mir-302d | 55680 | RUFY2 | RUN and FYVE domain containing 2 | 1 | | 2722 | hsa-mir-367 | 55680 | RUFY2 | RUN and FYVE domain containing 2 | 1 | | 2723 | hsa-mir-548b | 55680 | RUFY2 | RUN and FYVE domain containing 2 | 1 | | 2724 | hsa-mir-548d-1;hsa-mir-548d-2 | 55680 | RUFY2 | RUN and FYVE domain containing 2 | 1 | | 2725 | hsa-mir-938 | 55680 | RUFY2 | RUN and FYVE domain containing 2 | 1 | | 2726 | hsa-mir-1236 | 55680 | RUFY2 | RUN and FYVE domain containing 2 | 1 | | 2730 | hsa-mir-302b | 94235 | GNG8 | guanine nucleotide binding protein (G protein), gamma 8 | 1 | | 2731 | hsa-mir-302c | 94235 | GNG8 | guanine nucleotide binding protein (G protein), gamma 8 | 1 | | 2732 | hsa-mir-302d | 94235 | GNG8 | guanine nucleotide binding protein (G protein), gamma 8 | 1 | | 2733 | hsa-mir-367 | 94235 | GNG8 | guanine nucleotide binding protein (G protein), gamma 8 | 1 | | 2734 | hsa-mir-562 | 94235 | GNG8 | guanine nucleotide binding protein (G protein), gamma 8 | 1 | | 2735 | hsa-mir-604 | 94235 | GNG8 | guanine nucleotide binding protein (G protein), gamma 8 | 1 | | 2736 | hsa-mir-548d-1 | 94235 | GNG8 | guanine nucleotide binding protein (G protein), gamma 8 | 1 | | 2737 | hsa-mir-938 | 94235 | GNG8 | guanine nucleotide binding protein (G protein), gamma 8 | 1 | | 2738 | hsa-mir-548h-1 | 94235 | GNG8 | guanine nucleotide binding protein (G protein), gamma 8 | 1 | | 2739 | hsa-mir-3135a | 94235 | GNG8 | guanine nucleotide binding protein (G protein), gamma 8 | 1 | | 2740 | hsa-mir-3177 | 94235 | GNG8 | guanine nucleotide binding protein (G protein), gamma 8 | 1 | | 2741 | hsa-mir-3655 | 94235 | GNG8 | guanine nucleotide binding protein (G protein), gamma 8 | 1 | | 2742 | hsa-mir-4526 | 94235 | GNG8 | guanine nucleotide binding protein (G protein), gamma 8 | 1 | | 2743 | hsa-mir-4635 | 94235 | GNG8 | guanine nucleotide binding protein (G protein), gamma 8 | 1 | | 2744 | hsa-mir-4651 | 94235 | GNG8 | guanine nucleotide binding protein (G protein), gamma 8 | 1 | | 2745 | hsa-mir-4744 | 94235 | GNG8 | guanine nucleotide binding protein (G protein), gamma 8 | 1 | | 2746 | hsa-mir-5190 | 94235 | GNG8 | guanine nucleotide binding protein (G protein), gamma 8 | 1 | | 2747 | hsa-mir-101-2 | 94235 | GNG8 | guanine nucleotide binding protein (G protein), gamma 8 | 1 | | 2748 | hsa-mir-128-1;hsa-mir-128-2 | 94235 | GNG8 | guanine nucleotide binding protein (G protein), gamma 8 | 1 | | 2749 | hsa-mir-301a | 94235 | GNG8 | guanine nucleotide binding protein (G protein), gamma 8 | 1 | | 2750 | hsa-mir-511-1;hsa-mir-511-2 | 94235 | GNG8 | guanine nucleotide binding protein (G protein), gamma 8 | 1 | | 2751 | hsa-mir-586 | 94235 | GNG8 | guanine nucleotide binding protein (G protein), gamma 8 | 1 | | 2752 | hsa-mir-593 | 94235 | GNG8 | guanine nucleotide binding protein (G protein), gamma 8 | 1 | | 2753 | hsa-mir-620 | 94235 | GNG8 | guanine nucleotide binding protein (G protein), gamma 8 | 1 | | 2754 | hsa-mir-33b | 94235 | GNG8 | guanine nucleotide binding protein (G protein), gamma 8 | 1 | | 2755 | hsa-mir-942 | 94235 | GNG8 | guanine nucleotide binding protein (G protein), gamma 8 | 1 | | 2759 | hsa-mir-302b | 59269 | HIVEP3 | human immunodeficiency virus type I enhancer binding protein 3 | 1 | | 2760 | hsa-mir-302c | 59269 | HIVEP3 | human immunodeficiency virus type I enhancer binding protein 3 | 1 | | 2761 | hsa-mir-302d | 59269 | HIVEP3 | human immunodeficiency virus type I enhancer binding protein 3 | 1 | | 2762 | hsa-mir-367 | 59269 | HIVEP3 | human immunodeficiency virus type I enhancer binding protein 3 | 1 | | 2763 | hsa-mir-562 | 59269 | HIVEP3 | human immunodeficiency virus type I enhancer binding protein 3 | 1 | | 2764 | hsa-mir-604 | 59269 | HIVEP3 | human immunodeficiency virus type I enhancer binding protein 3 | 1 | | 2765 | hsa-mir-548d-1 | 59269 | HIVEP3 | human immunodeficiency virus type I enhancer binding protein 3 | 1 | | 2766 | hsa-mir-938 | 59269 | HIVEP3 | human immunodeficiency virus type I enhancer binding protein 3 | 1 | | 2767 | hsa-mir-548h-1 | 59269 | HIVEP3 | human immunodeficiency virus type I enhancer binding protein 3 | 1 | | 2768 | hsa-mir-3135a | 59269 | HIVEP3 | human immunodeficiency virus type I enhancer binding protein 3 | 1 | | 2769 | hsa-mir-3177 | 59269 | HIVEP3 | human immunodeficiency virus type I enhancer binding protein 3 | 1 | | 2770 | hsa-mir-3655 | 59269 | HIVEP3 | human immunodeficiency virus type I enhancer binding protein 3 | 1 | | 2771 | hsa-mir-4526 | 59269 | HIVEP3 | human immunodeficiency virus type I enhancer binding protein 3 | 1 | | 2772 | hsa-mir-4635 | 59269 | HIVEP3 | human immunodeficiency virus type I enhancer binding protein 3 | 1 | | 2773 | hsa-mir-4651 | 59269 | HIVEP3 | human immunodeficiency virus type I enhancer binding protein 3 | 1 | | 2774 | hsa-mir-4744 | 59269 | HIVEP3 | human immunodeficiency virus type I enhancer binding protein 3 | 1 | | 2775 | hsa-mir-5190 | 59269 | HIVEP3 | human immunodeficiency virus type I enhancer binding protein 3 | 1 | | 2776 | hsa-mir-101-2 | 59269 | HIVEP3 | human immunodeficiency virus type I enhancer binding protein 3 | 1 | | 2777 | hsa-mir-128-1;hsa-mir-128-2 | 59269 | HIVEP3 | human immunodeficiency virus type I enhancer binding protein 3 | 1 | | 2778 | hsa-mir-301a | 59269 | HIVEP3 | human immunodeficiency virus type I enhancer binding protein 3 | 1 | | 2779 | hsa-mir-511-1;hsa-mir-511-2 | 59269 | HIVEP3 | human immunodeficiency virus type I enhancer binding protein 3 | 1 | | 2780 | hsa-mir-586 | 59269 | HIVEP3 | human immunodeficiency virus type I enhancer binding protein 3 | 1 | | 2781 | hsa-mir-593 | 59269 | HIVEP3 | human immunodeficiency virus type I enhancer binding protein 3 | 1 | | 2782 | hsa-mir-620 | 59269 | HIVEP3 | human immunodeficiency virus type I enhancer binding protein 3 | 1 | | 2783 | hsa-mir-33b | 59269 | HIVEP3 | human immunodeficiency virus type I enhancer binding protein 3 | 1 | | 2784 | hsa-mir-942 | 59269 | HIVEP3 | human immunodeficiency virus type I enhancer binding protein 3 | 1 | | 2785 | hsa-mir-101-2 | 166824 | RASSF6 | Ras association (RalGDS/AF-6) domain family member 6 | 1 | | 2790 | hsa-mir-511-1 | 166824 | RASSF6 | Ras association (RalGDS/AF-6) domain family member 6 | 1 | | 2791 | hsa-mir-511-2 | 166824 | RASSF6 | Ras association (RalGDS/AF-6) domain family member 6 | 1 | | 2792 | hsa-mir-1284 | 166824 | RASSF6 | Ras association (RalGDS/AF-6) domain family member 6 | 1 | | 2793 | hsa-mir-3181 | 166824 | RASSF6 | Ras association (RalGDS/AF-6) domain family member 6 | 1 | | 2794 | hsa-mir-3651 | 166824 | RASSF6 | Ras association (RalGDS/AF-6) domain family member 6 | 1 | | 2795 | hsa-mir-4632 | 166824 | RASSF6 | Ras association (RalGDS/AF-6) domain family member 6 | 1 | | 2796 | hsa-mir-4648 | 166824 | RASSF6 | Ras association (RalGDS/AF-6) domain family member 6 | 1 | | 2797 | hsa-mir-4726 | 166824 | RASSF6 | Ras association (RalGDS/AF-6) domain family member 6 | 1 | | 2798 | hsa-mir-4785 | 166824 | RASSF6 | Ras association (RalGDS/AF-6) domain family member 6 | 1 | | 2799 | hsa-mir-4794 | 166824 | RASSF6 | Ras association (RalGDS/AF-6) domain family member 6 | 1 | | 2800 | hsa-mir-4802 | 166824 | RASSF6 | Ras association (RalGDS/AF-6) domain family member 6 | 1 | | 2801 | hsa-let-7i | 166824 | RASSF6 | Ras association (RalGDS/AF-6) domain family member 6 | 1 | | 2802 | hsa-mir-302b | 166824 | RASSF6 | Ras association (RalGDS/AF-6) domain family member 6 | 1 | | 2803 | hsa-mir-302c | 166824 | RASSF6 | Ras association (RalGDS/AF-6) domain family member 6 | 1 | | 2804 | hsa-mir-339 | 166824 | RASSF6 | Ras association (RalGDS/AF-6) domain family member 6 | 1 | | 2805 | hsa-mir-562 | 166824 | RASSF6 | Ras association (RalGDS/AF-6) domain family member 6 | 1 | | 2806 | hsa-mir-567 | 166824 | RASSF6 | Ras association (RalGDS/AF-6) domain family member 6 | 1 | | 2807 | hsa-mir-576 | 166824 | RASSF6 | Ras association (RalGDS/AF-6) domain family member 6 | 1 | | 2808 | hsa-mir-579 | 166824 | RASSF6 | Ras association (RalGDS/AF-6) domain family member 6 | 1 | | 2809 | hsa-mir-582 | 166824 | RASSF6 | Ras association (RalGDS/AF-6) domain family member 6 | 1 | | 2810 | hsa-mir-604 | 166824 | RASSF6 | Ras association (RalGDS/AF-6) domain family member 6 | 1 | | 2811 | hsa-mir-608 | 166824 | RASSF6 | Ras association (RalGDS/AF-6) domain family member 6 | 1 | | 2812 | hsa-mir-621 | 166824 | RASSF6 | Ras association (RalGDS/AF-6) domain family member 6 | 1 | | 2813 | hsa-mir-627 | 166824 | RASSF6 | Ras association (RalGDS/AF-6) domain family member 6 | 1 | | 2814 | hsa-mir-454 | 166824 | RASSF6 | Ras association (RalGDS/AF-6) domain family member 6 | 1 | | 2815 | hsa-mir-302d | 166824 | RASSF6 | Ras association (RalGDS/AF-6) domain family member 6 | 1 | | 2816 | hsa-mir-367 | 166824 | RASSF6 | Ras association (RalGDS/AF-6) domain family member 6 | 1 | | 2817 | hsa-mir-548b | 166824 | RASSF6 | Ras association (RalGDS/AF-6) domain family member 6 | 1 | | 2818 | hsa-mir-548d-1;hsa-mir-548d-2 | 166824 | RASSF6 | Ras association (RalGDS/AF-6) domain family member 6 | 1 | | 2819 | hsa-mir-938 | 166824 | RASSF6 | Ras association (RalGDS/AF-6) domain family member 6 | 1 | | 2820 | hsa-mir-1236 | 166824 | RASSF6 | Ras association (RalGDS/AF-6) domain family member 6 | 1 | | 2821 | hsa-mir-101-2 | 1105 | CHD1 | chromodomain helicase DNA binding protein 1 | 1 | | 2826 | hsa-mir-511-1 | 1105 | CHD1 | chromodomain helicase DNA binding protein 1 | 1 | | 2827 | hsa-mir-511-2 | 1105 | CHD1 | chromodomain helicase DNA binding protein 1 | 1 | | 2828 | hsa-mir-1284 | 1105 | CHD1 | chromodomain helicase DNA binding protein 1 | 1 | | 2829 | hsa-mir-3181 | 1105 | CHD1 | chromodomain helicase DNA binding protein 1 | 1 | | 2830 | hsa-mir-3651 | 1105 | CHD1 | chromodomain helicase DNA binding protein 1 | 1 | | 2831 | hsa-mir-4632 | 1105 | CHD1 | chromodomain helicase DNA binding protein 1 | 1 | | 2832 | hsa-mir-4648 | 1105 | CHD1 | chromodomain helicase DNA binding protein 1 | 1 | | 2833 | hsa-mir-4726 | 1105 | CHD1 | chromodomain helicase DNA binding protein 1 | 1 | | 2834 | hsa-mir-4785 | 1105 | CHD1 | chromodomain helicase DNA binding protein 1 | 1 | | 2835 | hsa-mir-4794 | 1105 | CHD1 | chromodomain helicase DNA binding protein 1 | 1 | | 2836 | hsa-mir-4802 | 1105 | CHD1 | chromodomain helicase DNA binding protein 1 | 1 | | 2837 | hsa-let-7i | 1105 | CHD1 | chromodomain helicase DNA binding protein 1 | 1 | | 2838 | hsa-mir-302b | 1105 | CHD1 | chromodomain helicase DNA binding protein 1 | 1 | | 2839 | hsa-mir-302c | 1105 | CHD1 | chromodomain helicase DNA binding protein 1 | 1 | | 2840 | hsa-mir-339 | 1105 | CHD1 | chromodomain helicase DNA binding protein 1 | 1 | | 2841 | hsa-mir-562 | 1105 | CHD1 | chromodomain helicase DNA binding protein 1 | 1 | | 2842 | hsa-mir-567 | 1105 | CHD1 | chromodomain helicase DNA binding protein 1 | 1 | | 2843 | hsa-mir-576 | 1105 | CHD1 | chromodomain helicase DNA binding protein 1 | 1 | | 2844 | hsa-mir-579 | 1105 | CHD1 | chromodomain helicase DNA binding protein 1 | 1 | | 2845 | hsa-mir-582 | 1105 | CHD1 | chromodomain helicase DNA binding protein 1 | 1 | | 2846 | hsa-mir-604 | 1105 | CHD1 | chromodomain helicase DNA binding protein 1 | 1 | | 2847 | hsa-mir-608 | 1105 | CHD1 | chromodomain helicase DNA binding protein 1 | 1 | | 2848 | hsa-mir-621 | 1105 | CHD1 | chromodomain helicase DNA binding protein 1 | 1 | | 2849 | hsa-mir-627 | 1105 | CHD1 | chromodomain helicase DNA binding protein 1 | 1 | | 2850 | hsa-mir-454 | 1105 | CHD1 | chromodomain helicase DNA binding protein 1 | 1 | | 2851 | hsa-mir-302d | 1105 | CHD1 | chromodomain helicase DNA binding protein 1 | 1 | | 2852 | hsa-mir-367 | 1105 | CHD1 | chromodomain helicase DNA binding protein 1 | 1 | | 2853 | hsa-mir-548b | 1105 | CHD1 | chromodomain helicase DNA binding protein 1 | 1 | | 2854 | hsa-mir-548d-1;hsa-mir-548d-2 | 1105 | CHD1 | chromodomain helicase DNA binding protein 1 | 1 | | 2855 | hsa-mir-938 | 1105 | CHD1 | chromodomain helicase DNA binding protein 1 | 1 | | 2856 | hsa-mir-1236 | 1105 | CHD1 | chromodomain helicase DNA binding protein 1 | 1 | | 2857 | hsa-mir-101-2 | 79703 | C11orf80 | chromosome 11 open reading frame 80 | 1 | | 2862 | hsa-mir-511-1 | 79703 | C11orf80 | chromosome 11 open reading frame 80 | 1 | | 2863 | hsa-mir-511-2 | 79703 | C11orf80 | chromosome 11 open reading frame 80 | 1 | | 2864 | hsa-mir-1284 | 79703 | C11orf80 | chromosome 11 open reading frame 80 | 1 | | 2865 | hsa-mir-3181 | 79703 | C11orf80 | chromosome 11 open reading frame 80 | 1 | | 2866 | hsa-mir-3651 | 79703 | C11orf80 | chromosome 11 open reading frame 80 | 1 | | 2867 | hsa-mir-4632 | 79703 | C11orf80 | chromosome 11 open reading frame 80 | 1 | | 2868 | hsa-mir-4648 | 79703 | C11orf80 | chromosome 11 open reading frame 80 | 1 | | 2869 | hsa-mir-4726 | 79703 | C11orf80 | chromosome 11 open reading frame 80 | 1 | | 2870 | hsa-mir-4785 | 79703 | C11orf80 | chromosome 11 open reading frame 80 | 1 | | 2871 | hsa-mir-4794 | 79703 | C11orf80 | chromosome 11 open reading frame 80 | 1 | | 2872 | hsa-mir-4802 | 79703 | C11orf80 | chromosome 11 open reading frame 80 | 1 | | 2873 | hsa-let-7i | 79703 | C11orf80 | chromosome 11 open reading frame 80 | 1 | | 2874 | hsa-mir-302b | 79703 | C11orf80 | chromosome 11 open reading frame 80 | 1 | | 2875 | hsa-mir-302c | 79703 | C11orf80 | chromosome 11 open reading frame 80 | 1 | | 2876 | hsa-mir-339 | 79703 | C11orf80 | chromosome 11 open reading frame 80 | 1 | | 2877 | hsa-mir-562 | 79703 | C11orf80 | chromosome 11 open reading frame 80 | 1 | | 2878 | hsa-mir-567 | 79703 | C11orf80 | chromosome 11 open reading frame 80 | 1 | | 2879 | hsa-mir-576 | 79703 | C11orf80 | chromosome 11 open reading frame 80 | 1 | | 2880 | hsa-mir-579 | 79703 | C11orf80 | chromosome 11 open reading frame 80 | 1 | | 2881 | hsa-mir-582 | 79703 | C11orf80 | chromosome 11 open reading frame 80 | 1 | | 2882 | hsa-mir-604 | 79703 | C11orf80 | chromosome 11 open reading frame 80 | 1 | | 2883 | hsa-mir-608 | 79703 | C11orf80 | chromosome 11 open reading frame 80 | 1 | | 2884 | hsa-mir-621 | 79703 | C11orf80 | chromosome 11 open reading frame 80 | 1 | | 2885 | hsa-mir-627 | 79703 | C11orf80 | chromosome 11 open reading frame 80 | 1 | | 2886 | hsa-mir-454 | 79703 | C11orf80 | chromosome 11 open reading frame 80 | 1 | | 2887 | hsa-mir-302d | 79703 | C11orf80 | chromosome 11 open reading frame 80 | 1 | | 2888 | hsa-mir-367 | 79703 | C11orf80 | chromosome 11 open reading frame 80 | 1 | | 2889 | hsa-mir-548b | 79703 | C11orf80 | chromosome 11 open reading frame 80 | 1 | | 2890 | hsa-mir-548d-1;hsa-mir-548d-2 | 79703 | C11orf80 | chromosome 11 open reading frame 80 | 1 | | 2891 | hsa-mir-938 | 79703 | C11orf80 | chromosome 11 open reading frame 80 | 1 | | 2892 | hsa-mir-1236 | 79703 | C11orf80 | chromosome 11 open reading frame 80 | 1 | | 2893 | hsa-mir-101-2 | 22995 | CEP152 | centrosomal protein 152kDa | 1 | | 2898 | hsa-mir-511-1 | 22995 | CEP152 | centrosomal protein 152kDa | 1 | | 2899 | hsa-mir-511-2 | 22995 | CEP152 | centrosomal protein 152kDa | 1 | | 2900 | hsa-mir-1284 | 22995 | CEP152 | centrosomal protein 152kDa | 1 | | 2901 | hsa-mir-3181 | 22995 | CEP152 | centrosomal protein 152kDa | 1 | | 2902 | hsa-mir-3651 | 22995 | CEP152 | centrosomal protein 152kDa | 1 | | 2903 | hsa-mir-4632 | 22995 | CEP152 | centrosomal protein 152kDa | 1 | | 2904 | hsa-mir-4648 | 22995 | CEP152 | centrosomal protein 152kDa | 1 | | 2905 | hsa-mir-4726 | 22995 | CEP152 | centrosomal protein 152kDa | 1 | | 2906 | hsa-mir-4785 | 22995 | CEP152 | centrosomal protein 152kDa | 1 | | 2907 | hsa-mir-4794 | 22995 | CEP152 | centrosomal protein 152kDa | 1 | | 2908 | hsa-mir-4802 | 22995 | CEP152 | centrosomal protein 152kDa | 1 | | 2909 | hsa-let-7i | 22995 | CEP152 | centrosomal protein 152kDa | 1 | | 2910 | hsa-mir-302b | 22995 | CEP152 | centrosomal protein 152kDa | 1 | | 2911 | hsa-mir-302c | 22995 | CEP152 | centrosomal protein 152kDa | 1 | | 2912 | hsa-mir-339 | 22995 | CEP152 | centrosomal protein 152kDa | 1 | | 2913 | hsa-mir-562 | 22995 | CEP152 | centrosomal protein 152kDa | 1 | | 2914 | hsa-mir-567 | 22995 | CEP152 | centrosomal protein 152kDa | 1 | | 2915 | hsa-mir-576 | 22995 | CEP152 | centrosomal protein 152kDa | 1 | | 2916 | hsa-mir-579 | 22995 | CEP152 | centrosomal protein 152kDa | 1 | | 2917 | hsa-mir-582 | 22995 | CEP152 | centrosomal protein 152kDa | 1 | | 2918 | hsa-mir-604 | 22995 | CEP152 | centrosomal protein 152kDa | 1 | | 2919 | hsa-mir-608 | 22995 | CEP152 | centrosomal protein 152kDa | 1 | | 2920 | hsa-mir-621 | 22995 | CEP152 | centrosomal protein 152kDa | 1 | | 2921 | hsa-mir-627 | 22995 | CEP152 | centrosomal protein 152kDa | 1 | | 2922 | hsa-mir-454 | 22995 | CEP152 | centrosomal protein 152kDa | 1 | | 2923 | hsa-mir-302d | 22995 | CEP152 | centrosomal protein 152kDa | 1 | | 2924 | hsa-mir-367 | 22995 | CEP152 | centrosomal protein 152kDa | 1 | | 2925 | hsa-mir-548b | 22995 | CEP152 | centrosomal protein 152kDa | 1 | | 2926 | hsa-mir-548d-1;hsa-mir-548d-2 | 22995 | CEP152 | centrosomal protein 152kDa | 1 | | 2927 | hsa-mir-938 | 22995 | CEP152 | centrosomal protein 152kDa | 1 | | 2928 | hsa-mir-1236 | 22995 | CEP152 | centrosomal protein 152kDa | 1 | | 2932 | hsa-mir-302b | 283551 | C14orf182 | chromosome 14 open reading frame 182 | 1 | | 2933 | hsa-mir-302c | 283551 | C14orf182 | chromosome 14 open reading frame 182 | 1 | | 2934 | hsa-mir-302d | 283551 | C14orf182 | chromosome 14 open reading frame 182 | 1 | | 2935 | hsa-mir-367 | 283551 | C14orf182 | chromosome 14 open reading frame 182 | 1 | | 2936 | hsa-mir-562 | 283551 | C14orf182 | chromosome 14 open reading frame 182 | 1 | | 2937 | hsa-mir-604 | 283551 | C14orf182 | chromosome 14 open reading frame 182 | 1 | | 2938 | hsa-mir-548d-1 | 283551 | C14orf182 | chromosome 14 open reading frame 182 | 1 | | 2939 | hsa-mir-938 | 283551 | C14orf182 | chromosome 14 open reading frame 182 | 1 | | 2940 | hsa-mir-548h-1 | 283551 | C14orf182 | chromosome 14 open reading frame 182 | 1 | | 2941 | hsa-mir-3135a | 283551 | C14orf182 | chromosome 14 open reading frame 182 | 1 | | 2942 | hsa-mir-3177 | 283551 | C14orf182 | chromosome 14 open reading frame 182 | 1 | | 2943 | hsa-mir-3655 | 283551 | C14orf182 | chromosome 14 open reading frame 182 | 1 | | 2944 | hsa-mir-4526 | 283551 | C14orf182 | chromosome 14 open reading frame 182 | 1 | | 2945 | hsa-mir-4635 | 283551 | C14orf182 | chromosome 14 open reading frame 182 | 1 | | 2946 | hsa-mir-4651 | 283551 | C14orf182 | chromosome 14 open reading frame 182 | 1 | | 2947 | hsa-mir-4744 | 283551 | C14orf182 | chromosome 14 open reading frame 182 | 1 | | 2948 | hsa-mir-5190 | 283551 | C14orf182 | chromosome 14 open reading frame 182 | 1 | | 2949 | hsa-mir-101-2 | 283551 | C14orf182 | chromosome 14 open reading frame 182 | 1 | | 2950 | hsa-mir-128-1;hsa-mir-128-2 | 283551 | C14orf182 | chromosome 14 open reading frame 182 | 1 | | 2951 | hsa-mir-301a | 283551 | C14orf182 | chromosome 14 open reading frame 182 | 1 | | 2952 | hsa-mir-511-1;hsa-mir-511-2 | 283551 | C14orf182 | chromosome 14 open reading frame 182 | 1 | | 2953 | hsa-mir-586 | 283551 | C14orf182 | chromosome 14 open reading frame 182 | 1 | | 2954 | hsa-mir-593 | 283551 | C14orf182 | chromosome 14 open reading frame 182 | 1 | | 2955 | hsa-mir-620 | 283551 | C14orf182 | chromosome 14 open reading frame 182 | 1 | | 2956 | hsa-mir-33b | 283551 | C14orf182 | chromosome 14 open reading frame 182 | 1 | | 2957 | hsa-mir-942 | 283551 | C14orf182 | chromosome 14 open reading frame 182 | 1 | |

---

Gene Ontology - Biological Process [Details: ]

| |  | genes in Category | percent in the observed List | percent in the genome | fold of overrepresents | odds ratio | p value | | --- | --- | --- | --- | --- | --- | --- | | regulation of immune response | 15 | 0.205 | 3.5e-02 | 5.8 | 7.3 | 2.9e-08 | | regulation of immune system process | 17 | 0.233 | 5.5e-02 | 4.2 | 5.3 | 3.0e-07 | | immune system process | 24 | 0.329 | 1.1e-01 | 2.9 | 3.9 | 6.3e-07 | | immune response-activating signal transduction | 9 | 0.123 | 1.5e-02 | 8.2 | 9.5 | 1.5e-06 | | immune response-regulating signaling pathway | 9 | 0.123 | 1.6e-02 | 7.8 | 9.1 | 2.1e-06 | | nucleotide-binding domain, leucine rich repeat containing receptor signaling pathway | 5 | 0.068 | 3.3e-03 | 21.0 | 25.0 | 3.8e-06 | | immune response | 17 | 0.233 | 6.8e-02 | 3.4 | 4.2 | 6.3e-06 | | innate immune response | 12 | 0.164 | 3.5e-02 | 4.7 | 5.5 | 7.8e-06 | | positive regulation of immune response | 10 | 0.137 | 2.4e-02 | 5.8 | 6.7 | 8.4e-06 | | positive regulation of immune system process | 12 | 0.164 | 3.5e-02 | 4.7 | 5.5 | 8.5e-06 | | regulation of apoptotic process | 18 | 0.247 | 7.9e-02 | 3.1 | 3.9 | 1.0e-05 | | regulation of programmed cell death | 18 | 0.247 | 7.9e-02 | 3.1 | 3.8 | 1.1e-05 | | activation of immune response | 9 | 0.123 | 1.9e-02 | 6.3 | 7.3 | 1.2e-05 | | regulation of cell death | 18 | 0.247 | 8.1e-02 | 3.0 | 3.7 | 1.6e-05 | | defense response | 16 | 0.219 | 7.0e-02 | 3.1 | 3.8 | 3.7e-05 | | intracellular receptor mediated signaling pathway | 7 | 0.096 | 1.4e-02 | 6.8 | 7.7 | 7.2e-05 | | negative regulation of NF-kappaB transcription factor activity | 4 | 0.055 | 3.1e-03 | 17.6 | 20.2 | 7.7e-05 | | response to cytokine stimulus | 10 | 0.137 | 3.2e-02 | 4.3 | 4.9 | 1.1e-04 | | regulation of response to stimulus | 23 | 0.315 | 1.4e-01 | 2.2 | 2.8 | 1.1e-04 | | cellular response to chemical stimulus | 18 | 0.247 | 9.6e-02 | 2.6 | 3.1 | 1.3e-04 | | positive regulation of cell activation | 7 | 0.096 | 1.6e-02 | 6.0 | 6.7 | 1.6e-04 | | apoptotic process | 18 | 0.247 | 1.0e-01 | 2.5 | 3.0 | 2.2e-04 | | response to chemical stimulus | 26 | 0.356 | 1.8e-01 | 2.0 | 2.5 | 2.3e-04 | | regulation of protein heterodimerization activity | 2 | 0.027 | 3.4e-04 | 80.8 | 137.7 | 2.4e-04 | | programmed cell death | 18 | 0.247 | 1.0e-01 | 2.5 | 3.0 | 2.5e-04 | | positive regulation of response to stimulus | 14 | 0.192 | 6.7e-02 | 2.9 | 3.3 | 2.9e-04 | | negative regulation of myeloid cell apoptosis | 2 | 0.027 | 4.1e-04 | 67.3 | 103.3 | 3.6e-04 | | positive regulation of defense response | 6 | 0.082 | 1.3e-02 | 6.1 | 6.8 | 4.4e-04 | | cellular response to organic substance | 14 | 0.192 | 7.0e-02 | 2.8 | 3.2 | 4.5e-04 | | immune response-activating cell surface receptor signaling pathway | 5 | 0.068 | 8.9e-03 | 7.7 | 8.5 | 4.8e-04 | | cellular response to cytokine stimulus | 8 | 0.110 | 2.5e-02 | 4.3 | 4.8 | 5.0e-04 | | response to stress | 26 | 0.356 | 1.9e-01 | 1.9 | 2.4 | 5.0e-04 | | cell-matrix adhesion | 5 | 0.068 | 9.2e-03 | 7.4 | 8.2 | 5.6e-04 | | immune response-regulating cell surface receptor signaling pathway | 5 | 0.068 | 9.4e-03 | 7.3 | 8.0 | 6.0e-04 | | induction of apoptosis | 8 | 0.110 | 2.6e-02 | 4.2 | 4.6 | 6.6e-04 | | induction of programmed cell death | 8 | 0.110 | 2.7e-02 | 4.1 | 4.6 | 6.8e-04 | | positive regulation of peptidyl-serine phosphorylation | 3 | 0.041 | 2.4e-03 | 16.8 | 19.0 | 7.4e-04 | | positive regulation of innate immune response | 5 | 0.068 | 9.8e-03 | 7.0 | 7.6 | 7.5e-04 | | cell death | 18 | 0.247 | 1.1e-01 | 2.2 | 2.7 | 7.7e-04 | | death | 18 | 0.247 | 1.1e-01 | 2.2 | 2.7 | 7.8e-04 | | cytokine-mediated signaling pathway | 7 | 0.096 | 2.1e-02 | 4.6 | 5.1 | 8.0e-04 | | focal adhesion assembly | 3 | 0.041 | 2.5e-03 | 16.4 | 18.4 | 8.0e-04 | | antigen processing and presentation of exogenous peptide antigen via MHC class I, TAP-independent | 2 | 0.027 | 6.1e-04 | 44.9 | 59.0 | 8.5e-04 | | positive regulation of leukocyte activation | 6 | 0.082 | 1.5e-02 | 5.3 | 5.8 | 9.2e-04 | | regulation of apoptotic signaling pathway | 3 | 0.041 | 2.6e-03 | 15.5 | 17.4 | 9.4e-04 | | regulation of response to stress | 10 | 0.137 | 4.3e-02 | 3.2 | 3.6 | 1.0e-03 | | regulation of mitochondrial membrane permeability | 2 | 0.027 | 6.8e-04 | 40.4 | 51.6 | 1.1e-03 | | regulation of myeloid cell apoptosis | 2 | 0.027 | 6.8e-04 | 40.4 | 51.6 | 1.1e-03 | | negative regulation of signal transduction | 9 | 0.123 | 3.5e-02 | 3.5 | 3.9 | 1.1e-03 | | regulation of cytokine production | 7 | 0.096 | 2.2e-02 | 4.3 | 4.8 | 1.2e-03 | | negative regulation of response to stimulus | 10 | 0.137 | 4.3e-02 | 3.2 | 3.5 | 1.2e-03 | | regulation of peptidyl-serine phosphorylation | 3 | 0.041 | 2.9e-03 | 14.1 | 15.7 | 1.2e-03 | | regulation of cell activation | 7 | 0.096 | 2.3e-02 | 4.2 | 4.6 | 1.4e-03 | | myeloid cell apoptosis | 2 | 0.027 | 8.1e-04 | 33.7 | 41.3 | 1.5e-03 | | negative regulation of sequence-specific DNA binding transcription factor activity | 4 | 0.055 | 6.9e-03 | 8.0 | 8.7 | 1.6e-03 | | apoptotic signaling pathway | 3 | 0.041 | 3.2e-03 | 12.9 | 14.2 | 1.6e-03 | | negative regulation of cell communication | 9 | 0.123 | 3.8e-02 | 3.2 | 3.6 | 1.8e-03 | | negative regulation of signaling | 9 | 0.123 | 3.8e-02 | 3.2 | 3.6 | 1.8e-03 | | negative regulation of apoptotic signaling pathway | 2 | 0.027 | 8.8e-04 | 31.1 | 37.5 | 1.8e-03 | | apoptotic mitochondrial changes | 3 | 0.041 | 3.4e-03 | 12.1 | 13.3 | 1.9e-03 | | response to organic substance | 17 | 0.233 | 1.1e-01 | 2.1 | 2.5 | 2.0e-03 | | cytokine production | 7 | 0.096 | 2.4e-02 | 3.9 | 4.3 | 2.0e-03 | | regulation of cell-matrix adhesion | 3 | 0.041 | 3.5e-03 | 11.7 | 12.8 | 2.2e-03 | | cell-substrate junction assembly | 3 | 0.041 | 3.6e-03 | 11.4 | 12.5 | 2.3e-03 | | pattern recognition receptor signaling pathway | 4 | 0.055 | 7.6e-03 | 7.2 | 7.8 | 2.3e-03 | | cell-substrate adhesion | 5 | 0.068 | 1.3e-02 | 5.3 | 5.7 | 2.6e-03 | | innate immune response-activating signal transduction | 4 | 0.055 | 7.9e-03 | 7.0 | 7.5 | 2.6e-03 | | actin filament organization | 5 | 0.068 | 1.3e-02 | 5.3 | 5.7 | 2.6e-03 | | activation of innate immune response | 4 | 0.055 | 8.0e-03 | 6.8 | 7.4 | 2.8e-03 | | antigen receptor-mediated signaling pathway | 4 | 0.055 | 8.2e-03 | 6.7 | 7.2 | 3.0e-03 | | regulation of interleukin-6 production | 3 | 0.041 | 4.1e-03 | 10.1 | 11.0 | 3.3e-03 | | regulation of innate immune response | 5 | 0.068 | 1.4e-02 | 4.9 | 5.3 | 3.4e-03 | | termination of RNA polymerase III transcription | 2 | 0.027 | 1.2e-03 | 22.4 | 25.8 | 3.5e-03 | | transcription elongation from RNA polymerase III promoter | 2 | 0.027 | 1.2e-03 | 22.4 | 25.8 | 3.5e-03 | | interleukin-6 production | 3 | 0.041 | 4.2e-03 | 9.8 | 10.6 | 3.6e-03 | | I-kappaB kinase/NF-kappaB cascade | 5 | 0.068 | 1.4e-02 | 4.8 | 5.2 | 3.8e-03 | | positive regulation of lymphocyte activation | 5 | 0.068 | 1.4e-02 | 4.8 | 5.2 | 3.8e-03 | | T cell activation | 6 | 0.082 | 2.1e-02 | 3.9 | 4.2 | 4.2e-03 | | response to other organism | 8 | 0.110 | 3.6e-02 | 3.1 | 3.4 | 4.3e-03 | | regulation of mitochondrial membrane potential | 2 | 0.027 | 1.4e-03 | 20.2 | 22.9 | 4.3e-03 | | B cell differentiation | 3 | 0.041 | 4.6e-03 | 8.9 | 9.6 | 4.6e-03 | | regulation of leukocyte activation | 6 | 0.082 | 2.1e-02 | 3.8 | 4.1 | 4.7e-03 | | positive regulation of neuron maturation | 1 | 0.014 | 6.8e-05 | 201.9 | Inf | 5.0e-03 | | cochlear nucleus development | 1 | 0.014 | 6.8e-05 | 201.9 | Inf | 5.0e-03 | | negative regulation of cellular pH reduction | 1 | 0.014 | 6.8e-05 | 201.9 | Inf | 5.0e-03 | | CD8-positive, alpha-beta T cell lineage commitment | 1 | 0.014 | 6.8e-05 | 201.9 | Inf | 5.0e-03 | | positive regulation of skeletal muscle fiber development | 1 | 0.014 | 6.8e-05 | 201.9 | Inf | 5.0e-03 | | positive regulation of type III hypersensitivity | 1 | 0.014 | 6.8e-05 | 201.9 | Inf | 5.0e-03 | | positive regulation of interleukin-6-mediated signaling pathway | 1 | 0.014 | 6.8e-05 | 201.9 | Inf | 5.0e-03 | | negative regulation of calcium-dependent cell-cell adhesion | 1 | 0.014 | 6.8e-05 | 201.9 | Inf | 5.0e-03 | | type III hypersensitivity | 1 | 0.014 | 6.8e-05 | 201.9 | Inf | 5.0e-03 | | regulation of type III hypersensitivity | 1 | 0.014 | 6.8e-05 | 201.9 | Inf | 5.0e-03 | | type I interferon-mediated signaling pathway | 3 | 0.041 | 4.8e-03 | 8.5 | 9.2 | 5.2e-03 | | cellular response to type I interferon | 3 | 0.041 | 4.8e-03 | 8.5 | 9.2 | 5.2e-03 | | regulation of survival gene product expression | 2 | 0.027 | 1.5e-03 | 18.4 | 20.6 | 5.2e-03 | | response to type I interferon | 3 | 0.041 | 4.9e-03 | 8.4 | 9.1 | 5.4e-03 | | response to biotic stimulus | 8 | 0.110 | 3.8e-02 | 2.9 | 3.2 | 5.9e-03 | | antigen processing and presentation of exogenous peptide antigen via MHC class I | 3 | 0.041 | 5.1e-03 | 8.1 | 8.7 | 6.1e-03 | | T cell costimulation | 3 | 0.041 | 5.1e-03 | 8.1 | 8.7 | 6.1e-03 | | lymphocyte costimulation | 3 | 0.041 | 5.1e-03 | 8.1 | 8.7 | 6.1e-03 | | T cell selection | 2 | 0.027 | 1.6e-03 | 16.8 | 18.8 | 6.2e-03 | | response to virus | 5 | 0.068 | 1.6e-02 | 4.3 | 4.6 | 6.3e-03 | | positive regulation of intracellular protein kinase cascade | 6 | 0.082 | 2.3e-02 | 3.6 | 3.9 | 6.6e-03 | | homeostasis of number of cells | 4 | 0.055 | 1.0e-02 | 5.3 | 5.7 | 6.7e-03 | | regulation of cell adhesion | 5 | 0.068 | 1.6e-02 | 4.2 | 4.5 | 6.7e-03 | | positive regulation of apoptotic process | 8 | 0.110 | 3.9e-02 | 2.8 | 3.1 | 7.0e-03 | | interferon-gamma-mediated signaling pathway | 3 | 0.041 | 5.4e-03 | 7.7 | 8.2 | 7.0e-03 | | regulation of protein binding | 3 | 0.041 | 5.4e-03 | 7.7 | 8.2 | 7.0e-03 | | negative regulation of apoptotic process | 8 | 0.110 | 3.9e-02 | 2.8 | 3.1 | 7.1e-03 | | hemopoiesis | 7 | 0.096 | 3.1e-02 | 3.1 | 3.4 | 7.2e-03 | | cell cycle cytokinesis | 2 | 0.027 | 1.8e-03 | 15.5 | 17.2 | 7.3e-03 | | response to inorganic substance | 6 | 0.082 | 2.3e-02 | 3.5 | 3.8 | 7.3e-03 | | negative regulation of neuron apoptosis | 3 | 0.041 | 5.4e-03 | 7.6 | 8.1 | 7.3e-03 | | antigen processing and presentation of exogenous peptide antigen | 3 | 0.041 | 5.4e-03 | 7.6 | 8.1 | 7.3e-03 | | positive regulation of programmed cell death | 8 | 0.110 | 3.9e-02 | 2.8 | 3.1 | 7.3e-03 | | negative regulation of programmed cell death | 8 | 0.110 | 3.9e-02 | 2.8 | 3.1 | 7.5e-03 | | post-embryonic development | 3 | 0.041 | 5.5e-03 | 7.5 | 8.0 | 7.5e-03 | | cell activation | 9 | 0.123 | 4.8e-02 | 2.6 | 2.8 | 7.7e-03 | | antigen processing and presentation of exogenous antigen | 3 | 0.041 | 5.6e-03 | 7.4 | 7.9 | 7.8e-03 | | positive regulation of cell death | 8 | 0.110 | 4.0e-02 | 2.8 | 3.0 | 8.2e-03 | | multi-organism process | 11 | 0.151 | 6.7e-02 | 2.3 | 2.5 | 8.8e-03 | | neuron maturation | 2 | 0.027 | 2.0e-03 | 13.9 | 15.3 | 9.0e-03 | | regulation of cell-substrate adhesion | 3 | 0.041 | 5.9e-03 | 7.0 | 7.4 | 9.2e-03 | | negative regulation of cell death | 8 | 0.110 | 4.1e-02 | 2.7 | 2.9 | 9.3e-03 | | cytokinesis | 3 | 0.041 | 6.0e-03 | 6.8 | 7.3 | 9.8e-03 | | intracellular protein kinase cascade | 9 | 0.123 | 5.0e-02 | 2.5 | 2.7 | 9.8e-03 | | positive regulation of T cell activation | 4 | 0.055 | 1.1e-02 | 4.8 | 5.1 | 9.8e-03 | | regulation of defense response | 6 | 0.082 | 2.5e-02 | 3.3 | 3.5 | 9.8e-03 | | regulation of nitrogen utilization | 1 | 0.014 | 1.4e-04 | 101.0 | 203.7 | 9.9e-03 | | negative regulation of retinal cell programmed cell death | 1 | 0.014 | 1.4e-04 | 101.0 | 203.7 | 9.9e-03 | | negative regulation of mitochondrial depolarization | 1 | 0.014 | 1.4e-04 | 101.0 | 203.7 | 9.9e-03 | | positive regulation of type IIa hypersensitivity | 1 | 0.014 | 1.4e-04 | 101.0 | 203.7 | 9.9e-03 | | positive regulation of type I hypersensitivity | 1 | 0.014 | 1.4e-04 | 101.0 | 203.7 | 9.9e-03 | | positive regulation of mast cell cytokine production | 1 | 0.014 | 1.4e-04 | 101.0 | 203.7 | 9.9e-03 | | isoleucyl-tRNA aminoacylation | 1 | 0.014 | 1.4e-04 | 101.0 | 203.7 | 9.9e-03 | | intestine smooth muscle contraction | 1 | 0.014 | 1.4e-04 | 101.0 | 203.7 | 9.9e-03 | | positive regulation of hair follicle cell proliferation | 1 | 0.014 | 1.4e-04 | 101.0 | 203.7 | 9.9e-03 | | negative regulation of intrinsic apoptotic signaling pathway | 1 | 0.014 | 1.4e-04 | 101.0 | 203.7 | 9.9e-03 | | habenula development | 1 | 0.014 | 1.4e-04 | 101.0 | 203.7 | 9.9e-03 | | nitrogen utilization | 1 | 0.014 | 1.4e-04 | 101.0 | 203.7 | 9.9e-03 | | CD4-positive or CD8-positive, alpha-beta T cell lineage commitment | 1 | 0.014 | 1.4e-04 | 101.0 | 203.7 | 9.9e-03 | | type IIa hypersensitivity | 1 | 0.014 | 1.4e-04 | 101.0 | 203.7 | 9.9e-03 | | regulation of type IIa hypersensitivity | 1 | 0.014 | 1.4e-04 | 101.0 | 203.7 | 9.9e-03 | | type II hypersensitivity | 1 | 0.014 | 1.4e-04 | 101.0 | 203.7 | 9.9e-03 | | positive regulation of myeloid leukocyte mediated immunity | 1 | 0.014 | 1.4e-04 | 101.0 | 203.7 | 9.9e-03 | | regulation of type II hypersensitivity | 1 | 0.014 | 1.4e-04 | 101.0 | 203.7 | 9.9e-03 | | positive regulation of type II hypersensitivity | 1 | 0.014 | 1.4e-04 | 101.0 | 203.7 | 9.9e-03 | | regulation of type I hypersensitivity | 1 | 0.014 | 1.4e-04 | 101.0 | 203.7 | 9.9e-03 | | type I hypersensitivity | 1 | 0.014 | 1.4e-04 | 101.0 | 203.7 | 9.9e-03 | | regulation of interleukin-6-mediated signaling pathway | 1 | 0.014 | 1.4e-04 | 101.0 | 203.7 | 9.9e-03 | | regulation of hair follicle cell proliferation | 1 | 0.014 | 1.4e-04 | 101.0 | 203.7 | 9.9e-03 | | epithalamus development | 1 | 0.014 | 1.4e-04 | 101.0 | 203.7 | 9.9e-03 | | regulation of calcium-dependent cell-cell adhesion | 1 | 0.014 | 1.4e-04 | 101.0 | 203.7 | 9.9e-03 | |

---

Gene Ontology - Biological Process, level II [Details: ]

| |  | genes in Category | percent in the observed List | percent in the genome | fold of overrepresents | odds ratio | p value | | --- | --- | --- | --- | --- | --- | --- | | regulation of immune response | 15 | 0.205 | 3.5e-02 | 5.8 | 7.3 | 2.9e-08 | | regulation of immune system process | 17 | 0.233 | 5.5e-02 | 4.2 | 5.3 | 3.0e-07 | | immune response-activating signal transduction | 9 | 0.123 | 1.5e-02 | 8.2 | 9.5 | 1.5e-06 | | immune response-regulating signaling pathway | 9 | 0.123 | 1.6e-02 | 7.8 | 9.1 | 2.1e-06 | | nucleotide-binding domain, leucine rich repeat containing receptor signaling pathway | 5 | 0.068 | 3.3e-03 | 21.0 | 25.0 | 3.8e-06 | | immune response | 17 | 0.233 | 6.8e-02 | 3.4 | 4.2 | 6.3e-06 | | innate immune response | 12 | 0.164 | 3.5e-02 | 4.7 | 5.5 | 7.8e-06 | | positive regulation of immune response | 10 | 0.137 | 2.4e-02 | 5.8 | 6.7 | 8.4e-06 | | positive regulation of immune system process | 12 | 0.164 | 3.5e-02 | 4.7 | 5.5 | 8.5e-06 | | regulation of apoptotic process | 18 | 0.247 | 7.9e-02 | 3.1 | 3.9 | 1.0e-05 | | regulation of programmed cell death | 18 | 0.247 | 7.9e-02 | 3.1 | 3.8 | 1.1e-05 | | activation of immune response | 9 | 0.123 | 1.9e-02 | 6.3 | 7.3 | 1.2e-05 | | regulation of cell death | 18 | 0.247 | 8.1e-02 | 3.0 | 3.7 | 1.6e-05 | | defense response | 16 | 0.219 | 7.0e-02 | 3.1 | 3.8 | 3.7e-05 | | intracellular receptor mediated signaling pathway | 7 | 0.096 | 1.4e-02 | 6.8 | 7.7 | 7.2e-05 | | negative regulation of NF-kappaB transcription factor activity | 4 | 0.055 | 3.1e-03 | 17.6 | 20.2 | 7.7e-05 | | response to cytokine stimulus | 10 | 0.137 | 3.2e-02 | 4.3 | 4.9 | 1.1e-04 | | regulation of response to stimulus | 23 | 0.315 | 1.4e-01 | 2.2 | 2.8 | 1.1e-04 | | cellular response to chemical stimulus | 18 | 0.247 | 9.6e-02 | 2.6 | 3.1 | 1.3e-04 | | positive regulation of cell activation | 7 | 0.096 | 1.6e-02 | 6.0 | 6.7 | 1.6e-04 | | apoptotic process | 18 | 0.247 | 1.0e-01 | 2.5 | 3.0 | 2.2e-04 | | response to chemical stimulus | 26 | 0.356 | 1.8e-01 | 2.0 | 2.5 | 2.3e-04 | | regulation of protein heterodimerization activity | 2 | 0.027 | 3.4e-04 | 80.8 | 137.7 | 2.4e-04 | | programmed cell death | 18 | 0.247 | 1.0e-01 | 2.5 | 3.0 | 2.5e-04 | | positive regulation of response to stimulus | 14 | 0.192 | 6.7e-02 | 2.9 | 3.3 | 2.9e-04 | | negative regulation of myeloid cell apoptosis | 2 | 0.027 | 4.1e-04 | 67.3 | 103.3 | 3.6e-04 | | positive regulation of defense response | 6 | 0.082 | 1.3e-02 | 6.1 | 6.8 | 4.4e-04 | | cellular response to organic substance | 14 | 0.192 | 7.0e-02 | 2.8 | 3.2 | 4.5e-04 | | immune response-activating cell surface receptor signaling pathway | 5 | 0.068 | 8.9e-03 | 7.7 | 8.5 | 4.8e-04 | | cellular response to cytokine stimulus | 8 | 0.110 | 2.5e-02 | 4.3 | 4.8 | 5.0e-04 | | response to stress | 26 | 0.356 | 1.9e-01 | 1.9 | 2.4 | 5.0e-04 | | cell-matrix adhesion | 5 | 0.068 | 9.2e-03 | 7.4 | 8.2 | 5.6e-04 | | immune response-regulating cell surface receptor signaling pathway | 5 | 0.068 | 9.4e-03 | 7.3 | 8.0 | 6.0e-04 | | induction of apoptosis | 8 | 0.110 | 2.6e-02 | 4.2 | 4.6 | 6.6e-04 | | induction of programmed cell death | 8 | 0.110 | 2.7e-02 | 4.1 | 4.6 | 6.8e-04 | | positive regulation of peptidyl-serine phosphorylation | 3 | 0.041 | 2.4e-03 | 16.8 | 19.0 | 7.4e-04 | | positive regulation of innate immune response | 5 | 0.068 | 9.8e-03 | 7.0 | 7.6 | 7.5e-04 | | cell death | 18 | 0.247 | 1.1e-01 | 2.2 | 2.7 | 7.7e-04 | | cytokine-mediated signaling pathway | 7 | 0.096 | 2.1e-02 | 4.6 | 5.1 | 8.0e-04 | | focal adhesion assembly | 3 | 0.041 | 2.5e-03 | 16.4 | 18.4 | 8.0e-04 | | antigen processing and presentation of exogenous peptide antigen via MHC class I, TAP-independent | 2 | 0.027 | 6.1e-04 | 44.9 | 59.0 | 8.5e-04 | | positive regulation of leukocyte activation | 6 | 0.082 | 1.5e-02 | 5.3 | 5.8 | 9.2e-04 | | regulation of apoptotic signaling pathway | 3 | 0.041 | 2.6e-03 | 15.5 | 17.4 | 9.4e-04 | | regulation of response to stress | 10 | 0.137 | 4.3e-02 | 3.2 | 3.6 | 1.0e-03 | | regulation of mitochondrial membrane permeability | 2 | 0.027 | 6.8e-04 | 40.4 | 51.6 | 1.1e-03 | | regulation of myeloid cell apoptosis | 2 | 0.027 | 6.8e-04 | 40.4 | 51.6 | 1.1e-03 | | negative regulation of signal transduction | 9 | 0.123 | 3.5e-02 | 3.5 | 3.9 | 1.1e-03 | | regulation of cytokine production | 7 | 0.096 | 2.2e-02 | 4.3 | 4.8 | 1.2e-03 | | negative regulation of response to stimulus | 10 | 0.137 | 4.3e-02 | 3.2 | 3.5 | 1.2e-03 | | regulation of peptidyl-serine phosphorylation | 3 | 0.041 | 2.9e-03 | 14.1 | 15.7 | 1.2e-03 | | regulation of cell activation | 7 | 0.096 | 2.3e-02 | 4.2 | 4.6 | 1.4e-03 | | myeloid cell apoptosis | 2 | 0.027 | 8.1e-04 | 33.7 | 41.3 | 1.5e-03 | | negative regulation of sequence-specific DNA binding transcription factor activity | 4 | 0.055 | 6.9e-03 | 8.0 | 8.7 | 1.6e-03 | | apoptotic signaling pathway | 3 | 0.041 | 3.2e-03 | 12.9 | 14.2 | 1.6e-03 | | negative regulation of cell communication | 9 | 0.123 | 3.8e-02 | 3.2 | 3.6 | 1.8e-03 | | negative regulation of signaling | 9 | 0.123 | 3.8e-02 | 3.2 | 3.6 | 1.8e-03 | | negative regulation of apoptotic signaling pathway | 2 | 0.027 | 8.8e-04 | 31.1 | 37.5 | 1.8e-03 | | apoptotic mitochondrial changes | 3 | 0.041 | 3.4e-03 | 12.1 | 13.3 | 1.9e-03 | | response to organic substance | 17 | 0.233 | 1.1e-01 | 2.1 | 2.5 | 2.0e-03 | | cytokine production | 7 | 0.096 | 2.4e-02 | 3.9 | 4.3 | 2.0e-03 | | regulation of cell-matrix adhesion | 3 | 0.041 | 3.5e-03 | 11.7 | 12.8 | 2.2e-03 | | cell-substrate junction assembly | 3 | 0.041 | 3.6e-03 | 11.4 | 12.5 | 2.3e-03 | | pattern recognition receptor signaling pathway | 4 | 0.055 | 7.6e-03 | 7.2 | 7.8 | 2.3e-03 | | cell-substrate adhesion | 5 | 0.068 | 1.3e-02 | 5.3 | 5.7 | 2.6e-03 | | innate immune response-activating signal transduction | 4 | 0.055 | 7.9e-03 | 7.0 | 7.5 | 2.6e-03 | | actin filament organization | 5 | 0.068 | 1.3e-02 | 5.3 | 5.7 | 2.6e-03 | | activation of innate immune response | 4 | 0.055 | 8.0e-03 | 6.8 | 7.4 | 2.8e-03 | | antigen receptor-mediated signaling pathway | 4 | 0.055 | 8.2e-03 | 6.7 | 7.2 | 3.0e-03 | | regulation of interleukin-6 production | 3 | 0.041 | 4.1e-03 | 10.1 | 11.0 | 3.3e-03 | | regulation of innate immune response | 5 | 0.068 | 1.4e-02 | 4.9 | 5.3 | 3.4e-03 | | termination of RNA polymerase III transcription | 2 | 0.027 | 1.2e-03 | 22.4 | 25.8 | 3.5e-03 | | transcription elongation from RNA polymerase III promoter | 2 | 0.027 | 1.2e-03 | 22.4 | 25.8 | 3.5e-03 | | interleukin-6 production | 3 | 0.041 | 4.2e-03 | 9.8 | 10.6 | 3.6e-03 | | I-kappaB kinase/NF-kappaB cascade | 5 | 0.068 | 1.4e-02 | 4.8 | 5.2 | 3.8e-03 | | positive regulation of lymphocyte activation | 5 | 0.068 | 1.4e-02 | 4.8 | 5.2 | 3.8e-03 | | T cell activation | 6 | 0.082 | 2.1e-02 | 3.9 | 4.2 | 4.2e-03 | | response to other organism | 8 | 0.110 | 3.6e-02 | 3.1 | 3.4 | 4.3e-03 | | regulation of mitochondrial membrane potential | 2 | 0.027 | 1.4e-03 | 20.2 | 22.9 | 4.3e-03 | | B cell differentiation | 3 | 0.041 | 4.6e-03 | 8.9 | 9.6 | 4.6e-03 | | regulation of leukocyte activation | 6 | 0.082 | 2.1e-02 | 3.8 | 4.1 | 4.7e-03 | | positive regulation of neuron maturation | 1 | 0.014 | 6.8e-05 | 201.9 | Inf | 5.0e-03 | | cochlear nucleus development | 1 | 0.014 | 6.8e-05 | 201.9 | Inf | 5.0e-03 | | negative regulation of cellular pH reduction | 1 | 0.014 | 6.8e-05 | 201.9 | Inf | 5.0e-03 | | CD8-positive, alpha-beta T cell lineage commitment | 1 | 0.014 | 6.8e-05 | 201.9 | Inf | 5.0e-03 | | positive regulation of skeletal muscle fiber development | 1 | 0.014 | 6.8e-05 | 201.9 | Inf | 5.0e-03 | | positive regulation of type III hypersensitivity | 1 | 0.014 | 6.8e-05 | 201.9 | Inf | 5.0e-03 | | positive regulation of interleukin-6-mediated signaling pathway | 1 | 0.014 | 6.8e-05 | 201.9 | Inf | 5.0e-03 | | negative regulation of calcium-dependent cell-cell adhesion | 1 | 0.014 | 6.8e-05 | 201.9 | Inf | 5.0e-03 | | type III hypersensitivity | 1 | 0.014 | 6.8e-05 | 201.9 | Inf | 5.0e-03 | | regulation of type III hypersensitivity | 1 | 0.014 | 6.8e-05 | 201.9 | Inf | 5.0e-03 | | type I interferon-mediated signaling pathway | 3 | 0.041 | 4.8e-03 | 8.5 | 9.2 | 5.2e-03 | | cellular response to type I interferon | 3 | 0.041 | 4.8e-03 | 8.5 | 9.2 | 5.2e-03 | | regulation of survival gene product expression | 2 | 0.027 | 1.5e-03 | 18.4 | 20.6 | 5.2e-03 | | response to type I interferon | 3 | 0.041 | 4.9e-03 | 8.4 | 9.1 | 5.4e-03 | | response to biotic stimulus | 8 | 0.110 | 3.8e-02 | 2.9 | 3.2 | 5.9e-03 | | antigen processing and presentation of exogenous peptide antigen via MHC class I | 3 | 0.041 | 5.1e-03 | 8.1 | 8.7 | 6.1e-03 | | T cell costimulation | 3 | 0.041 | 5.1e-03 | 8.1 | 8.7 | 6.1e-03 | | lymphocyte costimulation | 3 | 0.041 | 5.1e-03 | 8.1 | 8.7 | 6.1e-03 | | T cell selection | 2 | 0.027 | 1.6e-03 | 16.8 | 18.8 | 6.2e-03 | | response to virus | 5 | 0.068 | 1.6e-02 | 4.3 | 4.6 | 6.3e-03 | | positive regulation of intracellular protein kinase cascade | 6 | 0.082 | 2.3e-02 | 3.6 | 3.9 | 6.6e-03 | | homeostasis of number of cells | 4 | 0.055 | 1.0e-02 | 5.3 | 5.7 | 6.7e-03 | | regulation of cell adhesion | 5 | 0.068 | 1.6e-02 | 4.2 | 4.5 | 6.7e-03 | | positive regulation of apoptotic process | 8 | 0.110 | 3.9e-02 | 2.8 | 3.1 | 7.0e-03 | | interferon-gamma-mediated signaling pathway | 3 | 0.041 | 5.4e-03 | 7.7 | 8.2 | 7.0e-03 | | regulation of protein binding | 3 | 0.041 | 5.4e-03 | 7.7 | 8.2 | 7.0e-03 | | negative regulation of apoptotic process | 8 | 0.110 | 3.9e-02 | 2.8 | 3.1 | 7.1e-03 | | hemopoiesis | 7 | 0.096 | 3.1e-02 | 3.1 | 3.4 | 7.2e-03 | | cell cycle cytokinesis | 2 | 0.027 | 1.8e-03 | 15.5 | 17.2 | 7.3e-03 | | response to inorganic substance | 6 | 0.082 | 2.3e-02 | 3.5 | 3.8 | 7.3e-03 | | negative regulation of neuron apoptosis | 3 | 0.041 | 5.4e-03 | 7.6 | 8.1 | 7.3e-03 | | antigen processing and presentation of exogenous peptide antigen | 3 | 0.041 | 5.4e-03 | 7.6 | 8.1 | 7.3e-03 | | positive regulation of programmed cell death | 8 | 0.110 | 3.9e-02 | 2.8 | 3.1 | 7.3e-03 | | negative regulation of programmed cell death | 8 | 0.110 | 3.9e-02 | 2.8 | 3.1 | 7.5e-03 | | post-embryonic development | 3 | 0.041 | 5.5e-03 | 7.5 | 8.0 | 7.5e-03 | | cell activation | 9 | 0.123 | 4.8e-02 | 2.6 | 2.8 | 7.7e-03 | | antigen processing and presentation of exogenous antigen | 3 | 0.041 | 5.6e-03 | 7.4 | 7.9 | 7.8e-03 | | positive regulation of cell death | 8 | 0.110 | 4.0e-02 | 2.8 | 3.0 | 8.2e-03 | | neuron maturation | 2 | 0.027 | 2.0e-03 | 13.9 | 15.3 | 9.0e-03 | | regulation of cell-substrate adhesion | 3 | 0.041 | 5.9e-03 | 7.0 | 7.4 | 9.2e-03 | | negative regulation of cell death | 8 | 0.110 | 4.1e-02 | 2.7 | 2.9 | 9.3e-03 | | cytokinesis | 3 | 0.041 | 6.0e-03 | 6.8 | 7.3 | 9.8e-03 | | intracellular protein kinase cascade | 9 | 0.123 | 5.0e-02 | 2.5 | 2.7 | 9.8e-03 | | positive regulation of T cell activation | 4 | 0.055 | 1.1e-02 | 4.8 | 5.1 | 9.8e-03 | | regulation of defense response | 6 | 0.082 | 2.5e-02 | 3.3 | 3.5 | 9.8e-03 | | regulation of nitrogen utilization | 1 | 0.014 | 1.4e-04 | 101.0 | 203.7 | 9.9e-03 | | negative regulation of retinal cell programmed cell death | 1 | 0.014 | 1.4e-04 | 101.0 | 203.7 | 9.9e-03 | | negative regulation of mitochondrial depolarization | 1 | 0.014 | 1.4e-04 | 101.0 | 203.7 | 9.9e-03 | | positive regulation of type IIa hypersensitivity | 1 | 0.014 | 1.4e-04 | 101.0 | 203.7 | 9.9e-03 | | positive regulation of type I hypersensitivity | 1 | 0.014 | 1.4e-04 | 101.0 | 203.7 | 9.9e-03 | | positive regulation of mast cell cytokine production | 1 | 0.014 | 1.4e-04 | 101.0 | 203.7 | 9.9e-03 | | isoleucyl-tRNA aminoacylation | 1 | 0.014 | 1.4e-04 | 101.0 | 203.7 | 9.9e-03 | | intestine smooth muscle contraction | 1 | 0.014 | 1.4e-04 | 101.0 | 203.7 | 9.9e-03 | | positive regulation of hair follicle cell proliferation | 1 | 0.014 | 1.4e-04 | 101.0 | 203.7 | 9.9e-03 | | negative regulation of intrinsic apoptotic signaling pathway | 1 | 0.014 | 1.4e-04 | 101.0 | 203.7 | 9.9e-03 | | habenula development | 1 | 0.014 | 1.4e-04 | 101.0 | 203.7 | 9.9e-03 | | CD4-positive or CD8-positive, alpha-beta T cell lineage commitment | 1 | 0.014 | 1.4e-04 | 101.0 | 203.7 | 9.9e-03 | | type IIa hypersensitivity | 1 | 0.014 | 1.4e-04 | 101.0 | 203.7 | 9.9e-03 | | regulation of type IIa hypersensitivity | 1 | 0.014 | 1.4e-04 | 101.0 | 203.7 | 9.9e-03 | | type II hypersensitivity | 1 | 0.014 | 1.4e-04 | 101.0 | 203.7 | 9.9e-03 | | positive regulation of myeloid leukocyte mediated immunity | 1 | 0.014 | 1.4e-04 | 101.0 | 203.7 | 9.9e-03 | | regulation of type II hypersensitivity | 1 | 0.014 | 1.4e-04 | 101.0 | 203.7 | 9.9e-03 | | positive regulation of type II hypersensitivity | 1 | 0.014 | 1.4e-04 | 101.0 | 203.7 | 9.9e-03 | | regulation of type I hypersensitivity | 1 | 0.014 | 1.4e-04 | 101.0 | 203.7 | 9.9e-03 | | type I hypersensitivity | 1 | 0.014 | 1.4e-04 | 101.0 | 203.7 | 9.9e-03 | | regulation of interleukin-6-mediated signaling pathway | 1 | 0.014 | 1.4e-04 | 101.0 | 203.7 | 9.9e-03 | | regulation of hair follicle cell proliferation | 1 | 0.014 | 1.4e-04 | 101.0 | 203.7 | 9.9e-03 | | epithalamus development | 1 | 0.014 | 1.4e-04 | 101.0 | 203.7 | 9.9e-03 | | regulation of calcium-dependent cell-cell adhesion | 1 | 0.014 | 1.4e-04 | 101.0 | 203.7 | 9.9e-03 | |

---

KEGG pathways [Details: ]

| |  | genes in Category | percent in the observed List | percent in the genome | fold of overrepresents | odds ratio | p value | | --- | --- | --- | --- | --- | --- | --- | | Adherens junction | 4 | 0.105 | 0.01243 | 8.5 | 9.8 | 0.0012 | | Natural killer cell mediated cytotoxicity | 5 | 0.132 | 0.02316 | 5.7 | 6.6 | 0.0017 | | Amyotrophic lateral sclerosis (ALS) | 3 | 0.079 | 0.00903 | 8.7 | 9.9 | 0.0047 | | T cell receptor signaling pathway | 4 | 0.105 | 0.01840 | 5.7 | 6.5 | 0.0049 | | Shigellosis | 3 | 0.079 | 0.01039 | 7.6 | 8.5 | 0.0070 | | B cell receptor signaling pathway | 3 | 0.079 | 0.01277 | 6.2 | 6.9 | 0.0123 | | Small cell lung cancer | 3 | 0.079 | 0.01448 | 5.5 | 6.0 | 0.0172 | | Apoptosis | 3 | 0.079 | 0.01482 | 5.3 | 5.9 | 0.0183 | | Allograft rejection | 2 | 0.053 | 0.00630 | 8.4 | 9.2 | 0.0236 | | Graft-versus-host disease | 2 | 0.053 | 0.00698 | 7.5 | 8.3 | 0.0285 | | Type I diabetes mellitus | 2 | 0.053 | 0.00732 | 7.2 | 7.8 | 0.0312 | | Butirosin and neomycin biosynthesis | 1 | 0.026 | 0.00085 | 30.9 | 39.4 | 0.0320 | | Type II diabetes mellitus | 2 | 0.053 | 0.00818 | 6.4 | 7.0 | 0.0382 | | Autoimmune thyroid disease | 2 | 0.053 | 0.00886 | 5.9 | 6.4 | 0.0442 | | Neurotrophin signaling pathway | 3 | 0.079 | 0.02163 | 3.6 | 3.9 | 0.0481 | | Axon guidance | 3 | 0.079 | 0.02197 | 3.6 | 3.9 | 0.0500 | | Pathogenic Escherichia coli infection | 2 | 0.053 | 0.00954 | 5.5 | 5.9 | 0.0505 | | Cytosolic DNA-sensing pathway | 2 | 0.053 | 0.00954 | 5.5 | 5.9 | 0.0505 | | Tight junction | 3 | 0.079 | 0.02248 | 3.5 | 3.8 | 0.0529 | | Cell adhesion molecules (CAMs) | 3 | 0.079 | 0.02265 | 3.5 | 3.8 | 0.0539 | | Valine, leucine and isoleucine biosynthesis | 1 | 0.026 | 0.00187 | 14.0 | 15.7 | 0.0690 | | Viral myocarditis | 2 | 0.053 | 0.01192 | 4.4 | 4.7 | 0.0748 | | Bacterial invasion of epithelial cells | 2 | 0.053 | 0.01192 | 4.4 | 4.7 | 0.0748 | | Pancreatic cancer | 2 | 0.053 | 0.01192 | 4.4 | 4.7 | 0.0748 | | RIG-I-like receptor signaling pathway | 2 | 0.053 | 0.01209 | 4.4 | 4.6 | 0.0767 | | Antigen processing and presentation | 2 | 0.053 | 0.01294 | 4.1 | 4.3 | 0.0862 | | VEGF signaling pathway | 2 | 0.053 | 0.01294 | 4.1 | 4.3 | 0.0862 | | Protein processing in endoplasmic reticulum | 3 | 0.079 | 0.02810 | 2.8 | 3.0 | 0.0900 | | Pantothenate and CoA biosynthesis | 1 | 0.026 | 0.00273 | 9.7 | 10.5 | 0.0988 | |

---

Disease Ontology Lite terms [Details: ]

| |  | genes in Category | percent in the observed List | percent in the genome | fold of overrepresents | odds ratio | p value | | --- | --- | --- | --- | --- | --- | --- | | Epstein-Barr virus infection | 3 | 0.115 | 0.00518 | 22.3 | 29.0 | 0.00029 | | Neck cancer | 2 | 0.077 | 0.00247 | 31.2 | 41.8 | 0.00173 | | Wiskott-Aldrich syndrome | 2 | 0.077 | 0.00247 | 31.2 | 41.8 | 0.00173 | | Thyroid gland disease | 2 | 0.077 | 0.00469 | 16.4 | 19.6 | 0.00633 | | Hereditary nonpolyposis colorectal tumor | 1 | 0.038 | 0.00025 | 155.8 | Inf | 0.00642 | | Nasopharyngeal cancer | 2 | 0.077 | 0.00667 | 11.5 | 13.3 | 0.01260 | | Eye cancer | 1 | 0.038 | 0.00049 | 77.9 | 161.0 | 0.01280 | | Autoimmune disease | 3 | 0.115 | 0.02098 | 5.5 | 6.3 | 0.01637 | | Hodgkin's disease | 2 | 0.077 | 0.00790 | 9.7 | 11.1 | 0.01746 | | Infection by cryptococcus neoformans | 1 | 0.038 | 0.00074 | 51.9 | 80.5 | 0.01914 | | Glaucoma | 2 | 0.077 | 0.00889 | 8.7 | 9.8 | 0.02183 | | Adenoid cystic cancer | 1 | 0.038 | 0.00099 | 39.0 | 53.6 | 0.02544 | | Schistosomiasis | 1 | 0.038 | 0.00099 | 39.0 | 53.6 | 0.02544 | | Pseudoxanthoma elasticum | 1 | 0.038 | 0.00123 | 31.2 | 40.2 | 0.03170 | | Brain ischemia | 1 | 0.038 | 0.00123 | 31.2 | 40.2 | 0.03170 | | HIV infection | 3 | 0.115 | 0.02715 | 4.2 | 4.8 | 0.03217 | | Drug abuse | 3 | 0.115 | 0.02814 | 4.1 | 4.6 | 0.03525 | | Viremia | 1 | 0.038 | 0.00148 | 26.0 | 32.2 | 0.03792 | | Behcet syndrome | 2 | 0.077 | 0.01210 | 6.4 | 7.1 | 0.03873 | | Lymphopenia | 1 | 0.038 | 0.00173 | 22.3 | 26.8 | 0.04410 | | Tuberculosis | 2 | 0.077 | 0.01358 | 5.7 | 6.2 | 0.04778 | | Common variable immunodeficiency | 1 | 0.038 | 0.00197 | 19.5 | 23.0 | 0.05025 | | Lichen planus | 1 | 0.038 | 0.00197 | 19.5 | 23.0 | 0.05025 | | Uterine fibroids | 1 | 0.038 | 0.00197 | 19.5 | 23.0 | 0.05025 | | Multiple myeloma | 2 | 0.077 | 0.01432 | 5.4 | 5.9 | 0.05257 | | Ectodermal dysplasia | 1 | 0.038 | 0.00222 | 17.3 | 20.1 | 0.05636 | | Keratosis | 1 | 0.038 | 0.00222 | 17.3 | 20.1 | 0.05636 | | Spondylarthropathies | 1 | 0.038 | 0.00222 | 17.3 | 20.1 | 0.05636 | | Ischemia | 2 | 0.077 | 0.01506 | 5.1 | 5.6 | 0.05752 | | Lupus erythematosus | 3 | 0.115 | 0.03431 | 3.4 | 3.7 | 0.05779 | | Dental plaque | 2 | 0.077 | 0.01530 | 5.0 | 5.5 | 0.05921 | | "Pemphigoid, Bullous" | 1 | 0.038 | 0.00247 | 15.6 | 17.8 | 0.06243 | | Henoch-Schoenlein purpura | 1 | 0.038 | 0.00247 | 15.6 | 17.8 | 0.06243 | | Larynx cancer | 1 | 0.038 | 0.00247 | 15.6 | 17.8 | 0.06243 | | Severe acute respiratory syndrome | 1 | 0.038 | 0.00247 | 15.6 | 17.8 | 0.06243 | | Takayasu's arteritis | 1 | 0.038 | 0.00247 | 15.6 | 17.8 | 0.06243 | | Melanoma | 3 | 0.115 | 0.03555 | 3.2 | 3.6 | 0.06297 | | Brucellosis | 1 | 0.038 | 0.00272 | 14.2 | 16.1 | 0.06846 | | Esotropia | 1 | 0.038 | 0.00296 | 13.0 | 14.6 | 0.07445 | | Lymphoproliferative disorder | 1 | 0.038 | 0.00370 | 10.4 | 11.5 | 0.09222 | | Rheumatic fever | 1 | 0.038 | 0.00370 | 10.4 | 11.5 | 0.09222 | | Rabies | 2 | 0.077 | 0.02000 | 3.8 | 4.2 | 0.09427 | | Parkinson disease | 2 | 0.077 | 0.02024 | 3.8 | 4.1 | 0.09625 | |

---
