# Supplementary figures and images for: MMpred: functional miRNA – mRNA interaction analyses by miRNA expression prediction (part 3 of 3)
Source: BMC Genomics. 2012 Nov 14;13:620. doi: 10.1186/1471-2164-13-620 (PMC3562514; doi:10.1186/1471-2164-13-620)

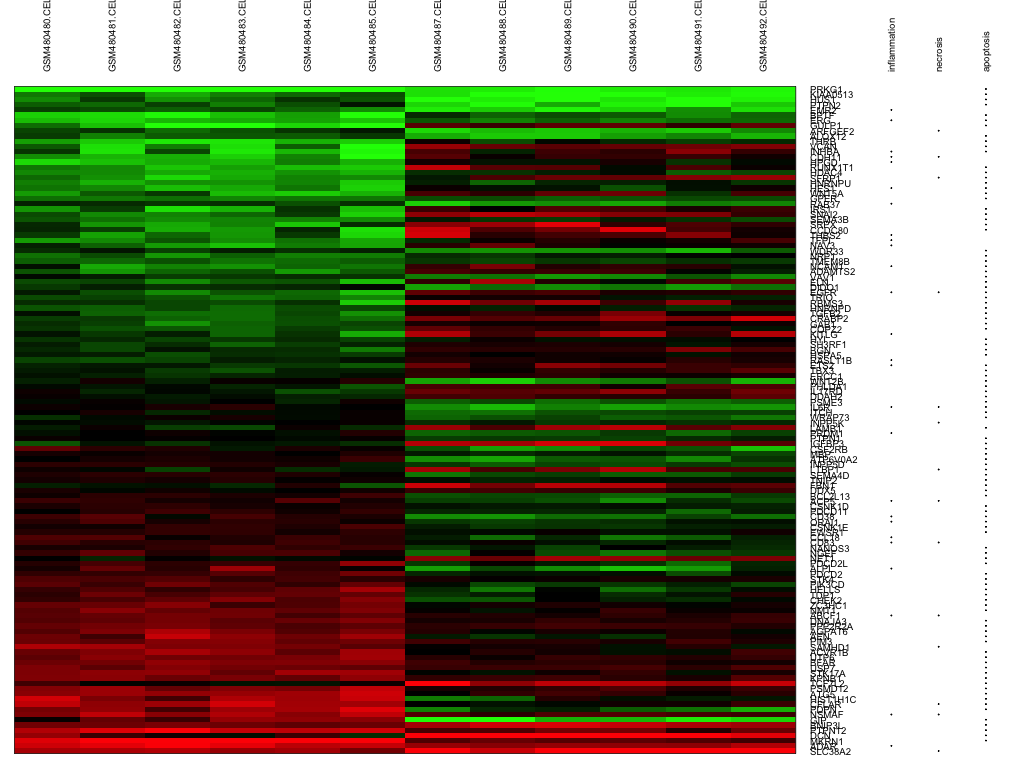

Supplement: Additional file 9 — Examples of MMpred predictions supported by experimental data and mapping against current databases. [file 1471-2164-13-620-S9.ZIP › Additional file 11 - Examples of MMpred predictions supported by experimental data and mapping against current databases/GSE19350/GRAPH_Sep 2_125718.png]

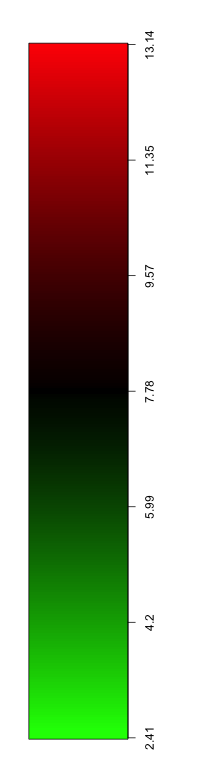

Supplement: Additional file 9 — Examples of MMpred predictions supported by experimental data and mapping against current databases. [file 1471-2164-13-620-S9.ZIP › Additional file 11 - Examples of MMpred predictions supported by experimental data and mapping against current databases/GSE19350/GRAPH_Sep 2_125720.png]

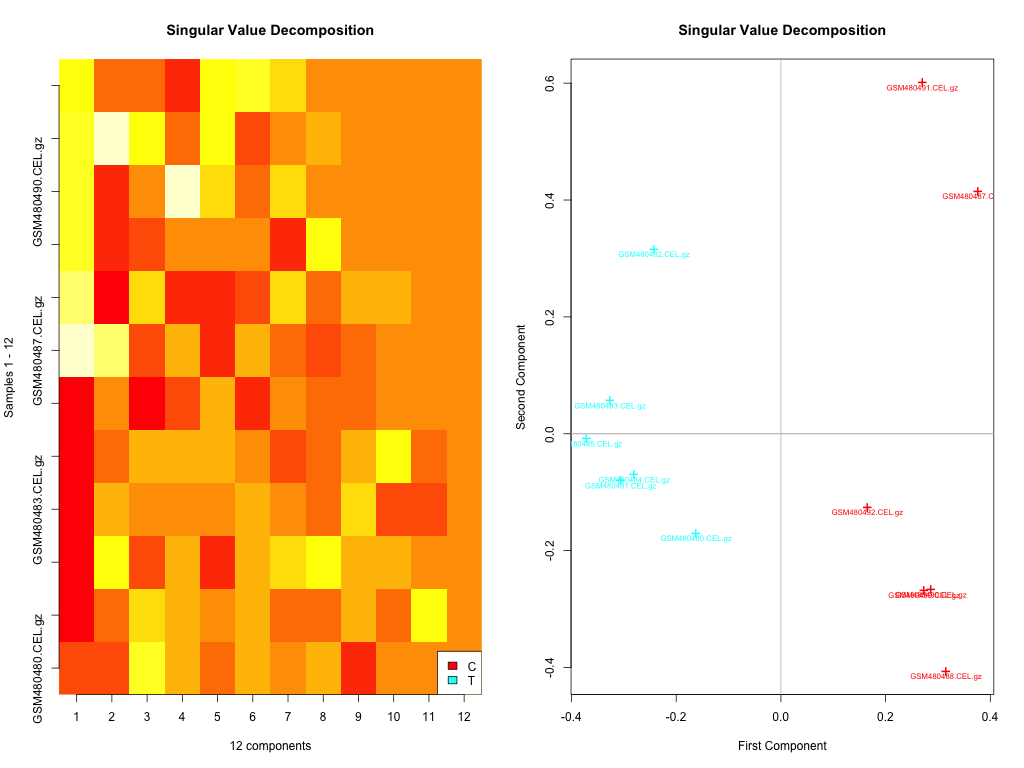

Supplement: Additional file 9 — Examples of MMpred predictions supported by experimental data and mapping against current databases. [file 1471-2164-13-620-S9.ZIP › Additional file 11 - Examples of MMpred predictions supported by experimental data and mapping against current databases/GSE19350/SVD_1346586074.52188.png]

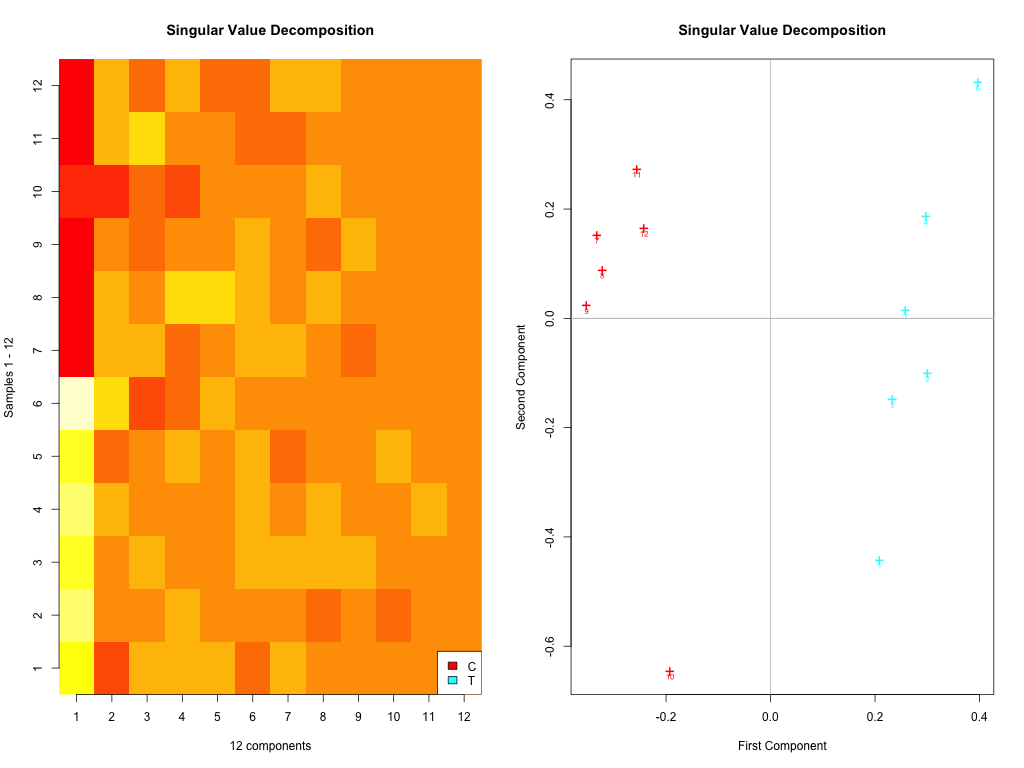

Supplement: Additional file 9 — Examples of MMpred predictions supported by experimental data and mapping against current databases. [file 1471-2164-13-620-S9.ZIP › Additional file 11 - Examples of MMpred predictions supported by experimental data and mapping against current databases/GSE19350/SVD_1346586283.28593.png]

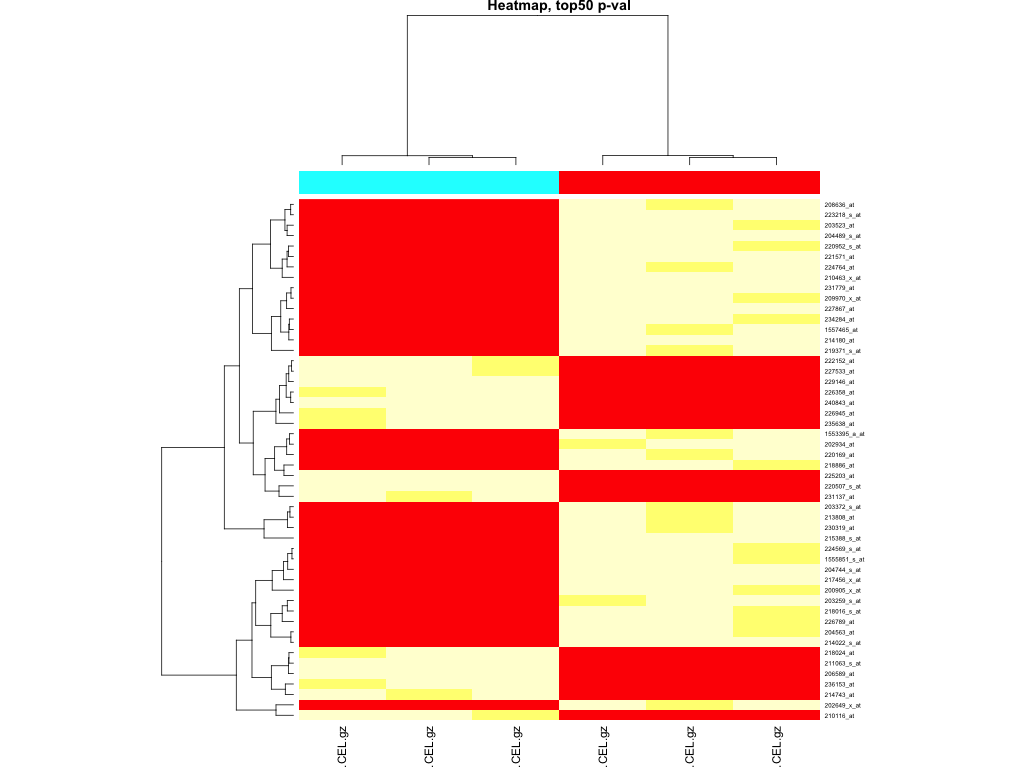

Supplement: Additional file 9 — Examples of MMpred predictions supported by experimental data and mapping against current databases. [file 1471-2164-13-620-S9.ZIP › Additional file 11 - Examples of MMpred predictions supported by experimental data and mapping against current databases/GSE26158/GRAPH_Sep 3_130154.png]

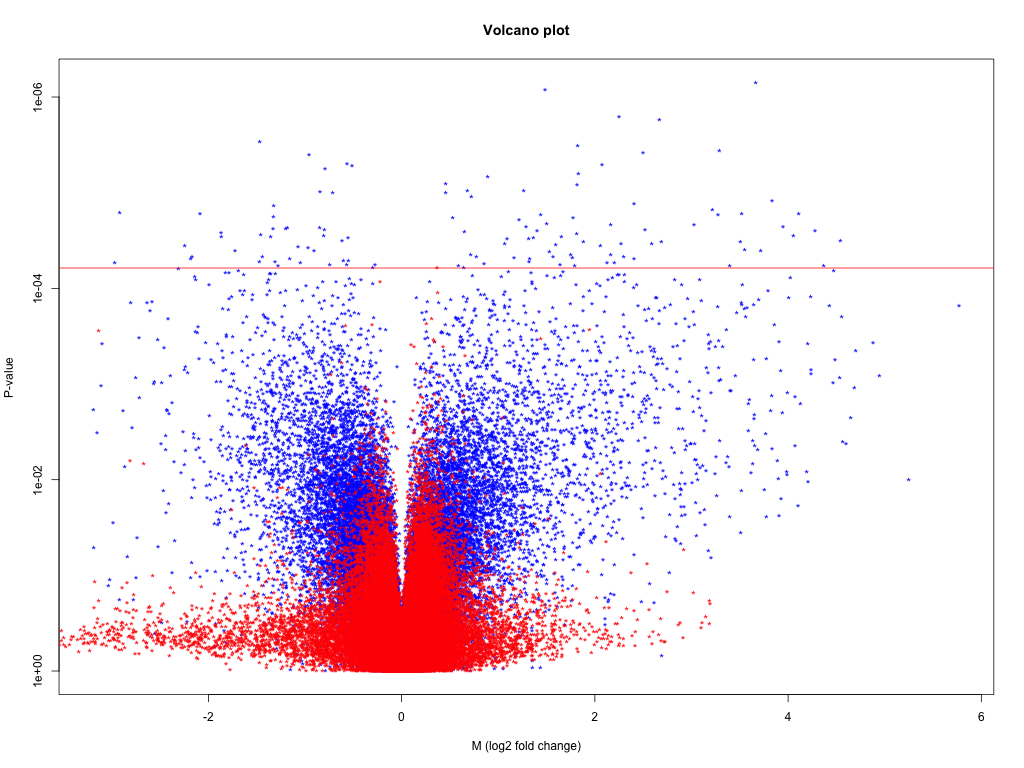

Supplement: Additional file 9 — Examples of MMpred predictions supported by experimental data and mapping against current databases. [file 1471-2164-13-620-S9.ZIP › Additional file 11 - Examples of MMpred predictions supported by experimental data and mapping against current databases/GSE26158/GRAPH_Sep 3_130155.png]

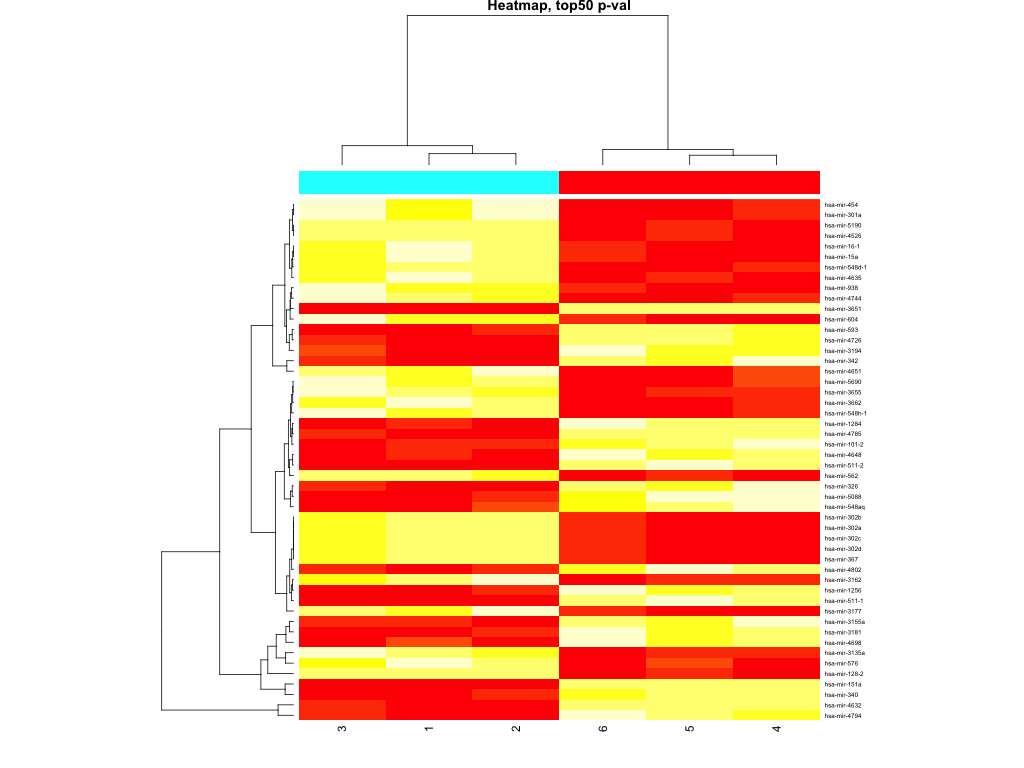

Supplement: Additional file 9 — Examples of MMpred predictions supported by experimental data and mapping against current databases. [file 1471-2164-13-620-S9.ZIP › Additional file 11 - Examples of MMpred predictions supported by experimental data and mapping against current databases/GSE26158/GRAPH_Sep 3_130426.png]

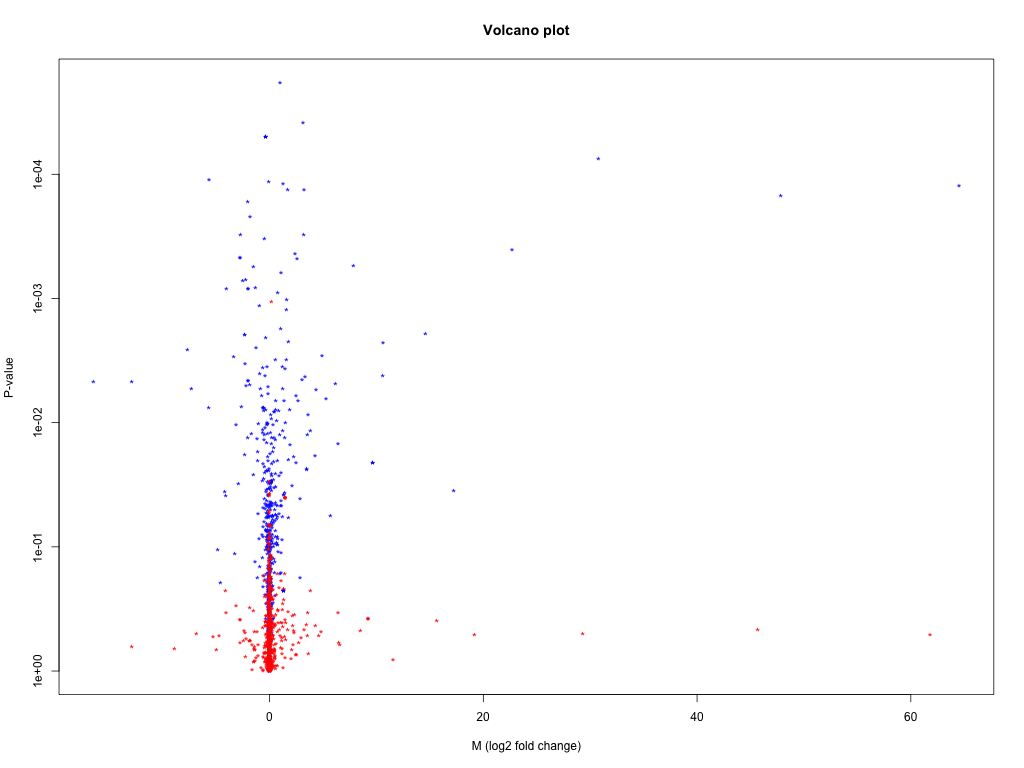

Supplement: Additional file 9 — Examples of MMpred predictions supported by experimental data and mapping against current databases. [file 1471-2164-13-620-S9.ZIP › Additional file 11 - Examples of MMpred predictions supported by experimental data and mapping against current databases/GSE26158/GRAPH_Sep 3_130428.png]

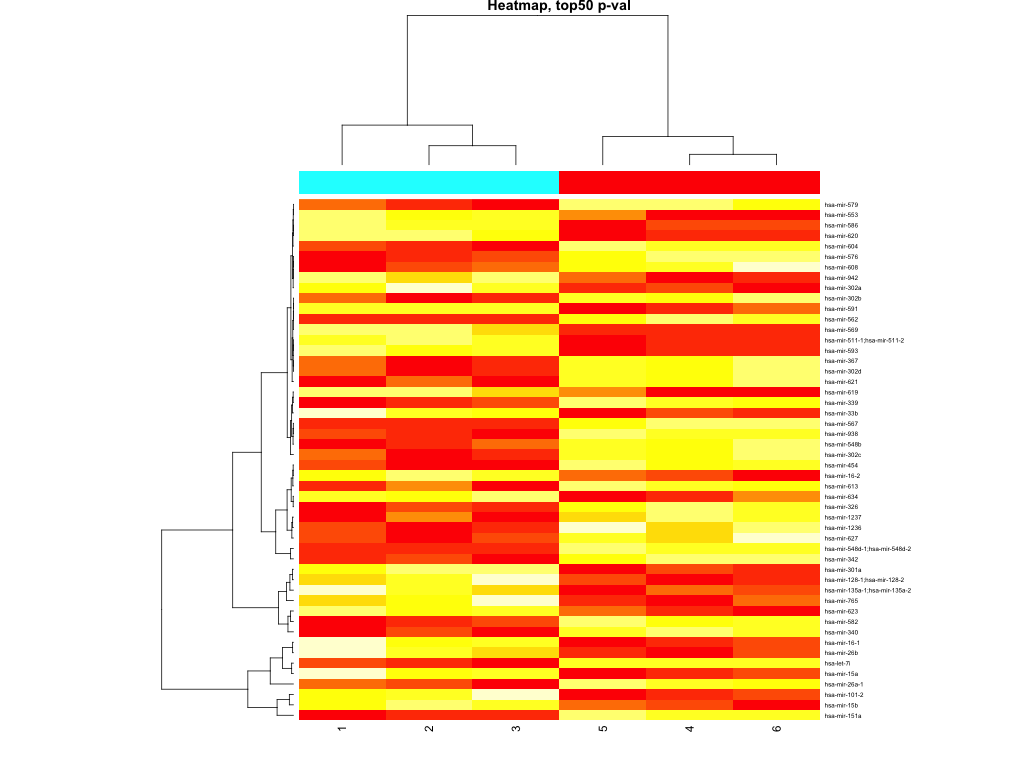

Supplement: Additional file 9 — Examples of MMpred predictions supported by experimental data and mapping against current databases. [file 1471-2164-13-620-S9.ZIP › Additional file 11 - Examples of MMpred predictions supported by experimental data and mapping against current databases/GSE26158/GRAPH_Sep 3_130430.png]

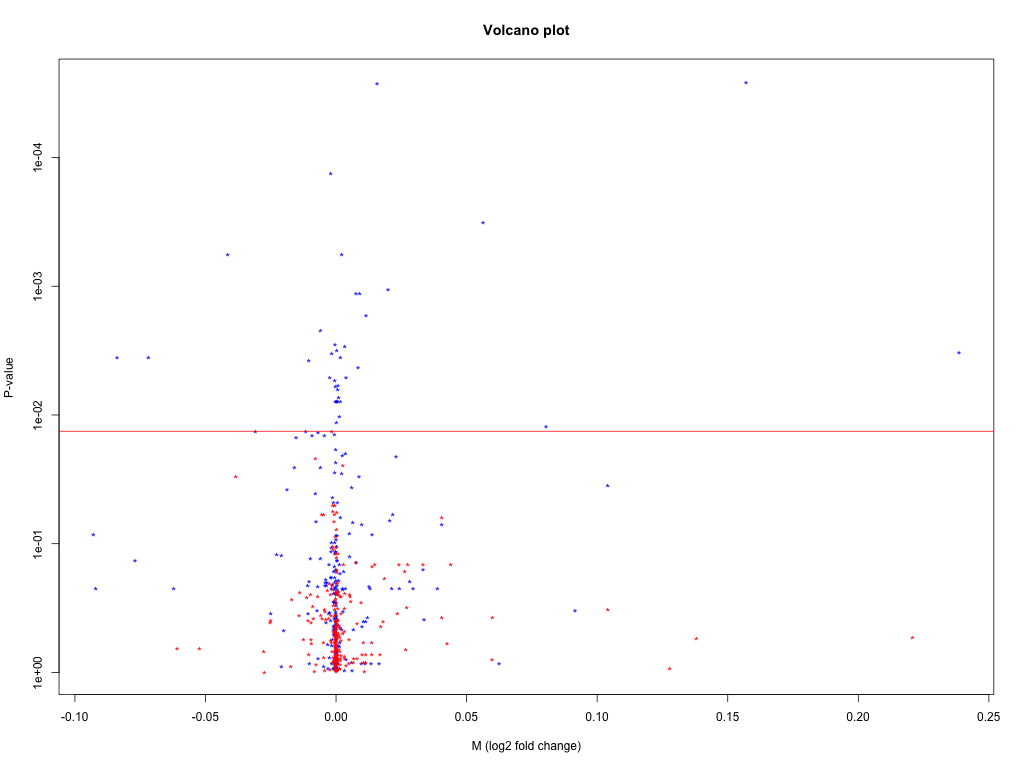

Supplement: Additional file 9 — Examples of MMpred predictions supported by experimental data and mapping against current databases. [file 1471-2164-13-620-S9.ZIP › Additional file 11 - Examples of MMpred predictions supported by experimental data and mapping against current databases/GSE26158/GRAPH_Sep 3_130432.png]

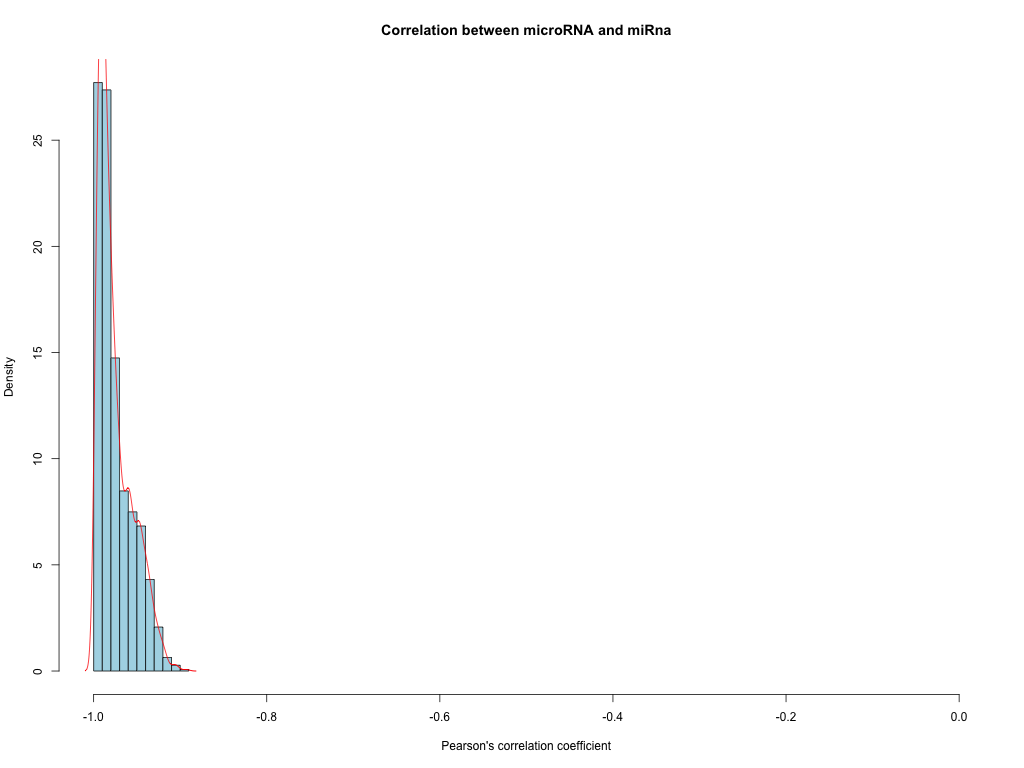

Supplement: Additional file 9 — Examples of MMpred predictions supported by experimental data and mapping against current databases. [file 1471-2164-13-620-S9.ZIP › Additional file 11 - Examples of MMpred predictions supported by experimental data and mapping against current databases/GSE26158/GRAPH_Sep 3_130434.png]

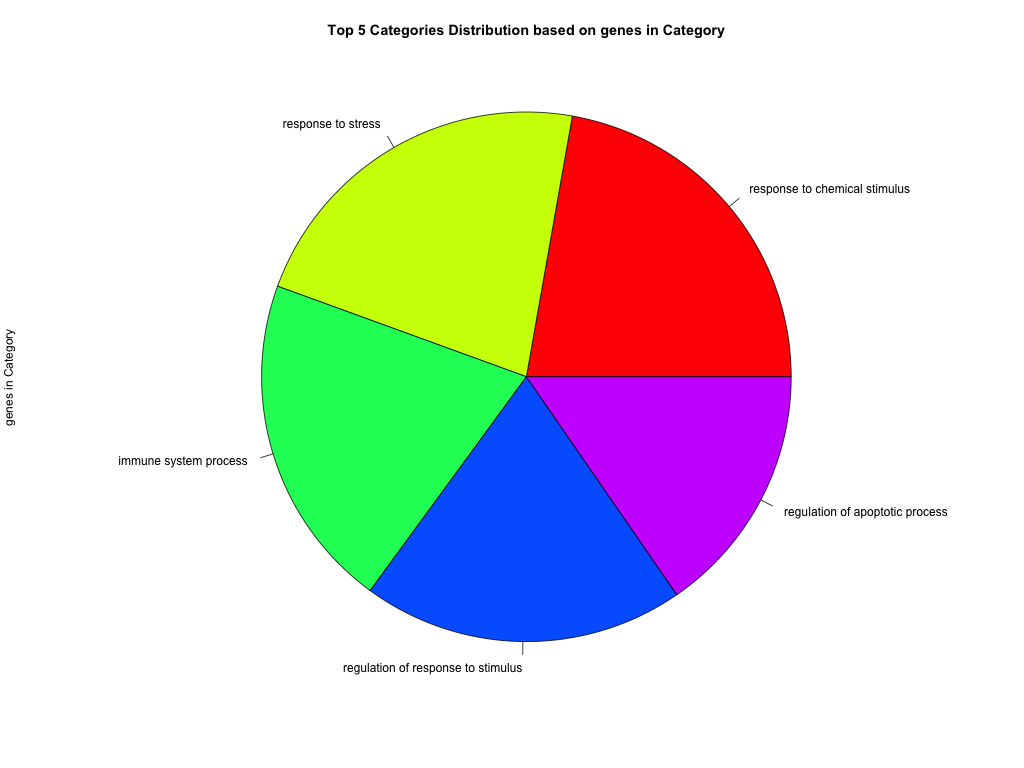

Supplement: Additional file 9 — Examples of MMpred predictions supported by experimental data and mapping against current databases. [file 1471-2164-13-620-S9.ZIP › Additional file 11 - Examples of MMpred predictions supported by experimental data and mapping against current databases/GSE26158/GRAPH_Sep 3_130527.png]

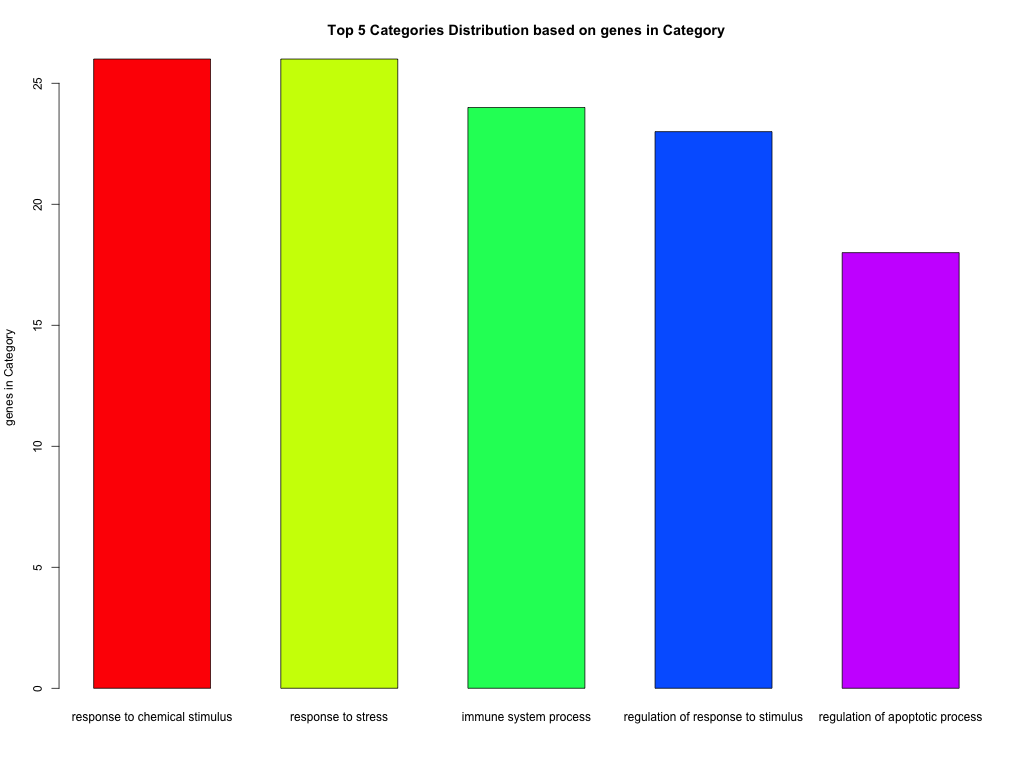

Supplement: Additional file 9 — Examples of MMpred predictions supported by experimental data and mapping against current databases. [file 1471-2164-13-620-S9.ZIP › Additional file 11 - Examples of MMpred predictions supported by experimental data and mapping against current databases/GSE26158/GRAPH_Sep 3_130528.png]

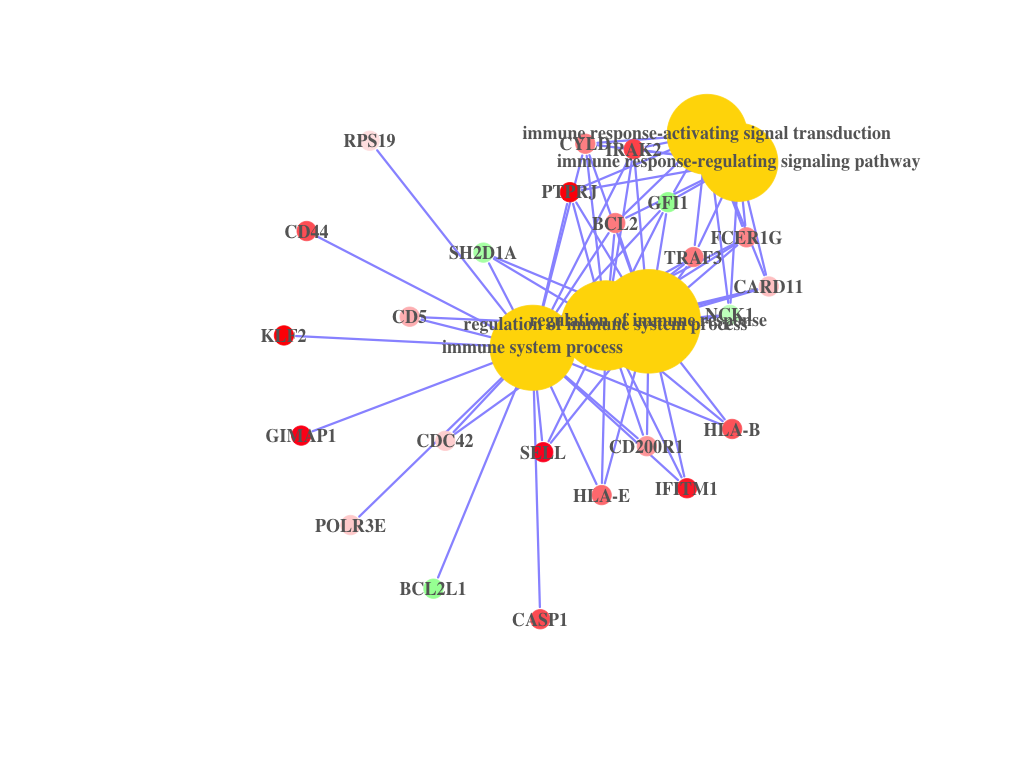

Supplement: Additional file 9 — Examples of MMpred predictions supported by experimental data and mapping against current databases. [file 1471-2164-13-620-S9.ZIP › Additional file 11 - Examples of MMpred predictions supported by experimental data and mapping against current databases/GSE26158/GRAPH_Sep 3_130530.png]

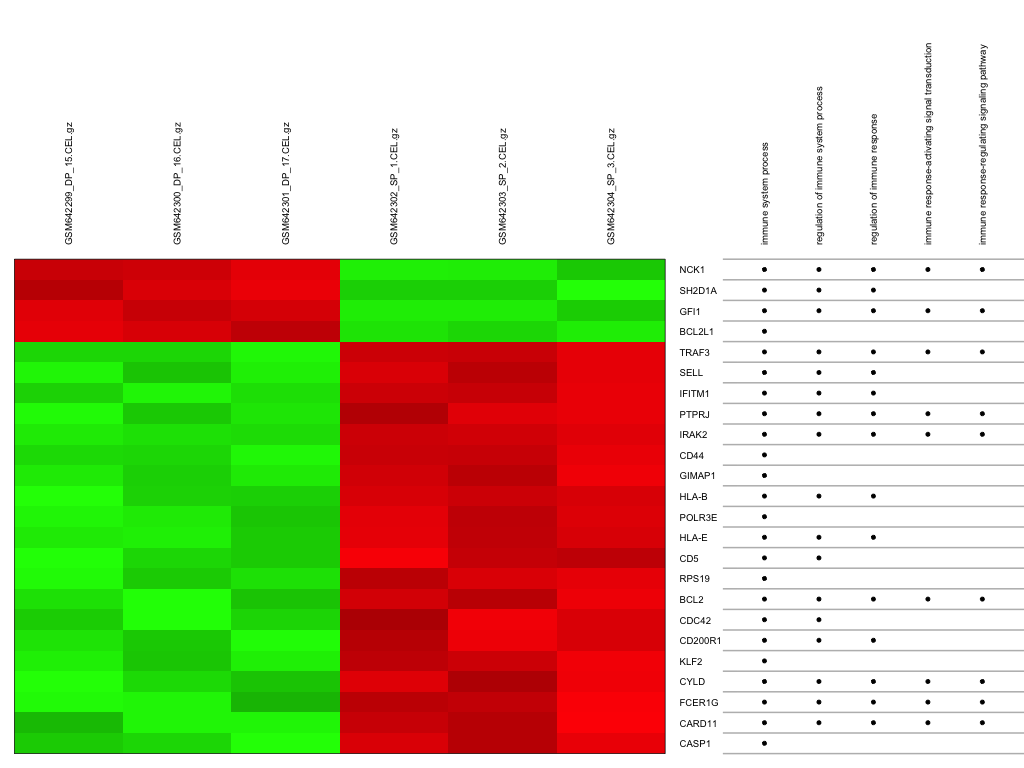

Supplement: Additional file 9 — Examples of MMpred predictions supported by experimental data and mapping against current databases. [file 1471-2164-13-620-S9.ZIP › Additional file 11 - Examples of MMpred predictions supported by experimental data and mapping against current databases/GSE26158/GRAPH_Sep 3_130531.png]

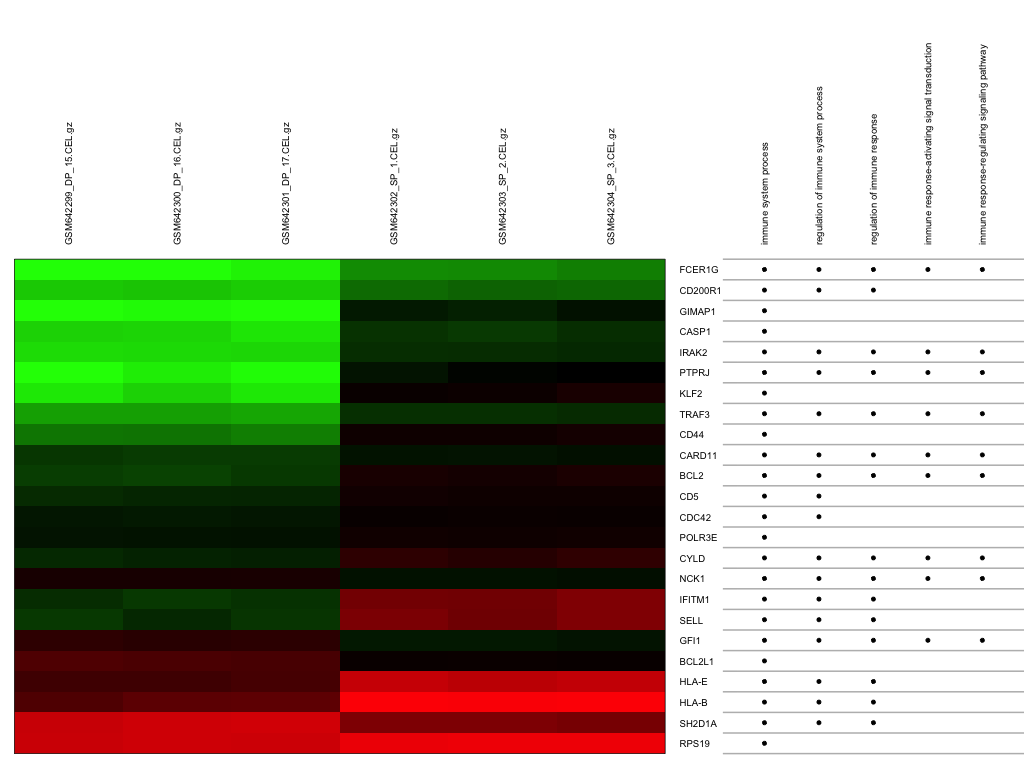

Supplement: Additional file 9 — Examples of MMpred predictions supported by experimental data and mapping against current databases. [file 1471-2164-13-620-S9.ZIP › Additional file 11 - Examples of MMpred predictions supported by experimental data and mapping against current databases/GSE26158/GRAPH_Sep 3_130532.png]

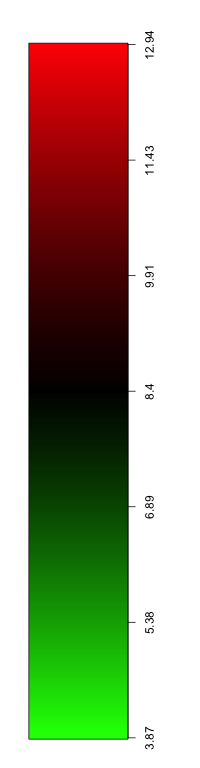

Supplement: Additional file 9 — Examples of MMpred predictions supported by experimental data and mapping against current databases. [file 1471-2164-13-620-S9.ZIP › Additional file 11 - Examples of MMpred predictions supported by experimental data and mapping against current databases/GSE26158/GRAPH_Sep 3_130533.png]

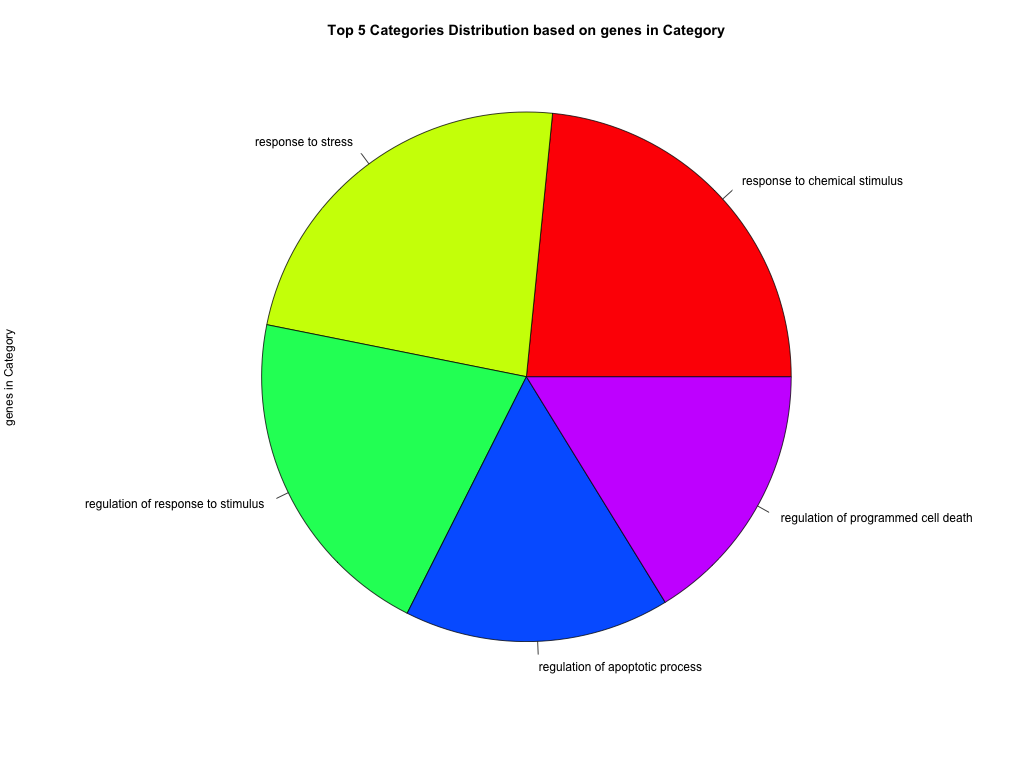

Supplement: Additional file 9 — Examples of MMpred predictions supported by experimental data and mapping against current databases. [file 1471-2164-13-620-S9.ZIP › Additional file 11 - Examples of MMpred predictions supported by experimental data and mapping against current databases/GSE26158/GRAPH_Sep 3_130611.png]

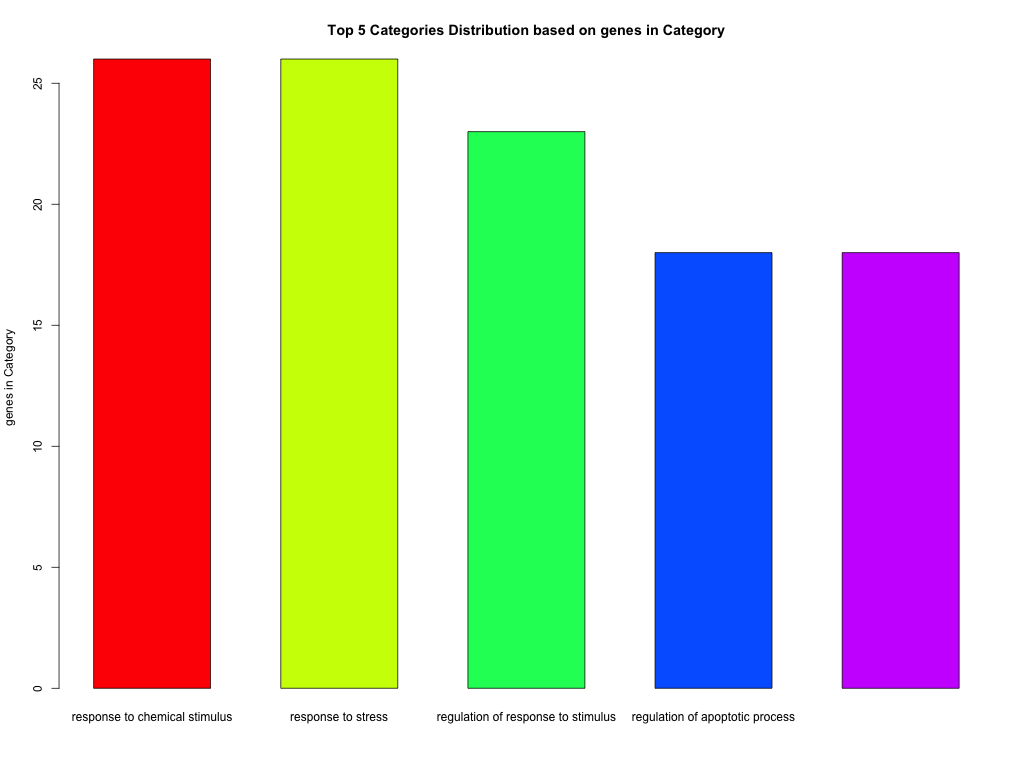

Supplement: Additional file 9 — Examples of MMpred predictions supported by experimental data and mapping against current databases. [file 1471-2164-13-620-S9.ZIP › Additional file 11 - Examples of MMpred predictions supported by experimental data and mapping against current databases/GSE26158/GRAPH_Sep 3_130612.png]

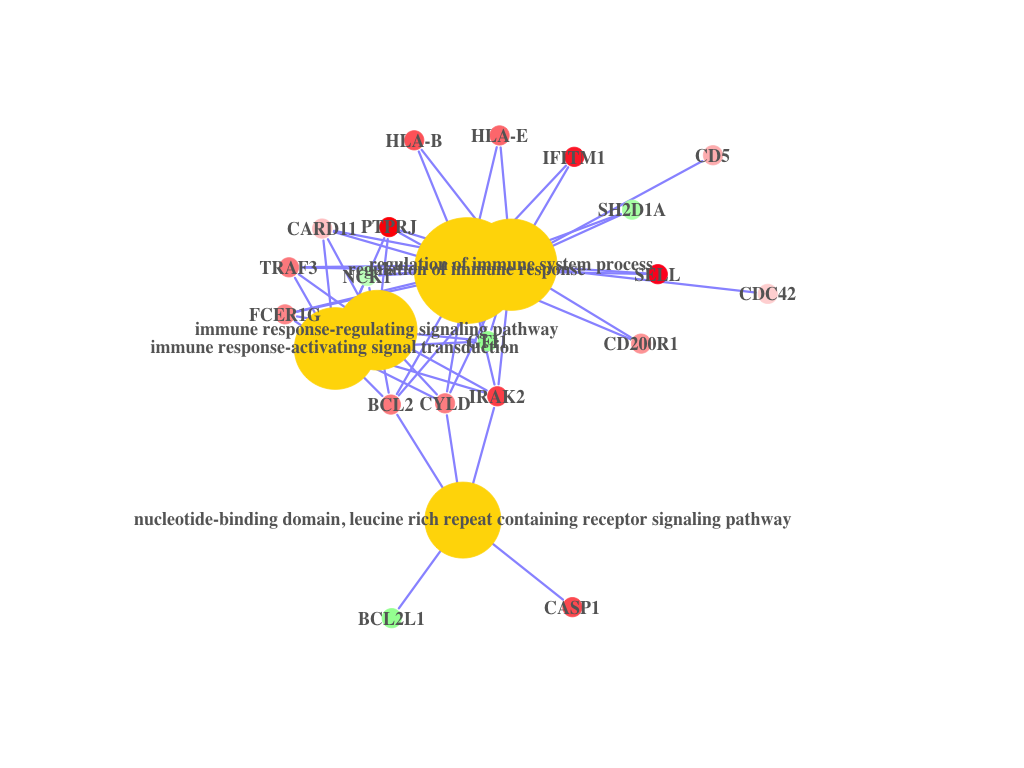

Supplement: Additional file 9 — Examples of MMpred predictions supported by experimental data and mapping against current databases. [file 1471-2164-13-620-S9.ZIP › Additional file 11 - Examples of MMpred predictions supported by experimental data and mapping against current databases/GSE26158/GRAPH_Sep 3_130613.png]

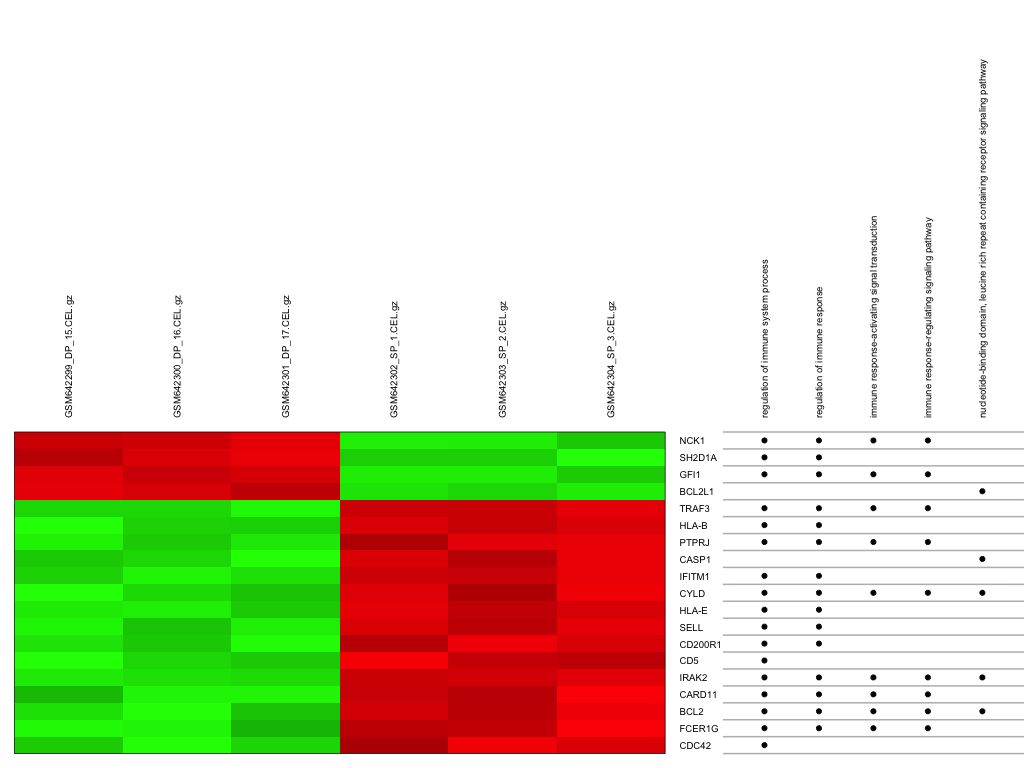

Supplement: Additional file 9 — Examples of MMpred predictions supported by experimental data and mapping against current databases. [file 1471-2164-13-620-S9.ZIP › Additional file 11 - Examples of MMpred predictions supported by experimental data and mapping against current databases/GSE26158/GRAPH_Sep 3_130615.png]

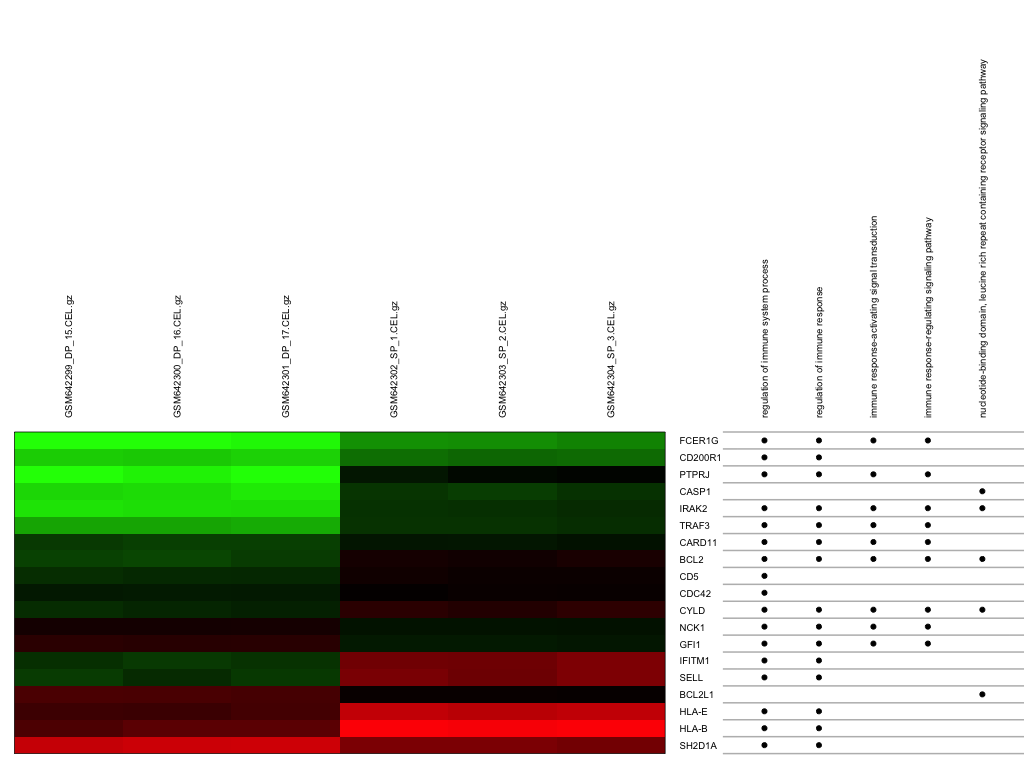

Supplement: Additional file 9 — Examples of MMpred predictions supported by experimental data and mapping against current databases. [file 1471-2164-13-620-S9.ZIP › Additional file 11 - Examples of MMpred predictions supported by experimental data and mapping against current databases/GSE26158/GRAPH_Sep 3_130616.png]

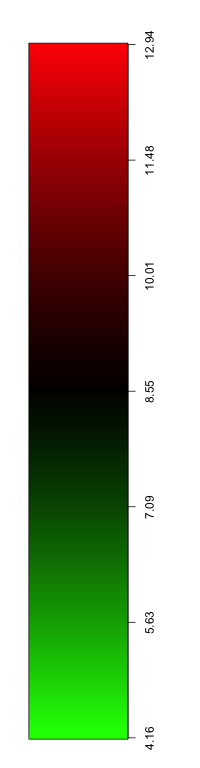

Supplement: Additional file 9 — Examples of MMpred predictions supported by experimental data and mapping against current databases. [file 1471-2164-13-620-S9.ZIP › Additional file 11 - Examples of MMpred predictions supported by experimental data and mapping against current databases/GSE26158/GRAPH_Sep 3_130617.png]

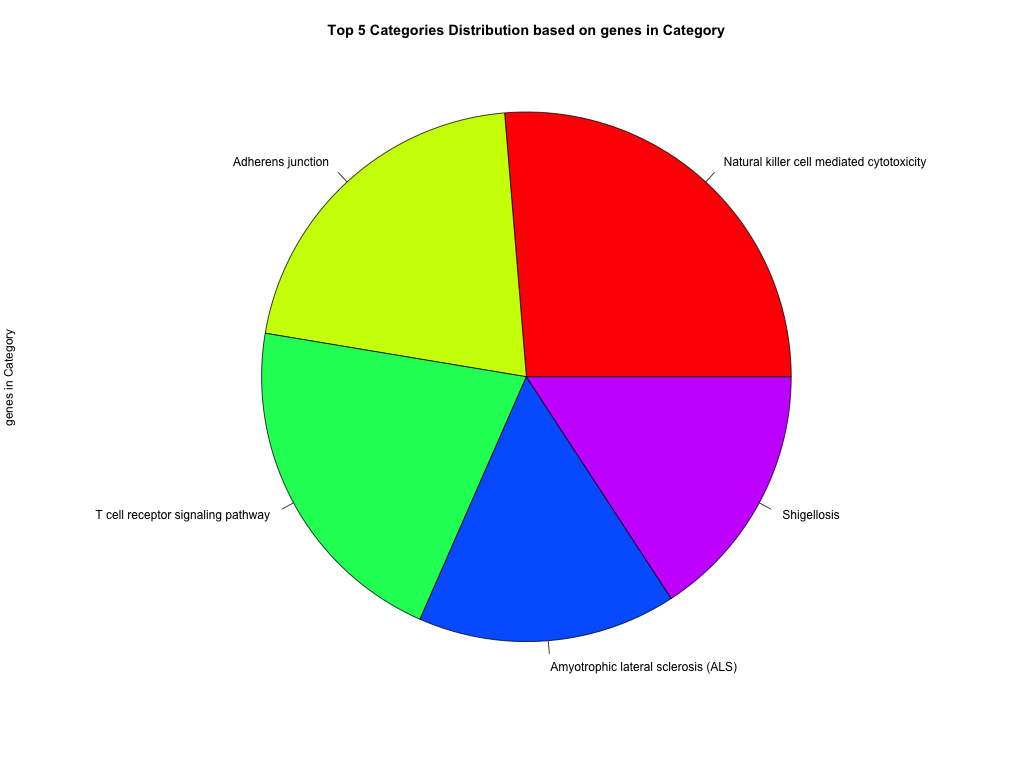

Supplement: Additional file 9 — Examples of MMpred predictions supported by experimental data and mapping against current databases. [file 1471-2164-13-620-S9.ZIP › Additional file 11 - Examples of MMpred predictions supported by experimental data and mapping against current databases/GSE26158/GRAPH_Sep 3_130618.png]

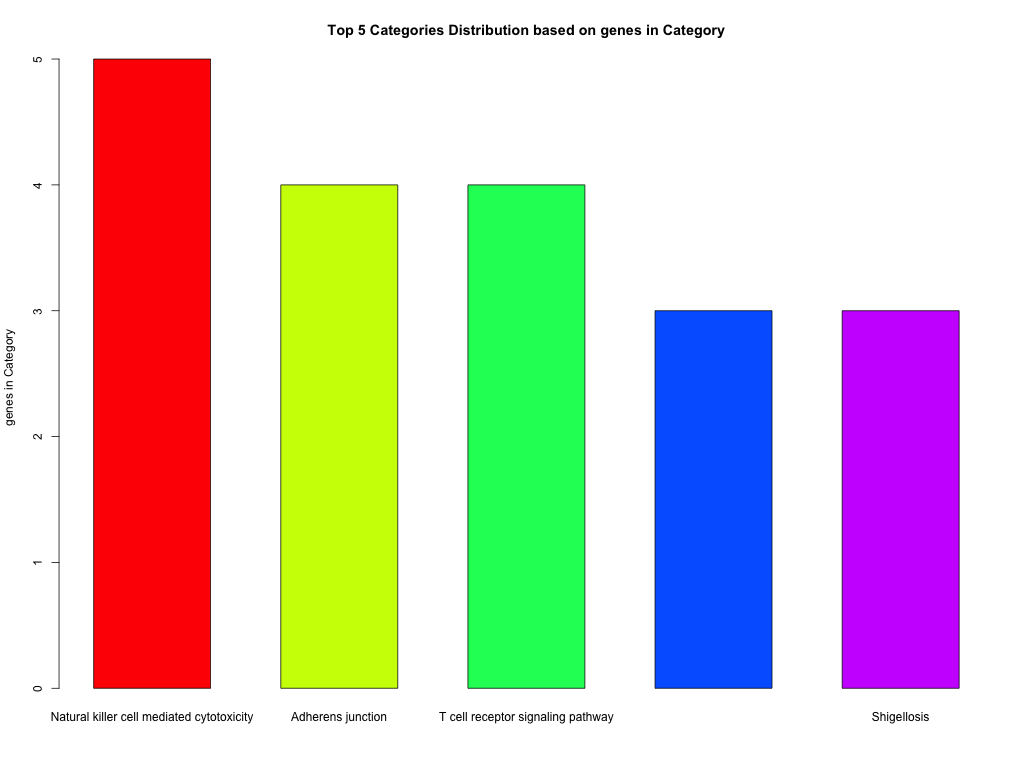

Supplement: Additional file 9 — Examples of MMpred predictions supported by experimental data and mapping against current databases. [file 1471-2164-13-620-S9.ZIP › Additional file 11 - Examples of MMpred predictions supported by experimental data and mapping against current databases/GSE26158/GRAPH_Sep 3_130619.png]

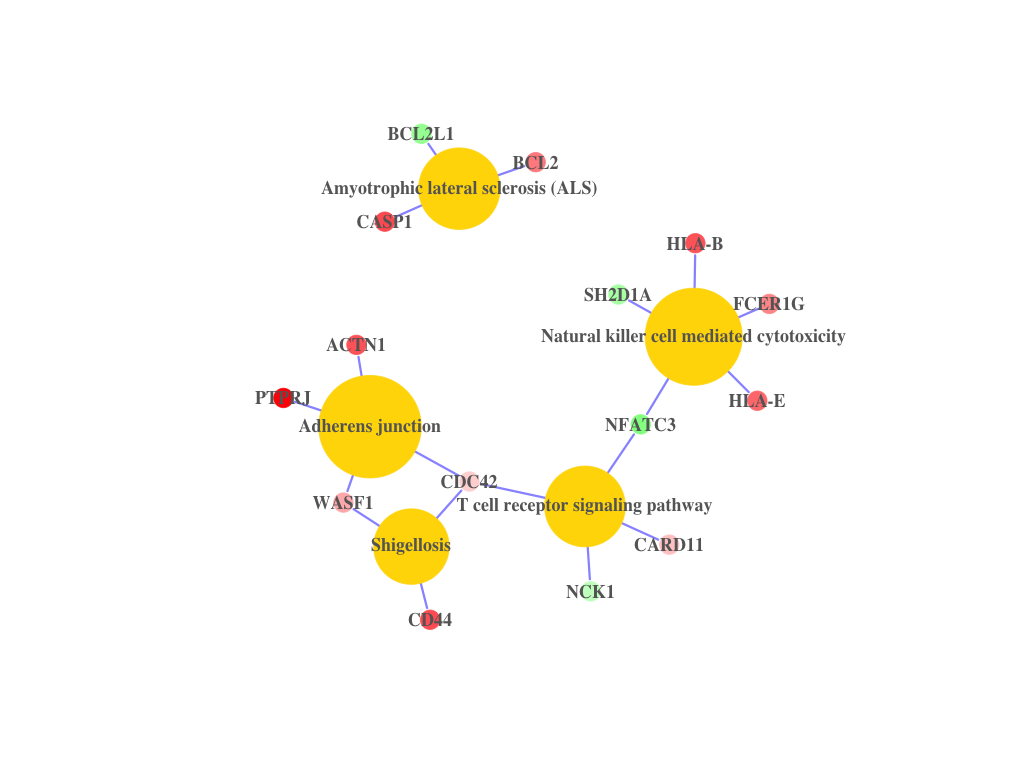

Supplement: Additional file 9 — Examples of MMpred predictions supported by experimental data and mapping against current databases. [file 1471-2164-13-620-S9.ZIP › Additional file 11 - Examples of MMpred predictions supported by experimental data and mapping against current databases/GSE26158/GRAPH_Sep 3_130621.png]

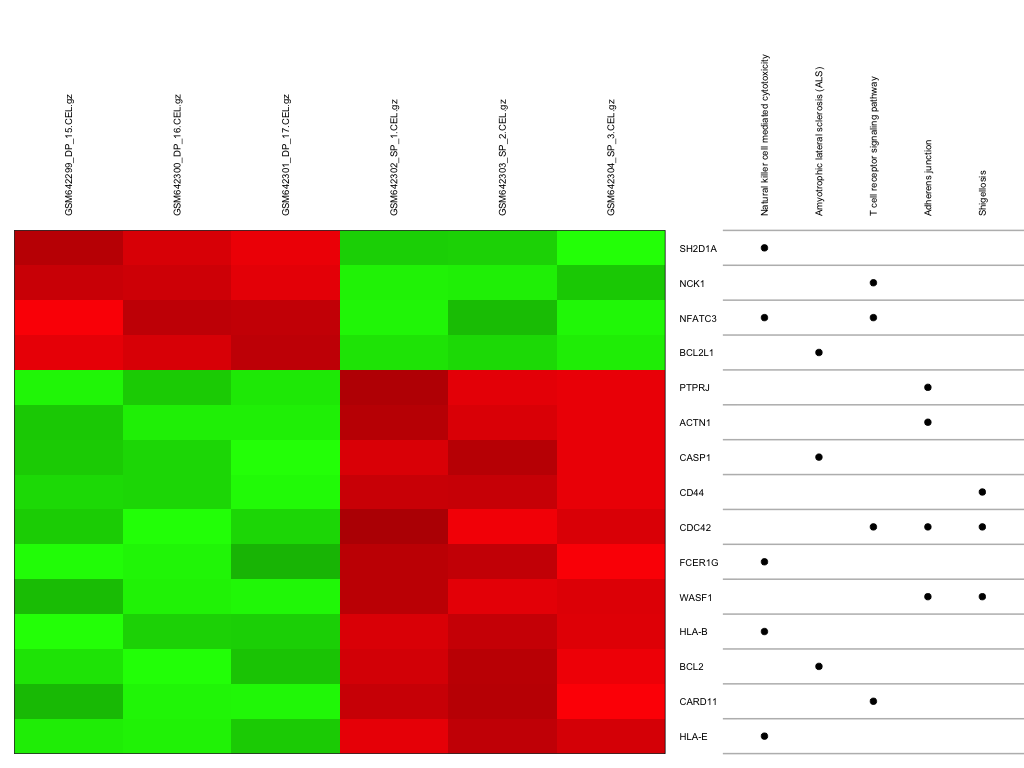

Supplement: Additional file 9 — Examples of MMpred predictions supported by experimental data and mapping against current databases. [file 1471-2164-13-620-S9.ZIP › Additional file 11 - Examples of MMpred predictions supported by experimental data and mapping against current databases/GSE26158/GRAPH_Sep 3_130622.png]

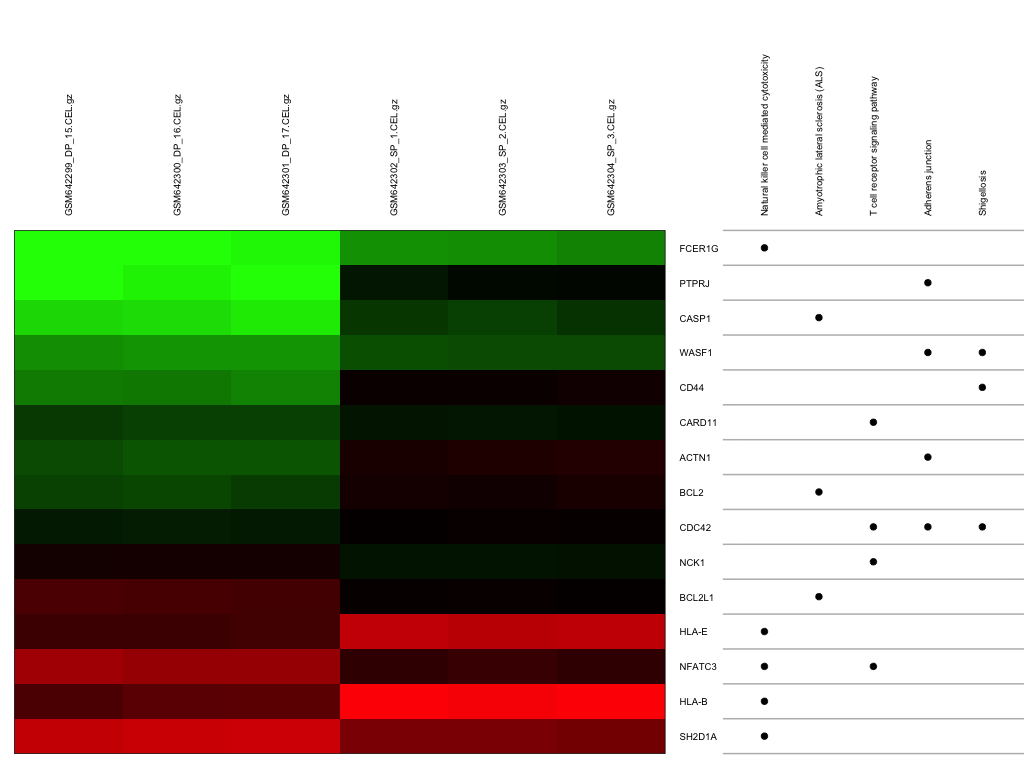

Supplement: Additional file 9 — Examples of MMpred predictions supported by experimental data and mapping against current databases. [file 1471-2164-13-620-S9.ZIP › Additional file 11 - Examples of MMpred predictions supported by experimental data and mapping against current databases/GSE26158/GRAPH_Sep 3_130623.png]

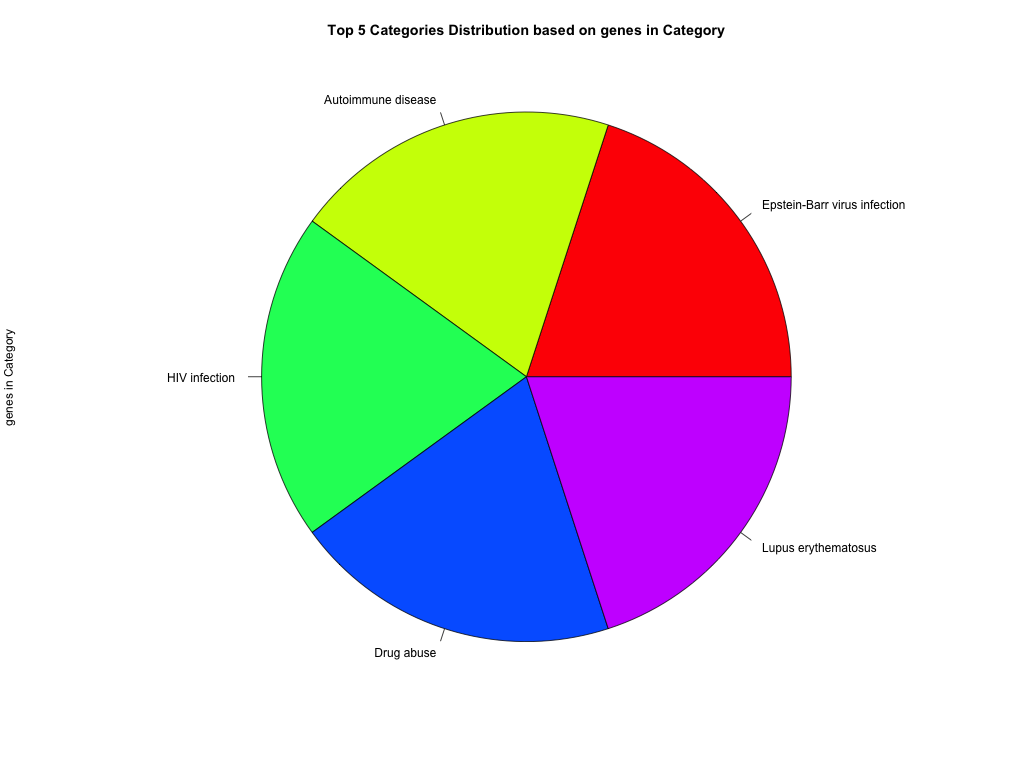

Supplement: Additional file 9 — Examples of MMpred predictions supported by experimental data and mapping against current databases. [file 1471-2164-13-620-S9.ZIP › Additional file 11 - Examples of MMpred predictions supported by experimental data and mapping against current databases/GSE26158/GRAPH_Sep 3_130626.png]

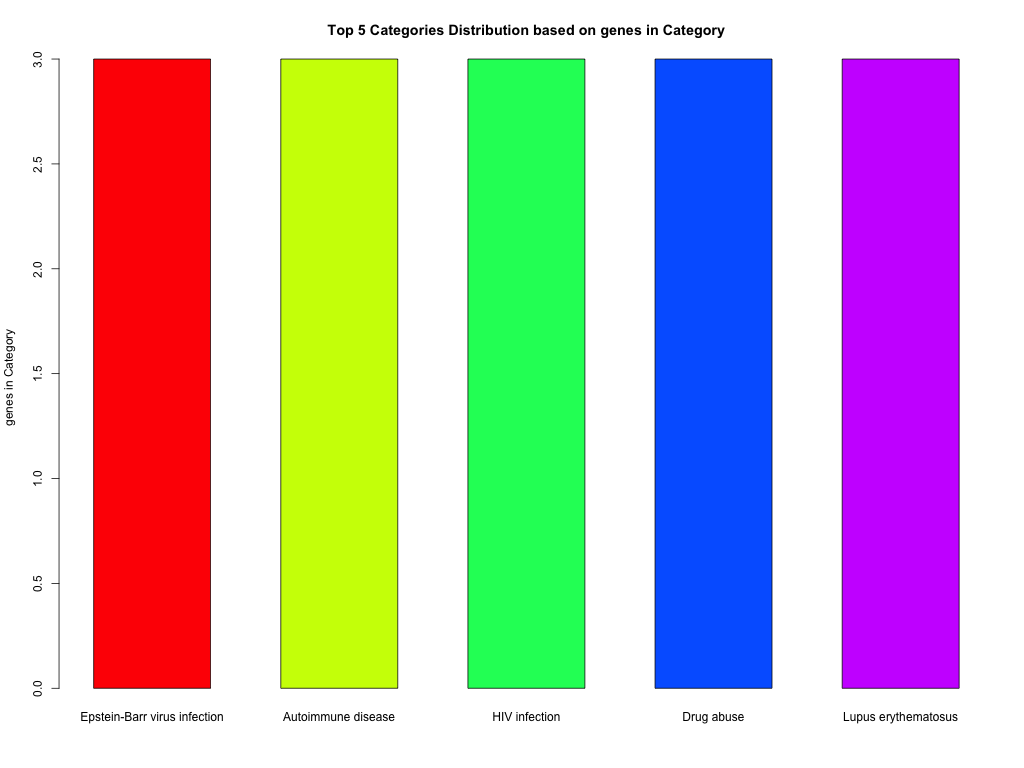

Supplement: Additional file 9 — Examples of MMpred predictions supported by experimental data and mapping against current databases. [file 1471-2164-13-620-S9.ZIP › Additional file 11 - Examples of MMpred predictions supported by experimental data and mapping against current databases/GSE26158/GRAPH_Sep 3_130627.png]

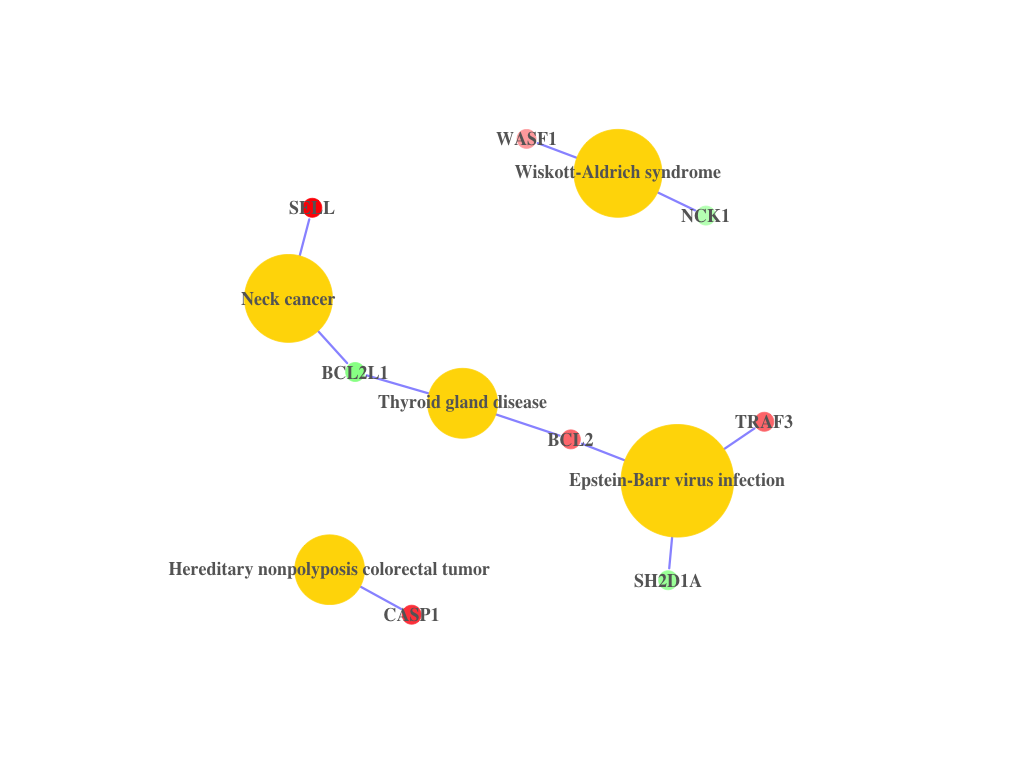

Supplement: Additional file 9 — Examples of MMpred predictions supported by experimental data and mapping against current databases. [file 1471-2164-13-620-S9.ZIP › Additional file 11 - Examples of MMpred predictions supported by experimental data and mapping against current databases/GSE26158/GRAPH_Sep 3_130628.png]

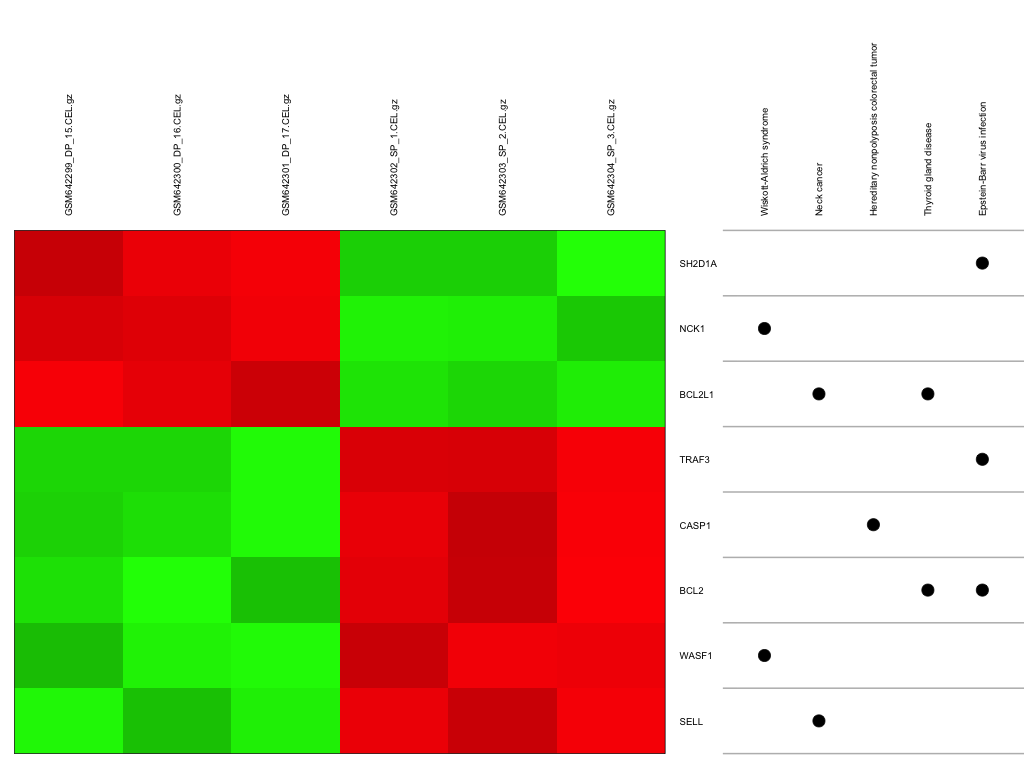

Supplement: Additional file 9 — Examples of MMpred predictions supported by experimental data and mapping against current databases. [file 1471-2164-13-620-S9.ZIP › Additional file 11 - Examples of MMpred predictions supported by experimental data and mapping against current databases/GSE26158/GRAPH_Sep 3_130629.png]

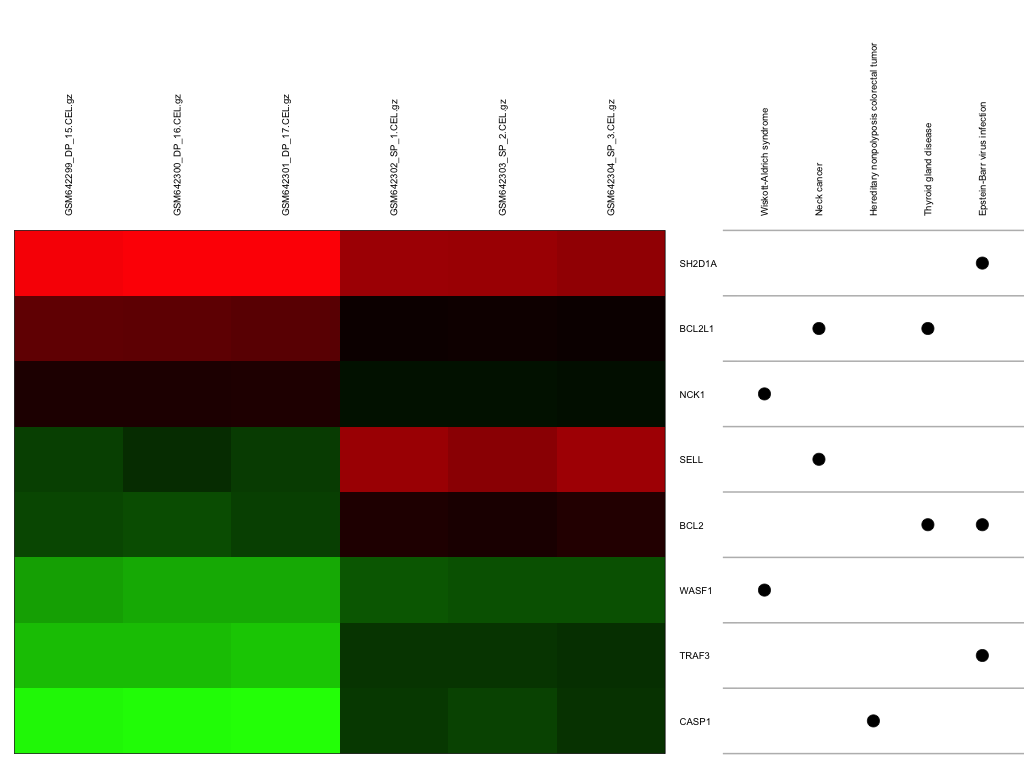

Supplement: Additional file 9 — Examples of MMpred predictions supported by experimental data and mapping against current databases. [file 1471-2164-13-620-S9.ZIP › Additional file 11 - Examples of MMpred predictions supported by experimental data and mapping against current databases/GSE26158/GRAPH_Sep 3_130630.png]

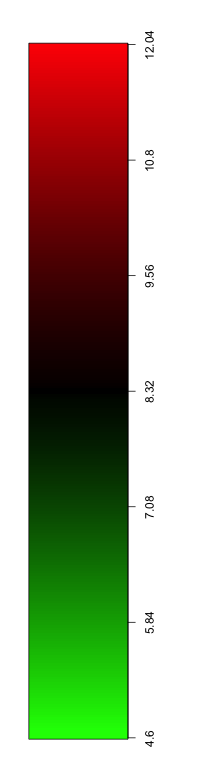

Supplement: Additional file 9 — Examples of MMpred predictions supported by experimental data and mapping against current databases. [file 1471-2164-13-620-S9.ZIP › Additional file 11 - Examples of MMpred predictions supported by experimental data and mapping against current databases/GSE26158/GRAPH_Sep 3_130631.png]

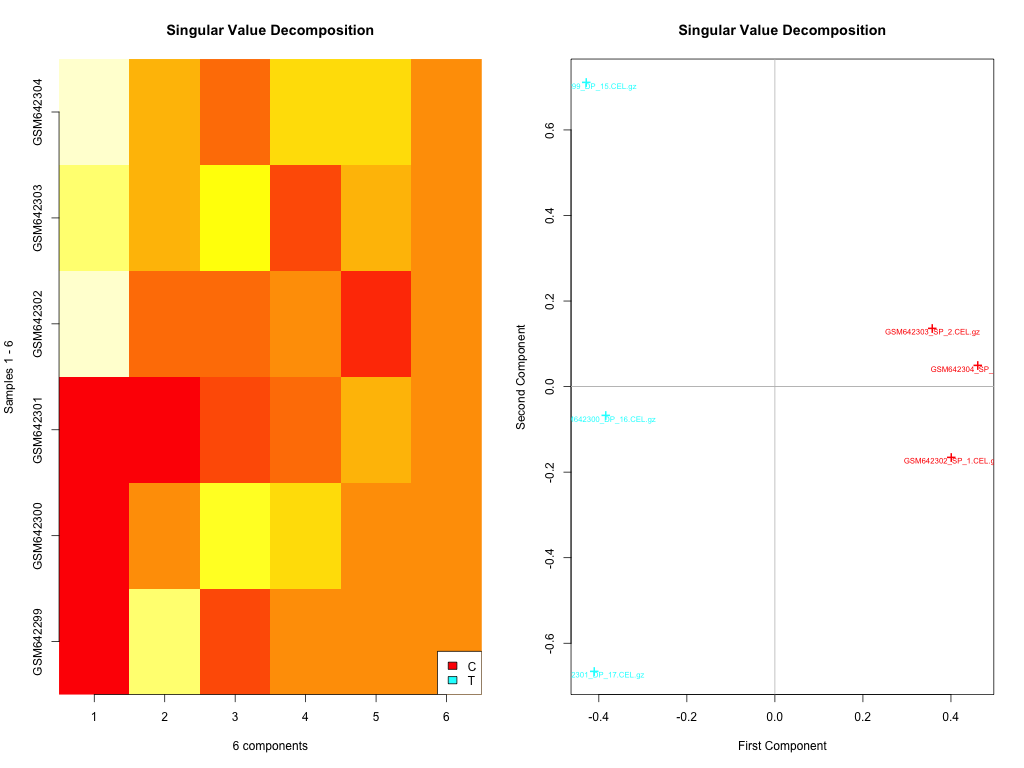

Supplement: Additional file 9 — Examples of MMpred predictions supported by experimental data and mapping against current databases. [file 1471-2164-13-620-S9.ZIP › Additional file 11 - Examples of MMpred predictions supported by experimental data and mapping against current databases/GSE26158/SVD_1346673714.37288.png]

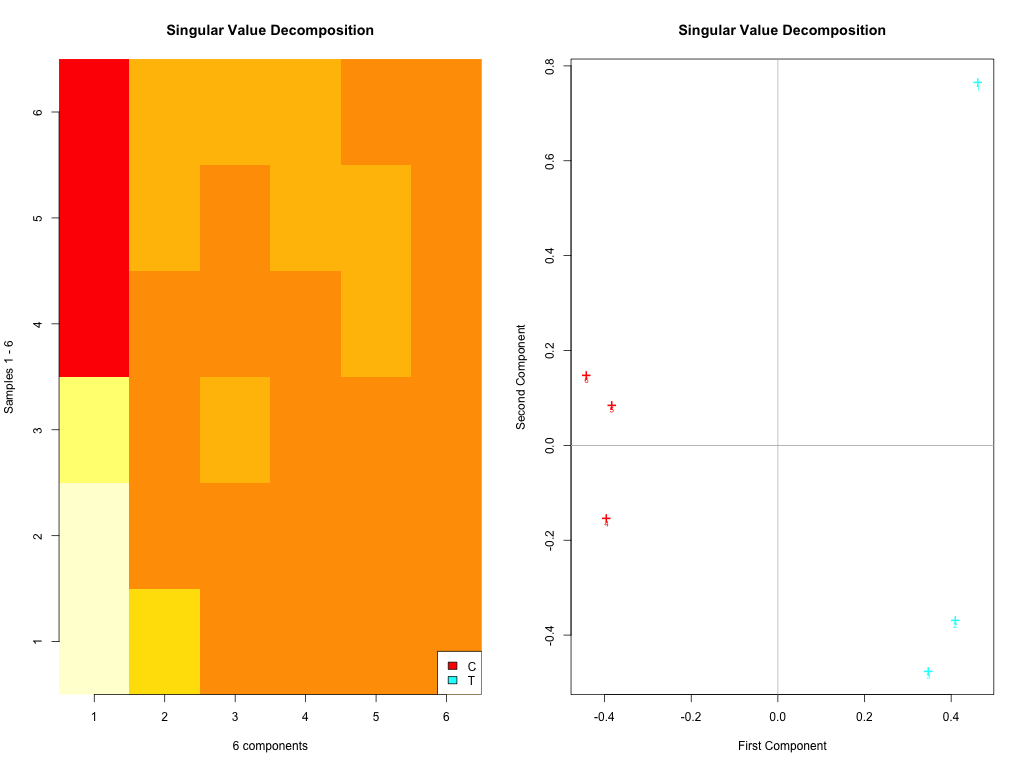

Supplement: Additional file 9 — Examples of MMpred predictions supported by experimental data and mapping against current databases. [file 1471-2164-13-620-S9.ZIP › Additional file 11 - Examples of MMpred predictions supported by experimental data and mapping against current databases/GSE26158/SVD_1346673866.81734.png]
