# Supplementary material for: MMpred: functional miRNA – mRNA interaction analyses by miRNA expression prediction
Source: BMC Genomics. 2012 Nov 14;13:620. doi: 10.1186/1471-2164-13-620 (PMC3562514; doi:10.1186/1471-2164-13-620)
Supplement: Additional file 10 — The detailed description of software implementation in R language. [file 1471-2164-13-620-S10.pdf]

Additional file 3 – The detailed software implementation of the model

The MMpred pipeline generates both graphical and text input allowing the user to quickly view the results and validate the output of each step. The method has been implemented in R language. The modular design of the pipeline and the full access to the scripts enables advanced users to customise the software accordingly to match their needs and requirements. For users with different requirements, unfamiliar with command line scripting the user friendly GUI has been developed. In addition to that the Java interface is being incorporated with the purpose to create a client-server infrastructure.

A.1 Detail program structure

From the earliest phase of development the MMpred pipeline has been planned in a modular way – every single component has been coded as different functions stored in separate R files. Moreover, each of those functions can be used separately.

Figure 0.1 shows a detailed flowchart presenting the design and structure of MMpred pipeline. Each component of the pipeline contains a name of the script responsible for its execution (printed in red). Between the elements labelled arrows indicate the variable(s) that are passed between the elements (functions, marked in green). Usually the output from a function becomes the input for the next, subsequent one. The flowchart also features pre-calculated files, user input and pipeline output.

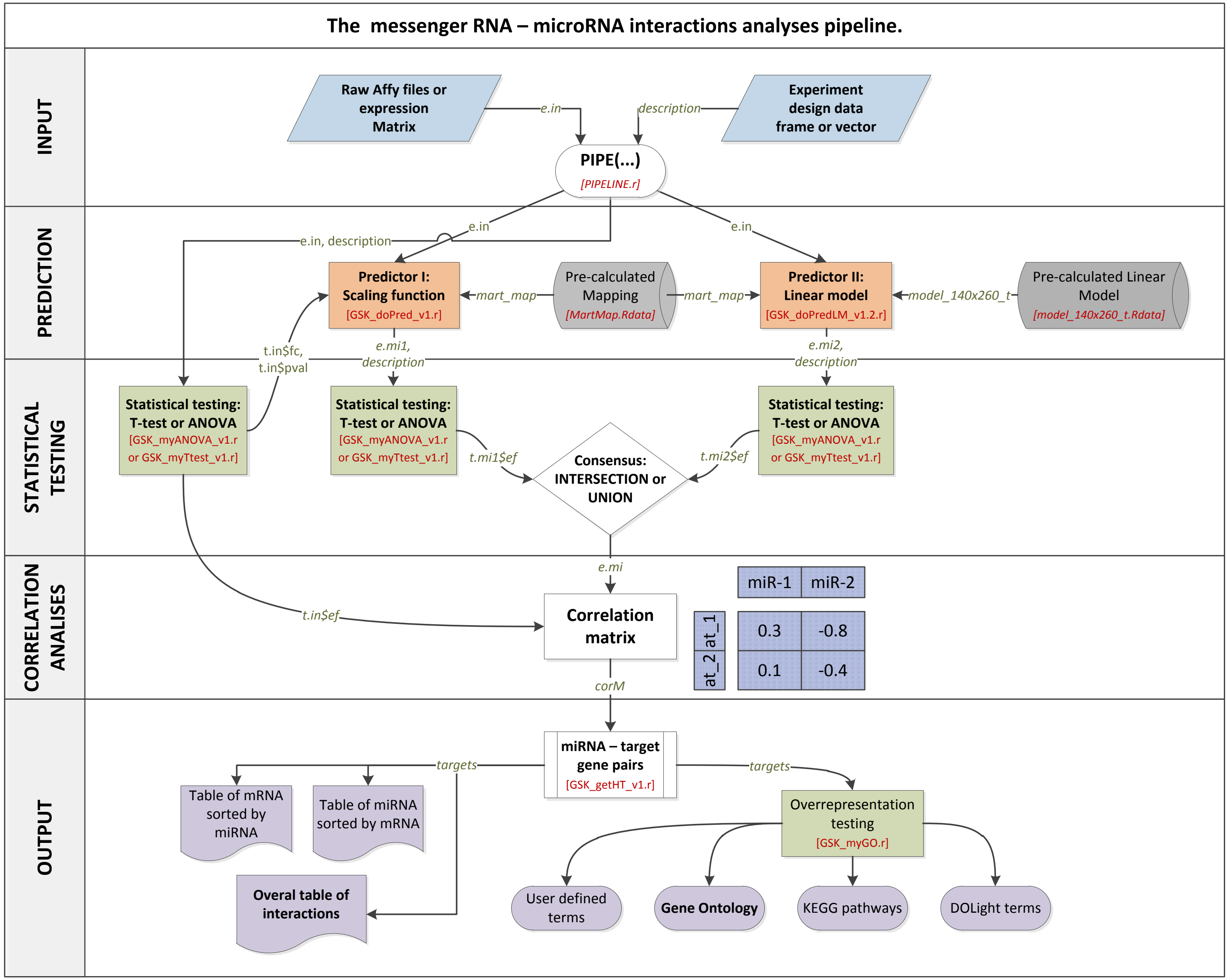

Figure 0.1 The flowchart presenting the structure of MMpred pipeline enriched with scripts, pre-calculated binaries and variables.

The ***"MMpred.R"*** file is the main script of the pipeline, contains MMpred(…) function - the wrapper calling other functions, controlling the flow and building HTML report. The R scripts containing R functions executing essential components of the pipeline are:

- ***"gMMpred.R"*** - GUI, called by gMMpred(…) function, require Gtk+ installed and usage of 32bit version of R;
- ***"doPred.R"*** – predictor I – scaling function – inputs mapping, mRNA expression matrix, fold change and p-value, outputs microRNA pseudo-expression matrix;
- ***"doPredLM.R"*** – predictor II – linear model – inputs mapping, mRNA expression matrix and pre-calculated model binary, outputs microRNA pseudo-expression matrix;
- ***"mySVD.R"*** – doing Singular Value Decomposition, plotting Principal Component Analyses plot. Used for information and input data quality assessing only. Does not return anything to the pipeline;
- ***"myANOVA.R"*** – ANOVA statistical analyses, inputs expression matrix and design vector/table, outputs list of p-values, fold changes and filtrated (according to auto-calculated p-value cutoff) expression matrix containing significantly up/down regulated genes only;
- ***"myANOVA_MC.R"*** – parallel, multi-core version of ANOVA statistical analyses. Usage of this script may significantly reduce the calculation time. Requires Linux doMC library or customized distribution of R from REvolution Computing (GPL for Linux, restricted for Windows). Input fully and output fully compatible with sequential implementation - expression matrix and design vector/table, outputs list of p-values, fold changes and filtrated (according to auto-calculated p-value cut-off) expression matrix containing significantly up/down regulated genes only. Usage requires editing ***"MMpred.R"*** script. Substituting sequential for parallel implementation recommended for input mRNA expression matrix only, since predictor generated pseudo-expression matrixes are much smaller and probably the time lost on distributing the tasks between the cores would exceed time gained on parallelization;
- ***"myTtest.R"*** – t-test statistical analyses, inputs expression matrix and design vector, outputs list of p-values, fold changes and filtrated (according to auto-calculated p-

value cut-off) expression matrix containing significantly up/down regulated genes only. Suitable for simple sample/control designed microarray experiments.

- ***"getHT.R"*** – function for extracting mRNA targets or host genes from correlation matrix, inputs correlation matrix, array type, cut-off value and parameter informing if targets or hosts should be outputted, outputs annotated lists of interactions ;
- ***"myGO.R"*** – this script uses BioConductor GeneAnswers library to test Gene Ontology, KEGG pathways, Disease Light ontology and user required terms overrepresentation in given list of genes and generate graphical output (pay charts, bar charts, concept maps, heatmaps). The input is gene list considered to be up/down-regulated thanks to miRNA differential expression (output of ***"getHT.R"*** *script*).

Beside function scripts the MMpred package provides the user with pre-calculated R binary files (not included into supplementary materials due to vast size). Those files contain mapping and linear model, which are loaded during the pipeline call. It should be noted, that the pipeline scripts provide methods to recalculate both mapping and linear model. However, in most of the cases those values should be loaded from binaries because of the significant reduction in both time and resources consumption of the pipeline instance. Those files are:

- ***"model_140x260_t.rda"*** – pre-calculated linear model
- ***"mart_map.rda"*** – pre-calculated mapping

A.2 The MMpred prerequisites and outside libraries

The pipeline utilises numerous third party libraries, of which the most is the part of BioConductor project. Before launching any of the pipeline's scripts the user must install the following R libraries (also known as packages):

- Affy
- Biostrings
- R2HTML
- GeneAnswers
- GenePlotter
- Fields
- HGU133plus2.db
- GO.db
- Heatplus
- MiRBase.db
- BiomaRt

The suggested method of installation is using biocLite() or install.packeges() function – this will ensure the current version is being installed. However, manual installation of those packages is also possible. Failing to load any of these libraries would terminate the pipeline with appropriate error informing the user which dependence is missing. MiRBase.db and BiomaRt libraries are required only for mapping matrix recalculation.

A.3 Interfaces to access the pipeline

A.3.1 R terminal

The basic interface for the pipeline is standard R command-line function. The function is the batch file which executes other functions responsible for each step of analyses and controls the flow of the pipeline according to user defined parameters. The HTML report generating is also handled by this function.

Summary

This function takes as input raw or processed mRNA microarray experiment and outputs miRNA-mRAN functional interaction report.

Usage

```
MMpred(e.in, description, ANOVA, READ_RAW, RELOAD, RAW_DIR,
HTML_dir, HTML_file, TERMS, CORR_CUTOFF = 0.8)
```

Arguments

- ***e.in*** **-** mRNA expression matrix driven from microarray experiment;
- ***description*** **-** Description vector of microarray experiment;
- ***ANOVA*** **-** *'TRUE'* to perform ANOVA or *'FALSE'* to perform Student's t-test statistics;
- ***READ_RAW*** **-** *'TRUE'* to load raw microarry experimnt files (CEL format) or *'FALSE'* to use pre-processed mRAN expression matrix given in ***e.in*** argument;
- ***RELOAD*** **-** *'TRUE'* to reload pre-calculated data (miRNA-mRAN mapping and linear model);
- ***RAW_DIR*** - CEL files directory (omitted if ***READ_RAW*** set to *'FALSE'*);
- ***HTML_dir*** **-** Specify directory to output HTML report;s
- ***HTML_file*** - Specify name of HTML report file;
- ***TERMS*** - ENTREZ terms to be tested for overrepresentation;
- ***CORR_CUTOFF*** **-** Case specific value of correlation cutoff (*default 0.8*).

Output

The MMpred outputs HTML report to the working directory. In addition the structure containing predictions of mRNA-miRNA interactions is returned to R workspace.

A.3.2 R graphical user interface (GUI)

The user friendly interface to run the pipeline is shown in **Figure 0.2**. This interface has been designed for less advanced users, not familiar with R terminal. However, it provides the same functionality as the command line interface – the input parameters may be passed to the pipeline in graphical manner. Additionally, all generated figures are available to view within the GUI, which gives users a quick overview of pipeline performance.

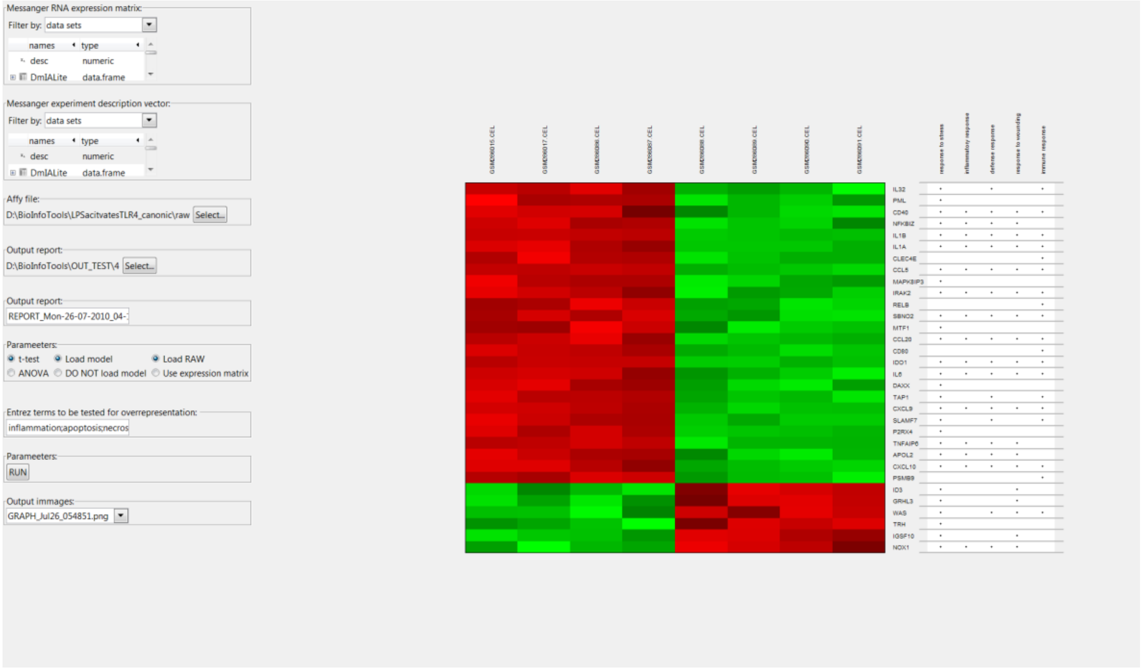

Figure 0.2 The screenshot presenting R Gtk+ graphical user interface.

A.3.3 R server - Java client application programming interface

The most advanced of the developed interfaces is intended to create a web-based implementation of the pipeline using R as the computational engine and Java Web as the way to communicate with the user. In this model the application server is running an

instance of R. The server functionality – handling incoming commands and returning output to remote location is handled by the Rserve library. The simplest model does not imply using Java at all. The client is handled by R running in client mode on the user's computer. The Application server running R in server mode connects to the file server in order to access the pre-calculated binaries and input files. The user must firstly import the data into the local instance of R or send them to the file server via FTP or SCP. This model is not recommended to be used via a public network, because of tremendous danger it creates for infrastructure running R sever. The connection and authentication is not encrypted, there is no real possibility to effectively protect the local network by firewall. The user would be able to run any R command including system(), which may be easily abused. For that reason this model is advised to be used only in the local network (**Figure 0.3**).

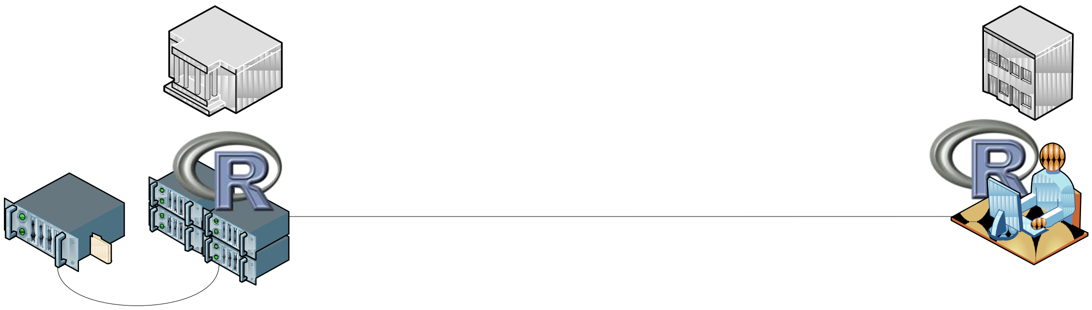

Figure 0.3 The simplest remote R model – R running on application server in server mode and on user's machine in client mode. The files server is also featured.

A more advanced model utilizes Java local application, Java web start application or web page placed applet. These approaches differ from the user point of view, but infrastructure and implementation is identical for all of them. The Java based client application is running on the user's local machine and connects to the application server running R in server mode. All file transfer operation, as well as input/output is handled by the client. This approach creates a much friendlier user environment, but still poses a threat for server infrastructure. In this case the firewall can be effectively used and the user is able to run only commands that have been implemented into the client application, the network traffic, as well as authentication remains unencrypted. The encrypted

connection can be achieved by adding intermediate application to application server and implementing suitable functionality to the client (**Figure 0.4**).

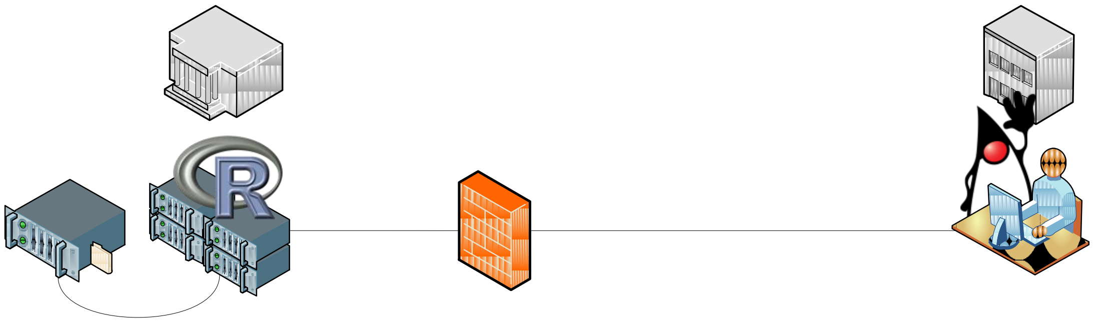

Figure 0.4 More advanced model – the client application is running on user's machine as desktop Java application, Java Web Start or Java applet. The usage of firewall is featured.

The most advanced model extends the previous model infrastructure by Java web application server. This server performs a double function – acting both as a client for R running in server mode on the application server, and a web and web services server processing users request and responding with output. Although those two applications may work on the same system it is strongly recommended to separate them although into two different physical or virtual machines. The suggested java application server is Tomcat or Glassfish, depending on what functionality would be required. This approach presents the most secure and user friendly interface (**Figure 0.5**)The basic users can access the pipeline via a web browser, while more advanced users are able to create their own programs and scripts using web services infracts, such as SOAP or REST. Both use the firewall and encryption is possible, and even in the case of attack the separation of application server from web server will minimize potential damages and data loss.

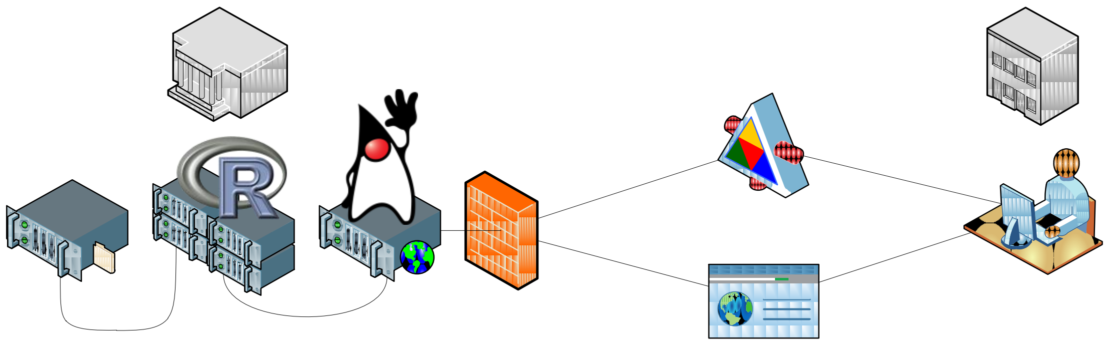

Figure 0.5 The most advanced infrastructure – both R server and java web server are running behind a firewall allowing user to access pipeline via web browser or web services.

A.4 Hardware and software specifications

Different software and hardware platforms have been tested during the development. Since R is universal and highly portable environment and fully backward compatible scripting language the pipeline can be used on any machine capable of running R client. The R software compiles and runs on a wide variety of UNIX platforms and Windows. However, it is suggested, that machine running the pipeline had at least 2GB of operating memory.

Most of the testing and development work have been done on different rack server class computers available at GlaxoSmithKline. All machines ware running Red Hat Enterprise Linux. The hardware specification differed from 8 to 32 Intel Xeon Quad-Core 3GHz (E5450) processors (32 to 128 operational cores) and from 32 to 128 GB of RAM. The machines used common file servers, so R and BioConductor version and configuration reminded the same. Also two desktop class machines have been used mostly for testing purposes:

- Lenovo T61p laptop (Intel Core 2 Duo T7500 @ 2,2 GHz, 4 GB RAM, Microsoft Windows 7 Professional 64-bit)
- HP Desktop Workstation (Intel Core 2 Duo, 2 GB RAM, Microsoft Windows XP 32-bit)

- Apple Mac Pro (2 x 3 GHz Quad-Core Intel Xeon, 32 GB RAM, Mac Os X 10.6 Snow Leopard)

All the machines were able to run the pipeline for simple cases and successfully accomplish the analyses. T61p laptop was able to run the analyses for the big burn and blunt datasets (187 and 184 arrays), however the pre-processing (CEL files processing, normalization, summarization and expression matrix extraction) had to be done on the machine 4.0 GB of RAM installed.

Both 32 bit and 64 bit version of R have been tested. For most of the purposes the 32 bit version is recommended (only this version can run GUI). However, if the user is working with extremely big detests (more than 250 arrays) the 64 bit version may be required, since there is a possibility of allocating variables bigger than 4.0 GB.

During the pipeline development the version of R has been changed R version from 2.10 to 2.11.1. Also the BioConductor have been updated from version 2.5 to 2.6. The parts of code developed using different versions work perfectly together – this proof the backward compatibility of R software. The software has been tested to run with every major R version up to current 2.14.0.
