# Supplementary material for: MMpred: functional miRNA – mRNA interaction analyses by miRNA expression prediction
Source: BMC Genomics. 2012 Nov 14;13:620. doi: 10.1186/1471-2164-13-620 (PMC3562514; doi:10.1186/1471-2164-13-620)
Supplement: Additional file 3 — Sample pipeline outputs in HTML format (compressed file). [file 1471-2164-13-620-S3.ZIP › TL4+LPS_activated/REPORT_LPSv4.html]

REPORT


## Report of miRNA-mRNA interactions for all arrays. [generated on 2010-07-21 19:03:11]

---

Statistical testing for messanger RNA arrays: 736 genes found significanltly up/downregulated . Details:

|  | ArrayFile | FunctionalGroup |
| --- | --- | --- |
| 1 | GSM286015.CEL | 1 |
| 2 | GSM286017.CEL | 1 |
| 3 | GSM286086.CEL | 1 |
| 4 | GSM286087.CEL | 1 |
| 5 | GSM286088.CEL | 2 |
| 6 | GSM286089.CEL | 2 |
| 7 | GSM286090.CEL | 2 |
| 8 | GSM286091.CEL | 2 |

  

Heatmap for top 50 geneses from statuistic analises (ordered by p-value):

Voolcano plot with for auto cutoff calculation audit (cutoff shown with red line):

---

Statistical testing for microRNA prediction method I - liniar modeling: 47 genes found significanltly up/downregulated . Details:

Heatmap for top 50 geneses from statuistic analises (ordered by p-value):

Voolcano plot with for auto cutoff calculation audit (cutoff shown with red line):

---

Statistical testing for microRNA prediction method II - scaling function: 26 genes found significanltly up/downregulated . Details:

Heatmap for top 50 geneses from statuistic analises (ordered by p-value):

Voolcano plot with for auto cutoff calculation audit (cutoff shown with red line):

---

Mean anti-correlation detected between mRNA and miRNA = -0.833773 . Details:

Histogram of most anti-correlated miRNA-mRNA pairs - potential mirNA-target intercations:

---

Total number of 73 miRNAs are predicted to have significantly downregulated targets. Expend:

|  | microRNA | NoSuppresedGenes |
| --- | --- | --- |
| 1 | hsa-miR-621 | 257 |
| 2 | hsa-miR-765 | 257 |
| 3 | hsa-mir-26a-1 | 257 |
| 4 | hsa-miR-449a | 256 |
| 5 | hsa-mir-449b | 256 |
| 6 | hsa-mir-449c | 256 |
| 7 | hsa-mir-194-2 | 254 |
| 8 | hsa-miR-770-5p | 253 |
| 9 | hsa-miR-449b | 251 |
| 10 | hsa-mir-155 | 251 |
| 11 | hsa-mir-22 | 251 |
| 12 | hsa-miR-340 | 250 |
| 13 | hsa-miR-608 | 250 |
| 14 | hsa-mir-340 | 250 |
| 15 | hsa-miR-1 | 249 |
| 16 | hsa-miR-133a | 249 |
| 17 | hsa-miR-186 | 249 |
| 18 | hsa-miR-26b | 249 |
| 19 | hsa-mir-10a | 249 |
| 20 | hsa-mir-186 | 249 |
| 21 | hsa-mir-26b | 249 |
| 22 | hsa-miR-152 | 244 |
| 23 | hsa-miR-499-5p | 240 |
| 24 | hsa-miR-877 | 238 |
| 25 | hsa-mir-877 | 233 |
| 26 | hsa-miR-31 | 232 |
| 27 | hsa-mir-31 | 232 |
| 28 | hsa-miR-10a | 230 |
| 29 | hsa-mir-499 | 227 |
| 30 | hsa-miR-326 | 225 |
| 31 | hsa-miR-575 | 223 |
| 32 | hsa-miR-338-5p | 220 |
| 33 | hsa-miR-657 | 220 |
| 34 | hsa-mir-3065 | 220 |
| 35 | hsa-mir-338 | 220 |
| 36 | hsa-miR-620 | 217 |
| 37 | hsa-miR-26a | 216 |
| 38 | hsa-miR-579 | 209 |
| 39 | hsa-miR-548b-5p | 199 |
| 40 | hsa-mir-548b | 199 |
| 41 | hsa-mir-2116 | 162 |
| 42 | hsa-miR-564 | 147 |
| 43 | hsa-mir-3190 | 146 |
| 44 | hsa-miR-139-5p | 136 |
| 45 | hsa-miR-490-5p | 128 |
| 46 | hsa-mir-490 | 128 |
| 47 | hsa-mir-423 | 127 |
| 48 | hsa-mir-550-1 | 127 |
| 49 | hsa-miR-551a | 126 |
| 50 | hsa-miR-885-5p | 124 |
| 51 | hsa-mir-885 | 124 |
| 52 | hsa-miR-562 | 118 |
| 53 | hsa-miR-550 | 109 |
| 54 | hsa-miR-423-5p | 108 |
| 55 | hsa-miR-637 | 105 |
| 56 | hsa-miR-383 | 103 |
| 57 | hsa-mir-548d-2 | 94 |
| 58 | hsa-miR-208a | 88 |
| 59 | hsa-let-7a-3 | 76 |
| 60 | hsa-let-7b | 76 |
| 61 | hsa-miR-663 | 71 |
| 62 | hsa-miR-584 | 68 |
| 63 | hsa-mir-3186 | 65 |
| 64 | hsa-miR-634 | 57 |
| 65 | hsa-miR-653 | 57 |
| 66 | hsa-miR-95 | 56 |
| 67 | hsa-miR-195 | 54 |
| 68 | hsa-miR-497 | 54 |
| 69 | hsa-miR-644 | 52 |
| 70 | hsa-miR-1228\* | 50 |
| 71 | hsa-miR-489 | 48 |
| 72 | hsa-miR-33a | 46 |
| 73 | hsa-miR-504 | 15 |

---

Total number of 507 genes are predicted to be undr miRNA repression. Expend:

|  | GenSymbols | GeneName | NoTargetingMicroRNA |
| --- | --- | --- | --- |
| 1 | FAS | Fas (TNF receptor superfamily, member 6) | 42 |
| 2 | PLXNC1 | plexin C1 | 36 |
| 3 | REL | signal-regulatory protein alpha | 36 |
| 4 | SIRPA | v-rel reticuloendotheliosis viral oncogene homolog (avian) | 36 |
| 5 | NUB1 | TAP binding protein (tapasin) | 35 |
| 6 | TAPBP | negative regulator of ubiquitin-like proteins 1 | 35 |
| 7 | BCL2A1 | BCL2-related protein A1 | 34 |
| 8 | DOT1L | DOT1-like, histone H3 methyltransferase (S. cerevisiae) | 34 |
| 9 | GPR114 | G protein-coupled receptor 114 | 34 |
| 10 | ISG20 | interferon stimulated exonuclease gene 20kDa | 34 |
| 11 | NFKB2 | nuclear factor of kappa light polypeptide gene enhancer in B-cells 2 (p49/p100) | 34 |
| 12 | NFKBIE | nuclear factor of kappa light polypeptide gene enhancer in B-cells inhibitor, epsilon | 34 |
| 13 | SERPINB9 | serpin peptidase inhibitor, clade B (ovalbumin), member 9 | 34 |
| 14 | SLC1A4 | solute carrier family 1 (glutamate/neutral amino acid transporter), member 4 | 34 |
| 15 | AIMP2 | BCL2-antagonist/killer 1 | 33 |
| 16 | BAK1 | CASP8 and FADD-like apoptosis regulator | 33 |
| 17 | CASP7 | EH-domain containing 1 | 33 |
| 18 | CFLAR | G protein-coupled receptor 132 | 33 |
| 19 | CXCL10 | GC-rich promoter binding protein 1 | 33 |
| 20 | EHD1 | OTU domain containing 4 | 33 |
| 21 | GBP1 | RAP2C, member of RAS oncogene family | 33 |
| 22 | GPBP1 | SLAM family member 7 | 33 |
| 23 | GPR132 | aminoacyl tRNA synthetase complex-interacting multifunctional protein 2 | 33 |
| 24 | INHBA | caspase 7, apoptosis-related cysteine peptidase | 33 |
| 25 | LACTB | chemokine (C-X-C motif) ligand 10 | 33 |
| 26 | MAP2K3 | guanylate binding protein 1, interferon-inducible, 67kDa | 33 |
| 27 | OTUD4 | inhibin, beta A | 33 |
| 28 | RAP2C | lactamase, beta | 33 |
| 29 | RARRES3 | mitogen-activated protein kinase kinase 3 | 33 |
| 30 | SLAMF7 | retinoic acid receptor responder (tazarotene induced) 3 | 33 |
| 31 | UBE2H | tryptophanyl-tRNA synthetase | 33 |
| 32 | WARS | ubiquitin-conjugating enzyme E2H (UBC8 homolog, yeast) | 33 |
| 33 | CCL5 | 5'-3' exoribonuclease 1 | 32 |
| 34 | CLEC4E | C-type lectin domain family 4, member E | 32 |
| 35 | DAXX | OTU domain containing 5 | 32 |
| 36 | GBP4 | Ras homolog enriched in brain like 1 | 32 |
| 37 | IL1A | chemokine (C-C motif) ligand 5 | 32 |
| 38 | JUN | death-domain associated protein | 32 |
| 39 | OTUD5 | guanylate binding protein 4 | 32 |
| 40 | PTGS2 | interleukin 1, alpha | 32 |
| 41 | RHEBL1 | jun oncogene | 32 |
| 42 | XRN1 | prostaglandin-endoperoxide synthase 2 (prostaglandin G/H synthase and cyclooxygenase) | 32 |
| 43 | ATP6V0E1 | ATPase, H+ transporting, lysosomal 9kDa, V0 subunit e1 | 31 |
| 44 | C9orf91 | CD40 molecule, TNF receptor superfamily member 5 | 31 |
| 45 | CCL20 | CDC42 small effector 1 | 31 |
| 46 | CD40 | RAB7, member RAS oncogene family-like 1 | 31 |
| 47 | CDC42SE1 | chemokine (C-C motif) ligand 20 | 31 |
| 48 | IFI27 | chromosome 9 open reading frame 91 | 31 |
| 49 | IL31RA | hypothetical protein LOC100287920 | 31 |
| 50 | IRAK2 | hypothetical protein LOC285758 | 31 |
| 51 | KDM4B | interferon, alpha-inducible protein 27 | 31 |
| 52 | LOC100133019 | interleukin 31 receptor A | 31 |
| 53 | LOC100287920 | interleukin-1 receptor-associated kinase 2 | 31 |
| 54 | LOC285758 | lysine (K)-specific demethylase 4B | 31 |
| 55 | PMAIP1 | phorbol-12-myristate-13-acetate-induced protein 1 | 31 |
| 56 | RAB7L1 | serine/threonine kinase 17b | 31 |
| 57 | SBNO2 | signal transducer and activator of transcription 1, 91kDa | 31 |
| 58 | SRD5A1 | similar to hCG1983765 | 31 |
| 59 | STAT1 | steroid-5-alpha-reductase, alpha polypeptide 1 (3-oxo-5 alpha-steroid delta 4-dehydrogenase alpha 1) | 31 |
| 60 | STK17B | strawberry notch homolog 2 (Drosophila) | 31 |
| 61 | TJP2 | tight junction protein 2 (zona occludens 2) | 31 |
| 62 | TNFAIP3 | tumor necrosis factor, alpha-induced protein 3 | 31 |
| 63 | TNFAIP6 | tumor necrosis factor, alpha-induced protein 6 | 31 |
| 64 | ZC3H12A | zinc finger CCCH-type containing 12A | 31 |
| 65 | ACSL4 | BCL2-like 14 (apoptosis facilitator) | 30 |
| 66 | B3GNT2 | CCR4 carbon catabolite repression 4-like (S. cerevisiae) | 30 |
| 67 | BAT2L2 | HLA-B associated transcript 2-like 2 | 30 |
| 68 | BCL2L14 | ISG15 ubiquitin-like modifier | 30 |
| 69 | CCRN4L | Janus kinase 1 | 30 |
| 70 | FAM49A | SCY1-like 3 (S. cerevisiae) | 30 |
| 71 | GBP5 | UDP-GlcNAc:betaGal beta-1,3-N-acetylglucosaminyltransferase 2 | 30 |
| 72 | HLA-F | Vac14 homolog (S. cerevisiae) | 30 |
| 73 | IL6 | acyl-CoA synthetase long-chain family member 4 | 30 |
| 74 | ISG15 | family with sequence similarity 49, member A | 30 |
| 75 | JAK1 | guanylate binding protein 5 | 30 |
| 76 | LOC100288387 | interleukin 6 (interferon, beta 2) | 30 |
| 77 | MREG | major histocompatibility complex, class I, F | 30 |
| 78 | NFKBIB | melanoregulin | 30 |
| 79 | P2RX4 | nuclear factor of kappa light polypeptide gene enhancer in B-cells inhibitor, beta | 30 |
| 80 | PPP3CC | protein phosphatase 3 (formerly 2B), catalytic subunit, gamma isoform | 30 |
| 81 | SCYL3 | purinergic receptor P2X, ligand-gated ion channel, 4 | 30 |
| 82 | SERPINB1 | serpin peptidase inhibitor, clade B (ovalbumin), member 1 | 30 |
| 83 | SLC15A4 | similar to c-jun | 30 |
| 84 | SLC22A6 | solute carrier family 15, member 4 | 30 |
| 85 | SYMPK | solute carrier family 22 (organic anion transporter), member 6 | 30 |
| 86 | TRIM56 | symplekin | 30 |
| 87 | VAC14 | tripartite motif-containing 56 | 30 |
| 88 | ARHGAP30 | C1q and tumor necrosis factor related protein 1 | 29 |
| 89 | B2M | CD274 molecule | 29 |
| 90 | BATF2 | CD80 molecule | 29 |
| 91 | C1QTNF1 | CDC42 effector protein (Rho GTPase binding) 2 | 29 |
| 92 | CCDC78 | ELOVL family member 7, elongation of long chain fatty acids (yeast) | 29 |
| 93 | CD274 | Rho GTPase activating protein 30 | 29 |
| 94 | CD80 | TNF receptor-associated factor 1 | 29 |
| 95 | CDC42EP2 | TNFAIP3 interacting protein 2 | 29 |
| 96 | CLDN5 | basic leucine zipper transcription factor, ATF-like 2 | 29 |
| 97 | CXCL9 | beta-2-microglobulin | 29 |
| 98 | DAPP1 | chemokine (C-X-C motif) ligand 9 | 29 |
| 99 | ELOVL7 | claudin 5 | 29 |
| 100 | FLT3LG | coiled-coil domain containing 78 | 29 |
| 101 | GBP3 | dual adaptor of phosphotyrosine and 3-phosphoinositides | 29 |
| 102 | IDO1 | fms-related tyrosine kinase 3 ligand | 29 |
| 103 | IDO2 | guanylate binding protein 3 | 29 |
| 104 | IFITM1 | hypothetical LOC100190939 | 29 |
| 105 | IL1B | indoleamine 2,3-dioxygenase 1 | 29 |
| 106 | LAD1 | indoleamine 2,3-dioxygenase 2 | 29 |
| 107 | LAMP3 | interferon induced transmembrane protein 1 (9-27) | 29 |
| 108 | LOC100190939 | interleukin 1, beta | 29 |
| 109 | MACF1 | ladinin 1 | 29 |
| 110 | MAP3K13 | lysosomal-associated membrane protein 3 | 29 |
| 111 | MT1F | metallothionein 1F | 29 |
| 112 | MT1X | metallothionein 1X | 29 |
| 113 | NFKBIZ | microtubule-actin crosslinking factor 1 | 29 |
| 114 | PIM3 | mitogen-activated protein kinase kinase kinase 13 | 29 |
| 115 | PML | nuclear factor of kappa light polypeptide gene enhancer in B-cells inhibitor, zeta | 29 |
| 116 | PPP4R1L | pim-3 oncogene | 29 |
| 117 | SLC37A1 | promyelocytic leukemia | 29 |
| 118 | SLC6A12 | protein phosphatase 4, regulatory subunit 1-like | 29 |
| 119 | TAP1 | solute carrier family 37 (glycerol-3-phosphate transporter), member 1 | 29 |
| 120 | TNIP2 | solute carrier family 6 (neurotransmitter transporter, betaine/GABA), member 12 | 29 |
| 121 | TRAF1 | trafficking protein particle complex 9 | 29 |
| 122 | TRAPPC9 | transporter 1, ATP-binding cassette, sub-family B (MDR/TAP) | 29 |
| 123 | ADAM19 | ADAM metallopeptidase domain 19 (meltrin beta) | 28 |
| 124 | ADAR | COX17 cytochrome c oxidase assembly homolog (S. cerevisiae) | 28 |
| 125 | ANKH | EH domain binding protein 1-like 1 | 28 |
| 126 | APOL2 | FK506 binding protein 6, 36kDa pseudogene | 28 |
| 127 | BRD4 | FtsJ methyltransferase domain containing 2 | 28 |
| 128 | CCNL1 | G protein-coupled receptor 137B | 28 |
| 129 | COX17 | MIR155 host gene (non-protein coding) | 28 |
| 130 | DMTF1 | Rap guanine nucleotide exchange factor (GEF) 2 | 28 |
| 131 | DND1 | TRAF-type zinc finger domain containing 1 | 28 |
| 132 | EHBP1L1 | UBX domain protein 1 | 28 |
| 133 | EIF4E2 | USP6 N-terminal like | 28 |
| 134 | FAM26F | WW domain binding protein 4 (formin binding protein 21) | 28 |
| 135 | FBRS | adenosine deaminase, RNA-specific | 28 |
| 136 | FTSJD2 | ankylosis, progressive homolog (mouse) | 28 |
| 137 | GBP2 | apolipoprotein L, 2 | 28 |
| 138 | GPR137B | bromodomain containing 4 | 28 |
| 139 | IL15 | cyclin D binding myb-like transcription factor 1 | 28 |
| 140 | IL32 | cyclin L1 | 28 |
| 141 | IRF1 | dead end homolog 1 (zebrafish) | 28 |
| 142 | LOC541473 | eukaryotic translation initiation factor 4E family member 2 | 28 |
| 143 | MAPRE3 | family with sequence similarity 26, member F | 28 |
| 144 | MGAT3 | fibrosin | 28 |
| 145 | MGC12916 | guanylate binding protein 2, interferon-inducible | 28 |
| 146 | MIR155HG | hypothetical protein MGC12916 | 28 |
| 147 | MT1H | interferon regulatory factor 1 | 28 |
| 148 | NFKBIA | interleukin 15 | 28 |
| 149 | PSMB9 | interleukin 32 | 28 |
| 150 | PSME2 | mannosyl (beta-1,4-)-glycoprotein beta-1,4-N-acetylglucosaminyltransferase | 28 |
| 151 | RAPGEF2 | metallothionein 1H | 28 |
| 152 | RHOF | microtubule-associated protein, RP/EB family, member 3 | 28 |
| 153 | RPL10 | nuclear factor of kappa light polypeptide gene enhancer in B-cells inhibitor, alpha | 28 |
| 154 | SDC4 | proteasome (prosome, macropain) activator subunit 2 (PA28 beta) | 28 |
| 155 | SLC12A3 | proteasome (prosome, macropain) subunit, beta type, 9 (large multifunctional peptidase 2) | 28 |
| 156 | TAP2 | ras homolog gene family, member F (in filopodia) | 28 |
| 157 | TCEB3 | ribosomal protein L10 | 28 |
| 158 | TNFAIP2 | solute carrier family 12 (sodium/chloride transporters), member 3 | 28 |
| 159 | TRAFD1 | syndecan 4 | 28 |
| 160 | UBXN1 | transcription elongation factor B (SIII), polypeptide 3 (110kDa, elongin A) | 28 |
| 161 | USP6NL | transporter 2, ATP-binding cassette, sub-family B (MDR/TAP) | 28 |
| 162 | WBP4 | tumor necrosis factor, alpha-induced protein 2 | 28 |
| 163 | ZNF548 | zinc finger protein 548 | 28 |
| 164 | APLP2 | DALR anticodon binding domain containing 3 | 27 |
| 165 | BBX | Epstein-Barr virus induced 3 | 27 |
| 166 | C19orf12 | GLI pathogenesis-related 2 | 27 |
| 167 | C5orf56 | GTP cyclohydrolase 1 | 27 |
| 168 | CCM2 | RNA binding motif protein 25 | 27 |
| 169 | COQ10B | RUN and FYVE domain containing 3 | 27 |
| 170 | CSRNP1 | SMAD specific E3 ubiquitin protein ligase 1 | 27 |
| 171 | DALRD3 | amyloid beta (A4) precursor-like protein 2 | 27 |
| 172 | EBI3 | bobby sox homolog (Drosophila) | 27 |
| 173 | FAM100B | cerebral cavernous malformation 2 | 27 |
| 174 | GCH1 | chromosome 19 open reading frame 12 | 27 |
| 175 | GLIPR2 | chromosome 5 open reading frame 56 | 27 |
| 176 | GUCY1B3 | coenzyme Q10 homolog B (S. cerevisiae) | 27 |
| 177 | HDAC10 | cysteine-serine-rich nuclear protein 1 | 27 |
| 178 | IFI6 | family with sequence similarity 100, member B | 27 |
| 179 | IL15RA | guanylate cyclase 1, soluble, beta 3 | 27 |
| 180 | IL2RG | histone deacetylase 10 | 27 |
| 181 | KREMEN1 | hypothetical LOC286359 | 27 |
| 182 | MAF | interferon, alpha-inducible protein 6 | 27 |
| 183 | MAPK8IP3 | interleukin 15 receptor, alpha | 27 |
| 184 | OPTN | interleukin 2 receptor, gamma (severe combined immunodeficiency) | 27 |
| 185 | PPM1K | kringle containing transmembrane protein 1 | 27 |
| 186 | RBM25 | mitogen-activated protein kinase 8 interacting protein 3 | 27 |
| 187 | RELB | optineurin | 27 |
| 188 | RUFY3 | protein phosphatase 1K (PP2C domain containing) | 27 |
| 189 | SMURF1 | sorting nexin 10 | 27 |
| 190 | SNX10 | unc-13 homolog A (C. elegans) | 27 |
| 191 | UNC13A | v-maf musculoaponeurotic fibrosarcoma oncogene homolog (avian) | 27 |
| 192 | ZNF646 | v-rel reticuloendotheliosis viral oncogene homolog B | 27 |
| 193 | ZNFX1 | zinc finger protein 646 | 27 |
| 194 | hCG\_2045089 | zinc finger, NFX1-type containing 1 | 27 |
| 195 | APOBEC3G | AT rich interactive domain 1B (SWI1-like) | 26 |
| 196 | ARID1B | COMM domain containing 6 | 26 |
| 197 | C11orf31 | RAB42, member RAS oncogene family | 26 |
| 198 | C1orf144 | SON DNA binding protein | 26 |
| 199 | C2orf85 | TNF receptor-associated factor 3 | 26 |
| 200 | COMMD6 | TNFAIP3 interacting protein 1 | 26 |
| 201 | CRTAP | apolipoprotein B mRNA editing enzyme, catalytic polypeptide-like 3G | 26 |
| 202 | CYB5R2 | cartilage associated protein | 26 |
| 203 | GALR3 | chromosome 1 open reading frame 144 | 26 |
| 204 | GNRHR | chromosome 11 open reading frame 31 | 26 |
| 205 | HES4 | chromosome 2 open reading frame 85 | 26 |
| 206 | HFE | cytochrome b5 reductase 2 | 26 |
| 207 | LHFP | galanin receptor 3 | 26 |
| 208 | LOC100128003 | gonadotropin-releasing hormone receptor | 26 |
| 209 | LRRFIP2 | hairy and enhancer of split 4 (Drosophila) | 26 |
| 210 | MAP4 | hemochromatosis | 26 |
| 211 | MGC39372 | hypothetical protein LOC100128003 | 26 |
| 212 | MT1E | hypothetical protein MGC39372 | 26 |
| 213 | MTF1 | leucine rich repeat (in FLII) interacting protein 2 | 26 |
| 214 | MYO1G | lipoma HMGIC fusion partner | 26 |
| 215 | PDK2 | metal-regulatory transcription factor 1 | 26 |
| 216 | RAB42 | metallothionein 1E | 26 |
| 217 | RELA | microtubule-associated protein 4 | 26 |
| 218 | RPL9 | myosin IG | 26 |
| 219 | SLC7A8 | pyruvate dehydrogenase kinase, isozyme 2 | 26 |
| 220 | SON | ribosomal protein L9 | 26 |
| 221 | TNIP1 | solute carrier family 7 (cationic amino acid transporter, y+ system), member 8 | 26 |
| 222 | TRAF3 | v-rel reticuloendotheliosis viral oncogene homolog A (avian) | 26 |
| 223 | VPS37C | vacuolar protein sorting 37 homolog C (S. cerevisiae) | 26 |
| 224 | YRDC | yrdC domain containing (E. coli) | 26 |
| 225 | ZGPAT | zinc finger, CCCH-type with G patch domain | 26 |
| 226 | APOL3 | CD97 molecule | 25 |
| 227 | B3GALT1 | FIP1 like 1 (S. cerevisiae) | 25 |
| 228 | C1orf100 | NLR family, CARD domain containing 5 | 25 |
| 229 | C20orf202 | PRO2055 | 25 |
| 230 | C6orf208 | Smg-7 homolog, nonsense mediated mRNA decay factor (C. elegans) | 25 |
| 231 | CAPRIN2 | TBC1 domain family, member 20 | 25 |
| 232 | CD97 | TROVE domain family, member 2 | 25 |
| 233 | CEACAM4 | UDP-Gal:betaGlcNAc beta 1,3-galactosyltransferase, polypeptide 1 | 25 |
| 234 | CELSR2 | WD repeat and SOCS box-containing 1 | 25 |
| 235 | CSF2 | apolipoprotein L, 3 | 25 |
| 236 | CTNNBIP1 | cadherin, EGF LAG seven-pass G-type receptor 2 (flamingo homolog, Drosophila) | 25 |
| 237 | CYB5A | caprin family member 2 | 25 |
| 238 | DUSP5 | carcinoembryonic antigen-related cell adhesion molecule 4 | 25 |
| 239 | FECH | catenin, beta interacting protein 1 | 25 |
| 240 | FIP1L1 | chromosome 1 open reading frame 100 | 25 |
| 241 | GADD45GIP1 | chromosome 20 open reading frame 202 | 25 |
| 242 | IGSF10 | chromosome 6 open reading frame 208 | 25 |
| 243 | KCNMB4 | colony stimulating factor 2 (granulocyte-macrophage) | 25 |
| 244 | KRTAP10-12 | cytochrome b5 type A (microsomal) | 25 |
| 245 | LOC100129449 | dual specificity phosphatase 5 | 25 |
| 246 | LOC100129637 | ferrochelatase (protoporphyria) | 25 |
| 247 | NBR2 | growth arrest and DNA-damage-inducible, gamma interacting protein 1 | 25 |
| 248 | NENF | hypothetical LOC100129637 | 25 |
| 249 | NLRC5 | immunoglobulin superfamily, member 10 | 25 |
| 250 | RPL27 | keratin associated protein 10-12 | 25 |
| 251 | RPS3A | neighbor of BRCA1 gene 2 (non-protein coding) | 25 |
| 252 | SLC12A4 | neuron derived neurotrophic factor | 25 |
| 253 | SLC24A4 | potassium large conductance calcium-activated channel, subfamily M, beta member 4 | 25 |
| 254 | SLC46A1 | ribosomal protein L27 | 25 |
| 255 | SMG7 | ribosomal protein S3A | 25 |
| 256 | STK33 | serine/threonine kinase 33 | 25 |
| 257 | TBC1D20 | solute carrier family 12 (potassium/chloride transporters), member 4 | 25 |
| 258 | TMEM41B | solute carrier family 24 (sodium/potassium/calcium exchanger), member 4 | 25 |
| 259 | TRIM13 | solute carrier family 46 (folate transporter), member 1 | 25 |
| 260 | TROVE2 | transmembrane protein 41B | 25 |
| 261 | UBE2L6 | tripartite motif-containing 13 | 25 |
| 262 | WSB1 | ubiquitin-conjugating enzyme E2L 6 | 25 |
| 263 | ZNF140 | zinc finger protein 140 | 25 |
| 264 | AGR2 | C-type lectin domain family 2, member D | 24 |
| 265 | C22orf37 | CD44 molecule (Indian blood group) | 24 |
| 266 | C8orf30A | CUB and zona pellucida-like domains 1 | 24 |
| 267 | CD44 | HCLS1 associated protein X-1 | 24 |
| 268 | CHAD | RAB12, member RAS oncogene family | 24 |
| 269 | CLEC2D | RER1 retention in endoplasmic reticulum 1 homolog (S. cerevisiae) | 24 |
| 270 | CUZD1 | TSC22 domain family, member 2 | 24 |
| 271 | FLJ20184 | anterior gradient homolog 2 (Xenopus laevis) | 24 |
| 272 | GRHL3 | chondroadherin | 24 |
| 273 | HAX1 | chromosome 22 open reading frame 37 | 24 |
| 274 | ID3 | chromosome 8 open reading frame 30A | 24 |
| 275 | IDH1 | grainyhead-like 3 (Drosophila) | 24 |
| 276 | IFT140 | hypothetical LOC285627 | 24 |
| 277 | IRS1 | hypothetical protein FLJ20184 | 24 |
| 278 | LOC285627 | hypothetical protein LOC286254 | 24 |
| 279 | LOC286254 | inhibitor of DNA binding 3, dominant negative helix-loop-helix protein | 24 |
| 280 | LRRC43 | insulin receptor substrate 1 | 24 |
| 281 | NFE2L1 | intraflagellar transport 140 homolog (Chlamydomonas) | 24 |
| 282 | NPHP1 | isocitrate dehydrogenase 1 (NADP+), soluble | 24 |
| 283 | NPHP4 | leucine rich repeat containing 43 | 24 |
| 284 | PAPPA | nephronophthisis 1 (juvenile) | 24 |
| 285 | PCNX | nephronophthisis 4 | 24 |
| 286 | PGBD3 | nuclear factor (erythroid-derived 2)-like 1 | 24 |
| 287 | PRLR | pecanex homolog (Drosophila) | 24 |
| 288 | PTGIR | piggyBac transposable element derived 3 | 24 |
| 289 | RAB12 | pregnancy-associated plasma protein A, pappalysin 1 | 24 |
| 290 | RER1 | prolactin receptor | 24 |
| 291 | RPL36A | prostaglandin I2 (prostacyclin) receptor (IP) | 24 |
| 292 | RTP3 | receptor (chemosensory) transporter protein 3 | 24 |
| 293 | RXRB | retinoid X receptor, beta | 24 |
| 294 | TRH | ribosomal protein L36a | 24 |
| 295 | TSC22D2 | thyrotropin-releasing hormone | 24 |
| 296 | ZNF419 | zinc finger protein 419 | 24 |
| 297 | AFF4 | AF4/FMR2 family, member 4 | 23 |
| 298 | ALDH3B1 | F-box protein 25 | 23 |
| 299 | C4orf12 | G protein-coupled receptor 115 | 23 |
| 300 | C6orf134 | GABA(A) receptor-associated protein | 23 |
| 301 | CCNB3 | M-phase phosphoprotein 9 | 23 |
| 302 | CXCR1 | RAS protein activator like 3 | 23 |
| 303 | CXorf40A | RAS-like, family 10, member A | 23 |
| 304 | DFNB59 | TSC22 domain family, member 3 | 23 |
| 305 | EYA1 | Wilms tumor 1 associated protein | 23 |
| 306 | FBXO25 | Wiskott-Aldrich syndrome (eczema-thrombocytopenia) | 23 |
| 307 | GABARAP | XK, Kell blood group complex subunit-related family, member 6 | 23 |
| 308 | GPR115 | aldehyde dehydrogenase 3 family, member B1 | 23 |
| 309 | HAPLN3 | chemokine (C-X-C motif) receptor 1 | 23 |
| 310 | IRS2 | chromosome 4 open reading frame 12 | 23 |
| 311 | KLC1 | chromosome 6 open reading frame 134 | 23 |
| 312 | LOC285796 | chromosome X open reading frame 40A | 23 |
| 313 | LOC338799 | cyclin B3 | 23 |
| 314 | LOC339894 | deafness, autosomal recessive 59 | 23 |
| 315 | MEI1 | eyes absent homolog 1 (Drosophila) | 23 |
| 316 | MFN2 | hyaluronan and proteoglycan link protein 3 | 23 |
| 317 | MPHOSPH9 | hypothetical LOC285796 | 23 |
| 318 | NBN | hypothetical LOC338799 | 23 |
| 319 | NEURL3 | hypothetical protein LOC339894 | 23 |
| 320 | OR2B6 | insulin receptor substrate 2 | 23 |
| 321 | PCSK2 | kinesin light chain 1 | 23 |
| 322 | POLH | meiosis inhibitor 1 | 23 |
| 323 | PROM2 | mitofusin 2 | 23 |
| 324 | RASAL3 | neuralized homolog 3 (Drosophila) pseudogene | 23 |
| 325 | RASL10A | nibrin | 23 |
| 326 | RMST | olfactory receptor, family 2, subfamily B, member 6 | 23 |
| 327 | RPS11 | polymerase (DNA directed), eta | 23 |
| 328 | RTN3 | prominin 2 | 23 |
| 329 | SCNN1G | proprotein convertase subtilisin/kexin type 2 | 23 |
| 330 | SLC26A7 | reticulon 3 | 23 |
| 331 | SPATA4 | rhabdomyosarcoma 2 associated transcript (non-protein coding) | 23 |
| 332 | TSC22D3 | ribosomal protein S11 | 23 |
| 333 | USP24 | sodium channel, nonvoltage-gated 1, gamma | 23 |
| 334 | WAS | solute carrier family 26, member 7 | 23 |
| 335 | WTAP | spermatogenesis associated 4 | 23 |
| 336 | XKR6 | ubiquitin specific peptidase 24 | 23 |
| 337 | ZNF571 | zinc finger protein 571 | 23 |
| 338 | ZNF573 | zinc finger protein 573 | 23 |
| 339 | ZNF615 | zinc finger protein 615 | 23 |
| 340 | ADCK2 | BTB (POZ) domain containing 9 | 22 |
| 341 | ANGEL1 | Cdc42 guanine nucleotide exchange factor (GEF) 9 | 22 |
| 342 | AP2A2 | F-box protein 31 | 22 |
| 343 | ARHGEF9 | LYR motif containing 1 | 22 |
| 344 | AZGP1P1 | NADPH oxidase 1 | 22 |
| 345 | BTBD9 | RAB26, member RAS oncogene family | 22 |
| 346 | C1orf128 | RALBP1 associated Eps domain containing 2 | 22 |
| 347 | CYP3A43 | Sad1 and UNC84 domain containing 1 | 22 |
| 348 | DLEU7 | WNK lysine deficient protein kinase 2 | 22 |
| 349 | EML2 | aarF domain containing kinase 2 | 22 |
| 350 | FBXO31 | adaptor-related protein complex 2, alpha 2 subunit | 22 |
| 351 | FOLH1 | alpha-2-glycoprotein 1, zinc-binding pseudogene 1 | 22 |
| 352 | FOXQ1 | angel homolog 1 (Drosophila) | 22 |
| 353 | GNAQ | chromosome 1 open reading frame 128 | 22 |
| 354 | HGSNAT | cytochrome P450, family 3, subfamily A, polypeptide 43 | 22 |
| 355 | ITGB5 | deleted in lymphocytic leukemia, 7 | 22 |
| 356 | KATNAL2 | echinoderm microtubule associated protein like 2 | 22 |
| 357 | LOC158960 | folate hydrolase (prostate-specific membrane antigen) 1 | 22 |
| 358 | LOC255480 | forkhead box Q1 | 22 |
| 359 | LYRM1 | guanine nucleotide binding protein (G protein), q polypeptide | 22 |
| 360 | MMAB | heparan-alpha-glucosaminide N-acetyltransferase | 22 |
| 361 | MYT1 | hypothetical protein BC009467 | 22 |
| 362 | NOX1 | hypothetical protein LOC255480 | 22 |
| 363 | OLFML2B | integrin, beta 5 | 22 |
| 364 | OR2F1 | katanin p60 subunit A-like 2 | 22 |
| 365 | PACS2 | methylmalonic aciduria (cobalamin deficiency) cblB type | 22 |
| 366 | PLA2G10 | myelin transcription factor 1 | 22 |
| 367 | PPAPDC3 | olfactomedin-like 2B | 22 |
| 368 | RAB26 | olfactory receptor, family 2, subfamily F, member 1 | 22 |
| 369 | REPS2 | phosphatidic acid phosphatase type 2 domain containing 3 | 22 |
| 370 | SLC38A11 | phosphofurin acidic cluster sorting protein 2 | 22 |
| 371 | SLC45A4 | phosphoinositide-interacting regulator of transient receptor potential channels | 22 |
| 372 | SUN1 | phospholipase A2, group X | 22 |
| 373 | TMEM49 | solute carrier family 38, member 11 | 22 |
| 374 | TTC12 | solute carrier family 45, member 4 | 22 |
| 375 | VAT1 | tetratricopeptide repeat domain 12 | 22 |
| 376 | WNK2 | transmembrane protein 49 | 22 |
| 377 | ZNF527 | vesicle amine transport protein 1 homolog (T. californica) | 22 |
| 378 | hCG\_1776018 | zinc finger protein 527 | 22 |
| 379 | ASB10 | BAI1-associated protein 2 | 21 |
| 380 | BAIAP2 | FH2 domain containing 1 | 21 |
| 381 | BPTF | G protein-coupled receptor 107 | 21 |
| 382 | C5orf4 | G protein-coupled receptor 157 | 21 |
| 383 | CRTAC1 | G protein-coupled receptor 81 | 21 |
| 384 | DACT2 | PDZ domain containing 3 | 21 |
| 385 | DOK7 | UDP-N-acetyl-alpha-D-galactosamine:polypeptide N-acetylgalactosaminyltransferase-like 2 | 21 |
| 386 | DUSP4 | ankyrin repeat and SOCS box-containing 10 | 21 |
| 387 | FHDC1 | bromodomain PHD finger transcription factor | 21 |
| 388 | FRAT2 | cartilage acidic protein 1 | 21 |
| 389 | GALNTL2 | chromosome 5 open reading frame 4 | 21 |
| 390 | GPR107 | dapper, antagonist of beta-catenin, homolog 2 (Xenopus laevis) | 21 |
| 391 | GPR157 | docking protein 7 | 21 |
| 392 | GPR81 | dual specificity phosphatase 4 | 21 |
| 393 | GSTM5 | frequently rearranged in advanced T-cell lymphomas 2 | 21 |
| 394 | IGSF3 | glutathione S-transferase mu 5 | 21 |
| 395 | OR52D1 | immunoglobulin superfamily, member 3 | 21 |
| 396 | PARP10 | olfactory receptor, family 52, subfamily D, member 1 | 21 |
| 397 | PDZD3 | poly (ADP-ribose) polymerase family, member 10 | 21 |
| 398 | SLCO2B1 | solute carrier organic anion transporter family, member 2B1 | 21 |
| 399 | SPAG11B | spectrin repeat containing, nuclear envelope 1 | 21 |
| 400 | SYNE1 | sperm associated antigen 11B | 21 |
| 401 | UMPS | uridine monophosphate synthetase | 21 |
| 402 | ZC3H7B | zinc finger CCCH-type containing 7B | 21 |
| 403 | ZNF780B | zinc finger protein 780B | 21 |
| 404 | AQP10 | ATPase, H+ transporting, lysosomal 34kDa, V1 subunit D | 20 |
| 405 | ATP6V1D | Dresden prostate cancer 2 | 20 |
| 406 | C15orf21 | GTP binding protein 1 | 20 |
| 407 | COL1A1 | aquaporin 10 | 20 |
| 408 | DPP7 | collagen, type I, alpha 1 | 20 |
| 409 | FMO5 | dipeptidyl-peptidase 7 | 20 |
| 410 | FOXN4 | flavin containing monooxygenase 5 | 20 |
| 411 | GTPBP1 | forkhead box N4 | 20 |
| 412 | HRSP12 | heat-responsive protein 12 | 20 |
| 413 | IFNA17 | hypothetical LOC400794 | 20 |
| 414 | KCNQ1 | hypothetical LOC414300 | 20 |
| 415 | LOC400794 | interferon, alpha 17 | 20 |
| 416 | LOC414300 | peptidase inhibitor 15 | 20 |
| 417 | P2RX1 | phospholipase A2, group IVC (cytosolic, calcium-independent) | 20 |
| 418 | PDK4 | potassium voltage-gated channel, KQT-like subfamily, member 1 | 20 |
| 419 | PI15 | purinergic receptor P2X, ligand-gated ion channel, 1 | 20 |
| 420 | PLA2G4C | pyruvate dehydrogenase kinase, isozyme 4 | 20 |
| 421 | RXFP3 | relaxin/insulin-like family peptide receptor 3 | 20 |
| 422 | SLC26A11 | slit homolog 1 (Drosophila) | 20 |
| 423 | SLIT1 | smu-1 suppressor of mec-8 and unc-52 homolog (C. elegans) | 20 |
| 424 | SMU1 | solute carrier family 26, member 11 | 20 |
| 425 | SUV39H1 | suppressor of variegation 3-9 homolog 1 (Drosophila) | 20 |
| 426 | TUB | tubby homolog (mouse) | 20 |
| 427 | ZC3H7A | zinc finger CCCH-type containing 7A | 20 |
| 428 | ACRBP | KIAA0391 | 19 |
| 429 | AKR1C2 | Kruppel-like factor 15 | 19 |
| 430 | ALDH3A2 | MPV17 mitochondrial membrane protein-like | 19 |
| 431 | BMP8A | NDRG family member 2 | 19 |
| 432 | C14orf133 | TBC1 domain family, member 5 | 19 |
| 433 | C20orf62 | acrosin binding protein | 19 |
| 434 | CPA6 | aldehyde dehydrogenase 3 family, member A2 | 19 |
| 435 | CSNK1A1 | aldo-keto reductase family 1, member C2 (dihydrodiol dehydrogenase 2; bile acid binding protein; 3-alpha hydroxysteroid dehydrogenase, type III) | 19 |
| 436 | CYP2B6 | bone morphogenetic protein 8a | 19 |
| 437 | FNBP1 | carboxypeptidase A6 | 19 |
| 438 | GHRHR | casein kinase 1, alpha 1 | 19 |
| 439 | IRF7 | chromosome 14 open reading frame 133 | 19 |
| 440 | KCNS2 | chromosome 20 open reading frame 62 | 19 |
| 441 | KIAA0391 | cytochrome P450, family 2, subfamily B, polypeptide 6 | 19 |
| 442 | KIF9 | formin binding protein 1 | 19 |
| 443 | KLF15 | growth hormone releasing hormone receptor | 19 |
| 444 | LOC257358 | hypothetical LOC257358 | 19 |
| 445 | MAP2K6 | interferon regulatory factor 7 | 19 |
| 446 | MPV17L | kinesin family member 9 | 19 |
| 447 | MRVI1 | mitogen-activated protein kinase kinase 6 | 19 |
| 448 | MYLK4 | murine retrovirus integration site 1 homolog | 19 |
| 449 | NDRG2 | myosin light chain kinase family, member 4 | 19 |
| 450 | SLC6A16 | potassium voltage-gated channel, delayed-rectifier, subfamily S, member 2 | 19 |
| 451 | ST7L | solute carrier family 6, member 16 | 19 |
| 452 | TBC1D5 | suppression of tumorigenicity 7 like | 19 |
| 453 | TLR5 | toll-like receptor 5 | 19 |
| 454 | TNFSF12 | tumor necrosis factor (ligand) superfamily, member 12 | 19 |
| 455 | AMICA1 | ADP-ribosylation factor-like 5C | 18 |
| 456 | APOM | BAI1-associated protein 2-like 2 | 18 |
| 457 | ARL5C | HIG1 hypoxia inducible domain family, member 2A | 18 |
| 458 | ASB16 | NEL-like 2 (chicken) | 18 |
| 459 | BAIAP2L2 | SEC14-like 3 (S. cerevisiae) | 18 |
| 460 | C21orf88 | Sep (O-phosphoserine) tRNA:Sec (selenocysteine) tRNA synthase | 18 |
| 461 | C6orf123 | adhesion molecule, interacts with CXADR antigen 1 | 18 |
| 462 | CAPN9 | ankyrin repeat and SOCS box-containing 16 | 18 |
| 463 | HIGD2A | apolipoprotein M | 18 |
| 464 | LOC283194 | calpain 9 | 18 |
| 465 | NELL2 | chromosome 21 open reading frame 88 | 18 |
| 466 | NR3C2 | chromosome 6 open reading frame 123 | 18 |
| 467 | SAMD3 | hypothetical protein LOC283194 | 18 |
| 468 | SEC14L3 | nuclear receptor subfamily 3, group C, member 2 | 18 |
| 469 | SEPSECS | signal peptide peptidase 3 | 18 |
| 470 | UNQ1887 | sterile alpha motif domain containing 3 | 18 |
| 471 | UPB1 | ureidopropionase, beta | 18 |
| 472 | ZFP36L2 | zinc finger protein 36, C3H type-like 2 | 18 |
| 473 | ZNF747 | zinc finger protein 747 | 18 |
| 474 | ACTL7A | ATPase, H+ transporting, lysosomal accessory protein 2 | 17 |
| 475 | ATP6AP2 | UDP glycosyltransferase 3 family, polypeptide A1 | 17 |
| 476 | CDCA2 | X-prolyl aminopeptidase (aminopeptidase P) 2, membrane-bound | 17 |
| 477 | COL24A1 | actin-like 7A | 17 |
| 478 | DEFB4 | cell division cycle associated 2 | 17 |
| 479 | ECM2 | collagen, type XXIV, alpha 1 | 17 |
| 480 | EEPD1 | defensin, beta 4 | 17 |
| 481 | KCNA1 | endonuclease/exonuclease/phosphatase family domain containing 1 | 17 |
| 482 | LOC338694 | extracellular matrix protein 2, female organ and adipocyte specific | 17 |
| 483 | RPL28 | hypothetical protein LOC338694 | 17 |
| 484 | TMEM18 | potassium voltage-gated channel, shaker-related subfamily, member 1 (episodic ataxia with myokymia) | 17 |
| 485 | TMEM95 | ribosomal protein L28 | 17 |
| 486 | UGT3A1 | transmembrane protein 18 | 17 |
| 487 | XPNPEP2 | transmembrane protein 95 | 17 |
| 488 | ABCB5 | ATP-binding cassette, sub-family B (MDR/TAP), member 5 | 16 |
| 489 | CEACAM7 | PWP2 periodic tryptophan protein homolog (yeast) | 16 |
| 490 | CXorf38 | S100 calcium binding protein Z | 16 |
| 491 | IL5RA | carcinoembryonic antigen-related cell adhesion molecule 7 | 16 |
| 492 | LOC100287676 | chromosome X open reading frame 38 | 16 |
| 493 | PWP2 | hypothetical protein LOC100287676 | 16 |
| 494 | S100Z | interleukin 5 receptor, alpha | 16 |
| 495 | SHMT1 | serine hydroxymethyltransferase 1 (soluble) | 16 |
| 496 | SLC4A1 | solute carrier family 4, anion exchanger, member 1 (erythrocyte membrane protein band 3, Diego blood group) | 16 |
| 497 | SNAP29 | synaptosomal-associated protein, 29kDa | 16 |
| 498 | UQCRB | ubiquinol-cytochrome c reductase binding protein | 16 |
| 499 | CHML | UBX domain protein 11 | 15 |
| 500 | FKSG2 | apoptosis inhibitor | 15 |
| 501 | PIN4 | choroideremia-like (Rab escort protein 2) | 15 |
| 502 | SIRPB2 | protein (peptidylprolyl cis/trans isomerase) NIMA-interacting, 4 (parvulin) | 15 |
| 503 | SYCN | signal-regulatory protein beta 2 | 15 |
| 504 | TRPM3 | syncollin | 15 |
| 505 | UBXN11 | transient receptor potential cation channel, subfamily M, member 3 | 15 |
| 506 | ZFP2 | zinc finger protein 2 homolog (mouse) | 15 |
| 507 | SYTL3 | synaptotagmin-like 3 | 14 |

---

Total number of miRNA-mRNA 12581 interactions for fiven cutoff is. Press for ALL:

|  | miR | EntrezID | Gene | Name | Score |
| --- | --- | --- | --- | --- | --- |
| 5575 | hsa-miR-10a | 8837 | CFLAR | CASP8 and FADD-like apoptosis regulator | 9 |
| 5576 | hsa-miR-139-5p | 8837 | CFLAR | CASP8 and FADD-like apoptosis regulator | 9 |
| 5577 | hsa-miR-152 | 8837 | CFLAR | CASP8 and FADD-like apoptosis regulator | 9 |
| 5578 | hsa-miR-26a | 8837 | CFLAR | CASP8 and FADD-like apoptosis regulator | 9 |
| 5579 | hsa-miR-26b | 8837 | CFLAR | CASP8 and FADD-like apoptosis regulator | 9 |
| 5580 | hsa-miR-31 | 8837 | CFLAR | CASP8 and FADD-like apoptosis regulator | 9 |
| 5581 | hsa-miR-338-5p | 8837 | CFLAR | CASP8 and FADD-like apoptosis regulator | 9 |
| 5582 | hsa-miR-340 | 8837 | CFLAR | CASP8 and FADD-like apoptosis regulator | 9 |
| 5583 | hsa-miR-449a | 8837 | CFLAR | CASP8 and FADD-like apoptosis regulator | 9 |
| 5584 | hsa-miR-499-5p | 8837 | CFLAR | CASP8 and FADD-like apoptosis regulator | 9 |
| 5585 | hsa-miR-548b-5p | 8837 | CFLAR | CASP8 and FADD-like apoptosis regulator | 9 |
| 5587 | hsa-miR-621 | 8837 | CFLAR | CASP8 and FADD-like apoptosis regulator | 9 |
| 5589 | hsa-miR-657 | 8837 | CFLAR | CASP8 and FADD-like apoptosis regulator | 9 |
| 5590 | hsa-miR-765 | 8837 | CFLAR | CASP8 and FADD-like apoptosis regulator | 9 |
| 5591 | hsa-miR-770-5p | 8837 | CFLAR | CASP8 and FADD-like apoptosis regulator | 9 |
| 5592 | hsa-miR-877 | 8837 | CFLAR | CASP8 and FADD-like apoptosis regulator | 9 |
| 5593 | hsa-mir-26a-1 | 8837 | CFLAR | CASP8 and FADD-like apoptosis regulator | 9 |
| 5594 | hsa-mir-26b | 8837 | CFLAR | CASP8 and FADD-like apoptosis regulator | 9 |
| 5595 | hsa-mir-31 | 8837 | CFLAR | CASP8 and FADD-like apoptosis regulator | 9 |
| 5596 | hsa-mir-194-2 | 8837 | CFLAR | CASP8 and FADD-like apoptosis regulator | 9 |
| 5597 | hsa-mir-340 | 8837 | CFLAR | CASP8 and FADD-like apoptosis regulator | 9 |
| 5598 | hsa-mir-338 | 8837 | CFLAR | CASP8 and FADD-like apoptosis regulator | 9 |
| 5599 | hsa-mir-548b | 8837 | CFLAR | CASP8 and FADD-like apoptosis regulator | 9 |
| 5601 | hsa-mir-449b | 8837 | CFLAR | CASP8 and FADD-like apoptosis regulator | 9 |
| 5602 | hsa-mir-449c | 8837 | CFLAR | CASP8 and FADD-like apoptosis regulator | 9 |
| 5603 | hsa-mir-3065 | 8837 | CFLAR | CASP8 and FADD-like apoptosis regulator | 9 |
| 5586 | hsa-miR-564 | 8837 | CFLAR | CASP8 and FADD-like apoptosis regulator | 8 |
| 5600 | hsa-mir-548d-2 | 8837 | CFLAR | CASP8 and FADD-like apoptosis regulator | 7 |
| 1906 | hsa-miR-1 | 4094 | MAF | v-maf musculoaponeurotic fibrosarcoma oncogene homolog (avian) | 5 |
| 1907 | hsa-miR-133a | 4094 | MAF | v-maf musculoaponeurotic fibrosarcoma oncogene homolog (avian) | 5 |
| 1908 | hsa-miR-186 | 4094 | MAF | v-maf musculoaponeurotic fibrosarcoma oncogene homolog (avian) | 5 |
| 1909 | hsa-miR-326 | 4094 | MAF | v-maf musculoaponeurotic fibrosarcoma oncogene homolog (avian) | 5 |
| 1910 | hsa-miR-449b | 4094 | MAF | v-maf musculoaponeurotic fibrosarcoma oncogene homolog (avian) | 5 |
| 1913 | hsa-miR-575 | 4094 | MAF | v-maf musculoaponeurotic fibrosarcoma oncogene homolog (avian) | 5 |
| 1914 | hsa-miR-579 | 4094 | MAF | v-maf musculoaponeurotic fibrosarcoma oncogene homolog (avian) | 5 |
| 1915 | hsa-miR-608 | 4094 | MAF | v-maf musculoaponeurotic fibrosarcoma oncogene homolog (avian) | 5 |
| 1916 | hsa-miR-620 | 4094 | MAF | v-maf musculoaponeurotic fibrosarcoma oncogene homolog (avian) | 5 |
| 1919 | hsa-mir-22 | 4094 | MAF | v-maf musculoaponeurotic fibrosarcoma oncogene homolog (avian) | 5 |
| 1920 | hsa-mir-10a | 4094 | MAF | v-maf musculoaponeurotic fibrosarcoma oncogene homolog (avian) | 5 |
| 1921 | hsa-mir-186 | 4094 | MAF | v-maf musculoaponeurotic fibrosarcoma oncogene homolog (avian) | 5 |
| 1922 | hsa-mir-155 | 4094 | MAF | v-maf musculoaponeurotic fibrosarcoma oncogene homolog (avian) | 5 |
| 1924 | hsa-mir-499 | 4094 | MAF | v-maf musculoaponeurotic fibrosarcoma oncogene homolog (avian) | 5 |
| 1926 | hsa-mir-877 | 4094 | MAF | v-maf musculoaponeurotic fibrosarcoma oncogene homolog (avian) | 5 |
| 1912 | hsa-miR-562 | 4094 | MAF | v-maf musculoaponeurotic fibrosarcoma oncogene homolog (avian) | 4 |
| 1917 | hsa-miR-637 | 4094 | MAF | v-maf musculoaponeurotic fibrosarcoma oncogene homolog (avian) | 4 |
| 1918 | hsa-miR-885-5p | 4094 | MAF | v-maf musculoaponeurotic fibrosarcoma oncogene homolog (avian) | 4 |
| 1925 | hsa-mir-885 | 4094 | MAF | v-maf musculoaponeurotic fibrosarcoma oncogene homolog (avian) | 4 |
| 5462 | hsa-miR-10a | 10938 | EHD1 | EH-domain containing 1 | 4 |
| 5464 | hsa-miR-152 | 10938 | EHD1 | EH-domain containing 1 | 4 |
| 5465 | hsa-miR-26a | 10938 | EHD1 | EH-domain containing 1 | 4 |
| 5466 | hsa-miR-26b | 10938 | EHD1 | EH-domain containing 1 | 4 |
| 5467 | hsa-miR-31 | 10938 | EHD1 | EH-domain containing 1 | 4 |
| 5468 | hsa-miR-338-5p | 10938 | EHD1 | EH-domain containing 1 | 4 |
| 5469 | hsa-miR-340 | 10938 | EHD1 | EH-domain containing 1 | 4 |
| 5470 | hsa-miR-449a | 10938 | EHD1 | EH-domain containing 1 | 4 |
| 5471 | hsa-miR-499-5p | 10938 | EHD1 | EH-domain containing 1 | 4 |
| 5472 | hsa-miR-548b-5p | 10938 | EHD1 | EH-domain containing 1 | 4 |
| 5473 | hsa-miR-564 | 10938 | EHD1 | EH-domain containing 1 | 4 |
| 5474 | hsa-miR-621 | 10938 | EHD1 | EH-domain containing 1 | 4 |
| 5475 | hsa-miR-634 | 10938 | EHD1 | EH-domain containing 1 | 4 |
| 5476 | hsa-miR-653 | 10938 | EHD1 | EH-domain containing 1 | 4 |
| 5477 | hsa-miR-657 | 10938 | EHD1 | EH-domain containing 1 | 4 |
| 5478 | hsa-miR-765 | 10938 | EHD1 | EH-domain containing 1 | 4 |
| 5479 | hsa-miR-770-5p | 10938 | EHD1 | EH-domain containing 1 | 4 |
| 5480 | hsa-miR-877 | 10938 | EHD1 | EH-domain containing 1 | 4 |
| 5481 | hsa-mir-26a-1 | 10938 | EHD1 | EH-domain containing 1 | 4 |
| 5482 | hsa-mir-26b | 10938 | EHD1 | EH-domain containing 1 | 4 |
| 5483 | hsa-mir-31 | 10938 | EHD1 | EH-domain containing 1 | 4 |
| 5484 | hsa-mir-194-2 | 10938 | EHD1 | EH-domain containing 1 | 4 |
| 5485 | hsa-mir-340 | 10938 | EHD1 | EH-domain containing 1 | 4 |
| 5486 | hsa-mir-338 | 10938 | EHD1 | EH-domain containing 1 | 4 |
| 5487 | hsa-mir-548b | 10938 | EHD1 | EH-domain containing 1 | 4 |
| 5488 | hsa-mir-449b | 10938 | EHD1 | EH-domain containing 1 | 4 |
| 5489 | hsa-mir-449c | 10938 | EHD1 | EH-domain containing 1 | 4 |
| 5490 | hsa-mir-3065 | 10938 | EHD1 | EH-domain containing 1 | 4 |
| 5896 | hsa-miR-10a | 6509 | SLC1A4 | solute carrier family 1 (glutamate/neutral amino acid transporter), member 4 | 4 |
| 5897 | hsa-miR-139-5p | 6509 | SLC1A4 | solute carrier family 1 (glutamate/neutral amino acid transporter), member 4 | 4 |
| 5898 | hsa-miR-152 | 6509 | SLC1A4 | solute carrier family 1 (glutamate/neutral amino acid transporter), member 4 | 4 |
| 5899 | hsa-miR-26a | 6509 | SLC1A4 | solute carrier family 1 (glutamate/neutral amino acid transporter), member 4 | 4 |
| 5900 | hsa-miR-26b | 6509 | SLC1A4 | solute carrier family 1 (glutamate/neutral amino acid transporter), member 4 | 4 |
| 5901 | hsa-miR-31 | 6509 | SLC1A4 | solute carrier family 1 (glutamate/neutral amino acid transporter), member 4 | 4 |
| 5902 | hsa-miR-338-5p | 6509 | SLC1A4 | solute carrier family 1 (glutamate/neutral amino acid transporter), member 4 | 4 |
| 5903 | hsa-miR-340 | 6509 | SLC1A4 | solute carrier family 1 (glutamate/neutral amino acid transporter), member 4 | 4 |
| 5906 | hsa-miR-449a | 6509 | SLC1A4 | solute carrier family 1 (glutamate/neutral amino acid transporter), member 4 | 4 |
| 5907 | hsa-miR-499-5p | 6509 | SLC1A4 | solute carrier family 1 (glutamate/neutral amino acid transporter), member 4 | 4 |
| 5908 | hsa-miR-548b-5p | 6509 | SLC1A4 | solute carrier family 1 (glutamate/neutral amino acid transporter), member 4 | 4 |
| 5909 | hsa-miR-621 | 6509 | SLC1A4 | solute carrier family 1 (glutamate/neutral amino acid transporter), member 4 | 4 |
| 5910 | hsa-miR-657 | 6509 | SLC1A4 | solute carrier family 1 (glutamate/neutral amino acid transporter), member 4 | 4 |
| 5912 | hsa-miR-765 | 6509 | SLC1A4 | solute carrier family 1 (glutamate/neutral amino acid transporter), member 4 | 4 |
| 5913 | hsa-miR-770-5p | 6509 | SLC1A4 | solute carrier family 1 (glutamate/neutral amino acid transporter), member 4 | 4 |
| 5914 | hsa-miR-877 | 6509 | SLC1A4 | solute carrier family 1 (glutamate/neutral amino acid transporter), member 4 | 4 |
| 5915 | hsa-mir-26a-1 | 6509 | SLC1A4 | solute carrier family 1 (glutamate/neutral amino acid transporter), member 4 | 4 |
| 5916 | hsa-mir-26b | 6509 | SLC1A4 | solute carrier family 1 (glutamate/neutral amino acid transporter), member 4 | 4 |
| 5917 | hsa-mir-31 | 6509 | SLC1A4 | solute carrier family 1 (glutamate/neutral amino acid transporter), member 4 | 4 |
| 5918 | hsa-mir-194-2 | 6509 | SLC1A4 | solute carrier family 1 (glutamate/neutral amino acid transporter), member 4 | 4 |
| 5919 | hsa-mir-340 | 6509 | SLC1A4 | solute carrier family 1 (glutamate/neutral amino acid transporter), member 4 | 4 |
| 5920 | hsa-mir-338 | 6509 | SLC1A4 | solute carrier family 1 (glutamate/neutral amino acid transporter), member 4 | 4 |
| 5921 | hsa-mir-548b | 6509 | SLC1A4 | solute carrier family 1 (glutamate/neutral amino acid transporter), member 4 | 4 |
| 5922 | hsa-mir-449b | 6509 | SLC1A4 | solute carrier family 1 (glutamate/neutral amino acid transporter), member 4 | 4 |
| 5923 | hsa-mir-449c | 6509 | SLC1A4 | solute carrier family 1 (glutamate/neutral amino acid transporter), member 4 | 4 |
| 5924 | hsa-mir-3065 | 6509 | SLC1A4 | solute carrier family 1 (glutamate/neutral amino acid transporter), member 4 | 4 |
| 1 | hsa-miR-10a | 6352 | CCL5 | chemokine (C-C motif) ligand 5 | 3 |
| 2 | hsa-miR-139-5p | 6352 | CCL5 | chemokine (C-C motif) ligand 5 | 3 |
| 3 | hsa-miR-152 | 6352 | CCL5 | chemokine (C-C motif) ligand 5 | 3 |
| 4 | hsa-miR-26a | 6352 | CCL5 | chemokine (C-C motif) ligand 5 | 3 |
| 5 | hsa-miR-26b | 6352 | CCL5 | chemokine (C-C motif) ligand 5 | 3 |
| 6 | hsa-miR-31 | 6352 | CCL5 | chemokine (C-C motif) ligand 5 | 3 |
| 7 | hsa-miR-338-5p | 6352 | CCL5 | chemokine (C-C motif) ligand 5 | 3 |
| 8 | hsa-miR-340 | 6352 | CCL5 | chemokine (C-C motif) ligand 5 | 3 |
| 10 | hsa-miR-449a | 6352 | CCL5 | chemokine (C-C motif) ligand 5 | 3 |
| 11 | hsa-miR-499-5p | 6352 | CCL5 | chemokine (C-C motif) ligand 5 | 3 |
| 12 | hsa-miR-548b-5p | 6352 | CCL5 | chemokine (C-C motif) ligand 5 | 3 |
| 13 | hsa-miR-621 | 6352 | CCL5 | chemokine (C-C motif) ligand 5 | 3 |
| 14 | hsa-miR-657 | 6352 | CCL5 | chemokine (C-C motif) ligand 5 | 3 |
| 15 | hsa-miR-765 | 6352 | CCL5 | chemokine (C-C motif) ligand 5 | 3 |
| 16 | hsa-miR-770-5p | 6352 | CCL5 | chemokine (C-C motif) ligand 5 | 3 |
| 17 | hsa-miR-877 | 6352 | CCL5 | chemokine (C-C motif) ligand 5 | 3 |
| 18 | hsa-mir-26a-1 | 6352 | CCL5 | chemokine (C-C motif) ligand 5 | 3 |
| 19 | hsa-mir-26b | 6352 | CCL5 | chemokine (C-C motif) ligand 5 | 3 |
| 20 | hsa-mir-31 | 6352 | CCL5 | chemokine (C-C motif) ligand 5 | 3 |
| 21 | hsa-mir-194-2 | 6352 | CCL5 | chemokine (C-C motif) ligand 5 | 3 |
| 22 | hsa-mir-340 | 6352 | CCL5 | chemokine (C-C motif) ligand 5 | 3 |
| 23 | hsa-mir-338 | 6352 | CCL5 | chemokine (C-C motif) ligand 5 | 3 |
| 24 | hsa-mir-548b | 6352 | CCL5 | chemokine (C-C motif) ligand 5 | 3 |
| 25 | hsa-mir-449b | 6352 | CCL5 | chemokine (C-C motif) ligand 5 | 3 |
| 26 | hsa-mir-449c | 6352 | CCL5 | chemokine (C-C motif) ligand 5 | 3 |
| 27 | hsa-mir-3065 | 6352 | CCL5 | chemokine (C-C motif) ligand 5 | 3 |
| 1072 | hsa-miR-10a | 51667 | NUB1 | negative regulator of ubiquitin-like proteins 1 | 3 |
| 1073 | hsa-miR-139-5p | 51667 | NUB1 | negative regulator of ubiquitin-like proteins 1 | 3 |
| 1074 | hsa-miR-152 | 51667 | NUB1 | negative regulator of ubiquitin-like proteins 1 | 3 |
| 1075 | hsa-miR-26a | 51667 | NUB1 | negative regulator of ubiquitin-like proteins 1 | 3 |
| 1076 | hsa-miR-26b | 51667 | NUB1 | negative regulator of ubiquitin-like proteins 1 | 3 |
| 1077 | hsa-miR-31 | 51667 | NUB1 | negative regulator of ubiquitin-like proteins 1 | 3 |
| 1078 | hsa-miR-338-5p | 51667 | NUB1 | negative regulator of ubiquitin-like proteins 1 | 3 |
| 1079 | hsa-miR-340 | 51667 | NUB1 | negative regulator of ubiquitin-like proteins 1 | 3 |
| 1081 | hsa-miR-449a | 51667 | NUB1 | negative regulator of ubiquitin-like proteins 1 | 3 |
| 1082 | hsa-miR-499-5p | 51667 | NUB1 | negative regulator of ubiquitin-like proteins 1 | 3 |
| 1083 | hsa-miR-548b-5p | 51667 | NUB1 | negative regulator of ubiquitin-like proteins 1 | 3 |
| 1084 | hsa-miR-621 | 51667 | NUB1 | negative regulator of ubiquitin-like proteins 1 | 3 |
| 1085 | hsa-miR-657 | 51667 | NUB1 | negative regulator of ubiquitin-like proteins 1 | 3 |
| 1086 | hsa-miR-765 | 51667 | NUB1 | negative regulator of ubiquitin-like proteins 1 | 3 |
| 1087 | hsa-miR-770-5p | 51667 | NUB1 | negative regulator of ubiquitin-like proteins 1 | 3 |
| 1088 | hsa-miR-877 | 51667 | NUB1 | negative regulator of ubiquitin-like proteins 1 | 3 |
| 1089 | hsa-mir-26a-1 | 51667 | NUB1 | negative regulator of ubiquitin-like proteins 1 | 3 |
| 1090 | hsa-mir-26b | 51667 | NUB1 | negative regulator of ubiquitin-like proteins 1 | 3 |
| 1091 | hsa-mir-31 | 51667 | NUB1 | negative regulator of ubiquitin-like proteins 1 | 3 |
| 1092 | hsa-mir-194-2 | 51667 | NUB1 | negative regulator of ubiquitin-like proteins 1 | 3 |
| 1093 | hsa-mir-340 | 51667 | NUB1 | negative regulator of ubiquitin-like proteins 1 | 3 |
| 1094 | hsa-mir-338 | 51667 | NUB1 | negative regulator of ubiquitin-like proteins 1 | 3 |
| 1095 | hsa-mir-548b | 51667 | NUB1 | negative regulator of ubiquitin-like proteins 1 | 3 |
| 1096 | hsa-mir-449b | 51667 | NUB1 | negative regulator of ubiquitin-like proteins 1 | 3 |
| 1097 | hsa-mir-449c | 51667 | NUB1 | negative regulator of ubiquitin-like proteins 1 | 3 |
| 1098 | hsa-mir-3065 | 51667 | NUB1 | negative regulator of ubiquitin-like proteins 1 | 3 |
| 1928 | hsa-mir-3190 | 4094 | MAF | v-maf musculoaponeurotic fibrosarcoma oncogene homolog (avian) | 3 |
| 2719 | hsa-miR-10a | 2633 | GBP1 | guanylate binding protein 1, interferon-inducible, 67kDa | 3 |
| 2720 | hsa-miR-139-5p | 2633 | GBP1 | guanylate binding protein 1, interferon-inducible, 67kDa | 3 |
| 2721 | hsa-miR-152 | 2633 | GBP1 | guanylate binding protein 1, interferon-inducible, 67kDa | 3 |
| 2722 | hsa-miR-26a | 2633 | GBP1 | guanylate binding protein 1, interferon-inducible, 67kDa | 3 |
| 2723 | hsa-miR-26b | 2633 | GBP1 | guanylate binding protein 1, interferon-inducible, 67kDa | 3 |
| 2724 | hsa-miR-31 | 2633 | GBP1 | guanylate binding protein 1, interferon-inducible, 67kDa | 3 |
| 2725 | hsa-miR-338-5p | 2633 | GBP1 | guanylate binding protein 1, interferon-inducible, 67kDa | 3 |
| 2726 | hsa-miR-340 | 2633 | GBP1 | guanylate binding protein 1, interferon-inducible, 67kDa | 3 |
| 2728 | hsa-miR-449a | 2633 | GBP1 | guanylate binding protein 1, interferon-inducible, 67kDa | 3 |
| 2730 | hsa-miR-499-5p | 2633 | GBP1 | guanylate binding protein 1, interferon-inducible, 67kDa | 3 |
| 2731 | hsa-miR-548b-5p | 2633 | GBP1 | guanylate binding protein 1, interferon-inducible, 67kDa | 3 |
| 2733 | hsa-miR-621 | 2633 | GBP1 | guanylate binding protein 1, interferon-inducible, 67kDa | 3 |
| 2734 | hsa-miR-657 | 2633 | GBP1 | guanylate binding protein 1, interferon-inducible, 67kDa | 3 |
| 2735 | hsa-miR-765 | 2633 | GBP1 | guanylate binding protein 1, interferon-inducible, 67kDa | 3 |
| 2736 | hsa-miR-770-5p | 2633 | GBP1 | guanylate binding protein 1, interferon-inducible, 67kDa | 3 |
| 2737 | hsa-miR-877 | 2633 | GBP1 | guanylate binding protein 1, interferon-inducible, 67kDa | 3 |
| 2738 | hsa-mir-26a-1 | 2633 | GBP1 | guanylate binding protein 1, interferon-inducible, 67kDa | 3 |
| 2739 | hsa-mir-26b | 2633 | GBP1 | guanylate binding protein 1, interferon-inducible, 67kDa | 3 |
| 2740 | hsa-mir-31 | 2633 | GBP1 | guanylate binding protein 1, interferon-inducible, 67kDa | 3 |
| 2741 | hsa-mir-194-2 | 2633 | GBP1 | guanylate binding protein 1, interferon-inducible, 67kDa | 3 |
| 2742 | hsa-mir-340 | 2633 | GBP1 | guanylate binding protein 1, interferon-inducible, 67kDa | 3 |
| 2743 | hsa-mir-338 | 2633 | GBP1 | guanylate binding protein 1, interferon-inducible, 67kDa | 3 |
| 2745 | hsa-mir-548b | 2633 | GBP1 | guanylate binding protein 1, interferon-inducible, 67kDa | 3 |
| 2746 | hsa-mir-548d-2 | 2633 | GBP1 | guanylate binding protein 1, interferon-inducible, 67kDa | 3 |
| 2747 | hsa-mir-449b | 2633 | GBP1 | guanylate binding protein 1, interferon-inducible, 67kDa | 3 |
| 2748 | hsa-mir-449c | 2633 | GBP1 | guanylate binding protein 1, interferon-inducible, 67kDa | 3 |
| 2749 | hsa-mir-3065 | 2633 | GBP1 | guanylate binding protein 1, interferon-inducible, 67kDa | 3 |
| 3022 | hsa-miR-1 | 23428 | SLC7A8 | solute carrier family 7 (cationic amino acid transporter, y+ system), member 8 | 3 |
| 3023 | hsa-miR-133a | 23428 | SLC7A8 | solute carrier family 7 (cationic amino acid transporter, y+ system), member 8 | 3 |
| 3024 | hsa-miR-186 | 23428 | SLC7A8 | solute carrier family 7 (cationic amino acid transporter, y+ system), member 8 | 3 |
| 3025 | hsa-miR-326 | 23428 | SLC7A8 | solute carrier family 7 (cationic amino acid transporter, y+ system), member 8 | 3 |
| 3026 | hsa-miR-449b | 23428 | SLC7A8 | solute carrier family 7 (cationic amino acid transporter, y+ system), member 8 | 3 |
| 3027 | hsa-miR-489 | 23428 | SLC7A8 | solute carrier family 7 (cationic amino acid transporter, y+ system), member 8 | 3 |
| 3029 | hsa-miR-575 | 23428 | SLC7A8 | solute carrier family 7 (cationic amino acid transporter, y+ system), member 8 | 3 |
| 3030 | hsa-miR-579 | 23428 | SLC7A8 | solute carrier family 7 (cationic amino acid transporter, y+ system), member 8 | 3 |
| 3031 | hsa-miR-608 | 23428 | SLC7A8 | solute carrier family 7 (cationic amino acid transporter, y+ system), member 8 | 3 |
| 3032 | hsa-miR-620 | 23428 | SLC7A8 | solute carrier family 7 (cationic amino acid transporter, y+ system), member 8 | 3 |
| 3034 | hsa-miR-885-5p | 23428 | SLC7A8 | solute carrier family 7 (cationic amino acid transporter, y+ system), member 8 | 3 |
| 3035 | hsa-mir-22 | 23428 | SLC7A8 | solute carrier family 7 (cationic amino acid transporter, y+ system), member 8 | 3 |
| 3036 | hsa-mir-10a | 23428 | SLC7A8 | solute carrier family 7 (cationic amino acid transporter, y+ system), member 8 | 3 |
| 3037 | hsa-mir-186 | 23428 | SLC7A8 | solute carrier family 7 (cationic amino acid transporter, y+ system), member 8 | 3 |
| 3038 | hsa-mir-155 | 23428 | SLC7A8 | solute carrier family 7 (cationic amino acid transporter, y+ system), member 8 | 3 |
| 3040 | hsa-mir-499 | 23428 | SLC7A8 | solute carrier family 7 (cationic amino acid transporter, y+ system), member 8 | 3 |
| 3041 | hsa-mir-885 | 23428 | SLC7A8 | solute carrier family 7 (cationic amino acid transporter, y+ system), member 8 | 3 |
| 3042 | hsa-mir-877 | 23428 | SLC7A8 | solute carrier family 7 (cationic amino acid transporter, y+ system), member 8 | 3 |
| 3043 | hsa-mir-3190 | 23428 | SLC7A8 | solute carrier family 7 (cationic amino acid transporter, y+ system), member 8 | 3 |
| 3072 | hsa-miR-10a | 10906 | TRAFD1 | TRAF-type zinc finger domain containing 1 | 3 |
| 3073 | hsa-miR-139-5p | 10906 | TRAFD1 | TRAF-type zinc finger domain containing 1 | 3 |
| 3074 | hsa-miR-152 | 10906 | TRAFD1 | TRAF-type zinc finger domain containing 1 | 3 |
| 3075 | hsa-miR-26b | 10906 | TRAFD1 | TRAF-type zinc finger domain containing 1 | 3 |
| 3076 | hsa-miR-31 | 10906 | TRAFD1 | TRAF-type zinc finger domain containing 1 | 3 |
| 3077 | hsa-miR-338-5p | 10906 | TRAFD1 | TRAF-type zinc finger domain containing 1 | 3 |
| 3078 | hsa-miR-340 | 10906 | TRAFD1 | TRAF-type zinc finger domain containing 1 | 3 |
| 3079 | hsa-miR-383 | 10906 | TRAFD1 | TRAF-type zinc finger domain containing 1 | 3 |
| 3080 | hsa-miR-449a | 10906 | TRAFD1 | TRAF-type zinc finger domain containing 1 | 3 |
| 3081 | hsa-miR-499-5p | 10906 | TRAFD1 | TRAF-type zinc finger domain containing 1 | 3 |
| 3082 | hsa-miR-548b-5p | 10906 | TRAFD1 | TRAF-type zinc finger domain containing 1 | 3 |
| 3083 | hsa-miR-621 | 10906 | TRAFD1 | TRAF-type zinc finger domain containing 1 | 3 |
| 3084 | hsa-miR-657 | 10906 | TRAFD1 | TRAF-type zinc finger domain containing 1 | 3 |
| 3085 | hsa-miR-663 | 10906 | TRAFD1 | TRAF-type zinc finger domain containing 1 | 3 |
| 3086 | hsa-miR-765 | 10906 | TRAFD1 | TRAF-type zinc finger domain containing 1 | 3 |
| 3087 | hsa-miR-770-5p | 10906 | TRAFD1 | TRAF-type zinc finger domain containing 1 | 3 |
| 3088 | hsa-miR-877 | 10906 | TRAFD1 | TRAF-type zinc finger domain containing 1 | 3 |
| 3089 | hsa-mir-26a-1 | 10906 | TRAFD1 | TRAF-type zinc finger domain containing 1 | 3 |
| 3090 | hsa-mir-26b | 10906 | TRAFD1 | TRAF-type zinc finger domain containing 1 | 3 |
| 3091 | hsa-mir-31 | 10906 | TRAFD1 | TRAF-type zinc finger domain containing 1 | 3 |
| 3092 | hsa-mir-194-2 | 10906 | TRAFD1 | TRAF-type zinc finger domain containing 1 | 3 |
| 3093 | hsa-mir-340 | 10906 | TRAFD1 | TRAF-type zinc finger domain containing 1 | 3 |
| 3094 | hsa-mir-338 | 10906 | TRAFD1 | TRAF-type zinc finger domain containing 1 | 3 |
| 3095 | hsa-mir-548b | 10906 | TRAFD1 | TRAF-type zinc finger domain containing 1 | 3 |
| 3096 | hsa-mir-548d-2 | 10906 | TRAFD1 | TRAF-type zinc finger domain containing 1 | 3 |
| 3097 | hsa-mir-449b | 10906 | TRAFD1 | TRAF-type zinc finger domain containing 1 | 3 |
| 3098 | hsa-mir-449c | 10906 | TRAFD1 | TRAF-type zinc finger domain containing 1 | 3 |
| 3099 | hsa-mir-3065 | 10906 | TRAFD1 | TRAF-type zinc finger domain containing 1 | 3 |
| 4795 | hsa-miR-489 | 4094 | MAF | v-maf musculoaponeurotic fibrosarcoma oncogene homolog (avian) | 3 |
| 5491 | hsa-miR-1 | 334 | APLP2 | amyloid beta (A4) precursor-like protein 2 | 3 |
| 5492 | hsa-miR-133a | 334 | APLP2 | amyloid beta (A4) precursor-like protein 2 | 3 |
| 5493 | hsa-miR-186 | 334 | APLP2 | amyloid beta (A4) precursor-like protein 2 | 3 |
| 5494 | hsa-miR-326 | 334 | APLP2 | amyloid beta (A4) precursor-like protein 2 | 3 |
| 5495 | hsa-miR-449b | 334 | APLP2 | amyloid beta (A4) precursor-like protein 2 | 3 |
| 5496 | hsa-miR-551a | 334 | APLP2 | amyloid beta (A4) precursor-like protein 2 | 3 |
| 5497 | hsa-miR-562 | 334 | APLP2 | amyloid beta (A4) precursor-like protein 2 | 3 |
| 5498 | hsa-miR-575 | 334 | APLP2 | amyloid beta (A4) precursor-like protein 2 | 3 |
| 5499 | hsa-miR-579 | 334 | APLP2 | amyloid beta (A4) precursor-like protein 2 | 3 |
| 5500 | hsa-miR-608 | 334 | APLP2 | amyloid beta (A4) precursor-like protein 2 | 3 |
| 5501 | hsa-miR-620 | 334 | APLP2 | amyloid beta (A4) precursor-like protein 2 | 3 |
| 5503 | hsa-mir-22 | 334 | APLP2 | amyloid beta (A4) precursor-like protein 2 | 3 |
| 5504 | hsa-mir-10a | 334 | APLP2 | amyloid beta (A4) precursor-like protein 2 | 3 |
| 5505 | hsa-mir-186 | 334 | APLP2 | amyloid beta (A4) precursor-like protein 2 | 3 |
| 5506 | hsa-mir-155 | 334 | APLP2 | amyloid beta (A4) precursor-like protein 2 | 3 |
| 5507 | hsa-mir-423 | 334 | APLP2 | amyloid beta (A4) precursor-like protein 2 | 3 |
| 5510 | hsa-mir-877 | 334 | APLP2 | amyloid beta (A4) precursor-like protein 2 | 3 |
| 5692 | hsa-miR-10a | 23499 | MACF1 | microtubule-actin crosslinking factor 1 | 3 |
| 5693 | hsa-miR-139-5p | 23499 | MACF1 | microtubule-actin crosslinking factor 1 | 3 |
| 5695 | hsa-miR-26a | 23499 | MACF1 | microtubule-actin crosslinking factor 1 | 3 |
| 5696 | hsa-miR-26b | 23499 | MACF1 | microtubule-actin crosslinking factor 1 | 3 |
| 5697 | hsa-miR-31 | 23499 | MACF1 | microtubule-actin crosslinking factor 1 | 3 |
| 5698 | hsa-miR-338-5p | 23499 | MACF1 | microtubule-actin crosslinking factor 1 | 3 |
| 5700 | hsa-miR-449a | 23499 | MACF1 | microtubule-actin crosslinking factor 1 | 3 |
| 5701 | hsa-miR-499-5p | 23499 | MACF1 | microtubule-actin crosslinking factor 1 | 3 |
| 5702 | hsa-miR-548b-5p | 23499 | MACF1 | microtubule-actin crosslinking factor 1 | 3 |
| 5703 | hsa-miR-564 | 23499 | MACF1 | microtubule-actin crosslinking factor 1 | 3 |
| 5704 | hsa-miR-621 | 23499 | MACF1 | microtubule-actin crosslinking factor 1 | 3 |
| 5705 | hsa-miR-657 | 23499 | MACF1 | microtubule-actin crosslinking factor 1 | 3 |
| 5706 | hsa-miR-765 | 23499 | MACF1 | microtubule-actin crosslinking factor 1 | 3 |
| 5707 | hsa-miR-770-5p | 23499 | MACF1 | microtubule-actin crosslinking factor 1 | 3 |
| 5709 | hsa-mir-26a-1 | 23499 | MACF1 | microtubule-actin crosslinking factor 1 | 3 |
| 5710 | hsa-mir-26b | 23499 | MACF1 | microtubule-actin crosslinking factor 1 | 3 |
| 5711 | hsa-mir-31 | 23499 | MACF1 | microtubule-actin crosslinking factor 1 | 3 |
| 5712 | hsa-mir-194-2 | 23499 | MACF1 | microtubule-actin crosslinking factor 1 | 3 |
| 5714 | hsa-mir-338 | 23499 | MACF1 | microtubule-actin crosslinking factor 1 | 3 |
| 5715 | hsa-mir-548b | 23499 | MACF1 | microtubule-actin crosslinking factor 1 | 3 |
| 5717 | hsa-mir-449b | 23499 | MACF1 | microtubule-actin crosslinking factor 1 | 3 |
| 5718 | hsa-mir-449c | 23499 | MACF1 | microtubule-actin crosslinking factor 1 | 3 |
| 5719 | hsa-mir-3065 | 23499 | MACF1 | microtubule-actin crosslinking factor 1 | 3 |
| 5725 | hsa-miR-490-5p | 10938 | EHD1 | EH-domain containing 1 | 3 |
| 5726 | hsa-miR-644 | 10938 | EHD1 | EH-domain containing 1 | 3 |
| 5727 | hsa-miR-95 | 10938 | EHD1 | EH-domain containing 1 | 3 |
| 5728 | hsa-mir-490 | 10938 | EHD1 | EH-domain containing 1 | 3 |
| 5954 | hsa-miR-10a | 5272 | SERPINB9 | serpin peptidase inhibitor, clade B (ovalbumin), member 9 | 3 |
| 5955 | hsa-miR-152 | 5272 | SERPINB9 | serpin peptidase inhibitor, clade B (ovalbumin), member 9 | 3 |
| 5956 | hsa-miR-26a | 5272 | SERPINB9 | serpin peptidase inhibitor, clade B (ovalbumin), member 9 | 3 |
| 5957 | hsa-miR-26b | 5272 | SERPINB9 | serpin peptidase inhibitor, clade B (ovalbumin), member 9 | 3 |
| 5958 | hsa-miR-31 | 5272 | SERPINB9 | serpin peptidase inhibitor, clade B (ovalbumin), member 9 | 3 |
| 5959 | hsa-miR-338-5p | 5272 | SERPINB9 | serpin peptidase inhibitor, clade B (ovalbumin), member 9 | 3 |
| 5960 | hsa-miR-340 | 5272 | SERPINB9 | serpin peptidase inhibitor, clade B (ovalbumin), member 9 | 3 |
| 5962 | hsa-miR-449a | 5272 | SERPINB9 | serpin peptidase inhibitor, clade B (ovalbumin), member 9 | 3 |
| 5963 | hsa-miR-490-5p | 5272 | SERPINB9 | serpin peptidase inhibitor, clade B (ovalbumin), member 9 | 3 |
| 5964 | hsa-miR-499-5p | 5272 | SERPINB9 | serpin peptidase inhibitor, clade B (ovalbumin), member 9 | 3 |
| 5968 | hsa-miR-621 | 5272 | SERPINB9 | serpin peptidase inhibitor, clade B (ovalbumin), member 9 | 3 |
| 5970 | hsa-miR-657 | 5272 | SERPINB9 | serpin peptidase inhibitor, clade B (ovalbumin), member 9 | 3 |
| 5971 | hsa-miR-765 | 5272 | SERPINB9 | serpin peptidase inhibitor, clade B (ovalbumin), member 9 | 3 |
| 5972 | hsa-miR-770-5p | 5272 | SERPINB9 | serpin peptidase inhibitor, clade B (ovalbumin), member 9 | 3 |
| 5973 | hsa-miR-877 | 5272 | SERPINB9 | serpin peptidase inhibitor, clade B (ovalbumin), member 9 | 3 |
| 5975 | hsa-mir-26a-1 | 5272 | SERPINB9 | serpin peptidase inhibitor, clade B (ovalbumin), member 9 | 3 |
| 5976 | hsa-mir-26b | 5272 | SERPINB9 | serpin peptidase inhibitor, clade B (ovalbumin), member 9 | 3 |
| 5977 | hsa-mir-31 | 5272 | SERPINB9 | serpin peptidase inhibitor, clade B (ovalbumin), member 9 | 3 |
| 5978 | hsa-mir-194-2 | 5272 | SERPINB9 | serpin peptidase inhibitor, clade B (ovalbumin), member 9 | 3 |
| 5979 | hsa-mir-340 | 5272 | SERPINB9 | serpin peptidase inhibitor, clade B (ovalbumin), member 9 | 3 |
| 5980 | hsa-mir-338 | 5272 | SERPINB9 | serpin peptidase inhibitor, clade B (ovalbumin), member 9 | 3 |
| 5981 | hsa-mir-490 | 5272 | SERPINB9 | serpin peptidase inhibitor, clade B (ovalbumin), member 9 | 3 |
| 5983 | hsa-mir-449b | 5272 | SERPINB9 | serpin peptidase inhibitor, clade B (ovalbumin), member 9 | 3 |
| 5984 | hsa-mir-449c | 5272 | SERPINB9 | serpin peptidase inhibitor, clade B (ovalbumin), member 9 | 3 |
| 5985 | hsa-mir-3065 | 5272 | SERPINB9 | serpin peptidase inhibitor, clade B (ovalbumin), member 9 | 3 |
| 6062 | hsa-miR-423-5p | 8837 | CFLAR | CASP8 and FADD-like apoptosis regulator | 3 |
| 6201 | hsa-miR-10a | 22902 | RUFY3 | RUN and FYVE domain containing 3 | 3 |
| 6203 | hsa-miR-152 | 22902 | RUFY3 | RUN and FYVE domain containing 3 | 3 |
| 6204 | hsa-miR-31 | 22902 | RUFY3 | RUN and FYVE domain containing 3 | 3 |
| 6205 | hsa-miR-338-5p | 22902 | RUFY3 | RUN and FYVE domain containing 3 | 3 |
| 6206 | hsa-miR-340 | 22902 | RUFY3 | RUN and FYVE domain containing 3 | 3 |
| 6207 | hsa-miR-383 | 22902 | RUFY3 | RUN and FYVE domain containing 3 | 3 |
| 6208 | hsa-miR-449a | 22902 | RUFY3 | RUN and FYVE domain containing 3 | 3 |
| 6209 | hsa-miR-548b-5p | 22902 | RUFY3 | RUN and FYVE domain containing 3 | 3 |
| 6210 | hsa-miR-621 | 22902 | RUFY3 | RUN and FYVE domain containing 3 | 3 |
| 6211 | hsa-miR-657 | 22902 | RUFY3 | RUN and FYVE domain containing 3 | 3 |
| 6212 | hsa-miR-663 | 22902 | RUFY3 | RUN and FYVE domain containing 3 | 3 |
| 6213 | hsa-miR-765 | 22902 | RUFY3 | RUN and FYVE domain containing 3 | 3 |
| 6214 | hsa-miR-770-5p | 22902 | RUFY3 | RUN and FYVE domain containing 3 | 3 |
| 6215 | hsa-miR-877 | 22902 | RUFY3 | RUN and FYVE domain containing 3 | 3 |
| 6216 | hsa-mir-26a-1 | 22902 | RUFY3 | RUN and FYVE domain containing 3 | 3 |
| 6217 | hsa-mir-31 | 22902 | RUFY3 | RUN and FYVE domain containing 3 | 3 |
| 6218 | hsa-mir-194-2 | 22902 | RUFY3 | RUN and FYVE domain containing 3 | 3 |
| 6219 | hsa-mir-340 | 22902 | RUFY3 | RUN and FYVE domain containing 3 | 3 |
| 6220 | hsa-mir-338 | 22902 | RUFY3 | RUN and FYVE domain containing 3 | 3 |
| 6221 | hsa-mir-548b | 22902 | RUFY3 | RUN and FYVE domain containing 3 | 3 |
| 6222 | hsa-mir-449b | 22902 | RUFY3 | RUN and FYVE domain containing 3 | 3 |
| 6223 | hsa-mir-449c | 22902 | RUFY3 | RUN and FYVE domain containing 3 | 3 |
| 6224 | hsa-mir-3065 | 22902 | RUFY3 | RUN and FYVE domain containing 3 | 3 |
| 7718 | hsa-miR-10a | 79155 | TNIP2 | TNFAIP3 interacting protein 2 | 3 |
| 7719 | hsa-miR-1228\* | 79155 | TNIP2 | TNFAIP3 interacting protein 2 | 3 |
| 7720 | hsa-miR-139-5p | 79155 | TNIP2 | TNFAIP3 interacting protein 2 | 3 |
| 7722 | hsa-miR-26a | 79155 | TNIP2 | TNFAIP3 interacting protein 2 | 3 |
| 7723 | hsa-miR-26b | 79155 | TNIP2 | TNFAIP3 interacting protein 2 | 3 |
| 7724 | hsa-miR-338-5p | 79155 | TNIP2 | TNFAIP3 interacting protein 2 | 3 |
| 7725 | hsa-miR-340 | 79155 | TNIP2 | TNFAIP3 interacting protein 2 | 3 |
| 7726 | hsa-miR-449a | 79155 | TNIP2 | TNFAIP3 interacting protein 2 | 3 |
| 7727 | hsa-miR-499-5p | 79155 | TNIP2 | TNFAIP3 interacting protein 2 | 3 |
| 7728 | hsa-miR-548b-5p | 79155 | TNIP2 | TNFAIP3 interacting protein 2 | 3 |
| 7729 | hsa-miR-564 | 79155 | TNIP2 | TNFAIP3 interacting protein 2 | 3 |
| 7730 | hsa-miR-584 | 79155 | TNIP2 | TNFAIP3 interacting protein 2 | 3 |
| 7731 | hsa-miR-621 | 79155 | TNIP2 | TNFAIP3 interacting protein 2 | 3 |
| 7732 | hsa-miR-657 | 79155 | TNIP2 | TNFAIP3 interacting protein 2 | 3 |
| 7733 | hsa-miR-765 | 79155 | TNIP2 | TNFAIP3 interacting protein 2 | 3 |
| 7734 | hsa-miR-770-5p | 79155 | TNIP2 | TNFAIP3 interacting protein 2 | 3 |
| 7735 | hsa-miR-877 | 79155 | TNIP2 | TNFAIP3 interacting protein 2 | 3 |
| 7736 | hsa-mir-26a-1 | 79155 | TNIP2 | TNFAIP3 interacting protein 2 | 3 |
| 7737 | hsa-mir-26b | 79155 | TNIP2 | TNFAIP3 interacting protein 2 | 3 |
| 7738 | hsa-mir-194-2 | 79155 | TNIP2 | TNFAIP3 interacting protein 2 | 3 |
| 7739 | hsa-mir-340 | 79155 | TNIP2 | TNFAIP3 interacting protein 2 | 3 |
| 7740 | hsa-mir-338 | 79155 | TNIP2 | TNFAIP3 interacting protein 2 | 3 |
| 7741 | hsa-mir-548b | 79155 | TNIP2 | TNFAIP3 interacting protein 2 | 3 |
| 7742 | hsa-mir-548d-2 | 79155 | TNIP2 | TNFAIP3 interacting protein 2 | 3 |
| 7743 | hsa-mir-449b | 79155 | TNIP2 | TNFAIP3 interacting protein 2 | 3 |
| 7744 | hsa-mir-449c | 79155 | TNIP2 | TNFAIP3 interacting protein 2 | 3 |
| 7745 | hsa-mir-3065 | 79155 | TNIP2 | TNFAIP3 interacting protein 2 | 3 |
| 8264 | hsa-miR-1 | 56172 | ANKH | ankylosis, progressive homolog (mouse) | 3 |
| 8265 | hsa-miR-133a | 56172 | ANKH | ankylosis, progressive homolog (mouse) | 3 |
| 8266 | hsa-miR-186 | 56172 | ANKH | ankylosis, progressive homolog (mouse) | 3 |
| 8267 | hsa-miR-326 | 56172 | ANKH | ankylosis, progressive homolog (mouse) | 3 |
| 8268 | hsa-miR-449b | 56172 | ANKH | ankylosis, progressive homolog (mouse) | 3 |
| 8270 | hsa-miR-562 | 56172 | ANKH | ankylosis, progressive homolog (mouse) | 3 |
| 8271 | hsa-miR-575 | 56172 | ANKH | ankylosis, progressive homolog (mouse) | 3 |
| 8272 | hsa-miR-579 | 56172 | ANKH | ankylosis, progressive homolog (mouse) | 3 |
| 8273 | hsa-miR-608 | 56172 | ANKH | ankylosis, progressive homolog (mouse) | 3 |
| 8274 | hsa-miR-620 | 56172 | ANKH | ankylosis, progressive homolog (mouse) | 3 |
| 8277 | hsa-mir-22 | 56172 | ANKH | ankylosis, progressive homolog (mouse) | 3 |
| 8278 | hsa-mir-10a | 56172 | ANKH | ankylosis, progressive homolog (mouse) | 3 |
| 8279 | hsa-mir-186 | 56172 | ANKH | ankylosis, progressive homolog (mouse) | 3 |
| 8280 | hsa-mir-155 | 56172 | ANKH | ankylosis, progressive homolog (mouse) | 3 |
| 8282 | hsa-mir-499 | 56172 | ANKH | ankylosis, progressive homolog (mouse) | 3 |
| 8284 | hsa-mir-877 | 56172 | ANKH | ankylosis, progressive homolog (mouse) | 3 |
| 9 | hsa-miR-383 | 6352 | CCL5 | chemokine (C-C motif) ligand 5 | 2 |
| 293 | hsa-miR-10a | 121268 | RHEBL1 | Ras homolog enriched in brain like 1 | 2 |
| 295 | hsa-miR-152 | 121268 | RHEBL1 | Ras homolog enriched in brain like 1 | 2 |
| 296 | hsa-miR-26a | 121268 | RHEBL1 | Ras homolog enriched in brain like 1 | 2 |
| 297 | hsa-miR-26b | 121268 | RHEBL1 | Ras homolog enriched in brain like 1 | 2 |
| 298 | hsa-miR-31 | 121268 | RHEBL1 | Ras homolog enriched in brain like 1 | 2 |
| 299 | hsa-miR-338-5p | 121268 | RHEBL1 | Ras homolog enriched in brain like 1 | 2 |
| 300 | hsa-miR-340 | 121268 | RHEBL1 | Ras homolog enriched in brain like 1 | 2 |
| 301 | hsa-miR-449a | 121268 | RHEBL1 | Ras homolog enriched in brain like 1 | 2 |
| 302 | hsa-miR-490-5p | 121268 | RHEBL1 | Ras homolog enriched in brain like 1 | 2 |
| 303 | hsa-miR-499-5p | 121268 | RHEBL1 | Ras homolog enriched in brain like 1 | 2 |
| 305 | hsa-miR-584 | 121268 | RHEBL1 | Ras homolog enriched in brain like 1 | 2 |
| 306 | hsa-miR-621 | 121268 | RHEBL1 | Ras homolog enriched in brain like 1 | 2 |
| 307 | hsa-miR-653 | 121268 | RHEBL1 | Ras homolog enriched in brain like 1 | 2 |
| 308 | hsa-miR-657 | 121268 | RHEBL1 | Ras homolog enriched in brain like 1 | 2 |
| 309 | hsa-miR-765 | 121268 | RHEBL1 | Ras homolog enriched in brain like 1 | 2 |
| 310 | hsa-miR-770-5p | 121268 | RHEBL1 | Ras homolog enriched in brain like 1 | 2 |
| 311 | hsa-miR-877 | 121268 | RHEBL1 | Ras homolog enriched in brain like 1 | 2 |
| 312 | hsa-mir-26a-1 | 121268 | RHEBL1 | Ras homolog enriched in brain like 1 | 2 |
| 313 | hsa-mir-26b | 121268 | RHEBL1 | Ras homolog enriched in brain like 1 | 2 |
| 314 | hsa-mir-31 | 121268 | RHEBL1 | Ras homolog enriched in brain like 1 | 2 |
| 315 | hsa-mir-194-2 | 121268 | RHEBL1 | Ras homolog enriched in brain like 1 | 2 |
| 316 | hsa-mir-340 | 121268 | RHEBL1 | Ras homolog enriched in brain like 1 | 2 |
| 317 | hsa-mir-338 | 121268 | RHEBL1 | Ras homolog enriched in brain like 1 | 2 |
| 318 | hsa-mir-490 | 121268 | RHEBL1 | Ras homolog enriched in brain like 1 | 2 |
| 320 | hsa-mir-449b | 121268 | RHEBL1 | Ras homolog enriched in brain like 1 | 2 |
| 321 | hsa-mir-449c | 121268 | RHEBL1 | Ras homolog enriched in brain like 1 | 2 |
| 322 | hsa-mir-3065 | 121268 | RHEBL1 | Ras homolog enriched in brain like 1 | 2 |
| 323 | hsa-miR-1 | 147694 | ZNF548 | zinc finger protein 548 | 2 |
| 324 | hsa-miR-133a | 147694 | ZNF548 | zinc finger protein 548 | 2 |
| 325 | hsa-miR-186 | 147694 | ZNF548 | zinc finger protein 548 | 2 |
| 327 | hsa-miR-326 | 147694 | ZNF548 | zinc finger protein 548 | 2 |
| 328 | hsa-miR-449b | 147694 | ZNF548 | zinc finger protein 548 | 2 |
| 332 | hsa-miR-575 | 147694 | ZNF548 | zinc finger protein 548 | 2 |
| 334 | hsa-miR-608 | 147694 | ZNF548 | zinc finger protein 548 | 2 |
| 335 | hsa-miR-620 | 147694 | ZNF548 | zinc finger protein 548 | 2 |
| 338 | hsa-mir-22 | 147694 | ZNF548 | zinc finger protein 548 | 2 |
| 339 | hsa-mir-10a | 147694 | ZNF548 | zinc finger protein 548 | 2 |
| 340 | hsa-mir-186 | 147694 | ZNF548 | zinc finger protein 548 | 2 |
| 341 | hsa-mir-155 | 147694 | ZNF548 | zinc finger protein 548 | 2 |
| 342 | hsa-mir-499 | 147694 | ZNF548 | zinc finger protein 548 | 2 |
| 344 | hsa-mir-877 | 147694 | ZNF548 | zinc finger protein 548 | 2 |
| 490 | hsa-miR-1 | 10491 | CRTAP | cartilage associated protein | 2 |
| 491 | hsa-miR-133a | 10491 | CRTAP | cartilage associated protein | 2 |
| 492 | hsa-miR-186 | 10491 | CRTAP | cartilage associated protein | 2 |
| 494 | hsa-miR-326 | 10491 | CRTAP | cartilage associated protein | 2 |
| 495 | hsa-miR-449b | 10491 | CRTAP | cartilage associated protein | 2 |
| 498 | hsa-miR-575 | 10491 | CRTAP | cartilage associated protein | 2 |
| 499 | hsa-miR-608 | 10491 | CRTAP | cartilage associated protein | 2 |
| 500 | hsa-miR-620 | 10491 | CRTAP | cartilage associated protein | 2 |
| 501 | hsa-mir-22 | 10491 | CRTAP | cartilage associated protein | 2 |
| 502 | hsa-mir-10a | 10491 | CRTAP | cartilage associated protein | 2 |
| 503 | hsa-mir-186 | 10491 | CRTAP | cartilage associated protein | 2 |
| 504 | hsa-mir-155 | 10491 | CRTAP | cartilage associated protein | 2 |
| 505 | hsa-mir-499 | 10491 | CRTAP | cartilage associated protein | 2 |
| 506 | hsa-mir-877 | 10491 | CRTAP | cartilage associated protein | 2 |
| 507 | hsa-miR-10a | 941 | CD80 | CD80 molecule | 2 |
| 508 | hsa-miR-139-5p | 941 | CD80 | CD80 molecule | 2 |
| 509 | hsa-miR-152 | 941 | CD80 | CD80 molecule | 2 |
| 511 | hsa-miR-26b | 941 | CD80 | CD80 molecule | 2 |
| 512 | hsa-miR-31 | 941 | CD80 | CD80 molecule | 2 |
| 513 | hsa-miR-338-5p | 941 | CD80 | CD80 molecule | 2 |
| 514 | hsa-miR-340 | 941 | CD80 | CD80 molecule | 2 |
| 515 | hsa-miR-449a | 941 | CD80 | CD80 molecule | 2 |
| 516 | hsa-miR-499-5p | 941 | CD80 | CD80 molecule | 2 |
| 517 | hsa-miR-548b-5p | 941 | CD80 | CD80 molecule | 2 |
| 519 | hsa-miR-621 | 941 | CD80 | CD80 molecule | 2 |
| 520 | hsa-miR-657 | 941 | CD80 | CD80 molecule | 2 |
| 521 | hsa-miR-765 | 941 | CD80 | CD80 molecule | 2 |
| 522 | hsa-miR-770-5p | 941 | CD80 | CD80 molecule | 2 |
| 523 | hsa-miR-877 | 941 | CD80 | CD80 molecule | 2 |
| 524 | hsa-mir-26a-1 | 941 | CD80 | CD80 molecule | 2 |
| 525 | hsa-mir-26b | 941 | CD80 | CD80 molecule | 2 |
| 526 | hsa-mir-31 | 941 | CD80 | CD80 molecule | 2 |
| 527 | hsa-mir-194-2 | 941 | CD80 | CD80 molecule | 2 |
| 528 | hsa-mir-340 | 941 | CD80 | CD80 molecule | 2 |
| 529 | hsa-mir-338 | 941 | CD80 | CD80 molecule | 2 |
| 530 | hsa-mir-548b | 941 | CD80 | CD80 molecule | 2 |
| 532 | hsa-mir-449b | 941 | CD80 | CD80 molecule | 2 |
| 533 | hsa-mir-449c | 941 | CD80 | CD80 molecule | 2 |
| 534 | hsa-mir-3065 | 941 | CD80 | CD80 molecule | 2 |
| 625 | hsa-miR-1 | 3417 | IDH1 | isocitrate dehydrogenase 1 (NADP+), soluble | 2 |
| 626 | hsa-miR-133a | 3417 | IDH1 | isocitrate dehydrogenase 1 (NADP+), soluble | 2 |
| 627 | hsa-miR-186 | 3417 | IDH1 | isocitrate dehydrogenase 1 (NADP+), soluble | 2 |
| 628 | hsa-miR-195 | 3417 | IDH1 | isocitrate dehydrogenase 1 (NADP+), soluble | 2 |
| 629 | hsa-miR-326 | 3417 | IDH1 | isocitrate dehydrogenase 1 (NADP+), soluble | 2 |
| 630 | hsa-miR-449b | 3417 | IDH1 | isocitrate dehydrogenase 1 (NADP+), soluble | 2 |
| 631 | hsa-miR-497 | 3417 | IDH1 | isocitrate dehydrogenase 1 (NADP+), soluble | 2 |
| 633 | hsa-miR-562 | 3417 | IDH1 | isocitrate dehydrogenase 1 (NADP+), soluble | 2 |
| 634 | hsa-miR-575 | 3417 | IDH1 | isocitrate dehydrogenase 1 (NADP+), soluble | 2 |
| 635 | hsa-miR-579 | 3417 | IDH1 | isocitrate dehydrogenase 1 (NADP+), soluble | 2 |
| 636 | hsa-miR-608 | 3417 | IDH1 | isocitrate dehydrogenase 1 (NADP+), soluble | 2 |
| 637 | hsa-miR-620 | 3417 | IDH1 | isocitrate dehydrogenase 1 (NADP+), soluble | 2 |
| 638 | hsa-mir-22 | 3417 | IDH1 | isocitrate dehydrogenase 1 (NADP+), soluble | 2 |
| 639 | hsa-mir-10a | 3417 | IDH1 | isocitrate dehydrogenase 1 (NADP+), soluble | 2 |
| 640 | hsa-mir-186 | 3417 | IDH1 | isocitrate dehydrogenase 1 (NADP+), soluble | 2 |
| 641 | hsa-mir-155 | 3417 | IDH1 | isocitrate dehydrogenase 1 (NADP+), soluble | 2 |
| 642 | hsa-mir-499 | 3417 | IDH1 | isocitrate dehydrogenase 1 (NADP+), soluble | 2 |
| 644 | hsa-mir-877 | 3417 | IDH1 | isocitrate dehydrogenase 1 (NADP+), soluble | 2 |
| 645 | hsa-mir-2116 | 3417 | IDH1 | isocitrate dehydrogenase 1 (NADP+), soluble | 2 |
| 699 | hsa-miR-10a | 57018 | CCNL1 | cyclin L1 | 2 |
| 700 | hsa-miR-152 | 57018 | CCNL1 | cyclin L1 | 2 |
| 701 | hsa-miR-26a | 57018 | CCNL1 | cyclin L1 | 2 |
| 702 | hsa-miR-26b | 57018 | CCNL1 | cyclin L1 | 2 |
| 703 | hsa-miR-31 | 57018 | CCNL1 | cyclin L1 | 2 |
| 704 | hsa-miR-340 | 57018 | CCNL1 | cyclin L1 | 2 |
| 706 | hsa-miR-449a | 57018 | CCNL1 | cyclin L1 | 2 |
| 707 | hsa-miR-490-5p | 57018 | CCNL1 | cyclin L1 | 2 |
| 708 | hsa-miR-499-5p | 57018 | CCNL1 | cyclin L1 | 2 |
| 710 | hsa-miR-564 | 57018 | CCNL1 | cyclin L1 | 2 |
| 711 | hsa-miR-621 | 57018 | CCNL1 | cyclin L1 | 2 |
| 712 | hsa-miR-765 | 57018 | CCNL1 | cyclin L1 | 2 |
| 713 | hsa-miR-770-5p | 57018 | CCNL1 | cyclin L1 | 2 |
| 714 | hsa-miR-877 | 57018 | CCNL1 | cyclin L1 | 2 |
| 716 | hsa-mir-26a-1 | 57018 | CCNL1 | cyclin L1 | 2 |
| 717 | hsa-mir-26b | 57018 | CCNL1 | cyclin L1 | 2 |
| 718 | hsa-mir-31 | 57018 | CCNL1 | cyclin L1 | 2 |
| 719 | hsa-mir-194-2 | 57018 | CCNL1 | cyclin L1 | 2 |
| 720 | hsa-mir-340 | 57018 | CCNL1 | cyclin L1 | 2 |
| 721 | hsa-mir-490 | 57018 | CCNL1 | cyclin L1 | 2 |
| 724 | hsa-mir-449b | 57018 | CCNL1 | cyclin L1 | 2 |
| 725 | hsa-mir-449c | 57018 | CCNL1 | cyclin L1 | 2 |
| 726 | hsa-miR-10a | 133396 | IL31RA | interleukin 31 receptor A | 2 |
| 727 | hsa-miR-152 | 133396 | IL31RA | interleukin 31 receptor A | 2 |
| 728 | hsa-miR-26a | 133396 | IL31RA | interleukin 31 receptor A | 2 |
| 729 | hsa-miR-26b | 133396 | IL31RA | interleukin 31 receptor A | 2 |
| 730 | hsa-miR-31 | 133396 | IL31RA | interleukin 31 receptor A | 2 |
| 731 | hsa-miR-340 | 133396 | IL31RA | interleukin 31 receptor A | 2 |
| 732 | hsa-miR-383 | 133396 | IL31RA | interleukin 31 receptor A | 2 |
| 733 | hsa-miR-449a | 133396 | IL31RA | interleukin 31 receptor A | 2 |
| 734 | hsa-miR-490-5p | 133396 | IL31RA | interleukin 31 receptor A | 2 |
| 735 | hsa-miR-499-5p | 133396 | IL31RA | interleukin 31 receptor A | 2 |
| 738 | hsa-miR-621 | 133396 | IL31RA | interleukin 31 receptor A | 2 |
| 739 | hsa-miR-653 | 133396 | IL31RA | interleukin 31 receptor A | 2 |
| 740 | hsa-miR-765 | 133396 | IL31RA | interleukin 31 receptor A | 2 |
| 741 | hsa-miR-770-5p | 133396 | IL31RA | interleukin 31 receptor A | 2 |
| 742 | hsa-miR-877 | 133396 | IL31RA | interleukin 31 receptor A | 2 |
| 744 | hsa-mir-26a-1 | 133396 | IL31RA | interleukin 31 receptor A | 2 |
| 745 | hsa-mir-26b | 133396 | IL31RA | interleukin 31 receptor A | 2 |
| 746 | hsa-mir-31 | 133396 | IL31RA | interleukin 31 receptor A | 2 |
| 747 | hsa-mir-194-2 | 133396 | IL31RA | interleukin 31 receptor A | 2 |
| 748 | hsa-mir-340 | 133396 | IL31RA | interleukin 31 receptor A | 2 |
| 749 | hsa-mir-490 | 133396 | IL31RA | interleukin 31 receptor A | 2 |
| 750 | hsa-mir-449b | 133396 | IL31RA | interleukin 31 receptor A | 2 |
| 751 | hsa-mir-449c | 133396 | IL31RA | interleukin 31 receptor A | 2 |
| 857 | hsa-miR-490-5p | 6352 | CCL5 | chemokine (C-C motif) ligand 5 | 2 |
| 858 | hsa-miR-564 | 6352 | CCL5 | chemokine (C-C motif) ligand 5 | 2 |
| 861 | hsa-mir-490 | 6352 | CCL5 | chemokine (C-C motif) ligand 5 | 2 |
| 862 | hsa-miR-10a | 54464 | XRN1 | 5'-3' exoribonuclease 1 | 2 |
| 863 | hsa-miR-152 | 54464 | XRN1 | 5'-3' exoribonuclease 1 | 2 |
| 864 | hsa-miR-26a | 54464 | XRN1 | 5'-3' exoribonuclease 1 | 2 |
| 865 | hsa-miR-26b | 54464 | XRN1 | 5'-3' exoribonuclease 1 | 2 |
| 866 | hsa-miR-31 | 54464 | XRN1 | 5'-3' exoribonuclease 1 | 2 |
| 867 | hsa-miR-338-5p | 54464 | XRN1 | 5'-3' exoribonuclease 1 | 2 |
| 869 | hsa-miR-423-5p | 54464 | XRN1 | 5'-3' exoribonuclease 1 | 2 |
| 870 | hsa-miR-449a | 54464 | XRN1 | 5'-3' exoribonuclease 1 | 2 |
| 872 | hsa-miR-499-5p | 54464 | XRN1 | 5'-3' exoribonuclease 1 | 2 |
| 873 | hsa-miR-548b-5p | 54464 | XRN1 | 5'-3' exoribonuclease 1 | 2 |
| 874 | hsa-miR-564 | 54464 | XRN1 | 5'-3' exoribonuclease 1 | 2 |
| 875 | hsa-miR-621 | 54464 | XRN1 | 5'-3' exoribonuclease 1 | 2 |
| 877 | hsa-miR-657 | 54464 | XRN1 | 5'-3' exoribonuclease 1 | 2 |
| 878 | hsa-miR-765 | 54464 | XRN1 | 5'-3' exoribonuclease 1 | 2 |
| 879 | hsa-miR-770-5p | 54464 | XRN1 | 5'-3' exoribonuclease 1 | 2 |
| 880 | hsa-miR-877 | 54464 | XRN1 | 5'-3' exoribonuclease 1 | 2 |
| 882 | hsa-mir-26a-1 | 54464 | XRN1 | 5'-3' exoribonuclease 1 | 2 |
| 883 | hsa-mir-26b | 54464 | XRN1 | 5'-3' exoribonuclease 1 | 2 |
| 884 | hsa-mir-31 | 54464 | XRN1 | 5'-3' exoribonuclease 1 | 2 |
| 885 | hsa-mir-194-2 | 54464 | XRN1 | 5'-3' exoribonuclease 1 | 2 |
| 887 | hsa-mir-338 | 54464 | XRN1 | 5'-3' exoribonuclease 1 | 2 |
| 889 | hsa-mir-548b | 54464 | XRN1 | 5'-3' exoribonuclease 1 | 2 |
| 890 | hsa-mir-449b | 54464 | XRN1 | 5'-3' exoribonuclease 1 | 2 |
| 891 | hsa-mir-449c | 54464 | XRN1 | 5'-3' exoribonuclease 1 | 2 |
| 892 | hsa-mir-3065 | 54464 | XRN1 | 5'-3' exoribonuclease 1 | 2 |
| 936 | hsa-miR-1 | 338799 | LOC338799 | hypothetical LOC338799 | 2 |
| 937 | hsa-miR-133a | 338799 | LOC338799 | hypothetical LOC338799 | 2 |
| 938 | hsa-miR-186 | 338799 | LOC338799 | hypothetical LOC338799 | 2 |
| 939 | hsa-miR-326 | 338799 | LOC338799 | hypothetical LOC338799 | 2 |
| 940 | hsa-miR-449b | 338799 | LOC338799 | hypothetical LOC338799 | 2 |
| 941 | hsa-miR-551a | 338799 | LOC338799 | hypothetical LOC338799 | 2 |
| 942 | hsa-miR-562 | 338799 | LOC338799 | hypothetical LOC338799 | 2 |
| 943 | hsa-miR-575 | 338799 | LOC338799 | hypothetical LOC338799 | 2 |
| 944 | hsa-miR-579 | 338799 | LOC338799 | hypothetical LOC338799 | 2 |
| 945 | hsa-miR-608 | 338799 | LOC338799 | hypothetical LOC338799 | 2 |
| 946 | hsa-miR-637 | 338799 | LOC338799 | hypothetical LOC338799 | 2 |
| 947 | hsa-mir-22 | 338799 | LOC338799 | hypothetical LOC338799 | 2 |
| 948 | hsa-mir-10a | 338799 | LOC338799 | hypothetical LOC338799 | 2 |
| 949 | hsa-mir-186 | 338799 | LOC338799 | hypothetical LOC338799 | 2 |
| 950 | hsa-mir-155 | 338799 | LOC338799 | hypothetical LOC338799 | 2 |
| 951 | hsa-mir-423 | 338799 | LOC338799 | hypothetical LOC338799 | 2 |
| 952 | hsa-mir-877 | 338799 | LOC338799 | hypothetical LOC338799 | 2 |
| 953 | hsa-mir-3190 | 338799 | LOC338799 | hypothetical LOC338799 | 2 |
| 1080 | hsa-miR-383 | 51667 | NUB1 | negative regulator of ubiquitin-like proteins 1 | 2 |
| 1241 | hsa-miR-10a | 254102 | EHBP1L1 | EH domain binding protein 1-like 1 | 2 |
| 1242 | hsa-miR-139-5p | 254102 | EHBP1L1 | EH domain binding protein 1-like 1 | 2 |
| 1243 | hsa-miR-152 | 254102 | EHBP1L1 | EH domain binding protein 1-like 1 | 2 |
| 1244 | hsa-miR-26b | 254102 | EHBP1L1 | EH domain binding protein 1-like 1 | 2 |
| 1245 | hsa-miR-31 | 254102 | EHBP1L1 | EH domain binding protein 1-like 1 | 2 |
| 1246 | hsa-miR-338-5p | 254102 | EHBP1L1 | EH domain binding protein 1-like 1 | 2 |
| 1247 | hsa-miR-340 | 254102 | EHBP1L1 | EH domain binding protein 1-like 1 | 2 |
| 1248 | hsa-miR-383 | 254102 | EHBP1L1 | EH domain binding protein 1-like 1 | 2 |
| 1249 | hsa-miR-449a | 254102 | EHBP1L1 | EH domain binding protein 1-like 1 | 2 |
| 1250 | hsa-miR-499-5p | 254102 | EHBP1L1 | EH domain binding protein 1-like 1 | 2 |
| 1251 | hsa-miR-548b-5p | 254102 | EHBP1L1 | EH domain binding protein 1-like 1 | 2 |
| 1252 | hsa-miR-621 | 254102 | EHBP1L1 | EH domain binding protein 1-like 1 | 2 |
| 1253 | hsa-miR-657 | 254102 | EHBP1L1 | EH domain binding protein 1-like 1 | 2 |
| 1254 | hsa-miR-663 | 254102 | EHBP1L1 | EH domain binding protein 1-like 1 | 2 |
| 1255 | hsa-miR-765 | 254102 | EHBP1L1 | EH domain binding protein 1-like 1 | 2 |
| 1256 | hsa-miR-770-5p | 254102 | EHBP1L1 | EH domain binding protein 1-like 1 | 2 |
| 1257 | hsa-miR-877 | 254102 | EHBP1L1 | EH domain binding protein 1-like 1 | 2 |
| 1258 | hsa-mir-26a-1 | 254102 | EHBP1L1 | EH domain binding protein 1-like 1 | 2 |
| 1259 | hsa-mir-26b | 254102 | EHBP1L1 | EH domain binding protein 1-like 1 | 2 |
| 1260 | hsa-mir-31 | 254102 | EHBP1L1 | EH domain binding protein 1-like 1 | 2 |
| 1261 | hsa-mir-194-2 | 254102 | EHBP1L1 | EH domain binding protein 1-like 1 | 2 |
| 1262 | hsa-mir-340 | 254102 | EHBP1L1 | EH domain binding protein 1-like 1 | 2 |
| 1263 | hsa-mir-338 | 254102 | EHBP1L1 | EH domain binding protein 1-like 1 | 2 |
| 1264 | hsa-mir-548b | 254102 | EHBP1L1 | EH domain binding protein 1-like 1 | 2 |
| 1266 | hsa-mir-449b | 254102 | EHBP1L1 | EH domain binding protein 1-like 1 | 2 |
| 1267 | hsa-mir-449c | 254102 | EHBP1L1 | EH domain binding protein 1-like 1 | 2 |
| 1268 | hsa-mir-3065 | 254102 | EHBP1L1 | EH domain binding protein 1-like 1 | 2 |
| 1927 | hsa-mir-2116 | 4094 | MAF | v-maf musculoaponeurotic fibrosarcoma oncogene homolog (avian) | 2 |
| 1929 | hsa-miR-1 | 57492 | ARID1B | AT rich interactive domain 1B (SWI1-like) | 2 |
| 1930 | hsa-miR-133a | 57492 | ARID1B | AT rich interactive domain 1B (SWI1-like) | 2 |
| 1931 | hsa-miR-186 | 57492 | ARID1B | AT rich interactive domain 1B (SWI1-like) | 2 |
| 1932 | hsa-miR-326 | 57492 | ARID1B | AT rich interactive domain 1B (SWI1-like) | 2 |
| 1933 | hsa-miR-449b | 57492 | ARID1B | AT rich interactive domain 1B (SWI1-like) | 2 |
| 1935 | hsa-miR-551a | 57492 | ARID1B | AT rich interactive domain 1B (SWI1-like) | 2 |
| 1936 | hsa-miR-562 | 57492 | ARID1B | AT rich interactive domain 1B (SWI1-like) | 2 |
| 1938 | hsa-miR-579 | 57492 | ARID1B | AT rich interactive domain 1B (SWI1-like) | 2 |
| 1939 | hsa-miR-608 | 57492 | ARID1B | AT rich interactive domain 1B (SWI1-like) | 2 |
| 1942 | hsa-mir-22 | 57492 | ARID1B | AT rich interactive domain 1B (SWI1-like) | 2 |
| 1943 | hsa-mir-10a | 57492 | ARID1B | AT rich interactive domain 1B (SWI1-like) | 2 |
| 1944 | hsa-mir-186 | 57492 | ARID1B | AT rich interactive domain 1B (SWI1-like) | 2 |
| 1945 | hsa-mir-155 | 57492 | ARID1B | AT rich interactive domain 1B (SWI1-like) | 2 |
| 1948 | hsa-mir-550-1 | 57492 | ARID1B | AT rich interactive domain 1B (SWI1-like) | 2 |
| 1950 | hsa-mir-877 | 57492 | ARID1B | AT rich interactive domain 1B (SWI1-like) | 2 |
| 1952 | hsa-mir-3186 | 57492 | ARID1B | AT rich interactive domain 1B (SWI1-like) | 2 |
| 2150 | hsa-miR-10a | 7453 | WARS | tryptophanyl-tRNA synthetase | 2 |
| 2151 | hsa-miR-152 | 7453 | WARS | tryptophanyl-tRNA synthetase | 2 |
| 2152 | hsa-miR-26a | 7453 | WARS | tryptophanyl-tRNA synthetase | 2 |
| 2153 | hsa-miR-26b | 7453 | WARS | tryptophanyl-tRNA synthetase | 2 |
| 2154 | hsa-miR-31 | 7453 | WARS | tryptophanyl-tRNA synthetase | 2 |
| 2155 | hsa-miR-338-5p | 7453 | WARS | tryptophanyl-tRNA synthetase | 2 |
| 2156 | hsa-miR-340 | 7453 | WARS | tryptophanyl-tRNA synthetase | 2 |
| 2157 | hsa-miR-423-5p | 7453 | WARS | tryptophanyl-tRNA synthetase | 2 |
| 2158 | hsa-miR-449a | 7453 | WARS | tryptophanyl-tRNA synthetase | 2 |
| 2159 | hsa-miR-490-5p | 7453 | WARS | tryptophanyl-tRNA synthetase | 2 |
| 2160 | hsa-miR-499-5p | 7453 | WARS | tryptophanyl-tRNA synthetase | 2 |
| 2161 | hsa-miR-548b-5p | 7453 | WARS | tryptophanyl-tRNA synthetase | 2 |
| 2162 | hsa-miR-564 | 7453 | WARS | tryptophanyl-tRNA synthetase | 2 |
| 2163 | hsa-miR-621 | 7453 | WARS | tryptophanyl-tRNA synthetase | 2 |
| 2164 | hsa-miR-644 | 7453 | WARS | tryptophanyl-tRNA synthetase | 2 |
| 2165 | hsa-miR-657 | 7453 | WARS | tryptophanyl-tRNA synthetase | 2 |
| 2166 | hsa-miR-765 | 7453 | WARS | tryptophanyl-tRNA synthetase | 2 |
| 2167 | hsa-miR-770-5p | 7453 | WARS | tryptophanyl-tRNA synthetase | 2 |
| 2168 | hsa-miR-877 | 7453 | WARS | tryptophanyl-tRNA synthetase | 2 |
| 2169 | hsa-miR-95 | 7453 | WARS | tryptophanyl-tRNA synthetase | 2 |
| 2170 | hsa-mir-26a-1 | 7453 | WARS | tryptophanyl-tRNA synthetase | 2 |
| 2171 | hsa-mir-26b | 7453 | WARS | tryptophanyl-tRNA synthetase | 2 |
| 2172 | hsa-mir-31 | 7453 | WARS | tryptophanyl-tRNA synthetase | 2 |
| 2173 | hsa-mir-194-2 | 7453 | WARS | tryptophanyl-tRNA synthetase | 2 |
| 2174 | hsa-mir-340 | 7453 | WARS | tryptophanyl-tRNA synthetase | 2 |
| 2175 | hsa-mir-338 | 7453 | WARS | tryptophanyl-tRNA synthetase | 2 |
| 2176 | hsa-mir-490 | 7453 | WARS | tryptophanyl-tRNA synthetase | 2 |
| 2177 | hsa-mir-548b | 7453 | WARS | tryptophanyl-tRNA synthetase | 2 |
| 2178 | hsa-mir-548d-2 | 7453 | WARS | tryptophanyl-tRNA synthetase | 2 |
| 2179 | hsa-mir-449b | 7453 | WARS | tryptophanyl-tRNA synthetase | 2 |
| 2180 | hsa-mir-449c | 7453 | WARS | tryptophanyl-tRNA synthetase | 2 |
| 2181 | hsa-mir-3065 | 7453 | WARS | tryptophanyl-tRNA synthetase | 2 |
| 2256 | hsa-miR-1 | 3693 | ITGB5 | integrin, beta 5 | 2 |
| 2257 | hsa-miR-133a | 3693 | ITGB5 | integrin, beta 5 | 2 |
| 2260 | hsa-miR-449b | 3693 | ITGB5 | integrin, beta 5 | 2 |
| 2261 | hsa-miR-562 | 3693 | ITGB5 | integrin, beta 5 | 2 |
| 2262 | hsa-miR-575 | 3693 | ITGB5 | integrin, beta 5 | 2 |
| 2264 | hsa-miR-608 | 3693 | ITGB5 | integrin, beta 5 | 2 |
| 2265 | hsa-miR-637 | 3693 | ITGB5 | integrin, beta 5 | 2 |
| 2267 | hsa-mir-22 | 3693 | ITGB5 | integrin, beta 5 | 2 |
| 2268 | hsa-mir-10a | 3693 | ITGB5 | integrin, beta 5 | 2 |
| 2270 | hsa-mir-155 | 3693 | ITGB5 | integrin, beta 5 | 2 |
| 2272 | hsa-mir-499 | 3693 | ITGB5 | integrin, beta 5 | 2 |
| 2274 | hsa-mir-877 | 3693 | ITGB5 | integrin, beta 5 | 2 |
| 2398 | hsa-miR-10a | 8519 | IFITM1 | interferon induced transmembrane protein 1 (9-27) | 2 |
| 2399 | hsa-miR-152 | 8519 | IFITM1 | interferon induced transmembrane protein 1 (9-27) | 2 |
| 2400 | hsa-miR-26a | 8519 | IFITM1 | interferon induced transmembrane protein 1 (9-27) | 2 |
| 2401 | hsa-miR-26b | 8519 | IFITM1 | interferon induced transmembrane protein 1 (9-27) | 2 |
| 2402 | hsa-miR-31 | 8519 | IFITM1 | interferon induced transmembrane protein 1 (9-27) | 2 |
| 2403 | hsa-miR-340 | 8519 | IFITM1 | interferon induced transmembrane protein 1 (9-27) | 2 |
| 2404 | hsa-miR-449a | 8519 | IFITM1 | interferon induced transmembrane protein 1 (9-27) | 2 |
| 2405 | hsa-miR-490-5p | 8519 | IFITM1 | interferon induced transmembrane protein 1 (9-27) | 2 |
| 2406 | hsa-miR-499-5p | 8519 | IFITM1 | interferon induced transmembrane protein 1 (9-27) | 2 |
| 2407 | hsa-miR-548b-5p | 8519 | IFITM1 | interferon induced transmembrane protein 1 (9-27) | 2 |
| 2408 | hsa-miR-564 | 8519 | IFITM1 | interferon induced transmembrane protein 1 (9-27) | 2 |
| 2410 | hsa-miR-621 | 8519 | IFITM1 | interferon induced transmembrane protein 1 (9-27) | 2 |
| 2411 | hsa-miR-634 | 8519 | IFITM1 | interferon induced transmembrane protein 1 (9-27) | 2 |
| 2412 | hsa-miR-644 | 8519 | IFITM1 | interferon induced transmembrane protein 1 (9-27) | 2 |
| 2413 | hsa-miR-765 | 8519 | IFITM1 | interferon induced transmembrane protein 1 (9-27) | 2 |
| 2414 | hsa-miR-770-5p | 8519 | IFITM1 | interferon induced transmembrane protein 1 (9-27) | 2 |
| 2415 | hsa-miR-877 | 8519 | IFITM1 | interferon induced transmembrane protein 1 (9-27) | 2 |
| 2416 | hsa-miR-95 | 8519 | IFITM1 | interferon induced transmembrane protein 1 (9-27) | 2 |
| 2417 | hsa-mir-26a-1 | 8519 | IFITM1 | interferon induced transmembrane protein 1 (9-27) | 2 |
| 2418 | hsa-mir-26b | 8519 | IFITM1 | interferon induced transmembrane protein 1 (9-27) | 2 |
| 2419 | hsa-mir-31 | 8519 | IFITM1 | interferon induced transmembrane protein 1 (9-27) | 2 |
| 2420 | hsa-mir-194-2 | 8519 | IFITM1 | interferon induced transmembrane protein 1 (9-27) | 2 |
| 2421 | hsa-mir-340 | 8519 | IFITM1 | interferon induced transmembrane protein 1 (9-27) | 2 |
| 2422 | hsa-mir-490 | 8519 | IFITM1 | interferon induced transmembrane protein 1 (9-27) | 2 |
| 2423 | hsa-mir-548b | 8519 | IFITM1 | interferon induced transmembrane protein 1 (9-27) | 2 |
| 2424 | hsa-mir-449b | 8519 | IFITM1 | interferon induced transmembrane protein 1 (9-27) | 2 |
| 2425 | hsa-mir-449c | 8519 | IFITM1 | interferon induced transmembrane protein 1 (9-27) | 2 |
| 2727 | hsa-miR-423-5p | 2633 | GBP1 | guanylate binding protein 1, interferon-inducible, 67kDa | 2 |
| 2729 | hsa-miR-490-5p | 2633 | GBP1 | guanylate binding protein 1, interferon-inducible, 67kDa | 2 |
| 2732 | hsa-miR-564 | 2633 | GBP1 | guanylate binding protein 1, interferon-inducible, 67kDa | 2 |
| 2744 | hsa-mir-490 | 2633 | GBP1 | guanylate binding protein 1, interferon-inducible, 67kDa | 2 |
| 2751 | hsa-miR-663 | 2633 | GBP1 | guanylate binding protein 1, interferon-inducible, 67kDa | 2 |
| 2870 | hsa-miR-10a | 3659 | IRF1 | interferon regulatory factor 1 | 2 |
| 2871 | hsa-miR-139-5p | 3659 | IRF1 | interferon regulatory factor 1 | 2 |
| 2872 | hsa-miR-152 | 3659 | IRF1 | interferon regulatory factor 1 | 2 |
| 2873 | hsa-miR-26a | 3659 | IRF1 | interferon regulatory factor 1 | 2 |
| 2874 | hsa-miR-26b | 3659 | IRF1 | interferon regulatory factor 1 | 2 |
| 2875 | hsa-miR-31 | 3659 | IRF1 | interferon regulatory factor 1 | 2 |
| 2876 | hsa-miR-338-5p | 3659 | IRF1 | interferon regulatory factor 1 | 2 |
| 2877 | hsa-miR-340 | 3659 | IRF1 | interferon regulatory factor 1 | 2 |
| 2878 | hsa-miR-383 | 3659 | IRF1 | interferon regulatory factor 1 | 2 |
| 2879 | hsa-miR-449a | 3659 | IRF1 | interferon regulatory factor 1 | 2 |
| 2880 | hsa-miR-499-5p | 3659 | IRF1 | interferon regulatory factor 1 | 2 |
| 2881 | hsa-miR-548b-5p | 3659 | IRF1 | interferon regulatory factor 1 | 2 |
| 2882 | hsa-miR-621 | 3659 | IRF1 | interferon regulatory factor 1 | 2 |
| 2883 | hsa-miR-657 | 3659 | IRF1 | interferon regulatory factor 1 | 2 |
| 2884 | hsa-miR-765 | 3659 | IRF1 | interferon regulatory factor 1 | 2 |
| 2885 | hsa-miR-770-5p | 3659 | IRF1 | interferon regulatory factor 1 | 2 |
| 2886 | hsa-miR-877 | 3659 | IRF1 | interferon regulatory factor 1 | 2 |
| 2887 | hsa-mir-26a-1 | 3659 | IRF1 | interferon regulatory factor 1 | 2 |
| 2888 | hsa-mir-26b | 3659 | IRF1 | interferon regulatory factor 1 | 2 |
| 2889 | hsa-mir-31 | 3659 | IRF1 | interferon regulatory factor 1 | 2 |
| 2890 | hsa-mir-194-2 | 3659 | IRF1 | interferon regulatory factor 1 | 2 |
| 2891 | hsa-mir-340 | 3659 | IRF1 | interferon regulatory factor 1 | 2 |
| 2892 | hsa-mir-338 | 3659 | IRF1 | interferon regulatory factor 1 | 2 |
| 2893 | hsa-mir-548b | 3659 | IRF1 | interferon regulatory factor 1 | 2 |
| 2894 | hsa-mir-449b | 3659 | IRF1 | interferon regulatory factor 1 | 2 |
| 2895 | hsa-mir-449c | 3659 | IRF1 | interferon regulatory factor 1 | 2 |
| 2896 | hsa-mir-3065 | 3659 | IRF1 | interferon regulatory factor 1 | 2 |
| 2923 | hsa-miR-10a | 7128 | TNFAIP3 | tumor necrosis factor, alpha-induced protein 3 | 2 |
| 2924 | hsa-miR-139-5p | 7128 | TNFAIP3 | tumor necrosis factor, alpha-induced protein 3 | 2 |
| 2925 | hsa-miR-152 | 7128 | TNFAIP3 | tumor necrosis factor, alpha-induced protein 3 | 2 |
| 2926 | hsa-miR-26a | 7128 | TNFAIP3 | tumor necrosis factor, alpha-induced protein 3 | 2 |
| 2927 | hsa-miR-26b | 7128 | TNFAIP3 | tumor necrosis factor, alpha-induced protein 3 | 2 |
| 2928 | hsa-miR-31 | 7128 | TNFAIP3 | tumor necrosis factor, alpha-induced protein 3 | 2 |
| 2929 | hsa-miR-338-5p | 7128 | TNFAIP3 | tumor necrosis factor, alpha-induced protein 3 | 2 |
| 2930 | hsa-miR-340 | 7128 | TNFAIP3 | tumor necrosis factor, alpha-induced protein 3 | 2 |
| 2931 | hsa-miR-449a | 7128 | TNFAIP3 | tumor necrosis factor, alpha-induced protein 3 | 2 |
| 2933 | hsa-miR-499-5p | 7128 | TNFAIP3 | tumor necrosis factor, alpha-induced protein 3 | 2 |
| 2934 | hsa-miR-548b-5p | 7128 | TNFAIP3 | tumor necrosis factor, alpha-induced protein 3 | 2 |
| 2936 | hsa-miR-621 | 7128 | TNFAIP3 | tumor necrosis factor, alpha-induced protein 3 | 2 |
| 2937 | hsa-miR-657 | 7128 | TNFAIP3 | tumor necrosis factor, alpha-induced protein 3 | 2 |
| 2938 | hsa-miR-663 | 7128 | TNFAIP3 | tumor necrosis factor, alpha-induced protein 3 | 2 |
| 2939 | hsa-miR-765 | 7128 | TNFAIP3 | tumor necrosis factor, alpha-induced protein 3 | 2 |
| 2940 | hsa-miR-770-5p | 7128 | TNFAIP3 | tumor necrosis factor, alpha-induced protein 3 | 2 |
| 2941 | hsa-miR-877 | 7128 | TNFAIP3 | tumor necrosis factor, alpha-induced protein 3 | 2 |
| 2942 | hsa-mir-26a-1 | 7128 | TNFAIP3 | tumor necrosis factor, alpha-induced protein 3 | 2 |
| 2943 | hsa-mir-26b | 7128 | TNFAIP3 | tumor necrosis factor, alpha-induced protein 3 | 2 |
| 2944 | hsa-mir-31 | 7128 | TNFAIP3 | tumor necrosis factor, alpha-induced protein 3 | 2 |
| 2945 | hsa-mir-194-2 | 7128 | TNFAIP3 | tumor necrosis factor, alpha-induced protein 3 | 2 |
| 2946 | hsa-mir-340 | 7128 | TNFAIP3 | tumor necrosis factor, alpha-induced protein 3 | 2 |
| 2947 | hsa-mir-338 | 7128 | TNFAIP3 | tumor necrosis factor, alpha-induced protein 3 | 2 |
| 2949 | hsa-mir-548b | 7128 | TNFAIP3 | tumor necrosis factor, alpha-induced protein 3 | 2 |
| 2950 | hsa-mir-449b | 7128 | TNFAIP3 | tumor necrosis factor, alpha-induced protein 3 | 2 |
| 2951 | hsa-mir-449c | 7128 | TNFAIP3 | tumor necrosis factor, alpha-induced protein 3 | 2 |
| 2952 | hsa-mir-3065 | 7128 | TNFAIP3 | tumor necrosis factor, alpha-induced protein 3 | 2 |
| 3028 | hsa-miR-562 | 23428 | SLC7A8 | solute carrier family 7 (cationic amino acid transporter, y+ system), member 8 | 2 |
| 3033 | hsa-miR-637 | 23428 | SLC7A8 | solute carrier family 7 (cationic amino acid transporter, y+ system), member 8 | 2 |
| 3100 | hsa-miR-10a | 140885 | SIRPA | signal-regulatory protein alpha | 2 |
| 3101 | hsa-miR-152 | 140885 | SIRPA | signal-regulatory protein alpha | 2 |
| 3103 | hsa-miR-26b | 140885 | SIRPA | signal-regulatory protein alpha | 2 |
| 3104 | hsa-miR-31 | 140885 | SIRPA | signal-regulatory protein alpha | 2 |
| 3105 | hsa-miR-340 | 140885 | SIRPA | signal-regulatory protein alpha | 2 |
| 3107 | hsa-miR-449a | 140885 | SIRPA | signal-regulatory protein alpha | 2 |
| 3109 | hsa-miR-499-5p | 140885 | SIRPA | signal-regulatory protein alpha | 2 |
| 3110 | hsa-miR-548b-5p | 140885 | SIRPA | signal-regulatory protein alpha | 2 |
| 3113 | hsa-miR-621 | 140885 | SIRPA | signal-regulatory protein alpha | 2 |
| 3117 | hsa-miR-765 | 140885 | SIRPA | signal-regulatory protein alpha | 2 |
| 3118 | hsa-miR-770-5p | 140885 | SIRPA | signal-regulatory protein alpha | 2 |
| 3119 | hsa-miR-877 | 140885 | SIRPA | signal-regulatory protein alpha | 2 |
| 3121 | hsa-mir-26a-1 | 140885 | SIRPA | signal-regulatory protein alpha | 2 |
| 3122 | hsa-mir-26b | 140885 | SIRPA | signal-regulatory protein alpha | 2 |
| 3123 | hsa-mir-31 | 140885 | SIRPA | signal-regulatory protein alpha | 2 |
| 3124 | hsa-mir-194-2 | 140885 | SIRPA | signal-regulatory protein alpha | 2 |
| 3125 | hsa-mir-340 | 140885 | SIRPA | signal-regulatory protein alpha | 2 |
| 3127 | hsa-mir-548b | 140885 | SIRPA | signal-regulatory protein alpha | 2 |
| 3128 | hsa-mir-449b | 140885 | SIRPA | signal-regulatory protein alpha | 2 |
| 3129 | hsa-mir-449c | 140885 | SIRPA | signal-regulatory protein alpha | 2 |
| 3186 | hsa-miR-10a | 9693 | RAPGEF2 | Rap guanine nucleotide exchange factor (GEF) 2 | 2 |
| 3188 | hsa-miR-139-5p | 9693 | RAPGEF2 | Rap guanine nucleotide exchange factor (GEF) 2 | 2 |
| 3190 | hsa-miR-26a | 9693 | RAPGEF2 | Rap guanine nucleotide exchange factor (GEF) 2 | 2 |
| 3191 | hsa-miR-26b | 9693 | RAPGEF2 | Rap guanine nucleotide exchange factor (GEF) 2 | 2 |
| 3192 | hsa-miR-338-5p | 9693 | RAPGEF2 | Rap guanine nucleotide exchange factor (GEF) 2 | 2 |
| 3193 | hsa-miR-340 | 9693 | RAPGEF2 | Rap guanine nucleotide exchange factor (GEF) 2 | 2 |
| 3195 | hsa-miR-449a | 9693 | RAPGEF2 | Rap guanine nucleotide exchange factor (GEF) 2 | 2 |
| 3196 | hsa-miR-499-5p | 9693 | RAPGEF2 | Rap guanine nucleotide exchange factor (GEF) 2 | 2 |
| 3197 | hsa-miR-548b-5p | 9693 | RAPGEF2 | Rap guanine nucleotide exchange factor (GEF) 2 | 2 |
| 3198 | hsa-miR-621 | 9693 | RAPGEF2 | Rap guanine nucleotide exchange factor (GEF) 2 | 2 |
| 3199 | hsa-miR-657 | 9693 | RAPGEF2 | Rap guanine nucleotide exchange factor (GEF) 2 | 2 |
| 3200 | hsa-miR-765 | 9693 | RAPGEF2 | Rap guanine nucleotide exchange factor (GEF) 2 | 2 |
| 3201 | hsa-miR-770-5p | 9693 | RAPGEF2 | Rap guanine nucleotide exchange factor (GEF) 2 | 2 |
| 3202 | hsa-miR-877 | 9693 | RAPGEF2 | Rap guanine nucleotide exchange factor (GEF) 2 | 2 |
| 3203 | hsa-mir-26a-1 | 9693 | RAPGEF2 | Rap guanine nucleotide exchange factor (GEF) 2 | 2 |
| 3204 | hsa-mir-26b | 9693 | RAPGEF2 | Rap guanine nucleotide exchange factor (GEF) 2 | 2 |
| 3205 | hsa-mir-194-2 | 9693 | RAPGEF2 | Rap guanine nucleotide exchange factor (GEF) 2 | 2 |
| 3206 | hsa-mir-340 | 9693 | RAPGEF2 | Rap guanine nucleotide exchange factor (GEF) 2 | 2 |
| 3207 | hsa-mir-338 | 9693 | RAPGEF2 | Rap guanine nucleotide exchange factor (GEF) 2 | 2 |
| 3208 | hsa-mir-548b | 9693 | RAPGEF2 | Rap guanine nucleotide exchange factor (GEF) 2 | 2 |
| 3209 | hsa-mir-548d-2 | 9693 | RAPGEF2 | Rap guanine nucleotide exchange factor (GEF) 2 | 2 |
| 3210 | hsa-mir-449b | 9693 | RAPGEF2 | Rap guanine nucleotide exchange factor (GEF) 2 | 2 |
| 3211 | hsa-mir-449c | 9693 | RAPGEF2 | Rap guanine nucleotide exchange factor (GEF) 2 | 2 |
| 3212 | hsa-mir-3065 | 9693 | RAPGEF2 | Rap guanine nucleotide exchange factor (GEF) 2 | 2 |
| 3924 | hsa-miR-152 | 960 | CD44 | CD44 molecule (Indian blood group) | 2 |
| 3925 | hsa-miR-26b | 960 | CD44 | CD44 molecule (Indian blood group) | 2 |
| 3926 | hsa-miR-31 | 960 | CD44 | CD44 molecule (Indian blood group) | 2 |
| 3927 | hsa-miR-340 | 960 | CD44 | CD44 molecule (Indian blood group) | 2 |
| 3929 | hsa-miR-449a | 960 | CD44 | CD44 molecule (Indian blood group) | 2 |
| 3930 | hsa-miR-621 | 960 | CD44 | CD44 molecule (Indian blood group) | 2 |
| 3931 | hsa-miR-653 | 960 | CD44 | CD44 molecule (Indian blood group) | 2 |
| 3932 | hsa-miR-765 | 960 | CD44 | CD44 molecule (Indian blood group) | 2 |
| 3933 | hsa-miR-770-5p | 960 | CD44 | CD44 molecule (Indian blood group) | 2 |
| 3934 | hsa-mir-26a-1 | 960 | CD44 | CD44 molecule (Indian blood group) | 2 |
| 3935 | hsa-mir-26b | 960 | CD44 | CD44 molecule (Indian blood group) | 2 |
| 3936 | hsa-mir-31 | 960 | CD44 | CD44 molecule (Indian blood group) | 2 |
| 3937 | hsa-mir-194-2 | 960 | CD44 | CD44 molecule (Indian blood group) | 2 |
| 3938 | hsa-mir-340 | 960 | CD44 | CD44 molecule (Indian blood group) | 2 |
| 3939 | hsa-mir-449b | 960 | CD44 | CD44 molecule (Indian blood group) | 2 |
| 3940 | hsa-mir-449c | 960 | CD44 | CD44 molecule (Indian blood group) | 2 |
| 3974 | hsa-miR-10a | 3669 | ISG20 | interferon stimulated exonuclease gene 20kDa | 2 |
| 3975 | hsa-miR-152 | 3669 | ISG20 | interferon stimulated exonuclease gene 20kDa | 2 |
| 3976 | hsa-miR-26a | 3669 | ISG20 | interferon stimulated exonuclease gene 20kDa | 2 |
| 3977 | hsa-miR-26b | 3669 | ISG20 | interferon stimulated exonuclease gene 20kDa | 2 |
| 3978 | hsa-miR-31 | 3669 | ISG20 | interferon stimulated exonuclease gene 20kDa | 2 |
| 3979 | hsa-miR-340 | 3669 | ISG20 | interferon stimulated exonuclease gene 20kDa | 2 |
| 3980 | hsa-miR-423-5p | 3669 | ISG20 | interferon stimulated exonuclease gene 20kDa | 2 |
| 3981 | hsa-miR-449a | 3669 | ISG20 | interferon stimulated exonuclease gene 20kDa | 2 |
| 3982 | hsa-miR-490-5p | 3669 | ISG20 | interferon stimulated exonuclease gene 20kDa | 2 |
| 3983 | hsa-miR-499-5p | 3669 | ISG20 | interferon stimulated exonuclease gene 20kDa | 2 |
| 3984 | hsa-miR-548b-5p | 3669 | ISG20 | interferon stimulated exonuclease gene 20kDa | 2 |
| 3985 | hsa-miR-564 | 3669 | ISG20 | interferon stimulated exonuclease gene 20kDa | 2 |
| 3987 | hsa-miR-621 | 3669 | ISG20 | interferon stimulated exonuclease gene 20kDa | 2 |
| 3988 | hsa-miR-634 | 3669 | ISG20 | interferon stimulated exonuclease gene 20kDa | 2 |
| 3989 | hsa-miR-644 | 3669 | ISG20 | interferon stimulated exonuclease gene 20kDa | 2 |
| 3991 | hsa-miR-765 | 3669 | ISG20 | interferon stimulated exonuclease gene 20kDa | 2 |
| 3992 | hsa-miR-770-5p | 3669 | ISG20 | interferon stimulated exonuclease gene 20kDa | 2 |
| 3993 | hsa-miR-877 | 3669 | ISG20 | interferon stimulated exonuclease gene 20kDa | 2 |
| 3994 | hsa-miR-95 | 3669 | ISG20 | interferon stimulated exonuclease gene 20kDa | 2 |
| 3995 | hsa-mir-26a-1 | 3669 | ISG20 | interferon stimulated exonuclease gene 20kDa | 2 |
| 3996 | hsa-mir-26b | 3669 | ISG20 | interferon stimulated exonuclease gene 20kDa | 2 |
| 3997 | hsa-mir-31 | 3669 | ISG20 | interferon stimulated exonuclease gene 20kDa | 2 |
| 3998 | hsa-mir-194-2 | 3669 | ISG20 | interferon stimulated exonuclease gene 20kDa | 2 |
| 3999 | hsa-mir-340 | 3669 | ISG20 | interferon stimulated exonuclease gene 20kDa | 2 |
| 4000 | hsa-mir-490 | 3669 | ISG20 | interferon stimulated exonuclease gene 20kDa | 2 |
| 4001 | hsa-mir-548b | 3669 | ISG20 | interferon stimulated exonuclease gene 20kDa | 2 |
| 4002 | hsa-mir-449b | 3669 | ISG20 | interferon stimulated exonuclease gene 20kDa | 2 |
| 4003 | hsa-mir-449c | 3669 | ISG20 | interferon stimulated exonuclease gene 20kDa | 2 |
| 4032 | hsa-miR-152 | 3134 | HLA-F | major histocompatibility complex, class I, F | 2 |
| 4033 | hsa-miR-26b | 3134 | HLA-F | major histocompatibility complex, class I, F | 2 |
| 4034 | hsa-miR-31 | 3134 | HLA-F | major histocompatibility complex, class I, F | 2 |
| 4035 | hsa-miR-338-5p | 3134 | HLA-F | major histocompatibility complex, class I, F | 2 |
| 4036 | hsa-miR-340 | 3134 | HLA-F | major histocompatibility complex, class I, F | 2 |
| 4037 | hsa-miR-383 | 3134 | HLA-F | major histocompatibility complex, class I, F | 2 |
| 4038 | hsa-miR-449a | 3134 | HLA-F | major histocompatibility complex, class I, F | 2 |
| 4039 | hsa-miR-621 | 3134 | HLA-F | major histocompatibility complex, class I, F | 2 |
| 4040 | hsa-miR-657 | 3134 | HLA-F | major histocompatibility complex, class I, F | 2 |
| 4041 | hsa-miR-765 | 3134 | HLA-F | major histocompatibility complex, class I, F | 2 |
| 4042 | hsa-miR-770-5p | 3134 | HLA-F | major histocompatibility complex, class I, F | 2 |
| 4043 | hsa-mir-26a-1 | 3134 | HLA-F | major histocompatibility complex, class I, F | 2 |
| 4044 | hsa-mir-26b | 3134 | HLA-F | major histocompatibility complex, class I, F | 2 |
| 4045 | hsa-mir-31 | 3134 | HLA-F | major histocompatibility complex, class I, F | 2 |
| 4046 | hsa-mir-194-2 | 3134 | HLA-F | major histocompatibility complex, class I, F | 2 |
| 4047 | hsa-mir-340 | 3134 | HLA-F | major histocompatibility complex, class I, F | 2 |
| 4048 | hsa-mir-338 | 3134 | HLA-F | major histocompatibility complex, class I, F | 2 |
| 4049 | hsa-mir-449b | 3134 | HLA-F | major histocompatibility complex, class I, F | 2 |
| 4050 | hsa-mir-449c | 3134 | HLA-F | major histocompatibility complex, class I, F | 2 |
| 4051 | hsa-mir-3065 | 3134 | HLA-F | major histocompatibility complex, class I, F | 2 |
| 4102 | hsa-miR-10a | 3553 | IL1B | interleukin 1, beta | 2 |
| 4103 | hsa-miR-152 | 3553 | IL1B | interleukin 1, beta | 2 |
| 4104 | hsa-miR-26a | 3553 | IL1B | interleukin 1, beta | 2 |
| 4105 | hsa-miR-26b | 3553 | IL1B | interleukin 1, beta | 2 |
| 4106 | hsa-miR-31 | 3553 | IL1B | interleukin 1, beta | 2 |
| 4107 | hsa-miR-338-5p | 3553 | IL1B | interleukin 1, beta | 2 |
| 4108 | hsa-miR-340 | 3553 | IL1B | interleukin 1, beta | 2 |
| 4109 | hsa-miR-449a | 3553 | IL1B | interleukin 1, beta | 2 |
| 4110 | hsa-miR-490-5p | 3553 | IL1B | interleukin 1, beta | 2 |
| 4111 | hsa-miR-499-5p | 3553 | IL1B | interleukin 1, beta | 2 |
| 4112 | hsa-miR-548b-5p | 3553 | IL1B | interleukin 1, beta | 2 |
| 4113 | hsa-miR-564 | 3553 | IL1B | interleukin 1, beta | 2 |
| 4114 | hsa-miR-621 | 3553 | IL1B | interleukin 1, beta | 2 |
| 4115 | hsa-miR-657 | 3553 | IL1B | interleukin 1, beta | 2 |
| 4116 | hsa-miR-765 | 3553 | IL1B | interleukin 1, beta | 2 |
| 4117 | hsa-miR-770-5p | 3553 | IL1B | interleukin 1, beta | 2 |
| 4118 | hsa-miR-877 | 3553 | IL1B | interleukin 1, beta | 2 |
| 4119 | hsa-mir-26a-1 | 3553 | IL1B | interleukin 1, beta | 2 |
| 4120 | hsa-mir-26b | 3553 | IL1B | interleukin 1, beta | 2 |
| 4121 | hsa-mir-31 | 3553 | IL1B | interleukin 1, beta | 2 |
| 4122 | hsa-mir-194-2 | 3553 | IL1B | interleukin 1, beta | 2 |
| 4123 | hsa-mir-340 | 3553 | IL1B | interleukin 1, beta | 2 |
| 4124 | hsa-mir-338 | 3553 | IL1B | interleukin 1, beta | 2 |
| 4125 | hsa-mir-490 | 3553 | IL1B | interleukin 1, beta | 2 |
| 4126 | hsa-mir-548b | 3553 | IL1B | interleukin 1, beta | 2 |
| 4127 | hsa-mir-449b | 3553 | IL1B | interleukin 1, beta | 2 |
| 4128 | hsa-mir-449c | 3553 | IL1B | interleukin 1, beta | 2 |
| 4129 | hsa-mir-3065 | 3553 | IL1B | interleukin 1, beta | 2 |
| 4130 | hsa-miR-10a | 958 | CD40 | CD40 molecule, TNF receptor superfamily member 5 | 2 |
| 4131 | hsa-miR-1228\* | 958 | CD40 | CD40 molecule, TNF receptor superfamily member 5 | 2 |
| 4132 | hsa-miR-139-5p | 958 | CD40 | CD40 molecule, TNF receptor superfamily member 5 | 2 |
| 4133 | hsa-miR-152 | 958 | CD40 | CD40 molecule, TNF receptor superfamily member 5 | 2 |
| 4134 | hsa-miR-26a | 958 | CD40 | CD40 molecule, TNF receptor superfamily member 5 | 2 |
| 4135 | hsa-miR-26b | 958 | CD40 | CD40 molecule, TNF receptor superfamily member 5 | 2 |
| 4136 | hsa-miR-338-5p | 958 | CD40 | CD40 molecule, TNF receptor superfamily member 5 | 2 |
| 4137 | hsa-miR-340 | 958 | CD40 | CD40 molecule, TNF receptor superfamily member 5 | 2 |
| 4138 | hsa-miR-423-5p | 958 | CD40 | CD40 molecule, TNF receptor superfamily member 5 | 2 |
| 4139 | hsa-miR-449a | 958 | CD40 | CD40 molecule, TNF receptor superfamily member 5 | 2 |
| 4140 | hsa-miR-499-5p | 958 | CD40 | CD40 molecule, TNF receptor superfamily member 5 | 2 |
| 4141 | hsa-miR-548b-5p | 958 | CD40 | CD40 molecule, TNF receptor superfamily member 5 | 2 |
| 4142 | hsa-miR-564 | 958 | CD40 | CD40 molecule, TNF receptor superfamily member 5 | 2 |
| 4143 | hsa-miR-621 | 958 | CD40 | CD40 molecule, TNF receptor superfamily member 5 | 2 |
| 4145 | hsa-miR-657 | 958 | CD40 | CD40 molecule, TNF receptor superfamily member 5 | 2 |
| 4146 | hsa-miR-765 | 958 | CD40 | CD40 molecule, TNF receptor superfamily member 5 | 2 |
| 4147 | hsa-miR-770-5p | 958 | CD40 | CD40 molecule, TNF receptor superfamily member 5 | 2 |
| 4148 | hsa-miR-877 | 958 | CD40 | CD40 molecule, TNF receptor superfamily member 5 | 2 |
| 4149 | hsa-mir-26a-1 | 958 | CD40 | CD40 molecule, TNF receptor superfamily member 5 | 2 |
| 4150 | hsa-mir-26b | 958 | CD40 | CD40 molecule, TNF receptor superfamily member 5 | 2 |
| 4151 | hsa-mir-194-2 | 958 | CD40 | CD40 molecule, TNF receptor superfamily member 5 | 2 |
| 4152 | hsa-mir-340 | 958 | CD40 | CD40 molecule, TNF receptor superfamily member 5 | 2 |
| 4153 | hsa-mir-338 | 958 | CD40 | CD40 molecule, TNF receptor superfamily member 5 | 2 |
| 4154 | hsa-mir-548b | 958 | CD40 | CD40 molecule, TNF receptor superfamily member 5 | 2 |
| 4155 | hsa-mir-548d-2 | 958 | CD40 | CD40 molecule, TNF receptor superfamily member 5 | 2 |
| 4156 | hsa-mir-449b | 958 | CD40 | CD40 molecule, TNF receptor superfamily member 5 | 2 |
| 4157 | hsa-mir-449c | 958 | CD40 | CD40 molecule, TNF receptor superfamily member 5 | 2 |
| 4158 | hsa-mir-3065 | 958 | CD40 | CD40 molecule, TNF receptor superfamily member 5 | 2 |
| 4286 | hsa-miR-10a | 4520 | MTF1 | metal-regulatory transcription factor 1 | 2 |
| 4287 | hsa-miR-139-5p | 4520 | MTF1 | metal-regulatory transcription factor 1 | 2 |
| 4288 | hsa-miR-152 | 4520 | MTF1 | metal-regulatory transcription factor 1 | 2 |
| 4289 | hsa-miR-26b | 4520 | MTF1 | metal-regulatory transcription factor 1 | 2 |
| 4290 | hsa-miR-31 | 4520 | MTF1 | metal-regulatory transcription factor 1 | 2 |
| 4291 | hsa-miR-338-5p | 4520 | MTF1 | metal-regulatory transcription factor 1 | 2 |
| 4292 | hsa-miR-340 | 4520 | MTF1 | metal-regulatory transcription factor 1 | 2 |
| 4293 | hsa-miR-383 | 4520 | MTF1 | metal-regulatory transcription factor 1 | 2 |
| 4294 | hsa-miR-449a | 4520 | MTF1 | metal-regulatory transcription factor 1 | 2 |
| 4295 | hsa-miR-499-5p | 4520 | MTF1 | metal-regulatory transcription factor 1 | 2 |
| 4296 | hsa-miR-548b-5p | 4520 | MTF1 | metal-regulatory transcription factor 1 | 2 |
| 4297 | hsa-miR-621 | 4520 | MTF1 | metal-regulatory transcription factor 1 | 2 |
| 4298 | hsa-miR-657 | 4520 | MTF1 | metal-regulatory transcription factor 1 | 2 |
| 4299 | hsa-miR-765 | 4520 | MTF1 | metal-regulatory transcription factor 1 | 2 |
| 4300 | hsa-miR-770-5p | 4520 | MTF1 | metal-regulatory transcription factor 1 | 2 |
| 4301 | hsa-miR-877 | 4520 | MTF1 | metal-regulatory transcription factor 1 | 2 |
| 4302 | hsa-mir-26a-1 | 4520 | MTF1 | metal-regulatory transcription factor 1 | 2 |
| 4303 | hsa-mir-26b | 4520 | MTF1 | metal-regulatory transcription factor 1 | 2 |
| 4304 | hsa-mir-31 | 4520 | MTF1 | metal-regulatory transcription factor 1 | 2 |
| 4305 | hsa-mir-194-2 | 4520 | MTF1 | metal-regulatory transcription factor 1 | 2 |
| 4306 | hsa-mir-340 | 4520 | MTF1 | metal-regulatory transcription factor 1 | 2 |
| 4307 | hsa-mir-338 | 4520 | MTF1 | metal-regulatory transcription factor 1 | 2 |
| 4308 | hsa-mir-548b | 4520 | MTF1 | metal-regulatory transcription factor 1 | 2 |
| 4309 | hsa-mir-449b | 4520 | MTF1 | metal-regulatory transcription factor 1 | 2 |
| 4310 | hsa-mir-449c | 4520 | MTF1 | metal-regulatory transcription factor 1 | 2 |
| 4311 | hsa-mir-3065 | 4520 | MTF1 | metal-regulatory transcription factor 1 | 2 |
| 4418 | hsa-miR-10a | 7185 | TRAF1 | TNF receptor-associated factor 1 | 2 |
| 4419 | hsa-miR-139-5p | 7185 | TRAF1 | TNF receptor-associated factor 1 | 2 |
| 4420 | hsa-miR-152 | 7185 | TRAF1 | TNF receptor-associated factor 1 | 2 |
| 4421 | hsa-miR-26a | 7185 | TRAF1 | TNF receptor-associated factor 1 | 2 |
| 4422 | hsa-miR-26b | 7185 | TRAF1 | TNF receptor-associated factor 1 | 2 |
| 4423 | hsa-miR-31 | 7185 | TRAF1 | TNF receptor-associated factor 1 | 2 |
| 4424 | hsa-miR-338-5p | 7185 | TRAF1 | TNF receptor-associated factor 1 | 2 |
| 4425 | hsa-miR-340 | 7185 | TRAF1 | TNF receptor-associated factor 1 | 2 |
| 4426 | hsa-miR-383 | 7185 | TRAF1 | TNF receptor-associated factor 1 | 2 |
| 4427 | hsa-miR-449a | 7185 | TRAF1 | TNF receptor-associated factor 1 | 2 |
| 4429 | hsa-miR-499-5p | 7185 | TRAF1 | TNF receptor-associated factor 1 | 2 |
| 4430 | hsa-miR-548b-5p | 7185 | TRAF1 | TNF receptor-associated factor 1 | 2 |
| 4431 | hsa-miR-621 | 7185 | TRAF1 | TNF receptor-associated factor 1 | 2 |
| 4432 | hsa-miR-657 | 7185 | TRAF1 | TNF receptor-associated factor 1 | 2 |
| 4433 | hsa-miR-765 | 7185 | TRAF1 | TNF receptor-associated factor 1 | 2 |
| 4434 | hsa-miR-770-5p | 7185 | TRAF1 | TNF receptor-associated factor 1 | 2 |
| 4435 | hsa-miR-877 | 7185 | TRAF1 | TNF receptor-associated factor 1 | 2 |
| 4436 | hsa-mir-26a-1 | 7185 | TRAF1 | TNF receptor-associated factor 1 | 2 |
| 4437 | hsa-mir-26b | 7185 | TRAF1 | TNF receptor-associated factor 1 | 2 |
| 4438 | hsa-mir-31 | 7185 | TRAF1 | TNF receptor-associated factor 1 | 2 |
| 4439 | hsa-mir-194-2 | 7185 | TRAF1 | TNF receptor-associated factor 1 | 2 |
| 4440 | hsa-mir-340 | 7185 | TRAF1 | TNF receptor-associated factor 1 | 2 |
| 4441 | hsa-mir-338 | 7185 | TRAF1 | TNF receptor-associated factor 1 | 2 |
| 4443 | hsa-mir-548b | 7185 | TRAF1 | TNF receptor-associated factor 1 | 2 |
| 4444 | hsa-mir-449b | 7185 | TRAF1 | TNF receptor-associated factor 1 | 2 |
| 4445 | hsa-mir-449c | 7185 | TRAF1 | TNF receptor-associated factor 1 | 2 |
| 4446 | hsa-mir-3065 | 7185 | TRAF1 | TNF receptor-associated factor 1 | 2 |
| 4447 | hsa-miR-10a | 57147 | SCYL3 | SCY1-like 3 (S. cerevisiae) | 2 |
| 4449 | hsa-miR-152 | 57147 | SCYL3 | SCY1-like 3 (S. cerevisiae) | 2 |
| 4450 | hsa-miR-26a | 57147 | SCYL3 | SCY1-like 3 (S. cerevisiae) | 2 |
| 4451 | hsa-miR-26b | 57147 | SCYL3 | SCY1-like 3 (S. cerevisiae) | 2 |
| 4452 | hsa-miR-31 | 57147 | SCYL3 | SCY1-like 3 (S. cerevisiae) | 2 |
| 4453 | hsa-miR-338-5p | 57147 | SCYL3 | SCY1-like 3 (S. cerevisiae) | 2 |
| 4454 | hsa-miR-340 | 57147 | SCYL3 | SCY1-like 3 (S. cerevisiae) | 2 |
| 4455 | hsa-miR-449a | 57147 | SCYL3 | SCY1-like 3 (S. cerevisiae) | 2 |
| 4456 | hsa-miR-499-5p | 57147 | SCYL3 | SCY1-like 3 (S. cerevisiae) | 2 |
| 4457 | hsa-miR-548b-5p | 57147 | SCYL3 | SCY1-like 3 (S. cerevisiae) | 2 |
| 4458 | hsa-miR-564 | 57147 | SCYL3 | SCY1-like 3 (S. cerevisiae) | 2 |
| 4459 | hsa-miR-621 | 57147 | SCYL3 | SCY1-like 3 (S. cerevisiae) | 2 |
| 4460 | hsa-miR-657 | 57147 | SCYL3 | SCY1-like 3 (S. cerevisiae) | 2 |
| 4461 | hsa-miR-765 | 57147 | SCYL3 | SCY1-like 3 (S. cerevisiae) | 2 |
| 4462 | hsa-miR-770-5p | 57147 | SCYL3 | SCY1-like 3 (S. cerevisiae) | 2 |
| 4463 | hsa-miR-877 | 57147 | SCYL3 | SCY1-like 3 (S. cerevisiae) | 2 |
| 4464 | hsa-mir-26a-1 | 57147 | SCYL3 | SCY1-like 3 (S. cerevisiae) | 2 |
| 4465 | hsa-mir-26b | 57147 | SCYL3 | SCY1-like 3 (S. cerevisiae) | 2 |
| 4466 | hsa-mir-31 | 57147 | SCYL3 | SCY1-like 3 (S. cerevisiae) | 2 |
| 4467 | hsa-mir-194-2 | 57147 | SCYL3 | SCY1-like 3 (S. cerevisiae) | 2 |
| 4468 | hsa-mir-340 | 57147 | SCYL3 | SCY1-like 3 (S. cerevisiae) | 2 |
| 4469 | hsa-mir-338 | 57147 | SCYL3 | SCY1-like 3 (S. cerevisiae) | 2 |
| 4470 | hsa-mir-548b | 57147 | SCYL3 | SCY1-like 3 (S. cerevisiae) | 2 |
| 4471 | hsa-mir-548d-2 | 57147 | SCYL3 | SCY1-like 3 (S. cerevisiae) | 2 |
| 4472 | hsa-mir-449b | 57147 | SCYL3 | SCY1-like 3 (S. cerevisiae) | 2 |
| 4473 | hsa-mir-449c | 57147 | SCYL3 | SCY1-like 3 (S. cerevisiae) | 2 |
| 4474 | hsa-mir-3065 | 57147 | SCYL3 | SCY1-like 3 (S. cerevisiae) | 2 |
| 4636 | hsa-miR-10a | 7130 | TNFAIP6 | tumor necrosis factor, alpha-induced protein 6 | 2 |
| 4637 | hsa-miR-152 | 7130 | TNFAIP6 | tumor necrosis factor, alpha-induced protein 6 | 2 |
| 4638 | hsa-miR-26a | 7130 | TNFAIP6 | tumor necrosis factor, alpha-induced protein 6 | 2 |
| 4639 | hsa-miR-26b | 7130 | TNFAIP6 | tumor necrosis factor, alpha-induced protein 6 | 2 |
| 4640 | hsa-miR-31 | 7130 | TNFAIP6 | tumor necrosis factor, alpha-induced protein 6 | 2 |
| 4641 | hsa-miR-338-5p | 7130 | TNFAIP6 | tumor necrosis factor, alpha-induced protein 6 | 2 |
| 4642 | hsa-miR-340 | 7130 | TNFAIP6 | tumor necrosis factor, alpha-induced protein 6 | 2 |
| 4643 | hsa-miR-383 | 7130 | TNFAIP6 | tumor necrosis factor, alpha-induced protein 6 | 2 |
| 4644 | hsa-miR-449a | 7130 | TNFAIP6 | tumor necrosis factor, alpha-induced protein 6 | 2 |
| 4645 | hsa-miR-490-5p | 7130 | TNFAIP6 | tumor necrosis factor, alpha-induced protein 6 | 2 |
| 4646 | hsa-miR-499-5p | 7130 | TNFAIP6 | tumor necrosis factor, alpha-induced protein 6 | 2 |
| 4647 | hsa-miR-548b-5p | 7130 | TNFAIP6 | tumor necrosis factor, alpha-induced protein 6 | 2 |
| 4648 | hsa-miR-564 | 7130 | TNFAIP6 | tumor necrosis factor, alpha-induced protein 6 | 2 |
| 4649 | hsa-miR-621 | 7130 | TNFAIP6 | tumor necrosis factor, alpha-induced protein 6 | 2 |
| 4651 | hsa-miR-657 | 7130 | TNFAIP6 | tumor necrosis factor, alpha-induced protein 6 | 2 |
| 4652 | hsa-miR-765 | 7130 | TNFAIP6 | tumor necrosis factor, alpha-induced protein 6 | 2 |
| 4653 | hsa-miR-770-5p | 7130 | TNFAIP6 | tumor necrosis factor, alpha-induced protein 6 | 2 |
| 4654 | hsa-miR-877 | 7130 | TNFAIP6 | tumor necrosis factor, alpha-induced protein 6 | 2 |
| 4655 | hsa-mir-26a-1 | 7130 | TNFAIP6 | tumor necrosis factor, alpha-induced protein 6 | 2 |
| 4656 | hsa-mir-26b | 7130 | TNFAIP6 | tumor necrosis factor, alpha-induced protein 6 | 2 |
| 4657 | hsa-mir-31 | 7130 | TNFAIP6 | tumor necrosis factor, alpha-induced protein 6 | 2 |
| 4658 | hsa-mir-194-2 | 7130 | TNFAIP6 | tumor necrosis factor, alpha-induced protein 6 | 2 |
| 4659 | hsa-mir-340 | 7130 | TNFAIP6 | tumor necrosis factor, alpha-induced protein 6 | 2 |
| 4660 | hsa-mir-338 | 7130 | TNFAIP6 | tumor necrosis factor, alpha-induced protein 6 | 2 |
| 4661 | hsa-mir-490 | 7130 | TNFAIP6 | tumor necrosis factor, alpha-induced protein 6 | 2 |
| 4662 | hsa-mir-548b | 7130 | TNFAIP6 | tumor necrosis factor, alpha-induced protein 6 | 2 |
| 4663 | hsa-mir-449b | 7130 | TNFAIP6 | tumor necrosis factor, alpha-induced protein 6 | 2 |
| 4664 | hsa-mir-449c | 7130 | TNFAIP6 | tumor necrosis factor, alpha-induced protein 6 | 2 |
| 4665 | hsa-mir-3065 | 7130 | TNFAIP6 | tumor necrosis factor, alpha-induced protein 6 | 2 |
| 4667 | hsa-miR-10a | 5966 | REL | v-rel reticuloendotheliosis viral oncogene homolog (avian) | 2 |
| 4670 | hsa-miR-152 | 5966 | REL | v-rel reticuloendotheliosis viral oncogene homolog (avian) | 2 |
| 4671 | hsa-miR-26a | 5966 | REL | v-rel reticuloendotheliosis viral oncogene homolog (avian) | 2 |
| 4672 | hsa-miR-26b | 5966 | REL | v-rel reticuloendotheliosis viral oncogene homolog (avian) | 2 |
| 4673 | hsa-miR-31 | 5966 | REL | v-rel reticuloendotheliosis viral oncogene homolog (avian) | 2 |
| 4675 | hsa-miR-340 | 5966 | REL | v-rel reticuloendotheliosis viral oncogene homolog (avian) | 2 |
| 4676 | hsa-miR-449a | 5966 | REL | v-rel reticuloendotheliosis viral oncogene homolog (avian) | 2 |
| 4677 | hsa-miR-490-5p | 5966 | REL | v-rel reticuloendotheliosis viral oncogene homolog (avian) | 2 |
| 4678 | hsa-miR-499-5p | 5966 | REL | v-rel reticuloendotheliosis viral oncogene homolog (avian) | 2 |
| 4679 | hsa-miR-548b-5p | 5966 | REL | v-rel reticuloendotheliosis viral oncogene homolog (avian) | 2 |
| 4680 | hsa-miR-584 | 5966 | REL | v-rel reticuloendotheliosis viral oncogene homolog (avian) | 2 |
| 4681 | hsa-miR-621 | 5966 | REL | v-rel reticuloendotheliosis viral oncogene homolog (avian) | 2 |
| 4684 | hsa-miR-765 | 5966 | REL | v-rel reticuloendotheliosis viral oncogene homolog (avian) | 2 |
| 4685 | hsa-miR-770-5p | 5966 | REL | v-rel reticuloendotheliosis viral oncogene homolog (avian) | 2 |
| 4686 | hsa-miR-877 | 5966 | REL | v-rel reticuloendotheliosis viral oncogene homolog (avian) | 2 |
| 4687 | hsa-mir-26a-1 | 5966 | REL | v-rel reticuloendotheliosis viral oncogene homolog (avian) | 2 |
| 4688 | hsa-mir-26b | 5966 | REL | v-rel reticuloendotheliosis viral oncogene homolog (avian) | 2 |
| 4689 | hsa-mir-31 | 5966 | REL | v-rel reticuloendotheliosis viral oncogene homolog (avian) | 2 |
| 4690 | hsa-mir-194-2 | 5966 | REL | v-rel reticuloendotheliosis viral oncogene homolog (avian) | 2 |
| 4691 | hsa-mir-340 | 5966 | REL | v-rel reticuloendotheliosis viral oncogene homolog (avian) | 2 |
| 4693 | hsa-mir-490 | 5966 | REL | v-rel reticuloendotheliosis viral oncogene homolog (avian) | 2 |
| 4694 | hsa-mir-548b | 5966 | REL | v-rel reticuloendotheliosis viral oncogene homolog (avian) | 2 |
| 4695 | hsa-mir-449b | 5966 | REL | v-rel reticuloendotheliosis viral oncogene homolog (avian) | 2 |
| 4696 | hsa-mir-449c | 5966 | REL | v-rel reticuloendotheliosis viral oncogene homolog (avian) | 2 |
| 4843 | hsa-miR-10a | 10154 | PLXNC1 | plexin C1 | 2 |
| 4845 | hsa-miR-152 | 10154 | PLXNC1 | plexin C1 | 2 |
| 4847 | hsa-miR-26b | 10154 | PLXNC1 | plexin C1 | 2 |
| 4848 | hsa-miR-340 | 10154 | PLXNC1 | plexin C1 | 2 |
| 4849 | hsa-miR-449a | 10154 | PLXNC1 | plexin C1 | 2 |
| 4851 | hsa-miR-499-5p | 10154 | PLXNC1 | plexin C1 | 2 |
| 4852 | hsa-miR-548b-5p | 10154 | PLXNC1 | plexin C1 | 2 |
| 4855 | hsa-miR-621 | 10154 | PLXNC1 | plexin C1 | 2 |
| 4858 | hsa-miR-765 | 10154 | PLXNC1 | plexin C1 | 2 |
| 4859 | hsa-miR-770-5p | 10154 | PLXNC1 | plexin C1 | 2 |
| 4860 | hsa-miR-877 | 10154 | PLXNC1 | plexin C1 | 2 |
| 4862 | hsa-mir-26a-1 | 10154 | PLXNC1 | plexin C1 | 2 |
| 4863 | hsa-mir-26b | 10154 | PLXNC1 | plexin C1 | 2 |
| 4864 | hsa-mir-194-2 | 10154 | PLXNC1 | plexin C1 | 2 |
| 4865 | hsa-mir-340 | 10154 | PLXNC1 | plexin C1 | 2 |
| 4867 | hsa-mir-548b | 10154 | PLXNC1 | plexin C1 | 2 |
| 4868 | hsa-mir-449b | 10154 | PLXNC1 | plexin C1 | 2 |
| 4869 | hsa-mir-449c | 10154 | PLXNC1 | plexin C1 | 2 |
| 4870 | hsa-miR-10a | 5371 | PML | promyelocytic leukemia | 2 |
| 4871 | hsa-miR-152 | 5371 | PML | promyelocytic leukemia | 2 |
| 4872 | hsa-miR-26a | 5371 | PML | promyelocytic leukemia | 2 |
| 4873 | hsa-miR-26b | 5371 | PML | promyelocytic leukemia | 2 |
| 4874 | hsa-miR-31 | 5371 | PML | promyelocytic leukemia | 2 |
| 4875 | hsa-miR-340 | 5371 | PML | promyelocytic leukemia | 2 |
| 4876 | hsa-miR-449a | 5371 | PML | promyelocytic leukemia | 2 |
| 4878 | hsa-miR-499-5p | 5371 | PML | promyelocytic leukemia | 2 |
| 4879 | hsa-miR-548b-5p | 5371 | PML | promyelocytic leukemia | 2 |
| 4880 | hsa-miR-564 | 5371 | PML | promyelocytic leukemia | 2 |
| 4881 | hsa-miR-621 | 5371 | PML | promyelocytic leukemia | 2 |
| 4882 | hsa-miR-634 | 5371 | PML | promyelocytic leukemia | 2 |
| 4883 | hsa-miR-765 | 5371 | PML | promyelocytic leukemia | 2 |
| 4884 | hsa-miR-770-5p | 5371 | PML | promyelocytic leukemia | 2 |
| 4885 | hsa-miR-877 | 5371 | PML | promyelocytic leukemia | 2 |
| 4886 | hsa-mir-26a-1 | 5371 | PML | promyelocytic leukemia | 2 |
| 4887 | hsa-mir-26b | 5371 | PML | promyelocytic leukemia | 2 |
| 4888 | hsa-mir-31 | 5371 | PML | promyelocytic leukemia | 2 |
| 4889 | hsa-mir-194-2 | 5371 | PML | promyelocytic leukemia | 2 |
| 4890 | hsa-mir-340 | 5371 | PML | promyelocytic leukemia | 2 |
| 4892 | hsa-mir-548b | 5371 | PML | promyelocytic leukemia | 2 |
| 4893 | hsa-mir-449b | 5371 | PML | promyelocytic leukemia | 2 |
| 4894 | hsa-mir-449c | 5371 | PML | promyelocytic leukemia | 2 |
| 5018 | hsa-miR-10a | 5533 | PPP3CC | protein phosphatase 3 (formerly 2B), catalytic subunit, gamma isoform | 2 |
| 5019 | hsa-miR-152 | 5533 | PPP3CC | protein phosphatase 3 (formerly 2B), catalytic subunit, gamma isoform | 2 |
| 5020 | hsa-miR-26a | 5533 | PPP3CC | protein phosphatase 3 (formerly 2B), catalytic subunit, gamma isoform | 2 |
| 5021 | hsa-miR-26b | 5533 | PPP3CC | protein phosphatase 3 (formerly 2B), catalytic subunit, gamma isoform | 2 |
| 5022 | hsa-miR-31 | 5533 | PPP3CC | protein phosphatase 3 (formerly 2B), catalytic subunit, gamma isoform | 2 |
| 5023 | hsa-miR-338-5p | 5533 | PPP3CC | protein phosphatase 3 (formerly 2B), catalytic subunit, gamma isoform | 2 |
| 5024 | hsa-miR-340 | 5533 | PPP3CC | protein phosphatase 3 (formerly 2B), catalytic subunit, gamma isoform | 2 |
| 5025 | hsa-miR-383 | 5533 | PPP3CC | protein phosphatase 3 (formerly 2B), catalytic subunit, gamma isoform | 2 |
| 5026 | hsa-miR-423-5p | 5533 | PPP3CC | protein phosphatase 3 (formerly 2B), catalytic subunit, gamma isoform | 2 |
| 5027 | hsa-miR-449a | 5533 | PPP3CC | protein phosphatase 3 (formerly 2B), catalytic subunit, gamma isoform | 2 |
| 5029 | hsa-miR-499-5p | 5533 | PPP3CC | protein phosphatase 3 (formerly 2B), catalytic subunit, gamma isoform | 2 |
| 5030 | hsa-miR-548b-5p | 5533 | PPP3CC | protein phosphatase 3 (formerly 2B), catalytic subunit, gamma isoform | 2 |
| 5031 | hsa-miR-621 | 5533 | PPP3CC | protein phosphatase 3 (formerly 2B), catalytic subunit, gamma isoform | 2 |
| 5032 | hsa-miR-657 | 5533 | PPP3CC | protein phosphatase 3 (formerly 2B), catalytic subunit, gamma isoform | 2 |
| 5033 | hsa-miR-663 | 5533 | PPP3CC | protein phosphatase 3 (formerly 2B), catalytic subunit, gamma isoform | 2 |
| 5034 | hsa-miR-765 | 5533 | PPP3CC | protein phosphatase 3 (formerly 2B), catalytic subunit, gamma isoform | 2 |
| 5035 | hsa-miR-770-5p | 5533 | PPP3CC | protein phosphatase 3 (formerly 2B), catalytic subunit, gamma isoform | 2 |
| 5036 | hsa-miR-877 | 5533 | PPP3CC | protein phosphatase 3 (formerly 2B), catalytic subunit, gamma isoform | 2 |
| 5037 | hsa-mir-26a-1 | 5533 | PPP3CC | protein phosphatase 3 (formerly 2B), catalytic subunit, gamma isoform | 2 |
| 5038 | hsa-mir-26b | 5533 | PPP3CC | protein phosphatase 3 (formerly 2B), catalytic subunit, gamma isoform | 2 |
| 5039 | hsa-mir-31 | 5533 | PPP3CC | protein phosphatase 3 (formerly 2B), catalytic subunit, gamma isoform | 2 |
| 5040 | hsa-mir-194-2 | 5533 | PPP3CC | protein phosphatase 3 (formerly 2B), catalytic subunit, gamma isoform | 2 |
| 5041 | hsa-mir-340 | 5533 | PPP3CC | protein phosphatase 3 (formerly 2B), catalytic subunit, gamma isoform | 2 |
| 5042 | hsa-mir-338 | 5533 | PPP3CC | protein phosphatase 3 (formerly 2B), catalytic subunit, gamma isoform | 2 |
| 5044 | hsa-mir-548b | 5533 | PPP3CC | protein phosphatase 3 (formerly 2B), catalytic subunit, gamma isoform | 2 |
| 5045 | hsa-mir-449b | 5533 | PPP3CC | protein phosphatase 3 (formerly 2B), catalytic subunit, gamma isoform | 2 |
| 5046 | hsa-mir-449c | 5533 | PPP3CC | protein phosphatase 3 (formerly 2B), catalytic subunit, gamma isoform | 2 |
| 5047 | hsa-mir-3065 | 5533 | PPP3CC | protein phosphatase 3 (formerly 2B), catalytic subunit, gamma isoform | 2 |
| 5267 | hsa-miR-10a | 4791 | NFKB2 | nuclear factor of kappa light polypeptide gene enhancer in B-cells 2 (p49/p100) | 2 |
| 5268 | hsa-miR-1228\* | 4791 | NFKB2 | nuclear factor of kappa light polypeptide gene enhancer in B-cells 2 (p49/p100) | 2 |
| 5269 | hsa-miR-139-5p | 4791 | NFKB2 | nuclear factor of kappa light polypeptide gene enhancer in B-cells 2 (p49/p100) | 2 |
| 5270 | hsa-miR-152 | 4791 | NFKB2 | nuclear factor of kappa light polypeptide gene enhancer in B-cells 2 (p49/p100) | 2 |
| 5271 | hsa-miR-26a | 4791 | NFKB2 | nuclear factor of kappa light polypeptide gene enhancer in B-cells 2 (p49/p100) | 2 |
| 5272 | hsa-miR-26b | 4791 | NFKB2 | nuclear factor of kappa light polypeptide gene enhancer in B-cells 2 (p49/p100) | 2 |
| 5273 | hsa-miR-338-5p | 4791 | NFKB2 | nuclear factor of kappa light polypeptide gene enhancer in B-cells 2 (p49/p100) | 2 |
| 5274 | hsa-miR-340 | 4791 | NFKB2 | nuclear factor of kappa light polypeptide gene enhancer in B-cells 2 (p49/p100) | 2 |
| 5275 | hsa-miR-449a | 4791 | NFKB2 | nuclear factor of kappa light polypeptide gene enhancer in B-cells 2 (p49/p100) | 2 |
| 5276 | hsa-miR-490-5p | 4791 | NFKB2 | nuclear factor of kappa light polypeptide gene enhancer in B-cells 2 (p49/p100) | 2 |
| 5277 | hsa-miR-499-5p | 4791 | NFKB2 | nuclear factor of kappa light polypeptide gene enhancer in B-cells 2 (p49/p100) | 2 |
| 5278 | hsa-miR-548b-5p | 4791 | NFKB2 | nuclear factor of kappa light polypeptide gene enhancer in B-cells 2 (p49/p100) | 2 |
| 5279 | hsa-miR-584 | 4791 | NFKB2 | nuclear factor of kappa light polypeptide gene enhancer in B-cells 2 (p49/p100) | 2 |
| 5280 | hsa-miR-621 | 4791 | NFKB2 | nuclear factor of kappa light polypeptide gene enhancer in B-cells 2 (p49/p100) | 2 |
| 5281 | hsa-miR-657 | 4791 | NFKB2 | nuclear factor of kappa light polypeptide gene enhancer in B-cells 2 (p49/p100) | 2 |
| 5282 | hsa-miR-765 | 4791 | NFKB2 | nuclear factor of kappa light polypeptide gene enhancer in B-cells 2 (p49/p100) | 2 |
| 5283 | hsa-miR-770-5p | 4791 | NFKB2 | nuclear factor of kappa light polypeptide gene enhancer in B-cells 2 (p49/p100) | 2 |
| 5284 | hsa-miR-877 | 4791 | NFKB2 | nuclear factor of kappa light polypeptide gene enhancer in B-cells 2 (p49/p100) | 2 |
| 5285 | hsa-mir-26a-1 | 4791 | NFKB2 | nuclear factor of kappa light polypeptide gene enhancer in B-cells 2 (p49/p100) | 2 |
| 5286 | hsa-mir-26b | 4791 | NFKB2 | nuclear factor of kappa light polypeptide gene enhancer in B-cells 2 (p49/p100) | 2 |
| 5287 | hsa-mir-194-2 | 4791 | NFKB2 | nuclear factor of kappa light polypeptide gene enhancer in B-cells 2 (p49/p100) | 2 |
| 5288 | hsa-mir-340 | 4791 | NFKB2 | nuclear factor of kappa light polypeptide gene enhancer in B-cells 2 (p49/p100) | 2 |
| 5289 | hsa-mir-338 | 4791 | NFKB2 | nuclear factor of kappa light polypeptide gene enhancer in B-cells 2 (p49/p100) | 2 |
| 5290 | hsa-mir-490 | 4791 | NFKB2 | nuclear factor of kappa light polypeptide gene enhancer in B-cells 2 (p49/p100) | 2 |
| 5291 | hsa-mir-548b | 4791 | NFKB2 | nuclear factor of kappa light polypeptide gene enhancer in B-cells 2 (p49/p100) | 2 |
| 5292 | hsa-mir-449b | 4791 | NFKB2 | nuclear factor of kappa light polypeptide gene enhancer in B-cells 2 (p49/p100) | 2 |
| 5293 | hsa-mir-449c | 4791 | NFKB2 | nuclear factor of kappa light polypeptide gene enhancer in B-cells 2 (p49/p100) | 2 |
| 5294 | hsa-mir-3065 | 4791 | NFKB2 | nuclear factor of kappa light polypeptide gene enhancer in B-cells 2 (p49/p100) | 2 |
| 5353 | hsa-miR-10a | 1528 | CYB5A | cytochrome b5 type A (microsomal) | 2 |
| 5354 | hsa-miR-139-5p | 1528 | CYB5A | cytochrome b5 type A (microsomal) | 2 |
| 5355 | hsa-miR-152 | 1528 | CYB5A | cytochrome b5 type A (microsomal) | 2 |
| 5356 | hsa-miR-31 | 1528 | CYB5A | cytochrome b5 type A (microsomal) | 2 |
| 5357 | hsa-miR-338-5p | 1528 | CYB5A | cytochrome b5 type A (microsomal) | 2 |
| 5358 | hsa-miR-340 | 1528 | CYB5A | cytochrome b5 type A (microsomal) | 2 |
| 5359 | hsa-miR-383 | 1528 | CYB5A | cytochrome b5 type A (microsomal) | 2 |
| 5360 | hsa-miR-449a | 1528 | CYB5A | cytochrome b5 type A (microsomal) | 2 |
| 5361 | hsa-miR-499-5p | 1528 | CYB5A | cytochrome b5 type A (microsomal) | 2 |
| 5362 | hsa-miR-548b-5p | 1528 | CYB5A | cytochrome b5 type A (microsomal) | 2 |
| 5363 | hsa-miR-621 | 1528 | CYB5A | cytochrome b5 type A (microsomal) | 2 |
| 5364 | hsa-miR-657 | 1528 | CYB5A | cytochrome b5 type A (microsomal) | 2 |
| 5365 | hsa-miR-663 | 1528 | CYB5A | cytochrome b5 type A (microsomal) | 2 |
| 5366 | hsa-miR-765 | 1528 | CYB5A | cytochrome b5 type A (microsomal) | 2 |
| 5367 | hsa-miR-770-5p | 1528 | CYB5A | cytochrome b5 type A (microsomal) | 2 |
| 5368 | hsa-miR-877 | 1528 | CYB5A | cytochrome b5 type A (microsomal) | 2 |
| 5369 | hsa-mir-26a-1 | 1528 | CYB5A | cytochrome b5 type A (microsomal) | 2 |
| 5370 | hsa-mir-31 | 1528 | CYB5A | cytochrome b5 type A (microsomal) | 2 |
| 5371 | hsa-mir-194-2 | 1528 | CYB5A | cytochrome b5 type A (microsomal) | 2 |
| 5372 | hsa-mir-340 | 1528 | CYB5A | cytochrome b5 type A (microsomal) | 2 |
| 5373 | hsa-mir-338 | 1528 | CYB5A | cytochrome b5 type A (microsomal) | 2 |
| 5374 | hsa-mir-548b | 1528 | CYB5A | cytochrome b5 type A (microsomal) | 2 |
| 5375 | hsa-mir-449b | 1528 | CYB5A | cytochrome b5 type A (microsomal) | 2 |
| 5376 | hsa-mir-449c | 1528 | CYB5A | cytochrome b5 type A (microsomal) | 2 |
| 5377 | hsa-mir-3065 | 1528 | CYB5A | cytochrome b5 type A (microsomal) | 2 |
| 5508 | hsa-mir-499 | 334 | APLP2 | amyloid beta (A4) precursor-like protein 2 | 2 |
| 5511 | hsa-mir-2116 | 334 | APLP2 | amyloid beta (A4) precursor-like protein 2 | 2 |
| 5512 | hsa-mir-3190 | 334 | APLP2 | amyloid beta (A4) precursor-like protein 2 | 2 |
| 5588 | hsa-miR-634 | 8837 | CFLAR | CASP8 and FADD-like apoptosis regulator | 2 |
| 5694 | hsa-miR-152 | 23499 | MACF1 | microtubule-actin crosslinking factor 1 | 2 |
| 5699 | hsa-miR-340 | 23499 | MACF1 | microtubule-actin crosslinking factor 1 | 2 |
| 5708 | hsa-miR-877 | 23499 | MACF1 | microtubule-actin crosslinking factor 1 | 2 |
| 5713 | hsa-mir-340 | 23499 | MACF1 | microtubule-actin crosslinking factor 1 | 2 |
| 5716 | hsa-mir-548d-2 | 23499 | MACF1 | microtubule-actin crosslinking factor 1 | 2 |
| 5720 | hsa-miR-195 | 334 | APLP2 | amyloid beta (A4) precursor-like protein 2 | 2 |
| 5721 | hsa-miR-497 | 334 | APLP2 | amyloid beta (A4) precursor-like protein 2 | 2 |
| 5847 | hsa-miR-208a | 4094 | MAF | v-maf musculoaponeurotic fibrosarcoma oncogene homolog (avian) | 2 |
| 5904 | hsa-miR-383 | 6509 | SLC1A4 | solute carrier family 1 (glutamate/neutral amino acid transporter), member 4 | 2 |
| 5911 | hsa-miR-663 | 6509 | SLC1A4 | solute carrier family 1 (glutamate/neutral amino acid transporter), member 4 | 2 |
| 5961 | hsa-miR-423-5p | 5272 | SERPINB9 | serpin peptidase inhibitor, clade B (ovalbumin), member 9 | 2 |
| 5965 | hsa-miR-548b-5p | 5272 | SERPINB9 | serpin peptidase inhibitor, clade B (ovalbumin), member 9 | 2 |
| 5966 | hsa-miR-564 | 5272 | SERPINB9 | serpin peptidase inhibitor, clade B (ovalbumin), member 9 | 2 |
| 5974 | hsa-miR-95 | 5272 | SERPINB9 | serpin peptidase inhibitor, clade B (ovalbumin), member 9 | 2 |
| 5982 | hsa-mir-548b | 5272 | SERPINB9 | serpin peptidase inhibitor, clade B (ovalbumin), member 9 | 2 |
| 6014 | hsa-miR-10a | 10435 | CDC42EP2 | CDC42 effector protein (Rho GTPase binding) 2 | 2 |
| 6015 | hsa-miR-139-5p | 10435 | CDC42EP2 | CDC42 effector protein (Rho GTPase binding) 2 | 2 |
| 6016 | hsa-miR-152 | 10435 | CDC42EP2 | CDC42 effector protein (Rho GTPase binding) 2 | 2 |
| 6017 | hsa-miR-26b | 10435 | CDC42EP2 | CDC42 effector protein (Rho GTPase binding) 2 | 2 |
| 6018 | hsa-miR-31 | 10435 | CDC42EP2 | CDC42 effector protein (Rho GTPase binding) 2 | 2 |
| 6019 | hsa-miR-338-5p | 10435 | CDC42EP2 | CDC42 effector protein (Rho GTPase binding) 2 | 2 |
| 6020 | hsa-miR-340 | 10435 | CDC42EP2 | CDC42 effector protein (Rho GTPase binding) 2 | 2 |
| 6022 | hsa-miR-449a | 10435 | CDC42EP2 | CDC42 effector protein (Rho GTPase binding) 2 | 2 |
| 6023 | hsa-miR-499-5p | 10435 | CDC42EP2 | CDC42 effector protein (Rho GTPase binding) 2 | 2 |
| 6024 | hsa-miR-548b-5p | 10435 | CDC42EP2 | CDC42 effector protein (Rho GTPase binding) 2 | 2 |
| 6025 | hsa-miR-621 | 10435 | CDC42EP2 | CDC42 effector protein (Rho GTPase binding) 2 | 2 |
| 6026 | hsa-miR-657 | 10435 | CDC42EP2 | CDC42 effector protein (Rho GTPase binding) 2 | 2 |
| 6027 | hsa-miR-663 | 10435 | CDC42EP2 | CDC42 effector protein (Rho GTPase binding) 2 | 2 |
| 6028 | hsa-miR-765 | 10435 | CDC42EP2 | CDC42 effector protein (Rho GTPase binding) 2 | 2 |
| 6029 | hsa-miR-770-5p | 10435 | CDC42EP2 | CDC42 effector protein (Rho GTPase binding) 2 | 2 |
| 6030 | hsa-miR-877 | 10435 | CDC42EP2 | CDC42 effector protein (Rho GTPase binding) 2 | 2 |
| 6031 | hsa-mir-26a-1 | 10435 | CDC42EP2 | CDC42 effector protein (Rho GTPase binding) 2 | 2 |
| 6032 | hsa-mir-26b | 10435 | CDC42EP2 | CDC42 effector protein (Rho GTPase binding) 2 | 2 |
| 6033 | hsa-mir-31 | 10435 | CDC42EP2 | CDC42 effector protein (Rho GTPase binding) 2 | 2 |
| 6034 | hsa-mir-194-2 | 10435 | CDC42EP2 | CDC42 effector protein (Rho GTPase binding) 2 | 2 |
| 6035 | hsa-mir-340 | 10435 | CDC42EP2 | CDC42 effector protein (Rho GTPase binding) 2 | 2 |
| 6036 | hsa-mir-338 | 10435 | CDC42EP2 | CDC42 effector protein (Rho GTPase binding) 2 | 2 |
| 6037 | hsa-mir-548b | 10435 | CDC42EP2 | CDC42 effector protein (Rho GTPase binding) 2 | 2 |
| 6038 | hsa-mir-449b | 10435 | CDC42EP2 | CDC42 effector protein (Rho GTPase binding) 2 | 2 |
| 6039 | hsa-mir-449c | 10435 | CDC42EP2 | CDC42 effector protein (Rho GTPase binding) 2 | 2 |
| 6040 | hsa-mir-3065 | 10435 | CDC42EP2 | CDC42 effector protein (Rho GTPase binding) 2 | 2 |
| 6202 | hsa-miR-139-5p | 22902 | RUFY3 | RUN and FYVE domain containing 3 | 2 |
| 6352 | hsa-miR-490-5p | 8837 | CFLAR | CASP8 and FADD-like apoptosis regulator | 2 |
| 6353 | hsa-mir-490 | 8837 | CFLAR | CASP8 and FADD-like apoptosis regulator | 2 |
| 6793 | hsa-miR-1 | 90480 | GADD45GIP1 | growth arrest and DNA-damage-inducible, gamma interacting protein 1 | 2 |
| 6794 | hsa-miR-133a | 90480 | GADD45GIP1 | growth arrest and DNA-damage-inducible, gamma interacting protein 1 | 2 |
| 6795 | hsa-miR-186 | 90480 | GADD45GIP1 | growth arrest and DNA-damage-inducible, gamma interacting protein 1 | 2 |
| 6797 | hsa-miR-326 | 90480 | GADD45GIP1 | growth arrest and DNA-damage-inducible, gamma interacting protein 1 | 2 |
| 6798 | hsa-miR-449b | 90480 | GADD45GIP1 | growth arrest and DNA-damage-inducible, gamma interacting protein 1 | 2 |
| 6799 | hsa-miR-550 | 90480 | GADD45GIP1 | growth arrest and DNA-damage-inducible, gamma interacting protein 1 | 2 |
| 6800 | hsa-miR-575 | 90480 | GADD45GIP1 | growth arrest and DNA-damage-inducible, gamma interacting protein 1 | 2 |
| 6802 | hsa-miR-608 | 90480 | GADD45GIP1 | growth arrest and DNA-damage-inducible, gamma interacting protein 1 | 2 |
| 6803 | hsa-miR-620 | 90480 | GADD45GIP1 | growth arrest and DNA-damage-inducible, gamma interacting protein 1 | 2 |
| 6804 | hsa-mir-22 | 90480 | GADD45GIP1 | growth arrest and DNA-damage-inducible, gamma interacting protein 1 | 2 |
| 6805 | hsa-mir-10a | 90480 | GADD45GIP1 | growth arrest and DNA-damage-inducible, gamma interacting protein 1 | 2 |
| 6806 | hsa-mir-186 | 90480 | GADD45GIP1 | growth arrest and DNA-damage-inducible, gamma interacting protein 1 | 2 |
| 6807 | hsa-mir-155 | 90480 | GADD45GIP1 | growth arrest and DNA-damage-inducible, gamma interacting protein 1 | 2 |
| 6810 | hsa-mir-550-1 | 90480 | GADD45GIP1 | growth arrest and DNA-damage-inducible, gamma interacting protein 1 | 2 |
| 7080 | hsa-miR-1 | 29937 | NENF | neuron derived neurotrophic factor | 2 |
| 7081 | hsa-miR-133a | 29937 | NENF | neuron derived neurotrophic factor | 2 |
| 7082 | hsa-miR-186 | 29937 | NENF | neuron derived neurotrophic factor | 2 |
| 7084 | hsa-miR-326 | 29937 | NENF | neuron derived neurotrophic factor | 2 |
| 7085 | hsa-miR-449b | 29937 | NENF | neuron derived neurotrophic factor | 2 |
| 7087 | hsa-miR-575 | 29937 | NENF | neuron derived neurotrophic factor | 2 |
| 7088 | hsa-miR-579 | 29937 | NENF | neuron derived neurotrophic factor | 2 |
| 7089 | hsa-miR-608 | 29937 | NENF | neuron derived neurotrophic factor | 2 |
| 7093 | hsa-mir-22 | 29937 | NENF | neuron derived neurotrophic factor | 2 |
| 7094 | hsa-mir-10a | 29937 | NENF | neuron derived neurotrophic factor | 2 |
| 7095 | hsa-mir-186 | 29937 | NENF | neuron derived neurotrophic factor | 2 |
| 7096 | hsa-mir-155 | 29937 | NENF | neuron derived neurotrophic factor | 2 |
| 7097 | hsa-mir-499 | 29937 | NENF | neuron derived neurotrophic factor | 2 |
| 7098 | hsa-mir-877 | 29937 | NENF | neuron derived neurotrophic factor | 2 |
| 7100 | hsa-mir-3190 | 29937 | NENF | neuron derived neurotrophic factor | 2 |
| 7101 | hsa-miR-10a | 8992 | ATP6V0E1 | ATPase, H+ transporting, lysosomal 9kDa, V0 subunit e1 | 2 |
| 7103 | hsa-miR-139-5p | 8992 | ATP6V0E1 | ATPase, H+ transporting, lysosomal 9kDa, V0 subunit e1 | 2 |
| 7105 | hsa-miR-26b | 8992 | ATP6V0E1 | ATPase, H+ transporting, lysosomal 9kDa, V0 subunit e1 | 2 |
| 7106 | hsa-miR-338-5p | 8992 | ATP6V0E1 | ATPase, H+ transporting, lysosomal 9kDa, V0 subunit e1 | 2 |
| 7107 | hsa-miR-340 | 8992 | ATP6V0E1 | ATPase, H+ transporting, lysosomal 9kDa, V0 subunit e1 | 2 |
| 7109 | hsa-miR-449a | 8992 | ATP6V0E1 | ATPase, H+ transporting, lysosomal 9kDa, V0 subunit e1 | 2 |
| 7110 | hsa-miR-499-5p | 8992 | ATP6V0E1 | ATPase, H+ transporting, lysosomal 9kDa, V0 subunit e1 | 2 |
| 7111 | hsa-miR-548b-5p | 8992 | ATP6V0E1 | ATPase, H+ transporting, lysosomal 9kDa, V0 subunit e1 | 2 |
| 7113 | hsa-miR-621 | 8992 | ATP6V0E1 | ATPase, H+ transporting, lysosomal 9kDa, V0 subunit e1 | 2 |
| 7114 | hsa-miR-657 | 8992 | ATP6V0E1 | ATPase, H+ transporting, lysosomal 9kDa, V0 subunit e1 | 2 |
| 7115 | hsa-miR-765 | 8992 | ATP6V0E1 | ATPase, H+ transporting, lysosomal 9kDa, V0 subunit e1 | 2 |
| 7116 | hsa-miR-770-5p | 8992 | ATP6V0E1 | ATPase, H+ transporting, lysosomal 9kDa, V0 subunit e1 | 2 |
| 7117 | hsa-miR-877 | 8992 | ATP6V0E1 | ATPase, H+ transporting, lysosomal 9kDa, V0 subunit e1 | 2 |
| 7118 | hsa-mir-26a-1 | 8992 | ATP6V0E1 | ATPase, H+ transporting, lysosomal 9kDa, V0 subunit e1 | 2 |
| 7119 | hsa-mir-26b | 8992 | ATP6V0E1 | ATPase, H+ transporting, lysosomal 9kDa, V0 subunit e1 | 2 |
| 7120 | hsa-mir-194-2 | 8992 | ATP6V0E1 | ATPase, H+ transporting, lysosomal 9kDa, V0 subunit e1 | 2 |
| 7121 | hsa-mir-340 | 8992 | ATP6V0E1 | ATPase, H+ transporting, lysosomal 9kDa, V0 subunit e1 | 2 |
| 7122 | hsa-mir-338 | 8992 | ATP6V0E1 | ATPase, H+ transporting, lysosomal 9kDa, V0 subunit e1 | 2 |
| 7123 | hsa-mir-548b | 8992 | ATP6V0E1 | ATPase, H+ transporting, lysosomal 9kDa, V0 subunit e1 | 2 |
| 7125 | hsa-mir-449b | 8992 | ATP6V0E1 | ATPase, H+ transporting, lysosomal 9kDa, V0 subunit e1 | 2 |
| 7126 | hsa-mir-449c | 8992 | ATP6V0E1 | ATPase, H+ transporting, lysosomal 9kDa, V0 subunit e1 | 2 |
| 7127 | hsa-mir-3065 | 8992 | ATP6V0E1 | ATPase, H+ transporting, lysosomal 9kDa, V0 subunit e1 | 2 |
| 7721 | hsa-miR-152 | 79155 | TNIP2 | TNFAIP3 interacting protein 2 | 2 |
| 7902 | hsa-miR-10a | 8934 | RAB7L1 | RAB7, member RAS oncogene family-like 1 | 2 |
| 7904 | hsa-miR-139-5p | 8934 | RAB7L1 | RAB7, member RAS oncogene family-like 1 | 2 |
| 7905 | hsa-miR-152 | 8934 | RAB7L1 | RAB7, member RAS oncogene family-like 1 | 2 |
| 7906 | hsa-miR-26a | 8934 | RAB7L1 | RAB7, member RAS oncogene family-like 1 | 2 |
| 7907 | hsa-miR-26b | 8934 | RAB7L1 | RAB7, member RAS oncogene family-like 1 | 2 |
| 7908 | hsa-miR-31 | 8934 | RAB7L1 | RAB7, member RAS oncogene family-like 1 | 2 |
| 7909 | hsa-miR-338-5p | 8934 | RAB7L1 | RAB7, member RAS oncogene family-like 1 | 2 |
| 7910 | hsa-miR-340 | 8934 | RAB7L1 | RAB7, member RAS oncogene family-like 1 | 2 |
| 7912 | hsa-miR-449a | 8934 | RAB7L1 | RAB7, member RAS oncogene family-like 1 | 2 |
| 7913 | hsa-miR-499-5p | 8934 | RAB7L1 | RAB7, member RAS oncogene family-like 1 | 2 |
| 7914 | hsa-miR-548b-5p | 8934 | RAB7L1 | RAB7, member RAS oncogene family-like 1 | 2 |
| 7915 | hsa-miR-564 | 8934 | RAB7L1 | RAB7, member RAS oncogene family-like 1 | 2 |
| 7916 | hsa-miR-621 | 8934 | RAB7L1 | RAB7, member RAS oncogene family-like 1 | 2 |
| 7917 | hsa-miR-644 | 8934 | RAB7L1 | RAB7, member RAS oncogene family-like 1 | 2 |
| 7918 | hsa-miR-657 | 8934 | RAB7L1 | RAB7, member RAS oncogene family-like 1 | 2 |
| 7919 | hsa-miR-765 | 8934 | RAB7L1 | RAB7, member RAS oncogene family-like 1 | 2 |
| 7920 | hsa-miR-770-5p | 8934 | RAB7L1 | RAB7, member RAS oncogene family-like 1 | 2 |
| 7921 | hsa-miR-877 | 8934 | RAB7L1 | RAB7, member RAS oncogene family-like 1 | 2 |
| 7922 | hsa-mir-26a-1 | 8934 | RAB7L1 | RAB7, member RAS oncogene family-like 1 | 2 |
| 7923 | hsa-mir-26b | 8934 | RAB7L1 | RAB7, member RAS oncogene family-like 1 | 2 |
| 7924 | hsa-mir-31 | 8934 | RAB7L1 | RAB7, member RAS oncogene family-like 1 | 2 |
| 7925 | hsa-mir-194-2 | 8934 | RAB7L1 | RAB7, member RAS oncogene family-like 1 | 2 |
| 7926 | hsa-mir-340 | 8934 | RAB7L1 | RAB7, member RAS oncogene family-like 1 | 2 |
| 7927 | hsa-mir-338 | 8934 | RAB7L1 | RAB7, member RAS oncogene family-like 1 | 2 |
| 7928 | hsa-mir-548b | 8934 | RAB7L1 | RAB7, member RAS oncogene family-like 1 | 2 |
| 7929 | hsa-mir-449b | 8934 | RAB7L1 | RAB7, member RAS oncogene family-like 1 | 2 |
| 7930 | hsa-mir-449c | 8934 | RAB7L1 | RAB7, member RAS oncogene family-like 1 | 2 |
| 7931 | hsa-mir-3065 | 8934 | RAB7L1 | RAB7, member RAS oncogene family-like 1 | 2 |
| 8071 | hsa-miR-10a | 57823 | SLAMF7 | SLAM family member 7 | 2 |
| 8072 | hsa-miR-139-5p | 57823 | SLAMF7 | SLAM family member 7 | 2 |
| 8073 | hsa-miR-152 | 57823 | SLAMF7 | SLAM family member 7 | 2 |
| 8074 | hsa-miR-26a | 57823 | SLAMF7 | SLAM family member 7 | 2 |
| 8075 | hsa-miR-26b | 57823 | SLAMF7 | SLAM family member 7 | 2 |
| 8076 | hsa-miR-31 | 57823 | SLAMF7 | SLAM family member 7 | 2 |
| 8077 | hsa-miR-338-5p | 57823 | SLAMF7 | SLAM family member 7 | 2 |
| 8078 | hsa-miR-340 | 57823 | SLAMF7 | SLAM family member 7 | 2 |
| 8079 | hsa-miR-449a | 57823 | SLAMF7 | SLAM family member 7 | 2 |
| 8080 | hsa-miR-490-5p | 57823 | SLAMF7 | SLAM family member 7 | 2 |
| 8081 | hsa-miR-499-5p | 57823 | SLAMF7 | SLAM family member 7 | 2 |
| 8082 | hsa-miR-548b-5p | 57823 | SLAMF7 | SLAM family member 7 | 2 |
| 8083 | hsa-miR-564 | 57823 | SLAMF7 | SLAM family member 7 | 2 |
| 8085 | hsa-miR-621 | 57823 | SLAMF7 | SLAM family member 7 | 2 |
| 8087 | hsa-miR-657 | 57823 | SLAMF7 | SLAM family member 7 | 2 |
| 8088 | hsa-miR-765 | 57823 | SLAMF7 | SLAM family member 7 | 2 |
| 8089 | hsa-miR-770-5p | 57823 | SLAMF7 | SLAM family member 7 | 2 |
| 8090 | hsa-miR-877 | 57823 | SLAMF7 | SLAM family member 7 | 2 |
| 8091 | hsa-mir-26a-1 | 57823 | SLAMF7 | SLAM family member 7 | 2 |
| 8092 | hsa-mir-26b | 57823 | SLAMF7 | SLAM family member 7 | 2 |
| 8093 | hsa-mir-31 | 57823 | SLAMF7 | SLAM family member 7 | 2 |
| 8094 | hsa-mir-194-2 | 57823 | SLAMF7 | SLAM family member 7 | 2 |
| 8095 | hsa-mir-340 | 57823 | SLAMF7 | SLAM family member 7 | 2 |
| 8096 | hsa-mir-338 | 57823 | SLAMF7 | SLAM family member 7 | 2 |
| 8097 | hsa-mir-490 | 57823 | SLAMF7 | SLAM family member 7 | 2 |
| 8098 | hsa-mir-548b | 57823 | SLAMF7 | SLAM family member 7 | 2 |
| 8099 | hsa-mir-548d-2 | 57823 | SLAMF7 | SLAM family member 7 | 2 |
| 8100 | hsa-mir-449b | 57823 | SLAMF7 | SLAM family member 7 | 2 |
| 8101 | hsa-mir-449c | 57823 | SLAMF7 | SLAM family member 7 | 2 |
| 8102 | hsa-mir-3065 | 57823 | SLAMF7 | SLAM family member 7 | 2 |
| 8269 | hsa-miR-551a | 56172 | ANKH | ankylosis, progressive homolog (mouse) | 2 |
| 8275 | hsa-miR-637 | 56172 | ANKH | ankylosis, progressive homolog (mouse) | 2 |
| 8276 | hsa-miR-885-5p | 56172 | ANKH | ankylosis, progressive homolog (mouse) | 2 |
| 8281 | hsa-mir-423 | 56172 | ANKH | ankylosis, progressive homolog (mouse) | 2 |
| 8283 | hsa-mir-885 | 56172 | ANKH | ankylosis, progressive homolog (mouse) | 2 |
| 8285 | hsa-mir-3190 | 56172 | ANKH | ankylosis, progressive homolog (mouse) | 2 |
| 8286 | hsa-miR-10a | 29121 | CLEC2D | C-type lectin domain family 2, member D | 2 |
| 8287 | hsa-miR-152 | 29121 | CLEC2D | C-type lectin domain family 2, member D | 2 |
| 8288 | hsa-miR-26a | 29121 | CLEC2D | C-type lectin domain family 2, member D | 2 |
| 8289 | hsa-miR-26b | 29121 | CLEC2D | C-type lectin domain family 2, member D | 2 |
| 8290 | hsa-miR-31 | 29121 | CLEC2D | C-type lectin domain family 2, member D | 2 |
| 8291 | hsa-miR-340 | 29121 | CLEC2D | C-type lectin domain family 2, member D | 2 |
| 8292 | hsa-miR-449a | 29121 | CLEC2D | C-type lectin domain family 2, member D | 2 |
| 8293 | hsa-miR-499-5p | 29121 | CLEC2D | C-type lectin domain family 2, member D | 2 |
| 8294 | hsa-miR-564 | 29121 | CLEC2D | C-type lectin domain family 2, member D | 2 |
| 8295 | hsa-miR-621 | 29121 | CLEC2D | C-type lectin domain family 2, member D | 2 |
| 8296 | hsa-miR-634 | 29121 | CLEC2D | C-type lectin domain family 2, member D | 2 |
| 8298 | hsa-miR-653 | 29121 | CLEC2D | C-type lectin domain family 2, member D | 2 |
| 8299 | hsa-miR-765 | 29121 | CLEC2D | C-type lectin domain family 2, member D | 2 |
| 8300 | hsa-miR-770-5p | 29121 | CLEC2D | C-type lectin domain family 2, member D | 2 |
| 8301 | hsa-miR-95 | 29121 | CLEC2D | C-type lectin domain family 2, member D | 2 |
| 8302 | hsa-mir-26a-1 | 29121 | CLEC2D | C-type lectin domain family 2, member D | 2 |
| 8303 | hsa-mir-26b | 29121 | CLEC2D | C-type lectin domain family 2, member D | 2 |
| 8304 | hsa-mir-31 | 29121 | CLEC2D | C-type lectin domain family 2, member D | 2 |
| 8305 | hsa-mir-194-2 | 29121 | CLEC2D | C-type lectin domain family 2, member D | 2 |
| 8306 | hsa-mir-340 | 29121 | CLEC2D | C-type lectin domain family 2, member D | 2 |
| 8307 | hsa-mir-449b | 29121 | CLEC2D | C-type lectin domain family 2, member D | 2 |
| 8308 | hsa-mir-449c | 29121 | CLEC2D | C-type lectin domain family 2, member D | 2 |
| 8467 | hsa-miR-10a | 114897 | C1QTNF1 | C1q and tumor necrosis factor related protein 1 | 2 |
| 8468 | hsa-miR-139-5p | 114897 | C1QTNF1 | C1q and tumor necrosis factor related protein 1 | 2 |
| 8469 | hsa-miR-152 | 114897 | C1QTNF1 | C1q and tumor necrosis factor related protein 1 | 2 |
| 8470 | hsa-miR-26b | 114897 | C1QTNF1 | C1q and tumor necrosis factor related protein 1 | 2 |
| 8471 | hsa-miR-31 | 114897 | C1QTNF1 | C1q and tumor necrosis factor related protein 1 | 2 |
| 8472 | hsa-miR-338-5p | 114897 | C1QTNF1 | C1q and tumor necrosis factor related protein 1 | 2 |
| 8473 | hsa-miR-340 | 114897 | C1QTNF1 | C1q and tumor necrosis factor related protein 1 | 2 |
| 8475 | hsa-miR-449a | 114897 | C1QTNF1 | C1q and tumor necrosis factor related protein 1 | 2 |
| 8476 | hsa-miR-499-5p | 114897 | C1QTNF1 | C1q and tumor necrosis factor related protein 1 | 2 |
| 8477 | hsa-miR-621 | 114897 | C1QTNF1 | C1q and tumor necrosis factor related protein 1 | 2 |
| 8479 | hsa-miR-657 | 114897 | C1QTNF1 | C1q and tumor necrosis factor related protein 1 | 2 |
| 8480 | hsa-miR-765 | 114897 | C1QTNF1 | C1q and tumor necrosis factor related protein 1 | 2 |
| 8481 | hsa-miR-770-5p | 114897 | C1QTNF1 | C1q and tumor necrosis factor related protein 1 | 2 |
| 8482 | hsa-miR-877 | 114897 | C1QTNF1 | C1q and tumor necrosis factor related protein 1 | 2 |
| 8483 | hsa-mir-26a-1 | 114897 | C1QTNF1 | C1q and tumor necrosis factor related protein 1 | 2 |
| 8484 | hsa-mir-26b | 114897 | C1QTNF1 | C1q and tumor necrosis factor related protein 1 | 2 |
| 8485 | hsa-mir-31 | 114897 | C1QTNF1 | C1q and tumor necrosis factor related protein 1 | 2 |
| 8486 | hsa-mir-194-2 | 114897 | C1QTNF1 | C1q and tumor necrosis factor related protein 1 | 2 |
| 8487 | hsa-mir-340 | 114897 | C1QTNF1 | C1q and tumor necrosis factor related protein 1 | 2 |
| 8488 | hsa-mir-338 | 114897 | C1QTNF1 | C1q and tumor necrosis factor related protein 1 | 2 |
| 8489 | hsa-mir-449b | 114897 | C1QTNF1 | C1q and tumor necrosis factor related protein 1 | 2 |
| 8490 | hsa-mir-449c | 114897 | C1QTNF1 | C1q and tumor necrosis factor related protein 1 | 2 |
| 8491 | hsa-mir-3065 | 114897 | C1QTNF1 | C1q and tumor necrosis factor related protein 1 | 2 |
| 8851 | hsa-miR-10a | 373863 | DND1 | dead end homolog 1 (zebrafish) | 2 |
| 8852 | hsa-miR-152 | 373863 | DND1 | dead end homolog 1 (zebrafish) | 2 |
| 8853 | hsa-miR-26b | 373863 | DND1 | dead end homolog 1 (zebrafish) | 2 |
| 8854 | hsa-miR-31 | 373863 | DND1 | dead end homolog 1 (zebrafish) | 2 |
| 8855 | hsa-miR-338-5p | 373863 | DND1 | dead end homolog 1 (zebrafish) | 2 |
| 8856 | hsa-miR-340 | 373863 | DND1 | dead end homolog 1 (zebrafish) | 2 |
| 8857 | hsa-miR-383 | 373863 | DND1 | dead end homolog 1 (zebrafish) | 2 |
| 8858 | hsa-miR-449a | 373863 | DND1 | dead end homolog 1 (zebrafish) | 2 |
| 8859 | hsa-miR-499-5p | 373863 | DND1 | dead end homolog 1 (zebrafish) | 2 |
| 8860 | hsa-miR-621 | 373863 | DND1 | dead end homolog 1 (zebrafish) | 2 |
| 8863 | hsa-miR-657 | 373863 | DND1 | dead end homolog 1 (zebrafish) | 2 |
| 8864 | hsa-miR-765 | 373863 | DND1 | dead end homolog 1 (zebrafish) | 2 |
| 8865 | hsa-miR-770-5p | 373863 | DND1 | dead end homolog 1 (zebrafish) | 2 |
| 8866 | hsa-miR-877 | 373863 | DND1 | dead end homolog 1 (zebrafish) | 2 |
| 8867 | hsa-mir-26a-1 | 373863 | DND1 | dead end homolog 1 (zebrafish) | 2 |
| 8868 | hsa-mir-26b | 373863 | DND1 | dead end homolog 1 (zebrafish) | 2 |
| 8869 | hsa-mir-31 | 373863 | DND1 | dead end homolog 1 (zebrafish) | 2 |
| 8870 | hsa-mir-194-2 | 373863 | DND1 | dead end homolog 1 (zebrafish) | 2 |
| 8871 | hsa-mir-340 | 373863 | DND1 | dead end homolog 1 (zebrafish) | 2 |
| 8872 | hsa-mir-338 | 373863 | DND1 | dead end homolog 1 (zebrafish) | 2 |
| 8873 | hsa-mir-449b | 373863 | DND1 | dead end homolog 1 (zebrafish) | 2 |
| 8874 | hsa-mir-449c | 373863 | DND1 | dead end homolog 1 (zebrafish) | 2 |
| 8875 | hsa-mir-3065 | 373863 | DND1 | dead end homolog 1 (zebrafish) | 2 |
| 8982 | hsa-miR-10a | 27071 | DAPP1 | dual adaptor of phosphotyrosine and 3-phosphoinositides | 2 |
| 8983 | hsa-miR-139-5p | 27071 | DAPP1 | dual adaptor of phosphotyrosine and 3-phosphoinositides | 2 |
| 8984 | hsa-miR-152 | 27071 | DAPP1 | dual adaptor of phosphotyrosine and 3-phosphoinositides | 2 |
| 8985 | hsa-miR-26b | 27071 | DAPP1 | dual adaptor of phosphotyrosine and 3-phosphoinositides | 2 |
| 8986 | hsa-miR-31 | 27071 | DAPP1 | dual adaptor of phosphotyrosine and 3-phosphoinositides | 2 |
| 8987 | hsa-miR-338-5p | 27071 | DAPP1 | dual adaptor of phosphotyrosine and 3-phosphoinositides | 2 |
| 8988 | hsa-miR-340 | 27071 | DAPP1 | dual adaptor of phosphotyrosine and 3-phosphoinositides | 2 |
| 8989 | hsa-miR-383 | 27071 | DAPP1 | dual adaptor of phosphotyrosine and 3-phosphoinositides | 2 |
| 8990 | hsa-miR-449a | 27071 | DAPP1 | dual adaptor of phosphotyrosine and 3-phosphoinositides | 2 |
| 8991 | hsa-miR-499-5p | 27071 | DAPP1 | dual adaptor of phosphotyrosine and 3-phosphoinositides | 2 |
| 8992 | hsa-miR-548b-5p | 27071 | DAPP1 | dual adaptor of phosphotyrosine and 3-phosphoinositides | 2 |
| 8993 | hsa-miR-621 | 27071 | DAPP1 | dual adaptor of phosphotyrosine and 3-phosphoinositides | 2 |
| 8994 | hsa-miR-657 | 27071 | DAPP1 | dual adaptor of phosphotyrosine and 3-phosphoinositides | 2 |
| 8995 | hsa-miR-765 | 27071 | DAPP1 | dual adaptor of phosphotyrosine and 3-phosphoinositides | 2 |
| 8996 | hsa-miR-770-5p | 27071 | DAPP1 | dual adaptor of phosphotyrosine and 3-phosphoinositides | 2 |
| 8997 | hsa-miR-877 | 27071 | DAPP1 | dual adaptor of phosphotyrosine and 3-phosphoinositides | 2 |
| 8998 | hsa-mir-26a-1 | 27071 | DAPP1 | dual adaptor of phosphotyrosine and 3-phosphoinositides | 2 |
| 8999 | hsa-mir-26b | 27071 | DAPP1 | dual adaptor of phosphotyrosine and 3-phosphoinositides | 2 |
| 9000 | hsa-mir-31 | 27071 | DAPP1 | dual adaptor of phosphotyrosine and 3-phosphoinositides | 2 |
| 9001 | hsa-mir-194-2 | 27071 | DAPP1 | dual adaptor of phosphotyrosine and 3-phosphoinositides | 2 |
| 9002 | hsa-mir-340 | 27071 | DAPP1 | dual adaptor of phosphotyrosine and 3-phosphoinositides | 2 |
| 9003 | hsa-mir-338 | 27071 | DAPP1 | dual adaptor of phosphotyrosine and 3-phosphoinositides | 2 |
| 9004 | hsa-mir-548b | 27071 | DAPP1 | dual adaptor of phosphotyrosine and 3-phosphoinositides | 2 |
| 9005 | hsa-mir-449b | 27071 | DAPP1 | dual adaptor of phosphotyrosine and 3-phosphoinositides | 2 |
| 9006 | hsa-mir-449c | 27071 | DAPP1 | dual adaptor of phosphotyrosine and 3-phosphoinositides | 2 |
| 9007 | hsa-mir-3065 | 27071 | DAPP1 | dual adaptor of phosphotyrosine and 3-phosphoinositides | 2 |
| 9102 | hsa-mir-550-1 | 56172 | ANKH | ankylosis, progressive homolog (mouse) | 2 |
| 9103 | hsa-mir-2116 | 56172 | ANKH | ankylosis, progressive homolog (mouse) | 2 |
| 9131 | hsa-miR-10a | 64332 | NFKBIZ | nuclear factor of kappa light polypeptide gene enhancer in B-cells inhibitor, zeta | 2 |
| 9133 | hsa-miR-152 | 64332 | NFKBIZ | nuclear factor of kappa light polypeptide gene enhancer in B-cells inhibitor, zeta | 2 |
| 9134 | hsa-miR-26a | 64332 | NFKBIZ | nuclear factor of kappa light polypeptide gene enhancer in B-cells inhibitor, zeta | 2 |
| 9135 | hsa-miR-26b | 64332 | NFKBIZ | nuclear factor of kappa light polypeptide gene enhancer in B-cells inhibitor, zeta | 2 |
| 9136 | hsa-miR-340 | 64332 | NFKBIZ | nuclear factor of kappa light polypeptide gene enhancer in B-cells inhibitor, zeta | 2 |
| 9137 | hsa-miR-449a | 64332 | NFKBIZ | nuclear factor of kappa light polypeptide gene enhancer in B-cells inhibitor, zeta | 2 |
| 9138 | hsa-miR-490-5p | 64332 | NFKBIZ | nuclear factor of kappa light polypeptide gene enhancer in B-cells inhibitor, zeta | 2 |
| 9139 | hsa-miR-499-5p | 64332 | NFKBIZ | nuclear factor of kappa light polypeptide gene enhancer in B-cells inhibitor, zeta | 2 |
| 9140 | hsa-miR-548b-5p | 64332 | NFKBIZ | nuclear factor of kappa light polypeptide gene enhancer in B-cells inhibitor, zeta | 2 |
| 9141 | hsa-miR-564 | 64332 | NFKBIZ | nuclear factor of kappa light polypeptide gene enhancer in B-cells inhibitor, zeta | 2 |
| 9142 | hsa-miR-584 | 64332 | NFKBIZ | nuclear factor of kappa light polypeptide gene enhancer in B-cells inhibitor, zeta | 2 |
| 9143 | hsa-miR-621 | 64332 | NFKBIZ | nuclear factor of kappa light polypeptide gene enhancer in B-cells inhibitor, zeta | 2 |
| 9144 | hsa-miR-634 | 64332 | NFKBIZ | nuclear factor of kappa light polypeptide gene enhancer in B-cells inhibitor, zeta | 2 |
| 9145 | hsa-miR-765 | 64332 | NFKBIZ | nuclear factor of kappa light polypeptide gene enhancer in B-cells inhibitor, zeta | 2 |
| 9146 | hsa-miR-770-5p | 64332 | NFKBIZ | nuclear factor of kappa light polypeptide gene enhancer in B-cells inhibitor, zeta | 2 |
| 9147 | hsa-miR-877 | 64332 | NFKBIZ | nuclear factor of kappa light polypeptide gene enhancer in B-cells inhibitor, zeta | 2 |
| 9148 | hsa-miR-95 | 64332 | NFKBIZ | nuclear factor of kappa light polypeptide gene enhancer in B-cells inhibitor, zeta | 2 |
| 9149 | hsa-mir-26a-1 | 64332 | NFKBIZ | nuclear factor of kappa light polypeptide gene enhancer in B-cells inhibitor, zeta | 2 |
| 9150 | hsa-mir-26b | 64332 | NFKBIZ | nuclear factor of kappa light polypeptide gene enhancer in B-cells inhibitor, zeta | 2 |
| 9151 | hsa-mir-194-2 | 64332 | NFKBIZ | nuclear factor of kappa light polypeptide gene enhancer in B-cells inhibitor, zeta | 2 |
| 9152 | hsa-mir-340 | 64332 | NFKBIZ | nuclear factor of kappa light polypeptide gene enhancer in B-cells inhibitor, zeta | 2 |
| 9153 | hsa-mir-490 | 64332 | NFKBIZ | nuclear factor of kappa light polypeptide gene enhancer in B-cells inhibitor, zeta | 2 |
| 9154 | hsa-mir-548b | 64332 | NFKBIZ | nuclear factor of kappa light polypeptide gene enhancer in B-cells inhibitor, zeta | 2 |
| 9155 | hsa-mir-449b | 64332 | NFKBIZ | nuclear factor of kappa light polypeptide gene enhancer in B-cells inhibitor, zeta | 2 |
| 9156 | hsa-mir-449c | 64332 | NFKBIZ | nuclear factor of kappa light polypeptide gene enhancer in B-cells inhibitor, zeta | 2 |
| 9562 | hsa-miR-10a | 55593 | OTUD5 | OTU domain containing 5 | 2 |
| 9563 | hsa-miR-152 | 55593 | OTUD5 | OTU domain containing 5 | 2 |
| 9564 | hsa-miR-26a | 55593 | OTUD5 | OTU domain containing 5 | 2 |
| 9565 | hsa-miR-26b | 55593 | OTUD5 | OTU domain containing 5 | 2 |
| 9568 | hsa-miR-340 | 55593 | OTUD5 | OTU domain containing 5 | 2 |
| 9570 | hsa-miR-449a | 55593 | OTUD5 | OTU domain containing 5 | 2 |
| 9571 | hsa-miR-499-5p | 55593 | OTUD5 | OTU domain containing 5 | 2 |
| 9572 | hsa-miR-621 | 55593 | OTUD5 | OTU domain containing 5 | 2 |
| 9573 | hsa-miR-634 | 55593 | OTUD5 | OTU domain containing 5 | 2 |
| 9576 | hsa-miR-765 | 55593 | OTUD5 | OTU domain containing 5 | 2 |
| 9577 | hsa-miR-770-5p | 55593 | OTUD5 | OTU domain containing 5 | 2 |
| 9578 | hsa-miR-877 | 55593 | OTUD5 | OTU domain containing 5 | 2 |
| 9579 | hsa-mir-26a-1 | 55593 | OTUD5 | OTU domain containing 5 | 2 |
| 9580 | hsa-mir-26b | 55593 | OTUD5 | OTU domain containing 5 | 2 |
| 9582 | hsa-mir-194-2 | 55593 | OTUD5 | OTU domain containing 5 | 2 |
| 9583 | hsa-mir-340 | 55593 | OTUD5 | OTU domain containing 5 | 2 |
| 9585 | hsa-mir-449b | 55593 | OTUD5 | OTU domain containing 5 | 2 |
| 9586 | hsa-mir-449c | 55593 | OTUD5 | OTU domain containing 5 | 2 |
| 9777 | hsa-miR-10a | 152007 | GLIPR2 | GLI pathogenesis-related 2 | 2 |
| 9778 | hsa-miR-152 | 152007 | GLIPR2 | GLI pathogenesis-related 2 | 2 |
| 9779 | hsa-miR-31 | 152007 | GLIPR2 | GLI pathogenesis-related 2 | 2 |
| 9780 | hsa-miR-338-5p | 152007 | GLIPR2 | GLI pathogenesis-related 2 | 2 |
| 9781 | hsa-miR-340 | 152007 | GLIPR2 | GLI pathogenesis-related 2 | 2 |
| 9782 | hsa-miR-383 | 152007 | GLIPR2 | GLI pathogenesis-related 2 | 2 |
| 9783 | hsa-miR-449a | 152007 | GLIPR2 | GLI pathogenesis-related 2 | 2 |
| 9784 | hsa-miR-621 | 152007 | GLIPR2 | GLI pathogenesis-related 2 | 2 |
| 9785 | hsa-miR-657 | 152007 | GLIPR2 | GLI pathogenesis-related 2 | 2 |
| 9786 | hsa-miR-663 | 152007 | GLIPR2 | GLI pathogenesis-related 2 | 2 |
| 9787 | hsa-miR-765 | 152007 | GLIPR2 | GLI pathogenesis-related 2 | 2 |
| 9788 | hsa-miR-770-5p | 152007 | GLIPR2 | GLI pathogenesis-related 2 | 2 |
| 9789 | hsa-miR-877 | 152007 | GLIPR2 | GLI pathogenesis-related 2 | 2 |
| 9790 | hsa-mir-26a-1 | 152007 | GLIPR2 | GLI pathogenesis-related 2 | 2 |
| 9791 | hsa-mir-31 | 152007 | GLIPR2 | GLI pathogenesis-related 2 | 2 |
| 9792 | hsa-mir-194-2 | 152007 | GLIPR2 | GLI pathogenesis-related 2 | 2 |
| 9793 | hsa-mir-340 | 152007 | GLIPR2 | GLI pathogenesis-related 2 | 2 |
| 9794 | hsa-mir-338 | 152007 | GLIPR2 | GLI pathogenesis-related 2 | 2 |
| 9795 | hsa-mir-449b | 152007 | GLIPR2 | GLI pathogenesis-related 2 | 2 |
| 9796 | hsa-mir-449c | 152007 | GLIPR2 | GLI pathogenesis-related 2 | 2 |
| 9797 | hsa-mir-3065 | 152007 | GLIPR2 | GLI pathogenesis-related 2 | 2 |
| 10237 | hsa-miR-10a | 441168 | FAM26F | family with sequence similarity 26, member F | 2 |
| 10238 | hsa-miR-139-5p | 441168 | FAM26F | family with sequence similarity 26, member F | 2 |
| 10239 | hsa-miR-152 | 441168 | FAM26F | family with sequence similarity 26, member F | 2 |
| 10240 | hsa-miR-26a | 441168 | FAM26F | family with sequence similarity 26, member F | 2 |
| 10241 | hsa-miR-26b | 441168 | FAM26F | family with sequence similarity 26, member F | 2 |
| 10242 | hsa-miR-31 | 441168 | FAM26F | family with sequence similarity 26, member F | 2 |
| 10243 | hsa-miR-338-5p | 441168 | FAM26F | family with sequence similarity 26, member F | 2 |
| 10244 | hsa-miR-340 | 441168 | FAM26F | family with sequence similarity 26, member F | 2 |
| 10245 | hsa-miR-449a | 441168 | FAM26F | family with sequence similarity 26, member F | 2 |
| 10246 | hsa-miR-499-5p | 441168 | FAM26F | family with sequence similarity 26, member F | 2 |
| 10247 | hsa-miR-548b-5p | 441168 | FAM26F | family with sequence similarity 26, member F | 2 |
| 10249 | hsa-miR-621 | 441168 | FAM26F | family with sequence similarity 26, member F | 2 |
| 10250 | hsa-miR-657 | 441168 | FAM26F | family with sequence similarity 26, member F | 2 |
| 10251 | hsa-miR-765 | 441168 | FAM26F | family with sequence similarity 26, member F | 2 |
| 10252 | hsa-miR-770-5p | 441168 | FAM26F | family with sequence similarity 26, member F | 2 |
| 10253 | hsa-miR-877 | 441168 | FAM26F | family with sequence similarity 26, member F | 2 |
| 10254 | hsa-mir-26a-1 | 441168 | FAM26F | family with sequence similarity 26, member F | 2 |
| 10255 | hsa-mir-26b | 441168 | FAM26F | family with sequence similarity 26, member F | 2 |
| 10256 | hsa-mir-31 | 441168 | FAM26F | family with sequence similarity 26, member F | 2 |
| 10257 | hsa-mir-194-2 | 441168 | FAM26F | family with sequence similarity 26, member F | 2 |
| 10258 | hsa-mir-340 | 441168 | FAM26F | family with sequence similarity 26, member F | 2 |
| 10259 | hsa-mir-338 | 441168 | FAM26F | family with sequence similarity 26, member F | 2 |
| 10260 | hsa-mir-548b | 441168 | FAM26F | family with sequence similarity 26, member F | 2 |
| 10261 | hsa-mir-548d-2 | 441168 | FAM26F | family with sequence similarity 26, member F | 2 |
| 10262 | hsa-mir-449b | 441168 | FAM26F | family with sequence similarity 26, member F | 2 |
| 10263 | hsa-mir-449c | 441168 | FAM26F | family with sequence similarity 26, member F | 2 |
| 10264 | hsa-mir-3065 | 441168 | FAM26F | family with sequence similarity 26, member F | 2 |
| 10552 | hsa-miR-10a | 115362 | GBP5 | guanylate binding protein 5 | 2 |
| 10554 | hsa-miR-152 | 115362 | GBP5 | guanylate binding protein 5 | 2 |
| 10555 | hsa-miR-26a | 115362 | GBP5 | guanylate binding protein 5 | 2 |
| 10556 | hsa-miR-26b | 115362 | GBP5 | guanylate binding protein 5 | 2 |
| 10557 | hsa-miR-31 | 115362 | GBP5 | guanylate binding protein 5 | 2 |
| 10558 | hsa-miR-338-5p | 115362 | GBP5 | guanylate binding protein 5 | 2 |
| 10559 | hsa-miR-340 | 115362 | GBP5 | guanylate binding protein 5 | 2 |
| 10560 | hsa-miR-449a | 115362 | GBP5 | guanylate binding protein 5 | 2 |
| 10561 | hsa-miR-490-5p | 115362 | GBP5 | guanylate binding protein 5 | 2 |
| 10562 | hsa-miR-499-5p | 115362 | GBP5 | guanylate binding protein 5 | 2 |
| 10563 | hsa-miR-548b-5p | 115362 | GBP5 | guanylate binding protein 5 | 2 |
| 10564 | hsa-miR-564 | 115362 | GBP5 | guanylate binding protein 5 | 2 |
| 10565 | hsa-miR-621 | 115362 | GBP5 | guanylate binding protein 5 | 2 |
| 10566 | hsa-miR-657 | 115362 | GBP5 | guanylate binding protein 5 | 2 |
| 10567 | hsa-miR-765 | 115362 | GBP5 | guanylate binding protein 5 | 2 |
| 10568 | hsa-miR-770-5p | 115362 | GBP5 | guanylate binding protein 5 | 2 |
| 10569 | hsa-miR-877 | 115362 | GBP5 | guanylate binding protein 5 | 2 |
| 10570 | hsa-mir-26a-1 | 115362 | GBP5 | guanylate binding protein 5 | 2 |
| 10571 | hsa-mir-26b | 115362 | GBP5 | guanylate binding protein 5 | 2 |
| 10572 | hsa-mir-31 | 115362 | GBP5 | guanylate binding protein 5 | 2 |
| 10573 | hsa-mir-194-2 | 115362 | GBP5 | guanylate binding protein 5 | 2 |
| 10574 | hsa-mir-340 | 115362 | GBP5 | guanylate binding protein 5 | 2 |
| 10575 | hsa-mir-338 | 115362 | GBP5 | guanylate binding protein 5 | 2 |
| 10576 | hsa-mir-490 | 115362 | GBP5 | guanylate binding protein 5 | 2 |
| 10577 | hsa-mir-548b | 115362 | GBP5 | guanylate binding protein 5 | 2 |
| 10579 | hsa-mir-449b | 115362 | GBP5 | guanylate binding protein 5 | 2 |
| 10580 | hsa-mir-449c | 115362 | GBP5 | guanylate binding protein 5 | 2 |
| 10581 | hsa-mir-3065 | 115362 | GBP5 | guanylate binding protein 5 | 2 |
| 11489 | hsa-miR-10a | 115361 | GBP4 | guanylate binding protein 4 | 2 |
| 11490 | hsa-miR-139-5p | 115361 | GBP4 | guanylate binding protein 4 | 2 |
| 11491 | hsa-miR-152 | 115361 | GBP4 | guanylate binding protein 4 | 2 |
| 11492 | hsa-miR-26a | 115361 | GBP4 | guanylate binding protein 4 | 2 |
| 11493 | hsa-miR-26b | 115361 | GBP4 | guanylate binding protein 4 | 2 |
| 11494 | hsa-miR-31 | 115361 | GBP4 | guanylate binding protein 4 | 2 |
| 11495 | hsa-miR-338-5p | 115361 | GBP4 | guanylate binding protein 4 | 2 |
| 11496 | hsa-miR-340 | 115361 | GBP4 | guanylate binding protein 4 | 2 |
| 11497 | hsa-miR-449a | 115361 | GBP4 | guanylate binding protein 4 | 2 |
| 11498 | hsa-miR-490-5p | 115361 | GBP4 | guanylate binding protein 4 | 2 |
| 11499 | hsa-miR-499-5p | 115361 | GBP4 | guanylate binding protein 4 | 2 |
| 11500 | hsa-miR-548b-5p | 115361 | GBP4 | guanylate binding protein 4 | 2 |
| 11501 | hsa-miR-564 | 115361 | GBP4 | guanylate binding protein 4 | 2 |
| 11502 | hsa-miR-584 | 115361 | GBP4 | guanylate binding protein 4 | 2 |
| 11503 | hsa-miR-621 | 115361 | GBP4 | guanylate binding protein 4 | 2 |
| 11504 | hsa-miR-657 | 115361 | GBP4 | guanylate binding protein 4 | 2 |
| 11506 | hsa-miR-765 | 115361 | GBP4 | guanylate binding protein 4 | 2 |
| 11507 | hsa-miR-770-5p | 115361 | GBP4 | guanylate binding protein 4 | 2 |
| 11508 | hsa-miR-877 | 115361 | GBP4 | guanylate binding protein 4 | 2 |
| 11509 | hsa-mir-26a-1 | 115361 | GBP4 | guanylate binding protein 4 | 2 |
| 11510 | hsa-mir-26b | 115361 | GBP4 | guanylate binding protein 4 | 2 |
| 11511 | hsa-mir-31 | 115361 | GBP4 | guanylate binding protein 4 | 2 |
| 11512 | hsa-mir-194-2 | 115361 | GBP4 | guanylate binding protein 4 | 2 |
| 11513 | hsa-mir-340 | 115361 | GBP4 | guanylate binding protein 4 | 2 |
| 11514 | hsa-mir-338 | 115361 | GBP4 | guanylate binding protein 4 | 2 |
| 11515 | hsa-mir-490 | 115361 | GBP4 | guanylate binding protein 4 | 2 |
| 11516 | hsa-mir-548b | 115361 | GBP4 | guanylate binding protein 4 | 2 |
| 11517 | hsa-mir-548d-2 | 115361 | GBP4 | guanylate binding protein 4 | 2 |
| 11518 | hsa-mir-449b | 115361 | GBP4 | guanylate binding protein 4 | 2 |
| 11519 | hsa-mir-449c | 115361 | GBP4 | guanylate binding protein 4 | 2 |
| 11520 | hsa-mir-3065 | 115361 | GBP4 | guanylate binding protein 4 | 2 |
| 28 | hsa-miR-1 | 113235 | SLC46A1 | solute carrier family 46 (folate transporter), member 1 | 1 |
| 29 | hsa-miR-133a | 113235 | SLC46A1 | solute carrier family 46 (folate transporter), member 1 | 1 |
| 30 | hsa-miR-186 | 113235 | SLC46A1 | solute carrier family 46 (folate transporter), member 1 | 1 |
| 31 | hsa-miR-208a | 113235 | SLC46A1 | solute carrier family 46 (folate transporter), member 1 | 1 |
| 32 | hsa-miR-326 | 113235 | SLC46A1 | solute carrier family 46 (folate transporter), member 1 | 1 |
| 33 | hsa-miR-33a | 113235 | SLC46A1 | solute carrier family 46 (folate transporter), member 1 | 1 |
| 34 | hsa-miR-449b | 113235 | SLC46A1 | solute carrier family 46 (folate transporter), member 1 | 1 |
| 35 | hsa-miR-550 | 113235 | SLC46A1 | solute carrier family 46 (folate transporter), member 1 | 1 |
| 36 | hsa-miR-551a | 113235 | SLC46A1 | solute carrier family 46 (folate transporter), member 1 | 1 |
| 37 | hsa-miR-575 | 113235 | SLC46A1 | solute carrier family 46 (folate transporter), member 1 | 1 |
| 38 | hsa-miR-579 | 113235 | SLC46A1 | solute carrier family 46 (folate transporter), member 1 | 1 |
| 39 | hsa-miR-608 | 113235 | SLC46A1 | solute carrier family 46 (folate transporter), member 1 | 1 |
| 40 | hsa-miR-620 | 113235 | SLC46A1 | solute carrier family 46 (folate transporter), member 1 | 1 |
| 41 | hsa-let-7a-3 | 113235 | SLC46A1 | solute carrier family 46 (folate transporter), member 1 | 1 |
| 42 | hsa-let-7b | 113235 | SLC46A1 | solute carrier family 46 (folate transporter), member 1 | 1 |
| 43 | hsa-mir-22 | 113235 | SLC46A1 | solute carrier family 46 (folate transporter), member 1 | 1 |
| 44 | hsa-mir-10a | 113235 | SLC46A1 | solute carrier family 46 (folate transporter), member 1 | 1 |
| 45 | hsa-mir-186 | 113235 | SLC46A1 | solute carrier family 46 (folate transporter), member 1 | 1 |
| 46 | hsa-mir-155 | 113235 | SLC46A1 | solute carrier family 46 (folate transporter), member 1 | 1 |
| 47 | hsa-mir-423 | 113235 | SLC46A1 | solute carrier family 46 (folate transporter), member 1 | 1 |
| 48 | hsa-mir-499 | 113235 | SLC46A1 | solute carrier family 46 (folate transporter), member 1 | 1 |
| 49 | hsa-mir-550-1 | 113235 | SLC46A1 | solute carrier family 46 (folate transporter), member 1 | 1 |
| 50 | hsa-mir-877 | 113235 | SLC46A1 | solute carrier family 46 (folate transporter), member 1 | 1 |
| 51 | hsa-mir-2116 | 113235 | SLC46A1 | solute carrier family 46 (folate transporter), member 1 | 1 |
| 52 | hsa-mir-3186 | 113235 | SLC46A1 | solute carrier family 46 (folate transporter), member 1 | 1 |
| 53 | hsa-miR-10a | 114294 | LACTB | lactamase, beta | 1 |
| 54 | hsa-miR-139-5p | 114294 | LACTB | lactamase, beta | 1 |
| 55 | hsa-miR-152 | 114294 | LACTB | lactamase, beta | 1 |
| 56 | hsa-miR-26a | 114294 | LACTB | lactamase, beta | 1 |
| 57 | hsa-miR-26b | 114294 | LACTB | lactamase, beta | 1 |
| 58 | hsa-miR-31 | 114294 | LACTB | lactamase, beta | 1 |
| 59 | hsa-miR-338-5p | 114294 | LACTB | lactamase, beta | 1 |
| 60 | hsa-miR-340 | 114294 | LACTB | lactamase, beta | 1 |
| 61 | hsa-miR-423-5p | 114294 | LACTB | lactamase, beta | 1 |
| 62 | hsa-miR-449a | 114294 | LACTB | lactamase, beta | 1 |
| 63 | hsa-miR-490-5p | 114294 | LACTB | lactamase, beta | 1 |
| 64 | hsa-miR-499-5p | 114294 | LACTB | lactamase, beta | 1 |
| 65 | hsa-miR-548b-5p | 114294 | LACTB | lactamase, beta | 1 |
| 66 | hsa-miR-564 | 114294 | LACTB | lactamase, beta | 1 |
| 67 | hsa-miR-584 | 114294 | LACTB | lactamase, beta | 1 |
| 68 | hsa-miR-621 | 114294 | LACTB | lactamase, beta | 1 |
| 69 | hsa-miR-657 | 114294 | LACTB | lactamase, beta | 1 |
| 70 | hsa-miR-663 | 114294 | LACTB | lactamase, beta | 1 |
| 71 | hsa-miR-765 | 114294 | LACTB | lactamase, beta | 1 |
| 72 | hsa-miR-770-5p | 114294 | LACTB | lactamase, beta | 1 |
| 73 | hsa-miR-877 | 114294 | LACTB | lactamase, beta | 1 |
| 74 | hsa-mir-26a-1 | 114294 | LACTB | lactamase, beta | 1 |
| 75 | hsa-mir-26b | 114294 | LACTB | lactamase, beta | 1 |
| 76 | hsa-mir-31 | 114294 | LACTB | lactamase, beta | 1 |
| 77 | hsa-mir-194-2 | 114294 | LACTB | lactamase, beta | 1 |
| 78 | hsa-mir-340 | 114294 | LACTB | lactamase, beta | 1 |
| 79 | hsa-mir-338 | 114294 | LACTB | lactamase, beta | 1 |
| 80 | hsa-mir-490 | 114294 | LACTB | lactamase, beta | 1 |
| 81 | hsa-mir-548b | 114294 | LACTB | lactamase, beta | 1 |
| 82 | hsa-mir-548d-2 | 114294 | LACTB | lactamase, beta | 1 |
| 83 | hsa-mir-449b | 114294 | LACTB | lactamase, beta | 1 |
| 84 | hsa-mir-449c | 114294 | LACTB | lactamase, beta | 1 |
| 85 | hsa-mir-3065 | 114294 | LACTB | lactamase, beta | 1 |
| 86 | hsa-miR-1 | 83597 | RTP3 | receptor (chemosensory) transporter protein 3 | 1 |
| 87 | hsa-miR-133a | 83597 | RTP3 | receptor (chemosensory) transporter protein 3 | 1 |
| 88 | hsa-miR-186 | 83597 | RTP3 | receptor (chemosensory) transporter protein 3 | 1 |
| 89 | hsa-miR-208a | 83597 | RTP3 | receptor (chemosensory) transporter protein 3 | 1 |
| 90 | hsa-miR-326 | 83597 | RTP3 | receptor (chemosensory) transporter protein 3 | 1 |
| 91 | hsa-miR-449b | 83597 | RTP3 | receptor (chemosensory) transporter protein 3 | 1 |
| 92 | hsa-miR-550 | 83597 | RTP3 | receptor (chemosensory) transporter protein 3 | 1 |
| 93 | hsa-miR-575 | 83597 | RTP3 | receptor (chemosensory) transporter protein 3 | 1 |
| 94 | hsa-miR-579 | 83597 | RTP3 | receptor (chemosensory) transporter protein 3 | 1 |
| 95 | hsa-miR-608 | 83597 | RTP3 | receptor (chemosensory) transporter protein 3 | 1 |
| 96 | hsa-miR-620 | 83597 | RTP3 | receptor (chemosensory) transporter protein 3 | 1 |
| 97 | hsa-miR-637 | 83597 | RTP3 | receptor (chemosensory) transporter protein 3 | 1 |
| 98 | hsa-miR-885-5p | 83597 | RTP3 | receptor (chemosensory) transporter protein 3 | 1 |
| 99 | hsa-mir-22 | 83597 | RTP3 | receptor (chemosensory) transporter protein 3 | 1 |
| 100 | hsa-mir-10a | 83597 | RTP3 | receptor (chemosensory) transporter protein 3 | 1 |
| 101 | hsa-mir-186 | 83597 | RTP3 | receptor (chemosensory) transporter protein 3 | 1 |
| 102 | hsa-mir-155 | 83597 | RTP3 | receptor (chemosensory) transporter protein 3 | 1 |
| 103 | hsa-mir-423 | 83597 | RTP3 | receptor (chemosensory) transporter protein 3 | 1 |
| 104 | hsa-mir-499 | 83597 | RTP3 | receptor (chemosensory) transporter protein 3 | 1 |
| 105 | hsa-mir-550-1 | 83597 | RTP3 | receptor (chemosensory) transporter protein 3 | 1 |
| 106 | hsa-mir-885 | 83597 | RTP3 | receptor (chemosensory) transporter protein 3 | 1 |
| 107 | hsa-mir-877 | 83597 | RTP3 | receptor (chemosensory) transporter protein 3 | 1 |
| 108 | hsa-mir-2116 | 83597 | RTP3 | receptor (chemosensory) transporter protein 3 | 1 |
| 109 | hsa-mir-3190 | 83597 | RTP3 | receptor (chemosensory) transporter protein 3 | 1 |
| 110 | hsa-miR-10a | 3716 | JAK1 | Janus kinase 1 | 1 |
| 111 | hsa-miR-152 | 3716 | JAK1 | Janus kinase 1 | 1 |
| 112 | hsa-miR-26a | 3716 | JAK1 | Janus kinase 1 | 1 |
| 113 | hsa-miR-26b | 3716 | JAK1 | Janus kinase 1 | 1 |
| 114 | hsa-miR-31 | 3716 | JAK1 | Janus kinase 1 | 1 |
| 115 | hsa-miR-338-5p | 3716 | JAK1 | Janus kinase 1 | 1 |
| 116 | hsa-miR-340 | 3716 | JAK1 | Janus kinase 1 | 1 |
| 117 | hsa-miR-423-5p | 3716 | JAK1 | Janus kinase 1 | 1 |
| 118 | hsa-miR-449a | 3716 | JAK1 | Janus kinase 1 | 1 |
| 119 | hsa-miR-499-5p | 3716 | JAK1 | Janus kinase 1 | 1 |
| 120 | hsa-miR-548b-5p | 3716 | JAK1 | Janus kinase 1 | 1 |
| 121 | hsa-miR-564 | 3716 | JAK1 | Janus kinase 1 | 1 |
| 122 | hsa-miR-621 | 3716 | JAK1 | Janus kinase 1 | 1 |
| 123 | hsa-miR-634 | 3716 | JAK1 | Janus kinase 1 | 1 |
| 124 | hsa-miR-644 | 3716 | JAK1 | Janus kinase 1 | 1 |
| 125 | hsa-miR-657 | 3716 | JAK1 | Janus kinase 1 | 1 |
| 126 | hsa-miR-765 | 3716 | JAK1 | Janus kinase 1 | 1 |
| 127 | hsa-miR-770-5p | 3716 | JAK1 | Janus kinase 1 | 1 |
| 128 | hsa-miR-877 | 3716 | JAK1 | Janus kinase 1 | 1 |
| 129 | hsa-miR-95 | 3716 | JAK1 | Janus kinase 1 | 1 |
| 130 | hsa-mir-26a-1 | 3716 | JAK1 | Janus kinase 1 | 1 |
| 131 | hsa-mir-26b | 3716 | JAK1 | Janus kinase 1 | 1 |
| 132 | hsa-mir-31 | 3716 | JAK1 | Janus kinase 1 | 1 |
| 133 | hsa-mir-194-2 | 3716 | JAK1 | Janus kinase 1 | 1 |
| 134 | hsa-mir-340 | 3716 | JAK1 | Janus kinase 1 | 1 |
| 135 | hsa-mir-338 | 3716 | JAK1 | Janus kinase 1 | 1 |
| 136 | hsa-mir-548b | 3716 | JAK1 | Janus kinase 1 | 1 |
| 137 | hsa-mir-449b | 3716 | JAK1 | Janus kinase 1 | 1 |
| 138 | hsa-mir-449c | 3716 | JAK1 | Janus kinase 1 | 1 |
| 139 | hsa-mir-3065 | 3716 | JAK1 | Janus kinase 1 | 1 |
| 140 | hsa-miR-1 | 54879 | ST7L | suppression of tumorigenicity 7 like | 1 |
| 141 | hsa-miR-133a | 54879 | ST7L | suppression of tumorigenicity 7 like | 1 |
| 142 | hsa-miR-186 | 54879 | ST7L | suppression of tumorigenicity 7 like | 1 |
| 143 | hsa-miR-326 | 54879 | ST7L | suppression of tumorigenicity 7 like | 1 |
| 144 | hsa-miR-449b | 54879 | ST7L | suppression of tumorigenicity 7 like | 1 |
| 145 | hsa-miR-489 | 54879 | ST7L | suppression of tumorigenicity 7 like | 1 |
| 146 | hsa-miR-562 | 54879 | ST7L | suppression of tumorigenicity 7 like | 1 |
| 147 | hsa-miR-575 | 54879 | ST7L | suppression of tumorigenicity 7 like | 1 |
| 148 | hsa-miR-579 | 54879 | ST7L | suppression of tumorigenicity 7 like | 1 |
| 149 | hsa-miR-608 | 54879 | ST7L | suppression of tumorigenicity 7 like | 1 |
| 150 | hsa-miR-620 | 54879 | ST7L | suppression of tumorigenicity 7 like | 1 |
| 151 | hsa-miR-637 | 54879 | ST7L | suppression of tumorigenicity 7 like | 1 |
| 152 | hsa-mir-22 | 54879 | ST7L | suppression of tumorigenicity 7 like | 1 |
| 153 | hsa-mir-10a | 54879 | ST7L | suppression of tumorigenicity 7 like | 1 |
| 154 | hsa-mir-186 | 54879 | ST7L | suppression of tumorigenicity 7 like | 1 |
| 155 | hsa-mir-155 | 54879 | ST7L | suppression of tumorigenicity 7 like | 1 |
| 156 | hsa-mir-499 | 54879 | ST7L | suppression of tumorigenicity 7 like | 1 |
| 157 | hsa-mir-877 | 54879 | ST7L | suppression of tumorigenicity 7 like | 1 |
| 158 | hsa-mir-3190 | 54879 | ST7L | suppression of tumorigenicity 7 like | 1 |
| 159 | hsa-miR-1 | 115111 | SLC26A7 | solute carrier family 26, member 7 | 1 |
| 160 | hsa-miR-133a | 115111 | SLC26A7 | solute carrier family 26, member 7 | 1 |
| 161 | hsa-miR-186 | 115111 | SLC26A7 | solute carrier family 26, member 7 | 1 |
| 162 | hsa-miR-195 | 115111 | SLC26A7 | solute carrier family 26, member 7 | 1 |
| 163 | hsa-miR-208a | 115111 | SLC26A7 | solute carrier family 26, member 7 | 1 |
| 164 | hsa-miR-326 | 115111 | SLC26A7 | solute carrier family 26, member 7 | 1 |
| 165 | hsa-miR-449b | 115111 | SLC26A7 | solute carrier family 26, member 7 | 1 |
| 166 | hsa-miR-497 | 115111 | SLC26A7 | solute carrier family 26, member 7 | 1 |
| 167 | hsa-miR-550 | 115111 | SLC26A7 | solute carrier family 26, member 7 | 1 |
| 168 | hsa-miR-575 | 115111 | SLC26A7 | solute carrier family 26, member 7 | 1 |
| 169 | hsa-miR-608 | 115111 | SLC26A7 | solute carrier family 26, member 7 | 1 |
| 170 | hsa-miR-620 | 115111 | SLC26A7 | solute carrier family 26, member 7 | 1 |
| 171 | hsa-let-7a-3 | 115111 | SLC26A7 | solute carrier family 26, member 7 | 1 |
| 172 | hsa-let-7b | 115111 | SLC26A7 | solute carrier family 26, member 7 | 1 |
| 173 | hsa-mir-22 | 115111 | SLC26A7 | solute carrier family 26, member 7 | 1 |
| 174 | hsa-mir-10a | 115111 | SLC26A7 | solute carrier family 26, member 7 | 1 |
| 175 | hsa-mir-186 | 115111 | SLC26A7 | solute carrier family 26, member 7 | 1 |
| 176 | hsa-mir-155 | 115111 | SLC26A7 | solute carrier family 26, member 7 | 1 |
| 177 | hsa-mir-499 | 115111 | SLC26A7 | solute carrier family 26, member 7 | 1 |
| 178 | hsa-mir-550-1 | 115111 | SLC26A7 | solute carrier family 26, member 7 | 1 |
| 179 | hsa-mir-877 | 115111 | SLC26A7 | solute carrier family 26, member 7 | 1 |
| 180 | hsa-mir-2116 | 115111 | SLC26A7 | solute carrier family 26, member 7 | 1 |
| 181 | hsa-mir-3190 | 115111 | SLC26A7 | solute carrier family 26, member 7 | 1 |
| 182 | hsa-miR-1 | 115273 | RAB42 | RAB42, member RAS oncogene family | 1 |
| 183 | hsa-miR-133a | 115273 | RAB42 | RAB42, member RAS oncogene family | 1 |
| 184 | hsa-miR-186 | 115273 | RAB42 | RAB42, member RAS oncogene family | 1 |
| 185 | hsa-miR-208a | 115273 | RAB42 | RAB42, member RAS oncogene family | 1 |
| 186 | hsa-miR-326 | 115273 | RAB42 | RAB42, member RAS oncogene family | 1 |
| 187 | hsa-miR-449b | 115273 | RAB42 | RAB42, member RAS oncogene family | 1 |
| 188 | hsa-miR-550 | 115273 | RAB42 | RAB42, member RAS oncogene family | 1 |
| 189 | hsa-miR-551a | 115273 | RAB42 | RAB42, member RAS oncogene family | 1 |
| 190 | hsa-miR-562 | 115273 | RAB42 | RAB42, member RAS oncogene family | 1 |
| 191 | hsa-miR-575 | 115273 | RAB42 | RAB42, member RAS oncogene family | 1 |
| 192 | hsa-miR-579 | 115273 | RAB42 | RAB42, member RAS oncogene family | 1 |
| 193 | hsa-miR-608 | 115273 | RAB42 | RAB42, member RAS oncogene family | 1 |
| 194 | hsa-miR-620 | 115273 | RAB42 | RAB42, member RAS oncogene family | 1 |
| 195 | hsa-miR-885-5p | 115273 | RAB42 | RAB42, member RAS oncogene family | 1 |
| 196 | hsa-mir-22 | 115273 | RAB42 | RAB42, member RAS oncogene family | 1 |
| 197 | hsa-mir-10a | 115273 | RAB42 | RAB42, member RAS oncogene family | 1 |
| 198 | hsa-mir-186 | 115273 | RAB42 | RAB42, member RAS oncogene family | 1 |
| 199 | hsa-mir-155 | 115273 | RAB42 | RAB42, member RAS oncogene family | 1 |
| 200 | hsa-mir-423 | 115273 | RAB42 | RAB42, member RAS oncogene family | 1 |
| 201 | hsa-mir-499 | 115273 | RAB42 | RAB42, member RAS oncogene family | 1 |
| 202 | hsa-mir-550-1 | 115273 | RAB42 | RAB42, member RAS oncogene family | 1 |
| 203 | hsa-mir-885 | 115273 | RAB42 | RAB42, member RAS oncogene family | 1 |
| 204 | hsa-mir-877 | 115273 | RAB42 | RAB42, member RAS oncogene family | 1 |
| 205 | hsa-mir-2116 | 115273 | RAB42 | RAB42, member RAS oncogene family | 1 |
| 206 | hsa-mir-3186 | 115273 | RAB42 | RAB42, member RAS oncogene family | 1 |
| 207 | hsa-mir-3190 | 115273 | RAB42 | RAB42, member RAS oncogene family | 1 |
| 208 | hsa-miR-1 | 221393 | GPR115 | G protein-coupled receptor 115 | 1 |
| 209 | hsa-miR-133a | 221393 | GPR115 | G protein-coupled receptor 115 | 1 |
| 210 | hsa-miR-186 | 221393 | GPR115 | G protein-coupled receptor 115 | 1 |
| 211 | hsa-miR-208a | 221393 | GPR115 | G protein-coupled receptor 115 | 1 |
| 212 | hsa-miR-326 | 221393 | GPR115 | G protein-coupled receptor 115 | 1 |
| 213 | hsa-miR-449b | 221393 | GPR115 | G protein-coupled receptor 115 | 1 |
| 214 | hsa-miR-550 | 221393 | GPR115 | G protein-coupled receptor 115 | 1 |
| 215 | hsa-miR-575 | 221393 | GPR115 | G protein-coupled receptor 115 | 1 |
| 216 | hsa-miR-579 | 221393 | GPR115 | G protein-coupled receptor 115 | 1 |
| 217 | hsa-miR-608 | 221393 | GPR115 | G protein-coupled receptor 115 | 1 |
| 218 | hsa-miR-620 | 221393 | GPR115 | G protein-coupled receptor 115 | 1 |
| 219 | hsa-miR-885-5p | 221393 | GPR115 | G protein-coupled receptor 115 | 1 |
| 220 | hsa-let-7a-3 | 221393 | GPR115 | G protein-coupled receptor 115 | 1 |
| 221 | hsa-let-7b | 221393 | GPR115 | G protein-coupled receptor 115 | 1 |
| 222 | hsa-mir-22 | 221393 | GPR115 | G protein-coupled receptor 115 | 1 |
| 223 | hsa-mir-10a | 221393 | GPR115 | G protein-coupled receptor 115 | 1 |
| 224 | hsa-mir-186 | 221393 | GPR115 | G protein-coupled receptor 115 | 1 |
| 225 | hsa-mir-155 | 221393 | GPR115 | G protein-coupled receptor 115 | 1 |
| 226 | hsa-mir-499 | 221393 | GPR115 | G protein-coupled receptor 115 | 1 |
| 227 | hsa-mir-885 | 221393 | GPR115 | G protein-coupled receptor 115 | 1 |
| 228 | hsa-mir-877 | 221393 | GPR115 | G protein-coupled receptor 115 | 1 |
| 229 | hsa-mir-2116 | 221393 | GPR115 | G protein-coupled receptor 115 | 1 |
| 230 | hsa-mir-3190 | 221393 | GPR115 | G protein-coupled receptor 115 | 1 |
| 231 | hsa-miR-1 | 136371 | ASB10 | ankyrin repeat and SOCS box-containing 10 | 1 |
| 232 | hsa-miR-133a | 136371 | ASB10 | ankyrin repeat and SOCS box-containing 10 | 1 |
| 233 | hsa-miR-186 | 136371 | ASB10 | ankyrin repeat and SOCS box-containing 10 | 1 |
| 234 | hsa-miR-326 | 136371 | ASB10 | ankyrin repeat and SOCS box-containing 10 | 1 |
| 235 | hsa-miR-449b | 136371 | ASB10 | ankyrin repeat and SOCS box-containing 10 | 1 |
| 236 | hsa-miR-551a | 136371 | ASB10 | ankyrin repeat and SOCS box-containing 10 | 1 |
| 237 | hsa-miR-575 | 136371 | ASB10 | ankyrin repeat and SOCS box-containing 10 | 1 |
| 238 | hsa-miR-579 | 136371 | ASB10 | ankyrin repeat and SOCS box-containing 10 | 1 |
| 239 | hsa-miR-608 | 136371 | ASB10 | ankyrin repeat and SOCS box-containing 10 | 1 |
| 240 | hsa-miR-620 | 136371 | ASB10 | ankyrin repeat and SOCS box-containing 10 | 1 |
| 241 | hsa-miR-637 | 136371 | ASB10 | ankyrin repeat and SOCS box-containing 10 | 1 |
| 242 | hsa-mir-22 | 136371 | ASB10 | ankyrin repeat and SOCS box-containing 10 | 1 |
| 243 | hsa-mir-10a | 136371 | ASB10 | ankyrin repeat and SOCS box-containing 10 | 1 |
| 244 | hsa-mir-186 | 136371 | ASB10 | ankyrin repeat and SOCS box-containing 10 | 1 |
| 245 | hsa-mir-155 | 136371 | ASB10 | ankyrin repeat and SOCS box-containing 10 | 1 |
| 246 | hsa-mir-423 | 136371 | ASB10 | ankyrin repeat and SOCS box-containing 10 | 1 |
| 247 | hsa-mir-499 | 136371 | ASB10 | ankyrin repeat and SOCS box-containing 10 | 1 |
| 248 | hsa-mir-550-1 | 136371 | ASB10 | ankyrin repeat and SOCS box-containing 10 | 1 |
| 249 | hsa-mir-877 | 136371 | ASB10 | ankyrin repeat and SOCS box-containing 10 | 1 |
| 250 | hsa-mir-2116 | 136371 | ASB10 | ankyrin repeat and SOCS box-containing 10 | 1 |
| 251 | hsa-mir-3190 | 136371 | ASB10 | ankyrin repeat and SOCS box-containing 10 | 1 |
| 252 | hsa-miR-1 | 92591 | ASB16 | ankyrin repeat and SOCS box-containing 16 | 1 |
| 253 | hsa-miR-133a | 92591 | ASB16 | ankyrin repeat and SOCS box-containing 16 | 1 |
| 254 | hsa-miR-186 | 92591 | ASB16 | ankyrin repeat and SOCS box-containing 16 | 1 |
| 255 | hsa-miR-449b | 92591 | ASB16 | ankyrin repeat and SOCS box-containing 16 | 1 |
| 256 | hsa-miR-562 | 92591 | ASB16 | ankyrin repeat and SOCS box-containing 16 | 1 |
| 257 | hsa-miR-579 | 92591 | ASB16 | ankyrin repeat and SOCS box-containing 16 | 1 |
| 258 | hsa-miR-608 | 92591 | ASB16 | ankyrin repeat and SOCS box-containing 16 | 1 |
| 259 | hsa-miR-637 | 92591 | ASB16 | ankyrin repeat and SOCS box-containing 16 | 1 |
| 260 | hsa-miR-885-5p | 92591 | ASB16 | ankyrin repeat and SOCS box-containing 16 | 1 |
| 261 | hsa-mir-22 | 92591 | ASB16 | ankyrin repeat and SOCS box-containing 16 | 1 |
| 262 | hsa-mir-10a | 92591 | ASB16 | ankyrin repeat and SOCS box-containing 16 | 1 |
| 263 | hsa-mir-186 | 92591 | ASB16 | ankyrin repeat and SOCS box-containing 16 | 1 |
| 264 | hsa-mir-155 | 92591 | ASB16 | ankyrin repeat and SOCS box-containing 16 | 1 |
| 265 | hsa-mir-423 | 92591 | ASB16 | ankyrin repeat and SOCS box-containing 16 | 1 |
| 266 | hsa-mir-499 | 92591 | ASB16 | ankyrin repeat and SOCS box-containing 16 | 1 |
| 267 | hsa-mir-885 | 92591 | ASB16 | ankyrin repeat and SOCS box-containing 16 | 1 |
| 268 | hsa-mir-877 | 92591 | ASB16 | ankyrin repeat and SOCS box-containing 16 | 1 |
| 269 | hsa-mir-3190 | 92591 | ASB16 | ankyrin repeat and SOCS box-containing 16 | 1 |
| 270 | hsa-miR-1 | 286046 | XKR6 | XK, Kell blood group complex subunit-related family, member 6 | 1 |
| 271 | hsa-miR-133a | 286046 | XKR6 | XK, Kell blood group complex subunit-related family, member 6 | 1 |
| 272 | hsa-miR-186 | 286046 | XKR6 | XK, Kell blood group complex subunit-related family, member 6 | 1 |
| 273 | hsa-miR-326 | 286046 | XKR6 | XK, Kell blood group complex subunit-related family, member 6 | 1 |
| 274 | hsa-miR-33a | 286046 | XKR6 | XK, Kell blood group complex subunit-related family, member 6 | 1 |
| 275 | hsa-miR-449b | 286046 | XKR6 | XK, Kell blood group complex subunit-related family, member 6 | 1 |
| 276 | hsa-miR-550 | 286046 | XKR6 | XK, Kell blood group complex subunit-related family, member 6 | 1 |
| 277 | hsa-miR-551a | 286046 | XKR6 | XK, Kell blood group complex subunit-related family, member 6 | 1 |
| 278 | hsa-miR-562 | 286046 | XKR6 | XK, Kell blood group complex subunit-related family, member 6 | 1 |
| 279 | hsa-miR-575 | 286046 | XKR6 | XK, Kell blood group complex subunit-related family, member 6 | 1 |
| 280 | hsa-miR-579 | 286046 | XKR6 | XK, Kell blood group complex subunit-related family, member 6 | 1 |
| 281 | hsa-miR-608 | 286046 | XKR6 | XK, Kell blood group complex subunit-related family, member 6 | 1 |
| 282 | hsa-miR-620 | 286046 | XKR6 | XK, Kell blood group complex subunit-related family, member 6 | 1 |
| 283 | hsa-mir-22 | 286046 | XKR6 | XK, Kell blood group complex subunit-related family, member 6 | 1 |
| 284 | hsa-mir-10a | 286046 | XKR6 | XK, Kell blood group complex subunit-related family, member 6 | 1 |
| 285 | hsa-mir-186 | 286046 | XKR6 | XK, Kell blood group complex subunit-related family, member 6 | 1 |
| 286 | hsa-mir-155 | 286046 | XKR6 | XK, Kell blood group complex subunit-related family, member 6 | 1 |
| 287 | hsa-mir-423 | 286046 | XKR6 | XK, Kell blood group complex subunit-related family, member 6 | 1 |
| 288 | hsa-mir-499 | 286046 | XKR6 | XK, Kell blood group complex subunit-related family, member 6 | 1 |
| 289 | hsa-mir-550-1 | 286046 | XKR6 | XK, Kell blood group complex subunit-related family, member 6 | 1 |
| 290 | hsa-mir-877 | 286046 | XKR6 | XK, Kell blood group complex subunit-related family, member 6 | 1 |
| 291 | hsa-mir-2116 | 286046 | XKR6 | XK, Kell blood group complex subunit-related family, member 6 | 1 |
| 292 | hsa-mir-3186 | 286046 | XKR6 | XK, Kell blood group complex subunit-related family, member 6 | 1 |
| 294 | hsa-miR-139-5p | 121268 | RHEBL1 | Ras homolog enriched in brain like 1 | 1 |
| 304 | hsa-miR-548b-5p | 121268 | RHEBL1 | Ras homolog enriched in brain like 1 | 1 |
| 319 | hsa-mir-548b | 121268 | RHEBL1 | Ras homolog enriched in brain like 1 | 1 |
| 326 | hsa-miR-195 | 147694 | ZNF548 | zinc finger protein 548 | 1 |
| 329 | hsa-miR-489 | 147694 | ZNF548 | zinc finger protein 548 | 1 |
| 330 | hsa-miR-497 | 147694 | ZNF548 | zinc finger protein 548 | 1 |
| 331 | hsa-miR-562 | 147694 | ZNF548 | zinc finger protein 548 | 1 |
| 333 | hsa-miR-579 | 147694 | ZNF548 | zinc finger protein 548 | 1 |
| 336 | hsa-miR-637 | 147694 | ZNF548 | zinc finger protein 548 | 1 |
| 337 | hsa-miR-885-5p | 147694 | ZNF548 | zinc finger protein 548 | 1 |
| 343 | hsa-mir-885 | 147694 | ZNF548 | zinc finger protein 548 | 1 |
| 345 | hsa-mir-3190 | 147694 | ZNF548 | zinc finger protein 548 | 1 |
| 346 | hsa-miR-1 | 254050 | LRRC43 | leucine rich repeat containing 43 | 1 |
| 347 | hsa-miR-133a | 254050 | LRRC43 | leucine rich repeat containing 43 | 1 |
| 348 | hsa-miR-186 | 254050 | LRRC43 | leucine rich repeat containing 43 | 1 |
| 349 | hsa-miR-326 | 254050 | LRRC43 | leucine rich repeat containing 43 | 1 |
| 350 | hsa-miR-449b | 254050 | LRRC43 | leucine rich repeat containing 43 | 1 |
| 351 | hsa-miR-550 | 254050 | LRRC43 | leucine rich repeat containing 43 | 1 |
| 352 | hsa-miR-551a | 254050 | LRRC43 | leucine rich repeat containing 43 | 1 |
| 353 | hsa-miR-575 | 254050 | LRRC43 | leucine rich repeat containing 43 | 1 |
| 354 | hsa-miR-579 | 254050 | LRRC43 | leucine rich repeat containing 43 | 1 |
| 355 | hsa-miR-608 | 254050 | LRRC43 | leucine rich repeat containing 43 | 1 |
| 356 | hsa-miR-620 | 254050 | LRRC43 | leucine rich repeat containing 43 | 1 |
| 357 | hsa-miR-885-5p | 254050 | LRRC43 | leucine rich repeat containing 43 | 1 |
| 358 | hsa-mir-22 | 254050 | LRRC43 | leucine rich repeat containing 43 | 1 |
| 359 | hsa-mir-10a | 254050 | LRRC43 | leucine rich repeat containing 43 | 1 |
| 360 | hsa-mir-186 | 254050 | LRRC43 | leucine rich repeat containing 43 | 1 |
| 361 | hsa-mir-155 | 254050 | LRRC43 | leucine rich repeat containing 43 | 1 |
| 362 | hsa-mir-423 | 254050 | LRRC43 | leucine rich repeat containing 43 | 1 |
| 363 | hsa-mir-499 | 254050 | LRRC43 | leucine rich repeat containing 43 | 1 |
| 364 | hsa-mir-550-1 | 254050 | LRRC43 | leucine rich repeat containing 43 | 1 |
| 365 | hsa-mir-885 | 254050 | LRRC43 | leucine rich repeat containing 43 | 1 |
| 366 | hsa-mir-877 | 254050 | LRRC43 | leucine rich repeat containing 43 | 1 |
| 367 | hsa-mir-2116 | 254050 | LRRC43 | leucine rich repeat containing 43 | 1 |
| 368 | hsa-mir-3186 | 254050 | LRRC43 | leucine rich repeat containing 43 | 1 |
| 369 | hsa-mir-3190 | 254050 | LRRC43 | leucine rich repeat containing 43 | 1 |
| 370 | hsa-miR-1 | 114041 | C21orf88 | chromosome 21 open reading frame 88 | 1 |
| 371 | hsa-miR-133a | 114041 | C21orf88 | chromosome 21 open reading frame 88 | 1 |
| 372 | hsa-miR-186 | 114041 | C21orf88 | chromosome 21 open reading frame 88 | 1 |
| 373 | hsa-miR-326 | 114041 | C21orf88 | chromosome 21 open reading frame 88 | 1 |
| 374 | hsa-miR-449b | 114041 | C21orf88 | chromosome 21 open reading frame 88 | 1 |
| 375 | hsa-miR-551a | 114041 | C21orf88 | chromosome 21 open reading frame 88 | 1 |
| 376 | hsa-miR-575 | 114041 | C21orf88 | chromosome 21 open reading frame 88 | 1 |
| 377 | hsa-miR-608 | 114041 | C21orf88 | chromosome 21 open reading frame 88 | 1 |
| 378 | hsa-miR-620 | 114041 | C21orf88 | chromosome 21 open reading frame 88 | 1 |
| 379 | hsa-mir-22 | 114041 | C21orf88 | chromosome 21 open reading frame 88 | 1 |
| 380 | hsa-mir-10a | 114041 | C21orf88 | chromosome 21 open reading frame 88 | 1 |
| 381 | hsa-mir-186 | 114041 | C21orf88 | chromosome 21 open reading frame 88 | 1 |
| 382 | hsa-mir-155 | 114041 | C21orf88 | chromosome 21 open reading frame 88 | 1 |
| 383 | hsa-mir-423 | 114041 | C21orf88 | chromosome 21 open reading frame 88 | 1 |
| 384 | hsa-mir-499 | 114041 | C21orf88 | chromosome 21 open reading frame 88 | 1 |
| 385 | hsa-mir-550-1 | 114041 | C21orf88 | chromosome 21 open reading frame 88 | 1 |
| 386 | hsa-mir-877 | 114041 | C21orf88 | chromosome 21 open reading frame 88 | 1 |
| 387 | hsa-mir-2116 | 114041 | C21orf88 | chromosome 21 open reading frame 88 | 1 |
| 388 | hsa-miR-1 | 10230 | NBR2 | neighbor of BRCA1 gene 2 (non-protein coding) | 1 |
| 389 | hsa-miR-133a | 10230 | NBR2 | neighbor of BRCA1 gene 2 (non-protein coding) | 1 |
| 390 | hsa-miR-186 | 10230 | NBR2 | neighbor of BRCA1 gene 2 (non-protein coding) | 1 |
| 391 | hsa-miR-208a | 10230 | NBR2 | neighbor of BRCA1 gene 2 (non-protein coding) | 1 |
| 392 | hsa-miR-326 | 10230 | NBR2 | neighbor of BRCA1 gene 2 (non-protein coding) | 1 |
| 393 | hsa-miR-449b | 10230 | NBR2 | neighbor of BRCA1 gene 2 (non-protein coding) | 1 |
| 394 | hsa-miR-550 | 10230 | NBR2 | neighbor of BRCA1 gene 2 (non-protein coding) | 1 |
| 395 | hsa-miR-575 | 10230 | NBR2 | neighbor of BRCA1 gene 2 (non-protein coding) | 1 |
| 396 | hsa-miR-579 | 10230 | NBR2 | neighbor of BRCA1 gene 2 (non-protein coding) | 1 |
| 397 | hsa-miR-608 | 10230 | NBR2 | neighbor of BRCA1 gene 2 (non-protein coding) | 1 |
| 398 | hsa-miR-620 | 10230 | NBR2 | neighbor of BRCA1 gene 2 (non-protein coding) | 1 |
| 399 | hsa-miR-885-5p | 10230 | NBR2 | neighbor of BRCA1 gene 2 (non-protein coding) | 1 |
| 400 | hsa-let-7a-3 | 10230 | NBR2 | neighbor of BRCA1 gene 2 (non-protein coding) | 1 |
| 401 | hsa-let-7b | 10230 | NBR2 | neighbor of BRCA1 gene 2 (non-protein coding) | 1 |
| 402 | hsa-mir-22 | 10230 | NBR2 | neighbor of BRCA1 gene 2 (non-protein coding) | 1 |
| 403 | hsa-mir-10a | 10230 | NBR2 | neighbor of BRCA1 gene 2 (non-protein coding) | 1 |
| 404 | hsa-mir-186 | 10230 | NBR2 | neighbor of BRCA1 gene 2 (non-protein coding) | 1 |
| 405 | hsa-mir-155 | 10230 | NBR2 | neighbor of BRCA1 gene 2 (non-protein coding) | 1 |
| 406 | hsa-mir-423 | 10230 | NBR2 | neighbor of BRCA1 gene 2 (non-protein coding) | 1 |
| 407 | hsa-mir-499 | 10230 | NBR2 | neighbor of BRCA1 gene 2 (non-protein coding) | 1 |
| 408 | hsa-mir-550-1 | 10230 | NBR2 | neighbor of BRCA1 gene 2 (non-protein coding) | 1 |
| 409 | hsa-mir-885 | 10230 | NBR2 | neighbor of BRCA1 gene 2 (non-protein coding) | 1 |
| 410 | hsa-mir-877 | 10230 | NBR2 | neighbor of BRCA1 gene 2 (non-protein coding) | 1 |
| 411 | hsa-mir-2116 | 10230 | NBR2 | neighbor of BRCA1 gene 2 (non-protein coding) | 1 |
| 412 | hsa-mir-3190 | 10230 | NBR2 | neighbor of BRCA1 gene 2 (non-protein coding) | 1 |
| 413 | hsa-miR-1 | 3788 | KCNS2 | potassium voltage-gated channel, delayed-rectifier, subfamily S, member 2 | 1 |
| 414 | hsa-miR-133a | 3788 | KCNS2 | potassium voltage-gated channel, delayed-rectifier, subfamily S, member 2 | 1 |
| 415 | hsa-miR-186 | 3788 | KCNS2 | potassium voltage-gated channel, delayed-rectifier, subfamily S, member 2 | 1 |
| 416 | hsa-miR-326 | 3788 | KCNS2 | potassium voltage-gated channel, delayed-rectifier, subfamily S, member 2 | 1 |
| 417 | hsa-miR-449b | 3788 | KCNS2 | potassium voltage-gated channel, delayed-rectifier, subfamily S, member 2 | 1 |
| 418 | hsa-miR-551a | 3788 | KCNS2 | potassium voltage-gated channel, delayed-rectifier, subfamily S, member 2 | 1 |
| 419 | hsa-miR-562 | 3788 | KCNS2 | potassium voltage-gated channel, delayed-rectifier, subfamily S, member 2 | 1 |
| 420 | hsa-miR-575 | 3788 | KCNS2 | potassium voltage-gated channel, delayed-rectifier, subfamily S, member 2 | 1 |
| 421 | hsa-miR-579 | 3788 | KCNS2 | potassium voltage-gated channel, delayed-rectifier, subfamily S, member 2 | 1 |
| 422 | hsa-miR-608 | 3788 | KCNS2 | potassium voltage-gated channel, delayed-rectifier, subfamily S, member 2 | 1 |
| 423 | hsa-miR-637 | 3788 | KCNS2 | potassium voltage-gated channel, delayed-rectifier, subfamily S, member 2 | 1 |
| 424 | hsa-mir-22 | 3788 | KCNS2 | potassium voltage-gated channel, delayed-rectifier, subfamily S, member 2 | 1 |
| 425 | hsa-mir-10a | 3788 | KCNS2 | potassium voltage-gated channel, delayed-rectifier, subfamily S, member 2 | 1 |
| 426 | hsa-mir-186 | 3788 | KCNS2 | potassium voltage-gated channel, delayed-rectifier, subfamily S, member 2 | 1 |
| 427 | hsa-mir-155 | 3788 | KCNS2 | potassium voltage-gated channel, delayed-rectifier, subfamily S, member 2 | 1 |
| 428 | hsa-mir-423 | 3788 | KCNS2 | potassium voltage-gated channel, delayed-rectifier, subfamily S, member 2 | 1 |
| 429 | hsa-mir-499 | 3788 | KCNS2 | potassium voltage-gated channel, delayed-rectifier, subfamily S, member 2 | 1 |
| 430 | hsa-mir-877 | 3788 | KCNS2 | potassium voltage-gated channel, delayed-rectifier, subfamily S, member 2 | 1 |
| 431 | hsa-mir-3190 | 3788 | KCNS2 | potassium voltage-gated channel, delayed-rectifier, subfamily S, member 2 | 1 |
| 432 | hsa-miR-10a | 25819 | CCRN4L | CCR4 carbon catabolite repression 4-like (S. cerevisiae) | 1 |
| 433 | hsa-miR-152 | 25819 | CCRN4L | CCR4 carbon catabolite repression 4-like (S. cerevisiae) | 1 |
| 434 | hsa-miR-26a | 25819 | CCRN4L | CCR4 carbon catabolite repression 4-like (S. cerevisiae) | 1 |
| 435 | hsa-miR-26b | 25819 | CCRN4L | CCR4 carbon catabolite repression 4-like (S. cerevisiae) | 1 |
| 436 | hsa-miR-31 | 25819 | CCRN4L | CCR4 carbon catabolite repression 4-like (S. cerevisiae) | 1 |
| 437 | hsa-miR-338-5p | 25819 | CCRN4L | CCR4 carbon catabolite repression 4-like (S. cerevisiae) | 1 |
| 438 | hsa-miR-340 | 25819 | CCRN4L | CCR4 carbon catabolite repression 4-like (S. cerevisiae) | 1 |
| 439 | hsa-miR-383 | 25819 | CCRN4L | CCR4 carbon catabolite repression 4-like (S. cerevisiae) | 1 |
| 440 | hsa-miR-423-5p | 25819 | CCRN4L | CCR4 carbon catabolite repression 4-like (S. cerevisiae) | 1 |
| 441 | hsa-miR-449a | 25819 | CCRN4L | CCR4 carbon catabolite repression 4-like (S. cerevisiae) | 1 |
| 442 | hsa-miR-490-5p | 25819 | CCRN4L | CCR4 carbon catabolite repression 4-like (S. cerevisiae) | 1 |
| 443 | hsa-miR-499-5p | 25819 | CCRN4L | CCR4 carbon catabolite repression 4-like (S. cerevisiae) | 1 |
| 444 | hsa-miR-548b-5p | 25819 | CCRN4L | CCR4 carbon catabolite repression 4-like (S. cerevisiae) | 1 |
| 445 | hsa-miR-621 | 25819 | CCRN4L | CCR4 carbon catabolite repression 4-like (S. cerevisiae) | 1 |
| 446 | hsa-miR-657 | 25819 | CCRN4L | CCR4 carbon catabolite repression 4-like (S. cerevisiae) | 1 |
| 447 | hsa-miR-663 | 25819 | CCRN4L | CCR4 carbon catabolite repression 4-like (S. cerevisiae) | 1 |
| 448 | hsa-miR-765 | 25819 | CCRN4L | CCR4 carbon catabolite repression 4-like (S. cerevisiae) | 1 |
| 449 | hsa-miR-770-5p | 25819 | CCRN4L | CCR4 carbon catabolite repression 4-like (S. cerevisiae) | 1 |
| 450 | hsa-miR-877 | 25819 | CCRN4L | CCR4 carbon catabolite repression 4-like (S. cerevisiae) | 1 |
| 451 | hsa-mir-26a-1 | 25819 | CCRN4L | CCR4 carbon catabolite repression 4-like (S. cerevisiae) | 1 |
| 452 | hsa-mir-26b | 25819 | CCRN4L | CCR4 carbon catabolite repression 4-like (S. cerevisiae) | 1 |
| 453 | hsa-mir-31 | 25819 | CCRN4L | CCR4 carbon catabolite repression 4-like (S. cerevisiae) | 1 |
| 454 | hsa-mir-194-2 | 25819 | CCRN4L | CCR4 carbon catabolite repression 4-like (S. cerevisiae) | 1 |
| 455 | hsa-mir-340 | 25819 | CCRN4L | CCR4 carbon catabolite repression 4-like (S. cerevisiae) | 1 |
| 456 | hsa-mir-338 | 25819 | CCRN4L | CCR4 carbon catabolite repression 4-like (S. cerevisiae) | 1 |
| 457 | hsa-mir-490 | 25819 | CCRN4L | CCR4 carbon catabolite repression 4-like (S. cerevisiae) | 1 |
| 458 | hsa-mir-548b | 25819 | CCRN4L | CCR4 carbon catabolite repression 4-like (S. cerevisiae) | 1 |
| 459 | hsa-mir-449b | 25819 | CCRN4L | CCR4 carbon catabolite repression 4-like (S. cerevisiae) | 1 |
| 460 | hsa-mir-449c | 25819 | CCRN4L | CCR4 carbon catabolite repression 4-like (S. cerevisiae) | 1 |
| 461 | hsa-mir-3065 | 25819 | CCRN4L | CCR4 carbon catabolite repression 4-like (S. cerevisiae) | 1 |
| 462 | hsa-miR-1 | 541473 | LOC541473 | FK506 binding protein 6, 36kDa pseudogene | 1 |
| 463 | hsa-miR-133a | 541473 | LOC541473 | FK506 binding protein 6, 36kDa pseudogene | 1 |
| 464 | hsa-miR-186 | 541473 | LOC541473 | FK506 binding protein 6, 36kDa pseudogene | 1 |
| 465 | hsa-miR-195 | 541473 | LOC541473 | FK506 binding protein 6, 36kDa pseudogene | 1 |
| 466 | hsa-miR-208a | 541473 | LOC541473 | FK506 binding protein 6, 36kDa pseudogene | 1 |
| 467 | hsa-miR-326 | 541473 | LOC541473 | FK506 binding protein 6, 36kDa pseudogene | 1 |
| 468 | hsa-miR-33a | 541473 | LOC541473 | FK506 binding protein 6, 36kDa pseudogene | 1 |
| 469 | hsa-miR-449b | 541473 | LOC541473 | FK506 binding protein 6, 36kDa pseudogene | 1 |
| 470 | hsa-miR-497 | 541473 | LOC541473 | FK506 binding protein 6, 36kDa pseudogene | 1 |
| 471 | hsa-miR-550 | 541473 | LOC541473 | FK506 binding protein 6, 36kDa pseudogene | 1 |
| 472 | hsa-miR-551a | 541473 | LOC541473 | FK506 binding protein 6, 36kDa pseudogene | 1 |
| 473 | hsa-miR-575 | 541473 | LOC541473 | FK506 binding protein 6, 36kDa pseudogene | 1 |
| 474 | hsa-miR-579 | 541473 | LOC541473 | FK506 binding protein 6, 36kDa pseudogene | 1 |
| 475 | hsa-miR-608 | 541473 | LOC541473 | FK506 binding protein 6, 36kDa pseudogene | 1 |
| 476 | hsa-miR-620 | 541473 | LOC541473 | FK506 binding protein 6, 36kDa pseudogene | 1 |
| 477 | hsa-miR-885-5p | 541473 | LOC541473 | FK506 binding protein 6, 36kDa pseudogene | 1 |
| 478 | hsa-let-7a-3 | 541473 | LOC541473 | FK506 binding protein 6, 36kDa pseudogene | 1 |
| 479 | hsa-let-7b | 541473 | LOC541473 | FK506 binding protein 6, 36kDa pseudogene | 1 |
| 480 | hsa-mir-22 | 541473 | LOC541473 | FK506 binding protein 6, 36kDa pseudogene | 1 |
| 481 | hsa-mir-10a | 541473 | LOC541473 | FK506 binding protein 6, 36kDa pseudogene | 1 |
| 482 | hsa-mir-186 | 541473 | LOC541473 | FK506 binding protein 6, 36kDa pseudogene | 1 |
| 483 | hsa-mir-155 | 541473 | LOC541473 | FK506 binding protein 6, 36kDa pseudogene | 1 |
| 484 | hsa-mir-499 | 541473 | LOC541473 | FK506 binding protein 6, 36kDa pseudogene | 1 |
| 485 | hsa-mir-550-1 | 541473 | LOC541473 | FK506 binding protein 6, 36kDa pseudogene | 1 |
| 486 | hsa-mir-885 | 541473 | LOC541473 | FK506 binding protein 6, 36kDa pseudogene | 1 |
| 487 | hsa-mir-877 | 541473 | LOC541473 | FK506 binding protein 6, 36kDa pseudogene | 1 |
| 488 | hsa-mir-2116 | 541473 | LOC541473 | FK506 binding protein 6, 36kDa pseudogene | 1 |
| 489 | hsa-mir-3186 | 541473 | LOC541473 | FK506 binding protein 6, 36kDa pseudogene | 1 |
| 493 | hsa-miR-195 | 10491 | CRTAP | cartilage associated protein | 1 |
| 496 | hsa-miR-489 | 10491 | CRTAP | cartilage associated protein | 1 |
| 497 | hsa-miR-497 | 10491 | CRTAP | cartilage associated protein | 1 |
| 510 | hsa-miR-26a | 941 | CD80 | CD80 molecule | 1 |
| 518 | hsa-miR-564 | 941 | CD80 | CD80 molecule | 1 |
| 531 | hsa-mir-548d-2 | 941 | CD80 | CD80 molecule | 1 |
| 535 | hsa-miR-1 | 54970 | TTC12 | tetratricopeptide repeat domain 12 | 1 |
| 536 | hsa-miR-133a | 54970 | TTC12 | tetratricopeptide repeat domain 12 | 1 |
| 537 | hsa-miR-186 | 54970 | TTC12 | tetratricopeptide repeat domain 12 | 1 |
| 538 | hsa-miR-326 | 54970 | TTC12 | tetratricopeptide repeat domain 12 | 1 |
| 539 | hsa-miR-449b | 54970 | TTC12 | tetratricopeptide repeat domain 12 | 1 |
| 540 | hsa-miR-551a | 54970 | TTC12 | tetratricopeptide repeat domain 12 | 1 |
| 541 | hsa-miR-575 | 54970 | TTC12 | tetratricopeptide repeat domain 12 | 1 |
| 542 | hsa-miR-579 | 54970 | TTC12 | tetratricopeptide repeat domain 12 | 1 |
| 543 | hsa-miR-608 | 54970 | TTC12 | tetratricopeptide repeat domain 12 | 1 |
| 544 | hsa-miR-620 | 54970 | TTC12 | tetratricopeptide repeat domain 12 | 1 |
| 545 | hsa-miR-637 | 54970 | TTC12 | tetratricopeptide repeat domain 12 | 1 |
| 546 | hsa-miR-885-5p | 54970 | TTC12 | tetratricopeptide repeat domain 12 | 1 |
| 547 | hsa-mir-22 | 54970 | TTC12 | tetratricopeptide repeat domain 12 | 1 |
| 548 | hsa-mir-10a | 54970 | TTC12 | tetratricopeptide repeat domain 12 | 1 |
| 549 | hsa-mir-186 | 54970 | TTC12 | tetratricopeptide repeat domain 12 | 1 |
| 550 | hsa-mir-155 | 54970 | TTC12 | tetratricopeptide repeat domain 12 | 1 |
| 551 | hsa-mir-423 | 54970 | TTC12 | tetratricopeptide repeat domain 12 | 1 |
| 552 | hsa-mir-499 | 54970 | TTC12 | tetratricopeptide repeat domain 12 | 1 |
| 553 | hsa-mir-885 | 54970 | TTC12 | tetratricopeptide repeat domain 12 | 1 |
| 554 | hsa-mir-877 | 54970 | TTC12 | tetratricopeptide repeat domain 12 | 1 |
| 555 | hsa-mir-2116 | 54970 | TTC12 | tetratricopeptide repeat domain 12 | 1 |
| 556 | hsa-mir-3190 | 54970 | TTC12 | tetratricopeptide repeat domain 12 | 1 |
| 557 | hsa-miR-10a | 8605 | PLA2G4C | phospholipase A2, group IVC (cytosolic, calcium-independent) | 1 |
| 558 | hsa-miR-139-5p | 8605 | PLA2G4C | phospholipase A2, group IVC (cytosolic, calcium-independent) | 1 |
| 559 | hsa-miR-26a | 8605 | PLA2G4C | phospholipase A2, group IVC (cytosolic, calcium-independent) | 1 |
| 560 | hsa-miR-26b | 8605 | PLA2G4C | phospholipase A2, group IVC (cytosolic, calcium-independent) | 1 |
| 561 | hsa-miR-340 | 8605 | PLA2G4C | phospholipase A2, group IVC (cytosolic, calcium-independent) | 1 |
| 562 | hsa-miR-449a | 8605 | PLA2G4C | phospholipase A2, group IVC (cytosolic, calcium-independent) | 1 |
| 563 | hsa-miR-499-5p | 8605 | PLA2G4C | phospholipase A2, group IVC (cytosolic, calcium-independent) | 1 |
| 564 | hsa-miR-548b-5p | 8605 | PLA2G4C | phospholipase A2, group IVC (cytosolic, calcium-independent) | 1 |
| 565 | hsa-miR-564 | 8605 | PLA2G4C | phospholipase A2, group IVC (cytosolic, calcium-independent) | 1 |
| 566 | hsa-miR-621 | 8605 | PLA2G4C | phospholipase A2, group IVC (cytosolic, calcium-independent) | 1 |
| 567 | hsa-miR-765 | 8605 | PLA2G4C | phospholipase A2, group IVC (cytosolic, calcium-independent) | 1 |
| 568 | hsa-miR-770-5p | 8605 | PLA2G4C | phospholipase A2, group IVC (cytosolic, calcium-independent) | 1 |
| 569 | hsa-miR-877 | 8605 | PLA2G4C | phospholipase A2, group IVC (cytosolic, calcium-independent) | 1 |
| 570 | hsa-mir-26a-1 | 8605 | PLA2G4C | phospholipase A2, group IVC (cytosolic, calcium-independent) | 1 |
| 571 | hsa-mir-26b | 8605 | PLA2G4C | phospholipase A2, group IVC (cytosolic, calcium-independent) | 1 |
| 572 | hsa-mir-340 | 8605 | PLA2G4C | phospholipase A2, group IVC (cytosolic, calcium-independent) | 1 |
| 573 | hsa-mir-548b | 8605 | PLA2G4C | phospholipase A2, group IVC (cytosolic, calcium-independent) | 1 |
| 574 | hsa-mir-548d-2 | 8605 | PLA2G4C | phospholipase A2, group IVC (cytosolic, calcium-independent) | 1 |
| 575 | hsa-mir-449b | 8605 | PLA2G4C | phospholipase A2, group IVC (cytosolic, calcium-independent) | 1 |
| 576 | hsa-mir-449c | 8605 | PLA2G4C | phospholipase A2, group IVC (cytosolic, calcium-independent) | 1 |
| 577 | hsa-miR-1 | 170591 | S100Z | S100 calcium binding protein Z | 1 |
| 578 | hsa-miR-133a | 170591 | S100Z | S100 calcium binding protein Z | 1 |
| 579 | hsa-miR-186 | 170591 | S100Z | S100 calcium binding protein Z | 1 |
| 580 | hsa-miR-326 | 170591 | S100Z | S100 calcium binding protein Z | 1 |
| 581 | hsa-miR-449b | 170591 | S100Z | S100 calcium binding protein Z | 1 |
| 582 | hsa-miR-575 | 170591 | S100Z | S100 calcium binding protein Z | 1 |
| 583 | hsa-miR-608 | 170591 | S100Z | S100 calcium binding protein Z | 1 |
| 584 | hsa-miR-620 | 170591 | S100Z | S100 calcium binding protein Z | 1 |
| 585 | hsa-miR-637 | 170591 | S100Z | S100 calcium binding protein Z | 1 |
| 586 | hsa-mir-22 | 170591 | S100Z | S100 calcium binding protein Z | 1 |
| 587 | hsa-mir-10a | 170591 | S100Z | S100 calcium binding protein Z | 1 |
| 588 | hsa-mir-186 | 170591 | S100Z | S100 calcium binding protein Z | 1 |
| 589 | hsa-mir-155 | 170591 | S100Z | S100 calcium binding protein Z | 1 |
| 590 | hsa-mir-499 | 170591 | S100Z | S100 calcium binding protein Z | 1 |
| 591 | hsa-mir-877 | 170591 | S100Z | S100 calcium binding protein Z | 1 |
| 592 | hsa-mir-2116 | 170591 | S100Z | S100 calcium binding protein Z | 1 |
| 593 | hsa-miR-10a | 5743 | PTGS2 | prostaglandin-endoperoxide synthase 2 (prostaglandin G/H synthase and cyclooxygenase) | 1 |
| 594 | hsa-miR-152 | 5743 | PTGS2 | prostaglandin-endoperoxide synthase 2 (prostaglandin G/H synthase and cyclooxygenase) | 1 |
| 595 | hsa-miR-26a | 5743 | PTGS2 | prostaglandin-endoperoxide synthase 2 (prostaglandin G/H synthase and cyclooxygenase) | 1 |
| 596 | hsa-miR-26b | 5743 | PTGS2 | prostaglandin-endoperoxide synthase 2 (prostaglandin G/H synthase and cyclooxygenase) | 1 |
| 597 | hsa-miR-31 | 5743 | PTGS2 | prostaglandin-endoperoxide synthase 2 (prostaglandin G/H synthase and cyclooxygenase) | 1 |
| 598 | hsa-miR-338-5p | 5743 | PTGS2 | prostaglandin-endoperoxide synthase 2 (prostaglandin G/H synthase and cyclooxygenase) | 1 |
| 599 | hsa-miR-340 | 5743 | PTGS2 | prostaglandin-endoperoxide synthase 2 (prostaglandin G/H synthase and cyclooxygenase) | 1 |
| 600 | hsa-miR-423-5p | 5743 | PTGS2 | prostaglandin-endoperoxide synthase 2 (prostaglandin G/H synthase and cyclooxygenase) | 1 |
| 601 | hsa-miR-449a | 5743 | PTGS2 | prostaglandin-endoperoxide synthase 2 (prostaglandin G/H synthase and cyclooxygenase) | 1 |
| 602 | hsa-miR-490-5p | 5743 | PTGS2 | prostaglandin-endoperoxide synthase 2 (prostaglandin G/H synthase and cyclooxygenase) | 1 |
| 603 | hsa-miR-499-5p | 5743 | PTGS2 | prostaglandin-endoperoxide synthase 2 (prostaglandin G/H synthase and cyclooxygenase) | 1 |
| 604 | hsa-miR-548b-5p | 5743 | PTGS2 | prostaglandin-endoperoxide synthase 2 (prostaglandin G/H synthase and cyclooxygenase) | 1 |
| 605 | hsa-miR-564 | 5743 | PTGS2 | prostaglandin-endoperoxide synthase 2 (prostaglandin G/H synthase and cyclooxygenase) | 1 |
| 606 | hsa-miR-584 | 5743 | PTGS2 | prostaglandin-endoperoxide synthase 2 (prostaglandin G/H synthase and cyclooxygenase) | 1 |
| 607 | hsa-miR-621 | 5743 | PTGS2 | prostaglandin-endoperoxide synthase 2 (prostaglandin G/H synthase and cyclooxygenase) | 1 |
| 608 | hsa-miR-644 | 5743 | PTGS2 | prostaglandin-endoperoxide synthase 2 (prostaglandin G/H synthase and cyclooxygenase) | 1 |
| 609 | hsa-miR-657 | 5743 | PTGS2 | prostaglandin-endoperoxide synthase 2 (prostaglandin G/H synthase and cyclooxygenase) | 1 |
| 610 | hsa-miR-765 | 5743 | PTGS2 | prostaglandin-endoperoxide synthase 2 (prostaglandin G/H synthase and cyclooxygenase) | 1 |
| 611 | hsa-miR-770-5p | 5743 | PTGS2 | prostaglandin-endoperoxide synthase 2 (prostaglandin G/H synthase and cyclooxygenase) | 1 |
| 612 | hsa-miR-877 | 5743 | PTGS2 | prostaglandin-endoperoxide synthase 2 (prostaglandin G/H synthase and cyclooxygenase) | 1 |
| 613 | hsa-miR-95 | 5743 | PTGS2 | prostaglandin-endoperoxide synthase 2 (prostaglandin G/H synthase and cyclooxygenase) | 1 |
| 614 | hsa-mir-26a-1 | 5743 | PTGS2 | prostaglandin-endoperoxide synthase 2 (prostaglandin G/H synthase and cyclooxygenase) | 1 |
| 615 | hsa-mir-26b | 5743 | PTGS2 | prostaglandin-endoperoxide synthase 2 (prostaglandin G/H synthase and cyclooxygenase) | 1 |
| 616 | hsa-mir-31 | 5743 | PTGS2 | prostaglandin-endoperoxide synthase 2 (prostaglandin G/H synthase and cyclooxygenase) | 1 |
| 617 | hsa-mir-194-2 | 5743 | PTGS2 | prostaglandin-endoperoxide synthase 2 (prostaglandin G/H synthase and cyclooxygenase) | 1 |
| 618 | hsa-mir-340 | 5743 | PTGS2 | prostaglandin-endoperoxide synthase 2 (prostaglandin G/H synthase and cyclooxygenase) | 1 |
| 619 | hsa-mir-338 | 5743 | PTGS2 | prostaglandin-endoperoxide synthase 2 (prostaglandin G/H synthase and cyclooxygenase) | 1 |
| 620 | hsa-mir-490 | 5743 | PTGS2 | prostaglandin-endoperoxide synthase 2 (prostaglandin G/H synthase and cyclooxygenase) | 1 |
| 621 | hsa-mir-548b | 5743 | PTGS2 | prostaglandin-endoperoxide synthase 2 (prostaglandin G/H synthase and cyclooxygenase) | 1 |
| 622 | hsa-mir-449b | 5743 | PTGS2 | prostaglandin-endoperoxide synthase 2 (prostaglandin G/H synthase and cyclooxygenase) | 1 |
| 623 | hsa-mir-449c | 5743 | PTGS2 | prostaglandin-endoperoxide synthase 2 (prostaglandin G/H synthase and cyclooxygenase) | 1 |
| 624 | hsa-mir-3065 | 5743 | PTGS2 | prostaglandin-endoperoxide synthase 2 (prostaglandin G/H synthase and cyclooxygenase) | 1 |
| 632 | hsa-miR-550 | 3417 | IDH1 | isocitrate dehydrogenase 1 (NADP+), soluble | 1 |
| 643 | hsa-mir-550-1 | 3417 | IDH1 | isocitrate dehydrogenase 1 (NADP+), soluble | 1 |
| 646 | hsa-miR-1 | 26238 | C6orf123 | chromosome 6 open reading frame 123 | 1 |
| 647 | hsa-miR-133a | 26238 | C6orf123 | chromosome 6 open reading frame 123 | 1 |
| 648 | hsa-miR-186 | 26238 | C6orf123 | chromosome 6 open reading frame 123 | 1 |
| 649 | hsa-miR-326 | 26238 | C6orf123 | chromosome 6 open reading frame 123 | 1 |
| 650 | hsa-miR-449b | 26238 | C6orf123 | chromosome 6 open reading frame 123 | 1 |
| 651 | hsa-miR-551a | 26238 | C6orf123 | chromosome 6 open reading frame 123 | 1 |
| 652 | hsa-miR-562 | 26238 | C6orf123 | chromosome 6 open reading frame 123 | 1 |
| 653 | hsa-miR-575 | 26238 | C6orf123 | chromosome 6 open reading frame 123 | 1 |
| 654 | hsa-miR-579 | 26238 | C6orf123 | chromosome 6 open reading frame 123 | 1 |
| 655 | hsa-miR-608 | 26238 | C6orf123 | chromosome 6 open reading frame 123 | 1 |
| 656 | hsa-miR-637 | 26238 | C6orf123 | chromosome 6 open reading frame 123 | 1 |
| 657 | hsa-mir-22 | 26238 | C6orf123 | chromosome 6 open reading frame 123 | 1 |
| 658 | hsa-mir-10a | 26238 | C6orf123 | chromosome 6 open reading frame 123 | 1 |
| 659 | hsa-mir-186 | 26238 | C6orf123 | chromosome 6 open reading frame 123 | 1 |
| 660 | hsa-mir-155 | 26238 | C6orf123 | chromosome 6 open reading frame 123 | 1 |
| 661 | hsa-mir-499 | 26238 | C6orf123 | chromosome 6 open reading frame 123 | 1 |
| 662 | hsa-mir-877 | 26238 | C6orf123 | chromosome 6 open reading frame 123 | 1 |
| 663 | hsa-mir-3190 | 26238 | C6orf123 | chromosome 6 open reading frame 123 | 1 |
| 664 | hsa-miR-1 | 80036 | TRPM3 | transient receptor potential cation channel, subfamily M, member 3 | 1 |
| 665 | hsa-miR-133a | 80036 | TRPM3 | transient receptor potential cation channel, subfamily M, member 3 | 1 |
| 666 | hsa-miR-186 | 80036 | TRPM3 | transient receptor potential cation channel, subfamily M, member 3 | 1 |
| 667 | hsa-miR-326 | 80036 | TRPM3 | transient receptor potential cation channel, subfamily M, member 3 | 1 |
| 668 | hsa-miR-449b | 80036 | TRPM3 | transient receptor potential cation channel, subfamily M, member 3 | 1 |
| 669 | hsa-miR-551a | 80036 | TRPM3 | transient receptor potential cation channel, subfamily M, member 3 | 1 |
| 670 | hsa-miR-562 | 80036 | TRPM3 | transient receptor potential cation channel, subfamily M, member 3 | 1 |
| 671 | hsa-miR-579 | 80036 | TRPM3 | transient receptor potential cation channel, subfamily M, member 3 | 1 |
| 672 | hsa-miR-608 | 80036 | TRPM3 | transient receptor potential cation channel, subfamily M, member 3 | 1 |
| 673 | hsa-miR-637 | 80036 | TRPM3 | transient receptor potential cation channel, subfamily M, member 3 | 1 |
| 674 | hsa-mir-22 | 80036 | TRPM3 | transient receptor potential cation channel, subfamily M, member 3 | 1 |
| 675 | hsa-mir-10a | 80036 | TRPM3 | transient receptor potential cation channel, subfamily M, member 3 | 1 |
| 676 | hsa-mir-186 | 80036 | TRPM3 | transient receptor potential cation channel, subfamily M, member 3 | 1 |
| 677 | hsa-mir-155 | 80036 | TRPM3 | transient receptor potential cation channel, subfamily M, member 3 | 1 |
| 678 | hsa-mir-877 | 80036 | TRPM3 | transient receptor potential cation channel, subfamily M, member 3 | 1 |
| 679 | hsa-miR-1 | 89872 | AQP10 | aquaporin 10 | 1 |
| 680 | hsa-miR-133a | 89872 | AQP10 | aquaporin 10 | 1 |
| 681 | hsa-miR-186 | 89872 | AQP10 | aquaporin 10 | 1 |
| 682 | hsa-miR-326 | 89872 | AQP10 | aquaporin 10 | 1 |
| 683 | hsa-miR-449b | 89872 | AQP10 | aquaporin 10 | 1 |
| 684 | hsa-miR-489 | 89872 | AQP10 | aquaporin 10 | 1 |
| 685 | hsa-miR-551a | 89872 | AQP10 | aquaporin 10 | 1 |
| 686 | hsa-miR-562 | 89872 | AQP10 | aquaporin 10 | 1 |
| 687 | hsa-miR-579 | 89872 | AQP10 | aquaporin 10 | 1 |
| 688 | hsa-miR-608 | 89872 | AQP10 | aquaporin 10 | 1 |
| 689 | hsa-miR-637 | 89872 | AQP10 | aquaporin 10 | 1 |
| 690 | hsa-miR-885-5p | 89872 | AQP10 | aquaporin 10 | 1 |
| 691 | hsa-mir-22 | 89872 | AQP10 | aquaporin 10 | 1 |
| 692 | hsa-mir-10a | 89872 | AQP10 | aquaporin 10 | 1 |
| 693 | hsa-mir-186 | 89872 | AQP10 | aquaporin 10 | 1 |
| 694 | hsa-mir-155 | 89872 | AQP10 | aquaporin 10 | 1 |
| 695 | hsa-mir-499 | 89872 | AQP10 | aquaporin 10 | 1 |
| 696 | hsa-mir-885 | 89872 | AQP10 | aquaporin 10 | 1 |
| 697 | hsa-mir-877 | 89872 | AQP10 | aquaporin 10 | 1 |
| 698 | hsa-mir-3190 | 89872 | AQP10 | aquaporin 10 | 1 |
| 705 | hsa-miR-423-5p | 57018 | CCNL1 | cyclin L1 | 1 |
| 709 | hsa-miR-548b-5p | 57018 | CCNL1 | cyclin L1 | 1 |
| 715 | hsa-miR-95 | 57018 | CCNL1 | cyclin L1 | 1 |
| 722 | hsa-mir-548b | 57018 | CCNL1 | cyclin L1 | 1 |
| 723 | hsa-mir-548d-2 | 57018 | CCNL1 | cyclin L1 | 1 |
| 736 | hsa-miR-504 | 133396 | IL31RA | interleukin 31 receptor A | 1 |
| 737 | hsa-miR-584 | 133396 | IL31RA | interleukin 31 receptor A | 1 |
| 743 | hsa-miR-95 | 133396 | IL31RA | interleukin 31 receptor A | 1 |
| 752 | hsa-miR-1 | 79849 | PDZD3 | PDZ domain containing 3 | 1 |
| 753 | hsa-miR-133a | 79849 | PDZD3 | PDZ domain containing 3 | 1 |
| 754 | hsa-miR-186 | 79849 | PDZD3 | PDZ domain containing 3 | 1 |
| 755 | hsa-miR-208a | 79849 | PDZD3 | PDZ domain containing 3 | 1 |
| 756 | hsa-miR-326 | 79849 | PDZD3 | PDZ domain containing 3 | 1 |
| 757 | hsa-miR-449b | 79849 | PDZD3 | PDZ domain containing 3 | 1 |
| 758 | hsa-miR-575 | 79849 | PDZD3 | PDZ domain containing 3 | 1 |
| 759 | hsa-miR-579 | 79849 | PDZD3 | PDZ domain containing 3 | 1 |
| 760 | hsa-miR-608 | 79849 | PDZD3 | PDZ domain containing 3 | 1 |
| 761 | hsa-miR-620 | 79849 | PDZD3 | PDZ domain containing 3 | 1 |
| 762 | hsa-miR-885-5p | 79849 | PDZD3 | PDZ domain containing 3 | 1 |
| 763 | hsa-let-7a-3 | 79849 | PDZD3 | PDZ domain containing 3 | 1 |
| 764 | hsa-let-7b | 79849 | PDZD3 | PDZ domain containing 3 | 1 |
| 765 | hsa-mir-22 | 79849 | PDZD3 | PDZ domain containing 3 | 1 |
| 766 | hsa-mir-10a | 79849 | PDZD3 | PDZ domain containing 3 | 1 |
| 767 | hsa-mir-186 | 79849 | PDZD3 | PDZ domain containing 3 | 1 |
| 768 | hsa-mir-155 | 79849 | PDZD3 | PDZ domain containing 3 | 1 |
| 769 | hsa-mir-499 | 79849 | PDZD3 | PDZ domain containing 3 | 1 |
| 770 | hsa-mir-885 | 79849 | PDZD3 | PDZ domain containing 3 | 1 |
| 771 | hsa-mir-877 | 79849 | PDZD3 | PDZ domain containing 3 | 1 |
| 772 | hsa-mir-2116 | 79849 | PDZD3 | PDZ domain containing 3 | 1 |
| 773 | hsa-miR-10a | 6892 | TAPBP | TAP binding protein (tapasin) | 1 |
| 774 | hsa-miR-1228\* | 6892 | TAPBP | TAP binding protein (tapasin) | 1 |
| 775 | hsa-miR-139-5p | 6892 | TAPBP | TAP binding protein (tapasin) | 1 |
| 776 | hsa-miR-152 | 6892 | TAPBP | TAP binding protein (tapasin) | 1 |
| 777 | hsa-miR-26a | 6892 | TAPBP | TAP binding protein (tapasin) | 1 |
| 778 | hsa-miR-26b | 6892 | TAPBP | TAP binding protein (tapasin) | 1 |
| 779 | hsa-miR-31 | 6892 | TAPBP | TAP binding protein (tapasin) | 1 |
| 780 | hsa-miR-338-5p | 6892 | TAPBP | TAP binding protein (tapasin) | 1 |
| 781 | hsa-miR-340 | 6892 | TAPBP | TAP binding protein (tapasin) | 1 |
| 782 | hsa-miR-423-5p | 6892 | TAPBP | TAP binding protein (tapasin) | 1 |
| 783 | hsa-miR-449a | 6892 | TAPBP | TAP binding protein (tapasin) | 1 |
| 784 | hsa-miR-490-5p | 6892 | TAPBP | TAP binding protein (tapasin) | 1 |
| 785 | hsa-miR-499-5p | 6892 | TAPBP | TAP binding protein (tapasin) | 1 |
| 786 | hsa-miR-548b-5p | 6892 | TAPBP | TAP binding protein (tapasin) | 1 |
| 787 | hsa-miR-564 | 6892 | TAPBP | TAP binding protein (tapasin) | 1 |
| 788 | hsa-miR-584 | 6892 | TAPBP | TAP binding protein (tapasin) | 1 |
| 789 | hsa-miR-621 | 6892 | TAPBP | TAP binding protein (tapasin) | 1 |
| 790 | hsa-miR-634 | 6892 | TAPBP | TAP binding protein (tapasin) | 1 |
| 791 | hsa-miR-644 | 6892 | TAPBP | TAP binding protein (tapasin) | 1 |
| 792 | hsa-miR-657 | 6892 | TAPBP | TAP binding protein (tapasin) | 1 |
| 793 | hsa-miR-765 | 6892 | TAPBP | TAP binding protein (tapasin) | 1 |
| 794 | hsa-miR-770-5p | 6892 | TAPBP | TAP binding protein (tapasin) | 1 |
| 795 | hsa-miR-877 | 6892 | TAPBP | TAP binding protein (tapasin) | 1 |
| 796 | hsa-miR-95 | 6892 | TAPBP | TAP binding protein (tapasin) | 1 |
| 797 | hsa-mir-26a-1 | 6892 | TAPBP | TAP binding protein (tapasin) | 1 |
| 798 | hsa-mir-26b | 6892 | TAPBP | TAP binding protein (tapasin) | 1 |
| 799 | hsa-mir-31 | 6892 | TAPBP | TAP binding protein (tapasin) | 1 |
| 800 | hsa-mir-194-2 | 6892 | TAPBP | TAP binding protein (tapasin) | 1 |
| 801 | hsa-mir-340 | 6892 | TAPBP | TAP binding protein (tapasin) | 1 |
| 802 | hsa-mir-338 | 6892 | TAPBP | TAP binding protein (tapasin) | 1 |
| 803 | hsa-mir-490 | 6892 | TAPBP | TAP binding protein (tapasin) | 1 |
| 804 | hsa-mir-548b | 6892 | TAPBP | TAP binding protein (tapasin) | 1 |
| 805 | hsa-mir-449b | 6892 | TAPBP | TAP binding protein (tapasin) | 1 |
| 806 | hsa-mir-449c | 6892 | TAPBP | TAP binding protein (tapasin) | 1 |
| 807 | hsa-mir-3065 | 6892 | TAPBP | TAP binding protein (tapasin) | 1 |
| 808 | hsa-miR-1 | 65981 | CAPRIN2 | caprin family member 2 | 1 |
| 809 | hsa-miR-133a | 65981 | CAPRIN2 | caprin family member 2 | 1 |
| 810 | hsa-miR-186 | 65981 | CAPRIN2 | caprin family member 2 | 1 |
| 811 | hsa-miR-195 | 65981 | CAPRIN2 | caprin family member 2 | 1 |
| 812 | hsa-miR-326 | 65981 | CAPRIN2 | caprin family member 2 | 1 |
| 813 | hsa-miR-449b | 65981 | CAPRIN2 | caprin family member 2 | 1 |
| 814 | hsa-miR-497 | 65981 | CAPRIN2 | caprin family member 2 | 1 |
| 815 | hsa-miR-550 | 65981 | CAPRIN2 | caprin family member 2 | 1 |
| 816 | hsa-miR-551a | 65981 | CAPRIN2 | caprin family member 2 | 1 |
| 817 | hsa-miR-562 | 65981 | CAPRIN2 | caprin family member 2 | 1 |
| 818 | hsa-miR-575 | 65981 | CAPRIN2 | caprin family member 2 | 1 |
| 819 | hsa-miR-579 | 65981 | CAPRIN2 | caprin family member 2 | 1 |
| 820 | hsa-miR-608 | 65981 | CAPRIN2 | caprin family member 2 | 1 |
| 821 | hsa-miR-620 | 65981 | CAPRIN2 | caprin family member 2 | 1 |
| 822 | hsa-miR-885-5p | 65981 | CAPRIN2 | caprin family member 2 | 1 |
| 823 | hsa-mir-22 | 65981 | CAPRIN2 | caprin family member 2 | 1 |
| 824 | hsa-mir-10a | 65981 | CAPRIN2 | caprin family member 2 | 1 |
| 825 | hsa-mir-186 | 65981 | CAPRIN2 | caprin family member 2 | 1 |
| 826 | hsa-mir-155 | 65981 | CAPRIN2 | caprin family member 2 | 1 |
| 827 | hsa-mir-423 | 65981 | CAPRIN2 | caprin family member 2 | 1 |
| 828 | hsa-mir-499 | 65981 | CAPRIN2 | caprin family member 2 | 1 |
| 829 | hsa-mir-550-1 | 65981 | CAPRIN2 | caprin family member 2 | 1 |
| 830 | hsa-mir-885 | 65981 | CAPRIN2 | caprin family member 2 | 1 |
| 831 | hsa-mir-2116 | 65981 | CAPRIN2 | caprin family member 2 | 1 |
| 832 | hsa-mir-3186 | 65981 | CAPRIN2 | caprin family member 2 | 1 |
| 833 | hsa-miR-10a | 5739 | PTGIR | prostaglandin I2 (prostacyclin) receptor (IP) | 1 |
| 834 | hsa-miR-139-5p | 5739 | PTGIR | prostaglandin I2 (prostacyclin) receptor (IP) | 1 |
| 835 | hsa-miR-152 | 5739 | PTGIR | prostaglandin I2 (prostacyclin) receptor (IP) | 1 |
| 836 | hsa-miR-26a | 5739 | PTGIR | prostaglandin I2 (prostacyclin) receptor (IP) | 1 |
| 837 | hsa-miR-26b | 5739 | PTGIR | prostaglandin I2 (prostacyclin) receptor (IP) | 1 |
| 838 | hsa-miR-340 | 5739 | PTGIR | prostaglandin I2 (prostacyclin) receptor (IP) | 1 |
| 839 | hsa-miR-449a | 5739 | PTGIR | prostaglandin I2 (prostacyclin) receptor (IP) | 1 |
| 840 | hsa-miR-490-5p | 5739 | PTGIR | prostaglandin I2 (prostacyclin) receptor (IP) | 1 |
| 841 | hsa-miR-499-5p | 5739 | PTGIR | prostaglandin I2 (prostacyclin) receptor (IP) | 1 |
| 842 | hsa-miR-548b-5p | 5739 | PTGIR | prostaglandin I2 (prostacyclin) receptor (IP) | 1 |
| 843 | hsa-miR-564 | 5739 | PTGIR | prostaglandin I2 (prostacyclin) receptor (IP) | 1 |
| 844 | hsa-miR-584 | 5739 | PTGIR | prostaglandin I2 (prostacyclin) receptor (IP) | 1 |
| 845 | hsa-miR-621 | 5739 | PTGIR | prostaglandin I2 (prostacyclin) receptor (IP) | 1 |
| 846 | hsa-miR-765 | 5739 | PTGIR | prostaglandin I2 (prostacyclin) receptor (IP) | 1 |
| 847 | hsa-miR-770-5p | 5739 | PTGIR | prostaglandin I2 (prostacyclin) receptor (IP) | 1 |
| 848 | hsa-miR-877 | 5739 | PTGIR | prostaglandin I2 (prostacyclin) receptor (IP) | 1 |
| 849 | hsa-mir-26a-1 | 5739 | PTGIR | prostaglandin I2 (prostacyclin) receptor (IP) | 1 |
| 850 | hsa-mir-26b | 5739 | PTGIR | prostaglandin I2 (prostacyclin) receptor (IP) | 1 |
| 851 | hsa-mir-194-2 | 5739 | PTGIR | prostaglandin I2 (prostacyclin) receptor (IP) | 1 |
| 852 | hsa-mir-340 | 5739 | PTGIR | prostaglandin I2 (prostacyclin) receptor (IP) | 1 |
| 853 | hsa-mir-490 | 5739 | PTGIR | prostaglandin I2 (prostacyclin) receptor (IP) | 1 |
| 854 | hsa-mir-548b | 5739 | PTGIR | prostaglandin I2 (prostacyclin) receptor (IP) | 1 |
| 855 | hsa-mir-449b | 5739 | PTGIR | prostaglandin I2 (prostacyclin) receptor (IP) | 1 |
| 856 | hsa-mir-449c | 5739 | PTGIR | prostaglandin I2 (prostacyclin) receptor (IP) | 1 |
| 859 | hsa-miR-634 | 6352 | CCL5 | chemokine (C-C motif) ligand 5 | 1 |
| 860 | hsa-miR-653 | 6352 | CCL5 | chemokine (C-C motif) ligand 5 | 1 |
| 868 | hsa-miR-340 | 54464 | XRN1 | 5'-3' exoribonuclease 1 | 1 |
| 871 | hsa-miR-490-5p | 54464 | XRN1 | 5'-3' exoribonuclease 1 | 1 |
| 876 | hsa-miR-644 | 54464 | XRN1 | 5'-3' exoribonuclease 1 | 1 |
| 881 | hsa-miR-95 | 54464 | XRN1 | 5'-3' exoribonuclease 1 | 1 |
| 886 | hsa-mir-340 | 54464 | XRN1 | 5'-3' exoribonuclease 1 | 1 |
| 888 | hsa-mir-490 | 54464 | XRN1 | 5'-3' exoribonuclease 1 | 1 |
| 893 | hsa-miR-1 | 23241 | PACS2 | phosphofurin acidic cluster sorting protein 2 | 1 |
| 894 | hsa-miR-133a | 23241 | PACS2 | phosphofurin acidic cluster sorting protein 2 | 1 |
| 895 | hsa-miR-186 | 23241 | PACS2 | phosphofurin acidic cluster sorting protein 2 | 1 |
| 896 | hsa-miR-326 | 23241 | PACS2 | phosphofurin acidic cluster sorting protein 2 | 1 |
| 897 | hsa-miR-449b | 23241 | PACS2 | phosphofurin acidic cluster sorting protein 2 | 1 |
| 898 | hsa-miR-550 | 23241 | PACS2 | phosphofurin acidic cluster sorting protein 2 | 1 |
| 899 | hsa-miR-551a | 23241 | PACS2 | phosphofurin acidic cluster sorting protein 2 | 1 |
| 900 | hsa-miR-575 | 23241 | PACS2 | phosphofurin acidic cluster sorting protein 2 | 1 |
| 901 | hsa-miR-579 | 23241 | PACS2 | phosphofurin acidic cluster sorting protein 2 | 1 |
| 902 | hsa-miR-608 | 23241 | PACS2 | phosphofurin acidic cluster sorting protein 2 | 1 |
| 903 | hsa-miR-620 | 23241 | PACS2 | phosphofurin acidic cluster sorting protein 2 | 1 |
| 904 | hsa-mir-22 | 23241 | PACS2 | phosphofurin acidic cluster sorting protein 2 | 1 |
| 905 | hsa-mir-10a | 23241 | PACS2 | phosphofurin acidic cluster sorting protein 2 | 1 |
| 906 | hsa-mir-186 | 23241 | PACS2 | phosphofurin acidic cluster sorting protein 2 | 1 |
| 907 | hsa-mir-155 | 23241 | PACS2 | phosphofurin acidic cluster sorting protein 2 | 1 |
| 908 | hsa-mir-423 | 23241 | PACS2 | phosphofurin acidic cluster sorting protein 2 | 1 |
| 909 | hsa-mir-499 | 23241 | PACS2 | phosphofurin acidic cluster sorting protein 2 | 1 |
| 910 | hsa-mir-550-1 | 23241 | PACS2 | phosphofurin acidic cluster sorting protein 2 | 1 |
| 911 | hsa-mir-877 | 23241 | PACS2 | phosphofurin acidic cluster sorting protein 2 | 1 |
| 912 | hsa-mir-2116 | 23241 | PACS2 | phosphofurin acidic cluster sorting protein 2 | 1 |
| 913 | hsa-mir-3186 | 23241 | PACS2 | phosphofurin acidic cluster sorting protein 2 | 1 |
| 914 | hsa-mir-3190 | 23241 | PACS2 | phosphofurin acidic cluster sorting protein 2 | 1 |
| 915 | hsa-miR-1 | 55118 | CRTAC1 | cartilage acidic protein 1 | 1 |
| 916 | hsa-miR-133a | 55118 | CRTAC1 | cartilage acidic protein 1 | 1 |
| 917 | hsa-miR-186 | 55118 | CRTAC1 | cartilage acidic protein 1 | 1 |
| 918 | hsa-miR-326 | 55118 | CRTAC1 | cartilage acidic protein 1 | 1 |
| 919 | hsa-miR-449b | 55118 | CRTAC1 | cartilage acidic protein 1 | 1 |
| 920 | hsa-miR-550 | 55118 | CRTAC1 | cartilage acidic protein 1 | 1 |
| 921 | hsa-miR-551a | 55118 | CRTAC1 | cartilage acidic protein 1 | 1 |
| 922 | hsa-miR-575 | 55118 | CRTAC1 | cartilage acidic protein 1 | 1 |
| 923 | hsa-miR-579 | 55118 | CRTAC1 | cartilage acidic protein 1 | 1 |
| 924 | hsa-miR-608 | 55118 | CRTAC1 | cartilage acidic protein 1 | 1 |
| 925 | hsa-miR-620 | 55118 | CRTAC1 | cartilage acidic protein 1 | 1 |
| 926 | hsa-mir-22 | 55118 | CRTAC1 | cartilage acidic protein 1 | 1 |
| 927 | hsa-mir-10a | 55118 | CRTAC1 | cartilage acidic protein 1 | 1 |
| 928 | hsa-mir-186 | 55118 | CRTAC1 | cartilage acidic protein 1 | 1 |
| 929 | hsa-mir-155 | 55118 | CRTAC1 | cartilage acidic protein 1 | 1 |
| 930 | hsa-mir-423 | 55118 | CRTAC1 | cartilage acidic protein 1 | 1 |
| 931 | hsa-mir-499 | 55118 | CRTAC1 | cartilage acidic protein 1 | 1 |
| 932 | hsa-mir-550-1 | 55118 | CRTAC1 | cartilage acidic protein 1 | 1 |
| 933 | hsa-mir-877 | 55118 | CRTAC1 | cartilage acidic protein 1 | 1 |
| 934 | hsa-mir-2116 | 55118 | CRTAC1 | cartilage acidic protein 1 | 1 |
| 935 | hsa-mir-3190 | 55118 | CRTAC1 | cartilage acidic protein 1 | 1 |
| 954 | hsa-miR-10a | 200298 | C22orf37 | chromosome 22 open reading frame 37 | 1 |
| 955 | hsa-miR-152 | 200298 | C22orf37 | chromosome 22 open reading frame 37 | 1 |
| 956 | hsa-miR-26b | 200298 | C22orf37 | chromosome 22 open reading frame 37 | 1 |
| 957 | hsa-miR-31 | 200298 | C22orf37 | chromosome 22 open reading frame 37 | 1 |
| 958 | hsa-miR-338-5p | 200298 | C22orf37 | chromosome 22 open reading frame 37 | 1 |
| 959 | hsa-miR-340 | 200298 | C22orf37 | chromosome 22 open reading frame 37 | 1 |
| 960 | hsa-miR-383 | 200298 | C22orf37 | chromosome 22 open reading frame 37 | 1 |
| 961 | hsa-miR-449a | 200298 | C22orf37 | chromosome 22 open reading frame 37 | 1 |
| 962 | hsa-miR-499-5p | 200298 | C22orf37 | chromosome 22 open reading frame 37 | 1 |
| 963 | hsa-miR-621 | 200298 | C22orf37 | chromosome 22 open reading frame 37 | 1 |
| 964 | hsa-miR-653 | 200298 | C22orf37 | chromosome 22 open reading frame 37 | 1 |
| 965 | hsa-miR-657 | 200298 | C22orf37 | chromosome 22 open reading frame 37 | 1 |
| 966 | hsa-miR-765 | 200298 | C22orf37 | chromosome 22 open reading frame 37 | 1 |
| 967 | hsa-miR-770-5p | 200298 | C22orf37 | chromosome 22 open reading frame 37 | 1 |
| 968 | hsa-miR-877 | 200298 | C22orf37 | chromosome 22 open reading frame 37 | 1 |
| 969 | hsa-mir-26a-1 | 200298 | C22orf37 | chromosome 22 open reading frame 37 | 1 |
| 970 | hsa-mir-26b | 200298 | C22orf37 | chromosome 22 open reading frame 37 | 1 |
| 971 | hsa-mir-31 | 200298 | C22orf37 | chromosome 22 open reading frame 37 | 1 |
| 972 | hsa-mir-194-2 | 200298 | C22orf37 | chromosome 22 open reading frame 37 | 1 |
| 973 | hsa-mir-340 | 200298 | C22orf37 | chromosome 22 open reading frame 37 | 1 |
| 974 | hsa-mir-338 | 200298 | C22orf37 | chromosome 22 open reading frame 37 | 1 |
| 975 | hsa-mir-449b | 200298 | C22orf37 | chromosome 22 open reading frame 37 | 1 |
| 976 | hsa-mir-449c | 200298 | C22orf37 | chromosome 22 open reading frame 37 | 1 |
| 977 | hsa-mir-3065 | 200298 | C22orf37 | chromosome 22 open reading frame 37 | 1 |
| 978 | hsa-miR-1 | 3321 | IGSF3 | immunoglobulin superfamily, member 3 | 1 |
| 979 | hsa-miR-133a | 3321 | IGSF3 | immunoglobulin superfamily, member 3 | 1 |
| 980 | hsa-miR-186 | 3321 | IGSF3 | immunoglobulin superfamily, member 3 | 1 |
| 981 | hsa-miR-326 | 3321 | IGSF3 | immunoglobulin superfamily, member 3 | 1 |
| 982 | hsa-miR-449b | 3321 | IGSF3 | immunoglobulin superfamily, member 3 | 1 |
| 983 | hsa-miR-550 | 3321 | IGSF3 | immunoglobulin superfamily, member 3 | 1 |
| 984 | hsa-miR-551a | 3321 | IGSF3 | immunoglobulin superfamily, member 3 | 1 |
| 985 | hsa-miR-562 | 3321 | IGSF3 | immunoglobulin superfamily, member 3 | 1 |
| 986 | hsa-miR-575 | 3321 | IGSF3 | immunoglobulin superfamily, member 3 | 1 |
| 987 | hsa-miR-579 | 3321 | IGSF3 | immunoglobulin superfamily, member 3 | 1 |
| 988 | hsa-miR-608 | 3321 | IGSF3 | immunoglobulin superfamily, member 3 | 1 |
| 989 | hsa-miR-620 | 3321 | IGSF3 | immunoglobulin superfamily, member 3 | 1 |
| 990 | hsa-miR-637 | 3321 | IGSF3 | immunoglobulin superfamily, member 3 | 1 |
| 991 | hsa-mir-22 | 3321 | IGSF3 | immunoglobulin superfamily, member 3 | 1 |
| 992 | hsa-mir-10a | 3321 | IGSF3 | immunoglobulin superfamily, member 3 | 1 |
| 993 | hsa-mir-186 | 3321 | IGSF3 | immunoglobulin superfamily, member 3 | 1 |
| 994 | hsa-mir-155 | 3321 | IGSF3 | immunoglobulin superfamily, member 3 | 1 |
| 995 | hsa-mir-423 | 3321 | IGSF3 | immunoglobulin superfamily, member 3 | 1 |
| 996 | hsa-mir-499 | 3321 | IGSF3 | immunoglobulin superfamily, member 3 | 1 |
| 997 | hsa-mir-550-1 | 3321 | IGSF3 | immunoglobulin superfamily, member 3 | 1 |
| 998 | hsa-mir-877 | 3321 | IGSF3 | immunoglobulin superfamily, member 3 | 1 |
| 999 | hsa-miR-1 | 400831 | C20orf202 | chromosome 20 open reading frame 202 | 1 |
| 1000 | hsa-miR-133a | 400831 | C20orf202 | chromosome 20 open reading frame 202 | 1 |
| 1001 | hsa-miR-186 | 400831 | C20orf202 | chromosome 20 open reading frame 202 | 1 |
| 1002 | hsa-miR-208a | 400831 | C20orf202 | chromosome 20 open reading frame 202 | 1 |
| 1003 | hsa-miR-326 | 400831 | C20orf202 | chromosome 20 open reading frame 202 | 1 |
| 1004 | hsa-miR-449b | 400831 | C20orf202 | chromosome 20 open reading frame 202 | 1 |
| 1005 | hsa-miR-550 | 400831 | C20orf202 | chromosome 20 open reading frame 202 | 1 |
| 1006 | hsa-miR-551a | 400831 | C20orf202 | chromosome 20 open reading frame 202 | 1 |
| 1007 | hsa-miR-562 | 400831 | C20orf202 | chromosome 20 open reading frame 202 | 1 |
| 1008 | hsa-miR-575 | 400831 | C20orf202 | chromosome 20 open reading frame 202 | 1 |
| 1009 | hsa-miR-579 | 400831 | C20orf202 | chromosome 20 open reading frame 202 | 1 |
| 1010 | hsa-miR-608 | 400831 | C20orf202 | chromosome 20 open reading frame 202 | 1 |
| 1011 | hsa-miR-620 | 400831 | C20orf202 | chromosome 20 open reading frame 202 | 1 |
| 1012 | hsa-miR-885-5p | 400831 | C20orf202 | chromosome 20 open reading frame 202 | 1 |
| 1013 | hsa-mir-22 | 400831 | C20orf202 | chromosome 20 open reading frame 202 | 1 |
| 1014 | hsa-mir-10a | 400831 | C20orf202 | chromosome 20 open reading frame 202 | 1 |
| 1015 | hsa-mir-186 | 400831 | C20orf202 | chromosome 20 open reading frame 202 | 1 |
| 1016 | hsa-mir-155 | 400831 | C20orf202 | chromosome 20 open reading frame 202 | 1 |
| 1017 | hsa-mir-423 | 400831 | C20orf202 | chromosome 20 open reading frame 202 | 1 |
| 1018 | hsa-mir-499 | 400831 | C20orf202 | chromosome 20 open reading frame 202 | 1 |
| 1019 | hsa-mir-550-1 | 400831 | C20orf202 | chromosome 20 open reading frame 202 | 1 |
| 1020 | hsa-mir-885 | 400831 | C20orf202 | chromosome 20 open reading frame 202 | 1 |
| 1021 | hsa-mir-877 | 400831 | C20orf202 | chromosome 20 open reading frame 202 | 1 |
| 1022 | hsa-mir-2116 | 400831 | C20orf202 | chromosome 20 open reading frame 202 | 1 |
| 1023 | hsa-mir-3190 | 400831 | C20orf202 | chromosome 20 open reading frame 202 | 1 |
| 1024 | hsa-miR-1 | 126231 | ZNF573 | zinc finger protein 573 | 1 |
| 1025 | hsa-miR-133a | 126231 | ZNF573 | zinc finger protein 573 | 1 |
| 1026 | hsa-miR-186 | 126231 | ZNF573 | zinc finger protein 573 | 1 |
| 1027 | hsa-miR-326 | 126231 | ZNF573 | zinc finger protein 573 | 1 |
| 1028 | hsa-miR-33a | 126231 | ZNF573 | zinc finger protein 573 | 1 |
| 1029 | hsa-miR-449b | 126231 | ZNF573 | zinc finger protein 573 | 1 |
| 1030 | hsa-miR-550 | 126231 | ZNF573 | zinc finger protein 573 | 1 |
| 1031 | hsa-miR-551a | 126231 | ZNF573 | zinc finger protein 573 | 1 |
| 1032 | hsa-miR-575 | 126231 | ZNF573 | zinc finger protein 573 | 1 |
| 1033 | hsa-miR-579 | 126231 | ZNF573 | zinc finger protein 573 | 1 |
| 1034 | hsa-miR-608 | 126231 | ZNF573 | zinc finger protein 573 | 1 |
| 1035 | hsa-miR-620 | 126231 | ZNF573 | zinc finger protein 573 | 1 |
| 1036 | hsa-mir-22 | 126231 | ZNF573 | zinc finger protein 573 | 1 |
| 1037 | hsa-mir-10a | 126231 | ZNF573 | zinc finger protein 573 | 1 |
| 1038 | hsa-mir-186 | 126231 | ZNF573 | zinc finger protein 573 | 1 |
| 1039 | hsa-mir-155 | 126231 | ZNF573 | zinc finger protein 573 | 1 |
| 1040 | hsa-mir-423 | 126231 | ZNF573 | zinc finger protein 573 | 1 |
| 1041 | hsa-mir-499 | 126231 | ZNF573 | zinc finger protein 573 | 1 |
| 1042 | hsa-mir-550-1 | 126231 | ZNF573 | zinc finger protein 573 | 1 |
| 1043 | hsa-mir-877 | 126231 | ZNF573 | zinc finger protein 573 | 1 |
| 1044 | hsa-mir-2116 | 126231 | ZNF573 | zinc finger protein 573 | 1 |
| 1045 | hsa-mir-3186 | 126231 | ZNF573 | zinc finger protein 573 | 1 |
| 1046 | hsa-mir-3190 | 126231 | ZNF573 | zinc finger protein 573 | 1 |
| 1047 | hsa-miR-1 | 285313 | IGSF10 | immunoglobulin superfamily, member 10 | 1 |
| 1048 | hsa-miR-133a | 285313 | IGSF10 | immunoglobulin superfamily, member 10 | 1 |
| 1049 | hsa-miR-186 | 285313 | IGSF10 | immunoglobulin superfamily, member 10 | 1 |
| 1050 | hsa-miR-195 | 285313 | IGSF10 | immunoglobulin superfamily, member 10 | 1 |
| 1051 | hsa-miR-208a | 285313 | IGSF10 | immunoglobulin superfamily, member 10 | 1 |
| 1052 | hsa-miR-326 | 285313 | IGSF10 | immunoglobulin superfamily, member 10 | 1 |
| 1053 | hsa-miR-449b | 285313 | IGSF10 | immunoglobulin superfamily, member 10 | 1 |
| 1054 | hsa-miR-489 | 285313 | IGSF10 | immunoglobulin superfamily, member 10 | 1 |
| 1055 | hsa-miR-497 | 285313 | IGSF10 | immunoglobulin superfamily, member 10 | 1 |
| 1056 | hsa-miR-562 | 285313 | IGSF10 | immunoglobulin superfamily, member 10 | 1 |
| 1057 | hsa-miR-575 | 285313 | IGSF10 | immunoglobulin superfamily, member 10 | 1 |
| 1058 | hsa-miR-579 | 285313 | IGSF10 | immunoglobulin superfamily, member 10 | 1 |
| 1059 | hsa-miR-608 | 285313 | IGSF10 | immunoglobulin superfamily, member 10 | 1 |
| 1060 | hsa-miR-620 | 285313 | IGSF10 | immunoglobulin superfamily, member 10 | 1 |
| 1061 | hsa-miR-637 | 285313 | IGSF10 | immunoglobulin superfamily, member 10 | 1 |
| 1062 | hsa-miR-885-5p | 285313 | IGSF10 | immunoglobulin superfamily, member 10 | 1 |
| 1063 | hsa-mir-22 | 285313 | IGSF10 | immunoglobulin superfamily, member 10 | 1 |
| 1064 | hsa-mir-10a | 285313 | IGSF10 | immunoglobulin superfamily, member 10 | 1 |
| 1065 | hsa-mir-186 | 285313 | IGSF10 | immunoglobulin superfamily, member 10 | 1 |
| 1066 | hsa-mir-155 | 285313 | IGSF10 | immunoglobulin superfamily, member 10 | 1 |
| 1067 | hsa-mir-499 | 285313 | IGSF10 | immunoglobulin superfamily, member 10 | 1 |
| 1068 | hsa-mir-885 | 285313 | IGSF10 | immunoglobulin superfamily, member 10 | 1 |
| 1069 | hsa-mir-877 | 285313 | IGSF10 | immunoglobulin superfamily, member 10 | 1 |
| 1070 | hsa-mir-2116 | 285313 | IGSF10 | immunoglobulin superfamily, member 10 | 1 |
| 1071 | hsa-mir-3190 | 285313 | IGSF10 | immunoglobulin superfamily, member 10 | 1 |
| 1099 | hsa-miR-10a | 285758 | LOC285758 | hypothetical protein LOC285758 | 1 |
| 1100 | hsa-miR-139-5p | 285758 | LOC285758 | hypothetical protein LOC285758 | 1 |
| 1101 | hsa-miR-152 | 285758 | LOC285758 | hypothetical protein LOC285758 | 1 |
| 1102 | hsa-miR-26a | 285758 | LOC285758 | hypothetical protein LOC285758 | 1 |
| 1103 | hsa-miR-26b | 285758 | LOC285758 | hypothetical protein LOC285758 | 1 |
| 1104 | hsa-miR-31 | 285758 | LOC285758 | hypothetical protein LOC285758 | 1 |
| 1105 | hsa-miR-338-5p | 285758 | LOC285758 | hypothetical protein LOC285758 | 1 |
| 1106 | hsa-miR-340 | 285758 | LOC285758 | hypothetical protein LOC285758 | 1 |
| 1107 | hsa-miR-383 | 285758 | LOC285758 | hypothetical protein LOC285758 | 1 |
| 1108 | hsa-miR-449a | 285758 | LOC285758 | hypothetical protein LOC285758 | 1 |
| 1109 | hsa-miR-490-5p | 285758 | LOC285758 | hypothetical protein LOC285758 | 1 |
| 1110 | hsa-miR-499-5p | 285758 | LOC285758 | hypothetical protein LOC285758 | 1 |
| 1111 | hsa-miR-548b-5p | 285758 | LOC285758 | hypothetical protein LOC285758 | 1 |
| 1112 | hsa-miR-621 | 285758 | LOC285758 | hypothetical protein LOC285758 | 1 |
| 1113 | hsa-miR-657 | 285758 | LOC285758 | hypothetical protein LOC285758 | 1 |
| 1114 | hsa-miR-663 | 285758 | LOC285758 | hypothetical protein LOC285758 | 1 |
| 1115 | hsa-miR-765 | 285758 | LOC285758 | hypothetical protein LOC285758 | 1 |
| 1116 | hsa-miR-770-5p | 285758 | LOC285758 | hypothetical protein LOC285758 | 1 |
| 1117 | hsa-miR-877 | 285758 | LOC285758 | hypothetical protein LOC285758 | 1 |
| 1118 | hsa-mir-26a-1 | 285758 | LOC285758 | hypothetical protein LOC285758 | 1 |
| 1119 | hsa-mir-26b | 285758 | LOC285758 | hypothetical protein LOC285758 | 1 |
| 1120 | hsa-mir-31 | 285758 | LOC285758 | hypothetical protein LOC285758 | 1 |
| 1121 | hsa-mir-194-2 | 285758 | LOC285758 | hypothetical protein LOC285758 | 1 |
| 1122 | hsa-mir-340 | 285758 | LOC285758 | hypothetical protein LOC285758 | 1 |
| 1123 | hsa-mir-338 | 285758 | LOC285758 | hypothetical protein LOC285758 | 1 |
| 1124 | hsa-mir-490 | 285758 | LOC285758 | hypothetical protein LOC285758 | 1 |
| 1125 | hsa-mir-548b | 285758 | LOC285758 | hypothetical protein LOC285758 | 1 |
| 1126 | hsa-mir-548d-2 | 285758 | LOC285758 | hypothetical protein LOC285758 | 1 |
| 1127 | hsa-mir-449b | 285758 | LOC285758 | hypothetical protein LOC285758 | 1 |
| 1128 | hsa-mir-449c | 285758 | LOC285758 | hypothetical protein LOC285758 | 1 |
| 1129 | hsa-mir-3065 | 285758 | LOC285758 | hypothetical protein LOC285758 | 1 |
| 1130 | hsa-miR-10a | 2182 | ACSL4 | acyl-CoA synthetase long-chain family member 4 | 1 |
| 1131 | hsa-miR-139-5p | 2182 | ACSL4 | acyl-CoA synthetase long-chain family member 4 | 1 |
| 1132 | hsa-miR-152 | 2182 | ACSL4 | acyl-CoA synthetase long-chain family member 4 | 1 |
| 1133 | hsa-miR-26a | 2182 | ACSL4 | acyl-CoA synthetase long-chain family member 4 | 1 |
| 1134 | hsa-miR-26b | 2182 | ACSL4 | acyl-CoA synthetase long-chain family member 4 | 1 |
| 1135 | hsa-miR-31 | 2182 | ACSL4 | acyl-CoA synthetase long-chain family member 4 | 1 |
| 1136 | hsa-miR-338-5p | 2182 | ACSL4 | acyl-CoA synthetase long-chain family member 4 | 1 |
| 1137 | hsa-miR-340 | 2182 | ACSL4 | acyl-CoA synthetase long-chain family member 4 | 1 |
| 1138 | hsa-miR-383 | 2182 | ACSL4 | acyl-CoA synthetase long-chain family member 4 | 1 |
| 1139 | hsa-miR-449a | 2182 | ACSL4 | acyl-CoA synthetase long-chain family member 4 | 1 |
| 1140 | hsa-miR-490-5p | 2182 | ACSL4 | acyl-CoA synthetase long-chain family member 4 | 1 |
| 1141 | hsa-miR-499-5p | 2182 | ACSL4 | acyl-CoA synthetase long-chain family member 4 | 1 |
| 1142 | hsa-miR-584 | 2182 | ACSL4 | acyl-CoA synthetase long-chain family member 4 | 1 |
| 1143 | hsa-miR-621 | 2182 | ACSL4 | acyl-CoA synthetase long-chain family member 4 | 1 |
| 1144 | hsa-miR-653 | 2182 | ACSL4 | acyl-CoA synthetase long-chain family member 4 | 1 |
| 1145 | hsa-miR-657 | 2182 | ACSL4 | acyl-CoA synthetase long-chain family member 4 | 1 |
| 1146 | hsa-miR-663 | 2182 | ACSL4 | acyl-CoA synthetase long-chain family member 4 | 1 |
| 1147 | hsa-miR-765 | 2182 | ACSL4 | acyl-CoA synthetase long-chain family member 4 | 1 |
| 1148 | hsa-miR-770-5p | 2182 | ACSL4 | acyl-CoA synthetase long-chain family member 4 | 1 |
| 1149 | hsa-miR-877 | 2182 | ACSL4 | acyl-CoA synthetase long-chain family member 4 | 1 |
| 1150 | hsa-mir-26a-1 | 2182 | ACSL4 | acyl-CoA synthetase long-chain family member 4 | 1 |
| 1151 | hsa-mir-26b | 2182 | ACSL4 | acyl-CoA synthetase long-chain family member 4 | 1 |
| 1152 | hsa-mir-31 | 2182 | ACSL4 | acyl-CoA synthetase long-chain family member 4 | 1 |
| 1153 | hsa-mir-194-2 | 2182 | ACSL4 | acyl-CoA synthetase long-chain family member 4 | 1 |
| 1154 | hsa-mir-340 | 2182 | ACSL4 | acyl-CoA synthetase long-chain family member 4 | 1 |
| 1155 | hsa-mir-338 | 2182 | ACSL4 | acyl-CoA synthetase long-chain family member 4 | 1 |
| 1156 | hsa-mir-490 | 2182 | ACSL4 | acyl-CoA synthetase long-chain family member 4 | 1 |
| 1157 | hsa-mir-449b | 2182 | ACSL4 | acyl-CoA synthetase long-chain family member 4 | 1 |
| 1158 | hsa-mir-449c | 2182 | ACSL4 | acyl-CoA synthetase long-chain family member 4 | 1 |
| 1159 | hsa-mir-3065 | 2182 | ACSL4 | acyl-CoA synthetase long-chain family member 4 | 1 |
| 1160 | hsa-miR-1 | 65268 | WNK2 | WNK lysine deficient protein kinase 2 | 1 |
| 1161 | hsa-miR-133a | 65268 | WNK2 | WNK lysine deficient protein kinase 2 | 1 |
| 1162 | hsa-miR-186 | 65268 | WNK2 | WNK lysine deficient protein kinase 2 | 1 |
| 1163 | hsa-miR-326 | 65268 | WNK2 | WNK lysine deficient protein kinase 2 | 1 |
| 1164 | hsa-miR-449b | 65268 | WNK2 | WNK lysine deficient protein kinase 2 | 1 |
| 1165 | hsa-miR-550 | 65268 | WNK2 | WNK lysine deficient protein kinase 2 | 1 |
| 1166 | hsa-miR-551a | 65268 | WNK2 | WNK lysine deficient protein kinase 2 | 1 |
| 1167 | hsa-miR-575 | 65268 | WNK2 | WNK lysine deficient protein kinase 2 | 1 |
| 1168 | hsa-miR-579 | 65268 | WNK2 | WNK lysine deficient protein kinase 2 | 1 |
| 1169 | hsa-miR-608 | 65268 | WNK2 | WNK lysine deficient protein kinase 2 | 1 |
| 1170 | hsa-miR-620 | 65268 | WNK2 | WNK lysine deficient protein kinase 2 | 1 |
| 1171 | hsa-miR-637 | 65268 | WNK2 | WNK lysine deficient protein kinase 2 | 1 |
| 1172 | hsa-mir-22 | 65268 | WNK2 | WNK lysine deficient protein kinase 2 | 1 |
| 1173 | hsa-mir-10a | 65268 | WNK2 | WNK lysine deficient protein kinase 2 | 1 |
| 1174 | hsa-mir-186 | 65268 | WNK2 | WNK lysine deficient protein kinase 2 | 1 |
| 1175 | hsa-mir-155 | 65268 | WNK2 | WNK lysine deficient protein kinase 2 | 1 |
| 1176 | hsa-mir-423 | 65268 | WNK2 | WNK lysine deficient protein kinase 2 | 1 |
| 1177 | hsa-mir-499 | 65268 | WNK2 | WNK lysine deficient protein kinase 2 | 1 |
| 1178 | hsa-mir-550-1 | 65268 | WNK2 | WNK lysine deficient protein kinase 2 | 1 |
| 1179 | hsa-mir-877 | 65268 | WNK2 | WNK lysine deficient protein kinase 2 | 1 |
| 1180 | hsa-mir-2116 | 65268 | WNK2 | WNK lysine deficient protein kinase 2 | 1 |
| 1181 | hsa-mir-3190 | 65268 | WNK2 | WNK lysine deficient protein kinase 2 | 1 |
| 1182 | hsa-miR-1 | 6585 | SLIT1 | slit homolog 1 (Drosophila) | 1 |
| 1183 | hsa-miR-133a | 6585 | SLIT1 | slit homolog 1 (Drosophila) | 1 |
| 1184 | hsa-miR-186 | 6585 | SLIT1 | slit homolog 1 (Drosophila) | 1 |
| 1185 | hsa-miR-208a | 6585 | SLIT1 | slit homolog 1 (Drosophila) | 1 |
| 1186 | hsa-miR-326 | 6585 | SLIT1 | slit homolog 1 (Drosophila) | 1 |
| 1187 | hsa-miR-449b | 6585 | SLIT1 | slit homolog 1 (Drosophila) | 1 |
| 1188 | hsa-miR-575 | 6585 | SLIT1 | slit homolog 1 (Drosophila) | 1 |
| 1189 | hsa-miR-579 | 6585 | SLIT1 | slit homolog 1 (Drosophila) | 1 |
| 1190 | hsa-miR-608 | 6585 | SLIT1 | slit homolog 1 (Drosophila) | 1 |
| 1191 | hsa-miR-620 | 6585 | SLIT1 | slit homolog 1 (Drosophila) | 1 |
| 1192 | hsa-miR-637 | 6585 | SLIT1 | slit homolog 1 (Drosophila) | 1 |
| 1193 | hsa-mir-22 | 6585 | SLIT1 | slit homolog 1 (Drosophila) | 1 |
| 1194 | hsa-mir-10a | 6585 | SLIT1 | slit homolog 1 (Drosophila) | 1 |
| 1195 | hsa-mir-186 | 6585 | SLIT1 | slit homolog 1 (Drosophila) | 1 |
| 1196 | hsa-mir-155 | 6585 | SLIT1 | slit homolog 1 (Drosophila) | 1 |
| 1197 | hsa-mir-423 | 6585 | SLIT1 | slit homolog 1 (Drosophila) | 1 |
| 1198 | hsa-mir-499 | 6585 | SLIT1 | slit homolog 1 (Drosophila) | 1 |
| 1199 | hsa-mir-877 | 6585 | SLIT1 | slit homolog 1 (Drosophila) | 1 |
| 1200 | hsa-mir-2116 | 6585 | SLIT1 | slit homolog 1 (Drosophila) | 1 |
| 1201 | hsa-mir-3190 | 6585 | SLIT1 | slit homolog 1 (Drosophila) | 1 |
| 1202 | hsa-miR-1 | 100287676 | LOC100287676 | hypothetical protein LOC100287676 | 1 |
| 1203 | hsa-miR-133a | 100287676 | LOC100287676 | hypothetical protein LOC100287676 | 1 |
| 1204 | hsa-miR-186 | 100287676 | LOC100287676 | hypothetical protein LOC100287676 | 1 |
| 1205 | hsa-miR-326 | 100287676 | LOC100287676 | hypothetical protein LOC100287676 | 1 |
| 1206 | hsa-miR-449b | 100287676 | LOC100287676 | hypothetical protein LOC100287676 | 1 |
| 1207 | hsa-miR-551a | 100287676 | LOC100287676 | hypothetical protein LOC100287676 | 1 |
| 1208 | hsa-miR-562 | 100287676 | LOC100287676 | hypothetical protein LOC100287676 | 1 |
| 1209 | hsa-miR-579 | 100287676 | LOC100287676 | hypothetical protein LOC100287676 | 1 |
| 1210 | hsa-miR-608 | 100287676 | LOC100287676 | hypothetical protein LOC100287676 | 1 |
| 1211 | hsa-miR-637 | 100287676 | LOC100287676 | hypothetical protein LOC100287676 | 1 |
| 1212 | hsa-mir-22 | 100287676 | LOC100287676 | hypothetical protein LOC100287676 | 1 |
| 1213 | hsa-mir-10a | 100287676 | LOC100287676 | hypothetical protein LOC100287676 | 1 |
| 1214 | hsa-mir-186 | 100287676 | LOC100287676 | hypothetical protein LOC100287676 | 1 |
| 1215 | hsa-mir-155 | 100287676 | LOC100287676 | hypothetical protein LOC100287676 | 1 |
| 1216 | hsa-mir-423 | 100287676 | LOC100287676 | hypothetical protein LOC100287676 | 1 |
| 1217 | hsa-mir-877 | 100287676 | LOC100287676 | hypothetical protein LOC100287676 | 1 |
| 1218 | hsa-miR-1 | 5429 | POLH | polymerase (DNA directed), eta | 1 |
| 1219 | hsa-miR-133a | 5429 | POLH | polymerase (DNA directed), eta | 1 |
| 1220 | hsa-miR-186 | 5429 | POLH | polymerase (DNA directed), eta | 1 |
| 1221 | hsa-miR-326 | 5429 | POLH | polymerase (DNA directed), eta | 1 |
| 1222 | hsa-miR-33a | 5429 | POLH | polymerase (DNA directed), eta | 1 |
| 1223 | hsa-miR-449b | 5429 | POLH | polymerase (DNA directed), eta | 1 |
| 1224 | hsa-miR-550 | 5429 | POLH | polymerase (DNA directed), eta | 1 |
| 1225 | hsa-miR-575 | 5429 | POLH | polymerase (DNA directed), eta | 1 |
| 1226 | hsa-miR-579 | 5429 | POLH | polymerase (DNA directed), eta | 1 |
| 1227 | hsa-miR-608 | 5429 | POLH | polymerase (DNA directed), eta | 1 |
| 1228 | hsa-miR-620 | 5429 | POLH | polymerase (DNA directed), eta | 1 |
| 1229 | hsa-let-7a-3 | 5429 | POLH | polymerase (DNA directed), eta | 1 |
| 1230 | hsa-let-7b | 5429 | POLH | polymerase (DNA directed), eta | 1 |
| 1231 | hsa-mir-22 | 5429 | POLH | polymerase (DNA directed), eta | 1 |
| 1232 | hsa-mir-10a | 5429 | POLH | polymerase (DNA directed), eta | 1 |
| 1233 | hsa-mir-186 | 5429 | POLH | polymerase (DNA directed), eta | 1 |
| 1234 | hsa-mir-155 | 5429 | POLH | polymerase (DNA directed), eta | 1 |
| 1235 | hsa-mir-499 | 5429 | POLH | polymerase (DNA directed), eta | 1 |
| 1236 | hsa-mir-550-1 | 5429 | POLH | polymerase (DNA directed), eta | 1 |
| 1237 | hsa-mir-877 | 5429 | POLH | polymerase (DNA directed), eta | 1 |
| 1238 | hsa-mir-2116 | 5429 | POLH | polymerase (DNA directed), eta | 1 |
| 1239 | hsa-mir-3186 | 5429 | POLH | polymerase (DNA directed), eta | 1 |
| 1240 | hsa-mir-3190 | 5429 | POLH | polymerase (DNA directed), eta | 1 |
| 1265 | hsa-mir-548d-2 | 254102 | EHBP1L1 | EH domain binding protein 1-like 1 | 1 |
| 1269 | hsa-miR-1 | 280636 | C11orf31 | chromosome 11 open reading frame 31 | 1 |
| 1270 | hsa-miR-133a | 280636 | C11orf31 | chromosome 11 open reading frame 31 | 1 |
| 1271 | hsa-miR-186 | 280636 | C11orf31 | chromosome 11 open reading frame 31 | 1 |
| 1272 | hsa-miR-195 | 280636 | C11orf31 | chromosome 11 open reading frame 31 | 1 |
| 1273 | hsa-miR-326 | 280636 | C11orf31 | chromosome 11 open reading frame 31 | 1 |
| 1274 | hsa-miR-33a | 280636 | C11orf31 | chromosome 11 open reading frame 31 | 1 |
| 1275 | hsa-miR-449b | 280636 | C11orf31 | chromosome 11 open reading frame 31 | 1 |
| 1276 | hsa-miR-497 | 280636 | C11orf31 | chromosome 11 open reading frame 31 | 1 |
| 1277 | hsa-miR-550 | 280636 | C11orf31 | chromosome 11 open reading frame 31 | 1 |
| 1278 | hsa-miR-551a | 280636 | C11orf31 | chromosome 11 open reading frame 31 | 1 |
| 1279 | hsa-miR-575 | 280636 | C11orf31 | chromosome 11 open reading frame 31 | 1 |
| 1280 | hsa-miR-579 | 280636 | C11orf31 | chromosome 11 open reading frame 31 | 1 |
| 1281 | hsa-miR-608 | 280636 | C11orf31 | chromosome 11 open reading frame 31 | 1 |
| 1282 | hsa-miR-620 | 280636 | C11orf31 | chromosome 11 open reading frame 31 | 1 |
| 1283 | hsa-let-7a-3 | 280636 | C11orf31 | chromosome 11 open reading frame 31 | 1 |
| 1284 | hsa-let-7b | 280636 | C11orf31 | chromosome 11 open reading frame 31 | 1 |
| 1285 | hsa-mir-22 | 280636 | C11orf31 | chromosome 11 open reading frame 31 | 1 |
| 1286 | hsa-mir-10a | 280636 | C11orf31 | chromosome 11 open reading frame 31 | 1 |
| 1287 | hsa-mir-186 | 280636 | C11orf31 | chromosome 11 open reading frame 31 | 1 |
| 1288 | hsa-mir-155 | 280636 | C11orf31 | chromosome 11 open reading frame 31 | 1 |
| 1289 | hsa-mir-423 | 280636 | C11orf31 | chromosome 11 open reading frame 31 | 1 |
| 1290 | hsa-mir-499 | 280636 | C11orf31 | chromosome 11 open reading frame 31 | 1 |
| 1291 | hsa-mir-550-1 | 280636 | C11orf31 | chromosome 11 open reading frame 31 | 1 |
| 1292 | hsa-mir-877 | 280636 | C11orf31 | chromosome 11 open reading frame 31 | 1 |
| 1293 | hsa-mir-2116 | 280636 | C11orf31 | chromosome 11 open reading frame 31 | 1 |
| 1294 | hsa-mir-3186 | 280636 | C11orf31 | chromosome 11 open reading frame 31 | 1 |
| 1295 | hsa-miR-33a | 147694 | ZNF548 | zinc finger protein 548 | 1 |
| 1296 | hsa-miR-551a | 147694 | ZNF548 | zinc finger protein 548 | 1 |
| 1297 | hsa-mir-423 | 147694 | ZNF548 | zinc finger protein 548 | 1 |
| 1298 | hsa-mir-550-1 | 147694 | ZNF548 | zinc finger protein 548 | 1 |
| 1299 | hsa-mir-2116 | 147694 | ZNF548 | zinc finger protein 548 | 1 |
| 1300 | hsa-miR-1 | 158960 | LOC158960 | hypothetical protein BC009467 | 1 |
| 1301 | hsa-miR-133a | 158960 | LOC158960 | hypothetical protein BC009467 | 1 |
| 1302 | hsa-miR-186 | 158960 | LOC158960 | hypothetical protein BC009467 | 1 |
| 1303 | hsa-miR-326 | 158960 | LOC158960 | hypothetical protein BC009467 | 1 |
| 1304 | hsa-miR-449b | 158960 | LOC158960 | hypothetical protein BC009467 | 1 |
| 1305 | hsa-miR-562 | 158960 | LOC158960 | hypothetical protein BC009467 | 1 |
| 1306 | hsa-miR-575 | 158960 | LOC158960 | hypothetical protein BC009467 | 1 |
| 1307 | hsa-miR-579 | 158960 | LOC158960 | hypothetical protein BC009467 | 1 |
| 1308 | hsa-miR-608 | 158960 | LOC158960 | hypothetical protein BC009467 | 1 |
| 1309 | hsa-miR-620 | 158960 | LOC158960 | hypothetical protein BC009467 | 1 |
| 1310 | hsa-miR-637 | 158960 | LOC158960 | hypothetical protein BC009467 | 1 |
| 1311 | hsa-miR-885-5p | 158960 | LOC158960 | hypothetical protein BC009467 | 1 |
| 1312 | hsa-mir-22 | 158960 | LOC158960 | hypothetical protein BC009467 | 1 |
| 1313 | hsa-mir-10a | 158960 | LOC158960 | hypothetical protein BC009467 | 1 |
| 1314 | hsa-mir-186 | 158960 | LOC158960 | hypothetical protein BC009467 | 1 |
| 1315 | hsa-mir-155 | 158960 | LOC158960 | hypothetical protein BC009467 | 1 |
| 1316 | hsa-mir-423 | 158960 | LOC158960 | hypothetical protein BC009467 | 1 |
| 1317 | hsa-mir-499 | 158960 | LOC158960 | hypothetical protein BC009467 | 1 |
| 1318 | hsa-mir-885 | 158960 | LOC158960 | hypothetical protein BC009467 | 1 |
| 1319 | hsa-mir-877 | 158960 | LOC158960 | hypothetical protein BC009467 | 1 |
| 1320 | hsa-mir-2116 | 158960 | LOC158960 | hypothetical protein BC009467 | 1 |
| 1321 | hsa-mir-3190 | 158960 | LOC158960 | hypothetical protein BC009467 | 1 |
| 1322 | hsa-miR-1 | 284759 | SIRPB2 | signal-regulatory protein beta 2 | 1 |
| 1323 | hsa-miR-133a | 284759 | SIRPB2 | signal-regulatory protein beta 2 | 1 |
| 1324 | hsa-miR-186 | 284759 | SIRPB2 | signal-regulatory protein beta 2 | 1 |
| 1325 | hsa-miR-326 | 284759 | SIRPB2 | signal-regulatory protein beta 2 | 1 |
| 1326 | hsa-miR-449b | 284759 | SIRPB2 | signal-regulatory protein beta 2 | 1 |
| 1327 | hsa-miR-551a | 284759 | SIRPB2 | signal-regulatory protein beta 2 | 1 |
| 1328 | hsa-miR-562 | 284759 | SIRPB2 | signal-regulatory protein beta 2 | 1 |
| 1329 | hsa-miR-579 | 284759 | SIRPB2 | signal-regulatory protein beta 2 | 1 |
| 1330 | hsa-miR-608 | 284759 | SIRPB2 | signal-regulatory protein beta 2 | 1 |
| 1331 | hsa-miR-637 | 284759 | SIRPB2 | signal-regulatory protein beta 2 | 1 |
| 1332 | hsa-mir-22 | 284759 | SIRPB2 | signal-regulatory protein beta 2 | 1 |
| 1333 | hsa-mir-10a | 284759 | SIRPB2 | signal-regulatory protein beta 2 | 1 |
| 1334 | hsa-mir-186 | 284759 | SIRPB2 | signal-regulatory protein beta 2 | 1 |
| 1335 | hsa-mir-155 | 284759 | SIRPB2 | signal-regulatory protein beta 2 | 1 |
| 1336 | hsa-mir-877 | 284759 | SIRPB2 | signal-regulatory protein beta 2 | 1 |
| 1337 | hsa-miR-10a | 100133019 | LOC100133019 | similar to hCG1983765 | 1 |
| 1338 | hsa-miR-139-5p | 100133019 | LOC100133019 | similar to hCG1983765 | 1 |
| 1339 | hsa-miR-152 | 100133019 | LOC100133019 | similar to hCG1983765 | 1 |
| 1340 | hsa-miR-26a | 100133019 | LOC100133019 | similar to hCG1983765 | 1 |
| 1341 | hsa-miR-26b | 100133019 | LOC100133019 | similar to hCG1983765 | 1 |
| 1342 | hsa-miR-31 | 100133019 | LOC100133019 | similar to hCG1983765 | 1 |
| 1343 | hsa-miR-338-5p | 100133019 | LOC100133019 | similar to hCG1983765 | 1 |
| 1344 | hsa-miR-340 | 100133019 | LOC100133019 | similar to hCG1983765 | 1 |
| 1345 | hsa-miR-383 | 100133019 | LOC100133019 | similar to hCG1983765 | 1 |
| 1346 | hsa-miR-449a | 100133019 | LOC100133019 | similar to hCG1983765 | 1 |
| 1347 | hsa-miR-490-5p | 100133019 | LOC100133019 | similar to hCG1983765 | 1 |
| 1348 | hsa-miR-499-5p | 100133019 | LOC100133019 | similar to hCG1983765 | 1 |
| 1349 | hsa-miR-548b-5p | 100133019 | LOC100133019 | similar to hCG1983765 | 1 |
| 1350 | hsa-miR-621 | 100133019 | LOC100133019 | similar to hCG1983765 | 1 |
| 1351 | hsa-miR-657 | 100133019 | LOC100133019 | similar to hCG1983765 | 1 |
| 1352 | hsa-miR-663 | 100133019 | LOC100133019 | similar to hCG1983765 | 1 |
| 1353 | hsa-miR-765 | 100133019 | LOC100133019 | similar to hCG1983765 | 1 |
| 1354 | hsa-miR-770-5p | 100133019 | LOC100133019 | similar to hCG1983765 | 1 |
| 1355 | hsa-miR-877 | 100133019 | LOC100133019 | similar to hCG1983765 | 1 |
| 1356 | hsa-mir-26a-1 | 100133019 | LOC100133019 | similar to hCG1983765 | 1 |
| 1357 | hsa-mir-26b | 100133019 | LOC100133019 | similar to hCG1983765 | 1 |
| 1358 | hsa-mir-31 | 100133019 | LOC100133019 | similar to hCG1983765 | 1 |
| 1359 | hsa-mir-194-2 | 100133019 | LOC100133019 | similar to hCG1983765 | 1 |
| 1360 | hsa-mir-340 | 100133019 | LOC100133019 | similar to hCG1983765 | 1 |
| 1361 | hsa-mir-338 | 100133019 | LOC100133019 | similar to hCG1983765 | 1 |
| 1362 | hsa-mir-490 | 100133019 | LOC100133019 | similar to hCG1983765 | 1 |
| 1363 | hsa-mir-548b | 100133019 | LOC100133019 | similar to hCG1983765 | 1 |
| 1364 | hsa-mir-548d-2 | 100133019 | LOC100133019 | similar to hCG1983765 | 1 |
| 1365 | hsa-mir-449b | 100133019 | LOC100133019 | similar to hCG1983765 | 1 |
| 1366 | hsa-mir-449c | 100133019 | LOC100133019 | similar to hCG1983765 | 1 |
| 1367 | hsa-mir-3065 | 100133019 | LOC100133019 | similar to hCG1983765 | 1 |
| 1368 | hsa-miR-1 | 255027 | MPV17L | MPV17 mitochondrial membrane protein-like | 1 |
| 1369 | hsa-miR-133a | 255027 | MPV17L | MPV17 mitochondrial membrane protein-like | 1 |
| 1370 | hsa-miR-186 | 255027 | MPV17L | MPV17 mitochondrial membrane protein-like | 1 |
| 1371 | hsa-miR-326 | 255027 | MPV17L | MPV17 mitochondrial membrane protein-like | 1 |
| 1372 | hsa-miR-33a | 255027 | MPV17L | MPV17 mitochondrial membrane protein-like | 1 |
| 1373 | hsa-miR-449b | 255027 | MPV17L | MPV17 mitochondrial membrane protein-like | 1 |
| 1374 | hsa-miR-550 | 255027 | MPV17L | MPV17 mitochondrial membrane protein-like | 1 |
| 1375 | hsa-miR-551a | 255027 | MPV17L | MPV17 mitochondrial membrane protein-like | 1 |
| 1376 | hsa-miR-562 | 255027 | MPV17L | MPV17 mitochondrial membrane protein-like | 1 |
| 1377 | hsa-miR-579 | 255027 | MPV17L | MPV17 mitochondrial membrane protein-like | 1 |
| 1378 | hsa-miR-608 | 255027 | MPV17L | MPV17 mitochondrial membrane protein-like | 1 |
| 1379 | hsa-mir-22 | 255027 | MPV17L | MPV17 mitochondrial membrane protein-like | 1 |
| 1380 | hsa-mir-10a | 255027 | MPV17L | MPV17 mitochondrial membrane protein-like | 1 |
| 1381 | hsa-mir-186 | 255027 | MPV17L | MPV17 mitochondrial membrane protein-like | 1 |
| 1382 | hsa-mir-155 | 255027 | MPV17L | MPV17 mitochondrial membrane protein-like | 1 |
| 1383 | hsa-mir-423 | 255027 | MPV17L | MPV17 mitochondrial membrane protein-like | 1 |
| 1384 | hsa-mir-550-1 | 255027 | MPV17L | MPV17 mitochondrial membrane protein-like | 1 |
| 1385 | hsa-mir-877 | 255027 | MPV17L | MPV17 mitochondrial membrane protein-like | 1 |
| 1386 | hsa-mir-3186 | 255027 | MPV17L | MPV17 mitochondrial membrane protein-like | 1 |
| 1387 | hsa-miR-1 | 257358 | LOC257358 | hypothetical LOC257358 | 1 |
| 1388 | hsa-miR-133a | 257358 | LOC257358 | hypothetical LOC257358 | 1 |
| 1389 | hsa-miR-186 | 257358 | LOC257358 | hypothetical LOC257358 | 1 |
| 1390 | hsa-miR-449b | 257358 | LOC257358 | hypothetical LOC257358 | 1 |
| 1391 | hsa-miR-551a | 257358 | LOC257358 | hypothetical LOC257358 | 1 |
| 1392 | hsa-miR-562 | 257358 | LOC257358 | hypothetical LOC257358 | 1 |
| 1393 | hsa-miR-575 | 257358 | LOC257358 | hypothetical LOC257358 | 1 |
| 1394 | hsa-miR-579 | 257358 | LOC257358 | hypothetical LOC257358 | 1 |
| 1395 | hsa-miR-608 | 257358 | LOC257358 | hypothetical LOC257358 | 1 |
| 1396 | hsa-miR-620 | 257358 | LOC257358 | hypothetical LOC257358 | 1 |
| 1397 | hsa-miR-637 | 257358 | LOC257358 | hypothetical LOC257358 | 1 |
| 1398 | hsa-miR-885-5p | 257358 | LOC257358 | hypothetical LOC257358 | 1 |
| 1399 | hsa-mir-22 | 257358 | LOC257358 | hypothetical LOC257358 | 1 |
| 1400 | hsa-mir-10a | 257358 | LOC257358 | hypothetical LOC257358 | 1 |
| 1401 | hsa-mir-186 | 257358 | LOC257358 | hypothetical LOC257358 | 1 |
| 1402 | hsa-mir-155 | 257358 | LOC257358 | hypothetical LOC257358 | 1 |
| 1403 | hsa-mir-423 | 257358 | LOC257358 | hypothetical LOC257358 | 1 |
| 1404 | hsa-mir-499 | 257358 | LOC257358 | hypothetical LOC257358 | 1 |
| 1405 | hsa-mir-885 | 257358 | LOC257358 | hypothetical LOC257358 | 1 |
| 1406 | hsa-miR-1 | 286254 | LOC286254 | hypothetical protein LOC286254 | 1 |
| 1407 | hsa-miR-133a | 286254 | LOC286254 | hypothetical protein LOC286254 | 1 |
| 1408 | hsa-miR-186 | 286254 | LOC286254 | hypothetical protein LOC286254 | 1 |
| 1409 | hsa-miR-195 | 286254 | LOC286254 | hypothetical protein LOC286254 | 1 |
| 1410 | hsa-miR-208a | 286254 | LOC286254 | hypothetical protein LOC286254 | 1 |
| 1411 | hsa-miR-326 | 286254 | LOC286254 | hypothetical protein LOC286254 | 1 |
| 1412 | hsa-miR-449b | 286254 | LOC286254 | hypothetical protein LOC286254 | 1 |
| 1413 | hsa-miR-497 | 286254 | LOC286254 | hypothetical protein LOC286254 | 1 |
| 1414 | hsa-miR-575 | 286254 | LOC286254 | hypothetical protein LOC286254 | 1 |
| 1415 | hsa-miR-579 | 286254 | LOC286254 | hypothetical protein LOC286254 | 1 |
| 1416 | hsa-miR-608 | 286254 | LOC286254 | hypothetical protein LOC286254 | 1 |
| 1417 | hsa-miR-620 | 286254 | LOC286254 | hypothetical protein LOC286254 | 1 |
| 1418 | hsa-miR-885-5p | 286254 | LOC286254 | hypothetical protein LOC286254 | 1 |
| 1419 | hsa-let-7a-3 | 286254 | LOC286254 | hypothetical protein LOC286254 | 1 |
| 1420 | hsa-let-7b | 286254 | LOC286254 | hypothetical protein LOC286254 | 1 |
| 1421 | hsa-mir-22 | 286254 | LOC286254 | hypothetical protein LOC286254 | 1 |
| 1422 | hsa-mir-10a | 286254 | LOC286254 | hypothetical protein LOC286254 | 1 |
| 1423 | hsa-mir-186 | 286254 | LOC286254 | hypothetical protein LOC286254 | 1 |
| 1424 | hsa-mir-155 | 286254 | LOC286254 | hypothetical protein LOC286254 | 1 |
| 1425 | hsa-mir-499 | 286254 | LOC286254 | hypothetical protein LOC286254 | 1 |
| 1426 | hsa-mir-885 | 286254 | LOC286254 | hypothetical protein LOC286254 | 1 |
| 1427 | hsa-mir-877 | 286254 | LOC286254 | hypothetical protein LOC286254 | 1 |
| 1428 | hsa-mir-2116 | 286254 | LOC286254 | hypothetical protein LOC286254 | 1 |
| 1429 | hsa-mir-3190 | 286254 | LOC286254 | hypothetical protein LOC286254 | 1 |
| 1430 | hsa-miR-1 | 285093 | C2orf85 | chromosome 2 open reading frame 85 | 1 |
| 1431 | hsa-miR-133a | 285093 | C2orf85 | chromosome 2 open reading frame 85 | 1 |
| 1432 | hsa-miR-186 | 285093 | C2orf85 | chromosome 2 open reading frame 85 | 1 |
| 1433 | hsa-miR-195 | 285093 | C2orf85 | chromosome 2 open reading frame 85 | 1 |
| 1434 | hsa-miR-326 | 285093 | C2orf85 | chromosome 2 open reading frame 85 | 1 |
| 1435 | hsa-miR-33a | 285093 | C2orf85 | chromosome 2 open reading frame 85 | 1 |
| 1436 | hsa-miR-449b | 285093 | C2orf85 | chromosome 2 open reading frame 85 | 1 |
| 1437 | hsa-miR-497 | 285093 | C2orf85 | chromosome 2 open reading frame 85 | 1 |
| 1438 | hsa-miR-550 | 285093 | C2orf85 | chromosome 2 open reading frame 85 | 1 |
| 1439 | hsa-miR-551a | 285093 | C2orf85 | chromosome 2 open reading frame 85 | 1 |
| 1440 | hsa-miR-575 | 285093 | C2orf85 | chromosome 2 open reading frame 85 | 1 |
| 1441 | hsa-miR-579 | 285093 | C2orf85 | chromosome 2 open reading frame 85 | 1 |
| 1442 | hsa-miR-608 | 285093 | C2orf85 | chromosome 2 open reading frame 85 | 1 |
| 1443 | hsa-miR-620 | 285093 | C2orf85 | chromosome 2 open reading frame 85 | 1 |
| 1444 | hsa-let-7a-3 | 285093 | C2orf85 | chromosome 2 open reading frame 85 | 1 |
| 1445 | hsa-let-7b | 285093 | C2orf85 | chromosome 2 open reading frame 85 | 1 |
| 1446 | hsa-mir-22 | 285093 | C2orf85 | chromosome 2 open reading frame 85 | 1 |
| 1447 | hsa-mir-10a | 285093 | C2orf85 | chromosome 2 open reading frame 85 | 1 |
| 1448 | hsa-mir-186 | 285093 | C2orf85 | chromosome 2 open reading frame 85 | 1 |
| 1449 | hsa-mir-155 | 285093 | C2orf85 | chromosome 2 open reading frame 85 | 1 |
| 1450 | hsa-mir-499 | 285093 | C2orf85 | chromosome 2 open reading frame 85 | 1 |
| 1451 | hsa-mir-550-1 | 285093 | C2orf85 | chromosome 2 open reading frame 85 | 1 |
| 1452 | hsa-mir-877 | 285093 | C2orf85 | chromosome 2 open reading frame 85 | 1 |
| 1453 | hsa-mir-2116 | 285093 | C2orf85 | chromosome 2 open reading frame 85 | 1 |
| 1454 | hsa-mir-3186 | 285093 | C2orf85 | chromosome 2 open reading frame 85 | 1 |
| 1455 | hsa-mir-3190 | 285093 | C2orf85 | chromosome 2 open reading frame 85 | 1 |
| 1456 | hsa-miR-152 | 55048 | VPS37C | vacuolar protein sorting 37 homolog C (S. cerevisiae) | 1 |
| 1457 | hsa-miR-26a | 55048 | VPS37C | vacuolar protein sorting 37 homolog C (S. cerevisiae) | 1 |
| 1458 | hsa-miR-26b | 55048 | VPS37C | vacuolar protein sorting 37 homolog C (S. cerevisiae) | 1 |
| 1459 | hsa-miR-31 | 55048 | VPS37C | vacuolar protein sorting 37 homolog C (S. cerevisiae) | 1 |
| 1460 | hsa-miR-338-5p | 55048 | VPS37C | vacuolar protein sorting 37 homolog C (S. cerevisiae) | 1 |
| 1461 | hsa-miR-340 | 55048 | VPS37C | vacuolar protein sorting 37 homolog C (S. cerevisiae) | 1 |
| 1462 | hsa-miR-383 | 55048 | VPS37C | vacuolar protein sorting 37 homolog C (S. cerevisiae) | 1 |
| 1463 | hsa-miR-449a | 55048 | VPS37C | vacuolar protein sorting 37 homolog C (S. cerevisiae) | 1 |
| 1464 | hsa-miR-490-5p | 55048 | VPS37C | vacuolar protein sorting 37 homolog C (S. cerevisiae) | 1 |
| 1465 | hsa-miR-499-5p | 55048 | VPS37C | vacuolar protein sorting 37 homolog C (S. cerevisiae) | 1 |
| 1466 | hsa-miR-621 | 55048 | VPS37C | vacuolar protein sorting 37 homolog C (S. cerevisiae) | 1 |
| 1467 | hsa-miR-657 | 55048 | VPS37C | vacuolar protein sorting 37 homolog C (S. cerevisiae) | 1 |
| 1468 | hsa-miR-663 | 55048 | VPS37C | vacuolar protein sorting 37 homolog C (S. cerevisiae) | 1 |
| 1469 | hsa-miR-765 | 55048 | VPS37C | vacuolar protein sorting 37 homolog C (S. cerevisiae) | 1 |
| 1470 | hsa-miR-770-5p | 55048 | VPS37C | vacuolar protein sorting 37 homolog C (S. cerevisiae) | 1 |
| 1471 | hsa-miR-877 | 55048 | VPS37C | vacuolar protein sorting 37 homolog C (S. cerevisiae) | 1 |
| 1472 | hsa-mir-26a-1 | 55048 | VPS37C | vacuolar protein sorting 37 homolog C (S. cerevisiae) | 1 |
| 1473 | hsa-mir-26b | 55048 | VPS37C | vacuolar protein sorting 37 homolog C (S. cerevisiae) | 1 |
| 1474 | hsa-mir-31 | 55048 | VPS37C | vacuolar protein sorting 37 homolog C (S. cerevisiae) | 1 |
| 1475 | hsa-mir-194-2 | 55048 | VPS37C | vacuolar protein sorting 37 homolog C (S. cerevisiae) | 1 |
| 1476 | hsa-mir-340 | 55048 | VPS37C | vacuolar protein sorting 37 homolog C (S. cerevisiae) | 1 |
| 1477 | hsa-mir-338 | 55048 | VPS37C | vacuolar protein sorting 37 homolog C (S. cerevisiae) | 1 |
| 1478 | hsa-mir-490 | 55048 | VPS37C | vacuolar protein sorting 37 homolog C (S. cerevisiae) | 1 |
| 1479 | hsa-mir-449b | 55048 | VPS37C | vacuolar protein sorting 37 homolog C (S. cerevisiae) | 1 |
| 1480 | hsa-mir-449c | 55048 | VPS37C | vacuolar protein sorting 37 homolog C (S. cerevisiae) | 1 |
| 1481 | hsa-mir-3065 | 55048 | VPS37C | vacuolar protein sorting 37 homolog C (S. cerevisiae) | 1 |
| 1482 | hsa-miR-1 | 390790 | ARL5C | ADP-ribosylation factor-like 5C | 1 |
| 1483 | hsa-miR-133a | 390790 | ARL5C | ADP-ribosylation factor-like 5C | 1 |
| 1484 | hsa-miR-186 | 390790 | ARL5C | ADP-ribosylation factor-like 5C | 1 |
| 1485 | hsa-miR-208a | 390790 | ARL5C | ADP-ribosylation factor-like 5C | 1 |
| 1486 | hsa-miR-326 | 390790 | ARL5C | ADP-ribosylation factor-like 5C | 1 |
| 1487 | hsa-miR-449b | 390790 | ARL5C | ADP-ribosylation factor-like 5C | 1 |
| 1488 | hsa-miR-575 | 390790 | ARL5C | ADP-ribosylation factor-like 5C | 1 |
| 1489 | hsa-miR-579 | 390790 | ARL5C | ADP-ribosylation factor-like 5C | 1 |
| 1490 | hsa-miR-608 | 390790 | ARL5C | ADP-ribosylation factor-like 5C | 1 |
| 1491 | hsa-miR-620 | 390790 | ARL5C | ADP-ribosylation factor-like 5C | 1 |
| 1492 | hsa-let-7a-3 | 390790 | ARL5C | ADP-ribosylation factor-like 5C | 1 |
| 1493 | hsa-let-7b | 390790 | ARL5C | ADP-ribosylation factor-like 5C | 1 |
| 1494 | hsa-mir-22 | 390790 | ARL5C | ADP-ribosylation factor-like 5C | 1 |
| 1495 | hsa-mir-10a | 390790 | ARL5C | ADP-ribosylation factor-like 5C | 1 |
| 1496 | hsa-mir-186 | 390790 | ARL5C | ADP-ribosylation factor-like 5C | 1 |
| 1497 | hsa-mir-155 | 390790 | ARL5C | ADP-ribosylation factor-like 5C | 1 |
| 1498 | hsa-mir-499 | 390790 | ARL5C | ADP-ribosylation factor-like 5C | 1 |
| 1499 | hsa-mir-877 | 390790 | ARL5C | ADP-ribosylation factor-like 5C | 1 |
| 1500 | hsa-miR-1 | 339168 | TMEM95 | transmembrane protein 95 | 1 |
| 1501 | hsa-miR-133a | 339168 | TMEM95 | transmembrane protein 95 | 1 |
| 1502 | hsa-miR-186 | 339168 | TMEM95 | transmembrane protein 95 | 1 |
| 1503 | hsa-miR-449b | 339168 | TMEM95 | transmembrane protein 95 | 1 |
| 1504 | hsa-miR-550 | 339168 | TMEM95 | transmembrane protein 95 | 1 |
| 1505 | hsa-miR-562 | 339168 | TMEM95 | transmembrane protein 95 | 1 |
| 1506 | hsa-miR-579 | 339168 | TMEM95 | transmembrane protein 95 | 1 |
| 1507 | hsa-miR-608 | 339168 | TMEM95 | transmembrane protein 95 | 1 |
| 1508 | hsa-miR-620 | 339168 | TMEM95 | transmembrane protein 95 | 1 |
| 1509 | hsa-miR-885-5p | 339168 | TMEM95 | transmembrane protein 95 | 1 |
| 1510 | hsa-mir-22 | 339168 | TMEM95 | transmembrane protein 95 | 1 |
| 1511 | hsa-mir-10a | 339168 | TMEM95 | transmembrane protein 95 | 1 |
| 1512 | hsa-mir-186 | 339168 | TMEM95 | transmembrane protein 95 | 1 |
| 1513 | hsa-mir-155 | 339168 | TMEM95 | transmembrane protein 95 | 1 |
| 1514 | hsa-mir-423 | 339168 | TMEM95 | transmembrane protein 95 | 1 |
| 1515 | hsa-mir-550-1 | 339168 | TMEM95 | transmembrane protein 95 | 1 |
| 1516 | hsa-mir-885 | 339168 | TMEM95 | transmembrane protein 95 | 1 |
| 1517 | hsa-miR-1 | 285796 | LOC285796 | hypothetical LOC285796 | 1 |
| 1518 | hsa-miR-133a | 285796 | LOC285796 | hypothetical LOC285796 | 1 |
| 1519 | hsa-miR-186 | 285796 | LOC285796 | hypothetical LOC285796 | 1 |
| 1520 | hsa-miR-326 | 285796 | LOC285796 | hypothetical LOC285796 | 1 |
| 1521 | hsa-miR-449b | 285796 | LOC285796 | hypothetical LOC285796 | 1 |
| 1522 | hsa-miR-551a | 285796 | LOC285796 | hypothetical LOC285796 | 1 |
| 1523 | hsa-miR-562 | 285796 | LOC285796 | hypothetical LOC285796 | 1 |
| 1524 | hsa-miR-575 | 285796 | LOC285796 | hypothetical LOC285796 | 1 |
| 1525 | hsa-miR-579 | 285796 | LOC285796 | hypothetical LOC285796 | 1 |
| 1526 | hsa-miR-608 | 285796 | LOC285796 | hypothetical LOC285796 | 1 |
| 1527 | hsa-miR-620 | 285796 | LOC285796 | hypothetical LOC285796 | 1 |
| 1528 | hsa-miR-637 | 285796 | LOC285796 | hypothetical LOC285796 | 1 |
| 1529 | hsa-miR-885-5p | 285796 | LOC285796 | hypothetical LOC285796 | 1 |
| 1530 | hsa-mir-22 | 285796 | LOC285796 | hypothetical LOC285796 | 1 |
| 1531 | hsa-mir-10a | 285796 | LOC285796 | hypothetical LOC285796 | 1 |
| 1532 | hsa-mir-186 | 285796 | LOC285796 | hypothetical LOC285796 | 1 |
| 1533 | hsa-mir-155 | 285796 | LOC285796 | hypothetical LOC285796 | 1 |
| 1534 | hsa-mir-423 | 285796 | LOC285796 | hypothetical LOC285796 | 1 |
| 1535 | hsa-mir-499 | 285796 | LOC285796 | hypothetical LOC285796 | 1 |
| 1536 | hsa-mir-885 | 285796 | LOC285796 | hypothetical LOC285796 | 1 |
| 1537 | hsa-mir-877 | 285796 | LOC285796 | hypothetical LOC285796 | 1 |
| 1538 | hsa-mir-2116 | 285796 | LOC285796 | hypothetical LOC285796 | 1 |
| 1539 | hsa-mir-3190 | 285796 | LOC285796 | hypothetical LOC285796 | 1 |
| 1540 | hsa-miR-1 | 285627 | LOC285627 | hypothetical LOC285627 | 1 |
| 1541 | hsa-miR-133a | 285627 | LOC285627 | hypothetical LOC285627 | 1 |
| 1542 | hsa-miR-186 | 285627 | LOC285627 | hypothetical LOC285627 | 1 |
| 1543 | hsa-miR-326 | 285627 | LOC285627 | hypothetical LOC285627 | 1 |
| 1544 | hsa-miR-449b | 285627 | LOC285627 | hypothetical LOC285627 | 1 |
| 1545 | hsa-miR-550 | 285627 | LOC285627 | hypothetical LOC285627 | 1 |
| 1546 | hsa-miR-551a | 285627 | LOC285627 | hypothetical LOC285627 | 1 |
| 1547 | hsa-miR-575 | 285627 | LOC285627 | hypothetical LOC285627 | 1 |
| 1548 | hsa-miR-579 | 285627 | LOC285627 | hypothetical LOC285627 | 1 |
| 1549 | hsa-miR-608 | 285627 | LOC285627 | hypothetical LOC285627 | 1 |
| 1550 | hsa-miR-620 | 285627 | LOC285627 | hypothetical LOC285627 | 1 |
| 1551 | hsa-miR-885-5p | 285627 | LOC285627 | hypothetical LOC285627 | 1 |
| 1552 | hsa-mir-22 | 285627 | LOC285627 | hypothetical LOC285627 | 1 |
| 1553 | hsa-mir-10a | 285627 | LOC285627 | hypothetical LOC285627 | 1 |
| 1554 | hsa-mir-186 | 285627 | LOC285627 | hypothetical LOC285627 | 1 |
| 1555 | hsa-mir-155 | 285627 | LOC285627 | hypothetical LOC285627 | 1 |
| 1556 | hsa-mir-423 | 285627 | LOC285627 | hypothetical LOC285627 | 1 |
| 1557 | hsa-mir-499 | 285627 | LOC285627 | hypothetical LOC285627 | 1 |
| 1558 | hsa-mir-550-1 | 285627 | LOC285627 | hypothetical LOC285627 | 1 |
| 1559 | hsa-mir-885 | 285627 | LOC285627 | hypothetical LOC285627 | 1 |
| 1560 | hsa-mir-877 | 285627 | LOC285627 | hypothetical LOC285627 | 1 |
| 1561 | hsa-mir-2116 | 285627 | LOC285627 | hypothetical LOC285627 | 1 |
| 1562 | hsa-mir-3186 | 285627 | LOC285627 | hypothetical LOC285627 | 1 |
| 1563 | hsa-mir-3190 | 285627 | LOC285627 | hypothetical LOC285627 | 1 |
| 1564 | hsa-miR-1 | 340156 | MYLK4 | myosin light chain kinase family, member 4 | 1 |
| 1565 | hsa-miR-133a | 340156 | MYLK4 | myosin light chain kinase family, member 4 | 1 |
| 1566 | hsa-miR-186 | 340156 | MYLK4 | myosin light chain kinase family, member 4 | 1 |
| 1567 | hsa-miR-326 | 340156 | MYLK4 | myosin light chain kinase family, member 4 | 1 |
| 1568 | hsa-miR-449b | 340156 | MYLK4 | myosin light chain kinase family, member 4 | 1 |
| 1569 | hsa-miR-551a | 340156 | MYLK4 | myosin light chain kinase family, member 4 | 1 |
| 1570 | hsa-miR-575 | 340156 | MYLK4 | myosin light chain kinase family, member 4 | 1 |
| 1571 | hsa-miR-608 | 340156 | MYLK4 | myosin light chain kinase family, member 4 | 1 |
| 1572 | hsa-miR-620 | 340156 | MYLK4 | myosin light chain kinase family, member 4 | 1 |
| 1573 | hsa-miR-637 | 340156 | MYLK4 | myosin light chain kinase family, member 4 | 1 |
| 1574 | hsa-mir-22 | 340156 | MYLK4 | myosin light chain kinase family, member 4 | 1 |
| 1575 | hsa-mir-10a | 340156 | MYLK4 | myosin light chain kinase family, member 4 | 1 |
| 1576 | hsa-mir-186 | 340156 | MYLK4 | myosin light chain kinase family, member 4 | 1 |
| 1577 | hsa-mir-155 | 340156 | MYLK4 | myosin light chain kinase family, member 4 | 1 |
| 1578 | hsa-mir-423 | 340156 | MYLK4 | myosin light chain kinase family, member 4 | 1 |
| 1579 | hsa-mir-499 | 340156 | MYLK4 | myosin light chain kinase family, member 4 | 1 |
| 1580 | hsa-mir-550-1 | 340156 | MYLK4 | myosin light chain kinase family, member 4 | 1 |
| 1581 | hsa-mir-877 | 340156 | MYLK4 | myosin light chain kinase family, member 4 | 1 |
| 1582 | hsa-mir-2116 | 340156 | MYLK4 | myosin light chain kinase family, member 4 | 1 |
| 1583 | hsa-miR-1 | 400794 | LOC400794 | hypothetical LOC400794 | 1 |
| 1584 | hsa-miR-133a | 400794 | LOC400794 | hypothetical LOC400794 | 1 |
| 1585 | hsa-miR-186 | 400794 | LOC400794 | hypothetical LOC400794 | 1 |
| 1586 | hsa-miR-326 | 400794 | LOC400794 | hypothetical LOC400794 | 1 |
| 1587 | hsa-miR-449b | 400794 | LOC400794 | hypothetical LOC400794 | 1 |
| 1588 | hsa-miR-550 | 400794 | LOC400794 | hypothetical LOC400794 | 1 |
| 1589 | hsa-miR-551a | 400794 | LOC400794 | hypothetical LOC400794 | 1 |
| 1590 | hsa-miR-562 | 400794 | LOC400794 | hypothetical LOC400794 | 1 |
| 1591 | hsa-miR-579 | 400794 | LOC400794 | hypothetical LOC400794 | 1 |
| 1592 | hsa-miR-608 | 400794 | LOC400794 | hypothetical LOC400794 | 1 |
| 1593 | hsa-miR-885-5p | 400794 | LOC400794 | hypothetical LOC400794 | 1 |
| 1594 | hsa-mir-22 | 400794 | LOC400794 | hypothetical LOC400794 | 1 |
| 1595 | hsa-mir-10a | 400794 | LOC400794 | hypothetical LOC400794 | 1 |
| 1596 | hsa-mir-186 | 400794 | LOC400794 | hypothetical LOC400794 | 1 |
| 1597 | hsa-mir-155 | 400794 | LOC400794 | hypothetical LOC400794 | 1 |
| 1598 | hsa-mir-499 | 400794 | LOC400794 | hypothetical LOC400794 | 1 |
| 1599 | hsa-mir-550-1 | 400794 | LOC400794 | hypothetical LOC400794 | 1 |
| 1600 | hsa-mir-885 | 400794 | LOC400794 | hypothetical LOC400794 | 1 |
| 1601 | hsa-mir-877 | 400794 | LOC400794 | hypothetical LOC400794 | 1 |
| 1602 | hsa-mir-2116 | 400794 | LOC400794 | hypothetical LOC400794 | 1 |
| 1603 | hsa-miR-1 | 339894 | LOC339894 | hypothetical protein LOC339894 | 1 |
| 1604 | hsa-miR-133a | 339894 | LOC339894 | hypothetical protein LOC339894 | 1 |
| 1605 | hsa-miR-186 | 339894 | LOC339894 | hypothetical protein LOC339894 | 1 |
| 1606 | hsa-miR-326 | 339894 | LOC339894 | hypothetical protein LOC339894 | 1 |
| 1607 | hsa-miR-33a | 339894 | LOC339894 | hypothetical protein LOC339894 | 1 |
| 1608 | hsa-miR-449b | 339894 | LOC339894 | hypothetical protein LOC339894 | 1 |
| 1609 | hsa-miR-550 | 339894 | LOC339894 | hypothetical protein LOC339894 | 1 |
| 1610 | hsa-miR-551a | 339894 | LOC339894 | hypothetical protein LOC339894 | 1 |
| 1611 | hsa-miR-575 | 339894 | LOC339894 | hypothetical protein LOC339894 | 1 |
| 1612 | hsa-miR-579 | 339894 | LOC339894 | hypothetical protein LOC339894 | 1 |
| 1613 | hsa-miR-608 | 339894 | LOC339894 | hypothetical protein LOC339894 | 1 |
| 1614 | hsa-miR-620 | 339894 | LOC339894 | hypothetical protein LOC339894 | 1 |
| 1615 | hsa-let-7a-3 | 339894 | LOC339894 | hypothetical protein LOC339894 | 1 |
| 1616 | hsa-let-7b | 339894 | LOC339894 | hypothetical protein LOC339894 | 1 |
| 1617 | hsa-mir-22 | 339894 | LOC339894 | hypothetical protein LOC339894 | 1 |
| 1618 | hsa-mir-10a | 339894 | LOC339894 | hypothetical protein LOC339894 | 1 |
| 1619 | hsa-mir-186 | 339894 | LOC339894 | hypothetical protein LOC339894 | 1 |
| 1620 | hsa-mir-155 | 339894 | LOC339894 | hypothetical protein LOC339894 | 1 |
| 1621 | hsa-mir-499 | 339894 | LOC339894 | hypothetical protein LOC339894 | 1 |
| 1622 | hsa-mir-550-1 | 339894 | LOC339894 | hypothetical protein LOC339894 | 1 |
| 1623 | hsa-mir-877 | 339894 | LOC339894 | hypothetical protein LOC339894 | 1 |
| 1624 | hsa-mir-2116 | 339894 | LOC339894 | hypothetical protein LOC339894 | 1 |
| 1625 | hsa-mir-3186 | 339894 | LOC339894 | hypothetical protein LOC339894 | 1 |
| 1626 | hsa-miR-1 | 150696 | PROM2 | prominin 2 | 1 |
| 1627 | hsa-miR-133a | 150696 | PROM2 | prominin 2 | 1 |
| 1628 | hsa-miR-186 | 150696 | PROM2 | prominin 2 | 1 |
| 1629 | hsa-miR-208a | 150696 | PROM2 | prominin 2 | 1 |
| 1630 | hsa-miR-326 | 150696 | PROM2 | prominin 2 | 1 |
| 1631 | hsa-miR-33a | 150696 | PROM2 | prominin 2 | 1 |
| 1632 | hsa-miR-449b | 150696 | PROM2 | prominin 2 | 1 |
| 1633 | hsa-miR-550 | 150696 | PROM2 | prominin 2 | 1 |
| 1634 | hsa-miR-575 | 150696 | PROM2 | prominin 2 | 1 |
| 1635 | hsa-miR-579 | 150696 | PROM2 | prominin 2 | 1 |
| 1636 | hsa-miR-608 | 150696 | PROM2 | prominin 2 | 1 |
| 1637 | hsa-miR-620 | 150696 | PROM2 | prominin 2 | 1 |
| 1638 | hsa-let-7a-3 | 150696 | PROM2 | prominin 2 | 1 |
| 1639 | hsa-let-7b | 150696 | PROM2 | prominin 2 | 1 |
| 1640 | hsa-mir-22 | 150696 | PROM2 | prominin 2 | 1 |
| 1641 | hsa-mir-10a | 150696 | PROM2 | prominin 2 | 1 |
| 1642 | hsa-mir-186 | 150696 | PROM2 | prominin 2 | 1 |
| 1643 | hsa-mir-155 | 150696 | PROM2 | prominin 2 | 1 |
| 1644 | hsa-mir-499 | 150696 | PROM2 | prominin 2 | 1 |
| 1645 | hsa-mir-550-1 | 150696 | PROM2 | prominin 2 | 1 |
| 1646 | hsa-mir-877 | 150696 | PROM2 | prominin 2 | 1 |
| 1647 | hsa-mir-2116 | 150696 | PROM2 | prominin 2 | 1 |
| 1648 | hsa-mir-3186 | 150696 | PROM2 | prominin 2 | 1 |
| 1649 | hsa-miR-1 | 196475 | RMST | rhabdomyosarcoma 2 associated transcript (non-protein coding) | 1 |
| 1650 | hsa-miR-133a | 196475 | RMST | rhabdomyosarcoma 2 associated transcript (non-protein coding) | 1 |
| 1651 | hsa-miR-186 | 196475 | RMST | rhabdomyosarcoma 2 associated transcript (non-protein coding) | 1 |
| 1652 | hsa-miR-326 | 196475 | RMST | rhabdomyosarcoma 2 associated transcript (non-protein coding) | 1 |
| 1653 | hsa-miR-449b | 196475 | RMST | rhabdomyosarcoma 2 associated transcript (non-protein coding) | 1 |
| 1654 | hsa-miR-551a | 196475 | RMST | rhabdomyosarcoma 2 associated transcript (non-protein coding) | 1 |
| 1655 | hsa-miR-575 | 196475 | RMST | rhabdomyosarcoma 2 associated transcript (non-protein coding) | 1 |
| 1656 | hsa-miR-579 | 196475 | RMST | rhabdomyosarcoma 2 associated transcript (non-protein coding) | 1 |
| 1657 | hsa-miR-608 | 196475 | RMST | rhabdomyosarcoma 2 associated transcript (non-protein coding) | 1 |
| 1658 | hsa-miR-620 | 196475 | RMST | rhabdomyosarcoma 2 associated transcript (non-protein coding) | 1 |
| 1659 | hsa-miR-637 | 196475 | RMST | rhabdomyosarcoma 2 associated transcript (non-protein coding) | 1 |
| 1660 | hsa-miR-885-5p | 196475 | RMST | rhabdomyosarcoma 2 associated transcript (non-protein coding) | 1 |
| 1661 | hsa-mir-22 | 196475 | RMST | rhabdomyosarcoma 2 associated transcript (non-protein coding) | 1 |
| 1662 | hsa-mir-10a | 196475 | RMST | rhabdomyosarcoma 2 associated transcript (non-protein coding) | 1 |
| 1663 | hsa-mir-186 | 196475 | RMST | rhabdomyosarcoma 2 associated transcript (non-protein coding) | 1 |
| 1664 | hsa-mir-155 | 196475 | RMST | rhabdomyosarcoma 2 associated transcript (non-protein coding) | 1 |
| 1665 | hsa-mir-423 | 196475 | RMST | rhabdomyosarcoma 2 associated transcript (non-protein coding) | 1 |
| 1666 | hsa-mir-499 | 196475 | RMST | rhabdomyosarcoma 2 associated transcript (non-protein coding) | 1 |
| 1667 | hsa-mir-550-1 | 196475 | RMST | rhabdomyosarcoma 2 associated transcript (non-protein coding) | 1 |
| 1668 | hsa-mir-885 | 196475 | RMST | rhabdomyosarcoma 2 associated transcript (non-protein coding) | 1 |
| 1669 | hsa-mir-877 | 196475 | RMST | rhabdomyosarcoma 2 associated transcript (non-protein coding) | 1 |
| 1670 | hsa-mir-2116 | 196475 | RMST | rhabdomyosarcoma 2 associated transcript (non-protein coding) | 1 |
| 1671 | hsa-mir-3190 | 196475 | RMST | rhabdomyosarcoma 2 associated transcript (non-protein coding) | 1 |
| 1672 | hsa-miR-1 | 100128003 | LOC100128003 | hypothetical protein LOC100128003 | 1 |
| 1673 | hsa-miR-133a | 100128003 | LOC100128003 | hypothetical protein LOC100128003 | 1 |
| 1674 | hsa-miR-186 | 100128003 | LOC100128003 | hypothetical protein LOC100128003 | 1 |
| 1675 | hsa-miR-208a | 100128003 | LOC100128003 | hypothetical protein LOC100128003 | 1 |
| 1676 | hsa-miR-326 | 100128003 | LOC100128003 | hypothetical protein LOC100128003 | 1 |
| 1677 | hsa-miR-33a | 100128003 | LOC100128003 | hypothetical protein LOC100128003 | 1 |
| 1678 | hsa-miR-449b | 100128003 | LOC100128003 | hypothetical protein LOC100128003 | 1 |
| 1679 | hsa-miR-550 | 100128003 | LOC100128003 | hypothetical protein LOC100128003 | 1 |
| 1680 | hsa-miR-551a | 100128003 | LOC100128003 | hypothetical protein LOC100128003 | 1 |
| 1681 | hsa-miR-575 | 100128003 | LOC100128003 | hypothetical protein LOC100128003 | 1 |
| 1682 | hsa-miR-579 | 100128003 | LOC100128003 | hypothetical protein LOC100128003 | 1 |
| 1683 | hsa-miR-608 | 100128003 | LOC100128003 | hypothetical protein LOC100128003 | 1 |
| 1684 | hsa-miR-620 | 100128003 | LOC100128003 | hypothetical protein LOC100128003 | 1 |
| 1685 | hsa-let-7a-3 | 100128003 | LOC100128003 | hypothetical protein LOC100128003 | 1 |
| 1686 | hsa-let-7b | 100128003 | LOC100128003 | hypothetical protein LOC100128003 | 1 |
| 1687 | hsa-mir-22 | 100128003 | LOC100128003 | hypothetical protein LOC100128003 | 1 |
| 1688 | hsa-mir-10a | 100128003 | LOC100128003 | hypothetical protein LOC100128003 | 1 |
| 1689 | hsa-mir-186 | 100128003 | LOC100128003 | hypothetical protein LOC100128003 | 1 |
| 1690 | hsa-mir-155 | 100128003 | LOC100128003 | hypothetical protein LOC100128003 | 1 |
| 1691 | hsa-mir-423 | 100128003 | LOC100128003 | hypothetical protein LOC100128003 | 1 |
| 1692 | hsa-mir-499 | 100128003 | LOC100128003 | hypothetical protein LOC100128003 | 1 |
| 1693 | hsa-mir-550-1 | 100128003 | LOC100128003 | hypothetical protein LOC100128003 | 1 |
| 1694 | hsa-mir-877 | 100128003 | LOC100128003 | hypothetical protein LOC100128003 | 1 |
| 1695 | hsa-mir-2116 | 100128003 | LOC100128003 | hypothetical protein LOC100128003 | 1 |
| 1696 | hsa-mir-3186 | 100128003 | LOC100128003 | hypothetical protein LOC100128003 | 1 |
| 1697 | hsa-mir-3190 | 100128003 | LOC100128003 | hypothetical protein LOC100128003 | 1 |
| 1698 | hsa-miR-1 | 121643 | FOXN4 | forkhead box N4 | 1 |
| 1699 | hsa-miR-133a | 121643 | FOXN4 | forkhead box N4 | 1 |
| 1700 | hsa-miR-186 | 121643 | FOXN4 | forkhead box N4 | 1 |
| 1701 | hsa-miR-208a | 121643 | FOXN4 | forkhead box N4 | 1 |
| 1702 | hsa-miR-449b | 121643 | FOXN4 | forkhead box N4 | 1 |
| 1703 | hsa-miR-562 | 121643 | FOXN4 | forkhead box N4 | 1 |
| 1704 | hsa-miR-575 | 121643 | FOXN4 | forkhead box N4 | 1 |
| 1705 | hsa-miR-579 | 121643 | FOXN4 | forkhead box N4 | 1 |
| 1706 | hsa-miR-608 | 121643 | FOXN4 | forkhead box N4 | 1 |
| 1707 | hsa-miR-620 | 121643 | FOXN4 | forkhead box N4 | 1 |
| 1708 | hsa-miR-637 | 121643 | FOXN4 | forkhead box N4 | 1 |
| 1709 | hsa-miR-885-5p | 121643 | FOXN4 | forkhead box N4 | 1 |
| 1710 | hsa-mir-22 | 121643 | FOXN4 | forkhead box N4 | 1 |
| 1711 | hsa-mir-10a | 121643 | FOXN4 | forkhead box N4 | 1 |
| 1712 | hsa-mir-186 | 121643 | FOXN4 | forkhead box N4 | 1 |
| 1713 | hsa-mir-155 | 121643 | FOXN4 | forkhead box N4 | 1 |
| 1714 | hsa-mir-423 | 121643 | FOXN4 | forkhead box N4 | 1 |
| 1715 | hsa-mir-499 | 121643 | FOXN4 | forkhead box N4 | 1 |
| 1716 | hsa-mir-885 | 121643 | FOXN4 | forkhead box N4 | 1 |
| 1717 | hsa-mir-877 | 121643 | FOXN4 | forkhead box N4 | 1 |
| 1718 | hsa-miR-1 | 338694 | LOC338694 | hypothetical protein LOC338694 | 1 |
| 1719 | hsa-miR-133a | 338694 | LOC338694 | hypothetical protein LOC338694 | 1 |
| 1720 | hsa-miR-186 | 338694 | LOC338694 | hypothetical protein LOC338694 | 1 |
| 1721 | hsa-miR-326 | 338694 | LOC338694 | hypothetical protein LOC338694 | 1 |
| 1722 | hsa-miR-449b | 338694 | LOC338694 | hypothetical protein LOC338694 | 1 |
| 1723 | hsa-miR-489 | 338694 | LOC338694 | hypothetical protein LOC338694 | 1 |
| 1724 | hsa-miR-575 | 338694 | LOC338694 | hypothetical protein LOC338694 | 1 |
| 1725 | hsa-miR-608 | 338694 | LOC338694 | hypothetical protein LOC338694 | 1 |
| 1726 | hsa-miR-620 | 338694 | LOC338694 | hypothetical protein LOC338694 | 1 |
| 1727 | hsa-let-7a-3 | 338694 | LOC338694 | hypothetical protein LOC338694 | 1 |
| 1728 | hsa-let-7b | 338694 | LOC338694 | hypothetical protein LOC338694 | 1 |
| 1729 | hsa-mir-22 | 338694 | LOC338694 | hypothetical protein LOC338694 | 1 |
| 1730 | hsa-mir-10a | 338694 | LOC338694 | hypothetical protein LOC338694 | 1 |
| 1731 | hsa-mir-186 | 338694 | LOC338694 | hypothetical protein LOC338694 | 1 |
| 1732 | hsa-mir-155 | 338694 | LOC338694 | hypothetical protein LOC338694 | 1 |
| 1733 | hsa-mir-499 | 338694 | LOC338694 | hypothetical protein LOC338694 | 1 |
| 1734 | hsa-mir-877 | 338694 | LOC338694 | hypothetical protein LOC338694 | 1 |
| 1735 | hsa-miR-1 | 414300 | LOC414300 | hypothetical LOC414300 | 1 |
| 1736 | hsa-miR-133a | 414300 | LOC414300 | hypothetical LOC414300 | 1 |
| 1737 | hsa-miR-186 | 414300 | LOC414300 | hypothetical LOC414300 | 1 |
| 1738 | hsa-miR-326 | 414300 | LOC414300 | hypothetical LOC414300 | 1 |
| 1739 | hsa-miR-449b | 414300 | LOC414300 | hypothetical LOC414300 | 1 |
| 1740 | hsa-miR-551a | 414300 | LOC414300 | hypothetical LOC414300 | 1 |
| 1741 | hsa-miR-575 | 414300 | LOC414300 | hypothetical LOC414300 | 1 |
| 1742 | hsa-miR-579 | 414300 | LOC414300 | hypothetical LOC414300 | 1 |
| 1743 | hsa-miR-608 | 414300 | LOC414300 | hypothetical LOC414300 | 1 |
| 1744 | hsa-miR-620 | 414300 | LOC414300 | hypothetical LOC414300 | 1 |
| 1745 | hsa-miR-637 | 414300 | LOC414300 | hypothetical LOC414300 | 1 |
| 1746 | hsa-mir-22 | 414300 | LOC414300 | hypothetical LOC414300 | 1 |
| 1747 | hsa-mir-10a | 414300 | LOC414300 | hypothetical LOC414300 | 1 |
| 1748 | hsa-mir-186 | 414300 | LOC414300 | hypothetical LOC414300 | 1 |
| 1749 | hsa-mir-155 | 414300 | LOC414300 | hypothetical LOC414300 | 1 |
| 1750 | hsa-mir-423 | 414300 | LOC414300 | hypothetical LOC414300 | 1 |
| 1751 | hsa-mir-499 | 414300 | LOC414300 | hypothetical LOC414300 | 1 |
| 1752 | hsa-mir-877 | 414300 | LOC414300 | hypothetical LOC414300 | 1 |
| 1753 | hsa-mir-2116 | 414300 | LOC414300 | hypothetical LOC414300 | 1 |
| 1754 | hsa-mir-3190 | 414300 | LOC414300 | hypothetical LOC414300 | 1 |
| 1755 | hsa-miR-10a | 286359 | hCG\_2045089 | hypothetical LOC286359 | 1 |
| 1756 | hsa-miR-139-5p | 286359 | hCG\_2045089 | hypothetical LOC286359 | 1 |
| 1757 | hsa-miR-26a | 286359 | hCG\_2045089 | hypothetical LOC286359 | 1 |
| 1758 | hsa-miR-26b | 286359 | hCG\_2045089 | hypothetical LOC286359 | 1 |
| 1759 | hsa-miR-31 | 286359 | hCG\_2045089 | hypothetical LOC286359 | 1 |
| 1760 | hsa-miR-338-5p | 286359 | hCG\_2045089 | hypothetical LOC286359 | 1 |
| 1761 | hsa-miR-340 | 286359 | hCG\_2045089 | hypothetical LOC286359 | 1 |
| 1762 | hsa-miR-449a | 286359 | hCG\_2045089 | hypothetical LOC286359 | 1 |
| 1763 | hsa-miR-499-5p | 286359 | hCG\_2045089 | hypothetical LOC286359 | 1 |
| 1764 | hsa-miR-548b-5p | 286359 | hCG\_2045089 | hypothetical LOC286359 | 1 |
| 1765 | hsa-miR-564 | 286359 | hCG\_2045089 | hypothetical LOC286359 | 1 |
| 1766 | hsa-miR-621 | 286359 | hCG\_2045089 | hypothetical LOC286359 | 1 |
| 1767 | hsa-miR-634 | 286359 | hCG\_2045089 | hypothetical LOC286359 | 1 |
| 1768 | hsa-miR-657 | 286359 | hCG\_2045089 | hypothetical LOC286359 | 1 |
| 1769 | hsa-miR-765 | 286359 | hCG\_2045089 | hypothetical LOC286359 | 1 |
| 1770 | hsa-miR-770-5p | 286359 | hCG\_2045089 | hypothetical LOC286359 | 1 |
| 1771 | hsa-miR-877 | 286359 | hCG\_2045089 | hypothetical LOC286359 | 1 |
| 1772 | hsa-mir-26a-1 | 286359 | hCG\_2045089 | hypothetical LOC286359 | 1 |
| 1773 | hsa-mir-26b | 286359 | hCG\_2045089 | hypothetical LOC286359 | 1 |
| 1774 | hsa-mir-31 | 286359 | hCG\_2045089 | hypothetical LOC286359 | 1 |
| 1775 | hsa-mir-194-2 | 286359 | hCG\_2045089 | hypothetical LOC286359 | 1 |
| 1776 | hsa-mir-340 | 286359 | hCG\_2045089 | hypothetical LOC286359 | 1 |
| 1777 | hsa-mir-338 | 286359 | hCG\_2045089 | hypothetical LOC286359 | 1 |
| 1778 | hsa-mir-548b | 286359 | hCG\_2045089 | hypothetical LOC286359 | 1 |
| 1779 | hsa-mir-449b | 286359 | hCG\_2045089 | hypothetical LOC286359 | 1 |
| 1780 | hsa-mir-449c | 286359 | hCG\_2045089 | hypothetical LOC286359 | 1 |
| 1781 | hsa-mir-3065 | 286359 | hCG\_2045089 | hypothetical LOC286359 | 1 |
| 1782 | hsa-miR-1 | 283194 | LOC283194 | hypothetical protein LOC283194 | 1 |
| 1783 | hsa-miR-133a | 283194 | LOC283194 | hypothetical protein LOC283194 | 1 |
| 1784 | hsa-miR-186 | 283194 | LOC283194 | hypothetical protein LOC283194 | 1 |
| 1785 | hsa-miR-208a | 283194 | LOC283194 | hypothetical protein LOC283194 | 1 |
| 1786 | hsa-miR-449b | 283194 | LOC283194 | hypothetical protein LOC283194 | 1 |
| 1787 | hsa-miR-562 | 283194 | LOC283194 | hypothetical protein LOC283194 | 1 |
| 1788 | hsa-miR-579 | 283194 | LOC283194 | hypothetical protein LOC283194 | 1 |
| 1789 | hsa-miR-608 | 283194 | LOC283194 | hypothetical protein LOC283194 | 1 |
| 1790 | hsa-miR-620 | 283194 | LOC283194 | hypothetical protein LOC283194 | 1 |
| 1791 | hsa-miR-637 | 283194 | LOC283194 | hypothetical protein LOC283194 | 1 |
| 1792 | hsa-miR-885-5p | 283194 | LOC283194 | hypothetical protein LOC283194 | 1 |
| 1793 | hsa-mir-22 | 283194 | LOC283194 | hypothetical protein LOC283194 | 1 |
| 1794 | hsa-mir-10a | 283194 | LOC283194 | hypothetical protein LOC283194 | 1 |
| 1795 | hsa-mir-186 | 283194 | LOC283194 | hypothetical protein LOC283194 | 1 |
| 1796 | hsa-mir-155 | 283194 | LOC283194 | hypothetical protein LOC283194 | 1 |
| 1797 | hsa-mir-423 | 283194 | LOC283194 | hypothetical protein LOC283194 | 1 |
| 1798 | hsa-mir-885 | 283194 | LOC283194 | hypothetical protein LOC283194 | 1 |
| 1799 | hsa-mir-877 | 283194 | LOC283194 | hypothetical protein LOC283194 | 1 |
| 1800 | hsa-miR-1 | 150365 | MEI1 | meiosis inhibitor 1 | 1 |
| 1801 | hsa-miR-133a | 150365 | MEI1 | meiosis inhibitor 1 | 1 |
| 1802 | hsa-miR-186 | 150365 | MEI1 | meiosis inhibitor 1 | 1 |
| 1803 | hsa-miR-208a | 150365 | MEI1 | meiosis inhibitor 1 | 1 |
| 1804 | hsa-miR-326 | 150365 | MEI1 | meiosis inhibitor 1 | 1 |
| 1805 | hsa-miR-449b | 150365 | MEI1 | meiosis inhibitor 1 | 1 |
| 1806 | hsa-miR-550 | 150365 | MEI1 | meiosis inhibitor 1 | 1 |
| 1807 | hsa-miR-551a | 150365 | MEI1 | meiosis inhibitor 1 | 1 |
| 1808 | hsa-miR-575 | 150365 | MEI1 | meiosis inhibitor 1 | 1 |
| 1809 | hsa-miR-579 | 150365 | MEI1 | meiosis inhibitor 1 | 1 |
| 1810 | hsa-miR-608 | 150365 | MEI1 | meiosis inhibitor 1 | 1 |
| 1811 | hsa-miR-620 | 150365 | MEI1 | meiosis inhibitor 1 | 1 |
| 1812 | hsa-mir-22 | 150365 | MEI1 | meiosis inhibitor 1 | 1 |
| 1813 | hsa-mir-10a | 150365 | MEI1 | meiosis inhibitor 1 | 1 |
| 1814 | hsa-mir-186 | 150365 | MEI1 | meiosis inhibitor 1 | 1 |
| 1815 | hsa-mir-155 | 150365 | MEI1 | meiosis inhibitor 1 | 1 |
| 1816 | hsa-mir-423 | 150365 | MEI1 | meiosis inhibitor 1 | 1 |
| 1817 | hsa-mir-499 | 150365 | MEI1 | meiosis inhibitor 1 | 1 |
| 1818 | hsa-mir-550-1 | 150365 | MEI1 | meiosis inhibitor 1 | 1 |
| 1819 | hsa-mir-877 | 150365 | MEI1 | meiosis inhibitor 1 | 1 |
| 1820 | hsa-mir-2116 | 150365 | MEI1 | meiosis inhibitor 1 | 1 |
| 1821 | hsa-mir-3186 | 150365 | MEI1 | meiosis inhibitor 1 | 1 |
| 1822 | hsa-mir-3190 | 150365 | MEI1 | meiosis inhibitor 1 | 1 |
| 1823 | hsa-miR-1 | 221 | ALDH3B1 | aldehyde dehydrogenase 3 family, member B1 | 1 |
| 1824 | hsa-miR-133a | 221 | ALDH3B1 | aldehyde dehydrogenase 3 family, member B1 | 1 |
| 1825 | hsa-miR-186 | 221 | ALDH3B1 | aldehyde dehydrogenase 3 family, member B1 | 1 |
| 1826 | hsa-miR-208a | 221 | ALDH3B1 | aldehyde dehydrogenase 3 family, member B1 | 1 |
| 1827 | hsa-miR-326 | 221 | ALDH3B1 | aldehyde dehydrogenase 3 family, member B1 | 1 |
| 1828 | hsa-miR-449b | 221 | ALDH3B1 | aldehyde dehydrogenase 3 family, member B1 | 1 |
| 1829 | hsa-miR-550 | 221 | ALDH3B1 | aldehyde dehydrogenase 3 family, member B1 | 1 |
| 1830 | hsa-miR-551a | 221 | ALDH3B1 | aldehyde dehydrogenase 3 family, member B1 | 1 |
| 1831 | hsa-miR-575 | 221 | ALDH3B1 | aldehyde dehydrogenase 3 family, member B1 | 1 |
| 1832 | hsa-miR-579 | 221 | ALDH3B1 | aldehyde dehydrogenase 3 family, member B1 | 1 |
| 1833 | hsa-miR-608 | 221 | ALDH3B1 | aldehyde dehydrogenase 3 family, member B1 | 1 |
| 1834 | hsa-miR-620 | 221 | ALDH3B1 | aldehyde dehydrogenase 3 family, member B1 | 1 |
| 1835 | hsa-mir-22 | 221 | ALDH3B1 | aldehyde dehydrogenase 3 family, member B1 | 1 |
| 1836 | hsa-mir-10a | 221 | ALDH3B1 | aldehyde dehydrogenase 3 family, member B1 | 1 |
| 1837 | hsa-mir-186 | 221 | ALDH3B1 | aldehyde dehydrogenase 3 family, member B1 | 1 |
| 1838 | hsa-mir-155 | 221 | ALDH3B1 | aldehyde dehydrogenase 3 family, member B1 | 1 |
| 1839 | hsa-mir-423 | 221 | ALDH3B1 | aldehyde dehydrogenase 3 family, member B1 | 1 |
| 1840 | hsa-mir-499 | 221 | ALDH3B1 | aldehyde dehydrogenase 3 family, member B1 | 1 |
| 1841 | hsa-mir-550-1 | 221 | ALDH3B1 | aldehyde dehydrogenase 3 family, member B1 | 1 |
| 1842 | hsa-mir-877 | 221 | ALDH3B1 | aldehyde dehydrogenase 3 family, member B1 | 1 |
| 1843 | hsa-mir-2116 | 221 | ALDH3B1 | aldehyde dehydrogenase 3 family, member B1 | 1 |
| 1844 | hsa-mir-3186 | 221 | ALDH3B1 | aldehyde dehydrogenase 3 family, member B1 | 1 |
| 1845 | hsa-mir-3190 | 221 | ALDH3B1 | aldehyde dehydrogenase 3 family, member B1 | 1 |
| 1846 | hsa-miR-1 | 1122 | CHML | choroideremia-like (Rab escort protein 2) | 1 |
| 1847 | hsa-miR-133a | 1122 | CHML | choroideremia-like (Rab escort protein 2) | 1 |
| 1848 | hsa-miR-449b | 1122 | CHML | choroideremia-like (Rab escort protein 2) | 1 |
| 1849 | hsa-miR-489 | 1122 | CHML | choroideremia-like (Rab escort protein 2) | 1 |
| 1850 | hsa-miR-562 | 1122 | CHML | choroideremia-like (Rab escort protein 2) | 1 |
| 1851 | hsa-miR-579 | 1122 | CHML | choroideremia-like (Rab escort protein 2) | 1 |
| 1852 | hsa-miR-608 | 1122 | CHML | choroideremia-like (Rab escort protein 2) | 1 |
| 1853 | hsa-miR-620 | 1122 | CHML | choroideremia-like (Rab escort protein 2) | 1 |
| 1854 | hsa-miR-637 | 1122 | CHML | choroideremia-like (Rab escort protein 2) | 1 |
| 1855 | hsa-miR-885-5p | 1122 | CHML | choroideremia-like (Rab escort protein 2) | 1 |
| 1856 | hsa-mir-22 | 1122 | CHML | choroideremia-like (Rab escort protein 2) | 1 |
| 1857 | hsa-mir-10a | 1122 | CHML | choroideremia-like (Rab escort protein 2) | 1 |
| 1858 | hsa-mir-155 | 1122 | CHML | choroideremia-like (Rab escort protein 2) | 1 |
| 1859 | hsa-mir-499 | 1122 | CHML | choroideremia-like (Rab escort protein 2) | 1 |
| 1860 | hsa-mir-885 | 1122 | CHML | choroideremia-like (Rab escort protein 2) | 1 |
| 1861 | hsa-miR-1 | 220107 | DLEU7 | deleted in lymphocytic leukemia, 7 | 1 |
| 1862 | hsa-miR-133a | 220107 | DLEU7 | deleted in lymphocytic leukemia, 7 | 1 |
| 1863 | hsa-miR-186 | 220107 | DLEU7 | deleted in lymphocytic leukemia, 7 | 1 |
| 1864 | hsa-miR-326 | 220107 | DLEU7 | deleted in lymphocytic leukemia, 7 | 1 |
| 1865 | hsa-miR-449b | 220107 | DLEU7 | deleted in lymphocytic leukemia, 7 | 1 |
| 1866 | hsa-miR-551a | 220107 | DLEU7 | deleted in lymphocytic leukemia, 7 | 1 |
| 1867 | hsa-miR-575 | 220107 | DLEU7 | deleted in lymphocytic leukemia, 7 | 1 |
| 1868 | hsa-miR-579 | 220107 | DLEU7 | deleted in lymphocytic leukemia, 7 | 1 |
| 1869 | hsa-miR-608 | 220107 | DLEU7 | deleted in lymphocytic leukemia, 7 | 1 |
| 1870 | hsa-miR-620 | 220107 | DLEU7 | deleted in lymphocytic leukemia, 7 | 1 |
| 1871 | hsa-miR-637 | 220107 | DLEU7 | deleted in lymphocytic leukemia, 7 | 1 |
| 1872 | hsa-miR-885-5p | 220107 | DLEU7 | deleted in lymphocytic leukemia, 7 | 1 |
| 1873 | hsa-mir-22 | 220107 | DLEU7 | deleted in lymphocytic leukemia, 7 | 1 |
| 1874 | hsa-mir-10a | 220107 | DLEU7 | deleted in lymphocytic leukemia, 7 | 1 |
| 1875 | hsa-mir-186 | 220107 | DLEU7 | deleted in lymphocytic leukemia, 7 | 1 |
| 1876 | hsa-mir-155 | 220107 | DLEU7 | deleted in lymphocytic leukemia, 7 | 1 |
| 1877 | hsa-mir-423 | 220107 | DLEU7 | deleted in lymphocytic leukemia, 7 | 1 |
| 1878 | hsa-mir-499 | 220107 | DLEU7 | deleted in lymphocytic leukemia, 7 | 1 |
| 1879 | hsa-mir-885 | 220107 | DLEU7 | deleted in lymphocytic leukemia, 7 | 1 |
| 1880 | hsa-mir-877 | 220107 | DLEU7 | deleted in lymphocytic leukemia, 7 | 1 |
| 1881 | hsa-mir-2116 | 220107 | DLEU7 | deleted in lymphocytic leukemia, 7 | 1 |
| 1882 | hsa-mir-3190 | 220107 | DLEU7 | deleted in lymphocytic leukemia, 7 | 1 |
| 1883 | hsa-miR-1 | 9927 | MFN2 | mitofusin 2 | 1 |
| 1884 | hsa-miR-133a | 9927 | MFN2 | mitofusin 2 | 1 |
| 1885 | hsa-miR-186 | 9927 | MFN2 | mitofusin 2 | 1 |
| 1886 | hsa-miR-208a | 9927 | MFN2 | mitofusin 2 | 1 |
| 1887 | hsa-miR-326 | 9927 | MFN2 | mitofusin 2 | 1 |
| 1888 | hsa-miR-449b | 9927 | MFN2 | mitofusin 2 | 1 |
| 1889 | hsa-miR-562 | 9927 | MFN2 | mitofusin 2 | 1 |
| 1890 | hsa-miR-575 | 9927 | MFN2 | mitofusin 2 | 1 |
| 1891 | hsa-miR-579 | 9927 | MFN2 | mitofusin 2 | 1 |
| 1892 | hsa-miR-608 | 9927 | MFN2 | mitofusin 2 | 1 |
| 1893 | hsa-miR-620 | 9927 | MFN2 | mitofusin 2 | 1 |
| 1894 | hsa-miR-885-5p | 9927 | MFN2 | mitofusin 2 | 1 |
| 1895 | hsa-let-7a-3 | 9927 | MFN2 | mitofusin 2 | 1 |
| 1896 | hsa-let-7b | 9927 | MFN2 | mitofusin 2 | 1 |
| 1897 | hsa-mir-22 | 9927 | MFN2 | mitofusin 2 | 1 |
| 1898 | hsa-mir-10a | 9927 | MFN2 | mitofusin 2 | 1 |
| 1899 | hsa-mir-186 | 9927 | MFN2 | mitofusin 2 | 1 |
| 1900 | hsa-mir-155 | 9927 | MFN2 | mitofusin 2 | 1 |
| 1901 | hsa-mir-499 | 9927 | MFN2 | mitofusin 2 | 1 |
| 1902 | hsa-mir-550-1 | 9927 | MFN2 | mitofusin 2 | 1 |
| 1903 | hsa-mir-885 | 9927 | MFN2 | mitofusin 2 | 1 |
| 1904 | hsa-mir-877 | 9927 | MFN2 | mitofusin 2 | 1 |
| 1905 | hsa-mir-2116 | 9927 | MFN2 | mitofusin 2 | 1 |
| 1911 | hsa-miR-551a | 4094 | MAF | v-maf musculoaponeurotic fibrosarcoma oncogene homolog (avian) | 1 |
| 1923 | hsa-mir-423 | 4094 | MAF | v-maf musculoaponeurotic fibrosarcoma oncogene homolog (avian) | 1 |
| 1934 | hsa-miR-550 | 57492 | ARID1B | AT rich interactive domain 1B (SWI1-like) | 1 |
| 1937 | hsa-miR-575 | 57492 | ARID1B | AT rich interactive domain 1B (SWI1-like) | 1 |
| 1940 | hsa-miR-620 | 57492 | ARID1B | AT rich interactive domain 1B (SWI1-like) | 1 |
| 1941 | hsa-miR-885-5p | 57492 | ARID1B | AT rich interactive domain 1B (SWI1-like) | 1 |
| 1946 | hsa-mir-423 | 57492 | ARID1B | AT rich interactive domain 1B (SWI1-like) | 1 |
| 1947 | hsa-mir-499 | 57492 | ARID1B | AT rich interactive domain 1B (SWI1-like) | 1 |
| 1949 | hsa-mir-885 | 57492 | ARID1B | AT rich interactive domain 1B (SWI1-like) | 1 |
| 1951 | hsa-mir-2116 | 57492 | ARID1B | AT rich interactive domain 1B (SWI1-like) | 1 |
| 1953 | hsa-mir-3190 | 57492 | ARID1B | AT rich interactive domain 1B (SWI1-like) | 1 |
| 1954 | hsa-miR-10a | 169355 | IDO2 | indoleamine 2,3-dioxygenase 2 | 1 |
| 1955 | hsa-miR-139-5p | 169355 | IDO2 | indoleamine 2,3-dioxygenase 2 | 1 |
| 1956 | hsa-miR-152 | 169355 | IDO2 | indoleamine 2,3-dioxygenase 2 | 1 |
| 1957 | hsa-miR-26a | 169355 | IDO2 | indoleamine 2,3-dioxygenase 2 | 1 |
| 1958 | hsa-miR-26b | 169355 | IDO2 | indoleamine 2,3-dioxygenase 2 | 1 |
| 1959 | hsa-miR-31 | 169355 | IDO2 | indoleamine 2,3-dioxygenase 2 | 1 |
| 1960 | hsa-miR-338-5p | 169355 | IDO2 | indoleamine 2,3-dioxygenase 2 | 1 |
| 1961 | hsa-miR-340 | 169355 | IDO2 | indoleamine 2,3-dioxygenase 2 | 1 |
| 1962 | hsa-miR-449a | 169355 | IDO2 | indoleamine 2,3-dioxygenase 2 | 1 |
| 1963 | hsa-miR-490-5p | 169355 | IDO2 | indoleamine 2,3-dioxygenase 2 | 1 |
| 1964 | hsa-miR-499-5p | 169355 | IDO2 | indoleamine 2,3-dioxygenase 2 | 1 |
| 1965 | hsa-miR-548b-5p | 169355 | IDO2 | indoleamine 2,3-dioxygenase 2 | 1 |
| 1966 | hsa-miR-621 | 169355 | IDO2 | indoleamine 2,3-dioxygenase 2 | 1 |
| 1967 | hsa-miR-657 | 169355 | IDO2 | indoleamine 2,3-dioxygenase 2 | 1 |
| 1968 | hsa-miR-765 | 169355 | IDO2 | indoleamine 2,3-dioxygenase 2 | 1 |
| 1969 | hsa-miR-770-5p | 169355 | IDO2 | indoleamine 2,3-dioxygenase 2 | 1 |
| 1970 | hsa-miR-877 | 169355 | IDO2 | indoleamine 2,3-dioxygenase 2 | 1 |
| 1971 | hsa-mir-26a-1 | 169355 | IDO2 | indoleamine 2,3-dioxygenase 2 | 1 |
| 1972 | hsa-mir-26b | 169355 | IDO2 | indoleamine 2,3-dioxygenase 2 | 1 |
| 1973 | hsa-mir-31 | 169355 | IDO2 | indoleamine 2,3-dioxygenase 2 | 1 |
| 1974 | hsa-mir-194-2 | 169355 | IDO2 | indoleamine 2,3-dioxygenase 2 | 1 |
| 1975 | hsa-mir-340 | 169355 | IDO2 | indoleamine 2,3-dioxygenase 2 | 1 |
| 1976 | hsa-mir-338 | 169355 | IDO2 | indoleamine 2,3-dioxygenase 2 | 1 |
| 1977 | hsa-mir-490 | 169355 | IDO2 | indoleamine 2,3-dioxygenase 2 | 1 |
| 1978 | hsa-mir-548b | 169355 | IDO2 | indoleamine 2,3-dioxygenase 2 | 1 |
| 1979 | hsa-mir-548d-2 | 169355 | IDO2 | indoleamine 2,3-dioxygenase 2 | 1 |
| 1980 | hsa-mir-449b | 169355 | IDO2 | indoleamine 2,3-dioxygenase 2 | 1 |
| 1981 | hsa-mir-449c | 169355 | IDO2 | indoleamine 2,3-dioxygenase 2 | 1 |
| 1982 | hsa-mir-3065 | 169355 | IDO2 | indoleamine 2,3-dioxygenase 2 | 1 |
| 1983 | hsa-miR-1 | 1842 | ECM2 | extracellular matrix protein 2, female organ and adipocyte specific | 1 |
| 1984 | hsa-miR-133a | 1842 | ECM2 | extracellular matrix protein 2, female organ and adipocyte specific | 1 |
| 1985 | hsa-miR-186 | 1842 | ECM2 | extracellular matrix protein 2, female organ and adipocyte specific | 1 |
| 1986 | hsa-miR-208a | 1842 | ECM2 | extracellular matrix protein 2, female organ and adipocyte specific | 1 |
| 1987 | hsa-miR-326 | 1842 | ECM2 | extracellular matrix protein 2, female organ and adipocyte specific | 1 |
| 1988 | hsa-miR-449b | 1842 | ECM2 | extracellular matrix protein 2, female organ and adipocyte specific | 1 |
| 1989 | hsa-miR-575 | 1842 | ECM2 | extracellular matrix protein 2, female organ and adipocyte specific | 1 |
| 1990 | hsa-miR-608 | 1842 | ECM2 | extracellular matrix protein 2, female organ and adipocyte specific | 1 |
| 1991 | hsa-miR-620 | 1842 | ECM2 | extracellular matrix protein 2, female organ and adipocyte specific | 1 |
| 1992 | hsa-mir-22 | 1842 | ECM2 | extracellular matrix protein 2, female organ and adipocyte specific | 1 |
| 1993 | hsa-mir-10a | 1842 | ECM2 | extracellular matrix protein 2, female organ and adipocyte specific | 1 |
| 1994 | hsa-mir-186 | 1842 | ECM2 | extracellular matrix protein 2, female organ and adipocyte specific | 1 |
| 1995 | hsa-mir-155 | 1842 | ECM2 | extracellular matrix protein 2, female organ and adipocyte specific | 1 |
| 1996 | hsa-mir-423 | 1842 | ECM2 | extracellular matrix protein 2, female organ and adipocyte specific | 1 |
| 1997 | hsa-mir-499 | 1842 | ECM2 | extracellular matrix protein 2, female organ and adipocyte specific | 1 |
| 1998 | hsa-mir-877 | 1842 | ECM2 | extracellular matrix protein 2, female organ and adipocyte specific | 1 |
| 1999 | hsa-mir-2116 | 1842 | ECM2 | extracellular matrix protein 2, female organ and adipocyte specific | 1 |
| 2000 | hsa-miR-1 | 340273 | ABCB5 | ATP-binding cassette, sub-family B (MDR/TAP), member 5 | 1 |
| 2001 | hsa-miR-133a | 340273 | ABCB5 | ATP-binding cassette, sub-family B (MDR/TAP), member 5 | 1 |
| 2002 | hsa-miR-186 | 340273 | ABCB5 | ATP-binding cassette, sub-family B (MDR/TAP), member 5 | 1 |
| 2003 | hsa-miR-326 | 340273 | ABCB5 | ATP-binding cassette, sub-family B (MDR/TAP), member 5 | 1 |
| 2004 | hsa-miR-449b | 340273 | ABCB5 | ATP-binding cassette, sub-family B (MDR/TAP), member 5 | 1 |
| 2005 | hsa-miR-550 | 340273 | ABCB5 | ATP-binding cassette, sub-family B (MDR/TAP), member 5 | 1 |
| 2006 | hsa-miR-551a | 340273 | ABCB5 | ATP-binding cassette, sub-family B (MDR/TAP), member 5 | 1 |
| 2007 | hsa-miR-562 | 340273 | ABCB5 | ATP-binding cassette, sub-family B (MDR/TAP), member 5 | 1 |
| 2008 | hsa-miR-579 | 340273 | ABCB5 | ATP-binding cassette, sub-family B (MDR/TAP), member 5 | 1 |
| 2009 | hsa-miR-608 | 340273 | ABCB5 | ATP-binding cassette, sub-family B (MDR/TAP), member 5 | 1 |
| 2010 | hsa-mir-22 | 340273 | ABCB5 | ATP-binding cassette, sub-family B (MDR/TAP), member 5 | 1 |
| 2011 | hsa-mir-10a | 340273 | ABCB5 | ATP-binding cassette, sub-family B (MDR/TAP), member 5 | 1 |
| 2012 | hsa-mir-186 | 340273 | ABCB5 | ATP-binding cassette, sub-family B (MDR/TAP), member 5 | 1 |
| 2013 | hsa-mir-155 | 340273 | ABCB5 | ATP-binding cassette, sub-family B (MDR/TAP), member 5 | 1 |
| 2014 | hsa-mir-423 | 340273 | ABCB5 | ATP-binding cassette, sub-family B (MDR/TAP), member 5 | 1 |
| 2015 | hsa-mir-550-1 | 340273 | ABCB5 | ATP-binding cassette, sub-family B (MDR/TAP), member 5 | 1 |
| 2016 | hsa-miR-1 | 51091 | SEPSECS | Sep (O-phosphoserine) tRNA:Sec (selenocysteine) tRNA synthase | 1 |
| 2017 | hsa-miR-133a | 51091 | SEPSECS | Sep (O-phosphoserine) tRNA:Sec (selenocysteine) tRNA synthase | 1 |
| 2018 | hsa-miR-186 | 51091 | SEPSECS | Sep (O-phosphoserine) tRNA:Sec (selenocysteine) tRNA synthase | 1 |
| 2019 | hsa-miR-208a | 51091 | SEPSECS | Sep (O-phosphoserine) tRNA:Sec (selenocysteine) tRNA synthase | 1 |
| 2020 | hsa-miR-326 | 51091 | SEPSECS | Sep (O-phosphoserine) tRNA:Sec (selenocysteine) tRNA synthase | 1 |
| 2021 | hsa-miR-449b | 51091 | SEPSECS | Sep (O-phosphoserine) tRNA:Sec (selenocysteine) tRNA synthase | 1 |
| 2022 | hsa-miR-575 | 51091 | SEPSECS | Sep (O-phosphoserine) tRNA:Sec (selenocysteine) tRNA synthase | 1 |
| 2023 | hsa-miR-608 | 51091 | SEPSECS | Sep (O-phosphoserine) tRNA:Sec (selenocysteine) tRNA synthase | 1 |
| 2024 | hsa-miR-620 | 51091 | SEPSECS | Sep (O-phosphoserine) tRNA:Sec (selenocysteine) tRNA synthase | 1 |
| 2025 | hsa-miR-637 | 51091 | SEPSECS | Sep (O-phosphoserine) tRNA:Sec (selenocysteine) tRNA synthase | 1 |
| 2026 | hsa-mir-22 | 51091 | SEPSECS | Sep (O-phosphoserine) tRNA:Sec (selenocysteine) tRNA synthase | 1 |
| 2027 | hsa-mir-10a | 51091 | SEPSECS | Sep (O-phosphoserine) tRNA:Sec (selenocysteine) tRNA synthase | 1 |
| 2028 | hsa-mir-186 | 51091 | SEPSECS | Sep (O-phosphoserine) tRNA:Sec (selenocysteine) tRNA synthase | 1 |
| 2029 | hsa-mir-155 | 51091 | SEPSECS | Sep (O-phosphoserine) tRNA:Sec (selenocysteine) tRNA synthase | 1 |
| 2030 | hsa-mir-499 | 51091 | SEPSECS | Sep (O-phosphoserine) tRNA:Sec (selenocysteine) tRNA synthase | 1 |
| 2031 | hsa-mir-877 | 51091 | SEPSECS | Sep (O-phosphoserine) tRNA:Sec (selenocysteine) tRNA synthase | 1 |
| 2032 | hsa-mir-2116 | 51091 | SEPSECS | Sep (O-phosphoserine) tRNA:Sec (selenocysteine) tRNA synthase | 1 |
| 2033 | hsa-mir-3190 | 51091 | SEPSECS | Sep (O-phosphoserine) tRNA:Sec (selenocysteine) tRNA synthase | 1 |
| 2034 | hsa-miR-1 | 168002 | DACT2 | dapper, antagonist of beta-catenin, homolog 2 (Xenopus laevis) | 1 |
| 2035 | hsa-miR-133a | 168002 | DACT2 | dapper, antagonist of beta-catenin, homolog 2 (Xenopus laevis) | 1 |
| 2036 | hsa-miR-186 | 168002 | DACT2 | dapper, antagonist of beta-catenin, homolog 2 (Xenopus laevis) | 1 |
| 2037 | hsa-miR-208a | 168002 | DACT2 | dapper, antagonist of beta-catenin, homolog 2 (Xenopus laevis) | 1 |
| 2038 | hsa-miR-326 | 168002 | DACT2 | dapper, antagonist of beta-catenin, homolog 2 (Xenopus laevis) | 1 |
| 2039 | hsa-miR-449b | 168002 | DACT2 | dapper, antagonist of beta-catenin, homolog 2 (Xenopus laevis) | 1 |
| 2040 | hsa-miR-550 | 168002 | DACT2 | dapper, antagonist of beta-catenin, homolog 2 (Xenopus laevis) | 1 |
| 2041 | hsa-miR-575 | 168002 | DACT2 | dapper, antagonist of beta-catenin, homolog 2 (Xenopus laevis) | 1 |
| 2042 | hsa-miR-579 | 168002 | DACT2 | dapper, antagonist of beta-catenin, homolog 2 (Xenopus laevis) | 1 |
| 2043 | hsa-miR-608 | 168002 | DACT2 | dapper, antagonist of beta-catenin, homolog 2 (Xenopus laevis) | 1 |
| 2044 | hsa-miR-620 | 168002 | DACT2 | dapper, antagonist of beta-catenin, homolog 2 (Xenopus laevis) | 1 |
| 2045 | hsa-mir-22 | 168002 | DACT2 | dapper, antagonist of beta-catenin, homolog 2 (Xenopus laevis) | 1 |
| 2046 | hsa-mir-10a | 168002 | DACT2 | dapper, antagonist of beta-catenin, homolog 2 (Xenopus laevis) | 1 |
| 2047 | hsa-mir-186 | 168002 | DACT2 | dapper, antagonist of beta-catenin, homolog 2 (Xenopus laevis) | 1 |
| 2048 | hsa-mir-155 | 168002 | DACT2 | dapper, antagonist of beta-catenin, homolog 2 (Xenopus laevis) | 1 |
| 2049 | hsa-mir-499 | 168002 | DACT2 | dapper, antagonist of beta-catenin, homolog 2 (Xenopus laevis) | 1 |
| 2050 | hsa-mir-550-1 | 168002 | DACT2 | dapper, antagonist of beta-catenin, homolog 2 (Xenopus laevis) | 1 |
| 2051 | hsa-mir-877 | 168002 | DACT2 | dapper, antagonist of beta-catenin, homolog 2 (Xenopus laevis) | 1 |
| 2052 | hsa-mir-2116 | 168002 | DACT2 | dapper, antagonist of beta-catenin, homolog 2 (Xenopus laevis) | 1 |
| 2053 | hsa-mir-3186 | 168002 | DACT2 | dapper, antagonist of beta-catenin, homolog 2 (Xenopus laevis) | 1 |
| 2054 | hsa-mir-3190 | 168002 | DACT2 | dapper, antagonist of beta-catenin, homolog 2 (Xenopus laevis) | 1 |
| 2055 | hsa-miR-383 | 121268 | RHEBL1 | Ras homolog enriched in brain like 1 | 1 |
| 2056 | hsa-miR-504 | 121268 | RHEBL1 | Ras homolog enriched in brain like 1 | 1 |
| 2057 | hsa-miR-1 | 140834 | C20orf62 | chromosome 20 open reading frame 62 | 1 |
| 2058 | hsa-miR-133a | 140834 | C20orf62 | chromosome 20 open reading frame 62 | 1 |
| 2059 | hsa-miR-186 | 140834 | C20orf62 | chromosome 20 open reading frame 62 | 1 |
| 2060 | hsa-miR-208a | 140834 | C20orf62 | chromosome 20 open reading frame 62 | 1 |
| 2061 | hsa-miR-326 | 140834 | C20orf62 | chromosome 20 open reading frame 62 | 1 |
| 2062 | hsa-miR-449b | 140834 | C20orf62 | chromosome 20 open reading frame 62 | 1 |
| 2063 | hsa-miR-575 | 140834 | C20orf62 | chromosome 20 open reading frame 62 | 1 |
| 2064 | hsa-miR-579 | 140834 | C20orf62 | chromosome 20 open reading frame 62 | 1 |
| 2065 | hsa-miR-608 | 140834 | C20orf62 | chromosome 20 open reading frame 62 | 1 |
| 2066 | hsa-miR-620 | 140834 | C20orf62 | chromosome 20 open reading frame 62 | 1 |
| 2067 | hsa-miR-637 | 140834 | C20orf62 | chromosome 20 open reading frame 62 | 1 |
| 2068 | hsa-mir-22 | 140834 | C20orf62 | chromosome 20 open reading frame 62 | 1 |
| 2069 | hsa-mir-10a | 140834 | C20orf62 | chromosome 20 open reading frame 62 | 1 |
| 2070 | hsa-mir-186 | 140834 | C20orf62 | chromosome 20 open reading frame 62 | 1 |
| 2071 | hsa-mir-155 | 140834 | C20orf62 | chromosome 20 open reading frame 62 | 1 |
| 2072 | hsa-mir-499 | 140834 | C20orf62 | chromosome 20 open reading frame 62 | 1 |
| 2073 | hsa-mir-877 | 140834 | C20orf62 | chromosome 20 open reading frame 62 | 1 |
| 2074 | hsa-mir-2116 | 140834 | C20orf62 | chromosome 20 open reading frame 62 | 1 |
| 2075 | hsa-mir-3190 | 140834 | C20orf62 | chromosome 20 open reading frame 62 | 1 |
| 2076 | hsa-miR-1 | 6155 | RPL27 | ribosomal protein L27 | 1 |
| 2077 | hsa-miR-133a | 6155 | RPL27 | ribosomal protein L27 | 1 |
| 2078 | hsa-miR-186 | 6155 | RPL27 | ribosomal protein L27 | 1 |
| 2079 | hsa-miR-195 | 6155 | RPL27 | ribosomal protein L27 | 1 |
| 2080 | hsa-miR-326 | 6155 | RPL27 | ribosomal protein L27 | 1 |
| 2081 | hsa-miR-449b | 6155 | RPL27 | ribosomal protein L27 | 1 |
| 2082 | hsa-miR-497 | 6155 | RPL27 | ribosomal protein L27 | 1 |
| 2083 | hsa-miR-550 | 6155 | RPL27 | ribosomal protein L27 | 1 |
| 2084 | hsa-miR-562 | 6155 | RPL27 | ribosomal protein L27 | 1 |
| 2085 | hsa-miR-575 | 6155 | RPL27 | ribosomal protein L27 | 1 |
| 2086 | hsa-miR-579 | 6155 | RPL27 | ribosomal protein L27 | 1 |
| 2087 | hsa-miR-608 | 6155 | RPL27 | ribosomal protein L27 | 1 |
| 2088 | hsa-miR-620 | 6155 | RPL27 | ribosomal protein L27 | 1 |
| 2089 | hsa-miR-885-5p | 6155 | RPL27 | ribosomal protein L27 | 1 |
| 2090 | hsa-let-7a-3 | 6155 | RPL27 | ribosomal protein L27 | 1 |
| 2091 | hsa-let-7b | 6155 | RPL27 | ribosomal protein L27 | 1 |
| 2092 | hsa-mir-22 | 6155 | RPL27 | ribosomal protein L27 | 1 |
| 2093 | hsa-mir-10a | 6155 | RPL27 | ribosomal protein L27 | 1 |
| 2094 | hsa-mir-186 | 6155 | RPL27 | ribosomal protein L27 | 1 |
| 2095 | hsa-mir-155 | 6155 | RPL27 | ribosomal protein L27 | 1 |
| 2096 | hsa-mir-499 | 6155 | RPL27 | ribosomal protein L27 | 1 |
| 2097 | hsa-mir-550-1 | 6155 | RPL27 | ribosomal protein L27 | 1 |
| 2098 | hsa-mir-885 | 6155 | RPL27 | ribosomal protein L27 | 1 |
| 2099 | hsa-mir-877 | 6155 | RPL27 | ribosomal protein L27 | 1 |
| 2100 | hsa-mir-2116 | 6155 | RPL27 | ribosomal protein L27 | 1 |
| 2101 | hsa-miR-1 | 6205 | RPS11 | ribosomal protein S11 | 1 |
| 2102 | hsa-miR-133a | 6205 | RPS11 | ribosomal protein S11 | 1 |
| 2103 | hsa-miR-186 | 6205 | RPS11 | ribosomal protein S11 | 1 |
| 2104 | hsa-miR-195 | 6205 | RPS11 | ribosomal protein S11 | 1 |
| 2105 | hsa-miR-208a | 6205 | RPS11 | ribosomal protein S11 | 1 |
| 2106 | hsa-miR-326 | 6205 | RPS11 | ribosomal protein S11 | 1 |
| 2107 | hsa-miR-449b | 6205 | RPS11 | ribosomal protein S11 | 1 |
| 2108 | hsa-miR-497 | 6205 | RPS11 | ribosomal protein S11 | 1 |
| 2109 | hsa-miR-550 | 6205 | RPS11 | ribosomal protein S11 | 1 |
| 2110 | hsa-miR-575 | 6205 | RPS11 | ribosomal protein S11 | 1 |
| 2111 | hsa-miR-608 | 6205 | RPS11 | ribosomal protein S11 | 1 |
| 2112 | hsa-miR-620 | 6205 | RPS11 | ribosomal protein S11 | 1 |
| 2113 | hsa-let-7a-3 | 6205 | RPS11 | ribosomal protein S11 | 1 |
| 2114 | hsa-let-7b | 6205 | RPS11 | ribosomal protein S11 | 1 |
| 2115 | hsa-mir-22 | 6205 | RPS11 | ribosomal protein S11 | 1 |
| 2116 | hsa-mir-10a | 6205 | RPS11 | ribosomal protein S11 | 1 |
| 2117 | hsa-mir-186 | 6205 | RPS11 | ribosomal protein S11 | 1 |
| 2118 | hsa-mir-155 | 6205 | RPS11 | ribosomal protein S11 | 1 |
| 2119 | hsa-mir-499 | 6205 | RPS11 | ribosomal protein S11 | 1 |
| 2120 | hsa-mir-550-1 | 6205 | RPS11 | ribosomal protein S11 | 1 |
| 2121 | hsa-mir-877 | 6205 | RPS11 | ribosomal protein S11 | 1 |
| 2122 | hsa-mir-2116 | 6205 | RPS11 | ribosomal protein S11 | 1 |
| 2123 | hsa-mir-3186 | 6205 | RPS11 | ribosomal protein S11 | 1 |
| 2124 | hsa-miR-1 | 6133 | RPL9 | ribosomal protein L9 | 1 |
| 2125 | hsa-miR-133a | 6133 | RPL9 | ribosomal protein L9 | 1 |
| 2126 | hsa-miR-186 | 6133 | RPL9 | ribosomal protein L9 | 1 |
| 2127 | hsa-miR-195 | 6133 | RPL9 | ribosomal protein L9 | 1 |
| 2128 | hsa-miR-208a | 6133 | RPL9 | ribosomal protein L9 | 1 |
| 2129 | hsa-miR-326 | 6133 | RPL9 | ribosomal protein L9 | 1 |
| 2130 | hsa-miR-449b | 6133 | RPL9 | ribosomal protein L9 | 1 |
| 2131 | hsa-miR-497 | 6133 | RPL9 | ribosomal protein L9 | 1 |
| 2132 | hsa-miR-550 | 6133 | RPL9 | ribosomal protein L9 | 1 |
| 2133 | hsa-miR-575 | 6133 | RPL9 | ribosomal protein L9 | 1 |
| 2134 | hsa-miR-579 | 6133 | RPL9 | ribosomal protein L9 | 1 |
| 2135 | hsa-miR-608 | 6133 | RPL9 | ribosomal protein L9 | 1 |
| 2136 | hsa-miR-620 | 6133 | RPL9 | ribosomal protein L9 | 1 |
| 2137 | hsa-miR-885-5p | 6133 | RPL9 | ribosomal protein L9 | 1 |
| 2138 | hsa-let-7a-3 | 6133 | RPL9 | ribosomal protein L9 | 1 |
| 2139 | hsa-let-7b | 6133 | RPL9 | ribosomal protein L9 | 1 |
| 2140 | hsa-mir-22 | 6133 | RPL9 | ribosomal protein L9 | 1 |
| 2141 | hsa-mir-10a | 6133 | RPL9 | ribosomal protein L9 | 1 |
| 2142 | hsa-mir-186 | 6133 | RPL9 | ribosomal protein L9 | 1 |
| 2143 | hsa-mir-155 | 6133 | RPL9 | ribosomal protein L9 | 1 |
| 2144 | hsa-mir-499 | 6133 | RPL9 | ribosomal protein L9 | 1 |
| 2145 | hsa-mir-550-1 | 6133 | RPL9 | ribosomal protein L9 | 1 |
| 2146 | hsa-mir-885 | 6133 | RPL9 | ribosomal protein L9 | 1 |
| 2147 | hsa-mir-877 | 6133 | RPL9 | ribosomal protein L9 | 1 |
| 2148 | hsa-mir-2116 | 6133 | RPL9 | ribosomal protein L9 | 1 |
| 2149 | hsa-mir-3190 | 6133 | RPL9 | ribosomal protein L9 | 1 |
| 2182 | hsa-miR-584 | 7453 | WARS | tryptophanyl-tRNA synthetase | 1 |
| 2183 | hsa-miR-1 | 11337 | GABARAP | GABA(A) receptor-associated protein | 1 |
| 2184 | hsa-miR-133a | 11337 | GABARAP | GABA(A) receptor-associated protein | 1 |
| 2185 | hsa-miR-186 | 11337 | GABARAP | GABA(A) receptor-associated protein | 1 |
| 2186 | hsa-miR-195 | 11337 | GABARAP | GABA(A) receptor-associated protein | 1 |
| 2187 | hsa-miR-326 | 11337 | GABARAP | GABA(A) receptor-associated protein | 1 |
| 2188 | hsa-miR-33a | 11337 | GABARAP | GABA(A) receptor-associated protein | 1 |
| 2189 | hsa-miR-449b | 11337 | GABARAP | GABA(A) receptor-associated protein | 1 |
| 2190 | hsa-miR-497 | 11337 | GABARAP | GABA(A) receptor-associated protein | 1 |
| 2191 | hsa-miR-550 | 11337 | GABARAP | GABA(A) receptor-associated protein | 1 |
| 2192 | hsa-miR-551a | 11337 | GABARAP | GABA(A) receptor-associated protein | 1 |
| 2193 | hsa-miR-575 | 11337 | GABARAP | GABA(A) receptor-associated protein | 1 |
| 2194 | hsa-miR-579 | 11337 | GABARAP | GABA(A) receptor-associated protein | 1 |
| 2195 | hsa-miR-608 | 11337 | GABARAP | GABA(A) receptor-associated protein | 1 |
| 2196 | hsa-miR-620 | 11337 | GABARAP | GABA(A) receptor-associated protein | 1 |
| 2197 | hsa-mir-22 | 11337 | GABARAP | GABA(A) receptor-associated protein | 1 |
| 2198 | hsa-mir-10a | 11337 | GABARAP | GABA(A) receptor-associated protein | 1 |
| 2199 | hsa-mir-186 | 11337 | GABARAP | GABA(A) receptor-associated protein | 1 |
| 2200 | hsa-mir-155 | 11337 | GABARAP | GABA(A) receptor-associated protein | 1 |
| 2201 | hsa-mir-499 | 11337 | GABARAP | GABA(A) receptor-associated protein | 1 |
| 2202 | hsa-mir-550-1 | 11337 | GABARAP | GABA(A) receptor-associated protein | 1 |
| 2203 | hsa-mir-877 | 11337 | GABARAP | GABA(A) receptor-associated protein | 1 |
| 2204 | hsa-mir-2116 | 11337 | GABARAP | GABA(A) receptor-associated protein | 1 |
| 2205 | hsa-mir-3190 | 11337 | GABARAP | GABA(A) receptor-associated protein | 1 |
| 2206 | hsa-miR-10a | 4779 | NFE2L1 | nuclear factor (erythroid-derived 2)-like 1 | 1 |
| 2207 | hsa-miR-152 | 4779 | NFE2L1 | nuclear factor (erythroid-derived 2)-like 1 | 1 |
| 2208 | hsa-miR-31 | 4779 | NFE2L1 | nuclear factor (erythroid-derived 2)-like 1 | 1 |
| 2209 | hsa-miR-338-5p | 4779 | NFE2L1 | nuclear factor (erythroid-derived 2)-like 1 | 1 |
| 2210 | hsa-miR-340 | 4779 | NFE2L1 | nuclear factor (erythroid-derived 2)-like 1 | 1 |
| 2211 | hsa-miR-449a | 4779 | NFE2L1 | nuclear factor (erythroid-derived 2)-like 1 | 1 |
| 2212 | hsa-miR-490-5p | 4779 | NFE2L1 | nuclear factor (erythroid-derived 2)-like 1 | 1 |
| 2213 | hsa-miR-499-5p | 4779 | NFE2L1 | nuclear factor (erythroid-derived 2)-like 1 | 1 |
| 2214 | hsa-miR-584 | 4779 | NFE2L1 | nuclear factor (erythroid-derived 2)-like 1 | 1 |
| 2215 | hsa-miR-621 | 4779 | NFE2L1 | nuclear factor (erythroid-derived 2)-like 1 | 1 |
| 2216 | hsa-miR-653 | 4779 | NFE2L1 | nuclear factor (erythroid-derived 2)-like 1 | 1 |
| 2217 | hsa-miR-657 | 4779 | NFE2L1 | nuclear factor (erythroid-derived 2)-like 1 | 1 |
| 2218 | hsa-miR-765 | 4779 | NFE2L1 | nuclear factor (erythroid-derived 2)-like 1 | 1 |
| 2219 | hsa-miR-770-5p | 4779 | NFE2L1 | nuclear factor (erythroid-derived 2)-like 1 | 1 |
| 2220 | hsa-miR-877 | 4779 | NFE2L1 | nuclear factor (erythroid-derived 2)-like 1 | 1 |
| 2221 | hsa-mir-26a-1 | 4779 | NFE2L1 | nuclear factor (erythroid-derived 2)-like 1 | 1 |
| 2222 | hsa-mir-31 | 4779 | NFE2L1 | nuclear factor (erythroid-derived 2)-like 1 | 1 |
| 2223 | hsa-mir-194-2 | 4779 | NFE2L1 | nuclear factor (erythroid-derived 2)-like 1 | 1 |
| 2224 | hsa-mir-340 | 4779 | NFE2L1 | nuclear factor (erythroid-derived 2)-like 1 | 1 |
| 2225 | hsa-mir-338 | 4779 | NFE2L1 | nuclear factor (erythroid-derived 2)-like 1 | 1 |
| 2226 | hsa-mir-490 | 4779 | NFE2L1 | nuclear factor (erythroid-derived 2)-like 1 | 1 |
| 2227 | hsa-mir-449b | 4779 | NFE2L1 | nuclear factor (erythroid-derived 2)-like 1 | 1 |
| 2228 | hsa-mir-449c | 4779 | NFE2L1 | nuclear factor (erythroid-derived 2)-like 1 | 1 |
| 2229 | hsa-mir-3065 | 4779 | NFE2L1 | nuclear factor (erythroid-derived 2)-like 1 | 1 |
| 2230 | hsa-miR-10a | 4134 | MAP4 | microtubule-associated protein 4 | 1 |
| 2231 | hsa-miR-139-5p | 4134 | MAP4 | microtubule-associated protein 4 | 1 |
| 2232 | hsa-miR-152 | 4134 | MAP4 | microtubule-associated protein 4 | 1 |
| 2233 | hsa-miR-26b | 4134 | MAP4 | microtubule-associated protein 4 | 1 |
| 2234 | hsa-miR-31 | 4134 | MAP4 | microtubule-associated protein 4 | 1 |
| 2235 | hsa-miR-338-5p | 4134 | MAP4 | microtubule-associated protein 4 | 1 |
| 2236 | hsa-miR-340 | 4134 | MAP4 | microtubule-associated protein 4 | 1 |
| 2237 | hsa-miR-449a | 4134 | MAP4 | microtubule-associated protein 4 | 1 |
| 2238 | hsa-miR-499-5p | 4134 | MAP4 | microtubule-associated protein 4 | 1 |
| 2239 | hsa-miR-548b-5p | 4134 | MAP4 | microtubule-associated protein 4 | 1 |
| 2240 | hsa-miR-621 | 4134 | MAP4 | microtubule-associated protein 4 | 1 |
| 2241 | hsa-miR-657 | 4134 | MAP4 | microtubule-associated protein 4 | 1 |
| 2242 | hsa-miR-765 | 4134 | MAP4 | microtubule-associated protein 4 | 1 |
| 2243 | hsa-miR-770-5p | 4134 | MAP4 | microtubule-associated protein 4 | 1 |
| 2244 | hsa-miR-877 | 4134 | MAP4 | microtubule-associated protein 4 | 1 |
| 2245 | hsa-mir-26a-1 | 4134 | MAP4 | microtubule-associated protein 4 | 1 |
| 2246 | hsa-mir-26b | 4134 | MAP4 | microtubule-associated protein 4 | 1 |
| 2247 | hsa-mir-31 | 4134 | MAP4 | microtubule-associated protein 4 | 1 |
| 2248 | hsa-mir-194-2 | 4134 | MAP4 | microtubule-associated protein 4 | 1 |
| 2249 | hsa-mir-340 | 4134 | MAP4 | microtubule-associated protein 4 | 1 |
| 2250 | hsa-mir-338 | 4134 | MAP4 | microtubule-associated protein 4 | 1 |
| 2251 | hsa-mir-548b | 4134 | MAP4 | microtubule-associated protein 4 | 1 |
| 2252 | hsa-mir-548d-2 | 4134 | MAP4 | microtubule-associated protein 4 | 1 |
| 2253 | hsa-mir-449b | 4134 | MAP4 | microtubule-associated protein 4 | 1 |
| 2254 | hsa-mir-449c | 4134 | MAP4 | microtubule-associated protein 4 | 1 |
| 2255 | hsa-mir-3065 | 4134 | MAP4 | microtubule-associated protein 4 | 1 |
| 2258 | hsa-miR-186 | 3693 | ITGB5 | integrin, beta 5 | 1 |
| 2259 | hsa-miR-326 | 3693 | ITGB5 | integrin, beta 5 | 1 |
| 2263 | hsa-miR-579 | 3693 | ITGB5 | integrin, beta 5 | 1 |
| 2266 | hsa-miR-885-5p | 3693 | ITGB5 | integrin, beta 5 | 1 |
| 2269 | hsa-mir-186 | 3693 | ITGB5 | integrin, beta 5 | 1 |
| 2271 | hsa-mir-423 | 3693 | ITGB5 | integrin, beta 5 | 1 |
| 2273 | hsa-mir-885 | 3693 | ITGB5 | integrin, beta 5 | 1 |
| 2275 | hsa-mir-3190 | 3693 | ITGB5 | integrin, beta 5 | 1 |
| 2276 | hsa-miR-489 | 3417 | IDH1 | isocitrate dehydrogenase 1 (NADP+), soluble | 1 |
| 2277 | hsa-miR-885-5p | 3417 | IDH1 | isocitrate dehydrogenase 1 (NADP+), soluble | 1 |
| 2278 | hsa-mir-885 | 3417 | IDH1 | isocitrate dehydrogenase 1 (NADP+), soluble | 1 |
| 2279 | hsa-miR-1 | 678 | ZFP36L2 | zinc finger protein 36, C3H type-like 2 | 1 |
| 2280 | hsa-miR-133a | 678 | ZFP36L2 | zinc finger protein 36, C3H type-like 2 | 1 |
| 2281 | hsa-miR-186 | 678 | ZFP36L2 | zinc finger protein 36, C3H type-like 2 | 1 |
| 2282 | hsa-miR-326 | 678 | ZFP36L2 | zinc finger protein 36, C3H type-like 2 | 1 |
| 2283 | hsa-miR-449b | 678 | ZFP36L2 | zinc finger protein 36, C3H type-like 2 | 1 |
| 2284 | hsa-miR-489 | 678 | ZFP36L2 | zinc finger protein 36, C3H type-like 2 | 1 |
| 2285 | hsa-miR-562 | 678 | ZFP36L2 | zinc finger protein 36, C3H type-like 2 | 1 |
| 2286 | hsa-miR-575 | 678 | ZFP36L2 | zinc finger protein 36, C3H type-like 2 | 1 |
| 2287 | hsa-miR-579 | 678 | ZFP36L2 | zinc finger protein 36, C3H type-like 2 | 1 |
| 2288 | hsa-miR-608 | 678 | ZFP36L2 | zinc finger protein 36, C3H type-like 2 | 1 |
| 2289 | hsa-miR-637 | 678 | ZFP36L2 | zinc finger protein 36, C3H type-like 2 | 1 |
| 2290 | hsa-mir-22 | 678 | ZFP36L2 | zinc finger protein 36, C3H type-like 2 | 1 |
| 2291 | hsa-mir-10a | 678 | ZFP36L2 | zinc finger protein 36, C3H type-like 2 | 1 |
| 2292 | hsa-mir-186 | 678 | ZFP36L2 | zinc finger protein 36, C3H type-like 2 | 1 |
| 2293 | hsa-mir-155 | 678 | ZFP36L2 | zinc finger protein 36, C3H type-like 2 | 1 |
| 2294 | hsa-mir-499 | 678 | ZFP36L2 | zinc finger protein 36, C3H type-like 2 | 1 |
| 2295 | hsa-mir-877 | 678 | ZFP36L2 | zinc finger protein 36, C3H type-like 2 | 1 |
| 2296 | hsa-mir-3190 | 678 | ZFP36L2 | zinc finger protein 36, C3H type-like 2 | 1 |
| 2297 | hsa-miR-1 | 6173 | RPL36A | ribosomal protein L36a | 1 |
| 2298 | hsa-miR-133a | 6173 | RPL36A | ribosomal protein L36a | 1 |
| 2299 | hsa-miR-186 | 6173 | RPL36A | ribosomal protein L36a | 1 |
| 2300 | hsa-miR-195 | 6173 | RPL36A | ribosomal protein L36a | 1 |
| 2301 | hsa-miR-208a | 6173 | RPL36A | ribosomal protein L36a | 1 |
| 2302 | hsa-miR-326 | 6173 | RPL36A | ribosomal protein L36a | 1 |
| 2303 | hsa-miR-33a | 6173 | RPL36A | ribosomal protein L36a | 1 |
| 2304 | hsa-miR-449b | 6173 | RPL36A | ribosomal protein L36a | 1 |
| 2305 | hsa-miR-497 | 6173 | RPL36A | ribosomal protein L36a | 1 |
| 2306 | hsa-miR-550 | 6173 | RPL36A | ribosomal protein L36a | 1 |
| 2307 | hsa-miR-575 | 6173 | RPL36A | ribosomal protein L36a | 1 |
| 2308 | hsa-miR-608 | 6173 | RPL36A | ribosomal protein L36a | 1 |
| 2309 | hsa-miR-620 | 6173 | RPL36A | ribosomal protein L36a | 1 |
| 2310 | hsa-let-7a-3 | 6173 | RPL36A | ribosomal protein L36a | 1 |
| 2311 | hsa-let-7b | 6173 | RPL36A | ribosomal protein L36a | 1 |
| 2312 | hsa-mir-22 | 6173 | RPL36A | ribosomal protein L36a | 1 |
| 2313 | hsa-mir-10a | 6173 | RPL36A | ribosomal protein L36a | 1 |
| 2314 | hsa-mir-186 | 6173 | RPL36A | ribosomal protein L36a | 1 |
| 2315 | hsa-mir-155 | 6173 | RPL36A | ribosomal protein L36a | 1 |
| 2316 | hsa-mir-499 | 6173 | RPL36A | ribosomal protein L36a | 1 |
| 2317 | hsa-mir-550-1 | 6173 | RPL36A | ribosomal protein L36a | 1 |
| 2318 | hsa-mir-877 | 6173 | RPL36A | ribosomal protein L36a | 1 |
| 2319 | hsa-mir-2116 | 6173 | RPL36A | ribosomal protein L36a | 1 |
| 2320 | hsa-mir-3186 | 6173 | RPL36A | ribosomal protein L36a | 1 |
| 2321 | hsa-miR-1 | 10159 | ATP6AP2 | ATPase, H+ transporting, lysosomal accessory protein 2 | 1 |
| 2322 | hsa-miR-133a | 10159 | ATP6AP2 | ATPase, H+ transporting, lysosomal accessory protein 2 | 1 |
| 2323 | hsa-miR-186 | 10159 | ATP6AP2 | ATPase, H+ transporting, lysosomal accessory protein 2 | 1 |
| 2324 | hsa-miR-326 | 10159 | ATP6AP2 | ATPase, H+ transporting, lysosomal accessory protein 2 | 1 |
| 2325 | hsa-miR-449b | 10159 | ATP6AP2 | ATPase, H+ transporting, lysosomal accessory protein 2 | 1 |
| 2326 | hsa-miR-489 | 10159 | ATP6AP2 | ATPase, H+ transporting, lysosomal accessory protein 2 | 1 |
| 2327 | hsa-miR-575 | 10159 | ATP6AP2 | ATPase, H+ transporting, lysosomal accessory protein 2 | 1 |
| 2328 | hsa-miR-579 | 10159 | ATP6AP2 | ATPase, H+ transporting, lysosomal accessory protein 2 | 1 |
| 2329 | hsa-miR-608 | 10159 | ATP6AP2 | ATPase, H+ transporting, lysosomal accessory protein 2 | 1 |
| 2330 | hsa-miR-637 | 10159 | ATP6AP2 | ATPase, H+ transporting, lysosomal accessory protein 2 | 1 |
| 2331 | hsa-mir-22 | 10159 | ATP6AP2 | ATPase, H+ transporting, lysosomal accessory protein 2 | 1 |
| 2332 | hsa-mir-10a | 10159 | ATP6AP2 | ATPase, H+ transporting, lysosomal accessory protein 2 | 1 |
| 2333 | hsa-mir-186 | 10159 | ATP6AP2 | ATPase, H+ transporting, lysosomal accessory protein 2 | 1 |
| 2334 | hsa-mir-155 | 10159 | ATP6AP2 | ATPase, H+ transporting, lysosomal accessory protein 2 | 1 |
| 2335 | hsa-mir-499 | 10159 | ATP6AP2 | ATPase, H+ transporting, lysosomal accessory protein 2 | 1 |
| 2336 | hsa-mir-877 | 10159 | ATP6AP2 | ATPase, H+ transporting, lysosomal accessory protein 2 | 1 |
| 2337 | hsa-mir-3190 | 10159 | ATP6AP2 | ATPase, H+ transporting, lysosomal accessory protein 2 | 1 |
| 2338 | hsa-miR-10a | 3725 | JUN | jun oncogene | 1 |
| 2339 | hsa-miR-139-5p | 3725 | JUN | jun oncogene | 1 |
| 2340 | hsa-miR-152 | 3725 | JUN | jun oncogene | 1 |
| 2341 | hsa-miR-26a | 3725 | JUN | jun oncogene | 1 |
| 2342 | hsa-miR-26b | 3725 | JUN | jun oncogene | 1 |
| 2343 | hsa-miR-31 | 3725 | JUN | jun oncogene | 1 |
| 2344 | hsa-miR-338-5p | 3725 | JUN | jun oncogene | 1 |
| 2345 | hsa-miR-340 | 3725 | JUN | jun oncogene | 1 |
| 2346 | hsa-miR-383 | 3725 | JUN | jun oncogene | 1 |
| 2347 | hsa-miR-423-5p | 3725 | JUN | jun oncogene | 1 |
| 2348 | hsa-miR-449a | 3725 | JUN | jun oncogene | 1 |
| 2349 | hsa-miR-490-5p | 3725 | JUN | jun oncogene | 1 |
| 2350 | hsa-miR-499-5p | 3725 | JUN | jun oncogene | 1 |
| 2351 | hsa-miR-548b-5p | 3725 | JUN | jun oncogene | 1 |
| 2352 | hsa-miR-564 | 3725 | JUN | jun oncogene | 1 |
| 2353 | hsa-miR-621 | 3725 | JUN | jun oncogene | 1 |
| 2354 | hsa-miR-657 | 3725 | JUN | jun oncogene | 1 |
| 2355 | hsa-miR-663 | 3725 | JUN | jun oncogene | 1 |
| 2356 | hsa-miR-765 | 3725 | JUN | jun oncogene | 1 |
| 2357 | hsa-miR-770-5p | 3725 | JUN | jun oncogene | 1 |
| 2358 | hsa-miR-877 | 3725 | JUN | jun oncogene | 1 |
| 2359 | hsa-mir-26a-1 | 3725 | JUN | jun oncogene | 1 |
| 2360 | hsa-mir-26b | 3725 | JUN | jun oncogene | 1 |
| 2361 | hsa-mir-31 | 3725 | JUN | jun oncogene | 1 |
| 2362 | hsa-mir-194-2 | 3725 | JUN | jun oncogene | 1 |
| 2363 | hsa-mir-340 | 3725 | JUN | jun oncogene | 1 |
| 2364 | hsa-mir-338 | 3725 | JUN | jun oncogene | 1 |
| 2365 | hsa-mir-490 | 3725 | JUN | jun oncogene | 1 |
| 2366 | hsa-mir-548b | 3725 | JUN | jun oncogene | 1 |
| 2367 | hsa-mir-449b | 3725 | JUN | jun oncogene | 1 |
| 2368 | hsa-mir-449c | 3725 | JUN | jun oncogene | 1 |
| 2369 | hsa-mir-3065 | 3725 | JUN | jun oncogene | 1 |
| 2370 | hsa-miR-10a | 4792 | NFKBIA | nuclear factor of kappa light polypeptide gene enhancer in B-cells inhibitor, alpha | 1 |
| 2371 | hsa-miR-139-5p | 4792 | NFKBIA | nuclear factor of kappa light polypeptide gene enhancer in B-cells inhibitor, alpha | 1 |
| 2372 | hsa-miR-152 | 4792 | NFKBIA | nuclear factor of kappa light polypeptide gene enhancer in B-cells inhibitor, alpha | 1 |
| 2373 | hsa-miR-26a | 4792 | NFKBIA | nuclear factor of kappa light polypeptide gene enhancer in B-cells inhibitor, alpha | 1 |
| 2374 | hsa-miR-26b | 4792 | NFKBIA | nuclear factor of kappa light polypeptide gene enhancer in B-cells inhibitor, alpha | 1 |
| 2375 | hsa-miR-31 | 4792 | NFKBIA | nuclear factor of kappa light polypeptide gene enhancer in B-cells inhibitor, alpha | 1 |
| 2376 | hsa-miR-338-5p | 4792 | NFKBIA | nuclear factor of kappa light polypeptide gene enhancer in B-cells inhibitor, alpha | 1 |
| 2377 | hsa-miR-340 | 4792 | NFKBIA | nuclear factor of kappa light polypeptide gene enhancer in B-cells inhibitor, alpha | 1 |
| 2378 | hsa-miR-449a | 4792 | NFKBIA | nuclear factor of kappa light polypeptide gene enhancer in B-cells inhibitor, alpha | 1 |
| 2379 | hsa-miR-490-5p | 4792 | NFKBIA | nuclear factor of kappa light polypeptide gene enhancer in B-cells inhibitor, alpha | 1 |
| 2380 | hsa-miR-499-5p | 4792 | NFKBIA | nuclear factor of kappa light polypeptide gene enhancer in B-cells inhibitor, alpha | 1 |
| 2381 | hsa-miR-548b-5p | 4792 | NFKBIA | nuclear factor of kappa light polypeptide gene enhancer in B-cells inhibitor, alpha | 1 |
| 2382 | hsa-miR-621 | 4792 | NFKBIA | nuclear factor of kappa light polypeptide gene enhancer in B-cells inhibitor, alpha | 1 |
| 2383 | hsa-miR-657 | 4792 | NFKBIA | nuclear factor of kappa light polypeptide gene enhancer in B-cells inhibitor, alpha | 1 |
| 2384 | hsa-miR-765 | 4792 | NFKBIA | nuclear factor of kappa light polypeptide gene enhancer in B-cells inhibitor, alpha | 1 |
| 2385 | hsa-miR-770-5p | 4792 | NFKBIA | nuclear factor of kappa light polypeptide gene enhancer in B-cells inhibitor, alpha | 1 |
| 2386 | hsa-miR-877 | 4792 | NFKBIA | nuclear factor of kappa light polypeptide gene enhancer in B-cells inhibitor, alpha | 1 |
| 2387 | hsa-mir-26a-1 | 4792 | NFKBIA | nuclear factor of kappa light polypeptide gene enhancer in B-cells inhibitor, alpha | 1 |
| 2388 | hsa-mir-26b | 4792 | NFKBIA | nuclear factor of kappa light polypeptide gene enhancer in B-cells inhibitor, alpha | 1 |
| 2389 | hsa-mir-31 | 4792 | NFKBIA | nuclear factor of kappa light polypeptide gene enhancer in B-cells inhibitor, alpha | 1 |
| 2390 | hsa-mir-194-2 | 4792 | NFKBIA | nuclear factor of kappa light polypeptide gene enhancer in B-cells inhibitor, alpha | 1 |
| 2391 | hsa-mir-340 | 4792 | NFKBIA | nuclear factor of kappa light polypeptide gene enhancer in B-cells inhibitor, alpha | 1 |
| 2392 | hsa-mir-338 | 4792 | NFKBIA | nuclear factor of kappa light polypeptide gene enhancer in B-cells inhibitor, alpha | 1 |
| 2393 | hsa-mir-490 | 4792 | NFKBIA | nuclear factor of kappa light polypeptide gene enhancer in B-cells inhibitor, alpha | 1 |
| 2394 | hsa-mir-548b | 4792 | NFKBIA | nuclear factor of kappa light polypeptide gene enhancer in B-cells inhibitor, alpha | 1 |
| 2395 | hsa-mir-449b | 4792 | NFKBIA | nuclear factor of kappa light polypeptide gene enhancer in B-cells inhibitor, alpha | 1 |
| 2396 | hsa-mir-449c | 4792 | NFKBIA | nuclear factor of kappa light polypeptide gene enhancer in B-cells inhibitor, alpha | 1 |
| 2397 | hsa-mir-3065 | 4792 | NFKBIA | nuclear factor of kappa light polypeptide gene enhancer in B-cells inhibitor, alpha | 1 |
| 2409 | hsa-miR-584 | 8519 | IFITM1 | interferon induced transmembrane protein 1 (9-27) | 1 |
| 2426 | hsa-miR-10a | 9246 | UBE2L6 | ubiquitin-conjugating enzyme E2L 6 | 1 |
| 2427 | hsa-miR-139-5p | 9246 | UBE2L6 | ubiquitin-conjugating enzyme E2L 6 | 1 |
| 2428 | hsa-miR-26a | 9246 | UBE2L6 | ubiquitin-conjugating enzyme E2L 6 | 1 |
| 2429 | hsa-miR-26b | 9246 | UBE2L6 | ubiquitin-conjugating enzyme E2L 6 | 1 |
| 2430 | hsa-miR-338-5p | 9246 | UBE2L6 | ubiquitin-conjugating enzyme E2L 6 | 1 |
| 2431 | hsa-miR-340 | 9246 | UBE2L6 | ubiquitin-conjugating enzyme E2L 6 | 1 |
| 2432 | hsa-miR-449a | 9246 | UBE2L6 | ubiquitin-conjugating enzyme E2L 6 | 1 |
| 2433 | hsa-miR-499-5p | 9246 | UBE2L6 | ubiquitin-conjugating enzyme E2L 6 | 1 |
| 2434 | hsa-miR-548b-5p | 9246 | UBE2L6 | ubiquitin-conjugating enzyme E2L 6 | 1 |
| 2435 | hsa-miR-564 | 9246 | UBE2L6 | ubiquitin-conjugating enzyme E2L 6 | 1 |
| 2436 | hsa-miR-621 | 9246 | UBE2L6 | ubiquitin-conjugating enzyme E2L 6 | 1 |
| 2437 | hsa-miR-657 | 9246 | UBE2L6 | ubiquitin-conjugating enzyme E2L 6 | 1 |
| 2438 | hsa-miR-765 | 9246 | UBE2L6 | ubiquitin-conjugating enzyme E2L 6 | 1 |
| 2439 | hsa-miR-770-5p | 9246 | UBE2L6 | ubiquitin-conjugating enzyme E2L 6 | 1 |
| 2440 | hsa-miR-877 | 9246 | UBE2L6 | ubiquitin-conjugating enzyme E2L 6 | 1 |
| 2441 | hsa-mir-26a-1 | 9246 | UBE2L6 | ubiquitin-conjugating enzyme E2L 6 | 1 |
| 2442 | hsa-mir-26b | 9246 | UBE2L6 | ubiquitin-conjugating enzyme E2L 6 | 1 |
| 2443 | hsa-mir-194-2 | 9246 | UBE2L6 | ubiquitin-conjugating enzyme E2L 6 | 1 |
| 2444 | hsa-mir-340 | 9246 | UBE2L6 | ubiquitin-conjugating enzyme E2L 6 | 1 |
| 2445 | hsa-mir-338 | 9246 | UBE2L6 | ubiquitin-conjugating enzyme E2L 6 | 1 |
| 2446 | hsa-mir-548b | 9246 | UBE2L6 | ubiquitin-conjugating enzyme E2L 6 | 1 |
| 2447 | hsa-mir-548d-2 | 9246 | UBE2L6 | ubiquitin-conjugating enzyme E2L 6 | 1 |
| 2448 | hsa-mir-449b | 9246 | UBE2L6 | ubiquitin-conjugating enzyme E2L 6 | 1 |
| 2449 | hsa-mir-449c | 9246 | UBE2L6 | ubiquitin-conjugating enzyme E2L 6 | 1 |
| 2450 | hsa-mir-3065 | 9246 | UBE2L6 | ubiquitin-conjugating enzyme E2L 6 | 1 |
| 2451 | hsa-miR-10a | 5721 | PSME2 | proteasome (prosome, macropain) activator subunit 2 (PA28 beta) | 1 |
| 2452 | hsa-miR-139-5p | 5721 | PSME2 | proteasome (prosome, macropain) activator subunit 2 (PA28 beta) | 1 |
| 2453 | hsa-miR-152 | 5721 | PSME2 | proteasome (prosome, macropain) activator subunit 2 (PA28 beta) | 1 |
| 2454 | hsa-miR-26a | 5721 | PSME2 | proteasome (prosome, macropain) activator subunit 2 (PA28 beta) | 1 |
| 2455 | hsa-miR-26b | 5721 | PSME2 | proteasome (prosome, macropain) activator subunit 2 (PA28 beta) | 1 |
| 2456 | hsa-miR-31 | 5721 | PSME2 | proteasome (prosome, macropain) activator subunit 2 (PA28 beta) | 1 |
| 2457 | hsa-miR-338-5p | 5721 | PSME2 | proteasome (prosome, macropain) activator subunit 2 (PA28 beta) | 1 |
| 2458 | hsa-miR-340 | 5721 | PSME2 | proteasome (prosome, macropain) activator subunit 2 (PA28 beta) | 1 |
| 2459 | hsa-miR-449a | 5721 | PSME2 | proteasome (prosome, macropain) activator subunit 2 (PA28 beta) | 1 |
| 2460 | hsa-miR-499-5p | 5721 | PSME2 | proteasome (prosome, macropain) activator subunit 2 (PA28 beta) | 1 |
| 2461 | hsa-miR-548b-5p | 5721 | PSME2 | proteasome (prosome, macropain) activator subunit 2 (PA28 beta) | 1 |
| 2462 | hsa-miR-621 | 5721 | PSME2 | proteasome (prosome, macropain) activator subunit 2 (PA28 beta) | 1 |
| 2463 | hsa-miR-657 | 5721 | PSME2 | proteasome (prosome, macropain) activator subunit 2 (PA28 beta) | 1 |
| 2464 | hsa-miR-663 | 5721 | PSME2 | proteasome (prosome, macropain) activator subunit 2 (PA28 beta) | 1 |
| 2465 | hsa-miR-765 | 5721 | PSME2 | proteasome (prosome, macropain) activator subunit 2 (PA28 beta) | 1 |
| 2466 | hsa-miR-770-5p | 5721 | PSME2 | proteasome (prosome, macropain) activator subunit 2 (PA28 beta) | 1 |
| 2467 | hsa-miR-877 | 5721 | PSME2 | proteasome (prosome, macropain) activator subunit 2 (PA28 beta) | 1 |
| 2468 | hsa-mir-26a-1 | 5721 | PSME2 | proteasome (prosome, macropain) activator subunit 2 (PA28 beta) | 1 |
| 2469 | hsa-mir-26b | 5721 | PSME2 | proteasome (prosome, macropain) activator subunit 2 (PA28 beta) | 1 |
| 2470 | hsa-mir-31 | 5721 | PSME2 | proteasome (prosome, macropain) activator subunit 2 (PA28 beta) | 1 |
| 2471 | hsa-mir-194-2 | 5721 | PSME2 | proteasome (prosome, macropain) activator subunit 2 (PA28 beta) | 1 |
| 2472 | hsa-mir-340 | 5721 | PSME2 | proteasome (prosome, macropain) activator subunit 2 (PA28 beta) | 1 |
| 2473 | hsa-mir-338 | 5721 | PSME2 | proteasome (prosome, macropain) activator subunit 2 (PA28 beta) | 1 |
| 2474 | hsa-mir-548b | 5721 | PSME2 | proteasome (prosome, macropain) activator subunit 2 (PA28 beta) | 1 |
| 2475 | hsa-mir-548d-2 | 5721 | PSME2 | proteasome (prosome, macropain) activator subunit 2 (PA28 beta) | 1 |
| 2476 | hsa-mir-449b | 5721 | PSME2 | proteasome (prosome, macropain) activator subunit 2 (PA28 beta) | 1 |
| 2477 | hsa-mir-449c | 5721 | PSME2 | proteasome (prosome, macropain) activator subunit 2 (PA28 beta) | 1 |
| 2478 | hsa-mir-3065 | 5721 | PSME2 | proteasome (prosome, macropain) activator subunit 2 (PA28 beta) | 1 |
| 2479 | hsa-miR-10a | 1616 | DAXX | death-domain associated protein | 1 |
| 2480 | hsa-miR-139-5p | 1616 | DAXX | death-domain associated protein | 1 |
| 2481 | hsa-miR-152 | 1616 | DAXX | death-domain associated protein | 1 |
| 2482 | hsa-miR-26a | 1616 | DAXX | death-domain associated protein | 1 |
| 2483 | hsa-miR-26b | 1616 | DAXX | death-domain associated protein | 1 |
| 2484 | hsa-miR-31 | 1616 | DAXX | death-domain associated protein | 1 |
| 2485 | hsa-miR-338-5p | 1616 | DAXX | death-domain associated protein | 1 |
| 2486 | hsa-miR-340 | 1616 | DAXX | death-domain associated protein | 1 |
| 2487 | hsa-miR-449a | 1616 | DAXX | death-domain associated protein | 1 |
| 2488 | hsa-miR-490-5p | 1616 | DAXX | death-domain associated protein | 1 |
| 2489 | hsa-miR-499-5p | 1616 | DAXX | death-domain associated protein | 1 |
| 2490 | hsa-miR-548b-5p | 1616 | DAXX | death-domain associated protein | 1 |
| 2491 | hsa-miR-564 | 1616 | DAXX | death-domain associated protein | 1 |
| 2492 | hsa-miR-584 | 1616 | DAXX | death-domain associated protein | 1 |
| 2493 | hsa-miR-621 | 1616 | DAXX | death-domain associated protein | 1 |
| 2494 | hsa-miR-634 | 1616 | DAXX | death-domain associated protein | 1 |
| 2495 | hsa-miR-657 | 1616 | DAXX | death-domain associated protein | 1 |
| 2496 | hsa-miR-765 | 1616 | DAXX | death-domain associated protein | 1 |
| 2497 | hsa-miR-770-5p | 1616 | DAXX | death-domain associated protein | 1 |
| 2498 | hsa-miR-877 | 1616 | DAXX | death-domain associated protein | 1 |
| 2499 | hsa-mir-26a-1 | 1616 | DAXX | death-domain associated protein | 1 |
| 2500 | hsa-mir-26b | 1616 | DAXX | death-domain associated protein | 1 |
| 2501 | hsa-mir-31 | 1616 | DAXX | death-domain associated protein | 1 |
| 2502 | hsa-mir-194-2 | 1616 | DAXX | death-domain associated protein | 1 |
| 2503 | hsa-mir-340 | 1616 | DAXX | death-domain associated protein | 1 |
| 2504 | hsa-mir-338 | 1616 | DAXX | death-domain associated protein | 1 |
| 2505 | hsa-mir-490 | 1616 | DAXX | death-domain associated protein | 1 |
| 2506 | hsa-mir-548b | 1616 | DAXX | death-domain associated protein | 1 |
| 2507 | hsa-mir-548d-2 | 1616 | DAXX | death-domain associated protein | 1 |
| 2508 | hsa-mir-449b | 1616 | DAXX | death-domain associated protein | 1 |
| 2509 | hsa-mir-449c | 1616 | DAXX | death-domain associated protein | 1 |
| 2510 | hsa-mir-3065 | 1616 | DAXX | death-domain associated protein | 1 |
| 2511 | hsa-miR-10a | 5970 | RELA | v-rel reticuloendotheliosis viral oncogene homolog A (avian) | 1 |
| 2512 | hsa-miR-152 | 5970 | RELA | v-rel reticuloendotheliosis viral oncogene homolog A (avian) | 1 |
| 2513 | hsa-miR-26a | 5970 | RELA | v-rel reticuloendotheliosis viral oncogene homolog A (avian) | 1 |
| 2514 | hsa-miR-26b | 5970 | RELA | v-rel reticuloendotheliosis viral oncogene homolog A (avian) | 1 |
| 2515 | hsa-miR-31 | 5970 | RELA | v-rel reticuloendotheliosis viral oncogene homolog A (avian) | 1 |
| 2516 | hsa-miR-340 | 5970 | RELA | v-rel reticuloendotheliosis viral oncogene homolog A (avian) | 1 |
| 2517 | hsa-miR-449a | 5970 | RELA | v-rel reticuloendotheliosis viral oncogene homolog A (avian) | 1 |
| 2518 | hsa-miR-490-5p | 5970 | RELA | v-rel reticuloendotheliosis viral oncogene homolog A (avian) | 1 |
| 2519 | hsa-miR-499-5p | 5970 | RELA | v-rel reticuloendotheliosis viral oncogene homolog A (avian) | 1 |
| 2520 | hsa-miR-504 | 5970 | RELA | v-rel reticuloendotheliosis viral oncogene homolog A (avian) | 1 |
| 2521 | hsa-miR-564 | 5970 | RELA | v-rel reticuloendotheliosis viral oncogene homolog A (avian) | 1 |
| 2522 | hsa-miR-621 | 5970 | RELA | v-rel reticuloendotheliosis viral oncogene homolog A (avian) | 1 |
| 2523 | hsa-miR-634 | 5970 | RELA | v-rel reticuloendotheliosis viral oncogene homolog A (avian) | 1 |
| 2524 | hsa-miR-653 | 5970 | RELA | v-rel reticuloendotheliosis viral oncogene homolog A (avian) | 1 |
| 2525 | hsa-miR-765 | 5970 | RELA | v-rel reticuloendotheliosis viral oncogene homolog A (avian) | 1 |
| 2526 | hsa-miR-770-5p | 5970 | RELA | v-rel reticuloendotheliosis viral oncogene homolog A (avian) | 1 |
| 2527 | hsa-miR-877 | 5970 | RELA | v-rel reticuloendotheliosis viral oncogene homolog A (avian) | 1 |
| 2528 | hsa-miR-95 | 5970 | RELA | v-rel reticuloendotheliosis viral oncogene homolog A (avian) | 1 |
| 2529 | hsa-mir-26a-1 | 5970 | RELA | v-rel reticuloendotheliosis viral oncogene homolog A (avian) | 1 |
| 2530 | hsa-mir-26b | 5970 | RELA | v-rel reticuloendotheliosis viral oncogene homolog A (avian) | 1 |
| 2531 | hsa-mir-31 | 5970 | RELA | v-rel reticuloendotheliosis viral oncogene homolog A (avian) | 1 |
| 2532 | hsa-mir-194-2 | 5970 | RELA | v-rel reticuloendotheliosis viral oncogene homolog A (avian) | 1 |
| 2533 | hsa-mir-340 | 5970 | RELA | v-rel reticuloendotheliosis viral oncogene homolog A (avian) | 1 |
| 2534 | hsa-mir-490 | 5970 | RELA | v-rel reticuloendotheliosis viral oncogene homolog A (avian) | 1 |
| 2535 | hsa-mir-449b | 5970 | RELA | v-rel reticuloendotheliosis viral oncogene homolog A (avian) | 1 |
| 2536 | hsa-mir-449c | 5970 | RELA | v-rel reticuloendotheliosis viral oncogene homolog A (avian) | 1 |
| 2537 | hsa-miR-10a | 103 | ADAR | adenosine deaminase, RNA-specific | 1 |
| 2538 | hsa-miR-139-5p | 103 | ADAR | adenosine deaminase, RNA-specific | 1 |
| 2539 | hsa-miR-152 | 103 | ADAR | adenosine deaminase, RNA-specific | 1 |
| 2540 | hsa-miR-26a | 103 | ADAR | adenosine deaminase, RNA-specific | 1 |
| 2541 | hsa-miR-26b | 103 | ADAR | adenosine deaminase, RNA-specific | 1 |
| 2542 | hsa-miR-31 | 103 | ADAR | adenosine deaminase, RNA-specific | 1 |
| 2543 | hsa-miR-338-5p | 103 | ADAR | adenosine deaminase, RNA-specific | 1 |
| 2544 | hsa-miR-340 | 103 | ADAR | adenosine deaminase, RNA-specific | 1 |
| 2545 | hsa-miR-449a | 103 | ADAR | adenosine deaminase, RNA-specific | 1 |
| 2546 | hsa-miR-499-5p | 103 | ADAR | adenosine deaminase, RNA-specific | 1 |
| 2547 | hsa-miR-548b-5p | 103 | ADAR | adenosine deaminase, RNA-specific | 1 |
| 2548 | hsa-miR-564 | 103 | ADAR | adenosine deaminase, RNA-specific | 1 |
| 2549 | hsa-miR-621 | 103 | ADAR | adenosine deaminase, RNA-specific | 1 |
| 2550 | hsa-miR-657 | 103 | ADAR | adenosine deaminase, RNA-specific | 1 |
| 2551 | hsa-miR-765 | 103 | ADAR | adenosine deaminase, RNA-specific | 1 |
| 2552 | hsa-miR-770-5p | 103 | ADAR | adenosine deaminase, RNA-specific | 1 |
| 2553 | hsa-miR-877 | 103 | ADAR | adenosine deaminase, RNA-specific | 1 |
| 2554 | hsa-mir-26a-1 | 103 | ADAR | adenosine deaminase, RNA-specific | 1 |
| 2555 | hsa-mir-26b | 103 | ADAR | adenosine deaminase, RNA-specific | 1 |
| 2556 | hsa-mir-31 | 103 | ADAR | adenosine deaminase, RNA-specific | 1 |
| 2557 | hsa-mir-194-2 | 103 | ADAR | adenosine deaminase, RNA-specific | 1 |
| 2558 | hsa-mir-340 | 103 | ADAR | adenosine deaminase, RNA-specific | 1 |
| 2559 | hsa-mir-338 | 103 | ADAR | adenosine deaminase, RNA-specific | 1 |
| 2560 | hsa-mir-548b | 103 | ADAR | adenosine deaminase, RNA-specific | 1 |
| 2561 | hsa-mir-548d-2 | 103 | ADAR | adenosine deaminase, RNA-specific | 1 |
| 2562 | hsa-mir-449b | 103 | ADAR | adenosine deaminase, RNA-specific | 1 |
| 2563 | hsa-mir-449c | 103 | ADAR | adenosine deaminase, RNA-specific | 1 |
| 2564 | hsa-mir-3065 | 103 | ADAR | adenosine deaminase, RNA-specific | 1 |
| 2565 | hsa-miR-10a | 9887 | SMG7 | Smg-7 homolog, nonsense mediated mRNA decay factor (C. elegans) | 1 |
| 2566 | hsa-miR-152 | 9887 | SMG7 | Smg-7 homolog, nonsense mediated mRNA decay factor (C. elegans) | 1 |
| 2567 | hsa-miR-26a | 9887 | SMG7 | Smg-7 homolog, nonsense mediated mRNA decay factor (C. elegans) | 1 |
| 2568 | hsa-miR-26b | 9887 | SMG7 | Smg-7 homolog, nonsense mediated mRNA decay factor (C. elegans) | 1 |
| 2569 | hsa-miR-31 | 9887 | SMG7 | Smg-7 homolog, nonsense mediated mRNA decay factor (C. elegans) | 1 |
| 2570 | hsa-miR-338-5p | 9887 | SMG7 | Smg-7 homolog, nonsense mediated mRNA decay factor (C. elegans) | 1 |
| 2571 | hsa-miR-340 | 9887 | SMG7 | Smg-7 homolog, nonsense mediated mRNA decay factor (C. elegans) | 1 |
| 2572 | hsa-miR-449a | 9887 | SMG7 | Smg-7 homolog, nonsense mediated mRNA decay factor (C. elegans) | 1 |
| 2573 | hsa-miR-499-5p | 9887 | SMG7 | Smg-7 homolog, nonsense mediated mRNA decay factor (C. elegans) | 1 |
| 2574 | hsa-miR-621 | 9887 | SMG7 | Smg-7 homolog, nonsense mediated mRNA decay factor (C. elegans) | 1 |
| 2575 | hsa-miR-634 | 9887 | SMG7 | Smg-7 homolog, nonsense mediated mRNA decay factor (C. elegans) | 1 |
| 2576 | hsa-miR-653 | 9887 | SMG7 | Smg-7 homolog, nonsense mediated mRNA decay factor (C. elegans) | 1 |
| 2577 | hsa-miR-657 | 9887 | SMG7 | Smg-7 homolog, nonsense mediated mRNA decay factor (C. elegans) | 1 |
| 2578 | hsa-miR-765 | 9887 | SMG7 | Smg-7 homolog, nonsense mediated mRNA decay factor (C. elegans) | 1 |
| 2579 | hsa-miR-770-5p | 9887 | SMG7 | Smg-7 homolog, nonsense mediated mRNA decay factor (C. elegans) | 1 |
| 2580 | hsa-miR-877 | 9887 | SMG7 | Smg-7 homolog, nonsense mediated mRNA decay factor (C. elegans) | 1 |
| 2581 | hsa-mir-26a-1 | 9887 | SMG7 | Smg-7 homolog, nonsense mediated mRNA decay factor (C. elegans) | 1 |
| 2582 | hsa-mir-26b | 9887 | SMG7 | Smg-7 homolog, nonsense mediated mRNA decay factor (C. elegans) | 1 |
| 2583 | hsa-mir-31 | 9887 | SMG7 | Smg-7 homolog, nonsense mediated mRNA decay factor (C. elegans) | 1 |
| 2584 | hsa-mir-194-2 | 9887 | SMG7 | Smg-7 homolog, nonsense mediated mRNA decay factor (C. elegans) | 1 |
| 2585 | hsa-mir-340 | 9887 | SMG7 | Smg-7 homolog, nonsense mediated mRNA decay factor (C. elegans) | 1 |
| 2586 | hsa-mir-338 | 9887 | SMG7 | Smg-7 homolog, nonsense mediated mRNA decay factor (C. elegans) | 1 |
| 2587 | hsa-mir-449b | 9887 | SMG7 | Smg-7 homolog, nonsense mediated mRNA decay factor (C. elegans) | 1 |
| 2588 | hsa-mir-449c | 9887 | SMG7 | Smg-7 homolog, nonsense mediated mRNA decay factor (C. elegans) | 1 |
| 2589 | hsa-mir-3065 | 9887 | SMG7 | Smg-7 homolog, nonsense mediated mRNA decay factor (C. elegans) | 1 |
| 2590 | hsa-miR-1 | 9779 | TBC1D5 | TBC1 domain family, member 5 | 1 |
| 2591 | hsa-miR-133a | 9779 | TBC1D5 | TBC1 domain family, member 5 | 1 |
| 2592 | hsa-miR-186 | 9779 | TBC1D5 | TBC1 domain family, member 5 | 1 |
| 2593 | hsa-miR-326 | 9779 | TBC1D5 | TBC1 domain family, member 5 | 1 |
| 2594 | hsa-miR-449b | 9779 | TBC1D5 | TBC1 domain family, member 5 | 1 |
| 2595 | hsa-miR-575 | 9779 | TBC1D5 | TBC1 domain family, member 5 | 1 |
| 2596 | hsa-miR-579 | 9779 | TBC1D5 | TBC1 domain family, member 5 | 1 |
| 2597 | hsa-miR-608 | 9779 | TBC1D5 | TBC1 domain family, member 5 | 1 |
| 2598 | hsa-miR-620 | 9779 | TBC1D5 | TBC1 domain family, member 5 | 1 |
| 2599 | hsa-miR-637 | 9779 | TBC1D5 | TBC1 domain family, member 5 | 1 |
| 2600 | hsa-let-7a-3 | 9779 | TBC1D5 | TBC1 domain family, member 5 | 1 |
| 2601 | hsa-let-7b | 9779 | TBC1D5 | TBC1 domain family, member 5 | 1 |
| 2602 | hsa-mir-22 | 9779 | TBC1D5 | TBC1 domain family, member 5 | 1 |
| 2603 | hsa-mir-10a | 9779 | TBC1D5 | TBC1 domain family, member 5 | 1 |
| 2604 | hsa-mir-186 | 9779 | TBC1D5 | TBC1 domain family, member 5 | 1 |
| 2605 | hsa-mir-155 | 9779 | TBC1D5 | TBC1 domain family, member 5 | 1 |
| 2606 | hsa-mir-499 | 9779 | TBC1D5 | TBC1 domain family, member 5 | 1 |
| 2607 | hsa-mir-877 | 9779 | TBC1D5 | TBC1 domain family, member 5 | 1 |
| 2608 | hsa-mir-2116 | 9779 | TBC1D5 | TBC1 domain family, member 5 | 1 |
| 2609 | hsa-miR-1 | 5069 | PAPPA | pregnancy-associated plasma protein A, pappalysin 1 | 1 |
| 2610 | hsa-miR-133a | 5069 | PAPPA | pregnancy-associated plasma protein A, pappalysin 1 | 1 |
| 2611 | hsa-miR-186 | 5069 | PAPPA | pregnancy-associated plasma protein A, pappalysin 1 | 1 |
| 2612 | hsa-miR-326 | 5069 | PAPPA | pregnancy-associated plasma protein A, pappalysin 1 | 1 |
| 2613 | hsa-miR-33a | 5069 | PAPPA | pregnancy-associated plasma protein A, pappalysin 1 | 1 |
| 2614 | hsa-miR-449b | 5069 | PAPPA | pregnancy-associated plasma protein A, pappalysin 1 | 1 |
| 2615 | hsa-miR-550 | 5069 | PAPPA | pregnancy-associated plasma protein A, pappalysin 1 | 1 |
| 2616 | hsa-miR-551a | 5069 | PAPPA | pregnancy-associated plasma protein A, pappalysin 1 | 1 |
| 2617 | hsa-miR-575 | 5069 | PAPPA | pregnancy-associated plasma protein A, pappalysin 1 | 1 |
| 2618 | hsa-miR-579 | 5069 | PAPPA | pregnancy-associated plasma protein A, pappalysin 1 | 1 |
| 2619 | hsa-miR-608 | 5069 | PAPPA | pregnancy-associated plasma protein A, pappalysin 1 | 1 |
| 2620 | hsa-miR-620 | 5069 | PAPPA | pregnancy-associated plasma protein A, pappalysin 1 | 1 |
| 2621 | hsa-let-7a-3 | 5069 | PAPPA | pregnancy-associated plasma protein A, pappalysin 1 | 1 |
| 2622 | hsa-let-7b | 5069 | PAPPA | pregnancy-associated plasma protein A, pappalysin 1 | 1 |
| 2623 | hsa-mir-22 | 5069 | PAPPA | pregnancy-associated plasma protein A, pappalysin 1 | 1 |
| 2624 | hsa-mir-10a | 5069 | PAPPA | pregnancy-associated plasma protein A, pappalysin 1 | 1 |
| 2625 | hsa-mir-186 | 5069 | PAPPA | pregnancy-associated plasma protein A, pappalysin 1 | 1 |
| 2626 | hsa-mir-155 | 5069 | PAPPA | pregnancy-associated plasma protein A, pappalysin 1 | 1 |
| 2627 | hsa-mir-423 | 5069 | PAPPA | pregnancy-associated plasma protein A, pappalysin 1 | 1 |
| 2628 | hsa-mir-499 | 5069 | PAPPA | pregnancy-associated plasma protein A, pappalysin 1 | 1 |
| 2629 | hsa-mir-550-1 | 5069 | PAPPA | pregnancy-associated plasma protein A, pappalysin 1 | 1 |
| 2630 | hsa-mir-877 | 5069 | PAPPA | pregnancy-associated plasma protein A, pappalysin 1 | 1 |
| 2631 | hsa-mir-2116 | 5069 | PAPPA | pregnancy-associated plasma protein A, pappalysin 1 | 1 |
| 2632 | hsa-mir-3186 | 5069 | PAPPA | pregnancy-associated plasma protein A, pappalysin 1 | 1 |
| 2633 | hsa-miR-10a | 6385 | SDC4 | syndecan 4 | 1 |
| 2634 | hsa-miR-152 | 6385 | SDC4 | syndecan 4 | 1 |
| 2635 | hsa-miR-26a | 6385 | SDC4 | syndecan 4 | 1 |
| 2636 | hsa-miR-26b | 6385 | SDC4 | syndecan 4 | 1 |
| 2637 | hsa-miR-31 | 6385 | SDC4 | syndecan 4 | 1 |
| 2638 | hsa-miR-340 | 6385 | SDC4 | syndecan 4 | 1 |
| 2639 | hsa-miR-423-5p | 6385 | SDC4 | syndecan 4 | 1 |
| 2640 | hsa-miR-449a | 6385 | SDC4 | syndecan 4 | 1 |
| 2641 | hsa-miR-490-5p | 6385 | SDC4 | syndecan 4 | 1 |
| 2642 | hsa-miR-499-5p | 6385 | SDC4 | syndecan 4 | 1 |
| 2643 | hsa-miR-548b-5p | 6385 | SDC4 | syndecan 4 | 1 |
| 2644 | hsa-miR-564 | 6385 | SDC4 | syndecan 4 | 1 |
| 2645 | hsa-miR-584 | 6385 | SDC4 | syndecan 4 | 1 |
| 2646 | hsa-miR-621 | 6385 | SDC4 | syndecan 4 | 1 |
| 2647 | hsa-miR-644 | 6385 | SDC4 | syndecan 4 | 1 |
| 2648 | hsa-miR-765 | 6385 | SDC4 | syndecan 4 | 1 |
| 2649 | hsa-miR-770-5p | 6385 | SDC4 | syndecan 4 | 1 |
| 2650 | hsa-miR-877 | 6385 | SDC4 | syndecan 4 | 1 |
| 2651 | hsa-miR-95 | 6385 | SDC4 | syndecan 4 | 1 |
| 2652 | hsa-mir-26a-1 | 6385 | SDC4 | syndecan 4 | 1 |
| 2653 | hsa-mir-26b | 6385 | SDC4 | syndecan 4 | 1 |
| 2654 | hsa-mir-31 | 6385 | SDC4 | syndecan 4 | 1 |
| 2655 | hsa-mir-194-2 | 6385 | SDC4 | syndecan 4 | 1 |
| 2656 | hsa-mir-340 | 6385 | SDC4 | syndecan 4 | 1 |
| 2657 | hsa-mir-490 | 6385 | SDC4 | syndecan 4 | 1 |
| 2658 | hsa-mir-548b | 6385 | SDC4 | syndecan 4 | 1 |
| 2659 | hsa-mir-449b | 6385 | SDC4 | syndecan 4 | 1 |
| 2660 | hsa-mir-449c | 6385 | SDC4 | syndecan 4 | 1 |
| 2661 | hsa-miR-10a | 10133 | OPTN | optineurin | 1 |
| 2662 | hsa-miR-1228\* | 10133 | OPTN | optineurin | 1 |
| 2663 | hsa-miR-139-5p | 10133 | OPTN | optineurin | 1 |
| 2664 | hsa-miR-26a | 10133 | OPTN | optineurin | 1 |
| 2665 | hsa-miR-26b | 10133 | OPTN | optineurin | 1 |
| 2666 | hsa-miR-338-5p | 10133 | OPTN | optineurin | 1 |
| 2667 | hsa-miR-340 | 10133 | OPTN | optineurin | 1 |
| 2668 | hsa-miR-423-5p | 10133 | OPTN | optineurin | 1 |
| 2669 | hsa-miR-449a | 10133 | OPTN | optineurin | 1 |
| 2670 | hsa-miR-499-5p | 10133 | OPTN | optineurin | 1 |
| 2671 | hsa-miR-548b-5p | 10133 | OPTN | optineurin | 1 |
| 2672 | hsa-miR-564 | 10133 | OPTN | optineurin | 1 |
| 2673 | hsa-miR-621 | 10133 | OPTN | optineurin | 1 |
| 2674 | hsa-miR-657 | 10133 | OPTN | optineurin | 1 |
| 2675 | hsa-miR-765 | 10133 | OPTN | optineurin | 1 |
| 2676 | hsa-miR-770-5p | 10133 | OPTN | optineurin | 1 |
| 2677 | hsa-miR-877 | 10133 | OPTN | optineurin | 1 |
| 2678 | hsa-mir-26a-1 | 10133 | OPTN | optineurin | 1 |
| 2679 | hsa-mir-26b | 10133 | OPTN | optineurin | 1 |
| 2680 | hsa-mir-194-2 | 10133 | OPTN | optineurin | 1 |
| 2681 | hsa-mir-340 | 10133 | OPTN | optineurin | 1 |
| 2682 | hsa-mir-338 | 10133 | OPTN | optineurin | 1 |
| 2683 | hsa-mir-548b | 10133 | OPTN | optineurin | 1 |
| 2684 | hsa-mir-548d-2 | 10133 | OPTN | optineurin | 1 |
| 2685 | hsa-mir-449b | 10133 | OPTN | optineurin | 1 |
| 2686 | hsa-mir-449c | 10133 | OPTN | optineurin | 1 |
| 2687 | hsa-mir-3065 | 10133 | OPTN | optineurin | 1 |
| 2688 | hsa-miR-10a | 9414 | TJP2 | tight junction protein 2 (zona occludens 2) | 1 |
| 2689 | hsa-miR-152 | 9414 | TJP2 | tight junction protein 2 (zona occludens 2) | 1 |
| 2690 | hsa-miR-26a | 9414 | TJP2 | tight junction protein 2 (zona occludens 2) | 1 |
| 2691 | hsa-miR-26b | 9414 | TJP2 | tight junction protein 2 (zona occludens 2) | 1 |
| 2692 | hsa-miR-31 | 9414 | TJP2 | tight junction protein 2 (zona occludens 2) | 1 |
| 2693 | hsa-miR-338-5p | 9414 | TJP2 | tight junction protein 2 (zona occludens 2) | 1 |
| 2694 | hsa-miR-340 | 9414 | TJP2 | tight junction protein 2 (zona occludens 2) | 1 |
| 2695 | hsa-miR-423-5p | 9414 | TJP2 | tight junction protein 2 (zona occludens 2) | 1 |
| 2696 | hsa-miR-449a | 9414 | TJP2 | tight junction protein 2 (zona occludens 2) | 1 |
| 2697 | hsa-miR-490-5p | 9414 | TJP2 | tight junction protein 2 (zona occludens 2) | 1 |
| 2698 | hsa-miR-499-5p | 9414 | TJP2 | tight junction protein 2 (zona occludens 2) | 1 |
| 2699 | hsa-miR-548b-5p | 9414 | TJP2 | tight junction protein 2 (zona occludens 2) | 1 |
| 2700 | hsa-miR-564 | 9414 | TJP2 | tight junction protein 2 (zona occludens 2) | 1 |
| 2701 | hsa-miR-621 | 9414 | TJP2 | tight junction protein 2 (zona occludens 2) | 1 |
| 2702 | hsa-miR-644 | 9414 | TJP2 | tight junction protein 2 (zona occludens 2) | 1 |
| 2703 | hsa-miR-657 | 9414 | TJP2 | tight junction protein 2 (zona occludens 2) | 1 |
| 2704 | hsa-miR-765 | 9414 | TJP2 | tight junction protein 2 (zona occludens 2) | 1 |
| 2705 | hsa-miR-770-5p | 9414 | TJP2 | tight junction protein 2 (zona occludens 2) | 1 |
| 2706 | hsa-miR-877 | 9414 | TJP2 | tight junction protein 2 (zona occludens 2) | 1 |
| 2707 | hsa-miR-95 | 9414 | TJP2 | tight junction protein 2 (zona occludens 2) | 1 |
| 2708 | hsa-mir-26a-1 | 9414 | TJP2 | tight junction protein 2 (zona occludens 2) | 1 |
| 2709 | hsa-mir-26b | 9414 | TJP2 | tight junction protein 2 (zona occludens 2) | 1 |
| 2710 | hsa-mir-31 | 9414 | TJP2 | tight junction protein 2 (zona occludens 2) | 1 |
| 2711 | hsa-mir-194-2 | 9414 | TJP2 | tight junction protein 2 (zona occludens 2) | 1 |
| 2712 | hsa-mir-340 | 9414 | TJP2 | tight junction protein 2 (zona occludens 2) | 1 |
| 2713 | hsa-mir-338 | 9414 | TJP2 | tight junction protein 2 (zona occludens 2) | 1 |
| 2714 | hsa-mir-490 | 9414 | TJP2 | tight junction protein 2 (zona occludens 2) | 1 |
| 2715 | hsa-mir-548b | 9414 | TJP2 | tight junction protein 2 (zona occludens 2) | 1 |
| 2716 | hsa-mir-449b | 9414 | TJP2 | tight junction protein 2 (zona occludens 2) | 1 |
| 2717 | hsa-mir-449c | 9414 | TJP2 | tight junction protein 2 (zona occludens 2) | 1 |
| 2718 | hsa-mir-3065 | 9414 | TJP2 | tight junction protein 2 (zona occludens 2) | 1 |
| 2750 | hsa-miR-383 | 2633 | GBP1 | guanylate binding protein 1, interferon-inducible, 67kDa | 1 |
| 2752 | hsa-miR-10a | 6890 | TAP1 | transporter 1, ATP-binding cassette, sub-family B (MDR/TAP) | 1 |
| 2753 | hsa-miR-139-5p | 6890 | TAP1 | transporter 1, ATP-binding cassette, sub-family B (MDR/TAP) | 1 |
| 2754 | hsa-miR-152 | 6890 | TAP1 | transporter 1, ATP-binding cassette, sub-family B (MDR/TAP) | 1 |
| 2755 | hsa-miR-26a | 6890 | TAP1 | transporter 1, ATP-binding cassette, sub-family B (MDR/TAP) | 1 |
| 2756 | hsa-miR-26b | 6890 | TAP1 | transporter 1, ATP-binding cassette, sub-family B (MDR/TAP) | 1 |
| 2757 | hsa-miR-31 | 6890 | TAP1 | transporter 1, ATP-binding cassette, sub-family B (MDR/TAP) | 1 |
| 2758 | hsa-miR-338-5p | 6890 | TAP1 | transporter 1, ATP-binding cassette, sub-family B (MDR/TAP) | 1 |
| 2759 | hsa-miR-340 | 6890 | TAP1 | transporter 1, ATP-binding cassette, sub-family B (MDR/TAP) | 1 |
| 2760 | hsa-miR-423-5p | 6890 | TAP1 | transporter 1, ATP-binding cassette, sub-family B (MDR/TAP) | 1 |
| 2761 | hsa-miR-449a | 6890 | TAP1 | transporter 1, ATP-binding cassette, sub-family B (MDR/TAP) | 1 |
| 2762 | hsa-miR-499-5p | 6890 | TAP1 | transporter 1, ATP-binding cassette, sub-family B (MDR/TAP) | 1 |
| 2763 | hsa-miR-548b-5p | 6890 | TAP1 | transporter 1, ATP-binding cassette, sub-family B (MDR/TAP) | 1 |
| 2764 | hsa-miR-564 | 6890 | TAP1 | transporter 1, ATP-binding cassette, sub-family B (MDR/TAP) | 1 |
| 2765 | hsa-miR-621 | 6890 | TAP1 | transporter 1, ATP-binding cassette, sub-family B (MDR/TAP) | 1 |
| 2766 | hsa-miR-657 | 6890 | TAP1 | transporter 1, ATP-binding cassette, sub-family B (MDR/TAP) | 1 |
| 2767 | hsa-miR-765 | 6890 | TAP1 | transporter 1, ATP-binding cassette, sub-family B (MDR/TAP) | 1 |
| 2768 | hsa-miR-770-5p | 6890 | TAP1 | transporter 1, ATP-binding cassette, sub-family B (MDR/TAP) | 1 |
| 2769 | hsa-miR-877 | 6890 | TAP1 | transporter 1, ATP-binding cassette, sub-family B (MDR/TAP) | 1 |
| 2770 | hsa-mir-26a-1 | 6890 | TAP1 | transporter 1, ATP-binding cassette, sub-family B (MDR/TAP) | 1 |
| 2771 | hsa-mir-26b | 6890 | TAP1 | transporter 1, ATP-binding cassette, sub-family B (MDR/TAP) | 1 |
| 2772 | hsa-mir-31 | 6890 | TAP1 | transporter 1, ATP-binding cassette, sub-family B (MDR/TAP) | 1 |
| 2773 | hsa-mir-194-2 | 6890 | TAP1 | transporter 1, ATP-binding cassette, sub-family B (MDR/TAP) | 1 |
| 2774 | hsa-mir-340 | 6890 | TAP1 | transporter 1, ATP-binding cassette, sub-family B (MDR/TAP) | 1 |
| 2775 | hsa-mir-338 | 6890 | TAP1 | transporter 1, ATP-binding cassette, sub-family B (MDR/TAP) | 1 |
| 2776 | hsa-mir-548b | 6890 | TAP1 | transporter 1, ATP-binding cassette, sub-family B (MDR/TAP) | 1 |
| 2777 | hsa-mir-548d-2 | 6890 | TAP1 | transporter 1, ATP-binding cassette, sub-family B (MDR/TAP) | 1 |
| 2778 | hsa-mir-449b | 6890 | TAP1 | transporter 1, ATP-binding cassette, sub-family B (MDR/TAP) | 1 |
| 2779 | hsa-mir-449c | 6890 | TAP1 | transporter 1, ATP-binding cassette, sub-family B (MDR/TAP) | 1 |
| 2780 | hsa-mir-3065 | 6890 | TAP1 | transporter 1, ATP-binding cassette, sub-family B (MDR/TAP) | 1 |
| 2781 | hsa-miR-10a | 8189 | SYMPK | symplekin | 1 |
| 2782 | hsa-miR-139-5p | 8189 | SYMPK | symplekin | 1 |
| 2783 | hsa-miR-152 | 8189 | SYMPK | symplekin | 1 |
| 2784 | hsa-miR-26a | 8189 | SYMPK | symplekin | 1 |
| 2785 | hsa-miR-26b | 8189 | SYMPK | symplekin | 1 |
| 2786 | hsa-miR-31 | 8189 | SYMPK | symplekin | 1 |
| 2787 | hsa-miR-338-5p | 8189 | SYMPK | symplekin | 1 |
| 2788 | hsa-miR-340 | 8189 | SYMPK | symplekin | 1 |
| 2789 | hsa-miR-423-5p | 8189 | SYMPK | symplekin | 1 |
| 2790 | hsa-miR-449a | 8189 | SYMPK | symplekin | 1 |
| 2791 | hsa-miR-499-5p | 8189 | SYMPK | symplekin | 1 |
| 2792 | hsa-miR-548b-5p | 8189 | SYMPK | symplekin | 1 |
| 2793 | hsa-miR-564 | 8189 | SYMPK | symplekin | 1 |
| 2794 | hsa-miR-621 | 8189 | SYMPK | symplekin | 1 |
| 2795 | hsa-miR-657 | 8189 | SYMPK | symplekin | 1 |
| 2796 | hsa-miR-663 | 8189 | SYMPK | symplekin | 1 |
| 2797 | hsa-miR-765 | 8189 | SYMPK | symplekin | 1 |
| 2798 | hsa-miR-770-5p | 8189 | SYMPK | symplekin | 1 |
| 2799 | hsa-miR-877 | 8189 | SYMPK | symplekin | 1 |
| 2800 | hsa-mir-26a-1 | 8189 | SYMPK | symplekin | 1 |
| 2801 | hsa-mir-26b | 8189 | SYMPK | symplekin | 1 |
| 2802 | hsa-mir-31 | 8189 | SYMPK | symplekin | 1 |
| 2803 | hsa-mir-194-2 | 8189 | SYMPK | symplekin | 1 |
| 2804 | hsa-mir-340 | 8189 | SYMPK | symplekin | 1 |
| 2805 | hsa-mir-338 | 8189 | SYMPK | symplekin | 1 |
| 2806 | hsa-mir-548b | 8189 | SYMPK | symplekin | 1 |
| 2807 | hsa-mir-548d-2 | 8189 | SYMPK | symplekin | 1 |
| 2808 | hsa-mir-449b | 8189 | SYMPK | symplekin | 1 |
| 2809 | hsa-mir-449c | 8189 | SYMPK | symplekin | 1 |
| 2810 | hsa-mir-3065 | 8189 | SYMPK | symplekin | 1 |
| 2811 | hsa-miR-10a | 3429 | IFI27 | interferon, alpha-inducible protein 27 | 1 |
| 2812 | hsa-miR-1228\* | 3429 | IFI27 | interferon, alpha-inducible protein 27 | 1 |
| 2813 | hsa-miR-139-5p | 3429 | IFI27 | interferon, alpha-inducible protein 27 | 1 |
| 2814 | hsa-miR-152 | 3429 | IFI27 | interferon, alpha-inducible protein 27 | 1 |
| 2815 | hsa-miR-26a | 3429 | IFI27 | interferon, alpha-inducible protein 27 | 1 |
| 2816 | hsa-miR-26b | 3429 | IFI27 | interferon, alpha-inducible protein 27 | 1 |
| 2817 | hsa-miR-31 | 3429 | IFI27 | interferon, alpha-inducible protein 27 | 1 |
| 2818 | hsa-miR-338-5p | 3429 | IFI27 | interferon, alpha-inducible protein 27 | 1 |
| 2819 | hsa-miR-340 | 3429 | IFI27 | interferon, alpha-inducible protein 27 | 1 |
| 2820 | hsa-miR-423-5p | 3429 | IFI27 | interferon, alpha-inducible protein 27 | 1 |
| 2821 | hsa-miR-449a | 3429 | IFI27 | interferon, alpha-inducible protein 27 | 1 |
| 2822 | hsa-miR-499-5p | 3429 | IFI27 | interferon, alpha-inducible protein 27 | 1 |
| 2823 | hsa-miR-548b-5p | 3429 | IFI27 | interferon, alpha-inducible protein 27 | 1 |
| 2824 | hsa-miR-564 | 3429 | IFI27 | interferon, alpha-inducible protein 27 | 1 |
| 2825 | hsa-miR-584 | 3429 | IFI27 | interferon, alpha-inducible protein 27 | 1 |
| 2826 | hsa-miR-621 | 3429 | IFI27 | interferon, alpha-inducible protein 27 | 1 |
| 2827 | hsa-miR-657 | 3429 | IFI27 | interferon, alpha-inducible protein 27 | 1 |
| 2828 | hsa-miR-765 | 3429 | IFI27 | interferon, alpha-inducible protein 27 | 1 |
| 2829 | hsa-miR-770-5p | 3429 | IFI27 | interferon, alpha-inducible protein 27 | 1 |
| 2830 | hsa-miR-877 | 3429 | IFI27 | interferon, alpha-inducible protein 27 | 1 |
| 2831 | hsa-mir-26a-1 | 3429 | IFI27 | interferon, alpha-inducible protein 27 | 1 |
| 2832 | hsa-mir-26b | 3429 | IFI27 | interferon, alpha-inducible protein 27 | 1 |
| 2833 | hsa-mir-31 | 3429 | IFI27 | interferon, alpha-inducible protein 27 | 1 |
| 2834 | hsa-mir-194-2 | 3429 | IFI27 | interferon, alpha-inducible protein 27 | 1 |
| 2835 | hsa-mir-340 | 3429 | IFI27 | interferon, alpha-inducible protein 27 | 1 |
| 2836 | hsa-mir-338 | 3429 | IFI27 | interferon, alpha-inducible protein 27 | 1 |
| 2837 | hsa-mir-548b | 3429 | IFI27 | interferon, alpha-inducible protein 27 | 1 |
| 2838 | hsa-mir-548d-2 | 3429 | IFI27 | interferon, alpha-inducible protein 27 | 1 |
| 2839 | hsa-mir-449b | 3429 | IFI27 | interferon, alpha-inducible protein 27 | 1 |
| 2840 | hsa-mir-449c | 3429 | IFI27 | interferon, alpha-inducible protein 27 | 1 |
| 2841 | hsa-mir-3065 | 3429 | IFI27 | interferon, alpha-inducible protein 27 | 1 |
| 2842 | hsa-miR-10a | 7127 | TNFAIP2 | tumor necrosis factor, alpha-induced protein 2 | 1 |
| 2843 | hsa-miR-139-5p | 7127 | TNFAIP2 | tumor necrosis factor, alpha-induced protein 2 | 1 |
| 2844 | hsa-miR-152 | 7127 | TNFAIP2 | tumor necrosis factor, alpha-induced protein 2 | 1 |
| 2845 | hsa-miR-26a | 7127 | TNFAIP2 | tumor necrosis factor, alpha-induced protein 2 | 1 |
| 2846 | hsa-miR-26b | 7127 | TNFAIP2 | tumor necrosis factor, alpha-induced protein 2 | 1 |
| 2847 | hsa-miR-31 | 7127 | TNFAIP2 | tumor necrosis factor, alpha-induced protein 2 | 1 |
| 2848 | hsa-miR-338-5p | 7127 | TNFAIP2 | tumor necrosis factor, alpha-induced protein 2 | 1 |
| 2849 | hsa-miR-340 | 7127 | TNFAIP2 | tumor necrosis factor, alpha-induced protein 2 | 1 |
| 2850 | hsa-miR-449a | 7127 | TNFAIP2 | tumor necrosis factor, alpha-induced protein 2 | 1 |
| 2851 | hsa-miR-499-5p | 7127 | TNFAIP2 | tumor necrosis factor, alpha-induced protein 2 | 1 |
| 2852 | hsa-miR-548b-5p | 7127 | TNFAIP2 | tumor necrosis factor, alpha-induced protein 2 | 1 |
| 2853 | hsa-miR-564 | 7127 | TNFAIP2 | tumor necrosis factor, alpha-induced protein 2 | 1 |
| 2854 | hsa-miR-621 | 7127 | TNFAIP2 | tumor necrosis factor, alpha-induced protein 2 | 1 |
| 2855 | hsa-miR-657 | 7127 | TNFAIP2 | tumor necrosis factor, alpha-induced protein 2 | 1 |
| 2856 | hsa-miR-765 | 7127 | TNFAIP2 | tumor necrosis factor, alpha-induced protein 2 | 1 |
| 2857 | hsa-miR-770-5p | 7127 | TNFAIP2 | tumor necrosis factor, alpha-induced protein 2 | 1 |
| 2858 | hsa-miR-877 | 7127 | TNFAIP2 | tumor necrosis factor, alpha-induced protein 2 | 1 |
| 2859 | hsa-mir-26a-1 | 7127 | TNFAIP2 | tumor necrosis factor, alpha-induced protein 2 | 1 |
| 2860 | hsa-mir-26b | 7127 | TNFAIP2 | tumor necrosis factor, alpha-induced protein 2 | 1 |
| 2861 | hsa-mir-31 | 7127 | TNFAIP2 | tumor necrosis factor, alpha-induced protein 2 | 1 |
| 2862 | hsa-mir-194-2 | 7127 | TNFAIP2 | tumor necrosis factor, alpha-induced protein 2 | 1 |
| 2863 | hsa-mir-340 | 7127 | TNFAIP2 | tumor necrosis factor, alpha-induced protein 2 | 1 |
| 2864 | hsa-mir-338 | 7127 | TNFAIP2 | tumor necrosis factor, alpha-induced protein 2 | 1 |
| 2865 | hsa-mir-548b | 7127 | TNFAIP2 | tumor necrosis factor, alpha-induced protein 2 | 1 |
| 2866 | hsa-mir-548d-2 | 7127 | TNFAIP2 | tumor necrosis factor, alpha-induced protein 2 | 1 |
| 2867 | hsa-mir-449b | 7127 | TNFAIP2 | tumor necrosis factor, alpha-induced protein 2 | 1 |
| 2868 | hsa-mir-449c | 7127 | TNFAIP2 | tumor necrosis factor, alpha-induced protein 2 | 1 |
| 2869 | hsa-mir-3065 | 7127 | TNFAIP2 | tumor necrosis factor, alpha-induced protein 2 | 1 |
| 2897 | hsa-miR-1 | 5164 | PDK2 | pyruvate dehydrogenase kinase, isozyme 2 | 1 |
| 2898 | hsa-miR-133a | 5164 | PDK2 | pyruvate dehydrogenase kinase, isozyme 2 | 1 |
| 2899 | hsa-miR-186 | 5164 | PDK2 | pyruvate dehydrogenase kinase, isozyme 2 | 1 |
| 2900 | hsa-miR-208a | 5164 | PDK2 | pyruvate dehydrogenase kinase, isozyme 2 | 1 |
| 2901 | hsa-miR-326 | 5164 | PDK2 | pyruvate dehydrogenase kinase, isozyme 2 | 1 |
| 2902 | hsa-miR-449b | 5164 | PDK2 | pyruvate dehydrogenase kinase, isozyme 2 | 1 |
| 2903 | hsa-miR-550 | 5164 | PDK2 | pyruvate dehydrogenase kinase, isozyme 2 | 1 |
| 2904 | hsa-miR-575 | 5164 | PDK2 | pyruvate dehydrogenase kinase, isozyme 2 | 1 |
| 2905 | hsa-miR-579 | 5164 | PDK2 | pyruvate dehydrogenase kinase, isozyme 2 | 1 |
| 2906 | hsa-miR-608 | 5164 | PDK2 | pyruvate dehydrogenase kinase, isozyme 2 | 1 |
| 2907 | hsa-miR-620 | 5164 | PDK2 | pyruvate dehydrogenase kinase, isozyme 2 | 1 |
| 2908 | hsa-miR-885-5p | 5164 | PDK2 | pyruvate dehydrogenase kinase, isozyme 2 | 1 |
| 2909 | hsa-let-7a-3 | 5164 | PDK2 | pyruvate dehydrogenase kinase, isozyme 2 | 1 |
| 2910 | hsa-let-7b | 5164 | PDK2 | pyruvate dehydrogenase kinase, isozyme 2 | 1 |
| 2911 | hsa-mir-22 | 5164 | PDK2 | pyruvate dehydrogenase kinase, isozyme 2 | 1 |
| 2912 | hsa-mir-10a | 5164 | PDK2 | pyruvate dehydrogenase kinase, isozyme 2 | 1 |
| 2913 | hsa-mir-186 | 5164 | PDK2 | pyruvate dehydrogenase kinase, isozyme 2 | 1 |
| 2914 | hsa-mir-155 | 5164 | PDK2 | pyruvate dehydrogenase kinase, isozyme 2 | 1 |
| 2915 | hsa-mir-423 | 5164 | PDK2 | pyruvate dehydrogenase kinase, isozyme 2 | 1 |
| 2916 | hsa-mir-499 | 5164 | PDK2 | pyruvate dehydrogenase kinase, isozyme 2 | 1 |
| 2917 | hsa-mir-550-1 | 5164 | PDK2 | pyruvate dehydrogenase kinase, isozyme 2 | 1 |
| 2918 | hsa-mir-885 | 5164 | PDK2 | pyruvate dehydrogenase kinase, isozyme 2 | 1 |
| 2919 | hsa-mir-877 | 5164 | PDK2 | pyruvate dehydrogenase kinase, isozyme 2 | 1 |
| 2920 | hsa-mir-2116 | 5164 | PDK2 | pyruvate dehydrogenase kinase, isozyme 2 | 1 |
| 2921 | hsa-mir-3186 | 5164 | PDK2 | pyruvate dehydrogenase kinase, isozyme 2 | 1 |
| 2922 | hsa-mir-3190 | 5164 | PDK2 | pyruvate dehydrogenase kinase, isozyme 2 | 1 |
| 2932 | hsa-miR-490-5p | 7128 | TNFAIP3 | tumor necrosis factor, alpha-induced protein 3 | 1 |
| 2935 | hsa-miR-584 | 7128 | TNFAIP3 | tumor necrosis factor, alpha-induced protein 3 | 1 |
| 2948 | hsa-mir-490 | 7128 | TNFAIP3 | tumor necrosis factor, alpha-induced protein 3 | 1 |
| 2953 | hsa-mir-548d-2 | 7128 | TNFAIP3 | tumor necrosis factor, alpha-induced protein 3 | 1 |
| 2954 | hsa-miR-1 | 7372 | UMPS | uridine monophosphate synthetase | 1 |
| 2955 | hsa-miR-133a | 7372 | UMPS | uridine monophosphate synthetase | 1 |
| 2956 | hsa-miR-186 | 7372 | UMPS | uridine monophosphate synthetase | 1 |
| 2957 | hsa-miR-208a | 7372 | UMPS | uridine monophosphate synthetase | 1 |
| 2958 | hsa-miR-326 | 7372 | UMPS | uridine monophosphate synthetase | 1 |
| 2959 | hsa-miR-449b | 7372 | UMPS | uridine monophosphate synthetase | 1 |
| 2960 | hsa-miR-489 | 7372 | UMPS | uridine monophosphate synthetase | 1 |
| 2961 | hsa-miR-575 | 7372 | UMPS | uridine monophosphate synthetase | 1 |
| 2962 | hsa-miR-579 | 7372 | UMPS | uridine monophosphate synthetase | 1 |
| 2963 | hsa-miR-608 | 7372 | UMPS | uridine monophosphate synthetase | 1 |
| 2964 | hsa-miR-620 | 7372 | UMPS | uridine monophosphate synthetase | 1 |
| 2965 | hsa-miR-637 | 7372 | UMPS | uridine monophosphate synthetase | 1 |
| 2966 | hsa-miR-885-5p | 7372 | UMPS | uridine monophosphate synthetase | 1 |
| 2967 | hsa-mir-22 | 7372 | UMPS | uridine monophosphate synthetase | 1 |
| 2968 | hsa-mir-10a | 7372 | UMPS | uridine monophosphate synthetase | 1 |
| 2969 | hsa-mir-186 | 7372 | UMPS | uridine monophosphate synthetase | 1 |
| 2970 | hsa-mir-155 | 7372 | UMPS | uridine monophosphate synthetase | 1 |
| 2971 | hsa-mir-499 | 7372 | UMPS | uridine monophosphate synthetase | 1 |
| 2972 | hsa-mir-885 | 7372 | UMPS | uridine monophosphate synthetase | 1 |
| 2973 | hsa-mir-877 | 7372 | UMPS | uridine monophosphate synthetase | 1 |
| 2974 | hsa-mir-3190 | 7372 | UMPS | uridine monophosphate synthetase | 1 |
| 2975 | hsa-miR-186 | 9692 | KIAA0391 | KIAA0391 | 1 |
| 2976 | hsa-miR-195 | 9692 | KIAA0391 | KIAA0391 | 1 |
| 2977 | hsa-miR-326 | 9692 | KIAA0391 | KIAA0391 | 1 |
| 2978 | hsa-miR-449b | 9692 | KIAA0391 | KIAA0391 | 1 |
| 2979 | hsa-miR-489 | 9692 | KIAA0391 | KIAA0391 | 1 |
| 2980 | hsa-miR-497 | 9692 | KIAA0391 | KIAA0391 | 1 |
| 2981 | hsa-miR-562 | 9692 | KIAA0391 | KIAA0391 | 1 |
| 2982 | hsa-miR-575 | 9692 | KIAA0391 | KIAA0391 | 1 |
| 2983 | hsa-miR-579 | 9692 | KIAA0391 | KIAA0391 | 1 |
| 2984 | hsa-miR-608 | 9692 | KIAA0391 | KIAA0391 | 1 |
| 2985 | hsa-miR-620 | 9692 | KIAA0391 | KIAA0391 | 1 |
| 2986 | hsa-miR-885-5p | 9692 | KIAA0391 | KIAA0391 | 1 |
| 2987 | hsa-mir-22 | 9692 | KIAA0391 | KIAA0391 | 1 |
| 2988 | hsa-mir-10a | 9692 | KIAA0391 | KIAA0391 | 1 |
| 2989 | hsa-mir-186 | 9692 | KIAA0391 | KIAA0391 | 1 |
| 2990 | hsa-mir-155 | 9692 | KIAA0391 | KIAA0391 | 1 |
| 2991 | hsa-mir-499 | 9692 | KIAA0391 | KIAA0391 | 1 |
| 2992 | hsa-mir-885 | 9692 | KIAA0391 | KIAA0391 | 1 |
| 2993 | hsa-mir-877 | 9692 | KIAA0391 | KIAA0391 | 1 |
| 2994 | hsa-miR-10a | 2634 | GBP2 | guanylate binding protein 2, interferon-inducible | 1 |
| 2995 | hsa-miR-152 | 2634 | GBP2 | guanylate binding protein 2, interferon-inducible | 1 |
| 2996 | hsa-miR-26a | 2634 | GBP2 | guanylate binding protein 2, interferon-inducible | 1 |
| 2997 | hsa-miR-26b | 2634 | GBP2 | guanylate binding protein 2, interferon-inducible | 1 |
| 2998 | hsa-miR-31 | 2634 | GBP2 | guanylate binding protein 2, interferon-inducible | 1 |
| 2999 | hsa-miR-338-5p | 2634 | GBP2 | guanylate binding protein 2, interferon-inducible | 1 |
| 3000 | hsa-miR-340 | 2634 | GBP2 | guanylate binding protein 2, interferon-inducible | 1 |
| 3001 | hsa-miR-383 | 2634 | GBP2 | guanylate binding protein 2, interferon-inducible | 1 |
| 3002 | hsa-miR-449a | 2634 | GBP2 | guanylate binding protein 2, interferon-inducible | 1 |
| 3003 | hsa-miR-490-5p | 2634 | GBP2 | guanylate binding protein 2, interferon-inducible | 1 |
| 3004 | hsa-miR-499-5p | 2634 | GBP2 | guanylate binding protein 2, interferon-inducible | 1 |
| 3005 | hsa-miR-504 | 2634 | GBP2 | guanylate binding protein 2, interferon-inducible | 1 |
| 3006 | hsa-miR-621 | 2634 | GBP2 | guanylate binding protein 2, interferon-inducible | 1 |
| 3007 | hsa-miR-653 | 2634 | GBP2 | guanylate binding protein 2, interferon-inducible | 1 |
| 3008 | hsa-miR-657 | 2634 | GBP2 | guanylate binding protein 2, interferon-inducible | 1 |
| 3009 | hsa-miR-765 | 2634 | GBP2 | guanylate binding protein 2, interferon-inducible | 1 |
| 3010 | hsa-miR-770-5p | 2634 | GBP2 | guanylate binding protein 2, interferon-inducible | 1 |
| 3011 | hsa-miR-877 | 2634 | GBP2 | guanylate binding protein 2, interferon-inducible | 1 |
| 3012 | hsa-mir-26a-1 | 2634 | GBP2 | guanylate binding protein 2, interferon-inducible | 1 |
| 3013 | hsa-mir-26b | 2634 | GBP2 | guanylate binding protein 2, interferon-inducible | 1 |
| 3014 | hsa-mir-31 | 2634 | GBP2 | guanylate binding protein 2, interferon-inducible | 1 |
| 3015 | hsa-mir-194-2 | 2634 | GBP2 | guanylate binding protein 2, interferon-inducible | 1 |
| 3016 | hsa-mir-340 | 2634 | GBP2 | guanylate binding protein 2, interferon-inducible | 1 |
| 3017 | hsa-mir-338 | 2634 | GBP2 | guanylate binding protein 2, interferon-inducible | 1 |
| 3018 | hsa-mir-490 | 2634 | GBP2 | guanylate binding protein 2, interferon-inducible | 1 |
| 3019 | hsa-mir-449b | 2634 | GBP2 | guanylate binding protein 2, interferon-inducible | 1 |
| 3020 | hsa-mir-449c | 2634 | GBP2 | guanylate binding protein 2, interferon-inducible | 1 |
| 3021 | hsa-mir-3065 | 2634 | GBP2 | guanylate binding protein 2, interferon-inducible | 1 |
| 3039 | hsa-mir-423 | 23428 | SLC7A8 | solute carrier family 7 (cationic amino acid transporter, y+ system), member 8 | 1 |
| 3044 | hsa-miR-10a | 6924 | TCEB3 | transcription elongation factor B (SIII), polypeptide 3 (110kDa, elongin A) | 1 |
| 3045 | hsa-miR-152 | 6924 | TCEB3 | transcription elongation factor B (SIII), polypeptide 3 (110kDa, elongin A) | 1 |
| 3046 | hsa-miR-26a | 6924 | TCEB3 | transcription elongation factor B (SIII), polypeptide 3 (110kDa, elongin A) | 1 |
| 3047 | hsa-miR-26b | 6924 | TCEB3 | transcription elongation factor B (SIII), polypeptide 3 (110kDa, elongin A) | 1 |
| 3048 | hsa-miR-31 | 6924 | TCEB3 | transcription elongation factor B (SIII), polypeptide 3 (110kDa, elongin A) | 1 |
| 3049 | hsa-miR-338-5p | 6924 | TCEB3 | transcription elongation factor B (SIII), polypeptide 3 (110kDa, elongin A) | 1 |
| 3050 | hsa-miR-340 | 6924 | TCEB3 | transcription elongation factor B (SIII), polypeptide 3 (110kDa, elongin A) | 1 |
| 3051 | hsa-miR-423-5p | 6924 | TCEB3 | transcription elongation factor B (SIII), polypeptide 3 (110kDa, elongin A) | 1 |
| 3052 | hsa-miR-449a | 6924 | TCEB3 | transcription elongation factor B (SIII), polypeptide 3 (110kDa, elongin A) | 1 |
| 3053 | hsa-miR-499-5p | 6924 | TCEB3 | transcription elongation factor B (SIII), polypeptide 3 (110kDa, elongin A) | 1 |
| 3054 | hsa-miR-548b-5p | 6924 | TCEB3 | transcription elongation factor B (SIII), polypeptide 3 (110kDa, elongin A) | 1 |
| 3055 | hsa-miR-564 | 6924 | TCEB3 | transcription elongation factor B (SIII), polypeptide 3 (110kDa, elongin A) | 1 |
| 3056 | hsa-miR-621 | 6924 | TCEB3 | transcription elongation factor B (SIII), polypeptide 3 (110kDa, elongin A) | 1 |
| 3057 | hsa-miR-657 | 6924 | TCEB3 | transcription elongation factor B (SIII), polypeptide 3 (110kDa, elongin A) | 1 |
| 3058 | hsa-miR-765 | 6924 | TCEB3 | transcription elongation factor B (SIII), polypeptide 3 (110kDa, elongin A) | 1 |
| 3059 | hsa-miR-770-5p | 6924 | TCEB3 | transcription elongation factor B (SIII), polypeptide 3 (110kDa, elongin A) | 1 |
| 3060 | hsa-miR-877 | 6924 | TCEB3 | transcription elongation factor B (SIII), polypeptide 3 (110kDa, elongin A) | 1 |
| 3061 | hsa-mir-26a-1 | 6924 | TCEB3 | transcription elongation factor B (SIII), polypeptide 3 (110kDa, elongin A) | 1 |
| 3062 | hsa-mir-26b | 6924 | TCEB3 | transcription elongation factor B (SIII), polypeptide 3 (110kDa, elongin A) | 1 |
| 3063 | hsa-mir-31 | 6924 | TCEB3 | transcription elongation factor B (SIII), polypeptide 3 (110kDa, elongin A) | 1 |
| 3064 | hsa-mir-194-2 | 6924 | TCEB3 | transcription elongation factor B (SIII), polypeptide 3 (110kDa, elongin A) | 1 |
| 3065 | hsa-mir-340 | 6924 | TCEB3 | transcription elongation factor B (SIII), polypeptide 3 (110kDa, elongin A) | 1 |
| 3066 | hsa-mir-338 | 6924 | TCEB3 | transcription elongation factor B (SIII), polypeptide 3 (110kDa, elongin A) | 1 |
| 3067 | hsa-mir-548b | 6924 | TCEB3 | transcription elongation factor B (SIII), polypeptide 3 (110kDa, elongin A) | 1 |
| 3068 | hsa-mir-548d-2 | 6924 | TCEB3 | transcription elongation factor B (SIII), polypeptide 3 (110kDa, elongin A) | 1 |
| 3069 | hsa-mir-449b | 6924 | TCEB3 | transcription elongation factor B (SIII), polypeptide 3 (110kDa, elongin A) | 1 |
| 3070 | hsa-mir-449c | 6924 | TCEB3 | transcription elongation factor B (SIII), polypeptide 3 (110kDa, elongin A) | 1 |
| 3071 | hsa-mir-3065 | 6924 | TCEB3 | transcription elongation factor B (SIII), polypeptide 3 (110kDa, elongin A) | 1 |
| 3102 | hsa-miR-26a | 140885 | SIRPA | signal-regulatory protein alpha | 1 |
| 3106 | hsa-miR-423-5p | 140885 | SIRPA | signal-regulatory protein alpha | 1 |
| 3108 | hsa-miR-490-5p | 140885 | SIRPA | signal-regulatory protein alpha | 1 |
| 3111 | hsa-miR-564 | 140885 | SIRPA | signal-regulatory protein alpha | 1 |
| 3112 | hsa-miR-584 | 140885 | SIRPA | signal-regulatory protein alpha | 1 |
| 3114 | hsa-miR-634 | 140885 | SIRPA | signal-regulatory protein alpha | 1 |
| 3115 | hsa-miR-644 | 140885 | SIRPA | signal-regulatory protein alpha | 1 |
| 3116 | hsa-miR-653 | 140885 | SIRPA | signal-regulatory protein alpha | 1 |
| 3120 | hsa-miR-95 | 140885 | SIRPA | signal-regulatory protein alpha | 1 |
| 3126 | hsa-mir-490 | 140885 | SIRPA | signal-regulatory protein alpha | 1 |
| 3130 | hsa-miR-139-5p | 140885 | SIRPA | signal-regulatory protein alpha | 1 |
| 3131 | hsa-miR-338-5p | 140885 | SIRPA | signal-regulatory protein alpha | 1 |
| 3132 | hsa-miR-383 | 140885 | SIRPA | signal-regulatory protein alpha | 1 |
| 3133 | hsa-miR-657 | 140885 | SIRPA | signal-regulatory protein alpha | 1 |
| 3134 | hsa-mir-338 | 140885 | SIRPA | signal-regulatory protein alpha | 1 |
| 3135 | hsa-mir-3065 | 140885 | SIRPA | signal-regulatory protein alpha | 1 |
| 3136 | hsa-miR-10a | 976 | CD97 | CD97 molecule | 1 |
| 3137 | hsa-miR-139-5p | 976 | CD97 | CD97 molecule | 1 |
| 3138 | hsa-miR-152 | 976 | CD97 | CD97 molecule | 1 |
| 3139 | hsa-miR-31 | 976 | CD97 | CD97 molecule | 1 |
| 3140 | hsa-miR-338-5p | 976 | CD97 | CD97 molecule | 1 |
| 3141 | hsa-miR-340 | 976 | CD97 | CD97 molecule | 1 |
| 3142 | hsa-miR-383 | 976 | CD97 | CD97 molecule | 1 |
| 3143 | hsa-miR-449a | 976 | CD97 | CD97 molecule | 1 |
| 3144 | hsa-miR-499-5p | 976 | CD97 | CD97 molecule | 1 |
| 3145 | hsa-miR-548b-5p | 976 | CD97 | CD97 molecule | 1 |
| 3146 | hsa-miR-621 | 976 | CD97 | CD97 molecule | 1 |
| 3147 | hsa-miR-657 | 976 | CD97 | CD97 molecule | 1 |
| 3148 | hsa-miR-663 | 976 | CD97 | CD97 molecule | 1 |
| 3149 | hsa-miR-765 | 976 | CD97 | CD97 molecule | 1 |
| 3150 | hsa-miR-770-5p | 976 | CD97 | CD97 molecule | 1 |
| 3151 | hsa-miR-877 | 976 | CD97 | CD97 molecule | 1 |
| 3152 | hsa-mir-26a-1 | 976 | CD97 | CD97 molecule | 1 |
| 3153 | hsa-mir-31 | 976 | CD97 | CD97 molecule | 1 |
| 3154 | hsa-mir-194-2 | 976 | CD97 | CD97 molecule | 1 |
| 3155 | hsa-mir-340 | 976 | CD97 | CD97 molecule | 1 |
| 3156 | hsa-mir-338 | 976 | CD97 | CD97 molecule | 1 |
| 3157 | hsa-mir-548b | 976 | CD97 | CD97 molecule | 1 |
| 3158 | hsa-mir-449b | 976 | CD97 | CD97 molecule | 1 |
| 3159 | hsa-mir-449c | 976 | CD97 | CD97 molecule | 1 |
| 3160 | hsa-mir-3065 | 976 | CD97 | CD97 molecule | 1 |
| 3161 | hsa-miR-1 | 56998 | CTNNBIP1 | catenin, beta interacting protein 1 | 1 |
| 3162 | hsa-miR-133a | 56998 | CTNNBIP1 | catenin, beta interacting protein 1 | 1 |
| 3163 | hsa-miR-186 | 56998 | CTNNBIP1 | catenin, beta interacting protein 1 | 1 |
| 3164 | hsa-miR-208a | 56998 | CTNNBIP1 | catenin, beta interacting protein 1 | 1 |
| 3165 | hsa-miR-326 | 56998 | CTNNBIP1 | catenin, beta interacting protein 1 | 1 |
| 3166 | hsa-miR-449b | 56998 | CTNNBIP1 | catenin, beta interacting protein 1 | 1 |
| 3167 | hsa-miR-550 | 56998 | CTNNBIP1 | catenin, beta interacting protein 1 | 1 |
| 3168 | hsa-miR-562 | 56998 | CTNNBIP1 | catenin, beta interacting protein 1 | 1 |
| 3169 | hsa-miR-575 | 56998 | CTNNBIP1 | catenin, beta interacting protein 1 | 1 |
| 3170 | hsa-miR-579 | 56998 | CTNNBIP1 | catenin, beta interacting protein 1 | 1 |
| 3171 | hsa-miR-608 | 56998 | CTNNBIP1 | catenin, beta interacting protein 1 | 1 |
| 3172 | hsa-miR-620 | 56998 | CTNNBIP1 | catenin, beta interacting protein 1 | 1 |
| 3173 | hsa-miR-637 | 56998 | CTNNBIP1 | catenin, beta interacting protein 1 | 1 |
| 3174 | hsa-miR-885-5p | 56998 | CTNNBIP1 | catenin, beta interacting protein 1 | 1 |
| 3175 | hsa-mir-22 | 56998 | CTNNBIP1 | catenin, beta interacting protein 1 | 1 |
| 3176 | hsa-mir-10a | 56998 | CTNNBIP1 | catenin, beta interacting protein 1 | 1 |
| 3177 | hsa-mir-186 | 56998 | CTNNBIP1 | catenin, beta interacting protein 1 | 1 |
| 3178 | hsa-mir-155 | 56998 | CTNNBIP1 | catenin, beta interacting protein 1 | 1 |
| 3179 | hsa-mir-423 | 56998 | CTNNBIP1 | catenin, beta interacting protein 1 | 1 |
| 3180 | hsa-mir-499 | 56998 | CTNNBIP1 | catenin, beta interacting protein 1 | 1 |
| 3181 | hsa-mir-550-1 | 56998 | CTNNBIP1 | catenin, beta interacting protein 1 | 1 |
| 3182 | hsa-mir-885 | 56998 | CTNNBIP1 | catenin, beta interacting protein 1 | 1 |
| 3183 | hsa-mir-877 | 56998 | CTNNBIP1 | catenin, beta interacting protein 1 | 1 |
| 3184 | hsa-mir-2116 | 56998 | CTNNBIP1 | catenin, beta interacting protein 1 | 1 |
| 3185 | hsa-mir-3190 | 56998 | CTNNBIP1 | catenin, beta interacting protein 1 | 1 |
| 3187 | hsa-miR-1228\* | 9693 | RAPGEF2 | Rap guanine nucleotide exchange factor (GEF) 2 | 1 |
| 3189 | hsa-miR-152 | 9693 | RAPGEF2 | Rap guanine nucleotide exchange factor (GEF) 2 | 1 |
| 3194 | hsa-miR-423-5p | 9693 | RAPGEF2 | Rap guanine nucleotide exchange factor (GEF) 2 | 1 |
| 3213 | hsa-miR-1 | 23229 | ARHGEF9 | Cdc42 guanine nucleotide exchange factor (GEF) 9 | 1 |
| 3214 | hsa-miR-133a | 23229 | ARHGEF9 | Cdc42 guanine nucleotide exchange factor (GEF) 9 | 1 |
| 3215 | hsa-miR-186 | 23229 | ARHGEF9 | Cdc42 guanine nucleotide exchange factor (GEF) 9 | 1 |
| 3216 | hsa-miR-326 | 23229 | ARHGEF9 | Cdc42 guanine nucleotide exchange factor (GEF) 9 | 1 |
| 3217 | hsa-miR-449b | 23229 | ARHGEF9 | Cdc42 guanine nucleotide exchange factor (GEF) 9 | 1 |
| 3218 | hsa-miR-489 | 23229 | ARHGEF9 | Cdc42 guanine nucleotide exchange factor (GEF) 9 | 1 |
| 3219 | hsa-miR-551a | 23229 | ARHGEF9 | Cdc42 guanine nucleotide exchange factor (GEF) 9 | 1 |
| 3220 | hsa-miR-562 | 23229 | ARHGEF9 | Cdc42 guanine nucleotide exchange factor (GEF) 9 | 1 |
| 3221 | hsa-miR-575 | 23229 | ARHGEF9 | Cdc42 guanine nucleotide exchange factor (GEF) 9 | 1 |
| 3222 | hsa-miR-579 | 23229 | ARHGEF9 | Cdc42 guanine nucleotide exchange factor (GEF) 9 | 1 |
| 3223 | hsa-miR-608 | 23229 | ARHGEF9 | Cdc42 guanine nucleotide exchange factor (GEF) 9 | 1 |
| 3224 | hsa-miR-637 | 23229 | ARHGEF9 | Cdc42 guanine nucleotide exchange factor (GEF) 9 | 1 |
| 3225 | hsa-miR-885-5p | 23229 | ARHGEF9 | Cdc42 guanine nucleotide exchange factor (GEF) 9 | 1 |
| 3226 | hsa-mir-22 | 23229 | ARHGEF9 | Cdc42 guanine nucleotide exchange factor (GEF) 9 | 1 |
| 3227 | hsa-mir-10a | 23229 | ARHGEF9 | Cdc42 guanine nucleotide exchange factor (GEF) 9 | 1 |
| 3228 | hsa-mir-186 | 23229 | ARHGEF9 | Cdc42 guanine nucleotide exchange factor (GEF) 9 | 1 |
| 3229 | hsa-mir-155 | 23229 | ARHGEF9 | Cdc42 guanine nucleotide exchange factor (GEF) 9 | 1 |
| 3230 | hsa-mir-423 | 23229 | ARHGEF9 | Cdc42 guanine nucleotide exchange factor (GEF) 9 | 1 |
| 3231 | hsa-mir-499 | 23229 | ARHGEF9 | Cdc42 guanine nucleotide exchange factor (GEF) 9 | 1 |
| 3232 | hsa-mir-885 | 23229 | ARHGEF9 | Cdc42 guanine nucleotide exchange factor (GEF) 9 | 1 |
| 3233 | hsa-mir-877 | 23229 | ARHGEF9 | Cdc42 guanine nucleotide exchange factor (GEF) 9 | 1 |
| 3234 | hsa-mir-3190 | 23229 | ARHGEF9 | Cdc42 guanine nucleotide exchange factor (GEF) 9 | 1 |
| 3235 | hsa-miR-152 | 9988 | DMTF1 | cyclin D binding myb-like transcription factor 1 | 1 |
| 3236 | hsa-miR-26a | 9988 | DMTF1 | cyclin D binding myb-like transcription factor 1 | 1 |
| 3237 | hsa-miR-26b | 9988 | DMTF1 | cyclin D binding myb-like transcription factor 1 | 1 |
| 3238 | hsa-miR-31 | 9988 | DMTF1 | cyclin D binding myb-like transcription factor 1 | 1 |
| 3239 | hsa-miR-338-5p | 9988 | DMTF1 | cyclin D binding myb-like transcription factor 1 | 1 |
| 3240 | hsa-miR-340 | 9988 | DMTF1 | cyclin D binding myb-like transcription factor 1 | 1 |
| 3241 | hsa-miR-383 | 9988 | DMTF1 | cyclin D binding myb-like transcription factor 1 | 1 |
| 3242 | hsa-miR-423-5p | 9988 | DMTF1 | cyclin D binding myb-like transcription factor 1 | 1 |
| 3243 | hsa-miR-449a | 9988 | DMTF1 | cyclin D binding myb-like transcription factor 1 | 1 |
| 3244 | hsa-miR-490-5p | 9988 | DMTF1 | cyclin D binding myb-like transcription factor 1 | 1 |
| 3245 | hsa-miR-499-5p | 9988 | DMTF1 | cyclin D binding myb-like transcription factor 1 | 1 |
| 3246 | hsa-miR-548b-5p | 9988 | DMTF1 | cyclin D binding myb-like transcription factor 1 | 1 |
| 3247 | hsa-miR-564 | 9988 | DMTF1 | cyclin D binding myb-like transcription factor 1 | 1 |
| 3248 | hsa-miR-621 | 9988 | DMTF1 | cyclin D binding myb-like transcription factor 1 | 1 |
| 3249 | hsa-miR-657 | 9988 | DMTF1 | cyclin D binding myb-like transcription factor 1 | 1 |
| 3250 | hsa-miR-765 | 9988 | DMTF1 | cyclin D binding myb-like transcription factor 1 | 1 |
| 3251 | hsa-miR-770-5p | 9988 | DMTF1 | cyclin D binding myb-like transcription factor 1 | 1 |
| 3252 | hsa-mir-26a-1 | 9988 | DMTF1 | cyclin D binding myb-like transcription factor 1 | 1 |
| 3253 | hsa-mir-26b | 9988 | DMTF1 | cyclin D binding myb-like transcription factor 1 | 1 |
| 3254 | hsa-mir-31 | 9988 | DMTF1 | cyclin D binding myb-like transcription factor 1 | 1 |
| 3255 | hsa-mir-194-2 | 9988 | DMTF1 | cyclin D binding myb-like transcription factor 1 | 1 |
| 3256 | hsa-mir-340 | 9988 | DMTF1 | cyclin D binding myb-like transcription factor 1 | 1 |
| 3257 | hsa-mir-338 | 9988 | DMTF1 | cyclin D binding myb-like transcription factor 1 | 1 |
| 3258 | hsa-mir-490 | 9988 | DMTF1 | cyclin D binding myb-like transcription factor 1 | 1 |
| 3259 | hsa-mir-548b | 9988 | DMTF1 | cyclin D binding myb-like transcription factor 1 | 1 |
| 3260 | hsa-mir-449b | 9988 | DMTF1 | cyclin D binding myb-like transcription factor 1 | 1 |
| 3261 | hsa-mir-449c | 9988 | DMTF1 | cyclin D binding myb-like transcription factor 1 | 1 |
| 3262 | hsa-mir-3065 | 9988 | DMTF1 | cyclin D binding myb-like transcription factor 1 | 1 |
| 3263 | hsa-miR-1 | 4753 | NELL2 | NEL-like 2 (chicken) | 1 |
| 3264 | hsa-miR-133a | 4753 | NELL2 | NEL-like 2 (chicken) | 1 |
| 3265 | hsa-miR-186 | 4753 | NELL2 | NEL-like 2 (chicken) | 1 |
| 3266 | hsa-miR-208a | 4753 | NELL2 | NEL-like 2 (chicken) | 1 |
| 3267 | hsa-miR-449b | 4753 | NELL2 | NEL-like 2 (chicken) | 1 |
| 3268 | hsa-miR-579 | 4753 | NELL2 | NEL-like 2 (chicken) | 1 |
| 3269 | hsa-miR-608 | 4753 | NELL2 | NEL-like 2 (chicken) | 1 |
| 3270 | hsa-miR-620 | 4753 | NELL2 | NEL-like 2 (chicken) | 1 |
| 3271 | hsa-miR-885-5p | 4753 | NELL2 | NEL-like 2 (chicken) | 1 |
| 3272 | hsa-let-7a-3 | 4753 | NELL2 | NEL-like 2 (chicken) | 1 |
| 3273 | hsa-let-7b | 4753 | NELL2 | NEL-like 2 (chicken) | 1 |
| 3274 | hsa-mir-22 | 4753 | NELL2 | NEL-like 2 (chicken) | 1 |
| 3275 | hsa-mir-10a | 4753 | NELL2 | NEL-like 2 (chicken) | 1 |
| 3276 | hsa-mir-186 | 4753 | NELL2 | NEL-like 2 (chicken) | 1 |
| 3277 | hsa-mir-155 | 4753 | NELL2 | NEL-like 2 (chicken) | 1 |
| 3278 | hsa-mir-499 | 4753 | NELL2 | NEL-like 2 (chicken) | 1 |
| 3279 | hsa-mir-885 | 4753 | NELL2 | NEL-like 2 (chicken) | 1 |
| 3280 | hsa-mir-877 | 4753 | NELL2 | NEL-like 2 (chicken) | 1 |
| 3281 | hsa-miR-1 | 11309 | SLCO2B1 | solute carrier organic anion transporter family, member 2B1 | 1 |
| 3282 | hsa-miR-133a | 11309 | SLCO2B1 | solute carrier organic anion transporter family, member 2B1 | 1 |
| 3283 | hsa-miR-186 | 11309 | SLCO2B1 | solute carrier organic anion transporter family, member 2B1 | 1 |
| 3284 | hsa-miR-326 | 11309 | SLCO2B1 | solute carrier organic anion transporter family, member 2B1 | 1 |
| 3285 | hsa-miR-449b | 11309 | SLCO2B1 | solute carrier organic anion transporter family, member 2B1 | 1 |
| 3286 | hsa-miR-489 | 11309 | SLCO2B1 | solute carrier organic anion transporter family, member 2B1 | 1 |
| 3287 | hsa-miR-562 | 11309 | SLCO2B1 | solute carrier organic anion transporter family, member 2B1 | 1 |
| 3288 | hsa-miR-575 | 11309 | SLCO2B1 | solute carrier organic anion transporter family, member 2B1 | 1 |
| 3289 | hsa-miR-579 | 11309 | SLCO2B1 | solute carrier organic anion transporter family, member 2B1 | 1 |
| 3290 | hsa-miR-608 | 11309 | SLCO2B1 | solute carrier organic anion transporter family, member 2B1 | 1 |
| 3291 | hsa-miR-620 | 11309 | SLCO2B1 | solute carrier organic anion transporter family, member 2B1 | 1 |
| 3292 | hsa-miR-637 | 11309 | SLCO2B1 | solute carrier organic anion transporter family, member 2B1 | 1 |
| 3293 | hsa-miR-885-5p | 11309 | SLCO2B1 | solute carrier organic anion transporter family, member 2B1 | 1 |
| 3294 | hsa-mir-22 | 11309 | SLCO2B1 | solute carrier organic anion transporter family, member 2B1 | 1 |
| 3295 | hsa-mir-10a | 11309 | SLCO2B1 | solute carrier organic anion transporter family, member 2B1 | 1 |
| 3296 | hsa-mir-186 | 11309 | SLCO2B1 | solute carrier organic anion transporter family, member 2B1 | 1 |
| 3297 | hsa-mir-155 | 11309 | SLCO2B1 | solute carrier organic anion transporter family, member 2B1 | 1 |
| 3298 | hsa-mir-499 | 11309 | SLCO2B1 | solute carrier organic anion transporter family, member 2B1 | 1 |
| 3299 | hsa-mir-885 | 11309 | SLCO2B1 | solute carrier organic anion transporter family, member 2B1 | 1 |
| 3300 | hsa-mir-877 | 11309 | SLCO2B1 | solute carrier organic anion transporter family, member 2B1 | 1 |
| 3301 | hsa-mir-3190 | 11309 | SLCO2B1 | solute carrier organic anion transporter family, member 2B1 | 1 |
| 3302 | hsa-miR-10a | 54726 | OTUD4 | OTU domain containing 4 | 1 |
| 3303 | hsa-miR-1228\* | 54726 | OTUD4 | OTU domain containing 4 | 1 |
| 3304 | hsa-miR-152 | 54726 | OTUD4 | OTU domain containing 4 | 1 |
| 3305 | hsa-miR-26a | 54726 | OTUD4 | OTU domain containing 4 | 1 |
| 3306 | hsa-miR-26b | 54726 | OTUD4 | OTU domain containing 4 | 1 |
| 3307 | hsa-miR-31 | 54726 | OTUD4 | OTU domain containing 4 | 1 |
| 3308 | hsa-miR-338-5p | 54726 | OTUD4 | OTU domain containing 4 | 1 |
| 3309 | hsa-miR-340 | 54726 | OTUD4 | OTU domain containing 4 | 1 |
| 3310 | hsa-miR-423-5p | 54726 | OTUD4 | OTU domain containing 4 | 1 |
| 3311 | hsa-miR-449a | 54726 | OTUD4 | OTU domain containing 4 | 1 |
| 3312 | hsa-miR-490-5p | 54726 | OTUD4 | OTU domain containing 4 | 1 |
| 3313 | hsa-miR-499-5p | 54726 | OTUD4 | OTU domain containing 4 | 1 |
| 3314 | hsa-miR-548b-5p | 54726 | OTUD4 | OTU domain containing 4 | 1 |
| 3315 | hsa-miR-564 | 54726 | OTUD4 | OTU domain containing 4 | 1 |
| 3316 | hsa-miR-621 | 54726 | OTUD4 | OTU domain containing 4 | 1 |
| 3317 | hsa-miR-644 | 54726 | OTUD4 | OTU domain containing 4 | 1 |
| 3318 | hsa-miR-657 | 54726 | OTUD4 | OTU domain containing 4 | 1 |
| 3319 | hsa-miR-663 | 54726 | OTUD4 | OTU domain containing 4 | 1 |
| 3320 | hsa-miR-765 | 54726 | OTUD4 | OTU domain containing 4 | 1 |
| 3321 | hsa-miR-770-5p | 54726 | OTUD4 | OTU domain containing 4 | 1 |
| 3322 | hsa-miR-877 | 54726 | OTUD4 | OTU domain containing 4 | 1 |
| 3323 | hsa-mir-26a-1 | 54726 | OTUD4 | OTU domain containing 4 | 1 |
| 3324 | hsa-mir-26b | 54726 | OTUD4 | OTU domain containing 4 | 1 |
| 3325 | hsa-mir-31 | 54726 | OTUD4 | OTU domain containing 4 | 1 |
| 3326 | hsa-mir-194-2 | 54726 | OTUD4 | OTU domain containing 4 | 1 |
| 3327 | hsa-mir-340 | 54726 | OTUD4 | OTU domain containing 4 | 1 |
| 3328 | hsa-mir-338 | 54726 | OTUD4 | OTU domain containing 4 | 1 |
| 3329 | hsa-mir-490 | 54726 | OTUD4 | OTU domain containing 4 | 1 |
| 3330 | hsa-mir-548b | 54726 | OTUD4 | OTU domain containing 4 | 1 |
| 3331 | hsa-mir-548d-2 | 54726 | OTUD4 | OTU domain containing 4 | 1 |
| 3332 | hsa-mir-449b | 54726 | OTUD4 | OTU domain containing 4 | 1 |
| 3333 | hsa-mir-449c | 54726 | OTUD4 | OTU domain containing 4 | 1 |
| 3334 | hsa-mir-3065 | 54726 | OTUD4 | OTU domain containing 4 | 1 |
| 3335 | hsa-miR-10a | 11193 | WBP4 | WW domain binding protein 4 (formin binding protein 21) | 1 |
| 3336 | hsa-miR-139-5p | 11193 | WBP4 | WW domain binding protein 4 (formin binding protein 21) | 1 |
| 3337 | hsa-miR-152 | 11193 | WBP4 | WW domain binding protein 4 (formin binding protein 21) | 1 |
| 3338 | hsa-miR-26a | 11193 | WBP4 | WW domain binding protein 4 (formin binding protein 21) | 1 |
| 3339 | hsa-miR-26b | 11193 | WBP4 | WW domain binding protein 4 (formin binding protein 21) | 1 |
| 3340 | hsa-miR-31 | 11193 | WBP4 | WW domain binding protein 4 (formin binding protein 21) | 1 |
| 3341 | hsa-miR-338-5p | 11193 | WBP4 | WW domain binding protein 4 (formin binding protein 21) | 1 |
| 3342 | hsa-miR-340 | 11193 | WBP4 | WW domain binding protein 4 (formin binding protein 21) | 1 |
| 3343 | hsa-miR-449a | 11193 | WBP4 | WW domain binding protein 4 (formin binding protein 21) | 1 |
| 3344 | hsa-miR-499-5p | 11193 | WBP4 | WW domain binding protein 4 (formin binding protein 21) | 1 |
| 3345 | hsa-miR-548b-5p | 11193 | WBP4 | WW domain binding protein 4 (formin binding protein 21) | 1 |
| 3346 | hsa-miR-564 | 11193 | WBP4 | WW domain binding protein 4 (formin binding protein 21) | 1 |
| 3347 | hsa-miR-621 | 11193 | WBP4 | WW domain binding protein 4 (formin binding protein 21) | 1 |
| 3348 | hsa-miR-634 | 11193 | WBP4 | WW domain binding protein 4 (formin binding protein 21) | 1 |
| 3349 | hsa-miR-657 | 11193 | WBP4 | WW domain binding protein 4 (formin binding protein 21) | 1 |
| 3350 | hsa-miR-765 | 11193 | WBP4 | WW domain binding protein 4 (formin binding protein 21) | 1 |
| 3351 | hsa-miR-770-5p | 11193 | WBP4 | WW domain binding protein 4 (formin binding protein 21) | 1 |
| 3352 | hsa-miR-877 | 11193 | WBP4 | WW domain binding protein 4 (formin binding protein 21) | 1 |
| 3353 | hsa-mir-26a-1 | 11193 | WBP4 | WW domain binding protein 4 (formin binding protein 21) | 1 |
| 3354 | hsa-mir-26b | 11193 | WBP4 | WW domain binding protein 4 (formin binding protein 21) | 1 |
| 3355 | hsa-mir-31 | 11193 | WBP4 | WW domain binding protein 4 (formin binding protein 21) | 1 |
| 3356 | hsa-mir-194-2 | 11193 | WBP4 | WW domain binding protein 4 (formin binding protein 21) | 1 |
| 3357 | hsa-mir-340 | 11193 | WBP4 | WW domain binding protein 4 (formin binding protein 21) | 1 |
| 3358 | hsa-mir-338 | 11193 | WBP4 | WW domain binding protein 4 (formin binding protein 21) | 1 |
| 3359 | hsa-mir-548b | 11193 | WBP4 | WW domain binding protein 4 (formin binding protein 21) | 1 |
| 3360 | hsa-mir-449b | 11193 | WBP4 | WW domain binding protein 4 (formin binding protein 21) | 1 |
| 3361 | hsa-mir-449c | 11193 | WBP4 | WW domain binding protein 4 (formin binding protein 21) | 1 |
| 3362 | hsa-mir-3065 | 11193 | WBP4 | WW domain binding protein 4 (formin binding protein 21) | 1 |
| 3363 | hsa-miR-10a | 10206 | TRIM13 | tripartite motif-containing 13 | 1 |
| 3364 | hsa-miR-152 | 10206 | TRIM13 | tripartite motif-containing 13 | 1 |
| 3365 | hsa-miR-31 | 10206 | TRIM13 | tripartite motif-containing 13 | 1 |
| 3366 | hsa-miR-338-5p | 10206 | TRIM13 | tripartite motif-containing 13 | 1 |
| 3367 | hsa-miR-340 | 10206 | TRIM13 | tripartite motif-containing 13 | 1 |
| 3368 | hsa-miR-383 | 10206 | TRIM13 | tripartite motif-containing 13 | 1 |
| 3369 | hsa-miR-449a | 10206 | TRIM13 | tripartite motif-containing 13 | 1 |
| 3370 | hsa-miR-490-5p | 10206 | TRIM13 | tripartite motif-containing 13 | 1 |
| 3371 | hsa-miR-504 | 10206 | TRIM13 | tripartite motif-containing 13 | 1 |
| 3372 | hsa-miR-621 | 10206 | TRIM13 | tripartite motif-containing 13 | 1 |
| 3373 | hsa-miR-653 | 10206 | TRIM13 | tripartite motif-containing 13 | 1 |
| 3374 | hsa-miR-657 | 10206 | TRIM13 | tripartite motif-containing 13 | 1 |
| 3375 | hsa-miR-765 | 10206 | TRIM13 | tripartite motif-containing 13 | 1 |
| 3376 | hsa-miR-770-5p | 10206 | TRIM13 | tripartite motif-containing 13 | 1 |
| 3377 | hsa-miR-877 | 10206 | TRIM13 | tripartite motif-containing 13 | 1 |
| 3378 | hsa-miR-95 | 10206 | TRIM13 | tripartite motif-containing 13 | 1 |
| 3379 | hsa-mir-26a-1 | 10206 | TRIM13 | tripartite motif-containing 13 | 1 |
| 3380 | hsa-mir-31 | 10206 | TRIM13 | tripartite motif-containing 13 | 1 |
| 3381 | hsa-mir-194-2 | 10206 | TRIM13 | tripartite motif-containing 13 | 1 |
| 3382 | hsa-mir-340 | 10206 | TRIM13 | tripartite motif-containing 13 | 1 |
| 3383 | hsa-mir-338 | 10206 | TRIM13 | tripartite motif-containing 13 | 1 |
| 3384 | hsa-mir-490 | 10206 | TRIM13 | tripartite motif-containing 13 | 1 |
| 3385 | hsa-mir-449b | 10206 | TRIM13 | tripartite motif-containing 13 | 1 |
| 3386 | hsa-mir-449c | 10206 | TRIM13 | tripartite motif-containing 13 | 1 |
| 3387 | hsa-mir-3065 | 10206 | TRIM13 | tripartite motif-containing 13 | 1 |
| 3388 | hsa-miR-10a | 578 | BAK1 | BCL2-antagonist/killer 1 | 1 |
| 3389 | hsa-miR-139-5p | 578 | BAK1 | BCL2-antagonist/killer 1 | 1 |
| 3390 | hsa-miR-152 | 578 | BAK1 | BCL2-antagonist/killer 1 | 1 |
| 3391 | hsa-miR-26a | 578 | BAK1 | BCL2-antagonist/killer 1 | 1 |
| 3392 | hsa-miR-26b | 578 | BAK1 | BCL2-antagonist/killer 1 | 1 |
| 3393 | hsa-miR-31 | 578 | BAK1 | BCL2-antagonist/killer 1 | 1 |
| 3394 | hsa-miR-338-5p | 578 | BAK1 | BCL2-antagonist/killer 1 | 1 |
| 3395 | hsa-miR-340 | 578 | BAK1 | BCL2-antagonist/killer 1 | 1 |
| 3396 | hsa-miR-423-5p | 578 | BAK1 | BCL2-antagonist/killer 1 | 1 |
| 3397 | hsa-miR-449a | 578 | BAK1 | BCL2-antagonist/killer 1 | 1 |
| 3398 | hsa-miR-490-5p | 578 | BAK1 | BCL2-antagonist/killer 1 | 1 |
| 3399 | hsa-miR-499-5p | 578 | BAK1 | BCL2-antagonist/killer 1 | 1 |
| 3400 | hsa-miR-548b-5p | 578 | BAK1 | BCL2-antagonist/killer 1 | 1 |
| 3401 | hsa-miR-564 | 578 | BAK1 | BCL2-antagonist/killer 1 | 1 |
| 3402 | hsa-miR-584 | 578 | BAK1 | BCL2-antagonist/killer 1 | 1 |
| 3403 | hsa-miR-621 | 578 | BAK1 | BCL2-antagonist/killer 1 | 1 |
| 3404 | hsa-miR-657 | 578 | BAK1 | BCL2-antagonist/killer 1 | 1 |
| 3405 | hsa-miR-663 | 578 | BAK1 | BCL2-antagonist/killer 1 | 1 |
| 3406 | hsa-miR-765 | 578 | BAK1 | BCL2-antagonist/killer 1 | 1 |
| 3407 | hsa-miR-770-5p | 578 | BAK1 | BCL2-antagonist/killer 1 | 1 |
| 3408 | hsa-miR-877 | 578 | BAK1 | BCL2-antagonist/killer 1 | 1 |
| 3409 | hsa-mir-26a-1 | 578 | BAK1 | BCL2-antagonist/killer 1 | 1 |
| 3410 | hsa-mir-26b | 578 | BAK1 | BCL2-antagonist/killer 1 | 1 |
| 3411 | hsa-mir-31 | 578 | BAK1 | BCL2-antagonist/killer 1 | 1 |
| 3412 | hsa-mir-194-2 | 578 | BAK1 | BCL2-antagonist/killer 1 | 1 |
| 3413 | hsa-mir-340 | 578 | BAK1 | BCL2-antagonist/killer 1 | 1 |
| 3414 | hsa-mir-338 | 578 | BAK1 | BCL2-antagonist/killer 1 | 1 |
| 3415 | hsa-mir-490 | 578 | BAK1 | BCL2-antagonist/killer 1 | 1 |
| 3416 | hsa-mir-548b | 578 | BAK1 | BCL2-antagonist/killer 1 | 1 |
| 3417 | hsa-mir-548d-2 | 578 | BAK1 | BCL2-antagonist/killer 1 | 1 |
| 3418 | hsa-mir-449b | 578 | BAK1 | BCL2-antagonist/killer 1 | 1 |
| 3419 | hsa-mir-449c | 578 | BAK1 | BCL2-antagonist/killer 1 | 1 |
| 3420 | hsa-mir-3065 | 578 | BAK1 | BCL2-antagonist/killer 1 | 1 |
| 3421 | hsa-miR-10a | 2983 | GUCY1B3 | guanylate cyclase 1, soluble, beta 3 | 1 |
| 3422 | hsa-miR-1228\* | 2983 | GUCY1B3 | guanylate cyclase 1, soluble, beta 3 | 1 |
| 3423 | hsa-miR-139-5p | 2983 | GUCY1B3 | guanylate cyclase 1, soluble, beta 3 | 1 |
| 3424 | hsa-miR-152 | 2983 | GUCY1B3 | guanylate cyclase 1, soluble, beta 3 | 1 |
| 3425 | hsa-miR-26a | 2983 | GUCY1B3 | guanylate cyclase 1, soluble, beta 3 | 1 |
| 3426 | hsa-miR-26b | 2983 | GUCY1B3 | guanylate cyclase 1, soluble, beta 3 | 1 |
| 3427 | hsa-miR-338-5p | 2983 | GUCY1B3 | guanylate cyclase 1, soluble, beta 3 | 1 |
| 3428 | hsa-miR-340 | 2983 | GUCY1B3 | guanylate cyclase 1, soluble, beta 3 | 1 |
| 3429 | hsa-miR-449a | 2983 | GUCY1B3 | guanylate cyclase 1, soluble, beta 3 | 1 |
| 3430 | hsa-miR-499-5p | 2983 | GUCY1B3 | guanylate cyclase 1, soluble, beta 3 | 1 |
| 3431 | hsa-miR-548b-5p | 2983 | GUCY1B3 | guanylate cyclase 1, soluble, beta 3 | 1 |
| 3432 | hsa-miR-584 | 2983 | GUCY1B3 | guanylate cyclase 1, soluble, beta 3 | 1 |
| 3433 | hsa-miR-621 | 2983 | GUCY1B3 | guanylate cyclase 1, soluble, beta 3 | 1 |
| 3434 | hsa-miR-657 | 2983 | GUCY1B3 | guanylate cyclase 1, soluble, beta 3 | 1 |
| 3435 | hsa-miR-663 | 2983 | GUCY1B3 | guanylate cyclase 1, soluble, beta 3 | 1 |
| 3436 | hsa-miR-765 | 2983 | GUCY1B3 | guanylate cyclase 1, soluble, beta 3 | 1 |
| 3437 | hsa-miR-770-5p | 2983 | GUCY1B3 | guanylate cyclase 1, soluble, beta 3 | 1 |
| 3438 | hsa-miR-877 | 2983 | GUCY1B3 | guanylate cyclase 1, soluble, beta 3 | 1 |
| 3439 | hsa-mir-26a-1 | 2983 | GUCY1B3 | guanylate cyclase 1, soluble, beta 3 | 1 |
| 3440 | hsa-mir-26b | 2983 | GUCY1B3 | guanylate cyclase 1, soluble, beta 3 | 1 |
| 3441 | hsa-mir-194-2 | 2983 | GUCY1B3 | guanylate cyclase 1, soluble, beta 3 | 1 |
| 3442 | hsa-mir-340 | 2983 | GUCY1B3 | guanylate cyclase 1, soluble, beta 3 | 1 |
| 3443 | hsa-mir-338 | 2983 | GUCY1B3 | guanylate cyclase 1, soluble, beta 3 | 1 |
| 3444 | hsa-mir-548b | 2983 | GUCY1B3 | guanylate cyclase 1, soluble, beta 3 | 1 |
| 3445 | hsa-mir-449b | 2983 | GUCY1B3 | guanylate cyclase 1, soluble, beta 3 | 1 |
| 3446 | hsa-mir-449c | 2983 | GUCY1B3 | guanylate cyclase 1, soluble, beta 3 | 1 |
| 3447 | hsa-mir-3065 | 2983 | GUCY1B3 | guanylate cyclase 1, soluble, beta 3 | 1 |
| 3448 | hsa-miR-10a | 9235 | IL32 | interleukin 32 | 1 |
| 3449 | hsa-miR-139-5p | 9235 | IL32 | interleukin 32 | 1 |
| 3450 | hsa-miR-152 | 9235 | IL32 | interleukin 32 | 1 |
| 3451 | hsa-miR-26a | 9235 | IL32 | interleukin 32 | 1 |
| 3452 | hsa-miR-26b | 9235 | IL32 | interleukin 32 | 1 |
| 3453 | hsa-miR-31 | 9235 | IL32 | interleukin 32 | 1 |
| 3454 | hsa-miR-338-5p | 9235 | IL32 | interleukin 32 | 1 |
| 3455 | hsa-miR-340 | 9235 | IL32 | interleukin 32 | 1 |
| 3456 | hsa-miR-423-5p | 9235 | IL32 | interleukin 32 | 1 |
| 3457 | hsa-miR-449a | 9235 | IL32 | interleukin 32 | 1 |
| 3458 | hsa-miR-499-5p | 9235 | IL32 | interleukin 32 | 1 |
| 3459 | hsa-miR-548b-5p | 9235 | IL32 | interleukin 32 | 1 |
| 3460 | hsa-miR-564 | 9235 | IL32 | interleukin 32 | 1 |
| 3461 | hsa-miR-621 | 9235 | IL32 | interleukin 32 | 1 |
| 3462 | hsa-miR-657 | 9235 | IL32 | interleukin 32 | 1 |
| 3463 | hsa-miR-765 | 9235 | IL32 | interleukin 32 | 1 |
| 3464 | hsa-miR-770-5p | 9235 | IL32 | interleukin 32 | 1 |
| 3465 | hsa-miR-877 | 9235 | IL32 | interleukin 32 | 1 |
| 3466 | hsa-mir-26a-1 | 9235 | IL32 | interleukin 32 | 1 |
| 3467 | hsa-mir-26b | 9235 | IL32 | interleukin 32 | 1 |
| 3468 | hsa-mir-31 | 9235 | IL32 | interleukin 32 | 1 |
| 3469 | hsa-mir-194-2 | 9235 | IL32 | interleukin 32 | 1 |
| 3470 | hsa-mir-340 | 9235 | IL32 | interleukin 32 | 1 |
| 3471 | hsa-mir-338 | 9235 | IL32 | interleukin 32 | 1 |
| 3472 | hsa-mir-548b | 9235 | IL32 | interleukin 32 | 1 |
| 3473 | hsa-mir-449b | 9235 | IL32 | interleukin 32 | 1 |
| 3474 | hsa-mir-449c | 9235 | IL32 | interleukin 32 | 1 |
| 3475 | hsa-mir-3065 | 9235 | IL32 | interleukin 32 | 1 |
| 3476 | hsa-miR-10a | 22924 | MAPRE3 | microtubule-associated protein, RP/EB family, member 3 | 1 |
| 3477 | hsa-miR-152 | 22924 | MAPRE3 | microtubule-associated protein, RP/EB family, member 3 | 1 |
| 3478 | hsa-miR-26a | 22924 | MAPRE3 | microtubule-associated protein, RP/EB family, member 3 | 1 |
| 3479 | hsa-miR-26b | 22924 | MAPRE3 | microtubule-associated protein, RP/EB family, member 3 | 1 |
| 3480 | hsa-miR-31 | 22924 | MAPRE3 | microtubule-associated protein, RP/EB family, member 3 | 1 |
| 3481 | hsa-miR-340 | 22924 | MAPRE3 | microtubule-associated protein, RP/EB family, member 3 | 1 |
| 3482 | hsa-miR-423-5p | 22924 | MAPRE3 | microtubule-associated protein, RP/EB family, member 3 | 1 |
| 3483 | hsa-miR-449a | 22924 | MAPRE3 | microtubule-associated protein, RP/EB family, member 3 | 1 |
| 3484 | hsa-miR-490-5p | 22924 | MAPRE3 | microtubule-associated protein, RP/EB family, member 3 | 1 |
| 3485 | hsa-miR-499-5p | 22924 | MAPRE3 | microtubule-associated protein, RP/EB family, member 3 | 1 |
| 3486 | hsa-miR-548b-5p | 22924 | MAPRE3 | microtubule-associated protein, RP/EB family, member 3 | 1 |
| 3487 | hsa-miR-564 | 22924 | MAPRE3 | microtubule-associated protein, RP/EB family, member 3 | 1 |
| 3488 | hsa-miR-621 | 22924 | MAPRE3 | microtubule-associated protein, RP/EB family, member 3 | 1 |
| 3489 | hsa-miR-634 | 22924 | MAPRE3 | microtubule-associated protein, RP/EB family, member 3 | 1 |
| 3490 | hsa-miR-644 | 22924 | MAPRE3 | microtubule-associated protein, RP/EB family, member 3 | 1 |
| 3491 | hsa-miR-765 | 22924 | MAPRE3 | microtubule-associated protein, RP/EB family, member 3 | 1 |
| 3492 | hsa-miR-770-5p | 22924 | MAPRE3 | microtubule-associated protein, RP/EB family, member 3 | 1 |
| 3493 | hsa-miR-877 | 22924 | MAPRE3 | microtubule-associated protein, RP/EB family, member 3 | 1 |
| 3494 | hsa-miR-95 | 22924 | MAPRE3 | microtubule-associated protein, RP/EB family, member 3 | 1 |
| 3495 | hsa-mir-26a-1 | 22924 | MAPRE3 | microtubule-associated protein, RP/EB family, member 3 | 1 |
| 3496 | hsa-mir-26b | 22924 | MAPRE3 | microtubule-associated protein, RP/EB family, member 3 | 1 |
| 3497 | hsa-mir-31 | 22924 | MAPRE3 | microtubule-associated protein, RP/EB family, member 3 | 1 |
| 3498 | hsa-mir-194-2 | 22924 | MAPRE3 | microtubule-associated protein, RP/EB family, member 3 | 1 |
| 3499 | hsa-mir-340 | 22924 | MAPRE3 | microtubule-associated protein, RP/EB family, member 3 | 1 |
| 3500 | hsa-mir-490 | 22924 | MAPRE3 | microtubule-associated protein, RP/EB family, member 3 | 1 |
| 3501 | hsa-mir-548b | 22924 | MAPRE3 | microtubule-associated protein, RP/EB family, member 3 | 1 |
| 3502 | hsa-mir-449b | 22924 | MAPRE3 | microtubule-associated protein, RP/EB family, member 3 | 1 |
| 3503 | hsa-mir-449c | 22924 | MAPRE3 | microtubule-associated protein, RP/EB family, member 3 | 1 |
| 3504 | hsa-miR-10a | 10063 | COX17 | COX17 cytochrome c oxidase assembly homolog (S. cerevisiae) | 1 |
| 3505 | hsa-miR-152 | 10063 | COX17 | COX17 cytochrome c oxidase assembly homolog (S. cerevisiae) | 1 |
| 3506 | hsa-miR-26a | 10063 | COX17 | COX17 cytochrome c oxidase assembly homolog (S. cerevisiae) | 1 |
| 3507 | hsa-miR-26b | 10063 | COX17 | COX17 cytochrome c oxidase assembly homolog (S. cerevisiae) | 1 |
| 3508 | hsa-miR-31 | 10063 | COX17 | COX17 cytochrome c oxidase assembly homolog (S. cerevisiae) | 1 |
| 3509 | hsa-miR-338-5p | 10063 | COX17 | COX17 cytochrome c oxidase assembly homolog (S. cerevisiae) | 1 |
| 3510 | hsa-miR-340 | 10063 | COX17 | COX17 cytochrome c oxidase assembly homolog (S. cerevisiae) | 1 |
| 3511 | hsa-miR-449a | 10063 | COX17 | COX17 cytochrome c oxidase assembly homolog (S. cerevisiae) | 1 |
| 3512 | hsa-miR-490-5p | 10063 | COX17 | COX17 cytochrome c oxidase assembly homolog (S. cerevisiae) | 1 |
| 3513 | hsa-miR-499-5p | 10063 | COX17 | COX17 cytochrome c oxidase assembly homolog (S. cerevisiae) | 1 |
| 3514 | hsa-miR-548b-5p | 10063 | COX17 | COX17 cytochrome c oxidase assembly homolog (S. cerevisiae) | 1 |
| 3515 | hsa-miR-584 | 10063 | COX17 | COX17 cytochrome c oxidase assembly homolog (S. cerevisiae) | 1 |
| 3516 | hsa-miR-621 | 10063 | COX17 | COX17 cytochrome c oxidase assembly homolog (S. cerevisiae) | 1 |
| 3517 | hsa-miR-657 | 10063 | COX17 | COX17 cytochrome c oxidase assembly homolog (S. cerevisiae) | 1 |
| 3518 | hsa-miR-765 | 10063 | COX17 | COX17 cytochrome c oxidase assembly homolog (S. cerevisiae) | 1 |
| 3519 | hsa-miR-770-5p | 10063 | COX17 | COX17 cytochrome c oxidase assembly homolog (S. cerevisiae) | 1 |
| 3520 | hsa-miR-877 | 10063 | COX17 | COX17 cytochrome c oxidase assembly homolog (S. cerevisiae) | 1 |
| 3521 | hsa-mir-26a-1 | 10063 | COX17 | COX17 cytochrome c oxidase assembly homolog (S. cerevisiae) | 1 |
| 3522 | hsa-mir-26b | 10063 | COX17 | COX17 cytochrome c oxidase assembly homolog (S. cerevisiae) | 1 |
| 3523 | hsa-mir-31 | 10063 | COX17 | COX17 cytochrome c oxidase assembly homolog (S. cerevisiae) | 1 |
| 3524 | hsa-mir-194-2 | 10063 | COX17 | COX17 cytochrome c oxidase assembly homolog (S. cerevisiae) | 1 |
| 3525 | hsa-mir-340 | 10063 | COX17 | COX17 cytochrome c oxidase assembly homolog (S. cerevisiae) | 1 |
| 3526 | hsa-mir-338 | 10063 | COX17 | COX17 cytochrome c oxidase assembly homolog (S. cerevisiae) | 1 |
| 3527 | hsa-mir-490 | 10063 | COX17 | COX17 cytochrome c oxidase assembly homolog (S. cerevisiae) | 1 |
| 3528 | hsa-mir-548b | 10063 | COX17 | COX17 cytochrome c oxidase assembly homolog (S. cerevisiae) | 1 |
| 3529 | hsa-mir-449b | 10063 | COX17 | COX17 cytochrome c oxidase assembly homolog (S. cerevisiae) | 1 |
| 3530 | hsa-mir-449c | 10063 | COX17 | COX17 cytochrome c oxidase assembly homolog (S. cerevisiae) | 1 |
| 3531 | hsa-mir-3065 | 10063 | COX17 | COX17 cytochrome c oxidase assembly homolog (S. cerevisiae) | 1 |
| 3532 | hsa-miR-152 | 57149 | LYRM1 | LYR motif containing 1 | 1 |
| 3533 | hsa-miR-26b | 57149 | LYRM1 | LYR motif containing 1 | 1 |
| 3534 | hsa-miR-31 | 57149 | LYRM1 | LYR motif containing 1 | 1 |
| 3535 | hsa-miR-338-5p | 57149 | LYRM1 | LYR motif containing 1 | 1 |
| 3536 | hsa-miR-340 | 57149 | LYRM1 | LYR motif containing 1 | 1 |
| 3537 | hsa-miR-383 | 57149 | LYRM1 | LYR motif containing 1 | 1 |
| 3538 | hsa-miR-449a | 57149 | LYRM1 | LYR motif containing 1 | 1 |
| 3539 | hsa-miR-621 | 57149 | LYRM1 | LYR motif containing 1 | 1 |
| 3540 | hsa-miR-634 | 57149 | LYRM1 | LYR motif containing 1 | 1 |
| 3541 | hsa-miR-653 | 57149 | LYRM1 | LYR motif containing 1 | 1 |
| 3542 | hsa-miR-657 | 57149 | LYRM1 | LYR motif containing 1 | 1 |
| 3543 | hsa-miR-765 | 57149 | LYRM1 | LYR motif containing 1 | 1 |
| 3544 | hsa-miR-770-5p | 57149 | LYRM1 | LYR motif containing 1 | 1 |
| 3545 | hsa-mir-26a-1 | 57149 | LYRM1 | LYR motif containing 1 | 1 |
| 3546 | hsa-mir-26b | 57149 | LYRM1 | LYR motif containing 1 | 1 |
| 3547 | hsa-mir-31 | 57149 | LYRM1 | LYR motif containing 1 | 1 |
| 3548 | hsa-mir-194-2 | 57149 | LYRM1 | LYR motif containing 1 | 1 |
| 3549 | hsa-mir-340 | 57149 | LYRM1 | LYR motif containing 1 | 1 |
| 3550 | hsa-mir-338 | 57149 | LYRM1 | LYR motif containing 1 | 1 |
| 3551 | hsa-mir-449b | 57149 | LYRM1 | LYR motif containing 1 | 1 |
| 3552 | hsa-mir-449c | 57149 | LYRM1 | LYR motif containing 1 | 1 |
| 3553 | hsa-mir-3065 | 57149 | LYRM1 | LYR motif containing 1 | 1 |
| 3554 | hsa-miR-10a | 4283 | CXCL9 | chemokine (C-X-C motif) ligand 9 | 1 |
| 3555 | hsa-miR-139-5p | 4283 | CXCL9 | chemokine (C-X-C motif) ligand 9 | 1 |
| 3556 | hsa-miR-152 | 4283 | CXCL9 | chemokine (C-X-C motif) ligand 9 | 1 |
| 3557 | hsa-miR-26a | 4283 | CXCL9 | chemokine (C-X-C motif) ligand 9 | 1 |
| 3558 | hsa-miR-26b | 4283 | CXCL9 | chemokine (C-X-C motif) ligand 9 | 1 |
| 3559 | hsa-miR-31 | 4283 | CXCL9 | chemokine (C-X-C motif) ligand 9 | 1 |
| 3560 | hsa-miR-338-5p | 4283 | CXCL9 | chemokine (C-X-C motif) ligand 9 | 1 |
| 3561 | hsa-miR-340 | 4283 | CXCL9 | chemokine (C-X-C motif) ligand 9 | 1 |
| 3562 | hsa-miR-449a | 4283 | CXCL9 | chemokine (C-X-C motif) ligand 9 | 1 |
| 3563 | hsa-miR-490-5p | 4283 | CXCL9 | chemokine (C-X-C motif) ligand 9 | 1 |
| 3564 | hsa-miR-499-5p | 4283 | CXCL9 | chemokine (C-X-C motif) ligand 9 | 1 |
| 3565 | hsa-miR-548b-5p | 4283 | CXCL9 | chemokine (C-X-C motif) ligand 9 | 1 |
| 3566 | hsa-miR-564 | 4283 | CXCL9 | chemokine (C-X-C motif) ligand 9 | 1 |
| 3567 | hsa-miR-621 | 4283 | CXCL9 | chemokine (C-X-C motif) ligand 9 | 1 |
| 3568 | hsa-miR-657 | 4283 | CXCL9 | chemokine (C-X-C motif) ligand 9 | 1 |
| 3569 | hsa-miR-765 | 4283 | CXCL9 | chemokine (C-X-C motif) ligand 9 | 1 |
| 3570 | hsa-miR-770-5p | 4283 | CXCL9 | chemokine (C-X-C motif) ligand 9 | 1 |
| 3571 | hsa-miR-877 | 4283 | CXCL9 | chemokine (C-X-C motif) ligand 9 | 1 |
| 3572 | hsa-mir-26a-1 | 4283 | CXCL9 | chemokine (C-X-C motif) ligand 9 | 1 |
| 3573 | hsa-mir-26b | 4283 | CXCL9 | chemokine (C-X-C motif) ligand 9 | 1 |
| 3574 | hsa-mir-31 | 4283 | CXCL9 | chemokine (C-X-C motif) ligand 9 | 1 |
| 3575 | hsa-mir-194-2 | 4283 | CXCL9 | chemokine (C-X-C motif) ligand 9 | 1 |
| 3576 | hsa-mir-340 | 4283 | CXCL9 | chemokine (C-X-C motif) ligand 9 | 1 |
| 3577 | hsa-mir-338 | 4283 | CXCL9 | chemokine (C-X-C motif) ligand 9 | 1 |
| 3578 | hsa-mir-490 | 4283 | CXCL9 | chemokine (C-X-C motif) ligand 9 | 1 |
| 3579 | hsa-mir-548b | 4283 | CXCL9 | chemokine (C-X-C motif) ligand 9 | 1 |
| 3580 | hsa-mir-449b | 4283 | CXCL9 | chemokine (C-X-C motif) ligand 9 | 1 |
| 3581 | hsa-mir-449c | 4283 | CXCL9 | chemokine (C-X-C motif) ligand 9 | 1 |
| 3582 | hsa-mir-3065 | 4283 | CXCL9 | chemokine (C-X-C motif) ligand 9 | 1 |
| 3583 | hsa-miR-10a | 4794 | NFKBIE | nuclear factor of kappa light polypeptide gene enhancer in B-cells inhibitor, epsilon | 1 |
| 3584 | hsa-miR-1228\* | 4794 | NFKBIE | nuclear factor of kappa light polypeptide gene enhancer in B-cells inhibitor, epsilon | 1 |
| 3585 | hsa-miR-139-5p | 4794 | NFKBIE | nuclear factor of kappa light polypeptide gene enhancer in B-cells inhibitor, epsilon | 1 |
| 3586 | hsa-miR-152 | 4794 | NFKBIE | nuclear factor of kappa light polypeptide gene enhancer in B-cells inhibitor, epsilon | 1 |
| 3587 | hsa-miR-26a | 4794 | NFKBIE | nuclear factor of kappa light polypeptide gene enhancer in B-cells inhibitor, epsilon | 1 |
| 3588 | hsa-miR-26b | 4794 | NFKBIE | nuclear factor of kappa light polypeptide gene enhancer in B-cells inhibitor, epsilon | 1 |
| 3589 | hsa-miR-31 | 4794 | NFKBIE | nuclear factor of kappa light polypeptide gene enhancer in B-cells inhibitor, epsilon | 1 |
| 3590 | hsa-miR-338-5p | 4794 | NFKBIE | nuclear factor of kappa light polypeptide gene enhancer in B-cells inhibitor, epsilon | 1 |
| 3591 | hsa-miR-340 | 4794 | NFKBIE | nuclear factor of kappa light polypeptide gene enhancer in B-cells inhibitor, epsilon | 1 |
| 3592 | hsa-miR-423-5p | 4794 | NFKBIE | nuclear factor of kappa light polypeptide gene enhancer in B-cells inhibitor, epsilon | 1 |
| 3593 | hsa-miR-449a | 4794 | NFKBIE | nuclear factor of kappa light polypeptide gene enhancer in B-cells inhibitor, epsilon | 1 |
| 3594 | hsa-miR-490-5p | 4794 | NFKBIE | nuclear factor of kappa light polypeptide gene enhancer in B-cells inhibitor, epsilon | 1 |
| 3595 | hsa-miR-499-5p | 4794 | NFKBIE | nuclear factor of kappa light polypeptide gene enhancer in B-cells inhibitor, epsilon | 1 |
| 3596 | hsa-miR-548b-5p | 4794 | NFKBIE | nuclear factor of kappa light polypeptide gene enhancer in B-cells inhibitor, epsilon | 1 |
| 3597 | hsa-miR-564 | 4794 | NFKBIE | nuclear factor of kappa light polypeptide gene enhancer in B-cells inhibitor, epsilon | 1 |
| 3598 | hsa-miR-584 | 4794 | NFKBIE | nuclear factor of kappa light polypeptide gene enhancer in B-cells inhibitor, epsilon | 1 |
| 3599 | hsa-miR-621 | 4794 | NFKBIE | nuclear factor of kappa light polypeptide gene enhancer in B-cells inhibitor, epsilon | 1 |
| 3600 | hsa-miR-644 | 4794 | NFKBIE | nuclear factor of kappa light polypeptide gene enhancer in B-cells inhibitor, epsilon | 1 |
| 3601 | hsa-miR-657 | 4794 | NFKBIE | nuclear factor of kappa light polypeptide gene enhancer in B-cells inhibitor, epsilon | 1 |
| 3602 | hsa-miR-765 | 4794 | NFKBIE | nuclear factor of kappa light polypeptide gene enhancer in B-cells inhibitor, epsilon | 1 |
| 3603 | hsa-miR-770-5p | 4794 | NFKBIE | nuclear factor of kappa light polypeptide gene enhancer in B-cells inhibitor, epsilon | 1 |
| 3604 | hsa-miR-877 | 4794 | NFKBIE | nuclear factor of kappa light polypeptide gene enhancer in B-cells inhibitor, epsilon | 1 |
| 3605 | hsa-mir-26a-1 | 4794 | NFKBIE | nuclear factor of kappa light polypeptide gene enhancer in B-cells inhibitor, epsilon | 1 |
| 3606 | hsa-mir-26b | 4794 | NFKBIE | nuclear factor of kappa light polypeptide gene enhancer in B-cells inhibitor, epsilon | 1 |
| 3607 | hsa-mir-31 | 4794 | NFKBIE | nuclear factor of kappa light polypeptide gene enhancer in B-cells inhibitor, epsilon | 1 |
| 3608 | hsa-mir-194-2 | 4794 | NFKBIE | nuclear factor of kappa light polypeptide gene enhancer in B-cells inhibitor, epsilon | 1 |
| 3609 | hsa-mir-340 | 4794 | NFKBIE | nuclear factor of kappa light polypeptide gene enhancer in B-cells inhibitor, epsilon | 1 |
| 3610 | hsa-mir-338 | 4794 | NFKBIE | nuclear factor of kappa light polypeptide gene enhancer in B-cells inhibitor, epsilon | 1 |
| 3611 | hsa-mir-490 | 4794 | NFKBIE | nuclear factor of kappa light polypeptide gene enhancer in B-cells inhibitor, epsilon | 1 |
| 3612 | hsa-mir-548b | 4794 | NFKBIE | nuclear factor of kappa light polypeptide gene enhancer in B-cells inhibitor, epsilon | 1 |
| 3613 | hsa-mir-548d-2 | 4794 | NFKBIE | nuclear factor of kappa light polypeptide gene enhancer in B-cells inhibitor, epsilon | 1 |
| 3614 | hsa-mir-449b | 4794 | NFKBIE | nuclear factor of kappa light polypeptide gene enhancer in B-cells inhibitor, epsilon | 1 |
| 3615 | hsa-mir-449c | 4794 | NFKBIE | nuclear factor of kappa light polypeptide gene enhancer in B-cells inhibitor, epsilon | 1 |
| 3616 | hsa-mir-3065 | 4794 | NFKBIE | nuclear factor of kappa light polypeptide gene enhancer in B-cells inhibitor, epsilon | 1 |
| 3617 | hsa-miR-10a | 1846 | DUSP4 | dual specificity phosphatase 4 | 1 |
| 3618 | hsa-miR-152 | 1846 | DUSP4 | dual specificity phosphatase 4 | 1 |
| 3619 | hsa-miR-26a | 1846 | DUSP4 | dual specificity phosphatase 4 | 1 |
| 3620 | hsa-miR-26b | 1846 | DUSP4 | dual specificity phosphatase 4 | 1 |
| 3621 | hsa-miR-340 | 1846 | DUSP4 | dual specificity phosphatase 4 | 1 |
| 3622 | hsa-miR-449a | 1846 | DUSP4 | dual specificity phosphatase 4 | 1 |
| 3623 | hsa-miR-490-5p | 1846 | DUSP4 | dual specificity phosphatase 4 | 1 |
| 3624 | hsa-miR-499-5p | 1846 | DUSP4 | dual specificity phosphatase 4 | 1 |
| 3625 | hsa-miR-564 | 1846 | DUSP4 | dual specificity phosphatase 4 | 1 |
| 3626 | hsa-miR-621 | 1846 | DUSP4 | dual specificity phosphatase 4 | 1 |
| 3627 | hsa-miR-634 | 1846 | DUSP4 | dual specificity phosphatase 4 | 1 |
| 3628 | hsa-miR-765 | 1846 | DUSP4 | dual specificity phosphatase 4 | 1 |
| 3629 | hsa-miR-770-5p | 1846 | DUSP4 | dual specificity phosphatase 4 | 1 |
| 3630 | hsa-miR-877 | 1846 | DUSP4 | dual specificity phosphatase 4 | 1 |
| 3631 | hsa-miR-95 | 1846 | DUSP4 | dual specificity phosphatase 4 | 1 |
| 3632 | hsa-mir-26a-1 | 1846 | DUSP4 | dual specificity phosphatase 4 | 1 |
| 3633 | hsa-mir-26b | 1846 | DUSP4 | dual specificity phosphatase 4 | 1 |
| 3634 | hsa-mir-340 | 1846 | DUSP4 | dual specificity phosphatase 4 | 1 |
| 3635 | hsa-mir-490 | 1846 | DUSP4 | dual specificity phosphatase 4 | 1 |
| 3636 | hsa-mir-449b | 1846 | DUSP4 | dual specificity phosphatase 4 | 1 |
| 3637 | hsa-mir-449c | 1846 | DUSP4 | dual specificity phosphatase 4 | 1 |
| 3638 | hsa-miR-10a | 5920 | RARRES3 | retinoic acid receptor responder (tazarotene induced) 3 | 1 |
| 3639 | hsa-miR-139-5p | 5920 | RARRES3 | retinoic acid receptor responder (tazarotene induced) 3 | 1 |
| 3640 | hsa-miR-152 | 5920 | RARRES3 | retinoic acid receptor responder (tazarotene induced) 3 | 1 |
| 3641 | hsa-miR-26a | 5920 | RARRES3 | retinoic acid receptor responder (tazarotene induced) 3 | 1 |
| 3642 | hsa-miR-26b | 5920 | RARRES3 | retinoic acid receptor responder (tazarotene induced) 3 | 1 |
| 3643 | hsa-miR-31 | 5920 | RARRES3 | retinoic acid receptor responder (tazarotene induced) 3 | 1 |
| 3644 | hsa-miR-338-5p | 5920 | RARRES3 | retinoic acid receptor responder (tazarotene induced) 3 | 1 |
| 3645 | hsa-miR-340 | 5920 | RARRES3 | retinoic acid receptor responder (tazarotene induced) 3 | 1 |
| 3646 | hsa-miR-383 | 5920 | RARRES3 | retinoic acid receptor responder (tazarotene induced) 3 | 1 |
| 3647 | hsa-miR-423-5p | 5920 | RARRES3 | retinoic acid receptor responder (tazarotene induced) 3 | 1 |
| 3648 | hsa-miR-449a | 5920 | RARRES3 | retinoic acid receptor responder (tazarotene induced) 3 | 1 |
| 3649 | hsa-miR-490-5p | 5920 | RARRES3 | retinoic acid receptor responder (tazarotene induced) 3 | 1 |
| 3650 | hsa-miR-499-5p | 5920 | RARRES3 | retinoic acid receptor responder (tazarotene induced) 3 | 1 |
| 3651 | hsa-miR-548b-5p | 5920 | RARRES3 | retinoic acid receptor responder (tazarotene induced) 3 | 1 |
| 3652 | hsa-miR-564 | 5920 | RARRES3 | retinoic acid receptor responder (tazarotene induced) 3 | 1 |
| 3653 | hsa-miR-621 | 5920 | RARRES3 | retinoic acid receptor responder (tazarotene induced) 3 | 1 |
| 3654 | hsa-miR-657 | 5920 | RARRES3 | retinoic acid receptor responder (tazarotene induced) 3 | 1 |
| 3655 | hsa-miR-663 | 5920 | RARRES3 | retinoic acid receptor responder (tazarotene induced) 3 | 1 |
| 3656 | hsa-miR-765 | 5920 | RARRES3 | retinoic acid receptor responder (tazarotene induced) 3 | 1 |
| 3657 | hsa-miR-770-5p | 5920 | RARRES3 | retinoic acid receptor responder (tazarotene induced) 3 | 1 |
| 3658 | hsa-miR-877 | 5920 | RARRES3 | retinoic acid receptor responder (tazarotene induced) 3 | 1 |
| 3659 | hsa-mir-26a-1 | 5920 | RARRES3 | retinoic acid receptor responder (tazarotene induced) 3 | 1 |
| 3660 | hsa-mir-26b | 5920 | RARRES3 | retinoic acid receptor responder (tazarotene induced) 3 | 1 |
| 3661 | hsa-mir-31 | 5920 | RARRES3 | retinoic acid receptor responder (tazarotene induced) 3 | 1 |
| 3662 | hsa-mir-194-2 | 5920 | RARRES3 | retinoic acid receptor responder (tazarotene induced) 3 | 1 |
| 3663 | hsa-mir-340 | 5920 | RARRES3 | retinoic acid receptor responder (tazarotene induced) 3 | 1 |
| 3664 | hsa-mir-338 | 5920 | RARRES3 | retinoic acid receptor responder (tazarotene induced) 3 | 1 |
| 3665 | hsa-mir-490 | 5920 | RARRES3 | retinoic acid receptor responder (tazarotene induced) 3 | 1 |
| 3666 | hsa-mir-548b | 5920 | RARRES3 | retinoic acid receptor responder (tazarotene induced) 3 | 1 |
| 3667 | hsa-mir-548d-2 | 5920 | RARRES3 | retinoic acid receptor responder (tazarotene induced) 3 | 1 |
| 3668 | hsa-mir-449b | 5920 | RARRES3 | retinoic acid receptor responder (tazarotene induced) 3 | 1 |
| 3669 | hsa-mir-449c | 5920 | RARRES3 | retinoic acid receptor responder (tazarotene induced) 3 | 1 |
| 3670 | hsa-mir-3065 | 5920 | RARRES3 | retinoic acid receptor responder (tazarotene induced) 3 | 1 |
| 3671 | hsa-miR-10a | 5025 | P2RX4 | purinergic receptor P2X, ligand-gated ion channel, 4 | 1 |
| 3672 | hsa-miR-152 | 5025 | P2RX4 | purinergic receptor P2X, ligand-gated ion channel, 4 | 1 |
| 3673 | hsa-miR-26a | 5025 | P2RX4 | purinergic receptor P2X, ligand-gated ion channel, 4 | 1 |
| 3674 | hsa-miR-26b | 5025 | P2RX4 | purinergic receptor P2X, ligand-gated ion channel, 4 | 1 |
| 3675 | hsa-miR-31 | 5025 | P2RX4 | purinergic receptor P2X, ligand-gated ion channel, 4 | 1 |
| 3676 | hsa-miR-338-5p | 5025 | P2RX4 | purinergic receptor P2X, ligand-gated ion channel, 4 | 1 |
| 3677 | hsa-miR-340 | 5025 | P2RX4 | purinergic receptor P2X, ligand-gated ion channel, 4 | 1 |
| 3678 | hsa-miR-449a | 5025 | P2RX4 | purinergic receptor P2X, ligand-gated ion channel, 4 | 1 |
| 3679 | hsa-miR-490-5p | 5025 | P2RX4 | purinergic receptor P2X, ligand-gated ion channel, 4 | 1 |
| 3680 | hsa-miR-499-5p | 5025 | P2RX4 | purinergic receptor P2X, ligand-gated ion channel, 4 | 1 |
| 3681 | hsa-miR-548b-5p | 5025 | P2RX4 | purinergic receptor P2X, ligand-gated ion channel, 4 | 1 |
| 3682 | hsa-miR-564 | 5025 | P2RX4 | purinergic receptor P2X, ligand-gated ion channel, 4 | 1 |
| 3683 | hsa-miR-621 | 5025 | P2RX4 | purinergic receptor P2X, ligand-gated ion channel, 4 | 1 |
| 3684 | hsa-miR-634 | 5025 | P2RX4 | purinergic receptor P2X, ligand-gated ion channel, 4 | 1 |
| 3685 | hsa-miR-653 | 5025 | P2RX4 | purinergic receptor P2X, ligand-gated ion channel, 4 | 1 |
| 3686 | hsa-miR-657 | 5025 | P2RX4 | purinergic receptor P2X, ligand-gated ion channel, 4 | 1 |
| 3687 | hsa-miR-765 | 5025 | P2RX4 | purinergic receptor P2X, ligand-gated ion channel, 4 | 1 |
| 3688 | hsa-miR-770-5p | 5025 | P2RX4 | purinergic receptor P2X, ligand-gated ion channel, 4 | 1 |
| 3689 | hsa-miR-877 | 5025 | P2RX4 | purinergic receptor P2X, ligand-gated ion channel, 4 | 1 |
| 3690 | hsa-mir-26a-1 | 5025 | P2RX4 | purinergic receptor P2X, ligand-gated ion channel, 4 | 1 |
| 3691 | hsa-mir-26b | 5025 | P2RX4 | purinergic receptor P2X, ligand-gated ion channel, 4 | 1 |
| 3692 | hsa-mir-31 | 5025 | P2RX4 | purinergic receptor P2X, ligand-gated ion channel, 4 | 1 |
| 3693 | hsa-mir-194-2 | 5025 | P2RX4 | purinergic receptor P2X, ligand-gated ion channel, 4 | 1 |
| 3694 | hsa-mir-340 | 5025 | P2RX4 | purinergic receptor P2X, ligand-gated ion channel, 4 | 1 |
| 3695 | hsa-mir-338 | 5025 | P2RX4 | purinergic receptor P2X, ligand-gated ion channel, 4 | 1 |
| 3696 | hsa-mir-490 | 5025 | P2RX4 | purinergic receptor P2X, ligand-gated ion channel, 4 | 1 |
| 3697 | hsa-mir-548b | 5025 | P2RX4 | purinergic receptor P2X, ligand-gated ion channel, 4 | 1 |
| 3698 | hsa-mir-449b | 5025 | P2RX4 | purinergic receptor P2X, ligand-gated ion channel, 4 | 1 |
| 3699 | hsa-mir-449c | 5025 | P2RX4 | purinergic receptor P2X, ligand-gated ion channel, 4 | 1 |
| 3700 | hsa-mir-3065 | 5025 | P2RX4 | purinergic receptor P2X, ligand-gated ion channel, 4 | 1 |
| 3701 | hsa-miR-10a | 3561 | IL2RG | interleukin 2 receptor, gamma (severe combined immunodeficiency) | 1 |
| 3702 | hsa-miR-139-5p | 3561 | IL2RG | interleukin 2 receptor, gamma (severe combined immunodeficiency) | 1 |
| 3703 | hsa-miR-152 | 3561 | IL2RG | interleukin 2 receptor, gamma (severe combined immunodeficiency) | 1 |
| 3704 | hsa-miR-26b | 3561 | IL2RG | interleukin 2 receptor, gamma (severe combined immunodeficiency) | 1 |
| 3705 | hsa-miR-31 | 3561 | IL2RG | interleukin 2 receptor, gamma (severe combined immunodeficiency) | 1 |
| 3706 | hsa-miR-338-5p | 3561 | IL2RG | interleukin 2 receptor, gamma (severe combined immunodeficiency) | 1 |
| 3707 | hsa-miR-340 | 3561 | IL2RG | interleukin 2 receptor, gamma (severe combined immunodeficiency) | 1 |
| 3708 | hsa-miR-383 | 3561 | IL2RG | interleukin 2 receptor, gamma (severe combined immunodeficiency) | 1 |
| 3709 | hsa-miR-449a | 3561 | IL2RG | interleukin 2 receptor, gamma (severe combined immunodeficiency) | 1 |
| 3710 | hsa-miR-499-5p | 3561 | IL2RG | interleukin 2 receptor, gamma (severe combined immunodeficiency) | 1 |
| 3711 | hsa-miR-548b-5p | 3561 | IL2RG | interleukin 2 receptor, gamma (severe combined immunodeficiency) | 1 |
| 3712 | hsa-miR-621 | 3561 | IL2RG | interleukin 2 receptor, gamma (severe combined immunodeficiency) | 1 |
| 3713 | hsa-miR-657 | 3561 | IL2RG | interleukin 2 receptor, gamma (severe combined immunodeficiency) | 1 |
| 3714 | hsa-miR-663 | 3561 | IL2RG | interleukin 2 receptor, gamma (severe combined immunodeficiency) | 1 |
| 3715 | hsa-miR-765 | 3561 | IL2RG | interleukin 2 receptor, gamma (severe combined immunodeficiency) | 1 |
| 3716 | hsa-miR-770-5p | 3561 | IL2RG | interleukin 2 receptor, gamma (severe combined immunodeficiency) | 1 |
| 3717 | hsa-miR-877 | 3561 | IL2RG | interleukin 2 receptor, gamma (severe combined immunodeficiency) | 1 |
| 3718 | hsa-mir-26a-1 | 3561 | IL2RG | interleukin 2 receptor, gamma (severe combined immunodeficiency) | 1 |
| 3719 | hsa-mir-26b | 3561 | IL2RG | interleukin 2 receptor, gamma (severe combined immunodeficiency) | 1 |
| 3720 | hsa-mir-31 | 3561 | IL2RG | interleukin 2 receptor, gamma (severe combined immunodeficiency) | 1 |
| 3721 | hsa-mir-194-2 | 3561 | IL2RG | interleukin 2 receptor, gamma (severe combined immunodeficiency) | 1 |
| 3722 | hsa-mir-340 | 3561 | IL2RG | interleukin 2 receptor, gamma (severe combined immunodeficiency) | 1 |
| 3723 | hsa-mir-338 | 3561 | IL2RG | interleukin 2 receptor, gamma (severe combined immunodeficiency) | 1 |
| 3724 | hsa-mir-548b | 3561 | IL2RG | interleukin 2 receptor, gamma (severe combined immunodeficiency) | 1 |
| 3725 | hsa-mir-449b | 3561 | IL2RG | interleukin 2 receptor, gamma (severe combined immunodeficiency) | 1 |
| 3726 | hsa-mir-449c | 3561 | IL2RG | interleukin 2 receptor, gamma (severe combined immunodeficiency) | 1 |
| 3727 | hsa-mir-3065 | 3561 | IL2RG | interleukin 2 receptor, gamma (severe combined immunodeficiency) | 1 |
| 3728 | hsa-miR-10a | 7107 | GPR137B | G protein-coupled receptor 137B | 1 |
| 3729 | hsa-miR-1228\* | 7107 | GPR137B | G protein-coupled receptor 137B | 1 |
| 3730 | hsa-miR-139-5p | 7107 | GPR137B | G protein-coupled receptor 137B | 1 |
| 3731 | hsa-miR-26a | 7107 | GPR137B | G protein-coupled receptor 137B | 1 |
| 3732 | hsa-miR-26b | 7107 | GPR137B | G protein-coupled receptor 137B | 1 |
| 3733 | hsa-miR-31 | 7107 | GPR137B | G protein-coupled receptor 137B | 1 |
| 3734 | hsa-miR-338-5p | 7107 | GPR137B | G protein-coupled receptor 137B | 1 |
| 3735 | hsa-miR-340 | 7107 | GPR137B | G protein-coupled receptor 137B | 1 |
| 3736 | hsa-miR-423-5p | 7107 | GPR137B | G protein-coupled receptor 137B | 1 |
| 3737 | hsa-miR-449a | 7107 | GPR137B | G protein-coupled receptor 137B | 1 |
| 3738 | hsa-miR-499-5p | 7107 | GPR137B | G protein-coupled receptor 137B | 1 |
| 3739 | hsa-miR-548b-5p | 7107 | GPR137B | G protein-coupled receptor 137B | 1 |
| 3740 | hsa-miR-564 | 7107 | GPR137B | G protein-coupled receptor 137B | 1 |
| 3741 | hsa-miR-621 | 7107 | GPR137B | G protein-coupled receptor 137B | 1 |
| 3742 | hsa-miR-657 | 7107 | GPR137B | G protein-coupled receptor 137B | 1 |
| 3743 | hsa-miR-765 | 7107 | GPR137B | G protein-coupled receptor 137B | 1 |
| 3744 | hsa-miR-770-5p | 7107 | GPR137B | G protein-coupled receptor 137B | 1 |
| 3745 | hsa-miR-877 | 7107 | GPR137B | G protein-coupled receptor 137B | 1 |
| 3746 | hsa-mir-26a-1 | 7107 | GPR137B | G protein-coupled receptor 137B | 1 |
| 3747 | hsa-mir-26b | 7107 | GPR137B | G protein-coupled receptor 137B | 1 |
| 3748 | hsa-mir-31 | 7107 | GPR137B | G protein-coupled receptor 137B | 1 |
| 3749 | hsa-mir-194-2 | 7107 | GPR137B | G protein-coupled receptor 137B | 1 |
| 3750 | hsa-mir-340 | 7107 | GPR137B | G protein-coupled receptor 137B | 1 |
| 3751 | hsa-mir-338 | 7107 | GPR137B | G protein-coupled receptor 137B | 1 |
| 3752 | hsa-mir-548b | 7107 | GPR137B | G protein-coupled receptor 137B | 1 |
| 3753 | hsa-mir-449b | 7107 | GPR137B | G protein-coupled receptor 137B | 1 |
| 3754 | hsa-mir-449c | 7107 | GPR137B | G protein-coupled receptor 137B | 1 |
| 3755 | hsa-mir-3065 | 7107 | GPR137B | G protein-coupled receptor 137B | 1 |
| 3756 | hsa-miR-152 | 60489 | APOBEC3G | apolipoprotein B mRNA editing enzyme, catalytic polypeptide-like 3G | 1 |
| 3757 | hsa-miR-26a | 60489 | APOBEC3G | apolipoprotein B mRNA editing enzyme, catalytic polypeptide-like 3G | 1 |
| 3758 | hsa-miR-26b | 60489 | APOBEC3G | apolipoprotein B mRNA editing enzyme, catalytic polypeptide-like 3G | 1 |
| 3759 | hsa-miR-31 | 60489 | APOBEC3G | apolipoprotein B mRNA editing enzyme, catalytic polypeptide-like 3G | 1 |
| 3760 | hsa-miR-338-5p | 60489 | APOBEC3G | apolipoprotein B mRNA editing enzyme, catalytic polypeptide-like 3G | 1 |
| 3761 | hsa-miR-340 | 60489 | APOBEC3G | apolipoprotein B mRNA editing enzyme, catalytic polypeptide-like 3G | 1 |
| 3762 | hsa-miR-383 | 60489 | APOBEC3G | apolipoprotein B mRNA editing enzyme, catalytic polypeptide-like 3G | 1 |
| 3763 | hsa-miR-449a | 60489 | APOBEC3G | apolipoprotein B mRNA editing enzyme, catalytic polypeptide-like 3G | 1 |
| 3764 | hsa-miR-499-5p | 60489 | APOBEC3G | apolipoprotein B mRNA editing enzyme, catalytic polypeptide-like 3G | 1 |
| 3765 | hsa-miR-548b-5p | 60489 | APOBEC3G | apolipoprotein B mRNA editing enzyme, catalytic polypeptide-like 3G | 1 |
| 3766 | hsa-miR-564 | 60489 | APOBEC3G | apolipoprotein B mRNA editing enzyme, catalytic polypeptide-like 3G | 1 |
| 3767 | hsa-miR-621 | 60489 | APOBEC3G | apolipoprotein B mRNA editing enzyme, catalytic polypeptide-like 3G | 1 |
| 3768 | hsa-miR-657 | 60489 | APOBEC3G | apolipoprotein B mRNA editing enzyme, catalytic polypeptide-like 3G | 1 |
| 3769 | hsa-miR-765 | 60489 | APOBEC3G | apolipoprotein B mRNA editing enzyme, catalytic polypeptide-like 3G | 1 |
| 3770 | hsa-miR-770-5p | 60489 | APOBEC3G | apolipoprotein B mRNA editing enzyme, catalytic polypeptide-like 3G | 1 |
| 3771 | hsa-mir-26a-1 | 60489 | APOBEC3G | apolipoprotein B mRNA editing enzyme, catalytic polypeptide-like 3G | 1 |
| 3772 | hsa-mir-26b | 60489 | APOBEC3G | apolipoprotein B mRNA editing enzyme, catalytic polypeptide-like 3G | 1 |
| 3773 | hsa-mir-31 | 60489 | APOBEC3G | apolipoprotein B mRNA editing enzyme, catalytic polypeptide-like 3G | 1 |
| 3774 | hsa-mir-194-2 | 60489 | APOBEC3G | apolipoprotein B mRNA editing enzyme, catalytic polypeptide-like 3G | 1 |
| 3775 | hsa-mir-340 | 60489 | APOBEC3G | apolipoprotein B mRNA editing enzyme, catalytic polypeptide-like 3G | 1 |
| 3776 | hsa-mir-338 | 60489 | APOBEC3G | apolipoprotein B mRNA editing enzyme, catalytic polypeptide-like 3G | 1 |
| 3777 | hsa-mir-548b | 60489 | APOBEC3G | apolipoprotein B mRNA editing enzyme, catalytic polypeptide-like 3G | 1 |
| 3778 | hsa-mir-548d-2 | 60489 | APOBEC3G | apolipoprotein B mRNA editing enzyme, catalytic polypeptide-like 3G | 1 |
| 3779 | hsa-mir-449b | 60489 | APOBEC3G | apolipoprotein B mRNA editing enzyme, catalytic polypeptide-like 3G | 1 |
| 3780 | hsa-mir-449c | 60489 | APOBEC3G | apolipoprotein B mRNA editing enzyme, catalytic polypeptide-like 3G | 1 |
| 3781 | hsa-mir-3065 | 60489 | APOBEC3G | apolipoprotein B mRNA editing enzyme, catalytic polypeptide-like 3G | 1 |
| 3782 | hsa-miR-10a | 2643 | GCH1 | GTP cyclohydrolase 1 | 1 |
| 3783 | hsa-miR-1228\* | 2643 | GCH1 | GTP cyclohydrolase 1 | 1 |
| 3784 | hsa-miR-139-5p | 2643 | GCH1 | GTP cyclohydrolase 1 | 1 |
| 3785 | hsa-miR-152 | 2643 | GCH1 | GTP cyclohydrolase 1 | 1 |
| 3786 | hsa-miR-26a | 2643 | GCH1 | GTP cyclohydrolase 1 | 1 |
| 3787 | hsa-miR-26b | 2643 | GCH1 | GTP cyclohydrolase 1 | 1 |
| 3788 | hsa-miR-338-5p | 2643 | GCH1 | GTP cyclohydrolase 1 | 1 |
| 3789 | hsa-miR-340 | 2643 | GCH1 | GTP cyclohydrolase 1 | 1 |
| 3790 | hsa-miR-449a | 2643 | GCH1 | GTP cyclohydrolase 1 | 1 |
| 3791 | hsa-miR-499-5p | 2643 | GCH1 | GTP cyclohydrolase 1 | 1 |
| 3792 | hsa-miR-548b-5p | 2643 | GCH1 | GTP cyclohydrolase 1 | 1 |
| 3793 | hsa-miR-564 | 2643 | GCH1 | GTP cyclohydrolase 1 | 1 |
| 3794 | hsa-miR-621 | 2643 | GCH1 | GTP cyclohydrolase 1 | 1 |
| 3795 | hsa-miR-657 | 2643 | GCH1 | GTP cyclohydrolase 1 | 1 |
| 3796 | hsa-miR-765 | 2643 | GCH1 | GTP cyclohydrolase 1 | 1 |
| 3797 | hsa-miR-770-5p | 2643 | GCH1 | GTP cyclohydrolase 1 | 1 |
| 3798 | hsa-miR-877 | 2643 | GCH1 | GTP cyclohydrolase 1 | 1 |
| 3799 | hsa-mir-26a-1 | 2643 | GCH1 | GTP cyclohydrolase 1 | 1 |
| 3800 | hsa-mir-26b | 2643 | GCH1 | GTP cyclohydrolase 1 | 1 |
| 3801 | hsa-mir-194-2 | 2643 | GCH1 | GTP cyclohydrolase 1 | 1 |
| 3802 | hsa-mir-340 | 2643 | GCH1 | GTP cyclohydrolase 1 | 1 |
| 3803 | hsa-mir-338 | 2643 | GCH1 | GTP cyclohydrolase 1 | 1 |
| 3804 | hsa-mir-548b | 2643 | GCH1 | GTP cyclohydrolase 1 | 1 |
| 3805 | hsa-mir-548d-2 | 2643 | GCH1 | GTP cyclohydrolase 1 | 1 |
| 3806 | hsa-mir-449b | 2643 | GCH1 | GTP cyclohydrolase 1 | 1 |
| 3807 | hsa-mir-449c | 2643 | GCH1 | GTP cyclohydrolase 1 | 1 |
| 3808 | hsa-mir-3065 | 2643 | GCH1 | GTP cyclohydrolase 1 | 1 |
| 3809 | hsa-miR-10a | 5698 | PSMB9 | proteasome (prosome, macropain) subunit, beta type, 9 (large multifunctional peptidase 2) | 1 |
| 3810 | hsa-miR-139-5p | 5698 | PSMB9 | proteasome (prosome, macropain) subunit, beta type, 9 (large multifunctional peptidase 2) | 1 |
| 3811 | hsa-miR-152 | 5698 | PSMB9 | proteasome (prosome, macropain) subunit, beta type, 9 (large multifunctional peptidase 2) | 1 |
| 3812 | hsa-miR-26a | 5698 | PSMB9 | proteasome (prosome, macropain) subunit, beta type, 9 (large multifunctional peptidase 2) | 1 |
| 3813 | hsa-miR-26b | 5698 | PSMB9 | proteasome (prosome, macropain) subunit, beta type, 9 (large multifunctional peptidase 2) | 1 |
| 3814 | hsa-miR-31 | 5698 | PSMB9 | proteasome (prosome, macropain) subunit, beta type, 9 (large multifunctional peptidase 2) | 1 |
| 3815 | hsa-miR-338-5p | 5698 | PSMB9 | proteasome (prosome, macropain) subunit, beta type, 9 (large multifunctional peptidase 2) | 1 |
| 3816 | hsa-miR-340 | 5698 | PSMB9 | proteasome (prosome, macropain) subunit, beta type, 9 (large multifunctional peptidase 2) | 1 |
| 3817 | hsa-miR-383 | 5698 | PSMB9 | proteasome (prosome, macropain) subunit, beta type, 9 (large multifunctional peptidase 2) | 1 |
| 3818 | hsa-miR-449a | 5698 | PSMB9 | proteasome (prosome, macropain) subunit, beta type, 9 (large multifunctional peptidase 2) | 1 |
| 3819 | hsa-miR-499-5p | 5698 | PSMB9 | proteasome (prosome, macropain) subunit, beta type, 9 (large multifunctional peptidase 2) | 1 |
| 3820 | hsa-miR-548b-5p | 5698 | PSMB9 | proteasome (prosome, macropain) subunit, beta type, 9 (large multifunctional peptidase 2) | 1 |
| 3821 | hsa-miR-621 | 5698 | PSMB9 | proteasome (prosome, macropain) subunit, beta type, 9 (large multifunctional peptidase 2) | 1 |
| 3822 | hsa-miR-657 | 5698 | PSMB9 | proteasome (prosome, macropain) subunit, beta type, 9 (large multifunctional peptidase 2) | 1 |
| 3823 | hsa-miR-663 | 5698 | PSMB9 | proteasome (prosome, macropain) subunit, beta type, 9 (large multifunctional peptidase 2) | 1 |
| 3824 | hsa-miR-765 | 5698 | PSMB9 | proteasome (prosome, macropain) subunit, beta type, 9 (large multifunctional peptidase 2) | 1 |
| 3825 | hsa-miR-770-5p | 5698 | PSMB9 | proteasome (prosome, macropain) subunit, beta type, 9 (large multifunctional peptidase 2) | 1 |
| 3826 | hsa-miR-877 | 5698 | PSMB9 | proteasome (prosome, macropain) subunit, beta type, 9 (large multifunctional peptidase 2) | 1 |
| 3827 | hsa-mir-26a-1 | 5698 | PSMB9 | proteasome (prosome, macropain) subunit, beta type, 9 (large multifunctional peptidase 2) | 1 |
| 3828 | hsa-mir-26b | 5698 | PSMB9 | proteasome (prosome, macropain) subunit, beta type, 9 (large multifunctional peptidase 2) | 1 |
| 3829 | hsa-mir-31 | 5698 | PSMB9 | proteasome (prosome, macropain) subunit, beta type, 9 (large multifunctional peptidase 2) | 1 |
| 3830 | hsa-mir-194-2 | 5698 | PSMB9 | proteasome (prosome, macropain) subunit, beta type, 9 (large multifunctional peptidase 2) | 1 |
| 3831 | hsa-mir-340 | 5698 | PSMB9 | proteasome (prosome, macropain) subunit, beta type, 9 (large multifunctional peptidase 2) | 1 |
| 3832 | hsa-mir-338 | 5698 | PSMB9 | proteasome (prosome, macropain) subunit, beta type, 9 (large multifunctional peptidase 2) | 1 |
| 3833 | hsa-mir-548b | 5698 | PSMB9 | proteasome (prosome, macropain) subunit, beta type, 9 (large multifunctional peptidase 2) | 1 |
| 3834 | hsa-mir-449b | 5698 | PSMB9 | proteasome (prosome, macropain) subunit, beta type, 9 (large multifunctional peptidase 2) | 1 |
| 3835 | hsa-mir-449c | 5698 | PSMB9 | proteasome (prosome, macropain) subunit, beta type, 9 (large multifunctional peptidase 2) | 1 |
| 3836 | hsa-mir-3065 | 5698 | PSMB9 | proteasome (prosome, macropain) subunit, beta type, 9 (large multifunctional peptidase 2) | 1 |
| 3837 | hsa-miR-10a | 5366 | PMAIP1 | phorbol-12-myristate-13-acetate-induced protein 1 | 1 |
| 3838 | hsa-miR-152 | 5366 | PMAIP1 | phorbol-12-myristate-13-acetate-induced protein 1 | 1 |
| 3839 | hsa-miR-26a | 5366 | PMAIP1 | phorbol-12-myristate-13-acetate-induced protein 1 | 1 |
| 3840 | hsa-miR-26b | 5366 | PMAIP1 | phorbol-12-myristate-13-acetate-induced protein 1 | 1 |
| 3841 | hsa-miR-31 | 5366 | PMAIP1 | phorbol-12-myristate-13-acetate-induced protein 1 | 1 |
| 3842 | hsa-miR-338-5p | 5366 | PMAIP1 | phorbol-12-myristate-13-acetate-induced protein 1 | 1 |
| 3843 | hsa-miR-340 | 5366 | PMAIP1 | phorbol-12-myristate-13-acetate-induced protein 1 | 1 |
| 3844 | hsa-miR-423-5p | 5366 | PMAIP1 | phorbol-12-myristate-13-acetate-induced protein 1 | 1 |
| 3845 | hsa-miR-449a | 5366 | PMAIP1 | phorbol-12-myristate-13-acetate-induced protein 1 | 1 |
| 3846 | hsa-miR-490-5p | 5366 | PMAIP1 | phorbol-12-myristate-13-acetate-induced protein 1 | 1 |
| 3847 | hsa-miR-499-5p | 5366 | PMAIP1 | phorbol-12-myristate-13-acetate-induced protein 1 | 1 |
| 3848 | hsa-miR-548b-5p | 5366 | PMAIP1 | phorbol-12-myristate-13-acetate-induced protein 1 | 1 |
| 3849 | hsa-miR-564 | 5366 | PMAIP1 | phorbol-12-myristate-13-acetate-induced protein 1 | 1 |
| 3850 | hsa-miR-621 | 5366 | PMAIP1 | phorbol-12-myristate-13-acetate-induced protein 1 | 1 |
| 3851 | hsa-miR-644 | 5366 | PMAIP1 | phorbol-12-myristate-13-acetate-induced protein 1 | 1 |
| 3852 | hsa-miR-657 | 5366 | PMAIP1 | phorbol-12-myristate-13-acetate-induced protein 1 | 1 |
| 3853 | hsa-miR-765 | 5366 | PMAIP1 | phorbol-12-myristate-13-acetate-induced protein 1 | 1 |
| 3854 | hsa-miR-770-5p | 5366 | PMAIP1 | phorbol-12-myristate-13-acetate-induced protein 1 | 1 |
| 3855 | hsa-miR-877 | 5366 | PMAIP1 | phorbol-12-myristate-13-acetate-induced protein 1 | 1 |
| 3856 | hsa-miR-95 | 5366 | PMAIP1 | phorbol-12-myristate-13-acetate-induced protein 1 | 1 |
| 3857 | hsa-mir-26a-1 | 5366 | PMAIP1 | phorbol-12-myristate-13-acetate-induced protein 1 | 1 |
| 3858 | hsa-mir-26b | 5366 | PMAIP1 | phorbol-12-myristate-13-acetate-induced protein 1 | 1 |
| 3859 | hsa-mir-31 | 5366 | PMAIP1 | phorbol-12-myristate-13-acetate-induced protein 1 | 1 |
| 3860 | hsa-mir-194-2 | 5366 | PMAIP1 | phorbol-12-myristate-13-acetate-induced protein 1 | 1 |
| 3861 | hsa-mir-340 | 5366 | PMAIP1 | phorbol-12-myristate-13-acetate-induced protein 1 | 1 |
| 3862 | hsa-mir-338 | 5366 | PMAIP1 | phorbol-12-myristate-13-acetate-induced protein 1 | 1 |
| 3863 | hsa-mir-490 | 5366 | PMAIP1 | phorbol-12-myristate-13-acetate-induced protein 1 | 1 |
| 3864 | hsa-mir-548b | 5366 | PMAIP1 | phorbol-12-myristate-13-acetate-induced protein 1 | 1 |
| 3865 | hsa-mir-449b | 5366 | PMAIP1 | phorbol-12-myristate-13-acetate-induced protein 1 | 1 |
| 3866 | hsa-mir-449c | 5366 | PMAIP1 | phorbol-12-myristate-13-acetate-induced protein 1 | 1 |
| 3867 | hsa-mir-3065 | 5366 | PMAIP1 | phorbol-12-myristate-13-acetate-induced protein 1 | 1 |
| 3868 | hsa-miR-10a | 2537 | IFI6 | interferon, alpha-inducible protein 6 | 1 |
| 3869 | hsa-miR-1228\* | 2537 | IFI6 | interferon, alpha-inducible protein 6 | 1 |
| 3870 | hsa-miR-139-5p | 2537 | IFI6 | interferon, alpha-inducible protein 6 | 1 |
| 3871 | hsa-miR-152 | 2537 | IFI6 | interferon, alpha-inducible protein 6 | 1 |
| 3872 | hsa-miR-26a | 2537 | IFI6 | interferon, alpha-inducible protein 6 | 1 |
| 3873 | hsa-miR-26b | 2537 | IFI6 | interferon, alpha-inducible protein 6 | 1 |
| 3874 | hsa-miR-338-5p | 2537 | IFI6 | interferon, alpha-inducible protein 6 | 1 |
| 3875 | hsa-miR-340 | 2537 | IFI6 | interferon, alpha-inducible protein 6 | 1 |
| 3876 | hsa-miR-449a | 2537 | IFI6 | interferon, alpha-inducible protein 6 | 1 |
| 3877 | hsa-miR-499-5p | 2537 | IFI6 | interferon, alpha-inducible protein 6 | 1 |
| 3878 | hsa-miR-548b-5p | 2537 | IFI6 | interferon, alpha-inducible protein 6 | 1 |
| 3879 | hsa-miR-564 | 2537 | IFI6 | interferon, alpha-inducible protein 6 | 1 |
| 3880 | hsa-miR-621 | 2537 | IFI6 | interferon, alpha-inducible protein 6 | 1 |
| 3881 | hsa-miR-657 | 2537 | IFI6 | interferon, alpha-inducible protein 6 | 1 |
| 3882 | hsa-miR-765 | 2537 | IFI6 | interferon, alpha-inducible protein 6 | 1 |
| 3883 | hsa-miR-770-5p | 2537 | IFI6 | interferon, alpha-inducible protein 6 | 1 |
| 3884 | hsa-miR-877 | 2537 | IFI6 | interferon, alpha-inducible protein 6 | 1 |
| 3885 | hsa-mir-26a-1 | 2537 | IFI6 | interferon, alpha-inducible protein 6 | 1 |
| 3886 | hsa-mir-26b | 2537 | IFI6 | interferon, alpha-inducible protein 6 | 1 |
| 3887 | hsa-mir-194-2 | 2537 | IFI6 | interferon, alpha-inducible protein 6 | 1 |
| 3888 | hsa-mir-340 | 2537 | IFI6 | interferon, alpha-inducible protein 6 | 1 |
| 3889 | hsa-mir-338 | 2537 | IFI6 | interferon, alpha-inducible protein 6 | 1 |
| 3890 | hsa-mir-548b | 2537 | IFI6 | interferon, alpha-inducible protein 6 | 1 |
| 3891 | hsa-mir-548d-2 | 2537 | IFI6 | interferon, alpha-inducible protein 6 | 1 |
| 3892 | hsa-mir-449b | 2537 | IFI6 | interferon, alpha-inducible protein 6 | 1 |
| 3893 | hsa-mir-449c | 2537 | IFI6 | interferon, alpha-inducible protein 6 | 1 |
| 3894 | hsa-mir-3065 | 2537 | IFI6 | interferon, alpha-inducible protein 6 | 1 |
| 3895 | hsa-miR-10a | 7122 | CLDN5 | claudin 5 | 1 |
| 3896 | hsa-miR-152 | 7122 | CLDN5 | claudin 5 | 1 |
| 3897 | hsa-miR-26a | 7122 | CLDN5 | claudin 5 | 1 |
| 3898 | hsa-miR-26b | 7122 | CLDN5 | claudin 5 | 1 |
| 3899 | hsa-miR-31 | 7122 | CLDN5 | claudin 5 | 1 |
| 3900 | hsa-miR-340 | 7122 | CLDN5 | claudin 5 | 1 |
| 3901 | hsa-miR-449a | 7122 | CLDN5 | claudin 5 | 1 |
| 3902 | hsa-miR-490-5p | 7122 | CLDN5 | claudin 5 | 1 |
| 3903 | hsa-miR-499-5p | 7122 | CLDN5 | claudin 5 | 1 |
| 3904 | hsa-miR-548b-5p | 7122 | CLDN5 | claudin 5 | 1 |
| 3905 | hsa-miR-564 | 7122 | CLDN5 | claudin 5 | 1 |
| 3906 | hsa-miR-584 | 7122 | CLDN5 | claudin 5 | 1 |
| 3907 | hsa-miR-621 | 7122 | CLDN5 | claudin 5 | 1 |
| 3908 | hsa-miR-634 | 7122 | CLDN5 | claudin 5 | 1 |
| 3909 | hsa-miR-644 | 7122 | CLDN5 | claudin 5 | 1 |
| 3910 | hsa-miR-653 | 7122 | CLDN5 | claudin 5 | 1 |
| 3911 | hsa-miR-765 | 7122 | CLDN5 | claudin 5 | 1 |
| 3912 | hsa-miR-770-5p | 7122 | CLDN5 | claudin 5 | 1 |
| 3913 | hsa-miR-877 | 7122 | CLDN5 | claudin 5 | 1 |
| 3914 | hsa-miR-95 | 7122 | CLDN5 | claudin 5 | 1 |
| 3915 | hsa-mir-26a-1 | 7122 | CLDN5 | claudin 5 | 1 |
| 3916 | hsa-mir-26b | 7122 | CLDN5 | claudin 5 | 1 |
| 3917 | hsa-mir-31 | 7122 | CLDN5 | claudin 5 | 1 |
| 3918 | hsa-mir-194-2 | 7122 | CLDN5 | claudin 5 | 1 |
| 3919 | hsa-mir-340 | 7122 | CLDN5 | claudin 5 | 1 |
| 3920 | hsa-mir-490 | 7122 | CLDN5 | claudin 5 | 1 |
| 3921 | hsa-mir-548b | 7122 | CLDN5 | claudin 5 | 1 |
| 3922 | hsa-mir-449b | 7122 | CLDN5 | claudin 5 | 1 |
| 3923 | hsa-mir-449c | 7122 | CLDN5 | claudin 5 | 1 |
| 3928 | hsa-miR-383 | 960 | CD44 | CD44 molecule (Indian blood group) | 1 |
| 3941 | hsa-miR-10a | 3627 | CXCL10 | chemokine (C-X-C motif) ligand 10 | 1 |
| 3942 | hsa-miR-1228\* | 3627 | CXCL10 | chemokine (C-X-C motif) ligand 10 | 1 |
| 3943 | hsa-miR-139-5p | 3627 | CXCL10 | chemokine (C-X-C motif) ligand 10 | 1 |
| 3944 | hsa-miR-152 | 3627 | CXCL10 | chemokine (C-X-C motif) ligand 10 | 1 |
| 3945 | hsa-miR-26a | 3627 | CXCL10 | chemokine (C-X-C motif) ligand 10 | 1 |
| 3946 | hsa-miR-26b | 3627 | CXCL10 | chemokine (C-X-C motif) ligand 10 | 1 |
| 3947 | hsa-miR-31 | 3627 | CXCL10 | chemokine (C-X-C motif) ligand 10 | 1 |
| 3948 | hsa-miR-338-5p | 3627 | CXCL10 | chemokine (C-X-C motif) ligand 10 | 1 |
| 3949 | hsa-miR-340 | 3627 | CXCL10 | chemokine (C-X-C motif) ligand 10 | 1 |
| 3950 | hsa-miR-423-5p | 3627 | CXCL10 | chemokine (C-X-C motif) ligand 10 | 1 |
| 3951 | hsa-miR-449a | 3627 | CXCL10 | chemokine (C-X-C motif) ligand 10 | 1 |
| 3952 | hsa-miR-490-5p | 3627 | CXCL10 | chemokine (C-X-C motif) ligand 10 | 1 |
| 3953 | hsa-miR-499-5p | 3627 | CXCL10 | chemokine (C-X-C motif) ligand 10 | 1 |
| 3954 | hsa-miR-548b-5p | 3627 | CXCL10 | chemokine (C-X-C motif) ligand 10 | 1 |
| 3955 | hsa-miR-564 | 3627 | CXCL10 | chemokine (C-X-C motif) ligand 10 | 1 |
| 3956 | hsa-miR-584 | 3627 | CXCL10 | chemokine (C-X-C motif) ligand 10 | 1 |
| 3957 | hsa-miR-621 | 3627 | CXCL10 | chemokine (C-X-C motif) ligand 10 | 1 |
| 3958 | hsa-miR-657 | 3627 | CXCL10 | chemokine (C-X-C motif) ligand 10 | 1 |
| 3959 | hsa-miR-765 | 3627 | CXCL10 | chemokine (C-X-C motif) ligand 10 | 1 |
| 3960 | hsa-miR-770-5p | 3627 | CXCL10 | chemokine (C-X-C motif) ligand 10 | 1 |
| 3961 | hsa-miR-877 | 3627 | CXCL10 | chemokine (C-X-C motif) ligand 10 | 1 |
| 3962 | hsa-mir-26a-1 | 3627 | CXCL10 | chemokine (C-X-C motif) ligand 10 | 1 |
| 3963 | hsa-mir-26b | 3627 | CXCL10 | chemokine (C-X-C motif) ligand 10 | 1 |
| 3964 | hsa-mir-31 | 3627 | CXCL10 | chemokine (C-X-C motif) ligand 10 | 1 |
| 3965 | hsa-mir-194-2 | 3627 | CXCL10 | chemokine (C-X-C motif) ligand 10 | 1 |
| 3966 | hsa-mir-340 | 3627 | CXCL10 | chemokine (C-X-C motif) ligand 10 | 1 |
| 3967 | hsa-mir-338 | 3627 | CXCL10 | chemokine (C-X-C motif) ligand 10 | 1 |
| 3968 | hsa-mir-490 | 3627 | CXCL10 | chemokine (C-X-C motif) ligand 10 | 1 |
| 3969 | hsa-mir-548b | 3627 | CXCL10 | chemokine (C-X-C motif) ligand 10 | 1 |
| 3970 | hsa-mir-548d-2 | 3627 | CXCL10 | chemokine (C-X-C motif) ligand 10 | 1 |
| 3971 | hsa-mir-449b | 3627 | CXCL10 | chemokine (C-X-C motif) ligand 10 | 1 |
| 3972 | hsa-mir-449c | 3627 | CXCL10 | chemokine (C-X-C motif) ligand 10 | 1 |
| 3973 | hsa-mir-3065 | 3627 | CXCL10 | chemokine (C-X-C motif) ligand 10 | 1 |
| 3986 | hsa-miR-584 | 3669 | ISG20 | interferon stimulated exonuclease gene 20kDa | 1 |
| 3990 | hsa-miR-653 | 3669 | ISG20 | interferon stimulated exonuclease gene 20kDa | 1 |
| 4004 | hsa-miR-10a | 9712 | USP6NL | USP6 N-terminal like | 1 |
| 4005 | hsa-miR-152 | 9712 | USP6NL | USP6 N-terminal like | 1 |
| 4006 | hsa-miR-26a | 9712 | USP6NL | USP6 N-terminal like | 1 |
| 4007 | hsa-miR-26b | 9712 | USP6NL | USP6 N-terminal like | 1 |
| 4008 | hsa-miR-31 | 9712 | USP6NL | USP6 N-terminal like | 1 |
| 4009 | hsa-miR-338-5p | 9712 | USP6NL | USP6 N-terminal like | 1 |
| 4010 | hsa-miR-340 | 9712 | USP6NL | USP6 N-terminal like | 1 |
| 4011 | hsa-miR-423-5p | 9712 | USP6NL | USP6 N-terminal like | 1 |
| 4012 | hsa-miR-449a | 9712 | USP6NL | USP6 N-terminal like | 1 |
| 4013 | hsa-miR-499-5p | 9712 | USP6NL | USP6 N-terminal like | 1 |
| 4014 | hsa-miR-548b-5p | 9712 | USP6NL | USP6 N-terminal like | 1 |
| 4015 | hsa-miR-564 | 9712 | USP6NL | USP6 N-terminal like | 1 |
| 4016 | hsa-miR-621 | 9712 | USP6NL | USP6 N-terminal like | 1 |
| 4017 | hsa-miR-657 | 9712 | USP6NL | USP6 N-terminal like | 1 |
| 4018 | hsa-miR-765 | 9712 | USP6NL | USP6 N-terminal like | 1 |
| 4019 | hsa-miR-770-5p | 9712 | USP6NL | USP6 N-terminal like | 1 |
| 4020 | hsa-miR-877 | 9712 | USP6NL | USP6 N-terminal like | 1 |
| 4021 | hsa-mir-26a-1 | 9712 | USP6NL | USP6 N-terminal like | 1 |
| 4022 | hsa-mir-26b | 9712 | USP6NL | USP6 N-terminal like | 1 |
| 4023 | hsa-mir-31 | 9712 | USP6NL | USP6 N-terminal like | 1 |
| 4024 | hsa-mir-194-2 | 9712 | USP6NL | USP6 N-terminal like | 1 |
| 4025 | hsa-mir-340 | 9712 | USP6NL | USP6 N-terminal like | 1 |
| 4026 | hsa-mir-338 | 9712 | USP6NL | USP6 N-terminal like | 1 |
| 4027 | hsa-mir-548b | 9712 | USP6NL | USP6 N-terminal like | 1 |
| 4028 | hsa-mir-548d-2 | 9712 | USP6NL | USP6 N-terminal like | 1 |
| 4029 | hsa-mir-449b | 9712 | USP6NL | USP6 N-terminal like | 1 |
| 4030 | hsa-mir-449c | 9712 | USP6NL | USP6 N-terminal like | 1 |
| 4031 | hsa-mir-3065 | 9712 | USP6NL | USP6 N-terminal like | 1 |
| 4052 | hsa-miR-1 | 5126 | PCSK2 | proprotein convertase subtilisin/kexin type 2 | 1 |
| 4053 | hsa-miR-133a | 5126 | PCSK2 | proprotein convertase subtilisin/kexin type 2 | 1 |
| 4054 | hsa-miR-186 | 5126 | PCSK2 | proprotein convertase subtilisin/kexin type 2 | 1 |
| 4055 | hsa-miR-195 | 5126 | PCSK2 | proprotein convertase subtilisin/kexin type 2 | 1 |
| 4056 | hsa-miR-208a | 5126 | PCSK2 | proprotein convertase subtilisin/kexin type 2 | 1 |
| 4057 | hsa-miR-326 | 5126 | PCSK2 | proprotein convertase subtilisin/kexin type 2 | 1 |
| 4058 | hsa-miR-449b | 5126 | PCSK2 | proprotein convertase subtilisin/kexin type 2 | 1 |
| 4059 | hsa-miR-497 | 5126 | PCSK2 | proprotein convertase subtilisin/kexin type 2 | 1 |
| 4060 | hsa-miR-575 | 5126 | PCSK2 | proprotein convertase subtilisin/kexin type 2 | 1 |
| 4061 | hsa-miR-608 | 5126 | PCSK2 | proprotein convertase subtilisin/kexin type 2 | 1 |
| 4062 | hsa-miR-620 | 5126 | PCSK2 | proprotein convertase subtilisin/kexin type 2 | 1 |
| 4063 | hsa-miR-885-5p | 5126 | PCSK2 | proprotein convertase subtilisin/kexin type 2 | 1 |
| 4064 | hsa-let-7a-3 | 5126 | PCSK2 | proprotein convertase subtilisin/kexin type 2 | 1 |
| 4065 | hsa-let-7b | 5126 | PCSK2 | proprotein convertase subtilisin/kexin type 2 | 1 |
| 4066 | hsa-mir-22 | 5126 | PCSK2 | proprotein convertase subtilisin/kexin type 2 | 1 |
| 4067 | hsa-mir-10a | 5126 | PCSK2 | proprotein convertase subtilisin/kexin type 2 | 1 |
| 4068 | hsa-mir-186 | 5126 | PCSK2 | proprotein convertase subtilisin/kexin type 2 | 1 |
| 4069 | hsa-mir-155 | 5126 | PCSK2 | proprotein convertase subtilisin/kexin type 2 | 1 |
| 4070 | hsa-mir-499 | 5126 | PCSK2 | proprotein convertase subtilisin/kexin type 2 | 1 |
| 4071 | hsa-mir-885 | 5126 | PCSK2 | proprotein convertase subtilisin/kexin type 2 | 1 |
| 4072 | hsa-mir-877 | 5126 | PCSK2 | proprotein convertase subtilisin/kexin type 2 | 1 |
| 4073 | hsa-mir-2116 | 5126 | PCSK2 | proprotein convertase subtilisin/kexin type 2 | 1 |
| 4074 | hsa-mir-3190 | 5126 | PCSK2 | proprotein convertase subtilisin/kexin type 2 | 1 |
| 4075 | hsa-miR-10a | 9726 | ZNF646 | zinc finger protein 646 | 1 |
| 4076 | hsa-miR-152 | 9726 | ZNF646 | zinc finger protein 646 | 1 |
| 4077 | hsa-miR-26a | 9726 | ZNF646 | zinc finger protein 646 | 1 |
| 4078 | hsa-miR-26b | 9726 | ZNF646 | zinc finger protein 646 | 1 |
| 4079 | hsa-miR-31 | 9726 | ZNF646 | zinc finger protein 646 | 1 |
| 4080 | hsa-miR-338-5p | 9726 | ZNF646 | zinc finger protein 646 | 1 |
| 4081 | hsa-miR-340 | 9726 | ZNF646 | zinc finger protein 646 | 1 |
| 4082 | hsa-miR-423-5p | 9726 | ZNF646 | zinc finger protein 646 | 1 |
| 4083 | hsa-miR-449a | 9726 | ZNF646 | zinc finger protein 646 | 1 |
| 4084 | hsa-miR-499-5p | 9726 | ZNF646 | zinc finger protein 646 | 1 |
| 4085 | hsa-miR-548b-5p | 9726 | ZNF646 | zinc finger protein 646 | 1 |
| 4086 | hsa-miR-564 | 9726 | ZNF646 | zinc finger protein 646 | 1 |
| 4087 | hsa-miR-621 | 9726 | ZNF646 | zinc finger protein 646 | 1 |
| 4088 | hsa-miR-657 | 9726 | ZNF646 | zinc finger protein 646 | 1 |
| 4089 | hsa-miR-765 | 9726 | ZNF646 | zinc finger protein 646 | 1 |
| 4090 | hsa-miR-770-5p | 9726 | ZNF646 | zinc finger protein 646 | 1 |
| 4091 | hsa-miR-877 | 9726 | ZNF646 | zinc finger protein 646 | 1 |
| 4092 | hsa-mir-26a-1 | 9726 | ZNF646 | zinc finger protein 646 | 1 |
| 4093 | hsa-mir-26b | 9726 | ZNF646 | zinc finger protein 646 | 1 |
| 4094 | hsa-mir-31 | 9726 | ZNF646 | zinc finger protein 646 | 1 |
| 4095 | hsa-mir-194-2 | 9726 | ZNF646 | zinc finger protein 646 | 1 |
| 4096 | hsa-mir-340 | 9726 | ZNF646 | zinc finger protein 646 | 1 |
| 4097 | hsa-mir-338 | 9726 | ZNF646 | zinc finger protein 646 | 1 |
| 4098 | hsa-mir-548b | 9726 | ZNF646 | zinc finger protein 646 | 1 |
| 4099 | hsa-mir-449b | 9726 | ZNF646 | zinc finger protein 646 | 1 |
| 4100 | hsa-mir-449c | 9726 | ZNF646 | zinc finger protein 646 | 1 |
| 4101 | hsa-mir-3065 | 9726 | ZNF646 | zinc finger protein 646 | 1 |
| 4144 | hsa-miR-644 | 958 | CD40 | CD40 molecule, TNF receptor superfamily member 5 | 1 |
| 4159 | hsa-miR-10a | 5971 | RELB | v-rel reticuloendotheliosis viral oncogene homolog B | 1 |
| 4160 | hsa-miR-139-5p | 5971 | RELB | v-rel reticuloendotheliosis viral oncogene homolog B | 1 |
| 4161 | hsa-miR-152 | 5971 | RELB | v-rel reticuloendotheliosis viral oncogene homolog B | 1 |
| 4162 | hsa-miR-26b | 5971 | RELB | v-rel reticuloendotheliosis viral oncogene homolog B | 1 |
| 4163 | hsa-miR-31 | 5971 | RELB | v-rel reticuloendotheliosis viral oncogene homolog B | 1 |
| 4164 | hsa-miR-338-5p | 5971 | RELB | v-rel reticuloendotheliosis viral oncogene homolog B | 1 |
| 4165 | hsa-miR-340 | 5971 | RELB | v-rel reticuloendotheliosis viral oncogene homolog B | 1 |
| 4166 | hsa-miR-383 | 5971 | RELB | v-rel reticuloendotheliosis viral oncogene homolog B | 1 |
| 4167 | hsa-miR-449a | 5971 | RELB | v-rel reticuloendotheliosis viral oncogene homolog B | 1 |
| 4168 | hsa-miR-499-5p | 5971 | RELB | v-rel reticuloendotheliosis viral oncogene homolog B | 1 |
| 4169 | hsa-miR-548b-5p | 5971 | RELB | v-rel reticuloendotheliosis viral oncogene homolog B | 1 |
| 4170 | hsa-miR-621 | 5971 | RELB | v-rel reticuloendotheliosis viral oncogene homolog B | 1 |
| 4171 | hsa-miR-657 | 5971 | RELB | v-rel reticuloendotheliosis viral oncogene homolog B | 1 |
| 4172 | hsa-miR-663 | 5971 | RELB | v-rel reticuloendotheliosis viral oncogene homolog B | 1 |
| 4173 | hsa-miR-765 | 5971 | RELB | v-rel reticuloendotheliosis viral oncogene homolog B | 1 |
| 4174 | hsa-miR-770-5p | 5971 | RELB | v-rel reticuloendotheliosis viral oncogene homolog B | 1 |
| 4175 | hsa-miR-877 | 5971 | RELB | v-rel reticuloendotheliosis viral oncogene homolog B | 1 |
| 4176 | hsa-mir-26a-1 | 5971 | RELB | v-rel reticuloendotheliosis viral oncogene homolog B | 1 |
| 4177 | hsa-mir-26b | 5971 | RELB | v-rel reticuloendotheliosis viral oncogene homolog B | 1 |
| 4178 | hsa-mir-31 | 5971 | RELB | v-rel reticuloendotheliosis viral oncogene homolog B | 1 |
| 4179 | hsa-mir-194-2 | 5971 | RELB | v-rel reticuloendotheliosis viral oncogene homolog B | 1 |
| 4180 | hsa-mir-340 | 5971 | RELB | v-rel reticuloendotheliosis viral oncogene homolog B | 1 |
| 4181 | hsa-mir-338 | 5971 | RELB | v-rel reticuloendotheliosis viral oncogene homolog B | 1 |
| 4182 | hsa-mir-548b | 5971 | RELB | v-rel reticuloendotheliosis viral oncogene homolog B | 1 |
| 4183 | hsa-mir-449b | 5971 | RELB | v-rel reticuloendotheliosis viral oncogene homolog B | 1 |
| 4184 | hsa-mir-449c | 5971 | RELB | v-rel reticuloendotheliosis viral oncogene homolog B | 1 |
| 4185 | hsa-mir-3065 | 5971 | RELB | v-rel reticuloendotheliosis viral oncogene homolog B | 1 |
| 4186 | hsa-miR-10a | 3569 | IL6 | interleukin 6 (interferon, beta 2) | 1 |
| 4187 | hsa-miR-1228\* | 3569 | IL6 | interleukin 6 (interferon, beta 2) | 1 |
| 4188 | hsa-miR-139-5p | 3569 | IL6 | interleukin 6 (interferon, beta 2) | 1 |
| 4189 | hsa-miR-152 | 3569 | IL6 | interleukin 6 (interferon, beta 2) | 1 |
| 4190 | hsa-miR-26a | 3569 | IL6 | interleukin 6 (interferon, beta 2) | 1 |
| 4191 | hsa-miR-26b | 3569 | IL6 | interleukin 6 (interferon, beta 2) | 1 |
| 4192 | hsa-miR-31 | 3569 | IL6 | interleukin 6 (interferon, beta 2) | 1 |
| 4193 | hsa-miR-338-5p | 3569 | IL6 | interleukin 6 (interferon, beta 2) | 1 |
| 4194 | hsa-miR-340 | 3569 | IL6 | interleukin 6 (interferon, beta 2) | 1 |
| 4195 | hsa-miR-423-5p | 3569 | IL6 | interleukin 6 (interferon, beta 2) | 1 |
| 4196 | hsa-miR-449a | 3569 | IL6 | interleukin 6 (interferon, beta 2) | 1 |
| 4197 | hsa-miR-499-5p | 3569 | IL6 | interleukin 6 (interferon, beta 2) | 1 |
| 4198 | hsa-miR-548b-5p | 3569 | IL6 | interleukin 6 (interferon, beta 2) | 1 |
| 4199 | hsa-miR-564 | 3569 | IL6 | interleukin 6 (interferon, beta 2) | 1 |
| 4200 | hsa-miR-621 | 3569 | IL6 | interleukin 6 (interferon, beta 2) | 1 |
| 4201 | hsa-miR-657 | 3569 | IL6 | interleukin 6 (interferon, beta 2) | 1 |
| 4202 | hsa-miR-765 | 3569 | IL6 | interleukin 6 (interferon, beta 2) | 1 |
| 4203 | hsa-miR-770-5p | 3569 | IL6 | interleukin 6 (interferon, beta 2) | 1 |
| 4204 | hsa-miR-877 | 3569 | IL6 | interleukin 6 (interferon, beta 2) | 1 |
| 4205 | hsa-mir-26a-1 | 3569 | IL6 | interleukin 6 (interferon, beta 2) | 1 |
| 4206 | hsa-mir-26b | 3569 | IL6 | interleukin 6 (interferon, beta 2) | 1 |
| 4207 | hsa-mir-31 | 3569 | IL6 | interleukin 6 (interferon, beta 2) | 1 |
| 4208 | hsa-mir-194-2 | 3569 | IL6 | interleukin 6 (interferon, beta 2) | 1 |
| 4209 | hsa-mir-340 | 3569 | IL6 | interleukin 6 (interferon, beta 2) | 1 |
| 4210 | hsa-mir-338 | 3569 | IL6 | interleukin 6 (interferon, beta 2) | 1 |
| 4211 | hsa-mir-548b | 3569 | IL6 | interleukin 6 (interferon, beta 2) | 1 |
| 4212 | hsa-mir-548d-2 | 3569 | IL6 | interleukin 6 (interferon, beta 2) | 1 |
| 4213 | hsa-mir-449b | 3569 | IL6 | interleukin 6 (interferon, beta 2) | 1 |
| 4214 | hsa-mir-449c | 3569 | IL6 | interleukin 6 (interferon, beta 2) | 1 |
| 4215 | hsa-mir-3065 | 3569 | IL6 | interleukin 6 (interferon, beta 2) | 1 |
| 4216 | hsa-miR-10a | 9262 | STK17B | serine/threonine kinase 17b | 1 |
| 4217 | hsa-miR-152 | 9262 | STK17B | serine/threonine kinase 17b | 1 |
| 4218 | hsa-miR-26a | 9262 | STK17B | serine/threonine kinase 17b | 1 |
| 4219 | hsa-miR-26b | 9262 | STK17B | serine/threonine kinase 17b | 1 |
| 4220 | hsa-miR-31 | 9262 | STK17B | serine/threonine kinase 17b | 1 |
| 4221 | hsa-miR-338-5p | 9262 | STK17B | serine/threonine kinase 17b | 1 |
| 4222 | hsa-miR-340 | 9262 | STK17B | serine/threonine kinase 17b | 1 |
| 4223 | hsa-miR-423-5p | 9262 | STK17B | serine/threonine kinase 17b | 1 |
| 4224 | hsa-miR-449a | 9262 | STK17B | serine/threonine kinase 17b | 1 |
| 4225 | hsa-miR-490-5p | 9262 | STK17B | serine/threonine kinase 17b | 1 |
| 4226 | hsa-miR-499-5p | 9262 | STK17B | serine/threonine kinase 17b | 1 |
| 4227 | hsa-miR-548b-5p | 9262 | STK17B | serine/threonine kinase 17b | 1 |
| 4228 | hsa-miR-564 | 9262 | STK17B | serine/threonine kinase 17b | 1 |
| 4229 | hsa-miR-621 | 9262 | STK17B | serine/threonine kinase 17b | 1 |
| 4230 | hsa-miR-644 | 9262 | STK17B | serine/threonine kinase 17b | 1 |
| 4231 | hsa-miR-657 | 9262 | STK17B | serine/threonine kinase 17b | 1 |
| 4232 | hsa-miR-765 | 9262 | STK17B | serine/threonine kinase 17b | 1 |
| 4233 | hsa-miR-770-5p | 9262 | STK17B | serine/threonine kinase 17b | 1 |
| 4234 | hsa-miR-877 | 9262 | STK17B | serine/threonine kinase 17b | 1 |
| 4235 | hsa-miR-95 | 9262 | STK17B | serine/threonine kinase 17b | 1 |
| 4236 | hsa-mir-26a-1 | 9262 | STK17B | serine/threonine kinase 17b | 1 |
| 4237 | hsa-mir-26b | 9262 | STK17B | serine/threonine kinase 17b | 1 |
| 4238 | hsa-mir-31 | 9262 | STK17B | serine/threonine kinase 17b | 1 |
| 4239 | hsa-mir-194-2 | 9262 | STK17B | serine/threonine kinase 17b | 1 |
| 4240 | hsa-mir-340 | 9262 | STK17B | serine/threonine kinase 17b | 1 |
| 4241 | hsa-mir-338 | 9262 | STK17B | serine/threonine kinase 17b | 1 |
| 4242 | hsa-mir-490 | 9262 | STK17B | serine/threonine kinase 17b | 1 |
| 4243 | hsa-mir-548b | 9262 | STK17B | serine/threonine kinase 17b | 1 |
| 4244 | hsa-mir-449b | 9262 | STK17B | serine/threonine kinase 17b | 1 |
| 4245 | hsa-mir-449c | 9262 | STK17B | serine/threonine kinase 17b | 1 |
| 4246 | hsa-mir-3065 | 9262 | STK17B | serine/threonine kinase 17b | 1 |
| 4247 | hsa-miR-1 | 4306 | NR3C2 | nuclear receptor subfamily 3, group C, member 2 | 1 |
| 4248 | hsa-miR-133a | 4306 | NR3C2 | nuclear receptor subfamily 3, group C, member 2 | 1 |
| 4249 | hsa-miR-186 | 4306 | NR3C2 | nuclear receptor subfamily 3, group C, member 2 | 1 |
| 4250 | hsa-miR-326 | 4306 | NR3C2 | nuclear receptor subfamily 3, group C, member 2 | 1 |
| 4251 | hsa-miR-449b | 4306 | NR3C2 | nuclear receptor subfamily 3, group C, member 2 | 1 |
| 4252 | hsa-miR-551a | 4306 | NR3C2 | nuclear receptor subfamily 3, group C, member 2 | 1 |
| 4253 | hsa-miR-575 | 4306 | NR3C2 | nuclear receptor subfamily 3, group C, member 2 | 1 |
| 4254 | hsa-miR-579 | 4306 | NR3C2 | nuclear receptor subfamily 3, group C, member 2 | 1 |
| 4255 | hsa-miR-608 | 4306 | NR3C2 | nuclear receptor subfamily 3, group C, member 2 | 1 |
| 4256 | hsa-miR-637 | 4306 | NR3C2 | nuclear receptor subfamily 3, group C, member 2 | 1 |
| 4257 | hsa-mir-22 | 4306 | NR3C2 | nuclear receptor subfamily 3, group C, member 2 | 1 |
| 4258 | hsa-mir-10a | 4306 | NR3C2 | nuclear receptor subfamily 3, group C, member 2 | 1 |
| 4259 | hsa-mir-186 | 4306 | NR3C2 | nuclear receptor subfamily 3, group C, member 2 | 1 |
| 4260 | hsa-mir-155 | 4306 | NR3C2 | nuclear receptor subfamily 3, group C, member 2 | 1 |
| 4261 | hsa-mir-423 | 4306 | NR3C2 | nuclear receptor subfamily 3, group C, member 2 | 1 |
| 4262 | hsa-mir-499 | 4306 | NR3C2 | nuclear receptor subfamily 3, group C, member 2 | 1 |
| 4263 | hsa-mir-877 | 4306 | NR3C2 | nuclear receptor subfamily 3, group C, member 2 | 1 |
| 4264 | hsa-mir-3190 | 4306 | NR3C2 | nuclear receptor subfamily 3, group C, member 2 | 1 |
| 4265 | hsa-miR-1 | 10458 | BAIAP2 | BAI1-associated protein 2 | 1 |
| 4266 | hsa-miR-133a | 10458 | BAIAP2 | BAI1-associated protein 2 | 1 |
| 4267 | hsa-miR-186 | 10458 | BAIAP2 | BAI1-associated protein 2 | 1 |
| 4268 | hsa-miR-195 | 10458 | BAIAP2 | BAI1-associated protein 2 | 1 |
| 4269 | hsa-miR-326 | 10458 | BAIAP2 | BAI1-associated protein 2 | 1 |
| 4270 | hsa-miR-449b | 10458 | BAIAP2 | BAI1-associated protein 2 | 1 |
| 4271 | hsa-miR-497 | 10458 | BAIAP2 | BAI1-associated protein 2 | 1 |
| 4272 | hsa-miR-550 | 10458 | BAIAP2 | BAI1-associated protein 2 | 1 |
| 4273 | hsa-miR-562 | 10458 | BAIAP2 | BAI1-associated protein 2 | 1 |
| 4274 | hsa-miR-575 | 10458 | BAIAP2 | BAI1-associated protein 2 | 1 |
| 4275 | hsa-miR-608 | 10458 | BAIAP2 | BAI1-associated protein 2 | 1 |
| 4276 | hsa-miR-620 | 10458 | BAIAP2 | BAI1-associated protein 2 | 1 |
| 4277 | hsa-let-7a-3 | 10458 | BAIAP2 | BAI1-associated protein 2 | 1 |
| 4278 | hsa-let-7b | 10458 | BAIAP2 | BAI1-associated protein 2 | 1 |
| 4279 | hsa-mir-22 | 10458 | BAIAP2 | BAI1-associated protein 2 | 1 |
| 4280 | hsa-mir-10a | 10458 | BAIAP2 | BAI1-associated protein 2 | 1 |
| 4281 | hsa-mir-186 | 10458 | BAIAP2 | BAI1-associated protein 2 | 1 |
| 4282 | hsa-mir-155 | 10458 | BAIAP2 | BAI1-associated protein 2 | 1 |
| 4283 | hsa-mir-499 | 10458 | BAIAP2 | BAI1-associated protein 2 | 1 |
| 4284 | hsa-mir-550-1 | 10458 | BAIAP2 | BAI1-associated protein 2 | 1 |
| 4285 | hsa-mir-2116 | 10458 | BAIAP2 | BAI1-associated protein 2 | 1 |
| 4312 | hsa-miR-10a | 6364 | CCL20 | chemokine (C-C motif) ligand 20 | 1 |
| 4313 | hsa-miR-152 | 6364 | CCL20 | chemokine (C-C motif) ligand 20 | 1 |
| 4314 | hsa-miR-26a | 6364 | CCL20 | chemokine (C-C motif) ligand 20 | 1 |
| 4315 | hsa-miR-26b | 6364 | CCL20 | chemokine (C-C motif) ligand 20 | 1 |
| 4316 | hsa-miR-31 | 6364 | CCL20 | chemokine (C-C motif) ligand 20 | 1 |
| 4317 | hsa-miR-338-5p | 6364 | CCL20 | chemokine (C-C motif) ligand 20 | 1 |
| 4318 | hsa-miR-340 | 6364 | CCL20 | chemokine (C-C motif) ligand 20 | 1 |
| 4319 | hsa-miR-449a | 6364 | CCL20 | chemokine (C-C motif) ligand 20 | 1 |
| 4320 | hsa-miR-490-5p | 6364 | CCL20 | chemokine (C-C motif) ligand 20 | 1 |
| 4321 | hsa-miR-499-5p | 6364 | CCL20 | chemokine (C-C motif) ligand 20 | 1 |
| 4322 | hsa-miR-548b-5p | 6364 | CCL20 | chemokine (C-C motif) ligand 20 | 1 |
| 4323 | hsa-miR-564 | 6364 | CCL20 | chemokine (C-C motif) ligand 20 | 1 |
| 4324 | hsa-miR-621 | 6364 | CCL20 | chemokine (C-C motif) ligand 20 | 1 |
| 4325 | hsa-miR-634 | 6364 | CCL20 | chemokine (C-C motif) ligand 20 | 1 |
| 4326 | hsa-miR-653 | 6364 | CCL20 | chemokine (C-C motif) ligand 20 | 1 |
| 4327 | hsa-miR-657 | 6364 | CCL20 | chemokine (C-C motif) ligand 20 | 1 |
| 4328 | hsa-miR-765 | 6364 | CCL20 | chemokine (C-C motif) ligand 20 | 1 |
| 4329 | hsa-miR-770-5p | 6364 | CCL20 | chemokine (C-C motif) ligand 20 | 1 |
| 4330 | hsa-miR-877 | 6364 | CCL20 | chemokine (C-C motif) ligand 20 | 1 |
| 4331 | hsa-miR-95 | 6364 | CCL20 | chemokine (C-C motif) ligand 20 | 1 |
| 4332 | hsa-mir-26a-1 | 6364 | CCL20 | chemokine (C-C motif) ligand 20 | 1 |
| 4333 | hsa-mir-26b | 6364 | CCL20 | chemokine (C-C motif) ligand 20 | 1 |
| 4334 | hsa-mir-31 | 6364 | CCL20 | chemokine (C-C motif) ligand 20 | 1 |
| 4335 | hsa-mir-194-2 | 6364 | CCL20 | chemokine (C-C motif) ligand 20 | 1 |
| 4336 | hsa-mir-340 | 6364 | CCL20 | chemokine (C-C motif) ligand 20 | 1 |
| 4337 | hsa-mir-338 | 6364 | CCL20 | chemokine (C-C motif) ligand 20 | 1 |
| 4338 | hsa-mir-490 | 6364 | CCL20 | chemokine (C-C motif) ligand 20 | 1 |
| 4339 | hsa-mir-548b | 6364 | CCL20 | chemokine (C-C motif) ligand 20 | 1 |
| 4340 | hsa-mir-449b | 6364 | CCL20 | chemokine (C-C motif) ligand 20 | 1 |
| 4341 | hsa-mir-449c | 6364 | CCL20 | chemokine (C-C motif) ligand 20 | 1 |
| 4342 | hsa-mir-3065 | 6364 | CCL20 | chemokine (C-C motif) ligand 20 | 1 |
| 4343 | hsa-miR-10a | 9636 | ISG15 | ISG15 ubiquitin-like modifier | 1 |
| 4344 | hsa-miR-1228\* | 9636 | ISG15 | ISG15 ubiquitin-like modifier | 1 |
| 4345 | hsa-miR-139-5p | 9636 | ISG15 | ISG15 ubiquitin-like modifier | 1 |
| 4346 | hsa-miR-152 | 9636 | ISG15 | ISG15 ubiquitin-like modifier | 1 |
| 4347 | hsa-miR-26a | 9636 | ISG15 | ISG15 ubiquitin-like modifier | 1 |
| 4348 | hsa-miR-26b | 9636 | ISG15 | ISG15 ubiquitin-like modifier | 1 |
| 4349 | hsa-miR-31 | 9636 | ISG15 | ISG15 ubiquitin-like modifier | 1 |
| 4350 | hsa-miR-338-5p | 9636 | ISG15 | ISG15 ubiquitin-like modifier | 1 |
| 4351 | hsa-miR-340 | 9636 | ISG15 | ISG15 ubiquitin-like modifier | 1 |
| 4352 | hsa-miR-423-5p | 9636 | ISG15 | ISG15 ubiquitin-like modifier | 1 |
| 4353 | hsa-miR-449a | 9636 | ISG15 | ISG15 ubiquitin-like modifier | 1 |
| 4354 | hsa-miR-499-5p | 9636 | ISG15 | ISG15 ubiquitin-like modifier | 1 |
| 4355 | hsa-miR-548b-5p | 9636 | ISG15 | ISG15 ubiquitin-like modifier | 1 |
| 4356 | hsa-miR-564 | 9636 | ISG15 | ISG15 ubiquitin-like modifier | 1 |
| 4357 | hsa-miR-621 | 9636 | ISG15 | ISG15 ubiquitin-like modifier | 1 |
| 4358 | hsa-miR-644 | 9636 | ISG15 | ISG15 ubiquitin-like modifier | 1 |
| 4359 | hsa-miR-657 | 9636 | ISG15 | ISG15 ubiquitin-like modifier | 1 |
| 4360 | hsa-miR-765 | 9636 | ISG15 | ISG15 ubiquitin-like modifier | 1 |
| 4361 | hsa-miR-770-5p | 9636 | ISG15 | ISG15 ubiquitin-like modifier | 1 |
| 4362 | hsa-miR-877 | 9636 | ISG15 | ISG15 ubiquitin-like modifier | 1 |
| 4363 | hsa-mir-26a-1 | 9636 | ISG15 | ISG15 ubiquitin-like modifier | 1 |
| 4364 | hsa-mir-26b | 9636 | ISG15 | ISG15 ubiquitin-like modifier | 1 |
| 4365 | hsa-mir-31 | 9636 | ISG15 | ISG15 ubiquitin-like modifier | 1 |
| 4366 | hsa-mir-194-2 | 9636 | ISG15 | ISG15 ubiquitin-like modifier | 1 |
| 4367 | hsa-mir-340 | 9636 | ISG15 | ISG15 ubiquitin-like modifier | 1 |
| 4368 | hsa-mir-338 | 9636 | ISG15 | ISG15 ubiquitin-like modifier | 1 |
| 4369 | hsa-mir-548b | 9636 | ISG15 | ISG15 ubiquitin-like modifier | 1 |
| 4370 | hsa-mir-449b | 9636 | ISG15 | ISG15 ubiquitin-like modifier | 1 |
| 4371 | hsa-mir-449c | 9636 | ISG15 | ISG15 ubiquitin-like modifier | 1 |
| 4372 | hsa-mir-3065 | 9636 | ISG15 | ISG15 ubiquitin-like modifier | 1 |
| 4373 | hsa-miR-10a | 27074 | LAMP3 | lysosomal-associated membrane protein 3 | 1 |
| 4374 | hsa-miR-139-5p | 27074 | LAMP3 | lysosomal-associated membrane protein 3 | 1 |
| 4375 | hsa-miR-152 | 27074 | LAMP3 | lysosomal-associated membrane protein 3 | 1 |
| 4376 | hsa-miR-26a | 27074 | LAMP3 | lysosomal-associated membrane protein 3 | 1 |
| 4377 | hsa-miR-26b | 27074 | LAMP3 | lysosomal-associated membrane protein 3 | 1 |
| 4378 | hsa-miR-31 | 27074 | LAMP3 | lysosomal-associated membrane protein 3 | 1 |
| 4379 | hsa-miR-338-5p | 27074 | LAMP3 | lysosomal-associated membrane protein 3 | 1 |
| 4380 | hsa-miR-340 | 27074 | LAMP3 | lysosomal-associated membrane protein 3 | 1 |
| 4381 | hsa-miR-383 | 27074 | LAMP3 | lysosomal-associated membrane protein 3 | 1 |
| 4382 | hsa-miR-449a | 27074 | LAMP3 | lysosomal-associated membrane protein 3 | 1 |
| 4383 | hsa-miR-499-5p | 27074 | LAMP3 | lysosomal-associated membrane protein 3 | 1 |
| 4384 | hsa-miR-548b-5p | 27074 | LAMP3 | lysosomal-associated membrane protein 3 | 1 |
| 4385 | hsa-miR-621 | 27074 | LAMP3 | lysosomal-associated membrane protein 3 | 1 |
| 4386 | hsa-miR-657 | 27074 | LAMP3 | lysosomal-associated membrane protein 3 | 1 |
| 4387 | hsa-miR-663 | 27074 | LAMP3 | lysosomal-associated membrane protein 3 | 1 |
| 4388 | hsa-miR-765 | 27074 | LAMP3 | lysosomal-associated membrane protein 3 | 1 |
| 4389 | hsa-miR-770-5p | 27074 | LAMP3 | lysosomal-associated membrane protein 3 | 1 |
| 4390 | hsa-miR-877 | 27074 | LAMP3 | lysosomal-associated membrane protein 3 | 1 |
| 4391 | hsa-mir-26a-1 | 27074 | LAMP3 | lysosomal-associated membrane protein 3 | 1 |
| 4392 | hsa-mir-26b | 27074 | LAMP3 | lysosomal-associated membrane protein 3 | 1 |
| 4393 | hsa-mir-31 | 27074 | LAMP3 | lysosomal-associated membrane protein 3 | 1 |
| 4394 | hsa-mir-194-2 | 27074 | LAMP3 | lysosomal-associated membrane protein 3 | 1 |
| 4395 | hsa-mir-340 | 27074 | LAMP3 | lysosomal-associated membrane protein 3 | 1 |
| 4396 | hsa-mir-338 | 27074 | LAMP3 | lysosomal-associated membrane protein 3 | 1 |
| 4397 | hsa-mir-548b | 27074 | LAMP3 | lysosomal-associated membrane protein 3 | 1 |
| 4398 | hsa-mir-548d-2 | 27074 | LAMP3 | lysosomal-associated membrane protein 3 | 1 |
| 4399 | hsa-mir-449b | 27074 | LAMP3 | lysosomal-associated membrane protein 3 | 1 |
| 4400 | hsa-mir-449c | 27074 | LAMP3 | lysosomal-associated membrane protein 3 | 1 |
| 4401 | hsa-mir-3065 | 27074 | LAMP3 | lysosomal-associated membrane protein 3 | 1 |
| 4402 | hsa-miR-1 | 6521 | SLC4A1 | solute carrier family 4, anion exchanger, member 1 (erythrocyte membrane protein band 3, Diego blood group) | 1 |
| 4403 | hsa-miR-133a | 6521 | SLC4A1 | solute carrier family 4, anion exchanger, member 1 (erythrocyte membrane protein band 3, Diego blood group) | 1 |
| 4404 | hsa-miR-186 | 6521 | SLC4A1 | solute carrier family 4, anion exchanger, member 1 (erythrocyte membrane protein band 3, Diego blood group) | 1 |
| 4405 | hsa-miR-449b | 6521 | SLC4A1 | solute carrier family 4, anion exchanger, member 1 (erythrocyte membrane protein band 3, Diego blood group) | 1 |
| 4406 | hsa-miR-489 | 6521 | SLC4A1 | solute carrier family 4, anion exchanger, member 1 (erythrocyte membrane protein band 3, Diego blood group) | 1 |
| 4407 | hsa-miR-562 | 6521 | SLC4A1 | solute carrier family 4, anion exchanger, member 1 (erythrocyte membrane protein band 3, Diego blood group) | 1 |
| 4408 | hsa-miR-579 | 6521 | SLC4A1 | solute carrier family 4, anion exchanger, member 1 (erythrocyte membrane protein band 3, Diego blood group) | 1 |
| 4409 | hsa-miR-608 | 6521 | SLC4A1 | solute carrier family 4, anion exchanger, member 1 (erythrocyte membrane protein band 3, Diego blood group) | 1 |
| 4410 | hsa-miR-637 | 6521 | SLC4A1 | solute carrier family 4, anion exchanger, member 1 (erythrocyte membrane protein band 3, Diego blood group) | 1 |
| 4411 | hsa-miR-885-5p | 6521 | SLC4A1 | solute carrier family 4, anion exchanger, member 1 (erythrocyte membrane protein band 3, Diego blood group) | 1 |
| 4412 | hsa-mir-22 | 6521 | SLC4A1 | solute carrier family 4, anion exchanger, member 1 (erythrocyte membrane protein band 3, Diego blood group) | 1 |
| 4413 | hsa-mir-10a | 6521 | SLC4A1 | solute carrier family 4, anion exchanger, member 1 (erythrocyte membrane protein band 3, Diego blood group) | 1 |
| 4414 | hsa-mir-186 | 6521 | SLC4A1 | solute carrier family 4, anion exchanger, member 1 (erythrocyte membrane protein band 3, Diego blood group) | 1 |
| 4415 | hsa-mir-155 | 6521 | SLC4A1 | solute carrier family 4, anion exchanger, member 1 (erythrocyte membrane protein band 3, Diego blood group) | 1 |
| 4416 | hsa-mir-499 | 6521 | SLC4A1 | solute carrier family 4, anion exchanger, member 1 (erythrocyte membrane protein band 3, Diego blood group) | 1 |
| 4417 | hsa-mir-885 | 6521 | SLC4A1 | solute carrier family 4, anion exchanger, member 1 (erythrocyte membrane protein band 3, Diego blood group) | 1 |
| 4428 | hsa-miR-490-5p | 7185 | TRAF1 | TNF receptor-associated factor 1 | 1 |
| 4442 | hsa-mir-490 | 7185 | TRAF1 | TNF receptor-associated factor 1 | 1 |
| 4448 | hsa-miR-139-5p | 57147 | SCYL3 | SCY1-like 3 (S. cerevisiae) | 1 |
| 4475 | hsa-miR-1 | 8742 | TNFSF12 | tumor necrosis factor (ligand) superfamily, member 12 | 1 |
| 4476 | hsa-miR-133a | 8742 | TNFSF12 | tumor necrosis factor (ligand) superfamily, member 12 | 1 |
| 4477 | hsa-miR-186 | 8742 | TNFSF12 | tumor necrosis factor (ligand) superfamily, member 12 | 1 |
| 4478 | hsa-miR-326 | 8742 | TNFSF12 | tumor necrosis factor (ligand) superfamily, member 12 | 1 |
| 4479 | hsa-miR-449b | 8742 | TNFSF12 | tumor necrosis factor (ligand) superfamily, member 12 | 1 |
| 4480 | hsa-miR-575 | 8742 | TNFSF12 | tumor necrosis factor (ligand) superfamily, member 12 | 1 |
| 4481 | hsa-miR-579 | 8742 | TNFSF12 | tumor necrosis factor (ligand) superfamily, member 12 | 1 |
| 4482 | hsa-miR-608 | 8742 | TNFSF12 | tumor necrosis factor (ligand) superfamily, member 12 | 1 |
| 4483 | hsa-miR-620 | 8742 | TNFSF12 | tumor necrosis factor (ligand) superfamily, member 12 | 1 |
| 4484 | hsa-miR-885-5p | 8742 | TNFSF12 | tumor necrosis factor (ligand) superfamily, member 12 | 1 |
| 4485 | hsa-mir-22 | 8742 | TNFSF12 | tumor necrosis factor (ligand) superfamily, member 12 | 1 |
| 4486 | hsa-mir-10a | 8742 | TNFSF12 | tumor necrosis factor (ligand) superfamily, member 12 | 1 |
| 4487 | hsa-mir-186 | 8742 | TNFSF12 | tumor necrosis factor (ligand) superfamily, member 12 | 1 |
| 4488 | hsa-mir-155 | 8742 | TNFSF12 | tumor necrosis factor (ligand) superfamily, member 12 | 1 |
| 4489 | hsa-mir-499 | 8742 | TNFSF12 | tumor necrosis factor (ligand) superfamily, member 12 | 1 |
| 4490 | hsa-mir-885 | 8742 | TNFSF12 | tumor necrosis factor (ligand) superfamily, member 12 | 1 |
| 4491 | hsa-mir-877 | 8742 | TNFSF12 | tumor necrosis factor (ligand) superfamily, member 12 | 1 |
| 4492 | hsa-mir-2116 | 8742 | TNFSF12 | tumor necrosis factor (ligand) superfamily, member 12 | 1 |
| 4493 | hsa-mir-3190 | 8742 | TNFSF12 | tumor necrosis factor (ligand) superfamily, member 12 | 1 |
| 4494 | hsa-miR-10a | 597 | BCL2A1 | BCL2-related protein A1 | 1 |
| 4495 | hsa-miR-139-5p | 597 | BCL2A1 | BCL2-related protein A1 | 1 |
| 4496 | hsa-miR-152 | 597 | BCL2A1 | BCL2-related protein A1 | 1 |
| 4497 | hsa-miR-26a | 597 | BCL2A1 | BCL2-related protein A1 | 1 |
| 4498 | hsa-miR-26b | 597 | BCL2A1 | BCL2-related protein A1 | 1 |
| 4499 | hsa-miR-31 | 597 | BCL2A1 | BCL2-related protein A1 | 1 |
| 4500 | hsa-miR-338-5p | 597 | BCL2A1 | BCL2-related protein A1 | 1 |
| 4501 | hsa-miR-340 | 597 | BCL2A1 | BCL2-related protein A1 | 1 |
| 4502 | hsa-miR-423-5p | 597 | BCL2A1 | BCL2-related protein A1 | 1 |
| 4503 | hsa-miR-449a | 597 | BCL2A1 | BCL2-related protein A1 | 1 |
| 4504 | hsa-miR-490-5p | 597 | BCL2A1 | BCL2-related protein A1 | 1 |
| 4505 | hsa-miR-499-5p | 597 | BCL2A1 | BCL2-related protein A1 | 1 |
| 4506 | hsa-miR-548b-5p | 597 | BCL2A1 | BCL2-related protein A1 | 1 |
| 4507 | hsa-miR-564 | 597 | BCL2A1 | BCL2-related protein A1 | 1 |
| 4508 | hsa-miR-584 | 597 | BCL2A1 | BCL2-related protein A1 | 1 |
| 4509 | hsa-miR-621 | 597 | BCL2A1 | BCL2-related protein A1 | 1 |
| 4510 | hsa-miR-634 | 597 | BCL2A1 | BCL2-related protein A1 | 1 |
| 4511 | hsa-miR-644 | 597 | BCL2A1 | BCL2-related protein A1 | 1 |
| 4512 | hsa-miR-657 | 597 | BCL2A1 | BCL2-related protein A1 | 1 |
| 4513 | hsa-miR-765 | 597 | BCL2A1 | BCL2-related protein A1 | 1 |
| 4514 | hsa-miR-770-5p | 597 | BCL2A1 | BCL2-related protein A1 | 1 |
| 4515 | hsa-miR-877 | 597 | BCL2A1 | BCL2-related protein A1 | 1 |
| 4516 | hsa-miR-95 | 597 | BCL2A1 | BCL2-related protein A1 | 1 |
| 4517 | hsa-mir-26a-1 | 597 | BCL2A1 | BCL2-related protein A1 | 1 |
| 4518 | hsa-mir-26b | 597 | BCL2A1 | BCL2-related protein A1 | 1 |
| 4519 | hsa-mir-31 | 597 | BCL2A1 | BCL2-related protein A1 | 1 |
| 4520 | hsa-mir-194-2 | 597 | BCL2A1 | BCL2-related protein A1 | 1 |
| 4521 | hsa-mir-340 | 597 | BCL2A1 | BCL2-related protein A1 | 1 |
| 4522 | hsa-mir-338 | 597 | BCL2A1 | BCL2-related protein A1 | 1 |
| 4523 | hsa-mir-490 | 597 | BCL2A1 | BCL2-related protein A1 | 1 |
| 4524 | hsa-mir-548b | 597 | BCL2A1 | BCL2-related protein A1 | 1 |
| 4525 | hsa-mir-449b | 597 | BCL2A1 | BCL2-related protein A1 | 1 |
| 4526 | hsa-mir-449c | 597 | BCL2A1 | BCL2-related protein A1 | 1 |
| 4527 | hsa-mir-3065 | 597 | BCL2A1 | BCL2-related protein A1 | 1 |
| 4528 | hsa-miR-1 | 5608 | MAP2K6 | mitogen-activated protein kinase kinase 6 | 1 |
| 4529 | hsa-miR-133a | 5608 | MAP2K6 | mitogen-activated protein kinase kinase 6 | 1 |
| 4530 | hsa-miR-186 | 5608 | MAP2K6 | mitogen-activated protein kinase kinase 6 | 1 |
| 4531 | hsa-miR-326 | 5608 | MAP2K6 | mitogen-activated protein kinase kinase 6 | 1 |
| 4532 | hsa-miR-449b | 5608 | MAP2K6 | mitogen-activated protein kinase kinase 6 | 1 |
| 4533 | hsa-miR-575 | 5608 | MAP2K6 | mitogen-activated protein kinase kinase 6 | 1 |
| 4534 | hsa-miR-579 | 5608 | MAP2K6 | mitogen-activated protein kinase kinase 6 | 1 |
| 4535 | hsa-miR-608 | 5608 | MAP2K6 | mitogen-activated protein kinase kinase 6 | 1 |
| 4536 | hsa-miR-620 | 5608 | MAP2K6 | mitogen-activated protein kinase kinase 6 | 1 |
| 4537 | hsa-miR-885-5p | 5608 | MAP2K6 | mitogen-activated protein kinase kinase 6 | 1 |
| 4538 | hsa-mir-22 | 5608 | MAP2K6 | mitogen-activated protein kinase kinase 6 | 1 |
| 4539 | hsa-mir-10a | 5608 | MAP2K6 | mitogen-activated protein kinase kinase 6 | 1 |
| 4540 | hsa-mir-186 | 5608 | MAP2K6 | mitogen-activated protein kinase kinase 6 | 1 |
| 4541 | hsa-mir-155 | 5608 | MAP2K6 | mitogen-activated protein kinase kinase 6 | 1 |
| 4542 | hsa-mir-499 | 5608 | MAP2K6 | mitogen-activated protein kinase kinase 6 | 1 |
| 4543 | hsa-mir-885 | 5608 | MAP2K6 | mitogen-activated protein kinase kinase 6 | 1 |
| 4544 | hsa-mir-877 | 5608 | MAP2K6 | mitogen-activated protein kinase kinase 6 | 1 |
| 4545 | hsa-mir-2116 | 5608 | MAP2K6 | mitogen-activated protein kinase kinase 6 | 1 |
| 4546 | hsa-mir-3190 | 5608 | MAP2K6 | mitogen-activated protein kinase kinase 6 | 1 |
| 4547 | hsa-miR-1 | 2949 | GSTM5 | glutathione S-transferase mu 5 | 1 |
| 4548 | hsa-miR-133a | 2949 | GSTM5 | glutathione S-transferase mu 5 | 1 |
| 4549 | hsa-miR-186 | 2949 | GSTM5 | glutathione S-transferase mu 5 | 1 |
| 4550 | hsa-miR-326 | 2949 | GSTM5 | glutathione S-transferase mu 5 | 1 |
| 4551 | hsa-miR-449b | 2949 | GSTM5 | glutathione S-transferase mu 5 | 1 |
| 4552 | hsa-miR-550 | 2949 | GSTM5 | glutathione S-transferase mu 5 | 1 |
| 4553 | hsa-miR-551a | 2949 | GSTM5 | glutathione S-transferase mu 5 | 1 |
| 4554 | hsa-miR-575 | 2949 | GSTM5 | glutathione S-transferase mu 5 | 1 |
| 4555 | hsa-miR-608 | 2949 | GSTM5 | glutathione S-transferase mu 5 | 1 |
| 4556 | hsa-miR-620 | 2949 | GSTM5 | glutathione S-transferase mu 5 | 1 |
| 4557 | hsa-mir-22 | 2949 | GSTM5 | glutathione S-transferase mu 5 | 1 |
| 4558 | hsa-mir-10a | 2949 | GSTM5 | glutathione S-transferase mu 5 | 1 |
| 4559 | hsa-mir-186 | 2949 | GSTM5 | glutathione S-transferase mu 5 | 1 |
| 4560 | hsa-mir-155 | 2949 | GSTM5 | glutathione S-transferase mu 5 | 1 |
| 4561 | hsa-mir-423 | 2949 | GSTM5 | glutathione S-transferase mu 5 | 1 |
| 4562 | hsa-mir-499 | 2949 | GSTM5 | glutathione S-transferase mu 5 | 1 |
| 4563 | hsa-mir-550-1 | 2949 | GSTM5 | glutathione S-transferase mu 5 | 1 |
| 4564 | hsa-mir-877 | 2949 | GSTM5 | glutathione S-transferase mu 5 | 1 |
| 4565 | hsa-mir-2116 | 2949 | GSTM5 | glutathione S-transferase mu 5 | 1 |
| 4566 | hsa-mir-3186 | 2949 | GSTM5 | glutathione S-transferase mu 5 | 1 |
| 4567 | hsa-mir-3190 | 2949 | GSTM5 | glutathione S-transferase mu 5 | 1 |
| 4568 | hsa-miR-1 | 2330 | FMO5 | flavin containing monooxygenase 5 | 1 |
| 4569 | hsa-miR-133a | 2330 | FMO5 | flavin containing monooxygenase 5 | 1 |
| 4570 | hsa-miR-186 | 2330 | FMO5 | flavin containing monooxygenase 5 | 1 |
| 4571 | hsa-miR-326 | 2330 | FMO5 | flavin containing monooxygenase 5 | 1 |
| 4572 | hsa-miR-449b | 2330 | FMO5 | flavin containing monooxygenase 5 | 1 |
| 4573 | hsa-miR-551a | 2330 | FMO5 | flavin containing monooxygenase 5 | 1 |
| 4574 | hsa-miR-575 | 2330 | FMO5 | flavin containing monooxygenase 5 | 1 |
| 4575 | hsa-miR-579 | 2330 | FMO5 | flavin containing monooxygenase 5 | 1 |
| 4576 | hsa-miR-608 | 2330 | FMO5 | flavin containing monooxygenase 5 | 1 |
| 4577 | hsa-miR-620 | 2330 | FMO5 | flavin containing monooxygenase 5 | 1 |
| 4578 | hsa-miR-637 | 2330 | FMO5 | flavin containing monooxygenase 5 | 1 |
| 4579 | hsa-mir-22 | 2330 | FMO5 | flavin containing monooxygenase 5 | 1 |
| 4580 | hsa-mir-10a | 2330 | FMO5 | flavin containing monooxygenase 5 | 1 |
| 4581 | hsa-mir-186 | 2330 | FMO5 | flavin containing monooxygenase 5 | 1 |
| 4582 | hsa-mir-155 | 2330 | FMO5 | flavin containing monooxygenase 5 | 1 |
| 4583 | hsa-mir-423 | 2330 | FMO5 | flavin containing monooxygenase 5 | 1 |
| 4584 | hsa-mir-499 | 2330 | FMO5 | flavin containing monooxygenase 5 | 1 |
| 4585 | hsa-mir-877 | 2330 | FMO5 | flavin containing monooxygenase 5 | 1 |
| 4586 | hsa-mir-2116 | 2330 | FMO5 | flavin containing monooxygenase 5 | 1 |
| 4587 | hsa-mir-3190 | 2330 | FMO5 | flavin containing monooxygenase 5 | 1 |
| 4588 | hsa-miR-1 | 5166 | PDK4 | pyruvate dehydrogenase kinase, isozyme 4 | 1 |
| 4589 | hsa-miR-133a | 5166 | PDK4 | pyruvate dehydrogenase kinase, isozyme 4 | 1 |
| 4590 | hsa-miR-186 | 5166 | PDK4 | pyruvate dehydrogenase kinase, isozyme 4 | 1 |
| 4591 | hsa-miR-195 | 5166 | PDK4 | pyruvate dehydrogenase kinase, isozyme 4 | 1 |
| 4592 | hsa-miR-326 | 5166 | PDK4 | pyruvate dehydrogenase kinase, isozyme 4 | 1 |
| 4593 | hsa-miR-449b | 5166 | PDK4 | pyruvate dehydrogenase kinase, isozyme 4 | 1 |
| 4594 | hsa-miR-497 | 5166 | PDK4 | pyruvate dehydrogenase kinase, isozyme 4 | 1 |
| 4595 | hsa-miR-562 | 5166 | PDK4 | pyruvate dehydrogenase kinase, isozyme 4 | 1 |
| 4596 | hsa-miR-575 | 5166 | PDK4 | pyruvate dehydrogenase kinase, isozyme 4 | 1 |
| 4597 | hsa-miR-608 | 5166 | PDK4 | pyruvate dehydrogenase kinase, isozyme 4 | 1 |
| 4598 | hsa-miR-620 | 5166 | PDK4 | pyruvate dehydrogenase kinase, isozyme 4 | 1 |
| 4599 | hsa-let-7a-3 | 5166 | PDK4 | pyruvate dehydrogenase kinase, isozyme 4 | 1 |
| 4600 | hsa-let-7b | 5166 | PDK4 | pyruvate dehydrogenase kinase, isozyme 4 | 1 |
| 4601 | hsa-mir-22 | 5166 | PDK4 | pyruvate dehydrogenase kinase, isozyme 4 | 1 |
| 4602 | hsa-mir-10a | 5166 | PDK4 | pyruvate dehydrogenase kinase, isozyme 4 | 1 |
| 4603 | hsa-mir-186 | 5166 | PDK4 | pyruvate dehydrogenase kinase, isozyme 4 | 1 |
| 4604 | hsa-mir-155 | 5166 | PDK4 | pyruvate dehydrogenase kinase, isozyme 4 | 1 |
| 4605 | hsa-mir-499 | 5166 | PDK4 | pyruvate dehydrogenase kinase, isozyme 4 | 1 |
| 4606 | hsa-mir-550-1 | 5166 | PDK4 | pyruvate dehydrogenase kinase, isozyme 4 | 1 |
| 4607 | hsa-mir-877 | 5166 | PDK4 | pyruvate dehydrogenase kinase, isozyme 4 | 1 |
| 4608 | hsa-miR-10a | 3600 | IL15 | interleukin 15 | 1 |
| 4609 | hsa-miR-139-5p | 3600 | IL15 | interleukin 15 | 1 |
| 4610 | hsa-miR-152 | 3600 | IL15 | interleukin 15 | 1 |
| 4611 | hsa-miR-26a | 3600 | IL15 | interleukin 15 | 1 |
| 4612 | hsa-miR-26b | 3600 | IL15 | interleukin 15 | 1 |
| 4613 | hsa-miR-31 | 3600 | IL15 | interleukin 15 | 1 |
| 4614 | hsa-miR-338-5p | 3600 | IL15 | interleukin 15 | 1 |
| 4615 | hsa-miR-340 | 3600 | IL15 | interleukin 15 | 1 |
| 4616 | hsa-miR-383 | 3600 | IL15 | interleukin 15 | 1 |
| 4617 | hsa-miR-449a | 3600 | IL15 | interleukin 15 | 1 |
| 4618 | hsa-miR-499-5p | 3600 | IL15 | interleukin 15 | 1 |
| 4619 | hsa-miR-548b-5p | 3600 | IL15 | interleukin 15 | 1 |
| 4620 | hsa-miR-621 | 3600 | IL15 | interleukin 15 | 1 |
| 4621 | hsa-miR-657 | 3600 | IL15 | interleukin 15 | 1 |
| 4622 | hsa-miR-765 | 3600 | IL15 | interleukin 15 | 1 |
| 4623 | hsa-miR-770-5p | 3600 | IL15 | interleukin 15 | 1 |
| 4624 | hsa-miR-877 | 3600 | IL15 | interleukin 15 | 1 |
| 4625 | hsa-mir-26a-1 | 3600 | IL15 | interleukin 15 | 1 |
| 4626 | hsa-mir-26b | 3600 | IL15 | interleukin 15 | 1 |
| 4627 | hsa-mir-31 | 3600 | IL15 | interleukin 15 | 1 |
| 4628 | hsa-mir-194-2 | 3600 | IL15 | interleukin 15 | 1 |
| 4629 | hsa-mir-340 | 3600 | IL15 | interleukin 15 | 1 |
| 4630 | hsa-mir-338 | 3600 | IL15 | interleukin 15 | 1 |
| 4631 | hsa-mir-548b | 3600 | IL15 | interleukin 15 | 1 |
| 4632 | hsa-mir-548d-2 | 3600 | IL15 | interleukin 15 | 1 |
| 4633 | hsa-mir-449b | 3600 | IL15 | interleukin 15 | 1 |
| 4634 | hsa-mir-449c | 3600 | IL15 | interleukin 15 | 1 |
| 4635 | hsa-mir-3065 | 3600 | IL15 | interleukin 15 | 1 |
| 4650 | hsa-miR-653 | 7130 | TNFAIP6 | tumor necrosis factor, alpha-induced protein 6 | 1 |
| 4666 | hsa-miR-423-5p | 7130 | TNFAIP6 | tumor necrosis factor, alpha-induced protein 6 | 1 |
| 4668 | hsa-miR-1228\* | 5966 | REL | v-rel reticuloendotheliosis viral oncogene homolog (avian) | 1 |
| 4669 | hsa-miR-139-5p | 5966 | REL | v-rel reticuloendotheliosis viral oncogene homolog (avian) | 1 |
| 4674 | hsa-miR-338-5p | 5966 | REL | v-rel reticuloendotheliosis viral oncogene homolog (avian) | 1 |
| 4682 | hsa-miR-657 | 5966 | REL | v-rel reticuloendotheliosis viral oncogene homolog (avian) | 1 |
| 4683 | hsa-miR-663 | 5966 | REL | v-rel reticuloendotheliosis viral oncogene homolog (avian) | 1 |
| 4692 | hsa-mir-338 | 5966 | REL | v-rel reticuloendotheliosis viral oncogene homolog (avian) | 1 |
| 4697 | hsa-mir-3065 | 5966 | REL | v-rel reticuloendotheliosis viral oncogene homolog (avian) | 1 |
| 4698 | hsa-miR-423-5p | 5966 | REL | v-rel reticuloendotheliosis viral oncogene homolog (avian) | 1 |
| 4699 | hsa-miR-504 | 5966 | REL | v-rel reticuloendotheliosis viral oncogene homolog (avian) | 1 |
| 4700 | hsa-miR-644 | 5966 | REL | v-rel reticuloendotheliosis viral oncogene homolog (avian) | 1 |
| 4701 | hsa-miR-653 | 5966 | REL | v-rel reticuloendotheliosis viral oncogene homolog (avian) | 1 |
| 4702 | hsa-miR-95 | 5966 | REL | v-rel reticuloendotheliosis viral oncogene homolog (avian) | 1 |
| 4703 | hsa-miR-10a | 6539 | SLC6A12 | solute carrier family 6 (neurotransmitter transporter, betaine/GABA), member 12 | 1 |
| 4704 | hsa-miR-152 | 6539 | SLC6A12 | solute carrier family 6 (neurotransmitter transporter, betaine/GABA), member 12 | 1 |
| 4705 | hsa-miR-26a | 6539 | SLC6A12 | solute carrier family 6 (neurotransmitter transporter, betaine/GABA), member 12 | 1 |
| 4706 | hsa-miR-26b | 6539 | SLC6A12 | solute carrier family 6 (neurotransmitter transporter, betaine/GABA), member 12 | 1 |
| 4707 | hsa-miR-31 | 6539 | SLC6A12 | solute carrier family 6 (neurotransmitter transporter, betaine/GABA), member 12 | 1 |
| 4708 | hsa-miR-338-5p | 6539 | SLC6A12 | solute carrier family 6 (neurotransmitter transporter, betaine/GABA), member 12 | 1 |
| 4709 | hsa-miR-340 | 6539 | SLC6A12 | solute carrier family 6 (neurotransmitter transporter, betaine/GABA), member 12 | 1 |
| 4710 | hsa-miR-383 | 6539 | SLC6A12 | solute carrier family 6 (neurotransmitter transporter, betaine/GABA), member 12 | 1 |
| 4711 | hsa-miR-449a | 6539 | SLC6A12 | solute carrier family 6 (neurotransmitter transporter, betaine/GABA), member 12 | 1 |
| 4712 | hsa-miR-490-5p | 6539 | SLC6A12 | solute carrier family 6 (neurotransmitter transporter, betaine/GABA), member 12 | 1 |
| 4713 | hsa-miR-499-5p | 6539 | SLC6A12 | solute carrier family 6 (neurotransmitter transporter, betaine/GABA), member 12 | 1 |
| 4714 | hsa-miR-621 | 6539 | SLC6A12 | solute carrier family 6 (neurotransmitter transporter, betaine/GABA), member 12 | 1 |
| 4715 | hsa-miR-634 | 6539 | SLC6A12 | solute carrier family 6 (neurotransmitter transporter, betaine/GABA), member 12 | 1 |
| 4716 | hsa-miR-653 | 6539 | SLC6A12 | solute carrier family 6 (neurotransmitter transporter, betaine/GABA), member 12 | 1 |
| 4717 | hsa-miR-657 | 6539 | SLC6A12 | solute carrier family 6 (neurotransmitter transporter, betaine/GABA), member 12 | 1 |
| 4718 | hsa-miR-765 | 6539 | SLC6A12 | solute carrier family 6 (neurotransmitter transporter, betaine/GABA), member 12 | 1 |
| 4719 | hsa-miR-770-5p | 6539 | SLC6A12 | solute carrier family 6 (neurotransmitter transporter, betaine/GABA), member 12 | 1 |
| 4720 | hsa-miR-877 | 6539 | SLC6A12 | solute carrier family 6 (neurotransmitter transporter, betaine/GABA), member 12 | 1 |
| 4721 | hsa-miR-95 | 6539 | SLC6A12 | solute carrier family 6 (neurotransmitter transporter, betaine/GABA), member 12 | 1 |
| 4722 | hsa-mir-26a-1 | 6539 | SLC6A12 | solute carrier family 6 (neurotransmitter transporter, betaine/GABA), member 12 | 1 |
| 4723 | hsa-mir-26b | 6539 | SLC6A12 | solute carrier family 6 (neurotransmitter transporter, betaine/GABA), member 12 | 1 |
| 4724 | hsa-mir-31 | 6539 | SLC6A12 | solute carrier family 6 (neurotransmitter transporter, betaine/GABA), member 12 | 1 |
| 4725 | hsa-mir-194-2 | 6539 | SLC6A12 | solute carrier family 6 (neurotransmitter transporter, betaine/GABA), member 12 | 1 |
| 4726 | hsa-mir-340 | 6539 | SLC6A12 | solute carrier family 6 (neurotransmitter transporter, betaine/GABA), member 12 | 1 |
| 4727 | hsa-mir-338 | 6539 | SLC6A12 | solute carrier family 6 (neurotransmitter transporter, betaine/GABA), member 12 | 1 |
| 4728 | hsa-mir-490 | 6539 | SLC6A12 | solute carrier family 6 (neurotransmitter transporter, betaine/GABA), member 12 | 1 |
| 4729 | hsa-mir-449b | 6539 | SLC6A12 | solute carrier family 6 (neurotransmitter transporter, betaine/GABA), member 12 | 1 |
| 4730 | hsa-mir-449c | 6539 | SLC6A12 | solute carrier family 6 (neurotransmitter transporter, betaine/GABA), member 12 | 1 |
| 4731 | hsa-mir-3065 | 6539 | SLC6A12 | solute carrier family 6 (neurotransmitter transporter, betaine/GABA), member 12 | 1 |
| 4732 | hsa-miR-1 | 23264 | ZC3H7B | zinc finger CCCH-type containing 7B | 1 |
| 4733 | hsa-miR-133a | 23264 | ZC3H7B | zinc finger CCCH-type containing 7B | 1 |
| 4734 | hsa-miR-186 | 23264 | ZC3H7B | zinc finger CCCH-type containing 7B | 1 |
| 4735 | hsa-miR-208a | 23264 | ZC3H7B | zinc finger CCCH-type containing 7B | 1 |
| 4736 | hsa-miR-326 | 23264 | ZC3H7B | zinc finger CCCH-type containing 7B | 1 |
| 4737 | hsa-miR-449b | 23264 | ZC3H7B | zinc finger CCCH-type containing 7B | 1 |
| 4738 | hsa-miR-575 | 23264 | ZC3H7B | zinc finger CCCH-type containing 7B | 1 |
| 4739 | hsa-miR-579 | 23264 | ZC3H7B | zinc finger CCCH-type containing 7B | 1 |
| 4740 | hsa-miR-608 | 23264 | ZC3H7B | zinc finger CCCH-type containing 7B | 1 |
| 4741 | hsa-miR-620 | 23264 | ZC3H7B | zinc finger CCCH-type containing 7B | 1 |
| 4742 | hsa-miR-637 | 23264 | ZC3H7B | zinc finger CCCH-type containing 7B | 1 |
| 4743 | hsa-miR-885-5p | 23264 | ZC3H7B | zinc finger CCCH-type containing 7B | 1 |
| 4744 | hsa-mir-22 | 23264 | ZC3H7B | zinc finger CCCH-type containing 7B | 1 |
| 4745 | hsa-mir-10a | 23264 | ZC3H7B | zinc finger CCCH-type containing 7B | 1 |
| 4746 | hsa-mir-186 | 23264 | ZC3H7B | zinc finger CCCH-type containing 7B | 1 |
| 4747 | hsa-mir-155 | 23264 | ZC3H7B | zinc finger CCCH-type containing 7B | 1 |
| 4748 | hsa-mir-499 | 23264 | ZC3H7B | zinc finger CCCH-type containing 7B | 1 |
| 4749 | hsa-mir-885 | 23264 | ZC3H7B | zinc finger CCCH-type containing 7B | 1 |
| 4750 | hsa-mir-877 | 23264 | ZC3H7B | zinc finger CCCH-type containing 7B | 1 |
| 4751 | hsa-mir-2116 | 23264 | ZC3H7B | zinc finger CCCH-type containing 7B | 1 |
| 4752 | hsa-mir-3190 | 23264 | ZC3H7B | zinc finger CCCH-type containing 7B | 1 |
| 4753 | hsa-miR-1 | 65988 | ZNF747 | zinc finger protein 747 | 1 |
| 4754 | hsa-miR-133a | 65988 | ZNF747 | zinc finger protein 747 | 1 |
| 4755 | hsa-miR-186 | 65988 | ZNF747 | zinc finger protein 747 | 1 |
| 4756 | hsa-miR-326 | 65988 | ZNF747 | zinc finger protein 747 | 1 |
| 4757 | hsa-miR-449b | 65988 | ZNF747 | zinc finger protein 747 | 1 |
| 4758 | hsa-miR-551a | 65988 | ZNF747 | zinc finger protein 747 | 1 |
| 4759 | hsa-miR-562 | 65988 | ZNF747 | zinc finger protein 747 | 1 |
| 4760 | hsa-miR-575 | 65988 | ZNF747 | zinc finger protein 747 | 1 |
| 4761 | hsa-miR-579 | 65988 | ZNF747 | zinc finger protein 747 | 1 |
| 4762 | hsa-miR-608 | 65988 | ZNF747 | zinc finger protein 747 | 1 |
| 4763 | hsa-miR-620 | 65988 | ZNF747 | zinc finger protein 747 | 1 |
| 4764 | hsa-mir-22 | 65988 | ZNF747 | zinc finger protein 747 | 1 |
| 4765 | hsa-mir-10a | 65988 | ZNF747 | zinc finger protein 747 | 1 |
| 4766 | hsa-mir-186 | 65988 | ZNF747 | zinc finger protein 747 | 1 |
| 4767 | hsa-mir-155 | 65988 | ZNF747 | zinc finger protein 747 | 1 |
| 4768 | hsa-mir-499 | 65988 | ZNF747 | zinc finger protein 747 | 1 |
| 4769 | hsa-mir-550-1 | 65988 | ZNF747 | zinc finger protein 747 | 1 |
| 4770 | hsa-mir-877 | 65988 | ZNF747 | zinc finger protein 747 | 1 |
| 4771 | hsa-miR-1 | 4867 | NPHP1 | nephronophthisis 1 (juvenile) | 1 |
| 4772 | hsa-miR-133a | 4867 | NPHP1 | nephronophthisis 1 (juvenile) | 1 |
| 4773 | hsa-miR-186 | 4867 | NPHP1 | nephronophthisis 1 (juvenile) | 1 |
| 4774 | hsa-miR-326 | 4867 | NPHP1 | nephronophthisis 1 (juvenile) | 1 |
| 4775 | hsa-miR-33a | 4867 | NPHP1 | nephronophthisis 1 (juvenile) | 1 |
| 4776 | hsa-miR-449b | 4867 | NPHP1 | nephronophthisis 1 (juvenile) | 1 |
| 4777 | hsa-miR-550 | 4867 | NPHP1 | nephronophthisis 1 (juvenile) | 1 |
| 4778 | hsa-miR-551a | 4867 | NPHP1 | nephronophthisis 1 (juvenile) | 1 |
| 4779 | hsa-miR-575 | 4867 | NPHP1 | nephronophthisis 1 (juvenile) | 1 |
| 4780 | hsa-miR-579 | 4867 | NPHP1 | nephronophthisis 1 (juvenile) | 1 |
| 4781 | hsa-miR-608 | 4867 | NPHP1 | nephronophthisis 1 (juvenile) | 1 |
| 4782 | hsa-miR-620 | 4867 | NPHP1 | nephronophthisis 1 (juvenile) | 1 |
| 4783 | hsa-let-7a-3 | 4867 | NPHP1 | nephronophthisis 1 (juvenile) | 1 |
| 4784 | hsa-let-7b | 4867 | NPHP1 | nephronophthisis 1 (juvenile) | 1 |
| 4785 | hsa-mir-22 | 4867 | NPHP1 | nephronophthisis 1 (juvenile) | 1 |
| 4786 | hsa-mir-10a | 4867 | NPHP1 | nephronophthisis 1 (juvenile) | 1 |
| 4787 | hsa-mir-186 | 4867 | NPHP1 | nephronophthisis 1 (juvenile) | 1 |
| 4788 | hsa-mir-155 | 4867 | NPHP1 | nephronophthisis 1 (juvenile) | 1 |
| 4789 | hsa-mir-423 | 4867 | NPHP1 | nephronophthisis 1 (juvenile) | 1 |
| 4790 | hsa-mir-499 | 4867 | NPHP1 | nephronophthisis 1 (juvenile) | 1 |
| 4791 | hsa-mir-550-1 | 4867 | NPHP1 | nephronophthisis 1 (juvenile) | 1 |
| 4792 | hsa-mir-877 | 4867 | NPHP1 | nephronophthisis 1 (juvenile) | 1 |
| 4793 | hsa-mir-2116 | 4867 | NPHP1 | nephronophthisis 1 (juvenile) | 1 |
| 4794 | hsa-mir-3186 | 4867 | NPHP1 | nephronophthisis 1 (juvenile) | 1 |
| 4796 | hsa-miR-1 | 57447 | NDRG2 | NDRG family member 2 | 1 |
| 4797 | hsa-miR-133a | 57447 | NDRG2 | NDRG family member 2 | 1 |
| 4798 | hsa-miR-186 | 57447 | NDRG2 | NDRG family member 2 | 1 |
| 4799 | hsa-miR-208a | 57447 | NDRG2 | NDRG family member 2 | 1 |
| 4800 | hsa-miR-326 | 57447 | NDRG2 | NDRG family member 2 | 1 |
| 4801 | hsa-miR-449b | 57447 | NDRG2 | NDRG family member 2 | 1 |
| 4802 | hsa-miR-575 | 57447 | NDRG2 | NDRG family member 2 | 1 |
| 4803 | hsa-miR-579 | 57447 | NDRG2 | NDRG family member 2 | 1 |
| 4804 | hsa-miR-608 | 57447 | NDRG2 | NDRG family member 2 | 1 |
| 4805 | hsa-miR-620 | 57447 | NDRG2 | NDRG family member 2 | 1 |
| 4806 | hsa-miR-637 | 57447 | NDRG2 | NDRG family member 2 | 1 |
| 4807 | hsa-mir-22 | 57447 | NDRG2 | NDRG family member 2 | 1 |
| 4808 | hsa-mir-10a | 57447 | NDRG2 | NDRG family member 2 | 1 |
| 4809 | hsa-mir-186 | 57447 | NDRG2 | NDRG family member 2 | 1 |
| 4810 | hsa-mir-155 | 57447 | NDRG2 | NDRG family member 2 | 1 |
| 4811 | hsa-mir-499 | 57447 | NDRG2 | NDRG family member 2 | 1 |
| 4812 | hsa-mir-877 | 57447 | NDRG2 | NDRG family member 2 | 1 |
| 4813 | hsa-mir-2116 | 57447 | NDRG2 | NDRG family member 2 | 1 |
| 4814 | hsa-mir-3190 | 57447 | NDRG2 | NDRG family member 2 | 1 |
| 4815 | hsa-miR-10a | 4496 | MT1H | metallothionein 1H | 1 |
| 4816 | hsa-miR-152 | 4496 | MT1H | metallothionein 1H | 1 |
| 4817 | hsa-miR-26a | 4496 | MT1H | metallothionein 1H | 1 |
| 4818 | hsa-miR-26b | 4496 | MT1H | metallothionein 1H | 1 |
| 4819 | hsa-miR-31 | 4496 | MT1H | metallothionein 1H | 1 |
| 4820 | hsa-miR-338-5p | 4496 | MT1H | metallothionein 1H | 1 |
| 4821 | hsa-miR-340 | 4496 | MT1H | metallothionein 1H | 1 |
| 4822 | hsa-miR-383 | 4496 | MT1H | metallothionein 1H | 1 |
| 4823 | hsa-miR-449a | 4496 | MT1H | metallothionein 1H | 1 |
| 4824 | hsa-miR-490-5p | 4496 | MT1H | metallothionein 1H | 1 |
| 4825 | hsa-miR-499-5p | 4496 | MT1H | metallothionein 1H | 1 |
| 4826 | hsa-miR-504 | 4496 | MT1H | metallothionein 1H | 1 |
| 4827 | hsa-miR-621 | 4496 | MT1H | metallothionein 1H | 1 |
| 4828 | hsa-miR-653 | 4496 | MT1H | metallothionein 1H | 1 |
| 4829 | hsa-miR-657 | 4496 | MT1H | metallothionein 1H | 1 |
| 4830 | hsa-miR-765 | 4496 | MT1H | metallothionein 1H | 1 |
| 4831 | hsa-miR-770-5p | 4496 | MT1H | metallothionein 1H | 1 |
| 4832 | hsa-miR-877 | 4496 | MT1H | metallothionein 1H | 1 |
| 4833 | hsa-mir-26a-1 | 4496 | MT1H | metallothionein 1H | 1 |
| 4834 | hsa-mir-26b | 4496 | MT1H | metallothionein 1H | 1 |
| 4835 | hsa-mir-31 | 4496 | MT1H | metallothionein 1H | 1 |
| 4836 | hsa-mir-194-2 | 4496 | MT1H | metallothionein 1H | 1 |
| 4837 | hsa-mir-340 | 4496 | MT1H | metallothionein 1H | 1 |
| 4838 | hsa-mir-338 | 4496 | MT1H | metallothionein 1H | 1 |
| 4839 | hsa-mir-490 | 4496 | MT1H | metallothionein 1H | 1 |
| 4840 | hsa-mir-449b | 4496 | MT1H | metallothionein 1H | 1 |
| 4841 | hsa-mir-449c | 4496 | MT1H | metallothionein 1H | 1 |
| 4842 | hsa-mir-3065 | 4496 | MT1H | metallothionein 1H | 1 |
| 4844 | hsa-miR-1228\* | 10154 | PLXNC1 | plexin C1 | 1 |
| 4846 | hsa-miR-26a | 10154 | PLXNC1 | plexin C1 | 1 |
| 4850 | hsa-miR-490-5p | 10154 | PLXNC1 | plexin C1 | 1 |
| 4853 | hsa-miR-564 | 10154 | PLXNC1 | plexin C1 | 1 |
| 4854 | hsa-miR-584 | 10154 | PLXNC1 | plexin C1 | 1 |
| 4856 | hsa-miR-634 | 10154 | PLXNC1 | plexin C1 | 1 |
| 4857 | hsa-miR-644 | 10154 | PLXNC1 | plexin C1 | 1 |
| 4861 | hsa-miR-95 | 10154 | PLXNC1 | plexin C1 | 1 |
| 4866 | hsa-mir-490 | 10154 | PLXNC1 | plexin C1 | 1 |
| 4877 | hsa-miR-490-5p | 5371 | PML | promyelocytic leukemia | 1 |
| 4891 | hsa-mir-490 | 5371 | PML | promyelocytic leukemia | 1 |
| 4895 | hsa-miR-1 | 7200 | TRH | thyrotropin-releasing hormone | 1 |
| 4896 | hsa-miR-133a | 7200 | TRH | thyrotropin-releasing hormone | 1 |
| 4897 | hsa-miR-186 | 7200 | TRH | thyrotropin-releasing hormone | 1 |
| 4898 | hsa-miR-195 | 7200 | TRH | thyrotropin-releasing hormone | 1 |
| 4899 | hsa-miR-326 | 7200 | TRH | thyrotropin-releasing hormone | 1 |
| 4900 | hsa-miR-33a | 7200 | TRH | thyrotropin-releasing hormone | 1 |
| 4901 | hsa-miR-449b | 7200 | TRH | thyrotropin-releasing hormone | 1 |
| 4902 | hsa-miR-497 | 7200 | TRH | thyrotropin-releasing hormone | 1 |
| 4903 | hsa-miR-550 | 7200 | TRH | thyrotropin-releasing hormone | 1 |
| 4904 | hsa-miR-551a | 7200 | TRH | thyrotropin-releasing hormone | 1 |
| 4905 | hsa-miR-562 | 7200 | TRH | thyrotropin-releasing hormone | 1 |
| 4906 | hsa-miR-575 | 7200 | TRH | thyrotropin-releasing hormone | 1 |
| 4907 | hsa-miR-608 | 7200 | TRH | thyrotropin-releasing hormone | 1 |
| 4908 | hsa-miR-620 | 7200 | TRH | thyrotropin-releasing hormone | 1 |
| 4909 | hsa-let-7a-3 | 7200 | TRH | thyrotropin-releasing hormone | 1 |
| 4910 | hsa-let-7b | 7200 | TRH | thyrotropin-releasing hormone | 1 |
| 4911 | hsa-mir-22 | 7200 | TRH | thyrotropin-releasing hormone | 1 |
| 4912 | hsa-mir-10a | 7200 | TRH | thyrotropin-releasing hormone | 1 |
| 4913 | hsa-mir-186 | 7200 | TRH | thyrotropin-releasing hormone | 1 |
| 4914 | hsa-mir-155 | 7200 | TRH | thyrotropin-releasing hormone | 1 |
| 4915 | hsa-mir-499 | 7200 | TRH | thyrotropin-releasing hormone | 1 |
| 4916 | hsa-mir-550-1 | 7200 | TRH | thyrotropin-releasing hormone | 1 |
| 4917 | hsa-mir-877 | 7200 | TRH | thyrotropin-releasing hormone | 1 |
| 4918 | hsa-mir-3186 | 7200 | TRH | thyrotropin-releasing hormone | 1 |
| 4919 | hsa-miR-1 | 51276 | ZNF571 | zinc finger protein 571 | 1 |
| 4920 | hsa-miR-133a | 51276 | ZNF571 | zinc finger protein 571 | 1 |
| 4921 | hsa-miR-186 | 51276 | ZNF571 | zinc finger protein 571 | 1 |
| 4922 | hsa-miR-195 | 51276 | ZNF571 | zinc finger protein 571 | 1 |
| 4923 | hsa-miR-326 | 51276 | ZNF571 | zinc finger protein 571 | 1 |
| 4924 | hsa-miR-449b | 51276 | ZNF571 | zinc finger protein 571 | 1 |
| 4925 | hsa-miR-497 | 51276 | ZNF571 | zinc finger protein 571 | 1 |
| 4926 | hsa-miR-562 | 51276 | ZNF571 | zinc finger protein 571 | 1 |
| 4927 | hsa-miR-575 | 51276 | ZNF571 | zinc finger protein 571 | 1 |
| 4928 | hsa-miR-579 | 51276 | ZNF571 | zinc finger protein 571 | 1 |
| 4929 | hsa-miR-608 | 51276 | ZNF571 | zinc finger protein 571 | 1 |
| 4930 | hsa-miR-620 | 51276 | ZNF571 | zinc finger protein 571 | 1 |
| 4931 | hsa-miR-885-5p | 51276 | ZNF571 | zinc finger protein 571 | 1 |
| 4932 | hsa-mir-22 | 51276 | ZNF571 | zinc finger protein 571 | 1 |
| 4933 | hsa-mir-10a | 51276 | ZNF571 | zinc finger protein 571 | 1 |
| 4934 | hsa-mir-186 | 51276 | ZNF571 | zinc finger protein 571 | 1 |
| 4935 | hsa-mir-155 | 51276 | ZNF571 | zinc finger protein 571 | 1 |
| 4936 | hsa-mir-423 | 51276 | ZNF571 | zinc finger protein 571 | 1 |
| 4937 | hsa-mir-499 | 51276 | ZNF571 | zinc finger protein 571 | 1 |
| 4938 | hsa-mir-885 | 51276 | ZNF571 | zinc finger protein 571 | 1 |
| 4939 | hsa-mir-877 | 51276 | ZNF571 | zinc finger protein 571 | 1 |
| 4940 | hsa-mir-2116 | 51276 | ZNF571 | zinc finger protein 571 | 1 |
| 4941 | hsa-mir-3190 | 51276 | ZNF571 | zinc finger protein 571 | 1 |
| 4942 | hsa-miR-1 | 10633 | RASL10A | RAS-like, family 10, member A | 1 |
| 4943 | hsa-miR-133a | 10633 | RASL10A | RAS-like, family 10, member A | 1 |
| 4944 | hsa-miR-186 | 10633 | RASL10A | RAS-like, family 10, member A | 1 |
| 4945 | hsa-miR-326 | 10633 | RASL10A | RAS-like, family 10, member A | 1 |
| 4946 | hsa-miR-449b | 10633 | RASL10A | RAS-like, family 10, member A | 1 |
| 4947 | hsa-miR-489 | 10633 | RASL10A | RAS-like, family 10, member A | 1 |
| 4948 | hsa-miR-551a | 10633 | RASL10A | RAS-like, family 10, member A | 1 |
| 4949 | hsa-miR-562 | 10633 | RASL10A | RAS-like, family 10, member A | 1 |
| 4950 | hsa-miR-575 | 10633 | RASL10A | RAS-like, family 10, member A | 1 |
| 4951 | hsa-miR-579 | 10633 | RASL10A | RAS-like, family 10, member A | 1 |
| 4952 | hsa-miR-608 | 10633 | RASL10A | RAS-like, family 10, member A | 1 |
| 4953 | hsa-miR-620 | 10633 | RASL10A | RAS-like, family 10, member A | 1 |
| 4954 | hsa-miR-637 | 10633 | RASL10A | RAS-like, family 10, member A | 1 |
| 4955 | hsa-miR-885-5p | 10633 | RASL10A | RAS-like, family 10, member A | 1 |
| 4956 | hsa-mir-22 | 10633 | RASL10A | RAS-like, family 10, member A | 1 |
| 4957 | hsa-mir-10a | 10633 | RASL10A | RAS-like, family 10, member A | 1 |
| 4958 | hsa-mir-186 | 10633 | RASL10A | RAS-like, family 10, member A | 1 |
| 4959 | hsa-mir-155 | 10633 | RASL10A | RAS-like, family 10, member A | 1 |
| 4960 | hsa-mir-423 | 10633 | RASL10A | RAS-like, family 10, member A | 1 |
| 4961 | hsa-mir-499 | 10633 | RASL10A | RAS-like, family 10, member A | 1 |
| 4962 | hsa-mir-885 | 10633 | RASL10A | RAS-like, family 10, member A | 1 |
| 4963 | hsa-mir-877 | 10633 | RASL10A | RAS-like, family 10, member A | 1 |
| 4964 | hsa-mir-3190 | 10633 | RASL10A | RAS-like, family 10, member A | 1 |
| 4965 | hsa-miR-1 | 1101 | CHAD | chondroadherin | 1 |
| 4966 | hsa-miR-133a | 1101 | CHAD | chondroadherin | 1 |
| 4967 | hsa-miR-186 | 1101 | CHAD | chondroadherin | 1 |
| 4968 | hsa-miR-326 | 1101 | CHAD | chondroadherin | 1 |
| 4969 | hsa-miR-449b | 1101 | CHAD | chondroadherin | 1 |
| 4970 | hsa-miR-550 | 1101 | CHAD | chondroadherin | 1 |
| 4971 | hsa-miR-551a | 1101 | CHAD | chondroadherin | 1 |
| 4972 | hsa-miR-575 | 1101 | CHAD | chondroadherin | 1 |
| 4973 | hsa-miR-579 | 1101 | CHAD | chondroadherin | 1 |
| 4974 | hsa-miR-608 | 1101 | CHAD | chondroadherin | 1 |
| 4975 | hsa-miR-620 | 1101 | CHAD | chondroadherin | 1 |
| 4976 | hsa-miR-885-5p | 1101 | CHAD | chondroadherin | 1 |
| 4977 | hsa-mir-22 | 1101 | CHAD | chondroadherin | 1 |
| 4978 | hsa-mir-10a | 1101 | CHAD | chondroadherin | 1 |
| 4979 | hsa-mir-186 | 1101 | CHAD | chondroadherin | 1 |
| 4980 | hsa-mir-155 | 1101 | CHAD | chondroadherin | 1 |
| 4981 | hsa-mir-423 | 1101 | CHAD | chondroadherin | 1 |
| 4982 | hsa-mir-499 | 1101 | CHAD | chondroadherin | 1 |
| 4983 | hsa-mir-550-1 | 1101 | CHAD | chondroadherin | 1 |
| 4984 | hsa-mir-885 | 1101 | CHAD | chondroadherin | 1 |
| 4985 | hsa-mir-877 | 1101 | CHAD | chondroadherin | 1 |
| 4986 | hsa-mir-2116 | 1101 | CHAD | chondroadherin | 1 |
| 4987 | hsa-mir-3186 | 1101 | CHAD | chondroadherin | 1 |
| 4988 | hsa-mir-3190 | 1101 | CHAD | chondroadherin | 1 |
| 4989 | hsa-miR-10a | 2323 | FLT3LG | fms-related tyrosine kinase 3 ligand | 1 |
| 4990 | hsa-miR-152 | 2323 | FLT3LG | fms-related tyrosine kinase 3 ligand | 1 |
| 4991 | hsa-miR-26a | 2323 | FLT3LG | fms-related tyrosine kinase 3 ligand | 1 |
| 4992 | hsa-miR-26b | 2323 | FLT3LG | fms-related tyrosine kinase 3 ligand | 1 |
| 4993 | hsa-miR-31 | 2323 | FLT3LG | fms-related tyrosine kinase 3 ligand | 1 |
| 4994 | hsa-miR-338-5p | 2323 | FLT3LG | fms-related tyrosine kinase 3 ligand | 1 |
| 4995 | hsa-miR-340 | 2323 | FLT3LG | fms-related tyrosine kinase 3 ligand | 1 |
| 4996 | hsa-miR-423-5p | 2323 | FLT3LG | fms-related tyrosine kinase 3 ligand | 1 |
| 4997 | hsa-miR-449a | 2323 | FLT3LG | fms-related tyrosine kinase 3 ligand | 1 |
| 4998 | hsa-miR-490-5p | 2323 | FLT3LG | fms-related tyrosine kinase 3 ligand | 1 |
| 4999 | hsa-miR-499-5p | 2323 | FLT3LG | fms-related tyrosine kinase 3 ligand | 1 |
| 5000 | hsa-miR-564 | 2323 | FLT3LG | fms-related tyrosine kinase 3 ligand | 1 |
| 5001 | hsa-miR-621 | 2323 | FLT3LG | fms-related tyrosine kinase 3 ligand | 1 |
| 5002 | hsa-miR-644 | 2323 | FLT3LG | fms-related tyrosine kinase 3 ligand | 1 |
| 5003 | hsa-miR-657 | 2323 | FLT3LG | fms-related tyrosine kinase 3 ligand | 1 |
| 5004 | hsa-miR-765 | 2323 | FLT3LG | fms-related tyrosine kinase 3 ligand | 1 |
| 5005 | hsa-miR-770-5p | 2323 | FLT3LG | fms-related tyrosine kinase 3 ligand | 1 |
| 5006 | hsa-miR-877 | 2323 | FLT3LG | fms-related tyrosine kinase 3 ligand | 1 |
| 5007 | hsa-miR-95 | 2323 | FLT3LG | fms-related tyrosine kinase 3 ligand | 1 |
| 5008 | hsa-mir-26a-1 | 2323 | FLT3LG | fms-related tyrosine kinase 3 ligand | 1 |
| 5009 | hsa-mir-26b | 2323 | FLT3LG | fms-related tyrosine kinase 3 ligand | 1 |
| 5010 | hsa-mir-31 | 2323 | FLT3LG | fms-related tyrosine kinase 3 ligand | 1 |
| 5011 | hsa-mir-194-2 | 2323 | FLT3LG | fms-related tyrosine kinase 3 ligand | 1 |
| 5012 | hsa-mir-340 | 2323 | FLT3LG | fms-related tyrosine kinase 3 ligand | 1 |
| 5013 | hsa-mir-338 | 2323 | FLT3LG | fms-related tyrosine kinase 3 ligand | 1 |
| 5014 | hsa-mir-490 | 2323 | FLT3LG | fms-related tyrosine kinase 3 ligand | 1 |
| 5015 | hsa-mir-449b | 2323 | FLT3LG | fms-related tyrosine kinase 3 ligand | 1 |
| 5016 | hsa-mir-449c | 2323 | FLT3LG | fms-related tyrosine kinase 3 ligand | 1 |
| 5017 | hsa-mir-3065 | 2323 | FLT3LG | fms-related tyrosine kinase 3 ligand | 1 |
| 5028 | hsa-miR-490-5p | 5533 | PPP3CC | protein phosphatase 3 (formerly 2B), catalytic subunit, gamma isoform | 1 |
| 5043 | hsa-mir-490 | 5533 | PPP3CC | protein phosphatase 3 (formerly 2B), catalytic subunit, gamma isoform | 1 |
| 5048 | hsa-miR-1 | 3577 | CXCR1 | chemokine (C-X-C motif) receptor 1 | 1 |
| 5049 | hsa-miR-133a | 3577 | CXCR1 | chemokine (C-X-C motif) receptor 1 | 1 |
| 5050 | hsa-miR-186 | 3577 | CXCR1 | chemokine (C-X-C motif) receptor 1 | 1 |
| 5051 | hsa-miR-208a | 3577 | CXCR1 | chemokine (C-X-C motif) receptor 1 | 1 |
| 5052 | hsa-miR-326 | 3577 | CXCR1 | chemokine (C-X-C motif) receptor 1 | 1 |
| 5053 | hsa-miR-449b | 3577 | CXCR1 | chemokine (C-X-C motif) receptor 1 | 1 |
| 5054 | hsa-miR-550 | 3577 | CXCR1 | chemokine (C-X-C motif) receptor 1 | 1 |
| 5055 | hsa-miR-575 | 3577 | CXCR1 | chemokine (C-X-C motif) receptor 1 | 1 |
| 5056 | hsa-miR-579 | 3577 | CXCR1 | chemokine (C-X-C motif) receptor 1 | 1 |
| 5057 | hsa-miR-608 | 3577 | CXCR1 | chemokine (C-X-C motif) receptor 1 | 1 |
| 5058 | hsa-miR-620 | 3577 | CXCR1 | chemokine (C-X-C motif) receptor 1 | 1 |
| 5059 | hsa-miR-885-5p | 3577 | CXCR1 | chemokine (C-X-C motif) receptor 1 | 1 |
| 5060 | hsa-mir-22 | 3577 | CXCR1 | chemokine (C-X-C motif) receptor 1 | 1 |
| 5061 | hsa-mir-10a | 3577 | CXCR1 | chemokine (C-X-C motif) receptor 1 | 1 |
| 5062 | hsa-mir-186 | 3577 | CXCR1 | chemokine (C-X-C motif) receptor 1 | 1 |
| 5063 | hsa-mir-155 | 3577 | CXCR1 | chemokine (C-X-C motif) receptor 1 | 1 |
| 5064 | hsa-mir-423 | 3577 | CXCR1 | chemokine (C-X-C motif) receptor 1 | 1 |
| 5065 | hsa-mir-499 | 3577 | CXCR1 | chemokine (C-X-C motif) receptor 1 | 1 |
| 5066 | hsa-mir-550-1 | 3577 | CXCR1 | chemokine (C-X-C motif) receptor 1 | 1 |
| 5067 | hsa-mir-885 | 3577 | CXCR1 | chemokine (C-X-C motif) receptor 1 | 1 |
| 5068 | hsa-mir-877 | 3577 | CXCR1 | chemokine (C-X-C motif) receptor 1 | 1 |
| 5069 | hsa-mir-2116 | 3577 | CXCR1 | chemokine (C-X-C motif) receptor 1 | 1 |
| 5070 | hsa-mir-3190 | 3577 | CXCR1 | chemokine (C-X-C motif) receptor 1 | 1 |
| 5071 | hsa-miR-383 | 941 | CD80 | CD80 molecule | 1 |
| 5072 | hsa-miR-10a | 840 | CASP7 | caspase 7, apoptosis-related cysteine peptidase | 1 |
| 5073 | hsa-miR-1228\* | 840 | CASP7 | caspase 7, apoptosis-related cysteine peptidase | 1 |
| 5074 | hsa-miR-139-5p | 840 | CASP7 | caspase 7, apoptosis-related cysteine peptidase | 1 |
| 5075 | hsa-miR-152 | 840 | CASP7 | caspase 7, apoptosis-related cysteine peptidase | 1 |
| 5076 | hsa-miR-26a | 840 | CASP7 | caspase 7, apoptosis-related cysteine peptidase | 1 |
| 5077 | hsa-miR-26b | 840 | CASP7 | caspase 7, apoptosis-related cysteine peptidase | 1 |
| 5078 | hsa-miR-338-5p | 840 | CASP7 | caspase 7, apoptosis-related cysteine peptidase | 1 |
| 5079 | hsa-miR-340 | 840 | CASP7 | caspase 7, apoptosis-related cysteine peptidase | 1 |
| 5080 | hsa-miR-423-5p | 840 | CASP7 | caspase 7, apoptosis-related cysteine peptidase | 1 |
| 5081 | hsa-miR-449a | 840 | CASP7 | caspase 7, apoptosis-related cysteine peptidase | 1 |
| 5082 | hsa-miR-490-5p | 840 | CASP7 | caspase 7, apoptosis-related cysteine peptidase | 1 |
| 5083 | hsa-miR-499-5p | 840 | CASP7 | caspase 7, apoptosis-related cysteine peptidase | 1 |
| 5084 | hsa-miR-548b-5p | 840 | CASP7 | caspase 7, apoptosis-related cysteine peptidase | 1 |
| 5085 | hsa-miR-564 | 840 | CASP7 | caspase 7, apoptosis-related cysteine peptidase | 1 |
| 5086 | hsa-miR-584 | 840 | CASP7 | caspase 7, apoptosis-related cysteine peptidase | 1 |
| 5087 | hsa-miR-621 | 840 | CASP7 | caspase 7, apoptosis-related cysteine peptidase | 1 |
| 5088 | hsa-miR-644 | 840 | CASP7 | caspase 7, apoptosis-related cysteine peptidase | 1 |
| 5089 | hsa-miR-657 | 840 | CASP7 | caspase 7, apoptosis-related cysteine peptidase | 1 |
| 5090 | hsa-miR-765 | 840 | CASP7 | caspase 7, apoptosis-related cysteine peptidase | 1 |
| 5091 | hsa-miR-770-5p | 840 | CASP7 | caspase 7, apoptosis-related cysteine peptidase | 1 |
| 5092 | hsa-miR-877 | 840 | CASP7 | caspase 7, apoptosis-related cysteine peptidase | 1 |
| 5093 | hsa-miR-95 | 840 | CASP7 | caspase 7, apoptosis-related cysteine peptidase | 1 |
| 5094 | hsa-mir-26a-1 | 840 | CASP7 | caspase 7, apoptosis-related cysteine peptidase | 1 |
| 5095 | hsa-mir-26b | 840 | CASP7 | caspase 7, apoptosis-related cysteine peptidase | 1 |
| 5096 | hsa-mir-194-2 | 840 | CASP7 | caspase 7, apoptosis-related cysteine peptidase | 1 |
| 5097 | hsa-mir-340 | 840 | CASP7 | caspase 7, apoptosis-related cysteine peptidase | 1 |
| 5098 | hsa-mir-338 | 840 | CASP7 | caspase 7, apoptosis-related cysteine peptidase | 1 |
| 5099 | hsa-mir-490 | 840 | CASP7 | caspase 7, apoptosis-related cysteine peptidase | 1 |
| 5100 | hsa-mir-548b | 840 | CASP7 | caspase 7, apoptosis-related cysteine peptidase | 1 |
| 5101 | hsa-mir-548d-2 | 840 | CASP7 | caspase 7, apoptosis-related cysteine peptidase | 1 |
| 5102 | hsa-mir-449b | 840 | CASP7 | caspase 7, apoptosis-related cysteine peptidase | 1 |
| 5103 | hsa-mir-449c | 840 | CASP7 | caspase 7, apoptosis-related cysteine peptidase | 1 |
| 5104 | hsa-mir-3065 | 840 | CASP7 | caspase 7, apoptosis-related cysteine peptidase | 1 |
| 5105 | hsa-miR-152 | 10318 | TNIP1 | TNFAIP3 interacting protein 1 | 1 |
| 5106 | hsa-miR-26a | 10318 | TNIP1 | TNFAIP3 interacting protein 1 | 1 |
| 5107 | hsa-miR-26b | 10318 | TNIP1 | TNFAIP3 interacting protein 1 | 1 |
| 5108 | hsa-miR-31 | 10318 | TNIP1 | TNFAIP3 interacting protein 1 | 1 |
| 5109 | hsa-miR-338-5p | 10318 | TNIP1 | TNFAIP3 interacting protein 1 | 1 |
| 5110 | hsa-miR-340 | 10318 | TNIP1 | TNFAIP3 interacting protein 1 | 1 |
| 5111 | hsa-miR-383 | 10318 | TNIP1 | TNFAIP3 interacting protein 1 | 1 |
| 5112 | hsa-miR-449a | 10318 | TNIP1 | TNFAIP3 interacting protein 1 | 1 |
| 5113 | hsa-miR-490-5p | 10318 | TNIP1 | TNFAIP3 interacting protein 1 | 1 |
| 5114 | hsa-miR-499-5p | 10318 | TNIP1 | TNFAIP3 interacting protein 1 | 1 |
| 5115 | hsa-miR-621 | 10318 | TNIP1 | TNFAIP3 interacting protein 1 | 1 |
| 5116 | hsa-miR-634 | 10318 | TNIP1 | TNFAIP3 interacting protein 1 | 1 |
| 5117 | hsa-miR-653 | 10318 | TNIP1 | TNFAIP3 interacting protein 1 | 1 |
| 5118 | hsa-miR-657 | 10318 | TNIP1 | TNFAIP3 interacting protein 1 | 1 |
| 5119 | hsa-miR-765 | 10318 | TNIP1 | TNFAIP3 interacting protein 1 | 1 |
| 5120 | hsa-miR-770-5p | 10318 | TNIP1 | TNFAIP3 interacting protein 1 | 1 |
| 5121 | hsa-mir-26a-1 | 10318 | TNIP1 | TNFAIP3 interacting protein 1 | 1 |
| 5122 | hsa-mir-26b | 10318 | TNIP1 | TNFAIP3 interacting protein 1 | 1 |
| 5123 | hsa-mir-31 | 10318 | TNIP1 | TNFAIP3 interacting protein 1 | 1 |
| 5124 | hsa-mir-194-2 | 10318 | TNIP1 | TNFAIP3 interacting protein 1 | 1 |
| 5125 | hsa-mir-340 | 10318 | TNIP1 | TNFAIP3 interacting protein 1 | 1 |
| 5126 | hsa-mir-338 | 10318 | TNIP1 | TNFAIP3 interacting protein 1 | 1 |
| 5127 | hsa-mir-490 | 10318 | TNIP1 | TNFAIP3 interacting protein 1 | 1 |
| 5128 | hsa-mir-449b | 10318 | TNIP1 | TNFAIP3 interacting protein 1 | 1 |
| 5129 | hsa-mir-449c | 10318 | TNIP1 | TNFAIP3 interacting protein 1 | 1 |
| 5130 | hsa-mir-3065 | 10318 | TNIP1 | TNFAIP3 interacting protein 1 | 1 |
| 5131 | hsa-miR-1 | 1089 | CEACAM4 | carcinoembryonic antigen-related cell adhesion molecule 4 | 1 |
| 5132 | hsa-miR-133a | 1089 | CEACAM4 | carcinoembryonic antigen-related cell adhesion molecule 4 | 1 |
| 5133 | hsa-miR-186 | 1089 | CEACAM4 | carcinoembryonic antigen-related cell adhesion molecule 4 | 1 |
| 5134 | hsa-miR-195 | 1089 | CEACAM4 | carcinoembryonic antigen-related cell adhesion molecule 4 | 1 |
| 5135 | hsa-miR-326 | 1089 | CEACAM4 | carcinoembryonic antigen-related cell adhesion molecule 4 | 1 |
| 5136 | hsa-miR-449b | 1089 | CEACAM4 | carcinoembryonic antigen-related cell adhesion molecule 4 | 1 |
| 5137 | hsa-miR-489 | 1089 | CEACAM4 | carcinoembryonic antigen-related cell adhesion molecule 4 | 1 |
| 5138 | hsa-miR-497 | 1089 | CEACAM4 | carcinoembryonic antigen-related cell adhesion molecule 4 | 1 |
| 5139 | hsa-miR-562 | 1089 | CEACAM4 | carcinoembryonic antigen-related cell adhesion molecule 4 | 1 |
| 5140 | hsa-miR-575 | 1089 | CEACAM4 | carcinoembryonic antigen-related cell adhesion molecule 4 | 1 |
| 5141 | hsa-miR-579 | 1089 | CEACAM4 | carcinoembryonic antigen-related cell adhesion molecule 4 | 1 |
| 5142 | hsa-miR-608 | 1089 | CEACAM4 | carcinoembryonic antigen-related cell adhesion molecule 4 | 1 |
| 5143 | hsa-miR-620 | 1089 | CEACAM4 | carcinoembryonic antigen-related cell adhesion molecule 4 | 1 |
| 5144 | hsa-miR-637 | 1089 | CEACAM4 | carcinoembryonic antigen-related cell adhesion molecule 4 | 1 |
| 5145 | hsa-miR-885-5p | 1089 | CEACAM4 | carcinoembryonic antigen-related cell adhesion molecule 4 | 1 |
| 5146 | hsa-mir-22 | 1089 | CEACAM4 | carcinoembryonic antigen-related cell adhesion molecule 4 | 1 |
| 5147 | hsa-mir-10a | 1089 | CEACAM4 | carcinoembryonic antigen-related cell adhesion molecule 4 | 1 |
| 5148 | hsa-mir-186 | 1089 | CEACAM4 | carcinoembryonic antigen-related cell adhesion molecule 4 | 1 |
| 5149 | hsa-mir-155 | 1089 | CEACAM4 | carcinoembryonic antigen-related cell adhesion molecule 4 | 1 |
| 5150 | hsa-mir-423 | 1089 | CEACAM4 | carcinoembryonic antigen-related cell adhesion molecule 4 | 1 |
| 5151 | hsa-mir-499 | 1089 | CEACAM4 | carcinoembryonic antigen-related cell adhesion molecule 4 | 1 |
| 5152 | hsa-mir-885 | 1089 | CEACAM4 | carcinoembryonic antigen-related cell adhesion molecule 4 | 1 |
| 5153 | hsa-mir-877 | 1089 | CEACAM4 | carcinoembryonic antigen-related cell adhesion molecule 4 | 1 |
| 5154 | hsa-mir-2116 | 1089 | CEACAM4 | carcinoembryonic antigen-related cell adhesion molecule 4 | 1 |
| 5155 | hsa-mir-3190 | 1089 | CEACAM4 | carcinoembryonic antigen-related cell adhesion molecule 4 | 1 |
| 5156 | hsa-miR-1 | 8399 | PLA2G10 | phospholipase A2, group X | 1 |
| 5157 | hsa-miR-133a | 8399 | PLA2G10 | phospholipase A2, group X | 1 |
| 5158 | hsa-miR-186 | 8399 | PLA2G10 | phospholipase A2, group X | 1 |
| 5159 | hsa-miR-326 | 8399 | PLA2G10 | phospholipase A2, group X | 1 |
| 5160 | hsa-miR-33a | 8399 | PLA2G10 | phospholipase A2, group X | 1 |
| 5161 | hsa-miR-449b | 8399 | PLA2G10 | phospholipase A2, group X | 1 |
| 5162 | hsa-miR-550 | 8399 | PLA2G10 | phospholipase A2, group X | 1 |
| 5163 | hsa-miR-551a | 8399 | PLA2G10 | phospholipase A2, group X | 1 |
| 5164 | hsa-miR-575 | 8399 | PLA2G10 | phospholipase A2, group X | 1 |
| 5165 | hsa-miR-608 | 8399 | PLA2G10 | phospholipase A2, group X | 1 |
| 5166 | hsa-miR-620 | 8399 | PLA2G10 | phospholipase A2, group X | 1 |
| 5167 | hsa-let-7a-3 | 8399 | PLA2G10 | phospholipase A2, group X | 1 |
| 5168 | hsa-let-7b | 8399 | PLA2G10 | phospholipase A2, group X | 1 |
| 5169 | hsa-mir-22 | 8399 | PLA2G10 | phospholipase A2, group X | 1 |
| 5170 | hsa-mir-10a | 8399 | PLA2G10 | phospholipase A2, group X | 1 |
| 5171 | hsa-mir-186 | 8399 | PLA2G10 | phospholipase A2, group X | 1 |
| 5172 | hsa-mir-155 | 8399 | PLA2G10 | phospholipase A2, group X | 1 |
| 5173 | hsa-mir-499 | 8399 | PLA2G10 | phospholipase A2, group X | 1 |
| 5174 | hsa-mir-550-1 | 8399 | PLA2G10 | phospholipase A2, group X | 1 |
| 5175 | hsa-mir-877 | 8399 | PLA2G10 | phospholipase A2, group X | 1 |
| 5176 | hsa-mir-2116 | 8399 | PLA2G10 | phospholipase A2, group X | 1 |
| 5177 | hsa-mir-3186 | 8399 | PLA2G10 | phospholipase A2, group X | 1 |
| 5178 | hsa-miR-1 | 6340 | SCNN1G | sodium channel, nonvoltage-gated 1, gamma | 1 |
| 5179 | hsa-miR-133a | 6340 | SCNN1G | sodium channel, nonvoltage-gated 1, gamma | 1 |
| 5180 | hsa-miR-186 | 6340 | SCNN1G | sodium channel, nonvoltage-gated 1, gamma | 1 |
| 5181 | hsa-miR-195 | 6340 | SCNN1G | sodium channel, nonvoltage-gated 1, gamma | 1 |
| 5182 | hsa-miR-326 | 6340 | SCNN1G | sodium channel, nonvoltage-gated 1, gamma | 1 |
| 5183 | hsa-miR-449b | 6340 | SCNN1G | sodium channel, nonvoltage-gated 1, gamma | 1 |
| 5184 | hsa-miR-489 | 6340 | SCNN1G | sodium channel, nonvoltage-gated 1, gamma | 1 |
| 5185 | hsa-miR-497 | 6340 | SCNN1G | sodium channel, nonvoltage-gated 1, gamma | 1 |
| 5186 | hsa-miR-550 | 6340 | SCNN1G | sodium channel, nonvoltage-gated 1, gamma | 1 |
| 5187 | hsa-miR-575 | 6340 | SCNN1G | sodium channel, nonvoltage-gated 1, gamma | 1 |
| 5188 | hsa-miR-579 | 6340 | SCNN1G | sodium channel, nonvoltage-gated 1, gamma | 1 |
| 5189 | hsa-miR-608 | 6340 | SCNN1G | sodium channel, nonvoltage-gated 1, gamma | 1 |
| 5190 | hsa-miR-620 | 6340 | SCNN1G | sodium channel, nonvoltage-gated 1, gamma | 1 |
| 5191 | hsa-let-7a-3 | 6340 | SCNN1G | sodium channel, nonvoltage-gated 1, gamma | 1 |
| 5192 | hsa-let-7b | 6340 | SCNN1G | sodium channel, nonvoltage-gated 1, gamma | 1 |
| 5193 | hsa-mir-22 | 6340 | SCNN1G | sodium channel, nonvoltage-gated 1, gamma | 1 |
| 5194 | hsa-mir-10a | 6340 | SCNN1G | sodium channel, nonvoltage-gated 1, gamma | 1 |
| 5195 | hsa-mir-186 | 6340 | SCNN1G | sodium channel, nonvoltage-gated 1, gamma | 1 |
| 5196 | hsa-mir-155 | 6340 | SCNN1G | sodium channel, nonvoltage-gated 1, gamma | 1 |
| 5197 | hsa-mir-499 | 6340 | SCNN1G | sodium channel, nonvoltage-gated 1, gamma | 1 |
| 5198 | hsa-mir-550-1 | 6340 | SCNN1G | sodium channel, nonvoltage-gated 1, gamma | 1 |
| 5199 | hsa-mir-877 | 6340 | SCNN1G | sodium channel, nonvoltage-gated 1, gamma | 1 |
| 5200 | hsa-mir-2116 | 6340 | SCNN1G | sodium channel, nonvoltage-gated 1, gamma | 1 |
| 5201 | hsa-miR-1 | 1673 | DEFB4 | defensin, beta 4 | 1 |
| 5202 | hsa-miR-133a | 1673 | DEFB4 | defensin, beta 4 | 1 |
| 5203 | hsa-miR-186 | 1673 | DEFB4 | defensin, beta 4 | 1 |
| 5204 | hsa-miR-449b | 1673 | DEFB4 | defensin, beta 4 | 1 |
| 5205 | hsa-miR-551a | 1673 | DEFB4 | defensin, beta 4 | 1 |
| 5206 | hsa-miR-562 | 1673 | DEFB4 | defensin, beta 4 | 1 |
| 5207 | hsa-miR-575 | 1673 | DEFB4 | defensin, beta 4 | 1 |
| 5208 | hsa-miR-579 | 1673 | DEFB4 | defensin, beta 4 | 1 |
| 5209 | hsa-miR-608 | 1673 | DEFB4 | defensin, beta 4 | 1 |
| 5210 | hsa-miR-637 | 1673 | DEFB4 | defensin, beta 4 | 1 |
| 5211 | hsa-mir-22 | 1673 | DEFB4 | defensin, beta 4 | 1 |
| 5212 | hsa-mir-10a | 1673 | DEFB4 | defensin, beta 4 | 1 |
| 5213 | hsa-mir-186 | 1673 | DEFB4 | defensin, beta 4 | 1 |
| 5214 | hsa-mir-155 | 1673 | DEFB4 | defensin, beta 4 | 1 |
| 5215 | hsa-mir-423 | 1673 | DEFB4 | defensin, beta 4 | 1 |
| 5216 | hsa-mir-877 | 1673 | DEFB4 | defensin, beta 4 | 1 |
| 5217 | hsa-mir-3190 | 1673 | DEFB4 | defensin, beta 4 | 1 |
| 5218 | hsa-miR-10a | 3601 | IL15RA | interleukin 15 receptor, alpha | 1 |
| 5219 | hsa-miR-152 | 3601 | IL15RA | interleukin 15 receptor, alpha | 1 |
| 5220 | hsa-miR-26a | 3601 | IL15RA | interleukin 15 receptor, alpha | 1 |
| 5221 | hsa-miR-26b | 3601 | IL15RA | interleukin 15 receptor, alpha | 1 |
| 5222 | hsa-miR-31 | 3601 | IL15RA | interleukin 15 receptor, alpha | 1 |
| 5223 | hsa-miR-338-5p | 3601 | IL15RA | interleukin 15 receptor, alpha | 1 |
| 5224 | hsa-miR-340 | 3601 | IL15RA | interleukin 15 receptor, alpha | 1 |
| 5225 | hsa-miR-383 | 3601 | IL15RA | interleukin 15 receptor, alpha | 1 |
| 5226 | hsa-miR-449a | 3601 | IL15RA | interleukin 15 receptor, alpha | 1 |
| 5227 | hsa-miR-490-5p | 3601 | IL15RA | interleukin 15 receptor, alpha | 1 |
| 5228 | hsa-miR-499-5p | 3601 | IL15RA | interleukin 15 receptor, alpha | 1 |
| 5229 | hsa-miR-621 | 3601 | IL15RA | interleukin 15 receptor, alpha | 1 |
| 5230 | hsa-miR-653 | 3601 | IL15RA | interleukin 15 receptor, alpha | 1 |
| 5231 | hsa-miR-657 | 3601 | IL15RA | interleukin 15 receptor, alpha | 1 |
| 5232 | hsa-miR-765 | 3601 | IL15RA | interleukin 15 receptor, alpha | 1 |
| 5233 | hsa-miR-770-5p | 3601 | IL15RA | interleukin 15 receptor, alpha | 1 |
| 5234 | hsa-miR-877 | 3601 | IL15RA | interleukin 15 receptor, alpha | 1 |
| 5235 | hsa-mir-26a-1 | 3601 | IL15RA | interleukin 15 receptor, alpha | 1 |
| 5236 | hsa-mir-26b | 3601 | IL15RA | interleukin 15 receptor, alpha | 1 |
| 5237 | hsa-mir-31 | 3601 | IL15RA | interleukin 15 receptor, alpha | 1 |
| 5238 | hsa-mir-194-2 | 3601 | IL15RA | interleukin 15 receptor, alpha | 1 |
| 5239 | hsa-mir-340 | 3601 | IL15RA | interleukin 15 receptor, alpha | 1 |
| 5240 | hsa-mir-338 | 3601 | IL15RA | interleukin 15 receptor, alpha | 1 |
| 5241 | hsa-mir-490 | 3601 | IL15RA | interleukin 15 receptor, alpha | 1 |
| 5242 | hsa-mir-449b | 3601 | IL15RA | interleukin 15 receptor, alpha | 1 |
| 5243 | hsa-mir-449c | 3601 | IL15RA | interleukin 15 receptor, alpha | 1 |
| 5244 | hsa-mir-3065 | 3601 | IL15RA | interleukin 15 receptor, alpha | 1 |
| 5245 | hsa-miR-1 | 27035 | NOX1 | NADPH oxidase 1 | 1 |
| 5246 | hsa-miR-133a | 27035 | NOX1 | NADPH oxidase 1 | 1 |
| 5247 | hsa-miR-186 | 27035 | NOX1 | NADPH oxidase 1 | 1 |
| 5248 | hsa-miR-208a | 27035 | NOX1 | NADPH oxidase 1 | 1 |
| 5249 | hsa-miR-326 | 27035 | NOX1 | NADPH oxidase 1 | 1 |
| 5250 | hsa-miR-449b | 27035 | NOX1 | NADPH oxidase 1 | 1 |
| 5251 | hsa-miR-489 | 27035 | NOX1 | NADPH oxidase 1 | 1 |
| 5252 | hsa-miR-562 | 27035 | NOX1 | NADPH oxidase 1 | 1 |
| 5253 | hsa-miR-575 | 27035 | NOX1 | NADPH oxidase 1 | 1 |
| 5254 | hsa-miR-579 | 27035 | NOX1 | NADPH oxidase 1 | 1 |
| 5255 | hsa-miR-608 | 27035 | NOX1 | NADPH oxidase 1 | 1 |
| 5256 | hsa-miR-620 | 27035 | NOX1 | NADPH oxidase 1 | 1 |
| 5257 | hsa-miR-637 | 27035 | NOX1 | NADPH oxidase 1 | 1 |
| 5258 | hsa-miR-885-5p | 27035 | NOX1 | NADPH oxidase 1 | 1 |
| 5259 | hsa-mir-22 | 27035 | NOX1 | NADPH oxidase 1 | 1 |
| 5260 | hsa-mir-10a | 27035 | NOX1 | NADPH oxidase 1 | 1 |
| 5261 | hsa-mir-186 | 27035 | NOX1 | NADPH oxidase 1 | 1 |
| 5262 | hsa-mir-155 | 27035 | NOX1 | NADPH oxidase 1 | 1 |
| 5263 | hsa-mir-499 | 27035 | NOX1 | NADPH oxidase 1 | 1 |
| 5264 | hsa-mir-885 | 27035 | NOX1 | NADPH oxidase 1 | 1 |
| 5265 | hsa-mir-877 | 27035 | NOX1 | NADPH oxidase 1 | 1 |
| 5266 | hsa-mir-3190 | 27035 | NOX1 | NADPH oxidase 1 | 1 |
| 5295 | hsa-miR-1 | 80108 | ZFP2 | zinc finger protein 2 homolog (mouse) | 1 |
| 5296 | hsa-miR-133a | 80108 | ZFP2 | zinc finger protein 2 homolog (mouse) | 1 |
| 5297 | hsa-miR-186 | 80108 | ZFP2 | zinc finger protein 2 homolog (mouse) | 1 |
| 5298 | hsa-miR-449b | 80108 | ZFP2 | zinc finger protein 2 homolog (mouse) | 1 |
| 5299 | hsa-miR-489 | 80108 | ZFP2 | zinc finger protein 2 homolog (mouse) | 1 |
| 5300 | hsa-miR-551a | 80108 | ZFP2 | zinc finger protein 2 homolog (mouse) | 1 |
| 5301 | hsa-miR-562 | 80108 | ZFP2 | zinc finger protein 2 homolog (mouse) | 1 |
| 5302 | hsa-miR-579 | 80108 | ZFP2 | zinc finger protein 2 homolog (mouse) | 1 |
| 5303 | hsa-miR-608 | 80108 | ZFP2 | zinc finger protein 2 homolog (mouse) | 1 |
| 5304 | hsa-miR-637 | 80108 | ZFP2 | zinc finger protein 2 homolog (mouse) | 1 |
| 5305 | hsa-mir-22 | 80108 | ZFP2 | zinc finger protein 2 homolog (mouse) | 1 |
| 5306 | hsa-mir-10a | 80108 | ZFP2 | zinc finger protein 2 homolog (mouse) | 1 |
| 5307 | hsa-mir-186 | 80108 | ZFP2 | zinc finger protein 2 homolog (mouse) | 1 |
| 5308 | hsa-mir-155 | 80108 | ZFP2 | zinc finger protein 2 homolog (mouse) | 1 |
| 5309 | hsa-mir-877 | 80108 | ZFP2 | zinc finger protein 2 homolog (mouse) | 1 |
| 5310 | hsa-miR-1 | 2692 | GHRHR | growth hormone releasing hormone receptor | 1 |
| 5311 | hsa-miR-133a | 2692 | GHRHR | growth hormone releasing hormone receptor | 1 |
| 5312 | hsa-miR-186 | 2692 | GHRHR | growth hormone releasing hormone receptor | 1 |
| 5313 | hsa-miR-449b | 2692 | GHRHR | growth hormone releasing hormone receptor | 1 |
| 5314 | hsa-miR-489 | 2692 | GHRHR | growth hormone releasing hormone receptor | 1 |
| 5315 | hsa-miR-562 | 2692 | GHRHR | growth hormone releasing hormone receptor | 1 |
| 5316 | hsa-miR-579 | 2692 | GHRHR | growth hormone releasing hormone receptor | 1 |
| 5317 | hsa-miR-608 | 2692 | GHRHR | growth hormone releasing hormone receptor | 1 |
| 5318 | hsa-miR-637 | 2692 | GHRHR | growth hormone releasing hormone receptor | 1 |
| 5319 | hsa-miR-885-5p | 2692 | GHRHR | growth hormone releasing hormone receptor | 1 |
| 5320 | hsa-mir-22 | 2692 | GHRHR | growth hormone releasing hormone receptor | 1 |
| 5321 | hsa-mir-10a | 2692 | GHRHR | growth hormone releasing hormone receptor | 1 |
| 5322 | hsa-mir-186 | 2692 | GHRHR | growth hormone releasing hormone receptor | 1 |
| 5323 | hsa-mir-155 | 2692 | GHRHR | growth hormone releasing hormone receptor | 1 |
| 5324 | hsa-mir-423 | 2692 | GHRHR | growth hormone releasing hormone receptor | 1 |
| 5325 | hsa-mir-499 | 2692 | GHRHR | growth hormone releasing hormone receptor | 1 |
| 5326 | hsa-mir-885 | 2692 | GHRHR | growth hormone releasing hormone receptor | 1 |
| 5327 | hsa-mir-877 | 2692 | GHRHR | growth hormone releasing hormone receptor | 1 |
| 5328 | hsa-mir-3190 | 2692 | GHRHR | growth hormone releasing hormone receptor | 1 |
| 5329 | hsa-miR-1 | 3399 | ID3 | inhibitor of DNA binding 3, dominant negative helix-loop-helix protein | 1 |
| 5330 | hsa-miR-133a | 3399 | ID3 | inhibitor of DNA binding 3, dominant negative helix-loop-helix protein | 1 |
| 5331 | hsa-miR-186 | 3399 | ID3 | inhibitor of DNA binding 3, dominant negative helix-loop-helix protein | 1 |
| 5332 | hsa-miR-195 | 3399 | ID3 | inhibitor of DNA binding 3, dominant negative helix-loop-helix protein | 1 |
| 5333 | hsa-miR-326 | 3399 | ID3 | inhibitor of DNA binding 3, dominant negative helix-loop-helix protein | 1 |
| 5334 | hsa-miR-33a | 3399 | ID3 | inhibitor of DNA binding 3, dominant negative helix-loop-helix protein | 1 |
| 5335 | hsa-miR-449b | 3399 | ID3 | inhibitor of DNA binding 3, dominant negative helix-loop-helix protein | 1 |
| 5336 | hsa-miR-497 | 3399 | ID3 | inhibitor of DNA binding 3, dominant negative helix-loop-helix protein | 1 |
| 5337 | hsa-miR-550 | 3399 | ID3 | inhibitor of DNA binding 3, dominant negative helix-loop-helix protein | 1 |
| 5338 | hsa-miR-562 | 3399 | ID3 | inhibitor of DNA binding 3, dominant negative helix-loop-helix protein | 1 |
| 5339 | hsa-miR-575 | 3399 | ID3 | inhibitor of DNA binding 3, dominant negative helix-loop-helix protein | 1 |
| 5340 | hsa-miR-608 | 3399 | ID3 | inhibitor of DNA binding 3, dominant negative helix-loop-helix protein | 1 |
| 5341 | hsa-miR-620 | 3399 | ID3 | inhibitor of DNA binding 3, dominant negative helix-loop-helix protein | 1 |
| 5342 | hsa-let-7a-3 | 3399 | ID3 | inhibitor of DNA binding 3, dominant negative helix-loop-helix protein | 1 |
| 5343 | hsa-let-7b | 3399 | ID3 | inhibitor of DNA binding 3, dominant negative helix-loop-helix protein | 1 |
| 5344 | hsa-mir-22 | 3399 | ID3 | inhibitor of DNA binding 3, dominant negative helix-loop-helix protein | 1 |
| 5345 | hsa-mir-10a | 3399 | ID3 | inhibitor of DNA binding 3, dominant negative helix-loop-helix protein | 1 |
| 5346 | hsa-mir-186 | 3399 | ID3 | inhibitor of DNA binding 3, dominant negative helix-loop-helix protein | 1 |
| 5347 | hsa-mir-155 | 3399 | ID3 | inhibitor of DNA binding 3, dominant negative helix-loop-helix protein | 1 |
| 5348 | hsa-mir-499 | 3399 | ID3 | inhibitor of DNA binding 3, dominant negative helix-loop-helix protein | 1 |
| 5349 | hsa-mir-550-1 | 3399 | ID3 | inhibitor of DNA binding 3, dominant negative helix-loop-helix protein | 1 |
| 5350 | hsa-mir-877 | 3399 | ID3 | inhibitor of DNA binding 3, dominant negative helix-loop-helix protein | 1 |
| 5351 | hsa-mir-2116 | 3399 | ID3 | inhibitor of DNA binding 3, dominant negative helix-loop-helix protein | 1 |
| 5352 | hsa-mir-3186 | 3399 | ID3 | inhibitor of DNA binding 3, dominant negative helix-loop-helix protein | 1 |
| 5378 | hsa-miR-1 | 3568 | IL5RA | interleukin 5 receptor, alpha | 1 |
| 5379 | hsa-miR-133a | 3568 | IL5RA | interleukin 5 receptor, alpha | 1 |
| 5380 | hsa-miR-186 | 3568 | IL5RA | interleukin 5 receptor, alpha | 1 |
| 5381 | hsa-miR-449b | 3568 | IL5RA | interleukin 5 receptor, alpha | 1 |
| 5382 | hsa-miR-550 | 3568 | IL5RA | interleukin 5 receptor, alpha | 1 |
| 5383 | hsa-miR-551a | 3568 | IL5RA | interleukin 5 receptor, alpha | 1 |
| 5384 | hsa-miR-562 | 3568 | IL5RA | interleukin 5 receptor, alpha | 1 |
| 5385 | hsa-miR-579 | 3568 | IL5RA | interleukin 5 receptor, alpha | 1 |
| 5386 | hsa-miR-608 | 3568 | IL5RA | interleukin 5 receptor, alpha | 1 |
| 5387 | hsa-mir-22 | 3568 | IL5RA | interleukin 5 receptor, alpha | 1 |
| 5388 | hsa-mir-10a | 3568 | IL5RA | interleukin 5 receptor, alpha | 1 |
| 5389 | hsa-mir-186 | 3568 | IL5RA | interleukin 5 receptor, alpha | 1 |
| 5390 | hsa-mir-155 | 3568 | IL5RA | interleukin 5 receptor, alpha | 1 |
| 5391 | hsa-mir-423 | 3568 | IL5RA | interleukin 5 receptor, alpha | 1 |
| 5392 | hsa-mir-550-1 | 3568 | IL5RA | interleukin 5 receptor, alpha | 1 |
| 5393 | hsa-mir-3186 | 3568 | IL5RA | interleukin 5 receptor, alpha | 1 |
| 5394 | hsa-miR-1 | 51050 | PI15 | peptidase inhibitor 15 | 1 |
| 5395 | hsa-miR-133a | 51050 | PI15 | peptidase inhibitor 15 | 1 |
| 5396 | hsa-miR-186 | 51050 | PI15 | peptidase inhibitor 15 | 1 |
| 5397 | hsa-miR-195 | 51050 | PI15 | peptidase inhibitor 15 | 1 |
| 5398 | hsa-miR-326 | 51050 | PI15 | peptidase inhibitor 15 | 1 |
| 5399 | hsa-miR-449b | 51050 | PI15 | peptidase inhibitor 15 | 1 |
| 5400 | hsa-miR-497 | 51050 | PI15 | peptidase inhibitor 15 | 1 |
| 5401 | hsa-miR-562 | 51050 | PI15 | peptidase inhibitor 15 | 1 |
| 5402 | hsa-miR-575 | 51050 | PI15 | peptidase inhibitor 15 | 1 |
| 5403 | hsa-miR-608 | 51050 | PI15 | peptidase inhibitor 15 | 1 |
| 5404 | hsa-miR-620 | 51050 | PI15 | peptidase inhibitor 15 | 1 |
| 5405 | hsa-mir-22 | 51050 | PI15 | peptidase inhibitor 15 | 1 |
| 5406 | hsa-mir-10a | 51050 | PI15 | peptidase inhibitor 15 | 1 |
| 5407 | hsa-mir-186 | 51050 | PI15 | peptidase inhibitor 15 | 1 |
| 5408 | hsa-mir-155 | 51050 | PI15 | peptidase inhibitor 15 | 1 |
| 5409 | hsa-mir-423 | 51050 | PI15 | peptidase inhibitor 15 | 1 |
| 5410 | hsa-mir-499 | 51050 | PI15 | peptidase inhibitor 15 | 1 |
| 5411 | hsa-mir-877 | 51050 | PI15 | peptidase inhibitor 15 | 1 |
| 5412 | hsa-mir-2116 | 51050 | PI15 | peptidase inhibitor 15 | 1 |
| 5413 | hsa-mir-3190 | 51050 | PI15 | peptidase inhibitor 15 | 1 |
| 5414 | hsa-miR-1 | 10753 | CAPN9 | calpain 9 | 1 |
| 5415 | hsa-miR-133a | 10753 | CAPN9 | calpain 9 | 1 |
| 5416 | hsa-miR-186 | 10753 | CAPN9 | calpain 9 | 1 |
| 5417 | hsa-miR-449b | 10753 | CAPN9 | calpain 9 | 1 |
| 5418 | hsa-miR-551a | 10753 | CAPN9 | calpain 9 | 1 |
| 5419 | hsa-miR-562 | 10753 | CAPN9 | calpain 9 | 1 |
| 5420 | hsa-miR-575 | 10753 | CAPN9 | calpain 9 | 1 |
| 5421 | hsa-miR-579 | 10753 | CAPN9 | calpain 9 | 1 |
| 5422 | hsa-miR-608 | 10753 | CAPN9 | calpain 9 | 1 |
| 5423 | hsa-miR-620 | 10753 | CAPN9 | calpain 9 | 1 |
| 5424 | hsa-miR-637 | 10753 | CAPN9 | calpain 9 | 1 |
| 5425 | hsa-mir-22 | 10753 | CAPN9 | calpain 9 | 1 |
| 5426 | hsa-mir-10a | 10753 | CAPN9 | calpain 9 | 1 |
| 5427 | hsa-mir-186 | 10753 | CAPN9 | calpain 9 | 1 |
| 5428 | hsa-mir-155 | 10753 | CAPN9 | calpain 9 | 1 |
| 5429 | hsa-mir-423 | 10753 | CAPN9 | calpain 9 | 1 |
| 5430 | hsa-mir-499 | 10753 | CAPN9 | calpain 9 | 1 |
| 5431 | hsa-mir-877 | 10753 | CAPN9 | calpain 9 | 1 |
| 5432 | hsa-miR-10a | 81553 | FAM49A | family with sequence similarity 49, member A | 1 |
| 5433 | hsa-miR-152 | 81553 | FAM49A | family with sequence similarity 49, member A | 1 |
| 5434 | hsa-miR-26a | 81553 | FAM49A | family with sequence similarity 49, member A | 1 |
| 5435 | hsa-miR-26b | 81553 | FAM49A | family with sequence similarity 49, member A | 1 |
| 5436 | hsa-miR-31 | 81553 | FAM49A | family with sequence similarity 49, member A | 1 |
| 5437 | hsa-miR-338-5p | 81553 | FAM49A | family with sequence similarity 49, member A | 1 |
| 5438 | hsa-miR-340 | 81553 | FAM49A | family with sequence similarity 49, member A | 1 |
| 5439 | hsa-miR-423-5p | 81553 | FAM49A | family with sequence similarity 49, member A | 1 |
| 5440 | hsa-miR-449a | 81553 | FAM49A | family with sequence similarity 49, member A | 1 |
| 5441 | hsa-miR-490-5p | 81553 | FAM49A | family with sequence similarity 49, member A | 1 |
| 5442 | hsa-miR-499-5p | 81553 | FAM49A | family with sequence similarity 49, member A | 1 |
| 5443 | hsa-miR-548b-5p | 81553 | FAM49A | family with sequence similarity 49, member A | 1 |
| 5444 | hsa-miR-564 | 81553 | FAM49A | family with sequence similarity 49, member A | 1 |
| 5445 | hsa-miR-621 | 81553 | FAM49A | family with sequence similarity 49, member A | 1 |
| 5446 | hsa-miR-657 | 81553 | FAM49A | family with sequence similarity 49, member A | 1 |
| 5447 | hsa-miR-765 | 81553 | FAM49A | family with sequence similarity 49, member A | 1 |
| 5448 | hsa-miR-770-5p | 81553 | FAM49A | family with sequence similarity 49, member A | 1 |
| 5449 | hsa-miR-877 | 81553 | FAM49A | family with sequence similarity 49, member A | 1 |
| 5450 | hsa-miR-95 | 81553 | FAM49A | family with sequence similarity 49, member A | 1 |
| 5451 | hsa-mir-26a-1 | 81553 | FAM49A | family with sequence similarity 49, member A | 1 |
| 5452 | hsa-mir-26b | 81553 | FAM49A | family with sequence similarity 49, member A | 1 |
| 5453 | hsa-mir-31 | 81553 | FAM49A | family with sequence similarity 49, member A | 1 |
| 5454 | hsa-mir-194-2 | 81553 | FAM49A | family with sequence similarity 49, member A | 1 |
| 5455 | hsa-mir-340 | 81553 | FAM49A | family with sequence similarity 49, member A | 1 |
| 5456 | hsa-mir-338 | 81553 | FAM49A | family with sequence similarity 49, member A | 1 |
| 5457 | hsa-mir-490 | 81553 | FAM49A | family with sequence similarity 49, member A | 1 |
| 5458 | hsa-mir-548b | 81553 | FAM49A | family with sequence similarity 49, member A | 1 |
| 5459 | hsa-mir-449b | 81553 | FAM49A | family with sequence similarity 49, member A | 1 |
| 5460 | hsa-mir-449c | 81553 | FAM49A | family with sequence similarity 49, member A | 1 |
| 5461 | hsa-mir-3065 | 81553 | FAM49A | family with sequence similarity 49, member A | 1 |
| 5463 | hsa-miR-139-5p | 10938 | EHD1 | EH-domain containing 1 | 1 |
| 5502 | hsa-miR-885-5p | 334 | APLP2 | amyloid beta (A4) precursor-like protein 2 | 1 |
| 5509 | hsa-mir-885 | 334 | APLP2 | amyloid beta (A4) precursor-like protein 2 | 1 |
| 5513 | hsa-miR-10a | 7187 | TRAF3 | TNF receptor-associated factor 3 | 1 |
| 5514 | hsa-miR-1228\* | 7187 | TRAF3 | TNF receptor-associated factor 3 | 1 |
| 5515 | hsa-miR-139-5p | 7187 | TRAF3 | TNF receptor-associated factor 3 | 1 |
| 5516 | hsa-miR-26a | 7187 | TRAF3 | TNF receptor-associated factor 3 | 1 |
| 5517 | hsa-miR-26b | 7187 | TRAF3 | TNF receptor-associated factor 3 | 1 |
| 5518 | hsa-miR-338-5p | 7187 | TRAF3 | TNF receptor-associated factor 3 | 1 |
| 5519 | hsa-miR-340 | 7187 | TRAF3 | TNF receptor-associated factor 3 | 1 |
| 5520 | hsa-miR-449a | 7187 | TRAF3 | TNF receptor-associated factor 3 | 1 |
| 5521 | hsa-miR-499-5p | 7187 | TRAF3 | TNF receptor-associated factor 3 | 1 |
| 5522 | hsa-miR-548b-5p | 7187 | TRAF3 | TNF receptor-associated factor 3 | 1 |
| 5523 | hsa-miR-564 | 7187 | TRAF3 | TNF receptor-associated factor 3 | 1 |
| 5524 | hsa-miR-621 | 7187 | TRAF3 | TNF receptor-associated factor 3 | 1 |
| 5525 | hsa-miR-657 | 7187 | TRAF3 | TNF receptor-associated factor 3 | 1 |
| 5526 | hsa-miR-765 | 7187 | TRAF3 | TNF receptor-associated factor 3 | 1 |
| 5527 | hsa-miR-770-5p | 7187 | TRAF3 | TNF receptor-associated factor 3 | 1 |
| 5528 | hsa-miR-877 | 7187 | TRAF3 | TNF receptor-associated factor 3 | 1 |
| 5529 | hsa-mir-26a-1 | 7187 | TRAF3 | TNF receptor-associated factor 3 | 1 |
| 5530 | hsa-mir-26b | 7187 | TRAF3 | TNF receptor-associated factor 3 | 1 |
| 5531 | hsa-mir-194-2 | 7187 | TRAF3 | TNF receptor-associated factor 3 | 1 |
| 5532 | hsa-mir-340 | 7187 | TRAF3 | TNF receptor-associated factor 3 | 1 |
| 5533 | hsa-mir-338 | 7187 | TRAF3 | TNF receptor-associated factor 3 | 1 |
| 5534 | hsa-mir-548b | 7187 | TRAF3 | TNF receptor-associated factor 3 | 1 |
| 5535 | hsa-mir-548d-2 | 7187 | TRAF3 | TNF receptor-associated factor 3 | 1 |
| 5536 | hsa-mir-449b | 7187 | TRAF3 | TNF receptor-associated factor 3 | 1 |
| 5537 | hsa-mir-449c | 7187 | TRAF3 | TNF receptor-associated factor 3 | 1 |
| 5538 | hsa-mir-3065 | 7187 | TRAF3 | TNF receptor-associated factor 3 | 1 |
| 5539 | hsa-miR-10a | 3665 | IRF7 | interferon regulatory factor 7 | 1 |
| 5540 | hsa-miR-152 | 3665 | IRF7 | interferon regulatory factor 7 | 1 |
| 5541 | hsa-miR-26a | 3665 | IRF7 | interferon regulatory factor 7 | 1 |
| 5542 | hsa-miR-26b | 3665 | IRF7 | interferon regulatory factor 7 | 1 |
| 5543 | hsa-miR-31 | 3665 | IRF7 | interferon regulatory factor 7 | 1 |
| 5544 | hsa-miR-449a | 3665 | IRF7 | interferon regulatory factor 7 | 1 |
| 5545 | hsa-miR-490-5p | 3665 | IRF7 | interferon regulatory factor 7 | 1 |
| 5546 | hsa-miR-499-5p | 3665 | IRF7 | interferon regulatory factor 7 | 1 |
| 5547 | hsa-miR-564 | 3665 | IRF7 | interferon regulatory factor 7 | 1 |
| 5548 | hsa-miR-621 | 3665 | IRF7 | interferon regulatory factor 7 | 1 |
| 5549 | hsa-miR-765 | 3665 | IRF7 | interferon regulatory factor 7 | 1 |
| 5550 | hsa-miR-770-5p | 3665 | IRF7 | interferon regulatory factor 7 | 1 |
| 5551 | hsa-mir-26a-1 | 3665 | IRF7 | interferon regulatory factor 7 | 1 |
| 5552 | hsa-mir-26b | 3665 | IRF7 | interferon regulatory factor 7 | 1 |
| 5553 | hsa-mir-31 | 3665 | IRF7 | interferon regulatory factor 7 | 1 |
| 5554 | hsa-mir-194-2 | 3665 | IRF7 | interferon regulatory factor 7 | 1 |
| 5555 | hsa-mir-490 | 3665 | IRF7 | interferon regulatory factor 7 | 1 |
| 5556 | hsa-mir-449b | 3665 | IRF7 | interferon regulatory factor 7 | 1 |
| 5557 | hsa-mir-449c | 3665 | IRF7 | interferon regulatory factor 7 | 1 |
| 5558 | hsa-miR-1 | 3736 | KCNA1 | potassium voltage-gated channel, shaker-related subfamily, member 1 (episodic ataxia with myokymia) | 1 |
| 5559 | hsa-miR-133a | 3736 | KCNA1 | potassium voltage-gated channel, shaker-related subfamily, member 1 (episodic ataxia with myokymia) | 1 |
| 5560 | hsa-miR-186 | 3736 | KCNA1 | potassium voltage-gated channel, shaker-related subfamily, member 1 (episodic ataxia with myokymia) | 1 |
| 5561 | hsa-miR-326 | 3736 | KCNA1 | potassium voltage-gated channel, shaker-related subfamily, member 1 (episodic ataxia with myokymia) | 1 |
| 5562 | hsa-miR-33a | 3736 | KCNA1 | potassium voltage-gated channel, shaker-related subfamily, member 1 (episodic ataxia with myokymia) | 1 |
| 5563 | hsa-miR-449b | 3736 | KCNA1 | potassium voltage-gated channel, shaker-related subfamily, member 1 (episodic ataxia with myokymia) | 1 |
| 5564 | hsa-miR-551a | 3736 | KCNA1 | potassium voltage-gated channel, shaker-related subfamily, member 1 (episodic ataxia with myokymia) | 1 |
| 5565 | hsa-miR-562 | 3736 | KCNA1 | potassium voltage-gated channel, shaker-related subfamily, member 1 (episodic ataxia with myokymia) | 1 |
| 5566 | hsa-miR-579 | 3736 | KCNA1 | potassium voltage-gated channel, shaker-related subfamily, member 1 (episodic ataxia with myokymia) | 1 |
| 5567 | hsa-miR-608 | 3736 | KCNA1 | potassium voltage-gated channel, shaker-related subfamily, member 1 (episodic ataxia with myokymia) | 1 |
| 5568 | hsa-miR-637 | 3736 | KCNA1 | potassium voltage-gated channel, shaker-related subfamily, member 1 (episodic ataxia with myokymia) | 1 |
| 5569 | hsa-mir-22 | 3736 | KCNA1 | potassium voltage-gated channel, shaker-related subfamily, member 1 (episodic ataxia with myokymia) | 1 |
| 5570 | hsa-mir-10a | 3736 | KCNA1 | potassium voltage-gated channel, shaker-related subfamily, member 1 (episodic ataxia with myokymia) | 1 |
| 5571 | hsa-mir-186 | 3736 | KCNA1 | potassium voltage-gated channel, shaker-related subfamily, member 1 (episodic ataxia with myokymia) | 1 |
| 5572 | hsa-mir-155 | 3736 | KCNA1 | potassium voltage-gated channel, shaker-related subfamily, member 1 (episodic ataxia with myokymia) | 1 |
| 5573 | hsa-mir-550-1 | 3736 | KCNA1 | potassium voltage-gated channel, shaker-related subfamily, member 1 (episodic ataxia with myokymia) | 1 |
| 5574 | hsa-mir-877 | 3736 | KCNA1 | potassium voltage-gated channel, shaker-related subfamily, member 1 (episodic ataxia with myokymia) | 1 |
| 5604 | hsa-miR-1 | 26211 | OR2F1 | olfactory receptor, family 2, subfamily F, member 1 | 1 |
| 5605 | hsa-miR-133a | 26211 | OR2F1 | olfactory receptor, family 2, subfamily F, member 1 | 1 |
| 5606 | hsa-miR-186 | 26211 | OR2F1 | olfactory receptor, family 2, subfamily F, member 1 | 1 |
| 5607 | hsa-miR-195 | 26211 | OR2F1 | olfactory receptor, family 2, subfamily F, member 1 | 1 |
| 5608 | hsa-miR-326 | 26211 | OR2F1 | olfactory receptor, family 2, subfamily F, member 1 | 1 |
| 5609 | hsa-miR-33a | 26211 | OR2F1 | olfactory receptor, family 2, subfamily F, member 1 | 1 |
| 5610 | hsa-miR-449b | 26211 | OR2F1 | olfactory receptor, family 2, subfamily F, member 1 | 1 |
| 5611 | hsa-miR-497 | 26211 | OR2F1 | olfactory receptor, family 2, subfamily F, member 1 | 1 |
| 5612 | hsa-miR-551a | 26211 | OR2F1 | olfactory receptor, family 2, subfamily F, member 1 | 1 |
| 5613 | hsa-miR-562 | 26211 | OR2F1 | olfactory receptor, family 2, subfamily F, member 1 | 1 |
| 5614 | hsa-miR-575 | 26211 | OR2F1 | olfactory receptor, family 2, subfamily F, member 1 | 1 |
| 5615 | hsa-miR-579 | 26211 | OR2F1 | olfactory receptor, family 2, subfamily F, member 1 | 1 |
| 5616 | hsa-miR-608 | 26211 | OR2F1 | olfactory receptor, family 2, subfamily F, member 1 | 1 |
| 5617 | hsa-miR-620 | 26211 | OR2F1 | olfactory receptor, family 2, subfamily F, member 1 | 1 |
| 5618 | hsa-mir-22 | 26211 | OR2F1 | olfactory receptor, family 2, subfamily F, member 1 | 1 |
| 5619 | hsa-mir-10a | 26211 | OR2F1 | olfactory receptor, family 2, subfamily F, member 1 | 1 |
| 5620 | hsa-mir-186 | 26211 | OR2F1 | olfactory receptor, family 2, subfamily F, member 1 | 1 |
| 5621 | hsa-mir-155 | 26211 | OR2F1 | olfactory receptor, family 2, subfamily F, member 1 | 1 |
| 5622 | hsa-mir-499 | 26211 | OR2F1 | olfactory receptor, family 2, subfamily F, member 1 | 1 |
| 5623 | hsa-mir-550-1 | 26211 | OR2F1 | olfactory receptor, family 2, subfamily F, member 1 | 1 |
| 5624 | hsa-mir-877 | 26211 | OR2F1 | olfactory receptor, family 2, subfamily F, member 1 | 1 |
| 5625 | hsa-mir-2116 | 26211 | OR2F1 | olfactory receptor, family 2, subfamily F, member 1 | 1 |
| 5626 | hsa-miR-10a | 4501 | MT1X | metallothionein 1X | 1 |
| 5627 | hsa-miR-152 | 4501 | MT1X | metallothionein 1X | 1 |
| 5628 | hsa-miR-26a | 4501 | MT1X | metallothionein 1X | 1 |
| 5629 | hsa-miR-26b | 4501 | MT1X | metallothionein 1X | 1 |
| 5630 | hsa-miR-31 | 4501 | MT1X | metallothionein 1X | 1 |
| 5631 | hsa-miR-338-5p | 4501 | MT1X | metallothionein 1X | 1 |
| 5632 | hsa-miR-340 | 4501 | MT1X | metallothionein 1X | 1 |
| 5633 | hsa-miR-383 | 4501 | MT1X | metallothionein 1X | 1 |
| 5634 | hsa-miR-449a | 4501 | MT1X | metallothionein 1X | 1 |
| 5635 | hsa-miR-490-5p | 4501 | MT1X | metallothionein 1X | 1 |
| 5636 | hsa-miR-499-5p | 4501 | MT1X | metallothionein 1X | 1 |
| 5637 | hsa-miR-504 | 4501 | MT1X | metallothionein 1X | 1 |
| 5638 | hsa-miR-621 | 4501 | MT1X | metallothionein 1X | 1 |
| 5639 | hsa-miR-653 | 4501 | MT1X | metallothionein 1X | 1 |
| 5640 | hsa-miR-657 | 4501 | MT1X | metallothionein 1X | 1 |
| 5641 | hsa-miR-765 | 4501 | MT1X | metallothionein 1X | 1 |
| 5642 | hsa-miR-770-5p | 4501 | MT1X | metallothionein 1X | 1 |
| 5643 | hsa-miR-877 | 4501 | MT1X | metallothionein 1X | 1 |
| 5644 | hsa-miR-95 | 4501 | MT1X | metallothionein 1X | 1 |
| 5645 | hsa-mir-26a-1 | 4501 | MT1X | metallothionein 1X | 1 |
| 5646 | hsa-mir-26b | 4501 | MT1X | metallothionein 1X | 1 |
| 5647 | hsa-mir-31 | 4501 | MT1X | metallothionein 1X | 1 |
| 5648 | hsa-mir-194-2 | 4501 | MT1X | metallothionein 1X | 1 |
| 5649 | hsa-mir-340 | 4501 | MT1X | metallothionein 1X | 1 |
| 5650 | hsa-mir-338 | 4501 | MT1X | metallothionein 1X | 1 |
| 5651 | hsa-mir-490 | 4501 | MT1X | metallothionein 1X | 1 |
| 5652 | hsa-mir-449b | 4501 | MT1X | metallothionein 1X | 1 |
| 5653 | hsa-mir-449c | 4501 | MT1X | metallothionein 1X | 1 |
| 5654 | hsa-mir-3065 | 4501 | MT1X | metallothionein 1X | 1 |
| 5655 | hsa-miR-1 | 59347 | FKSG2 | apoptosis inhibitor | 1 |
| 5656 | hsa-miR-133a | 59347 | FKSG2 | apoptosis inhibitor | 1 |
| 5657 | hsa-miR-186 | 59347 | FKSG2 | apoptosis inhibitor | 1 |
| 5658 | hsa-miR-326 | 59347 | FKSG2 | apoptosis inhibitor | 1 |
| 5659 | hsa-miR-449b | 59347 | FKSG2 | apoptosis inhibitor | 1 |
| 5660 | hsa-miR-575 | 59347 | FKSG2 | apoptosis inhibitor | 1 |
| 5661 | hsa-miR-608 | 59347 | FKSG2 | apoptosis inhibitor | 1 |
| 5662 | hsa-miR-620 | 59347 | FKSG2 | apoptosis inhibitor | 1 |
| 5663 | hsa-miR-637 | 59347 | FKSG2 | apoptosis inhibitor | 1 |
| 5664 | hsa-mir-22 | 59347 | FKSG2 | apoptosis inhibitor | 1 |
| 5665 | hsa-mir-10a | 59347 | FKSG2 | apoptosis inhibitor | 1 |
| 5666 | hsa-mir-186 | 59347 | FKSG2 | apoptosis inhibitor | 1 |
| 5667 | hsa-mir-155 | 59347 | FKSG2 | apoptosis inhibitor | 1 |
| 5668 | hsa-mir-499 | 59347 | FKSG2 | apoptosis inhibitor | 1 |
| 5669 | hsa-mir-877 | 59347 | FKSG2 | apoptosis inhibitor | 1 |
| 5670 | hsa-miR-1 | 10493 | VAT1 | vesicle amine transport protein 1 homolog (T. californica) | 1 |
| 5671 | hsa-miR-133a | 10493 | VAT1 | vesicle amine transport protein 1 homolog (T. californica) | 1 |
| 5672 | hsa-miR-186 | 10493 | VAT1 | vesicle amine transport protein 1 homolog (T. californica) | 1 |
| 5673 | hsa-miR-208a | 10493 | VAT1 | vesicle amine transport protein 1 homolog (T. californica) | 1 |
| 5674 | hsa-miR-326 | 10493 | VAT1 | vesicle amine transport protein 1 homolog (T. californica) | 1 |
| 5675 | hsa-miR-449b | 10493 | VAT1 | vesicle amine transport protein 1 homolog (T. californica) | 1 |
| 5676 | hsa-miR-489 | 10493 | VAT1 | vesicle amine transport protein 1 homolog (T. californica) | 1 |
| 5677 | hsa-miR-575 | 10493 | VAT1 | vesicle amine transport protein 1 homolog (T. californica) | 1 |
| 5678 | hsa-miR-579 | 10493 | VAT1 | vesicle amine transport protein 1 homolog (T. californica) | 1 |
| 5679 | hsa-miR-608 | 10493 | VAT1 | vesicle amine transport protein 1 homolog (T. californica) | 1 |
| 5680 | hsa-miR-620 | 10493 | VAT1 | vesicle amine transport protein 1 homolog (T. californica) | 1 |
| 5681 | hsa-miR-637 | 10493 | VAT1 | vesicle amine transport protein 1 homolog (T. californica) | 1 |
| 5682 | hsa-miR-885-5p | 10493 | VAT1 | vesicle amine transport protein 1 homolog (T. californica) | 1 |
| 5683 | hsa-mir-22 | 10493 | VAT1 | vesicle amine transport protein 1 homolog (T. californica) | 1 |
| 5684 | hsa-mir-10a | 10493 | VAT1 | vesicle amine transport protein 1 homolog (T. californica) | 1 |
| 5685 | hsa-mir-186 | 10493 | VAT1 | vesicle amine transport protein 1 homolog (T. californica) | 1 |
| 5686 | hsa-mir-155 | 10493 | VAT1 | vesicle amine transport protein 1 homolog (T. californica) | 1 |
| 5687 | hsa-mir-423 | 10493 | VAT1 | vesicle amine transport protein 1 homolog (T. californica) | 1 |
| 5688 | hsa-mir-499 | 10493 | VAT1 | vesicle amine transport protein 1 homolog (T. californica) | 1 |
| 5689 | hsa-mir-885 | 10493 | VAT1 | vesicle amine transport protein 1 homolog (T. californica) | 1 |
| 5690 | hsa-mir-877 | 10493 | VAT1 | vesicle amine transport protein 1 homolog (T. californica) | 1 |
| 5691 | hsa-mir-3190 | 10493 | VAT1 | vesicle amine transport protein 1 homolog (T. californica) | 1 |
| 5722 | hsa-miR-550 | 334 | APLP2 | amyloid beta (A4) precursor-like protein 2 | 1 |
| 5723 | hsa-mir-550-1 | 334 | APLP2 | amyloid beta (A4) precursor-like protein 2 | 1 |
| 5724 | hsa-miR-637 | 334 | APLP2 | amyloid beta (A4) precursor-like protein 2 | 1 |
| 5729 | hsa-miR-1 | 7381 | UQCRB | ubiquinol-cytochrome c reductase binding protein | 1 |
| 5730 | hsa-miR-133a | 7381 | UQCRB | ubiquinol-cytochrome c reductase binding protein | 1 |
| 5731 | hsa-miR-195 | 7381 | UQCRB | ubiquinol-cytochrome c reductase binding protein | 1 |
| 5732 | hsa-miR-326 | 7381 | UQCRB | ubiquinol-cytochrome c reductase binding protein | 1 |
| 5733 | hsa-miR-449b | 7381 | UQCRB | ubiquinol-cytochrome c reductase binding protein | 1 |
| 5734 | hsa-miR-489 | 7381 | UQCRB | ubiquinol-cytochrome c reductase binding protein | 1 |
| 5735 | hsa-miR-497 | 7381 | UQCRB | ubiquinol-cytochrome c reductase binding protein | 1 |
| 5736 | hsa-miR-562 | 7381 | UQCRB | ubiquinol-cytochrome c reductase binding protein | 1 |
| 5737 | hsa-miR-575 | 7381 | UQCRB | ubiquinol-cytochrome c reductase binding protein | 1 |
| 5738 | hsa-miR-608 | 7381 | UQCRB | ubiquinol-cytochrome c reductase binding protein | 1 |
| 5739 | hsa-miR-620 | 7381 | UQCRB | ubiquinol-cytochrome c reductase binding protein | 1 |
| 5740 | hsa-mir-22 | 7381 | UQCRB | ubiquinol-cytochrome c reductase binding protein | 1 |
| 5741 | hsa-mir-10a | 7381 | UQCRB | ubiquinol-cytochrome c reductase binding protein | 1 |
| 5742 | hsa-mir-155 | 7381 | UQCRB | ubiquinol-cytochrome c reductase binding protein | 1 |
| 5743 | hsa-mir-499 | 7381 | UQCRB | ubiquinol-cytochrome c reductase binding protein | 1 |
| 5744 | hsa-mir-877 | 7381 | UQCRB | ubiquinol-cytochrome c reductase binding protein | 1 |
| 5745 | hsa-miR-1 | 10551 | AGR2 | anterior gradient homolog 2 (Xenopus laevis) | 1 |
| 5746 | hsa-miR-133a | 10551 | AGR2 | anterior gradient homolog 2 (Xenopus laevis) | 1 |
| 5747 | hsa-miR-186 | 10551 | AGR2 | anterior gradient homolog 2 (Xenopus laevis) | 1 |
| 5748 | hsa-miR-326 | 10551 | AGR2 | anterior gradient homolog 2 (Xenopus laevis) | 1 |
| 5749 | hsa-miR-449b | 10551 | AGR2 | anterior gradient homolog 2 (Xenopus laevis) | 1 |
| 5750 | hsa-miR-550 | 10551 | AGR2 | anterior gradient homolog 2 (Xenopus laevis) | 1 |
| 5751 | hsa-miR-551a | 10551 | AGR2 | anterior gradient homolog 2 (Xenopus laevis) | 1 |
| 5752 | hsa-miR-562 | 10551 | AGR2 | anterior gradient homolog 2 (Xenopus laevis) | 1 |
| 5753 | hsa-miR-575 | 10551 | AGR2 | anterior gradient homolog 2 (Xenopus laevis) | 1 |
| 5754 | hsa-miR-579 | 10551 | AGR2 | anterior gradient homolog 2 (Xenopus laevis) | 1 |
| 5755 | hsa-miR-608 | 10551 | AGR2 | anterior gradient homolog 2 (Xenopus laevis) | 1 |
| 5756 | hsa-miR-620 | 10551 | AGR2 | anterior gradient homolog 2 (Xenopus laevis) | 1 |
| 5757 | hsa-miR-637 | 10551 | AGR2 | anterior gradient homolog 2 (Xenopus laevis) | 1 |
| 5758 | hsa-miR-885-5p | 10551 | AGR2 | anterior gradient homolog 2 (Xenopus laevis) | 1 |
| 5759 | hsa-mir-22 | 10551 | AGR2 | anterior gradient homolog 2 (Xenopus laevis) | 1 |
| 5760 | hsa-mir-10a | 10551 | AGR2 | anterior gradient homolog 2 (Xenopus laevis) | 1 |
| 5761 | hsa-mir-186 | 10551 | AGR2 | anterior gradient homolog 2 (Xenopus laevis) | 1 |
| 5762 | hsa-mir-155 | 10551 | AGR2 | anterior gradient homolog 2 (Xenopus laevis) | 1 |
| 5763 | hsa-mir-423 | 10551 | AGR2 | anterior gradient homolog 2 (Xenopus laevis) | 1 |
| 5764 | hsa-mir-499 | 10551 | AGR2 | anterior gradient homolog 2 (Xenopus laevis) | 1 |
| 5765 | hsa-mir-550-1 | 10551 | AGR2 | anterior gradient homolog 2 (Xenopus laevis) | 1 |
| 5766 | hsa-mir-885 | 10551 | AGR2 | anterior gradient homolog 2 (Xenopus laevis) | 1 |
| 5767 | hsa-mir-877 | 10551 | AGR2 | anterior gradient homolog 2 (Xenopus laevis) | 1 |
| 5768 | hsa-mir-2116 | 10551 | AGR2 | anterior gradient homolog 2 (Xenopus laevis) | 1 |
| 5769 | hsa-miR-1 | 8660 | IRS2 | insulin receptor substrate 2 | 1 |
| 5770 | hsa-miR-133a | 8660 | IRS2 | insulin receptor substrate 2 | 1 |
| 5771 | hsa-miR-186 | 8660 | IRS2 | insulin receptor substrate 2 | 1 |
| 5772 | hsa-miR-208a | 8660 | IRS2 | insulin receptor substrate 2 | 1 |
| 5773 | hsa-miR-326 | 8660 | IRS2 | insulin receptor substrate 2 | 1 |
| 5774 | hsa-miR-449b | 8660 | IRS2 | insulin receptor substrate 2 | 1 |
| 5775 | hsa-miR-550 | 8660 | IRS2 | insulin receptor substrate 2 | 1 |
| 5776 | hsa-miR-575 | 8660 | IRS2 | insulin receptor substrate 2 | 1 |
| 5777 | hsa-miR-579 | 8660 | IRS2 | insulin receptor substrate 2 | 1 |
| 5778 | hsa-miR-608 | 8660 | IRS2 | insulin receptor substrate 2 | 1 |
| 5779 | hsa-miR-620 | 8660 | IRS2 | insulin receptor substrate 2 | 1 |
| 5780 | hsa-miR-885-5p | 8660 | IRS2 | insulin receptor substrate 2 | 1 |
| 5781 | hsa-mir-22 | 8660 | IRS2 | insulin receptor substrate 2 | 1 |
| 5782 | hsa-mir-10a | 8660 | IRS2 | insulin receptor substrate 2 | 1 |
| 5783 | hsa-mir-186 | 8660 | IRS2 | insulin receptor substrate 2 | 1 |
| 5784 | hsa-mir-155 | 8660 | IRS2 | insulin receptor substrate 2 | 1 |
| 5785 | hsa-mir-423 | 8660 | IRS2 | insulin receptor substrate 2 | 1 |
| 5786 | hsa-mir-499 | 8660 | IRS2 | insulin receptor substrate 2 | 1 |
| 5787 | hsa-mir-550-1 | 8660 | IRS2 | insulin receptor substrate 2 | 1 |
| 5788 | hsa-mir-885 | 8660 | IRS2 | insulin receptor substrate 2 | 1 |
| 5789 | hsa-mir-877 | 8660 | IRS2 | insulin receptor substrate 2 | 1 |
| 5790 | hsa-mir-2116 | 8660 | IRS2 | insulin receptor substrate 2 | 1 |
| 5791 | hsa-mir-3190 | 8660 | IRS2 | insulin receptor substrate 2 | 1 |
| 5792 | hsa-miR-1 | 2186 | BPTF | bromodomain PHD finger transcription factor | 1 |
| 5793 | hsa-miR-133a | 2186 | BPTF | bromodomain PHD finger transcription factor | 1 |
| 5794 | hsa-miR-186 | 2186 | BPTF | bromodomain PHD finger transcription factor | 1 |
| 5795 | hsa-miR-195 | 2186 | BPTF | bromodomain PHD finger transcription factor | 1 |
| 5796 | hsa-miR-208a | 2186 | BPTF | bromodomain PHD finger transcription factor | 1 |
| 5797 | hsa-miR-326 | 2186 | BPTF | bromodomain PHD finger transcription factor | 1 |
| 5798 | hsa-miR-449b | 2186 | BPTF | bromodomain PHD finger transcription factor | 1 |
| 5799 | hsa-miR-497 | 2186 | BPTF | bromodomain PHD finger transcription factor | 1 |
| 5800 | hsa-miR-575 | 2186 | BPTF | bromodomain PHD finger transcription factor | 1 |
| 5801 | hsa-miR-608 | 2186 | BPTF | bromodomain PHD finger transcription factor | 1 |
| 5802 | hsa-miR-620 | 2186 | BPTF | bromodomain PHD finger transcription factor | 1 |
| 5803 | hsa-miR-885-5p | 2186 | BPTF | bromodomain PHD finger transcription factor | 1 |
| 5804 | hsa-let-7a-3 | 2186 | BPTF | bromodomain PHD finger transcription factor | 1 |
| 5805 | hsa-let-7b | 2186 | BPTF | bromodomain PHD finger transcription factor | 1 |
| 5806 | hsa-mir-22 | 2186 | BPTF | bromodomain PHD finger transcription factor | 1 |
| 5807 | hsa-mir-186 | 2186 | BPTF | bromodomain PHD finger transcription factor | 1 |
| 5808 | hsa-mir-155 | 2186 | BPTF | bromodomain PHD finger transcription factor | 1 |
| 5809 | hsa-mir-499 | 2186 | BPTF | bromodomain PHD finger transcription factor | 1 |
| 5810 | hsa-mir-885 | 2186 | BPTF | bromodomain PHD finger transcription factor | 1 |
| 5811 | hsa-mir-877 | 2186 | BPTF | bromodomain PHD finger transcription factor | 1 |
| 5812 | hsa-mir-2116 | 2186 | BPTF | bromodomain PHD finger transcription factor | 1 |
| 5813 | hsa-miR-1 | 192286 | HIGD2A | HIG1 hypoxia inducible domain family, member 2A | 1 |
| 5814 | hsa-miR-133a | 192286 | HIGD2A | HIG1 hypoxia inducible domain family, member 2A | 1 |
| 5815 | hsa-miR-186 | 192286 | HIGD2A | HIG1 hypoxia inducible domain family, member 2A | 1 |
| 5816 | hsa-miR-208a | 192286 | HIGD2A | HIG1 hypoxia inducible domain family, member 2A | 1 |
| 5817 | hsa-miR-449b | 192286 | HIGD2A | HIG1 hypoxia inducible domain family, member 2A | 1 |
| 5818 | hsa-miR-489 | 192286 | HIGD2A | HIG1 hypoxia inducible domain family, member 2A | 1 |
| 5819 | hsa-miR-562 | 192286 | HIGD2A | HIG1 hypoxia inducible domain family, member 2A | 1 |
| 5820 | hsa-miR-575 | 192286 | HIGD2A | HIG1 hypoxia inducible domain family, member 2A | 1 |
| 5821 | hsa-miR-579 | 192286 | HIGD2A | HIG1 hypoxia inducible domain family, member 2A | 1 |
| 5822 | hsa-miR-608 | 192286 | HIGD2A | HIG1 hypoxia inducible domain family, member 2A | 1 |
| 5823 | hsa-miR-620 | 192286 | HIGD2A | HIG1 hypoxia inducible domain family, member 2A | 1 |
| 5824 | hsa-miR-885-5p | 192286 | HIGD2A | HIG1 hypoxia inducible domain family, member 2A | 1 |
| 5825 | hsa-mir-22 | 192286 | HIGD2A | HIG1 hypoxia inducible domain family, member 2A | 1 |
| 5826 | hsa-mir-10a | 192286 | HIGD2A | HIG1 hypoxia inducible domain family, member 2A | 1 |
| 5827 | hsa-mir-186 | 192286 | HIGD2A | HIG1 hypoxia inducible domain family, member 2A | 1 |
| 5828 | hsa-mir-155 | 192286 | HIGD2A | HIG1 hypoxia inducible domain family, member 2A | 1 |
| 5829 | hsa-mir-499 | 192286 | HIGD2A | HIG1 hypoxia inducible domain family, member 2A | 1 |
| 5830 | hsa-mir-885 | 192286 | HIGD2A | HIG1 hypoxia inducible domain family, member 2A | 1 |
| 5831 | hsa-miR-1 | 5822 | PWP2 | PWP2 periodic tryptophan protein homolog (yeast) | 1 |
| 5832 | hsa-miR-133a | 5822 | PWP2 | PWP2 periodic tryptophan protein homolog (yeast) | 1 |
| 5833 | hsa-miR-186 | 5822 | PWP2 | PWP2 periodic tryptophan protein homolog (yeast) | 1 |
| 5834 | hsa-miR-449b | 5822 | PWP2 | PWP2 periodic tryptophan protein homolog (yeast) | 1 |
| 5835 | hsa-miR-489 | 5822 | PWP2 | PWP2 periodic tryptophan protein homolog (yeast) | 1 |
| 5836 | hsa-miR-562 | 5822 | PWP2 | PWP2 periodic tryptophan protein homolog (yeast) | 1 |
| 5837 | hsa-miR-579 | 5822 | PWP2 | PWP2 periodic tryptophan protein homolog (yeast) | 1 |
| 5838 | hsa-miR-608 | 5822 | PWP2 | PWP2 periodic tryptophan protein homolog (yeast) | 1 |
| 5839 | hsa-miR-620 | 5822 | PWP2 | PWP2 periodic tryptophan protein homolog (yeast) | 1 |
| 5840 | hsa-miR-637 | 5822 | PWP2 | PWP2 periodic tryptophan protein homolog (yeast) | 1 |
| 5841 | hsa-miR-885-5p | 5822 | PWP2 | PWP2 periodic tryptophan protein homolog (yeast) | 1 |
| 5842 | hsa-mir-22 | 5822 | PWP2 | PWP2 periodic tryptophan protein homolog (yeast) | 1 |
| 5843 | hsa-mir-10a | 5822 | PWP2 | PWP2 periodic tryptophan protein homolog (yeast) | 1 |
| 5844 | hsa-mir-186 | 5822 | PWP2 | PWP2 periodic tryptophan protein homolog (yeast) | 1 |
| 5845 | hsa-mir-155 | 5822 | PWP2 | PWP2 periodic tryptophan protein homolog (yeast) | 1 |
| 5846 | hsa-mir-885 | 5822 | PWP2 | PWP2 periodic tryptophan protein homolog (yeast) | 1 |
| 5848 | hsa-let-7a-3 | 4094 | MAF | v-maf musculoaponeurotic fibrosarcoma oncogene homolog (avian) | 1 |
| 5849 | hsa-let-7b | 4094 | MAF | v-maf musculoaponeurotic fibrosarcoma oncogene homolog (avian) | 1 |
| 5850 | hsa-miR-1 | 23345 | SYNE1 | spectrin repeat containing, nuclear envelope 1 | 1 |
| 5851 | hsa-miR-133a | 23345 | SYNE1 | spectrin repeat containing, nuclear envelope 1 | 1 |
| 5852 | hsa-miR-186 | 23345 | SYNE1 | spectrin repeat containing, nuclear envelope 1 | 1 |
| 5853 | hsa-miR-326 | 23345 | SYNE1 | spectrin repeat containing, nuclear envelope 1 | 1 |
| 5854 | hsa-miR-449b | 23345 | SYNE1 | spectrin repeat containing, nuclear envelope 1 | 1 |
| 5855 | hsa-miR-489 | 23345 | SYNE1 | spectrin repeat containing, nuclear envelope 1 | 1 |
| 5856 | hsa-miR-562 | 23345 | SYNE1 | spectrin repeat containing, nuclear envelope 1 | 1 |
| 5857 | hsa-miR-575 | 23345 | SYNE1 | spectrin repeat containing, nuclear envelope 1 | 1 |
| 5858 | hsa-miR-579 | 23345 | SYNE1 | spectrin repeat containing, nuclear envelope 1 | 1 |
| 5859 | hsa-miR-608 | 23345 | SYNE1 | spectrin repeat containing, nuclear envelope 1 | 1 |
| 5860 | hsa-miR-637 | 23345 | SYNE1 | spectrin repeat containing, nuclear envelope 1 | 1 |
| 5861 | hsa-miR-885-5p | 23345 | SYNE1 | spectrin repeat containing, nuclear envelope 1 | 1 |
| 5862 | hsa-mir-22 | 23345 | SYNE1 | spectrin repeat containing, nuclear envelope 1 | 1 |
| 5863 | hsa-mir-10a | 23345 | SYNE1 | spectrin repeat containing, nuclear envelope 1 | 1 |
| 5864 | hsa-mir-186 | 23345 | SYNE1 | spectrin repeat containing, nuclear envelope 1 | 1 |
| 5865 | hsa-mir-155 | 23345 | SYNE1 | spectrin repeat containing, nuclear envelope 1 | 1 |
| 5866 | hsa-mir-423 | 23345 | SYNE1 | spectrin repeat containing, nuclear envelope 1 | 1 |
| 5867 | hsa-mir-499 | 23345 | SYNE1 | spectrin repeat containing, nuclear envelope 1 | 1 |
| 5868 | hsa-mir-885 | 23345 | SYNE1 | spectrin repeat containing, nuclear envelope 1 | 1 |
| 5869 | hsa-mir-877 | 23345 | SYNE1 | spectrin repeat containing, nuclear envelope 1 | 1 |
| 5870 | hsa-mir-3190 | 23345 | SYNE1 | spectrin repeat containing, nuclear envelope 1 | 1 |
| 5871 | hsa-miR-10a | 1847 | DUSP5 | dual specificity phosphatase 5 | 1 |
| 5872 | hsa-miR-139-5p | 1847 | DUSP5 | dual specificity phosphatase 5 | 1 |
| 5873 | hsa-miR-152 | 1847 | DUSP5 | dual specificity phosphatase 5 | 1 |
| 5874 | hsa-miR-26b | 1847 | DUSP5 | dual specificity phosphatase 5 | 1 |
| 5875 | hsa-miR-31 | 1847 | DUSP5 | dual specificity phosphatase 5 | 1 |
| 5876 | hsa-miR-338-5p | 1847 | DUSP5 | dual specificity phosphatase 5 | 1 |
| 5877 | hsa-miR-340 | 1847 | DUSP5 | dual specificity phosphatase 5 | 1 |
| 5878 | hsa-miR-383 | 1847 | DUSP5 | dual specificity phosphatase 5 | 1 |
| 5879 | hsa-miR-449a | 1847 | DUSP5 | dual specificity phosphatase 5 | 1 |
| 5880 | hsa-miR-499-5p | 1847 | DUSP5 | dual specificity phosphatase 5 | 1 |
| 5881 | hsa-miR-621 | 1847 | DUSP5 | dual specificity phosphatase 5 | 1 |
| 5882 | hsa-miR-653 | 1847 | DUSP5 | dual specificity phosphatase 5 | 1 |
| 5883 | hsa-miR-657 | 1847 | DUSP5 | dual specificity phosphatase 5 | 1 |
| 5884 | hsa-miR-765 | 1847 | DUSP5 | dual specificity phosphatase 5 | 1 |
| 5885 | hsa-miR-770-5p | 1847 | DUSP5 | dual specificity phosphatase 5 | 1 |
| 5886 | hsa-miR-877 | 1847 | DUSP5 | dual specificity phosphatase 5 | 1 |
| 5887 | hsa-mir-26a-1 | 1847 | DUSP5 | dual specificity phosphatase 5 | 1 |
| 5888 | hsa-mir-26b | 1847 | DUSP5 | dual specificity phosphatase 5 | 1 |
| 5889 | hsa-mir-31 | 1847 | DUSP5 | dual specificity phosphatase 5 | 1 |
| 5890 | hsa-mir-194-2 | 1847 | DUSP5 | dual specificity phosphatase 5 | 1 |
| 5891 | hsa-mir-340 | 1847 | DUSP5 | dual specificity phosphatase 5 | 1 |
| 5892 | hsa-mir-338 | 1847 | DUSP5 | dual specificity phosphatase 5 | 1 |
| 5893 | hsa-mir-449b | 1847 | DUSP5 | dual specificity phosphatase 5 | 1 |
| 5894 | hsa-mir-449c | 1847 | DUSP5 | dual specificity phosphatase 5 | 1 |
| 5895 | hsa-mir-3065 | 1847 | DUSP5 | dual specificity phosphatase 5 | 1 |
| 5905 | hsa-miR-423-5p | 6509 | SLC1A4 | solute carrier family 1 (glutamate/neutral amino acid transporter), member 4 | 1 |
| 5925 | hsa-miR-1228\* | 6509 | SLC1A4 | solute carrier family 1 (glutamate/neutral amino acid transporter), member 4 | 1 |
| 5926 | hsa-miR-490-5p | 6509 | SLC1A4 | solute carrier family 1 (glutamate/neutral amino acid transporter), member 4 | 1 |
| 5927 | hsa-miR-584 | 6509 | SLC1A4 | solute carrier family 1 (glutamate/neutral amino acid transporter), member 4 | 1 |
| 5928 | hsa-mir-490 | 6509 | SLC1A4 | solute carrier family 1 (glutamate/neutral amino acid transporter), member 4 | 1 |
| 5929 | hsa-miR-31 | 4791 | NFKB2 | nuclear factor of kappa light polypeptide gene enhancer in B-cells 2 (p49/p100) | 1 |
| 5930 | hsa-miR-423-5p | 4791 | NFKB2 | nuclear factor of kappa light polypeptide gene enhancer in B-cells 2 (p49/p100) | 1 |
| 5931 | hsa-miR-564 | 4791 | NFKB2 | nuclear factor of kappa light polypeptide gene enhancer in B-cells 2 (p49/p100) | 1 |
| 5932 | hsa-miR-644 | 4791 | NFKB2 | nuclear factor of kappa light polypeptide gene enhancer in B-cells 2 (p49/p100) | 1 |
| 5933 | hsa-miR-95 | 4791 | NFKB2 | nuclear factor of kappa light polypeptide gene enhancer in B-cells 2 (p49/p100) | 1 |
| 5934 | hsa-mir-31 | 4791 | NFKB2 | nuclear factor of kappa light polypeptide gene enhancer in B-cells 2 (p49/p100) | 1 |
| 5935 | hsa-miR-1 | 1646 | AKR1C2 | aldo-keto reductase family 1, member C2 (dihydrodiol dehydrogenase 2; bile acid binding protein; 3-alpha hydroxysteroid dehydrogenase, type III) | 1 |
| 5936 | hsa-miR-133a | 1646 | AKR1C2 | aldo-keto reductase family 1, member C2 (dihydrodiol dehydrogenase 2; bile acid binding protein; 3-alpha hydroxysteroid dehydrogenase, type III) | 1 |
| 5937 | hsa-miR-186 | 1646 | AKR1C2 | aldo-keto reductase family 1, member C2 (dihydrodiol dehydrogenase 2; bile acid binding protein; 3-alpha hydroxysteroid dehydrogenase, type III) | 1 |
| 5938 | hsa-miR-195 | 1646 | AKR1C2 | aldo-keto reductase family 1, member C2 (dihydrodiol dehydrogenase 2; bile acid binding protein; 3-alpha hydroxysteroid dehydrogenase, type III) | 1 |
| 5939 | hsa-miR-326 | 1646 | AKR1C2 | aldo-keto reductase family 1, member C2 (dihydrodiol dehydrogenase 2; bile acid binding protein; 3-alpha hydroxysteroid dehydrogenase, type III) | 1 |
| 5940 | hsa-miR-449b | 1646 | AKR1C2 | aldo-keto reductase family 1, member C2 (dihydrodiol dehydrogenase 2; bile acid binding protein; 3-alpha hydroxysteroid dehydrogenase, type III) | 1 |
| 5941 | hsa-miR-497 | 1646 | AKR1C2 | aldo-keto reductase family 1, member C2 (dihydrodiol dehydrogenase 2; bile acid binding protein; 3-alpha hydroxysteroid dehydrogenase, type III) | 1 |
| 5942 | hsa-miR-551a | 1646 | AKR1C2 | aldo-keto reductase family 1, member C2 (dihydrodiol dehydrogenase 2; bile acid binding protein; 3-alpha hydroxysteroid dehydrogenase, type III) | 1 |
| 5943 | hsa-miR-562 | 1646 | AKR1C2 | aldo-keto reductase family 1, member C2 (dihydrodiol dehydrogenase 2; bile acid binding protein; 3-alpha hydroxysteroid dehydrogenase, type III) | 1 |
| 5944 | hsa-miR-575 | 1646 | AKR1C2 | aldo-keto reductase family 1, member C2 (dihydrodiol dehydrogenase 2; bile acid binding protein; 3-alpha hydroxysteroid dehydrogenase, type III) | 1 |
| 5945 | hsa-miR-608 | 1646 | AKR1C2 | aldo-keto reductase family 1, member C2 (dihydrodiol dehydrogenase 2; bile acid binding protein; 3-alpha hydroxysteroid dehydrogenase, type III) | 1 |
| 5946 | hsa-miR-620 | 1646 | AKR1C2 | aldo-keto reductase family 1, member C2 (dihydrodiol dehydrogenase 2; bile acid binding protein; 3-alpha hydroxysteroid dehydrogenase, type III) | 1 |
| 5947 | hsa-mir-22 | 1646 | AKR1C2 | aldo-keto reductase family 1, member C2 (dihydrodiol dehydrogenase 2; bile acid binding protein; 3-alpha hydroxysteroid dehydrogenase, type III) | 1 |
| 5948 | hsa-mir-10a | 1646 | AKR1C2 | aldo-keto reductase family 1, member C2 (dihydrodiol dehydrogenase 2; bile acid binding protein; 3-alpha hydroxysteroid dehydrogenase, type III) | 1 |
| 5949 | hsa-mir-186 | 1646 | AKR1C2 | aldo-keto reductase family 1, member C2 (dihydrodiol dehydrogenase 2; bile acid binding protein; 3-alpha hydroxysteroid dehydrogenase, type III) | 1 |
| 5950 | hsa-mir-155 | 1646 | AKR1C2 | aldo-keto reductase family 1, member C2 (dihydrodiol dehydrogenase 2; bile acid binding protein; 3-alpha hydroxysteroid dehydrogenase, type III) | 1 |
| 5951 | hsa-mir-499 | 1646 | AKR1C2 | aldo-keto reductase family 1, member C2 (dihydrodiol dehydrogenase 2; bile acid binding protein; 3-alpha hydroxysteroid dehydrogenase, type III) | 1 |
| 5952 | hsa-mir-550-1 | 1646 | AKR1C2 | aldo-keto reductase family 1, member C2 (dihydrodiol dehydrogenase 2; bile acid binding protein; 3-alpha hydroxysteroid dehydrogenase, type III) | 1 |
| 5953 | hsa-mir-877 | 1646 | AKR1C2 | aldo-keto reductase family 1, member C2 (dihydrodiol dehydrogenase 2; bile acid binding protein; 3-alpha hydroxysteroid dehydrogenase, type III) | 1 |
| 5967 | hsa-miR-584 | 5272 | SERPINB9 | serpin peptidase inhibitor, clade B (ovalbumin), member 9 | 1 |
| 5969 | hsa-miR-644 | 5272 | SERPINB9 | serpin peptidase inhibitor, clade B (ovalbumin), member 9 | 1 |
| 5986 | hsa-miR-10a | 8728 | ADAM19 | ADAM metallopeptidase domain 19 (meltrin beta) | 1 |
| 5987 | hsa-miR-139-5p | 8728 | ADAM19 | ADAM metallopeptidase domain 19 (meltrin beta) | 1 |
| 5988 | hsa-miR-152 | 8728 | ADAM19 | ADAM metallopeptidase domain 19 (meltrin beta) | 1 |
| 5989 | hsa-miR-26b | 8728 | ADAM19 | ADAM metallopeptidase domain 19 (meltrin beta) | 1 |
| 5990 | hsa-miR-31 | 8728 | ADAM19 | ADAM metallopeptidase domain 19 (meltrin beta) | 1 |
| 5991 | hsa-miR-338-5p | 8728 | ADAM19 | ADAM metallopeptidase domain 19 (meltrin beta) | 1 |
| 5992 | hsa-miR-340 | 8728 | ADAM19 | ADAM metallopeptidase domain 19 (meltrin beta) | 1 |
| 5993 | hsa-miR-383 | 8728 | ADAM19 | ADAM metallopeptidase domain 19 (meltrin beta) | 1 |
| 5994 | hsa-miR-449a | 8728 | ADAM19 | ADAM metallopeptidase domain 19 (meltrin beta) | 1 |
| 5995 | hsa-miR-499-5p | 8728 | ADAM19 | ADAM metallopeptidase domain 19 (meltrin beta) | 1 |
| 5996 | hsa-miR-548b-5p | 8728 | ADAM19 | ADAM metallopeptidase domain 19 (meltrin beta) | 1 |
| 5997 | hsa-miR-621 | 8728 | ADAM19 | ADAM metallopeptidase domain 19 (meltrin beta) | 1 |
| 5998 | hsa-miR-657 | 8728 | ADAM19 | ADAM metallopeptidase domain 19 (meltrin beta) | 1 |
| 5999 | hsa-miR-663 | 8728 | ADAM19 | ADAM metallopeptidase domain 19 (meltrin beta) | 1 |
| 6000 | hsa-miR-765 | 8728 | ADAM19 | ADAM metallopeptidase domain 19 (meltrin beta) | 1 |
| 6001 | hsa-miR-770-5p | 8728 | ADAM19 | ADAM metallopeptidase domain 19 (meltrin beta) | 1 |
| 6002 | hsa-miR-877 | 8728 | ADAM19 | ADAM metallopeptidase domain 19 (meltrin beta) | 1 |
| 6003 | hsa-mir-26a-1 | 8728 | ADAM19 | ADAM metallopeptidase domain 19 (meltrin beta) | 1 |
| 6004 | hsa-mir-26b | 8728 | ADAM19 | ADAM metallopeptidase domain 19 (meltrin beta) | 1 |
| 6005 | hsa-mir-31 | 8728 | ADAM19 | ADAM metallopeptidase domain 19 (meltrin beta) | 1 |
| 6006 | hsa-mir-194-2 | 8728 | ADAM19 | ADAM metallopeptidase domain 19 (meltrin beta) | 1 |
| 6007 | hsa-mir-340 | 8728 | ADAM19 | ADAM metallopeptidase domain 19 (meltrin beta) | 1 |
| 6008 | hsa-mir-338 | 8728 | ADAM19 | ADAM metallopeptidase domain 19 (meltrin beta) | 1 |
| 6009 | hsa-mir-548b | 8728 | ADAM19 | ADAM metallopeptidase domain 19 (meltrin beta) | 1 |
| 6010 | hsa-mir-548d-2 | 8728 | ADAM19 | ADAM metallopeptidase domain 19 (meltrin beta) | 1 |
| 6011 | hsa-mir-449b | 8728 | ADAM19 | ADAM metallopeptidase domain 19 (meltrin beta) | 1 |
| 6012 | hsa-mir-449c | 8728 | ADAM19 | ADAM metallopeptidase domain 19 (meltrin beta) | 1 |
| 6013 | hsa-mir-3065 | 8728 | ADAM19 | ADAM metallopeptidase domain 19 (meltrin beta) | 1 |
| 6021 | hsa-miR-383 | 10435 | CDC42EP2 | CDC42 effector protein (Rho GTPase binding) 2 | 1 |
| 6041 | hsa-miR-1 | 23401 | FRAT2 | frequently rearranged in advanced T-cell lymphomas 2 | 1 |
| 6042 | hsa-miR-133a | 23401 | FRAT2 | frequently rearranged in advanced T-cell lymphomas 2 | 1 |
| 6043 | hsa-miR-186 | 23401 | FRAT2 | frequently rearranged in advanced T-cell lymphomas 2 | 1 |
| 6044 | hsa-miR-326 | 23401 | FRAT2 | frequently rearranged in advanced T-cell lymphomas 2 | 1 |
| 6045 | hsa-miR-449b | 23401 | FRAT2 | frequently rearranged in advanced T-cell lymphomas 2 | 1 |
| 6046 | hsa-miR-575 | 23401 | FRAT2 | frequently rearranged in advanced T-cell lymphomas 2 | 1 |
| 6047 | hsa-miR-579 | 23401 | FRAT2 | frequently rearranged in advanced T-cell lymphomas 2 | 1 |
| 6048 | hsa-miR-608 | 23401 | FRAT2 | frequently rearranged in advanced T-cell lymphomas 2 | 1 |
| 6049 | hsa-miR-620 | 23401 | FRAT2 | frequently rearranged in advanced T-cell lymphomas 2 | 1 |
| 6050 | hsa-miR-637 | 23401 | FRAT2 | frequently rearranged in advanced T-cell lymphomas 2 | 1 |
| 6051 | hsa-miR-885-5p | 23401 | FRAT2 | frequently rearranged in advanced T-cell lymphomas 2 | 1 |
| 6052 | hsa-mir-22 | 23401 | FRAT2 | frequently rearranged in advanced T-cell lymphomas 2 | 1 |
| 6053 | hsa-mir-10a | 23401 | FRAT2 | frequently rearranged in advanced T-cell lymphomas 2 | 1 |
| 6054 | hsa-mir-186 | 23401 | FRAT2 | frequently rearranged in advanced T-cell lymphomas 2 | 1 |
| 6055 | hsa-mir-155 | 23401 | FRAT2 | frequently rearranged in advanced T-cell lymphomas 2 | 1 |
| 6056 | hsa-mir-423 | 23401 | FRAT2 | frequently rearranged in advanced T-cell lymphomas 2 | 1 |
| 6057 | hsa-mir-499 | 23401 | FRAT2 | frequently rearranged in advanced T-cell lymphomas 2 | 1 |
| 6058 | hsa-mir-885 | 23401 | FRAT2 | frequently rearranged in advanced T-cell lymphomas 2 | 1 |
| 6059 | hsa-mir-877 | 23401 | FRAT2 | frequently rearranged in advanced T-cell lymphomas 2 | 1 |
| 6060 | hsa-mir-2116 | 23401 | FRAT2 | frequently rearranged in advanced T-cell lymphomas 2 | 1 |
| 6061 | hsa-mir-3190 | 23401 | FRAT2 | frequently rearranged in advanced T-cell lymphomas 2 | 1 |
| 6063 | hsa-miR-10a | 7965 | AIMP2 | aminoacyl tRNA synthetase complex-interacting multifunctional protein 2 | 1 |
| 6064 | hsa-miR-1228\* | 7965 | AIMP2 | aminoacyl tRNA synthetase complex-interacting multifunctional protein 2 | 1 |
| 6065 | hsa-miR-139-5p | 7965 | AIMP2 | aminoacyl tRNA synthetase complex-interacting multifunctional protein 2 | 1 |
| 6066 | hsa-miR-152 | 7965 | AIMP2 | aminoacyl tRNA synthetase complex-interacting multifunctional protein 2 | 1 |
| 6067 | hsa-miR-26a | 7965 | AIMP2 | aminoacyl tRNA synthetase complex-interacting multifunctional protein 2 | 1 |
| 6068 | hsa-miR-26b | 7965 | AIMP2 | aminoacyl tRNA synthetase complex-interacting multifunctional protein 2 | 1 |
| 6069 | hsa-miR-31 | 7965 | AIMP2 | aminoacyl tRNA synthetase complex-interacting multifunctional protein 2 | 1 |
| 6070 | hsa-miR-338-5p | 7965 | AIMP2 | aminoacyl tRNA synthetase complex-interacting multifunctional protein 2 | 1 |
| 6071 | hsa-miR-340 | 7965 | AIMP2 | aminoacyl tRNA synthetase complex-interacting multifunctional protein 2 | 1 |
| 6072 | hsa-miR-423-5p | 7965 | AIMP2 | aminoacyl tRNA synthetase complex-interacting multifunctional protein 2 | 1 |
| 6073 | hsa-miR-449a | 7965 | AIMP2 | aminoacyl tRNA synthetase complex-interacting multifunctional protein 2 | 1 |
| 6074 | hsa-miR-490-5p | 7965 | AIMP2 | aminoacyl tRNA synthetase complex-interacting multifunctional protein 2 | 1 |
| 6075 | hsa-miR-499-5p | 7965 | AIMP2 | aminoacyl tRNA synthetase complex-interacting multifunctional protein 2 | 1 |
| 6076 | hsa-miR-548b-5p | 7965 | AIMP2 | aminoacyl tRNA synthetase complex-interacting multifunctional protein 2 | 1 |
| 6077 | hsa-miR-584 | 7965 | AIMP2 | aminoacyl tRNA synthetase complex-interacting multifunctional protein 2 | 1 |
| 6078 | hsa-miR-621 | 7965 | AIMP2 | aminoacyl tRNA synthetase complex-interacting multifunctional protein 2 | 1 |
| 6079 | hsa-miR-657 | 7965 | AIMP2 | aminoacyl tRNA synthetase complex-interacting multifunctional protein 2 | 1 |
| 6080 | hsa-miR-663 | 7965 | AIMP2 | aminoacyl tRNA synthetase complex-interacting multifunctional protein 2 | 1 |
| 6081 | hsa-miR-765 | 7965 | AIMP2 | aminoacyl tRNA synthetase complex-interacting multifunctional protein 2 | 1 |
| 6082 | hsa-miR-770-5p | 7965 | AIMP2 | aminoacyl tRNA synthetase complex-interacting multifunctional protein 2 | 1 |
| 6083 | hsa-miR-877 | 7965 | AIMP2 | aminoacyl tRNA synthetase complex-interacting multifunctional protein 2 | 1 |
| 6084 | hsa-mir-26a-1 | 7965 | AIMP2 | aminoacyl tRNA synthetase complex-interacting multifunctional protein 2 | 1 |
| 6085 | hsa-mir-26b | 7965 | AIMP2 | aminoacyl tRNA synthetase complex-interacting multifunctional protein 2 | 1 |
| 6086 | hsa-mir-31 | 7965 | AIMP2 | aminoacyl tRNA synthetase complex-interacting multifunctional protein 2 | 1 |
| 6087 | hsa-mir-194-2 | 7965 | AIMP2 | aminoacyl tRNA synthetase complex-interacting multifunctional protein 2 | 1 |
| 6088 | hsa-mir-340 | 7965 | AIMP2 | aminoacyl tRNA synthetase complex-interacting multifunctional protein 2 | 1 |
| 6089 | hsa-mir-338 | 7965 | AIMP2 | aminoacyl tRNA synthetase complex-interacting multifunctional protein 2 | 1 |
| 6090 | hsa-mir-490 | 7965 | AIMP2 | aminoacyl tRNA synthetase complex-interacting multifunctional protein 2 | 1 |
| 6091 | hsa-mir-548b | 7965 | AIMP2 | aminoacyl tRNA synthetase complex-interacting multifunctional protein 2 | 1 |
| 6092 | hsa-mir-548d-2 | 7965 | AIMP2 | aminoacyl tRNA synthetase complex-interacting multifunctional protein 2 | 1 |
| 6093 | hsa-mir-449b | 7965 | AIMP2 | aminoacyl tRNA synthetase complex-interacting multifunctional protein 2 | 1 |
| 6094 | hsa-mir-449c | 7965 | AIMP2 | aminoacyl tRNA synthetase complex-interacting multifunctional protein 2 | 1 |
| 6095 | hsa-mir-3065 | 7965 | AIMP2 | aminoacyl tRNA synthetase complex-interacting multifunctional protein 2 | 1 |
| 6096 | hsa-miR-10a | 3620 | IDO1 | indoleamine 2,3-dioxygenase 1 | 1 |
| 6097 | hsa-miR-152 | 3620 | IDO1 | indoleamine 2,3-dioxygenase 1 | 1 |
| 6098 | hsa-miR-26a | 3620 | IDO1 | indoleamine 2,3-dioxygenase 1 | 1 |
| 6099 | hsa-miR-26b | 3620 | IDO1 | indoleamine 2,3-dioxygenase 1 | 1 |
| 6100 | hsa-miR-31 | 3620 | IDO1 | indoleamine 2,3-dioxygenase 1 | 1 |
| 6101 | hsa-miR-338-5p | 3620 | IDO1 | indoleamine 2,3-dioxygenase 1 | 1 |
| 6102 | hsa-miR-340 | 3620 | IDO1 | indoleamine 2,3-dioxygenase 1 | 1 |
| 6103 | hsa-miR-383 | 3620 | IDO1 | indoleamine 2,3-dioxygenase 1 | 1 |
| 6104 | hsa-miR-449a | 3620 | IDO1 | indoleamine 2,3-dioxygenase 1 | 1 |
| 6105 | hsa-miR-490-5p | 3620 | IDO1 | indoleamine 2,3-dioxygenase 1 | 1 |
| 6106 | hsa-miR-499-5p | 3620 | IDO1 | indoleamine 2,3-dioxygenase 1 | 1 |
| 6107 | hsa-miR-548b-5p | 3620 | IDO1 | indoleamine 2,3-dioxygenase 1 | 1 |
| 6108 | hsa-miR-564 | 3620 | IDO1 | indoleamine 2,3-dioxygenase 1 | 1 |
| 6109 | hsa-miR-621 | 3620 | IDO1 | indoleamine 2,3-dioxygenase 1 | 1 |
| 6110 | hsa-miR-657 | 3620 | IDO1 | indoleamine 2,3-dioxygenase 1 | 1 |
| 6111 | hsa-miR-765 | 3620 | IDO1 | indoleamine 2,3-dioxygenase 1 | 1 |
| 6112 | hsa-miR-770-5p | 3620 | IDO1 | indoleamine 2,3-dioxygenase 1 | 1 |
| 6113 | hsa-miR-877 | 3620 | IDO1 | indoleamine 2,3-dioxygenase 1 | 1 |
| 6114 | hsa-mir-26a-1 | 3620 | IDO1 | indoleamine 2,3-dioxygenase 1 | 1 |
| 6115 | hsa-mir-26b | 3620 | IDO1 | indoleamine 2,3-dioxygenase 1 | 1 |
| 6116 | hsa-mir-31 | 3620 | IDO1 | indoleamine 2,3-dioxygenase 1 | 1 |
| 6117 | hsa-mir-194-2 | 3620 | IDO1 | indoleamine 2,3-dioxygenase 1 | 1 |
| 6118 | hsa-mir-340 | 3620 | IDO1 | indoleamine 2,3-dioxygenase 1 | 1 |
| 6119 | hsa-mir-338 | 3620 | IDO1 | indoleamine 2,3-dioxygenase 1 | 1 |
| 6120 | hsa-mir-490 | 3620 | IDO1 | indoleamine 2,3-dioxygenase 1 | 1 |
| 6121 | hsa-mir-548b | 3620 | IDO1 | indoleamine 2,3-dioxygenase 1 | 1 |
| 6122 | hsa-mir-449b | 3620 | IDO1 | indoleamine 2,3-dioxygenase 1 | 1 |
| 6123 | hsa-mir-449c | 3620 | IDO1 | indoleamine 2,3-dioxygenase 1 | 1 |
| 6124 | hsa-mir-3065 | 3620 | IDO1 | indoleamine 2,3-dioxygenase 1 | 1 |
| 6125 | hsa-miR-10a | 3552 | IL1A | interleukin 1, alpha | 1 |
| 6126 | hsa-miR-139-5p | 3552 | IL1A | interleukin 1, alpha | 1 |
| 6127 | hsa-miR-152 | 3552 | IL1A | interleukin 1, alpha | 1 |
| 6128 | hsa-miR-26a | 3552 | IL1A | interleukin 1, alpha | 1 |
| 6129 | hsa-miR-26b | 3552 | IL1A | interleukin 1, alpha | 1 |
| 6130 | hsa-miR-31 | 3552 | IL1A | interleukin 1, alpha | 1 |
| 6131 | hsa-miR-338-5p | 3552 | IL1A | interleukin 1, alpha | 1 |
| 6132 | hsa-miR-340 | 3552 | IL1A | interleukin 1, alpha | 1 |
| 6133 | hsa-miR-449a | 3552 | IL1A | interleukin 1, alpha | 1 |
| 6134 | hsa-miR-490-5p | 3552 | IL1A | interleukin 1, alpha | 1 |
| 6135 | hsa-miR-499-5p | 3552 | IL1A | interleukin 1, alpha | 1 |
| 6136 | hsa-miR-548b-5p | 3552 | IL1A | interleukin 1, alpha | 1 |
| 6137 | hsa-miR-564 | 3552 | IL1A | interleukin 1, alpha | 1 |
| 6138 | hsa-miR-584 | 3552 | IL1A | interleukin 1, alpha | 1 |
| 6139 | hsa-miR-621 | 3552 | IL1A | interleukin 1, alpha | 1 |
| 6140 | hsa-miR-634 | 3552 | IL1A | interleukin 1, alpha | 1 |
| 6141 | hsa-miR-657 | 3552 | IL1A | interleukin 1, alpha | 1 |
| 6142 | hsa-miR-765 | 3552 | IL1A | interleukin 1, alpha | 1 |
| 6143 | hsa-miR-770-5p | 3552 | IL1A | interleukin 1, alpha | 1 |
| 6144 | hsa-miR-877 | 3552 | IL1A | interleukin 1, alpha | 1 |
| 6145 | hsa-miR-95 | 3552 | IL1A | interleukin 1, alpha | 1 |
| 6146 | hsa-mir-26a-1 | 3552 | IL1A | interleukin 1, alpha | 1 |
| 6147 | hsa-mir-26b | 3552 | IL1A | interleukin 1, alpha | 1 |
| 6148 | hsa-mir-31 | 3552 | IL1A | interleukin 1, alpha | 1 |
| 6149 | hsa-mir-194-2 | 3552 | IL1A | interleukin 1, alpha | 1 |
| 6150 | hsa-mir-340 | 3552 | IL1A | interleukin 1, alpha | 1 |
| 6151 | hsa-mir-338 | 3552 | IL1A | interleukin 1, alpha | 1 |
| 6152 | hsa-mir-490 | 3552 | IL1A | interleukin 1, alpha | 1 |
| 6153 | hsa-mir-548b | 3552 | IL1A | interleukin 1, alpha | 1 |
| 6154 | hsa-mir-449b | 3552 | IL1A | interleukin 1, alpha | 1 |
| 6155 | hsa-mir-449c | 3552 | IL1A | interleukin 1, alpha | 1 |
| 6156 | hsa-mir-3065 | 3552 | IL1A | interleukin 1, alpha | 1 |
| 6157 | hsa-miR-1 | 7100 | TLR5 | toll-like receptor 5 | 1 |
| 6158 | hsa-miR-133a | 7100 | TLR5 | toll-like receptor 5 | 1 |
| 6159 | hsa-miR-186 | 7100 | TLR5 | toll-like receptor 5 | 1 |
| 6160 | hsa-miR-326 | 7100 | TLR5 | toll-like receptor 5 | 1 |
| 6161 | hsa-miR-33a | 7100 | TLR5 | toll-like receptor 5 | 1 |
| 6162 | hsa-miR-449b | 7100 | TLR5 | toll-like receptor 5 | 1 |
| 6163 | hsa-miR-551a | 7100 | TLR5 | toll-like receptor 5 | 1 |
| 6164 | hsa-miR-575 | 7100 | TLR5 | toll-like receptor 5 | 1 |
| 6165 | hsa-miR-579 | 7100 | TLR5 | toll-like receptor 5 | 1 |
| 6166 | hsa-miR-608 | 7100 | TLR5 | toll-like receptor 5 | 1 |
| 6167 | hsa-miR-620 | 7100 | TLR5 | toll-like receptor 5 | 1 |
| 6168 | hsa-mir-22 | 7100 | TLR5 | toll-like receptor 5 | 1 |
| 6169 | hsa-mir-10a | 7100 | TLR5 | toll-like receptor 5 | 1 |
| 6170 | hsa-mir-186 | 7100 | TLR5 | toll-like receptor 5 | 1 |
| 6171 | hsa-mir-155 | 7100 | TLR5 | toll-like receptor 5 | 1 |
| 6172 | hsa-mir-499 | 7100 | TLR5 | toll-like receptor 5 | 1 |
| 6173 | hsa-mir-550-1 | 7100 | TLR5 | toll-like receptor 5 | 1 |
| 6174 | hsa-mir-877 | 7100 | TLR5 | toll-like receptor 5 | 1 |
| 6175 | hsa-mir-2116 | 7100 | TLR5 | toll-like receptor 5 | 1 |
| 6176 | hsa-miR-10a | 1437 | CSF2 | colony stimulating factor 2 (granulocyte-macrophage) | 1 |
| 6177 | hsa-miR-152 | 1437 | CSF2 | colony stimulating factor 2 (granulocyte-macrophage) | 1 |
| 6178 | hsa-miR-26a | 1437 | CSF2 | colony stimulating factor 2 (granulocyte-macrophage) | 1 |
| 6179 | hsa-miR-26b | 1437 | CSF2 | colony stimulating factor 2 (granulocyte-macrophage) | 1 |
| 6180 | hsa-miR-31 | 1437 | CSF2 | colony stimulating factor 2 (granulocyte-macrophage) | 1 |
| 6181 | hsa-miR-340 | 1437 | CSF2 | colony stimulating factor 2 (granulocyte-macrophage) | 1 |
| 6182 | hsa-miR-449a | 1437 | CSF2 | colony stimulating factor 2 (granulocyte-macrophage) | 1 |
| 6183 | hsa-miR-490-5p | 1437 | CSF2 | colony stimulating factor 2 (granulocyte-macrophage) | 1 |
| 6184 | hsa-miR-499-5p | 1437 | CSF2 | colony stimulating factor 2 (granulocyte-macrophage) | 1 |
| 6185 | hsa-miR-564 | 1437 | CSF2 | colony stimulating factor 2 (granulocyte-macrophage) | 1 |
| 6186 | hsa-miR-621 | 1437 | CSF2 | colony stimulating factor 2 (granulocyte-macrophage) | 1 |
| 6187 | hsa-miR-634 | 1437 | CSF2 | colony stimulating factor 2 (granulocyte-macrophage) | 1 |
| 6188 | hsa-miR-644 | 1437 | CSF2 | colony stimulating factor 2 (granulocyte-macrophage) | 1 |
| 6189 | hsa-miR-653 | 1437 | CSF2 | colony stimulating factor 2 (granulocyte-macrophage) | 1 |
| 6190 | hsa-miR-765 | 1437 | CSF2 | colony stimulating factor 2 (granulocyte-macrophage) | 1 |
| 6191 | hsa-miR-770-5p | 1437 | CSF2 | colony stimulating factor 2 (granulocyte-macrophage) | 1 |
| 6192 | hsa-miR-877 | 1437 | CSF2 | colony stimulating factor 2 (granulocyte-macrophage) | 1 |
| 6193 | hsa-miR-95 | 1437 | CSF2 | colony stimulating factor 2 (granulocyte-macrophage) | 1 |
| 6194 | hsa-mir-26a-1 | 1437 | CSF2 | colony stimulating factor 2 (granulocyte-macrophage) | 1 |
| 6195 | hsa-mir-26b | 1437 | CSF2 | colony stimulating factor 2 (granulocyte-macrophage) | 1 |
| 6196 | hsa-mir-31 | 1437 | CSF2 | colony stimulating factor 2 (granulocyte-macrophage) | 1 |
| 6197 | hsa-mir-340 | 1437 | CSF2 | colony stimulating factor 2 (granulocyte-macrophage) | 1 |
| 6198 | hsa-mir-490 | 1437 | CSF2 | colony stimulating factor 2 (granulocyte-macrophage) | 1 |
| 6199 | hsa-mir-449b | 1437 | CSF2 | colony stimulating factor 2 (granulocyte-macrophage) | 1 |
| 6200 | hsa-mir-449c | 1437 | CSF2 | colony stimulating factor 2 (granulocyte-macrophage) | 1 |
| 6225 | hsa-miR-10a | 9356 | SLC22A6 | solute carrier family 22 (organic anion transporter), member 6 | 1 |
| 6226 | hsa-miR-139-5p | 9356 | SLC22A6 | solute carrier family 22 (organic anion transporter), member 6 | 1 |
| 6227 | hsa-miR-152 | 9356 | SLC22A6 | solute carrier family 22 (organic anion transporter), member 6 | 1 |
| 6228 | hsa-miR-26a | 9356 | SLC22A6 | solute carrier family 22 (organic anion transporter), member 6 | 1 |
| 6229 | hsa-miR-26b | 9356 | SLC22A6 | solute carrier family 22 (organic anion transporter), member 6 | 1 |
| 6230 | hsa-miR-31 | 9356 | SLC22A6 | solute carrier family 22 (organic anion transporter), member 6 | 1 |
| 6231 | hsa-miR-338-5p | 9356 | SLC22A6 | solute carrier family 22 (organic anion transporter), member 6 | 1 |
| 6232 | hsa-miR-340 | 9356 | SLC22A6 | solute carrier family 22 (organic anion transporter), member 6 | 1 |
| 6233 | hsa-miR-449a | 9356 | SLC22A6 | solute carrier family 22 (organic anion transporter), member 6 | 1 |
| 6234 | hsa-miR-499-5p | 9356 | SLC22A6 | solute carrier family 22 (organic anion transporter), member 6 | 1 |
| 6235 | hsa-miR-548b-5p | 9356 | SLC22A6 | solute carrier family 22 (organic anion transporter), member 6 | 1 |
| 6236 | hsa-miR-564 | 9356 | SLC22A6 | solute carrier family 22 (organic anion transporter), member 6 | 1 |
| 6237 | hsa-miR-584 | 9356 | SLC22A6 | solute carrier family 22 (organic anion transporter), member 6 | 1 |
| 6238 | hsa-miR-621 | 9356 | SLC22A6 | solute carrier family 22 (organic anion transporter), member 6 | 1 |
| 6239 | hsa-miR-634 | 9356 | SLC22A6 | solute carrier family 22 (organic anion transporter), member 6 | 1 |
| 6240 | hsa-miR-644 | 9356 | SLC22A6 | solute carrier family 22 (organic anion transporter), member 6 | 1 |
| 6241 | hsa-miR-657 | 9356 | SLC22A6 | solute carrier family 22 (organic anion transporter), member 6 | 1 |
| 6242 | hsa-miR-765 | 9356 | SLC22A6 | solute carrier family 22 (organic anion transporter), member 6 | 1 |
| 6243 | hsa-miR-770-5p | 9356 | SLC22A6 | solute carrier family 22 (organic anion transporter), member 6 | 1 |
| 6244 | hsa-miR-877 | 9356 | SLC22A6 | solute carrier family 22 (organic anion transporter), member 6 | 1 |
| 6245 | hsa-mir-26a-1 | 9356 | SLC22A6 | solute carrier family 22 (organic anion transporter), member 6 | 1 |
| 6246 | hsa-mir-26b | 9356 | SLC22A6 | solute carrier family 22 (organic anion transporter), member 6 | 1 |
| 6247 | hsa-mir-31 | 9356 | SLC22A6 | solute carrier family 22 (organic anion transporter), member 6 | 1 |
| 6248 | hsa-mir-194-2 | 9356 | SLC22A6 | solute carrier family 22 (organic anion transporter), member 6 | 1 |
| 6249 | hsa-mir-340 | 9356 | SLC22A6 | solute carrier family 22 (organic anion transporter), member 6 | 1 |
| 6250 | hsa-mir-338 | 9356 | SLC22A6 | solute carrier family 22 (organic anion transporter), member 6 | 1 |
| 6251 | hsa-mir-548b | 9356 | SLC22A6 | solute carrier family 22 (organic anion transporter), member 6 | 1 |
| 6252 | hsa-mir-449b | 9356 | SLC22A6 | solute carrier family 22 (organic anion transporter), member 6 | 1 |
| 6253 | hsa-mir-449c | 9356 | SLC22A6 | solute carrier family 22 (organic anion transporter), member 6 | 1 |
| 6254 | hsa-mir-3065 | 9356 | SLC22A6 | solute carrier family 22 (organic anion transporter), member 6 | 1 |
| 6255 | hsa-miR-1 | 5023 | P2RX1 | purinergic receptor P2X, ligand-gated ion channel, 1 | 1 |
| 6256 | hsa-miR-133a | 5023 | P2RX1 | purinergic receptor P2X, ligand-gated ion channel, 1 | 1 |
| 6257 | hsa-miR-186 | 5023 | P2RX1 | purinergic receptor P2X, ligand-gated ion channel, 1 | 1 |
| 6258 | hsa-miR-195 | 5023 | P2RX1 | purinergic receptor P2X, ligand-gated ion channel, 1 | 1 |
| 6259 | hsa-miR-326 | 5023 | P2RX1 | purinergic receptor P2X, ligand-gated ion channel, 1 | 1 |
| 6260 | hsa-miR-33a | 5023 | P2RX1 | purinergic receptor P2X, ligand-gated ion channel, 1 | 1 |
| 6261 | hsa-miR-449b | 5023 | P2RX1 | purinergic receptor P2X, ligand-gated ion channel, 1 | 1 |
| 6262 | hsa-miR-497 | 5023 | P2RX1 | purinergic receptor P2X, ligand-gated ion channel, 1 | 1 |
| 6263 | hsa-miR-551a | 5023 | P2RX1 | purinergic receptor P2X, ligand-gated ion channel, 1 | 1 |
| 6264 | hsa-miR-575 | 5023 | P2RX1 | purinergic receptor P2X, ligand-gated ion channel, 1 | 1 |
| 6265 | hsa-miR-608 | 5023 | P2RX1 | purinergic receptor P2X, ligand-gated ion channel, 1 | 1 |
| 6266 | hsa-miR-620 | 5023 | P2RX1 | purinergic receptor P2X, ligand-gated ion channel, 1 | 1 |
| 6267 | hsa-mir-22 | 5023 | P2RX1 | purinergic receptor P2X, ligand-gated ion channel, 1 | 1 |
| 6268 | hsa-mir-10a | 5023 | P2RX1 | purinergic receptor P2X, ligand-gated ion channel, 1 | 1 |
| 6269 | hsa-mir-186 | 5023 | P2RX1 | purinergic receptor P2X, ligand-gated ion channel, 1 | 1 |
| 6270 | hsa-mir-155 | 5023 | P2RX1 | purinergic receptor P2X, ligand-gated ion channel, 1 | 1 |
| 6271 | hsa-mir-499 | 5023 | P2RX1 | purinergic receptor P2X, ligand-gated ion channel, 1 | 1 |
| 6272 | hsa-mir-550-1 | 5023 | P2RX1 | purinergic receptor P2X, ligand-gated ion channel, 1 | 1 |
| 6273 | hsa-mir-877 | 5023 | P2RX1 | purinergic receptor P2X, ligand-gated ion channel, 1 | 1 |
| 6274 | hsa-mir-2116 | 5023 | P2RX1 | purinergic receptor P2X, ligand-gated ion channel, 1 | 1 |
| 6275 | hsa-miR-10a | 6738 | TROVE2 | TROVE domain family, member 2 | 1 |
| 6276 | hsa-miR-139-5p | 6738 | TROVE2 | TROVE domain family, member 2 | 1 |
| 6277 | hsa-miR-152 | 6738 | TROVE2 | TROVE domain family, member 2 | 1 |
| 6278 | hsa-miR-26b | 6738 | TROVE2 | TROVE domain family, member 2 | 1 |
| 6279 | hsa-miR-31 | 6738 | TROVE2 | TROVE domain family, member 2 | 1 |
| 6280 | hsa-miR-338-5p | 6738 | TROVE2 | TROVE domain family, member 2 | 1 |
| 6281 | hsa-miR-340 | 6738 | TROVE2 | TROVE domain family, member 2 | 1 |
| 6282 | hsa-miR-383 | 6738 | TROVE2 | TROVE domain family, member 2 | 1 |
| 6283 | hsa-miR-449a | 6738 | TROVE2 | TROVE domain family, member 2 | 1 |
| 6284 | hsa-miR-499-5p | 6738 | TROVE2 | TROVE domain family, member 2 | 1 |
| 6285 | hsa-miR-621 | 6738 | TROVE2 | TROVE domain family, member 2 | 1 |
| 6286 | hsa-miR-653 | 6738 | TROVE2 | TROVE domain family, member 2 | 1 |
| 6287 | hsa-miR-657 | 6738 | TROVE2 | TROVE domain family, member 2 | 1 |
| 6288 | hsa-miR-765 | 6738 | TROVE2 | TROVE domain family, member 2 | 1 |
| 6289 | hsa-miR-770-5p | 6738 | TROVE2 | TROVE domain family, member 2 | 1 |
| 6290 | hsa-miR-877 | 6738 | TROVE2 | TROVE domain family, member 2 | 1 |
| 6291 | hsa-mir-26a-1 | 6738 | TROVE2 | TROVE domain family, member 2 | 1 |
| 6292 | hsa-mir-26b | 6738 | TROVE2 | TROVE domain family, member 2 | 1 |
| 6293 | hsa-mir-31 | 6738 | TROVE2 | TROVE domain family, member 2 | 1 |
| 6294 | hsa-mir-194-2 | 6738 | TROVE2 | TROVE domain family, member 2 | 1 |
| 6295 | hsa-mir-340 | 6738 | TROVE2 | TROVE domain family, member 2 | 1 |
| 6296 | hsa-mir-338 | 6738 | TROVE2 | TROVE domain family, member 2 | 1 |
| 6297 | hsa-mir-449b | 6738 | TROVE2 | TROVE domain family, member 2 | 1 |
| 6298 | hsa-mir-449c | 6738 | TROVE2 | TROVE domain family, member 2 | 1 |
| 6299 | hsa-mir-3065 | 6738 | TROVE2 | TROVE domain family, member 2 | 1 |
| 6300 | hsa-miR-10a | 3624 | INHBA | inhibin, beta A | 1 |
| 6301 | hsa-miR-139-5p | 3624 | INHBA | inhibin, beta A | 1 |
| 6302 | hsa-miR-152 | 3624 | INHBA | inhibin, beta A | 1 |
| 6303 | hsa-miR-26a | 3624 | INHBA | inhibin, beta A | 1 |
| 6304 | hsa-miR-26b | 3624 | INHBA | inhibin, beta A | 1 |
| 6305 | hsa-miR-31 | 3624 | INHBA | inhibin, beta A | 1 |
| 6306 | hsa-miR-338-5p | 3624 | INHBA | inhibin, beta A | 1 |
| 6307 | hsa-miR-340 | 3624 | INHBA | inhibin, beta A | 1 |
| 6308 | hsa-miR-423-5p | 3624 | INHBA | inhibin, beta A | 1 |
| 6309 | hsa-miR-449a | 3624 | INHBA | inhibin, beta A | 1 |
| 6310 | hsa-miR-490-5p | 3624 | INHBA | inhibin, beta A | 1 |
| 6311 | hsa-miR-499-5p | 3624 | INHBA | inhibin, beta A | 1 |
| 6312 | hsa-miR-548b-5p | 3624 | INHBA | inhibin, beta A | 1 |
| 6313 | hsa-miR-564 | 3624 | INHBA | inhibin, beta A | 1 |
| 6314 | hsa-miR-584 | 3624 | INHBA | inhibin, beta A | 1 |
| 6315 | hsa-miR-621 | 3624 | INHBA | inhibin, beta A | 1 |
| 6316 | hsa-miR-657 | 3624 | INHBA | inhibin, beta A | 1 |
| 6317 | hsa-miR-765 | 3624 | INHBA | inhibin, beta A | 1 |
| 6318 | hsa-miR-770-5p | 3624 | INHBA | inhibin, beta A | 1 |
| 6319 | hsa-miR-877 | 3624 | INHBA | inhibin, beta A | 1 |
| 6320 | hsa-miR-95 | 3624 | INHBA | inhibin, beta A | 1 |
| 6321 | hsa-mir-26a-1 | 3624 | INHBA | inhibin, beta A | 1 |
| 6322 | hsa-mir-26b | 3624 | INHBA | inhibin, beta A | 1 |
| 6323 | hsa-mir-31 | 3624 | INHBA | inhibin, beta A | 1 |
| 6324 | hsa-mir-194-2 | 3624 | INHBA | inhibin, beta A | 1 |
| 6325 | hsa-mir-340 | 3624 | INHBA | inhibin, beta A | 1 |
| 6326 | hsa-mir-338 | 3624 | INHBA | inhibin, beta A | 1 |
| 6327 | hsa-mir-490 | 3624 | INHBA | inhibin, beta A | 1 |
| 6328 | hsa-mir-548b | 3624 | INHBA | inhibin, beta A | 1 |
| 6329 | hsa-mir-548d-2 | 3624 | INHBA | inhibin, beta A | 1 |
| 6330 | hsa-mir-449b | 3624 | INHBA | inhibin, beta A | 1 |
| 6331 | hsa-mir-449c | 3624 | INHBA | inhibin, beta A | 1 |
| 6332 | hsa-mir-3065 | 3624 | INHBA | inhibin, beta A | 1 |
| 6333 | hsa-miR-1 | 224 | ALDH3A2 | aldehyde dehydrogenase 3 family, member A2 | 1 |
| 6334 | hsa-miR-133a | 224 | ALDH3A2 | aldehyde dehydrogenase 3 family, member A2 | 1 |
| 6335 | hsa-miR-186 | 224 | ALDH3A2 | aldehyde dehydrogenase 3 family, member A2 | 1 |
| 6336 | hsa-miR-195 | 224 | ALDH3A2 | aldehyde dehydrogenase 3 family, member A2 | 1 |
| 6337 | hsa-miR-326 | 224 | ALDH3A2 | aldehyde dehydrogenase 3 family, member A2 | 1 |
| 6338 | hsa-miR-449b | 224 | ALDH3A2 | aldehyde dehydrogenase 3 family, member A2 | 1 |
| 6339 | hsa-miR-497 | 224 | ALDH3A2 | aldehyde dehydrogenase 3 family, member A2 | 1 |
| 6340 | hsa-miR-550 | 224 | ALDH3A2 | aldehyde dehydrogenase 3 family, member A2 | 1 |
| 6341 | hsa-miR-551a | 224 | ALDH3A2 | aldehyde dehydrogenase 3 family, member A2 | 1 |
| 6342 | hsa-miR-562 | 224 | ALDH3A2 | aldehyde dehydrogenase 3 family, member A2 | 1 |
| 6343 | hsa-miR-575 | 224 | ALDH3A2 | aldehyde dehydrogenase 3 family, member A2 | 1 |
| 6344 | hsa-miR-579 | 224 | ALDH3A2 | aldehyde dehydrogenase 3 family, member A2 | 1 |
| 6345 | hsa-miR-608 | 224 | ALDH3A2 | aldehyde dehydrogenase 3 family, member A2 | 1 |
| 6346 | hsa-miR-620 | 224 | ALDH3A2 | aldehyde dehydrogenase 3 family, member A2 | 1 |
| 6347 | hsa-mir-22 | 224 | ALDH3A2 | aldehyde dehydrogenase 3 family, member A2 | 1 |
| 6348 | hsa-mir-10a | 224 | ALDH3A2 | aldehyde dehydrogenase 3 family, member A2 | 1 |
| 6349 | hsa-mir-186 | 224 | ALDH3A2 | aldehyde dehydrogenase 3 family, member A2 | 1 |
| 6350 | hsa-mir-155 | 224 | ALDH3A2 | aldehyde dehydrogenase 3 family, member A2 | 1 |
| 6351 | hsa-mir-550-1 | 224 | ALDH3A2 | aldehyde dehydrogenase 3 family, member A2 | 1 |
| 6354 | hsa-miR-10a | 51035 | UBXN1 | UBX domain protein 1 | 1 |
| 6355 | hsa-miR-152 | 51035 | UBXN1 | UBX domain protein 1 | 1 |
| 6356 | hsa-miR-26a | 51035 | UBXN1 | UBX domain protein 1 | 1 |
| 6357 | hsa-miR-26b | 51035 | UBXN1 | UBX domain protein 1 | 1 |
| 6358 | hsa-miR-31 | 51035 | UBXN1 | UBX domain protein 1 | 1 |
| 6359 | hsa-miR-340 | 51035 | UBXN1 | UBX domain protein 1 | 1 |
| 6360 | hsa-miR-423-5p | 51035 | UBXN1 | UBX domain protein 1 | 1 |
| 6361 | hsa-miR-449a | 51035 | UBXN1 | UBX domain protein 1 | 1 |
| 6362 | hsa-miR-490-5p | 51035 | UBXN1 | UBX domain protein 1 | 1 |
| 6363 | hsa-miR-499-5p | 51035 | UBXN1 | UBX domain protein 1 | 1 |
| 6364 | hsa-miR-548b-5p | 51035 | UBXN1 | UBX domain protein 1 | 1 |
| 6365 | hsa-miR-564 | 51035 | UBXN1 | UBX domain protein 1 | 1 |
| 6366 | hsa-miR-584 | 51035 | UBXN1 | UBX domain protein 1 | 1 |
| 6367 | hsa-miR-621 | 51035 | UBXN1 | UBX domain protein 1 | 1 |
| 6368 | hsa-miR-644 | 51035 | UBXN1 | UBX domain protein 1 | 1 |
| 6369 | hsa-miR-765 | 51035 | UBXN1 | UBX domain protein 1 | 1 |
| 6370 | hsa-miR-770-5p | 51035 | UBXN1 | UBX domain protein 1 | 1 |
| 6371 | hsa-miR-877 | 51035 | UBXN1 | UBX domain protein 1 | 1 |
| 6372 | hsa-miR-95 | 51035 | UBXN1 | UBX domain protein 1 | 1 |
| 6373 | hsa-mir-26a-1 | 51035 | UBXN1 | UBX domain protein 1 | 1 |
| 6374 | hsa-mir-26b | 51035 | UBXN1 | UBX domain protein 1 | 1 |
| 6375 | hsa-mir-31 | 51035 | UBXN1 | UBX domain protein 1 | 1 |
| 6376 | hsa-mir-194-2 | 51035 | UBXN1 | UBX domain protein 1 | 1 |
| 6377 | hsa-mir-340 | 51035 | UBXN1 | UBX domain protein 1 | 1 |
| 6378 | hsa-mir-490 | 51035 | UBXN1 | UBX domain protein 1 | 1 |
| 6379 | hsa-mir-548b | 51035 | UBXN1 | UBX domain protein 1 | 1 |
| 6380 | hsa-mir-449b | 51035 | UBXN1 | UBX domain protein 1 | 1 |
| 6381 | hsa-mir-449c | 51035 | UBXN1 | UBX domain protein 1 | 1 |
| 6382 | hsa-miR-10a | 6715 | SRD5A1 | steroid-5-alpha-reductase, alpha polypeptide 1 (3-oxo-5 alpha-steroid delta 4-dehydrogenase alpha 1) | 1 |
| 6383 | hsa-miR-152 | 6715 | SRD5A1 | steroid-5-alpha-reductase, alpha polypeptide 1 (3-oxo-5 alpha-steroid delta 4-dehydrogenase alpha 1) | 1 |
| 6384 | hsa-miR-26a | 6715 | SRD5A1 | steroid-5-alpha-reductase, alpha polypeptide 1 (3-oxo-5 alpha-steroid delta 4-dehydrogenase alpha 1) | 1 |
| 6385 | hsa-miR-26b | 6715 | SRD5A1 | steroid-5-alpha-reductase, alpha polypeptide 1 (3-oxo-5 alpha-steroid delta 4-dehydrogenase alpha 1) | 1 |
| 6386 | hsa-miR-31 | 6715 | SRD5A1 | steroid-5-alpha-reductase, alpha polypeptide 1 (3-oxo-5 alpha-steroid delta 4-dehydrogenase alpha 1) | 1 |
| 6387 | hsa-miR-338-5p | 6715 | SRD5A1 | steroid-5-alpha-reductase, alpha polypeptide 1 (3-oxo-5 alpha-steroid delta 4-dehydrogenase alpha 1) | 1 |
| 6388 | hsa-miR-340 | 6715 | SRD5A1 | steroid-5-alpha-reductase, alpha polypeptide 1 (3-oxo-5 alpha-steroid delta 4-dehydrogenase alpha 1) | 1 |
| 6389 | hsa-miR-423-5p | 6715 | SRD5A1 | steroid-5-alpha-reductase, alpha polypeptide 1 (3-oxo-5 alpha-steroid delta 4-dehydrogenase alpha 1) | 1 |
| 6390 | hsa-miR-449a | 6715 | SRD5A1 | steroid-5-alpha-reductase, alpha polypeptide 1 (3-oxo-5 alpha-steroid delta 4-dehydrogenase alpha 1) | 1 |
| 6391 | hsa-miR-490-5p | 6715 | SRD5A1 | steroid-5-alpha-reductase, alpha polypeptide 1 (3-oxo-5 alpha-steroid delta 4-dehydrogenase alpha 1) | 1 |
| 6392 | hsa-miR-499-5p | 6715 | SRD5A1 | steroid-5-alpha-reductase, alpha polypeptide 1 (3-oxo-5 alpha-steroid delta 4-dehydrogenase alpha 1) | 1 |
| 6393 | hsa-miR-564 | 6715 | SRD5A1 | steroid-5-alpha-reductase, alpha polypeptide 1 (3-oxo-5 alpha-steroid delta 4-dehydrogenase alpha 1) | 1 |
| 6394 | hsa-miR-621 | 6715 | SRD5A1 | steroid-5-alpha-reductase, alpha polypeptide 1 (3-oxo-5 alpha-steroid delta 4-dehydrogenase alpha 1) | 1 |
| 6395 | hsa-miR-634 | 6715 | SRD5A1 | steroid-5-alpha-reductase, alpha polypeptide 1 (3-oxo-5 alpha-steroid delta 4-dehydrogenase alpha 1) | 1 |
| 6396 | hsa-miR-644 | 6715 | SRD5A1 | steroid-5-alpha-reductase, alpha polypeptide 1 (3-oxo-5 alpha-steroid delta 4-dehydrogenase alpha 1) | 1 |
| 6397 | hsa-miR-653 | 6715 | SRD5A1 | steroid-5-alpha-reductase, alpha polypeptide 1 (3-oxo-5 alpha-steroid delta 4-dehydrogenase alpha 1) | 1 |
| 6398 | hsa-miR-657 | 6715 | SRD5A1 | steroid-5-alpha-reductase, alpha polypeptide 1 (3-oxo-5 alpha-steroid delta 4-dehydrogenase alpha 1) | 1 |
| 6399 | hsa-miR-765 | 6715 | SRD5A1 | steroid-5-alpha-reductase, alpha polypeptide 1 (3-oxo-5 alpha-steroid delta 4-dehydrogenase alpha 1) | 1 |
| 6400 | hsa-miR-770-5p | 6715 | SRD5A1 | steroid-5-alpha-reductase, alpha polypeptide 1 (3-oxo-5 alpha-steroid delta 4-dehydrogenase alpha 1) | 1 |
| 6401 | hsa-miR-877 | 6715 | SRD5A1 | steroid-5-alpha-reductase, alpha polypeptide 1 (3-oxo-5 alpha-steroid delta 4-dehydrogenase alpha 1) | 1 |
| 6402 | hsa-miR-95 | 6715 | SRD5A1 | steroid-5-alpha-reductase, alpha polypeptide 1 (3-oxo-5 alpha-steroid delta 4-dehydrogenase alpha 1) | 1 |
| 6403 | hsa-mir-26a-1 | 6715 | SRD5A1 | steroid-5-alpha-reductase, alpha polypeptide 1 (3-oxo-5 alpha-steroid delta 4-dehydrogenase alpha 1) | 1 |
| 6404 | hsa-mir-26b | 6715 | SRD5A1 | steroid-5-alpha-reductase, alpha polypeptide 1 (3-oxo-5 alpha-steroid delta 4-dehydrogenase alpha 1) | 1 |
| 6405 | hsa-mir-31 | 6715 | SRD5A1 | steroid-5-alpha-reductase, alpha polypeptide 1 (3-oxo-5 alpha-steroid delta 4-dehydrogenase alpha 1) | 1 |
| 6406 | hsa-mir-194-2 | 6715 | SRD5A1 | steroid-5-alpha-reductase, alpha polypeptide 1 (3-oxo-5 alpha-steroid delta 4-dehydrogenase alpha 1) | 1 |
| 6407 | hsa-mir-340 | 6715 | SRD5A1 | steroid-5-alpha-reductase, alpha polypeptide 1 (3-oxo-5 alpha-steroid delta 4-dehydrogenase alpha 1) | 1 |
| 6408 | hsa-mir-338 | 6715 | SRD5A1 | steroid-5-alpha-reductase, alpha polypeptide 1 (3-oxo-5 alpha-steroid delta 4-dehydrogenase alpha 1) | 1 |
| 6409 | hsa-mir-490 | 6715 | SRD5A1 | steroid-5-alpha-reductase, alpha polypeptide 1 (3-oxo-5 alpha-steroid delta 4-dehydrogenase alpha 1) | 1 |
| 6410 | hsa-mir-449b | 6715 | SRD5A1 | steroid-5-alpha-reductase, alpha polypeptide 1 (3-oxo-5 alpha-steroid delta 4-dehydrogenase alpha 1) | 1 |
| 6411 | hsa-mir-449c | 6715 | SRD5A1 | steroid-5-alpha-reductase, alpha polypeptide 1 (3-oxo-5 alpha-steroid delta 4-dehydrogenase alpha 1) | 1 |
| 6412 | hsa-mir-3065 | 6715 | SRD5A1 | steroid-5-alpha-reductase, alpha polypeptide 1 (3-oxo-5 alpha-steroid delta 4-dehydrogenase alpha 1) | 1 |
| 6413 | hsa-miR-338-5p | 5371 | PML | promyelocytic leukemia | 1 |
| 6414 | hsa-miR-657 | 5371 | PML | promyelocytic leukemia | 1 |
| 6415 | hsa-mir-338 | 5371 | PML | promyelocytic leukemia | 1 |
| 6416 | hsa-mir-3065 | 5371 | PML | promyelocytic leukemia | 1 |
| 6417 | hsa-miR-1 | 3784 | KCNQ1 | potassium voltage-gated channel, KQT-like subfamily, member 1 | 1 |
| 6418 | hsa-miR-133a | 3784 | KCNQ1 | potassium voltage-gated channel, KQT-like subfamily, member 1 | 1 |
| 6419 | hsa-miR-186 | 3784 | KCNQ1 | potassium voltage-gated channel, KQT-like subfamily, member 1 | 1 |
| 6420 | hsa-miR-326 | 3784 | KCNQ1 | potassium voltage-gated channel, KQT-like subfamily, member 1 | 1 |
| 6421 | hsa-miR-449b | 3784 | KCNQ1 | potassium voltage-gated channel, KQT-like subfamily, member 1 | 1 |
| 6422 | hsa-miR-551a | 3784 | KCNQ1 | potassium voltage-gated channel, KQT-like subfamily, member 1 | 1 |
| 6423 | hsa-miR-562 | 3784 | KCNQ1 | potassium voltage-gated channel, KQT-like subfamily, member 1 | 1 |
| 6424 | hsa-miR-575 | 3784 | KCNQ1 | potassium voltage-gated channel, KQT-like subfamily, member 1 | 1 |
| 6425 | hsa-miR-579 | 3784 | KCNQ1 | potassium voltage-gated channel, KQT-like subfamily, member 1 | 1 |
| 6426 | hsa-miR-608 | 3784 | KCNQ1 | potassium voltage-gated channel, KQT-like subfamily, member 1 | 1 |
| 6427 | hsa-miR-620 | 3784 | KCNQ1 | potassium voltage-gated channel, KQT-like subfamily, member 1 | 1 |
| 6428 | hsa-miR-637 | 3784 | KCNQ1 | potassium voltage-gated channel, KQT-like subfamily, member 1 | 1 |
| 6429 | hsa-miR-885-5p | 3784 | KCNQ1 | potassium voltage-gated channel, KQT-like subfamily, member 1 | 1 |
| 6430 | hsa-mir-22 | 3784 | KCNQ1 | potassium voltage-gated channel, KQT-like subfamily, member 1 | 1 |
| 6431 | hsa-mir-10a | 3784 | KCNQ1 | potassium voltage-gated channel, KQT-like subfamily, member 1 | 1 |
| 6432 | hsa-mir-186 | 3784 | KCNQ1 | potassium voltage-gated channel, KQT-like subfamily, member 1 | 1 |
| 6433 | hsa-mir-155 | 3784 | KCNQ1 | potassium voltage-gated channel, KQT-like subfamily, member 1 | 1 |
| 6434 | hsa-mir-499 | 3784 | KCNQ1 | potassium voltage-gated channel, KQT-like subfamily, member 1 | 1 |
| 6435 | hsa-mir-885 | 3784 | KCNQ1 | potassium voltage-gated channel, KQT-like subfamily, member 1 | 1 |
| 6436 | hsa-mir-877 | 3784 | KCNQ1 | potassium voltage-gated channel, KQT-like subfamily, member 1 | 1 |
| 6437 | hsa-miR-1 | 3077 | HFE | hemochromatosis | 1 |
| 6438 | hsa-miR-133a | 3077 | HFE | hemochromatosis | 1 |
| 6439 | hsa-miR-186 | 3077 | HFE | hemochromatosis | 1 |
| 6440 | hsa-miR-195 | 3077 | HFE | hemochromatosis | 1 |
| 6441 | hsa-miR-208a | 3077 | HFE | hemochromatosis | 1 |
| 6442 | hsa-miR-326 | 3077 | HFE | hemochromatosis | 1 |
| 6443 | hsa-miR-449b | 3077 | HFE | hemochromatosis | 1 |
| 6444 | hsa-miR-489 | 3077 | HFE | hemochromatosis | 1 |
| 6445 | hsa-miR-497 | 3077 | HFE | hemochromatosis | 1 |
| 6446 | hsa-miR-562 | 3077 | HFE | hemochromatosis | 1 |
| 6447 | hsa-miR-575 | 3077 | HFE | hemochromatosis | 1 |
| 6448 | hsa-miR-579 | 3077 | HFE | hemochromatosis | 1 |
| 6449 | hsa-miR-608 | 3077 | HFE | hemochromatosis | 1 |
| 6450 | hsa-miR-620 | 3077 | HFE | hemochromatosis | 1 |
| 6451 | hsa-miR-885-5p | 3077 | HFE | hemochromatosis | 1 |
| 6452 | hsa-let-7a-3 | 3077 | HFE | hemochromatosis | 1 |
| 6453 | hsa-let-7b | 3077 | HFE | hemochromatosis | 1 |
| 6454 | hsa-mir-22 | 3077 | HFE | hemochromatosis | 1 |
| 6455 | hsa-mir-10a | 3077 | HFE | hemochromatosis | 1 |
| 6456 | hsa-mir-186 | 3077 | HFE | hemochromatosis | 1 |
| 6457 | hsa-mir-155 | 3077 | HFE | hemochromatosis | 1 |
| 6458 | hsa-mir-499 | 3077 | HFE | hemochromatosis | 1 |
| 6459 | hsa-mir-885 | 3077 | HFE | hemochromatosis | 1 |
| 6460 | hsa-mir-877 | 3077 | HFE | hemochromatosis | 1 |
| 6461 | hsa-mir-2116 | 3077 | HFE | hemochromatosis | 1 |
| 6462 | hsa-mir-3190 | 3077 | HFE | hemochromatosis | 1 |
| 6463 | hsa-miR-1 | 10407 | SPAG11B | sperm associated antigen 11B | 1 |
| 6464 | hsa-miR-133a | 10407 | SPAG11B | sperm associated antigen 11B | 1 |
| 6465 | hsa-miR-186 | 10407 | SPAG11B | sperm associated antigen 11B | 1 |
| 6466 | hsa-miR-326 | 10407 | SPAG11B | sperm associated antigen 11B | 1 |
| 6467 | hsa-miR-449b | 10407 | SPAG11B | sperm associated antigen 11B | 1 |
| 6468 | hsa-miR-562 | 10407 | SPAG11B | sperm associated antigen 11B | 1 |
| 6469 | hsa-miR-575 | 10407 | SPAG11B | sperm associated antigen 11B | 1 |
| 6470 | hsa-miR-579 | 10407 | SPAG11B | sperm associated antigen 11B | 1 |
| 6471 | hsa-miR-608 | 10407 | SPAG11B | sperm associated antigen 11B | 1 |
| 6472 | hsa-miR-620 | 10407 | SPAG11B | sperm associated antigen 11B | 1 |
| 6473 | hsa-miR-637 | 10407 | SPAG11B | sperm associated antigen 11B | 1 |
| 6474 | hsa-miR-885-5p | 10407 | SPAG11B | sperm associated antigen 11B | 1 |
| 6475 | hsa-mir-22 | 10407 | SPAG11B | sperm associated antigen 11B | 1 |
| 6476 | hsa-mir-10a | 10407 | SPAG11B | sperm associated antigen 11B | 1 |
| 6477 | hsa-mir-186 | 10407 | SPAG11B | sperm associated antigen 11B | 1 |
| 6478 | hsa-mir-155 | 10407 | SPAG11B | sperm associated antigen 11B | 1 |
| 6479 | hsa-mir-423 | 10407 | SPAG11B | sperm associated antigen 11B | 1 |
| 6480 | hsa-mir-499 | 10407 | SPAG11B | sperm associated antigen 11B | 1 |
| 6481 | hsa-mir-885 | 10407 | SPAG11B | sperm associated antigen 11B | 1 |
| 6482 | hsa-mir-877 | 10407 | SPAG11B | sperm associated antigen 11B | 1 |
| 6483 | hsa-mir-3190 | 10407 | SPAG11B | sperm associated antigen 11B | 1 |
| 6484 | hsa-miR-1 | 3451 | IFNA17 | interferon, alpha 17 | 1 |
| 6485 | hsa-miR-133a | 3451 | IFNA17 | interferon, alpha 17 | 1 |
| 6486 | hsa-miR-186 | 3451 | IFNA17 | interferon, alpha 17 | 1 |
| 6487 | hsa-miR-208a | 3451 | IFNA17 | interferon, alpha 17 | 1 |
| 6488 | hsa-miR-326 | 3451 | IFNA17 | interferon, alpha 17 | 1 |
| 6489 | hsa-miR-449b | 3451 | IFNA17 | interferon, alpha 17 | 1 |
| 6490 | hsa-miR-550 | 3451 | IFNA17 | interferon, alpha 17 | 1 |
| 6491 | hsa-miR-575 | 3451 | IFNA17 | interferon, alpha 17 | 1 |
| 6492 | hsa-miR-579 | 3451 | IFNA17 | interferon, alpha 17 | 1 |
| 6493 | hsa-miR-608 | 3451 | IFNA17 | interferon, alpha 17 | 1 |
| 6494 | hsa-miR-620 | 3451 | IFNA17 | interferon, alpha 17 | 1 |
| 6495 | hsa-mir-22 | 3451 | IFNA17 | interferon, alpha 17 | 1 |
| 6496 | hsa-mir-10a | 3451 | IFNA17 | interferon, alpha 17 | 1 |
| 6497 | hsa-mir-186 | 3451 | IFNA17 | interferon, alpha 17 | 1 |
| 6498 | hsa-mir-155 | 3451 | IFNA17 | interferon, alpha 17 | 1 |
| 6499 | hsa-mir-423 | 3451 | IFNA17 | interferon, alpha 17 | 1 |
| 6500 | hsa-mir-499 | 3451 | IFNA17 | interferon, alpha 17 | 1 |
| 6501 | hsa-mir-550-1 | 3451 | IFNA17 | interferon, alpha 17 | 1 |
| 6502 | hsa-mir-877 | 3451 | IFNA17 | interferon, alpha 17 | 1 |
| 6503 | hsa-mir-2116 | 3451 | IFNA17 | interferon, alpha 17 | 1 |
| 6504 | hsa-miR-1 | 2776 | GNAQ | guanine nucleotide binding protein (G protein), q polypeptide | 1 |
| 6505 | hsa-miR-133a | 2776 | GNAQ | guanine nucleotide binding protein (G protein), q polypeptide | 1 |
| 6506 | hsa-miR-186 | 2776 | GNAQ | guanine nucleotide binding protein (G protein), q polypeptide | 1 |
| 6507 | hsa-miR-195 | 2776 | GNAQ | guanine nucleotide binding protein (G protein), q polypeptide | 1 |
| 6508 | hsa-miR-326 | 2776 | GNAQ | guanine nucleotide binding protein (G protein), q polypeptide | 1 |
| 6509 | hsa-miR-449b | 2776 | GNAQ | guanine nucleotide binding protein (G protein), q polypeptide | 1 |
| 6510 | hsa-miR-497 | 2776 | GNAQ | guanine nucleotide binding protein (G protein), q polypeptide | 1 |
| 6511 | hsa-miR-550 | 2776 | GNAQ | guanine nucleotide binding protein (G protein), q polypeptide | 1 |
| 6512 | hsa-miR-575 | 2776 | GNAQ | guanine nucleotide binding protein (G protein), q polypeptide | 1 |
| 6513 | hsa-miR-608 | 2776 | GNAQ | guanine nucleotide binding protein (G protein), q polypeptide | 1 |
| 6514 | hsa-miR-620 | 2776 | GNAQ | guanine nucleotide binding protein (G protein), q polypeptide | 1 |
| 6515 | hsa-let-7a-3 | 2776 | GNAQ | guanine nucleotide binding protein (G protein), q polypeptide | 1 |
| 6516 | hsa-let-7b | 2776 | GNAQ | guanine nucleotide binding protein (G protein), q polypeptide | 1 |
| 6517 | hsa-mir-22 | 2776 | GNAQ | guanine nucleotide binding protein (G protein), q polypeptide | 1 |
| 6518 | hsa-mir-10a | 2776 | GNAQ | guanine nucleotide binding protein (G protein), q polypeptide | 1 |
| 6519 | hsa-mir-186 | 2776 | GNAQ | guanine nucleotide binding protein (G protein), q polypeptide | 1 |
| 6520 | hsa-mir-155 | 2776 | GNAQ | guanine nucleotide binding protein (G protein), q polypeptide | 1 |
| 6521 | hsa-mir-499 | 2776 | GNAQ | guanine nucleotide binding protein (G protein), q polypeptide | 1 |
| 6522 | hsa-mir-550-1 | 2776 | GNAQ | guanine nucleotide binding protein (G protein), q polypeptide | 1 |
| 6523 | hsa-mir-877 | 2776 | GNAQ | guanine nucleotide binding protein (G protein), q polypeptide | 1 |
| 6524 | hsa-mir-2116 | 2776 | GNAQ | guanine nucleotide binding protein (G protein), q polypeptide | 1 |
| 6525 | hsa-mir-3190 | 2776 | GNAQ | guanine nucleotide binding protein (G protein), q polypeptide | 1 |
| 6526 | hsa-miR-1 | 64816 | CYP3A43 | cytochrome P450, family 3, subfamily A, polypeptide 43 | 1 |
| 6527 | hsa-miR-133a | 64816 | CYP3A43 | cytochrome P450, family 3, subfamily A, polypeptide 43 | 1 |
| 6528 | hsa-miR-186 | 64816 | CYP3A43 | cytochrome P450, family 3, subfamily A, polypeptide 43 | 1 |
| 6529 | hsa-miR-326 | 64816 | CYP3A43 | cytochrome P450, family 3, subfamily A, polypeptide 43 | 1 |
| 6530 | hsa-miR-449b | 64816 | CYP3A43 | cytochrome P450, family 3, subfamily A, polypeptide 43 | 1 |
| 6531 | hsa-miR-551a | 64816 | CYP3A43 | cytochrome P450, family 3, subfamily A, polypeptide 43 | 1 |
| 6532 | hsa-miR-562 | 64816 | CYP3A43 | cytochrome P450, family 3, subfamily A, polypeptide 43 | 1 |
| 6533 | hsa-miR-575 | 64816 | CYP3A43 | cytochrome P450, family 3, subfamily A, polypeptide 43 | 1 |
| 6534 | hsa-miR-579 | 64816 | CYP3A43 | cytochrome P450, family 3, subfamily A, polypeptide 43 | 1 |
| 6535 | hsa-miR-608 | 64816 | CYP3A43 | cytochrome P450, family 3, subfamily A, polypeptide 43 | 1 |
| 6536 | hsa-miR-620 | 64816 | CYP3A43 | cytochrome P450, family 3, subfamily A, polypeptide 43 | 1 |
| 6537 | hsa-miR-637 | 64816 | CYP3A43 | cytochrome P450, family 3, subfamily A, polypeptide 43 | 1 |
| 6538 | hsa-miR-885-5p | 64816 | CYP3A43 | cytochrome P450, family 3, subfamily A, polypeptide 43 | 1 |
| 6539 | hsa-mir-22 | 64816 | CYP3A43 | cytochrome P450, family 3, subfamily A, polypeptide 43 | 1 |
| 6540 | hsa-mir-10a | 64816 | CYP3A43 | cytochrome P450, family 3, subfamily A, polypeptide 43 | 1 |
| 6541 | hsa-mir-186 | 64816 | CYP3A43 | cytochrome P450, family 3, subfamily A, polypeptide 43 | 1 |
| 6542 | hsa-mir-155 | 64816 | CYP3A43 | cytochrome P450, family 3, subfamily A, polypeptide 43 | 1 |
| 6543 | hsa-mir-423 | 64816 | CYP3A43 | cytochrome P450, family 3, subfamily A, polypeptide 43 | 1 |
| 6544 | hsa-mir-499 | 64816 | CYP3A43 | cytochrome P450, family 3, subfamily A, polypeptide 43 | 1 |
| 6545 | hsa-mir-885 | 64816 | CYP3A43 | cytochrome P450, family 3, subfamily A, polypeptide 43 | 1 |
| 6546 | hsa-mir-877 | 64816 | CYP3A43 | cytochrome P450, family 3, subfamily A, polypeptide 43 | 1 |
| 6547 | hsa-mir-3190 | 64816 | CYP3A43 | cytochrome P450, family 3, subfamily A, polypeptide 43 | 1 |
| 6548 | hsa-miR-1 | 161 | AP2A2 | adaptor-related protein complex 2, alpha 2 subunit | 1 |
| 6549 | hsa-miR-133a | 161 | AP2A2 | adaptor-related protein complex 2, alpha 2 subunit | 1 |
| 6550 | hsa-miR-186 | 161 | AP2A2 | adaptor-related protein complex 2, alpha 2 subunit | 1 |
| 6551 | hsa-miR-208a | 161 | AP2A2 | adaptor-related protein complex 2, alpha 2 subunit | 1 |
| 6552 | hsa-miR-326 | 161 | AP2A2 | adaptor-related protein complex 2, alpha 2 subunit | 1 |
| 6553 | hsa-miR-449b | 161 | AP2A2 | adaptor-related protein complex 2, alpha 2 subunit | 1 |
| 6554 | hsa-miR-575 | 161 | AP2A2 | adaptor-related protein complex 2, alpha 2 subunit | 1 |
| 6555 | hsa-miR-579 | 161 | AP2A2 | adaptor-related protein complex 2, alpha 2 subunit | 1 |
| 6556 | hsa-miR-608 | 161 | AP2A2 | adaptor-related protein complex 2, alpha 2 subunit | 1 |
| 6557 | hsa-miR-620 | 161 | AP2A2 | adaptor-related protein complex 2, alpha 2 subunit | 1 |
| 6558 | hsa-miR-637 | 161 | AP2A2 | adaptor-related protein complex 2, alpha 2 subunit | 1 |
| 6559 | hsa-miR-885-5p | 161 | AP2A2 | adaptor-related protein complex 2, alpha 2 subunit | 1 |
| 6560 | hsa-mir-22 | 161 | AP2A2 | adaptor-related protein complex 2, alpha 2 subunit | 1 |
| 6561 | hsa-mir-10a | 161 | AP2A2 | adaptor-related protein complex 2, alpha 2 subunit | 1 |
| 6562 | hsa-mir-186 | 161 | AP2A2 | adaptor-related protein complex 2, alpha 2 subunit | 1 |
| 6563 | hsa-mir-155 | 161 | AP2A2 | adaptor-related protein complex 2, alpha 2 subunit | 1 |
| 6564 | hsa-mir-423 | 161 | AP2A2 | adaptor-related protein complex 2, alpha 2 subunit | 1 |
| 6565 | hsa-mir-499 | 161 | AP2A2 | adaptor-related protein complex 2, alpha 2 subunit | 1 |
| 6566 | hsa-mir-885 | 161 | AP2A2 | adaptor-related protein complex 2, alpha 2 subunit | 1 |
| 6567 | hsa-mir-877 | 161 | AP2A2 | adaptor-related protein complex 2, alpha 2 subunit | 1 |
| 6568 | hsa-mir-2116 | 161 | AP2A2 | adaptor-related protein complex 2, alpha 2 subunit | 1 |
| 6569 | hsa-mir-3190 | 161 | AP2A2 | adaptor-related protein complex 2, alpha 2 subunit | 1 |
| 6570 | hsa-miR-1 | 1087 | CEACAM7 | carcinoembryonic antigen-related cell adhesion molecule 7 | 1 |
| 6571 | hsa-miR-133a | 1087 | CEACAM7 | carcinoembryonic antigen-related cell adhesion molecule 7 | 1 |
| 6572 | hsa-miR-186 | 1087 | CEACAM7 | carcinoembryonic antigen-related cell adhesion molecule 7 | 1 |
| 6573 | hsa-miR-326 | 1087 | CEACAM7 | carcinoembryonic antigen-related cell adhesion molecule 7 | 1 |
| 6574 | hsa-miR-449b | 1087 | CEACAM7 | carcinoembryonic antigen-related cell adhesion molecule 7 | 1 |
| 6575 | hsa-miR-551a | 1087 | CEACAM7 | carcinoembryonic antigen-related cell adhesion molecule 7 | 1 |
| 6576 | hsa-miR-575 | 1087 | CEACAM7 | carcinoembryonic antigen-related cell adhesion molecule 7 | 1 |
| 6577 | hsa-miR-608 | 1087 | CEACAM7 | carcinoembryonic antigen-related cell adhesion molecule 7 | 1 |
| 6578 | hsa-miR-620 | 1087 | CEACAM7 | carcinoembryonic antigen-related cell adhesion molecule 7 | 1 |
| 6579 | hsa-mir-22 | 1087 | CEACAM7 | carcinoembryonic antigen-related cell adhesion molecule 7 | 1 |
| 6580 | hsa-mir-10a | 1087 | CEACAM7 | carcinoembryonic antigen-related cell adhesion molecule 7 | 1 |
| 6581 | hsa-mir-186 | 1087 | CEACAM7 | carcinoembryonic antigen-related cell adhesion molecule 7 | 1 |
| 6582 | hsa-mir-155 | 1087 | CEACAM7 | carcinoembryonic antigen-related cell adhesion molecule 7 | 1 |
| 6583 | hsa-mir-499 | 1087 | CEACAM7 | carcinoembryonic antigen-related cell adhesion molecule 7 | 1 |
| 6584 | hsa-mir-877 | 1087 | CEACAM7 | carcinoembryonic antigen-related cell adhesion molecule 7 | 1 |
| 6585 | hsa-mir-2116 | 1087 | CEACAM7 | carcinoembryonic antigen-related cell adhesion molecule 7 | 1 |
| 6586 | hsa-miR-152 | 57720 | GPR107 | G protein-coupled receptor 107 | 1 |
| 6587 | hsa-miR-26b | 57720 | GPR107 | G protein-coupled receptor 107 | 1 |
| 6588 | hsa-miR-31 | 57720 | GPR107 | G protein-coupled receptor 107 | 1 |
| 6589 | hsa-miR-338-5p | 57720 | GPR107 | G protein-coupled receptor 107 | 1 |
| 6590 | hsa-miR-340 | 57720 | GPR107 | G protein-coupled receptor 107 | 1 |
| 6591 | hsa-miR-383 | 57720 | GPR107 | G protein-coupled receptor 107 | 1 |
| 6592 | hsa-miR-423-5p | 57720 | GPR107 | G protein-coupled receptor 107 | 1 |
| 6593 | hsa-miR-449a | 57720 | GPR107 | G protein-coupled receptor 107 | 1 |
| 6594 | hsa-miR-621 | 57720 | GPR107 | G protein-coupled receptor 107 | 1 |
| 6595 | hsa-miR-657 | 57720 | GPR107 | G protein-coupled receptor 107 | 1 |
| 6596 | hsa-miR-765 | 57720 | GPR107 | G protein-coupled receptor 107 | 1 |
| 6597 | hsa-miR-770-5p | 57720 | GPR107 | G protein-coupled receptor 107 | 1 |
| 6598 | hsa-mir-26a-1 | 57720 | GPR107 | G protein-coupled receptor 107 | 1 |
| 6599 | hsa-mir-26b | 57720 | GPR107 | G protein-coupled receptor 107 | 1 |
| 6600 | hsa-mir-31 | 57720 | GPR107 | G protein-coupled receptor 107 | 1 |
| 6601 | hsa-mir-194-2 | 57720 | GPR107 | G protein-coupled receptor 107 | 1 |
| 6602 | hsa-mir-340 | 57720 | GPR107 | G protein-coupled receptor 107 | 1 |
| 6603 | hsa-mir-338 | 57720 | GPR107 | G protein-coupled receptor 107 | 1 |
| 6604 | hsa-mir-449b | 57720 | GPR107 | G protein-coupled receptor 107 | 1 |
| 6605 | hsa-mir-449c | 57720 | GPR107 | G protein-coupled receptor 107 | 1 |
| 6606 | hsa-mir-3065 | 57720 | GPR107 | G protein-coupled receptor 107 | 1 |
| 6607 | hsa-miR-10a | 26099 | C1orf144 | chromosome 1 open reading frame 144 | 1 |
| 6608 | hsa-miR-152 | 26099 | C1orf144 | chromosome 1 open reading frame 144 | 1 |
| 6609 | hsa-miR-26a | 26099 | C1orf144 | chromosome 1 open reading frame 144 | 1 |
| 6610 | hsa-miR-26b | 26099 | C1orf144 | chromosome 1 open reading frame 144 | 1 |
| 6611 | hsa-miR-31 | 26099 | C1orf144 | chromosome 1 open reading frame 144 | 1 |
| 6612 | hsa-miR-340 | 26099 | C1orf144 | chromosome 1 open reading frame 144 | 1 |
| 6613 | hsa-miR-449a | 26099 | C1orf144 | chromosome 1 open reading frame 144 | 1 |
| 6614 | hsa-miR-499-5p | 26099 | C1orf144 | chromosome 1 open reading frame 144 | 1 |
| 6615 | hsa-miR-548b-5p | 26099 | C1orf144 | chromosome 1 open reading frame 144 | 1 |
| 6616 | hsa-miR-564 | 26099 | C1orf144 | chromosome 1 open reading frame 144 | 1 |
| 6617 | hsa-miR-621 | 26099 | C1orf144 | chromosome 1 open reading frame 144 | 1 |
| 6618 | hsa-miR-634 | 26099 | C1orf144 | chromosome 1 open reading frame 144 | 1 |
| 6619 | hsa-miR-644 | 26099 | C1orf144 | chromosome 1 open reading frame 144 | 1 |
| 6620 | hsa-miR-653 | 26099 | C1orf144 | chromosome 1 open reading frame 144 | 1 |
| 6621 | hsa-miR-765 | 26099 | C1orf144 | chromosome 1 open reading frame 144 | 1 |
| 6622 | hsa-miR-770-5p | 26099 | C1orf144 | chromosome 1 open reading frame 144 | 1 |
| 6623 | hsa-miR-877 | 26099 | C1orf144 | chromosome 1 open reading frame 144 | 1 |
| 6624 | hsa-miR-95 | 26099 | C1orf144 | chromosome 1 open reading frame 144 | 1 |
| 6625 | hsa-mir-26a-1 | 26099 | C1orf144 | chromosome 1 open reading frame 144 | 1 |
| 6626 | hsa-mir-26b | 26099 | C1orf144 | chromosome 1 open reading frame 144 | 1 |
| 6627 | hsa-mir-31 | 26099 | C1orf144 | chromosome 1 open reading frame 144 | 1 |
| 6628 | hsa-mir-194-2 | 26099 | C1orf144 | chromosome 1 open reading frame 144 | 1 |
| 6629 | hsa-mir-340 | 26099 | C1orf144 | chromosome 1 open reading frame 144 | 1 |
| 6630 | hsa-mir-548b | 26099 | C1orf144 | chromosome 1 open reading frame 144 | 1 |
| 6631 | hsa-mir-449b | 26099 | C1orf144 | chromosome 1 open reading frame 144 | 1 |
| 6632 | hsa-mir-449c | 26099 | C1orf144 | chromosome 1 open reading frame 144 | 1 |
| 6633 | hsa-miR-26a | 960 | CD44 | CD44 molecule (Indian blood group) | 1 |
| 6634 | hsa-miR-490-5p | 960 | CD44 | CD44 molecule (Indian blood group) | 1 |
| 6635 | hsa-miR-499-5p | 960 | CD44 | CD44 molecule (Indian blood group) | 1 |
| 6636 | hsa-miR-564 | 960 | CD44 | CD44 molecule (Indian blood group) | 1 |
| 6637 | hsa-miR-634 | 960 | CD44 | CD44 molecule (Indian blood group) | 1 |
| 6638 | hsa-miR-95 | 960 | CD44 | CD44 molecule (Indian blood group) | 1 |
| 6639 | hsa-mir-490 | 960 | CD44 | CD44 molecule (Indian blood group) | 1 |
| 6640 | hsa-miR-10a | 58517 | RBM25 | RNA binding motif protein 25 | 1 |
| 6641 | hsa-miR-152 | 58517 | RBM25 | RNA binding motif protein 25 | 1 |
| 6642 | hsa-miR-26a | 58517 | RBM25 | RNA binding motif protein 25 | 1 |
| 6643 | hsa-miR-26b | 58517 | RBM25 | RNA binding motif protein 25 | 1 |
| 6644 | hsa-miR-31 | 58517 | RBM25 | RNA binding motif protein 25 | 1 |
| 6645 | hsa-miR-338-5p | 58517 | RBM25 | RNA binding motif protein 25 | 1 |
| 6646 | hsa-miR-340 | 58517 | RBM25 | RNA binding motif protein 25 | 1 |
| 6647 | hsa-miR-383 | 58517 | RBM25 | RNA binding motif protein 25 | 1 |
| 6648 | hsa-miR-449a | 58517 | RBM25 | RNA binding motif protein 25 | 1 |
| 6649 | hsa-miR-490-5p | 58517 | RBM25 | RNA binding motif protein 25 | 1 |
| 6650 | hsa-miR-499-5p | 58517 | RBM25 | RNA binding motif protein 25 | 1 |
| 6651 | hsa-miR-621 | 58517 | RBM25 | RNA binding motif protein 25 | 1 |
| 6652 | hsa-miR-653 | 58517 | RBM25 | RNA binding motif protein 25 | 1 |
| 6653 | hsa-miR-657 | 58517 | RBM25 | RNA binding motif protein 25 | 1 |
| 6654 | hsa-miR-765 | 58517 | RBM25 | RNA binding motif protein 25 | 1 |
| 6655 | hsa-miR-770-5p | 58517 | RBM25 | RNA binding motif protein 25 | 1 |
| 6656 | hsa-miR-877 | 58517 | RBM25 | RNA binding motif protein 25 | 1 |
| 6657 | hsa-mir-26a-1 | 58517 | RBM25 | RNA binding motif protein 25 | 1 |
| 6658 | hsa-mir-26b | 58517 | RBM25 | RNA binding motif protein 25 | 1 |
| 6659 | hsa-mir-31 | 58517 | RBM25 | RNA binding motif protein 25 | 1 |
| 6660 | hsa-mir-194-2 | 58517 | RBM25 | RNA binding motif protein 25 | 1 |
| 6661 | hsa-mir-340 | 58517 | RBM25 | RNA binding motif protein 25 | 1 |
| 6662 | hsa-mir-338 | 58517 | RBM25 | RNA binding motif protein 25 | 1 |
| 6663 | hsa-mir-490 | 58517 | RBM25 | RNA binding motif protein 25 | 1 |
| 6664 | hsa-mir-449b | 58517 | RBM25 | RNA binding motif protein 25 | 1 |
| 6665 | hsa-mir-449c | 58517 | RBM25 | RNA binding motif protein 25 | 1 |
| 6666 | hsa-mir-3065 | 58517 | RBM25 | RNA binding motif protein 25 | 1 |
| 6667 | hsa-miR-10a | 23070 | FTSJD2 | FtsJ methyltransferase domain containing 2 | 1 |
| 6668 | hsa-miR-152 | 23070 | FTSJD2 | FtsJ methyltransferase domain containing 2 | 1 |
| 6669 | hsa-miR-26a | 23070 | FTSJD2 | FtsJ methyltransferase domain containing 2 | 1 |
| 6670 | hsa-miR-26b | 23070 | FTSJD2 | FtsJ methyltransferase domain containing 2 | 1 |
| 6671 | hsa-miR-31 | 23070 | FTSJD2 | FtsJ methyltransferase domain containing 2 | 1 |
| 6672 | hsa-miR-338-5p | 23070 | FTSJD2 | FtsJ methyltransferase domain containing 2 | 1 |
| 6673 | hsa-miR-340 | 23070 | FTSJD2 | FtsJ methyltransferase domain containing 2 | 1 |
| 6674 | hsa-miR-423-5p | 23070 | FTSJD2 | FtsJ methyltransferase domain containing 2 | 1 |
| 6675 | hsa-miR-449a | 23070 | FTSJD2 | FtsJ methyltransferase domain containing 2 | 1 |
| 6676 | hsa-miR-499-5p | 23070 | FTSJD2 | FtsJ methyltransferase domain containing 2 | 1 |
| 6677 | hsa-miR-548b-5p | 23070 | FTSJD2 | FtsJ methyltransferase domain containing 2 | 1 |
| 6678 | hsa-miR-564 | 23070 | FTSJD2 | FtsJ methyltransferase domain containing 2 | 1 |
| 6679 | hsa-miR-621 | 23070 | FTSJD2 | FtsJ methyltransferase domain containing 2 | 1 |
| 6680 | hsa-miR-657 | 23070 | FTSJD2 | FtsJ methyltransferase domain containing 2 | 1 |
| 6681 | hsa-miR-765 | 23070 | FTSJD2 | FtsJ methyltransferase domain containing 2 | 1 |
| 6682 | hsa-miR-770-5p | 23070 | FTSJD2 | FtsJ methyltransferase domain containing 2 | 1 |
| 6683 | hsa-miR-877 | 23070 | FTSJD2 | FtsJ methyltransferase domain containing 2 | 1 |
| 6684 | hsa-mir-26a-1 | 23070 | FTSJD2 | FtsJ methyltransferase domain containing 2 | 1 |
| 6685 | hsa-mir-26b | 23070 | FTSJD2 | FtsJ methyltransferase domain containing 2 | 1 |
| 6686 | hsa-mir-31 | 23070 | FTSJD2 | FtsJ methyltransferase domain containing 2 | 1 |
| 6687 | hsa-mir-194-2 | 23070 | FTSJD2 | FtsJ methyltransferase domain containing 2 | 1 |
| 6688 | hsa-mir-340 | 23070 | FTSJD2 | FtsJ methyltransferase domain containing 2 | 1 |
| 6689 | hsa-mir-338 | 23070 | FTSJD2 | FtsJ methyltransferase domain containing 2 | 1 |
| 6690 | hsa-mir-548b | 23070 | FTSJD2 | FtsJ methyltransferase domain containing 2 | 1 |
| 6691 | hsa-mir-548d-2 | 23070 | FTSJD2 | FtsJ methyltransferase domain containing 2 | 1 |
| 6692 | hsa-mir-449b | 23070 | FTSJD2 | FtsJ methyltransferase domain containing 2 | 1 |
| 6693 | hsa-mir-449c | 23070 | FTSJD2 | FtsJ methyltransferase domain containing 2 | 1 |
| 6694 | hsa-mir-3065 | 23070 | FTSJD2 | FtsJ methyltransferase domain containing 2 | 1 |
| 6695 | hsa-miR-152 | 23358 | USP24 | ubiquitin specific peptidase 24 | 1 |
| 6696 | hsa-miR-26b | 23358 | USP24 | ubiquitin specific peptidase 24 | 1 |
| 6697 | hsa-miR-31 | 23358 | USP24 | ubiquitin specific peptidase 24 | 1 |
| 6698 | hsa-miR-338-5p | 23358 | USP24 | ubiquitin specific peptidase 24 | 1 |
| 6699 | hsa-miR-340 | 23358 | USP24 | ubiquitin specific peptidase 24 | 1 |
| 6700 | hsa-miR-383 | 23358 | USP24 | ubiquitin specific peptidase 24 | 1 |
| 6701 | hsa-miR-423-5p | 23358 | USP24 | ubiquitin specific peptidase 24 | 1 |
| 6702 | hsa-miR-449a | 23358 | USP24 | ubiquitin specific peptidase 24 | 1 |
| 6703 | hsa-miR-548b-5p | 23358 | USP24 | ubiquitin specific peptidase 24 | 1 |
| 6704 | hsa-miR-621 | 23358 | USP24 | ubiquitin specific peptidase 24 | 1 |
| 6705 | hsa-miR-657 | 23358 | USP24 | ubiquitin specific peptidase 24 | 1 |
| 6706 | hsa-miR-765 | 23358 | USP24 | ubiquitin specific peptidase 24 | 1 |
| 6707 | hsa-miR-770-5p | 23358 | USP24 | ubiquitin specific peptidase 24 | 1 |
| 6708 | hsa-mir-26a-1 | 23358 | USP24 | ubiquitin specific peptidase 24 | 1 |
| 6709 | hsa-mir-26b | 23358 | USP24 | ubiquitin specific peptidase 24 | 1 |
| 6710 | hsa-mir-31 | 23358 | USP24 | ubiquitin specific peptidase 24 | 1 |
| 6711 | hsa-mir-194-2 | 23358 | USP24 | ubiquitin specific peptidase 24 | 1 |
| 6712 | hsa-mir-340 | 23358 | USP24 | ubiquitin specific peptidase 24 | 1 |
| 6713 | hsa-mir-338 | 23358 | USP24 | ubiquitin specific peptidase 24 | 1 |
| 6714 | hsa-mir-548b | 23358 | USP24 | ubiquitin specific peptidase 24 | 1 |
| 6715 | hsa-mir-449b | 23358 | USP24 | ubiquitin specific peptidase 24 | 1 |
| 6716 | hsa-mir-449c | 23358 | USP24 | ubiquitin specific peptidase 24 | 1 |
| 6717 | hsa-mir-3065 | 23358 | USP24 | ubiquitin specific peptidase 24 | 1 |
| 6718 | hsa-miR-139-5p | 440026 | TMEM41B | transmembrane protein 41B | 1 |
| 6719 | hsa-miR-152 | 440026 | TMEM41B | transmembrane protein 41B | 1 |
| 6720 | hsa-miR-26b | 440026 | TMEM41B | transmembrane protein 41B | 1 |
| 6721 | hsa-miR-31 | 440026 | TMEM41B | transmembrane protein 41B | 1 |
| 6722 | hsa-miR-338-5p | 440026 | TMEM41B | transmembrane protein 41B | 1 |
| 6723 | hsa-miR-340 | 440026 | TMEM41B | transmembrane protein 41B | 1 |
| 6724 | hsa-miR-383 | 440026 | TMEM41B | transmembrane protein 41B | 1 |
| 6725 | hsa-miR-449a | 440026 | TMEM41B | transmembrane protein 41B | 1 |
| 6726 | hsa-miR-548b-5p | 440026 | TMEM41B | transmembrane protein 41B | 1 |
| 6727 | hsa-miR-621 | 440026 | TMEM41B | transmembrane protein 41B | 1 |
| 6728 | hsa-miR-657 | 440026 | TMEM41B | transmembrane protein 41B | 1 |
| 6729 | hsa-miR-663 | 440026 | TMEM41B | transmembrane protein 41B | 1 |
| 6730 | hsa-miR-765 | 440026 | TMEM41B | transmembrane protein 41B | 1 |
| 6731 | hsa-miR-770-5p | 440026 | TMEM41B | transmembrane protein 41B | 1 |
| 6732 | hsa-miR-877 | 440026 | TMEM41B | transmembrane protein 41B | 1 |
| 6733 | hsa-mir-26a-1 | 440026 | TMEM41B | transmembrane protein 41B | 1 |
| 6734 | hsa-mir-26b | 440026 | TMEM41B | transmembrane protein 41B | 1 |
| 6735 | hsa-mir-31 | 440026 | TMEM41B | transmembrane protein 41B | 1 |
| 6736 | hsa-mir-194-2 | 440026 | TMEM41B | transmembrane protein 41B | 1 |
| 6737 | hsa-mir-340 | 440026 | TMEM41B | transmembrane protein 41B | 1 |
| 6738 | hsa-mir-338 | 440026 | TMEM41B | transmembrane protein 41B | 1 |
| 6739 | hsa-mir-548b | 440026 | TMEM41B | transmembrane protein 41B | 1 |
| 6740 | hsa-mir-449b | 440026 | TMEM41B | transmembrane protein 41B | 1 |
| 6741 | hsa-mir-449c | 440026 | TMEM41B | transmembrane protein 41B | 1 |
| 6742 | hsa-mir-3065 | 440026 | TMEM41B | transmembrane protein 41B | 1 |
| 6743 | hsa-mir-548d-2 | 6509 | SLC1A4 | solute carrier family 1 (glutamate/neutral amino acid transporter), member 4 | 1 |
| 6744 | hsa-miR-10a | 4493 | MT1E | metallothionein 1E | 1 |
| 6745 | hsa-miR-152 | 4493 | MT1E | metallothionein 1E | 1 |
| 6746 | hsa-miR-26b | 4493 | MT1E | metallothionein 1E | 1 |
| 6747 | hsa-miR-31 | 4493 | MT1E | metallothionein 1E | 1 |
| 6748 | hsa-miR-338-5p | 4493 | MT1E | metallothionein 1E | 1 |
| 6749 | hsa-miR-340 | 4493 | MT1E | metallothionein 1E | 1 |
| 6750 | hsa-miR-383 | 4493 | MT1E | metallothionein 1E | 1 |
| 6751 | hsa-miR-449a | 4493 | MT1E | metallothionein 1E | 1 |
| 6752 | hsa-miR-490-5p | 4493 | MT1E | metallothionein 1E | 1 |
| 6753 | hsa-miR-499-5p | 4493 | MT1E | metallothionein 1E | 1 |
| 6754 | hsa-miR-621 | 4493 | MT1E | metallothionein 1E | 1 |
| 6755 | hsa-miR-653 | 4493 | MT1E | metallothionein 1E | 1 |
| 6756 | hsa-miR-657 | 4493 | MT1E | metallothionein 1E | 1 |
| 6757 | hsa-miR-765 | 4493 | MT1E | metallothionein 1E | 1 |
| 6758 | hsa-miR-770-5p | 4493 | MT1E | metallothionein 1E | 1 |
| 6759 | hsa-miR-877 | 4493 | MT1E | metallothionein 1E | 1 |
| 6760 | hsa-mir-26a-1 | 4493 | MT1E | metallothionein 1E | 1 |
| 6761 | hsa-mir-26b | 4493 | MT1E | metallothionein 1E | 1 |
| 6762 | hsa-mir-31 | 4493 | MT1E | metallothionein 1E | 1 |
| 6763 | hsa-mir-194-2 | 4493 | MT1E | metallothionein 1E | 1 |
| 6764 | hsa-mir-340 | 4493 | MT1E | metallothionein 1E | 1 |
| 6765 | hsa-mir-338 | 4493 | MT1E | metallothionein 1E | 1 |
| 6766 | hsa-mir-490 | 4493 | MT1E | metallothionein 1E | 1 |
| 6767 | hsa-mir-449b | 4493 | MT1E | metallothionein 1E | 1 |
| 6768 | hsa-mir-449c | 4493 | MT1E | metallothionein 1E | 1 |
| 6769 | hsa-mir-3065 | 4493 | MT1E | metallothionein 1E | 1 |
| 6770 | hsa-miR-10a | 3831 | KLC1 | kinesin light chain 1 | 1 |
| 6771 | hsa-miR-152 | 3831 | KLC1 | kinesin light chain 1 | 1 |
| 6772 | hsa-miR-26a | 3831 | KLC1 | kinesin light chain 1 | 1 |
| 6773 | hsa-miR-26b | 3831 | KLC1 | kinesin light chain 1 | 1 |
| 6774 | hsa-miR-31 | 3831 | KLC1 | kinesin light chain 1 | 1 |
| 6775 | hsa-miR-338-5p | 3831 | KLC1 | kinesin light chain 1 | 1 |
| 6776 | hsa-miR-340 | 3831 | KLC1 | kinesin light chain 1 | 1 |
| 6777 | hsa-miR-449a | 3831 | KLC1 | kinesin light chain 1 | 1 |
| 6778 | hsa-miR-499-5p | 3831 | KLC1 | kinesin light chain 1 | 1 |
| 6779 | hsa-miR-621 | 3831 | KLC1 | kinesin light chain 1 | 1 |
| 6780 | hsa-miR-657 | 3831 | KLC1 | kinesin light chain 1 | 1 |
| 6781 | hsa-miR-765 | 3831 | KLC1 | kinesin light chain 1 | 1 |
| 6782 | hsa-miR-770-5p | 3831 | KLC1 | kinesin light chain 1 | 1 |
| 6783 | hsa-miR-877 | 3831 | KLC1 | kinesin light chain 1 | 1 |
| 6784 | hsa-mir-26a-1 | 3831 | KLC1 | kinesin light chain 1 | 1 |
| 6785 | hsa-mir-26b | 3831 | KLC1 | kinesin light chain 1 | 1 |
| 6786 | hsa-mir-31 | 3831 | KLC1 | kinesin light chain 1 | 1 |
| 6787 | hsa-mir-194-2 | 3831 | KLC1 | kinesin light chain 1 | 1 |
| 6788 | hsa-mir-340 | 3831 | KLC1 | kinesin light chain 1 | 1 |
| 6789 | hsa-mir-338 | 3831 | KLC1 | kinesin light chain 1 | 1 |
| 6790 | hsa-mir-449b | 3831 | KLC1 | kinesin light chain 1 | 1 |
| 6791 | hsa-mir-449c | 3831 | KLC1 | kinesin light chain 1 | 1 |
| 6792 | hsa-mir-3065 | 3831 | KLC1 | kinesin light chain 1 | 1 |
| 6796 | hsa-miR-208a | 90480 | GADD45GIP1 | growth arrest and DNA-damage-inducible, gamma interacting protein 1 | 1 |
| 6801 | hsa-miR-579 | 90480 | GADD45GIP1 | growth arrest and DNA-damage-inducible, gamma interacting protein 1 | 1 |
| 6808 | hsa-mir-423 | 90480 | GADD45GIP1 | growth arrest and DNA-damage-inducible, gamma interacting protein 1 | 1 |
| 6809 | hsa-mir-499 | 90480 | GADD45GIP1 | growth arrest and DNA-damage-inducible, gamma interacting protein 1 | 1 |
| 6811 | hsa-mir-877 | 90480 | GADD45GIP1 | growth arrest and DNA-damage-inducible, gamma interacting protein 1 | 1 |
| 6812 | hsa-mir-2116 | 90480 | GADD45GIP1 | growth arrest and DNA-damage-inducible, gamma interacting protein 1 | 1 |
| 6813 | hsa-mir-3190 | 90480 | GADD45GIP1 | growth arrest and DNA-damage-inducible, gamma interacting protein 1 | 1 |
| 6814 | hsa-miR-10a | 56987 | BBX | bobby sox homolog (Drosophila) | 1 |
| 6815 | hsa-miR-152 | 56987 | BBX | bobby sox homolog (Drosophila) | 1 |
| 6816 | hsa-miR-26a | 56987 | BBX | bobby sox homolog (Drosophila) | 1 |
| 6817 | hsa-miR-26b | 56987 | BBX | bobby sox homolog (Drosophila) | 1 |
| 6818 | hsa-miR-31 | 56987 | BBX | bobby sox homolog (Drosophila) | 1 |
| 6819 | hsa-miR-338-5p | 56987 | BBX | bobby sox homolog (Drosophila) | 1 |
| 6820 | hsa-miR-423-5p | 56987 | BBX | bobby sox homolog (Drosophila) | 1 |
| 6821 | hsa-miR-449a | 56987 | BBX | bobby sox homolog (Drosophila) | 1 |
| 6822 | hsa-miR-499-5p | 56987 | BBX | bobby sox homolog (Drosophila) | 1 |
| 6823 | hsa-miR-548b-5p | 56987 | BBX | bobby sox homolog (Drosophila) | 1 |
| 6824 | hsa-miR-564 | 56987 | BBX | bobby sox homolog (Drosophila) | 1 |
| 6825 | hsa-miR-621 | 56987 | BBX | bobby sox homolog (Drosophila) | 1 |
| 6826 | hsa-miR-657 | 56987 | BBX | bobby sox homolog (Drosophila) | 1 |
| 6827 | hsa-miR-663 | 56987 | BBX | bobby sox homolog (Drosophila) | 1 |
| 6828 | hsa-miR-765 | 56987 | BBX | bobby sox homolog (Drosophila) | 1 |
| 6829 | hsa-miR-770-5p | 56987 | BBX | bobby sox homolog (Drosophila) | 1 |
| 6830 | hsa-miR-877 | 56987 | BBX | bobby sox homolog (Drosophila) | 1 |
| 6831 | hsa-mir-26a-1 | 56987 | BBX | bobby sox homolog (Drosophila) | 1 |
| 6832 | hsa-mir-26b | 56987 | BBX | bobby sox homolog (Drosophila) | 1 |
| 6833 | hsa-mir-31 | 56987 | BBX | bobby sox homolog (Drosophila) | 1 |
| 6834 | hsa-mir-194-2 | 56987 | BBX | bobby sox homolog (Drosophila) | 1 |
| 6835 | hsa-mir-338 | 56987 | BBX | bobby sox homolog (Drosophila) | 1 |
| 6836 | hsa-mir-548b | 56987 | BBX | bobby sox homolog (Drosophila) | 1 |
| 6837 | hsa-mir-548d-2 | 56987 | BBX | bobby sox homolog (Drosophila) | 1 |
| 6838 | hsa-mir-449b | 56987 | BBX | bobby sox homolog (Drosophila) | 1 |
| 6839 | hsa-mir-449c | 56987 | BBX | bobby sox homolog (Drosophila) | 1 |
| 6840 | hsa-mir-3065 | 56987 | BBX | bobby sox homolog (Drosophila) | 1 |
| 6841 | hsa-miR-1 | 25903 | OLFML2B | olfactomedin-like 2B | 1 |
| 6842 | hsa-miR-133a | 25903 | OLFML2B | olfactomedin-like 2B | 1 |
| 6843 | hsa-miR-186 | 25903 | OLFML2B | olfactomedin-like 2B | 1 |
| 6844 | hsa-miR-208a | 25903 | OLFML2B | olfactomedin-like 2B | 1 |
| 6845 | hsa-miR-326 | 25903 | OLFML2B | olfactomedin-like 2B | 1 |
| 6846 | hsa-miR-449b | 25903 | OLFML2B | olfactomedin-like 2B | 1 |
| 6847 | hsa-miR-575 | 25903 | OLFML2B | olfactomedin-like 2B | 1 |
| 6848 | hsa-miR-579 | 25903 | OLFML2B | olfactomedin-like 2B | 1 |
| 6849 | hsa-miR-608 | 25903 | OLFML2B | olfactomedin-like 2B | 1 |
| 6850 | hsa-miR-620 | 25903 | OLFML2B | olfactomedin-like 2B | 1 |
| 6851 | hsa-miR-637 | 25903 | OLFML2B | olfactomedin-like 2B | 1 |
| 6852 | hsa-miR-885-5p | 25903 | OLFML2B | olfactomedin-like 2B | 1 |
| 6853 | hsa-mir-22 | 25903 | OLFML2B | olfactomedin-like 2B | 1 |
| 6854 | hsa-mir-10a | 25903 | OLFML2B | olfactomedin-like 2B | 1 |
| 6855 | hsa-mir-186 | 25903 | OLFML2B | olfactomedin-like 2B | 1 |
| 6856 | hsa-mir-155 | 25903 | OLFML2B | olfactomedin-like 2B | 1 |
| 6857 | hsa-mir-423 | 25903 | OLFML2B | olfactomedin-like 2B | 1 |
| 6858 | hsa-mir-499 | 25903 | OLFML2B | olfactomedin-like 2B | 1 |
| 6859 | hsa-mir-885 | 25903 | OLFML2B | olfactomedin-like 2B | 1 |
| 6860 | hsa-mir-877 | 25903 | OLFML2B | olfactomedin-like 2B | 1 |
| 6861 | hsa-mir-2116 | 25903 | OLFML2B | olfactomedin-like 2B | 1 |
| 6862 | hsa-mir-3190 | 25903 | OLFML2B | olfactomedin-like 2B | 1 |
| 6863 | hsa-miR-10a | 23162 | MAPK8IP3 | mitogen-activated protein kinase 8 interacting protein 3 | 1 |
| 6864 | hsa-miR-152 | 23162 | MAPK8IP3 | mitogen-activated protein kinase 8 interacting protein 3 | 1 |
| 6865 | hsa-miR-26a | 23162 | MAPK8IP3 | mitogen-activated protein kinase 8 interacting protein 3 | 1 |
| 6866 | hsa-miR-26b | 23162 | MAPK8IP3 | mitogen-activated protein kinase 8 interacting protein 3 | 1 |
| 6867 | hsa-miR-31 | 23162 | MAPK8IP3 | mitogen-activated protein kinase 8 interacting protein 3 | 1 |
| 6868 | hsa-miR-340 | 23162 | MAPK8IP3 | mitogen-activated protein kinase 8 interacting protein 3 | 1 |
| 6869 | hsa-miR-449a | 23162 | MAPK8IP3 | mitogen-activated protein kinase 8 interacting protein 3 | 1 |
| 6870 | hsa-miR-490-5p | 23162 | MAPK8IP3 | mitogen-activated protein kinase 8 interacting protein 3 | 1 |
| 6871 | hsa-miR-499-5p | 23162 | MAPK8IP3 | mitogen-activated protein kinase 8 interacting protein 3 | 1 |
| 6872 | hsa-miR-548b-5p | 23162 | MAPK8IP3 | mitogen-activated protein kinase 8 interacting protein 3 | 1 |
| 6873 | hsa-miR-564 | 23162 | MAPK8IP3 | mitogen-activated protein kinase 8 interacting protein 3 | 1 |
| 6874 | hsa-miR-621 | 23162 | MAPK8IP3 | mitogen-activated protein kinase 8 interacting protein 3 | 1 |
| 6875 | hsa-miR-634 | 23162 | MAPK8IP3 | mitogen-activated protein kinase 8 interacting protein 3 | 1 |
| 6876 | hsa-miR-653 | 23162 | MAPK8IP3 | mitogen-activated protein kinase 8 interacting protein 3 | 1 |
| 6877 | hsa-miR-765 | 23162 | MAPK8IP3 | mitogen-activated protein kinase 8 interacting protein 3 | 1 |
| 6878 | hsa-miR-770-5p | 23162 | MAPK8IP3 | mitogen-activated protein kinase 8 interacting protein 3 | 1 |
| 6879 | hsa-miR-877 | 23162 | MAPK8IP3 | mitogen-activated protein kinase 8 interacting protein 3 | 1 |
| 6880 | hsa-miR-95 | 23162 | MAPK8IP3 | mitogen-activated protein kinase 8 interacting protein 3 | 1 |
| 6881 | hsa-mir-26a-1 | 23162 | MAPK8IP3 | mitogen-activated protein kinase 8 interacting protein 3 | 1 |
| 6882 | hsa-mir-26b | 23162 | MAPK8IP3 | mitogen-activated protein kinase 8 interacting protein 3 | 1 |
| 6883 | hsa-mir-31 | 23162 | MAPK8IP3 | mitogen-activated protein kinase 8 interacting protein 3 | 1 |
| 6884 | hsa-mir-194-2 | 23162 | MAPK8IP3 | mitogen-activated protein kinase 8 interacting protein 3 | 1 |
| 6885 | hsa-mir-340 | 23162 | MAPK8IP3 | mitogen-activated protein kinase 8 interacting protein 3 | 1 |
| 6886 | hsa-mir-490 | 23162 | MAPK8IP3 | mitogen-activated protein kinase 8 interacting protein 3 | 1 |
| 6887 | hsa-mir-548b | 23162 | MAPK8IP3 | mitogen-activated protein kinase 8 interacting protein 3 | 1 |
| 6888 | hsa-mir-449b | 23162 | MAPK8IP3 | mitogen-activated protein kinase 8 interacting protein 3 | 1 |
| 6889 | hsa-mir-449c | 23162 | MAPK8IP3 | mitogen-activated protein kinase 8 interacting protein 3 | 1 |
| 6890 | hsa-miR-1 | 6158 | RPL28 | ribosomal protein L28 | 1 |
| 6891 | hsa-miR-133a | 6158 | RPL28 | ribosomal protein L28 | 1 |
| 6892 | hsa-miR-186 | 6158 | RPL28 | ribosomal protein L28 | 1 |
| 6893 | hsa-miR-326 | 6158 | RPL28 | ribosomal protein L28 | 1 |
| 6894 | hsa-miR-449b | 6158 | RPL28 | ribosomal protein L28 | 1 |
| 6895 | hsa-miR-575 | 6158 | RPL28 | ribosomal protein L28 | 1 |
| 6896 | hsa-miR-579 | 6158 | RPL28 | ribosomal protein L28 | 1 |
| 6897 | hsa-miR-608 | 6158 | RPL28 | ribosomal protein L28 | 1 |
| 6898 | hsa-miR-620 | 6158 | RPL28 | ribosomal protein L28 | 1 |
| 6899 | hsa-mir-22 | 6158 | RPL28 | ribosomal protein L28 | 1 |
| 6900 | hsa-mir-10a | 6158 | RPL28 | ribosomal protein L28 | 1 |
| 6901 | hsa-mir-186 | 6158 | RPL28 | ribosomal protein L28 | 1 |
| 6902 | hsa-mir-155 | 6158 | RPL28 | ribosomal protein L28 | 1 |
| 6903 | hsa-mir-499 | 6158 | RPL28 | ribosomal protein L28 | 1 |
| 6904 | hsa-mir-877 | 6158 | RPL28 | ribosomal protein L28 | 1 |
| 6905 | hsa-mir-2116 | 6158 | RPL28 | ribosomal protein L28 | 1 |
| 6906 | hsa-mir-3190 | 6158 | RPL28 | ribosomal protein L28 | 1 |
| 6907 | hsa-miR-139-5p | 10154 | PLXNC1 | plexin C1 | 1 |
| 6908 | hsa-miR-31 | 10154 | PLXNC1 | plexin C1 | 1 |
| 6909 | hsa-miR-338-5p | 10154 | PLXNC1 | plexin C1 | 1 |
| 6910 | hsa-miR-383 | 10154 | PLXNC1 | plexin C1 | 1 |
| 6911 | hsa-miR-657 | 10154 | PLXNC1 | plexin C1 | 1 |
| 6912 | hsa-miR-663 | 10154 | PLXNC1 | plexin C1 | 1 |
| 6913 | hsa-mir-31 | 10154 | PLXNC1 | plexin C1 | 1 |
| 6914 | hsa-mir-338 | 10154 | PLXNC1 | plexin C1 | 1 |
| 6915 | hsa-mir-3065 | 10154 | PLXNC1 | plexin C1 | 1 |
| 6916 | hsa-miR-10a | 100288387 | LOC100288387 | similar to c-jun | 1 |
| 6917 | hsa-miR-1228\* | 100288387 | LOC100288387 | similar to c-jun | 1 |
| 6918 | hsa-miR-152 | 100288387 | LOC100288387 | similar to c-jun | 1 |
| 6919 | hsa-miR-26a | 100288387 | LOC100288387 | similar to c-jun | 1 |
| 6920 | hsa-miR-26b | 100288387 | LOC100288387 | similar to c-jun | 1 |
| 6921 | hsa-miR-31 | 100288387 | LOC100288387 | similar to c-jun | 1 |
| 6922 | hsa-miR-338-5p | 100288387 | LOC100288387 | similar to c-jun | 1 |
| 6923 | hsa-miR-340 | 100288387 | LOC100288387 | similar to c-jun | 1 |
| 6924 | hsa-miR-449a | 100288387 | LOC100288387 | similar to c-jun | 1 |
| 6925 | hsa-miR-499-5p | 100288387 | LOC100288387 | similar to c-jun | 1 |
| 6926 | hsa-miR-548b-5p | 100288387 | LOC100288387 | similar to c-jun | 1 |
| 6927 | hsa-miR-584 | 100288387 | LOC100288387 | similar to c-jun | 1 |
| 6928 | hsa-miR-621 | 100288387 | LOC100288387 | similar to c-jun | 1 |
| 6929 | hsa-miR-634 | 100288387 | LOC100288387 | similar to c-jun | 1 |
| 6930 | hsa-miR-644 | 100288387 | LOC100288387 | similar to c-jun | 1 |
| 6931 | hsa-miR-653 | 100288387 | LOC100288387 | similar to c-jun | 1 |
| 6932 | hsa-miR-657 | 100288387 | LOC100288387 | similar to c-jun | 1 |
| 6933 | hsa-miR-765 | 100288387 | LOC100288387 | similar to c-jun | 1 |
| 6934 | hsa-miR-770-5p | 100288387 | LOC100288387 | similar to c-jun | 1 |
| 6935 | hsa-miR-877 | 100288387 | LOC100288387 | similar to c-jun | 1 |
| 6936 | hsa-mir-26a-1 | 100288387 | LOC100288387 | similar to c-jun | 1 |
| 6937 | hsa-mir-26b | 100288387 | LOC100288387 | similar to c-jun | 1 |
| 6938 | hsa-mir-31 | 100288387 | LOC100288387 | similar to c-jun | 1 |
| 6939 | hsa-mir-194-2 | 100288387 | LOC100288387 | similar to c-jun | 1 |
| 6940 | hsa-mir-340 | 100288387 | LOC100288387 | similar to c-jun | 1 |
| 6941 | hsa-mir-338 | 100288387 | LOC100288387 | similar to c-jun | 1 |
| 6942 | hsa-mir-548b | 100288387 | LOC100288387 | similar to c-jun | 1 |
| 6943 | hsa-mir-449b | 100288387 | LOC100288387 | similar to c-jun | 1 |
| 6944 | hsa-mir-449c | 100288387 | LOC100288387 | similar to c-jun | 1 |
| 6945 | hsa-mir-3065 | 100288387 | LOC100288387 | similar to c-jun | 1 |
| 6946 | hsa-miR-1 | 11079 | RER1 | RER1 retention in endoplasmic reticulum 1 homolog (S. cerevisiae) | 1 |
| 6947 | hsa-miR-133a | 11079 | RER1 | RER1 retention in endoplasmic reticulum 1 homolog (S. cerevisiae) | 1 |
| 6948 | hsa-miR-186 | 11079 | RER1 | RER1 retention in endoplasmic reticulum 1 homolog (S. cerevisiae) | 1 |
| 6949 | hsa-miR-208a | 11079 | RER1 | RER1 retention in endoplasmic reticulum 1 homolog (S. cerevisiae) | 1 |
| 6950 | hsa-miR-326 | 11079 | RER1 | RER1 retention in endoplasmic reticulum 1 homolog (S. cerevisiae) | 1 |
| 6951 | hsa-miR-449b | 11079 | RER1 | RER1 retention in endoplasmic reticulum 1 homolog (S. cerevisiae) | 1 |
| 6952 | hsa-miR-489 | 11079 | RER1 | RER1 retention in endoplasmic reticulum 1 homolog (S. cerevisiae) | 1 |
| 6953 | hsa-miR-562 | 11079 | RER1 | RER1 retention in endoplasmic reticulum 1 homolog (S. cerevisiae) | 1 |
| 6954 | hsa-miR-575 | 11079 | RER1 | RER1 retention in endoplasmic reticulum 1 homolog (S. cerevisiae) | 1 |
| 6955 | hsa-miR-579 | 11079 | RER1 | RER1 retention in endoplasmic reticulum 1 homolog (S. cerevisiae) | 1 |
| 6956 | hsa-miR-608 | 11079 | RER1 | RER1 retention in endoplasmic reticulum 1 homolog (S. cerevisiae) | 1 |
| 6957 | hsa-miR-620 | 11079 | RER1 | RER1 retention in endoplasmic reticulum 1 homolog (S. cerevisiae) | 1 |
| 6958 | hsa-miR-637 | 11079 | RER1 | RER1 retention in endoplasmic reticulum 1 homolog (S. cerevisiae) | 1 |
| 6959 | hsa-miR-885-5p | 11079 | RER1 | RER1 retention in endoplasmic reticulum 1 homolog (S. cerevisiae) | 1 |
| 6960 | hsa-mir-22 | 11079 | RER1 | RER1 retention in endoplasmic reticulum 1 homolog (S. cerevisiae) | 1 |
| 6961 | hsa-mir-10a | 11079 | RER1 | RER1 retention in endoplasmic reticulum 1 homolog (S. cerevisiae) | 1 |
| 6962 | hsa-mir-186 | 11079 | RER1 | RER1 retention in endoplasmic reticulum 1 homolog (S. cerevisiae) | 1 |
| 6963 | hsa-mir-155 | 11079 | RER1 | RER1 retention in endoplasmic reticulum 1 homolog (S. cerevisiae) | 1 |
| 6964 | hsa-mir-423 | 11079 | RER1 | RER1 retention in endoplasmic reticulum 1 homolog (S. cerevisiae) | 1 |
| 6965 | hsa-mir-499 | 11079 | RER1 | RER1 retention in endoplasmic reticulum 1 homolog (S. cerevisiae) | 1 |
| 6966 | hsa-mir-885 | 11079 | RER1 | RER1 retention in endoplasmic reticulum 1 homolog (S. cerevisiae) | 1 |
| 6967 | hsa-mir-877 | 11079 | RER1 | RER1 retention in endoplasmic reticulum 1 homolog (S. cerevisiae) | 1 |
| 6968 | hsa-mir-2116 | 11079 | RER1 | RER1 retention in endoplasmic reticulum 1 homolog (S. cerevisiae) | 1 |
| 6969 | hsa-mir-3190 | 11079 | RER1 | RER1 retention in endoplasmic reticulum 1 homolog (S. cerevisiae) | 1 |
| 6970 | hsa-miR-10a | 91966 | CXorf40A | chromosome X open reading frame 40A | 1 |
| 6971 | hsa-miR-139-5p | 91966 | CXorf40A | chromosome X open reading frame 40A | 1 |
| 6972 | hsa-miR-152 | 91966 | CXorf40A | chromosome X open reading frame 40A | 1 |
| 6973 | hsa-miR-31 | 91966 | CXorf40A | chromosome X open reading frame 40A | 1 |
| 6974 | hsa-miR-338-5p | 91966 | CXorf40A | chromosome X open reading frame 40A | 1 |
| 6975 | hsa-miR-340 | 91966 | CXorf40A | chromosome X open reading frame 40A | 1 |
| 6976 | hsa-miR-383 | 91966 | CXorf40A | chromosome X open reading frame 40A | 1 |
| 6977 | hsa-miR-449a | 91966 | CXorf40A | chromosome X open reading frame 40A | 1 |
| 6978 | hsa-miR-499-5p | 91966 | CXorf40A | chromosome X open reading frame 40A | 1 |
| 6979 | hsa-miR-621 | 91966 | CXorf40A | chromosome X open reading frame 40A | 1 |
| 6980 | hsa-miR-653 | 91966 | CXorf40A | chromosome X open reading frame 40A | 1 |
| 6981 | hsa-miR-657 | 91966 | CXorf40A | chromosome X open reading frame 40A | 1 |
| 6982 | hsa-miR-765 | 91966 | CXorf40A | chromosome X open reading frame 40A | 1 |
| 6983 | hsa-miR-770-5p | 91966 | CXorf40A | chromosome X open reading frame 40A | 1 |
| 6984 | hsa-miR-877 | 91966 | CXorf40A | chromosome X open reading frame 40A | 1 |
| 6985 | hsa-mir-26a-1 | 91966 | CXorf40A | chromosome X open reading frame 40A | 1 |
| 6986 | hsa-mir-31 | 91966 | CXorf40A | chromosome X open reading frame 40A | 1 |
| 6987 | hsa-mir-194-2 | 91966 | CXorf40A | chromosome X open reading frame 40A | 1 |
| 6988 | hsa-mir-340 | 91966 | CXorf40A | chromosome X open reading frame 40A | 1 |
| 6989 | hsa-mir-338 | 91966 | CXorf40A | chromosome X open reading frame 40A | 1 |
| 6990 | hsa-mir-449b | 91966 | CXorf40A | chromosome X open reading frame 40A | 1 |
| 6991 | hsa-mir-449c | 91966 | CXorf40A | chromosome X open reading frame 40A | 1 |
| 6992 | hsa-mir-3065 | 91966 | CXorf40A | chromosome X open reading frame 40A | 1 |
| 6993 | hsa-miR-10a | 26118 | WSB1 | WD repeat and SOCS box-containing 1 | 1 |
| 6994 | hsa-miR-152 | 26118 | WSB1 | WD repeat and SOCS box-containing 1 | 1 |
| 6995 | hsa-miR-26a | 26118 | WSB1 | WD repeat and SOCS box-containing 1 | 1 |
| 6996 | hsa-miR-26b | 26118 | WSB1 | WD repeat and SOCS box-containing 1 | 1 |
| 6997 | hsa-miR-340 | 26118 | WSB1 | WD repeat and SOCS box-containing 1 | 1 |
| 6998 | hsa-miR-449a | 26118 | WSB1 | WD repeat and SOCS box-containing 1 | 1 |
| 6999 | hsa-miR-490-5p | 26118 | WSB1 | WD repeat and SOCS box-containing 1 | 1 |
| 7000 | hsa-miR-499-5p | 26118 | WSB1 | WD repeat and SOCS box-containing 1 | 1 |
| 7001 | hsa-miR-548b-5p | 26118 | WSB1 | WD repeat and SOCS box-containing 1 | 1 |
| 7002 | hsa-miR-564 | 26118 | WSB1 | WD repeat and SOCS box-containing 1 | 1 |
| 7003 | hsa-miR-584 | 26118 | WSB1 | WD repeat and SOCS box-containing 1 | 1 |
| 7004 | hsa-miR-621 | 26118 | WSB1 | WD repeat and SOCS box-containing 1 | 1 |
| 7005 | hsa-miR-634 | 26118 | WSB1 | WD repeat and SOCS box-containing 1 | 1 |
| 7006 | hsa-miR-644 | 26118 | WSB1 | WD repeat and SOCS box-containing 1 | 1 |
| 7007 | hsa-miR-765 | 26118 | WSB1 | WD repeat and SOCS box-containing 1 | 1 |
| 7008 | hsa-miR-770-5p | 26118 | WSB1 | WD repeat and SOCS box-containing 1 | 1 |
| 7009 | hsa-miR-877 | 26118 | WSB1 | WD repeat and SOCS box-containing 1 | 1 |
| 7010 | hsa-mir-26a-1 | 26118 | WSB1 | WD repeat and SOCS box-containing 1 | 1 |
| 7011 | hsa-mir-26b | 26118 | WSB1 | WD repeat and SOCS box-containing 1 | 1 |
| 7012 | hsa-mir-194-2 | 26118 | WSB1 | WD repeat and SOCS box-containing 1 | 1 |
| 7013 | hsa-mir-340 | 26118 | WSB1 | WD repeat and SOCS box-containing 1 | 1 |
| 7014 | hsa-mir-490 | 26118 | WSB1 | WD repeat and SOCS box-containing 1 | 1 |
| 7015 | hsa-mir-548b | 26118 | WSB1 | WD repeat and SOCS box-containing 1 | 1 |
| 7016 | hsa-mir-449b | 26118 | WSB1 | WD repeat and SOCS box-containing 1 | 1 |
| 7017 | hsa-mir-449c | 26118 | WSB1 | WD repeat and SOCS box-containing 1 | 1 |
| 7018 | hsa-miR-499-5p | 22902 | RUFY3 | RUN and FYVE domain containing 3 | 1 |
| 7019 | hsa-miR-584 | 22902 | RUFY3 | RUN and FYVE domain containing 3 | 1 |
| 7020 | hsa-miR-423-5p | 22902 | RUFY3 | RUN and FYVE domain containing 3 | 1 |
| 7021 | hsa-miR-10a | 6651 | SON | SON DNA binding protein | 1 |
| 7022 | hsa-miR-139-5p | 6651 | SON | SON DNA binding protein | 1 |
| 7023 | hsa-miR-152 | 6651 | SON | SON DNA binding protein | 1 |
| 7024 | hsa-miR-26a | 6651 | SON | SON DNA binding protein | 1 |
| 7025 | hsa-miR-26b | 6651 | SON | SON DNA binding protein | 1 |
| 7026 | hsa-miR-338-5p | 6651 | SON | SON DNA binding protein | 1 |
| 7027 | hsa-miR-340 | 6651 | SON | SON DNA binding protein | 1 |
| 7028 | hsa-miR-449a | 6651 | SON | SON DNA binding protein | 1 |
| 7029 | hsa-miR-499-5p | 6651 | SON | SON DNA binding protein | 1 |
| 7030 | hsa-miR-548b-5p | 6651 | SON | SON DNA binding protein | 1 |
| 7031 | hsa-miR-621 | 6651 | SON | SON DNA binding protein | 1 |
| 7032 | hsa-miR-657 | 6651 | SON | SON DNA binding protein | 1 |
| 7033 | hsa-miR-663 | 6651 | SON | SON DNA binding protein | 1 |
| 7034 | hsa-miR-765 | 6651 | SON | SON DNA binding protein | 1 |
| 7035 | hsa-miR-770-5p | 6651 | SON | SON DNA binding protein | 1 |
| 7036 | hsa-miR-877 | 6651 | SON | SON DNA binding protein | 1 |
| 7037 | hsa-mir-26a-1 | 6651 | SON | SON DNA binding protein | 1 |
| 7038 | hsa-mir-26b | 6651 | SON | SON DNA binding protein | 1 |
| 7039 | hsa-mir-194-2 | 6651 | SON | SON DNA binding protein | 1 |
| 7040 | hsa-mir-340 | 6651 | SON | SON DNA binding protein | 1 |
| 7041 | hsa-mir-338 | 6651 | SON | SON DNA binding protein | 1 |
| 7042 | hsa-mir-548b | 6651 | SON | SON DNA binding protein | 1 |
| 7043 | hsa-mir-548d-2 | 6651 | SON | SON DNA binding protein | 1 |
| 7044 | hsa-mir-449b | 6651 | SON | SON DNA binding protein | 1 |
| 7045 | hsa-mir-449c | 6651 | SON | SON DNA binding protein | 1 |
| 7046 | hsa-mir-3065 | 6651 | SON | SON DNA binding protein | 1 |
| 7047 | hsa-miR-10a | 9470 | EIF4E2 | eukaryotic translation initiation factor 4E family member 2 | 1 |
| 7048 | hsa-miR-139-5p | 9470 | EIF4E2 | eukaryotic translation initiation factor 4E family member 2 | 1 |
| 7049 | hsa-miR-26a | 9470 | EIF4E2 | eukaryotic translation initiation factor 4E family member 2 | 1 |
| 7050 | hsa-miR-26b | 9470 | EIF4E2 | eukaryotic translation initiation factor 4E family member 2 | 1 |
| 7051 | hsa-miR-31 | 9470 | EIF4E2 | eukaryotic translation initiation factor 4E family member 2 | 1 |
| 7052 | hsa-miR-338-5p | 9470 | EIF4E2 | eukaryotic translation initiation factor 4E family member 2 | 1 |
| 7053 | hsa-miR-340 | 9470 | EIF4E2 | eukaryotic translation initiation factor 4E family member 2 | 1 |
| 7054 | hsa-miR-423-5p | 9470 | EIF4E2 | eukaryotic translation initiation factor 4E family member 2 | 1 |
| 7055 | hsa-miR-449a | 9470 | EIF4E2 | eukaryotic translation initiation factor 4E family member 2 | 1 |
| 7056 | hsa-miR-499-5p | 9470 | EIF4E2 | eukaryotic translation initiation factor 4E family member 2 | 1 |
| 7057 | hsa-miR-548b-5p | 9470 | EIF4E2 | eukaryotic translation initiation factor 4E family member 2 | 1 |
| 7058 | hsa-miR-564 | 9470 | EIF4E2 | eukaryotic translation initiation factor 4E family member 2 | 1 |
| 7059 | hsa-miR-621 | 9470 | EIF4E2 | eukaryotic translation initiation factor 4E family member 2 | 1 |
| 7060 | hsa-miR-657 | 9470 | EIF4E2 | eukaryotic translation initiation factor 4E family member 2 | 1 |
| 7061 | hsa-miR-765 | 9470 | EIF4E2 | eukaryotic translation initiation factor 4E family member 2 | 1 |
| 7062 | hsa-miR-770-5p | 9470 | EIF4E2 | eukaryotic translation initiation factor 4E family member 2 | 1 |
| 7063 | hsa-miR-877 | 9470 | EIF4E2 | eukaryotic translation initiation factor 4E family member 2 | 1 |
| 7064 | hsa-mir-26a-1 | 9470 | EIF4E2 | eukaryotic translation initiation factor 4E family member 2 | 1 |
| 7065 | hsa-mir-26b | 9470 | EIF4E2 | eukaryotic translation initiation factor 4E family member 2 | 1 |
| 7066 | hsa-mir-31 | 9470 | EIF4E2 | eukaryotic translation initiation factor 4E family member 2 | 1 |
| 7067 | hsa-mir-194-2 | 9470 | EIF4E2 | eukaryotic translation initiation factor 4E family member 2 | 1 |
| 7068 | hsa-mir-340 | 9470 | EIF4E2 | eukaryotic translation initiation factor 4E family member 2 | 1 |
| 7069 | hsa-mir-338 | 9470 | EIF4E2 | eukaryotic translation initiation factor 4E family member 2 | 1 |
| 7070 | hsa-mir-548b | 9470 | EIF4E2 | eukaryotic translation initiation factor 4E family member 2 | 1 |
| 7071 | hsa-mir-548d-2 | 9470 | EIF4E2 | eukaryotic translation initiation factor 4E family member 2 | 1 |
| 7072 | hsa-mir-449b | 9470 | EIF4E2 | eukaryotic translation initiation factor 4E family member 2 | 1 |
| 7073 | hsa-mir-449c | 9470 | EIF4E2 | eukaryotic translation initiation factor 4E family member 2 | 1 |
| 7074 | hsa-mir-3065 | 9470 | EIF4E2 | eukaryotic translation initiation factor 4E family member 2 | 1 |
| 7075 | hsa-miR-26a | 10435 | CDC42EP2 | CDC42 effector protein (Rho GTPase binding) 2 | 1 |
| 7076 | hsa-mir-548d-2 | 10435 | CDC42EP2 | CDC42 effector protein (Rho GTPase binding) 2 | 1 |
| 7077 | hsa-miR-489 | 3693 | ITGB5 | integrin, beta 5 | 1 |
| 7078 | hsa-miR-620 | 3693 | ITGB5 | integrin, beta 5 | 1 |
| 7079 | hsa-miR-653 | 8519 | IFITM1 | interferon induced transmembrane protein 1 (9-27) | 1 |
| 7083 | hsa-miR-195 | 29937 | NENF | neuron derived neurotrophic factor | 1 |
| 7086 | hsa-miR-497 | 29937 | NENF | neuron derived neurotrophic factor | 1 |
| 7090 | hsa-miR-620 | 29937 | NENF | neuron derived neurotrophic factor | 1 |
| 7091 | hsa-let-7a-3 | 29937 | NENF | neuron derived neurotrophic factor | 1 |
| 7092 | hsa-let-7b | 29937 | NENF | neuron derived neurotrophic factor | 1 |
| 7099 | hsa-mir-2116 | 29937 | NENF | neuron derived neurotrophic factor | 1 |
| 7102 | hsa-miR-1228\* | 8992 | ATP6V0E1 | ATPase, H+ transporting, lysosomal 9kDa, V0 subunit e1 | 1 |
| 7104 | hsa-miR-26a | 8992 | ATP6V0E1 | ATPase, H+ transporting, lysosomal 9kDa, V0 subunit e1 | 1 |
| 7108 | hsa-miR-423-5p | 8992 | ATP6V0E1 | ATPase, H+ transporting, lysosomal 9kDa, V0 subunit e1 | 1 |
| 7112 | hsa-miR-564 | 8992 | ATP6V0E1 | ATPase, H+ transporting, lysosomal 9kDa, V0 subunit e1 | 1 |
| 7124 | hsa-mir-548d-2 | 8992 | ATP6V0E1 | ATPase, H+ transporting, lysosomal 9kDa, V0 subunit e1 | 1 |
| 7128 | hsa-miR-1 | 5303 | PIN4 | protein (peptidylprolyl cis/trans isomerase) NIMA-interacting, 4 (parvulin) | 1 |
| 7129 | hsa-miR-133a | 5303 | PIN4 | protein (peptidylprolyl cis/trans isomerase) NIMA-interacting, 4 (parvulin) | 1 |
| 7130 | hsa-miR-186 | 5303 | PIN4 | protein (peptidylprolyl cis/trans isomerase) NIMA-interacting, 4 (parvulin) | 1 |
| 7131 | hsa-miR-326 | 5303 | PIN4 | protein (peptidylprolyl cis/trans isomerase) NIMA-interacting, 4 (parvulin) | 1 |
| 7132 | hsa-miR-449b | 5303 | PIN4 | protein (peptidylprolyl cis/trans isomerase) NIMA-interacting, 4 (parvulin) | 1 |
| 7133 | hsa-miR-551a | 5303 | PIN4 | protein (peptidylprolyl cis/trans isomerase) NIMA-interacting, 4 (parvulin) | 1 |
| 7134 | hsa-miR-562 | 5303 | PIN4 | protein (peptidylprolyl cis/trans isomerase) NIMA-interacting, 4 (parvulin) | 1 |
| 7135 | hsa-miR-579 | 5303 | PIN4 | protein (peptidylprolyl cis/trans isomerase) NIMA-interacting, 4 (parvulin) | 1 |
| 7136 | hsa-miR-608 | 5303 | PIN4 | protein (peptidylprolyl cis/trans isomerase) NIMA-interacting, 4 (parvulin) | 1 |
| 7137 | hsa-mir-22 | 5303 | PIN4 | protein (peptidylprolyl cis/trans isomerase) NIMA-interacting, 4 (parvulin) | 1 |
| 7138 | hsa-mir-10a | 5303 | PIN4 | protein (peptidylprolyl cis/trans isomerase) NIMA-interacting, 4 (parvulin) | 1 |
| 7139 | hsa-mir-186 | 5303 | PIN4 | protein (peptidylprolyl cis/trans isomerase) NIMA-interacting, 4 (parvulin) | 1 |
| 7140 | hsa-mir-155 | 5303 | PIN4 | protein (peptidylprolyl cis/trans isomerase) NIMA-interacting, 4 (parvulin) | 1 |
| 7141 | hsa-mir-423 | 5303 | PIN4 | protein (peptidylprolyl cis/trans isomerase) NIMA-interacting, 4 (parvulin) | 1 |
| 7142 | hsa-mir-550-1 | 5303 | PIN4 | protein (peptidylprolyl cis/trans isomerase) NIMA-interacting, 4 (parvulin) | 1 |
| 7143 | hsa-miR-152 | 8992 | ATP6V0E1 | ATPase, H+ transporting, lysosomal 9kDa, V0 subunit e1 | 1 |
| 7144 | hsa-miR-31 | 8992 | ATP6V0E1 | ATPase, H+ transporting, lysosomal 9kDa, V0 subunit e1 | 1 |
| 7145 | hsa-miR-634 | 8992 | ATP6V0E1 | ATPase, H+ transporting, lysosomal 9kDa, V0 subunit e1 | 1 |
| 7146 | hsa-mir-31 | 8992 | ATP6V0E1 | ATPase, H+ transporting, lysosomal 9kDa, V0 subunit e1 | 1 |
| 7147 | hsa-miR-383 | 8837 | CFLAR | CASP8 and FADD-like apoptosis regulator | 1 |
| 7148 | hsa-miR-1 | 2138 | EYA1 | eyes absent homolog 1 (Drosophila) | 1 |
| 7149 | hsa-miR-133a | 2138 | EYA1 | eyes absent homolog 1 (Drosophila) | 1 |
| 7150 | hsa-miR-186 | 2138 | EYA1 | eyes absent homolog 1 (Drosophila) | 1 |
| 7151 | hsa-miR-326 | 2138 | EYA1 | eyes absent homolog 1 (Drosophila) | 1 |
| 7152 | hsa-miR-449b | 2138 | EYA1 | eyes absent homolog 1 (Drosophila) | 1 |
| 7153 | hsa-miR-550 | 2138 | EYA1 | eyes absent homolog 1 (Drosophila) | 1 |
| 7154 | hsa-miR-551a | 2138 | EYA1 | eyes absent homolog 1 (Drosophila) | 1 |
| 7155 | hsa-miR-562 | 2138 | EYA1 | eyes absent homolog 1 (Drosophila) | 1 |
| 7156 | hsa-miR-579 | 2138 | EYA1 | eyes absent homolog 1 (Drosophila) | 1 |
| 7157 | hsa-miR-608 | 2138 | EYA1 | eyes absent homolog 1 (Drosophila) | 1 |
| 7158 | hsa-miR-637 | 2138 | EYA1 | eyes absent homolog 1 (Drosophila) | 1 |
| 7159 | hsa-miR-885-5p | 2138 | EYA1 | eyes absent homolog 1 (Drosophila) | 1 |
| 7160 | hsa-mir-22 | 2138 | EYA1 | eyes absent homolog 1 (Drosophila) | 1 |
| 7161 | hsa-mir-10a | 2138 | EYA1 | eyes absent homolog 1 (Drosophila) | 1 |
| 7162 | hsa-mir-186 | 2138 | EYA1 | eyes absent homolog 1 (Drosophila) | 1 |
| 7163 | hsa-mir-155 | 2138 | EYA1 | eyes absent homolog 1 (Drosophila) | 1 |
| 7164 | hsa-mir-423 | 2138 | EYA1 | eyes absent homolog 1 (Drosophila) | 1 |
| 7165 | hsa-mir-499 | 2138 | EYA1 | eyes absent homolog 1 (Drosophila) | 1 |
| 7166 | hsa-mir-550-1 | 2138 | EYA1 | eyes absent homolog 1 (Drosophila) | 1 |
| 7167 | hsa-mir-885 | 2138 | EYA1 | eyes absent homolog 1 (Drosophila) | 1 |
| 7168 | hsa-mir-877 | 2138 | EYA1 | eyes absent homolog 1 (Drosophila) | 1 |
| 7169 | hsa-mir-2116 | 2138 | EYA1 | eyes absent homolog 1 (Drosophila) | 1 |
| 7170 | hsa-mir-3190 | 2138 | EYA1 | eyes absent homolog 1 (Drosophila) | 1 |
| 7171 | hsa-miR-10a | 23025 | UNC13A | unc-13 homolog A (C. elegans) | 1 |
| 7172 | hsa-miR-152 | 23025 | UNC13A | unc-13 homolog A (C. elegans) | 1 |
| 7173 | hsa-miR-26a | 23025 | UNC13A | unc-13 homolog A (C. elegans) | 1 |
| 7174 | hsa-miR-26b | 23025 | UNC13A | unc-13 homolog A (C. elegans) | 1 |
| 7175 | hsa-miR-31 | 23025 | UNC13A | unc-13 homolog A (C. elegans) | 1 |
| 7176 | hsa-miR-338-5p | 23025 | UNC13A | unc-13 homolog A (C. elegans) | 1 |
| 7177 | hsa-miR-340 | 23025 | UNC13A | unc-13 homolog A (C. elegans) | 1 |
| 7178 | hsa-miR-383 | 23025 | UNC13A | unc-13 homolog A (C. elegans) | 1 |
| 7179 | hsa-miR-423-5p | 23025 | UNC13A | unc-13 homolog A (C. elegans) | 1 |
| 7180 | hsa-miR-449a | 23025 | UNC13A | unc-13 homolog A (C. elegans) | 1 |
| 7181 | hsa-miR-499-5p | 23025 | UNC13A | unc-13 homolog A (C. elegans) | 1 |
| 7182 | hsa-miR-548b-5p | 23025 | UNC13A | unc-13 homolog A (C. elegans) | 1 |
| 7183 | hsa-miR-621 | 23025 | UNC13A | unc-13 homolog A (C. elegans) | 1 |
| 7184 | hsa-miR-657 | 23025 | UNC13A | unc-13 homolog A (C. elegans) | 1 |
| 7185 | hsa-miR-765 | 23025 | UNC13A | unc-13 homolog A (C. elegans) | 1 |
| 7186 | hsa-miR-770-5p | 23025 | UNC13A | unc-13 homolog A (C. elegans) | 1 |
| 7187 | hsa-miR-877 | 23025 | UNC13A | unc-13 homolog A (C. elegans) | 1 |
| 7188 | hsa-mir-26a-1 | 23025 | UNC13A | unc-13 homolog A (C. elegans) | 1 |
| 7189 | hsa-mir-26b | 23025 | UNC13A | unc-13 homolog A (C. elegans) | 1 |
| 7190 | hsa-mir-31 | 23025 | UNC13A | unc-13 homolog A (C. elegans) | 1 |
| 7191 | hsa-mir-194-2 | 23025 | UNC13A | unc-13 homolog A (C. elegans) | 1 |
| 7192 | hsa-mir-340 | 23025 | UNC13A | unc-13 homolog A (C. elegans) | 1 |
| 7193 | hsa-mir-338 | 23025 | UNC13A | unc-13 homolog A (C. elegans) | 1 |
| 7194 | hsa-mir-548b | 23025 | UNC13A | unc-13 homolog A (C. elegans) | 1 |
| 7195 | hsa-mir-449b | 23025 | UNC13A | unc-13 homolog A (C. elegans) | 1 |
| 7196 | hsa-mir-449c | 23025 | UNC13A | unc-13 homolog A (C. elegans) | 1 |
| 7197 | hsa-mir-3065 | 23025 | UNC13A | unc-13 homolog A (C. elegans) | 1 |
| 7198 | hsa-miR-1 | 55937 | APOM | apolipoprotein M | 1 |
| 7199 | hsa-miR-133a | 55937 | APOM | apolipoprotein M | 1 |
| 7200 | hsa-miR-186 | 55937 | APOM | apolipoprotein M | 1 |
| 7201 | hsa-miR-326 | 55937 | APOM | apolipoprotein M | 1 |
| 7202 | hsa-miR-33a | 55937 | APOM | apolipoprotein M | 1 |
| 7203 | hsa-miR-449b | 55937 | APOM | apolipoprotein M | 1 |
| 7204 | hsa-miR-551a | 55937 | APOM | apolipoprotein M | 1 |
| 7205 | hsa-miR-562 | 55937 | APOM | apolipoprotein M | 1 |
| 7206 | hsa-miR-575 | 55937 | APOM | apolipoprotein M | 1 |
| 7207 | hsa-miR-579 | 55937 | APOM | apolipoprotein M | 1 |
| 7208 | hsa-miR-608 | 55937 | APOM | apolipoprotein M | 1 |
| 7209 | hsa-miR-620 | 55937 | APOM | apolipoprotein M | 1 |
| 7210 | hsa-mir-22 | 55937 | APOM | apolipoprotein M | 1 |
| 7211 | hsa-mir-10a | 55937 | APOM | apolipoprotein M | 1 |
| 7212 | hsa-mir-186 | 55937 | APOM | apolipoprotein M | 1 |
| 7213 | hsa-mir-155 | 55937 | APOM | apolipoprotein M | 1 |
| 7214 | hsa-mir-499 | 55937 | APOM | apolipoprotein M | 1 |
| 7215 | hsa-mir-877 | 55937 | APOM | apolipoprotein M | 1 |
| 7216 | hsa-miR-1 | 51382 | ATP6V1D | ATPase, H+ transporting, lysosomal 34kDa, V1 subunit D | 1 |
| 7217 | hsa-miR-133a | 51382 | ATP6V1D | ATPase, H+ transporting, lysosomal 34kDa, V1 subunit D | 1 |
| 7218 | hsa-miR-186 | 51382 | ATP6V1D | ATPase, H+ transporting, lysosomal 34kDa, V1 subunit D | 1 |
| 7219 | hsa-miR-326 | 51382 | ATP6V1D | ATPase, H+ transporting, lysosomal 34kDa, V1 subunit D | 1 |
| 7220 | hsa-miR-33a | 51382 | ATP6V1D | ATPase, H+ transporting, lysosomal 34kDa, V1 subunit D | 1 |
| 7221 | hsa-miR-449b | 51382 | ATP6V1D | ATPase, H+ transporting, lysosomal 34kDa, V1 subunit D | 1 |
| 7222 | hsa-miR-550 | 51382 | ATP6V1D | ATPase, H+ transporting, lysosomal 34kDa, V1 subunit D | 1 |
| 7223 | hsa-miR-551a | 51382 | ATP6V1D | ATPase, H+ transporting, lysosomal 34kDa, V1 subunit D | 1 |
| 7224 | hsa-miR-562 | 51382 | ATP6V1D | ATPase, H+ transporting, lysosomal 34kDa, V1 subunit D | 1 |
| 7225 | hsa-miR-579 | 51382 | ATP6V1D | ATPase, H+ transporting, lysosomal 34kDa, V1 subunit D | 1 |
| 7226 | hsa-miR-608 | 51382 | ATP6V1D | ATPase, H+ transporting, lysosomal 34kDa, V1 subunit D | 1 |
| 7227 | hsa-miR-620 | 51382 | ATP6V1D | ATPase, H+ transporting, lysosomal 34kDa, V1 subunit D | 1 |
| 7228 | hsa-mir-22 | 51382 | ATP6V1D | ATPase, H+ transporting, lysosomal 34kDa, V1 subunit D | 1 |
| 7229 | hsa-mir-10a | 51382 | ATP6V1D | ATPase, H+ transporting, lysosomal 34kDa, V1 subunit D | 1 |
| 7230 | hsa-mir-186 | 51382 | ATP6V1D | ATPase, H+ transporting, lysosomal 34kDa, V1 subunit D | 1 |
| 7231 | hsa-mir-155 | 51382 | ATP6V1D | ATPase, H+ transporting, lysosomal 34kDa, V1 subunit D | 1 |
| 7232 | hsa-mir-499 | 51382 | ATP6V1D | ATPase, H+ transporting, lysosomal 34kDa, V1 subunit D | 1 |
| 7233 | hsa-mir-550-1 | 51382 | ATP6V1D | ATPase, H+ transporting, lysosomal 34kDa, V1 subunit D | 1 |
| 7234 | hsa-mir-2116 | 51382 | ATP6V1D | ATPase, H+ transporting, lysosomal 34kDa, V1 subunit D | 1 |
| 7235 | hsa-mir-3186 | 51382 | ATP6V1D | ATPase, H+ transporting, lysosomal 34kDa, V1 subunit D | 1 |
| 7236 | hsa-miR-1 | 6257 | RXRB | retinoid X receptor, beta | 1 |
| 7237 | hsa-miR-133a | 6257 | RXRB | retinoid X receptor, beta | 1 |
| 7238 | hsa-miR-186 | 6257 | RXRB | retinoid X receptor, beta | 1 |
| 7239 | hsa-miR-208a | 6257 | RXRB | retinoid X receptor, beta | 1 |
| 7240 | hsa-miR-326 | 6257 | RXRB | retinoid X receptor, beta | 1 |
| 7241 | hsa-miR-449b | 6257 | RXRB | retinoid X receptor, beta | 1 |
| 7242 | hsa-miR-550 | 6257 | RXRB | retinoid X receptor, beta | 1 |
| 7243 | hsa-miR-575 | 6257 | RXRB | retinoid X receptor, beta | 1 |
| 7244 | hsa-miR-579 | 6257 | RXRB | retinoid X receptor, beta | 1 |
| 7245 | hsa-miR-608 | 6257 | RXRB | retinoid X receptor, beta | 1 |
| 7246 | hsa-miR-620 | 6257 | RXRB | retinoid X receptor, beta | 1 |
| 7247 | hsa-miR-885-5p | 6257 | RXRB | retinoid X receptor, beta | 1 |
| 7248 | hsa-mir-22 | 6257 | RXRB | retinoid X receptor, beta | 1 |
| 7249 | hsa-mir-10a | 6257 | RXRB | retinoid X receptor, beta | 1 |
| 7250 | hsa-mir-186 | 6257 | RXRB | retinoid X receptor, beta | 1 |
| 7251 | hsa-mir-155 | 6257 | RXRB | retinoid X receptor, beta | 1 |
| 7252 | hsa-mir-423 | 6257 | RXRB | retinoid X receptor, beta | 1 |
| 7253 | hsa-mir-499 | 6257 | RXRB | retinoid X receptor, beta | 1 |
| 7254 | hsa-mir-550-1 | 6257 | RXRB | retinoid X receptor, beta | 1 |
| 7255 | hsa-mir-885 | 6257 | RXRB | retinoid X receptor, beta | 1 |
| 7256 | hsa-mir-877 | 6257 | RXRB | retinoid X receptor, beta | 1 |
| 7257 | hsa-mir-2116 | 6257 | RXRB | retinoid X receptor, beta | 1 |
| 7258 | hsa-mir-3186 | 6257 | RXRB | retinoid X receptor, beta | 1 |
| 7259 | hsa-mir-3190 | 6257 | RXRB | retinoid X receptor, beta | 1 |
| 7260 | hsa-miR-383 | 23499 | MACF1 | microtubule-actin crosslinking factor 1 | 1 |
| 7261 | hsa-miR-31 | 958 | CD40 | CD40 molecule, TNF receptor superfamily member 5 | 1 |
| 7262 | hsa-mir-31 | 958 | CD40 | CD40 molecule, TNF receptor superfamily member 5 | 1 |
| 7263 | hsa-miR-10a | 57154 | SMURF1 | SMAD specific E3 ubiquitin protein ligase 1 | 1 |
| 7264 | hsa-miR-152 | 57154 | SMURF1 | SMAD specific E3 ubiquitin protein ligase 1 | 1 |
| 7265 | hsa-miR-26a | 57154 | SMURF1 | SMAD specific E3 ubiquitin protein ligase 1 | 1 |
| 7266 | hsa-miR-26b | 57154 | SMURF1 | SMAD specific E3 ubiquitin protein ligase 1 | 1 |
| 7267 | hsa-miR-31 | 57154 | SMURF1 | SMAD specific E3 ubiquitin protein ligase 1 | 1 |
| 7268 | hsa-miR-338-5p | 57154 | SMURF1 | SMAD specific E3 ubiquitin protein ligase 1 | 1 |
| 7269 | hsa-miR-340 | 57154 | SMURF1 | SMAD specific E3 ubiquitin protein ligase 1 | 1 |
| 7270 | hsa-miR-383 | 57154 | SMURF1 | SMAD specific E3 ubiquitin protein ligase 1 | 1 |
| 7271 | hsa-miR-423-5p | 57154 | SMURF1 | SMAD specific E3 ubiquitin protein ligase 1 | 1 |
| 7272 | hsa-miR-449a | 57154 | SMURF1 | SMAD specific E3 ubiquitin protein ligase 1 | 1 |
| 7273 | hsa-miR-499-5p | 57154 | SMURF1 | SMAD specific E3 ubiquitin protein ligase 1 | 1 |
| 7274 | hsa-miR-548b-5p | 57154 | SMURF1 | SMAD specific E3 ubiquitin protein ligase 1 | 1 |
| 7275 | hsa-miR-564 | 57154 | SMURF1 | SMAD specific E3 ubiquitin protein ligase 1 | 1 |
| 7276 | hsa-miR-621 | 57154 | SMURF1 | SMAD specific E3 ubiquitin protein ligase 1 | 1 |
| 7277 | hsa-miR-657 | 57154 | SMURF1 | SMAD specific E3 ubiquitin protein ligase 1 | 1 |
| 7278 | hsa-miR-765 | 57154 | SMURF1 | SMAD specific E3 ubiquitin protein ligase 1 | 1 |
| 7279 | hsa-miR-770-5p | 57154 | SMURF1 | SMAD specific E3 ubiquitin protein ligase 1 | 1 |
| 7280 | hsa-mir-26a-1 | 57154 | SMURF1 | SMAD specific E3 ubiquitin protein ligase 1 | 1 |
| 7281 | hsa-mir-26b | 57154 | SMURF1 | SMAD specific E3 ubiquitin protein ligase 1 | 1 |
| 7282 | hsa-mir-31 | 57154 | SMURF1 | SMAD specific E3 ubiquitin protein ligase 1 | 1 |
| 7283 | hsa-mir-194-2 | 57154 | SMURF1 | SMAD specific E3 ubiquitin protein ligase 1 | 1 |
| 7284 | hsa-mir-340 | 57154 | SMURF1 | SMAD specific E3 ubiquitin protein ligase 1 | 1 |
| 7285 | hsa-mir-338 | 57154 | SMURF1 | SMAD specific E3 ubiquitin protein ligase 1 | 1 |
| 7286 | hsa-mir-548b | 57154 | SMURF1 | SMAD specific E3 ubiquitin protein ligase 1 | 1 |
| 7287 | hsa-mir-449b | 57154 | SMURF1 | SMAD specific E3 ubiquitin protein ligase 1 | 1 |
| 7288 | hsa-mir-449c | 57154 | SMURF1 | SMAD specific E3 ubiquitin protein ligase 1 | 1 |
| 7289 | hsa-mir-3065 | 57154 | SMURF1 | SMAD specific E3 ubiquitin protein ligase 1 | 1 |
| 7290 | hsa-miR-10a | 5606 | MAP2K3 | mitogen-activated protein kinase kinase 3 | 1 |
| 7291 | hsa-miR-1228\* | 5606 | MAP2K3 | mitogen-activated protein kinase kinase 3 | 1 |
| 7292 | hsa-miR-139-5p | 5606 | MAP2K3 | mitogen-activated protein kinase kinase 3 | 1 |
| 7293 | hsa-miR-152 | 5606 | MAP2K3 | mitogen-activated protein kinase kinase 3 | 1 |
| 7294 | hsa-miR-26a | 5606 | MAP2K3 | mitogen-activated protein kinase kinase 3 | 1 |
| 7295 | hsa-miR-26b | 5606 | MAP2K3 | mitogen-activated protein kinase kinase 3 | 1 |
| 7296 | hsa-miR-31 | 5606 | MAP2K3 | mitogen-activated protein kinase kinase 3 | 1 |
| 7297 | hsa-miR-338-5p | 5606 | MAP2K3 | mitogen-activated protein kinase kinase 3 | 1 |
| 7298 | hsa-miR-340 | 5606 | MAP2K3 | mitogen-activated protein kinase kinase 3 | 1 |
| 7299 | hsa-miR-423-5p | 5606 | MAP2K3 | mitogen-activated protein kinase kinase 3 | 1 |
| 7300 | hsa-miR-449a | 5606 | MAP2K3 | mitogen-activated protein kinase kinase 3 | 1 |
| 7301 | hsa-miR-499-5p | 5606 | MAP2K3 | mitogen-activated protein kinase kinase 3 | 1 |
| 7302 | hsa-miR-548b-5p | 5606 | MAP2K3 | mitogen-activated protein kinase kinase 3 | 1 |
| 7303 | hsa-miR-564 | 5606 | MAP2K3 | mitogen-activated protein kinase kinase 3 | 1 |
| 7304 | hsa-miR-584 | 5606 | MAP2K3 | mitogen-activated protein kinase kinase 3 | 1 |
| 7305 | hsa-miR-621 | 5606 | MAP2K3 | mitogen-activated protein kinase kinase 3 | 1 |
| 7306 | hsa-miR-644 | 5606 | MAP2K3 | mitogen-activated protein kinase kinase 3 | 1 |
| 7307 | hsa-miR-657 | 5606 | MAP2K3 | mitogen-activated protein kinase kinase 3 | 1 |
| 7308 | hsa-miR-765 | 5606 | MAP2K3 | mitogen-activated protein kinase kinase 3 | 1 |
| 7309 | hsa-miR-770-5p | 5606 | MAP2K3 | mitogen-activated protein kinase kinase 3 | 1 |
| 7310 | hsa-miR-877 | 5606 | MAP2K3 | mitogen-activated protein kinase kinase 3 | 1 |
| 7311 | hsa-miR-95 | 5606 | MAP2K3 | mitogen-activated protein kinase kinase 3 | 1 |
| 7312 | hsa-mir-26a-1 | 5606 | MAP2K3 | mitogen-activated protein kinase kinase 3 | 1 |
| 7313 | hsa-mir-26b | 5606 | MAP2K3 | mitogen-activated protein kinase kinase 3 | 1 |
| 7314 | hsa-mir-31 | 5606 | MAP2K3 | mitogen-activated protein kinase kinase 3 | 1 |
| 7315 | hsa-mir-194-2 | 5606 | MAP2K3 | mitogen-activated protein kinase kinase 3 | 1 |
| 7316 | hsa-mir-340 | 5606 | MAP2K3 | mitogen-activated protein kinase kinase 3 | 1 |
| 7317 | hsa-mir-338 | 5606 | MAP2K3 | mitogen-activated protein kinase kinase 3 | 1 |
| 7318 | hsa-mir-548b | 5606 | MAP2K3 | mitogen-activated protein kinase kinase 3 | 1 |
| 7319 | hsa-mir-548d-2 | 5606 | MAP2K3 | mitogen-activated protein kinase kinase 3 | 1 |
| 7320 | hsa-mir-449b | 5606 | MAP2K3 | mitogen-activated protein kinase kinase 3 | 1 |
| 7321 | hsa-mir-449c | 5606 | MAP2K3 | mitogen-activated protein kinase kinase 3 | 1 |
| 7322 | hsa-mir-3065 | 5606 | MAP2K3 | mitogen-activated protein kinase kinase 3 | 1 |
| 7323 | hsa-miR-152 | 9819 | TSC22D2 | TSC22 domain family, member 2 | 1 |
| 7324 | hsa-miR-26b | 9819 | TSC22D2 | TSC22 domain family, member 2 | 1 |
| 7325 | hsa-miR-31 | 9819 | TSC22D2 | TSC22 domain family, member 2 | 1 |
| 7326 | hsa-miR-338-5p | 9819 | TSC22D2 | TSC22 domain family, member 2 | 1 |
| 7327 | hsa-miR-340 | 9819 | TSC22D2 | TSC22 domain family, member 2 | 1 |
| 7328 | hsa-miR-383 | 9819 | TSC22D2 | TSC22 domain family, member 2 | 1 |
| 7329 | hsa-miR-449a | 9819 | TSC22D2 | TSC22 domain family, member 2 | 1 |
| 7330 | hsa-miR-548b-5p | 9819 | TSC22D2 | TSC22 domain family, member 2 | 1 |
| 7331 | hsa-miR-621 | 9819 | TSC22D2 | TSC22 domain family, member 2 | 1 |
| 7332 | hsa-miR-657 | 9819 | TSC22D2 | TSC22 domain family, member 2 | 1 |
| 7333 | hsa-miR-663 | 9819 | TSC22D2 | TSC22 domain family, member 2 | 1 |
| 7334 | hsa-miR-765 | 9819 | TSC22D2 | TSC22 domain family, member 2 | 1 |
| 7335 | hsa-miR-770-5p | 9819 | TSC22D2 | TSC22 domain family, member 2 | 1 |
| 7336 | hsa-miR-877 | 9819 | TSC22D2 | TSC22 domain family, member 2 | 1 |
| 7337 | hsa-mir-26a-1 | 9819 | TSC22D2 | TSC22 domain family, member 2 | 1 |
| 7338 | hsa-mir-26b | 9819 | TSC22D2 | TSC22 domain family, member 2 | 1 |
| 7339 | hsa-mir-31 | 9819 | TSC22D2 | TSC22 domain family, member 2 | 1 |
| 7340 | hsa-mir-194-2 | 9819 | TSC22D2 | TSC22 domain family, member 2 | 1 |
| 7341 | hsa-mir-340 | 9819 | TSC22D2 | TSC22 domain family, member 2 | 1 |
| 7342 | hsa-mir-338 | 9819 | TSC22D2 | TSC22 domain family, member 2 | 1 |
| 7343 | hsa-mir-548b | 9819 | TSC22D2 | TSC22 domain family, member 2 | 1 |
| 7344 | hsa-mir-449b | 9819 | TSC22D2 | TSC22 domain family, member 2 | 1 |
| 7345 | hsa-mir-449c | 9819 | TSC22D2 | TSC22 domain family, member 2 | 1 |
| 7346 | hsa-mir-3065 | 9819 | TSC22D2 | TSC22 domain family, member 2 | 1 |
| 7347 | hsa-miR-10a | 355 | FAS | Fas (TNF receptor superfamily, member 6) | 1 |
| 7348 | hsa-miR-152 | 355 | FAS | Fas (TNF receptor superfamily, member 6) | 1 |
| 7349 | hsa-miR-26b | 355 | FAS | Fas (TNF receptor superfamily, member 6) | 1 |
| 7350 | hsa-miR-31 | 355 | FAS | Fas (TNF receptor superfamily, member 6) | 1 |
| 7351 | hsa-miR-338-5p | 355 | FAS | Fas (TNF receptor superfamily, member 6) | 1 |
| 7352 | hsa-miR-340 | 355 | FAS | Fas (TNF receptor superfamily, member 6) | 1 |
| 7353 | hsa-miR-383 | 355 | FAS | Fas (TNF receptor superfamily, member 6) | 1 |
| 7354 | hsa-miR-449a | 355 | FAS | Fas (TNF receptor superfamily, member 6) | 1 |
| 7355 | hsa-miR-499-5p | 355 | FAS | Fas (TNF receptor superfamily, member 6) | 1 |
| 7356 | hsa-miR-548b-5p | 355 | FAS | Fas (TNF receptor superfamily, member 6) | 1 |
| 7357 | hsa-miR-621 | 355 | FAS | Fas (TNF receptor superfamily, member 6) | 1 |
| 7358 | hsa-miR-657 | 355 | FAS | Fas (TNF receptor superfamily, member 6) | 1 |
| 7359 | hsa-miR-663 | 355 | FAS | Fas (TNF receptor superfamily, member 6) | 1 |
| 7360 | hsa-miR-765 | 355 | FAS | Fas (TNF receptor superfamily, member 6) | 1 |
| 7361 | hsa-miR-770-5p | 355 | FAS | Fas (TNF receptor superfamily, member 6) | 1 |
| 7362 | hsa-miR-877 | 355 | FAS | Fas (TNF receptor superfamily, member 6) | 1 |
| 7363 | hsa-mir-26a-1 | 355 | FAS | Fas (TNF receptor superfamily, member 6) | 1 |
| 7364 | hsa-mir-26b | 355 | FAS | Fas (TNF receptor superfamily, member 6) | 1 |
| 7365 | hsa-mir-31 | 355 | FAS | Fas (TNF receptor superfamily, member 6) | 1 |
| 7366 | hsa-mir-194-2 | 355 | FAS | Fas (TNF receptor superfamily, member 6) | 1 |
| 7367 | hsa-mir-340 | 355 | FAS | Fas (TNF receptor superfamily, member 6) | 1 |
| 7368 | hsa-mir-338 | 355 | FAS | Fas (TNF receptor superfamily, member 6) | 1 |
| 7369 | hsa-mir-548b | 355 | FAS | Fas (TNF receptor superfamily, member 6) | 1 |
| 7370 | hsa-mir-449b | 355 | FAS | Fas (TNF receptor superfamily, member 6) | 1 |
| 7371 | hsa-mir-449c | 355 | FAS | Fas (TNF receptor superfamily, member 6) | 1 |
| 7372 | hsa-mir-3065 | 355 | FAS | Fas (TNF receptor superfamily, member 6) | 1 |
| 7373 | hsa-miR-10a | 22904 | SBNO2 | strawberry notch homolog 2 (Drosophila) | 1 |
| 7374 | hsa-miR-139-5p | 22904 | SBNO2 | strawberry notch homolog 2 (Drosophila) | 1 |
| 7375 | hsa-miR-152 | 22904 | SBNO2 | strawberry notch homolog 2 (Drosophila) | 1 |
| 7376 | hsa-miR-26a | 22904 | SBNO2 | strawberry notch homolog 2 (Drosophila) | 1 |
| 7377 | hsa-miR-26b | 22904 | SBNO2 | strawberry notch homolog 2 (Drosophila) | 1 |
| 7378 | hsa-miR-31 | 22904 | SBNO2 | strawberry notch homolog 2 (Drosophila) | 1 |
| 7379 | hsa-miR-338-5p | 22904 | SBNO2 | strawberry notch homolog 2 (Drosophila) | 1 |
| 7380 | hsa-miR-340 | 22904 | SBNO2 | strawberry notch homolog 2 (Drosophila) | 1 |
| 7381 | hsa-miR-383 | 22904 | SBNO2 | strawberry notch homolog 2 (Drosophila) | 1 |
| 7382 | hsa-miR-449a | 22904 | SBNO2 | strawberry notch homolog 2 (Drosophila) | 1 |
| 7383 | hsa-miR-490-5p | 22904 | SBNO2 | strawberry notch homolog 2 (Drosophila) | 1 |
| 7384 | hsa-miR-499-5p | 22904 | SBNO2 | strawberry notch homolog 2 (Drosophila) | 1 |
| 7385 | hsa-miR-548b-5p | 22904 | SBNO2 | strawberry notch homolog 2 (Drosophila) | 1 |
| 7386 | hsa-miR-621 | 22904 | SBNO2 | strawberry notch homolog 2 (Drosophila) | 1 |
| 7387 | hsa-miR-657 | 22904 | SBNO2 | strawberry notch homolog 2 (Drosophila) | 1 |
| 7388 | hsa-miR-663 | 22904 | SBNO2 | strawberry notch homolog 2 (Drosophila) | 1 |
| 7389 | hsa-miR-765 | 22904 | SBNO2 | strawberry notch homolog 2 (Drosophila) | 1 |
| 7390 | hsa-miR-770-5p | 22904 | SBNO2 | strawberry notch homolog 2 (Drosophila) | 1 |
| 7391 | hsa-miR-877 | 22904 | SBNO2 | strawberry notch homolog 2 (Drosophila) | 1 |
| 7392 | hsa-mir-26a-1 | 22904 | SBNO2 | strawberry notch homolog 2 (Drosophila) | 1 |
| 7393 | hsa-mir-26b | 22904 | SBNO2 | strawberry notch homolog 2 (Drosophila) | 1 |
| 7394 | hsa-mir-31 | 22904 | SBNO2 | strawberry notch homolog 2 (Drosophila) | 1 |
| 7395 | hsa-mir-194-2 | 22904 | SBNO2 | strawberry notch homolog 2 (Drosophila) | 1 |
| 7396 | hsa-mir-340 | 22904 | SBNO2 | strawberry notch homolog 2 (Drosophila) | 1 |
| 7397 | hsa-mir-338 | 22904 | SBNO2 | strawberry notch homolog 2 (Drosophila) | 1 |
| 7398 | hsa-mir-490 | 22904 | SBNO2 | strawberry notch homolog 2 (Drosophila) | 1 |
| 7399 | hsa-mir-548b | 22904 | SBNO2 | strawberry notch homolog 2 (Drosophila) | 1 |
| 7400 | hsa-mir-548d-2 | 22904 | SBNO2 | strawberry notch homolog 2 (Drosophila) | 1 |
| 7401 | hsa-mir-449b | 22904 | SBNO2 | strawberry notch homolog 2 (Drosophila) | 1 |
| 7402 | hsa-mir-449c | 22904 | SBNO2 | strawberry notch homolog 2 (Drosophila) | 1 |
| 7403 | hsa-mir-3065 | 22904 | SBNO2 | strawberry notch homolog 2 (Drosophila) | 1 |
| 7404 | hsa-miR-1 | 4661 | MYT1 | myelin transcription factor 1 | 1 |
| 7405 | hsa-miR-133a | 4661 | MYT1 | myelin transcription factor 1 | 1 |
| 7406 | hsa-miR-186 | 4661 | MYT1 | myelin transcription factor 1 | 1 |
| 7407 | hsa-miR-208a | 4661 | MYT1 | myelin transcription factor 1 | 1 |
| 7408 | hsa-miR-326 | 4661 | MYT1 | myelin transcription factor 1 | 1 |
| 7409 | hsa-miR-449b | 4661 | MYT1 | myelin transcription factor 1 | 1 |
| 7410 | hsa-miR-575 | 4661 | MYT1 | myelin transcription factor 1 | 1 |
| 7411 | hsa-miR-579 | 4661 | MYT1 | myelin transcription factor 1 | 1 |
| 7412 | hsa-miR-608 | 4661 | MYT1 | myelin transcription factor 1 | 1 |
| 7413 | hsa-miR-620 | 4661 | MYT1 | myelin transcription factor 1 | 1 |
| 7414 | hsa-miR-885-5p | 4661 | MYT1 | myelin transcription factor 1 | 1 |
| 7415 | hsa-let-7a-3 | 4661 | MYT1 | myelin transcription factor 1 | 1 |
| 7416 | hsa-let-7b | 4661 | MYT1 | myelin transcription factor 1 | 1 |
| 7417 | hsa-mir-22 | 4661 | MYT1 | myelin transcription factor 1 | 1 |
| 7418 | hsa-mir-10a | 4661 | MYT1 | myelin transcription factor 1 | 1 |
| 7419 | hsa-mir-186 | 4661 | MYT1 | myelin transcription factor 1 | 1 |
| 7420 | hsa-mir-155 | 4661 | MYT1 | myelin transcription factor 1 | 1 |
| 7421 | hsa-mir-499 | 4661 | MYT1 | myelin transcription factor 1 | 1 |
| 7422 | hsa-mir-885 | 4661 | MYT1 | myelin transcription factor 1 | 1 |
| 7423 | hsa-mir-877 | 4661 | MYT1 | myelin transcription factor 1 | 1 |
| 7424 | hsa-mir-2116 | 4661 | MYT1 | myelin transcription factor 1 | 1 |
| 7425 | hsa-mir-3190 | 4661 | MYT1 | myelin transcription factor 1 | 1 |
| 7426 | hsa-miR-564 | 9693 | RAPGEF2 | Rap guanine nucleotide exchange factor (GEF) 2 | 1 |
| 7427 | hsa-miR-1 | 2798 | GNRHR | gonadotropin-releasing hormone receptor | 1 |
| 7428 | hsa-miR-133a | 2798 | GNRHR | gonadotropin-releasing hormone receptor | 1 |
| 7429 | hsa-miR-186 | 2798 | GNRHR | gonadotropin-releasing hormone receptor | 1 |
| 7430 | hsa-miR-195 | 2798 | GNRHR | gonadotropin-releasing hormone receptor | 1 |
| 7431 | hsa-miR-208a | 2798 | GNRHR | gonadotropin-releasing hormone receptor | 1 |
| 7432 | hsa-miR-326 | 2798 | GNRHR | gonadotropin-releasing hormone receptor | 1 |
| 7433 | hsa-miR-449b | 2798 | GNRHR | gonadotropin-releasing hormone receptor | 1 |
| 7434 | hsa-miR-497 | 2798 | GNRHR | gonadotropin-releasing hormone receptor | 1 |
| 7435 | hsa-miR-550 | 2798 | GNRHR | gonadotropin-releasing hormone receptor | 1 |
| 7436 | hsa-miR-575 | 2798 | GNRHR | gonadotropin-releasing hormone receptor | 1 |
| 7437 | hsa-miR-579 | 2798 | GNRHR | gonadotropin-releasing hormone receptor | 1 |
| 7438 | hsa-miR-608 | 2798 | GNRHR | gonadotropin-releasing hormone receptor | 1 |
| 7439 | hsa-miR-620 | 2798 | GNRHR | gonadotropin-releasing hormone receptor | 1 |
| 7440 | hsa-miR-885-5p | 2798 | GNRHR | gonadotropin-releasing hormone receptor | 1 |
| 7441 | hsa-let-7a-3 | 2798 | GNRHR | gonadotropin-releasing hormone receptor | 1 |
| 7442 | hsa-let-7b | 2798 | GNRHR | gonadotropin-releasing hormone receptor | 1 |
| 7443 | hsa-mir-22 | 2798 | GNRHR | gonadotropin-releasing hormone receptor | 1 |
| 7444 | hsa-mir-10a | 2798 | GNRHR | gonadotropin-releasing hormone receptor | 1 |
| 7445 | hsa-mir-186 | 2798 | GNRHR | gonadotropin-releasing hormone receptor | 1 |
| 7446 | hsa-mir-155 | 2798 | GNRHR | gonadotropin-releasing hormone receptor | 1 |
| 7447 | hsa-mir-499 | 2798 | GNRHR | gonadotropin-releasing hormone receptor | 1 |
| 7448 | hsa-mir-550-1 | 2798 | GNRHR | gonadotropin-releasing hormone receptor | 1 |
| 7449 | hsa-mir-885 | 2798 | GNRHR | gonadotropin-releasing hormone receptor | 1 |
| 7450 | hsa-mir-877 | 2798 | GNRHR | gonadotropin-releasing hormone receptor | 1 |
| 7451 | hsa-mir-2116 | 2798 | GNRHR | gonadotropin-releasing hormone receptor | 1 |
| 7452 | hsa-mir-3190 | 2798 | GNRHR | gonadotropin-releasing hormone receptor | 1 |
| 7453 | hsa-miR-1 | 261734 | NPHP4 | nephronophthisis 4 | 1 |
| 7454 | hsa-miR-133a | 261734 | NPHP4 | nephronophthisis 4 | 1 |
| 7455 | hsa-miR-186 | 261734 | NPHP4 | nephronophthisis 4 | 1 |
| 7456 | hsa-miR-208a | 261734 | NPHP4 | nephronophthisis 4 | 1 |
| 7457 | hsa-miR-326 | 261734 | NPHP4 | nephronophthisis 4 | 1 |
| 7458 | hsa-miR-449b | 261734 | NPHP4 | nephronophthisis 4 | 1 |
| 7459 | hsa-miR-550 | 261734 | NPHP4 | nephronophthisis 4 | 1 |
| 7460 | hsa-miR-575 | 261734 | NPHP4 | nephronophthisis 4 | 1 |
| 7461 | hsa-miR-579 | 261734 | NPHP4 | nephronophthisis 4 | 1 |
| 7462 | hsa-miR-608 | 261734 | NPHP4 | nephronophthisis 4 | 1 |
| 7463 | hsa-miR-620 | 261734 | NPHP4 | nephronophthisis 4 | 1 |
| 7464 | hsa-miR-885-5p | 261734 | NPHP4 | nephronophthisis 4 | 1 |
| 7465 | hsa-let-7a-3 | 261734 | NPHP4 | nephronophthisis 4 | 1 |
| 7466 | hsa-let-7b | 261734 | NPHP4 | nephronophthisis 4 | 1 |
| 7467 | hsa-mir-22 | 261734 | NPHP4 | nephronophthisis 4 | 1 |
| 7468 | hsa-mir-10a | 261734 | NPHP4 | nephronophthisis 4 | 1 |
| 7469 | hsa-mir-186 | 261734 | NPHP4 | nephronophthisis 4 | 1 |
| 7470 | hsa-mir-155 | 261734 | NPHP4 | nephronophthisis 4 | 1 |
| 7471 | hsa-mir-499 | 261734 | NPHP4 | nephronophthisis 4 | 1 |
| 7472 | hsa-mir-550-1 | 261734 | NPHP4 | nephronophthisis 4 | 1 |
| 7473 | hsa-mir-885 | 261734 | NPHP4 | nephronophthisis 4 | 1 |
| 7474 | hsa-mir-877 | 261734 | NPHP4 | nephronophthisis 4 | 1 |
| 7475 | hsa-mir-2116 | 261734 | NPHP4 | nephronophthisis 4 | 1 |
| 7476 | hsa-mir-3186 | 261734 | NPHP4 | nephronophthisis 4 | 1 |
| 7477 | hsa-miR-1 | 26212 | OR2B6 | olfactory receptor, family 2, subfamily B, member 6 | 1 |
| 7478 | hsa-miR-133a | 26212 | OR2B6 | olfactory receptor, family 2, subfamily B, member 6 | 1 |
| 7479 | hsa-miR-186 | 26212 | OR2B6 | olfactory receptor, family 2, subfamily B, member 6 | 1 |
| 7480 | hsa-miR-326 | 26212 | OR2B6 | olfactory receptor, family 2, subfamily B, member 6 | 1 |
| 7481 | hsa-miR-33a | 26212 | OR2B6 | olfactory receptor, family 2, subfamily B, member 6 | 1 |
| 7482 | hsa-miR-449b | 26212 | OR2B6 | olfactory receptor, family 2, subfamily B, member 6 | 1 |
| 7483 | hsa-miR-550 | 26212 | OR2B6 | olfactory receptor, family 2, subfamily B, member 6 | 1 |
| 7484 | hsa-miR-551a | 26212 | OR2B6 | olfactory receptor, family 2, subfamily B, member 6 | 1 |
| 7485 | hsa-miR-575 | 26212 | OR2B6 | olfactory receptor, family 2, subfamily B, member 6 | 1 |
| 7486 | hsa-miR-579 | 26212 | OR2B6 | olfactory receptor, family 2, subfamily B, member 6 | 1 |
| 7487 | hsa-miR-608 | 26212 | OR2B6 | olfactory receptor, family 2, subfamily B, member 6 | 1 |
| 7488 | hsa-miR-620 | 26212 | OR2B6 | olfactory receptor, family 2, subfamily B, member 6 | 1 |
| 7489 | hsa-let-7a-3 | 26212 | OR2B6 | olfactory receptor, family 2, subfamily B, member 6 | 1 |
| 7490 | hsa-let-7b | 26212 | OR2B6 | olfactory receptor, family 2, subfamily B, member 6 | 1 |
| 7491 | hsa-mir-22 | 26212 | OR2B6 | olfactory receptor, family 2, subfamily B, member 6 | 1 |
| 7492 | hsa-mir-10a | 26212 | OR2B6 | olfactory receptor, family 2, subfamily B, member 6 | 1 |
| 7493 | hsa-mir-186 | 26212 | OR2B6 | olfactory receptor, family 2, subfamily B, member 6 | 1 |
| 7494 | hsa-mir-155 | 26212 | OR2B6 | olfactory receptor, family 2, subfamily B, member 6 | 1 |
| 7495 | hsa-mir-499 | 26212 | OR2B6 | olfactory receptor, family 2, subfamily B, member 6 | 1 |
| 7496 | hsa-mir-550-1 | 26212 | OR2B6 | olfactory receptor, family 2, subfamily B, member 6 | 1 |
| 7497 | hsa-mir-877 | 26212 | OR2B6 | olfactory receptor, family 2, subfamily B, member 6 | 1 |
| 7498 | hsa-mir-2116 | 26212 | OR2B6 | olfactory receptor, family 2, subfamily B, member 6 | 1 |
| 7499 | hsa-mir-3186 | 26212 | OR2B6 | olfactory receptor, family 2, subfamily B, member 6 | 1 |
| 7500 | hsa-miR-208a | 23428 | SLC7A8 | solute carrier family 7 (cationic amino acid transporter, y+ system), member 8 | 1 |
| 7501 | hsa-let-7a-3 | 23428 | SLC7A8 | solute carrier family 7 (cationic amino acid transporter, y+ system), member 8 | 1 |
| 7502 | hsa-let-7b | 23428 | SLC7A8 | solute carrier family 7 (cationic amino acid transporter, y+ system), member 8 | 1 |
| 7503 | hsa-mir-2116 | 23428 | SLC7A8 | solute carrier family 7 (cationic amino acid transporter, y+ system), member 8 | 1 |
| 7504 | hsa-miR-10a | 3898 | LAD1 | ladinin 1 | 1 |
| 7505 | hsa-miR-152 | 3898 | LAD1 | ladinin 1 | 1 |
| 7506 | hsa-miR-26a | 3898 | LAD1 | ladinin 1 | 1 |
| 7507 | hsa-miR-26b | 3898 | LAD1 | ladinin 1 | 1 |
| 7508 | hsa-miR-31 | 3898 | LAD1 | ladinin 1 | 1 |
| 7509 | hsa-miR-338-5p | 3898 | LAD1 | ladinin 1 | 1 |
| 7510 | hsa-miR-340 | 3898 | LAD1 | ladinin 1 | 1 |
| 7511 | hsa-miR-383 | 3898 | LAD1 | ladinin 1 | 1 |
| 7512 | hsa-miR-423-5p | 3898 | LAD1 | ladinin 1 | 1 |
| 7513 | hsa-miR-449a | 3898 | LAD1 | ladinin 1 | 1 |
| 7514 | hsa-miR-490-5p | 3898 | LAD1 | ladinin 1 | 1 |
| 7515 | hsa-miR-499-5p | 3898 | LAD1 | ladinin 1 | 1 |
| 7516 | hsa-miR-548b-5p | 3898 | LAD1 | ladinin 1 | 1 |
| 7517 | hsa-miR-621 | 3898 | LAD1 | ladinin 1 | 1 |
| 7518 | hsa-miR-657 | 3898 | LAD1 | ladinin 1 | 1 |
| 7519 | hsa-miR-765 | 3898 | LAD1 | ladinin 1 | 1 |
| 7520 | hsa-miR-770-5p | 3898 | LAD1 | ladinin 1 | 1 |
| 7521 | hsa-miR-877 | 3898 | LAD1 | ladinin 1 | 1 |
| 7522 | hsa-mir-26a-1 | 3898 | LAD1 | ladinin 1 | 1 |
| 7523 | hsa-mir-26b | 3898 | LAD1 | ladinin 1 | 1 |
| 7524 | hsa-mir-31 | 3898 | LAD1 | ladinin 1 | 1 |
| 7525 | hsa-mir-194-2 | 3898 | LAD1 | ladinin 1 | 1 |
| 7526 | hsa-mir-340 | 3898 | LAD1 | ladinin 1 | 1 |
| 7527 | hsa-mir-338 | 3898 | LAD1 | ladinin 1 | 1 |
| 7528 | hsa-mir-490 | 3898 | LAD1 | ladinin 1 | 1 |
| 7529 | hsa-mir-548b | 3898 | LAD1 | ladinin 1 | 1 |
| 7530 | hsa-mir-449b | 3898 | LAD1 | ladinin 1 | 1 |
| 7531 | hsa-mir-449c | 3898 | LAD1 | ladinin 1 | 1 |
| 7532 | hsa-mir-3065 | 3898 | LAD1 | ladinin 1 | 1 |
| 7533 | hsa-miR-1 | 6189 | RPS3A | ribosomal protein S3A | 1 |
| 7534 | hsa-miR-133a | 6189 | RPS3A | ribosomal protein S3A | 1 |
| 7535 | hsa-miR-186 | 6189 | RPS3A | ribosomal protein S3A | 1 |
| 7536 | hsa-miR-208a | 6189 | RPS3A | ribosomal protein S3A | 1 |
| 7537 | hsa-miR-326 | 6189 | RPS3A | ribosomal protein S3A | 1 |
| 7538 | hsa-miR-449b | 6189 | RPS3A | ribosomal protein S3A | 1 |
| 7539 | hsa-miR-550 | 6189 | RPS3A | ribosomal protein S3A | 1 |
| 7540 | hsa-miR-575 | 6189 | RPS3A | ribosomal protein S3A | 1 |
| 7541 | hsa-miR-579 | 6189 | RPS3A | ribosomal protein S3A | 1 |
| 7542 | hsa-miR-608 | 6189 | RPS3A | ribosomal protein S3A | 1 |
| 7543 | hsa-miR-620 | 6189 | RPS3A | ribosomal protein S3A | 1 |
| 7544 | hsa-miR-885-5p | 6189 | RPS3A | ribosomal protein S3A | 1 |
| 7545 | hsa-let-7a-3 | 6189 | RPS3A | ribosomal protein S3A | 1 |
| 7546 | hsa-let-7b | 6189 | RPS3A | ribosomal protein S3A | 1 |
| 7547 | hsa-mir-22 | 6189 | RPS3A | ribosomal protein S3A | 1 |
| 7548 | hsa-mir-10a | 6189 | RPS3A | ribosomal protein S3A | 1 |
| 7549 | hsa-mir-186 | 6189 | RPS3A | ribosomal protein S3A | 1 |
| 7550 | hsa-mir-155 | 6189 | RPS3A | ribosomal protein S3A | 1 |
| 7551 | hsa-mir-499 | 6189 | RPS3A | ribosomal protein S3A | 1 |
| 7552 | hsa-mir-550-1 | 6189 | RPS3A | ribosomal protein S3A | 1 |
| 7553 | hsa-mir-885 | 6189 | RPS3A | ribosomal protein S3A | 1 |
| 7554 | hsa-mir-877 | 6189 | RPS3A | ribosomal protein S3A | 1 |
| 7555 | hsa-mir-2116 | 6189 | RPS3A | ribosomal protein S3A | 1 |
| 7556 | hsa-mir-3186 | 6189 | RPS3A | ribosomal protein S3A | 1 |
| 7557 | hsa-mir-3190 | 6189 | RPS3A | ribosomal protein S3A | 1 |
| 7558 | hsa-miR-1 | 7512 | XPNPEP2 | X-prolyl aminopeptidase (aminopeptidase P) 2, membrane-bound | 1 |
| 7559 | hsa-miR-133a | 7512 | XPNPEP2 | X-prolyl aminopeptidase (aminopeptidase P) 2, membrane-bound | 1 |
| 7560 | hsa-miR-186 | 7512 | XPNPEP2 | X-prolyl aminopeptidase (aminopeptidase P) 2, membrane-bound | 1 |
| 7561 | hsa-miR-208a | 7512 | XPNPEP2 | X-prolyl aminopeptidase (aminopeptidase P) 2, membrane-bound | 1 |
| 7562 | hsa-miR-449b | 7512 | XPNPEP2 | X-prolyl aminopeptidase (aminopeptidase P) 2, membrane-bound | 1 |
| 7563 | hsa-miR-489 | 7512 | XPNPEP2 | X-prolyl aminopeptidase (aminopeptidase P) 2, membrane-bound | 1 |
| 7564 | hsa-miR-575 | 7512 | XPNPEP2 | X-prolyl aminopeptidase (aminopeptidase P) 2, membrane-bound | 1 |
| 7565 | hsa-miR-579 | 7512 | XPNPEP2 | X-prolyl aminopeptidase (aminopeptidase P) 2, membrane-bound | 1 |
| 7566 | hsa-miR-608 | 7512 | XPNPEP2 | X-prolyl aminopeptidase (aminopeptidase P) 2, membrane-bound | 1 |
| 7567 | hsa-miR-637 | 7512 | XPNPEP2 | X-prolyl aminopeptidase (aminopeptidase P) 2, membrane-bound | 1 |
| 7568 | hsa-mir-22 | 7512 | XPNPEP2 | X-prolyl aminopeptidase (aminopeptidase P) 2, membrane-bound | 1 |
| 7569 | hsa-mir-10a | 7512 | XPNPEP2 | X-prolyl aminopeptidase (aminopeptidase P) 2, membrane-bound | 1 |
| 7570 | hsa-mir-186 | 7512 | XPNPEP2 | X-prolyl aminopeptidase (aminopeptidase P) 2, membrane-bound | 1 |
| 7571 | hsa-mir-155 | 7512 | XPNPEP2 | X-prolyl aminopeptidase (aminopeptidase P) 2, membrane-bound | 1 |
| 7572 | hsa-mir-499 | 7512 | XPNPEP2 | X-prolyl aminopeptidase (aminopeptidase P) 2, membrane-bound | 1 |
| 7573 | hsa-mir-877 | 7512 | XPNPEP2 | X-prolyl aminopeptidase (aminopeptidase P) 2, membrane-bound | 1 |
| 7574 | hsa-mir-3190 | 7512 | XPNPEP2 | X-prolyl aminopeptidase (aminopeptidase P) 2, membrane-bound | 1 |
| 7575 | hsa-miR-1 | 1555 | CYP2B6 | cytochrome P450, family 2, subfamily B, polypeptide 6 | 1 |
| 7576 | hsa-miR-133a | 1555 | CYP2B6 | cytochrome P450, family 2, subfamily B, polypeptide 6 | 1 |
| 7577 | hsa-miR-186 | 1555 | CYP2B6 | cytochrome P450, family 2, subfamily B, polypeptide 6 | 1 |
| 7578 | hsa-miR-195 | 1555 | CYP2B6 | cytochrome P450, family 2, subfamily B, polypeptide 6 | 1 |
| 7579 | hsa-miR-208a | 1555 | CYP2B6 | cytochrome P450, family 2, subfamily B, polypeptide 6 | 1 |
| 7580 | hsa-miR-326 | 1555 | CYP2B6 | cytochrome P450, family 2, subfamily B, polypeptide 6 | 1 |
| 7581 | hsa-miR-449b | 1555 | CYP2B6 | cytochrome P450, family 2, subfamily B, polypeptide 6 | 1 |
| 7582 | hsa-miR-497 | 1555 | CYP2B6 | cytochrome P450, family 2, subfamily B, polypeptide 6 | 1 |
| 7583 | hsa-miR-575 | 1555 | CYP2B6 | cytochrome P450, family 2, subfamily B, polypeptide 6 | 1 |
| 7584 | hsa-miR-608 | 1555 | CYP2B6 | cytochrome P450, family 2, subfamily B, polypeptide 6 | 1 |
| 7585 | hsa-miR-620 | 1555 | CYP2B6 | cytochrome P450, family 2, subfamily B, polypeptide 6 | 1 |
| 7586 | hsa-miR-637 | 1555 | CYP2B6 | cytochrome P450, family 2, subfamily B, polypeptide 6 | 1 |
| 7587 | hsa-mir-22 | 1555 | CYP2B6 | cytochrome P450, family 2, subfamily B, polypeptide 6 | 1 |
| 7588 | hsa-mir-10a | 1555 | CYP2B6 | cytochrome P450, family 2, subfamily B, polypeptide 6 | 1 |
| 7589 | hsa-mir-186 | 1555 | CYP2B6 | cytochrome P450, family 2, subfamily B, polypeptide 6 | 1 |
| 7590 | hsa-mir-155 | 1555 | CYP2B6 | cytochrome P450, family 2, subfamily B, polypeptide 6 | 1 |
| 7591 | hsa-mir-499 | 1555 | CYP2B6 | cytochrome P450, family 2, subfamily B, polypeptide 6 | 1 |
| 7592 | hsa-mir-877 | 1555 | CYP2B6 | cytochrome P450, family 2, subfamily B, polypeptide 6 | 1 |
| 7593 | hsa-mir-3190 | 1555 | CYP2B6 | cytochrome P450, family 2, subfamily B, polypeptide 6 | 1 |
| 7594 | hsa-miR-10a | 4494 | MT1F | metallothionein 1F | 1 |
| 7595 | hsa-miR-152 | 4494 | MT1F | metallothionein 1F | 1 |
| 7596 | hsa-miR-26a | 4494 | MT1F | metallothionein 1F | 1 |
| 7597 | hsa-miR-26b | 4494 | MT1F | metallothionein 1F | 1 |
| 7598 | hsa-miR-31 | 4494 | MT1F | metallothionein 1F | 1 |
| 7599 | hsa-miR-338-5p | 4494 | MT1F | metallothionein 1F | 1 |
| 7600 | hsa-miR-340 | 4494 | MT1F | metallothionein 1F | 1 |
| 7601 | hsa-miR-383 | 4494 | MT1F | metallothionein 1F | 1 |
| 7602 | hsa-miR-449a | 4494 | MT1F | metallothionein 1F | 1 |
| 7603 | hsa-miR-490-5p | 4494 | MT1F | metallothionein 1F | 1 |
| 7604 | hsa-miR-499-5p | 4494 | MT1F | metallothionein 1F | 1 |
| 7605 | hsa-miR-504 | 4494 | MT1F | metallothionein 1F | 1 |
| 7606 | hsa-miR-621 | 4494 | MT1F | metallothionein 1F | 1 |
| 7607 | hsa-miR-653 | 4494 | MT1F | metallothionein 1F | 1 |
| 7608 | hsa-miR-657 | 4494 | MT1F | metallothionein 1F | 1 |
| 7609 | hsa-miR-663 | 4494 | MT1F | metallothionein 1F | 1 |
| 7610 | hsa-miR-765 | 4494 | MT1F | metallothionein 1F | 1 |
| 7611 | hsa-miR-770-5p | 4494 | MT1F | metallothionein 1F | 1 |
| 7612 | hsa-miR-877 | 4494 | MT1F | metallothionein 1F | 1 |
| 7613 | hsa-mir-26a-1 | 4494 | MT1F | metallothionein 1F | 1 |
| 7614 | hsa-mir-26b | 4494 | MT1F | metallothionein 1F | 1 |
| 7615 | hsa-mir-31 | 4494 | MT1F | metallothionein 1F | 1 |
| 7616 | hsa-mir-194-2 | 4494 | MT1F | metallothionein 1F | 1 |
| 7617 | hsa-mir-340 | 4494 | MT1F | metallothionein 1F | 1 |
| 7618 | hsa-mir-338 | 4494 | MT1F | metallothionein 1F | 1 |
| 7619 | hsa-mir-490 | 4494 | MT1F | metallothionein 1F | 1 |
| 7620 | hsa-mir-449b | 4494 | MT1F | metallothionein 1F | 1 |
| 7621 | hsa-mir-449c | 4494 | MT1F | metallothionein 1F | 1 |
| 7622 | hsa-mir-3065 | 4494 | MT1F | metallothionein 1F | 1 |
| 7623 | hsa-miR-152 | 4683 | NBN | nibrin | 1 |
| 7624 | hsa-miR-31 | 4683 | NBN | nibrin | 1 |
| 7625 | hsa-miR-338-5p | 4683 | NBN | nibrin | 1 |
| 7626 | hsa-miR-340 | 4683 | NBN | nibrin | 1 |
| 7627 | hsa-miR-383 | 4683 | NBN | nibrin | 1 |
| 7628 | hsa-miR-423-5p | 4683 | NBN | nibrin | 1 |
| 7629 | hsa-miR-449a | 4683 | NBN | nibrin | 1 |
| 7630 | hsa-miR-490-5p | 4683 | NBN | nibrin | 1 |
| 7631 | hsa-miR-621 | 4683 | NBN | nibrin | 1 |
| 7632 | hsa-miR-657 | 4683 | NBN | nibrin | 1 |
| 7633 | hsa-miR-663 | 4683 | NBN | nibrin | 1 |
| 7634 | hsa-miR-765 | 4683 | NBN | nibrin | 1 |
| 7635 | hsa-miR-770-5p | 4683 | NBN | nibrin | 1 |
| 7636 | hsa-miR-877 | 4683 | NBN | nibrin | 1 |
| 7637 | hsa-mir-26a-1 | 4683 | NBN | nibrin | 1 |
| 7638 | hsa-mir-31 | 4683 | NBN | nibrin | 1 |
| 7639 | hsa-mir-194-2 | 4683 | NBN | nibrin | 1 |
| 7640 | hsa-mir-340 | 4683 | NBN | nibrin | 1 |
| 7641 | hsa-mir-338 | 4683 | NBN | nibrin | 1 |
| 7642 | hsa-mir-490 | 4683 | NBN | nibrin | 1 |
| 7643 | hsa-mir-449b | 4683 | NBN | nibrin | 1 |
| 7644 | hsa-mir-449c | 4683 | NBN | nibrin | 1 |
| 7645 | hsa-mir-3065 | 4683 | NBN | nibrin | 1 |
| 7646 | hsa-miR-1 | 1277 | COL1A1 | collagen, type I, alpha 1 | 1 |
| 7647 | hsa-miR-133a | 1277 | COL1A1 | collagen, type I, alpha 1 | 1 |
| 7648 | hsa-miR-186 | 1277 | COL1A1 | collagen, type I, alpha 1 | 1 |
| 7649 | hsa-miR-208a | 1277 | COL1A1 | collagen, type I, alpha 1 | 1 |
| 7650 | hsa-miR-449b | 1277 | COL1A1 | collagen, type I, alpha 1 | 1 |
| 7651 | hsa-miR-489 | 1277 | COL1A1 | collagen, type I, alpha 1 | 1 |
| 7652 | hsa-miR-575 | 1277 | COL1A1 | collagen, type I, alpha 1 | 1 |
| 7653 | hsa-miR-579 | 1277 | COL1A1 | collagen, type I, alpha 1 | 1 |
| 7654 | hsa-miR-608 | 1277 | COL1A1 | collagen, type I, alpha 1 | 1 |
| 7655 | hsa-miR-620 | 1277 | COL1A1 | collagen, type I, alpha 1 | 1 |
| 7656 | hsa-miR-885-5p | 1277 | COL1A1 | collagen, type I, alpha 1 | 1 |
| 7657 | hsa-let-7a-3 | 1277 | COL1A1 | collagen, type I, alpha 1 | 1 |
| 7658 | hsa-let-7b | 1277 | COL1A1 | collagen, type I, alpha 1 | 1 |
| 7659 | hsa-mir-22 | 1277 | COL1A1 | collagen, type I, alpha 1 | 1 |
| 7660 | hsa-mir-10a | 1277 | COL1A1 | collagen, type I, alpha 1 | 1 |
| 7661 | hsa-mir-186 | 1277 | COL1A1 | collagen, type I, alpha 1 | 1 |
| 7662 | hsa-mir-155 | 1277 | COL1A1 | collagen, type I, alpha 1 | 1 |
| 7663 | hsa-mir-499 | 1277 | COL1A1 | collagen, type I, alpha 1 | 1 |
| 7664 | hsa-mir-885 | 1277 | COL1A1 | collagen, type I, alpha 1 | 1 |
| 7665 | hsa-mir-877 | 1277 | COL1A1 | collagen, type I, alpha 1 | 1 |
| 7666 | hsa-miR-1 | 2346 | FOLH1 | folate hydrolase (prostate-specific membrane antigen) 1 | 1 |
| 7667 | hsa-miR-133a | 2346 | FOLH1 | folate hydrolase (prostate-specific membrane antigen) 1 | 1 |
| 7668 | hsa-miR-186 | 2346 | FOLH1 | folate hydrolase (prostate-specific membrane antigen) 1 | 1 |
| 7669 | hsa-miR-208a | 2346 | FOLH1 | folate hydrolase (prostate-specific membrane antigen) 1 | 1 |
| 7670 | hsa-miR-326 | 2346 | FOLH1 | folate hydrolase (prostate-specific membrane antigen) 1 | 1 |
| 7671 | hsa-miR-449b | 2346 | FOLH1 | folate hydrolase (prostate-specific membrane antigen) 1 | 1 |
| 7672 | hsa-miR-575 | 2346 | FOLH1 | folate hydrolase (prostate-specific membrane antigen) 1 | 1 |
| 7673 | hsa-miR-579 | 2346 | FOLH1 | folate hydrolase (prostate-specific membrane antigen) 1 | 1 |
| 7674 | hsa-miR-608 | 2346 | FOLH1 | folate hydrolase (prostate-specific membrane antigen) 1 | 1 |
| 7675 | hsa-miR-620 | 2346 | FOLH1 | folate hydrolase (prostate-specific membrane antigen) 1 | 1 |
| 7676 | hsa-miR-637 | 2346 | FOLH1 | folate hydrolase (prostate-specific membrane antigen) 1 | 1 |
| 7677 | hsa-miR-885-5p | 2346 | FOLH1 | folate hydrolase (prostate-specific membrane antigen) 1 | 1 |
| 7678 | hsa-mir-22 | 2346 | FOLH1 | folate hydrolase (prostate-specific membrane antigen) 1 | 1 |
| 7679 | hsa-mir-10a | 2346 | FOLH1 | folate hydrolase (prostate-specific membrane antigen) 1 | 1 |
| 7680 | hsa-mir-186 | 2346 | FOLH1 | folate hydrolase (prostate-specific membrane antigen) 1 | 1 |
| 7681 | hsa-mir-155 | 2346 | FOLH1 | folate hydrolase (prostate-specific membrane antigen) 1 | 1 |
| 7682 | hsa-mir-423 | 2346 | FOLH1 | folate hydrolase (prostate-specific membrane antigen) 1 | 1 |
| 7683 | hsa-mir-499 | 2346 | FOLH1 | folate hydrolase (prostate-specific membrane antigen) 1 | 1 |
| 7684 | hsa-mir-885 | 2346 | FOLH1 | folate hydrolase (prostate-specific membrane antigen) 1 | 1 |
| 7685 | hsa-mir-877 | 2346 | FOLH1 | folate hydrolase (prostate-specific membrane antigen) 1 | 1 |
| 7686 | hsa-mir-2116 | 2346 | FOLH1 | folate hydrolase (prostate-specific membrane antigen) 1 | 1 |
| 7687 | hsa-mir-3190 | 2346 | FOLH1 | folate hydrolase (prostate-specific membrane antigen) 1 | 1 |
| 7688 | hsa-miR-10a | 55697 | VAC14 | Vac14 homolog (S. cerevisiae) | 1 |
| 7689 | hsa-miR-1228\* | 55697 | VAC14 | Vac14 homolog (S. cerevisiae) | 1 |
| 7690 | hsa-miR-139-5p | 55697 | VAC14 | Vac14 homolog (S. cerevisiae) | 1 |
| 7691 | hsa-miR-152 | 55697 | VAC14 | Vac14 homolog (S. cerevisiae) | 1 |
| 7692 | hsa-miR-26a | 55697 | VAC14 | Vac14 homolog (S. cerevisiae) | 1 |
| 7693 | hsa-miR-26b | 55697 | VAC14 | Vac14 homolog (S. cerevisiae) | 1 |
| 7694 | hsa-miR-31 | 55697 | VAC14 | Vac14 homolog (S. cerevisiae) | 1 |
| 7695 | hsa-miR-338-5p | 55697 | VAC14 | Vac14 homolog (S. cerevisiae) | 1 |
| 7696 | hsa-miR-340 | 55697 | VAC14 | Vac14 homolog (S. cerevisiae) | 1 |
| 7697 | hsa-miR-383 | 55697 | VAC14 | Vac14 homolog (S. cerevisiae) | 1 |
| 7698 | hsa-miR-449a | 55697 | VAC14 | Vac14 homolog (S. cerevisiae) | 1 |
| 7699 | hsa-miR-499-5p | 55697 | VAC14 | Vac14 homolog (S. cerevisiae) | 1 |
| 7700 | hsa-miR-548b-5p | 55697 | VAC14 | Vac14 homolog (S. cerevisiae) | 1 |
| 7701 | hsa-miR-584 | 55697 | VAC14 | Vac14 homolog (S. cerevisiae) | 1 |
| 7702 | hsa-miR-621 | 55697 | VAC14 | Vac14 homolog (S. cerevisiae) | 1 |
| 7703 | hsa-miR-657 | 55697 | VAC14 | Vac14 homolog (S. cerevisiae) | 1 |
| 7704 | hsa-miR-663 | 55697 | VAC14 | Vac14 homolog (S. cerevisiae) | 1 |
| 7705 | hsa-miR-765 | 55697 | VAC14 | Vac14 homolog (S. cerevisiae) | 1 |
| 7706 | hsa-miR-770-5p | 55697 | VAC14 | Vac14 homolog (S. cerevisiae) | 1 |
| 7707 | hsa-miR-877 | 55697 | VAC14 | Vac14 homolog (S. cerevisiae) | 1 |
| 7708 | hsa-mir-26a-1 | 55697 | VAC14 | Vac14 homolog (S. cerevisiae) | 1 |
| 7709 | hsa-mir-26b | 55697 | VAC14 | Vac14 homolog (S. cerevisiae) | 1 |
| 7710 | hsa-mir-31 | 55697 | VAC14 | Vac14 homolog (S. cerevisiae) | 1 |
| 7711 | hsa-mir-194-2 | 55697 | VAC14 | Vac14 homolog (S. cerevisiae) | 1 |
| 7712 | hsa-mir-340 | 55697 | VAC14 | Vac14 homolog (S. cerevisiae) | 1 |
| 7713 | hsa-mir-338 | 55697 | VAC14 | Vac14 homolog (S. cerevisiae) | 1 |
| 7714 | hsa-mir-548b | 55697 | VAC14 | Vac14 homolog (S. cerevisiae) | 1 |
| 7715 | hsa-mir-449b | 55697 | VAC14 | Vac14 homolog (S. cerevisiae) | 1 |
| 7716 | hsa-mir-449c | 55697 | VAC14 | Vac14 homolog (S. cerevisiae) | 1 |
| 7717 | hsa-mir-3065 | 55697 | VAC14 | Vac14 homolog (S. cerevisiae) | 1 |
| 7746 | hsa-miR-152 | 29066 | ZC3H7A | zinc finger CCCH-type containing 7A | 1 |
| 7747 | hsa-miR-26a | 29066 | ZC3H7A | zinc finger CCCH-type containing 7A | 1 |
| 7748 | hsa-miR-26b | 29066 | ZC3H7A | zinc finger CCCH-type containing 7A | 1 |
| 7749 | hsa-miR-31 | 29066 | ZC3H7A | zinc finger CCCH-type containing 7A | 1 |
| 7750 | hsa-miR-340 | 29066 | ZC3H7A | zinc finger CCCH-type containing 7A | 1 |
| 7751 | hsa-miR-383 | 29066 | ZC3H7A | zinc finger CCCH-type containing 7A | 1 |
| 7752 | hsa-miR-423-5p | 29066 | ZC3H7A | zinc finger CCCH-type containing 7A | 1 |
| 7753 | hsa-miR-449a | 29066 | ZC3H7A | zinc finger CCCH-type containing 7A | 1 |
| 7754 | hsa-miR-564 | 29066 | ZC3H7A | zinc finger CCCH-type containing 7A | 1 |
| 7755 | hsa-miR-621 | 29066 | ZC3H7A | zinc finger CCCH-type containing 7A | 1 |
| 7756 | hsa-miR-653 | 29066 | ZC3H7A | zinc finger CCCH-type containing 7A | 1 |
| 7757 | hsa-miR-765 | 29066 | ZC3H7A | zinc finger CCCH-type containing 7A | 1 |
| 7758 | hsa-miR-770-5p | 29066 | ZC3H7A | zinc finger CCCH-type containing 7A | 1 |
| 7759 | hsa-mir-26a-1 | 29066 | ZC3H7A | zinc finger CCCH-type containing 7A | 1 |
| 7760 | hsa-mir-26b | 29066 | ZC3H7A | zinc finger CCCH-type containing 7A | 1 |
| 7761 | hsa-mir-31 | 29066 | ZC3H7A | zinc finger CCCH-type containing 7A | 1 |
| 7762 | hsa-mir-194-2 | 29066 | ZC3H7A | zinc finger CCCH-type containing 7A | 1 |
| 7763 | hsa-mir-340 | 29066 | ZC3H7A | zinc finger CCCH-type containing 7A | 1 |
| 7764 | hsa-mir-449b | 29066 | ZC3H7A | zinc finger CCCH-type containing 7A | 1 |
| 7765 | hsa-mir-449c | 29066 | ZC3H7A | zinc finger CCCH-type containing 7A | 1 |
| 7766 | hsa-miR-10a | 29887 | SNX10 | sorting nexin 10 | 1 |
| 7767 | hsa-miR-152 | 29887 | SNX10 | sorting nexin 10 | 1 |
| 7768 | hsa-miR-26a | 29887 | SNX10 | sorting nexin 10 | 1 |
| 7769 | hsa-miR-26b | 29887 | SNX10 | sorting nexin 10 | 1 |
| 7770 | hsa-miR-31 | 29887 | SNX10 | sorting nexin 10 | 1 |
| 7771 | hsa-miR-338-5p | 29887 | SNX10 | sorting nexin 10 | 1 |
| 7772 | hsa-miR-423-5p | 29887 | SNX10 | sorting nexin 10 | 1 |
| 7773 | hsa-miR-449a | 29887 | SNX10 | sorting nexin 10 | 1 |
| 7774 | hsa-miR-499-5p | 29887 | SNX10 | sorting nexin 10 | 1 |
| 7775 | hsa-miR-548b-5p | 29887 | SNX10 | sorting nexin 10 | 1 |
| 7776 | hsa-miR-564 | 29887 | SNX10 | sorting nexin 10 | 1 |
| 7777 | hsa-miR-621 | 29887 | SNX10 | sorting nexin 10 | 1 |
| 7778 | hsa-miR-657 | 29887 | SNX10 | sorting nexin 10 | 1 |
| 7779 | hsa-miR-663 | 29887 | SNX10 | sorting nexin 10 | 1 |
| 7780 | hsa-miR-765 | 29887 | SNX10 | sorting nexin 10 | 1 |
| 7781 | hsa-miR-770-5p | 29887 | SNX10 | sorting nexin 10 | 1 |
| 7782 | hsa-miR-877 | 29887 | SNX10 | sorting nexin 10 | 1 |
| 7783 | hsa-mir-26a-1 | 29887 | SNX10 | sorting nexin 10 | 1 |
| 7784 | hsa-mir-26b | 29887 | SNX10 | sorting nexin 10 | 1 |
| 7785 | hsa-mir-31 | 29887 | SNX10 | sorting nexin 10 | 1 |
| 7786 | hsa-mir-194-2 | 29887 | SNX10 | sorting nexin 10 | 1 |
| 7787 | hsa-mir-338 | 29887 | SNX10 | sorting nexin 10 | 1 |
| 7788 | hsa-mir-548b | 29887 | SNX10 | sorting nexin 10 | 1 |
| 7789 | hsa-mir-548d-2 | 29887 | SNX10 | sorting nexin 10 | 1 |
| 7790 | hsa-mir-449b | 29887 | SNX10 | sorting nexin 10 | 1 |
| 7791 | hsa-mir-449c | 29887 | SNX10 | sorting nexin 10 | 1 |
| 7792 | hsa-mir-3065 | 29887 | SNX10 | sorting nexin 10 | 1 |
| 7793 | hsa-miR-489 | 29937 | NENF | neuron derived neurotrophic factor | 1 |
| 7794 | hsa-miR-637 | 29937 | NENF | neuron derived neurotrophic factor | 1 |
| 7795 | hsa-miR-885-5p | 29937 | NENF | neuron derived neurotrophic factor | 1 |
| 7796 | hsa-mir-885 | 29937 | NENF | neuron derived neurotrophic factor | 1 |
| 7797 | hsa-miR-1 | 6839 | SUV39H1 | suppressor of variegation 3-9 homolog 1 (Drosophila) | 1 |
| 7798 | hsa-miR-133a | 6839 | SUV39H1 | suppressor of variegation 3-9 homolog 1 (Drosophila) | 1 |
| 7799 | hsa-miR-186 | 6839 | SUV39H1 | suppressor of variegation 3-9 homolog 1 (Drosophila) | 1 |
| 7800 | hsa-miR-195 | 6839 | SUV39H1 | suppressor of variegation 3-9 homolog 1 (Drosophila) | 1 |
| 7801 | hsa-miR-326 | 6839 | SUV39H1 | suppressor of variegation 3-9 homolog 1 (Drosophila) | 1 |
| 7802 | hsa-miR-449b | 6839 | SUV39H1 | suppressor of variegation 3-9 homolog 1 (Drosophila) | 1 |
| 7803 | hsa-miR-497 | 6839 | SUV39H1 | suppressor of variegation 3-9 homolog 1 (Drosophila) | 1 |
| 7804 | hsa-miR-551a | 6839 | SUV39H1 | suppressor of variegation 3-9 homolog 1 (Drosophila) | 1 |
| 7805 | hsa-miR-575 | 6839 | SUV39H1 | suppressor of variegation 3-9 homolog 1 (Drosophila) | 1 |
| 7806 | hsa-miR-579 | 6839 | SUV39H1 | suppressor of variegation 3-9 homolog 1 (Drosophila) | 1 |
| 7807 | hsa-miR-608 | 6839 | SUV39H1 | suppressor of variegation 3-9 homolog 1 (Drosophila) | 1 |
| 7808 | hsa-miR-620 | 6839 | SUV39H1 | suppressor of variegation 3-9 homolog 1 (Drosophila) | 1 |
| 7809 | hsa-mir-22 | 6839 | SUV39H1 | suppressor of variegation 3-9 homolog 1 (Drosophila) | 1 |
| 7810 | hsa-mir-10a | 6839 | SUV39H1 | suppressor of variegation 3-9 homolog 1 (Drosophila) | 1 |
| 7811 | hsa-mir-186 | 6839 | SUV39H1 | suppressor of variegation 3-9 homolog 1 (Drosophila) | 1 |
| 7812 | hsa-mir-155 | 6839 | SUV39H1 | suppressor of variegation 3-9 homolog 1 (Drosophila) | 1 |
| 7813 | hsa-mir-499 | 6839 | SUV39H1 | suppressor of variegation 3-9 homolog 1 (Drosophila) | 1 |
| 7814 | hsa-mir-877 | 6839 | SUV39H1 | suppressor of variegation 3-9 homolog 1 (Drosophila) | 1 |
| 7815 | hsa-mir-2116 | 6839 | SUV39H1 | suppressor of variegation 3-9 homolog 1 (Drosophila) | 1 |
| 7816 | hsa-mir-3190 | 6839 | SUV39H1 | suppressor of variegation 3-9 homolog 1 (Drosophila) | 1 |
| 7817 | hsa-miR-10a | 79693 | YRDC | yrdC domain containing (E. coli) | 1 |
| 7818 | hsa-miR-139-5p | 79693 | YRDC | yrdC domain containing (E. coli) | 1 |
| 7819 | hsa-miR-152 | 79693 | YRDC | yrdC domain containing (E. coli) | 1 |
| 7820 | hsa-miR-26a | 79693 | YRDC | yrdC domain containing (E. coli) | 1 |
| 7821 | hsa-miR-26b | 79693 | YRDC | yrdC domain containing (E. coli) | 1 |
| 7822 | hsa-miR-340 | 79693 | YRDC | yrdC domain containing (E. coli) | 1 |
| 7823 | hsa-miR-449a | 79693 | YRDC | yrdC domain containing (E. coli) | 1 |
| 7824 | hsa-miR-490-5p | 79693 | YRDC | yrdC domain containing (E. coli) | 1 |
| 7825 | hsa-miR-499-5p | 79693 | YRDC | yrdC domain containing (E. coli) | 1 |
| 7826 | hsa-miR-548b-5p | 79693 | YRDC | yrdC domain containing (E. coli) | 1 |
| 7827 | hsa-miR-564 | 79693 | YRDC | yrdC domain containing (E. coli) | 1 |
| 7828 | hsa-miR-584 | 79693 | YRDC | yrdC domain containing (E. coli) | 1 |
| 7829 | hsa-miR-621 | 79693 | YRDC | yrdC domain containing (E. coli) | 1 |
| 7830 | hsa-miR-634 | 79693 | YRDC | yrdC domain containing (E. coli) | 1 |
| 7831 | hsa-miR-765 | 79693 | YRDC | yrdC domain containing (E. coli) | 1 |
| 7832 | hsa-miR-770-5p | 79693 | YRDC | yrdC domain containing (E. coli) | 1 |
| 7833 | hsa-miR-877 | 79693 | YRDC | yrdC domain containing (E. coli) | 1 |
| 7834 | hsa-mir-26a-1 | 79693 | YRDC | yrdC domain containing (E. coli) | 1 |
| 7835 | hsa-mir-26b | 79693 | YRDC | yrdC domain containing (E. coli) | 1 |
| 7836 | hsa-mir-194-2 | 79693 | YRDC | yrdC domain containing (E. coli) | 1 |
| 7837 | hsa-mir-340 | 79693 | YRDC | yrdC domain containing (E. coli) | 1 |
| 7838 | hsa-mir-490 | 79693 | YRDC | yrdC domain containing (E. coli) | 1 |
| 7839 | hsa-mir-548b | 79693 | YRDC | yrdC domain containing (E. coli) | 1 |
| 7840 | hsa-mir-548d-2 | 79693 | YRDC | yrdC domain containing (E. coli) | 1 |
| 7841 | hsa-mir-449b | 79693 | YRDC | yrdC domain containing (E. coli) | 1 |
| 7842 | hsa-mir-449c | 79693 | YRDC | yrdC domain containing (E. coli) | 1 |
| 7843 | hsa-miR-10a | 10186 | LHFP | lipoma HMGIC fusion partner | 1 |
| 7844 | hsa-miR-139-5p | 10186 | LHFP | lipoma HMGIC fusion partner | 1 |
| 7845 | hsa-miR-152 | 10186 | LHFP | lipoma HMGIC fusion partner | 1 |
| 7846 | hsa-miR-26b | 10186 | LHFP | lipoma HMGIC fusion partner | 1 |
| 7847 | hsa-miR-31 | 10186 | LHFP | lipoma HMGIC fusion partner | 1 |
| 7848 | hsa-miR-338-5p | 10186 | LHFP | lipoma HMGIC fusion partner | 1 |
| 7849 | hsa-miR-340 | 10186 | LHFP | lipoma HMGIC fusion partner | 1 |
| 7850 | hsa-miR-383 | 10186 | LHFP | lipoma HMGIC fusion partner | 1 |
| 7851 | hsa-miR-449a | 10186 | LHFP | lipoma HMGIC fusion partner | 1 |
| 7852 | hsa-miR-499-5p | 10186 | LHFP | lipoma HMGIC fusion partner | 1 |
| 7853 | hsa-miR-548b-5p | 10186 | LHFP | lipoma HMGIC fusion partner | 1 |
| 7854 | hsa-miR-621 | 10186 | LHFP | lipoma HMGIC fusion partner | 1 |
| 7855 | hsa-miR-657 | 10186 | LHFP | lipoma HMGIC fusion partner | 1 |
| 7856 | hsa-miR-765 | 10186 | LHFP | lipoma HMGIC fusion partner | 1 |
| 7857 | hsa-miR-770-5p | 10186 | LHFP | lipoma HMGIC fusion partner | 1 |
| 7858 | hsa-miR-877 | 10186 | LHFP | lipoma HMGIC fusion partner | 1 |
| 7859 | hsa-mir-26a-1 | 10186 | LHFP | lipoma HMGIC fusion partner | 1 |
| 7860 | hsa-mir-26b | 10186 | LHFP | lipoma HMGIC fusion partner | 1 |
| 7861 | hsa-mir-31 | 10186 | LHFP | lipoma HMGIC fusion partner | 1 |
| 7862 | hsa-mir-194-2 | 10186 | LHFP | lipoma HMGIC fusion partner | 1 |
| 7863 | hsa-mir-340 | 10186 | LHFP | lipoma HMGIC fusion partner | 1 |
| 7864 | hsa-mir-338 | 10186 | LHFP | lipoma HMGIC fusion partner | 1 |
| 7865 | hsa-mir-548b | 10186 | LHFP | lipoma HMGIC fusion partner | 1 |
| 7866 | hsa-mir-449b | 10186 | LHFP | lipoma HMGIC fusion partner | 1 |
| 7867 | hsa-mir-449c | 10186 | LHFP | lipoma HMGIC fusion partner | 1 |
| 7868 | hsa-mir-3065 | 10186 | LHFP | lipoma HMGIC fusion partner | 1 |
| 7869 | hsa-miR-10a | 57826 | RAP2C | RAP2C, member of RAS oncogene family | 1 |
| 7870 | hsa-miR-1228\* | 57826 | RAP2C | RAP2C, member of RAS oncogene family | 1 |
| 7871 | hsa-miR-152 | 57826 | RAP2C | RAP2C, member of RAS oncogene family | 1 |
| 7872 | hsa-miR-26a | 57826 | RAP2C | RAP2C, member of RAS oncogene family | 1 |
| 7873 | hsa-miR-26b | 57826 | RAP2C | RAP2C, member of RAS oncogene family | 1 |
| 7874 | hsa-miR-31 | 57826 | RAP2C | RAP2C, member of RAS oncogene family | 1 |
| 7875 | hsa-miR-338-5p | 57826 | RAP2C | RAP2C, member of RAS oncogene family | 1 |
| 7876 | hsa-miR-340 | 57826 | RAP2C | RAP2C, member of RAS oncogene family | 1 |
| 7877 | hsa-miR-423-5p | 57826 | RAP2C | RAP2C, member of RAS oncogene family | 1 |
| 7878 | hsa-miR-449a | 57826 | RAP2C | RAP2C, member of RAS oncogene family | 1 |
| 7879 | hsa-miR-490-5p | 57826 | RAP2C | RAP2C, member of RAS oncogene family | 1 |
| 7880 | hsa-miR-499-5p | 57826 | RAP2C | RAP2C, member of RAS oncogene family | 1 |
| 7881 | hsa-miR-548b-5p | 57826 | RAP2C | RAP2C, member of RAS oncogene family | 1 |
| 7882 | hsa-miR-564 | 57826 | RAP2C | RAP2C, member of RAS oncogene family | 1 |
| 7883 | hsa-miR-584 | 57826 | RAP2C | RAP2C, member of RAS oncogene family | 1 |
| 7884 | hsa-miR-621 | 57826 | RAP2C | RAP2C, member of RAS oncogene family | 1 |
| 7885 | hsa-miR-644 | 57826 | RAP2C | RAP2C, member of RAS oncogene family | 1 |
| 7886 | hsa-miR-657 | 57826 | RAP2C | RAP2C, member of RAS oncogene family | 1 |
| 7887 | hsa-miR-765 | 57826 | RAP2C | RAP2C, member of RAS oncogene family | 1 |
| 7888 | hsa-miR-770-5p | 57826 | RAP2C | RAP2C, member of RAS oncogene family | 1 |
| 7889 | hsa-miR-877 | 57826 | RAP2C | RAP2C, member of RAS oncogene family | 1 |
| 7890 | hsa-miR-95 | 57826 | RAP2C | RAP2C, member of RAS oncogene family | 1 |
| 7891 | hsa-mir-26a-1 | 57826 | RAP2C | RAP2C, member of RAS oncogene family | 1 |
| 7892 | hsa-mir-26b | 57826 | RAP2C | RAP2C, member of RAS oncogene family | 1 |
| 7893 | hsa-mir-31 | 57826 | RAP2C | RAP2C, member of RAS oncogene family | 1 |
| 7894 | hsa-mir-194-2 | 57826 | RAP2C | RAP2C, member of RAS oncogene family | 1 |
| 7895 | hsa-mir-340 | 57826 | RAP2C | RAP2C, member of RAS oncogene family | 1 |
| 7896 | hsa-mir-338 | 57826 | RAP2C | RAP2C, member of RAS oncogene family | 1 |
| 7897 | hsa-mir-490 | 57826 | RAP2C | RAP2C, member of RAS oncogene family | 1 |
| 7898 | hsa-mir-548b | 57826 | RAP2C | RAP2C, member of RAS oncogene family | 1 |
| 7899 | hsa-mir-449b | 57826 | RAP2C | RAP2C, member of RAS oncogene family | 1 |
| 7900 | hsa-mir-449c | 57826 | RAP2C | RAP2C, member of RAS oncogene family | 1 |
| 7901 | hsa-mir-3065 | 57826 | RAP2C | RAP2C, member of RAS oncogene family | 1 |
| 7903 | hsa-miR-1228\* | 8934 | RAB7L1 | RAB7, member RAS oncogene family-like 1 | 1 |
| 7911 | hsa-miR-423-5p | 8934 | RAB7L1 | RAB7, member RAS oncogene family-like 1 | 1 |
| 7932 | hsa-miR-634 | 8934 | RAB7L1 | RAB7, member RAS oncogene family-like 1 | 1 |
| 7933 | hsa-miR-10a | 55152 | DALRD3 | DALR anticodon binding domain containing 3 | 1 |
| 7934 | hsa-miR-139-5p | 55152 | DALRD3 | DALR anticodon binding domain containing 3 | 1 |
| 7935 | hsa-miR-152 | 55152 | DALRD3 | DALR anticodon binding domain containing 3 | 1 |
| 7936 | hsa-miR-26b | 55152 | DALRD3 | DALR anticodon binding domain containing 3 | 1 |
| 7937 | hsa-miR-31 | 55152 | DALRD3 | DALR anticodon binding domain containing 3 | 1 |
| 7938 | hsa-miR-338-5p | 55152 | DALRD3 | DALR anticodon binding domain containing 3 | 1 |
| 7939 | hsa-miR-340 | 55152 | DALRD3 | DALR anticodon binding domain containing 3 | 1 |
| 7940 | hsa-miR-383 | 55152 | DALRD3 | DALR anticodon binding domain containing 3 | 1 |
| 7941 | hsa-miR-449a | 55152 | DALRD3 | DALR anticodon binding domain containing 3 | 1 |
| 7942 | hsa-miR-499-5p | 55152 | DALRD3 | DALR anticodon binding domain containing 3 | 1 |
| 7943 | hsa-miR-548b-5p | 55152 | DALRD3 | DALR anticodon binding domain containing 3 | 1 |
| 7944 | hsa-miR-621 | 55152 | DALRD3 | DALR anticodon binding domain containing 3 | 1 |
| 7945 | hsa-miR-657 | 55152 | DALRD3 | DALR anticodon binding domain containing 3 | 1 |
| 7946 | hsa-miR-663 | 55152 | DALRD3 | DALR anticodon binding domain containing 3 | 1 |
| 7947 | hsa-miR-765 | 55152 | DALRD3 | DALR anticodon binding domain containing 3 | 1 |
| 7948 | hsa-miR-770-5p | 55152 | DALRD3 | DALR anticodon binding domain containing 3 | 1 |
| 7949 | hsa-miR-877 | 55152 | DALRD3 | DALR anticodon binding domain containing 3 | 1 |
| 7950 | hsa-mir-26a-1 | 55152 | DALRD3 | DALR anticodon binding domain containing 3 | 1 |
| 7951 | hsa-mir-26b | 55152 | DALRD3 | DALR anticodon binding domain containing 3 | 1 |
| 7952 | hsa-mir-31 | 55152 | DALRD3 | DALR anticodon binding domain containing 3 | 1 |
| 7953 | hsa-mir-194-2 | 55152 | DALRD3 | DALR anticodon binding domain containing 3 | 1 |
| 7954 | hsa-mir-340 | 55152 | DALRD3 | DALR anticodon binding domain containing 3 | 1 |
| 7955 | hsa-mir-338 | 55152 | DALRD3 | DALR anticodon binding domain containing 3 | 1 |
| 7956 | hsa-mir-548b | 55152 | DALRD3 | DALR anticodon binding domain containing 3 | 1 |
| 7957 | hsa-mir-449b | 55152 | DALRD3 | DALR anticodon binding domain containing 3 | 1 |
| 7958 | hsa-mir-449c | 55152 | DALRD3 | DALR anticodon binding domain containing 3 | 1 |
| 7959 | hsa-mir-3065 | 55152 | DALRD3 | DALR anticodon binding domain containing 3 | 1 |
| 7960 | hsa-miR-10a | 80149 | ZC3H12A | zinc finger CCCH-type containing 12A | 1 |
| 7961 | hsa-miR-139-5p | 80149 | ZC3H12A | zinc finger CCCH-type containing 12A | 1 |
| 7962 | hsa-miR-152 | 80149 | ZC3H12A | zinc finger CCCH-type containing 12A | 1 |
| 7963 | hsa-miR-26a | 80149 | ZC3H12A | zinc finger CCCH-type containing 12A | 1 |
| 7964 | hsa-miR-26b | 80149 | ZC3H12A | zinc finger CCCH-type containing 12A | 1 |
| 7965 | hsa-miR-31 | 80149 | ZC3H12A | zinc finger CCCH-type containing 12A | 1 |
| 7966 | hsa-miR-338-5p | 80149 | ZC3H12A | zinc finger CCCH-type containing 12A | 1 |
| 7967 | hsa-miR-340 | 80149 | ZC3H12A | zinc finger CCCH-type containing 12A | 1 |
| 7968 | hsa-miR-449a | 80149 | ZC3H12A | zinc finger CCCH-type containing 12A | 1 |
| 7969 | hsa-miR-490-5p | 80149 | ZC3H12A | zinc finger CCCH-type containing 12A | 1 |
| 7970 | hsa-miR-499-5p | 80149 | ZC3H12A | zinc finger CCCH-type containing 12A | 1 |
| 7971 | hsa-miR-548b-5p | 80149 | ZC3H12A | zinc finger CCCH-type containing 12A | 1 |
| 7972 | hsa-miR-584 | 80149 | ZC3H12A | zinc finger CCCH-type containing 12A | 1 |
| 7973 | hsa-miR-621 | 80149 | ZC3H12A | zinc finger CCCH-type containing 12A | 1 |
| 7974 | hsa-miR-657 | 80149 | ZC3H12A | zinc finger CCCH-type containing 12A | 1 |
| 7975 | hsa-miR-663 | 80149 | ZC3H12A | zinc finger CCCH-type containing 12A | 1 |
| 7976 | hsa-miR-765 | 80149 | ZC3H12A | zinc finger CCCH-type containing 12A | 1 |
| 7977 | hsa-miR-770-5p | 80149 | ZC3H12A | zinc finger CCCH-type containing 12A | 1 |
| 7978 | hsa-miR-877 | 80149 | ZC3H12A | zinc finger CCCH-type containing 12A | 1 |
| 7979 | hsa-mir-26a-1 | 80149 | ZC3H12A | zinc finger CCCH-type containing 12A | 1 |
| 7980 | hsa-mir-26b | 80149 | ZC3H12A | zinc finger CCCH-type containing 12A | 1 |
| 7981 | hsa-mir-31 | 80149 | ZC3H12A | zinc finger CCCH-type containing 12A | 1 |
| 7982 | hsa-mir-194-2 | 80149 | ZC3H12A | zinc finger CCCH-type containing 12A | 1 |
| 7983 | hsa-mir-340 | 80149 | ZC3H12A | zinc finger CCCH-type containing 12A | 1 |
| 7984 | hsa-mir-338 | 80149 | ZC3H12A | zinc finger CCCH-type containing 12A | 1 |
| 7985 | hsa-mir-490 | 80149 | ZC3H12A | zinc finger CCCH-type containing 12A | 1 |
| 7986 | hsa-mir-548b | 80149 | ZC3H12A | zinc finger CCCH-type containing 12A | 1 |
| 7987 | hsa-mir-548d-2 | 80149 | ZC3H12A | zinc finger CCCH-type containing 12A | 1 |
| 7988 | hsa-mir-449b | 80149 | ZC3H12A | zinc finger CCCH-type containing 12A | 1 |
| 7989 | hsa-mir-449c | 80149 | ZC3H12A | zinc finger CCCH-type containing 12A | 1 |
| 7990 | hsa-mir-3065 | 80149 | ZC3H12A | zinc finger CCCH-type containing 12A | 1 |
| 7991 | hsa-miR-1 | 79969 | C6orf134 | chromosome 6 open reading frame 134 | 1 |
| 7992 | hsa-miR-133a | 79969 | C6orf134 | chromosome 6 open reading frame 134 | 1 |
| 7993 | hsa-miR-186 | 79969 | C6orf134 | chromosome 6 open reading frame 134 | 1 |
| 7994 | hsa-miR-195 | 79969 | C6orf134 | chromosome 6 open reading frame 134 | 1 |
| 7995 | hsa-miR-326 | 79969 | C6orf134 | chromosome 6 open reading frame 134 | 1 |
| 7996 | hsa-miR-33a | 79969 | C6orf134 | chromosome 6 open reading frame 134 | 1 |
| 7997 | hsa-miR-449b | 79969 | C6orf134 | chromosome 6 open reading frame 134 | 1 |
| 7998 | hsa-miR-497 | 79969 | C6orf134 | chromosome 6 open reading frame 134 | 1 |
| 7999 | hsa-miR-550 | 79969 | C6orf134 | chromosome 6 open reading frame 134 | 1 |
| 8000 | hsa-miR-575 | 79969 | C6orf134 | chromosome 6 open reading frame 134 | 1 |
| 8001 | hsa-miR-608 | 79969 | C6orf134 | chromosome 6 open reading frame 134 | 1 |
| 8002 | hsa-miR-620 | 79969 | C6orf134 | chromosome 6 open reading frame 134 | 1 |
| 8003 | hsa-let-7a-3 | 79969 | C6orf134 | chromosome 6 open reading frame 134 | 1 |
| 8004 | hsa-let-7b | 79969 | C6orf134 | chromosome 6 open reading frame 134 | 1 |
| 8005 | hsa-mir-22 | 79969 | C6orf134 | chromosome 6 open reading frame 134 | 1 |
| 8006 | hsa-mir-10a | 79969 | C6orf134 | chromosome 6 open reading frame 134 | 1 |
| 8007 | hsa-mir-186 | 79969 | C6orf134 | chromosome 6 open reading frame 134 | 1 |
| 8008 | hsa-mir-155 | 79969 | C6orf134 | chromosome 6 open reading frame 134 | 1 |
| 8009 | hsa-mir-499 | 79969 | C6orf134 | chromosome 6 open reading frame 134 | 1 |
| 8010 | hsa-mir-550-1 | 79969 | C6orf134 | chromosome 6 open reading frame 134 | 1 |
| 8011 | hsa-mir-877 | 79969 | C6orf134 | chromosome 6 open reading frame 134 | 1 |
| 8012 | hsa-mir-2116 | 79969 | C6orf134 | chromosome 6 open reading frame 134 | 1 |
| 8013 | hsa-mir-3186 | 79969 | C6orf134 | chromosome 6 open reading frame 134 | 1 |
| 8014 | hsa-miR-10a | 54020 | SLC37A1 | solute carrier family 37 (glycerol-3-phosphate transporter), member 1 | 1 |
| 8015 | hsa-miR-1228\* | 54020 | SLC37A1 | solute carrier family 37 (glycerol-3-phosphate transporter), member 1 | 1 |
| 8016 | hsa-miR-139-5p | 54020 | SLC37A1 | solute carrier family 37 (glycerol-3-phosphate transporter), member 1 | 1 |
| 8017 | hsa-miR-152 | 54020 | SLC37A1 | solute carrier family 37 (glycerol-3-phosphate transporter), member 1 | 1 |
| 8018 | hsa-miR-26a | 54020 | SLC37A1 | solute carrier family 37 (glycerol-3-phosphate transporter), member 1 | 1 |
| 8019 | hsa-miR-26b | 54020 | SLC37A1 | solute carrier family 37 (glycerol-3-phosphate transporter), member 1 | 1 |
| 8020 | hsa-miR-338-5p | 54020 | SLC37A1 | solute carrier family 37 (glycerol-3-phosphate transporter), member 1 | 1 |
| 8021 | hsa-miR-340 | 54020 | SLC37A1 | solute carrier family 37 (glycerol-3-phosphate transporter), member 1 | 1 |
| 8022 | hsa-miR-423-5p | 54020 | SLC37A1 | solute carrier family 37 (glycerol-3-phosphate transporter), member 1 | 1 |
| 8023 | hsa-miR-449a | 54020 | SLC37A1 | solute carrier family 37 (glycerol-3-phosphate transporter), member 1 | 1 |
| 8024 | hsa-miR-499-5p | 54020 | SLC37A1 | solute carrier family 37 (glycerol-3-phosphate transporter), member 1 | 1 |
| 8025 | hsa-miR-548b-5p | 54020 | SLC37A1 | solute carrier family 37 (glycerol-3-phosphate transporter), member 1 | 1 |
| 8026 | hsa-miR-584 | 54020 | SLC37A1 | solute carrier family 37 (glycerol-3-phosphate transporter), member 1 | 1 |
| 8027 | hsa-miR-621 | 54020 | SLC37A1 | solute carrier family 37 (glycerol-3-phosphate transporter), member 1 | 1 |
| 8028 | hsa-miR-657 | 54020 | SLC37A1 | solute carrier family 37 (glycerol-3-phosphate transporter), member 1 | 1 |
| 8029 | hsa-miR-663 | 54020 | SLC37A1 | solute carrier family 37 (glycerol-3-phosphate transporter), member 1 | 1 |
| 8030 | hsa-miR-765 | 54020 | SLC37A1 | solute carrier family 37 (glycerol-3-phosphate transporter), member 1 | 1 |
| 8031 | hsa-miR-770-5p | 54020 | SLC37A1 | solute carrier family 37 (glycerol-3-phosphate transporter), member 1 | 1 |
| 8032 | hsa-miR-877 | 54020 | SLC37A1 | solute carrier family 37 (glycerol-3-phosphate transporter), member 1 | 1 |
| 8033 | hsa-mir-26a-1 | 54020 | SLC37A1 | solute carrier family 37 (glycerol-3-phosphate transporter), member 1 | 1 |
| 8034 | hsa-mir-26b | 54020 | SLC37A1 | solute carrier family 37 (glycerol-3-phosphate transporter), member 1 | 1 |
| 8035 | hsa-mir-194-2 | 54020 | SLC37A1 | solute carrier family 37 (glycerol-3-phosphate transporter), member 1 | 1 |
| 8036 | hsa-mir-340 | 54020 | SLC37A1 | solute carrier family 37 (glycerol-3-phosphate transporter), member 1 | 1 |
| 8037 | hsa-mir-338 | 54020 | SLC37A1 | solute carrier family 37 (glycerol-3-phosphate transporter), member 1 | 1 |
| 8038 | hsa-mir-548b | 54020 | SLC37A1 | solute carrier family 37 (glycerol-3-phosphate transporter), member 1 | 1 |
| 8039 | hsa-mir-548d-2 | 54020 | SLC37A1 | solute carrier family 37 (glycerol-3-phosphate transporter), member 1 | 1 |
| 8040 | hsa-mir-449b | 54020 | SLC37A1 | solute carrier family 37 (glycerol-3-phosphate transporter), member 1 | 1 |
| 8041 | hsa-mir-449c | 54020 | SLC37A1 | solute carrier family 37 (glycerol-3-phosphate transporter), member 1 | 1 |
| 8042 | hsa-mir-3065 | 54020 | SLC37A1 | solute carrier family 37 (glycerol-3-phosphate transporter), member 1 | 1 |
| 8043 | hsa-miR-10a | 54509 | RHOF | ras homolog gene family, member F (in filopodia) | 1 |
| 8044 | hsa-miR-152 | 54509 | RHOF | ras homolog gene family, member F (in filopodia) | 1 |
| 8045 | hsa-miR-26a | 54509 | RHOF | ras homolog gene family, member F (in filopodia) | 1 |
| 8046 | hsa-miR-26b | 54509 | RHOF | ras homolog gene family, member F (in filopodia) | 1 |
| 8047 | hsa-miR-31 | 54509 | RHOF | ras homolog gene family, member F (in filopodia) | 1 |
| 8048 | hsa-miR-338-5p | 54509 | RHOF | ras homolog gene family, member F (in filopodia) | 1 |
| 8049 | hsa-miR-340 | 54509 | RHOF | ras homolog gene family, member F (in filopodia) | 1 |
| 8050 | hsa-miR-383 | 54509 | RHOF | ras homolog gene family, member F (in filopodia) | 1 |
| 8051 | hsa-miR-449a | 54509 | RHOF | ras homolog gene family, member F (in filopodia) | 1 |
| 8052 | hsa-miR-490-5p | 54509 | RHOF | ras homolog gene family, member F (in filopodia) | 1 |
| 8053 | hsa-miR-499-5p | 54509 | RHOF | ras homolog gene family, member F (in filopodia) | 1 |
| 8054 | hsa-miR-621 | 54509 | RHOF | ras homolog gene family, member F (in filopodia) | 1 |
| 8055 | hsa-miR-653 | 54509 | RHOF | ras homolog gene family, member F (in filopodia) | 1 |
| 8056 | hsa-miR-657 | 54509 | RHOF | ras homolog gene family, member F (in filopodia) | 1 |
| 8057 | hsa-miR-663 | 54509 | RHOF | ras homolog gene family, member F (in filopodia) | 1 |
| 8058 | hsa-miR-765 | 54509 | RHOF | ras homolog gene family, member F (in filopodia) | 1 |
| 8059 | hsa-miR-770-5p | 54509 | RHOF | ras homolog gene family, member F (in filopodia) | 1 |
| 8060 | hsa-miR-877 | 54509 | RHOF | ras homolog gene family, member F (in filopodia) | 1 |
| 8061 | hsa-mir-26a-1 | 54509 | RHOF | ras homolog gene family, member F (in filopodia) | 1 |
| 8062 | hsa-mir-26b | 54509 | RHOF | ras homolog gene family, member F (in filopodia) | 1 |
| 8063 | hsa-mir-31 | 54509 | RHOF | ras homolog gene family, member F (in filopodia) | 1 |
| 8064 | hsa-mir-194-2 | 54509 | RHOF | ras homolog gene family, member F (in filopodia) | 1 |
| 8065 | hsa-mir-340 | 54509 | RHOF | ras homolog gene family, member F (in filopodia) | 1 |
| 8066 | hsa-mir-338 | 54509 | RHOF | ras homolog gene family, member F (in filopodia) | 1 |
| 8067 | hsa-mir-490 | 54509 | RHOF | ras homolog gene family, member F (in filopodia) | 1 |
| 8068 | hsa-mir-449b | 54509 | RHOF | ras homolog gene family, member F (in filopodia) | 1 |
| 8069 | hsa-mir-449c | 54509 | RHOF | ras homolog gene family, member F (in filopodia) | 1 |
| 8070 | hsa-mir-3065 | 54509 | RHOF | ras homolog gene family, member F (in filopodia) | 1 |
| 8084 | hsa-miR-584 | 57823 | SLAMF7 | SLAM family member 7 | 1 |
| 8086 | hsa-miR-634 | 57823 | SLAMF7 | SLAM family member 7 | 1 |
| 8103 | hsa-miR-152 | 9567 | GTPBP1 | GTP binding protein 1 | 1 |
| 8104 | hsa-miR-26a | 9567 | GTPBP1 | GTP binding protein 1 | 1 |
| 8105 | hsa-miR-26b | 9567 | GTPBP1 | GTP binding protein 1 | 1 |
| 8106 | hsa-miR-31 | 9567 | GTPBP1 | GTP binding protein 1 | 1 |
| 8107 | hsa-miR-340 | 9567 | GTPBP1 | GTP binding protein 1 | 1 |
| 8108 | hsa-miR-383 | 9567 | GTPBP1 | GTP binding protein 1 | 1 |
| 8109 | hsa-miR-449a | 9567 | GTPBP1 | GTP binding protein 1 | 1 |
| 8110 | hsa-miR-499-5p | 9567 | GTPBP1 | GTP binding protein 1 | 1 |
| 8111 | hsa-miR-621 | 9567 | GTPBP1 | GTP binding protein 1 | 1 |
| 8112 | hsa-miR-634 | 9567 | GTPBP1 | GTP binding protein 1 | 1 |
| 8113 | hsa-miR-653 | 9567 | GTPBP1 | GTP binding protein 1 | 1 |
| 8114 | hsa-miR-765 | 9567 | GTPBP1 | GTP binding protein 1 | 1 |
| 8115 | hsa-miR-770-5p | 9567 | GTPBP1 | GTP binding protein 1 | 1 |
| 8116 | hsa-mir-26a-1 | 9567 | GTPBP1 | GTP binding protein 1 | 1 |
| 8117 | hsa-mir-26b | 9567 | GTPBP1 | GTP binding protein 1 | 1 |
| 8118 | hsa-mir-31 | 9567 | GTPBP1 | GTP binding protein 1 | 1 |
| 8119 | hsa-mir-194-2 | 9567 | GTPBP1 | GTP binding protein 1 | 1 |
| 8120 | hsa-mir-340 | 9567 | GTPBP1 | GTP binding protein 1 | 1 |
| 8121 | hsa-mir-449b | 9567 | GTPBP1 | GTP binding protein 1 | 1 |
| 8122 | hsa-mir-449c | 9567 | GTPBP1 | GTP binding protein 1 | 1 |
| 8123 | hsa-miR-10a | 80219 | COQ10B | coenzyme Q10 homolog B (S. cerevisiae) | 1 |
| 8124 | hsa-miR-1228\* | 80219 | COQ10B | coenzyme Q10 homolog B (S. cerevisiae) | 1 |
| 8125 | hsa-miR-139-5p | 80219 | COQ10B | coenzyme Q10 homolog B (S. cerevisiae) | 1 |
| 8126 | hsa-miR-26a | 80219 | COQ10B | coenzyme Q10 homolog B (S. cerevisiae) | 1 |
| 8127 | hsa-miR-26b | 80219 | COQ10B | coenzyme Q10 homolog B (S. cerevisiae) | 1 |
| 8128 | hsa-miR-338-5p | 80219 | COQ10B | coenzyme Q10 homolog B (S. cerevisiae) | 1 |
| 8129 | hsa-miR-340 | 80219 | COQ10B | coenzyme Q10 homolog B (S. cerevisiae) | 1 |
| 8130 | hsa-miR-449a | 80219 | COQ10B | coenzyme Q10 homolog B (S. cerevisiae) | 1 |
| 8131 | hsa-miR-499-5p | 80219 | COQ10B | coenzyme Q10 homolog B (S. cerevisiae) | 1 |
| 8132 | hsa-miR-548b-5p | 80219 | COQ10B | coenzyme Q10 homolog B (S. cerevisiae) | 1 |
| 8133 | hsa-miR-564 | 80219 | COQ10B | coenzyme Q10 homolog B (S. cerevisiae) | 1 |
| 8134 | hsa-miR-584 | 80219 | COQ10B | coenzyme Q10 homolog B (S. cerevisiae) | 1 |
| 8135 | hsa-miR-621 | 80219 | COQ10B | coenzyme Q10 homolog B (S. cerevisiae) | 1 |
| 8136 | hsa-miR-657 | 80219 | COQ10B | coenzyme Q10 homolog B (S. cerevisiae) | 1 |
| 8137 | hsa-miR-765 | 80219 | COQ10B | coenzyme Q10 homolog B (S. cerevisiae) | 1 |
| 8138 | hsa-miR-770-5p | 80219 | COQ10B | coenzyme Q10 homolog B (S. cerevisiae) | 1 |
| 8139 | hsa-miR-877 | 80219 | COQ10B | coenzyme Q10 homolog B (S. cerevisiae) | 1 |
| 8140 | hsa-mir-26a-1 | 80219 | COQ10B | coenzyme Q10 homolog B (S. cerevisiae) | 1 |
| 8141 | hsa-mir-26b | 80219 | COQ10B | coenzyme Q10 homolog B (S. cerevisiae) | 1 |
| 8142 | hsa-mir-194-2 | 80219 | COQ10B | coenzyme Q10 homolog B (S. cerevisiae) | 1 |
| 8143 | hsa-mir-340 | 80219 | COQ10B | coenzyme Q10 homolog B (S. cerevisiae) | 1 |
| 8144 | hsa-mir-338 | 80219 | COQ10B | coenzyme Q10 homolog B (S. cerevisiae) | 1 |
| 8145 | hsa-mir-548b | 80219 | COQ10B | coenzyme Q10 homolog B (S. cerevisiae) | 1 |
| 8146 | hsa-mir-548d-2 | 80219 | COQ10B | coenzyme Q10 homolog B (S. cerevisiae) | 1 |
| 8147 | hsa-mir-449b | 80219 | COQ10B | coenzyme Q10 homolog B (S. cerevisiae) | 1 |
| 8148 | hsa-mir-449c | 80219 | COQ10B | coenzyme Q10 homolog B (S. cerevisiae) | 1 |
| 8149 | hsa-mir-3065 | 80219 | COQ10B | coenzyme Q10 homolog B (S. cerevisiae) | 1 |
| 8150 | hsa-miR-10a | 10148 | EBI3 | Epstein-Barr virus induced 3 | 1 |
| 8151 | hsa-miR-152 | 10148 | EBI3 | Epstein-Barr virus induced 3 | 1 |
| 8152 | hsa-miR-26a | 10148 | EBI3 | Epstein-Barr virus induced 3 | 1 |
| 8153 | hsa-miR-26b | 10148 | EBI3 | Epstein-Barr virus induced 3 | 1 |
| 8154 | hsa-miR-31 | 10148 | EBI3 | Epstein-Barr virus induced 3 | 1 |
| 8155 | hsa-miR-338-5p | 10148 | EBI3 | Epstein-Barr virus induced 3 | 1 |
| 8156 | hsa-miR-340 | 10148 | EBI3 | Epstein-Barr virus induced 3 | 1 |
| 8157 | hsa-miR-383 | 10148 | EBI3 | Epstein-Barr virus induced 3 | 1 |
| 8158 | hsa-miR-449a | 10148 | EBI3 | Epstein-Barr virus induced 3 | 1 |
| 8159 | hsa-miR-490-5p | 10148 | EBI3 | Epstein-Barr virus induced 3 | 1 |
| 8160 | hsa-miR-499-5p | 10148 | EBI3 | Epstein-Barr virus induced 3 | 1 |
| 8161 | hsa-miR-621 | 10148 | EBI3 | Epstein-Barr virus induced 3 | 1 |
| 8162 | hsa-miR-653 | 10148 | EBI3 | Epstein-Barr virus induced 3 | 1 |
| 8163 | hsa-miR-657 | 10148 | EBI3 | Epstein-Barr virus induced 3 | 1 |
| 8164 | hsa-miR-765 | 10148 | EBI3 | Epstein-Barr virus induced 3 | 1 |
| 8165 | hsa-miR-770-5p | 10148 | EBI3 | Epstein-Barr virus induced 3 | 1 |
| 8166 | hsa-miR-877 | 10148 | EBI3 | Epstein-Barr virus induced 3 | 1 |
| 8167 | hsa-mir-26a-1 | 10148 | EBI3 | Epstein-Barr virus induced 3 | 1 |
| 8168 | hsa-mir-26b | 10148 | EBI3 | Epstein-Barr virus induced 3 | 1 |
| 8169 | hsa-mir-31 | 10148 | EBI3 | Epstein-Barr virus induced 3 | 1 |
| 8170 | hsa-mir-194-2 | 10148 | EBI3 | Epstein-Barr virus induced 3 | 1 |
| 8171 | hsa-mir-340 | 10148 | EBI3 | Epstein-Barr virus induced 3 | 1 |
| 8172 | hsa-mir-338 | 10148 | EBI3 | Epstein-Barr virus induced 3 | 1 |
| 8173 | hsa-mir-490 | 10148 | EBI3 | Epstein-Barr virus induced 3 | 1 |
| 8174 | hsa-mir-449b | 10148 | EBI3 | Epstein-Barr virus induced 3 | 1 |
| 8175 | hsa-mir-449c | 10148 | EBI3 | Epstein-Barr virus induced 3 | 1 |
| 8176 | hsa-mir-3065 | 10148 | EBI3 | Epstein-Barr virus induced 3 | 1 |
| 8177 | hsa-miR-1 | 10313 | RTN3 | reticulon 3 | 1 |
| 8178 | hsa-miR-133a | 10313 | RTN3 | reticulon 3 | 1 |
| 8179 | hsa-miR-186 | 10313 | RTN3 | reticulon 3 | 1 |
| 8180 | hsa-miR-195 | 10313 | RTN3 | reticulon 3 | 1 |
| 8181 | hsa-miR-326 | 10313 | RTN3 | reticulon 3 | 1 |
| 8182 | hsa-miR-33a | 10313 | RTN3 | reticulon 3 | 1 |
| 8183 | hsa-miR-449b | 10313 | RTN3 | reticulon 3 | 1 |
| 8184 | hsa-miR-497 | 10313 | RTN3 | reticulon 3 | 1 |
| 8185 | hsa-miR-550 | 10313 | RTN3 | reticulon 3 | 1 |
| 8186 | hsa-miR-551a | 10313 | RTN3 | reticulon 3 | 1 |
| 8187 | hsa-miR-562 | 10313 | RTN3 | reticulon 3 | 1 |
| 8188 | hsa-miR-575 | 10313 | RTN3 | reticulon 3 | 1 |
| 8189 | hsa-miR-579 | 10313 | RTN3 | reticulon 3 | 1 |
| 8190 | hsa-miR-608 | 10313 | RTN3 | reticulon 3 | 1 |
| 8191 | hsa-miR-620 | 10313 | RTN3 | reticulon 3 | 1 |
| 8192 | hsa-mir-22 | 10313 | RTN3 | reticulon 3 | 1 |
| 8193 | hsa-mir-10a | 10313 | RTN3 | reticulon 3 | 1 |
| 8194 | hsa-mir-186 | 10313 | RTN3 | reticulon 3 | 1 |
| 8195 | hsa-mir-155 | 10313 | RTN3 | reticulon 3 | 1 |
| 8196 | hsa-mir-499 | 10313 | RTN3 | reticulon 3 | 1 |
| 8197 | hsa-mir-550-1 | 10313 | RTN3 | reticulon 3 | 1 |
| 8198 | hsa-mir-877 | 10313 | RTN3 | reticulon 3 | 1 |
| 8199 | hsa-mir-2116 | 10313 | RTN3 | reticulon 3 | 1 |
| 8200 | hsa-miR-1 | 25837 | RAB26 | RAB26, member RAS oncogene family | 1 |
| 8201 | hsa-miR-133a | 25837 | RAB26 | RAB26, member RAS oncogene family | 1 |
| 8202 | hsa-miR-186 | 25837 | RAB26 | RAB26, member RAS oncogene family | 1 |
| 8203 | hsa-miR-326 | 25837 | RAB26 | RAB26, member RAS oncogene family | 1 |
| 8204 | hsa-miR-449b | 25837 | RAB26 | RAB26, member RAS oncogene family | 1 |
| 8205 | hsa-miR-550 | 25837 | RAB26 | RAB26, member RAS oncogene family | 1 |
| 8206 | hsa-miR-551a | 25837 | RAB26 | RAB26, member RAS oncogene family | 1 |
| 8207 | hsa-miR-575 | 25837 | RAB26 | RAB26, member RAS oncogene family | 1 |
| 8208 | hsa-miR-579 | 25837 | RAB26 | RAB26, member RAS oncogene family | 1 |
| 8209 | hsa-miR-608 | 25837 | RAB26 | RAB26, member RAS oncogene family | 1 |
| 8210 | hsa-miR-620 | 25837 | RAB26 | RAB26, member RAS oncogene family | 1 |
| 8211 | hsa-mir-22 | 25837 | RAB26 | RAB26, member RAS oncogene family | 1 |
| 8212 | hsa-mir-10a | 25837 | RAB26 | RAB26, member RAS oncogene family | 1 |
| 8213 | hsa-mir-186 | 25837 | RAB26 | RAB26, member RAS oncogene family | 1 |
| 8214 | hsa-mir-155 | 25837 | RAB26 | RAB26, member RAS oncogene family | 1 |
| 8215 | hsa-mir-423 | 25837 | RAB26 | RAB26, member RAS oncogene family | 1 |
| 8216 | hsa-mir-499 | 25837 | RAB26 | RAB26, member RAS oncogene family | 1 |
| 8217 | hsa-mir-550-1 | 25837 | RAB26 | RAB26, member RAS oncogene family | 1 |
| 8218 | hsa-mir-877 | 25837 | RAB26 | RAB26, member RAS oncogene family | 1 |
| 8219 | hsa-mir-2116 | 25837 | RAB26 | RAB26, member RAS oncogene family | 1 |
| 8220 | hsa-mir-3186 | 25837 | RAB26 | RAB26, member RAS oncogene family | 1 |
| 8221 | hsa-mir-3190 | 25837 | RAB26 | RAB26, member RAS oncogene family | 1 |
| 8222 | hsa-miR-1 | 79791 | FBXO31 | F-box protein 31 | 1 |
| 8223 | hsa-miR-133a | 79791 | FBXO31 | F-box protein 31 | 1 |
| 8224 | hsa-miR-186 | 79791 | FBXO31 | F-box protein 31 | 1 |
| 8225 | hsa-miR-208a | 79791 | FBXO31 | F-box protein 31 | 1 |
| 8226 | hsa-miR-326 | 79791 | FBXO31 | F-box protein 31 | 1 |
| 8227 | hsa-miR-33a | 79791 | FBXO31 | F-box protein 31 | 1 |
| 8228 | hsa-miR-449b | 79791 | FBXO31 | F-box protein 31 | 1 |
| 8229 | hsa-miR-550 | 79791 | FBXO31 | F-box protein 31 | 1 |
| 8230 | hsa-miR-575 | 79791 | FBXO31 | F-box protein 31 | 1 |
| 8231 | hsa-miR-608 | 79791 | FBXO31 | F-box protein 31 | 1 |
| 8232 | hsa-miR-620 | 79791 | FBXO31 | F-box protein 31 | 1 |
| 8233 | hsa-let-7a-3 | 79791 | FBXO31 | F-box protein 31 | 1 |
| 8234 | hsa-let-7b | 79791 | FBXO31 | F-box protein 31 | 1 |
| 8235 | hsa-mir-22 | 79791 | FBXO31 | F-box protein 31 | 1 |
| 8236 | hsa-mir-10a | 79791 | FBXO31 | F-box protein 31 | 1 |
| 8237 | hsa-mir-186 | 79791 | FBXO31 | F-box protein 31 | 1 |
| 8238 | hsa-mir-155 | 79791 | FBXO31 | F-box protein 31 | 1 |
| 8239 | hsa-mir-499 | 79791 | FBXO31 | F-box protein 31 | 1 |
| 8240 | hsa-mir-550-1 | 79791 | FBXO31 | F-box protein 31 | 1 |
| 8241 | hsa-mir-877 | 79791 | FBXO31 | F-box protein 31 | 1 |
| 8242 | hsa-mir-2116 | 79791 | FBXO31 | F-box protein 31 | 1 |
| 8243 | hsa-mir-3186 | 79791 | FBXO31 | F-box protein 31 | 1 |
| 8244 | hsa-miR-1 | 28968 | SLC6A16 | solute carrier family 6, member 16 | 1 |
| 8245 | hsa-miR-133a | 28968 | SLC6A16 | solute carrier family 6, member 16 | 1 |
| 8246 | hsa-miR-186 | 28968 | SLC6A16 | solute carrier family 6, member 16 | 1 |
| 8247 | hsa-miR-195 | 28968 | SLC6A16 | solute carrier family 6, member 16 | 1 |
| 8248 | hsa-miR-326 | 28968 | SLC6A16 | solute carrier family 6, member 16 | 1 |
| 8249 | hsa-miR-449b | 28968 | SLC6A16 | solute carrier family 6, member 16 | 1 |
| 8250 | hsa-miR-497 | 28968 | SLC6A16 | solute carrier family 6, member 16 | 1 |
| 8251 | hsa-miR-551a | 28968 | SLC6A16 | solute carrier family 6, member 16 | 1 |
| 8252 | hsa-miR-562 | 28968 | SLC6A16 | solute carrier family 6, member 16 | 1 |
| 8253 | hsa-miR-575 | 28968 | SLC6A16 | solute carrier family 6, member 16 | 1 |
| 8254 | hsa-miR-608 | 28968 | SLC6A16 | solute carrier family 6, member 16 | 1 |
| 8255 | hsa-miR-620 | 28968 | SLC6A16 | solute carrier family 6, member 16 | 1 |
| 8256 | hsa-mir-22 | 28968 | SLC6A16 | solute carrier family 6, member 16 | 1 |
| 8257 | hsa-mir-10a | 28968 | SLC6A16 | solute carrier family 6, member 16 | 1 |
| 8258 | hsa-mir-186 | 28968 | SLC6A16 | solute carrier family 6, member 16 | 1 |
| 8259 | hsa-mir-155 | 28968 | SLC6A16 | solute carrier family 6, member 16 | 1 |
| 8260 | hsa-mir-423 | 28968 | SLC6A16 | solute carrier family 6, member 16 | 1 |
| 8261 | hsa-mir-499 | 28968 | SLC6A16 | solute carrier family 6, member 16 | 1 |
| 8262 | hsa-mir-877 | 28968 | SLC6A16 | solute carrier family 6, member 16 | 1 |
| 8263 | hsa-miR-634 | 57018 | CCNL1 | cyclin L1 | 1 |
| 8297 | hsa-miR-644 | 29121 | CLEC2D | C-type lectin domain family 2, member D | 1 |
| 8309 | hsa-miR-1 | 353500 | BMP8A | bone morphogenetic protein 8a | 1 |
| 8310 | hsa-miR-133a | 353500 | BMP8A | bone morphogenetic protein 8a | 1 |
| 8311 | hsa-miR-186 | 353500 | BMP8A | bone morphogenetic protein 8a | 1 |
| 8312 | hsa-miR-326 | 353500 | BMP8A | bone morphogenetic protein 8a | 1 |
| 8313 | hsa-miR-449b | 353500 | BMP8A | bone morphogenetic protein 8a | 1 |
| 8314 | hsa-miR-562 | 353500 | BMP8A | bone morphogenetic protein 8a | 1 |
| 8315 | hsa-miR-575 | 353500 | BMP8A | bone morphogenetic protein 8a | 1 |
| 8316 | hsa-miR-579 | 353500 | BMP8A | bone morphogenetic protein 8a | 1 |
| 8317 | hsa-miR-608 | 353500 | BMP8A | bone morphogenetic protein 8a | 1 |
| 8318 | hsa-miR-620 | 353500 | BMP8A | bone morphogenetic protein 8a | 1 |
| 8319 | hsa-miR-637 | 353500 | BMP8A | bone morphogenetic protein 8a | 1 |
| 8320 | hsa-let-7a-3 | 353500 | BMP8A | bone morphogenetic protein 8a | 1 |
| 8321 | hsa-let-7b | 353500 | BMP8A | bone morphogenetic protein 8a | 1 |
| 8322 | hsa-mir-22 | 353500 | BMP8A | bone morphogenetic protein 8a | 1 |
| 8323 | hsa-mir-10a | 353500 | BMP8A | bone morphogenetic protein 8a | 1 |
| 8324 | hsa-mir-186 | 353500 | BMP8A | bone morphogenetic protein 8a | 1 |
| 8325 | hsa-mir-155 | 353500 | BMP8A | bone morphogenetic protein 8a | 1 |
| 8326 | hsa-mir-499 | 353500 | BMP8A | bone morphogenetic protein 8a | 1 |
| 8327 | hsa-mir-877 | 353500 | BMP8A | bone morphogenetic protein 8a | 1 |
| 8328 | hsa-miR-10a | 51700 | CYB5R2 | cytochrome b5 reductase 2 | 1 |
| 8329 | hsa-miR-152 | 51700 | CYB5R2 | cytochrome b5 reductase 2 | 1 |
| 8330 | hsa-miR-26a | 51700 | CYB5R2 | cytochrome b5 reductase 2 | 1 |
| 8331 | hsa-miR-26b | 51700 | CYB5R2 | cytochrome b5 reductase 2 | 1 |
| 8332 | hsa-miR-31 | 51700 | CYB5R2 | cytochrome b5 reductase 2 | 1 |
| 8333 | hsa-miR-338-5p | 51700 | CYB5R2 | cytochrome b5 reductase 2 | 1 |
| 8334 | hsa-miR-340 | 51700 | CYB5R2 | cytochrome b5 reductase 2 | 1 |
| 8335 | hsa-miR-383 | 51700 | CYB5R2 | cytochrome b5 reductase 2 | 1 |
| 8336 | hsa-miR-449a | 51700 | CYB5R2 | cytochrome b5 reductase 2 | 1 |
| 8337 | hsa-miR-499-5p | 51700 | CYB5R2 | cytochrome b5 reductase 2 | 1 |
| 8338 | hsa-miR-548b-5p | 51700 | CYB5R2 | cytochrome b5 reductase 2 | 1 |
| 8339 | hsa-miR-621 | 51700 | CYB5R2 | cytochrome b5 reductase 2 | 1 |
| 8340 | hsa-miR-657 | 51700 | CYB5R2 | cytochrome b5 reductase 2 | 1 |
| 8341 | hsa-miR-765 | 51700 | CYB5R2 | cytochrome b5 reductase 2 | 1 |
| 8342 | hsa-miR-770-5p | 51700 | CYB5R2 | cytochrome b5 reductase 2 | 1 |
| 8343 | hsa-miR-877 | 51700 | CYB5R2 | cytochrome b5 reductase 2 | 1 |
| 8344 | hsa-mir-26a-1 | 51700 | CYB5R2 | cytochrome b5 reductase 2 | 1 |
| 8345 | hsa-mir-26b | 51700 | CYB5R2 | cytochrome b5 reductase 2 | 1 |
| 8346 | hsa-mir-31 | 51700 | CYB5R2 | cytochrome b5 reductase 2 | 1 |
| 8347 | hsa-mir-194-2 | 51700 | CYB5R2 | cytochrome b5 reductase 2 | 1 |
| 8348 | hsa-mir-340 | 51700 | CYB5R2 | cytochrome b5 reductase 2 | 1 |
| 8349 | hsa-mir-338 | 51700 | CYB5R2 | cytochrome b5 reductase 2 | 1 |
| 8350 | hsa-mir-548b | 51700 | CYB5R2 | cytochrome b5 reductase 2 | 1 |
| 8351 | hsa-mir-449b | 51700 | CYB5R2 | cytochrome b5 reductase 2 | 1 |
| 8352 | hsa-mir-449c | 51700 | CYB5R2 | cytochrome b5 reductase 2 | 1 |
| 8353 | hsa-mir-3065 | 51700 | CYB5R2 | cytochrome b5 reductase 2 | 1 |
| 8354 | hsa-miR-1 | 50624 | CUZD1 | CUB and zona pellucida-like domains 1 | 1 |
| 8355 | hsa-miR-133a | 50624 | CUZD1 | CUB and zona pellucida-like domains 1 | 1 |
| 8356 | hsa-miR-186 | 50624 | CUZD1 | CUB and zona pellucida-like domains 1 | 1 |
| 8357 | hsa-miR-326 | 50624 | CUZD1 | CUB and zona pellucida-like domains 1 | 1 |
| 8358 | hsa-miR-449b | 50624 | CUZD1 | CUB and zona pellucida-like domains 1 | 1 |
| 8359 | hsa-miR-550 | 50624 | CUZD1 | CUB and zona pellucida-like domains 1 | 1 |
| 8360 | hsa-miR-551a | 50624 | CUZD1 | CUB and zona pellucida-like domains 1 | 1 |
| 8361 | hsa-miR-575 | 50624 | CUZD1 | CUB and zona pellucida-like domains 1 | 1 |
| 8362 | hsa-miR-579 | 50624 | CUZD1 | CUB and zona pellucida-like domains 1 | 1 |
| 8363 | hsa-miR-608 | 50624 | CUZD1 | CUB and zona pellucida-like domains 1 | 1 |
| 8364 | hsa-miR-620 | 50624 | CUZD1 | CUB and zona pellucida-like domains 1 | 1 |
| 8365 | hsa-miR-885-5p | 50624 | CUZD1 | CUB and zona pellucida-like domains 1 | 1 |
| 8366 | hsa-mir-22 | 50624 | CUZD1 | CUB and zona pellucida-like domains 1 | 1 |
| 8367 | hsa-mir-10a | 50624 | CUZD1 | CUB and zona pellucida-like domains 1 | 1 |
| 8368 | hsa-mir-186 | 50624 | CUZD1 | CUB and zona pellucida-like domains 1 | 1 |
| 8369 | hsa-mir-155 | 50624 | CUZD1 | CUB and zona pellucida-like domains 1 | 1 |
| 8370 | hsa-mir-423 | 50624 | CUZD1 | CUB and zona pellucida-like domains 1 | 1 |
| 8371 | hsa-mir-499 | 50624 | CUZD1 | CUB and zona pellucida-like domains 1 | 1 |
| 8372 | hsa-mir-550-1 | 50624 | CUZD1 | CUB and zona pellucida-like domains 1 | 1 |
| 8373 | hsa-mir-885 | 50624 | CUZD1 | CUB and zona pellucida-like domains 1 | 1 |
| 8374 | hsa-mir-877 | 50624 | CUZD1 | CUB and zona pellucida-like domains 1 | 1 |
| 8375 | hsa-mir-2116 | 50624 | CUZD1 | CUB and zona pellucida-like domains 1 | 1 |
| 8376 | hsa-mir-3186 | 50624 | CUZD1 | CUB and zona pellucida-like domains 1 | 1 |
| 8377 | hsa-mir-3190 | 50624 | CUZD1 | CUB and zona pellucida-like domains 1 | 1 |
| 8378 | hsa-miR-1 | 10881 | ACTL7A | actin-like 7A | 1 |
| 8379 | hsa-miR-133a | 10881 | ACTL7A | actin-like 7A | 1 |
| 8380 | hsa-miR-186 | 10881 | ACTL7A | actin-like 7A | 1 |
| 8381 | hsa-miR-449b | 10881 | ACTL7A | actin-like 7A | 1 |
| 8382 | hsa-miR-551a | 10881 | ACTL7A | actin-like 7A | 1 |
| 8383 | hsa-miR-562 | 10881 | ACTL7A | actin-like 7A | 1 |
| 8384 | hsa-miR-575 | 10881 | ACTL7A | actin-like 7A | 1 |
| 8385 | hsa-miR-579 | 10881 | ACTL7A | actin-like 7A | 1 |
| 8386 | hsa-miR-608 | 10881 | ACTL7A | actin-like 7A | 1 |
| 8387 | hsa-miR-620 | 10881 | ACTL7A | actin-like 7A | 1 |
| 8388 | hsa-miR-637 | 10881 | ACTL7A | actin-like 7A | 1 |
| 8389 | hsa-mir-22 | 10881 | ACTL7A | actin-like 7A | 1 |
| 8390 | hsa-mir-10a | 10881 | ACTL7A | actin-like 7A | 1 |
| 8391 | hsa-mir-186 | 10881 | ACTL7A | actin-like 7A | 1 |
| 8392 | hsa-mir-155 | 10881 | ACTL7A | actin-like 7A | 1 |
| 8393 | hsa-mir-423 | 10881 | ACTL7A | actin-like 7A | 1 |
| 8394 | hsa-mir-877 | 10881 | ACTL7A | actin-like 7A | 1 |
| 8395 | hsa-miR-10a | 9209 | LRRFIP2 | leucine rich repeat (in FLII) interacting protein 2 | 1 |
| 8396 | hsa-miR-152 | 9209 | LRRFIP2 | leucine rich repeat (in FLII) interacting protein 2 | 1 |
| 8397 | hsa-miR-26a | 9209 | LRRFIP2 | leucine rich repeat (in FLII) interacting protein 2 | 1 |
| 8398 | hsa-miR-26b | 9209 | LRRFIP2 | leucine rich repeat (in FLII) interacting protein 2 | 1 |
| 8399 | hsa-miR-31 | 9209 | LRRFIP2 | leucine rich repeat (in FLII) interacting protein 2 | 1 |
| 8400 | hsa-miR-338-5p | 9209 | LRRFIP2 | leucine rich repeat (in FLII) interacting protein 2 | 1 |
| 8401 | hsa-miR-340 | 9209 | LRRFIP2 | leucine rich repeat (in FLII) interacting protein 2 | 1 |
| 8402 | hsa-miR-383 | 9209 | LRRFIP2 | leucine rich repeat (in FLII) interacting protein 2 | 1 |
| 8403 | hsa-miR-449a | 9209 | LRRFIP2 | leucine rich repeat (in FLII) interacting protein 2 | 1 |
| 8404 | hsa-miR-499-5p | 9209 | LRRFIP2 | leucine rich repeat (in FLII) interacting protein 2 | 1 |
| 8405 | hsa-miR-548b-5p | 9209 | LRRFIP2 | leucine rich repeat (in FLII) interacting protein 2 | 1 |
| 8406 | hsa-miR-621 | 9209 | LRRFIP2 | leucine rich repeat (in FLII) interacting protein 2 | 1 |
| 8407 | hsa-miR-657 | 9209 | LRRFIP2 | leucine rich repeat (in FLII) interacting protein 2 | 1 |
| 8408 | hsa-miR-765 | 9209 | LRRFIP2 | leucine rich repeat (in FLII) interacting protein 2 | 1 |
| 8409 | hsa-miR-770-5p | 9209 | LRRFIP2 | leucine rich repeat (in FLII) interacting protein 2 | 1 |
| 8410 | hsa-miR-877 | 9209 | LRRFIP2 | leucine rich repeat (in FLII) interacting protein 2 | 1 |
| 8411 | hsa-mir-26a-1 | 9209 | LRRFIP2 | leucine rich repeat (in FLII) interacting protein 2 | 1 |
| 8412 | hsa-mir-26b | 9209 | LRRFIP2 | leucine rich repeat (in FLII) interacting protein 2 | 1 |
| 8413 | hsa-mir-31 | 9209 | LRRFIP2 | leucine rich repeat (in FLII) interacting protein 2 | 1 |
| 8414 | hsa-mir-194-2 | 9209 | LRRFIP2 | leucine rich repeat (in FLII) interacting protein 2 | 1 |
| 8415 | hsa-mir-340 | 9209 | LRRFIP2 | leucine rich repeat (in FLII) interacting protein 2 | 1 |
| 8416 | hsa-mir-338 | 9209 | LRRFIP2 | leucine rich repeat (in FLII) interacting protein 2 | 1 |
| 8417 | hsa-mir-548b | 9209 | LRRFIP2 | leucine rich repeat (in FLII) interacting protein 2 | 1 |
| 8418 | hsa-mir-449b | 9209 | LRRFIP2 | leucine rich repeat (in FLII) interacting protein 2 | 1 |
| 8419 | hsa-mir-449c | 9209 | LRRFIP2 | leucine rich repeat (in FLII) interacting protein 2 | 1 |
| 8420 | hsa-mir-3065 | 9209 | LRRFIP2 | leucine rich repeat (in FLII) interacting protein 2 | 1 |
| 8421 | hsa-miR-10a | 80045 | GPR157 | G protein-coupled receptor 157 | 1 |
| 8422 | hsa-miR-152 | 80045 | GPR157 | G protein-coupled receptor 157 | 1 |
| 8423 | hsa-miR-26a | 80045 | GPR157 | G protein-coupled receptor 157 | 1 |
| 8424 | hsa-miR-26b | 80045 | GPR157 | G protein-coupled receptor 157 | 1 |
| 8425 | hsa-miR-31 | 80045 | GPR157 | G protein-coupled receptor 157 | 1 |
| 8426 | hsa-miR-340 | 80045 | GPR157 | G protein-coupled receptor 157 | 1 |
| 8427 | hsa-miR-383 | 80045 | GPR157 | G protein-coupled receptor 157 | 1 |
| 8428 | hsa-miR-449a | 80045 | GPR157 | G protein-coupled receptor 157 | 1 |
| 8429 | hsa-miR-490-5p | 80045 | GPR157 | G protein-coupled receptor 157 | 1 |
| 8430 | hsa-miR-499-5p | 80045 | GPR157 | G protein-coupled receptor 157 | 1 |
| 8431 | hsa-miR-621 | 80045 | GPR157 | G protein-coupled receptor 157 | 1 |
| 8432 | hsa-miR-765 | 80045 | GPR157 | G protein-coupled receptor 157 | 1 |
| 8433 | hsa-miR-877 | 80045 | GPR157 | G protein-coupled receptor 157 | 1 |
| 8434 | hsa-mir-26a-1 | 80045 | GPR157 | G protein-coupled receptor 157 | 1 |
| 8435 | hsa-mir-26b | 80045 | GPR157 | G protein-coupled receptor 157 | 1 |
| 8436 | hsa-mir-31 | 80045 | GPR157 | G protein-coupled receptor 157 | 1 |
| 8437 | hsa-mir-194-2 | 80045 | GPR157 | G protein-coupled receptor 157 | 1 |
| 8438 | hsa-mir-340 | 80045 | GPR157 | G protein-coupled receptor 157 | 1 |
| 8439 | hsa-mir-490 | 80045 | GPR157 | G protein-coupled receptor 157 | 1 |
| 8440 | hsa-mir-449b | 80045 | GPR157 | G protein-coupled receptor 157 | 1 |
| 8441 | hsa-mir-449c | 80045 | GPR157 | G protein-coupled receptor 157 | 1 |
| 8442 | hsa-miR-1 | 80069 | C6orf208 | chromosome 6 open reading frame 208 | 1 |
| 8443 | hsa-miR-133a | 80069 | C6orf208 | chromosome 6 open reading frame 208 | 1 |
| 8444 | hsa-miR-186 | 80069 | C6orf208 | chromosome 6 open reading frame 208 | 1 |
| 8445 | hsa-miR-208a | 80069 | C6orf208 | chromosome 6 open reading frame 208 | 1 |
| 8446 | hsa-miR-326 | 80069 | C6orf208 | chromosome 6 open reading frame 208 | 1 |
| 8447 | hsa-miR-33a | 80069 | C6orf208 | chromosome 6 open reading frame 208 | 1 |
| 8448 | hsa-miR-449b | 80069 | C6orf208 | chromosome 6 open reading frame 208 | 1 |
| 8449 | hsa-miR-550 | 80069 | C6orf208 | chromosome 6 open reading frame 208 | 1 |
| 8450 | hsa-miR-551a | 80069 | C6orf208 | chromosome 6 open reading frame 208 | 1 |
| 8451 | hsa-miR-575 | 80069 | C6orf208 | chromosome 6 open reading frame 208 | 1 |
| 8452 | hsa-miR-579 | 80069 | C6orf208 | chromosome 6 open reading frame 208 | 1 |
| 8453 | hsa-miR-608 | 80069 | C6orf208 | chromosome 6 open reading frame 208 | 1 |
| 8454 | hsa-miR-620 | 80069 | C6orf208 | chromosome 6 open reading frame 208 | 1 |
| 8455 | hsa-let-7a-3 | 80069 | C6orf208 | chromosome 6 open reading frame 208 | 1 |
| 8456 | hsa-let-7b | 80069 | C6orf208 | chromosome 6 open reading frame 208 | 1 |
| 8457 | hsa-mir-22 | 80069 | C6orf208 | chromosome 6 open reading frame 208 | 1 |
| 8458 | hsa-mir-10a | 80069 | C6orf208 | chromosome 6 open reading frame 208 | 1 |
| 8459 | hsa-mir-186 | 80069 | C6orf208 | chromosome 6 open reading frame 208 | 1 |
| 8460 | hsa-mir-155 | 80069 | C6orf208 | chromosome 6 open reading frame 208 | 1 |
| 8461 | hsa-mir-423 | 80069 | C6orf208 | chromosome 6 open reading frame 208 | 1 |
| 8462 | hsa-mir-499 | 80069 | C6orf208 | chromosome 6 open reading frame 208 | 1 |
| 8463 | hsa-mir-550-1 | 80069 | C6orf208 | chromosome 6 open reading frame 208 | 1 |
| 8464 | hsa-mir-877 | 80069 | C6orf208 | chromosome 6 open reading frame 208 | 1 |
| 8465 | hsa-mir-2116 | 80069 | C6orf208 | chromosome 6 open reading frame 208 | 1 |
| 8466 | hsa-mir-3186 | 80069 | C6orf208 | chromosome 6 open reading frame 208 | 1 |
| 8474 | hsa-miR-383 | 114897 | C1QTNF1 | C1q and tumor necrosis factor related protein 1 | 1 |
| 8478 | hsa-miR-653 | 114897 | C1QTNF1 | C1q and tumor necrosis factor related protein 1 | 1 |
| 8492 | hsa-miR-10a | 81608 | FIP1L1 | FIP1 like 1 (S. cerevisiae) | 1 |
| 8493 | hsa-miR-152 | 81608 | FIP1L1 | FIP1 like 1 (S. cerevisiae) | 1 |
| 8494 | hsa-miR-26a | 81608 | FIP1L1 | FIP1 like 1 (S. cerevisiae) | 1 |
| 8495 | hsa-miR-31 | 81608 | FIP1L1 | FIP1 like 1 (S. cerevisiae) | 1 |
| 8496 | hsa-miR-338-5p | 81608 | FIP1L1 | FIP1 like 1 (S. cerevisiae) | 1 |
| 8497 | hsa-miR-340 | 81608 | FIP1L1 | FIP1 like 1 (S. cerevisiae) | 1 |
| 8498 | hsa-miR-449a | 81608 | FIP1L1 | FIP1 like 1 (S. cerevisiae) | 1 |
| 8499 | hsa-miR-490-5p | 81608 | FIP1L1 | FIP1 like 1 (S. cerevisiae) | 1 |
| 8500 | hsa-miR-499-5p | 81608 | FIP1L1 | FIP1 like 1 (S. cerevisiae) | 1 |
| 8501 | hsa-miR-584 | 81608 | FIP1L1 | FIP1 like 1 (S. cerevisiae) | 1 |
| 8502 | hsa-miR-621 | 81608 | FIP1L1 | FIP1 like 1 (S. cerevisiae) | 1 |
| 8503 | hsa-miR-657 | 81608 | FIP1L1 | FIP1 like 1 (S. cerevisiae) | 1 |
| 8504 | hsa-miR-663 | 81608 | FIP1L1 | FIP1 like 1 (S. cerevisiae) | 1 |
| 8505 | hsa-miR-765 | 81608 | FIP1L1 | FIP1 like 1 (S. cerevisiae) | 1 |
| 8506 | hsa-miR-770-5p | 81608 | FIP1L1 | FIP1 like 1 (S. cerevisiae) | 1 |
| 8507 | hsa-miR-877 | 81608 | FIP1L1 | FIP1 like 1 (S. cerevisiae) | 1 |
| 8508 | hsa-mir-26a-1 | 81608 | FIP1L1 | FIP1 like 1 (S. cerevisiae) | 1 |
| 8509 | hsa-mir-31 | 81608 | FIP1L1 | FIP1 like 1 (S. cerevisiae) | 1 |
| 8510 | hsa-mir-194-2 | 81608 | FIP1L1 | FIP1 like 1 (S. cerevisiae) | 1 |
| 8511 | hsa-mir-340 | 81608 | FIP1L1 | FIP1 like 1 (S. cerevisiae) | 1 |
| 8512 | hsa-mir-338 | 81608 | FIP1L1 | FIP1 like 1 (S. cerevisiae) | 1 |
| 8513 | hsa-mir-490 | 81608 | FIP1L1 | FIP1 like 1 (S. cerevisiae) | 1 |
| 8514 | hsa-mir-449b | 81608 | FIP1L1 | FIP1 like 1 (S. cerevisiae) | 1 |
| 8515 | hsa-mir-449c | 81608 | FIP1L1 | FIP1 like 1 (S. cerevisiae) | 1 |
| 8516 | hsa-mir-3065 | 81608 | FIP1L1 | FIP1 like 1 (S. cerevisiae) | 1 |
| 8517 | hsa-miR-1 | 51289 | RXFP3 | relaxin/insulin-like family peptide receptor 3 | 1 |
| 8518 | hsa-miR-133a | 51289 | RXFP3 | relaxin/insulin-like family peptide receptor 3 | 1 |
| 8519 | hsa-miR-186 | 51289 | RXFP3 | relaxin/insulin-like family peptide receptor 3 | 1 |
| 8520 | hsa-miR-326 | 51289 | RXFP3 | relaxin/insulin-like family peptide receptor 3 | 1 |
| 8521 | hsa-miR-449b | 51289 | RXFP3 | relaxin/insulin-like family peptide receptor 3 | 1 |
| 8522 | hsa-miR-551a | 51289 | RXFP3 | relaxin/insulin-like family peptide receptor 3 | 1 |
| 8523 | hsa-miR-562 | 51289 | RXFP3 | relaxin/insulin-like family peptide receptor 3 | 1 |
| 8524 | hsa-miR-575 | 51289 | RXFP3 | relaxin/insulin-like family peptide receptor 3 | 1 |
| 8525 | hsa-miR-579 | 51289 | RXFP3 | relaxin/insulin-like family peptide receptor 3 | 1 |
| 8526 | hsa-miR-608 | 51289 | RXFP3 | relaxin/insulin-like family peptide receptor 3 | 1 |
| 8527 | hsa-miR-620 | 51289 | RXFP3 | relaxin/insulin-like family peptide receptor 3 | 1 |
| 8528 | hsa-miR-637 | 51289 | RXFP3 | relaxin/insulin-like family peptide receptor 3 | 1 |
| 8529 | hsa-mir-22 | 51289 | RXFP3 | relaxin/insulin-like family peptide receptor 3 | 1 |
| 8530 | hsa-mir-10a | 51289 | RXFP3 | relaxin/insulin-like family peptide receptor 3 | 1 |
| 8531 | hsa-mir-186 | 51289 | RXFP3 | relaxin/insulin-like family peptide receptor 3 | 1 |
| 8532 | hsa-mir-155 | 51289 | RXFP3 | relaxin/insulin-like family peptide receptor 3 | 1 |
| 8533 | hsa-mir-423 | 51289 | RXFP3 | relaxin/insulin-like family peptide receptor 3 | 1 |
| 8534 | hsa-mir-499 | 51289 | RXFP3 | relaxin/insulin-like family peptide receptor 3 | 1 |
| 8535 | hsa-mir-877 | 51289 | RXFP3 | relaxin/insulin-like family peptide receptor 3 | 1 |
| 8536 | hsa-mir-3190 | 51289 | RXFP3 | relaxin/insulin-like family peptide receptor 3 | 1 |
| 8537 | hsa-miR-10a | 80833 | APOL3 | apolipoprotein L, 3 | 1 |
| 8538 | hsa-miR-139-5p | 80833 | APOL3 | apolipoprotein L, 3 | 1 |
| 8539 | hsa-miR-26a | 80833 | APOL3 | apolipoprotein L, 3 | 1 |
| 8540 | hsa-miR-26b | 80833 | APOL3 | apolipoprotein L, 3 | 1 |
| 8541 | hsa-miR-338-5p | 80833 | APOL3 | apolipoprotein L, 3 | 1 |
| 8542 | hsa-miR-340 | 80833 | APOL3 | apolipoprotein L, 3 | 1 |
| 8543 | hsa-miR-449a | 80833 | APOL3 | apolipoprotein L, 3 | 1 |
| 8544 | hsa-miR-499-5p | 80833 | APOL3 | apolipoprotein L, 3 | 1 |
| 8545 | hsa-miR-548b-5p | 80833 | APOL3 | apolipoprotein L, 3 | 1 |
| 8546 | hsa-miR-564 | 80833 | APOL3 | apolipoprotein L, 3 | 1 |
| 8547 | hsa-miR-621 | 80833 | APOL3 | apolipoprotein L, 3 | 1 |
| 8548 | hsa-miR-657 | 80833 | APOL3 | apolipoprotein L, 3 | 1 |
| 8549 | hsa-miR-765 | 80833 | APOL3 | apolipoprotein L, 3 | 1 |
| 8550 | hsa-miR-770-5p | 80833 | APOL3 | apolipoprotein L, 3 | 1 |
| 8551 | hsa-miR-877 | 80833 | APOL3 | apolipoprotein L, 3 | 1 |
| 8552 | hsa-mir-26a-1 | 80833 | APOL3 | apolipoprotein L, 3 | 1 |
| 8553 | hsa-mir-26b | 80833 | APOL3 | apolipoprotein L, 3 | 1 |
| 8554 | hsa-mir-194-2 | 80833 | APOL3 | apolipoprotein L, 3 | 1 |
| 8555 | hsa-mir-340 | 80833 | APOL3 | apolipoprotein L, 3 | 1 |
| 8556 | hsa-mir-338 | 80833 | APOL3 | apolipoprotein L, 3 | 1 |
| 8557 | hsa-mir-548b | 80833 | APOL3 | apolipoprotein L, 3 | 1 |
| 8558 | hsa-mir-548d-2 | 80833 | APOL3 | apolipoprotein L, 3 | 1 |
| 8559 | hsa-mir-449b | 80833 | APOL3 | apolipoprotein L, 3 | 1 |
| 8560 | hsa-mir-449c | 80833 | APOL3 | apolipoprotein L, 3 | 1 |
| 8561 | hsa-mir-3065 | 80833 | APOL3 | apolipoprotein L, 3 | 1 |
| 8562 | hsa-miR-1 | 54848 | FLJ20184 | hypothetical protein FLJ20184 | 1 |
| 8563 | hsa-miR-133a | 54848 | FLJ20184 | hypothetical protein FLJ20184 | 1 |
| 8564 | hsa-miR-186 | 54848 | FLJ20184 | hypothetical protein FLJ20184 | 1 |
| 8565 | hsa-miR-326 | 54848 | FLJ20184 | hypothetical protein FLJ20184 | 1 |
| 8566 | hsa-miR-33a | 54848 | FLJ20184 | hypothetical protein FLJ20184 | 1 |
| 8567 | hsa-miR-449b | 54848 | FLJ20184 | hypothetical protein FLJ20184 | 1 |
| 8568 | hsa-miR-550 | 54848 | FLJ20184 | hypothetical protein FLJ20184 | 1 |
| 8569 | hsa-miR-551a | 54848 | FLJ20184 | hypothetical protein FLJ20184 | 1 |
| 8570 | hsa-miR-575 | 54848 | FLJ20184 | hypothetical protein FLJ20184 | 1 |
| 8571 | hsa-miR-579 | 54848 | FLJ20184 | hypothetical protein FLJ20184 | 1 |
| 8572 | hsa-miR-608 | 54848 | FLJ20184 | hypothetical protein FLJ20184 | 1 |
| 8573 | hsa-miR-620 | 54848 | FLJ20184 | hypothetical protein FLJ20184 | 1 |
| 8574 | hsa-let-7a-3 | 54848 | FLJ20184 | hypothetical protein FLJ20184 | 1 |
| 8575 | hsa-let-7b | 54848 | FLJ20184 | hypothetical protein FLJ20184 | 1 |
| 8576 | hsa-mir-22 | 54848 | FLJ20184 | hypothetical protein FLJ20184 | 1 |
| 8577 | hsa-mir-10a | 54848 | FLJ20184 | hypothetical protein FLJ20184 | 1 |
| 8578 | hsa-mir-186 | 54848 | FLJ20184 | hypothetical protein FLJ20184 | 1 |
| 8579 | hsa-mir-155 | 54848 | FLJ20184 | hypothetical protein FLJ20184 | 1 |
| 8580 | hsa-mir-423 | 54848 | FLJ20184 | hypothetical protein FLJ20184 | 1 |
| 8581 | hsa-mir-499 | 54848 | FLJ20184 | hypothetical protein FLJ20184 | 1 |
| 8582 | hsa-mir-550-1 | 54848 | FLJ20184 | hypothetical protein FLJ20184 | 1 |
| 8583 | hsa-mir-877 | 54848 | FLJ20184 | hypothetical protein FLJ20184 | 1 |
| 8584 | hsa-mir-2116 | 54848 | FLJ20184 | hypothetical protein FLJ20184 | 1 |
| 8585 | hsa-mir-3186 | 54848 | FLJ20184 | hypothetical protein FLJ20184 | 1 |
| 8586 | hsa-miR-1 | 80115 | BAIAP2L2 | BAI1-associated protein 2-like 2 | 1 |
| 8587 | hsa-miR-133a | 80115 | BAIAP2L2 | BAI1-associated protein 2-like 2 | 1 |
| 8588 | hsa-miR-186 | 80115 | BAIAP2L2 | BAI1-associated protein 2-like 2 | 1 |
| 8589 | hsa-miR-195 | 80115 | BAIAP2L2 | BAI1-associated protein 2-like 2 | 1 |
| 8590 | hsa-miR-326 | 80115 | BAIAP2L2 | BAI1-associated protein 2-like 2 | 1 |
| 8591 | hsa-miR-449b | 80115 | BAIAP2L2 | BAI1-associated protein 2-like 2 | 1 |
| 8592 | hsa-miR-497 | 80115 | BAIAP2L2 | BAI1-associated protein 2-like 2 | 1 |
| 8593 | hsa-miR-551a | 80115 | BAIAP2L2 | BAI1-associated protein 2-like 2 | 1 |
| 8594 | hsa-miR-562 | 80115 | BAIAP2L2 | BAI1-associated protein 2-like 2 | 1 |
| 8595 | hsa-miR-575 | 80115 | BAIAP2L2 | BAI1-associated protein 2-like 2 | 1 |
| 8596 | hsa-miR-608 | 80115 | BAIAP2L2 | BAI1-associated protein 2-like 2 | 1 |
| 8597 | hsa-miR-620 | 80115 | BAIAP2L2 | BAI1-associated protein 2-like 2 | 1 |
| 8598 | hsa-mir-22 | 80115 | BAIAP2L2 | BAI1-associated protein 2-like 2 | 1 |
| 8599 | hsa-mir-10a | 80115 | BAIAP2L2 | BAI1-associated protein 2-like 2 | 1 |
| 8600 | hsa-mir-186 | 80115 | BAIAP2L2 | BAI1-associated protein 2-like 2 | 1 |
| 8601 | hsa-mir-155 | 80115 | BAIAP2L2 | BAI1-associated protein 2-like 2 | 1 |
| 8602 | hsa-mir-499 | 80115 | BAIAP2L2 | BAI1-associated protein 2-like 2 | 1 |
| 8603 | hsa-mir-877 | 80115 | BAIAP2L2 | BAI1-associated protein 2-like 2 | 1 |
| 8604 | hsa-miR-10a | 79370 | BCL2L14 | BCL2-like 14 (apoptosis facilitator) | 1 |
| 8605 | hsa-miR-1228\* | 79370 | BCL2L14 | BCL2-like 14 (apoptosis facilitator) | 1 |
| 8606 | hsa-miR-139-5p | 79370 | BCL2L14 | BCL2-like 14 (apoptosis facilitator) | 1 |
| 8607 | hsa-miR-152 | 79370 | BCL2L14 | BCL2-like 14 (apoptosis facilitator) | 1 |
| 8608 | hsa-miR-26a | 79370 | BCL2L14 | BCL2-like 14 (apoptosis facilitator) | 1 |
| 8609 | hsa-miR-26b | 79370 | BCL2L14 | BCL2-like 14 (apoptosis facilitator) | 1 |
| 8610 | hsa-miR-31 | 79370 | BCL2L14 | BCL2-like 14 (apoptosis facilitator) | 1 |
| 8611 | hsa-miR-338-5p | 79370 | BCL2L14 | BCL2-like 14 (apoptosis facilitator) | 1 |
| 8612 | hsa-miR-340 | 79370 | BCL2L14 | BCL2-like 14 (apoptosis facilitator) | 1 |
| 8613 | hsa-miR-449a | 79370 | BCL2L14 | BCL2-like 14 (apoptosis facilitator) | 1 |
| 8614 | hsa-miR-490-5p | 79370 | BCL2L14 | BCL2-like 14 (apoptosis facilitator) | 1 |
| 8615 | hsa-miR-499-5p | 79370 | BCL2L14 | BCL2-like 14 (apoptosis facilitator) | 1 |
| 8616 | hsa-miR-548b-5p | 79370 | BCL2L14 | BCL2-like 14 (apoptosis facilitator) | 1 |
| 8617 | hsa-miR-584 | 79370 | BCL2L14 | BCL2-like 14 (apoptosis facilitator) | 1 |
| 8618 | hsa-miR-621 | 79370 | BCL2L14 | BCL2-like 14 (apoptosis facilitator) | 1 |
| 8619 | hsa-miR-657 | 79370 | BCL2L14 | BCL2-like 14 (apoptosis facilitator) | 1 |
| 8620 | hsa-miR-765 | 79370 | BCL2L14 | BCL2-like 14 (apoptosis facilitator) | 1 |
| 8621 | hsa-miR-770-5p | 79370 | BCL2L14 | BCL2-like 14 (apoptosis facilitator) | 1 |
| 8622 | hsa-miR-877 | 79370 | BCL2L14 | BCL2-like 14 (apoptosis facilitator) | 1 |
| 8623 | hsa-mir-26a-1 | 79370 | BCL2L14 | BCL2-like 14 (apoptosis facilitator) | 1 |
| 8624 | hsa-mir-26b | 79370 | BCL2L14 | BCL2-like 14 (apoptosis facilitator) | 1 |
| 8625 | hsa-mir-31 | 79370 | BCL2L14 | BCL2-like 14 (apoptosis facilitator) | 1 |
| 8626 | hsa-mir-194-2 | 79370 | BCL2L14 | BCL2-like 14 (apoptosis facilitator) | 1 |
| 8627 | hsa-mir-340 | 79370 | BCL2L14 | BCL2-like 14 (apoptosis facilitator) | 1 |
| 8628 | hsa-mir-338 | 79370 | BCL2L14 | BCL2-like 14 (apoptosis facilitator) | 1 |
| 8629 | hsa-mir-490 | 79370 | BCL2L14 | BCL2-like 14 (apoptosis facilitator) | 1 |
| 8630 | hsa-mir-548b | 79370 | BCL2L14 | BCL2-like 14 (apoptosis facilitator) | 1 |
| 8631 | hsa-mir-449b | 79370 | BCL2L14 | BCL2-like 14 (apoptosis facilitator) | 1 |
| 8632 | hsa-mir-449c | 79370 | BCL2L14 | BCL2-like 14 (apoptosis facilitator) | 1 |
| 8633 | hsa-mir-3065 | 79370 | BCL2L14 | BCL2-like 14 (apoptosis facilitator) | 1 |
| 8634 | hsa-miR-1 | 28999 | KLF15 | Kruppel-like factor 15 | 1 |
| 8635 | hsa-miR-133a | 28999 | KLF15 | Kruppel-like factor 15 | 1 |
| 8636 | hsa-miR-186 | 28999 | KLF15 | Kruppel-like factor 15 | 1 |
| 8637 | hsa-miR-326 | 28999 | KLF15 | Kruppel-like factor 15 | 1 |
| 8638 | hsa-miR-449b | 28999 | KLF15 | Kruppel-like factor 15 | 1 |
| 8639 | hsa-miR-551a | 28999 | KLF15 | Kruppel-like factor 15 | 1 |
| 8640 | hsa-miR-562 | 28999 | KLF15 | Kruppel-like factor 15 | 1 |
| 8641 | hsa-miR-579 | 28999 | KLF15 | Kruppel-like factor 15 | 1 |
| 8642 | hsa-miR-608 | 28999 | KLF15 | Kruppel-like factor 15 | 1 |
| 8643 | hsa-miR-637 | 28999 | KLF15 | Kruppel-like factor 15 | 1 |
| 8644 | hsa-miR-885-5p | 28999 | KLF15 | Kruppel-like factor 15 | 1 |
| 8645 | hsa-mir-22 | 28999 | KLF15 | Kruppel-like factor 15 | 1 |
| 8646 | hsa-mir-10a | 28999 | KLF15 | Kruppel-like factor 15 | 1 |
| 8647 | hsa-mir-186 | 28999 | KLF15 | Kruppel-like factor 15 | 1 |
| 8648 | hsa-mir-155 | 28999 | KLF15 | Kruppel-like factor 15 | 1 |
| 8649 | hsa-mir-423 | 28999 | KLF15 | Kruppel-like factor 15 | 1 |
| 8650 | hsa-mir-885 | 28999 | KLF15 | Kruppel-like factor 15 | 1 |
| 8651 | hsa-mir-877 | 28999 | KLF15 | Kruppel-like factor 15 | 1 |
| 8652 | hsa-mir-3190 | 28999 | KLF15 | Kruppel-like factor 15 | 1 |
| 8653 | hsa-miR-1 | 51236 | C8orf30A | chromosome 8 open reading frame 30A | 1 |
| 8654 | hsa-miR-133a | 51236 | C8orf30A | chromosome 8 open reading frame 30A | 1 |
| 8655 | hsa-miR-186 | 51236 | C8orf30A | chromosome 8 open reading frame 30A | 1 |
| 8656 | hsa-miR-326 | 51236 | C8orf30A | chromosome 8 open reading frame 30A | 1 |
| 8657 | hsa-miR-449b | 51236 | C8orf30A | chromosome 8 open reading frame 30A | 1 |
| 8658 | hsa-miR-550 | 51236 | C8orf30A | chromosome 8 open reading frame 30A | 1 |
| 8659 | hsa-miR-551a | 51236 | C8orf30A | chromosome 8 open reading frame 30A | 1 |
| 8660 | hsa-miR-562 | 51236 | C8orf30A | chromosome 8 open reading frame 30A | 1 |
| 8661 | hsa-miR-575 | 51236 | C8orf30A | chromosome 8 open reading frame 30A | 1 |
| 8662 | hsa-miR-579 | 51236 | C8orf30A | chromosome 8 open reading frame 30A | 1 |
| 8663 | hsa-miR-608 | 51236 | C8orf30A | chromosome 8 open reading frame 30A | 1 |
| 8664 | hsa-miR-620 | 51236 | C8orf30A | chromosome 8 open reading frame 30A | 1 |
| 8665 | hsa-miR-885-5p | 51236 | C8orf30A | chromosome 8 open reading frame 30A | 1 |
| 8666 | hsa-mir-22 | 51236 | C8orf30A | chromosome 8 open reading frame 30A | 1 |
| 8667 | hsa-mir-10a | 51236 | C8orf30A | chromosome 8 open reading frame 30A | 1 |
| 8668 | hsa-mir-186 | 51236 | C8orf30A | chromosome 8 open reading frame 30A | 1 |
| 8669 | hsa-mir-155 | 51236 | C8orf30A | chromosome 8 open reading frame 30A | 1 |
| 8670 | hsa-mir-423 | 51236 | C8orf30A | chromosome 8 open reading frame 30A | 1 |
| 8671 | hsa-mir-499 | 51236 | C8orf30A | chromosome 8 open reading frame 30A | 1 |
| 8672 | hsa-mir-550-1 | 51236 | C8orf30A | chromosome 8 open reading frame 30A | 1 |
| 8673 | hsa-mir-885 | 51236 | C8orf30A | chromosome 8 open reading frame 30A | 1 |
| 8674 | hsa-mir-877 | 51236 | C8orf30A | chromosome 8 open reading frame 30A | 1 |
| 8675 | hsa-mir-2116 | 51236 | C8orf30A | chromosome 8 open reading frame 30A | 1 |
| 8676 | hsa-mir-3190 | 51236 | C8orf30A | chromosome 8 open reading frame 30A | 1 |
| 8677 | hsa-miR-10a | 23780 | APOL2 | apolipoprotein L, 2 | 1 |
| 8678 | hsa-miR-139-5p | 23780 | APOL2 | apolipoprotein L, 2 | 1 |
| 8679 | hsa-miR-152 | 23780 | APOL2 | apolipoprotein L, 2 | 1 |
| 8680 | hsa-miR-26a | 23780 | APOL2 | apolipoprotein L, 2 | 1 |
| 8681 | hsa-miR-26b | 23780 | APOL2 | apolipoprotein L, 2 | 1 |
| 8682 | hsa-miR-31 | 23780 | APOL2 | apolipoprotein L, 2 | 1 |
| 8683 | hsa-miR-338-5p | 23780 | APOL2 | apolipoprotein L, 2 | 1 |
| 8684 | hsa-miR-340 | 23780 | APOL2 | apolipoprotein L, 2 | 1 |
| 8685 | hsa-miR-449a | 23780 | APOL2 | apolipoprotein L, 2 | 1 |
| 8686 | hsa-miR-499-5p | 23780 | APOL2 | apolipoprotein L, 2 | 1 |
| 8687 | hsa-miR-548b-5p | 23780 | APOL2 | apolipoprotein L, 2 | 1 |
| 8688 | hsa-miR-564 | 23780 | APOL2 | apolipoprotein L, 2 | 1 |
| 8689 | hsa-miR-621 | 23780 | APOL2 | apolipoprotein L, 2 | 1 |
| 8690 | hsa-miR-657 | 23780 | APOL2 | apolipoprotein L, 2 | 1 |
| 8691 | hsa-miR-765 | 23780 | APOL2 | apolipoprotein L, 2 | 1 |
| 8692 | hsa-miR-770-5p | 23780 | APOL2 | apolipoprotein L, 2 | 1 |
| 8693 | hsa-miR-877 | 23780 | APOL2 | apolipoprotein L, 2 | 1 |
| 8694 | hsa-mir-26a-1 | 23780 | APOL2 | apolipoprotein L, 2 | 1 |
| 8695 | hsa-mir-26b | 23780 | APOL2 | apolipoprotein L, 2 | 1 |
| 8696 | hsa-mir-31 | 23780 | APOL2 | apolipoprotein L, 2 | 1 |
| 8697 | hsa-mir-194-2 | 23780 | APOL2 | apolipoprotein L, 2 | 1 |
| 8698 | hsa-mir-340 | 23780 | APOL2 | apolipoprotein L, 2 | 1 |
| 8699 | hsa-mir-338 | 23780 | APOL2 | apolipoprotein L, 2 | 1 |
| 8700 | hsa-mir-548b | 23780 | APOL2 | apolipoprotein L, 2 | 1 |
| 8701 | hsa-mir-548d-2 | 23780 | APOL2 | apolipoprotein L, 2 | 1 |
| 8702 | hsa-mir-449b | 23780 | APOL2 | apolipoprotein L, 2 | 1 |
| 8703 | hsa-mir-449c | 23780 | APOL2 | apolipoprotein L, 2 | 1 |
| 8704 | hsa-mir-3065 | 23780 | APOL2 | apolipoprotein L, 2 | 1 |
| 8705 | hsa-miR-10a | 83696 | TRAPPC9 | trafficking protein particle complex 9 | 1 |
| 8706 | hsa-miR-152 | 83696 | TRAPPC9 | trafficking protein particle complex 9 | 1 |
| 8707 | hsa-miR-26a | 83696 | TRAPPC9 | trafficking protein particle complex 9 | 1 |
| 8708 | hsa-miR-26b | 83696 | TRAPPC9 | trafficking protein particle complex 9 | 1 |
| 8709 | hsa-miR-31 | 83696 | TRAPPC9 | trafficking protein particle complex 9 | 1 |
| 8710 | hsa-miR-338-5p | 83696 | TRAPPC9 | trafficking protein particle complex 9 | 1 |
| 8711 | hsa-miR-340 | 83696 | TRAPPC9 | trafficking protein particle complex 9 | 1 |
| 8712 | hsa-miR-423-5p | 83696 | TRAPPC9 | trafficking protein particle complex 9 | 1 |
| 8713 | hsa-miR-449a | 83696 | TRAPPC9 | trafficking protein particle complex 9 | 1 |
| 8714 | hsa-miR-499-5p | 83696 | TRAPPC9 | trafficking protein particle complex 9 | 1 |
| 8715 | hsa-miR-548b-5p | 83696 | TRAPPC9 | trafficking protein particle complex 9 | 1 |
| 8716 | hsa-miR-564 | 83696 | TRAPPC9 | trafficking protein particle complex 9 | 1 |
| 8717 | hsa-miR-621 | 83696 | TRAPPC9 | trafficking protein particle complex 9 | 1 |
| 8718 | hsa-miR-644 | 83696 | TRAPPC9 | trafficking protein particle complex 9 | 1 |
| 8719 | hsa-miR-657 | 83696 | TRAPPC9 | trafficking protein particle complex 9 | 1 |
| 8720 | hsa-miR-765 | 83696 | TRAPPC9 | trafficking protein particle complex 9 | 1 |
| 8721 | hsa-miR-770-5p | 83696 | TRAPPC9 | trafficking protein particle complex 9 | 1 |
| 8722 | hsa-miR-877 | 83696 | TRAPPC9 | trafficking protein particle complex 9 | 1 |
| 8723 | hsa-miR-95 | 83696 | TRAPPC9 | trafficking protein particle complex 9 | 1 |
| 8724 | hsa-mir-26a-1 | 83696 | TRAPPC9 | trafficking protein particle complex 9 | 1 |
| 8725 | hsa-mir-26b | 83696 | TRAPPC9 | trafficking protein particle complex 9 | 1 |
| 8726 | hsa-mir-31 | 83696 | TRAPPC9 | trafficking protein particle complex 9 | 1 |
| 8727 | hsa-mir-194-2 | 83696 | TRAPPC9 | trafficking protein particle complex 9 | 1 |
| 8728 | hsa-mir-340 | 83696 | TRAPPC9 | trafficking protein particle complex 9 | 1 |
| 8729 | hsa-mir-338 | 83696 | TRAPPC9 | trafficking protein particle complex 9 | 1 |
| 8730 | hsa-mir-548b | 83696 | TRAPPC9 | trafficking protein particle complex 9 | 1 |
| 8731 | hsa-mir-449b | 83696 | TRAPPC9 | trafficking protein particle complex 9 | 1 |
| 8732 | hsa-mir-449c | 83696 | TRAPPC9 | trafficking protein particle complex 9 | 1 |
| 8733 | hsa-mir-3065 | 83696 | TRAPPC9 | trafficking protein particle complex 9 | 1 |
| 8734 | hsa-miR-10a | 84619 | ZGPAT | zinc finger, CCCH-type with G patch domain | 1 |
| 8735 | hsa-miR-1228\* | 84619 | ZGPAT | zinc finger, CCCH-type with G patch domain | 1 |
| 8736 | hsa-miR-139-5p | 84619 | ZGPAT | zinc finger, CCCH-type with G patch domain | 1 |
| 8737 | hsa-miR-152 | 84619 | ZGPAT | zinc finger, CCCH-type with G patch domain | 1 |
| 8738 | hsa-miR-26a | 84619 | ZGPAT | zinc finger, CCCH-type with G patch domain | 1 |
| 8739 | hsa-miR-26b | 84619 | ZGPAT | zinc finger, CCCH-type with G patch domain | 1 |
| 8740 | hsa-miR-338-5p | 84619 | ZGPAT | zinc finger, CCCH-type with G patch domain | 1 |
| 8741 | hsa-miR-340 | 84619 | ZGPAT | zinc finger, CCCH-type with G patch domain | 1 |
| 8742 | hsa-miR-449a | 84619 | ZGPAT | zinc finger, CCCH-type with G patch domain | 1 |
| 8743 | hsa-miR-499-5p | 84619 | ZGPAT | zinc finger, CCCH-type with G patch domain | 1 |
| 8744 | hsa-miR-548b-5p | 84619 | ZGPAT | zinc finger, CCCH-type with G patch domain | 1 |
| 8745 | hsa-miR-584 | 84619 | ZGPAT | zinc finger, CCCH-type with G patch domain | 1 |
| 8746 | hsa-miR-621 | 84619 | ZGPAT | zinc finger, CCCH-type with G patch domain | 1 |
| 8747 | hsa-miR-657 | 84619 | ZGPAT | zinc finger, CCCH-type with G patch domain | 1 |
| 8748 | hsa-miR-765 | 84619 | ZGPAT | zinc finger, CCCH-type with G patch domain | 1 |
| 8749 | hsa-miR-770-5p | 84619 | ZGPAT | zinc finger, CCCH-type with G patch domain | 1 |
| 8750 | hsa-miR-877 | 84619 | ZGPAT | zinc finger, CCCH-type with G patch domain | 1 |
| 8751 | hsa-mir-26a-1 | 84619 | ZGPAT | zinc finger, CCCH-type with G patch domain | 1 |
| 8752 | hsa-mir-26b | 84619 | ZGPAT | zinc finger, CCCH-type with G patch domain | 1 |
| 8753 | hsa-mir-194-2 | 84619 | ZGPAT | zinc finger, CCCH-type with G patch domain | 1 |
| 8754 | hsa-mir-340 | 84619 | ZGPAT | zinc finger, CCCH-type with G patch domain | 1 |
| 8755 | hsa-mir-338 | 84619 | ZGPAT | zinc finger, CCCH-type with G patch domain | 1 |
| 8756 | hsa-mir-548b | 84619 | ZGPAT | zinc finger, CCCH-type with G patch domain | 1 |
| 8757 | hsa-mir-449b | 84619 | ZGPAT | zinc finger, CCCH-type with G patch domain | 1 |
| 8758 | hsa-mir-449c | 84619 | ZGPAT | zinc finger, CCCH-type with G patch domain | 1 |
| 8759 | hsa-mir-3065 | 84619 | ZGPAT | zinc finger, CCCH-type with G patch domain | 1 |
| 8760 | hsa-miR-10a | 203197 | C9orf91 | chromosome 9 open reading frame 91 | 1 |
| 8761 | hsa-miR-152 | 203197 | C9orf91 | chromosome 9 open reading frame 91 | 1 |
| 8762 | hsa-miR-26a | 203197 | C9orf91 | chromosome 9 open reading frame 91 | 1 |
| 8763 | hsa-miR-26b | 203197 | C9orf91 | chromosome 9 open reading frame 91 | 1 |
| 8764 | hsa-miR-31 | 203197 | C9orf91 | chromosome 9 open reading frame 91 | 1 |
| 8765 | hsa-miR-338-5p | 203197 | C9orf91 | chromosome 9 open reading frame 91 | 1 |
| 8766 | hsa-miR-340 | 203197 | C9orf91 | chromosome 9 open reading frame 91 | 1 |
| 8767 | hsa-miR-423-5p | 203197 | C9orf91 | chromosome 9 open reading frame 91 | 1 |
| 8768 | hsa-miR-449a | 203197 | C9orf91 | chromosome 9 open reading frame 91 | 1 |
| 8769 | hsa-miR-490-5p | 203197 | C9orf91 | chromosome 9 open reading frame 91 | 1 |
| 8770 | hsa-miR-499-5p | 203197 | C9orf91 | chromosome 9 open reading frame 91 | 1 |
| 8771 | hsa-miR-548b-5p | 203197 | C9orf91 | chromosome 9 open reading frame 91 | 1 |
| 8772 | hsa-miR-564 | 203197 | C9orf91 | chromosome 9 open reading frame 91 | 1 |
| 8773 | hsa-miR-621 | 203197 | C9orf91 | chromosome 9 open reading frame 91 | 1 |
| 8774 | hsa-miR-657 | 203197 | C9orf91 | chromosome 9 open reading frame 91 | 1 |
| 8775 | hsa-miR-663 | 203197 | C9orf91 | chromosome 9 open reading frame 91 | 1 |
| 8776 | hsa-miR-765 | 203197 | C9orf91 | chromosome 9 open reading frame 91 | 1 |
| 8777 | hsa-miR-770-5p | 203197 | C9orf91 | chromosome 9 open reading frame 91 | 1 |
| 8778 | hsa-miR-877 | 203197 | C9orf91 | chromosome 9 open reading frame 91 | 1 |
| 8779 | hsa-mir-26a-1 | 203197 | C9orf91 | chromosome 9 open reading frame 91 | 1 |
| 8780 | hsa-mir-26b | 203197 | C9orf91 | chromosome 9 open reading frame 91 | 1 |
| 8781 | hsa-mir-31 | 203197 | C9orf91 | chromosome 9 open reading frame 91 | 1 |
| 8782 | hsa-mir-194-2 | 203197 | C9orf91 | chromosome 9 open reading frame 91 | 1 |
| 8783 | hsa-mir-340 | 203197 | C9orf91 | chromosome 9 open reading frame 91 | 1 |
| 8784 | hsa-mir-338 | 203197 | C9orf91 | chromosome 9 open reading frame 91 | 1 |
| 8785 | hsa-mir-490 | 203197 | C9orf91 | chromosome 9 open reading frame 91 | 1 |
| 8786 | hsa-mir-548b | 203197 | C9orf91 | chromosome 9 open reading frame 91 | 1 |
| 8787 | hsa-mir-548d-2 | 203197 | C9orf91 | chromosome 9 open reading frame 91 | 1 |
| 8788 | hsa-mir-449b | 203197 | C9orf91 | chromosome 9 open reading frame 91 | 1 |
| 8789 | hsa-mir-449c | 203197 | C9orf91 | chromosome 9 open reading frame 91 | 1 |
| 8790 | hsa-mir-3065 | 203197 | C9orf91 | chromosome 9 open reading frame 91 | 1 |
| 8791 | hsa-miR-1 | 90956 | ADCK2 | aarF domain containing kinase 2 | 1 |
| 8792 | hsa-miR-133a | 90956 | ADCK2 | aarF domain containing kinase 2 | 1 |
| 8793 | hsa-miR-186 | 90956 | ADCK2 | aarF domain containing kinase 2 | 1 |
| 8794 | hsa-miR-208a | 90956 | ADCK2 | aarF domain containing kinase 2 | 1 |
| 8795 | hsa-miR-326 | 90956 | ADCK2 | aarF domain containing kinase 2 | 1 |
| 8796 | hsa-miR-449b | 90956 | ADCK2 | aarF domain containing kinase 2 | 1 |
| 8797 | hsa-miR-550 | 90956 | ADCK2 | aarF domain containing kinase 2 | 1 |
| 8798 | hsa-miR-575 | 90956 | ADCK2 | aarF domain containing kinase 2 | 1 |
| 8799 | hsa-miR-608 | 90956 | ADCK2 | aarF domain containing kinase 2 | 1 |
| 8800 | hsa-miR-620 | 90956 | ADCK2 | aarF domain containing kinase 2 | 1 |
| 8801 | hsa-let-7a-3 | 90956 | ADCK2 | aarF domain containing kinase 2 | 1 |
| 8802 | hsa-let-7b | 90956 | ADCK2 | aarF domain containing kinase 2 | 1 |
| 8803 | hsa-mir-22 | 90956 | ADCK2 | aarF domain containing kinase 2 | 1 |
| 8804 | hsa-mir-10a | 90956 | ADCK2 | aarF domain containing kinase 2 | 1 |
| 8805 | hsa-mir-186 | 90956 | ADCK2 | aarF domain containing kinase 2 | 1 |
| 8806 | hsa-mir-155 | 90956 | ADCK2 | aarF domain containing kinase 2 | 1 |
| 8807 | hsa-mir-499 | 90956 | ADCK2 | aarF domain containing kinase 2 | 1 |
| 8808 | hsa-mir-550-1 | 90956 | ADCK2 | aarF domain containing kinase 2 | 1 |
| 8809 | hsa-mir-877 | 90956 | ADCK2 | aarF domain containing kinase 2 | 1 |
| 8810 | hsa-mir-2116 | 90956 | ADCK2 | aarF domain containing kinase 2 | 1 |
| 8811 | hsa-mir-3186 | 90956 | ADCK2 | aarF domain containing kinase 2 | 1 |
| 8812 | hsa-mir-3190 | 90956 | ADCK2 | aarF domain containing kinase 2 | 1 |
| 8813 | hsa-miR-10a | 3134 | HLA-F | major histocompatibility complex, class I, F | 1 |
| 8814 | hsa-miR-139-5p | 3134 | HLA-F | major histocompatibility complex, class I, F | 1 |
| 8815 | hsa-miR-26a | 3134 | HLA-F | major histocompatibility complex, class I, F | 1 |
| 8816 | hsa-miR-490-5p | 3134 | HLA-F | major histocompatibility complex, class I, F | 1 |
| 8817 | hsa-miR-499-5p | 3134 | HLA-F | major histocompatibility complex, class I, F | 1 |
| 8818 | hsa-miR-548b-5p | 3134 | HLA-F | major histocompatibility complex, class I, F | 1 |
| 8819 | hsa-miR-663 | 3134 | HLA-F | major histocompatibility complex, class I, F | 1 |
| 8820 | hsa-miR-877 | 3134 | HLA-F | major histocompatibility complex, class I, F | 1 |
| 8821 | hsa-mir-490 | 3134 | HLA-F | major histocompatibility complex, class I, F | 1 |
| 8822 | hsa-mir-548b | 3134 | HLA-F | major histocompatibility complex, class I, F | 1 |
| 8823 | hsa-miR-10a | 6134 | RPL10 | ribosomal protein L10 | 1 |
| 8824 | hsa-miR-1228\* | 6134 | RPL10 | ribosomal protein L10 | 1 |
| 8825 | hsa-miR-139-5p | 6134 | RPL10 | ribosomal protein L10 | 1 |
| 8826 | hsa-miR-152 | 6134 | RPL10 | ribosomal protein L10 | 1 |
| 8827 | hsa-miR-26a | 6134 | RPL10 | ribosomal protein L10 | 1 |
| 8828 | hsa-miR-26b | 6134 | RPL10 | ribosomal protein L10 | 1 |
| 8829 | hsa-miR-338-5p | 6134 | RPL10 | ribosomal protein L10 | 1 |
| 8830 | hsa-miR-340 | 6134 | RPL10 | ribosomal protein L10 | 1 |
| 8831 | hsa-miR-449a | 6134 | RPL10 | ribosomal protein L10 | 1 |
| 8832 | hsa-miR-499-5p | 6134 | RPL10 | ribosomal protein L10 | 1 |
| 8833 | hsa-miR-548b-5p | 6134 | RPL10 | ribosomal protein L10 | 1 |
| 8834 | hsa-miR-564 | 6134 | RPL10 | ribosomal protein L10 | 1 |
| 8835 | hsa-miR-584 | 6134 | RPL10 | ribosomal protein L10 | 1 |
| 8836 | hsa-miR-621 | 6134 | RPL10 | ribosomal protein L10 | 1 |
| 8837 | hsa-miR-657 | 6134 | RPL10 | ribosomal protein L10 | 1 |
| 8838 | hsa-miR-765 | 6134 | RPL10 | ribosomal protein L10 | 1 |
| 8839 | hsa-miR-770-5p | 6134 | RPL10 | ribosomal protein L10 | 1 |
| 8840 | hsa-miR-877 | 6134 | RPL10 | ribosomal protein L10 | 1 |
| 8841 | hsa-mir-26a-1 | 6134 | RPL10 | ribosomal protein L10 | 1 |
| 8842 | hsa-mir-26b | 6134 | RPL10 | ribosomal protein L10 | 1 |
| 8843 | hsa-mir-194-2 | 6134 | RPL10 | ribosomal protein L10 | 1 |
| 8844 | hsa-mir-340 | 6134 | RPL10 | ribosomal protein L10 | 1 |
| 8845 | hsa-mir-338 | 6134 | RPL10 | ribosomal protein L10 | 1 |
| 8846 | hsa-mir-548b | 6134 | RPL10 | ribosomal protein L10 | 1 |
| 8847 | hsa-mir-548d-2 | 6134 | RPL10 | ribosomal protein L10 | 1 |
| 8848 | hsa-mir-449b | 6134 | RPL10 | ribosomal protein L10 | 1 |
| 8849 | hsa-mir-449c | 6134 | RPL10 | ribosomal protein L10 | 1 |
| 8850 | hsa-mir-3065 | 6134 | RPL10 | ribosomal protein L10 | 1 |
| 8861 | hsa-miR-634 | 373863 | DND1 | dead end homolog 1 (zebrafish) | 1 |
| 8862 | hsa-miR-653 | 373863 | DND1 | dead end homolog 1 (zebrafish) | 1 |
| 8876 | hsa-miR-10a | 7328 | UBE2H | ubiquitin-conjugating enzyme E2H (UBC8 homolog, yeast) | 1 |
| 8877 | hsa-miR-1228\* | 7328 | UBE2H | ubiquitin-conjugating enzyme E2H (UBC8 homolog, yeast) | 1 |
| 8878 | hsa-miR-152 | 7328 | UBE2H | ubiquitin-conjugating enzyme E2H (UBC8 homolog, yeast) | 1 |
| 8879 | hsa-miR-26a | 7328 | UBE2H | ubiquitin-conjugating enzyme E2H (UBC8 homolog, yeast) | 1 |
| 8880 | hsa-miR-26b | 7328 | UBE2H | ubiquitin-conjugating enzyme E2H (UBC8 homolog, yeast) | 1 |
| 8881 | hsa-miR-31 | 7328 | UBE2H | ubiquitin-conjugating enzyme E2H (UBC8 homolog, yeast) | 1 |
| 8882 | hsa-miR-338-5p | 7328 | UBE2H | ubiquitin-conjugating enzyme E2H (UBC8 homolog, yeast) | 1 |
| 8883 | hsa-miR-340 | 7328 | UBE2H | ubiquitin-conjugating enzyme E2H (UBC8 homolog, yeast) | 1 |
| 8884 | hsa-miR-423-5p | 7328 | UBE2H | ubiquitin-conjugating enzyme E2H (UBC8 homolog, yeast) | 1 |
| 8885 | hsa-miR-449a | 7328 | UBE2H | ubiquitin-conjugating enzyme E2H (UBC8 homolog, yeast) | 1 |
| 8886 | hsa-miR-490-5p | 7328 | UBE2H | ubiquitin-conjugating enzyme E2H (UBC8 homolog, yeast) | 1 |
| 8887 | hsa-miR-499-5p | 7328 | UBE2H | ubiquitin-conjugating enzyme E2H (UBC8 homolog, yeast) | 1 |
| 8888 | hsa-miR-548b-5p | 7328 | UBE2H | ubiquitin-conjugating enzyme E2H (UBC8 homolog, yeast) | 1 |
| 8889 | hsa-miR-564 | 7328 | UBE2H | ubiquitin-conjugating enzyme E2H (UBC8 homolog, yeast) | 1 |
| 8890 | hsa-miR-584 | 7328 | UBE2H | ubiquitin-conjugating enzyme E2H (UBC8 homolog, yeast) | 1 |
| 8891 | hsa-miR-621 | 7328 | UBE2H | ubiquitin-conjugating enzyme E2H (UBC8 homolog, yeast) | 1 |
| 8892 | hsa-miR-644 | 7328 | UBE2H | ubiquitin-conjugating enzyme E2H (UBC8 homolog, yeast) | 1 |
| 8893 | hsa-miR-657 | 7328 | UBE2H | ubiquitin-conjugating enzyme E2H (UBC8 homolog, yeast) | 1 |
| 8894 | hsa-miR-765 | 7328 | UBE2H | ubiquitin-conjugating enzyme E2H (UBC8 homolog, yeast) | 1 |
| 8895 | hsa-miR-770-5p | 7328 | UBE2H | ubiquitin-conjugating enzyme E2H (UBC8 homolog, yeast) | 1 |
| 8896 | hsa-miR-877 | 7328 | UBE2H | ubiquitin-conjugating enzyme E2H (UBC8 homolog, yeast) | 1 |
| 8897 | hsa-miR-95 | 7328 | UBE2H | ubiquitin-conjugating enzyme E2H (UBC8 homolog, yeast) | 1 |
| 8898 | hsa-mir-26a-1 | 7328 | UBE2H | ubiquitin-conjugating enzyme E2H (UBC8 homolog, yeast) | 1 |
| 8899 | hsa-mir-26b | 7328 | UBE2H | ubiquitin-conjugating enzyme E2H (UBC8 homolog, yeast) | 1 |
| 8900 | hsa-mir-31 | 7328 | UBE2H | ubiquitin-conjugating enzyme E2H (UBC8 homolog, yeast) | 1 |
| 8901 | hsa-mir-194-2 | 7328 | UBE2H | ubiquitin-conjugating enzyme E2H (UBC8 homolog, yeast) | 1 |
| 8902 | hsa-mir-340 | 7328 | UBE2H | ubiquitin-conjugating enzyme E2H (UBC8 homolog, yeast) | 1 |
| 8903 | hsa-mir-338 | 7328 | UBE2H | ubiquitin-conjugating enzyme E2H (UBC8 homolog, yeast) | 1 |
| 8904 | hsa-mir-490 | 7328 | UBE2H | ubiquitin-conjugating enzyme E2H (UBC8 homolog, yeast) | 1 |
| 8905 | hsa-mir-548b | 7328 | UBE2H | ubiquitin-conjugating enzyme E2H (UBC8 homolog, yeast) | 1 |
| 8906 | hsa-mir-449b | 7328 | UBE2H | ubiquitin-conjugating enzyme E2H (UBC8 homolog, yeast) | 1 |
| 8907 | hsa-mir-449c | 7328 | UBE2H | ubiquitin-conjugating enzyme E2H (UBC8 homolog, yeast) | 1 |
| 8908 | hsa-mir-3065 | 7328 | UBE2H | ubiquitin-conjugating enzyme E2H (UBC8 homolog, yeast) | 1 |
| 8909 | hsa-miR-10a | 56882 | CDC42SE1 | CDC42 small effector 1 | 1 |
| 8910 | hsa-miR-1228\* | 56882 | CDC42SE1 | CDC42 small effector 1 | 1 |
| 8911 | hsa-miR-139-5p | 56882 | CDC42SE1 | CDC42 small effector 1 | 1 |
| 8912 | hsa-miR-152 | 56882 | CDC42SE1 | CDC42 small effector 1 | 1 |
| 8913 | hsa-miR-26a | 56882 | CDC42SE1 | CDC42 small effector 1 | 1 |
| 8914 | hsa-miR-26b | 56882 | CDC42SE1 | CDC42 small effector 1 | 1 |
| 8915 | hsa-miR-31 | 56882 | CDC42SE1 | CDC42 small effector 1 | 1 |
| 8916 | hsa-miR-338-5p | 56882 | CDC42SE1 | CDC42 small effector 1 | 1 |
| 8917 | hsa-miR-340 | 56882 | CDC42SE1 | CDC42 small effector 1 | 1 |
| 8918 | hsa-miR-423-5p | 56882 | CDC42SE1 | CDC42 small effector 1 | 1 |
| 8919 | hsa-miR-449a | 56882 | CDC42SE1 | CDC42 small effector 1 | 1 |
| 8920 | hsa-miR-499-5p | 56882 | CDC42SE1 | CDC42 small effector 1 | 1 |
| 8921 | hsa-miR-548b-5p | 56882 | CDC42SE1 | CDC42 small effector 1 | 1 |
| 8922 | hsa-miR-584 | 56882 | CDC42SE1 | CDC42 small effector 1 | 1 |
| 8923 | hsa-miR-621 | 56882 | CDC42SE1 | CDC42 small effector 1 | 1 |
| 8924 | hsa-miR-657 | 56882 | CDC42SE1 | CDC42 small effector 1 | 1 |
| 8925 | hsa-miR-663 | 56882 | CDC42SE1 | CDC42 small effector 1 | 1 |
| 8926 | hsa-miR-765 | 56882 | CDC42SE1 | CDC42 small effector 1 | 1 |
| 8927 | hsa-miR-770-5p | 56882 | CDC42SE1 | CDC42 small effector 1 | 1 |
| 8928 | hsa-miR-877 | 56882 | CDC42SE1 | CDC42 small effector 1 | 1 |
| 8929 | hsa-mir-26a-1 | 56882 | CDC42SE1 | CDC42 small effector 1 | 1 |
| 8930 | hsa-mir-26b | 56882 | CDC42SE1 | CDC42 small effector 1 | 1 |
| 8931 | hsa-mir-31 | 56882 | CDC42SE1 | CDC42 small effector 1 | 1 |
| 8932 | hsa-mir-194-2 | 56882 | CDC42SE1 | CDC42 small effector 1 | 1 |
| 8933 | hsa-mir-340 | 56882 | CDC42SE1 | CDC42 small effector 1 | 1 |
| 8934 | hsa-mir-338 | 56882 | CDC42SE1 | CDC42 small effector 1 | 1 |
| 8935 | hsa-mir-548b | 56882 | CDC42SE1 | CDC42 small effector 1 | 1 |
| 8936 | hsa-mir-548d-2 | 56882 | CDC42SE1 | CDC42 small effector 1 | 1 |
| 8937 | hsa-mir-449b | 56882 | CDC42SE1 | CDC42 small effector 1 | 1 |
| 8938 | hsa-mir-449c | 56882 | CDC42SE1 | CDC42 small effector 1 | 1 |
| 8939 | hsa-mir-3065 | 56882 | CDC42SE1 | CDC42 small effector 1 | 1 |
| 8940 | hsa-miR-1 | 9342 | SNAP29 | synaptosomal-associated protein, 29kDa | 1 |
| 8941 | hsa-miR-133a | 9342 | SNAP29 | synaptosomal-associated protein, 29kDa | 1 |
| 8942 | hsa-miR-186 | 9342 | SNAP29 | synaptosomal-associated protein, 29kDa | 1 |
| 8943 | hsa-miR-326 | 9342 | SNAP29 | synaptosomal-associated protein, 29kDa | 1 |
| 8944 | hsa-miR-449b | 9342 | SNAP29 | synaptosomal-associated protein, 29kDa | 1 |
| 8945 | hsa-miR-489 | 9342 | SNAP29 | synaptosomal-associated protein, 29kDa | 1 |
| 8946 | hsa-miR-562 | 9342 | SNAP29 | synaptosomal-associated protein, 29kDa | 1 |
| 8947 | hsa-miR-579 | 9342 | SNAP29 | synaptosomal-associated protein, 29kDa | 1 |
| 8948 | hsa-miR-608 | 9342 | SNAP29 | synaptosomal-associated protein, 29kDa | 1 |
| 8949 | hsa-miR-637 | 9342 | SNAP29 | synaptosomal-associated protein, 29kDa | 1 |
| 8950 | hsa-miR-885-5p | 9342 | SNAP29 | synaptosomal-associated protein, 29kDa | 1 |
| 8951 | hsa-mir-22 | 9342 | SNAP29 | synaptosomal-associated protein, 29kDa | 1 |
| 8952 | hsa-mir-10a | 9342 | SNAP29 | synaptosomal-associated protein, 29kDa | 1 |
| 8953 | hsa-mir-186 | 9342 | SNAP29 | synaptosomal-associated protein, 29kDa | 1 |
| 8954 | hsa-mir-155 | 9342 | SNAP29 | synaptosomal-associated protein, 29kDa | 1 |
| 8955 | hsa-mir-885 | 9342 | SNAP29 | synaptosomal-associated protein, 29kDa | 1 |
| 8956 | hsa-miR-423-5p | 57823 | SLAMF7 | SLAM family member 7 | 1 |
| 8957 | hsa-miR-1 | 27345 | KCNMB4 | potassium large conductance calcium-activated channel, subfamily M, beta member 4 | 1 |
| 8958 | hsa-miR-133a | 27345 | KCNMB4 | potassium large conductance calcium-activated channel, subfamily M, beta member 4 | 1 |
| 8959 | hsa-miR-186 | 27345 | KCNMB4 | potassium large conductance calcium-activated channel, subfamily M, beta member 4 | 1 |
| 8960 | hsa-miR-326 | 27345 | KCNMB4 | potassium large conductance calcium-activated channel, subfamily M, beta member 4 | 1 |
| 8961 | hsa-miR-33a | 27345 | KCNMB4 | potassium large conductance calcium-activated channel, subfamily M, beta member 4 | 1 |
| 8962 | hsa-miR-449b | 27345 | KCNMB4 | potassium large conductance calcium-activated channel, subfamily M, beta member 4 | 1 |
| 8963 | hsa-miR-550 | 27345 | KCNMB4 | potassium large conductance calcium-activated channel, subfamily M, beta member 4 | 1 |
| 8964 | hsa-miR-551a | 27345 | KCNMB4 | potassium large conductance calcium-activated channel, subfamily M, beta member 4 | 1 |
| 8965 | hsa-miR-575 | 27345 | KCNMB4 | potassium large conductance calcium-activated channel, subfamily M, beta member 4 | 1 |
| 8966 | hsa-miR-579 | 27345 | KCNMB4 | potassium large conductance calcium-activated channel, subfamily M, beta member 4 | 1 |
| 8967 | hsa-miR-608 | 27345 | KCNMB4 | potassium large conductance calcium-activated channel, subfamily M, beta member 4 | 1 |
| 8968 | hsa-miR-620 | 27345 | KCNMB4 | potassium large conductance calcium-activated channel, subfamily M, beta member 4 | 1 |
| 8969 | hsa-let-7a-3 | 27345 | KCNMB4 | potassium large conductance calcium-activated channel, subfamily M, beta member 4 | 1 |
| 8970 | hsa-let-7b | 27345 | KCNMB4 | potassium large conductance calcium-activated channel, subfamily M, beta member 4 | 1 |
| 8971 | hsa-mir-22 | 27345 | KCNMB4 | potassium large conductance calcium-activated channel, subfamily M, beta member 4 | 1 |
| 8972 | hsa-mir-10a | 27345 | KCNMB4 | potassium large conductance calcium-activated channel, subfamily M, beta member 4 | 1 |
| 8973 | hsa-mir-186 | 27345 | KCNMB4 | potassium large conductance calcium-activated channel, subfamily M, beta member 4 | 1 |
| 8974 | hsa-mir-155 | 27345 | KCNMB4 | potassium large conductance calcium-activated channel, subfamily M, beta member 4 | 1 |
| 8975 | hsa-mir-423 | 27345 | KCNMB4 | potassium large conductance calcium-activated channel, subfamily M, beta member 4 | 1 |
| 8976 | hsa-mir-499 | 27345 | KCNMB4 | potassium large conductance calcium-activated channel, subfamily M, beta member 4 | 1 |
| 8977 | hsa-mir-550-1 | 27345 | KCNMB4 | potassium large conductance calcium-activated channel, subfamily M, beta member 4 | 1 |
| 8978 | hsa-mir-877 | 27345 | KCNMB4 | potassium large conductance calcium-activated channel, subfamily M, beta member 4 | 1 |
| 8979 | hsa-mir-2116 | 27345 | KCNMB4 | potassium large conductance calcium-activated channel, subfamily M, beta member 4 | 1 |
| 8980 | hsa-mir-3186 | 27345 | KCNMB4 | potassium large conductance calcium-activated channel, subfamily M, beta member 4 | 1 |
| 8981 | hsa-mir-3190 | 27345 | KCNMB4 | potassium large conductance calcium-activated channel, subfamily M, beta member 4 | 1 |
| 9008 | hsa-miR-26a | 27071 | DAPP1 | dual adaptor of phosphotyrosine and 3-phosphoinositides | 1 |
| 9009 | hsa-miR-423-5p | 27071 | DAPP1 | dual adaptor of phosphotyrosine and 3-phosphoinositides | 1 |
| 9010 | hsa-miR-663 | 27071 | DAPP1 | dual adaptor of phosphotyrosine and 3-phosphoinositides | 1 |
| 9011 | hsa-miR-10a | 10678 | B3GNT2 | UDP-GlcNAc:betaGal beta-1,3-N-acetylglucosaminyltransferase 2 | 1 |
| 9012 | hsa-miR-1228\* | 10678 | B3GNT2 | UDP-GlcNAc:betaGal beta-1,3-N-acetylglucosaminyltransferase 2 | 1 |
| 9013 | hsa-miR-139-5p | 10678 | B3GNT2 | UDP-GlcNAc:betaGal beta-1,3-N-acetylglucosaminyltransferase 2 | 1 |
| 9014 | hsa-miR-152 | 10678 | B3GNT2 | UDP-GlcNAc:betaGal beta-1,3-N-acetylglucosaminyltransferase 2 | 1 |
| 9015 | hsa-miR-26a | 10678 | B3GNT2 | UDP-GlcNAc:betaGal beta-1,3-N-acetylglucosaminyltransferase 2 | 1 |
| 9016 | hsa-miR-26b | 10678 | B3GNT2 | UDP-GlcNAc:betaGal beta-1,3-N-acetylglucosaminyltransferase 2 | 1 |
| 9017 | hsa-miR-31 | 10678 | B3GNT2 | UDP-GlcNAc:betaGal beta-1,3-N-acetylglucosaminyltransferase 2 | 1 |
| 9018 | hsa-miR-338-5p | 10678 | B3GNT2 | UDP-GlcNAc:betaGal beta-1,3-N-acetylglucosaminyltransferase 2 | 1 |
| 9019 | hsa-miR-340 | 10678 | B3GNT2 | UDP-GlcNAc:betaGal beta-1,3-N-acetylglucosaminyltransferase 2 | 1 |
| 9020 | hsa-miR-423-5p | 10678 | B3GNT2 | UDP-GlcNAc:betaGal beta-1,3-N-acetylglucosaminyltransferase 2 | 1 |
| 9021 | hsa-miR-449a | 10678 | B3GNT2 | UDP-GlcNAc:betaGal beta-1,3-N-acetylglucosaminyltransferase 2 | 1 |
| 9022 | hsa-miR-499-5p | 10678 | B3GNT2 | UDP-GlcNAc:betaGal beta-1,3-N-acetylglucosaminyltransferase 2 | 1 |
| 9023 | hsa-miR-548b-5p | 10678 | B3GNT2 | UDP-GlcNAc:betaGal beta-1,3-N-acetylglucosaminyltransferase 2 | 1 |
| 9024 | hsa-miR-564 | 10678 | B3GNT2 | UDP-GlcNAc:betaGal beta-1,3-N-acetylglucosaminyltransferase 2 | 1 |
| 9025 | hsa-miR-621 | 10678 | B3GNT2 | UDP-GlcNAc:betaGal beta-1,3-N-acetylglucosaminyltransferase 2 | 1 |
| 9026 | hsa-miR-644 | 10678 | B3GNT2 | UDP-GlcNAc:betaGal beta-1,3-N-acetylglucosaminyltransferase 2 | 1 |
| 9027 | hsa-miR-657 | 10678 | B3GNT2 | UDP-GlcNAc:betaGal beta-1,3-N-acetylglucosaminyltransferase 2 | 1 |
| 9028 | hsa-miR-765 | 10678 | B3GNT2 | UDP-GlcNAc:betaGal beta-1,3-N-acetylglucosaminyltransferase 2 | 1 |
| 9029 | hsa-miR-770-5p | 10678 | B3GNT2 | UDP-GlcNAc:betaGal beta-1,3-N-acetylglucosaminyltransferase 2 | 1 |
| 9030 | hsa-miR-877 | 10678 | B3GNT2 | UDP-GlcNAc:betaGal beta-1,3-N-acetylglucosaminyltransferase 2 | 1 |
| 9031 | hsa-mir-26a-1 | 10678 | B3GNT2 | UDP-GlcNAc:betaGal beta-1,3-N-acetylglucosaminyltransferase 2 | 1 |
| 9032 | hsa-mir-26b | 10678 | B3GNT2 | UDP-GlcNAc:betaGal beta-1,3-N-acetylglucosaminyltransferase 2 | 1 |
| 9033 | hsa-mir-31 | 10678 | B3GNT2 | UDP-GlcNAc:betaGal beta-1,3-N-acetylglucosaminyltransferase 2 | 1 |
| 9034 | hsa-mir-194-2 | 10678 | B3GNT2 | UDP-GlcNAc:betaGal beta-1,3-N-acetylglucosaminyltransferase 2 | 1 |
| 9035 | hsa-mir-340 | 10678 | B3GNT2 | UDP-GlcNAc:betaGal beta-1,3-N-acetylglucosaminyltransferase 2 | 1 |
| 9036 | hsa-mir-338 | 10678 | B3GNT2 | UDP-GlcNAc:betaGal beta-1,3-N-acetylglucosaminyltransferase 2 | 1 |
| 9037 | hsa-mir-548b | 10678 | B3GNT2 | UDP-GlcNAc:betaGal beta-1,3-N-acetylglucosaminyltransferase 2 | 1 |
| 9038 | hsa-mir-449b | 10678 | B3GNT2 | UDP-GlcNAc:betaGal beta-1,3-N-acetylglucosaminyltransferase 2 | 1 |
| 9039 | hsa-mir-449c | 10678 | B3GNT2 | UDP-GlcNAc:betaGal beta-1,3-N-acetylglucosaminyltransferase 2 | 1 |
| 9040 | hsa-mir-3065 | 10678 | B3GNT2 | UDP-GlcNAc:betaGal beta-1,3-N-acetylglucosaminyltransferase 2 | 1 |
| 9041 | hsa-miR-10a | 26253 | CLEC4E | C-type lectin domain family 4, member E | 1 |
| 9042 | hsa-miR-152 | 26253 | CLEC4E | C-type lectin domain family 4, member E | 1 |
| 9043 | hsa-miR-26a | 26253 | CLEC4E | C-type lectin domain family 4, member E | 1 |
| 9044 | hsa-miR-26b | 26253 | CLEC4E | C-type lectin domain family 4, member E | 1 |
| 9045 | hsa-miR-31 | 26253 | CLEC4E | C-type lectin domain family 4, member E | 1 |
| 9046 | hsa-miR-338-5p | 26253 | CLEC4E | C-type lectin domain family 4, member E | 1 |
| 9047 | hsa-miR-340 | 26253 | CLEC4E | C-type lectin domain family 4, member E | 1 |
| 9048 | hsa-miR-423-5p | 26253 | CLEC4E | C-type lectin domain family 4, member E | 1 |
| 9049 | hsa-miR-449a | 26253 | CLEC4E | C-type lectin domain family 4, member E | 1 |
| 9050 | hsa-miR-490-5p | 26253 | CLEC4E | C-type lectin domain family 4, member E | 1 |
| 9051 | hsa-miR-499-5p | 26253 | CLEC4E | C-type lectin domain family 4, member E | 1 |
| 9052 | hsa-miR-504 | 26253 | CLEC4E | C-type lectin domain family 4, member E | 1 |
| 9053 | hsa-miR-548b-5p | 26253 | CLEC4E | C-type lectin domain family 4, member E | 1 |
| 9054 | hsa-miR-564 | 26253 | CLEC4E | C-type lectin domain family 4, member E | 1 |
| 9055 | hsa-miR-584 | 26253 | CLEC4E | C-type lectin domain family 4, member E | 1 |
| 9056 | hsa-miR-621 | 26253 | CLEC4E | C-type lectin domain family 4, member E | 1 |
| 9057 | hsa-miR-657 | 26253 | CLEC4E | C-type lectin domain family 4, member E | 1 |
| 9058 | hsa-miR-765 | 26253 | CLEC4E | C-type lectin domain family 4, member E | 1 |
| 9059 | hsa-miR-770-5p | 26253 | CLEC4E | C-type lectin domain family 4, member E | 1 |
| 9060 | hsa-miR-877 | 26253 | CLEC4E | C-type lectin domain family 4, member E | 1 |
| 9061 | hsa-miR-95 | 26253 | CLEC4E | C-type lectin domain family 4, member E | 1 |
| 9062 | hsa-mir-26a-1 | 26253 | CLEC4E | C-type lectin domain family 4, member E | 1 |
| 9063 | hsa-mir-26b | 26253 | CLEC4E | C-type lectin domain family 4, member E | 1 |
| 9064 | hsa-mir-31 | 26253 | CLEC4E | C-type lectin domain family 4, member E | 1 |
| 9065 | hsa-mir-194-2 | 26253 | CLEC4E | C-type lectin domain family 4, member E | 1 |
| 9066 | hsa-mir-340 | 26253 | CLEC4E | C-type lectin domain family 4, member E | 1 |
| 9067 | hsa-mir-338 | 26253 | CLEC4E | C-type lectin domain family 4, member E | 1 |
| 9068 | hsa-mir-490 | 26253 | CLEC4E | C-type lectin domain family 4, member E | 1 |
| 9069 | hsa-mir-548b | 26253 | CLEC4E | C-type lectin domain family 4, member E | 1 |
| 9070 | hsa-mir-449b | 26253 | CLEC4E | C-type lectin domain family 4, member E | 1 |
| 9071 | hsa-mir-449c | 26253 | CLEC4E | C-type lectin domain family 4, member E | 1 |
| 9072 | hsa-mir-3065 | 26253 | CLEC4E | C-type lectin domain family 4, member E | 1 |
| 9073 | hsa-miR-1 | 8708 | B3GALT1 | UDP-Gal:betaGlcNAc beta 1,3-galactosyltransferase, polypeptide 1 | 1 |
| 9074 | hsa-miR-133a | 8708 | B3GALT1 | UDP-Gal:betaGlcNAc beta 1,3-galactosyltransferase, polypeptide 1 | 1 |
| 9075 | hsa-miR-186 | 8708 | B3GALT1 | UDP-Gal:betaGlcNAc beta 1,3-galactosyltransferase, polypeptide 1 | 1 |
| 9076 | hsa-miR-195 | 8708 | B3GALT1 | UDP-Gal:betaGlcNAc beta 1,3-galactosyltransferase, polypeptide 1 | 1 |
| 9077 | hsa-miR-208a | 8708 | B3GALT1 | UDP-Gal:betaGlcNAc beta 1,3-galactosyltransferase, polypeptide 1 | 1 |
| 9078 | hsa-miR-326 | 8708 | B3GALT1 | UDP-Gal:betaGlcNAc beta 1,3-galactosyltransferase, polypeptide 1 | 1 |
| 9079 | hsa-miR-449b | 8708 | B3GALT1 | UDP-Gal:betaGlcNAc beta 1,3-galactosyltransferase, polypeptide 1 | 1 |
| 9080 | hsa-miR-497 | 8708 | B3GALT1 | UDP-Gal:betaGlcNAc beta 1,3-galactosyltransferase, polypeptide 1 | 1 |
| 9081 | hsa-miR-550 | 8708 | B3GALT1 | UDP-Gal:betaGlcNAc beta 1,3-galactosyltransferase, polypeptide 1 | 1 |
| 9082 | hsa-miR-575 | 8708 | B3GALT1 | UDP-Gal:betaGlcNAc beta 1,3-galactosyltransferase, polypeptide 1 | 1 |
| 9083 | hsa-miR-579 | 8708 | B3GALT1 | UDP-Gal:betaGlcNAc beta 1,3-galactosyltransferase, polypeptide 1 | 1 |
| 9084 | hsa-miR-608 | 8708 | B3GALT1 | UDP-Gal:betaGlcNAc beta 1,3-galactosyltransferase, polypeptide 1 | 1 |
| 9085 | hsa-miR-620 | 8708 | B3GALT1 | UDP-Gal:betaGlcNAc beta 1,3-galactosyltransferase, polypeptide 1 | 1 |
| 9086 | hsa-let-7a-3 | 8708 | B3GALT1 | UDP-Gal:betaGlcNAc beta 1,3-galactosyltransferase, polypeptide 1 | 1 |
| 9087 | hsa-let-7b | 8708 | B3GALT1 | UDP-Gal:betaGlcNAc beta 1,3-galactosyltransferase, polypeptide 1 | 1 |
| 9088 | hsa-mir-22 | 8708 | B3GALT1 | UDP-Gal:betaGlcNAc beta 1,3-galactosyltransferase, polypeptide 1 | 1 |
| 9089 | hsa-mir-10a | 8708 | B3GALT1 | UDP-Gal:betaGlcNAc beta 1,3-galactosyltransferase, polypeptide 1 | 1 |
| 9090 | hsa-mir-186 | 8708 | B3GALT1 | UDP-Gal:betaGlcNAc beta 1,3-galactosyltransferase, polypeptide 1 | 1 |
| 9091 | hsa-mir-155 | 8708 | B3GALT1 | UDP-Gal:betaGlcNAc beta 1,3-galactosyltransferase, polypeptide 1 | 1 |
| 9092 | hsa-mir-499 | 8708 | B3GALT1 | UDP-Gal:betaGlcNAc beta 1,3-galactosyltransferase, polypeptide 1 | 1 |
| 9093 | hsa-mir-550-1 | 8708 | B3GALT1 | UDP-Gal:betaGlcNAc beta 1,3-galactosyltransferase, polypeptide 1 | 1 |
| 9094 | hsa-mir-877 | 8708 | B3GALT1 | UDP-Gal:betaGlcNAc beta 1,3-galactosyltransferase, polypeptide 1 | 1 |
| 9095 | hsa-mir-2116 | 8708 | B3GALT1 | UDP-Gal:betaGlcNAc beta 1,3-galactosyltransferase, polypeptide 1 | 1 |
| 9096 | hsa-mir-3186 | 8708 | B3GALT1 | UDP-Gal:betaGlcNAc beta 1,3-galactosyltransferase, polypeptide 1 | 1 |
| 9097 | hsa-mir-3190 | 8708 | B3GALT1 | UDP-Gal:betaGlcNAc beta 1,3-galactosyltransferase, polypeptide 1 | 1 |
| 9098 | hsa-miR-208a | 56172 | ANKH | ankylosis, progressive homolog (mouse) | 1 |
| 9099 | hsa-miR-550 | 56172 | ANKH | ankylosis, progressive homolog (mouse) | 1 |
| 9100 | hsa-let-7a-3 | 56172 | ANKH | ankylosis, progressive homolog (mouse) | 1 |
| 9101 | hsa-let-7b | 56172 | ANKH | ankylosis, progressive homolog (mouse) | 1 |
| 9104 | hsa-miR-10a | 83605 | CCM2 | cerebral cavernous malformation 2 | 1 |
| 9105 | hsa-miR-139-5p | 83605 | CCM2 | cerebral cavernous malformation 2 | 1 |
| 9106 | hsa-miR-152 | 83605 | CCM2 | cerebral cavernous malformation 2 | 1 |
| 9107 | hsa-miR-26b | 83605 | CCM2 | cerebral cavernous malformation 2 | 1 |
| 9108 | hsa-miR-31 | 83605 | CCM2 | cerebral cavernous malformation 2 | 1 |
| 9109 | hsa-miR-338-5p | 83605 | CCM2 | cerebral cavernous malformation 2 | 1 |
| 9110 | hsa-miR-340 | 83605 | CCM2 | cerebral cavernous malformation 2 | 1 |
| 9111 | hsa-miR-383 | 83605 | CCM2 | cerebral cavernous malformation 2 | 1 |
| 9112 | hsa-miR-449a | 83605 | CCM2 | cerebral cavernous malformation 2 | 1 |
| 9113 | hsa-miR-499-5p | 83605 | CCM2 | cerebral cavernous malformation 2 | 1 |
| 9114 | hsa-miR-548b-5p | 83605 | CCM2 | cerebral cavernous malformation 2 | 1 |
| 9115 | hsa-miR-621 | 83605 | CCM2 | cerebral cavernous malformation 2 | 1 |
| 9116 | hsa-miR-657 | 83605 | CCM2 | cerebral cavernous malformation 2 | 1 |
| 9117 | hsa-miR-663 | 83605 | CCM2 | cerebral cavernous malformation 2 | 1 |
| 9118 | hsa-miR-765 | 83605 | CCM2 | cerebral cavernous malformation 2 | 1 |
| 9119 | hsa-miR-770-5p | 83605 | CCM2 | cerebral cavernous malformation 2 | 1 |
| 9120 | hsa-miR-877 | 83605 | CCM2 | cerebral cavernous malformation 2 | 1 |
| 9121 | hsa-mir-26a-1 | 83605 | CCM2 | cerebral cavernous malformation 2 | 1 |
| 9122 | hsa-mir-26b | 83605 | CCM2 | cerebral cavernous malformation 2 | 1 |
| 9123 | hsa-mir-31 | 83605 | CCM2 | cerebral cavernous malformation 2 | 1 |
| 9124 | hsa-mir-194-2 | 83605 | CCM2 | cerebral cavernous malformation 2 | 1 |
| 9125 | hsa-mir-340 | 83605 | CCM2 | cerebral cavernous malformation 2 | 1 |
| 9126 | hsa-mir-338 | 83605 | CCM2 | cerebral cavernous malformation 2 | 1 |
| 9127 | hsa-mir-548b | 83605 | CCM2 | cerebral cavernous malformation 2 | 1 |
| 9128 | hsa-mir-449b | 83605 | CCM2 | cerebral cavernous malformation 2 | 1 |
| 9129 | hsa-mir-449c | 83605 | CCM2 | cerebral cavernous malformation 2 | 1 |
| 9130 | hsa-mir-3065 | 83605 | CCM2 | cerebral cavernous malformation 2 | 1 |
| 9132 | hsa-miR-139-5p | 64332 | NFKBIZ | nuclear factor of kappa light polypeptide gene enhancer in B-cells inhibitor, zeta | 1 |
| 9157 | hsa-miR-31 | 64332 | NFKBIZ | nuclear factor of kappa light polypeptide gene enhancer in B-cells inhibitor, zeta | 1 |
| 9158 | hsa-miR-653 | 64332 | NFKBIZ | nuclear factor of kappa light polypeptide gene enhancer in B-cells inhibitor, zeta | 1 |
| 9159 | hsa-mir-31 | 64332 | NFKBIZ | nuclear factor of kappa light polypeptide gene enhancer in B-cells inhibitor, zeta | 1 |
| 9160 | hsa-miR-10a | 2635 | GBP3 | guanylate binding protein 3 | 1 |
| 9161 | hsa-miR-139-5p | 2635 | GBP3 | guanylate binding protein 3 | 1 |
| 9162 | hsa-miR-152 | 2635 | GBP3 | guanylate binding protein 3 | 1 |
| 9163 | hsa-miR-26a | 2635 | GBP3 | guanylate binding protein 3 | 1 |
| 9164 | hsa-miR-26b | 2635 | GBP3 | guanylate binding protein 3 | 1 |
| 9165 | hsa-miR-31 | 2635 | GBP3 | guanylate binding protein 3 | 1 |
| 9166 | hsa-miR-338-5p | 2635 | GBP3 | guanylate binding protein 3 | 1 |
| 9167 | hsa-miR-340 | 2635 | GBP3 | guanylate binding protein 3 | 1 |
| 9168 | hsa-miR-423-5p | 2635 | GBP3 | guanylate binding protein 3 | 1 |
| 9169 | hsa-miR-449a | 2635 | GBP3 | guanylate binding protein 3 | 1 |
| 9170 | hsa-miR-499-5p | 2635 | GBP3 | guanylate binding protein 3 | 1 |
| 9171 | hsa-miR-548b-5p | 2635 | GBP3 | guanylate binding protein 3 | 1 |
| 9172 | hsa-miR-564 | 2635 | GBP3 | guanylate binding protein 3 | 1 |
| 9173 | hsa-miR-621 | 2635 | GBP3 | guanylate binding protein 3 | 1 |
| 9174 | hsa-miR-657 | 2635 | GBP3 | guanylate binding protein 3 | 1 |
| 9175 | hsa-miR-765 | 2635 | GBP3 | guanylate binding protein 3 | 1 |
| 9176 | hsa-miR-770-5p | 2635 | GBP3 | guanylate binding protein 3 | 1 |
| 9177 | hsa-miR-877 | 2635 | GBP3 | guanylate binding protein 3 | 1 |
| 9178 | hsa-mir-26a-1 | 2635 | GBP3 | guanylate binding protein 3 | 1 |
| 9179 | hsa-mir-26b | 2635 | GBP3 | guanylate binding protein 3 | 1 |
| 9180 | hsa-mir-31 | 2635 | GBP3 | guanylate binding protein 3 | 1 |
| 9181 | hsa-mir-194-2 | 2635 | GBP3 | guanylate binding protein 3 | 1 |
| 9182 | hsa-mir-340 | 2635 | GBP3 | guanylate binding protein 3 | 1 |
| 9183 | hsa-mir-338 | 2635 | GBP3 | guanylate binding protein 3 | 1 |
| 9184 | hsa-mir-548b | 2635 | GBP3 | guanylate binding protein 3 | 1 |
| 9185 | hsa-mir-548d-2 | 2635 | GBP3 | guanylate binding protein 3 | 1 |
| 9186 | hsa-mir-449b | 2635 | GBP3 | guanylate binding protein 3 | 1 |
| 9187 | hsa-mir-449c | 2635 | GBP3 | guanylate binding protein 3 | 1 |
| 9188 | hsa-mir-3065 | 2635 | GBP3 | guanylate binding protein 3 | 1 |
| 9189 | hsa-miR-1 | 84519 | ACRBP | acrosin binding protein | 1 |
| 9190 | hsa-miR-133a | 84519 | ACRBP | acrosin binding protein | 1 |
| 9191 | hsa-miR-186 | 84519 | ACRBP | acrosin binding protein | 1 |
| 9192 | hsa-miR-326 | 84519 | ACRBP | acrosin binding protein | 1 |
| 9193 | hsa-miR-33a | 84519 | ACRBP | acrosin binding protein | 1 |
| 9194 | hsa-miR-449b | 84519 | ACRBP | acrosin binding protein | 1 |
| 9195 | hsa-miR-551a | 84519 | ACRBP | acrosin binding protein | 1 |
| 9196 | hsa-miR-562 | 84519 | ACRBP | acrosin binding protein | 1 |
| 9197 | hsa-miR-575 | 84519 | ACRBP | acrosin binding protein | 1 |
| 9198 | hsa-miR-608 | 84519 | ACRBP | acrosin binding protein | 1 |
| 9199 | hsa-miR-620 | 84519 | ACRBP | acrosin binding protein | 1 |
| 9200 | hsa-mir-22 | 84519 | ACRBP | acrosin binding protein | 1 |
| 9201 | hsa-mir-10a | 84519 | ACRBP | acrosin binding protein | 1 |
| 9202 | hsa-mir-186 | 84519 | ACRBP | acrosin binding protein | 1 |
| 9203 | hsa-mir-155 | 84519 | ACRBP | acrosin binding protein | 1 |
| 9204 | hsa-mir-499 | 84519 | ACRBP | acrosin binding protein | 1 |
| 9205 | hsa-mir-550-1 | 84519 | ACRBP | acrosin binding protein | 1 |
| 9206 | hsa-mir-877 | 84519 | ACRBP | acrosin binding protein | 1 |
| 9207 | hsa-mir-2116 | 84519 | ACRBP | acrosin binding protein | 1 |
| 9208 | hsa-miR-10a | 55370 | PPP4R1L | protein phosphatase 4, regulatory subunit 1-like | 1 |
| 9209 | hsa-miR-152 | 55370 | PPP4R1L | protein phosphatase 4, regulatory subunit 1-like | 1 |
| 9210 | hsa-miR-26a | 55370 | PPP4R1L | protein phosphatase 4, regulatory subunit 1-like | 1 |
| 9211 | hsa-miR-26b | 55370 | PPP4R1L | protein phosphatase 4, regulatory subunit 1-like | 1 |
| 9212 | hsa-miR-31 | 55370 | PPP4R1L | protein phosphatase 4, regulatory subunit 1-like | 1 |
| 9213 | hsa-miR-340 | 55370 | PPP4R1L | protein phosphatase 4, regulatory subunit 1-like | 1 |
| 9214 | hsa-miR-423-5p | 55370 | PPP4R1L | protein phosphatase 4, regulatory subunit 1-like | 1 |
| 9215 | hsa-miR-449a | 55370 | PPP4R1L | protein phosphatase 4, regulatory subunit 1-like | 1 |
| 9216 | hsa-miR-490-5p | 55370 | PPP4R1L | protein phosphatase 4, regulatory subunit 1-like | 1 |
| 9217 | hsa-miR-499-5p | 55370 | PPP4R1L | protein phosphatase 4, regulatory subunit 1-like | 1 |
| 9218 | hsa-miR-548b-5p | 55370 | PPP4R1L | protein phosphatase 4, regulatory subunit 1-like | 1 |
| 9219 | hsa-miR-564 | 55370 | PPP4R1L | protein phosphatase 4, regulatory subunit 1-like | 1 |
| 9220 | hsa-miR-584 | 55370 | PPP4R1L | protein phosphatase 4, regulatory subunit 1-like | 1 |
| 9221 | hsa-miR-621 | 55370 | PPP4R1L | protein phosphatase 4, regulatory subunit 1-like | 1 |
| 9222 | hsa-miR-634 | 55370 | PPP4R1L | protein phosphatase 4, regulatory subunit 1-like | 1 |
| 9223 | hsa-miR-644 | 55370 | PPP4R1L | protein phosphatase 4, regulatory subunit 1-like | 1 |
| 9224 | hsa-miR-765 | 55370 | PPP4R1L | protein phosphatase 4, regulatory subunit 1-like | 1 |
| 9225 | hsa-miR-770-5p | 55370 | PPP4R1L | protein phosphatase 4, regulatory subunit 1-like | 1 |
| 9226 | hsa-miR-877 | 55370 | PPP4R1L | protein phosphatase 4, regulatory subunit 1-like | 1 |
| 9227 | hsa-miR-95 | 55370 | PPP4R1L | protein phosphatase 4, regulatory subunit 1-like | 1 |
| 9228 | hsa-mir-26a-1 | 55370 | PPP4R1L | protein phosphatase 4, regulatory subunit 1-like | 1 |
| 9229 | hsa-mir-26b | 55370 | PPP4R1L | protein phosphatase 4, regulatory subunit 1-like | 1 |
| 9230 | hsa-mir-31 | 55370 | PPP4R1L | protein phosphatase 4, regulatory subunit 1-like | 1 |
| 9231 | hsa-mir-194-2 | 55370 | PPP4R1L | protein phosphatase 4, regulatory subunit 1-like | 1 |
| 9232 | hsa-mir-340 | 55370 | PPP4R1L | protein phosphatase 4, regulatory subunit 1-like | 1 |
| 9233 | hsa-mir-490 | 55370 | PPP4R1L | protein phosphatase 4, regulatory subunit 1-like | 1 |
| 9234 | hsa-mir-548b | 55370 | PPP4R1L | protein phosphatase 4, regulatory subunit 1-like | 1 |
| 9235 | hsa-mir-449b | 55370 | PPP4R1L | protein phosphatase 4, regulatory subunit 1-like | 1 |
| 9236 | hsa-mir-449c | 55370 | PPP4R1L | protein phosphatase 4, regulatory subunit 1-like | 1 |
| 9237 | hsa-miR-1 | 64147 | KIF9 | kinesin family member 9 | 1 |
| 9238 | hsa-miR-133a | 64147 | KIF9 | kinesin family member 9 | 1 |
| 9239 | hsa-miR-186 | 64147 | KIF9 | kinesin family member 9 | 1 |
| 9240 | hsa-miR-326 | 64147 | KIF9 | kinesin family member 9 | 1 |
| 9241 | hsa-miR-449b | 64147 | KIF9 | kinesin family member 9 | 1 |
| 9242 | hsa-miR-550 | 64147 | KIF9 | kinesin family member 9 | 1 |
| 9243 | hsa-miR-551a | 64147 | KIF9 | kinesin family member 9 | 1 |
| 9244 | hsa-miR-562 | 64147 | KIF9 | kinesin family member 9 | 1 |
| 9245 | hsa-miR-579 | 64147 | KIF9 | kinesin family member 9 | 1 |
| 9246 | hsa-miR-608 | 64147 | KIF9 | kinesin family member 9 | 1 |
| 9247 | hsa-miR-885-5p | 64147 | KIF9 | kinesin family member 9 | 1 |
| 9248 | hsa-mir-22 | 64147 | KIF9 | kinesin family member 9 | 1 |
| 9249 | hsa-mir-10a | 64147 | KIF9 | kinesin family member 9 | 1 |
| 9250 | hsa-mir-186 | 64147 | KIF9 | kinesin family member 9 | 1 |
| 9251 | hsa-mir-155 | 64147 | KIF9 | kinesin family member 9 | 1 |
| 9252 | hsa-mir-499 | 64147 | KIF9 | kinesin family member 9 | 1 |
| 9253 | hsa-mir-550-1 | 64147 | KIF9 | kinesin family member 9 | 1 |
| 9254 | hsa-mir-885 | 64147 | KIF9 | kinesin family member 9 | 1 |
| 9255 | hsa-mir-877 | 64147 | KIF9 | kinesin family member 9 | 1 |
| 9256 | hsa-miR-10a | 29126 | CD274 | CD274 molecule | 1 |
| 9257 | hsa-miR-139-5p | 29126 | CD274 | CD274 molecule | 1 |
| 9258 | hsa-miR-152 | 29126 | CD274 | CD274 molecule | 1 |
| 9259 | hsa-miR-26a | 29126 | CD274 | CD274 molecule | 1 |
| 9260 | hsa-miR-26b | 29126 | CD274 | CD274 molecule | 1 |
| 9261 | hsa-miR-31 | 29126 | CD274 | CD274 molecule | 1 |
| 9262 | hsa-miR-338-5p | 29126 | CD274 | CD274 molecule | 1 |
| 9263 | hsa-miR-340 | 29126 | CD274 | CD274 molecule | 1 |
| 9264 | hsa-miR-383 | 29126 | CD274 | CD274 molecule | 1 |
| 9265 | hsa-miR-449a | 29126 | CD274 | CD274 molecule | 1 |
| 9266 | hsa-miR-499-5p | 29126 | CD274 | CD274 molecule | 1 |
| 9267 | hsa-miR-548b-5p | 29126 | CD274 | CD274 molecule | 1 |
| 9268 | hsa-miR-621 | 29126 | CD274 | CD274 molecule | 1 |
| 9269 | hsa-miR-657 | 29126 | CD274 | CD274 molecule | 1 |
| 9270 | hsa-miR-663 | 29126 | CD274 | CD274 molecule | 1 |
| 9271 | hsa-miR-765 | 29126 | CD274 | CD274 molecule | 1 |
| 9272 | hsa-miR-770-5p | 29126 | CD274 | CD274 molecule | 1 |
| 9273 | hsa-miR-877 | 29126 | CD274 | CD274 molecule | 1 |
| 9274 | hsa-mir-26a-1 | 29126 | CD274 | CD274 molecule | 1 |
| 9275 | hsa-mir-26b | 29126 | CD274 | CD274 molecule | 1 |
| 9276 | hsa-mir-31 | 29126 | CD274 | CD274 molecule | 1 |
| 9277 | hsa-mir-194-2 | 29126 | CD274 | CD274 molecule | 1 |
| 9278 | hsa-mir-340 | 29126 | CD274 | CD274 molecule | 1 |
| 9279 | hsa-mir-338 | 29126 | CD274 | CD274 molecule | 1 |
| 9280 | hsa-mir-548b | 29126 | CD274 | CD274 molecule | 1 |
| 9281 | hsa-mir-548d-2 | 29126 | CD274 | CD274 molecule | 1 |
| 9282 | hsa-mir-449b | 29126 | CD274 | CD274 molecule | 1 |
| 9283 | hsa-mir-449c | 29126 | CD274 | CD274 molecule | 1 |
| 9284 | hsa-mir-3065 | 29126 | CD274 | CD274 molecule | 1 |
| 9285 | hsa-miR-10a | 29933 | GPR132 | G protein-coupled receptor 132 | 1 |
| 9286 | hsa-miR-152 | 29933 | GPR132 | G protein-coupled receptor 132 | 1 |
| 9287 | hsa-miR-26a | 29933 | GPR132 | G protein-coupled receptor 132 | 1 |
| 9288 | hsa-miR-26b | 29933 | GPR132 | G protein-coupled receptor 132 | 1 |
| 9289 | hsa-miR-31 | 29933 | GPR132 | G protein-coupled receptor 132 | 1 |
| 9290 | hsa-miR-338-5p | 29933 | GPR132 | G protein-coupled receptor 132 | 1 |
| 9291 | hsa-miR-340 | 29933 | GPR132 | G protein-coupled receptor 132 | 1 |
| 9292 | hsa-miR-423-5p | 29933 | GPR132 | G protein-coupled receptor 132 | 1 |
| 9293 | hsa-miR-449a | 29933 | GPR132 | G protein-coupled receptor 132 | 1 |
| 9294 | hsa-miR-490-5p | 29933 | GPR132 | G protein-coupled receptor 132 | 1 |
| 9295 | hsa-miR-499-5p | 29933 | GPR132 | G protein-coupled receptor 132 | 1 |
| 9296 | hsa-miR-548b-5p | 29933 | GPR132 | G protein-coupled receptor 132 | 1 |
| 9297 | hsa-miR-564 | 29933 | GPR132 | G protein-coupled receptor 132 | 1 |
| 9298 | hsa-miR-584 | 29933 | GPR132 | G protein-coupled receptor 132 | 1 |
| 9299 | hsa-miR-621 | 29933 | GPR132 | G protein-coupled receptor 132 | 1 |
| 9300 | hsa-miR-634 | 29933 | GPR132 | G protein-coupled receptor 132 | 1 |
| 9301 | hsa-miR-644 | 29933 | GPR132 | G protein-coupled receptor 132 | 1 |
| 9302 | hsa-miR-657 | 29933 | GPR132 | G protein-coupled receptor 132 | 1 |
| 9303 | hsa-miR-765 | 29933 | GPR132 | G protein-coupled receptor 132 | 1 |
| 9304 | hsa-miR-770-5p | 29933 | GPR132 | G protein-coupled receptor 132 | 1 |
| 9305 | hsa-miR-877 | 29933 | GPR132 | G protein-coupled receptor 132 | 1 |
| 9306 | hsa-miR-95 | 29933 | GPR132 | G protein-coupled receptor 132 | 1 |
| 9307 | hsa-mir-26a-1 | 29933 | GPR132 | G protein-coupled receptor 132 | 1 |
| 9308 | hsa-mir-26b | 29933 | GPR132 | G protein-coupled receptor 132 | 1 |
| 9309 | hsa-mir-31 | 29933 | GPR132 | G protein-coupled receptor 132 | 1 |
| 9310 | hsa-mir-194-2 | 29933 | GPR132 | G protein-coupled receptor 132 | 1 |
| 9311 | hsa-mir-340 | 29933 | GPR132 | G protein-coupled receptor 132 | 1 |
| 9312 | hsa-mir-338 | 29933 | GPR132 | G protein-coupled receptor 132 | 1 |
| 9313 | hsa-mir-490 | 29933 | GPR132 | G protein-coupled receptor 132 | 1 |
| 9314 | hsa-mir-548b | 29933 | GPR132 | G protein-coupled receptor 132 | 1 |
| 9315 | hsa-mir-449b | 29933 | GPR132 | G protein-coupled receptor 132 | 1 |
| 9316 | hsa-mir-449c | 29933 | GPR132 | G protein-coupled receptor 132 | 1 |
| 9317 | hsa-mir-3065 | 29933 | GPR132 | G protein-coupled receptor 132 | 1 |
| 9318 | hsa-miR-10a | 83636 | C19orf12 | chromosome 19 open reading frame 12 | 1 |
| 9319 | hsa-miR-139-5p | 83636 | C19orf12 | chromosome 19 open reading frame 12 | 1 |
| 9320 | hsa-miR-152 | 83636 | C19orf12 | chromosome 19 open reading frame 12 | 1 |
| 9321 | hsa-miR-26a | 83636 | C19orf12 | chromosome 19 open reading frame 12 | 1 |
| 9322 | hsa-miR-26b | 83636 | C19orf12 | chromosome 19 open reading frame 12 | 1 |
| 9323 | hsa-miR-31 | 83636 | C19orf12 | chromosome 19 open reading frame 12 | 1 |
| 9324 | hsa-miR-338-5p | 83636 | C19orf12 | chromosome 19 open reading frame 12 | 1 |
| 9325 | hsa-miR-383 | 83636 | C19orf12 | chromosome 19 open reading frame 12 | 1 |
| 9326 | hsa-miR-449a | 83636 | C19orf12 | chromosome 19 open reading frame 12 | 1 |
| 9327 | hsa-miR-499-5p | 83636 | C19orf12 | chromosome 19 open reading frame 12 | 1 |
| 9328 | hsa-miR-548b-5p | 83636 | C19orf12 | chromosome 19 open reading frame 12 | 1 |
| 9329 | hsa-miR-621 | 83636 | C19orf12 | chromosome 19 open reading frame 12 | 1 |
| 9330 | hsa-miR-657 | 83636 | C19orf12 | chromosome 19 open reading frame 12 | 1 |
| 9331 | hsa-miR-663 | 83636 | C19orf12 | chromosome 19 open reading frame 12 | 1 |
| 9332 | hsa-miR-765 | 83636 | C19orf12 | chromosome 19 open reading frame 12 | 1 |
| 9333 | hsa-miR-770-5p | 83636 | C19orf12 | chromosome 19 open reading frame 12 | 1 |
| 9334 | hsa-miR-877 | 83636 | C19orf12 | chromosome 19 open reading frame 12 | 1 |
| 9335 | hsa-mir-26a-1 | 83636 | C19orf12 | chromosome 19 open reading frame 12 | 1 |
| 9336 | hsa-mir-26b | 83636 | C19orf12 | chromosome 19 open reading frame 12 | 1 |
| 9337 | hsa-mir-31 | 83636 | C19orf12 | chromosome 19 open reading frame 12 | 1 |
| 9338 | hsa-mir-194-2 | 83636 | C19orf12 | chromosome 19 open reading frame 12 | 1 |
| 9339 | hsa-mir-338 | 83636 | C19orf12 | chromosome 19 open reading frame 12 | 1 |
| 9340 | hsa-mir-548b | 83636 | C19orf12 | chromosome 19 open reading frame 12 | 1 |
| 9341 | hsa-mir-548d-2 | 83636 | C19orf12 | chromosome 19 open reading frame 12 | 1 |
| 9342 | hsa-mir-449b | 83636 | C19orf12 | chromosome 19 open reading frame 12 | 1 |
| 9343 | hsa-mir-449c | 83636 | C19orf12 | chromosome 19 open reading frame 12 | 1 |
| 9344 | hsa-mir-3065 | 83636 | C19orf12 | chromosome 19 open reading frame 12 | 1 |
| 9345 | hsa-miR-1 | 57094 | CPA6 | carboxypeptidase A6 | 1 |
| 9346 | hsa-miR-133a | 57094 | CPA6 | carboxypeptidase A6 | 1 |
| 9347 | hsa-miR-186 | 57094 | CPA6 | carboxypeptidase A6 | 1 |
| 9348 | hsa-miR-208a | 57094 | CPA6 | carboxypeptidase A6 | 1 |
| 9349 | hsa-miR-449b | 57094 | CPA6 | carboxypeptidase A6 | 1 |
| 9350 | hsa-miR-562 | 57094 | CPA6 | carboxypeptidase A6 | 1 |
| 9351 | hsa-miR-579 | 57094 | CPA6 | carboxypeptidase A6 | 1 |
| 9352 | hsa-miR-608 | 57094 | CPA6 | carboxypeptidase A6 | 1 |
| 9353 | hsa-miR-620 | 57094 | CPA6 | carboxypeptidase A6 | 1 |
| 9354 | hsa-miR-637 | 57094 | CPA6 | carboxypeptidase A6 | 1 |
| 9355 | hsa-miR-885-5p | 57094 | CPA6 | carboxypeptidase A6 | 1 |
| 9356 | hsa-let-7a-3 | 57094 | CPA6 | carboxypeptidase A6 | 1 |
| 9357 | hsa-let-7b | 57094 | CPA6 | carboxypeptidase A6 | 1 |
| 9358 | hsa-mir-22 | 57094 | CPA6 | carboxypeptidase A6 | 1 |
| 9359 | hsa-mir-10a | 57094 | CPA6 | carboxypeptidase A6 | 1 |
| 9360 | hsa-mir-186 | 57094 | CPA6 | carboxypeptidase A6 | 1 |
| 9361 | hsa-mir-155 | 57094 | CPA6 | carboxypeptidase A6 | 1 |
| 9362 | hsa-mir-499 | 57094 | CPA6 | carboxypeptidase A6 | 1 |
| 9363 | hsa-mir-885 | 57094 | CPA6 | carboxypeptidase A6 | 1 |
| 9364 | hsa-miR-1 | 51733 | UPB1 | ureidopropionase, beta | 1 |
| 9365 | hsa-miR-133a | 51733 | UPB1 | ureidopropionase, beta | 1 |
| 9366 | hsa-miR-186 | 51733 | UPB1 | ureidopropionase, beta | 1 |
| 9367 | hsa-miR-326 | 51733 | UPB1 | ureidopropionase, beta | 1 |
| 9368 | hsa-miR-449b | 51733 | UPB1 | ureidopropionase, beta | 1 |
| 9369 | hsa-miR-551a | 51733 | UPB1 | ureidopropionase, beta | 1 |
| 9370 | hsa-miR-562 | 51733 | UPB1 | ureidopropionase, beta | 1 |
| 9371 | hsa-miR-575 | 51733 | UPB1 | ureidopropionase, beta | 1 |
| 9372 | hsa-miR-579 | 51733 | UPB1 | ureidopropionase, beta | 1 |
| 9373 | hsa-miR-608 | 51733 | UPB1 | ureidopropionase, beta | 1 |
| 9374 | hsa-miR-637 | 51733 | UPB1 | ureidopropionase, beta | 1 |
| 9375 | hsa-mir-22 | 51733 | UPB1 | ureidopropionase, beta | 1 |
| 9376 | hsa-mir-10a | 51733 | UPB1 | ureidopropionase, beta | 1 |
| 9377 | hsa-mir-186 | 51733 | UPB1 | ureidopropionase, beta | 1 |
| 9378 | hsa-mir-155 | 51733 | UPB1 | ureidopropionase, beta | 1 |
| 9379 | hsa-mir-423 | 51733 | UPB1 | ureidopropionase, beta | 1 |
| 9380 | hsa-mir-877 | 51733 | UPB1 | ureidopropionase, beta | 1 |
| 9381 | hsa-mir-3190 | 51733 | UPB1 | ureidopropionase, beta | 1 |
| 9382 | hsa-miR-1 | 27198 | GPR81 | G protein-coupled receptor 81 | 1 |
| 9383 | hsa-miR-133a | 27198 | GPR81 | G protein-coupled receptor 81 | 1 |
| 9384 | hsa-miR-186 | 27198 | GPR81 | G protein-coupled receptor 81 | 1 |
| 9385 | hsa-miR-195 | 27198 | GPR81 | G protein-coupled receptor 81 | 1 |
| 9386 | hsa-miR-208a | 27198 | GPR81 | G protein-coupled receptor 81 | 1 |
| 9387 | hsa-miR-326 | 27198 | GPR81 | G protein-coupled receptor 81 | 1 |
| 9388 | hsa-miR-449b | 27198 | GPR81 | G protein-coupled receptor 81 | 1 |
| 9389 | hsa-miR-497 | 27198 | GPR81 | G protein-coupled receptor 81 | 1 |
| 9390 | hsa-miR-575 | 27198 | GPR81 | G protein-coupled receptor 81 | 1 |
| 9391 | hsa-miR-608 | 27198 | GPR81 | G protein-coupled receptor 81 | 1 |
| 9392 | hsa-miR-620 | 27198 | GPR81 | G protein-coupled receptor 81 | 1 |
| 9393 | hsa-miR-885-5p | 27198 | GPR81 | G protein-coupled receptor 81 | 1 |
| 9394 | hsa-let-7a-3 | 27198 | GPR81 | G protein-coupled receptor 81 | 1 |
| 9395 | hsa-let-7b | 27198 | GPR81 | G protein-coupled receptor 81 | 1 |
| 9396 | hsa-mir-22 | 27198 | GPR81 | G protein-coupled receptor 81 | 1 |
| 9397 | hsa-mir-186 | 27198 | GPR81 | G protein-coupled receptor 81 | 1 |
| 9398 | hsa-mir-155 | 27198 | GPR81 | G protein-coupled receptor 81 | 1 |
| 9399 | hsa-mir-499 | 27198 | GPR81 | G protein-coupled receptor 81 | 1 |
| 9400 | hsa-mir-885 | 27198 | GPR81 | G protein-coupled receptor 81 | 1 |
| 9401 | hsa-mir-877 | 27198 | GPR81 | G protein-coupled receptor 81 | 1 |
| 9402 | hsa-mir-2116 | 27198 | GPR81 | G protein-coupled receptor 81 | 1 |
| 9403 | hsa-miR-26a | 114897 | C1QTNF1 | C1q and tumor necrosis factor related protein 1 | 1 |
| 9404 | hsa-miR-548b-5p | 114897 | C1QTNF1 | C1q and tumor necrosis factor related protein 1 | 1 |
| 9405 | hsa-miR-564 | 114897 | C1QTNF1 | C1q and tumor necrosis factor related protein 1 | 1 |
| 9406 | hsa-mir-548b | 114897 | C1QTNF1 | C1q and tumor necrosis factor related protein 1 | 1 |
| 9407 | hsa-miR-1 | 100129449 | LOC100129449 | PRO2055 | 1 |
| 9408 | hsa-miR-133a | 100129449 | LOC100129449 | PRO2055 | 1 |
| 9409 | hsa-miR-186 | 100129449 | LOC100129449 | PRO2055 | 1 |
| 9410 | hsa-miR-326 | 100129449 | LOC100129449 | PRO2055 | 1 |
| 9411 | hsa-miR-449b | 100129449 | LOC100129449 | PRO2055 | 1 |
| 9412 | hsa-miR-550 | 100129449 | LOC100129449 | PRO2055 | 1 |
| 9413 | hsa-miR-551a | 100129449 | LOC100129449 | PRO2055 | 1 |
| 9414 | hsa-miR-562 | 100129449 | LOC100129449 | PRO2055 | 1 |
| 9415 | hsa-miR-575 | 100129449 | LOC100129449 | PRO2055 | 1 |
| 9416 | hsa-miR-579 | 100129449 | LOC100129449 | PRO2055 | 1 |
| 9417 | hsa-miR-608 | 100129449 | LOC100129449 | PRO2055 | 1 |
| 9418 | hsa-miR-620 | 100129449 | LOC100129449 | PRO2055 | 1 |
| 9419 | hsa-miR-637 | 100129449 | LOC100129449 | PRO2055 | 1 |
| 9420 | hsa-miR-885-5p | 100129449 | LOC100129449 | PRO2055 | 1 |
| 9421 | hsa-mir-22 | 100129449 | LOC100129449 | PRO2055 | 1 |
| 9422 | hsa-mir-10a | 100129449 | LOC100129449 | PRO2055 | 1 |
| 9423 | hsa-mir-186 | 100129449 | LOC100129449 | PRO2055 | 1 |
| 9424 | hsa-mir-155 | 100129449 | LOC100129449 | PRO2055 | 1 |
| 9425 | hsa-mir-423 | 100129449 | LOC100129449 | PRO2055 | 1 |
| 9426 | hsa-mir-499 | 100129449 | LOC100129449 | PRO2055 | 1 |
| 9427 | hsa-mir-550-1 | 100129449 | LOC100129449 | PRO2055 | 1 |
| 9428 | hsa-mir-885 | 100129449 | LOC100129449 | PRO2055 | 1 |
| 9429 | hsa-mir-877 | 100129449 | LOC100129449 | PRO2055 | 1 |
| 9430 | hsa-mir-2116 | 100129449 | LOC100129449 | PRO2055 | 1 |
| 9431 | hsa-mir-3190 | 100129449 | LOC100129449 | PRO2055 | 1 |
| 9432 | hsa-miR-1 | 84814 | PPAPDC3 | phosphatidic acid phosphatase type 2 domain containing 3 | 1 |
| 9433 | hsa-miR-133a | 84814 | PPAPDC3 | phosphatidic acid phosphatase type 2 domain containing 3 | 1 |
| 9434 | hsa-miR-186 | 84814 | PPAPDC3 | phosphatidic acid phosphatase type 2 domain containing 3 | 1 |
| 9435 | hsa-miR-326 | 84814 | PPAPDC3 | phosphatidic acid phosphatase type 2 domain containing 3 | 1 |
| 9436 | hsa-miR-449b | 84814 | PPAPDC3 | phosphatidic acid phosphatase type 2 domain containing 3 | 1 |
| 9437 | hsa-miR-550 | 84814 | PPAPDC3 | phosphatidic acid phosphatase type 2 domain containing 3 | 1 |
| 9438 | hsa-miR-551a | 84814 | PPAPDC3 | phosphatidic acid phosphatase type 2 domain containing 3 | 1 |
| 9439 | hsa-miR-575 | 84814 | PPAPDC3 | phosphatidic acid phosphatase type 2 domain containing 3 | 1 |
| 9440 | hsa-miR-579 | 84814 | PPAPDC3 | phosphatidic acid phosphatase type 2 domain containing 3 | 1 |
| 9441 | hsa-miR-608 | 84814 | PPAPDC3 | phosphatidic acid phosphatase type 2 domain containing 3 | 1 |
| 9442 | hsa-miR-620 | 84814 | PPAPDC3 | phosphatidic acid phosphatase type 2 domain containing 3 | 1 |
| 9443 | hsa-mir-22 | 84814 | PPAPDC3 | phosphatidic acid phosphatase type 2 domain containing 3 | 1 |
| 9444 | hsa-mir-10a | 84814 | PPAPDC3 | phosphatidic acid phosphatase type 2 domain containing 3 | 1 |
| 9445 | hsa-mir-186 | 84814 | PPAPDC3 | phosphatidic acid phosphatase type 2 domain containing 3 | 1 |
| 9446 | hsa-mir-155 | 84814 | PPAPDC3 | phosphatidic acid phosphatase type 2 domain containing 3 | 1 |
| 9447 | hsa-mir-423 | 84814 | PPAPDC3 | phosphatidic acid phosphatase type 2 domain containing 3 | 1 |
| 9448 | hsa-mir-499 | 84814 | PPAPDC3 | phosphatidic acid phosphatase type 2 domain containing 3 | 1 |
| 9449 | hsa-mir-550-1 | 84814 | PPAPDC3 | phosphatidic acid phosphatase type 2 domain containing 3 | 1 |
| 9450 | hsa-mir-877 | 84814 | PPAPDC3 | phosphatidic acid phosphatase type 2 domain containing 3 | 1 |
| 9451 | hsa-mir-2116 | 84814 | PPAPDC3 | phosphatidic acid phosphatase type 2 domain containing 3 | 1 |
| 9452 | hsa-mir-3186 | 84814 | PPAPDC3 | phosphatidic acid phosphatase type 2 domain containing 3 | 1 |
| 9453 | hsa-mir-3190 | 84814 | PPAPDC3 | phosphatidic acid phosphatase type 2 domain containing 3 | 1 |
| 9454 | hsa-miR-10a | 84815 | MGC12916 | hypothetical protein MGC12916 | 1 |
| 9455 | hsa-miR-152 | 84815 | MGC12916 | hypothetical protein MGC12916 | 1 |
| 9456 | hsa-miR-26a | 84815 | MGC12916 | hypothetical protein MGC12916 | 1 |
| 9457 | hsa-miR-26b | 84815 | MGC12916 | hypothetical protein MGC12916 | 1 |
| 9458 | hsa-miR-31 | 84815 | MGC12916 | hypothetical protein MGC12916 | 1 |
| 9459 | hsa-miR-338-5p | 84815 | MGC12916 | hypothetical protein MGC12916 | 1 |
| 9460 | hsa-miR-340 | 84815 | MGC12916 | hypothetical protein MGC12916 | 1 |
| 9461 | hsa-miR-449a | 84815 | MGC12916 | hypothetical protein MGC12916 | 1 |
| 9462 | hsa-miR-490-5p | 84815 | MGC12916 | hypothetical protein MGC12916 | 1 |
| 9463 | hsa-miR-499-5p | 84815 | MGC12916 | hypothetical protein MGC12916 | 1 |
| 9464 | hsa-miR-548b-5p | 84815 | MGC12916 | hypothetical protein MGC12916 | 1 |
| 9465 | hsa-miR-564 | 84815 | MGC12916 | hypothetical protein MGC12916 | 1 |
| 9466 | hsa-miR-621 | 84815 | MGC12916 | hypothetical protein MGC12916 | 1 |
| 9467 | hsa-miR-657 | 84815 | MGC12916 | hypothetical protein MGC12916 | 1 |
| 9468 | hsa-miR-765 | 84815 | MGC12916 | hypothetical protein MGC12916 | 1 |
| 9469 | hsa-miR-770-5p | 84815 | MGC12916 | hypothetical protein MGC12916 | 1 |
| 9470 | hsa-miR-877 | 84815 | MGC12916 | hypothetical protein MGC12916 | 1 |
| 9471 | hsa-mir-26a-1 | 84815 | MGC12916 | hypothetical protein MGC12916 | 1 |
| 9472 | hsa-mir-26b | 84815 | MGC12916 | hypothetical protein MGC12916 | 1 |
| 9473 | hsa-mir-31 | 84815 | MGC12916 | hypothetical protein MGC12916 | 1 |
| 9474 | hsa-mir-194-2 | 84815 | MGC12916 | hypothetical protein MGC12916 | 1 |
| 9475 | hsa-mir-340 | 84815 | MGC12916 | hypothetical protein MGC12916 | 1 |
| 9476 | hsa-mir-338 | 84815 | MGC12916 | hypothetical protein MGC12916 | 1 |
| 9477 | hsa-mir-490 | 84815 | MGC12916 | hypothetical protein MGC12916 | 1 |
| 9478 | hsa-mir-548b | 84815 | MGC12916 | hypothetical protein MGC12916 | 1 |
| 9479 | hsa-mir-449b | 84815 | MGC12916 | hypothetical protein MGC12916 | 1 |
| 9480 | hsa-mir-449c | 84815 | MGC12916 | hypothetical protein MGC12916 | 1 |
| 9481 | hsa-mir-3065 | 84815 | MGC12916 | hypothetical protein MGC12916 | 1 |
| 9482 | hsa-miR-186 | 121665 | UNQ1887 | signal peptide peptidase 3 | 1 |
| 9483 | hsa-miR-195 | 121665 | UNQ1887 | signal peptide peptidase 3 | 1 |
| 9484 | hsa-miR-326 | 121665 | UNQ1887 | signal peptide peptidase 3 | 1 |
| 9485 | hsa-miR-449b | 121665 | UNQ1887 | signal peptide peptidase 3 | 1 |
| 9486 | hsa-miR-489 | 121665 | UNQ1887 | signal peptide peptidase 3 | 1 |
| 9487 | hsa-miR-497 | 121665 | UNQ1887 | signal peptide peptidase 3 | 1 |
| 9488 | hsa-miR-562 | 121665 | UNQ1887 | signal peptide peptidase 3 | 1 |
| 9489 | hsa-miR-575 | 121665 | UNQ1887 | signal peptide peptidase 3 | 1 |
| 9490 | hsa-miR-608 | 121665 | UNQ1887 | signal peptide peptidase 3 | 1 |
| 9491 | hsa-miR-620 | 121665 | UNQ1887 | signal peptide peptidase 3 | 1 |
| 9492 | hsa-let-7a-3 | 121665 | UNQ1887 | signal peptide peptidase 3 | 1 |
| 9493 | hsa-let-7b | 121665 | UNQ1887 | signal peptide peptidase 3 | 1 |
| 9494 | hsa-mir-22 | 121665 | UNQ1887 | signal peptide peptidase 3 | 1 |
| 9495 | hsa-mir-10a | 121665 | UNQ1887 | signal peptide peptidase 3 | 1 |
| 9496 | hsa-mir-186 | 121665 | UNQ1887 | signal peptide peptidase 3 | 1 |
| 9497 | hsa-mir-155 | 121665 | UNQ1887 | signal peptide peptidase 3 | 1 |
| 9498 | hsa-mir-499 | 121665 | UNQ1887 | signal peptide peptidase 3 | 1 |
| 9499 | hsa-mir-877 | 121665 | UNQ1887 | signal peptide peptidase 3 | 1 |
| 9500 | hsa-miR-10a | 65056 | GPBP1 | GC-rich promoter binding protein 1 | 1 |
| 9501 | hsa-miR-1228\* | 65056 | GPBP1 | GC-rich promoter binding protein 1 | 1 |
| 9502 | hsa-miR-152 | 65056 | GPBP1 | GC-rich promoter binding protein 1 | 1 |
| 9503 | hsa-miR-26a | 65056 | GPBP1 | GC-rich promoter binding protein 1 | 1 |
| 9504 | hsa-miR-26b | 65056 | GPBP1 | GC-rich promoter binding protein 1 | 1 |
| 9505 | hsa-miR-31 | 65056 | GPBP1 | GC-rich promoter binding protein 1 | 1 |
| 9506 | hsa-miR-338-5p | 65056 | GPBP1 | GC-rich promoter binding protein 1 | 1 |
| 9507 | hsa-miR-340 | 65056 | GPBP1 | GC-rich promoter binding protein 1 | 1 |
| 9508 | hsa-miR-423-5p | 65056 | GPBP1 | GC-rich promoter binding protein 1 | 1 |
| 9509 | hsa-miR-449a | 65056 | GPBP1 | GC-rich promoter binding protein 1 | 1 |
| 9510 | hsa-miR-490-5p | 65056 | GPBP1 | GC-rich promoter binding protein 1 | 1 |
| 9511 | hsa-miR-499-5p | 65056 | GPBP1 | GC-rich promoter binding protein 1 | 1 |
| 9512 | hsa-miR-548b-5p | 65056 | GPBP1 | GC-rich promoter binding protein 1 | 1 |
| 9513 | hsa-miR-564 | 65056 | GPBP1 | GC-rich promoter binding protein 1 | 1 |
| 9514 | hsa-miR-584 | 65056 | GPBP1 | GC-rich promoter binding protein 1 | 1 |
| 9515 | hsa-miR-621 | 65056 | GPBP1 | GC-rich promoter binding protein 1 | 1 |
| 9516 | hsa-miR-644 | 65056 | GPBP1 | GC-rich promoter binding protein 1 | 1 |
| 9517 | hsa-miR-657 | 65056 | GPBP1 | GC-rich promoter binding protein 1 | 1 |
| 9518 | hsa-miR-765 | 65056 | GPBP1 | GC-rich promoter binding protein 1 | 1 |
| 9519 | hsa-miR-770-5p | 65056 | GPBP1 | GC-rich promoter binding protein 1 | 1 |
| 9520 | hsa-miR-877 | 65056 | GPBP1 | GC-rich promoter binding protein 1 | 1 |
| 9521 | hsa-miR-95 | 65056 | GPBP1 | GC-rich promoter binding protein 1 | 1 |
| 9522 | hsa-mir-26a-1 | 65056 | GPBP1 | GC-rich promoter binding protein 1 | 1 |
| 9523 | hsa-mir-26b | 65056 | GPBP1 | GC-rich promoter binding protein 1 | 1 |
| 9524 | hsa-mir-31 | 65056 | GPBP1 | GC-rich promoter binding protein 1 | 1 |
| 9525 | hsa-mir-194-2 | 65056 | GPBP1 | GC-rich promoter binding protein 1 | 1 |
| 9526 | hsa-mir-340 | 65056 | GPBP1 | GC-rich promoter binding protein 1 | 1 |
| 9527 | hsa-mir-338 | 65056 | GPBP1 | GC-rich promoter binding protein 1 | 1 |
| 9528 | hsa-mir-490 | 65056 | GPBP1 | GC-rich promoter binding protein 1 | 1 |
| 9529 | hsa-mir-548b | 65056 | GPBP1 | GC-rich promoter binding protein 1 | 1 |
| 9530 | hsa-mir-449b | 65056 | GPBP1 | GC-rich promoter binding protein 1 | 1 |
| 9531 | hsa-mir-449c | 65056 | GPBP1 | GC-rich promoter binding protein 1 | 1 |
| 9532 | hsa-mir-3065 | 65056 | GPBP1 | GC-rich promoter binding protein 1 | 1 |
| 9533 | hsa-miR-10a | 415116 | PIM3 | pim-3 oncogene | 1 |
| 9534 | hsa-miR-1228\* | 415116 | PIM3 | pim-3 oncogene | 1 |
| 9535 | hsa-miR-139-5p | 415116 | PIM3 | pim-3 oncogene | 1 |
| 9536 | hsa-miR-152 | 415116 | PIM3 | pim-3 oncogene | 1 |
| 9537 | hsa-miR-26b | 415116 | PIM3 | pim-3 oncogene | 1 |
| 9538 | hsa-miR-31 | 415116 | PIM3 | pim-3 oncogene | 1 |
| 9539 | hsa-miR-338-5p | 415116 | PIM3 | pim-3 oncogene | 1 |
| 9540 | hsa-miR-340 | 415116 | PIM3 | pim-3 oncogene | 1 |
| 9541 | hsa-miR-383 | 415116 | PIM3 | pim-3 oncogene | 1 |
| 9542 | hsa-miR-449a | 415116 | PIM3 | pim-3 oncogene | 1 |
| 9543 | hsa-miR-499-5p | 415116 | PIM3 | pim-3 oncogene | 1 |
| 9544 | hsa-miR-548b-5p | 415116 | PIM3 | pim-3 oncogene | 1 |
| 9545 | hsa-miR-584 | 415116 | PIM3 | pim-3 oncogene | 1 |
| 9546 | hsa-miR-621 | 415116 | PIM3 | pim-3 oncogene | 1 |
| 9547 | hsa-miR-657 | 415116 | PIM3 | pim-3 oncogene | 1 |
| 9548 | hsa-miR-663 | 415116 | PIM3 | pim-3 oncogene | 1 |
| 9549 | hsa-miR-765 | 415116 | PIM3 | pim-3 oncogene | 1 |
| 9550 | hsa-miR-770-5p | 415116 | PIM3 | pim-3 oncogene | 1 |
| 9551 | hsa-miR-877 | 415116 | PIM3 | pim-3 oncogene | 1 |
| 9552 | hsa-mir-26a-1 | 415116 | PIM3 | pim-3 oncogene | 1 |
| 9553 | hsa-mir-26b | 415116 | PIM3 | pim-3 oncogene | 1 |
| 9554 | hsa-mir-31 | 415116 | PIM3 | pim-3 oncogene | 1 |
| 9555 | hsa-mir-194-2 | 415116 | PIM3 | pim-3 oncogene | 1 |
| 9556 | hsa-mir-340 | 415116 | PIM3 | pim-3 oncogene | 1 |
| 9557 | hsa-mir-338 | 415116 | PIM3 | pim-3 oncogene | 1 |
| 9558 | hsa-mir-548b | 415116 | PIM3 | pim-3 oncogene | 1 |
| 9559 | hsa-mir-449b | 415116 | PIM3 | pim-3 oncogene | 1 |
| 9560 | hsa-mir-449c | 415116 | PIM3 | pim-3 oncogene | 1 |
| 9561 | hsa-mir-3065 | 415116 | PIM3 | pim-3 oncogene | 1 |
| 9566 | hsa-miR-31 | 55593 | OTUD5 | OTU domain containing 5 | 1 |
| 9567 | hsa-miR-338-5p | 55593 | OTUD5 | OTU domain containing 5 | 1 |
| 9569 | hsa-miR-383 | 55593 | OTUD5 | OTU domain containing 5 | 1 |
| 9574 | hsa-miR-653 | 55593 | OTUD5 | OTU domain containing 5 | 1 |
| 9575 | hsa-miR-657 | 55593 | OTUD5 | OTU domain containing 5 | 1 |
| 9581 | hsa-mir-31 | 55593 | OTUD5 | OTU domain containing 5 | 1 |
| 9584 | hsa-mir-338 | 55593 | OTUD5 | OTU domain containing 5 | 1 |
| 9587 | hsa-mir-3065 | 55593 | OTUD5 | OTU domain containing 5 | 1 |
| 9588 | hsa-miR-10a | 283991 | FAM100B | family with sequence similarity 100, member B | 1 |
| 9589 | hsa-miR-152 | 283991 | FAM100B | family with sequence similarity 100, member B | 1 |
| 9590 | hsa-miR-26a | 283991 | FAM100B | family with sequence similarity 100, member B | 1 |
| 9591 | hsa-miR-26b | 283991 | FAM100B | family with sequence similarity 100, member B | 1 |
| 9592 | hsa-miR-31 | 283991 | FAM100B | family with sequence similarity 100, member B | 1 |
| 9593 | hsa-miR-340 | 283991 | FAM100B | family with sequence similarity 100, member B | 1 |
| 9594 | hsa-miR-423-5p | 283991 | FAM100B | family with sequence similarity 100, member B | 1 |
| 9595 | hsa-miR-449a | 283991 | FAM100B | family with sequence similarity 100, member B | 1 |
| 9596 | hsa-miR-490-5p | 283991 | FAM100B | family with sequence similarity 100, member B | 1 |
| 9597 | hsa-miR-499-5p | 283991 | FAM100B | family with sequence similarity 100, member B | 1 |
| 9598 | hsa-miR-504 | 283991 | FAM100B | family with sequence similarity 100, member B | 1 |
| 9599 | hsa-miR-564 | 283991 | FAM100B | family with sequence similarity 100, member B | 1 |
| 9600 | hsa-miR-621 | 283991 | FAM100B | family with sequence similarity 100, member B | 1 |
| 9601 | hsa-miR-644 | 283991 | FAM100B | family with sequence similarity 100, member B | 1 |
| 9602 | hsa-miR-653 | 283991 | FAM100B | family with sequence similarity 100, member B | 1 |
| 9603 | hsa-miR-765 | 283991 | FAM100B | family with sequence similarity 100, member B | 1 |
| 9604 | hsa-miR-770-5p | 283991 | FAM100B | family with sequence similarity 100, member B | 1 |
| 9605 | hsa-miR-877 | 283991 | FAM100B | family with sequence similarity 100, member B | 1 |
| 9606 | hsa-miR-95 | 283991 | FAM100B | family with sequence similarity 100, member B | 1 |
| 9607 | hsa-mir-26a-1 | 283991 | FAM100B | family with sequence similarity 100, member B | 1 |
| 9608 | hsa-mir-26b | 283991 | FAM100B | family with sequence similarity 100, member B | 1 |
| 9609 | hsa-mir-31 | 283991 | FAM100B | family with sequence similarity 100, member B | 1 |
| 9610 | hsa-mir-194-2 | 283991 | FAM100B | family with sequence similarity 100, member B | 1 |
| 9611 | hsa-mir-340 | 283991 | FAM100B | family with sequence similarity 100, member B | 1 |
| 9612 | hsa-mir-490 | 283991 | FAM100B | family with sequence similarity 100, member B | 1 |
| 9613 | hsa-mir-449b | 283991 | FAM100B | family with sequence similarity 100, member B | 1 |
| 9614 | hsa-mir-449c | 283991 | FAM100B | family with sequence similarity 100, member B | 1 |
| 9615 | hsa-miR-1 | 6470 | SHMT1 | serine hydroxymethyltransferase 1 (soluble) | 1 |
| 9616 | hsa-miR-133a | 6470 | SHMT1 | serine hydroxymethyltransferase 1 (soluble) | 1 |
| 9617 | hsa-miR-186 | 6470 | SHMT1 | serine hydroxymethyltransferase 1 (soluble) | 1 |
| 9618 | hsa-miR-449b | 6470 | SHMT1 | serine hydroxymethyltransferase 1 (soluble) | 1 |
| 9619 | hsa-miR-489 | 6470 | SHMT1 | serine hydroxymethyltransferase 1 (soluble) | 1 |
| 9620 | hsa-miR-579 | 6470 | SHMT1 | serine hydroxymethyltransferase 1 (soluble) | 1 |
| 9621 | hsa-miR-620 | 6470 | SHMT1 | serine hydroxymethyltransferase 1 (soluble) | 1 |
| 9622 | hsa-miR-885-5p | 6470 | SHMT1 | serine hydroxymethyltransferase 1 (soluble) | 1 |
| 9623 | hsa-let-7a-3 | 6470 | SHMT1 | serine hydroxymethyltransferase 1 (soluble) | 1 |
| 9624 | hsa-let-7b | 6470 | SHMT1 | serine hydroxymethyltransferase 1 (soluble) | 1 |
| 9625 | hsa-mir-22 | 6470 | SHMT1 | serine hydroxymethyltransferase 1 (soluble) | 1 |
| 9626 | hsa-mir-10a | 6470 | SHMT1 | serine hydroxymethyltransferase 1 (soluble) | 1 |
| 9627 | hsa-mir-186 | 6470 | SHMT1 | serine hydroxymethyltransferase 1 (soluble) | 1 |
| 9628 | hsa-mir-155 | 6470 | SHMT1 | serine hydroxymethyltransferase 1 (soluble) | 1 |
| 9629 | hsa-mir-499 | 6470 | SHMT1 | serine hydroxymethyltransferase 1 (soluble) | 1 |
| 9630 | hsa-mir-885 | 6470 | SHMT1 | serine hydroxymethyltransferase 1 (soluble) | 1 |
| 9631 | hsa-miR-10a | 57169 | ZNFX1 | zinc finger, NFX1-type containing 1 | 1 |
| 9632 | hsa-miR-139-5p | 57169 | ZNFX1 | zinc finger, NFX1-type containing 1 | 1 |
| 9633 | hsa-miR-26a | 57169 | ZNFX1 | zinc finger, NFX1-type containing 1 | 1 |
| 9634 | hsa-miR-26b | 57169 | ZNFX1 | zinc finger, NFX1-type containing 1 | 1 |
| 9635 | hsa-miR-31 | 57169 | ZNFX1 | zinc finger, NFX1-type containing 1 | 1 |
| 9636 | hsa-miR-338-5p | 57169 | ZNFX1 | zinc finger, NFX1-type containing 1 | 1 |
| 9637 | hsa-miR-340 | 57169 | ZNFX1 | zinc finger, NFX1-type containing 1 | 1 |
| 9638 | hsa-miR-449a | 57169 | ZNFX1 | zinc finger, NFX1-type containing 1 | 1 |
| 9639 | hsa-miR-499-5p | 57169 | ZNFX1 | zinc finger, NFX1-type containing 1 | 1 |
| 9640 | hsa-miR-548b-5p | 57169 | ZNFX1 | zinc finger, NFX1-type containing 1 | 1 |
| 9641 | hsa-miR-564 | 57169 | ZNFX1 | zinc finger, NFX1-type containing 1 | 1 |
| 9642 | hsa-miR-621 | 57169 | ZNFX1 | zinc finger, NFX1-type containing 1 | 1 |
| 9643 | hsa-miR-657 | 57169 | ZNFX1 | zinc finger, NFX1-type containing 1 | 1 |
| 9644 | hsa-miR-765 | 57169 | ZNFX1 | zinc finger, NFX1-type containing 1 | 1 |
| 9645 | hsa-miR-770-5p | 57169 | ZNFX1 | zinc finger, NFX1-type containing 1 | 1 |
| 9646 | hsa-miR-877 | 57169 | ZNFX1 | zinc finger, NFX1-type containing 1 | 1 |
| 9647 | hsa-mir-26a-1 | 57169 | ZNFX1 | zinc finger, NFX1-type containing 1 | 1 |
| 9648 | hsa-mir-26b | 57169 | ZNFX1 | zinc finger, NFX1-type containing 1 | 1 |
| 9649 | hsa-mir-31 | 57169 | ZNFX1 | zinc finger, NFX1-type containing 1 | 1 |
| 9650 | hsa-mir-194-2 | 57169 | ZNFX1 | zinc finger, NFX1-type containing 1 | 1 |
| 9651 | hsa-mir-340 | 57169 | ZNFX1 | zinc finger, NFX1-type containing 1 | 1 |
| 9652 | hsa-mir-338 | 57169 | ZNFX1 | zinc finger, NFX1-type containing 1 | 1 |
| 9653 | hsa-mir-548b | 57169 | ZNFX1 | zinc finger, NFX1-type containing 1 | 1 |
| 9654 | hsa-mir-548d-2 | 57169 | ZNFX1 | zinc finger, NFX1-type containing 1 | 1 |
| 9655 | hsa-mir-449b | 57169 | ZNFX1 | zinc finger, NFX1-type containing 1 | 1 |
| 9656 | hsa-mir-449c | 57169 | ZNFX1 | zinc finger, NFX1-type containing 1 | 1 |
| 9657 | hsa-mir-3065 | 57169 | ZNFX1 | zinc finger, NFX1-type containing 1 | 1 |
| 9658 | hsa-miR-1 | 170622 | COMMD6 | COMM domain containing 6 | 1 |
| 9659 | hsa-miR-133a | 170622 | COMMD6 | COMM domain containing 6 | 1 |
| 9660 | hsa-miR-186 | 170622 | COMMD6 | COMM domain containing 6 | 1 |
| 9661 | hsa-miR-326 | 170622 | COMMD6 | COMM domain containing 6 | 1 |
| 9662 | hsa-miR-449b | 170622 | COMMD6 | COMM domain containing 6 | 1 |
| 9663 | hsa-miR-550 | 170622 | COMMD6 | COMM domain containing 6 | 1 |
| 9664 | hsa-miR-551a | 170622 | COMMD6 | COMM domain containing 6 | 1 |
| 9665 | hsa-miR-575 | 170622 | COMMD6 | COMM domain containing 6 | 1 |
| 9666 | hsa-miR-579 | 170622 | COMMD6 | COMM domain containing 6 | 1 |
| 9667 | hsa-miR-608 | 170622 | COMMD6 | COMM domain containing 6 | 1 |
| 9668 | hsa-miR-620 | 170622 | COMMD6 | COMM domain containing 6 | 1 |
| 9669 | hsa-miR-885-5p | 170622 | COMMD6 | COMM domain containing 6 | 1 |
| 9670 | hsa-let-7a-3 | 170622 | COMMD6 | COMM domain containing 6 | 1 |
| 9671 | hsa-let-7b | 170622 | COMMD6 | COMM domain containing 6 | 1 |
| 9672 | hsa-mir-22 | 170622 | COMMD6 | COMM domain containing 6 | 1 |
| 9673 | hsa-mir-10a | 170622 | COMMD6 | COMM domain containing 6 | 1 |
| 9674 | hsa-mir-186 | 170622 | COMMD6 | COMM domain containing 6 | 1 |
| 9675 | hsa-mir-155 | 170622 | COMMD6 | COMM domain containing 6 | 1 |
| 9676 | hsa-mir-423 | 170622 | COMMD6 | COMM domain containing 6 | 1 |
| 9677 | hsa-mir-499 | 170622 | COMMD6 | COMM domain containing 6 | 1 |
| 9678 | hsa-mir-550-1 | 170622 | COMMD6 | COMM domain containing 6 | 1 |
| 9679 | hsa-mir-885 | 170622 | COMMD6 | COMM domain containing 6 | 1 |
| 9680 | hsa-mir-877 | 170622 | COMMD6 | COMM domain containing 6 | 1 |
| 9681 | hsa-mir-2116 | 170622 | COMMD6 | COMM domain containing 6 | 1 |
| 9682 | hsa-mir-3186 | 170622 | COMMD6 | COMM domain containing 6 | 1 |
| 9683 | hsa-mir-3190 | 170622 | COMMD6 | COMM domain containing 6 | 1 |
| 9684 | hsa-miR-1 | 129787 | TMEM18 | transmembrane protein 18 | 1 |
| 9685 | hsa-miR-133a | 129787 | TMEM18 | transmembrane protein 18 | 1 |
| 9686 | hsa-miR-186 | 129787 | TMEM18 | transmembrane protein 18 | 1 |
| 9687 | hsa-miR-326 | 129787 | TMEM18 | transmembrane protein 18 | 1 |
| 9688 | hsa-miR-449b | 129787 | TMEM18 | transmembrane protein 18 | 1 |
| 9689 | hsa-miR-489 | 129787 | TMEM18 | transmembrane protein 18 | 1 |
| 9690 | hsa-miR-575 | 129787 | TMEM18 | transmembrane protein 18 | 1 |
| 9691 | hsa-miR-608 | 129787 | TMEM18 | transmembrane protein 18 | 1 |
| 9692 | hsa-miR-620 | 129787 | TMEM18 | transmembrane protein 18 | 1 |
| 9693 | hsa-let-7a-3 | 129787 | TMEM18 | transmembrane protein 18 | 1 |
| 9694 | hsa-let-7b | 129787 | TMEM18 | transmembrane protein 18 | 1 |
| 9695 | hsa-mir-22 | 129787 | TMEM18 | transmembrane protein 18 | 1 |
| 9696 | hsa-mir-10a | 129787 | TMEM18 | transmembrane protein 18 | 1 |
| 9697 | hsa-mir-186 | 129787 | TMEM18 | transmembrane protein 18 | 1 |
| 9698 | hsa-mir-155 | 129787 | TMEM18 | transmembrane protein 18 | 1 |
| 9699 | hsa-mir-499 | 129787 | TMEM18 | transmembrane protein 18 | 1 |
| 9700 | hsa-mir-877 | 129787 | TMEM18 | transmembrane protein 18 | 1 |
| 9701 | hsa-miR-33a | 90480 | GADD45GIP1 | growth arrest and DNA-damage-inducible, gamma interacting protein 1 | 1 |
| 9702 | hsa-miR-551a | 90480 | GADD45GIP1 | growth arrest and DNA-damage-inducible, gamma interacting protein 1 | 1 |
| 9703 | hsa-miR-562 | 90480 | GADD45GIP1 | growth arrest and DNA-damage-inducible, gamma interacting protein 1 | 1 |
| 9704 | hsa-mir-3186 | 90480 | GADD45GIP1 | growth arrest and DNA-damage-inducible, gamma interacting protein 1 | 1 |
| 9705 | hsa-miR-10a | 64651 | CSRNP1 | cysteine-serine-rich nuclear protein 1 | 1 |
| 9706 | hsa-miR-152 | 64651 | CSRNP1 | cysteine-serine-rich nuclear protein 1 | 1 |
| 9707 | hsa-miR-26a | 64651 | CSRNP1 | cysteine-serine-rich nuclear protein 1 | 1 |
| 9708 | hsa-miR-26b | 64651 | CSRNP1 | cysteine-serine-rich nuclear protein 1 | 1 |
| 9709 | hsa-miR-31 | 64651 | CSRNP1 | cysteine-serine-rich nuclear protein 1 | 1 |
| 9710 | hsa-miR-340 | 64651 | CSRNP1 | cysteine-serine-rich nuclear protein 1 | 1 |
| 9711 | hsa-miR-423-5p | 64651 | CSRNP1 | cysteine-serine-rich nuclear protein 1 | 1 |
| 9712 | hsa-miR-449a | 64651 | CSRNP1 | cysteine-serine-rich nuclear protein 1 | 1 |
| 9713 | hsa-miR-490-5p | 64651 | CSRNP1 | cysteine-serine-rich nuclear protein 1 | 1 |
| 9714 | hsa-miR-499-5p | 64651 | CSRNP1 | cysteine-serine-rich nuclear protein 1 | 1 |
| 9715 | hsa-miR-564 | 64651 | CSRNP1 | cysteine-serine-rich nuclear protein 1 | 1 |
| 9716 | hsa-miR-621 | 64651 | CSRNP1 | cysteine-serine-rich nuclear protein 1 | 1 |
| 9717 | hsa-miR-634 | 64651 | CSRNP1 | cysteine-serine-rich nuclear protein 1 | 1 |
| 9718 | hsa-miR-644 | 64651 | CSRNP1 | cysteine-serine-rich nuclear protein 1 | 1 |
| 9719 | hsa-miR-653 | 64651 | CSRNP1 | cysteine-serine-rich nuclear protein 1 | 1 |
| 9720 | hsa-miR-765 | 64651 | CSRNP1 | cysteine-serine-rich nuclear protein 1 | 1 |
| 9721 | hsa-miR-770-5p | 64651 | CSRNP1 | cysteine-serine-rich nuclear protein 1 | 1 |
| 9722 | hsa-miR-877 | 64651 | CSRNP1 | cysteine-serine-rich nuclear protein 1 | 1 |
| 9723 | hsa-miR-95 | 64651 | CSRNP1 | cysteine-serine-rich nuclear protein 1 | 1 |
| 9724 | hsa-mir-26a-1 | 64651 | CSRNP1 | cysteine-serine-rich nuclear protein 1 | 1 |
| 9725 | hsa-mir-26b | 64651 | CSRNP1 | cysteine-serine-rich nuclear protein 1 | 1 |
| 9726 | hsa-mir-31 | 64651 | CSRNP1 | cysteine-serine-rich nuclear protein 1 | 1 |
| 9727 | hsa-mir-194-2 | 64651 | CSRNP1 | cysteine-serine-rich nuclear protein 1 | 1 |
| 9728 | hsa-mir-340 | 64651 | CSRNP1 | cysteine-serine-rich nuclear protein 1 | 1 |
| 9729 | hsa-mir-490 | 64651 | CSRNP1 | cysteine-serine-rich nuclear protein 1 | 1 |
| 9730 | hsa-mir-449b | 64651 | CSRNP1 | cysteine-serine-rich nuclear protein 1 | 1 |
| 9731 | hsa-mir-449c | 64651 | CSRNP1 | cysteine-serine-rich nuclear protein 1 | 1 |
| 9732 | hsa-miR-1 | 26260 | FBXO25 | F-box protein 25 | 1 |
| 9733 | hsa-miR-133a | 26260 | FBXO25 | F-box protein 25 | 1 |
| 9734 | hsa-miR-186 | 26260 | FBXO25 | F-box protein 25 | 1 |
| 9735 | hsa-miR-208a | 26260 | FBXO25 | F-box protein 25 | 1 |
| 9736 | hsa-miR-449b | 26260 | FBXO25 | F-box protein 25 | 1 |
| 9737 | hsa-miR-489 | 26260 | FBXO25 | F-box protein 25 | 1 |
| 9738 | hsa-miR-562 | 26260 | FBXO25 | F-box protein 25 | 1 |
| 9739 | hsa-miR-575 | 26260 | FBXO25 | F-box protein 25 | 1 |
| 9740 | hsa-miR-579 | 26260 | FBXO25 | F-box protein 25 | 1 |
| 9741 | hsa-miR-608 | 26260 | FBXO25 | F-box protein 25 | 1 |
| 9742 | hsa-miR-620 | 26260 | FBXO25 | F-box protein 25 | 1 |
| 9743 | hsa-miR-885-5p | 26260 | FBXO25 | F-box protein 25 | 1 |
| 9744 | hsa-let-7a-3 | 26260 | FBXO25 | F-box protein 25 | 1 |
| 9745 | hsa-let-7b | 26260 | FBXO25 | F-box protein 25 | 1 |
| 9746 | hsa-mir-22 | 26260 | FBXO25 | F-box protein 25 | 1 |
| 9747 | hsa-mir-10a | 26260 | FBXO25 | F-box protein 25 | 1 |
| 9748 | hsa-mir-186 | 26260 | FBXO25 | F-box protein 25 | 1 |
| 9749 | hsa-mir-155 | 26260 | FBXO25 | F-box protein 25 | 1 |
| 9750 | hsa-mir-499 | 26260 | FBXO25 | F-box protein 25 | 1 |
| 9751 | hsa-mir-885 | 26260 | FBXO25 | F-box protein 25 | 1 |
| 9752 | hsa-mir-877 | 26260 | FBXO25 | F-box protein 25 | 1 |
| 9753 | hsa-mir-2116 | 26260 | FBXO25 | F-box protein 25 | 1 |
| 9754 | hsa-mir-3190 | 26260 | FBXO25 | F-box protein 25 | 1 |
| 9755 | hsa-miR-1 | 57210 | SLC45A4 | solute carrier family 45, member 4 | 1 |
| 9756 | hsa-miR-133a | 57210 | SLC45A4 | solute carrier family 45, member 4 | 1 |
| 9757 | hsa-miR-186 | 57210 | SLC45A4 | solute carrier family 45, member 4 | 1 |
| 9758 | hsa-miR-326 | 57210 | SLC45A4 | solute carrier family 45, member 4 | 1 |
| 9759 | hsa-miR-449b | 57210 | SLC45A4 | solute carrier family 45, member 4 | 1 |
| 9760 | hsa-miR-489 | 57210 | SLC45A4 | solute carrier family 45, member 4 | 1 |
| 9761 | hsa-miR-562 | 57210 | SLC45A4 | solute carrier family 45, member 4 | 1 |
| 9762 | hsa-miR-575 | 57210 | SLC45A4 | solute carrier family 45, member 4 | 1 |
| 9763 | hsa-miR-579 | 57210 | SLC45A4 | solute carrier family 45, member 4 | 1 |
| 9764 | hsa-miR-608 | 57210 | SLC45A4 | solute carrier family 45, member 4 | 1 |
| 9765 | hsa-miR-620 | 57210 | SLC45A4 | solute carrier family 45, member 4 | 1 |
| 9766 | hsa-miR-637 | 57210 | SLC45A4 | solute carrier family 45, member 4 | 1 |
| 9767 | hsa-miR-885-5p | 57210 | SLC45A4 | solute carrier family 45, member 4 | 1 |
| 9768 | hsa-mir-22 | 57210 | SLC45A4 | solute carrier family 45, member 4 | 1 |
| 9769 | hsa-mir-10a | 57210 | SLC45A4 | solute carrier family 45, member 4 | 1 |
| 9770 | hsa-mir-186 | 57210 | SLC45A4 | solute carrier family 45, member 4 | 1 |
| 9771 | hsa-mir-155 | 57210 | SLC45A4 | solute carrier family 45, member 4 | 1 |
| 9772 | hsa-mir-423 | 57210 | SLC45A4 | solute carrier family 45, member 4 | 1 |
| 9773 | hsa-mir-499 | 57210 | SLC45A4 | solute carrier family 45, member 4 | 1 |
| 9774 | hsa-mir-885 | 57210 | SLC45A4 | solute carrier family 45, member 4 | 1 |
| 9775 | hsa-mir-877 | 57210 | SLC45A4 | solute carrier family 45, member 4 | 1 |
| 9776 | hsa-mir-3190 | 57210 | SLC45A4 | solute carrier family 45, member 4 | 1 |
| 9798 | hsa-miR-26a | 152007 | GLIPR2 | GLI pathogenesis-related 2 | 1 |
| 9799 | hsa-miR-26b | 152007 | GLIPR2 | GLI pathogenesis-related 2 | 1 |
| 9800 | hsa-miR-423-5p | 152007 | GLIPR2 | GLI pathogenesis-related 2 | 1 |
| 9801 | hsa-miR-490-5p | 152007 | GLIPR2 | GLI pathogenesis-related 2 | 1 |
| 9802 | hsa-mir-26b | 152007 | GLIPR2 | GLI pathogenesis-related 2 | 1 |
| 9803 | hsa-mir-490 | 152007 | GLIPR2 | GLI pathogenesis-related 2 | 1 |
| 9804 | hsa-miR-1 | 80820 | EEPD1 | endonuclease/exonuclease/phosphatase family domain containing 1 | 1 |
| 9805 | hsa-miR-133a | 80820 | EEPD1 | endonuclease/exonuclease/phosphatase family domain containing 1 | 1 |
| 9806 | hsa-miR-186 | 80820 | EEPD1 | endonuclease/exonuclease/phosphatase family domain containing 1 | 1 |
| 9807 | hsa-miR-208a | 80820 | EEPD1 | endonuclease/exonuclease/phosphatase family domain containing 1 | 1 |
| 9808 | hsa-miR-449b | 80820 | EEPD1 | endonuclease/exonuclease/phosphatase family domain containing 1 | 1 |
| 9809 | hsa-miR-575 | 80820 | EEPD1 | endonuclease/exonuclease/phosphatase family domain containing 1 | 1 |
| 9810 | hsa-miR-579 | 80820 | EEPD1 | endonuclease/exonuclease/phosphatase family domain containing 1 | 1 |
| 9811 | hsa-miR-608 | 80820 | EEPD1 | endonuclease/exonuclease/phosphatase family domain containing 1 | 1 |
| 9812 | hsa-miR-620 | 80820 | EEPD1 | endonuclease/exonuclease/phosphatase family domain containing 1 | 1 |
| 9813 | hsa-let-7a-3 | 80820 | EEPD1 | endonuclease/exonuclease/phosphatase family domain containing 1 | 1 |
| 9814 | hsa-let-7b | 80820 | EEPD1 | endonuclease/exonuclease/phosphatase family domain containing 1 | 1 |
| 9815 | hsa-mir-22 | 80820 | EEPD1 | endonuclease/exonuclease/phosphatase family domain containing 1 | 1 |
| 9816 | hsa-mir-10a | 80820 | EEPD1 | endonuclease/exonuclease/phosphatase family domain containing 1 | 1 |
| 9817 | hsa-mir-186 | 80820 | EEPD1 | endonuclease/exonuclease/phosphatase family domain containing 1 | 1 |
| 9818 | hsa-mir-155 | 80820 | EEPD1 | endonuclease/exonuclease/phosphatase family domain containing 1 | 1 |
| 9819 | hsa-mir-499 | 80820 | EEPD1 | endonuclease/exonuclease/phosphatase family domain containing 1 | 1 |
| 9820 | hsa-mir-877 | 80820 | EEPD1 | endonuclease/exonuclease/phosphatase family domain containing 1 | 1 |
| 9821 | hsa-miR-10a | 6891 | TAP2 | transporter 2, ATP-binding cassette, sub-family B (MDR/TAP) | 1 |
| 9822 | hsa-miR-139-5p | 6891 | TAP2 | transporter 2, ATP-binding cassette, sub-family B (MDR/TAP) | 1 |
| 9823 | hsa-miR-152 | 6891 | TAP2 | transporter 2, ATP-binding cassette, sub-family B (MDR/TAP) | 1 |
| 9824 | hsa-miR-26a | 6891 | TAP2 | transporter 2, ATP-binding cassette, sub-family B (MDR/TAP) | 1 |
| 9825 | hsa-miR-26b | 6891 | TAP2 | transporter 2, ATP-binding cassette, sub-family B (MDR/TAP) | 1 |
| 9826 | hsa-miR-31 | 6891 | TAP2 | transporter 2, ATP-binding cassette, sub-family B (MDR/TAP) | 1 |
| 9827 | hsa-miR-338-5p | 6891 | TAP2 | transporter 2, ATP-binding cassette, sub-family B (MDR/TAP) | 1 |
| 9828 | hsa-miR-340 | 6891 | TAP2 | transporter 2, ATP-binding cassette, sub-family B (MDR/TAP) | 1 |
| 9829 | hsa-miR-449a | 6891 | TAP2 | transporter 2, ATP-binding cassette, sub-family B (MDR/TAP) | 1 |
| 9830 | hsa-miR-499-5p | 6891 | TAP2 | transporter 2, ATP-binding cassette, sub-family B (MDR/TAP) | 1 |
| 9831 | hsa-miR-548b-5p | 6891 | TAP2 | transporter 2, ATP-binding cassette, sub-family B (MDR/TAP) | 1 |
| 9832 | hsa-miR-621 | 6891 | TAP2 | transporter 2, ATP-binding cassette, sub-family B (MDR/TAP) | 1 |
| 9833 | hsa-miR-657 | 6891 | TAP2 | transporter 2, ATP-binding cassette, sub-family B (MDR/TAP) | 1 |
| 9834 | hsa-miR-663 | 6891 | TAP2 | transporter 2, ATP-binding cassette, sub-family B (MDR/TAP) | 1 |
| 9835 | hsa-miR-765 | 6891 | TAP2 | transporter 2, ATP-binding cassette, sub-family B (MDR/TAP) | 1 |
| 9836 | hsa-miR-770-5p | 6891 | TAP2 | transporter 2, ATP-binding cassette, sub-family B (MDR/TAP) | 1 |
| 9837 | hsa-miR-877 | 6891 | TAP2 | transporter 2, ATP-binding cassette, sub-family B (MDR/TAP) | 1 |
| 9838 | hsa-mir-26a-1 | 6891 | TAP2 | transporter 2, ATP-binding cassette, sub-family B (MDR/TAP) | 1 |
| 9839 | hsa-mir-26b | 6891 | TAP2 | transporter 2, ATP-binding cassette, sub-family B (MDR/TAP) | 1 |
| 9840 | hsa-mir-31 | 6891 | TAP2 | transporter 2, ATP-binding cassette, sub-family B (MDR/TAP) | 1 |
| 9841 | hsa-mir-194-2 | 6891 | TAP2 | transporter 2, ATP-binding cassette, sub-family B (MDR/TAP) | 1 |
| 9842 | hsa-mir-340 | 6891 | TAP2 | transporter 2, ATP-binding cassette, sub-family B (MDR/TAP) | 1 |
| 9843 | hsa-mir-338 | 6891 | TAP2 | transporter 2, ATP-binding cassette, sub-family B (MDR/TAP) | 1 |
| 9844 | hsa-mir-548b | 6891 | TAP2 | transporter 2, ATP-binding cassette, sub-family B (MDR/TAP) | 1 |
| 9845 | hsa-mir-548d-2 | 6891 | TAP2 | transporter 2, ATP-binding cassette, sub-family B (MDR/TAP) | 1 |
| 9846 | hsa-mir-449b | 6891 | TAP2 | transporter 2, ATP-binding cassette, sub-family B (MDR/TAP) | 1 |
| 9847 | hsa-mir-449c | 6891 | TAP2 | transporter 2, ATP-binding cassette, sub-family B (MDR/TAP) | 1 |
| 9848 | hsa-mir-3065 | 6891 | TAP2 | transporter 2, ATP-binding cassette, sub-family B (MDR/TAP) | 1 |
| 9849 | hsa-miR-10a | 23476 | BRD4 | bromodomain containing 4 | 1 |
| 9850 | hsa-miR-139-5p | 23476 | BRD4 | bromodomain containing 4 | 1 |
| 9851 | hsa-miR-152 | 23476 | BRD4 | bromodomain containing 4 | 1 |
| 9852 | hsa-miR-26b | 23476 | BRD4 | bromodomain containing 4 | 1 |
| 9853 | hsa-miR-31 | 23476 | BRD4 | bromodomain containing 4 | 1 |
| 9854 | hsa-miR-338-5p | 23476 | BRD4 | bromodomain containing 4 | 1 |
| 9855 | hsa-miR-340 | 23476 | BRD4 | bromodomain containing 4 | 1 |
| 9856 | hsa-miR-383 | 23476 | BRD4 | bromodomain containing 4 | 1 |
| 9857 | hsa-miR-449a | 23476 | BRD4 | bromodomain containing 4 | 1 |
| 9858 | hsa-miR-499-5p | 23476 | BRD4 | bromodomain containing 4 | 1 |
| 9859 | hsa-miR-548b-5p | 23476 | BRD4 | bromodomain containing 4 | 1 |
| 9860 | hsa-miR-621 | 23476 | BRD4 | bromodomain containing 4 | 1 |
| 9861 | hsa-miR-657 | 23476 | BRD4 | bromodomain containing 4 | 1 |
| 9862 | hsa-miR-663 | 23476 | BRD4 | bromodomain containing 4 | 1 |
| 9863 | hsa-miR-765 | 23476 | BRD4 | bromodomain containing 4 | 1 |
| 9864 | hsa-miR-770-5p | 23476 | BRD4 | bromodomain containing 4 | 1 |
| 9865 | hsa-miR-877 | 23476 | BRD4 | bromodomain containing 4 | 1 |
| 9866 | hsa-mir-26a-1 | 23476 | BRD4 | bromodomain containing 4 | 1 |
| 9867 | hsa-mir-26b | 23476 | BRD4 | bromodomain containing 4 | 1 |
| 9868 | hsa-mir-31 | 23476 | BRD4 | bromodomain containing 4 | 1 |
| 9869 | hsa-mir-194-2 | 23476 | BRD4 | bromodomain containing 4 | 1 |
| 9870 | hsa-mir-340 | 23476 | BRD4 | bromodomain containing 4 | 1 |
| 9871 | hsa-mir-338 | 23476 | BRD4 | bromodomain containing 4 | 1 |
| 9872 | hsa-mir-548b | 23476 | BRD4 | bromodomain containing 4 | 1 |
| 9873 | hsa-mir-548d-2 | 23476 | BRD4 | bromodomain containing 4 | 1 |
| 9874 | hsa-mir-449b | 23476 | BRD4 | bromodomain containing 4 | 1 |
| 9875 | hsa-mir-449c | 23476 | BRD4 | bromodomain containing 4 | 1 |
| 9876 | hsa-mir-3065 | 23476 | BRD4 | bromodomain containing 4 | 1 |
| 9877 | hsa-miR-10a | 9175 | MAP3K13 | mitogen-activated protein kinase kinase kinase 13 | 1 |
| 9878 | hsa-miR-139-5p | 9175 | MAP3K13 | mitogen-activated protein kinase kinase kinase 13 | 1 |
| 9879 | hsa-miR-152 | 9175 | MAP3K13 | mitogen-activated protein kinase kinase kinase 13 | 1 |
| 9880 | hsa-miR-26a | 9175 | MAP3K13 | mitogen-activated protein kinase kinase kinase 13 | 1 |
| 9881 | hsa-miR-26b | 9175 | MAP3K13 | mitogen-activated protein kinase kinase kinase 13 | 1 |
| 9882 | hsa-miR-31 | 9175 | MAP3K13 | mitogen-activated protein kinase kinase kinase 13 | 1 |
| 9883 | hsa-miR-338-5p | 9175 | MAP3K13 | mitogen-activated protein kinase kinase kinase 13 | 1 |
| 9884 | hsa-miR-340 | 9175 | MAP3K13 | mitogen-activated protein kinase kinase kinase 13 | 1 |
| 9885 | hsa-miR-423-5p | 9175 | MAP3K13 | mitogen-activated protein kinase kinase kinase 13 | 1 |
| 9886 | hsa-miR-449a | 9175 | MAP3K13 | mitogen-activated protein kinase kinase kinase 13 | 1 |
| 9887 | hsa-miR-499-5p | 9175 | MAP3K13 | mitogen-activated protein kinase kinase kinase 13 | 1 |
| 9888 | hsa-miR-548b-5p | 9175 | MAP3K13 | mitogen-activated protein kinase kinase kinase 13 | 1 |
| 9889 | hsa-miR-621 | 9175 | MAP3K13 | mitogen-activated protein kinase kinase kinase 13 | 1 |
| 9890 | hsa-miR-657 | 9175 | MAP3K13 | mitogen-activated protein kinase kinase kinase 13 | 1 |
| 9891 | hsa-miR-663 | 9175 | MAP3K13 | mitogen-activated protein kinase kinase kinase 13 | 1 |
| 9892 | hsa-miR-765 | 9175 | MAP3K13 | mitogen-activated protein kinase kinase kinase 13 | 1 |
| 9893 | hsa-miR-770-5p | 9175 | MAP3K13 | mitogen-activated protein kinase kinase kinase 13 | 1 |
| 9894 | hsa-miR-877 | 9175 | MAP3K13 | mitogen-activated protein kinase kinase kinase 13 | 1 |
| 9895 | hsa-mir-26a-1 | 9175 | MAP3K13 | mitogen-activated protein kinase kinase kinase 13 | 1 |
| 9896 | hsa-mir-26b | 9175 | MAP3K13 | mitogen-activated protein kinase kinase kinase 13 | 1 |
| 9897 | hsa-mir-31 | 9175 | MAP3K13 | mitogen-activated protein kinase kinase kinase 13 | 1 |
| 9898 | hsa-mir-194-2 | 9175 | MAP3K13 | mitogen-activated protein kinase kinase kinase 13 | 1 |
| 9899 | hsa-mir-340 | 9175 | MAP3K13 | mitogen-activated protein kinase kinase kinase 13 | 1 |
| 9900 | hsa-mir-338 | 9175 | MAP3K13 | mitogen-activated protein kinase kinase kinase 13 | 1 |
| 9901 | hsa-mir-548b | 9175 | MAP3K13 | mitogen-activated protein kinase kinase kinase 13 | 1 |
| 9902 | hsa-mir-548d-2 | 9175 | MAP3K13 | mitogen-activated protein kinase kinase kinase 13 | 1 |
| 9903 | hsa-mir-449b | 9175 | MAP3K13 | mitogen-activated protein kinase kinase kinase 13 | 1 |
| 9904 | hsa-mir-449c | 9175 | MAP3K13 | mitogen-activated protein kinase kinase kinase 13 | 1 |
| 9905 | hsa-mir-3065 | 9175 | MAP3K13 | mitogen-activated protein kinase kinase kinase 13 | 1 |
| 9906 | hsa-miR-620 | 338799 | LOC338799 | hypothetical LOC338799 | 1 |
| 9907 | hsa-miR-885-5p | 338799 | LOC338799 | hypothetical LOC338799 | 1 |
| 9908 | hsa-mir-499 | 338799 | LOC338799 | hypothetical LOC338799 | 1 |
| 9909 | hsa-mir-885 | 338799 | LOC338799 | hypothetical LOC338799 | 1 |
| 9910 | hsa-mir-2116 | 338799 | LOC338799 | hypothetical LOC338799 | 1 |
| 9911 | hsa-miR-10a | 128637 | TBC1D20 | TBC1 domain family, member 20 | 1 |
| 9912 | hsa-miR-139-5p | 128637 | TBC1D20 | TBC1 domain family, member 20 | 1 |
| 9913 | hsa-miR-26a | 128637 | TBC1D20 | TBC1 domain family, member 20 | 1 |
| 9914 | hsa-miR-26b | 128637 | TBC1D20 | TBC1 domain family, member 20 | 1 |
| 9915 | hsa-miR-338-5p | 128637 | TBC1D20 | TBC1 domain family, member 20 | 1 |
| 9916 | hsa-miR-340 | 128637 | TBC1D20 | TBC1 domain family, member 20 | 1 |
| 9917 | hsa-miR-449a | 128637 | TBC1D20 | TBC1 domain family, member 20 | 1 |
| 9918 | hsa-miR-499-5p | 128637 | TBC1D20 | TBC1 domain family, member 20 | 1 |
| 9919 | hsa-miR-548b-5p | 128637 | TBC1D20 | TBC1 domain family, member 20 | 1 |
| 9920 | hsa-miR-564 | 128637 | TBC1D20 | TBC1 domain family, member 20 | 1 |
| 9921 | hsa-miR-621 | 128637 | TBC1D20 | TBC1 domain family, member 20 | 1 |
| 9922 | hsa-miR-657 | 128637 | TBC1D20 | TBC1 domain family, member 20 | 1 |
| 9923 | hsa-miR-765 | 128637 | TBC1D20 | TBC1 domain family, member 20 | 1 |
| 9924 | hsa-miR-770-5p | 128637 | TBC1D20 | TBC1 domain family, member 20 | 1 |
| 9925 | hsa-miR-877 | 128637 | TBC1D20 | TBC1 domain family, member 20 | 1 |
| 9926 | hsa-mir-26a-1 | 128637 | TBC1D20 | TBC1 domain family, member 20 | 1 |
| 9927 | hsa-mir-26b | 128637 | TBC1D20 | TBC1 domain family, member 20 | 1 |
| 9928 | hsa-mir-194-2 | 128637 | TBC1D20 | TBC1 domain family, member 20 | 1 |
| 9929 | hsa-mir-340 | 128637 | TBC1D20 | TBC1 domain family, member 20 | 1 |
| 9930 | hsa-mir-338 | 128637 | TBC1D20 | TBC1 domain family, member 20 | 1 |
| 9931 | hsa-mir-548b | 128637 | TBC1D20 | TBC1 domain family, member 20 | 1 |
| 9932 | hsa-mir-548d-2 | 128637 | TBC1D20 | TBC1 domain family, member 20 | 1 |
| 9933 | hsa-mir-449b | 128637 | TBC1D20 | TBC1 domain family, member 20 | 1 |
| 9934 | hsa-mir-449c | 128637 | TBC1D20 | TBC1 domain family, member 20 | 1 |
| 9935 | hsa-mir-3065 | 128637 | TBC1D20 | TBC1 domain family, member 20 | 1 |
| 9936 | hsa-miR-10a | 84166 | NLRC5 | NLR family, CARD domain containing 5 | 1 |
| 9937 | hsa-miR-139-5p | 84166 | NLRC5 | NLR family, CARD domain containing 5 | 1 |
| 9938 | hsa-miR-26a | 84166 | NLRC5 | NLR family, CARD domain containing 5 | 1 |
| 9939 | hsa-miR-26b | 84166 | NLRC5 | NLR family, CARD domain containing 5 | 1 |
| 9940 | hsa-miR-338-5p | 84166 | NLRC5 | NLR family, CARD domain containing 5 | 1 |
| 9941 | hsa-miR-340 | 84166 | NLRC5 | NLR family, CARD domain containing 5 | 1 |
| 9942 | hsa-miR-449a | 84166 | NLRC5 | NLR family, CARD domain containing 5 | 1 |
| 9943 | hsa-miR-499-5p | 84166 | NLRC5 | NLR family, CARD domain containing 5 | 1 |
| 9944 | hsa-miR-548b-5p | 84166 | NLRC5 | NLR family, CARD domain containing 5 | 1 |
| 9945 | hsa-miR-564 | 84166 | NLRC5 | NLR family, CARD domain containing 5 | 1 |
| 9946 | hsa-miR-621 | 84166 | NLRC5 | NLR family, CARD domain containing 5 | 1 |
| 9947 | hsa-miR-657 | 84166 | NLRC5 | NLR family, CARD domain containing 5 | 1 |
| 9948 | hsa-miR-765 | 84166 | NLRC5 | NLR family, CARD domain containing 5 | 1 |
| 9949 | hsa-miR-770-5p | 84166 | NLRC5 | NLR family, CARD domain containing 5 | 1 |
| 9950 | hsa-miR-877 | 84166 | NLRC5 | NLR family, CARD domain containing 5 | 1 |
| 9951 | hsa-mir-26a-1 | 84166 | NLRC5 | NLR family, CARD domain containing 5 | 1 |
| 9952 | hsa-mir-26b | 84166 | NLRC5 | NLR family, CARD domain containing 5 | 1 |
| 9953 | hsa-mir-194-2 | 84166 | NLRC5 | NLR family, CARD domain containing 5 | 1 |
| 9954 | hsa-mir-340 | 84166 | NLRC5 | NLR family, CARD domain containing 5 | 1 |
| 9955 | hsa-mir-338 | 84166 | NLRC5 | NLR family, CARD domain containing 5 | 1 |
| 9956 | hsa-mir-548b | 84166 | NLRC5 | NLR family, CARD domain containing 5 | 1 |
| 9957 | hsa-mir-548d-2 | 84166 | NLRC5 | NLR family, CARD domain containing 5 | 1 |
| 9958 | hsa-mir-449b | 84166 | NLRC5 | NLR family, CARD domain containing 5 | 1 |
| 9959 | hsa-mir-449c | 84166 | NLRC5 | NLR family, CARD domain containing 5 | 1 |
| 9960 | hsa-mir-3065 | 84166 | NLRC5 | NLR family, CARD domain containing 5 | 1 |
| 9961 | hsa-miR-1 | 85462 | FHDC1 | FH2 domain containing 1 | 1 |
| 9962 | hsa-miR-133a | 85462 | FHDC1 | FH2 domain containing 1 | 1 |
| 9963 | hsa-miR-186 | 85462 | FHDC1 | FH2 domain containing 1 | 1 |
| 9964 | hsa-miR-326 | 85462 | FHDC1 | FH2 domain containing 1 | 1 |
| 9965 | hsa-miR-449b | 85462 | FHDC1 | FH2 domain containing 1 | 1 |
| 9966 | hsa-miR-551a | 85462 | FHDC1 | FH2 domain containing 1 | 1 |
| 9967 | hsa-miR-575 | 85462 | FHDC1 | FH2 domain containing 1 | 1 |
| 9968 | hsa-miR-579 | 85462 | FHDC1 | FH2 domain containing 1 | 1 |
| 9969 | hsa-miR-608 | 85462 | FHDC1 | FH2 domain containing 1 | 1 |
| 9970 | hsa-miR-620 | 85462 | FHDC1 | FH2 domain containing 1 | 1 |
| 9971 | hsa-miR-637 | 85462 | FHDC1 | FH2 domain containing 1 | 1 |
| 9972 | hsa-mir-22 | 85462 | FHDC1 | FH2 domain containing 1 | 1 |
| 9973 | hsa-mir-10a | 85462 | FHDC1 | FH2 domain containing 1 | 1 |
| 9974 | hsa-mir-186 | 85462 | FHDC1 | FH2 domain containing 1 | 1 |
| 9975 | hsa-mir-155 | 85462 | FHDC1 | FH2 domain containing 1 | 1 |
| 9976 | hsa-mir-423 | 85462 | FHDC1 | FH2 domain containing 1 | 1 |
| 9977 | hsa-mir-499 | 85462 | FHDC1 | FH2 domain containing 1 | 1 |
| 9978 | hsa-mir-550-1 | 85462 | FHDC1 | FH2 domain containing 1 | 1 |
| 9979 | hsa-mir-877 | 85462 | FHDC1 | FH2 domain containing 1 | 1 |
| 9980 | hsa-mir-2116 | 85462 | FHDC1 | FH2 domain containing 1 | 1 |
| 9981 | hsa-mir-3190 | 85462 | FHDC1 | FH2 domain containing 1 | 1 |
| 9982 | hsa-miR-33a | 10491 | CRTAP | cartilage associated protein | 1 |
| 9983 | hsa-miR-550 | 10491 | CRTAP | cartilage associated protein | 1 |
| 9984 | hsa-miR-551a | 10491 | CRTAP | cartilage associated protein | 1 |
| 9985 | hsa-miR-579 | 10491 | CRTAP | cartilage associated protein | 1 |
| 9986 | hsa-mir-423 | 10491 | CRTAP | cartilage associated protein | 1 |
| 9987 | hsa-mir-550-1 | 10491 | CRTAP | cartilage associated protein | 1 |
| 9988 | hsa-mir-2116 | 10491 | CRTAP | cartilage associated protein | 1 |
| 9989 | hsa-mir-3186 | 10491 | CRTAP | cartilage associated protein | 1 |
| 9990 | hsa-mir-3190 | 10491 | CRTAP | cartilage associated protein | 1 |
| 9991 | hsa-miR-1 | 284129 | SLC26A11 | solute carrier family 26, member 11 | 1 |
| 9992 | hsa-miR-133a | 284129 | SLC26A11 | solute carrier family 26, member 11 | 1 |
| 9993 | hsa-miR-186 | 284129 | SLC26A11 | solute carrier family 26, member 11 | 1 |
| 9994 | hsa-miR-208a | 284129 | SLC26A11 | solute carrier family 26, member 11 | 1 |
| 9995 | hsa-miR-326 | 284129 | SLC26A11 | solute carrier family 26, member 11 | 1 |
| 9996 | hsa-miR-449b | 284129 | SLC26A11 | solute carrier family 26, member 11 | 1 |
| 9997 | hsa-miR-575 | 284129 | SLC26A11 | solute carrier family 26, member 11 | 1 |
| 9998 | hsa-miR-579 | 284129 | SLC26A11 | solute carrier family 26, member 11 | 1 |
| 9999 | hsa-miR-608 | 284129 | SLC26A11 | solute carrier family 26, member 11 | 1 |
| 10000 | hsa-miR-620 | 284129 | SLC26A11 | solute carrier family 26, member 11 | 1 |
| 10001 | hsa-miR-885-5p | 284129 | SLC26A11 | solute carrier family 26, member 11 | 1 |
| 10002 | hsa-mir-22 | 284129 | SLC26A11 | solute carrier family 26, member 11 | 1 |
| 10003 | hsa-mir-10a | 284129 | SLC26A11 | solute carrier family 26, member 11 | 1 |
| 10004 | hsa-mir-186 | 284129 | SLC26A11 | solute carrier family 26, member 11 | 1 |
| 10005 | hsa-mir-155 | 284129 | SLC26A11 | solute carrier family 26, member 11 | 1 |
| 10006 | hsa-mir-499 | 284129 | SLC26A11 | solute carrier family 26, member 11 | 1 |
| 10007 | hsa-mir-885 | 284129 | SLC26A11 | solute carrier family 26, member 11 | 1 |
| 10008 | hsa-mir-877 | 284129 | SLC26A11 | solute carrier family 26, member 11 | 1 |
| 10009 | hsa-mir-2116 | 284129 | SLC26A11 | solute carrier family 26, member 11 | 1 |
| 10010 | hsa-mir-3190 | 284129 | SLC26A11 | solute carrier family 26, member 11 | 1 |
| 10011 | hsa-miR-10a | 79993 | ELOVL7 | ELOVL family member 7, elongation of long chain fatty acids (yeast) | 1 |
| 10012 | hsa-miR-1228\* | 79993 | ELOVL7 | ELOVL family member 7, elongation of long chain fatty acids (yeast) | 1 |
| 10013 | hsa-miR-139-5p | 79993 | ELOVL7 | ELOVL family member 7, elongation of long chain fatty acids (yeast) | 1 |
| 10014 | hsa-miR-152 | 79993 | ELOVL7 | ELOVL family member 7, elongation of long chain fatty acids (yeast) | 1 |
| 10015 | hsa-miR-26a | 79993 | ELOVL7 | ELOVL family member 7, elongation of long chain fatty acids (yeast) | 1 |
| 10016 | hsa-miR-26b | 79993 | ELOVL7 | ELOVL family member 7, elongation of long chain fatty acids (yeast) | 1 |
| 10017 | hsa-miR-31 | 79993 | ELOVL7 | ELOVL family member 7, elongation of long chain fatty acids (yeast) | 1 |
| 10018 | hsa-miR-338-5p | 79993 | ELOVL7 | ELOVL family member 7, elongation of long chain fatty acids (yeast) | 1 |
| 10019 | hsa-miR-340 | 79993 | ELOVL7 | ELOVL family member 7, elongation of long chain fatty acids (yeast) | 1 |
| 10020 | hsa-miR-423-5p | 79993 | ELOVL7 | ELOVL family member 7, elongation of long chain fatty acids (yeast) | 1 |
| 10021 | hsa-miR-449a | 79993 | ELOVL7 | ELOVL family member 7, elongation of long chain fatty acids (yeast) | 1 |
| 10022 | hsa-miR-499-5p | 79993 | ELOVL7 | ELOVL family member 7, elongation of long chain fatty acids (yeast) | 1 |
| 10023 | hsa-miR-548b-5p | 79993 | ELOVL7 | ELOVL family member 7, elongation of long chain fatty acids (yeast) | 1 |
| 10024 | hsa-miR-564 | 79993 | ELOVL7 | ELOVL family member 7, elongation of long chain fatty acids (yeast) | 1 |
| 10025 | hsa-miR-621 | 79993 | ELOVL7 | ELOVL family member 7, elongation of long chain fatty acids (yeast) | 1 |
| 10026 | hsa-miR-657 | 79993 | ELOVL7 | ELOVL family member 7, elongation of long chain fatty acids (yeast) | 1 |
| 10027 | hsa-miR-765 | 79993 | ELOVL7 | ELOVL family member 7, elongation of long chain fatty acids (yeast) | 1 |
| 10028 | hsa-miR-770-5p | 79993 | ELOVL7 | ELOVL family member 7, elongation of long chain fatty acids (yeast) | 1 |
| 10029 | hsa-miR-877 | 79993 | ELOVL7 | ELOVL family member 7, elongation of long chain fatty acids (yeast) | 1 |
| 10030 | hsa-mir-26a-1 | 79993 | ELOVL7 | ELOVL family member 7, elongation of long chain fatty acids (yeast) | 1 |
| 10031 | hsa-mir-26b | 79993 | ELOVL7 | ELOVL family member 7, elongation of long chain fatty acids (yeast) | 1 |
| 10032 | hsa-mir-31 | 79993 | ELOVL7 | ELOVL family member 7, elongation of long chain fatty acids (yeast) | 1 |
| 10033 | hsa-mir-194-2 | 79993 | ELOVL7 | ELOVL family member 7, elongation of long chain fatty acids (yeast) | 1 |
| 10034 | hsa-mir-340 | 79993 | ELOVL7 | ELOVL family member 7, elongation of long chain fatty acids (yeast) | 1 |
| 10035 | hsa-mir-338 | 79993 | ELOVL7 | ELOVL family member 7, elongation of long chain fatty acids (yeast) | 1 |
| 10036 | hsa-mir-548b | 79993 | ELOVL7 | ELOVL family member 7, elongation of long chain fatty acids (yeast) | 1 |
| 10037 | hsa-mir-449b | 79993 | ELOVL7 | ELOVL family member 7, elongation of long chain fatty acids (yeast) | 1 |
| 10038 | hsa-mir-449c | 79993 | ELOVL7 | ELOVL family member 7, elongation of long chain fatty acids (yeast) | 1 |
| 10039 | hsa-mir-3065 | 79993 | ELOVL7 | ELOVL family member 7, elongation of long chain fatty acids (yeast) | 1 |
| 10040 | hsa-miR-10a | 145864 | HAPLN3 | hyaluronan and proteoglycan link protein 3 | 1 |
| 10041 | hsa-miR-152 | 145864 | HAPLN3 | hyaluronan and proteoglycan link protein 3 | 1 |
| 10042 | hsa-miR-26a | 145864 | HAPLN3 | hyaluronan and proteoglycan link protein 3 | 1 |
| 10043 | hsa-miR-26b | 145864 | HAPLN3 | hyaluronan and proteoglycan link protein 3 | 1 |
| 10044 | hsa-miR-31 | 145864 | HAPLN3 | hyaluronan and proteoglycan link protein 3 | 1 |
| 10045 | hsa-miR-340 | 145864 | HAPLN3 | hyaluronan and proteoglycan link protein 3 | 1 |
| 10046 | hsa-miR-449a | 145864 | HAPLN3 | hyaluronan and proteoglycan link protein 3 | 1 |
| 10047 | hsa-miR-490-5p | 145864 | HAPLN3 | hyaluronan and proteoglycan link protein 3 | 1 |
| 10048 | hsa-miR-499-5p | 145864 | HAPLN3 | hyaluronan and proteoglycan link protein 3 | 1 |
| 10049 | hsa-miR-504 | 145864 | HAPLN3 | hyaluronan and proteoglycan link protein 3 | 1 |
| 10050 | hsa-miR-621 | 145864 | HAPLN3 | hyaluronan and proteoglycan link protein 3 | 1 |
| 10051 | hsa-miR-653 | 145864 | HAPLN3 | hyaluronan and proteoglycan link protein 3 | 1 |
| 10052 | hsa-miR-765 | 145864 | HAPLN3 | hyaluronan and proteoglycan link protein 3 | 1 |
| 10053 | hsa-miR-770-5p | 145864 | HAPLN3 | hyaluronan and proteoglycan link protein 3 | 1 |
| 10054 | hsa-miR-877 | 145864 | HAPLN3 | hyaluronan and proteoglycan link protein 3 | 1 |
| 10055 | hsa-mir-26a-1 | 145864 | HAPLN3 | hyaluronan and proteoglycan link protein 3 | 1 |
| 10056 | hsa-mir-26b | 145864 | HAPLN3 | hyaluronan and proteoglycan link protein 3 | 1 |
| 10057 | hsa-mir-31 | 145864 | HAPLN3 | hyaluronan and proteoglycan link protein 3 | 1 |
| 10058 | hsa-mir-194-2 | 145864 | HAPLN3 | hyaluronan and proteoglycan link protein 3 | 1 |
| 10059 | hsa-mir-340 | 145864 | HAPLN3 | hyaluronan and proteoglycan link protein 3 | 1 |
| 10060 | hsa-mir-490 | 145864 | HAPLN3 | hyaluronan and proteoglycan link protein 3 | 1 |
| 10061 | hsa-mir-449b | 145864 | HAPLN3 | hyaluronan and proteoglycan link protein 3 | 1 |
| 10062 | hsa-mir-449c | 145864 | HAPLN3 | hyaluronan and proteoglycan link protein 3 | 1 |
| 10063 | hsa-miR-10a | 57801 | HES4 | hairy and enhancer of split 4 (Drosophila) | 1 |
| 10064 | hsa-miR-152 | 57801 | HES4 | hairy and enhancer of split 4 (Drosophila) | 1 |
| 10065 | hsa-miR-26a | 57801 | HES4 | hairy and enhancer of split 4 (Drosophila) | 1 |
| 10066 | hsa-miR-26b | 57801 | HES4 | hairy and enhancer of split 4 (Drosophila) | 1 |
| 10067 | hsa-miR-31 | 57801 | HES4 | hairy and enhancer of split 4 (Drosophila) | 1 |
| 10068 | hsa-miR-338-5p | 57801 | HES4 | hairy and enhancer of split 4 (Drosophila) | 1 |
| 10069 | hsa-miR-340 | 57801 | HES4 | hairy and enhancer of split 4 (Drosophila) | 1 |
| 10070 | hsa-miR-423-5p | 57801 | HES4 | hairy and enhancer of split 4 (Drosophila) | 1 |
| 10071 | hsa-miR-449a | 57801 | HES4 | hairy and enhancer of split 4 (Drosophila) | 1 |
| 10072 | hsa-miR-499-5p | 57801 | HES4 | hairy and enhancer of split 4 (Drosophila) | 1 |
| 10073 | hsa-miR-564 | 57801 | HES4 | hairy and enhancer of split 4 (Drosophila) | 1 |
| 10074 | hsa-miR-621 | 57801 | HES4 | hairy and enhancer of split 4 (Drosophila) | 1 |
| 10075 | hsa-miR-634 | 57801 | HES4 | hairy and enhancer of split 4 (Drosophila) | 1 |
| 10076 | hsa-miR-644 | 57801 | HES4 | hairy and enhancer of split 4 (Drosophila) | 1 |
| 10077 | hsa-miR-657 | 57801 | HES4 | hairy and enhancer of split 4 (Drosophila) | 1 |
| 10078 | hsa-miR-765 | 57801 | HES4 | hairy and enhancer of split 4 (Drosophila) | 1 |
| 10079 | hsa-miR-770-5p | 57801 | HES4 | hairy and enhancer of split 4 (Drosophila) | 1 |
| 10080 | hsa-mir-26a-1 | 57801 | HES4 | hairy and enhancer of split 4 (Drosophila) | 1 |
| 10081 | hsa-mir-26b | 57801 | HES4 | hairy and enhancer of split 4 (Drosophila) | 1 |
| 10082 | hsa-mir-31 | 57801 | HES4 | hairy and enhancer of split 4 (Drosophila) | 1 |
| 10083 | hsa-mir-194-2 | 57801 | HES4 | hairy and enhancer of split 4 (Drosophila) | 1 |
| 10084 | hsa-mir-340 | 57801 | HES4 | hairy and enhancer of split 4 (Drosophila) | 1 |
| 10085 | hsa-mir-338 | 57801 | HES4 | hairy and enhancer of split 4 (Drosophila) | 1 |
| 10086 | hsa-mir-449b | 57801 | HES4 | hairy and enhancer of split 4 (Drosophila) | 1 |
| 10087 | hsa-mir-449c | 57801 | HES4 | hairy and enhancer of split 4 (Drosophila) | 1 |
| 10088 | hsa-mir-3065 | 57801 | HES4 | hairy and enhancer of split 4 (Drosophila) | 1 |
| 10089 | hsa-miR-1 | 9185 | REPS2 | RALBP1 associated Eps domain containing 2 | 1 |
| 10090 | hsa-miR-133a | 9185 | REPS2 | RALBP1 associated Eps domain containing 2 | 1 |
| 10091 | hsa-miR-186 | 9185 | REPS2 | RALBP1 associated Eps domain containing 2 | 1 |
| 10092 | hsa-miR-326 | 9185 | REPS2 | RALBP1 associated Eps domain containing 2 | 1 |
| 10093 | hsa-miR-449b | 9185 | REPS2 | RALBP1 associated Eps domain containing 2 | 1 |
| 10094 | hsa-miR-489 | 9185 | REPS2 | RALBP1 associated Eps domain containing 2 | 1 |
| 10095 | hsa-miR-562 | 9185 | REPS2 | RALBP1 associated Eps domain containing 2 | 1 |
| 10096 | hsa-miR-575 | 9185 | REPS2 | RALBP1 associated Eps domain containing 2 | 1 |
| 10097 | hsa-miR-579 | 9185 | REPS2 | RALBP1 associated Eps domain containing 2 | 1 |
| 10098 | hsa-miR-608 | 9185 | REPS2 | RALBP1 associated Eps domain containing 2 | 1 |
| 10099 | hsa-miR-620 | 9185 | REPS2 | RALBP1 associated Eps domain containing 2 | 1 |
| 10100 | hsa-miR-637 | 9185 | REPS2 | RALBP1 associated Eps domain containing 2 | 1 |
| 10101 | hsa-miR-885-5p | 9185 | REPS2 | RALBP1 associated Eps domain containing 2 | 1 |
| 10102 | hsa-mir-22 | 9185 | REPS2 | RALBP1 associated Eps domain containing 2 | 1 |
| 10103 | hsa-mir-10a | 9185 | REPS2 | RALBP1 associated Eps domain containing 2 | 1 |
| 10104 | hsa-mir-186 | 9185 | REPS2 | RALBP1 associated Eps domain containing 2 | 1 |
| 10105 | hsa-mir-155 | 9185 | REPS2 | RALBP1 associated Eps domain containing 2 | 1 |
| 10106 | hsa-mir-423 | 9185 | REPS2 | RALBP1 associated Eps domain containing 2 | 1 |
| 10107 | hsa-mir-499 | 9185 | REPS2 | RALBP1 associated Eps domain containing 2 | 1 |
| 10108 | hsa-mir-885 | 9185 | REPS2 | RALBP1 associated Eps domain containing 2 | 1 |
| 10109 | hsa-mir-877 | 9185 | REPS2 | RALBP1 associated Eps domain containing 2 | 1 |
| 10110 | hsa-mir-3190 | 9185 | REPS2 | RALBP1 associated Eps domain containing 2 | 1 |
| 10111 | hsa-miR-1 | 94234 | FOXQ1 | forkhead box Q1 | 1 |
| 10112 | hsa-miR-133a | 94234 | FOXQ1 | forkhead box Q1 | 1 |
| 10113 | hsa-miR-186 | 94234 | FOXQ1 | forkhead box Q1 | 1 |
| 10114 | hsa-miR-195 | 94234 | FOXQ1 | forkhead box Q1 | 1 |
| 10115 | hsa-miR-326 | 94234 | FOXQ1 | forkhead box Q1 | 1 |
| 10116 | hsa-miR-449b | 94234 | FOXQ1 | forkhead box Q1 | 1 |
| 10117 | hsa-miR-497 | 94234 | FOXQ1 | forkhead box Q1 | 1 |
| 10118 | hsa-miR-550 | 94234 | FOXQ1 | forkhead box Q1 | 1 |
| 10119 | hsa-miR-551a | 94234 | FOXQ1 | forkhead box Q1 | 1 |
| 10120 | hsa-miR-575 | 94234 | FOXQ1 | forkhead box Q1 | 1 |
| 10121 | hsa-miR-579 | 94234 | FOXQ1 | forkhead box Q1 | 1 |
| 10122 | hsa-miR-608 | 94234 | FOXQ1 | forkhead box Q1 | 1 |
| 10123 | hsa-miR-620 | 94234 | FOXQ1 | forkhead box Q1 | 1 |
| 10124 | hsa-mir-22 | 94234 | FOXQ1 | forkhead box Q1 | 1 |
| 10125 | hsa-mir-10a | 94234 | FOXQ1 | forkhead box Q1 | 1 |
| 10126 | hsa-mir-186 | 94234 | FOXQ1 | forkhead box Q1 | 1 |
| 10127 | hsa-mir-155 | 94234 | FOXQ1 | forkhead box Q1 | 1 |
| 10128 | hsa-mir-499 | 94234 | FOXQ1 | forkhead box Q1 | 1 |
| 10129 | hsa-mir-550-1 | 94234 | FOXQ1 | forkhead box Q1 | 1 |
| 10130 | hsa-mir-877 | 94234 | FOXQ1 | forkhead box Q1 | 1 |
| 10131 | hsa-mir-2116 | 94234 | FOXQ1 | forkhead box Q1 | 1 |
| 10132 | hsa-mir-3190 | 94234 | FOXQ1 | forkhead box Q1 | 1 |
| 10133 | hsa-miR-1 | 138050 | HGSNAT | heparan-alpha-glucosaminide N-acetyltransferase | 1 |
| 10134 | hsa-miR-133a | 138050 | HGSNAT | heparan-alpha-glucosaminide N-acetyltransferase | 1 |
| 10135 | hsa-miR-186 | 138050 | HGSNAT | heparan-alpha-glucosaminide N-acetyltransferase | 1 |
| 10136 | hsa-miR-208a | 138050 | HGSNAT | heparan-alpha-glucosaminide N-acetyltransferase | 1 |
| 10137 | hsa-miR-326 | 138050 | HGSNAT | heparan-alpha-glucosaminide N-acetyltransferase | 1 |
| 10138 | hsa-miR-449b | 138050 | HGSNAT | heparan-alpha-glucosaminide N-acetyltransferase | 1 |
| 10139 | hsa-miR-575 | 138050 | HGSNAT | heparan-alpha-glucosaminide N-acetyltransferase | 1 |
| 10140 | hsa-miR-579 | 138050 | HGSNAT | heparan-alpha-glucosaminide N-acetyltransferase | 1 |
| 10141 | hsa-miR-608 | 138050 | HGSNAT | heparan-alpha-glucosaminide N-acetyltransferase | 1 |
| 10142 | hsa-miR-620 | 138050 | HGSNAT | heparan-alpha-glucosaminide N-acetyltransferase | 1 |
| 10143 | hsa-miR-637 | 138050 | HGSNAT | heparan-alpha-glucosaminide N-acetyltransferase | 1 |
| 10144 | hsa-miR-885-5p | 138050 | HGSNAT | heparan-alpha-glucosaminide N-acetyltransferase | 1 |
| 10145 | hsa-mir-22 | 138050 | HGSNAT | heparan-alpha-glucosaminide N-acetyltransferase | 1 |
| 10146 | hsa-mir-10a | 138050 | HGSNAT | heparan-alpha-glucosaminide N-acetyltransferase | 1 |
| 10147 | hsa-mir-186 | 138050 | HGSNAT | heparan-alpha-glucosaminide N-acetyltransferase | 1 |
| 10148 | hsa-mir-155 | 138050 | HGSNAT | heparan-alpha-glucosaminide N-acetyltransferase | 1 |
| 10149 | hsa-mir-423 | 138050 | HGSNAT | heparan-alpha-glucosaminide N-acetyltransferase | 1 |
| 10150 | hsa-mir-499 | 138050 | HGSNAT | heparan-alpha-glucosaminide N-acetyltransferase | 1 |
| 10151 | hsa-mir-885 | 138050 | HGSNAT | heparan-alpha-glucosaminide N-acetyltransferase | 1 |
| 10152 | hsa-mir-877 | 138050 | HGSNAT | heparan-alpha-glucosaminide N-acetyltransferase | 1 |
| 10153 | hsa-mir-2116 | 138050 | HGSNAT | heparan-alpha-glucosaminide N-acetyltransferase | 1 |
| 10154 | hsa-mir-3190 | 138050 | HGSNAT | heparan-alpha-glucosaminide N-acetyltransferase | 1 |
| 10155 | hsa-miR-139-5p | 5618 | PRLR | prolactin receptor | 1 |
| 10156 | hsa-miR-152 | 5618 | PRLR | prolactin receptor | 1 |
| 10157 | hsa-miR-26b | 5618 | PRLR | prolactin receptor | 1 |
| 10158 | hsa-miR-31 | 5618 | PRLR | prolactin receptor | 1 |
| 10159 | hsa-miR-338-5p | 5618 | PRLR | prolactin receptor | 1 |
| 10160 | hsa-miR-340 | 5618 | PRLR | prolactin receptor | 1 |
| 10161 | hsa-miR-383 | 5618 | PRLR | prolactin receptor | 1 |
| 10162 | hsa-miR-449a | 5618 | PRLR | prolactin receptor | 1 |
| 10163 | hsa-miR-548b-5p | 5618 | PRLR | prolactin receptor | 1 |
| 10164 | hsa-miR-621 | 5618 | PRLR | prolactin receptor | 1 |
| 10165 | hsa-miR-657 | 5618 | PRLR | prolactin receptor | 1 |
| 10166 | hsa-miR-765 | 5618 | PRLR | prolactin receptor | 1 |
| 10167 | hsa-miR-770-5p | 5618 | PRLR | prolactin receptor | 1 |
| 10168 | hsa-miR-877 | 5618 | PRLR | prolactin receptor | 1 |
| 10169 | hsa-mir-26a-1 | 5618 | PRLR | prolactin receptor | 1 |
| 10170 | hsa-mir-26b | 5618 | PRLR | prolactin receptor | 1 |
| 10171 | hsa-mir-31 | 5618 | PRLR | prolactin receptor | 1 |
| 10172 | hsa-mir-194-2 | 5618 | PRLR | prolactin receptor | 1 |
| 10173 | hsa-mir-340 | 5618 | PRLR | prolactin receptor | 1 |
| 10174 | hsa-mir-338 | 5618 | PRLR | prolactin receptor | 1 |
| 10175 | hsa-mir-548b | 5618 | PRLR | prolactin receptor | 1 |
| 10176 | hsa-mir-449b | 5618 | PRLR | prolactin receptor | 1 |
| 10177 | hsa-mir-449c | 5618 | PRLR | prolactin receptor | 1 |
| 10178 | hsa-mir-3065 | 5618 | PRLR | prolactin receptor | 1 |
| 10179 | hsa-miR-1 | 91544 | UBXN11 | UBX domain protein 11 | 1 |
| 10180 | hsa-miR-133a | 91544 | UBXN11 | UBX domain protein 11 | 1 |
| 10181 | hsa-miR-186 | 91544 | UBXN11 | UBX domain protein 11 | 1 |
| 10182 | hsa-miR-326 | 91544 | UBXN11 | UBX domain protein 11 | 1 |
| 10183 | hsa-miR-449b | 91544 | UBXN11 | UBX domain protein 11 | 1 |
| 10184 | hsa-miR-551a | 91544 | UBXN11 | UBX domain protein 11 | 1 |
| 10185 | hsa-miR-562 | 91544 | UBXN11 | UBX domain protein 11 | 1 |
| 10186 | hsa-miR-575 | 91544 | UBXN11 | UBX domain protein 11 | 1 |
| 10187 | hsa-miR-608 | 91544 | UBXN11 | UBX domain protein 11 | 1 |
| 10188 | hsa-miR-637 | 91544 | UBXN11 | UBX domain protein 11 | 1 |
| 10189 | hsa-mir-22 | 91544 | UBXN11 | UBX domain protein 11 | 1 |
| 10190 | hsa-mir-10a | 91544 | UBXN11 | UBX domain protein 11 | 1 |
| 10191 | hsa-mir-186 | 91544 | UBXN11 | UBX domain protein 11 | 1 |
| 10192 | hsa-mir-155 | 91544 | UBXN11 | UBX domain protein 11 | 1 |
| 10193 | hsa-mir-877 | 91544 | UBXN11 | UBX domain protein 11 | 1 |
| 10194 | hsa-miR-1 | 65975 | STK33 | serine/threonine kinase 33 | 1 |
| 10195 | hsa-miR-133a | 65975 | STK33 | serine/threonine kinase 33 | 1 |
| 10196 | hsa-miR-186 | 65975 | STK33 | serine/threonine kinase 33 | 1 |
| 10197 | hsa-miR-208a | 65975 | STK33 | serine/threonine kinase 33 | 1 |
| 10198 | hsa-miR-326 | 65975 | STK33 | serine/threonine kinase 33 | 1 |
| 10199 | hsa-miR-449b | 65975 | STK33 | serine/threonine kinase 33 | 1 |
| 10200 | hsa-miR-550 | 65975 | STK33 | serine/threonine kinase 33 | 1 |
| 10201 | hsa-miR-575 | 65975 | STK33 | serine/threonine kinase 33 | 1 |
| 10202 | hsa-miR-579 | 65975 | STK33 | serine/threonine kinase 33 | 1 |
| 10203 | hsa-miR-608 | 65975 | STK33 | serine/threonine kinase 33 | 1 |
| 10204 | hsa-miR-620 | 65975 | STK33 | serine/threonine kinase 33 | 1 |
| 10205 | hsa-miR-885-5p | 65975 | STK33 | serine/threonine kinase 33 | 1 |
| 10206 | hsa-let-7a-3 | 65975 | STK33 | serine/threonine kinase 33 | 1 |
| 10207 | hsa-let-7b | 65975 | STK33 | serine/threonine kinase 33 | 1 |
| 10208 | hsa-mir-22 | 65975 | STK33 | serine/threonine kinase 33 | 1 |
| 10209 | hsa-mir-10a | 65975 | STK33 | serine/threonine kinase 33 | 1 |
| 10210 | hsa-mir-186 | 65975 | STK33 | serine/threonine kinase 33 | 1 |
| 10211 | hsa-mir-155 | 65975 | STK33 | serine/threonine kinase 33 | 1 |
| 10212 | hsa-mir-423 | 65975 | STK33 | serine/threonine kinase 33 | 1 |
| 10213 | hsa-mir-499 | 65975 | STK33 | serine/threonine kinase 33 | 1 |
| 10214 | hsa-mir-550-1 | 65975 | STK33 | serine/threonine kinase 33 | 1 |
| 10215 | hsa-mir-885 | 65975 | STK33 | serine/threonine kinase 33 | 1 |
| 10216 | hsa-mir-877 | 65975 | STK33 | serine/threonine kinase 33 | 1 |
| 10217 | hsa-mir-2116 | 65975 | STK33 | serine/threonine kinase 33 | 1 |
| 10218 | hsa-mir-3190 | 65975 | STK33 | serine/threonine kinase 33 | 1 |
| 10219 | hsa-miR-1 | 120425 | AMICA1 | adhesion molecule, interacts with CXADR antigen 1 | 1 |
| 10220 | hsa-miR-133a | 120425 | AMICA1 | adhesion molecule, interacts with CXADR antigen 1 | 1 |
| 10221 | hsa-miR-186 | 120425 | AMICA1 | adhesion molecule, interacts with CXADR antigen 1 | 1 |
| 10222 | hsa-miR-326 | 120425 | AMICA1 | adhesion molecule, interacts with CXADR antigen 1 | 1 |
| 10223 | hsa-miR-449b | 120425 | AMICA1 | adhesion molecule, interacts with CXADR antigen 1 | 1 |
| 10224 | hsa-miR-489 | 120425 | AMICA1 | adhesion molecule, interacts with CXADR antigen 1 | 1 |
| 10225 | hsa-miR-562 | 120425 | AMICA1 | adhesion molecule, interacts with CXADR antigen 1 | 1 |
| 10226 | hsa-miR-575 | 120425 | AMICA1 | adhesion molecule, interacts with CXADR antigen 1 | 1 |
| 10227 | hsa-miR-579 | 120425 | AMICA1 | adhesion molecule, interacts with CXADR antigen 1 | 1 |
| 10228 | hsa-miR-608 | 120425 | AMICA1 | adhesion molecule, interacts with CXADR antigen 1 | 1 |
| 10229 | hsa-miR-637 | 120425 | AMICA1 | adhesion molecule, interacts with CXADR antigen 1 | 1 |
| 10230 | hsa-mir-22 | 120425 | AMICA1 | adhesion molecule, interacts with CXADR antigen 1 | 1 |
| 10231 | hsa-mir-10a | 120425 | AMICA1 | adhesion molecule, interacts with CXADR antigen 1 | 1 |
| 10232 | hsa-mir-186 | 120425 | AMICA1 | adhesion molecule, interacts with CXADR antigen 1 | 1 |
| 10233 | hsa-mir-155 | 120425 | AMICA1 | adhesion molecule, interacts with CXADR antigen 1 | 1 |
| 10234 | hsa-mir-499 | 120425 | AMICA1 | adhesion molecule, interacts with CXADR antigen 1 | 1 |
| 10235 | hsa-mir-877 | 120425 | AMICA1 | adhesion molecule, interacts with CXADR antigen 1 | 1 |
| 10236 | hsa-mir-3190 | 120425 | AMICA1 | adhesion molecule, interacts with CXADR antigen 1 | 1 |
| 10248 | hsa-miR-564 | 441168 | FAM26F | family with sequence similarity 26, member F | 1 |
| 10265 | hsa-miR-10a | 4793 | NFKBIB | nuclear factor of kappa light polypeptide gene enhancer in B-cells inhibitor, beta | 1 |
| 10266 | hsa-miR-1228\* | 4793 | NFKBIB | nuclear factor of kappa light polypeptide gene enhancer in B-cells inhibitor, beta | 1 |
| 10267 | hsa-miR-139-5p | 4793 | NFKBIB | nuclear factor of kappa light polypeptide gene enhancer in B-cells inhibitor, beta | 1 |
| 10268 | hsa-miR-152 | 4793 | NFKBIB | nuclear factor of kappa light polypeptide gene enhancer in B-cells inhibitor, beta | 1 |
| 10269 | hsa-miR-26a | 4793 | NFKBIB | nuclear factor of kappa light polypeptide gene enhancer in B-cells inhibitor, beta | 1 |
| 10270 | hsa-miR-26b | 4793 | NFKBIB | nuclear factor of kappa light polypeptide gene enhancer in B-cells inhibitor, beta | 1 |
| 10271 | hsa-miR-31 | 4793 | NFKBIB | nuclear factor of kappa light polypeptide gene enhancer in B-cells inhibitor, beta | 1 |
| 10272 | hsa-miR-338-5p | 4793 | NFKBIB | nuclear factor of kappa light polypeptide gene enhancer in B-cells inhibitor, beta | 1 |
| 10273 | hsa-miR-340 | 4793 | NFKBIB | nuclear factor of kappa light polypeptide gene enhancer in B-cells inhibitor, beta | 1 |
| 10274 | hsa-miR-423-5p | 4793 | NFKBIB | nuclear factor of kappa light polypeptide gene enhancer in B-cells inhibitor, beta | 1 |
| 10275 | hsa-miR-449a | 4793 | NFKBIB | nuclear factor of kappa light polypeptide gene enhancer in B-cells inhibitor, beta | 1 |
| 10276 | hsa-miR-499-5p | 4793 | NFKBIB | nuclear factor of kappa light polypeptide gene enhancer in B-cells inhibitor, beta | 1 |
| 10277 | hsa-miR-548b-5p | 4793 | NFKBIB | nuclear factor of kappa light polypeptide gene enhancer in B-cells inhibitor, beta | 1 |
| 10278 | hsa-miR-564 | 4793 | NFKBIB | nuclear factor of kappa light polypeptide gene enhancer in B-cells inhibitor, beta | 1 |
| 10279 | hsa-miR-621 | 4793 | NFKBIB | nuclear factor of kappa light polypeptide gene enhancer in B-cells inhibitor, beta | 1 |
| 10280 | hsa-miR-657 | 4793 | NFKBIB | nuclear factor of kappa light polypeptide gene enhancer in B-cells inhibitor, beta | 1 |
| 10281 | hsa-miR-765 | 4793 | NFKBIB | nuclear factor of kappa light polypeptide gene enhancer in B-cells inhibitor, beta | 1 |
| 10282 | hsa-miR-770-5p | 4793 | NFKBIB | nuclear factor of kappa light polypeptide gene enhancer in B-cells inhibitor, beta | 1 |
| 10283 | hsa-miR-877 | 4793 | NFKBIB | nuclear factor of kappa light polypeptide gene enhancer in B-cells inhibitor, beta | 1 |
| 10284 | hsa-mir-26a-1 | 4793 | NFKBIB | nuclear factor of kappa light polypeptide gene enhancer in B-cells inhibitor, beta | 1 |
| 10285 | hsa-mir-26b | 4793 | NFKBIB | nuclear factor of kappa light polypeptide gene enhancer in B-cells inhibitor, beta | 1 |
| 10286 | hsa-mir-31 | 4793 | NFKBIB | nuclear factor of kappa light polypeptide gene enhancer in B-cells inhibitor, beta | 1 |
| 10287 | hsa-mir-194-2 | 4793 | NFKBIB | nuclear factor of kappa light polypeptide gene enhancer in B-cells inhibitor, beta | 1 |
| 10288 | hsa-mir-340 | 4793 | NFKBIB | nuclear factor of kappa light polypeptide gene enhancer in B-cells inhibitor, beta | 1 |
| 10289 | hsa-mir-338 | 4793 | NFKBIB | nuclear factor of kappa light polypeptide gene enhancer in B-cells inhibitor, beta | 1 |
| 10290 | hsa-mir-548b | 4793 | NFKBIB | nuclear factor of kappa light polypeptide gene enhancer in B-cells inhibitor, beta | 1 |
| 10291 | hsa-mir-548d-2 | 4793 | NFKBIB | nuclear factor of kappa light polypeptide gene enhancer in B-cells inhibitor, beta | 1 |
| 10292 | hsa-mir-449b | 4793 | NFKBIB | nuclear factor of kappa light polypeptide gene enhancer in B-cells inhibitor, beta | 1 |
| 10293 | hsa-mir-449c | 4793 | NFKBIB | nuclear factor of kappa light polypeptide gene enhancer in B-cells inhibitor, beta | 1 |
| 10294 | hsa-mir-3065 | 4793 | NFKBIB | nuclear factor of kappa light polypeptide gene enhancer in B-cells inhibitor, beta | 1 |
| 10295 | hsa-miR-10a | 116071 | BATF2 | basic leucine zipper transcription factor, ATF-like 2 | 1 |
| 10296 | hsa-miR-139-5p | 116071 | BATF2 | basic leucine zipper transcription factor, ATF-like 2 | 1 |
| 10297 | hsa-miR-152 | 116071 | BATF2 | basic leucine zipper transcription factor, ATF-like 2 | 1 |
| 10298 | hsa-miR-26a | 116071 | BATF2 | basic leucine zipper transcription factor, ATF-like 2 | 1 |
| 10299 | hsa-miR-26b | 116071 | BATF2 | basic leucine zipper transcription factor, ATF-like 2 | 1 |
| 10300 | hsa-miR-31 | 116071 | BATF2 | basic leucine zipper transcription factor, ATF-like 2 | 1 |
| 10301 | hsa-miR-338-5p | 116071 | BATF2 | basic leucine zipper transcription factor, ATF-like 2 | 1 |
| 10302 | hsa-miR-340 | 116071 | BATF2 | basic leucine zipper transcription factor, ATF-like 2 | 1 |
| 10303 | hsa-miR-423-5p | 116071 | BATF2 | basic leucine zipper transcription factor, ATF-like 2 | 1 |
| 10304 | hsa-miR-449a | 116071 | BATF2 | basic leucine zipper transcription factor, ATF-like 2 | 1 |
| 10305 | hsa-miR-499-5p | 116071 | BATF2 | basic leucine zipper transcription factor, ATF-like 2 | 1 |
| 10306 | hsa-miR-548b-5p | 116071 | BATF2 | basic leucine zipper transcription factor, ATF-like 2 | 1 |
| 10307 | hsa-miR-564 | 116071 | BATF2 | basic leucine zipper transcription factor, ATF-like 2 | 1 |
| 10308 | hsa-miR-621 | 116071 | BATF2 | basic leucine zipper transcription factor, ATF-like 2 | 1 |
| 10309 | hsa-miR-657 | 116071 | BATF2 | basic leucine zipper transcription factor, ATF-like 2 | 1 |
| 10310 | hsa-miR-765 | 116071 | BATF2 | basic leucine zipper transcription factor, ATF-like 2 | 1 |
| 10311 | hsa-miR-770-5p | 116071 | BATF2 | basic leucine zipper transcription factor, ATF-like 2 | 1 |
| 10312 | hsa-miR-877 | 116071 | BATF2 | basic leucine zipper transcription factor, ATF-like 2 | 1 |
| 10313 | hsa-mir-26a-1 | 116071 | BATF2 | basic leucine zipper transcription factor, ATF-like 2 | 1 |
| 10314 | hsa-mir-26b | 116071 | BATF2 | basic leucine zipper transcription factor, ATF-like 2 | 1 |
| 10315 | hsa-mir-31 | 116071 | BATF2 | basic leucine zipper transcription factor, ATF-like 2 | 1 |
| 10316 | hsa-mir-194-2 | 116071 | BATF2 | basic leucine zipper transcription factor, ATF-like 2 | 1 |
| 10317 | hsa-mir-340 | 116071 | BATF2 | basic leucine zipper transcription factor, ATF-like 2 | 1 |
| 10318 | hsa-mir-338 | 116071 | BATF2 | basic leucine zipper transcription factor, ATF-like 2 | 1 |
| 10319 | hsa-mir-548b | 116071 | BATF2 | basic leucine zipper transcription factor, ATF-like 2 | 1 |
| 10320 | hsa-mir-548d-2 | 116071 | BATF2 | basic leucine zipper transcription factor, ATF-like 2 | 1 |
| 10321 | hsa-mir-449b | 116071 | BATF2 | basic leucine zipper transcription factor, ATF-like 2 | 1 |
| 10322 | hsa-mir-449c | 116071 | BATF2 | basic leucine zipper transcription factor, ATF-like 2 | 1 |
| 10323 | hsa-mir-3065 | 116071 | BATF2 | basic leucine zipper transcription factor, ATF-like 2 | 1 |
| 10324 | hsa-miR-1 | 117248 | GALNTL2 | UDP-N-acetyl-alpha-D-galactosamine:polypeptide N-acetylgalactosaminyltransferase-like 2 | 1 |
| 10325 | hsa-miR-133a | 117248 | GALNTL2 | UDP-N-acetyl-alpha-D-galactosamine:polypeptide N-acetylgalactosaminyltransferase-like 2 | 1 |
| 10326 | hsa-miR-186 | 117248 | GALNTL2 | UDP-N-acetyl-alpha-D-galactosamine:polypeptide N-acetylgalactosaminyltransferase-like 2 | 1 |
| 10327 | hsa-miR-208a | 117248 | GALNTL2 | UDP-N-acetyl-alpha-D-galactosamine:polypeptide N-acetylgalactosaminyltransferase-like 2 | 1 |
| 10328 | hsa-miR-449b | 117248 | GALNTL2 | UDP-N-acetyl-alpha-D-galactosamine:polypeptide N-acetylgalactosaminyltransferase-like 2 | 1 |
| 10329 | hsa-miR-489 | 117248 | GALNTL2 | UDP-N-acetyl-alpha-D-galactosamine:polypeptide N-acetylgalactosaminyltransferase-like 2 | 1 |
| 10330 | hsa-miR-562 | 117248 | GALNTL2 | UDP-N-acetyl-alpha-D-galactosamine:polypeptide N-acetylgalactosaminyltransferase-like 2 | 1 |
| 10331 | hsa-miR-575 | 117248 | GALNTL2 | UDP-N-acetyl-alpha-D-galactosamine:polypeptide N-acetylgalactosaminyltransferase-like 2 | 1 |
| 10332 | hsa-miR-579 | 117248 | GALNTL2 | UDP-N-acetyl-alpha-D-galactosamine:polypeptide N-acetylgalactosaminyltransferase-like 2 | 1 |
| 10333 | hsa-miR-608 | 117248 | GALNTL2 | UDP-N-acetyl-alpha-D-galactosamine:polypeptide N-acetylgalactosaminyltransferase-like 2 | 1 |
| 10334 | hsa-miR-620 | 117248 | GALNTL2 | UDP-N-acetyl-alpha-D-galactosamine:polypeptide N-acetylgalactosaminyltransferase-like 2 | 1 |
| 10335 | hsa-miR-637 | 117248 | GALNTL2 | UDP-N-acetyl-alpha-D-galactosamine:polypeptide N-acetylgalactosaminyltransferase-like 2 | 1 |
| 10336 | hsa-miR-885-5p | 117248 | GALNTL2 | UDP-N-acetyl-alpha-D-galactosamine:polypeptide N-acetylgalactosaminyltransferase-like 2 | 1 |
| 10337 | hsa-mir-22 | 117248 | GALNTL2 | UDP-N-acetyl-alpha-D-galactosamine:polypeptide N-acetylgalactosaminyltransferase-like 2 | 1 |
| 10338 | hsa-mir-10a | 117248 | GALNTL2 | UDP-N-acetyl-alpha-D-galactosamine:polypeptide N-acetylgalactosaminyltransferase-like 2 | 1 |
| 10339 | hsa-mir-186 | 117248 | GALNTL2 | UDP-N-acetyl-alpha-D-galactosamine:polypeptide N-acetylgalactosaminyltransferase-like 2 | 1 |
| 10340 | hsa-mir-155 | 117248 | GALNTL2 | UDP-N-acetyl-alpha-D-galactosamine:polypeptide N-acetylgalactosaminyltransferase-like 2 | 1 |
| 10341 | hsa-mir-499 | 117248 | GALNTL2 | UDP-N-acetyl-alpha-D-galactosamine:polypeptide N-acetylgalactosaminyltransferase-like 2 | 1 |
| 10342 | hsa-mir-885 | 117248 | GALNTL2 | UDP-N-acetyl-alpha-D-galactosamine:polypeptide N-acetylgalactosaminyltransferase-like 2 | 1 |
| 10343 | hsa-mir-877 | 117248 | GALNTL2 | UDP-N-acetyl-alpha-D-galactosamine:polypeptide N-acetylgalactosaminyltransferase-like 2 | 1 |
| 10344 | hsa-mir-3190 | 117248 | GALNTL2 | UDP-N-acetyl-alpha-D-galactosamine:polypeptide N-acetylgalactosaminyltransferase-like 2 | 1 |
| 10345 | hsa-miR-152 | 100287920 | LOC100287920 | hypothetical protein LOC100287920 | 1 |
| 10346 | hsa-miR-26a | 100287920 | LOC100287920 | hypothetical protein LOC100287920 | 1 |
| 10347 | hsa-miR-26b | 100287920 | LOC100287920 | hypothetical protein LOC100287920 | 1 |
| 10348 | hsa-miR-31 | 100287920 | LOC100287920 | hypothetical protein LOC100287920 | 1 |
| 10349 | hsa-miR-338-5p | 100287920 | LOC100287920 | hypothetical protein LOC100287920 | 1 |
| 10350 | hsa-miR-340 | 100287920 | LOC100287920 | hypothetical protein LOC100287920 | 1 |
| 10351 | hsa-miR-383 | 100287920 | LOC100287920 | hypothetical protein LOC100287920 | 1 |
| 10352 | hsa-miR-423-5p | 100287920 | LOC100287920 | hypothetical protein LOC100287920 | 1 |
| 10353 | hsa-miR-449a | 100287920 | LOC100287920 | hypothetical protein LOC100287920 | 1 |
| 10354 | hsa-miR-490-5p | 100287920 | LOC100287920 | hypothetical protein LOC100287920 | 1 |
| 10355 | hsa-miR-499-5p | 100287920 | LOC100287920 | hypothetical protein LOC100287920 | 1 |
| 10356 | hsa-miR-548b-5p | 100287920 | LOC100287920 | hypothetical protein LOC100287920 | 1 |
| 10357 | hsa-miR-564 | 100287920 | LOC100287920 | hypothetical protein LOC100287920 | 1 |
| 10358 | hsa-miR-621 | 100287920 | LOC100287920 | hypothetical protein LOC100287920 | 1 |
| 10359 | hsa-miR-657 | 100287920 | LOC100287920 | hypothetical protein LOC100287920 | 1 |
| 10360 | hsa-miR-663 | 100287920 | LOC100287920 | hypothetical protein LOC100287920 | 1 |
| 10361 | hsa-miR-765 | 100287920 | LOC100287920 | hypothetical protein LOC100287920 | 1 |
| 10362 | hsa-miR-770-5p | 100287920 | LOC100287920 | hypothetical protein LOC100287920 | 1 |
| 10363 | hsa-miR-877 | 100287920 | LOC100287920 | hypothetical protein LOC100287920 | 1 |
| 10364 | hsa-mir-26a-1 | 100287920 | LOC100287920 | hypothetical protein LOC100287920 | 1 |
| 10365 | hsa-mir-26b | 100287920 | LOC100287920 | hypothetical protein LOC100287920 | 1 |
| 10366 | hsa-mir-31 | 100287920 | LOC100287920 | hypothetical protein LOC100287920 | 1 |
| 10367 | hsa-mir-194-2 | 100287920 | LOC100287920 | hypothetical protein LOC100287920 | 1 |
| 10368 | hsa-mir-340 | 100287920 | LOC100287920 | hypothetical protein LOC100287920 | 1 |
| 10369 | hsa-mir-338 | 100287920 | LOC100287920 | hypothetical protein LOC100287920 | 1 |
| 10370 | hsa-mir-490 | 100287920 | LOC100287920 | hypothetical protein LOC100287920 | 1 |
| 10371 | hsa-mir-548b | 100287920 | LOC100287920 | hypothetical protein LOC100287920 | 1 |
| 10372 | hsa-mir-548d-2 | 100287920 | LOC100287920 | hypothetical protein LOC100287920 | 1 |
| 10373 | hsa-mir-449b | 100287920 | LOC100287920 | hypothetical protein LOC100287920 | 1 |
| 10374 | hsa-mir-449c | 100287920 | LOC100287920 | hypothetical protein LOC100287920 | 1 |
| 10375 | hsa-mir-3065 | 100287920 | LOC100287920 | hypothetical protein LOC100287920 | 1 |
| 10376 | hsa-miR-1 | 6560 | SLC12A4 | solute carrier family 12 (potassium/chloride transporters), member 4 | 1 |
| 10377 | hsa-miR-133a | 6560 | SLC12A4 | solute carrier family 12 (potassium/chloride transporters), member 4 | 1 |
| 10378 | hsa-miR-186 | 6560 | SLC12A4 | solute carrier family 12 (potassium/chloride transporters), member 4 | 1 |
| 10379 | hsa-miR-326 | 6560 | SLC12A4 | solute carrier family 12 (potassium/chloride transporters), member 4 | 1 |
| 10380 | hsa-miR-33a | 6560 | SLC12A4 | solute carrier family 12 (potassium/chloride transporters), member 4 | 1 |
| 10381 | hsa-miR-449b | 6560 | SLC12A4 | solute carrier family 12 (potassium/chloride transporters), member 4 | 1 |
| 10382 | hsa-miR-550 | 6560 | SLC12A4 | solute carrier family 12 (potassium/chloride transporters), member 4 | 1 |
| 10383 | hsa-miR-551a | 6560 | SLC12A4 | solute carrier family 12 (potassium/chloride transporters), member 4 | 1 |
| 10384 | hsa-miR-575 | 6560 | SLC12A4 | solute carrier family 12 (potassium/chloride transporters), member 4 | 1 |
| 10385 | hsa-miR-579 | 6560 | SLC12A4 | solute carrier family 12 (potassium/chloride transporters), member 4 | 1 |
| 10386 | hsa-miR-608 | 6560 | SLC12A4 | solute carrier family 12 (potassium/chloride transporters), member 4 | 1 |
| 10387 | hsa-miR-620 | 6560 | SLC12A4 | solute carrier family 12 (potassium/chloride transporters), member 4 | 1 |
| 10388 | hsa-miR-885-5p | 6560 | SLC12A4 | solute carrier family 12 (potassium/chloride transporters), member 4 | 1 |
| 10389 | hsa-mir-22 | 6560 | SLC12A4 | solute carrier family 12 (potassium/chloride transporters), member 4 | 1 |
| 10390 | hsa-mir-10a | 6560 | SLC12A4 | solute carrier family 12 (potassium/chloride transporters), member 4 | 1 |
| 10391 | hsa-mir-186 | 6560 | SLC12A4 | solute carrier family 12 (potassium/chloride transporters), member 4 | 1 |
| 10392 | hsa-mir-155 | 6560 | SLC12A4 | solute carrier family 12 (potassium/chloride transporters), member 4 | 1 |
| 10393 | hsa-mir-423 | 6560 | SLC12A4 | solute carrier family 12 (potassium/chloride transporters), member 4 | 1 |
| 10394 | hsa-mir-499 | 6560 | SLC12A4 | solute carrier family 12 (potassium/chloride transporters), member 4 | 1 |
| 10395 | hsa-mir-550-1 | 6560 | SLC12A4 | solute carrier family 12 (potassium/chloride transporters), member 4 | 1 |
| 10396 | hsa-mir-885 | 6560 | SLC12A4 | solute carrier family 12 (potassium/chloride transporters), member 4 | 1 |
| 10397 | hsa-mir-877 | 6560 | SLC12A4 | solute carrier family 12 (potassium/chloride transporters), member 4 | 1 |
| 10398 | hsa-mir-2116 | 6560 | SLC12A4 | solute carrier family 12 (potassium/chloride transporters), member 4 | 1 |
| 10399 | hsa-mir-3186 | 6560 | SLC12A4 | solute carrier family 12 (potassium/chloride transporters), member 4 | 1 |
| 10400 | hsa-mir-3190 | 6560 | SLC12A4 | solute carrier family 12 (potassium/chloride transporters), member 4 | 1 |
| 10401 | hsa-miR-10a | 64926 | RASAL3 | RAS protein activator like 3 | 1 |
| 10402 | hsa-miR-152 | 64926 | RASAL3 | RAS protein activator like 3 | 1 |
| 10403 | hsa-miR-26b | 64926 | RASAL3 | RAS protein activator like 3 | 1 |
| 10404 | hsa-miR-31 | 64926 | RASAL3 | RAS protein activator like 3 | 1 |
| 10405 | hsa-miR-338-5p | 64926 | RASAL3 | RAS protein activator like 3 | 1 |
| 10406 | hsa-miR-340 | 64926 | RASAL3 | RAS protein activator like 3 | 1 |
| 10407 | hsa-miR-449a | 64926 | RASAL3 | RAS protein activator like 3 | 1 |
| 10408 | hsa-miR-499-5p | 64926 | RASAL3 | RAS protein activator like 3 | 1 |
| 10409 | hsa-miR-621 | 64926 | RASAL3 | RAS protein activator like 3 | 1 |
| 10410 | hsa-miR-634 | 64926 | RASAL3 | RAS protein activator like 3 | 1 |
| 10411 | hsa-miR-657 | 64926 | RASAL3 | RAS protein activator like 3 | 1 |
| 10412 | hsa-miR-765 | 64926 | RASAL3 | RAS protein activator like 3 | 1 |
| 10413 | hsa-miR-770-5p | 64926 | RASAL3 | RAS protein activator like 3 | 1 |
| 10414 | hsa-miR-877 | 64926 | RASAL3 | RAS protein activator like 3 | 1 |
| 10415 | hsa-mir-26a-1 | 64926 | RASAL3 | RAS protein activator like 3 | 1 |
| 10416 | hsa-mir-26b | 64926 | RASAL3 | RAS protein activator like 3 | 1 |
| 10417 | hsa-mir-31 | 64926 | RASAL3 | RAS protein activator like 3 | 1 |
| 10418 | hsa-mir-194-2 | 64926 | RASAL3 | RAS protein activator like 3 | 1 |
| 10419 | hsa-mir-340 | 64926 | RASAL3 | RAS protein activator like 3 | 1 |
| 10420 | hsa-mir-338 | 64926 | RASAL3 | RAS protein activator like 3 | 1 |
| 10421 | hsa-mir-449b | 64926 | RASAL3 | RAS protein activator like 3 | 1 |
| 10422 | hsa-mir-449c | 64926 | RASAL3 | RAS protein activator like 3 | 1 |
| 10423 | hsa-mir-3065 | 64926 | RASAL3 | RAS protein activator like 3 | 1 |
| 10424 | hsa-miR-1 | 7275 | TUB | tubby homolog (mouse) | 1 |
| 10425 | hsa-miR-133a | 7275 | TUB | tubby homolog (mouse) | 1 |
| 10426 | hsa-miR-186 | 7275 | TUB | tubby homolog (mouse) | 1 |
| 10427 | hsa-miR-326 | 7275 | TUB | tubby homolog (mouse) | 1 |
| 10428 | hsa-miR-449b | 7275 | TUB | tubby homolog (mouse) | 1 |
| 10429 | hsa-miR-551a | 7275 | TUB | tubby homolog (mouse) | 1 |
| 10430 | hsa-miR-562 | 7275 | TUB | tubby homolog (mouse) | 1 |
| 10431 | hsa-miR-575 | 7275 | TUB | tubby homolog (mouse) | 1 |
| 10432 | hsa-miR-579 | 7275 | TUB | tubby homolog (mouse) | 1 |
| 10433 | hsa-miR-608 | 7275 | TUB | tubby homolog (mouse) | 1 |
| 10434 | hsa-miR-637 | 7275 | TUB | tubby homolog (mouse) | 1 |
| 10435 | hsa-miR-885-5p | 7275 | TUB | tubby homolog (mouse) | 1 |
| 10436 | hsa-mir-22 | 7275 | TUB | tubby homolog (mouse) | 1 |
| 10437 | hsa-mir-10a | 7275 | TUB | tubby homolog (mouse) | 1 |
| 10438 | hsa-mir-186 | 7275 | TUB | tubby homolog (mouse) | 1 |
| 10439 | hsa-mir-155 | 7275 | TUB | tubby homolog (mouse) | 1 |
| 10440 | hsa-mir-423 | 7275 | TUB | tubby homolog (mouse) | 1 |
| 10441 | hsa-mir-885 | 7275 | TUB | tubby homolog (mouse) | 1 |
| 10442 | hsa-mir-877 | 7275 | TUB | tubby homolog (mouse) | 1 |
| 10443 | hsa-mir-3190 | 7275 | TUB | tubby homolog (mouse) | 1 |
| 10444 | hsa-miR-10a | 100190939 | LOC100190939 | hypothetical LOC100190939 | 1 |
| 10445 | hsa-miR-152 | 100190939 | LOC100190939 | hypothetical LOC100190939 | 1 |
| 10446 | hsa-miR-26a | 100190939 | LOC100190939 | hypothetical LOC100190939 | 1 |
| 10447 | hsa-miR-26b | 100190939 | LOC100190939 | hypothetical LOC100190939 | 1 |
| 10448 | hsa-miR-31 | 100190939 | LOC100190939 | hypothetical LOC100190939 | 1 |
| 10449 | hsa-miR-338-5p | 100190939 | LOC100190939 | hypothetical LOC100190939 | 1 |
| 10450 | hsa-miR-340 | 100190939 | LOC100190939 | hypothetical LOC100190939 | 1 |
| 10451 | hsa-miR-383 | 100190939 | LOC100190939 | hypothetical LOC100190939 | 1 |
| 10452 | hsa-miR-449a | 100190939 | LOC100190939 | hypothetical LOC100190939 | 1 |
| 10453 | hsa-miR-490-5p | 100190939 | LOC100190939 | hypothetical LOC100190939 | 1 |
| 10454 | hsa-miR-499-5p | 100190939 | LOC100190939 | hypothetical LOC100190939 | 1 |
| 10455 | hsa-miR-584 | 100190939 | LOC100190939 | hypothetical LOC100190939 | 1 |
| 10456 | hsa-miR-621 | 100190939 | LOC100190939 | hypothetical LOC100190939 | 1 |
| 10457 | hsa-miR-634 | 100190939 | LOC100190939 | hypothetical LOC100190939 | 1 |
| 10458 | hsa-miR-653 | 100190939 | LOC100190939 | hypothetical LOC100190939 | 1 |
| 10459 | hsa-miR-657 | 100190939 | LOC100190939 | hypothetical LOC100190939 | 1 |
| 10460 | hsa-miR-765 | 100190939 | LOC100190939 | hypothetical LOC100190939 | 1 |
| 10461 | hsa-miR-770-5p | 100190939 | LOC100190939 | hypothetical LOC100190939 | 1 |
| 10462 | hsa-miR-877 | 100190939 | LOC100190939 | hypothetical LOC100190939 | 1 |
| 10463 | hsa-mir-26a-1 | 100190939 | LOC100190939 | hypothetical LOC100190939 | 1 |
| 10464 | hsa-mir-26b | 100190939 | LOC100190939 | hypothetical LOC100190939 | 1 |
| 10465 | hsa-mir-31 | 100190939 | LOC100190939 | hypothetical LOC100190939 | 1 |
| 10466 | hsa-mir-194-2 | 100190939 | LOC100190939 | hypothetical LOC100190939 | 1 |
| 10467 | hsa-mir-340 | 100190939 | LOC100190939 | hypothetical LOC100190939 | 1 |
| 10468 | hsa-mir-338 | 100190939 | LOC100190939 | hypothetical LOC100190939 | 1 |
| 10469 | hsa-mir-490 | 100190939 | LOC100190939 | hypothetical LOC100190939 | 1 |
| 10470 | hsa-mir-449b | 100190939 | LOC100190939 | hypothetical LOC100190939 | 1 |
| 10471 | hsa-mir-449c | 100190939 | LOC100190939 | hypothetical LOC100190939 | 1 |
| 10472 | hsa-mir-3065 | 100190939 | LOC100190939 | hypothetical LOC100190939 | 1 |
| 10473 | hsa-miR-152 | 84875 | PARP10 | poly (ADP-ribose) polymerase family, member 10 | 1 |
| 10474 | hsa-miR-26a | 84875 | PARP10 | poly (ADP-ribose) polymerase family, member 10 | 1 |
| 10475 | hsa-miR-26b | 84875 | PARP10 | poly (ADP-ribose) polymerase family, member 10 | 1 |
| 10476 | hsa-miR-31 | 84875 | PARP10 | poly (ADP-ribose) polymerase family, member 10 | 1 |
| 10477 | hsa-miR-340 | 84875 | PARP10 | poly (ADP-ribose) polymerase family, member 10 | 1 |
| 10478 | hsa-miR-383 | 84875 | PARP10 | poly (ADP-ribose) polymerase family, member 10 | 1 |
| 10479 | hsa-miR-449a | 84875 | PARP10 | poly (ADP-ribose) polymerase family, member 10 | 1 |
| 10480 | hsa-miR-490-5p | 84875 | PARP10 | poly (ADP-ribose) polymerase family, member 10 | 1 |
| 10481 | hsa-miR-499-5p | 84875 | PARP10 | poly (ADP-ribose) polymerase family, member 10 | 1 |
| 10482 | hsa-miR-621 | 84875 | PARP10 | poly (ADP-ribose) polymerase family, member 10 | 1 |
| 10483 | hsa-miR-653 | 84875 | PARP10 | poly (ADP-ribose) polymerase family, member 10 | 1 |
| 10484 | hsa-miR-765 | 84875 | PARP10 | poly (ADP-ribose) polymerase family, member 10 | 1 |
| 10485 | hsa-miR-770-5p | 84875 | PARP10 | poly (ADP-ribose) polymerase family, member 10 | 1 |
| 10486 | hsa-mir-26a-1 | 84875 | PARP10 | poly (ADP-ribose) polymerase family, member 10 | 1 |
| 10487 | hsa-mir-26b | 84875 | PARP10 | poly (ADP-ribose) polymerase family, member 10 | 1 |
| 10488 | hsa-mir-31 | 84875 | PARP10 | poly (ADP-ribose) polymerase family, member 10 | 1 |
| 10489 | hsa-mir-194-2 | 84875 | PARP10 | poly (ADP-ribose) polymerase family, member 10 | 1 |
| 10490 | hsa-mir-340 | 84875 | PARP10 | poly (ADP-ribose) polymerase family, member 10 | 1 |
| 10491 | hsa-mir-490 | 84875 | PARP10 | poly (ADP-ribose) polymerase family, member 10 | 1 |
| 10492 | hsa-mir-449b | 84875 | PARP10 | poly (ADP-ribose) polymerase family, member 10 | 1 |
| 10493 | hsa-mir-449c | 84875 | PARP10 | poly (ADP-ribose) polymerase family, member 10 | 1 |
| 10494 | hsa-miR-10a | 114614 | MIR155HG | MIR155 host gene (non-protein coding) | 1 |
| 10495 | hsa-miR-139-5p | 114614 | MIR155HG | MIR155 host gene (non-protein coding) | 1 |
| 10496 | hsa-miR-152 | 114614 | MIR155HG | MIR155 host gene (non-protein coding) | 1 |
| 10497 | hsa-miR-26a | 114614 | MIR155HG | MIR155 host gene (non-protein coding) | 1 |
| 10498 | hsa-miR-26b | 114614 | MIR155HG | MIR155 host gene (non-protein coding) | 1 |
| 10499 | hsa-miR-31 | 114614 | MIR155HG | MIR155 host gene (non-protein coding) | 1 |
| 10500 | hsa-miR-338-5p | 114614 | MIR155HG | MIR155 host gene (non-protein coding) | 1 |
| 10501 | hsa-miR-340 | 114614 | MIR155HG | MIR155 host gene (non-protein coding) | 1 |
| 10502 | hsa-miR-449a | 114614 | MIR155HG | MIR155 host gene (non-protein coding) | 1 |
| 10503 | hsa-miR-490-5p | 114614 | MIR155HG | MIR155 host gene (non-protein coding) | 1 |
| 10504 | hsa-miR-499-5p | 114614 | MIR155HG | MIR155 host gene (non-protein coding) | 1 |
| 10505 | hsa-miR-548b-5p | 114614 | MIR155HG | MIR155 host gene (non-protein coding) | 1 |
| 10506 | hsa-miR-621 | 114614 | MIR155HG | MIR155 host gene (non-protein coding) | 1 |
| 10507 | hsa-miR-657 | 114614 | MIR155HG | MIR155 host gene (non-protein coding) | 1 |
| 10508 | hsa-miR-765 | 114614 | MIR155HG | MIR155 host gene (non-protein coding) | 1 |
| 10509 | hsa-miR-770-5p | 114614 | MIR155HG | MIR155 host gene (non-protein coding) | 1 |
| 10510 | hsa-miR-877 | 114614 | MIR155HG | MIR155 host gene (non-protein coding) | 1 |
| 10511 | hsa-mir-26a-1 | 114614 | MIR155HG | MIR155 host gene (non-protein coding) | 1 |
| 10512 | hsa-mir-26b | 114614 | MIR155HG | MIR155 host gene (non-protein coding) | 1 |
| 10513 | hsa-mir-31 | 114614 | MIR155HG | MIR155 host gene (non-protein coding) | 1 |
| 10514 | hsa-mir-194-2 | 114614 | MIR155HG | MIR155 host gene (non-protein coding) | 1 |
| 10515 | hsa-mir-340 | 114614 | MIR155HG | MIR155 host gene (non-protein coding) | 1 |
| 10516 | hsa-mir-338 | 114614 | MIR155HG | MIR155 host gene (non-protein coding) | 1 |
| 10517 | hsa-mir-490 | 114614 | MIR155HG | MIR155 host gene (non-protein coding) | 1 |
| 10518 | hsa-mir-548b | 114614 | MIR155HG | MIR155 host gene (non-protein coding) | 1 |
| 10519 | hsa-mir-449b | 114614 | MIR155HG | MIR155 host gene (non-protein coding) | 1 |
| 10520 | hsa-mir-449c | 114614 | MIR155HG | MIR155 host gene (non-protein coding) | 1 |
| 10521 | hsa-mir-3065 | 114614 | MIR155HG | MIR155 host gene (non-protein coding) | 1 |
| 10522 | hsa-miR-10a | 121260 | SLC15A4 | solute carrier family 15, member 4 | 1 |
| 10523 | hsa-miR-1228\* | 121260 | SLC15A4 | solute carrier family 15, member 4 | 1 |
| 10524 | hsa-miR-152 | 121260 | SLC15A4 | solute carrier family 15, member 4 | 1 |
| 10525 | hsa-miR-26a | 121260 | SLC15A4 | solute carrier family 15, member 4 | 1 |
| 10526 | hsa-miR-26b | 121260 | SLC15A4 | solute carrier family 15, member 4 | 1 |
| 10527 | hsa-miR-338-5p | 121260 | SLC15A4 | solute carrier family 15, member 4 | 1 |
| 10528 | hsa-miR-340 | 121260 | SLC15A4 | solute carrier family 15, member 4 | 1 |
| 10529 | hsa-miR-423-5p | 121260 | SLC15A4 | solute carrier family 15, member 4 | 1 |
| 10530 | hsa-miR-449a | 121260 | SLC15A4 | solute carrier family 15, member 4 | 1 |
| 10531 | hsa-miR-499-5p | 121260 | SLC15A4 | solute carrier family 15, member 4 | 1 |
| 10532 | hsa-miR-548b-5p | 121260 | SLC15A4 | solute carrier family 15, member 4 | 1 |
| 10533 | hsa-miR-564 | 121260 | SLC15A4 | solute carrier family 15, member 4 | 1 |
| 10534 | hsa-miR-584 | 121260 | SLC15A4 | solute carrier family 15, member 4 | 1 |
| 10535 | hsa-miR-621 | 121260 | SLC15A4 | solute carrier family 15, member 4 | 1 |
| 10536 | hsa-miR-634 | 121260 | SLC15A4 | solute carrier family 15, member 4 | 1 |
| 10537 | hsa-miR-644 | 121260 | SLC15A4 | solute carrier family 15, member 4 | 1 |
| 10538 | hsa-miR-657 | 121260 | SLC15A4 | solute carrier family 15, member 4 | 1 |
| 10539 | hsa-miR-765 | 121260 | SLC15A4 | solute carrier family 15, member 4 | 1 |
| 10540 | hsa-miR-770-5p | 121260 | SLC15A4 | solute carrier family 15, member 4 | 1 |
| 10541 | hsa-miR-877 | 121260 | SLC15A4 | solute carrier family 15, member 4 | 1 |
| 10542 | hsa-miR-95 | 121260 | SLC15A4 | solute carrier family 15, member 4 | 1 |
| 10543 | hsa-mir-26a-1 | 121260 | SLC15A4 | solute carrier family 15, member 4 | 1 |
| 10544 | hsa-mir-26b | 121260 | SLC15A4 | solute carrier family 15, member 4 | 1 |
| 10545 | hsa-mir-194-2 | 121260 | SLC15A4 | solute carrier family 15, member 4 | 1 |
| 10546 | hsa-mir-340 | 121260 | SLC15A4 | solute carrier family 15, member 4 | 1 |
| 10547 | hsa-mir-338 | 121260 | SLC15A4 | solute carrier family 15, member 4 | 1 |
| 10548 | hsa-mir-548b | 121260 | SLC15A4 | solute carrier family 15, member 4 | 1 |
| 10549 | hsa-mir-449b | 121260 | SLC15A4 | solute carrier family 15, member 4 | 1 |
| 10550 | hsa-mir-449c | 121260 | SLC15A4 | solute carrier family 15, member 4 | 1 |
| 10551 | hsa-mir-3065 | 121260 | SLC15A4 | solute carrier family 15, member 4 | 1 |
| 10553 | hsa-miR-139-5p | 115362 | GBP5 | guanylate binding protein 5 | 1 |
| 10578 | hsa-mir-548d-2 | 115362 | GBP5 | guanylate binding protein 5 | 1 |
| 10582 | hsa-miR-152 | 9589 | WTAP | Wilms tumor 1 associated protein | 1 |
| 10583 | hsa-miR-26a | 9589 | WTAP | Wilms tumor 1 associated protein | 1 |
| 10584 | hsa-miR-26b | 9589 | WTAP | Wilms tumor 1 associated protein | 1 |
| 10585 | hsa-miR-31 | 9589 | WTAP | Wilms tumor 1 associated protein | 1 |
| 10586 | hsa-miR-423-5p | 9589 | WTAP | Wilms tumor 1 associated protein | 1 |
| 10587 | hsa-miR-449a | 9589 | WTAP | Wilms tumor 1 associated protein | 1 |
| 10588 | hsa-miR-490-5p | 9589 | WTAP | Wilms tumor 1 associated protein | 1 |
| 10589 | hsa-miR-499-5p | 9589 | WTAP | Wilms tumor 1 associated protein | 1 |
| 10590 | hsa-miR-548b-5p | 9589 | WTAP | Wilms tumor 1 associated protein | 1 |
| 10591 | hsa-miR-564 | 9589 | WTAP | Wilms tumor 1 associated protein | 1 |
| 10592 | hsa-miR-621 | 9589 | WTAP | Wilms tumor 1 associated protein | 1 |
| 10593 | hsa-miR-765 | 9589 | WTAP | Wilms tumor 1 associated protein | 1 |
| 10594 | hsa-miR-770-5p | 9589 | WTAP | Wilms tumor 1 associated protein | 1 |
| 10595 | hsa-miR-877 | 9589 | WTAP | Wilms tumor 1 associated protein | 1 |
| 10596 | hsa-mir-26a-1 | 9589 | WTAP | Wilms tumor 1 associated protein | 1 |
| 10597 | hsa-mir-26b | 9589 | WTAP | Wilms tumor 1 associated protein | 1 |
| 10598 | hsa-mir-31 | 9589 | WTAP | Wilms tumor 1 associated protein | 1 |
| 10599 | hsa-mir-194-2 | 9589 | WTAP | Wilms tumor 1 associated protein | 1 |
| 10600 | hsa-mir-490 | 9589 | WTAP | Wilms tumor 1 associated protein | 1 |
| 10601 | hsa-mir-548b | 9589 | WTAP | Wilms tumor 1 associated protein | 1 |
| 10602 | hsa-mir-548d-2 | 9589 | WTAP | Wilms tumor 1 associated protein | 1 |
| 10603 | hsa-mir-449b | 9589 | WTAP | Wilms tumor 1 associated protein | 1 |
| 10604 | hsa-mir-449c | 9589 | WTAP | Wilms tumor 1 associated protein | 1 |
| 10605 | hsa-miR-1 | 2235 | FECH | ferrochelatase (protoporphyria) | 1 |
| 10606 | hsa-miR-133a | 2235 | FECH | ferrochelatase (protoporphyria) | 1 |
| 10607 | hsa-miR-186 | 2235 | FECH | ferrochelatase (protoporphyria) | 1 |
| 10608 | hsa-miR-208a | 2235 | FECH | ferrochelatase (protoporphyria) | 1 |
| 10609 | hsa-miR-326 | 2235 | FECH | ferrochelatase (protoporphyria) | 1 |
| 10610 | hsa-miR-449b | 2235 | FECH | ferrochelatase (protoporphyria) | 1 |
| 10611 | hsa-miR-550 | 2235 | FECH | ferrochelatase (protoporphyria) | 1 |
| 10612 | hsa-miR-551a | 2235 | FECH | ferrochelatase (protoporphyria) | 1 |
| 10613 | hsa-miR-575 | 2235 | FECH | ferrochelatase (protoporphyria) | 1 |
| 10614 | hsa-miR-579 | 2235 | FECH | ferrochelatase (protoporphyria) | 1 |
| 10615 | hsa-miR-608 | 2235 | FECH | ferrochelatase (protoporphyria) | 1 |
| 10616 | hsa-miR-620 | 2235 | FECH | ferrochelatase (protoporphyria) | 1 |
| 10617 | hsa-miR-637 | 2235 | FECH | ferrochelatase (protoporphyria) | 1 |
| 10618 | hsa-miR-885-5p | 2235 | FECH | ferrochelatase (protoporphyria) | 1 |
| 10619 | hsa-mir-22 | 2235 | FECH | ferrochelatase (protoporphyria) | 1 |
| 10620 | hsa-mir-10a | 2235 | FECH | ferrochelatase (protoporphyria) | 1 |
| 10621 | hsa-mir-186 | 2235 | FECH | ferrochelatase (protoporphyria) | 1 |
| 10622 | hsa-mir-155 | 2235 | FECH | ferrochelatase (protoporphyria) | 1 |
| 10623 | hsa-mir-423 | 2235 | FECH | ferrochelatase (protoporphyria) | 1 |
| 10624 | hsa-mir-499 | 2235 | FECH | ferrochelatase (protoporphyria) | 1 |
| 10625 | hsa-mir-550-1 | 2235 | FECH | ferrochelatase (protoporphyria) | 1 |
| 10626 | hsa-mir-885 | 2235 | FECH | ferrochelatase (protoporphyria) | 1 |
| 10627 | hsa-mir-877 | 2235 | FECH | ferrochelatase (protoporphyria) | 1 |
| 10628 | hsa-mir-2116 | 2235 | FECH | ferrochelatase (protoporphyria) | 1 |
| 10629 | hsa-mir-3190 | 2235 | FECH | ferrochelatase (protoporphyria) | 1 |
| 10630 | hsa-miR-1 | 57095 | C1orf128 | chromosome 1 open reading frame 128 | 1 |
| 10631 | hsa-miR-133a | 57095 | C1orf128 | chromosome 1 open reading frame 128 | 1 |
| 10632 | hsa-miR-186 | 57095 | C1orf128 | chromosome 1 open reading frame 128 | 1 |
| 10633 | hsa-miR-326 | 57095 | C1orf128 | chromosome 1 open reading frame 128 | 1 |
| 10634 | hsa-miR-449b | 57095 | C1orf128 | chromosome 1 open reading frame 128 | 1 |
| 10635 | hsa-miR-550 | 57095 | C1orf128 | chromosome 1 open reading frame 128 | 1 |
| 10636 | hsa-miR-562 | 57095 | C1orf128 | chromosome 1 open reading frame 128 | 1 |
| 10637 | hsa-miR-575 | 57095 | C1orf128 | chromosome 1 open reading frame 128 | 1 |
| 10638 | hsa-miR-579 | 57095 | C1orf128 | chromosome 1 open reading frame 128 | 1 |
| 10639 | hsa-miR-608 | 57095 | C1orf128 | chromosome 1 open reading frame 128 | 1 |
| 10640 | hsa-miR-620 | 57095 | C1orf128 | chromosome 1 open reading frame 128 | 1 |
| 10641 | hsa-miR-885-5p | 57095 | C1orf128 | chromosome 1 open reading frame 128 | 1 |
| 10642 | hsa-mir-22 | 57095 | C1orf128 | chromosome 1 open reading frame 128 | 1 |
| 10643 | hsa-mir-10a | 57095 | C1orf128 | chromosome 1 open reading frame 128 | 1 |
| 10644 | hsa-mir-186 | 57095 | C1orf128 | chromosome 1 open reading frame 128 | 1 |
| 10645 | hsa-mir-155 | 57095 | C1orf128 | chromosome 1 open reading frame 128 | 1 |
| 10646 | hsa-mir-423 | 57095 | C1orf128 | chromosome 1 open reading frame 128 | 1 |
| 10647 | hsa-mir-499 | 57095 | C1orf128 | chromosome 1 open reading frame 128 | 1 |
| 10648 | hsa-mir-550-1 | 57095 | C1orf128 | chromosome 1 open reading frame 128 | 1 |
| 10649 | hsa-mir-885 | 57095 | C1orf128 | chromosome 1 open reading frame 128 | 1 |
| 10650 | hsa-mir-2116 | 57095 | C1orf128 | chromosome 1 open reading frame 128 | 1 |
| 10651 | hsa-mir-3186 | 57095 | C1orf128 | chromosome 1 open reading frame 128 | 1 |
| 10652 | hsa-miR-10a | 221188 | GPR114 | G protein-coupled receptor 114 | 1 |
| 10653 | hsa-miR-1228\* | 221188 | GPR114 | G protein-coupled receptor 114 | 1 |
| 10654 | hsa-miR-152 | 221188 | GPR114 | G protein-coupled receptor 114 | 1 |
| 10655 | hsa-miR-26a | 221188 | GPR114 | G protein-coupled receptor 114 | 1 |
| 10656 | hsa-miR-26b | 221188 | GPR114 | G protein-coupled receptor 114 | 1 |
| 10657 | hsa-miR-31 | 221188 | GPR114 | G protein-coupled receptor 114 | 1 |
| 10658 | hsa-miR-338-5p | 221188 | GPR114 | G protein-coupled receptor 114 | 1 |
| 10659 | hsa-miR-340 | 221188 | GPR114 | G protein-coupled receptor 114 | 1 |
| 10660 | hsa-miR-423-5p | 221188 | GPR114 | G protein-coupled receptor 114 | 1 |
| 10661 | hsa-miR-449a | 221188 | GPR114 | G protein-coupled receptor 114 | 1 |
| 10662 | hsa-miR-490-5p | 221188 | GPR114 | G protein-coupled receptor 114 | 1 |
| 10663 | hsa-miR-499-5p | 221188 | GPR114 | G protein-coupled receptor 114 | 1 |
| 10664 | hsa-miR-548b-5p | 221188 | GPR114 | G protein-coupled receptor 114 | 1 |
| 10665 | hsa-miR-564 | 221188 | GPR114 | G protein-coupled receptor 114 | 1 |
| 10666 | hsa-miR-584 | 221188 | GPR114 | G protein-coupled receptor 114 | 1 |
| 10667 | hsa-miR-621 | 221188 | GPR114 | G protein-coupled receptor 114 | 1 |
| 10668 | hsa-miR-634 | 221188 | GPR114 | G protein-coupled receptor 114 | 1 |
| 10669 | hsa-miR-644 | 221188 | GPR114 | G protein-coupled receptor 114 | 1 |
| 10670 | hsa-miR-657 | 221188 | GPR114 | G protein-coupled receptor 114 | 1 |
| 10671 | hsa-miR-765 | 221188 | GPR114 | G protein-coupled receptor 114 | 1 |
| 10672 | hsa-miR-770-5p | 221188 | GPR114 | G protein-coupled receptor 114 | 1 |
| 10673 | hsa-miR-877 | 221188 | GPR114 | G protein-coupled receptor 114 | 1 |
| 10674 | hsa-miR-95 | 221188 | GPR114 | G protein-coupled receptor 114 | 1 |
| 10675 | hsa-mir-26a-1 | 221188 | GPR114 | G protein-coupled receptor 114 | 1 |
| 10676 | hsa-mir-26b | 221188 | GPR114 | G protein-coupled receptor 114 | 1 |
| 10677 | hsa-mir-31 | 221188 | GPR114 | G protein-coupled receptor 114 | 1 |
| 10678 | hsa-mir-194-2 | 221188 | GPR114 | G protein-coupled receptor 114 | 1 |
| 10679 | hsa-mir-340 | 221188 | GPR114 | G protein-coupled receptor 114 | 1 |
| 10680 | hsa-mir-338 | 221188 | GPR114 | G protein-coupled receptor 114 | 1 |
| 10681 | hsa-mir-490 | 221188 | GPR114 | G protein-coupled receptor 114 | 1 |
| 10682 | hsa-mir-548b | 221188 | GPR114 | G protein-coupled receptor 114 | 1 |
| 10683 | hsa-mir-449b | 221188 | GPR114 | G protein-coupled receptor 114 | 1 |
| 10684 | hsa-mir-449c | 221188 | GPR114 | G protein-coupled receptor 114 | 1 |
| 10685 | hsa-mir-3065 | 221188 | GPR114 | G protein-coupled receptor 114 | 1 |
| 10686 | hsa-miR-1 | 342898 | SYCN | syncollin | 1 |
| 10687 | hsa-miR-133a | 342898 | SYCN | syncollin | 1 |
| 10688 | hsa-miR-186 | 342898 | SYCN | syncollin | 1 |
| 10689 | hsa-miR-449b | 342898 | SYCN | syncollin | 1 |
| 10690 | hsa-miR-551a | 342898 | SYCN | syncollin | 1 |
| 10691 | hsa-miR-562 | 342898 | SYCN | syncollin | 1 |
| 10692 | hsa-miR-579 | 342898 | SYCN | syncollin | 1 |
| 10693 | hsa-miR-608 | 342898 | SYCN | syncollin | 1 |
| 10694 | hsa-miR-637 | 342898 | SYCN | syncollin | 1 |
| 10695 | hsa-mir-22 | 342898 | SYCN | syncollin | 1 |
| 10696 | hsa-mir-10a | 342898 | SYCN | syncollin | 1 |
| 10697 | hsa-mir-186 | 342898 | SYCN | syncollin | 1 |
| 10698 | hsa-mir-155 | 342898 | SYCN | syncollin | 1 |
| 10699 | hsa-mir-423 | 342898 | SYCN | syncollin | 1 |
| 10700 | hsa-mir-877 | 342898 | SYCN | syncollin | 1 |
| 10701 | hsa-miR-10a | 23030 | KDM4B | lysine (K)-specific demethylase 4B | 1 |
| 10702 | hsa-miR-1228\* | 23030 | KDM4B | lysine (K)-specific demethylase 4B | 1 |
| 10703 | hsa-miR-139-5p | 23030 | KDM4B | lysine (K)-specific demethylase 4B | 1 |
| 10704 | hsa-miR-152 | 23030 | KDM4B | lysine (K)-specific demethylase 4B | 1 |
| 10705 | hsa-miR-26a | 23030 | KDM4B | lysine (K)-specific demethylase 4B | 1 |
| 10706 | hsa-miR-26b | 23030 | KDM4B | lysine (K)-specific demethylase 4B | 1 |
| 10707 | hsa-miR-31 | 23030 | KDM4B | lysine (K)-specific demethylase 4B | 1 |
| 10708 | hsa-miR-338-5p | 23030 | KDM4B | lysine (K)-specific demethylase 4B | 1 |
| 10709 | hsa-miR-340 | 23030 | KDM4B | lysine (K)-specific demethylase 4B | 1 |
| 10710 | hsa-miR-423-5p | 23030 | KDM4B | lysine (K)-specific demethylase 4B | 1 |
| 10711 | hsa-miR-449a | 23030 | KDM4B | lysine (K)-specific demethylase 4B | 1 |
| 10712 | hsa-miR-499-5p | 23030 | KDM4B | lysine (K)-specific demethylase 4B | 1 |
| 10713 | hsa-miR-548b-5p | 23030 | KDM4B | lysine (K)-specific demethylase 4B | 1 |
| 10714 | hsa-miR-584 | 23030 | KDM4B | lysine (K)-specific demethylase 4B | 1 |
| 10715 | hsa-miR-621 | 23030 | KDM4B | lysine (K)-specific demethylase 4B | 1 |
| 10716 | hsa-miR-644 | 23030 | KDM4B | lysine (K)-specific demethylase 4B | 1 |
| 10717 | hsa-miR-657 | 23030 | KDM4B | lysine (K)-specific demethylase 4B | 1 |
| 10718 | hsa-miR-663 | 23030 | KDM4B | lysine (K)-specific demethylase 4B | 1 |
| 10719 | hsa-miR-765 | 23030 | KDM4B | lysine (K)-specific demethylase 4B | 1 |
| 10720 | hsa-miR-770-5p | 23030 | KDM4B | lysine (K)-specific demethylase 4B | 1 |
| 10721 | hsa-miR-877 | 23030 | KDM4B | lysine (K)-specific demethylase 4B | 1 |
| 10722 | hsa-mir-26a-1 | 23030 | KDM4B | lysine (K)-specific demethylase 4B | 1 |
| 10723 | hsa-mir-26b | 23030 | KDM4B | lysine (K)-specific demethylase 4B | 1 |
| 10724 | hsa-mir-31 | 23030 | KDM4B | lysine (K)-specific demethylase 4B | 1 |
| 10725 | hsa-mir-194-2 | 23030 | KDM4B | lysine (K)-specific demethylase 4B | 1 |
| 10726 | hsa-mir-340 | 23030 | KDM4B | lysine (K)-specific demethylase 4B | 1 |
| 10727 | hsa-mir-338 | 23030 | KDM4B | lysine (K)-specific demethylase 4B | 1 |
| 10728 | hsa-mir-548b | 23030 | KDM4B | lysine (K)-specific demethylase 4B | 1 |
| 10729 | hsa-mir-449b | 23030 | KDM4B | lysine (K)-specific demethylase 4B | 1 |
| 10730 | hsa-mir-449c | 23030 | KDM4B | lysine (K)-specific demethylase 4B | 1 |
| 10731 | hsa-mir-3065 | 23030 | KDM4B | lysine (K)-specific demethylase 4B | 1 |
| 10732 | hsa-miR-1 | 23353 | SUN1 | Sad1 and UNC84 domain containing 1 | 1 |
| 10733 | hsa-miR-133a | 23353 | SUN1 | Sad1 and UNC84 domain containing 1 | 1 |
| 10734 | hsa-miR-186 | 23353 | SUN1 | Sad1 and UNC84 domain containing 1 | 1 |
| 10735 | hsa-miR-326 | 23353 | SUN1 | Sad1 and UNC84 domain containing 1 | 1 |
| 10736 | hsa-miR-449b | 23353 | SUN1 | Sad1 and UNC84 domain containing 1 | 1 |
| 10737 | hsa-miR-550 | 23353 | SUN1 | Sad1 and UNC84 domain containing 1 | 1 |
| 10738 | hsa-miR-551a | 23353 | SUN1 | Sad1 and UNC84 domain containing 1 | 1 |
| 10739 | hsa-miR-562 | 23353 | SUN1 | Sad1 and UNC84 domain containing 1 | 1 |
| 10740 | hsa-miR-575 | 23353 | SUN1 | Sad1 and UNC84 domain containing 1 | 1 |
| 10741 | hsa-miR-579 | 23353 | SUN1 | Sad1 and UNC84 domain containing 1 | 1 |
| 10742 | hsa-miR-608 | 23353 | SUN1 | Sad1 and UNC84 domain containing 1 | 1 |
| 10743 | hsa-miR-620 | 23353 | SUN1 | Sad1 and UNC84 domain containing 1 | 1 |
| 10744 | hsa-miR-885-5p | 23353 | SUN1 | Sad1 and UNC84 domain containing 1 | 1 |
| 10745 | hsa-mir-22 | 23353 | SUN1 | Sad1 and UNC84 domain containing 1 | 1 |
| 10746 | hsa-mir-10a | 23353 | SUN1 | Sad1 and UNC84 domain containing 1 | 1 |
| 10747 | hsa-mir-186 | 23353 | SUN1 | Sad1 and UNC84 domain containing 1 | 1 |
| 10748 | hsa-mir-155 | 23353 | SUN1 | Sad1 and UNC84 domain containing 1 | 1 |
| 10749 | hsa-mir-499 | 23353 | SUN1 | Sad1 and UNC84 domain containing 1 | 1 |
| 10750 | hsa-mir-550-1 | 23353 | SUN1 | Sad1 and UNC84 domain containing 1 | 1 |
| 10751 | hsa-mir-885 | 23353 | SUN1 | Sad1 and UNC84 domain containing 1 | 1 |
| 10752 | hsa-mir-877 | 23353 | SUN1 | Sad1 and UNC84 domain containing 1 | 1 |
| 10753 | hsa-mir-2116 | 23353 | SUN1 | Sad1 and UNC84 domain containing 1 | 1 |
| 10754 | hsa-miR-1 | 10335 | MRVI1 | murine retrovirus integration site 1 homolog | 1 |
| 10755 | hsa-miR-133a | 10335 | MRVI1 | murine retrovirus integration site 1 homolog | 1 |
| 10756 | hsa-miR-186 | 10335 | MRVI1 | murine retrovirus integration site 1 homolog | 1 |
| 10757 | hsa-miR-326 | 10335 | MRVI1 | murine retrovirus integration site 1 homolog | 1 |
| 10758 | hsa-miR-449b | 10335 | MRVI1 | murine retrovirus integration site 1 homolog | 1 |
| 10759 | hsa-miR-551a | 10335 | MRVI1 | murine retrovirus integration site 1 homolog | 1 |
| 10760 | hsa-miR-575 | 10335 | MRVI1 | murine retrovirus integration site 1 homolog | 1 |
| 10761 | hsa-miR-579 | 10335 | MRVI1 | murine retrovirus integration site 1 homolog | 1 |
| 10762 | hsa-miR-608 | 10335 | MRVI1 | murine retrovirus integration site 1 homolog | 1 |
| 10763 | hsa-miR-637 | 10335 | MRVI1 | murine retrovirus integration site 1 homolog | 1 |
| 10764 | hsa-mir-22 | 10335 | MRVI1 | murine retrovirus integration site 1 homolog | 1 |
| 10765 | hsa-mir-10a | 10335 | MRVI1 | murine retrovirus integration site 1 homolog | 1 |
| 10766 | hsa-mir-186 | 10335 | MRVI1 | murine retrovirus integration site 1 homolog | 1 |
| 10767 | hsa-mir-155 | 10335 | MRVI1 | murine retrovirus integration site 1 homolog | 1 |
| 10768 | hsa-mir-423 | 10335 | MRVI1 | murine retrovirus integration site 1 homolog | 1 |
| 10769 | hsa-mir-499 | 10335 | MRVI1 | murine retrovirus integration site 1 homolog | 1 |
| 10770 | hsa-mir-877 | 10335 | MRVI1 | murine retrovirus integration site 1 homolog | 1 |
| 10771 | hsa-mir-2116 | 10335 | MRVI1 | murine retrovirus integration site 1 homolog | 1 |
| 10772 | hsa-mir-3190 | 10335 | MRVI1 | murine retrovirus integration site 1 homolog | 1 |
| 10773 | hsa-miR-152 | 23048 | FNBP1 | formin binding protein 1 | 1 |
| 10774 | hsa-miR-26b | 23048 | FNBP1 | formin binding protein 1 | 1 |
| 10775 | hsa-miR-31 | 23048 | FNBP1 | formin binding protein 1 | 1 |
| 10776 | hsa-miR-338-5p | 23048 | FNBP1 | formin binding protein 1 | 1 |
| 10777 | hsa-miR-340 | 23048 | FNBP1 | formin binding protein 1 | 1 |
| 10778 | hsa-miR-383 | 23048 | FNBP1 | formin binding protein 1 | 1 |
| 10779 | hsa-miR-449a | 23048 | FNBP1 | formin binding protein 1 | 1 |
| 10780 | hsa-miR-621 | 23048 | FNBP1 | formin binding protein 1 | 1 |
| 10781 | hsa-miR-657 | 23048 | FNBP1 | formin binding protein 1 | 1 |
| 10782 | hsa-miR-765 | 23048 | FNBP1 | formin binding protein 1 | 1 |
| 10783 | hsa-mir-26a-1 | 23048 | FNBP1 | formin binding protein 1 | 1 |
| 10784 | hsa-mir-26b | 23048 | FNBP1 | formin binding protein 1 | 1 |
| 10785 | hsa-mir-31 | 23048 | FNBP1 | formin binding protein 1 | 1 |
| 10786 | hsa-mir-194-2 | 23048 | FNBP1 | formin binding protein 1 | 1 |
| 10787 | hsa-mir-340 | 23048 | FNBP1 | formin binding protein 1 | 1 |
| 10788 | hsa-mir-338 | 23048 | FNBP1 | formin binding protein 1 | 1 |
| 10789 | hsa-mir-449b | 23048 | FNBP1 | formin binding protein 1 | 1 |
| 10790 | hsa-mir-449c | 23048 | FNBP1 | formin binding protein 1 | 1 |
| 10791 | hsa-mir-3065 | 23048 | FNBP1 | formin binding protein 1 | 1 |
| 10792 | hsa-miR-10a | 441108 | C5orf56 | chromosome 5 open reading frame 56 | 1 |
| 10793 | hsa-miR-139-5p | 441108 | C5orf56 | chromosome 5 open reading frame 56 | 1 |
| 10794 | hsa-miR-152 | 441108 | C5orf56 | chromosome 5 open reading frame 56 | 1 |
| 10795 | hsa-miR-26a | 441108 | C5orf56 | chromosome 5 open reading frame 56 | 1 |
| 10796 | hsa-miR-26b | 441108 | C5orf56 | chromosome 5 open reading frame 56 | 1 |
| 10797 | hsa-miR-31 | 441108 | C5orf56 | chromosome 5 open reading frame 56 | 1 |
| 10798 | hsa-miR-338-5p | 441108 | C5orf56 | chromosome 5 open reading frame 56 | 1 |
| 10799 | hsa-miR-340 | 441108 | C5orf56 | chromosome 5 open reading frame 56 | 1 |
| 10800 | hsa-miR-383 | 441108 | C5orf56 | chromosome 5 open reading frame 56 | 1 |
| 10801 | hsa-miR-449a | 441108 | C5orf56 | chromosome 5 open reading frame 56 | 1 |
| 10802 | hsa-miR-499-5p | 441108 | C5orf56 | chromosome 5 open reading frame 56 | 1 |
| 10803 | hsa-miR-548b-5p | 441108 | C5orf56 | chromosome 5 open reading frame 56 | 1 |
| 10804 | hsa-miR-621 | 441108 | C5orf56 | chromosome 5 open reading frame 56 | 1 |
| 10805 | hsa-miR-657 | 441108 | C5orf56 | chromosome 5 open reading frame 56 | 1 |
| 10806 | hsa-miR-765 | 441108 | C5orf56 | chromosome 5 open reading frame 56 | 1 |
| 10807 | hsa-miR-770-5p | 441108 | C5orf56 | chromosome 5 open reading frame 56 | 1 |
| 10808 | hsa-miR-877 | 441108 | C5orf56 | chromosome 5 open reading frame 56 | 1 |
| 10809 | hsa-mir-26a-1 | 441108 | C5orf56 | chromosome 5 open reading frame 56 | 1 |
| 10810 | hsa-mir-26b | 441108 | C5orf56 | chromosome 5 open reading frame 56 | 1 |
| 10811 | hsa-mir-31 | 441108 | C5orf56 | chromosome 5 open reading frame 56 | 1 |
| 10812 | hsa-mir-194-2 | 441108 | C5orf56 | chromosome 5 open reading frame 56 | 1 |
| 10813 | hsa-mir-340 | 441108 | C5orf56 | chromosome 5 open reading frame 56 | 1 |
| 10814 | hsa-mir-338 | 441108 | C5orf56 | chromosome 5 open reading frame 56 | 1 |
| 10815 | hsa-mir-548b | 441108 | C5orf56 | chromosome 5 open reading frame 56 | 1 |
| 10816 | hsa-mir-449b | 441108 | C5orf56 | chromosome 5 open reading frame 56 | 1 |
| 10817 | hsa-mir-449c | 441108 | C5orf56 | chromosome 5 open reading frame 56 | 1 |
| 10818 | hsa-mir-3065 | 441108 | C5orf56 | chromosome 5 open reading frame 56 | 1 |
| 10819 | hsa-miR-152 | 159013 | CXorf38 | chromosome X open reading frame 38 | 1 |
| 10820 | hsa-miR-26a | 159013 | CXorf38 | chromosome X open reading frame 38 | 1 |
| 10821 | hsa-miR-26b | 159013 | CXorf38 | chromosome X open reading frame 38 | 1 |
| 10822 | hsa-miR-31 | 159013 | CXorf38 | chromosome X open reading frame 38 | 1 |
| 10823 | hsa-miR-449a | 159013 | CXorf38 | chromosome X open reading frame 38 | 1 |
| 10824 | hsa-miR-499-5p | 159013 | CXorf38 | chromosome X open reading frame 38 | 1 |
| 10825 | hsa-miR-564 | 159013 | CXorf38 | chromosome X open reading frame 38 | 1 |
| 10826 | hsa-miR-621 | 159013 | CXorf38 | chromosome X open reading frame 38 | 1 |
| 10827 | hsa-miR-765 | 159013 | CXorf38 | chromosome X open reading frame 38 | 1 |
| 10828 | hsa-miR-770-5p | 159013 | CXorf38 | chromosome X open reading frame 38 | 1 |
| 10829 | hsa-mir-26a-1 | 159013 | CXorf38 | chromosome X open reading frame 38 | 1 |
| 10830 | hsa-mir-26b | 159013 | CXorf38 | chromosome X open reading frame 38 | 1 |
| 10831 | hsa-mir-31 | 159013 | CXorf38 | chromosome X open reading frame 38 | 1 |
| 10832 | hsa-mir-194-2 | 159013 | CXorf38 | chromosome X open reading frame 38 | 1 |
| 10833 | hsa-mir-449b | 159013 | CXorf38 | chromosome X open reading frame 38 | 1 |
| 10834 | hsa-mir-449c | 159013 | CXorf38 | chromosome X open reading frame 38 | 1 |
| 10835 | hsa-miR-10a | 23215 | BAT2L2 | HLA-B associated transcript 2-like 2 | 1 |
| 10836 | hsa-miR-1228\* | 23215 | BAT2L2 | HLA-B associated transcript 2-like 2 | 1 |
| 10837 | hsa-miR-139-5p | 23215 | BAT2L2 | HLA-B associated transcript 2-like 2 | 1 |
| 10838 | hsa-miR-152 | 23215 | BAT2L2 | HLA-B associated transcript 2-like 2 | 1 |
| 10839 | hsa-miR-26a | 23215 | BAT2L2 | HLA-B associated transcript 2-like 2 | 1 |
| 10840 | hsa-miR-26b | 23215 | BAT2L2 | HLA-B associated transcript 2-like 2 | 1 |
| 10841 | hsa-miR-31 | 23215 | BAT2L2 | HLA-B associated transcript 2-like 2 | 1 |
| 10842 | hsa-miR-338-5p | 23215 | BAT2L2 | HLA-B associated transcript 2-like 2 | 1 |
| 10843 | hsa-miR-340 | 23215 | BAT2L2 | HLA-B associated transcript 2-like 2 | 1 |
| 10844 | hsa-miR-423-5p | 23215 | BAT2L2 | HLA-B associated transcript 2-like 2 | 1 |
| 10845 | hsa-miR-449a | 23215 | BAT2L2 | HLA-B associated transcript 2-like 2 | 1 |
| 10846 | hsa-miR-499-5p | 23215 | BAT2L2 | HLA-B associated transcript 2-like 2 | 1 |
| 10847 | hsa-miR-548b-5p | 23215 | BAT2L2 | HLA-B associated transcript 2-like 2 | 1 |
| 10848 | hsa-miR-564 | 23215 | BAT2L2 | HLA-B associated transcript 2-like 2 | 1 |
| 10849 | hsa-miR-621 | 23215 | BAT2L2 | HLA-B associated transcript 2-like 2 | 1 |
| 10850 | hsa-miR-657 | 23215 | BAT2L2 | HLA-B associated transcript 2-like 2 | 1 |
| 10851 | hsa-miR-765 | 23215 | BAT2L2 | HLA-B associated transcript 2-like 2 | 1 |
| 10852 | hsa-miR-770-5p | 23215 | BAT2L2 | HLA-B associated transcript 2-like 2 | 1 |
| 10853 | hsa-miR-877 | 23215 | BAT2L2 | HLA-B associated transcript 2-like 2 | 1 |
| 10854 | hsa-mir-26a-1 | 23215 | BAT2L2 | HLA-B associated transcript 2-like 2 | 1 |
| 10855 | hsa-mir-26b | 23215 | BAT2L2 | HLA-B associated transcript 2-like 2 | 1 |
| 10856 | hsa-mir-31 | 23215 | BAT2L2 | HLA-B associated transcript 2-like 2 | 1 |
| 10857 | hsa-mir-194-2 | 23215 | BAT2L2 | HLA-B associated transcript 2-like 2 | 1 |
| 10858 | hsa-mir-340 | 23215 | BAT2L2 | HLA-B associated transcript 2-like 2 | 1 |
| 10859 | hsa-mir-338 | 23215 | BAT2L2 | HLA-B associated transcript 2-like 2 | 1 |
| 10860 | hsa-mir-548b | 23215 | BAT2L2 | HLA-B associated transcript 2-like 2 | 1 |
| 10861 | hsa-mir-548d-2 | 23215 | BAT2L2 | HLA-B associated transcript 2-like 2 | 1 |
| 10862 | hsa-mir-449b | 23215 | BAT2L2 | HLA-B associated transcript 2-like 2 | 1 |
| 10863 | hsa-mir-449c | 23215 | BAT2L2 | HLA-B associated transcript 2-like 2 | 1 |
| 10864 | hsa-mir-3065 | 23215 | BAT2L2 | HLA-B associated transcript 2-like 2 | 1 |
| 10865 | hsa-miR-1 | 132851 | SPATA4 | spermatogenesis associated 4 | 1 |
| 10866 | hsa-miR-133a | 132851 | SPATA4 | spermatogenesis associated 4 | 1 |
| 10867 | hsa-miR-186 | 132851 | SPATA4 | spermatogenesis associated 4 | 1 |
| 10868 | hsa-miR-208a | 132851 | SPATA4 | spermatogenesis associated 4 | 1 |
| 10869 | hsa-miR-326 | 132851 | SPATA4 | spermatogenesis associated 4 | 1 |
| 10870 | hsa-miR-449b | 132851 | SPATA4 | spermatogenesis associated 4 | 1 |
| 10871 | hsa-miR-550 | 132851 | SPATA4 | spermatogenesis associated 4 | 1 |
| 10872 | hsa-miR-575 | 132851 | SPATA4 | spermatogenesis associated 4 | 1 |
| 10873 | hsa-miR-579 | 132851 | SPATA4 | spermatogenesis associated 4 | 1 |
| 10874 | hsa-miR-608 | 132851 | SPATA4 | spermatogenesis associated 4 | 1 |
| 10875 | hsa-miR-620 | 132851 | SPATA4 | spermatogenesis associated 4 | 1 |
| 10876 | hsa-miR-885-5p | 132851 | SPATA4 | spermatogenesis associated 4 | 1 |
| 10877 | hsa-mir-22 | 132851 | SPATA4 | spermatogenesis associated 4 | 1 |
| 10878 | hsa-mir-10a | 132851 | SPATA4 | spermatogenesis associated 4 | 1 |
| 10879 | hsa-mir-186 | 132851 | SPATA4 | spermatogenesis associated 4 | 1 |
| 10880 | hsa-mir-155 | 132851 | SPATA4 | spermatogenesis associated 4 | 1 |
| 10881 | hsa-mir-423 | 132851 | SPATA4 | spermatogenesis associated 4 | 1 |
| 10882 | hsa-mir-499 | 132851 | SPATA4 | spermatogenesis associated 4 | 1 |
| 10883 | hsa-mir-550-1 | 132851 | SPATA4 | spermatogenesis associated 4 | 1 |
| 10884 | hsa-mir-885 | 132851 | SPATA4 | spermatogenesis associated 4 | 1 |
| 10885 | hsa-mir-877 | 132851 | SPATA4 | spermatogenesis associated 4 | 1 |
| 10886 | hsa-mir-2116 | 132851 | SPATA4 | spermatogenesis associated 4 | 1 |
| 10887 | hsa-mir-3190 | 132851 | SPATA4 | spermatogenesis associated 4 | 1 |
| 10888 | hsa-miR-10a | 84444 | DOT1L | DOT1-like, histone H3 methyltransferase (S. cerevisiae) | 1 |
| 10889 | hsa-miR-1228\* | 84444 | DOT1L | DOT1-like, histone H3 methyltransferase (S. cerevisiae) | 1 |
| 10890 | hsa-miR-152 | 84444 | DOT1L | DOT1-like, histone H3 methyltransferase (S. cerevisiae) | 1 |
| 10891 | hsa-miR-26a | 84444 | DOT1L | DOT1-like, histone H3 methyltransferase (S. cerevisiae) | 1 |
| 10892 | hsa-miR-26b | 84444 | DOT1L | DOT1-like, histone H3 methyltransferase (S. cerevisiae) | 1 |
| 10893 | hsa-miR-31 | 84444 | DOT1L | DOT1-like, histone H3 methyltransferase (S. cerevisiae) | 1 |
| 10894 | hsa-miR-338-5p | 84444 | DOT1L | DOT1-like, histone H3 methyltransferase (S. cerevisiae) | 1 |
| 10895 | hsa-miR-340 | 84444 | DOT1L | DOT1-like, histone H3 methyltransferase (S. cerevisiae) | 1 |
| 10896 | hsa-miR-423-5p | 84444 | DOT1L | DOT1-like, histone H3 methyltransferase (S. cerevisiae) | 1 |
| 10897 | hsa-miR-449a | 84444 | DOT1L | DOT1-like, histone H3 methyltransferase (S. cerevisiae) | 1 |
| 10898 | hsa-miR-490-5p | 84444 | DOT1L | DOT1-like, histone H3 methyltransferase (S. cerevisiae) | 1 |
| 10899 | hsa-miR-499-5p | 84444 | DOT1L | DOT1-like, histone H3 methyltransferase (S. cerevisiae) | 1 |
| 10900 | hsa-miR-548b-5p | 84444 | DOT1L | DOT1-like, histone H3 methyltransferase (S. cerevisiae) | 1 |
| 10901 | hsa-miR-564 | 84444 | DOT1L | DOT1-like, histone H3 methyltransferase (S. cerevisiae) | 1 |
| 10902 | hsa-miR-584 | 84444 | DOT1L | DOT1-like, histone H3 methyltransferase (S. cerevisiae) | 1 |
| 10903 | hsa-miR-621 | 84444 | DOT1L | DOT1-like, histone H3 methyltransferase (S. cerevisiae) | 1 |
| 10904 | hsa-miR-644 | 84444 | DOT1L | DOT1-like, histone H3 methyltransferase (S. cerevisiae) | 1 |
| 10905 | hsa-miR-657 | 84444 | DOT1L | DOT1-like, histone H3 methyltransferase (S. cerevisiae) | 1 |
| 10906 | hsa-miR-765 | 84444 | DOT1L | DOT1-like, histone H3 methyltransferase (S. cerevisiae) | 1 |
| 10907 | hsa-miR-770-5p | 84444 | DOT1L | DOT1-like, histone H3 methyltransferase (S. cerevisiae) | 1 |
| 10908 | hsa-miR-877 | 84444 | DOT1L | DOT1-like, histone H3 methyltransferase (S. cerevisiae) | 1 |
| 10909 | hsa-miR-95 | 84444 | DOT1L | DOT1-like, histone H3 methyltransferase (S. cerevisiae) | 1 |
| 10910 | hsa-mir-26a-1 | 84444 | DOT1L | DOT1-like, histone H3 methyltransferase (S. cerevisiae) | 1 |
| 10911 | hsa-mir-26b | 84444 | DOT1L | DOT1-like, histone H3 methyltransferase (S. cerevisiae) | 1 |
| 10912 | hsa-mir-31 | 84444 | DOT1L | DOT1-like, histone H3 methyltransferase (S. cerevisiae) | 1 |
| 10913 | hsa-mir-194-2 | 84444 | DOT1L | DOT1-like, histone H3 methyltransferase (S. cerevisiae) | 1 |
| 10914 | hsa-mir-340 | 84444 | DOT1L | DOT1-like, histone H3 methyltransferase (S. cerevisiae) | 1 |
| 10915 | hsa-mir-338 | 84444 | DOT1L | DOT1-like, histone H3 methyltransferase (S. cerevisiae) | 1 |
| 10916 | hsa-mir-490 | 84444 | DOT1L | DOT1-like, histone H3 methyltransferase (S. cerevisiae) | 1 |
| 10917 | hsa-mir-548b | 84444 | DOT1L | DOT1-like, histone H3 methyltransferase (S. cerevisiae) | 1 |
| 10918 | hsa-mir-548d-2 | 84444 | DOT1L | DOT1-like, histone H3 methyltransferase (S. cerevisiae) | 1 |
| 10919 | hsa-mir-449b | 84444 | DOT1L | DOT1-like, histone H3 methyltransferase (S. cerevisiae) | 1 |
| 10920 | hsa-mir-449c | 84444 | DOT1L | DOT1-like, histone H3 methyltransferase (S. cerevisiae) | 1 |
| 10921 | hsa-mir-3065 | 84444 | DOT1L | DOT1-like, histone H3 methyltransferase (S. cerevisiae) | 1 |
| 10922 | hsa-miR-1 | 85417 | CCNB3 | cyclin B3 | 1 |
| 10923 | hsa-miR-133a | 85417 | CCNB3 | cyclin B3 | 1 |
| 10924 | hsa-miR-186 | 85417 | CCNB3 | cyclin B3 | 1 |
| 10925 | hsa-miR-326 | 85417 | CCNB3 | cyclin B3 | 1 |
| 10926 | hsa-miR-449b | 85417 | CCNB3 | cyclin B3 | 1 |
| 10927 | hsa-miR-551a | 85417 | CCNB3 | cyclin B3 | 1 |
| 10928 | hsa-miR-562 | 85417 | CCNB3 | cyclin B3 | 1 |
| 10929 | hsa-miR-575 | 85417 | CCNB3 | cyclin B3 | 1 |
| 10930 | hsa-miR-579 | 85417 | CCNB3 | cyclin B3 | 1 |
| 10931 | hsa-miR-608 | 85417 | CCNB3 | cyclin B3 | 1 |
| 10932 | hsa-miR-620 | 85417 | CCNB3 | cyclin B3 | 1 |
| 10933 | hsa-miR-637 | 85417 | CCNB3 | cyclin B3 | 1 |
| 10934 | hsa-miR-885-5p | 85417 | CCNB3 | cyclin B3 | 1 |
| 10935 | hsa-mir-22 | 85417 | CCNB3 | cyclin B3 | 1 |
| 10936 | hsa-mir-10a | 85417 | CCNB3 | cyclin B3 | 1 |
| 10937 | hsa-mir-186 | 85417 | CCNB3 | cyclin B3 | 1 |
| 10938 | hsa-mir-155 | 85417 | CCNB3 | cyclin B3 | 1 |
| 10939 | hsa-mir-499 | 85417 | CCNB3 | cyclin B3 | 1 |
| 10940 | hsa-mir-550-1 | 85417 | CCNB3 | cyclin B3 | 1 |
| 10941 | hsa-mir-885 | 85417 | CCNB3 | cyclin B3 | 1 |
| 10942 | hsa-mir-877 | 85417 | CCNB3 | cyclin B3 | 1 |
| 10943 | hsa-mir-2116 | 85417 | CCNB3 | cyclin B3 | 1 |
| 10944 | hsa-mir-3190 | 85417 | CCNB3 | cyclin B3 | 1 |
| 10945 | hsa-miR-152 | 81671 | TMEM49 | transmembrane protein 49 | 1 |
| 10946 | hsa-miR-26b | 81671 | TMEM49 | transmembrane protein 49 | 1 |
| 10947 | hsa-miR-31 | 81671 | TMEM49 | transmembrane protein 49 | 1 |
| 10948 | hsa-miR-338-5p | 81671 | TMEM49 | transmembrane protein 49 | 1 |
| 10949 | hsa-miR-340 | 81671 | TMEM49 | transmembrane protein 49 | 1 |
| 10950 | hsa-miR-383 | 81671 | TMEM49 | transmembrane protein 49 | 1 |
| 10951 | hsa-miR-449a | 81671 | TMEM49 | transmembrane protein 49 | 1 |
| 10952 | hsa-miR-621 | 81671 | TMEM49 | transmembrane protein 49 | 1 |
| 10953 | hsa-miR-657 | 81671 | TMEM49 | transmembrane protein 49 | 1 |
| 10954 | hsa-miR-663 | 81671 | TMEM49 | transmembrane protein 49 | 1 |
| 10955 | hsa-miR-765 | 81671 | TMEM49 | transmembrane protein 49 | 1 |
| 10956 | hsa-miR-770-5p | 81671 | TMEM49 | transmembrane protein 49 | 1 |
| 10957 | hsa-miR-877 | 81671 | TMEM49 | transmembrane protein 49 | 1 |
| 10958 | hsa-mir-26a-1 | 81671 | TMEM49 | transmembrane protein 49 | 1 |
| 10959 | hsa-mir-26b | 81671 | TMEM49 | transmembrane protein 49 | 1 |
| 10960 | hsa-mir-31 | 81671 | TMEM49 | transmembrane protein 49 | 1 |
| 10961 | hsa-mir-194-2 | 81671 | TMEM49 | transmembrane protein 49 | 1 |
| 10962 | hsa-mir-340 | 81671 | TMEM49 | transmembrane protein 49 | 1 |
| 10963 | hsa-mir-338 | 81671 | TMEM49 | transmembrane protein 49 | 1 |
| 10964 | hsa-mir-449b | 81671 | TMEM49 | transmembrane protein 49 | 1 |
| 10965 | hsa-mir-449c | 81671 | TMEM49 | transmembrane protein 49 | 1 |
| 10966 | hsa-mir-3065 | 81671 | TMEM49 | transmembrane protein 49 | 1 |
| 10967 | hsa-miR-1 | 10247 | HRSP12 | heat-responsive protein 12 | 1 |
| 10968 | hsa-miR-133a | 10247 | HRSP12 | heat-responsive protein 12 | 1 |
| 10969 | hsa-miR-186 | 10247 | HRSP12 | heat-responsive protein 12 | 1 |
| 10970 | hsa-miR-326 | 10247 | HRSP12 | heat-responsive protein 12 | 1 |
| 10971 | hsa-miR-449b | 10247 | HRSP12 | heat-responsive protein 12 | 1 |
| 10972 | hsa-miR-551a | 10247 | HRSP12 | heat-responsive protein 12 | 1 |
| 10973 | hsa-miR-562 | 10247 | HRSP12 | heat-responsive protein 12 | 1 |
| 10974 | hsa-miR-575 | 10247 | HRSP12 | heat-responsive protein 12 | 1 |
| 10975 | hsa-miR-579 | 10247 | HRSP12 | heat-responsive protein 12 | 1 |
| 10976 | hsa-miR-608 | 10247 | HRSP12 | heat-responsive protein 12 | 1 |
| 10977 | hsa-miR-620 | 10247 | HRSP12 | heat-responsive protein 12 | 1 |
| 10978 | hsa-miR-637 | 10247 | HRSP12 | heat-responsive protein 12 | 1 |
| 10979 | hsa-mir-22 | 10247 | HRSP12 | heat-responsive protein 12 | 1 |
| 10980 | hsa-mir-10a | 10247 | HRSP12 | heat-responsive protein 12 | 1 |
| 10981 | hsa-mir-186 | 10247 | HRSP12 | heat-responsive protein 12 | 1 |
| 10982 | hsa-mir-155 | 10247 | HRSP12 | heat-responsive protein 12 | 1 |
| 10983 | hsa-mir-423 | 10247 | HRSP12 | heat-responsive protein 12 | 1 |
| 10984 | hsa-mir-499 | 10247 | HRSP12 | heat-responsive protein 12 | 1 |
| 10985 | hsa-mir-877 | 10247 | HRSP12 | heat-responsive protein 12 | 1 |
| 10986 | hsa-mir-3190 | 10247 | HRSP12 | heat-responsive protein 12 | 1 |
| 10987 | hsa-miR-10a | 3656 | IRAK2 | interleukin-1 receptor-associated kinase 2 | 1 |
| 10988 | hsa-miR-152 | 3656 | IRAK2 | interleukin-1 receptor-associated kinase 2 | 1 |
| 10989 | hsa-miR-26a | 3656 | IRAK2 | interleukin-1 receptor-associated kinase 2 | 1 |
| 10990 | hsa-miR-26b | 3656 | IRAK2 | interleukin-1 receptor-associated kinase 2 | 1 |
| 10991 | hsa-miR-31 | 3656 | IRAK2 | interleukin-1 receptor-associated kinase 2 | 1 |
| 10992 | hsa-miR-338-5p | 3656 | IRAK2 | interleukin-1 receptor-associated kinase 2 | 1 |
| 10993 | hsa-miR-340 | 3656 | IRAK2 | interleukin-1 receptor-associated kinase 2 | 1 |
| 10994 | hsa-miR-423-5p | 3656 | IRAK2 | interleukin-1 receptor-associated kinase 2 | 1 |
| 10995 | hsa-miR-449a | 3656 | IRAK2 | interleukin-1 receptor-associated kinase 2 | 1 |
| 10996 | hsa-miR-490-5p | 3656 | IRAK2 | interleukin-1 receptor-associated kinase 2 | 1 |
| 10997 | hsa-miR-499-5p | 3656 | IRAK2 | interleukin-1 receptor-associated kinase 2 | 1 |
| 10998 | hsa-miR-548b-5p | 3656 | IRAK2 | interleukin-1 receptor-associated kinase 2 | 1 |
| 10999 | hsa-miR-564 | 3656 | IRAK2 | interleukin-1 receptor-associated kinase 2 | 1 |
| 11000 | hsa-miR-621 | 3656 | IRAK2 | interleukin-1 receptor-associated kinase 2 | 1 |
| 11001 | hsa-miR-644 | 3656 | IRAK2 | interleukin-1 receptor-associated kinase 2 | 1 |
| 11002 | hsa-miR-657 | 3656 | IRAK2 | interleukin-1 receptor-associated kinase 2 | 1 |
| 11003 | hsa-miR-765 | 3656 | IRAK2 | interleukin-1 receptor-associated kinase 2 | 1 |
| 11004 | hsa-miR-770-5p | 3656 | IRAK2 | interleukin-1 receptor-associated kinase 2 | 1 |
| 11005 | hsa-miR-877 | 3656 | IRAK2 | interleukin-1 receptor-associated kinase 2 | 1 |
| 11006 | hsa-miR-95 | 3656 | IRAK2 | interleukin-1 receptor-associated kinase 2 | 1 |
| 11007 | hsa-mir-26a-1 | 3656 | IRAK2 | interleukin-1 receptor-associated kinase 2 | 1 |
| 11008 | hsa-mir-26b | 3656 | IRAK2 | interleukin-1 receptor-associated kinase 2 | 1 |
| 11009 | hsa-mir-31 | 3656 | IRAK2 | interleukin-1 receptor-associated kinase 2 | 1 |
| 11010 | hsa-mir-194-2 | 3656 | IRAK2 | interleukin-1 receptor-associated kinase 2 | 1 |
| 11011 | hsa-mir-340 | 3656 | IRAK2 | interleukin-1 receptor-associated kinase 2 | 1 |
| 11012 | hsa-mir-338 | 3656 | IRAK2 | interleukin-1 receptor-associated kinase 2 | 1 |
| 11013 | hsa-mir-490 | 3656 | IRAK2 | interleukin-1 receptor-associated kinase 2 | 1 |
| 11014 | hsa-mir-548b | 3656 | IRAK2 | interleukin-1 receptor-associated kinase 2 | 1 |
| 11015 | hsa-mir-449b | 3656 | IRAK2 | interleukin-1 receptor-associated kinase 2 | 1 |
| 11016 | hsa-mir-449c | 3656 | IRAK2 | interleukin-1 receptor-associated kinase 2 | 1 |
| 11017 | hsa-mir-3065 | 3656 | IRAK2 | interleukin-1 receptor-associated kinase 2 | 1 |
| 11018 | hsa-miR-10a | 81844 | TRIM56 | tripartite motif-containing 56 | 1 |
| 11019 | hsa-miR-152 | 81844 | TRIM56 | tripartite motif-containing 56 | 1 |
| 11020 | hsa-miR-26a | 81844 | TRIM56 | tripartite motif-containing 56 | 1 |
| 11021 | hsa-miR-26b | 81844 | TRIM56 | tripartite motif-containing 56 | 1 |
| 11022 | hsa-miR-31 | 81844 | TRIM56 | tripartite motif-containing 56 | 1 |
| 11023 | hsa-miR-338-5p | 81844 | TRIM56 | tripartite motif-containing 56 | 1 |
| 11024 | hsa-miR-340 | 81844 | TRIM56 | tripartite motif-containing 56 | 1 |
| 11025 | hsa-miR-423-5p | 81844 | TRIM56 | tripartite motif-containing 56 | 1 |
| 11026 | hsa-miR-449a | 81844 | TRIM56 | tripartite motif-containing 56 | 1 |
| 11027 | hsa-miR-490-5p | 81844 | TRIM56 | tripartite motif-containing 56 | 1 |
| 11028 | hsa-miR-499-5p | 81844 | TRIM56 | tripartite motif-containing 56 | 1 |
| 11029 | hsa-miR-548b-5p | 81844 | TRIM56 | tripartite motif-containing 56 | 1 |
| 11030 | hsa-miR-564 | 81844 | TRIM56 | tripartite motif-containing 56 | 1 |
| 11031 | hsa-miR-621 | 81844 | TRIM56 | tripartite motif-containing 56 | 1 |
| 11032 | hsa-miR-657 | 81844 | TRIM56 | tripartite motif-containing 56 | 1 |
| 11033 | hsa-miR-765 | 81844 | TRIM56 | tripartite motif-containing 56 | 1 |
| 11034 | hsa-miR-770-5p | 81844 | TRIM56 | tripartite motif-containing 56 | 1 |
| 11035 | hsa-miR-877 | 81844 | TRIM56 | tripartite motif-containing 56 | 1 |
| 11036 | hsa-mir-26a-1 | 81844 | TRIM56 | tripartite motif-containing 56 | 1 |
| 11037 | hsa-mir-26b | 81844 | TRIM56 | tripartite motif-containing 56 | 1 |
| 11038 | hsa-mir-31 | 81844 | TRIM56 | tripartite motif-containing 56 | 1 |
| 11039 | hsa-mir-194-2 | 81844 | TRIM56 | tripartite motif-containing 56 | 1 |
| 11040 | hsa-mir-340 | 81844 | TRIM56 | tripartite motif-containing 56 | 1 |
| 11041 | hsa-mir-338 | 81844 | TRIM56 | tripartite motif-containing 56 | 1 |
| 11042 | hsa-mir-490 | 81844 | TRIM56 | tripartite motif-containing 56 | 1 |
| 11043 | hsa-mir-548b | 81844 | TRIM56 | tripartite motif-containing 56 | 1 |
| 11044 | hsa-mir-548d-2 | 81844 | TRIM56 | tripartite motif-containing 56 | 1 |
| 11045 | hsa-mir-449b | 81844 | TRIM56 | tripartite motif-containing 56 | 1 |
| 11046 | hsa-mir-449c | 81844 | TRIM56 | tripartite motif-containing 56 | 1 |
| 11047 | hsa-mir-3065 | 81844 | TRIM56 | tripartite motif-containing 56 | 1 |
| 11048 | hsa-miR-1 | 57822 | GRHL3 | grainyhead-like 3 (Drosophila) | 1 |
| 11049 | hsa-miR-133a | 57822 | GRHL3 | grainyhead-like 3 (Drosophila) | 1 |
| 11050 | hsa-miR-186 | 57822 | GRHL3 | grainyhead-like 3 (Drosophila) | 1 |
| 11051 | hsa-miR-195 | 57822 | GRHL3 | grainyhead-like 3 (Drosophila) | 1 |
| 11052 | hsa-miR-326 | 57822 | GRHL3 | grainyhead-like 3 (Drosophila) | 1 |
| 11053 | hsa-miR-33a | 57822 | GRHL3 | grainyhead-like 3 (Drosophila) | 1 |
| 11054 | hsa-miR-449b | 57822 | GRHL3 | grainyhead-like 3 (Drosophila) | 1 |
| 11055 | hsa-miR-497 | 57822 | GRHL3 | grainyhead-like 3 (Drosophila) | 1 |
| 11056 | hsa-miR-550 | 57822 | GRHL3 | grainyhead-like 3 (Drosophila) | 1 |
| 11057 | hsa-miR-562 | 57822 | GRHL3 | grainyhead-like 3 (Drosophila) | 1 |
| 11058 | hsa-miR-575 | 57822 | GRHL3 | grainyhead-like 3 (Drosophila) | 1 |
| 11059 | hsa-miR-579 | 57822 | GRHL3 | grainyhead-like 3 (Drosophila) | 1 |
| 11060 | hsa-miR-608 | 57822 | GRHL3 | grainyhead-like 3 (Drosophila) | 1 |
| 11061 | hsa-miR-620 | 57822 | GRHL3 | grainyhead-like 3 (Drosophila) | 1 |
| 11062 | hsa-let-7a-3 | 57822 | GRHL3 | grainyhead-like 3 (Drosophila) | 1 |
| 11063 | hsa-let-7b | 57822 | GRHL3 | grainyhead-like 3 (Drosophila) | 1 |
| 11064 | hsa-mir-22 | 57822 | GRHL3 | grainyhead-like 3 (Drosophila) | 1 |
| 11065 | hsa-mir-10a | 57822 | GRHL3 | grainyhead-like 3 (Drosophila) | 1 |
| 11066 | hsa-mir-186 | 57822 | GRHL3 | grainyhead-like 3 (Drosophila) | 1 |
| 11067 | hsa-mir-155 | 57822 | GRHL3 | grainyhead-like 3 (Drosophila) | 1 |
| 11068 | hsa-mir-499 | 57822 | GRHL3 | grainyhead-like 3 (Drosophila) | 1 |
| 11069 | hsa-mir-550-1 | 57822 | GRHL3 | grainyhead-like 3 (Drosophila) | 1 |
| 11070 | hsa-mir-877 | 57822 | GRHL3 | grainyhead-like 3 (Drosophila) | 1 |
| 11071 | hsa-mir-2116 | 57822 | GRHL3 | grainyhead-like 3 (Drosophila) | 1 |
| 11072 | hsa-miR-1 | 100129637 | LOC100129637 | hypothetical LOC100129637 | 1 |
| 11073 | hsa-miR-133a | 100129637 | LOC100129637 | hypothetical LOC100129637 | 1 |
| 11074 | hsa-miR-186 | 100129637 | LOC100129637 | hypothetical LOC100129637 | 1 |
| 11075 | hsa-miR-195 | 100129637 | LOC100129637 | hypothetical LOC100129637 | 1 |
| 11076 | hsa-miR-208a | 100129637 | LOC100129637 | hypothetical LOC100129637 | 1 |
| 11077 | hsa-miR-326 | 100129637 | LOC100129637 | hypothetical LOC100129637 | 1 |
| 11078 | hsa-miR-449b | 100129637 | LOC100129637 | hypothetical LOC100129637 | 1 |
| 11079 | hsa-miR-497 | 100129637 | LOC100129637 | hypothetical LOC100129637 | 1 |
| 11080 | hsa-miR-550 | 100129637 | LOC100129637 | hypothetical LOC100129637 | 1 |
| 11081 | hsa-miR-575 | 100129637 | LOC100129637 | hypothetical LOC100129637 | 1 |
| 11082 | hsa-miR-579 | 100129637 | LOC100129637 | hypothetical LOC100129637 | 1 |
| 11083 | hsa-miR-608 | 100129637 | LOC100129637 | hypothetical LOC100129637 | 1 |
| 11084 | hsa-miR-620 | 100129637 | LOC100129637 | hypothetical LOC100129637 | 1 |
| 11085 | hsa-miR-885-5p | 100129637 | LOC100129637 | hypothetical LOC100129637 | 1 |
| 11086 | hsa-let-7a-3 | 100129637 | LOC100129637 | hypothetical LOC100129637 | 1 |
| 11087 | hsa-let-7b | 100129637 | LOC100129637 | hypothetical LOC100129637 | 1 |
| 11088 | hsa-mir-22 | 100129637 | LOC100129637 | hypothetical LOC100129637 | 1 |
| 11089 | hsa-mir-10a | 100129637 | LOC100129637 | hypothetical LOC100129637 | 1 |
| 11090 | hsa-mir-186 | 100129637 | LOC100129637 | hypothetical LOC100129637 | 1 |
| 11091 | hsa-mir-155 | 100129637 | LOC100129637 | hypothetical LOC100129637 | 1 |
| 11092 | hsa-mir-499 | 100129637 | LOC100129637 | hypothetical LOC100129637 | 1 |
| 11093 | hsa-mir-885 | 100129637 | LOC100129637 | hypothetical LOC100129637 | 1 |
| 11094 | hsa-mir-877 | 100129637 | LOC100129637 | hypothetical LOC100129637 | 1 |
| 11095 | hsa-mir-2116 | 100129637 | LOC100129637 | hypothetical LOC100129637 | 1 |
| 11096 | hsa-mir-3190 | 100129637 | LOC100129637 | hypothetical LOC100129637 | 1 |
| 11097 | hsa-miR-1 | 267004 | PGBD3 | piggyBac transposable element derived 3 | 1 |
| 11098 | hsa-miR-133a | 267004 | PGBD3 | piggyBac transposable element derived 3 | 1 |
| 11099 | hsa-miR-186 | 267004 | PGBD3 | piggyBac transposable element derived 3 | 1 |
| 11100 | hsa-miR-195 | 267004 | PGBD3 | piggyBac transposable element derived 3 | 1 |
| 11101 | hsa-miR-326 | 267004 | PGBD3 | piggyBac transposable element derived 3 | 1 |
| 11102 | hsa-miR-33a | 267004 | PGBD3 | piggyBac transposable element derived 3 | 1 |
| 11103 | hsa-miR-449b | 267004 | PGBD3 | piggyBac transposable element derived 3 | 1 |
| 11104 | hsa-miR-497 | 267004 | PGBD3 | piggyBac transposable element derived 3 | 1 |
| 11105 | hsa-miR-550 | 267004 | PGBD3 | piggyBac transposable element derived 3 | 1 |
| 11106 | hsa-miR-551a | 267004 | PGBD3 | piggyBac transposable element derived 3 | 1 |
| 11107 | hsa-miR-575 | 267004 | PGBD3 | piggyBac transposable element derived 3 | 1 |
| 11108 | hsa-miR-608 | 267004 | PGBD3 | piggyBac transposable element derived 3 | 1 |
| 11109 | hsa-miR-620 | 267004 | PGBD3 | piggyBac transposable element derived 3 | 1 |
| 11110 | hsa-let-7a-3 | 267004 | PGBD3 | piggyBac transposable element derived 3 | 1 |
| 11111 | hsa-let-7b | 267004 | PGBD3 | piggyBac transposable element derived 3 | 1 |
| 11112 | hsa-mir-22 | 267004 | PGBD3 | piggyBac transposable element derived 3 | 1 |
| 11113 | hsa-mir-10a | 267004 | PGBD3 | piggyBac transposable element derived 3 | 1 |
| 11114 | hsa-mir-186 | 267004 | PGBD3 | piggyBac transposable element derived 3 | 1 |
| 11115 | hsa-mir-155 | 267004 | PGBD3 | piggyBac transposable element derived 3 | 1 |
| 11116 | hsa-mir-499 | 267004 | PGBD3 | piggyBac transposable element derived 3 | 1 |
| 11117 | hsa-mir-550-1 | 267004 | PGBD3 | piggyBac transposable element derived 3 | 1 |
| 11118 | hsa-mir-877 | 267004 | PGBD3 | piggyBac transposable element derived 3 | 1 |
| 11119 | hsa-mir-2116 | 267004 | PGBD3 | piggyBac transposable element derived 3 | 1 |
| 11120 | hsa-mir-3186 | 267004 | PGBD3 | piggyBac transposable element derived 3 | 1 |
| 11121 | hsa-miR-10a | 567 | B2M | beta-2-microglobulin | 1 |
| 11122 | hsa-miR-139-5p | 567 | B2M | beta-2-microglobulin | 1 |
| 11123 | hsa-miR-152 | 567 | B2M | beta-2-microglobulin | 1 |
| 11124 | hsa-miR-26a | 567 | B2M | beta-2-microglobulin | 1 |
| 11125 | hsa-miR-26b | 567 | B2M | beta-2-microglobulin | 1 |
| 11126 | hsa-miR-31 | 567 | B2M | beta-2-microglobulin | 1 |
| 11127 | hsa-miR-338-5p | 567 | B2M | beta-2-microglobulin | 1 |
| 11128 | hsa-miR-340 | 567 | B2M | beta-2-microglobulin | 1 |
| 11129 | hsa-miR-383 | 567 | B2M | beta-2-microglobulin | 1 |
| 11130 | hsa-miR-449a | 567 | B2M | beta-2-microglobulin | 1 |
| 11131 | hsa-miR-499-5p | 567 | B2M | beta-2-microglobulin | 1 |
| 11132 | hsa-miR-548b-5p | 567 | B2M | beta-2-microglobulin | 1 |
| 11133 | hsa-miR-621 | 567 | B2M | beta-2-microglobulin | 1 |
| 11134 | hsa-miR-657 | 567 | B2M | beta-2-microglobulin | 1 |
| 11135 | hsa-miR-663 | 567 | B2M | beta-2-microglobulin | 1 |
| 11136 | hsa-miR-765 | 567 | B2M | beta-2-microglobulin | 1 |
| 11137 | hsa-miR-770-5p | 567 | B2M | beta-2-microglobulin | 1 |
| 11138 | hsa-miR-877 | 567 | B2M | beta-2-microglobulin | 1 |
| 11139 | hsa-mir-26a-1 | 567 | B2M | beta-2-microglobulin | 1 |
| 11140 | hsa-mir-26b | 567 | B2M | beta-2-microglobulin | 1 |
| 11141 | hsa-mir-31 | 567 | B2M | beta-2-microglobulin | 1 |
| 11142 | hsa-mir-194-2 | 567 | B2M | beta-2-microglobulin | 1 |
| 11143 | hsa-mir-340 | 567 | B2M | beta-2-microglobulin | 1 |
| 11144 | hsa-mir-338 | 567 | B2M | beta-2-microglobulin | 1 |
| 11145 | hsa-mir-548b | 567 | B2M | beta-2-microglobulin | 1 |
| 11146 | hsa-mir-548d-2 | 567 | B2M | beta-2-microglobulin | 1 |
| 11147 | hsa-mir-449b | 567 | B2M | beta-2-microglobulin | 1 |
| 11148 | hsa-mir-449c | 567 | B2M | beta-2-microglobulin | 1 |
| 11149 | hsa-mir-3065 | 567 | B2M | beta-2-microglobulin | 1 |
| 11150 | hsa-miR-139-5p | 93082 | NEURL3 | neuralized homolog 3 (Drosophila) pseudogene | 1 |
| 11151 | hsa-miR-152 | 93082 | NEURL3 | neuralized homolog 3 (Drosophila) pseudogene | 1 |
| 11152 | hsa-miR-26b | 93082 | NEURL3 | neuralized homolog 3 (Drosophila) pseudogene | 1 |
| 11153 | hsa-miR-31 | 93082 | NEURL3 | neuralized homolog 3 (Drosophila) pseudogene | 1 |
| 11154 | hsa-miR-338-5p | 93082 | NEURL3 | neuralized homolog 3 (Drosophila) pseudogene | 1 |
| 11155 | hsa-miR-340 | 93082 | NEURL3 | neuralized homolog 3 (Drosophila) pseudogene | 1 |
| 11156 | hsa-miR-383 | 93082 | NEURL3 | neuralized homolog 3 (Drosophila) pseudogene | 1 |
| 11157 | hsa-miR-449a | 93082 | NEURL3 | neuralized homolog 3 (Drosophila) pseudogene | 1 |
| 11158 | hsa-miR-621 | 93082 | NEURL3 | neuralized homolog 3 (Drosophila) pseudogene | 1 |
| 11159 | hsa-miR-657 | 93082 | NEURL3 | neuralized homolog 3 (Drosophila) pseudogene | 1 |
| 11160 | hsa-miR-663 | 93082 | NEURL3 | neuralized homolog 3 (Drosophila) pseudogene | 1 |
| 11161 | hsa-miR-765 | 93082 | NEURL3 | neuralized homolog 3 (Drosophila) pseudogene | 1 |
| 11162 | hsa-miR-770-5p | 93082 | NEURL3 | neuralized homolog 3 (Drosophila) pseudogene | 1 |
| 11163 | hsa-miR-877 | 93082 | NEURL3 | neuralized homolog 3 (Drosophila) pseudogene | 1 |
| 11164 | hsa-mir-26a-1 | 93082 | NEURL3 | neuralized homolog 3 (Drosophila) pseudogene | 1 |
| 11165 | hsa-mir-26b | 93082 | NEURL3 | neuralized homolog 3 (Drosophila) pseudogene | 1 |
| 11166 | hsa-mir-31 | 93082 | NEURL3 | neuralized homolog 3 (Drosophila) pseudogene | 1 |
| 11167 | hsa-mir-194-2 | 93082 | NEURL3 | neuralized homolog 3 (Drosophila) pseudogene | 1 |
| 11168 | hsa-mir-340 | 93082 | NEURL3 | neuralized homolog 3 (Drosophila) pseudogene | 1 |
| 11169 | hsa-mir-338 | 93082 | NEURL3 | neuralized homolog 3 (Drosophila) pseudogene | 1 |
| 11170 | hsa-mir-449b | 93082 | NEURL3 | neuralized homolog 3 (Drosophila) pseudogene | 1 |
| 11171 | hsa-mir-449c | 93082 | NEURL3 | neuralized homolog 3 (Drosophila) pseudogene | 1 |
| 11172 | hsa-mir-3065 | 93082 | NEURL3 | neuralized homolog 3 (Drosophila) pseudogene | 1 |
| 11173 | hsa-miR-10a | 55686 | MREG | melanoregulin | 1 |
| 11174 | hsa-miR-152 | 55686 | MREG | melanoregulin | 1 |
| 11175 | hsa-miR-26a | 55686 | MREG | melanoregulin | 1 |
| 11176 | hsa-miR-26b | 55686 | MREG | melanoregulin | 1 |
| 11177 | hsa-miR-31 | 55686 | MREG | melanoregulin | 1 |
| 11178 | hsa-miR-338-5p | 55686 | MREG | melanoregulin | 1 |
| 11179 | hsa-miR-340 | 55686 | MREG | melanoregulin | 1 |
| 11180 | hsa-miR-449a | 55686 | MREG | melanoregulin | 1 |
| 11181 | hsa-miR-490-5p | 55686 | MREG | melanoregulin | 1 |
| 11182 | hsa-miR-499-5p | 55686 | MREG | melanoregulin | 1 |
| 11183 | hsa-miR-548b-5p | 55686 | MREG | melanoregulin | 1 |
| 11184 | hsa-miR-584 | 55686 | MREG | melanoregulin | 1 |
| 11185 | hsa-miR-621 | 55686 | MREG | melanoregulin | 1 |
| 11186 | hsa-miR-634 | 55686 | MREG | melanoregulin | 1 |
| 11187 | hsa-miR-653 | 55686 | MREG | melanoregulin | 1 |
| 11188 | hsa-miR-657 | 55686 | MREG | melanoregulin | 1 |
| 11189 | hsa-miR-765 | 55686 | MREG | melanoregulin | 1 |
| 11190 | hsa-miR-770-5p | 55686 | MREG | melanoregulin | 1 |
| 11191 | hsa-miR-877 | 55686 | MREG | melanoregulin | 1 |
| 11192 | hsa-mir-26a-1 | 55686 | MREG | melanoregulin | 1 |
| 11193 | hsa-mir-26b | 55686 | MREG | melanoregulin | 1 |
| 11194 | hsa-mir-31 | 55686 | MREG | melanoregulin | 1 |
| 11195 | hsa-mir-194-2 | 55686 | MREG | melanoregulin | 1 |
| 11196 | hsa-mir-340 | 55686 | MREG | melanoregulin | 1 |
| 11197 | hsa-mir-338 | 55686 | MREG | melanoregulin | 1 |
| 11198 | hsa-mir-490 | 55686 | MREG | melanoregulin | 1 |
| 11199 | hsa-mir-548b | 55686 | MREG | melanoregulin | 1 |
| 11200 | hsa-mir-449b | 55686 | MREG | melanoregulin | 1 |
| 11201 | hsa-mir-449c | 55686 | MREG | melanoregulin | 1 |
| 11202 | hsa-mir-3065 | 55686 | MREG | melanoregulin | 1 |
| 11203 | hsa-miR-10a | 27125 | AFF4 | AF4/FMR2 family, member 4 | 1 |
| 11204 | hsa-miR-152 | 27125 | AFF4 | AF4/FMR2 family, member 4 | 1 |
| 11205 | hsa-miR-26a | 27125 | AFF4 | AF4/FMR2 family, member 4 | 1 |
| 11206 | hsa-miR-26b | 27125 | AFF4 | AF4/FMR2 family, member 4 | 1 |
| 11207 | hsa-miR-31 | 27125 | AFF4 | AF4/FMR2 family, member 4 | 1 |
| 11208 | hsa-miR-340 | 27125 | AFF4 | AF4/FMR2 family, member 4 | 1 |
| 11209 | hsa-miR-449a | 27125 | AFF4 | AF4/FMR2 family, member 4 | 1 |
| 11210 | hsa-miR-490-5p | 27125 | AFF4 | AF4/FMR2 family, member 4 | 1 |
| 11211 | hsa-miR-499-5p | 27125 | AFF4 | AF4/FMR2 family, member 4 | 1 |
| 11212 | hsa-miR-564 | 27125 | AFF4 | AF4/FMR2 family, member 4 | 1 |
| 11213 | hsa-miR-621 | 27125 | AFF4 | AF4/FMR2 family, member 4 | 1 |
| 11214 | hsa-miR-634 | 27125 | AFF4 | AF4/FMR2 family, member 4 | 1 |
| 11215 | hsa-miR-765 | 27125 | AFF4 | AF4/FMR2 family, member 4 | 1 |
| 11216 | hsa-miR-770-5p | 27125 | AFF4 | AF4/FMR2 family, member 4 | 1 |
| 11217 | hsa-miR-877 | 27125 | AFF4 | AF4/FMR2 family, member 4 | 1 |
| 11218 | hsa-mir-26a-1 | 27125 | AFF4 | AF4/FMR2 family, member 4 | 1 |
| 11219 | hsa-mir-26b | 27125 | AFF4 | AF4/FMR2 family, member 4 | 1 |
| 11220 | hsa-mir-31 | 27125 | AFF4 | AF4/FMR2 family, member 4 | 1 |
| 11221 | hsa-mir-194-2 | 27125 | AFF4 | AF4/FMR2 family, member 4 | 1 |
| 11222 | hsa-mir-340 | 27125 | AFF4 | AF4/FMR2 family, member 4 | 1 |
| 11223 | hsa-mir-490 | 27125 | AFF4 | AF4/FMR2 family, member 4 | 1 |
| 11224 | hsa-mir-449b | 27125 | AFF4 | AF4/FMR2 family, member 4 | 1 |
| 11225 | hsa-mir-449c | 27125 | AFF4 | AF4/FMR2 family, member 4 | 1 |
| 11226 | hsa-miR-10a | 83933 | HDAC10 | histone deacetylase 10 | 1 |
| 11227 | hsa-miR-152 | 83933 | HDAC10 | histone deacetylase 10 | 1 |
| 11228 | hsa-miR-26a | 83933 | HDAC10 | histone deacetylase 10 | 1 |
| 11229 | hsa-miR-26b | 83933 | HDAC10 | histone deacetylase 10 | 1 |
| 11230 | hsa-miR-31 | 83933 | HDAC10 | histone deacetylase 10 | 1 |
| 11231 | hsa-miR-338-5p | 83933 | HDAC10 | histone deacetylase 10 | 1 |
| 11232 | hsa-miR-340 | 83933 | HDAC10 | histone deacetylase 10 | 1 |
| 11233 | hsa-miR-423-5p | 83933 | HDAC10 | histone deacetylase 10 | 1 |
| 11234 | hsa-miR-449a | 83933 | HDAC10 | histone deacetylase 10 | 1 |
| 11235 | hsa-miR-499-5p | 83933 | HDAC10 | histone deacetylase 10 | 1 |
| 11236 | hsa-miR-548b-5p | 83933 | HDAC10 | histone deacetylase 10 | 1 |
| 11237 | hsa-miR-564 | 83933 | HDAC10 | histone deacetylase 10 | 1 |
| 11238 | hsa-miR-621 | 83933 | HDAC10 | histone deacetylase 10 | 1 |
| 11239 | hsa-miR-657 | 83933 | HDAC10 | histone deacetylase 10 | 1 |
| 11240 | hsa-miR-765 | 83933 | HDAC10 | histone deacetylase 10 | 1 |
| 11241 | hsa-miR-770-5p | 83933 | HDAC10 | histone deacetylase 10 | 1 |
| 11242 | hsa-miR-877 | 83933 | HDAC10 | histone deacetylase 10 | 1 |
| 11243 | hsa-mir-26a-1 | 83933 | HDAC10 | histone deacetylase 10 | 1 |
| 11244 | hsa-mir-26b | 83933 | HDAC10 | histone deacetylase 10 | 1 |
| 11245 | hsa-mir-31 | 83933 | HDAC10 | histone deacetylase 10 | 1 |
| 11246 | hsa-mir-194-2 | 83933 | HDAC10 | histone deacetylase 10 | 1 |
| 11247 | hsa-mir-340 | 83933 | HDAC10 | histone deacetylase 10 | 1 |
| 11248 | hsa-mir-338 | 83933 | HDAC10 | histone deacetylase 10 | 1 |
| 11249 | hsa-mir-548b | 83933 | HDAC10 | histone deacetylase 10 | 1 |
| 11250 | hsa-mir-449b | 83933 | HDAC10 | histone deacetylase 10 | 1 |
| 11251 | hsa-mir-449c | 83933 | HDAC10 | histone deacetylase 10 | 1 |
| 11252 | hsa-mir-3065 | 83933 | HDAC10 | histone deacetylase 10 | 1 |
| 11253 | hsa-miR-1 | 644139 | hCG\_1776018 | phosphoinositide-interacting regulator of transient receptor potential channels | 1 |
| 11254 | hsa-miR-133a | 644139 | hCG\_1776018 | phosphoinositide-interacting regulator of transient receptor potential channels | 1 |
| 11255 | hsa-miR-186 | 644139 | hCG\_1776018 | phosphoinositide-interacting regulator of transient receptor potential channels | 1 |
| 11256 | hsa-miR-326 | 644139 | hCG\_1776018 | phosphoinositide-interacting regulator of transient receptor potential channels | 1 |
| 11257 | hsa-miR-33a | 644139 | hCG\_1776018 | phosphoinositide-interacting regulator of transient receptor potential channels | 1 |
| 11258 | hsa-miR-449b | 644139 | hCG\_1776018 | phosphoinositide-interacting regulator of transient receptor potential channels | 1 |
| 11259 | hsa-miR-550 | 644139 | hCG\_1776018 | phosphoinositide-interacting regulator of transient receptor potential channels | 1 |
| 11260 | hsa-miR-551a | 644139 | hCG\_1776018 | phosphoinositide-interacting regulator of transient receptor potential channels | 1 |
| 11261 | hsa-miR-562 | 644139 | hCG\_1776018 | phosphoinositide-interacting regulator of transient receptor potential channels | 1 |
| 11262 | hsa-miR-575 | 644139 | hCG\_1776018 | phosphoinositide-interacting regulator of transient receptor potential channels | 1 |
| 11263 | hsa-miR-579 | 644139 | hCG\_1776018 | phosphoinositide-interacting regulator of transient receptor potential channels | 1 |
| 11264 | hsa-miR-608 | 644139 | hCG\_1776018 | phosphoinositide-interacting regulator of transient receptor potential channels | 1 |
| 11265 | hsa-miR-620 | 644139 | hCG\_1776018 | phosphoinositide-interacting regulator of transient receptor potential channels | 1 |
| 11266 | hsa-mir-22 | 644139 | hCG\_1776018 | phosphoinositide-interacting regulator of transient receptor potential channels | 1 |
| 11267 | hsa-mir-10a | 644139 | hCG\_1776018 | phosphoinositide-interacting regulator of transient receptor potential channels | 1 |
| 11268 | hsa-mir-186 | 644139 | hCG\_1776018 | phosphoinositide-interacting regulator of transient receptor potential channels | 1 |
| 11269 | hsa-mir-155 | 644139 | hCG\_1776018 | phosphoinositide-interacting regulator of transient receptor potential channels | 1 |
| 11270 | hsa-mir-423 | 644139 | hCG\_1776018 | phosphoinositide-interacting regulator of transient receptor potential channels | 1 |
| 11271 | hsa-mir-499 | 644139 | hCG\_1776018 | phosphoinositide-interacting regulator of transient receptor potential channels | 1 |
| 11272 | hsa-mir-550-1 | 644139 | hCG\_1776018 | phosphoinositide-interacting regulator of transient receptor potential channels | 1 |
| 11273 | hsa-mir-877 | 644139 | hCG\_1776018 | phosphoinositide-interacting regulator of transient receptor potential channels | 1 |
| 11274 | hsa-mir-2116 | 644139 | hCG\_1776018 | phosphoinositide-interacting regulator of transient receptor potential channels | 1 |
| 11275 | hsa-miR-1 | 63894 | C14orf133 | chromosome 14 open reading frame 133 | 1 |
| 11276 | hsa-miR-133a | 63894 | C14orf133 | chromosome 14 open reading frame 133 | 1 |
| 11277 | hsa-miR-186 | 63894 | C14orf133 | chromosome 14 open reading frame 133 | 1 |
| 11278 | hsa-miR-208a | 63894 | C14orf133 | chromosome 14 open reading frame 133 | 1 |
| 11279 | hsa-miR-326 | 63894 | C14orf133 | chromosome 14 open reading frame 133 | 1 |
| 11280 | hsa-miR-449b | 63894 | C14orf133 | chromosome 14 open reading frame 133 | 1 |
| 11281 | hsa-miR-575 | 63894 | C14orf133 | chromosome 14 open reading frame 133 | 1 |
| 11282 | hsa-miR-608 | 63894 | C14orf133 | chromosome 14 open reading frame 133 | 1 |
| 11283 | hsa-miR-620 | 63894 | C14orf133 | chromosome 14 open reading frame 133 | 1 |
| 11284 | hsa-let-7a-3 | 63894 | C14orf133 | chromosome 14 open reading frame 133 | 1 |
| 11285 | hsa-let-7b | 63894 | C14orf133 | chromosome 14 open reading frame 133 | 1 |
| 11286 | hsa-mir-22 | 63894 | C14orf133 | chromosome 14 open reading frame 133 | 1 |
| 11287 | hsa-mir-10a | 63894 | C14orf133 | chromosome 14 open reading frame 133 | 1 |
| 11288 | hsa-mir-186 | 63894 | C14orf133 | chromosome 14 open reading frame 133 | 1 |
| 11289 | hsa-mir-155 | 63894 | C14orf133 | chromosome 14 open reading frame 133 | 1 |
| 11290 | hsa-mir-499 | 63894 | C14orf133 | chromosome 14 open reading frame 133 | 1 |
| 11291 | hsa-mir-877 | 63894 | C14orf133 | chromosome 14 open reading frame 133 | 1 |
| 11292 | hsa-mir-2116 | 63894 | C14orf133 | chromosome 14 open reading frame 133 | 1 |
| 11293 | hsa-mir-3190 | 63894 | C14orf133 | chromosome 14 open reading frame 133 | 1 |
| 11294 | hsa-miR-33a | 57492 | ARID1B | AT rich interactive domain 1B (SWI1-like) | 1 |
| 11295 | hsa-miR-877 | 29121 | CLEC2D | C-type lectin domain family 2, member D | 1 |
| 11296 | hsa-mir-548d-2 | 54464 | XRN1 | 5'-3' exoribonuclease 1 | 1 |
| 11297 | hsa-miR-1 | 83473 | KATNAL2 | katanin p60 subunit A-like 2 | 1 |
| 11298 | hsa-miR-133a | 83473 | KATNAL2 | katanin p60 subunit A-like 2 | 1 |
| 11299 | hsa-miR-186 | 83473 | KATNAL2 | katanin p60 subunit A-like 2 | 1 |
| 11300 | hsa-miR-208a | 83473 | KATNAL2 | katanin p60 subunit A-like 2 | 1 |
| 11301 | hsa-miR-326 | 83473 | KATNAL2 | katanin p60 subunit A-like 2 | 1 |
| 11302 | hsa-miR-449b | 83473 | KATNAL2 | katanin p60 subunit A-like 2 | 1 |
| 11303 | hsa-miR-575 | 83473 | KATNAL2 | katanin p60 subunit A-like 2 | 1 |
| 11304 | hsa-miR-579 | 83473 | KATNAL2 | katanin p60 subunit A-like 2 | 1 |
| 11305 | hsa-miR-608 | 83473 | KATNAL2 | katanin p60 subunit A-like 2 | 1 |
| 11306 | hsa-miR-620 | 83473 | KATNAL2 | katanin p60 subunit A-like 2 | 1 |
| 11307 | hsa-miR-885-5p | 83473 | KATNAL2 | katanin p60 subunit A-like 2 | 1 |
| 11308 | hsa-let-7a-3 | 83473 | KATNAL2 | katanin p60 subunit A-like 2 | 1 |
| 11309 | hsa-let-7b | 83473 | KATNAL2 | katanin p60 subunit A-like 2 | 1 |
| 11310 | hsa-mir-22 | 83473 | KATNAL2 | katanin p60 subunit A-like 2 | 1 |
| 11311 | hsa-mir-10a | 83473 | KATNAL2 | katanin p60 subunit A-like 2 | 1 |
| 11312 | hsa-mir-186 | 83473 | KATNAL2 | katanin p60 subunit A-like 2 | 1 |
| 11313 | hsa-mir-155 | 83473 | KATNAL2 | katanin p60 subunit A-like 2 | 1 |
| 11314 | hsa-mir-499 | 83473 | KATNAL2 | katanin p60 subunit A-like 2 | 1 |
| 11315 | hsa-mir-885 | 83473 | KATNAL2 | katanin p60 subunit A-like 2 | 1 |
| 11316 | hsa-mir-877 | 83473 | KATNAL2 | katanin p60 subunit A-like 2 | 1 |
| 11317 | hsa-mir-2116 | 83473 | KATNAL2 | katanin p60 subunit A-like 2 | 1 |
| 11318 | hsa-mir-3190 | 83473 | KATNAL2 | katanin p60 subunit A-like 2 | 1 |
| 11319 | hsa-miR-490-5p | 55593 | OTUD5 | OTU domain containing 5 | 1 |
| 11320 | hsa-miR-548b-5p | 55593 | OTUD5 | OTU domain containing 5 | 1 |
| 11321 | hsa-miR-564 | 55593 | OTUD5 | OTU domain containing 5 | 1 |
| 11322 | hsa-mir-490 | 55593 | OTUD5 | OTU domain containing 5 | 1 |
| 11323 | hsa-mir-548b | 55593 | OTUD5 | OTU domain containing 5 | 1 |
| 11324 | hsa-mir-548d-2 | 55593 | OTUD5 | OTU domain containing 5 | 1 |
| 11325 | hsa-miR-1 | 55234 | SMU1 | smu-1 suppressor of mec-8 and unc-52 homolog (C. elegans) | 1 |
| 11326 | hsa-miR-133a | 55234 | SMU1 | smu-1 suppressor of mec-8 and unc-52 homolog (C. elegans) | 1 |
| 11327 | hsa-miR-186 | 55234 | SMU1 | smu-1 suppressor of mec-8 and unc-52 homolog (C. elegans) | 1 |
| 11328 | hsa-miR-195 | 55234 | SMU1 | smu-1 suppressor of mec-8 and unc-52 homolog (C. elegans) | 1 |
| 11329 | hsa-miR-326 | 55234 | SMU1 | smu-1 suppressor of mec-8 and unc-52 homolog (C. elegans) | 1 |
| 11330 | hsa-miR-449b | 55234 | SMU1 | smu-1 suppressor of mec-8 and unc-52 homolog (C. elegans) | 1 |
| 11331 | hsa-miR-489 | 55234 | SMU1 | smu-1 suppressor of mec-8 and unc-52 homolog (C. elegans) | 1 |
| 11332 | hsa-miR-497 | 55234 | SMU1 | smu-1 suppressor of mec-8 and unc-52 homolog (C. elegans) | 1 |
| 11333 | hsa-miR-575 | 55234 | SMU1 | smu-1 suppressor of mec-8 and unc-52 homolog (C. elegans) | 1 |
| 11334 | hsa-miR-579 | 55234 | SMU1 | smu-1 suppressor of mec-8 and unc-52 homolog (C. elegans) | 1 |
| 11335 | hsa-miR-608 | 55234 | SMU1 | smu-1 suppressor of mec-8 and unc-52 homolog (C. elegans) | 1 |
| 11336 | hsa-miR-620 | 55234 | SMU1 | smu-1 suppressor of mec-8 and unc-52 homolog (C. elegans) | 1 |
| 11337 | hsa-mir-22 | 55234 | SMU1 | smu-1 suppressor of mec-8 and unc-52 homolog (C. elegans) | 1 |
| 11338 | hsa-mir-10a | 55234 | SMU1 | smu-1 suppressor of mec-8 and unc-52 homolog (C. elegans) | 1 |
| 11339 | hsa-mir-186 | 55234 | SMU1 | smu-1 suppressor of mec-8 and unc-52 homolog (C. elegans) | 1 |
| 11340 | hsa-mir-155 | 55234 | SMU1 | smu-1 suppressor of mec-8 and unc-52 homolog (C. elegans) | 1 |
| 11341 | hsa-mir-499 | 55234 | SMU1 | smu-1 suppressor of mec-8 and unc-52 homolog (C. elegans) | 1 |
| 11342 | hsa-mir-877 | 55234 | SMU1 | smu-1 suppressor of mec-8 and unc-52 homolog (C. elegans) | 1 |
| 11343 | hsa-mir-2116 | 55234 | SMU1 | smu-1 suppressor of mec-8 and unc-52 homolog (C. elegans) | 1 |
| 11344 | hsa-mir-3190 | 55234 | SMU1 | smu-1 suppressor of mec-8 and unc-52 homolog (C. elegans) | 1 |
| 11345 | hsa-miR-1 | 255480 | LOC255480 | hypothetical protein LOC255480 | 1 |
| 11346 | hsa-miR-133a | 255480 | LOC255480 | hypothetical protein LOC255480 | 1 |
| 11347 | hsa-miR-186 | 255480 | LOC255480 | hypothetical protein LOC255480 | 1 |
| 11348 | hsa-miR-208a | 255480 | LOC255480 | hypothetical protein LOC255480 | 1 |
| 11349 | hsa-miR-326 | 255480 | LOC255480 | hypothetical protein LOC255480 | 1 |
| 11350 | hsa-miR-33a | 255480 | LOC255480 | hypothetical protein LOC255480 | 1 |
| 11351 | hsa-miR-449b | 255480 | LOC255480 | hypothetical protein LOC255480 | 1 |
| 11352 | hsa-miR-550 | 255480 | LOC255480 | hypothetical protein LOC255480 | 1 |
| 11353 | hsa-miR-575 | 255480 | LOC255480 | hypothetical protein LOC255480 | 1 |
| 11354 | hsa-miR-608 | 255480 | LOC255480 | hypothetical protein LOC255480 | 1 |
| 11355 | hsa-miR-620 | 255480 | LOC255480 | hypothetical protein LOC255480 | 1 |
| 11356 | hsa-let-7a-3 | 255480 | LOC255480 | hypothetical protein LOC255480 | 1 |
| 11357 | hsa-let-7b | 255480 | LOC255480 | hypothetical protein LOC255480 | 1 |
| 11358 | hsa-mir-22 | 255480 | LOC255480 | hypothetical protein LOC255480 | 1 |
| 11359 | hsa-mir-10a | 255480 | LOC255480 | hypothetical protein LOC255480 | 1 |
| 11360 | hsa-mir-186 | 255480 | LOC255480 | hypothetical protein LOC255480 | 1 |
| 11361 | hsa-mir-155 | 255480 | LOC255480 | hypothetical protein LOC255480 | 1 |
| 11362 | hsa-mir-499 | 255480 | LOC255480 | hypothetical protein LOC255480 | 1 |
| 11363 | hsa-mir-550-1 | 255480 | LOC255480 | hypothetical protein LOC255480 | 1 |
| 11364 | hsa-mir-877 | 255480 | LOC255480 | hypothetical protein LOC255480 | 1 |
| 11365 | hsa-mir-2116 | 255480 | LOC255480 | hypothetical protein LOC255480 | 1 |
| 11366 | hsa-mir-3186 | 255480 | LOC255480 | hypothetical protein LOC255480 | 1 |
| 11367 | hsa-miR-10a | 24139 | EML2 | echinoderm microtubule associated protein like 2 | 1 |
| 11368 | hsa-miR-139-5p | 24139 | EML2 | echinoderm microtubule associated protein like 2 | 1 |
| 11369 | hsa-miR-26a | 24139 | EML2 | echinoderm microtubule associated protein like 2 | 1 |
| 11370 | hsa-miR-26b | 24139 | EML2 | echinoderm microtubule associated protein like 2 | 1 |
| 11371 | hsa-miR-340 | 24139 | EML2 | echinoderm microtubule associated protein like 2 | 1 |
| 11372 | hsa-miR-449a | 24139 | EML2 | echinoderm microtubule associated protein like 2 | 1 |
| 11373 | hsa-miR-499-5p | 24139 | EML2 | echinoderm microtubule associated protein like 2 | 1 |
| 11374 | hsa-miR-548b-5p | 24139 | EML2 | echinoderm microtubule associated protein like 2 | 1 |
| 11375 | hsa-miR-564 | 24139 | EML2 | echinoderm microtubule associated protein like 2 | 1 |
| 11376 | hsa-miR-584 | 24139 | EML2 | echinoderm microtubule associated protein like 2 | 1 |
| 11377 | hsa-miR-621 | 24139 | EML2 | echinoderm microtubule associated protein like 2 | 1 |
| 11378 | hsa-miR-634 | 24139 | EML2 | echinoderm microtubule associated protein like 2 | 1 |
| 11379 | hsa-miR-765 | 24139 | EML2 | echinoderm microtubule associated protein like 2 | 1 |
| 11380 | hsa-miR-770-5p | 24139 | EML2 | echinoderm microtubule associated protein like 2 | 1 |
| 11381 | hsa-miR-877 | 24139 | EML2 | echinoderm microtubule associated protein like 2 | 1 |
| 11382 | hsa-mir-26a-1 | 24139 | EML2 | echinoderm microtubule associated protein like 2 | 1 |
| 11383 | hsa-mir-26b | 24139 | EML2 | echinoderm microtubule associated protein like 2 | 1 |
| 11384 | hsa-mir-194-2 | 24139 | EML2 | echinoderm microtubule associated protein like 2 | 1 |
| 11385 | hsa-mir-340 | 24139 | EML2 | echinoderm microtubule associated protein like 2 | 1 |
| 11386 | hsa-mir-548b | 24139 | EML2 | echinoderm microtubule associated protein like 2 | 1 |
| 11387 | hsa-mir-449b | 24139 | EML2 | echinoderm microtubule associated protein like 2 | 1 |
| 11388 | hsa-mir-449c | 24139 | EML2 | echinoderm microtubule associated protein like 2 | 1 |
| 11389 | hsa-miR-1228\* | 51667 | NUB1 | negative regulator of ubiquitin-like proteins 1 | 1 |
| 11390 | hsa-miR-423-5p | 51667 | NUB1 | negative regulator of ubiquitin-like proteins 1 | 1 |
| 11391 | hsa-miR-490-5p | 51667 | NUB1 | negative regulator of ubiquitin-like proteins 1 | 1 |
| 11392 | hsa-miR-564 | 51667 | NUB1 | negative regulator of ubiquitin-like proteins 1 | 1 |
| 11393 | hsa-miR-584 | 51667 | NUB1 | negative regulator of ubiquitin-like proteins 1 | 1 |
| 11394 | hsa-miR-663 | 51667 | NUB1 | negative regulator of ubiquitin-like proteins 1 | 1 |
| 11395 | hsa-mir-490 | 51667 | NUB1 | negative regulator of ubiquitin-like proteins 1 | 1 |
| 11396 | hsa-mir-548d-2 | 51667 | NUB1 | negative regulator of ubiquitin-like proteins 1 | 1 |
| 11397 | hsa-miR-1 | 390066 | OR52D1 | olfactory receptor, family 52, subfamily D, member 1 | 1 |
| 11398 | hsa-miR-133a | 390066 | OR52D1 | olfactory receptor, family 52, subfamily D, member 1 | 1 |
| 11399 | hsa-miR-186 | 390066 | OR52D1 | olfactory receptor, family 52, subfamily D, member 1 | 1 |
| 11400 | hsa-miR-208a | 390066 | OR52D1 | olfactory receptor, family 52, subfamily D, member 1 | 1 |
| 11401 | hsa-miR-326 | 390066 | OR52D1 | olfactory receptor, family 52, subfamily D, member 1 | 1 |
| 11402 | hsa-miR-449b | 390066 | OR52D1 | olfactory receptor, family 52, subfamily D, member 1 | 1 |
| 11403 | hsa-miR-489 | 390066 | OR52D1 | olfactory receptor, family 52, subfamily D, member 1 | 1 |
| 11404 | hsa-miR-575 | 390066 | OR52D1 | olfactory receptor, family 52, subfamily D, member 1 | 1 |
| 11405 | hsa-miR-579 | 390066 | OR52D1 | olfactory receptor, family 52, subfamily D, member 1 | 1 |
| 11406 | hsa-miR-608 | 390066 | OR52D1 | olfactory receptor, family 52, subfamily D, member 1 | 1 |
| 11407 | hsa-miR-620 | 390066 | OR52D1 | olfactory receptor, family 52, subfamily D, member 1 | 1 |
| 11408 | hsa-miR-885-5p | 390066 | OR52D1 | olfactory receptor, family 52, subfamily D, member 1 | 1 |
| 11409 | hsa-let-7a-3 | 390066 | OR52D1 | olfactory receptor, family 52, subfamily D, member 1 | 1 |
| 11410 | hsa-let-7b | 390066 | OR52D1 | olfactory receptor, family 52, subfamily D, member 1 | 1 |
| 11411 | hsa-mir-22 | 390066 | OR52D1 | olfactory receptor, family 52, subfamily D, member 1 | 1 |
| 11412 | hsa-mir-10a | 390066 | OR52D1 | olfactory receptor, family 52, subfamily D, member 1 | 1 |
| 11413 | hsa-mir-186 | 390066 | OR52D1 | olfactory receptor, family 52, subfamily D, member 1 | 1 |
| 11414 | hsa-mir-155 | 390066 | OR52D1 | olfactory receptor, family 52, subfamily D, member 1 | 1 |
| 11415 | hsa-mir-499 | 390066 | OR52D1 | olfactory receptor, family 52, subfamily D, member 1 | 1 |
| 11416 | hsa-mir-885 | 390066 | OR52D1 | olfactory receptor, family 52, subfamily D, member 1 | 1 |
| 11417 | hsa-mir-877 | 390066 | OR52D1 | olfactory receptor, family 52, subfamily D, member 1 | 1 |
| 11418 | hsa-miR-1 | 114781 | BTBD9 | BTB (POZ) domain containing 9 | 1 |
| 11419 | hsa-miR-133a | 114781 | BTBD9 | BTB (POZ) domain containing 9 | 1 |
| 11420 | hsa-miR-186 | 114781 | BTBD9 | BTB (POZ) domain containing 9 | 1 |
| 11421 | hsa-miR-208a | 114781 | BTBD9 | BTB (POZ) domain containing 9 | 1 |
| 11422 | hsa-miR-326 | 114781 | BTBD9 | BTB (POZ) domain containing 9 | 1 |
| 11423 | hsa-miR-449b | 114781 | BTBD9 | BTB (POZ) domain containing 9 | 1 |
| 11424 | hsa-miR-551a | 114781 | BTBD9 | BTB (POZ) domain containing 9 | 1 |
| 11425 | hsa-miR-575 | 114781 | BTBD9 | BTB (POZ) domain containing 9 | 1 |
| 11426 | hsa-miR-579 | 114781 | BTBD9 | BTB (POZ) domain containing 9 | 1 |
| 11427 | hsa-miR-608 | 114781 | BTBD9 | BTB (POZ) domain containing 9 | 1 |
| 11428 | hsa-miR-620 | 114781 | BTBD9 | BTB (POZ) domain containing 9 | 1 |
| 11429 | hsa-miR-637 | 114781 | BTBD9 | BTB (POZ) domain containing 9 | 1 |
| 11430 | hsa-mir-22 | 114781 | BTBD9 | BTB (POZ) domain containing 9 | 1 |
| 11431 | hsa-mir-10a | 114781 | BTBD9 | BTB (POZ) domain containing 9 | 1 |
| 11432 | hsa-mir-186 | 114781 | BTBD9 | BTB (POZ) domain containing 9 | 1 |
| 11433 | hsa-mir-155 | 114781 | BTBD9 | BTB (POZ) domain containing 9 | 1 |
| 11434 | hsa-mir-423 | 114781 | BTBD9 | BTB (POZ) domain containing 9 | 1 |
| 11435 | hsa-mir-499 | 114781 | BTBD9 | BTB (POZ) domain containing 9 | 1 |
| 11436 | hsa-mir-550-1 | 114781 | BTBD9 | BTB (POZ) domain containing 9 | 1 |
| 11437 | hsa-mir-877 | 114781 | BTBD9 | BTB (POZ) domain containing 9 | 1 |
| 11438 | hsa-mir-2116 | 114781 | BTBD9 | BTB (POZ) domain containing 9 | 1 |
| 11439 | hsa-mir-3190 | 114781 | BTBD9 | BTB (POZ) domain containing 9 | 1 |
| 11440 | hsa-miR-1 | 646282 | AZGP1P1 | alpha-2-glycoprotein 1, zinc-binding pseudogene 1 | 1 |
| 11441 | hsa-miR-133a | 646282 | AZGP1P1 | alpha-2-glycoprotein 1, zinc-binding pseudogene 1 | 1 |
| 11442 | hsa-miR-186 | 646282 | AZGP1P1 | alpha-2-glycoprotein 1, zinc-binding pseudogene 1 | 1 |
| 11443 | hsa-miR-326 | 646282 | AZGP1P1 | alpha-2-glycoprotein 1, zinc-binding pseudogene 1 | 1 |
| 11444 | hsa-miR-33a | 646282 | AZGP1P1 | alpha-2-glycoprotein 1, zinc-binding pseudogene 1 | 1 |
| 11445 | hsa-miR-449b | 646282 | AZGP1P1 | alpha-2-glycoprotein 1, zinc-binding pseudogene 1 | 1 |
| 11446 | hsa-miR-550 | 646282 | AZGP1P1 | alpha-2-glycoprotein 1, zinc-binding pseudogene 1 | 1 |
| 11447 | hsa-miR-551a | 646282 | AZGP1P1 | alpha-2-glycoprotein 1, zinc-binding pseudogene 1 | 1 |
| 11448 | hsa-miR-562 | 646282 | AZGP1P1 | alpha-2-glycoprotein 1, zinc-binding pseudogene 1 | 1 |
| 11449 | hsa-miR-575 | 646282 | AZGP1P1 | alpha-2-glycoprotein 1, zinc-binding pseudogene 1 | 1 |
| 11450 | hsa-miR-579 | 646282 | AZGP1P1 | alpha-2-glycoprotein 1, zinc-binding pseudogene 1 | 1 |
| 11451 | hsa-miR-608 | 646282 | AZGP1P1 | alpha-2-glycoprotein 1, zinc-binding pseudogene 1 | 1 |
| 11452 | hsa-miR-620 | 646282 | AZGP1P1 | alpha-2-glycoprotein 1, zinc-binding pseudogene 1 | 1 |
| 11453 | hsa-mir-22 | 646282 | AZGP1P1 | alpha-2-glycoprotein 1, zinc-binding pseudogene 1 | 1 |
| 11454 | hsa-mir-10a | 646282 | AZGP1P1 | alpha-2-glycoprotein 1, zinc-binding pseudogene 1 | 1 |
| 11455 | hsa-mir-186 | 646282 | AZGP1P1 | alpha-2-glycoprotein 1, zinc-binding pseudogene 1 | 1 |
| 11456 | hsa-mir-155 | 646282 | AZGP1P1 | alpha-2-glycoprotein 1, zinc-binding pseudogene 1 | 1 |
| 11457 | hsa-mir-499 | 646282 | AZGP1P1 | alpha-2-glycoprotein 1, zinc-binding pseudogene 1 | 1 |
| 11458 | hsa-mir-550-1 | 646282 | AZGP1P1 | alpha-2-glycoprotein 1, zinc-binding pseudogene 1 | 1 |
| 11459 | hsa-mir-877 | 646282 | AZGP1P1 | alpha-2-glycoprotein 1, zinc-binding pseudogene 1 | 1 |
| 11460 | hsa-mir-2116 | 646282 | AZGP1P1 | alpha-2-glycoprotein 1, zinc-binding pseudogene 1 | 1 |
| 11461 | hsa-mir-3186 | 646282 | AZGP1P1 | alpha-2-glycoprotein 1, zinc-binding pseudogene 1 | 1 |
| 11462 | hsa-miR-10a | 152926 | PPM1K | protein phosphatase 1K (PP2C domain containing) | 1 |
| 11463 | hsa-miR-1228\* | 152926 | PPM1K | protein phosphatase 1K (PP2C domain containing) | 1 |
| 11464 | hsa-miR-139-5p | 152926 | PPM1K | protein phosphatase 1K (PP2C domain containing) | 1 |
| 11465 | hsa-miR-152 | 152926 | PPM1K | protein phosphatase 1K (PP2C domain containing) | 1 |
| 11466 | hsa-miR-26a | 152926 | PPM1K | protein phosphatase 1K (PP2C domain containing) | 1 |
| 11467 | hsa-miR-26b | 152926 | PPM1K | protein phosphatase 1K (PP2C domain containing) | 1 |
| 11468 | hsa-miR-338-5p | 152926 | PPM1K | protein phosphatase 1K (PP2C domain containing) | 1 |
| 11469 | hsa-miR-340 | 152926 | PPM1K | protein phosphatase 1K (PP2C domain containing) | 1 |
| 11470 | hsa-miR-423-5p | 152926 | PPM1K | protein phosphatase 1K (PP2C domain containing) | 1 |
| 11471 | hsa-miR-449a | 152926 | PPM1K | protein phosphatase 1K (PP2C domain containing) | 1 |
| 11472 | hsa-miR-499-5p | 152926 | PPM1K | protein phosphatase 1K (PP2C domain containing) | 1 |
| 11473 | hsa-miR-548b-5p | 152926 | PPM1K | protein phosphatase 1K (PP2C domain containing) | 1 |
| 11474 | hsa-miR-564 | 152926 | PPM1K | protein phosphatase 1K (PP2C domain containing) | 1 |
| 11475 | hsa-miR-621 | 152926 | PPM1K | protein phosphatase 1K (PP2C domain containing) | 1 |
| 11476 | hsa-miR-657 | 152926 | PPM1K | protein phosphatase 1K (PP2C domain containing) | 1 |
| 11477 | hsa-miR-765 | 152926 | PPM1K | protein phosphatase 1K (PP2C domain containing) | 1 |
| 11478 | hsa-miR-770-5p | 152926 | PPM1K | protein phosphatase 1K (PP2C domain containing) | 1 |
| 11479 | hsa-miR-877 | 152926 | PPM1K | protein phosphatase 1K (PP2C domain containing) | 1 |
| 11480 | hsa-mir-26a-1 | 152926 | PPM1K | protein phosphatase 1K (PP2C domain containing) | 1 |
| 11481 | hsa-mir-26b | 152926 | PPM1K | protein phosphatase 1K (PP2C domain containing) | 1 |
| 11482 | hsa-mir-194-2 | 152926 | PPM1K | protein phosphatase 1K (PP2C domain containing) | 1 |
| 11483 | hsa-mir-340 | 152926 | PPM1K | protein phosphatase 1K (PP2C domain containing) | 1 |
| 11484 | hsa-mir-338 | 152926 | PPM1K | protein phosphatase 1K (PP2C domain containing) | 1 |
| 11485 | hsa-mir-548b | 152926 | PPM1K | protein phosphatase 1K (PP2C domain containing) | 1 |
| 11486 | hsa-mir-449b | 152926 | PPM1K | protein phosphatase 1K (PP2C domain containing) | 1 |
| 11487 | hsa-mir-449c | 152926 | PPM1K | protein phosphatase 1K (PP2C domain containing) | 1 |
| 11488 | hsa-mir-3065 | 152926 | PPM1K | protein phosphatase 1K (PP2C domain containing) | 1 |
| 11505 | hsa-miR-663 | 115361 | GBP4 | guanylate binding protein 4 | 1 |
| 11521 | hsa-miR-1 | 1831 | TSC22D3 | TSC22 domain family, member 3 | 1 |
| 11522 | hsa-miR-133a | 1831 | TSC22D3 | TSC22 domain family, member 3 | 1 |
| 11523 | hsa-miR-186 | 1831 | TSC22D3 | TSC22 domain family, member 3 | 1 |
| 11524 | hsa-miR-326 | 1831 | TSC22D3 | TSC22 domain family, member 3 | 1 |
| 11525 | hsa-miR-33a | 1831 | TSC22D3 | TSC22 domain family, member 3 | 1 |
| 11526 | hsa-miR-449b | 1831 | TSC22D3 | TSC22 domain family, member 3 | 1 |
| 11527 | hsa-miR-550 | 1831 | TSC22D3 | TSC22 domain family, member 3 | 1 |
| 11528 | hsa-miR-551a | 1831 | TSC22D3 | TSC22 domain family, member 3 | 1 |
| 11529 | hsa-miR-575 | 1831 | TSC22D3 | TSC22 domain family, member 3 | 1 |
| 11530 | hsa-miR-579 | 1831 | TSC22D3 | TSC22 domain family, member 3 | 1 |
| 11531 | hsa-miR-608 | 1831 | TSC22D3 | TSC22 domain family, member 3 | 1 |
| 11532 | hsa-miR-620 | 1831 | TSC22D3 | TSC22 domain family, member 3 | 1 |
| 11533 | hsa-mir-22 | 1831 | TSC22D3 | TSC22 domain family, member 3 | 1 |
| 11534 | hsa-mir-10a | 1831 | TSC22D3 | TSC22 domain family, member 3 | 1 |
| 11535 | hsa-mir-186 | 1831 | TSC22D3 | TSC22 domain family, member 3 | 1 |
| 11536 | hsa-mir-155 | 1831 | TSC22D3 | TSC22 domain family, member 3 | 1 |
| 11537 | hsa-mir-423 | 1831 | TSC22D3 | TSC22 domain family, member 3 | 1 |
| 11538 | hsa-mir-499 | 1831 | TSC22D3 | TSC22 domain family, member 3 | 1 |
| 11539 | hsa-mir-550-1 | 1831 | TSC22D3 | TSC22 domain family, member 3 | 1 |
| 11540 | hsa-mir-877 | 1831 | TSC22D3 | TSC22 domain family, member 3 | 1 |
| 11541 | hsa-mir-2116 | 1831 | TSC22D3 | TSC22 domain family, member 3 | 1 |
| 11542 | hsa-mir-3186 | 1831 | TSC22D3 | TSC22 domain family, member 3 | 1 |
| 11543 | hsa-mir-3190 | 1831 | TSC22D3 | TSC22 domain family, member 3 | 1 |
| 11544 | hsa-miR-1 | 494513 | DFNB59 | deafness, autosomal recessive 59 | 1 |
| 11545 | hsa-miR-133a | 494513 | DFNB59 | deafness, autosomal recessive 59 | 1 |
| 11546 | hsa-miR-186 | 494513 | DFNB59 | deafness, autosomal recessive 59 | 1 |
| 11547 | hsa-miR-208a | 494513 | DFNB59 | deafness, autosomal recessive 59 | 1 |
| 11548 | hsa-miR-326 | 494513 | DFNB59 | deafness, autosomal recessive 59 | 1 |
| 11549 | hsa-miR-449b | 494513 | DFNB59 | deafness, autosomal recessive 59 | 1 |
| 11550 | hsa-miR-489 | 494513 | DFNB59 | deafness, autosomal recessive 59 | 1 |
| 11551 | hsa-miR-562 | 494513 | DFNB59 | deafness, autosomal recessive 59 | 1 |
| 11552 | hsa-miR-575 | 494513 | DFNB59 | deafness, autosomal recessive 59 | 1 |
| 11553 | hsa-miR-579 | 494513 | DFNB59 | deafness, autosomal recessive 59 | 1 |
| 11554 | hsa-miR-608 | 494513 | DFNB59 | deafness, autosomal recessive 59 | 1 |
| 11555 | hsa-miR-620 | 494513 | DFNB59 | deafness, autosomal recessive 59 | 1 |
| 11556 | hsa-miR-885-5p | 494513 | DFNB59 | deafness, autosomal recessive 59 | 1 |
| 11557 | hsa-mir-22 | 494513 | DFNB59 | deafness, autosomal recessive 59 | 1 |
| 11558 | hsa-mir-10a | 494513 | DFNB59 | deafness, autosomal recessive 59 | 1 |
| 11559 | hsa-mir-186 | 494513 | DFNB59 | deafness, autosomal recessive 59 | 1 |
| 11560 | hsa-mir-155 | 494513 | DFNB59 | deafness, autosomal recessive 59 | 1 |
| 11561 | hsa-mir-423 | 494513 | DFNB59 | deafness, autosomal recessive 59 | 1 |
| 11562 | hsa-mir-499 | 494513 | DFNB59 | deafness, autosomal recessive 59 | 1 |
| 11563 | hsa-mir-885 | 494513 | DFNB59 | deafness, autosomal recessive 59 | 1 |
| 11564 | hsa-mir-877 | 494513 | DFNB59 | deafness, autosomal recessive 59 | 1 |
| 11565 | hsa-mir-2116 | 494513 | DFNB59 | deafness, autosomal recessive 59 | 1 |
| 11566 | hsa-mir-3190 | 494513 | DFNB59 | deafness, autosomal recessive 59 | 1 |
| 11567 | hsa-miR-10a | 83999 | KREMEN1 | kringle containing transmembrane protein 1 | 1 |
| 11568 | hsa-miR-139-5p | 83999 | KREMEN1 | kringle containing transmembrane protein 1 | 1 |
| 11569 | hsa-miR-152 | 83999 | KREMEN1 | kringle containing transmembrane protein 1 | 1 |
| 11570 | hsa-miR-26b | 83999 | KREMEN1 | kringle containing transmembrane protein 1 | 1 |
| 11571 | hsa-miR-31 | 83999 | KREMEN1 | kringle containing transmembrane protein 1 | 1 |
| 11572 | hsa-miR-338-5p | 83999 | KREMEN1 | kringle containing transmembrane protein 1 | 1 |
| 11573 | hsa-miR-340 | 83999 | KREMEN1 | kringle containing transmembrane protein 1 | 1 |
| 11574 | hsa-miR-383 | 83999 | KREMEN1 | kringle containing transmembrane protein 1 | 1 |
| 11575 | hsa-miR-449a | 83999 | KREMEN1 | kringle containing transmembrane protein 1 | 1 |
| 11576 | hsa-miR-499-5p | 83999 | KREMEN1 | kringle containing transmembrane protein 1 | 1 |
| 11577 | hsa-miR-548b-5p | 83999 | KREMEN1 | kringle containing transmembrane protein 1 | 1 |
| 11578 | hsa-miR-621 | 83999 | KREMEN1 | kringle containing transmembrane protein 1 | 1 |
| 11579 | hsa-miR-657 | 83999 | KREMEN1 | kringle containing transmembrane protein 1 | 1 |
| 11580 | hsa-miR-663 | 83999 | KREMEN1 | kringle containing transmembrane protein 1 | 1 |
| 11581 | hsa-miR-765 | 83999 | KREMEN1 | kringle containing transmembrane protein 1 | 1 |
| 11582 | hsa-miR-770-5p | 83999 | KREMEN1 | kringle containing transmembrane protein 1 | 1 |
| 11583 | hsa-miR-877 | 83999 | KREMEN1 | kringle containing transmembrane protein 1 | 1 |
| 11584 | hsa-mir-26a-1 | 83999 | KREMEN1 | kringle containing transmembrane protein 1 | 1 |
| 11585 | hsa-mir-26b | 83999 | KREMEN1 | kringle containing transmembrane protein 1 | 1 |
| 11586 | hsa-mir-31 | 83999 | KREMEN1 | kringle containing transmembrane protein 1 | 1 |
| 11587 | hsa-mir-194-2 | 83999 | KREMEN1 | kringle containing transmembrane protein 1 | 1 |
| 11588 | hsa-mir-340 | 83999 | KREMEN1 | kringle containing transmembrane protein 1 | 1 |
| 11589 | hsa-mir-338 | 83999 | KREMEN1 | kringle containing transmembrane protein 1 | 1 |
| 11590 | hsa-mir-548b | 83999 | KREMEN1 | kringle containing transmembrane protein 1 | 1 |
| 11591 | hsa-mir-449b | 83999 | KREMEN1 | kringle containing transmembrane protein 1 | 1 |
| 11592 | hsa-mir-449c | 83999 | KREMEN1 | kringle containing transmembrane protein 1 | 1 |
| 11593 | hsa-mir-3065 | 83999 | KREMEN1 | kringle containing transmembrane protein 1 | 1 |
| 11594 | hsa-miR-1 | 133688 | UGT3A1 | UDP glycosyltransferase 3 family, polypeptide A1 | 1 |
| 11595 | hsa-miR-133a | 133688 | UGT3A1 | UDP glycosyltransferase 3 family, polypeptide A1 | 1 |
| 11596 | hsa-miR-186 | 133688 | UGT3A1 | UDP glycosyltransferase 3 family, polypeptide A1 | 1 |
| 11597 | hsa-miR-449b | 133688 | UGT3A1 | UDP glycosyltransferase 3 family, polypeptide A1 | 1 |
| 11598 | hsa-miR-550 | 133688 | UGT3A1 | UDP glycosyltransferase 3 family, polypeptide A1 | 1 |
| 11599 | hsa-miR-551a | 133688 | UGT3A1 | UDP glycosyltransferase 3 family, polypeptide A1 | 1 |
| 11600 | hsa-miR-562 | 133688 | UGT3A1 | UDP glycosyltransferase 3 family, polypeptide A1 | 1 |
| 11601 | hsa-miR-579 | 133688 | UGT3A1 | UDP glycosyltransferase 3 family, polypeptide A1 | 1 |
| 11602 | hsa-miR-608 | 133688 | UGT3A1 | UDP glycosyltransferase 3 family, polypeptide A1 | 1 |
| 11603 | hsa-miR-620 | 133688 | UGT3A1 | UDP glycosyltransferase 3 family, polypeptide A1 | 1 |
| 11604 | hsa-mir-22 | 133688 | UGT3A1 | UDP glycosyltransferase 3 family, polypeptide A1 | 1 |
| 11605 | hsa-mir-10a | 133688 | UGT3A1 | UDP glycosyltransferase 3 family, polypeptide A1 | 1 |
| 11606 | hsa-mir-186 | 133688 | UGT3A1 | UDP glycosyltransferase 3 family, polypeptide A1 | 1 |
| 11607 | hsa-mir-155 | 133688 | UGT3A1 | UDP glycosyltransferase 3 family, polypeptide A1 | 1 |
| 11608 | hsa-mir-423 | 133688 | UGT3A1 | UDP glycosyltransferase 3 family, polypeptide A1 | 1 |
| 11609 | hsa-mir-550-1 | 133688 | UGT3A1 | UDP glycosyltransferase 3 family, polypeptide A1 | 1 |
| 11610 | hsa-mir-3186 | 133688 | UGT3A1 | UDP glycosyltransferase 3 family, polypeptide A1 | 1 |
| 11611 | hsa-miR-152 | 201475 | RAB12 | RAB12, member RAS oncogene family | 1 |
| 11612 | hsa-miR-26b | 201475 | RAB12 | RAB12, member RAS oncogene family | 1 |
| 11613 | hsa-miR-31 | 201475 | RAB12 | RAB12, member RAS oncogene family | 1 |
| 11614 | hsa-miR-338-5p | 201475 | RAB12 | RAB12, member RAS oncogene family | 1 |
| 11615 | hsa-miR-340 | 201475 | RAB12 | RAB12, member RAS oncogene family | 1 |
| 11616 | hsa-miR-383 | 201475 | RAB12 | RAB12, member RAS oncogene family | 1 |
| 11617 | hsa-miR-449a | 201475 | RAB12 | RAB12, member RAS oncogene family | 1 |
| 11618 | hsa-miR-490-5p | 201475 | RAB12 | RAB12, member RAS oncogene family | 1 |
| 11619 | hsa-miR-504 | 201475 | RAB12 | RAB12, member RAS oncogene family | 1 |
| 11620 | hsa-miR-621 | 201475 | RAB12 | RAB12, member RAS oncogene family | 1 |
| 11621 | hsa-miR-657 | 201475 | RAB12 | RAB12, member RAS oncogene family | 1 |
| 11622 | hsa-miR-765 | 201475 | RAB12 | RAB12, member RAS oncogene family | 1 |
| 11623 | hsa-miR-770-5p | 201475 | RAB12 | RAB12, member RAS oncogene family | 1 |
| 11624 | hsa-miR-877 | 201475 | RAB12 | RAB12, member RAS oncogene family | 1 |
| 11625 | hsa-mir-26a-1 | 201475 | RAB12 | RAB12, member RAS oncogene family | 1 |
| 11626 | hsa-mir-26b | 201475 | RAB12 | RAB12, member RAS oncogene family | 1 |
| 11627 | hsa-mir-31 | 201475 | RAB12 | RAB12, member RAS oncogene family | 1 |
| 11628 | hsa-mir-194-2 | 201475 | RAB12 | RAB12, member RAS oncogene family | 1 |
| 11629 | hsa-mir-340 | 201475 | RAB12 | RAB12, member RAS oncogene family | 1 |
| 11630 | hsa-mir-338 | 201475 | RAB12 | RAB12, member RAS oncogene family | 1 |
| 11631 | hsa-mir-490 | 201475 | RAB12 | RAB12, member RAS oncogene family | 1 |
| 11632 | hsa-mir-449b | 201475 | RAB12 | RAB12, member RAS oncogene family | 1 |
| 11633 | hsa-mir-449c | 201475 | RAB12 | RAB12, member RAS oncogene family | 1 |
| 11634 | hsa-mir-3065 | 201475 | RAB12 | RAB12, member RAS oncogene family | 1 |
| 11635 | hsa-miR-10a | 124093 | CCDC78 | coiled-coil domain containing 78 | 1 |
| 11636 | hsa-miR-1228\* | 124093 | CCDC78 | coiled-coil domain containing 78 | 1 |
| 11637 | hsa-miR-139-5p | 124093 | CCDC78 | coiled-coil domain containing 78 | 1 |
| 11638 | hsa-miR-152 | 124093 | CCDC78 | coiled-coil domain containing 78 | 1 |
| 11639 | hsa-miR-26a | 124093 | CCDC78 | coiled-coil domain containing 78 | 1 |
| 11640 | hsa-miR-26b | 124093 | CCDC78 | coiled-coil domain containing 78 | 1 |
| 11641 | hsa-miR-31 | 124093 | CCDC78 | coiled-coil domain containing 78 | 1 |
| 11642 | hsa-miR-338-5p | 124093 | CCDC78 | coiled-coil domain containing 78 | 1 |
| 11643 | hsa-miR-340 | 124093 | CCDC78 | coiled-coil domain containing 78 | 1 |
| 11644 | hsa-miR-423-5p | 124093 | CCDC78 | coiled-coil domain containing 78 | 1 |
| 11645 | hsa-miR-449a | 124093 | CCDC78 | coiled-coil domain containing 78 | 1 |
| 11646 | hsa-miR-499-5p | 124093 | CCDC78 | coiled-coil domain containing 78 | 1 |
| 11647 | hsa-miR-548b-5p | 124093 | CCDC78 | coiled-coil domain containing 78 | 1 |
| 11648 | hsa-miR-564 | 124093 | CCDC78 | coiled-coil domain containing 78 | 1 |
| 11649 | hsa-miR-621 | 124093 | CCDC78 | coiled-coil domain containing 78 | 1 |
| 11650 | hsa-miR-657 | 124093 | CCDC78 | coiled-coil domain containing 78 | 1 |
| 11651 | hsa-miR-765 | 124093 | CCDC78 | coiled-coil domain containing 78 | 1 |
| 11652 | hsa-miR-770-5p | 124093 | CCDC78 | coiled-coil domain containing 78 | 1 |
| 11653 | hsa-miR-877 | 124093 | CCDC78 | coiled-coil domain containing 78 | 1 |
| 11654 | hsa-mir-26a-1 | 124093 | CCDC78 | coiled-coil domain containing 78 | 1 |
| 11655 | hsa-mir-26b | 124093 | CCDC78 | coiled-coil domain containing 78 | 1 |
| 11656 | hsa-mir-31 | 124093 | CCDC78 | coiled-coil domain containing 78 | 1 |
| 11657 | hsa-mir-194-2 | 124093 | CCDC78 | coiled-coil domain containing 78 | 1 |
| 11658 | hsa-mir-340 | 124093 | CCDC78 | coiled-coil domain containing 78 | 1 |
| 11659 | hsa-mir-338 | 124093 | CCDC78 | coiled-coil domain containing 78 | 1 |
| 11660 | hsa-mir-548b | 124093 | CCDC78 | coiled-coil domain containing 78 | 1 |
| 11661 | hsa-mir-449b | 124093 | CCDC78 | coiled-coil domain containing 78 | 1 |
| 11662 | hsa-mir-449c | 124093 | CCDC78 | coiled-coil domain containing 78 | 1 |
| 11663 | hsa-mir-3065 | 124093 | CCDC78 | coiled-coil domain containing 78 | 1 |
| 11664 | hsa-miR-152 | 154075 | SAMD3 | sterile alpha motif domain containing 3 | 1 |
| 11665 | hsa-miR-26a | 154075 | SAMD3 | sterile alpha motif domain containing 3 | 1 |
| 11666 | hsa-miR-26b | 154075 | SAMD3 | sterile alpha motif domain containing 3 | 1 |
| 11667 | hsa-miR-31 | 154075 | SAMD3 | sterile alpha motif domain containing 3 | 1 |
| 11668 | hsa-miR-449a | 154075 | SAMD3 | sterile alpha motif domain containing 3 | 1 |
| 11669 | hsa-miR-490-5p | 154075 | SAMD3 | sterile alpha motif domain containing 3 | 1 |
| 11670 | hsa-miR-499-5p | 154075 | SAMD3 | sterile alpha motif domain containing 3 | 1 |
| 11671 | hsa-miR-564 | 154075 | SAMD3 | sterile alpha motif domain containing 3 | 1 |
| 11672 | hsa-miR-621 | 154075 | SAMD3 | sterile alpha motif domain containing 3 | 1 |
| 11673 | hsa-miR-765 | 154075 | SAMD3 | sterile alpha motif domain containing 3 | 1 |
| 11674 | hsa-miR-770-5p | 154075 | SAMD3 | sterile alpha motif domain containing 3 | 1 |
| 11675 | hsa-mir-26a-1 | 154075 | SAMD3 | sterile alpha motif domain containing 3 | 1 |
| 11676 | hsa-mir-26b | 154075 | SAMD3 | sterile alpha motif domain containing 3 | 1 |
| 11677 | hsa-mir-31 | 154075 | SAMD3 | sterile alpha motif domain containing 3 | 1 |
| 11678 | hsa-mir-194-2 | 154075 | SAMD3 | sterile alpha motif domain containing 3 | 1 |
| 11679 | hsa-mir-490 | 154075 | SAMD3 | sterile alpha motif domain containing 3 | 1 |
| 11680 | hsa-mir-449b | 154075 | SAMD3 | sterile alpha motif domain containing 3 | 1 |
| 11681 | hsa-mir-449c | 154075 | SAMD3 | sterile alpha motif domain containing 3 | 1 |
| 11682 | hsa-miR-1 | 157313 | CDCA2 | cell division cycle associated 2 | 1 |
| 11683 | hsa-miR-133a | 157313 | CDCA2 | cell division cycle associated 2 | 1 |
| 11684 | hsa-miR-186 | 157313 | CDCA2 | cell division cycle associated 2 | 1 |
| 11685 | hsa-miR-208a | 157313 | CDCA2 | cell division cycle associated 2 | 1 |
| 11686 | hsa-miR-326 | 157313 | CDCA2 | cell division cycle associated 2 | 1 |
| 11687 | hsa-miR-449b | 157313 | CDCA2 | cell division cycle associated 2 | 1 |
| 11688 | hsa-miR-562 | 157313 | CDCA2 | cell division cycle associated 2 | 1 |
| 11689 | hsa-miR-575 | 157313 | CDCA2 | cell division cycle associated 2 | 1 |
| 11690 | hsa-miR-608 | 157313 | CDCA2 | cell division cycle associated 2 | 1 |
| 11691 | hsa-miR-620 | 157313 | CDCA2 | cell division cycle associated 2 | 1 |
| 11692 | hsa-miR-637 | 157313 | CDCA2 | cell division cycle associated 2 | 1 |
| 11693 | hsa-mir-22 | 157313 | CDCA2 | cell division cycle associated 2 | 1 |
| 11694 | hsa-mir-10a | 157313 | CDCA2 | cell division cycle associated 2 | 1 |
| 11695 | hsa-mir-186 | 157313 | CDCA2 | cell division cycle associated 2 | 1 |
| 11696 | hsa-mir-155 | 157313 | CDCA2 | cell division cycle associated 2 | 1 |
| 11697 | hsa-mir-499 | 157313 | CDCA2 | cell division cycle associated 2 | 1 |
| 11698 | hsa-mir-877 | 157313 | CDCA2 | cell division cycle associated 2 | 1 |
| 11699 | hsa-miR-1 | 10198 | MPHOSPH9 | M-phase phosphoprotein 9 | 1 |
| 11700 | hsa-miR-133a | 10198 | MPHOSPH9 | M-phase phosphoprotein 9 | 1 |
| 11701 | hsa-miR-186 | 10198 | MPHOSPH9 | M-phase phosphoprotein 9 | 1 |
| 11702 | hsa-miR-195 | 10198 | MPHOSPH9 | M-phase phosphoprotein 9 | 1 |
| 11703 | hsa-miR-208a | 10198 | MPHOSPH9 | M-phase phosphoprotein 9 | 1 |
| 11704 | hsa-miR-326 | 10198 | MPHOSPH9 | M-phase phosphoprotein 9 | 1 |
| 11705 | hsa-miR-449b | 10198 | MPHOSPH9 | M-phase phosphoprotein 9 | 1 |
| 11706 | hsa-miR-497 | 10198 | MPHOSPH9 | M-phase phosphoprotein 9 | 1 |
| 11707 | hsa-miR-550 | 10198 | MPHOSPH9 | M-phase phosphoprotein 9 | 1 |
| 11708 | hsa-miR-575 | 10198 | MPHOSPH9 | M-phase phosphoprotein 9 | 1 |
| 11709 | hsa-miR-608 | 10198 | MPHOSPH9 | M-phase phosphoprotein 9 | 1 |
| 11710 | hsa-miR-620 | 10198 | MPHOSPH9 | M-phase phosphoprotein 9 | 1 |
| 11711 | hsa-let-7a-3 | 10198 | MPHOSPH9 | M-phase phosphoprotein 9 | 1 |
| 11712 | hsa-let-7b | 10198 | MPHOSPH9 | M-phase phosphoprotein 9 | 1 |
| 11713 | hsa-mir-22 | 10198 | MPHOSPH9 | M-phase phosphoprotein 9 | 1 |
| 11714 | hsa-mir-10a | 10198 | MPHOSPH9 | M-phase phosphoprotein 9 | 1 |
| 11715 | hsa-mir-186 | 10198 | MPHOSPH9 | M-phase phosphoprotein 9 | 1 |
| 11716 | hsa-mir-155 | 10198 | MPHOSPH9 | M-phase phosphoprotein 9 | 1 |
| 11717 | hsa-mir-499 | 10198 | MPHOSPH9 | M-phase phosphoprotein 9 | 1 |
| 11718 | hsa-mir-550-1 | 10198 | MPHOSPH9 | M-phase phosphoprotein 9 | 1 |
| 11719 | hsa-mir-877 | 10198 | MPHOSPH9 | M-phase phosphoprotein 9 | 1 |
| 11720 | hsa-mir-2116 | 10198 | MPHOSPH9 | M-phase phosphoprotein 9 | 1 |
| 11721 | hsa-mir-3186 | 10198 | MPHOSPH9 | M-phase phosphoprotein 9 | 1 |
| 11722 | hsa-miR-1 | 355 | FAS | Fas (TNF receptor superfamily, member 6) | 1 |
| 11723 | hsa-miR-133a | 355 | FAS | Fas (TNF receptor superfamily, member 6) | 1 |
| 11724 | hsa-miR-186 | 355 | FAS | Fas (TNF receptor superfamily, member 6) | 1 |
| 11725 | hsa-miR-326 | 355 | FAS | Fas (TNF receptor superfamily, member 6) | 1 |
| 11726 | hsa-miR-449b | 355 | FAS | Fas (TNF receptor superfamily, member 6) | 1 |
| 11727 | hsa-miR-551a | 355 | FAS | Fas (TNF receptor superfamily, member 6) | 1 |
| 11728 | hsa-miR-562 | 355 | FAS | Fas (TNF receptor superfamily, member 6) | 1 |
| 11729 | hsa-miR-575 | 355 | FAS | Fas (TNF receptor superfamily, member 6) | 1 |
| 11730 | hsa-miR-579 | 355 | FAS | Fas (TNF receptor superfamily, member 6) | 1 |
| 11731 | hsa-miR-608 | 355 | FAS | Fas (TNF receptor superfamily, member 6) | 1 |
| 11732 | hsa-miR-637 | 355 | FAS | Fas (TNF receptor superfamily, member 6) | 1 |
| 11733 | hsa-mir-22 | 355 | FAS | Fas (TNF receptor superfamily, member 6) | 1 |
| 11734 | hsa-mir-10a | 355 | FAS | Fas (TNF receptor superfamily, member 6) | 1 |
| 11735 | hsa-mir-186 | 355 | FAS | Fas (TNF receptor superfamily, member 6) | 1 |
| 11736 | hsa-mir-155 | 355 | FAS | Fas (TNF receptor superfamily, member 6) | 1 |
| 11737 | hsa-mir-877 | 355 | FAS | Fas (TNF receptor superfamily, member 6) | 1 |
| 11738 | hsa-miR-1 | 200159 | C1orf100 | chromosome 1 open reading frame 100 | 1 |
| 11739 | hsa-miR-133a | 200159 | C1orf100 | chromosome 1 open reading frame 100 | 1 |
| 11740 | hsa-miR-186 | 200159 | C1orf100 | chromosome 1 open reading frame 100 | 1 |
| 11741 | hsa-miR-326 | 200159 | C1orf100 | chromosome 1 open reading frame 100 | 1 |
| 11742 | hsa-miR-33a | 200159 | C1orf100 | chromosome 1 open reading frame 100 | 1 |
| 11743 | hsa-miR-449b | 200159 | C1orf100 | chromosome 1 open reading frame 100 | 1 |
| 11744 | hsa-miR-550 | 200159 | C1orf100 | chromosome 1 open reading frame 100 | 1 |
| 11745 | hsa-miR-551a | 200159 | C1orf100 | chromosome 1 open reading frame 100 | 1 |
| 11746 | hsa-miR-562 | 200159 | C1orf100 | chromosome 1 open reading frame 100 | 1 |
| 11747 | hsa-miR-575 | 200159 | C1orf100 | chromosome 1 open reading frame 100 | 1 |
| 11748 | hsa-miR-579 | 200159 | C1orf100 | chromosome 1 open reading frame 100 | 1 |
| 11749 | hsa-miR-608 | 200159 | C1orf100 | chromosome 1 open reading frame 100 | 1 |
| 11750 | hsa-miR-620 | 200159 | C1orf100 | chromosome 1 open reading frame 100 | 1 |
| 11751 | hsa-miR-885-5p | 200159 | C1orf100 | chromosome 1 open reading frame 100 | 1 |
| 11752 | hsa-mir-22 | 200159 | C1orf100 | chromosome 1 open reading frame 100 | 1 |
| 11753 | hsa-mir-10a | 200159 | C1orf100 | chromosome 1 open reading frame 100 | 1 |
| 11754 | hsa-mir-186 | 200159 | C1orf100 | chromosome 1 open reading frame 100 | 1 |
| 11755 | hsa-mir-155 | 200159 | C1orf100 | chromosome 1 open reading frame 100 | 1 |
| 11756 | hsa-mir-423 | 200159 | C1orf100 | chromosome 1 open reading frame 100 | 1 |
| 11757 | hsa-mir-499 | 200159 | C1orf100 | chromosome 1 open reading frame 100 | 1 |
| 11758 | hsa-mir-550-1 | 200159 | C1orf100 | chromosome 1 open reading frame 100 | 1 |
| 11759 | hsa-mir-885 | 200159 | C1orf100 | chromosome 1 open reading frame 100 | 1 |
| 11760 | hsa-mir-877 | 200159 | C1orf100 | chromosome 1 open reading frame 100 | 1 |
| 11761 | hsa-mir-2116 | 200159 | C1orf100 | chromosome 1 open reading frame 100 | 1 |
| 11762 | hsa-mir-3186 | 200159 | C1orf100 | chromosome 1 open reading frame 100 | 1 |
| 11763 | hsa-miR-1 | 386685 | KRTAP10-12 | keratin associated protein 10-12 | 1 |
| 11764 | hsa-miR-133a | 386685 | KRTAP10-12 | keratin associated protein 10-12 | 1 |
| 11765 | hsa-miR-186 | 386685 | KRTAP10-12 | keratin associated protein 10-12 | 1 |
| 11766 | hsa-miR-208a | 386685 | KRTAP10-12 | keratin associated protein 10-12 | 1 |
| 11767 | hsa-miR-326 | 386685 | KRTAP10-12 | keratin associated protein 10-12 | 1 |
| 11768 | hsa-miR-449b | 386685 | KRTAP10-12 | keratin associated protein 10-12 | 1 |
| 11769 | hsa-miR-550 | 386685 | KRTAP10-12 | keratin associated protein 10-12 | 1 |
| 11770 | hsa-miR-575 | 386685 | KRTAP10-12 | keratin associated protein 10-12 | 1 |
| 11771 | hsa-miR-579 | 386685 | KRTAP10-12 | keratin associated protein 10-12 | 1 |
| 11772 | hsa-miR-608 | 386685 | KRTAP10-12 | keratin associated protein 10-12 | 1 |
| 11773 | hsa-miR-620 | 386685 | KRTAP10-12 | keratin associated protein 10-12 | 1 |
| 11774 | hsa-miR-885-5p | 386685 | KRTAP10-12 | keratin associated protein 10-12 | 1 |
| 11775 | hsa-let-7a-3 | 386685 | KRTAP10-12 | keratin associated protein 10-12 | 1 |
| 11776 | hsa-let-7b | 386685 | KRTAP10-12 | keratin associated protein 10-12 | 1 |
| 11777 | hsa-mir-22 | 386685 | KRTAP10-12 | keratin associated protein 10-12 | 1 |
| 11778 | hsa-mir-10a | 386685 | KRTAP10-12 | keratin associated protein 10-12 | 1 |
| 11779 | hsa-mir-186 | 386685 | KRTAP10-12 | keratin associated protein 10-12 | 1 |
| 11780 | hsa-mir-155 | 386685 | KRTAP10-12 | keratin associated protein 10-12 | 1 |
| 11781 | hsa-mir-499 | 386685 | KRTAP10-12 | keratin associated protein 10-12 | 1 |
| 11782 | hsa-mir-550-1 | 386685 | KRTAP10-12 | keratin associated protein 10-12 | 1 |
| 11783 | hsa-mir-885 | 386685 | KRTAP10-12 | keratin associated protein 10-12 | 1 |
| 11784 | hsa-mir-877 | 386685 | KRTAP10-12 | keratin associated protein 10-12 | 1 |
| 11785 | hsa-mir-2116 | 386685 | KRTAP10-12 | keratin associated protein 10-12 | 1 |
| 11786 | hsa-mir-3186 | 386685 | KRTAP10-12 | keratin associated protein 10-12 | 1 |
| 11787 | hsa-mir-3190 | 386685 | KRTAP10-12 | keratin associated protein 10-12 | 1 |
| 11788 | hsa-miR-1 | 404201 | C4orf12 | chromosome 4 open reading frame 12 | 1 |
| 11789 | hsa-miR-133a | 404201 | C4orf12 | chromosome 4 open reading frame 12 | 1 |
| 11790 | hsa-miR-186 | 404201 | C4orf12 | chromosome 4 open reading frame 12 | 1 |
| 11791 | hsa-miR-326 | 404201 | C4orf12 | chromosome 4 open reading frame 12 | 1 |
| 11792 | hsa-miR-449b | 404201 | C4orf12 | chromosome 4 open reading frame 12 | 1 |
| 11793 | hsa-miR-550 | 404201 | C4orf12 | chromosome 4 open reading frame 12 | 1 |
| 11794 | hsa-miR-551a | 404201 | C4orf12 | chromosome 4 open reading frame 12 | 1 |
| 11795 | hsa-miR-562 | 404201 | C4orf12 | chromosome 4 open reading frame 12 | 1 |
| 11796 | hsa-miR-575 | 404201 | C4orf12 | chromosome 4 open reading frame 12 | 1 |
| 11797 | hsa-miR-579 | 404201 | C4orf12 | chromosome 4 open reading frame 12 | 1 |
| 11798 | hsa-miR-608 | 404201 | C4orf12 | chromosome 4 open reading frame 12 | 1 |
| 11799 | hsa-miR-620 | 404201 | C4orf12 | chromosome 4 open reading frame 12 | 1 |
| 11800 | hsa-miR-885-5p | 404201 | C4orf12 | chromosome 4 open reading frame 12 | 1 |
| 11801 | hsa-mir-22 | 404201 | C4orf12 | chromosome 4 open reading frame 12 | 1 |
| 11802 | hsa-mir-10a | 404201 | C4orf12 | chromosome 4 open reading frame 12 | 1 |
| 11803 | hsa-mir-186 | 404201 | C4orf12 | chromosome 4 open reading frame 12 | 1 |
| 11804 | hsa-mir-155 | 404201 | C4orf12 | chromosome 4 open reading frame 12 | 1 |
| 11805 | hsa-mir-499 | 404201 | C4orf12 | chromosome 4 open reading frame 12 | 1 |
| 11806 | hsa-mir-550-1 | 404201 | C4orf12 | chromosome 4 open reading frame 12 | 1 |
| 11807 | hsa-mir-885 | 404201 | C4orf12 | chromosome 4 open reading frame 12 | 1 |
| 11808 | hsa-mir-877 | 404201 | C4orf12 | chromosome 4 open reading frame 12 | 1 |
| 11809 | hsa-mir-2116 | 404201 | C4orf12 | chromosome 4 open reading frame 12 | 1 |
| 11810 | hsa-mir-3190 | 404201 | C4orf12 | chromosome 4 open reading frame 12 | 1 |
| 11811 | hsa-miR-1 | 7699 | ZNF140 | zinc finger protein 140 | 1 |
| 11812 | hsa-miR-133a | 7699 | ZNF140 | zinc finger protein 140 | 1 |
| 11813 | hsa-miR-186 | 7699 | ZNF140 | zinc finger protein 140 | 1 |
| 11814 | hsa-miR-208a | 7699 | ZNF140 | zinc finger protein 140 | 1 |
| 11815 | hsa-miR-326 | 7699 | ZNF140 | zinc finger protein 140 | 1 |
| 11816 | hsa-miR-449b | 7699 | ZNF140 | zinc finger protein 140 | 1 |
| 11817 | hsa-miR-550 | 7699 | ZNF140 | zinc finger protein 140 | 1 |
| 11818 | hsa-miR-551a | 7699 | ZNF140 | zinc finger protein 140 | 1 |
| 11819 | hsa-miR-575 | 7699 | ZNF140 | zinc finger protein 140 | 1 |
| 11820 | hsa-miR-579 | 7699 | ZNF140 | zinc finger protein 140 | 1 |
| 11821 | hsa-miR-608 | 7699 | ZNF140 | zinc finger protein 140 | 1 |
| 11822 | hsa-miR-620 | 7699 | ZNF140 | zinc finger protein 140 | 1 |
| 11823 | hsa-miR-885-5p | 7699 | ZNF140 | zinc finger protein 140 | 1 |
| 11824 | hsa-mir-22 | 7699 | ZNF140 | zinc finger protein 140 | 1 |
| 11825 | hsa-mir-10a | 7699 | ZNF140 | zinc finger protein 140 | 1 |
| 11826 | hsa-mir-186 | 7699 | ZNF140 | zinc finger protein 140 | 1 |
| 11827 | hsa-mir-155 | 7699 | ZNF140 | zinc finger protein 140 | 1 |
| 11828 | hsa-mir-423 | 7699 | ZNF140 | zinc finger protein 140 | 1 |
| 11829 | hsa-mir-499 | 7699 | ZNF140 | zinc finger protein 140 | 1 |
| 11830 | hsa-mir-550-1 | 7699 | ZNF140 | zinc finger protein 140 | 1 |
| 11831 | hsa-mir-885 | 7699 | ZNF140 | zinc finger protein 140 | 1 |
| 11832 | hsa-mir-877 | 7699 | ZNF140 | zinc finger protein 140 | 1 |
| 11833 | hsa-mir-2116 | 7699 | ZNF140 | zinc finger protein 140 | 1 |
| 11834 | hsa-mir-3186 | 7699 | ZNF140 | zinc finger protein 140 | 1 |
| 11835 | hsa-mir-3190 | 7699 | ZNF140 | zinc finger protein 140 | 1 |
| 11836 | hsa-mir-548d-2 | 3659 | IRF1 | interferon regulatory factor 1 | 1 |
| 11837 | hsa-miR-1 | 255631 | COL24A1 | collagen, type XXIV, alpha 1 | 1 |
| 11838 | hsa-miR-133a | 255631 | COL24A1 | collagen, type XXIV, alpha 1 | 1 |
| 11839 | hsa-miR-186 | 255631 | COL24A1 | collagen, type XXIV, alpha 1 | 1 |
| 11840 | hsa-miR-326 | 255631 | COL24A1 | collagen, type XXIV, alpha 1 | 1 |
| 11841 | hsa-miR-449b | 255631 | COL24A1 | collagen, type XXIV, alpha 1 | 1 |
| 11842 | hsa-miR-575 | 255631 | COL24A1 | collagen, type XXIV, alpha 1 | 1 |
| 11843 | hsa-miR-579 | 255631 | COL24A1 | collagen, type XXIV, alpha 1 | 1 |
| 11844 | hsa-miR-608 | 255631 | COL24A1 | collagen, type XXIV, alpha 1 | 1 |
| 11845 | hsa-miR-620 | 255631 | COL24A1 | collagen, type XXIV, alpha 1 | 1 |
| 11846 | hsa-miR-637 | 255631 | COL24A1 | collagen, type XXIV, alpha 1 | 1 |
| 11847 | hsa-mir-22 | 255631 | COL24A1 | collagen, type XXIV, alpha 1 | 1 |
| 11848 | hsa-mir-10a | 255631 | COL24A1 | collagen, type XXIV, alpha 1 | 1 |
| 11849 | hsa-mir-186 | 255631 | COL24A1 | collagen, type XXIV, alpha 1 | 1 |
| 11850 | hsa-mir-155 | 255631 | COL24A1 | collagen, type XXIV, alpha 1 | 1 |
| 11851 | hsa-mir-499 | 255631 | COL24A1 | collagen, type XXIV, alpha 1 | 1 |
| 11852 | hsa-mir-877 | 255631 | COL24A1 | collagen, type XXIV, alpha 1 | 1 |
| 11853 | hsa-mir-3190 | 255631 | COL24A1 | collagen, type XXIV, alpha 1 | 1 |
| 11854 | hsa-miR-1 | 3667 | IRS1 | insulin receptor substrate 1 | 1 |
| 11855 | hsa-miR-133a | 3667 | IRS1 | insulin receptor substrate 1 | 1 |
| 11856 | hsa-miR-186 | 3667 | IRS1 | insulin receptor substrate 1 | 1 |
| 11857 | hsa-miR-326 | 3667 | IRS1 | insulin receptor substrate 1 | 1 |
| 11858 | hsa-miR-449b | 3667 | IRS1 | insulin receptor substrate 1 | 1 |
| 11859 | hsa-miR-551a | 3667 | IRS1 | insulin receptor substrate 1 | 1 |
| 11860 | hsa-miR-562 | 3667 | IRS1 | insulin receptor substrate 1 | 1 |
| 11861 | hsa-miR-575 | 3667 | IRS1 | insulin receptor substrate 1 | 1 |
| 11862 | hsa-miR-579 | 3667 | IRS1 | insulin receptor substrate 1 | 1 |
| 11863 | hsa-miR-608 | 3667 | IRS1 | insulin receptor substrate 1 | 1 |
| 11864 | hsa-miR-620 | 3667 | IRS1 | insulin receptor substrate 1 | 1 |
| 11865 | hsa-miR-637 | 3667 | IRS1 | insulin receptor substrate 1 | 1 |
| 11866 | hsa-miR-885-5p | 3667 | IRS1 | insulin receptor substrate 1 | 1 |
| 11867 | hsa-mir-22 | 3667 | IRS1 | insulin receptor substrate 1 | 1 |
| 11868 | hsa-mir-10a | 3667 | IRS1 | insulin receptor substrate 1 | 1 |
| 11869 | hsa-mir-186 | 3667 | IRS1 | insulin receptor substrate 1 | 1 |
| 11870 | hsa-mir-155 | 3667 | IRS1 | insulin receptor substrate 1 | 1 |
| 11871 | hsa-mir-423 | 3667 | IRS1 | insulin receptor substrate 1 | 1 |
| 11872 | hsa-mir-499 | 3667 | IRS1 | insulin receptor substrate 1 | 1 |
| 11873 | hsa-mir-550-1 | 3667 | IRS1 | insulin receptor substrate 1 | 1 |
| 11874 | hsa-mir-885 | 3667 | IRS1 | insulin receptor substrate 1 | 1 |
| 11875 | hsa-mir-877 | 3667 | IRS1 | insulin receptor substrate 1 | 1 |
| 11876 | hsa-mir-2116 | 3667 | IRS1 | insulin receptor substrate 1 | 1 |
| 11877 | hsa-mir-3190 | 3667 | IRS1 | insulin receptor substrate 1 | 1 |
| 11878 | hsa-miR-1 | 326625 | MMAB | methylmalonic aciduria (cobalamin deficiency) cblB type | 1 |
| 11879 | hsa-miR-133a | 326625 | MMAB | methylmalonic aciduria (cobalamin deficiency) cblB type | 1 |
| 11880 | hsa-miR-186 | 326625 | MMAB | methylmalonic aciduria (cobalamin deficiency) cblB type | 1 |
| 11881 | hsa-miR-195 | 326625 | MMAB | methylmalonic aciduria (cobalamin deficiency) cblB type | 1 |
| 11882 | hsa-miR-326 | 326625 | MMAB | methylmalonic aciduria (cobalamin deficiency) cblB type | 1 |
| 11883 | hsa-miR-449b | 326625 | MMAB | methylmalonic aciduria (cobalamin deficiency) cblB type | 1 |
| 11884 | hsa-miR-489 | 326625 | MMAB | methylmalonic aciduria (cobalamin deficiency) cblB type | 1 |
| 11885 | hsa-miR-497 | 326625 | MMAB | methylmalonic aciduria (cobalamin deficiency) cblB type | 1 |
| 11886 | hsa-miR-562 | 326625 | MMAB | methylmalonic aciduria (cobalamin deficiency) cblB type | 1 |
| 11887 | hsa-miR-575 | 326625 | MMAB | methylmalonic aciduria (cobalamin deficiency) cblB type | 1 |
| 11888 | hsa-miR-579 | 326625 | MMAB | methylmalonic aciduria (cobalamin deficiency) cblB type | 1 |
| 11889 | hsa-miR-608 | 326625 | MMAB | methylmalonic aciduria (cobalamin deficiency) cblB type | 1 |
| 11890 | hsa-miR-620 | 326625 | MMAB | methylmalonic aciduria (cobalamin deficiency) cblB type | 1 |
| 11891 | hsa-miR-637 | 326625 | MMAB | methylmalonic aciduria (cobalamin deficiency) cblB type | 1 |
| 11892 | hsa-miR-885-5p | 326625 | MMAB | methylmalonic aciduria (cobalamin deficiency) cblB type | 1 |
| 11893 | hsa-mir-22 | 326625 | MMAB | methylmalonic aciduria (cobalamin deficiency) cblB type | 1 |
| 11894 | hsa-mir-10a | 326625 | MMAB | methylmalonic aciduria (cobalamin deficiency) cblB type | 1 |
| 11895 | hsa-mir-186 | 326625 | MMAB | methylmalonic aciduria (cobalamin deficiency) cblB type | 1 |
| 11896 | hsa-mir-155 | 326625 | MMAB | methylmalonic aciduria (cobalamin deficiency) cblB type | 1 |
| 11897 | hsa-mir-499 | 326625 | MMAB | methylmalonic aciduria (cobalamin deficiency) cblB type | 1 |
| 11898 | hsa-mir-885 | 326625 | MMAB | methylmalonic aciduria (cobalamin deficiency) cblB type | 1 |
| 11899 | hsa-mir-877 | 326625 | MMAB | methylmalonic aciduria (cobalamin deficiency) cblB type | 1 |
| 11900 | hsa-miR-152 | 22990 | PCNX | pecanex homolog (Drosophila) | 1 |
| 11901 | hsa-miR-26a | 22990 | PCNX | pecanex homolog (Drosophila) | 1 |
| 11902 | hsa-miR-26b | 22990 | PCNX | pecanex homolog (Drosophila) | 1 |
| 11903 | hsa-miR-31 | 22990 | PCNX | pecanex homolog (Drosophila) | 1 |
| 11904 | hsa-miR-338-5p | 22990 | PCNX | pecanex homolog (Drosophila) | 1 |
| 11905 | hsa-miR-340 | 22990 | PCNX | pecanex homolog (Drosophila) | 1 |
| 11906 | hsa-miR-383 | 22990 | PCNX | pecanex homolog (Drosophila) | 1 |
| 11907 | hsa-miR-449a | 22990 | PCNX | pecanex homolog (Drosophila) | 1 |
| 11908 | hsa-miR-499-5p | 22990 | PCNX | pecanex homolog (Drosophila) | 1 |
| 11909 | hsa-miR-621 | 22990 | PCNX | pecanex homolog (Drosophila) | 1 |
| 11910 | hsa-miR-657 | 22990 | PCNX | pecanex homolog (Drosophila) | 1 |
| 11911 | hsa-miR-663 | 22990 | PCNX | pecanex homolog (Drosophila) | 1 |
| 11912 | hsa-miR-765 | 22990 | PCNX | pecanex homolog (Drosophila) | 1 |
| 11913 | hsa-miR-770-5p | 22990 | PCNX | pecanex homolog (Drosophila) | 1 |
| 11914 | hsa-miR-877 | 22990 | PCNX | pecanex homolog (Drosophila) | 1 |
| 11915 | hsa-mir-26a-1 | 22990 | PCNX | pecanex homolog (Drosophila) | 1 |
| 11916 | hsa-mir-26b | 22990 | PCNX | pecanex homolog (Drosophila) | 1 |
| 11917 | hsa-mir-31 | 22990 | PCNX | pecanex homolog (Drosophila) | 1 |
| 11918 | hsa-mir-194-2 | 22990 | PCNX | pecanex homolog (Drosophila) | 1 |
| 11919 | hsa-mir-340 | 22990 | PCNX | pecanex homolog (Drosophila) | 1 |
| 11920 | hsa-mir-338 | 22990 | PCNX | pecanex homolog (Drosophila) | 1 |
| 11921 | hsa-mir-449b | 22990 | PCNX | pecanex homolog (Drosophila) | 1 |
| 11922 | hsa-mir-449c | 22990 | PCNX | pecanex homolog (Drosophila) | 1 |
| 11923 | hsa-mir-3065 | 22990 | PCNX | pecanex homolog (Drosophila) | 1 |
| 11924 | hsa-miR-10a | 221756 | MGC39372 | hypothetical protein MGC39372 | 1 |
| 11925 | hsa-miR-152 | 221756 | MGC39372 | hypothetical protein MGC39372 | 1 |
| 11926 | hsa-miR-26a | 221756 | MGC39372 | hypothetical protein MGC39372 | 1 |
| 11927 | hsa-miR-26b | 221756 | MGC39372 | hypothetical protein MGC39372 | 1 |
| 11928 | hsa-miR-31 | 221756 | MGC39372 | hypothetical protein MGC39372 | 1 |
| 11929 | hsa-miR-340 | 221756 | MGC39372 | hypothetical protein MGC39372 | 1 |
| 11930 | hsa-miR-449a | 221756 | MGC39372 | hypothetical protein MGC39372 | 1 |
| 11931 | hsa-miR-490-5p | 221756 | MGC39372 | hypothetical protein MGC39372 | 1 |
| 11932 | hsa-miR-499-5p | 221756 | MGC39372 | hypothetical protein MGC39372 | 1 |
| 11933 | hsa-miR-548b-5p | 221756 | MGC39372 | hypothetical protein MGC39372 | 1 |
| 11934 | hsa-miR-564 | 221756 | MGC39372 | hypothetical protein MGC39372 | 1 |
| 11935 | hsa-miR-621 | 221756 | MGC39372 | hypothetical protein MGC39372 | 1 |
| 11936 | hsa-miR-634 | 221756 | MGC39372 | hypothetical protein MGC39372 | 1 |
| 11937 | hsa-miR-653 | 221756 | MGC39372 | hypothetical protein MGC39372 | 1 |
| 11938 | hsa-miR-765 | 221756 | MGC39372 | hypothetical protein MGC39372 | 1 |
| 11939 | hsa-miR-770-5p | 221756 | MGC39372 | hypothetical protein MGC39372 | 1 |
| 11940 | hsa-miR-877 | 221756 | MGC39372 | hypothetical protein MGC39372 | 1 |
| 11941 | hsa-mir-26a-1 | 221756 | MGC39372 | hypothetical protein MGC39372 | 1 |
| 11942 | hsa-mir-26b | 221756 | MGC39372 | hypothetical protein MGC39372 | 1 |
| 11943 | hsa-mir-31 | 221756 | MGC39372 | hypothetical protein MGC39372 | 1 |
| 11944 | hsa-mir-194-2 | 221756 | MGC39372 | hypothetical protein MGC39372 | 1 |
| 11945 | hsa-mir-340 | 221756 | MGC39372 | hypothetical protein MGC39372 | 1 |
| 11946 | hsa-mir-490 | 221756 | MGC39372 | hypothetical protein MGC39372 | 1 |
| 11947 | hsa-mir-548b | 221756 | MGC39372 | hypothetical protein MGC39372 | 1 |
| 11948 | hsa-mir-449b | 221756 | MGC39372 | hypothetical protein MGC39372 | 1 |
| 11949 | hsa-mir-449c | 221756 | MGC39372 | hypothetical protein MGC39372 | 1 |
| 11950 | hsa-miR-10a | 1992 | SERPINB1 | serpin peptidase inhibitor, clade B (ovalbumin), member 1 | 1 |
| 11951 | hsa-miR-139-5p | 1992 | SERPINB1 | serpin peptidase inhibitor, clade B (ovalbumin), member 1 | 1 |
| 11952 | hsa-miR-152 | 1992 | SERPINB1 | serpin peptidase inhibitor, clade B (ovalbumin), member 1 | 1 |
| 11953 | hsa-miR-26a | 1992 | SERPINB1 | serpin peptidase inhibitor, clade B (ovalbumin), member 1 | 1 |
| 11954 | hsa-miR-26b | 1992 | SERPINB1 | serpin peptidase inhibitor, clade B (ovalbumin), member 1 | 1 |
| 11955 | hsa-miR-31 | 1992 | SERPINB1 | serpin peptidase inhibitor, clade B (ovalbumin), member 1 | 1 |
| 11956 | hsa-miR-338-5p | 1992 | SERPINB1 | serpin peptidase inhibitor, clade B (ovalbumin), member 1 | 1 |
| 11957 | hsa-miR-340 | 1992 | SERPINB1 | serpin peptidase inhibitor, clade B (ovalbumin), member 1 | 1 |
| 11958 | hsa-miR-449a | 1992 | SERPINB1 | serpin peptidase inhibitor, clade B (ovalbumin), member 1 | 1 |
| 11959 | hsa-miR-490-5p | 1992 | SERPINB1 | serpin peptidase inhibitor, clade B (ovalbumin), member 1 | 1 |
| 11960 | hsa-miR-499-5p | 1992 | SERPINB1 | serpin peptidase inhibitor, clade B (ovalbumin), member 1 | 1 |
| 11961 | hsa-miR-548b-5p | 1992 | SERPINB1 | serpin peptidase inhibitor, clade B (ovalbumin), member 1 | 1 |
| 11962 | hsa-miR-564 | 1992 | SERPINB1 | serpin peptidase inhibitor, clade B (ovalbumin), member 1 | 1 |
| 11963 | hsa-miR-621 | 1992 | SERPINB1 | serpin peptidase inhibitor, clade B (ovalbumin), member 1 | 1 |
| 11964 | hsa-miR-657 | 1992 | SERPINB1 | serpin peptidase inhibitor, clade B (ovalbumin), member 1 | 1 |
| 11965 | hsa-miR-765 | 1992 | SERPINB1 | serpin peptidase inhibitor, clade B (ovalbumin), member 1 | 1 |
| 11966 | hsa-miR-770-5p | 1992 | SERPINB1 | serpin peptidase inhibitor, clade B (ovalbumin), member 1 | 1 |
| 11967 | hsa-miR-877 | 1992 | SERPINB1 | serpin peptidase inhibitor, clade B (ovalbumin), member 1 | 1 |
| 11968 | hsa-mir-26a-1 | 1992 | SERPINB1 | serpin peptidase inhibitor, clade B (ovalbumin), member 1 | 1 |
| 11969 | hsa-mir-26b | 1992 | SERPINB1 | serpin peptidase inhibitor, clade B (ovalbumin), member 1 | 1 |
| 11970 | hsa-mir-31 | 1992 | SERPINB1 | serpin peptidase inhibitor, clade B (ovalbumin), member 1 | 1 |
| 11971 | hsa-mir-194-2 | 1992 | SERPINB1 | serpin peptidase inhibitor, clade B (ovalbumin), member 1 | 1 |
| 11972 | hsa-mir-340 | 1992 | SERPINB1 | serpin peptidase inhibitor, clade B (ovalbumin), member 1 | 1 |
| 11973 | hsa-mir-338 | 1992 | SERPINB1 | serpin peptidase inhibitor, clade B (ovalbumin), member 1 | 1 |
| 11974 | hsa-mir-490 | 1992 | SERPINB1 | serpin peptidase inhibitor, clade B (ovalbumin), member 1 | 1 |
| 11975 | hsa-mir-548b | 1992 | SERPINB1 | serpin peptidase inhibitor, clade B (ovalbumin), member 1 | 1 |
| 11976 | hsa-mir-548d-2 | 1992 | SERPINB1 | serpin peptidase inhibitor, clade B (ovalbumin), member 1 | 1 |
| 11977 | hsa-mir-449b | 1992 | SERPINB1 | serpin peptidase inhibitor, clade B (ovalbumin), member 1 | 1 |
| 11978 | hsa-mir-449c | 1992 | SERPINB1 | serpin peptidase inhibitor, clade B (ovalbumin), member 1 | 1 |
| 11979 | hsa-mir-3065 | 1992 | SERPINB1 | serpin peptidase inhibitor, clade B (ovalbumin), member 1 | 1 |
| 11980 | hsa-miR-1 | 285489 | DOK7 | docking protein 7 | 1 |
| 11981 | hsa-miR-133a | 285489 | DOK7 | docking protein 7 | 1 |
| 11982 | hsa-miR-186 | 285489 | DOK7 | docking protein 7 | 1 |
| 11983 | hsa-miR-326 | 285489 | DOK7 | docking protein 7 | 1 |
| 11984 | hsa-miR-449b | 285489 | DOK7 | docking protein 7 | 1 |
| 11985 | hsa-miR-550 | 285489 | DOK7 | docking protein 7 | 1 |
| 11986 | hsa-miR-551a | 285489 | DOK7 | docking protein 7 | 1 |
| 11987 | hsa-miR-575 | 285489 | DOK7 | docking protein 7 | 1 |
| 11988 | hsa-miR-579 | 285489 | DOK7 | docking protein 7 | 1 |
| 11989 | hsa-miR-608 | 285489 | DOK7 | docking protein 7 | 1 |
| 11990 | hsa-miR-620 | 285489 | DOK7 | docking protein 7 | 1 |
| 11991 | hsa-mir-22 | 285489 | DOK7 | docking protein 7 | 1 |
| 11992 | hsa-mir-10a | 285489 | DOK7 | docking protein 7 | 1 |
| 11993 | hsa-mir-186 | 285489 | DOK7 | docking protein 7 | 1 |
| 11994 | hsa-mir-155 | 285489 | DOK7 | docking protein 7 | 1 |
| 11995 | hsa-mir-423 | 285489 | DOK7 | docking protein 7 | 1 |
| 11996 | hsa-mir-499 | 285489 | DOK7 | docking protein 7 | 1 |
| 11997 | hsa-mir-550-1 | 285489 | DOK7 | docking protein 7 | 1 |
| 11998 | hsa-mir-877 | 285489 | DOK7 | docking protein 7 | 1 |
| 11999 | hsa-mir-2116 | 285489 | DOK7 | docking protein 7 | 1 |
| 12000 | hsa-mir-3186 | 285489 | DOK7 | docking protein 7 | 1 |
| 12001 | hsa-miR-1 | 266629 | SEC14L3 | SEC14-like 3 (S. cerevisiae) | 1 |
| 12002 | hsa-miR-133a | 266629 | SEC14L3 | SEC14-like 3 (S. cerevisiae) | 1 |
| 12003 | hsa-miR-186 | 266629 | SEC14L3 | SEC14-like 3 (S. cerevisiae) | 1 |
| 12004 | hsa-miR-326 | 266629 | SEC14L3 | SEC14-like 3 (S. cerevisiae) | 1 |
| 12005 | hsa-miR-449b | 266629 | SEC14L3 | SEC14-like 3 (S. cerevisiae) | 1 |
| 12006 | hsa-miR-551a | 266629 | SEC14L3 | SEC14-like 3 (S. cerevisiae) | 1 |
| 12007 | hsa-miR-562 | 266629 | SEC14L3 | SEC14-like 3 (S. cerevisiae) | 1 |
| 12008 | hsa-miR-575 | 266629 | SEC14L3 | SEC14-like 3 (S. cerevisiae) | 1 |
| 12009 | hsa-miR-579 | 266629 | SEC14L3 | SEC14-like 3 (S. cerevisiae) | 1 |
| 12010 | hsa-miR-608 | 266629 | SEC14L3 | SEC14-like 3 (S. cerevisiae) | 1 |
| 12011 | hsa-miR-620 | 266629 | SEC14L3 | SEC14-like 3 (S. cerevisiae) | 1 |
| 12012 | hsa-miR-637 | 266629 | SEC14L3 | SEC14-like 3 (S. cerevisiae) | 1 |
| 12013 | hsa-mir-22 | 266629 | SEC14L3 | SEC14-like 3 (S. cerevisiae) | 1 |
| 12014 | hsa-mir-10a | 266629 | SEC14L3 | SEC14-like 3 (S. cerevisiae) | 1 |
| 12015 | hsa-mir-186 | 266629 | SEC14L3 | SEC14-like 3 (S. cerevisiae) | 1 |
| 12016 | hsa-mir-155 | 266629 | SEC14L3 | SEC14-like 3 (S. cerevisiae) | 1 |
| 12017 | hsa-mir-423 | 266629 | SEC14L3 | SEC14-like 3 (S. cerevisiae) | 1 |
| 12018 | hsa-mir-877 | 266629 | SEC14L3 | SEC14-like 3 (S. cerevisiae) | 1 |
| 12019 | hsa-miR-10a | 257106 | ARHGAP30 | Rho GTPase activating protein 30 | 1 |
| 12020 | hsa-miR-1228\* | 257106 | ARHGAP30 | Rho GTPase activating protein 30 | 1 |
| 12021 | hsa-miR-152 | 257106 | ARHGAP30 | Rho GTPase activating protein 30 | 1 |
| 12022 | hsa-miR-26a | 257106 | ARHGAP30 | Rho GTPase activating protein 30 | 1 |
| 12023 | hsa-miR-26b | 257106 | ARHGAP30 | Rho GTPase activating protein 30 | 1 |
| 12024 | hsa-miR-31 | 257106 | ARHGAP30 | Rho GTPase activating protein 30 | 1 |
| 12025 | hsa-miR-338-5p | 257106 | ARHGAP30 | Rho GTPase activating protein 30 | 1 |
| 12026 | hsa-miR-340 | 257106 | ARHGAP30 | Rho GTPase activating protein 30 | 1 |
| 12027 | hsa-miR-423-5p | 257106 | ARHGAP30 | Rho GTPase activating protein 30 | 1 |
| 12028 | hsa-miR-449a | 257106 | ARHGAP30 | Rho GTPase activating protein 30 | 1 |
| 12029 | hsa-miR-499-5p | 257106 | ARHGAP30 | Rho GTPase activating protein 30 | 1 |
| 12030 | hsa-miR-548b-5p | 257106 | ARHGAP30 | Rho GTPase activating protein 30 | 1 |
| 12031 | hsa-miR-564 | 257106 | ARHGAP30 | Rho GTPase activating protein 30 | 1 |
| 12032 | hsa-miR-621 | 257106 | ARHGAP30 | Rho GTPase activating protein 30 | 1 |
| 12033 | hsa-miR-657 | 257106 | ARHGAP30 | Rho GTPase activating protein 30 | 1 |
| 12034 | hsa-miR-765 | 257106 | ARHGAP30 | Rho GTPase activating protein 30 | 1 |
| 12035 | hsa-miR-770-5p | 257106 | ARHGAP30 | Rho GTPase activating protein 30 | 1 |
| 12036 | hsa-miR-877 | 257106 | ARHGAP30 | Rho GTPase activating protein 30 | 1 |
| 12037 | hsa-mir-26a-1 | 257106 | ARHGAP30 | Rho GTPase activating protein 30 | 1 |
| 12038 | hsa-mir-26b | 257106 | ARHGAP30 | Rho GTPase activating protein 30 | 1 |
| 12039 | hsa-mir-31 | 257106 | ARHGAP30 | Rho GTPase activating protein 30 | 1 |
| 12040 | hsa-mir-194-2 | 257106 | ARHGAP30 | Rho GTPase activating protein 30 | 1 |
| 12041 | hsa-mir-340 | 257106 | ARHGAP30 | Rho GTPase activating protein 30 | 1 |
| 12042 | hsa-mir-338 | 257106 | ARHGAP30 | Rho GTPase activating protein 30 | 1 |
| 12043 | hsa-mir-548b | 257106 | ARHGAP30 | Rho GTPase activating protein 30 | 1 |
| 12044 | hsa-mir-548d-2 | 257106 | ARHGAP30 | Rho GTPase activating protein 30 | 1 |
| 12045 | hsa-mir-449b | 257106 | ARHGAP30 | Rho GTPase activating protein 30 | 1 |
| 12046 | hsa-mir-449c | 257106 | ARHGAP30 | Rho GTPase activating protein 30 | 1 |
| 12047 | hsa-mir-3065 | 257106 | ARHGAP30 | Rho GTPase activating protein 30 | 1 |
| 12048 | hsa-miR-1 | 284370 | ZNF615 | zinc finger protein 615 | 1 |
| 12049 | hsa-miR-133a | 284370 | ZNF615 | zinc finger protein 615 | 1 |
| 12050 | hsa-miR-186 | 284370 | ZNF615 | zinc finger protein 615 | 1 |
| 12051 | hsa-miR-208a | 284370 | ZNF615 | zinc finger protein 615 | 1 |
| 12052 | hsa-miR-326 | 284370 | ZNF615 | zinc finger protein 615 | 1 |
| 12053 | hsa-miR-449b | 284370 | ZNF615 | zinc finger protein 615 | 1 |
| 12054 | hsa-miR-575 | 284370 | ZNF615 | zinc finger protein 615 | 1 |
| 12055 | hsa-miR-579 | 284370 | ZNF615 | zinc finger protein 615 | 1 |
| 12056 | hsa-miR-608 | 284370 | ZNF615 | zinc finger protein 615 | 1 |
| 12057 | hsa-miR-620 | 284370 | ZNF615 | zinc finger protein 615 | 1 |
| 12058 | hsa-miR-637 | 284370 | ZNF615 | zinc finger protein 615 | 1 |
| 12059 | hsa-miR-885-5p | 284370 | ZNF615 | zinc finger protein 615 | 1 |
| 12060 | hsa-mir-22 | 284370 | ZNF615 | zinc finger protein 615 | 1 |
| 12061 | hsa-mir-10a | 284370 | ZNF615 | zinc finger protein 615 | 1 |
| 12062 | hsa-mir-186 | 284370 | ZNF615 | zinc finger protein 615 | 1 |
| 12063 | hsa-mir-155 | 284370 | ZNF615 | zinc finger protein 615 | 1 |
| 12064 | hsa-mir-423 | 284370 | ZNF615 | zinc finger protein 615 | 1 |
| 12065 | hsa-mir-499 | 284370 | ZNF615 | zinc finger protein 615 | 1 |
| 12066 | hsa-mir-550-1 | 284370 | ZNF615 | zinc finger protein 615 | 1 |
| 12067 | hsa-mir-885 | 284370 | ZNF615 | zinc finger protein 615 | 1 |
| 12068 | hsa-mir-877 | 284370 | ZNF615 | zinc finger protein 615 | 1 |
| 12069 | hsa-mir-2116 | 284370 | ZNF615 | zinc finger protein 615 | 1 |
| 12070 | hsa-mir-3190 | 284370 | ZNF615 | zinc finger protein 615 | 1 |
| 12071 | hsa-miR-1 | 29952 | DPP7 | dipeptidyl-peptidase 7 | 1 |
| 12072 | hsa-miR-133a | 29952 | DPP7 | dipeptidyl-peptidase 7 | 1 |
| 12073 | hsa-miR-186 | 29952 | DPP7 | dipeptidyl-peptidase 7 | 1 |
| 12074 | hsa-miR-326 | 29952 | DPP7 | dipeptidyl-peptidase 7 | 1 |
| 12075 | hsa-miR-33a | 29952 | DPP7 | dipeptidyl-peptidase 7 | 1 |
| 12076 | hsa-miR-449b | 29952 | DPP7 | dipeptidyl-peptidase 7 | 1 |
| 12077 | hsa-miR-550 | 29952 | DPP7 | dipeptidyl-peptidase 7 | 1 |
| 12078 | hsa-miR-575 | 29952 | DPP7 | dipeptidyl-peptidase 7 | 1 |
| 12079 | hsa-miR-608 | 29952 | DPP7 | dipeptidyl-peptidase 7 | 1 |
| 12080 | hsa-miR-620 | 29952 | DPP7 | dipeptidyl-peptidase 7 | 1 |
| 12081 | hsa-let-7a-3 | 29952 | DPP7 | dipeptidyl-peptidase 7 | 1 |
| 12082 | hsa-let-7b | 29952 | DPP7 | dipeptidyl-peptidase 7 | 1 |
| 12083 | hsa-mir-22 | 29952 | DPP7 | dipeptidyl-peptidase 7 | 1 |
| 12084 | hsa-mir-10a | 29952 | DPP7 | dipeptidyl-peptidase 7 | 1 |
| 12085 | hsa-mir-186 | 29952 | DPP7 | dipeptidyl-peptidase 7 | 1 |
| 12086 | hsa-mir-155 | 29952 | DPP7 | dipeptidyl-peptidase 7 | 1 |
| 12087 | hsa-mir-499 | 29952 | DPP7 | dipeptidyl-peptidase 7 | 1 |
| 12088 | hsa-mir-550-1 | 29952 | DPP7 | dipeptidyl-peptidase 7 | 1 |
| 12089 | hsa-mir-877 | 29952 | DPP7 | dipeptidyl-peptidase 7 | 1 |
| 12090 | hsa-mir-3186 | 29952 | DPP7 | dipeptidyl-peptidase 7 | 1 |
| 12091 | hsa-miR-152 | 94120 | SYTL3 | synaptotagmin-like 3 | 1 |
| 12092 | hsa-miR-26b | 94120 | SYTL3 | synaptotagmin-like 3 | 1 |
| 12093 | hsa-miR-31 | 94120 | SYTL3 | synaptotagmin-like 3 | 1 |
| 12094 | hsa-miR-340 | 94120 | SYTL3 | synaptotagmin-like 3 | 1 |
| 12095 | hsa-miR-383 | 94120 | SYTL3 | synaptotagmin-like 3 | 1 |
| 12096 | hsa-miR-621 | 94120 | SYTL3 | synaptotagmin-like 3 | 1 |
| 12097 | hsa-miR-634 | 94120 | SYTL3 | synaptotagmin-like 3 | 1 |
| 12098 | hsa-miR-653 | 94120 | SYTL3 | synaptotagmin-like 3 | 1 |
| 12099 | hsa-miR-765 | 94120 | SYTL3 | synaptotagmin-like 3 | 1 |
| 12100 | hsa-mir-26a-1 | 94120 | SYTL3 | synaptotagmin-like 3 | 1 |
| 12101 | hsa-mir-26b | 94120 | SYTL3 | synaptotagmin-like 3 | 1 |
| 12102 | hsa-mir-31 | 94120 | SYTL3 | synaptotagmin-like 3 | 1 |
| 12103 | hsa-mir-194-2 | 94120 | SYTL3 | synaptotagmin-like 3 | 1 |
| 12104 | hsa-mir-340 | 94120 | SYTL3 | synaptotagmin-like 3 | 1 |
| 12105 | hsa-miR-10a | 64319 | FBRS | fibrosin | 1 |
| 12106 | hsa-miR-1228\* | 64319 | FBRS | fibrosin | 1 |
| 12107 | hsa-miR-139-5p | 64319 | FBRS | fibrosin | 1 |
| 12108 | hsa-miR-152 | 64319 | FBRS | fibrosin | 1 |
| 12109 | hsa-miR-26a | 64319 | FBRS | fibrosin | 1 |
| 12110 | hsa-miR-26b | 64319 | FBRS | fibrosin | 1 |
| 12111 | hsa-miR-31 | 64319 | FBRS | fibrosin | 1 |
| 12112 | hsa-miR-338-5p | 64319 | FBRS | fibrosin | 1 |
| 12113 | hsa-miR-340 | 64319 | FBRS | fibrosin | 1 |
| 12114 | hsa-miR-423-5p | 64319 | FBRS | fibrosin | 1 |
| 12115 | hsa-miR-449a | 64319 | FBRS | fibrosin | 1 |
| 12116 | hsa-miR-499-5p | 64319 | FBRS | fibrosin | 1 |
| 12117 | hsa-miR-548b-5p | 64319 | FBRS | fibrosin | 1 |
| 12118 | hsa-miR-621 | 64319 | FBRS | fibrosin | 1 |
| 12119 | hsa-miR-657 | 64319 | FBRS | fibrosin | 1 |
| 12120 | hsa-miR-765 | 64319 | FBRS | fibrosin | 1 |
| 12121 | hsa-miR-770-5p | 64319 | FBRS | fibrosin | 1 |
| 12122 | hsa-miR-877 | 64319 | FBRS | fibrosin | 1 |
| 12123 | hsa-mir-26a-1 | 64319 | FBRS | fibrosin | 1 |
| 12124 | hsa-mir-26b | 64319 | FBRS | fibrosin | 1 |
| 12125 | hsa-mir-31 | 64319 | FBRS | fibrosin | 1 |
| 12126 | hsa-mir-194-2 | 64319 | FBRS | fibrosin | 1 |
| 12127 | hsa-mir-340 | 64319 | FBRS | fibrosin | 1 |
| 12128 | hsa-mir-338 | 64319 | FBRS | fibrosin | 1 |
| 12129 | hsa-mir-548b | 64319 | FBRS | fibrosin | 1 |
| 12130 | hsa-mir-449b | 64319 | FBRS | fibrosin | 1 |
| 12131 | hsa-mir-449c | 64319 | FBRS | fibrosin | 1 |
| 12132 | hsa-mir-3065 | 64319 | FBRS | fibrosin | 1 |
| 12133 | hsa-miR-152 | 283651 | C15orf21 | Dresden prostate cancer 2 | 1 |
| 12134 | hsa-miR-26b | 283651 | C15orf21 | Dresden prostate cancer 2 | 1 |
| 12135 | hsa-miR-31 | 283651 | C15orf21 | Dresden prostate cancer 2 | 1 |
| 12136 | hsa-miR-340 | 283651 | C15orf21 | Dresden prostate cancer 2 | 1 |
| 12137 | hsa-miR-383 | 283651 | C15orf21 | Dresden prostate cancer 2 | 1 |
| 12138 | hsa-miR-449a | 283651 | C15orf21 | Dresden prostate cancer 2 | 1 |
| 12139 | hsa-miR-490-5p | 283651 | C15orf21 | Dresden prostate cancer 2 | 1 |
| 12140 | hsa-miR-504 | 283651 | C15orf21 | Dresden prostate cancer 2 | 1 |
| 12141 | hsa-miR-621 | 283651 | C15orf21 | Dresden prostate cancer 2 | 1 |
| 12142 | hsa-miR-653 | 283651 | C15orf21 | Dresden prostate cancer 2 | 1 |
| 12143 | hsa-miR-765 | 283651 | C15orf21 | Dresden prostate cancer 2 | 1 |
| 12144 | hsa-miR-770-5p | 283651 | C15orf21 | Dresden prostate cancer 2 | 1 |
| 12145 | hsa-mir-26a-1 | 283651 | C15orf21 | Dresden prostate cancer 2 | 1 |
| 12146 | hsa-mir-26b | 283651 | C15orf21 | Dresden prostate cancer 2 | 1 |
| 12147 | hsa-mir-31 | 283651 | C15orf21 | Dresden prostate cancer 2 | 1 |
| 12148 | hsa-mir-194-2 | 283651 | C15orf21 | Dresden prostate cancer 2 | 1 |
| 12149 | hsa-mir-340 | 283651 | C15orf21 | Dresden prostate cancer 2 | 1 |
| 12150 | hsa-mir-490 | 283651 | C15orf21 | Dresden prostate cancer 2 | 1 |
| 12151 | hsa-mir-449b | 283651 | C15orf21 | Dresden prostate cancer 2 | 1 |
| 12152 | hsa-mir-449c | 283651 | C15orf21 | Dresden prostate cancer 2 | 1 |
| 12153 | hsa-miR-383 | 5272 | SERPINB9 | serpin peptidase inhibitor, clade B (ovalbumin), member 9 | 1 |
| 12154 | hsa-miR-653 | 5272 | SERPINB9 | serpin peptidase inhibitor, clade B (ovalbumin), member 9 | 1 |
| 12155 | hsa-miR-1 | 151258 | SLC38A11 | solute carrier family 38, member 11 | 1 |
| 12156 | hsa-miR-133a | 151258 | SLC38A11 | solute carrier family 38, member 11 | 1 |
| 12157 | hsa-miR-186 | 151258 | SLC38A11 | solute carrier family 38, member 11 | 1 |
| 12158 | hsa-miR-326 | 151258 | SLC38A11 | solute carrier family 38, member 11 | 1 |
| 12159 | hsa-miR-449b | 151258 | SLC38A11 | solute carrier family 38, member 11 | 1 |
| 12160 | hsa-miR-551a | 151258 | SLC38A11 | solute carrier family 38, member 11 | 1 |
| 12161 | hsa-miR-562 | 151258 | SLC38A11 | solute carrier family 38, member 11 | 1 |
| 12162 | hsa-miR-575 | 151258 | SLC38A11 | solute carrier family 38, member 11 | 1 |
| 12163 | hsa-miR-579 | 151258 | SLC38A11 | solute carrier family 38, member 11 | 1 |
| 12164 | hsa-miR-608 | 151258 | SLC38A11 | solute carrier family 38, member 11 | 1 |
| 12165 | hsa-miR-620 | 151258 | SLC38A11 | solute carrier family 38, member 11 | 1 |
| 12166 | hsa-miR-637 | 151258 | SLC38A11 | solute carrier family 38, member 11 | 1 |
| 12167 | hsa-miR-885-5p | 151258 | SLC38A11 | solute carrier family 38, member 11 | 1 |
| 12168 | hsa-mir-22 | 151258 | SLC38A11 | solute carrier family 38, member 11 | 1 |
| 12169 | hsa-mir-10a | 151258 | SLC38A11 | solute carrier family 38, member 11 | 1 |
| 12170 | hsa-mir-186 | 151258 | SLC38A11 | solute carrier family 38, member 11 | 1 |
| 12171 | hsa-mir-155 | 151258 | SLC38A11 | solute carrier family 38, member 11 | 1 |
| 12172 | hsa-mir-423 | 151258 | SLC38A11 | solute carrier family 38, member 11 | 1 |
| 12173 | hsa-mir-499 | 151258 | SLC38A11 | solute carrier family 38, member 11 | 1 |
| 12174 | hsa-mir-885 | 151258 | SLC38A11 | solute carrier family 38, member 11 | 1 |
| 12175 | hsa-mir-877 | 151258 | SLC38A11 | solute carrier family 38, member 11 | 1 |
| 12176 | hsa-mir-3190 | 151258 | SLC38A11 | solute carrier family 38, member 11 | 1 |
| 12177 | hsa-miR-152 | 1452 | CSNK1A1 | casein kinase 1, alpha 1 | 1 |
| 12178 | hsa-miR-26b | 1452 | CSNK1A1 | casein kinase 1, alpha 1 | 1 |
| 12179 | hsa-miR-31 | 1452 | CSNK1A1 | casein kinase 1, alpha 1 | 1 |
| 12180 | hsa-miR-340 | 1452 | CSNK1A1 | casein kinase 1, alpha 1 | 1 |
| 12181 | hsa-miR-383 | 1452 | CSNK1A1 | casein kinase 1, alpha 1 | 1 |
| 12182 | hsa-miR-449a | 1452 | CSNK1A1 | casein kinase 1, alpha 1 | 1 |
| 12183 | hsa-miR-490-5p | 1452 | CSNK1A1 | casein kinase 1, alpha 1 | 1 |
| 12184 | hsa-miR-504 | 1452 | CSNK1A1 | casein kinase 1, alpha 1 | 1 |
| 12185 | hsa-miR-621 | 1452 | CSNK1A1 | casein kinase 1, alpha 1 | 1 |
| 12186 | hsa-miR-765 | 1452 | CSNK1A1 | casein kinase 1, alpha 1 | 1 |
| 12187 | hsa-miR-877 | 1452 | CSNK1A1 | casein kinase 1, alpha 1 | 1 |
| 12188 | hsa-mir-26a-1 | 1452 | CSNK1A1 | casein kinase 1, alpha 1 | 1 |
| 12189 | hsa-mir-26b | 1452 | CSNK1A1 | casein kinase 1, alpha 1 | 1 |
| 12190 | hsa-mir-31 | 1452 | CSNK1A1 | casein kinase 1, alpha 1 | 1 |
| 12191 | hsa-mir-194-2 | 1452 | CSNK1A1 | casein kinase 1, alpha 1 | 1 |
| 12192 | hsa-mir-340 | 1452 | CSNK1A1 | casein kinase 1, alpha 1 | 1 |
| 12193 | hsa-mir-490 | 1452 | CSNK1A1 | casein kinase 1, alpha 1 | 1 |
| 12194 | hsa-mir-449b | 1452 | CSNK1A1 | casein kinase 1, alpha 1 | 1 |
| 12195 | hsa-mir-449c | 1452 | CSNK1A1 | casein kinase 1, alpha 1 | 1 |
| 12196 | hsa-miR-338-5p | 133396 | IL31RA | interleukin 31 receptor A | 1 |
| 12197 | hsa-miR-657 | 133396 | IL31RA | interleukin 31 receptor A | 1 |
| 12198 | hsa-miR-663 | 133396 | IL31RA | interleukin 31 receptor A | 1 |
| 12199 | hsa-mir-338 | 133396 | IL31RA | interleukin 31 receptor A | 1 |
| 12200 | hsa-mir-3065 | 133396 | IL31RA | interleukin 31 receptor A | 1 |
| 12201 | hsa-miR-1 | 123041 | SLC24A4 | solute carrier family 24 (sodium/potassium/calcium exchanger), member 4 | 1 |
| 12202 | hsa-miR-133a | 123041 | SLC24A4 | solute carrier family 24 (sodium/potassium/calcium exchanger), member 4 | 1 |
| 12203 | hsa-miR-186 | 123041 | SLC24A4 | solute carrier family 24 (sodium/potassium/calcium exchanger), member 4 | 1 |
| 12204 | hsa-miR-208a | 123041 | SLC24A4 | solute carrier family 24 (sodium/potassium/calcium exchanger), member 4 | 1 |
| 12205 | hsa-miR-326 | 123041 | SLC24A4 | solute carrier family 24 (sodium/potassium/calcium exchanger), member 4 | 1 |
| 12206 | hsa-miR-449b | 123041 | SLC24A4 | solute carrier family 24 (sodium/potassium/calcium exchanger), member 4 | 1 |
| 12207 | hsa-miR-550 | 123041 | SLC24A4 | solute carrier family 24 (sodium/potassium/calcium exchanger), member 4 | 1 |
| 12208 | hsa-miR-575 | 123041 | SLC24A4 | solute carrier family 24 (sodium/potassium/calcium exchanger), member 4 | 1 |
| 12209 | hsa-miR-579 | 123041 | SLC24A4 | solute carrier family 24 (sodium/potassium/calcium exchanger), member 4 | 1 |
| 12210 | hsa-miR-608 | 123041 | SLC24A4 | solute carrier family 24 (sodium/potassium/calcium exchanger), member 4 | 1 |
| 12211 | hsa-miR-620 | 123041 | SLC24A4 | solute carrier family 24 (sodium/potassium/calcium exchanger), member 4 | 1 |
| 12212 | hsa-miR-885-5p | 123041 | SLC24A4 | solute carrier family 24 (sodium/potassium/calcium exchanger), member 4 | 1 |
| 12213 | hsa-let-7a-3 | 123041 | SLC24A4 | solute carrier family 24 (sodium/potassium/calcium exchanger), member 4 | 1 |
| 12214 | hsa-let-7b | 123041 | SLC24A4 | solute carrier family 24 (sodium/potassium/calcium exchanger), member 4 | 1 |
| 12215 | hsa-mir-22 | 123041 | SLC24A4 | solute carrier family 24 (sodium/potassium/calcium exchanger), member 4 | 1 |
| 12216 | hsa-mir-10a | 123041 | SLC24A4 | solute carrier family 24 (sodium/potassium/calcium exchanger), member 4 | 1 |
| 12217 | hsa-mir-186 | 123041 | SLC24A4 | solute carrier family 24 (sodium/potassium/calcium exchanger), member 4 | 1 |
| 12218 | hsa-mir-155 | 123041 | SLC24A4 | solute carrier family 24 (sodium/potassium/calcium exchanger), member 4 | 1 |
| 12219 | hsa-mir-499 | 123041 | SLC24A4 | solute carrier family 24 (sodium/potassium/calcium exchanger), member 4 | 1 |
| 12220 | hsa-mir-550-1 | 123041 | SLC24A4 | solute carrier family 24 (sodium/potassium/calcium exchanger), member 4 | 1 |
| 12221 | hsa-mir-885 | 123041 | SLC24A4 | solute carrier family 24 (sodium/potassium/calcium exchanger), member 4 | 1 |
| 12222 | hsa-mir-877 | 123041 | SLC24A4 | solute carrier family 24 (sodium/potassium/calcium exchanger), member 4 | 1 |
| 12223 | hsa-mir-2116 | 123041 | SLC24A4 | solute carrier family 24 (sodium/potassium/calcium exchanger), member 4 | 1 |
| 12224 | hsa-mir-3186 | 123041 | SLC24A4 | solute carrier family 24 (sodium/potassium/calcium exchanger), member 4 | 1 |
| 12225 | hsa-mir-3190 | 123041 | SLC24A4 | solute carrier family 24 (sodium/potassium/calcium exchanger), member 4 | 1 |
| 12226 | hsa-miR-10a | 4248 | MGAT3 | mannosyl (beta-1,4-)-glycoprotein beta-1,4-N-acetylglucosaminyltransferase | 1 |
| 12227 | hsa-miR-139-5p | 4248 | MGAT3 | mannosyl (beta-1,4-)-glycoprotein beta-1,4-N-acetylglucosaminyltransferase | 1 |
| 12228 | hsa-miR-152 | 4248 | MGAT3 | mannosyl (beta-1,4-)-glycoprotein beta-1,4-N-acetylglucosaminyltransferase | 1 |
| 12229 | hsa-miR-26a | 4248 | MGAT3 | mannosyl (beta-1,4-)-glycoprotein beta-1,4-N-acetylglucosaminyltransferase | 1 |
| 12230 | hsa-miR-26b | 4248 | MGAT3 | mannosyl (beta-1,4-)-glycoprotein beta-1,4-N-acetylglucosaminyltransferase | 1 |
| 12231 | hsa-miR-31 | 4248 | MGAT3 | mannosyl (beta-1,4-)-glycoprotein beta-1,4-N-acetylglucosaminyltransferase | 1 |
| 12232 | hsa-miR-338-5p | 4248 | MGAT3 | mannosyl (beta-1,4-)-glycoprotein beta-1,4-N-acetylglucosaminyltransferase | 1 |
| 12233 | hsa-miR-340 | 4248 | MGAT3 | mannosyl (beta-1,4-)-glycoprotein beta-1,4-N-acetylglucosaminyltransferase | 1 |
| 12234 | hsa-miR-449a | 4248 | MGAT3 | mannosyl (beta-1,4-)-glycoprotein beta-1,4-N-acetylglucosaminyltransferase | 1 |
| 12235 | hsa-miR-499-5p | 4248 | MGAT3 | mannosyl (beta-1,4-)-glycoprotein beta-1,4-N-acetylglucosaminyltransferase | 1 |
| 12236 | hsa-miR-548b-5p | 4248 | MGAT3 | mannosyl (beta-1,4-)-glycoprotein beta-1,4-N-acetylglucosaminyltransferase | 1 |
| 12237 | hsa-miR-564 | 4248 | MGAT3 | mannosyl (beta-1,4-)-glycoprotein beta-1,4-N-acetylglucosaminyltransferase | 1 |
| 12238 | hsa-miR-621 | 4248 | MGAT3 | mannosyl (beta-1,4-)-glycoprotein beta-1,4-N-acetylglucosaminyltransferase | 1 |
| 12239 | hsa-miR-657 | 4248 | MGAT3 | mannosyl (beta-1,4-)-glycoprotein beta-1,4-N-acetylglucosaminyltransferase | 1 |
| 12240 | hsa-miR-765 | 4248 | MGAT3 | mannosyl (beta-1,4-)-glycoprotein beta-1,4-N-acetylglucosaminyltransferase | 1 |
| 12241 | hsa-miR-770-5p | 4248 | MGAT3 | mannosyl (beta-1,4-)-glycoprotein beta-1,4-N-acetylglucosaminyltransferase | 1 |
| 12242 | hsa-miR-877 | 4248 | MGAT3 | mannosyl (beta-1,4-)-glycoprotein beta-1,4-N-acetylglucosaminyltransferase | 1 |
| 12243 | hsa-mir-26a-1 | 4248 | MGAT3 | mannosyl (beta-1,4-)-glycoprotein beta-1,4-N-acetylglucosaminyltransferase | 1 |
| 12244 | hsa-mir-26b | 4248 | MGAT3 | mannosyl (beta-1,4-)-glycoprotein beta-1,4-N-acetylglucosaminyltransferase | 1 |
| 12245 | hsa-mir-31 | 4248 | MGAT3 | mannosyl (beta-1,4-)-glycoprotein beta-1,4-N-acetylglucosaminyltransferase | 1 |
| 12246 | hsa-mir-194-2 | 4248 | MGAT3 | mannosyl (beta-1,4-)-glycoprotein beta-1,4-N-acetylglucosaminyltransferase | 1 |
| 12247 | hsa-mir-340 | 4248 | MGAT3 | mannosyl (beta-1,4-)-glycoprotein beta-1,4-N-acetylglucosaminyltransferase | 1 |
| 12248 | hsa-mir-338 | 4248 | MGAT3 | mannosyl (beta-1,4-)-glycoprotein beta-1,4-N-acetylglucosaminyltransferase | 1 |
| 12249 | hsa-mir-548b | 4248 | MGAT3 | mannosyl (beta-1,4-)-glycoprotein beta-1,4-N-acetylglucosaminyltransferase | 1 |
| 12250 | hsa-mir-548d-2 | 4248 | MGAT3 | mannosyl (beta-1,4-)-glycoprotein beta-1,4-N-acetylglucosaminyltransferase | 1 |
| 12251 | hsa-mir-449b | 4248 | MGAT3 | mannosyl (beta-1,4-)-glycoprotein beta-1,4-N-acetylglucosaminyltransferase | 1 |
| 12252 | hsa-mir-449c | 4248 | MGAT3 | mannosyl (beta-1,4-)-glycoprotein beta-1,4-N-acetylglucosaminyltransferase | 1 |
| 12253 | hsa-mir-3065 | 4248 | MGAT3 | mannosyl (beta-1,4-)-glycoprotein beta-1,4-N-acetylglucosaminyltransferase | 1 |
| 12254 | hsa-miR-10a | 6559 | SLC12A3 | solute carrier family 12 (sodium/chloride transporters), member 3 | 1 |
| 12255 | hsa-miR-152 | 6559 | SLC12A3 | solute carrier family 12 (sodium/chloride transporters), member 3 | 1 |
| 12256 | hsa-miR-26a | 6559 | SLC12A3 | solute carrier family 12 (sodium/chloride transporters), member 3 | 1 |
| 12257 | hsa-miR-26b | 6559 | SLC12A3 | solute carrier family 12 (sodium/chloride transporters), member 3 | 1 |
| 12258 | hsa-miR-31 | 6559 | SLC12A3 | solute carrier family 12 (sodium/chloride transporters), member 3 | 1 |
| 12259 | hsa-miR-340 | 6559 | SLC12A3 | solute carrier family 12 (sodium/chloride transporters), member 3 | 1 |
| 12260 | hsa-miR-423-5p | 6559 | SLC12A3 | solute carrier family 12 (sodium/chloride transporters), member 3 | 1 |
| 12261 | hsa-miR-449a | 6559 | SLC12A3 | solute carrier family 12 (sodium/chloride transporters), member 3 | 1 |
| 12262 | hsa-miR-490-5p | 6559 | SLC12A3 | solute carrier family 12 (sodium/chloride transporters), member 3 | 1 |
| 12263 | hsa-miR-499-5p | 6559 | SLC12A3 | solute carrier family 12 (sodium/chloride transporters), member 3 | 1 |
| 12264 | hsa-miR-548b-5p | 6559 | SLC12A3 | solute carrier family 12 (sodium/chloride transporters), member 3 | 1 |
| 12265 | hsa-miR-564 | 6559 | SLC12A3 | solute carrier family 12 (sodium/chloride transporters), member 3 | 1 |
| 12266 | hsa-miR-584 | 6559 | SLC12A3 | solute carrier family 12 (sodium/chloride transporters), member 3 | 1 |
| 12267 | hsa-miR-621 | 6559 | SLC12A3 | solute carrier family 12 (sodium/chloride transporters), member 3 | 1 |
| 12268 | hsa-miR-644 | 6559 | SLC12A3 | solute carrier family 12 (sodium/chloride transporters), member 3 | 1 |
| 12269 | hsa-miR-765 | 6559 | SLC12A3 | solute carrier family 12 (sodium/chloride transporters), member 3 | 1 |
| 12270 | hsa-miR-770-5p | 6559 | SLC12A3 | solute carrier family 12 (sodium/chloride transporters), member 3 | 1 |
| 12271 | hsa-miR-877 | 6559 | SLC12A3 | solute carrier family 12 (sodium/chloride transporters), member 3 | 1 |
| 12272 | hsa-miR-95 | 6559 | SLC12A3 | solute carrier family 12 (sodium/chloride transporters), member 3 | 1 |
| 12273 | hsa-mir-26a-1 | 6559 | SLC12A3 | solute carrier family 12 (sodium/chloride transporters), member 3 | 1 |
| 12274 | hsa-mir-26b | 6559 | SLC12A3 | solute carrier family 12 (sodium/chloride transporters), member 3 | 1 |
| 12275 | hsa-mir-31 | 6559 | SLC12A3 | solute carrier family 12 (sodium/chloride transporters), member 3 | 1 |
| 12276 | hsa-mir-194-2 | 6559 | SLC12A3 | solute carrier family 12 (sodium/chloride transporters), member 3 | 1 |
| 12277 | hsa-mir-340 | 6559 | SLC12A3 | solute carrier family 12 (sodium/chloride transporters), member 3 | 1 |
| 12278 | hsa-mir-490 | 6559 | SLC12A3 | solute carrier family 12 (sodium/chloride transporters), member 3 | 1 |
| 12279 | hsa-mir-548b | 6559 | SLC12A3 | solute carrier family 12 (sodium/chloride transporters), member 3 | 1 |
| 12280 | hsa-mir-449b | 6559 | SLC12A3 | solute carrier family 12 (sodium/chloride transporters), member 3 | 1 |
| 12281 | hsa-mir-449c | 6559 | SLC12A3 | solute carrier family 12 (sodium/chloride transporters), member 3 | 1 |
| 12282 | hsa-miR-1 | 9742 | IFT140 | intraflagellar transport 140 homolog (Chlamydomonas) | 1 |
| 12283 | hsa-miR-133a | 9742 | IFT140 | intraflagellar transport 140 homolog (Chlamydomonas) | 1 |
| 12284 | hsa-miR-186 | 9742 | IFT140 | intraflagellar transport 140 homolog (Chlamydomonas) | 1 |
| 12285 | hsa-miR-326 | 9742 | IFT140 | intraflagellar transport 140 homolog (Chlamydomonas) | 1 |
| 12286 | hsa-miR-449b | 9742 | IFT140 | intraflagellar transport 140 homolog (Chlamydomonas) | 1 |
| 12287 | hsa-miR-550 | 9742 | IFT140 | intraflagellar transport 140 homolog (Chlamydomonas) | 1 |
| 12288 | hsa-miR-551a | 9742 | IFT140 | intraflagellar transport 140 homolog (Chlamydomonas) | 1 |
| 12289 | hsa-miR-575 | 9742 | IFT140 | intraflagellar transport 140 homolog (Chlamydomonas) | 1 |
| 12290 | hsa-miR-579 | 9742 | IFT140 | intraflagellar transport 140 homolog (Chlamydomonas) | 1 |
| 12291 | hsa-miR-608 | 9742 | IFT140 | intraflagellar transport 140 homolog (Chlamydomonas) | 1 |
| 12292 | hsa-miR-620 | 9742 | IFT140 | intraflagellar transport 140 homolog (Chlamydomonas) | 1 |
| 12293 | hsa-miR-885-5p | 9742 | IFT140 | intraflagellar transport 140 homolog (Chlamydomonas) | 1 |
| 12294 | hsa-mir-22 | 9742 | IFT140 | intraflagellar transport 140 homolog (Chlamydomonas) | 1 |
| 12295 | hsa-mir-10a | 9742 | IFT140 | intraflagellar transport 140 homolog (Chlamydomonas) | 1 |
| 12296 | hsa-mir-186 | 9742 | IFT140 | intraflagellar transport 140 homolog (Chlamydomonas) | 1 |
| 12297 | hsa-mir-155 | 9742 | IFT140 | intraflagellar transport 140 homolog (Chlamydomonas) | 1 |
| 12298 | hsa-mir-423 | 9742 | IFT140 | intraflagellar transport 140 homolog (Chlamydomonas) | 1 |
| 12299 | hsa-mir-499 | 9742 | IFT140 | intraflagellar transport 140 homolog (Chlamydomonas) | 1 |
| 12300 | hsa-mir-550-1 | 9742 | IFT140 | intraflagellar transport 140 homolog (Chlamydomonas) | 1 |
| 12301 | hsa-mir-885 | 9742 | IFT140 | intraflagellar transport 140 homolog (Chlamydomonas) | 1 |
| 12302 | hsa-mir-877 | 9742 | IFT140 | intraflagellar transport 140 homolog (Chlamydomonas) | 1 |
| 12303 | hsa-mir-2116 | 9742 | IFT140 | intraflagellar transport 140 homolog (Chlamydomonas) | 1 |
| 12304 | hsa-mir-3186 | 9742 | IFT140 | intraflagellar transport 140 homolog (Chlamydomonas) | 1 |
| 12305 | hsa-mir-3190 | 9742 | IFT140 | intraflagellar transport 140 homolog (Chlamydomonas) | 1 |
| 12306 | hsa-miR-10a | 64005 | MYO1G | myosin IG | 1 |
| 12307 | hsa-miR-139-5p | 64005 | MYO1G | myosin IG | 1 |
| 12308 | hsa-miR-152 | 64005 | MYO1G | myosin IG | 1 |
| 12309 | hsa-miR-26b | 64005 | MYO1G | myosin IG | 1 |
| 12310 | hsa-miR-31 | 64005 | MYO1G | myosin IG | 1 |
| 12311 | hsa-miR-338-5p | 64005 | MYO1G | myosin IG | 1 |
| 12312 | hsa-miR-340 | 64005 | MYO1G | myosin IG | 1 |
| 12313 | hsa-miR-383 | 64005 | MYO1G | myosin IG | 1 |
| 12314 | hsa-miR-449a | 64005 | MYO1G | myosin IG | 1 |
| 12315 | hsa-miR-499-5p | 64005 | MYO1G | myosin IG | 1 |
| 12316 | hsa-miR-548b-5p | 64005 | MYO1G | myosin IG | 1 |
| 12317 | hsa-miR-621 | 64005 | MYO1G | myosin IG | 1 |
| 12318 | hsa-miR-657 | 64005 | MYO1G | myosin IG | 1 |
| 12319 | hsa-miR-765 | 64005 | MYO1G | myosin IG | 1 |
| 12320 | hsa-miR-770-5p | 64005 | MYO1G | myosin IG | 1 |
| 12321 | hsa-miR-877 | 64005 | MYO1G | myosin IG | 1 |
| 12322 | hsa-mir-26a-1 | 64005 | MYO1G | myosin IG | 1 |
| 12323 | hsa-mir-26b | 64005 | MYO1G | myosin IG | 1 |
| 12324 | hsa-mir-31 | 64005 | MYO1G | myosin IG | 1 |
| 12325 | hsa-mir-194-2 | 64005 | MYO1G | myosin IG | 1 |
| 12326 | hsa-mir-340 | 64005 | MYO1G | myosin IG | 1 |
| 12327 | hsa-mir-338 | 64005 | MYO1G | myosin IG | 1 |
| 12328 | hsa-mir-548b | 64005 | MYO1G | myosin IG | 1 |
| 12329 | hsa-mir-449b | 64005 | MYO1G | myosin IG | 1 |
| 12330 | hsa-mir-449c | 64005 | MYO1G | myosin IG | 1 |
| 12331 | hsa-mir-3065 | 64005 | MYO1G | myosin IG | 1 |
| 12332 | hsa-miR-1 | 23357 | ANGEL1 | angel homolog 1 (Drosophila) | 1 |
| 12333 | hsa-miR-133a | 23357 | ANGEL1 | angel homolog 1 (Drosophila) | 1 |
| 12334 | hsa-miR-186 | 23357 | ANGEL1 | angel homolog 1 (Drosophila) | 1 |
| 12335 | hsa-miR-326 | 23357 | ANGEL1 | angel homolog 1 (Drosophila) | 1 |
| 12336 | hsa-miR-449b | 23357 | ANGEL1 | angel homolog 1 (Drosophila) | 1 |
| 12337 | hsa-miR-551a | 23357 | ANGEL1 | angel homolog 1 (Drosophila) | 1 |
| 12338 | hsa-miR-562 | 23357 | ANGEL1 | angel homolog 1 (Drosophila) | 1 |
| 12339 | hsa-miR-575 | 23357 | ANGEL1 | angel homolog 1 (Drosophila) | 1 |
| 12340 | hsa-miR-579 | 23357 | ANGEL1 | angel homolog 1 (Drosophila) | 1 |
| 12341 | hsa-miR-608 | 23357 | ANGEL1 | angel homolog 1 (Drosophila) | 1 |
| 12342 | hsa-miR-620 | 23357 | ANGEL1 | angel homolog 1 (Drosophila) | 1 |
| 12343 | hsa-miR-637 | 23357 | ANGEL1 | angel homolog 1 (Drosophila) | 1 |
| 12344 | hsa-miR-885-5p | 23357 | ANGEL1 | angel homolog 1 (Drosophila) | 1 |
| 12345 | hsa-mir-22 | 23357 | ANGEL1 | angel homolog 1 (Drosophila) | 1 |
| 12346 | hsa-mir-10a | 23357 | ANGEL1 | angel homolog 1 (Drosophila) | 1 |
| 12347 | hsa-mir-186 | 23357 | ANGEL1 | angel homolog 1 (Drosophila) | 1 |
| 12348 | hsa-mir-155 | 23357 | ANGEL1 | angel homolog 1 (Drosophila) | 1 |
| 12349 | hsa-mir-423 | 23357 | ANGEL1 | angel homolog 1 (Drosophila) | 1 |
| 12350 | hsa-mir-499 | 23357 | ANGEL1 | angel homolog 1 (Drosophila) | 1 |
| 12351 | hsa-mir-885 | 23357 | ANGEL1 | angel homolog 1 (Drosophila) | 1 |
| 12352 | hsa-mir-877 | 23357 | ANGEL1 | angel homolog 1 (Drosophila) | 1 |
| 12353 | hsa-mir-3190 | 23357 | ANGEL1 | angel homolog 1 (Drosophila) | 1 |
| 12354 | hsa-miR-1 | 163131 | ZNF780B | zinc finger protein 780B | 1 |
| 12355 | hsa-miR-133a | 163131 | ZNF780B | zinc finger protein 780B | 1 |
| 12356 | hsa-miR-186 | 163131 | ZNF780B | zinc finger protein 780B | 1 |
| 12357 | hsa-miR-208a | 163131 | ZNF780B | zinc finger protein 780B | 1 |
| 12358 | hsa-miR-449b | 163131 | ZNF780B | zinc finger protein 780B | 1 |
| 12359 | hsa-miR-489 | 163131 | ZNF780B | zinc finger protein 780B | 1 |
| 12360 | hsa-miR-575 | 163131 | ZNF780B | zinc finger protein 780B | 1 |
| 12361 | hsa-miR-579 | 163131 | ZNF780B | zinc finger protein 780B | 1 |
| 12362 | hsa-miR-608 | 163131 | ZNF780B | zinc finger protein 780B | 1 |
| 12363 | hsa-miR-620 | 163131 | ZNF780B | zinc finger protein 780B | 1 |
| 12364 | hsa-miR-885-5p | 163131 | ZNF780B | zinc finger protein 780B | 1 |
| 12365 | hsa-let-7a-3 | 163131 | ZNF780B | zinc finger protein 780B | 1 |
| 12366 | hsa-let-7b | 163131 | ZNF780B | zinc finger protein 780B | 1 |
| 12367 | hsa-mir-22 | 163131 | ZNF780B | zinc finger protein 780B | 1 |
| 12368 | hsa-mir-10a | 163131 | ZNF780B | zinc finger protein 780B | 1 |
| 12369 | hsa-mir-186 | 163131 | ZNF780B | zinc finger protein 780B | 1 |
| 12370 | hsa-mir-155 | 163131 | ZNF780B | zinc finger protein 780B | 1 |
| 12371 | hsa-mir-499 | 163131 | ZNF780B | zinc finger protein 780B | 1 |
| 12372 | hsa-mir-885 | 163131 | ZNF780B | zinc finger protein 780B | 1 |
| 12373 | hsa-mir-877 | 163131 | ZNF780B | zinc finger protein 780B | 1 |
| 12374 | hsa-mir-3190 | 163131 | ZNF780B | zinc finger protein 780B | 1 |
| 12375 | hsa-miR-1 | 84503 | ZNF527 | zinc finger protein 527 | 1 |
| 12376 | hsa-miR-133a | 84503 | ZNF527 | zinc finger protein 527 | 1 |
| 12377 | hsa-miR-186 | 84503 | ZNF527 | zinc finger protein 527 | 1 |
| 12378 | hsa-miR-326 | 84503 | ZNF527 | zinc finger protein 527 | 1 |
| 12379 | hsa-miR-449b | 84503 | ZNF527 | zinc finger protein 527 | 1 |
| 12380 | hsa-miR-551a | 84503 | ZNF527 | zinc finger protein 527 | 1 |
| 12381 | hsa-miR-562 | 84503 | ZNF527 | zinc finger protein 527 | 1 |
| 12382 | hsa-miR-575 | 84503 | ZNF527 | zinc finger protein 527 | 1 |
| 12383 | hsa-miR-579 | 84503 | ZNF527 | zinc finger protein 527 | 1 |
| 12384 | hsa-miR-608 | 84503 | ZNF527 | zinc finger protein 527 | 1 |
| 12385 | hsa-miR-620 | 84503 | ZNF527 | zinc finger protein 527 | 1 |
| 12386 | hsa-miR-637 | 84503 | ZNF527 | zinc finger protein 527 | 1 |
| 12387 | hsa-miR-885-5p | 84503 | ZNF527 | zinc finger protein 527 | 1 |
| 12388 | hsa-mir-22 | 84503 | ZNF527 | zinc finger protein 527 | 1 |
| 12389 | hsa-mir-10a | 84503 | ZNF527 | zinc finger protein 527 | 1 |
| 12390 | hsa-mir-186 | 84503 | ZNF527 | zinc finger protein 527 | 1 |
| 12391 | hsa-mir-155 | 84503 | ZNF527 | zinc finger protein 527 | 1 |
| 12392 | hsa-mir-423 | 84503 | ZNF527 | zinc finger protein 527 | 1 |
| 12393 | hsa-mir-499 | 84503 | ZNF527 | zinc finger protein 527 | 1 |
| 12394 | hsa-mir-885 | 84503 | ZNF527 | zinc finger protein 527 | 1 |
| 12395 | hsa-mir-877 | 84503 | ZNF527 | zinc finger protein 527 | 1 |
| 12396 | hsa-mir-3190 | 84503 | ZNF527 | zinc finger protein 527 | 1 |
| 12397 | hsa-miR-10a | 10456 | HAX1 | HCLS1 associated protein X-1 | 1 |
| 12398 | hsa-miR-152 | 10456 | HAX1 | HCLS1 associated protein X-1 | 1 |
| 12399 | hsa-miR-26b | 10456 | HAX1 | HCLS1 associated protein X-1 | 1 |
| 12400 | hsa-miR-31 | 10456 | HAX1 | HCLS1 associated protein X-1 | 1 |
| 12401 | hsa-miR-338-5p | 10456 | HAX1 | HCLS1 associated protein X-1 | 1 |
| 12402 | hsa-miR-340 | 10456 | HAX1 | HCLS1 associated protein X-1 | 1 |
| 12403 | hsa-miR-383 | 10456 | HAX1 | HCLS1 associated protein X-1 | 1 |
| 12404 | hsa-miR-449a | 10456 | HAX1 | HCLS1 associated protein X-1 | 1 |
| 12405 | hsa-miR-499-5p | 10456 | HAX1 | HCLS1 associated protein X-1 | 1 |
| 12406 | hsa-miR-621 | 10456 | HAX1 | HCLS1 associated protein X-1 | 1 |
| 12407 | hsa-miR-634 | 10456 | HAX1 | HCLS1 associated protein X-1 | 1 |
| 12408 | hsa-miR-653 | 10456 | HAX1 | HCLS1 associated protein X-1 | 1 |
| 12409 | hsa-miR-657 | 10456 | HAX1 | HCLS1 associated protein X-1 | 1 |
| 12410 | hsa-miR-765 | 10456 | HAX1 | HCLS1 associated protein X-1 | 1 |
| 12411 | hsa-miR-770-5p | 10456 | HAX1 | HCLS1 associated protein X-1 | 1 |
| 12412 | hsa-mir-26a-1 | 10456 | HAX1 | HCLS1 associated protein X-1 | 1 |
| 12413 | hsa-mir-26b | 10456 | HAX1 | HCLS1 associated protein X-1 | 1 |
| 12414 | hsa-mir-31 | 10456 | HAX1 | HCLS1 associated protein X-1 | 1 |
| 12415 | hsa-mir-194-2 | 10456 | HAX1 | HCLS1 associated protein X-1 | 1 |
| 12416 | hsa-mir-340 | 10456 | HAX1 | HCLS1 associated protein X-1 | 1 |
| 12417 | hsa-mir-338 | 10456 | HAX1 | HCLS1 associated protein X-1 | 1 |
| 12418 | hsa-mir-449b | 10456 | HAX1 | HCLS1 associated protein X-1 | 1 |
| 12419 | hsa-mir-449c | 10456 | HAX1 | HCLS1 associated protein X-1 | 1 |
| 12420 | hsa-mir-3065 | 10456 | HAX1 | HCLS1 associated protein X-1 | 1 |
| 12421 | hsa-miR-338-5p | 3669 | ISG20 | interferon stimulated exonuclease gene 20kDa | 1 |
| 12422 | hsa-miR-657 | 3669 | ISG20 | interferon stimulated exonuclease gene 20kDa | 1 |
| 12423 | hsa-mir-338 | 3669 | ISG20 | interferon stimulated exonuclease gene 20kDa | 1 |
| 12424 | hsa-mir-3065 | 3669 | ISG20 | interferon stimulated exonuclease gene 20kDa | 1 |
| 12425 | hsa-miR-1 | 8484 | GALR3 | galanin receptor 3 | 1 |
| 12426 | hsa-miR-133a | 8484 | GALR3 | galanin receptor 3 | 1 |
| 12427 | hsa-miR-186 | 8484 | GALR3 | galanin receptor 3 | 1 |
| 12428 | hsa-miR-208a | 8484 | GALR3 | galanin receptor 3 | 1 |
| 12429 | hsa-miR-326 | 8484 | GALR3 | galanin receptor 3 | 1 |
| 12430 | hsa-miR-449b | 8484 | GALR3 | galanin receptor 3 | 1 |
| 12431 | hsa-miR-550 | 8484 | GALR3 | galanin receptor 3 | 1 |
| 12432 | hsa-miR-575 | 8484 | GALR3 | galanin receptor 3 | 1 |
| 12433 | hsa-miR-579 | 8484 | GALR3 | galanin receptor 3 | 1 |
| 12434 | hsa-miR-608 | 8484 | GALR3 | galanin receptor 3 | 1 |
| 12435 | hsa-miR-620 | 8484 | GALR3 | galanin receptor 3 | 1 |
| 12436 | hsa-miR-885-5p | 8484 | GALR3 | galanin receptor 3 | 1 |
| 12437 | hsa-let-7a-3 | 8484 | GALR3 | galanin receptor 3 | 1 |
| 12438 | hsa-let-7b | 8484 | GALR3 | galanin receptor 3 | 1 |
| 12439 | hsa-mir-22 | 8484 | GALR3 | galanin receptor 3 | 1 |
| 12440 | hsa-mir-10a | 8484 | GALR3 | galanin receptor 3 | 1 |
| 12441 | hsa-mir-186 | 8484 | GALR3 | galanin receptor 3 | 1 |
| 12442 | hsa-mir-155 | 8484 | GALR3 | galanin receptor 3 | 1 |
| 12443 | hsa-mir-423 | 8484 | GALR3 | galanin receptor 3 | 1 |
| 12444 | hsa-mir-499 | 8484 | GALR3 | galanin receptor 3 | 1 |
| 12445 | hsa-mir-550-1 | 8484 | GALR3 | galanin receptor 3 | 1 |
| 12446 | hsa-mir-885 | 8484 | GALR3 | galanin receptor 3 | 1 |
| 12447 | hsa-mir-877 | 8484 | GALR3 | galanin receptor 3 | 1 |
| 12448 | hsa-mir-2116 | 8484 | GALR3 | galanin receptor 3 | 1 |
| 12449 | hsa-mir-3186 | 8484 | GALR3 | galanin receptor 3 | 1 |
| 12450 | hsa-mir-3190 | 8484 | GALR3 | galanin receptor 3 | 1 |
| 12451 | hsa-miR-1 | 1952 | CELSR2 | cadherin, EGF LAG seven-pass G-type receptor 2 (flamingo homolog, Drosophila) | 1 |
| 12452 | hsa-miR-133a | 1952 | CELSR2 | cadherin, EGF LAG seven-pass G-type receptor 2 (flamingo homolog, Drosophila) | 1 |
| 12453 | hsa-miR-186 | 1952 | CELSR2 | cadherin, EGF LAG seven-pass G-type receptor 2 (flamingo homolog, Drosophila) | 1 |
| 12454 | hsa-miR-195 | 1952 | CELSR2 | cadherin, EGF LAG seven-pass G-type receptor 2 (flamingo homolog, Drosophila) | 1 |
| 12455 | hsa-miR-208a | 1952 | CELSR2 | cadherin, EGF LAG seven-pass G-type receptor 2 (flamingo homolog, Drosophila) | 1 |
| 12456 | hsa-miR-326 | 1952 | CELSR2 | cadherin, EGF LAG seven-pass G-type receptor 2 (flamingo homolog, Drosophila) | 1 |
| 12457 | hsa-miR-449b | 1952 | CELSR2 | cadherin, EGF LAG seven-pass G-type receptor 2 (flamingo homolog, Drosophila) | 1 |
| 12458 | hsa-miR-497 | 1952 | CELSR2 | cadherin, EGF LAG seven-pass G-type receptor 2 (flamingo homolog, Drosophila) | 1 |
| 12459 | hsa-miR-550 | 1952 | CELSR2 | cadherin, EGF LAG seven-pass G-type receptor 2 (flamingo homolog, Drosophila) | 1 |
| 12460 | hsa-miR-575 | 1952 | CELSR2 | cadherin, EGF LAG seven-pass G-type receptor 2 (flamingo homolog, Drosophila) | 1 |
| 12461 | hsa-miR-608 | 1952 | CELSR2 | cadherin, EGF LAG seven-pass G-type receptor 2 (flamingo homolog, Drosophila) | 1 |
| 12462 | hsa-miR-620 | 1952 | CELSR2 | cadherin, EGF LAG seven-pass G-type receptor 2 (flamingo homolog, Drosophila) | 1 |
| 12463 | hsa-miR-885-5p | 1952 | CELSR2 | cadherin, EGF LAG seven-pass G-type receptor 2 (flamingo homolog, Drosophila) | 1 |
| 12464 | hsa-let-7a-3 | 1952 | CELSR2 | cadherin, EGF LAG seven-pass G-type receptor 2 (flamingo homolog, Drosophila) | 1 |
| 12465 | hsa-let-7b | 1952 | CELSR2 | cadherin, EGF LAG seven-pass G-type receptor 2 (flamingo homolog, Drosophila) | 1 |
| 12466 | hsa-mir-22 | 1952 | CELSR2 | cadherin, EGF LAG seven-pass G-type receptor 2 (flamingo homolog, Drosophila) | 1 |
| 12467 | hsa-mir-10a | 1952 | CELSR2 | cadherin, EGF LAG seven-pass G-type receptor 2 (flamingo homolog, Drosophila) | 1 |
| 12468 | hsa-mir-186 | 1952 | CELSR2 | cadherin, EGF LAG seven-pass G-type receptor 2 (flamingo homolog, Drosophila) | 1 |
| 12469 | hsa-mir-155 | 1952 | CELSR2 | cadherin, EGF LAG seven-pass G-type receptor 2 (flamingo homolog, Drosophila) | 1 |
| 12470 | hsa-mir-499 | 1952 | CELSR2 | cadherin, EGF LAG seven-pass G-type receptor 2 (flamingo homolog, Drosophila) | 1 |
| 12471 | hsa-mir-550-1 | 1952 | CELSR2 | cadherin, EGF LAG seven-pass G-type receptor 2 (flamingo homolog, Drosophila) | 1 |
| 12472 | hsa-mir-885 | 1952 | CELSR2 | cadherin, EGF LAG seven-pass G-type receptor 2 (flamingo homolog, Drosophila) | 1 |
| 12473 | hsa-mir-877 | 1952 | CELSR2 | cadherin, EGF LAG seven-pass G-type receptor 2 (flamingo homolog, Drosophila) | 1 |
| 12474 | hsa-mir-2116 | 1952 | CELSR2 | cadherin, EGF LAG seven-pass G-type receptor 2 (flamingo homolog, Drosophila) | 1 |
| 12475 | hsa-mir-3190 | 1952 | CELSR2 | cadherin, EGF LAG seven-pass G-type receptor 2 (flamingo homolog, Drosophila) | 1 |
| 12476 | hsa-miR-1 | 7454 | WAS | Wiskott-Aldrich syndrome (eczema-thrombocytopenia) | 1 |
| 12477 | hsa-miR-133a | 7454 | WAS | Wiskott-Aldrich syndrome (eczema-thrombocytopenia) | 1 |
| 12478 | hsa-miR-186 | 7454 | WAS | Wiskott-Aldrich syndrome (eczema-thrombocytopenia) | 1 |
| 12479 | hsa-miR-326 | 7454 | WAS | Wiskott-Aldrich syndrome (eczema-thrombocytopenia) | 1 |
| 12480 | hsa-miR-449b | 7454 | WAS | Wiskott-Aldrich syndrome (eczema-thrombocytopenia) | 1 |
| 12481 | hsa-miR-551a | 7454 | WAS | Wiskott-Aldrich syndrome (eczema-thrombocytopenia) | 1 |
| 12482 | hsa-miR-562 | 7454 | WAS | Wiskott-Aldrich syndrome (eczema-thrombocytopenia) | 1 |
| 12483 | hsa-miR-575 | 7454 | WAS | Wiskott-Aldrich syndrome (eczema-thrombocytopenia) | 1 |
| 12484 | hsa-miR-579 | 7454 | WAS | Wiskott-Aldrich syndrome (eczema-thrombocytopenia) | 1 |
| 12485 | hsa-miR-608 | 7454 | WAS | Wiskott-Aldrich syndrome (eczema-thrombocytopenia) | 1 |
| 12486 | hsa-miR-620 | 7454 | WAS | Wiskott-Aldrich syndrome (eczema-thrombocytopenia) | 1 |
| 12487 | hsa-miR-637 | 7454 | WAS | Wiskott-Aldrich syndrome (eczema-thrombocytopenia) | 1 |
| 12488 | hsa-miR-885-5p | 7454 | WAS | Wiskott-Aldrich syndrome (eczema-thrombocytopenia) | 1 |
| 12489 | hsa-mir-22 | 7454 | WAS | Wiskott-Aldrich syndrome (eczema-thrombocytopenia) | 1 |
| 12490 | hsa-mir-10a | 7454 | WAS | Wiskott-Aldrich syndrome (eczema-thrombocytopenia) | 1 |
| 12491 | hsa-mir-186 | 7454 | WAS | Wiskott-Aldrich syndrome (eczema-thrombocytopenia) | 1 |
| 12492 | hsa-mir-155 | 7454 | WAS | Wiskott-Aldrich syndrome (eczema-thrombocytopenia) | 1 |
| 12493 | hsa-mir-423 | 7454 | WAS | Wiskott-Aldrich syndrome (eczema-thrombocytopenia) | 1 |
| 12494 | hsa-mir-499 | 7454 | WAS | Wiskott-Aldrich syndrome (eczema-thrombocytopenia) | 1 |
| 12495 | hsa-mir-885 | 7454 | WAS | Wiskott-Aldrich syndrome (eczema-thrombocytopenia) | 1 |
| 12496 | hsa-mir-877 | 7454 | WAS | Wiskott-Aldrich syndrome (eczema-thrombocytopenia) | 1 |
| 12497 | hsa-mir-2116 | 7454 | WAS | Wiskott-Aldrich syndrome (eczema-thrombocytopenia) | 1 |
| 12498 | hsa-mir-3190 | 7454 | WAS | Wiskott-Aldrich syndrome (eczema-thrombocytopenia) | 1 |
| 12499 | hsa-miR-423-5p | 3553 | IL1B | interleukin 1, beta | 1 |
| 12500 | hsa-miR-423-5p | 57147 | SCYL3 | SCY1-like 3 (S. cerevisiae) | 1 |
| 12501 | hsa-miR-663 | 57147 | SCYL3 | SCY1-like 3 (S. cerevisiae) | 1 |
| 12502 | hsa-miR-1 | 10826 | C5orf4 | chromosome 5 open reading frame 4 | 1 |
| 12503 | hsa-miR-133a | 10826 | C5orf4 | chromosome 5 open reading frame 4 | 1 |
| 12504 | hsa-miR-186 | 10826 | C5orf4 | chromosome 5 open reading frame 4 | 1 |
| 12505 | hsa-miR-326 | 10826 | C5orf4 | chromosome 5 open reading frame 4 | 1 |
| 12506 | hsa-miR-449b | 10826 | C5orf4 | chromosome 5 open reading frame 4 | 1 |
| 12507 | hsa-miR-550 | 10826 | C5orf4 | chromosome 5 open reading frame 4 | 1 |
| 12508 | hsa-miR-551a | 10826 | C5orf4 | chromosome 5 open reading frame 4 | 1 |
| 12509 | hsa-miR-575 | 10826 | C5orf4 | chromosome 5 open reading frame 4 | 1 |
| 12510 | hsa-miR-579 | 10826 | C5orf4 | chromosome 5 open reading frame 4 | 1 |
| 12511 | hsa-miR-608 | 10826 | C5orf4 | chromosome 5 open reading frame 4 | 1 |
| 12512 | hsa-miR-620 | 10826 | C5orf4 | chromosome 5 open reading frame 4 | 1 |
| 12513 | hsa-mir-22 | 10826 | C5orf4 | chromosome 5 open reading frame 4 | 1 |
| 12514 | hsa-mir-10a | 10826 | C5orf4 | chromosome 5 open reading frame 4 | 1 |
| 12515 | hsa-mir-186 | 10826 | C5orf4 | chromosome 5 open reading frame 4 | 1 |
| 12516 | hsa-mir-155 | 10826 | C5orf4 | chromosome 5 open reading frame 4 | 1 |
| 12517 | hsa-mir-423 | 10826 | C5orf4 | chromosome 5 open reading frame 4 | 1 |
| 12518 | hsa-mir-499 | 10826 | C5orf4 | chromosome 5 open reading frame 4 | 1 |
| 12519 | hsa-mir-550-1 | 10826 | C5orf4 | chromosome 5 open reading frame 4 | 1 |
| 12520 | hsa-mir-877 | 10826 | C5orf4 | chromosome 5 open reading frame 4 | 1 |
| 12521 | hsa-mir-2116 | 10826 | C5orf4 | chromosome 5 open reading frame 4 | 1 |
| 12522 | hsa-mir-3190 | 10826 | C5orf4 | chromosome 5 open reading frame 4 | 1 |
| 12523 | hsa-miR-423-5p | 79155 | TNIP2 | TNFAIP3 interacting protein 2 | 1 |
| 12524 | hsa-miR-139-5p | 373863 | DND1 | dead end homolog 1 (zebrafish) | 1 |
| 12525 | hsa-miR-26a | 373863 | DND1 | dead end homolog 1 (zebrafish) | 1 |
| 12526 | hsa-miR-663 | 373863 | DND1 | dead end homolog 1 (zebrafish) | 1 |
| 12527 | hsa-miR-1 | 79744 | ZNF419 | zinc finger protein 419 | 1 |
| 12528 | hsa-miR-133a | 79744 | ZNF419 | zinc finger protein 419 | 1 |
| 12529 | hsa-miR-186 | 79744 | ZNF419 | zinc finger protein 419 | 1 |
| 12530 | hsa-miR-195 | 79744 | ZNF419 | zinc finger protein 419 | 1 |
| 12531 | hsa-miR-326 | 79744 | ZNF419 | zinc finger protein 419 | 1 |
| 12532 | hsa-miR-33a | 79744 | ZNF419 | zinc finger protein 419 | 1 |
| 12533 | hsa-miR-449b | 79744 | ZNF419 | zinc finger protein 419 | 1 |
| 12534 | hsa-miR-497 | 79744 | ZNF419 | zinc finger protein 419 | 1 |
| 12535 | hsa-miR-550 | 79744 | ZNF419 | zinc finger protein 419 | 1 |
| 12536 | hsa-miR-551a | 79744 | ZNF419 | zinc finger protein 419 | 1 |
| 12537 | hsa-miR-575 | 79744 | ZNF419 | zinc finger protein 419 | 1 |
| 12538 | hsa-miR-579 | 79744 | ZNF419 | zinc finger protein 419 | 1 |
| 12539 | hsa-miR-608 | 79744 | ZNF419 | zinc finger protein 419 | 1 |
| 12540 | hsa-miR-620 | 79744 | ZNF419 | zinc finger protein 419 | 1 |
| 12541 | hsa-mir-22 | 79744 | ZNF419 | zinc finger protein 419 | 1 |
| 12542 | hsa-mir-10a | 79744 | ZNF419 | zinc finger protein 419 | 1 |
| 12543 | hsa-mir-186 | 79744 | ZNF419 | zinc finger protein 419 | 1 |
| 12544 | hsa-mir-155 | 79744 | ZNF419 | zinc finger protein 419 | 1 |
| 12545 | hsa-mir-423 | 79744 | ZNF419 | zinc finger protein 419 | 1 |
| 12546 | hsa-mir-499 | 79744 | ZNF419 | zinc finger protein 419 | 1 |
| 12547 | hsa-mir-550-1 | 79744 | ZNF419 | zinc finger protein 419 | 1 |
| 12548 | hsa-mir-877 | 79744 | ZNF419 | zinc finger protein 419 | 1 |
| 12549 | hsa-mir-2116 | 79744 | ZNF419 | zinc finger protein 419 | 1 |
| 12550 | hsa-mir-3190 | 79744 | ZNF419 | zinc finger protein 419 | 1 |
| 12551 | hsa-miR-10a | 6772 | STAT1 | signal transducer and activator of transcription 1, 91kDa | 1 |
| 12552 | hsa-miR-152 | 6772 | STAT1 | signal transducer and activator of transcription 1, 91kDa | 1 |
| 12553 | hsa-miR-26a | 6772 | STAT1 | signal transducer and activator of transcription 1, 91kDa | 1 |
| 12554 | hsa-miR-26b | 6772 | STAT1 | signal transducer and activator of transcription 1, 91kDa | 1 |
| 12555 | hsa-miR-31 | 6772 | STAT1 | signal transducer and activator of transcription 1, 91kDa | 1 |
| 12556 | hsa-miR-338-5p | 6772 | STAT1 | signal transducer and activator of transcription 1, 91kDa | 1 |
| 12557 | hsa-miR-340 | 6772 | STAT1 | signal transducer and activator of transcription 1, 91kDa | 1 |
| 12558 | hsa-miR-423-5p | 6772 | STAT1 | signal transducer and activator of transcription 1, 91kDa | 1 |
| 12559 | hsa-miR-449a | 6772 | STAT1 | signal transducer and activator of transcription 1, 91kDa | 1 |
| 12560 | hsa-miR-490-5p | 6772 | STAT1 | signal transducer and activator of transcription 1, 91kDa | 1 |
| 12561 | hsa-miR-499-5p | 6772 | STAT1 | signal transducer and activator of transcription 1, 91kDa | 1 |
| 12562 | hsa-miR-548b-5p | 6772 | STAT1 | signal transducer and activator of transcription 1, 91kDa | 1 |
| 12563 | hsa-miR-564 | 6772 | STAT1 | signal transducer and activator of transcription 1, 91kDa | 1 |
| 12564 | hsa-miR-621 | 6772 | STAT1 | signal transducer and activator of transcription 1, 91kDa | 1 |
| 12565 | hsa-miR-657 | 6772 | STAT1 | signal transducer and activator of transcription 1, 91kDa | 1 |
| 12566 | hsa-miR-663 | 6772 | STAT1 | signal transducer and activator of transcription 1, 91kDa | 1 |
| 12567 | hsa-miR-765 | 6772 | STAT1 | signal transducer and activator of transcription 1, 91kDa | 1 |
| 12568 | hsa-miR-770-5p | 6772 | STAT1 | signal transducer and activator of transcription 1, 91kDa | 1 |
| 12569 | hsa-miR-877 | 6772 | STAT1 | signal transducer and activator of transcription 1, 91kDa | 1 |
| 12570 | hsa-mir-26a-1 | 6772 | STAT1 | signal transducer and activator of transcription 1, 91kDa | 1 |
| 12571 | hsa-mir-26b | 6772 | STAT1 | signal transducer and activator of transcription 1, 91kDa | 1 |
| 12572 | hsa-mir-31 | 6772 | STAT1 | signal transducer and activator of transcription 1, 91kDa | 1 |
| 12573 | hsa-mir-194-2 | 6772 | STAT1 | signal transducer and activator of transcription 1, 91kDa | 1 |
| 12574 | hsa-mir-340 | 6772 | STAT1 | signal transducer and activator of transcription 1, 91kDa | 1 |
| 12575 | hsa-mir-338 | 6772 | STAT1 | signal transducer and activator of transcription 1, 91kDa | 1 |
| 12576 | hsa-mir-490 | 6772 | STAT1 | signal transducer and activator of transcription 1, 91kDa | 1 |
| 12577 | hsa-mir-548b | 6772 | STAT1 | signal transducer and activator of transcription 1, 91kDa | 1 |
| 12578 | hsa-mir-548d-2 | 6772 | STAT1 | signal transducer and activator of transcription 1, 91kDa | 1 |
| 12579 | hsa-mir-449b | 6772 | STAT1 | signal transducer and activator of transcription 1, 91kDa | 1 |
| 12580 | hsa-mir-449c | 6772 | STAT1 | signal transducer and activator of transcription 1, 91kDa | 1 |
| 12581 | hsa-mir-3065 | 6772 | STAT1 | signal transducer and activator of transcription 1, 91kDa | 1 |

---

Gene Ontology - Biological Process [Details: ]

|  | genes in Category | percent in the observed List | percent in the genome | fold of overrepresents | odds ratio | p value |
| --- | --- | --- | --- | --- | --- | --- |
| immune response | 45 | 0.1178 | 0.05288 | 2.2 | 2.5 | 3.7e-07 |
| defense response | 39 | 0.1021 | 0.04683 | 2.2 | 2.4 | 4.0e-06 |
| inflammatory response | 26 | 0.0681 | 0.02531 | 2.7 | 3.0 | 4.8e-06 |
| protein kinase cascade | 33 | 0.0864 | 0.03783 | 2.3 | 2.5 | 8.9e-06 |
| immune system process | 53 | 0.1387 | 0.07615 | 1.8 | 2.0 | 1.4e-05 |
| multi-organism process | 39 | 0.1021 | 0.05042 | 2.0 | 2.2 | 2.2e-05 |
| I-kappaB kinase/NF-kappaB cascade | 15 | 0.0393 | 0.01111 | 3.5 | 3.9 | 2.3e-05 |
| apoptosis | 51 | 0.1335 | 0.07419 | 1.8 | 2.0 | 2.9e-05 |
| programmed cell death | 51 | 0.1335 | 0.07482 | 1.8 | 2.0 | 3.6e-05 |
| regulation of apoptosis | 42 | 0.1099 | 0.05780 | 1.9 | 2.1 | 4.5e-05 |
| regulation of interleukin-12 production | 5 | 0.0131 | 0.00113 | 11.6 | 16.7 | 4.7e-05 |
| intracellular signaling cascade | 65 | 0.1702 | 0.10442 | 1.6 | 1.8 | 4.8e-05 |
| regulation of programmed cell death | 42 | 0.1099 | 0.05836 | 1.9 | 2.0 | 5.7e-05 |
| regulation of cell death | 42 | 0.1099 | 0.05858 | 1.9 | 2.0 | 6.2e-05 |
| cell activation | 24 | 0.0628 | 0.02602 | 2.4 | 2.6 | 6.4e-05 |
| antigen processing and presentation of peptide antigen via MHC class I | 5 | 0.0131 | 0.00120 | 10.9 | 15.3 | 6.5e-05 |
| interleukin-12 production | 5 | 0.0131 | 0.00120 | 10.9 | 15.3 | 6.5e-05 |
| response to molecule of bacterial origin | 10 | 0.0262 | 0.00584 | 4.5 | 5.1 | 7.4e-05 |
| response to wounding | 32 | 0.0838 | 0.04029 | 2.1 | 2.2 | 7.6e-05 |
| cell death | 53 | 0.1387 | 0.08157 | 1.7 | 1.9 | 9.0e-05 |
| regulation of smooth muscle cell proliferation | 7 | 0.0183 | 0.00288 | 6.4 | 7.6 | 9.8e-05 |
| death | 53 | 0.1387 | 0.08185 | 1.7 | 1.8 | 9.8e-05 |
| smooth muscle cell proliferation | 7 | 0.0183 | 0.00302 | 6.1 | 7.2 | 1.3e-04 |
| regulation of cytokine biosynthetic process | 9 | 0.0236 | 0.00513 | 4.6 | 5.2 | 1.4e-04 |
| regulation of vascular endothelial growth factor production | 4 | 0.0105 | 0.00077 | 13.5 | 20.9 | 1.5e-04 |
| vascular endothelial growth factor production | 4 | 0.0105 | 0.00077 | 13.5 | 20.9 | 1.5e-04 |
| response to lipopolysaccharide | 9 | 0.0236 | 0.00527 | 4.5 | 5.0 | 1.7e-04 |
| fever | 3 | 0.0079 | 0.00035 | 22.3 | 54.8 | 1.8e-04 |
| positive regulation of organic acid transport | 3 | 0.0079 | 0.00035 | 22.3 | 54.8 | 1.8e-04 |
| release of cytochrome c from mitochondria | 5 | 0.0131 | 0.00148 | 8.9 | 11.5 | 1.9e-04 |
| leukocyte activation | 21 | 0.0550 | 0.02299 | 2.4 | 2.6 | 2.1e-04 |
| signaling | 142 | 0.3717 | 0.28817 | 1.3 | 1.5 | 2.2e-04 |
| positive regulation of cytokine biosynthetic process | 7 | 0.0183 | 0.00338 | 5.4 | 6.3 | 2.7e-04 |
| signal transduction | 113 | 0.2958 | 0.22017 | 1.3 | 1.5 | 2.9e-04 |
| regulation of organic acid transport | 4 | 0.0105 | 0.00091 | 11.5 | 16.3 | 3.0e-04 |
| regulation of defense response | 12 | 0.0314 | 0.00963 | 3.3 | 3.6 | 3.2e-04 |
| negative regulation of viral genome replication | 3 | 0.0079 | 0.00042 | 18.6 | 36.5 | 3.6e-04 |
| arachidonic acid metabolic process | 3 | 0.0079 | 0.00042 | 18.6 | 36.5 | 3.6e-04 |
| regulation of cytokine production | 14 | 0.0366 | 0.01266 | 2.9 | 3.1 | 3.6e-04 |
| cytokine biosynthetic process | 9 | 0.0236 | 0.00584 | 4.0 | 4.5 | 3.8e-04 |
| regulation of response to stress | 18 | 0.0471 | 0.01913 | 2.5 | 2.6 | 4.1e-04 |
| cytokine metabolic process | 9 | 0.0236 | 0.00591 | 4.0 | 4.4 | 4.1e-04 |
| antigen processing and presentation of peptide antigen | 5 | 0.0131 | 0.00176 | 7.4 | 9.2 | 4.6e-04 |
| interspecies interaction between organisms | 19 | 0.0497 | 0.02124 | 2.3 | 2.5 | 5.4e-04 |
| signal transmission | 123 | 0.3220 | 0.24808 | 1.3 | 1.5 | 5.8e-04 |
| signaling process | 123 | 0.3220 | 0.24815 | 1.3 | 1.5 | 5.9e-04 |
| chronic inflammatory response | 3 | 0.0079 | 0.00049 | 16.0 | 27.4 | 6.2e-04 |
| positive regulation of interleukin-12 biosynthetic process | 3 | 0.0079 | 0.00049 | 16.0 | 27.4 | 6.2e-04 |
| negative regulation of viral reproduction | 3 | 0.0079 | 0.00049 | 16.0 | 27.4 | 6.2e-04 |
| heat generation | 3 | 0.0079 | 0.00049 | 16.0 | 27.4 | 6.2e-04 |
| positive regulation of smooth muscle cell proliferation | 5 | 0.0131 | 0.00190 | 6.9 | 8.3 | 6.8e-04 |
| positive regulation of NF-kappaB transcription factor activity | 6 | 0.0157 | 0.00288 | 5.4 | 6.3 | 7.3e-04 |
| muscle cell proliferation | 7 | 0.0183 | 0.00401 | 4.6 | 5.1 | 7.9e-04 |
| regulation of protein kinase cascade | 17 | 0.0445 | 0.01870 | 2.4 | 2.5 | 8.7e-04 |
| cytokine-mediated signaling pathway | 8 | 0.0209 | 0.00527 | 4.0 | 4.4 | 8.8e-04 |
| initiation of signal transduction | 8 | 0.0209 | 0.00527 | 4.0 | 4.4 | 8.8e-04 |
| signal initiation by diffusible mediator | 8 | 0.0209 | 0.00527 | 4.0 | 4.4 | 8.8e-04 |
| signal initiation by protein/peptide mediator | 8 | 0.0209 | 0.00527 | 4.0 | 4.4 | 8.8e-04 |
| interleukin-12 biosynthetic process | 3 | 0.0079 | 0.00056 | 14.0 | 21.9 | 9.7e-04 |
| regulation of interleukin-12 biosynthetic process | 3 | 0.0079 | 0.00056 | 14.0 | 21.9 | 9.7e-04 |
| response to other organism | 19 | 0.0497 | 0.02257 | 2.2 | 2.3 | 1.1e-03 |
| apoptotic mitochondrial changes | 5 | 0.0131 | 0.00211 | 6.2 | 7.3 | 1.1e-03 |
| regulation of immune system process | 21 | 0.0550 | 0.02616 | 2.1 | 2.2 | 1.1e-03 |
| carboxylic acid transport | 12 | 0.0314 | 0.01111 | 2.8 | 3.0 | 1.2e-03 |
| anti-apoptosis | 15 | 0.0393 | 0.01589 | 2.5 | 2.6 | 1.2e-03 |
| antigen processing and presentation | 7 | 0.0183 | 0.00429 | 4.3 | 4.8 | 1.2e-03 |
| organic acid transport | 12 | 0.0314 | 0.01118 | 2.8 | 3.0 | 1.2e-03 |
| lymphocyte activation | 17 | 0.0445 | 0.01934 | 2.3 | 2.5 | 1.3e-03 |
| regulation of T cell proliferation | 7 | 0.0183 | 0.00436 | 4.2 | 4.7 | 1.3e-03 |
| response to cytokine stimulus | 8 | 0.0209 | 0.00563 | 3.7 | 4.1 | 1.4e-03 |
| cytokine production | 14 | 0.0366 | 0.01456 | 2.5 | 2.7 | 1.4e-03 |
| pattern recognition receptor signaling pathway | 4 | 0.0105 | 0.00134 | 7.8 | 9.8 | 1.4e-03 |
| regulation of leukocyte activation | 12 | 0.0314 | 0.01146 | 2.7 | 2.9 | 1.5e-03 |
| positive regulation of protein kinase cascade | 13 | 0.0340 | 0.01315 | 2.6 | 2.8 | 1.6e-03 |
| regulation of lymphocyte proliferation | 8 | 0.0209 | 0.00591 | 3.5 | 3.9 | 1.9e-03 |
| alpha-beta T cell activation | 6 | 0.0157 | 0.00345 | 4.6 | 5.1 | 1.9e-03 |
| regulation of mononuclear cell proliferation | 8 | 0.0209 | 0.00598 | 3.5 | 3.8 | 2.0e-03 |
| regulation of leukocyte proliferation | 8 | 0.0209 | 0.00598 | 3.5 | 3.8 | 2.0e-03 |
| positive regulation vascular endothelial growth factor production | 3 | 0.0079 | 0.00070 | 11.2 | 15.6 | 2.0e-03 |
| activation of innate immune response | 4 | 0.0105 | 0.00148 | 7.1 | 8.6 | 2.1e-03 |
| innate immune response-activating signal transduction | 4 | 0.0105 | 0.00148 | 7.1 | 8.6 | 2.1e-03 |
| biological regulation | 227 | 0.5942 | 0.52120 | 1.1 | 1.4 | 2.2e-03 |
| response to stimulus | 120 | 0.3141 | 0.24921 | 1.3 | 1.4 | 2.2e-03 |
| peptide transport | 8 | 0.0209 | 0.00612 | 3.4 | 3.7 | 2.3e-03 |
| positive regulation of signal transduction | 18 | 0.0471 | 0.02229 | 2.1 | 2.2 | 2.4e-03 |
| positive regulation of signaling process | 18 | 0.0471 | 0.02229 | 2.1 | 2.2 | 2.4e-03 |
| regulation of cell activation | 12 | 0.0314 | 0.01209 | 2.6 | 2.8 | 2.4e-03 |
| positive regulation of cell communication | 19 | 0.0497 | 0.02412 | 2.1 | 2.2 | 2.4e-03 |
| response to virus | 11 | 0.0288 | 0.01055 | 2.7 | 2.9 | 2.4e-03 |
| cytoplasmic sequestering of transcription factor | 3 | 0.0079 | 0.00077 | 10.2 | 13.7 | 2.7e-03 |
| positive regulation of lipid transport | 3 | 0.0079 | 0.00077 | 10.2 | 13.7 | 2.7e-03 |
| positive regulation of cytokine production | 8 | 0.0209 | 0.00633 | 3.3 | 3.6 | 2.9e-03 |
| positive regulation of DNA binding | 7 | 0.0183 | 0.00499 | 3.7 | 4.0 | 2.9e-03 |
| positive regulation of apoptosis | 22 | 0.0576 | 0.03017 | 1.9 | 2.0 | 2.9e-03 |
| defense response to virus | 5 | 0.0131 | 0.00260 | 5.0 | 5.7 | 2.9e-03 |
| regulation of biological process | 216 | 0.5654 | 0.49476 | 1.1 | 1.3 | 3.0e-03 |
| positive regulation of defense response | 7 | 0.0183 | 0.00506 | 3.6 | 4.0 | 3.1e-03 |
| positive regulation of programmed cell death | 22 | 0.0576 | 0.03038 | 1.9 | 2.0 | 3.2e-03 |
| regulation of I-kappaB kinase/NF-kappaB cascade | 9 | 0.0236 | 0.00788 | 3.0 | 3.2 | 3.2e-03 |
| positive regulation of cell death | 22 | 0.0576 | 0.03045 | 1.9 | 2.0 | 3.3e-03 |
| acute-phase response | 5 | 0.0131 | 0.00267 | 4.9 | 5.5 | 3.3e-03 |
| negative regulation of transport | 10 | 0.0262 | 0.00942 | 2.8 | 3.0 | 3.3e-03 |
| response to organic cyclic substance | 9 | 0.0236 | 0.00795 | 3.0 | 3.2 | 3.4e-03 |
| lipopolysaccharide-mediated signaling pathway | 3 | 0.0079 | 0.00084 | 9.3 | 12.2 | 3.5e-03 |
| response to stress | 65 | 0.1702 | 0.12264 | 1.4 | 1.5 | 3.7e-03 |
| positive regulation of T cell proliferation | 5 | 0.0131 | 0.00274 | 4.8 | 5.4 | 3.7e-03 |
| positive regulation of lymphocyte proliferation | 6 | 0.0157 | 0.00394 | 4.0 | 4.4 | 3.8e-03 |
| positive regulation of mononuclear cell proliferation | 6 | 0.0157 | 0.00401 | 3.9 | 4.3 | 4.1e-03 |
| positive regulation of leukocyte proliferation | 6 | 0.0157 | 0.00401 | 3.9 | 4.3 | 4.1e-03 |
| base conversion or substitution editing | 2 | 0.0052 | 0.00028 | 18.6 | 36.4 | 4.2e-03 |
| positive regulation of prostaglandin secretion | 2 | 0.0052 | 0.00028 | 18.6 | 36.4 | 4.2e-03 |
| neutrophil apoptosis | 2 | 0.0052 | 0.00028 | 18.6 | 36.4 | 4.2e-03 |
| serine transport | 2 | 0.0052 | 0.00028 | 18.6 | 36.4 | 4.2e-03 |
| regulation of icosanoid secretion | 2 | 0.0052 | 0.00028 | 18.6 | 36.4 | 4.2e-03 |
| positive regulation of icosanoid secretion | 2 | 0.0052 | 0.00028 | 18.6 | 36.4 | 4.2e-03 |
| regulation of prostaglandin secretion | 2 | 0.0052 | 0.00028 | 18.6 | 36.4 | 4.2e-03 |
| prostaglandin secretion | 2 | 0.0052 | 0.00028 | 18.6 | 36.4 | 4.2e-03 |
| neutrophil homeostasis | 2 | 0.0052 | 0.00028 | 18.6 | 36.4 | 4.2e-03 |
| response to bacterium | 12 | 0.0314 | 0.01308 | 2.4 | 2.5 | 4.5e-03 |
| positive regulation of interleukin-2 biosynthetic process | 3 | 0.0079 | 0.00091 | 8.6 | 10.9 | 4.5e-03 |
| response to ATP | 3 | 0.0079 | 0.00091 | 8.6 | 10.9 | 4.5e-03 |
| response to chemical stimulus | 50 | 0.1309 | 0.09029 | 1.4 | 1.5 | 4.8e-03 |
| regulation of signal transduction | 38 | 0.0995 | 0.06420 | 1.5 | 1.6 | 4.8e-03 |
| regulation of signaling process | 38 | 0.0995 | 0.06420 | 1.5 | 1.6 | 4.8e-03 |
| regulation of DNA binding | 9 | 0.0236 | 0.00851 | 2.8 | 3.0 | 5.3e-03 |
| negative regulation of apoptosis | 19 | 0.0497 | 0.02602 | 1.9 | 2.0 | 5.4e-03 |
| positive regulation of lipid catabolic process | 3 | 0.0079 | 0.00098 | 8.0 | 10.0 | 5.6e-03 |
| negative regulation of transcription factor import into nucleus | 3 | 0.0079 | 0.00098 | 8.0 | 10.0 | 5.6e-03 |
| cytoplasmic sequestering of protein | 3 | 0.0079 | 0.00098 | 8.0 | 10.0 | 5.6e-03 |
| cellular response to insulin stimulus | 7 | 0.0183 | 0.00563 | 3.3 | 3.5 | 5.6e-03 |
| regulation of lymphocyte activation | 10 | 0.0262 | 0.01020 | 2.6 | 2.7 | 5.8e-03 |
| cell communication | 58 | 0.1518 | 0.10928 | 1.4 | 1.5 | 5.9e-03 |
| positive regulation of binding | 7 | 0.0183 | 0.00570 | 3.2 | 3.5 | 6.0e-03 |
| negative regulation of programmed cell death | 19 | 0.0497 | 0.02637 | 1.9 | 2.0 | 6.2e-03 |
| positive regulation of transcription factor activity | 6 | 0.0157 | 0.00436 | 3.6 | 3.9 | 6.3e-03 |
| positive regulation of transcription regulator activity | 6 | 0.0157 | 0.00436 | 3.6 | 3.9 | 6.3e-03 |
| regulation of cell communication | 42 | 0.1099 | 0.07398 | 1.5 | 1.6 | 6.3e-03 |
| response to corticosteroid stimulus | 7 | 0.0183 | 0.00577 | 3.2 | 3.4 | 6.5e-03 |
| T cell proliferation | 7 | 0.0183 | 0.00577 | 3.2 | 3.4 | 6.5e-03 |
| regulation of cellular process | 206 | 0.5393 | 0.47528 | 1.1 | 1.3 | 6.5e-03 |
| T cell activation | 12 | 0.0314 | 0.01371 | 2.3 | 2.4 | 6.5e-03 |
| peptidyl-tyrosine phosphorylation | 8 | 0.0209 | 0.00724 | 2.9 | 3.1 | 6.5e-03 |
| positive regulation of I-kappaB kinase/NF-kappaB cascade | 8 | 0.0209 | 0.00724 | 2.9 | 3.1 | 6.5e-03 |
| secretion | 21 | 0.0550 | 0.03038 | 1.8 | 1.9 | 6.6e-03 |
| cytoplasmic sequestering of NF-kappaB | 2 | 0.0052 | 0.00035 | 14.9 | 24.3 | 6.8e-03 |
| nucleotide-binding oligomerization domain containing 2 signaling pathway | 2 | 0.0052 | 0.00035 | 14.9 | 24.3 | 6.8e-03 |
| ISG15-protein conjugation | 2 | 0.0052 | 0.00035 | 14.9 | 24.3 | 6.8e-03 |
| peptidyl-histidine phosphorylation | 2 | 0.0052 | 0.00035 | 14.9 | 24.3 | 6.8e-03 |
| positive regulation of interleukin-12 production | 2 | 0.0052 | 0.00035 | 14.9 | 24.3 | 6.8e-03 |
| positive regulation of fatty acid beta-oxidation | 2 | 0.0052 | 0.00035 | 14.9 | 24.3 | 6.8e-03 |
| nucleotide-binding oligomerization domain containing signaling pathway | 2 | 0.0052 | 0.00035 | 14.9 | 24.3 | 6.8e-03 |
| prostaglandin transport | 2 | 0.0052 | 0.00035 | 14.9 | 24.3 | 6.8e-03 |
| dicarboxylic acid transport | 3 | 0.0079 | 0.00105 | 7.4 | 9.1 | 6.9e-03 |
| regulation of viral genome replication | 3 | 0.0079 | 0.00105 | 7.4 | 9.1 | 6.9e-03 |
| regulation of transcription factor activity | 8 | 0.0209 | 0.00731 | 2.9 | 3.1 | 6.9e-03 |
| regulation of transcription regulator activity | 8 | 0.0209 | 0.00731 | 2.9 | 3.1 | 6.9e-03 |
| negative regulation of cell death | 19 | 0.0497 | 0.02665 | 1.9 | 2.0 | 6.9e-03 |
| positive regulation of cell proliferation | 20 | 0.0524 | 0.02862 | 1.8 | 1.9 | 7.0e-03 |
| peptidyl-tyrosine modification | 8 | 0.0209 | 0.00738 | 2.8 | 3.0 | 7.3e-03 |
| regulation of secretion | 12 | 0.0314 | 0.01392 | 2.3 | 2.4 | 7.3e-03 |
| response to biotic stimulus | 20 | 0.0524 | 0.02897 | 1.8 | 1.9 | 8.0e-03 |
| negative regulation of insulin receptor signaling pathway | 3 | 0.0079 | 0.00113 | 7.0 | 8.4 | 8.3e-03 |
| alpha-beta T cell proliferation | 3 | 0.0079 | 0.00113 | 7.0 | 8.4 | 8.3e-03 |
| negative regulation of protein import into nucleus | 3 | 0.0079 | 0.00113 | 7.0 | 8.4 | 8.3e-03 |
| toll-like receptor signaling pathway | 3 | 0.0079 | 0.00113 | 7.0 | 8.4 | 8.3e-03 |
| cellular response to peptide hormone stimulus | 7 | 0.0183 | 0.00612 | 3.0 | 3.2 | 8.9e-03 |
| positive regulation of cellular process | 65 | 0.1702 | 0.12770 | 1.3 | 1.4 | 9.0e-03 |
| positive regulation of peptidyl-tyrosine phosphorylation | 5 | 0.0131 | 0.00338 | 3.9 | 4.3 | 9.0e-03 |
| regulation of angiogenesis | 6 | 0.0157 | 0.00471 | 3.3 | 3.6 | 9.1e-03 |
| regulation of lipid transport | 4 | 0.0105 | 0.00218 | 4.8 | 5.4 | 9.1e-03 |
| regulation of binding | 10 | 0.0262 | 0.01097 | 2.4 | 2.5 | 9.5e-03 |
| response to external stimulus | 37 | 0.0969 | 0.06497 | 1.5 | 1.6 | 9.7e-03 |
| response to organic substance | 30 | 0.0785 | 0.05000 | 1.6 | 1.6 | 9.7e-03 |
| G1/S transition checkpoint | 3 | 0.0079 | 0.00120 | 6.6 | 7.8 | 9.9e-03 |
| T-helper 1 type immune response | 3 | 0.0079 | 0.00120 | 6.6 | 7.8 | 9.9e-03 |
| positive regulation of biological process | 70 | 0.1832 | 0.13993 | 1.3 | 1.4 | 9.9e-03 |

---

Gene Ontology - Biological Process, level II [Details: ]

|  | genes in Category | percent in the observed List | percent in the genome | fold of overrepresents | odds ratio | p value |
| --- | --- | --- | --- | --- | --- | --- |
| immune response | 45 | 0.1187 | 0.05288 | 2.2 | 2.5 | 3.0e-07 |
| defense response | 39 | 0.1029 | 0.04683 | 2.2 | 2.4 | 3.3e-06 |
| inflammatory response | 26 | 0.0686 | 0.02531 | 2.7 | 3.0 | 4.2e-06 |
| protein kinase cascade | 33 | 0.0871 | 0.03783 | 2.3 | 2.5 | 7.5e-06 |
| I-kappaB kinase/NF-kappaB cascade | 15 | 0.0396 | 0.01111 | 3.6 | 3.9 | 2.1e-05 |
| apoptosis | 51 | 0.1346 | 0.07419 | 1.8 | 2.0 | 2.4e-05 |
| programmed cell death | 51 | 0.1346 | 0.07482 | 1.8 | 2.0 | 3.0e-05 |
| intracellular signaling cascade | 65 | 0.1715 | 0.10442 | 1.6 | 1.8 | 3.8e-05 |
| regulation of apoptosis | 42 | 0.1108 | 0.05780 | 1.9 | 2.1 | 3.8e-05 |
| regulation of interleukin-12 production | 5 | 0.0132 | 0.00113 | 11.7 | 16.8 | 4.5e-05 |
| regulation of programmed cell death | 42 | 0.1108 | 0.05836 | 1.9 | 2.1 | 4.7e-05 |
| regulation of cell death | 42 | 0.1108 | 0.05858 | 1.9 | 2.1 | 5.1e-05 |
| cell activation | 24 | 0.0633 | 0.02602 | 2.4 | 2.6 | 5.7e-05 |
| antigen processing and presentation of peptide antigen via MHC class I | 5 | 0.0132 | 0.00120 | 11.0 | 15.4 | 6.2e-05 |
| interleukin-12 production | 5 | 0.0132 | 0.00120 | 11.0 | 15.4 | 6.2e-05 |
| response to wounding | 32 | 0.0844 | 0.04029 | 2.1 | 2.3 | 6.5e-05 |
| response to molecule of bacterial origin | 10 | 0.0264 | 0.00584 | 4.5 | 5.1 | 6.9e-05 |
| cell death | 53 | 0.1398 | 0.08157 | 1.7 | 1.9 | 7.3e-05 |
| regulation of smooth muscle cell proliferation | 7 | 0.0185 | 0.00288 | 6.4 | 7.6 | 9.3e-05 |
| smooth muscle cell proliferation | 7 | 0.0185 | 0.00302 | 6.1 | 7.2 | 1.3e-04 |
| regulation of cytokine biosynthetic process | 9 | 0.0237 | 0.00513 | 4.6 | 5.2 | 1.3e-04 |
| regulation of vascular endothelial growth factor production | 4 | 0.0106 | 0.00077 | 13.6 | 21.1 | 1.4e-04 |
| vascular endothelial growth factor production | 4 | 0.0106 | 0.00077 | 13.6 | 21.1 | 1.4e-04 |
| response to lipopolysaccharide | 9 | 0.0237 | 0.00527 | 4.5 | 5.1 | 1.6e-04 |
| fever | 3 | 0.0079 | 0.00035 | 22.5 | 55.2 | 1.8e-04 |
| positive regulation of organic acid transport | 3 | 0.0079 | 0.00035 | 22.5 | 55.2 | 1.8e-04 |
| release of cytochrome c from mitochondria | 5 | 0.0132 | 0.00148 | 8.9 | 11.6 | 1.9e-04 |
| leukocyte activation | 21 | 0.0554 | 0.02299 | 2.4 | 2.6 | 1.9e-04 |
| signal transduction | 113 | 0.2982 | 0.22017 | 1.4 | 1.5 | 2.0e-04 |
| positive regulation of cytokine biosynthetic process | 7 | 0.0185 | 0.00338 | 5.5 | 6.3 | 2.6e-04 |
| regulation of organic acid transport | 4 | 0.0106 | 0.00091 | 11.5 | 16.4 | 2.9e-04 |
| regulation of defense response | 12 | 0.0317 | 0.00963 | 3.3 | 3.6 | 3.0e-04 |
| regulation of cytokine production | 14 | 0.0369 | 0.01266 | 2.9 | 3.2 | 3.3e-04 |
| negative regulation of viral genome replication | 3 | 0.0079 | 0.00042 | 18.8 | 36.8 | 3.5e-04 |
| arachidonic acid metabolic process | 3 | 0.0079 | 0.00042 | 18.8 | 36.8 | 3.5e-04 |
| cytokine biosynthetic process | 9 | 0.0237 | 0.00584 | 4.1 | 4.5 | 3.6e-04 |
| regulation of response to stress | 18 | 0.0475 | 0.01913 | 2.5 | 2.7 | 3.7e-04 |
| cytokine metabolic process | 9 | 0.0237 | 0.00591 | 4.0 | 4.5 | 3.9e-04 |
| signal transmission | 123 | 0.3245 | 0.24808 | 1.3 | 1.5 | 4.1e-04 |
| antigen processing and presentation of peptide antigen | 5 | 0.0132 | 0.00176 | 7.5 | 9.2 | 4.5e-04 |
| interspecies interaction between organisms | 19 | 0.0501 | 0.02124 | 2.4 | 2.5 | 4.9e-04 |
| chronic inflammatory response | 3 | 0.0079 | 0.00049 | 16.1 | 27.6 | 6.1e-04 |
| positive regulation of interleukin-12 biosynthetic process | 3 | 0.0079 | 0.00049 | 16.1 | 27.6 | 6.1e-04 |
| negative regulation of viral reproduction | 3 | 0.0079 | 0.00049 | 16.1 | 27.6 | 6.1e-04 |
| heat generation | 3 | 0.0079 | 0.00049 | 16.1 | 27.6 | 6.1e-04 |
| positive regulation of smooth muscle cell proliferation | 5 | 0.0132 | 0.00190 | 6.9 | 8.4 | 6.5e-04 |
| positive regulation of NF-kappaB transcription factor activity | 6 | 0.0158 | 0.00288 | 5.5 | 6.3 | 7.0e-04 |
| muscle cell proliferation | 7 | 0.0185 | 0.00401 | 4.6 | 5.2 | 7.6e-04 |
| regulation of protein kinase cascade | 17 | 0.0449 | 0.01870 | 2.4 | 2.6 | 8.0e-04 |
| cytokine-mediated signaling pathway | 8 | 0.0211 | 0.00527 | 4.0 | 4.4 | 8.4e-04 |
| initiation of signal transduction | 8 | 0.0211 | 0.00527 | 4.0 | 4.4 | 8.4e-04 |
| signal initiation by diffusible mediator | 8 | 0.0211 | 0.00527 | 4.0 | 4.4 | 8.4e-04 |
| signal initiation by protein/peptide mediator | 8 | 0.0211 | 0.00527 | 4.0 | 4.4 | 8.4e-04 |
| interleukin-12 biosynthetic process | 3 | 0.0079 | 0.00056 | 14.1 | 22.1 | 9.5e-04 |
| regulation of interleukin-12 biosynthetic process | 3 | 0.0079 | 0.00056 | 14.1 | 22.1 | 9.5e-04 |
| response to other organism | 19 | 0.0501 | 0.02257 | 2.2 | 2.4 | 1.0e-03 |
| regulation of immune system process | 21 | 0.0554 | 0.02616 | 2.1 | 2.3 | 1.0e-03 |
| apoptotic mitochondrial changes | 5 | 0.0132 | 0.00211 | 6.3 | 7.4 | 1.1e-03 |
| carboxylic acid transport | 12 | 0.0317 | 0.01111 | 2.8 | 3.1 | 1.1e-03 |
| anti-apoptosis | 15 | 0.0396 | 0.01589 | 2.5 | 2.7 | 1.1e-03 |
| organic acid transport | 12 | 0.0317 | 0.01118 | 2.8 | 3.0 | 1.1e-03 |
| antigen processing and presentation | 7 | 0.0185 | 0.00429 | 4.3 | 4.8 | 1.1e-03 |
| lymphocyte activation | 17 | 0.0449 | 0.01934 | 2.3 | 2.5 | 1.2e-03 |
| regulation of T cell proliferation | 7 | 0.0185 | 0.00436 | 4.2 | 4.7 | 1.3e-03 |
| response to cytokine stimulus | 8 | 0.0211 | 0.00563 | 3.8 | 4.1 | 1.3e-03 |
| cytokine production | 14 | 0.0369 | 0.01456 | 2.5 | 2.7 | 1.3e-03 |
| pattern recognition receptor signaling pathway | 4 | 0.0106 | 0.00134 | 7.9 | 9.8 | 1.4e-03 |
| regulation of leukocyte activation | 12 | 0.0317 | 0.01146 | 2.8 | 3.0 | 1.4e-03 |
| positive regulation of protein kinase cascade | 13 | 0.0343 | 0.01315 | 2.6 | 2.8 | 1.5e-03 |
| regulation of lymphocyte proliferation | 8 | 0.0211 | 0.00591 | 3.6 | 3.9 | 1.8e-03 |
| alpha-beta T cell activation | 6 | 0.0158 | 0.00345 | 4.6 | 5.2 | 1.8e-03 |
| regulation of mononuclear cell proliferation | 8 | 0.0211 | 0.00598 | 3.5 | 3.9 | 1.9e-03 |
| regulation of leukocyte proliferation | 8 | 0.0211 | 0.00598 | 3.5 | 3.9 | 1.9e-03 |
| positive regulation vascular endothelial growth factor production | 3 | 0.0079 | 0.00070 | 11.3 | 15.8 | 2.0e-03 |
| activation of innate immune response | 4 | 0.0106 | 0.00148 | 7.1 | 8.7 | 2.1e-03 |
| innate immune response-activating signal transduction | 4 | 0.0106 | 0.00148 | 7.1 | 8.7 | 2.1e-03 |
| positive regulation of signal transduction | 18 | 0.0475 | 0.02229 | 2.1 | 2.3 | 2.2e-03 |
| positive regulation of signaling process | 18 | 0.0475 | 0.02229 | 2.1 | 2.3 | 2.2e-03 |
| positive regulation of cell communication | 19 | 0.0501 | 0.02412 | 2.1 | 2.2 | 2.2e-03 |
| peptide transport | 8 | 0.0211 | 0.00612 | 3.5 | 3.8 | 2.2e-03 |
| regulation of cell activation | 12 | 0.0317 | 0.01209 | 2.6 | 2.8 | 2.2e-03 |
| response to virus | 11 | 0.0290 | 0.01055 | 2.8 | 2.9 | 2.3e-03 |
| cytoplasmic sequestering of transcription factor | 3 | 0.0079 | 0.00077 | 10.2 | 13.8 | 2.6e-03 |
| positive regulation of lipid transport | 3 | 0.0079 | 0.00077 | 10.2 | 13.8 | 2.6e-03 |
| positive regulation of apoptosis | 22 | 0.0580 | 0.03017 | 1.9 | 2.0 | 2.7e-03 |
| positive regulation of cytokine production | 8 | 0.0211 | 0.00633 | 3.3 | 3.6 | 2.7e-03 |
| positive regulation of DNA binding | 7 | 0.0185 | 0.00499 | 3.7 | 4.1 | 2.8e-03 |
| defense response to virus | 5 | 0.0132 | 0.00260 | 5.1 | 5.8 | 2.8e-03 |
| positive regulation of programmed cell death | 22 | 0.0580 | 0.03038 | 1.9 | 2.0 | 2.9e-03 |
| positive regulation of cell death | 22 | 0.0580 | 0.03045 | 1.9 | 2.0 | 3.0e-03 |
| positive regulation of defense response | 7 | 0.0185 | 0.00506 | 3.6 | 4.0 | 3.0e-03 |
| response to stress | 65 | 0.1715 | 0.12264 | 1.4 | 1.5 | 3.0e-03 |
| regulation of I-kappaB kinase/NF-kappaB cascade | 9 | 0.0237 | 0.00788 | 3.0 | 3.2 | 3.0e-03 |
| negative regulation of transport | 10 | 0.0264 | 0.00942 | 2.8 | 3.0 | 3.1e-03 |
| acute-phase response | 5 | 0.0132 | 0.00267 | 4.9 | 5.6 | 3.2e-03 |
| response to organic cyclic substance | 9 | 0.0237 | 0.00795 | 3.0 | 3.2 | 3.2e-03 |
| lipopolysaccharide-mediated signaling pathway | 3 | 0.0079 | 0.00084 | 9.4 | 12.3 | 3.5e-03 |
| positive regulation of T cell proliferation | 5 | 0.0132 | 0.00274 | 4.8 | 5.4 | 3.6e-03 |
| positive regulation of lymphocyte proliferation | 6 | 0.0158 | 0.00394 | 4.0 | 4.4 | 3.6e-03 |
| positive regulation of mononuclear cell proliferation | 6 | 0.0158 | 0.00401 | 3.9 | 4.3 | 4.0e-03 |
| positive regulation of leukocyte proliferation | 6 | 0.0158 | 0.00401 | 3.9 | 4.3 | 4.0e-03 |
| response to chemical stimulus | 50 | 0.1319 | 0.09029 | 1.5 | 1.6 | 4.1e-03 |
| base conversion or substitution editing | 2 | 0.0053 | 0.00028 | 18.8 | 36.7 | 4.1e-03 |
| positive regulation of prostaglandin secretion | 2 | 0.0053 | 0.00028 | 18.8 | 36.7 | 4.1e-03 |
| neutrophil apoptosis | 2 | 0.0053 | 0.00028 | 18.8 | 36.7 | 4.1e-03 |
| serine transport | 2 | 0.0053 | 0.00028 | 18.8 | 36.7 | 4.1e-03 |
| regulation of icosanoid secretion | 2 | 0.0053 | 0.00028 | 18.8 | 36.7 | 4.1e-03 |
| positive regulation of icosanoid secretion | 2 | 0.0053 | 0.00028 | 18.8 | 36.7 | 4.1e-03 |
| regulation of prostaglandin secretion | 2 | 0.0053 | 0.00028 | 18.8 | 36.7 | 4.1e-03 |
| prostaglandin secretion | 2 | 0.0053 | 0.00028 | 18.8 | 36.7 | 4.1e-03 |
| neutrophil homeostasis | 2 | 0.0053 | 0.00028 | 18.8 | 36.7 | 4.1e-03 |
| regulation of cellular process | 206 | 0.5435 | 0.47528 | 1.1 | 1.3 | 4.1e-03 |
| regulation of signal transduction | 38 | 0.1003 | 0.06420 | 1.6 | 1.7 | 4.2e-03 |
| regulation of signaling process | 38 | 0.1003 | 0.06420 | 1.6 | 1.7 | 4.2e-03 |
| response to bacterium | 12 | 0.0317 | 0.01308 | 2.4 | 2.6 | 4.2e-03 |
| positive regulation of interleukin-2 biosynthetic process | 3 | 0.0079 | 0.00091 | 8.7 | 11.0 | 4.4e-03 |
| response to ATP | 3 | 0.0079 | 0.00091 | 8.7 | 11.0 | 4.4e-03 |
| negative regulation of apoptosis | 19 | 0.0501 | 0.02602 | 1.9 | 2.0 | 5.0e-03 |
| cell communication | 58 | 0.1530 | 0.10928 | 1.4 | 1.5 | 5.0e-03 |
| regulation of DNA binding | 9 | 0.0237 | 0.00851 | 2.8 | 3.0 | 5.1e-03 |
| cellular response to insulin stimulus | 7 | 0.0185 | 0.00563 | 3.3 | 3.5 | 5.4e-03 |
| regulation of lymphocyte activation | 10 | 0.0264 | 0.01020 | 2.6 | 2.8 | 5.5e-03 |
| positive regulation of lipid catabolic process | 3 | 0.0079 | 0.00098 | 8.0 | 10.0 | 5.5e-03 |
| negative regulation of transcription factor import into nucleus | 3 | 0.0079 | 0.00098 | 8.0 | 10.0 | 5.5e-03 |
| cytoplasmic sequestering of protein | 3 | 0.0079 | 0.00098 | 8.0 | 10.0 | 5.5e-03 |
| regulation of cell communication | 42 | 0.1108 | 0.07398 | 1.5 | 1.6 | 5.5e-03 |
| negative regulation of programmed cell death | 19 | 0.0501 | 0.02637 | 1.9 | 2.0 | 5.7e-03 |
| positive regulation of binding | 7 | 0.0185 | 0.00570 | 3.2 | 3.5 | 5.8e-03 |
| positive regulation of transcription factor activity | 6 | 0.0158 | 0.00436 | 3.6 | 4.0 | 6.0e-03 |
| positive regulation of transcription regulator activity | 6 | 0.0158 | 0.00436 | 3.6 | 4.0 | 6.0e-03 |
| secretion | 21 | 0.0554 | 0.03038 | 1.8 | 1.9 | 6.0e-03 |
| T cell activation | 12 | 0.0317 | 0.01371 | 2.3 | 2.4 | 6.1e-03 |
| response to corticosteroid stimulus | 7 | 0.0185 | 0.00577 | 3.2 | 3.5 | 6.2e-03 |
| T cell proliferation | 7 | 0.0185 | 0.00577 | 3.2 | 3.5 | 6.2e-03 |
| peptidyl-tyrosine phosphorylation | 8 | 0.0211 | 0.00724 | 2.9 | 3.1 | 6.2e-03 |
| positive regulation of I-kappaB kinase/NF-kappaB cascade | 8 | 0.0211 | 0.00724 | 2.9 | 3.1 | 6.2e-03 |
| negative regulation of cell death | 19 | 0.0501 | 0.02665 | 1.9 | 2.0 | 6.4e-03 |
| positive regulation of cell proliferation | 20 | 0.0528 | 0.02862 | 1.8 | 1.9 | 6.5e-03 |
| regulation of transcription factor activity | 8 | 0.0211 | 0.00731 | 2.9 | 3.1 | 6.6e-03 |
| regulation of transcription regulator activity | 8 | 0.0211 | 0.00731 | 2.9 | 3.1 | 6.6e-03 |
| cytoplasmic sequestering of NF-kappaB | 2 | 0.0053 | 0.00035 | 15.0 | 24.5 | 6.7e-03 |
| nucleotide-binding oligomerization domain containing 2 signaling pathway | 2 | 0.0053 | 0.00035 | 15.0 | 24.5 | 6.7e-03 |
| ISG15-protein conjugation | 2 | 0.0053 | 0.00035 | 15.0 | 24.5 | 6.7e-03 |
| peptidyl-histidine phosphorylation | 2 | 0.0053 | 0.00035 | 15.0 | 24.5 | 6.7e-03 |
| positive regulation of interleukin-12 production | 2 | 0.0053 | 0.00035 | 15.0 | 24.5 | 6.7e-03 |
| positive regulation of fatty acid beta-oxidation | 2 | 0.0053 | 0.00035 | 15.0 | 24.5 | 6.7e-03 |
| nucleotide-binding oligomerization domain containing signaling pathway | 2 | 0.0053 | 0.00035 | 15.0 | 24.5 | 6.7e-03 |
| prostaglandin transport | 2 | 0.0053 | 0.00035 | 15.0 | 24.5 | 6.7e-03 |
| dicarboxylic acid transport | 3 | 0.0079 | 0.00105 | 7.5 | 9.2 | 6.7e-03 |
| regulation of viral genome replication | 3 | 0.0079 | 0.00105 | 7.5 | 9.2 | 6.7e-03 |
| regulation of secretion | 12 | 0.0317 | 0.01392 | 2.3 | 2.4 | 6.9e-03 |
| peptidyl-tyrosine modification | 8 | 0.0211 | 0.00738 | 2.9 | 3.1 | 7.0e-03 |
| response to biotic stimulus | 20 | 0.0528 | 0.02897 | 1.8 | 1.9 | 7.4e-03 |
| positive regulation of cellular process | 65 | 0.1715 | 0.12770 | 1.3 | 1.4 | 7.6e-03 |
| negative regulation of insulin receptor signaling pathway | 3 | 0.0079 | 0.00113 | 7.0 | 8.5 | 8.1e-03 |
| alpha-beta T cell proliferation | 3 | 0.0079 | 0.00113 | 7.0 | 8.5 | 8.1e-03 |
| negative regulation of protein import into nucleus | 3 | 0.0079 | 0.00113 | 7.0 | 8.5 | 8.1e-03 |
| toll-like receptor signaling pathway | 3 | 0.0079 | 0.00113 | 7.0 | 8.5 | 8.1e-03 |
| cellular response to peptide hormone stimulus | 7 | 0.0185 | 0.00612 | 3.0 | 3.2 | 8.5e-03 |
| response to external stimulus | 37 | 0.0976 | 0.06497 | 1.5 | 1.6 | 8.6e-03 |
| regulation of angiogenesis | 6 | 0.0158 | 0.00471 | 3.4 | 3.6 | 8.8e-03 |
| positive regulation of peptidyl-tyrosine phosphorylation | 5 | 0.0132 | 0.00338 | 3.9 | 4.3 | 8.8e-03 |
| response to organic substance | 30 | 0.0792 | 0.05000 | 1.6 | 1.7 | 8.8e-03 |
| regulation of lipid transport | 4 | 0.0106 | 0.00218 | 4.8 | 5.5 | 8.8e-03 |
| regulation of binding | 10 | 0.0264 | 0.01097 | 2.4 | 2.5 | 9.0e-03 |
| intracellular signaling pathway | 35 | 0.0923 | 0.06097 | 1.5 | 1.6 | 9.3e-03 |
| response to insulin stimulus | 8 | 0.0211 | 0.00781 | 2.7 | 2.9 | 9.6e-03 |
| G1/S transition checkpoint | 3 | 0.0079 | 0.00120 | 6.6 | 7.9 | 9.7e-03 |
| T-helper 1 type immune response | 3 | 0.0079 | 0.00120 | 6.6 | 7.9 | 9.7e-03 |
| antigen processing and presentation of endogenous peptide antigen via MHC class I | 2 | 0.0053 | 0.00042 | 12.5 | 18.4 | 9.9e-03 |
| tryptophan catabolic process to kynurenine | 2 | 0.0053 | 0.00042 | 12.5 | 18.4 | 9.9e-03 |
| negative regulation of glutamate secretion | 2 | 0.0053 | 0.00042 | 12.5 | 18.4 | 9.9e-03 |
| T-helper 1 cell differentiation | 2 | 0.0053 | 0.00042 | 12.5 | 18.4 | 9.9e-03 |
| response to peptidoglycan | 2 | 0.0053 | 0.00042 | 12.5 | 18.4 | 9.9e-03 |
| antigen processing and presentation of endogenous peptide antigen | 2 | 0.0053 | 0.00042 | 12.5 | 18.4 | 9.9e-03 |
| tryptophan catabolic process | 2 | 0.0053 | 0.00042 | 12.5 | 18.4 | 9.9e-03 |
| indole derivative catabolic process | 2 | 0.0053 | 0.00042 | 12.5 | 18.4 | 9.9e-03 |
| indolalkylamine catabolic process | 2 | 0.0053 | 0.00042 | 12.5 | 18.4 | 9.9e-03 |

---

KEGG pathways [Details: ]

|  | genes in Category | percent in the observed List | percent in the genome | fold of overrepresents | odds ratio | p value |
| --- | --- | --- | --- | --- | --- | --- |
| Toll-like receptor signaling pathway | 17 | 0.108 | 0.0200 | 5.4 | 7.0 | 8.2e-09 |
| Cytosolic DNA-sensing pathway | 10 | 0.064 | 0.0111 | 5.8 | 7.2 | 6.5e-06 |
| Cytokine-cytokine receptor interaction | 20 | 0.127 | 0.0528 | 2.4 | 2.7 | 1.9e-04 |
| RIG-I-like receptor signaling pathway | 9 | 0.057 | 0.0140 | 4.1 | 4.7 | 3.1e-04 |
| Adipocytokine signaling pathway | 8 | 0.051 | 0.0133 | 3.8 | 4.4 | 9.9e-04 |
| Apoptosis | 9 | 0.057 | 0.0174 | 3.3 | 3.7 | 1.5e-03 |
| Graft-versus-host disease | 6 | 0.038 | 0.0083 | 4.6 | 5.4 | 1.7e-03 |
| B cell receptor signaling pathway | 8 | 0.051 | 0.0148 | 3.4 | 3.9 | 2.1e-03 |
| NOD-like receptor signaling pathway | 7 | 0.045 | 0.0123 | 3.6 | 4.1 | 2.9e-03 |
| MAPK signaling pathway | 17 | 0.108 | 0.0532 | 2.0 | 2.2 | 3.8e-03 |
| Epithelial cell signaling in Helicobacter pylori infection | 7 | 0.045 | 0.0134 | 3.3 | 3.7 | 4.8e-03 |
| Type I diabetes mellitus | 5 | 0.032 | 0.0087 | 3.7 | 4.1 | 1.1e-02 |
| Intestinal immune network for IgA production | 5 | 0.032 | 0.0095 | 3.4 | 3.7 | 1.6e-02 |
| Ribosome | 7 | 0.045 | 0.0174 | 2.6 | 2.8 | 1.9e-02 |
| Hematopoietic cell lineage | 7 | 0.045 | 0.0174 | 2.6 | 2.8 | 1.9e-02 |
| Antigen processing and presentation | 7 | 0.045 | 0.0176 | 2.5 | 2.7 | 2.0e-02 |
| Jak-STAT signaling pathway | 10 | 0.064 | 0.0307 | 2.1 | 2.2 | 2.2e-02 |
| Prion diseases | 4 | 0.025 | 0.0069 | 3.7 | 4.1 | 2.2e-02 |
| Primary immunodeficiency | 4 | 0.025 | 0.0069 | 3.7 | 4.1 | 2.2e-02 |
| Autoimmune thyroid disease | 5 | 0.032 | 0.0105 | 3.0 | 3.3 | 2.3e-02 |
| Allograft rejection | 4 | 0.025 | 0.0075 | 3.4 | 3.7 | 2.9e-02 |
| Tryptophan metabolism | 4 | 0.025 | 0.0079 | 3.2 | 3.5 | 3.4e-02 |
| Aldosterone-regulated sodium reabsorption | 4 | 0.025 | 0.0083 | 3.1 | 3.3 | 4.0e-02 |
| Neurotrophin signaling pathway | 8 | 0.051 | 0.0249 | 2.0 | 2.2 | 4.1e-02 |
| Small cell lung cancer | 6 | 0.038 | 0.0166 | 2.3 | 2.5 | 4.5e-02 |
| T cell receptor signaling pathway | 7 | 0.045 | 0.0214 | 2.1 | 2.2 | 5.0e-02 |
| Metabolism of xenobiotics by cytochrome P450 | 5 | 0.032 | 0.0138 | 2.3 | 2.4 | 6.5e-02 |
| Chemokine signaling pathway | 10 | 0.064 | 0.0376 | 1.7 | 1.8 | 7.0e-02 |
| Drug metabolism - cytochrome P450 | 5 | 0.032 | 0.0142 | 2.2 | 2.4 | 7.2e-02 |
| Amyotrophic lateral sclerosis (ALS) | 4 | 0.025 | 0.0105 | 2.4 | 2.6 | 8.1e-02 |
| GnRH signaling pathway | 6 | 0.038 | 0.0200 | 1.9 | 2.0 | 9.3e-02 |
| Vibrio cholerae infection | 4 | 0.025 | 0.0111 | 2.3 | 2.4 | 9.5e-02 |

---

DOLite desease terms [Details: ]

|  | genes in Category | percent in the observed List | percent in the genome | fold of overrepresents | odds ratio | p value |
| --- | --- | --- | --- | --- | --- | --- |
| Hepatitis C | 9 | 0.0698 | 0.01160 | 6.0 | 7.7 | 1.2e-05 |
| HTLV-I infection | 4 | 0.0310 | 0.00222 | 14.0 | 25.1 | 1.1e-04 |
| Melanoma | 14 | 0.1085 | 0.03555 | 3.1 | 3.6 | 1.6e-04 |
| Cervical cancer | 9 | 0.0698 | 0.01851 | 3.8 | 4.4 | 5.4e-04 |
| Hepatitis | 4 | 0.0310 | 0.00370 | 8.4 | 11.4 | 1.0e-03 |
| Sicca syndrome | 5 | 0.0388 | 0.00642 | 6.0 | 7.5 | 1.2e-03 |
| Thrombocytopenia | 4 | 0.0310 | 0.00395 | 7.9 | 10.4 | 1.3e-03 |
| Thyroid gland disease | 4 | 0.0310 | 0.00469 | 6.6 | 8.3 | 2.6e-03 |
| Lymphoma | 8 | 0.0620 | 0.01901 | 3.3 | 3.7 | 2.8e-03 |
| Multiple sclerosis | 9 | 0.0698 | 0.02345 | 3.0 | 3.3 | 3.0e-03 |
| Graves' disease | 5 | 0.0388 | 0.00790 | 4.9 | 5.8 | 3.1e-03 |
| Embryoma | 17 | 0.1318 | 0.06517 | 2.0 | 2.3 | 3.8e-03 |
| Epstein-Barr virus infection | 4 | 0.0310 | 0.00518 | 6.0 | 7.4 | 3.9e-03 |
| Autoimmune disease | 8 | 0.0620 | 0.02098 | 3.0 | 3.3 | 5.2e-03 |
| Infertility | 6 | 0.0465 | 0.01308 | 3.6 | 4.0 | 6.2e-03 |
| Liver metastases | 3 | 0.0233 | 0.00321 | 7.2 | 9.3 | 7.1e-03 |
| Respiratory distress syndrome | 3 | 0.0233 | 0.00321 | 7.2 | 9.3 | 7.1e-03 |
| Esophagitis | 2 | 0.0155 | 0.00123 | 12.6 | 20.6 | 9.4e-03 |
| Multiple myeloma | 6 | 0.0465 | 0.01432 | 3.2 | 3.6 | 9.7e-03 |
| Rheumatoid arthritis | 16 | 0.1240 | 0.06616 | 1.9 | 2.1 | 1.0e-02 |
| Gastritis | 4 | 0.0310 | 0.00691 | 4.5 | 5.2 | 1.1e-02 |
| Chronic rejection of renal transplant | 3 | 0.0233 | 0.00395 | 5.9 | 7.2 | 1.3e-02 |
| Dental plaque | 6 | 0.0465 | 0.01530 | 3.0 | 3.4 | 1.3e-02 |
| Bronchiectasis | 2 | 0.0155 | 0.00148 | 10.5 | 15.4 | 1.4e-02 |
| Dermatitis | 9 | 0.0698 | 0.02987 | 2.3 | 2.6 | 1.4e-02 |
| Primary biliary cirrhosis | 4 | 0.0310 | 0.00765 | 4.1 | 4.6 | 1.6e-02 |
| Lymphopenia | 2 | 0.0155 | 0.00173 | 9.0 | 12.3 | 1.9e-02 |
| Helicobacter infection | 3 | 0.0233 | 0.00494 | 4.7 | 5.5 | 2.4e-02 |
| Nephrosis | 3 | 0.0233 | 0.00494 | 4.7 | 5.5 | 2.4e-02 |
| Bronchiolitis | 2 | 0.0155 | 0.00197 | 7.9 | 10.3 | 2.5e-02 |
| Glaucoma | 4 | 0.0310 | 0.00889 | 3.5 | 3.9 | 2.6e-02 |
| Virus disease | 4 | 0.0310 | 0.00889 | 3.5 | 3.9 | 2.6e-02 |
| Celiac disease | 4 | 0.0310 | 0.00913 | 3.4 | 3.8 | 2.9e-02 |
| Ulcerative colitis | 7 | 0.0543 | 0.02320 | 2.3 | 2.5 | 2.9e-02 |
| Tuberculosis | 5 | 0.0388 | 0.01358 | 2.9 | 3.1 | 3.0e-02 |
| Keratoconjunctivitis Sicca | 2 | 0.0155 | 0.00222 | 7.0 | 8.8 | 3.1e-02 |
| Lyme disease | 2 | 0.0155 | 0.00222 | 7.0 | 8.8 | 3.1e-02 |
| Renal tubular acidosis | 2 | 0.0155 | 0.00222 | 7.0 | 8.8 | 3.1e-02 |
| Tumor virus infection | 1 | 0.0078 | 0.00025 | 31.4 | Inf | 3.2e-02 |
| Lupus erythematosus | 9 | 0.0698 | 0.03431 | 2.0 | 2.2 | 3.2e-02 |
| Henoch-Schoenlein purpura | 2 | 0.0155 | 0.00247 | 6.3 | 7.7 | 3.8e-02 |
| Advanced cancer | 2 | 0.0155 | 0.00247 | 6.3 | 7.7 | 3.8e-02 |
| Adenovirus infection | 5 | 0.0388 | 0.01456 | 2.7 | 2.9 | 3.8e-02 |
| Endometriosis | 9 | 0.0698 | 0.03579 | 1.9 | 2.1 | 4.0e-02 |
| Bladder cancer | 5 | 0.0388 | 0.01481 | 2.6 | 2.8 | 4.1e-02 |
| Vaccinia | 2 | 0.0155 | 0.00272 | 5.7 | 6.8 | 4.6e-02 |
| Periodontitis | 4 | 0.0310 | 0.01061 | 2.9 | 3.2 | 4.7e-02 |
| Hyperlipidemia | 3 | 0.0233 | 0.00642 | 3.6 | 4.0 | 4.8e-02 |
| Nasopharyngeal cancer | 3 | 0.0233 | 0.00667 | 3.5 | 3.9 | 5.3e-02 |
| Arthritis | 5 | 0.0388 | 0.01605 | 2.4 | 2.6 | 5.5e-02 |
| Lupus vulgaris | 3 | 0.0233 | 0.00691 | 3.4 | 3.7 | 5.8e-02 |
| HIV infection | 7 | 0.0543 | 0.02715 | 2.0 | 2.1 | 6.0e-02 |
| Atherosclerosis | 11 | 0.0853 | 0.05060 | 1.7 | 1.8 | 6.0e-02 |
| Gouts | 2 | 0.0155 | 0.00321 | 4.8 | 5.6 | 6.2e-02 |
| Amnionitis | 2 | 0.0155 | 0.00321 | 4.8 | 5.6 | 6.2e-02 |
| Basal cell carcinoma | 2 | 0.0155 | 0.00321 | 4.8 | 5.6 | 6.2e-02 |
| Chorioretinitis | 1 | 0.0078 | 0.00049 | 15.7 | 30.6 | 6.3e-02 |
| Fibroadenoma | 1 | 0.0078 | 0.00049 | 15.7 | 30.6 | 6.3e-02 |
| Thyroiditis | 1 | 0.0078 | 0.00049 | 15.7 | 30.6 | 6.3e-02 |
| Uterine cancer | 1 | 0.0078 | 0.00049 | 15.7 | 30.6 | 6.3e-02 |
| Cystic fibrosis | 4 | 0.0310 | 0.01210 | 2.6 | 2.8 | 6.9e-02 |
| Systemic scleroderma | 6 | 0.0465 | 0.02271 | 2.0 | 2.2 | 7.1e-02 |
| Prostate cancer | 17 | 0.1318 | 0.09084 | 1.5 | 1.5 | 7.3e-02 |
| "Skin disease, Genetic" | 3 | 0.0233 | 0.00765 | 3.0 | 3.3 | 7.4e-02 |
| Immunologic deficiency syndrome | 3 | 0.0233 | 0.00765 | 3.0 | 3.3 | 7.4e-02 |
| Leukemia | 15 | 0.1163 | 0.07801 | 1.5 | 1.6 | 7.5e-02 |
| Stomach cancer | 8 | 0.0620 | 0.03481 | 1.8 | 1.9 | 7.9e-02 |
| Hodgkin's disease | 3 | 0.0233 | 0.00790 | 2.9 | 3.2 | 8.0e-02 |
| Lung disease | 3 | 0.0233 | 0.00790 | 2.9 | 3.2 | 8.0e-02 |
| Cytomegalovirus infection | 2 | 0.0155 | 0.00370 | 4.2 | 4.7 | 8.1e-02 |
| Polymyositis | 2 | 0.0155 | 0.00370 | 4.2 | 4.7 | 8.1e-02 |
| Squamous cell cancer | 6 | 0.0465 | 0.02370 | 2.0 | 2.1 | 8.4e-02 |
| IGA glomerulonephritis | 3 | 0.0233 | 0.00839 | 2.8 | 3.0 | 9.2e-02 |
| Myeloid metaplasia | 1 | 0.0078 | 0.00074 | 10.5 | 15.3 | 9.3e-02 |
| Retinoblastoma | 1 | 0.0078 | 0.00074 | 10.5 | 15.3 | 9.3e-02 |
| Polyarthritis | 5 | 0.0388 | 0.01901 | 2.0 | 2.2 | 9.7e-02 |

---
